# Twenty-Nine New Limonoids with Skeletal Diversity from the Mangrove Plant, *Xylocarpus moluccensis*

**DOI:** 10.3390/md16010038

**Published:** 2018-01-19

**Authors:** Jianzhi Zhang, Wanshan Li, Yiguo Dai, Li Shen, Jun Wu

**Affiliations:** 1 Marine Drugs Research Center, College of Pharmacy, Jinan University, 601 Huangpu Avenue West, Guangzhou 510632, China; jierzhang1990@163.com (J.Z.); mimowanshan@163.com (W.L.); 15521337878@163.com (Y.D.); 2 School of Pharmaceutical Sciences, Southern Medical University, 1838 Guangzhou Avenue North, Guangzhou 510515, China

**Keywords:** mangrove, *Xylocarpus moluccensis*, limonoid, xylomolins, skeletal diversity, antitumor, anti-HIV

## Abstract

Twenty-nine new limonoids—named xylomolins A_1_–A_7_, B_1_–B_2_, C_1_–C_2_, D–F, G_1_–G_5_, H–I, J_1_–J_2_, K_1_–K_2_, L_1_–L_2_, and M–N, were isolated from the seeds of the mangrove plant, *Xylocarpus moluccensis*. Compounds **1**–**13** are mexicanolides with one double bond or two conjugated double bonds, while **14** belongs to a small group of mexicanolides with an oxygen bridge between C1 and C8. Compounds **15**–**19** are khayanolides containing a Δ^8,14^ double bond, whereas **20** and **21** are rare khayanolides containing a Δ^14,15^ double bond and Δ^8,9^, Δ^14,15^ conjugated double bonds, respectively. Compounds **22** and **23** are unusual limonoids possessing a (*Z*)-bicyclo[5.2.1]dec-3-en-8-one motif, while **24** and **25** are 30-ketophragmalins with Δ^8,9^, Δ^14,15^ conjugated double bonds. Compounds **26** and **27** are phragmalin 8,9,30-*ortho* esters, whereas **28** and **29** are azadirone and andirobin derivatives, respectively. The structures of these compounds, including absolute configurations of **15**–**19**, **21**–**23**, and **26**, were established by HRESIMS, extensive 1D and 2D NMR investigations, and the comparison of experimental electronic circular dichroism (ECD) spectra. The absolute configuration of **1** was unequivocally established by single-crystal X-ray diffraction analysis, obtained with Cu Kα radiation. The diverse cyclization patterns of **1**–**29** reveal the strong flexibility of skeletal plasticity in the limonoid biosynthesis of *X. moluccensis*. Compound **23** exhibited weak antitumor activity against human triple-negative breast MD-MBA-231 cancer cells with an IC_50_ value of 37.7 μM. Anti-HIV activities of **1**, **3**, **8**, **10**, **11**, **14**, **20**, **23**–**25**, and **27** were tested in vitro. However, no compounds showed potent inhibitory activity.

## 1. Introduction

Limonoids are highly oxidized tetranortriterpenoids from a biosynthetic precursor with a 4,4,8-trimethyl-17-furanylsteroid skeleton. This group of natural products has attracted considerable attention because of its abundance, fascinating structural diversity, and various biological activities [1,2,3,4]. *Xylocarpus* is a well-known genus of mangrove plants which has been found to produce various types of limonoids with a broad range of bioactivities, such as insect antifeedant, antitumor, neuroprotective, gastroprotective, and antidepressant-like activities [5,6,7,8,9,10,11]. *X. moluccensis*, a true mangrove tree, is mainly distributed in Bangladesh, India, Indochina, Malesia, and tropical Australia. Previous chemical investigations of *X. moluccensis* resulted in the isolation of more than 100 limonoids with diverse carbon skeletons, such as mexicanolide, phragmalin, gedunin, andirobin, and khayanolide compounds [12,13,14,15,16,17,18]. Limonoids with diverse skeletons from *X. moluccensis* revealed structural plasticity in the limonoid biosynthesis of this mangrove species. This inference drove us to obtain and identify more novel and bioactive limonoids from this mangrove plant. 

**Figure 1:**
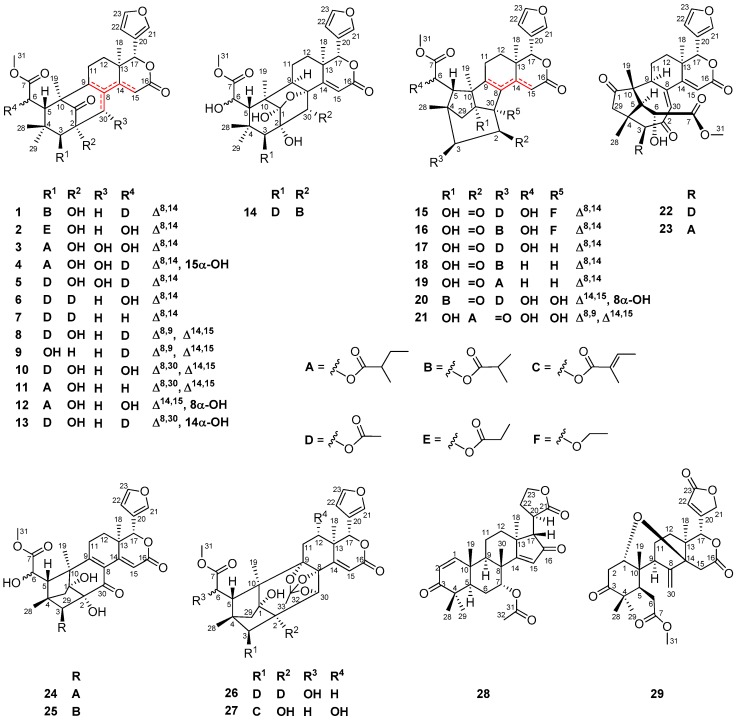
Structures of compounds **1**–**29**.

Further investigation of the seeds of *X. moluccensis* afforded fourteen mexicanolides (**1**–**14**), seven khayanolides (**15**–**21**), two unusual limonoids with a (*Z*)-bicyclo[5.2.1]dec-3-en-8-one substructure (**22**, **23**), two 30-ketophragmalins (**24**, **25**), two phragmalin 8,9,30-*ortho* esters (**26**, **27**), an azadirone derivative (**28**), and an andirobin derivative (**29**) (Figure 1). Herein, we report the isolation, structural identification, antitumor, and anti-HIV activities of these new limonoids.

## 2. Results and Discussion

In this paper, 16 compounds (*viz*. **1**, **3**–**6**, **8**, **9**, **12**–**14**, **20**, **21**, **24**, **26**, **27**, and **29**) were obtained from seeds of the Thai *X. moluccensis*, whereas 13 compounds (*viz*. **2**, **7**, **10**, **11**, **15**–**19**, **22**, **23**, **25**, and **28**) were isolated from those of the Indian *X. moluccensis*.

Compound **1** was obtained as a colorless crystal. The molecular formula of **1** was established from the positive HRESIMS ion peak at *m*/*z* 615.2785 (calcd. for [M + H]^+^, 615.2800) to be C_33_H_42_O_11_, implying thirteen degrees of unsaturation. According to the ^1^H and ^13^C NMR spectroscopic data (Table 1 and Table 2), five elements of unsaturation were due to four ester groups, a keto carbonyl function, and three carbon-carbon double bonds; thus, the molecule was pentacyclic. The ^1^H and ^13^C NMR spectroscopic data (Table 1 and Table 2) showed the presence of a β-substituted furan ring [δ_H_ 7.52 br d (*J* = 0.8 Hz H-21), 6.45 br d (*J* = 1.2 Hz H-22), 7.44 t (*J* = 1.6 Hz H-23)], four tertiary methyl groups (δ_H_ 1.27 s H_3_-19, 1.04 s H_3_-29, 1.05 s H_3_-18, 0.84 s H_3_-28), a methoxy (δ_H_ 3.73 s H_3_-31), and a keto function (δ_C_ 216.7 qC), indicating a mexicanolide-type limonoid for **1**.

**Figure 2:**
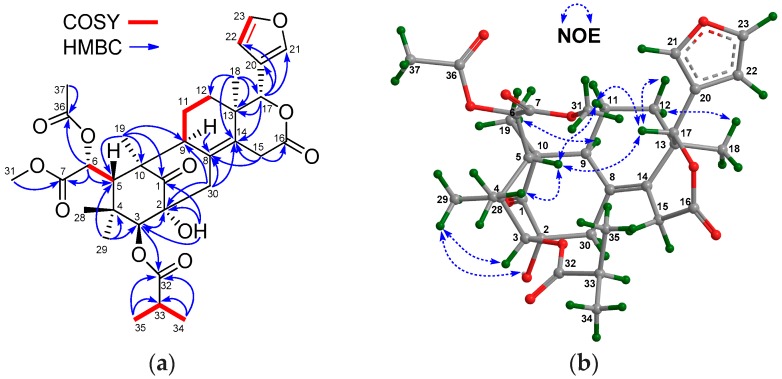
(**a**) Selected ^1^H-^1^H COSY and HMBC correlations for compound **1** (measured in CDCl_3_); (**b**) Diagnostic NOE interactions for compound **1** (measured in CDCl_3_, crystal structure of X-ray diffraction).

The NMR spectroscopic data of **1** (Table 1 and Table 2) were similar to those of khayalenoid H [19], except for the replacement of the 3-*O*-acetyl group in khayalenoid H by an isobutyryloxy group [δ_H_ 2.64 m 1H, 1.22 d (*J* = 7.2 Hz 3H), 1.24 d (*J* = 7.2 Hz 3H); δ_C_ 175.9 qC, 34.5 CH, 19.9 CH_3_, 18.4 CH_3_] in **1**. The presence of the isobutyryloxy group was corroborated by ^1^H-^1^H COSY correlations between H_3_-34/H-33 and H_3_-35/H-33. The significant HMBC correlation from H-3 (δ_H_ 4.97 s) to the carbonyl carbon (δ_C_ 175.9 qC) of the isobutyryloxy group placed it at C-3 (Figure 2a). 

The relative configuration of **1** was established by diagnostic NOE interactions (Figure 2b). Those between H-17/H-11β, H-17/H-12β, H-17/H-5, and H-5/H_3_-28 revealed their cofacial relationship, and were arbitrarily assigned as the β-oriented H-17 and H-5. NOE interactions between H-9/H-12α, H-9/H_3_-19, H_3_-18/H-12α, H-3/H_3_-29, and 2-OH/H_3_-29 assigned the α-orientation for H-9, H_3_-18, H_3_-19, H-3, and 2-OH. 

**Figure 3:**
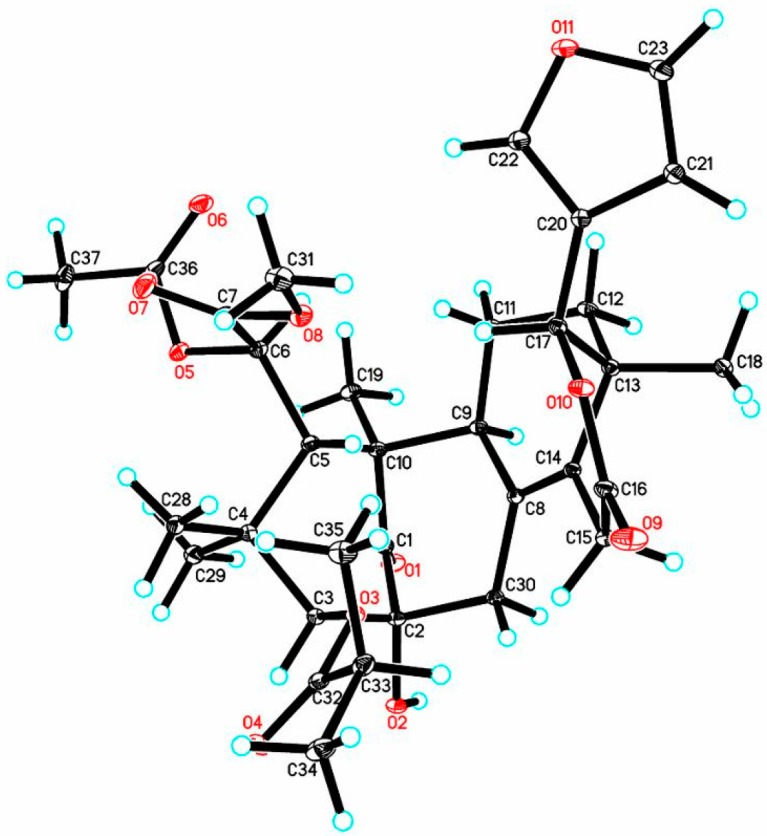
Oak Ridge Thermal-Ellipsoid Plot Program (ORTEP) illustration of the X-ray structure of compound **1**. Ellipsoids are given at the 30% probability level.

In order to establish the absolute configuration of **1**, single-crystal X-ray diffraction analysis with Cu Kα radiation (Flack parameter of −0.06 (10), Flack x of −0.14 (11), and Hooft y of −0.04 (3); suitable crystals of **1** were obtained from acetone/methanol (1:2) at room temperature) was employed, which unequivocally assigned the absolute configuration of **1** as 2*R*,3*S*,5*S*,6*R*,9*S*,10*R*,13*R*,17*R*. The computer-generated perspective drawing of the X-ray structure of **1** is shown in Figure 3. Therefore, the absolute configuration of **1** named xylomolin A_1_ was assigned as shown (Figure 2a).

Compound **2** was obtained as an amorphous white powder. The molecular formula was determined to be C_30_H_38_O_10_ by the negative HRESIMS ion peak at *m*/*z* 593.2143 (calcd. for [M + Cl]^−^, 593.2159). The NMR spectroscopic data of **2** resembled those of **1** (Table 1 and Table 2), except for the replacement of the 3-*O*-isobutyryl moiety and the 6-*O*-acetyl group in **1** by a 3-*O*-propionyl moiety (δ_H_ 2.45 m 1H, 2.43 m 1H, 1.20 t (*J* = 7.6 Hz 3H); δ_C_ 173.3 qC, 28.0 CH_2_, 9.4 CH_3_) and a 6-OH group in **2**, respectively. The presence of a 3-*O*-propionyl moiety was confirmed by ^1^H-^1^H COSY correlations between H_3_-34/H_2_-33 and HMBC cross-peaks between H_3_-34/C-33, H_3_-34/C-32, H_2_-33/C-32, and H-3/C-32. The relative configuration of **2** (except for that of C-6) was determined to be the same as that of **1** by NOE interactions between H-17/H-15β, H-15β/H-30β, H-17/H-12β, H-17/H-11β, H-11β/H-5, H-17/H-5, and H-5/H_3_-28, and those between H-9/H_3_-19, H_3_-19/H_3_-29, H_3_-29/H-3, and H_3_-18/H-15α. Thus, the structure of **2**—named xylomolin A_2_—was assigned as shown in Figure 1. 

Compound **3** gave the molecular formula C_32_H_42_O_11_ as established by the HRESIMS ion peak at *m*/*z* 625.2620 (calcd. for [M + Na]^+^, 625.2619). The NMR spectroscopic data of **3** (Table 1 and Table 2) were similar to those of moluccensin S [20], except for the presence of an additional 30-OH group, which was supported by the downshifted C-30 signal (δ_C_ 73.0 CH in **3**, whereas δ_C_ 44.6 CH_2_ in moluccensin S) and HMBC correlations from the proton of 30-OH to C-30, C-2, and C-8. NOE interactions between H-17/H-12β, H-17/H-11β, H-5/H-12β, H-5/H-17, H-5/H_3_-28, and H-5/H-30 revealed the β-orientation for H-5, H-17, H-30, and H_3_-28, and the corresponding 30α-OH. Similarly, those between H-3/2-OH, H_3_-29/2-OH, H-9/H_3_-19, H_3_-29/H_3_-19, and H_3_-18/H-11α assigned the α-orientation for H-3, H-9, H_3_-18, H_3_-19, H_3_-29, and 2-OH. Thus, the structure of **3**—named xylomolin A_3_—was determined to be 30α-hydroxy-moluccensin S.

Compound **4** provided the molecular formula of C_34_H_44_O_13_ as determined from the positive HRESIMS ion peak at *m*/*z* 683.2675 (calcd. for [M + Na]^+^, 683.2674). The ^1^H and ^13^C NMR spectroscopic data of **4** resembled those of **3** (Table 1 and Table 2), the difference being the presence of a 15-OH function (δ_H_ 3.66 br s) and a 6-acetoxy group (δ_H_ 2.18 s 3H; δ_C_ 169.8 qC, 21.0 CH_3_) in **4**. The existence of a 15-OH function was corroborated by the downshifted C-15 signal (δ_C_ 65.4 CH in **4**, whereas δ_C_ 32.8 CH_2_ in **3**) and HMBC correlations from the proton of 15-OH to C-14, C-15, and C-16. The HMBC correlation from H-6 to the carbonyl carbon (C-37) of the acetoxy group placed it at C-6. The relative configuration of **4** (except that of C-15) was assigned as the same as that of **3** on the basis of NOE interactions. Those between H-17/H-15 and H-5/H-30 assigned the β-oriented H-15 and H-30, and the corresponding 15α-OH group. Consequently, the structure of **4**—named xylomolin A_4_—was identified as 15α-hydroxy-6-acetoxy-xylomolin A_3_.

The molecular formula of **5** was determined to be C_31_H_38_O_12_ by the positive HRESIMS ion peak at *m*/*z* 625.2252 (calcd. for [M + Na]^+^, 625.2255). The ^1^H and ^13^C NMR spectroscopic data of **5** (Table 1 and Table 2) were closely related to those of khayalenoid H, the difference being the existence of a 30-OH function, which was supported by the downshifted C-30 signal (δ_C_ 72.9 CH in **5**, whereas δ_C_ 44.4 CH_2_ in khayalenoid H). HMBC correlations from the proton of 30-OH (δ_H_ 2.67 br s) to C-30 and C-8 demonstrated the above deduction. The NOE interaction between H-5/H-30 assigned the β-oriented H-30 and the corresponding 30α-OH. Thus, the structure of **5**—named xylomolin A_5_—was assigned as 30α-hydroxy-khayalenoid H.

Compound **6** was isolated as a white and amorphous powder. Its molecular formula was determined to be C_31_H_38_O_11_ from the positive HRESIMS ion peak at *m*/*z* 609.2311 (calcd. for [M + Na]^+^, 609.2306). The ^1^H and ^13^C NMR spectroscopic data of **6** (Table 1 and Table 2) closely resembled those of khayalenoid H [19], except for the replacement of the 2-OH function and the 6-*O*-acetyl group in khayalenoid H by a 2-*O*-acetyl (δ_H_ 2.13 s 3H; δ_C_ 169.1 qC, 21.8 CH_3_) and a 6-OH group (δ_H_ 2.80 br s) in **6**, respectively. HMBC correlations from the proton of the 6-OH group to C-5, C-6,and C-7 placed it at C-6. The presence of the 2-*O*-acetyl group in **6** was corroborated by the downshifted C-2 in **6** (δ_C_ 86.5 qC in **6**, whereas δ_C_ 77.9 qC in khayalenoid H). HMBC correlations from H_2_-30 and H-3 to C-2 confirmed the above deduction. The relative configuration of **6** was determined to be the same as that of khayalenoid H based on diagnostic NOE interactions between H-17/H-15β, H-17/H-12β, H-17/H-5, and H-5/H_3_-28 and those between H-9/H_3_-19, H_3_-19/H_3_-29, and H_3_-18/H-15α. Therefore, the structure of **6**—named xylomolin A_6_—was determined to be 2-*O*-acetyl-6-*O*-deacetyl-khayalenoid H.

The molecular formula of **7** was determined to be C_31_H_38_O_10_ by the positive HRESIMS ion peak at *m*/*z* 593.2358 (calcd. for [M + Na]^+^, 593.2357). The ^1^H and ^13^C NMR spectroscopic data of **7** (Table 1 and Table 2) were closely related to those of **6**, except for the absence of the 6-OH group, which was corroborated by the upshifted CH_2_-6 signal (δ_H_ 2.32 dd (*J* = 16.5, 1.6 Hz), 2.42 dd (*J* = 16.5, 10.7 Hz), δ_C_ 33.3) in **7**. ^1^H-^1^H COSY correlations between H_2_-6/H-5 and HMBC cross-peaks from H_2_-6 and C-5 and C-7 confirmed the above result. The analysis of diagnostic NOE interactions revealed that **7** possessed the same relative configuration as that of **6**. Therefore, the structure of **7**—named xylomolin A_7_—was assigned as 6-dehydroxy-xylomolin A_6_.

Compound **8** was isolated as a white and amorphous powder. It had a molecular formula of C_31_H_36_O_11_ as deduced from the positive HRESIMS ion peak at *m*/*z* 607.2151 (calcd. for [M + Na]^+^, 607.2150). The ^1^H and ^13^C NMR spectroscopic data of **8** (Table 3 and Table 4) were similar to those of heytrijunolide E [21] with Δ^8,9^ and Δ^14,15^ conjugated double bonds, except for the absence of the 15-OH group and the presence of an acetoxy group at C-3 in **8**. The absence of the 15-OH group was confirmed by the upshifted C-15 signal (δ_C_ 111.6 qC in **8**, whereas δ_C_ 134.7 qC in heytrijunolide E) and HMBC correlations between H-15/C-14, H-15/C-16, H-15/C-8, and H-15/C-13. The HMBC correlation from H-3 (δ_H_ 5.51 s) to the carbonyl carbon (δ_C_ 170.2 qC) of the acetoxy group placed it at C-3. The relative configuration of **8** was established to be the same as that of heytrijunolide E based on diagnostic NOE interactions between H_3_-28/H-5, H-5/H-11β, H-12β/H-17, H_3_-29/H-3, H-12α/H_3_-18, and H-11α/H_3_-19. Therefore, the structure of **8**—named xylomolin B_1_—was assigned as 15-dehydroxy-3β-acetoxy-heytrijunolide E.

Compound **9** provided the molecular formula C_29_H_3__4_O_9_ as established by the positive HRESIMS ion peak at *m*/*z* 527.2273 (calcd. for [M + H]^+^, 527.2276). The ^1^H and ^13^C NMR spectroscopic data of **9** (Table 3 and Table 4) were similar to those of heytrijunolide E [21], the difference being the absence of the 2-OH and 15-OH groups in **9**, which was corroborated by the upshifted C-15 (δ_C_ 110.6 CH in **9**, whereas δ_C_ 134.7 qC in heytrijunolide E) and C-2 (δ_C_ 50.5 CH in **9**, whereas δ_C_ 78.2 qC in heytrijunolide E) signals. A proton (δ_H_ 3.02 t (*J* = 6.0 Hz)) exhibiting ^1^H-^1^H COSY correlations to H-3 and H-30 and HMBC cross-peaks to C-1, C-3, C-4, and C-30 was assigned as H-2. The existence of H-15 (δ_H_ 5.89 s) was further confirmed by its HMBC correlations to C-8, C-13, C-14, and C-16. The relative configuration of **9** was determined to be the same as that of heytrijunolide E based on diagnostic NOE interactions between H_3_-28/H-5, H-5/H-11β, H-12β/H-17, H_3_-29/H-3, H-12α/H_3_-18, and H-11α/H_3_-19. Thus, the structure of **9**—named xylomolin B_2_—was assigned as 2,15-dedihydroxy-heytrijunolide E.

Compound **10** had a molecular formula of C_29_H_34_O_1__0_ as determined from the positive HRESIMS ion peak at *m*/*z* 543.2238 (calcd. for [M + H]^+^, 543.2230). The NMR data of **10** (Table 3 and Table 4) closely resembled those of moluccensin U [20], except for the replacement of the 3-*O*-(2-methyl)butyryl group in moluccensin U by an acetoxy group (δ_H_ 2.22 s 3H; δ_C_ 169.9 qC, 20.7 CH_3_) in **10**. The significant HMBC cross-peak from H-3 (δ_H_ 4.83 s) to the carbonyl carbon of the above acetoxy group placed it at C-3. NOE interactions between H-11β/H-17, H-12β/H-17, H-11β/H-5, H-5/H_3_-28, H-9/H_3_-18, H-9/H_3_-19, and H_3_-29/H-3 revealed the same relative configuration of **10** as that of moluccensin U. Therefore, the structure of **10**—named xylomolin C_1_—was assigned as 3-*O*-acetyl-3-de(2-methyl) butyryloxy-moluccensin U.

Compound **11** afforded the molecular formula C_32_H_40_O_9_ as established by the positive HRESIMS ion peak at *m*/*z* 591.2570 (calcd. for [M + Na]^+^, 591.2565). The ^1^H and ^13^C NMR spectroscopic data of **11** (Table 3 and Table 4) closely resembled those of swietmanin G [22], the difference being the replacement of the 3-isobutyryloxy group in swietmanin G by a 2-methylbutyryloxy group (δ_H_ 2.57 m 1H, 1.61 m 1H, 1.81 m 1H, 1.04 t (*J* = 7.2 Hz 3H), 1.25 d (*J* = 6.8 Hz 3H); δ_C_ 176.0 qC, 41.4 CH, 26.9 CH_2_, 11.9 CH_3_, 17.0 CH_3_) in **11**. The deduction was confirmed by ^1^H-^1^H COSY correlations between H_3_-36/H-33, H_3_-35/H_2_-34, and H_2_-34/H-33, and HMBC correlations between H-3/C-32, H_3_-35/C-34, H_3_-35/C-33, H_3_-36/C-33, H_3_-36/C-32, and H_3_-36/C-34. NOE interactions between H-12β/H-17, H-17/H-11β, H-5/H-11β, H-5/H_3_-28, H-9/H_3_-18, H-9/H_3_-19, and H_3_-29/H-3 assigned the α-orientation for H-9, H_3_-18, H_3_-19, and H-3. Thus, the structure of **11**—named xylomolin C_2_—was assigned as 3-*O*-(2-methyl)butyryl-3-deisobutyryloxy-swietmanin G.

Compound **12** had the molecular formula C_32_H_42_O_11_ as determined from the positive HRESIMS ion peak at *m*/*z* 625.2612 (calcd. for [M + Na]^+^, 625.2619). The ^1^H and ^13^C NMR spectroscopic data of **12** (Table 3 and Table 4) were similar to those of moluccensin U [20], except for the absence of the Δ^8,30^ double bond and the presence of an 8-OH group. This finding was verified by the upshifted C-8 (δ_C_ 71.3 qC) and C-30 (δ_C_ 45.9 CH_2_) signals as compared with those (δ_C_ 134.5 qC and 133.6 CH) of moluccensin U, respectively. The presence of the 8-OH group was confirmed by HMBC correlations from its proton to C-8, C-9, and C-30. NOE interactions between 8-OH/H-9 and 8-OH/H_3_-18 assigned the α-orientation for the 8-OH group. The relative configuration of **12** (except that of C-8) was established to be identical to that of moluccensin U based on diagnostic NOE interactions between H-12β/H-17, H-5/H_3_-28, H-9/H_3_-19, and H_3_-29/H-3. Thus, the structure of **12**—named xylomolin D—was assigned as 8,30-dihydrogen-8α-hydroxy-moluccensin U.

**Figure 4:**
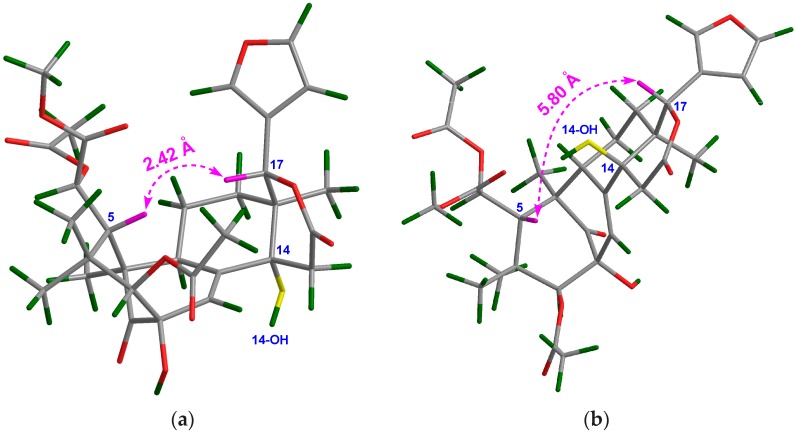
Molecular Mechanics, Allinger Force Field version 2 (MM2)-optimized two possible 3D structures for compound **13**. (**a**) The structure with a 14α-OH group; (**b**) The structure with a 14β-OH group.

Compound **13** was obtained as an amorphous white power. The molecular formula was determined to be C_31_H_38_O_12_ by the positive HRESIMS ion peak at *m*/*z* 625.2250 (calcd. for [M + Na]^+^, 625.2255). The ^1^H and ^13^C NMR spectroscopic data of **13** (Table 3 and Table 4) were similar to those of **8**, except for the replacement of Δ^8,9^ and Δ^14,15^ conjugated double bonds in **8** by a Δ^8,^^30^ double bond (δ_C_ 138.8 qC C-8, 130.2 CH C-30) in **13**, and the presence of an additional 14-OH group. HMBC correlations between H_2_-15/C-14, H_2_-15/C-16, H_2_-15/C-13, H-30/C-14, H-30/C-9, and H-9/C-8 demonstrated the above deduction. In order to establish the relative configuration of the 14-OH group in **13**, two possible 3D structures with a 14α-OH and 14β-OH groups, respectively, were simulated by using the ChemBio3D software (Figure 4). When the 14-OH group occupies α-orientation, the space distance between H-5/H-17 is around 2.4 Å (Figure 4a), implying the presence of a strong NOE interaction between these protons. On the contrary, when the 14-OH group occupies the β-orientation, the space distance between H-5/H-17 is around 5.8 Å (Figure 4b), indicating the absence of a NOE interaction between these protons. Quite evidently, the NOE interaction between H-5/H-17 could be utilized as an effective criterion to resolve the relative configuration of the 14-OH group. Thus, the orientation of the 14-OH group in **13** was assigned as α based on the strong NOE interaction between H-5/H-17. Furthermore, the relative configuration of the whole molecule of **13** (except that of C-14) was determined to be the same as that of **8** on the basis of NOE interactions between H_3_-28/H-5, H-5/H-11β, H-12β/H-17, H_3_-29/H-3, H-12α/H_3_-18, and H-11α/H_3_-19. Thus, the structure of **13**—named xylomolin E—was assigned as depicted.

Compounds **2**–**13** are analogues of **1**. From the point of view of biogenetic origins, these mexicanolides should possess the same absolute configurations of carbon skeletons as that of **1**. The absolute sterostructures of **2**–**13** are shown as in Figure 1.

The molecular formula of **14** was determined to be C_33_H_42_O_13_ by the positive HRESIMS ion peak at *m*/*z* 669.2521 (calcd. for [M + Na]^+^, 669.2518). The NMR spectroscopic data of **14** (Table 3 and Table 4) were similar to those of xylorumphiin H [23], being a mexicanolide containing a C1-*O*-C8 bridge, except for the presence of an additional Δ^14,15^ double bond (δ_H_ 6.08 s 1H; δ_C_ 158.3 qC, 118.4 CH) and an additional 6-OH function (δ_H_ 2.93 s) in **14**. The existence of the Δ^14,15^ double bond was corroborated by HMBC correlations between H_3_-18/C-14, H-15/C-8, and H-15/C-16. The downshifted C-6 signal (δ_C_ 71.7 CH in **14**, whereas δ_C_ 32.3 CH_2_ in xylorumphiin H), along with HMBC cross-peaks from H-6 to C-5 and C-7, supported the location of a hydroxy group at C-6. Similar NOE interactions of **14** as those of xylorumphiin H suggested that both mexicanolides possessed the same relative configuration. Thus, the structure of **14**—named xylomolin F—was assigned as 6-hydroxy-14,15-dedihydrogen-xylorumphiin H.

**Figure 5:**
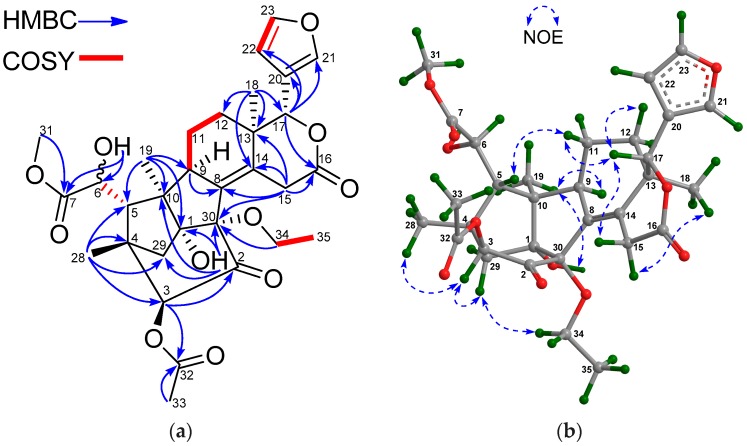
(**a**) Selected ^1^H-^1^H COSY and HMBC correlations for compound **15** (measured in CDCl_3_); (**b**) Diagnostic NOE interactions for compound **15** (measured in CDCl_3_, MM2-optimized structure).

The molecular formula of **15** was established by the positive HRESIMS ion peak at *m*/*z* 587.2494 (calcd. for [M + H]^+^, 587.2492) to be C_31_H_38_O_11_, implying thirteen degrees of unsaturation. According to the NMR spectroscopic data of **15** (Table 5 and Table 6), seven elements of unsaturation were due to three carbon-carbon double bonds, one carbonyl group, and three ester functionalities; thus, **15** should be hexacyclic. The NMR spectroscopic data of **15** resembled those of thaixylomolin L [18], being a khayanolide isolated from seeds of the Thai *X. moluccensis*, except for the presence of an additional 6-OH group in **15**. Strong ^3^*J* HMBC correlations from H_2_-29 to C-30 further confirmed a khayanolide for **15** instead of a phragmalin, which should exhibit weak ^4^*J* HMBC correlations between H_2_-29/C-30. HMBC cross-peaks from an active proton (δ_H_ 2.91 d (*J* = 3.4 Hz)) to C-5 (δ_C_ 45.4, CH), C-6 (δ_C_ 72.0, CH), and C-7 (δ_C_ 175.3, qC) (Figure 5a) revealed the existence of a 6-OH group in **15**.

**Figure 6:**
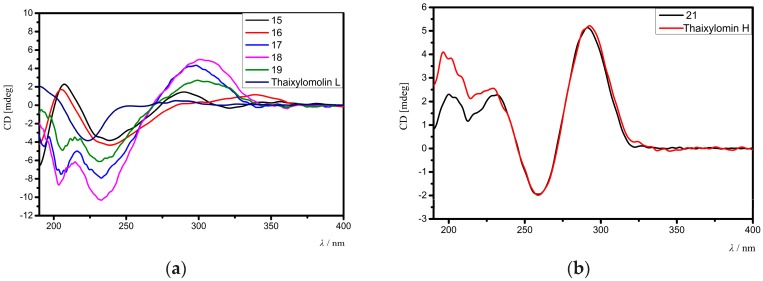
(**a**) Comparison of the experimental electronic circular dichroism (ECD) spectra of compounds **15**–**19** with that of the known compound, thaixylomolin L, containing a Δ^8,^^14^ double bonds; (**b**) Comparison of the experimental ECD spectra of compound **21** with that of the known compound, thaixylomolin H, containing Δ^8,9^, Δ^14^^,^^15^ conjugated double bonds.

The relative configuration of **15** was assigned by analysis of NOE interactions (Figure 5b). Those between H-17/H-12β, H-17/H-15β, H-17/H-11β, and H-11β/H-5 revealed their cofacial relationship and were assigned as β-oriented. In turn, NOE interactions between H_3_-18/H-15α, H-9/H_3_-19, H_3_-19/1-OH, and H-34/H_pro__-*R*_-29 indicated the α-orientation for H-9, H_3_-18, H_3_-19, 1-OH, and 30-OEt. The NOE interaction between H-3/H_pro__-*R*_-29 established the 3α-H and the corresponding 3β-acetoxy function. Therefore, the relative configuration of **15** was determined. Comparison of the electronic circular dichroism (ECD) spectrum of **15** with that of thaixylomolin L [18] showed that **15** had the same 1*R*,3*S*,4*R*,5*S*,9*R*,10*S*,13*R*,17*R*,30*S*-absolute configuration as that of thaixylomolin L (Figure 6a). Thus, the structure of **15**—named xylomolin G_1_—was assigned as depicted.

Compound **16** had a molecular formula of C_33_H_42_O_11_ as determined from the positive HRESIMS ion peak at *m*/*z* 615.2809 (calcd. for [M + H]^+^, 615.2805). The NMR spectroscopic data of **16** (Table 5 and Table 6) were closely related to those of **15**, the difference being the replacement of 3-acetoxy group in **15** by an isobutyryloxy group (δ_H_ 2.57 m, 1.20 d (*J* = 7.2 Hz), 1.22 d (*J* = 7.2 Hz); δ_C_ 176.0 qC, 33.8 CH, 19.0 CH_3_, 19.1 CH_3_) in **16**. The presence of the above isobutyryloxy group was further supported by ^1^H-^1^H COSY correlations between H_3_-34/H-33 and H_3_-35/H-33, and HMBC correlations between H-33/C-32, H_3_-34/C-32, and H_3_-35/C-32. The significant HMBC cross-peak from H-3 to the carbonyl carbon (C-32) of the isobutyryloxy group confirmed its location at C-3. NOE interactions between H-17/H-12β, H-17/H-15β, H-17/H-11β, H-11β/H-5, H-17/H-5, H-5/H_3_-28, H-3/H_pro_-*_R_*-29, H-15α/H_3_-18, H_3_-18/H-9, H-9/H_3_-19, and 1-OH/H-9 indicated the same relative configuration of **16** as that of **15**. Comparison of the ECD spectrum of **16** with that of thaixylomolin L concluded that **16** had the same 1*R*,3*S*,4*R*,5*S*,9*R*,10*S*,13*R*,17*R*,30*S*-absolute configuration as that of thaixylomolin L (Figure 6a). Thus, the structure of **16**—named xylomolin G_2_—was assigned as 3-*O*-isobutyryl-3-deacetoxy-xylomolin G_1_.

Compound **17** was isolated as an amorphous yellow solid. Its molecular formula was determined to be C_29_H_34_O_10_ by the positive HRESIMS ion peak at *m*/*z* 543.2246 (calcd. for [M + H]^+^, 543.2230). The similarities between the NMR spectroscopic data of **17** (Table 5 and Table 6) and **15** revealed their close structural resemblance, except for the absence of the ethoxyl group at C-30, which was confirmed by the upshifted C-30 signal (δ_C_ 63.0 CH in **17**, whereas δ_C_ 92.2 qC in **15**) and HMBC cross-peaks between H-30/C-1, H-30/C-2, H-30/C-8, and H-30/C-10. The relative configuration of **17** was determined to be identical to that of **15** by analysis of NOE interactions. Comparison of ECD spectra of **17** and **15** concluded that both compounds had the same 1*R*,3*S*,4*R*,5*S*,9*R*,10*S*,13*R*,17*R*,30*S*-absolute configuration (Figure 6a). Thus, the structure of **17**—named xylomolin G_3_—was assigned as 30-deethoxyl-xylomolin G_1_.

Compound **18** provided the molecular formula C_31_H_38_O_9_ as established by the positive HRESIMS ion peak at *m*/*z* 555.2593 (calcd. for [M + H]^+^, 555.2594). The NMR spectroscopic data of **18** (Table 5 and Table 6) were similar to those of **15**, the difference being the absence of the 30-ethoxyl group and the 6-OH function in **18**. The upshifted C-30 (δ_C_ 63.4 CH in **18**, whereas δ_C_ 92.2 qC in **15**) and C-6 (δ_C_ 34.2 CH_2_ in **18**, whereas δ_C_ 72.1 CH in **15**) signals and HMBC cross-peaks between H-30/C-1, H-30/C-2, H-30/C-8, H-30/C-10, H-6/C-5, and H-6/C-7 supported the above deduction. The relative and absolute configurations of **18** were determined to be the same as that of **15** by analysis of their NOE interactions and ECD spectra (Figure 6a). Thus, the structure of **18**—named xylomolin G_4_—was concluded to be 30-deethoxyl-6-dehydroxy-xylomolin G_1_.

Compound **19** gave the molecular formula C_32_H_40_O_9_ as determined from the positive HRESIMS ion peak at *m*/*z* 569.2753 (calcd. for [M + H]^+^, 569.2751). The NMR data of **19** (Table 5 and Table 6) were closely related to those of **18**, except for the replacement of the 3-isobutyryloxy group in **18** by a 2-methylbutyryloxy group (δ_H_ 2.47 m 1H, 1.73 m 1H, 1.52 m 1H, 0.94 t (*J* = 7.2 Hz 3H), 1.19 d (*J* = 7.2 Hz 3H); δ_C_ 176.0 qC, 40.8 CH, 26.7 CH_2_, 11.5 CH_3_, 16.5 CH_3_) in **19**. The presence of the 2-methylbutyryloxy group was supported by the ^1^H-^1^H COSY correlations between H-33/H-34, H-33/H_3_-36, and H-34/H_3_-35 and HMBC cross-peaks between H-33/C-32, H_2_-34/C-32, H_3_-36/C-32, H_3_-36/C-34, and H_3_-35/C-33. The significant HMBC cross-peak from H-3 to the carbonyl carbon (C-32) of the above 2-methylbutyryloxy group assigned its location at C-3. The relative and absolute configurations of **19** were determined to be the same as those of **18** by analysis of their NOE interactions and ECD spectra (Figure 6a). Thus, the structure of **19**—named xylomolin G_5_—was assigned as 3-*O*-(2-methyl)butyryl-3-deisobutyryloxy-xylomolin G_4_.

Compound **20** was isolated as an amorphous white powder. Its molecular formula was determined to be C_33_H_40_O_13_ by the positive HRESIMS ion peak at *m*/*z* 667.2359 (calcd. for [M + Na]^+^, 667.2361). The NMR spectroscopic data of **20** (Table 5 and Table 6) resembled those of **15**, except for the presence of an additional 8-OH group (δ_H_ 5.00 s) and the replacement of the Δ^8,^^14^ double bond, 1-OH function, and 30-ethoxyl group in **15** by a Δ^14^^,^^15^ double bond (δ_H_ 5.72 br s H-15; δ_C_ 160.0 qC C-14, 120.2 CH C-15), a 1-*O*-isobutyryl moiety (δ_H_ 2.55 m H-35, 1.11 d (*J* = 7.2 Hz H_3_-36), 1.10 d (*J* = 7.2 Hz H_3_-37); δ_C_ 176.0 qC C-34, 35.1 CH C-35, 19.4 CH_3_ C-36, 19.3 CH_3_ C-37), and a 30-OH group (δ_H_ 4.87 s) in **20**, respectively (Table 5 and Table 6, recorded in CDCl_3_). HMBC correlations between H-15/C-14, H-15/C-16, H-15/C-13, H-15/C-8, 8-OH/C-8, and 30-OH/C-30 confirmed the presence of a Δ^14^^,^^15^ double bond and the existence of two hydroxy groups at C-8 and C-30, respectively. The presence of the isobutyryloxy group was supported by ^1^H-^1^H COSY cross-peaks between H-35/H_3_-36 and H-35/H_3_-37 and HMBC correlations between H-35/C-34, H_3_-36/C-34, and H_3_-37/C-34. Its location at C-1 was corroborated by the downshifted C-1 signal (δ_C_ 92.6 qC in **20**, whereas δ_C_ 84.5 qC in **15**). The relative configuration of **20** was established by NOE interactions, in which those between H-17/H-12β, H-12β/H-5, H-5/H_3_-28 assigned the β-orientation for H-17, H-5, H_3_-28, whereas those between H-11α/H_3_-18, H_3_-18/8-OH, 8-OH/H-9, H_pro__-*R*_-29/H-3, H-9/H_3_-19, and H-3/30-OH concluded the α-orientation for H_3_-18, 8-OH, H-9, H-3, H_3_-19, and 30-OH. Thus, the structure of **20**—named xylomolin H—was assigned as depicted.

Compound **21** afforded the molecular formula C_32_H_38_O_11_ as deduced from the positive HRESIMS ion peak at *m*/*z* 621.2304 (calcd. for [M + Na]^+^, 621.2306). The NMR spectroscopic data of **21** (Table 5 and Table 6) were similar to those of thaixylomolin H [18], except for the presence of an additional 6-OH group (δ_H_ 3.19 s) and the replacement of the 2-acetoxy group by a 2-methylbutyryloxy moiety (δ_H_ 2.42 m 1H, 1.73 m 1H, 1.48 m 1H, 0.97 t (*J* = 7.6 Hz 3H), 1.22 d (*J* = 7.2 Hz 3H); δ_C_ 176.0 qC, 41.3 CH, 26.5 CH_2_, 11.7 CH_3_, 17.2 CH_3_) in **21**. The presence of the 6-OH group was supported by the downshifted C-6 signal (δ_C_ 71.0 CH in **21**, whereas δ_C_ 31.6 CH_2_ in thaixylomolin H), the ^1^H-^1^H COSY cross-peak between H-5/H-6, and HMBC correlations between H-6/C-5 and H-6/C-7. The existence of the 2-methylbutyryloxy group was corroborated by ^1^H-^1^H COSY cross-peaks between H-33/H_3_-36, H-33/H-34, and H_2_-34/H_3_-35 and HMBC correlations between H-33/C-32, H-34/C-32, H_3_-36/C-32, H-33/C-34, H_3_-36/C-34, H_3_-35/C-34, H-34/C-33, H_3_-35/C-33, and H_3_-36/C-33. The significant HMBC correlation from H-2 to the carbonyl carbon (C-32) of the 2-methylbutyryloxy group placed it at C-2. NOE interactions between H-17/H-12β, H-11α/H_3_-19, H-11α/H_3_-18, H-11β/H-5, H-5/H_3_-28, H_3_-19/1-OH, H_pro__-*R*_-29/H-2, and H-2/30-OH assigned the β-orientation for H-17, H_3_-28, and H-5, and the α-orientation for H-2, H_3_-18, H_3_-19, 30-OH, and 1-OH. The ECD spectrum of **21** was identical to that of thaixylomolin H (Figure 6b), concluding that **21** had the same 1*R*,2*R*,4*R*,5*R*,10*S*,13*R*,17*R*,30*R*-absolute configuration as that of thaixylomolin H. Thus, the structure of **21**—named xylomolin I—was identified as 6-hydroxy-2-*O*-(2-methyl)butyryl-2-deacetoxy-thaixylomolin H.

**Figure 7:**
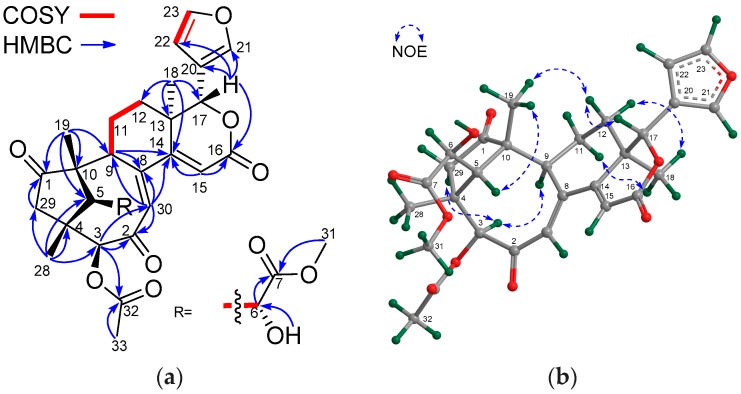
(**a**) Selected ^1^H-^1^H COSY and HMBC correlations for compound **22** (measured in CDCl_3_); (**b**) Diagnostic NOE interactions for compound **22** (measured in CDCl_3_, MM2-optimized structure).

**Figure 8:**
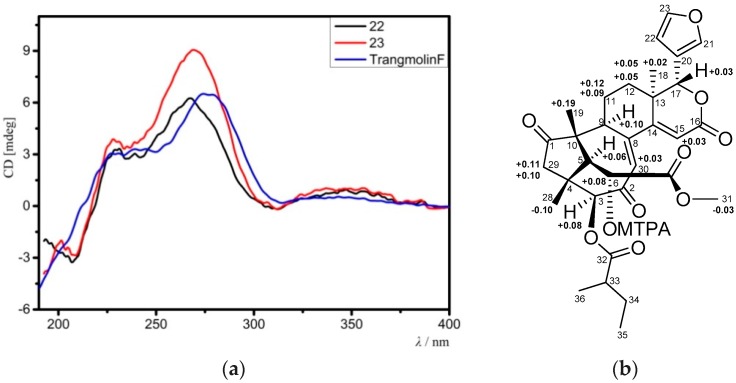
(**a**) Comparison of the experimental ECD spectra of compounds **22**, **23** with that of the known compound, trangmolin F; (**b**) Δδ values (Δδ [ppm] = [δ*_S_* − δ*_R_*]) obtained for the (6*S*) and (6*R*)-MTPA esters of **23**. MTPA: α-methoxy-α-(trifluoromethyl)phenylacetyl.

Compound **22** had the molecular formula C_29_H_32_O_10_ as determined from the positive HRESIMS ion peak at *m*/*z* 541.2077 (calcd. for [M + H]^+^, 541.2074). The similarities between the NMR spectroscopic data of **22** (Table 7 and Table 8) and those of trangmolin F [16], containing a (*Z*)-bicyclo[5.2.1]dec-3-en-8-one substructure, revealed their close structural resemblance. However, the 3-*O*-isobutyryl function in trangmolin F was replaced by an acetoxy group (δ_H_ 2.17 s 3H; δ_C_ 170.4 qC, 20.6 CH_3_) in **22**, being unambiguously confirmed by HMBC cross-peaks between H-3/C-32 and H_3_-33/C-32 (Figure 7a). The relative configuration of **22** was assigned by NOE interactions (Figure 7b). Those between H-17/H-12β, H-12β/H_3_-19, and H_3_-19/H-5 revealed their cofacial relationship and were determined as β-oriented, whereas those between H-3/H-9, H_pro-*R*_-29/H-3, and H-12α/H_3_-18 indicated the α-orientation for H-3, H-9, and H_3_-18, and the corresponding 3β-acetoxy function. The ECD spectrum of **22** was nicely matched with that of trangmolin F (Figure 8a), concluding that the absolute configuration of **22** was the same as that of trangmolin F. Thus, the structure of **22**—named xylomolin J_1_—was assigned as 3-*O*-acetyl-3-deisobutyryloxy-trangmolin F.

Compound **23** provided the molecular formula of C_32_H_38_O_10_ as established by the positive HRESIMS ion peak at *m*/*z* 583.2548 (calcd. for [M + H]^+^, 583.2543). The NMR spectroscopic data of **23** (Table 7 and Table 8) resembled those of **22**, except for the replacement of the 3-acetoxy group in **22** by a 2-methylbutyryloxy moiety (δ_H_ 2.51 m 1H, 1.53 m 1H, 1.74 m 1H, 0.96 t (*J* = 7.2 Hz, 3H), 1.18 d (*J* = 7.2 Hz, 3H); δ_C_ 176.2 qC, 40.7 CH, 26.6 CH_2_, 11.5 CH_3_, 16.4 CH_3_) in **23**. The presence of the 2-methylbutyryloxy group was further evidenced by ^1^H-^1^H COSY cross-peaks between H-33/H_3_-36, H-33/H_2_-34, and H_2_-34/H_3_-35 and HMBC correlations between H-33/C-32, H-34/C-32, and H_3_-36/C-32. The HMBC correlation from H-3 to the carbonyl carbon (C-32) of the above 2-methylbutyryloxy group placed it at C-3. The relative configuration of **23** was confirmed to be the same as that of **22** by analysis of NOE interactions. Comparison of ECD spectra of compounds **23**, **22**, and trangmolin F (Figure 8a) revealed that these compounds had the same absolute configuration. The absolute configuration of C-6 was further determined by the modified Mosher α-methoxy-α-(trifluoromethyl)phenylacetyl (MTPA) ester method [24]. The ∆δ values of H-5, H_3_-19, and H_3_-29 were positive, while that of H_3_-31 was negative (Figure 8b). This regular arrangement concluded the *R-*absolute configuration for C-6. Finally, the absolute configuration of **23**—named xylomolin J_2_—was unequivocally established as 3*S*,4*R*,5*S*,6*R*,9*S*,10*R*,13*R*,17*R.*

**Figure 9:**
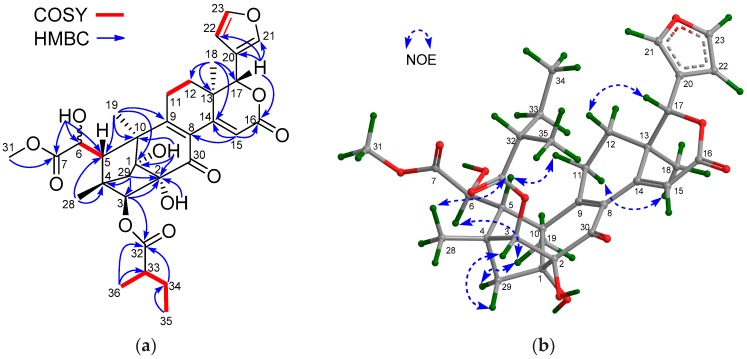
(**a**) Selected ^1^H-^1^H COSY and HMBC correlations for compound **24** (measured in CDCl_3_); (**b**) Diagnostic NOE interactions for compound **24** (measured in CDCl_3_, MM2-optimized structure).

Compound **24** had the molecular formula C_32_H_38_O_1__1_ as determined from the positive HRESIMS ion peak at *m*/*z* 621.2306 (calcd. for [M + Na]^+^, 621.2306). The NMR spectroscopic data of **24** (Table 7 and Table 8) were similar to those of moluccensin I [25], except for the presence of an additional 6-OH group (δ_H_ 3.12 br s) and the replacement of the 1-*O*-isobutyryl group in moluccensin I by a 1-OH function (δ_H_ 2.81 s) in **24**. The downshifted C-6 signal (δ_C_ 71.5 CH in **24**, whereas δ_C_ 33.2 CH_2_ in moluccensin I) and HMBC correlations from the active proton (δ_H_ 3.12 br s) to C-5, C-6, and C-7 revealed the presence of the 6-OH group (Figure 9a). The existence of the 1-OH function was confirmed by the upshifted C-1 signal (δ_C_ 85.8 qC in **24**, whereas δ_C_ 90.8 qC in moluccensin I) and strong HMBC cross-peaks from the active proton (δ_H_ 2.81 s) to C-1, C-2, and C-10 (Figure 9a). The relative configuration of **24** was identified as the same as that of moluccensin I based on NOE interactions between H-17/H-12β, H-5/H-11β, H-5/H-28, H_3_-18/H-11α, H_3_-19/H*_pro-__S_*-29, H-3/H*_pro-R_*-29, and 2-OH/H*_pro-R_*-29 (Figure 9b). Therefore, the structure of **24**—named xylomolin K_1_—was assigned as 6-hydroxy-1-*O*-deisobutyryl-moluccensin I.

Compound **25** provided the molecular formula C_31_H_36_O_11_ as determined from the positive HRESIMS ion peak at *m*/*z* 607.2164 (calcd. for [M + Na]^+^, 607.2150). The NMR spectroscopic data of **25** (Table 7 and Table 8) were similar to those of **24**, except for the replacement of 3-*O*-(2-methyl)butyryl in **24** by an isobutyryloxy group (δ_H_ 2.45 m 1H, 1.11 d (*J* = 6.8 Hz, 3H), 1.11 d (*J* = 6.8 Hz, 3H); δ_C_ 174.8 qC, 34.2 CH, 18.8 CH_3_, 19.0 CH_3_) in **25**. The HMBC correlation from H-3 to the carbonyl carbon (C-32) of the isobutyryloxy group placed it at C-3. The relative configuration of **25** was determined to be the same as that of **24** based on NOE interactions between H-17/H-12β, H-11β/H-5, H-5/H_3_-28, H-12α/H_3_-18, H_3_-18/H-11α, H*_pro-R_*-29/H-3, and H*_pro-S_*-29/H_3_-19. Thus, the structure of **25**—named xylomolin K_2_—was identified as 3-*O*-isobutyryl-3-de(2-methyl)butyryloxy-xylomolin K_1_.

**Figure 10:**
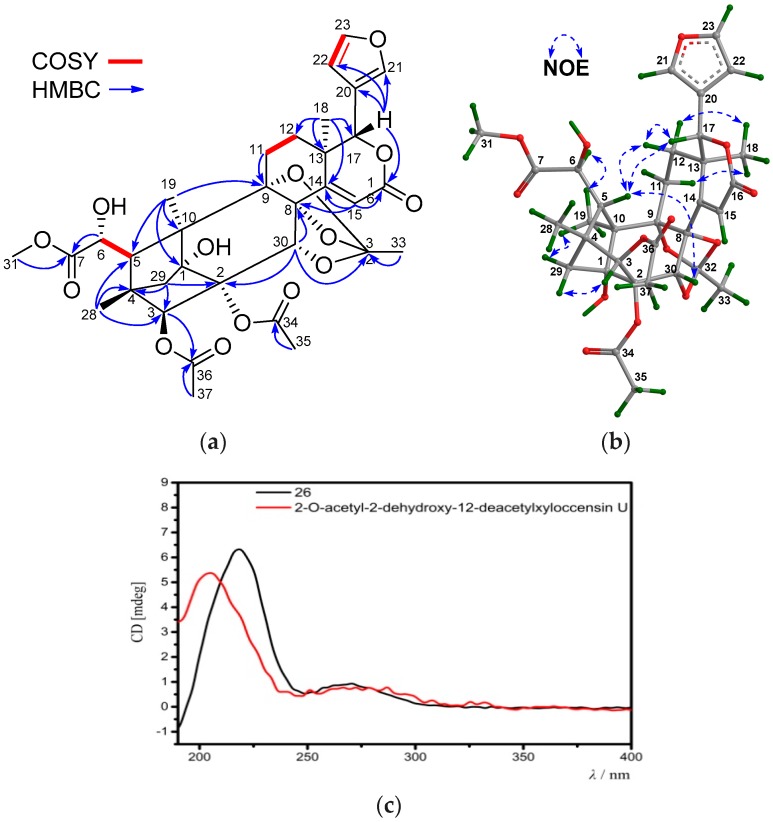
(**a**) Selected ^1^H-^1^H COSY and HMBC correlations for compound **26** (measured in CDCl_3_); (**b**) Diagnostic NOE interactions for compound **26** (measured in CDCl_3_, MM2-optimized structure); (**c**) Comparison of the experimental ECD spectrum of compound **26** with that of the known compound, 2-*O*-acetyl-2-dehydroxy-12-deacetylxyloccensin U.

Compound **26** was obtained as an amorphous white powder. Its molecular formula was determined to be C_33_H_38_O_14_ by the positive HRESIMS ion peak at *m*/*z* 681.2149 (calcd. for [M + Na]^+^, 681.2154). The NMR spectroscopic data of **26** (Table 7 and Table 8) resembled those of 2-*O*-acetyl-2-dehydroxy-12-deacetylxyloccensin U [18], being a phragmalin 8,9,30-*ortho* ester isolated from *X. moluccensis*, except for the presence of an additional 6-OH group and the absence of the 12-OH group in **26**. The presence of the 6-OH group was supported by the downshifted C-6 signal (δ_C_ 71.5 CH in **26**, whereas δ_C_ 33.7 CH_2_ in 2-*O*-acetyl-2-dehydroxy-12-deacetylxyloccensin U), the ^1^H-^1^H COSY cross-peak between H-5/H-6 and HMBC correlations between H-6/C-5 and H-6/C-7. The upshifted C-12 signal (δ_C_ 29.5, CH_2_ in **26**, whereas δ_C_ 66.6 CH in 2-*O*-acetyl-2-dehydroxy-12-deacetylxyloccensin U), ^1^H-^1^H COSY cross-peaks between H_2_-11/H_2_-12, and HMBC correlations between H_3_-18/C-12 (Figure 10a) confirmed the absence of the 12-OH group in **26**. NOE correlations between H-17/H-12β, H-12β/H-5, H-17/H-5, H-5/H-30, H_3_-18/H-12α, H_3_-18/H-11α, H_3_-19/H*_pro-S_*-29, and H-3/H*_pro-R_*-29 revealed the same relative configuration of **26** as that of 2-*O*-acetyl-2-dehydroxy-12-deacetylxyloccensin U (Figure 10b). The ECD spectrum of **26** was nicely matched with that of 2-*O*-acetyl-2-dehydroxy-12-deacetylxyloccensin U (Figure 10c), revealing the same absolute configuration for the two compounds. Thus, the structure of **26**—named xylomolin L_1_—was assigned as 6-hydroxy-12-dehydroxy-2-*O*-acetyl-2-dehydroxy-12-deacetylxyloccensin U.

The molecular formula of **27** was determined to be C_34_H_40_O_13_ by the positive HRESIMS ion peak at *m*/*z* 679.2374 (calcd. for [M + Na]^+^, 679.2361). The NMR spectroscopic data of **27** (Table 7 and Table 8) closely resembled those of swietephragmin G [26], being a phragmalin 8,9,30-*ortho* ester, except for the presence of an additional 12-OH group, which was supported by the downshifted C-12 signal (δ_C_ 66.5 CH in **27**, whereas δ_C_ 29.2 CH_2_ in swietephragmin G), ^1^H-^1^H COSY cross-peaks between H_2_-11/H-12, and HMBC correlations between H_3_-18/C-12. The strong NOE interaction between H-17/H-12 assigned the β-oriented H-12 and the corresponding α-orientation for the 12-OH group. The NOE interaction between H_3_-37/H_3_-38 assigned the *E* configuration for the double bond of 3-tigloyloxy group. Therefore, the structure of **27**—named xylomolin L_2_—was identified as 12α-hydroxy-swietephragmin G.

**Figure 11:**
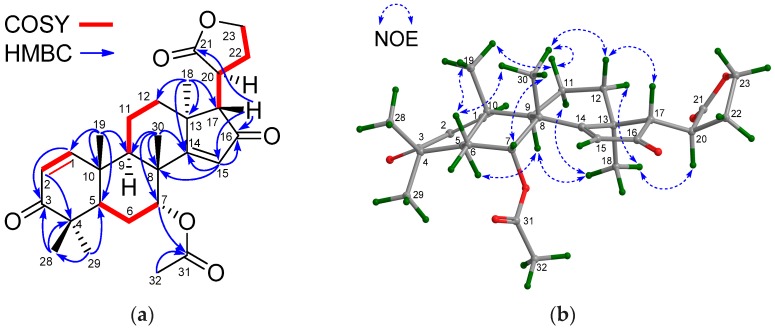
(**a**) Selected ^1^H-^1^H COSY and HMBC correlations for compound **28** (measured in CDCl_3_); (**b**) Diagnostic NOE interactions for compound **28** (measured in CDCl_3_, MM2-optimized structure).

Compound **28** had the molecular formula C_28_H_37_O_6_ as determined from the positive HRESIMS ion peak at *m*/*z* 469.2603 (calcd. for [M + H]^+^, 469.2585). The NMR spectroscopic data of **28** (Table 7 and Table 8) were closely related to those of andirolide Q [27], except for the different positions of the ester carbonyl carbon of the C-17 attached five-membered γ-lactone ring, *viz.* C-21 in **28** instead of C-23 in andirolide Q. Significant ^1^H-^1^H COSY correlations between H-17/H-20, H-20/H_2_-22, H_2_-22/H_2_-23 and the HMBC correlation between H-17/C-21 confirmed the above deduction (Figure 11a). NOE interactions between H-17/H-12β, H-12β/H_3_-30, H_3_-30/H-7, H_3_-30/H-11β, H-11β/H_3_-19, and H_3_-18/H-11α, H_3_-18/H-12α, H_3_-18/H-20, H_3_-18/H-9, and H-9/H-5 indicated the β-orientation for H-7, H-17, H_3_-19, and H_3_-30, and the α-orientation for H-5, H-9, H_3_-18, and H-20 (Figure 11b). Hence, the relative configuration of **28**—named xylomolin M—was assigned as depicted.

Compound **29** afforded the molecular formula C_27_H_34_O_8_ as deduced from the positive HRESIMS ion peak at *m*/*z* 509.2147 (calcd. for [M + Na]^+^, 509.2146). The NMR spectroscopic data of **29** (Table 7 and Table 8) closely resembled those of moluccensin O [25], except for the absence of the 21-OH group, which was corroborated by the upshifted C-21 signal (δc 72.4 CH_2_ in **29**, δc 98.1 CH in moluccensin O) and HMBC correlations from H_2_-21 (δ_H_ 4.85 br d (*J* = 18.3 Hz), 5.03 dd (*J* = 18.3, 2.0 Hz)) to C-20 and C-22. The relative configuration of **29** was assigned as the same as that of moluccensin O based on NOE correlations. Thus, the structure of compound **29**—named xylomolin N—was assigned as 21-dehydroxy-moluccensin O.

The antitumor activities of **1**, **3**, **8**, **10**, **11**, **14**–**16**, **20**, **23**, **25**, and **27** were tested by the MTT cytotoxity assay against five human tumor cell lines, including human colorectal HCT-8 and HCT-8/T, human ovarian A2780 and A2780/T, and human breast MD-MBA-231 (Table S1) [28]. Cisplatin was used as the positive control. Compounds **11** and **23** showed weak activities against the tested cancer cell lines, whereas the other ten compounds were inactive at 100 μM. Compound **23** exhibited selective antitumor activity against human breast MD-MBA-231 cancer cells with an IC_50_ value of 37.7 μM.

Anti-HIV activities of **1**, **3**, **8**, **10**, **11**, **14**, **20**, **23**–**25**, and **27** were tested in vitro with the HIV-1 virus transfected 293 T cells [29]. At the concentration of 20 μM, **1**, **11**, **23**, and **24** showed inhibitory rates of 17.49 ± 6.93%, 24.47 ± 5.04%, 14.77 ± 5.91%, and 14.34 ± 3.92%, respectively (Table S2). Efavirenz was used as the positive control with an inhibitory rate of 88.54 ± 0.45% at the same concentration. 

## 3. Materials and Methods

### 3.1. General Methods

Optical rotations were recorded on a MCP500 modular circular polarimeter (Anton Paar Opto Tec GmbH, Seelze, Germany). UV spectra were obtained on a GENESYS 10S UV-Vis spectrophotometer (Thermo Fisher Scientific, Shanghai, China). HRESIMS were measured on a Bruker maXis ESI-QTOF mass spectrometer (Bruker Daltonics, Bremen, Germany). NMR spectra were recorded on a Bruker AV-400 spectrometer with TMS as the internal standard. Single-crystal X-ray diffraction analyses were made on an Agilent Xcalibur Atlas Gemini Ultra-diffractometer (Agilent Technologies, Santa Clara, CA, USA) with mirror monochromated CuKα radiation (λ = 1.54178 Å) at 150 K. Semi-preparative HPLC (Waters Corporation, Milford, MA, USA) was performed on a Waters 2535 pump equipped with a waters 2998 photodiode array detector and YMC C18 reverse-phased columns (250 mm × 10 mm i.d., 5 μm). For column chromatography, silica gel (100–200 mesh) (Qingdao Mar. Chem. Ind. Co. Ltd., Qingdao, China) and C_18_ reverse-phased silica gel (ODS-A-HG 12 nm, 50 µm, YMC Co. Ltd., Kyoto, Japan) were used. ECD spectra were measured on a Jasco J-810 spectropolarimeter (JASCO Corporation, Tokyo, Japan) in MeCN.

### 3.2. Plant Material

A batch of seeds of *Xylocarpus moluccensis* were collected at the mangrove swamp of Trang Province, Thailand, in June 2013, whereas another batch of seeds of the same mangrove plant were collected in Godavari estuary, Andhra Pradesh, India, in October 2009, respectively. The identification of the plant was performed by one of the authors (J.W.) and Mr. Tirumani Satyanandamurty (Government Degree College at Amadala Valasa, India). Voucher samples (No. ThaiXM-03 and No. IXM200901, respectively) were maintained in the Marine Drugs Research Center, College of Pharmacy, Jinan University.

### 3.3. Extraction and Isolation

The dried seeds (10.0 kg, ThaiXM-03) of *X. moluccensis* were extracted with 95% (*v*/*v*) EtOH at room temperature (5 × 20 L) to afford the EtOH extract (680.0 g), which was suspended in water and extracted with EtOAc. The resulting EtOAc extract (296.0 g) was chromatographed on silica gel column and eluted with CHCl_3_/MeOH (100:0 to 5:1) to yield 160 fractions. 

Fractions 26–28 (31.6 g) were combined and further purified with an RP-18 column (acetone/H_2_O, 50:50 to 100:0) to afford 57 subfractions. Subfraction 23 was purified by preparative HPLC (YMC-Pack ODS-5-A, 250 mm × 10 mm i.d., MeOH/H_2_O, 50:50) to afford compound **1** (45.0 mg).

Fractions 29–40 (111.0 g) were combined and further purified with an RP-18 column (acetone/H_2_O, 50:50 to 100:0) to afford 360 subfractions. The combination of subfractions 90–95 was subjected to preparative HPLC (YMC-Pack ODS-5-A, 250 mm × 10 mm i.d., MeCN/H_2_O, 38:62) to give compound **8** (72 mg), along with five other subfractions (SFr.90-95-1 to SFr.90-95-5). Recrystallization of SFr.90-95-1 afforded compound **20** (15.3 mg). Further purification of SFr.90-95-2 with preparative HPLC (YMC-Pack ODS-5-A, 250 mm × 10 mm i.d., MeOH/MeCN/H_2_O, 10:40:50) yielded compounds **3** (7.1 mg), **5** (4.3 mg), **12** (37 mg), **13** (2.8 mg), and **21** (1.2 mg), whereas that of SFr.90-95-3 with preparative HPLC (YMC-Pack ODS-5-A, 250 mm × 10 mm i.d., MeOH/H_2_O, 60:40 or MeCN/H_2_O, 35:65) afforded compounds **6** (4.3 mg) and **9** (3.1 mg). SFr.90-95-4 was further purified with preparative HPLC (YMC-Pack ODS-5-A, 250 mm × 10 mm i.d., MeCN/H_2_O, 46:64) to afford compound **14** (57.0 mg). Subfractions 78–83 were combined and further purified by preparative HPLC (YMC-Pack ODS-5-A, 250 × 10 mm i.d., MeOH/H_2_O, 50:50) to give compounds **24** (3.5 mg), **26** (2.0 mg), **27** (7.0 mg), and **29** (2.5 mg).

Fractions 157–175 (14.5 g) were combined and further purified with an RP-18 column (acetone/H_2_O, 40:60 to 100:0) to afford 45 subfractions. Subfraction 27 was purified by preparative HPLC (YMC-Pack 250 mm × 10 mm i.d., MeCN/MeOH/H_2_O, 50:15:35) to afford compound **4** (1.2 mg).

The air-dried seeds (15.0 kg, IXM200901) were powdered and extracted with 95% (*v*/*v*) EtOH (5 × 20 L) at room temperature to afford the EtOH extract (1.1 kg), which was partitioned between EtOAc and water to afford the EtOAc portion (572.0 g). Then, 252.0 g of the EtOAc extract was further subjected to a silica gel column (105.0 cm × 9.5 cm i.d.) and eluted with a gradient mixture of CHCl_3_/MeOH (100:1 to 5:1) to afford 184 fractions. 

Fractions 38–43 (26.0 g) were combined and further separated on an RP-18 column (64.0 cm × 6.3 cm i.d.), and eluted with a gradient mixture of acetone/H_2_O (40:60 to 100:0) to afford 74 subfractions. 

Subfractions 12–16 (1.5 g) were combined and separated by preparative HPLC (YMC-Pack ODS-5-A, 250 mm × 10 mm i.d., MeCN/H_2_O, 36:64) to afford seven parts (SFr.12-16-1 to SFr.12-16-7). SFr.12-16-2 was further purified by preparative HPLC (YMC-Pack ODS-5-A, 250 mm × 10 mm i.d., MeOH/H_2_O, 49:51) to yield compounds **10** (49.3 mg) and **25** (25.7 mg). SFr.12-16-4 and SFr.12-16-5 were further purified by preparative HPLC (YMC-Pack ODS-5-A, 250 mm × 10 mm i.d., MeOH/H_2_O, 52:48) to afford compounds **15** (13 mg) and **22** (3.0 mg), respectively. SFr.12-16-6 was further subjected to preparative HPLC (MeOH/H_2_O, 51:49) to yield compound **2** (2.0 mg), whereas SFr.12-16-7 was further purified by preparative HPLC (MeOH/H_2_O, 60:40, subsequently with MeCN/H_2_O, 60:40, and then MeCN/MeOH/H_2_O, 60:20:20) to yield compound **28** (1.5 mg).

Preparative HPLC (YMC-Pack ODS-5-A, 250 mm × 10 mm i.d., MeOH/H_2_O, 58:42) was performed on subfraction 27 (2.9 g) to gain compound **16** (4.0 mg). Subfractions 32–35 (1.5 g) were combined and separated by preparative HPLC (YMC-Pack ODS-5-A, 250 mm × 10 mm i.d., MeCN/H_2_O, 55:45) to give four parts (SFr.32-35-1 to SFr.32-35-4). SFr.32-35-2 and SFr.32-35-3 were purified by preparative HPLC (MeOH/H_2_O, 65:35, MeOH/H_2_O, 60:40, respectively) to yield compounds **23** (27.0 mg) and **11** (27.5 mg), respectively, whereas recrystallization (acetone) of SFr.32-35-4 afforded compound **7** (23.8 mg).

Fractions 48–86 (43.9 g) were combined and further performed on an RP-18 column (46.7 cm × 6.4 cm i.d.), and eluted with a gradient mixture of MeCN/H_2_O (50:50 to 100:0), to afford 81 subfractions. Subfraction 4 (6.5 g) was purified by preparative HPLC (MeOH/H_2_O, 53:47) to yield compound **17** (5.5 mg). Subfractions 8–14 (10.4 g) were combined and further separated on an RP-18 column (62.0 cm × 6.5 cm i.d.) and eluted with a gradient mixture of acetone/H_2_O (50:50 to 100:0) to afford 37 subsubfractions, among which subsubfractions 12–15 (2.1 g) were combined and separated on the preparative HPLC (MeOH/MeCN/H_2_O, 50:15:35) to give compound **18** (2.1 mg). Subfraction 17 (1.78 g) was subjected to preparative HPLC (MeOH/H_2_O, 80:20) to yield compound **19** (1.2 mg). 

Xylomolin A_1_ (**1**): Colorless crystal; ${\left[\alpha \right]}_{D}^{25}$ −62.0 (*c* = 0.06, acetone); UV (MeCN) λ_max_ (log ε) 199.7 (3.7) nm; ECD (*c* 0.41 mM, MeCN) λ_max_ (Δε) 190.0 (−12.0), 289.6 (−2.2) nm; ^1^H and ^13^C NMR spectroscopic data see Table 1 and Table 2; HRESIMS *m*/*z* 615.2785 [M + H]^+^ (calcd. for C_33_H_43_O_11_, 615.2800).

Xylomolin A_2_ (**2**): White, amorphous powder; ${\left[\alpha \right]}_{D}^{25}$ −74.7 (*c* 0.04, acetone); UV (MeCN) λ_max_ (log ε) 194.0 (4.5), 279.0 (3.3) nm; ^1^H and ^13^C NMR spectroscopic data see Table 1 and Table 2; HRESIMS *m*/*z* 593.2143 [M + Cl]^−^ (calcd. for C_3__0_H_38_O_10_Cl, 593.2159).

Xylomolin A_3_ (**3**): White, amorphous solid; ${\left[\alpha \right]}_{D}^{25}$ −96.0 (*c* 0.05, acetone); UV (MeCN) λ_max_ (log ε) 198.9 (3.9) nm; ECD (*c* 0.16 mM, MeCN) λ_max_ (Δε) 191.0 (−14.5) nm; ^1^H and ^13^C NMR spectroscopic data see Table 1 and Table 2; HRESIMS *m*/*z* 625.2620 [M + Na]^+^ (calcd. for C_32_H_42_NaO_11_, 625.2619).

Xylomolin A_4_ (**4**): White, amorphous powder; ${\left[\alpha \right]}_{D}^{25}$ −24.0 (*c* 0.08, acetone); UV (MeCN) λ_max_ (log ε) 197.0 (3.9), 201.8 (3.8) nm; ECD (*c* 0.15 mM, MeCN) λ_max_ (Δε) 190(−8.1), 200.4 (−5.0), 211.6 (−6.6) nm; ^1^H and ^13^C NMR spectroscopic data see Table 1 and Table 2; HRESIMS *m*/*z* 683.2675 [M + Na]^+^ (calcd. for C_34_H_44_NaO_13_, 683.2674).

Xylomolin A_5_ (**5**): White, amorphous powder; ${\left[\alpha \right]}_{D}^{25}$ −52.0 (*c* 0.04, acetone); UV (MeCN) λ_max_ (log ε) 201.0 (3.8) nm; ECD (*c* 0.16 mM, MeCN) λ_max_ (Δε) 191.0 (−8.6) nm; ^1^H and ^13^C NMR spectroscopic data see Table 1 and Table 2; HRESIMS *m*/*z* 625.2252 [M + Na]^+^ (calcd. for C_31_H_38_NaO_12_, 625.2255).

Xylomolin A_6_ (**6**): White, amorphous powder; ${\left[\alpha \right]}_{D}^{25}$ −216.0 (*c* 0.10, acetone); UV (MeCN) λ_max_ (log ε) 197.8 (4.0) nm; ECD (*c* 0.16 mM, MeCN) λ_max_ (Δε) 190.0 (−12.8), 216.4 (+1.5), 294.2 (−2.3) nm; ^1^H and ^13^C NMR spectroscopic data see Table 1 and Table 2; HRESIMS *m*/*z* 609.2311 [M + Na]^+^ (calcd. for C_31_H_38_NaO_11_, 609.2306).

Xylomolin A_7_ (**7**): White powder; ${\left[\alpha \right]}_{D}^{25}$ −250.7 (*c* 0.08, acetone); UV (MeCN) λ_max_ (log ε) 193.0 (4.5) nm; ^1^H and ^13^C NMR spectroscopic data see Table 1 and Table 2; HRESIMS *m*/*z* 593.2358 [M + Na]^+^ (calcd. for C_3__1_H_38_NaO_10_, 593.2357).

Xylomolin B_1_ (**8**): White, amorphous powder; ${\left[\alpha \right]}_{D}^{25}$ +212.0 (*c* 0.08, acetone); UV (MeCN) λ_max_ (log ε) 204.0 (3.9), 285.4 (4.0) nm; ECD (*c* 0.10 mM, MeCN) λ_max_ (Δε) 252.1 (−1.6), 278.9 (+8.6) nm; ^1^H and ^13^C NMR spectroscopic data see Table 3 and Table 4; HRESIMS *m*/*z* 607.2151 [M + Na]^+^ (calcd. for C_31_H_36_NaO_11_, 607.2150).

Xylomolin B_2_ (**9**): White, amorphous solid; ${\left[\alpha \right]}_{D}^{25}$ +137.0 (*c* 0.10, acetone); UV (MeCN) λ_max_ (log ε) 207.2 (3.7), 285.2 (4.0) nm; ECD (*c* 0.19 mM, MeCN) λ_max_ (Δε) 190.0 (+3.6), 213.7 (−1.1), 282.2 (+9.5) nm; ^1^H and ^13^C NMR spectroscopic data see Table 3 and Table 4; HRESIMS *m*/*z* 527.2273 [M + H]^+^ (calcd. for C_29_H_35_O_9_, 527.2276).

Xylomolin C_1_ (**10**): White, amorphous powder; ${\left[\alpha \right]}_{D}^{25}$ +156.0 (*c* 0.07, acetone); UV (MeCN) λ_max_ (log ε) 191.0 (4.3), 275 (4.1) nm; ^1^H and ^13^C NMR spectroscopic data see Table 3 and Table 4; HRESIMS *m*/*z* 543.2238) [M + H]^+^ (calcd. for C_29_H_3__5_O_10_, 543.2230).

Xylomolin C_2_ (**11**): White, amorphous powder; ${\left[\alpha \right]}_{D}^{25}$ +125.7 (*c* 0.09, acetone); UV (MeCN) λ_max_ (log ε) 194.0 (4.7), 277 (4.6) nm; ^1^H and ^13^C NMR spectroscopic data see Table 3 and Table 4; HRESIMS *m*/*z* 591.2570 [M + Na]^+^ (calcd. for C_32_H_40_NaO_9_, 591.2565).

Xylomolin D (**12**): White, amorphous powder; ${\left[\alpha \right]}_{D}^{25}$ −34.0 (*c* 0.05, acetone); UV (MeCN) λ_max_ (log ε) 208.2 (3.8) nm; ECD (*c* 0.16 mM, MeCN) λ_max_ (Δε) 190.0 (−2.6), 217.5 (+13.7), 244.5 (−0.3), 261 (+ 1.5), 291.2 (−3.8) nm; ^1^H and ^13^C NMR spectroscopic data see Table 3 and Table 4; HRESIMS *m*/*z* 625.2612 [M + Na]^+^ (calcd. for C_32_H_42_NaO_11_, 625.2619).

Xylomolin E (**13**): White, amorphous powder; ${\left[\alpha \right]}_{D}^{25}$ −68.0 (*c* 0.04, acetone); UV (MeCN) λ_max_ (log ε) 207.2 (3.9) nm; ECD (*c* 0.15 mM, MeCN) λ_max_ (Δε) 190.0 (−9.5), 213.7 (+2.5) nm; ^1^H and ^13^C NMR spectroscopic data see Table 3 and Table 4; HRESIMS *m*/*z* 625.2250 [M + Na]^+^ (calcd. for C_31_H_38_NaO_12_, 625.2255).

Xylomolin F (**14**): White, amorphous powder; ${\left[\alpha \right]}_{D}^{25}$ +4.0 (*c* 0.03, acetone); UV (MeCN) λ_max_ (log ε) 211.0 (4.1) nm; ECD (*c* 0.21 mM, MeCN) λ_max_ (Δε) 192.0 (−7.1), 219.0 (+4.0), 265.0 (+3.2) nm; ^1^H and ^13^C NMR spectroscopic data see Table 3 and Table 4; HRESIMS *m*/*z* 669.2521 [M + Na]^+^ (calcd. for C_33_H_42_NaO_13_, 669.2518).

Xylomolin G_1_ (**15**): White, amorphous solid; ${\left[\alpha \right]}_{D}^{25}$ −65.0 (*c* 0.1, acetone); UV (MeCN) λ_max_ (log ε) 190.2 (4.20) nm; ECD (*c* 0.14 mM, MeCN) λ_max_ (Δε) 207 (+2.3), 238 (−3.8), 290 (+1.4), 321 (−0.32), 344 (+0.32) nm; ^1^H and ^13^C NMR spectroscopic data see Table 5 and Table 6; HRESIMS *m*/*z* 587.2494 [M + H]^+^ (calcd. for C_31_H_3__9_O_11_, 587.2492).

Xylomolin G_2_ (**16**): White, amorphous solid; ${\left[\alpha \right]}_{D}^{25}$ −72.0 (*c* 0.05,acetone); UV (MeCN) λ_max_ (log ε) 190.2 (4.03); ECD (*c* 0.13 mM, MeCN) λ_max_ (Δε) 205.0 (+1.7), 239.0 (−4.3), 302.0 (+0.34), 339.0 (+1.1) nm; ^1^H and ^13^C NMR spectroscopic data see Table 5 and Table 6; HRESIMS *m*/*z* 615.2809 [M + H]^+^ (calcd. for C_33_H_4__3_O_11_, 615.2805).

Xylomolin G_3_ (**17**): White, amorphous solid; ${\left[\alpha \right]}_{D}^{25}$ −43.5 (*c* 0.04, acetone); UV (MeCN) λ_max_ (log ε) 196.6 (4.03), 285.0 (2.93) nm; ECD (*c* 0.15 mM, MeCN) λ_max_ (Δε) 193.0 (−4.5), 197.0 (−3.4), 205.0 (−7.5), 216.0 (−5.0), 233.0 (−7.9), 298 (+4.3) nm; ^1^H and^13^C NMR spectroscopic data see Table 5 and Table 6; HRESIMS *m*/*z* 543.2246 [M + H]^+^ (calcd. for C_29_H_3__5_O_10_, 543.2230).

Xylomolin G_4_ (**18**): White, amorphous solid; ${\left[\alpha \right]}_{D}^{25}$ −44.0 (*c* 0.01,acetone); UV (MeCN) λ_max_ (log ε) 196.6 (4.08) nm; ECD (*c* 0.18 mM, MeCN) λ_max_ (Δε) 203.0 (−8.7), 215.0 (−6.2), 233.0 (−10.3), 301.0 (+5.0) nm; ^1^H and^13^C NMR spectroscopic data see Table 5 and Table 6; HRESIMS *m*/*z* 555.2593 [M + H]^+^ (calcd. for C_31_H_3__9_O_9_, 555.2594).

Xylomolin G_5_ (**19**): White, amorphous solid; ${\left[\alpha \right]}_{D}^{25}$ −37.5 (*c* 0.024, acetone); UV (MeCN) λ_max_ (log ε) 195.0 (4.27), 284.2 (2.86) nm; ECD (*c* 0.11 mM, MeCN) λ_max_ (Δε) 195.0 (+ 0.70), 206.0 (−4.9), 214.0 (−3.5), 231.0 (−6.1), 299.0 (+2.7) nm; ^1^H and^13^C NMR spectroscopic data see Table 5 and Table 6; HRESIMS *m*/*z* 569.2753 [M + H]^+^ (calcd. for C_32_H_4__1_O_9_, 569.2751).

Xylomolin H (**20**): White, amorphous powder; ${\left[\alpha \right]}_{D}^{25}$ +65.0 (*c* 0.06, acetone); UV (MeCN) λ_max_ (log ε) 212.3 (4.0) nm; ECD (c 0.16 mM, MeCN) λ_max_ (Δε) 223 (+10.9), 245 (+0.19),270 (+8.1) nm; ^1^H and ^13^C NMR spectroscopic data see Table 5 and Table 6; HRESIMS *m*/*z* 667.2359 [M + Na]^+^ (calcd. for C_33_H_40_NaO_13_, 667.2361).

Xylomolin I (**21**): Light yellow, amorphous gum; ${\left[\alpha \right]}_{D}^{25}$ +129.0 (*c* 0.08, acetone); UV (MeCN) λ_max_ (log ε) 208.4 (3.8), 287.6 (4.1) nm; ECD (*c* 0.17 mM, MeCN) λ_max_ (Δε) 200.0 (+4.6), 213.0 (+2.3), 232.0 (+4.6), 259.0 (−3.9), 291.0 (+10.3) nm; ^1^H and ^13^C NMR spectroscopic data see Table 5 and Table 6; HRESIMS *m*/*z* 621.2304 [M + Na]^+^ (calcd. for C_32_H_38_NaO_11_, 621.2306)

Xylomolin J_1_ (**22**): White, amorphous powder; ${\left[\alpha \right]}_{D}^{25}$ −135.0 (*c* 0.11, acetone); UV (MeCN) λ_max_ (log ε) 197.0 (4.9), 213.4 (4.8), 260.8 (4.6) nm; ECD (*c* 0.039 mM, MeCN) λ_max_ (Δε) 204.0 (−3.3), 229.0 (+3.3), 266.0 (+6.3), 310.0 (−0.19), 346.0 (+0.95) nm; ^1^H and ^13^C NMR spectroscopic data see Table 7 and Table 8; HRESIMS *m*/*z* 541.2077 [M + H]^+^ (calcd. for C_29_H_33_O_10_, 541.2074).

Xylomolin J_2_ (**23**): White, amorphous powder; ${\left[\alpha \right]}_{D}^{25}$ +232.0 (*c* 0.1, acetone); UV (MeCN) λ_max_ (log ε) 196.8 (5.2), 211.0 (5.1), 260.8 (5.0) nm; ECD (*c* 0.039 mM, MeCN) λ_max_ (Δε) 190.0 (−3.9), 199.0 (−2.0), 206.0 (−2.9), 225.0 (+3.9), 267.0 (+9.1), 310.0 (−0.17), 335.0 (+1.0) nm; ^1^H and ^13^C NMR spectroscopic data see Table 7 and Table 8; HRESIMS *m*/*z* 583.2548 [M + H]^+^ (calcd. for C_32_H_39_O_10_, 583.2543).

Xylomolin K_1_ (**24**): White, amorphous powder; ${\left[\alpha \right]}_{D}^{25}$ +98.0 (*c* 0.1, acetone); UV (MeCN) λ_max_ (log ε) 194.0 (4.3), 271.0 (4.3) nm; ECD (*c* 0.33 mM, MeCN) λ_max_ (Δε) 209.0 (+3.0), 211.0 (+2.9), 232.0 (+8.5), 275.0 (−2.8), 300.0 (+6.4) nm; ^1^H and ^13^C NMR spectroscopic data see Table 7 and Table 8; HRESIMS *m*/*z* 621.2306 [M + Na]^+^ (calcd. for C_32_H_38_NaO_11_, 621.2306).

Xylomolin K_2_ (**25**): White, amorphous powder; ${\left[\alpha \right]}_{D}^{25}$ +120.4 (*c* 0.07, acetone); UV (MeCN) λ_max_ (log ε) 191.0 (4.3), 272.0 (4.5) nm; ^1^H and ^13^C NMR spectroscopic data see Table 7 and Table 8; HRESIMS *m*/*z* 607.2164 [M + Na]^+^ (calcd. for C_31_H_36_NaO_11_, 607.2150).

Xylomolin L_1_ (**26**) : White, amorphous powder; ${\left[\alpha \right]}_{D}^{25}$ +64.0 (*c* 0.09, acetone); UV (MeCN) λ_max_ (log ε) 213.0 (4.0) nm; ECD (*c* 0.30 mM, MeCN) λ_max_ (Δε) 220.0 (+12.5), 249.0 (+ 1.1), 270.0 (+ 0.8), 236.0 (+ 1.9) nm; ^1^H and ^13^C NMR spectroscopic data see Table 7 and Table 8; HRESIMS *m*/*z* 681.2149 [M + Na]^+^ (calcd. for C_33_H_38_NaO_14_, 681.2154).

Xylomolin L_2_ (**27**): White, amorphous powder; ${\left[\alpha \right]}_{D}^{25}$ +42.0 (*c* 0.06, acetone); UV (MeCN) λ_max_ (log ε) 214 (4.5) nm; ECD (*c* 0.15 mM, MeCN) λ_max_ (Δε) 198.0 (+3.2), 213.0 (−3.6), 234.0 (+11.6) nm; ^1^H and ^13^C NMR spectroscopic data see Table 7 and Table 8; HRESIMS *m*/*z* 679.2374 [M + Na]^+^ (calcd. for C_34_H_40_NaO_13_, 679.2361).

Xylomolin M (**28**): White, amorphous powder; ${\left[\alpha \right]}_{D}^{25}$ −252.0 (*c* 0.03, acetone); UV (MeCN) λ_max_ 200.0 (4.0), 232 (4.1) nm; ^1^H and ^13^C NMR spectroscopic data see Table 7 and Table 8; HRESIMS *m*/*z* 469.2603 [M + H]^+^ (calcd. for C_28_H_37_O_6_, 469.2585).

Xylomolin N (**29**): White, amorphous powder; ${\left[\alpha \right]}_{D}^{25}$ −17.0 (*c* 0.05, acetone); UV (MeCN) λ_max_ (log ε) 196 (3.7), 211 (3.7) nm; ECD (*c* 0.21 mM, MeCN) λ_max_ (Δε) 200.0 (−7.0), 224.0 (+2.1), 252.0 (−0.3) nm; ^1^H and ^13^C NMR spectroscopic data see Table 7 and Table 8; HRESIMS *m*/*z* 509.2147 [M + Na]^+^ (calcd. for C_27_H_34_NaO_8_, 509.2146). 

### 3.4. X-ray Crystal Data for Xylomolin A_1_ *(**1**)*

Orthorhombic, C_3__4_H_46_O_12_ (C_3__3_H_4__2_O_1__1_·CH_3_OH), space group *P*2(1)2(1)2(1), a = 8.82730 (5) Å, b = 17.93740 (10) Å, c = 20.84108 (13) Å, α = 90°, β = 90°, γ = 90°, V = 3299.95 (3) Å^3^, Z = 4, D_calcd._ = 1.302 Mg/m^3^, μ = 0.816 mm^−1^. Crystal size: 0.40 × 0.40 × 0.28 mm^3^. 47,329 measured reflections, 5877 [*R*_int_ = 0.0378] independent reflections, 426 parameters, 0 restraints, *F*(000) = 1384.0, *R*_1_ = 0.0296, *wR*_2_ = 0.0773(all data), *R*_1_ = 0.0286, *wR*_2_ = 0.0763 [*I* > 2σ(*I*)], and goodness-of-fit (*F*^2^) = 1.065. The absolute structural parameter is −0.06(10), Flack x is −0.14(11), and Hooft y is −0.04(3). 

CCDC-1590301 (**1**) contains the supplementary crystallographic data for this paper (excluding structure factors). These data are provided free of charge by The Cambridge Crystallographic Data Centre.

### 3.5. MTT Cytotoxicity Assay

Compounds **1**, **3**, **8**, **10**, **11**, **14**–**16**, **20**, **23**, **25**, and **27** were evaluated by the MTT method for cytotoxicities against human colorectal HCT-8 and HCT-8/T, ovarian A2780 and A2780/T, and breast MD-MBA-231 cancer cell lines. All cell lines were cultured as adherent monolayers in flasks in DMEM culture medium with 10% fetal bovine serum, benzylpenicillin (50 kU/L), and streptomycin (50 mg/L) at 37 °C in a humidified atmosphere of 5% CO_2_. Cells were collected with trypsin and resuspended in a final concentration of 5 × 10^4^/mL. One hundred microliter aliquots for each cell suspension were distributed evenly into 96-well multiplates (number of cells per well is 5 × 10^3^). Different concentrations of the compounds were added into the designated wells. After 72 h, a 10 μL MTT solution (5 mg/mL) was added to each well, and the plate was further incubated for 4 h, allowing viable cells to reduce the yellow MTT into dark-blue formazan crystals which were dissolved in DMSO 100 μL. The absorbance in individual wells was determined at 490 nm by a microplate reader (Biotek, VT, USA) [28]. The concentrations required to inhibit the growth of cancer cells by 50% (IC_50_ values) were calculated from cytotoxicity curves by Bliss method. The positive control was cisplatin. The IC_50_ values of cisplatin in human colorectal HCT-8 and HCT-8/T, ovarian A2780 and A2780/T, and breast MD-MBA-231, were 15.43, 21.98, 8.54, 9.26, and 6.25 μM, respectively.

### 3.6. HIV-Inhibitory Bioassay

For the assay, 293 T cells (2 × 10^5^) were co-transfected with 0.6 μg of pNL-Luc-E^−^-Vpu^−^ and 0.4 μg of vesicular stomatitis virus glycoprotein (VSV-G) expression vector pHIT/G. After 48 h, the VSV-G pseudotyped viral supernatant (HIV-1) was harvested by filtration through a 0.45-μm filter and the concentration of viral capsid protein was determined by p24 antigen capture ELISA (Biomerieux, Shanghai, China). SupT1 cells were exposed to VSV-G pseudotyped HIV-1 (multiplicity of infection (MOI) = 1) at 37 °C for 48 h in the absence or presence of the test compounds (**1**, **3**, **8**, **10**, **11**, **14**, **20**, **23**–**25**, and **27**). Efavirenz was used as the positive control. The inhibition rates were determined by using a firefly Luciferase Assay System (Promega, Madison, WI, USA) [29].

## 4. Conclusions

Twenty-nine new limonoids were isolated from the seeds of the mangrove plant, *Xylocarpus moluccensis*, collected in Thailand and India. The structures of these limonoids, including absolute configurations of ten compounds, *viz.* **1**, **15**–**19**, **21**–**23**, and **26**, were established by HRESIMS, extensive NMR investigations, single-crystal X-ray diffraction analysis conducted with Cu Kα radiation, and the comparison of experimental ECD spectra. Compounds **1**–**14** are mexicanolides, whereas **15**–**21** are khayanolides. Compounds **22** and **23** are unusual limonoids possessing a (*Z*)-bicyclo[5.2.1]dec-3-en-8-one motif, while **24** and **25** are 30-ketophragmalins. Compounds **26** and **27** are phragmalin 8,9,30-*ortho* esters, whereas **28** and **29** are azadirone and andirobin derivatives. These results demonstrate that *X. moluccensis* continues to be an abundant resource for the production of novel limonoids with structural diversity. Compound **23** exhibited selective antitumor activity against human triple-negative breast MD-MBA-231 cancer cells with an IC_50_ value of 37.7 μM, whereas compounds **1**, **11**, **23**, and **24** showed inhibitory rates of 17.49 ± 6.93%, 24.47 ± 5.04%, 14.77 ± 5.91%, and 14.34 ± 3.92% against HIV-I virus, respectively, at the concentration of 20 μM.

## Figures and Tables

**Table 1:** ^1^H NMR spectroscopic data (400 MHz, in CDCl_3_) of compounds **1**–**7** (δ in ppm, *J* in Hz).

Position	1	2	3	4	5	6	7
3	4.97 s	4.93 s	5.04 s	5.12 s	5.06 s	5.19 s	5.52 s
5	3.35 s	3.13 s	3.28 s	3.47 s	3.45 s	3.09 s	3.10 br d (10.8)
6α	5.47 s	4.52 br s	4.54 s	5.44 s	5.47 s	4.52 s	2.32 dd (16.5, 1.6)
6β							2.42 dd (16.5, 10.7)
9	2.10 br s	2.14 br s	2.46 br s	2.49 br d (6.0)	2.42 br s	2.17 br s	2.08 m
11α	1.80 m	1.84 m	1.85 m	1.84 m	1.84 m	1.82 m	1.74 m
11β	1.92m	1.84 m	1.91 m	1.99 m	1.94 m	1.82 ^1^	1.81 m
12β	1.81 m	1.72 m	1.74 m	1.86 m	1.85 m	1.71 m	1.75 m
12α	1.18 m	1.24 m	1.23 m	1.13 m	1.22 m	1.17 m	1.13 m
15α	3.46 dt (20.8, 2.8)	3.47 dt (20.8, 3.2)	3.64 dd (20.8, 3.2)	5.23 d (2.4)	3.65 dd (20.8, 3.2)	3.51 dt (20.8, 3.2)	3.52 dt (20.8, 2.8)
15β	3.79 d (20.8)	3.77 d (20.8)	3.85 br d (20.8)		3.81 br d (20.8)	3.88 br d (20.8)	3.88 d (20.8)
17	5.58 s	5.53 s	5.50 s	5.50 s	5.57 s	5.56 s	5.73 s
18	1.05 s	1.03 s	1.09 s	1.06 s	1.12 s	1.05 s	1.09 s
19	1.27 s	1.52 s	1.51 s	1.26 s	1.26 s	1.49 s	1.23 s
21	7.52 br d (0.8)	7.47 br d (0.8)	7.50 br s	7.59 t (0.8)	7.57 br s	7.47 br d (0.8)	7.54 br s
22	6.45 br d (1.2)	6.41 br d (0.8)	6.42 br d (1.2)	6.50 br d (1.2)	6.48 br d (1.2)	6.41 br t (0.8)	6.47 br d (1.2)
23	7.44 t (1.6)	7.43 t (1.6)	7.44 t (1.6)	7.45 t (1.6)	7.44 t (1.6)	7.43 t (1.6)	7.42 br d (1.6)
28	0.84 s	0.78 s	0.78 s	0.82 s	0.82 s	0.79 s	0.71 s
29	1.04 s	1.05 s	1.06 s	1.09 s	1.05 s	1.21 s	0.97 s
30β	3.23 d (14.4)	3.21 d (14.8)	4.69 s	5.00 s	4.69 s	3.72 d (14.4)	3.47 d (14.8)
30α	1.79 ^1^	1.78 m				2.06 br d (14.4)	2.07 ^1^
7-OMe-31	3.73 s	3.83 s	3.83 br s	3.75 s	3.76 s	3.84 s	3.70 s
3-OAcyl							
33	2.64 m	2.45 m	2.38 m	2.27 m	2.17 s	2.20 s	2.22 s
		2.43 m					
34	1.22 d (7.2)	1.20 t (7.6)	1.50 m	1.49 m	6-Acyl	2-Acyl	2-Acyl
			1.80 m	1.80 m			
35	1.24 d (7.2)		0.99 t (7.2)	0.95 t (7.2)	2.18 s	2.13 s	2.12 s
36	6-Acyl		1.19 d (7.2)	1.20 d (7.2)			
37	2.19 s			6-Acyl			
38				2.18 s			
2-OH	4.12 br s	4.16 s	4.62 s	4.22 s	4.57 br s		
6-OH		2.80 br s	2.85 s			2.80 br s	
15-OH				3.66 br s			
30-OH			2.77 br s	2.58 br s	2.67 br s		

^1^ Overlapped signals assigned by ^1^H-^1^H COSY, HSQC, and HMBC spectra without designating multiplicity.

**Table 2:** ^13^C NMR spectroscopic data (100 MHz, in CDCl_3_) of compounds **1**–**7** (δ in ppm).

Position	1	2	3	4	5	6	7
1	216.7 qC	217.2 qC	213.8 qC	212.7 qC	213.1 qC	209.4 qC	209.6 qC
2	78.0 qC	77.8 qC	79.4 qC	79.3 qC	79.5 qC	85.6 qC	85.8 qC
3	86.1 CH	86.7 CH	86.3 CH	85.2 CH	86.4 CH	83.4 CH	81.3 CH
4	39.7 qC	39.5 qC	39.9 qC	39.9 qC	39.8 qC	40.4 qC	40.1 qC
5	44.2 CH	45.1 CH	45.7 CH	44.7 CH	44.8 CH	44.7 CH	40.7 CH
6	72.7 CH	73.1 CH	73.0 CH	72.6 CH	72.7 CH	73.2 CH	33.3 CH_2_
7	171.1 qC	175.2 qC	175.1 qC	171.5 qC	171.2 qC	175.2 qC	174.1 qC
8	125.7 qC	126.6 qC	128.6 qC	135.4 qC	127.6 qC	126.2 qC	125.5 qC
9	52.9 CH	53.3 CH	47.4 CH	46.0 CH	46.9 CH	53.1 CH	52.3 CH
10	52.4 qC	52.4 qC	51.8 qC	52.5 qC	51.9 qC	53.4 qC	53.2 qC
11	18.7 CH_2_	18.9 CH_2_	18.4 CH_2_	17.9 CH_2_	18.2 CH_2_	18.9 CH_2_	18.7 CH_2_
12	29.4 CH_2_	29.6 CH_2_	29.1 CH_2_	27.8 CH_2_	28.8 CH_2_	29.5 CH_2_	29.1 CH_2_
13	38.3 qC	38.2 qC	38.5 qC	39.4 qC	38.6 qC	38.2 qC	38.3 qC
14	133.5 qC	132.6 qC	137.8 qC	140.1 qC	138.5 qC	133.2 qC	133.4 qC
15	33.4 CH_2_	33.6 CH_2_	32.8 CH_2_	65.4 CH	32.8 CH_2_	33.6 CH_2_	33.5 CH_2_
16	169.2 qC	169.3 qC	168.9 qC	173.6 qC	169.2 qC	169.3 qC	169.9 qC
17	80.8 CH	80.8 CH	80.7 CH	81.1 CH	80.6 CH	80.6 CH	80.3 CH
18	18.0 CH_3_	18.3 CH_3_	17.7 CH_3_	16.1 CH_3_	17.6 CH_3_	18.4 CH_3_	18.1 CH_3_
19	16.7 CH_3_	17.6 CH_3_	17.6 CH_3_	17.0 CH_3_	16.8 CH_3_	17.8 CH_3_	16.9 CH_3_
20	120.5 qC	120.6 qC	120.5 qC	119.8 qC	120.3 qC	120.6 qC	120.5 qC
21	141.5 CH	141.1 CH	141.2 CH	141.9 CH	141.7 CH	141.2 CH	141.8 CH
22	109.8 CH	109.7 CH	109.7 CH	109.8 CH	109.9 CH	109.7 CH	109.9 CH
23	143.1 CH	143.2 CH	143.2 CH	143.2 CH	143.1 CH	143.2 CH	142.9 CH
28	22.5 CH_3_	22.6 CH_3_	22.8 CH_3_	22.5 CH_3_	22.7 CH_3_	22.6 CH_3_	22.8 CH_3_
29	22.7 CH_3_	22.6 CH_3_	22.7 CH_3_	22.7 CH_3_	22.6 CH_3_	23.5 CH_3_	21.1 CH_3_
30	44.4 CH_2_	44.6 CH_2_	73.0 CH	73.6 CH	72.9 CH	40.1 CH_2_	40.3 CH_2_
31	53.2 CH_3_	53.2 CH_3_	53.3 CH_3_	53.3 CH_3_	53.3 CH_3_	53.3 CH_3_	52.2 CH_3_
	3-Acyl	3-Acyl	3-Acyl	3-Acyl	3-Acyl	3-Acyl	3-Acyl
32	175.9 qC	173.3 qC	175.5 qC	175.3 qC	169.7 qC	169.6 qC	169.6 qC
33	34.5 CH	28.0 CH_2_	41.2 CH	41.8 CH	21.2 CH_3_	21.2 CH_3_	21.3 CH_3_
					6-Acyl	2-Acyl	2-Acyl
34	19.9 CH_3_	9.4 CH_3_	26.1 CH_2_	26.5 CH_2_	169.7 qC	169.1 qC	169.0 qC
35	18.4 CH_3_		11.6 CH_3_	11.8 CH_3_	21.0 CH_3_	21.8 CH_3_	21.7 CH_3_
36			17.5 CH_3_	17.5 CH_3_			
	6-Acyl			6-Acyl			
37	169.7 qC			169.8 qC			
38	21.0 CH_3_			21.0 CH_3_			

**Table 3:** ^1^H NMR spectroscopic data (400 MHz) of compounds **8**–**14** (δ in ppm, *J* in Hz).

Position	8 ^1^	9 ^1^	10 ^1^	11 ^1^	12 ^2^	13 ^1^	14 ^1^
2		3.02 t (6.0)					
3	5.51 s	4.26 d (6.0)	4.83 s	4.99 s	4.55 s	4.76 s	4.55 s
5	2.98 s	2.94 br s	3.12 s	3.20 dd (9.2, 3.2)	3.22 s	3.49 s	2.67 s
6	5.51 s	5.47 s	4.38 s	2.32 m	4.40 d (4.8)	5.55 s	4.20 br s
				2.35 m			
9			2.29 dt (12.4, 2.8)	2.29 m	1.71 t (13.2)	2.81 m	2.30 ^3^
11α	2.38 m	2.35 m	1.84 m	1.77 m	1.68 m	1.78 m	1.83 m
11β	2.38 ^3^	2.35 ^3^	1.38 qd (12.8, 4.4)	1.56 m	1.33 m	2.18 m	2.30 m
12β	1.46 m	1.45 m	2.00 m	1.93 m	1.85 d (14.0)	1.44 m	1.95 m
12α	1.65 m	1.61 m	1.24 td (13.6, 4.4)	1.29 m	1.19 m	2.05 m	1.41 m
15α	5.90 s	5.89 s	6.28 s	6.32 s	5.99 s	2.93 d (18.0)	6.08 s
15β						3.05 d (18.0)	
17	5.10 s	5.09 s	5.12 s	5.16 s	5.25 s	5.61 s	4.84 s
18	1.01 s	1.00 s	1.06 s	1.06 s	1.16 s	1.05 s	1.24 s
19	1.30 s	1.17 s	1.54 s	1.28 s	1.29 s	1.28 s	1.43 s
21	7.50 br s	7.49 br s	7.52 br s	7.51 br s	7.85 br s	7.76 br s	7.48 br t (0.8)
22	6.45 br d (1.2)	6.45 br d (1.2)	6.50 br d (1.2)	6.49 br d (1.2)	6.60 br d (1.2)	6.48 br s	6.42 br d (1.2)
23	7.46 t (1.6)	7.45 t (1.6)	7.45 t (1.6)	7.44 t (1.6)	7.70 t (1.6)	7.45 br s	7.42 t (1.6)
28	1.20 s	1.17 s	0.81 s	0.74 s	0.69 s	0.92 s	0.84 s
29	1.01 s	1.13 s	1.09 s	0.82 s	1.04 s	1.07 s	1.59 s
30β	3.55 d (17.6)	3.29 d (17.6)	6.29 d (4.4)	6.31 d (4.4)	3.38 d (15.6)	5.60 d (2.0)	5.49 s
30α	2.42 br d (17.6)	2.52 br d (17.6)			2.31 d (15.6)		
31	3.74 s	3.75 s	3.84 s	3.69 s	3.65 s	3.73 s	3.88 s
	3-Acyl	6-Acyl	3-Acyl	3-Acyl	3-Acyl	3-Acyl	3-Acyl
33	2.21 s	2.07 s	2.22 s	2.57 m	2.54 m	2.11 s	2.02 s
34	6-Acyl			1.61 m	1.60 m	6-Acyl	30-Acyl
				1.81 m	1.54 m		
35	2.11 s			1.04 t (7.2)	0.93 t (7.2)	2.19 s	2.59 m
36				1.25 d (6.8)	1.12 d (6.8)		1.13 d (6.8)
37							1.17 d (6.8)
1-OH							4.55 ^3^
2-OH			4.07 br s		5.08 s	4.17 br s	4.11 br s
6-OH			2.97 br s		5.58 d (4.8)		2.93 s
8-OH					5.05 s		

^1^ Recorded in CDCl_3_; ^2^ Recorded in DMSO-*d*_6_; ^3^ Overlapped signals assigned by ^1^H-^1^H COSY, HSQC, and HMBC spectra without designating multiplicity.

**Table 4:** ^13^C NMR spectroscopic data (100 MHz) of compounds **8**–**14** (δ in ppm).

Position	8 ^1^	9 ^1^	10 ^1^	11 ^1^	12 ^2^	13 ^1^	14 ^1^
1	211.1 qC	212.5 qC	212.0 qC	212.7 qC	215.9 qC	213.9 qC	108.4 qC
2	76.9 qC	50.5 CH	77.5 qC	77.4 qC	76.2 qC	76.9 qC	81.1 qC
3	81.6 CH	76.7 CH	87.2 CH	85.5 CH	86.5 CH	85.8 CH	85.6 CH
4	39.6 qC	39.7 qC	39.9 qC	39.7 qC	39.5 qC	39.4 qC	38.8 qC
5	55.0 CH	55.4 CH	44.8 CH	40.8 CH	44.6 CH	44.8 CH	44.6 CH
6	69.8 CH	70.2 CH	72.0 CH	32.7 CH_2_	71.2 CH	72.4 CH	71.7 CH
7	170.6 qC	170.9 qC	175.5 qC	173.5 qC	175.7 qC	171.0 qC	175.9 qC
8	126.1 qC	127.1 qC	134.7 qC	134.1 qC	71.3 qC	138.8 qC	80.0 qC
9	148.4 qC	150.3 qC	55.3 CH	53.8 CH	61.6 CH	53.3 CH	51.5 CH
10	51.9 qC	51.2 qC	53.2 qC	52.6 qC	48.4 qC	49.8 qC	43.1 qC
11	22.3 CH_2_	22.2 CH_2_	22.5 CH_2_	21.5 CH_2_	20.7 CH_2_	20.3 CH_2_	15.6 CH_2_
12	29.7 CH_2_	29.7 CH_2_	33.3 CH_2_	32.4 CH_2_	32.5 CH_2_	28.3 CH_2_	25.2 CH_2_
13	36.8 qC	36.8 qC	37.6 qC	37.5 qC	37.8 qC	41.0 qC	38.8 qC
14	156.4 qC	157.4 qC	160.7 qC	160.0 qC	168.3 qC	72.9 qC	158.3 qC
15	111.6 CH	110.6 CH	113.4 CH	113.2 CH	114.6 CH_2_	38.9 CH_2_	118.4 CH
16	165.2 qC	165.7 qC	164.8 qC	164.6 qC	164.3 qC	168.4 qC	163.1 qC
17	80.6 CH	80.7 CH	79.7 CH	79.7 CH	78.5 CH	77.2 CH	81.4 CH
18	16.2 CH_3_	16.1 CH_3_	22.5 CH_3_	21.9 CH_3_	22.6 CH_3_	15.7 CH_3_	19.5 CH_3_
19	18.2 CH_3_	18.3 CH_3_	16.2 CH_3_	15.6 CH_3_	18.5 CH_3_	15.7 CH_3_	22.1 CH_3_
20	119.8 qC	119.9 qC	120.0 qC	120.1 qC	120.0 qC	120.1 qC	119.9 qC
21	141.2 CH	141.2 CH	141.4 CH	141.5 CH	142.3 CH	141.7 CH	141.3 CH
22	109.9 CH	110.0 CH	110.2 CH	110.2 CH	110.6 CH	109.8 CH	109.9 CH
23	143.2 CH	143.1 CH	143.3 CH	143.2 CH	143.2 CH	143.1 CH	143.0 CH
28	26.3 CH_3_	25.6 CH_3_	21.4 CH_3_	21.5 CH_3_	21.7 CH_3_	22.0 CH_3_	24.0 CH_3_
29	28.0 CH_3_	28.3 CH_3_	23.0 CH_3_	20.8 CH_3_	24.8 CH_3_	22.1 CH_3_	24.5 CH_3_
30	39.2 CH_2_	28.8 CH_2_	133.5 CH	133.0 CH	45.9 CH_2_	130.2 CH	74.9 CH
31	53.0 CH_3_	52.8 CH_3_	53.4 CH_3_	52.1 CH_3_	52.0 CH_3_	53.4 CH_3_	53.4 CH_3_
	3-Acyl	6-Acyl	3-Acyl	3-Acyl	3-Acyl	3-Acyl	3-Acyl
32	170.2 qC	169.2 qC	169.9 qC	176.0 qC	174.7 qC	170.7 qC	171.5 qC
33	20.9 CH_3_	20.5 CH_3_	20.7 CH_3_	41.4 CH	40.4 CH	20.4 CH	20.7 CH_3_
	6-Acyl					6-Acyl	30-Acyl
34	169.1 qC			26.9 CH_2_	26.4 CH	169.6 qC	176.0 qC
35	20.5 CH_3_			11.9 CH_3_	11.2 CH_3_	21.0 CH_3_	34.2 CH
36				17.0 CH_3_	16.5 CH_3_		19.0 CH_3_
37							19.1 CH_3_

^1^ Recorded in CDCl_3_; ^2^ Recorded in DMSO-*d*_6_.

**Table 5:** ^1^H NMR spectroscopic data (400 MHz) of compounds **15**–**21** (δ in ppm, *J* in Hz).

Position	15 ^1^	16 ^1^	17 ^1^	18 ^1^	19 ^1^	20 ^2^	21 ^1^
2							5.77 s
3	4.87 s	4.86 s	4.94 s	5.01 s	5.02 s	4.97 s	
5	2.24 br s	2.22 s	2.13 ^3^	2.24 dd (11.2, 2.4)	2.26 ^3^	2.87 s	2.19 br s
6	4.36 br s	4.37 br d (1.6)	4.33 s	2.27 dd (16.0, 2.8)	2.28 dd (16.0, 2.8)	4.83 br d (3.6)	4.42 s
				2.43 dd (16.0, 11.2)	2.43 dd (16.0, 11.2)		
9	2.52 m	2.51 m	2.51 m	2.45 m	2.46 m	2.49 dd (6.4, 2.8)	
11α	1.81 m	1.80 m	1.84 m	1.77 m	1.79 m	2.20 ^3^	2.25 m
11β	1.40 m	1.40 m	1.40 m	1.46 m	1.48 m	2.07 m	2.03 dd (18.8, 4.0)
12β	1.58 m	1.53 m	1.60 m	1.54 m	1.55 m	1.76 m	1.36 m
12α	1.43 m	1.43 m	1.47 m	1.43 m	1.44 m	1.27 m	1.51 overlapped
15α	3.71 ^3^	3.73 dd (20.0,3.2)	3.43 dd (19.2, 1.6)	3.43 br d (18.4)	3.43 dd (19.6, 1.2)	5.72 br s	6.55 s
15β	4.07 dd (20.0, 2.8)	4.09 dd (20.0,3.2)	3.98 dd (19.2, 3.2)	3.94 ddd (19.6, 3.6, 2.0)	3.93 ddd (19.6, 3.6, 2.0)		
17	5.13 s	5.06 s	5.18 s	5.17 s	5.14 s	5.66 s	5.09 s
18	1.05 s	1.04 s	1.04 s	1.01 s	1.01 s	1.38 s	1.00 s
19	1.34 s	1.34 s	1.29 s	1.01 s	1.01 s	1.45 s	1.33 s
21	7.45 br s	7.43 br s	7.46 s	7.47 br s	7.47 br s	7.64 br s	7.49 br s
22	6.41 br d (1.2)	6.41 br d (0.8)	6.43 br d (0.8)	6.43 br d (1.2)	6.43 br d (0.8)	6.58 br d (1.2)	6.45 br d (1.2)
23	7.43 t (1.6)	7.42 t (1.6)	7.43 t (1.6)	7.42 t (1.6)	7.42 t (1.6)	7.60 t (1.6)	7.44 t (1.6)
28	1.19 s	1.20 s	1.20 s	1.00 s	0.99 s	1.01 s	1.13 s
29*_pro-R_*	1.77 d (13.2)	1.80 d (12.8)	1.97 d (12.6)	1.99 d (12.8)	1.98 d (12.4)	2.21 d (11.2)	2.35 d (12.8)
29*_pro-S_*	2.43 d (13.2)	2.45 d (12.8)	2.31 d (12.6)	2.15 d (12.8)	2.15 d (12.4)	2.88 d (11.2)	2.70 d (12.4)
30			3.46 br s	3.44 br s	3.45 br s		
31	3.86 s	3.85 s	3.87 s	3.67 s	3.67 s	3.80 s	3.86 s
	3-Acyl	3-Acyl	3-Acyl	3-Acyl	3-Acyl	3-Acyl	2-Acyl
33	2.15 s	2.57 m	2.13 s	2.66 m	2.47 m	1.94 s	2.42 m
	30-Acyl						
34	3.50 m	1.20 d (7.2)		1.21 d (6.8)	1.73 m	1-Acyl	1.48 m
	3.69 m				1.52 m		1.73 m
35	1.27 t (6.8)	1.22 d (7.2)		1.19 d (7.2)	0.94 t (7.2)	2.55 m	0.97 t (7.6)
		30-Acyl					
36		3.51 m			1.17 d (7.2)	1.11 d (7.2)	1.22 d (7.2)
		3.67 m					
37		1.27 t (6.8)			5.08 s	1.10 d (7.2)	
1-OH	2.91 br s	2.85 s					2.74 s
6-OH	2.92 d (3.2)	2.88 d (3.2)	2.94 br d (2.7)		5.58 d (4.8)	4.69 br d (3.6)	3.19 s
8-OH					5.05 s	5.00 s	
30-OH						4.87 s	3.43 s

^1^ Recorded in CDCl_3_; ^2^ Recorded in acetone-*d*_6_; ^3^ Overlapped signals assigned by ^1^H-^1^H COSY, HSQC, and HMBC spectra without designating multiplicity.

**Table 6:** ^13^C NMR spectroscopic data (100 MHz) of compounds **15**–**21** (δ in ppm).

Position	15 ^1^	16 ^1^	17 ^1^	18 ^1^	19 ^2^	20 ^1^	21 ^1^
1	84.5 qC	84.3 qC	85.2 qC	84.8 qC	84.8 qC	92.6 qC	85.5 qC
2	204.3 qC	204.1 qC	204.3 qC	204.1 qC	203.9 qC	209.1 qC	82.4 CH
3	84.8 CH	84.7 CH	85.0 CH	83.3 CH	83.0 CH	90.6 CH	207.8 qC
4	40.6 qC	40.6 qC	41.2 qC	40.6 qC	40.5 qC	45.1 qC	52.8 qC
5	45.4 CH	45.5 CH	46.0 CH	40.7 CH	40.5 CH	43.4 CH	51.4 CH
6	72.0 CH	72.1 CH	71.9 CH	34.2 CH_2_	34.2 CH_2_	71.9 CH	71.0 CH
7	175.3 qC	175.3 qC	175.4 qC	172.7 qC	172.7 qC	176.2 qC	175.2 qC
8	134.9 qC	135.0 qC	135.2 qC	134.5 qC	134.4 qC	76.8 qC	130.4 qC
9	48.3 CH	48.1 CH	49.4 CH	47.9 CH	48.0 CH	48.4 CH	156.6 qC
10	55.9 qC	56.0 qC	57.0 qC	56.4 qC	56.4 qC	48.0 qC	58.8 qC
11	19.5 CH_2_	19.5 CH_2_	20.2 CH_2_	19.4 CH_2_	19.4 CH_2_	17.8 CH_2_	20.2 CH_2_
12	31.9 CH_2_	32.0 CH_2_	32.2 CH_2_	31.9 CH_2_	31.9 CH_2_	29.5 CH_2_	30.0 CH_2_
13	40.7 qC	40.7 qC	40.4 qC	40.5 qC	40.6 qC	38.7 qC	37.8 qC
14	138.4 qC	138.5 qC	132.5 qC	133.0 qC	133.0 qC	160.0 qC	154.9 qC
15	33.1 CH_2_	33.0 CH_2_	35.4 CH_2_	35.4 CH_2_	35.3 CH_2_	120.2 CH	113.4 CH
16	169.6 qC	169.9 qC	169.8 qC	170.3 qC	170.3 qC	164.9 qC	165.7 qC
17	80.5 CH	80.5 CH	80.8 CH	80.7 CH	80.7 CH	81.2 CH	80.4 CH
18	17.7 CH_3_	17.4 CH_3_	17.5 CH_3_	16.8 CH_3_	16.8 CH_3_	21.4 CH_3_	16.2 CH_3_
19	17.0 CH_3_	17.1 CH_3_	16.7 CH_3_	14.9 CH_3_	14.9 CH_3_	22.2 CH_3_	15.7 CH_3_
20	120.6 qC	120.6 qC	120.6 qC	120.5 qC	120.5 qC	121.1 qC	120.3 qC
21	141.1 CH	141.1 CH	141.1 CH	141.2 CH	141.1 CH	142.7 CH	141.2 CH
22	110.0 CH	110.0 CH	110.1 CH	110.1 CH	110.1 CH	111.1 CH	110.1 CH
23	143.1 CH	143.1 CH	143.0 CH	142.9 CH	142.9 CH	144.0 CH	143.0 CH
28	19.8 CH_3_	19.7 CH_3_	19.5 CH_3_	19.2 CH_3_	19.2 CH_3_	15.7 CH_3_	16.3 CH_3_
29	44.7 CH_2_	44.9 CH_2_	45.4 CH_2_	44.6 CH_2_	44.5 CH_2_	41.5 CH_2_	39.3 CH_2_
30	92.2 qC	92.3 qC	63.0 CH	63.4CH	63.5 CH	83.6 qC	82.4 qC
31	53.2 CH_3_	53.1 CH_3_	53.3 CH_3_	51.8 CH_3_	51.7 CH_3_	53.0 CH_3_	53.3 CH_3_
	3-Acyl	3-Acyl	3-Acyl	3-Acyl	3-Acyl	3-Acyl	2-Acyl
32	169.9 qC	176.0 qC	170.1 qC	176.5 qC	176.0 qC	170.1 qC	176.0 qC
33	20.6 CH_3_	33.8 CH	20.7 CH_3_	33.9 CH	40.8 CH	21.3 CH_3_	41.3 CH
	30-Acyl					1-Acyl	
34	63.3 CH_2_	19.0 CH_3_		18.9 CH_3_	26.7 CH_2_	176.0 qC	26.5 CH_2_
35	15.7 CH_3_	19.1 CH_3_		18.9CH_3_	11.5 CH_3_	35.1 CH	11.7 CH_3_
		30-Acyl					
36		63.4 CH_2_		17.0 CH_3_	16.5 CH_3_	19.4 CH_3_	17.2 CH_3_
37		15.7 CH_3_				19.3 CH_3_	

^1^ Recorded in CDCl_3_; ^2^ Recorded in acetone-*d*_6_.

**Table 7:** ^1^H NMR spectroscopic data (400 MHz, in CDCl_3_) of compounds **22**–**29** (δ in ppm, *J* in Hz).

Position	22	23	24	25	26	27	28	29
1							7.14 d (10.4)	3.52 m
2β							5.90 d (10.4)	2.92 dd (14.4,6.4)
2α								2.46 dd (14.4,3.6)
3	5.54 s	5.50 s	4.88 s	4.86 s	5.17 s	4.82 s		
5	2.92 s	2.94 s	2.80 br s	2.82 s	2.40 s	2.37 d (13.6)	2.22 dd (12.4, 2.8)	2.82 d (10.4)
6a	4.26 br s	4.27 br s	4.58 s	4.58 s	4.51 s	2.40 br d (23.2)	1.93 m (α)	2.24 d (16.4)
6b						2.42 dd (23.2, 13.6)	1.99 m (β)	2.62 dd (16.4, 10.4)
7							5.30 t (2.8)	
9	3.23 m	3.25 m					2.54 m	2.22 m
11α	1.89 m	1.88 m	2.63 m	2.62 m	1.94 m	1.98 t (14.4)	2.10 m	1.65 m
11β	1.75 m	1.74 m	2.37 m	2.37 dd (20.0, 3.6)	2.26 m	2.19 dd (14.4, 4.0)	1.83 m	2.30 m
12β	1.83 m	1.83 m	1.39 m	1.40 td (12.8, 4.8)	1.56 m	3.87 br d (13.6)	1.70 m	1.96 m
12α	1.25 m	1.24 m	1.72 m	1.72 dd (12.8, 4.0)	1.25 m		2.20 m	1.35 dd (17.2, 4.4)
15α	6.00 s	5.99 s	7.23 s	7.18 s	6.56 s	6.00 s	5.87 s	2.86 d (18.0)
15β								2.60 d (18.0)
17	5.33 s	5.34 s	5.01 s	5.01 s	5.61 s	5.80 s	2.53 overlapped	5.66 s
18	1.08 s	1.07 s	1.04 s	1.04 s	1.38 s	1.43 s	1.43 s	0.93 s
19	1.21 s	1.22 s	1.54 s	1.54 s	1.54 s	1.33 s	1.24 s	0.97 s
20							2.97 ddd (12.0, 9.2, 2.8)	
21α	7.53 br s	7.53 br s	7.51 br s	7.51 s	7.45 br s	7.67 br s		4.85 br d (18.3)
21β								5.03 dd (18.3, 2.0)
22α	6.49 d (1.2)	6.49 d (1.2)	6.48 br d (1.2)	6.47 br d (1.2)	6.41 br s	6.61 br s	2.44 m	6.08 br s
22β							2.49 m	
23α	7.47 t (1.6)	7.47 t (1.6)	7.46 t (1.6)	7.45 t (1.6)	7.43 br s	7.53 br s	4.23 ddd (10.4, 9.2, 6.8)	
23β							4.46 td (8.8, 2.0)	
28	1.21 s	1.22 s	1.00 s	1.00 s	0.87 s	0.82 s	1.10 s	1.03 s
29*_pro-R_*	2.49 d (19.2)	2.46 br d (18.8)	1.81 dd (10.8, 2.0)	1.81 dd (10.4, 2.0)	1.67 br d (11.0)	1.80 d (11.2)	1.09 s	1.20 s
29*_pro-S_*	2.56 d (19.2)	2.58 d (18.8)	2.71 d (10.8)	2.70 d (10.4)	2.36 d (11.0)	1.86 d (11.2)		
30α	6.59 br d (1.6)	6.56 br d (1.6)			5.34 s	4.49 s	1.29 s	4.92 s (30a)
30β								5.22 s (30b)
31	3.81 s	3.81 s	3.81 s	3.82 s	3.80 s	3.76 s	7-Acyl	3.73 s
32	3-Acyl	3-Acyl	3-Acyl	3-Acyl			1.97 s	
33	2.17 s	2.51 m	2.27 m	2.45 m	1.70 s	1.68 s		
34		1.53 m	1.42 m		3-Acyl	3-Acyl		
		1.74 m	1.58 m					
35		0.96 t (7.2)	0.87 t (7.6)	1.11 d (6.8)	2.07 s			
36		1.18 d (7.2)	1.07 d (6.8)	1.11 d (6.8)	2-Acyl	6.88 q (6.8)		
37					2.17 s	1.73 br d (6.8)		
38						1.84 s		
1-OH			2.81 s	2.84 br s		3.45 s		
2-OH			4.93 s	4.93 br s		3.57 s		
6-OH	3.01 s	3.06 br s	3.12 br s	3.13 s				

**Table 8:** ^13^C NMR spectroscopic data (100 MHz, in CDCl_3_.) of compounds **22**–**29** (δ in ppm).

Position	22	23	24	25	26	27	28	29
1	215.1 qC	215.4 qC	85.8 qC	85.9 qC	84.4 qC	84.4 qC	156.7 CH	77.5 CH
2	193.6 qC	193.5 qC	80.7 qC	80.8 qC	84.0 qC	75.7 qC	126.0 CH	39.2 CH_2_
3	82.9 CH	82.4 CH	87.2 CH	87.2 CH	85.6 CH	86.6 CH	204.0 qC	212.4 qC
4	41.1 qC	41.2 qC	45.6 qC	45.6 qC	44.4 qC	43.7 qC	44.1 qC	48.1 qC
5	49.2 CH	49.2 CH	48.2 CH	48.1 CH	44.8 CH	40.5 CH	46.2 CH	42.9 CH
6	70.9 CH	70.9 CH	71.5 CH	71.5 CH	71.5 CH	33.8 CH_2_	23.5 CH_2_	32.6 CH_2_
7	175.1 qC	175.2 qC	175.0 qC	174.9 qC	174.1 qC	174.1 qC	73.9 CH	173.7 qC
8	149.7 qC	149.6 qC	121.0 qC	121.1 qC	84.0 qC	83.7 qC	44.8 qC	144.8 qC
9	43.7 CH	44.1 CH	169.8 qC	169.7 qC	86.8 qC	87.1 qC	37.8 CH	49.6 CH
10	52.7 qC	52.6 qC	48.8 qC	48.8 qC	49.0 qC	47.4 qC	39.9 qC	44.0 qC
11	21.0 CH_2_	21.2 CH_2_	25.6 CH_2_	25.6 CH_2_	26.3 CH_2_	34.3 CH_2_	15.8 CH_2_	23.7 CH_2_
12	30.8 CH_2_	30.8 CH_2_	30.1 CH_2_	30.1 CH_2_	29.5 CH_2_	66.5 CH	31.5 CH_2_	29.2 CH_2_
13	40.0 qC	40.0 qC	36.5 qC	36.5 qC	38.1 qC	44.7 qC	47.9 qC	41.8 qC
14	165.9 qC	166.2 qC	152.1 qC	152.2 qC	154.6 qC	152.3 qC	193.6 qC	80.1 qC
15	115.1 CH	115.0 CH	115.8 CH	115.7 CH	122.3 CH	123.7 CH	123.8 CH	33.5 CH_2_
16	163.8 qC	163.8 qC	165.4 qC	165.5 qC	163.9 qC	162.4 qC	205.1 qC	168.2 qC
17	79.2 CH	79.1 CH	80.3 CH	80.3 CH	80.6 CH	78.2 qC	61.8 CH	81.2 CH
18	19.6 CH_3_	19.6 CH_3_	15.8 CH_3_	15.8 CH_3_	19.6 CH_3_	13.2 CH_3_	24.2 CH_3_	14.1 CH_3_
19	15.3 CH_3_	15.4 CH_3_	16.1 CH_3_	16.0 CH_3_	17.4 CH_3_	15.6 CH_3_	19.1 CH_3_	21.7 CH_3_
20	119.6 qC	119.7 qC	119.9 qC	119.9 qC	119.6 qC	121.5 qC	36.8 CH	164.3 qC
21	141.5 CH	141.5 CH	141.3 qC	141.3 CH	141.5 CH	142.3 CH	177.5 qC	72.4 CH_2_
22	110.0 CH	110.1 CH	110.0 CH	110.0 CH	109.8 CH	109.7 CH	29.2 CH_2_	117.8 CH
23	143.4 CH	143.4 CH	143.2 CH	143.2 CH	143.2 CH	144.9 CH	66.5 CH_2_	172.5 qC
28	16.7 CH_3_	16.5 CH_3_	16.6 CH_3_	16.6 CH_3_	15.4 CH_3_	14.5 CH_3_	27.0 CH_3_	25.5 CH_3_
29	49.7 CH_2_	49.6 CH_2_	43.1 CH_2_	43.1 CH_2_	40.5 CH_3_	39.0 CH_3_	21.3 CH_3_	21.4 CH_3_
30	134.7 CH	134.8 CH	194.9 qC	195.0 qC	74.0 CH	78.2 CH	26.8 CH_3_	112.5 CH_2_
							7-Acyl	
31	53.2 CH_3_	53.2 CH_3_	53.3 CH_3_	53.3 CH_3_	53.2 CH_3_	52.3 CH_3_	169.8 qC	52.2 CH_3_
	3-Acyl	3-Acyl	3-Acyl	3-Acyl				
32	170.4 qC	176.2 qC	174.3 qC	174.8 qC	119.5 qC	119.4 qC	21.0 CH_3_	
33	20.6 CH_3_	40.7 CH	41.3 CH	34.2 CH	16.6 CH_3_	16.5 CH_3_		
					3-Acyl	3-Acyl		
34		26.6 CH_2_	26.5 CH_2_	18.8 CH_3_	169.0 qC	167.8 qC		
35		11.5 CH_3_	11.8 CH_3_	19.0 CH_3_	21.7 CH_3_	130.1 qC		
					2-Acyl			
36		16.4 CH_3_	16.7 CH_3_		170.7 qC	139.7 CH		
37					21.9 CH_3_	14.4 CH_3_		
38						12.5 CH_3_		

## Supplementary Materials

The following are available online at www.mdpi.com/1660-3397/16/1/38/s1. Table S1: Cytotoxic assay results for compounds against human cancer cells; Table S2: HIV-inhibitory bioassay results for tested compounds; Copies of HRESIMS of compounds **1**–**29**, and 1D and 2D NMR spectra of compounds **1**–**29**.
