# Supplementary material for: Twenty-Nine New Limonoids with Skeletal Diversity from the Mangrove Plant, Xylocarpus moluccensis
Source: Mar Drugs. 2018 Jan 19;16(1):38. doi: 10.3390/md16010038 (PMC5793086; doi:10.3390/md16010038)

Supporting Information

Copies of HR-ESIMS, and 1D and 2D NMR spectra of Compounds 1-14.

Table of Contents:

1. Table S1: Cytotoxic assay results for compounds against human cancer cells [IC_{50} (μM)] ··········S8
2. Table S2: HIV-inhibitory bioassay results for tested compounds ··········S9
3. HR-ESIMS for compound **1** ··········S10-S11
4. ^{1}H NMR (400 MHz) spectrum of compound **1** in $CDCl_3$··········S12-S14
5. ^{13}C NMR (100 MHz) spectrum of compound **1** in $CDCl_3$··········S15-S18
6. DEPT135 (100 MHz) spectrum of compound **1** in $CDCl_3$ ··········S19-S21
7. ^{1}H-^{1}H COSY (400 MHz) spectrum of compound **1** in $CDCl_3$··········S22-S24
8. HSQC (400 MHz) spectrum of compound **1** in $CDCl_3$··········S25-S28
9. HMBC (400 MHz) spectrum of compound **1** in $CDCl_3$··········S29-S37
10. NOESY (400 MHz) spectrum of compound **1** in $CDCl_3$··········S38-S43
11. HR-ESIMS for compound **2**··········S44-S45
12. ^{1}H NMR (400 MHz) spectrum of compound **2** in $CDCl_3$··········S46-S48
13. ^{13}C NMR (100 MHz) spectrum of compound **2** in $CDCl_3$··········S49-S53
14. DEPT135 (100 MHz) spectrum of compound **2** in $CDCl_3$ ··········S54-S59
15. ^{1}H-^{1}H COSY (400 MHz) spectrum of compound **2** in $CDCl_3$··········S60-S62
16. HSQC (400 MHz) spectrum of compound **2** in $CDCl_3$··········S63-S65
17. HMBC (400 MHz) spectrum of compound **2** in $CDCl_3$··········S66-S70
18. NOESY (400 MHz) spectrum of compound **2** in $CDCl_3$··········S71-S75
19. HR-ESIMS for compound **3**··········S76-S77

20. ^{1}H NMR (400 MHz) spectrum of compound **3** in $CDCl_3$ ··················S78-S80
21. ^{13}C NMR (100 MHz) spectrum of compound **3** in $CDCl_3$ ················S81-S84
22. DEPT135 (100 MHz) spectrum of compound **3** in $CDCl_3$ ·················S85-S88
23. ^{1}H-^{1}H COSY (400 MHz) spectrum of compound **3** in $CDCl_3$ ·············S89-S92
24. HSQC (400 MHz) spectrum of compound **3** in $CDCl_3$ ·····················S93-S96
25. HMBC (400 MHz) spectrum of compound **3** in $CDCl_3$ ···················S97-S102
26. NOESY (400 MHz) spectrum of compound **3** in $CDCl_3$ ················S103-S108
27. HR-ESIMS for compound **4**···S109-S110
28. ^{1}H NMR (400 MHz) spectrum of compound **4** in $CDCl_3$················S111-S113
29. ^{13}C NMR (100 MHz) spectrum of compound **4** in $CDCl_3$···············S114-S116
30. DEPT135 (100 MHz) spectrum of compound **4** in $CDCl_3$ ··············S117-S119
31. ^{1}H-^{1}H COSY (400 MHz) spectrum of compound **4** in $CDCl_3$ ··········S120-S121
32. HSQC (400 MHz) spectrum of compound **4** in $CDCl_3$ ··················S122-S124
33. HMBC (400 MHz) spectrum of compound **4** in $CDCl_3$ ·················S125-S129
34. NOESY (400 MHz) spectrum of compound **4** in $CDCl_3$ ················S130-S134
35. HR-ESIMS for compound **5**···S135-S136
36. ^{1}H NMR (400 MHz) spectrum of compound **5** in $CDCl_3$················S137-S139
37. ^{13}C NMR (100 MHz) spectrum of compound **5** in $CDCl_3$···············S140-S142
38. DEPT135 (100 MHz) spectrum of compound **5** in $CDCl_3$ ··············S143-S145
39. ^{1}H-^{1}H COSY (400 MHz) spectrum of compound **5** in $CDCl_3$ ··········S146-S147
40. HSQC (400 MHz) spectrum of compound **5** in $CDCl_3$ ··················S148-S150
41. HMBC (400 MHz) spectrum of compound **5** in $CDCl_3$ ·················S151-S157

42. NOESY (400 MHz) spectrum of compound **5** in $CDCl_3$ ···············S158-S162
43. HR-ESIMS for compound **6**···S163-S164
44. ^{1}H NMR (400 MHz) spectrum of compound **6** in $CDCl_3$···············S165-S167
45. ^{13}C NMR (100 MHz) spectrum of compound **6** in $CDCl_3$··············S168-S170
46. DEPT135 (100 MHz) spectrum of compound **6** in $CDCl_3$ ·············S171-S174
47. ^{1}H-^{1}H COSY (400 MHz) spectrum of compound **6** in $CDCl_3$ ·········S175-S176
48. HSQC (400 MHz) spectrum of compound **6** in $CDCl_3$ ··················S177-S179
49. HMBC (400 MHz) spectrum of compound **6** in $CDCl_3$ ·················S180-S185
50. NOESY (400 MHz) spectrum of compound **6** in $CDCl_3$ ···············S186-S189
51. HR-ESIMS for compound **7**···S190-S191
52. ^{1}H NMR (400 MHz) spectrum of compound **7** in $CDCl_3$···············S192-S194
53. ^{13}C NMR (100 MHz) spectrum of compound **7** in $CDCl_3$··············S195-S198
54. DEPT135 (100 MHz) spectrum of compound **7** in $CDCl_3$ ·············S199-S201
55. ^{1}H-^{1}H COSY (400 MHz) spectrum of compound **7** in $CDCl_3$ ·········S202-S203
56. HSQC (400 MHz) spectrum of compound **7** in $CDCl_3$ ··················S204-S208
57. HMBC (400 MHz) spectrum of compound **7** in $CDCl_3$ ·················S209-S217
58. NOESY (400 MHz) spectrum of compound **7** in $CDCl_3$ ···············S218-S220
59. HR-ESIMS for compound **8**···S221-S223
60. ^{1}H NMR (400 MHz) spectrum of compound **8** in $CDCl_3$ ··············S224-S226
61. ^{13}C NMR (100 MHz) spectrum of compound **8** in $CDCl_3$ ············S227-S229
62. DEPT135 (100 MHz) spectrum of compound **8** in $CDCl_3$ ············S230-S232
63. ^{1}H-^{1}H COSY (400 MHz) spectrum of compound **8** in $CDCl_3$ ·········S233-S234

64. HSQC (400 MHz) spectrum of compound **8** in $CDCl_3$ ············S235-S237
65. HMBC (400 MHz) spectrum of compound **8** in $CDCl_3$ ············S238-S243
66. NOESY (400 MHz) spectrum of compound **8** in $CDCl_3$ ················S244-S246
67. HR-ESIMS for compound **9**···S247-S248
68. ^{1}H NMR (400 MHz) spectrum of compound **9** in $CDCl_3$················S249-S251
69. ^{13}C NMR (100 MHz) spectrum of compound **9** in $CDCl_3$···············S252-S254
70. DEPT135 (100 MHz) spectrum of compound **9** in $CDCl_3$ ···············S255-S257
71. ^{1}H-^{1}H COSY (400 MHz) spectrum of compound **9** in $CDCl_3$ ··········S258-S259
72. HSQC (400 MHz) spectrum of compound **9** in $CDCl_3$ ··················S260-S262
73. HMBC (400 MHz) spectrum of compound **9** in $CDCl_3$ ·················S263-S268
74. NOESY (400 MHz) spectrum of compound **9** in $CDCl_3$ ················S269-S271
75. HR-ESIMS for compound **10**·······································S272
76. ^{1}H NMR (400 MHz) spectrum of compound **10** in $CDCl_3$···············S273-S276
77. ^{13}C NMR (100 MHz) spectrum of compound **10** in $CDCl_3$··············S277-S279
78. DEPT135 (100 MHz) spectrum of compound **10** in $CDCl_3$ ·············S281-S285
79. ^{1}H-^{1}H COSY (400 MHz) spectrum of compound **10** in $CDCl_3$ ·········S286-S288
80. HSQC (400 MHz) spectrum of compound **10** in $CDCl_3$ ················S289-S291
81. HMBC (400 MHz) spectrum of compound **10** in $CDCl_3$ ················S292-S297
82. NOESY (400 MHz) spectrum of compound **10** in $CDCl_3$ ··············S298-S302
83. HR-ESIMS for compound **11**·······································S303-S304
84. ^{1}H NMR (400 MHz) spectrum of compound **11** in $CDCl_3$···············S305-S308
85. ^{13}C NMR (100 MHz) spectrum of compound **11** in $CDCl_3$··············S309-S313
86. DEPT135 (100 MHz) spectrum of compound **11** in $CDCl_3$ ·············S314-S318

87. ^{1}H-^{1}H COSY (400 MHz) spectrum of compound **11** in $CDCl_3$ ··········S319-S322
88. HSQC (400 MHz) spectrum of compound **11** in $CDCl_3$ ··················S323-S326
89. HMBC (400 MHz) spectrum of compound **11** in $CDCl_3$ ················S327-S332
90. NOESY (400 MHz) spectrum of compound **11** in $CDCl_3$ ···············S333-S336
91. HR-ESIMS for compound **12**···S337-S338
92. ^{1}H NMR (400 MHz) spectrum of compound **12** in DMSO-*d6* ··········S339-S341
93. ^{13}C NMR (100 MHz) spectrum of compound **12** in DMSO-*d6* ·········S342-S344
94. DEPT135 (100 MHz) spectrum of compound **12** in DMSO-*d6* ········S345-S347
95. ^{1}H-^{1}H COSY (400 MHz) spectrum of compound **12** in DMSO-*d6* ····S348-S349
96. HSQC (400 MHz) spectrum of compound **12** in DMSO-*d6*·············S350-S353
97. HMBC (400 MHz) spectrum of compound **12** in DMSO-*d6*············S354-S361
98. NOESY (400 MHz) spectrum of compound **12** in DMSO-*d6* ··········S362-S365
99. HR-ESIMS for compound **13**···S366-S367
100. ^{1}H NMR (400 MHz) spectrum of compound **13** in $CDCl_3$··············S368-S371
101. ^{13}C NMR (100 MHz) spectrum of compound **13** in $CDCl_3$·············S372-S374
102. DEPT135 (100 MHz) spectrum of compound **13** in $CDCl_3$ ············S375-S377
103. ^{1}H-^{1}H COSY (400 MHz) spectrum of compound **13** in $CDCl_3$········S378-S380
104. HSQC (400 MHz) spectrum of compound **13** in $CDCl_3$ ················S381-S384
105. HMBC (400 MHz) spectrum of compound **13** in $CDCl_3$ ···············S385-S391
106. NOESY (400 MHz) spectrum of compound **13** in $CDCl_3$ ··············S392-S396
107. HR-ESIMS for compound **14**··S397-S398

108. ^{1}H NMR (400 MHz) spectrum of compound **14** in $CDCl_3$·············S399-S401
109. ^{13}C NMR (100 MHz) spectrum of compound **14** in $CDCl_3$·············S402-S405
110. DEPT135 (100 MHz) spectrum of compound **14** in $CDCl_3$ ···········S406-S408
111. ^{1}H-^{1}H COSY (400 MHz) spectrum of compound **14** in $CDCl_3$········S409-S410
112. HSQC (400 MHz) spectrum of compound **14** in $CDCl_3$················S411-S413
113. HMBC (400 MHz) spectrum of compound **14** in $CDCl_3$···············S414-S420
114. NOESY (400 MHz) spectrum of compound **14** in $CDCl_3$··············S421-S423

Table S**1:** Cytotoxic assay results for compounds against human cancer cells [**IC$_{50}$** (μM)]

Compound	HCT-8	HCT-8/T	A2780	A2780/T	MDA-MB-231
**1**	>100	>100	>100	>100	>100
**3**	>100	>100	>100	>100	>100
**8**	>100	>100	>100	>100	>100
**10**	>100	>100	>100	>100	>100
**11**	70.14	64.14	62.04	82.17	>100
**14**	>100	>100	>100	>100	>100
**15**	>100	>100	>100	>100	>100
**16**	>100	>100	>100	>100	94.51
**20**	>100	>100	>100	>100	>100
**23**	68.84	>100	64.18	77.80	37.68
**25**	>100	>100	>100	>100	>100
**27**	>100	>100	>100	>100	>100
**Cisplatin**	15.43	21.98	8.54	9.26	6.25

Table S**2:** HIV-inhibitory bioassay results for tested compounds

**Compound**	**HIV-1 inhibitory rate** (100μM)	**HIV-1 inhibitory rate** (20μM)	**IC$_{50}$** (μM)	CC$_{50}$ (μM)
**1**	53.74 ± 7.31	17.49 ± 6.93	NA	NT
**3**	15.15 ± 19.49	NA	NA	NT
**8**	NA	NA	NA	NT
**10**	40.69 ± 2.25	NA	NA	NT
**11**	98.56 ± 0.81	24.47 ± 5.04	NA	NT
**14**	59.33 ± 1.54	NA	NA	NT
**20**	31.83 ± 3.20	NA	NA	NT
**23**	97.57 ± 2.67	14.77 ± 5.91	NA	NT
**24**	31.83 ± 2.58	14.34 ± 3.92	NA	NT
**25**	99.44 ± 0.77	NA	NA	NT
**27**	23.86 ± 2.89	NA	NA	NT

NA = No activity；NT = None tested

HR-ESIMS for compound **1**

Mass Spectrum SmartFormula Report

Analysis Info

Analysis Name	D:\Data\MS\data\201504\liwanshan_LWS-46_pos.d	Acquisition Date	4/27/2015 10:36:39 AM
Method	POS_100-2000_Dirrect Infusion.m	Operator	SCSIO
Sample Name	SCSIO	Instrument / Ser#	maXis 29
Comment			

Acquisition Parameter

Source Type	ESI	Ion Polarity	Positive	Set Nebulizer	0.3 Bar
Focus	Not active	Set Capillary	4500 V	Set Dry Heater	180 °C
Scan Begin	100 m/z	Set End Plate Offset	-500 V	Set Dry Gas	4.0 l/min
Scan End	2000 m/z	Set Collision Cell RF	2000.0 Vpp	Set Divert Valve	Waste

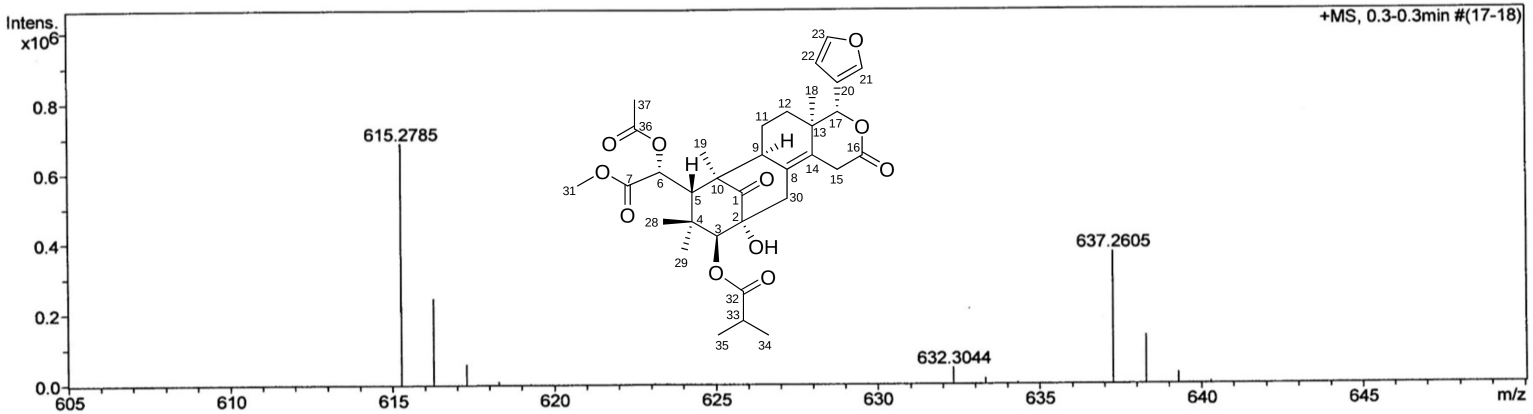

Meas. m/z	#	Formula	Score	m/z	err [mDa]	err [ppm]	mSigma	rdb	e⁻ Conf	N-Rule
615.2785	1	C 33 H 43 O 11	100.00	615.2800	1.5	2.4	5.9	12.5	even	ok
637.2605	1	C 33 H 42 Na O 11	100.00	637.2619	1.5	2.3	3.7	12.5	even	ok

Bruker Compass DataAnalysis 4.0 printed: 4/27/2015 10:41:10 AM Page 1 of 1

HR-ESIMS for compound **1**

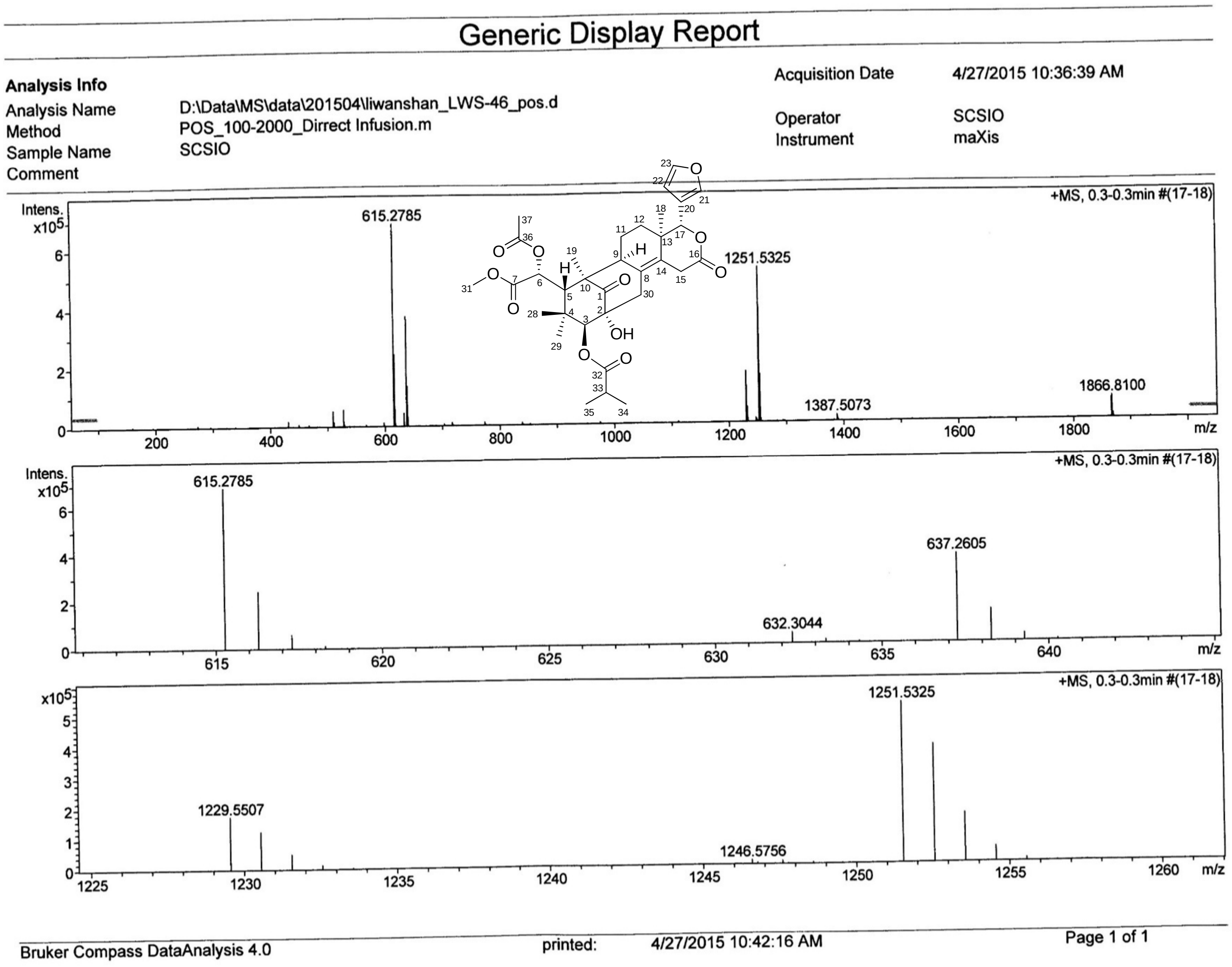

^{1}H NMR (400 MHz) spectrum of compound **1** in $CDCl_3$

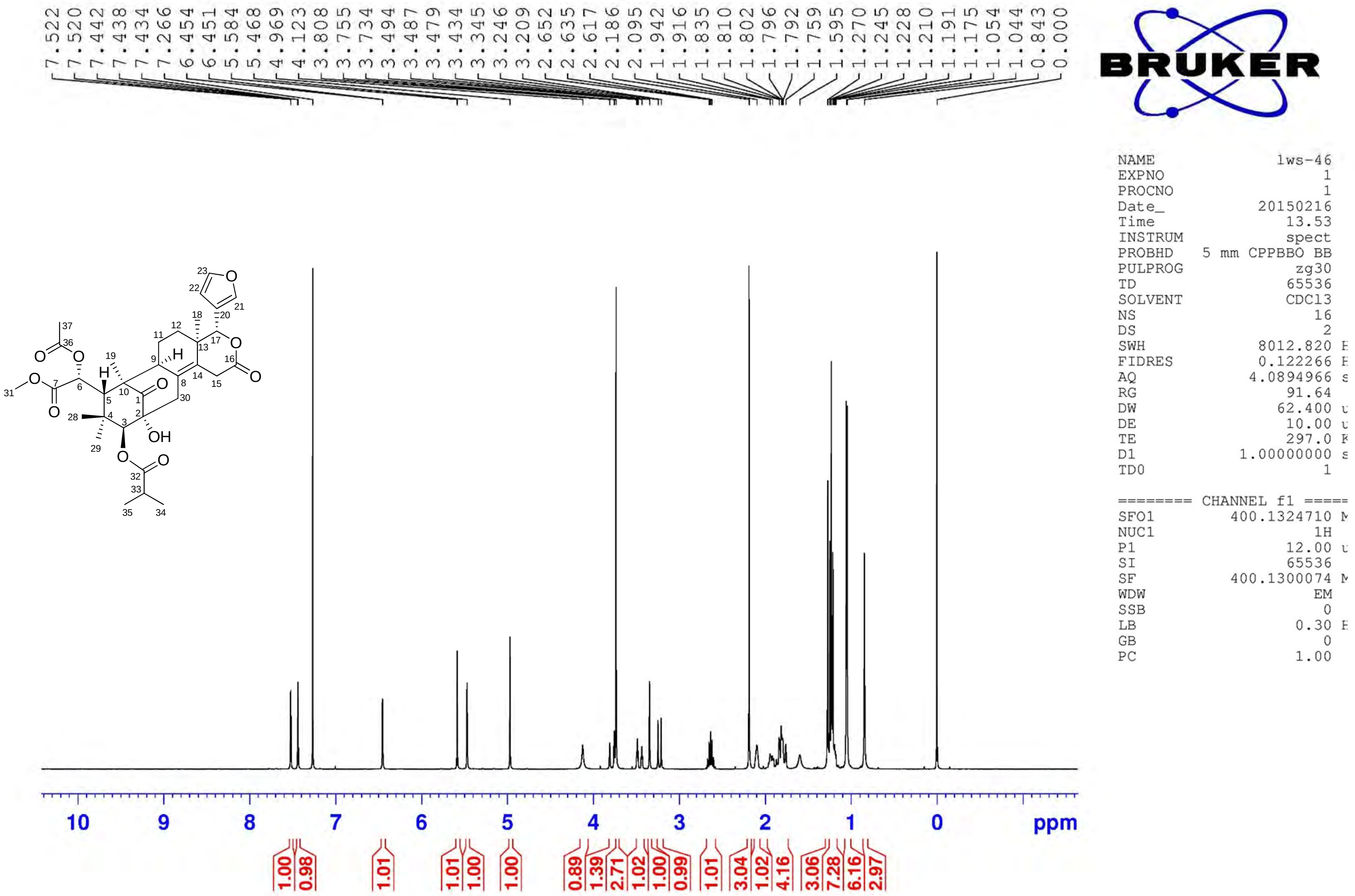

^{1}H NMR (400 MHz) spectrum of compound **1** in $CDCl_3$

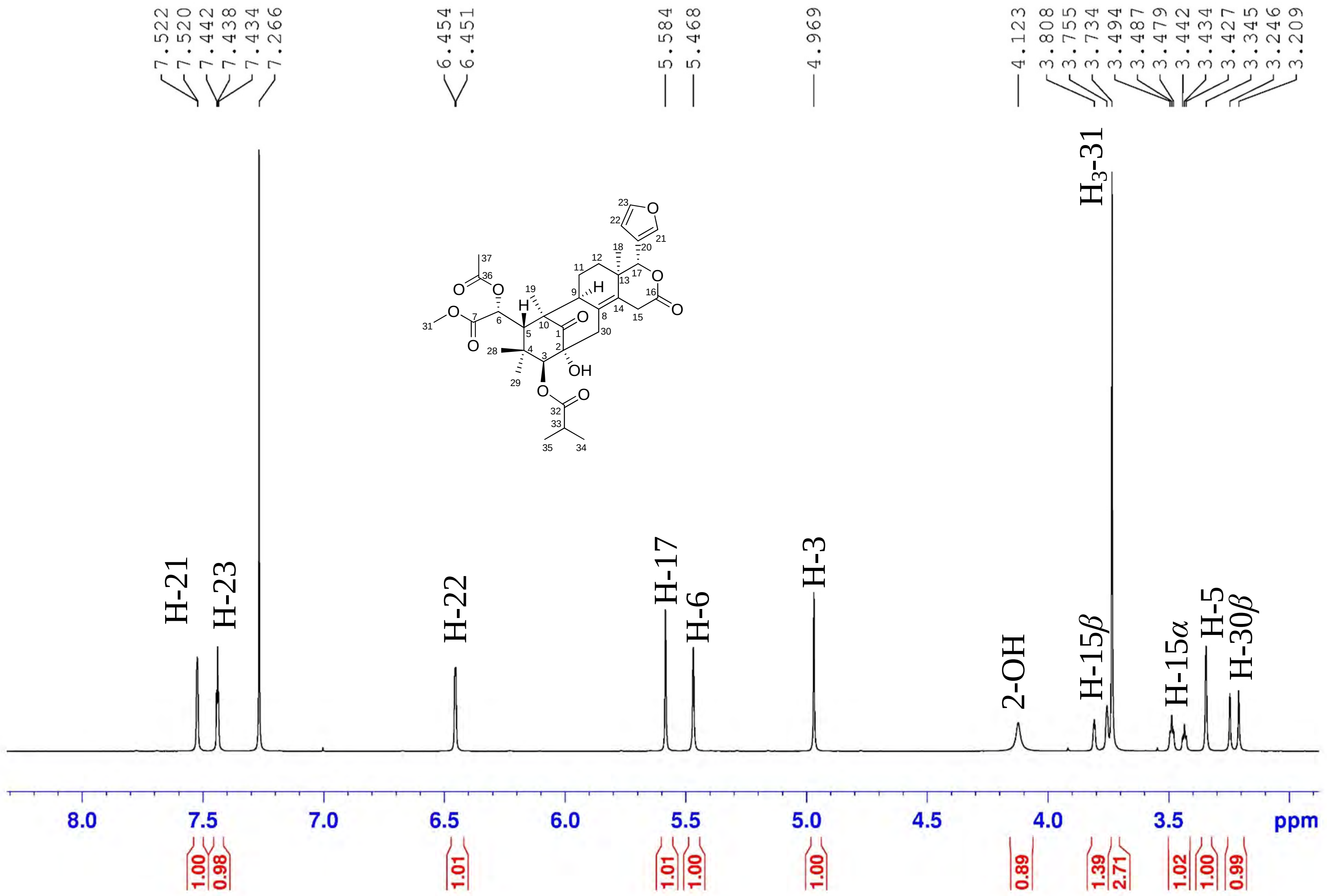

^{1}H NMR (400 MHz) spectrum of compound **1** in $CDCl_3$

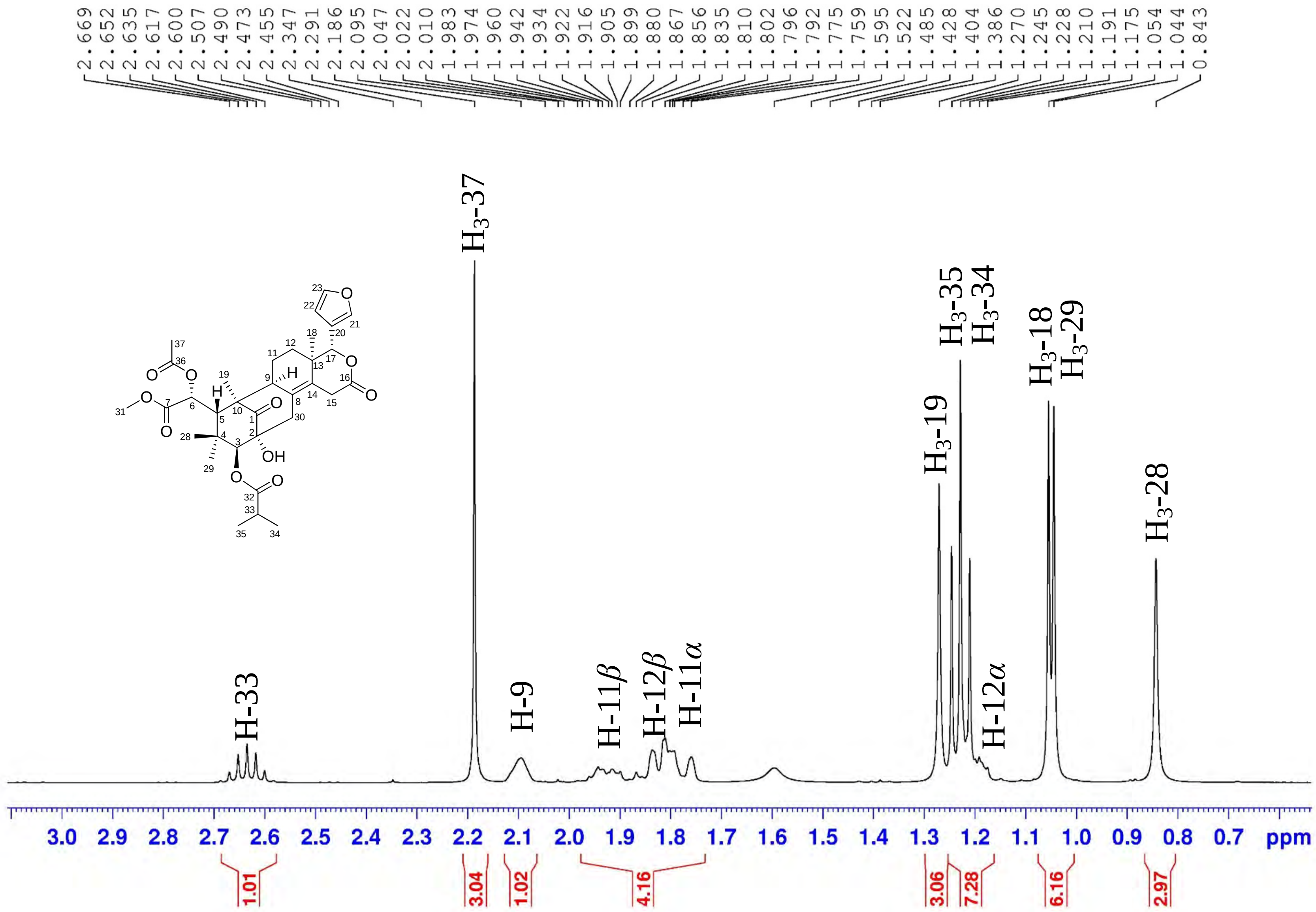

^{13}C NMR (100 MHz) spectrum of compound **1** in $CDCl_3$

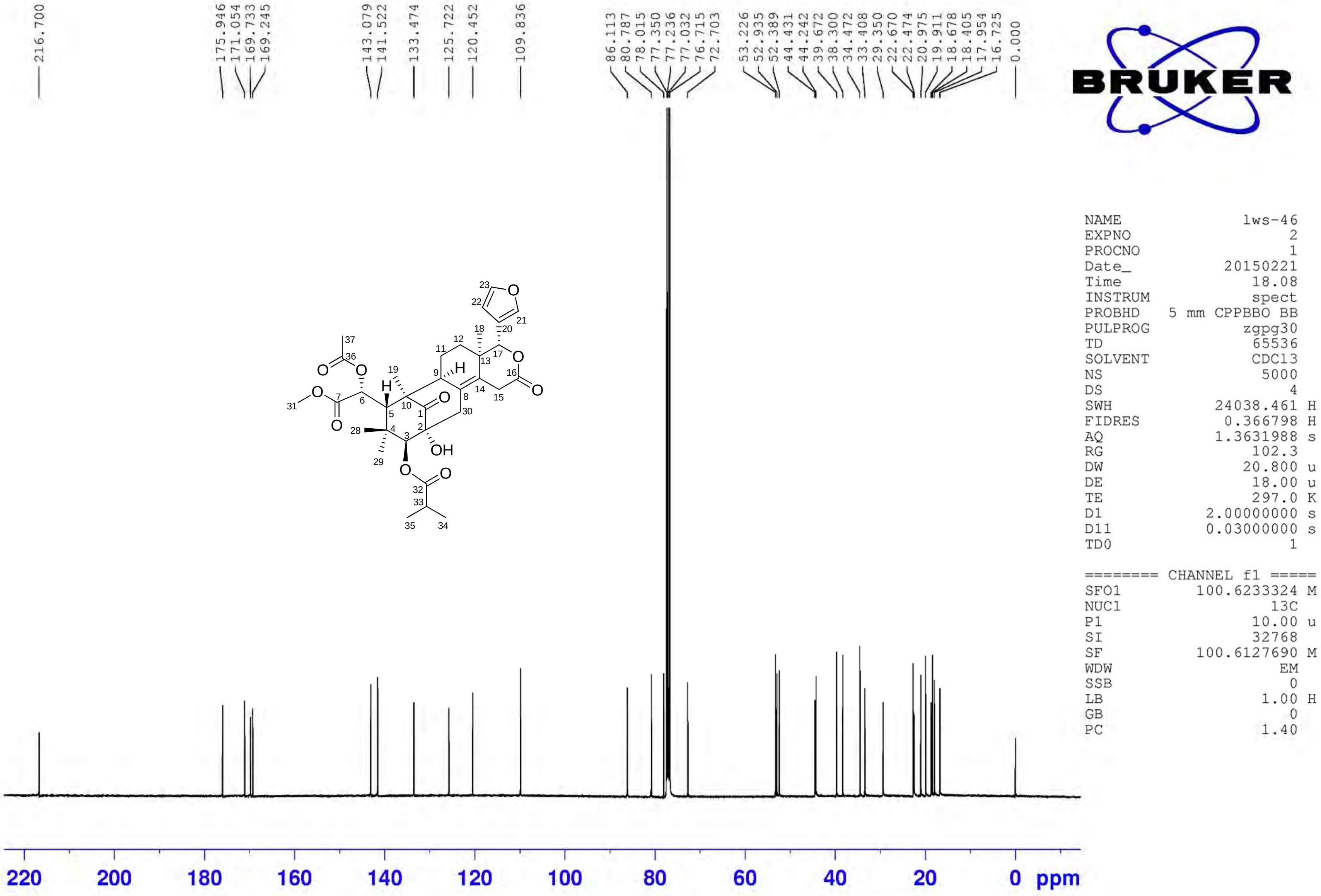

^{13}C NMR (100 MHz) spectrum of compound **1** in $CDCl_3$

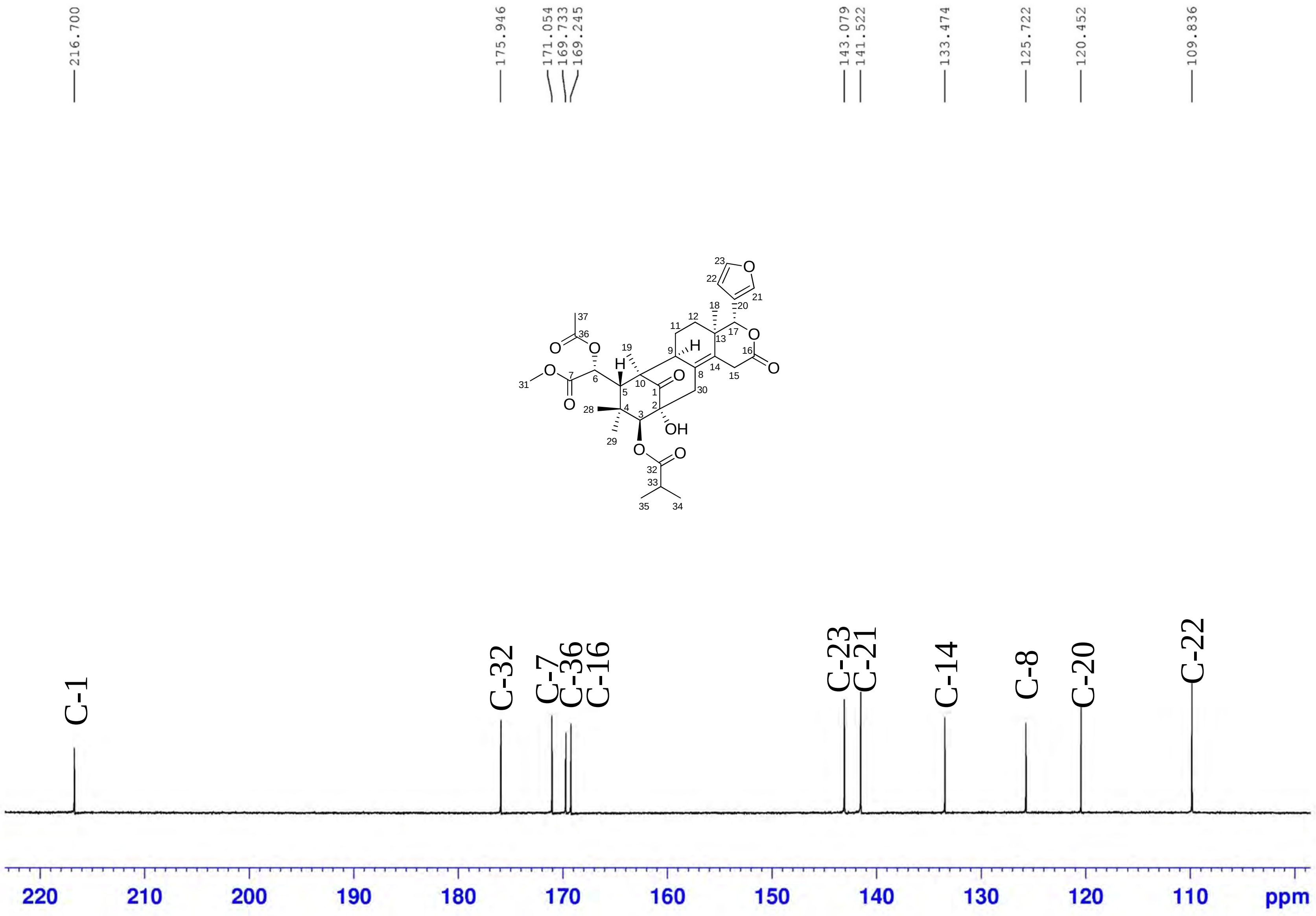

^{13}C NMR (100 MHz) spectrum of compound **1** in $CDCl_3$

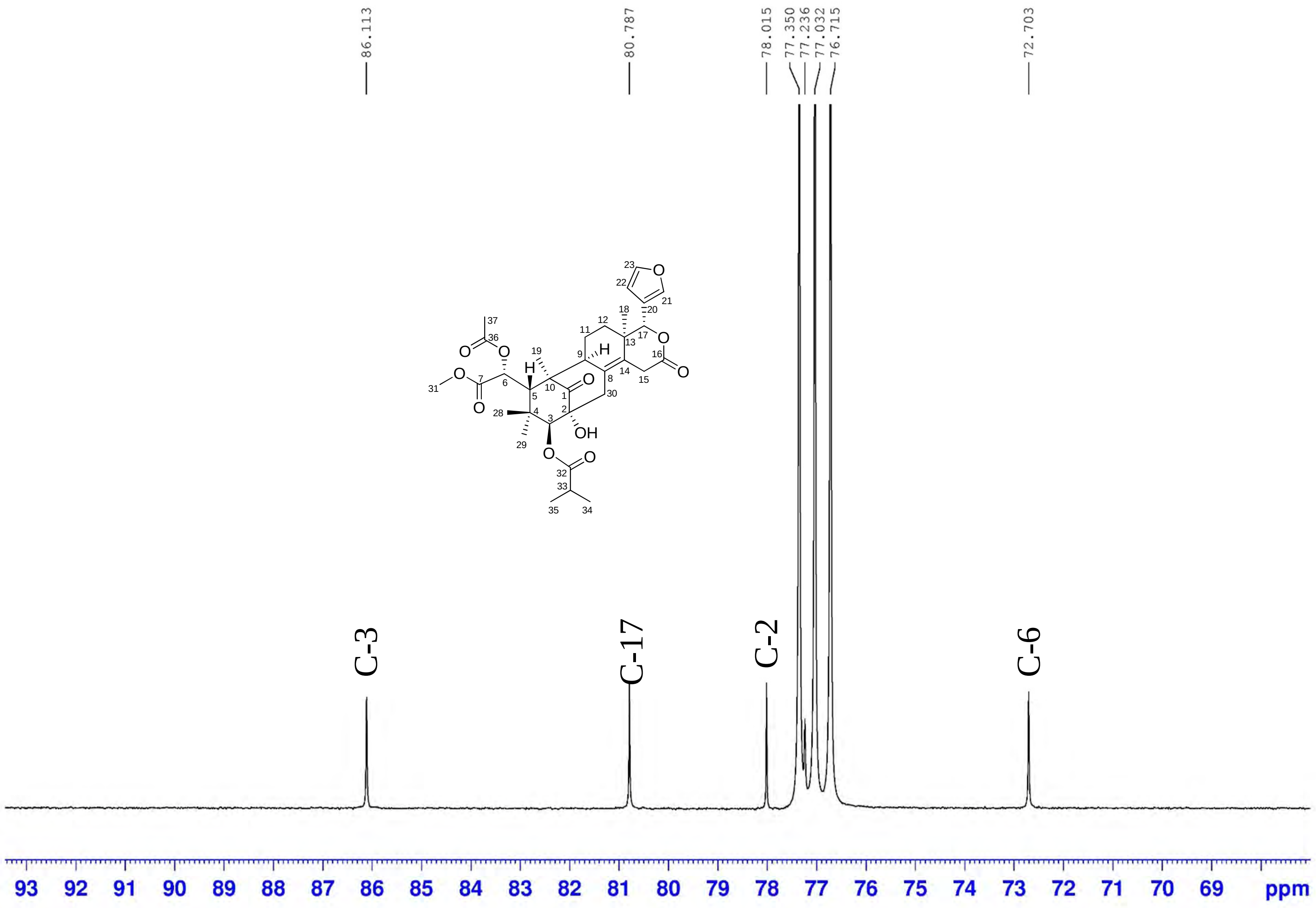

^{13}C NMR (100 MHz) spectrum of compound **1** in $CDCl_3$

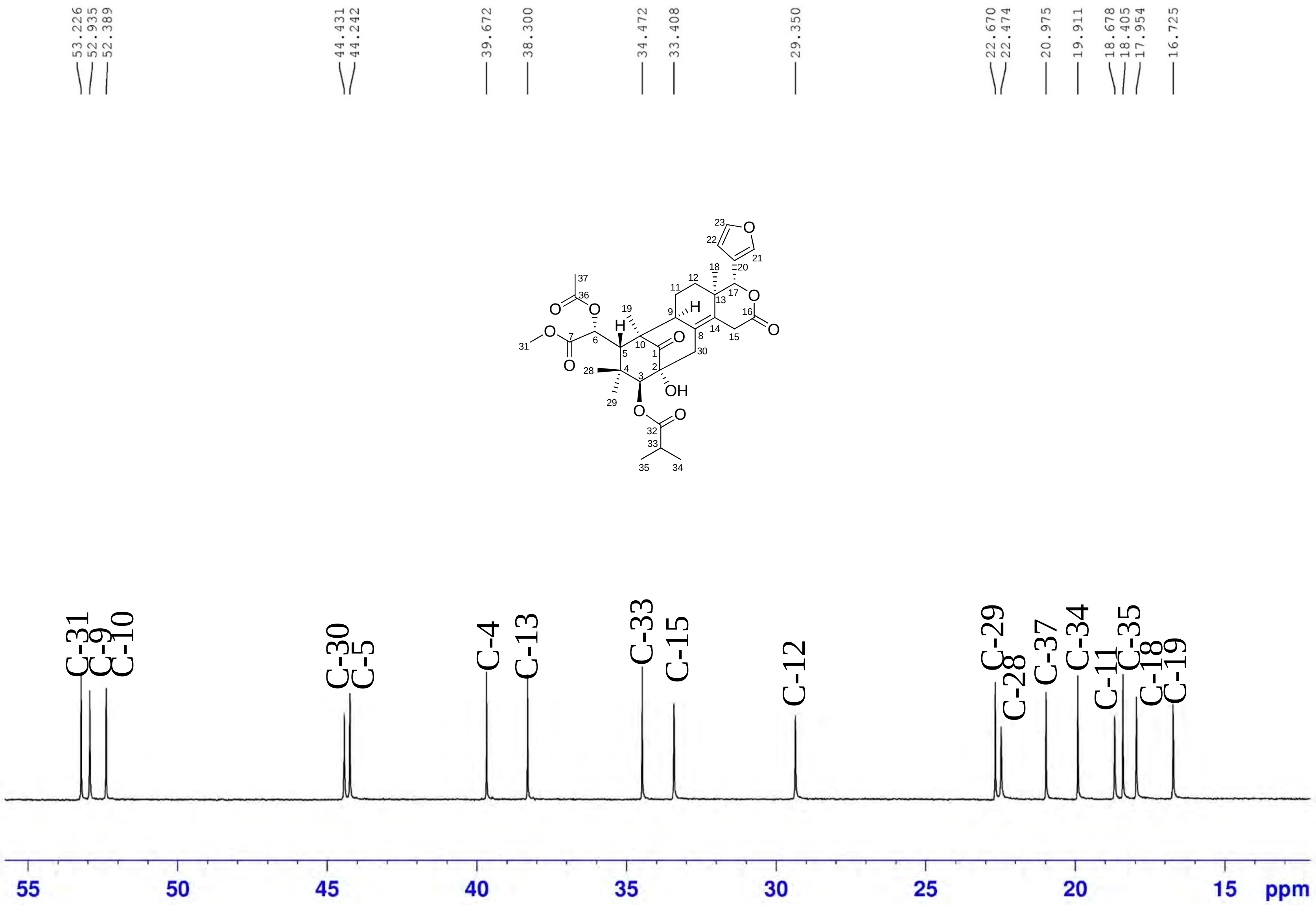

DEPT135 (100 MHz) spectrum of compound **1** in $CDCl_3$

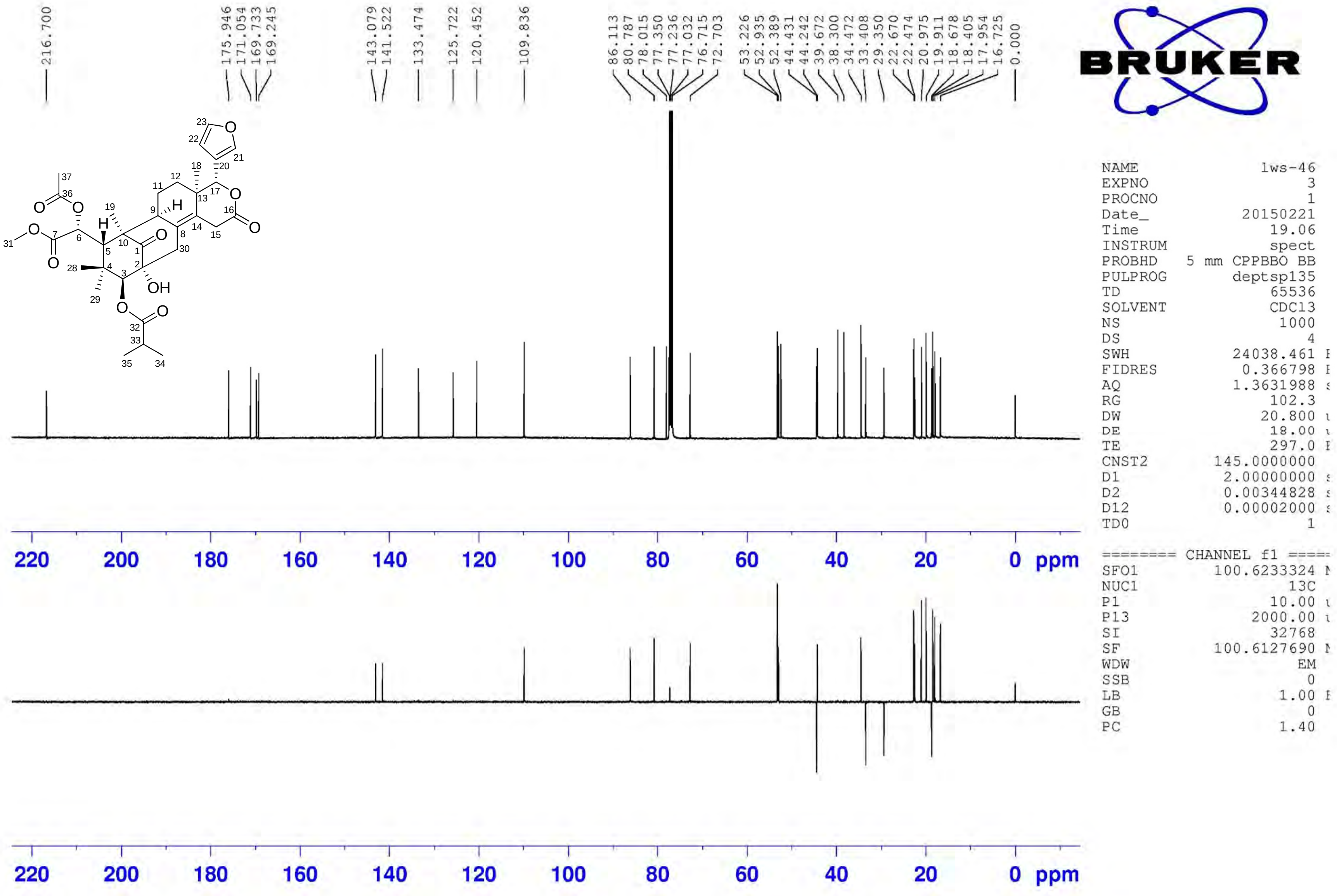

DEPT135 (100 MHz) spectrum of compound **1** in $CDCl_3$

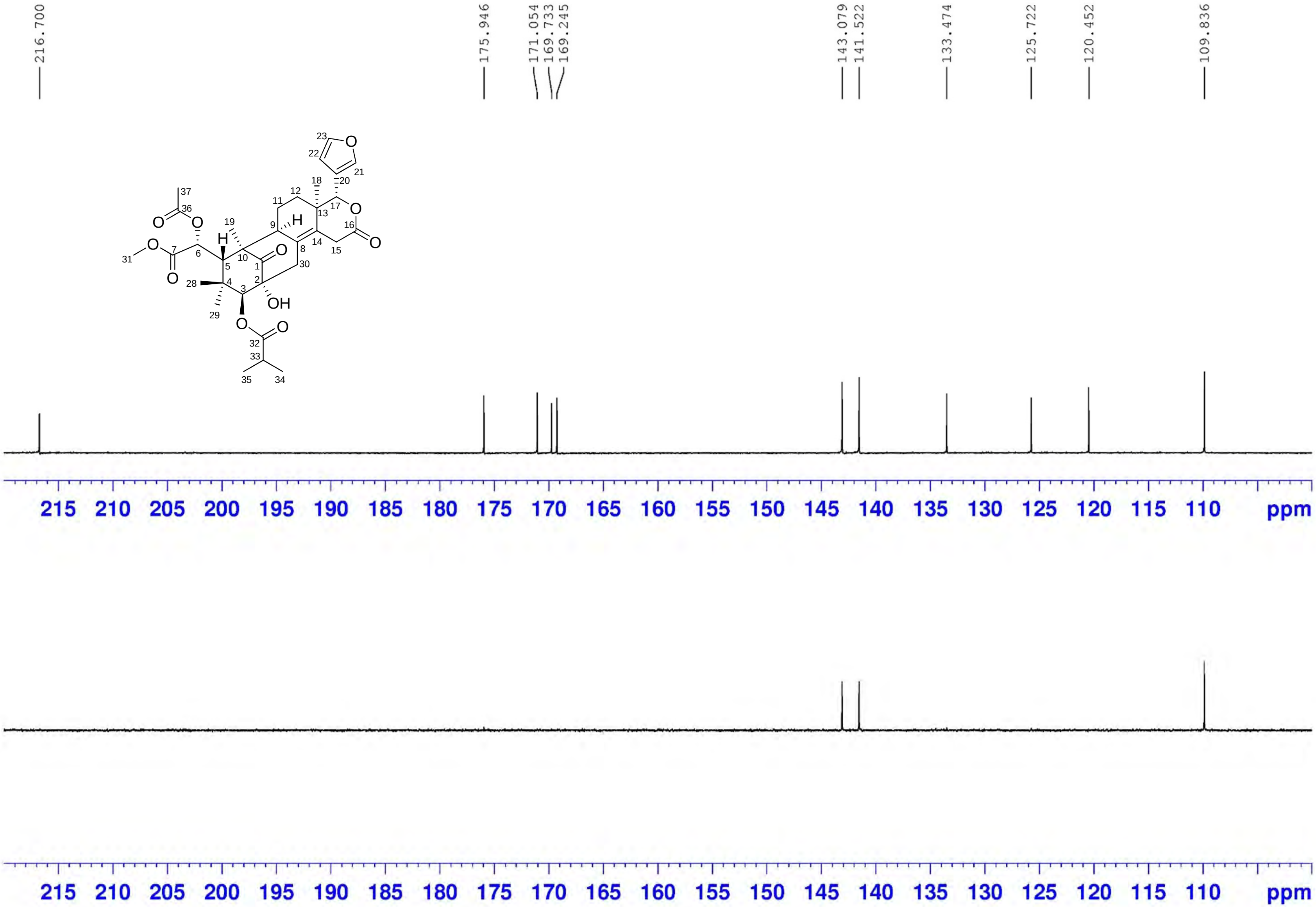

DEPT135 (100 MHz) spectrum of compound **1** in $CDCl_3$

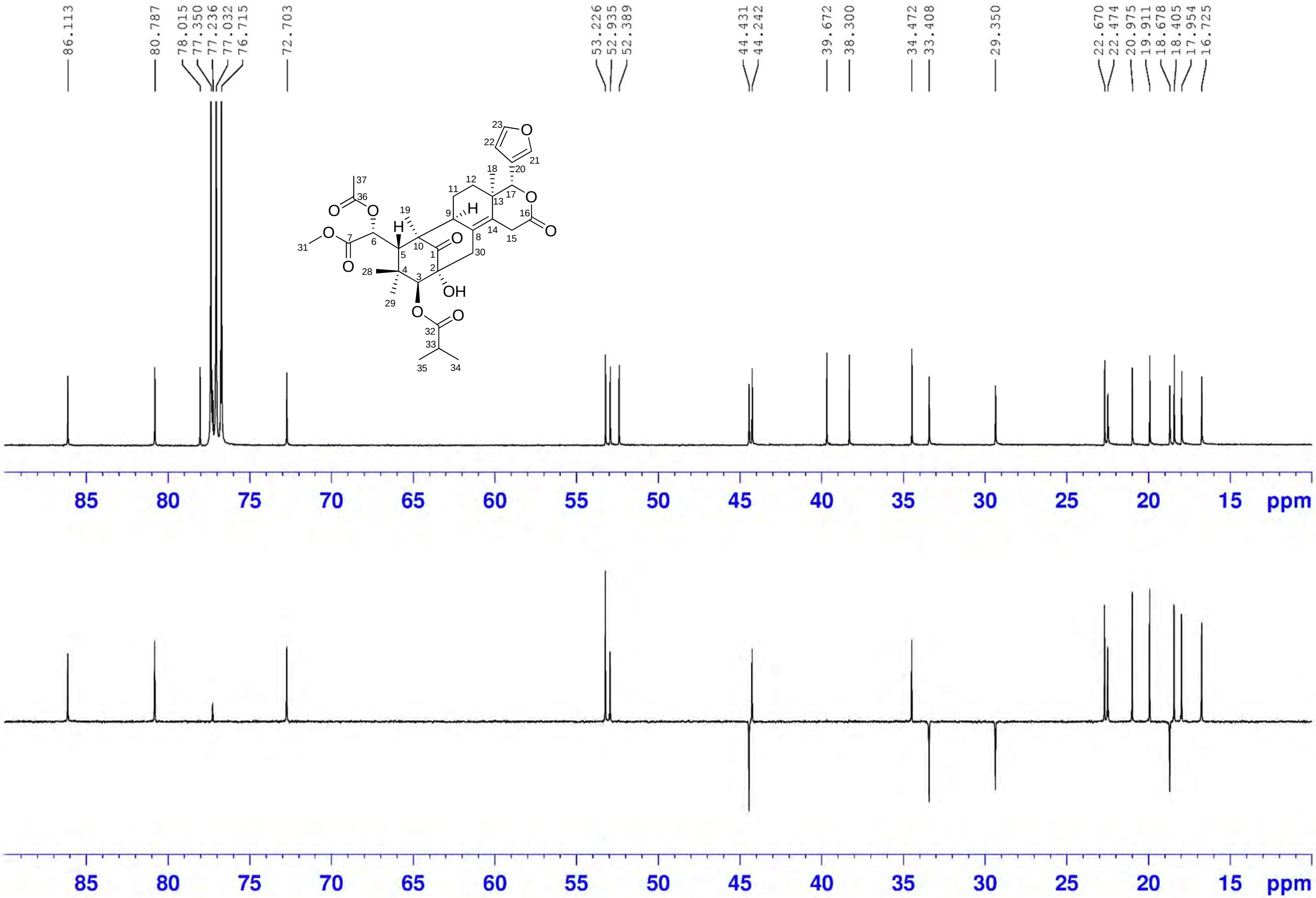

^{1}H-^{1}H COSY (400 MHz) spectrum of compound **1** in $CDCl_3$

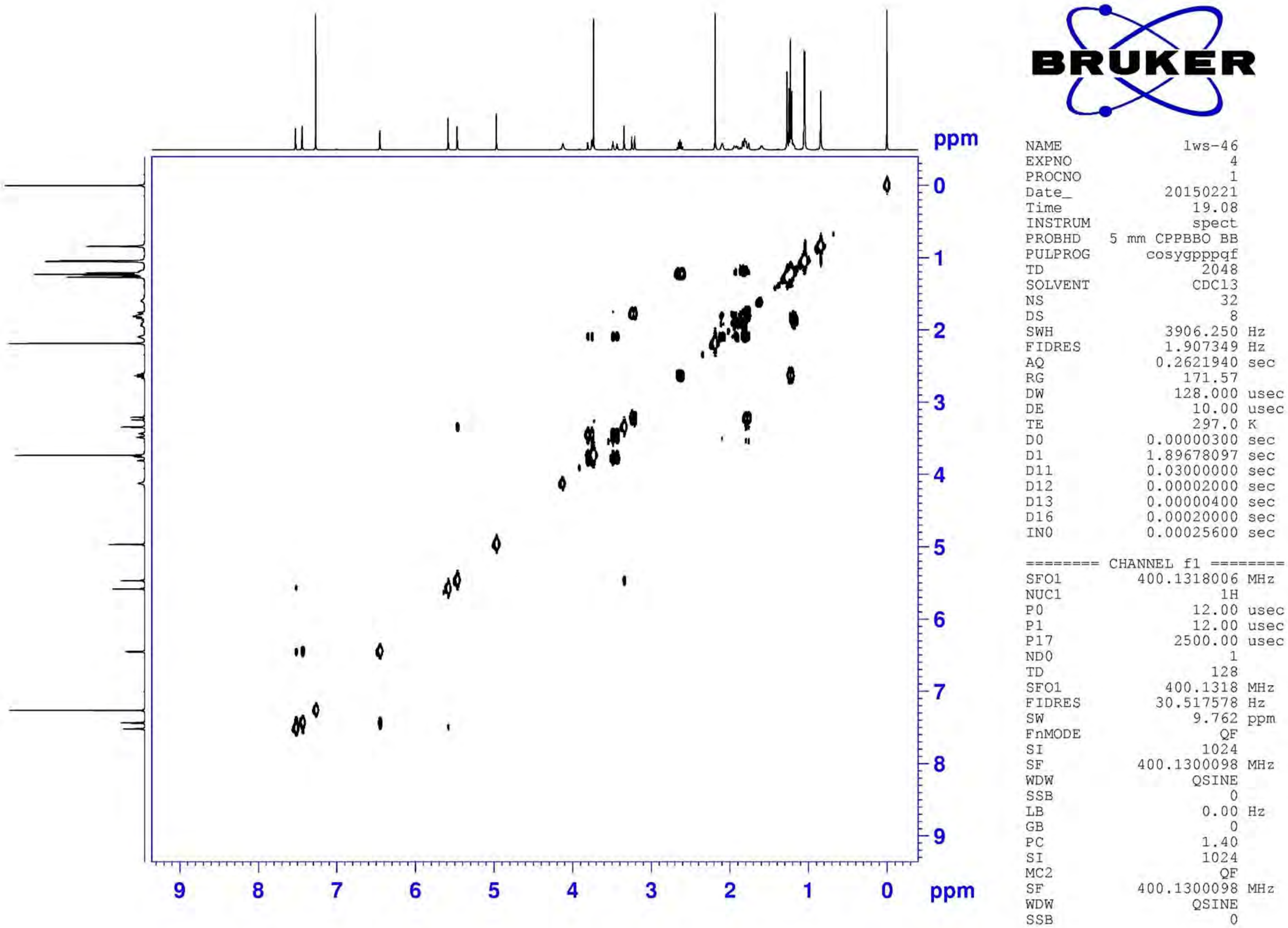

^{1}H-^{1}H COSY (400 MHz) spectrum of compound **1** in $CDCl_3$

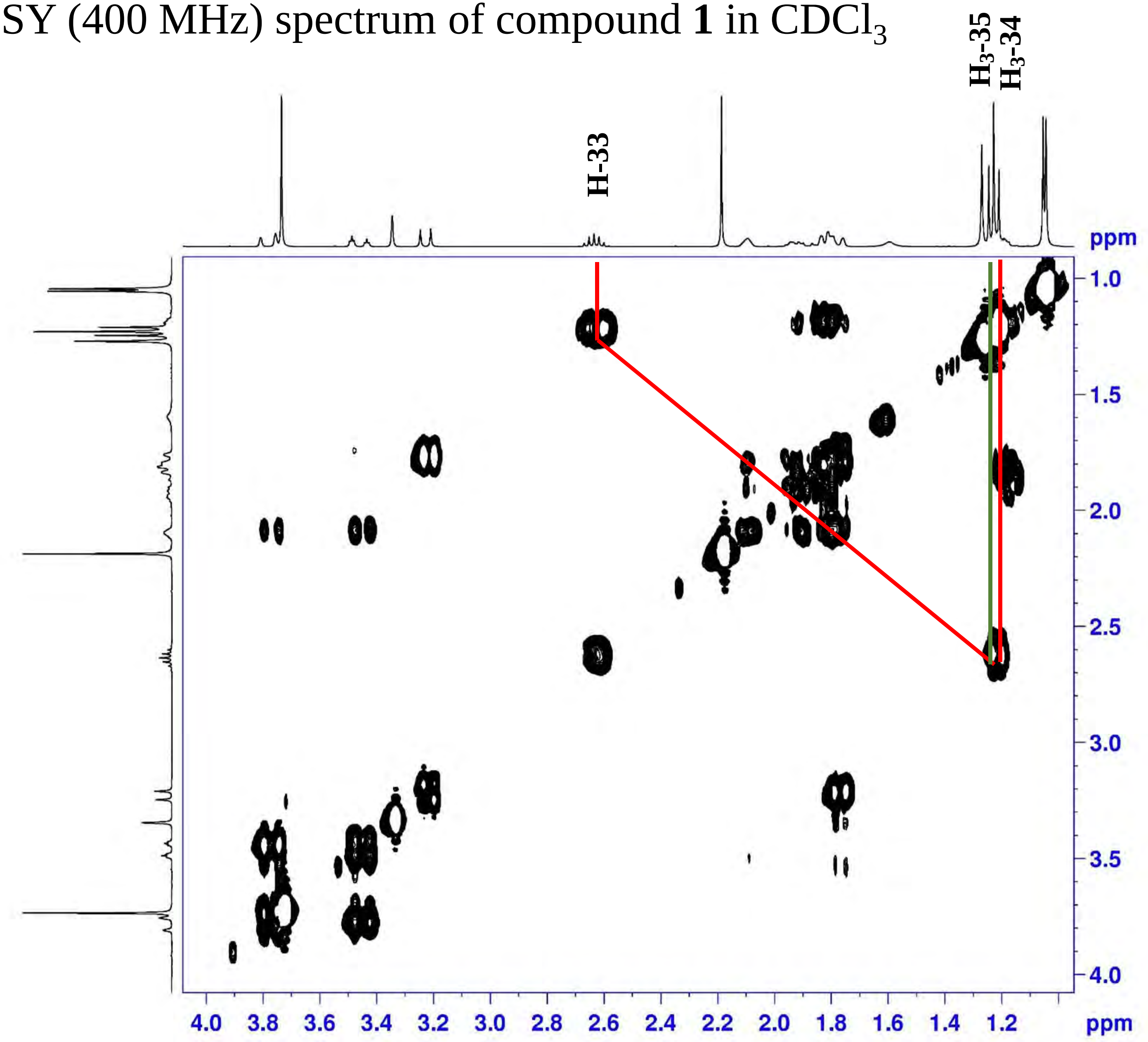

^1H-^1H COSY (400 MHz) spectrum of compound **1** in $CDCl_3$

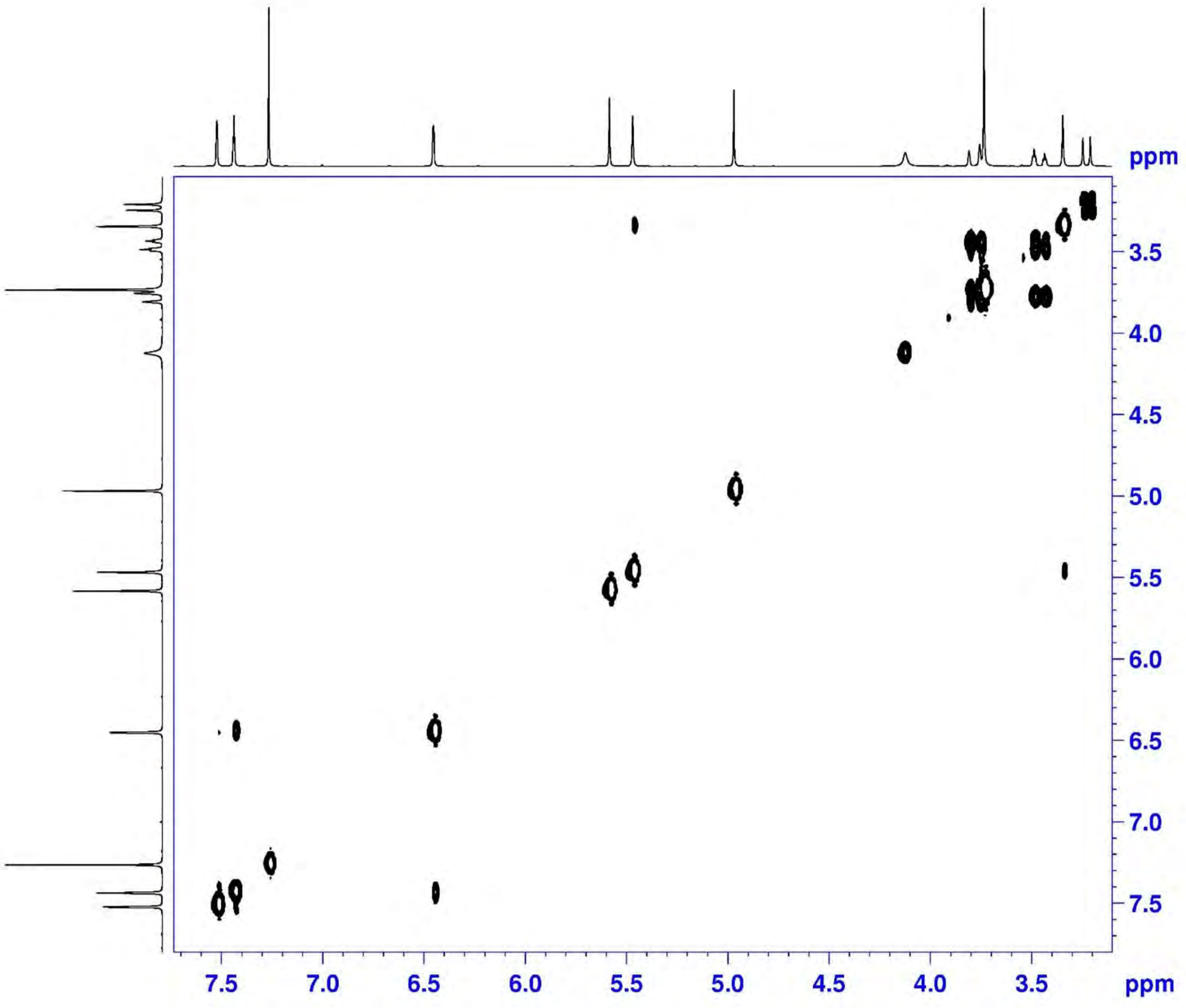

HSQC (400 MHz) spectrum of compound **1** in $CDCl_3$

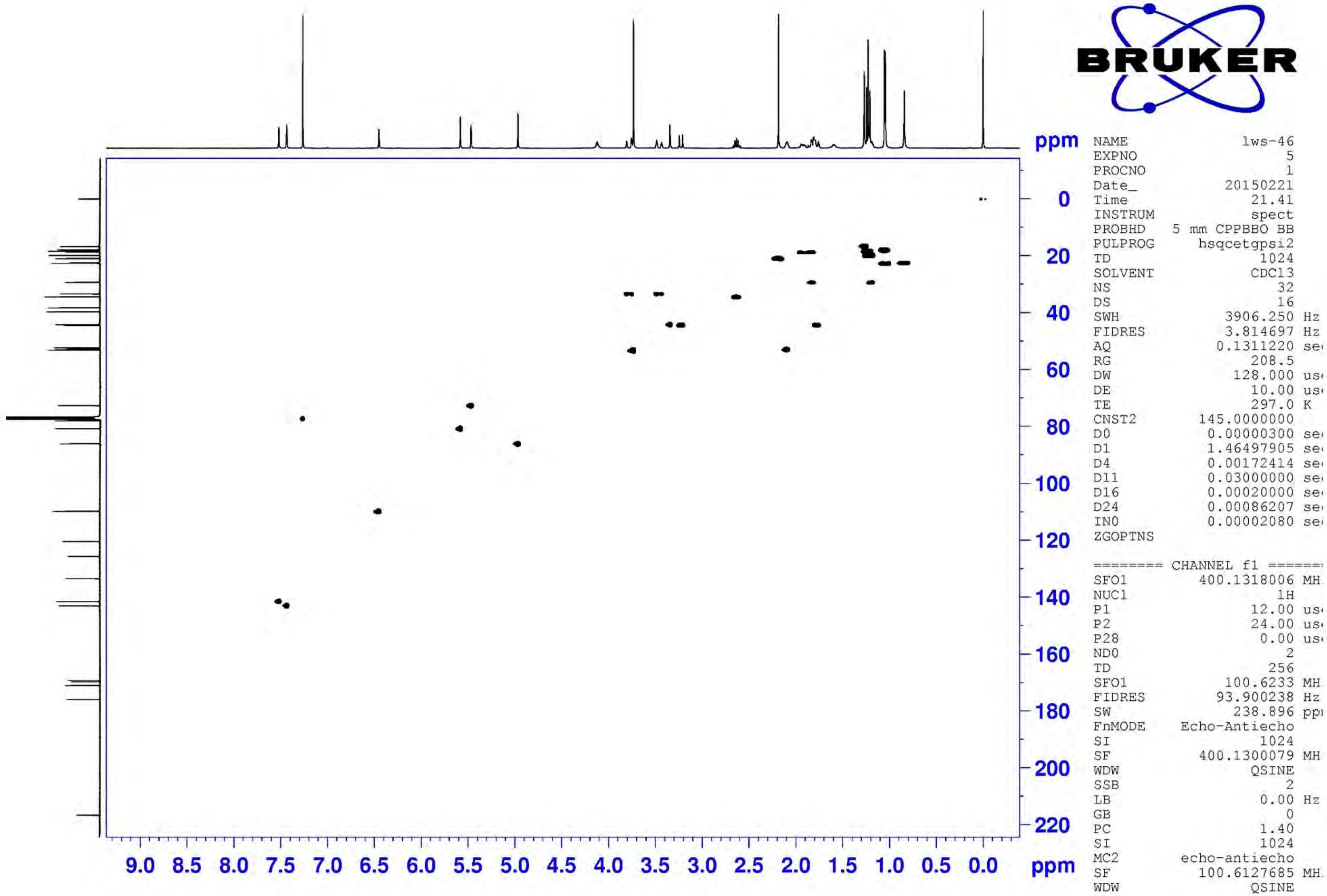

NAME	lws-46
EXPNO	5
PROCNO	1
Date_	20150221
Time	21.41
INSTRUM	spect
PROBHD	5 mm CPPBBO BB
PULPROG	hsqcetgpsi2
TD	1024
SOLVENT	CDCl3
NS	32
DS	16
SWH	3906.250 Hz
FIDRES	3.814697 Hz
AQ	0.1311220 se
RG	208.5
DW	128.000 us
DE	10.00 us
TE	297.0 K
CNST2	145.0000000
D0	0.00000300 se
D1	1.46497905 se
D4	0.00172414 se
D11	0.03000000 se
D16	0.00020000 se
D24	0.00086207 se
IN0	0.00002080 se
ZGOPTNS	
======== CHANNEL f1 ======	
SFO1	400.1318006 MH
NUC1	1H
P1	12.00 us
P2	24.00 us
P28	0.00 us
ND0	2
TD	256
SFO1	100.6233 MH
FIDRES	93.900238 Hz
SW	238.896 pp
FnMODE	Echo-Antiecho
SI	1024
SF	400.1300079 MH
WDW	QSINE
SSB	2
LB	0.00 Hz
GB	0
PC	1.40
SI	1024
MC2	echo-antiecho
SF	100.6127685 MH
WDW	QSINE
SSB	2

HSQC (400 MHz) spectrum of compound **1** in $CDCl_3$

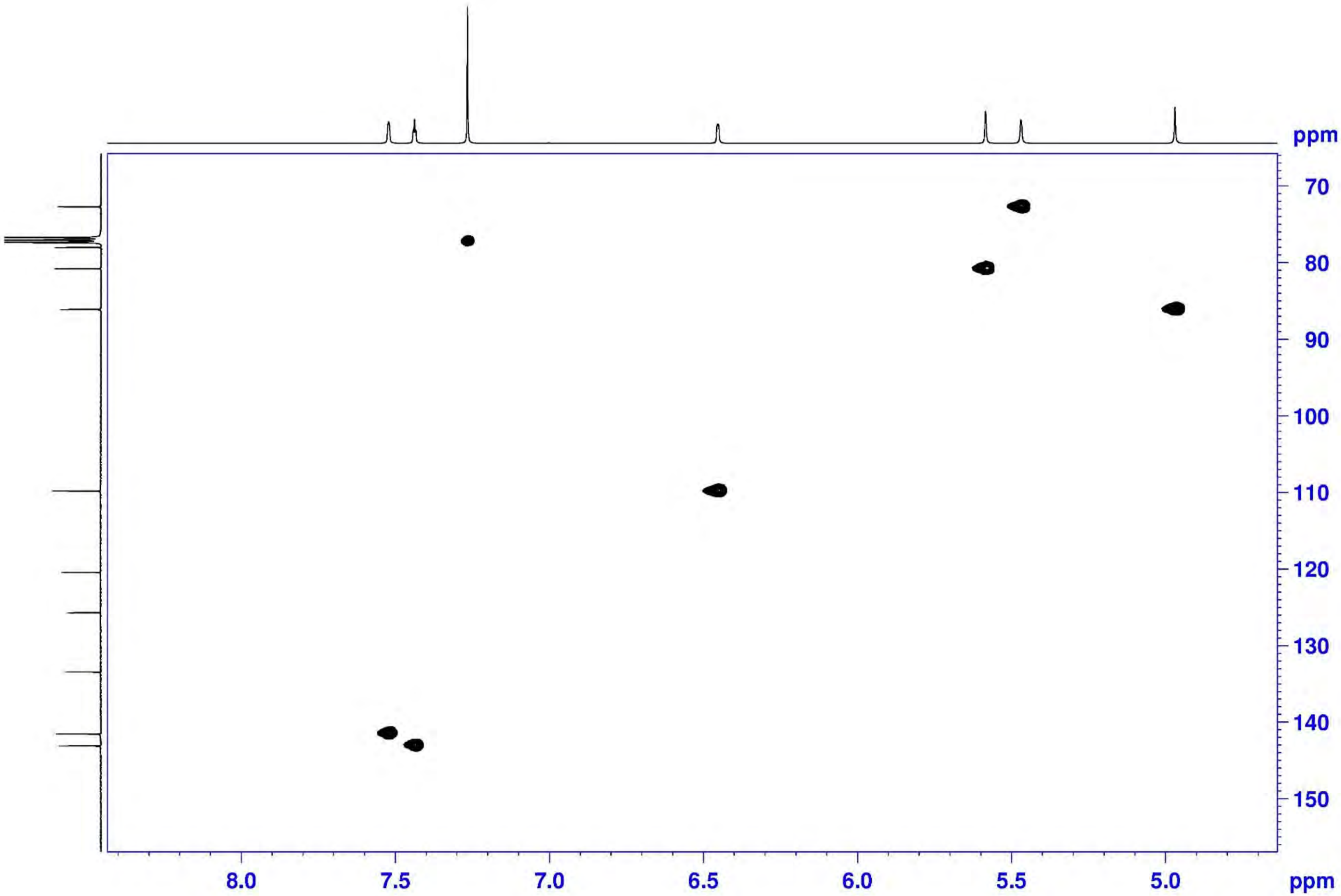

HSQC (400 MHz) spectrum of compound **1** in $CDCl_3$

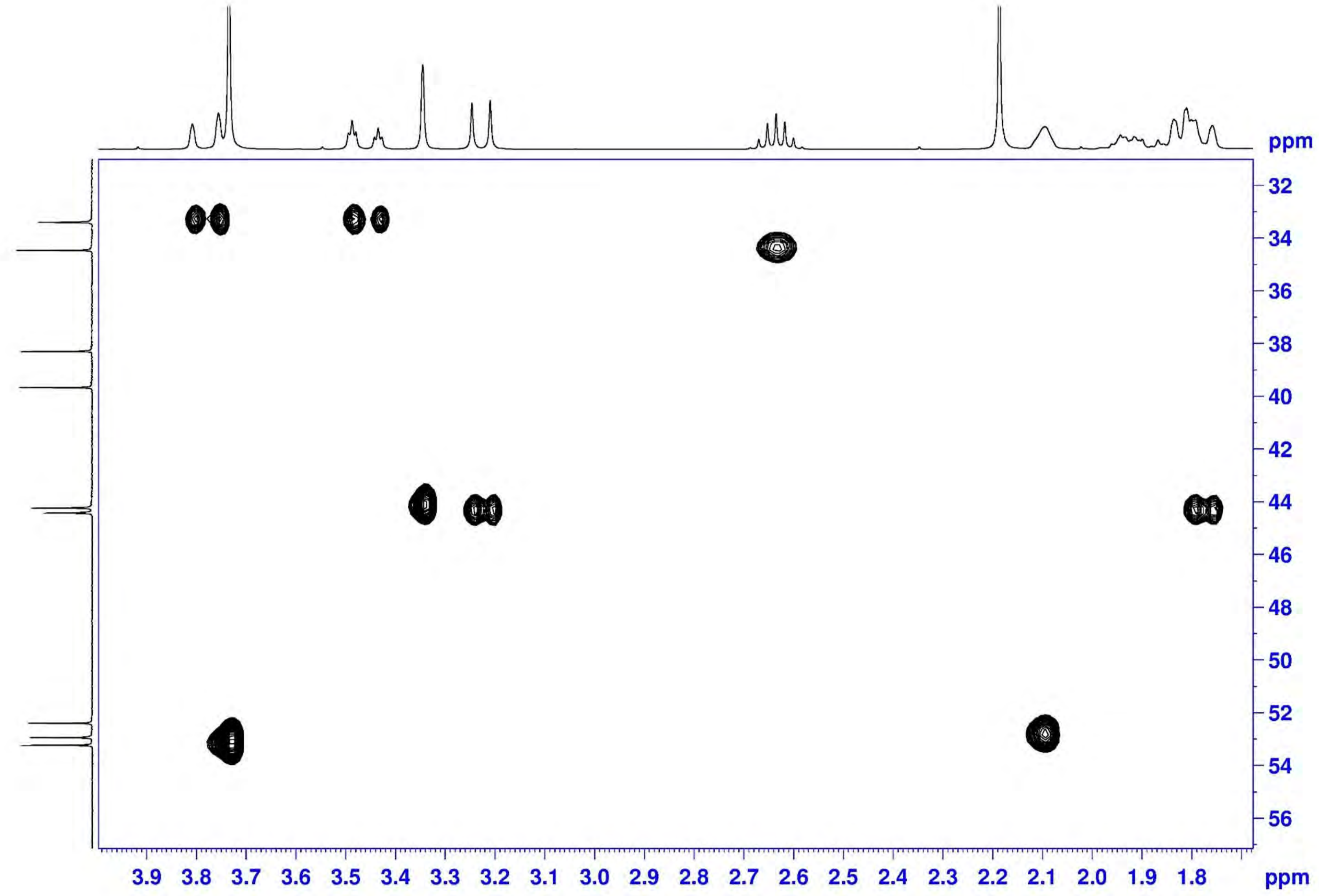

HSQC (400 MHz) spectrum of compound **1** in $CDCl_3$

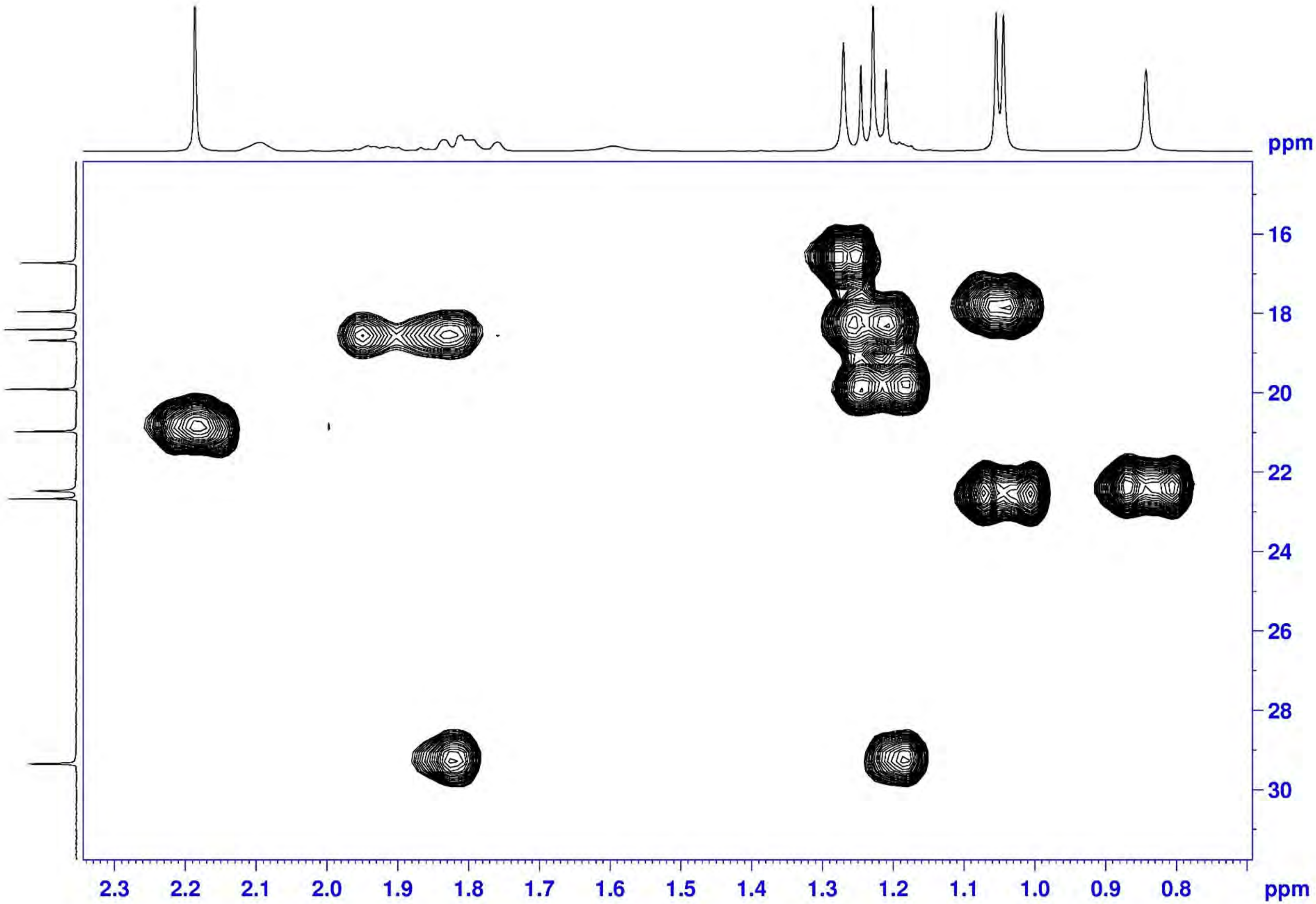

HMBC (400 MHz) spectrum of compound **1** in $CDCl_3$

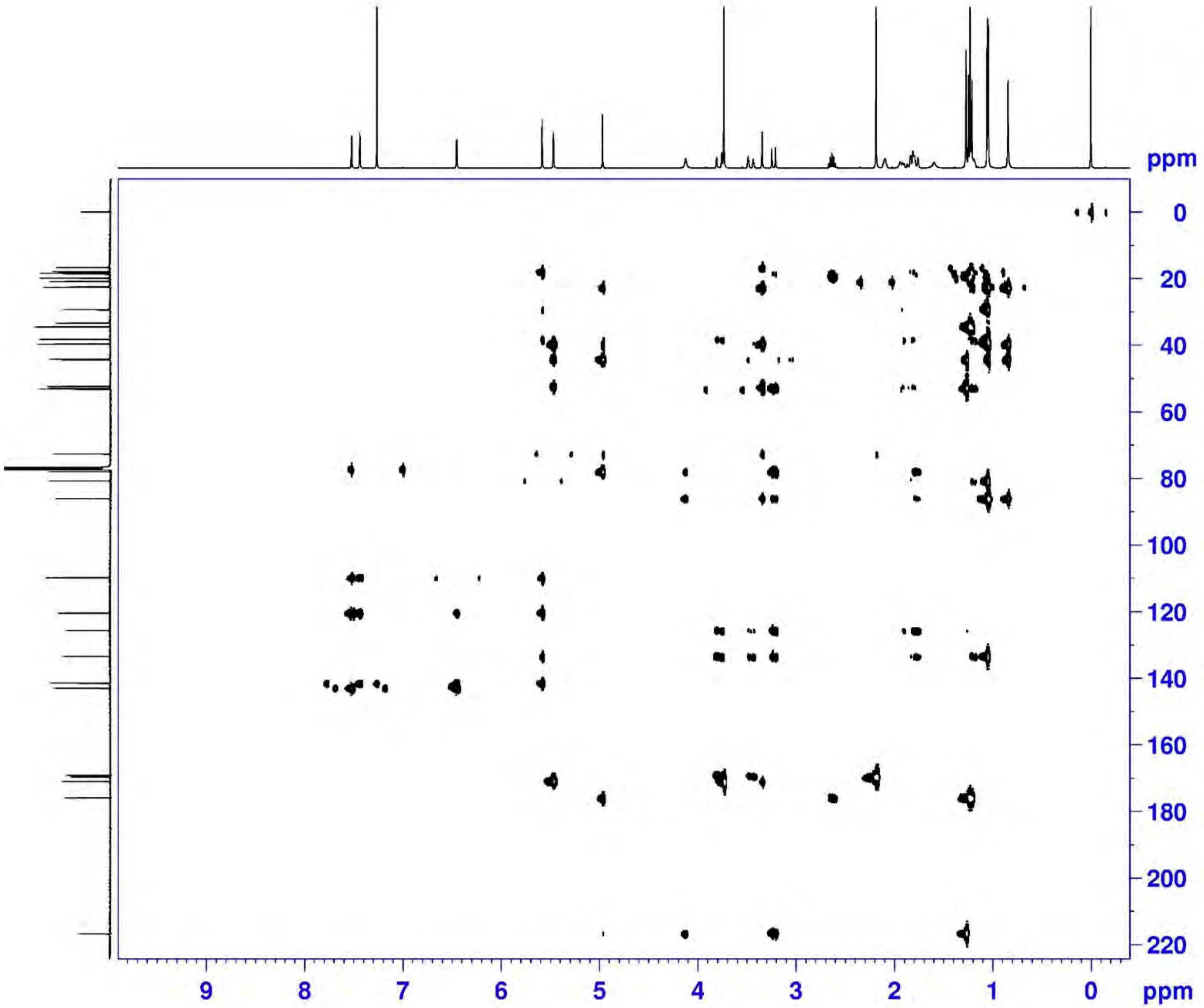


```
NAME            lws-46
EXPNO                6
PROCNO               1
Date_         20150222
Time              1.26
INSTRUM          spect
PROBHD   5 mm CPPBBO BB
PULPROG   hmbcgplpndqf
TD                4096
SOLVENT          CDCl3
NS                 128
DS                  16
SWH           5197.505 Hz
FIDRES        1.268922 Hz
AQ           0.3940852 se
RG               208.5
DW              96.200 us
DE               10.00 us
TE               297.0 K
CNST2      145.0000000
CNST13      10.0000000
D0          0.00000300 se
D1          1.50000000 se
D2          0.00344828 se
D6          0.05000000 se
D16         0.00020000 se
IN0         0.00002080 se

======== CHANNEL f1 ======
SFO1       400.1323208 MH
NUC1                1H
P1               12.00 us
P2               24.00 us
ND0                  2
TD                 128
SFO1       100.6233 MH
FIDRES      187.800476 Hz
SW             238.896 pp
FnMODE              QF
SI                2048
SF         400.1300096 MH
WDW               SINE
SSB                  0
LB                0.00 Hz
GB                   0
PC                1.40
SI                1024
MC2                 QF
SF         100.6127692 MH
WDW               SINE
SSB                  0
```

HMBC (400 MHz) spectrum of compound **1** in $CDCl_3$

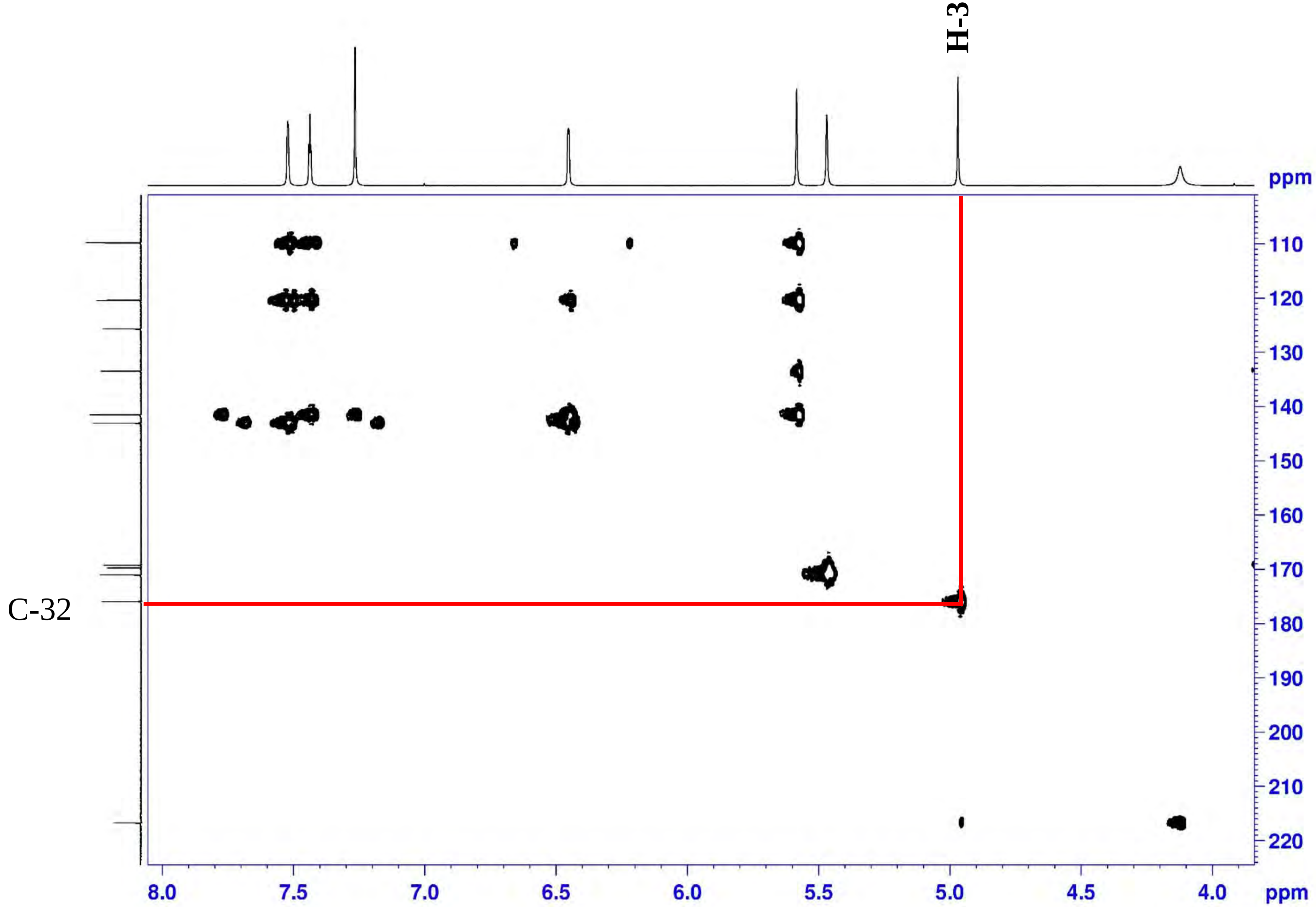

HMBC (400 MHz) spectrum of compound **1** in $CDCl_3$

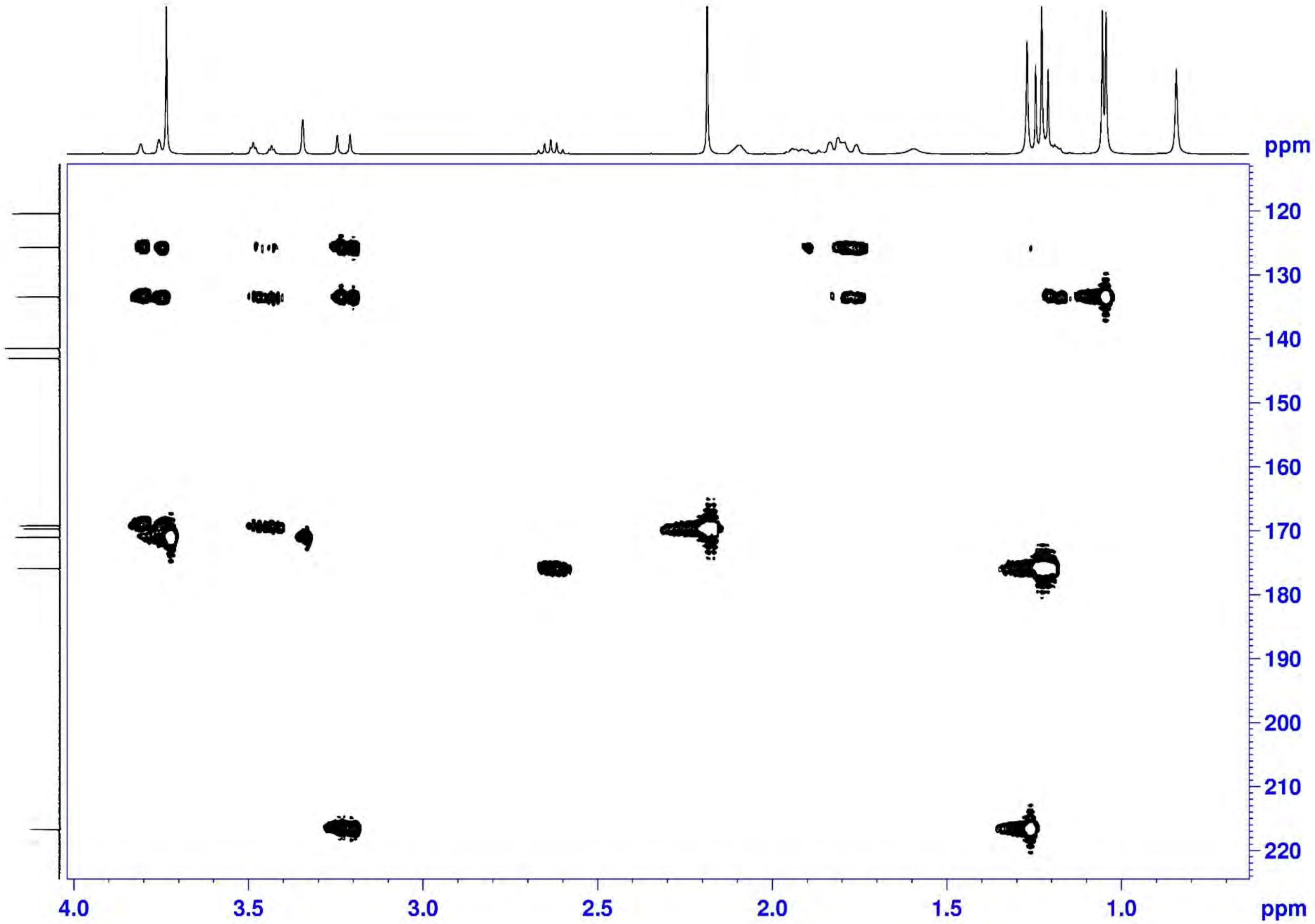

HMBC (400 MHz) spectrum of compound **1** in $CDCl_3$

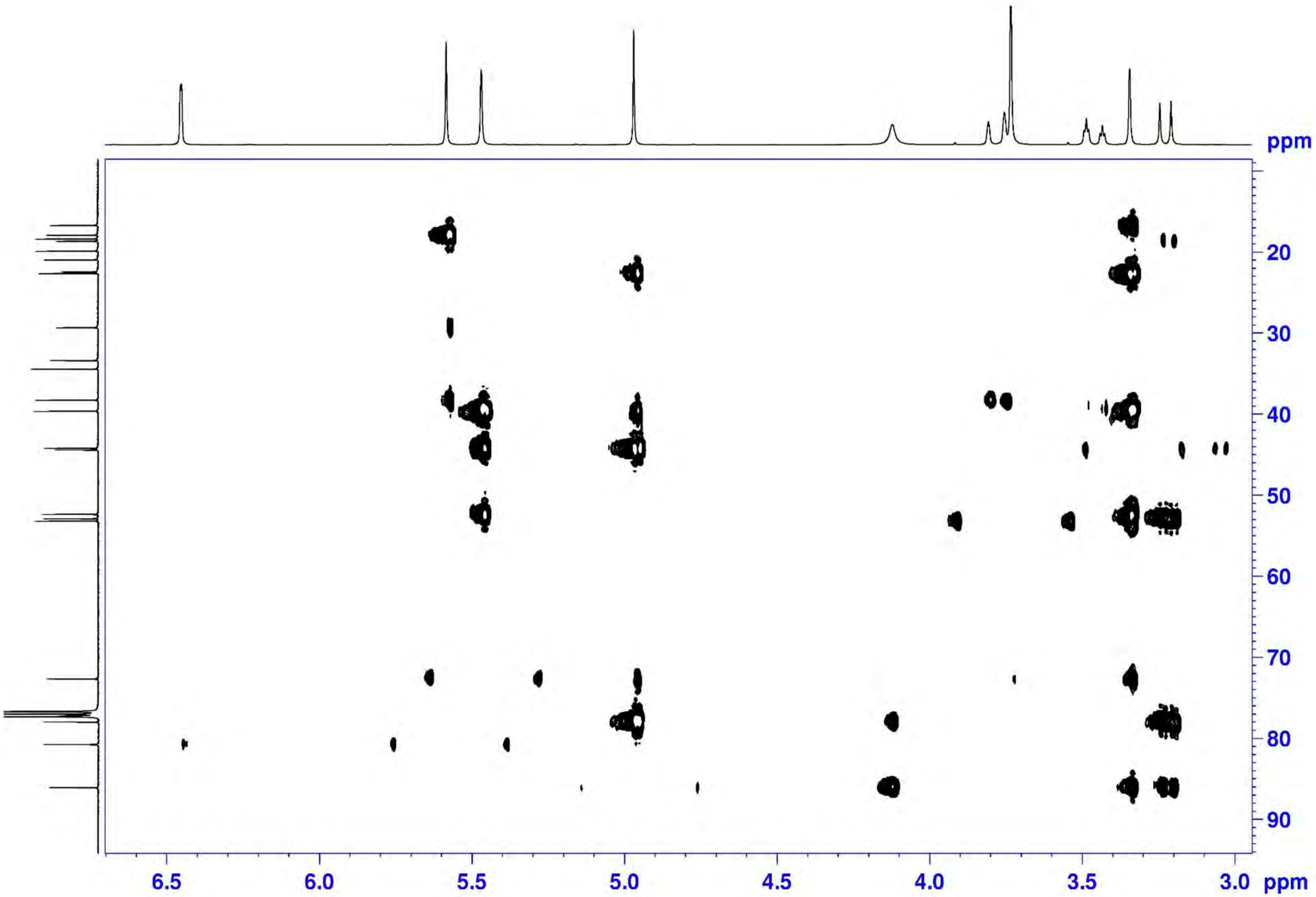

HMBC (400 MHz) spectrum of compound **1** in $CDCl_3$

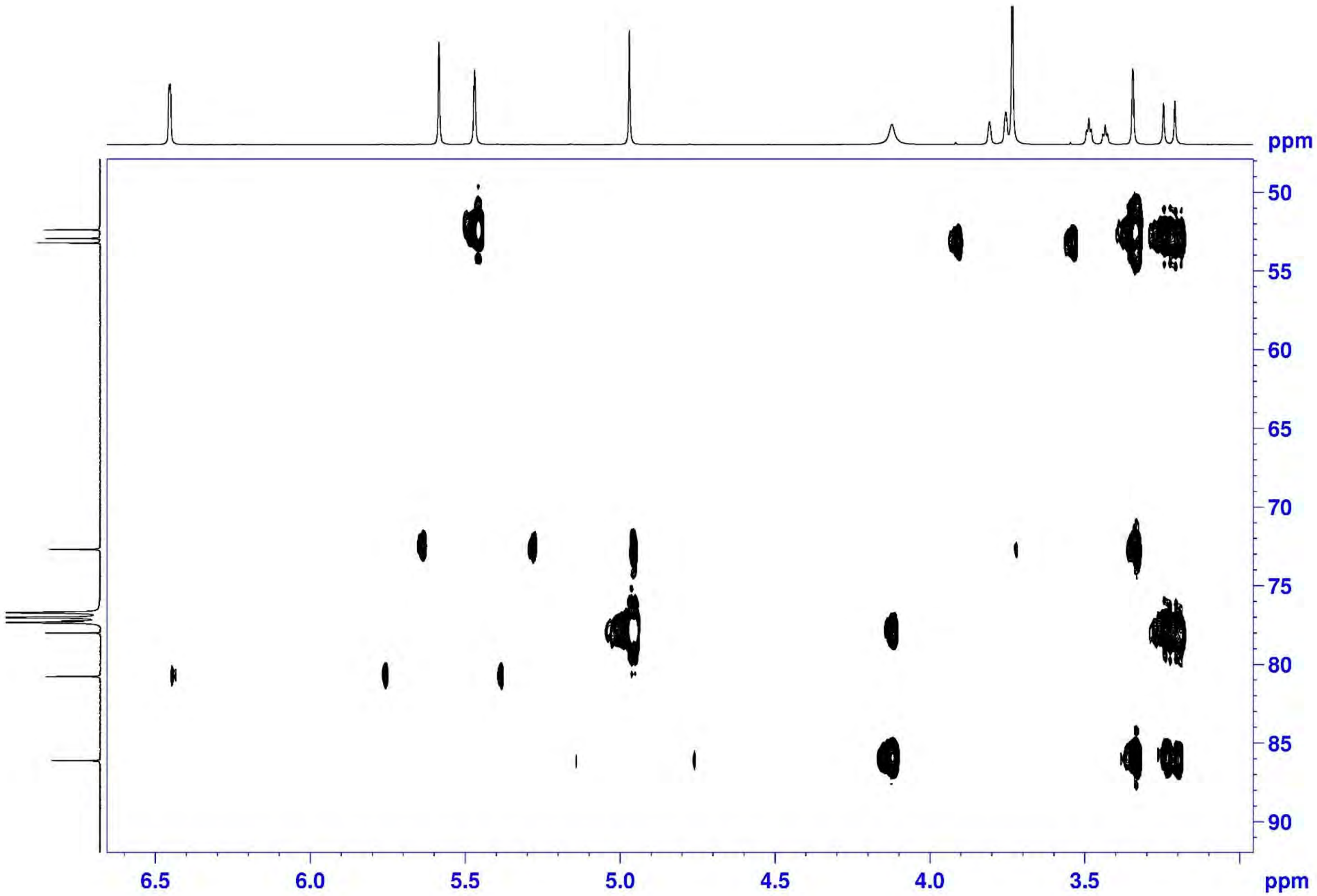

HMBC (400 MHz) spectrum of compound **1** in $CDCl_3$

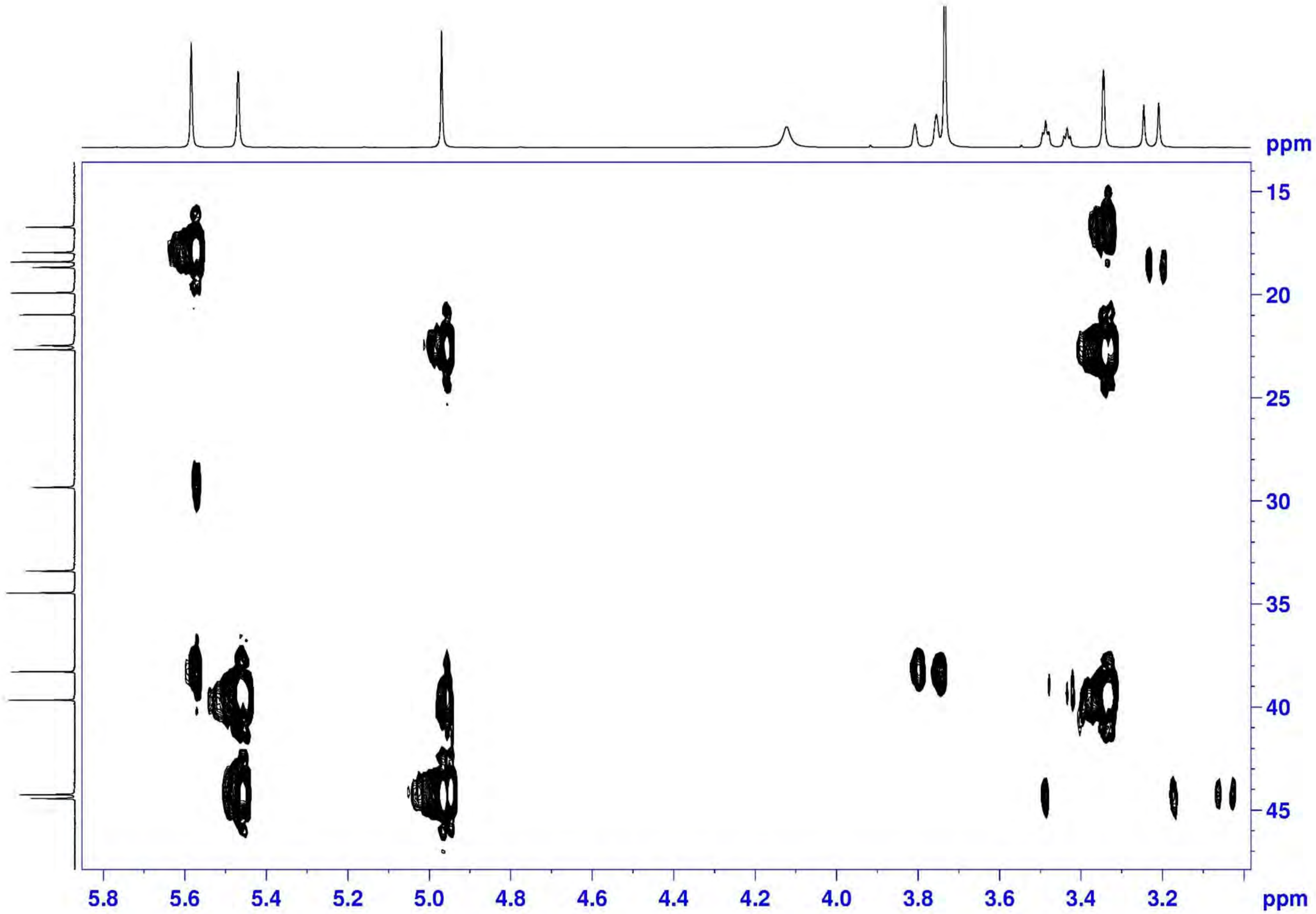

HMBC (400 MHz) spectrum of compound **1** in $CDCl_3$

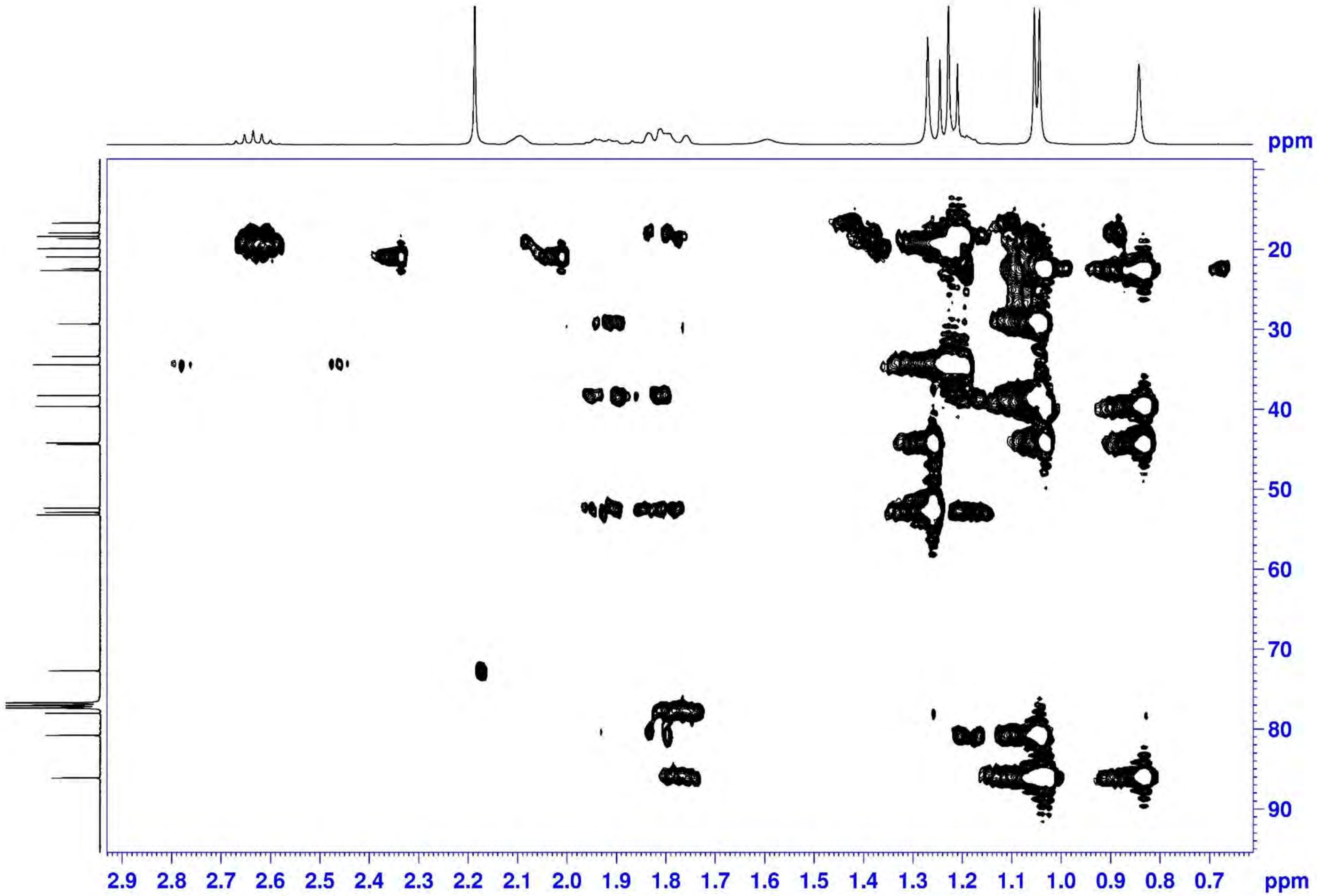

HMBC (400 MHz) spectrum of compound **1** in $CDCl_3$

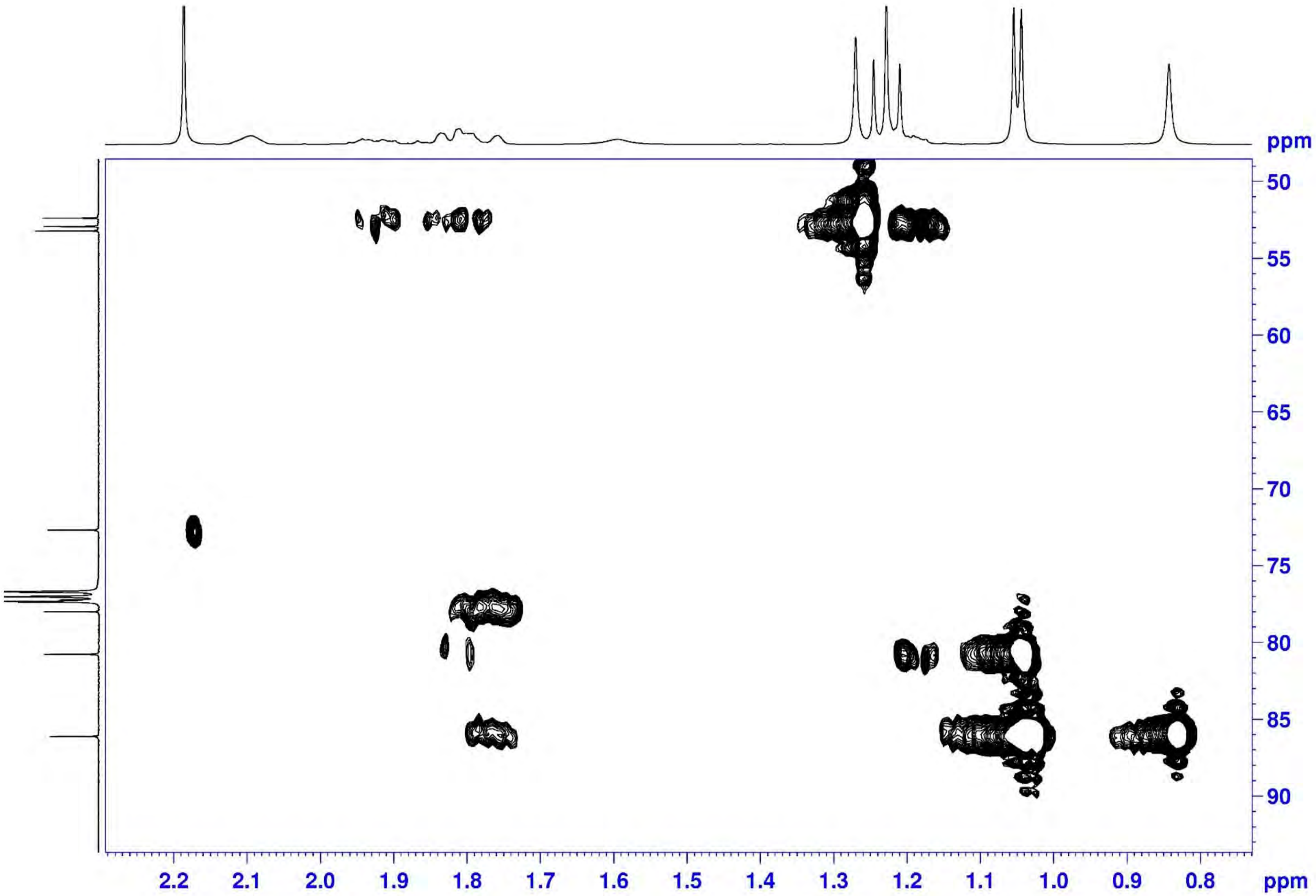

HMBC (400 MHz) spectrum of compound **1** in $CDCl_3$

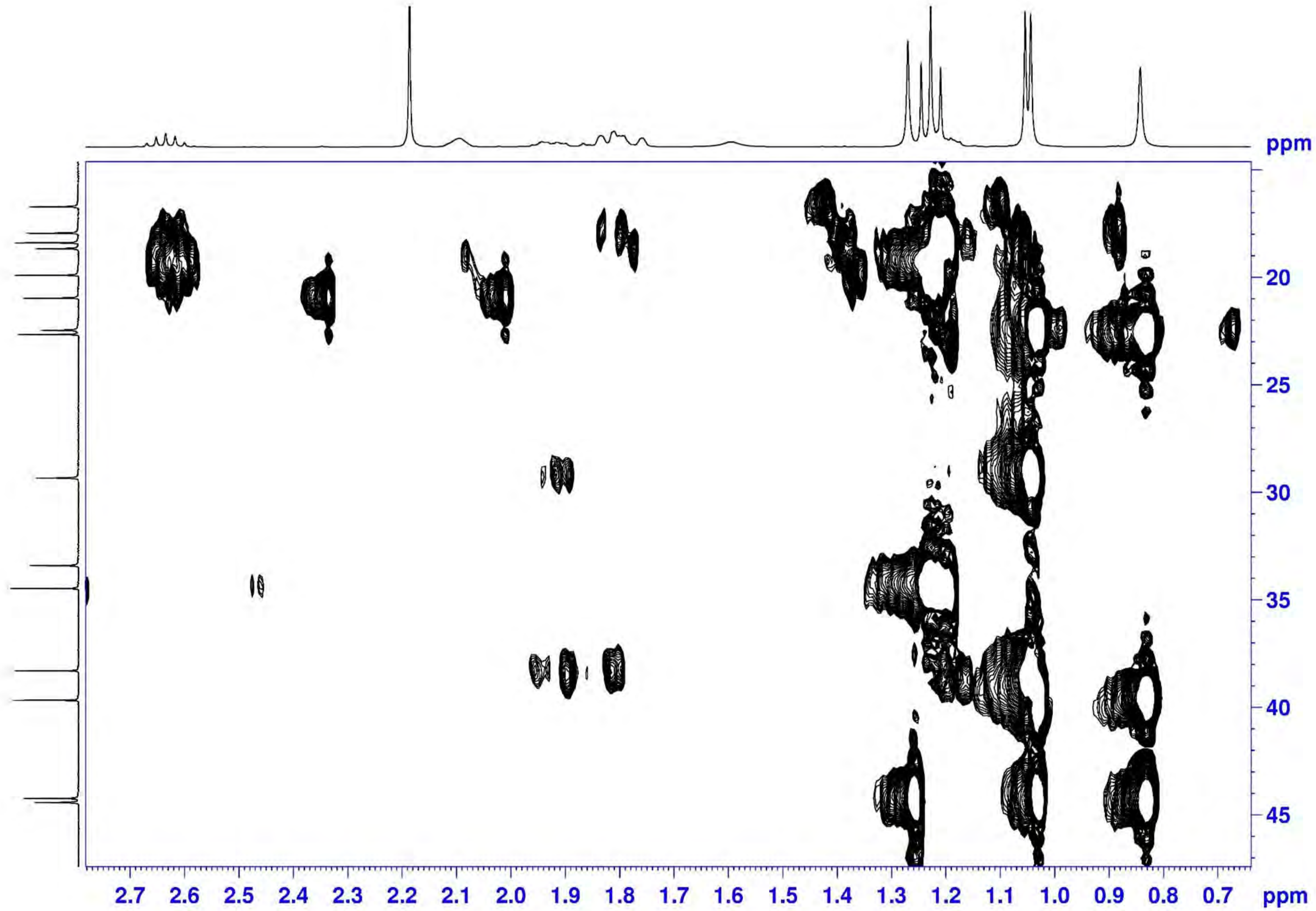

NOESY (400 MHz) spectrum of compound **1** in $CDCl_3$

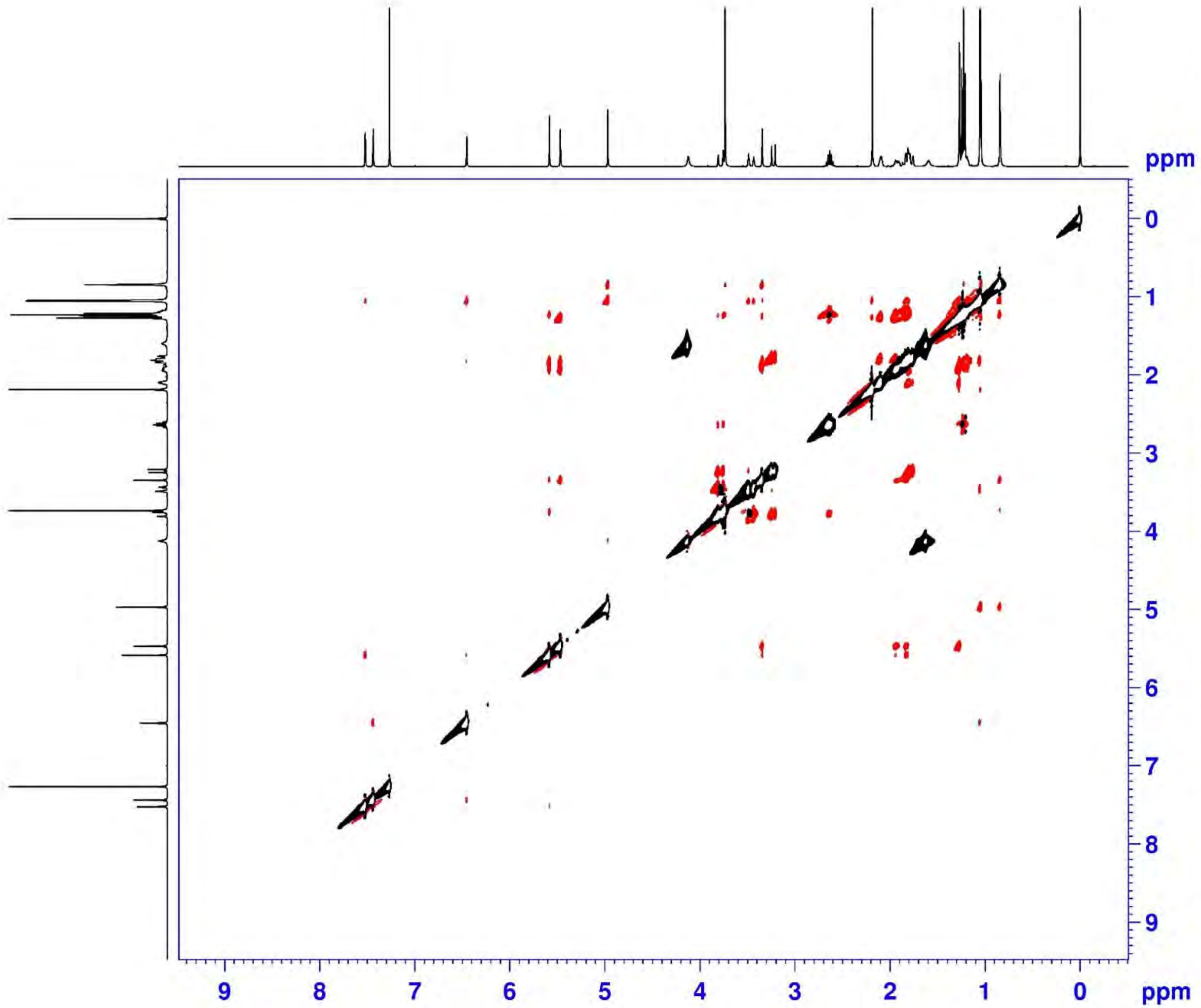

NAME	lws-46
EXPNO	7
PROCNO	1
Date_	20150222
Time	10.24
INSTRUM	spect
PROBHD	5 mm CPPBBO BB
PULPROG	noesygpphpp
TD	2048
SOLVENT	CDCl3
NS	128
DS	32
SWH	4000.000 Hz
FIDRES	1.953125 Hz
AQ	0.2560500 se
RG	208.5
DW	125.000 us
DE	10.00 us
TE	297.0 K
D0	0.00010972 se
D1	1.99385595 se
D8	0.30000001 se
D11	0.03000000 se
D12	0.00002000 se
D16	0.00020000 se
IN0	0.00025000 se

======== CHANNEL f1 ======

SFO1	400.1318006 MH
NUC1	1H
P1	12.00 us
P2	24.00 us
P17	2500.00 us
ND0	1
TD	256
SFO1	400.1318 MH
FIDRES	15.625000 Hz
SW	9.997 pp
FnMODE	States-TPPI
SI	1024
SF	400.1300074 MH
WDW	QSINE
SSB	2
LB	0.00 Hz
GB	0
PC	1.00
SI	1024
MC2	States-TPPI
SF	400.1300074 MH
WDW	QSINE
SSB	2

NOESY (400 MHz) spectrum of compound **1** in $CDCl_3$

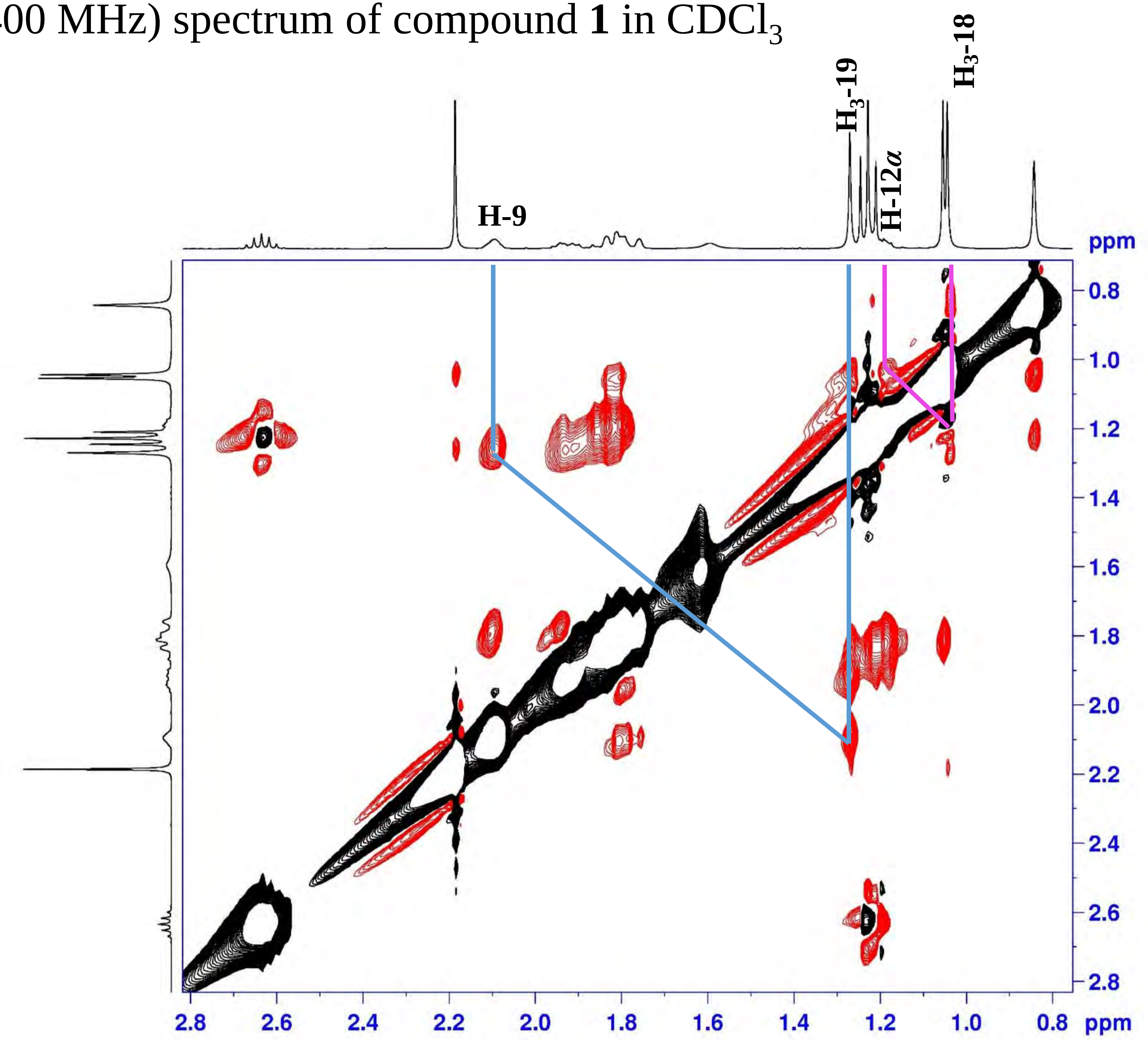

NOESY (400 MHz) spectrum of compound **1** in $CDCl_3$

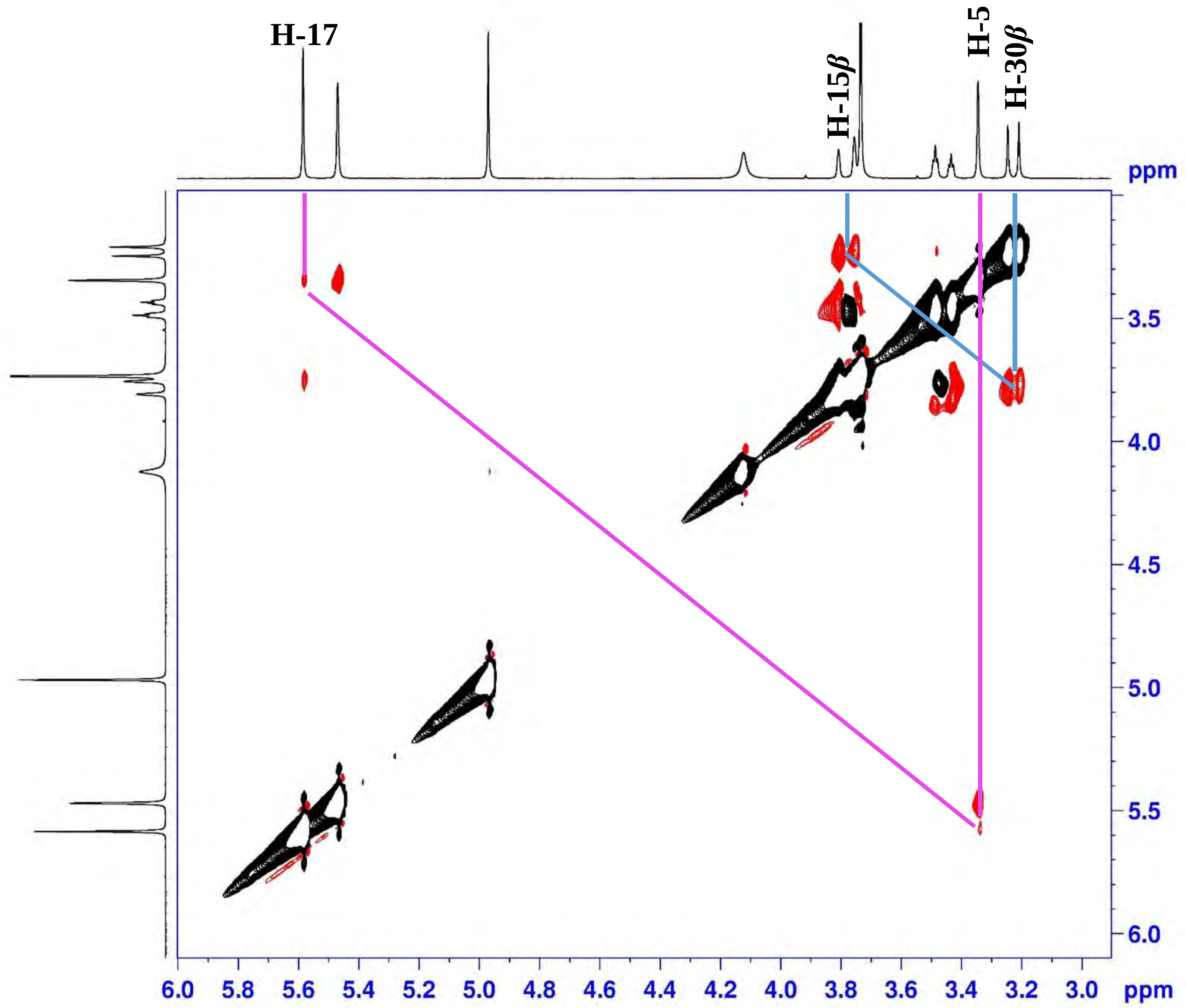

NOESY (400 MHz) spectrum of compound **1** in $CDCl_3$

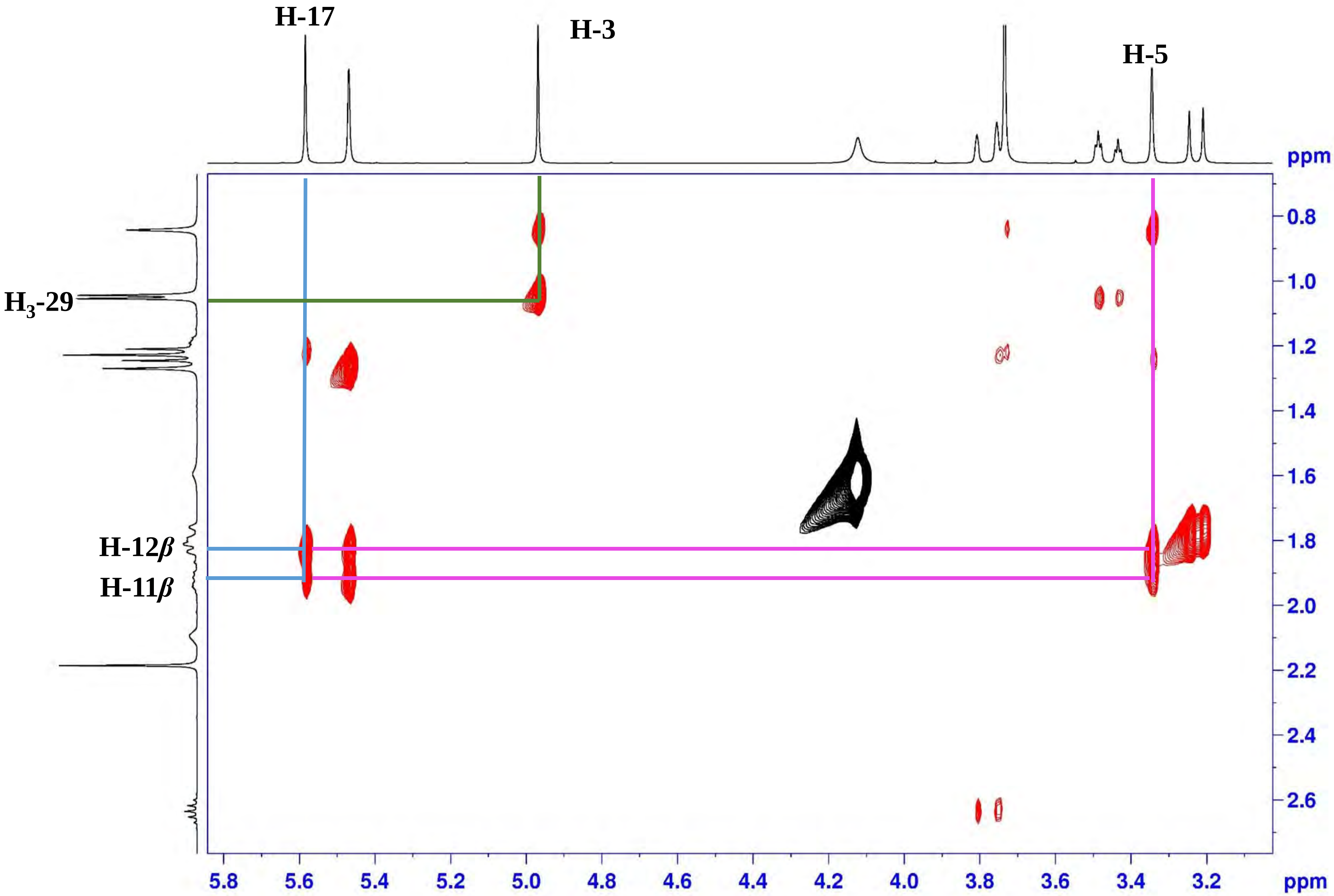

NOESY (400 MHz) spectrum of compound **1** in $CDCl_3$

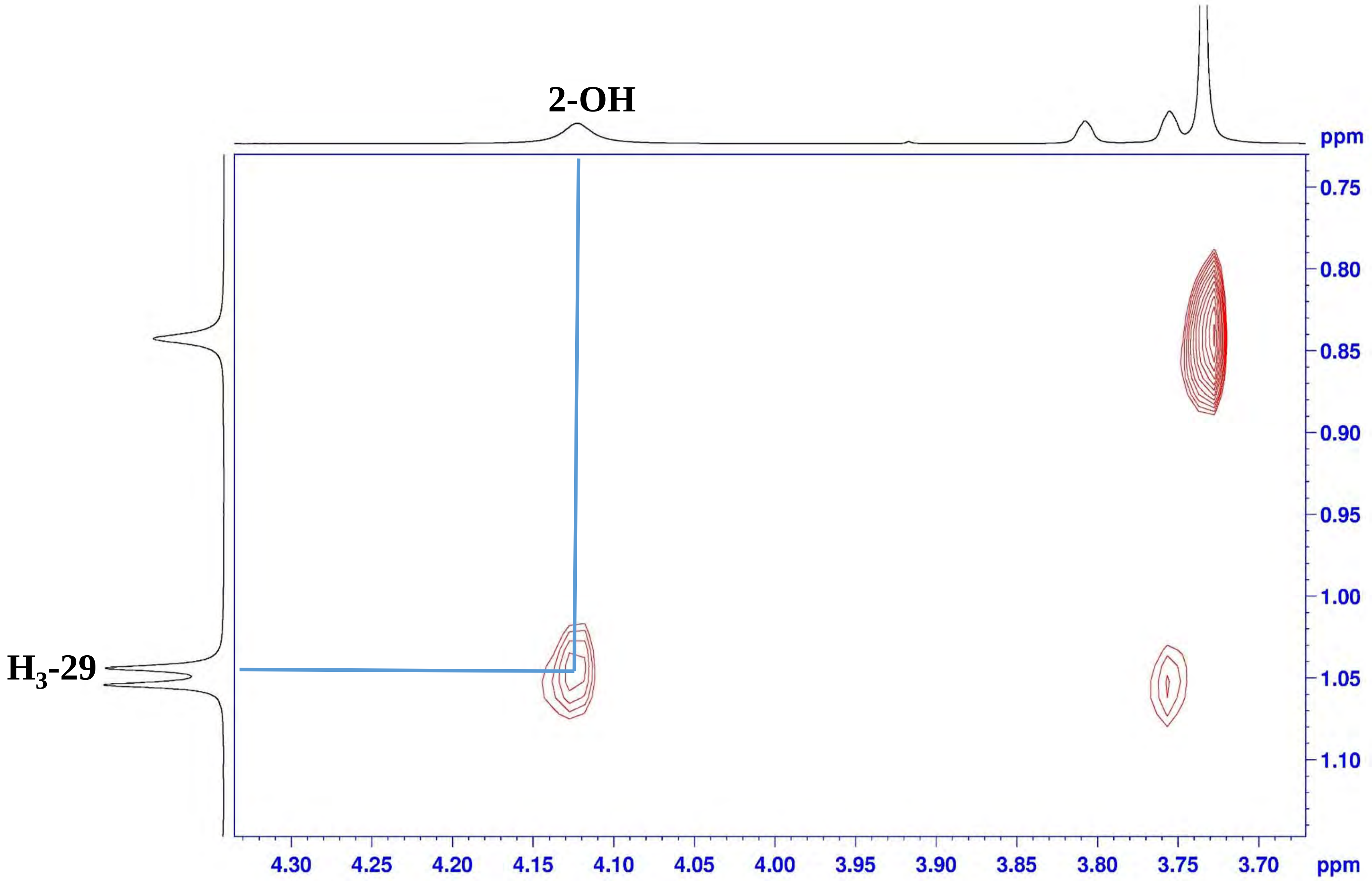

NOESY (400 MHz) spectrum of compound **1** in $CDCl_3$

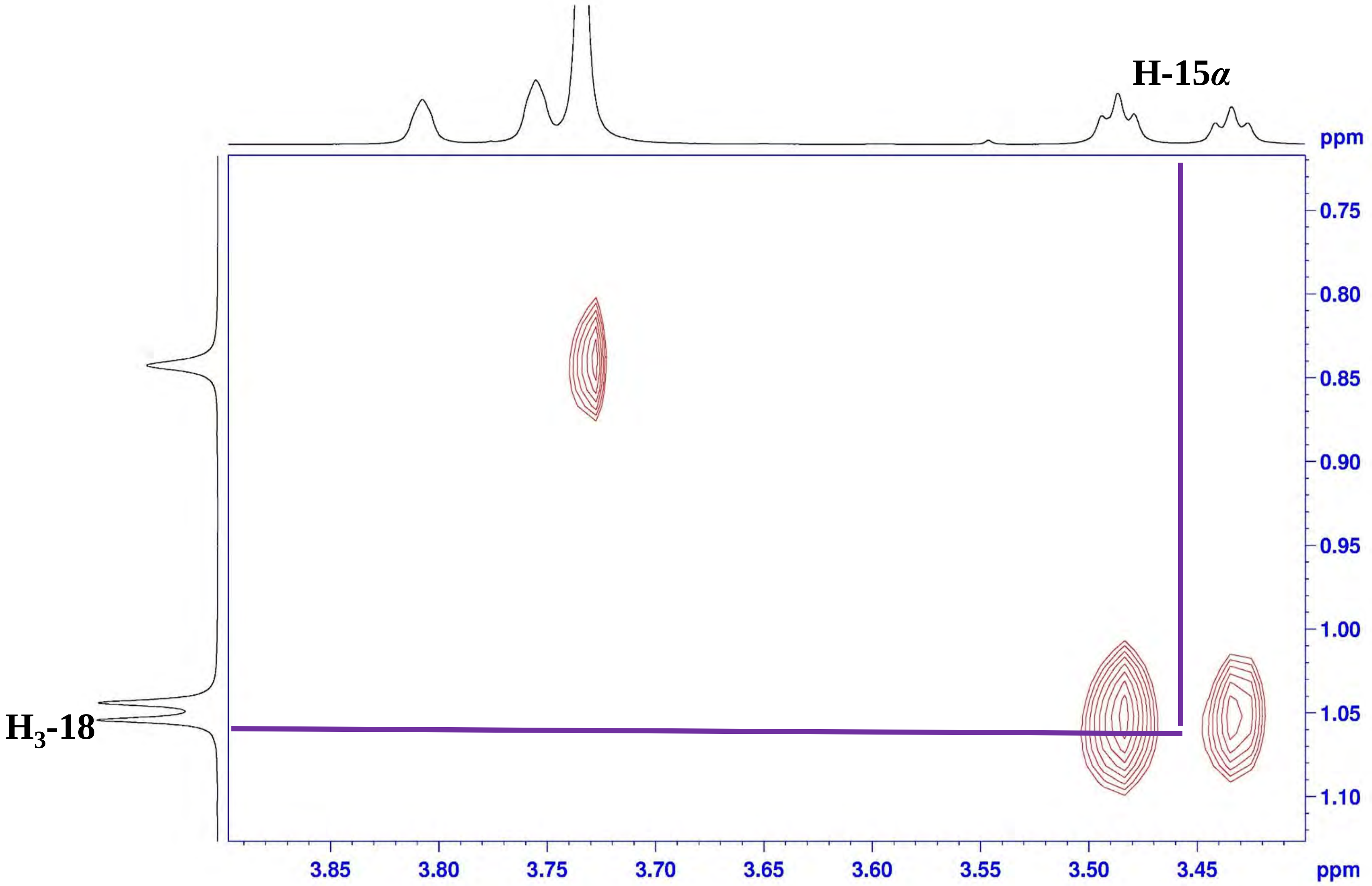

HRESIMS for compound **2**

Mass Spectrum SmartFormula Report

Analysis Info

Analysis Name	D:\Data\MS\data\201612\zhangjianzhi_zjz-26_neg_83_01_2736.d	Acquisition Date	12/12/2016 3:15:13 PM
Method	LC_Direct Infusion_neg_100-1000mz.m	Operator	SCSIO
Sample Name	zhangjianzhi_zjz-26_neg	Instrument	maXis 255552.00029
Comment			

Acquisition Parameter

Source Type	ESI	Ion Polarity	Negative	Set Nebulizer	0.4 Bar
Focus	Active	Set Capillary	4000 V	Set Dry Heater	180 °C
Scan Begin	100 m/z	Set End Plate Offset	-500 V	Set Dry Gas	4.0 l/min
Scan End	2000 m/z	Set Charging Voltage	0 V	Set Divert Valve	Waste
		Set Corona	0 nA	Set APCI Heater	0 °C

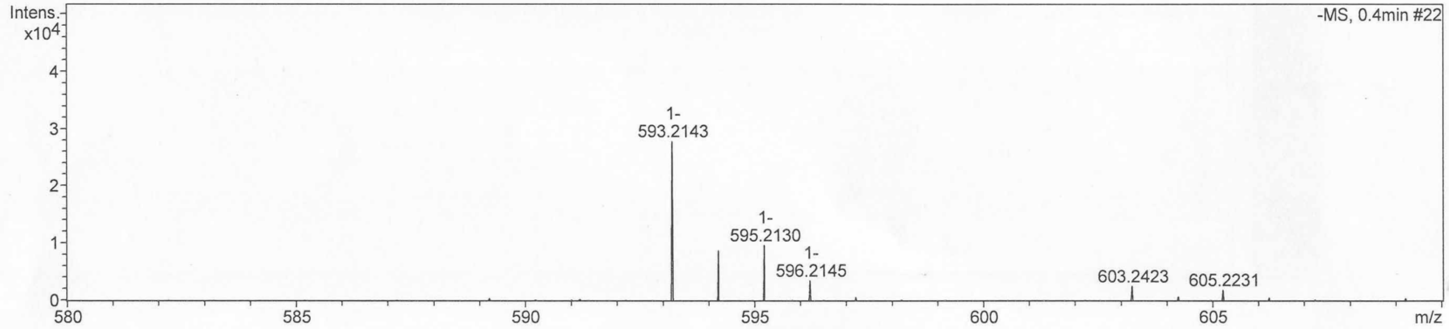

Meas. m/z	#	Ion Formula	Score	m/z	err [ppm]	err [mDa]	mSigma	rdb	e⁻ Conf	N-Rule
593.214270	1	C30H38ClO10	100.00	593.215899	2.7	1.6	21.1	11.5	even	ok
1151.459232	1	C60H76ClO20	100.00	1151.462396	-2.7	-3.2	23.0	22.5	even	ok

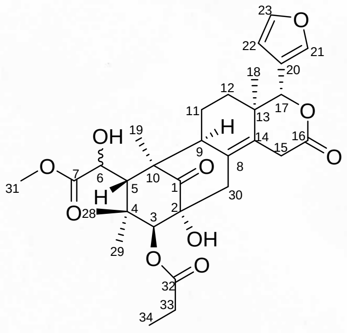

zjz-26

zhangjianzhi_zjz-26_neg_83_01_2736.d

Bruker Compass DataAnalysis 4.1 printed: 12/12/2016 3:24:22 PM by: SCSIO Page 1 of 1

HRESIMS for compound **2**

Generic Display Report

Analysis Info

Analysis Name	D:\Data\MS\data\201612\zhangjianzhi_zjz-26_neg_83_01_2736.d
Method	LC_Direct Infusion_neg_100-1000mz.m
Sample Name	zhangjianzhi_zjz-26_neg
Comment	

Acquisition Date	12/12/2016 3:15:13 PM
Operator	SCSIO
Instrument	maXis

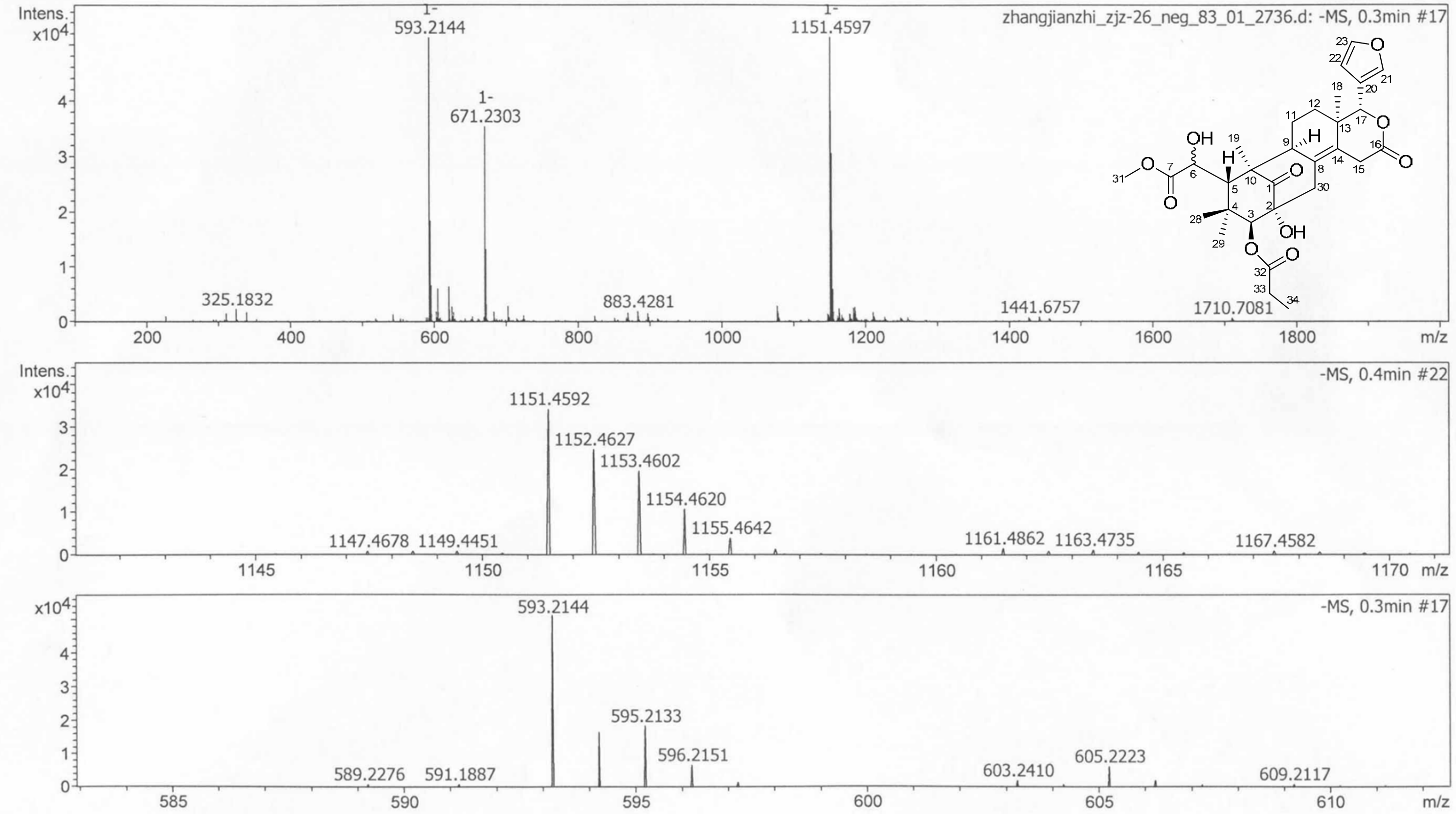

Bruker Compass DataAnalysis 4.1 printed: 12/12/2016 3:25:08 PM by: SCSIO Page 1 of 1

^{1}H NMR (400 MHz) spectrum of compound **2** in $CDCl_3$

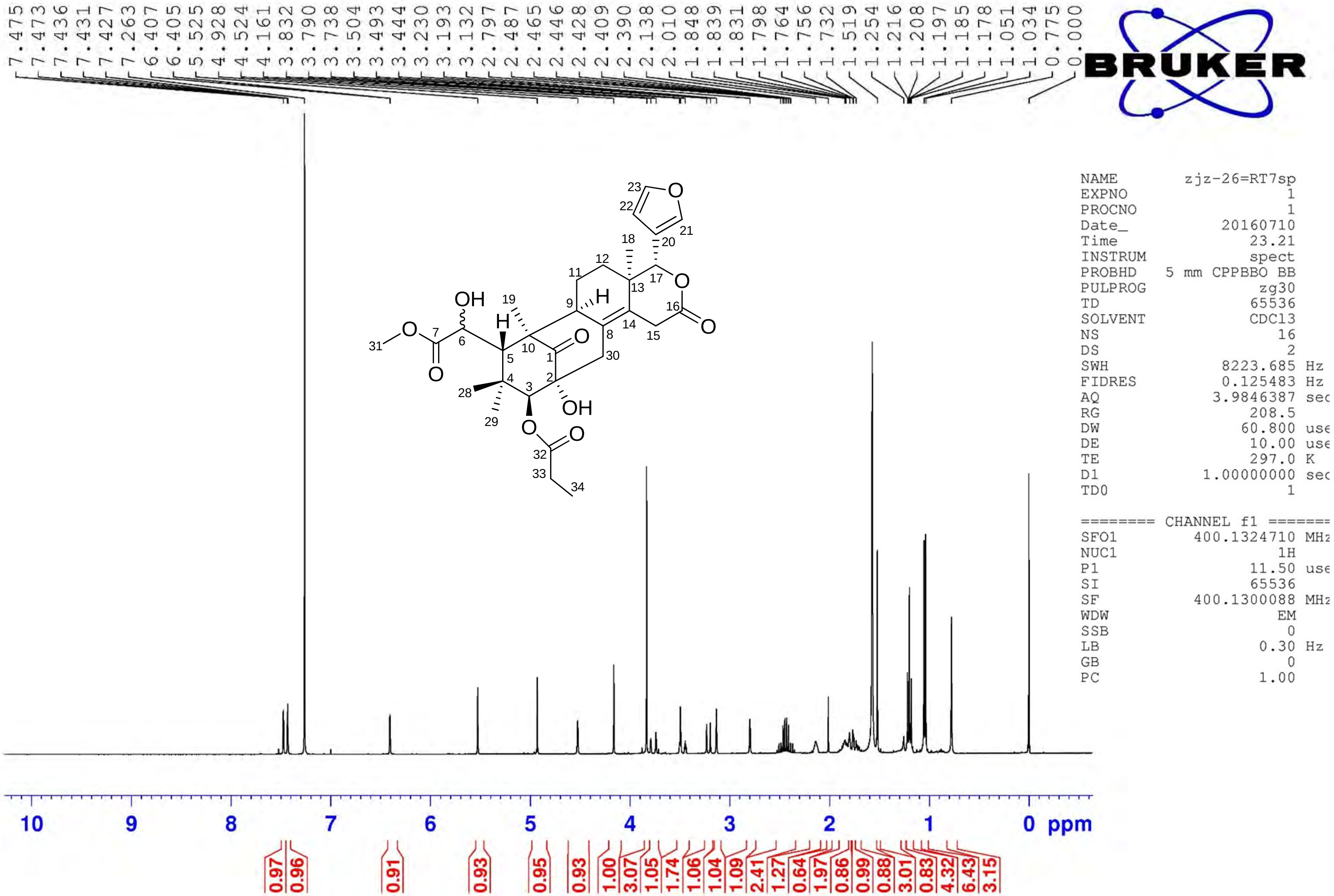

NAME	zjz-26=RT7sp
EXPNO	1
PROCNO	1
Date_	20160710
Time	23.21
INSTRUM	spect
PROBHD	5 mm CPPBBO BB
PULPROG	zg30
TD	65536
SOLVENT	CDCl3
NS	16
DS	2
SWH	8223.685 Hz
FIDRES	0.125483 Hz
AQ	3.9846387 sec
RG	208.5
DW	60.800 use
DE	10.00 use
TE	297.0 K
D1	1.00000000 sec
TD0	1

======== CHANNEL f1 ========

SFO1	400.1324710 MHz
NUC1	1H
P1	11.50 use
SI	65536
SF	400.1300088 MHz
WDW	EM
SSB	0
LB	0.30 Hz
GB	0
PC	1.00

^{1}H NMR (400 MHz) spectrum of compound **2** in $CDCl_3$

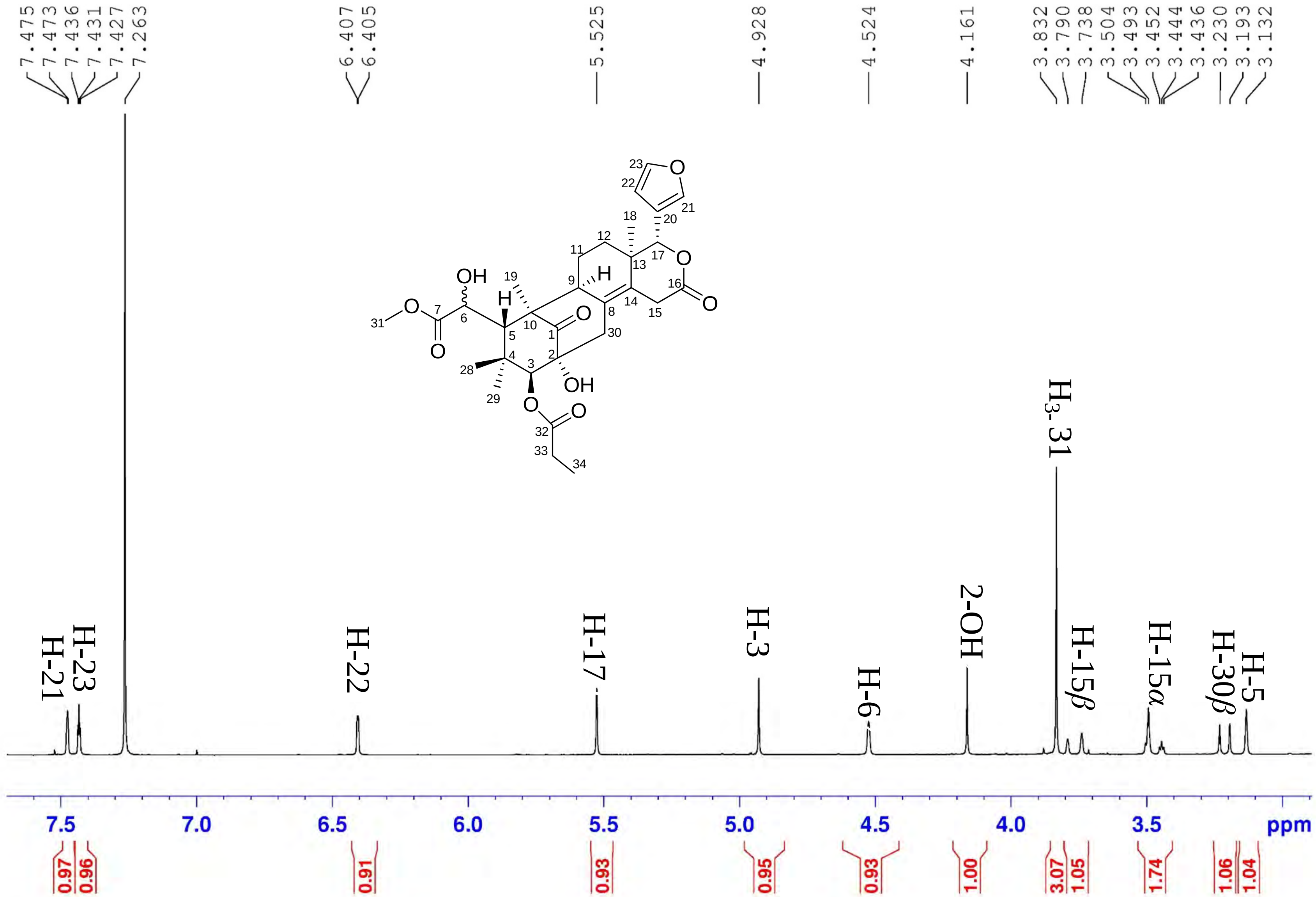

^{1}H NMR (400 MHz) spectrum of compound **2** in $CDCl_3$

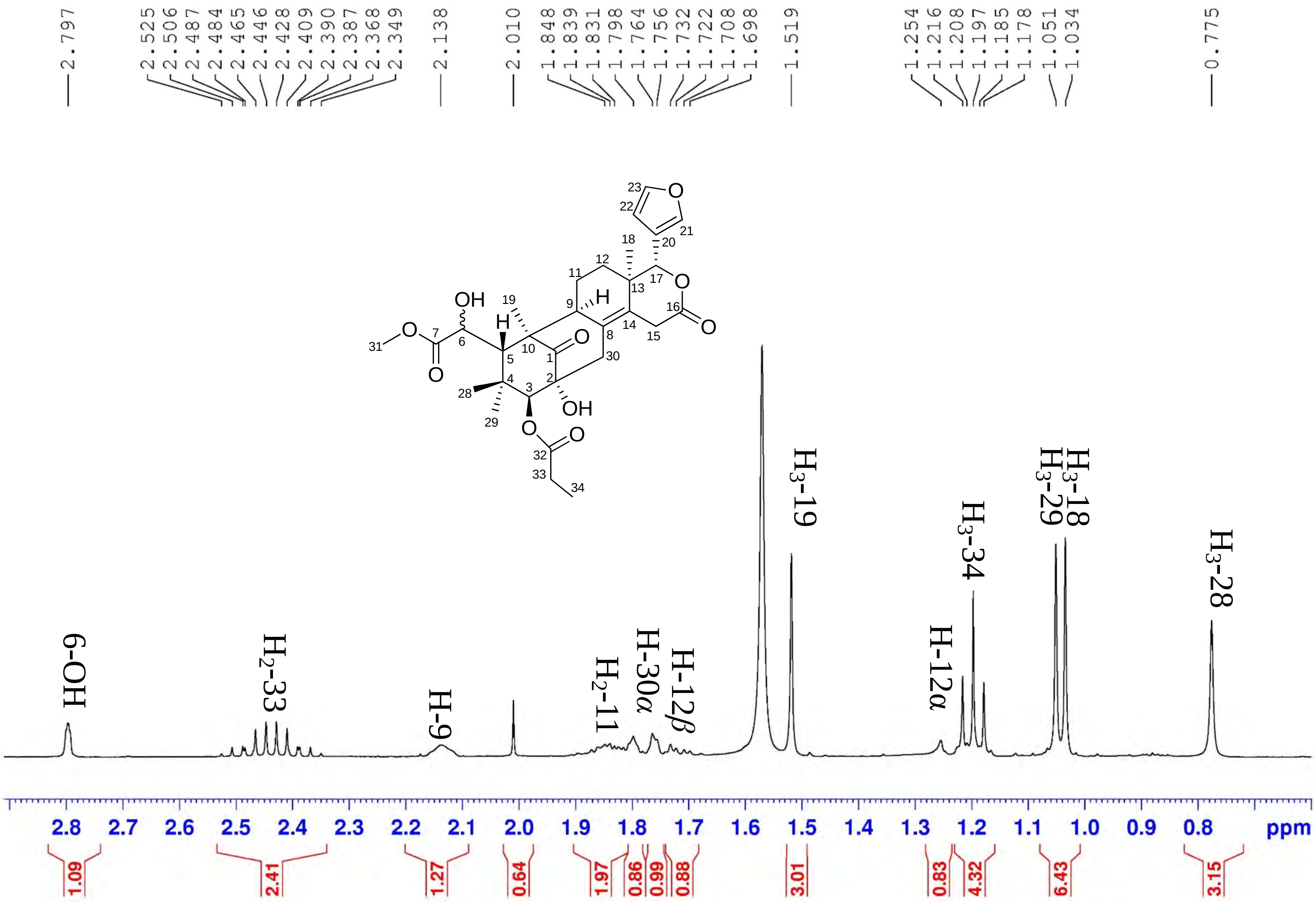

^{13}C NMR (100 MHz) spectrum of compound **2** in $CDCl_3$

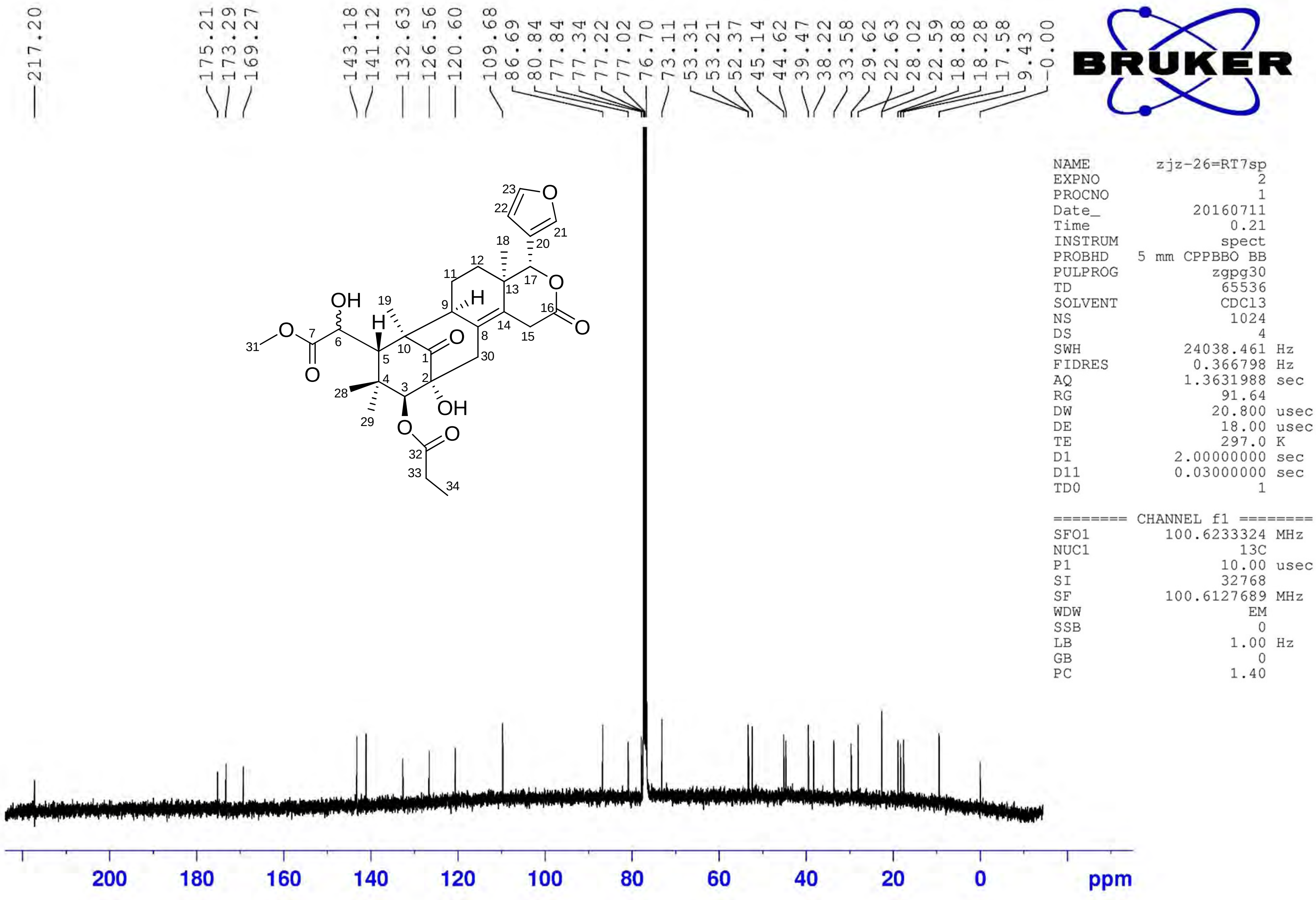

^{13}C NMR (100 MHz) spectrum of compound **2** in $CDCl_3$

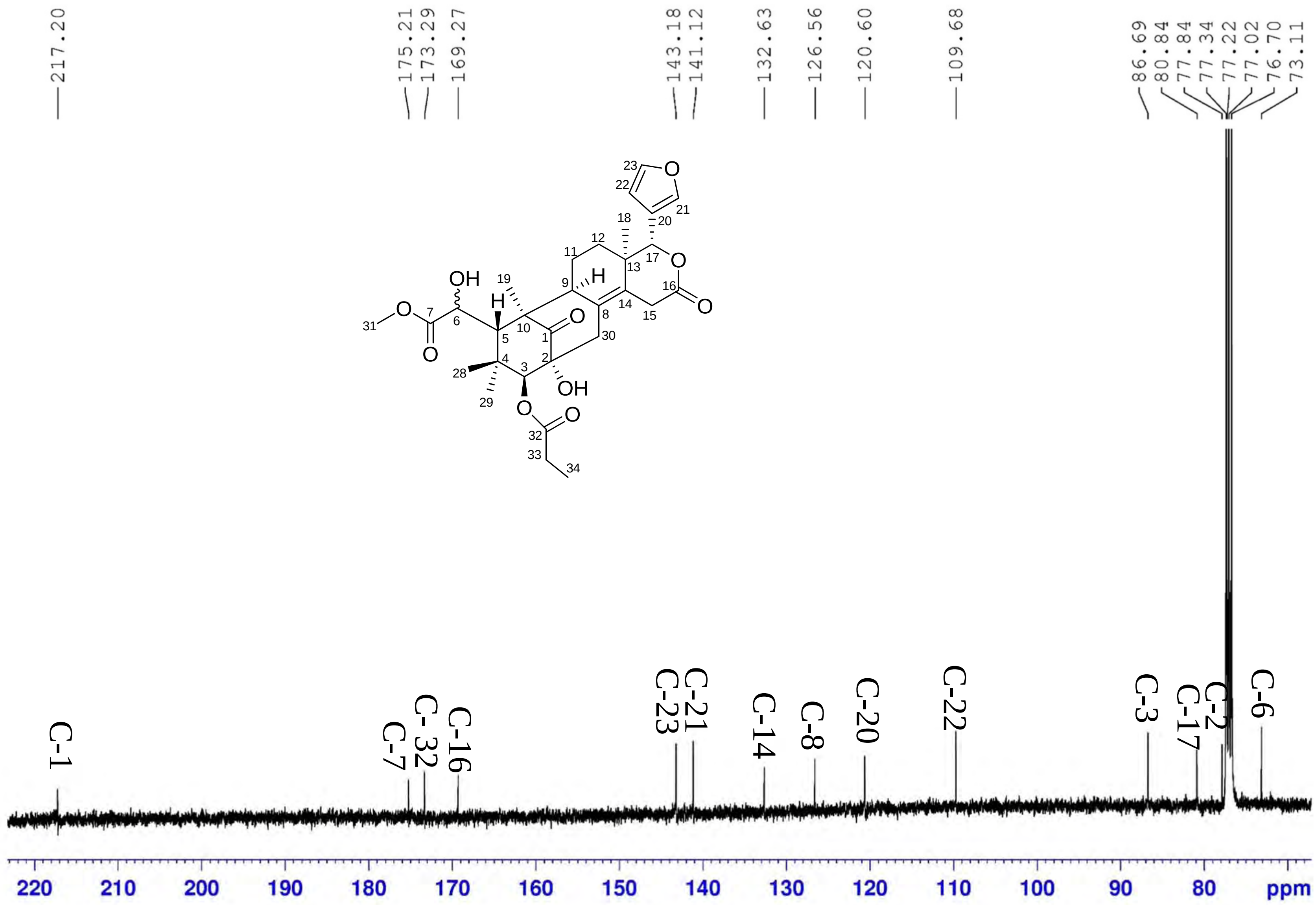

^{13}C NMR (100 MHz) spectrum of compound **2** in $CDCl_3$

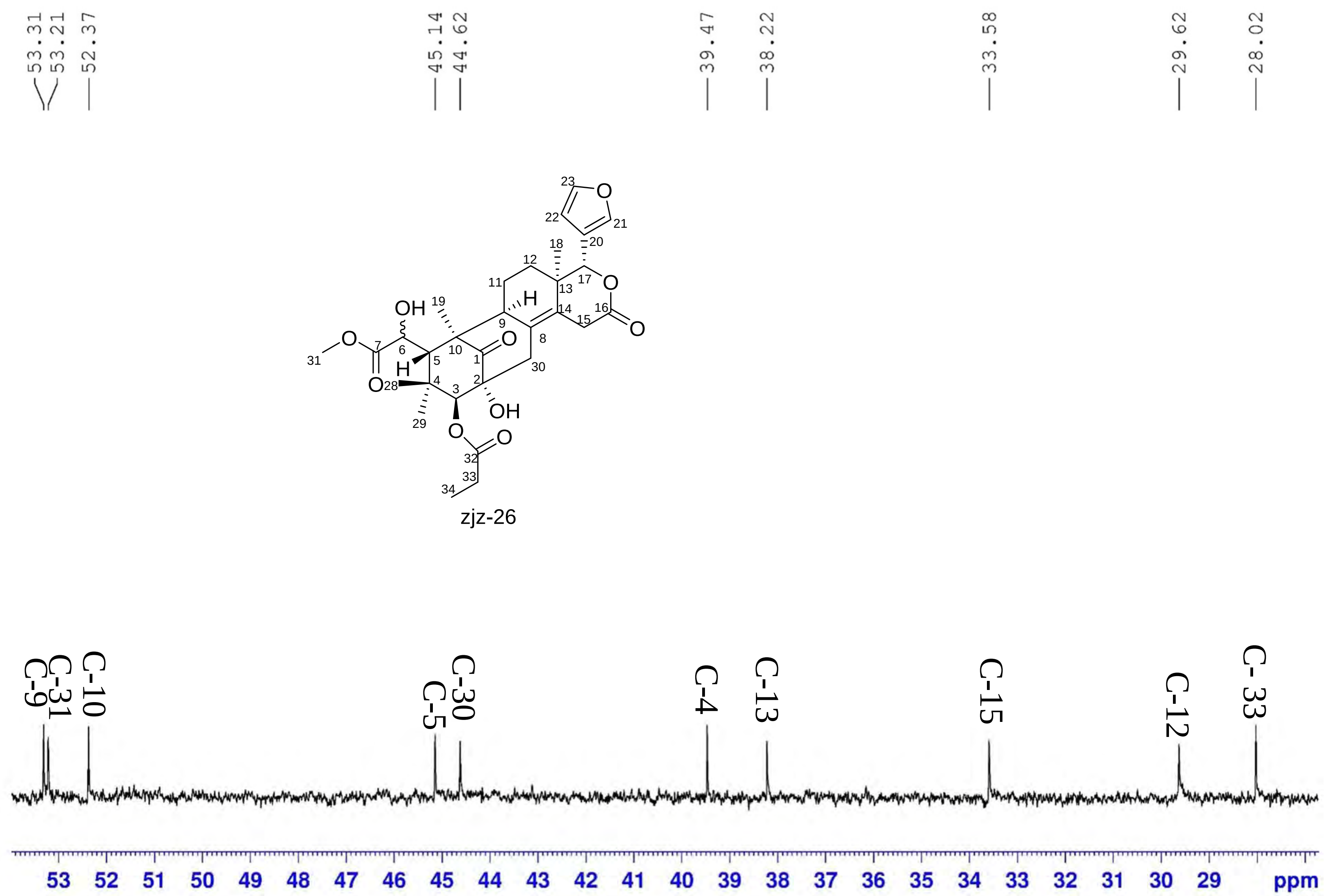

^{13}C NMR (100 MHz) spectrum of compound **2** in $CDCl_3$

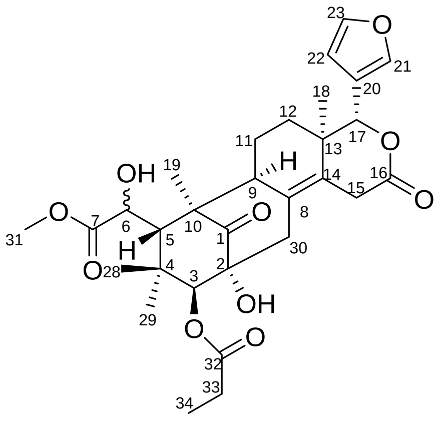

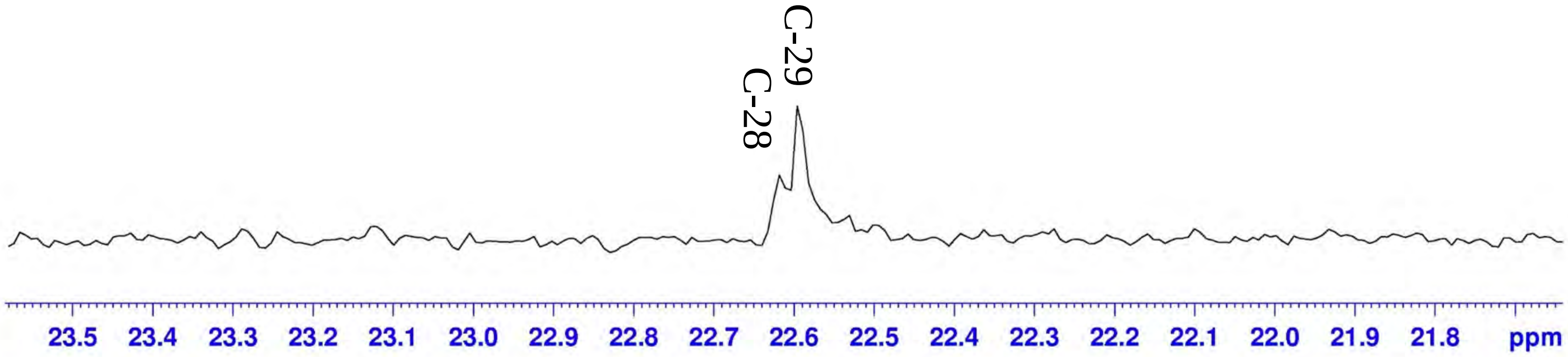

^{13}C NMR (100 MHz) spectrum of compound **2** in $CDCl_3$

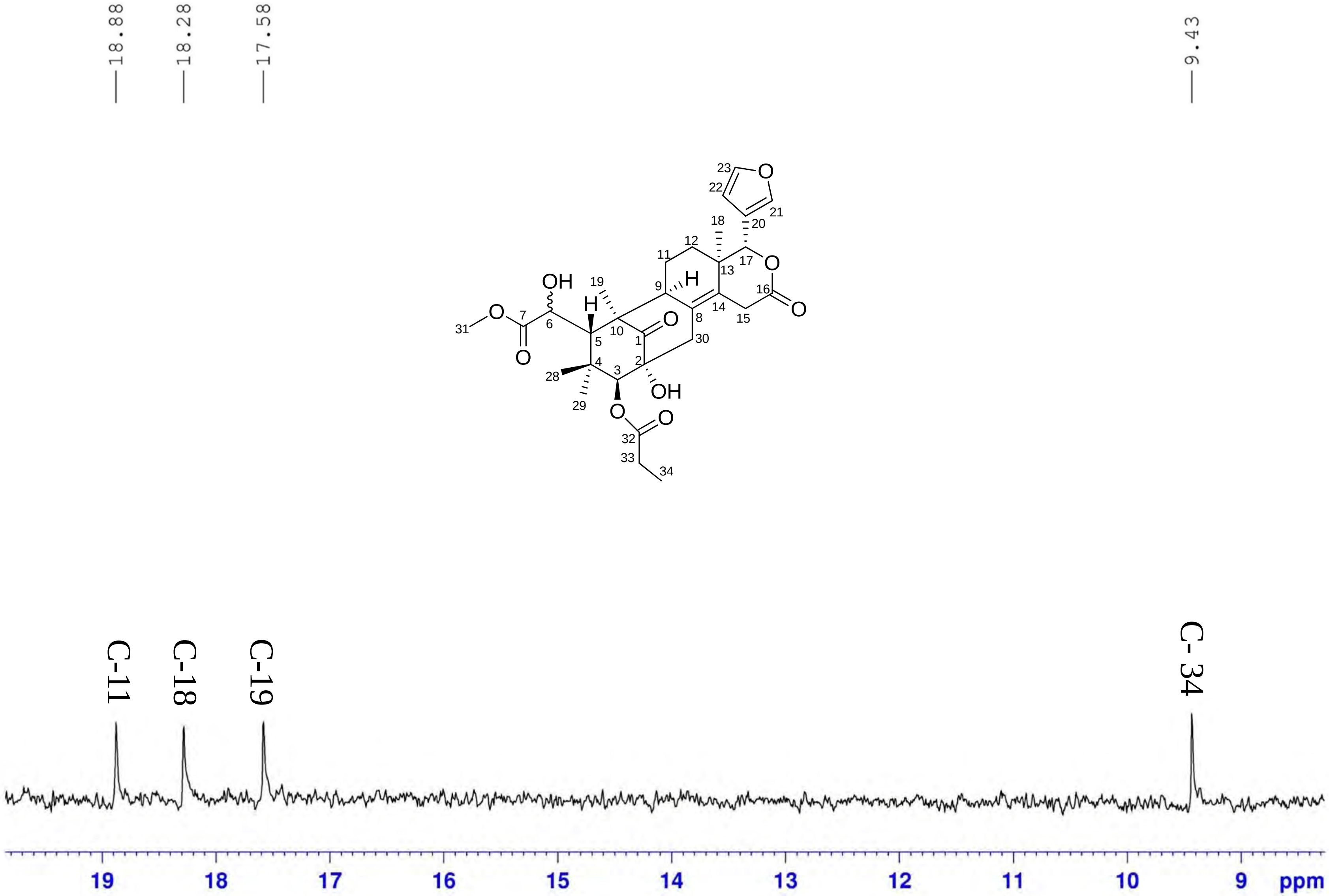

DEPT135 (100 MHz) spectrum of compound **2** in $CDCl_3$

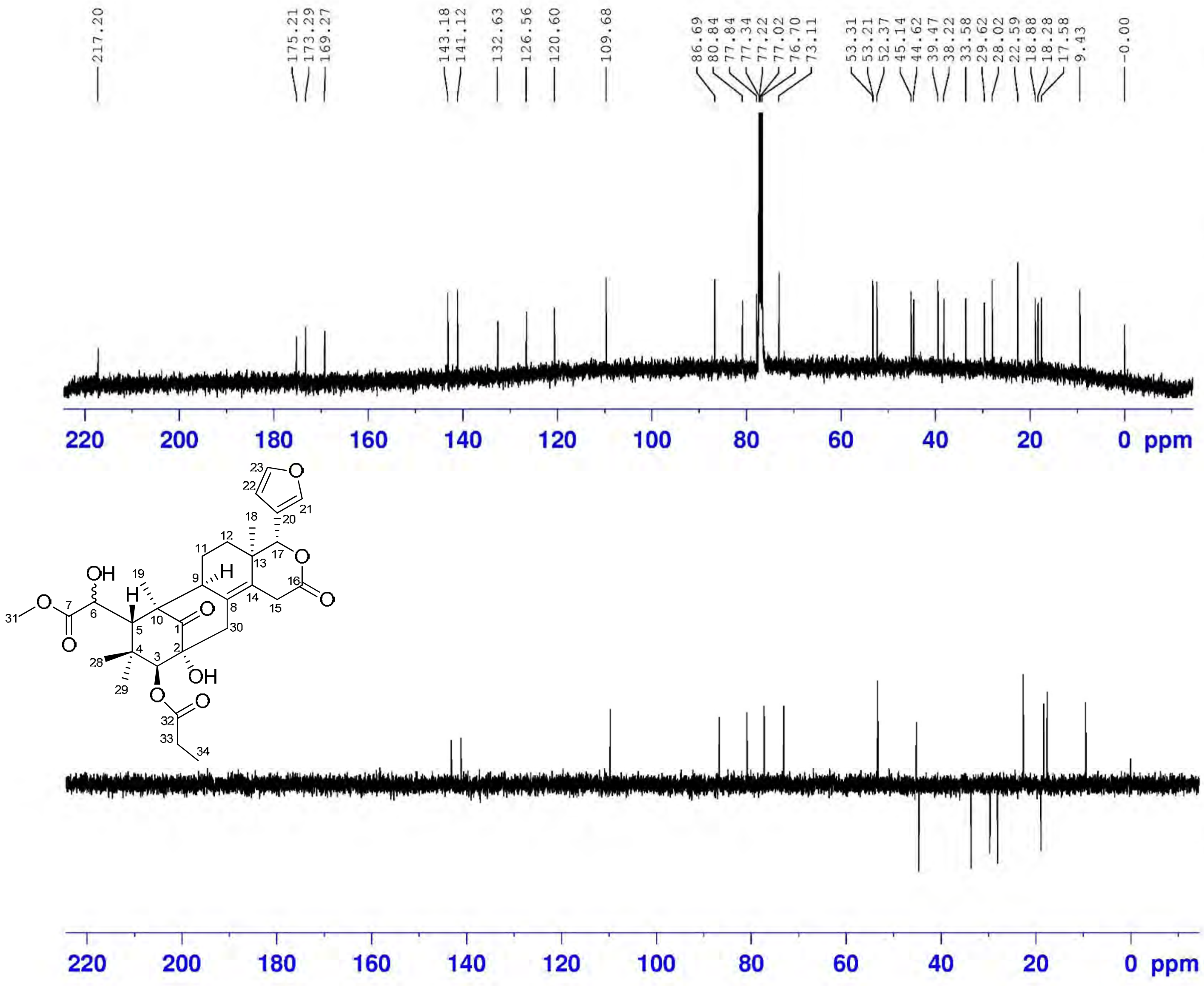

NAME	zjz-26=RT7sp
EXPNO	3
PROCNO	1
Date_	20160711
Time	0.39
INSTRUM	spect
PROBHD	5 mm CPPBBO BB
PULPROG	deptsp135
TD	65536
SOLVENT	CDCl3
NS	300
DS	4
SWH	24038.461 Hz
FIDRES	0.366798 Hz
AQ	1.3631988 sec
RG	130.26
DW	20.800 usec
DE	18.00 usec
TE	297.0 K
CNST2	145.0000000
D1	2.00000000 sec
D2	0.00344828 sec
D12	0.00002000 sec
TD0	1

======== CHANNEL f1 ========

SFO1	100.6233324 MHz
NUC1	13C
P1	10.00 usec
P13	2000.00 usec
SI	32768
SF	100.6127689 MHz
WDW	EM
SSB	0
LB	1.00 Hz
GB	0
PC	1.40

DEPT135 (100 MHz) spectrum of compound **2** in $CDCl_3$

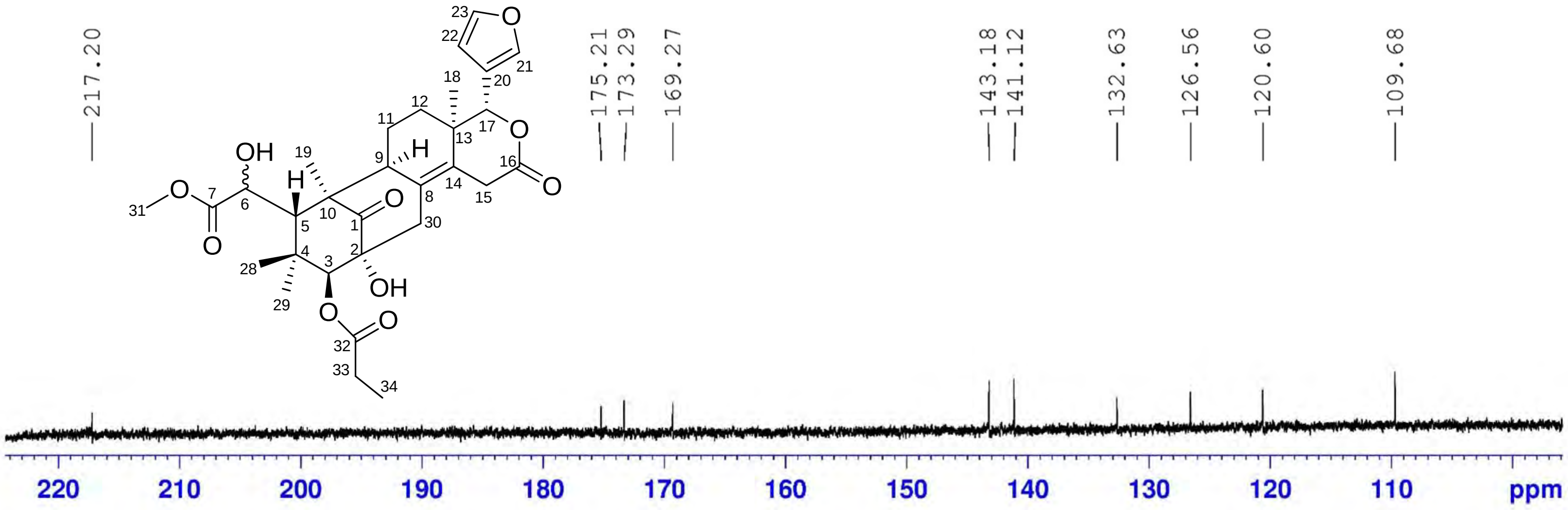

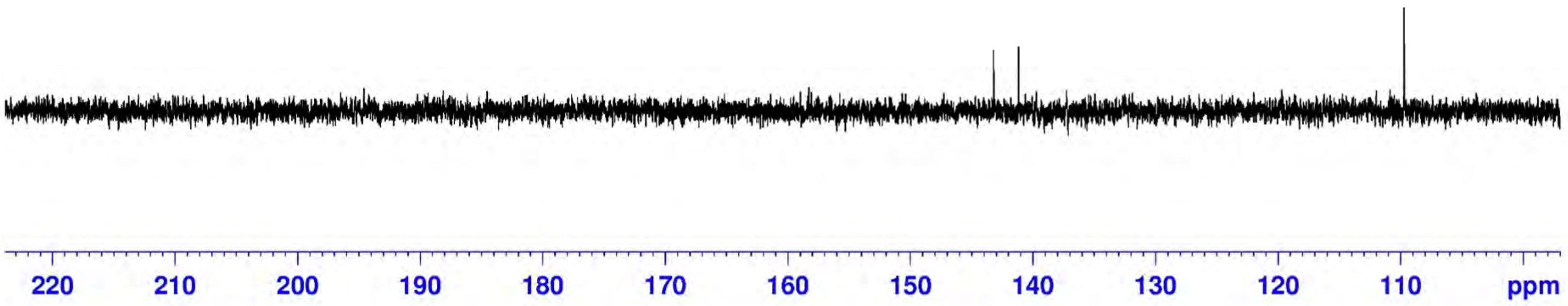

DEPT135 (100 MHz) spectrum of compound **2** in $CDCl_3$

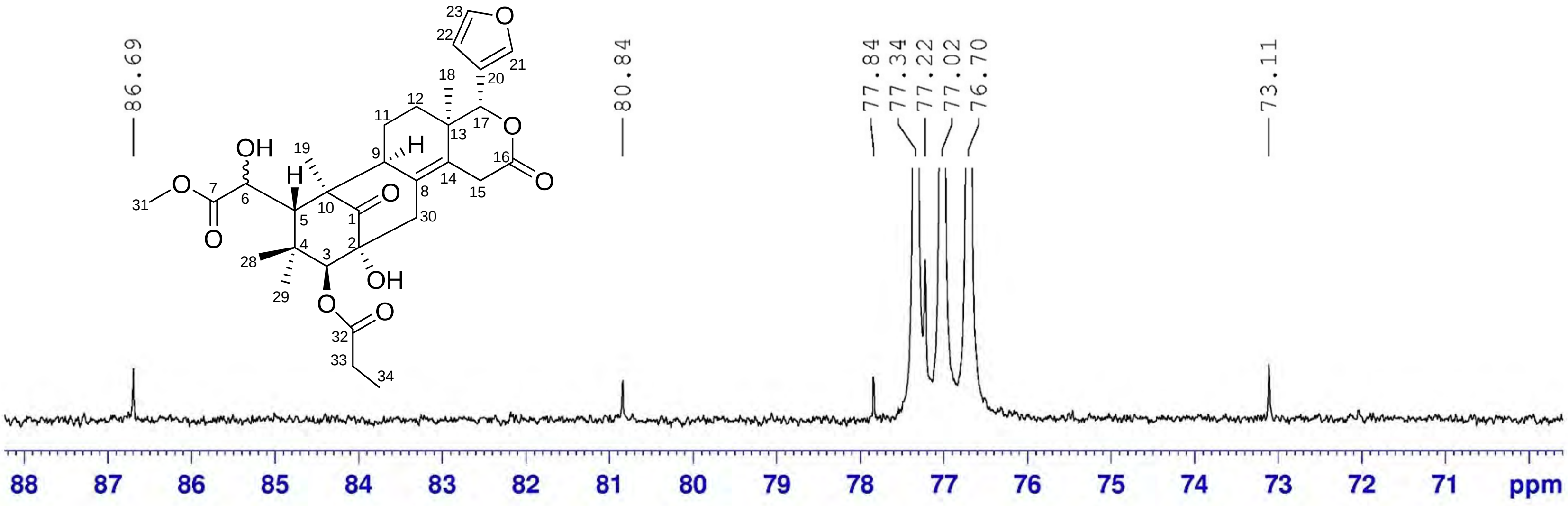

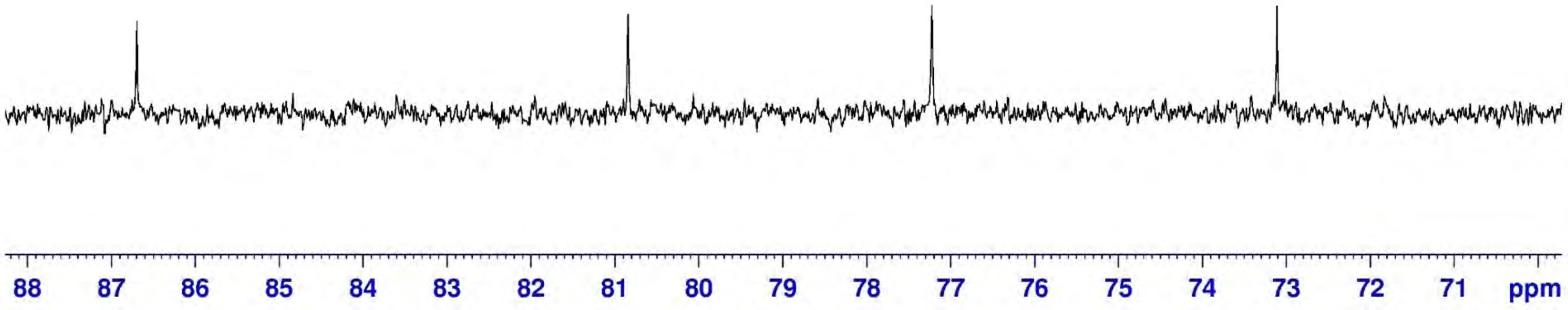

DEPT135 (100 MHz) spectrum of compound **2** in $CDCl_3$

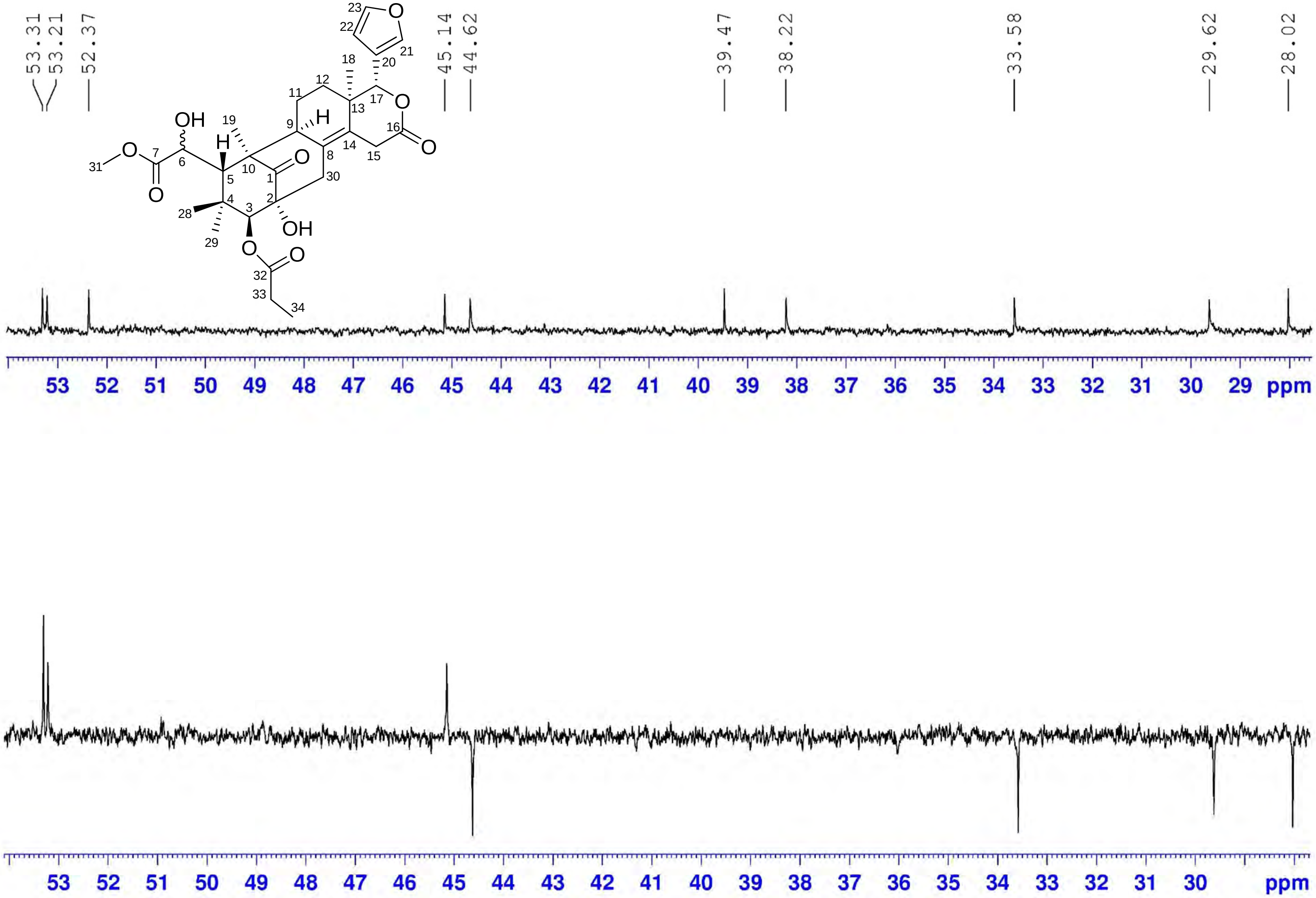

DEPT135 (100 MHz) spectrum of compound **2** in $CDCl_3$

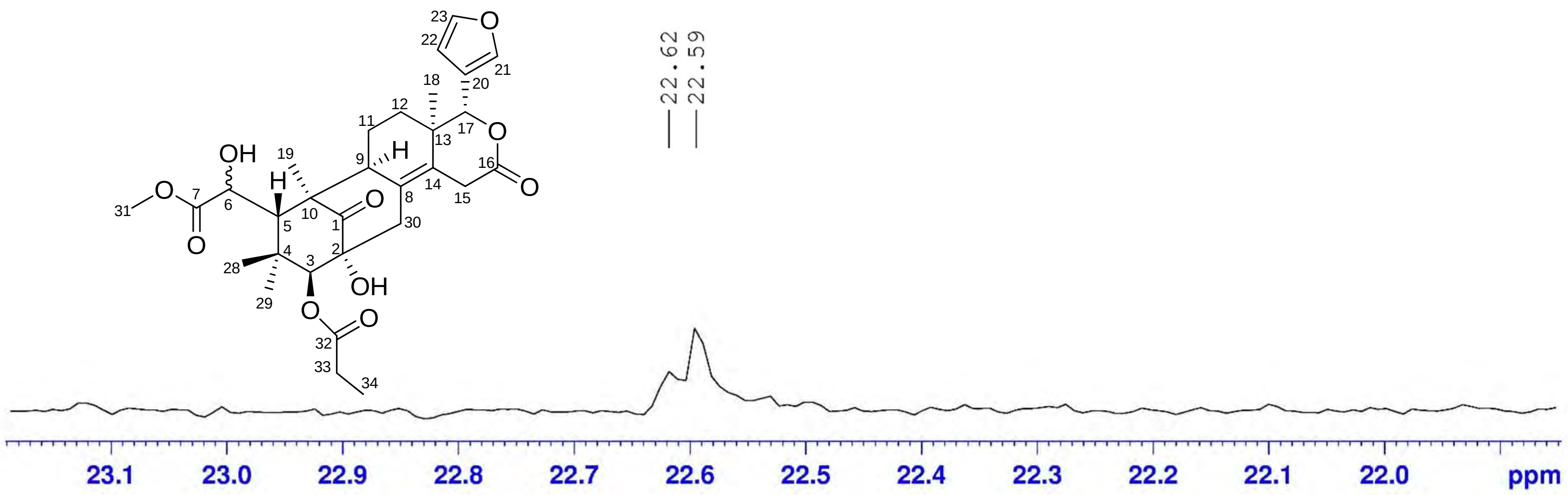

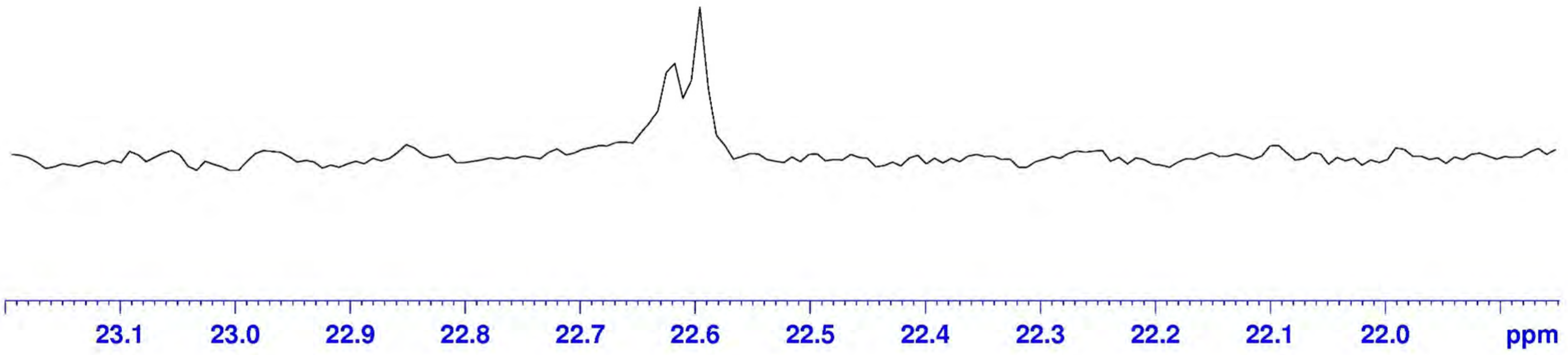

DEPT135 (100 MHz) spectrum of compound **2** in $CDCl_3$

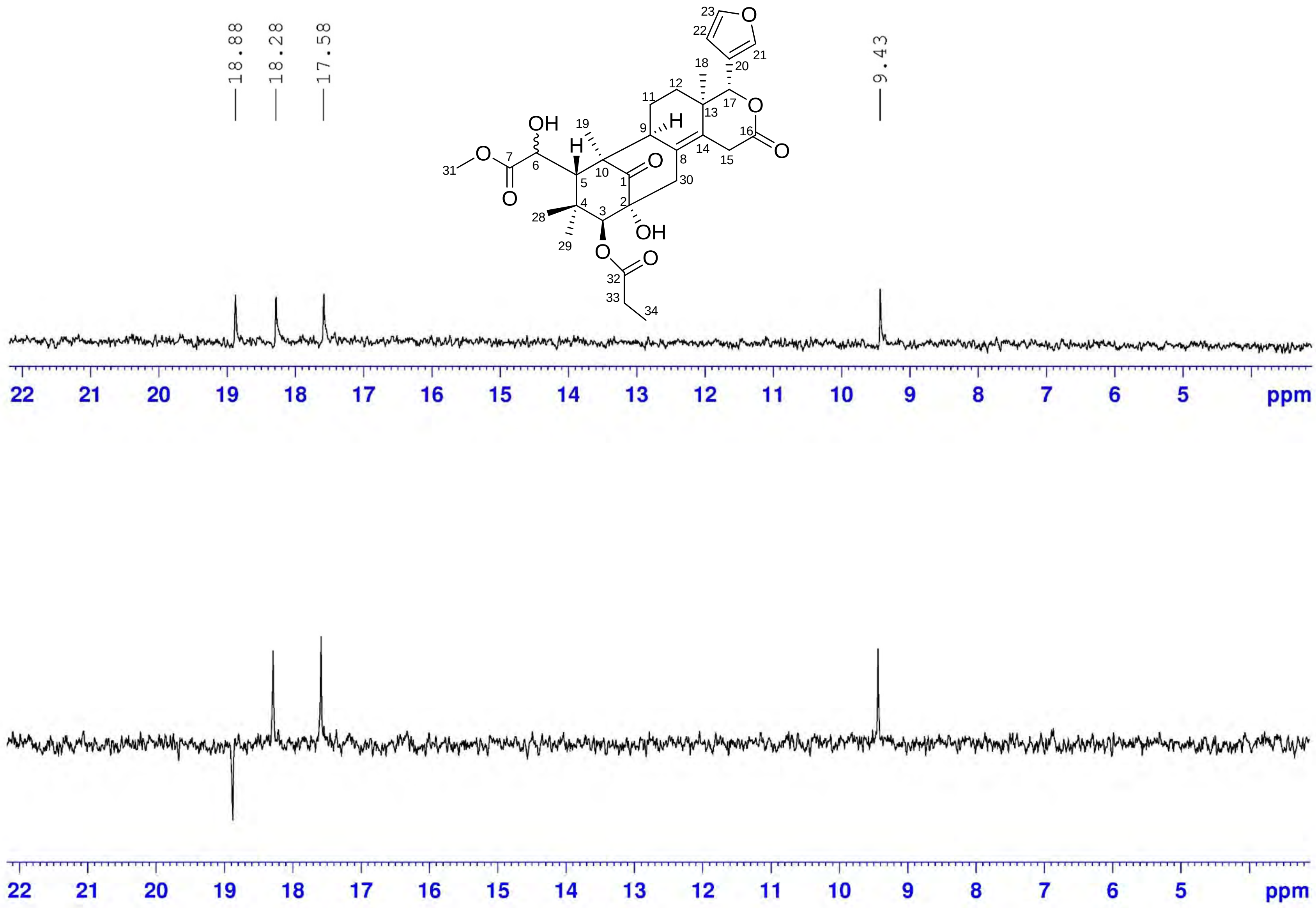

^{1}H-^{1}H COSY (400 MHz) spectrum of compound **2** in $CDCl_3$

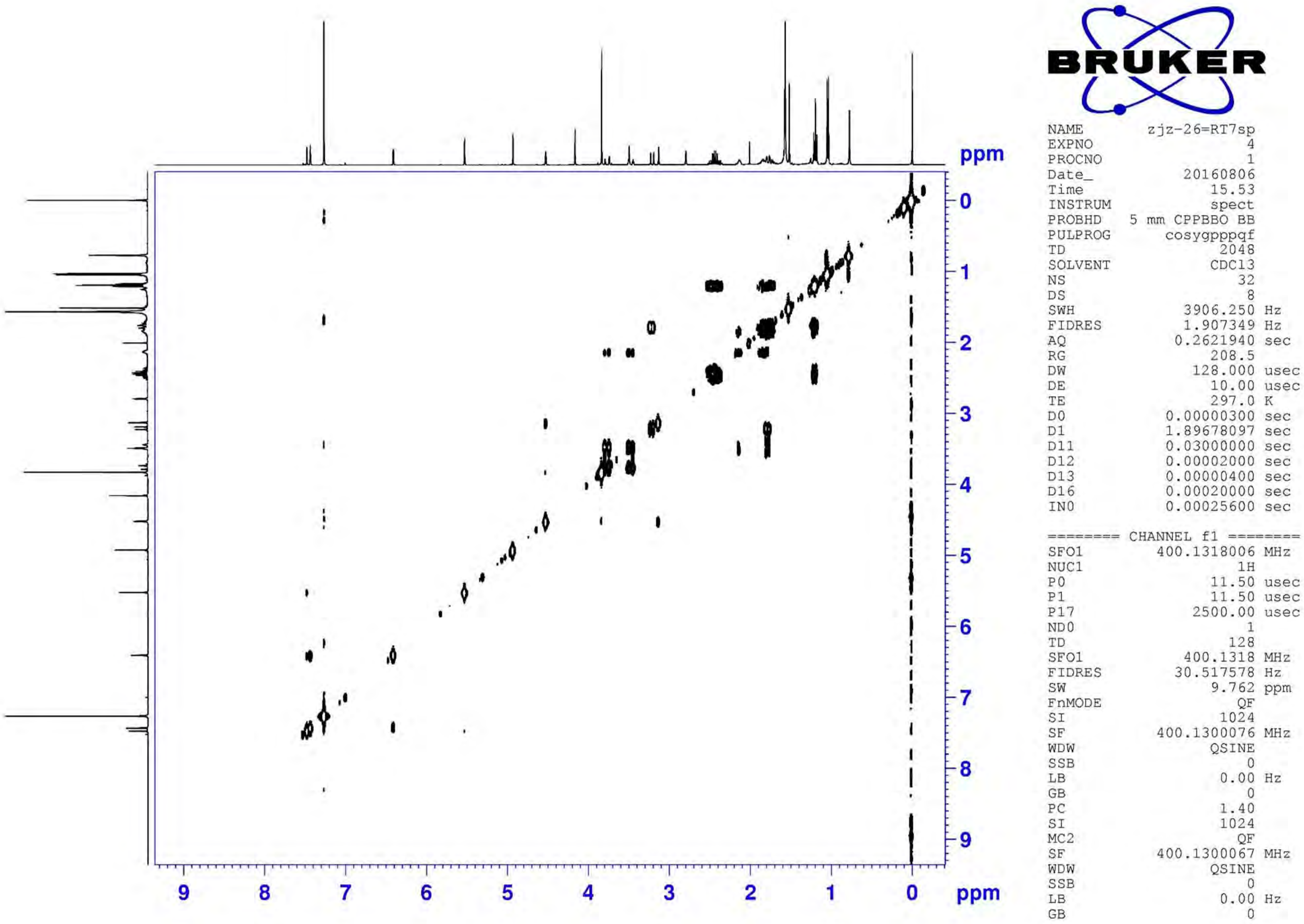

^{1}H-^{1}H COSY (400 MHz) spectrum of compound **2** in $CDCl_3$

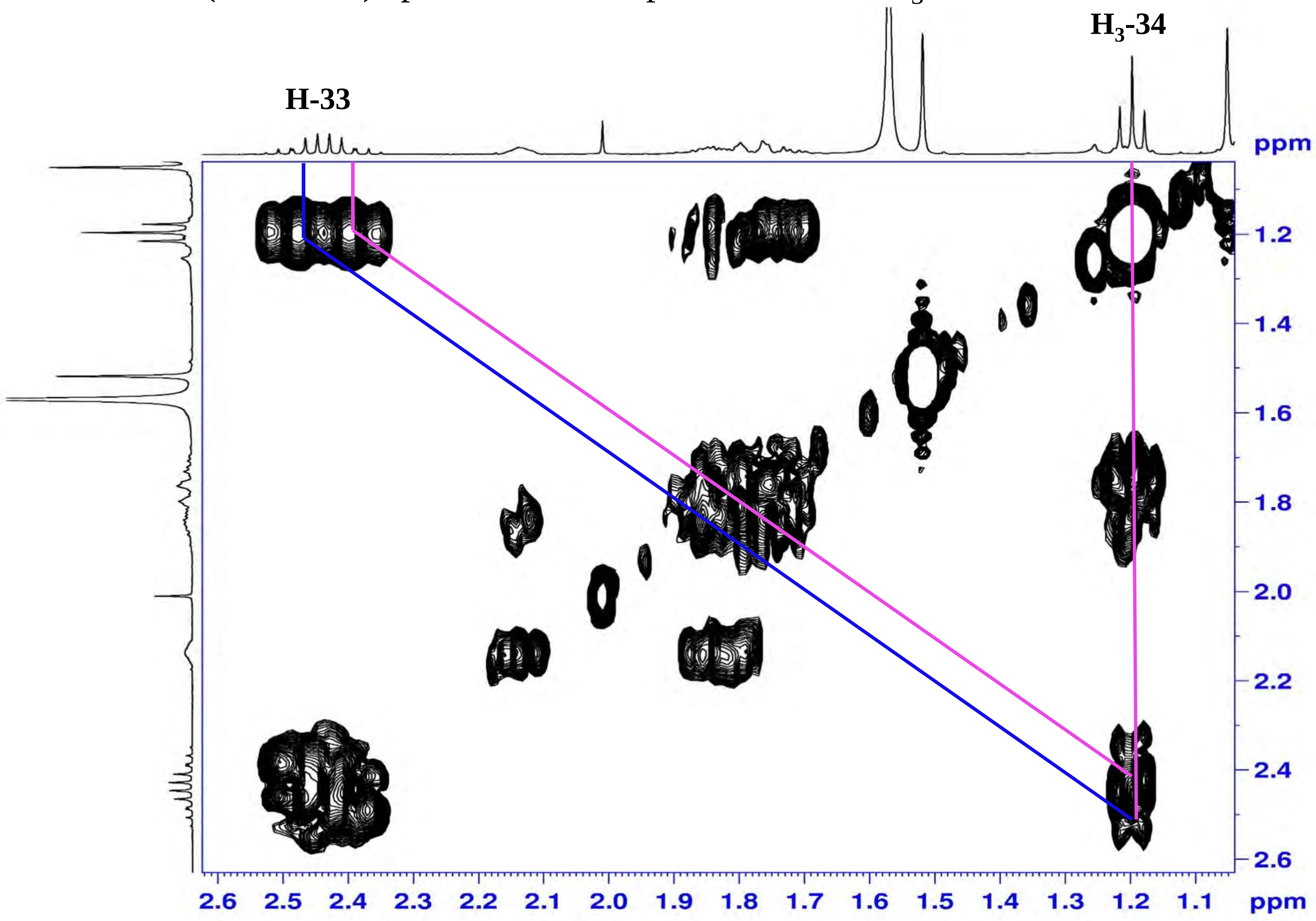

^1H-^1H COSY (400 MHz) spectrum of compound **2** in $CDCl_3$

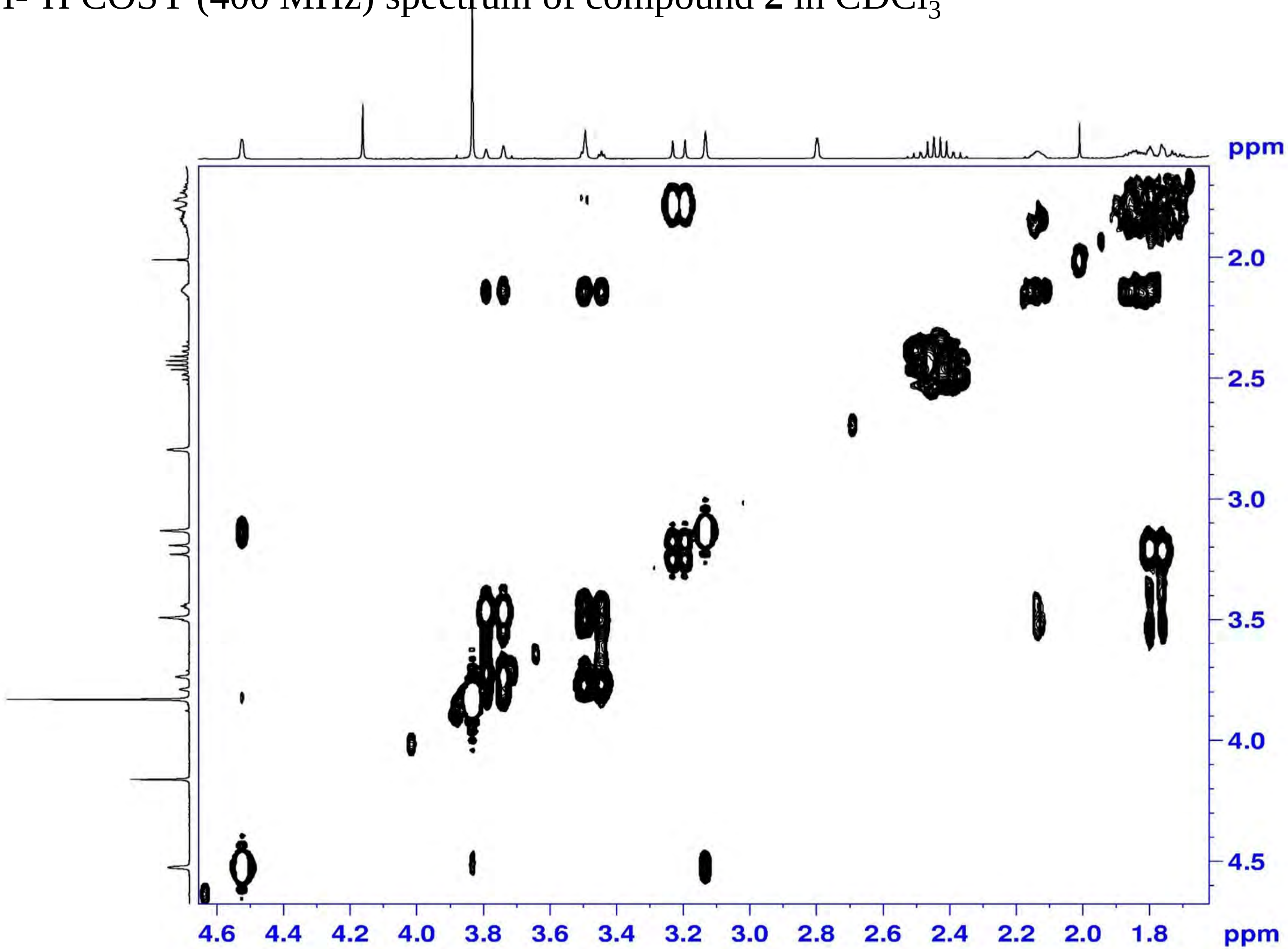

HSQC (400 MHz) spectrum of compound **2** in $CDCl_3$

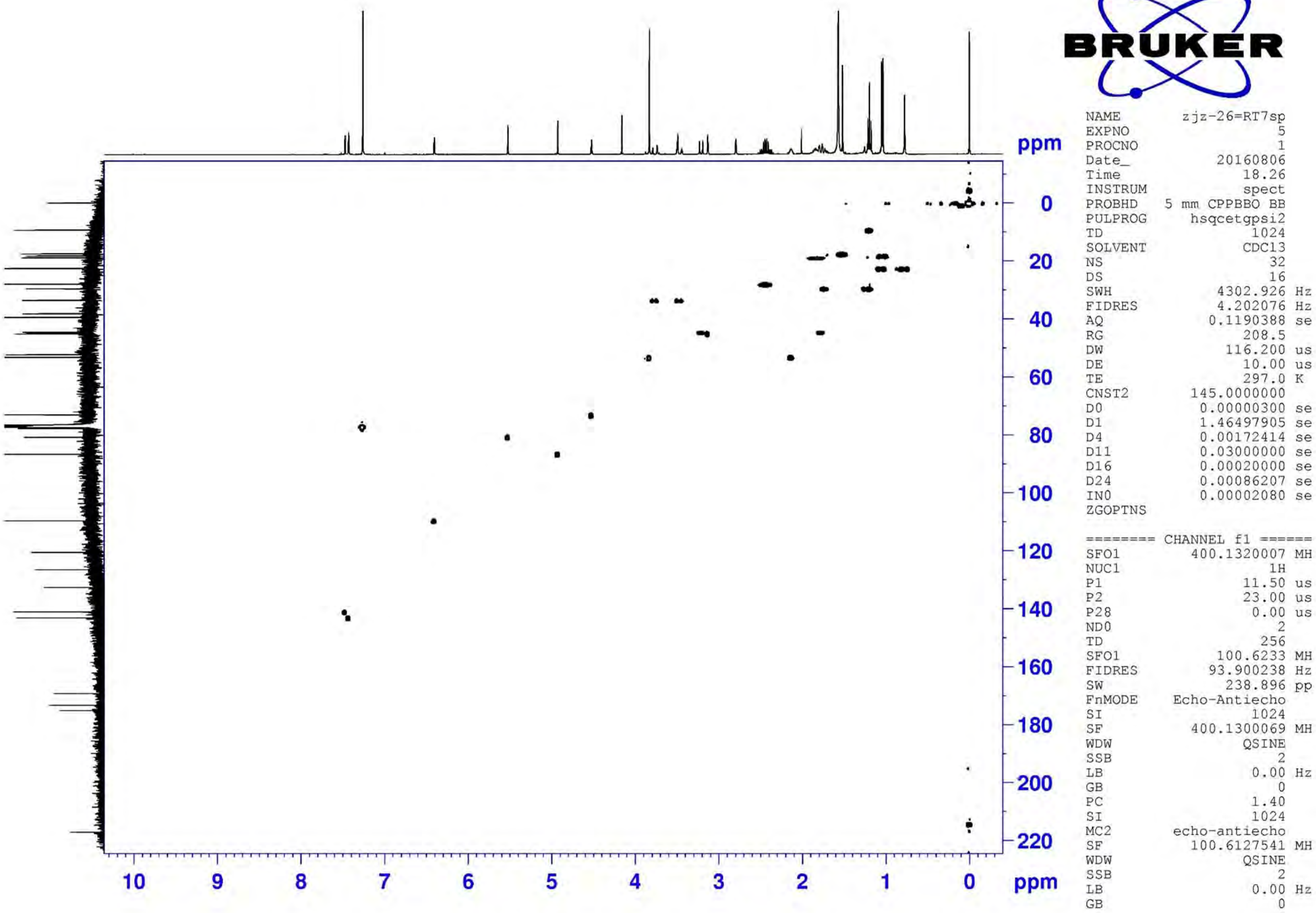

HSQC (400 MHz) spectrum of compound **2** in $CDCl_3$

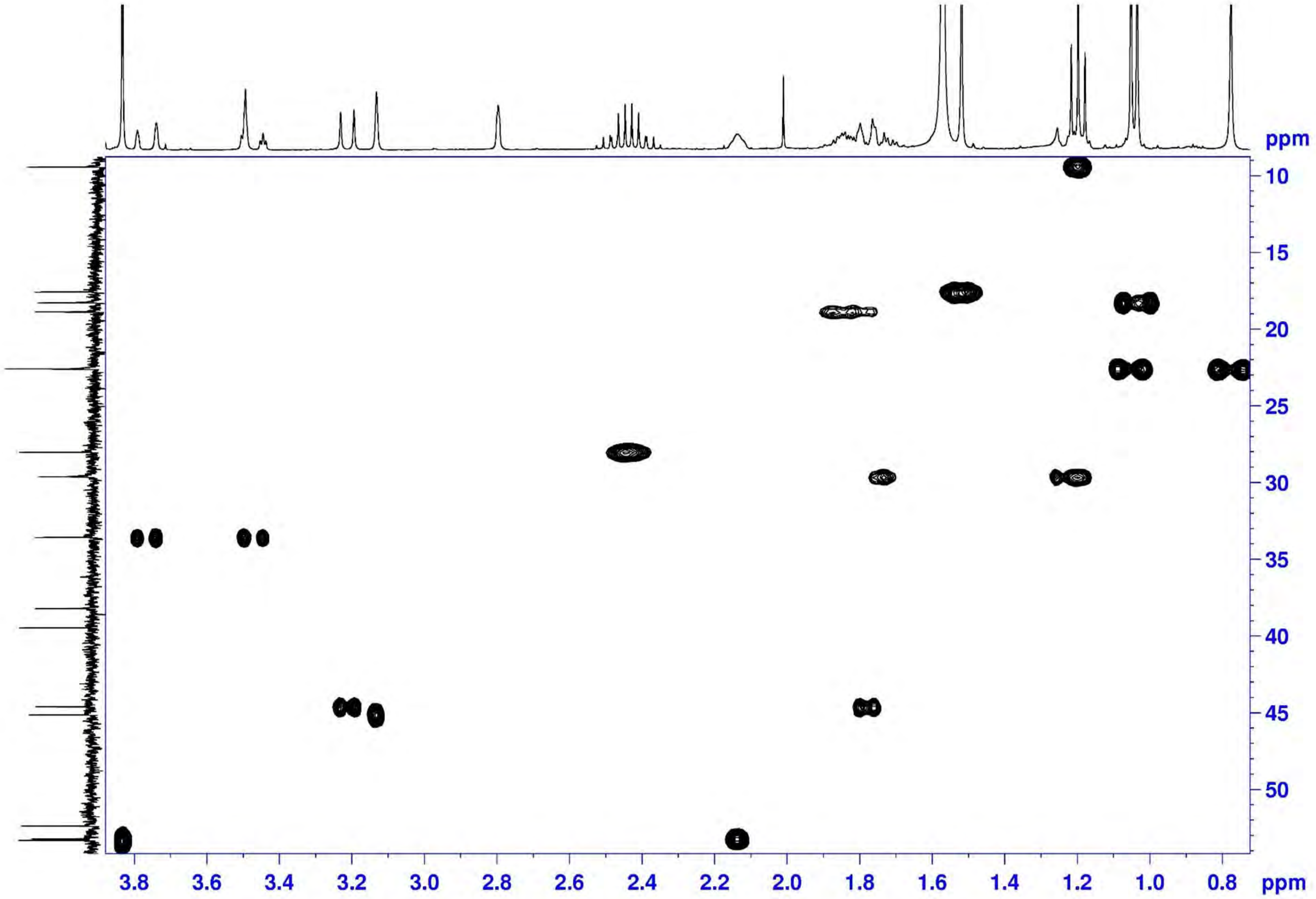

HSQC (400 MHz) spectrum of compound **2** in $CDCl_3$

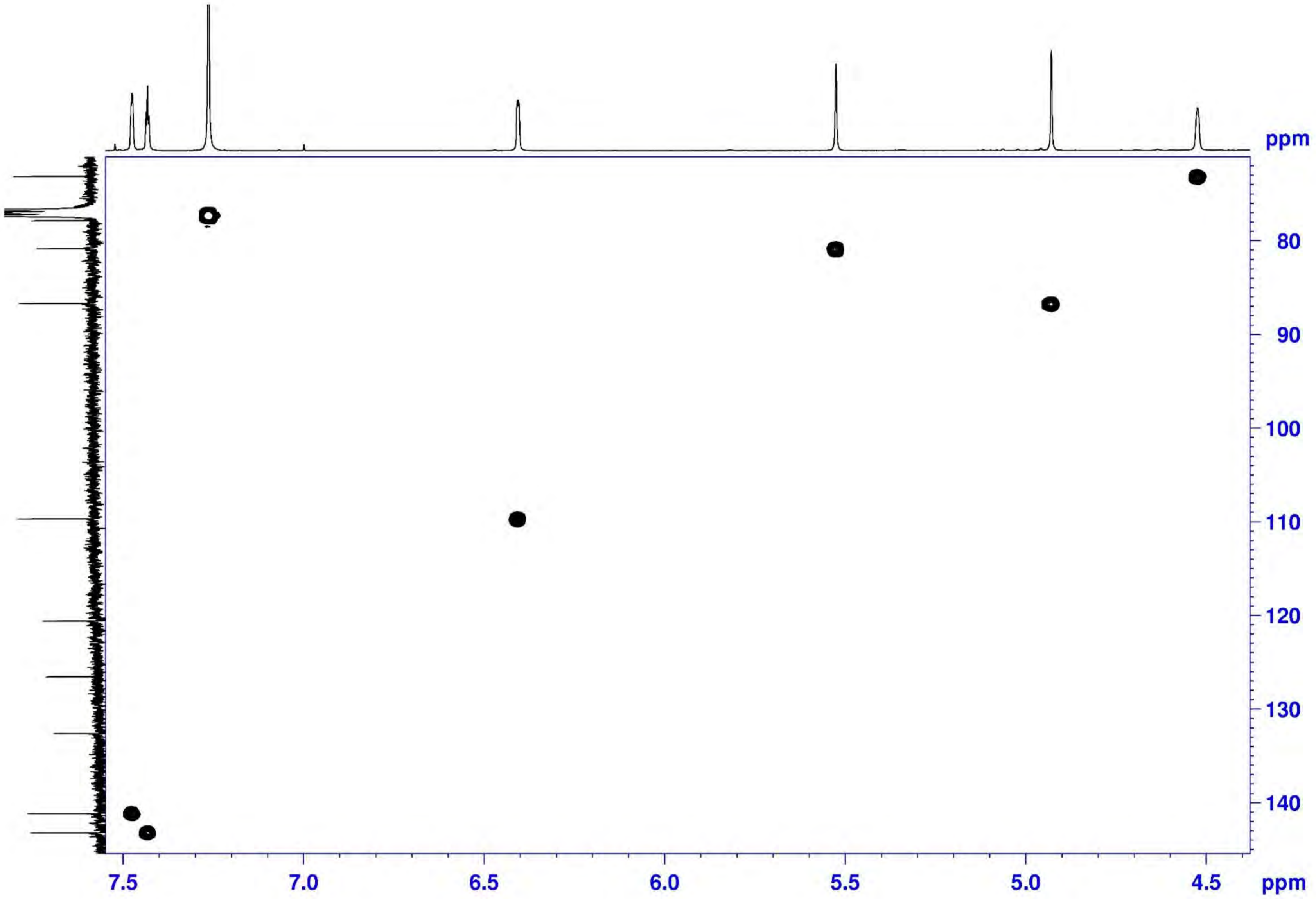

HMBC (400 MHz) spectrum of compound **2** in $CDCl_3$

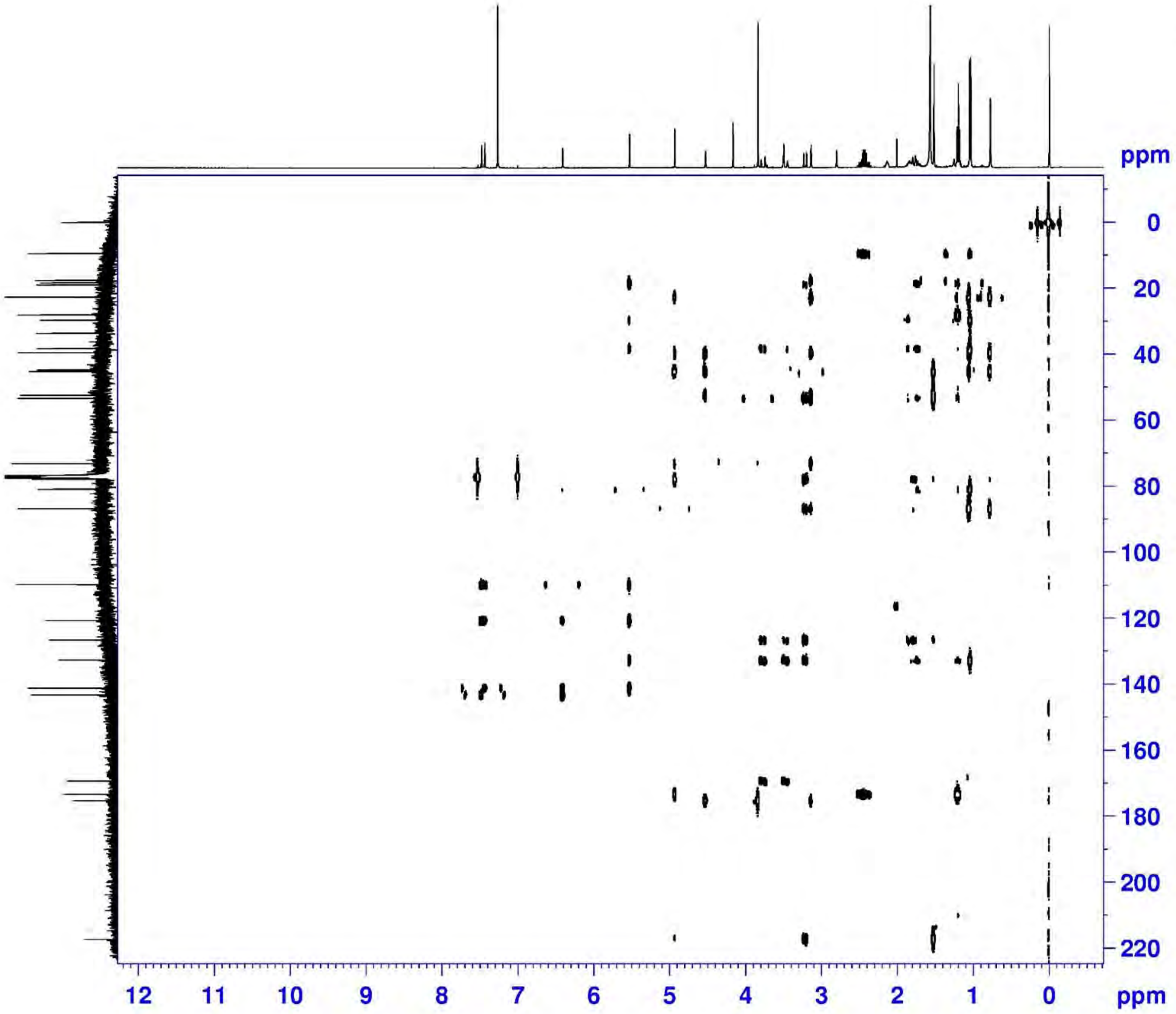

NAME	zjz-26=RT7sp
EXPNO	6
PROCNO	1
Date_	20160806
Time	22.09
INSTRUM	spect
PROBHD	5 mm CPPBBO BB
PULPROG	hmbcgplpndqf
TD	4096
SOLVENT	CDCl3
NS	128
DS	16
SWH	5197.505 Hz
FIDRES	1.268922 Hz
AQ	0.3940852 sec
RG	208.5
DW	96.200 usec
DE	10.00 usec
TE	297.0 K
CNST2	145.0000000
CNST13	10.0000000
D0	0.00000300 sec
D1	1.50000000 sec
D2	0.00344828 sec
D6	0.05000000 sec
D16	0.00020000 sec
IN0	0.00002080 sec
======== CHANNEL f1 ========	
SFO1	400.1323208 MHz
NUC1	1H
P1	11.50 usec
P2	23.00 usec
ND0	2
TD	128
SFO1	100.6233 MHz
FIDRES	187.800476 Hz
SW	238.896 ppm
FnMODE	QF
SI	2048
SF	400.1300081 MHz
WDW	SINE
SSB	0
LB	0.00 Hz
GB	0
PC	1.40
SI	1024
MC2	QF
SF	100.6127568 MHz
WDW	SINE
SSB	0
LB	0.00 Hz
GB	0

HMBC (400 MHz) spectrum of compound **2** in $CDCl_3$

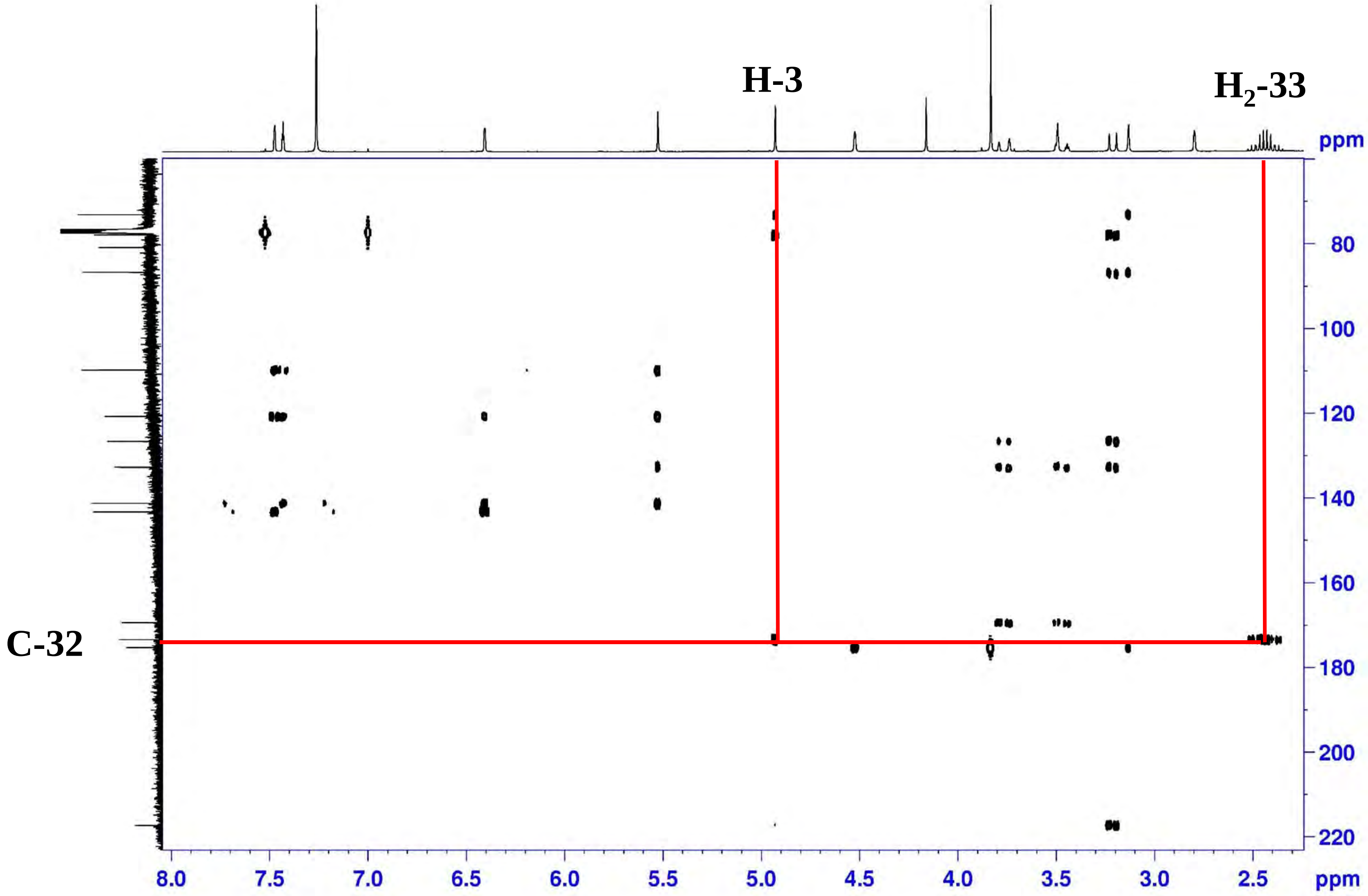

HMBC (400 MHz) spectrum of compound **2** in $CDCl_3$

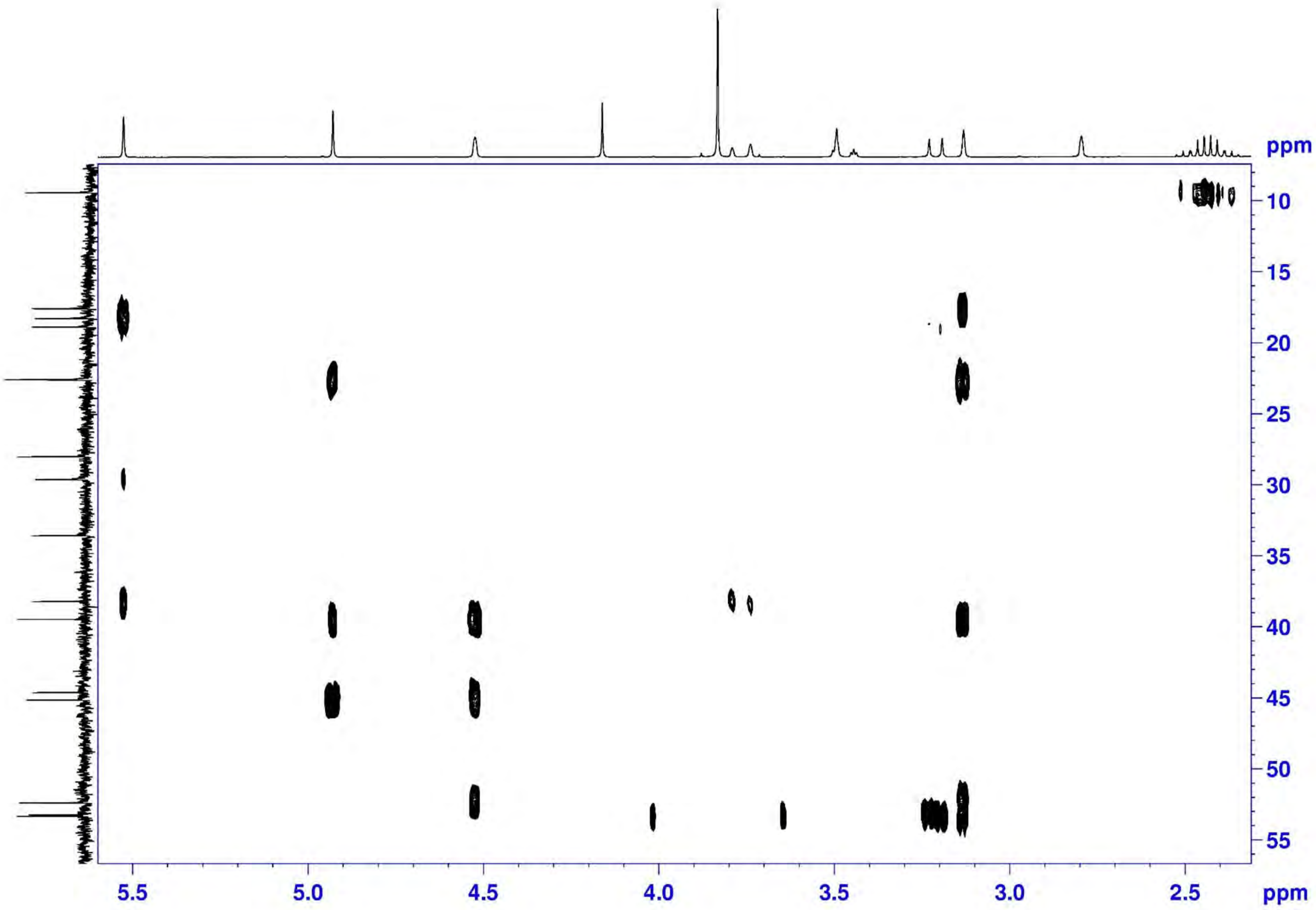

HMBC (400 MHz) spectrum of compound **2** in $CDCl_3$

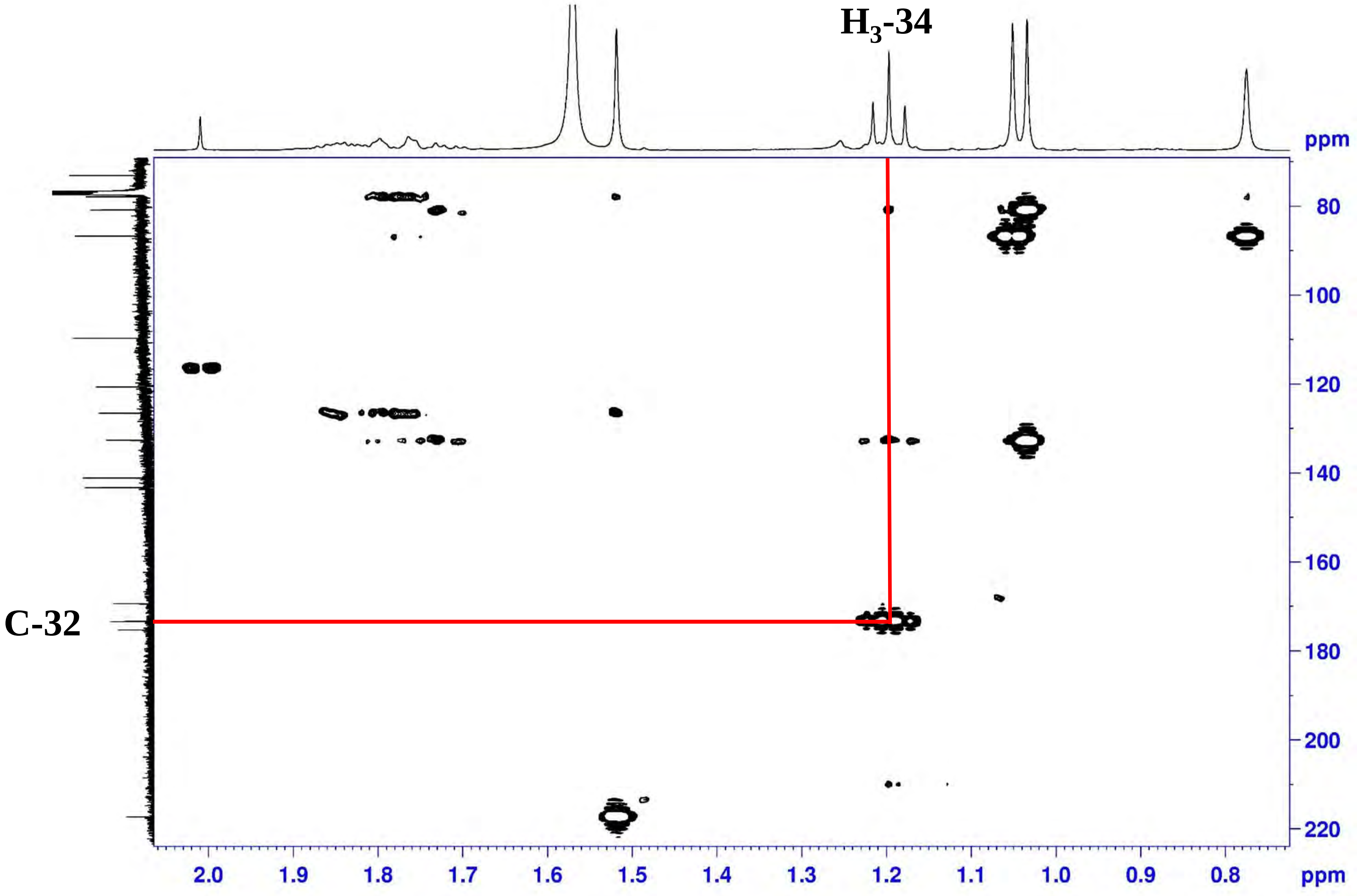

HMBC (400 MHz) spectrum of compound **2** in $CDCl_3$

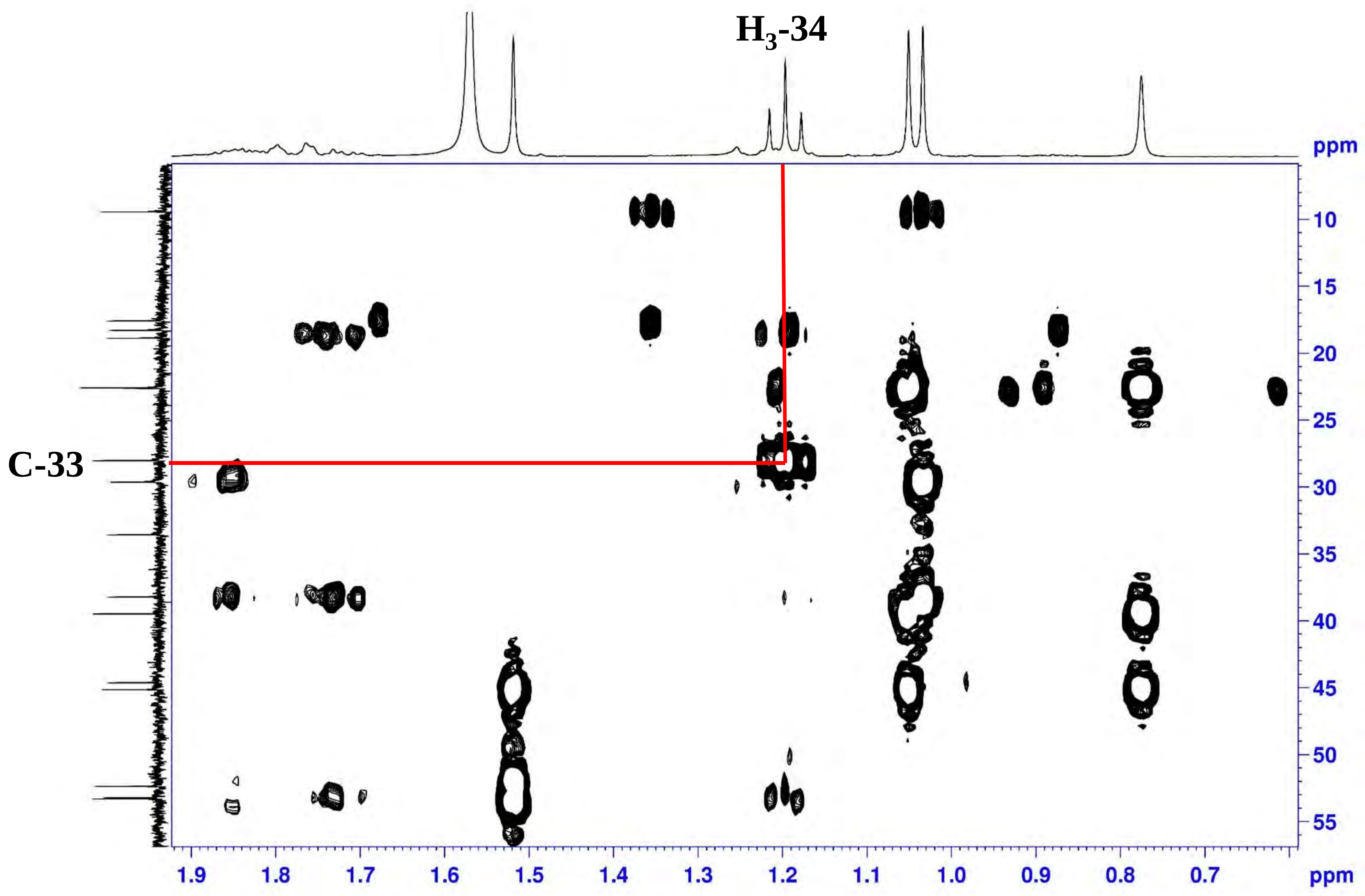

NOESY (400 MHz) spectrum of compound **2** in $CDCl_3$

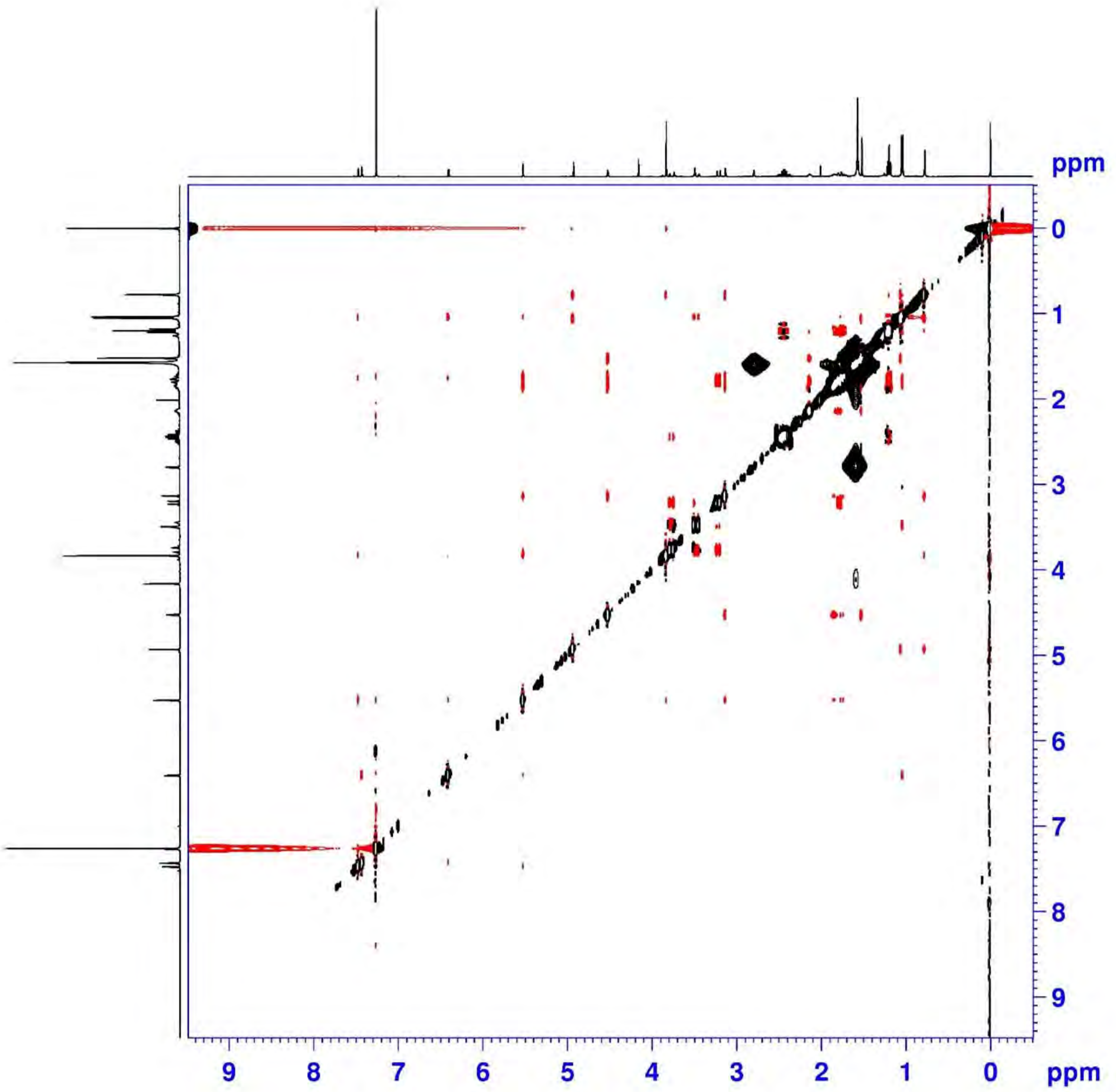


```
NAME          zjz-26=RT7sp
EXPNO                    7
PROCNO                   1
Date_             20160807
Time                  7.04
INSTRUM              spect
PROBHD   5 mm CPPBBO BB
PULPROG        noesygpphpp
TD                    2048
SOLVENT              CDCl3
NS                      64
DS                      32
SWH               4000.000 Hz
FIDRES            1.953125 Hz
AQ               0.2560500 sec
RG                   208.5
DW                 125.000 usec
DE                   10.00 usec
TE                   297.0 K
D0              0.00011036 sec
D1              1.99385595 sec
D8              0.30000001 sec
D11             0.03000000 sec
D12             0.00002000 sec
D16             0.00020000 sec
IN0             0.00025000 sec

======== CHANNEL f1 ========
SFO1           400.1318006 MHz
NUC1                    1H
P1                   11.50 usec
P2                   23.00 usec
P17                2500.00 usec
ND0                      1
TD                     256
SFO1              400.1318 MHz
FIDRES           15.625000 Hz
SW                   9.997 ppm
FnMODE          States-TPPI
SI                    1024
SF             400.1300064 MHz
WDW                  QSINE
SSB                      2
LB                    0.00 Hz
GB                       0
PC                    1.00
SI                    1024
MC2             States-TPPI
SF             400.1300079 MHz
WDW                  QSINE
SSB                      2
LB                    0.00 Hz
GB                       0
```

NOESY (400 MHz) spectrum of compound **2** in $CDCl_3$

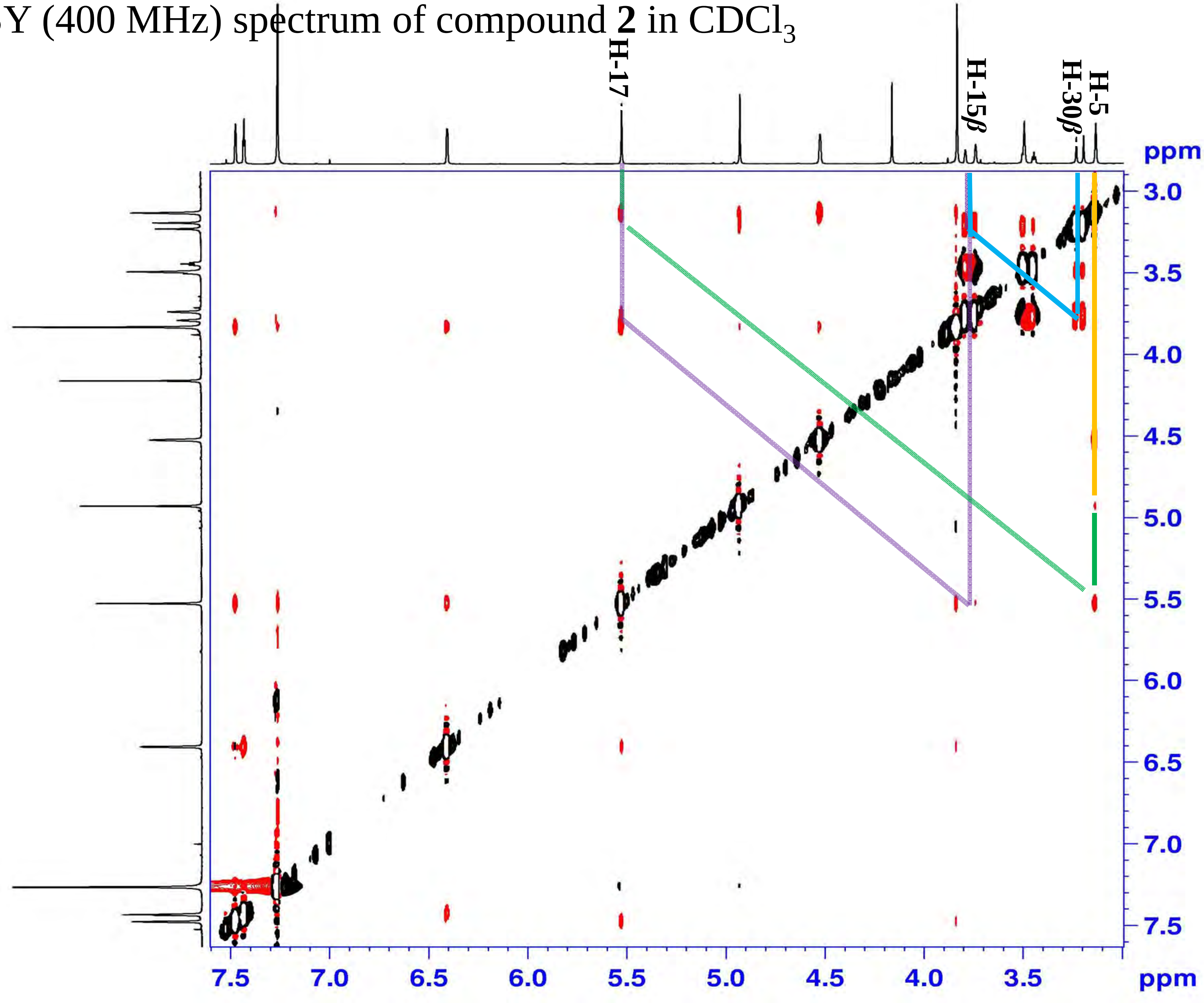

NOESY (400 MHz) spectrum of compound **2** in $CDCl_3$

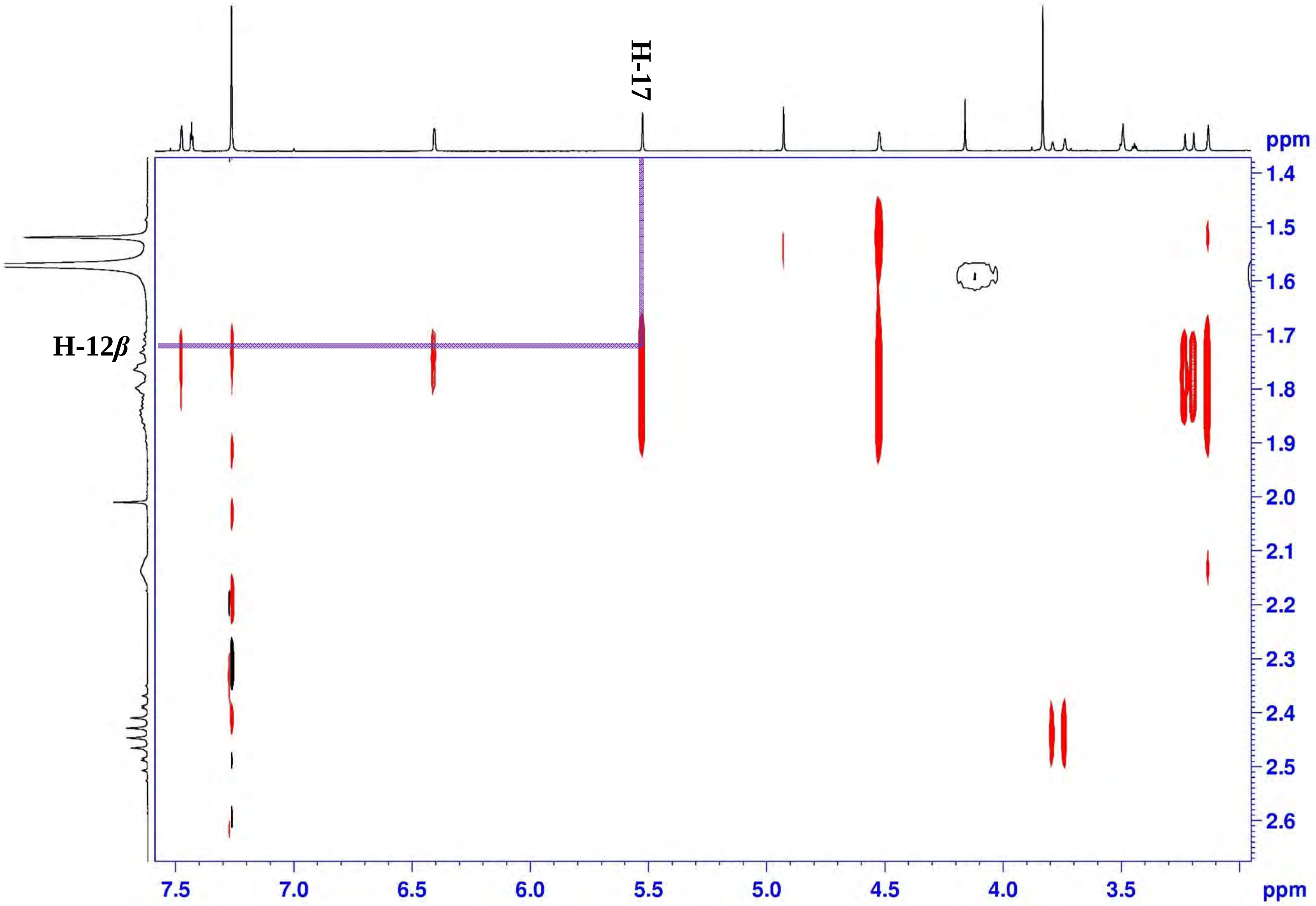

NOESY (400 MHz) spectrum of compound **2** in $CDCl_3$

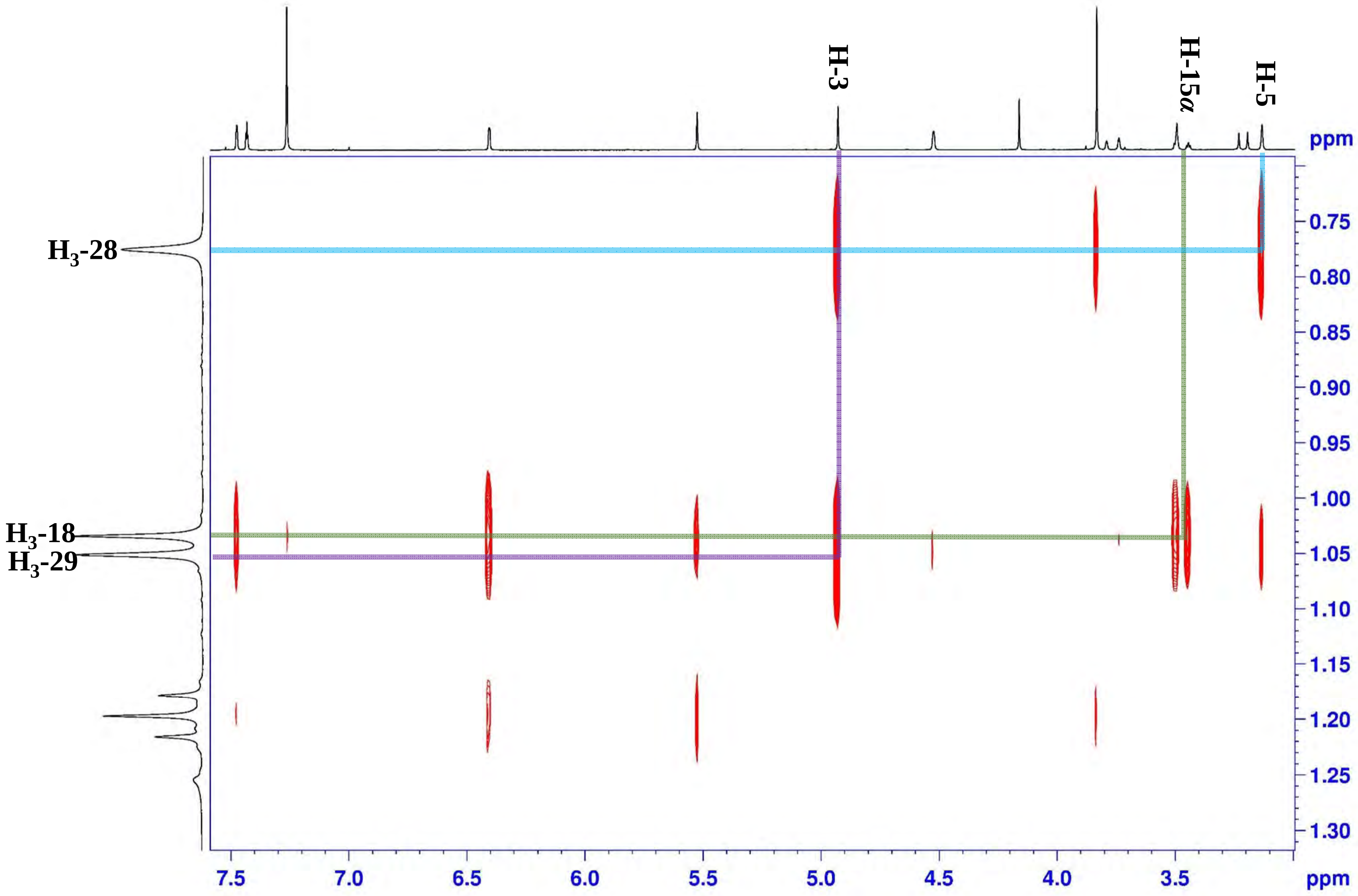

NOESY (400 MHz) spectrum of compound **2** in $CDCl_3$

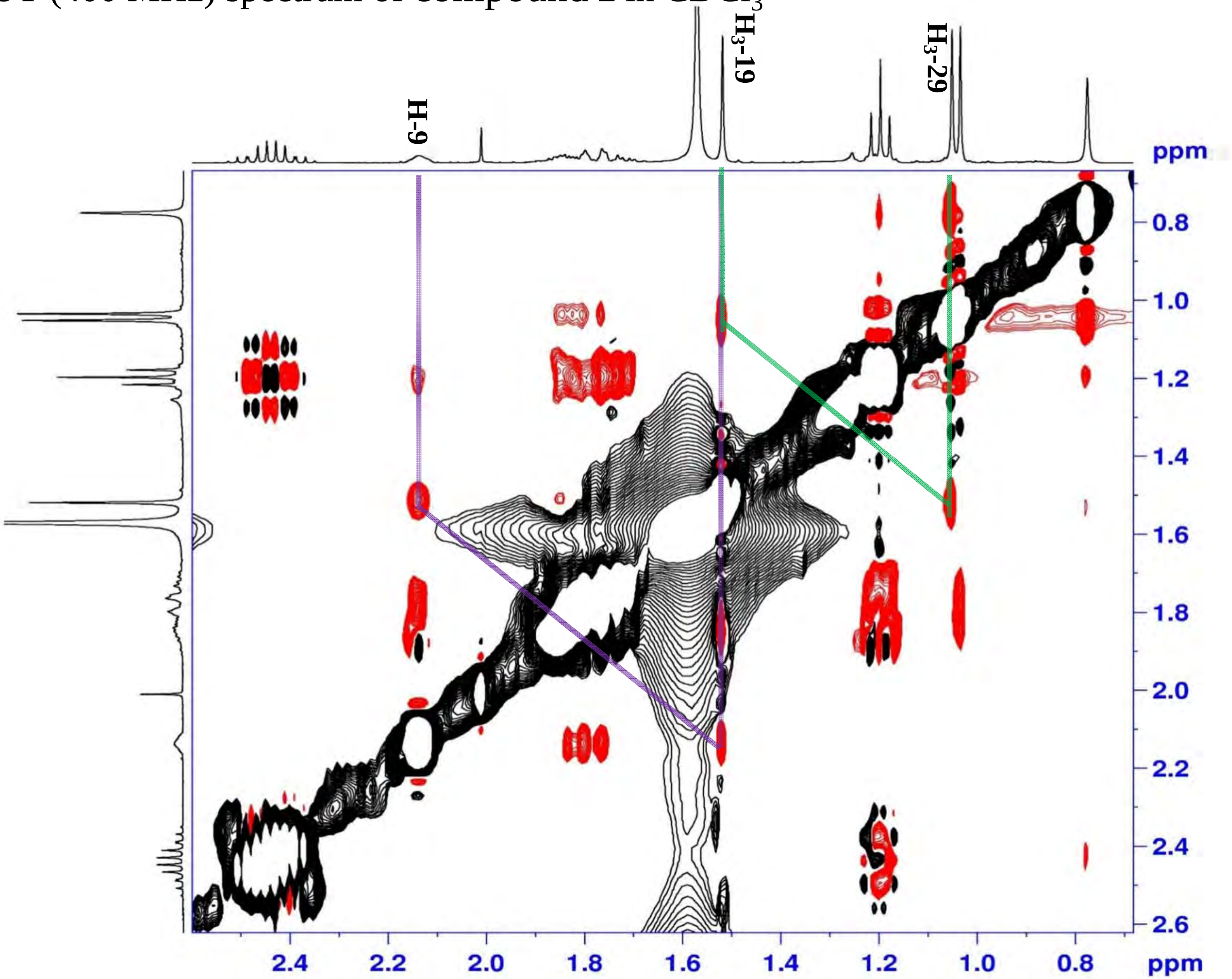

HR-ESIMS for compound **3**

Mass Spectrum SmartFormula Report

Analysis Info

		Acquisition Date	5/19/2016 9:01:46 AM
Analysis Name	D:\Data\MS\data\201605\liwanshan_lws-63-1_pos_14_01_1861.d		
Method	LC_Direct Infusion_pos_100-1000mz.m	Operator	SCSIO
Sample Name	liwanshan_lws-63-1_pos	Instrument / Ser#	maXis 29
Comment			

Acquisition Parameter

Source Type	ESI	Ion Polarity	Positive	Set Nebulizer	0.4 Bar
Focus	Active	Set Capillary	4500 V	Set Dry Heater	180 °C
Scan Begin	100 m/z	Set End Plate Offset	-500 V	Set Dry Gas	4.0 l/min
Scan End	2000 m/z	Set Collision Cell RF	800.0 Vpp	Set Divert Valve	Waste

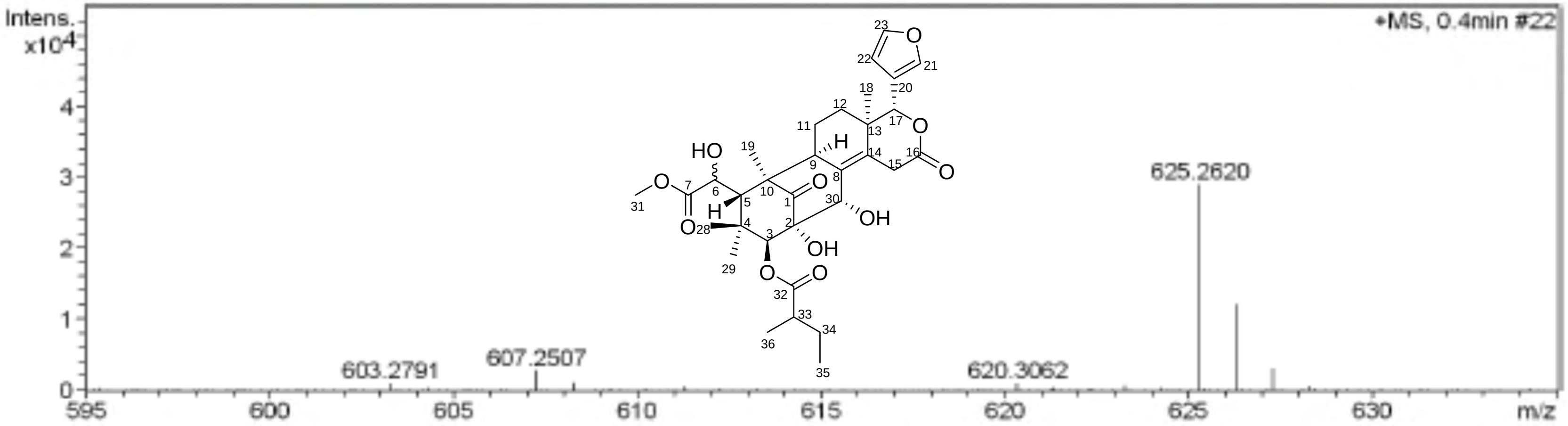

Meas. m/z	#	Formula	Score	m/z	err [ppm]	err [mDa]	mSigma	rdb	e⁻ Conf	N-Rule
603.2791	1	C 32 H 43 O 11	100.00	603.2800	1.4	0.9	47.9	11.5	even	ok
625.2620	1	C 32 H 42 Na O 11	100.00	625.2619	-0.1	-0.1	31.7	11.5	even	ok
1227.5357	1	C 64 H 84 Na O 22	90.63	1227.5346	-0.9	-1.1	20.7	22.5	even	ok

HR-ESIMS for compound **3**

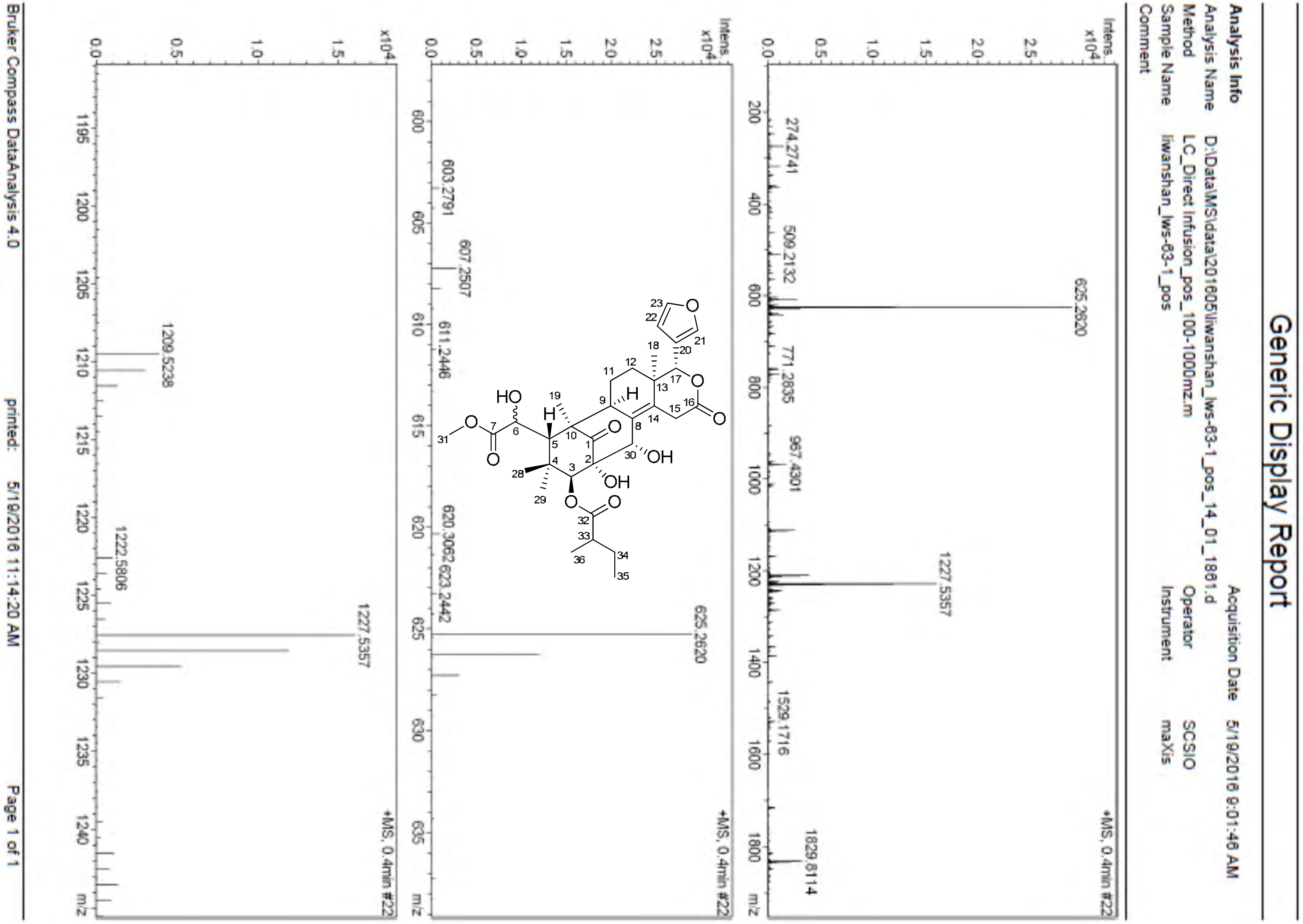

^{1}H NMR (400 MHz) spectrum of compound **3** in $CDCl_3$

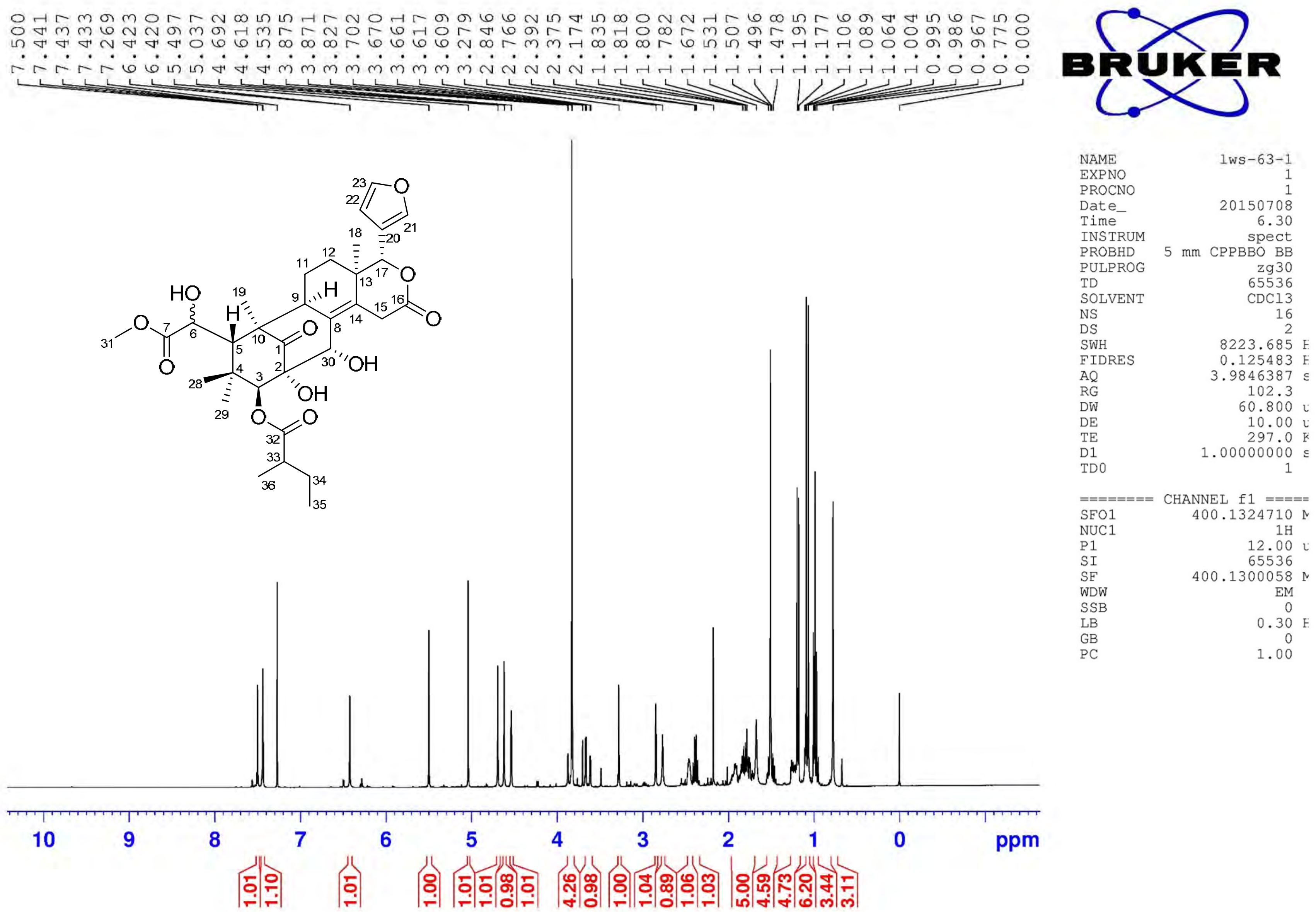

^{1}H NMR (400 MHz) spectrum of compound **3** in $CDCl_3$

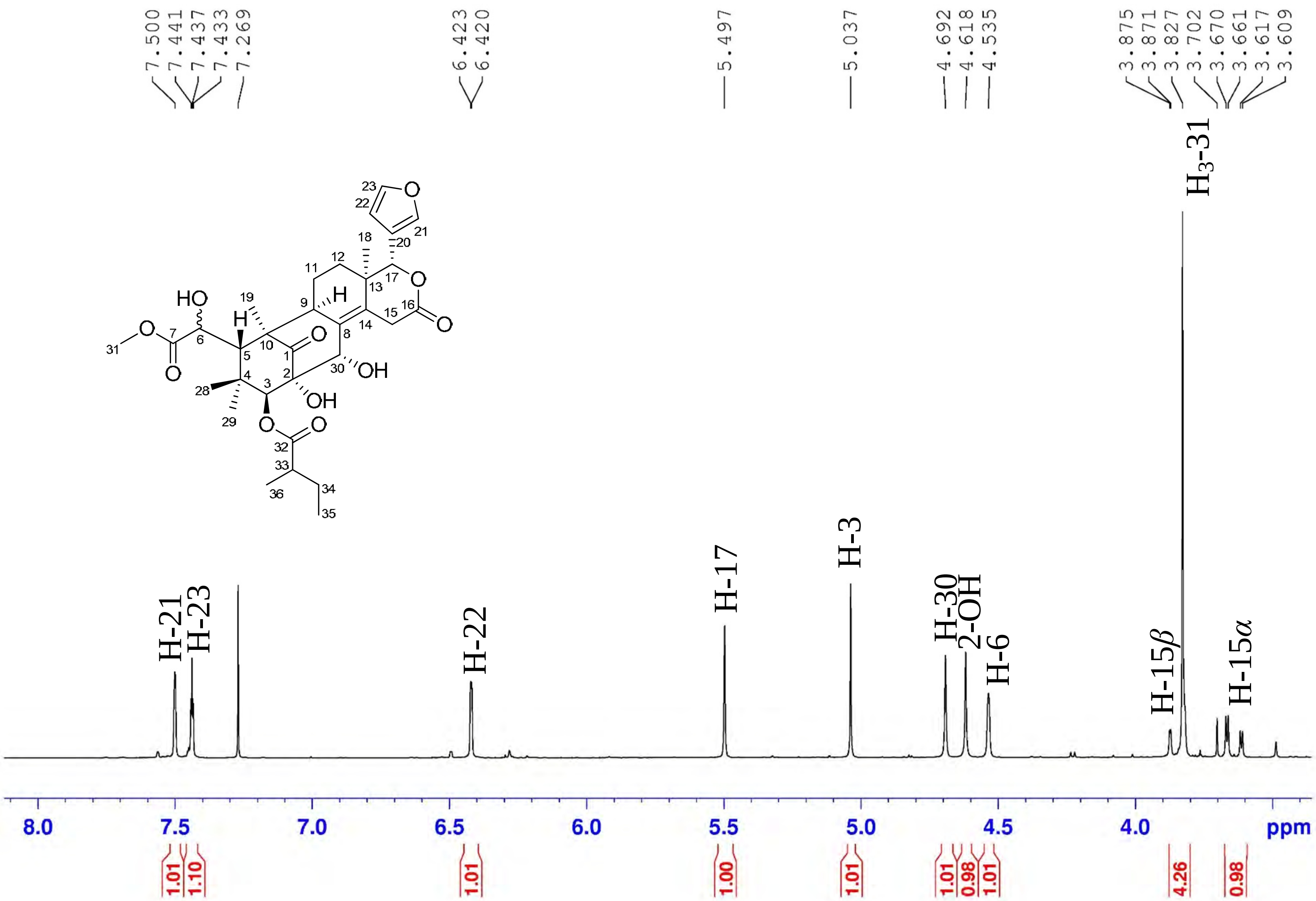

^{1}H NMR (400 MHz) spectrum of compound **3** in $CDCl_3$

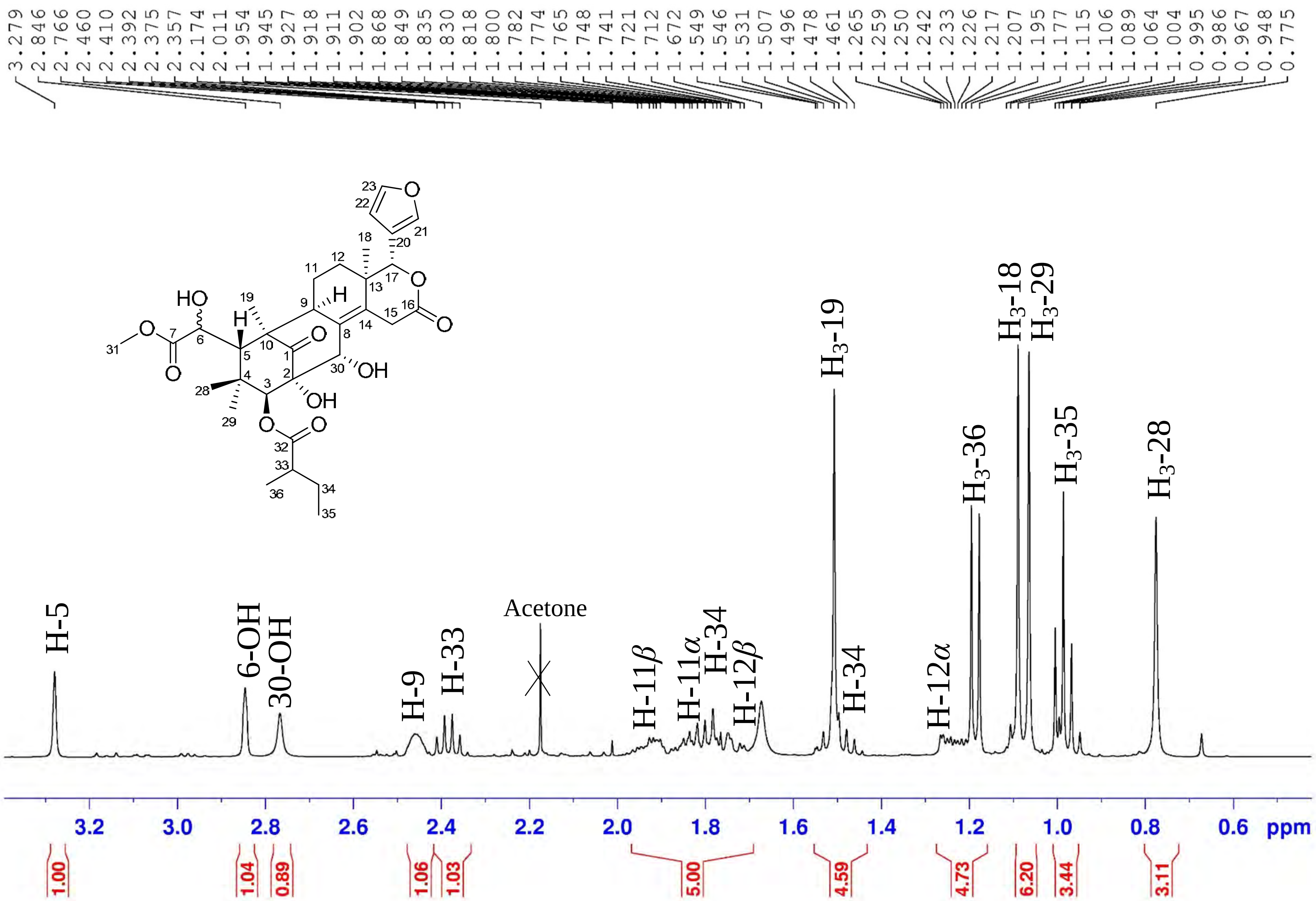

^{13}C NMR (100 MHz) spectrum of compound **3** in $CDCl_3$

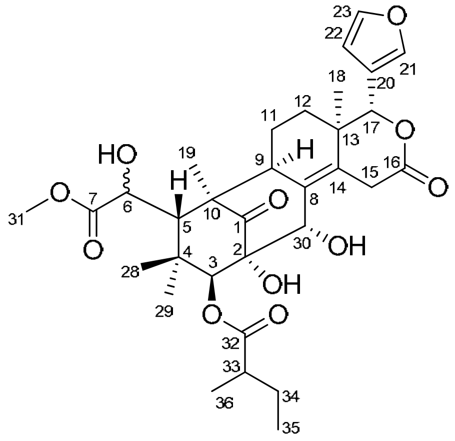

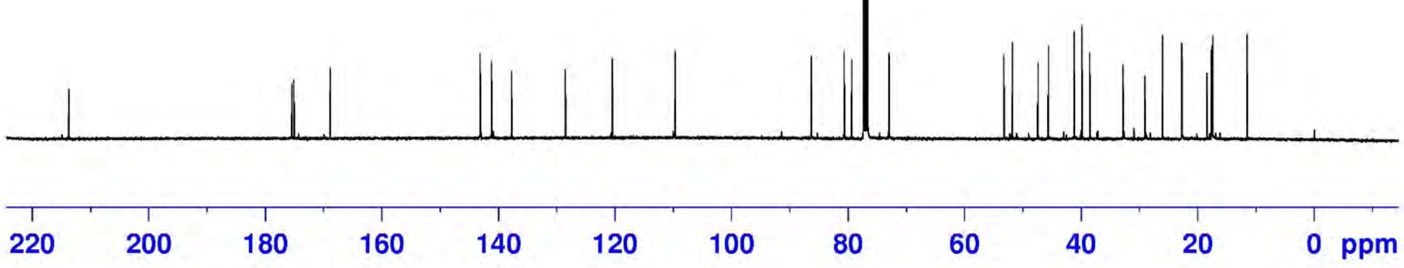

^{13}C NMR (100 MHz) spectrum of compound **3** in $CDCl_3$

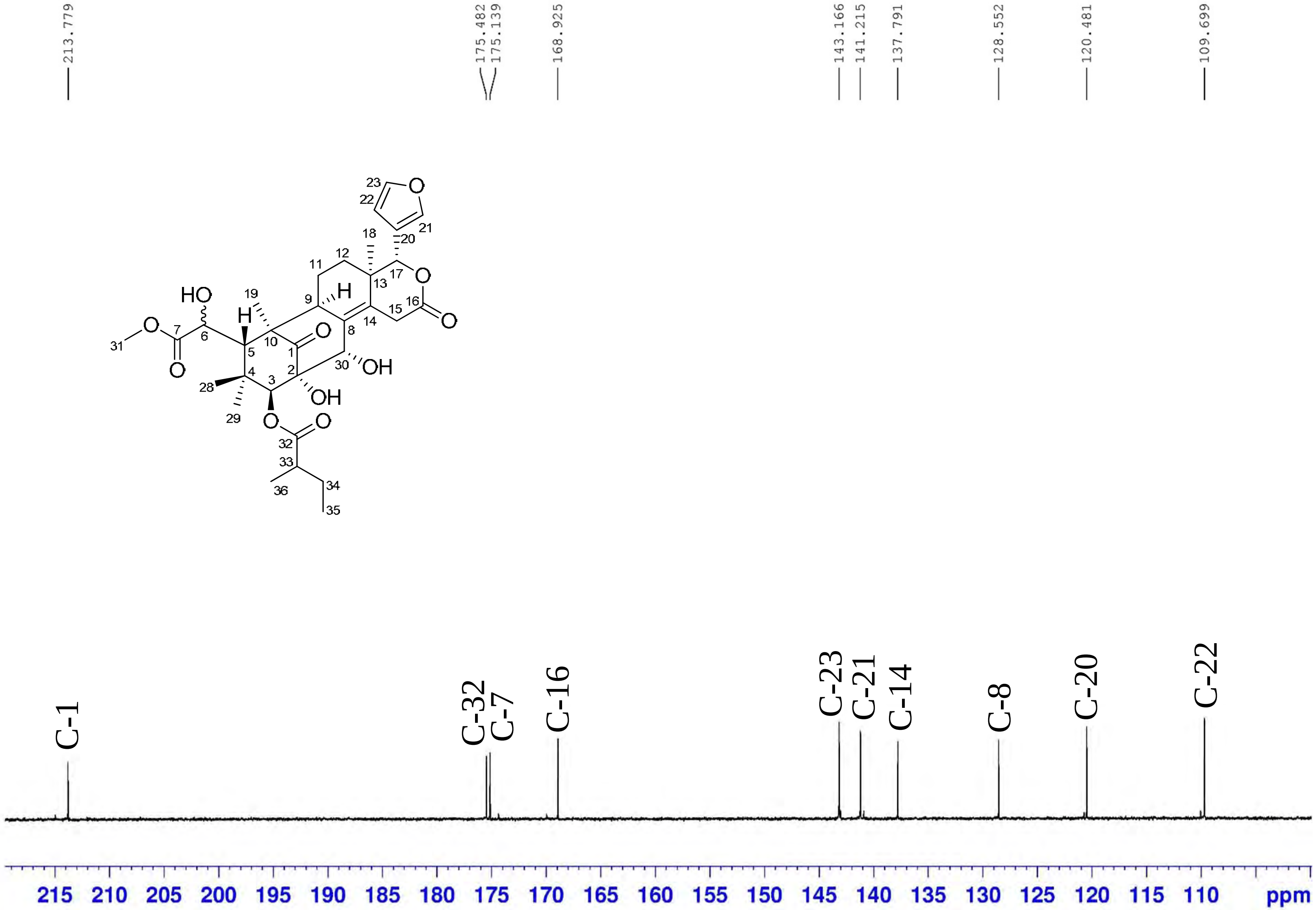

^{13}C NMR (100 MHz) spectrum of compound **3** in $CDCl_3$

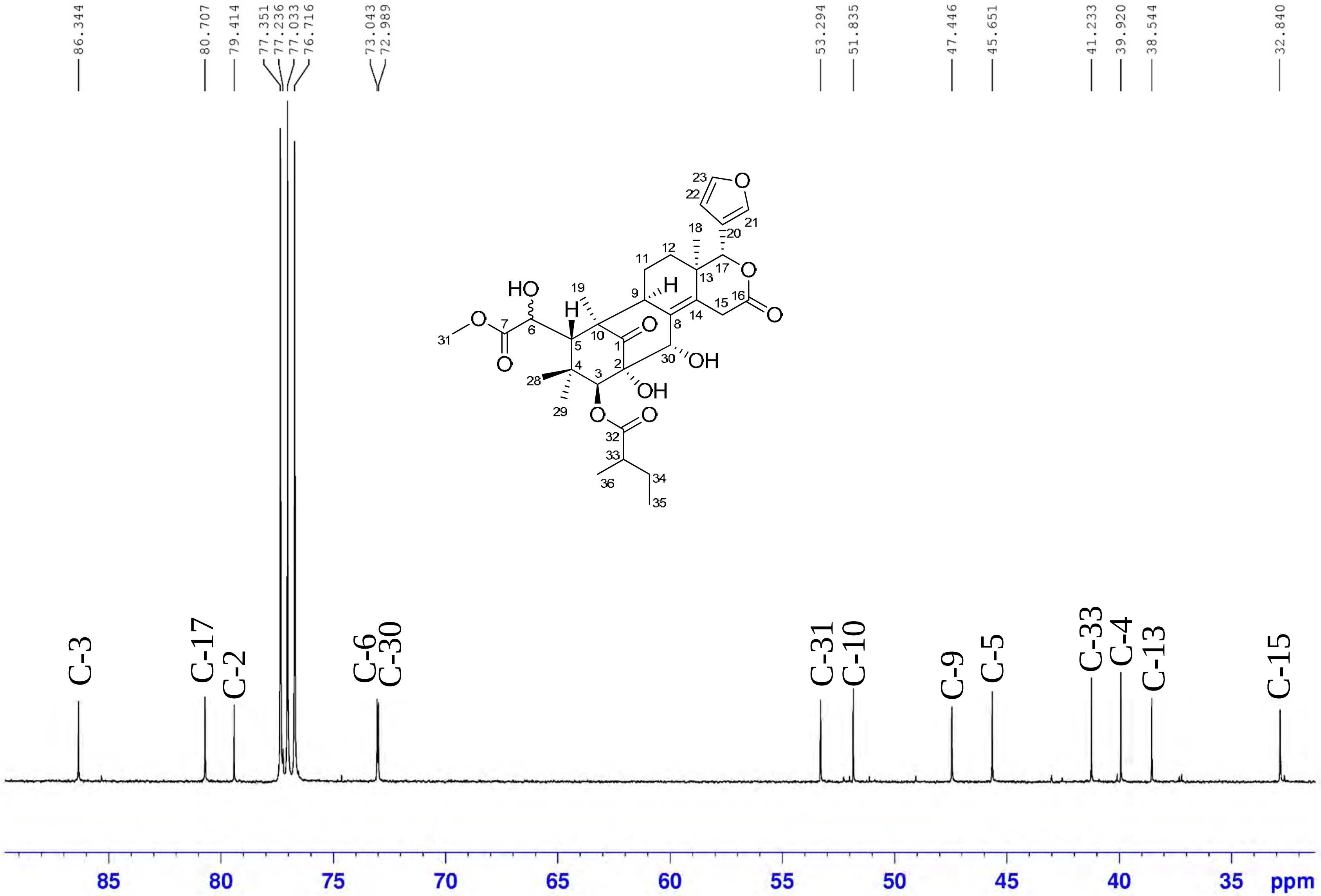

^{13}C NMR (100 MHz) spectrum of compound **3** in $CDCl_3$

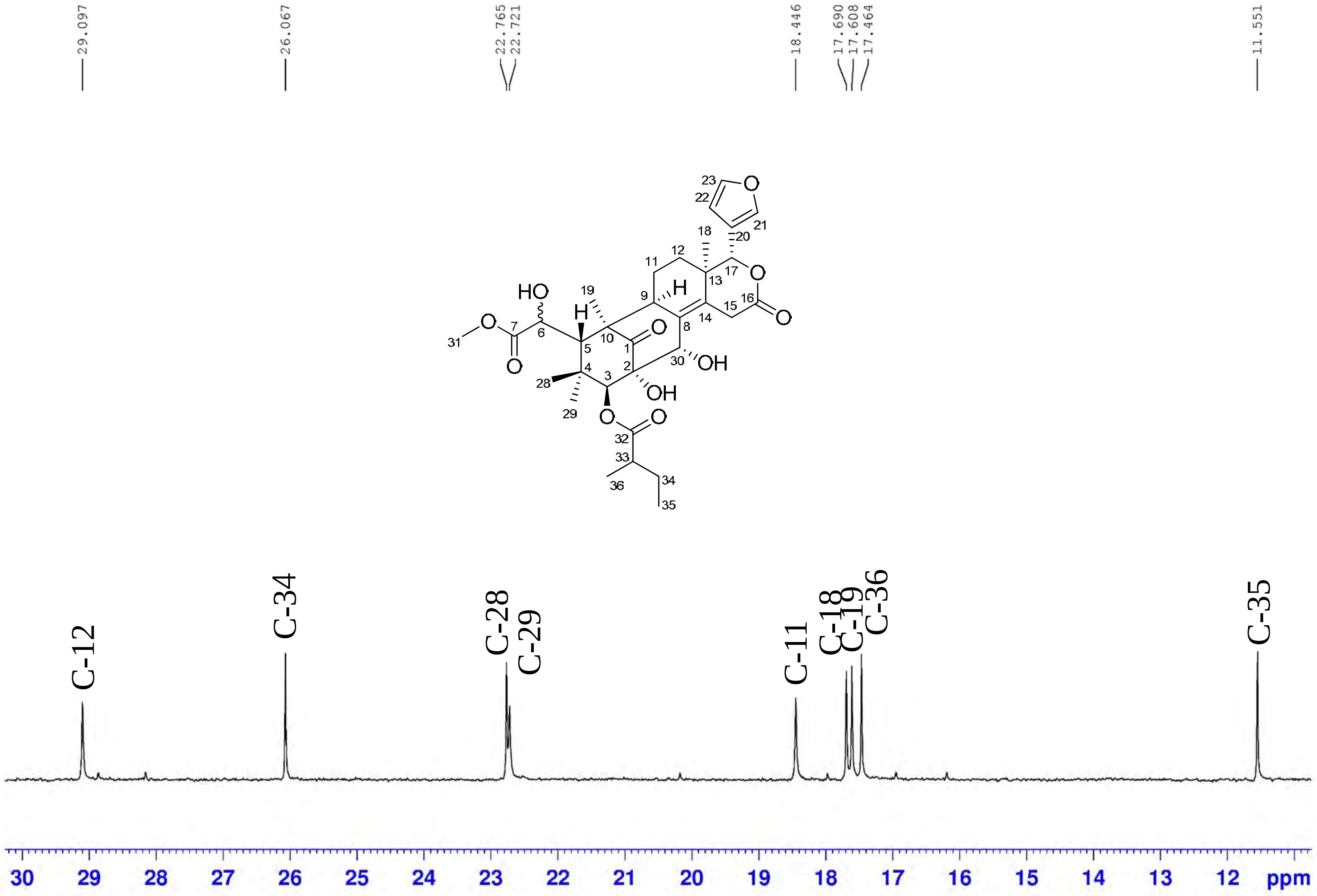

DEPT135 (100 MHz) spectrum of compound **3** in $CDCl_3$

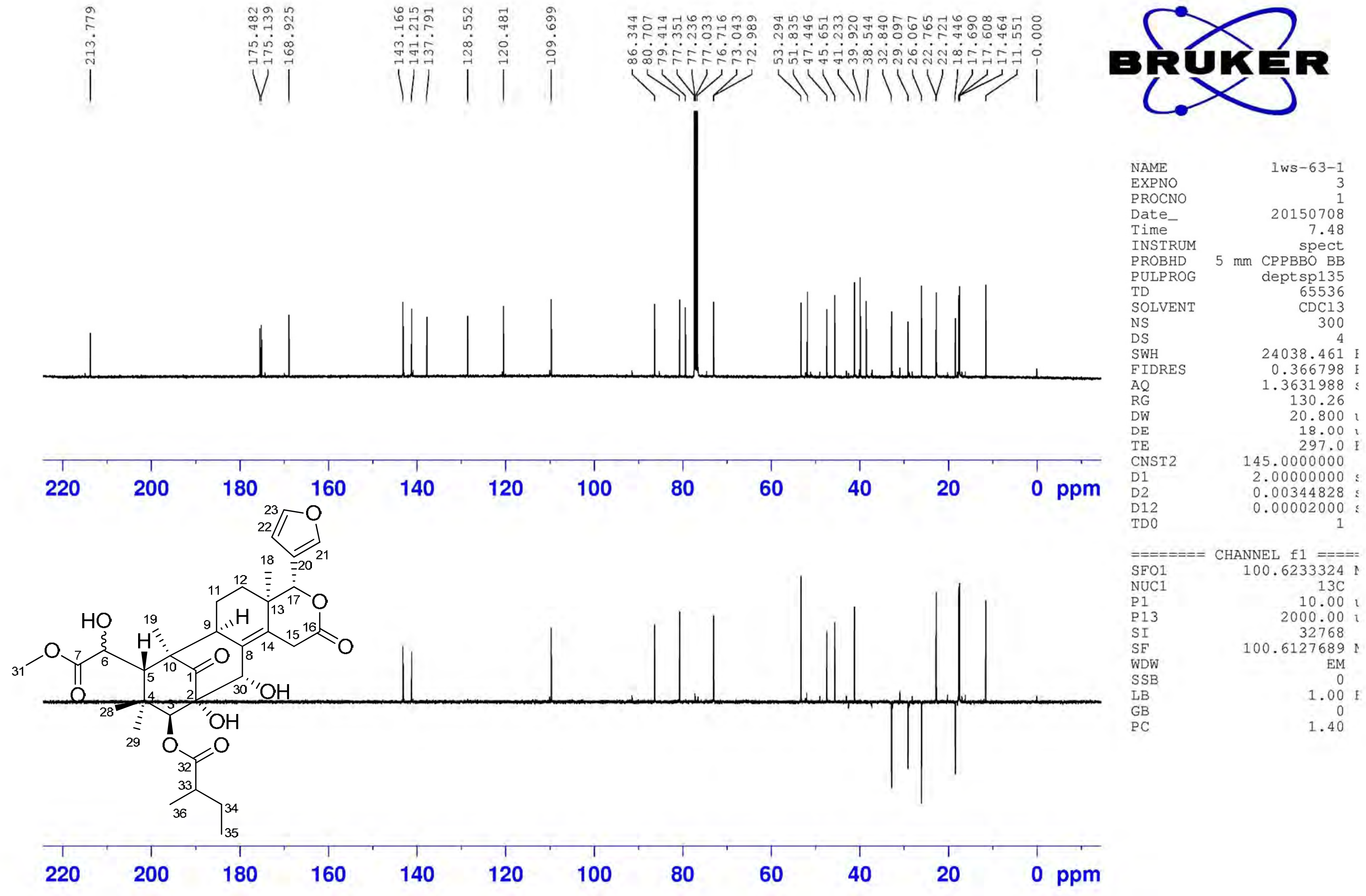

DEPT135 (100 MHz) spectrum of compound **3** in $CDCl_3$

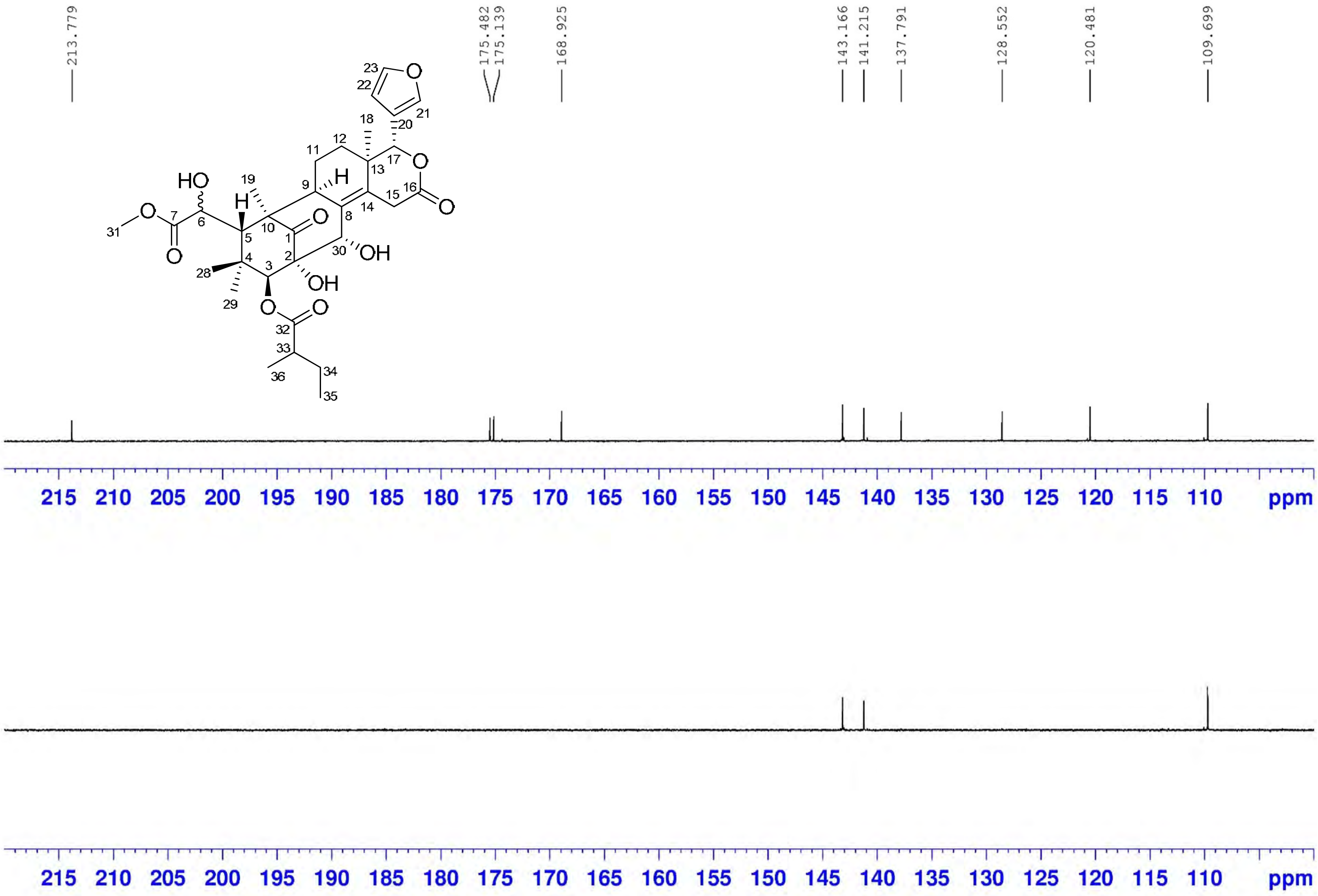

DEPT135 (100 MHz) spectrum of compound **3** in $CDCl_3$

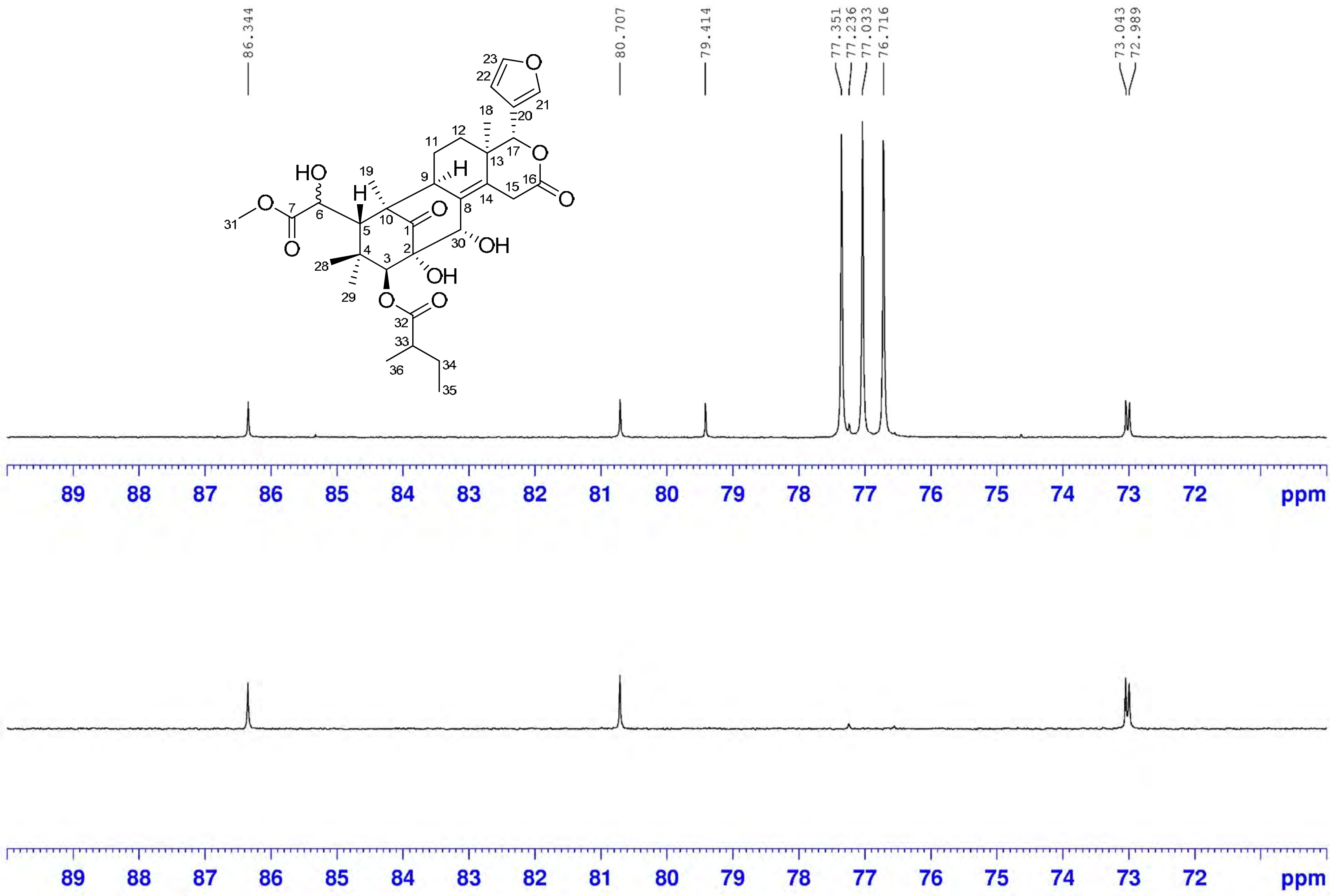

DEPT135 (100 MHz) spectrum of compound **3** in $CDCl_3$

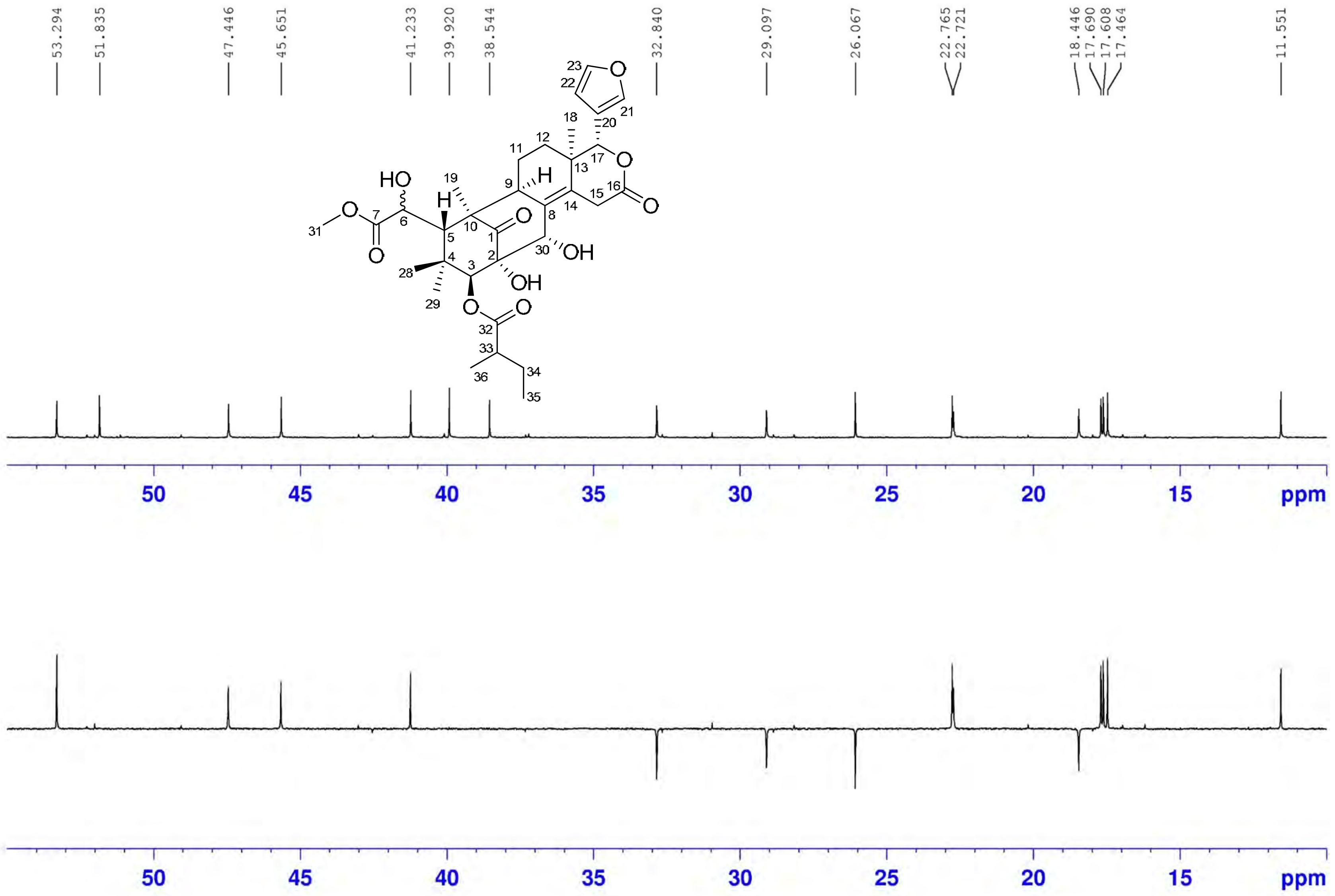

^{1}H-^{1}H COSY (400 MHz) spectrum of compound **3** in $CDCl_3$

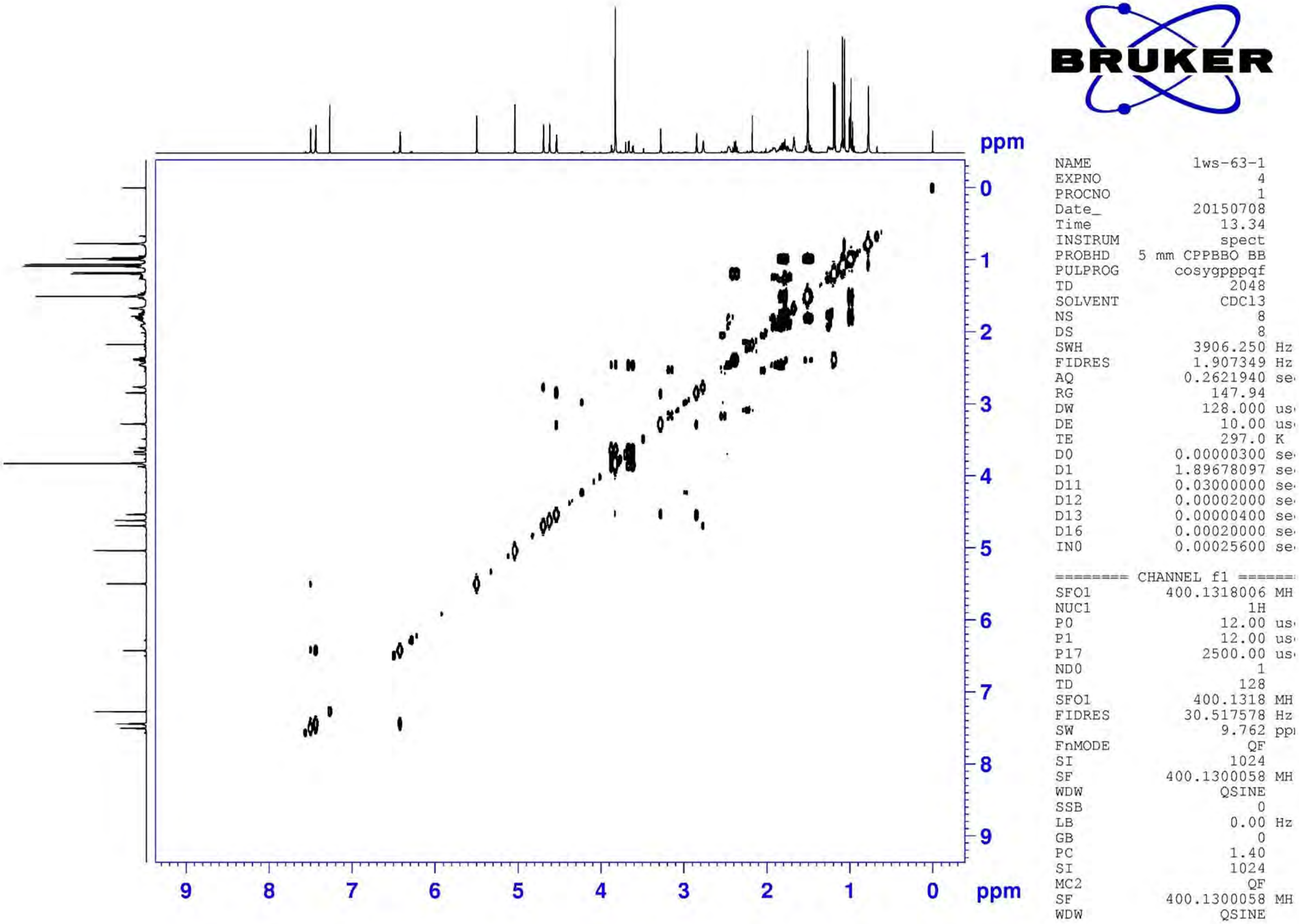

^{1}H-^{1}H COSY (400 MHz) spectrum of compound **3** in $CDCl_3$

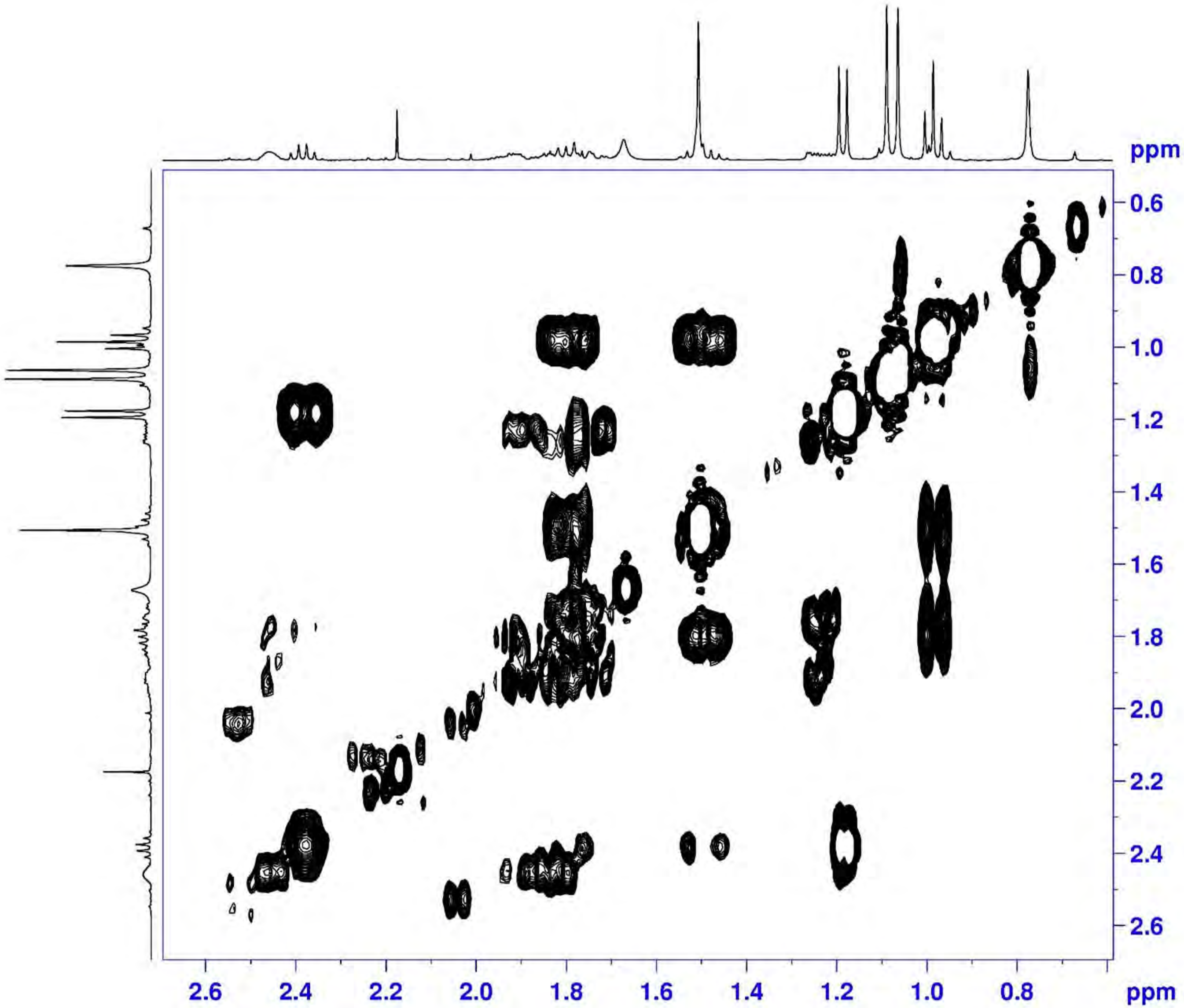

^1H-^1H COSY (400 MHz) spectrum of compound **3** in $CDCl_3$

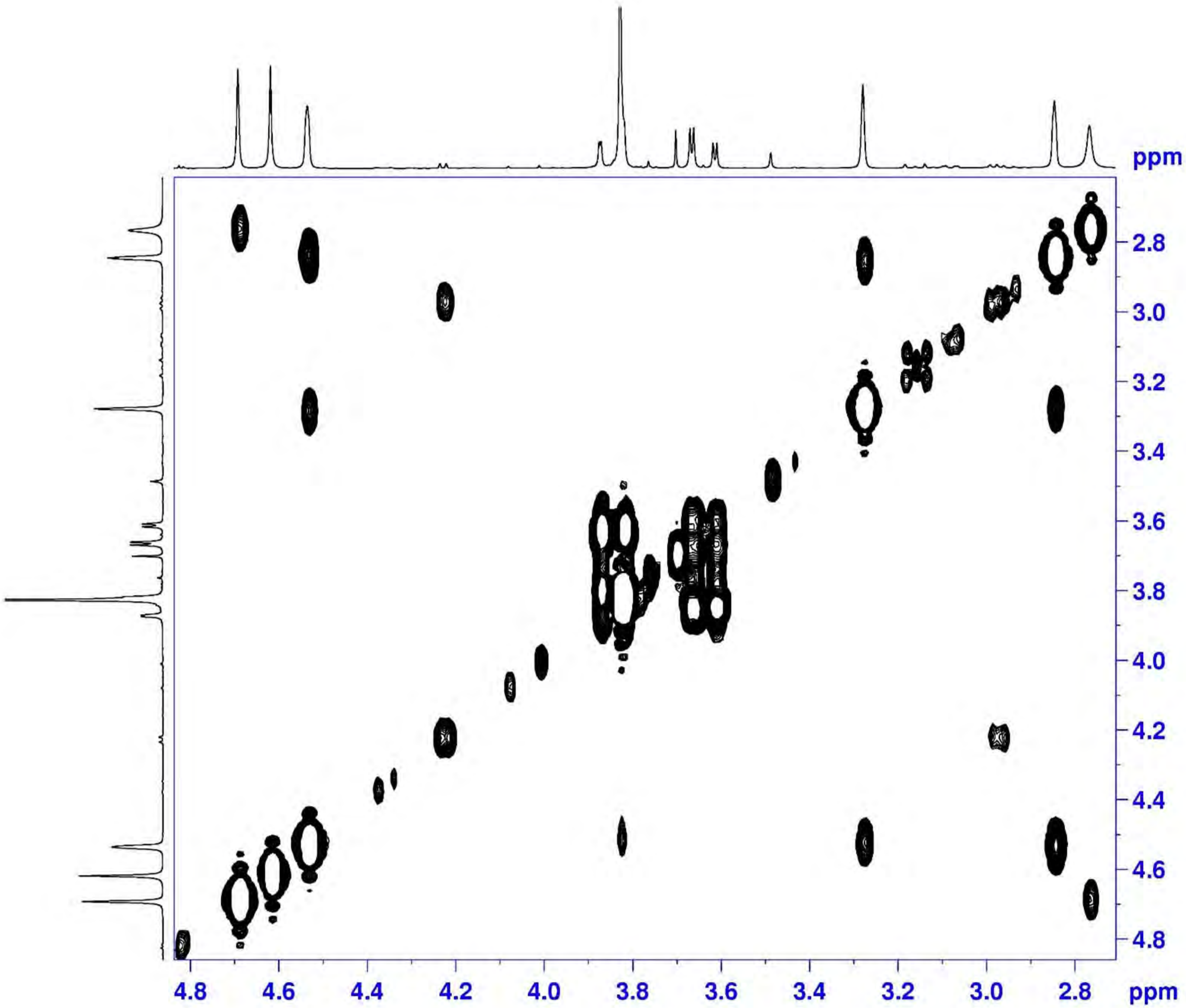

^1H-^1H COSY (400 MHz) spectrum of compound **3** in $CDCl_3$

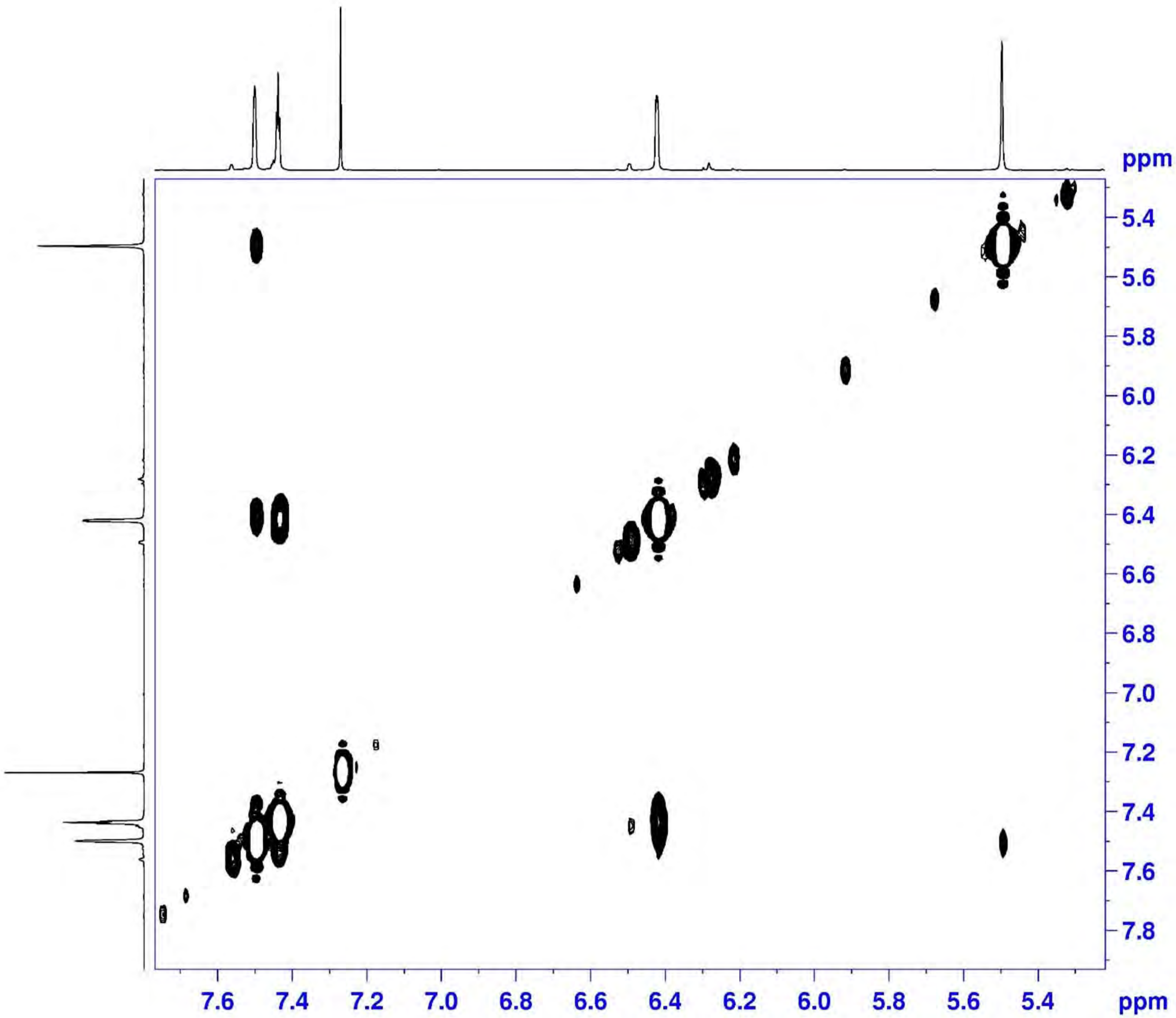

HSQC (400 MHz) spectrum of compound **3** in $CDCl_3$

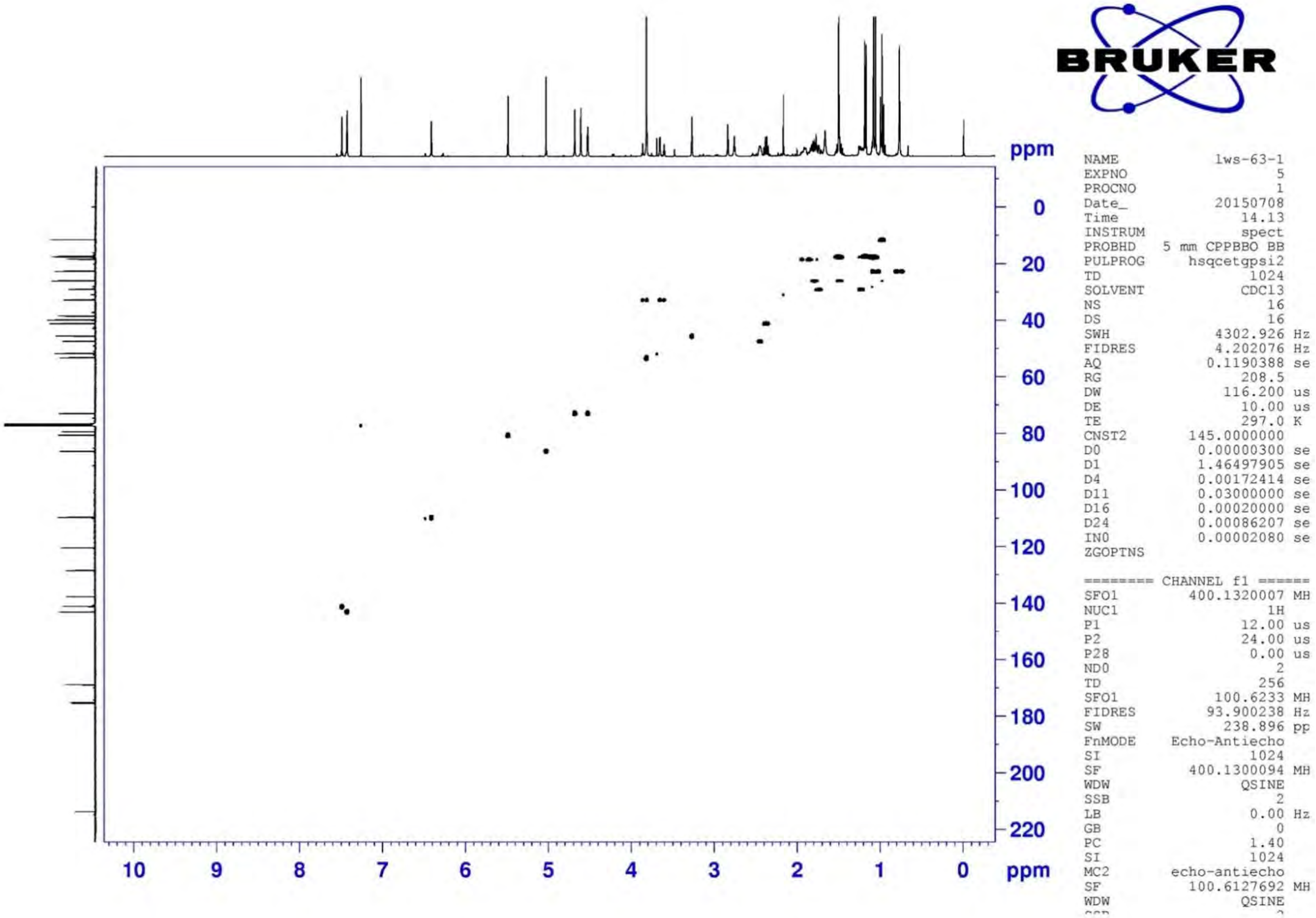

HSQC (400 MHz) spectrum of compound **3** in $CDCl_3$

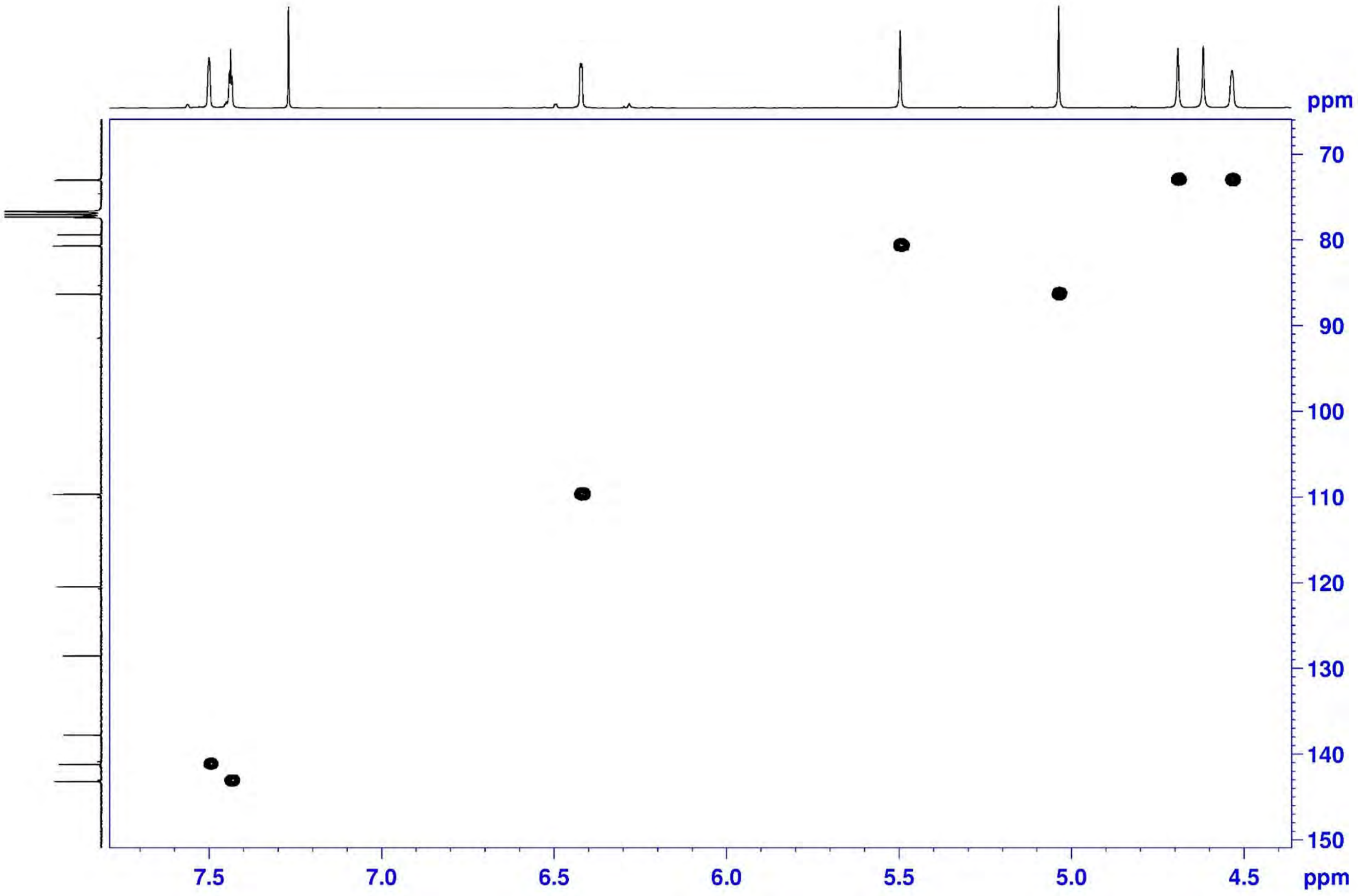

HSQC (400 MHz) spectrum of compound **3** in $CDCl_3$

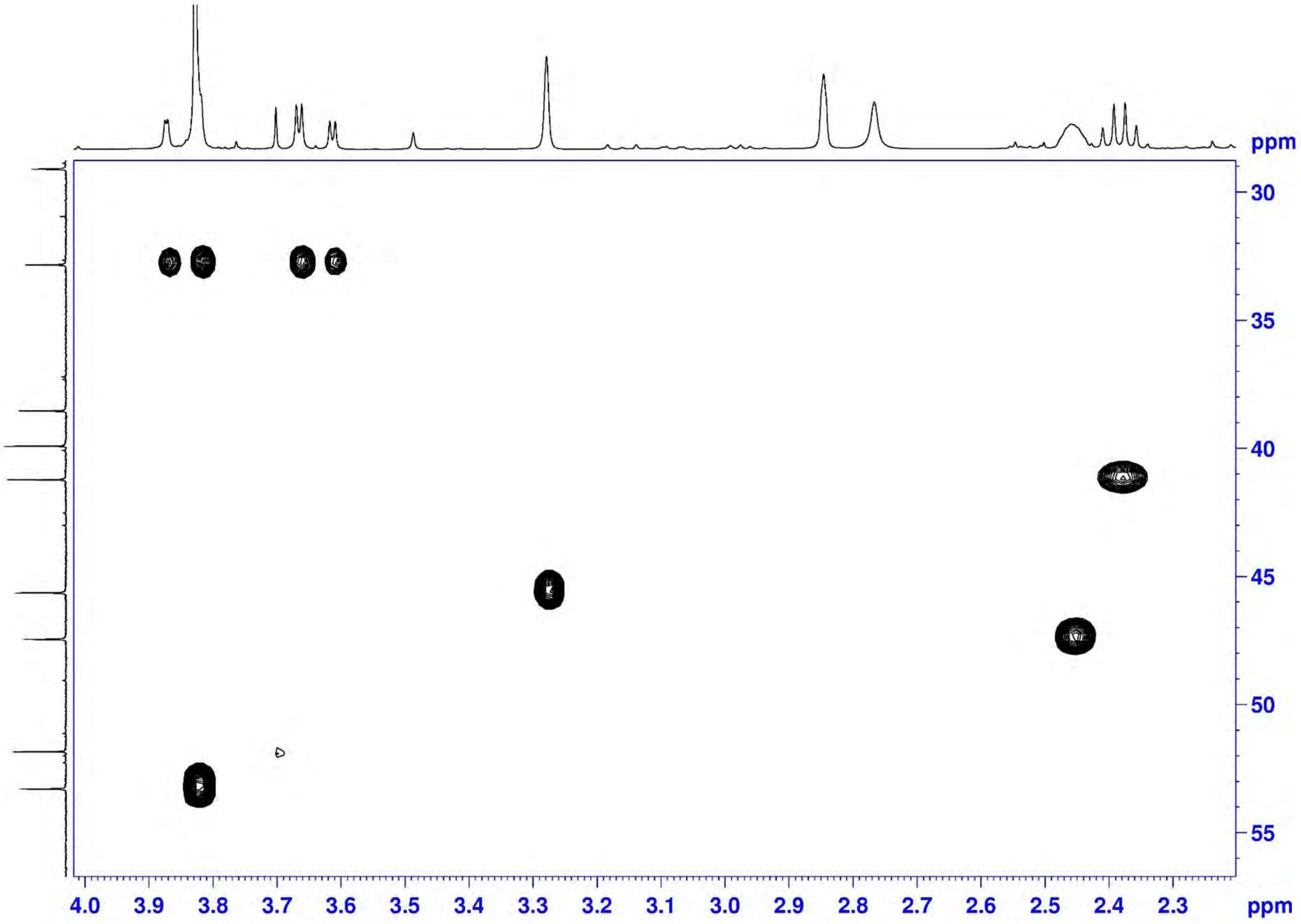

HSQC (400 MHz) spectrum of compound **3** in $CDCl_3$

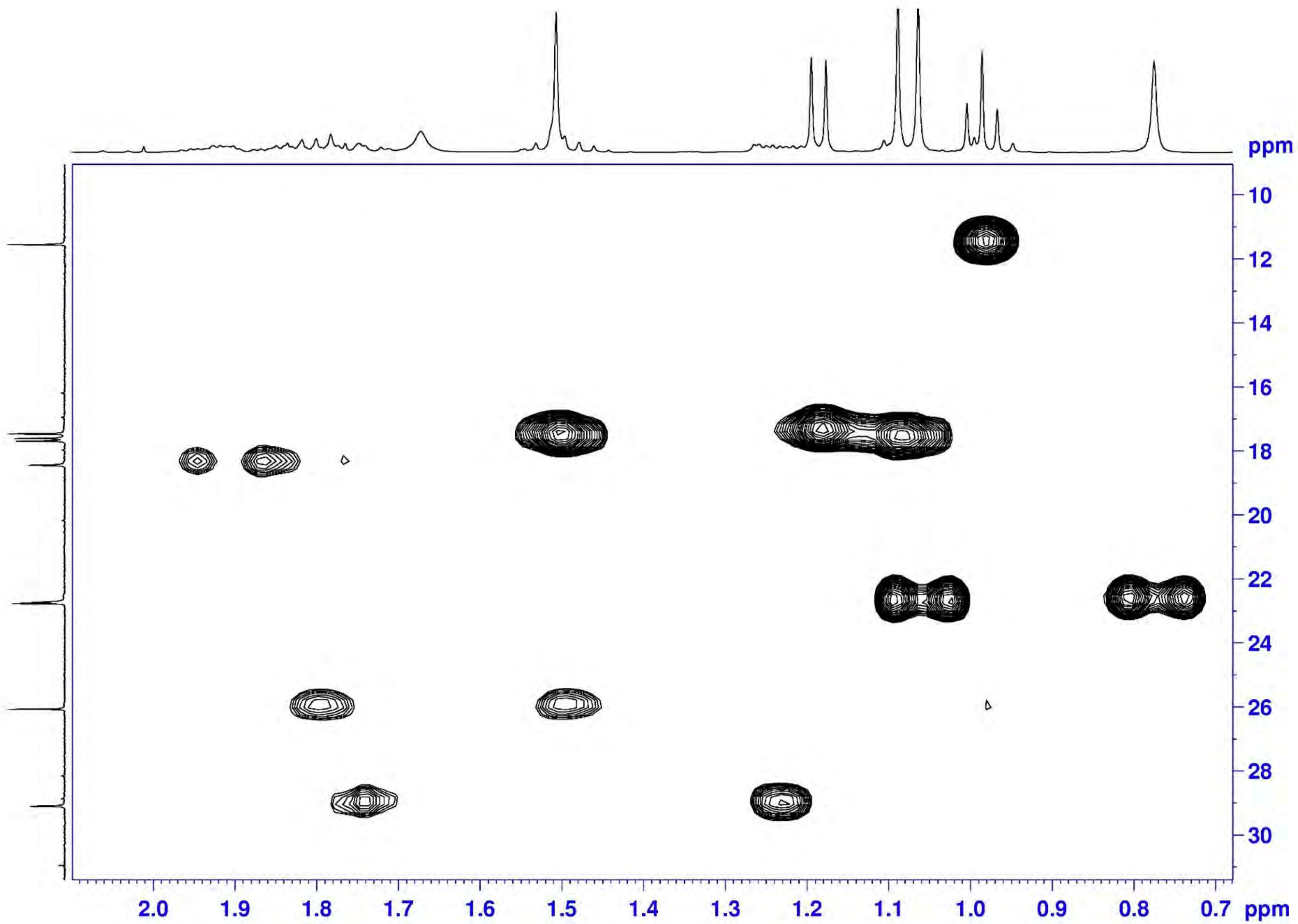

HMBC (400 MHz) spectrum of compound **3** in $CDCl_3$

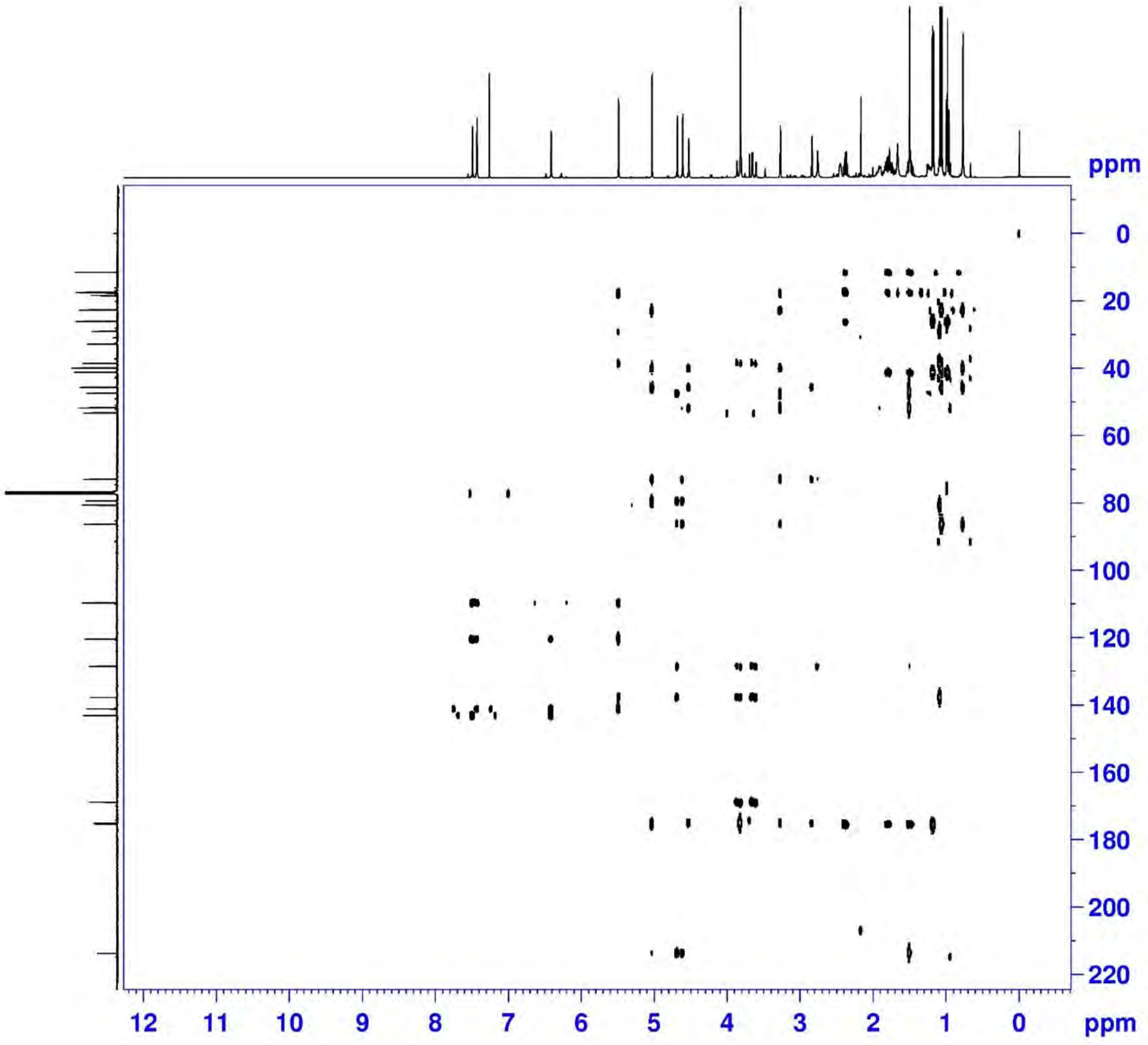

HMBC (400 MHz) spectrum of compound **3** in $CDCl_3$

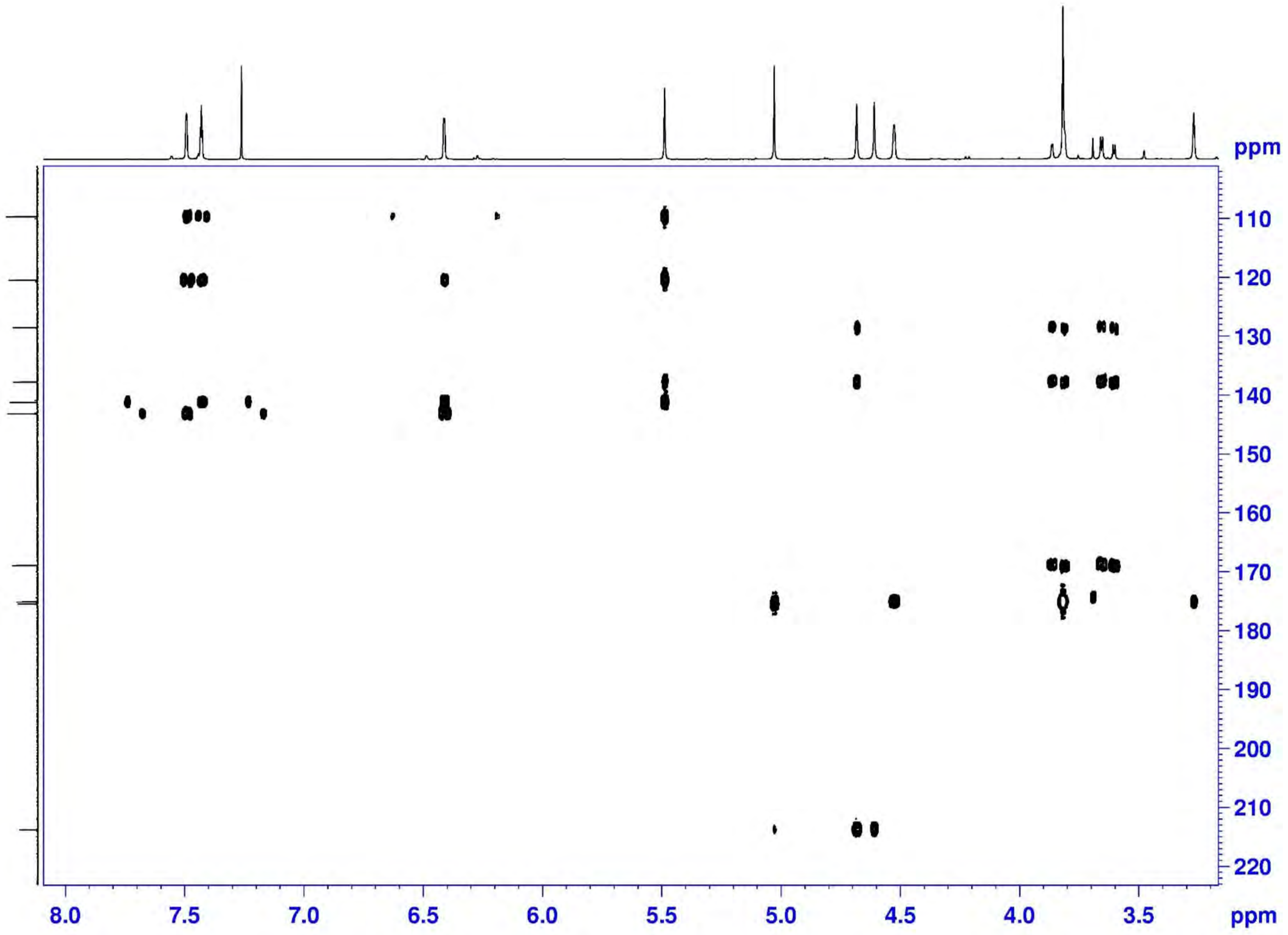

HMBC (400 MHz) spectrum of compound **3** in $CDCl_3$

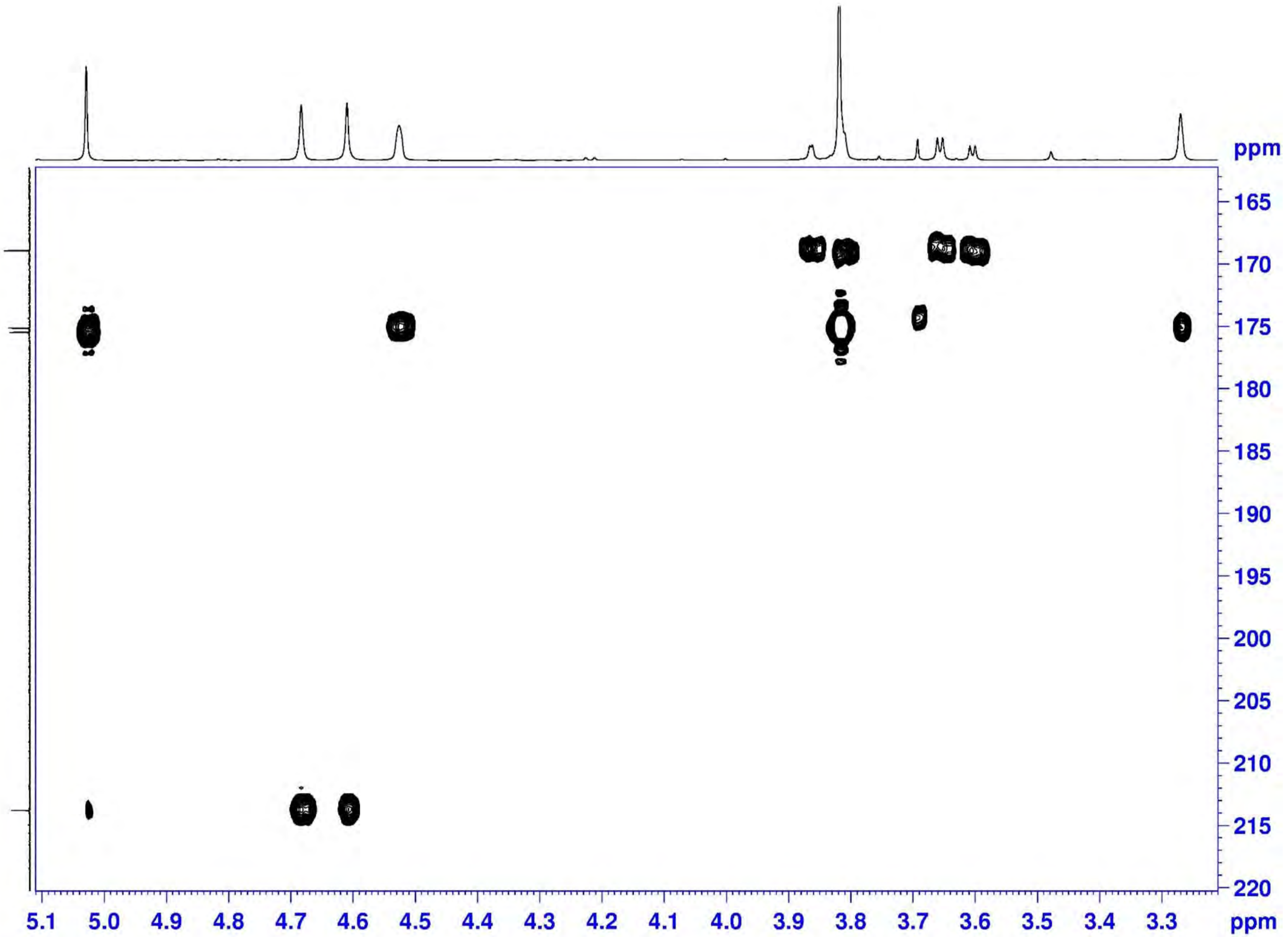

HMBC (400 MHz) spectrum of compound **3** in $CDCl_3$

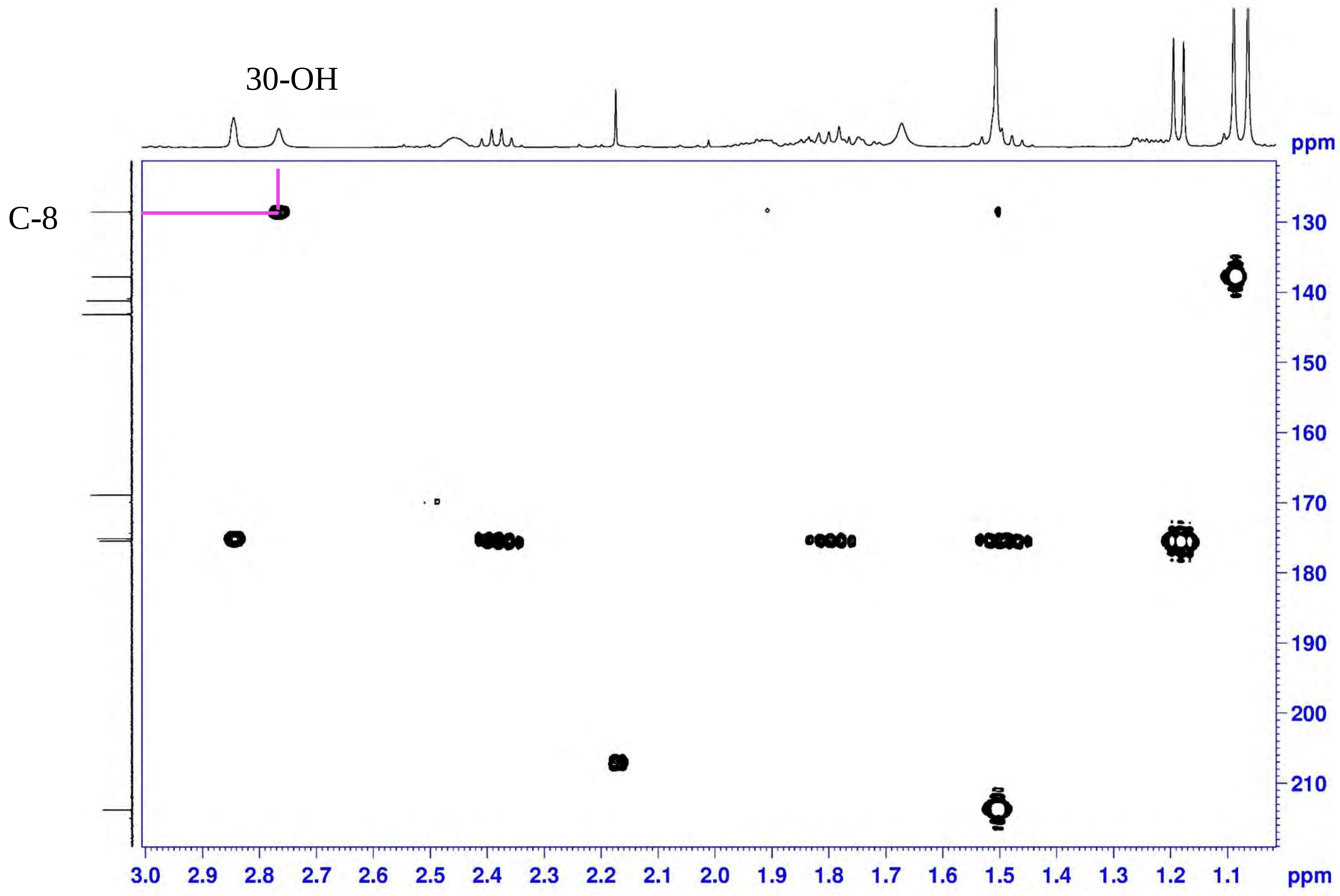

HMBC (400 MHz) spectrum of compound **3** in $CDCl_3$

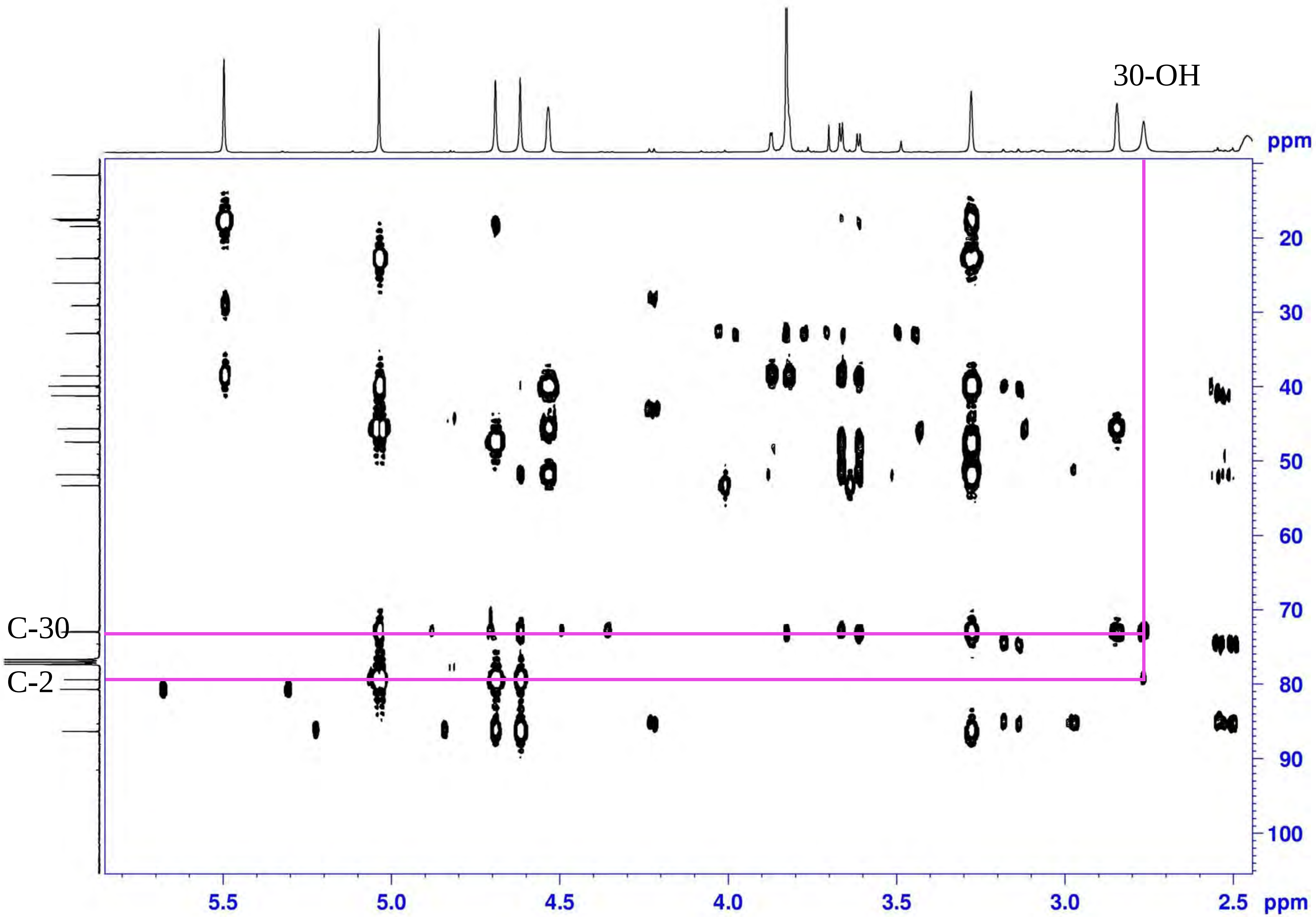

HMBC (400 MHz) spectrum of compound **3** in $CDCl_3$

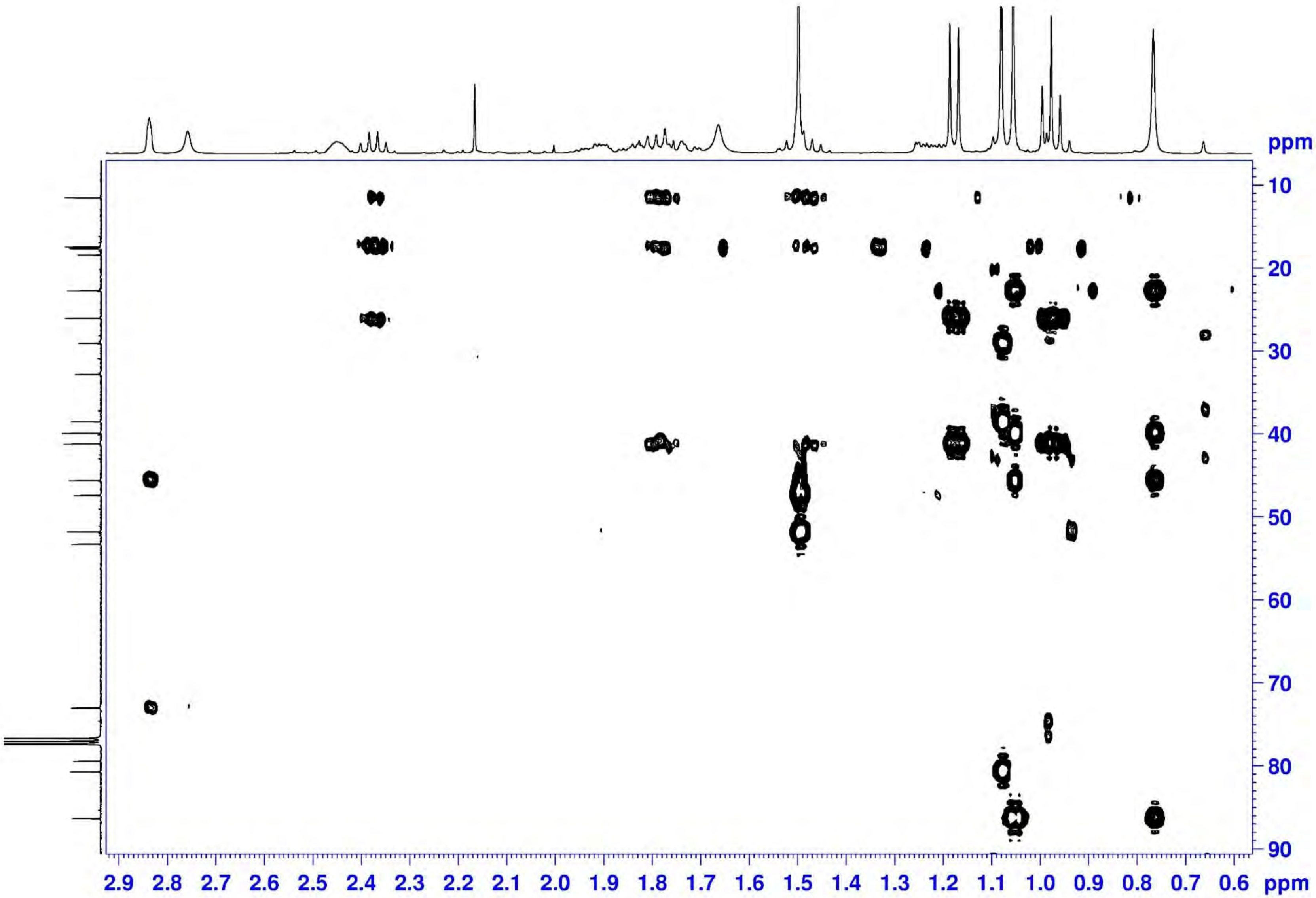

NOESY (400 MHz) spectrum of compound **3** in $CDCl_3$

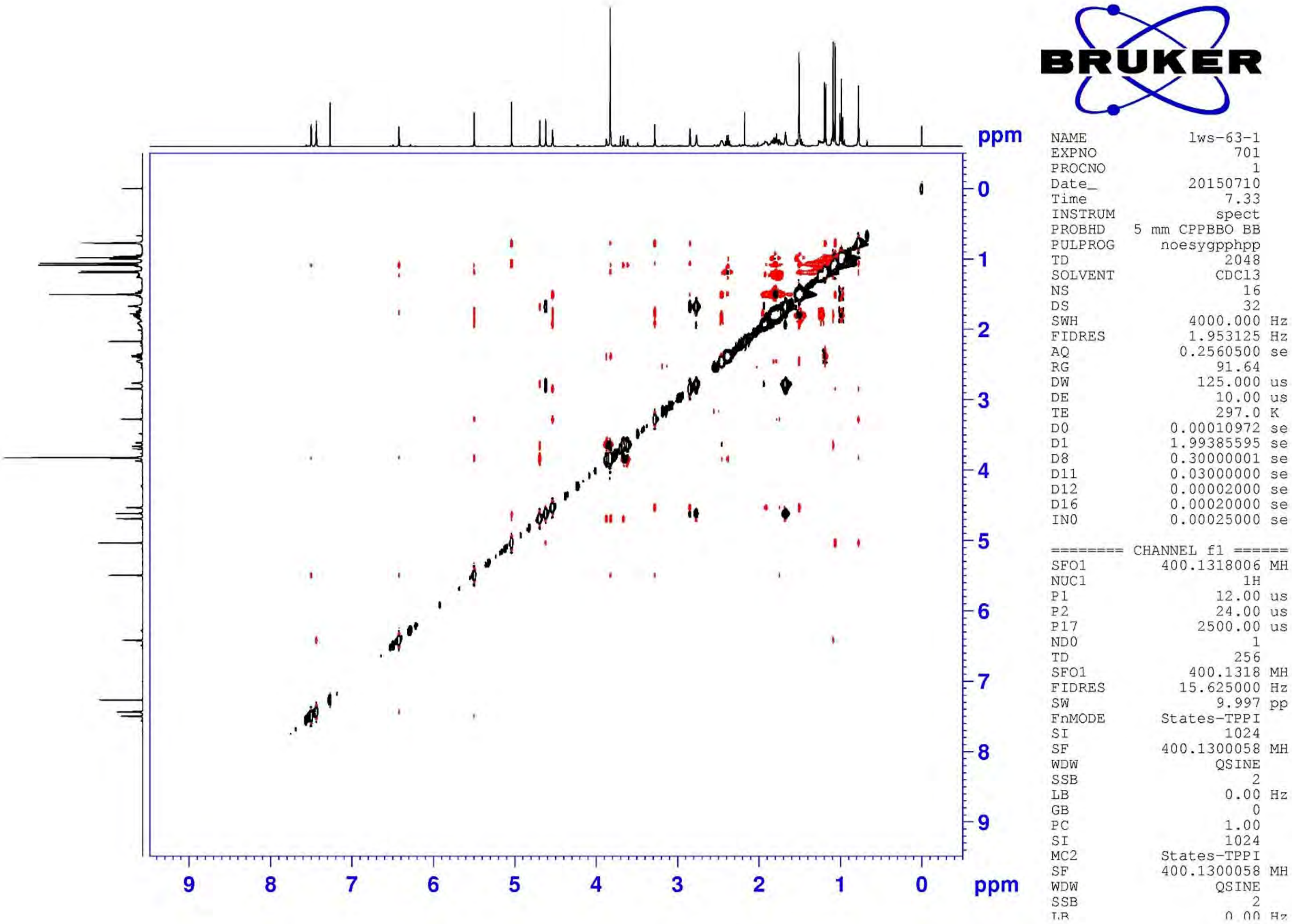

NOESY (400 MHz) spectrum of compound **3** in $CDCl_3$

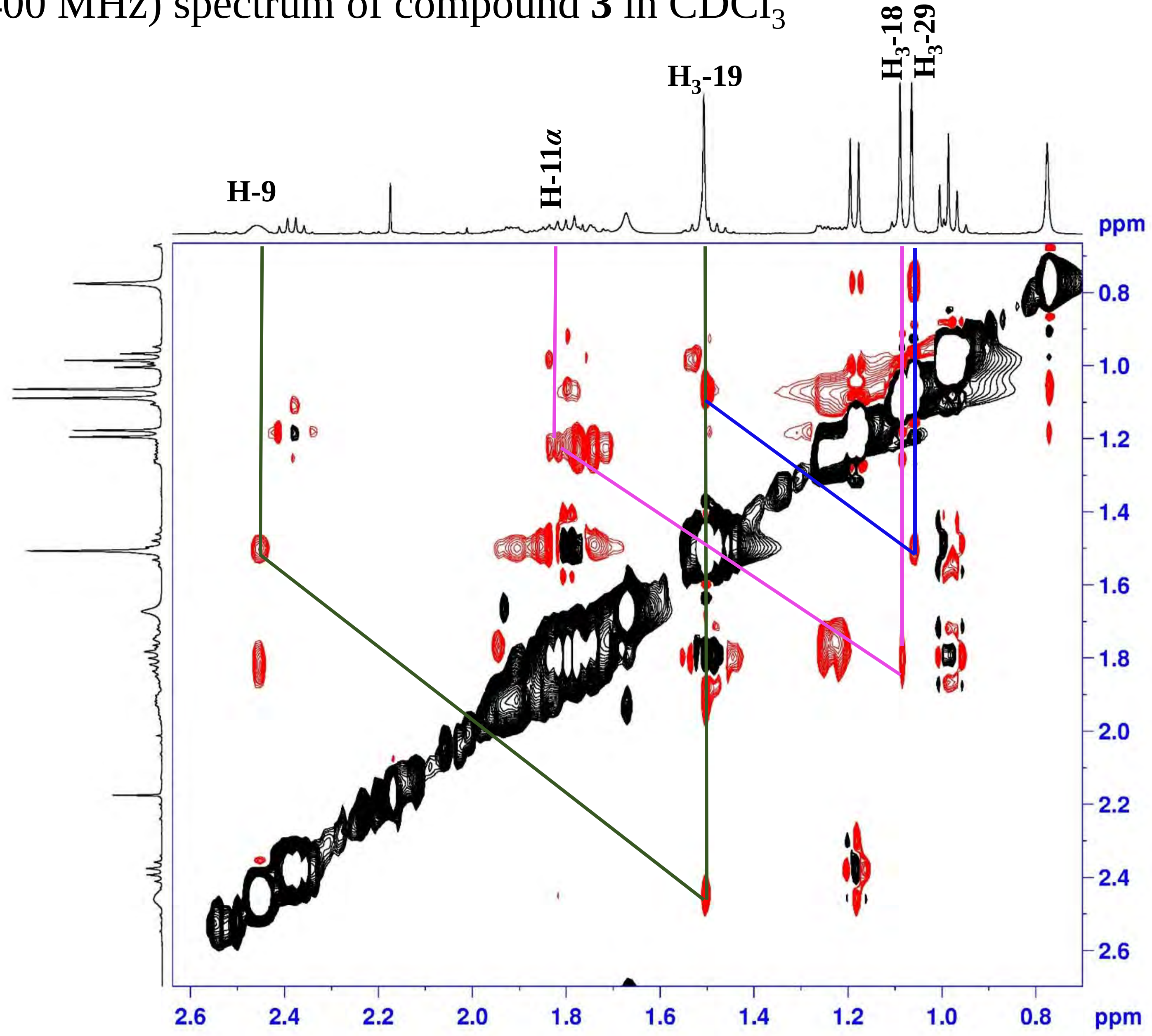

NOESY (400 MHz) spectrum of compound **3** in $CDCl_3$

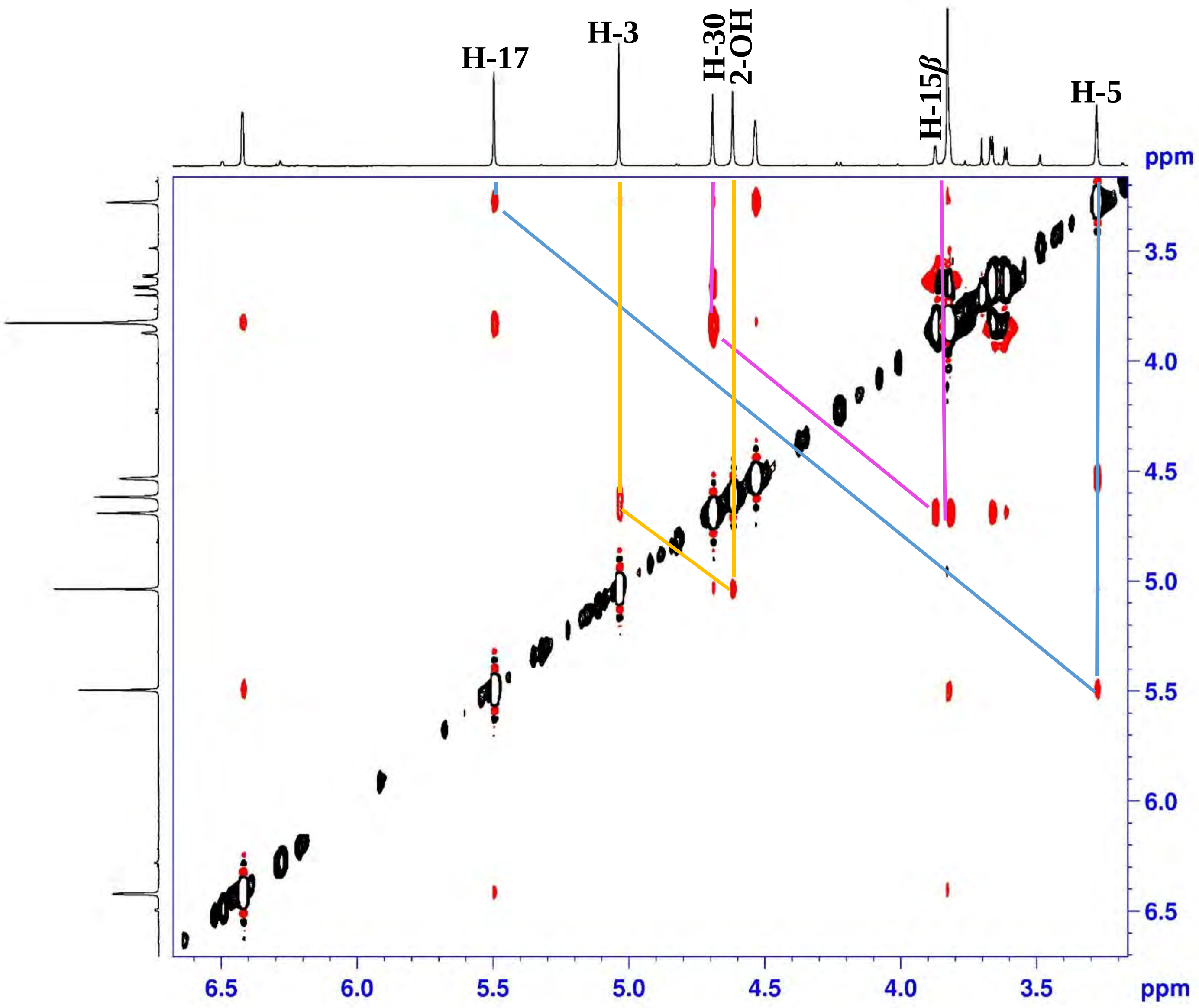

NOESY (400 MHz) spectrum of compound **3** in $CDCl_3$

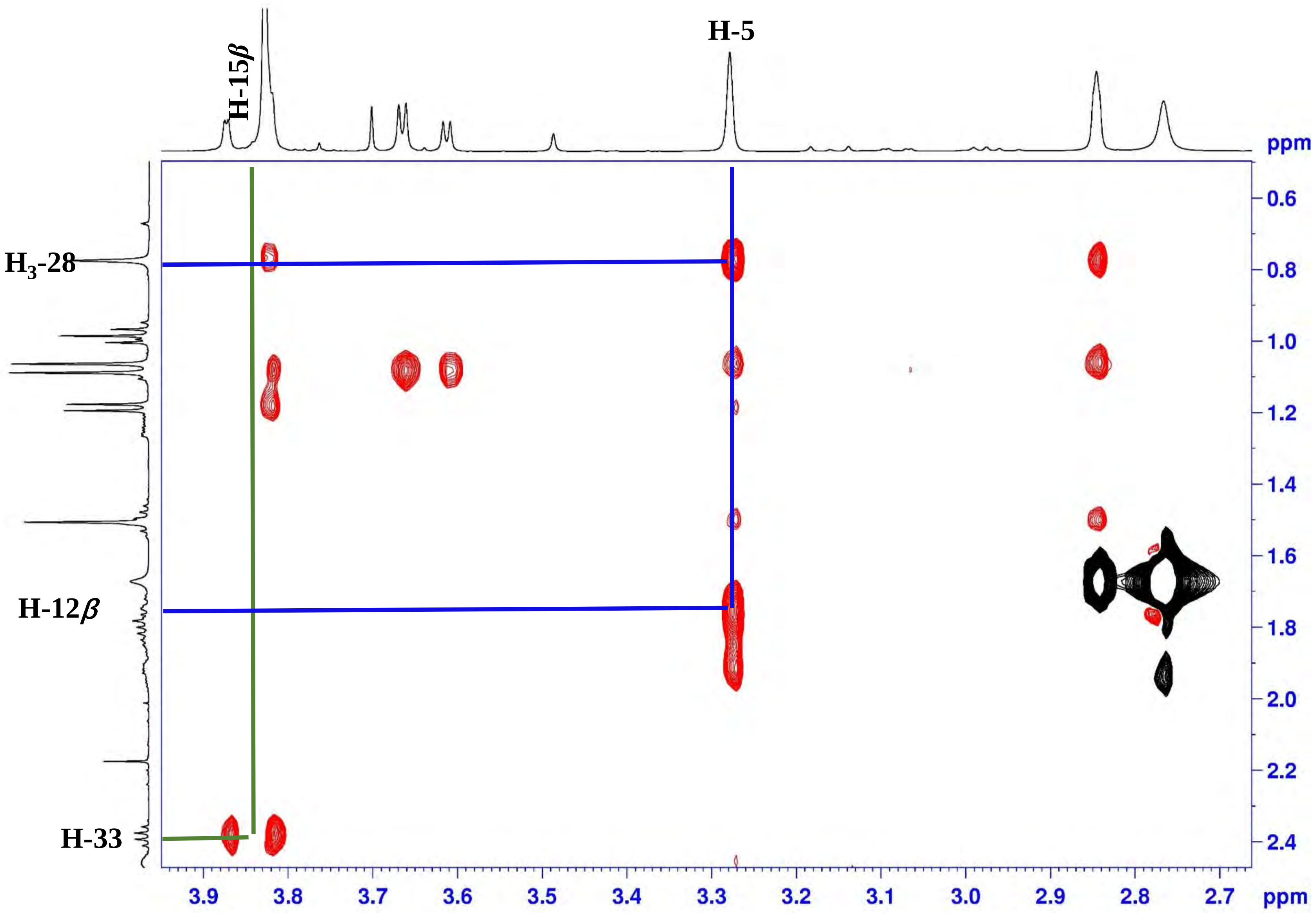

NOESY (400 MHz) spectrum of compound **3** in $CDCl_3$

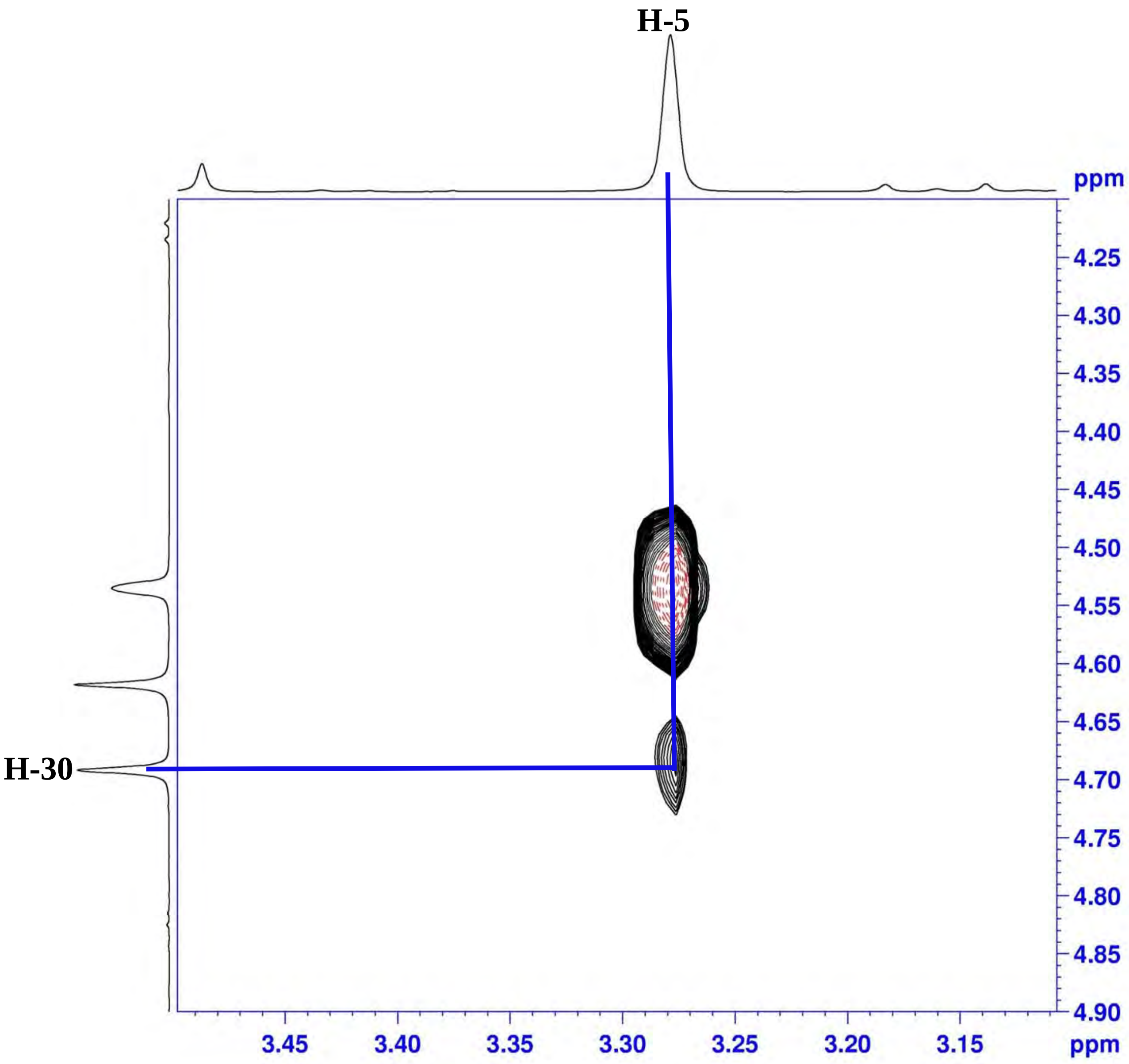

NOESY (400 MHz) spectrum of compound **3** in $CDCl_3$

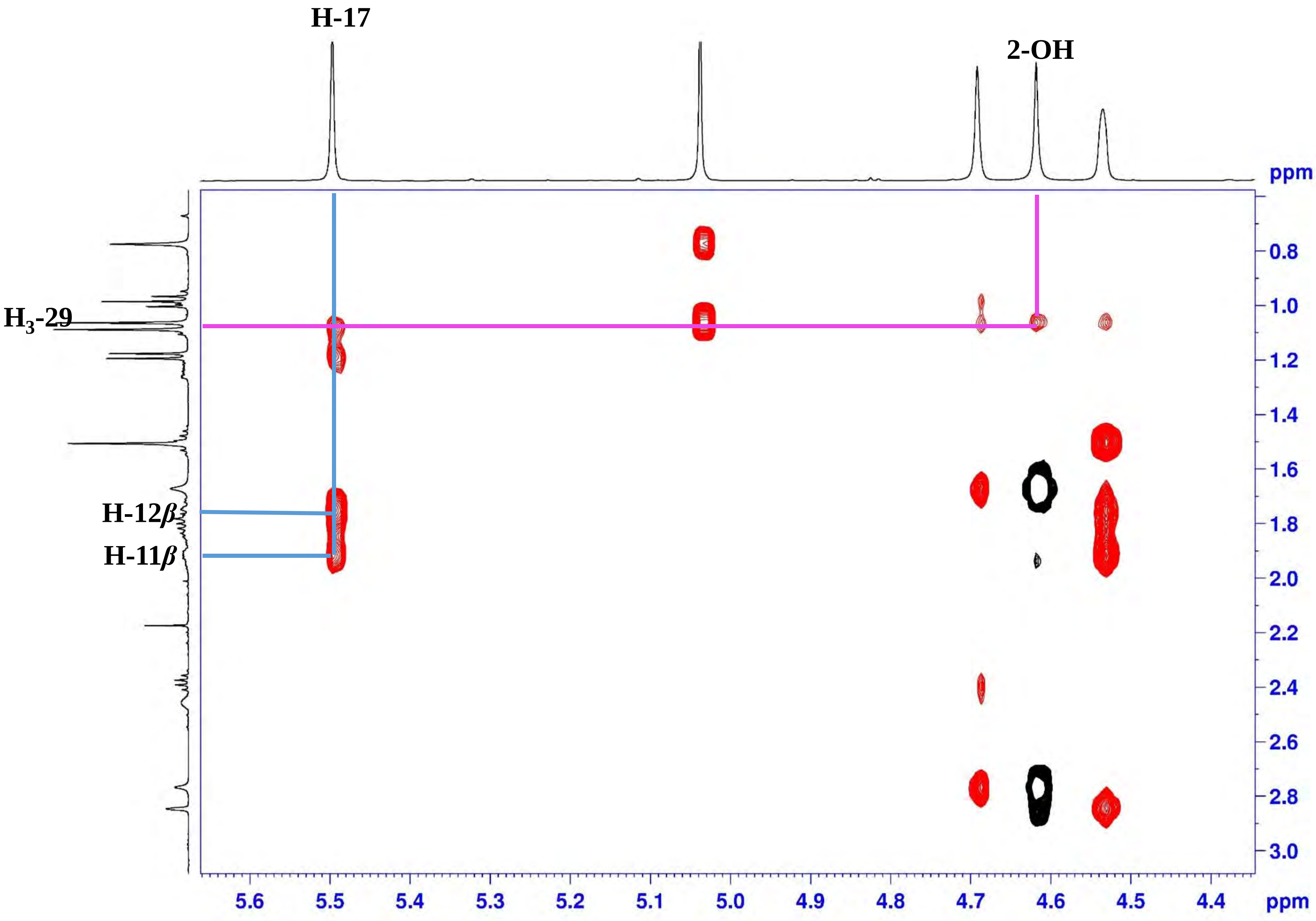

HR-ESIMS for compound **4**

Mass Spectrum SmartFormula Report

Analysis Info

Analysis Name	D:\Data\MS\data\201605\liwanshan_lws-91_pos_11_01_1858.d	Acquisition Date	5/19/2016 8:51:32 AM
Method	LC_Direct Infusion_pos_100-1000mz.m	Operator	SCSIO
Sample Name	liwanshan_lws-91_pos	Instrument / Ser#	maXis 29
Comment			

Acquisition Parameter

Source Type	ESI	Ion Polarity	Positive	Set Nebulizer	0.4 Bar
Focus	Active	Set Capillary	4500 V	Set Dry Heater	180 °C
Scan Begin	100 m/z	Set End Plate Offset	-500 V	Set Dry Gas	4.0 l/min
Scan End	2000 m/z	Set Collision Cell RF	800.0 Vpp	Set Divert Valve	Waste

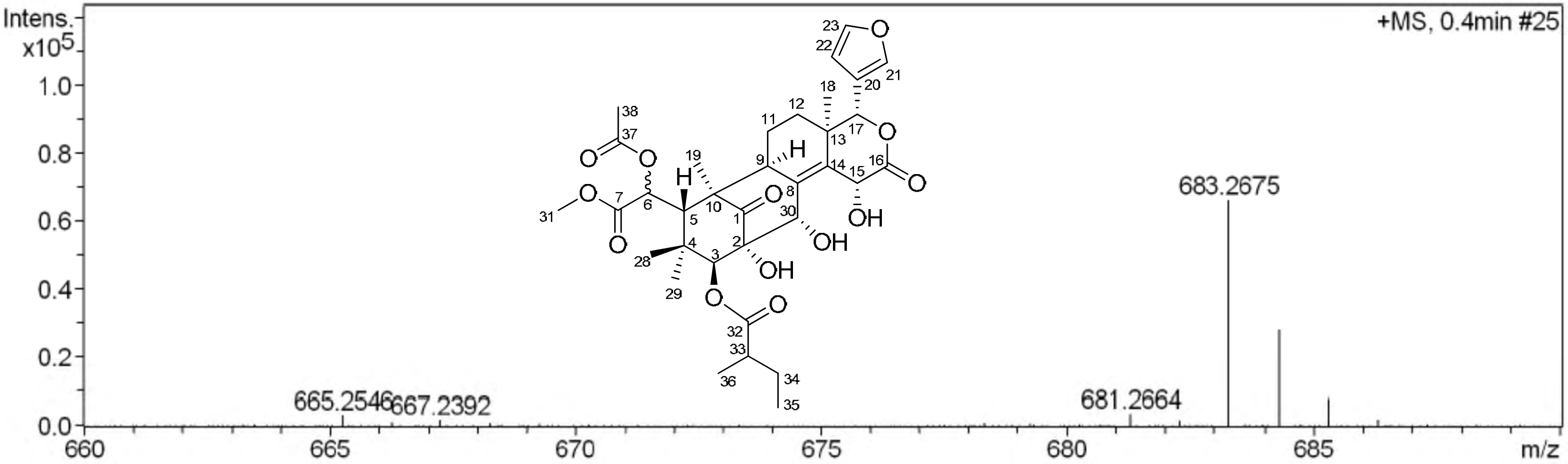

Meas. m/z	#	Formula	Score	m/z	err [ppm]	err [mDa]	mSigma	rdb	e⁻ Conf	N-Rule
683.2675	1	C 34 H 44 Na O 13	100.00	683.2674	-0.1	-0.1	24.3	12.5	even	ok
1343.5491	1	C 68 H 88 Na O 26	8.87	1343.5456	-2.6	-3.5	48.9	24.5	even	ok

HR-ESIMS for compound **4**

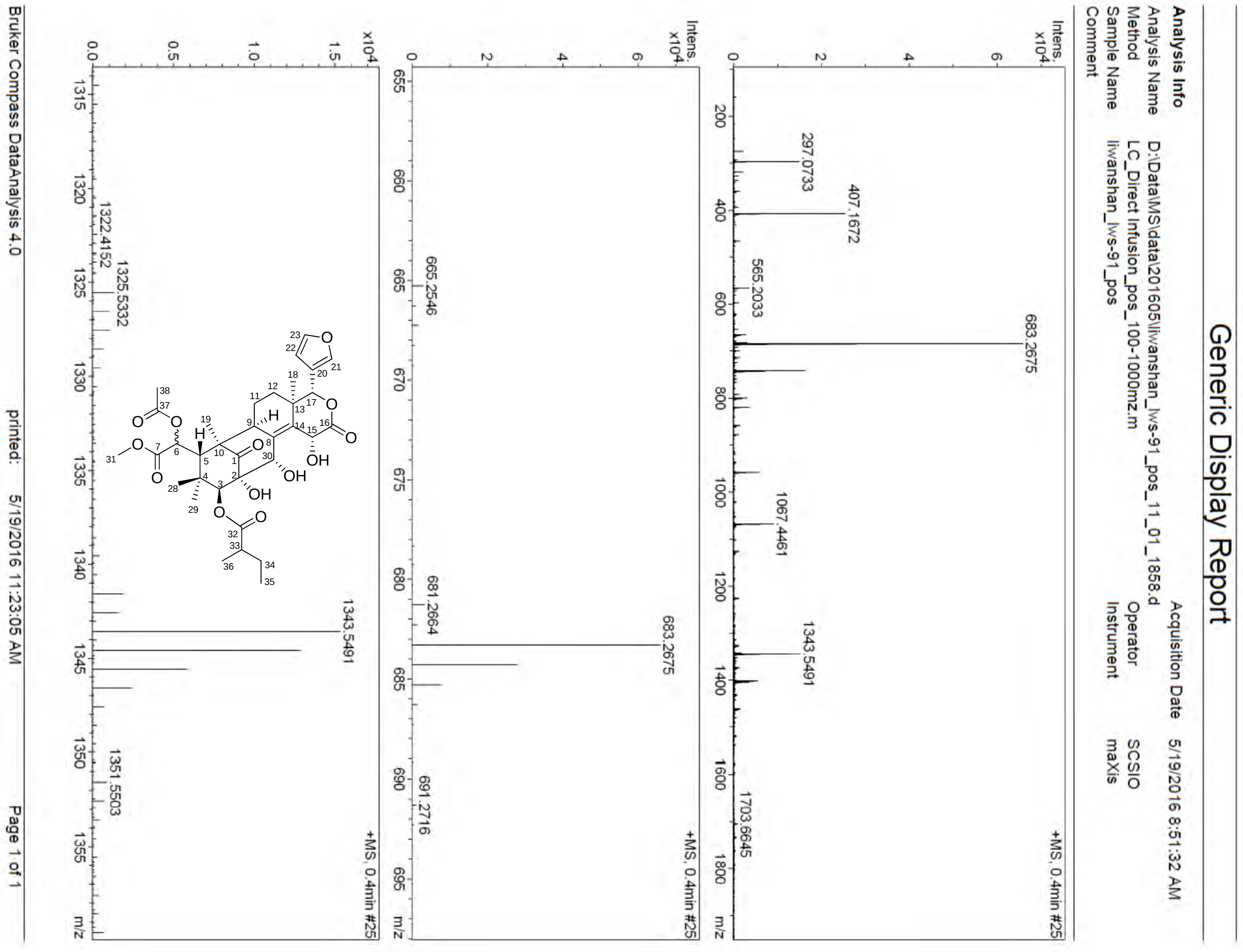

^{1}H NMR (400 MHz) spectrum of compound **4** in $CDCl_3$

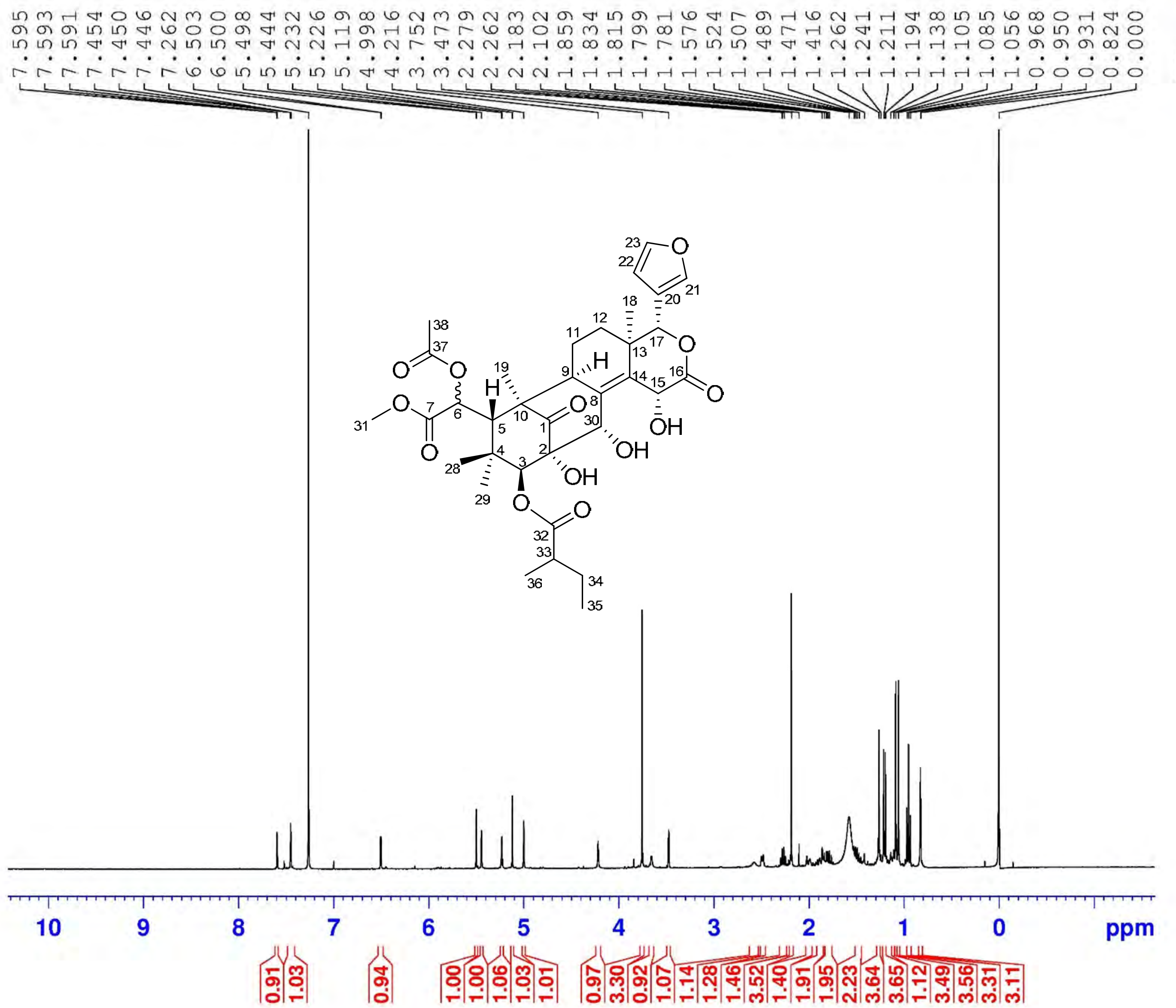

NAME	lws-93
EXPNO	1
PROCNO	1
Date_	20160204
Time	16.55
INSTRUM	spect
PROBHD	5 mm CPPBBO BB
PULPROG	zg30
TD	65536
SOLVENT	CDCl3
NS	16
DS	2
SWH	8223.685 H
FIDRES	0.125483 H
AQ	3.9846387 s
RG	208.5
DW	60.800 u
DE	10.00 u
TE	297.0 K
D1	1.00000000 s
TD0	1

======== CHANNEL f1 =====

SFO1	400.1324710 M
NUC1	1H
P1	11.50 u
SI	65536
SF	400.1300090 M
WDW	EM
SSB	0
LB	0.30 H
GB	0
PC	1.00

^{1}H NMR (400 MHz) spectrum of compound **4** in $CDCl_3$

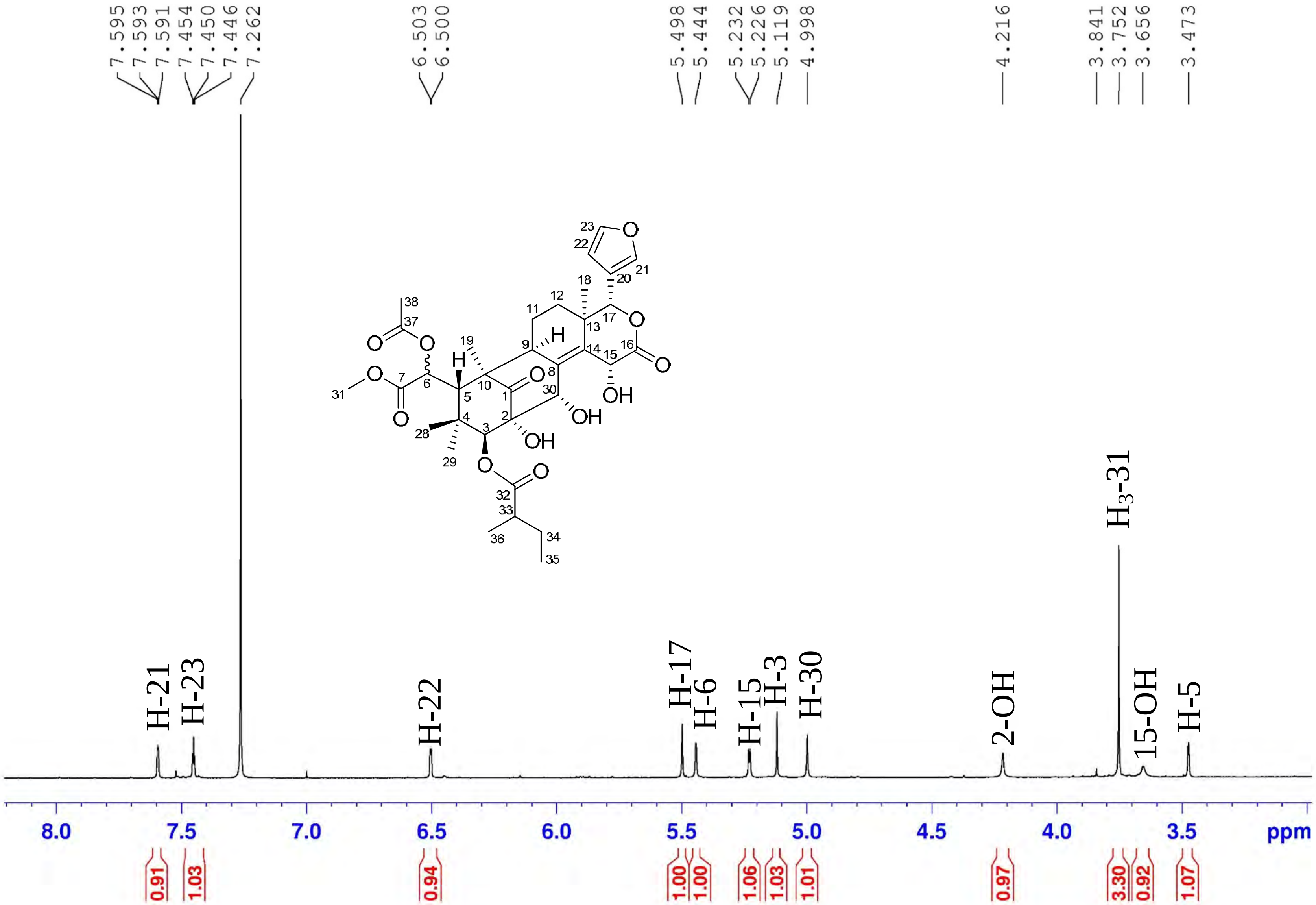

^{1}H NMR (400 MHz) spectrum of compound **4** in $CDCl_3$

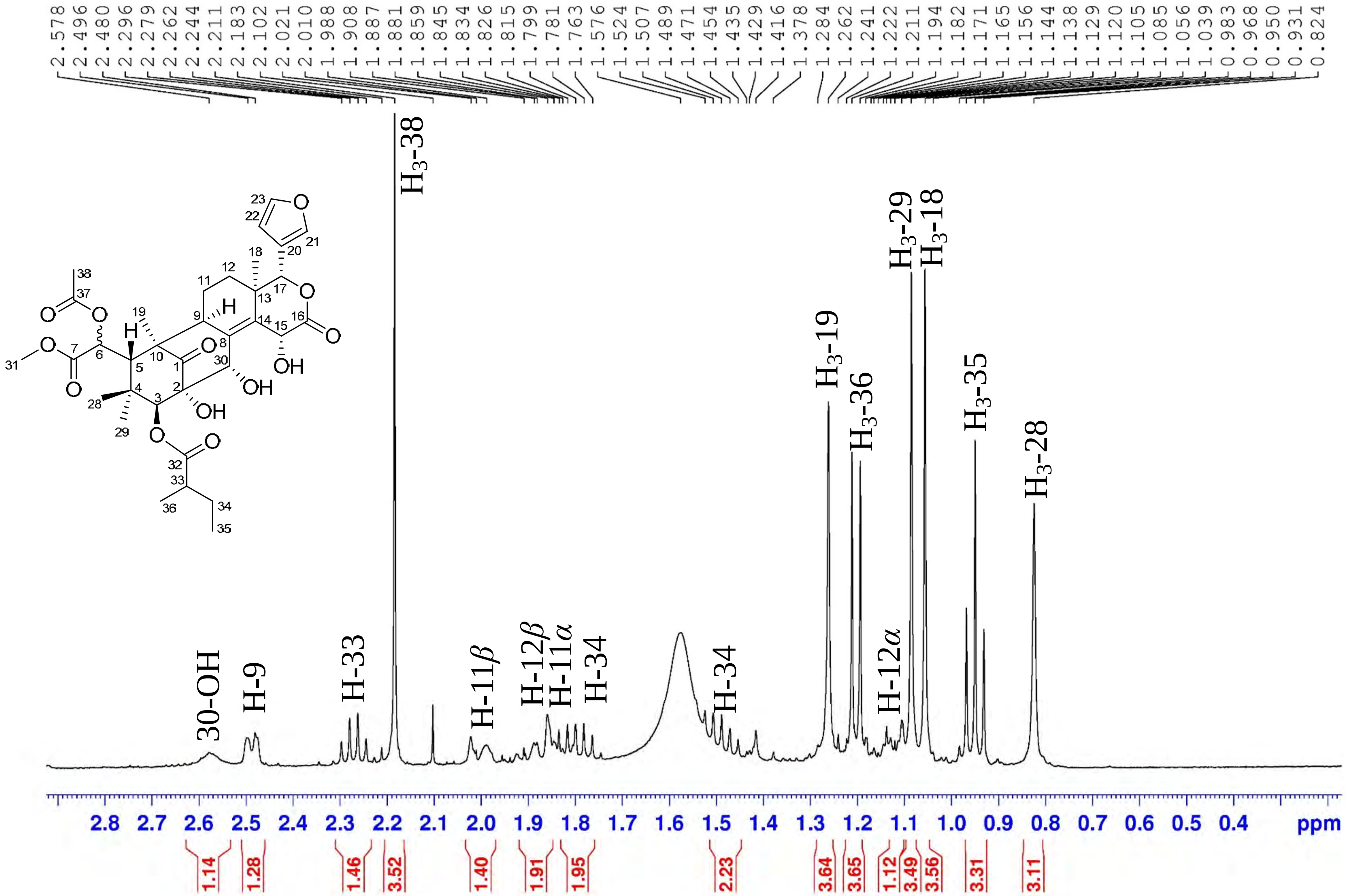

^{13}C NMR (100 MHz) spectrum of compound **4** in $CDCl_3$

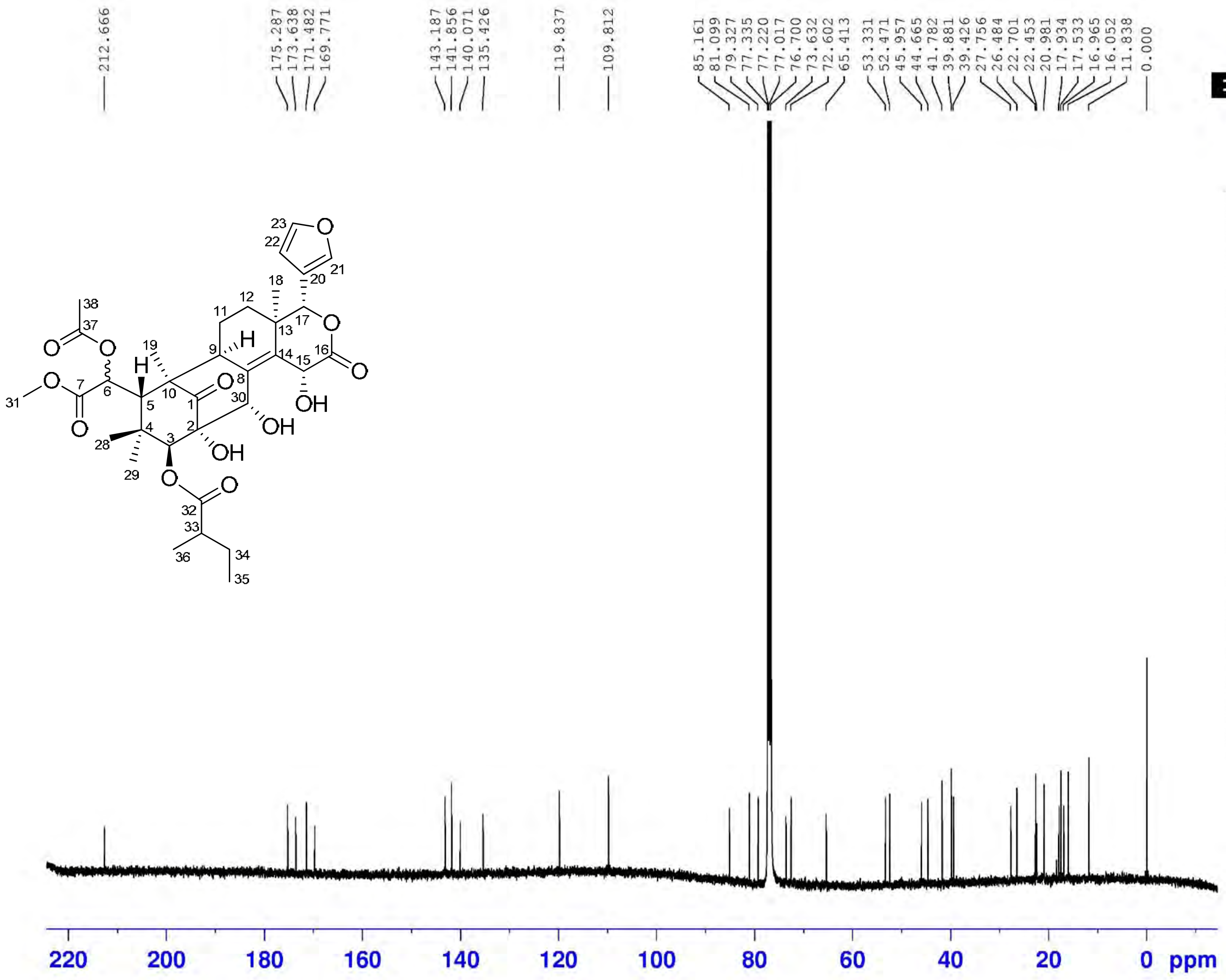


```
NAME             lws-93
EXPNO                 2
PROCNO                1
Date_          20160205
Time              12.24
INSTRUM           spect
PROBHD   5 mm CPPBBO BB
PULPROG         zgpg30
TD               65536
SOLVENT           CDCl3
NS               20000
DS                    4
SWH          24038.461 H
FIDRES        0.366798 H
AQ           1.3631988 s
RG               102.3
DW              20.800 u
DE               18.00 u
TE               297.0 K
D1          2.00000000 s
D11         0.03000000 s
TD0                   1

======== CHANNEL f1 =====
SFO1       100.6233324 M
NUC1                13C
P1                10.00 u
SI                32768
SF         100.6127692 M
WDW                  EM
SSB                   0
LB                 1.00 H
GB                    0
PC                 1.40
```

^{13}C NMR (100 MHz) spectrum of compound **4** in $CDCl_3$

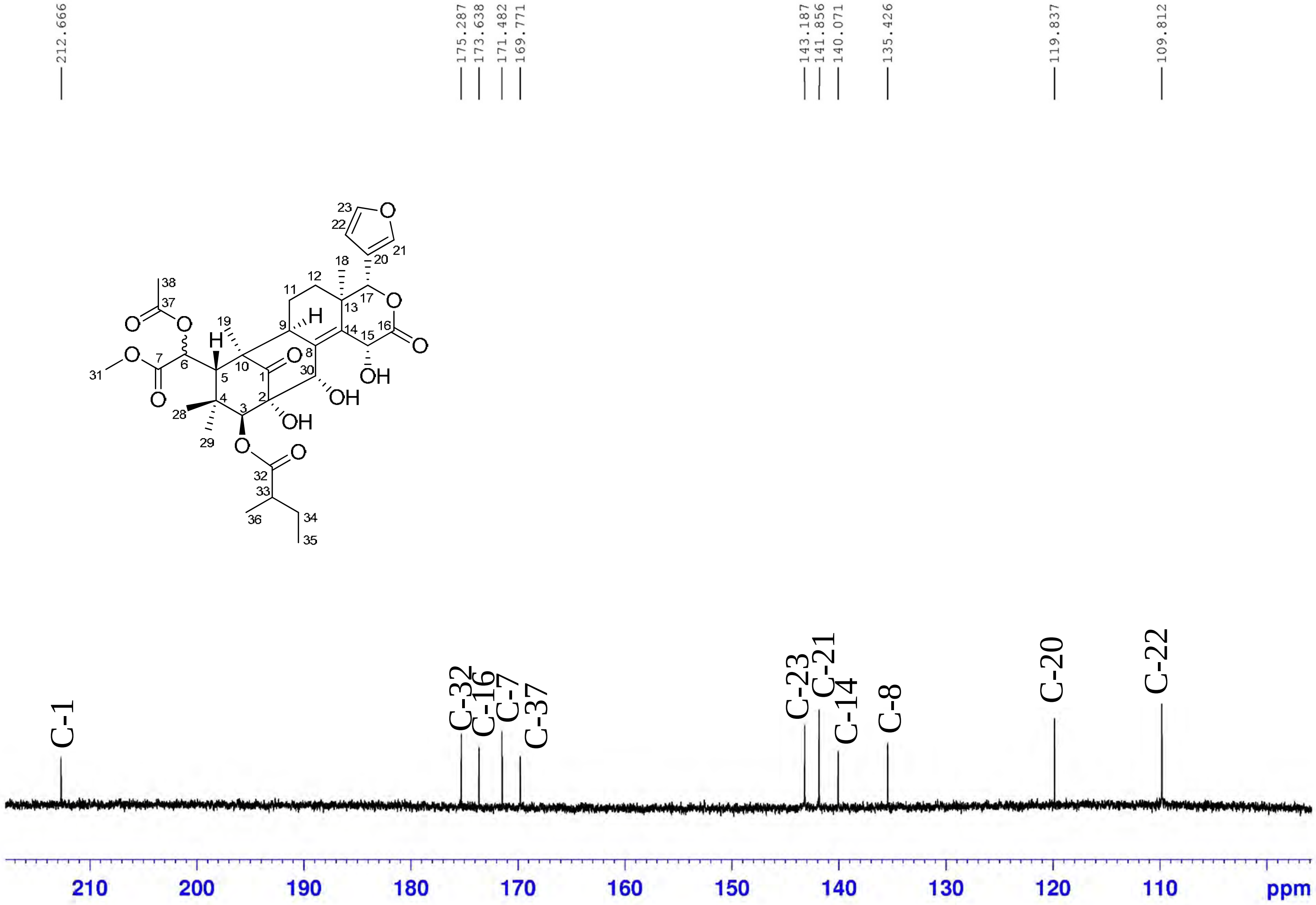

^{13}C NMR (100 MHz) spectrum of compound **4** in $CDCl_3$

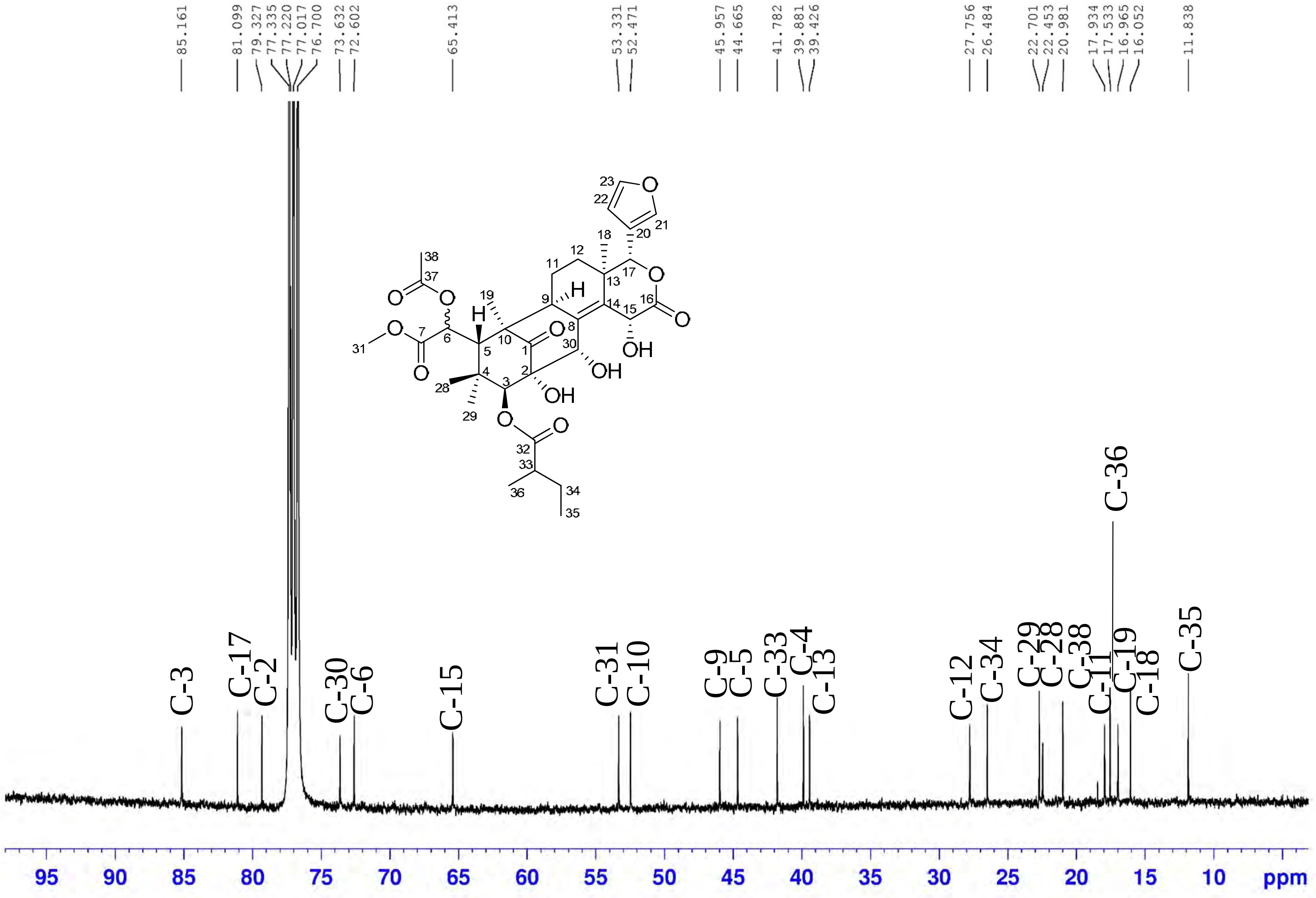

DEPT135 (100 MHz) spectrum of compound **4** in $CDCl_3$

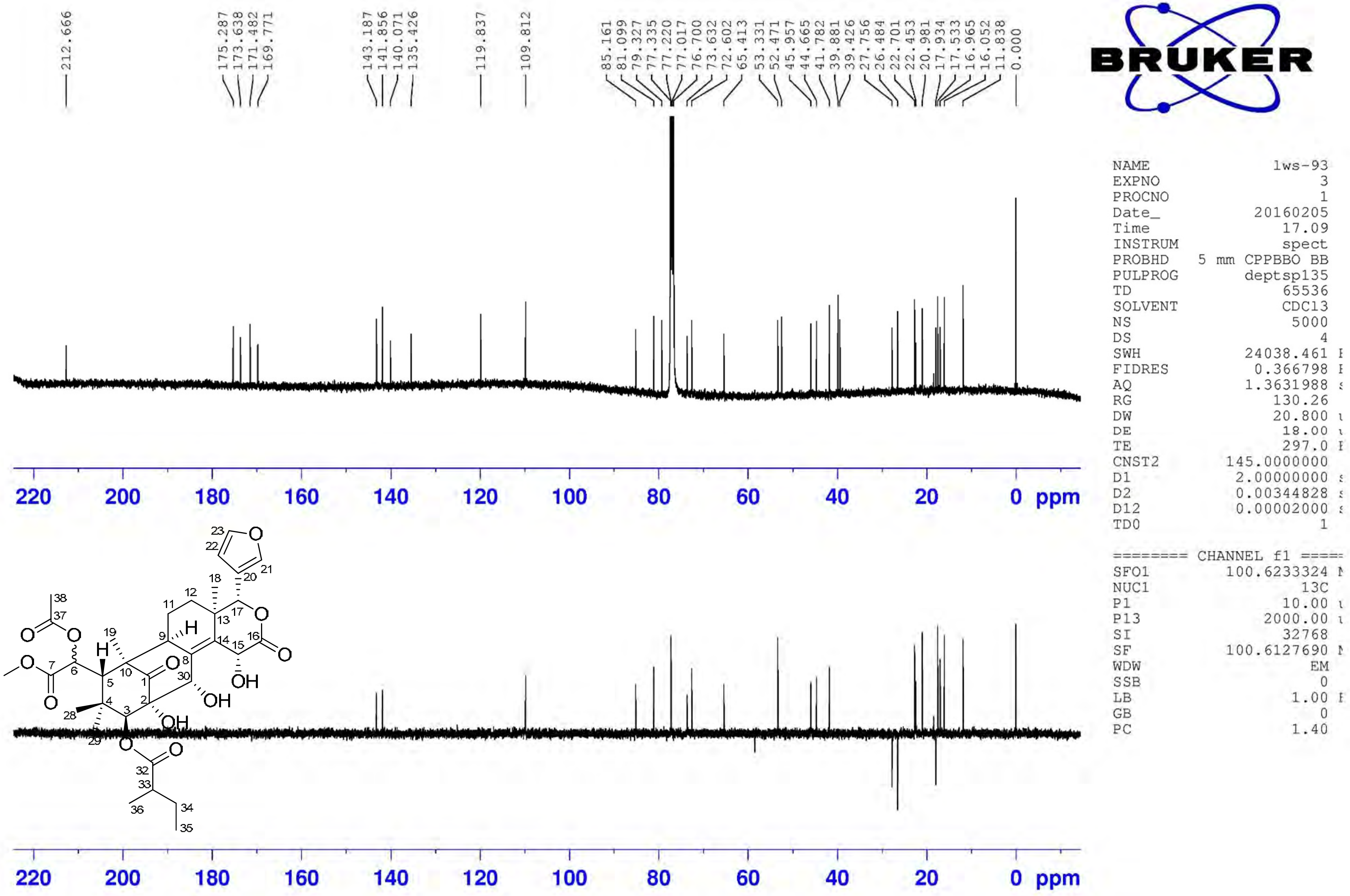

DEPT135 (100 MHz) spectrum of compound **4** in $CDCl_3$

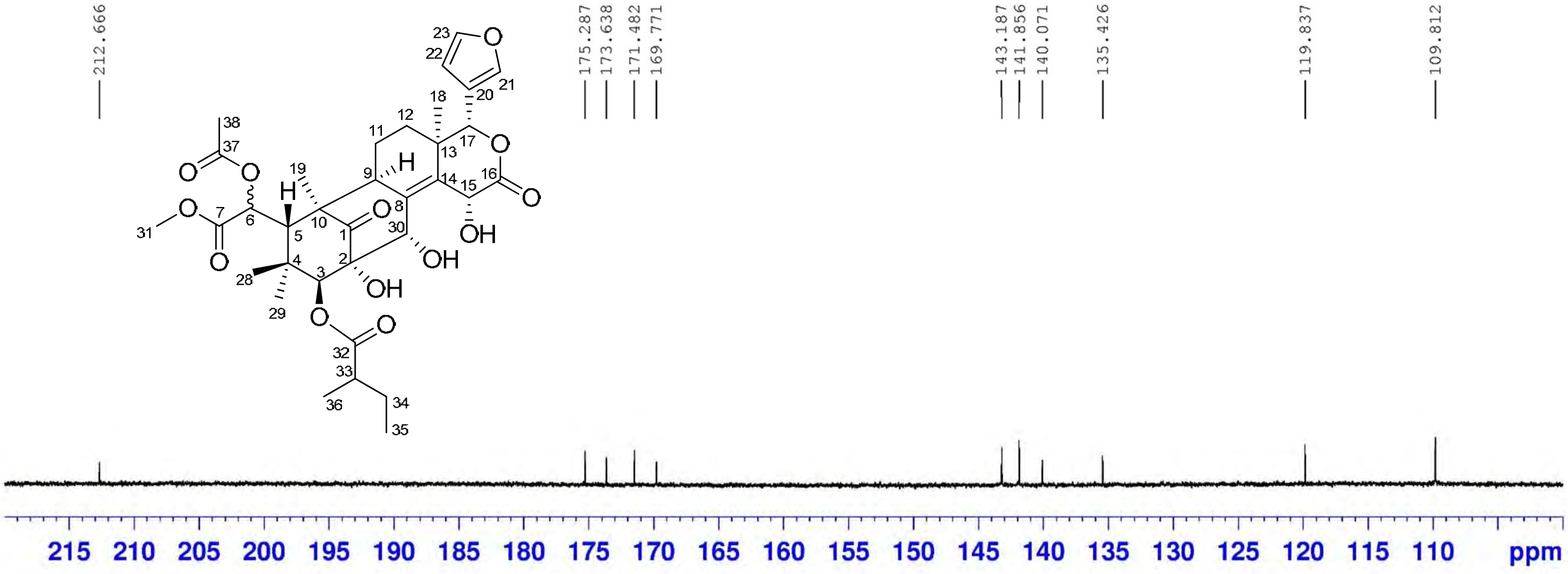

DEPT135 (100 MHz) spectrum of compound **4** in $CDCl_3$

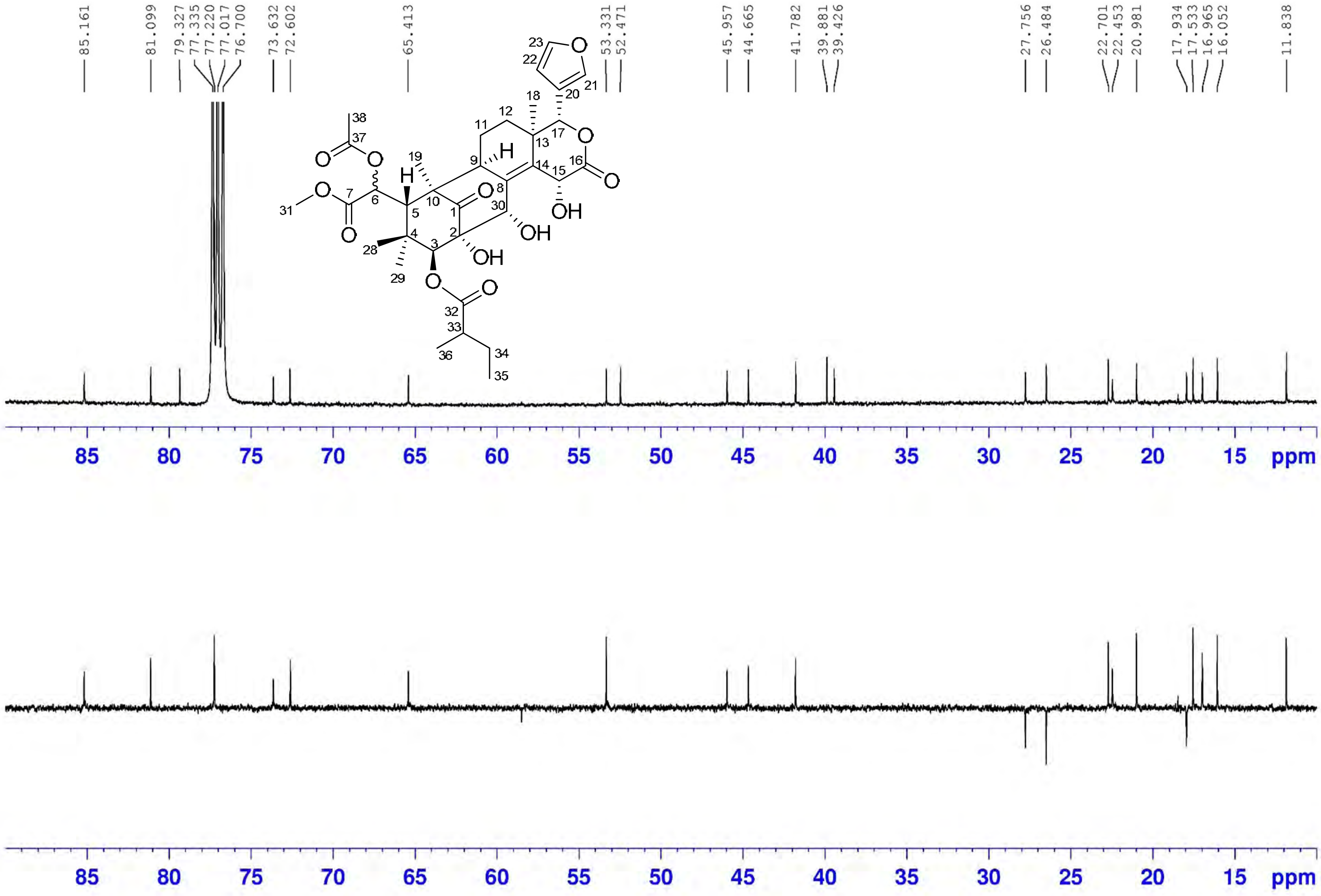

^{1}H-^{1}H COSY (400 MHz) spectrum of compound **4** in $CDCl_3$

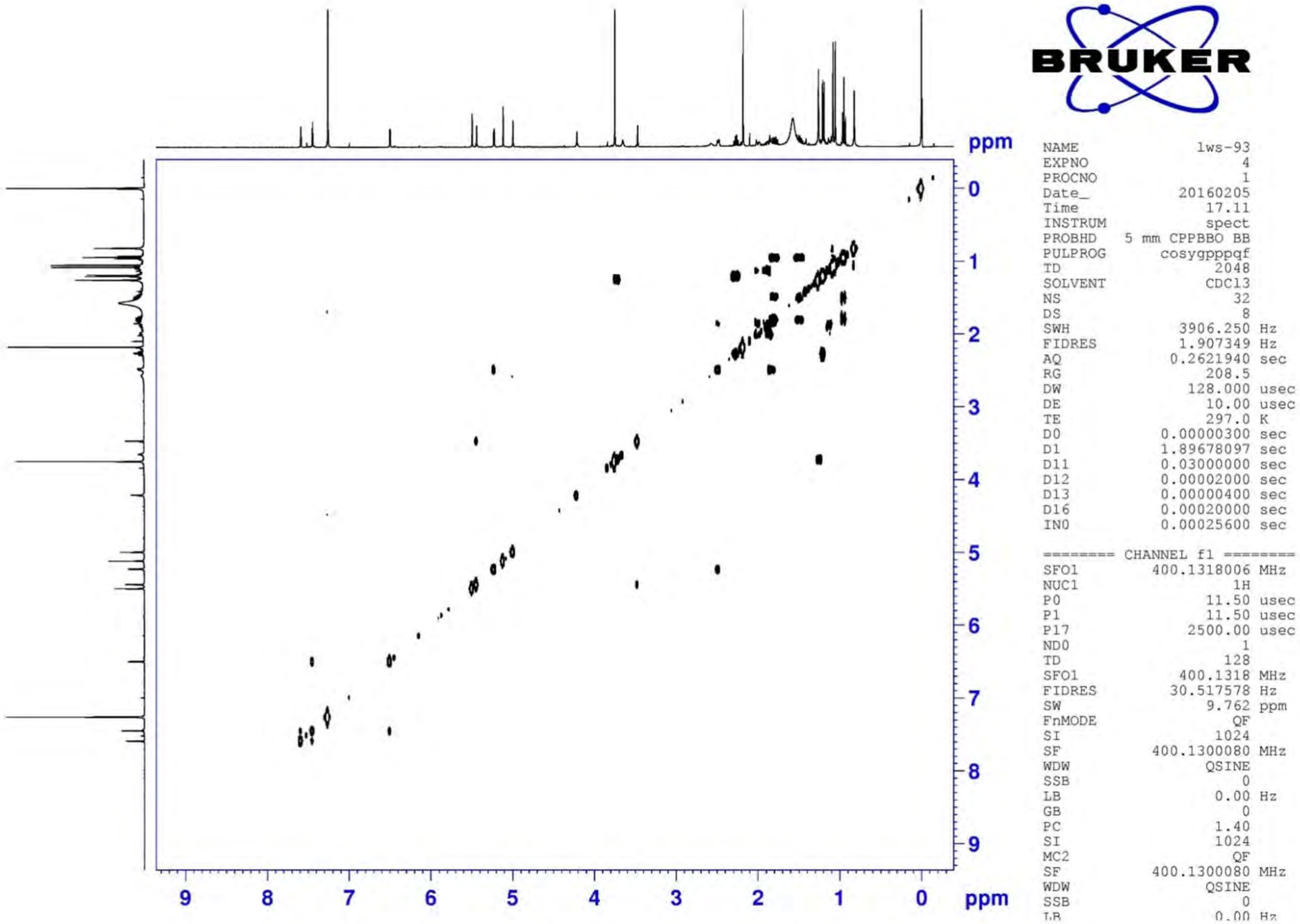

^{1}H-^{1}H COSY (400 MHz) spectrum of compound **4** in $CDCl_3$

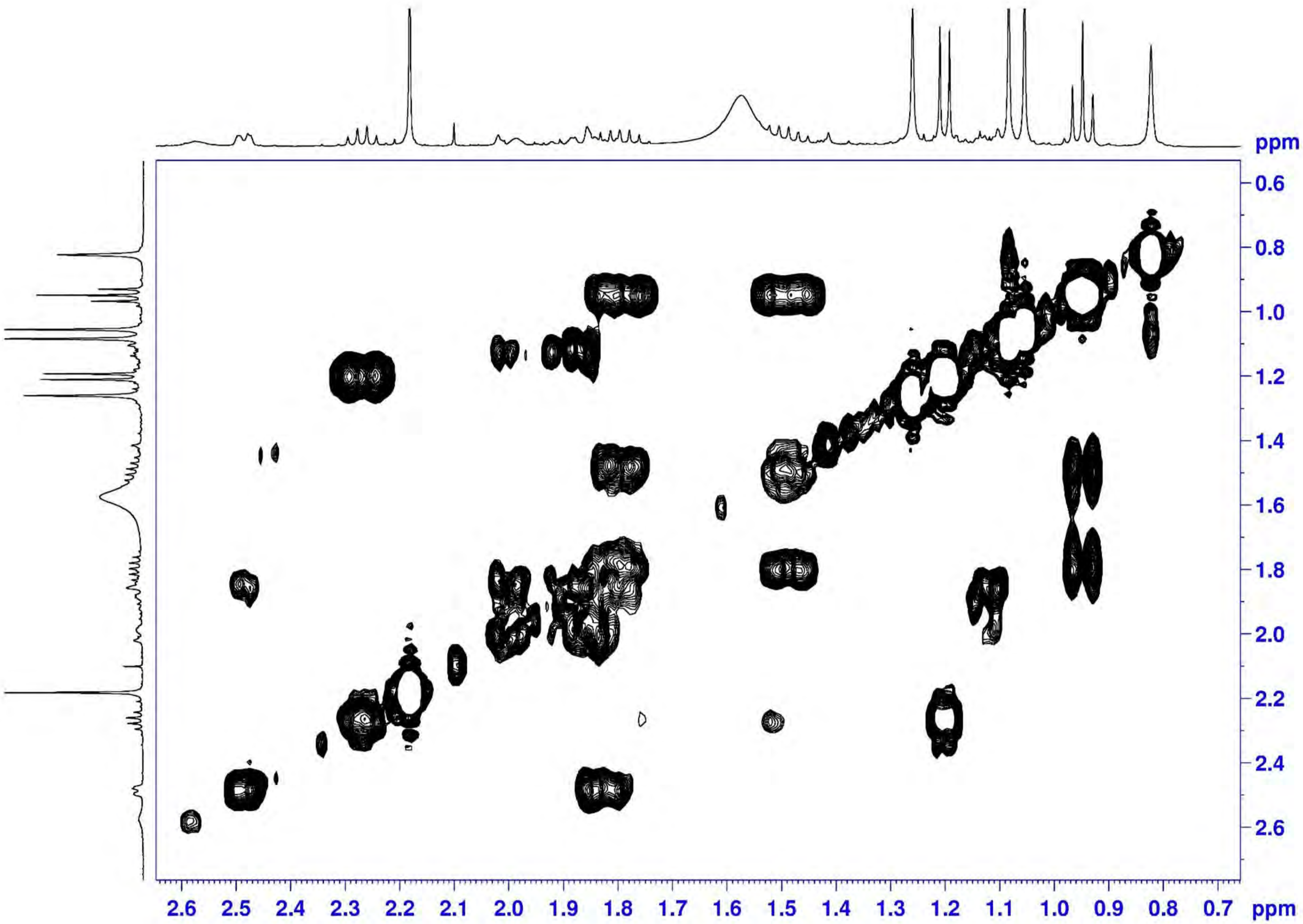

HSQC (400 MHz) spectrum of compound **4** in $CDCl_3$

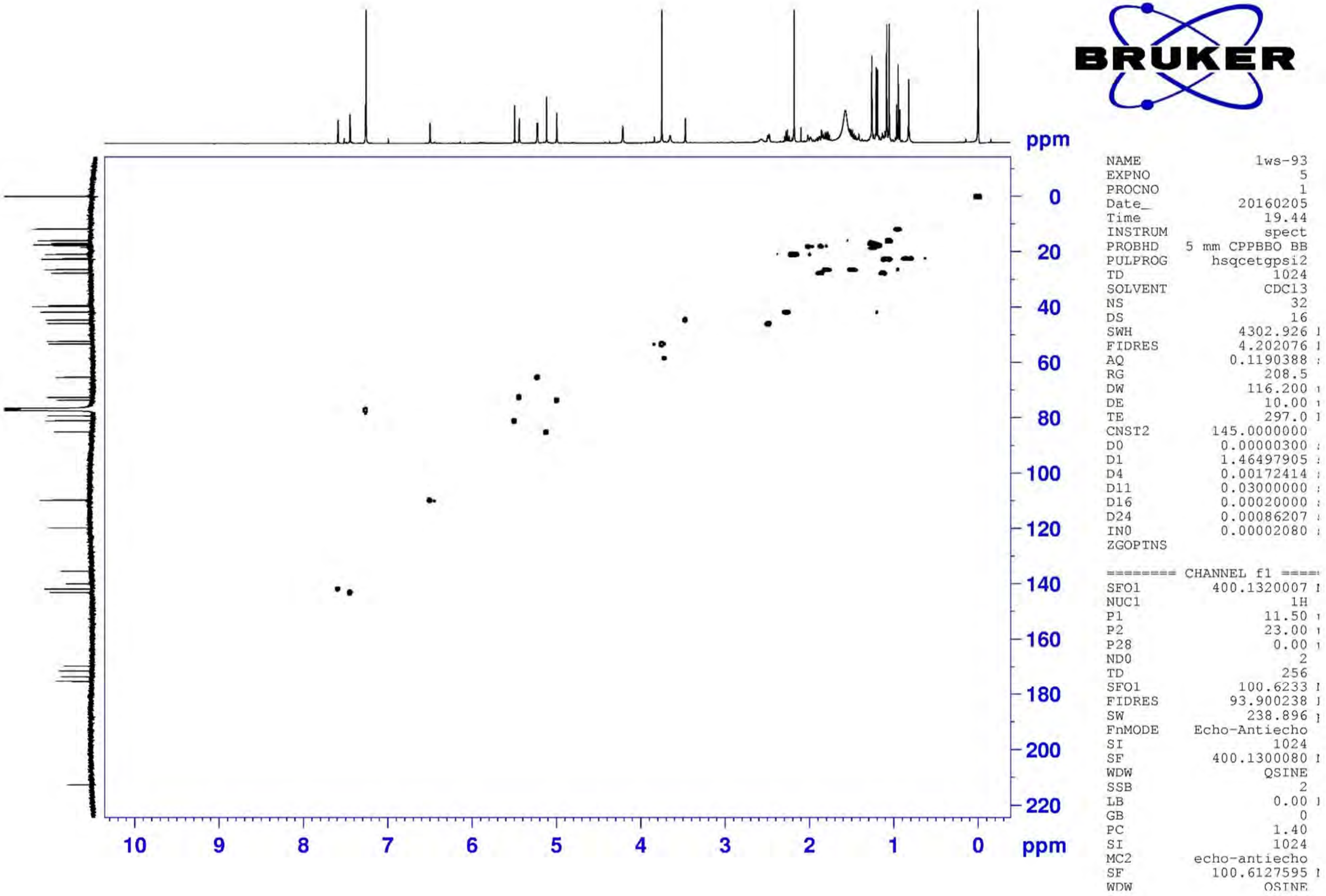

HSQC (400 MHz) spectrum of compound **4** in $CDCl_3$

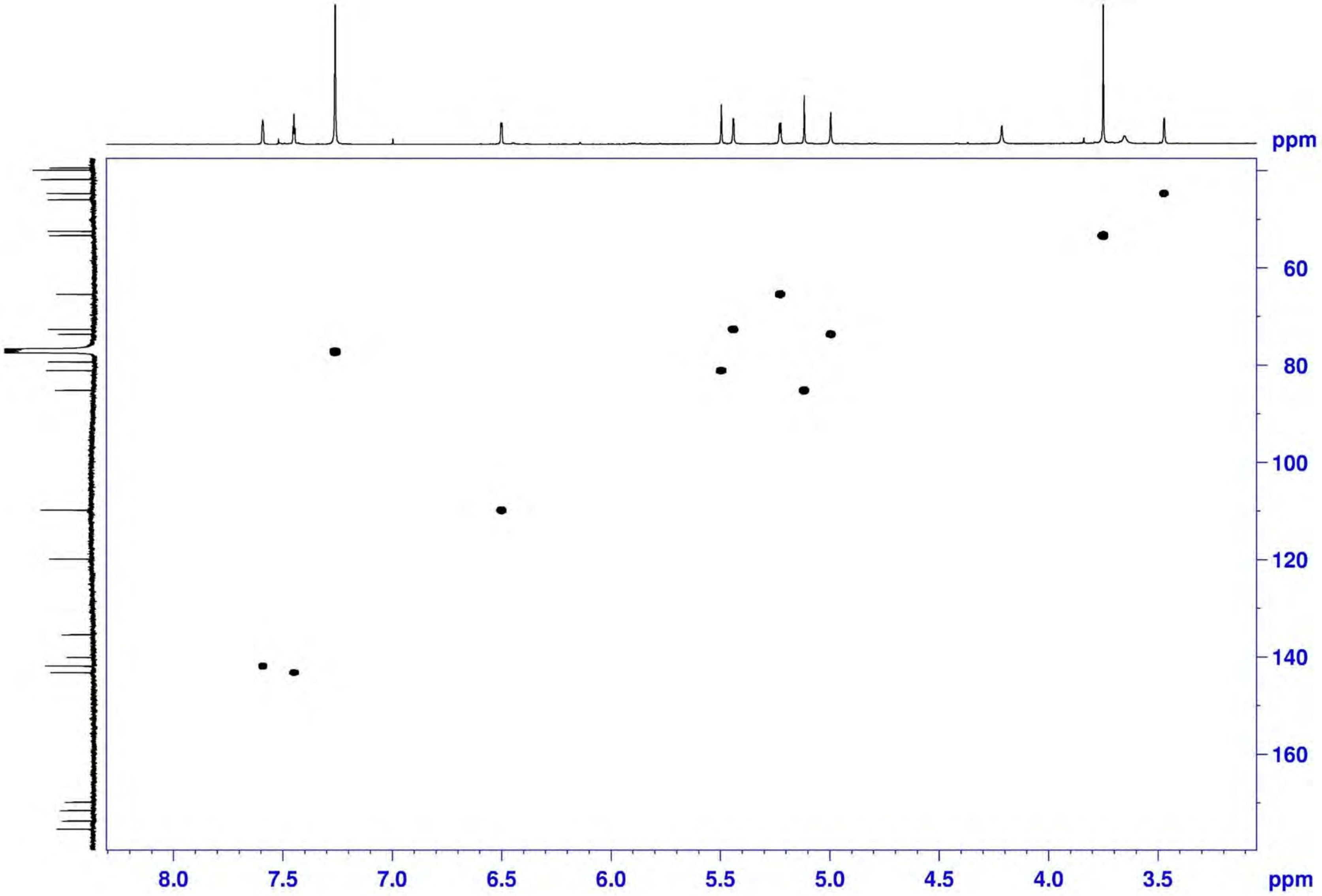

HSQC (400 MHz) spectrum of compound **4** in $CDCl_3$

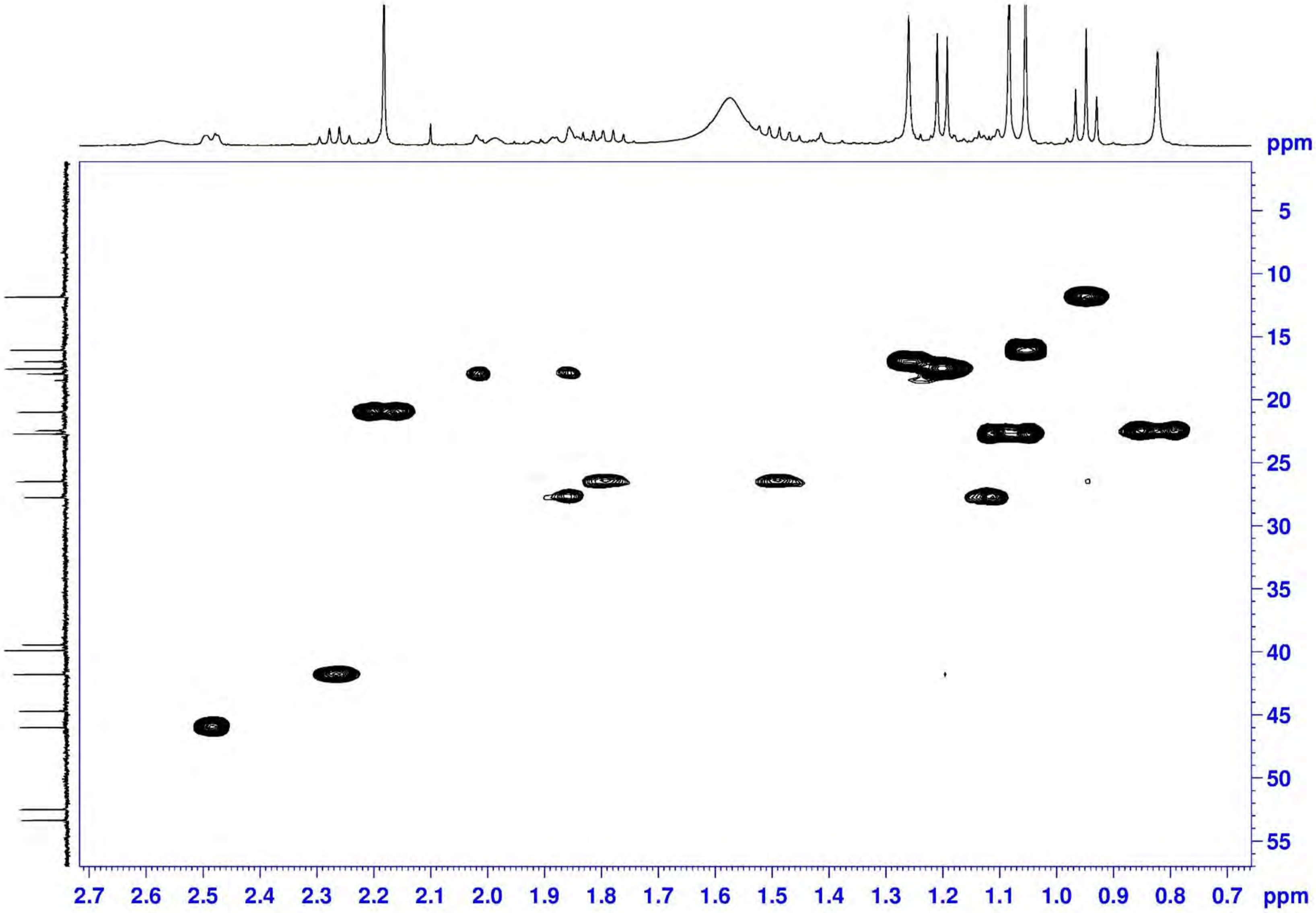

HMBC (400 MHz) spectrum of compound **4** in $CDCl_3$

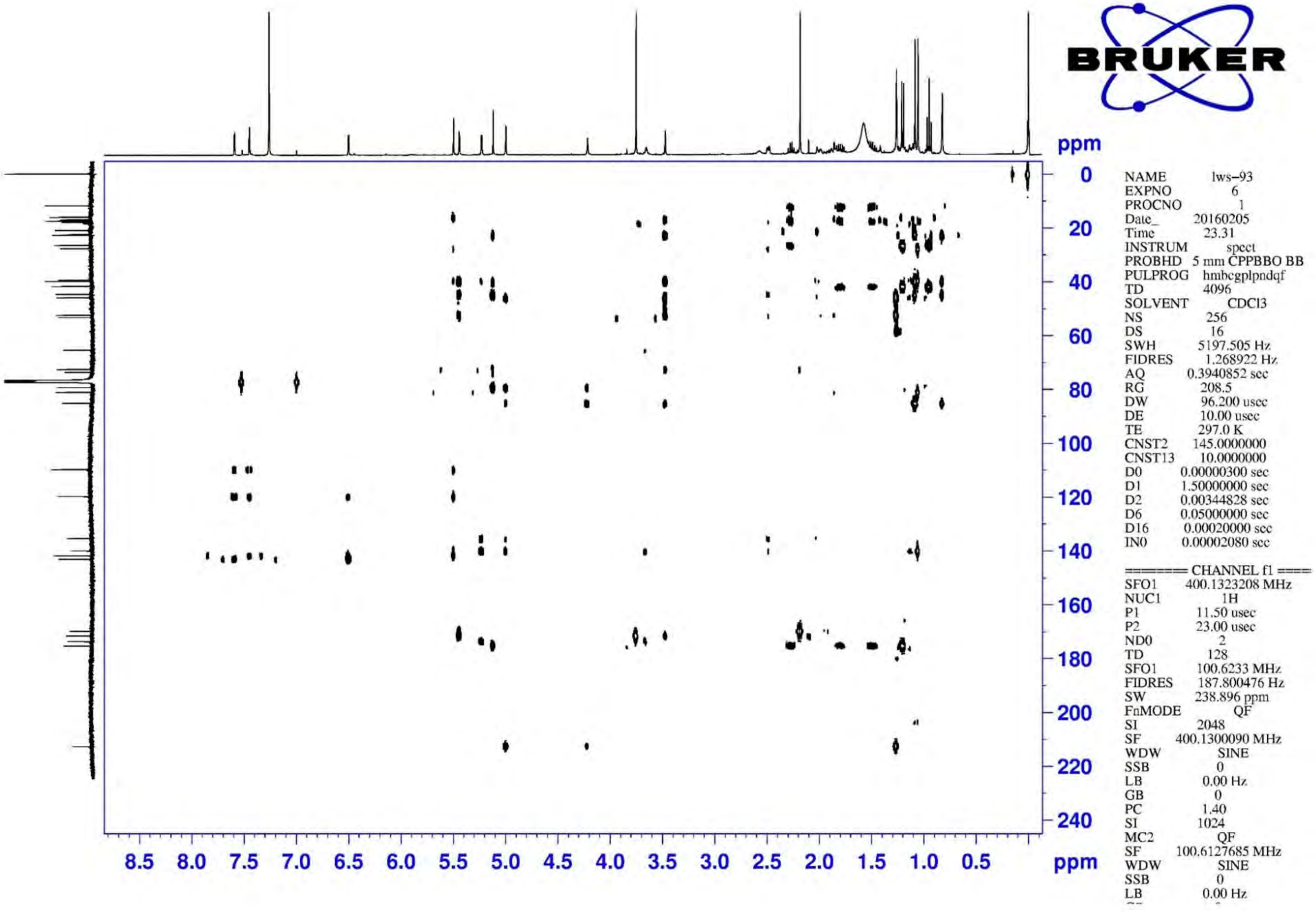

HMBC (400 MHz) spectrum of compound **4** in $CDCl_3$

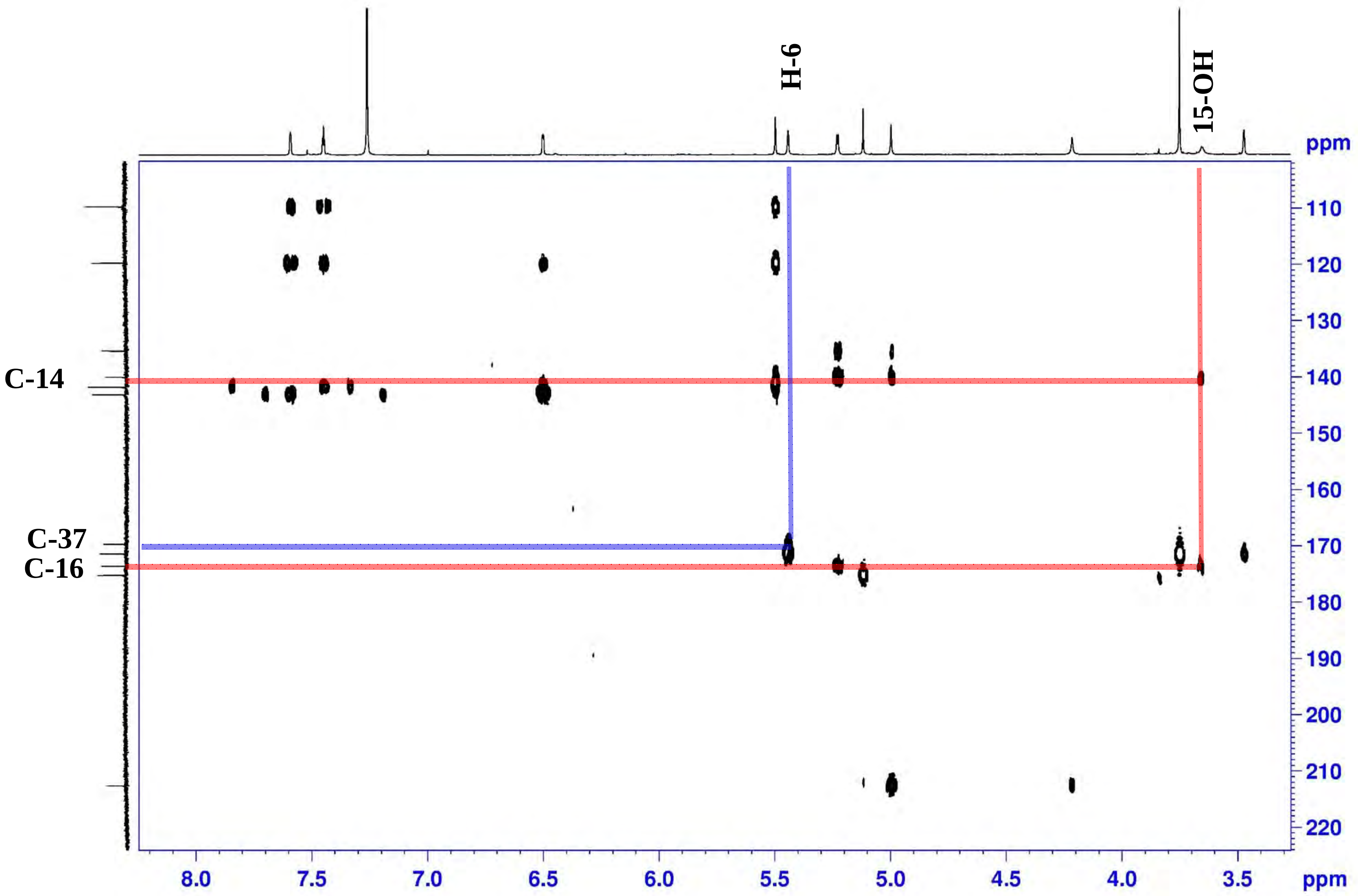

HMBC (400 MHz) spectrum of compound **4** in $CDCl_3$

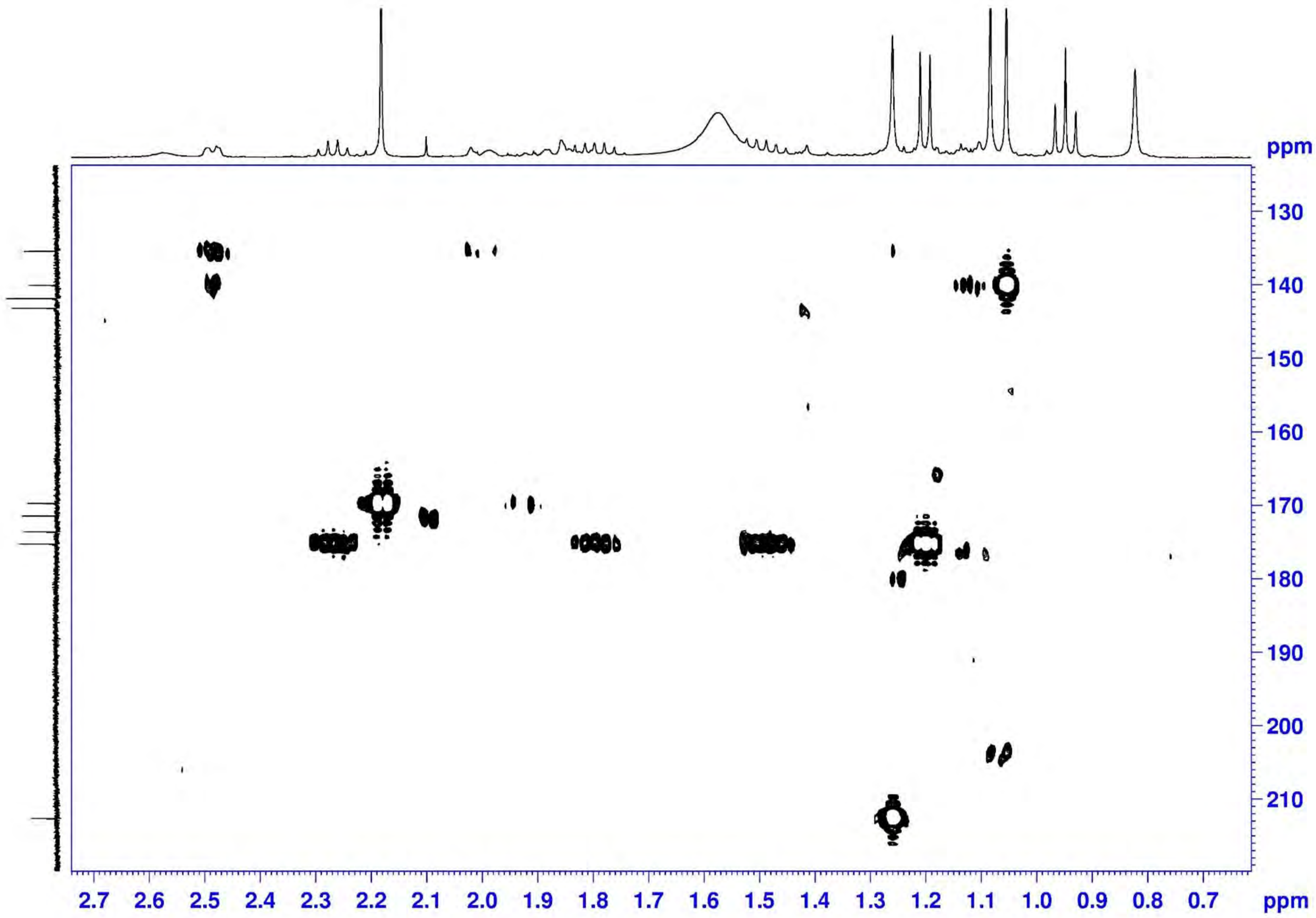

HMBC (400 MHz) spectrum of compound **4** in $CDCl_3$

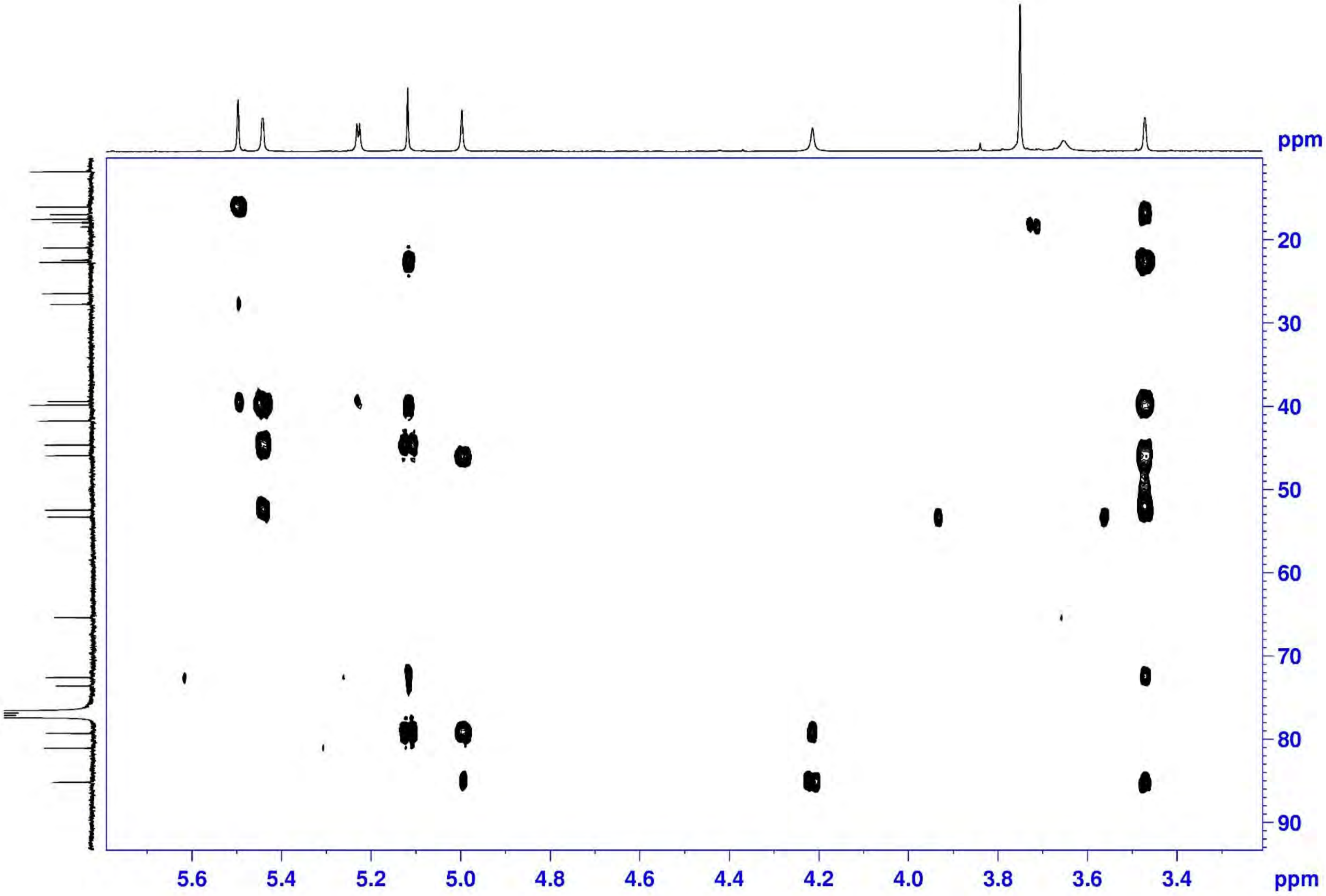

HMBC (400 MHz) spectrum of compound **4** in $CDCl_3$

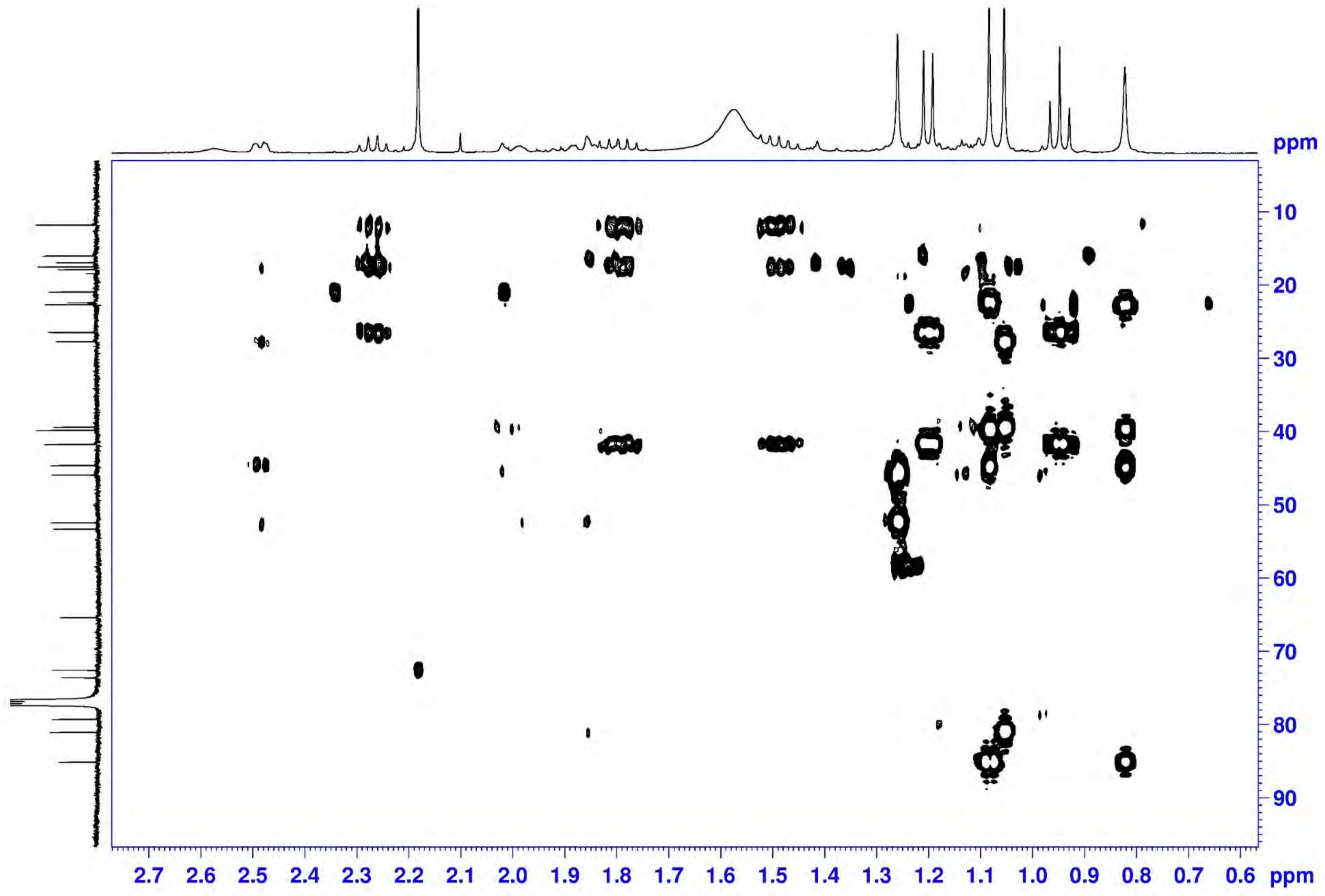

NOESY (400 MHz) spectrum of compound **4** in $CDCl_3$

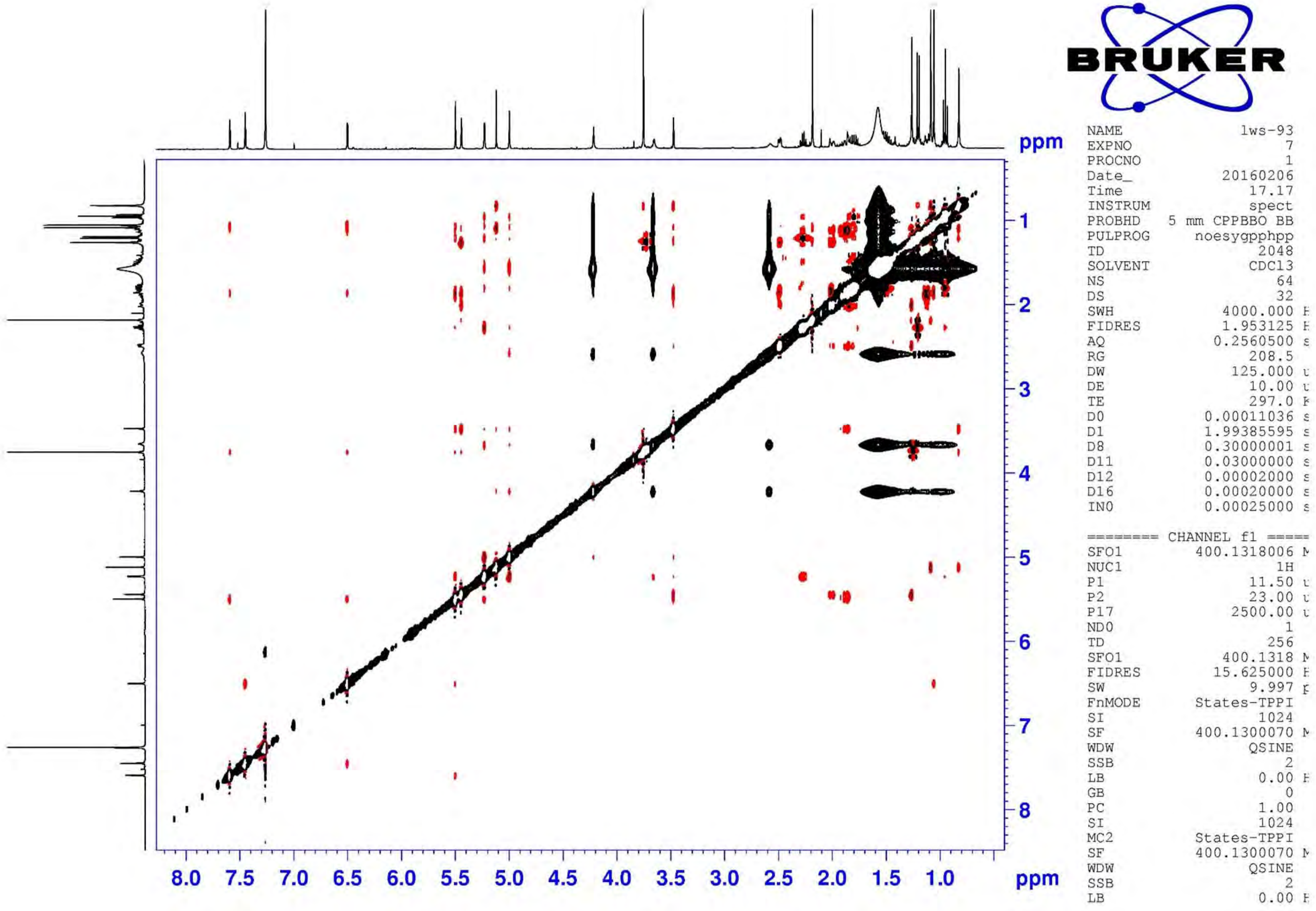

NOESY (400 MHz) spectrum of compound **4** in $CDCl_3$

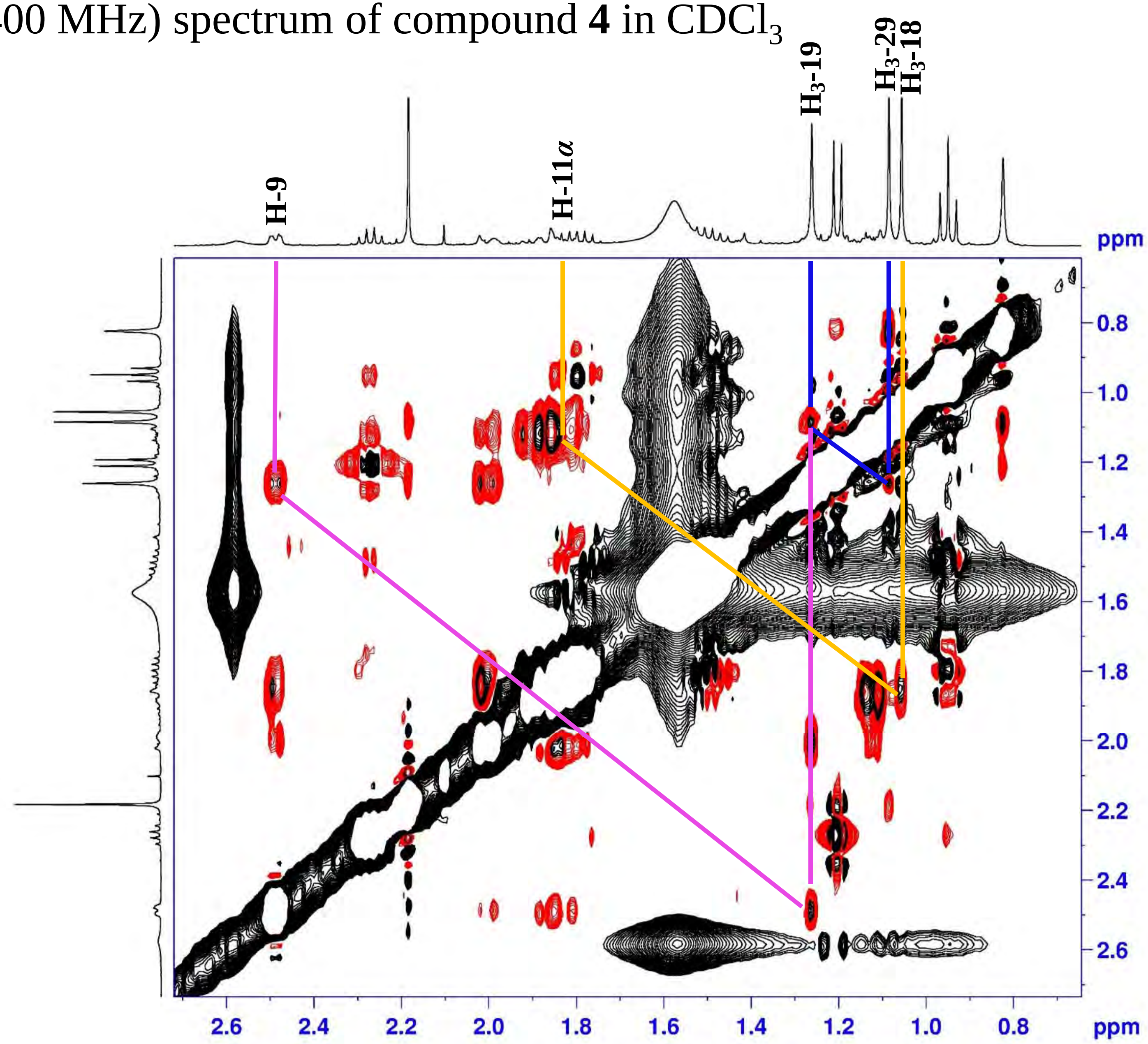

NOESY (400 MHz) spectrum of compound **4** in $CDCl_3$

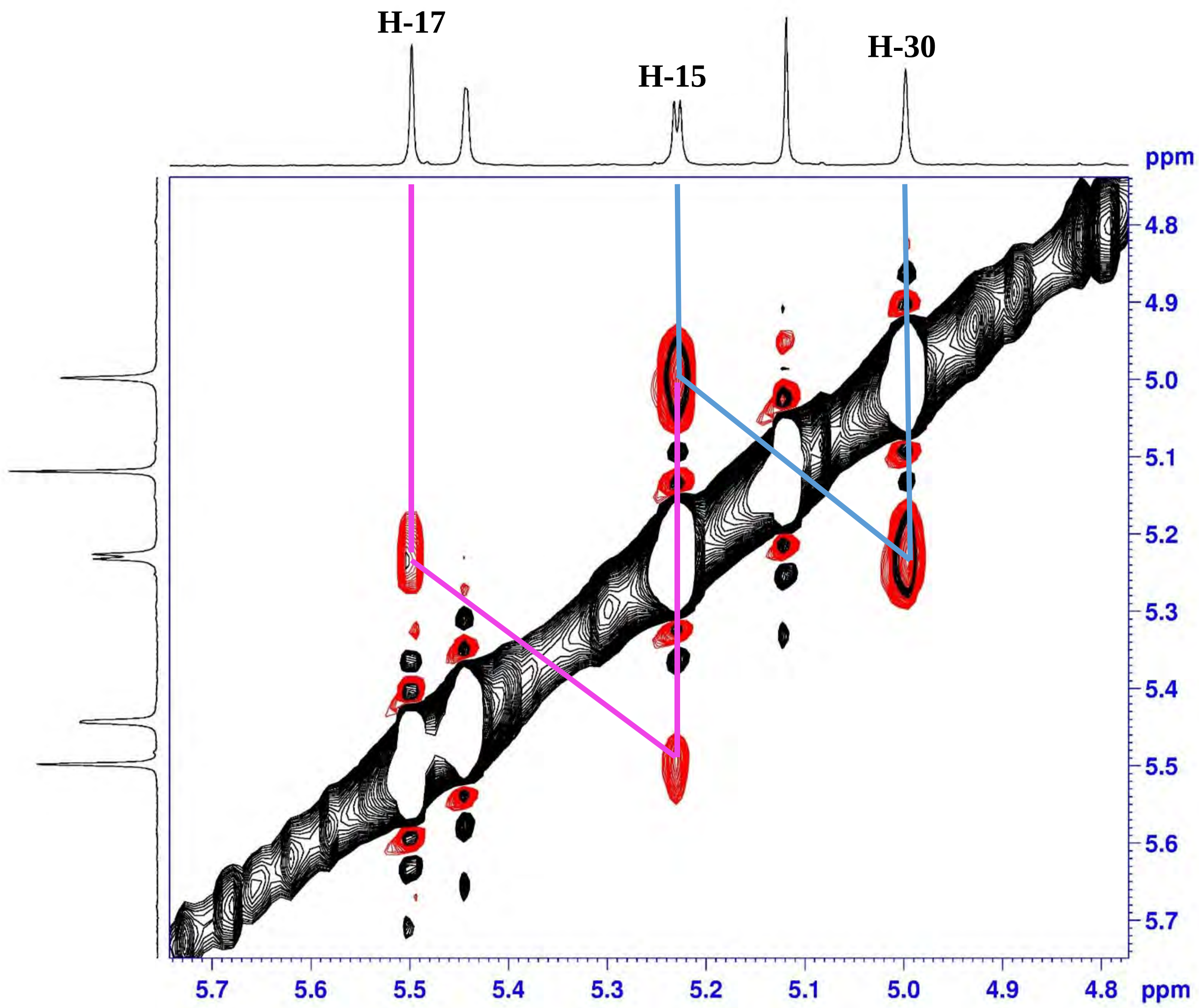

NOESY (400 MHz) spectrum of compound **4** in $CDCl_3$

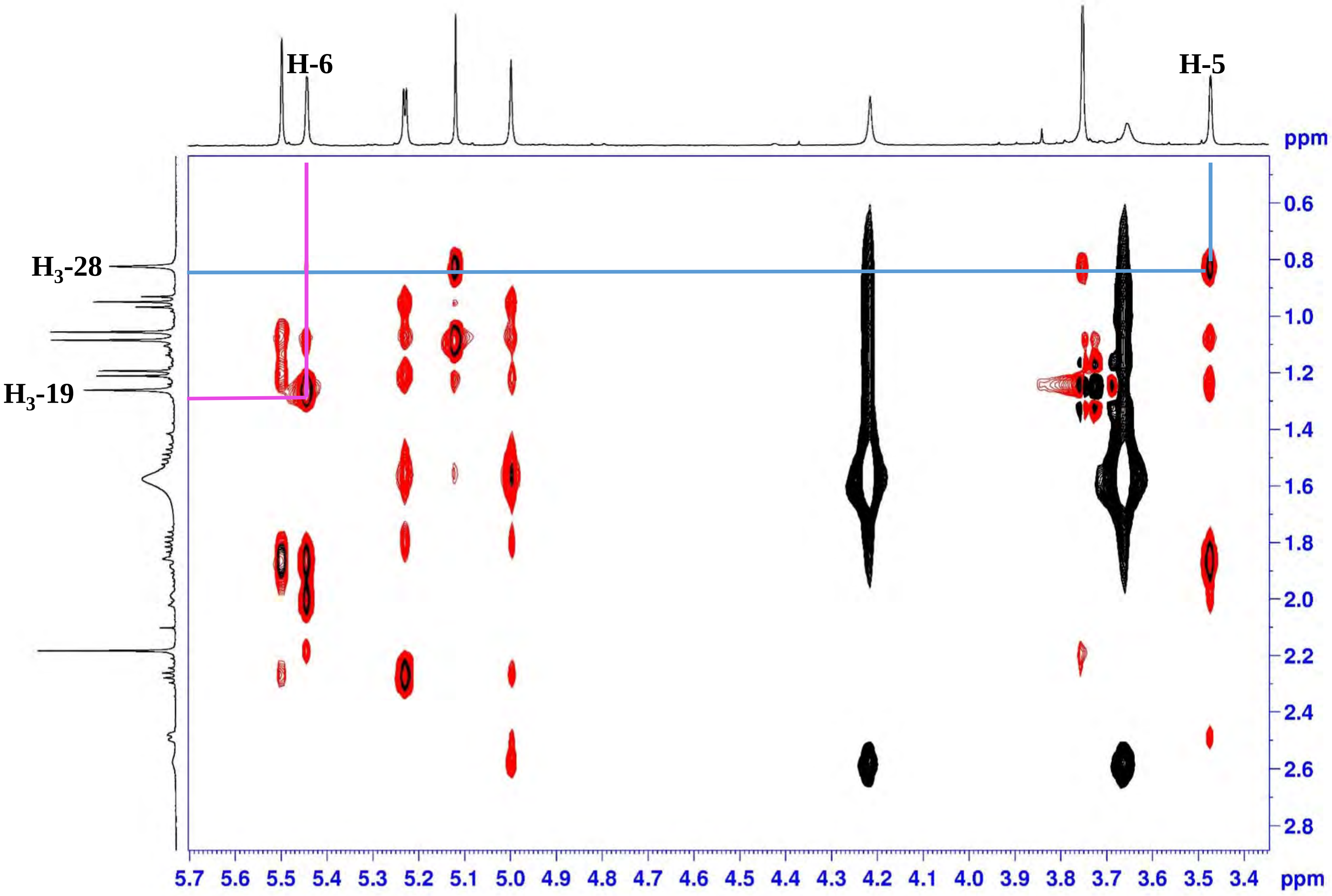

NOESY (400 MHz) spectrum of compound **4** in $CDCl_3$

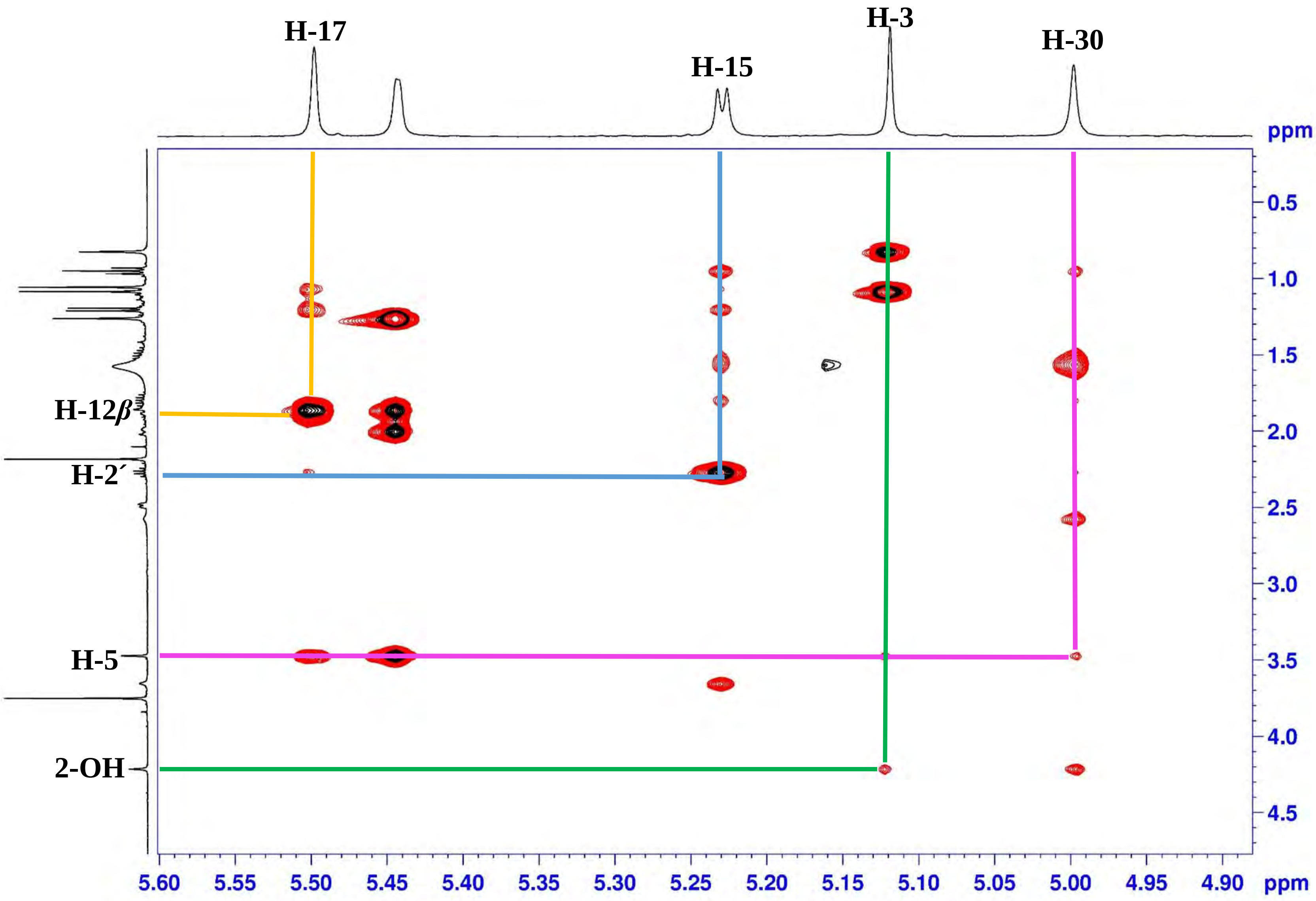

HR-ESIMS for compound **5**

Mass Spectrum SmartFormula Report

Analysis Info

Analysis Name	D:\Data\MS\data\201605\liwanshan_lws-65_pos_15_01_1862.d	Acquisition Date	5/19/2016 9:05:13 AM
Method	LC_Direct Infusion_pos_100-1000mz.m	Operator	SCSIO
Sample Name	liwanshan_lws-65_pos	Instrument / Ser#	maXis 29
Comment			

Acquisition Parameter

Source Type	ESI	Ion Polarity	Positive	Set Nebulizer	0.4 Bar
Focus	Active	Set Capillary	4500 V	Set Dry Heater	180 °C
Scan Begin	100 m/z	Set End Plate Offset	-500 V	Set Dry Gas	4.0 l/min
Scan End	2000 m/z	Set Collision Cell RF	800.0 Vpp	Set Divert Valve	Waste

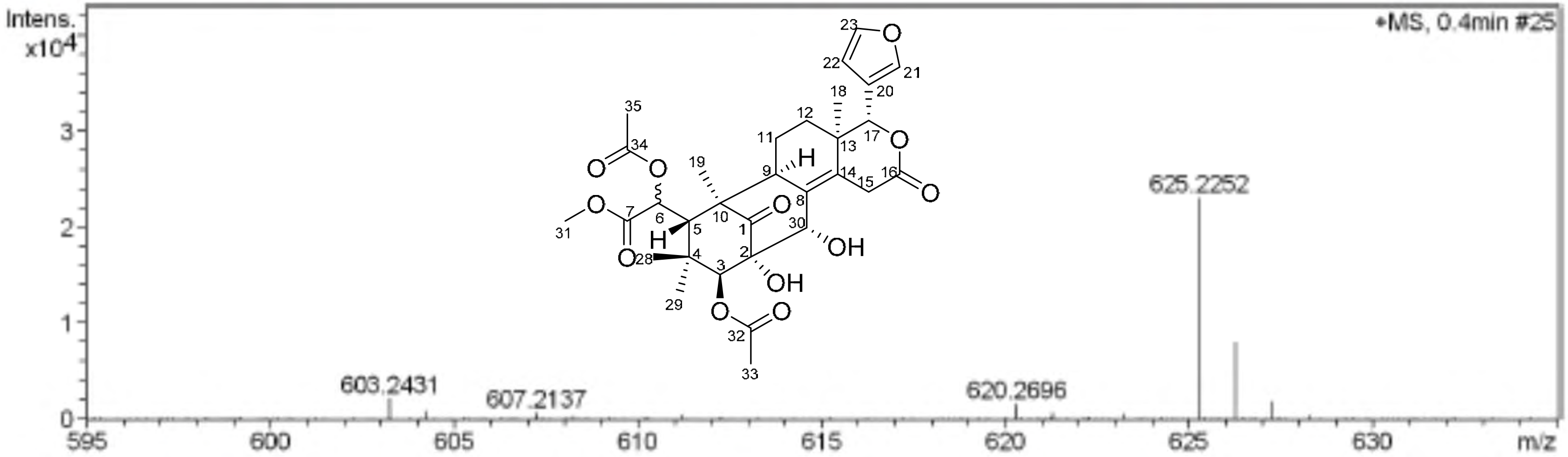

Meas. m/z	#	Formula	Score	m/z	err [ppm]	err [mDa]	mSigma	rdb	e⁻ Conf	N-Rule
603.2431	1	C 31 H 39 O 12	100.00	603.2436	0.9	0.5	18.0	12.5	even	ok
625.2252	1	C 31 H 38 Na O 12	100.00	625.2255	0.5	0.3	2.4	12.5	even	ok
1227.4629	1	C 62 H 76 Na O 24	90.59	1227.4619	-0.8	-1.0	45.6	24.5	even	ok

HR-ESIMS for compound **5**

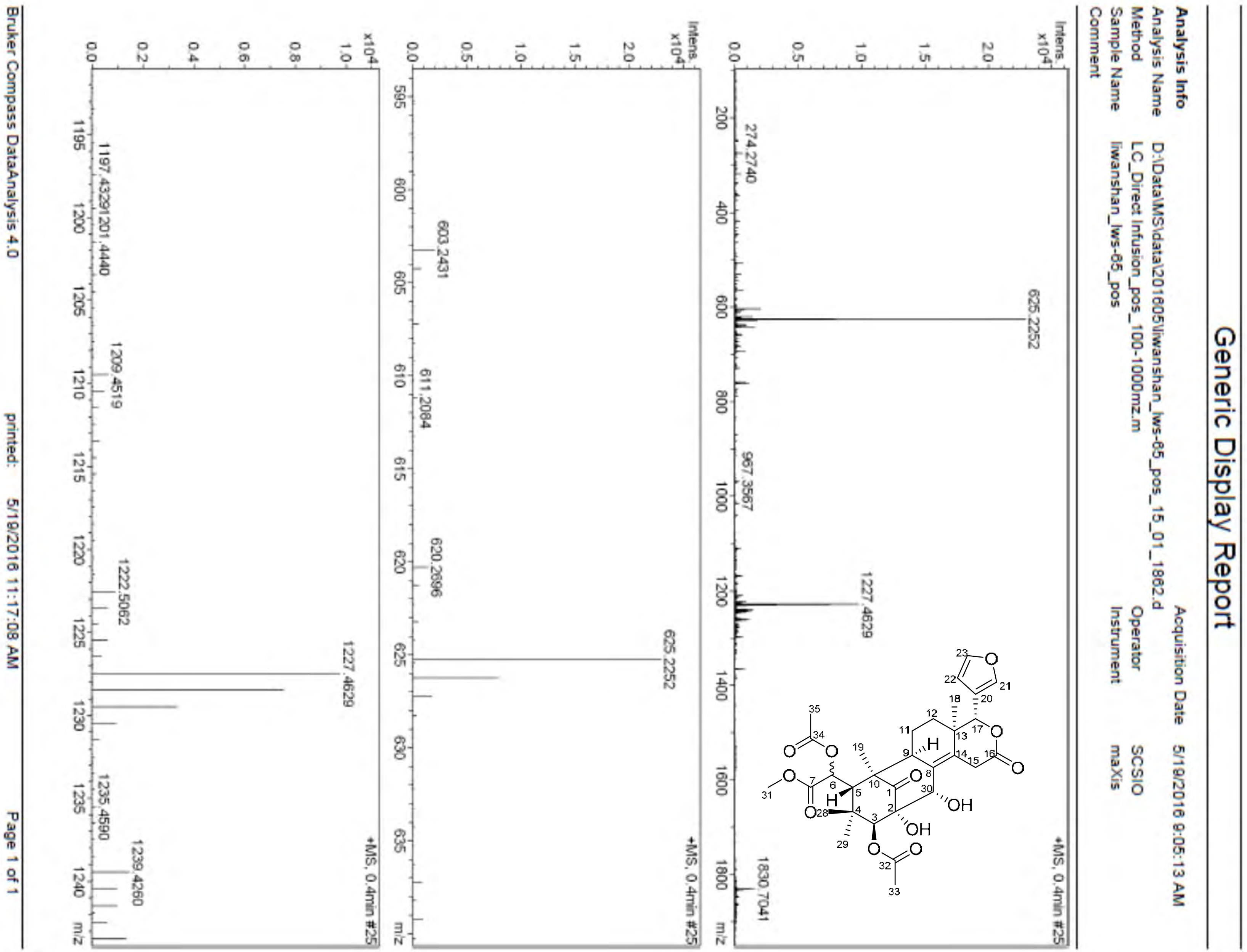

^{1}H NMR (400 MHz) spectrum of compound **5** in $CDCl_3$

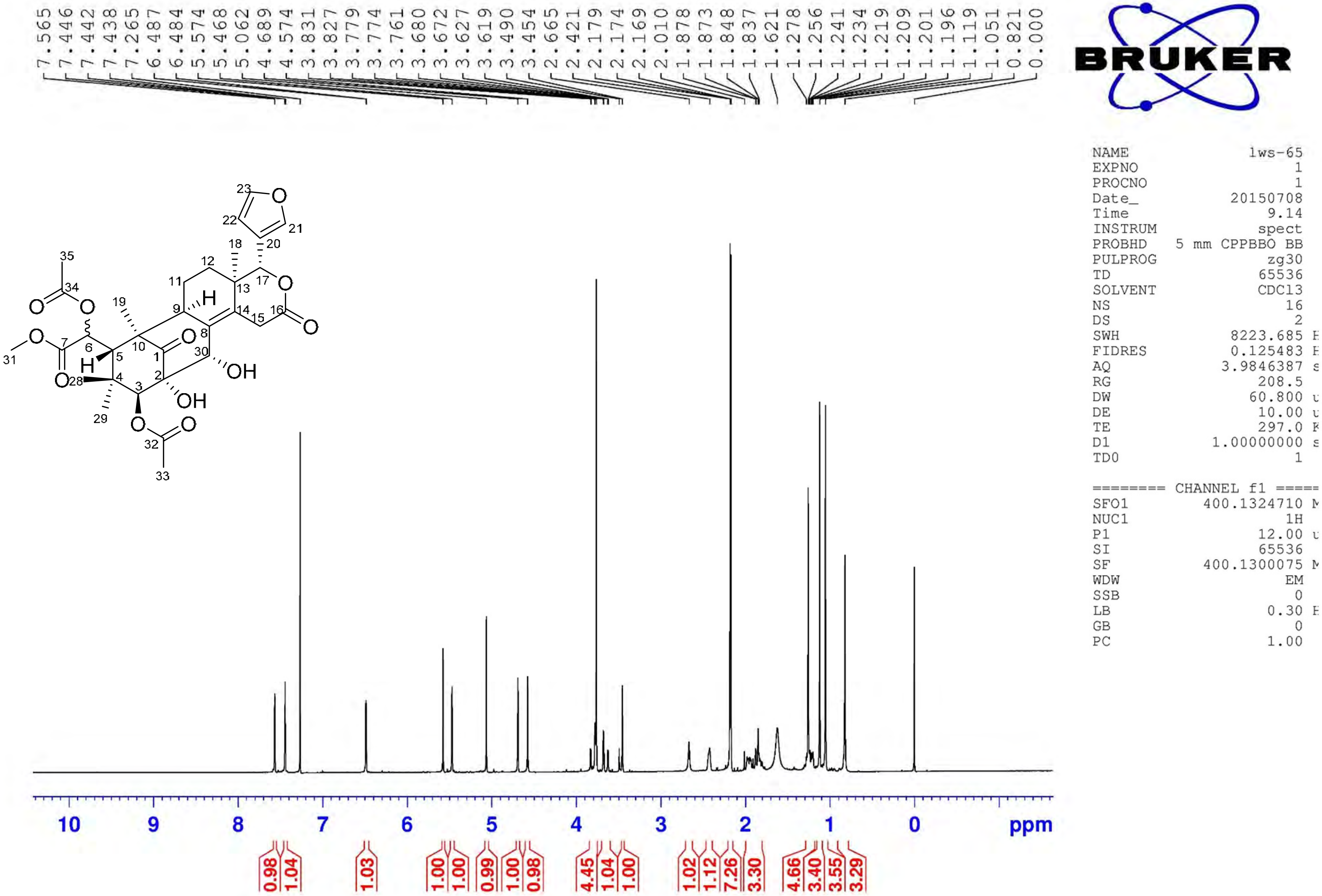

^{1}H NMR (400 MHz) spectrum of compound **5** in $CDCl_3$

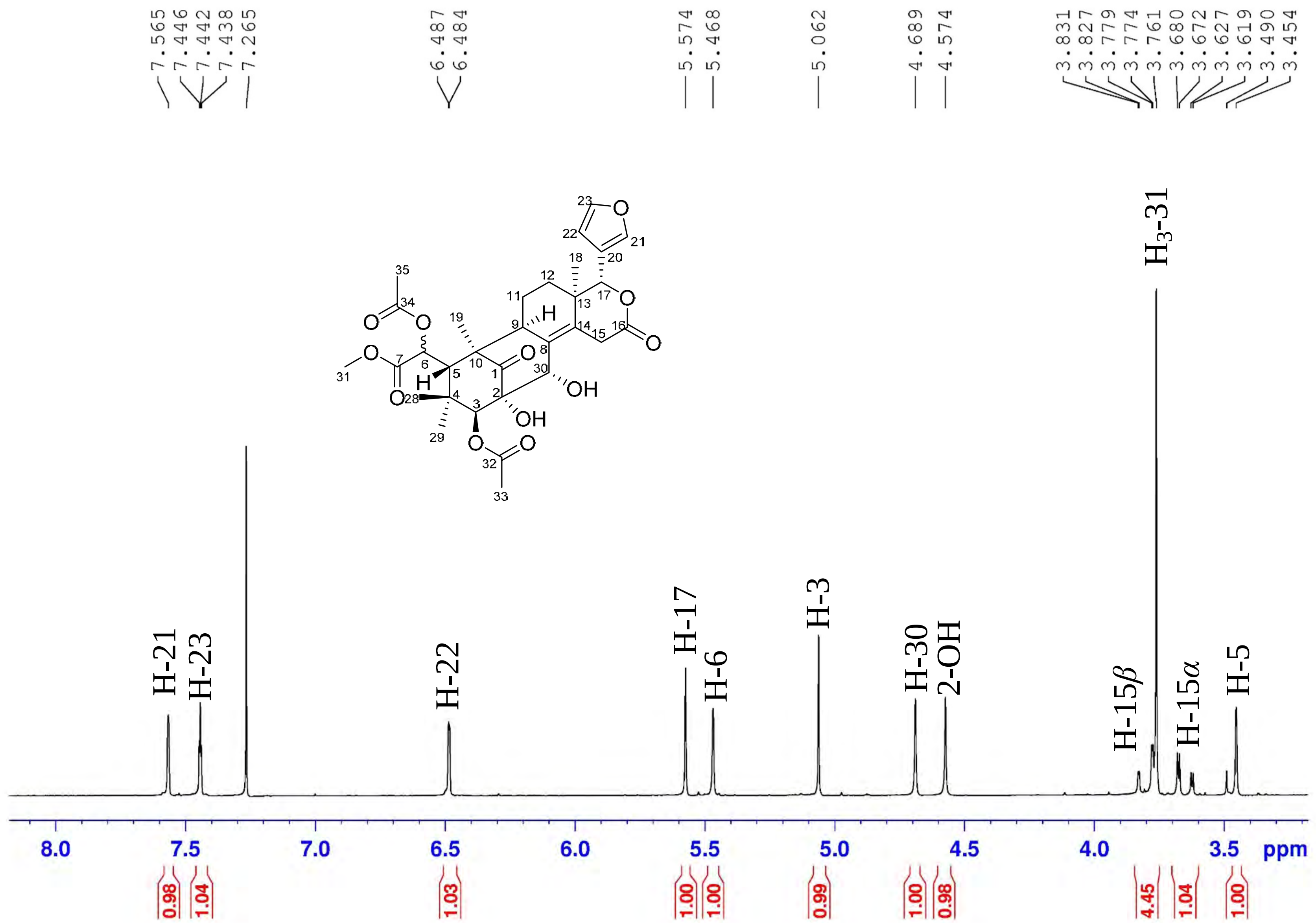

^{1}H NMR (400 MHz) spectrum of compound **5** in $CDCl_3$

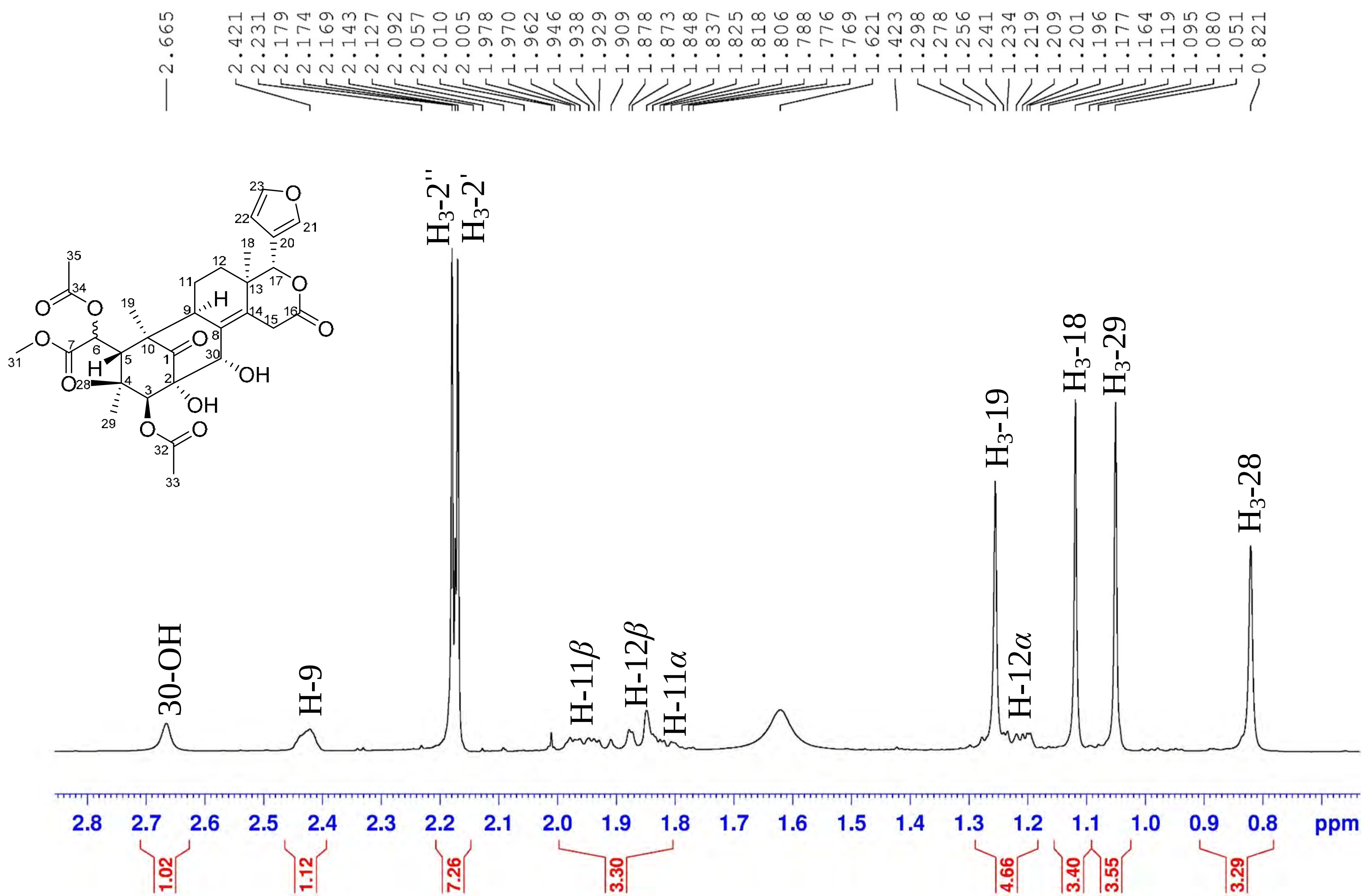

^{13}C NMR (100 MHz) spectrum of compound **5** in $CDCl_3$

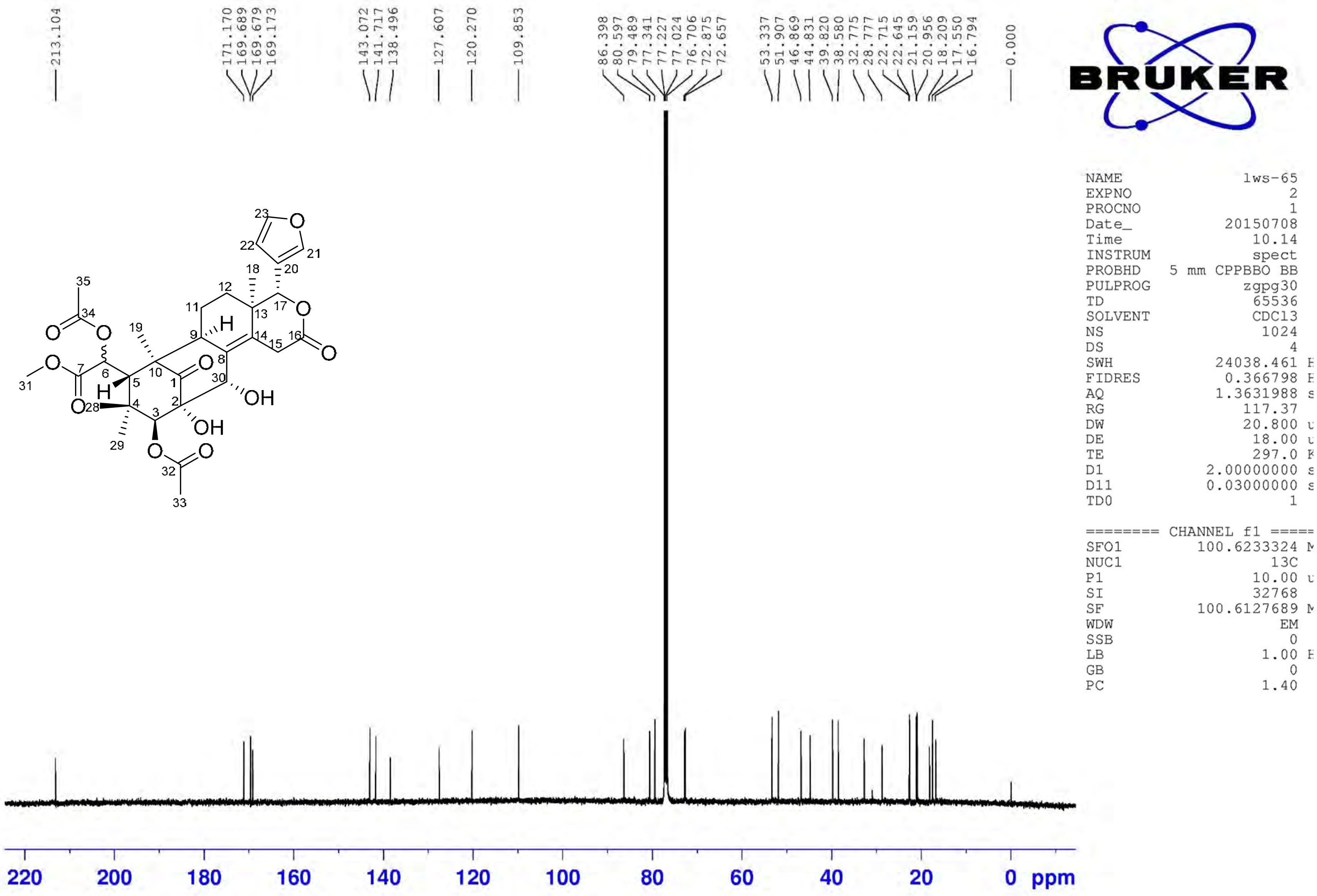

^{13}C NMR (100 MHz) spectrum of compound **5** in $CDCl_3$

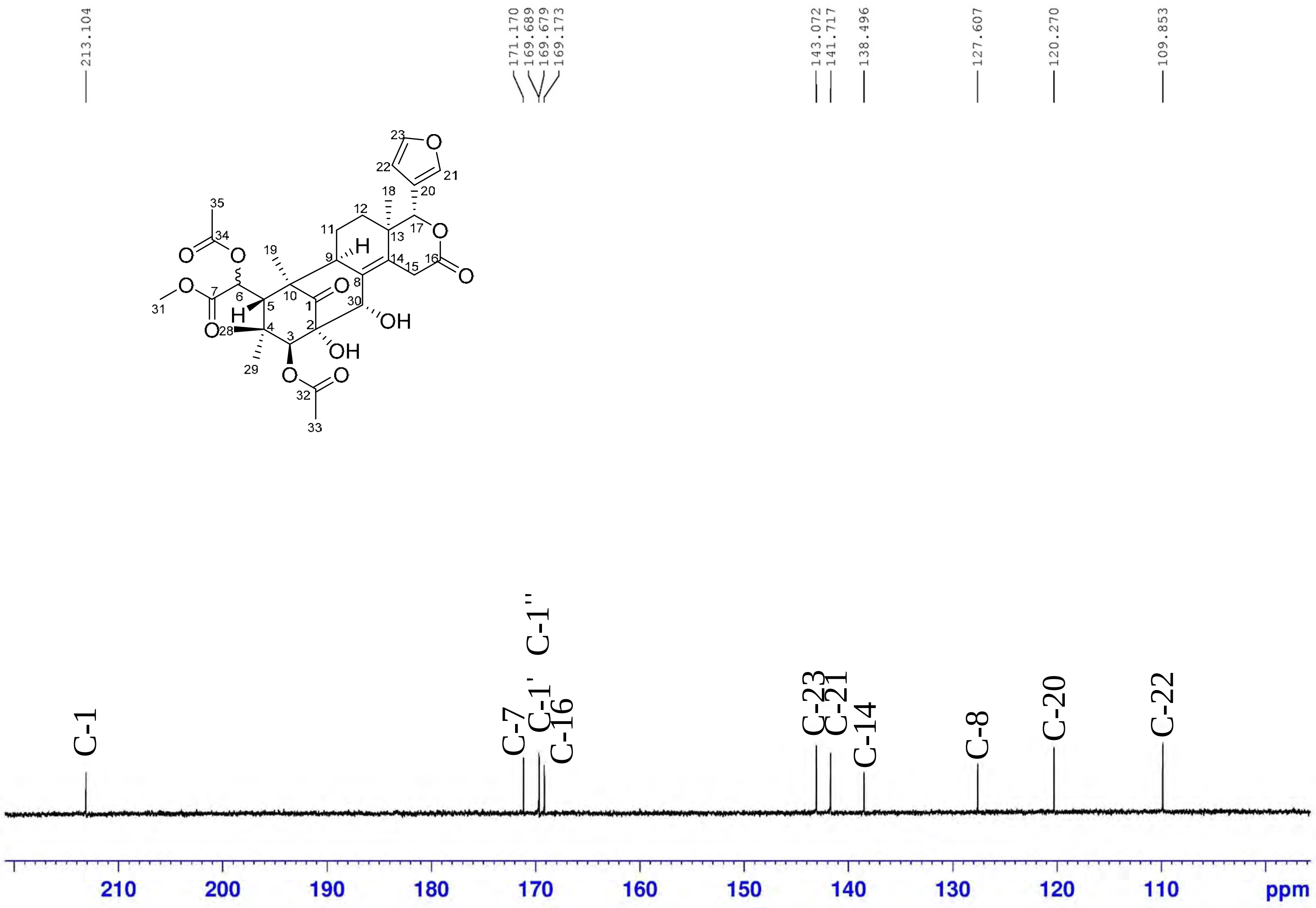

^{13}C NMR (100 MHz) spectrum of compound **5** in $CDCl_3$

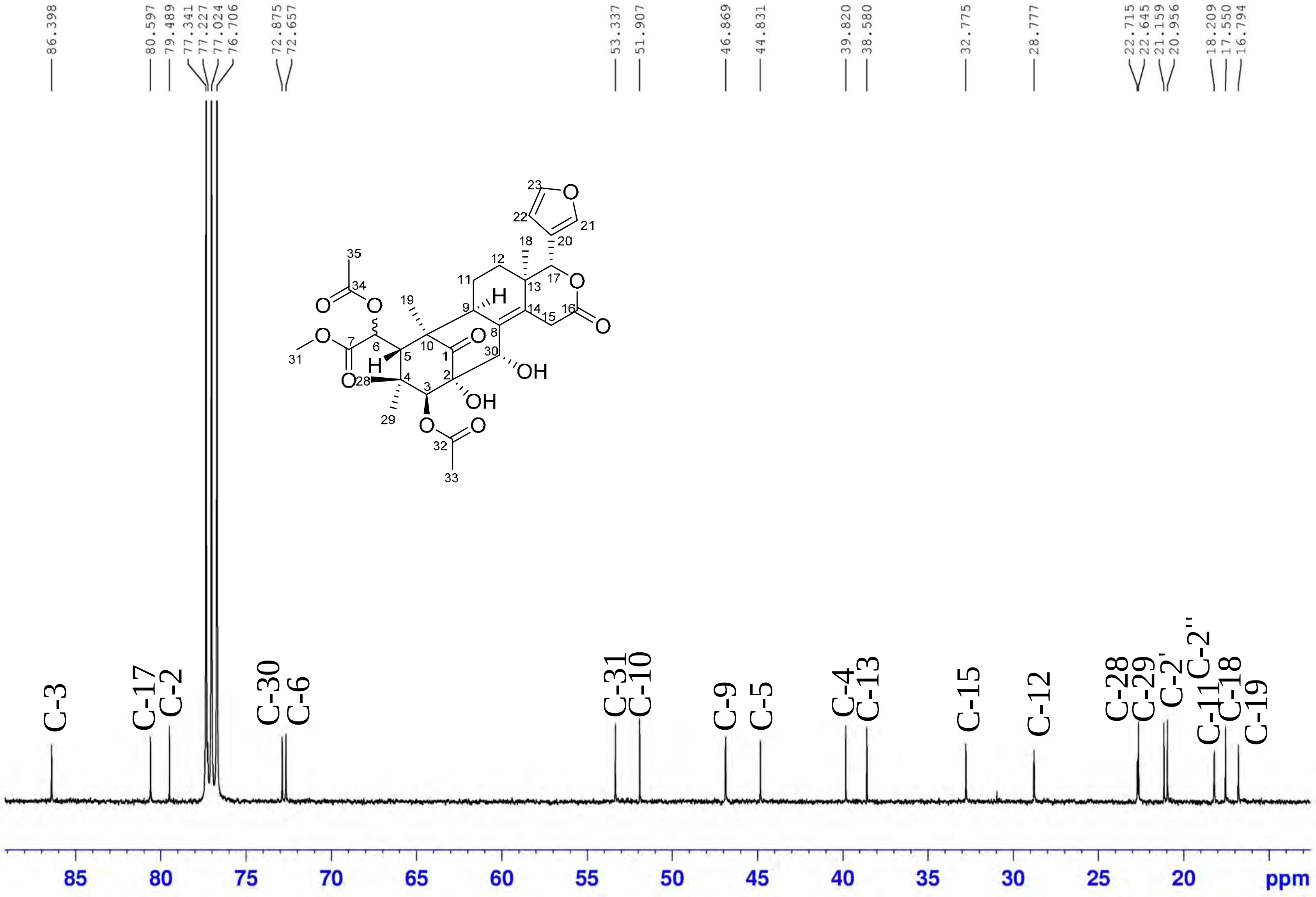

DEPT135 (100 MHz) spectrum of compound **5** in $CDCl_3$

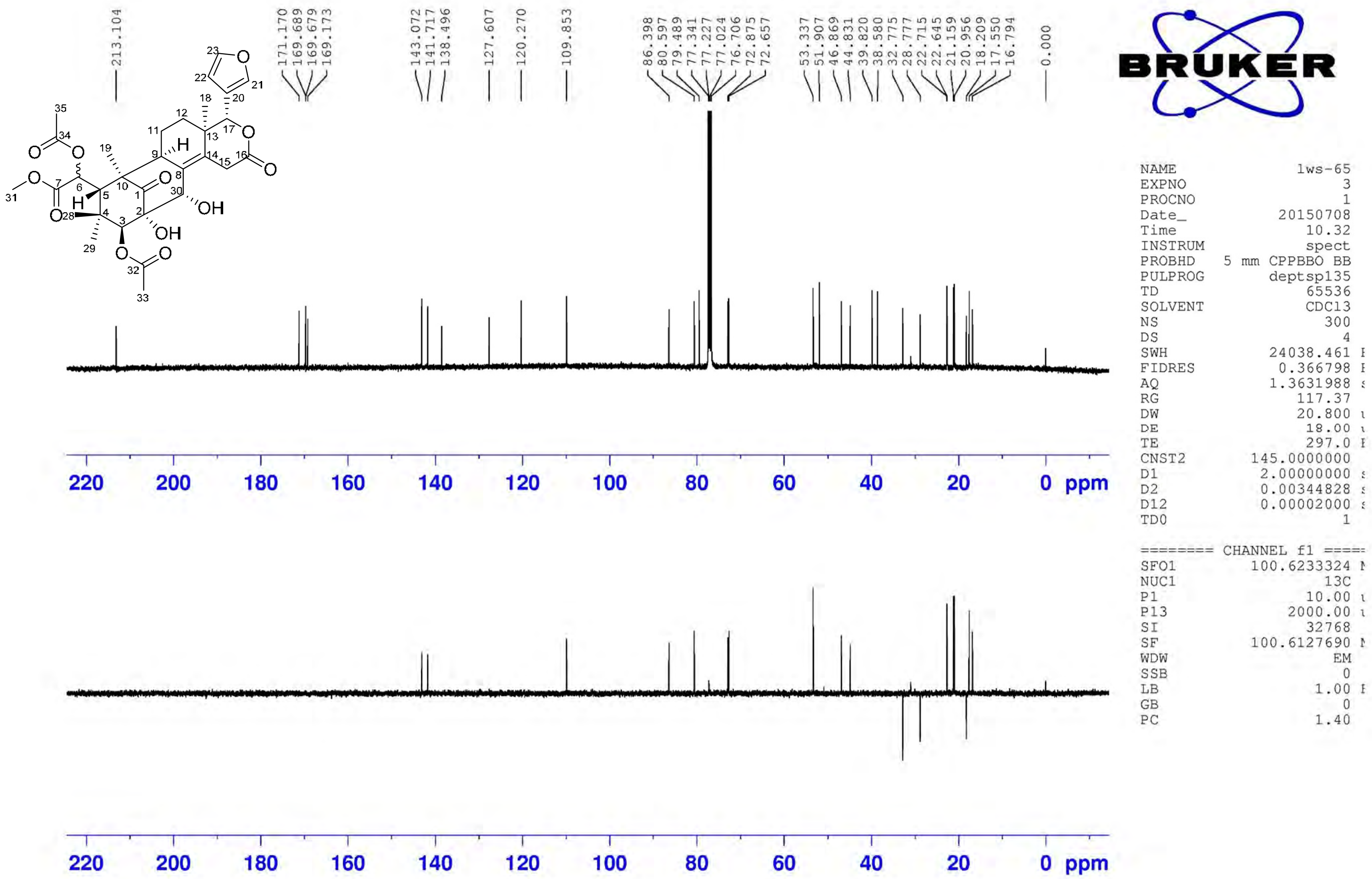

DEPT135 (100 MHz) spectrum of compound **5** in $CDCl_3$

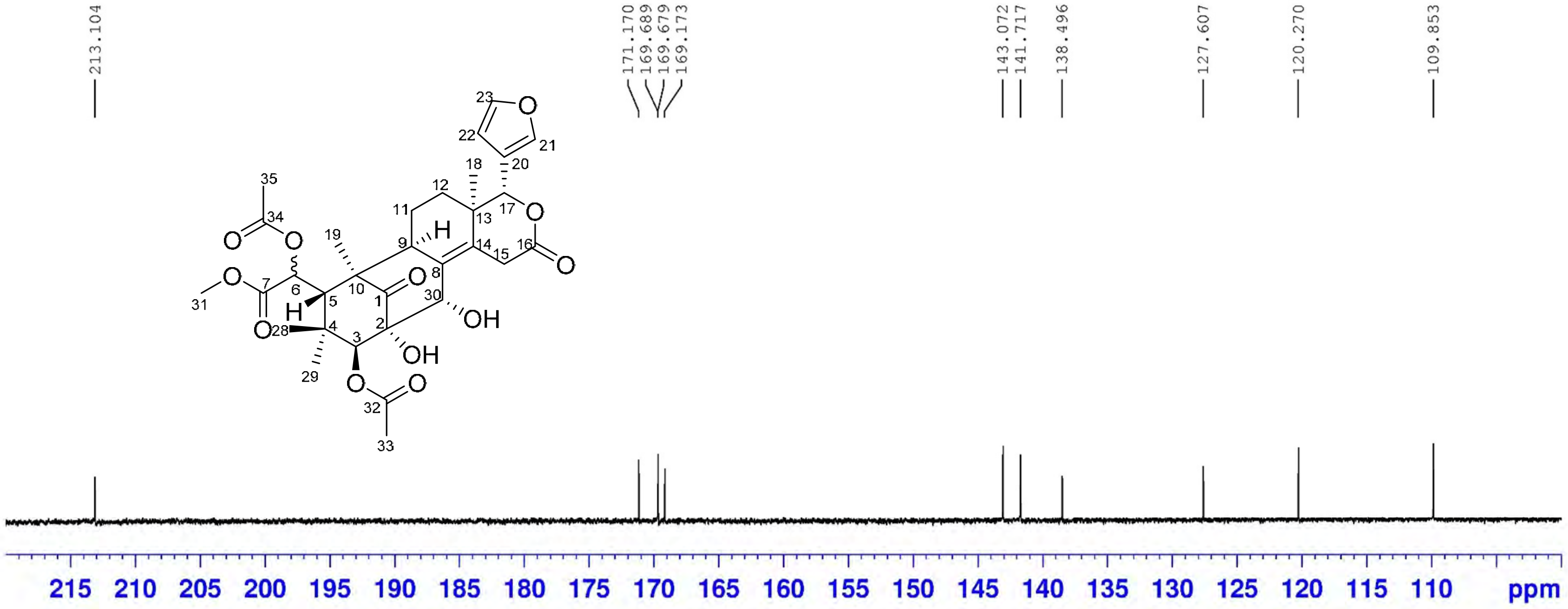

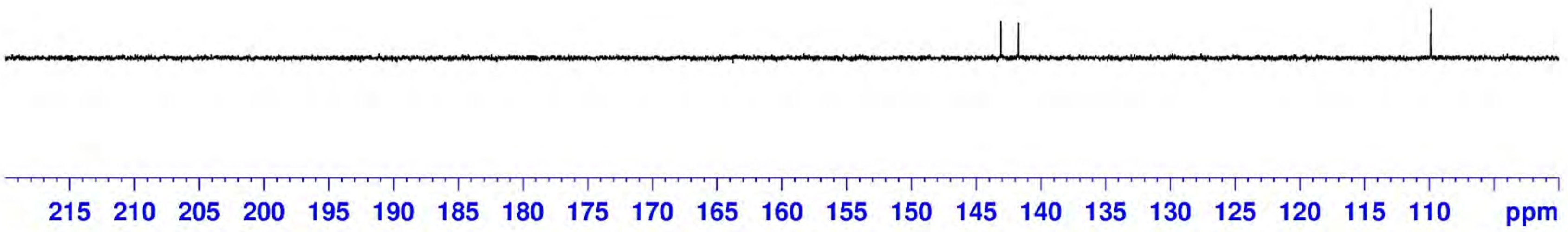

DEPT135 (100 MHz) spectrum of compound **5** in $CDCl_3$

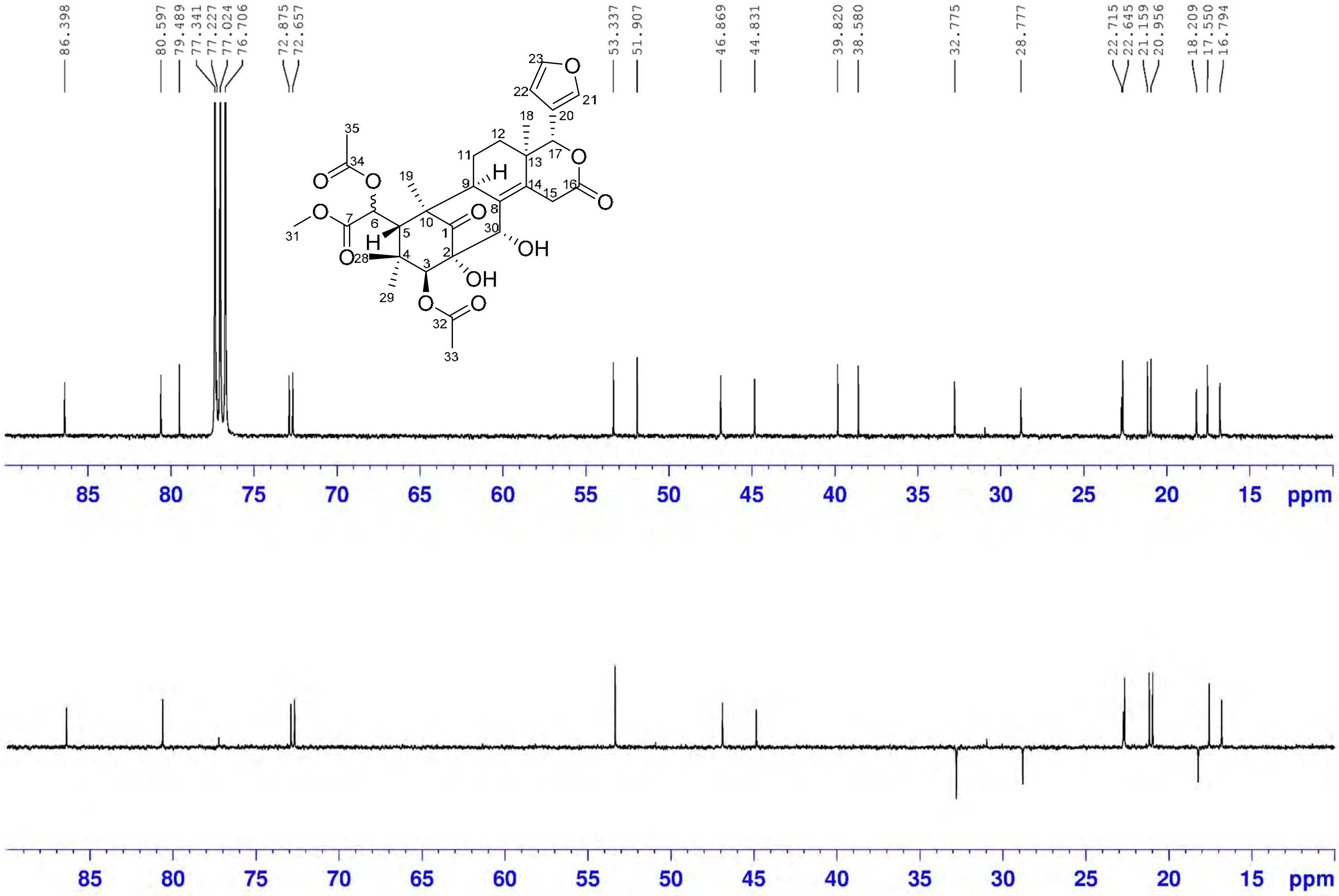

^{1}H-^{1}H COSY (400 MHz) spectrum of compound **5** in $CDCl_3$

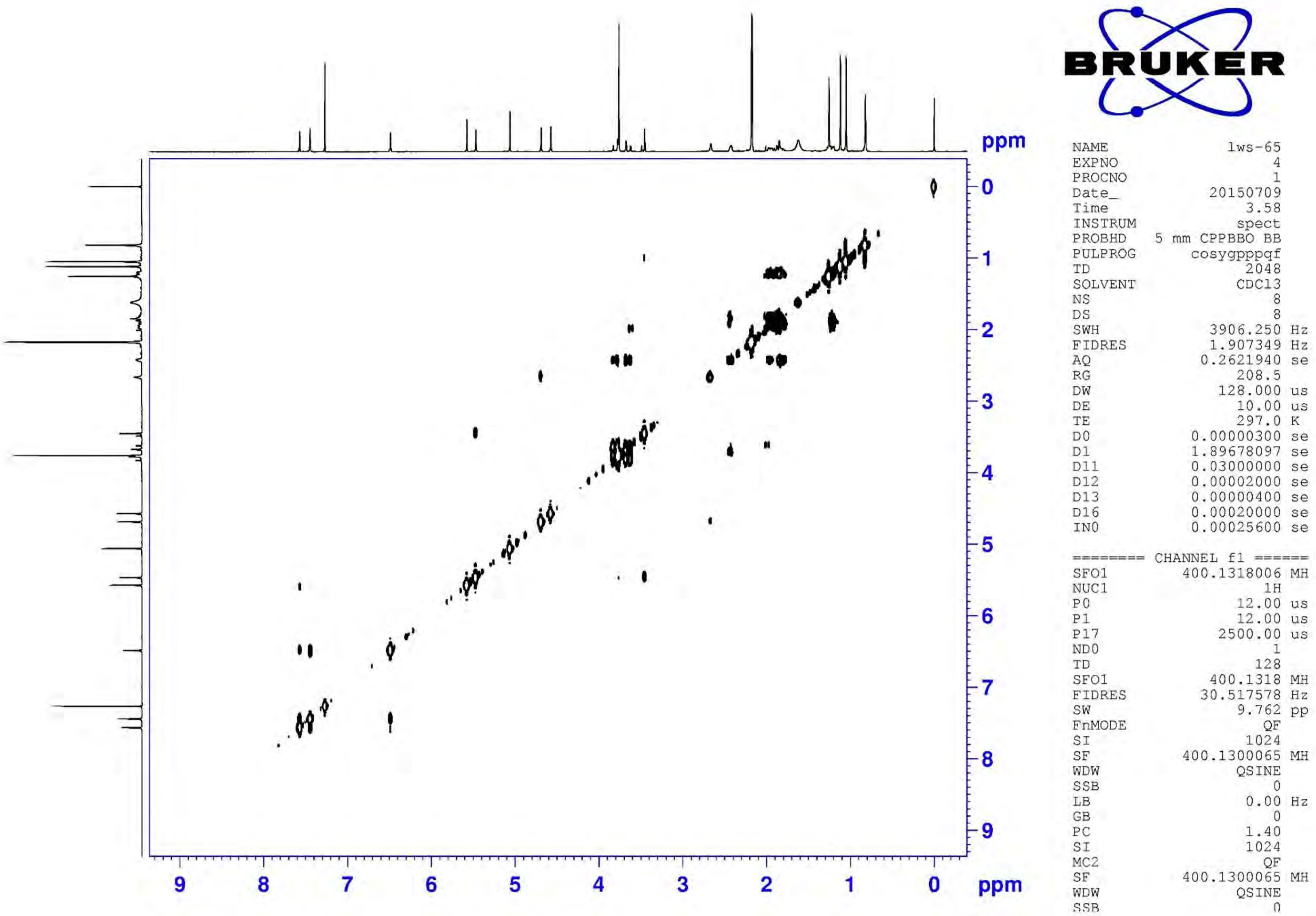

^1H-^1H COSY (400 MHz) spectrum of compound **5** in $CDCl_3$

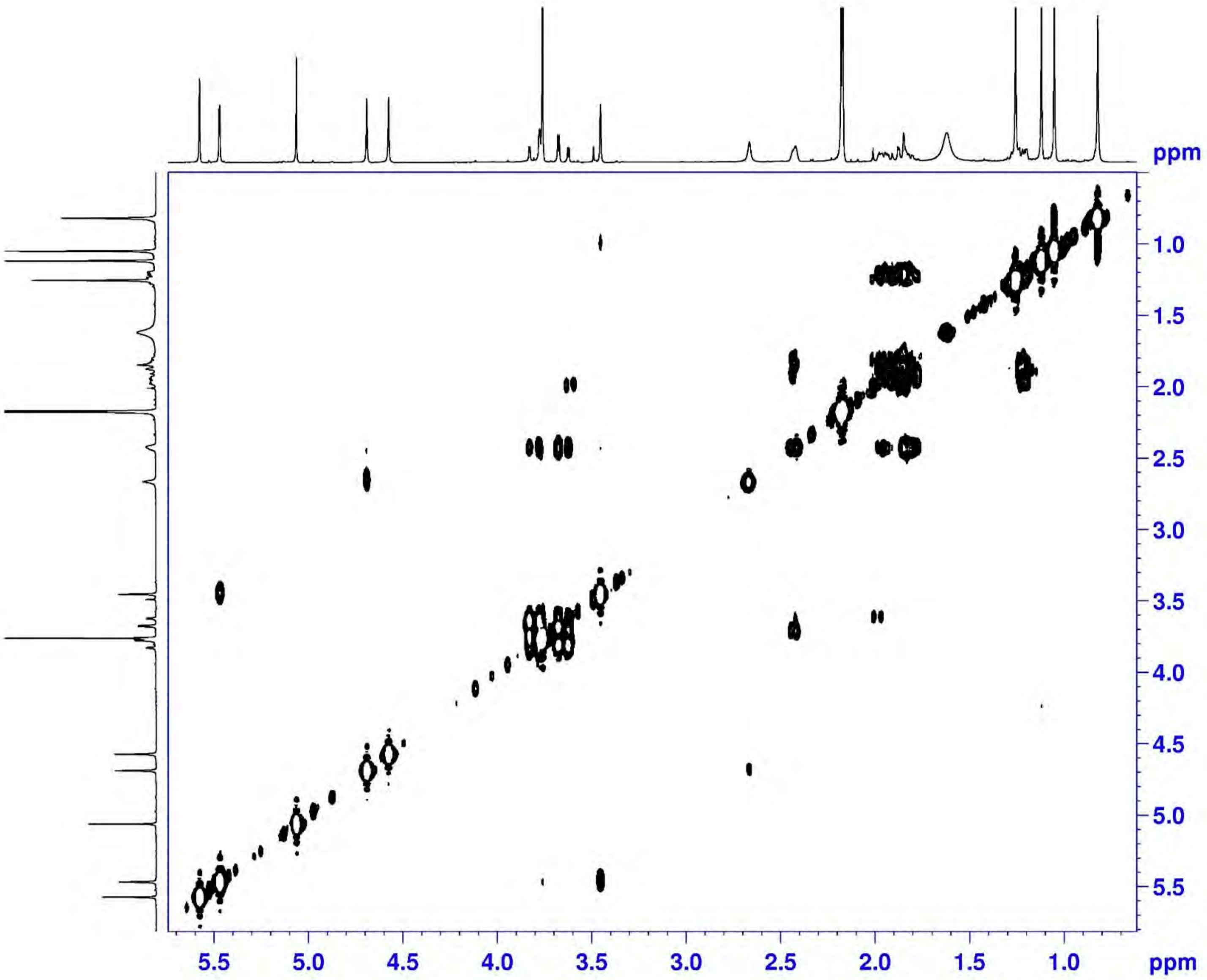

HSQC (400 MHz) spectrum of compound **5** in $CDCl_3$

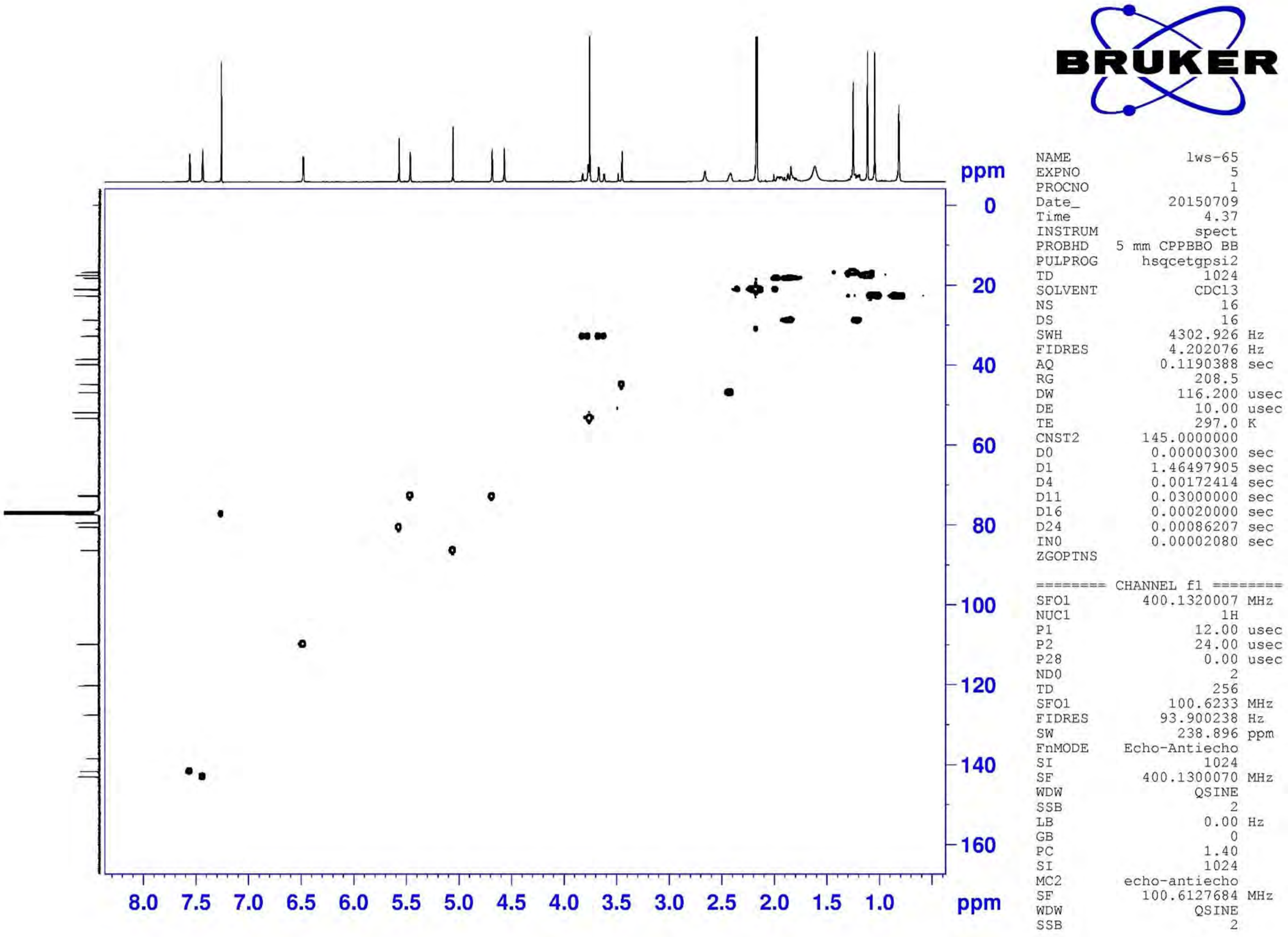

HSQC (400 MHz) spectrum of compound **5** in $CDCl_3$

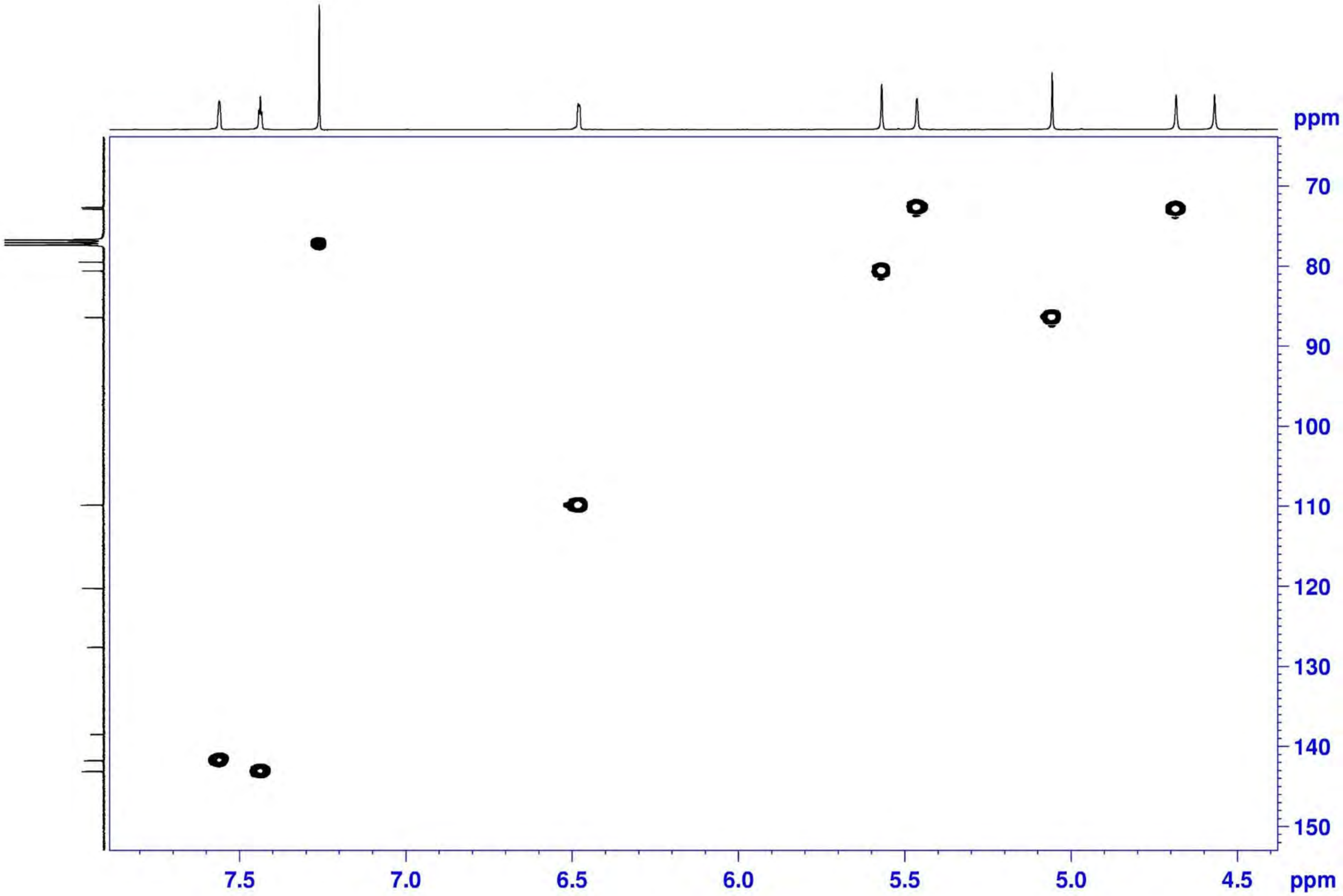

HSQC (400 MHz) spectrum of compound **5** in $CDCl_3$

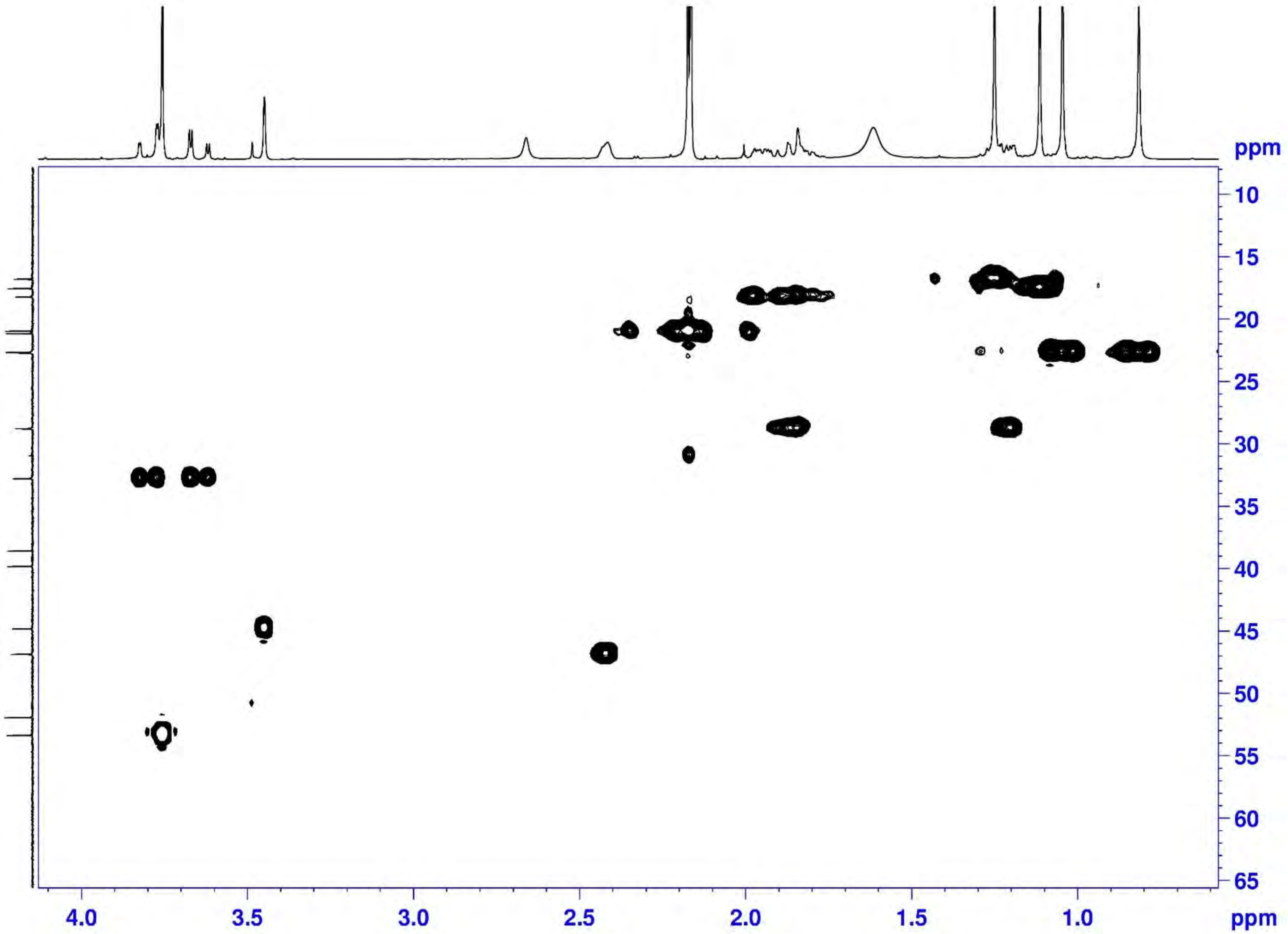

HMBC (400 MHz) spectrum of compound **5** in $CDCl_3$

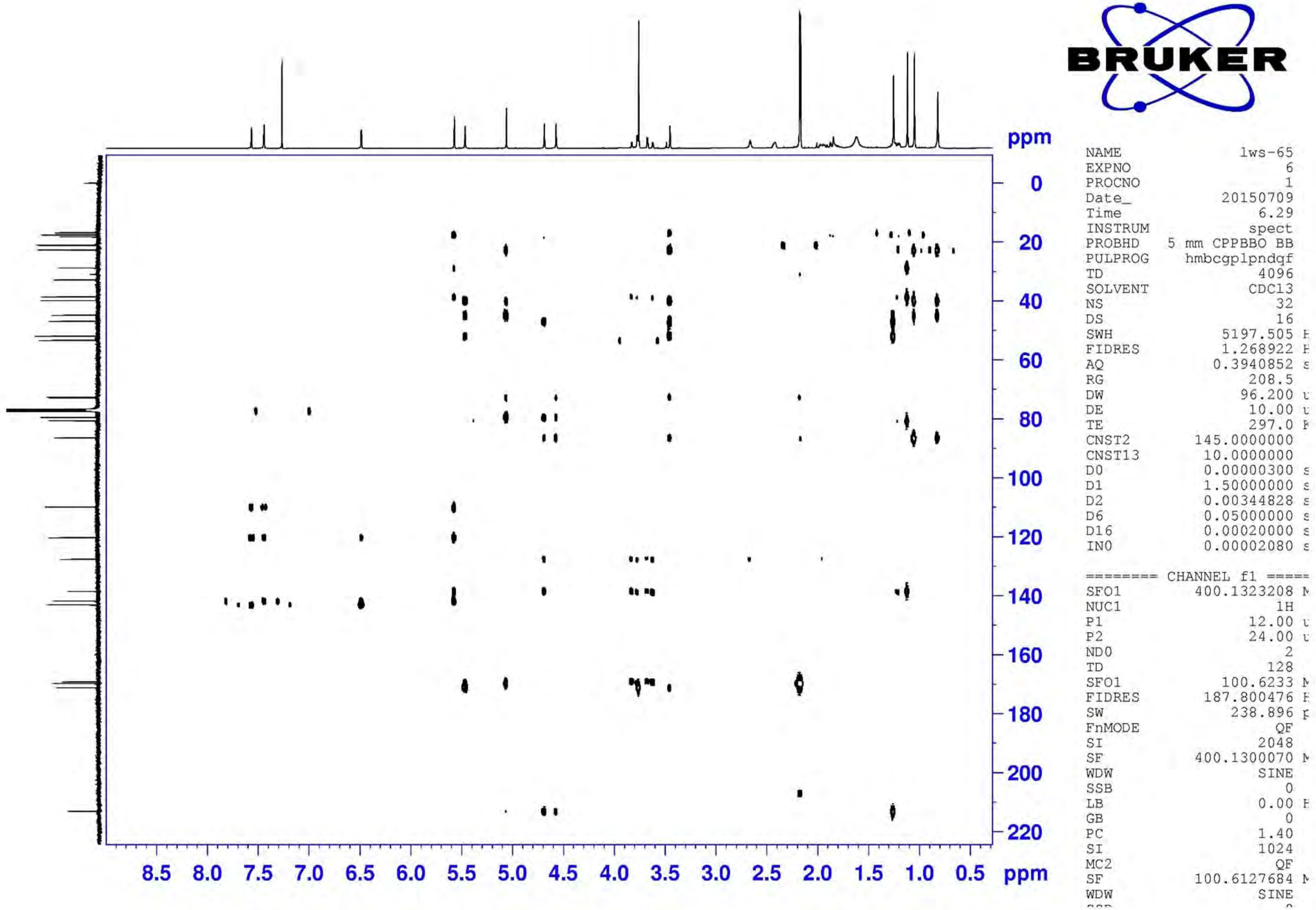

HMBC (400 MHz) spectrum of compound **5** in $CDCl_3$

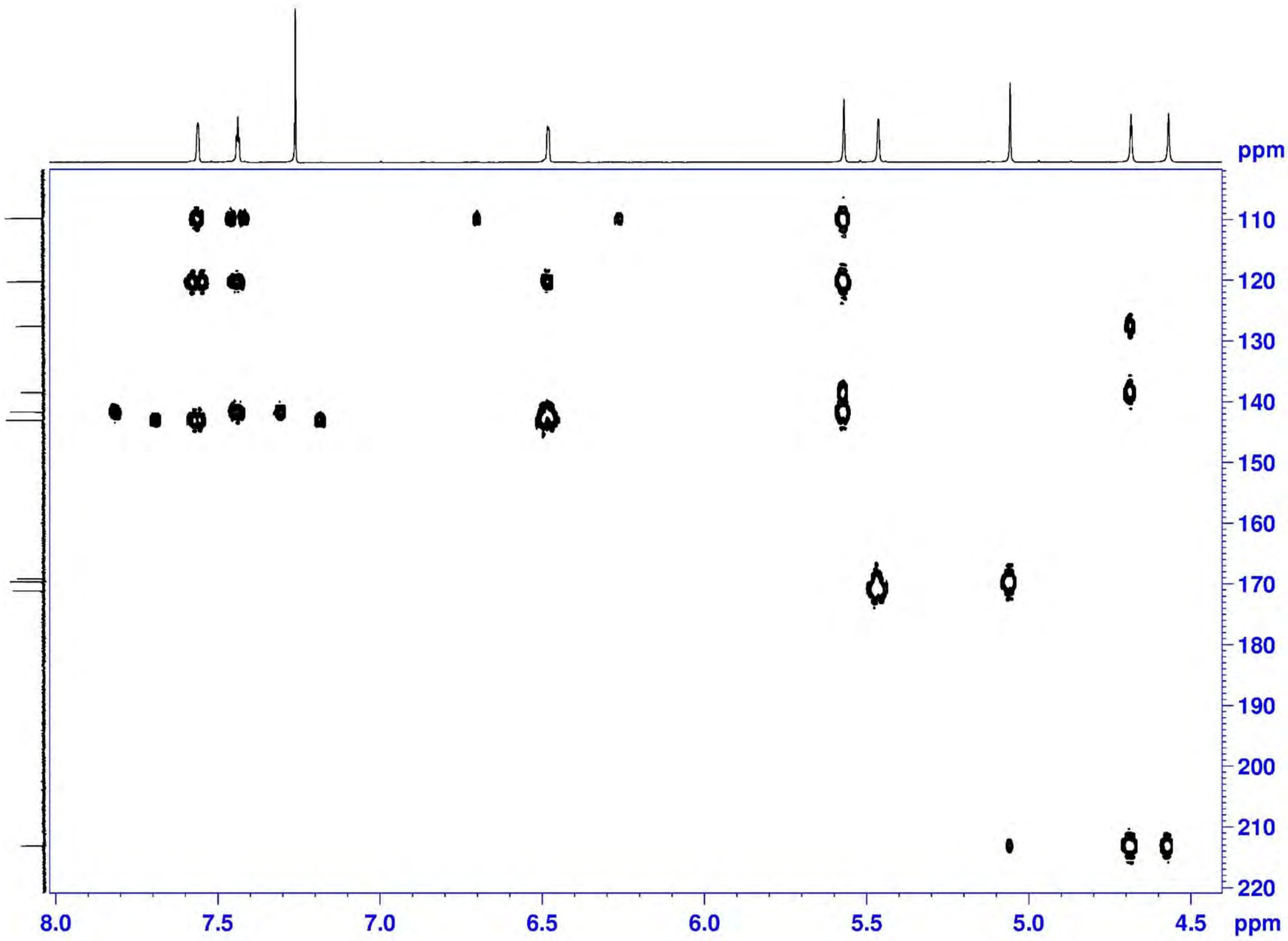

HMBC (400 MHz) spectrum of compound **5** in $CDCl_3$

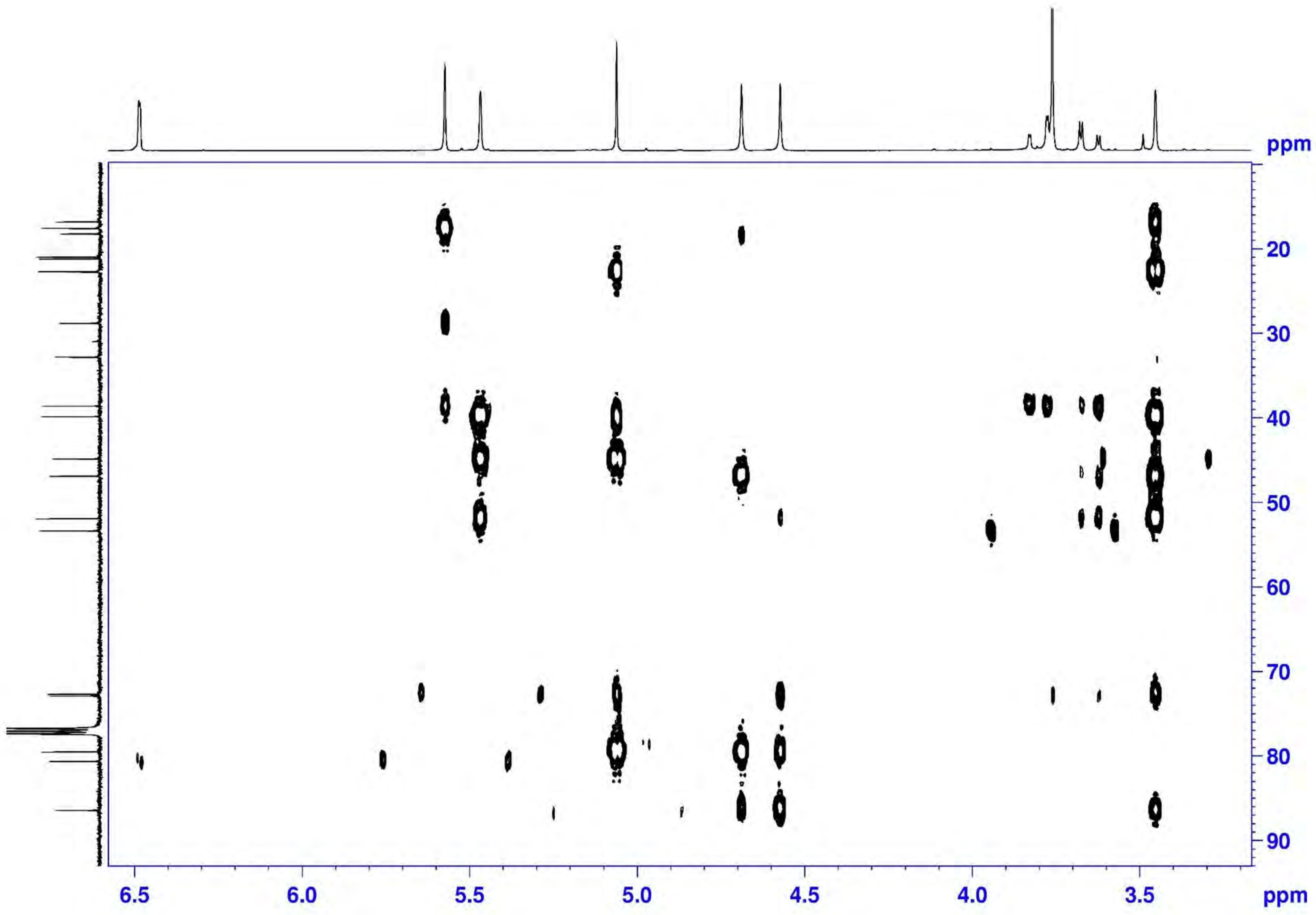

HMBC (400 MHz) spectrum of compound **5** in $CDCl_3$

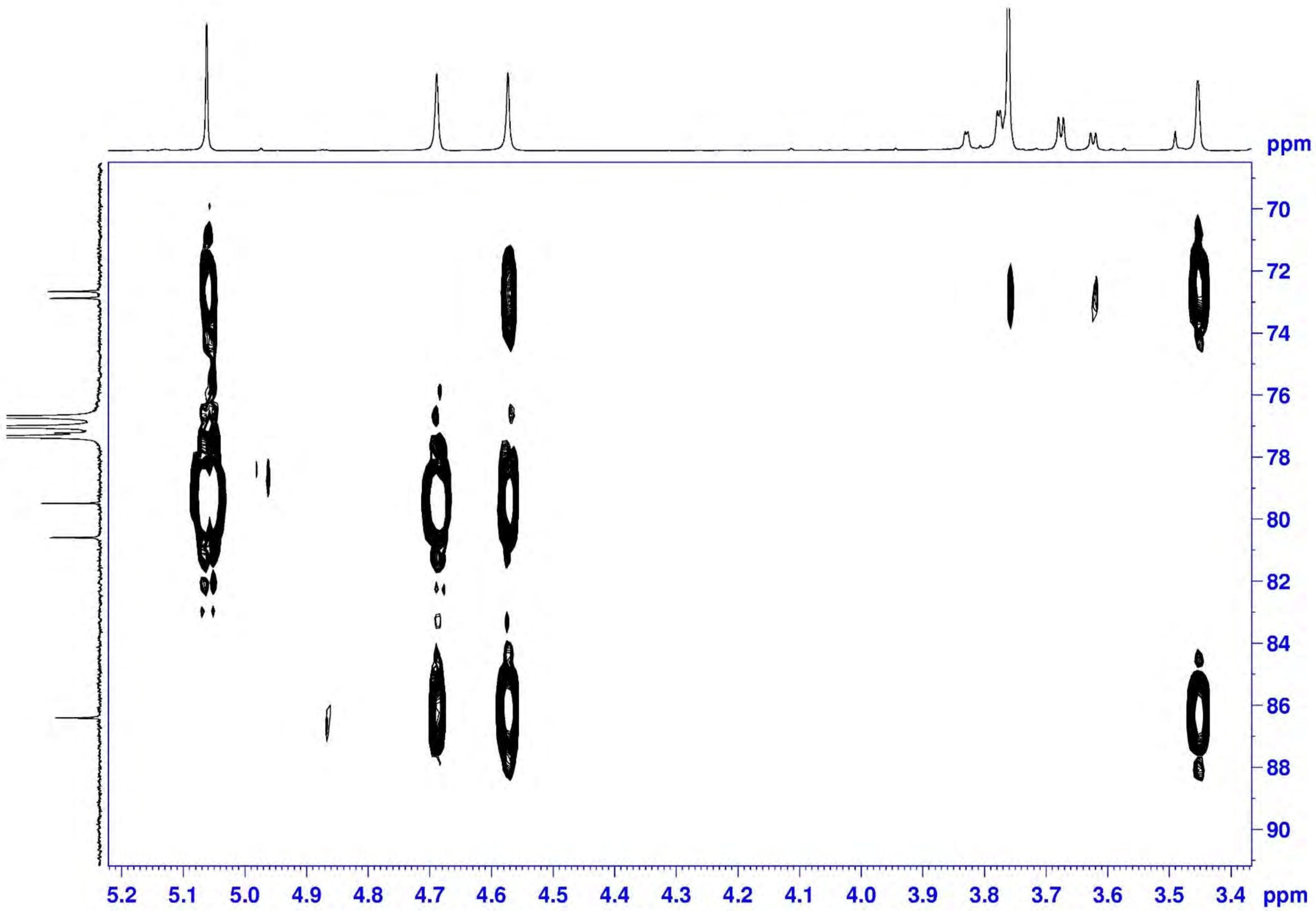

HMBC (400 MHz) spectrum of compound **5** in $CDCl_3$

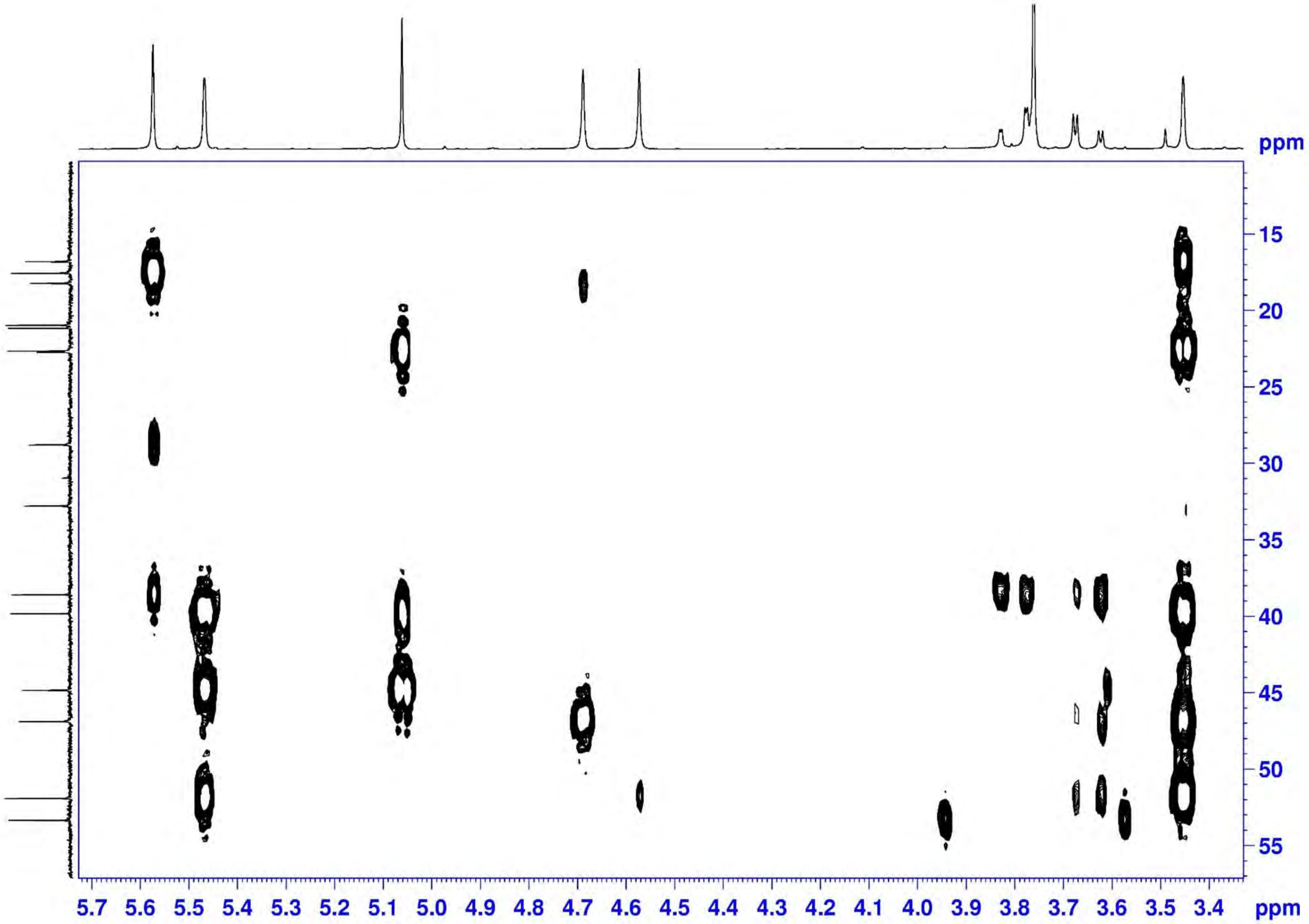

HMBC (400 MHz) spectrum of compound **5** in $CDCl_3$

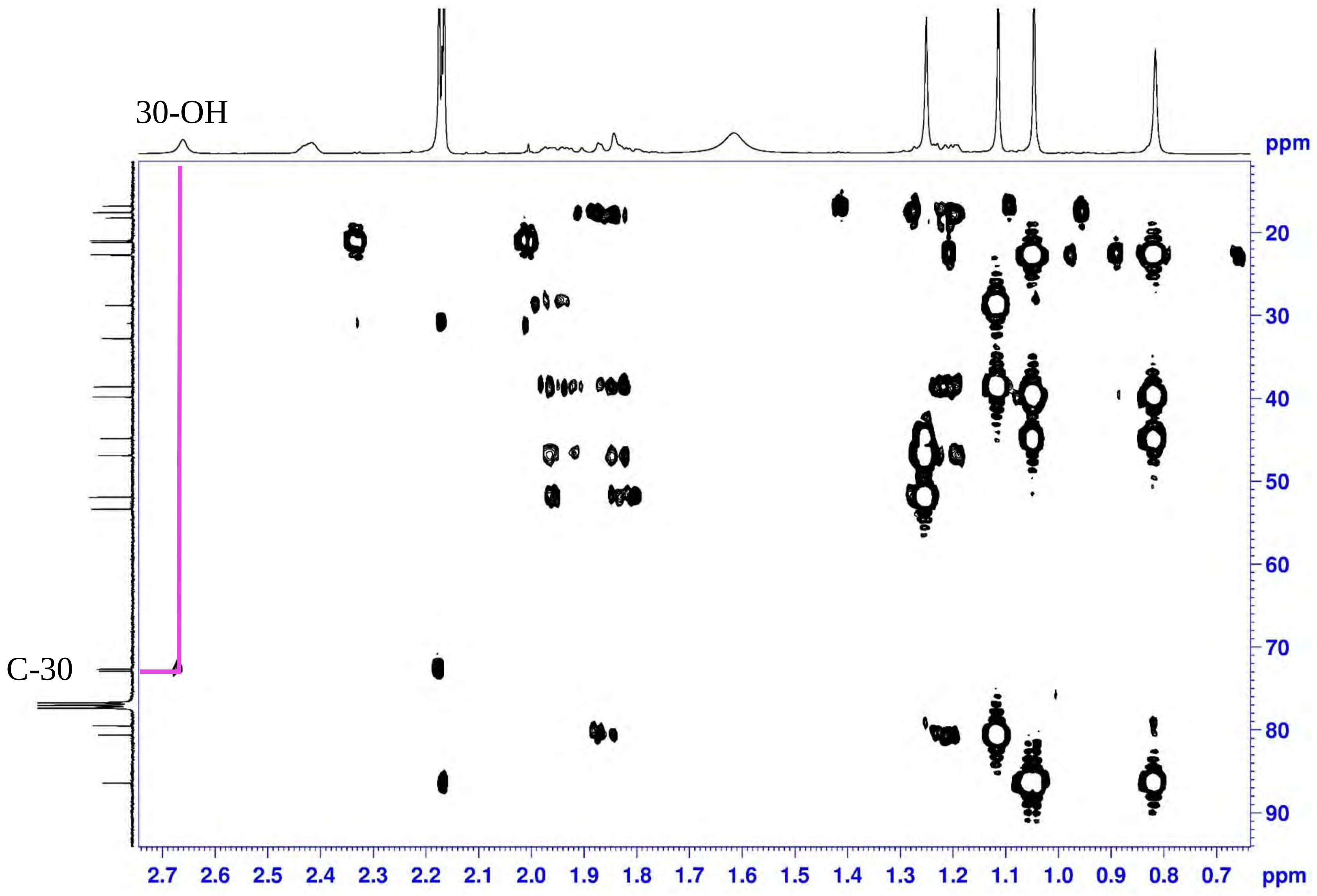

HMBC (400 MHz) spectrum of compound **5** in $CDCl_3$

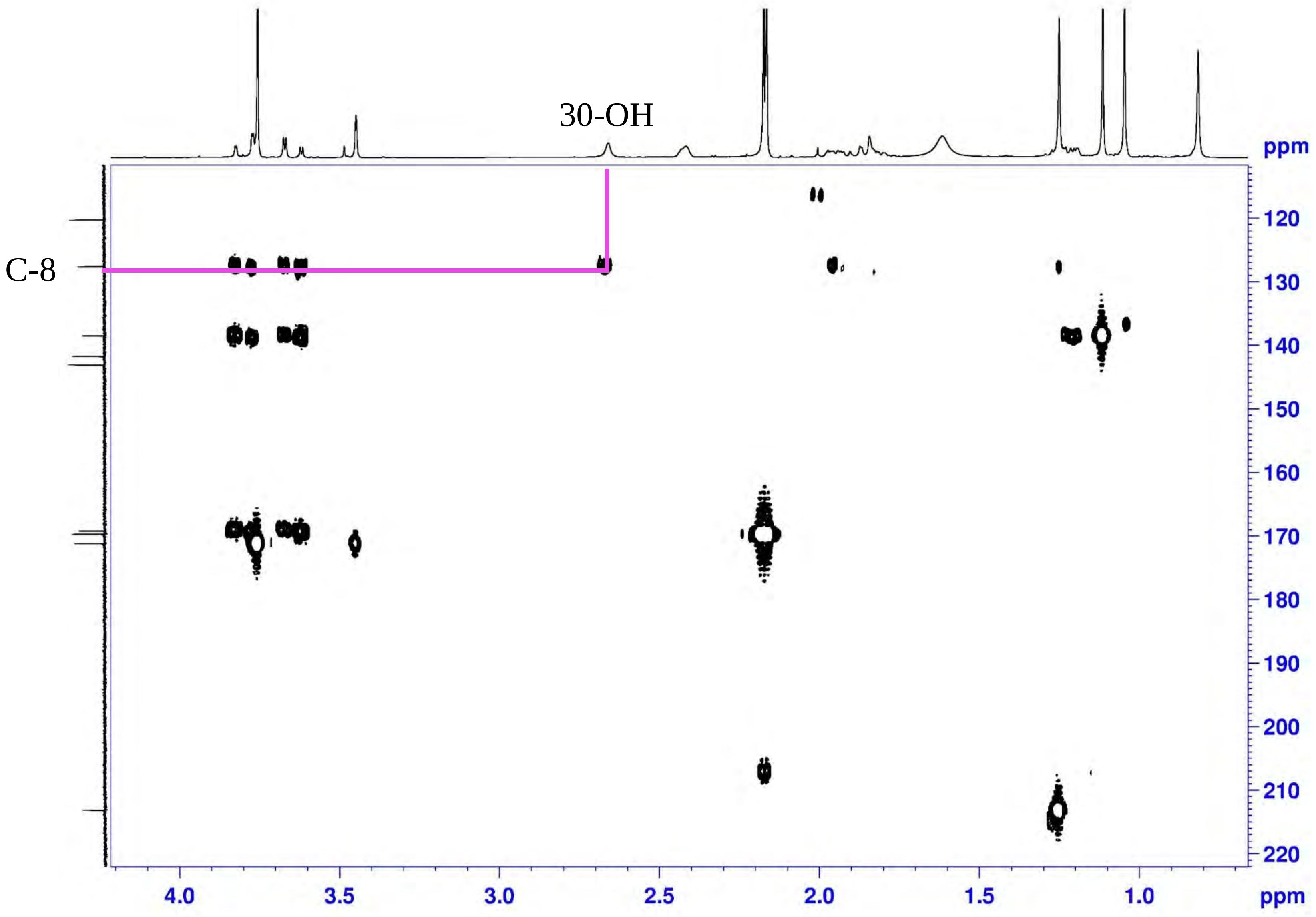

NOESY (400 MHz) spectrum of compound **5** in $CDCl_3$

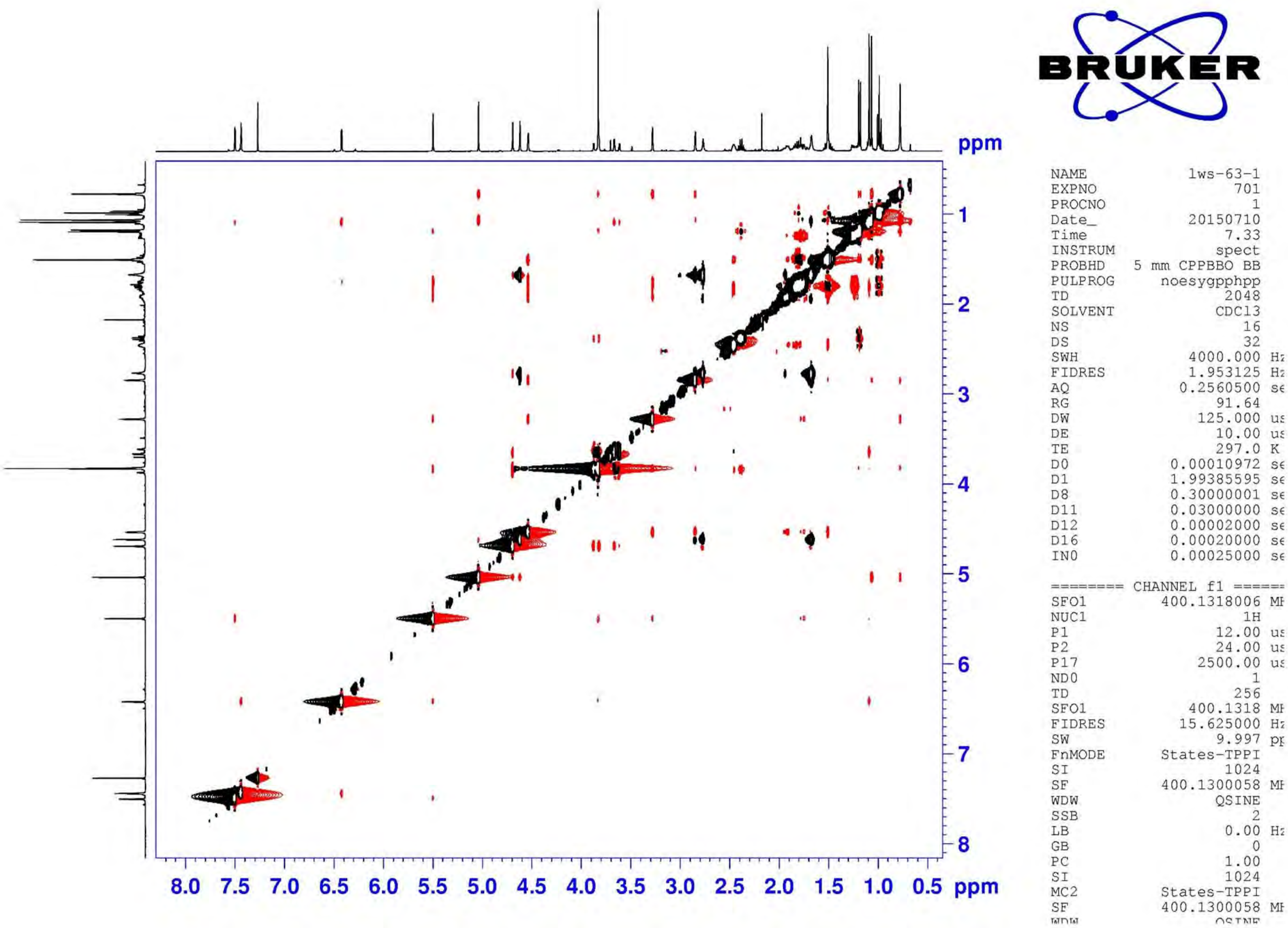

NOESY (400 MHz) spectrum of compound **5** in $CDCl_3$

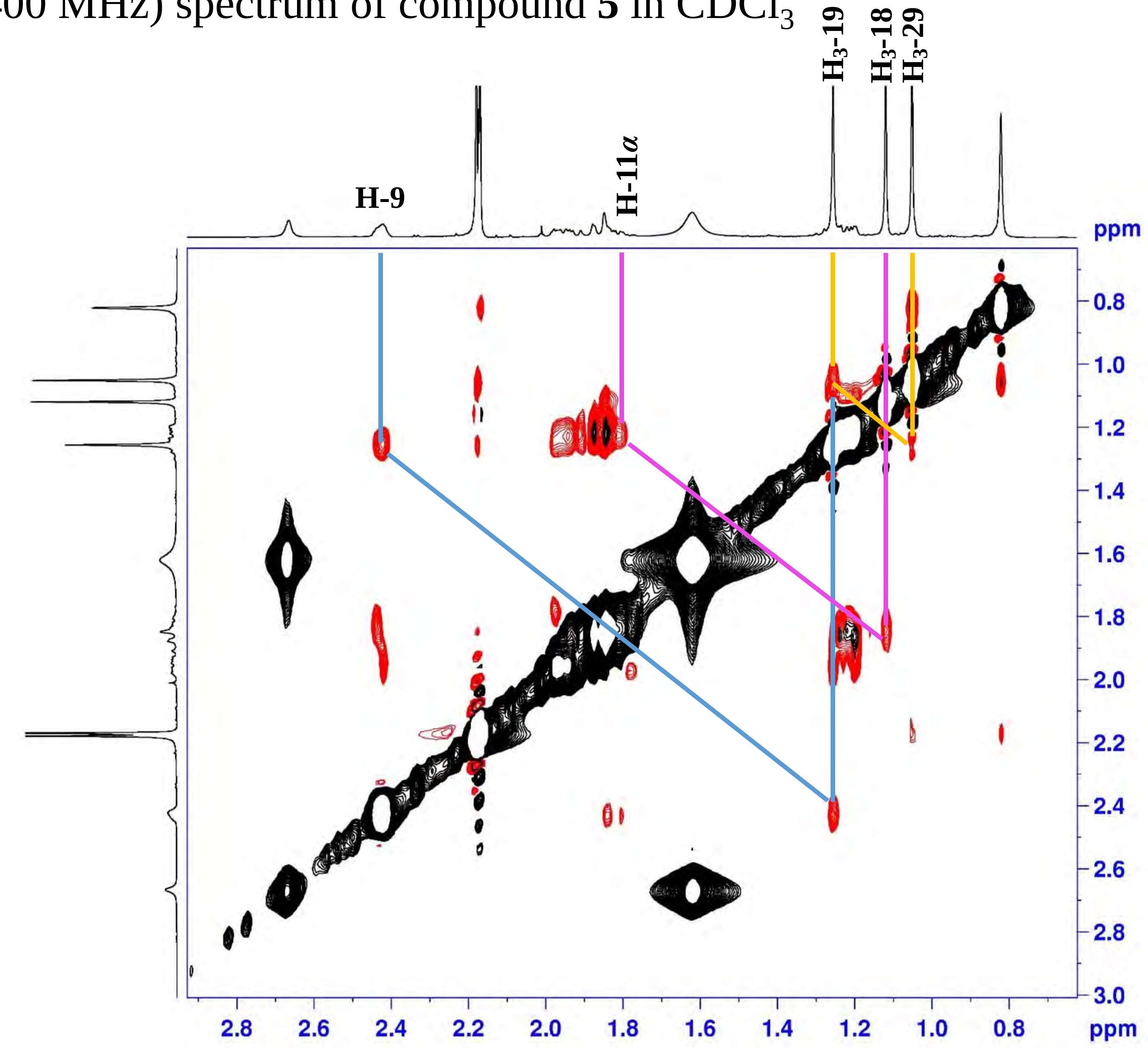

NOESY (400 MHz) spectrum of compound **5** in $CDCl_3$

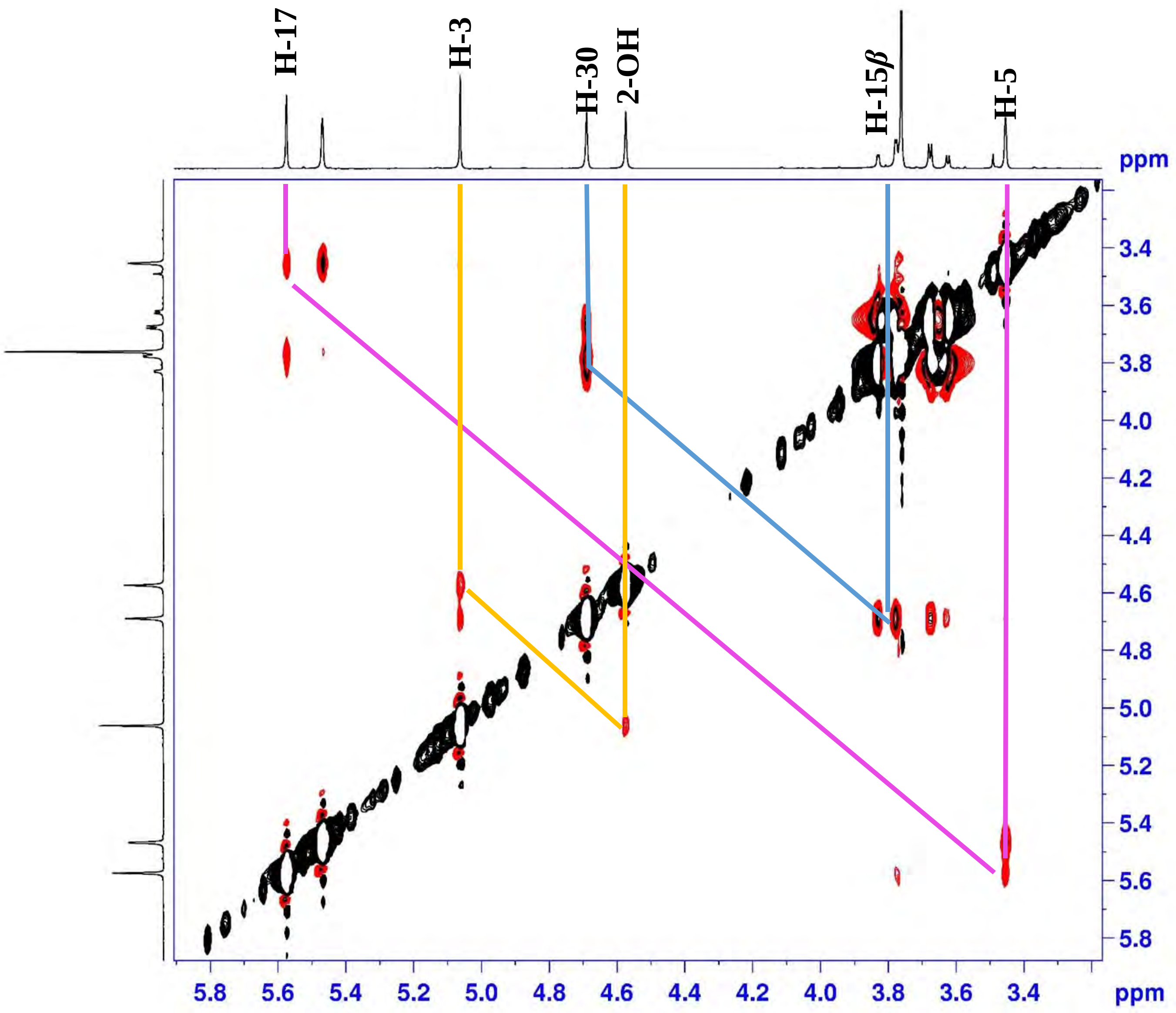

NOESY (400 MHz) spectrum of compound **5** in $CDCl_3$

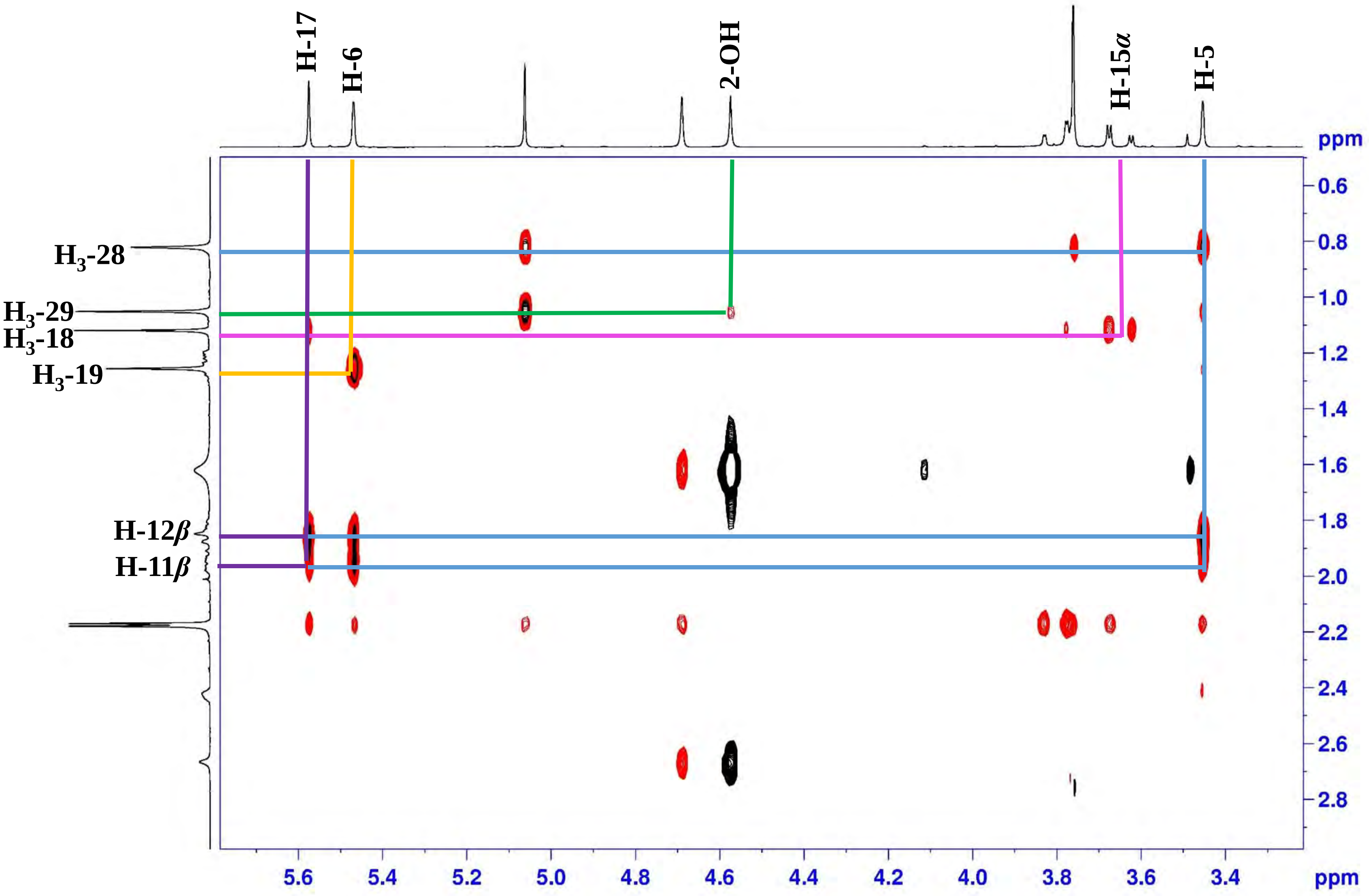

NOESY (400 MHz) spectrum of compound **5** in $CDCl_3$

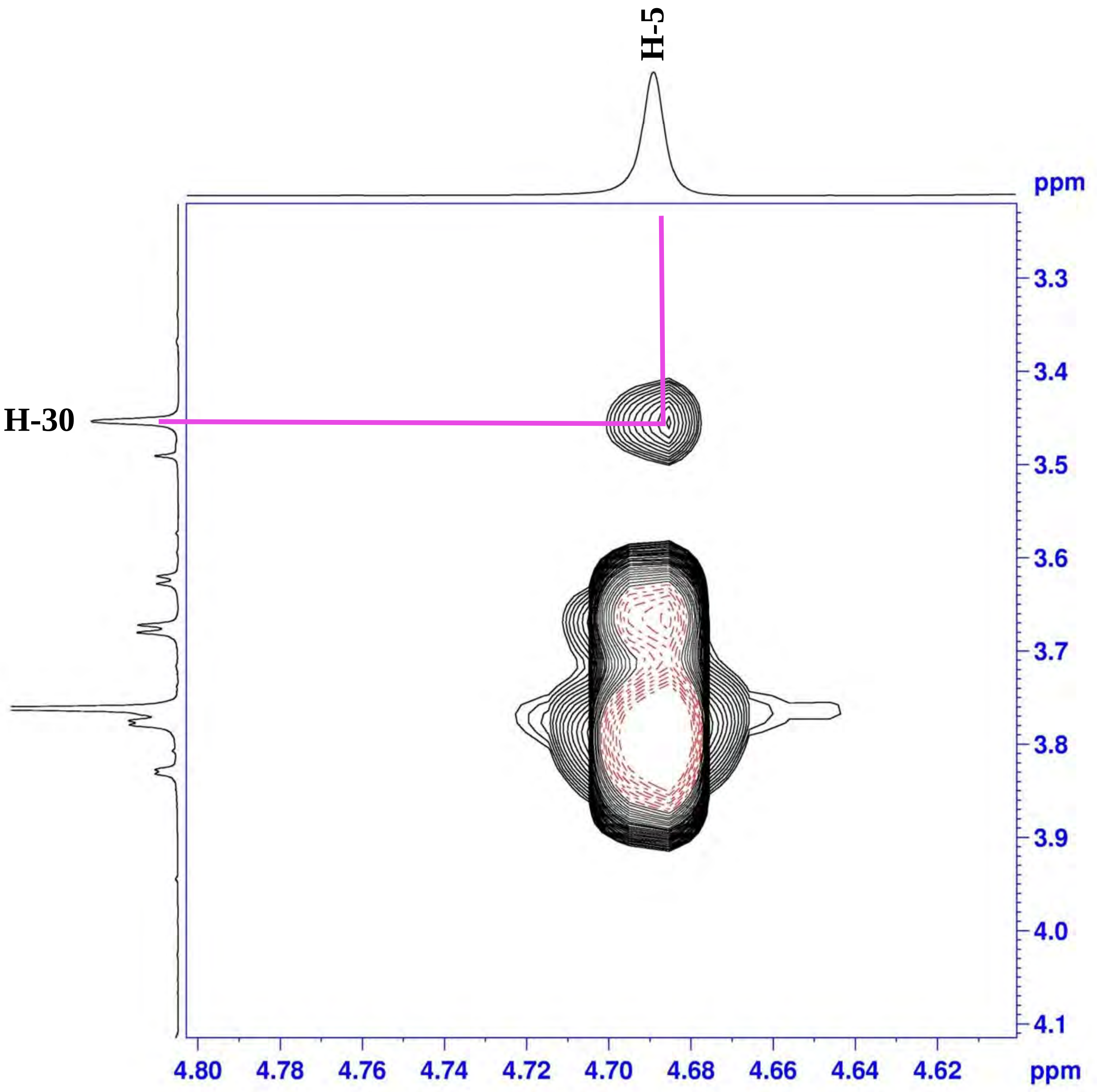

HR-ESIMS for compound **6**

Mass Spectrum SmartFormula Report

Analysis Info

Analysis Name	D:\Data\MS\data\201511\liwanshan_lws-80-1_pos_23_01_833.d
Method	LC_Direct Infusion_pos_100-1000mz.m
Sample Name	liwanshan_lws-80-1_pos
Comment	
Acquisition Date	11/18/2015 10:07:03 PM
Operator	SCSIO
Instrument / Ser#	maXis 29

Acquisition Parameter

Source Type	ESI	Ion Polarity	Positive	Set Nebulizer	0.4 Bar
Focus	Active	Set Capillary	4500 V	Set Dry Heater	180 °C
Scan Begin	100 m/z	Set End Plate Offset	-500 V	Set Dry Gas	4.0 l/min
Scan End	2000 m/z	Set Collision Cell RF	800.0 Vpp	Set Divert Valve	Waste

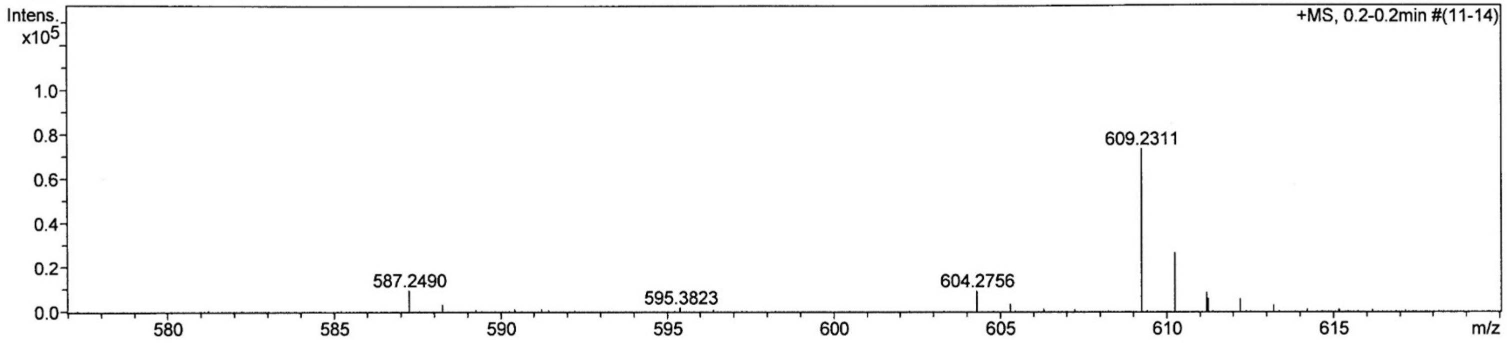

Meas. m/z	#	Formula	Score	m/z	err [ppm]	err [mDa]	mSigma	rdb	e⁻ Conf	N-Rule
587.2490	1	C 31 H 39 O 11	100.00	587.2487	-0.6	-0.4	4.3	12.5	even	ok
609.2311	1	C 31 H 38 Na O 11	100.00	609.2306	-0.7	-0.4	11.0	12.5	even	ok

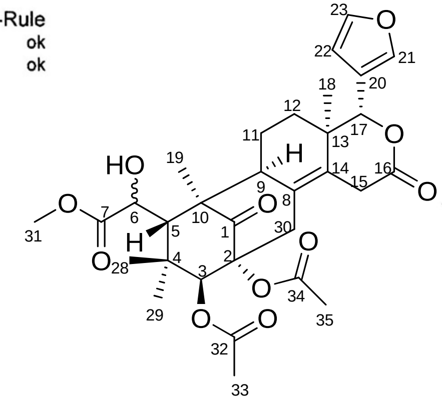

Bruker Compass DataAnalysis 4.0 printed: 11/18/2015 10:13:59 PM Page 1 of 1

HR-ESIMS for compound **6**

Generic Display Report

Analysis Info			
Analysis Name	D:\Data\MS\data\201511\liwanshan_lws-80-1_pos_23_01_833.d	Acquisition Date	11/18/2015 10:07:03 PM
Method	LC_Direct Infusion_pos_100-1000mz.m	Operator	SCSIO
Sample Name	liwanshan_lws-80-1_pos	Instrument	maXis
Comment			

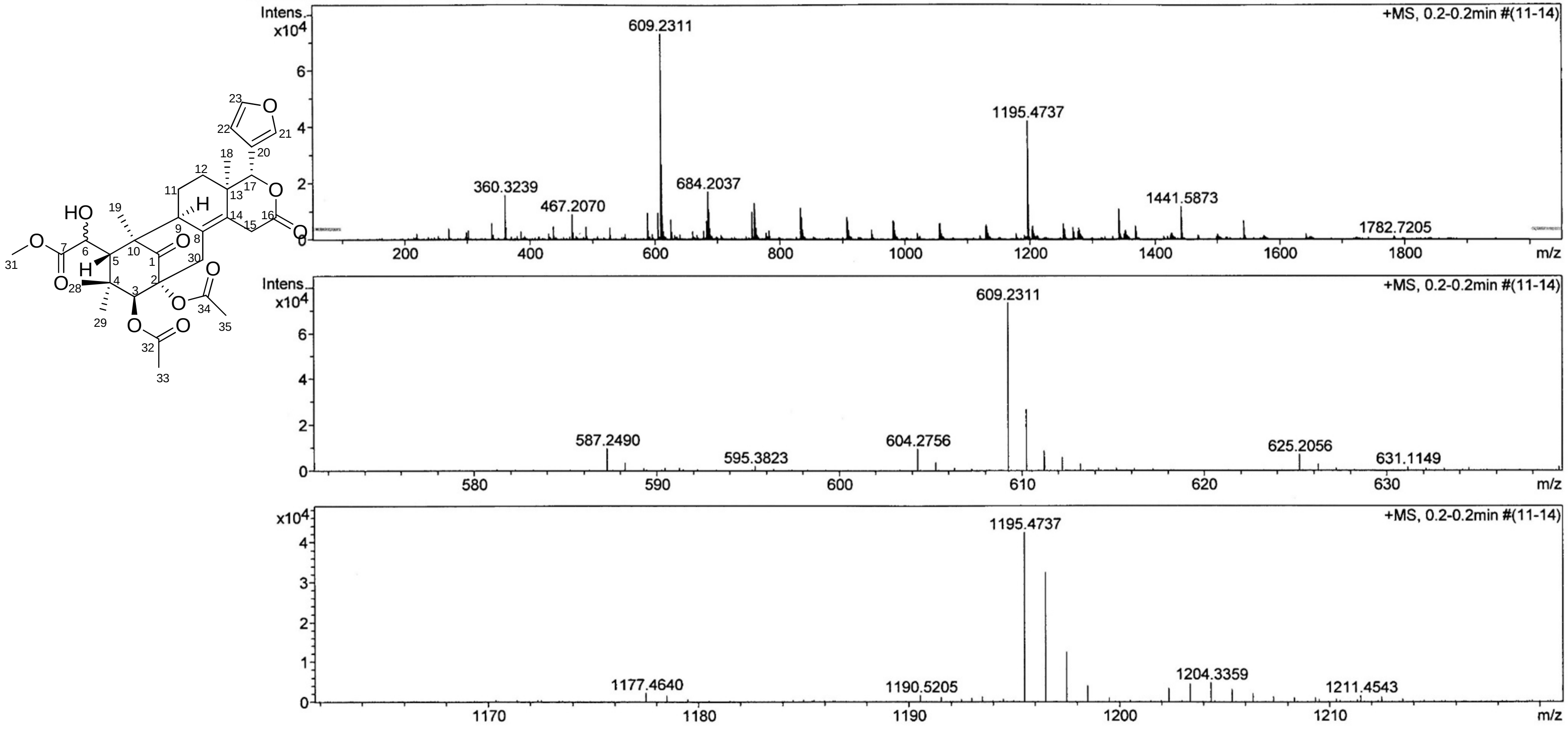

^{1}H NMR (400 MHz) spectrum of compound **6** in $CDCl_3$

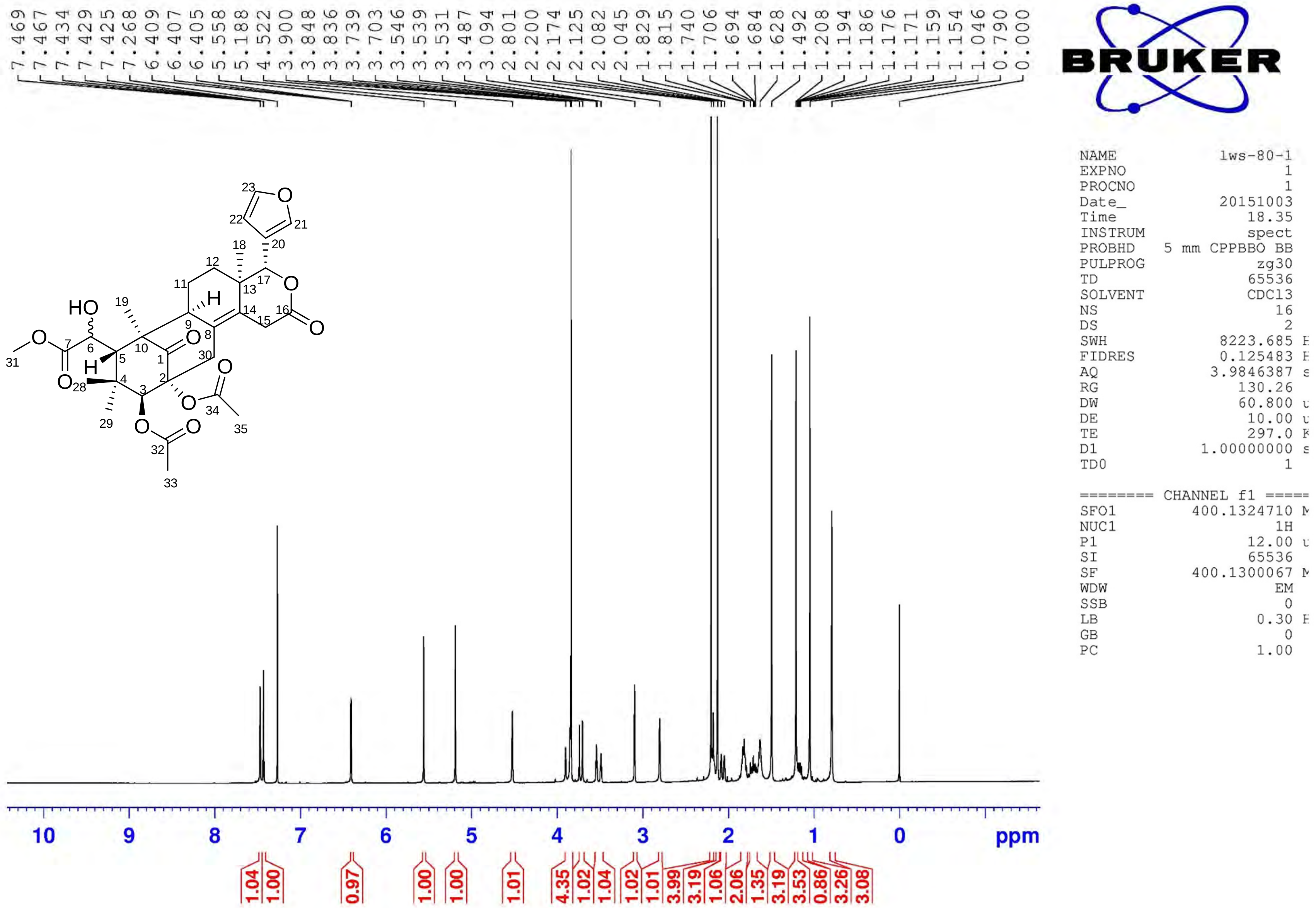

^{1}H NMR (400 MHz) spectrum of compound **6** in $CDCl_3$

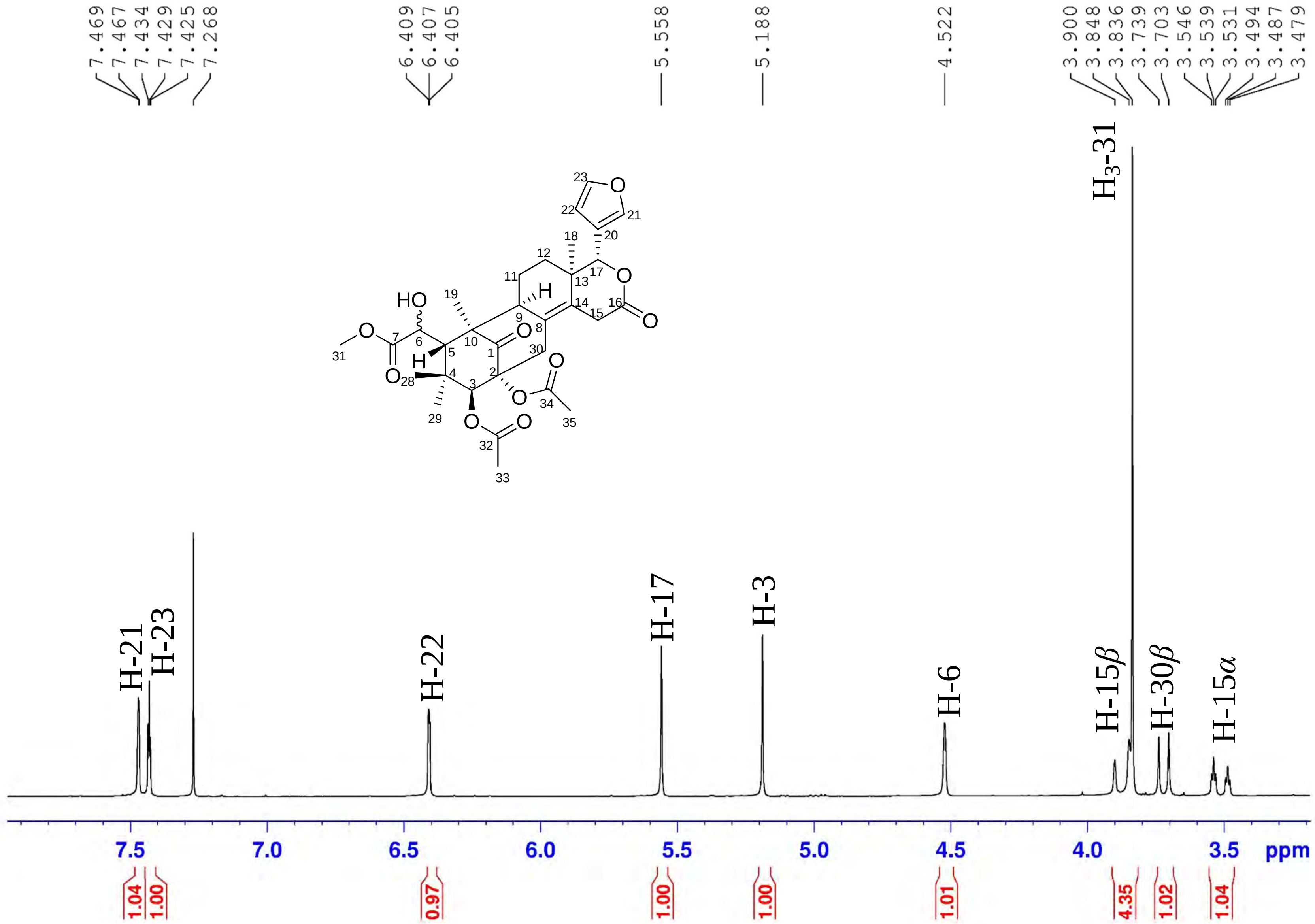

^{1}H NMR (400 MHz) spectrum of compound **6** in $CDCl_3$

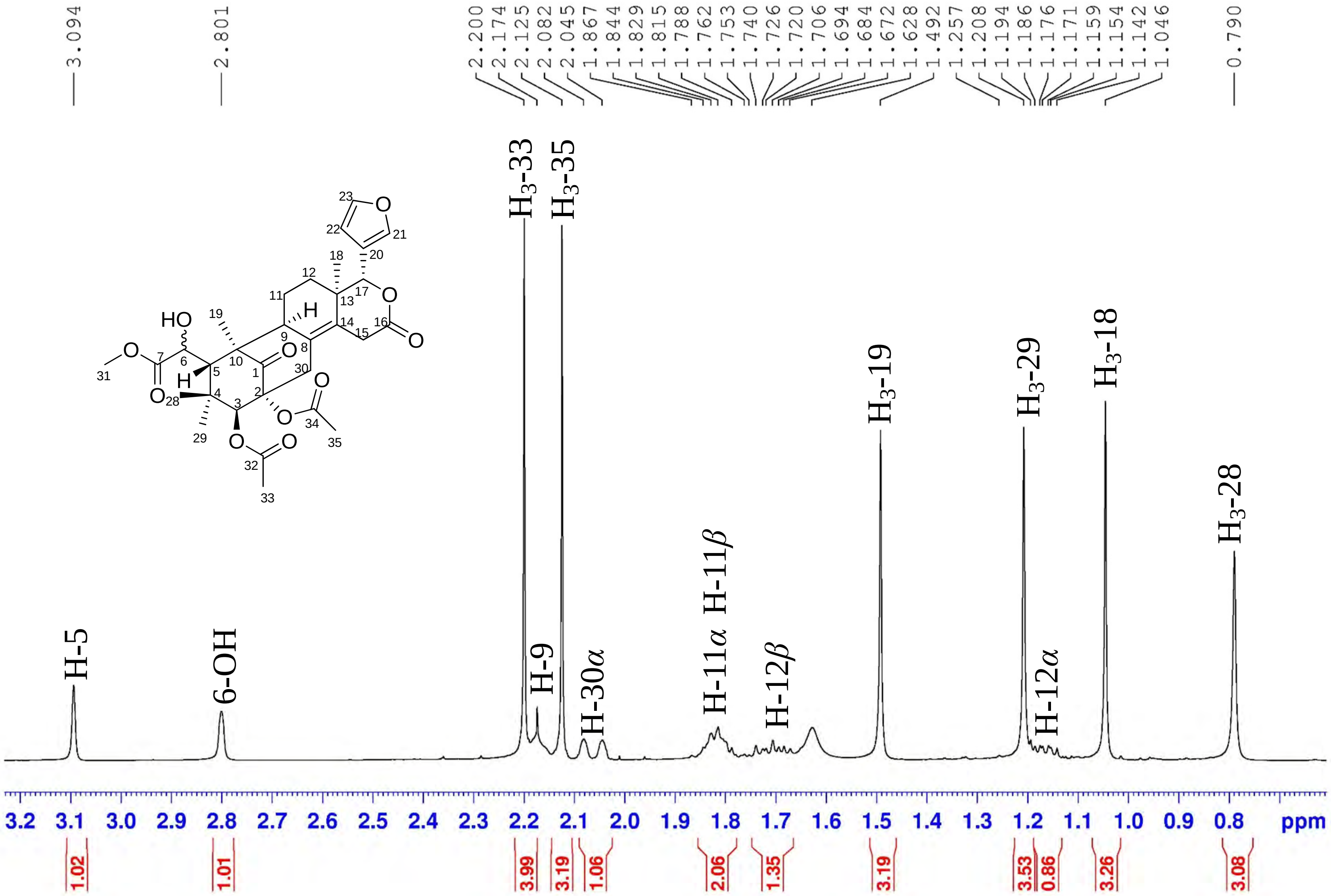

^{13}C NMR (100 MHz) spectrum of compound **6** in $CDCl_3$

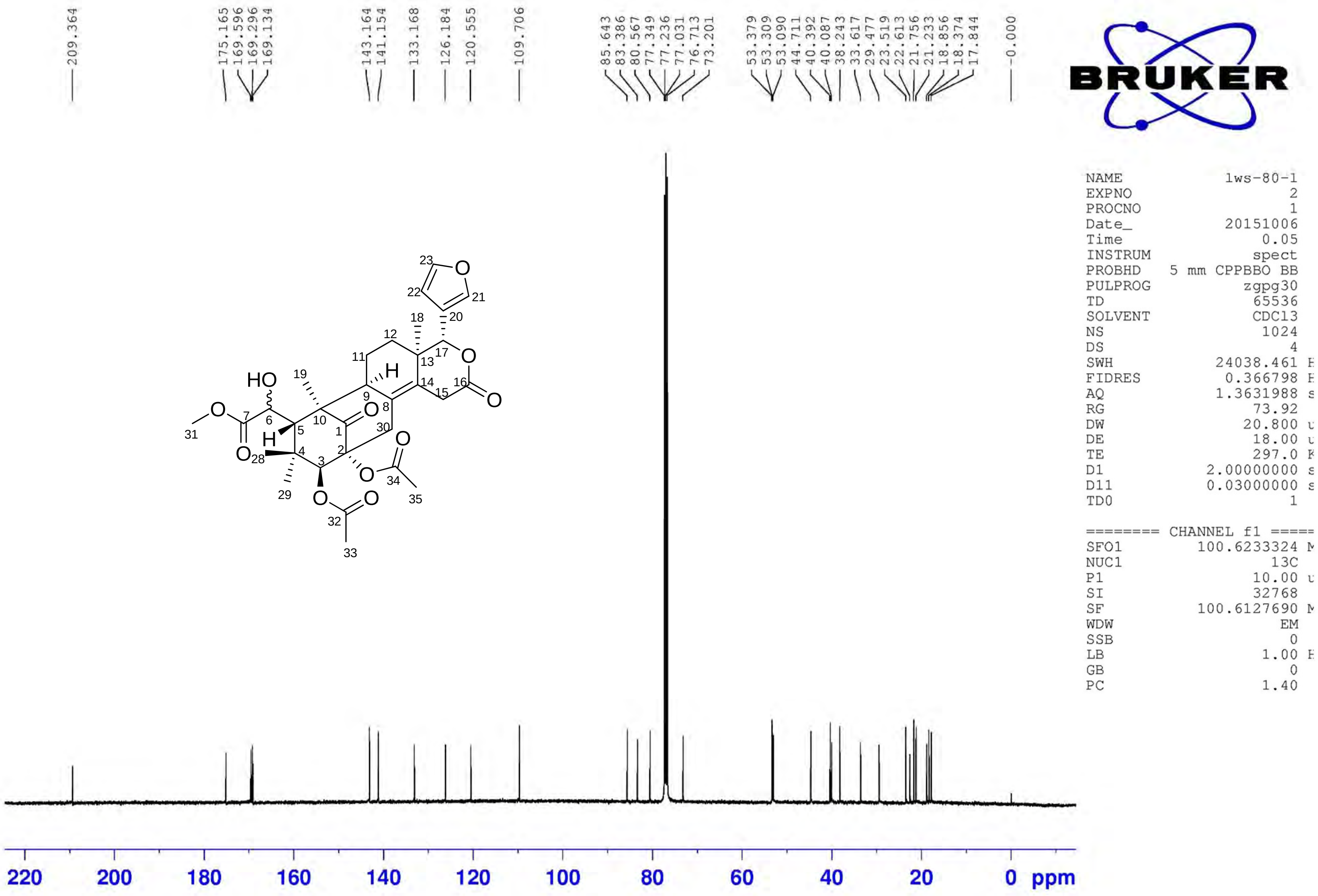

NAME	lws-80-1
EXPNO	2
PROCNO	1
Date_	20151006
Time	0.05
INSTRUM	spect
PROBHD	5 mm CPPBBO BB
PULPROG	zgpg30
TD	65536
SOLVENT	CDCl3
NS	1024
DS	4
SWH	24038.461 H
FIDRES	0.366798 H
AQ	1.3631988 s
RG	73.92
DW	20.800 u
DE	18.00 u
TE	297.0 K
D1	2.00000000 s
D11	0.03000000 s
TD0	1

======== CHANNEL f1 =====

SFO1	100.6233324 M
NUC1	13C
P1	10.00 u
SI	32768
SF	100.6127690 M
WDW	EM
SSB	0
LB	1.00 H
GB	0
PC	1.40

^{13}C NMR (100 MHz) spectrum of compound **6** in $CDCl_3$

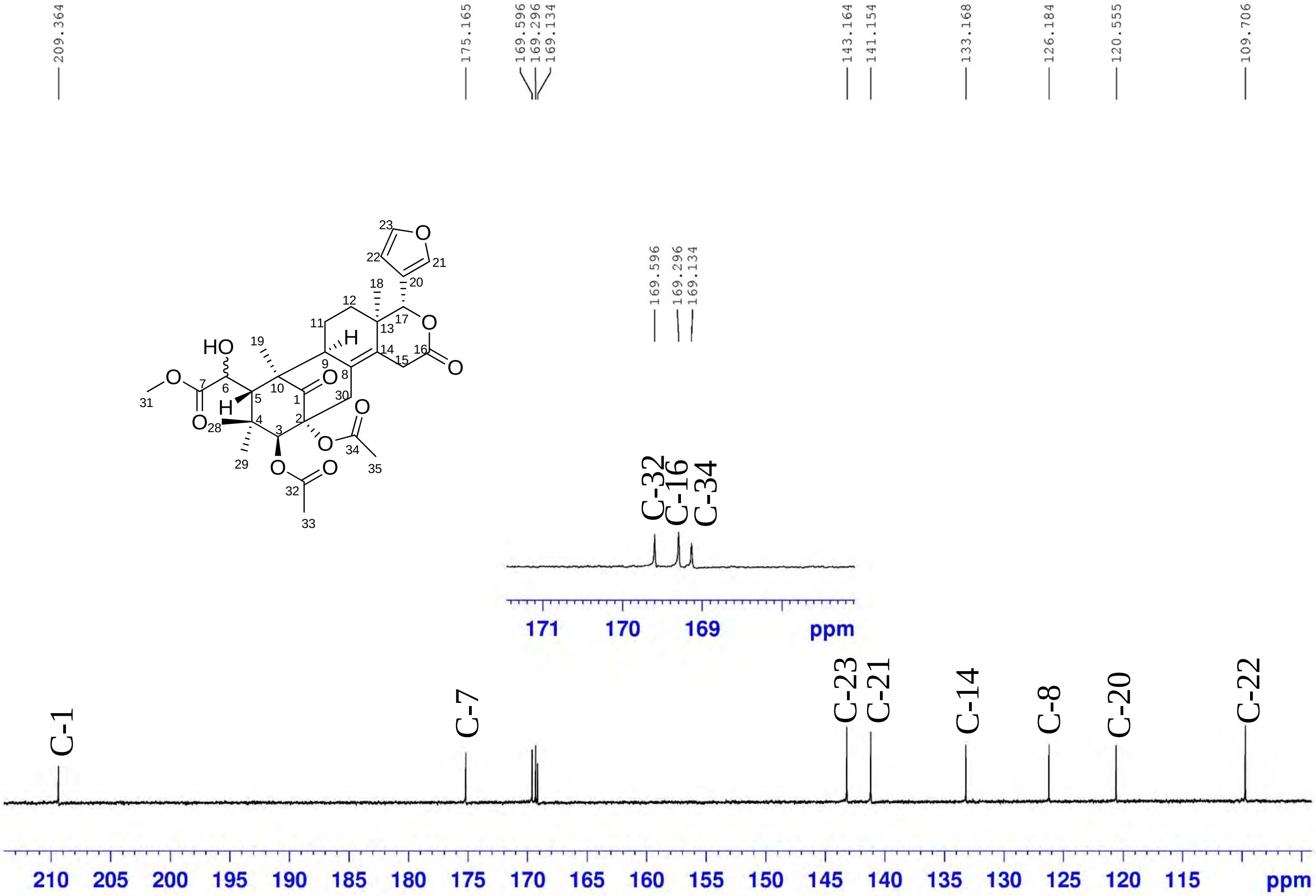

^{13}C NMR (100 MHz) spectrum of compound **6** in $CDCl_3$

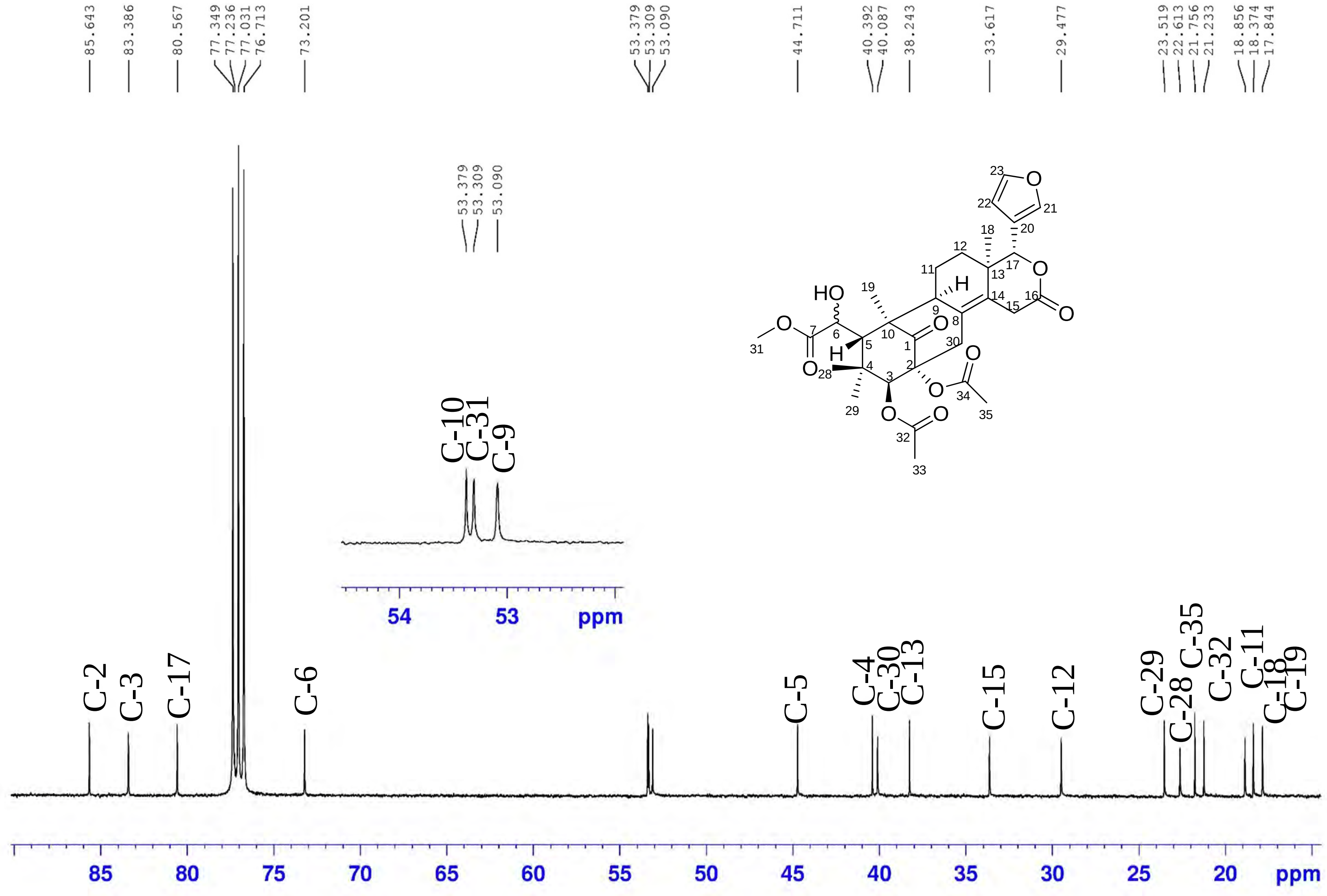

DEPT135 (100 MHz) spectrum of compound **6** in $CDCl_3$

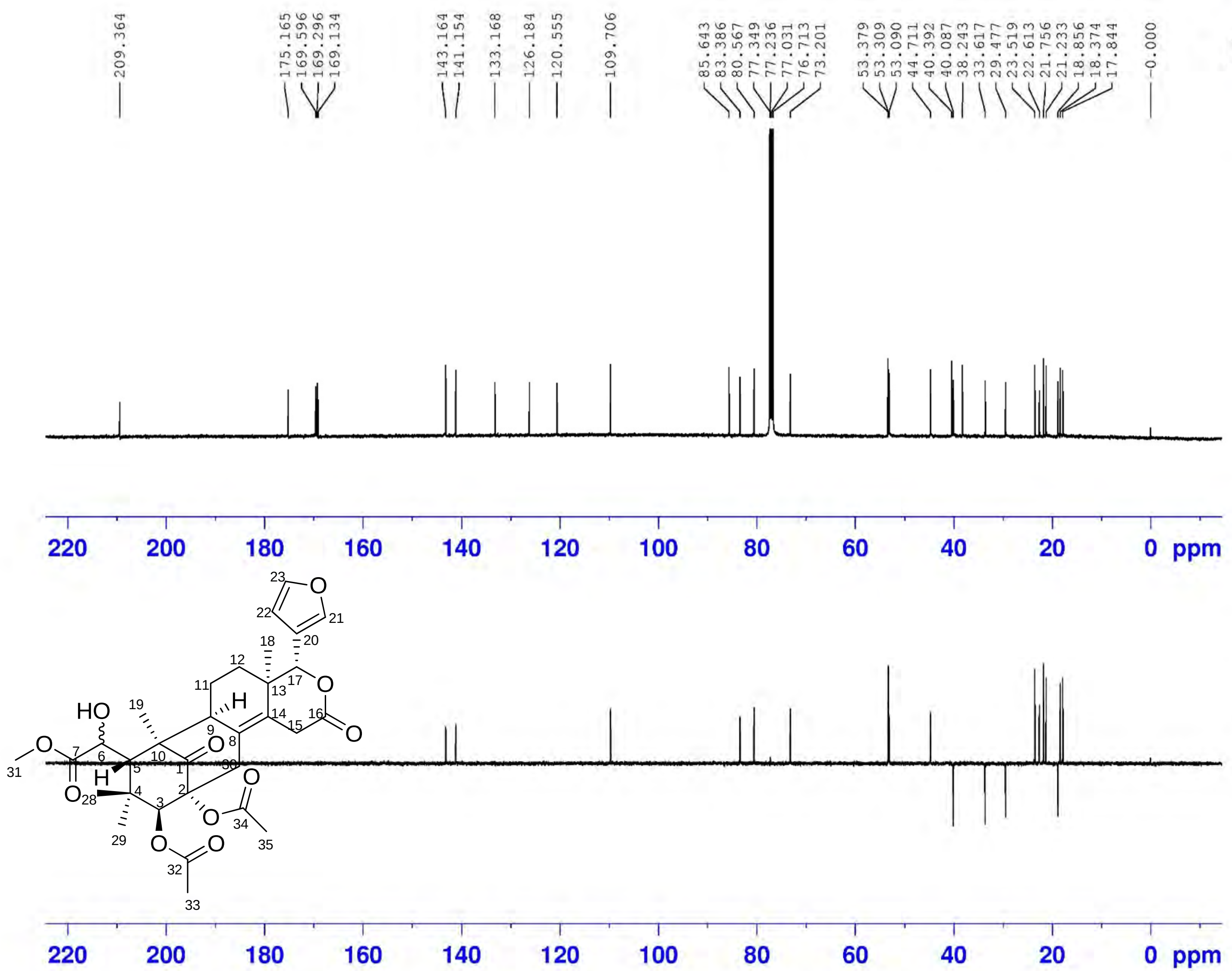

NAME	lws-80-1
EXPNO	3
PROCNO	1
Date_	20151006
Time	0.23
INSTRUM	spect
PROBHD	5 mm CPPBBO BB
PULPROG	deptsp135
TD	65536
SOLVENT	CDCl3
NS	300
DS	4
SWH	24038.461 H
FIDRES	0.366798 H
AQ	1.3631988 s
RG	130.26
DW	20.800 u
DE	18.00 u
TE	297.0 K
CNST2	145.0000000
D1	2.00000000 s
D2	0.00344828 s
D12	0.00002000 s
TD0	1

======== CHANNEL f1 ========

SFO1	100.6233324 M
NUC1	13C
P1	10.00 u
P13	2000.00 u
SI	32768
SF	100.6127690 M
WDW	EM
SSB	0
LB	1.00 H
GB	0
PC	1.40

DEPT135 (100 MHz) spectrum of compound **6** in $CDCl_3$

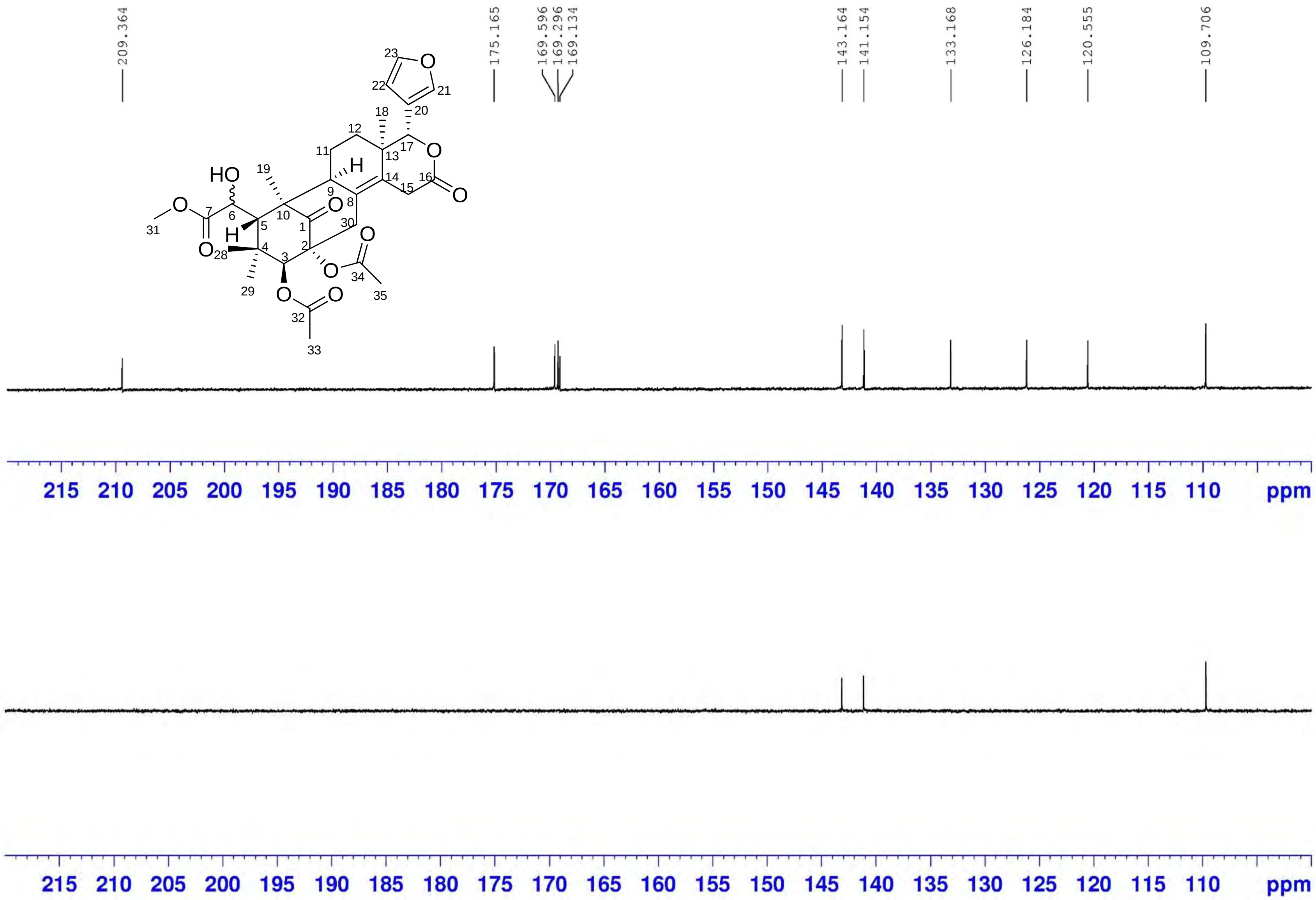

DEPT135 (100 MHz) spectrum of compound **6** in $CDCl_3$

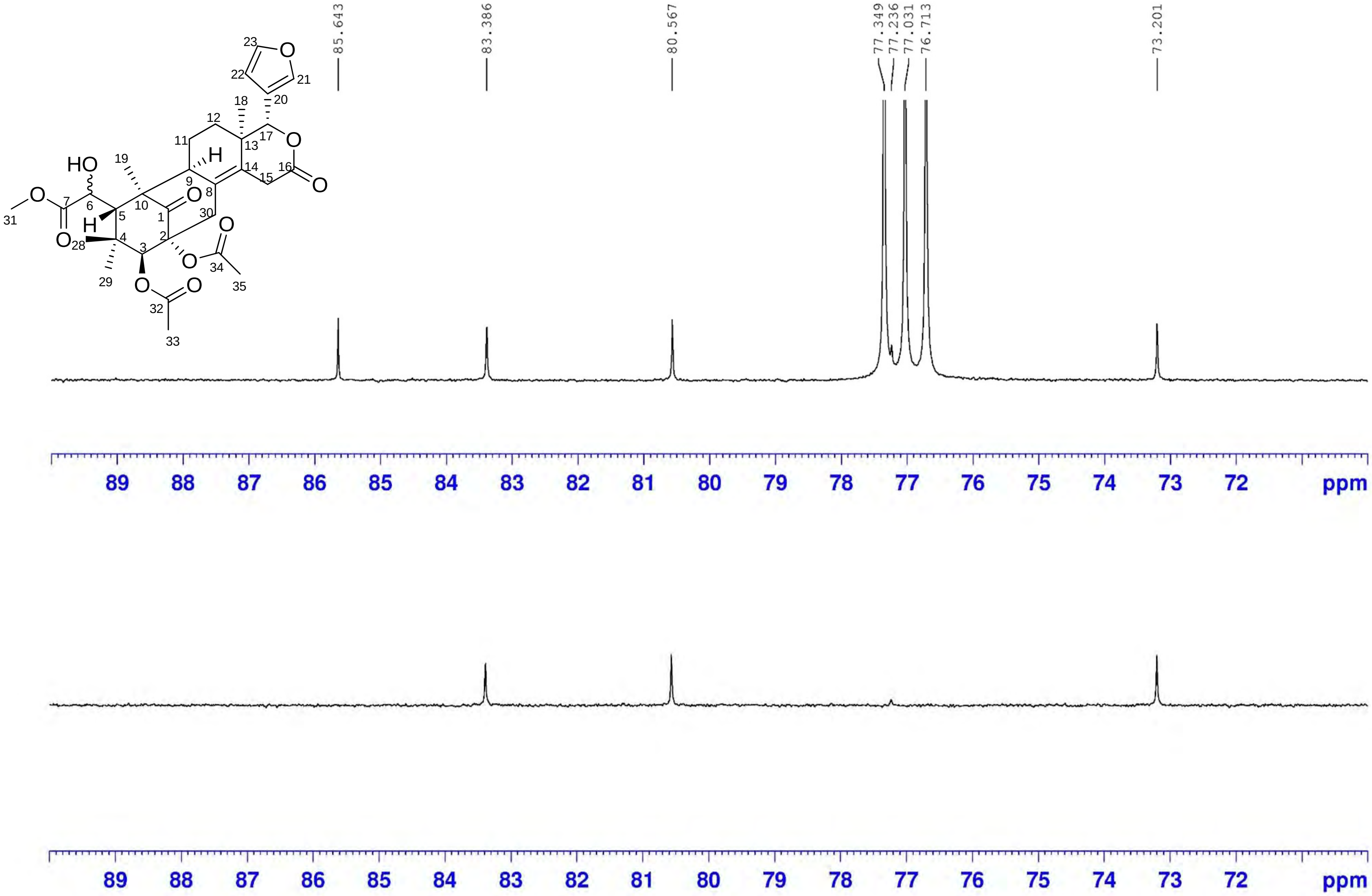

DEPT135 (100 MHz) spectrum of compound **6** in $CDCl_3$

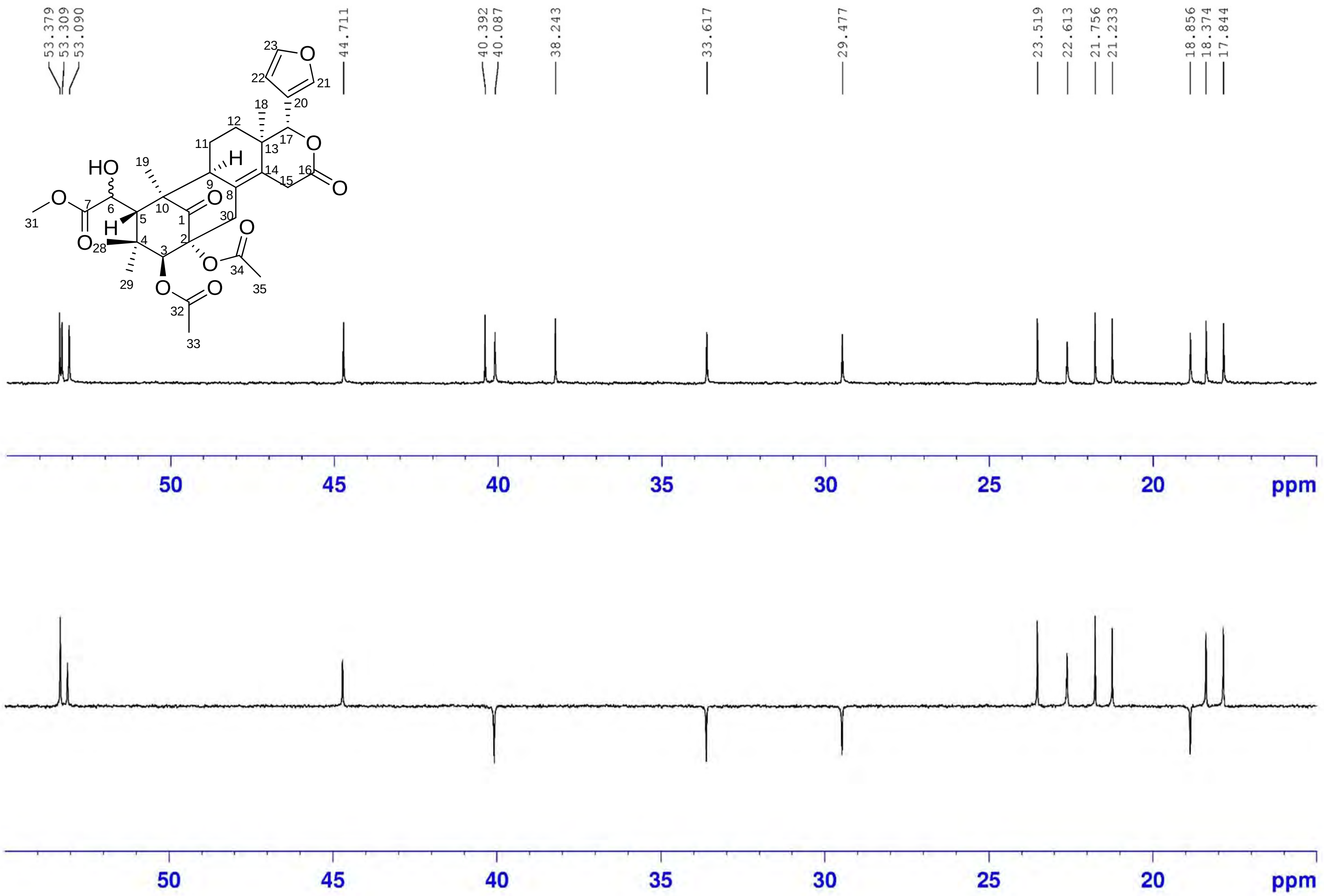

^1H-^1H COSY (400 MHz) spectrum of compound **6** in $CDCl_3$

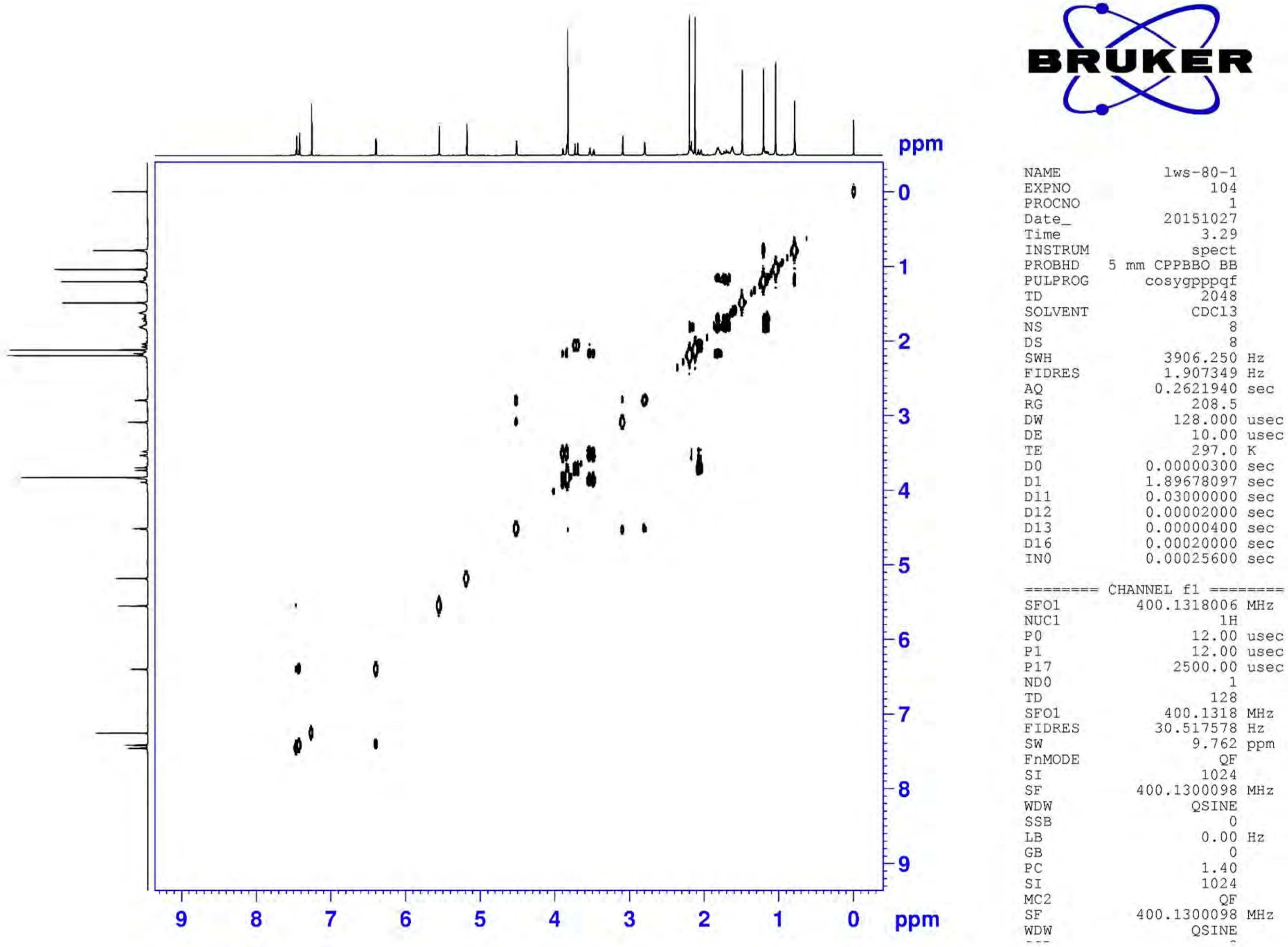

^1H-^1H COSY (400 MHz) spectrum of compound **6** in $CDCl_3$

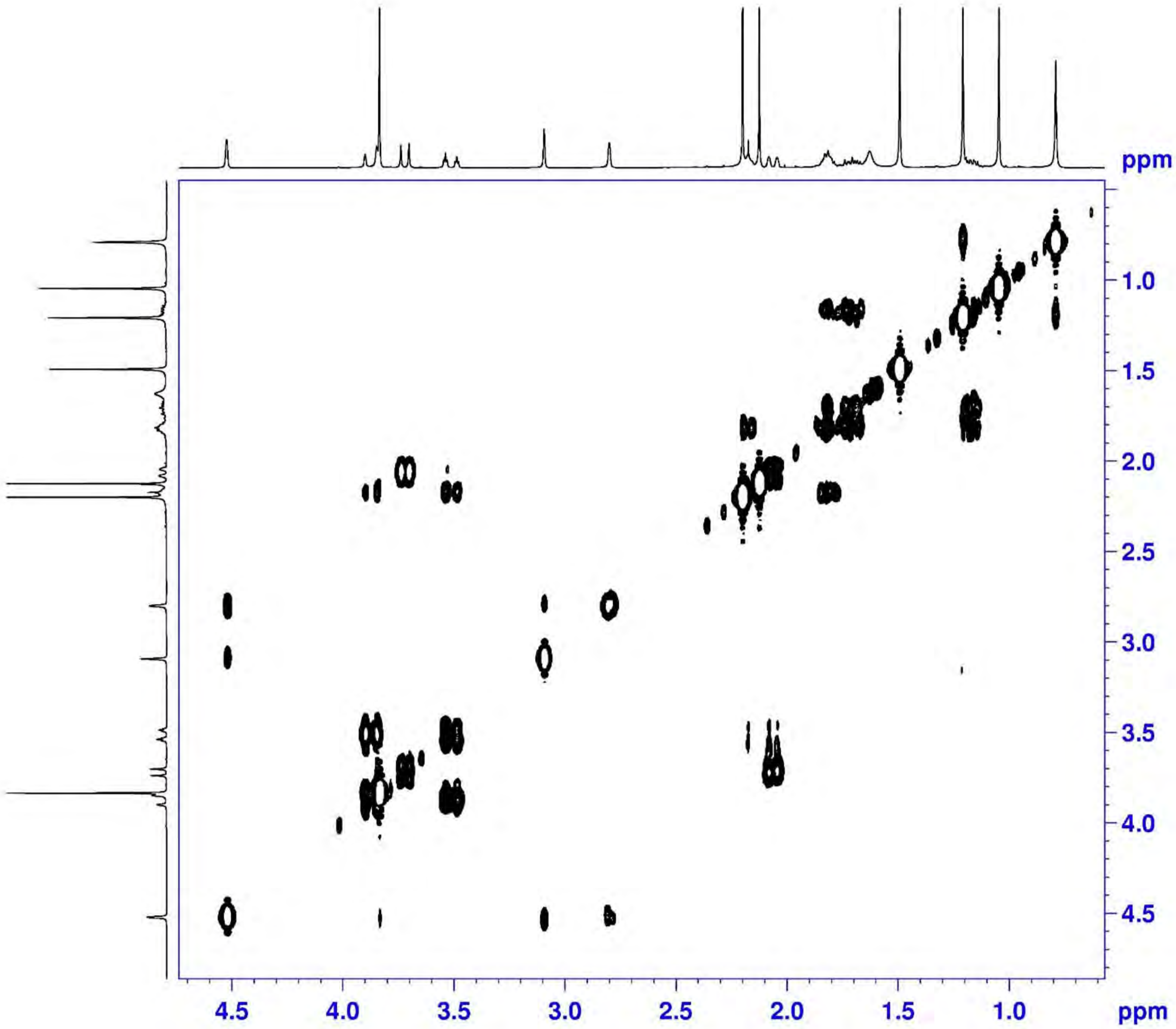

HSQC (400 MHz) spectrum of compound **6** in $CDCl_3$

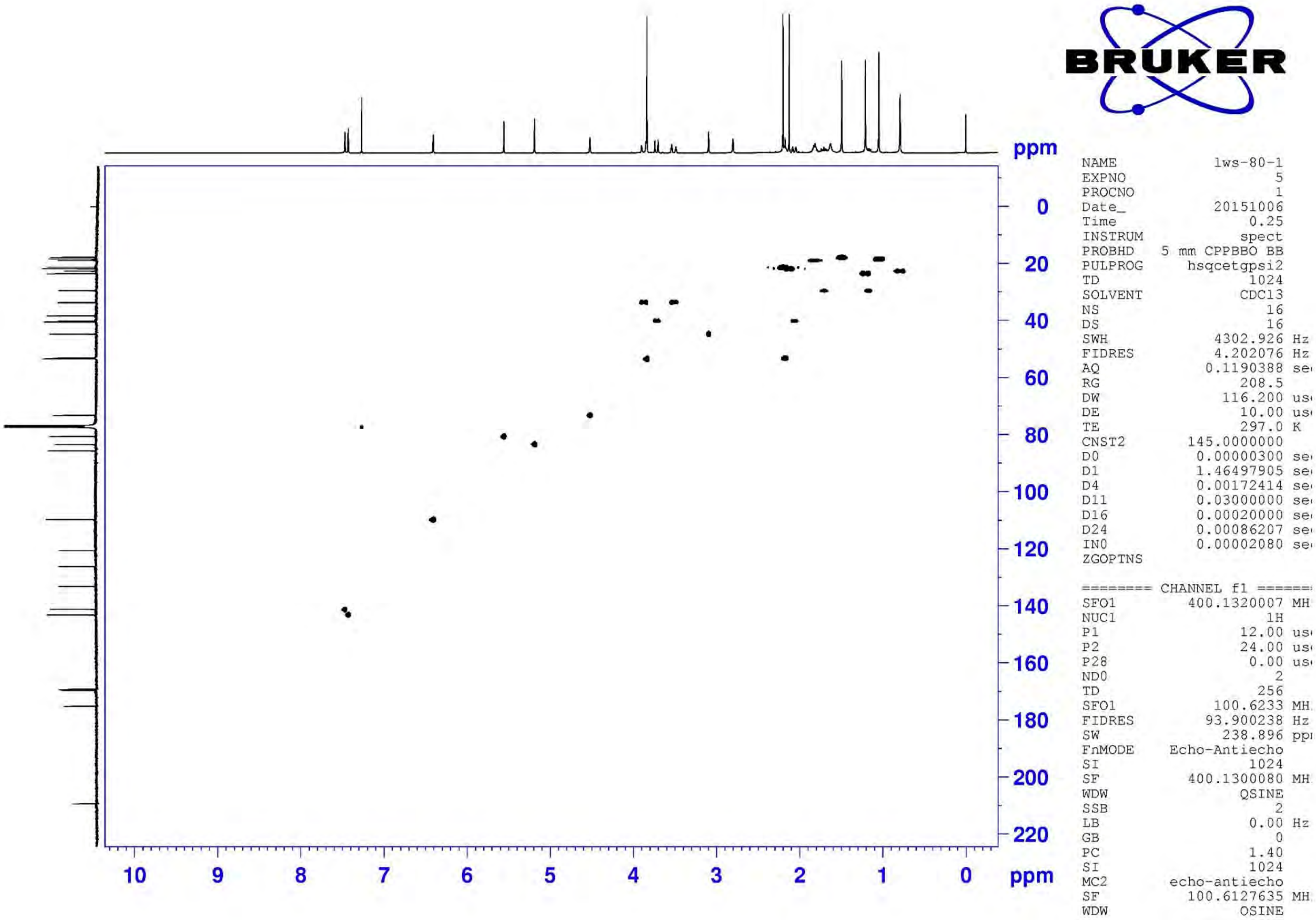

HSQC (400 MHz) spectrum of compound **6** in $CDCl_3$

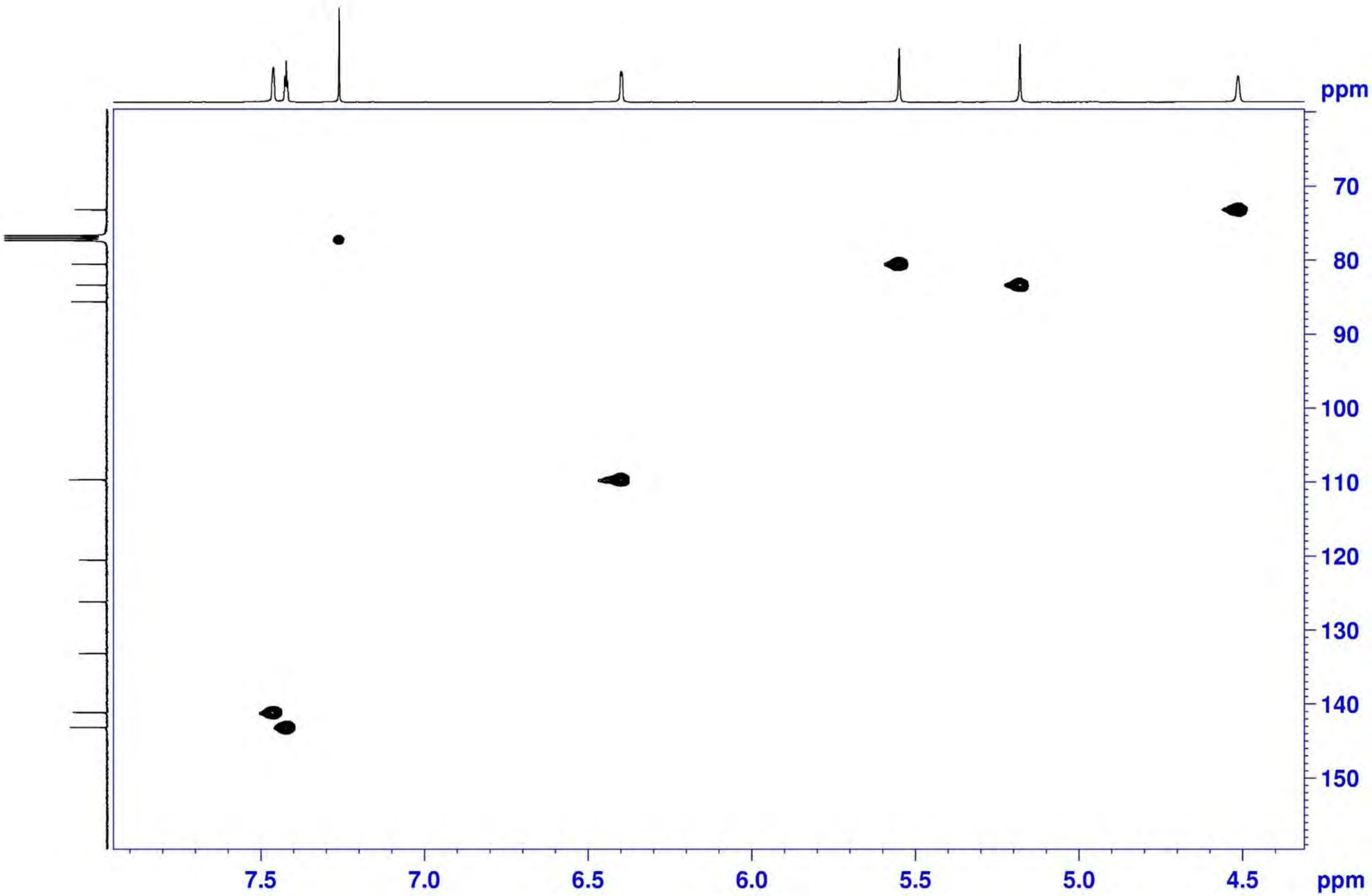

HSQC (400 MHz) spectrum of compound **6** in $CDCl_3$

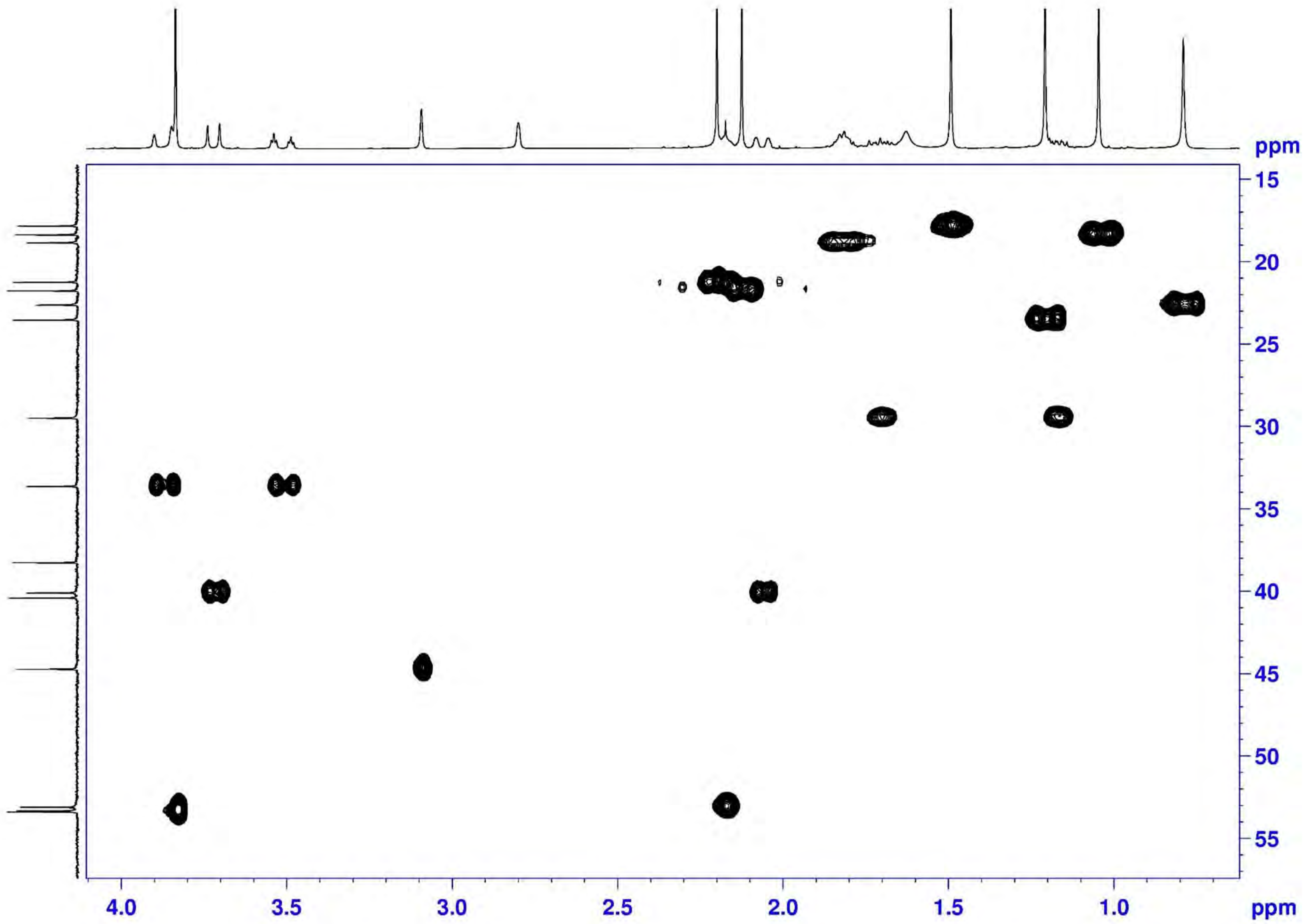

HMBC (400 MHz) spectrum of compound **6** in $CDCl_3$

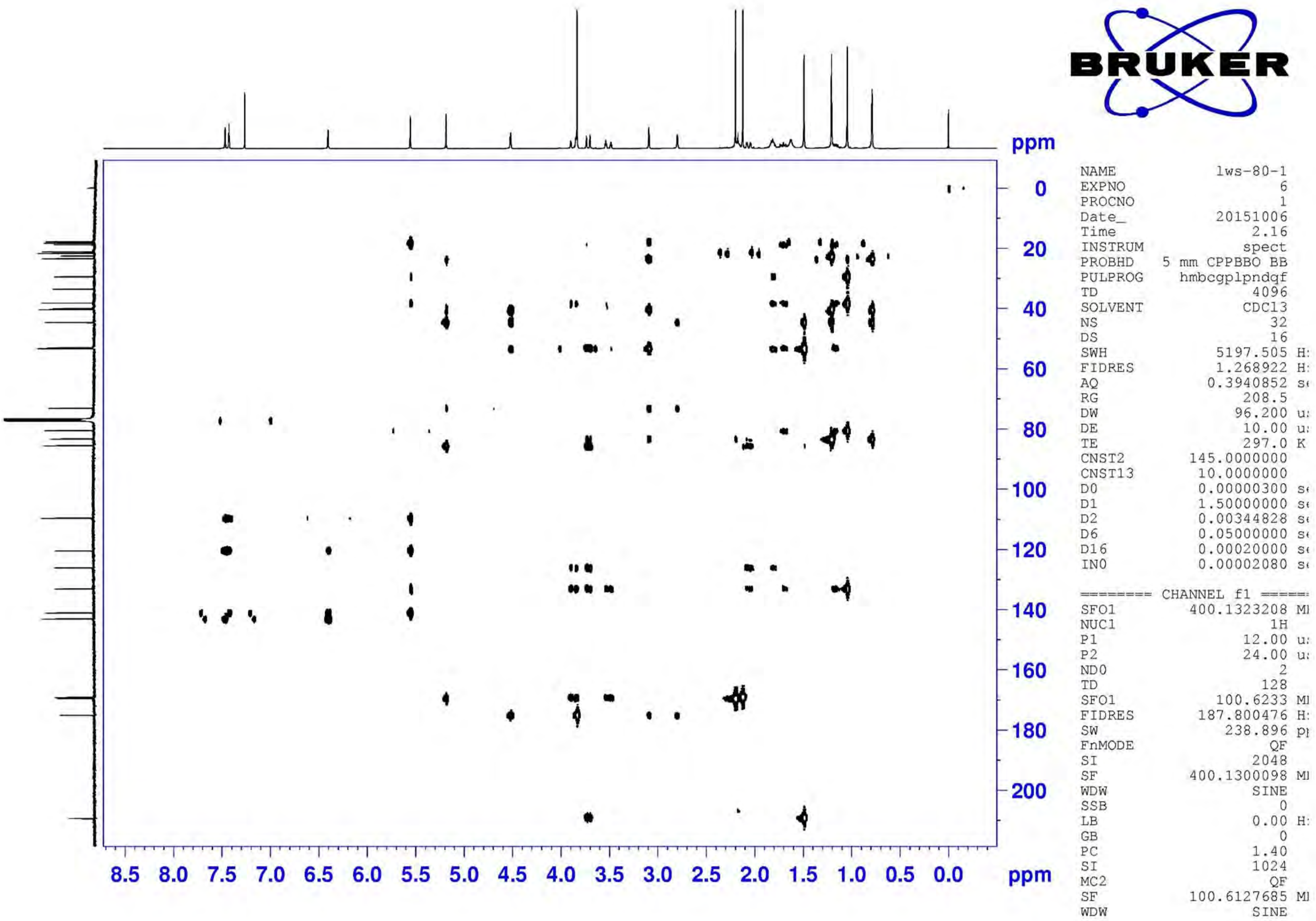

HMBC (400 MHz) spectrum of compound **6** in $CDCl_3$

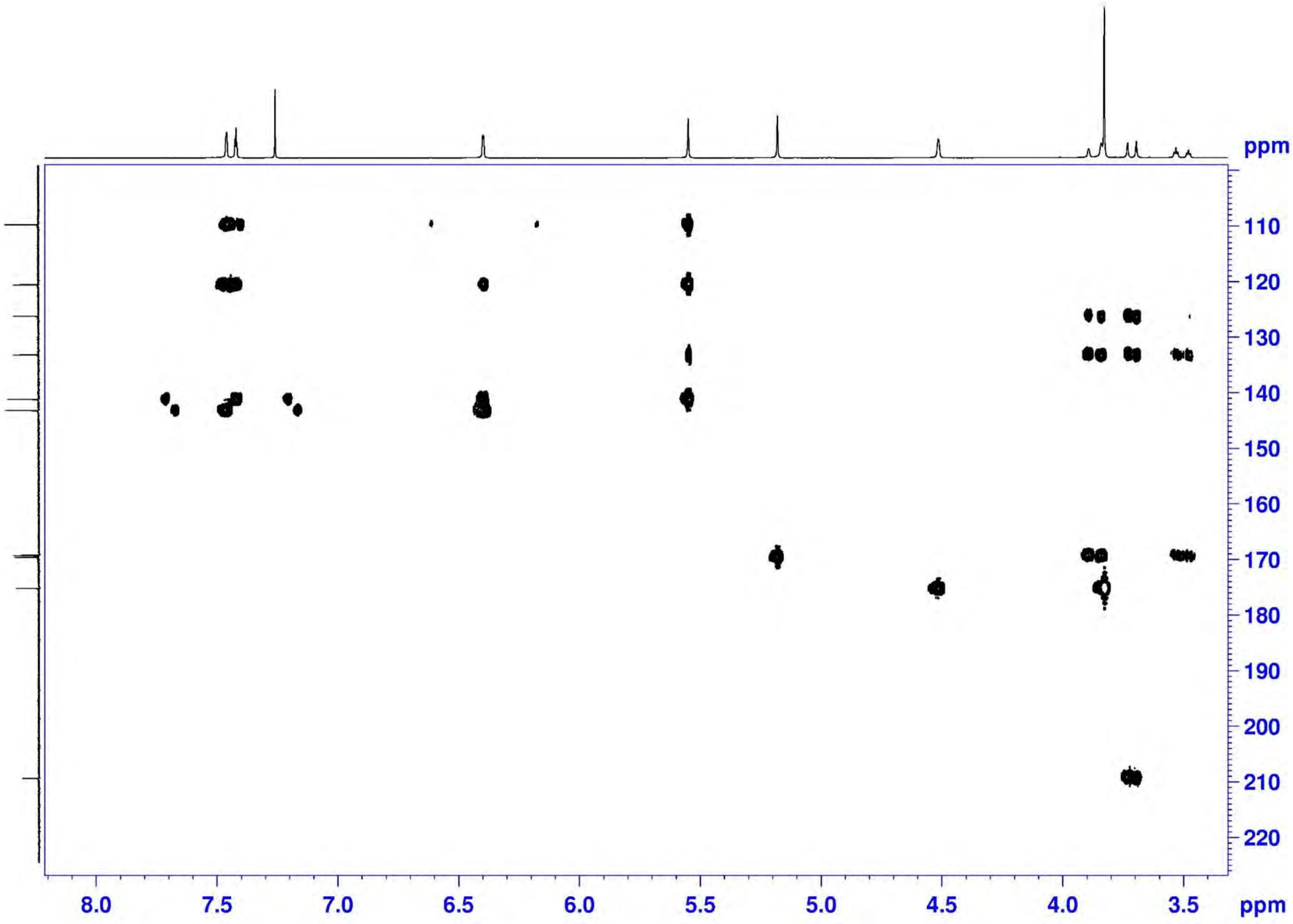

HMBC (400 MHz) spectrum of compound **6** in $CDCl_3$

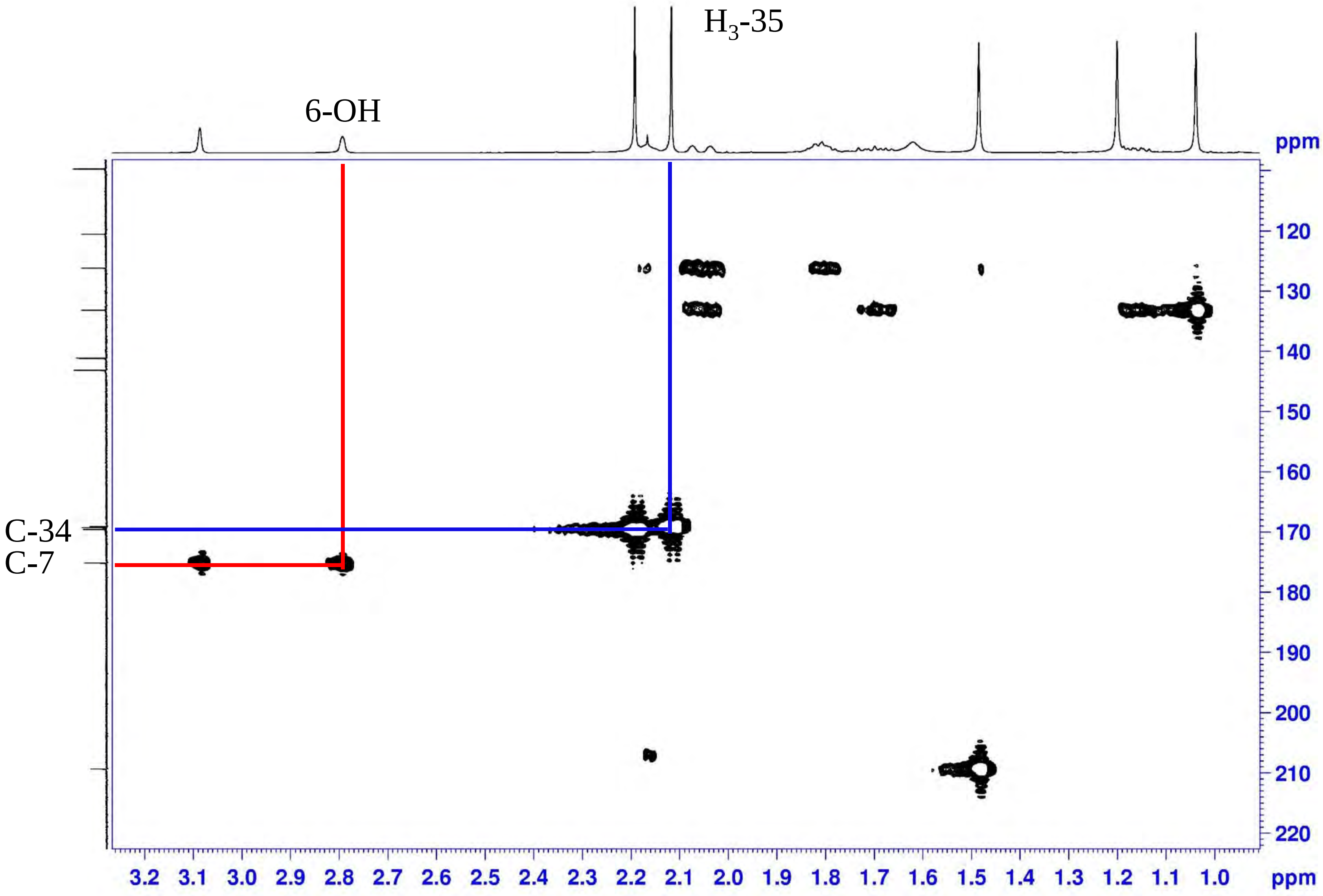

HMBC (400 MHz) spectrum of compound **6** in $CDCl_3$

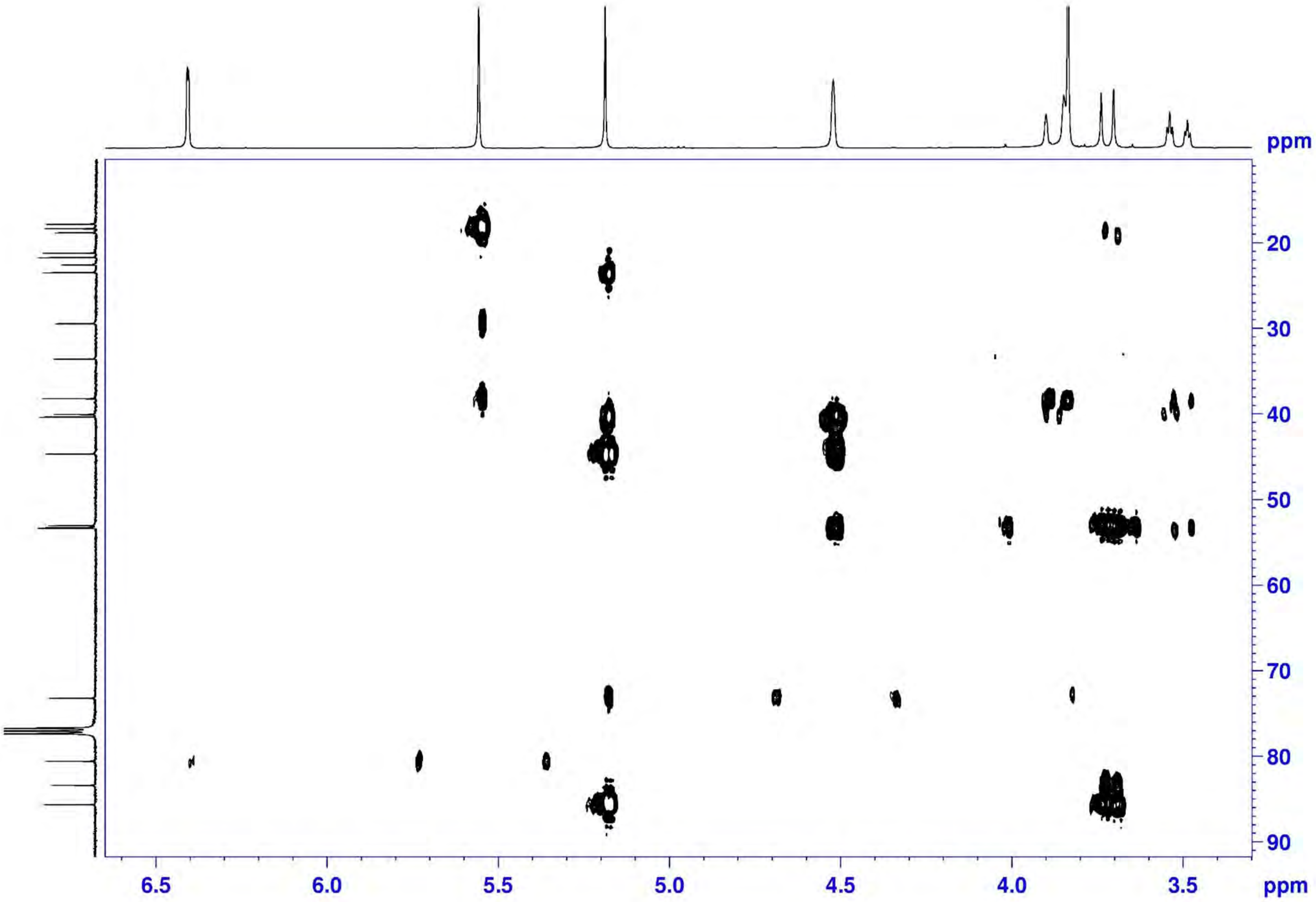

HMBC (400 MHz) spectrum of compound **6** in $CDCl_3$

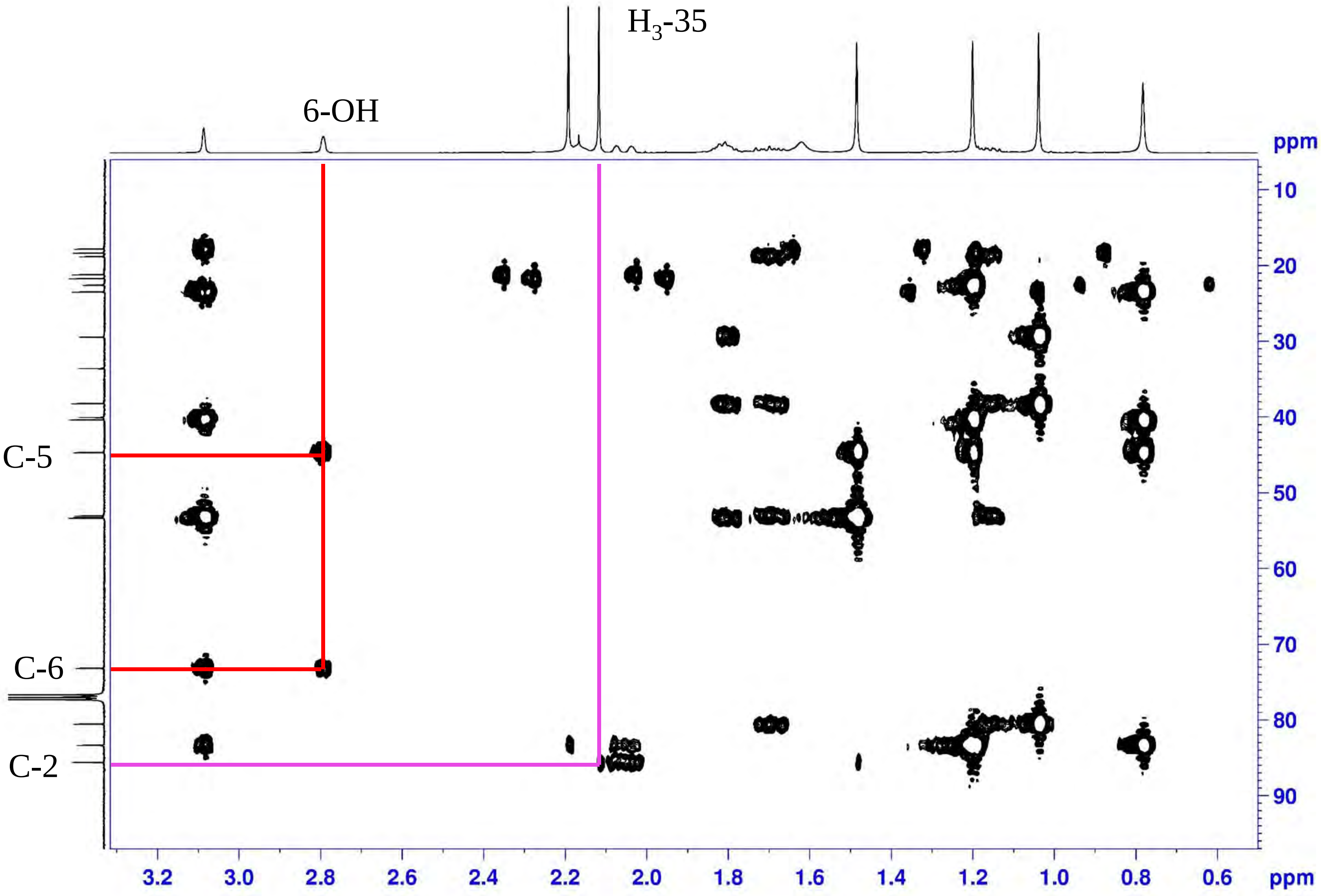

HMBC (400 MHz) spectrum of compound **6** in $CDCl_3$

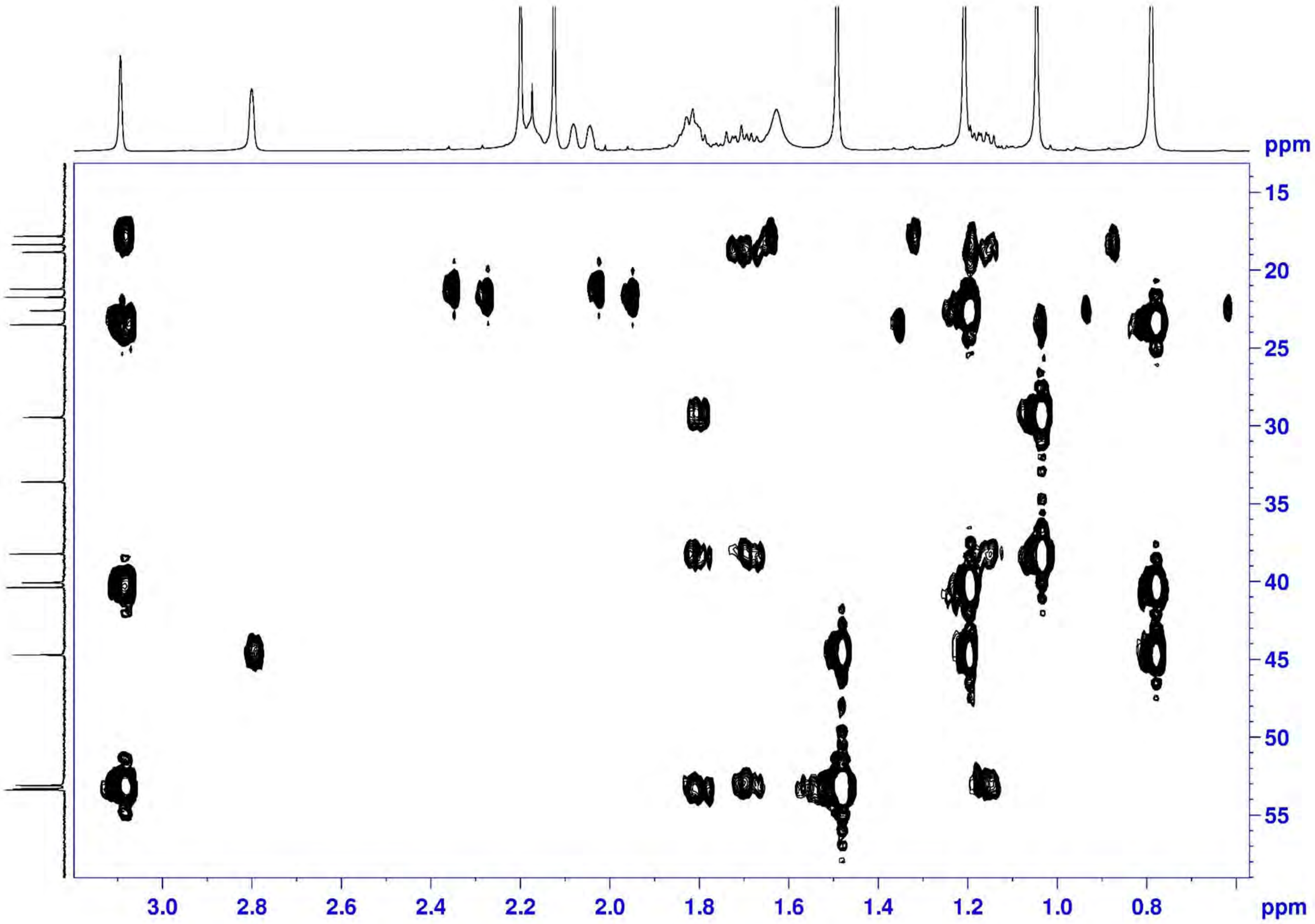

NOESY (400 MHz) spectrum of compound **6** in $CDCl_3$

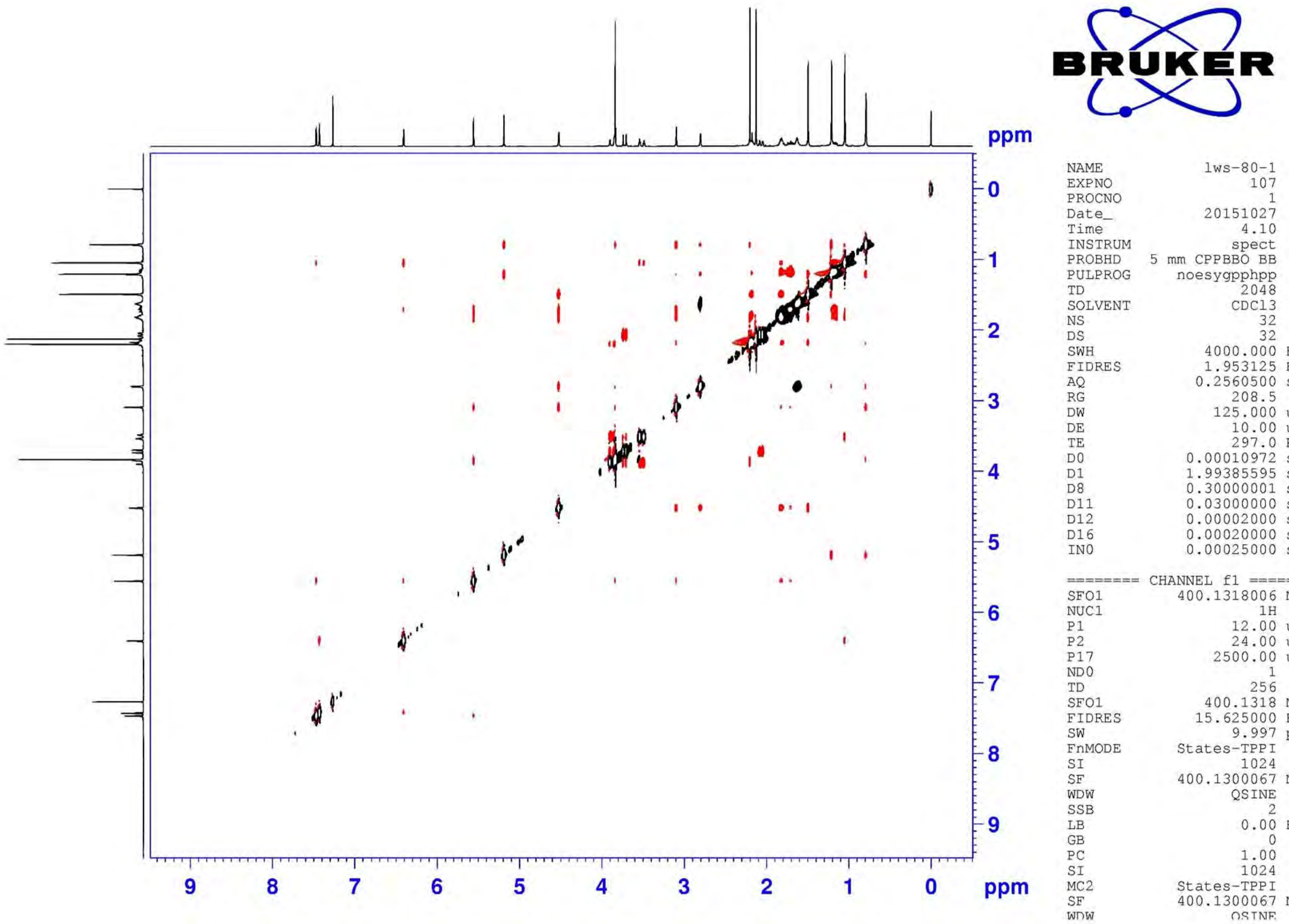

NOESY (400 MHz) spectrum of compound **6** in $CDCl_3$

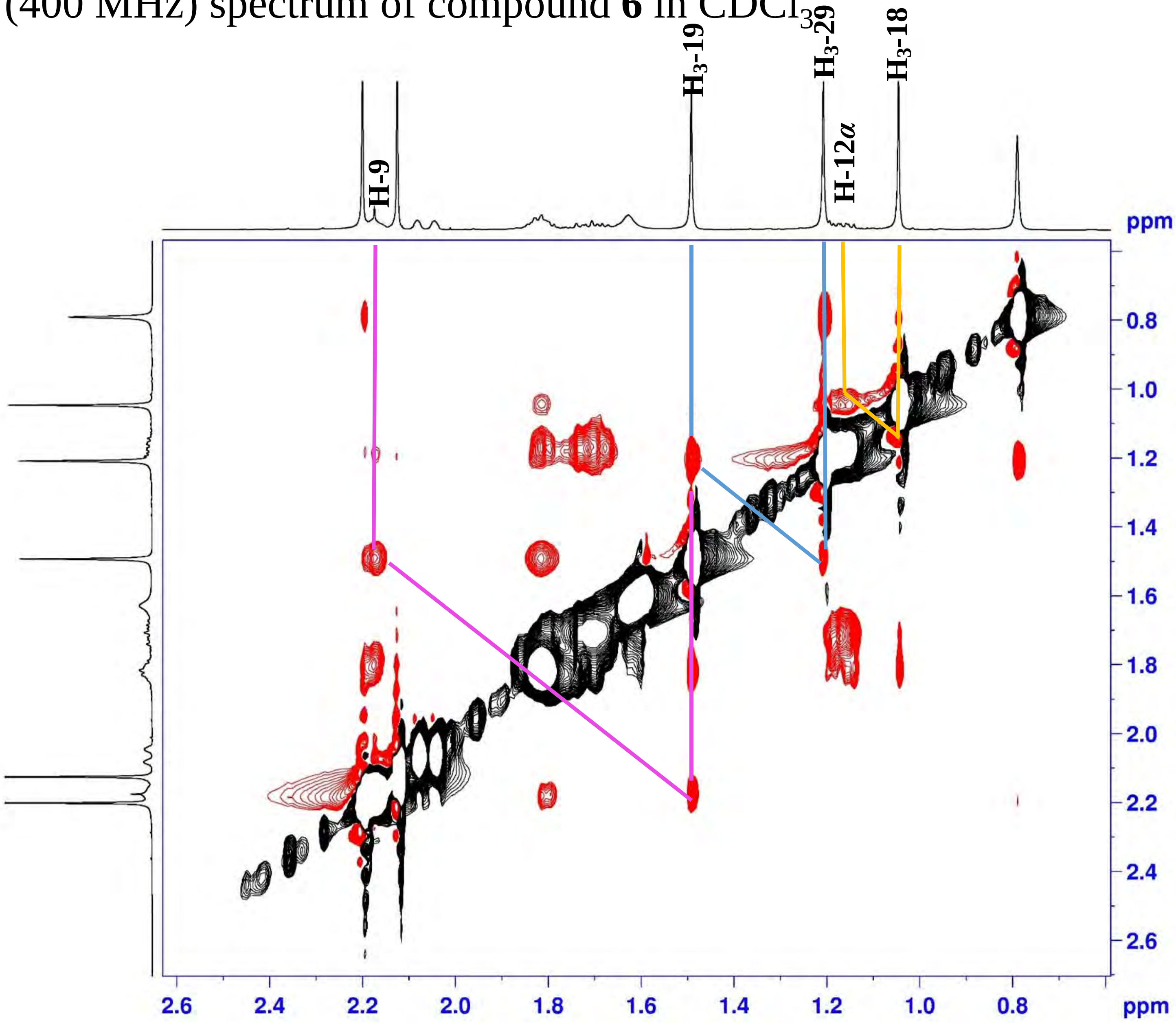

NOESY (400 MHz) spectrum of compound **6** in $CDCl_3$

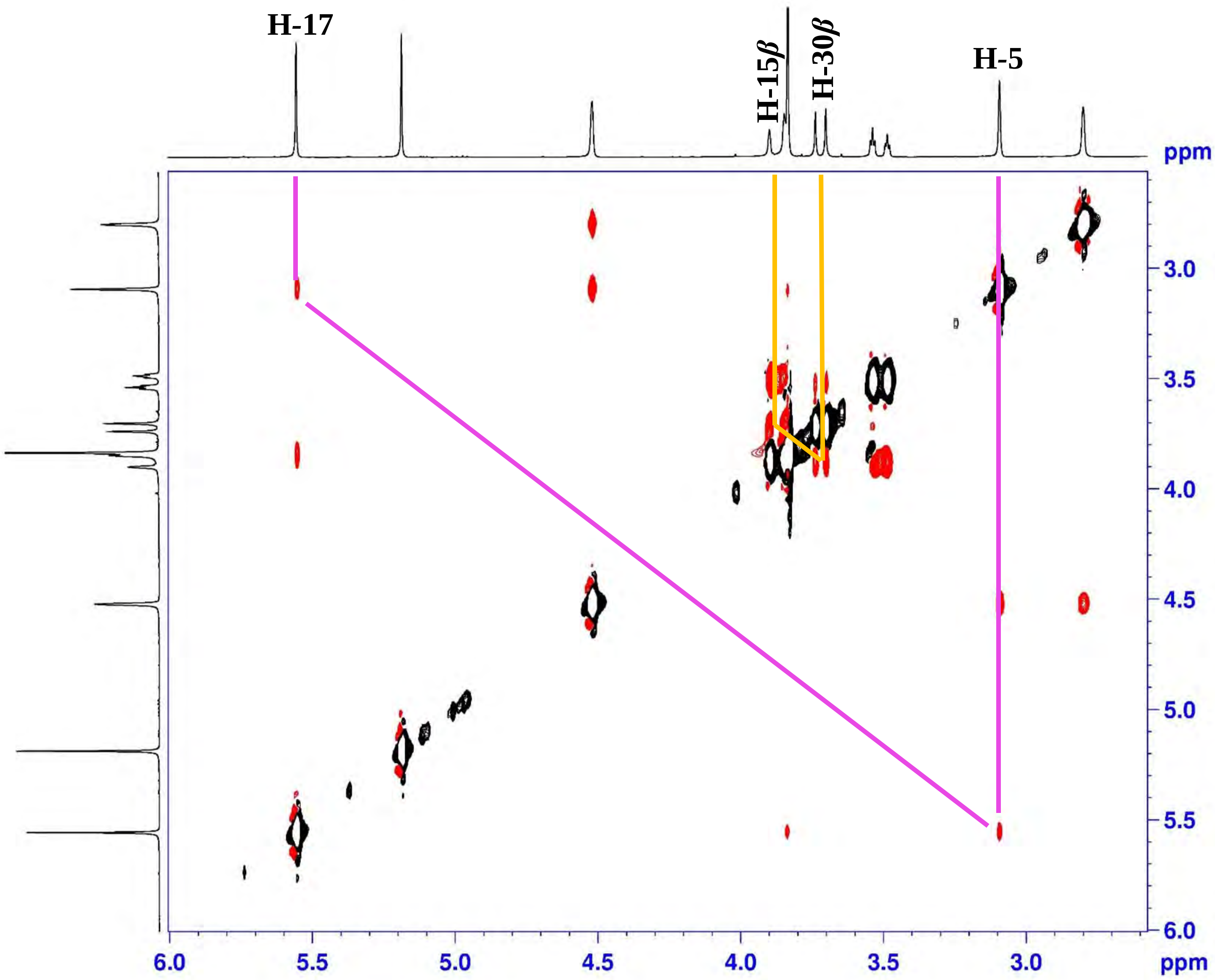

NOESY (400 MHz) spectrum of compound **6** in $CDCl_3$

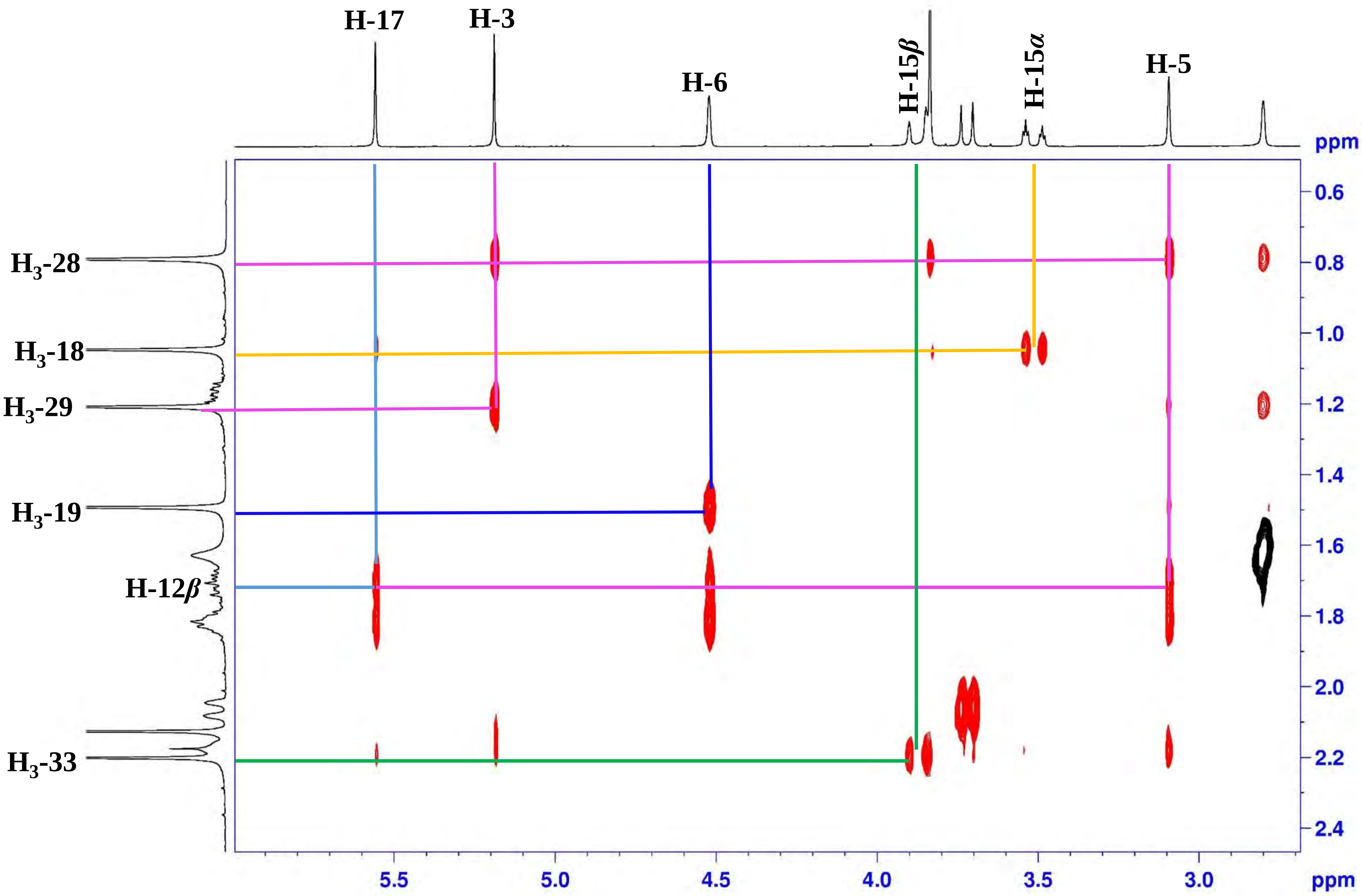

HRESIMS for compound **7**

Mass Spectrum SmartFormula Report

Analysis Info

Analysis Name	D:\Data\MS\data\201612\zhangjianzhi_zjz-34_pos_83_01_2759.d	Acquisition Date	12/14/2016 2:51:51 PM
Method	LC MS_Direct Infusion_pos_100-1000mz.m	Operator	SCSIO
Sample Name	zhangjianzhi_zjz-34_pos	Instrument	maXis 255552.00029
Comment			

Acquisition Parameter

Source Type	ESI	Ion Polarity	Positive	Set Nebulizer	0.4 Bar
Focus	Active	Set Capillary	4500 V	Set Dry Heater	180 °C
Scan Begin	100 m/z	Set End Plate Offset	-500 V	Set Dry Gas	4.0 l/min
Scan End	2000 m/z	Set Charging Voltage	0 V	Set Divert Valve	Waste
		Set Corona	0 nA	Set APCI Heater	0 °C

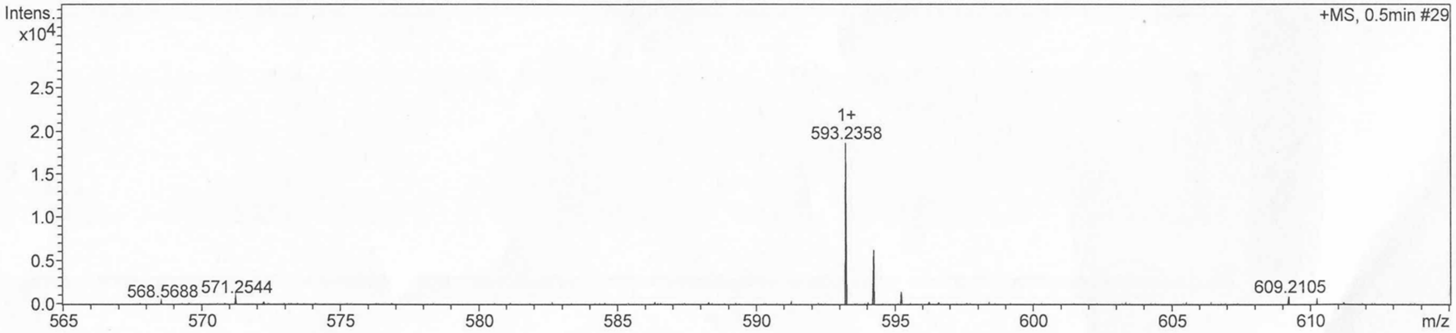

Meas. m/z	#	Ion Formula	Score	m/z	err [ppm]	err [mDa]	mSigma	rdb	e⁻ Conf	N-Rule
571.254411	1	C31H39O10	100.00	571.253774	-1.1	-0.6	47.1	12.5	even	ok
593.235801	1	C31H38NaO10	100.00	593.235718	-0.1	-0.1	3.4	12.5	even	ok
1163.482868	1	C62H76NaO20	100.00	1163.482216	-0.6	-0.7	35.0	24.5	even	ok

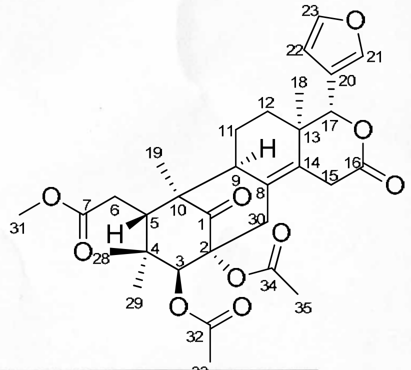

zhangjianzhi_zjz-34_pos_83_01_2759.d

Bruker Compass DataAnalysis 4.1 printed: 12/14/2016 2:58:29 PM by: SCSIO Page 1 of 1

HRESIMS for compound **7**

Generic Display Report

Analysis Info

Analysis Name	D:\Data\MS\data\201612\zhangjianzhi_zjz-34_pos_83_01_2759.d	Acquisition Date	12/14/2016 2:51:51 PM
Method	LC MS_Direct Infusion_pos_100-1000mz.m	Operator	SCSIO
Sample Name	zhangjianzhi_zjz-34_pos	Instrument	maXis
Comment			

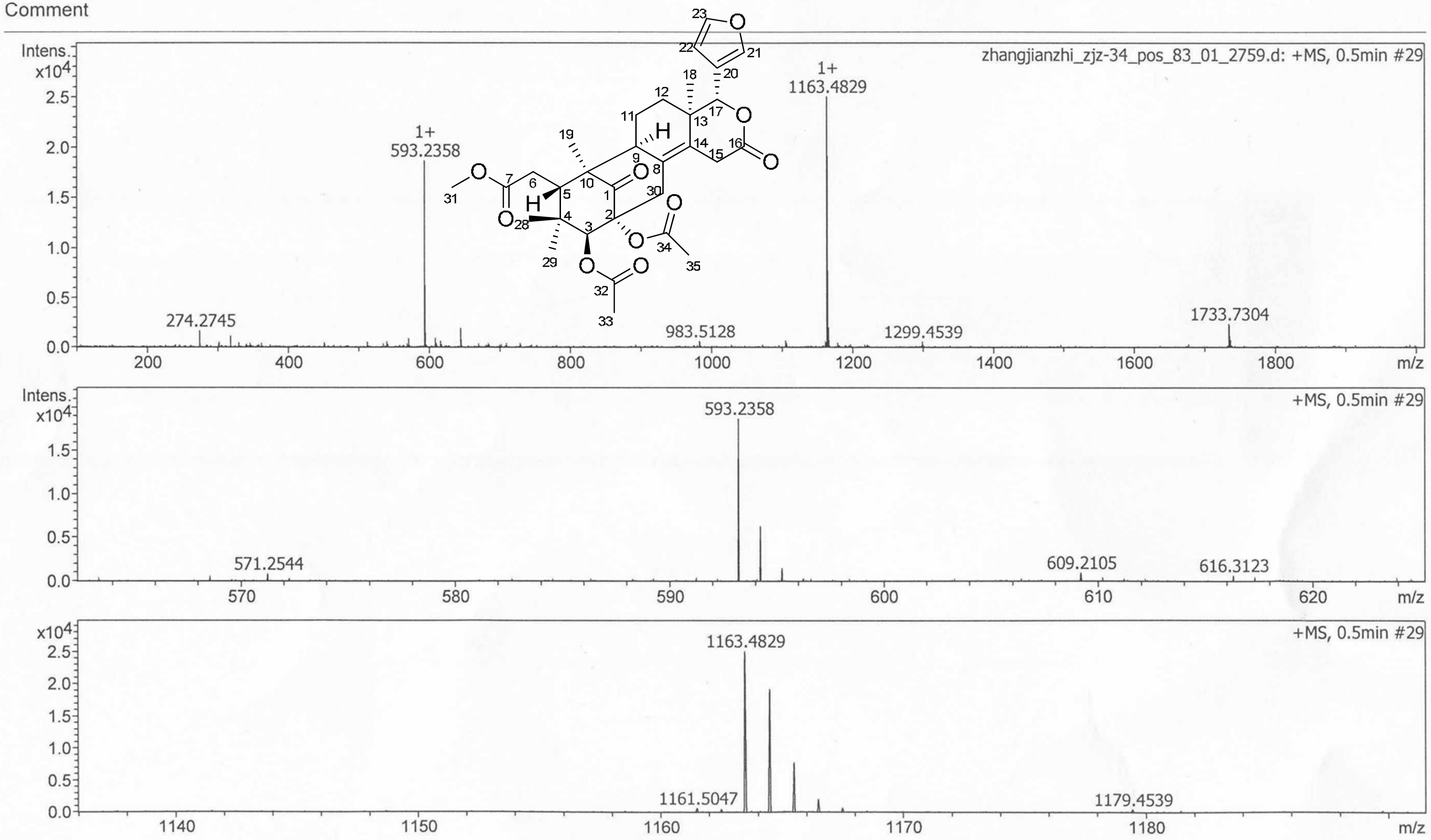

^{1}H NMR (400 MHz) spectrum of compound **7** in $CDCl_3$

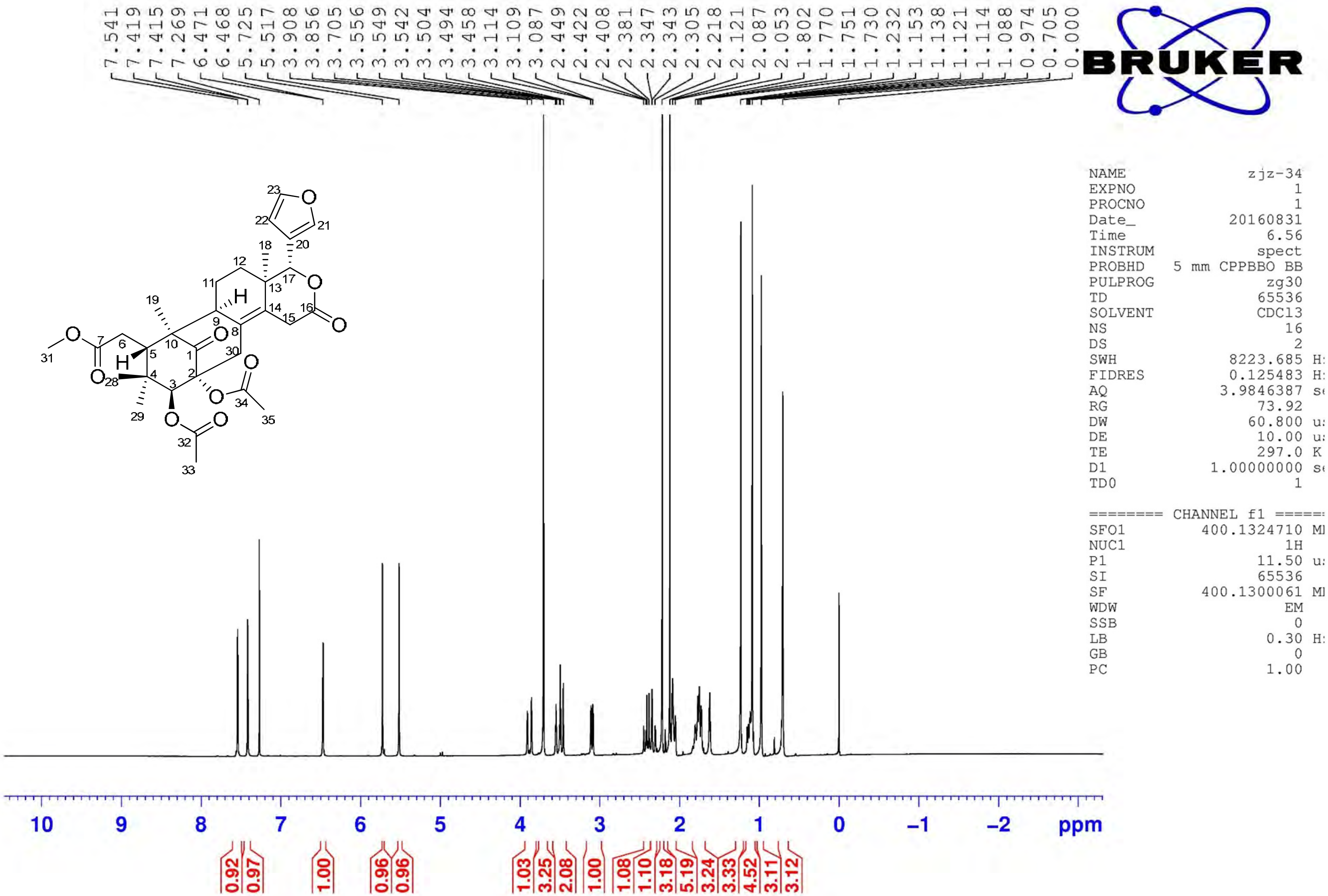

^{1}H NMR (400 MHz) spectrum of compound **7** in $CDCl_3$

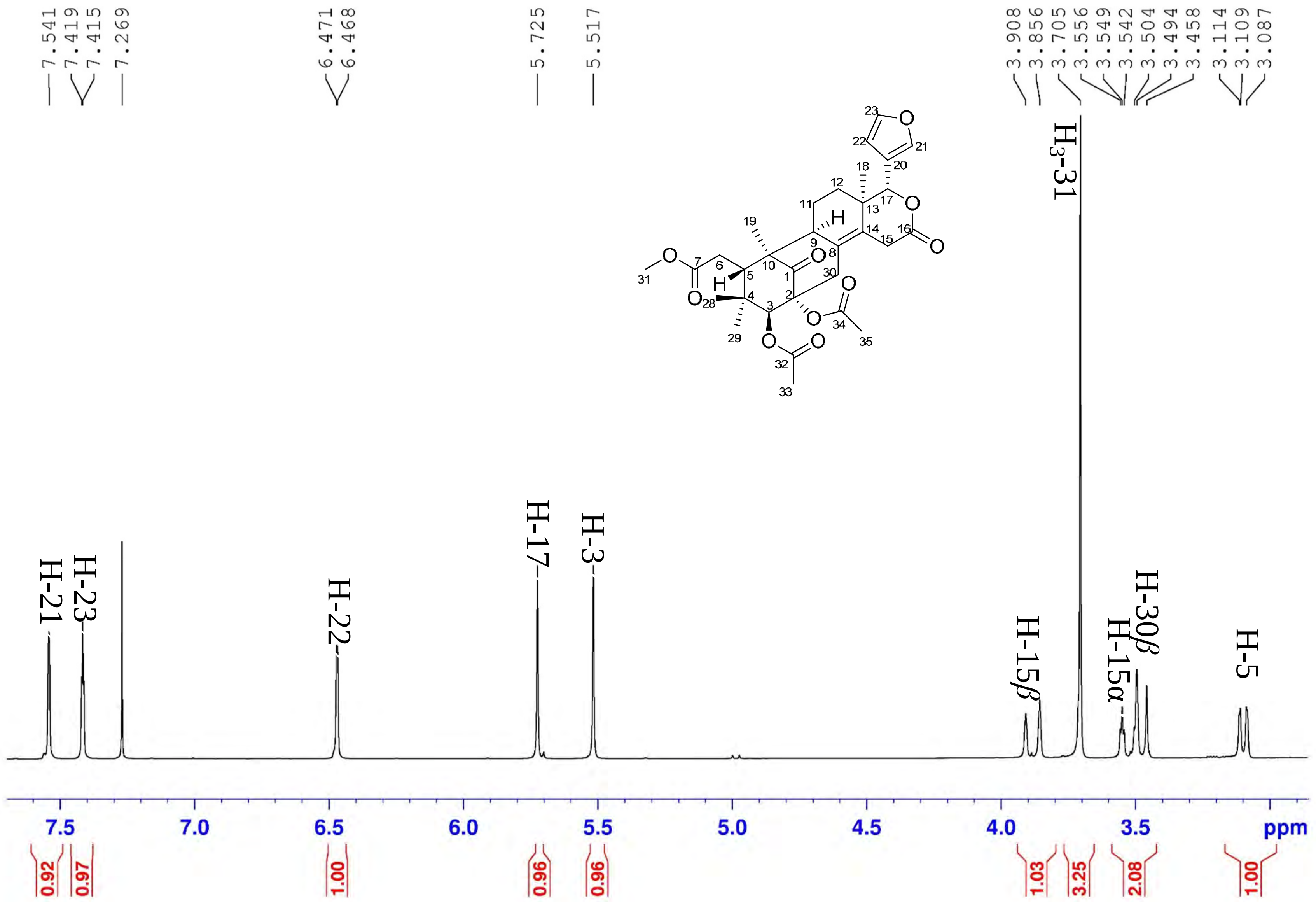

^{1}H NMR (400 MHz) spectrum of compound **7** in $CDCl_3$

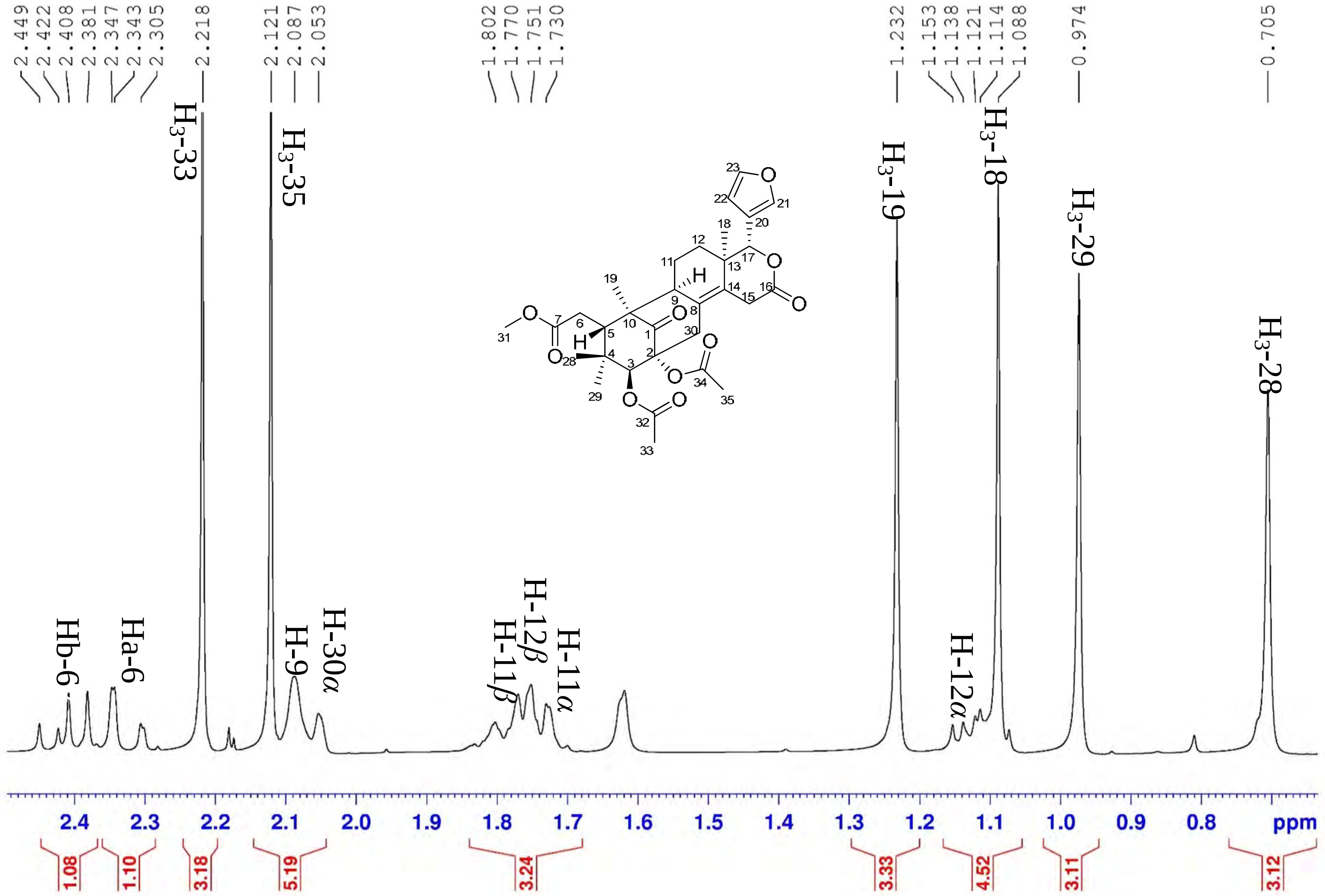

^{13}C NMR (100 MHz) spectrum of compound **7** in $CDCl_3$

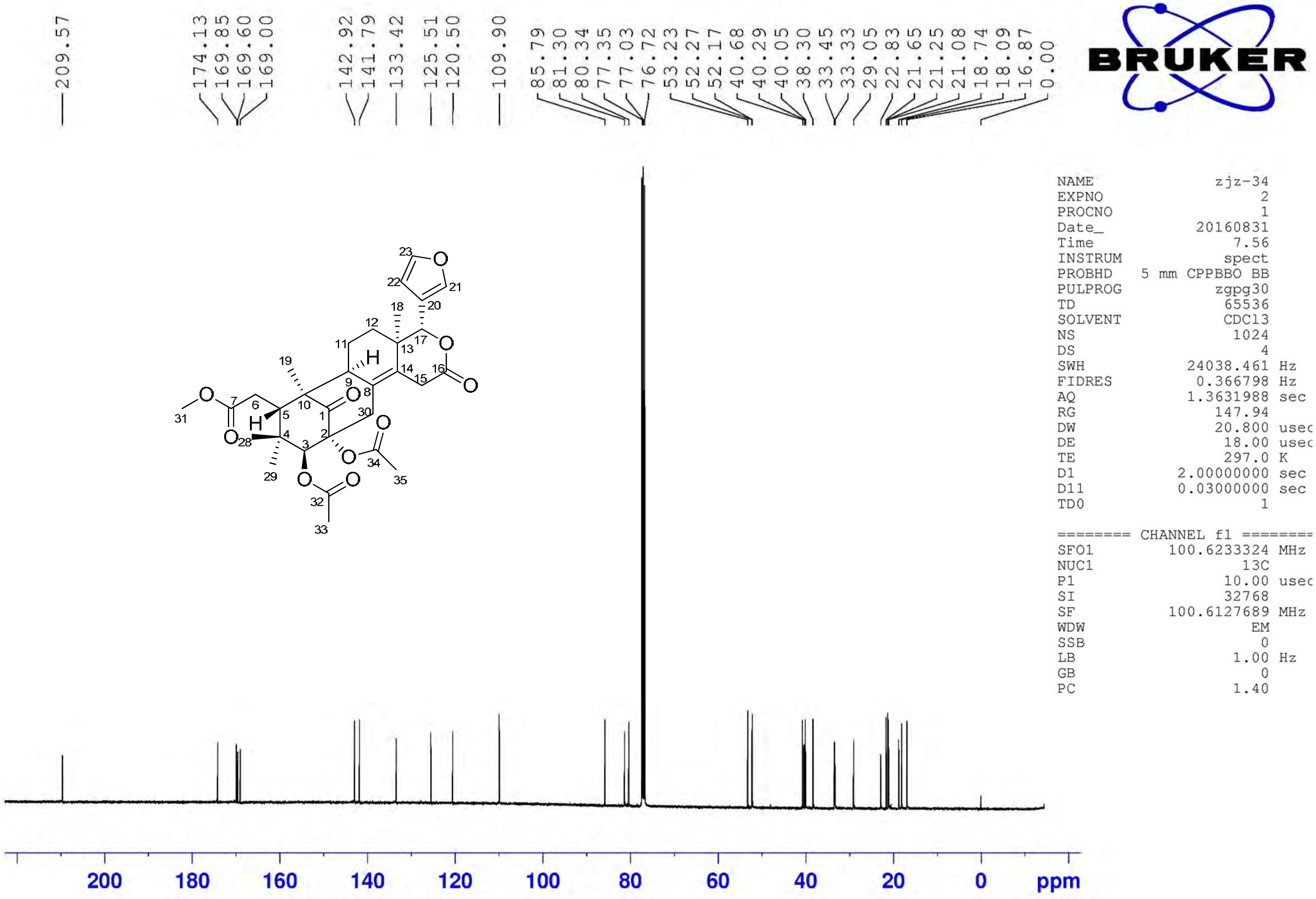

^{13}C NMR (100 MHz) spectrum of compound **7** in $CDCl_3$

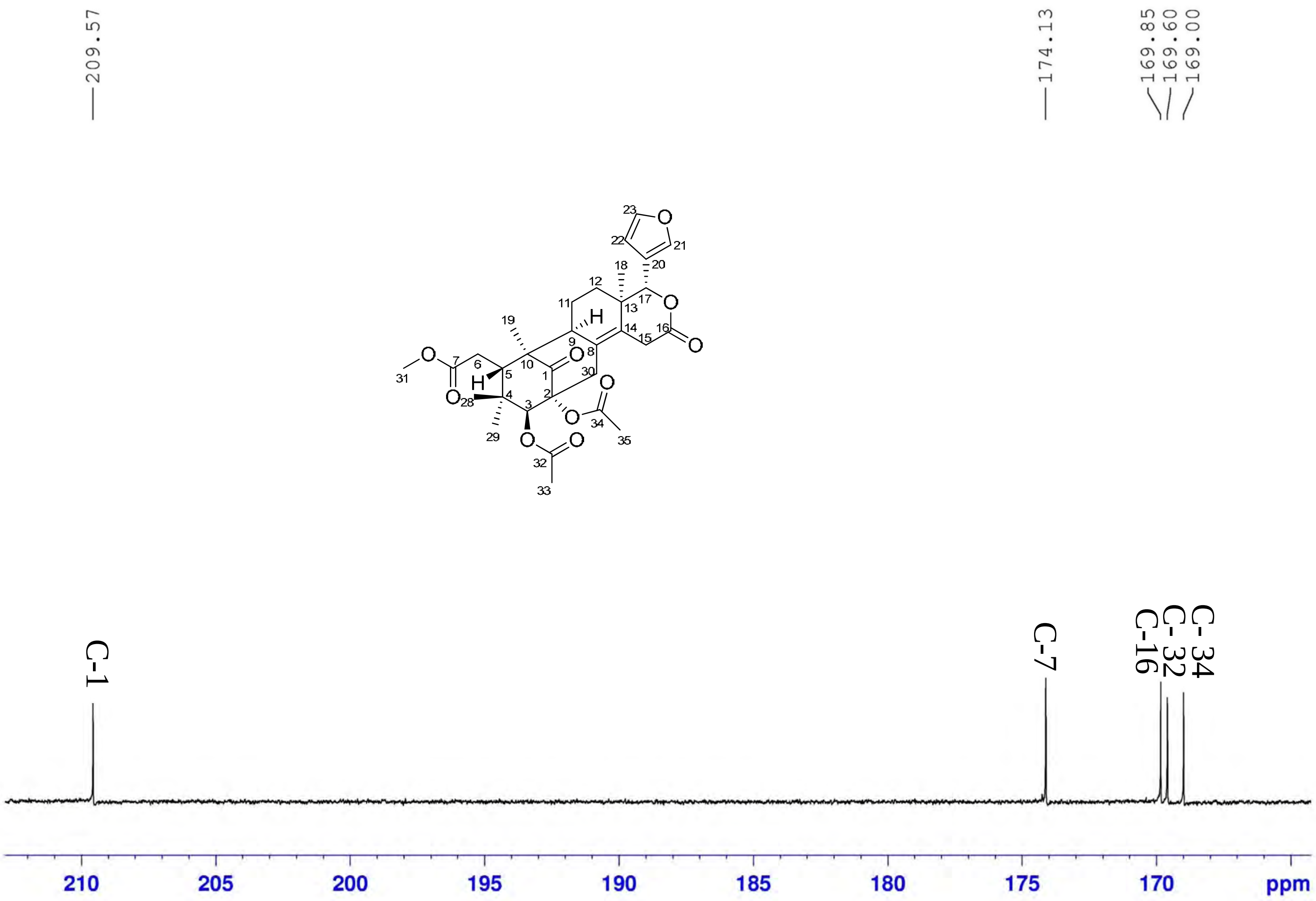

^{13}C NMR (100 MHz) spectrum of compound **7** in $CDCl_3$

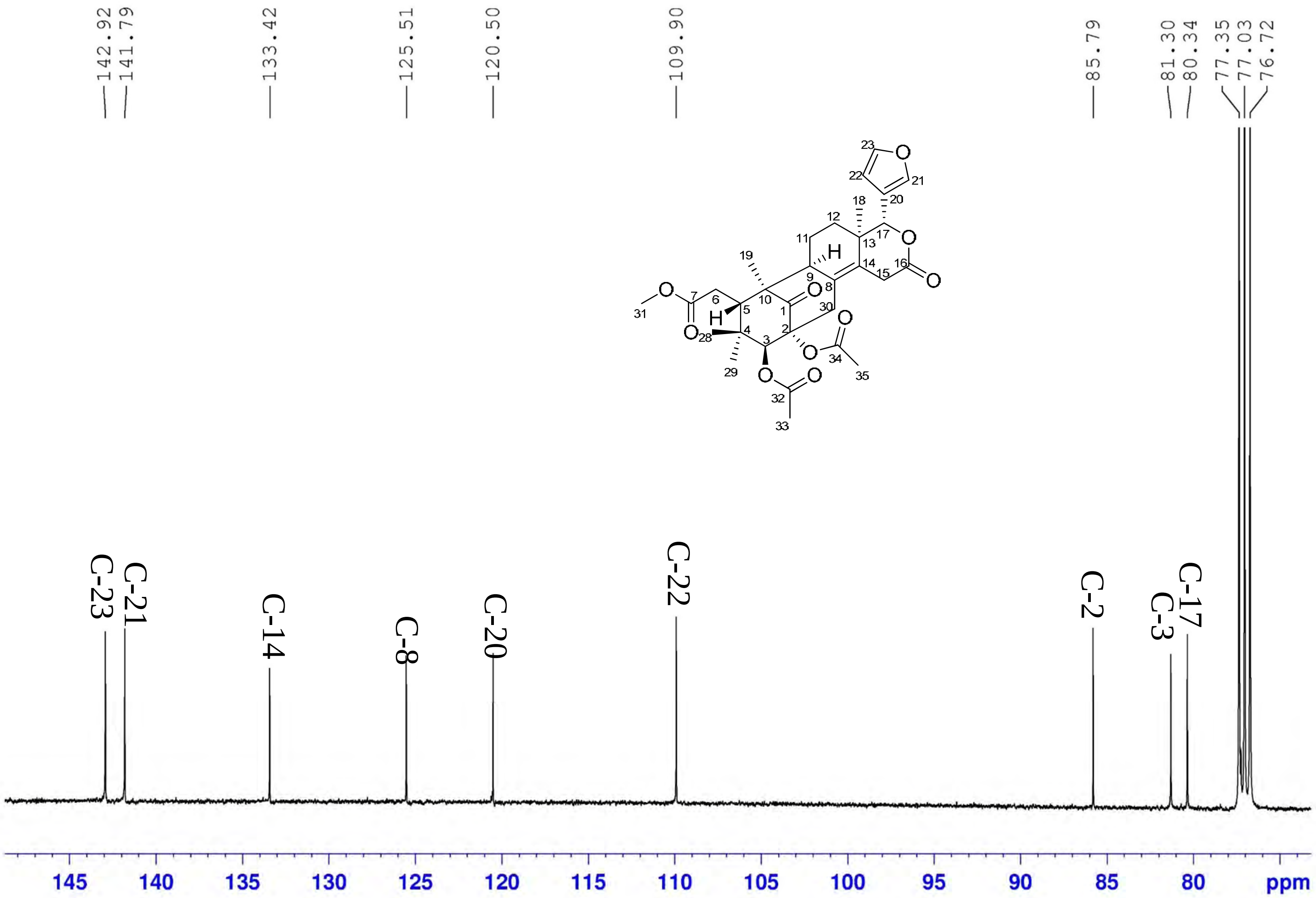

^{13}C NMR (100 MHz) spectrum of compound **7** in $CDCl_3$

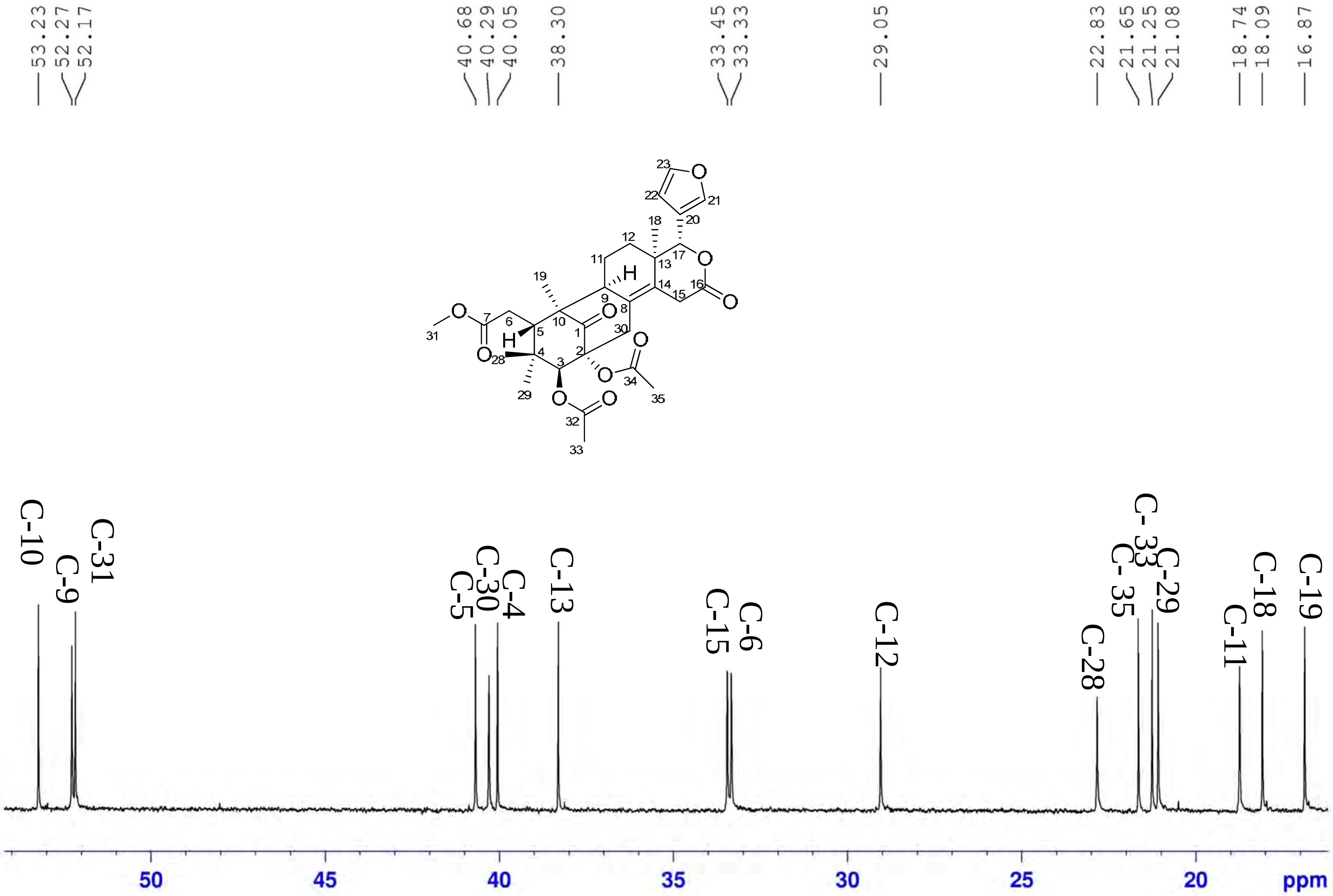

DEPT135 (100 MHz) spectrum of compound **7** in $CDCl_3$

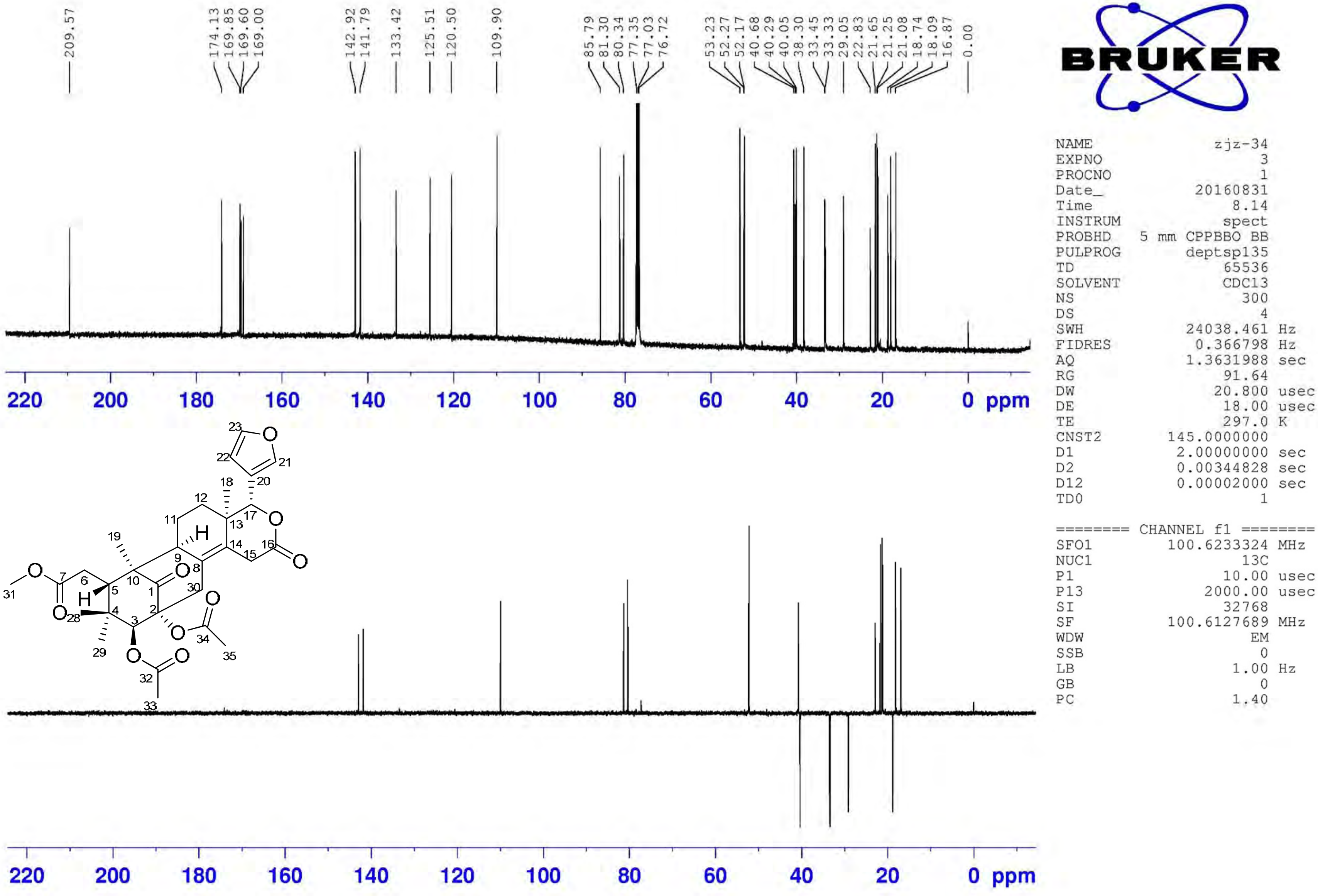

DEPT135 (100 MHz) spectrum of compound **7** in $CDCl_3$

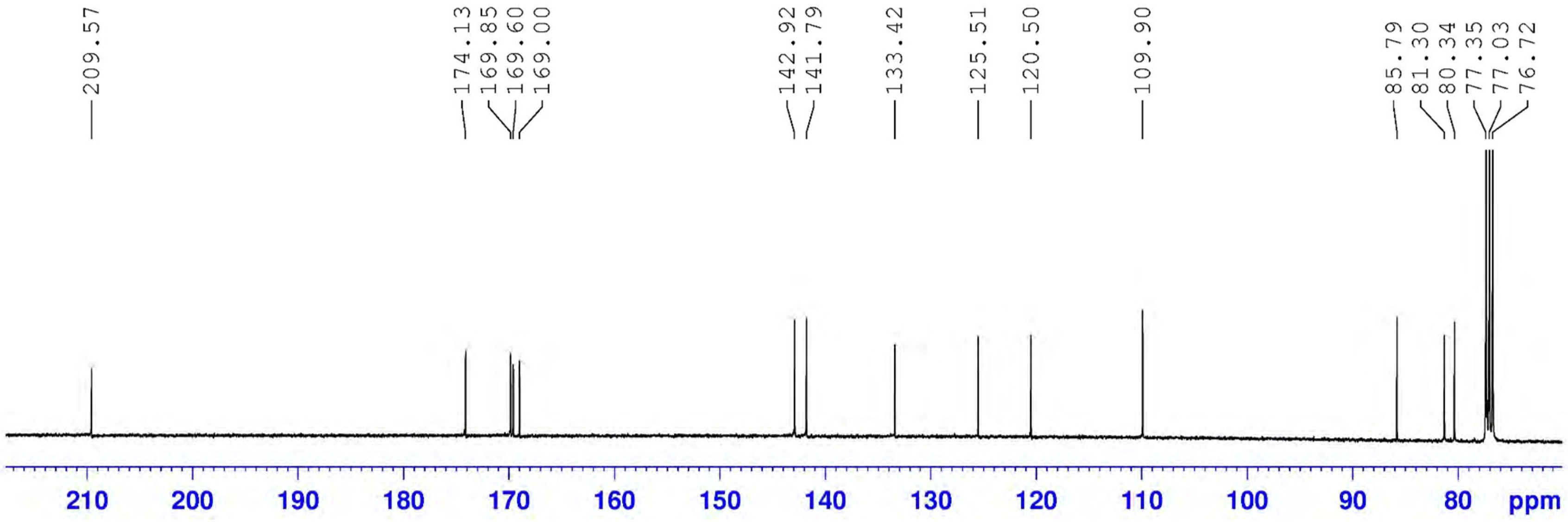

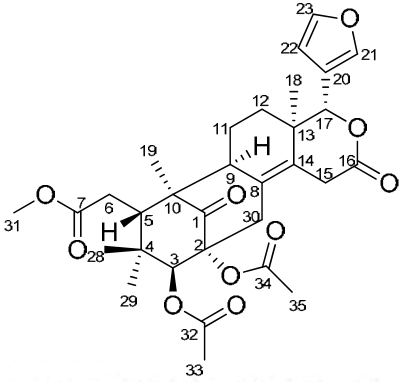

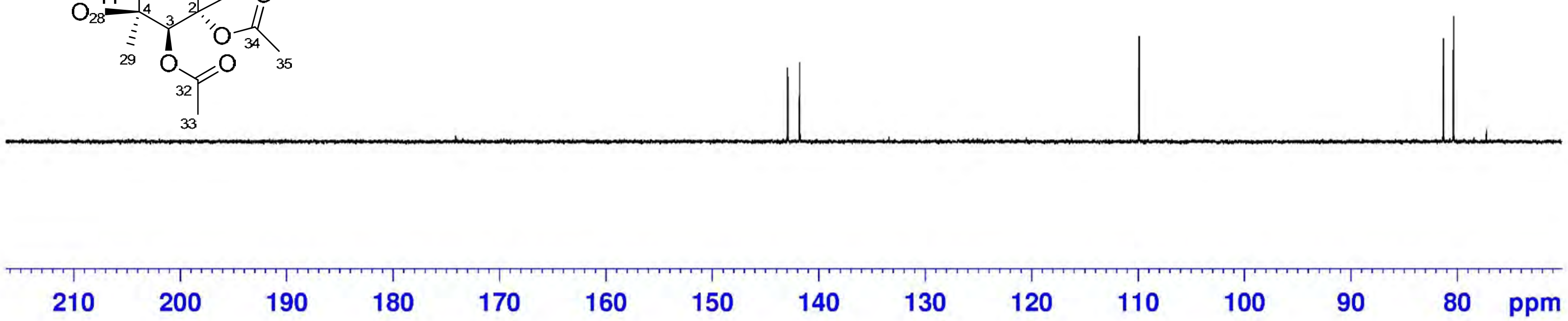

DEPT135 (100 MHz) spectrum of compound **7** in $CDCl_3$

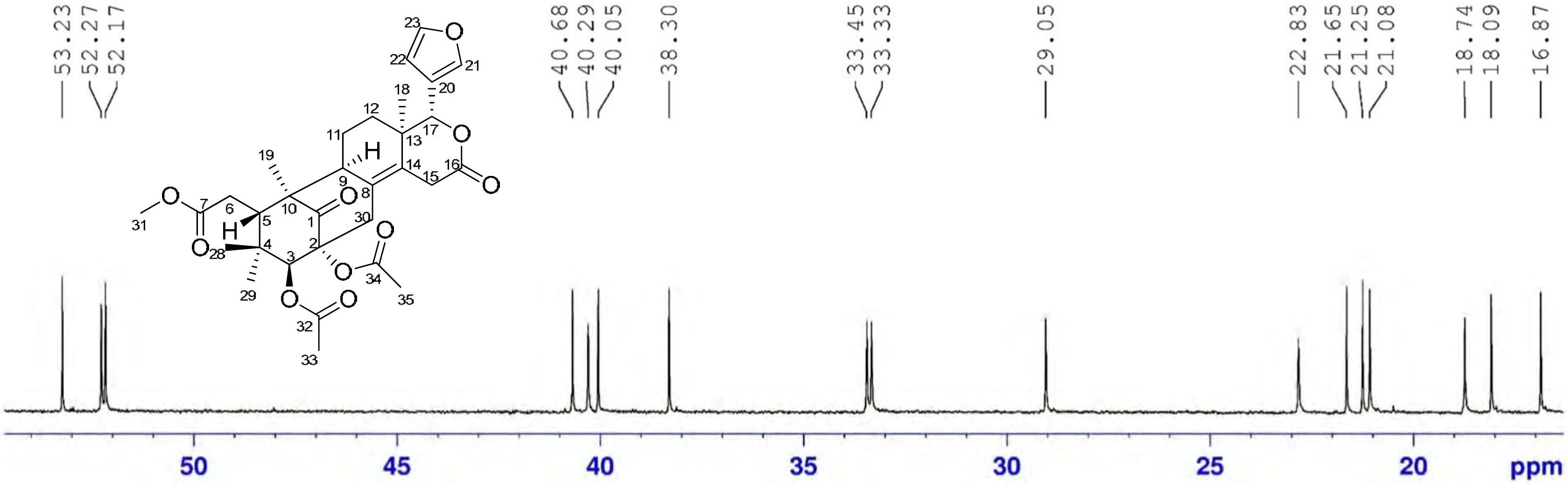

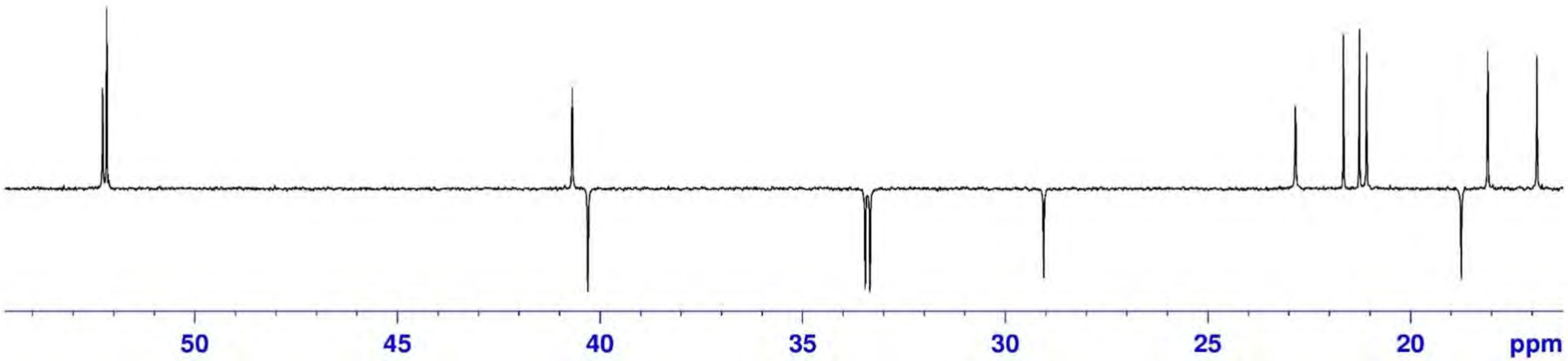

^{1}H-^{1}H COSY (400 MHz) spectrum of compound **7** in $CDCl_3$

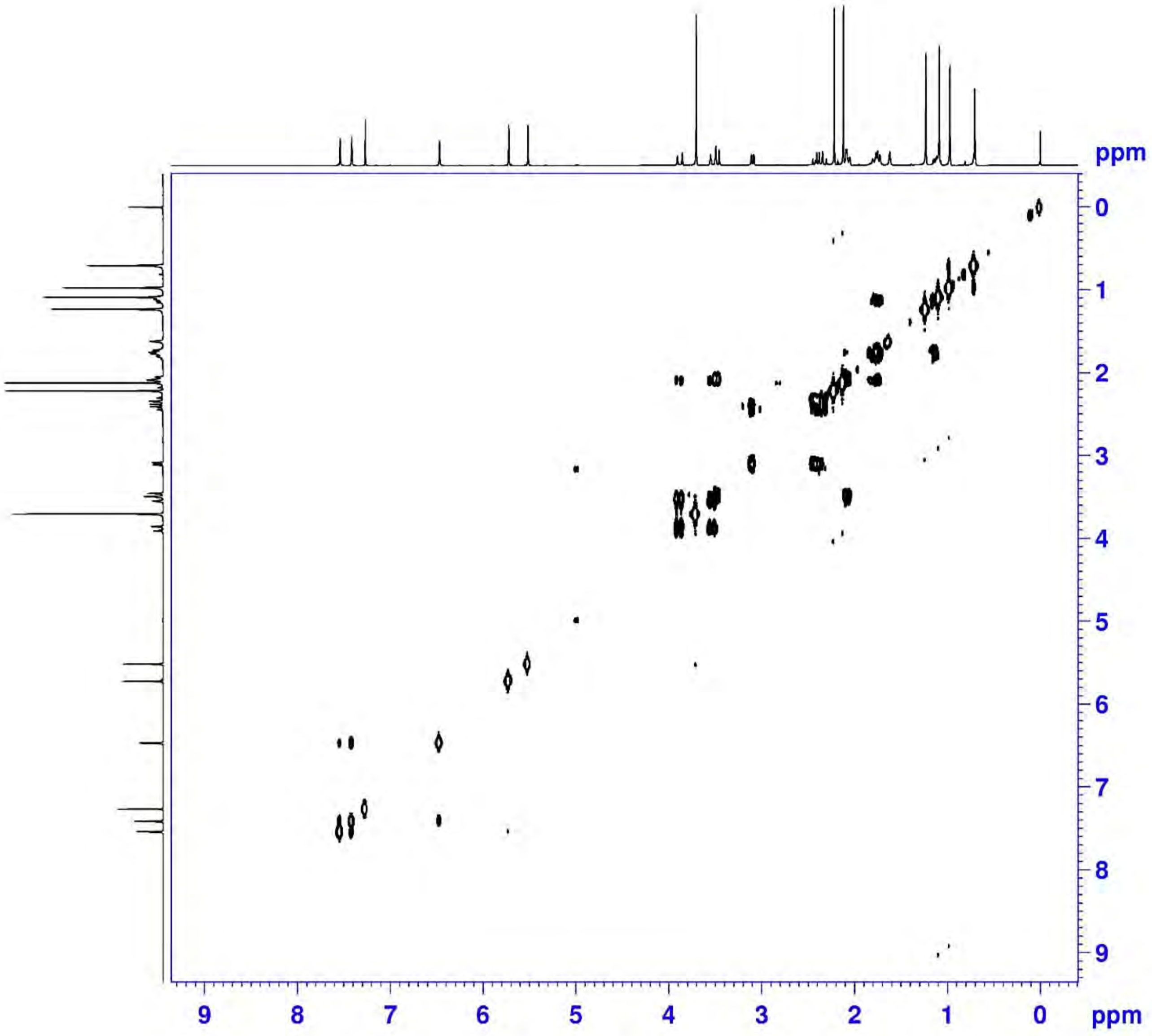

NAME	zjz-34	
EXPNO	4	
PROCNO	1	
Date_	20160913	
Time	22.31	
INSTRUM	spect	
PROBHD	5 mm CPPBBO BB	
PULPROG	cosygpppqf	
TD	2048	
SOLVENT	CDCl3	
NS	8	
DS	8	
SWH	3906.250	Hz
FIDRES	1.907349	Hz
AQ	0.2621940	sec
RG	171.57	
DW	128.000	usec
DE	10.00	usec
TE	297.0	K
D0	0.00000300	sec
D1	1.89678097	sec
D11	0.03000000	sec
D12	0.00002000	sec
D13	0.00000400	sec
D16	0.00020000	sec
IN0	0.00025600	sec
======== CHANNEL f1 ========		
SFO1	400.1318006	MHz
NUC1	1H	
P0	11.50	usec
P1	11.50	usec
P17	2500.00	usec
ND0	1	
TD	128	
SFO1	400.1318	MHz
FIDRES	30.517578	Hz
SW	9.762	ppm
FnMODE	QF	
SI	1024	
SF	400.1300040	MHz
WDW	QSINE	
SSB	0	
LB	0.00	Hz
GB	0	
PC	1.40	
SI	1024	
MC2	QF	
SF	400.1300051	MHz
WDW	QSINE	
SSB	0	
LB	0.00	Hz
GB	0	

^{1}H-^{1}H COSY (400 MHz) spectrum of compound **7** in $CDCl_3$

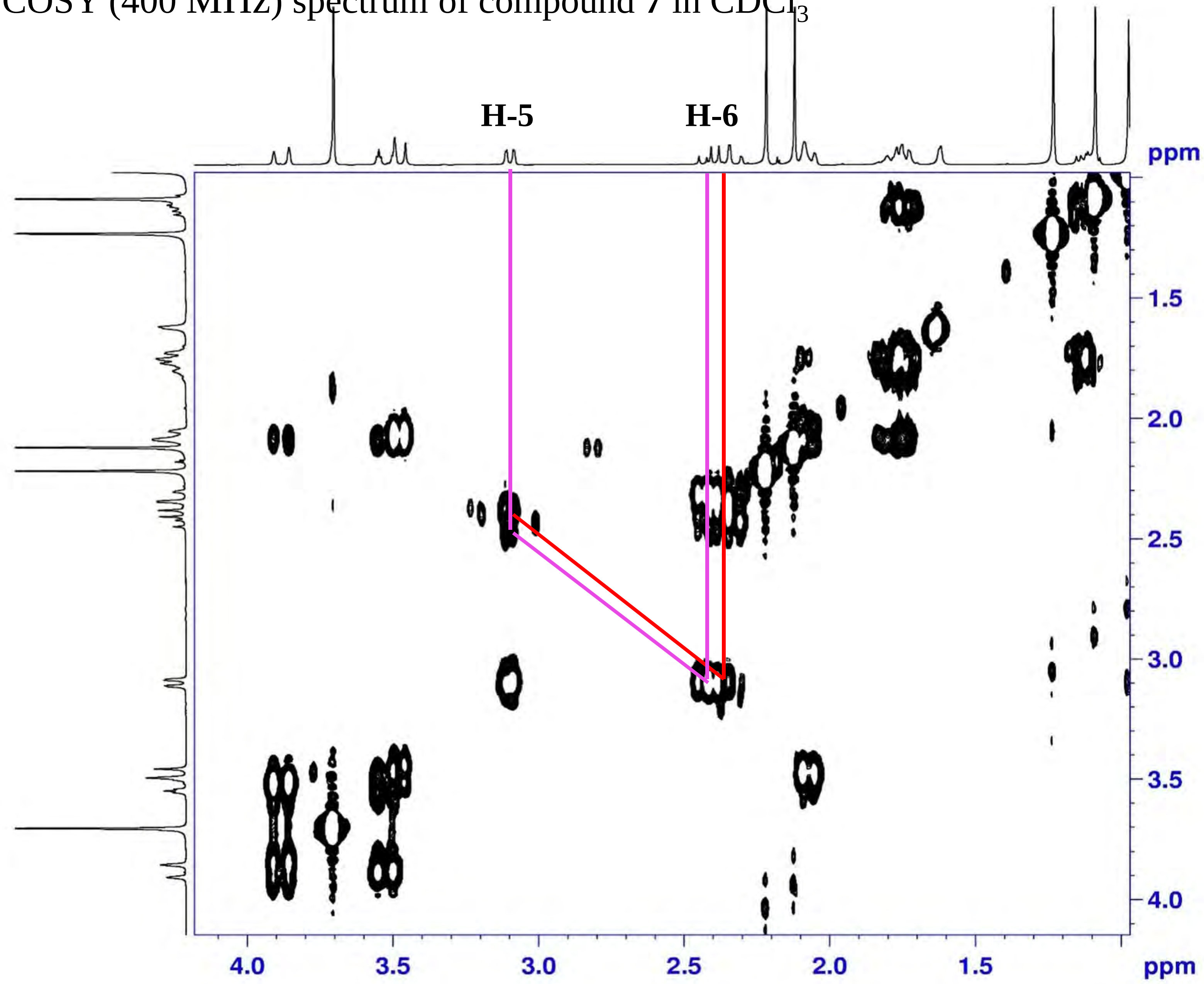

HSQC (400 MHz) spectrum of compound **7** in $CDCl_3$

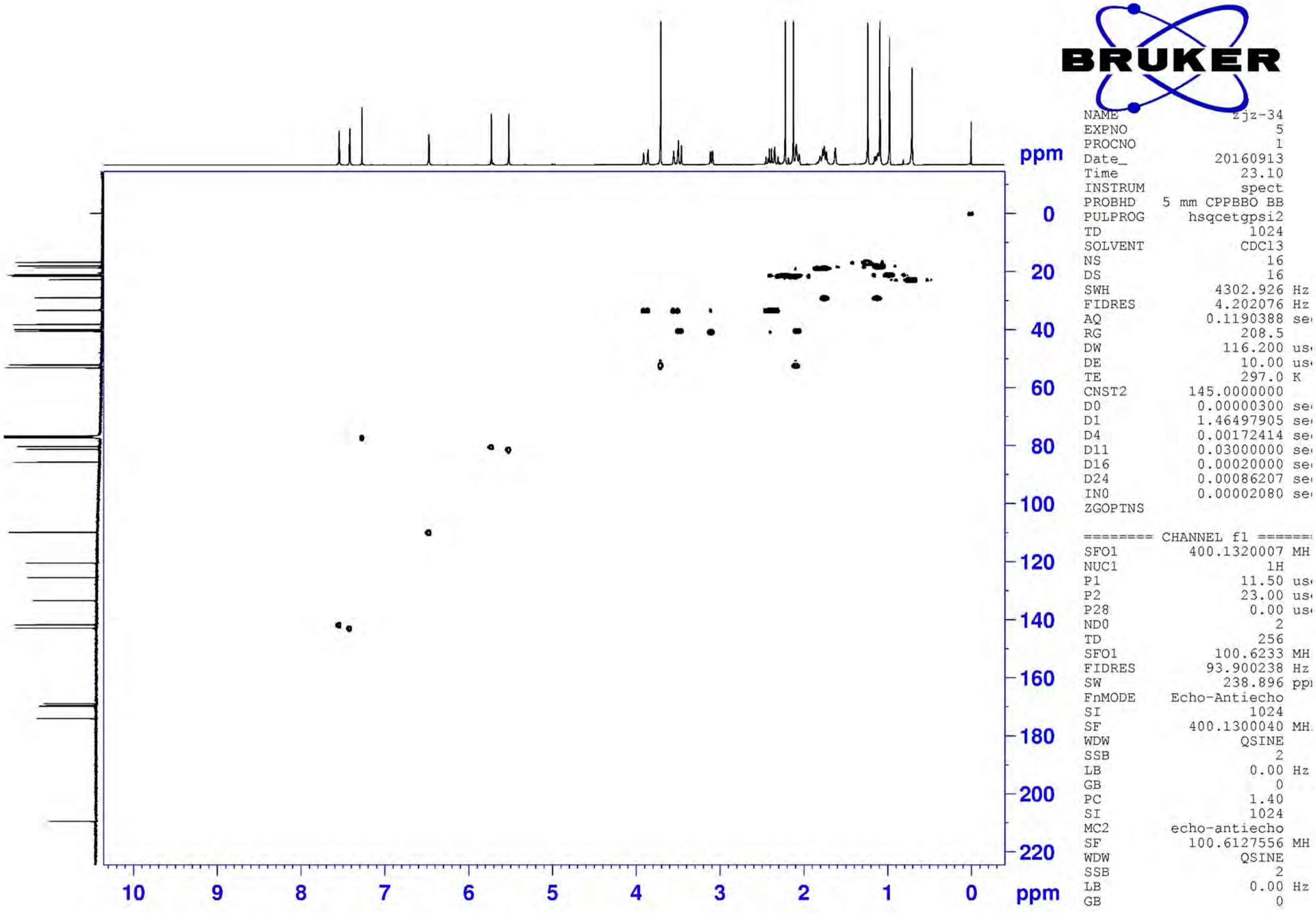

HSQC (400 MHz) spectrum of compound **7** in $CDCl_3$

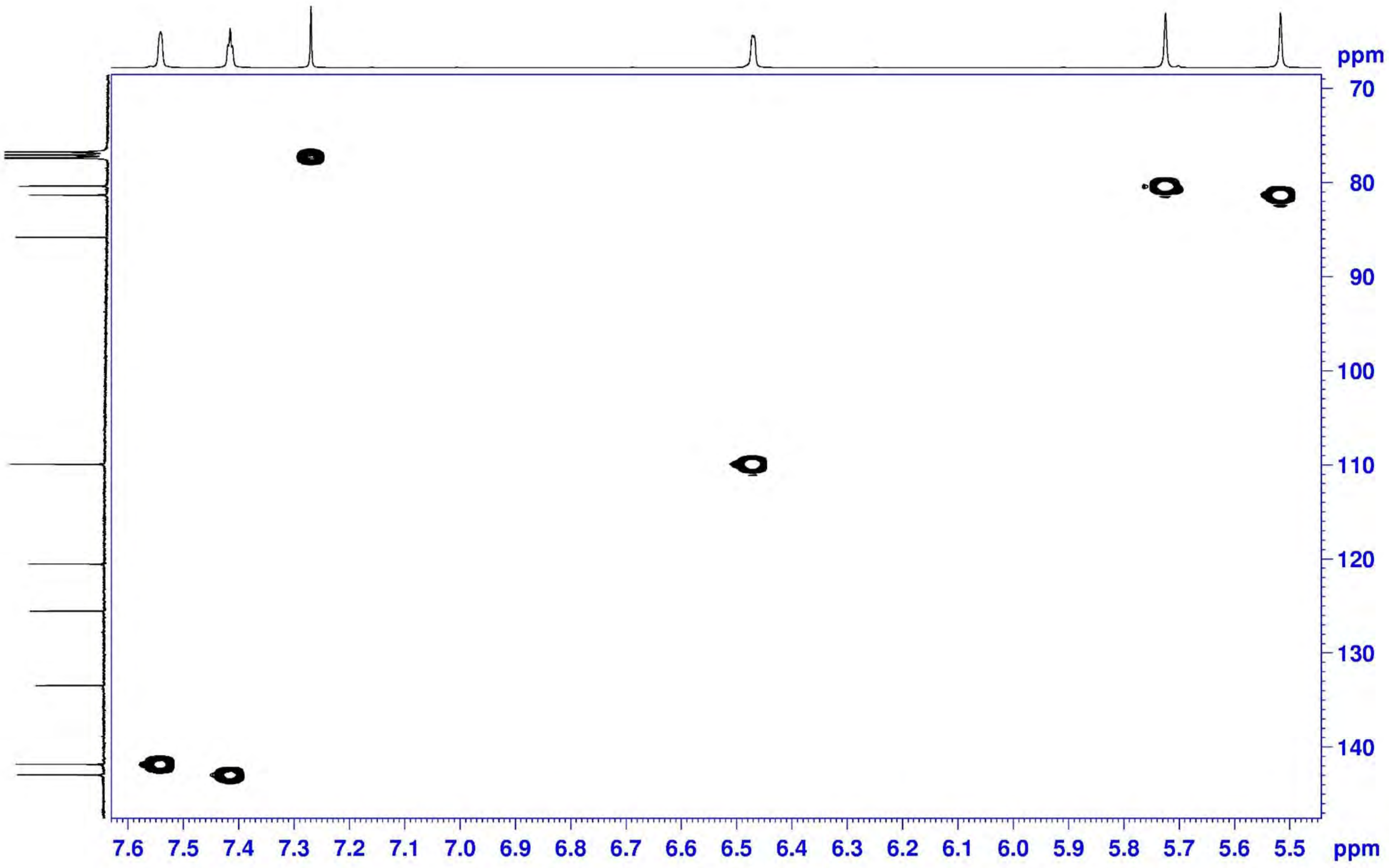

HSQC (400 MHz) spectrum of compound **7** in $CDCl_3$

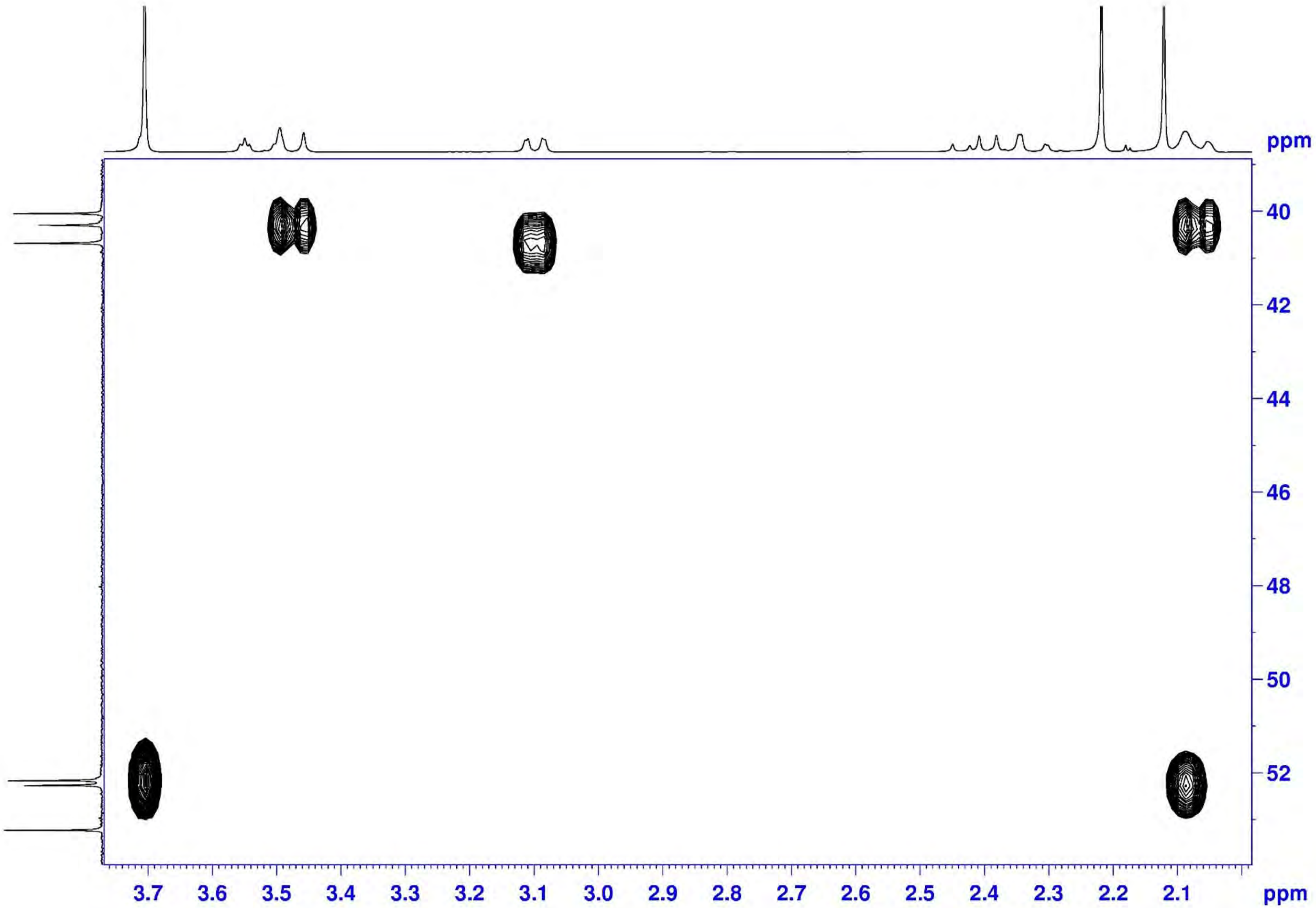

HSQC (400 MHz) spectrum of compound **7** in $CDCl_3$

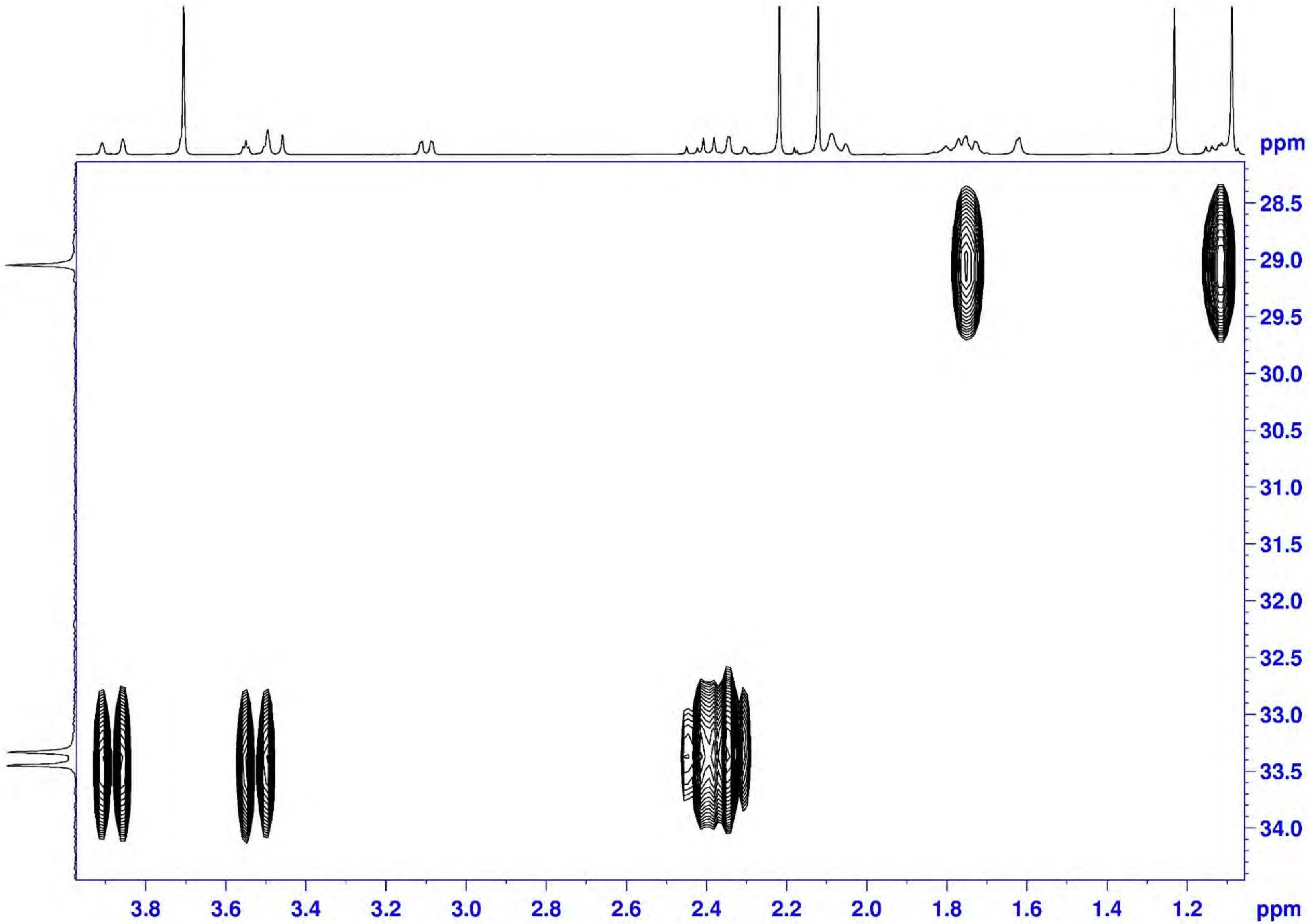

HSQC (400 MHz) spectrum of compound **7** in $CDCl_3$

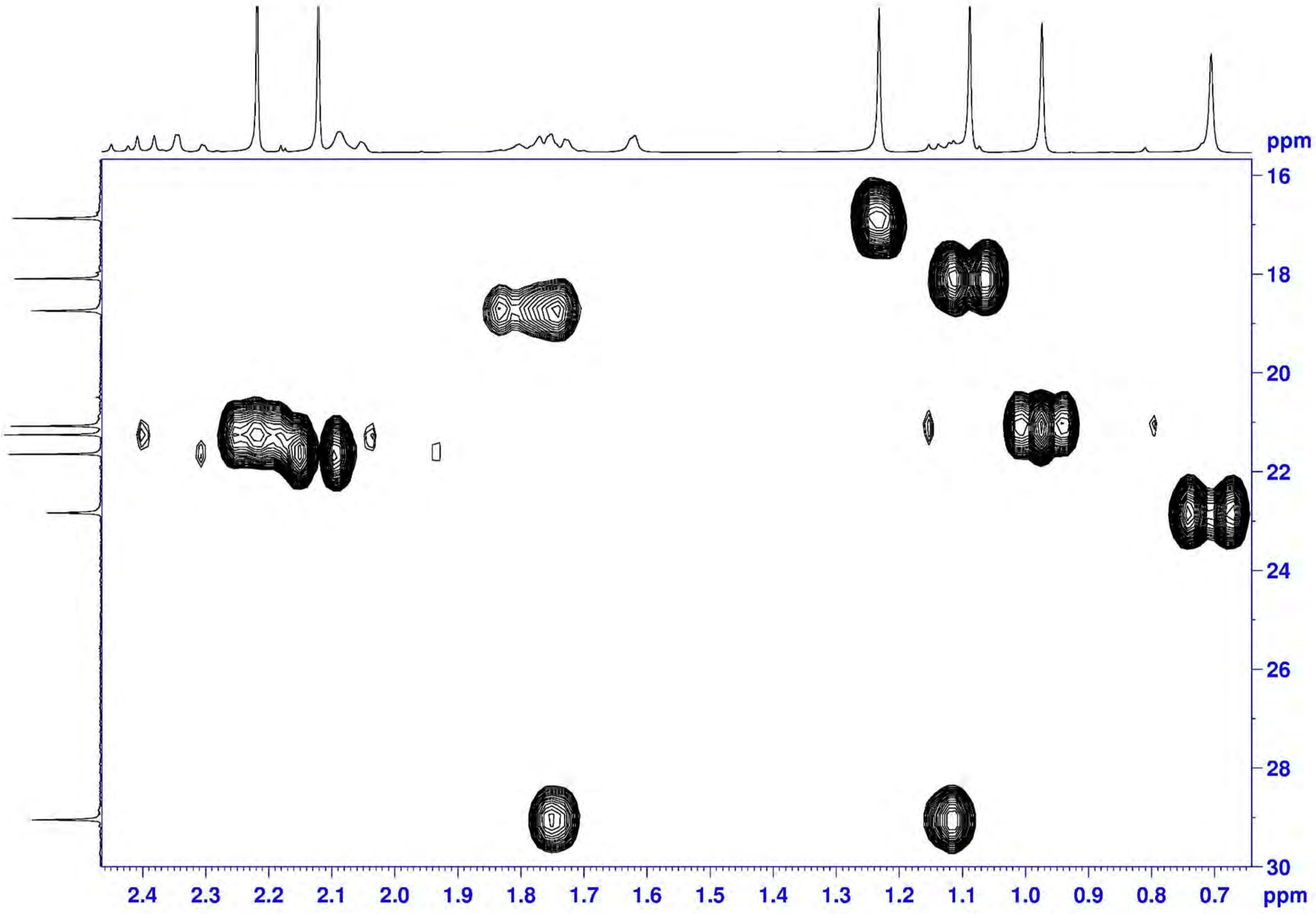

HMBC (400 MHz) spectrum of compound **7** in $CDCl_3$

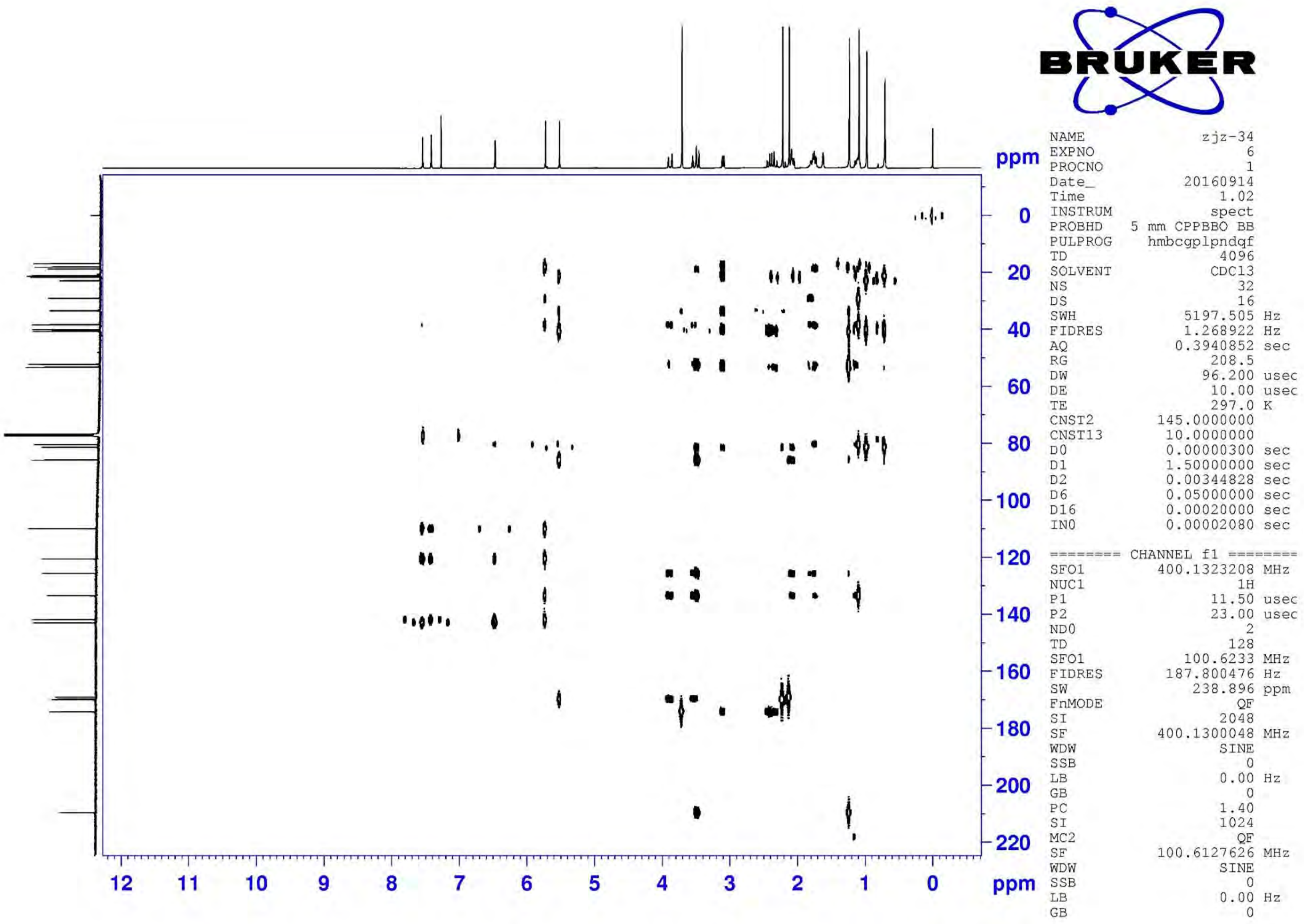

HMBC (400 MHz) spectrum of compound **7** in $CDCl_3$

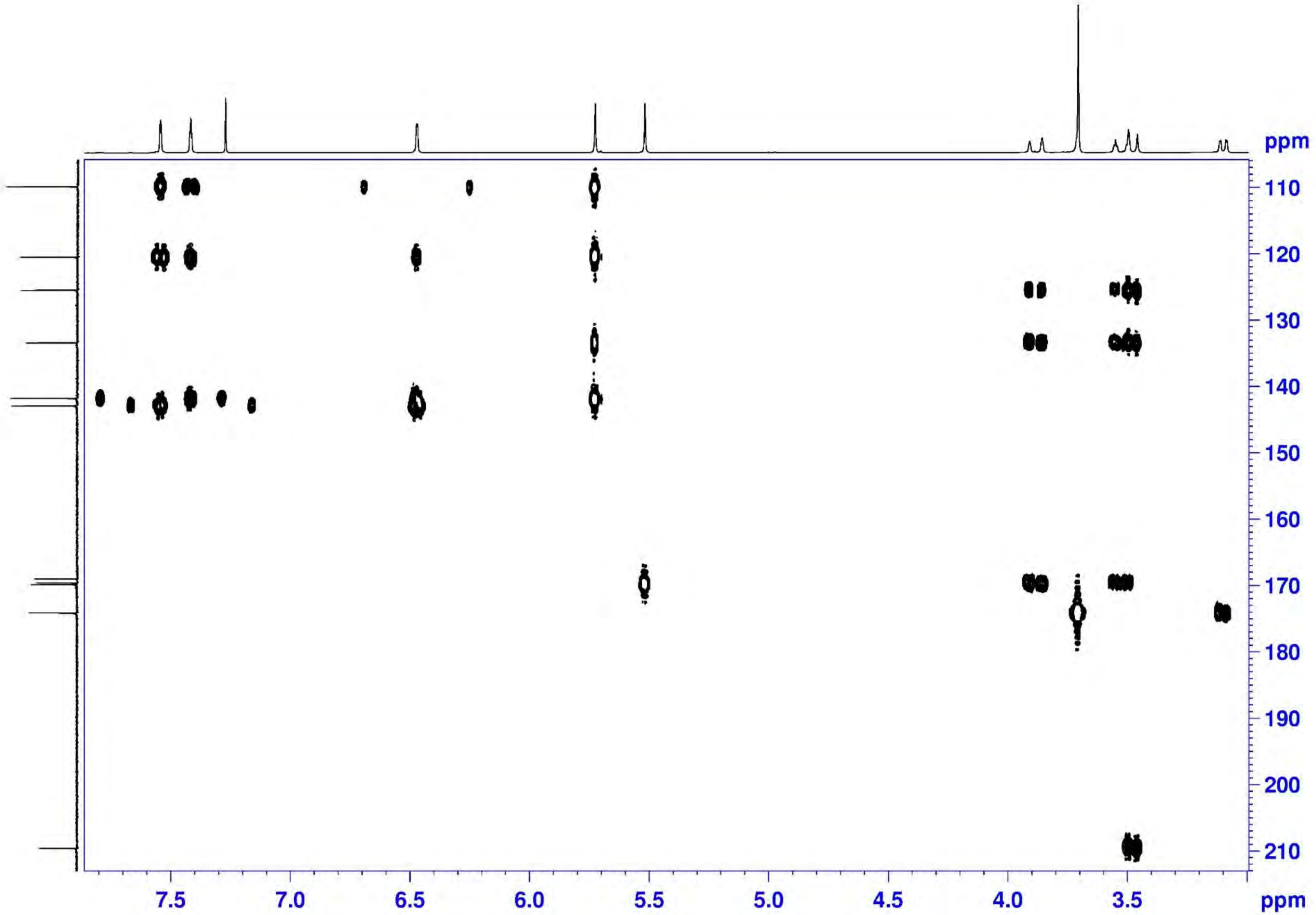

HMBC (400 MHz) spectrum of compound **7** in $CDCl_3$

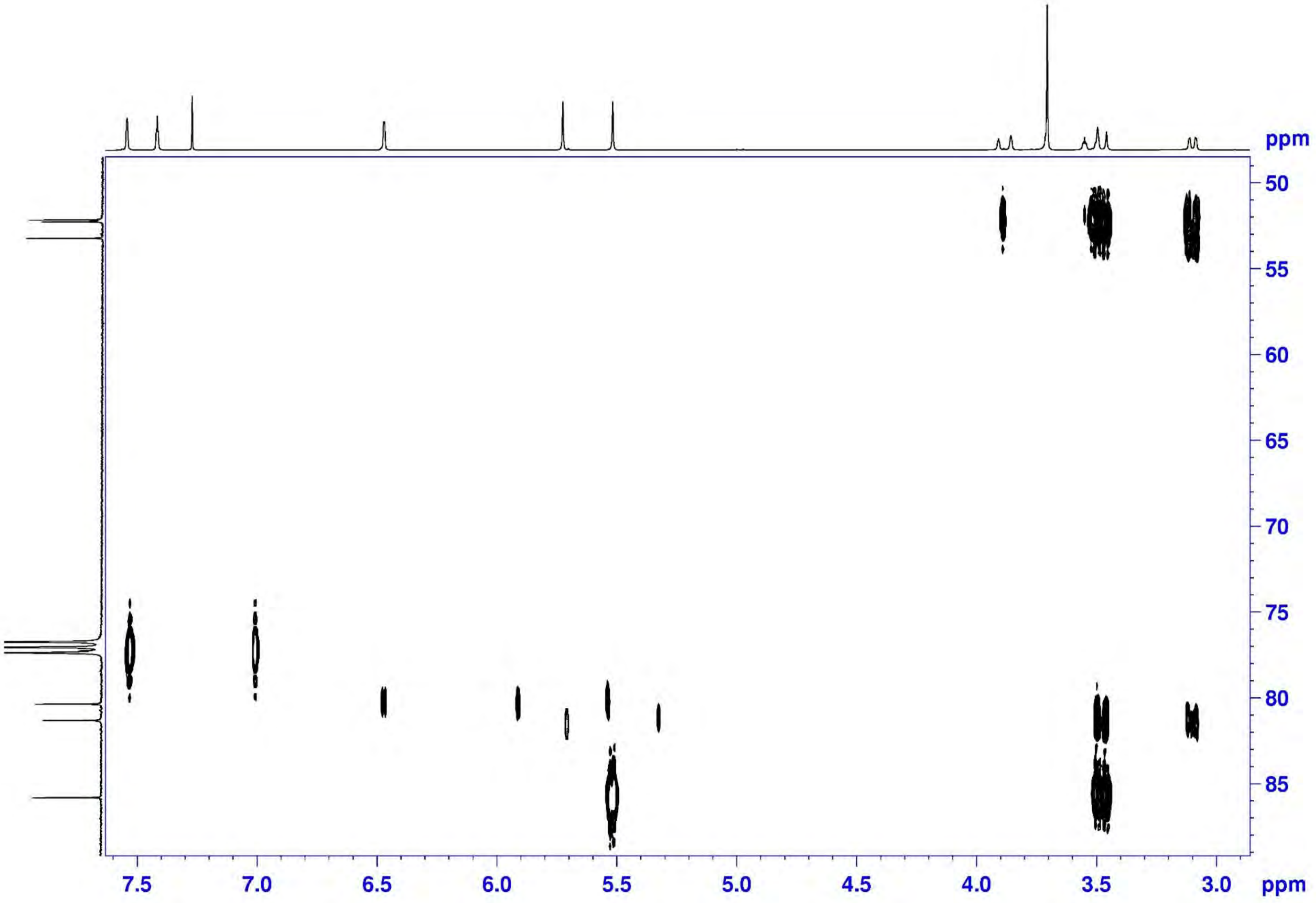

HMBC (400 MHz) spectrum of compound **7** in $CDCl_3$

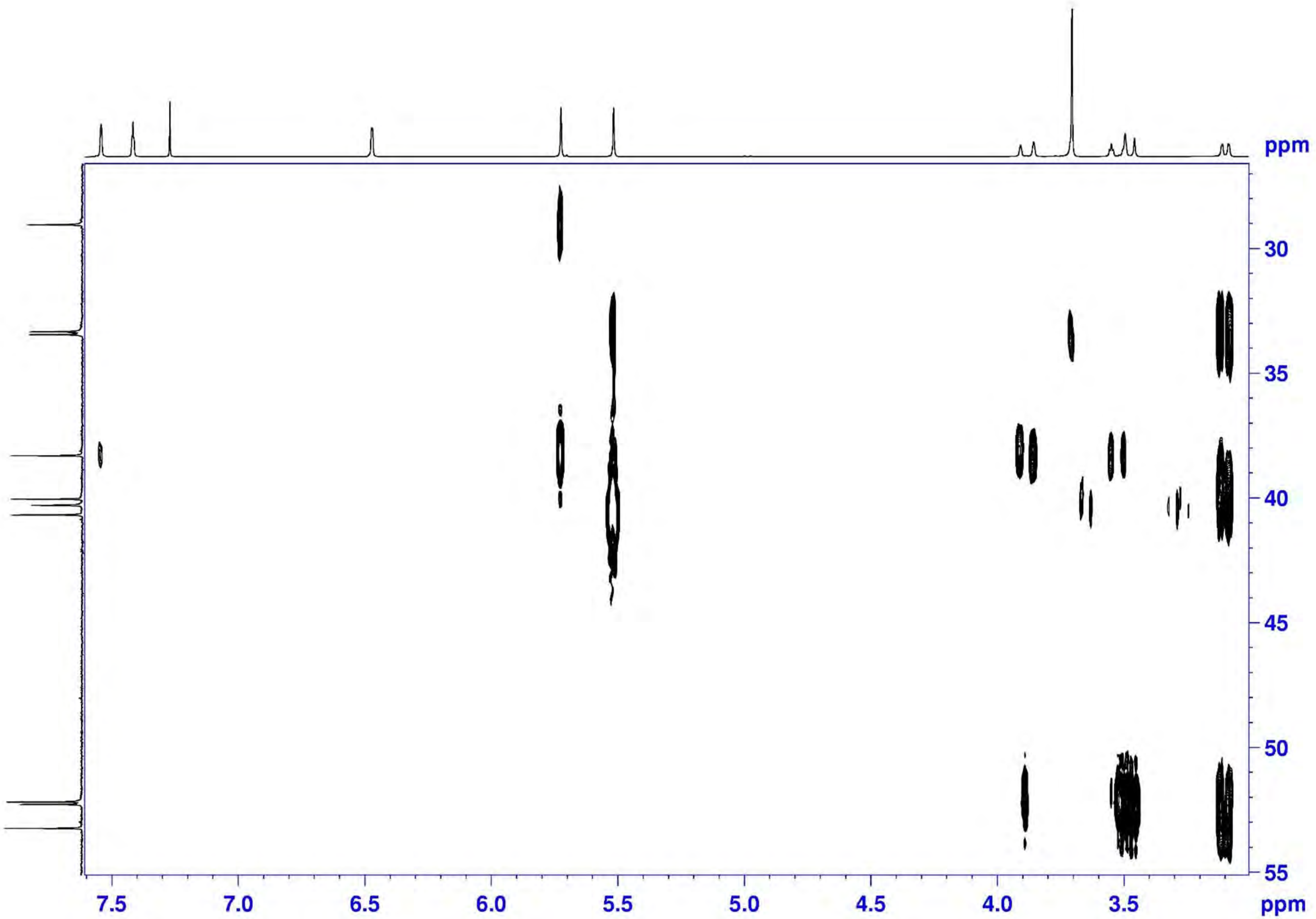

HMBC (400 MHz) spectrum of compound **7** in $CDCl_3$

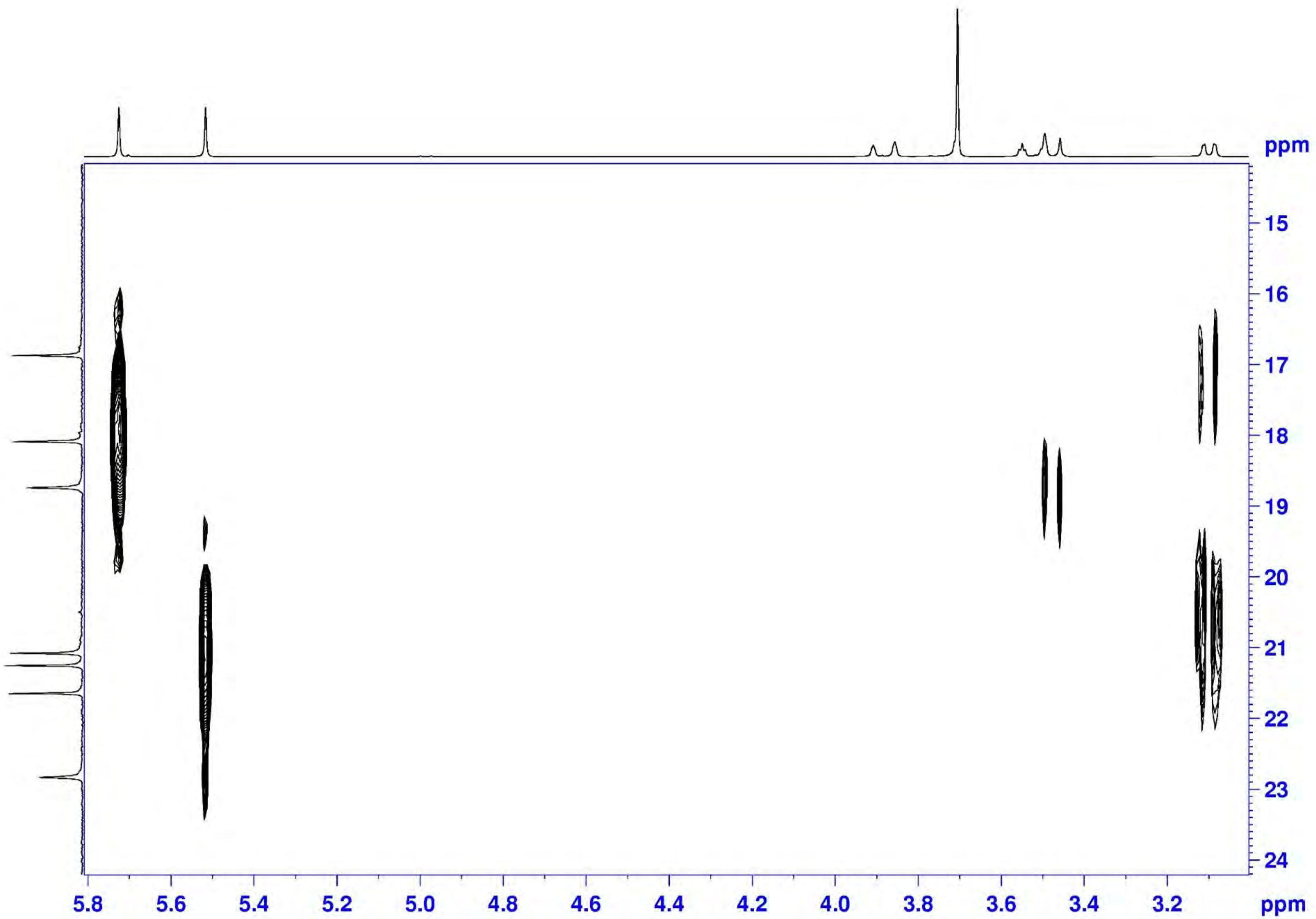

HMBC (400 MHz) spectrum of compound **7** in $CDCl_3$

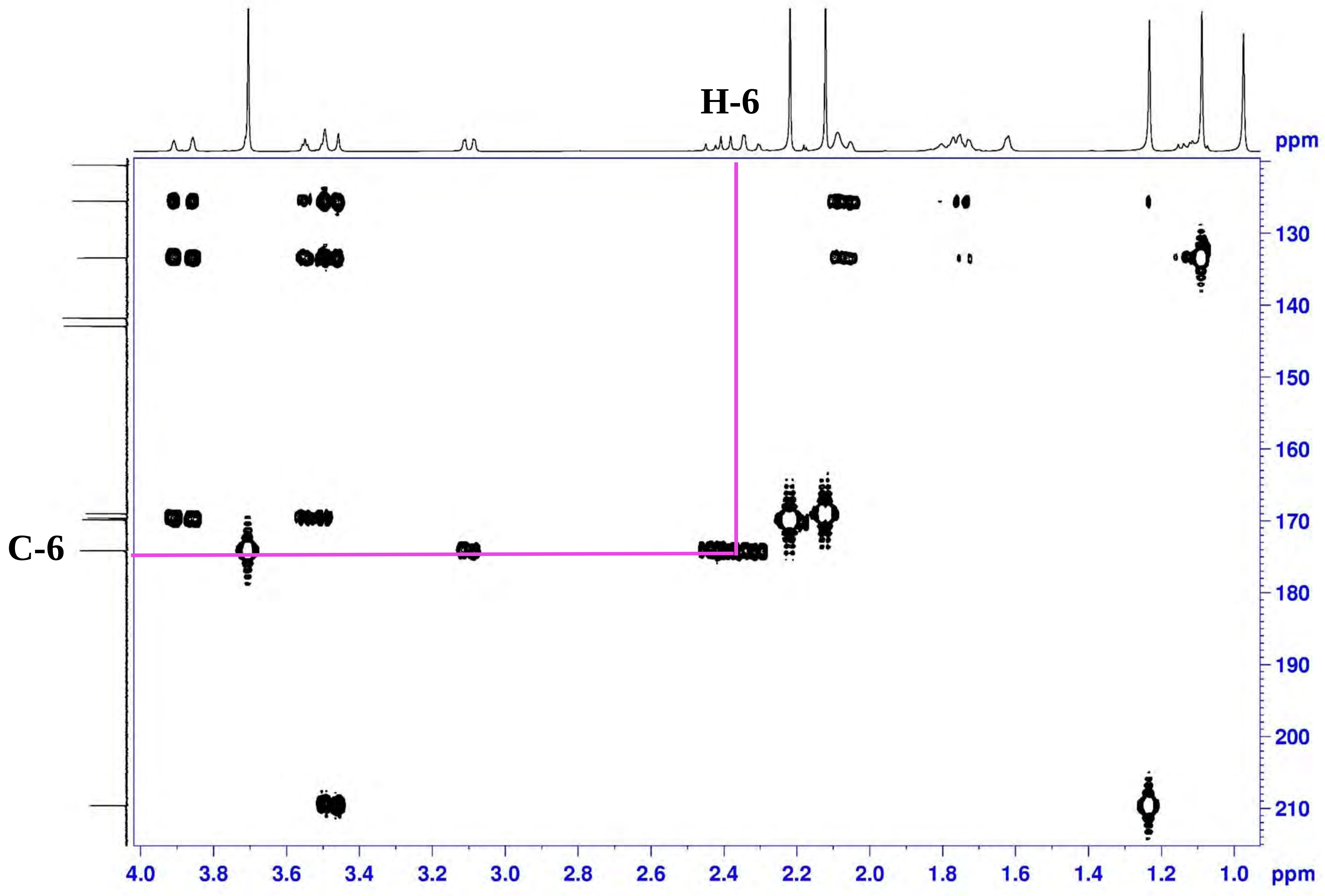

HMBC (400 MHz) spectrum of compound **7** in $CDCl_3$

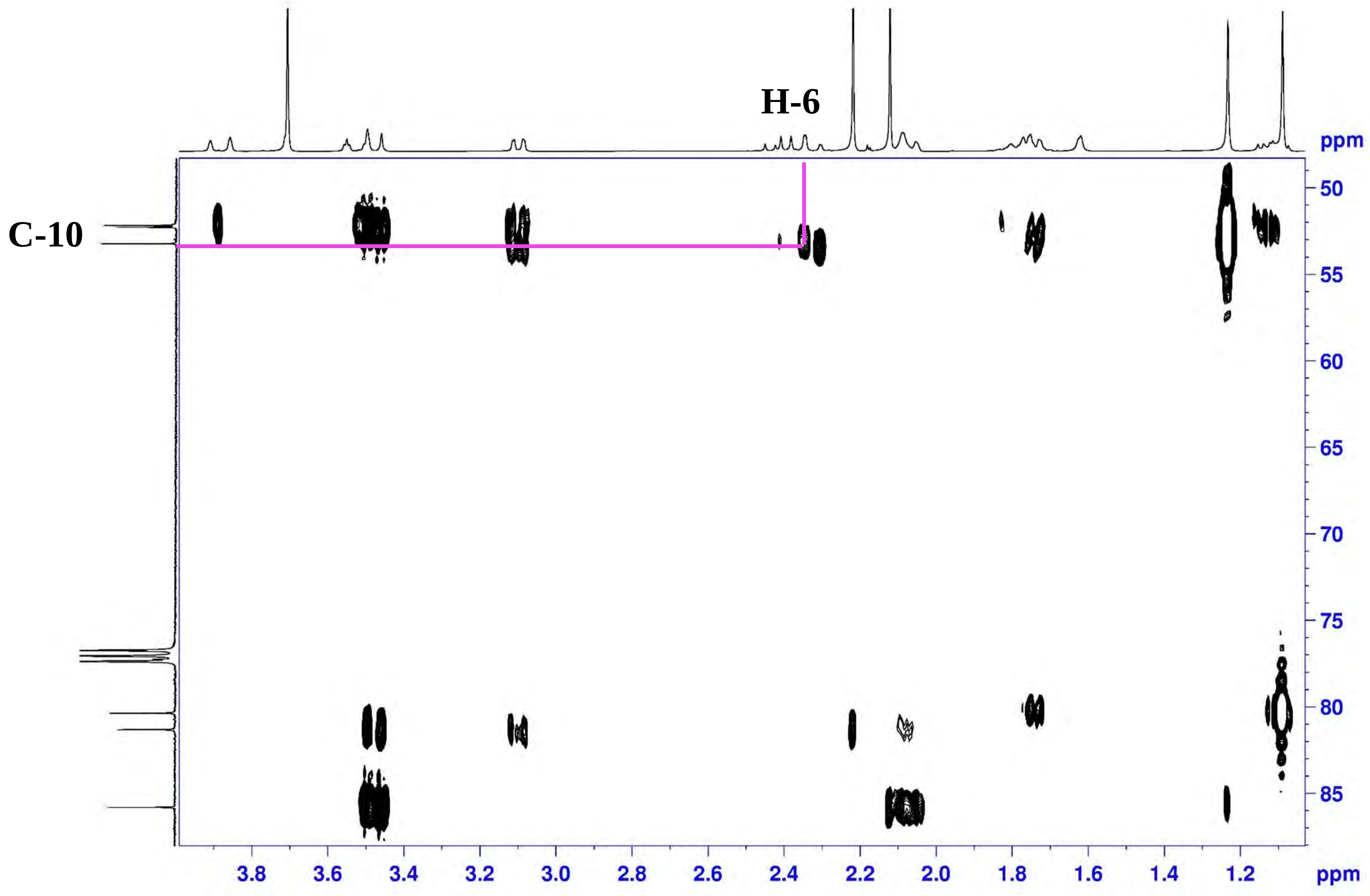

HMBC (400 MHz) spectrum of compound **7** in $CDCl_3$

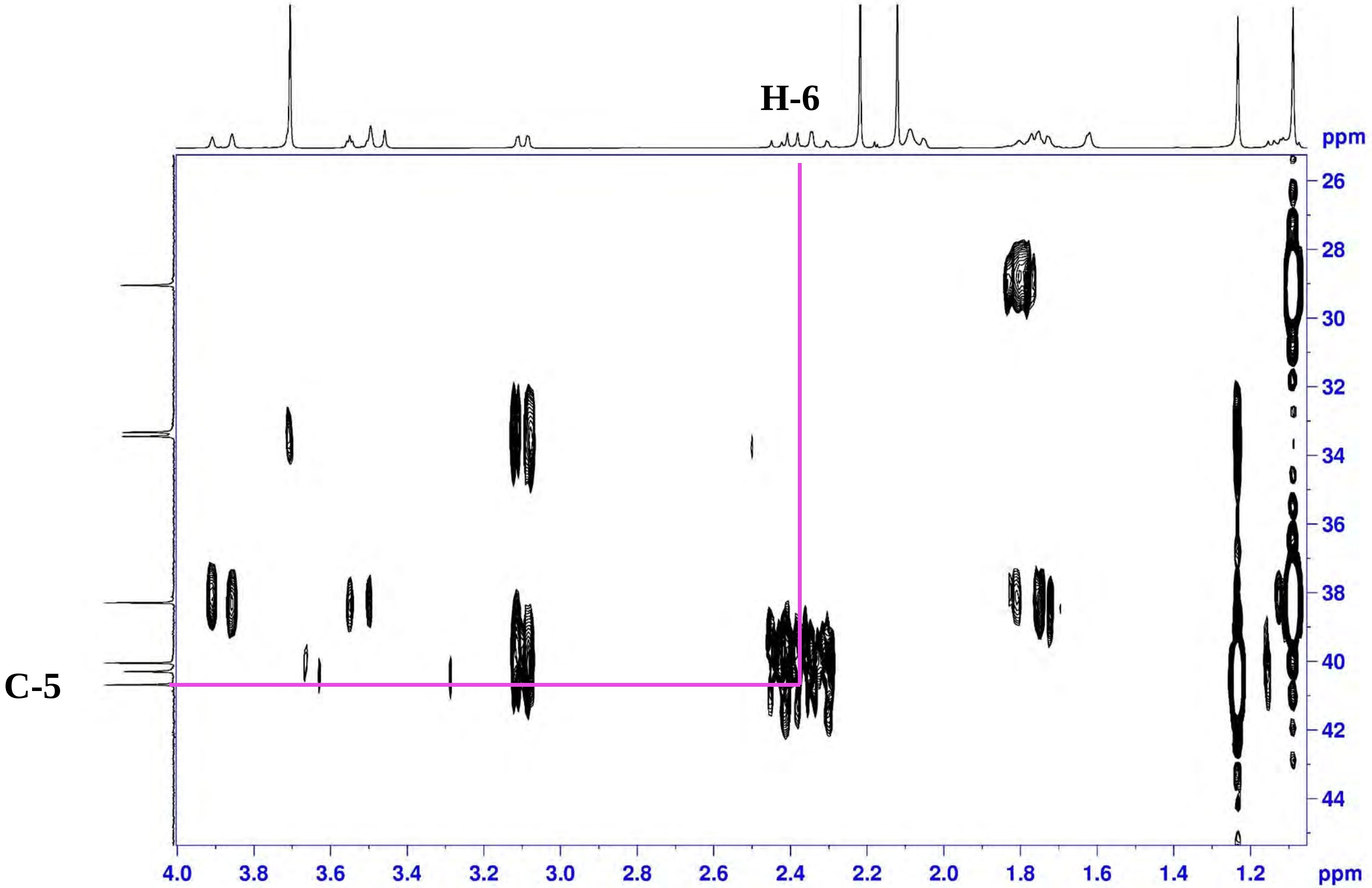

HMBC (400 MHz) spectrum of compound **7** in $CDCl_3$

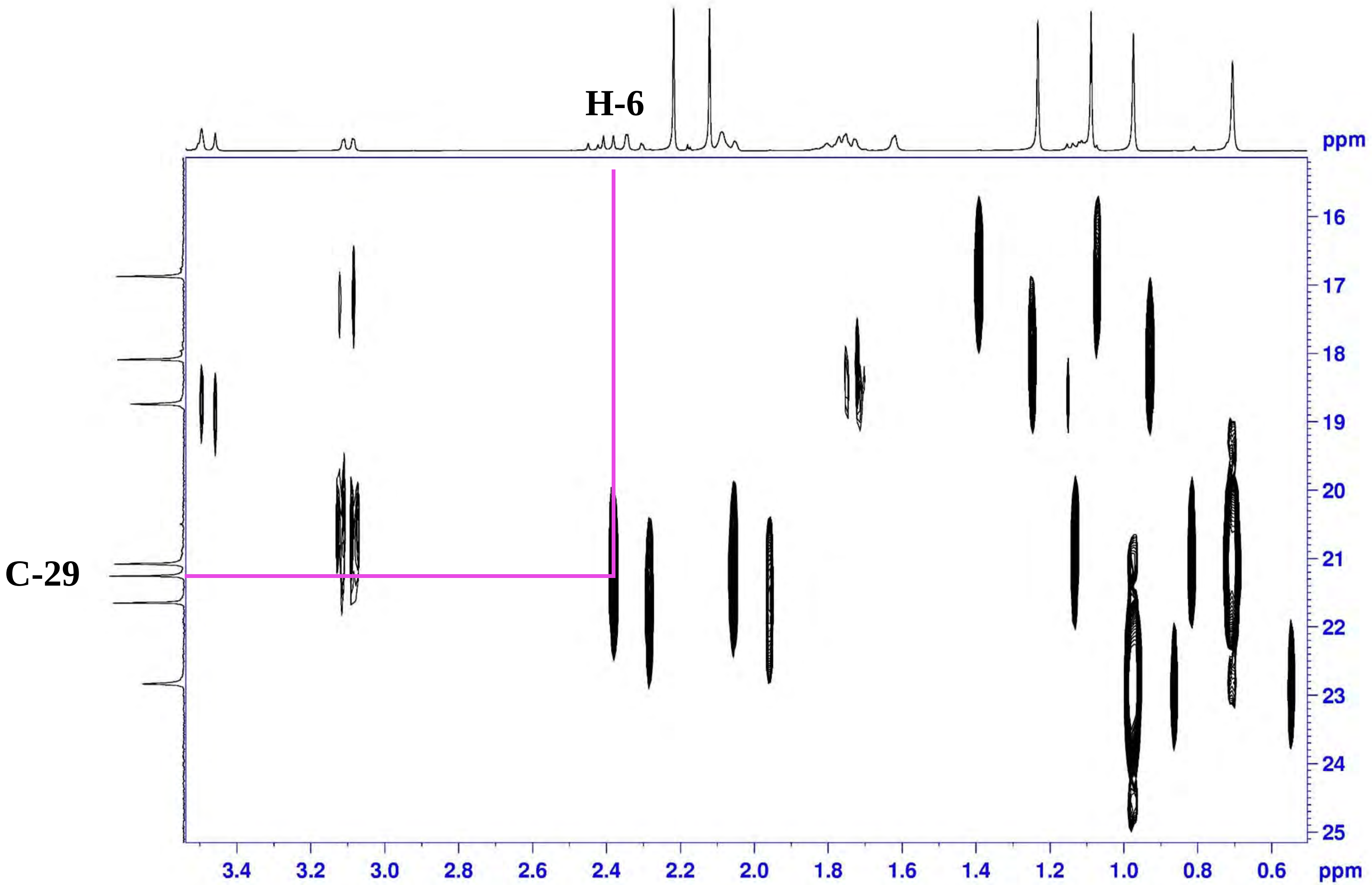

NOESY (400 MHz) spectrum of compound **7** in $CDCl_3$

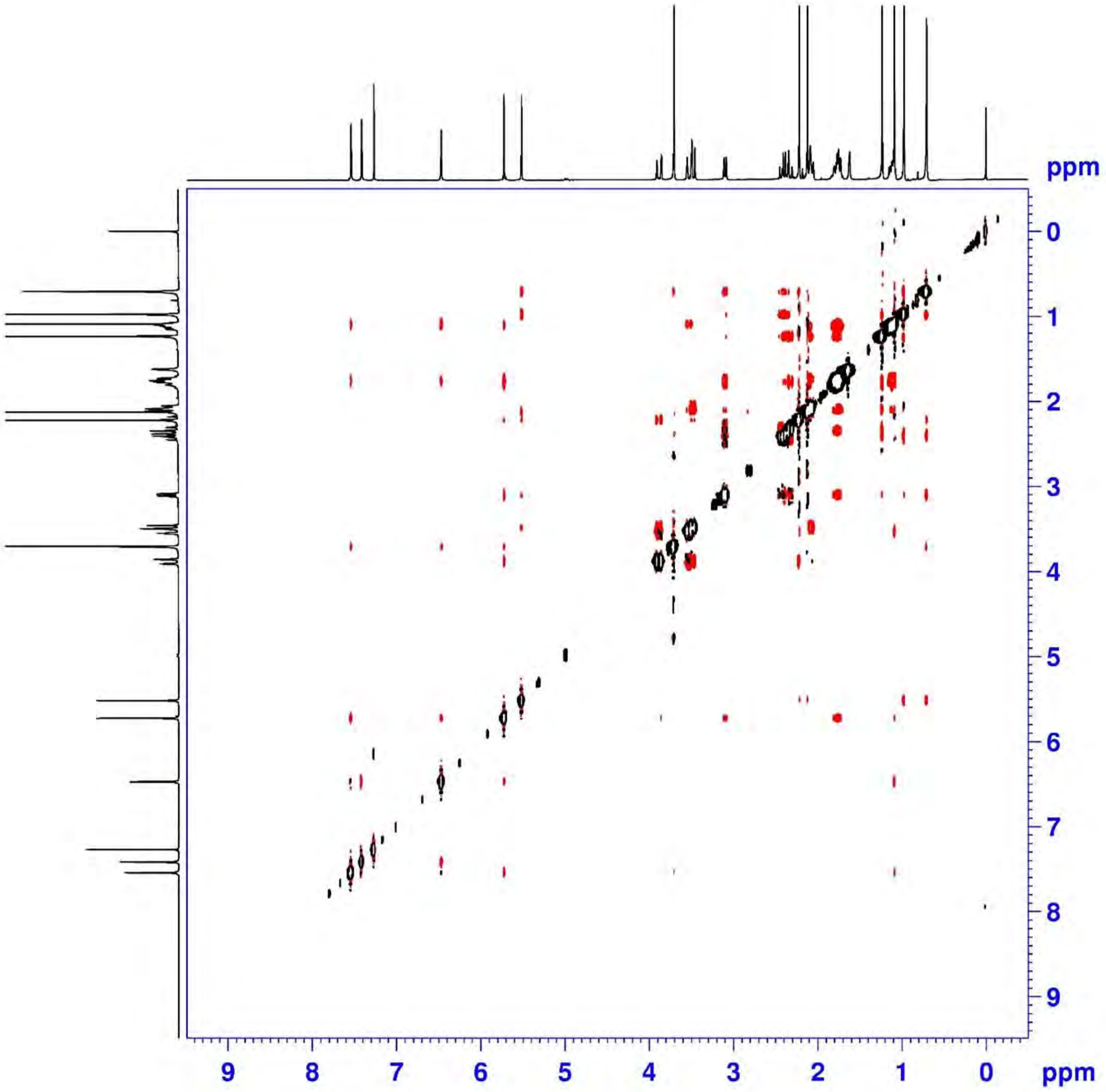


```
NAME             zjz-34
EXPNO                 7
PROCNO                1
Date_          20160914
Time               3.17
INSTRUM           spect
PROBHD   5 mm CPPBBO BB
PULPROG      noesygpphpp
TD                 2048
SOLVENT           CDCl3
NS                   16
DS                   32
SWH            4000.000 Hz
FIDRES         1.953125 Hz
AQ            0.2560500 sec
RG                102.3
DW              125.000 usec
DE                10.00 usec
TE                297.0 K
D0           0.00011036 sec
D1           1.99385595 sec
D8           0.30000001 sec
D11          0.03000000 sec
D12          0.00002000 sec
D16          0.00020000 sec
IN0          0.00025000 sec

======== CHANNEL f1 ========
SFO1        400.1318006 MHz
NUC1                 1H
P1                11.50 usec
P2                23.00 usec
P17             2500.00 usec
ND0                   1
TD                  256
SFO1           400.1318 MHz
FIDRES        15.625000 Hz
SW                9.997 ppm
FnMODE      States-TPPI
SI                 1024
SF          400.1300044 MHz
WDW               QSINE
SSB                   2
LB                 0.00 Hz
GB                    0
PC                 1.00
SI                 1024
MC2         States-TPPI
SF          400.1300044 MHz
WDW               QSINE
SSB                   2
LB                 0.00 Hz
GB                    0
```

NOESY (400 MHz) spectrum of compound **7** in $CDCl_3$

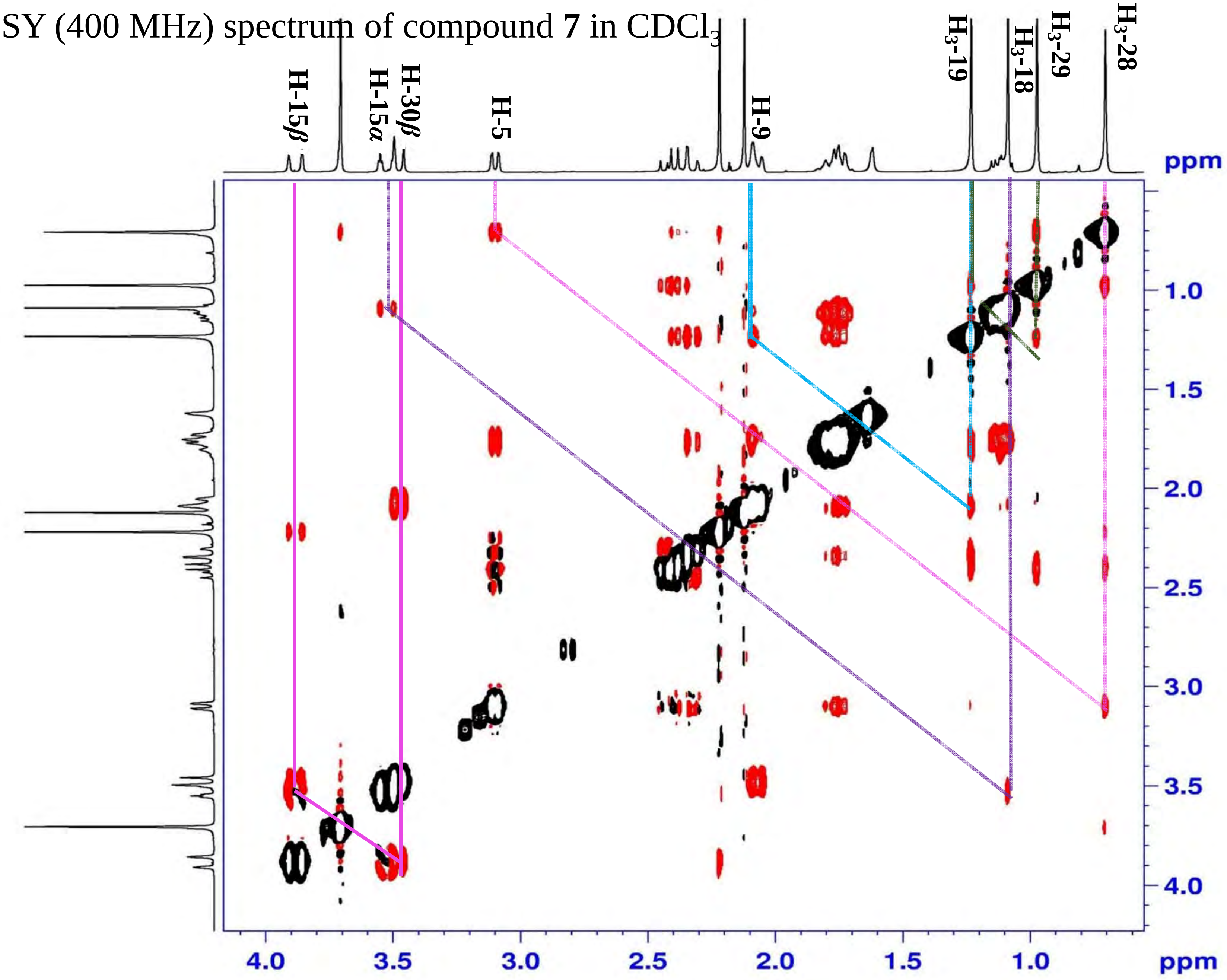

NOESY (400 MHz) spectrum of compound **7** in $CDCl_3$

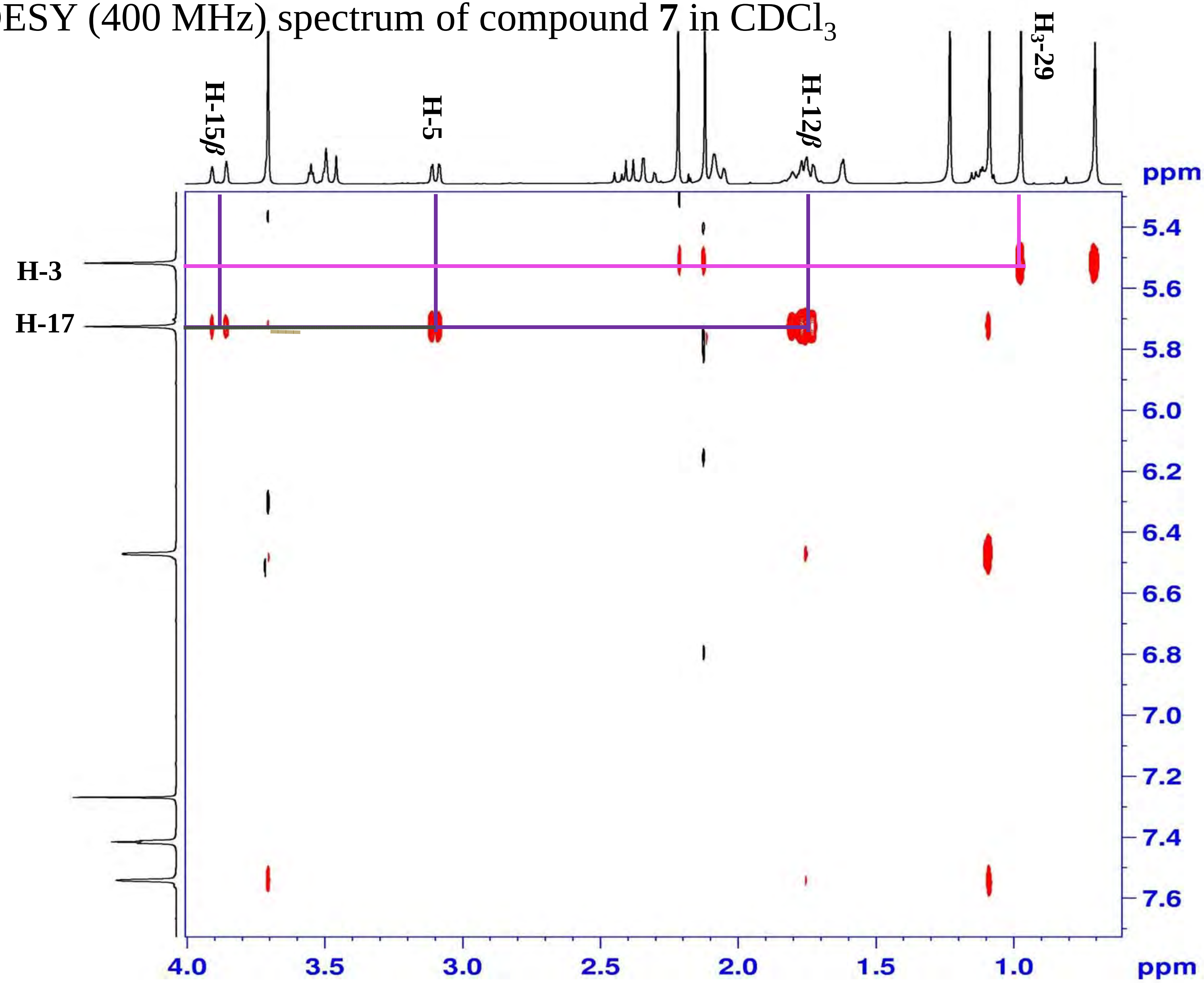

NOESY (400 MHz) spectrum of compound **7** in $CDCl_3$

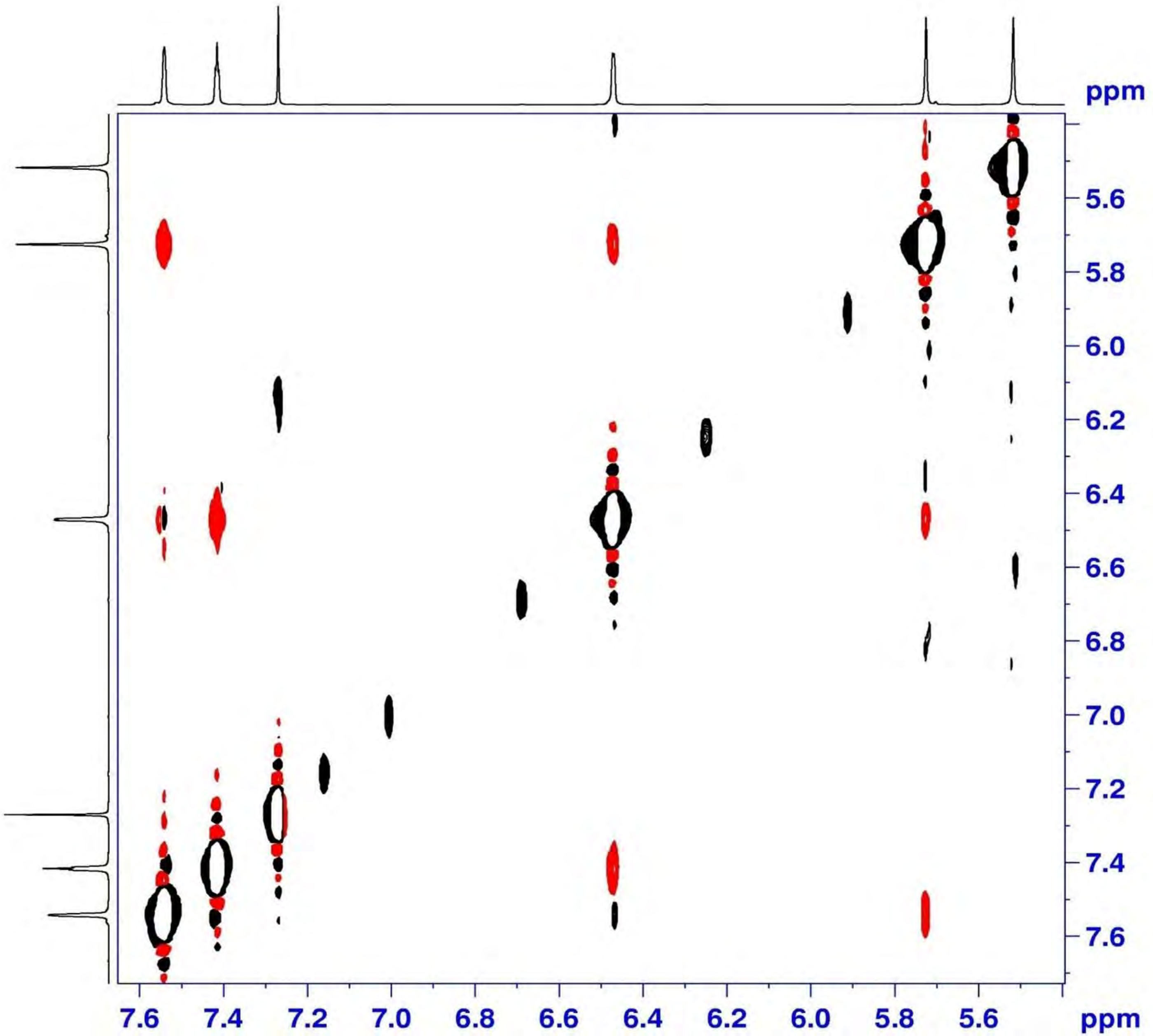

HR-ESIMS for compound **8**

Mass Spectrum SmartFormula Report

Analysis Info

Analysis Name	D:\Data\MS\data\201605\liwanshan_lws-58_pos_12_01_1859.d	Acquisition Date	5/19/2016 8:54:55 AM
Method	LC_Direct Infusion_pos_100-1000mz.m	Operator	SCSIO
Sample Name	liwanshan_lws-58_pos	Instrument / Ser#	maXis 29
Comment			

Acquisition Parameter

Source Type	ESI	Ion Polarity	Positive	Set Nebulizer	0.4 Bar
Focus	Active	Set Capillary	4500 V	Set Dry Heater	180 °C
Scan Begin	100 m/z	Set End Plate Offset	-500 V	Set Dry Gas	4.0 l/min
Scan End	2000 m/z	Set Collision Cell RF	800.0 Vpp	Set Divert Valve	Waste

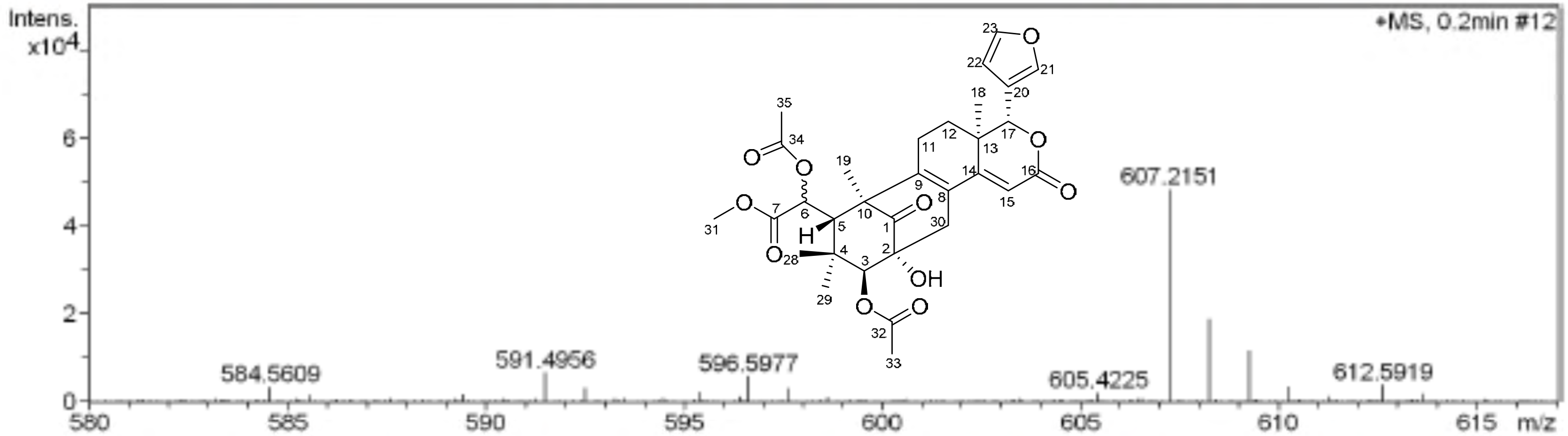

Meas. m/z	#	Formula	Score	m/z	err [ppm]	err [mDa]	mSigma	rdb	e⁻ Conf	N-Rule
585.2295	1	C 31 H 37 O 11	7.04	585.2330	6.0	3.5	128.8	13.5	even	ok
607.2151	1	C 31 H 36 Na O 11	100.00	607.2150	-0.3	-0.2	85.5	13.5	even	ok
1191.4416	1	C 62 H 72 Na O 22	100.00	1191.4407	-0.7	-0.8	50.9	26.5	even	ok

HR-ESIMS for compound **8**

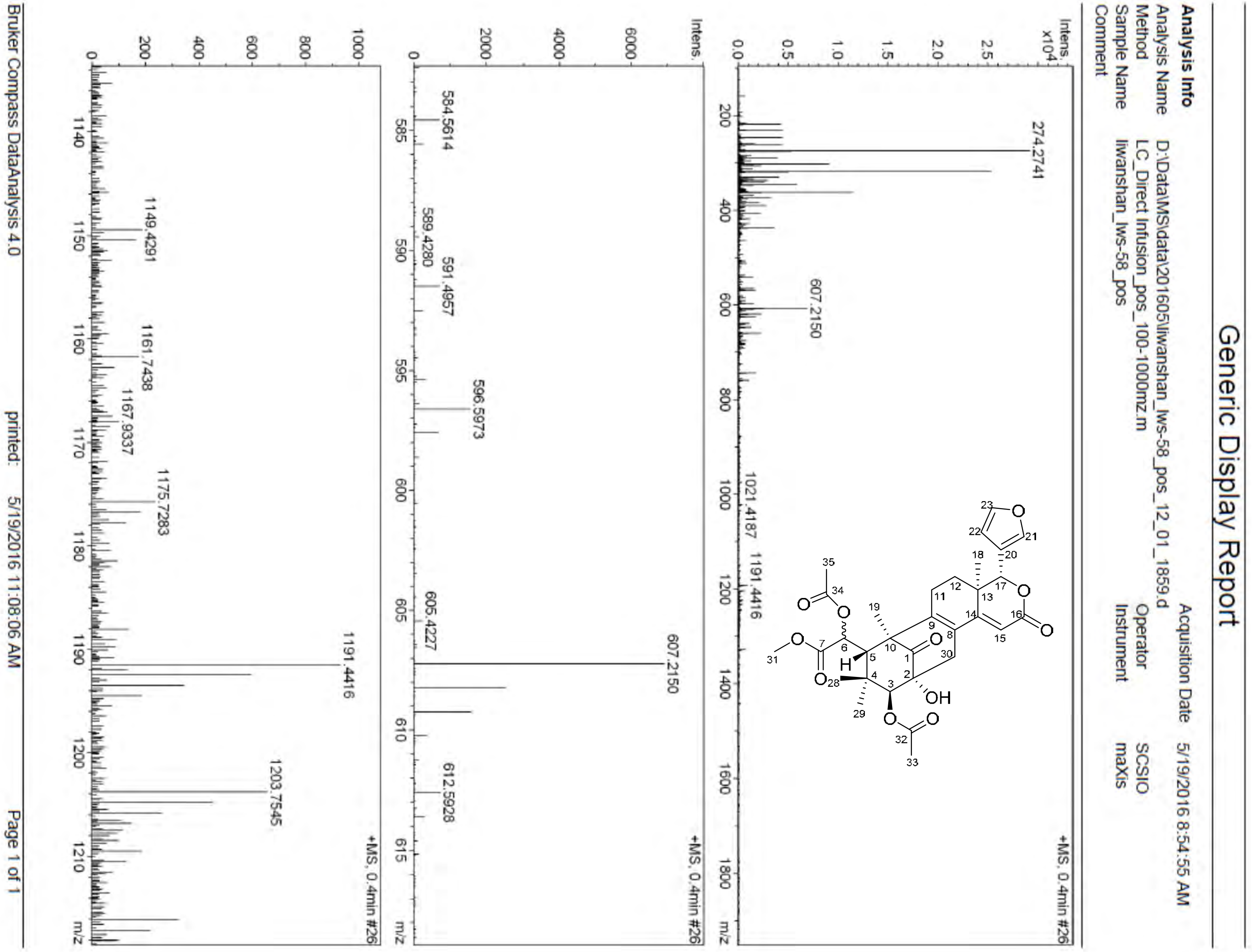

^{1}H NMR (400 MHz) spectrum of compound **8** in $CDCl_3$

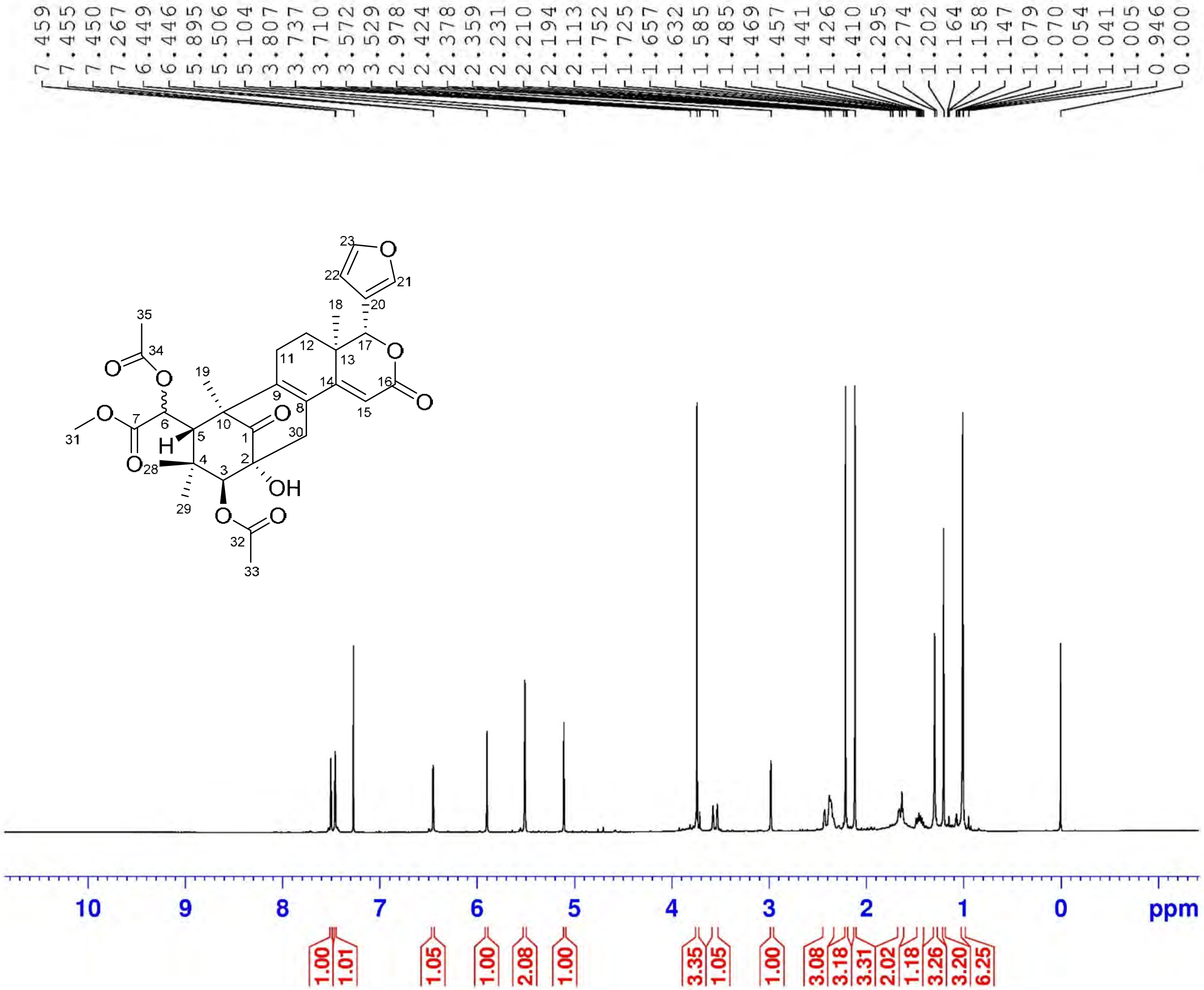

NAME	lws-58
EXPNO	1
PROCNO	1
Date_	20150611
Time	1.24
INSTRUM	spect
PROBHD	5 mm CPPBBO BB
PULPROG	zg30
TD	65536
SOLVENT	CDCl3
NS	16
DS	2
SWH	8223.685 H
FIDRES	0.125483 H
AQ	3.9846387 s
RG	147.94
DW	60.800 u
DE	10.00 u
TE	297.0 K
D1	1.00000000 s
TD0	1

======== CHANNEL f1 =====

SFO1	400.1324710 M
NUC1	1H
P1	12.00 u
SI	65536
SF	400.1300065 M
WDW	EM
SSB	0
LB	0.30 H
GB	0
PC	1.00

^{1}H NMR (400 MHz) spectrum of compound **8** in $CDCl_3$

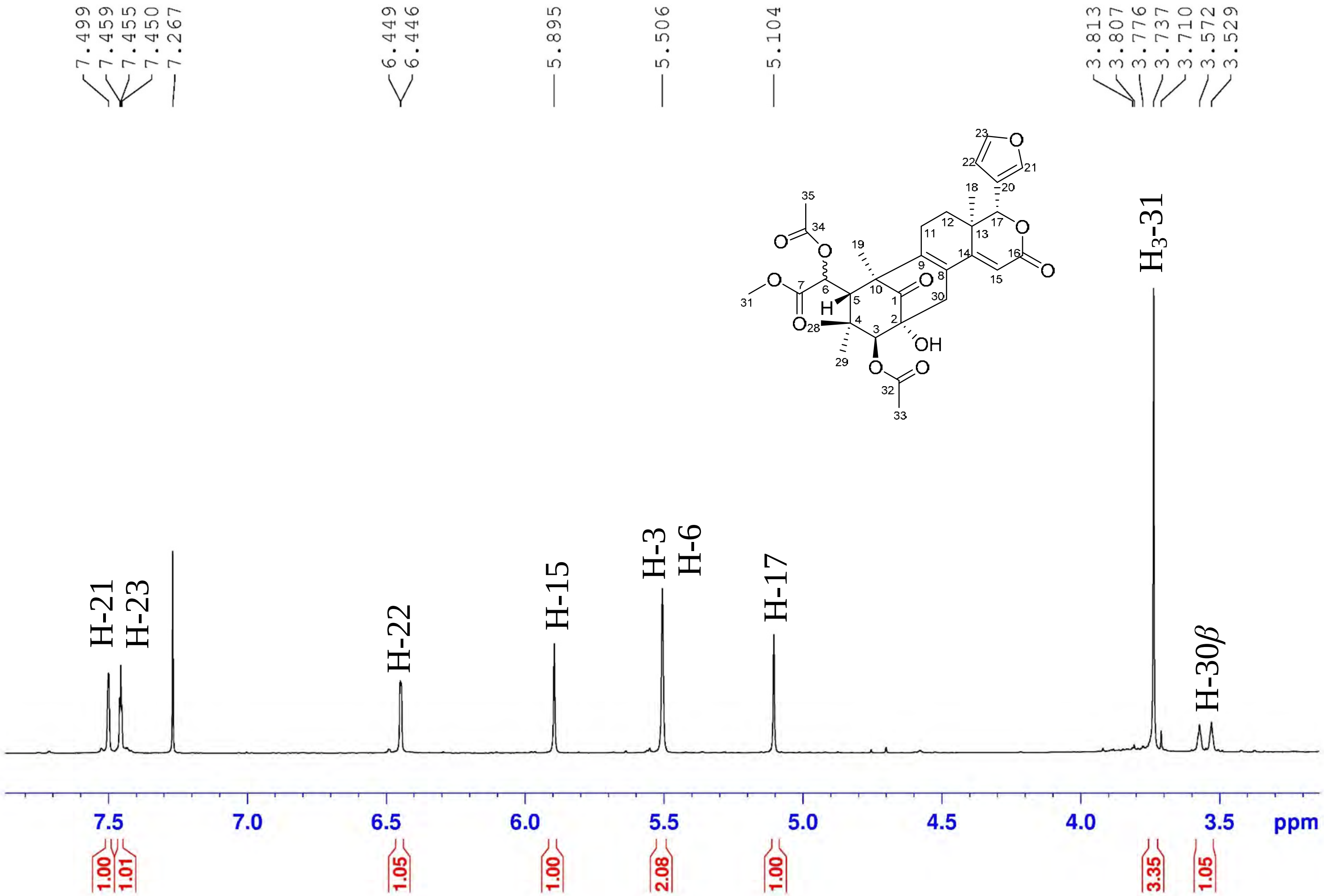

^{1}H NMR (400 MHz) spectrum of compound **8** in $CDCl_3$

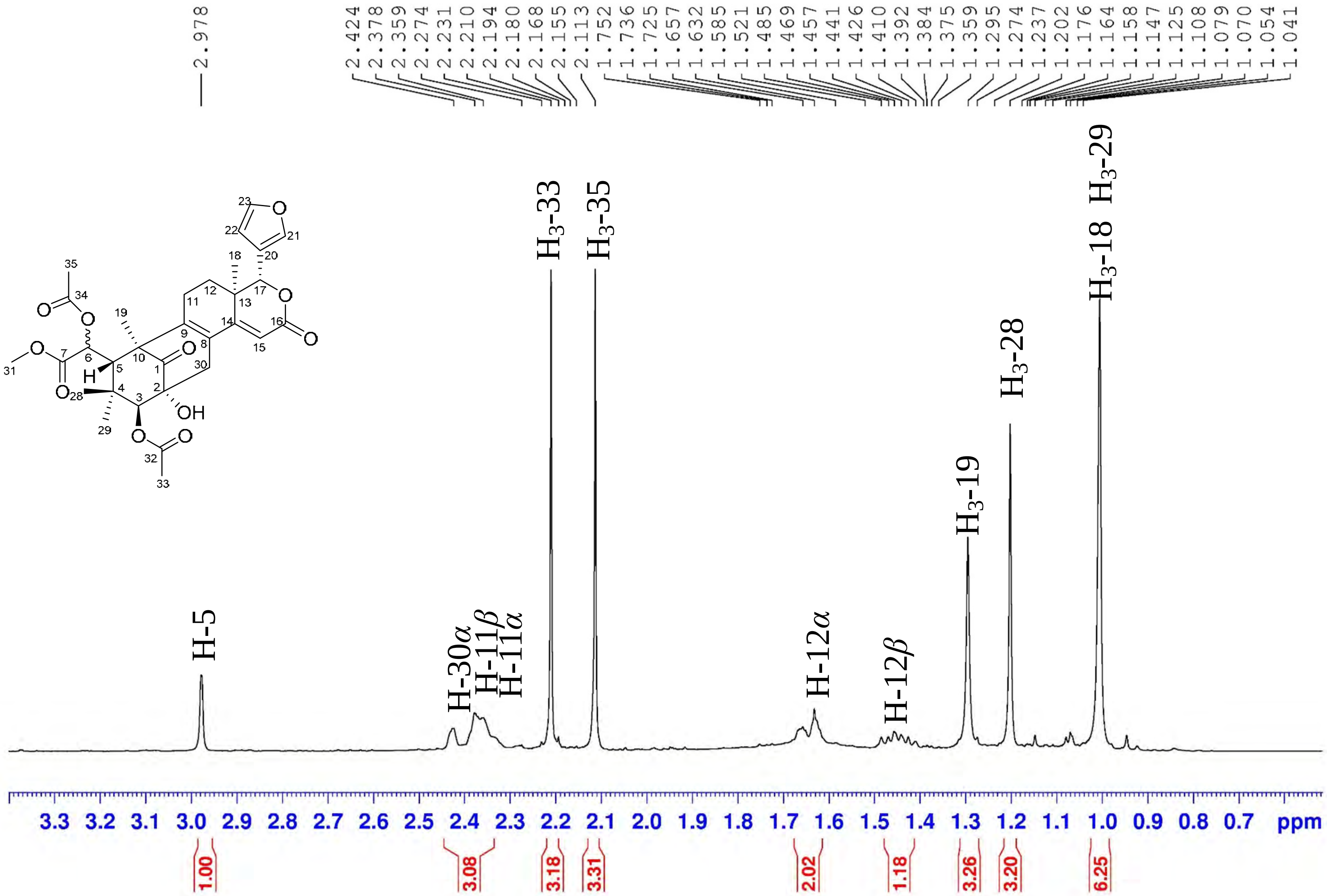

^{13}C NMR (100 MHz) spectrum of compound **8** in $CDCl_3$

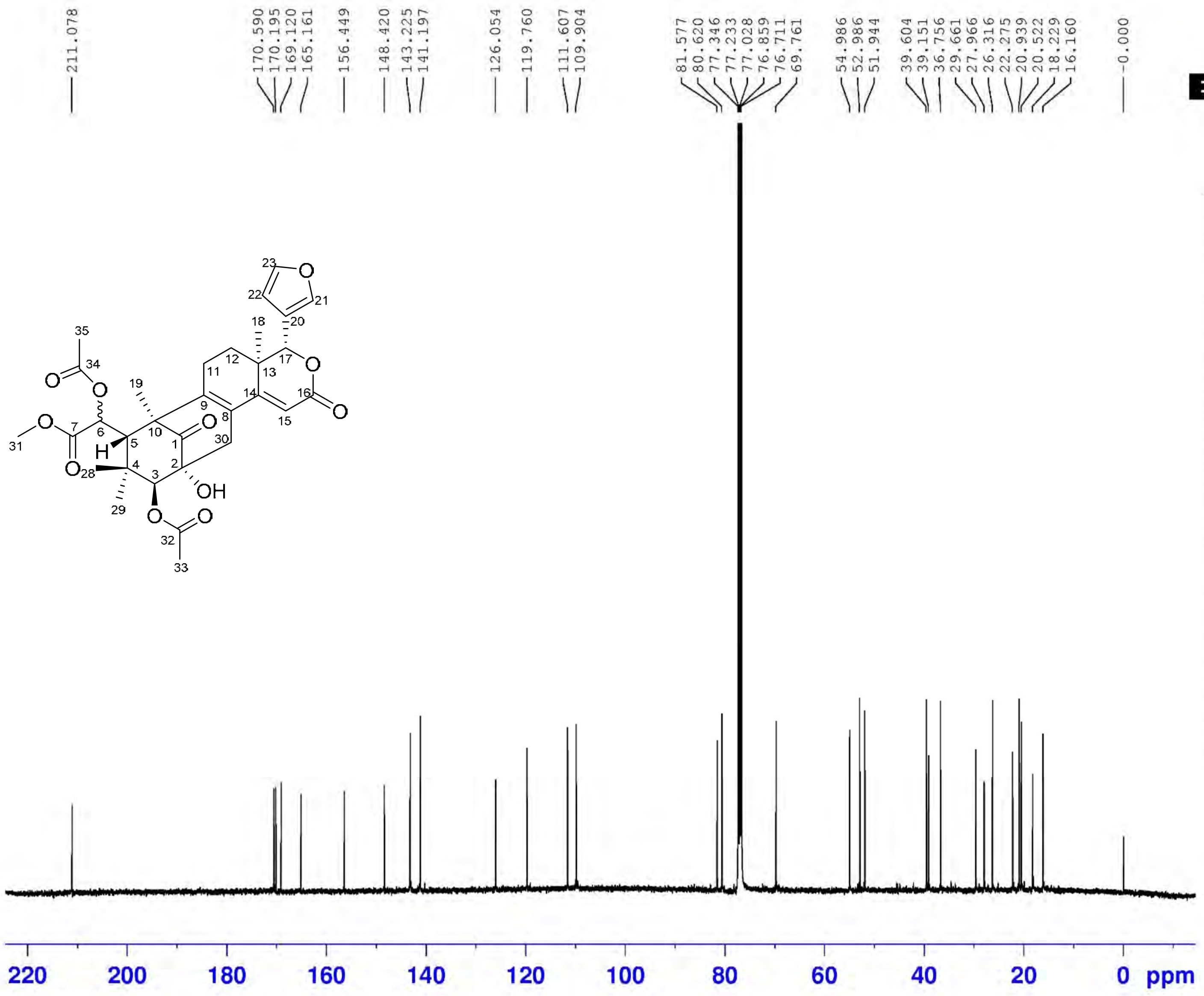

NAME	lws-58
EXPNO	2
PROCNO	1
Date_	20150611
Time	17.57
INSTRUM	spect
PROBHD	5 mm CPPBBO BB
PULPROG	zgpg30
TD	65536
SOLVENT	CDCl3
NS	3000
DS	4
SWH	24038.461 H
FIDRES	0.366798 H
AQ	1.3631988 s
RG	102.3
DW	20.800 u
DE	18.00 u
TE	297.0 K
D1	2.00000000 s
D11	0.03000000 s
TD0	1

======== CHANNEL f1 =====

SFO1	100.6233324 M
NUC1	13C
P1	10.00 u
SI	32768
SF	100.6127690 M
WDW	EM
SSB	0
LB	1.00 H
GB	0
PC	1.40

^{13}C NMR (100 MHz) spectrum of compound **8** in $CDCl_3$

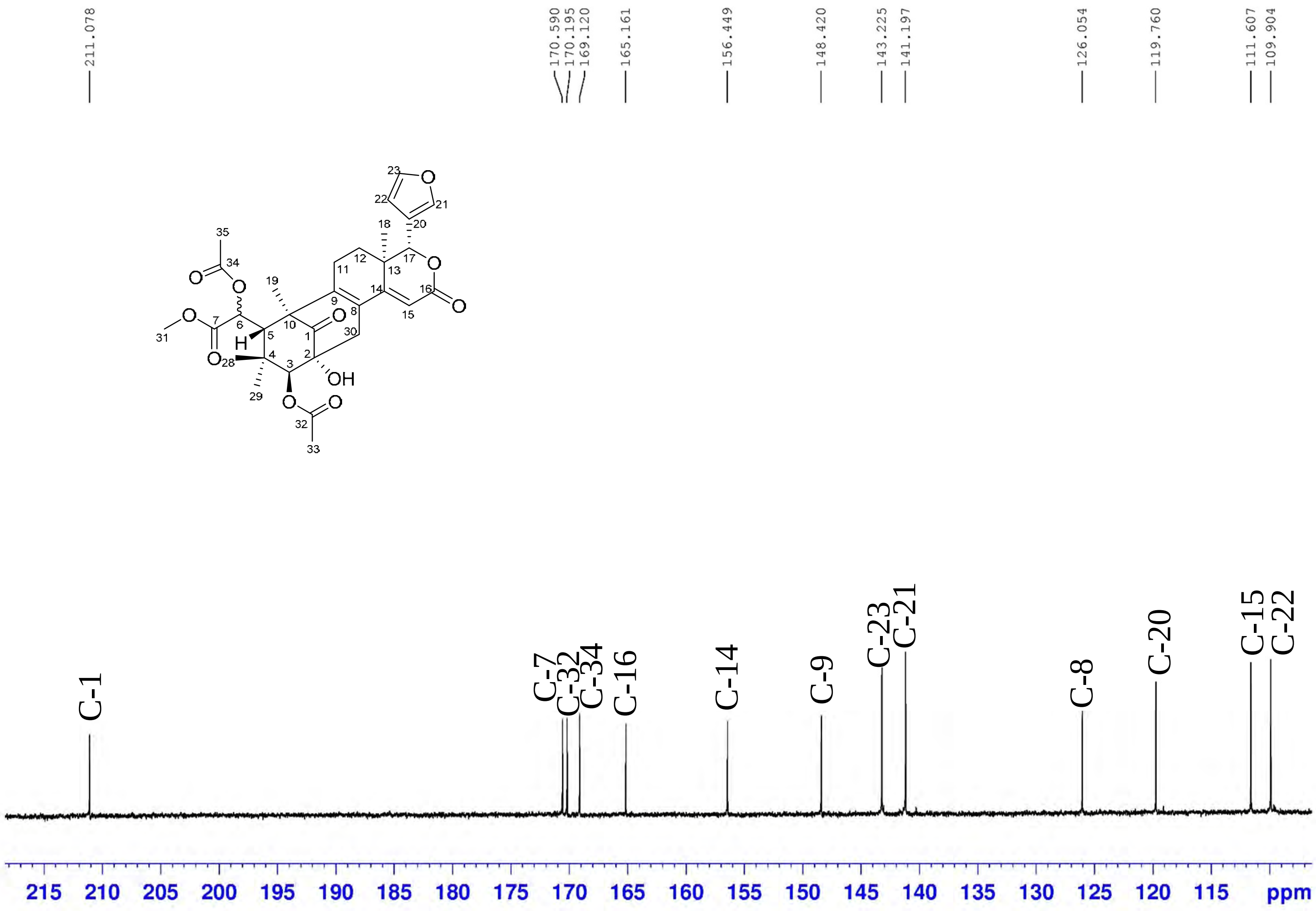

^{13}C NMR (100 MHz) spectrum of compound **8** in $CDCl_3$

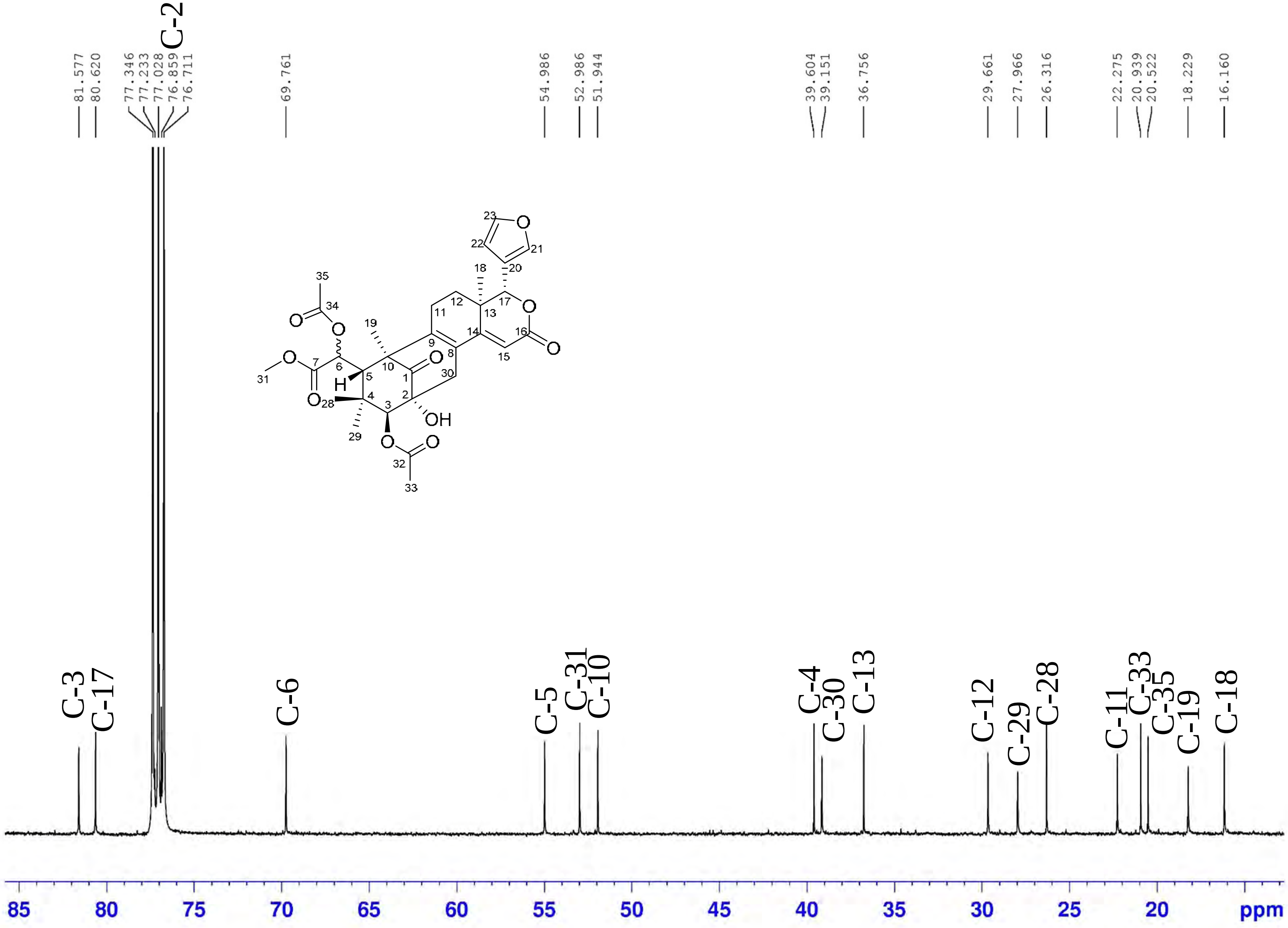

DEPT135 (100 MHz) spectrum of compound **8** in $CDCl_3$

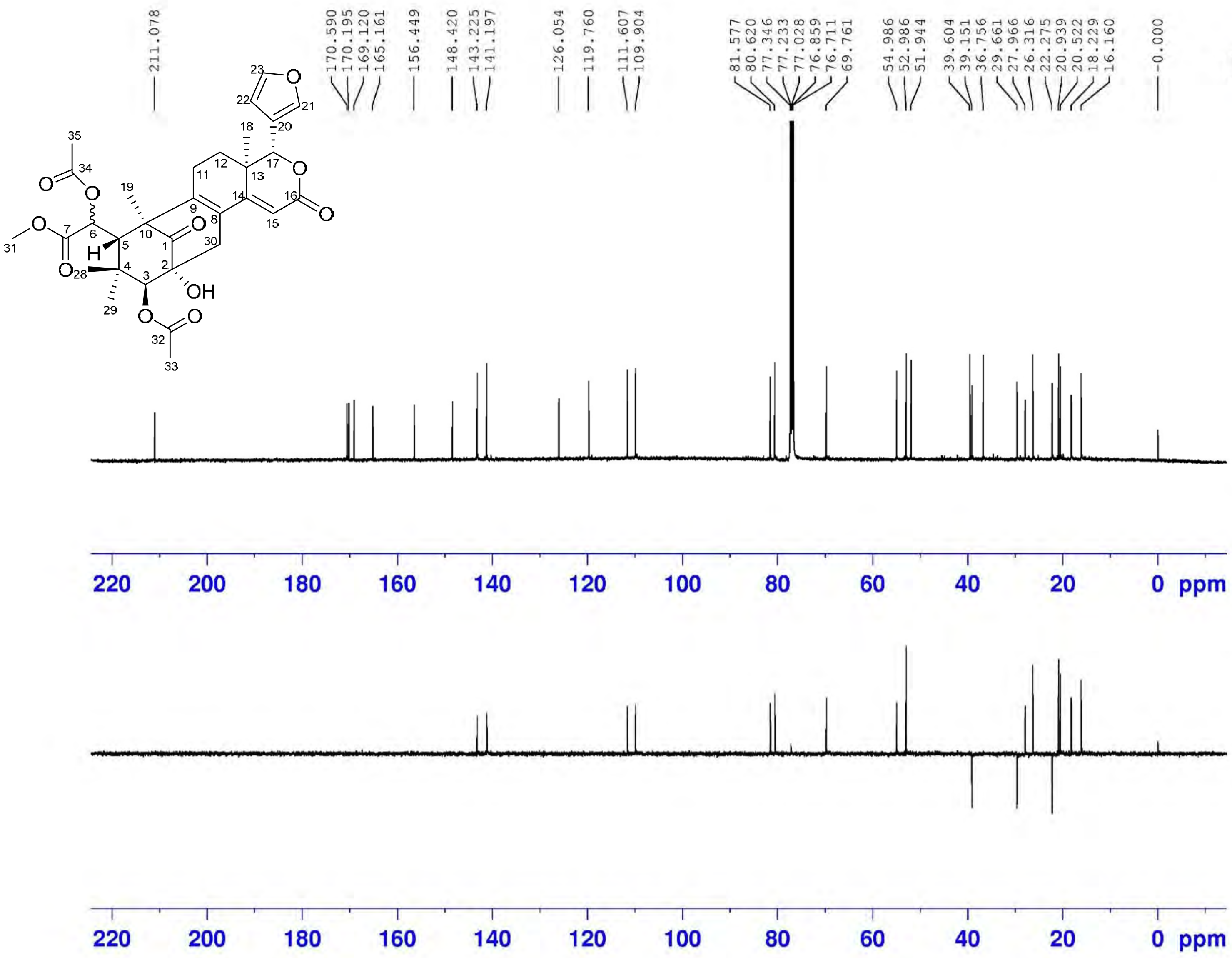


```
NAME            lws-58
EXPNO           103
PROCNO          1
Date_           20150613
Time            17.26
INSTRUM         spect
PROBHD          5 mm CPPBBO BB
PULPROG         deptsp135
TD              65536
SOLVENT         CDCl3
NS              300
DS              4
SWH             24038.461
FIDRES          0.366798
AQ              1.3631988
RG              91.64
DW              20.800
DE              18.00
TE              297.0
CNST2           145.0000000
D1              2.00000000
D2              0.00344828
D12             0.00002000
TD0             1

======== CHANNEL f1 ====
SFO1            100.6233324
NUC1            13C
P1              10.00
P13             2000.00
SI              32768
SF              100.6127690
WDW             EM
SSB             0
LB              1.00
GB              0
PC              1.40
```

DEPT135 (100 MHz) spectrum of compound **8** in $CDCl_3$

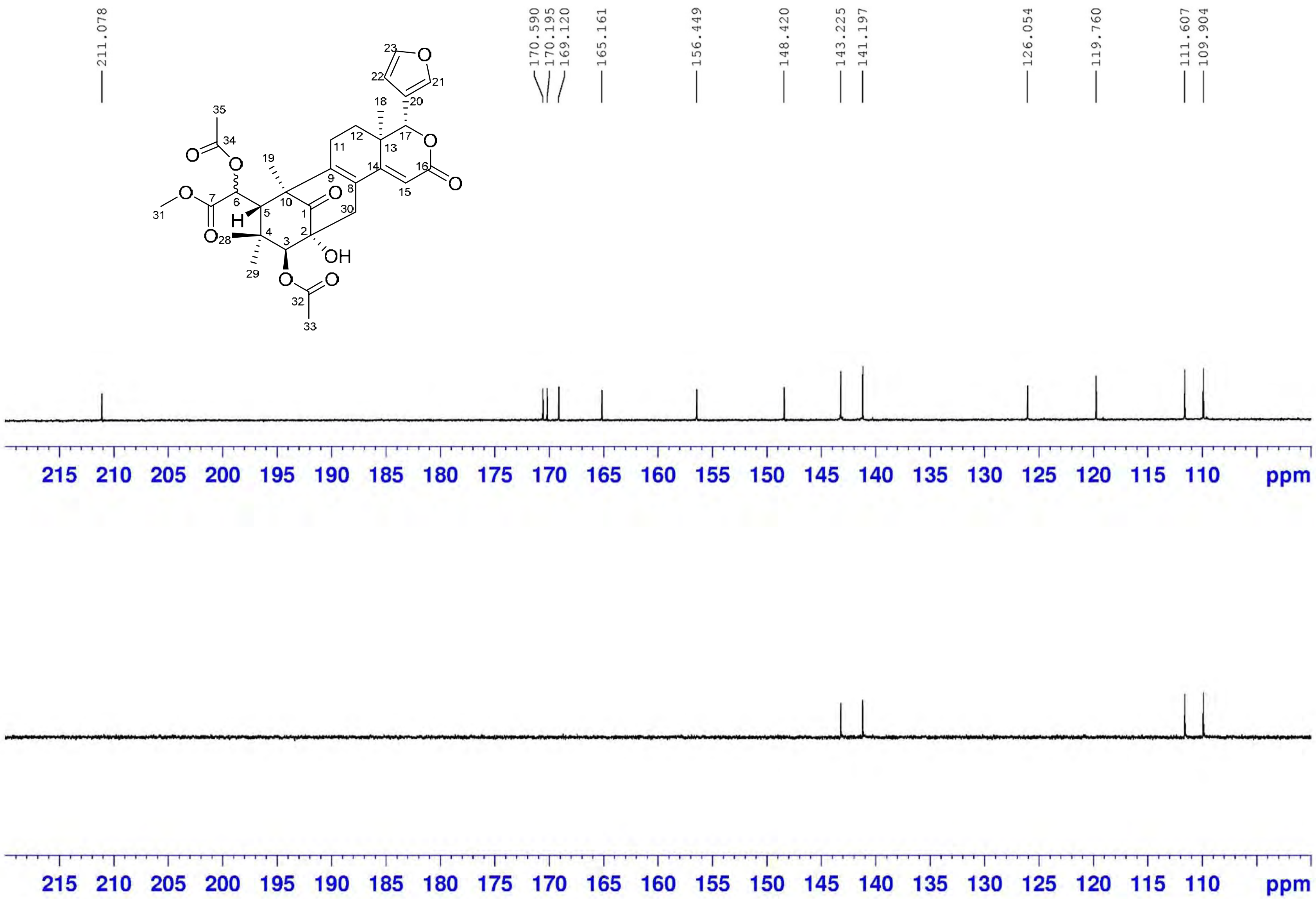

DEPT135 (100 MHz) spectrum of compound **8** in $CDCl_3$

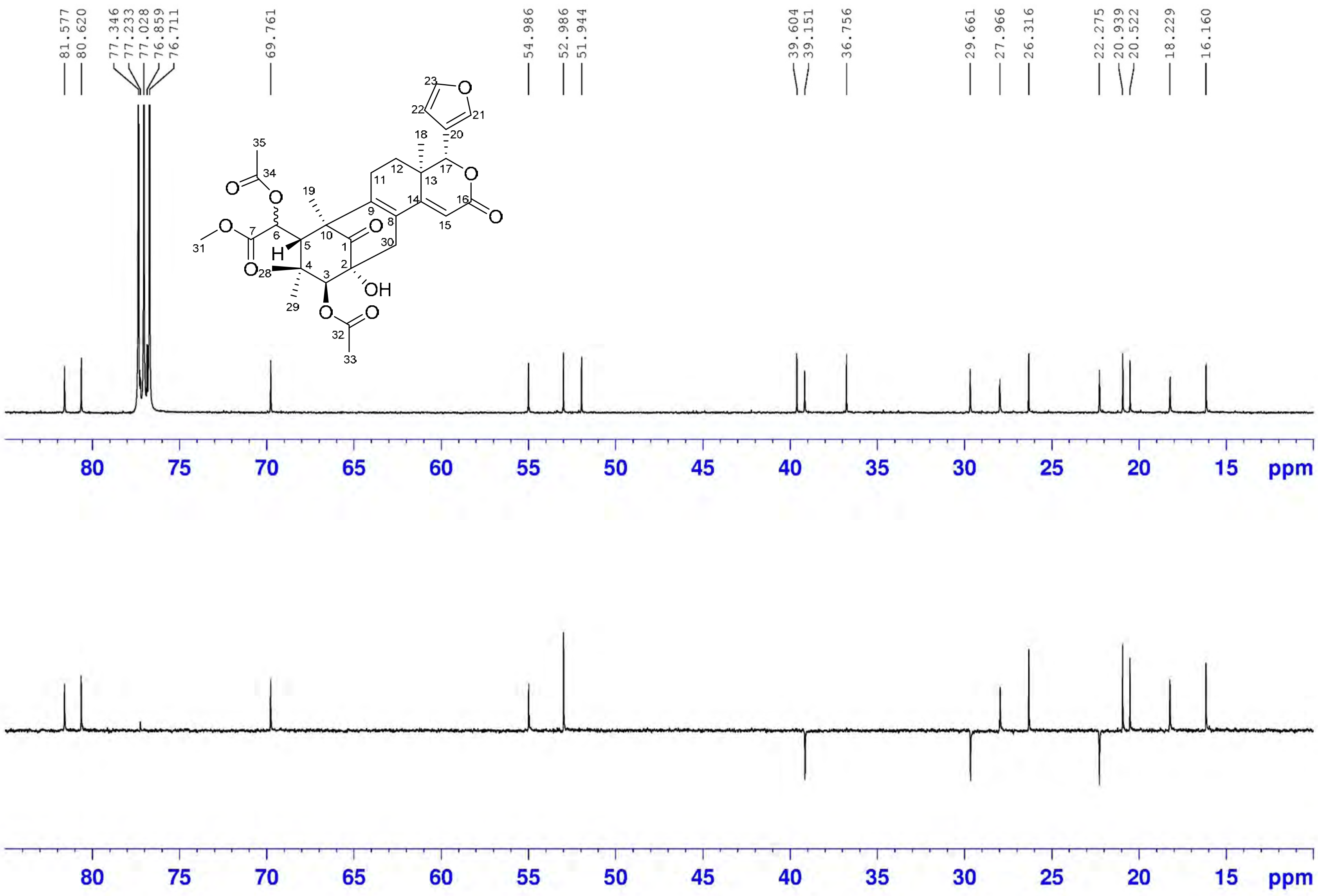

^1H-^1H COSY (400 MHz) spectrum of compound **8** in $CDCl_3$

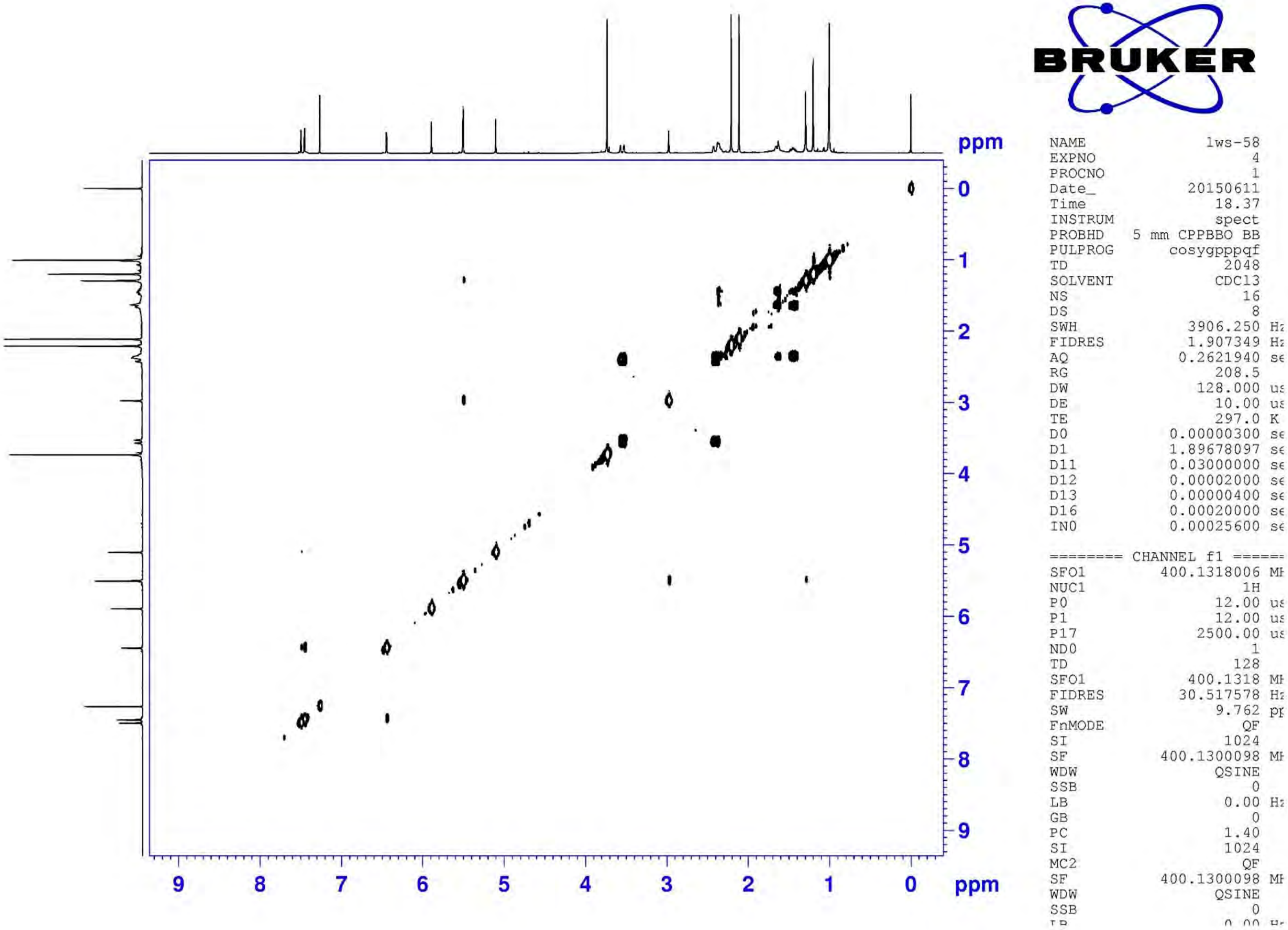

^1H-^1H COSY (400 MHz) spectrum of compound **8** in $CDCl_3$

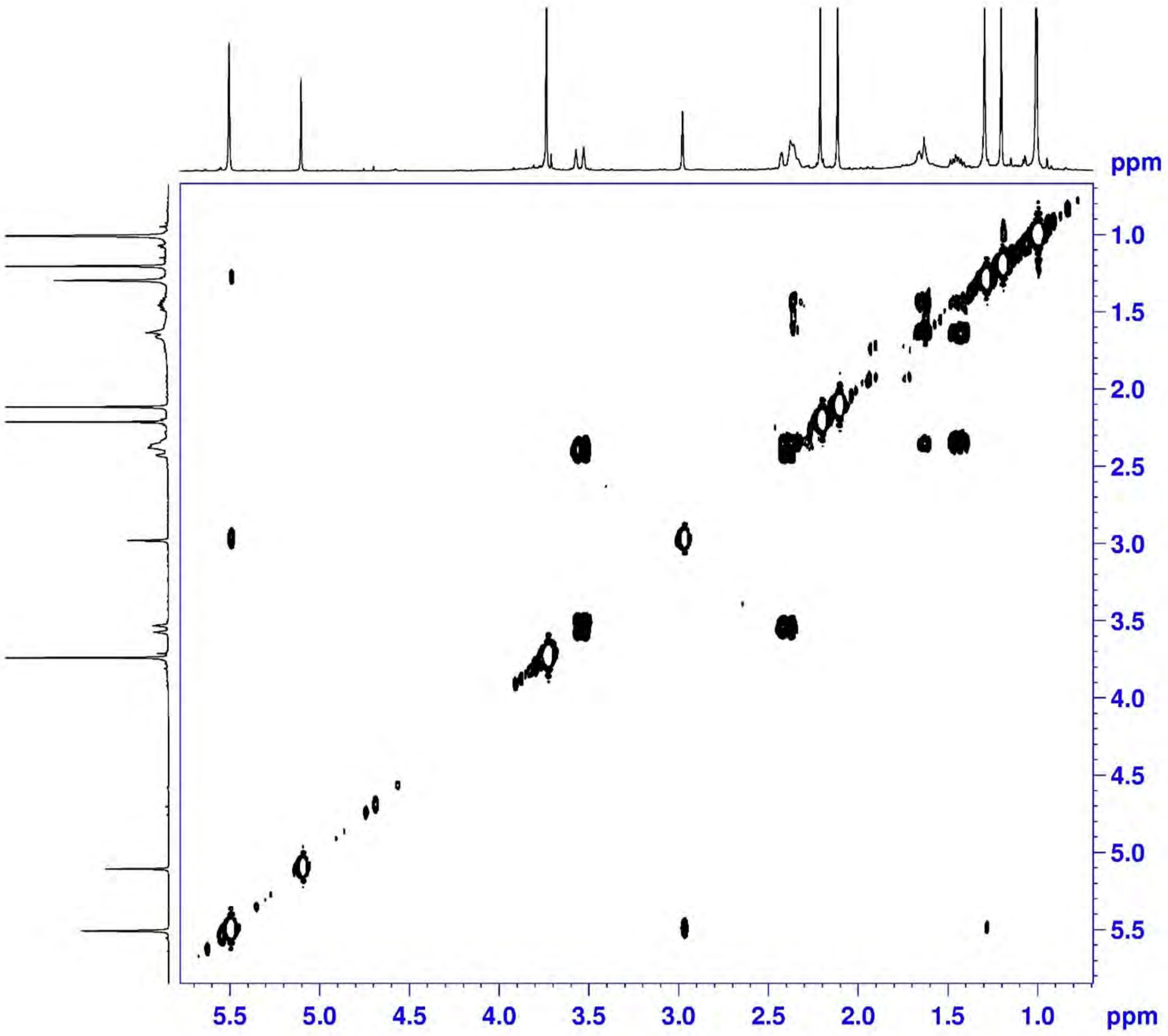

HSQC (400 MHz) spectrum of compound **8** in $CDCl_3$

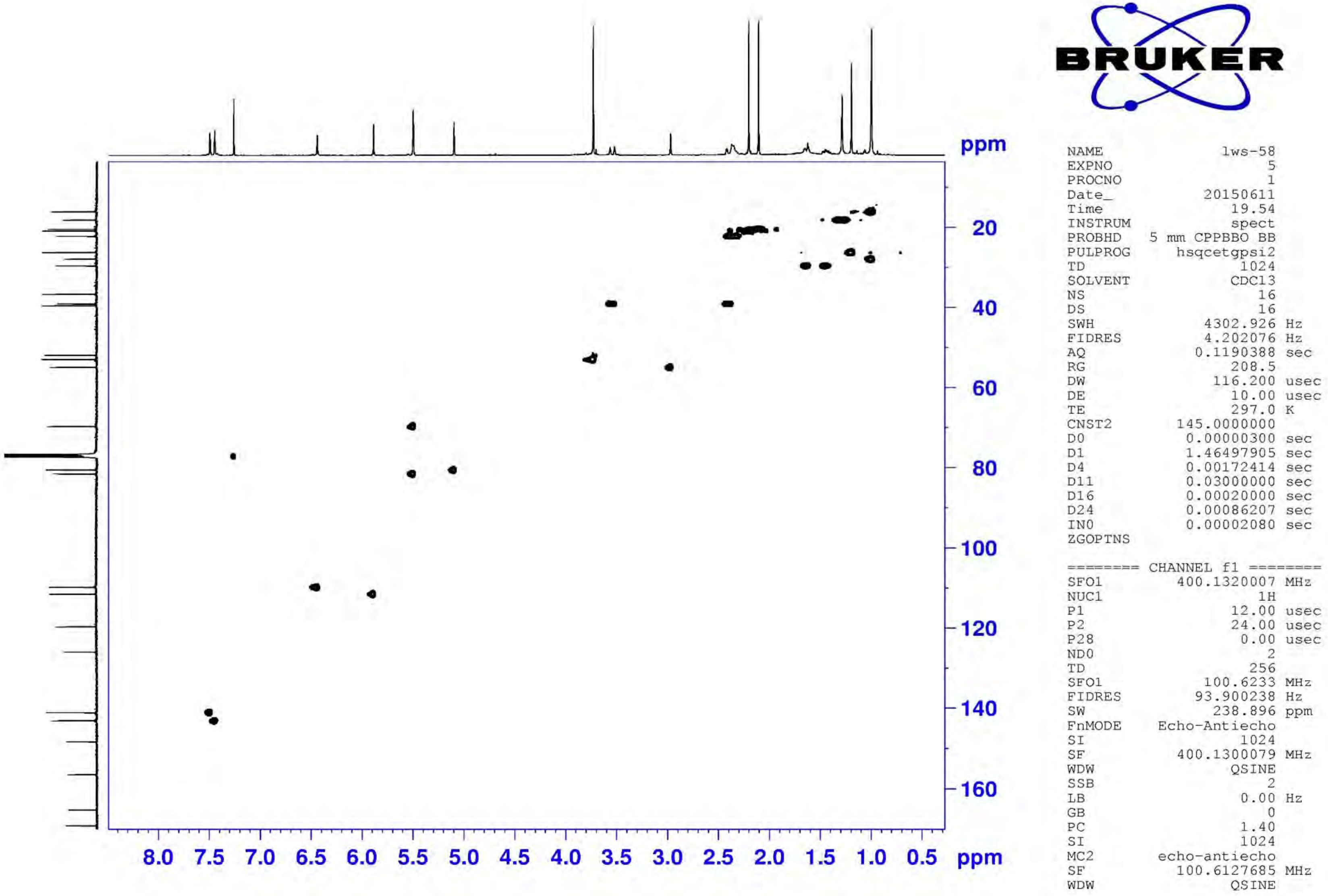

HSQC (400 MHz) spectrum of compound **8** in $CDCl_3$

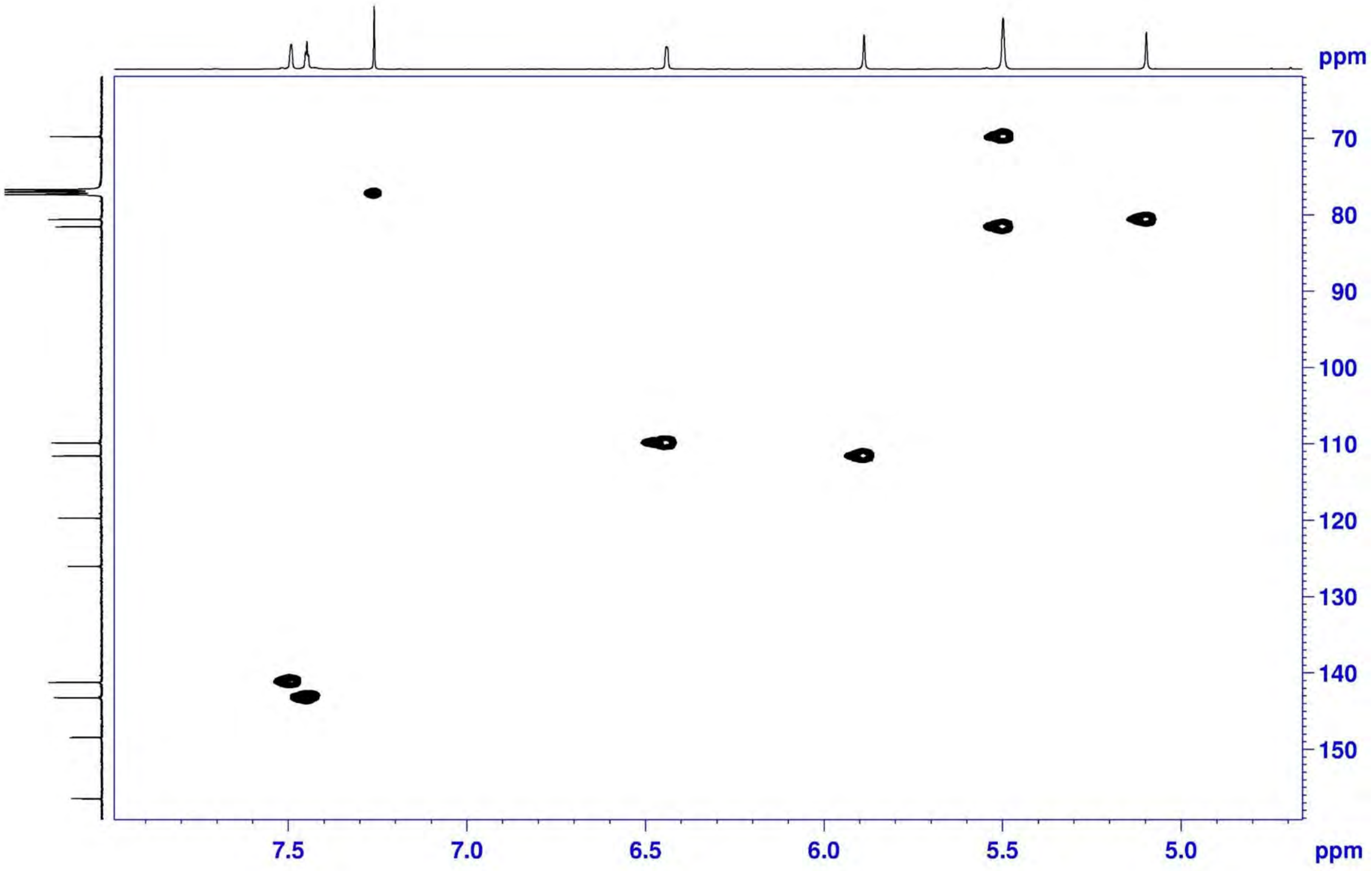

HSQC (400 MHz) spectrum of compound **8** in $CDCl_3$

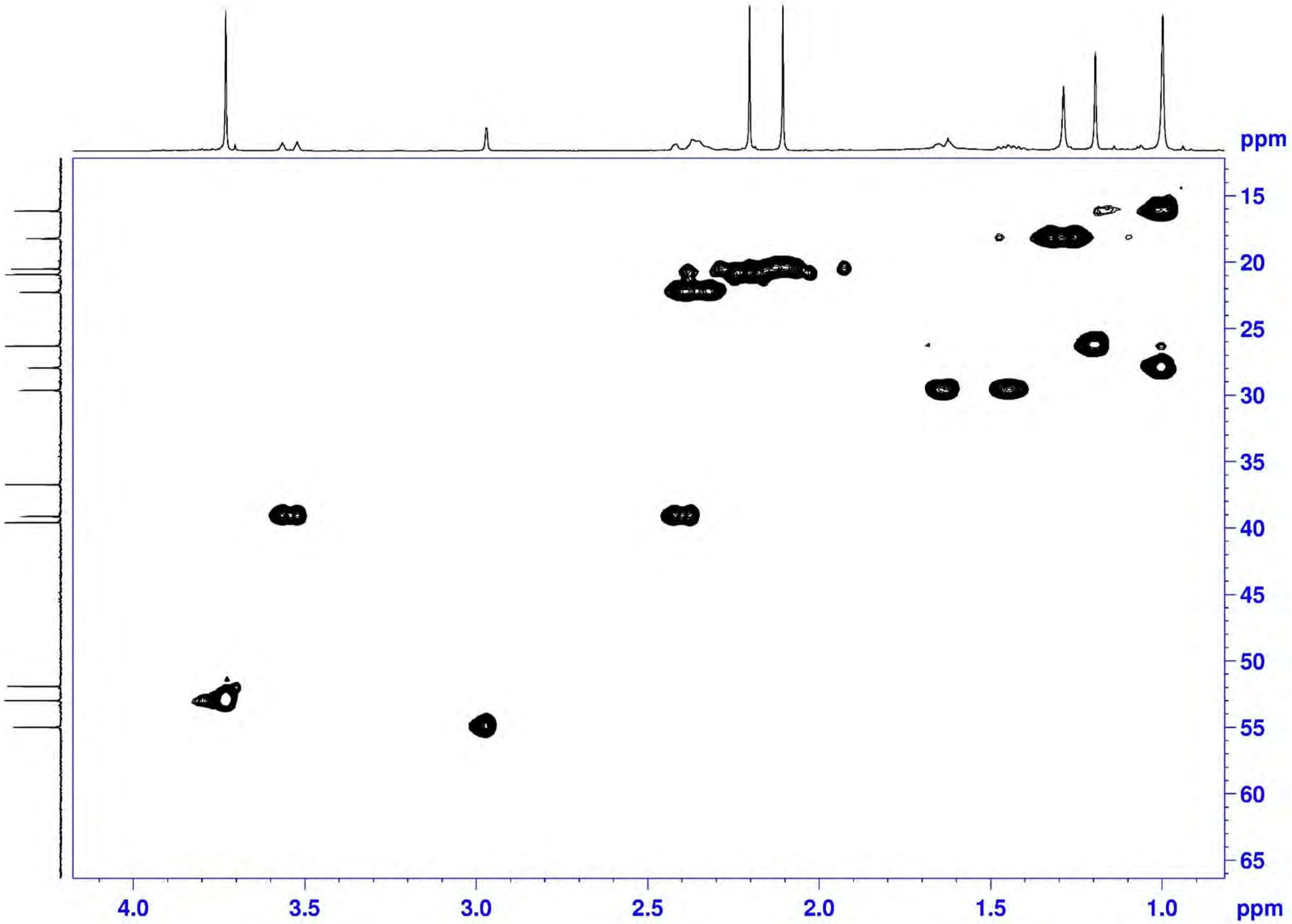

HMBC (400 MHz) spectrum of compound **8** in $CDCl_3$

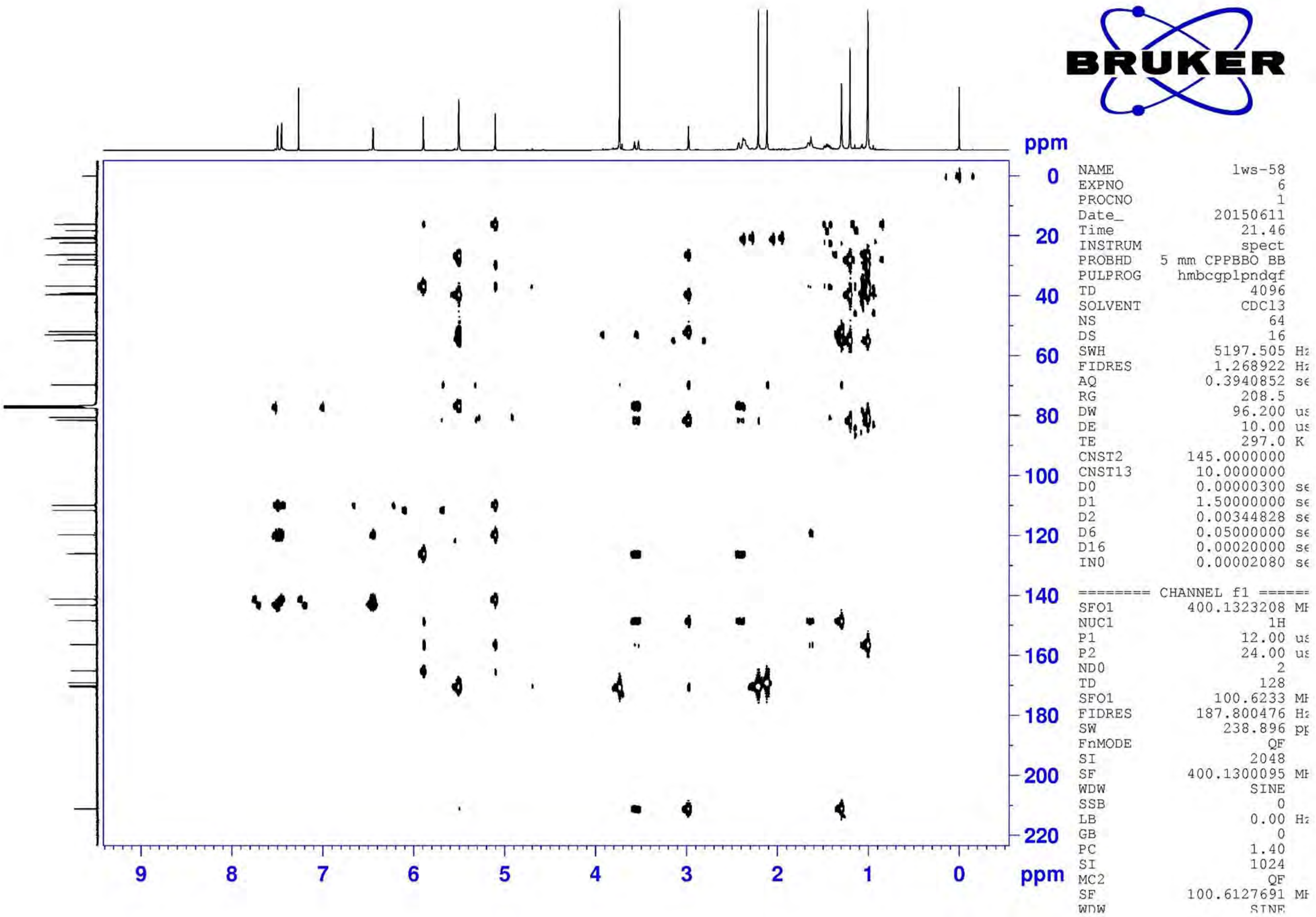

HMBC (400 MHz) spectrum of compound **8** in $CDCl_3$

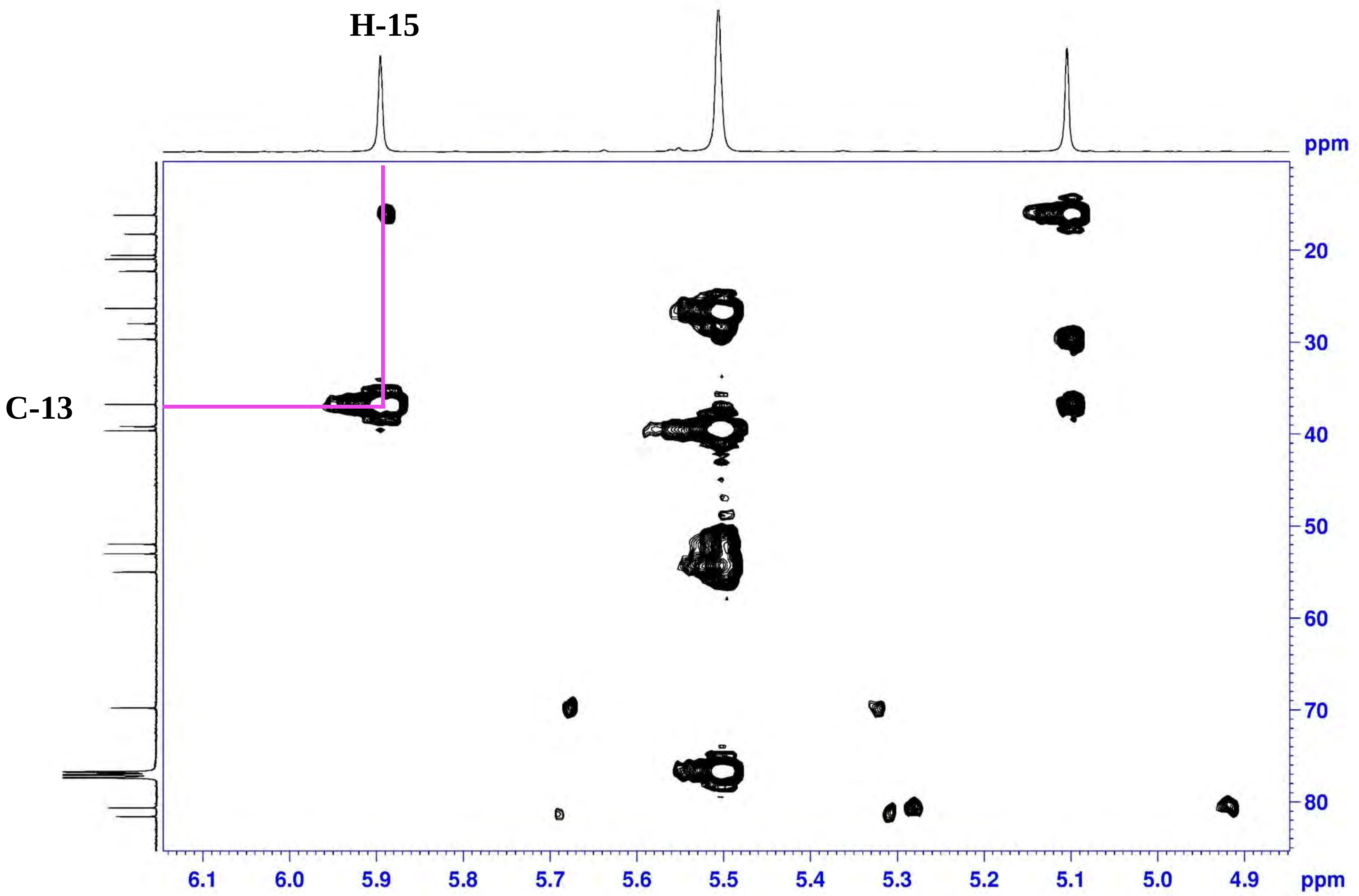

HMBC (400 MHz) spectrum of compound **8** in $CDCl_3$

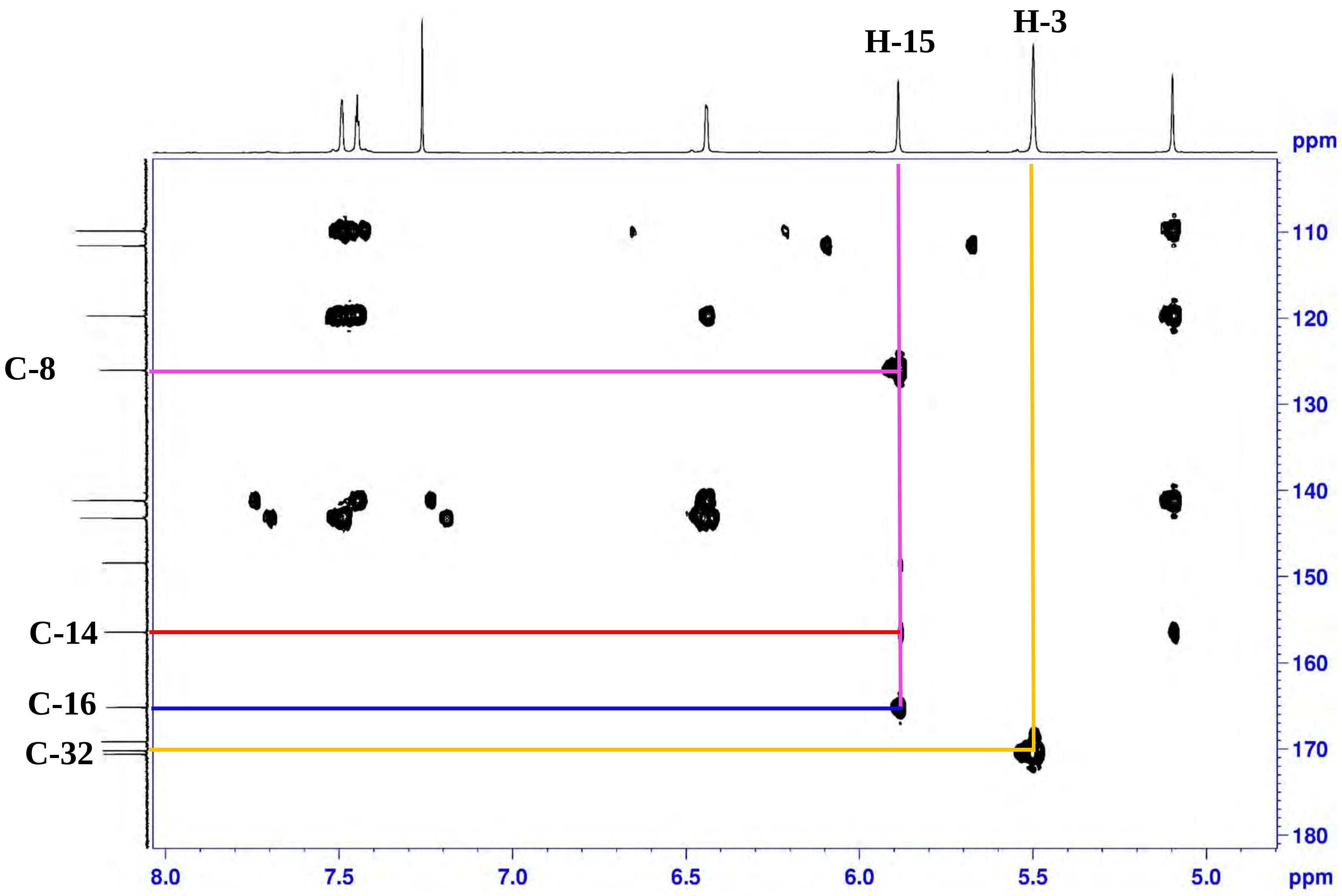

HMBC (400 MHz) spectrum of compound **8** in $CDCl_3$

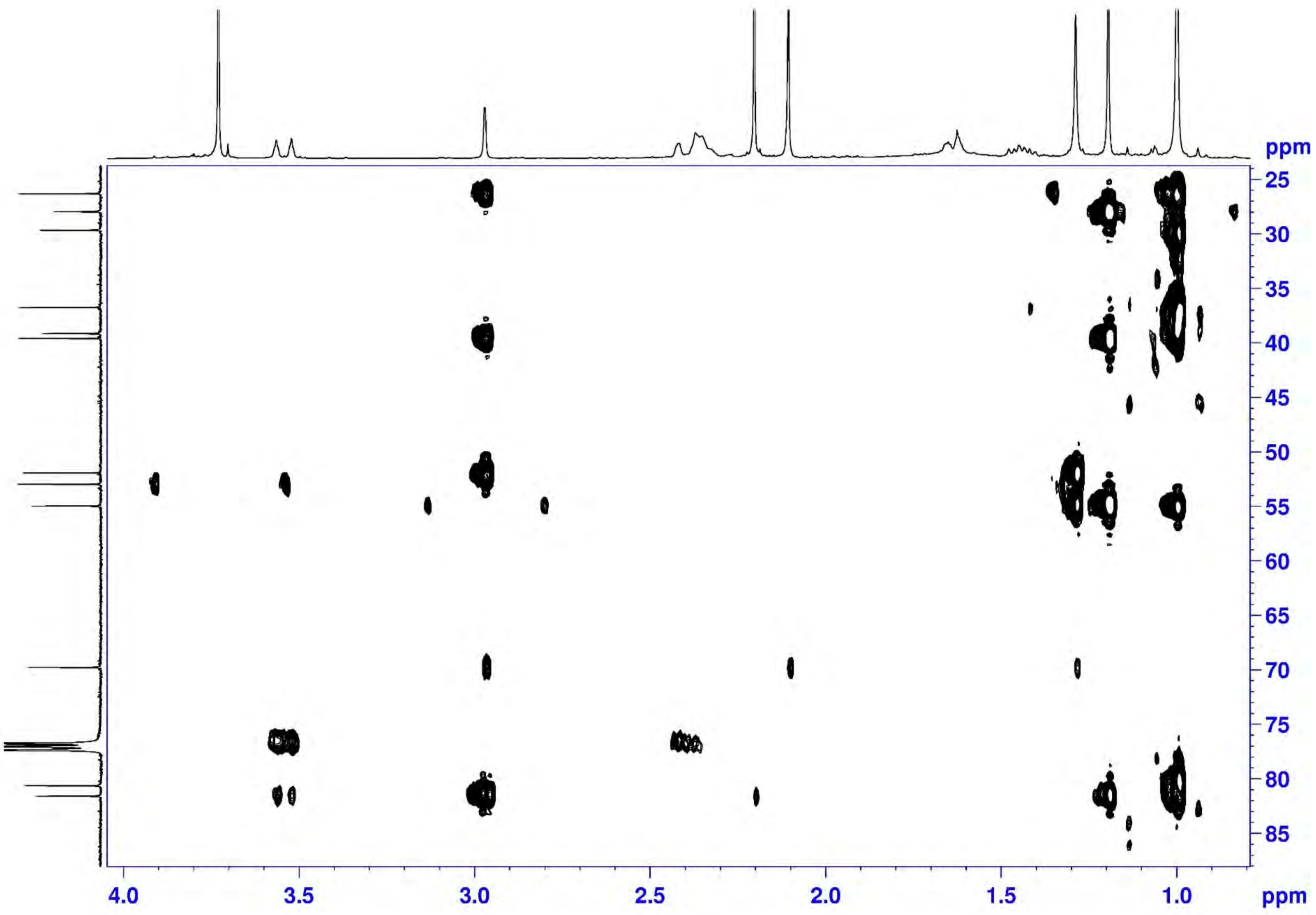

HMBC (400 MHz) spectrum of compound **8** in $CDCl_3$

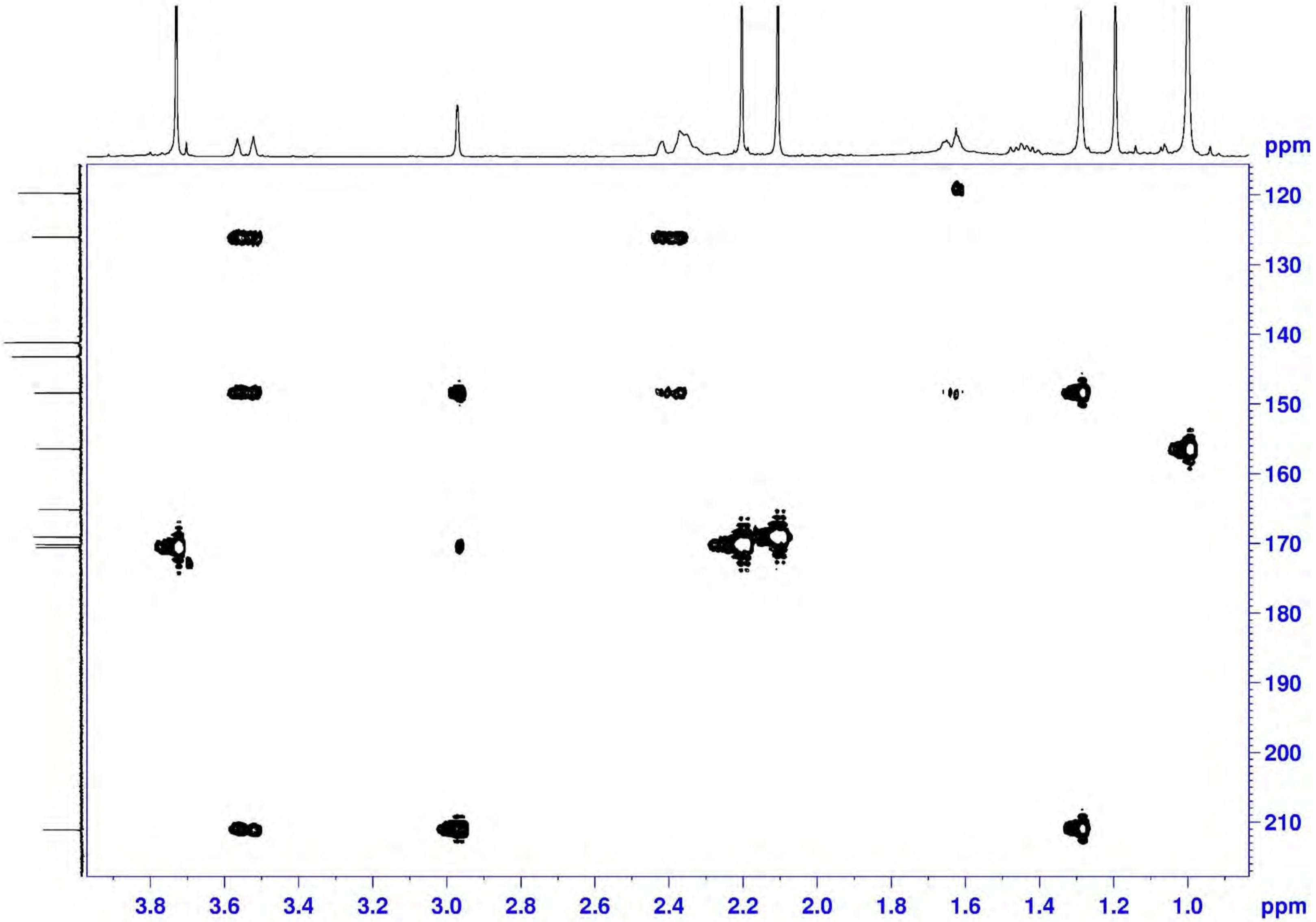

HMBC (400 MHz) spectrum of compound **8** in $CDCl_3$

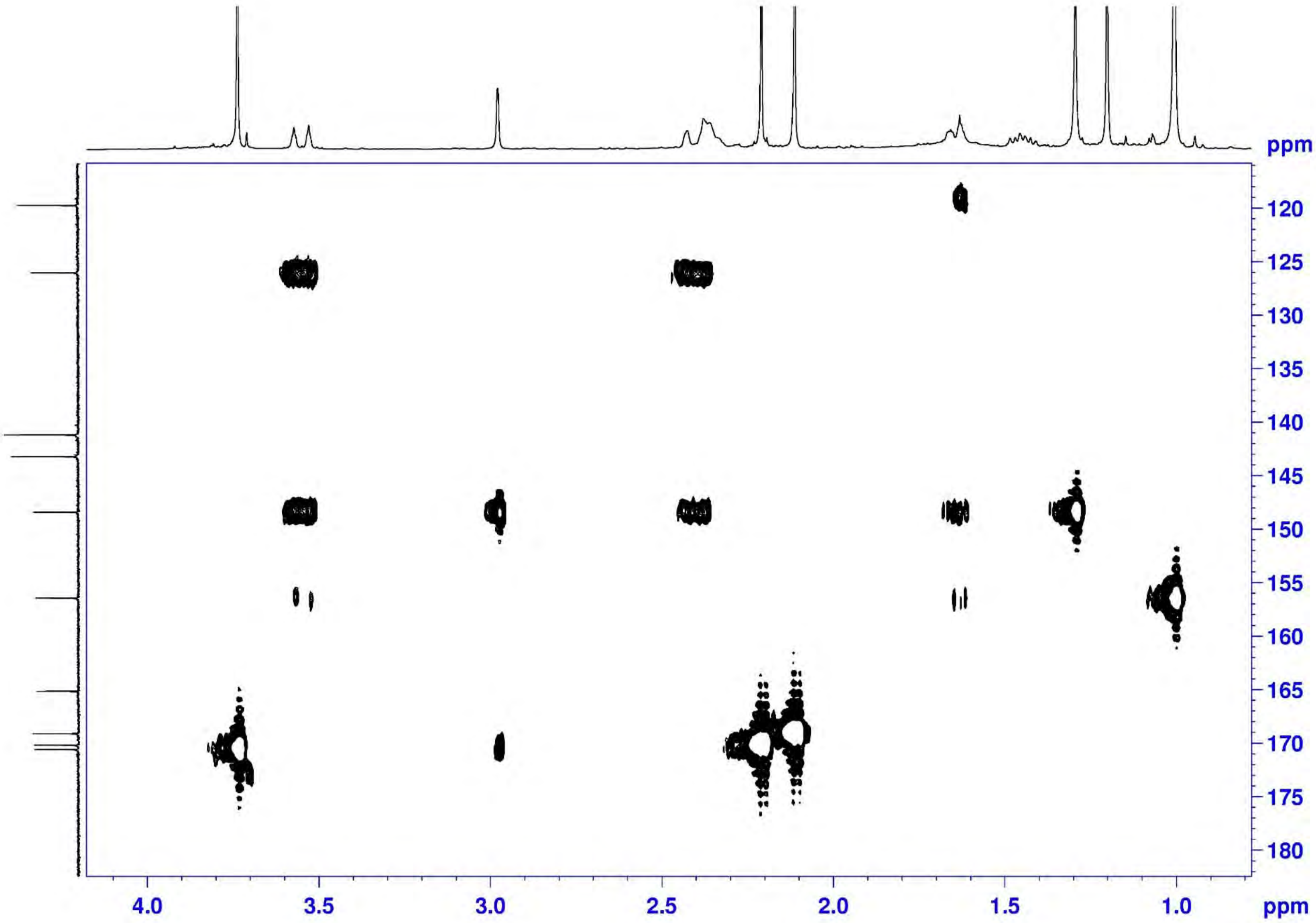

NOESY (400 MHz) spectrum of compound **8** in $CDCl_3$

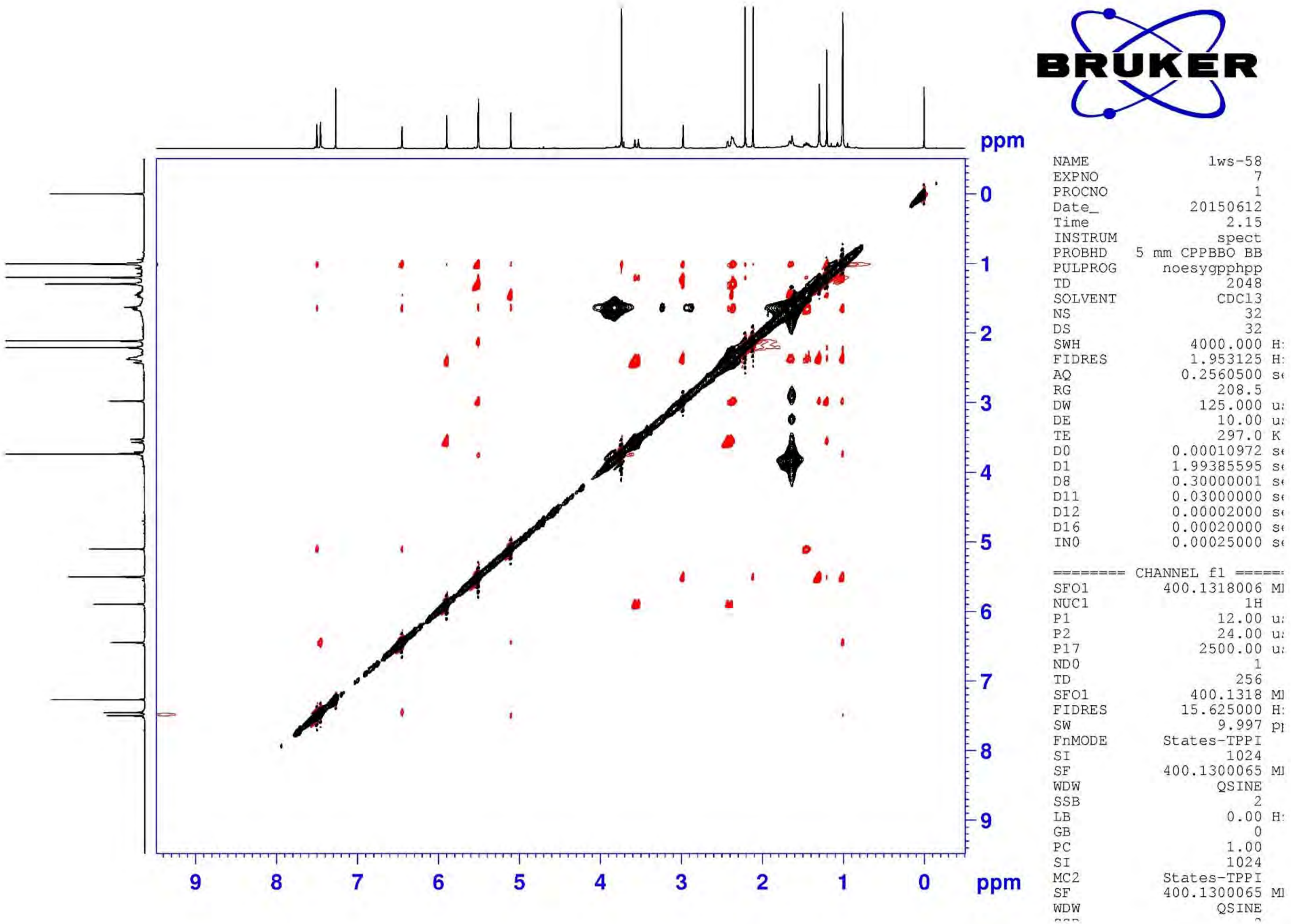

NOESY (400 MHz) spectrum of compound **8** in $CDCl_3$

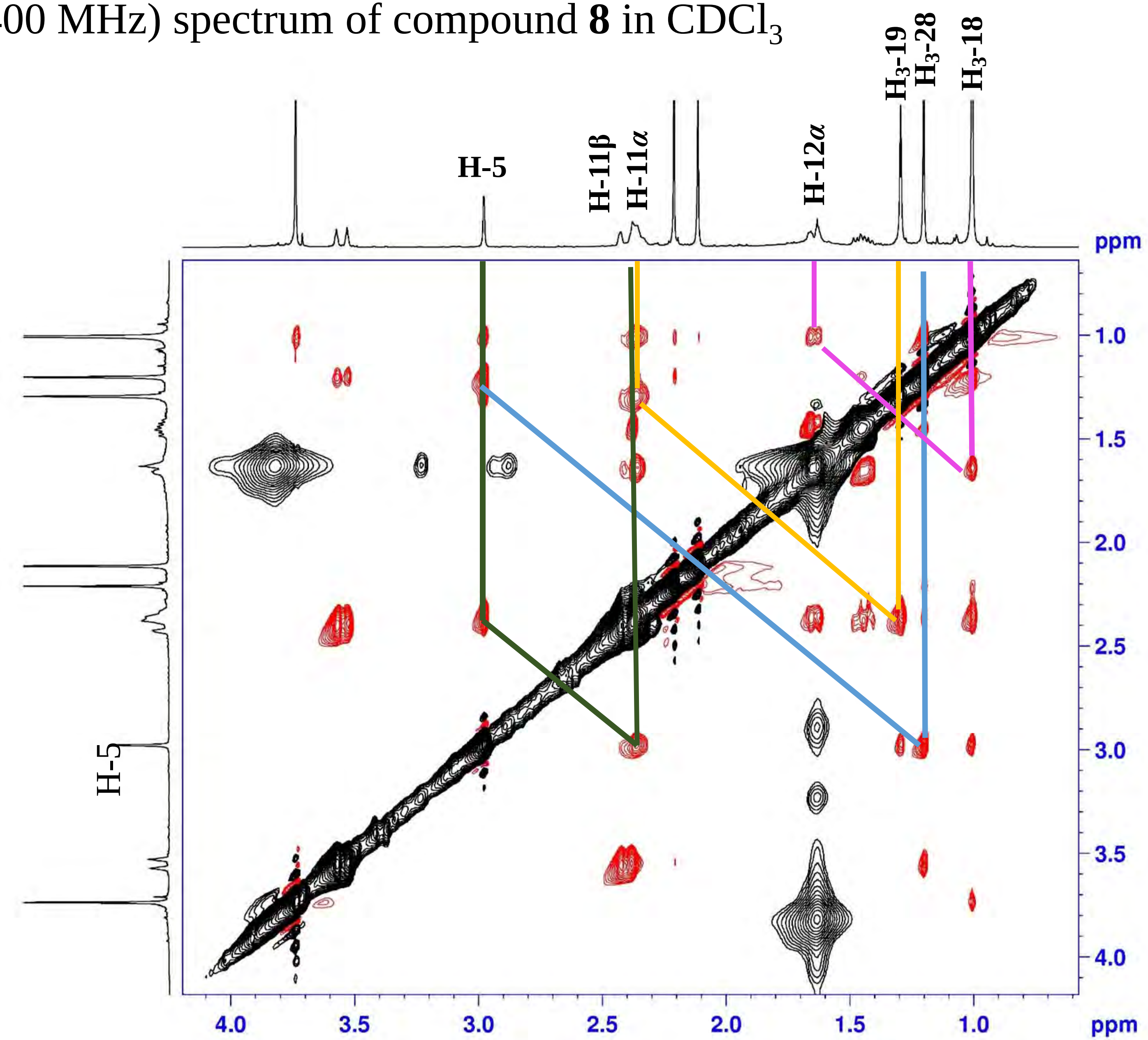

NOESY (400 MHz) spectrum of compound **8** in $CDCl_3$

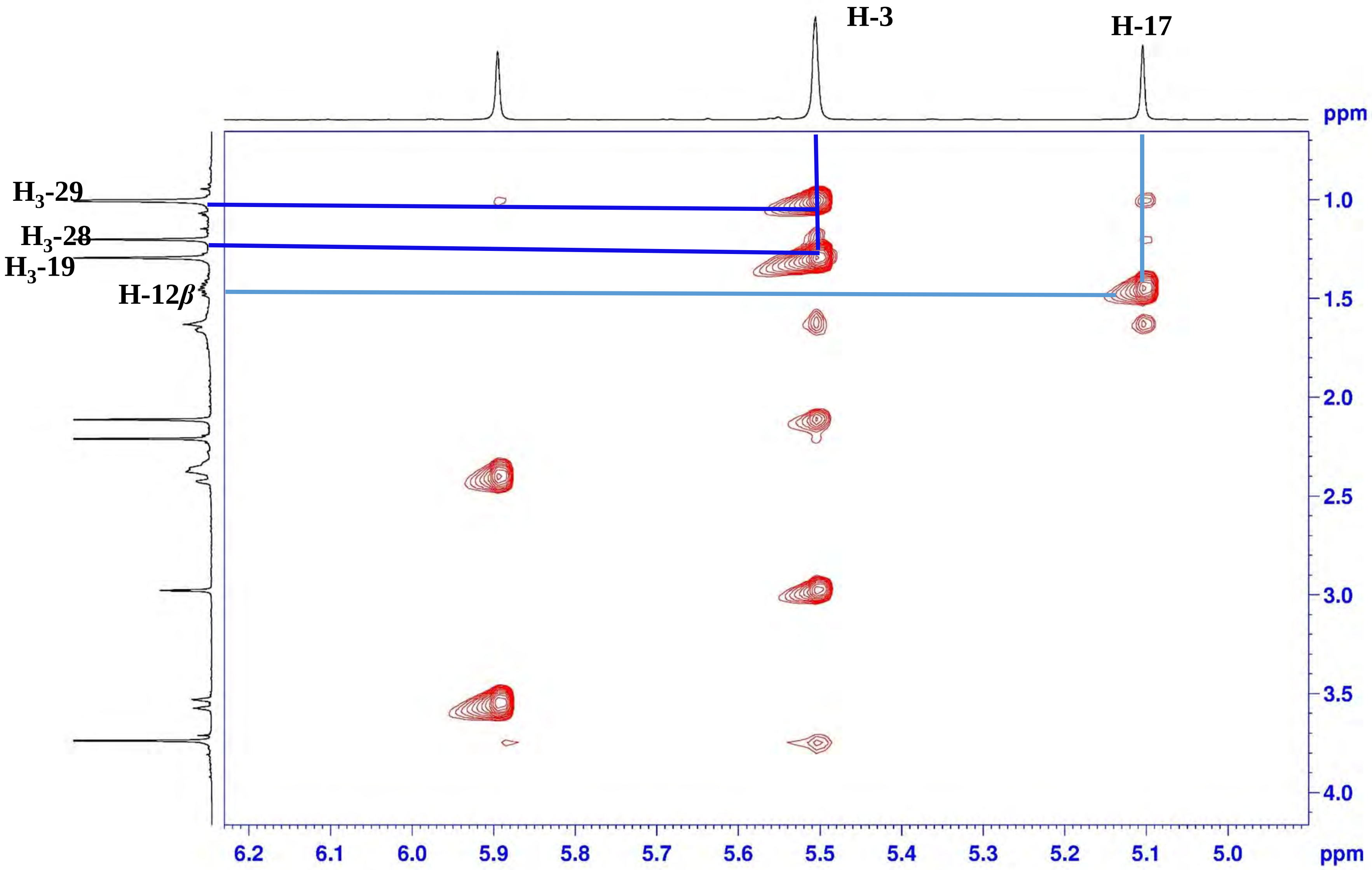

HR-ESIMS for compound **9**

Mass Spectrum SmartFormula Report

Analysis Info

Analysis Name	D:\Data\MS\data\201511\liwanshan_lws-86_pos_24_01_834.d	Acquisition Date	11/18/2015 10:10:28 PM
Method	LC_Direct Infusion_pos_100-1000mz.m	Operator	SCSIO
Sample Name	liwanshan_lws-86_pos	Instrument / Ser#	maXis 29
Comment			

Acquisition Parameter

Source Type	ESI	Ion Polarity	Positive	Set Nebulizer	0.4 Bar		
Focus	Active	Set Capillary	4500 V	Set Dry Heater	180 °C		
Scan Begin	100 m/z	Set End Plate Offset	-500 V	Set Dry Gas	4.0 l/min		
Scan End	2000 m/z	Set Collision Cell RF	800.0 Vpp	Set Divert Valve	Waste		

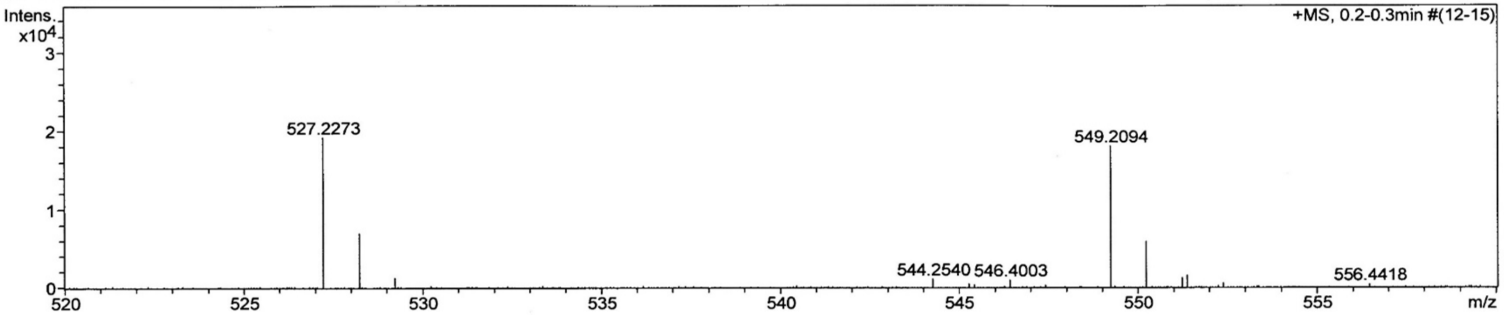

Meas. m/z	#	Formula	Score	m/z	err [ppm]	err [mDa]	mSigma	rdb	e⁻ Conf	N-Rule
527.2273	1	C 29 H 35 O 9	100.00	527.2276	0.5	0.2	23.5	12.5	even	ok
549.2094	1	C 29 H 34 Na O 9	100.00	549.2095	0.1	0.1	4.3	12.5	even	ok

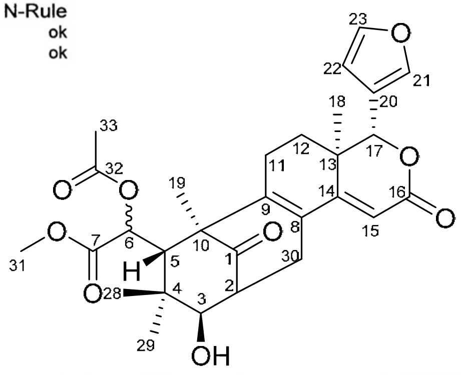

Bruker Compass DataAnalysis 4.0 printed: 11/18/2015 10:16:35 PM Page 1 of 1

HR-ESIMS for compound **9**

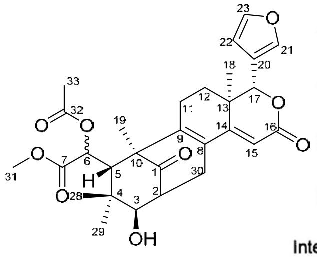

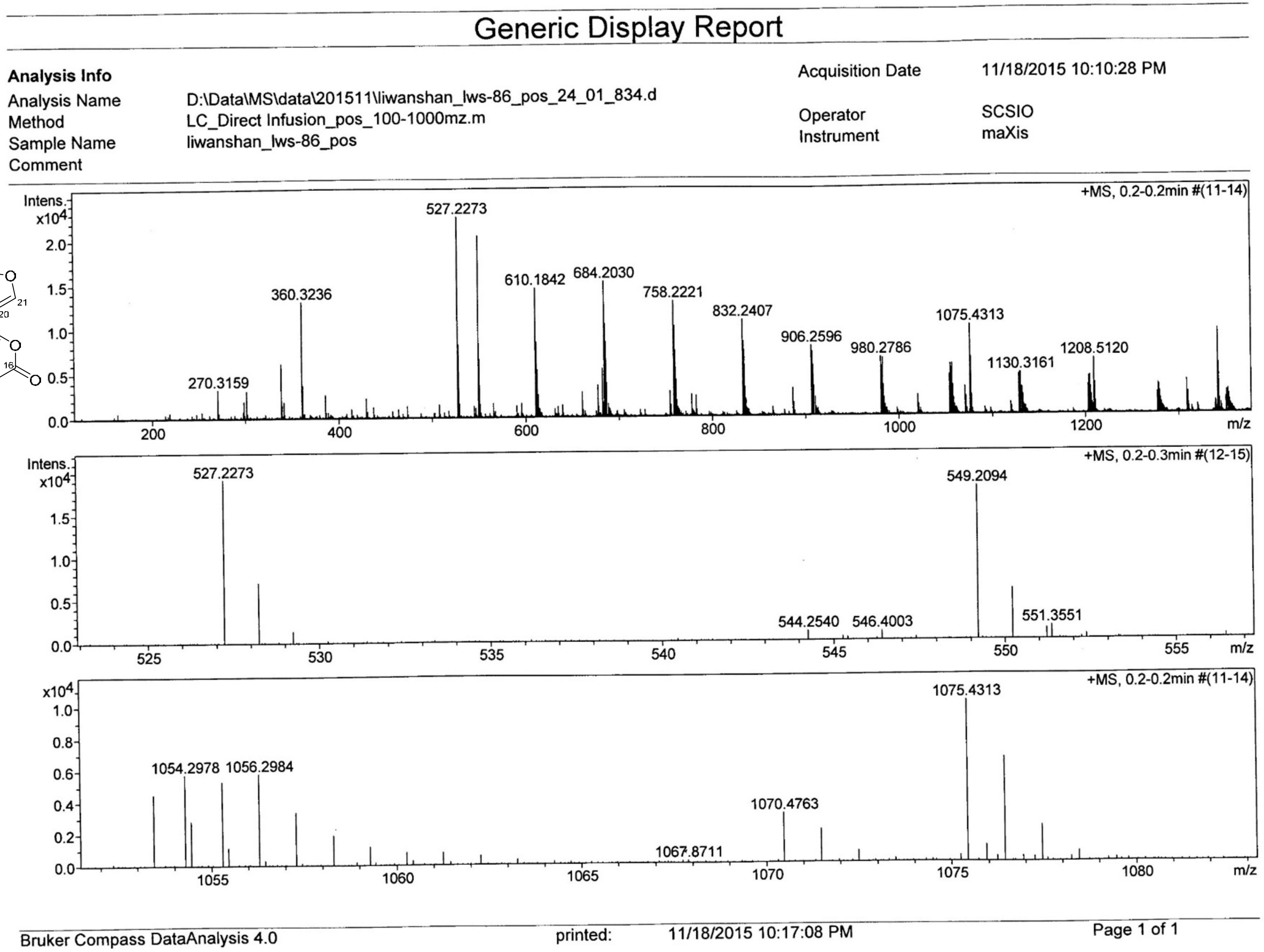

^{1}H NMR (400 MHz) spectrum of compound **9** in $CDCl_3$

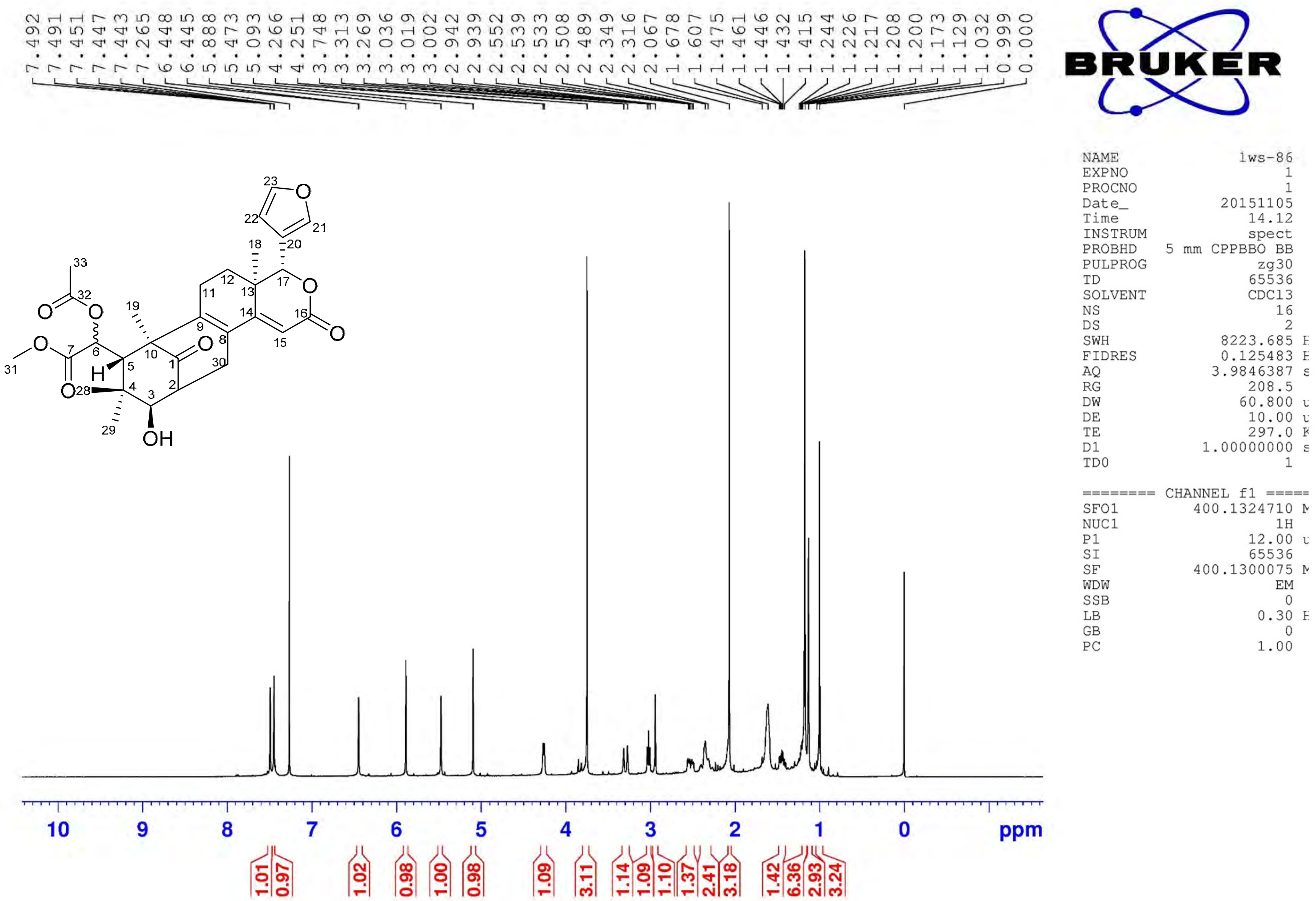

^{1}H NMR (400 MHz) spectrum of compound **9** in $CDCl_3$

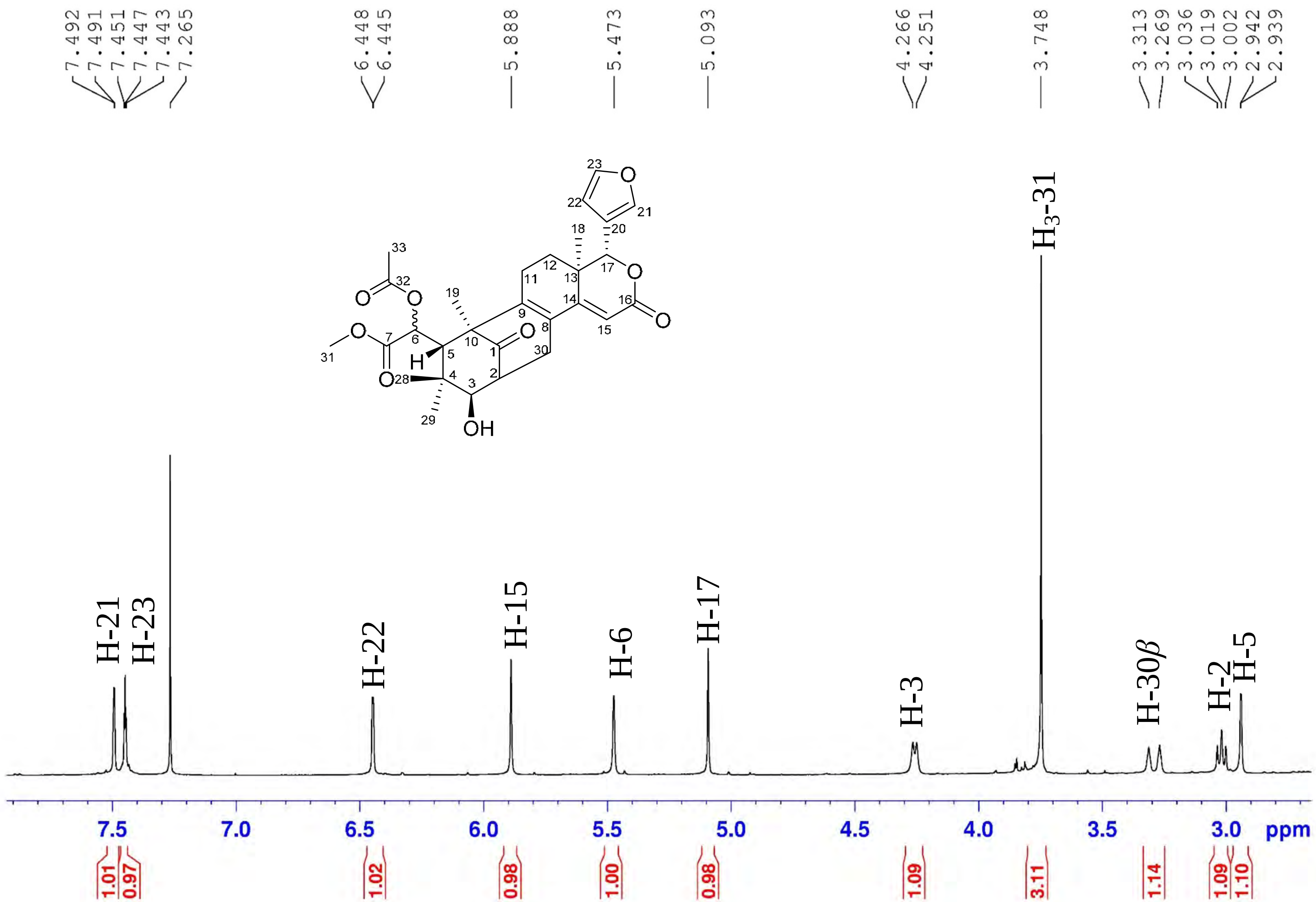

^{1}H NMR (400 MHz) spectrum of compound **9** in $CDCl_3$

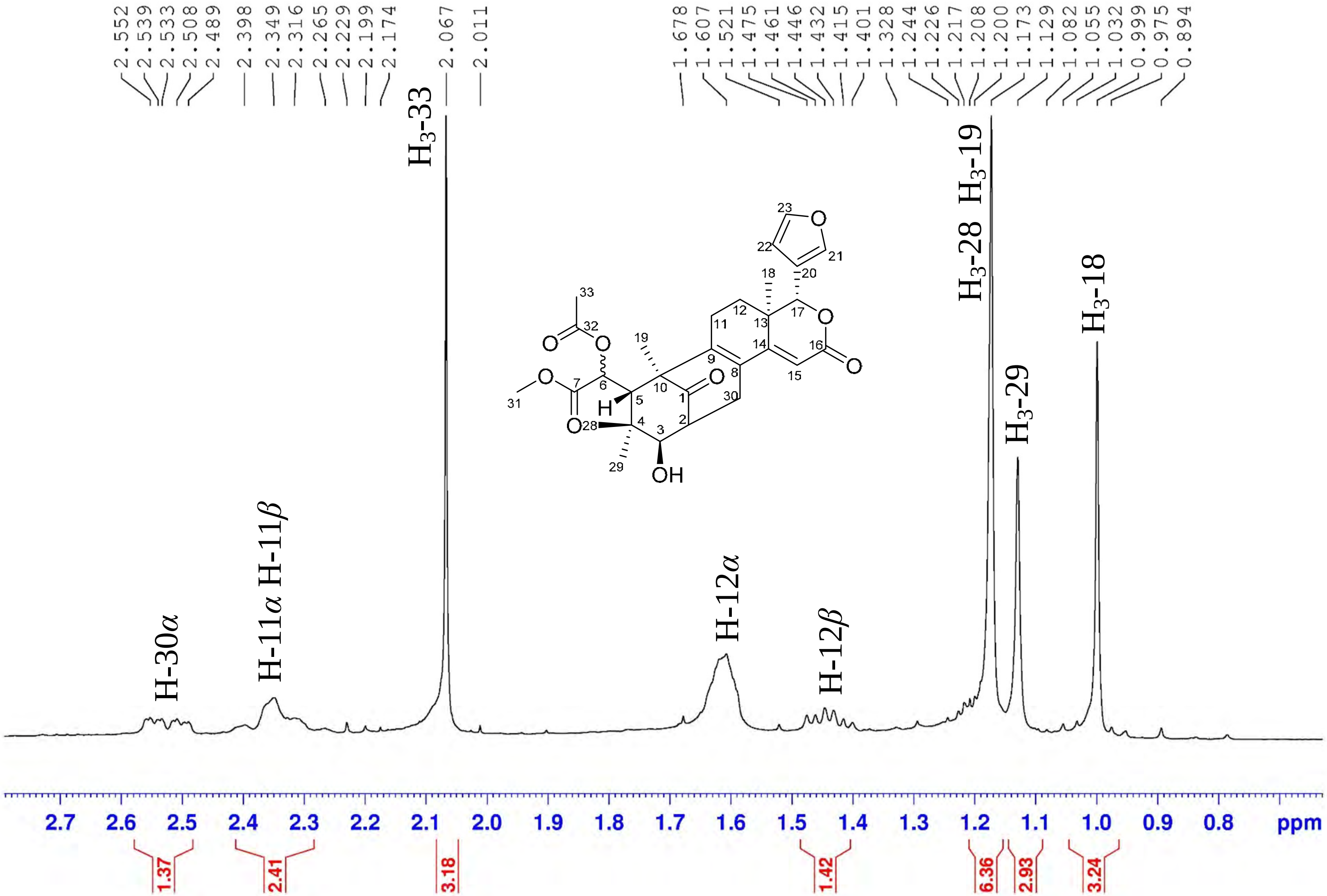

^{13}C NMR (100 MHz) spectrum of compound **9** in $CDCl_3$

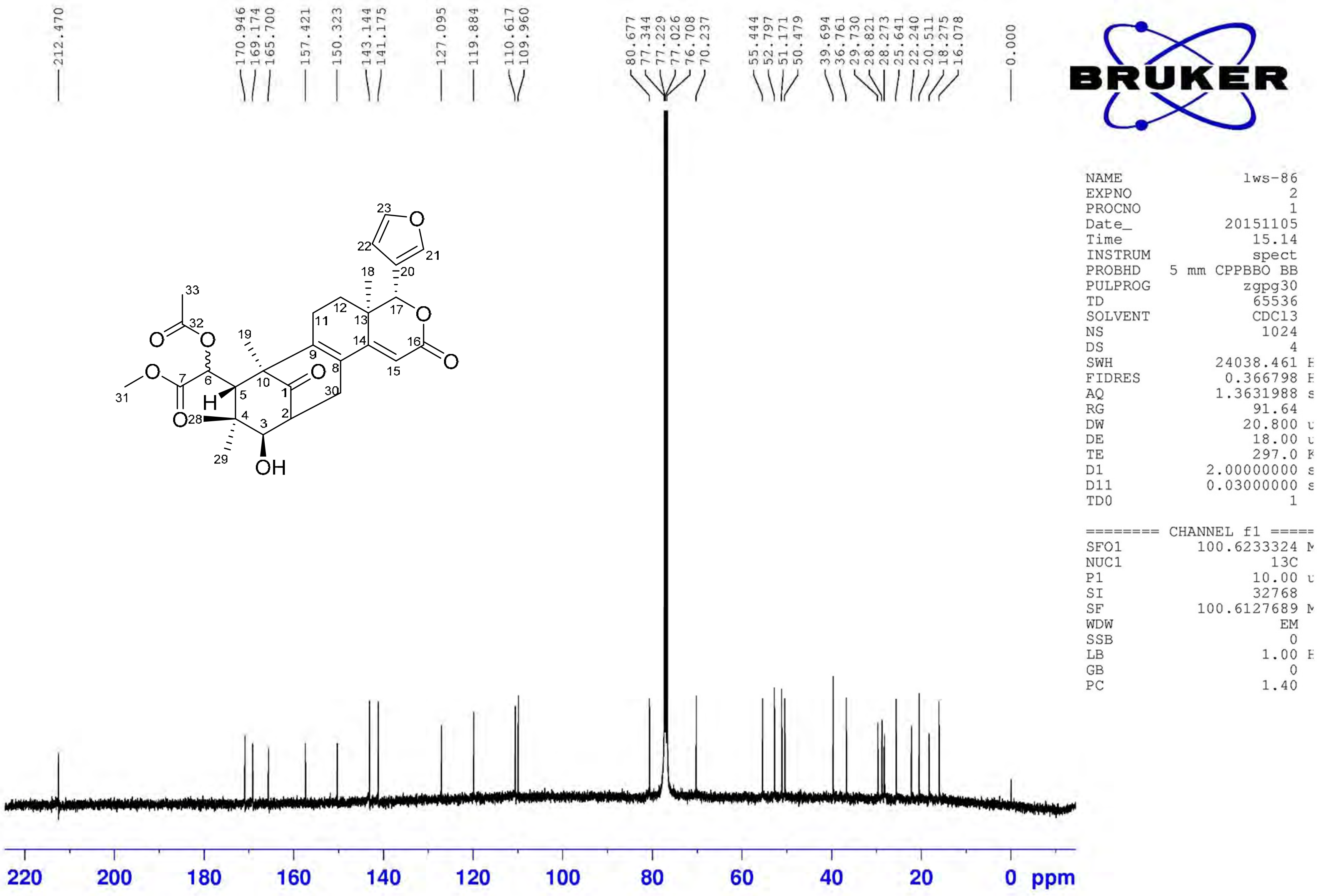

^{13}C NMR (100 MHz) spectrum of compound **9** in $CDCl_3$

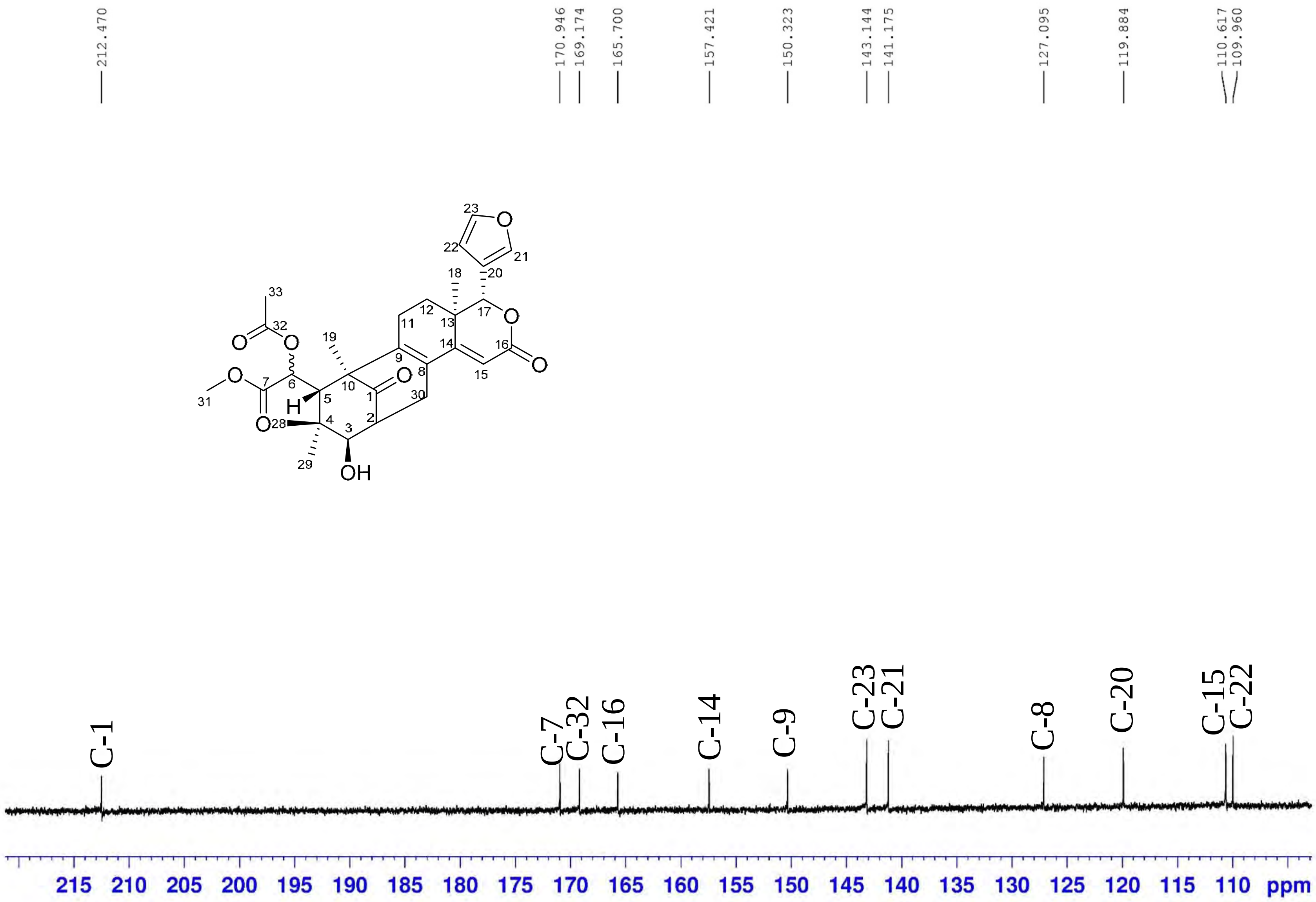

^{13}C NMR (100 MHz) spectrum of compound **9** in $CDCl_3$

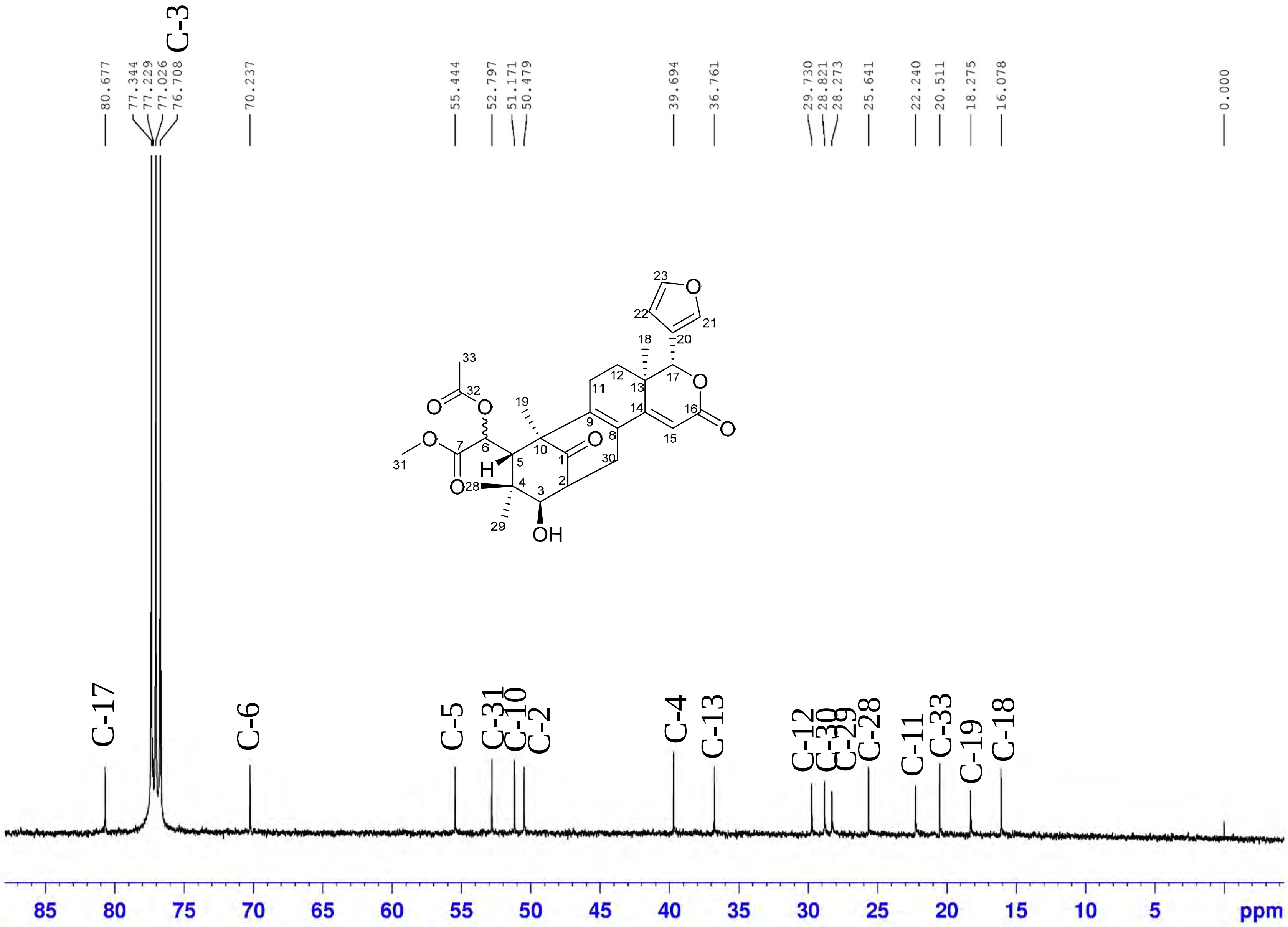

DEPT135 (100 MHz) spectrum of compound **9** in $CDCl_3$

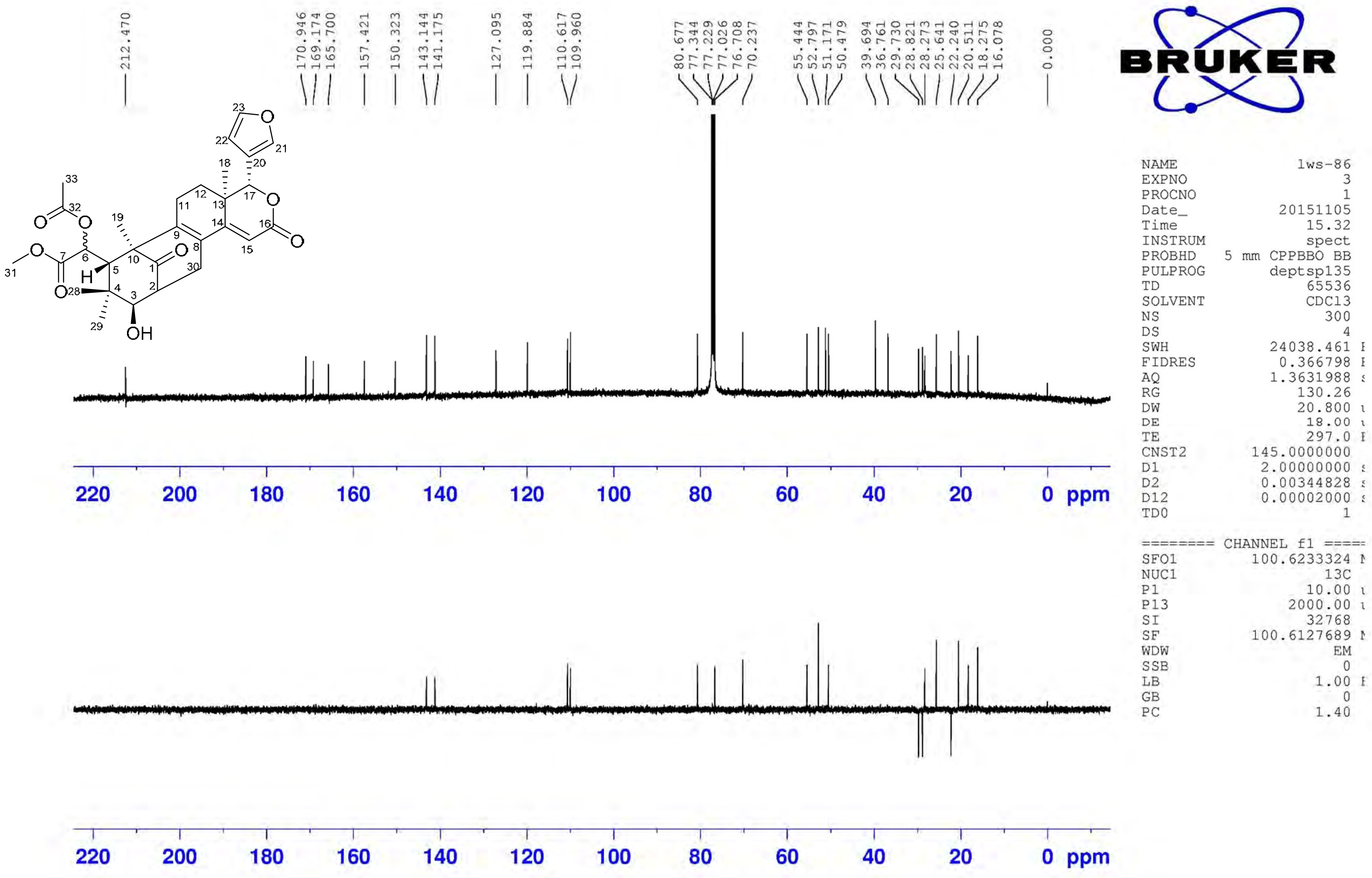

DEPT135 (100 MHz) spectrum of compound **9** in $CDCl_3$

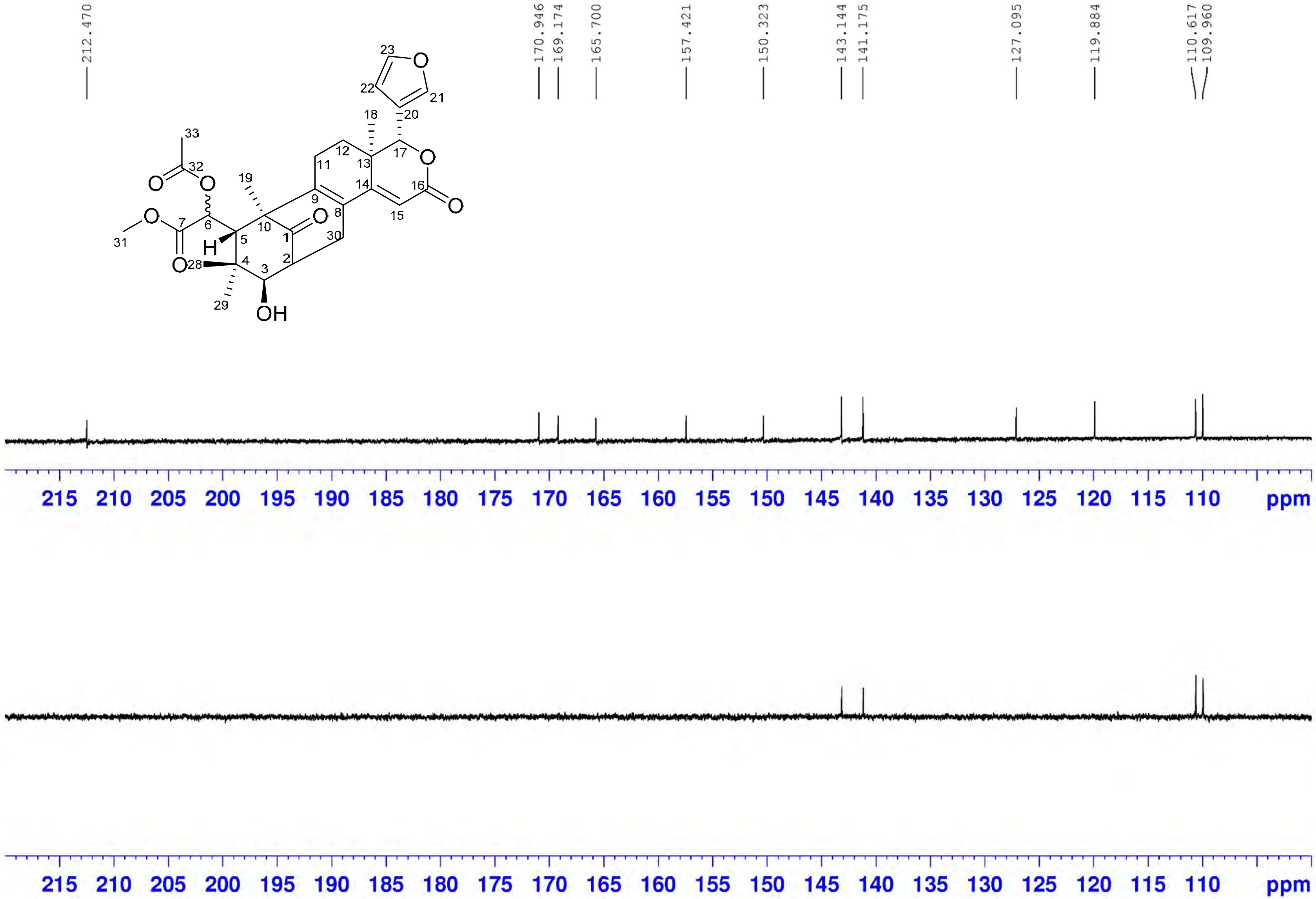

DEPT135 (100 MHz) spectrum of compound **9** in $CDCl_3$

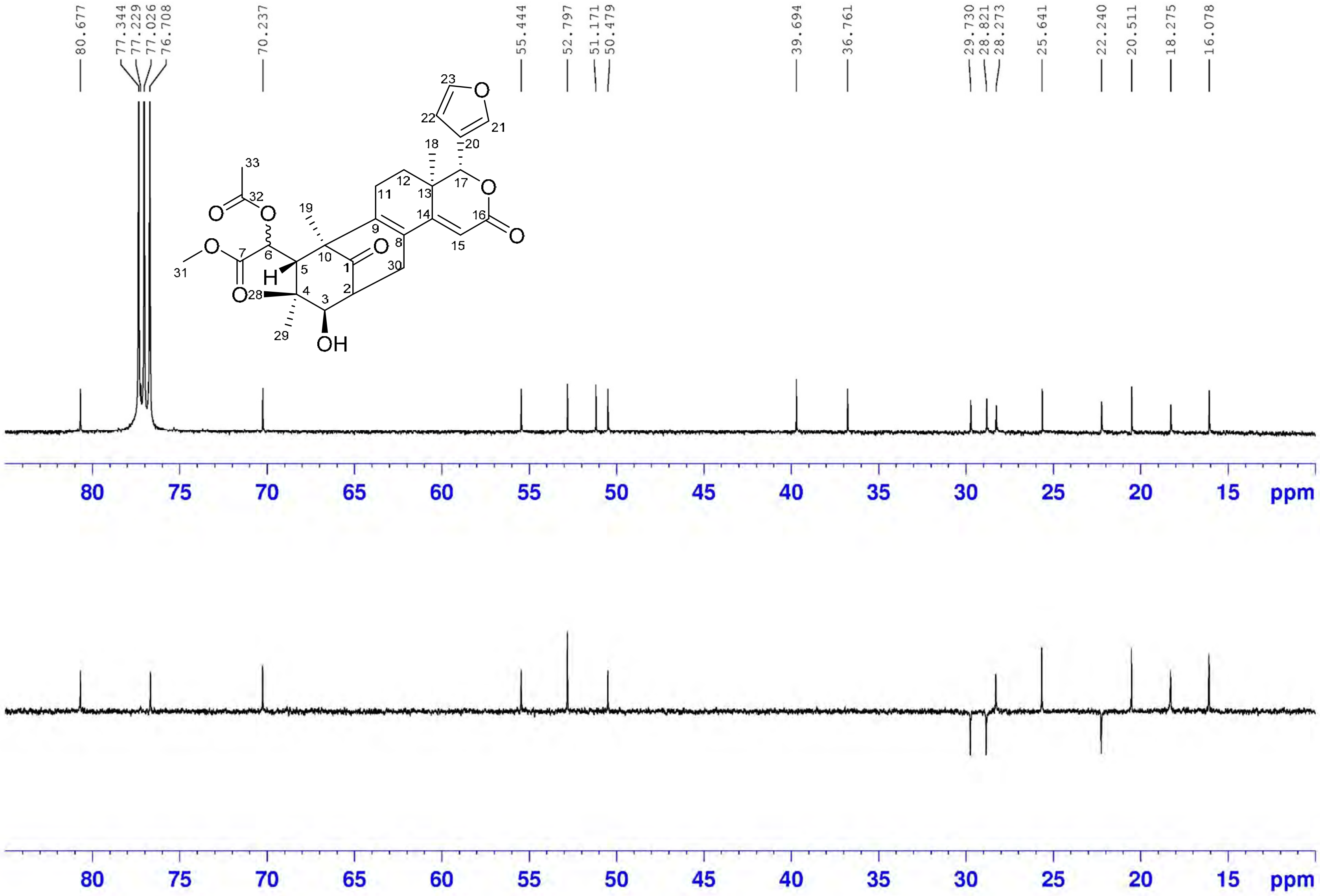

^{1}H-^{1}H COSY (400 MHz) spectrum of compound **9** in $CDCl_3$

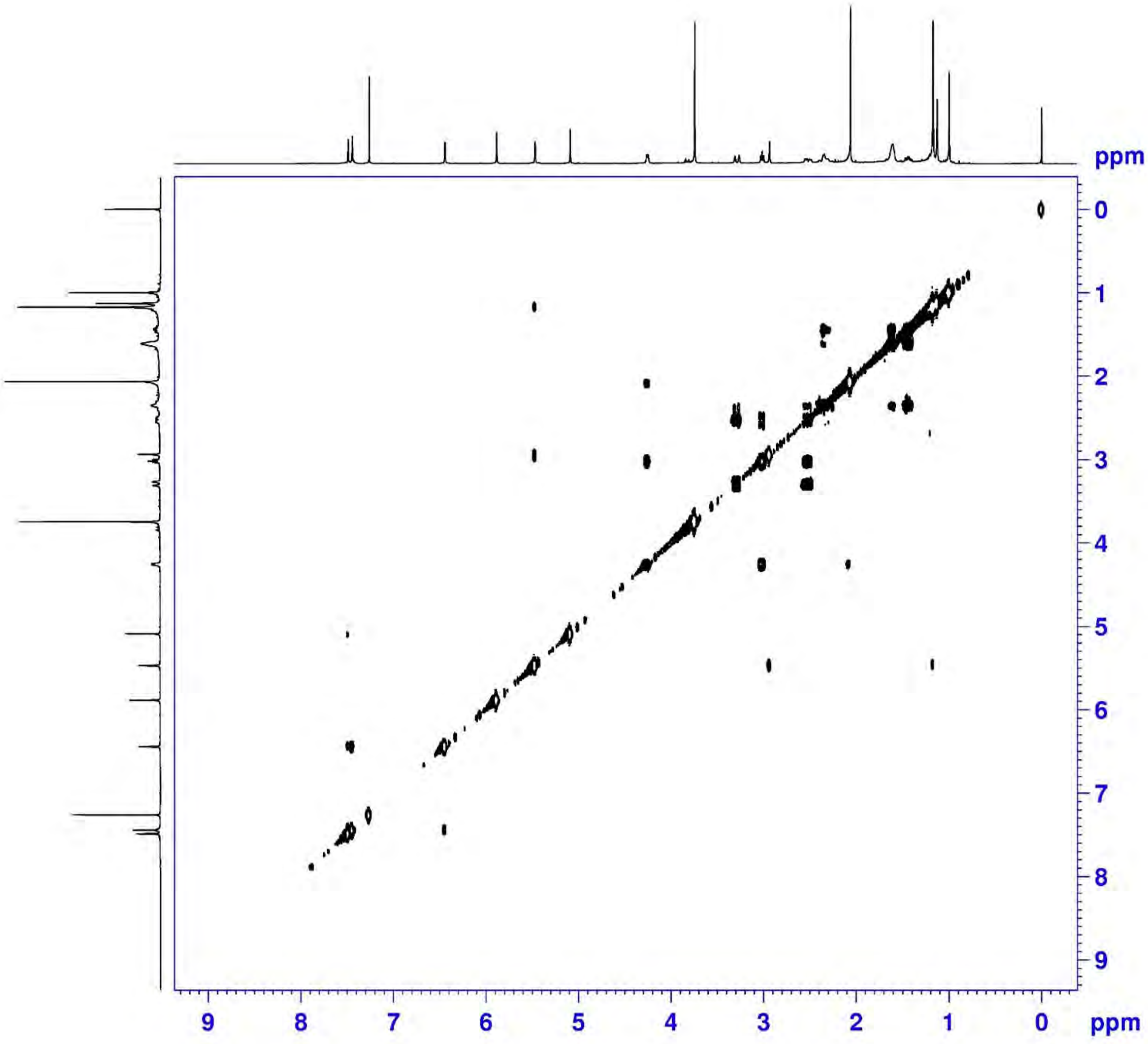

NAME	lws-86	
EXPNO	4	
PROCNO	1	
Date_	20151105	
Time	15.33	
INSTRUM	spect	
PROBHD	5 mm CPPBBO BB	
PULPROG	cosygpppqf	
TD	2048	
SOLVENT	CDCl3	
NS	8	
DS	8	
SWH	3906.250	Hz
FIDRES	1.907349	Hz
AQ	0.2621940	sec
RG	208.5	
DW	128.000	usec
DE	10.00	usec
TE	297.0	K
D0	0.00000300	sec
D1	1.89678097	sec
D11	0.03000000	sec
D12	0.00002000	sec
D13	0.00000400	sec
D16	0.00020000	sec
IN0	0.00025600	sec
======== CHANNEL f1 ========		
SFO1	400.1318006	MHz
NUC1	1H	
P0	12.00	usec
P1	12.00	usec
P17	2500.00	usec
ND0	1	
TD	128	
SFO1	400.1318	MHz
FIDRES	30.517578	Hz
SW	9.762	ppm
FnMODE	QF	
SI	1024	
SF	400.1300070	MHz
WDW	QSINE	
SSB	0	
LB	0.00	Hz
GB	0	
PC	1.40	
SI	1024	
MC2	QF	
SF	400.1300070	MHz

^{1}H-^{1}H COSY (400 MHz) spectrum of compound **9** in $CDCl_3$

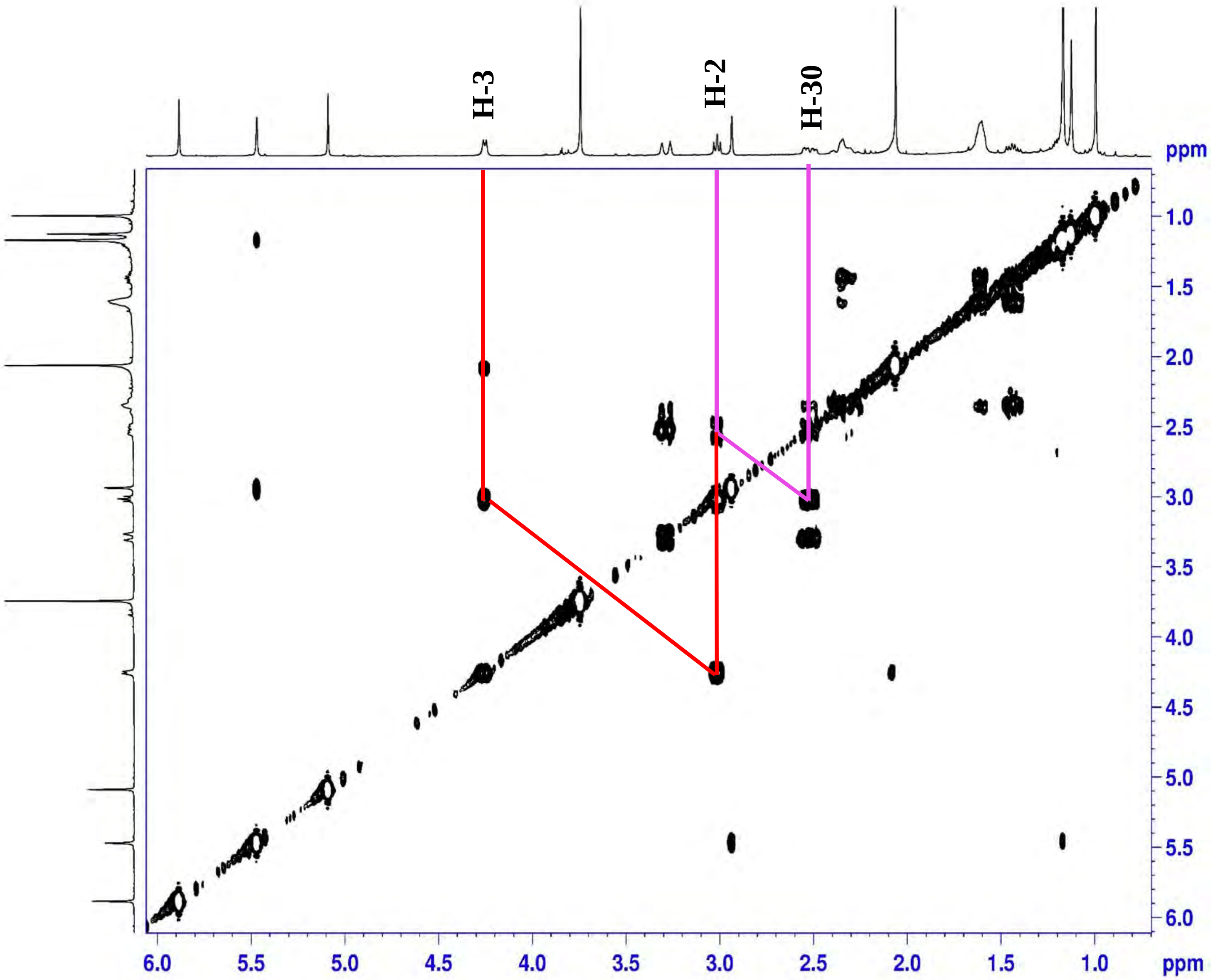

HSQC (400 MHz) spectrum of compound **9** in $CDCl_3$

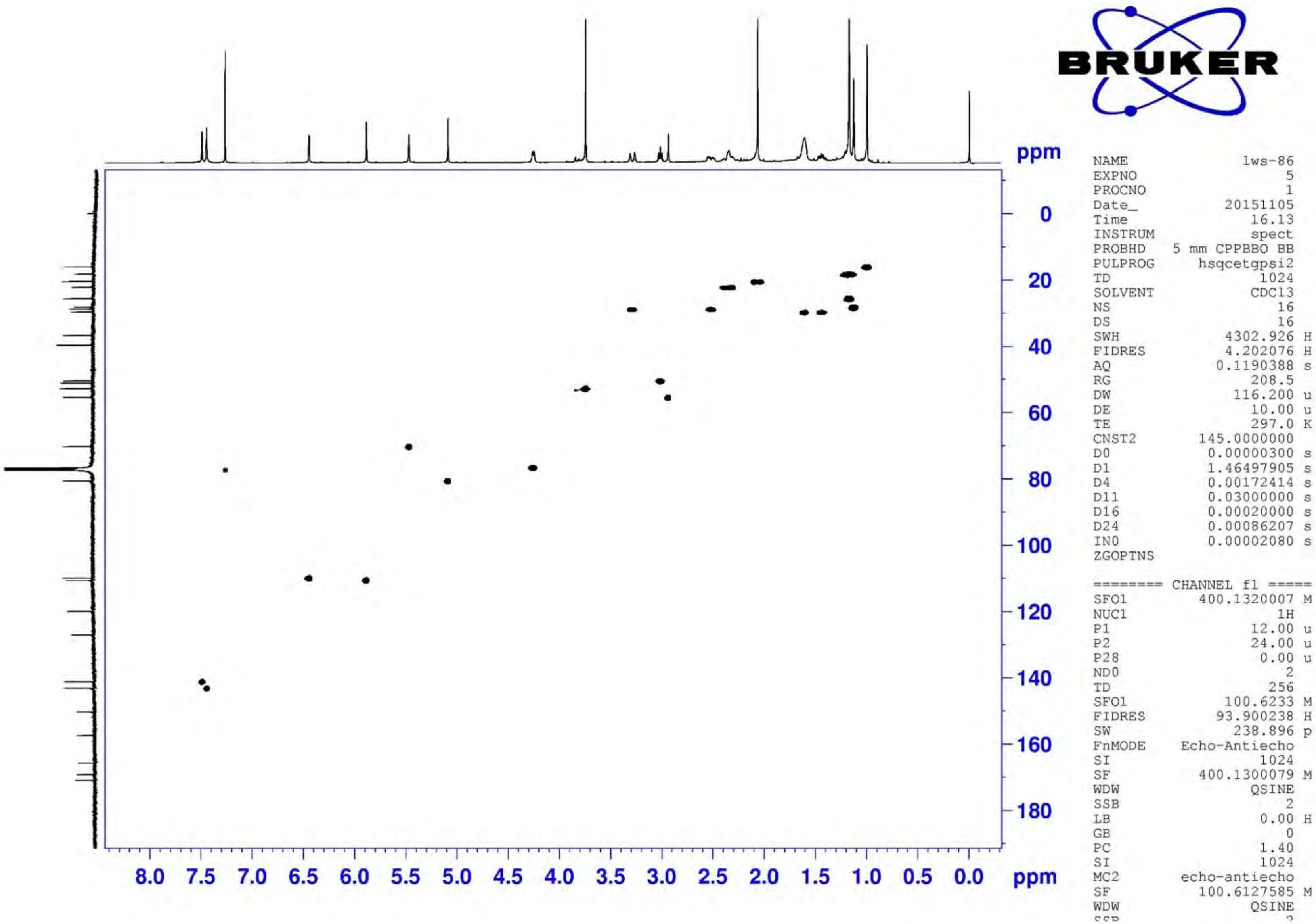

HSQC (400 MHz) spectrum of compound **9** in $CDCl_3$

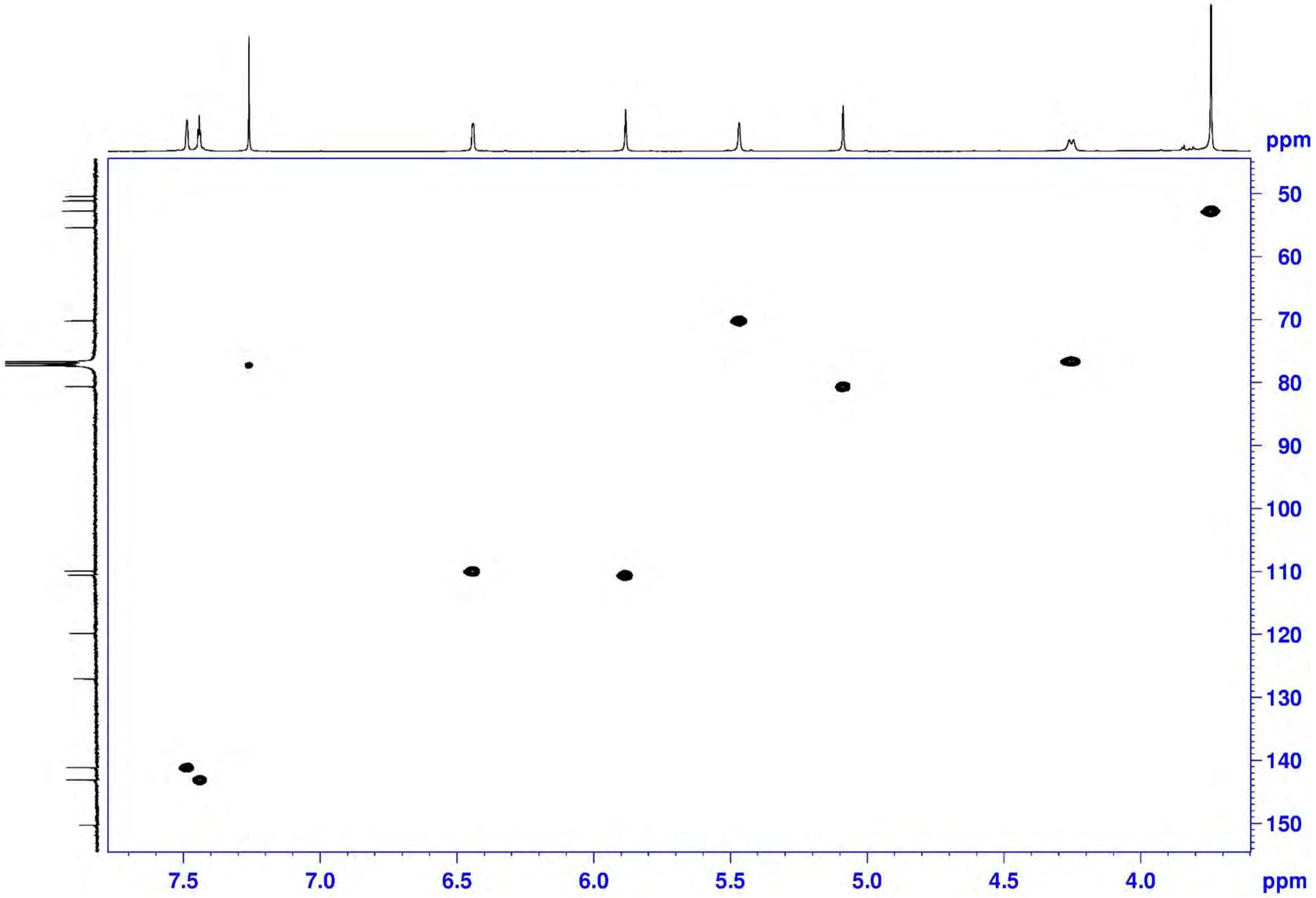

HSQC (400 MHz) spectrum of compound **9** in $CDCl_3$

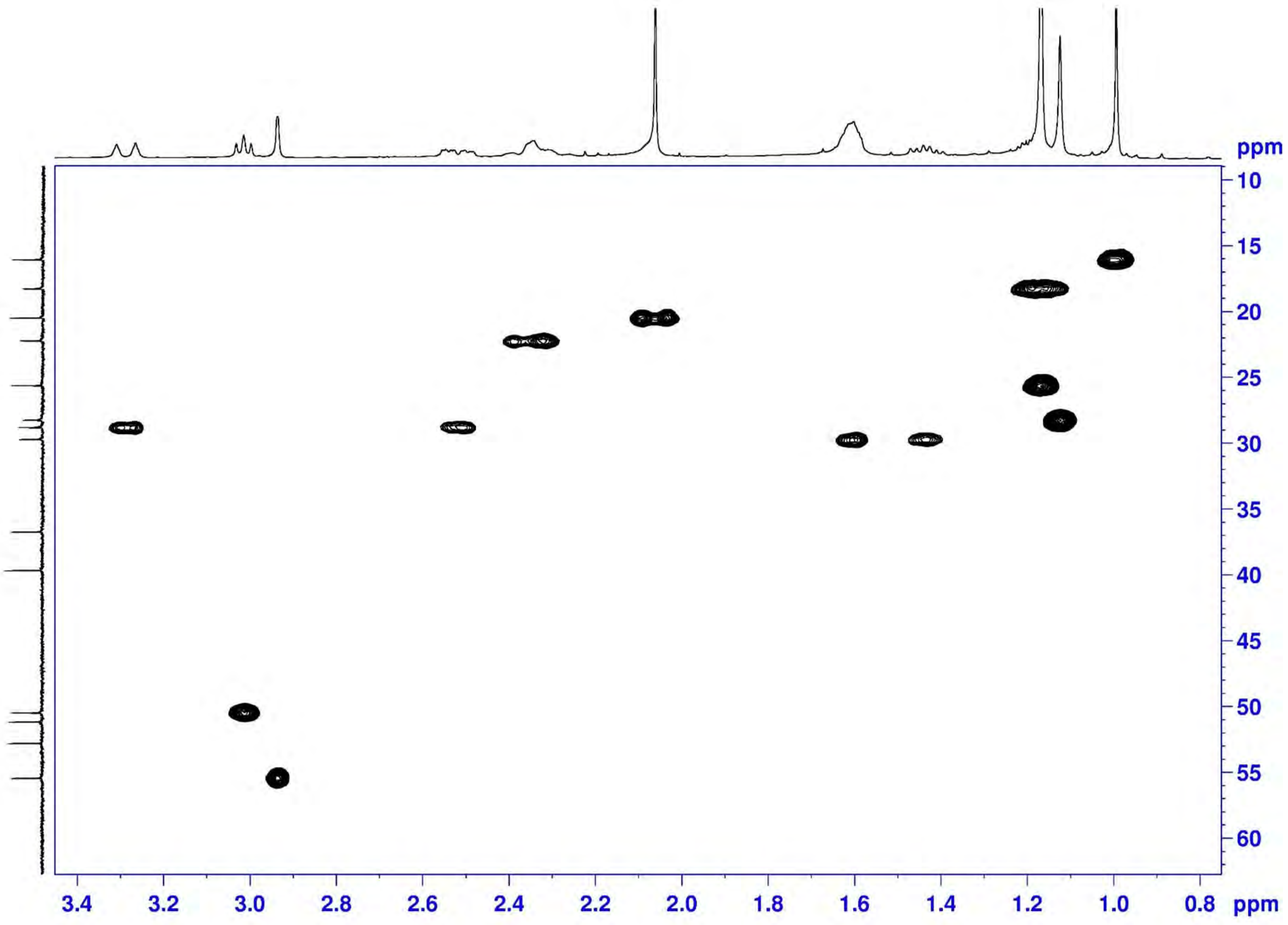

HMBC (400 MHz) spectrum of compound **9** in $CDCl_3$

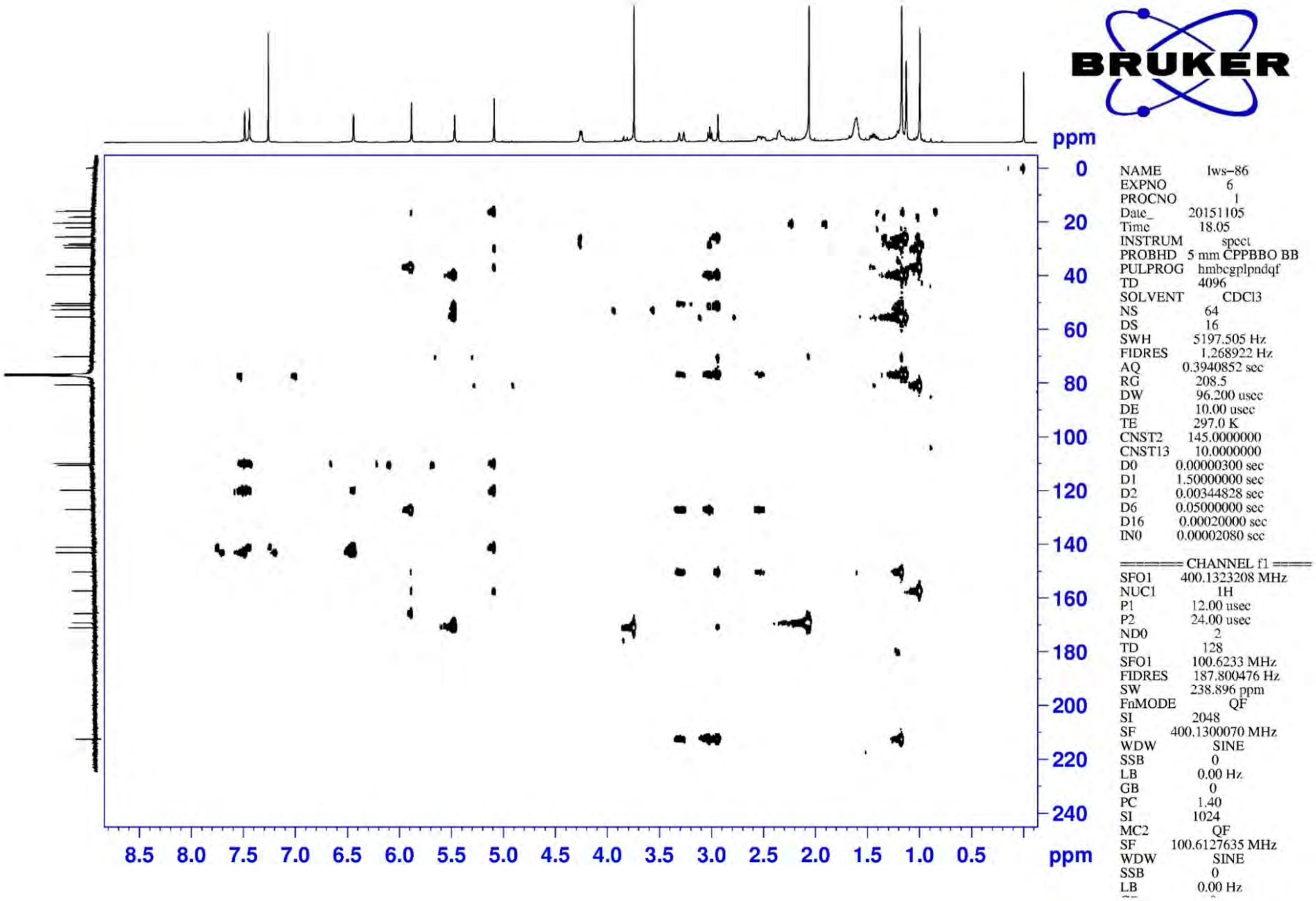

HMBC (400 MHz) spectrum of compound **9** in $CDCl_3$

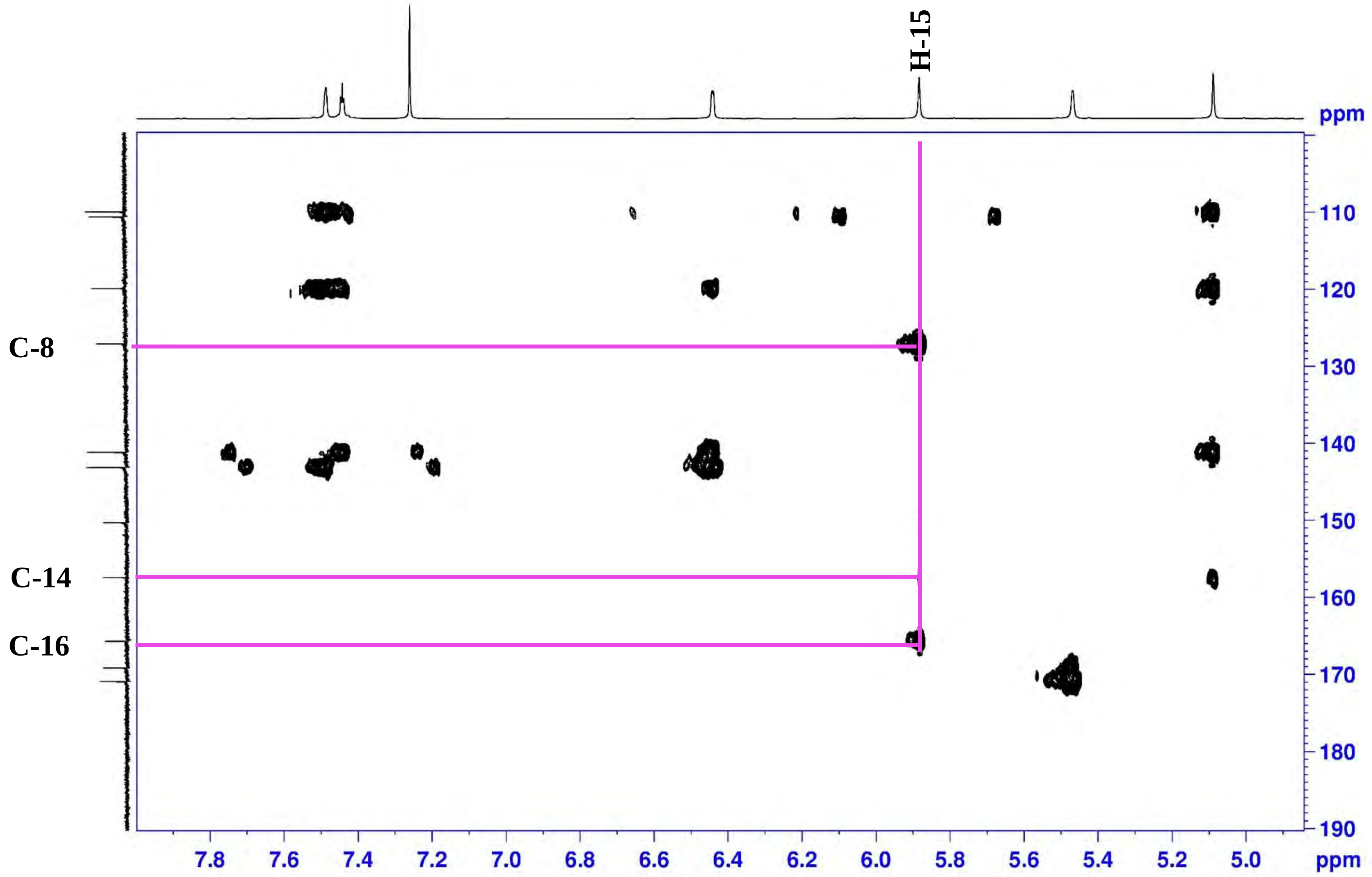

HMBC (400 MHz) spectrum of compound **9** in $CDCl_3$

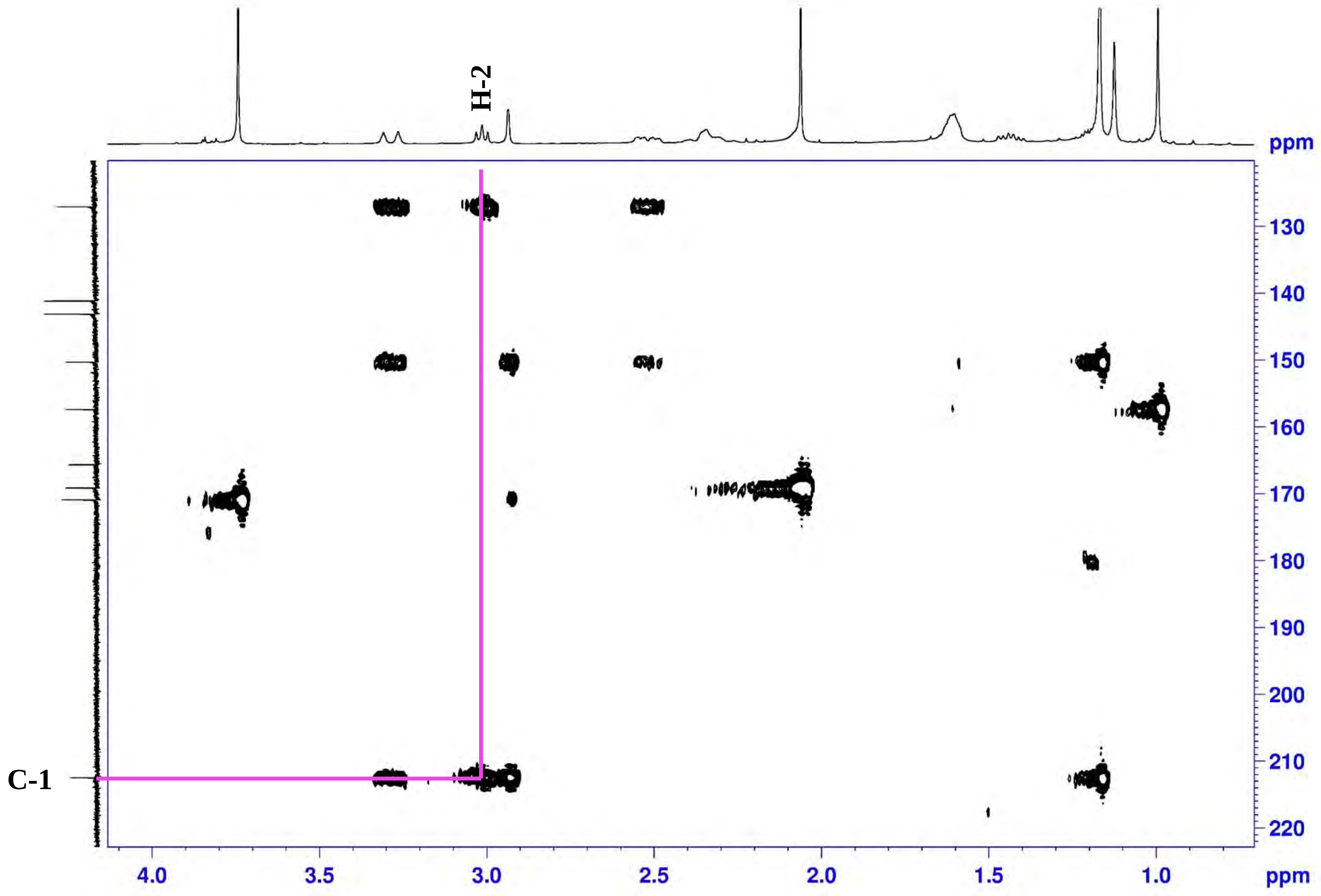

HMBC (400 MHz) spectrum of compound **9** in $CDCl_3$

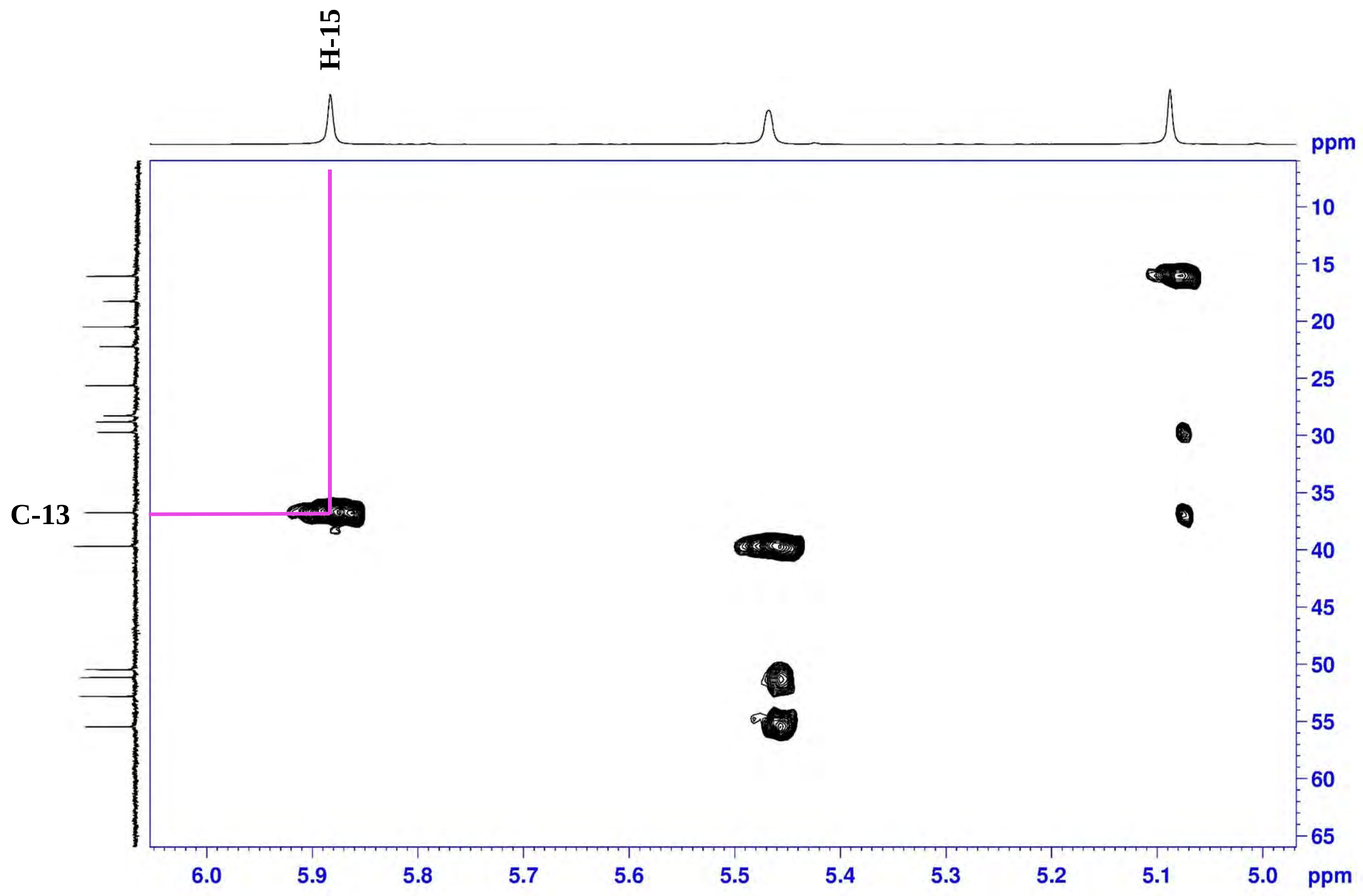

HMBC (400 MHz) spectrum of compound **9** in $CDCl_3$

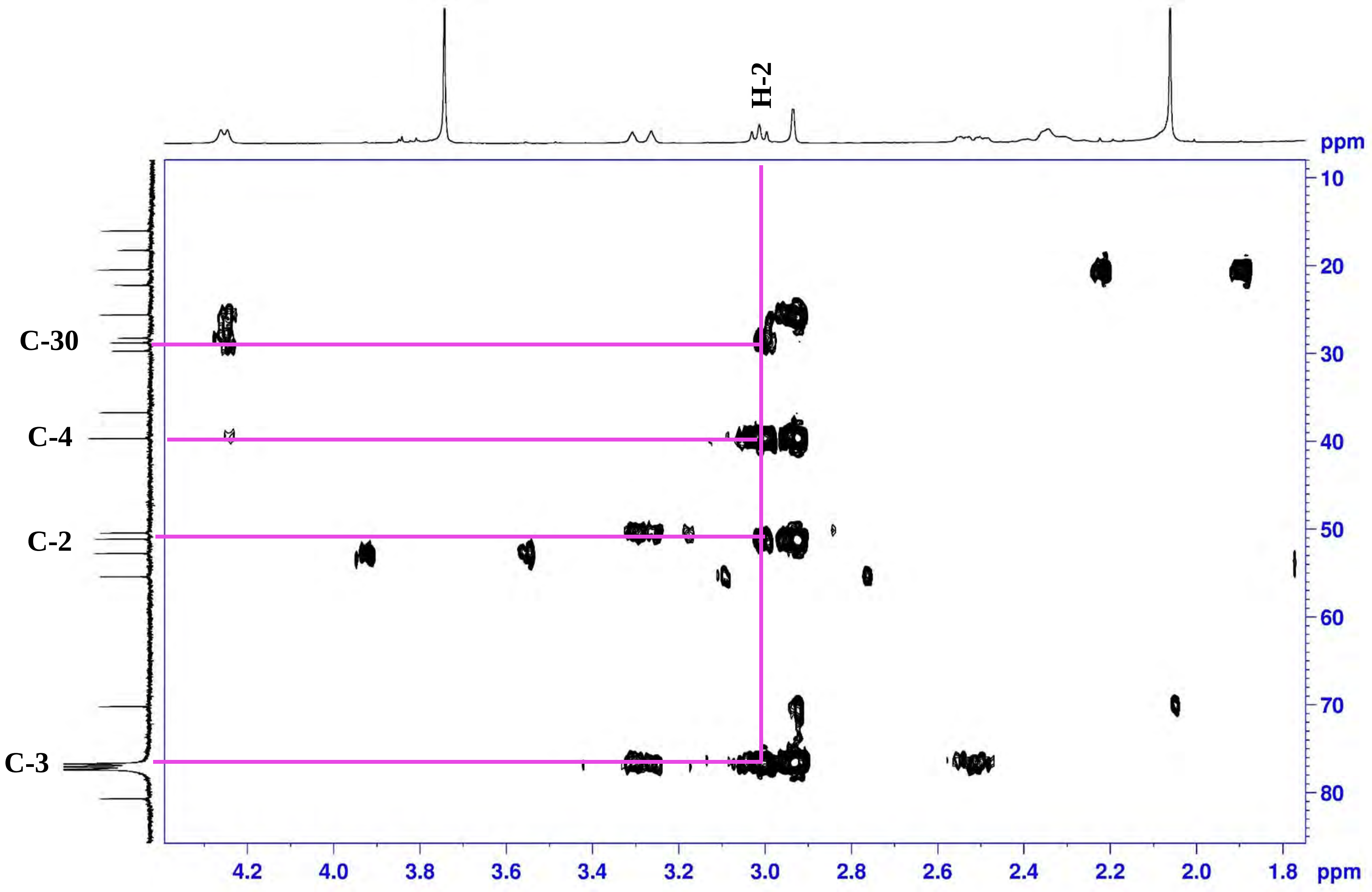

HMBC (400 MHz) spectrum of compound **9** in $CDCl_3$

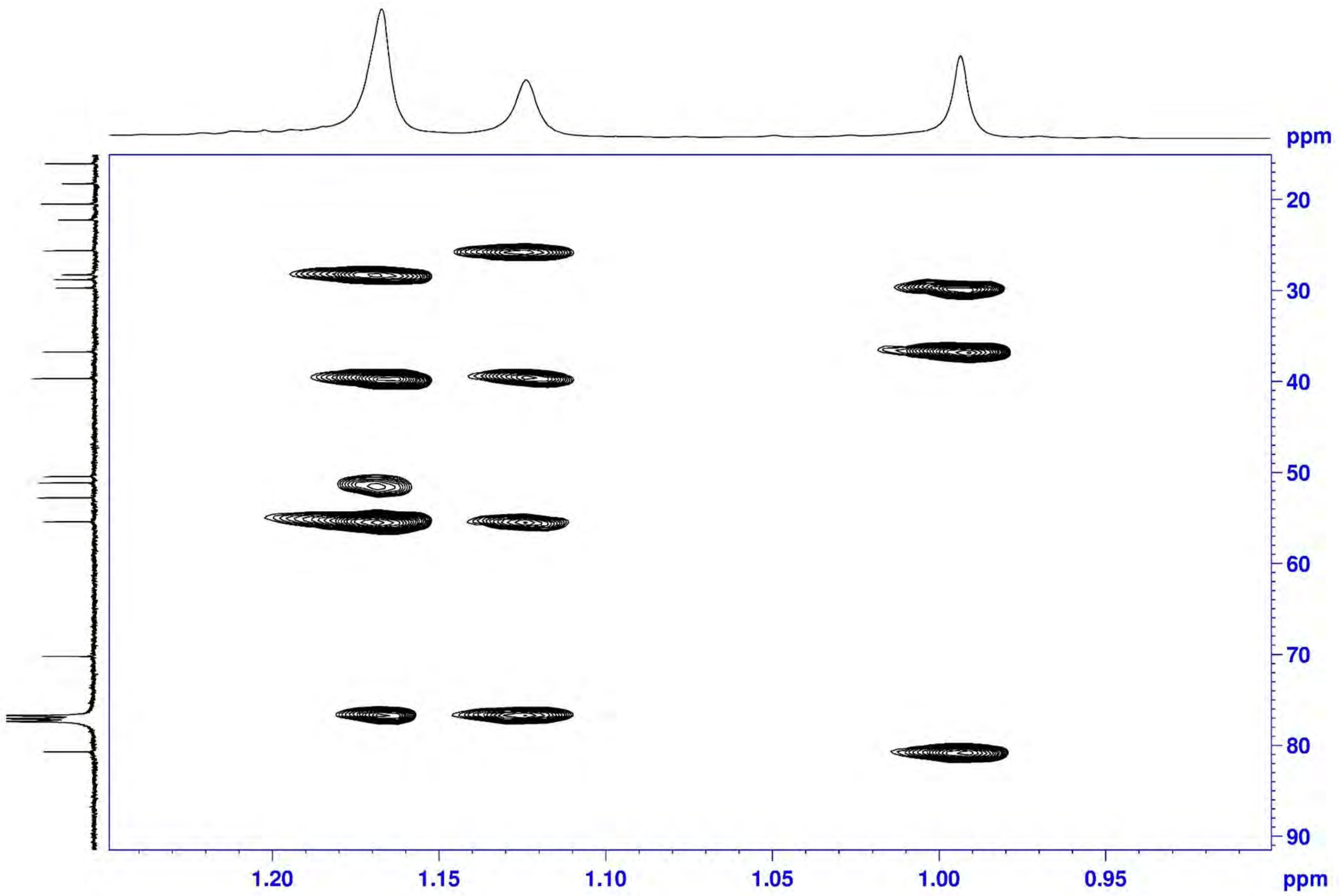

NOESY (400 MHz) spectrum of compound **9** in $CDCl_3$

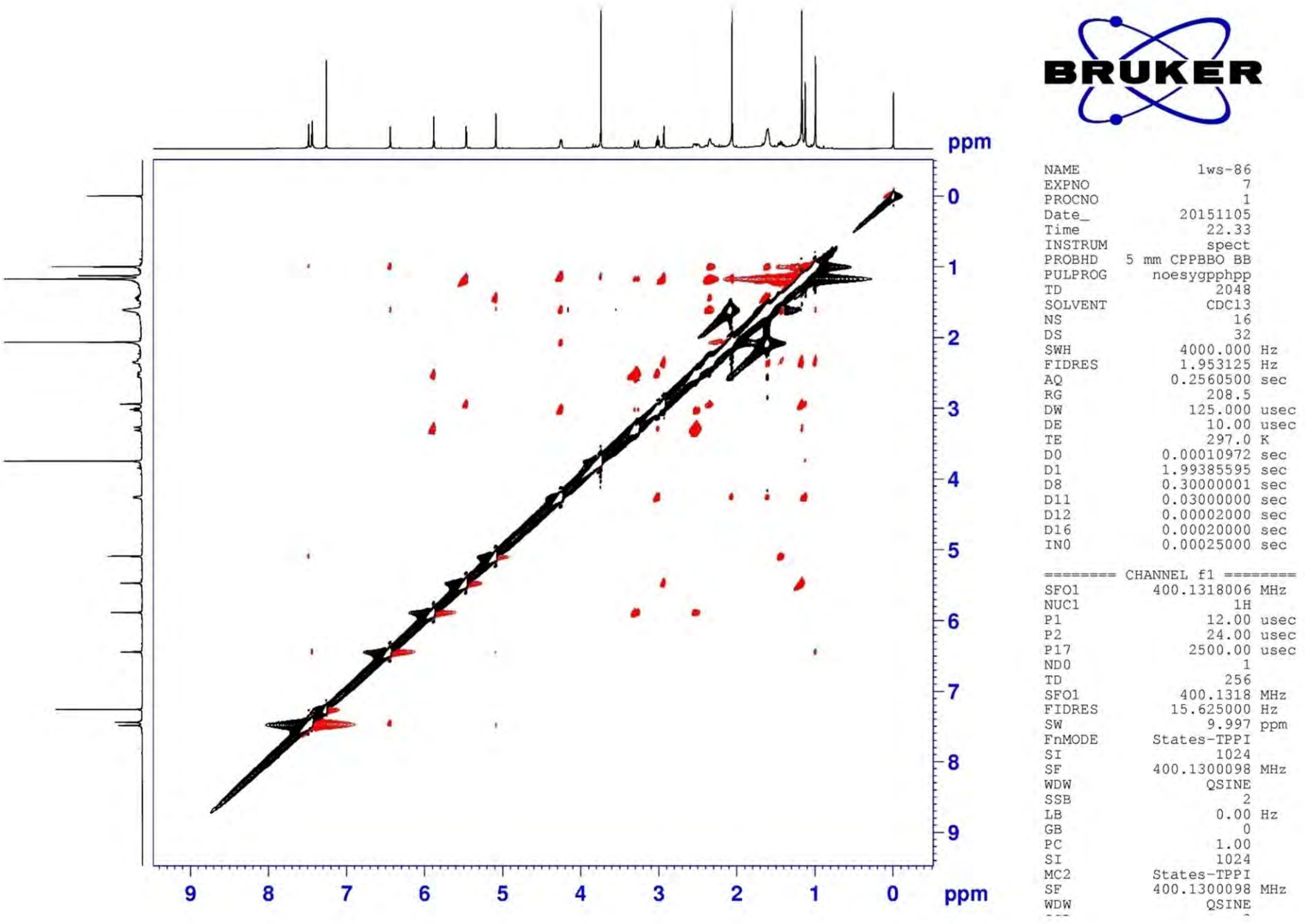

NOESY (400 MHz) spectrum of compound **9** in $CDCl_3$

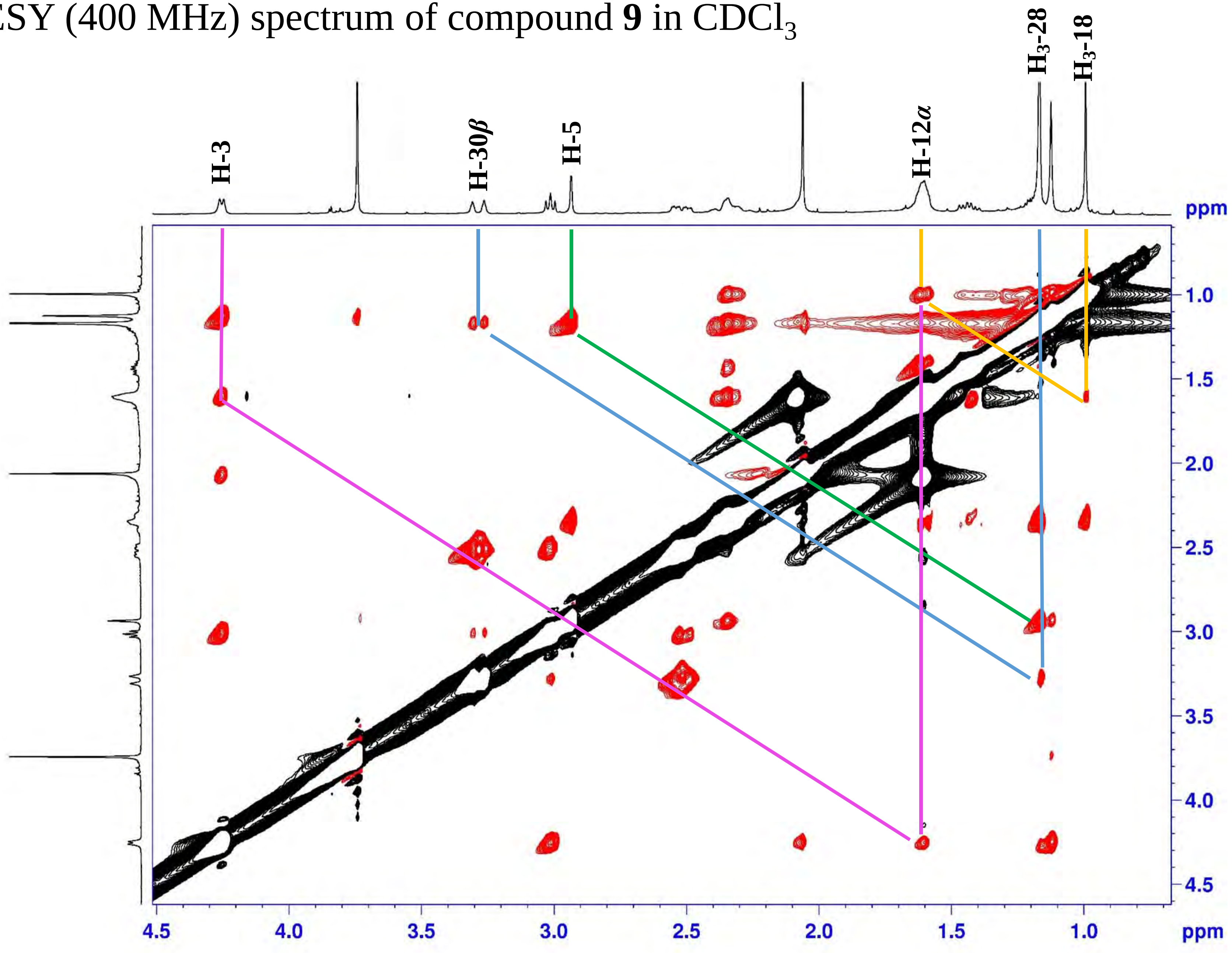

NOESY (400 MHz) spectrum of compound **9** in $CDCl_3$

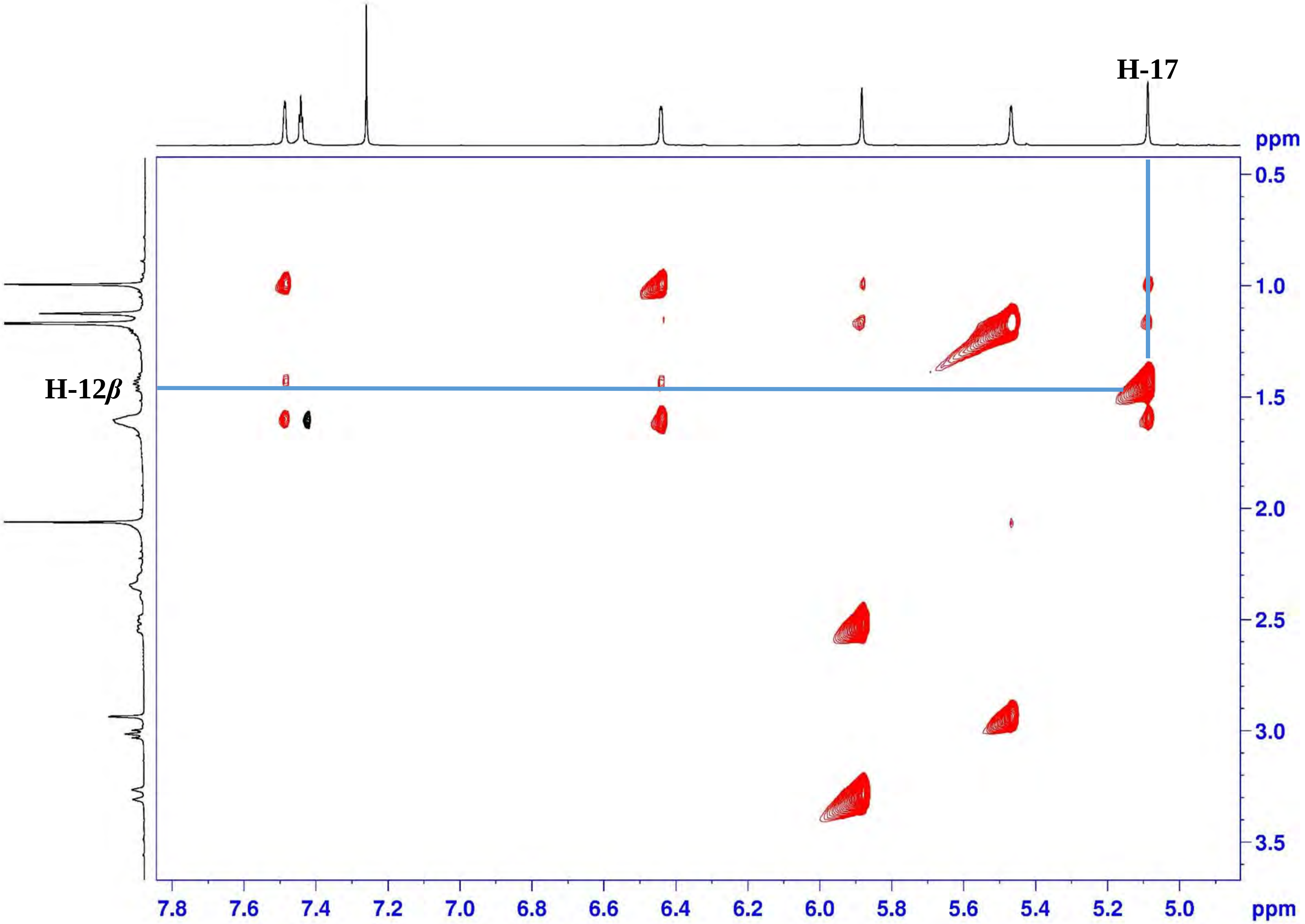

HRESIMS for compound **10**

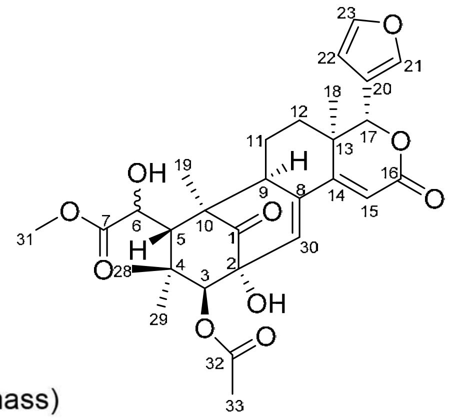

Elemental Composition Report

Single Mass Analysis

Tolerance = 5.0 mDa / DBE: min = -1.5, max = 50.0
Element prediction: Off
Number of isotope peaks used for i-FIT = 3

Monoisotopic Mass, Even Electron Ions
151 formula(e) evaluated with 1 results within limits (up to 50 best isotopic matches for each mass)
Elements Used:
C: 0-100 H: 0-200 O: 0-100
2j2
20161212-27 144 (1.167) Cm (143:146)

1: TOF MS ES+
2.90e+006

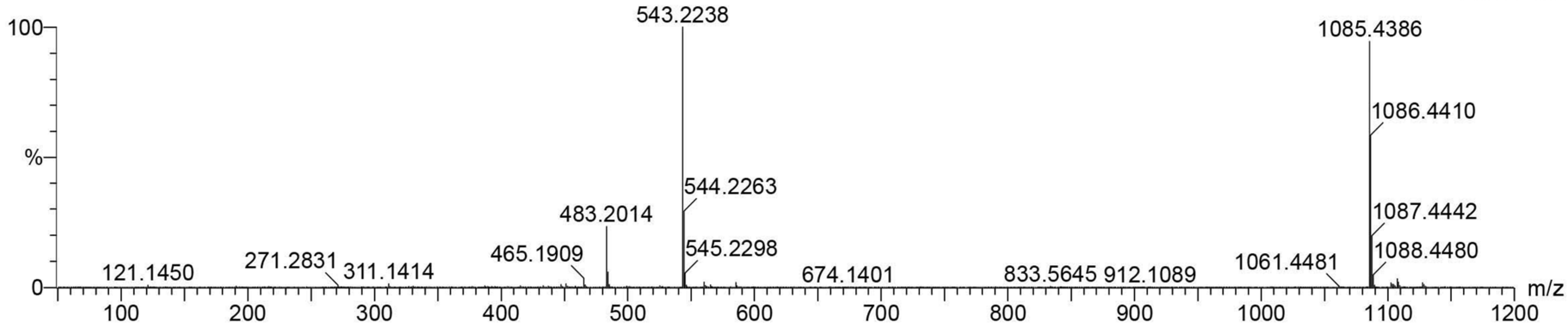

Minimum:				-1.5				
Maximum:		5.0	10.0	50.0				
Mass	Calc. Mass	mDa	PPM	DBE	i-FIT	Norm	Conf(%)	Formula
543.2238	543.2230	0.8	1.5	12.5	34.7	n/a	n/a	C29 H35 O10

^{1}H NMR (400 MHz) spectrum of compound **10** in $CDCl_3$

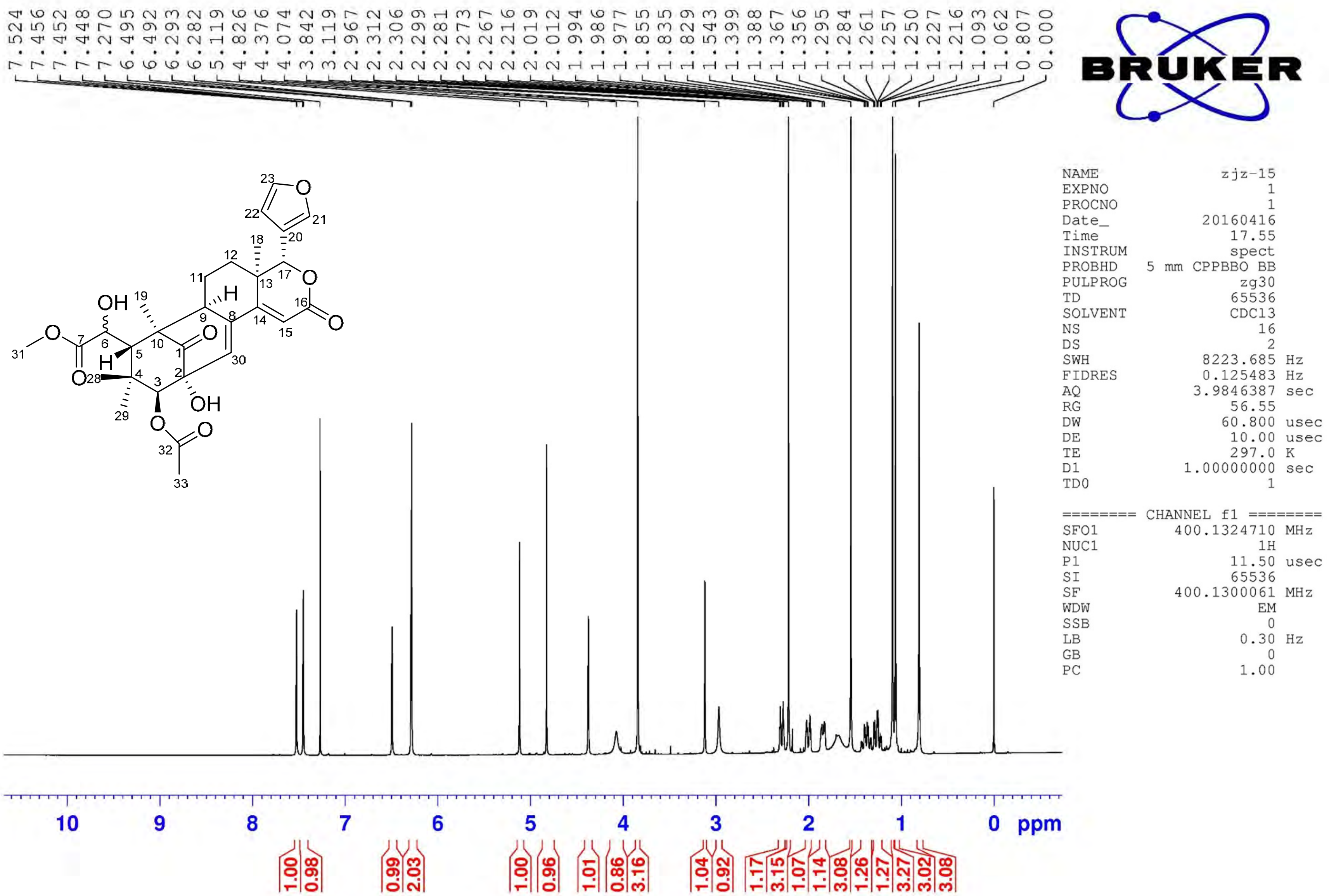

^{1}H NMR (400 MHz) spectrum of compound **10** in $CDCl_3$

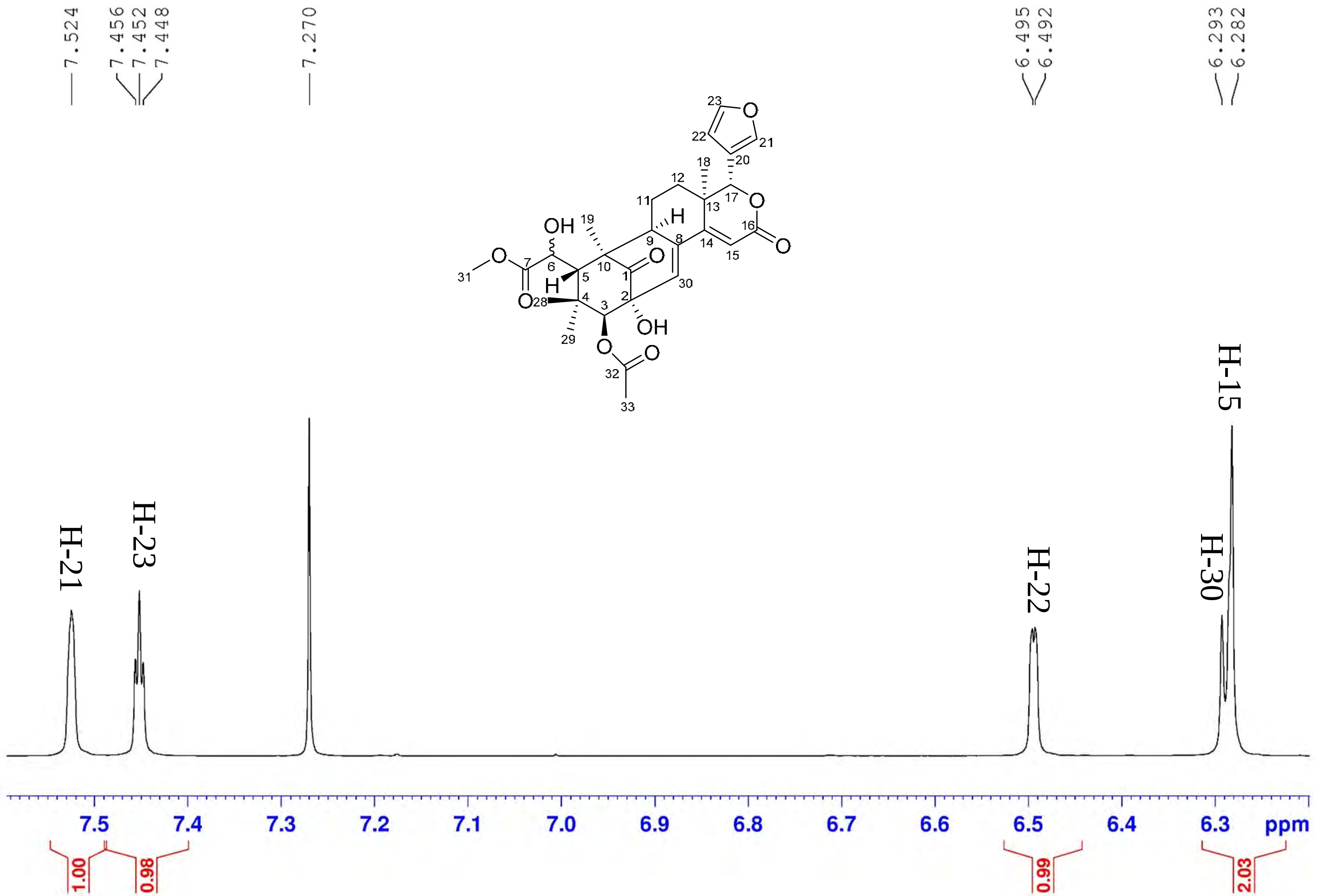

^{1}H NMR (400 MHz) spectrum of compound **10** in $CDCl_3$

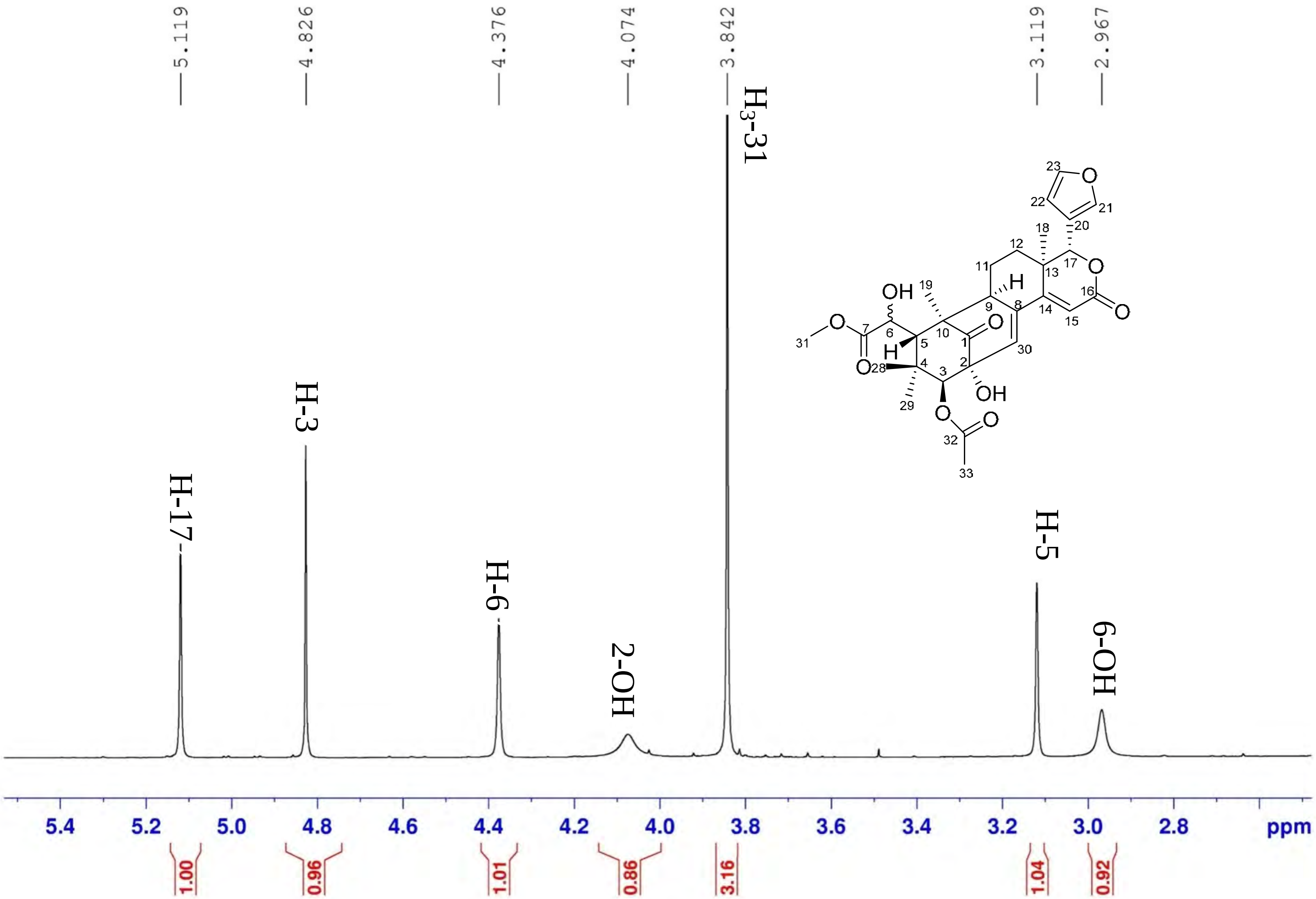

^{1}H NMR (400 MHz) spectrum of compound **10** in $CDCl_3$

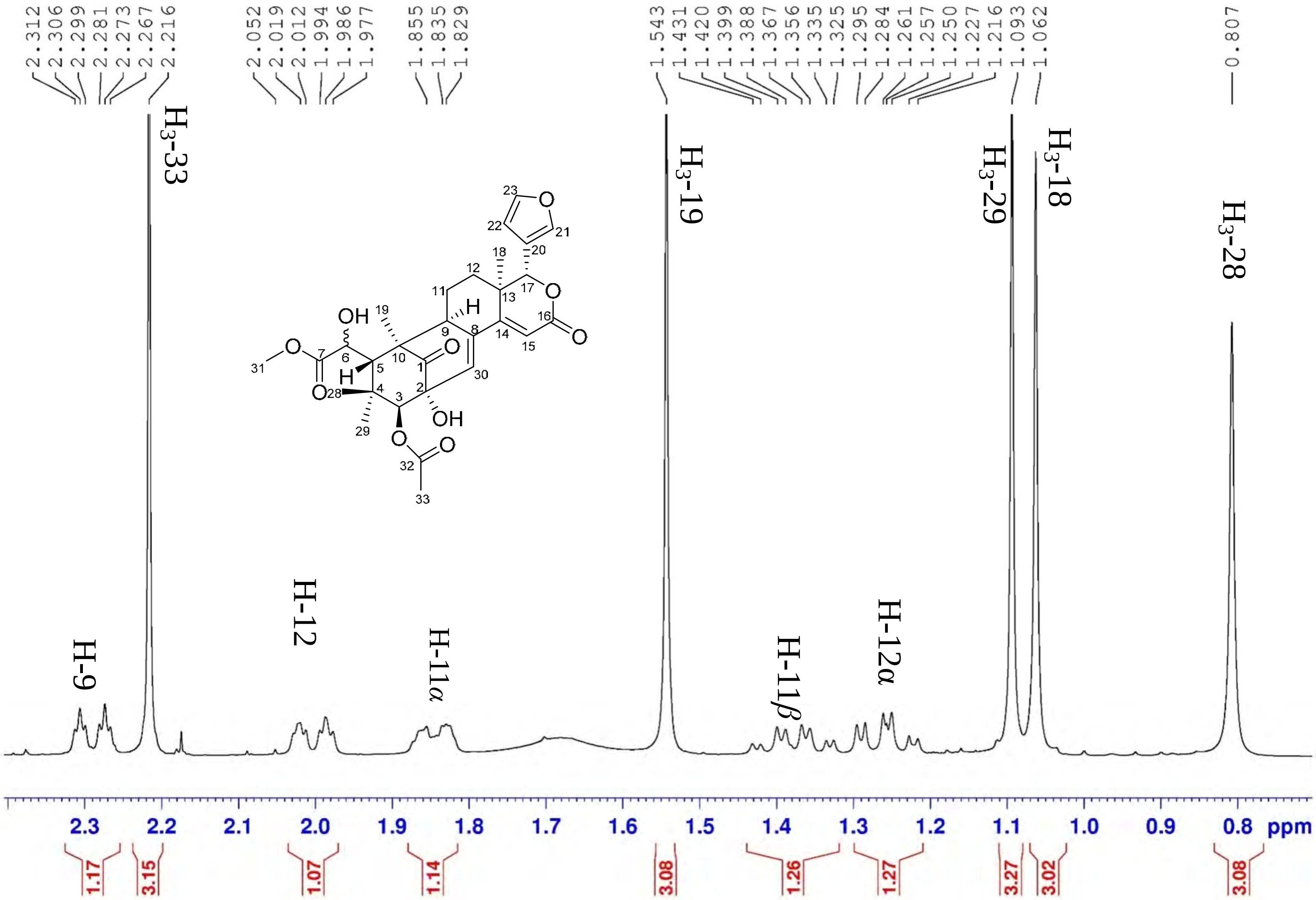

^{13}C NMR (100 MHz) spectrum of compound **10** in $CDCl_3$

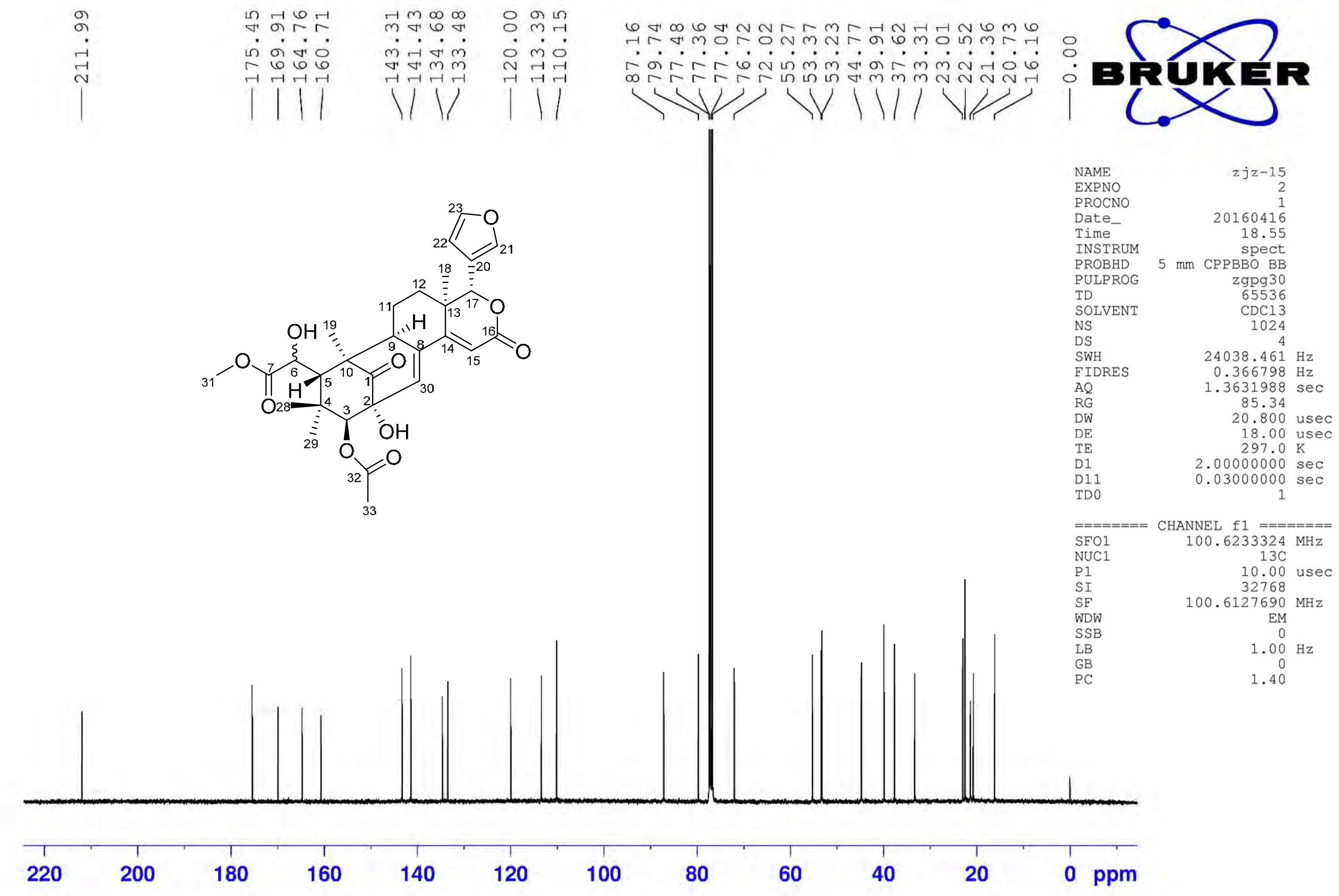

^{13}C NMR (100 MHz) spectrum of compound **10** in $CDCl_3$

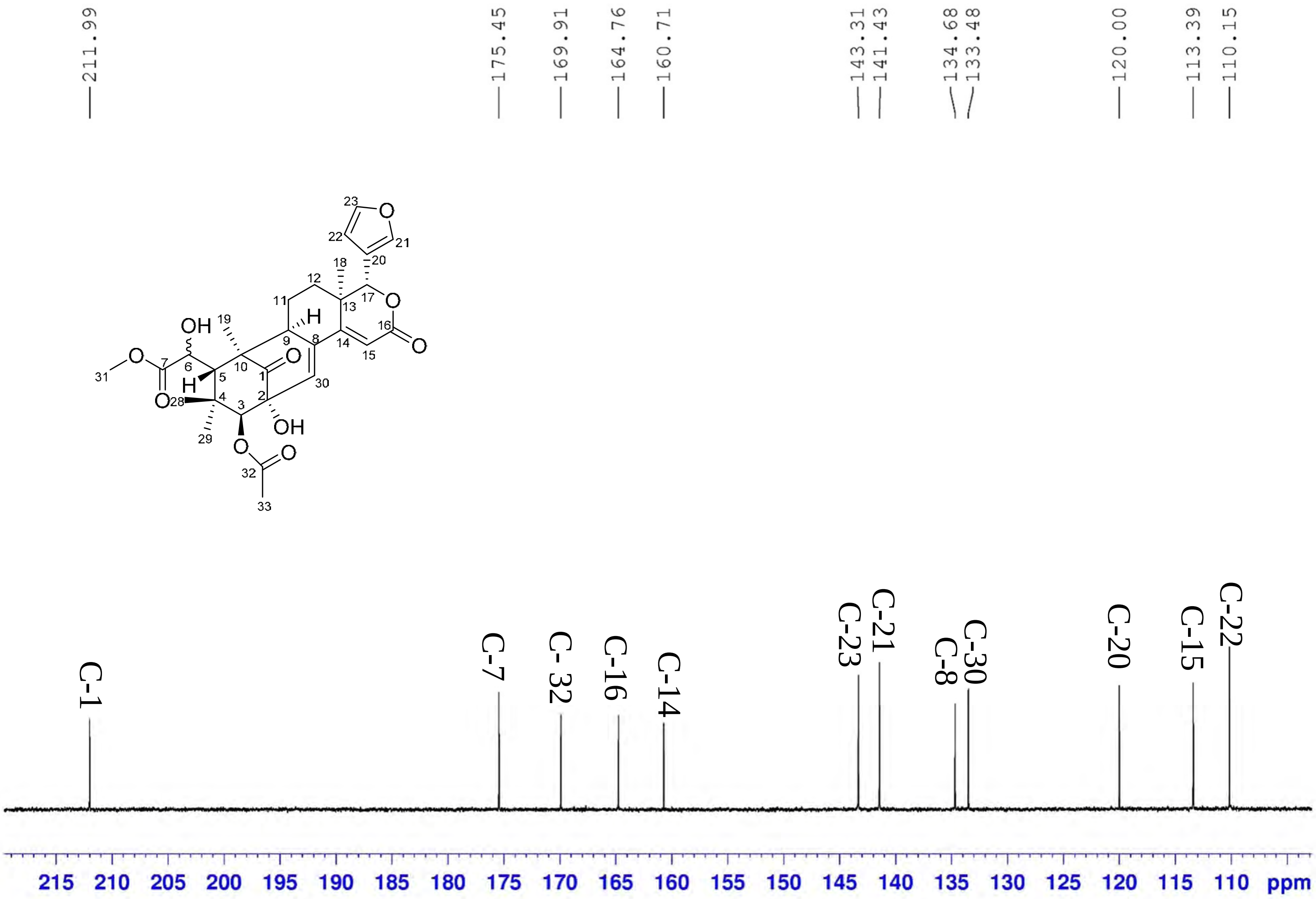

^{13}C NMR (100 MHz) spectrum of compound **10** in $CDCl_3$

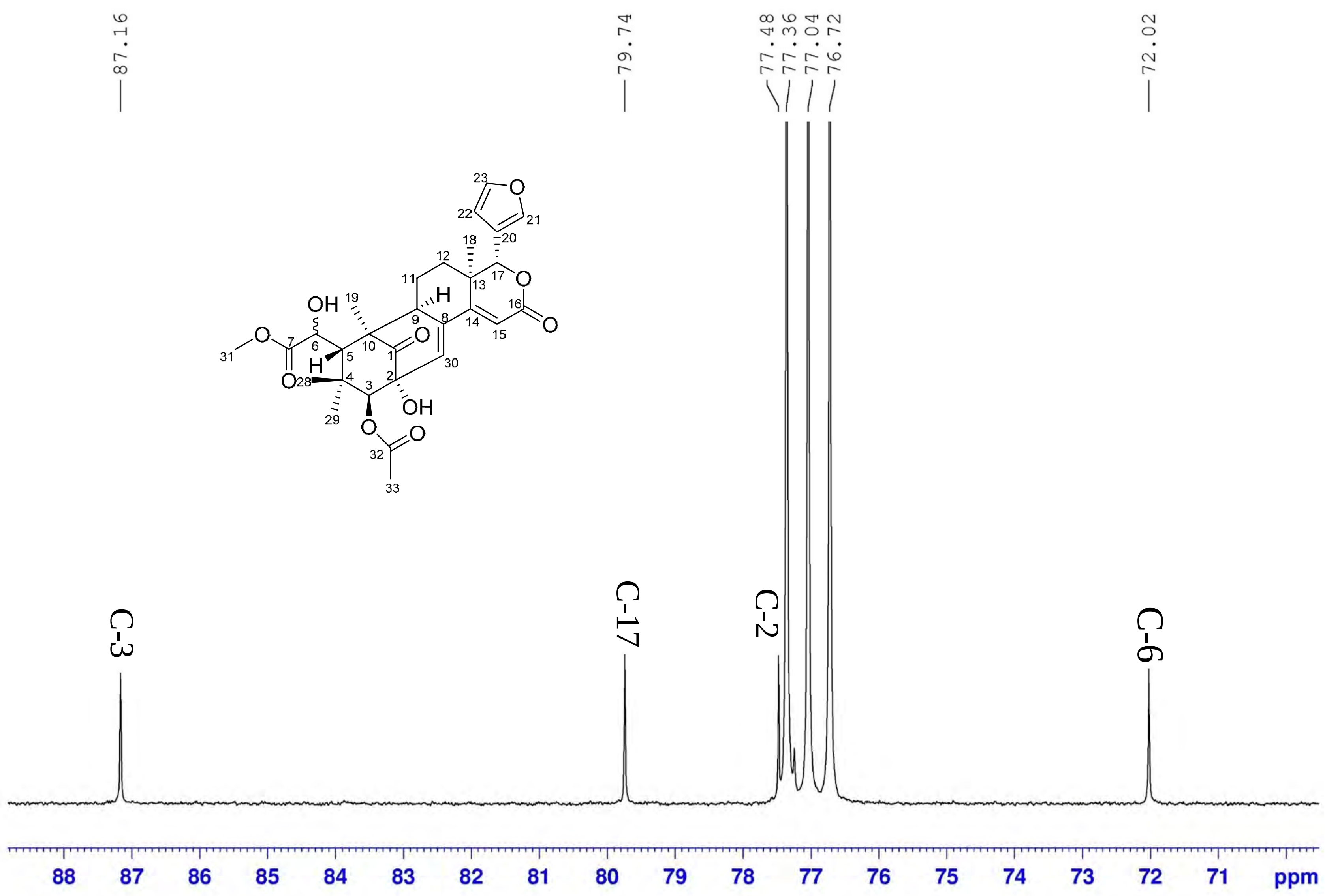

^{13}C NMR (100 MHz) spectrum of compound **10** in $CDCl_3$

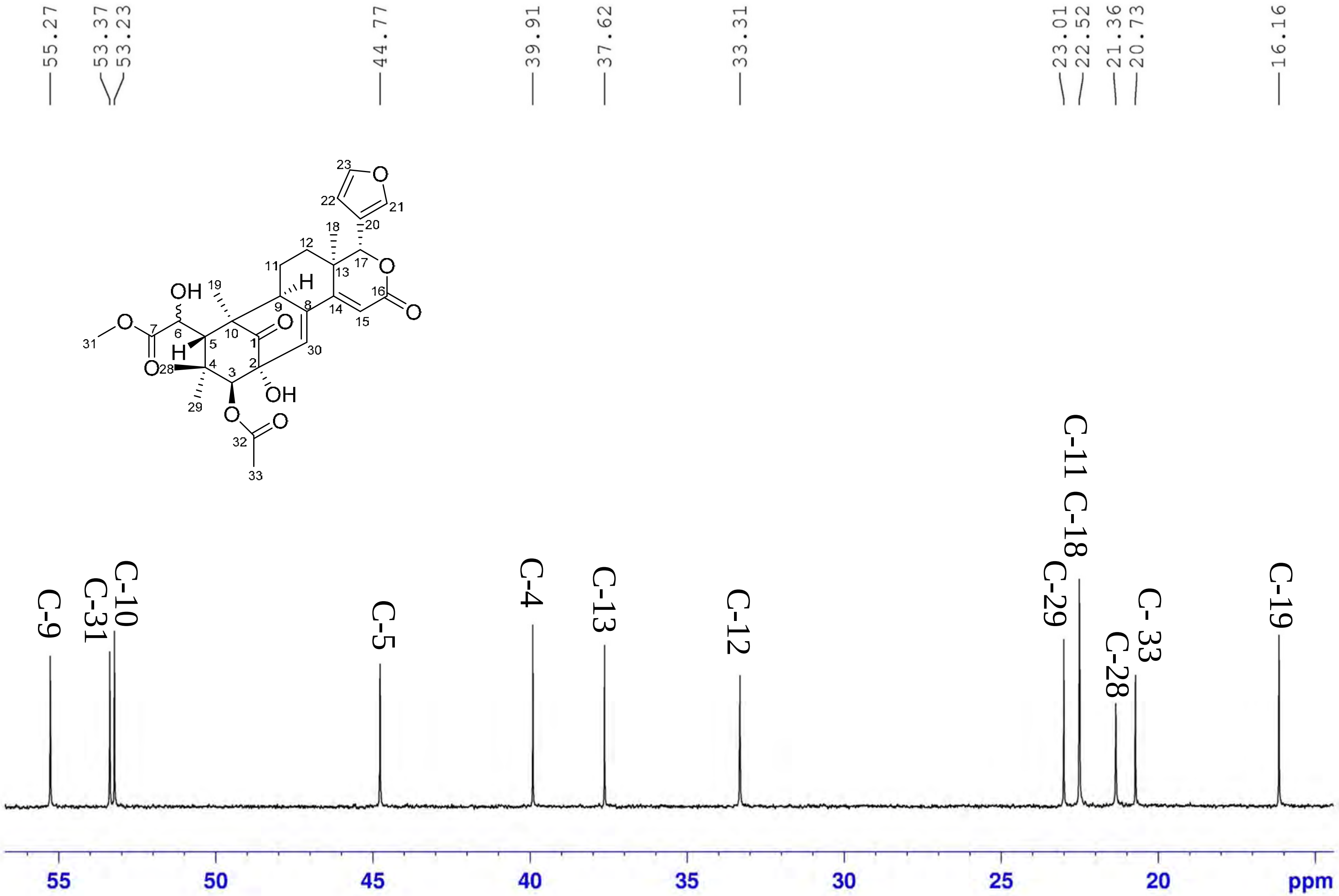

DEPT135 (100 MHz) spectrum of compound **10** in $CDCl_3$

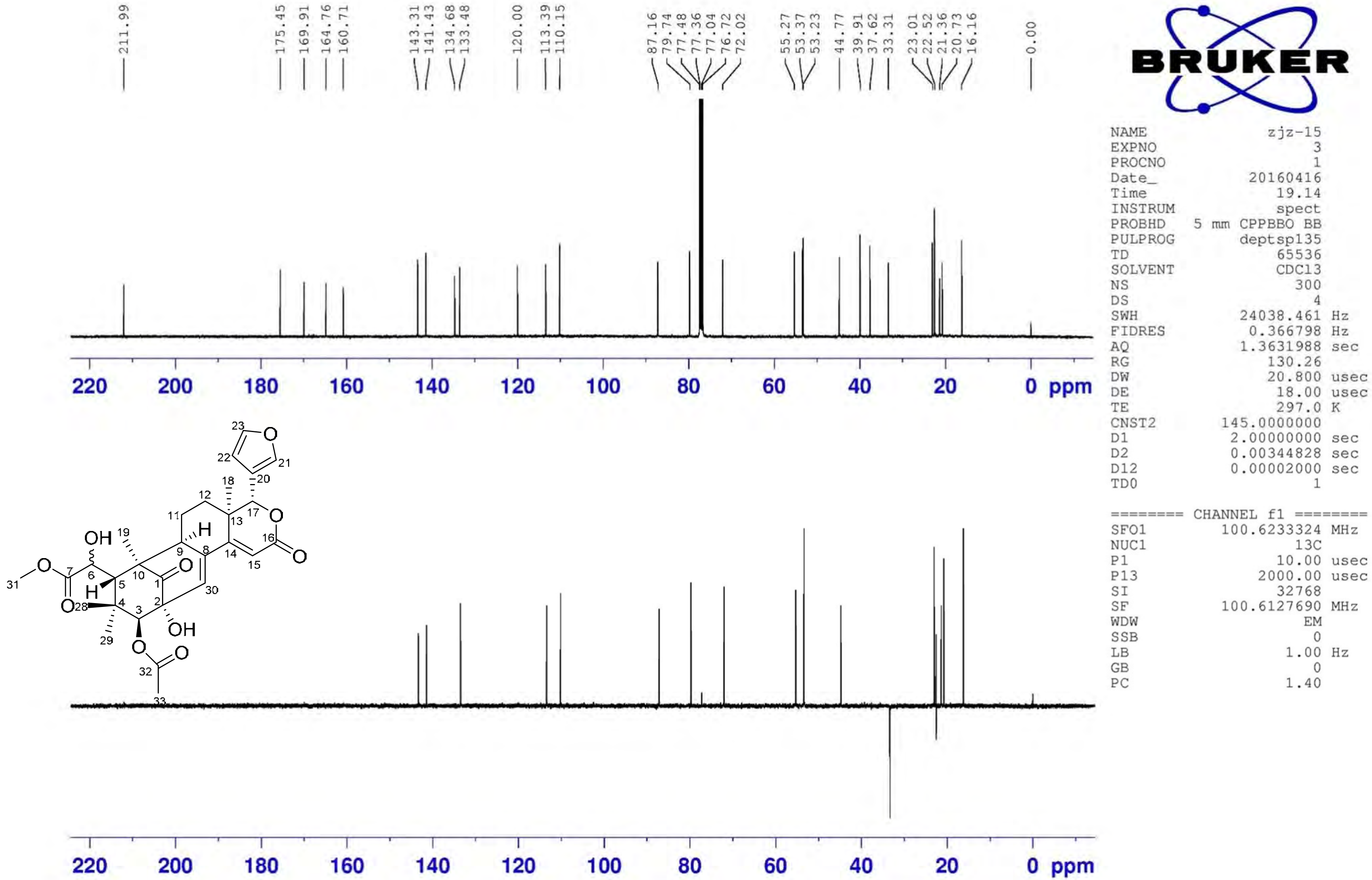

DEPT135 (100 MHz) spectrum of compound **10** in $CDCl_3$

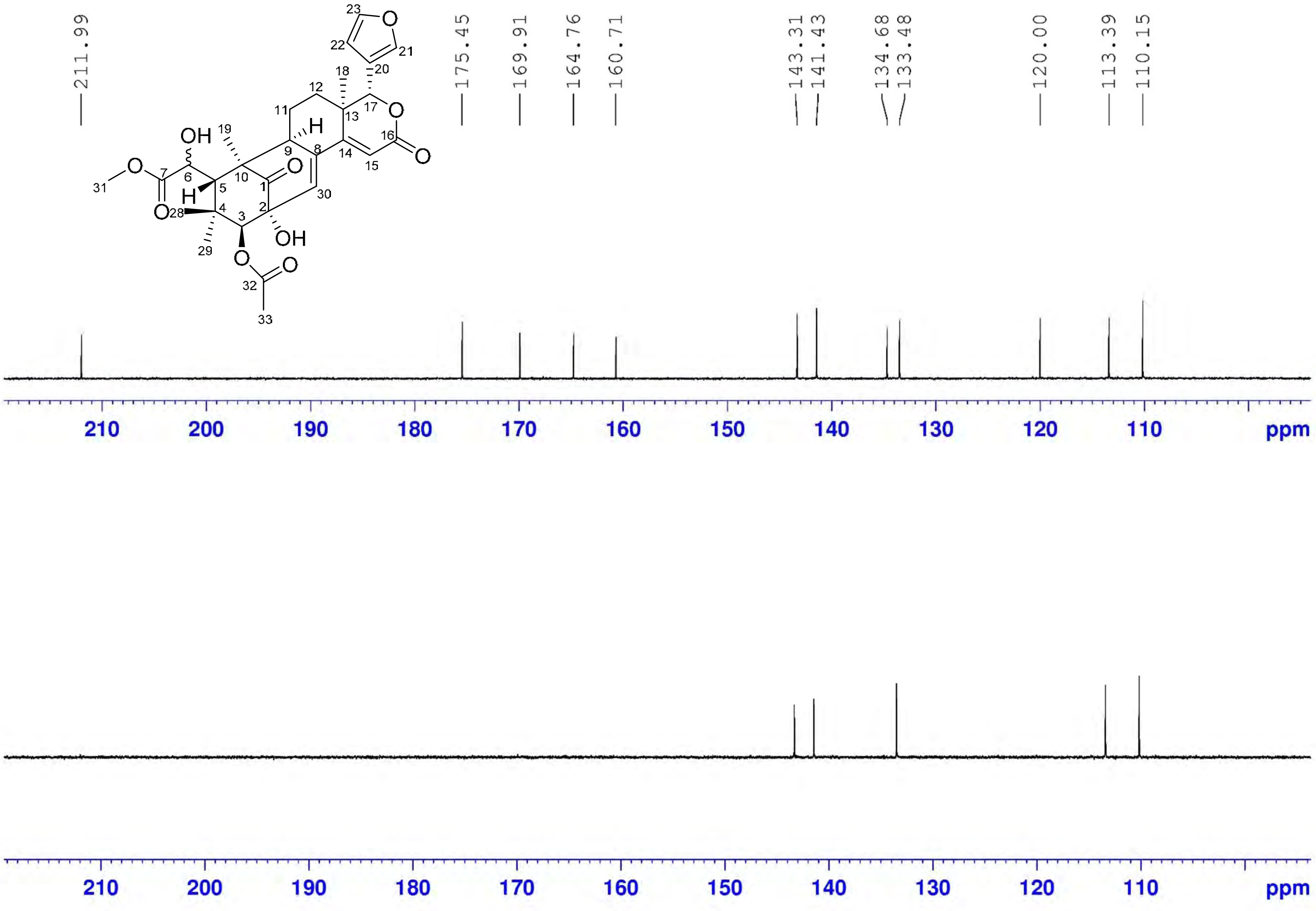

DEPT135 (100 MHz) spectrum of compound **10** in $CDCl_3$

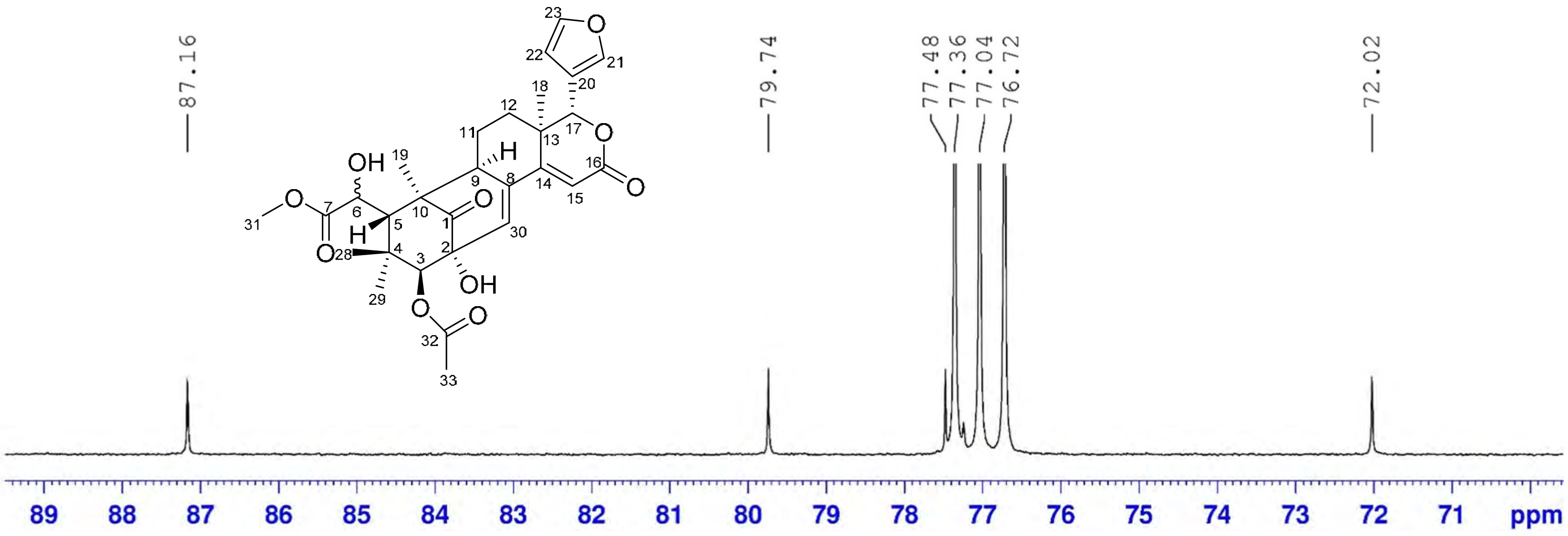

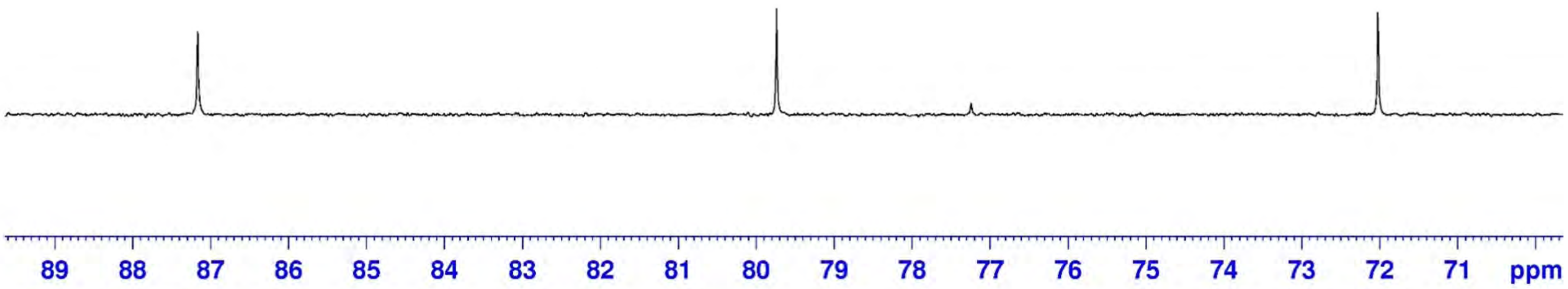

DEPT135 (100 MHz) spectrum of compound **10** in $CDCl_3$

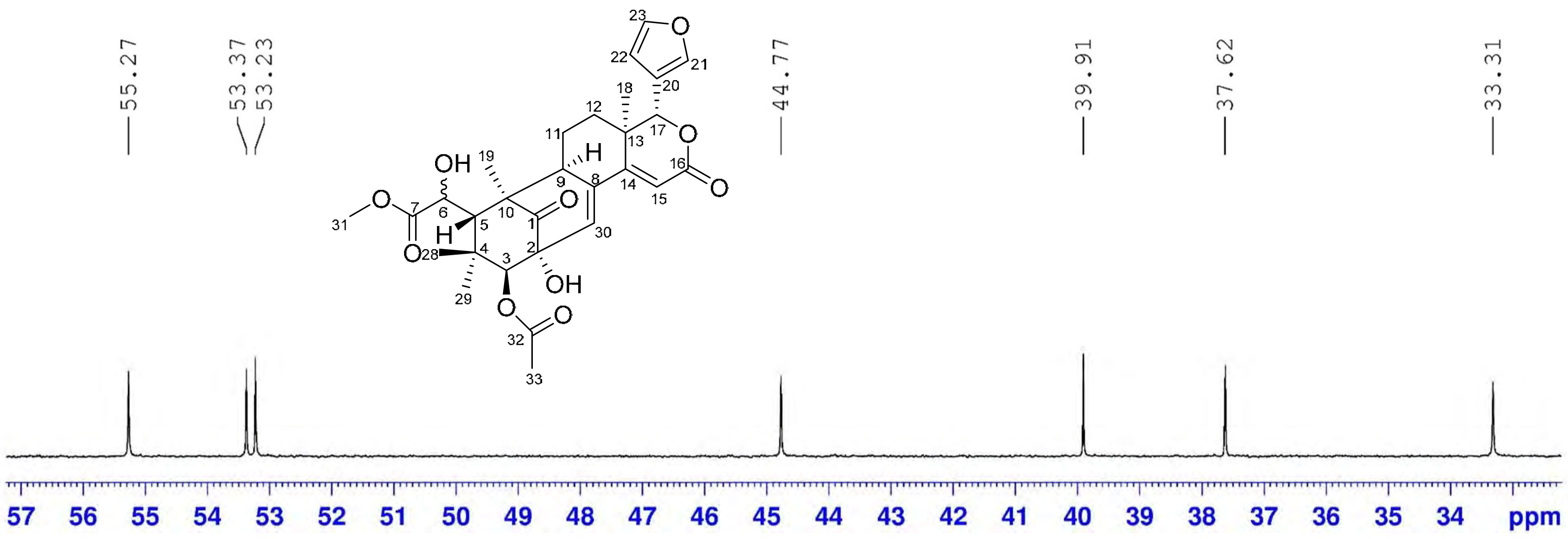

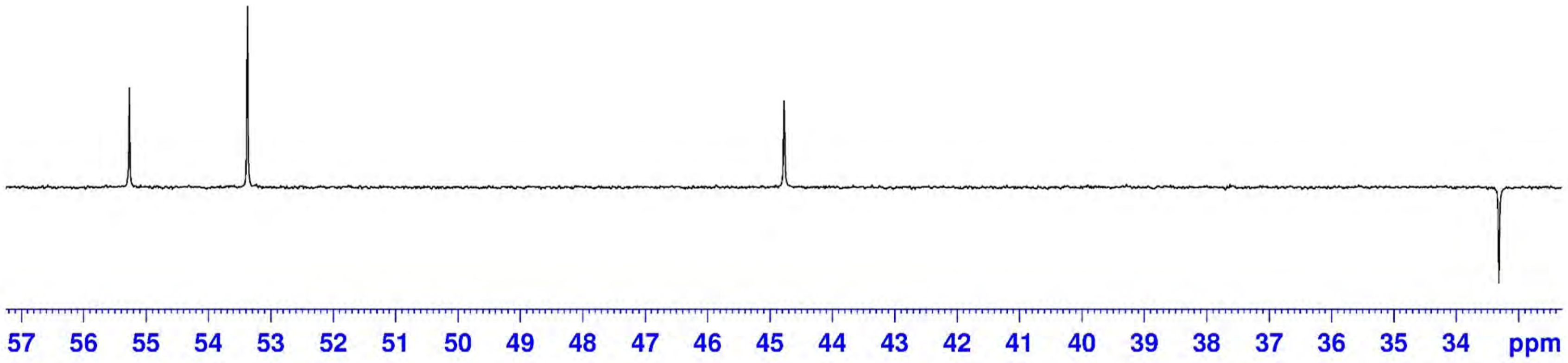

DEPT135 (100 MHz) spectrum of compound **10** in $CDCl_3$

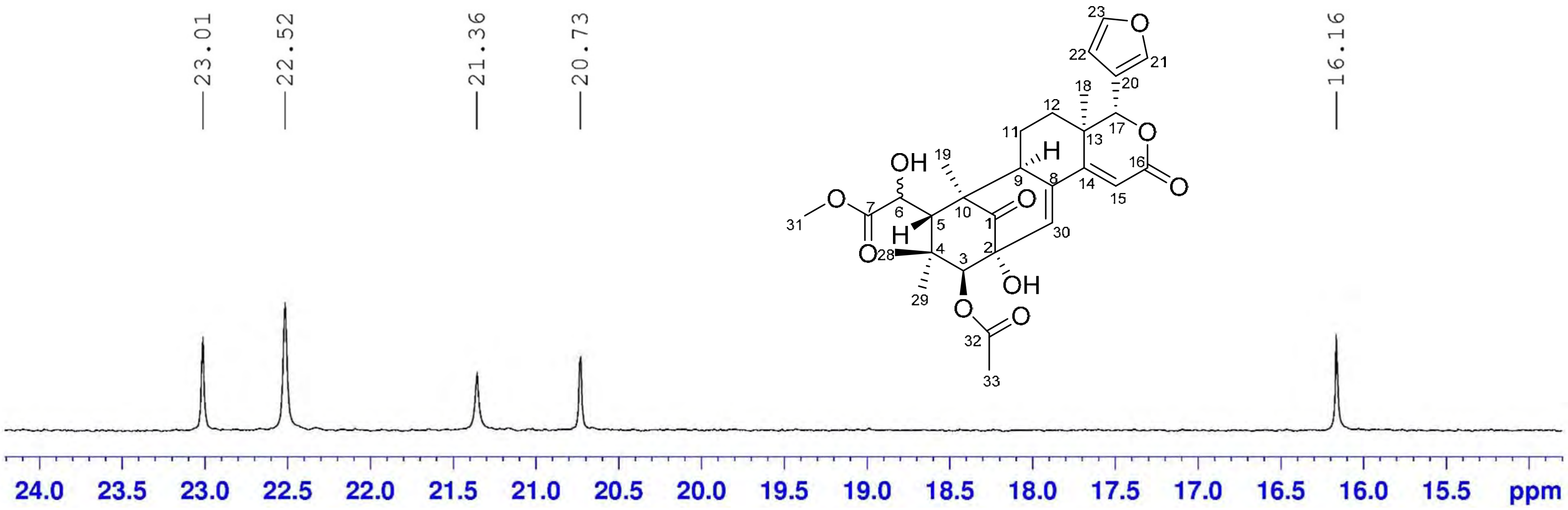

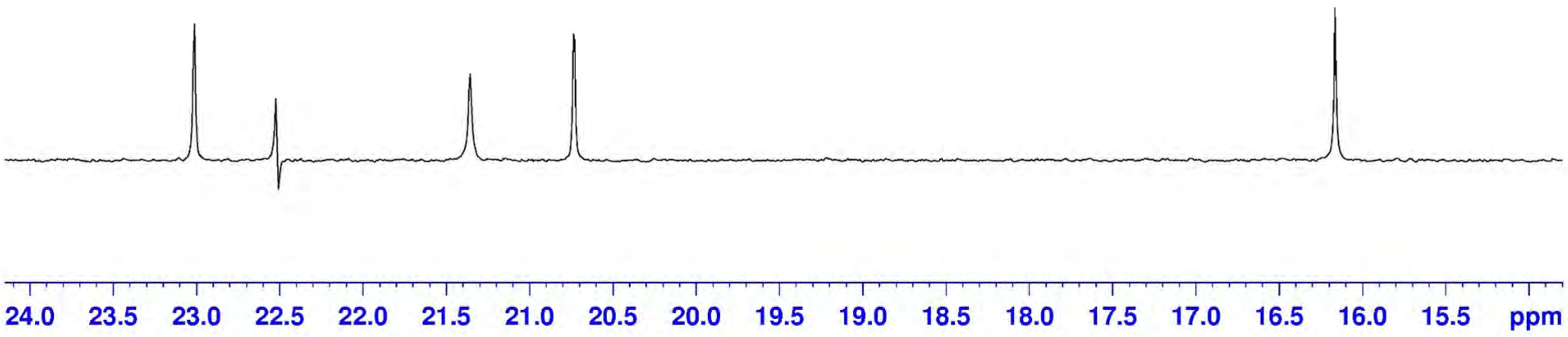

^1H-^1H COSY (400 MHz) spectrum of compound **10** in $CDCl_3$

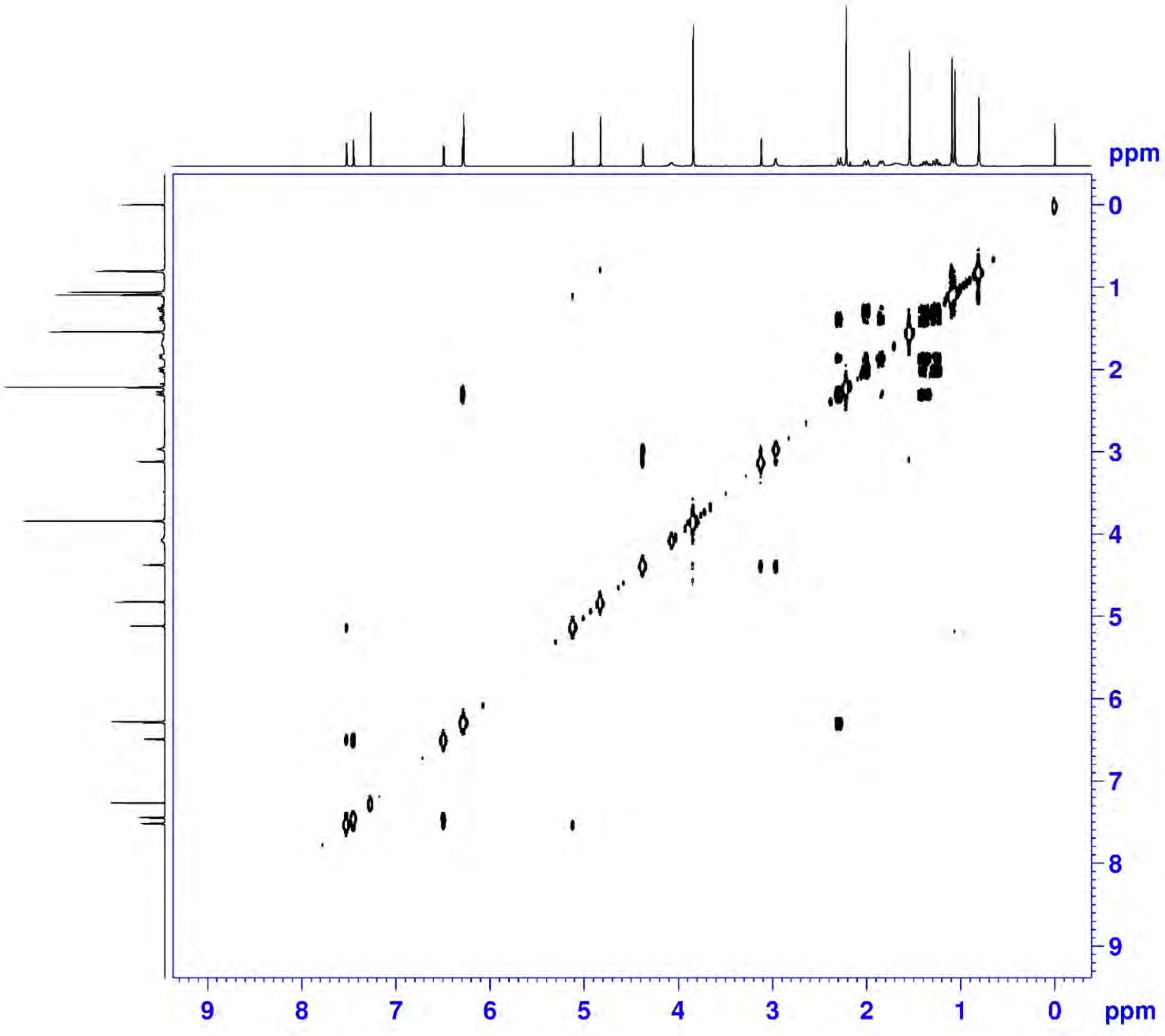

NAME	zjz-15	
EXPNO	4	
PROCNO	1	
Date_	20160517	
Time	9.25	
INSTRUM	spect	
PROBHD	5 mm CPPBBO BB	
PULPROG	cosygpppqf	
TD	2048	
SOLVENT	CDCl3	
NS	8	
DS	8	
SWH	3906.250	Hz
FIDRES	1.907349	Hz
AQ	0.2621940	sec
RG	208.5	
DW	128.000	usec
DE	10.00	usec
TE	297.0	K
D0	0.00000300	sec
D1	1.89678097	sec
D11	0.03000000	sec
D12	0.00002000	sec
D13	0.00000400	sec
D16	0.00020000	sec
IN0	0.00025600	sec
======== CHANNEL f1 ========		
SFO1	400.1318006	MHz
NUC1	1H	
P0	11.50	usec
P1	11.50	usec
P17	2500.00	usec
ND0	1	
TD	128	
SFO1	400.1318	MHz
FIDRES	30.517578	Hz
SW	9.762	ppm
FnMODE	QF	
SI	1024	
SF	400.1300061	MHz
WDW	QSINE	
SSB	0	
LB	0.00	Hz
GB	0	
PC	1.40	
SI	1024	
MC2	QF	
SF	400.1300005	MHz
WDW	QSINE	
SSB	0	
LB	0.00	Hz
GB	0	

^{1}H-^{1}H COSY (400 MHz) spectrum of compound **10** in $CDCl_3$

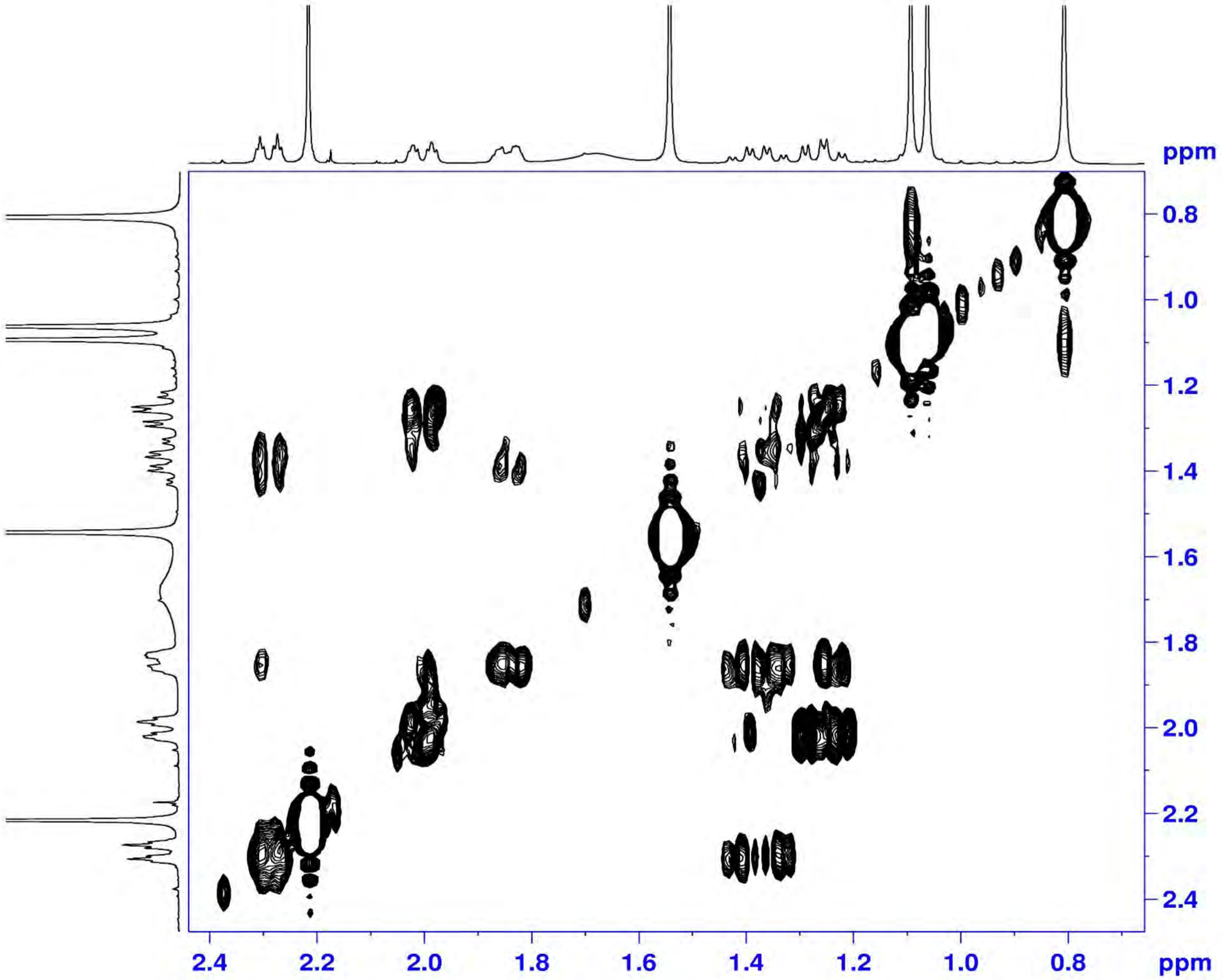

^1H-^1H COSY (400 MHz) spectrum of compound **10** in $CDCl_3$

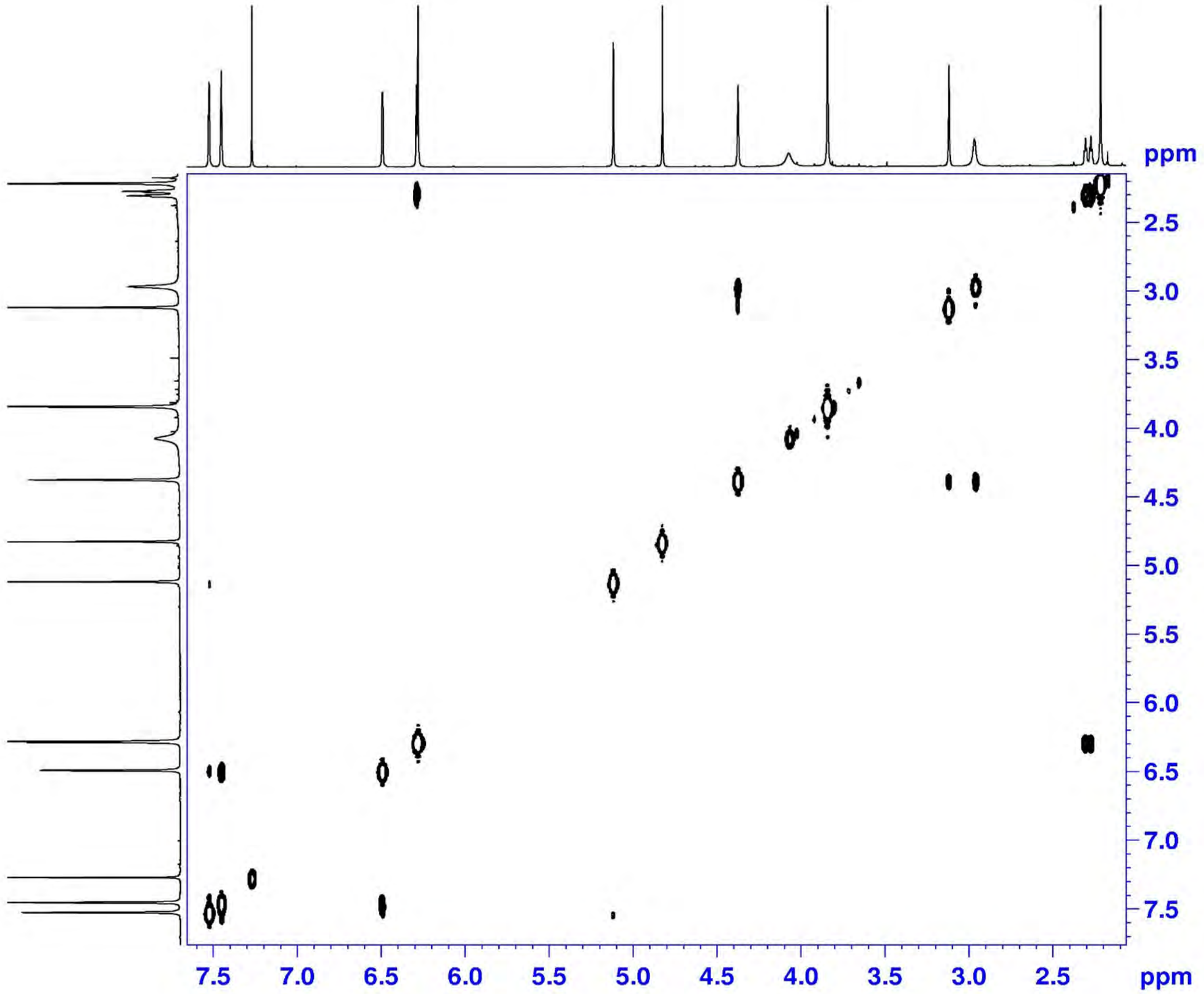

HSQC (400 MHz) spectrum of compound **10** in $CDCl_3$

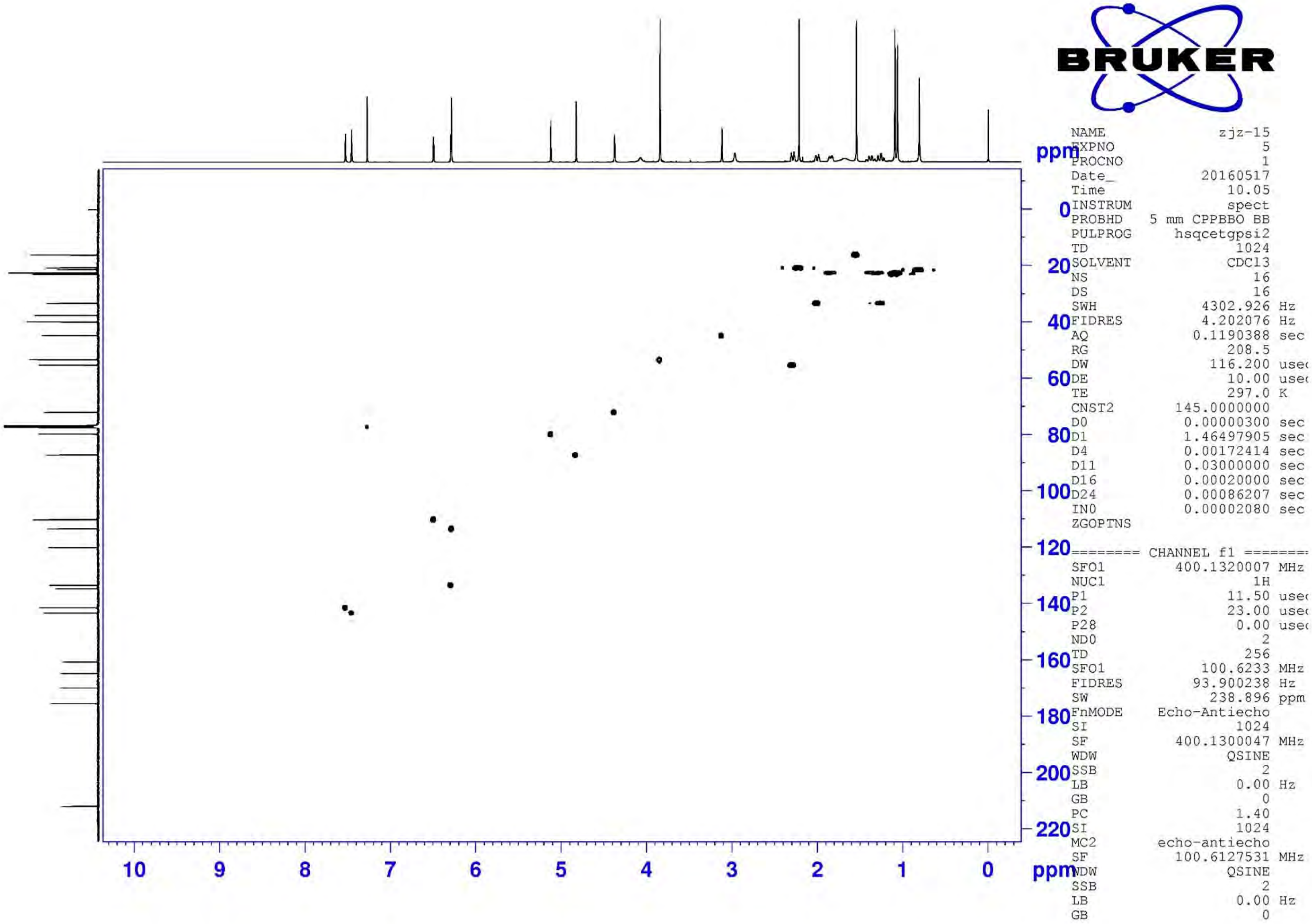

HSQC (400 MHz) spectrum of compound **10** in $CDCl_3$

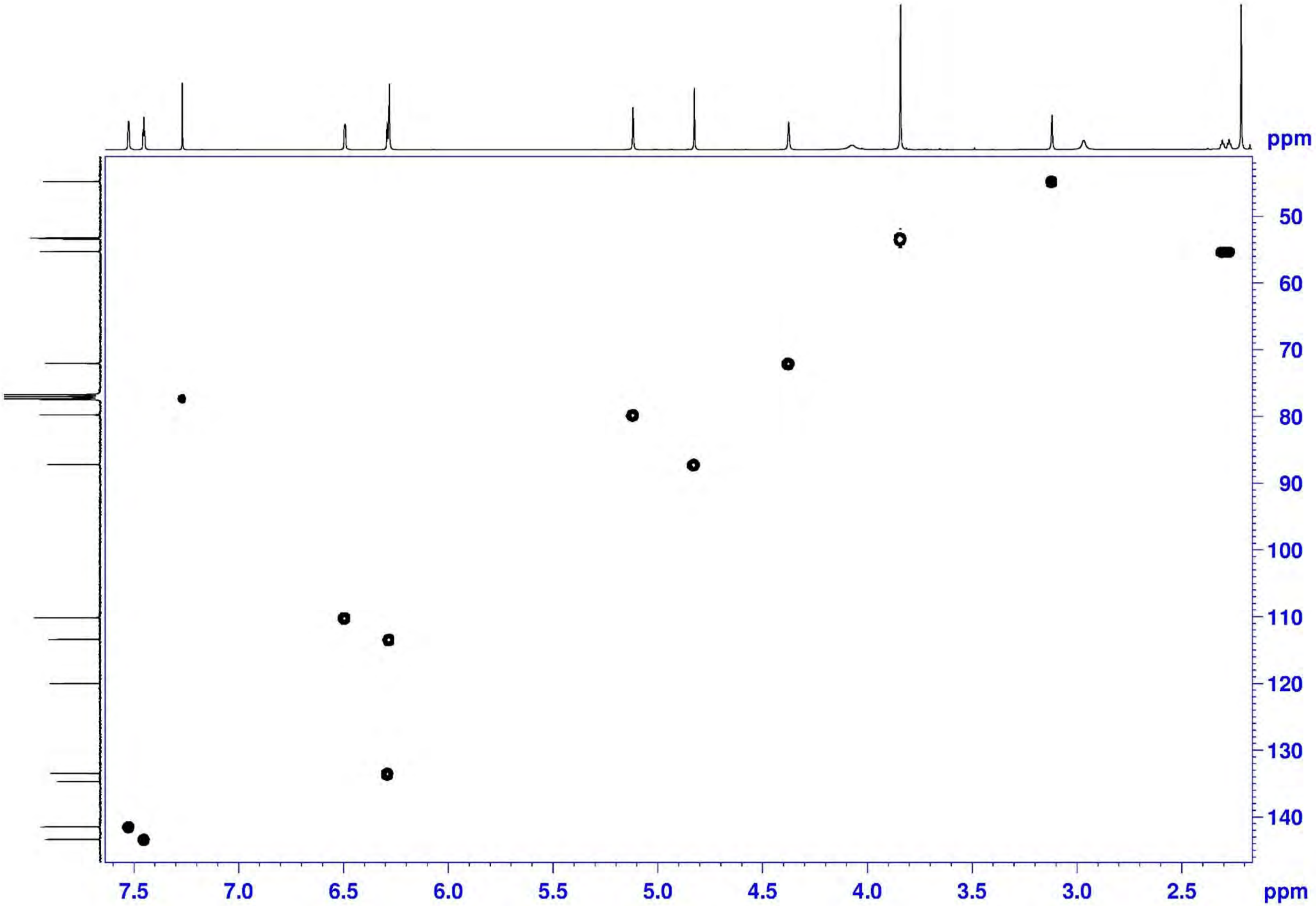

HSQC (400 MHz) spectrum of compound **10** in $CDCl_3$

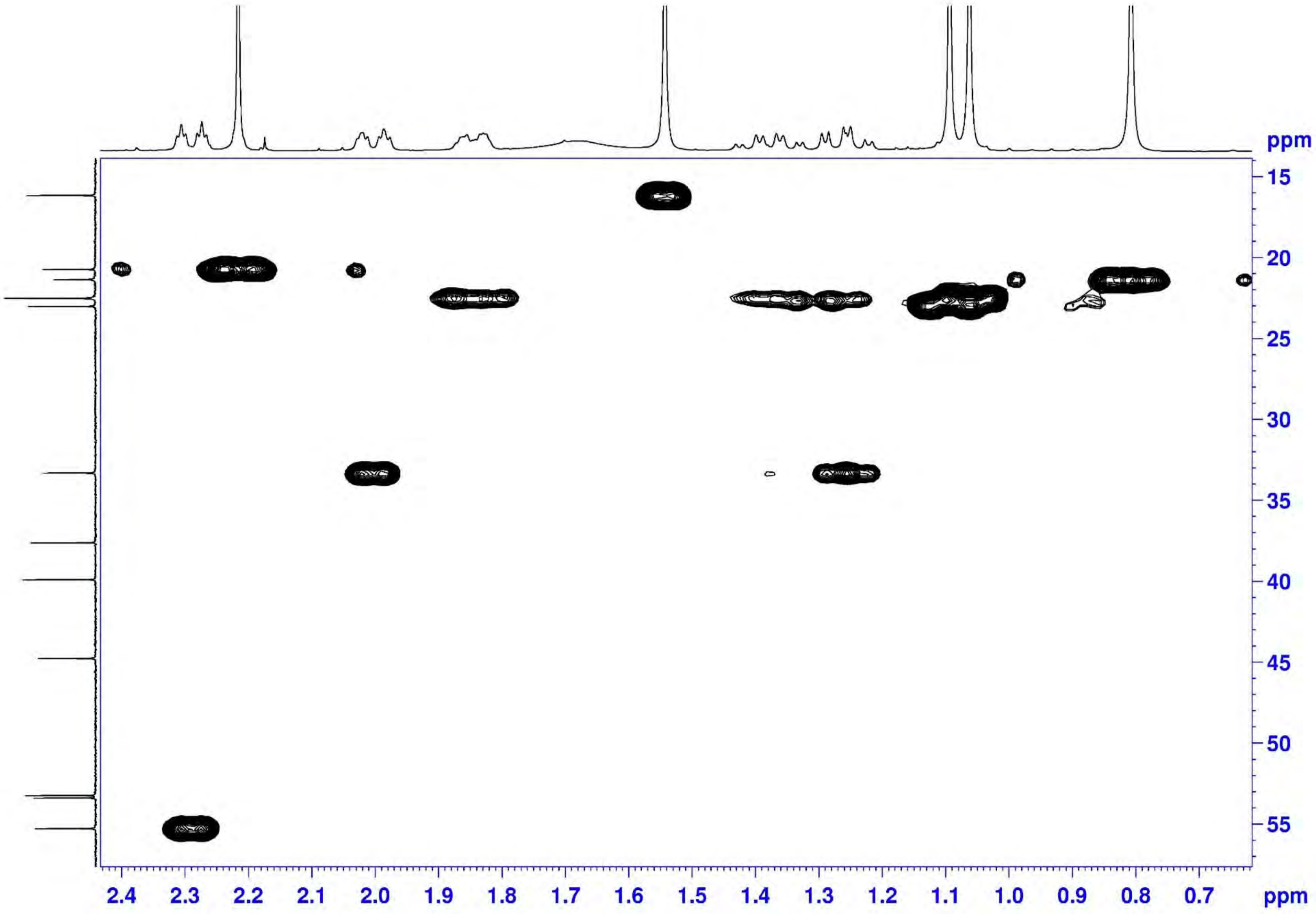

HMBC (400 MHz) spectrum of compound **10** in $CDCl_3$

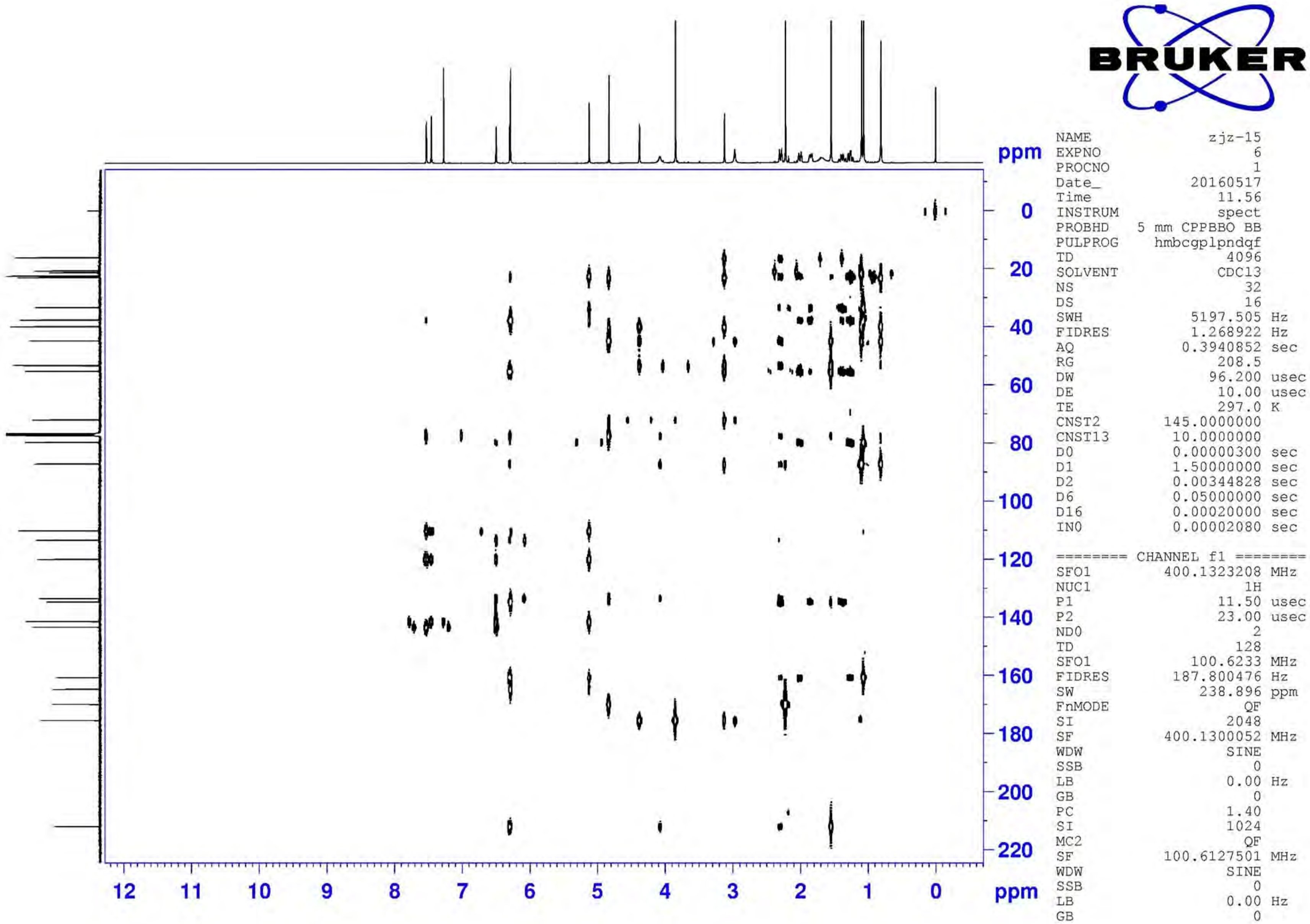

HMBC (400 MHz) spectrum of compound **10** in $CDCl_3$

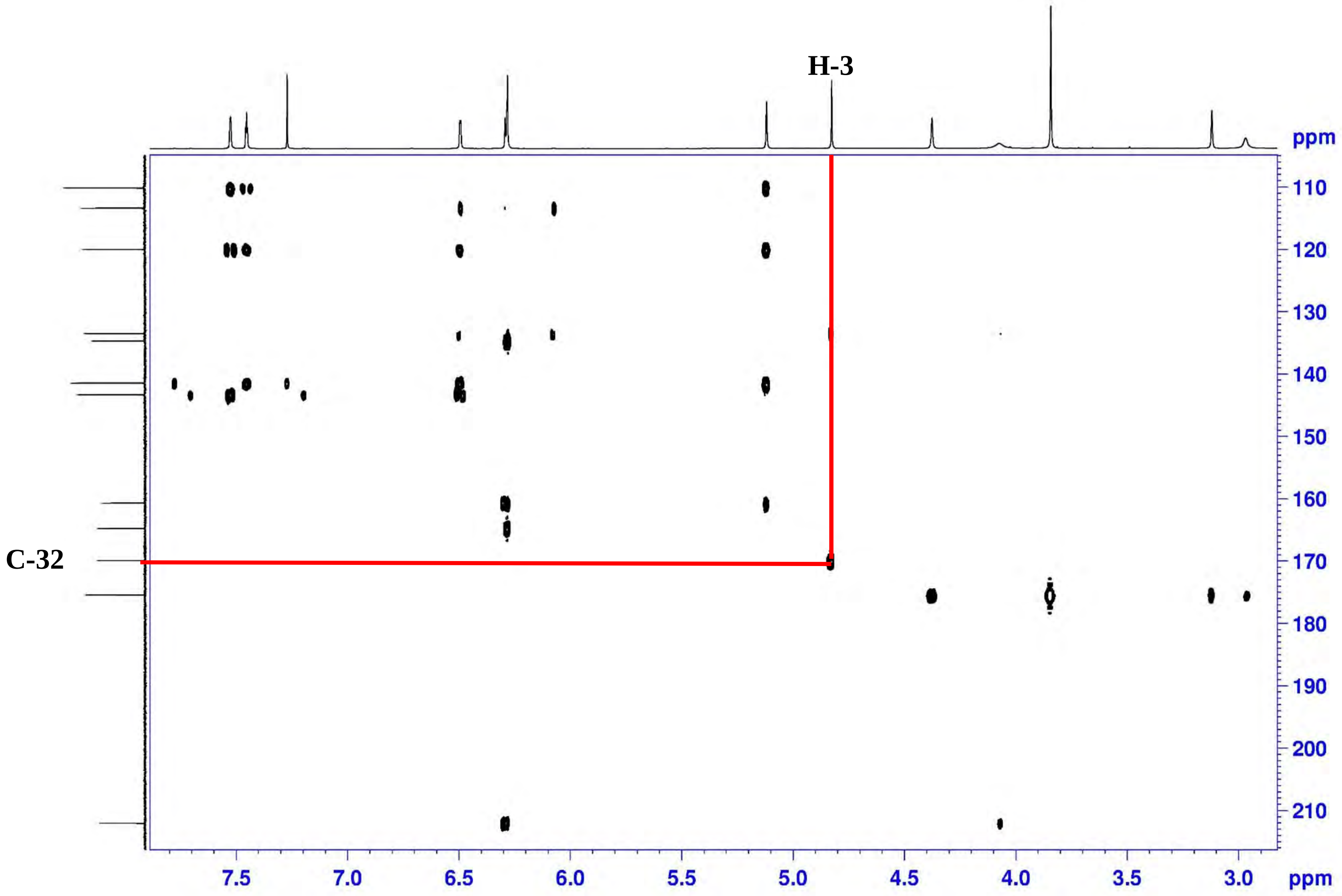

HMBC (400 MHz) spectrum of compound **10** in $CDCl_3$

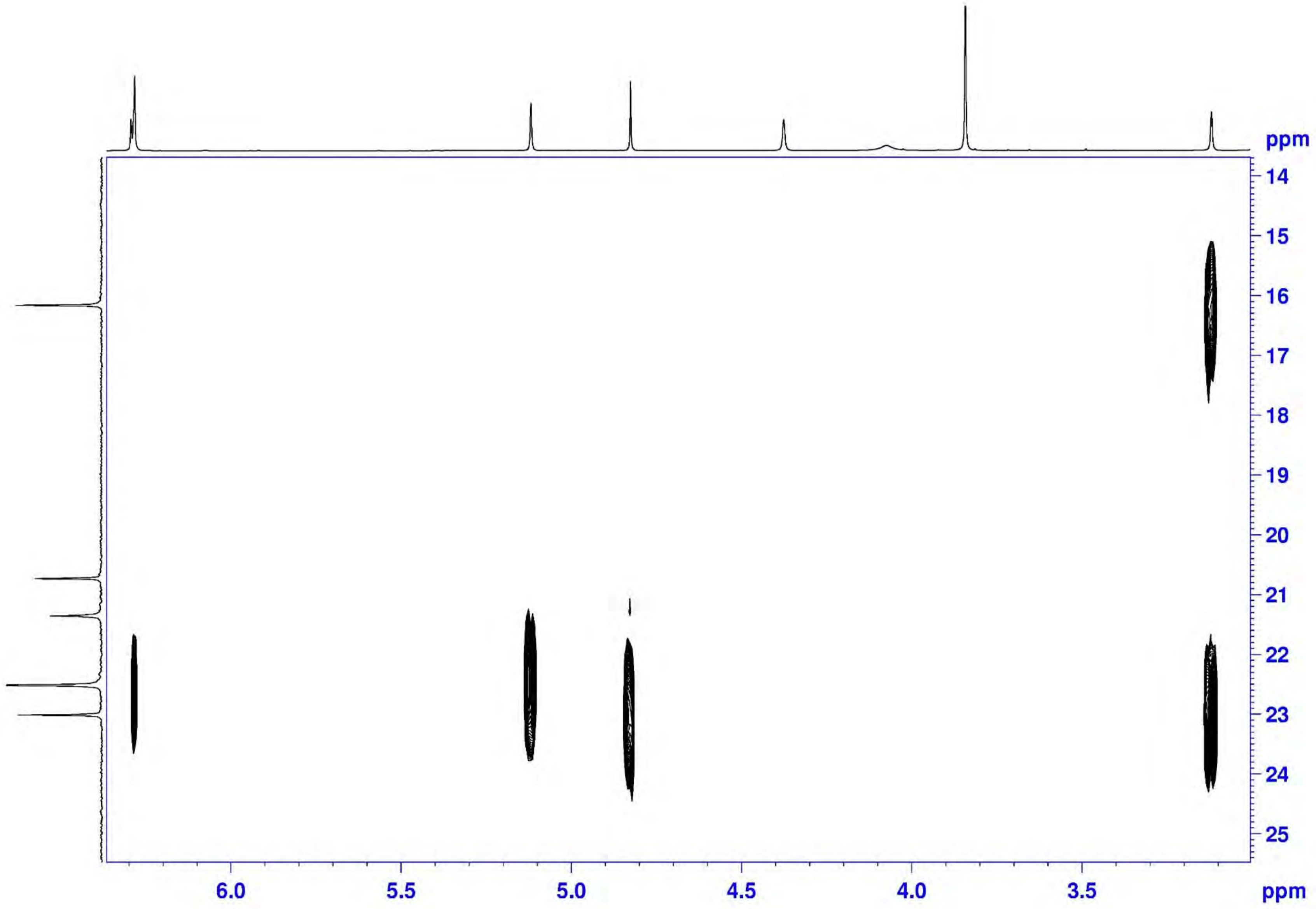

HMBC (400 MHz) spectrum of compound **10** in $CDCl_3$

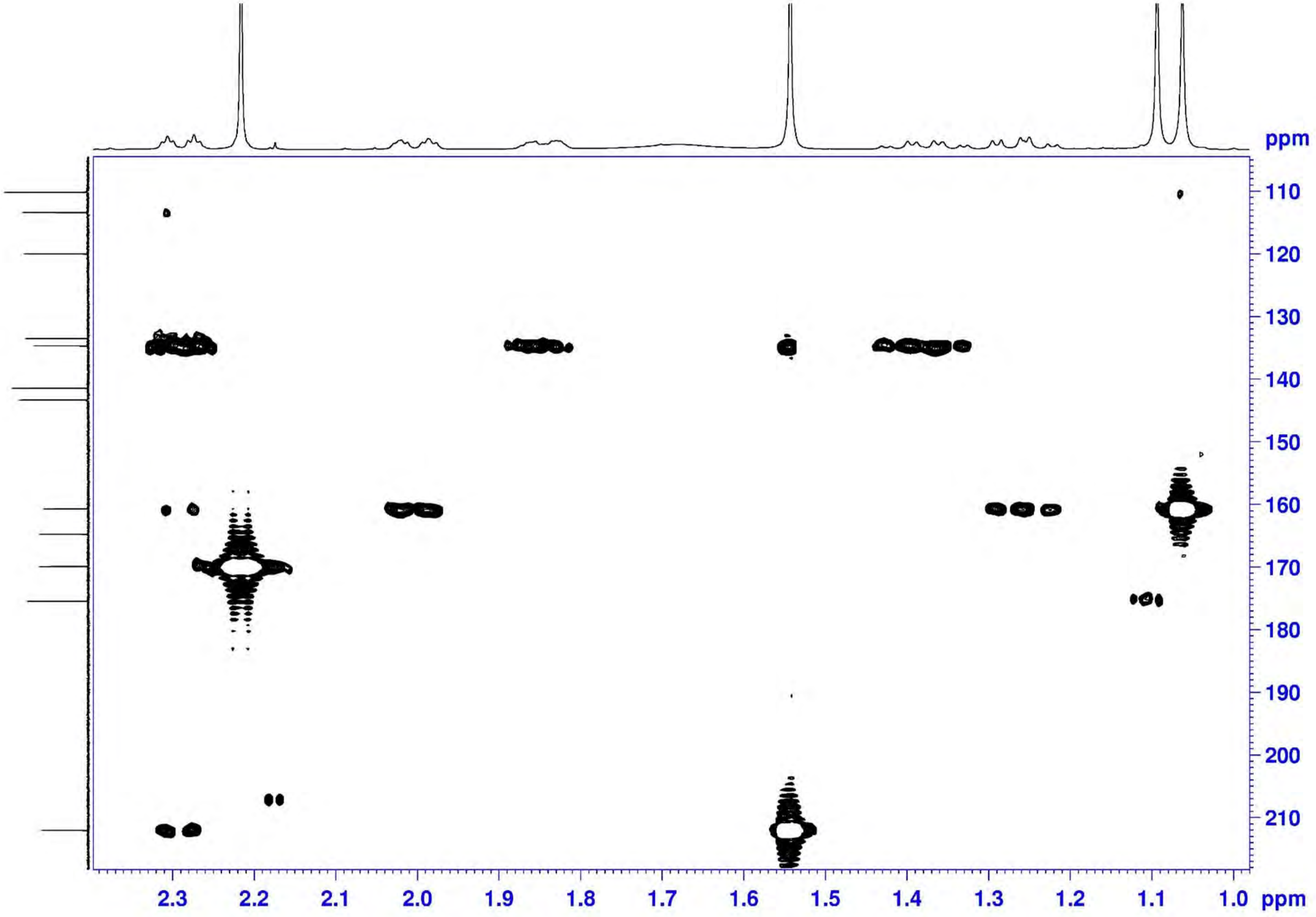

HMBC (400 MHz) spectrum of compound **10** in $CDCl_3$

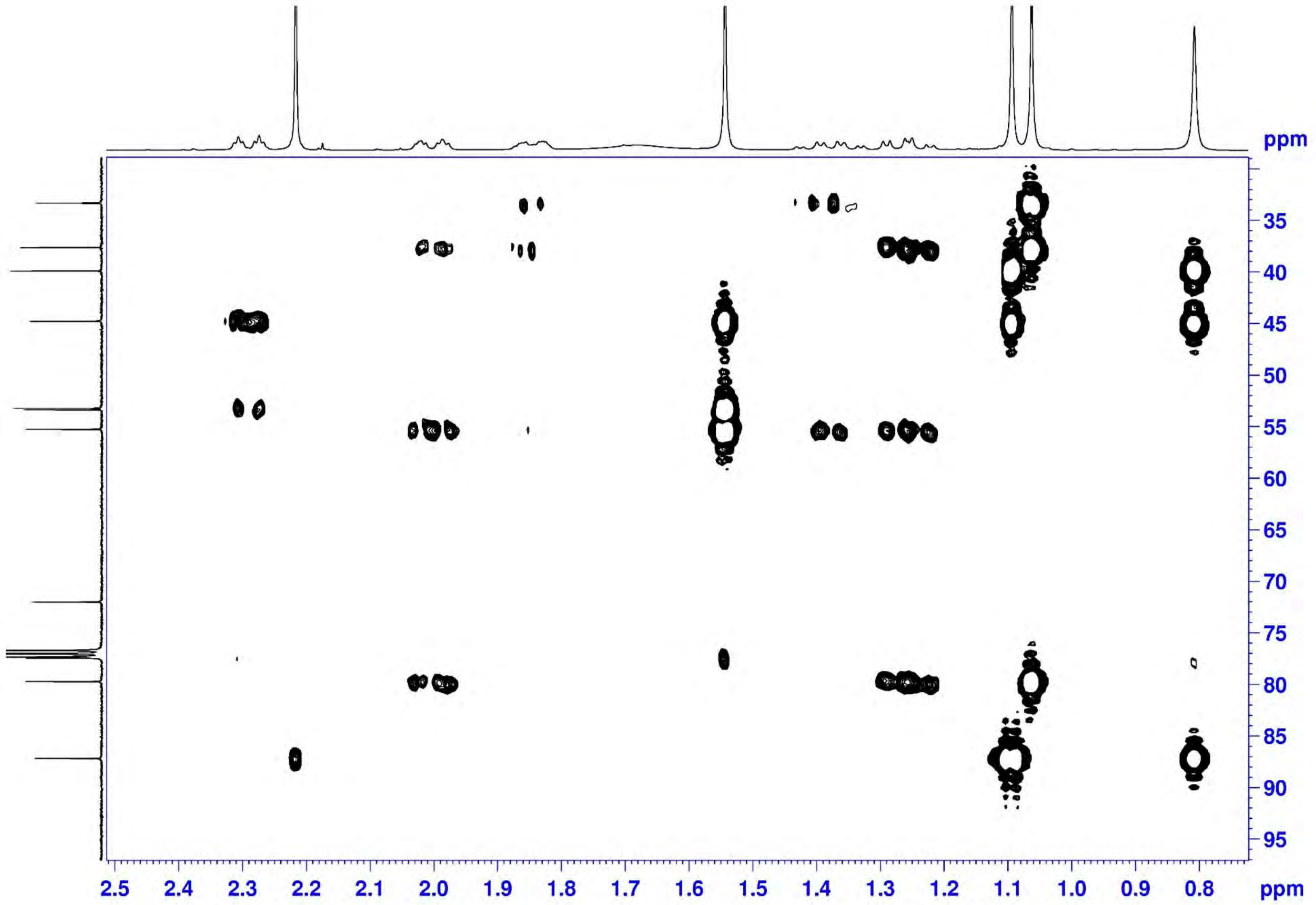

HMBC (400 MHz) spectrum of compound **10** in $CDCl_3$

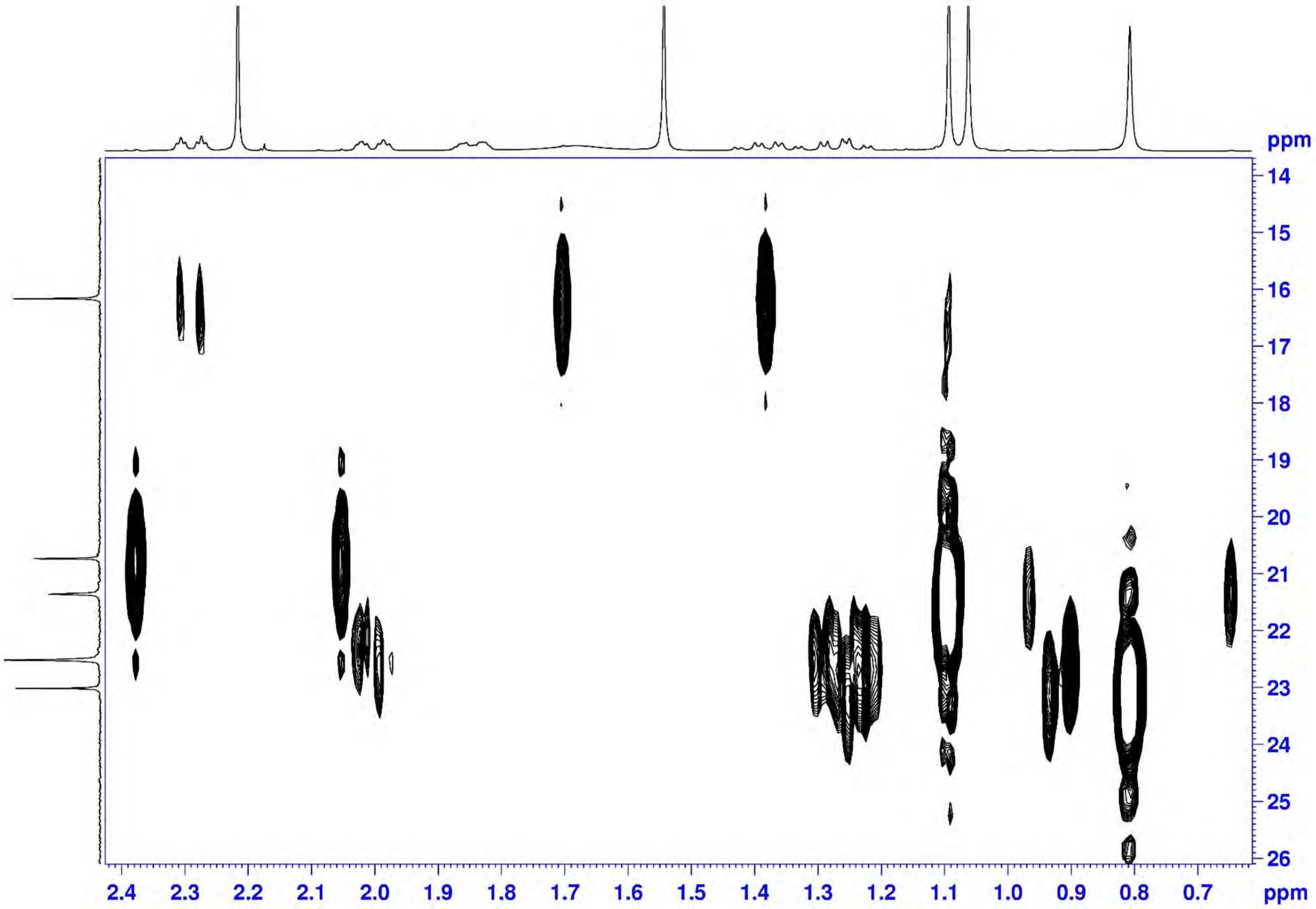

NOESY (400 MHz) spectrum of compound **10** in $CDCl_3$

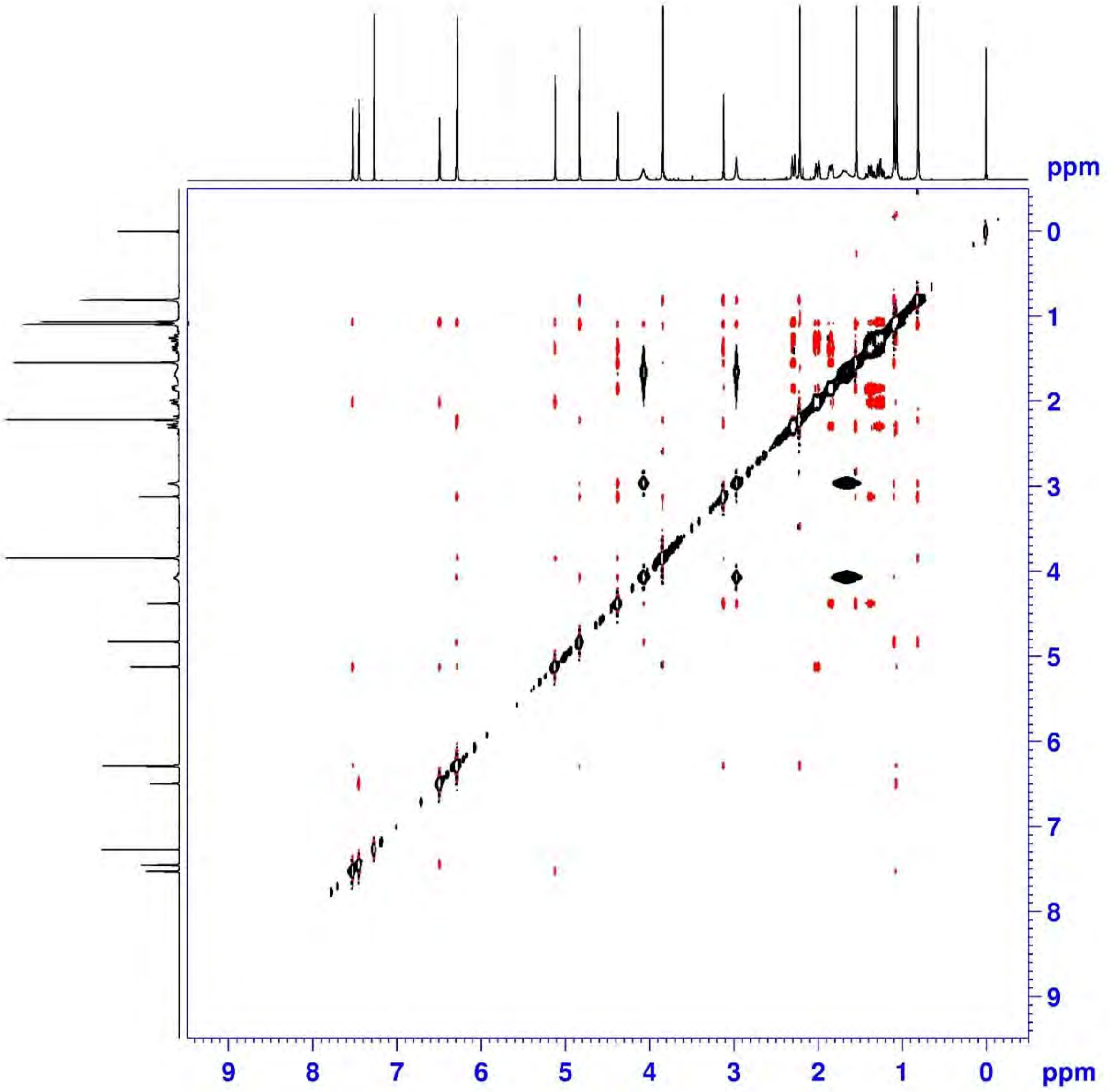

NAME	zjz-15	
EXPNO	7	
PROCNO	1	
Date_	20160517	
Time	14.12	
INSTRUM	spect	
PROBHD	5 mm CPPBBO BB	
PULPROG	noesygpphpp	
TD	2048	
SOLVENT	CDCl3	
NS	16	
DS	32	
SWH	4000.000	Hz
FIDRES	1.953125	Hz
AQ	0.2560500	sec
RG	208.5	
DW	125.000	usec
DE	10.00	usec
TE	297.0	K
D0	0.00011036	sec
D1	1.99385595	sec
D8	0.30000001	sec
D11	0.03000000	sec
D12	0.00002000	sec
D16	0.00020000	sec
IN0	0.00025000	sec
======== CHANNEL f1 ========		
SFO1	400.1318006	MHz
NUC1	1H	
P1	11.50	usec
P2	23.00	usec
P17	2500.00	usec
ND0	1	
TD	256	
SFO1	400.1318	MHz
FIDRES	15.625000	Hz
SW	9.997	ppm
FnMODE	States-TPPI	
SI	1024	
SF	400.1300049	MHz
WDW	QSINE	
SSB	2	
LB	0.00	Hz
GB	0	
PC	1.00	
SI	1024	
MC2	States-TPPI	
SF	400.1300039	MHz
WDW	QSINE	
SSB	2	
LB	0.00	Hz
GB	0	

NOESY (400 MHz) spectrum of compound **10** in $CDCl_3$

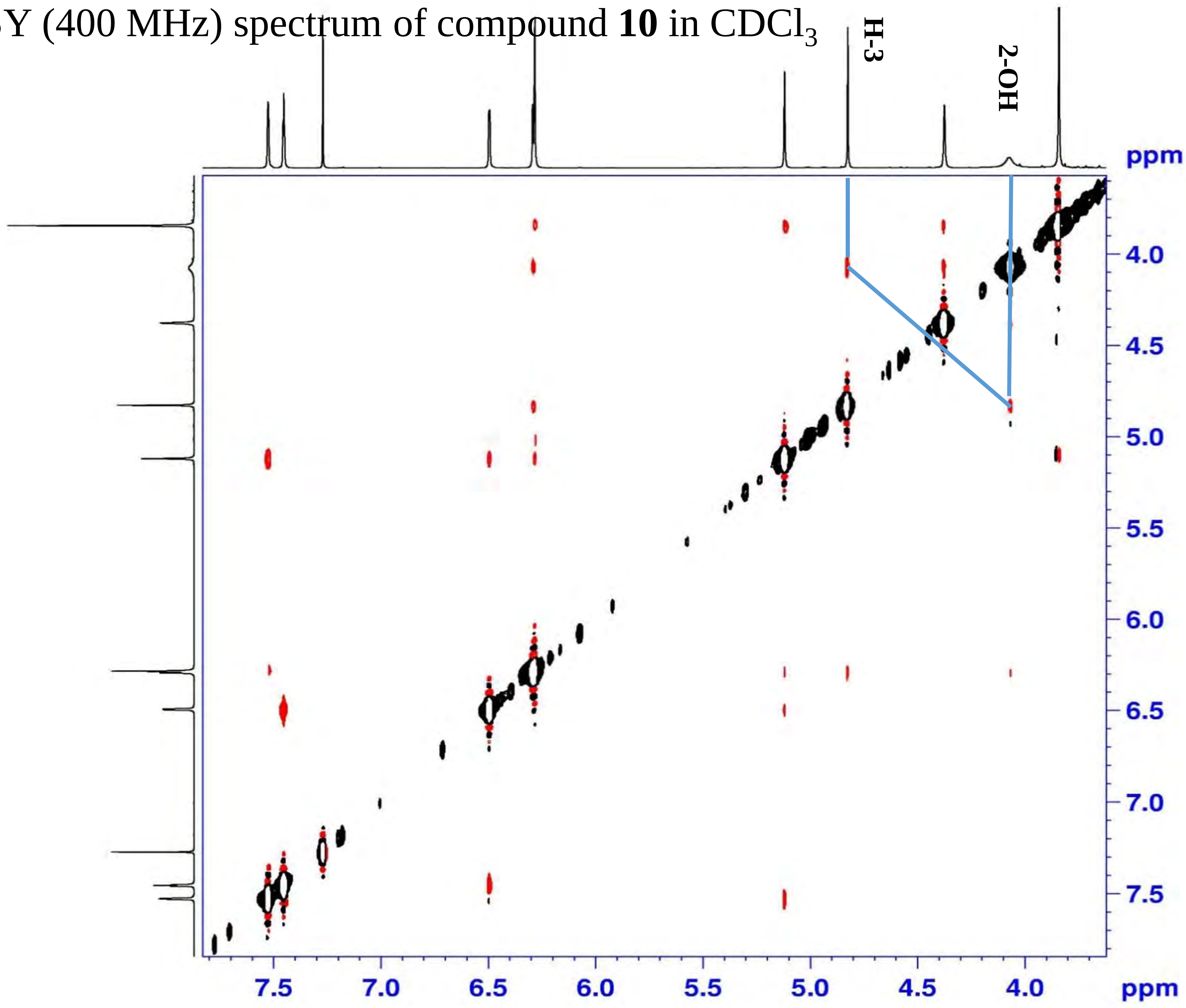

NOESY (400 MHz) spectrum of compound **10** in $CDCl_3$

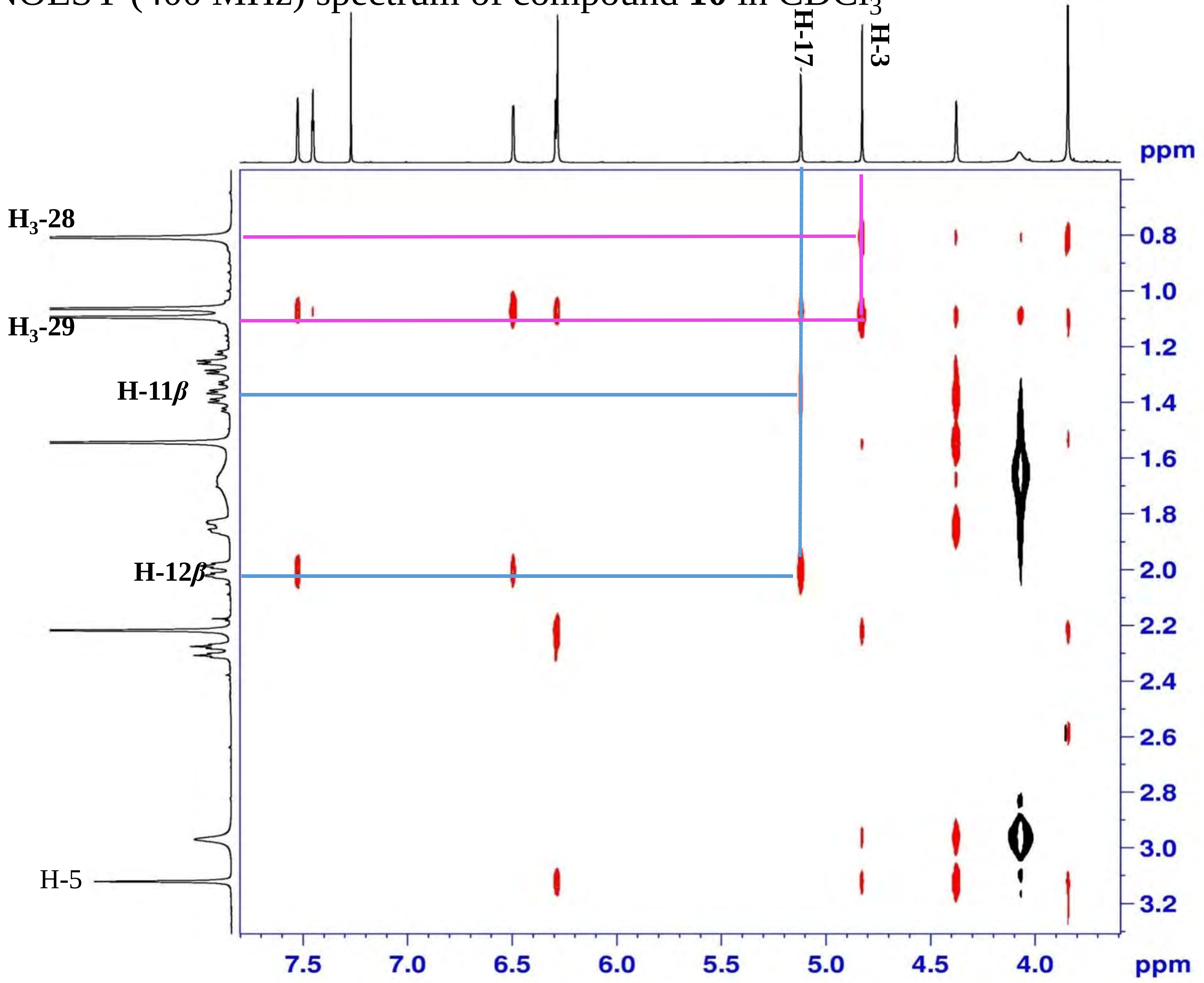

NOESY (400 MHz) spectrum of compound **10** in $CDCl_3$

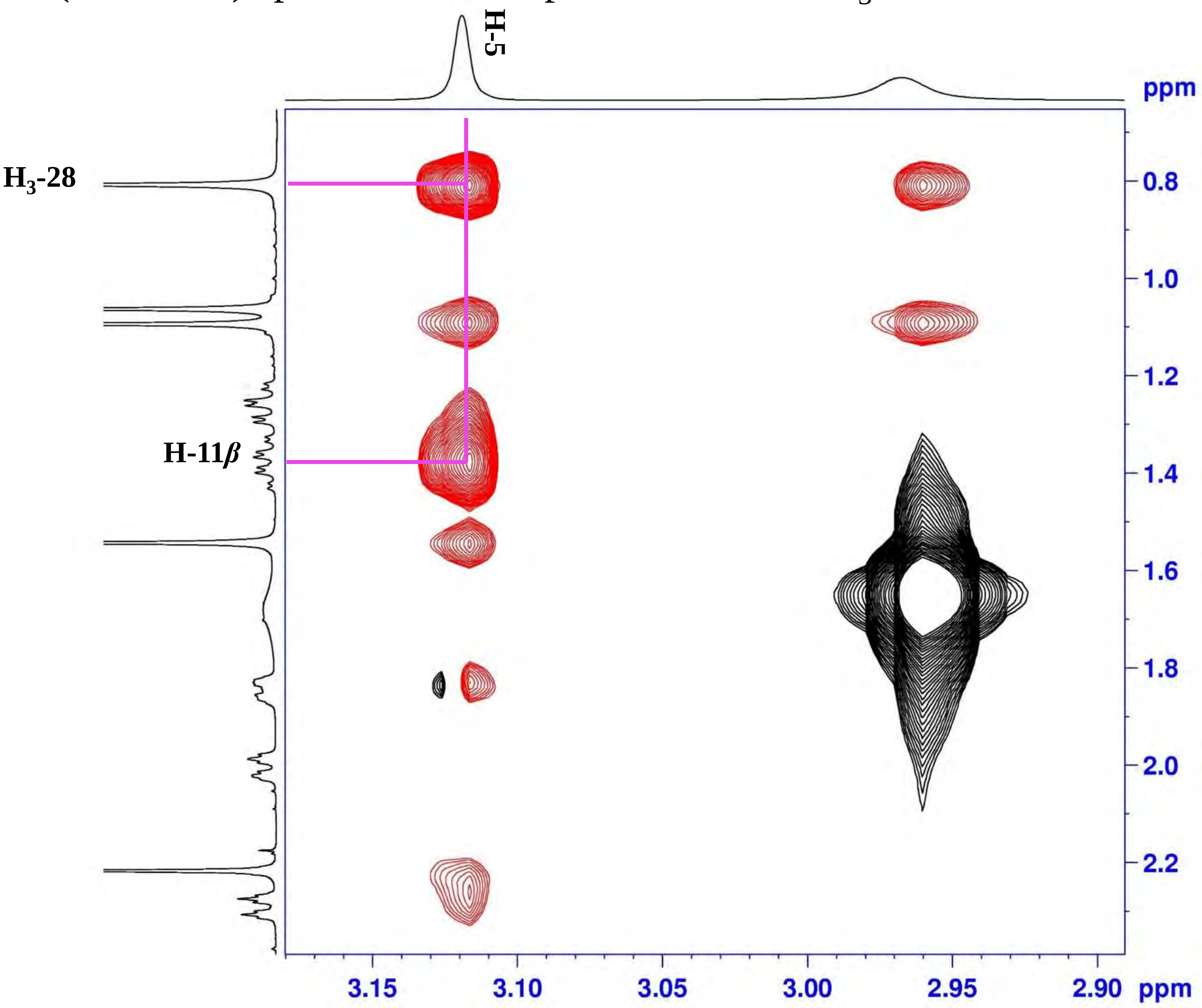

NOESY (400 MHz) spectrum of compound **10** in $CDCl_3$

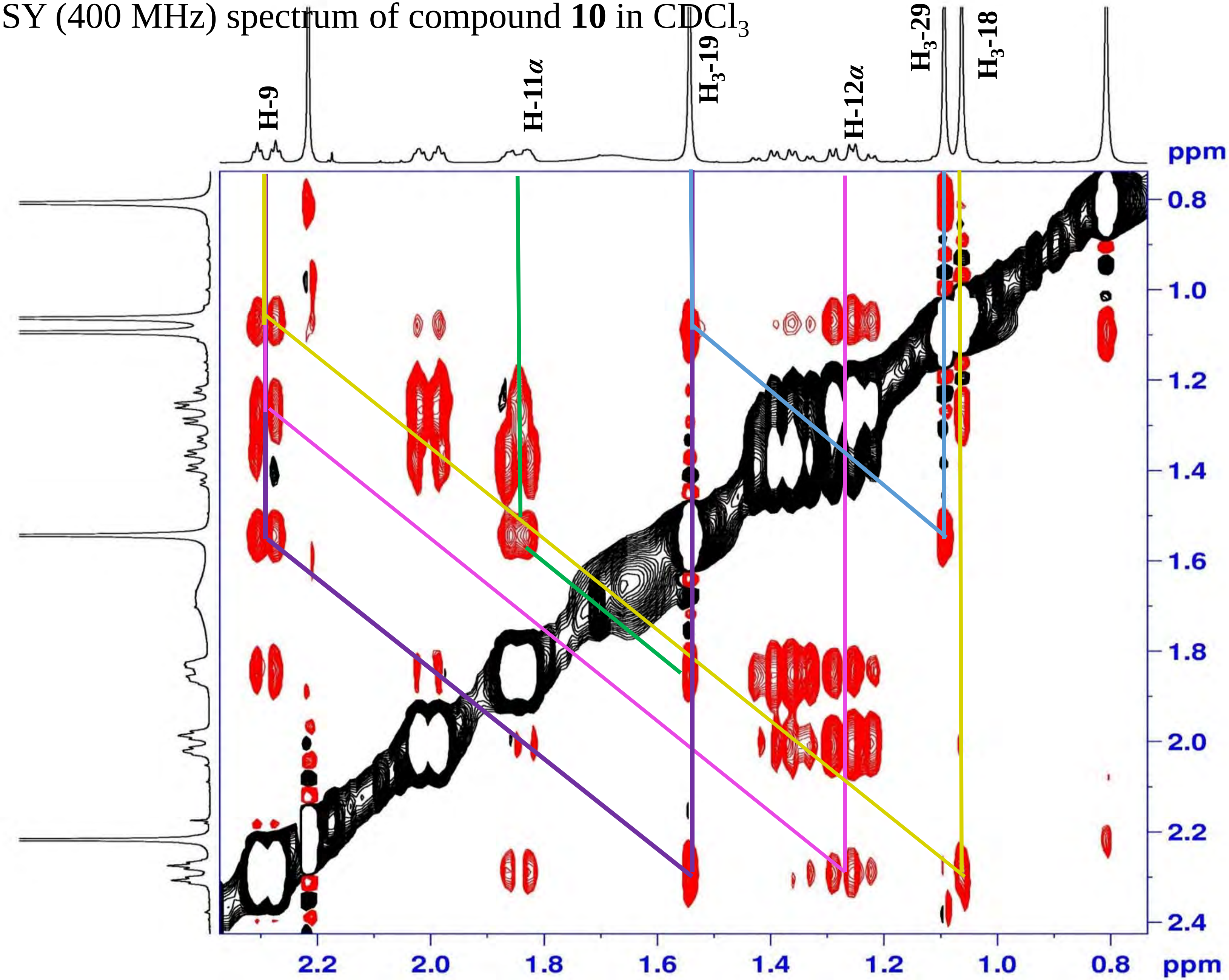

HRESIMS for compound **11**

Mass Spectrum SmartFormula Report

Analysis Info

Analysis Name	D:\Data\MS\data\201612\zhangjianzhi_zjz-31_pos_82_01_2758.d	Acquisition Date	12/14/2016 2:48:30 PM
Method	LC MS_Direct Infusion_pos_100-1000mz.m	Operator	SCSIO
Sample Name	zhangjianzhi_zjz-31_pos	Instrument	maXis 255552.00029
Comment			

Acquisition Parameter

Source Type	ESI	Ion Polarity	Positive	Set Nebulizer	0.4 Bar
Focus	Active	Set Capillary	4500 V	Set Dry Heater	180 °C
Scan Begin	100 m/z	Set End Plate Offset	-500 V	Set Dry Gas	4.0 l/min
Scan End	2000 m/z	Set Charging Voltage	0 V	Set Divert Valve	Waste
		Set Corona	0 nA	Set APCI Heater	0 °C

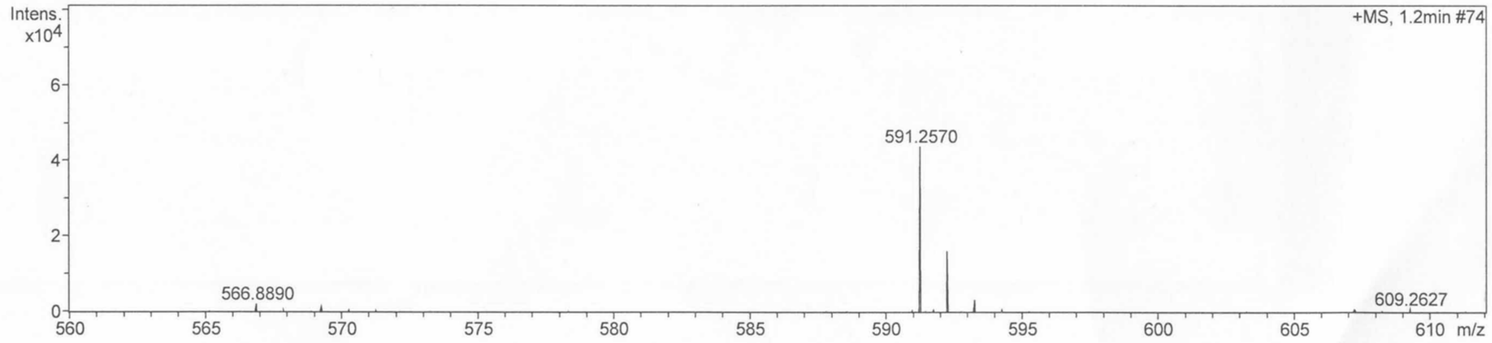

Meas. m/z	#	Ion Formula	Score	m/z	err [ppm]	err [mDa]	mSigma	rdb	e¯ Conf	N-Rule
569.274225	1	C32H41O9	100.00	569.274509	-0.5	-0.3	42.9	12.5	even	ok
591.256980	1	C32H40NaO9	100.00	591.256454	0.9	0.5	7.3	12.5	even	ok
1159.523732	1	C64H80NaO18	100.00	1159.523686	0.0	0.0	48.1	24.5	even	ok

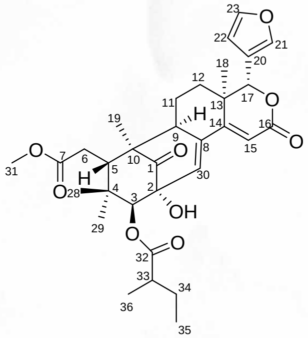

zhangjianzhi_zjz-31_pos_82_01_2758.d

Bruker Compass DataAnalysis 4.1 printed: 12/14/2016 2:56:09 PM by: SCSIO Page 1 of 1

HRESIMS for compound **11**

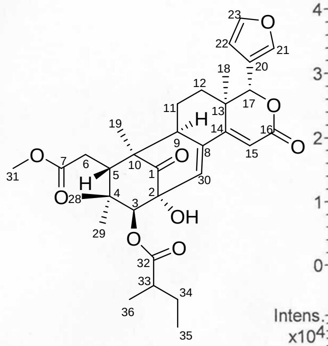

Generic Display Report

Analysis Info		Acquisition Date	12/14/2016 2:48:30 PM
Analysis Name	D:\Data\MS\data\201612\zhangjianzhi_zjz-31_pos_82_01_2758.d		
Method	LC MS_Direct Infusion_pos_100-1000mz.m	Operator	SCSIO
Sample Name	zhangjianzhi_zjz-31_pos	Instrument	maXis
Comment			

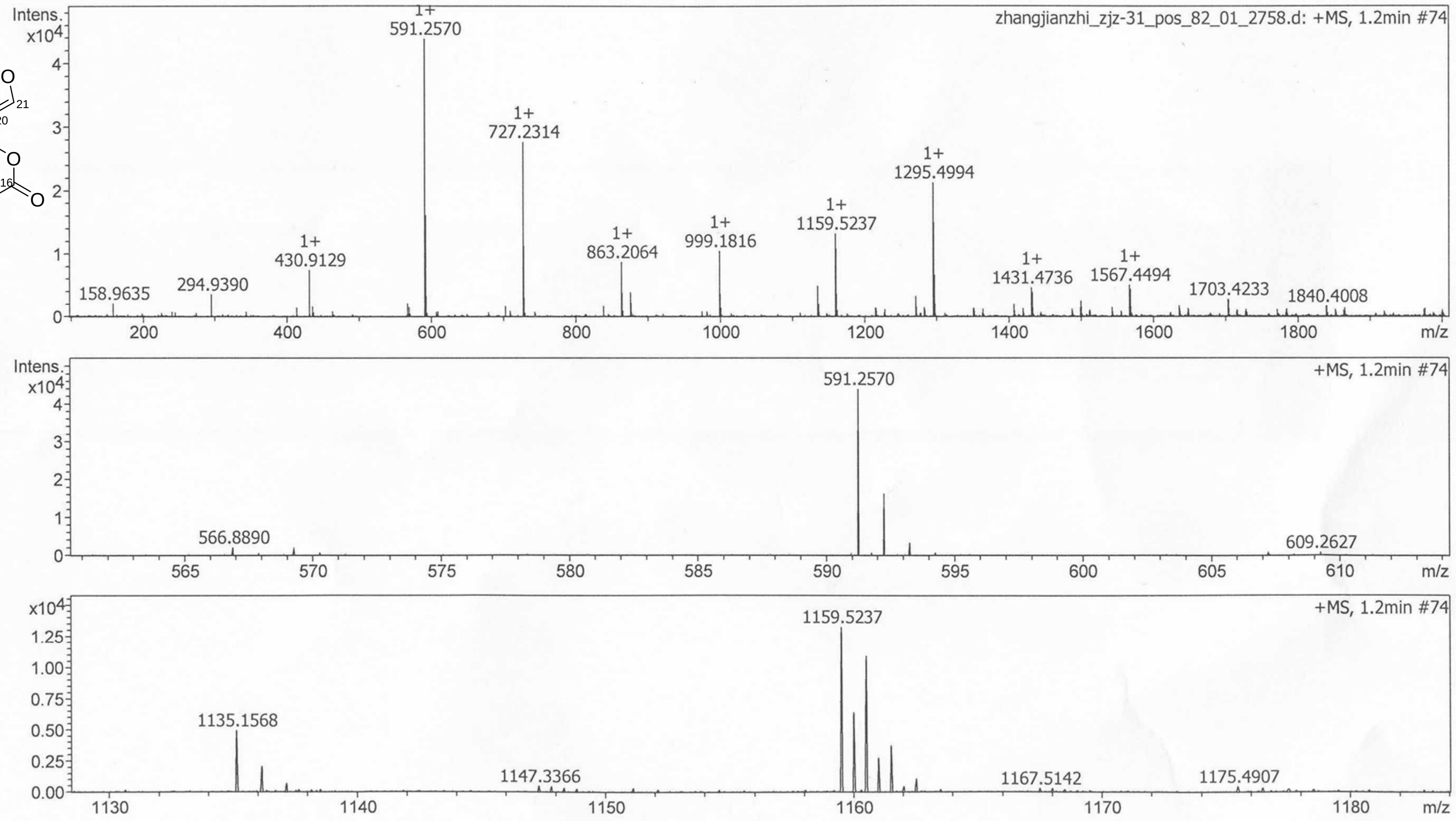

Bruker Compass DataAnalysis 4.1 printed: 12/14/2016 2:57:02 PM by: SCSIO Page 1 of 1

^{1}H NMR (400 MHz) spectrum of compound **11** in $CDCl_3$

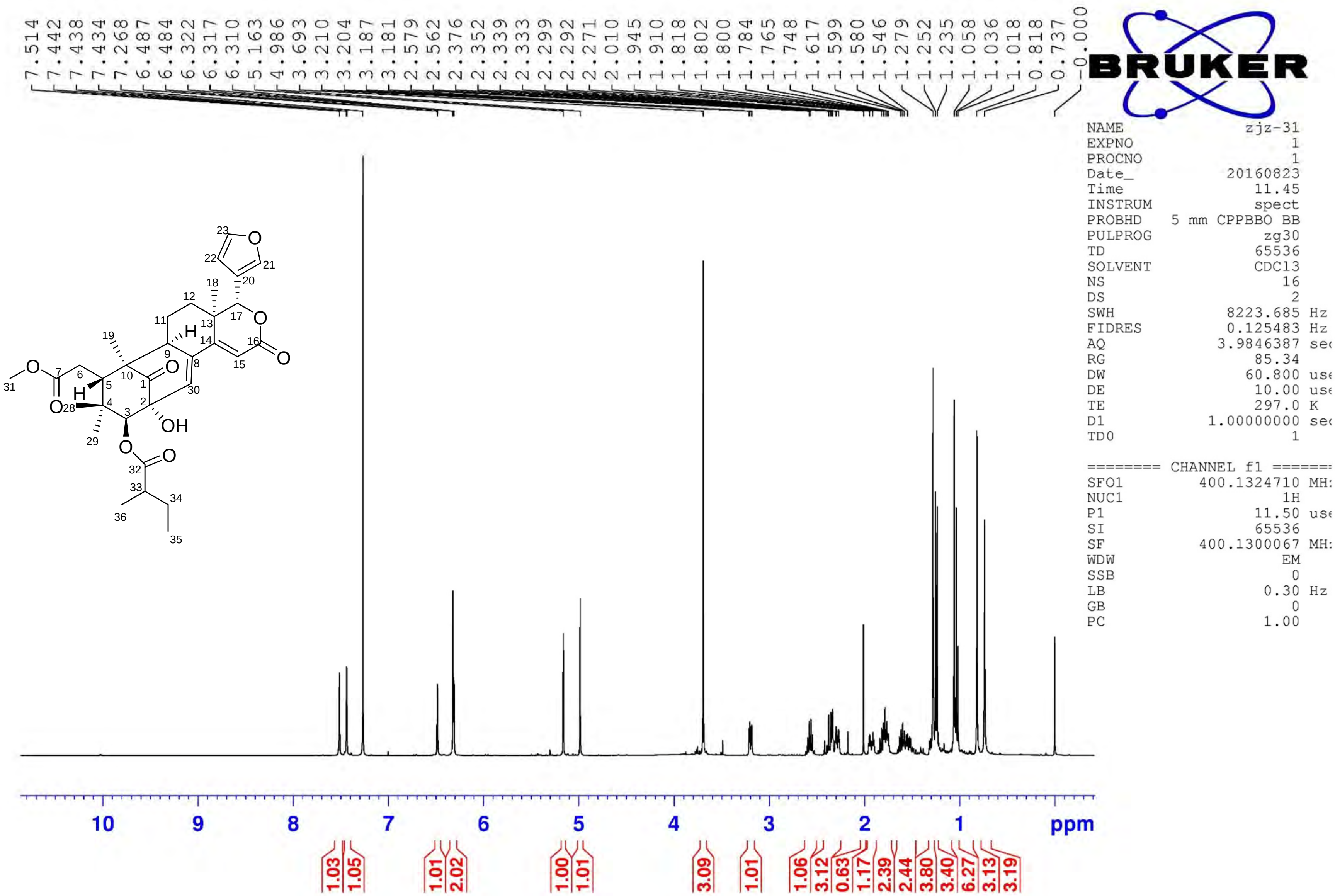

^{1}H NMR (400 MHz) spectrum of compound **11** in $CDCl_3$

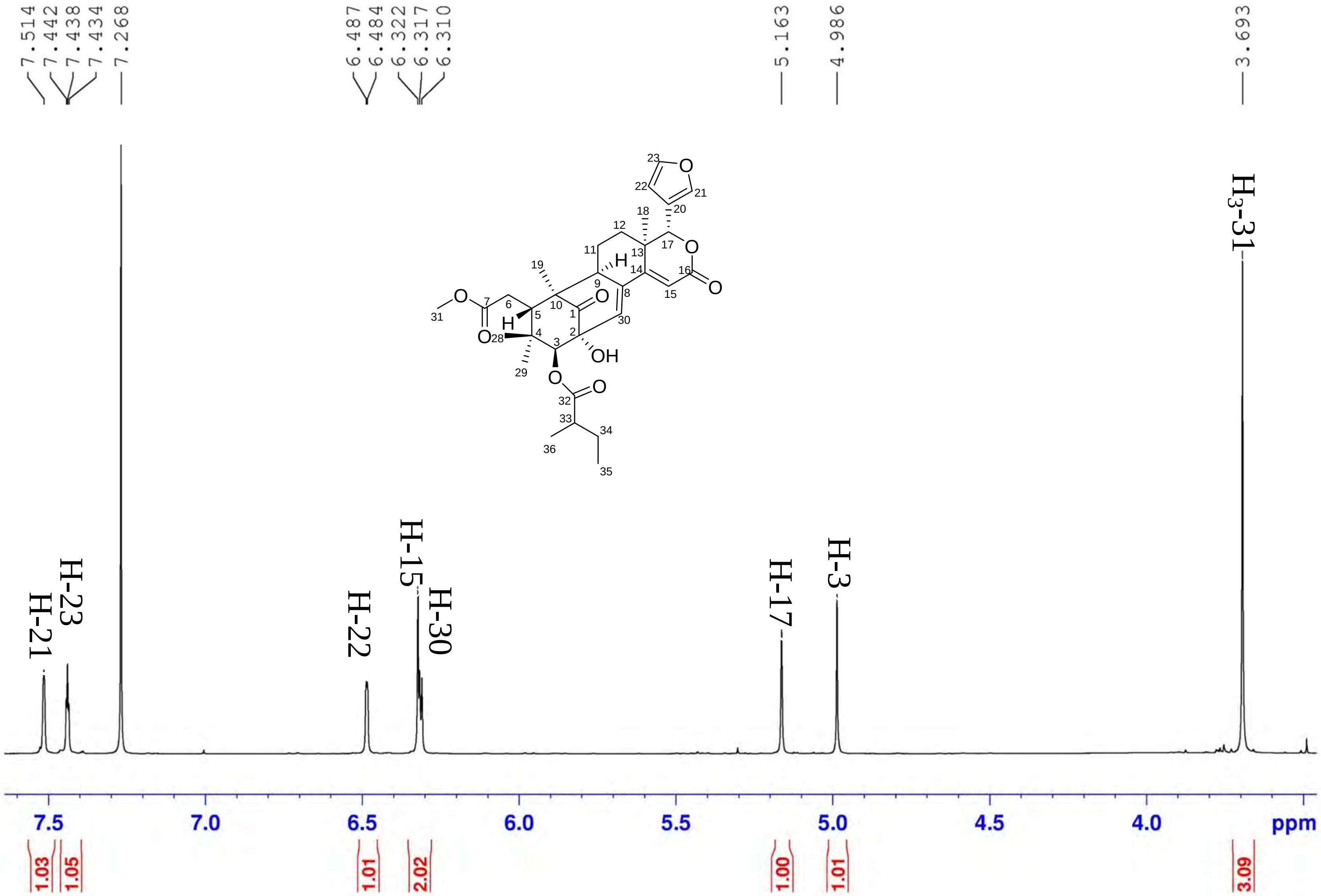

^{1}H NMR (400 MHz) spectrum of compound **11** in $CDCl_3$

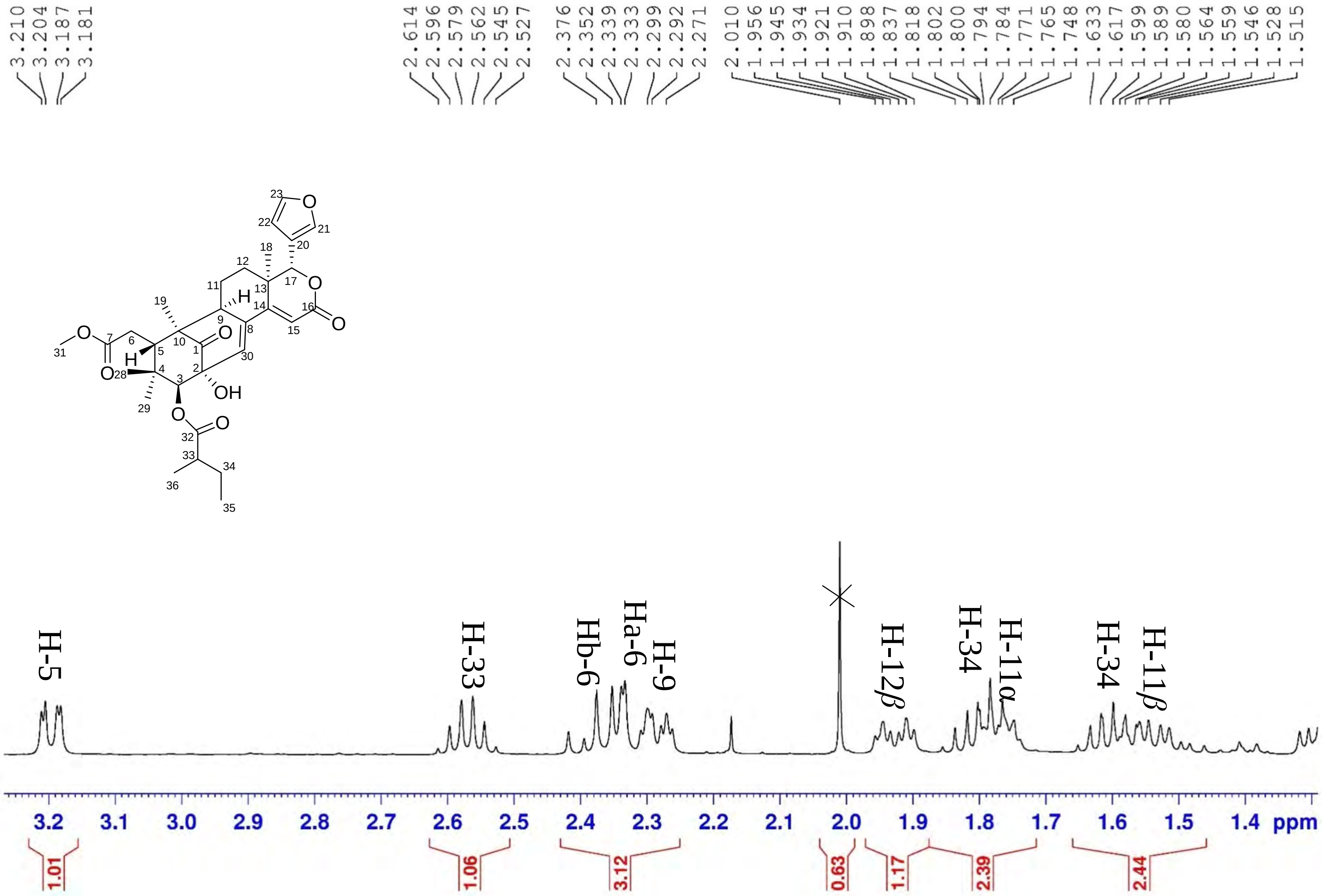

^{1}H NMR (400 MHz) spectrum of compound **11** in $CDCl_3$

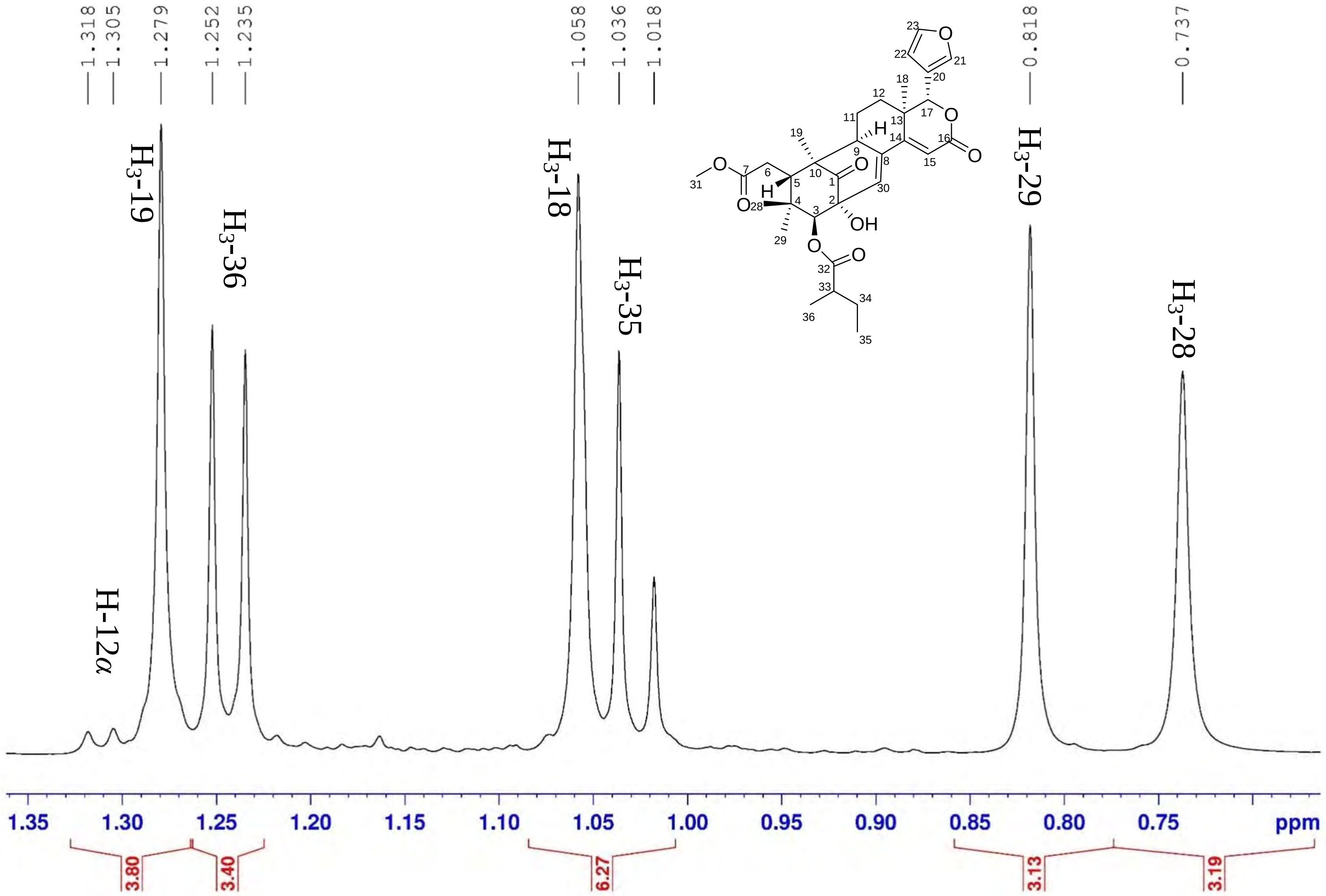

^{13}C NMR (100 MHz) spectrum of compound **11** in $CDCl_3$

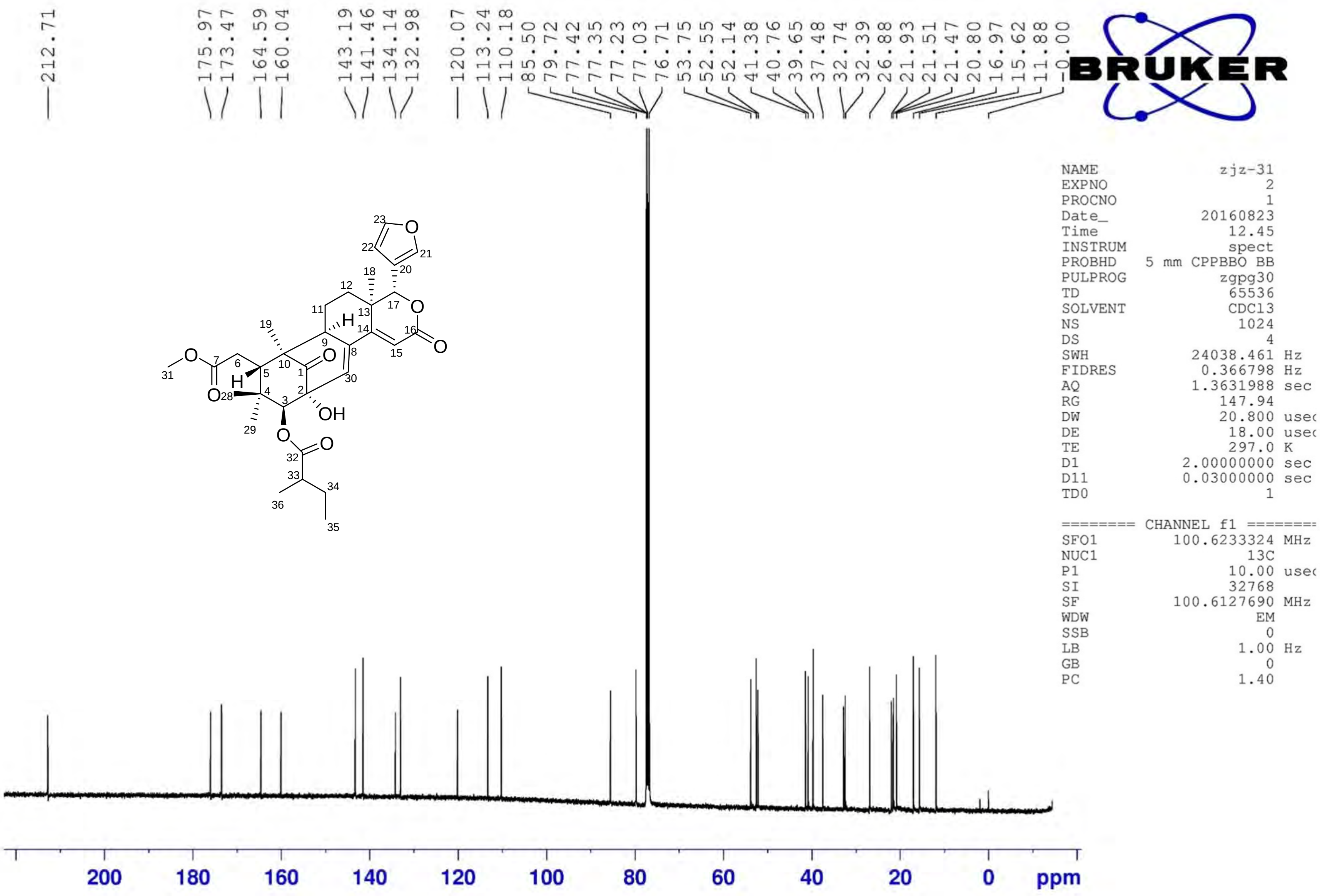

NAME	zjz-31
EXPNO	2
PROCNO	1
Date_	20160823
Time	12.45
INSTRUM	spect
PROBHD	5 mm CPPBBO BB
PULPROG	zgpg30
TD	65536
SOLVENT	CDCl3
NS	1024
DS	4
SWH	24038.461 Hz
FIDRES	0.366798 Hz
AQ	1.3631988 sec
RG	147.94
DW	20.800 usec
DE	18.00 usec
TE	297.0 K
D1	2.00000000 sec
D11	0.03000000 sec
TD0	1

======== CHANNEL f1 ========

SFO1	100.6233324 MHz
NUC1	13C
P1	10.00 usec
SI	32768
SF	100.6127690 MHz
WDW	EM
SSB	0
LB	1.00 Hz
GB	0
PC	1.40

^{13}C NMR (100 MHz) spectrum of compound **11** in $CDCl_3$

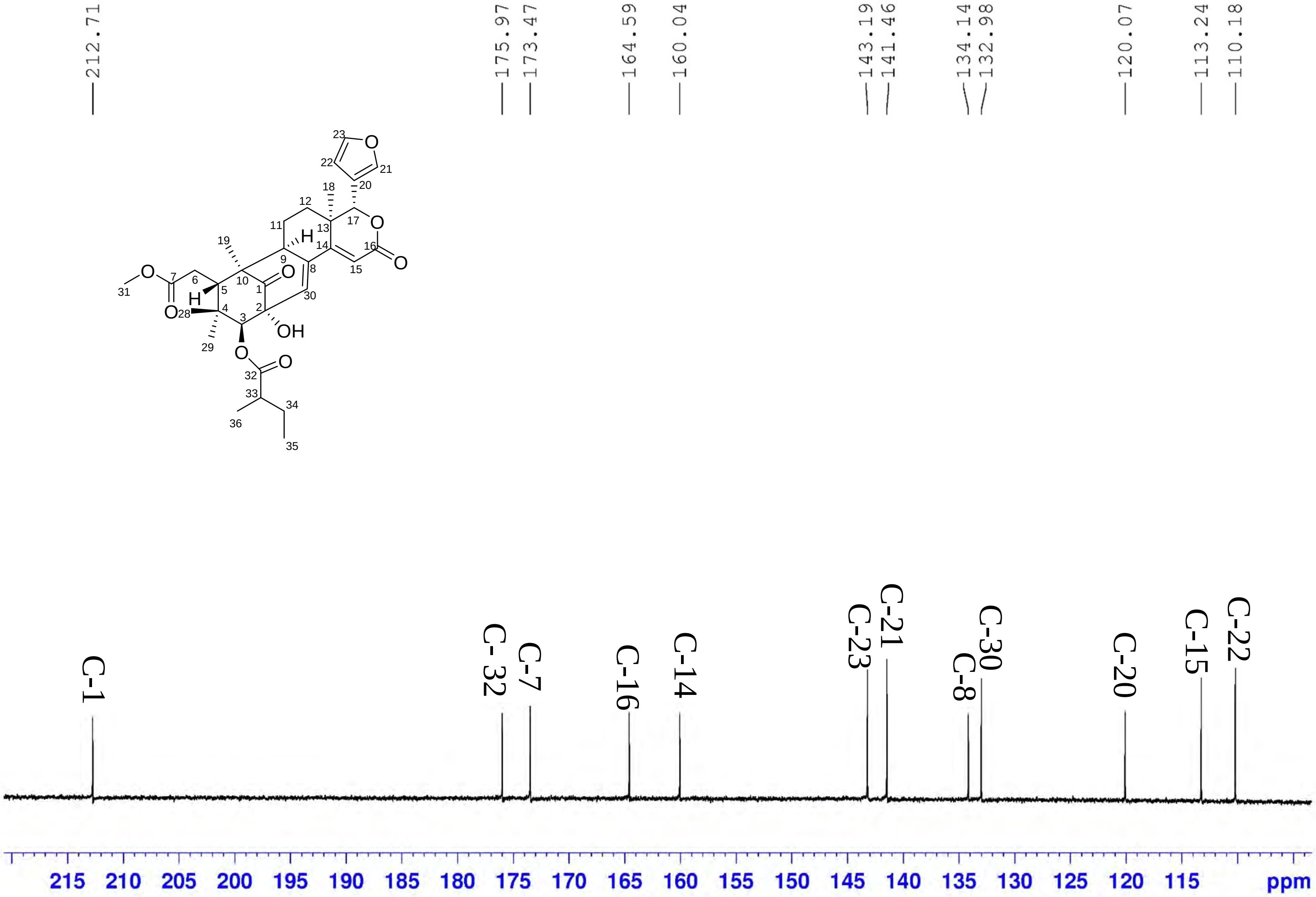

^{13}C NMR (100 MHz) spectrum of compound **11** in $CDCl_3$

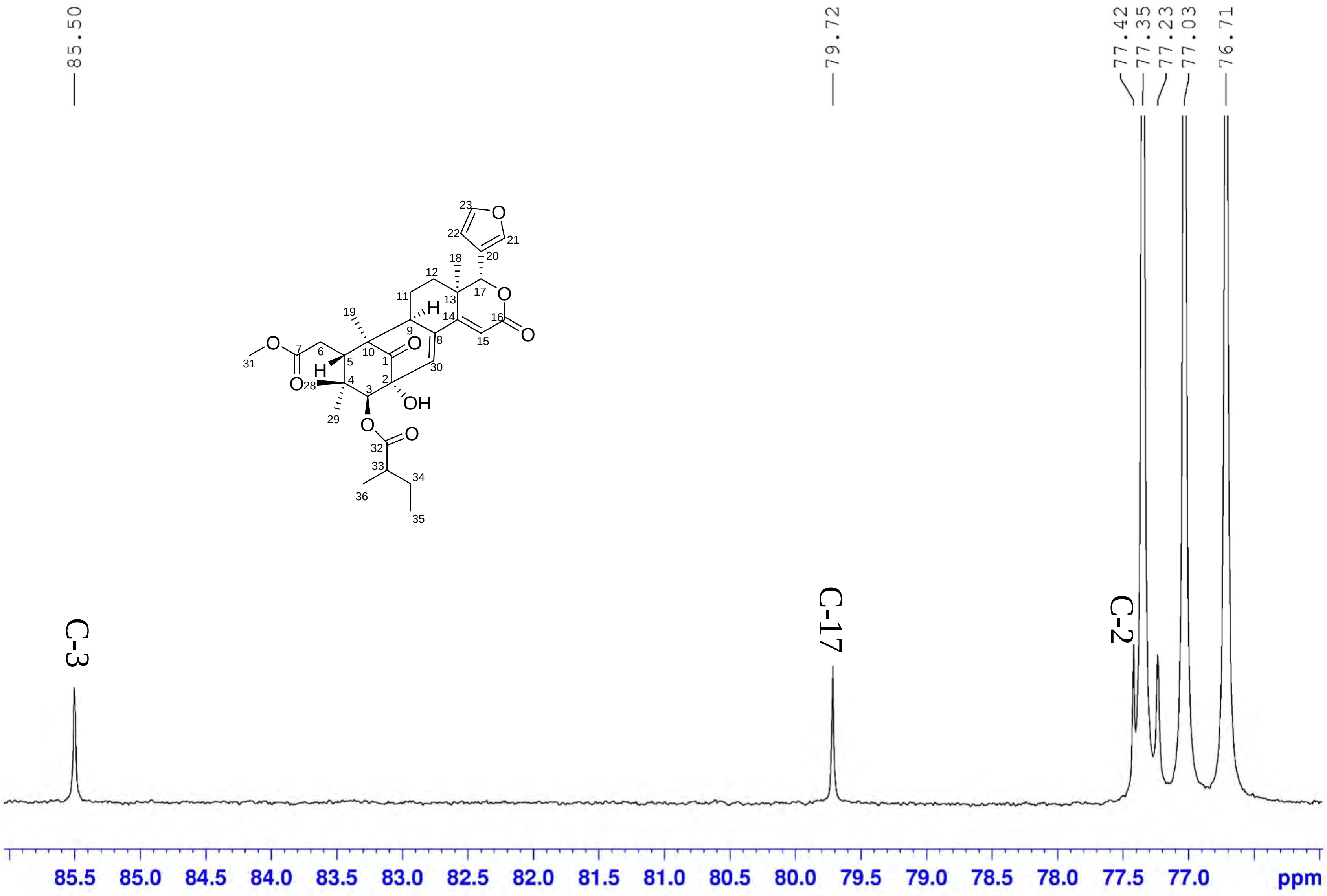

^{13}C NMR (100 MHz) spectrum of compound **11** in $CDCl_3$

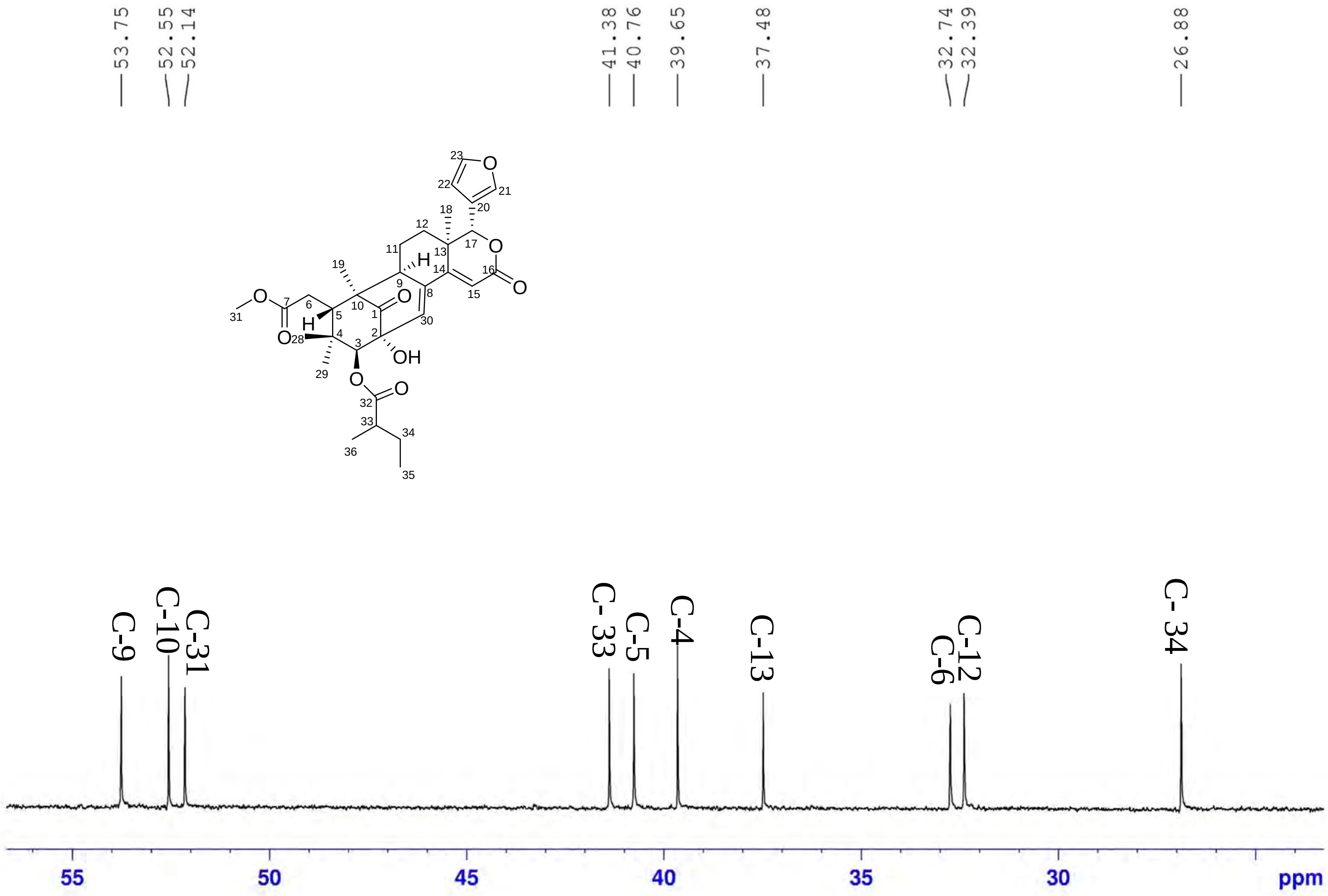

^{13}C NMR (100 MHz) spectrum of compound **11** in $CDCl_3$

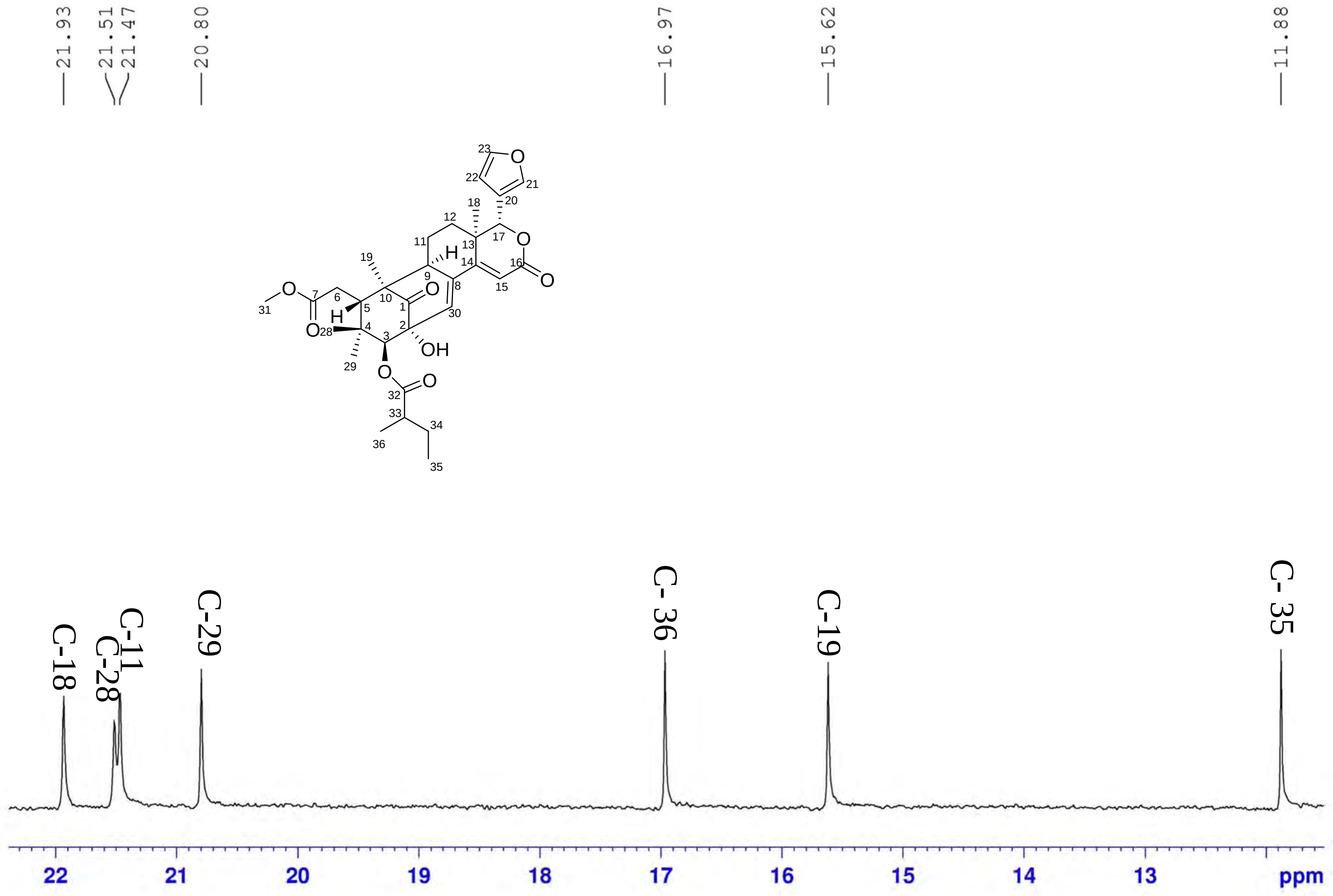

DEPT135 (100 MHz) spectrum of compound **11** in $CDCl_3$

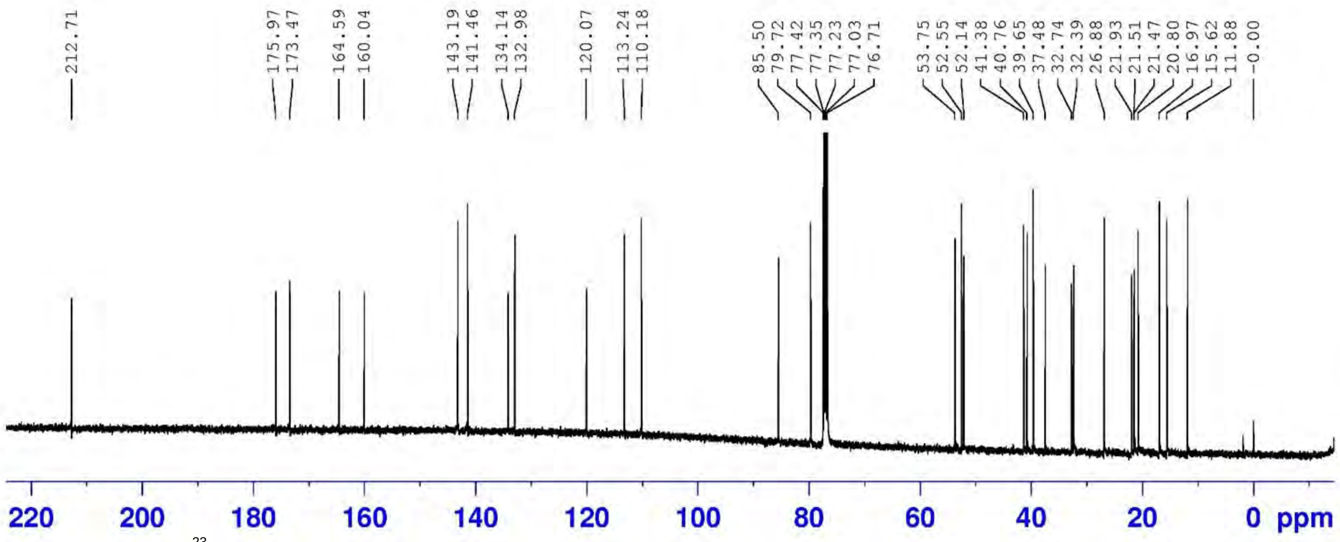

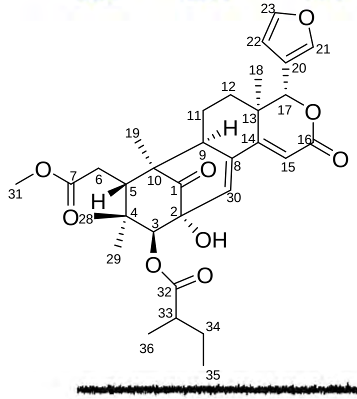

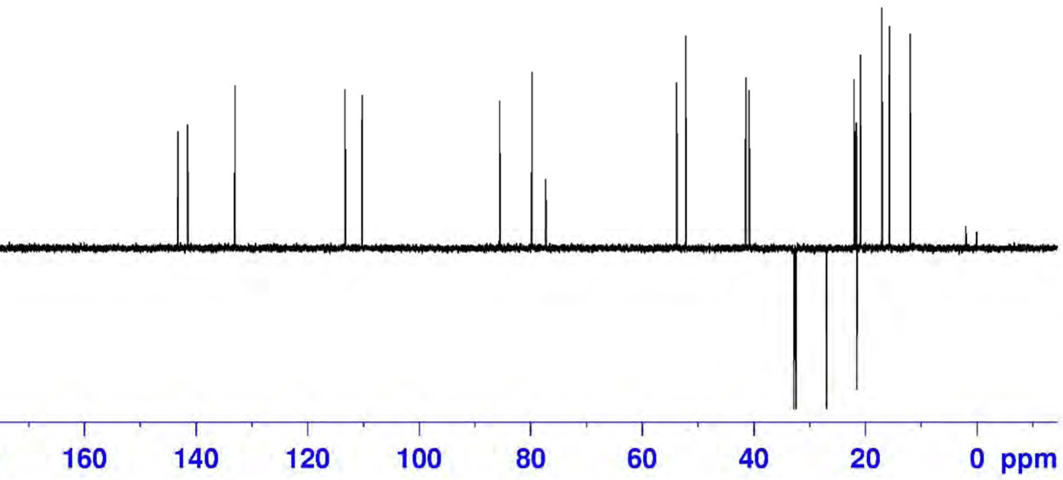

NAME	zjz-31
EXPNO	3
PROCNO	1
Date_	20160823
Time	13.03
INSTRUM	spect
PROBHD	5 mm CPPBBO BB
PULPROG	deptsp135
TD	65536
SOLVENT	CDCl3
NS	300
DS	4
SWH	24038.461 Hz
FIDRES	0.366798 Hz
AQ	1.3631988 sec
RG	130.26
DW	20.800 usec
DE	18.00 usec
TE	297.0 K
CNST2	145.0000000
D1	2.00000000 sec
D2	0.00344828 sec
D12	0.00002000 sec
TD0	1

======== CHANNEL f1 ========

SFO1	100.6233324 MHz
NUC1	13C
P1	10.00 usec
P13	2000.00 usec
SI	32768
SF	100.6127690 MHz
WDW	EM
SSB	0
LB	1.00 Hz
GB	0
PC	1.40

DEPT135 (100 MHz) spectrum of compound **11** in $CDCl_3$

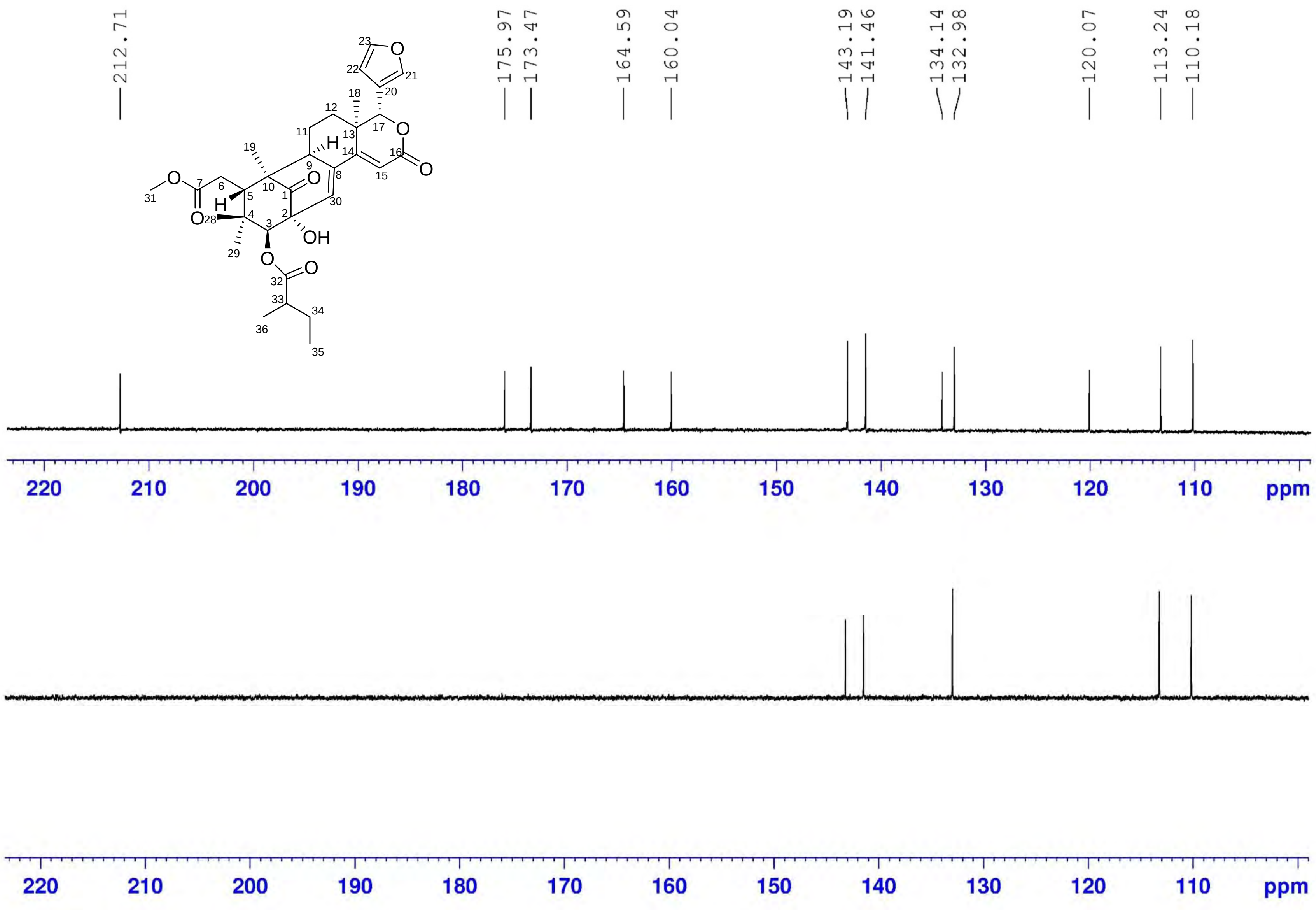

DEPT135 (100 MHz) spectrum of compound **11** in $CDCl_3$

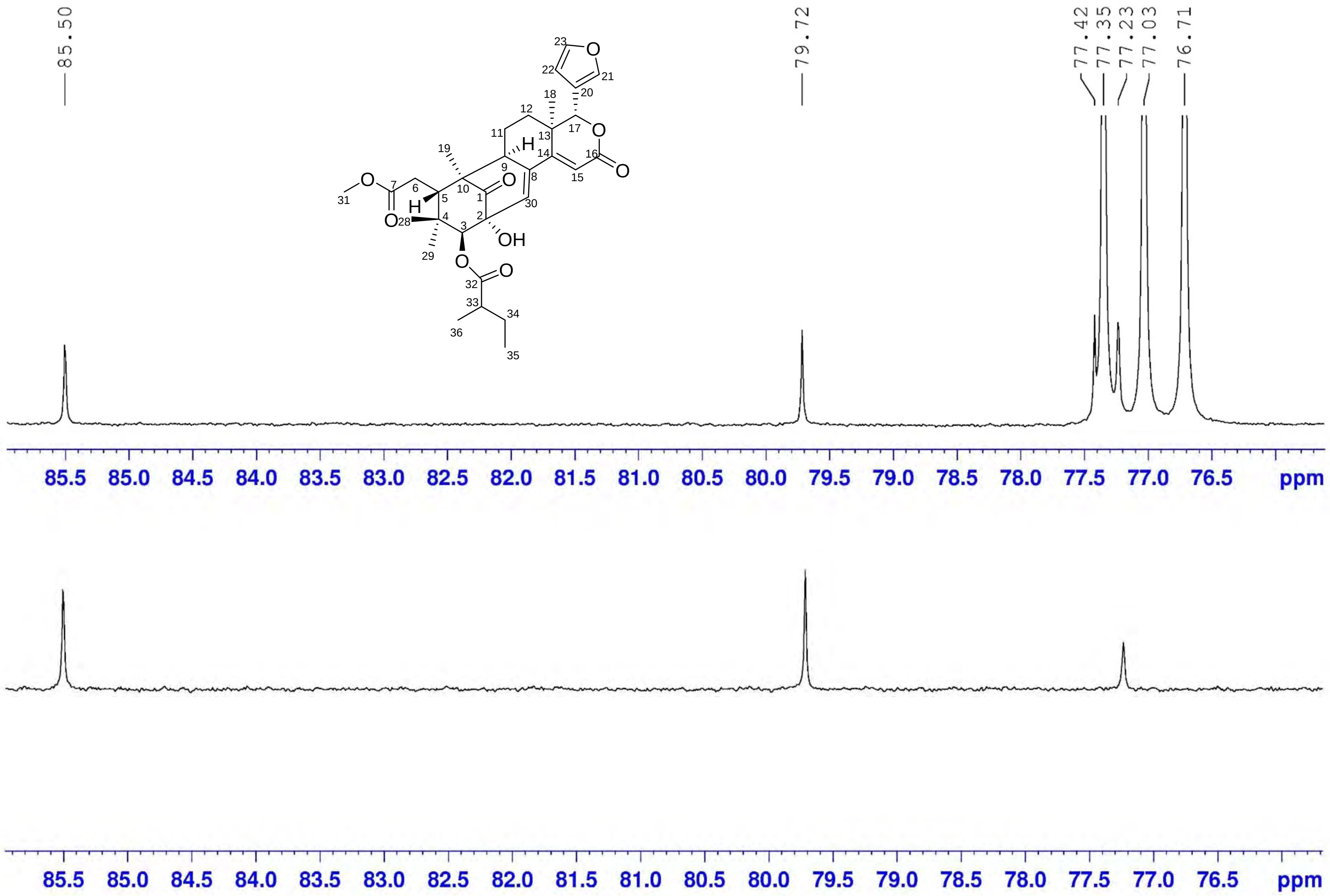

DEPT135 (100 MHz) spectrum of compound **11** in $CDCl_3$

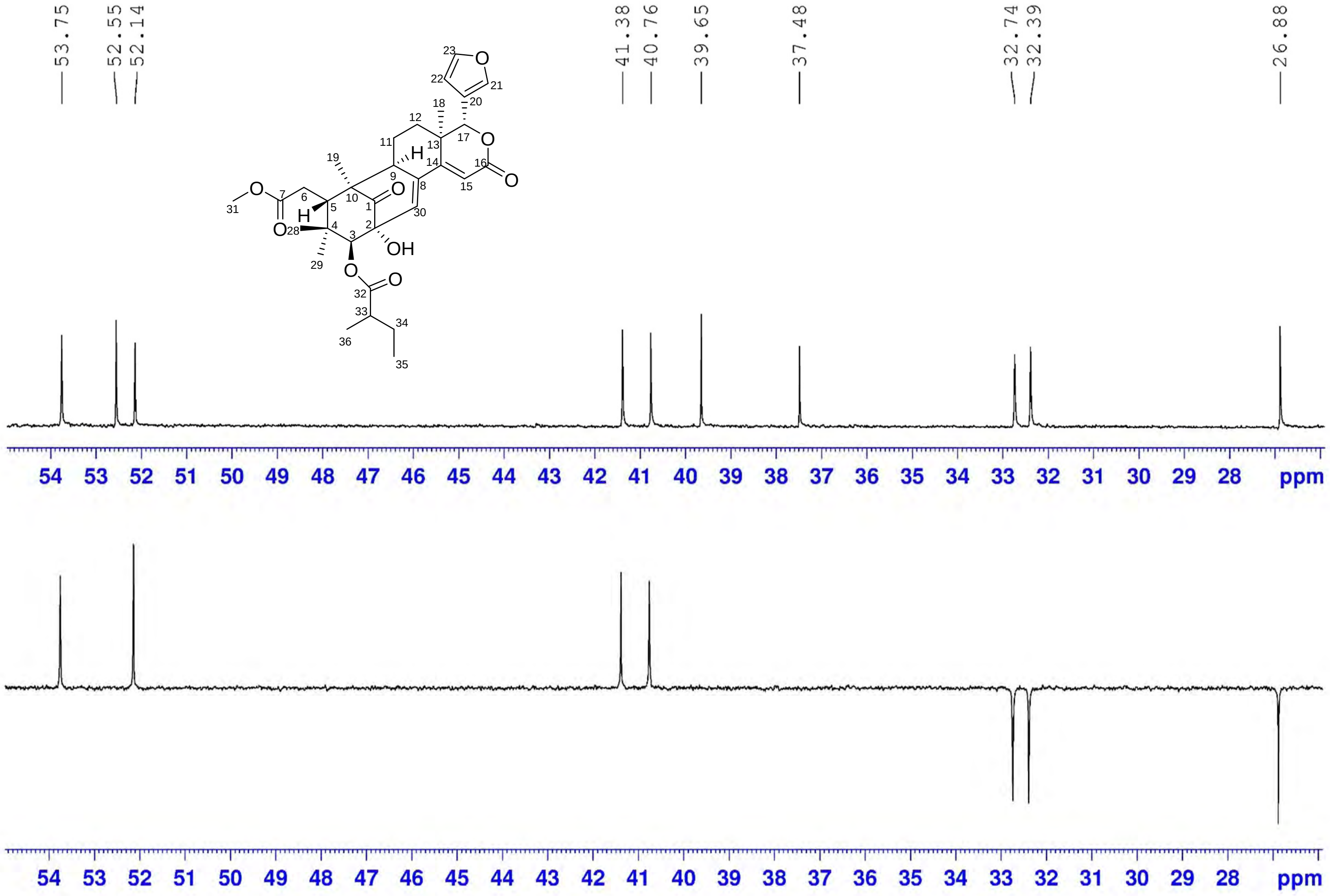

DEPT135 (100 MHz) spectrum of compound **11** in $CDCl_3$

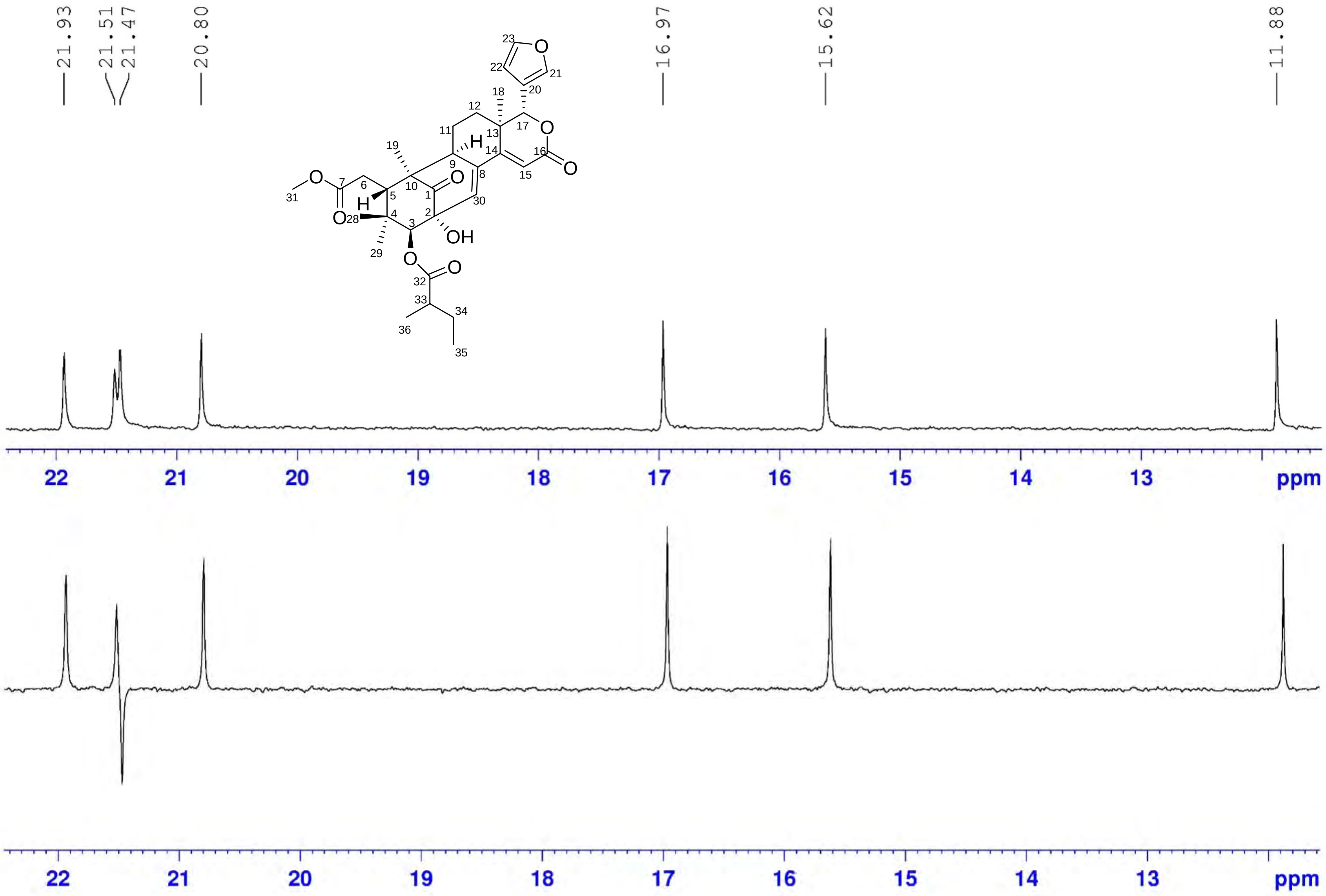

^{1}H-^{1}H COSY (400 MHz) spectrum of compound **11** in $CDCl_3$

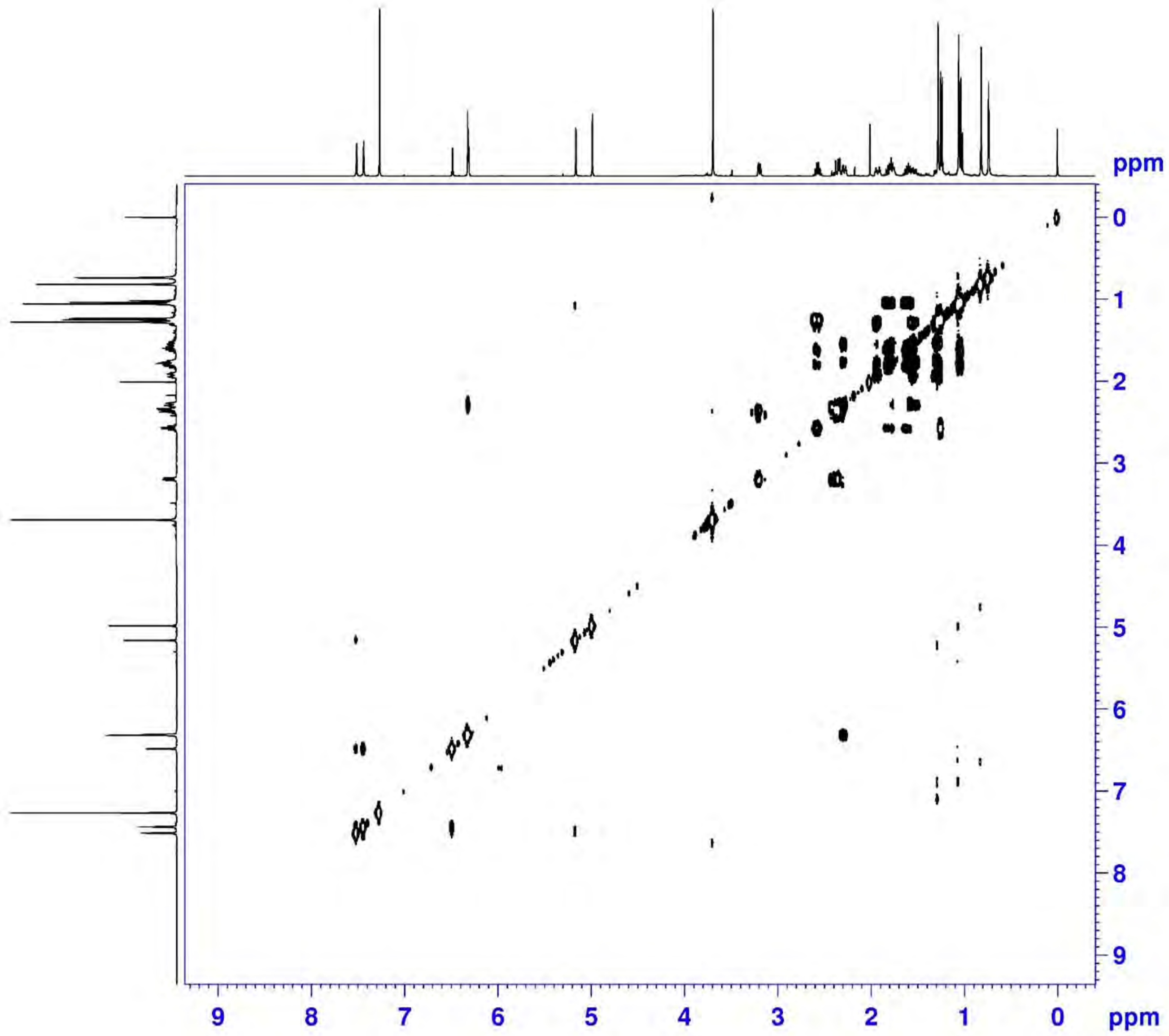


```
NAME              zjz-31
EXPNO                  4
PROCNO                 1
Date_           20160824
Time               16.31
INSTRUM            spect
PROBHD   5 mm CPPBBO BB
PULPROG       cosygpppqf
TD                  2048
SOLVENT            CDCl3
NS                     8
DS                     8
SWH             3906.250 Hz
FIDRES          1.907349 Hz
AQ             0.2621940 sec
RG                 208.5
DW               128.000 usec
DE                 10.00 usec
TE                 297.0 K
D0            0.00000300 sec
D1            1.89678097 sec
D11           0.03000000 sec
D12           0.00002000 sec
D13           0.00000400 sec
D16           0.00020000 sec
IN0           0.00025600 sec

======== CHANNEL f1 ========
SFO1         400.1318006 MHz
NUC1                  1H
P0                 11.50 usec
P1                 11.50 usec
P17              2500.00 usec
ND0                    1
TD                   128
SFO1            400.1318 MHz
FIDRES         30.517578 Hz
SW                 9.762 ppm
FnMODE                QF
SI                  1024
SF           400.1300049 MHz
WDW                QSINE
SSB                    0
LB                  0.00 Hz
GB                     0
PC                  1.40
SI                  1024
MC2                   QF
SF           400.1300044 MHz
WDW                QSINE
SSB                    0
LB                  0.00 Hz
GB                     0
```

^{1}H-^{1}H COSY (400 MHz) spectrum of compound **11** in $CDCl_3$

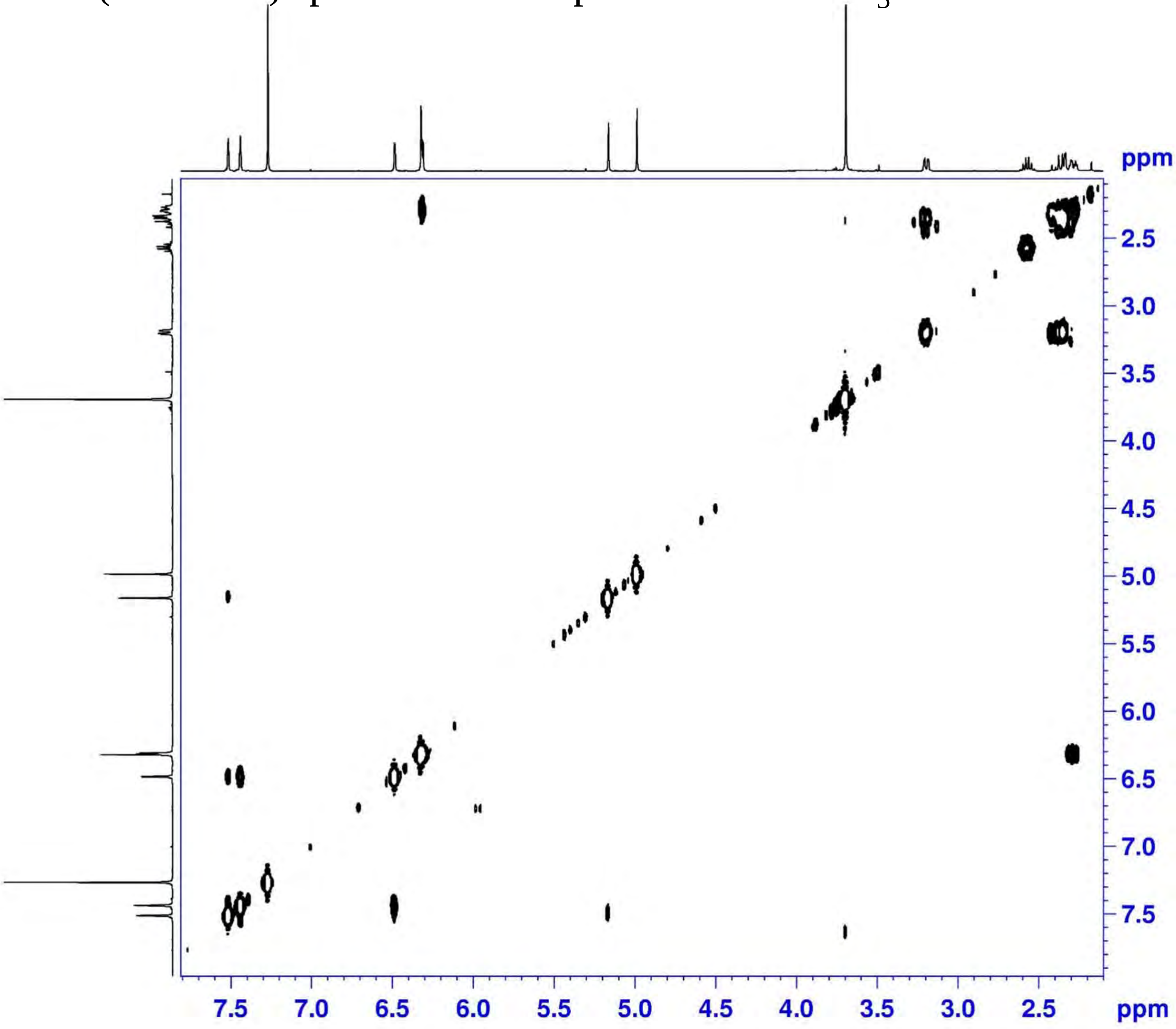

^1H-^1H COSY (400 MHz) spectrum of compound **11** in $CDCl_3$

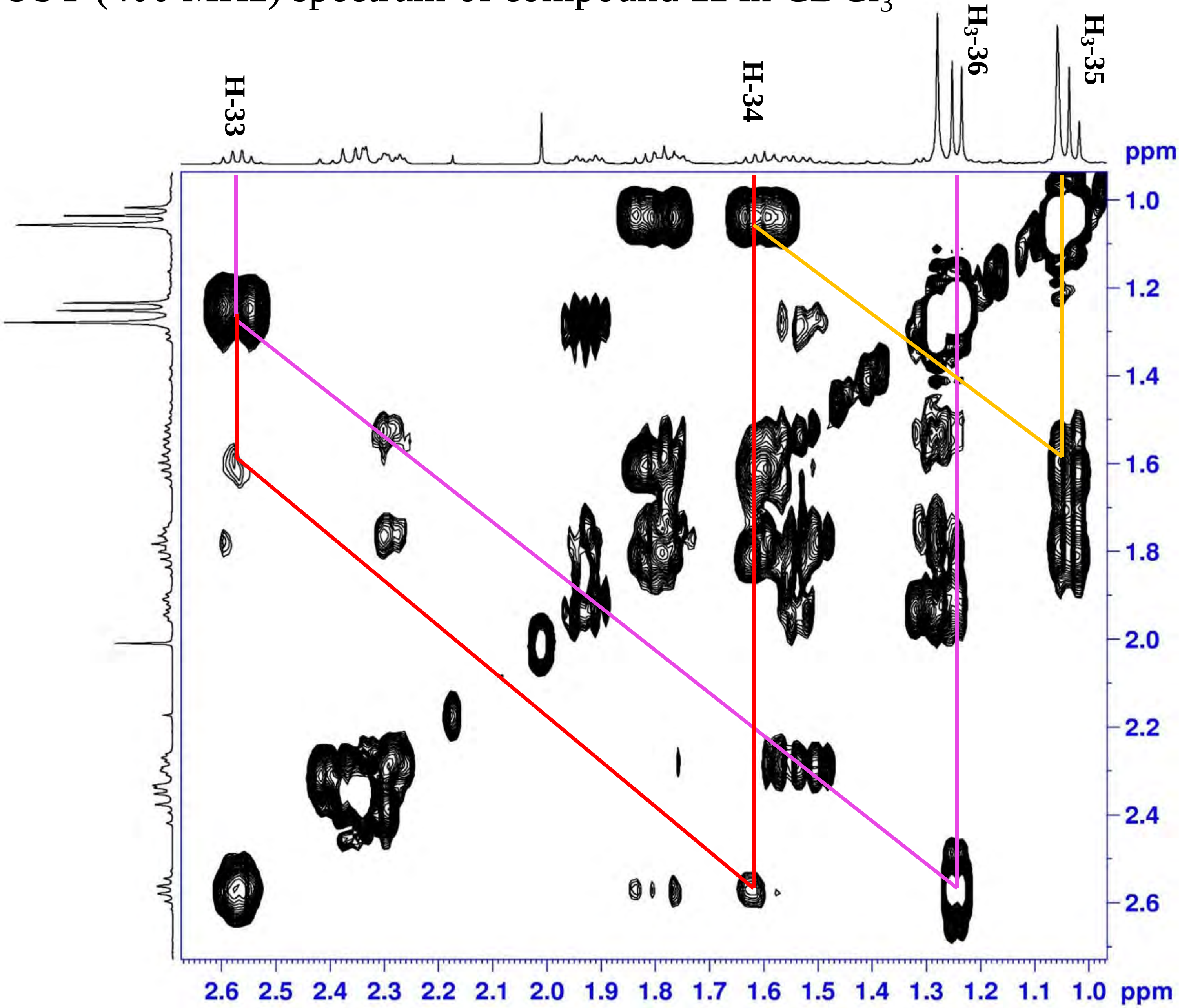

^{1}H-^{1}H COSY (400 MHz) spectrum of compound **11** in $CDCl_3$

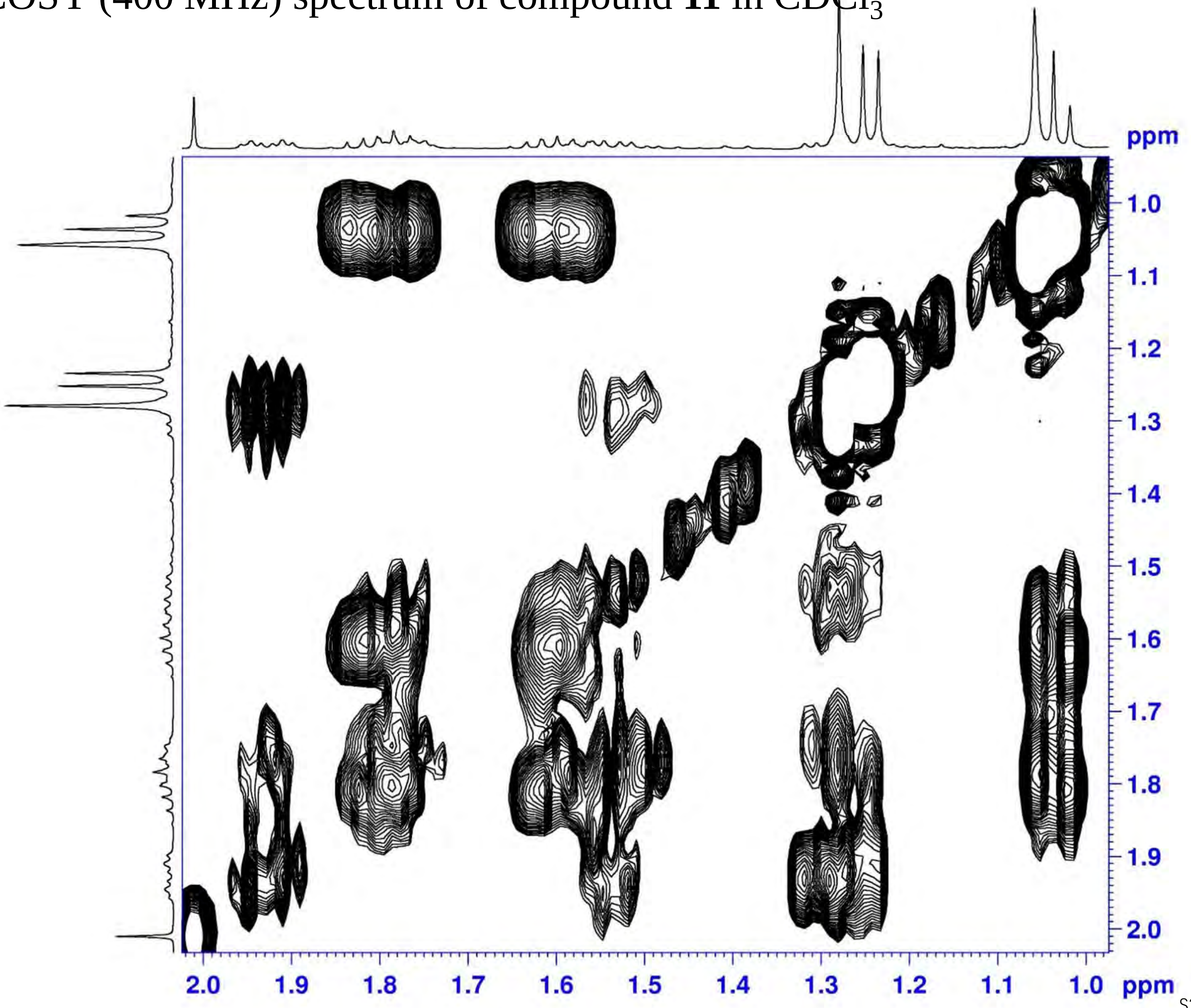

HSQC (400 MHz) spectrum of compound **11** in $CDCl_3$

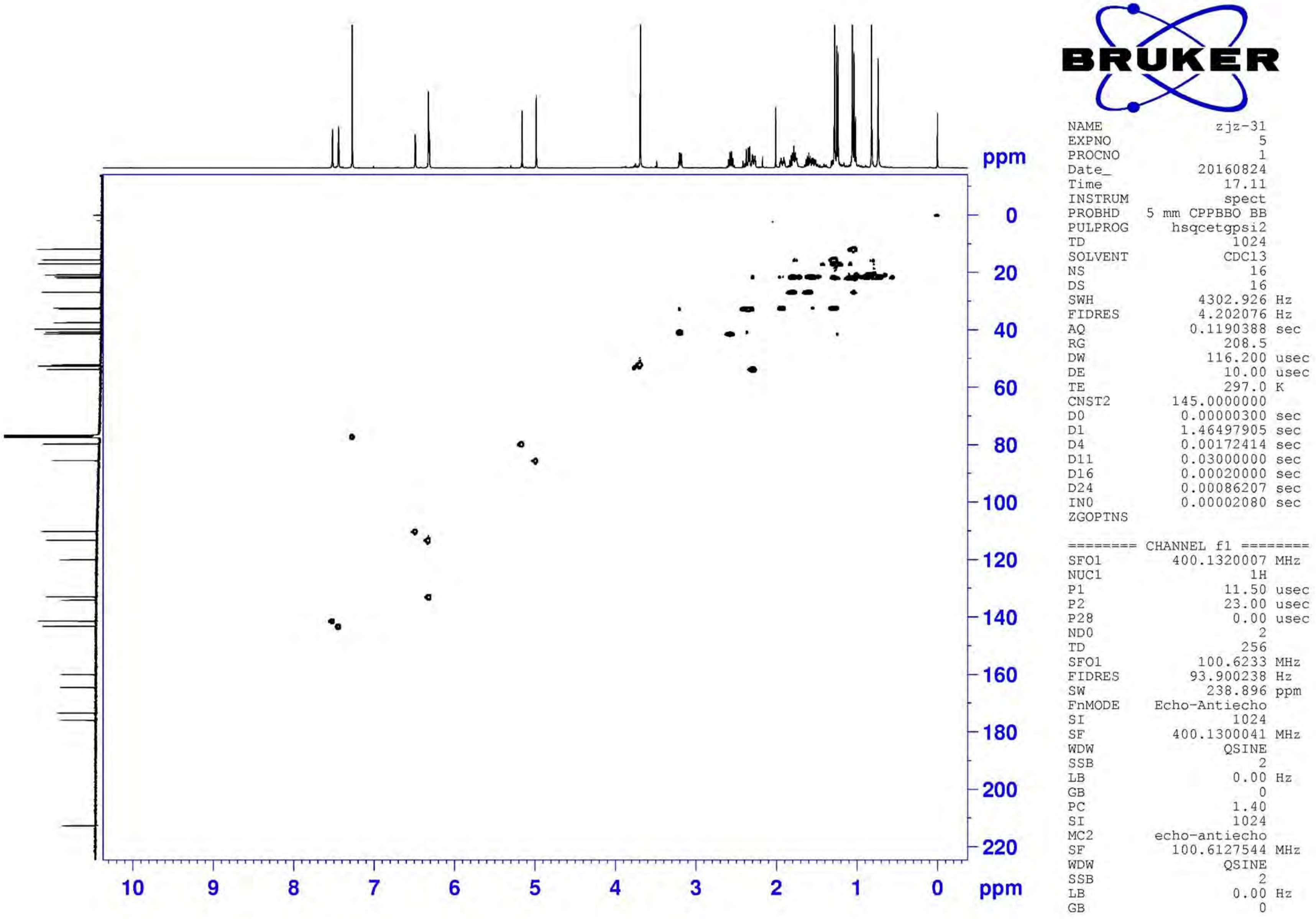

HSQC (400 MHz) spectrum of compound **11** in $CDCl_3$

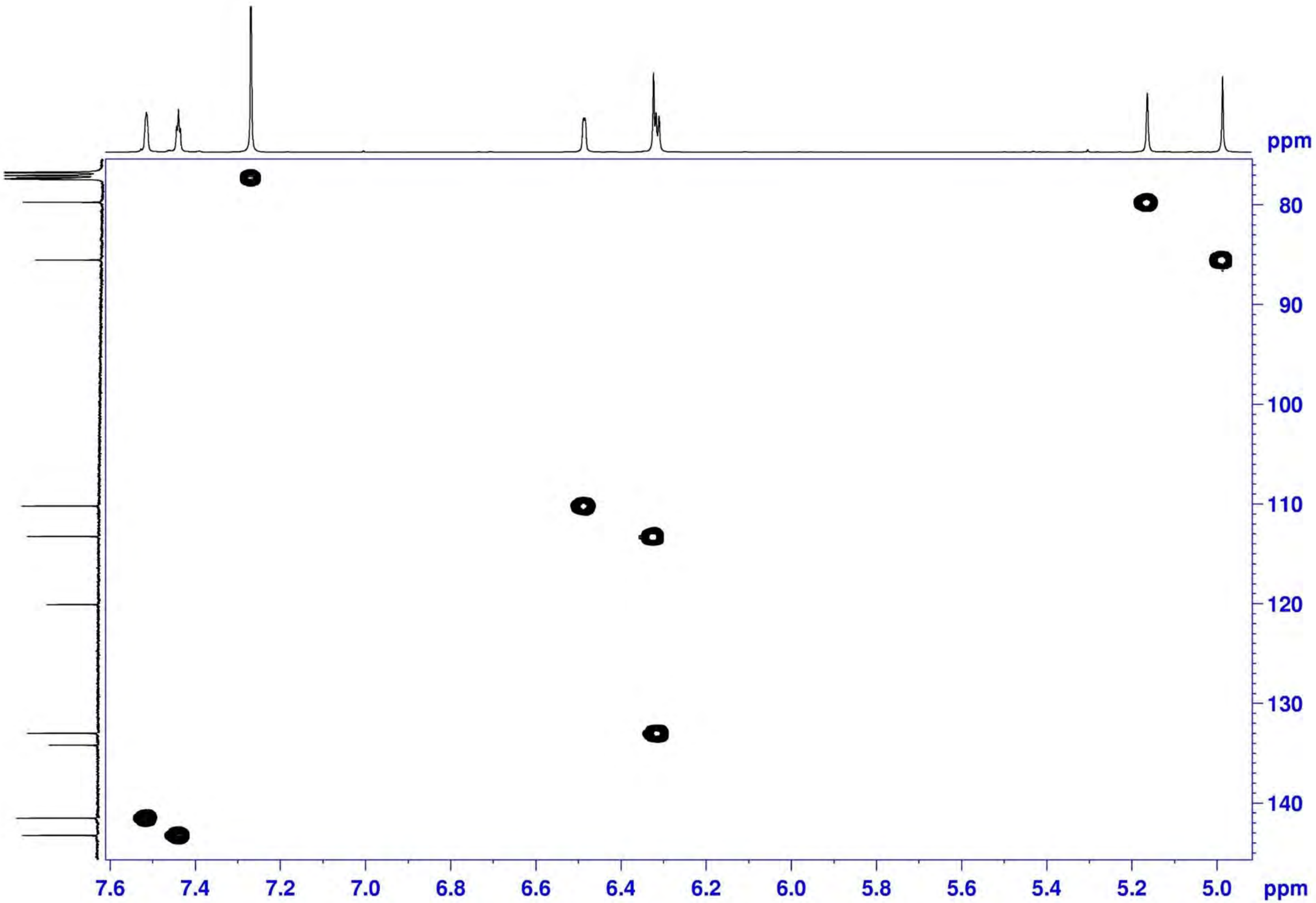

HSQC (400 MHz) spectrum of compound **11** in $CDCl_3$

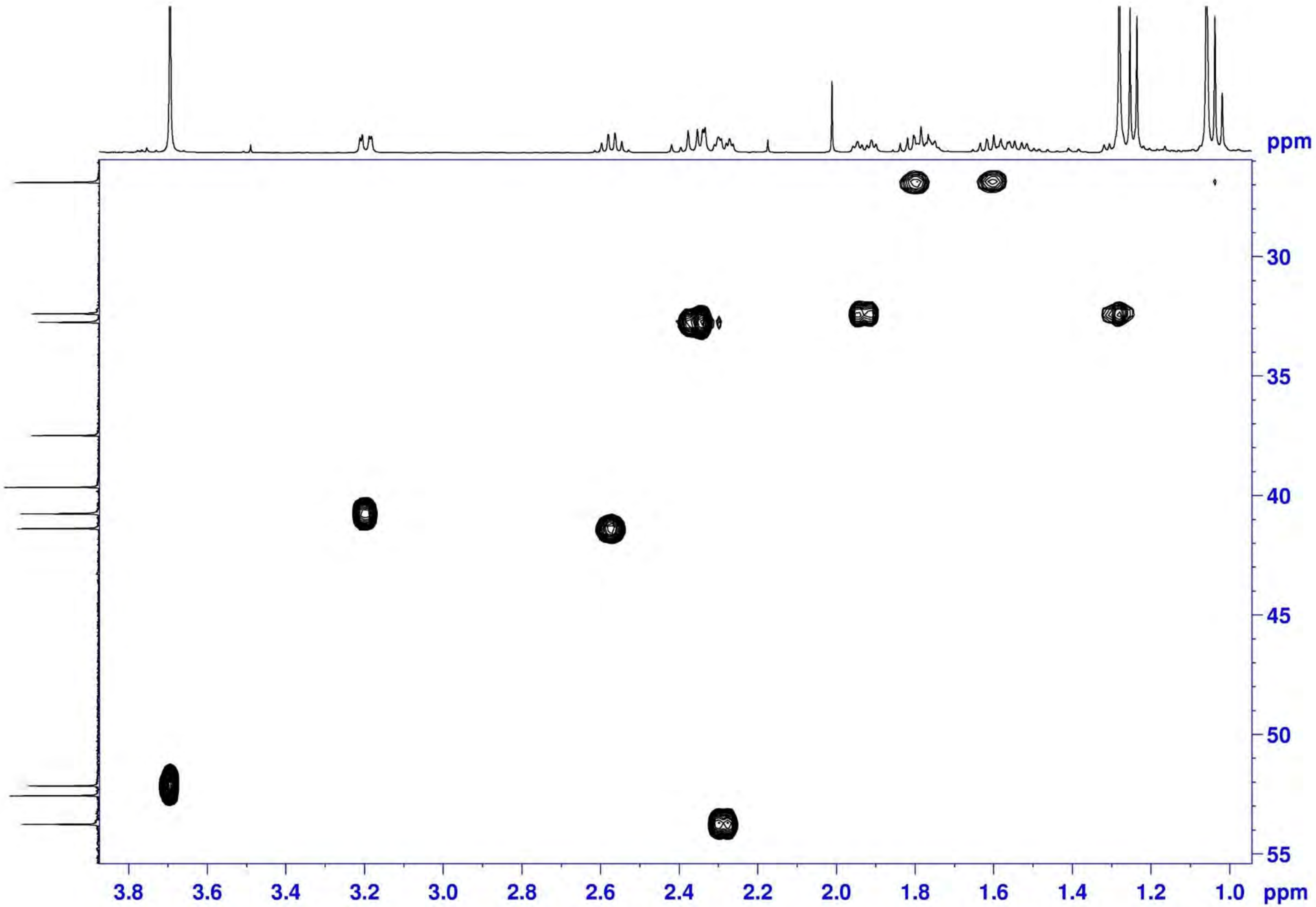

HSQC (400 MHz) spectrum of compound **11** in $CDCl_3$

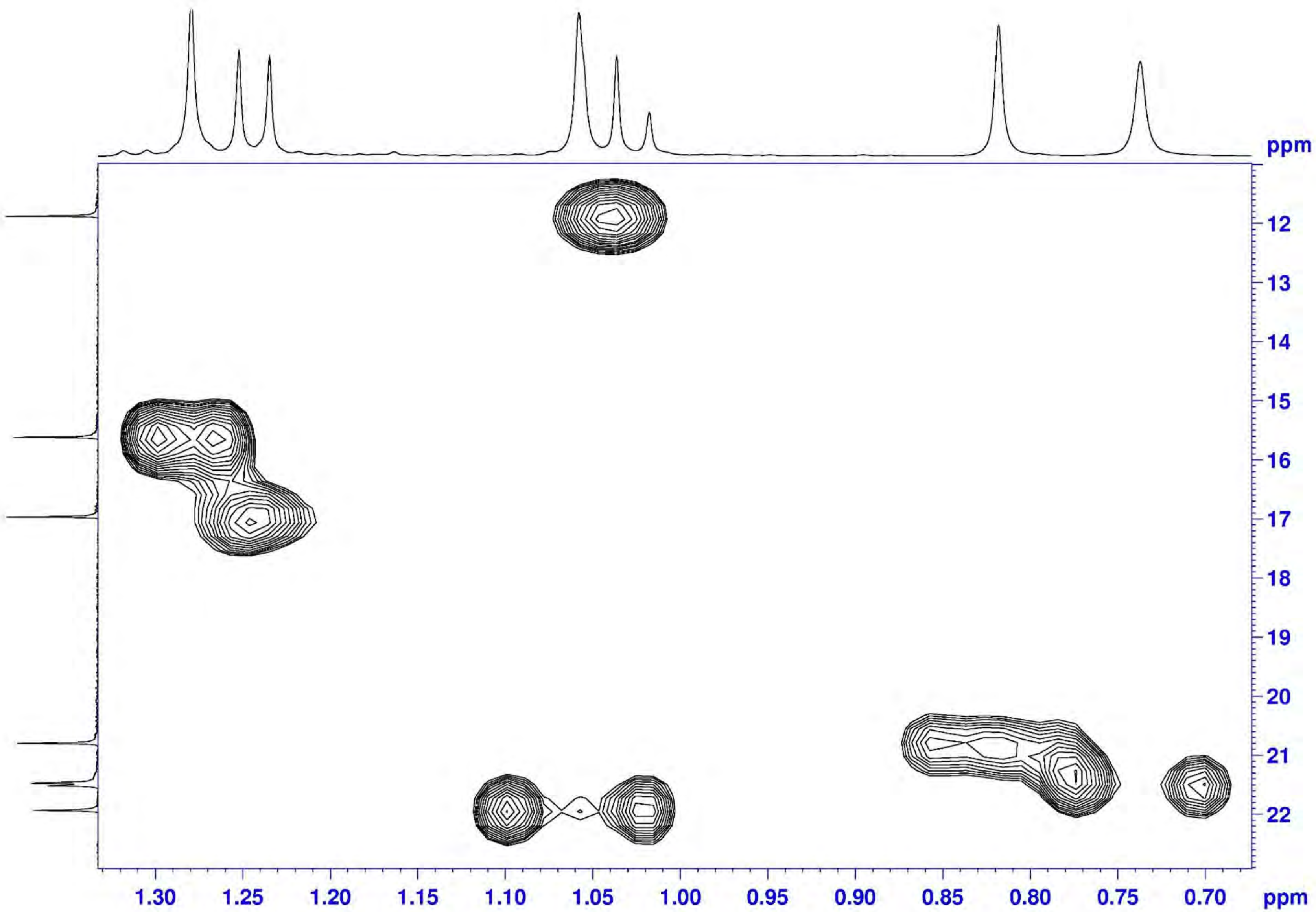

HMBC (400 MHz) spectrum of compound **11** in $CDCl_3$

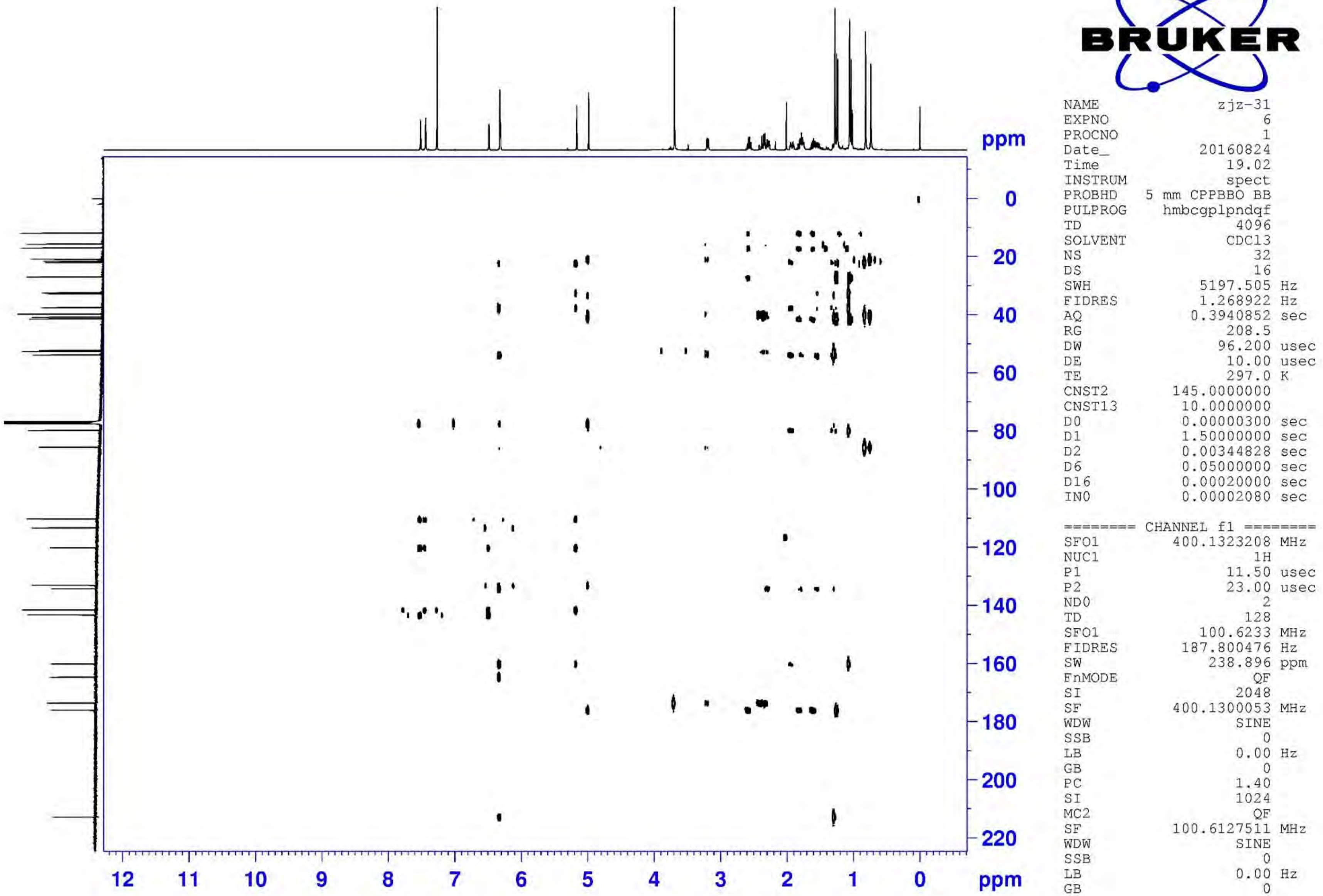

HMBC (400 MHz) spectrum of compound **11** in $CDCl_3$

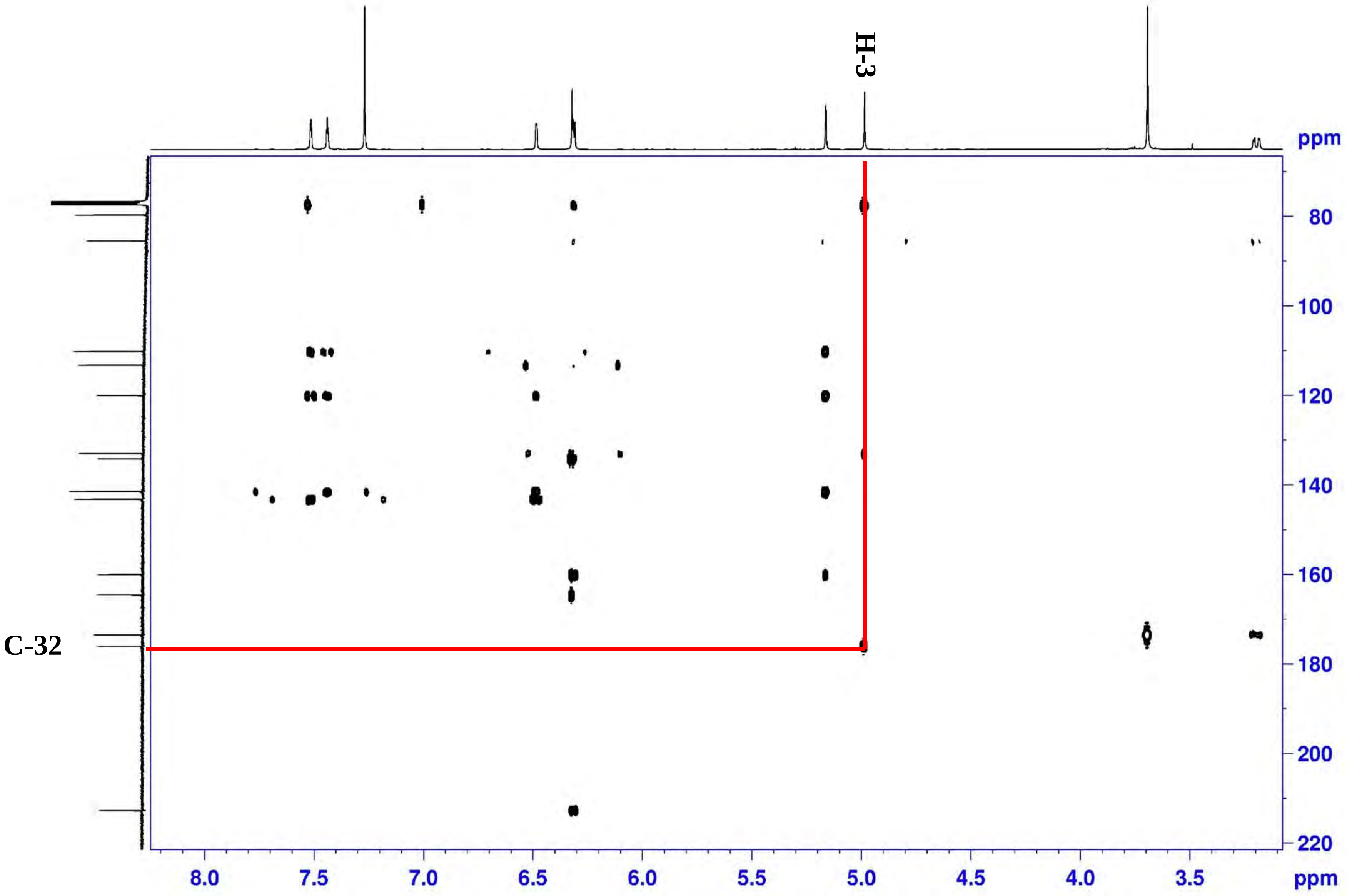

HMBC (400 MHz) spectrum of compound **11** in $CDCl_3$

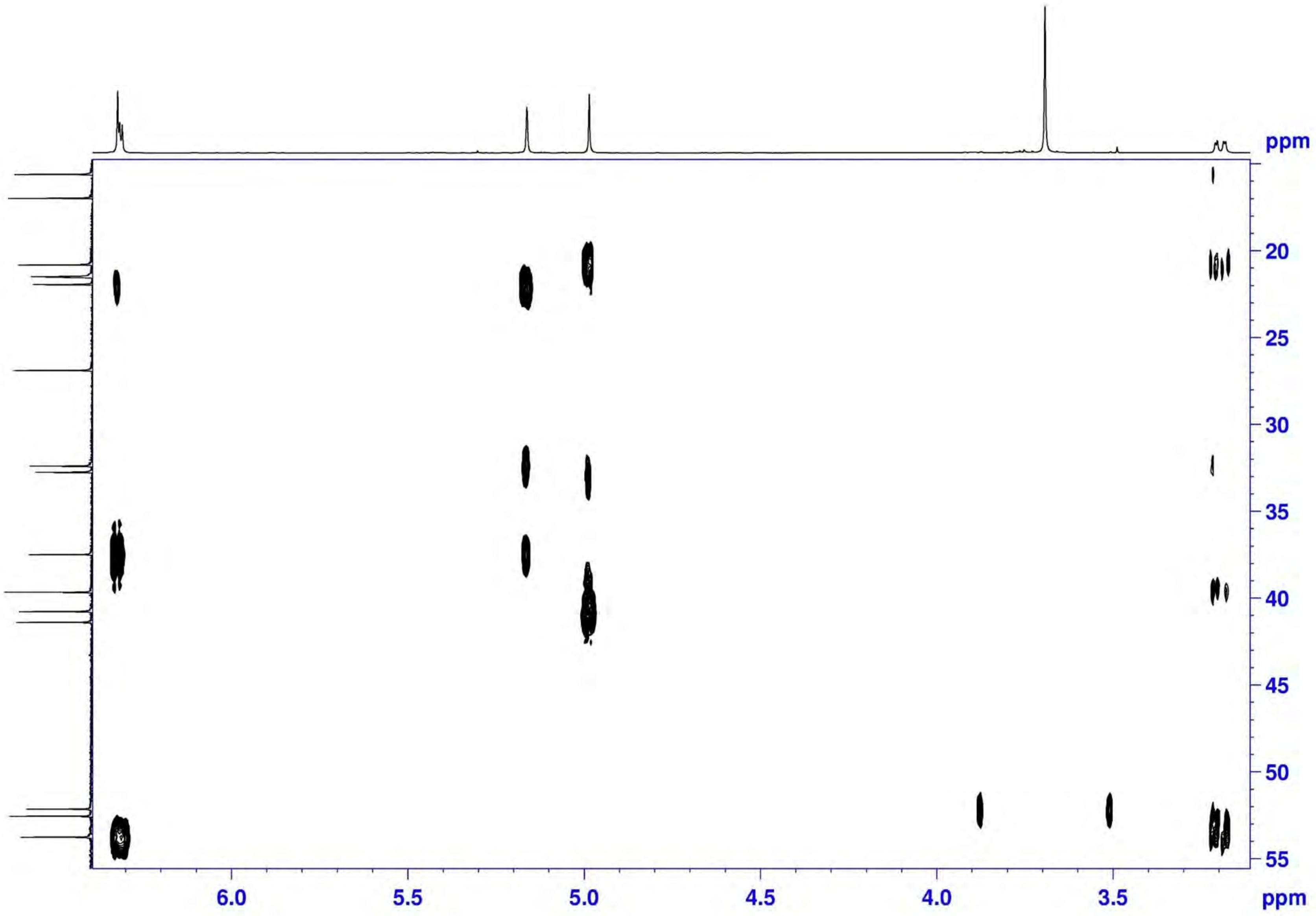

HMBC (400 MHz) spectrum of compound **11** in $CDCl_3$

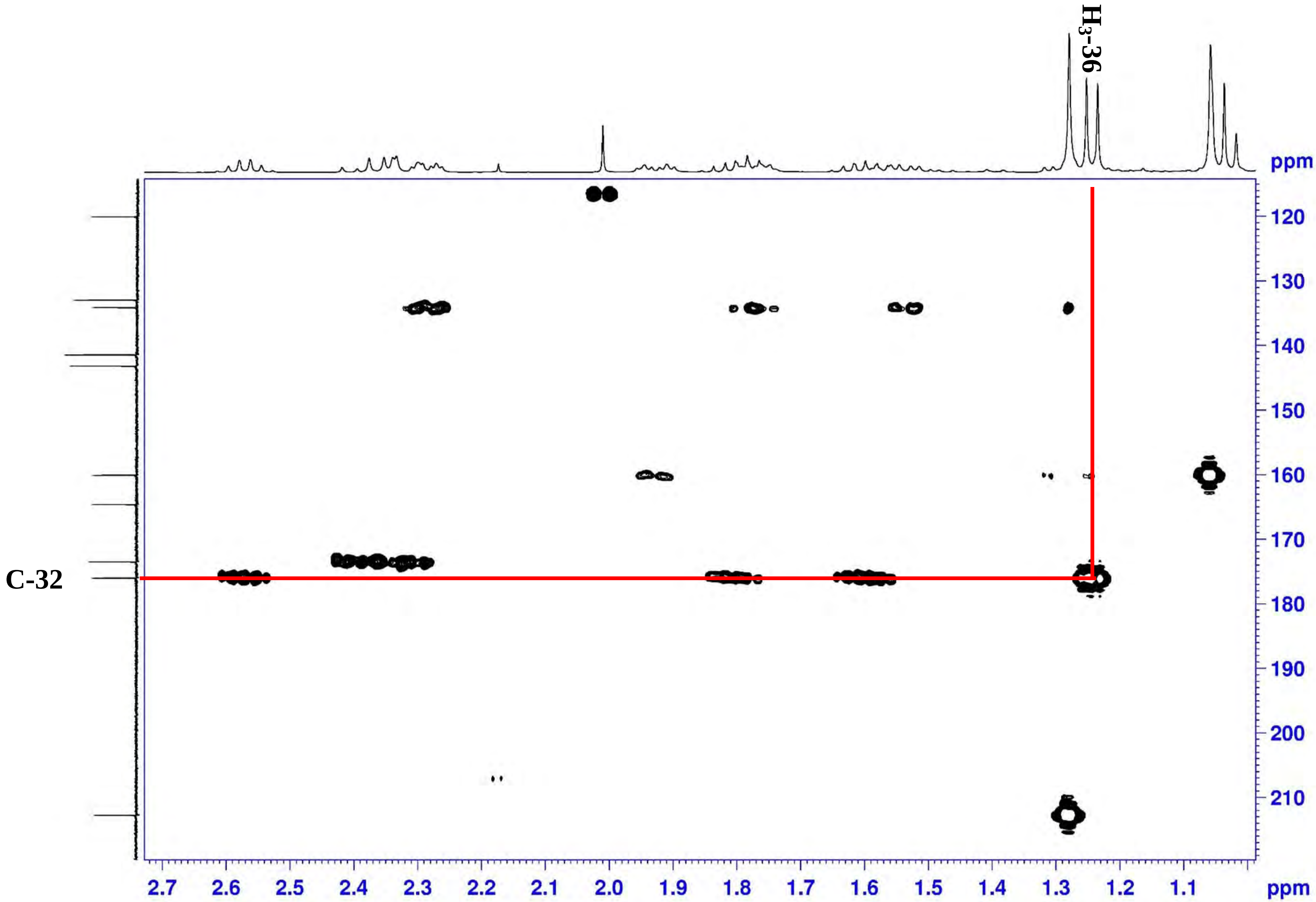

HMBC (400 MHz) spectrum of compound **11** in $CDCl_3$

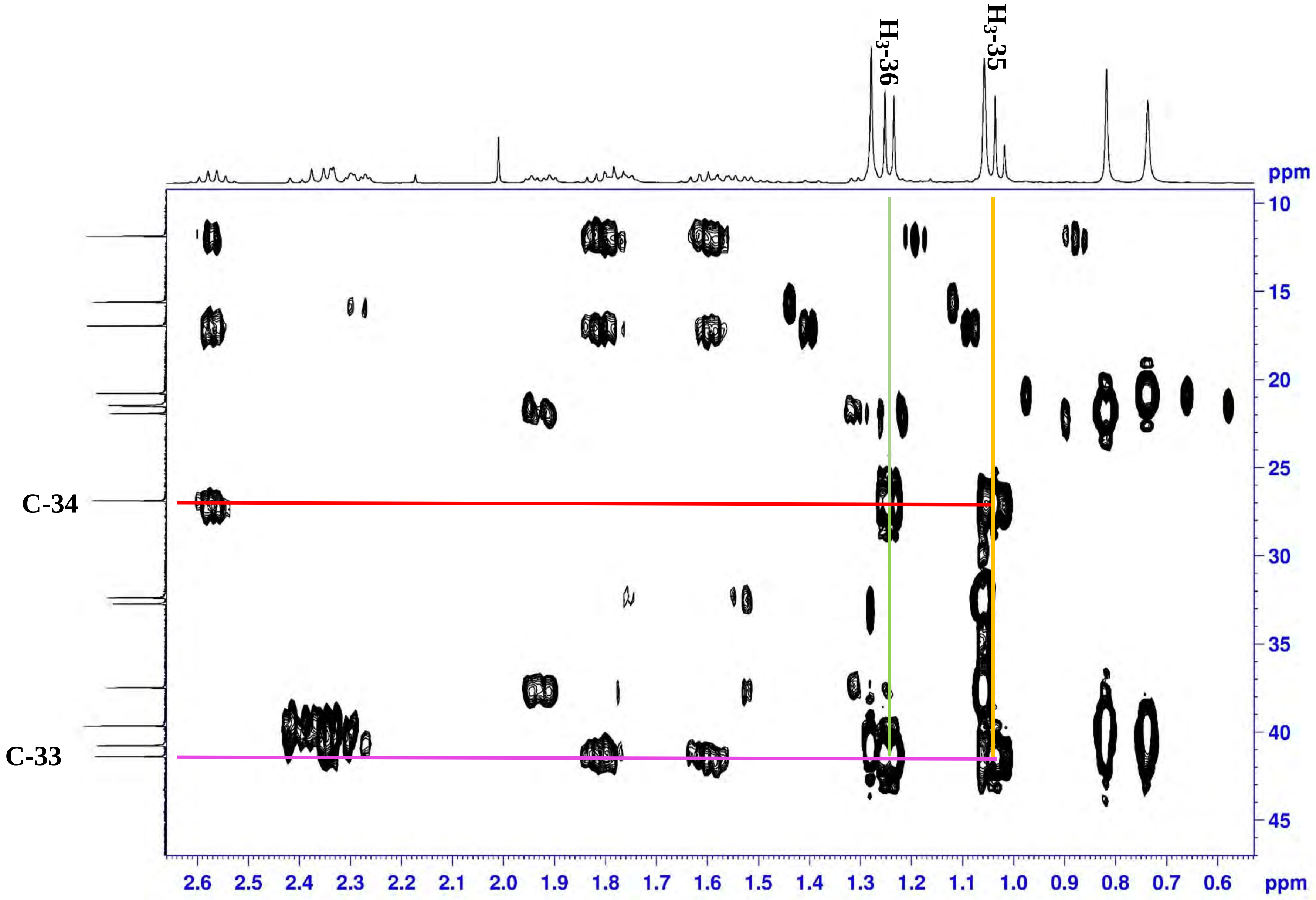

HMBC (400 MHz) spectrum of compound **11** in $CDCl_3$

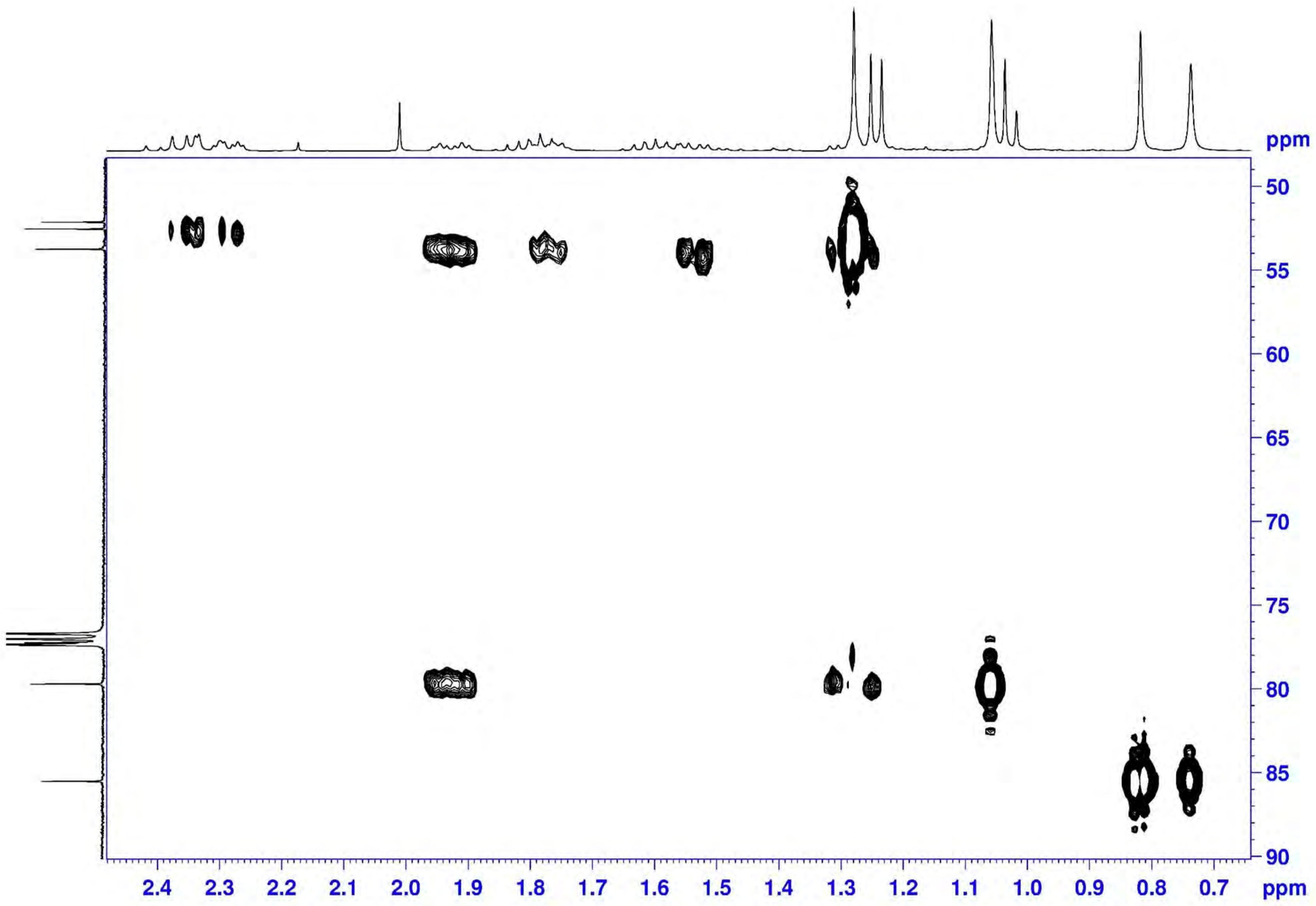

NOESY (400 MHz) spectrum of compound **11** in $CDCl_3$

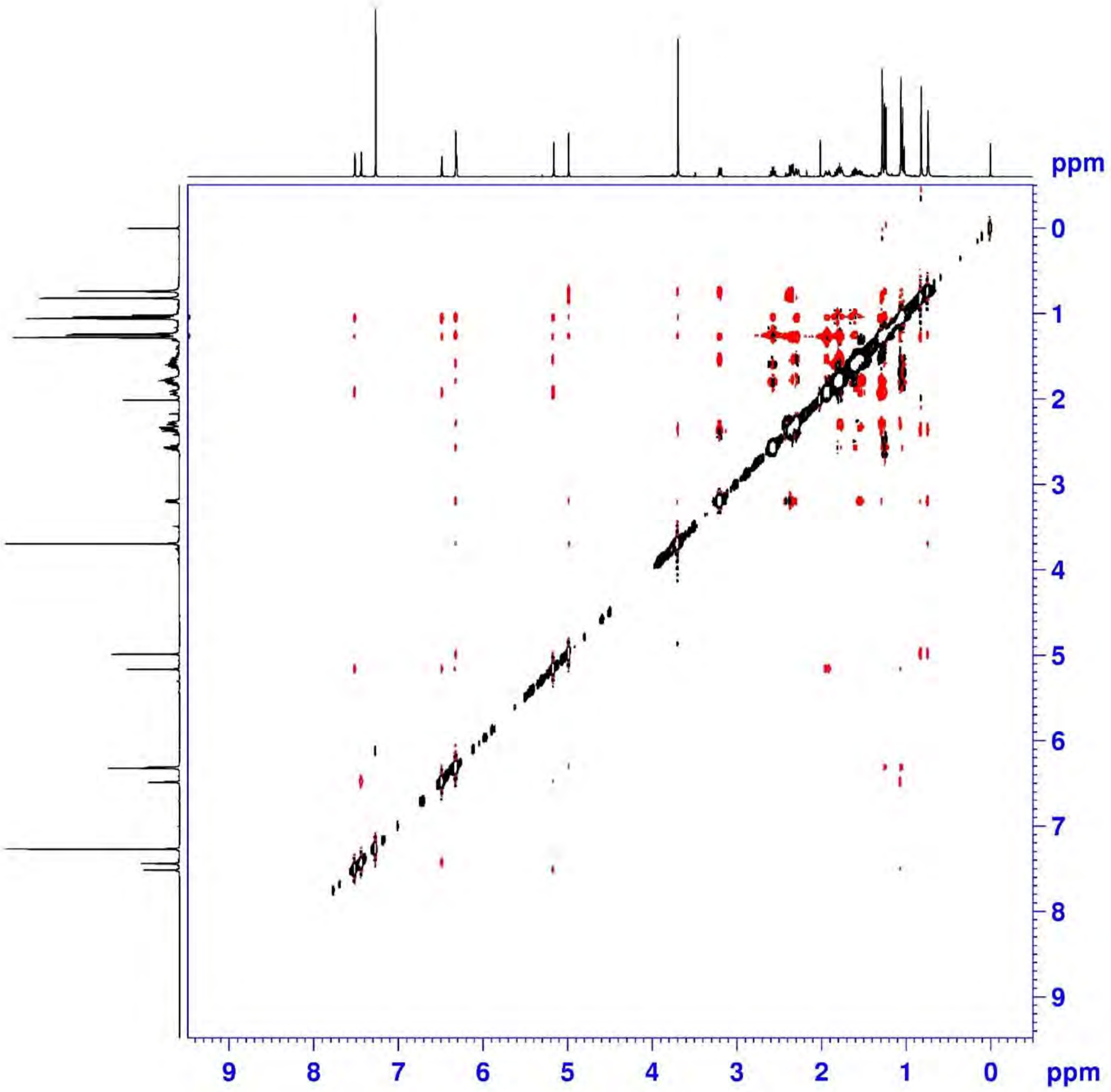

NAME	zjz-31	
EXPNO	7	
PROCNO	1	
Date_	20160824	
Time	21.18	
INSTRUM	spect	
PROBHD	5 mm CPPBBO BB	
PULPROG	noesygpphpp	
TD	2048	
SOLVENT	CDCl3	
NS	16	
DS	32	
SWH	4000.000	Hz
FIDRES	1.953125	Hz
AQ	0.2560500	sec
RG	208.5	
DW	125.000	usec
DE	10.00	usec
TE	297.0	K
D0	0.00011036	sec
D1	1.99385595	sec
D8	0.30000001	sec
D11	0.03000000	sec
D12	0.00002000	sec
D16	0.00020000	sec
IN0	0.00025000	sec
======== CHANNEL f1 ========		
SFO1	400.1318006	MHz
NUC1	1H	
P1	11.50	usec
P2	23.00	usec
P17	2500.00	usec
ND0	1	
TD	256	
SFO1	400.1318	MHz
FIDRES	15.625000	Hz
SW	9.997	ppm
FnMODE	States-TPPI	
SI	1024	
SF	400.1300046	MHz
WDW	QSINE	
SSB	2	
LB	0.00	Hz
GB	0	
PC	1.00	
SI	1024	
MC2	States-TPPI	
SF	400.1300069	MHz
WDW	QSINE	
SSB	2	
LB	0.00	Hz
GB	0	

NOESY (400 MHz) spectrum of compound **11** in $CDCl_3$

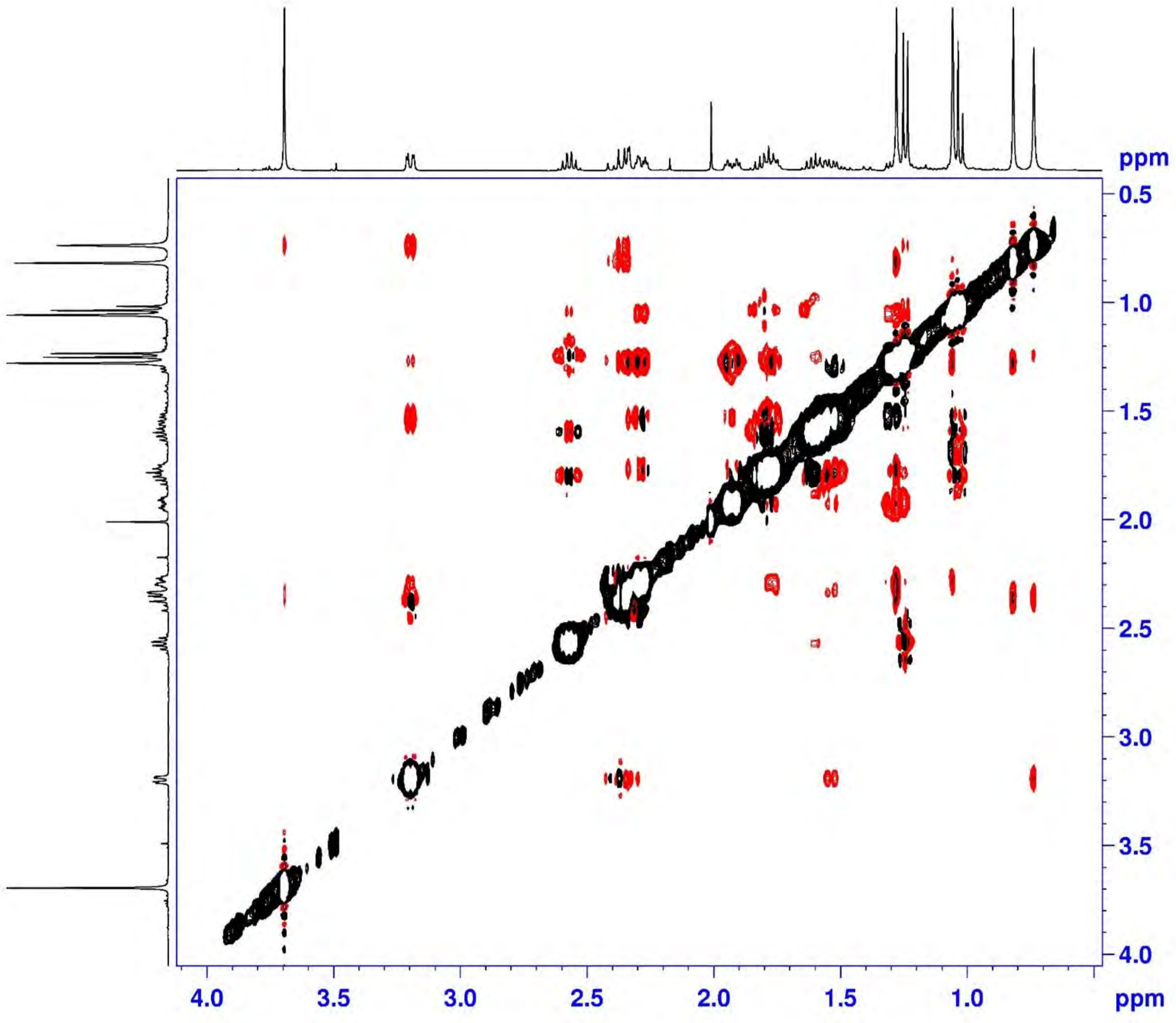

NOESY (400 MHz) spectrum of compound **11** in $CDCl_3$

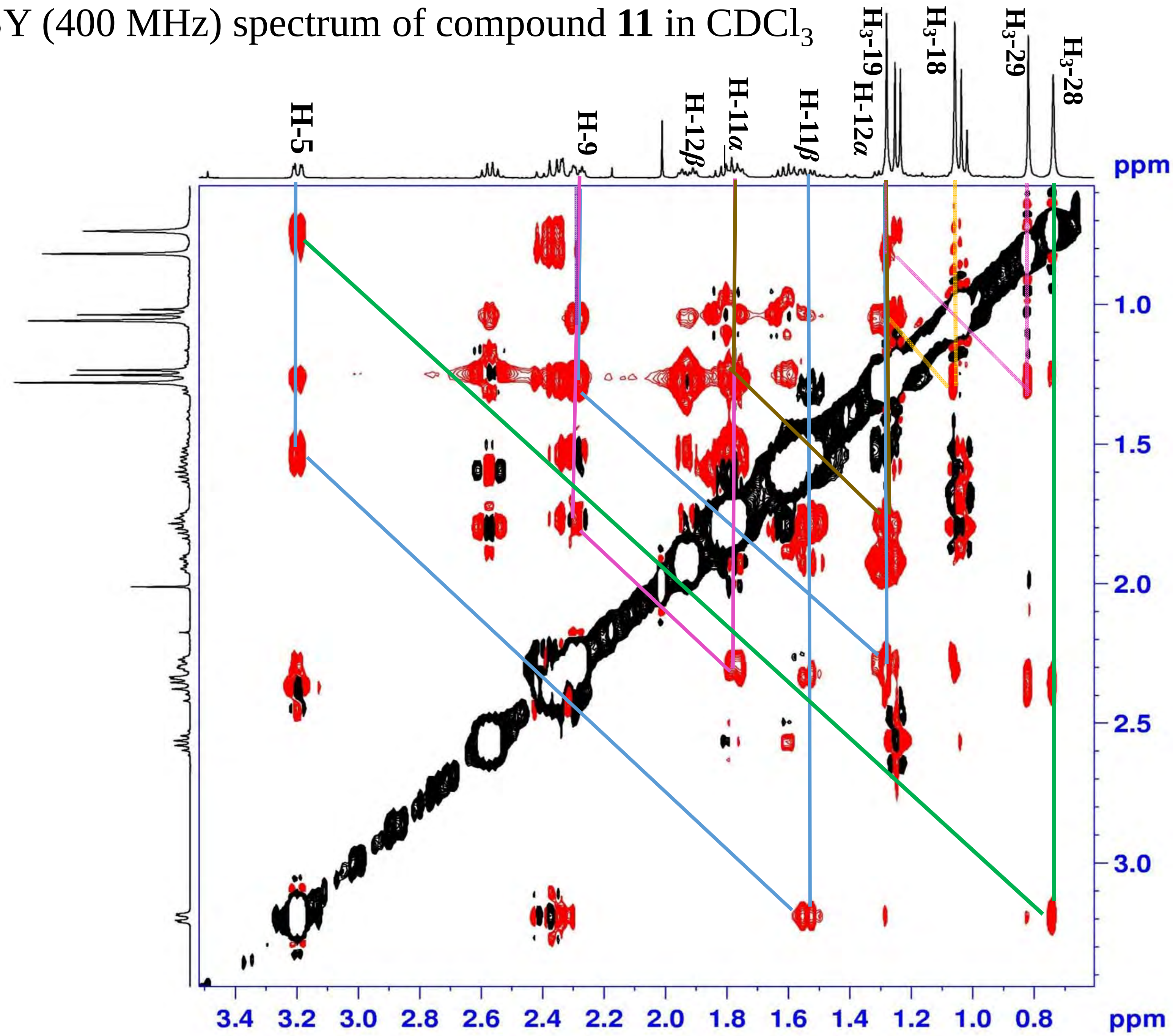

NOESY (400 MHz) spectrum of compound **11** in $CDCl_3$

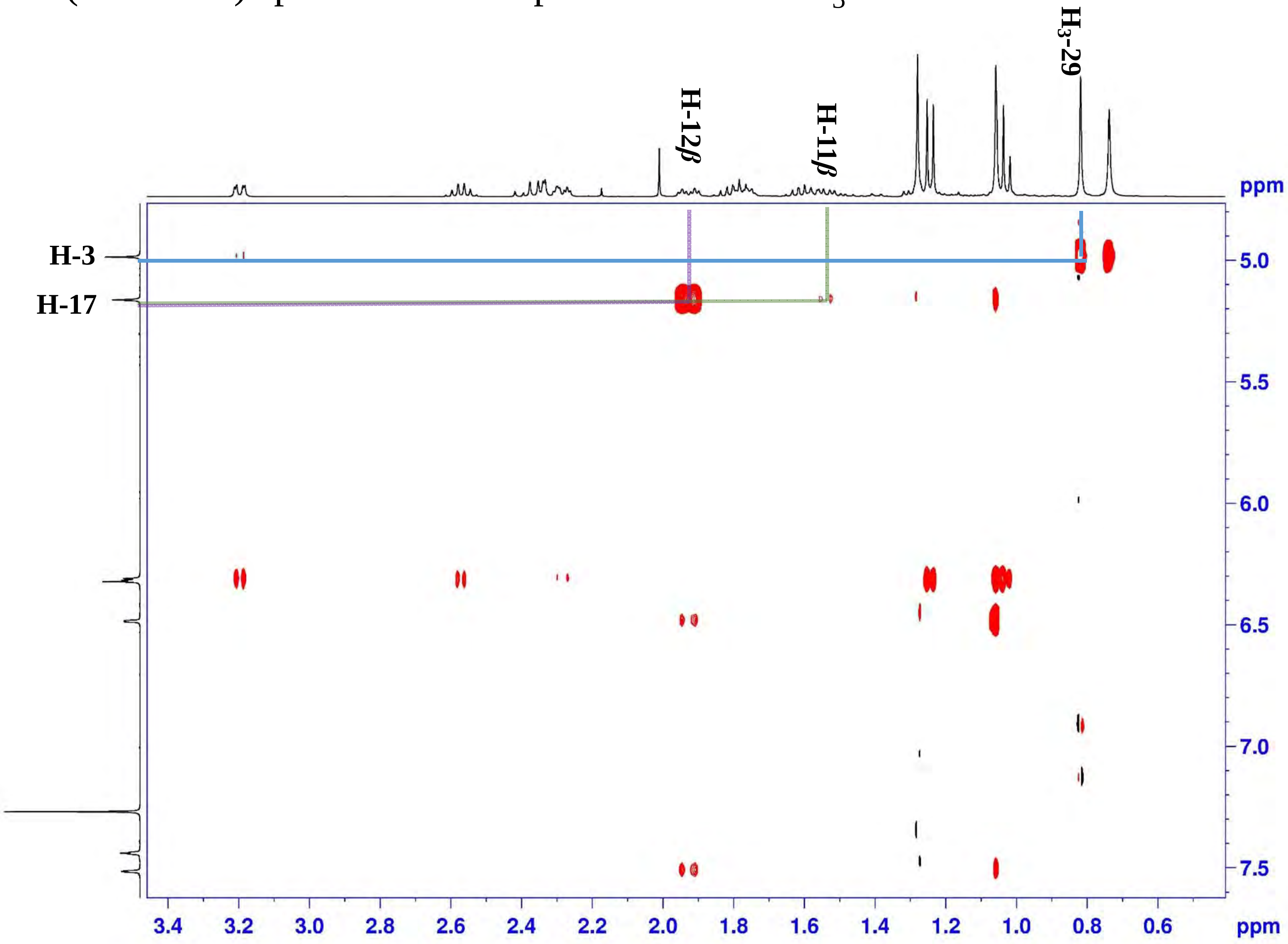

HR-ESIMS for compound **12**

Mass Spectrum SmartFormula Report

Analysis Info

Analysis Name	D:\Data\MS\data\201605\liwanshan_lws-67_pos_16_01_1863.d	Acquisition Date	5/19/2016 9:08:38 AM
Method	LC_Direct Infusion_pos_100-1000mz.m	Operator	SCSIO
Sample Name	liwanshan_lws-67_pos	Instrument / Ser#	maXis 29
Comment			

Acquisition Parameter

Source Type	ESI	Ion Polarity	Positive	Set Nebulizer	0.4 Bar
Focus	Active	Set Capillary	4500 V	Set Dry Heater	180 °C
Scan Begin	100 m/z	Set End Plate Offset	-500 V	Set Dry Gas	4.0 l/min
Scan End	2000 m/z	Set Collision Cell RF	800.0 Vpp	Set Divert Valve	Waste

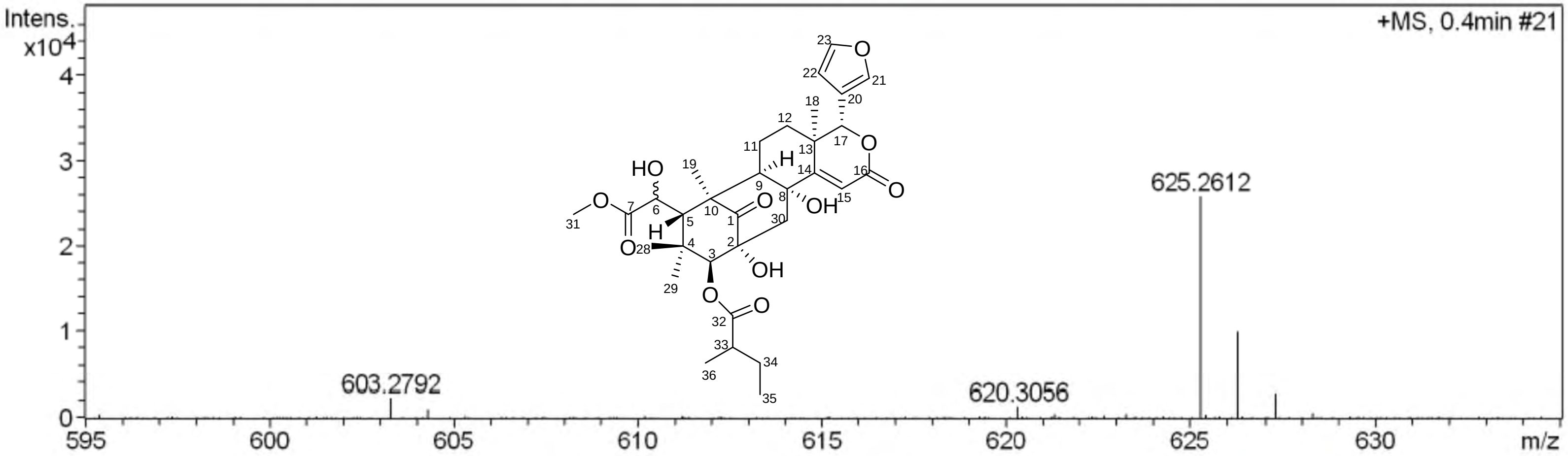

Meas. m/z	#	Formula	Score	m/z	err [ppm]	err [mDa]	mSigma	rdb	e⁻ Conf	N-Rule
603.2792	1	C 32 H 43 O 11	100.00	603.2800	1.3	0.8	8.8	11.5	even	ok
625.2612	1	C 32 H 42 Na O 11	100.00	625.2619	1.1	0.7	18.4	11.5	even	ok
1227.5350	1	C 64 H 84 Na O 22	100.00	1227.5346	-0.3	-0.3	59.9	22.5	even	ok

HR-ESIMS for compound **12**

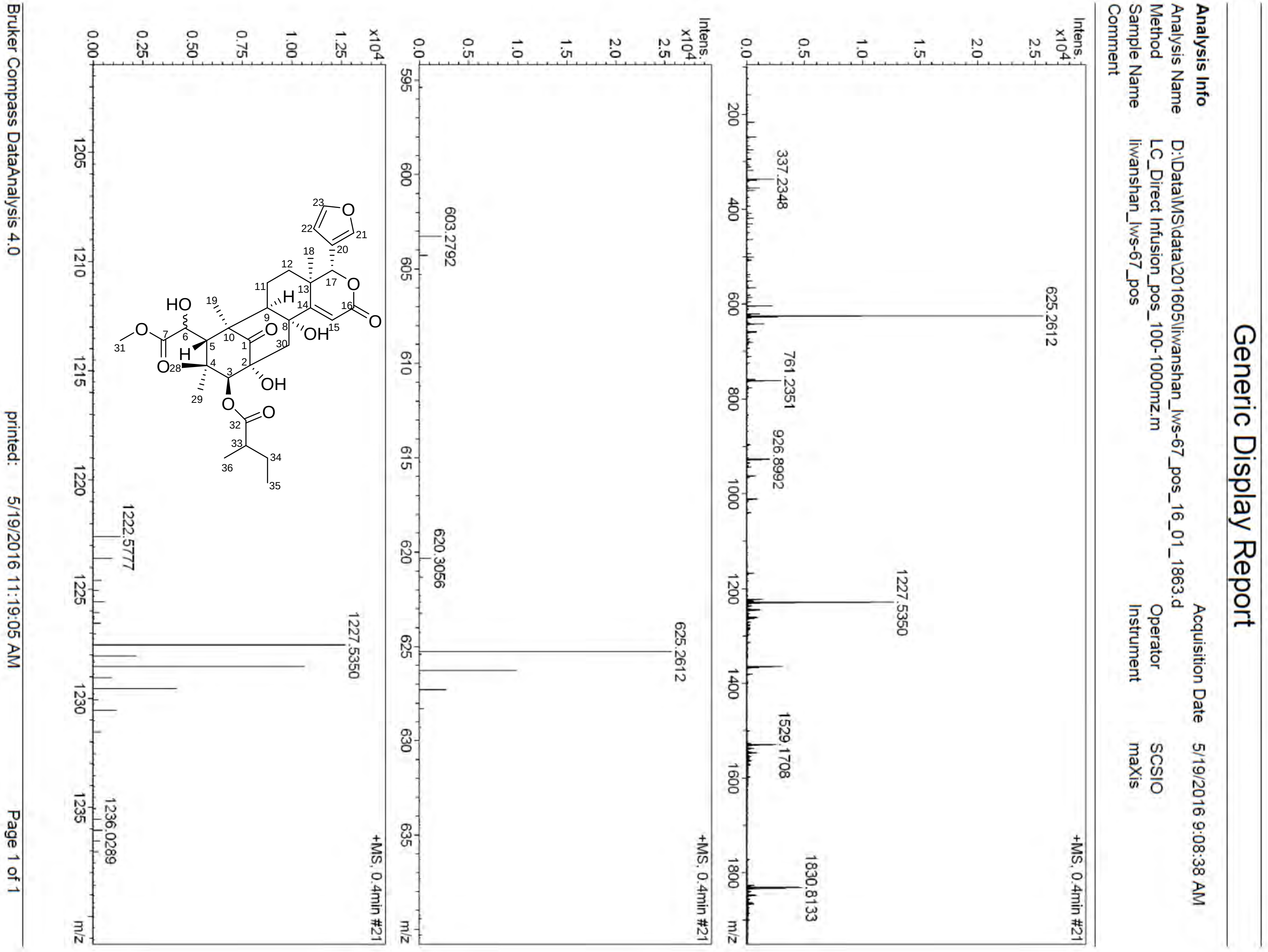

^{1}H NMR (400 MHz) spectrum of compound **12** in DMSO-*d6*

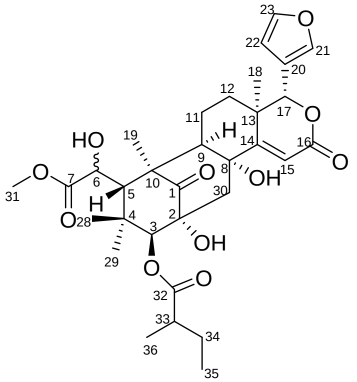

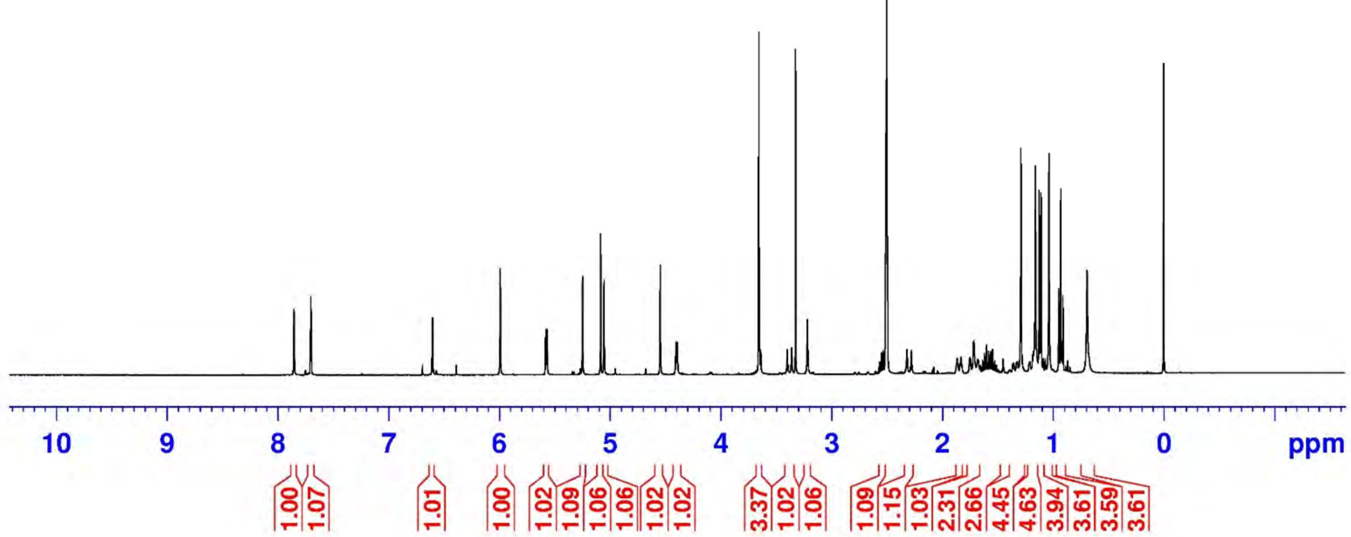

NAME lws-67-DMSO
EXPNO 1
PROCNO 1
Date_ 20160627
Time 17.22
INSTRUM spect
PROBHD 5 mm CPPBBO BB
PULPROG zg30
TD 65536
SOLVENT DMSO
NS 16
DS 2
SWH 8223.685 H
FIDRES 0.125483 H
AQ 3.9846387 s
RG 208.5
DW 60.800 u
DE 10.00 u
TE 297.0 K
D1 1.00000000 s
TD0 1

======== CHANNEL f1 =====
SFO1 400.1324710 M
NUC1 1H
P1 11.50 u
SI 65536
SF 400.1300030 M
WDW EM
SSB 0
LB 0.30 H
GB 0
PC 1.00

^{1}H NMR (400 MHz) spectrum of compound **12** in DMSO-*d6*

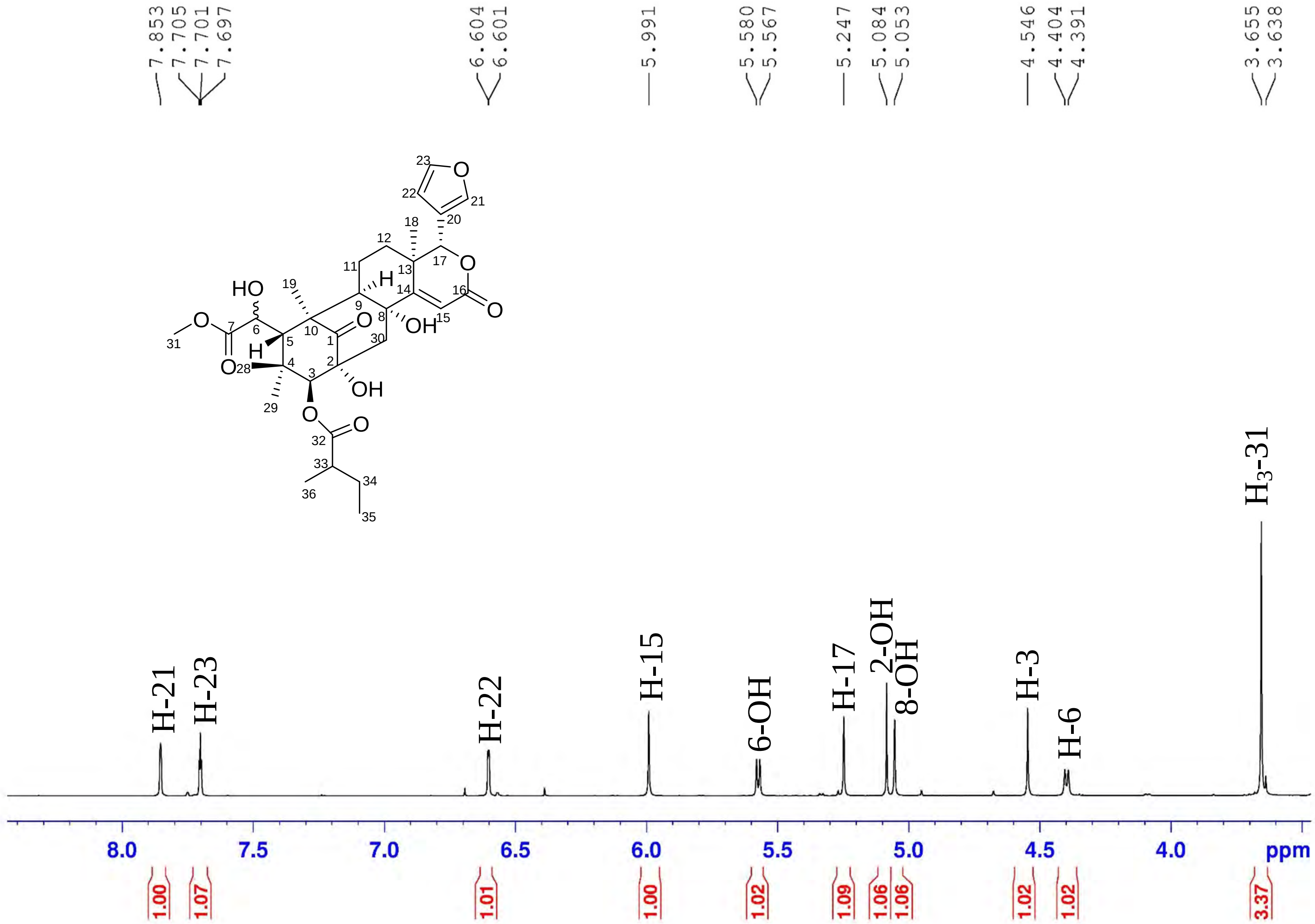

^{1}H NMR (400 MHz) spectrum of compound **12** in DMSO-*d6*

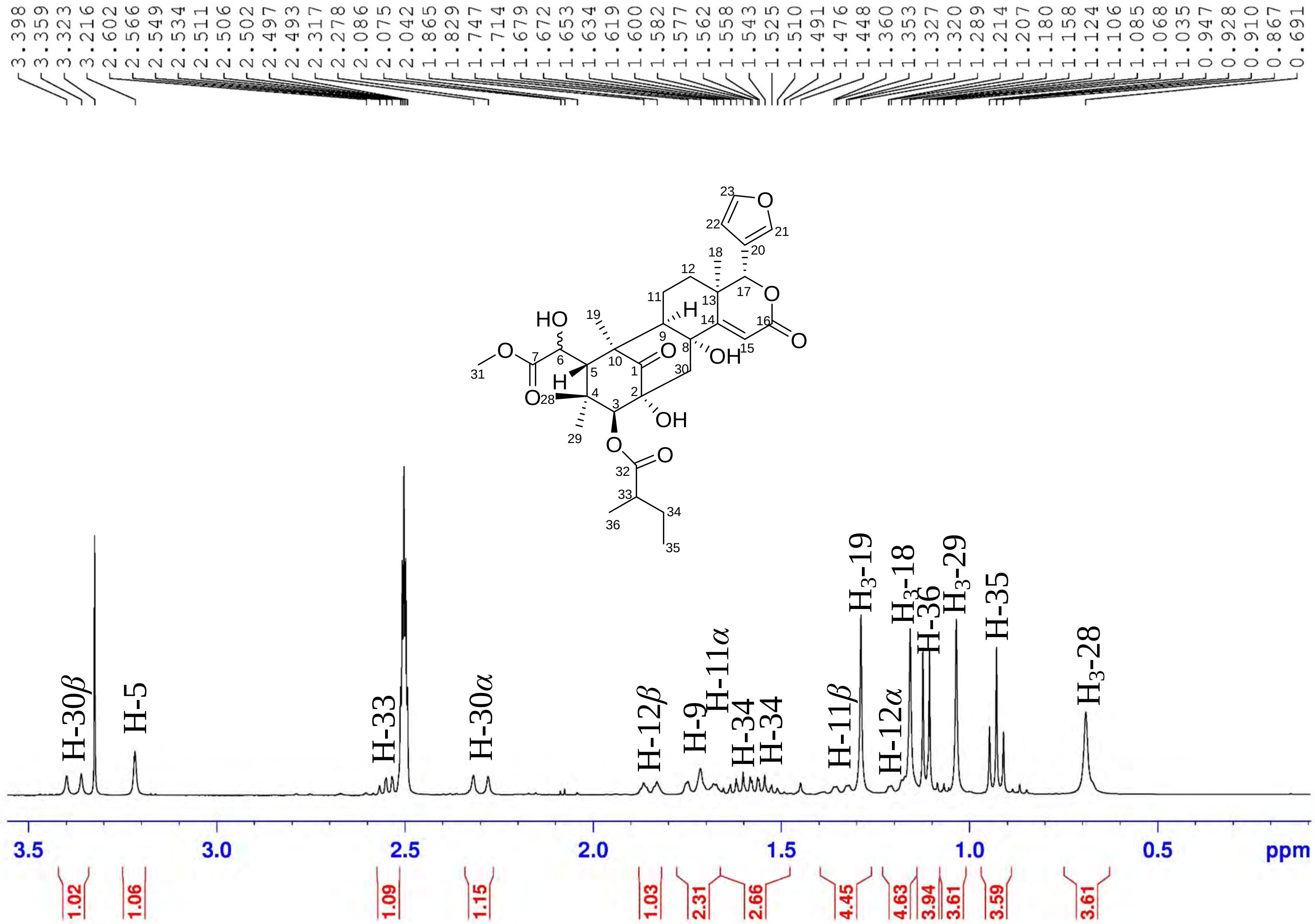

^{13}C NMR (100 MHz) spectrum of compound **12** in DMSO-*d6*

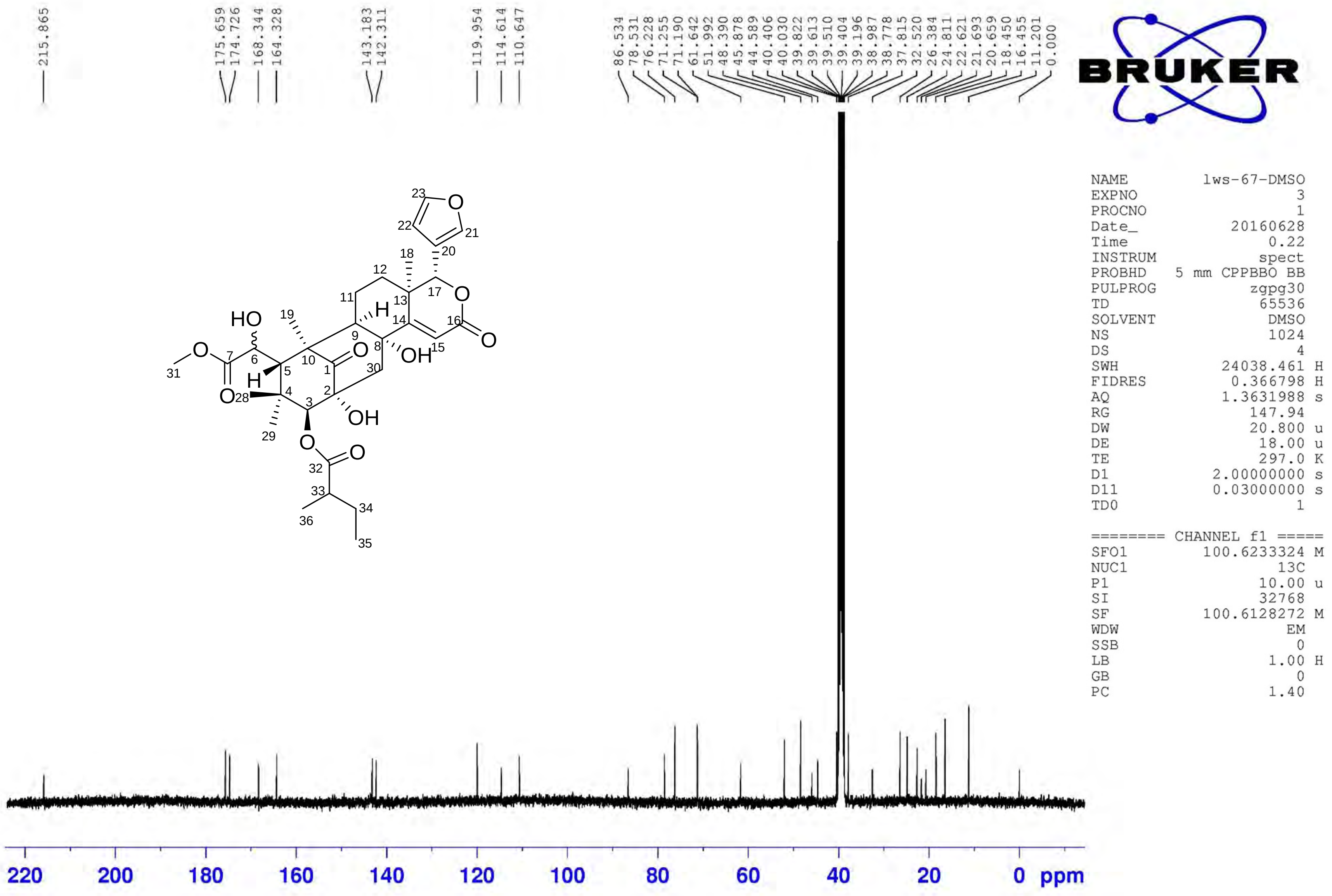

^{13}C NMR (100 MHz) spectrum of compound **12** in DMSO-*d6*

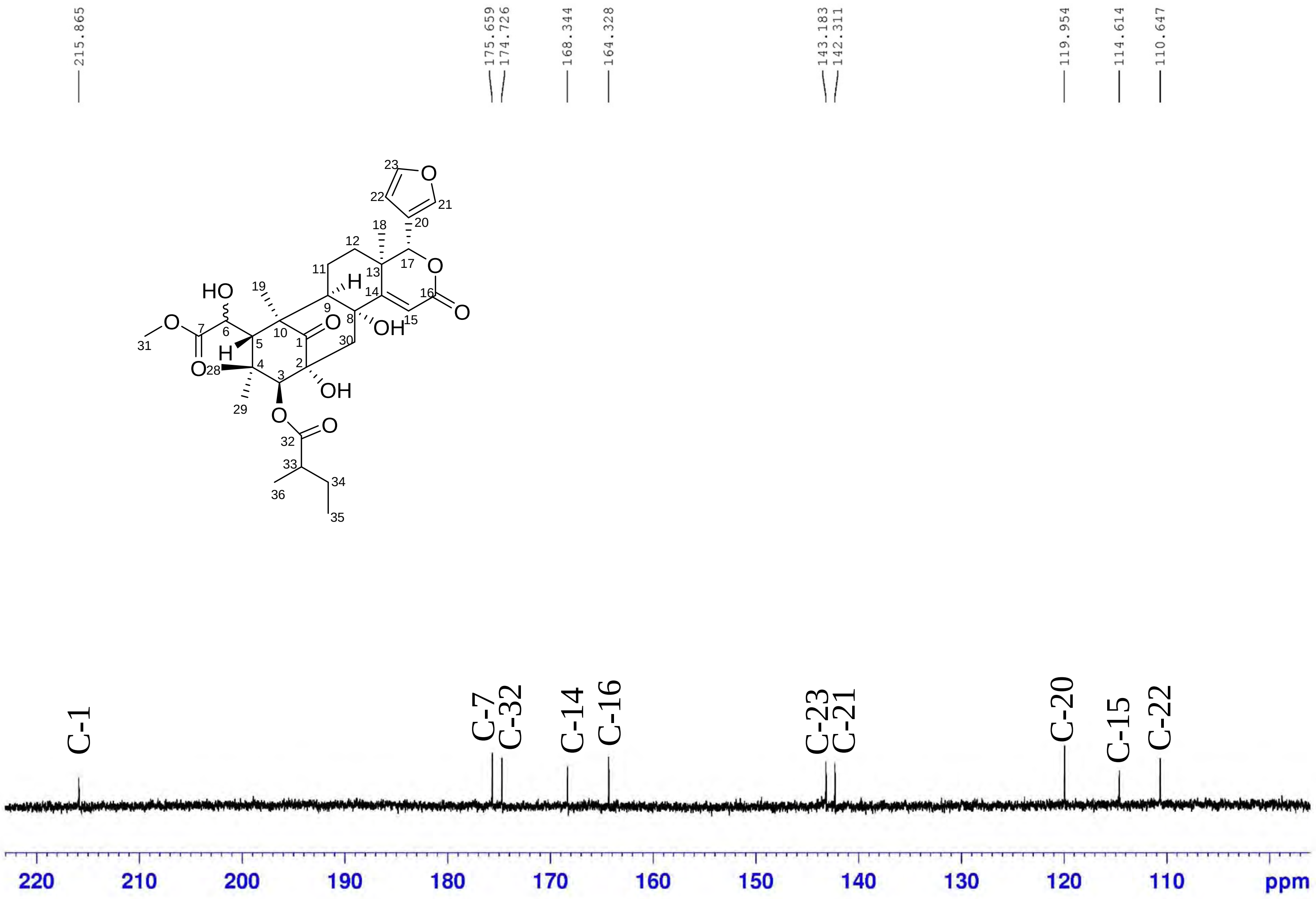

^{13}C NMR (100 MHz) spectrum of compound **12** in DMSO-*d6*

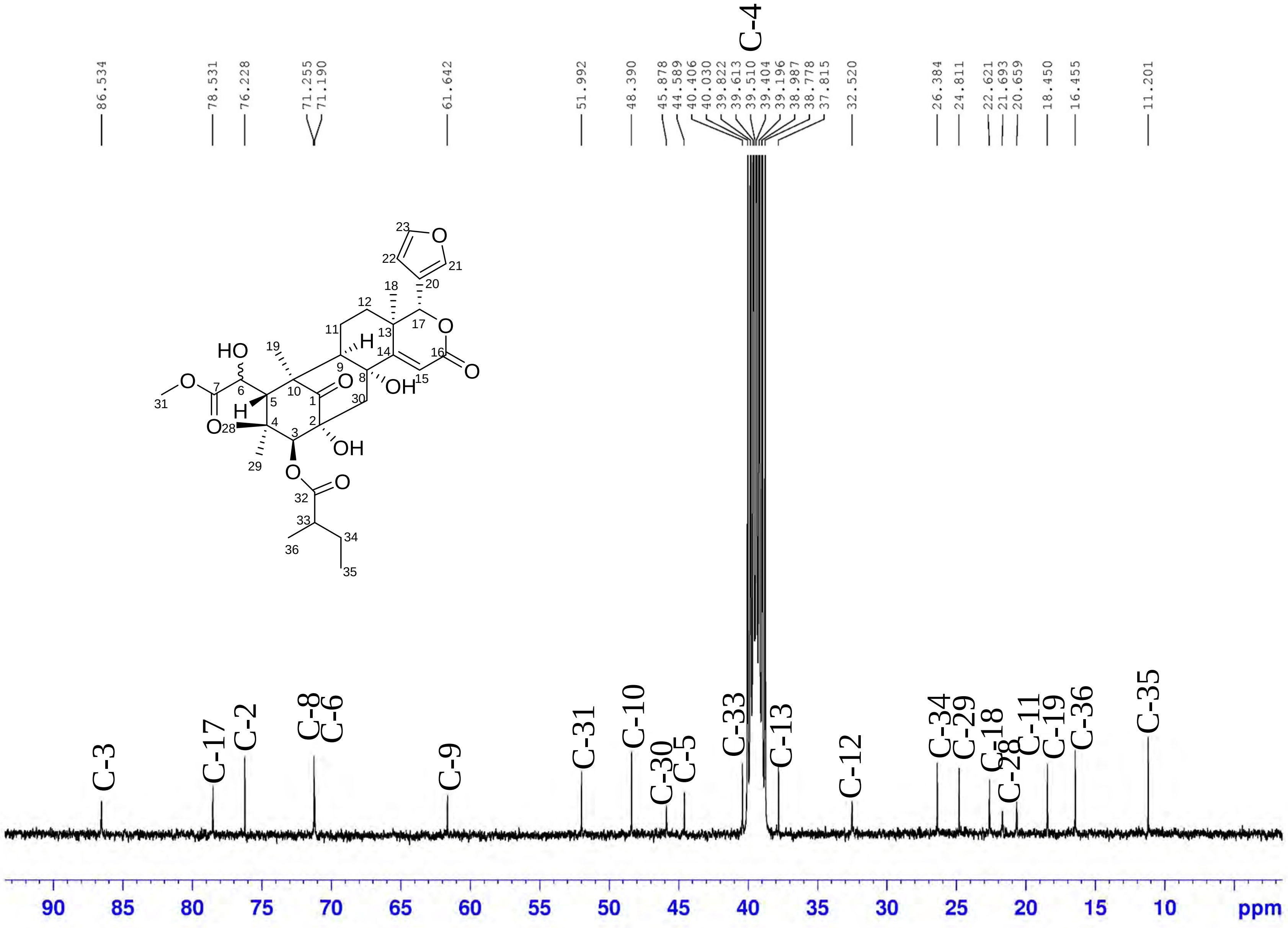

DEPT135 (100 MHz) spectrum of compound **12** in DMSO-*d6*

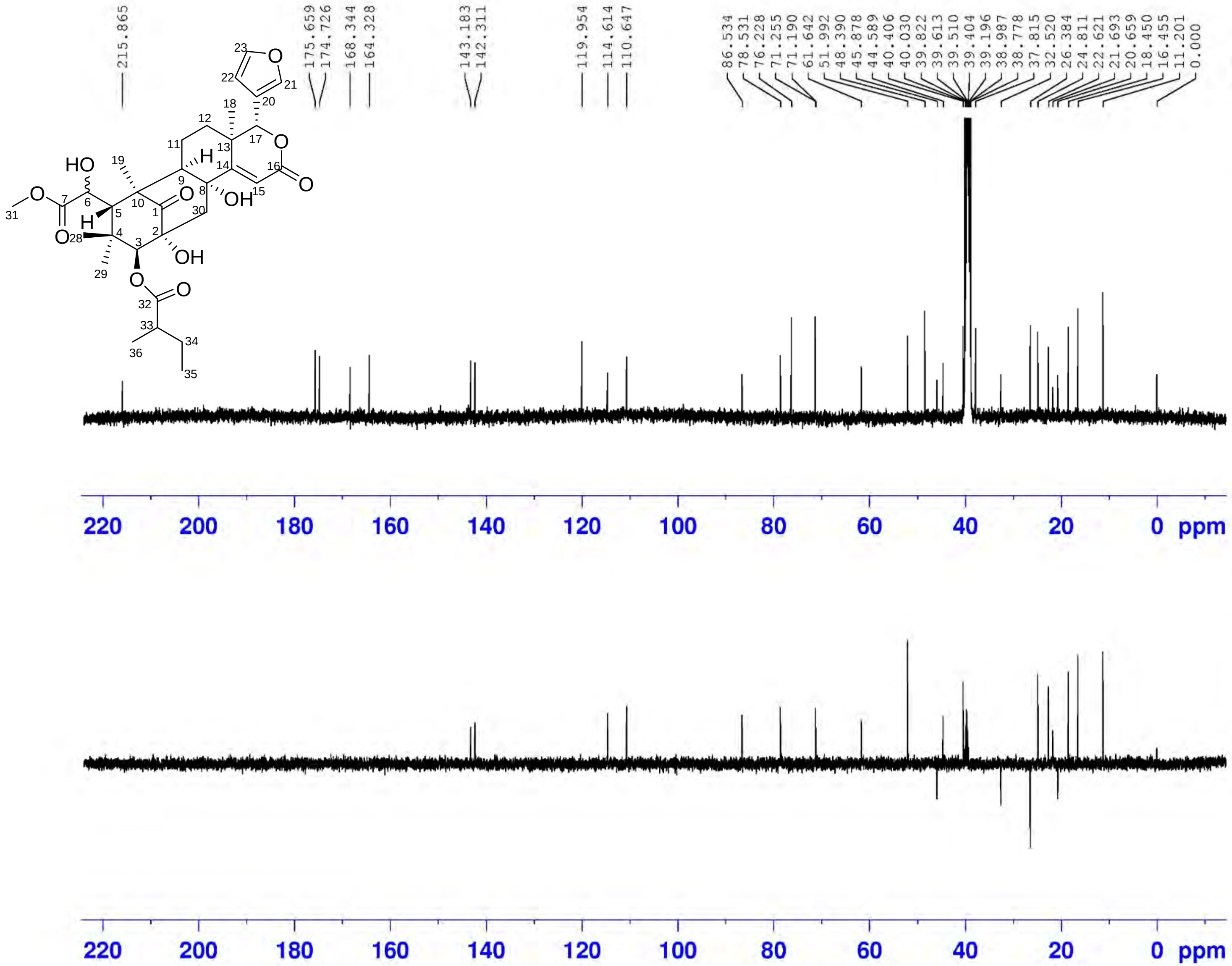


```
NAME          lws-67-DMSO
EXPNO                   4
PROCNO                  1
Date_            20160628
Time                 0.40
INSTRUM             spect
PROBHD   5 mm CPPBBO BB
PULPROG         deptsp135
TD                  65536
SOLVENT              DMSO
NS                    300
DS                      4
SWH             24038.461
FIDRES           0.366798
AQ              1.3631988
RG                 130.26
DW                 20.800
DE                  18.00
TE                  297.0
CNST2         145.0000000
D1             2.00000000
D2             0.00344828
D12            0.00002000
TD0                     1

======== CHANNEL f1 ====
SFO1          100.6233324
NUC1                  13C
P1                  10.00
P13               2000.00
SI                  32768
SF            100.6128273
WDW                    EM
SSB                     0
LB                   1.00
GB                      0
PC                   1.40
```

DEPT135 (100 MHz) spectrum of compound **12** in DMSO-*d6*

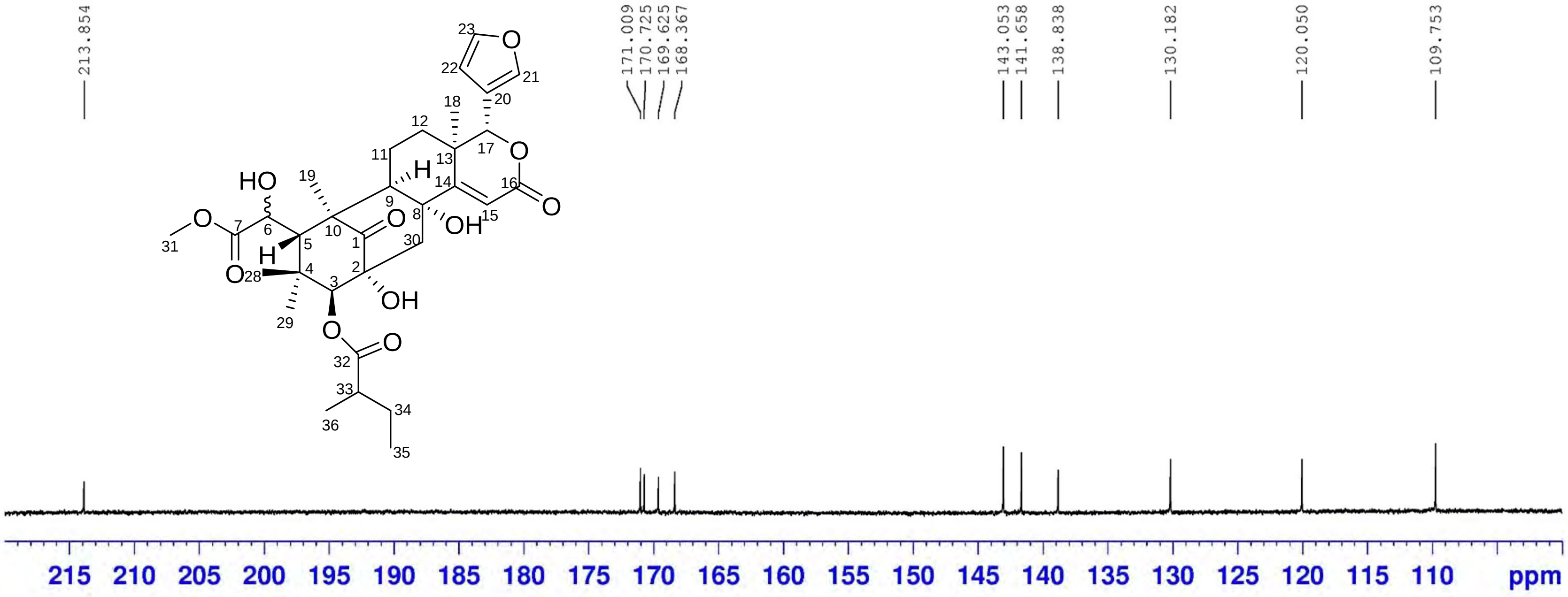

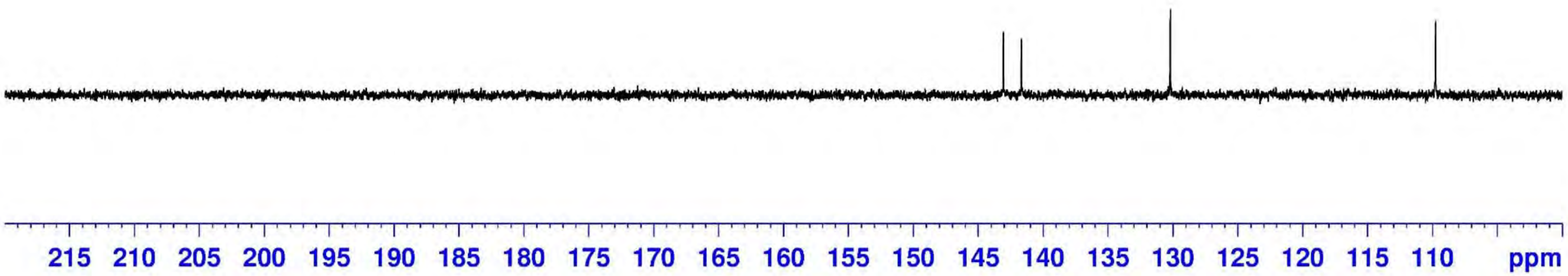

DEPT135 (100 MHz) spectrum of compound **12** in DMSO-*d6*

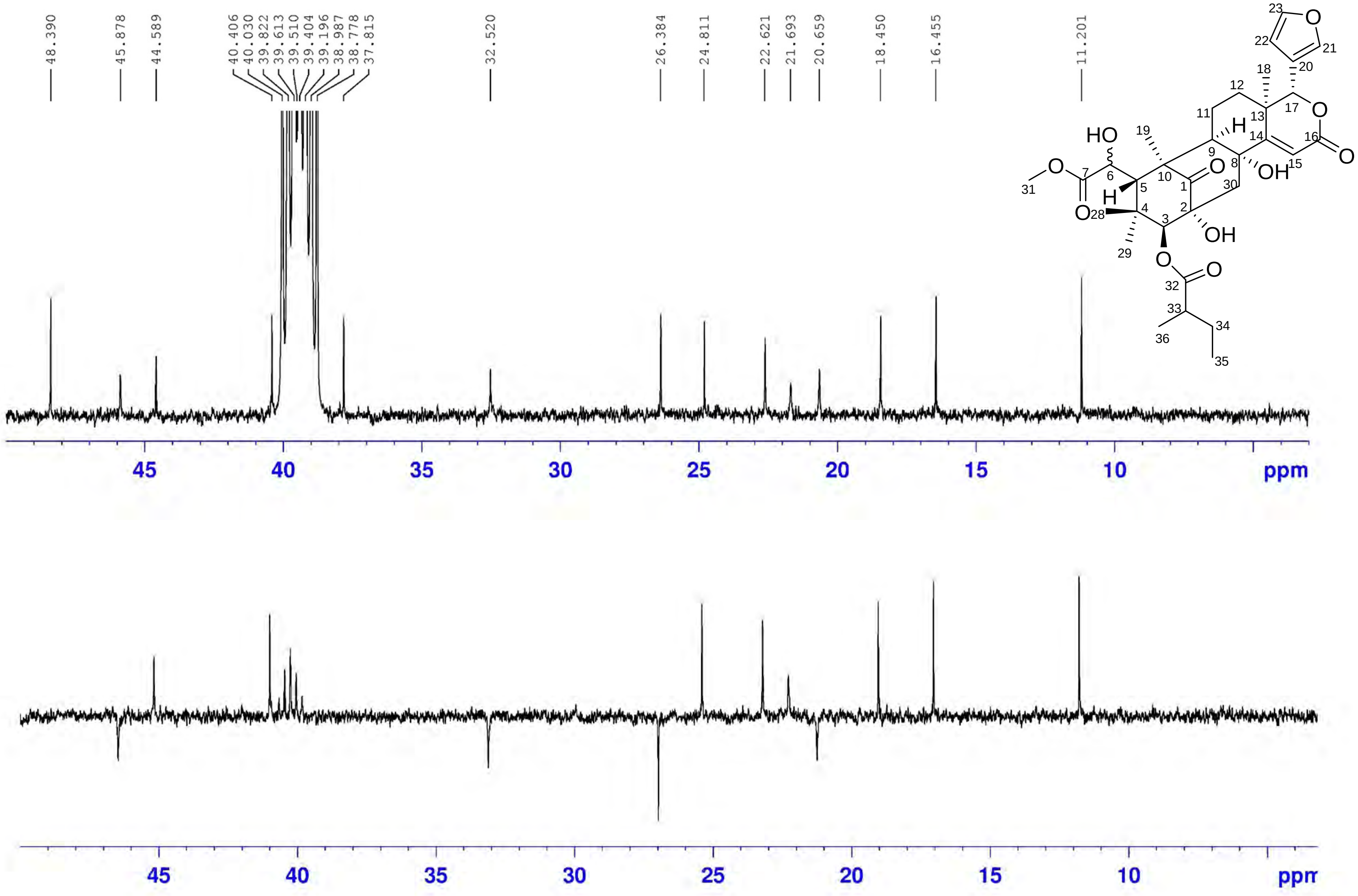

^{1}H-^{1}H COSY (400 MHz) spectrum of compound **12** in DMSO-*d6*

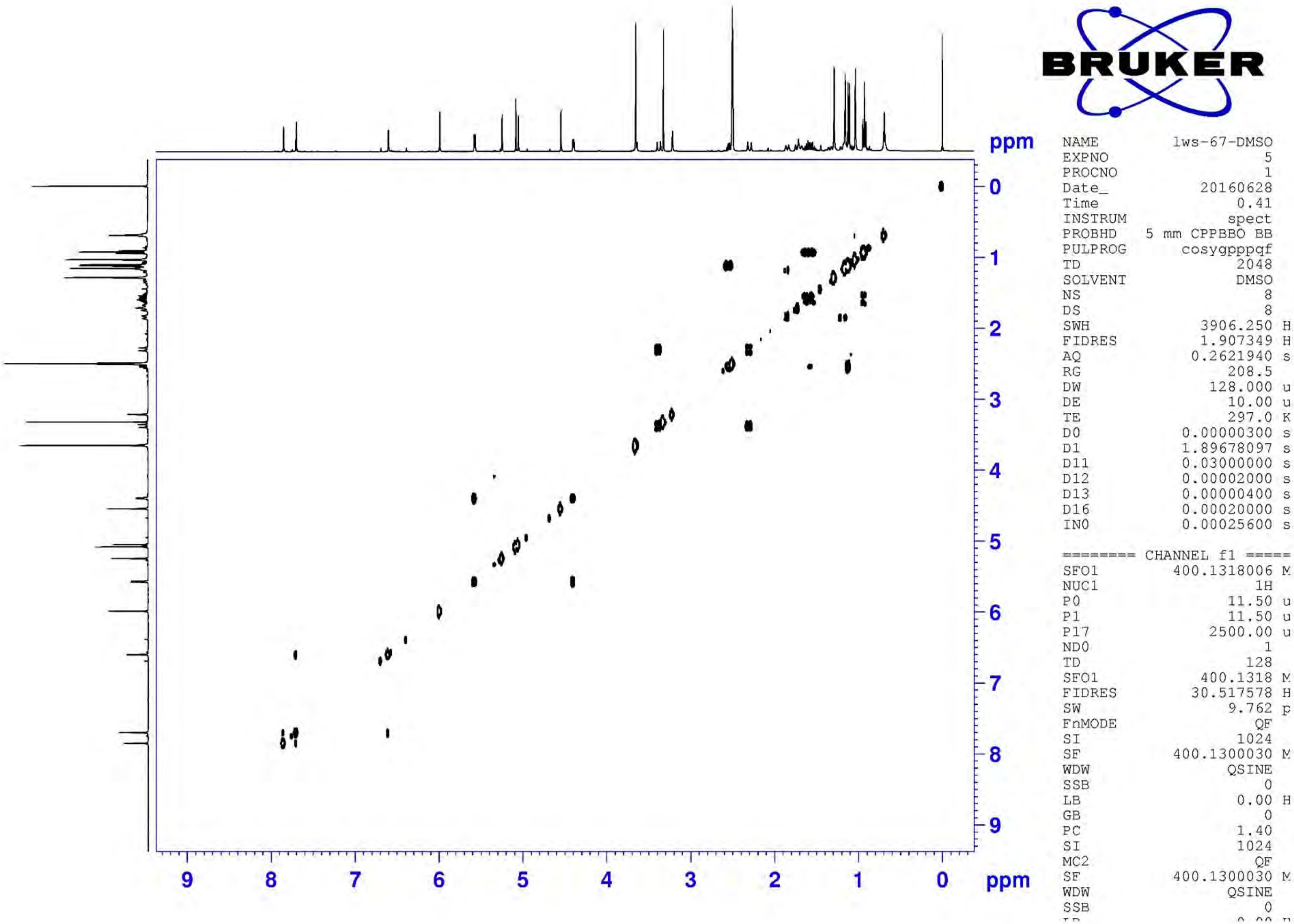

^{1}H-^{1}H COSY (400 MHz) spectrum of compound **12** in DMSO-*d6*

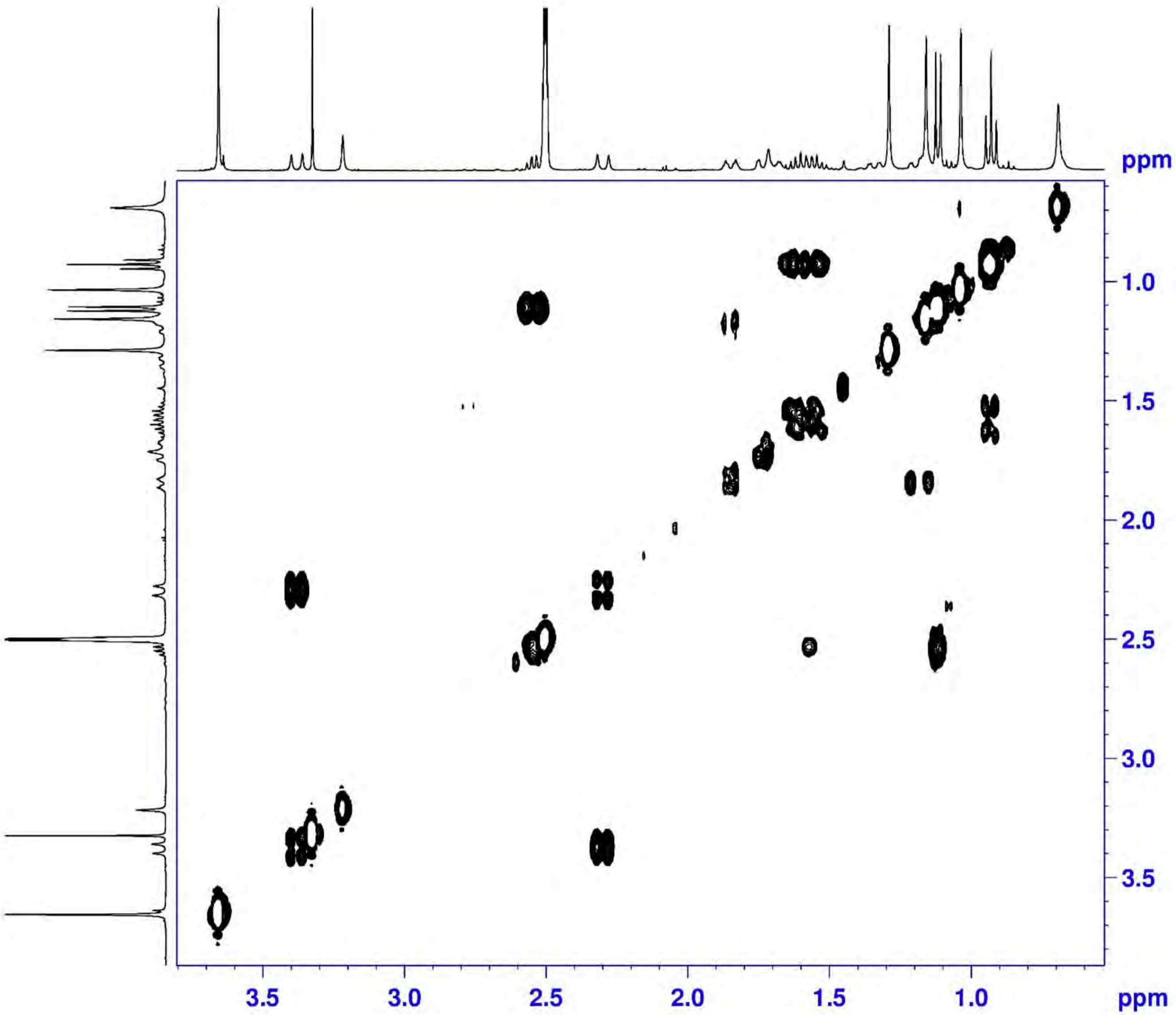

HSQC (400 MHz) spectrum of compound **12** in DMSO-*d6*

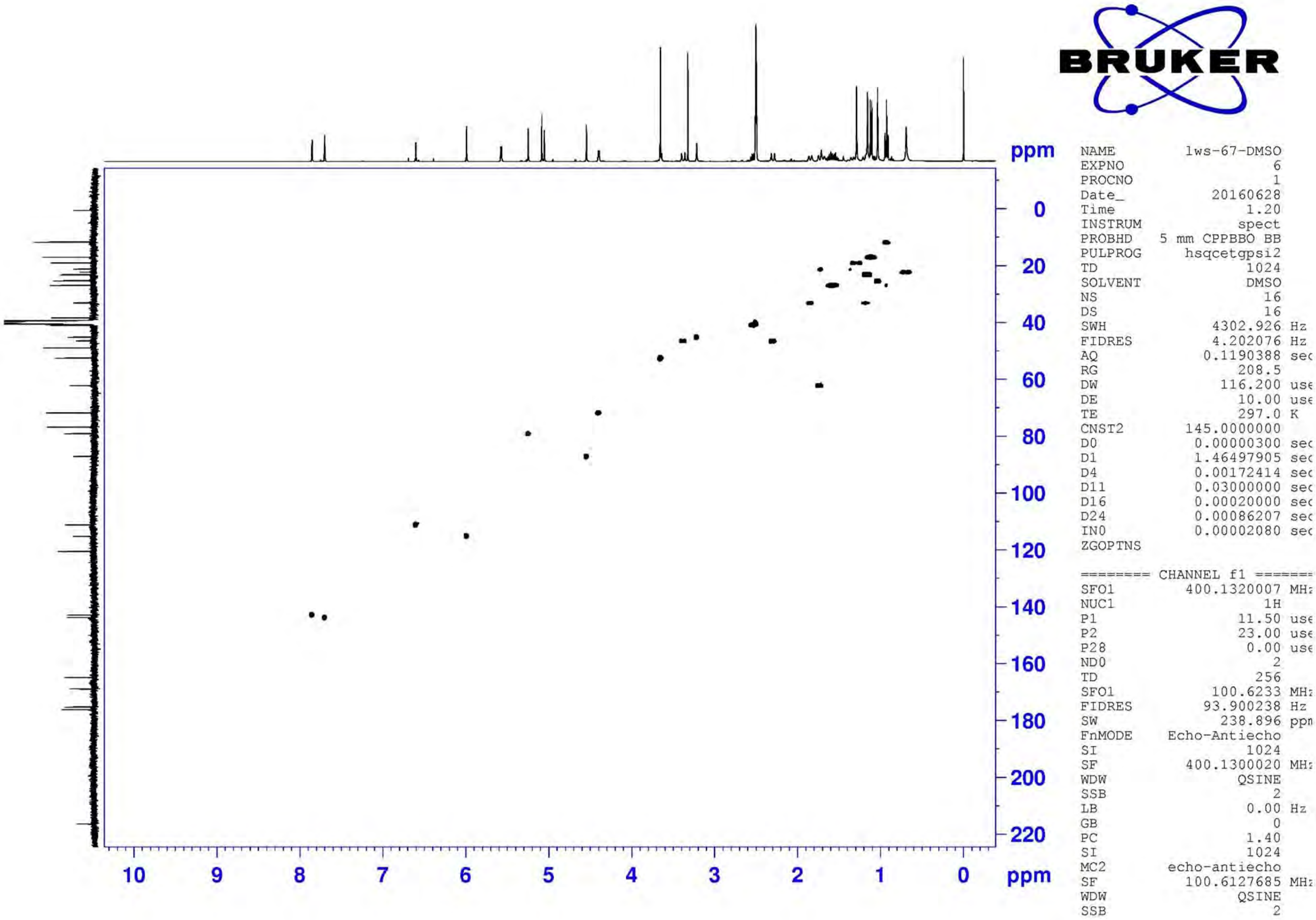

HSQC (400 MHz) spectrum of compound **12** in DMSO-*d6*

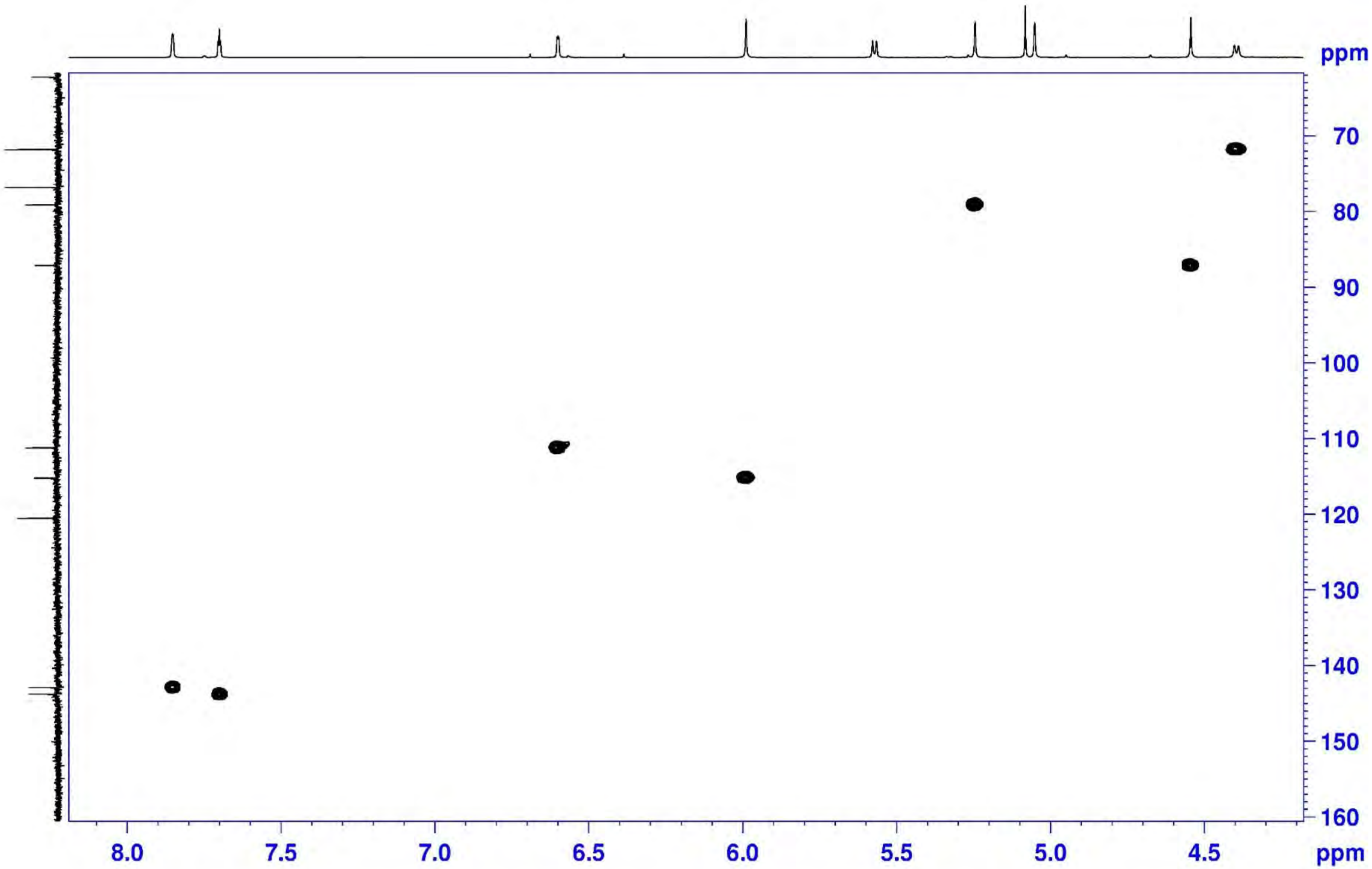

HSQC (400 MHz) spectrum of compound **12** in DMSO-*d6*

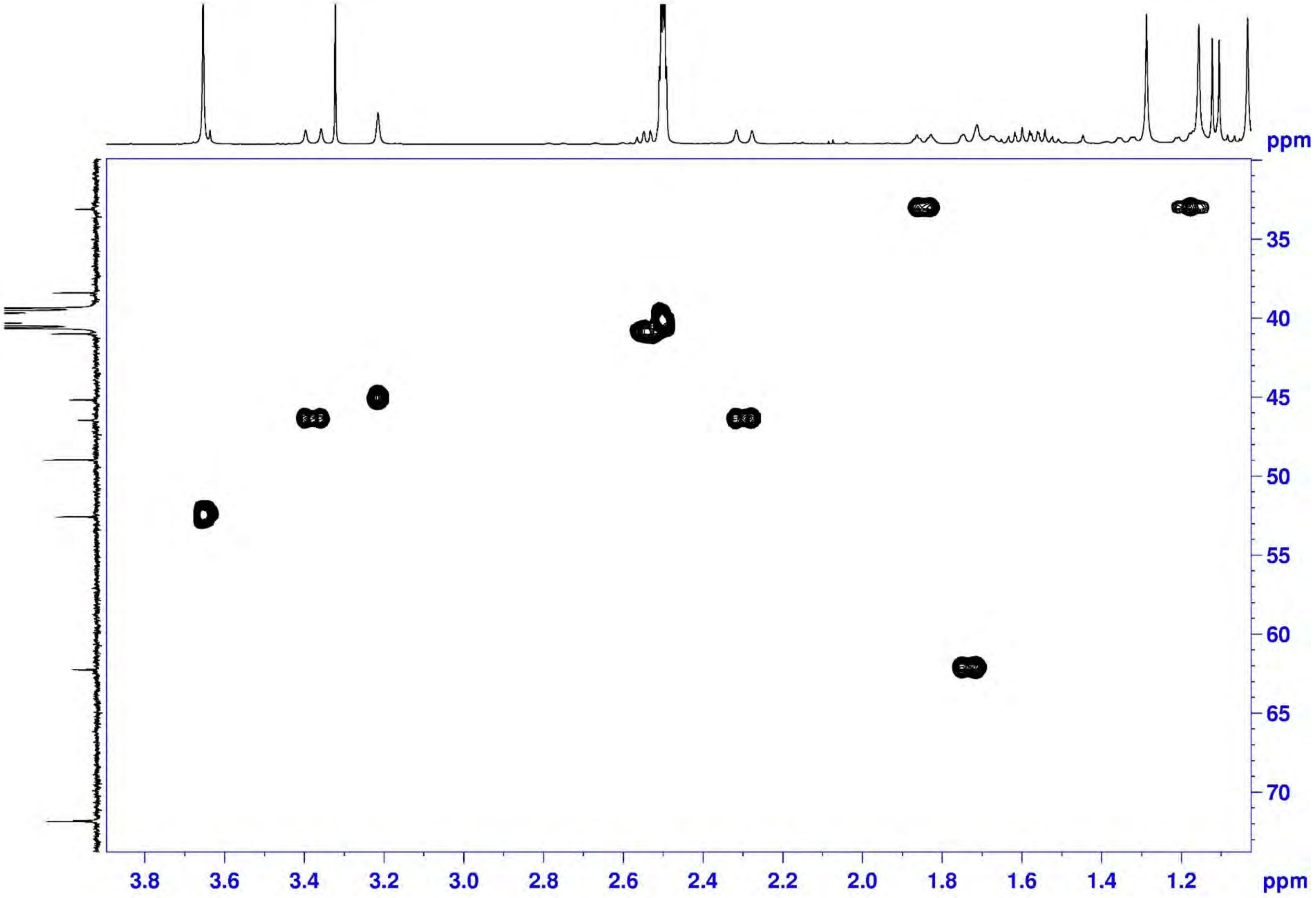

HSQC (400 MHz) spectrum of compound **12** in DMSO-*d6*

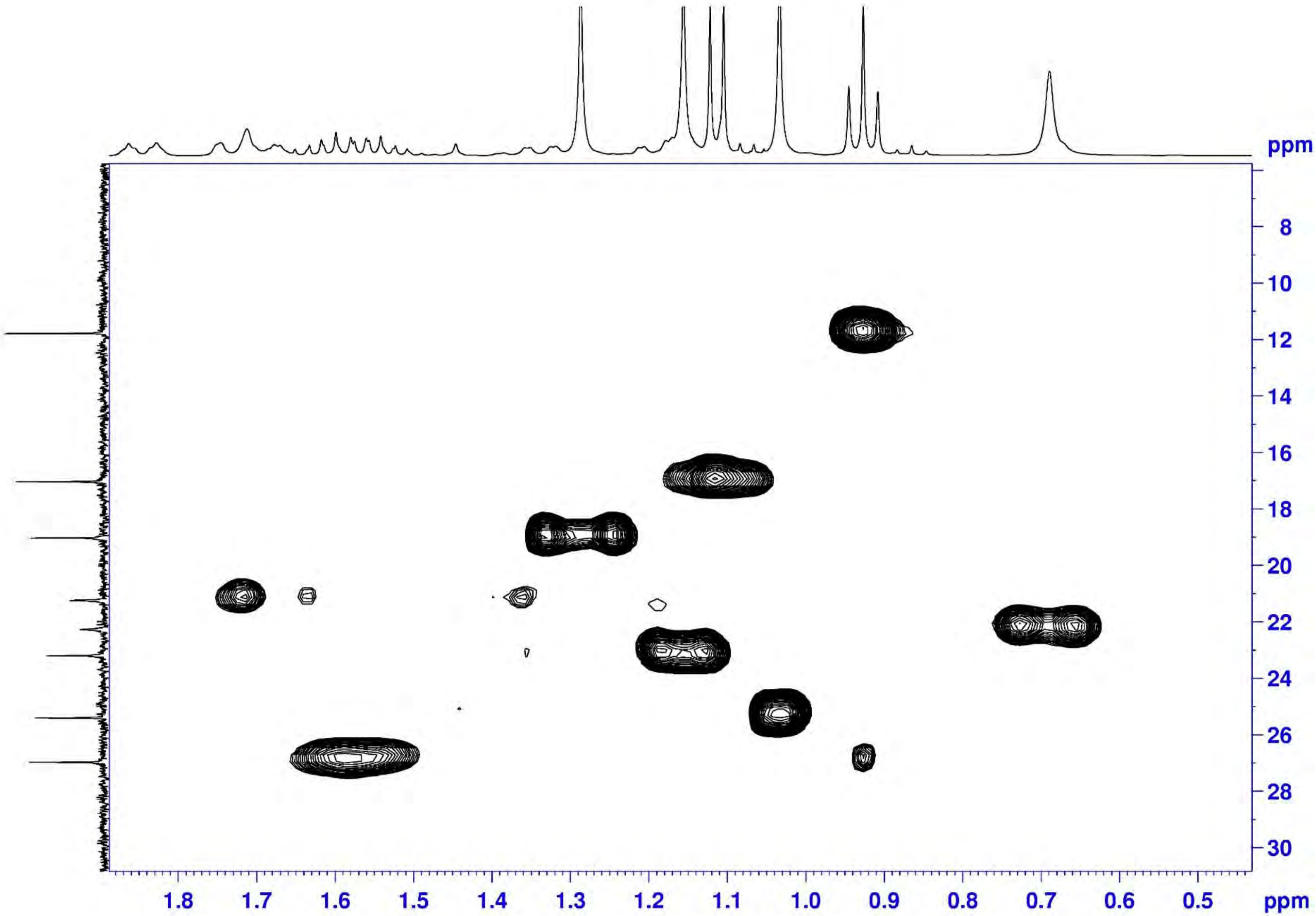

HMBC (400 MHz) spectrum of compound **12** in DMSO-*d6*

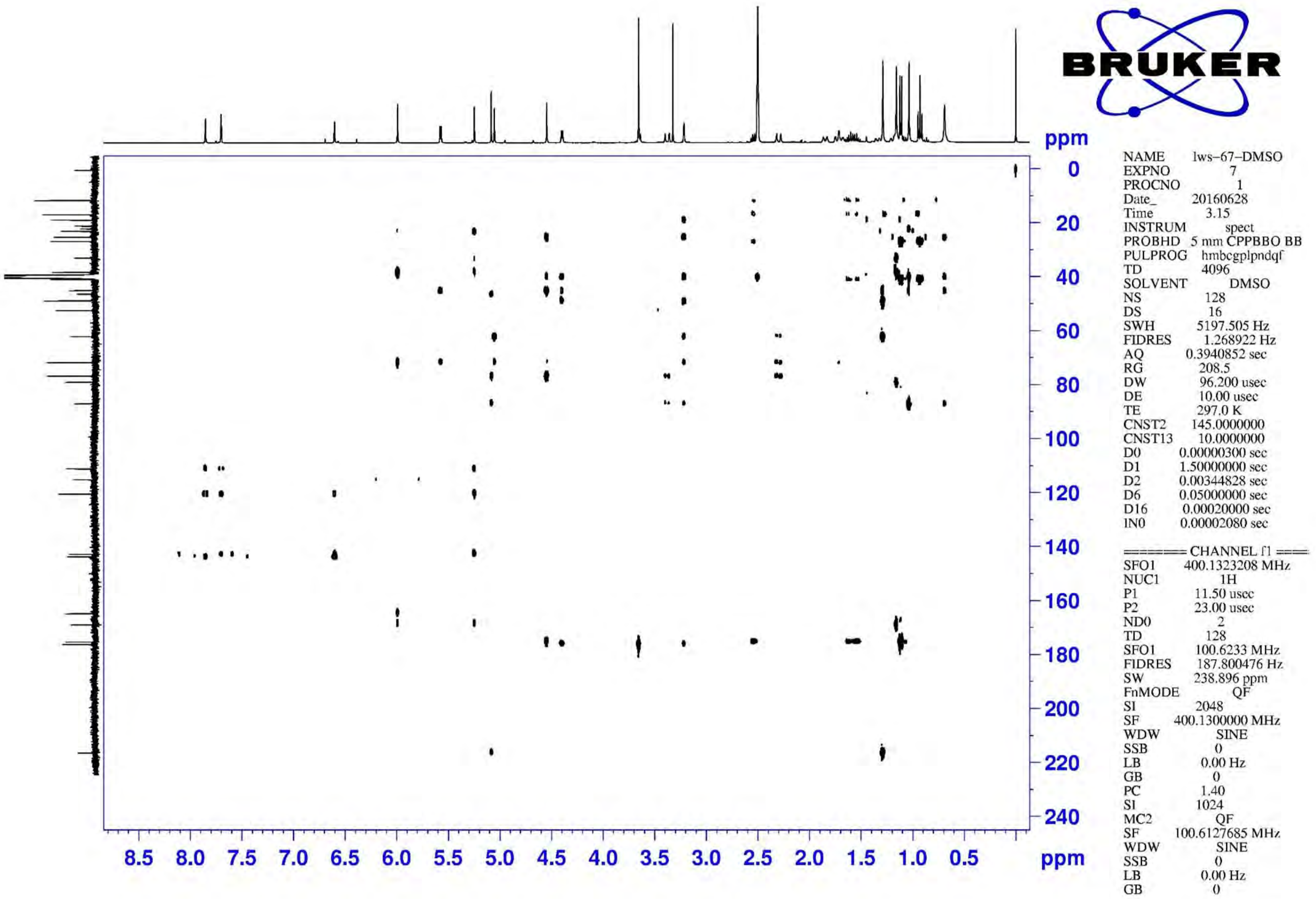

HMBC (400 MHz) spectrum of compound **12** in DMSO-*d6*

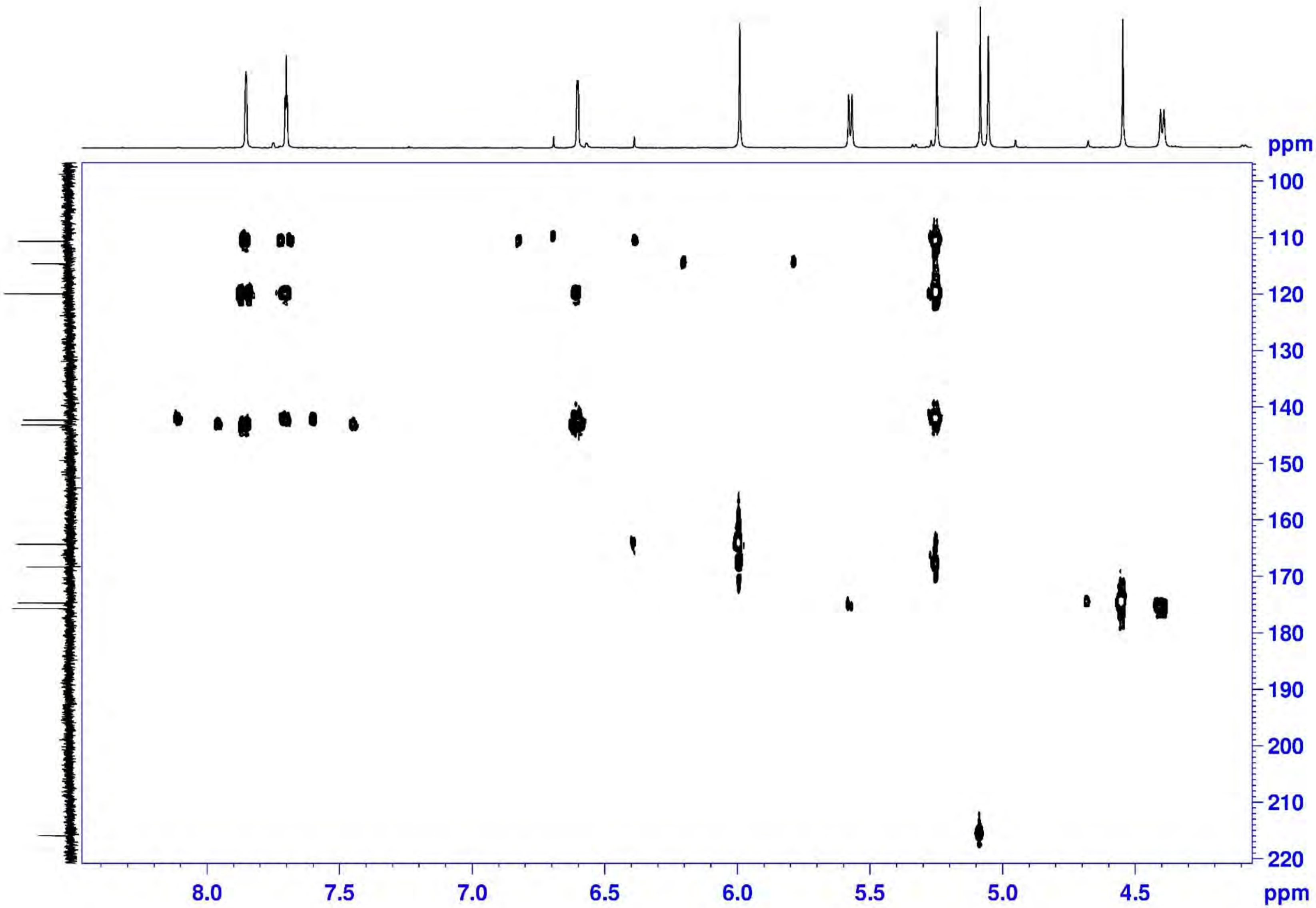

HMBC (400 MHz) spectrum of compound **12** in DMSO-*d6*

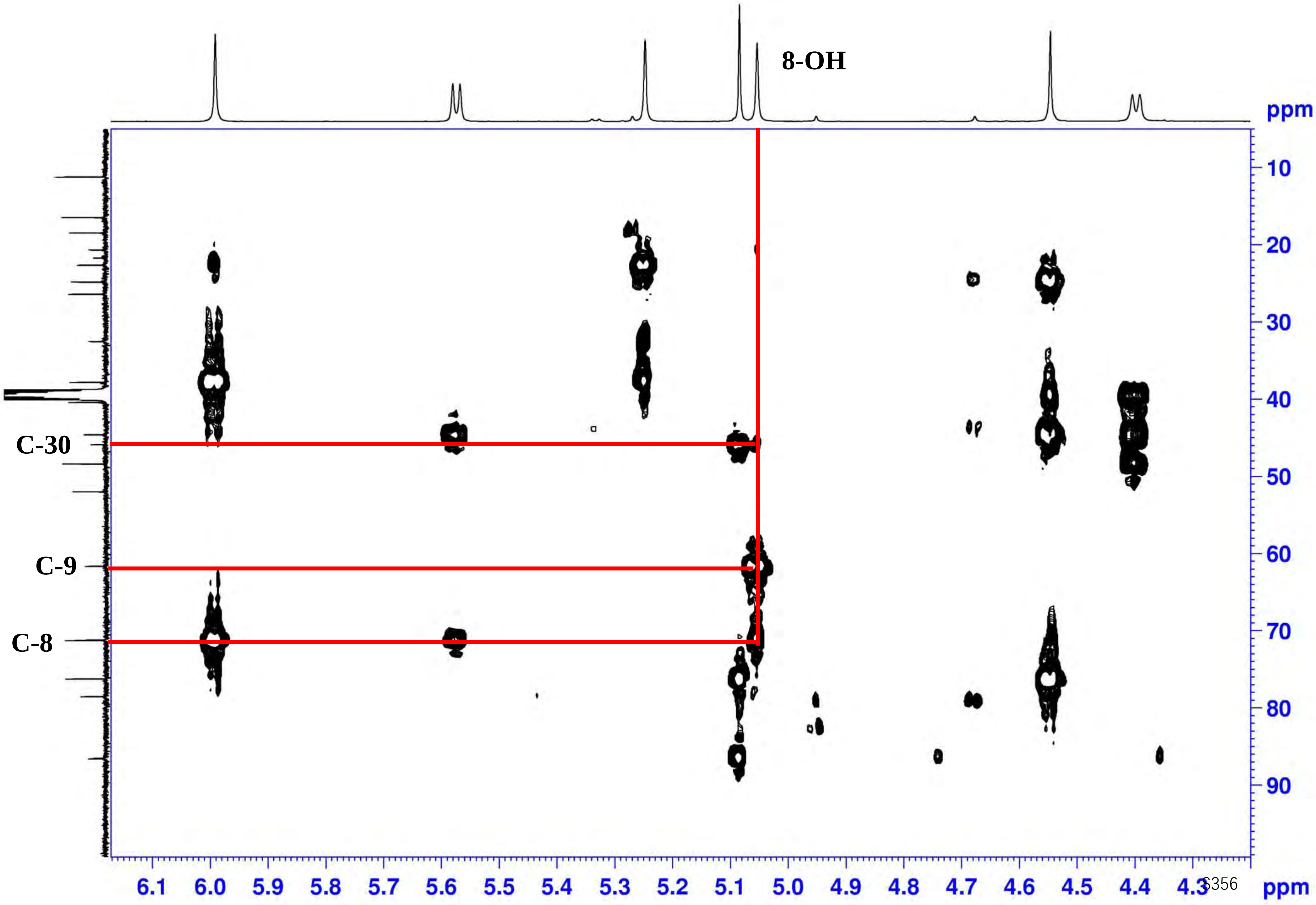

HMBC (400 MHz) spectrum of compound **12** in DMSO-*d6*

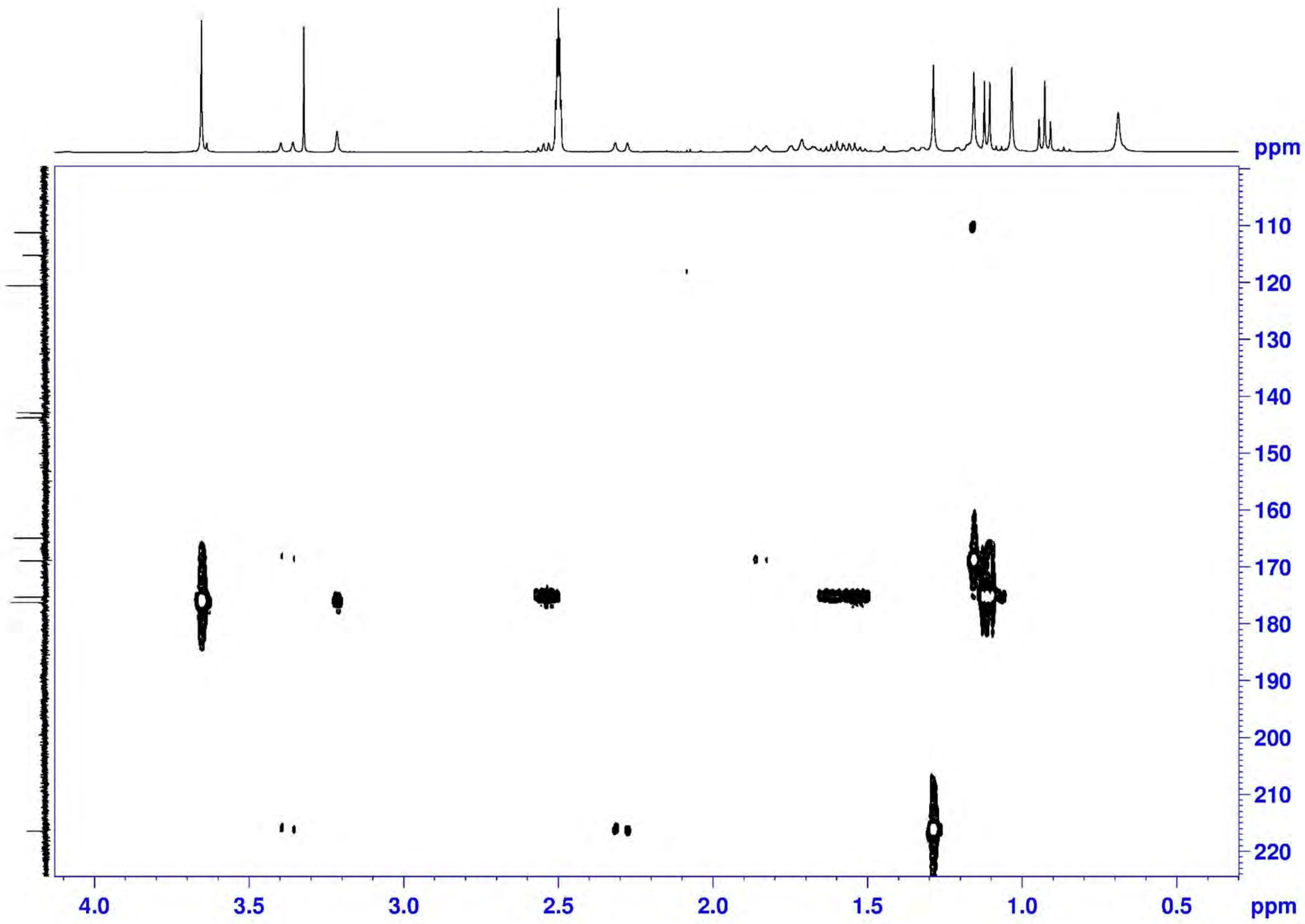

HMBC (400 MHz) spectrum of compound **12** in DMSO-*d6*

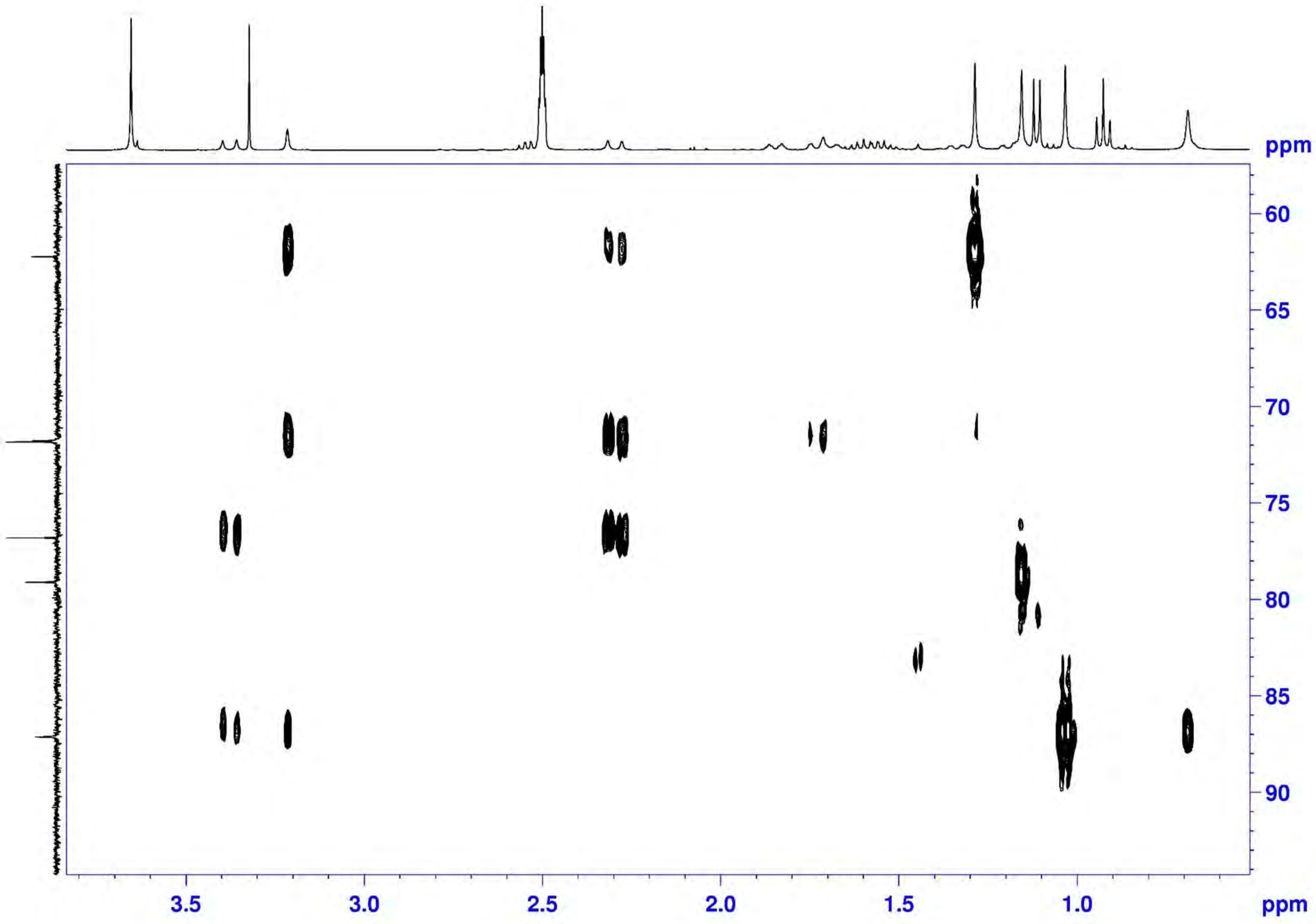

HMBC (400 MHz) spectrum of compound **12** in DMSO-*d6*

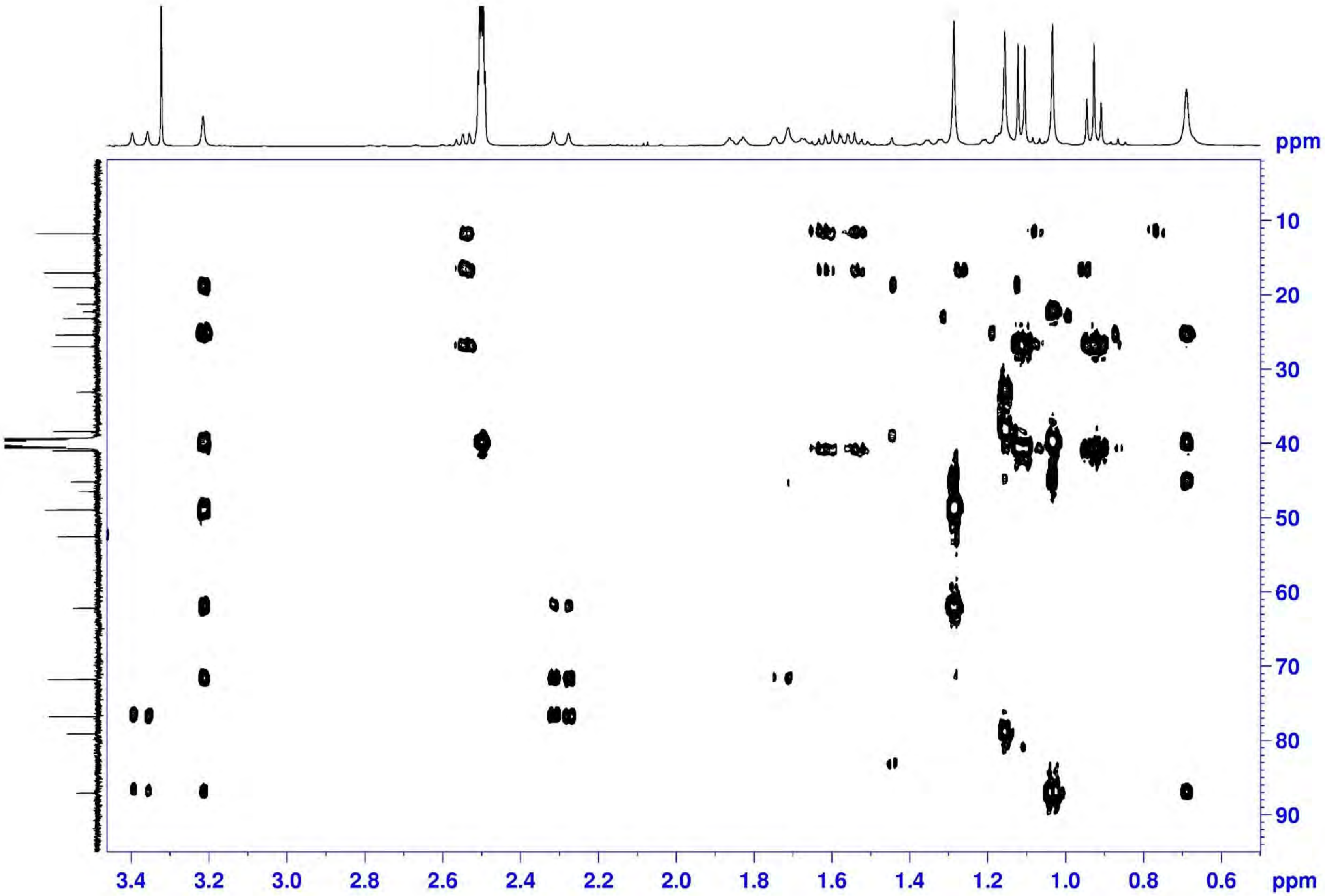

HMBC (400 MHz) spectrum of compound **12** in DMSO-*d6*

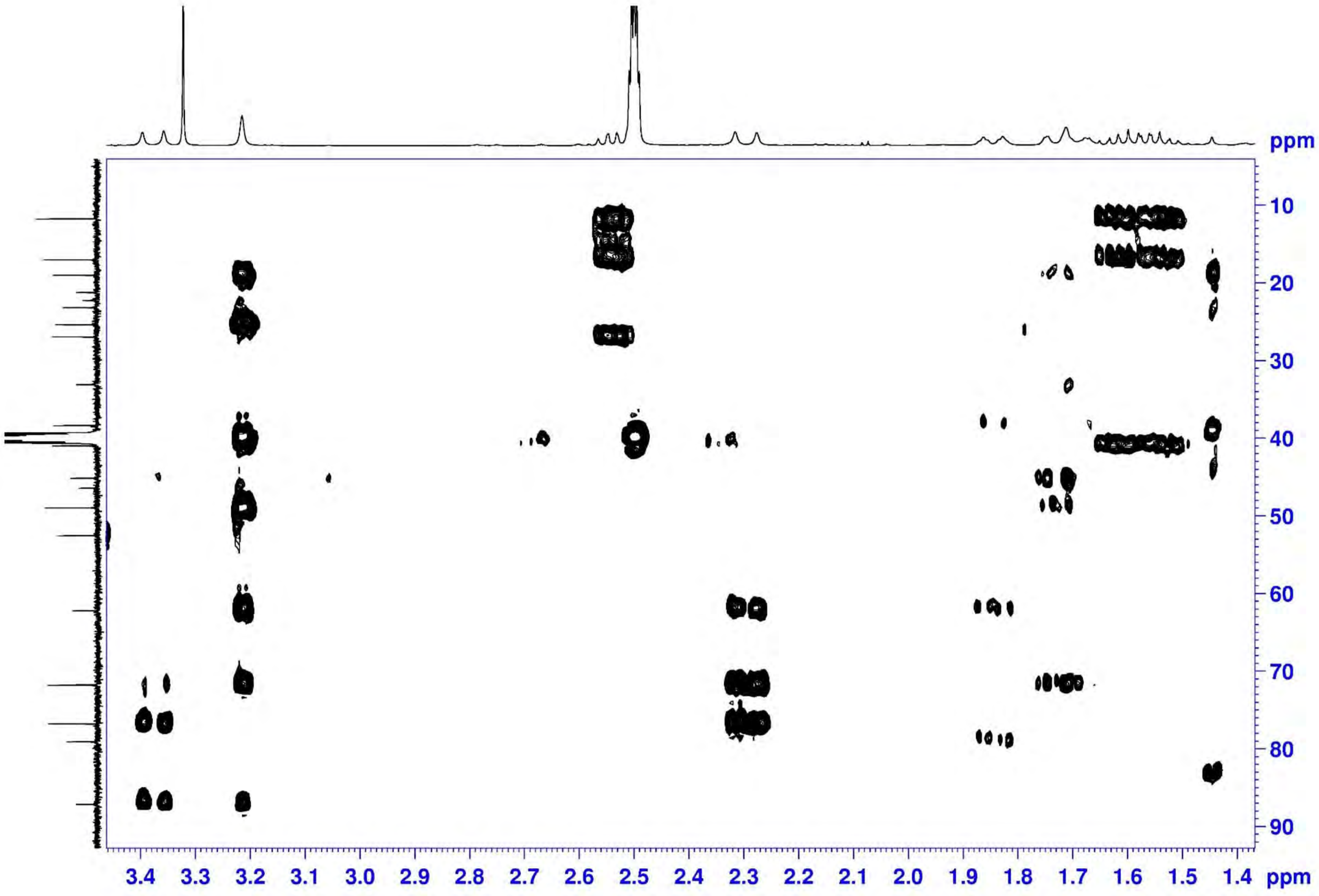

HMBC (400 MHz) spectrum of compound **12** in DMSO-*d6*

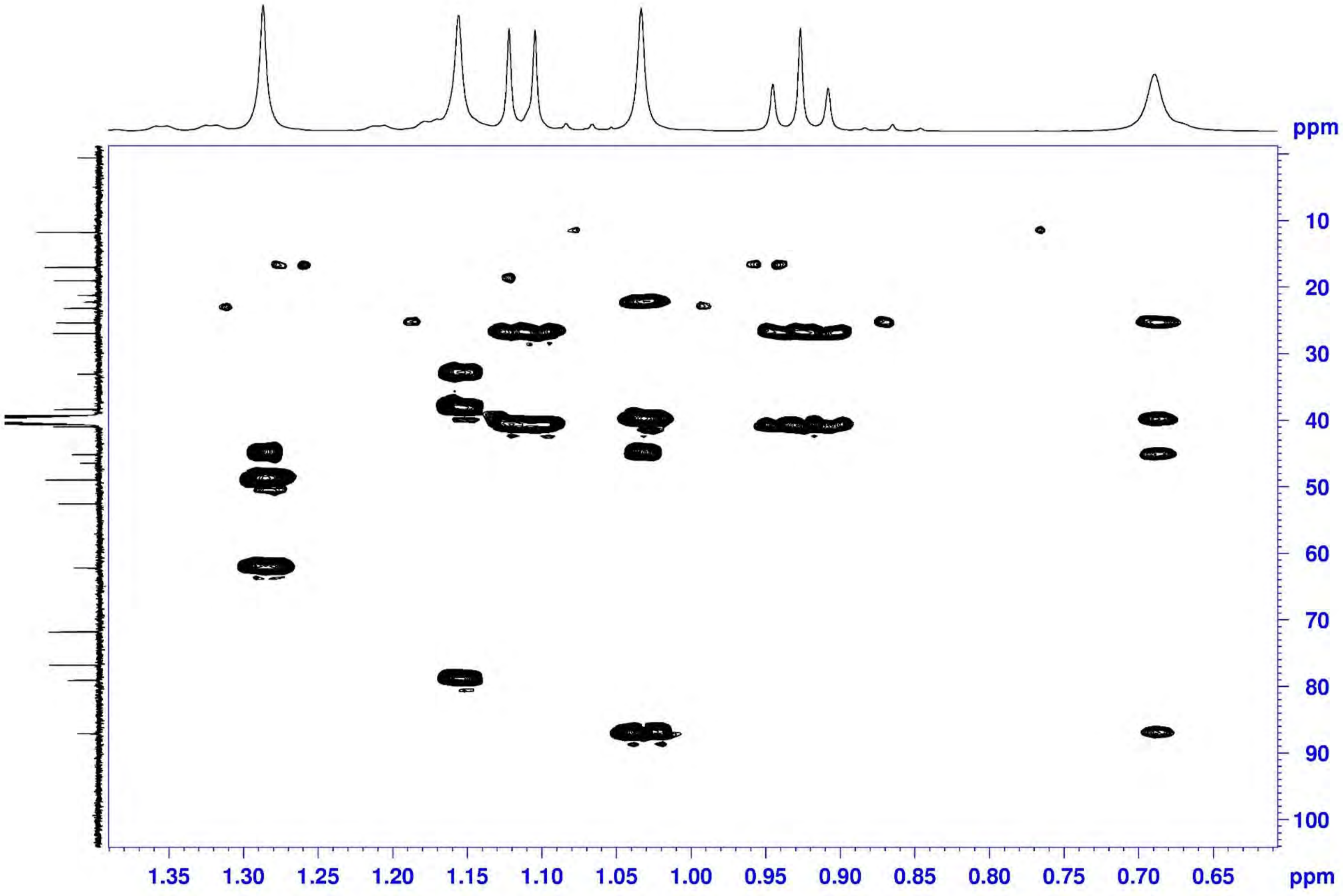

NOESY (400 MHz) spectrum of compound **12** in DMSO-*d6*

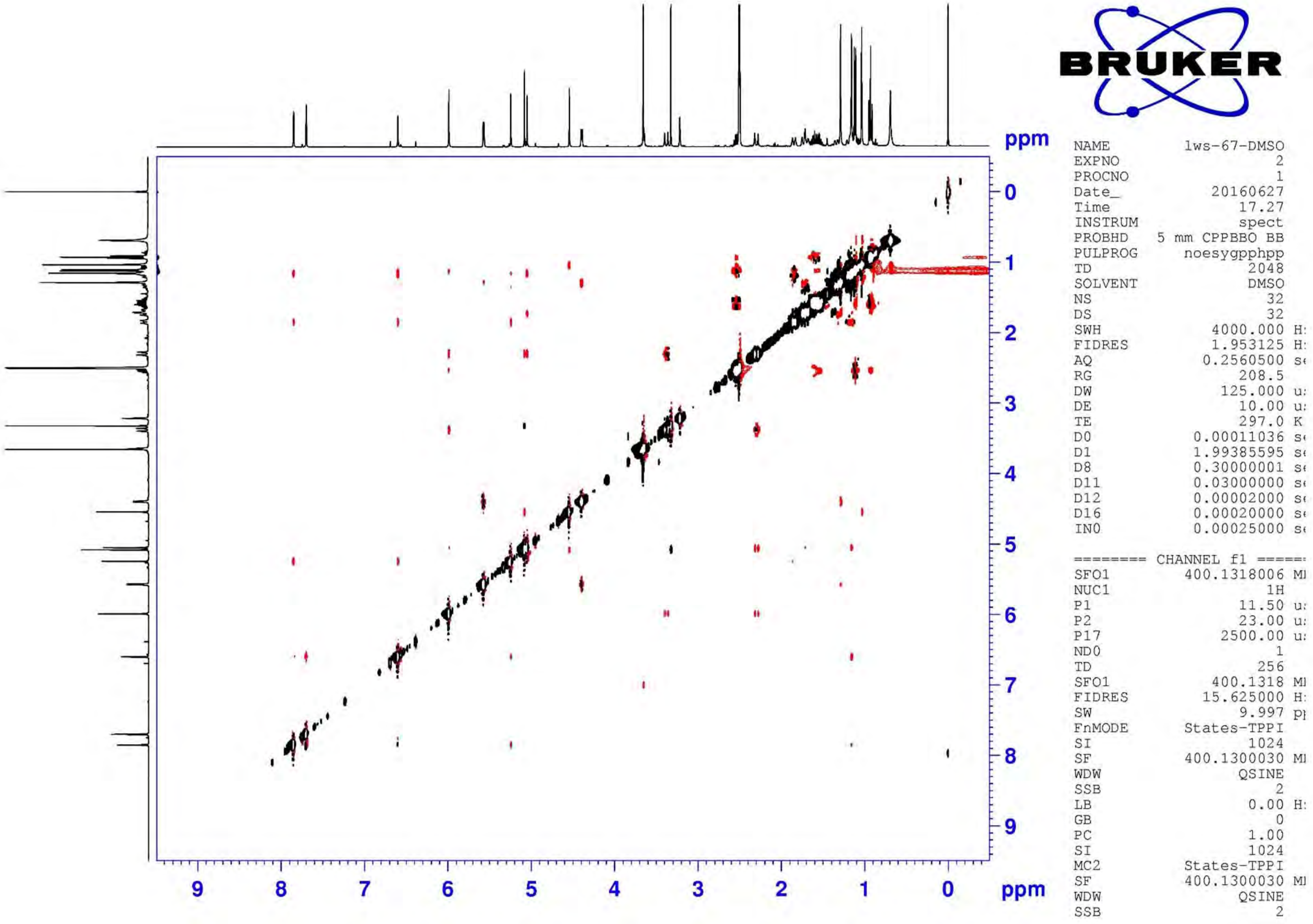

NOESY (400 MHz) spectrum of compound **12** in DMSO-*d6*

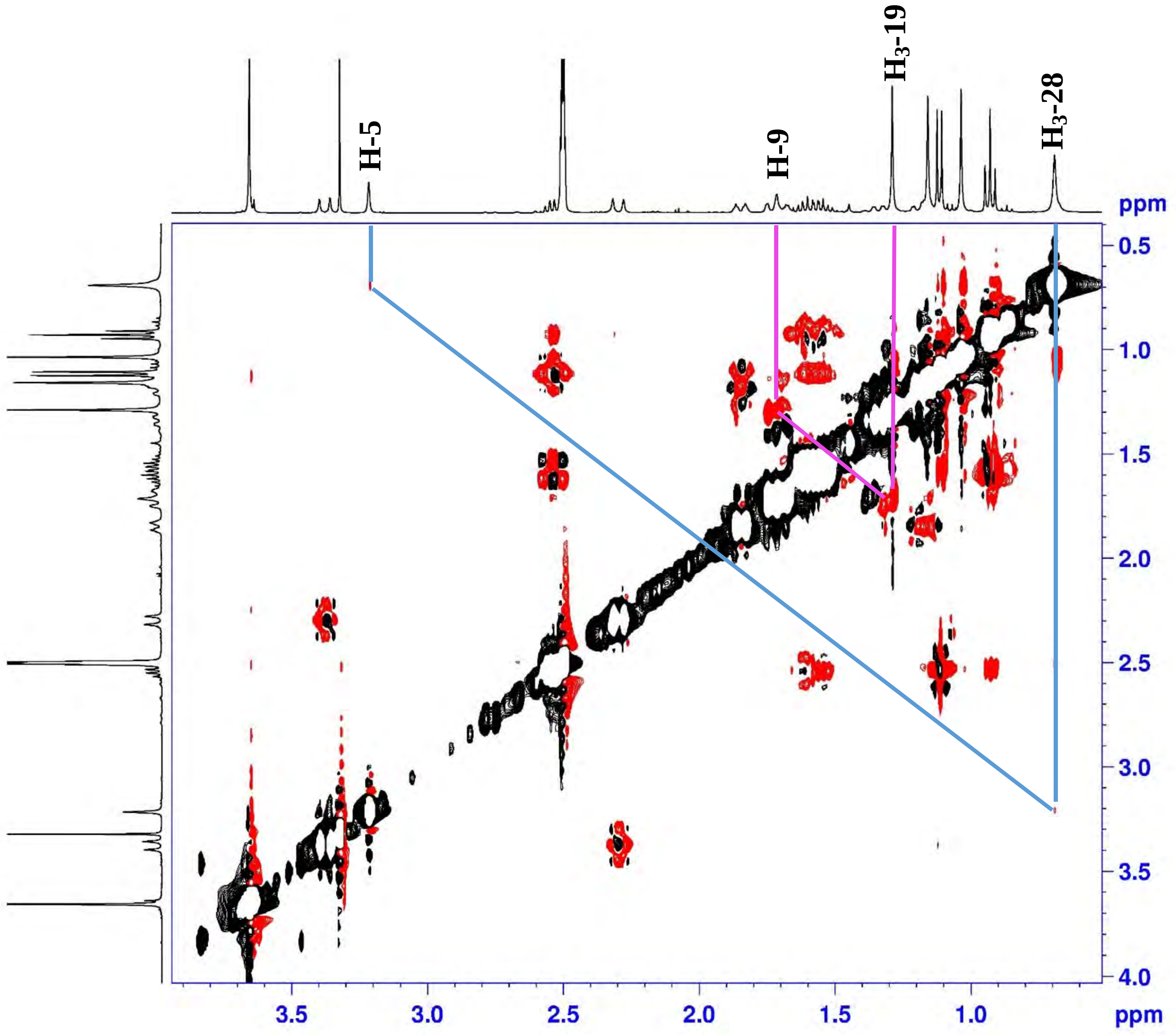

NOESY (400 MHz) spectrum of compound **12** in DMSO-*d6*

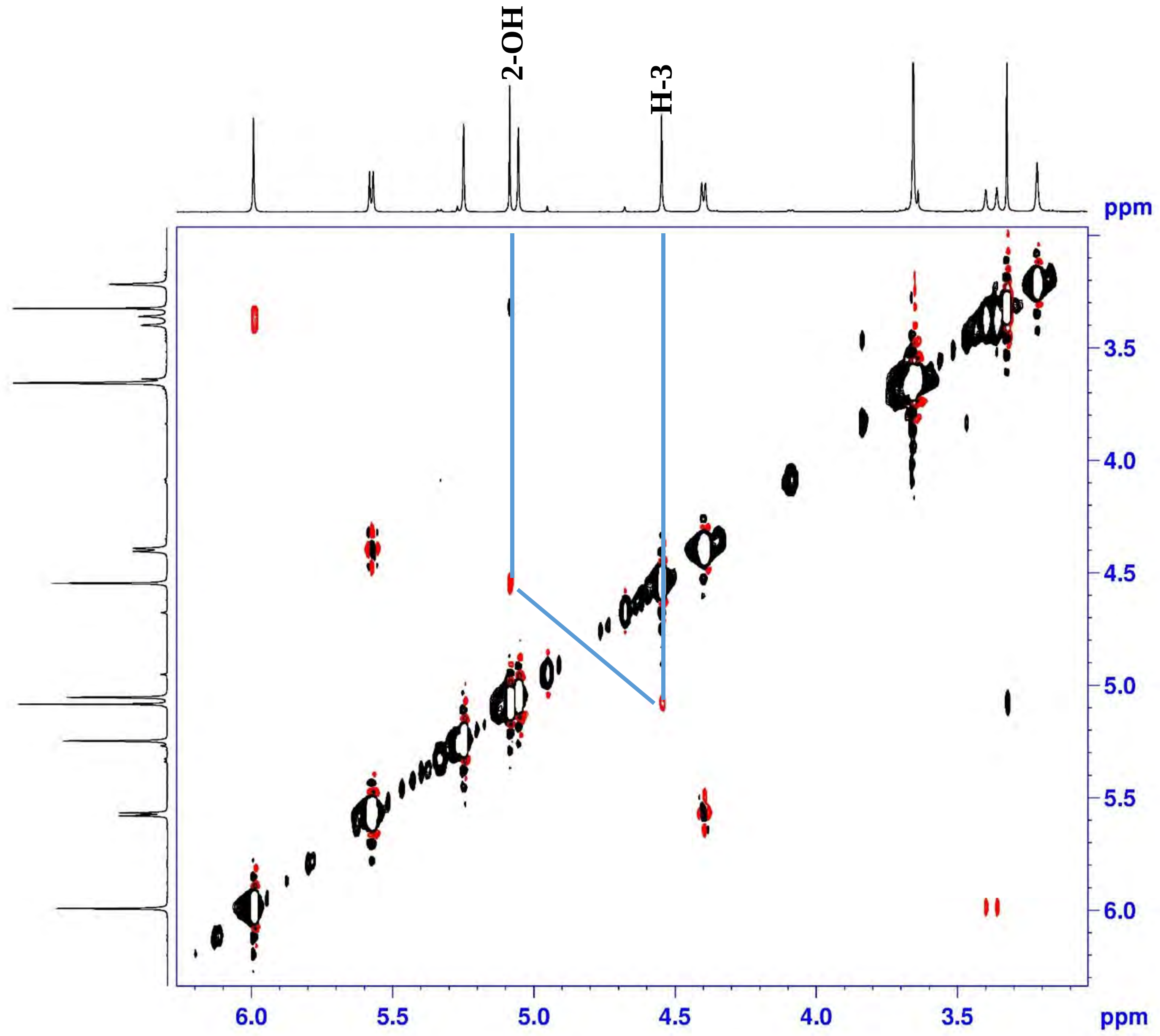

NOESY (400 MHz) spectrum of compound **12** in DMSO-*d6*

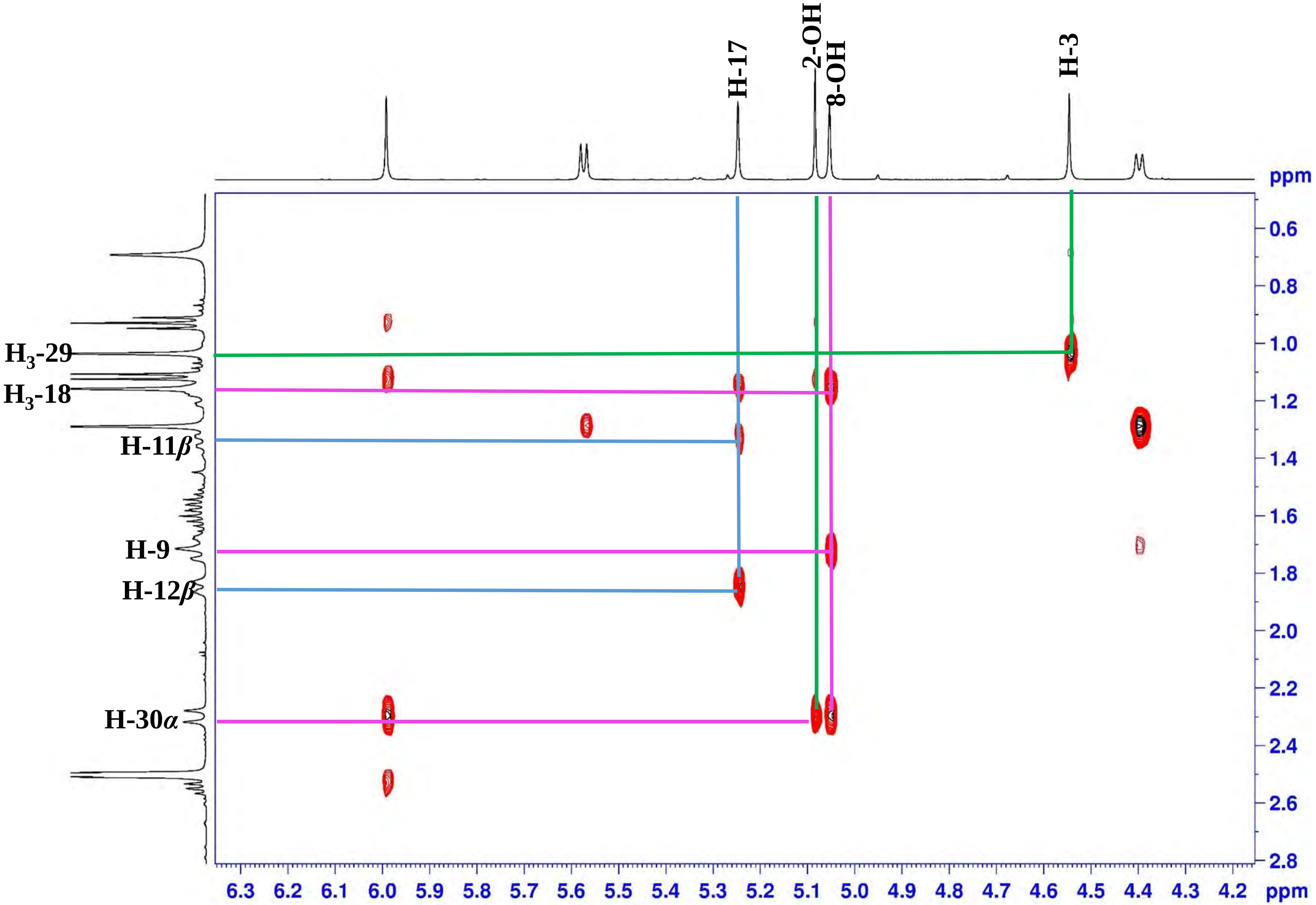

HR-ESIMS for compound **13**

Mass Spectrum SmartFormula Report

Analysis Info

Analysis Name	D:\Data\MS\data\201511\liwanshan_lws-74_pos_22_01_832.d	Acquisition Date	11/18/2015 10:03:37 PM
Method	LC_Direct Infusion_pos_100-1000mz.m	Operator	SCSIO
Sample Name	liwanshan_lws-74_pos	Instrument / Ser#	maXis 29
Comment			

Acquisition Parameter

Source Type	ESI	Ion Polarity	Positive	Set Nebulizer	0.4 Bar
Focus	Active	Set Capillary	4500 V	Set Dry Heater	180 °C
Scan Begin	100 m/z	Set End Plate Offset	-500 V	Set Dry Gas	4.0 l/min
Scan End	2000 m/z	Set Collision Cell RF	800.0 Vpp	Set Divert Valve	Waste

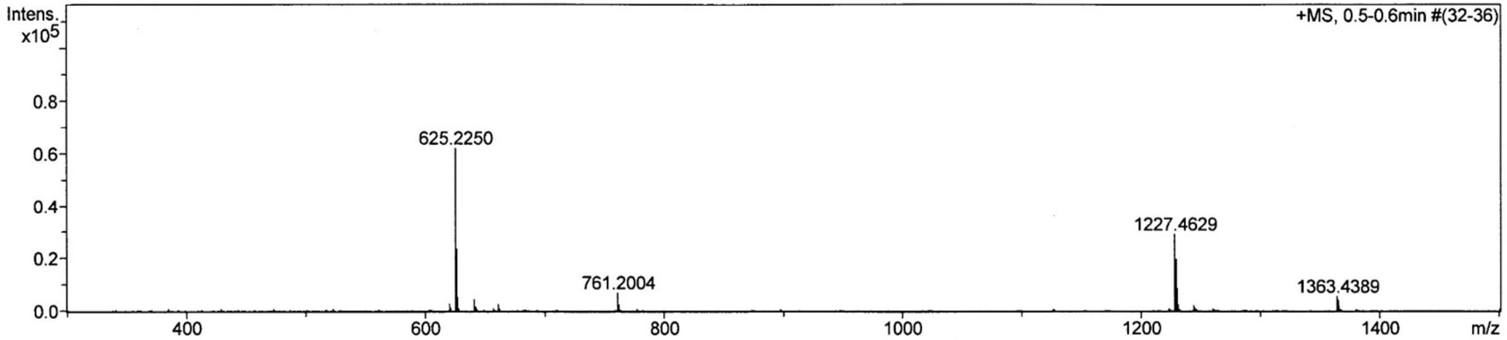

Meas. m/z	#	Formula	Score	m/z	err [ppm]	err [mDa]	mSigma	rdb	e⁻ Conf	N-Rule
603.2433	1	C 31 H 39 O 12	100.00	603.2436	0.5	0.3	46.5	12.5	even	ok
625.2250	1	C 31 H 38 Na O 12	100.00	625.2255	0.9	0.5	20.2	12.5	even	ok
1227.4629	1	C 62 H 76 Na O 24	100.00	1227.4619	-0.9	-1.1	8.0	24.5	even	ok

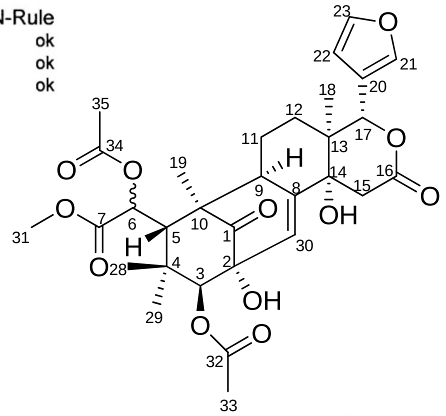

Bruker Compass DataAnalysis 4.0 printed: 11/18/2015 10:11:49 PM Page 1 of 1

HR-ESIMS for compound **13**

Generic Display Report

Analysis Info

Analysis Name	D:\Data\MS\data\201511\liwanshan_lws-74_pos_22_01_832.d	Acquisition Date	11/18/2015 10:03:37 PM
Method	LC_Direct Infusion_pos_100-1000mz.m	Operator	SCSIO
Sample Name	liwanshan_lws-74_pos	Instrument	maXis
Comment			

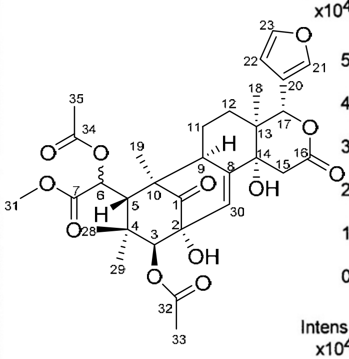

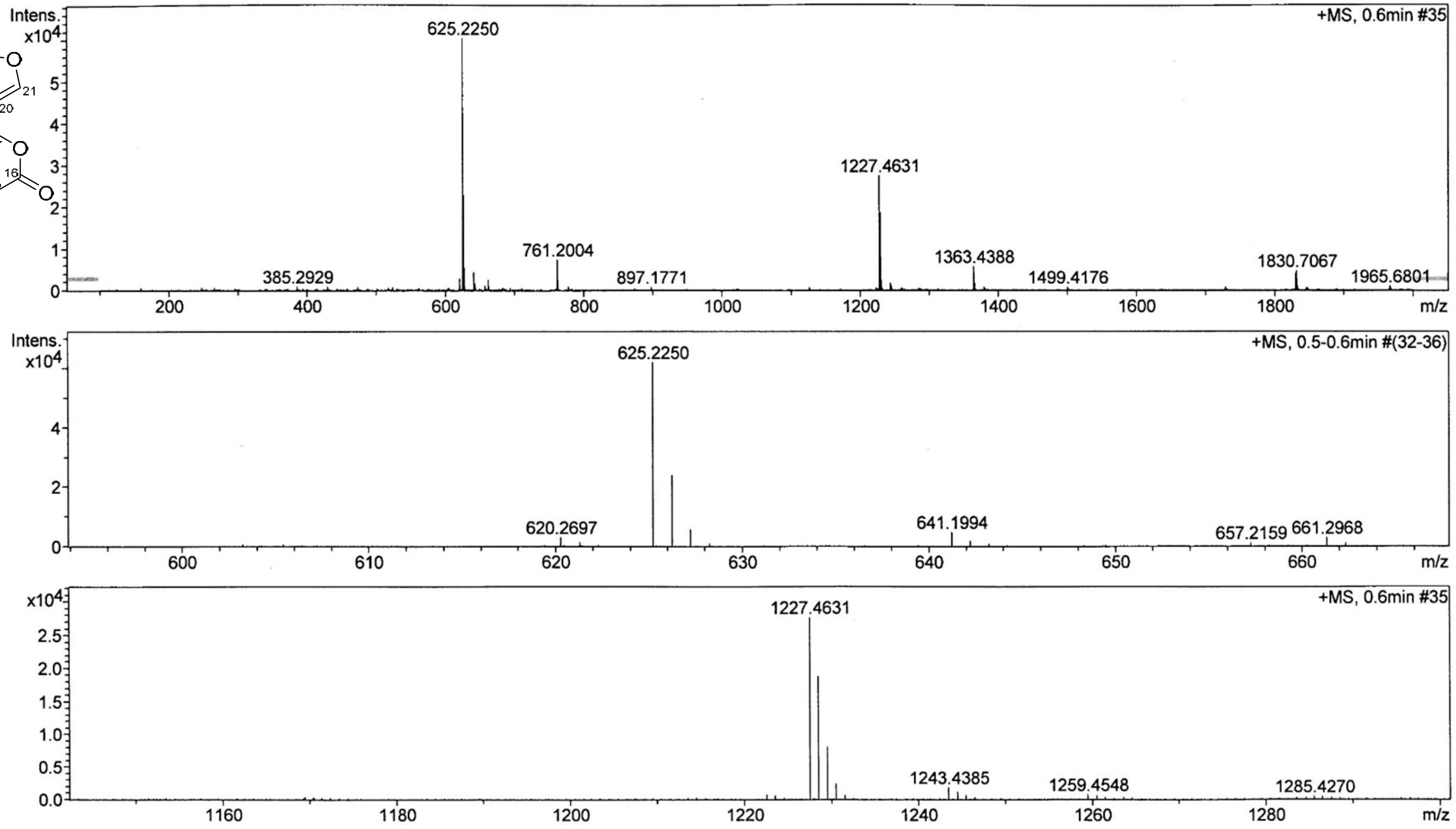

^{1}H NMR (400 MHz) spectrum of compound **13** in $CDCl_3$

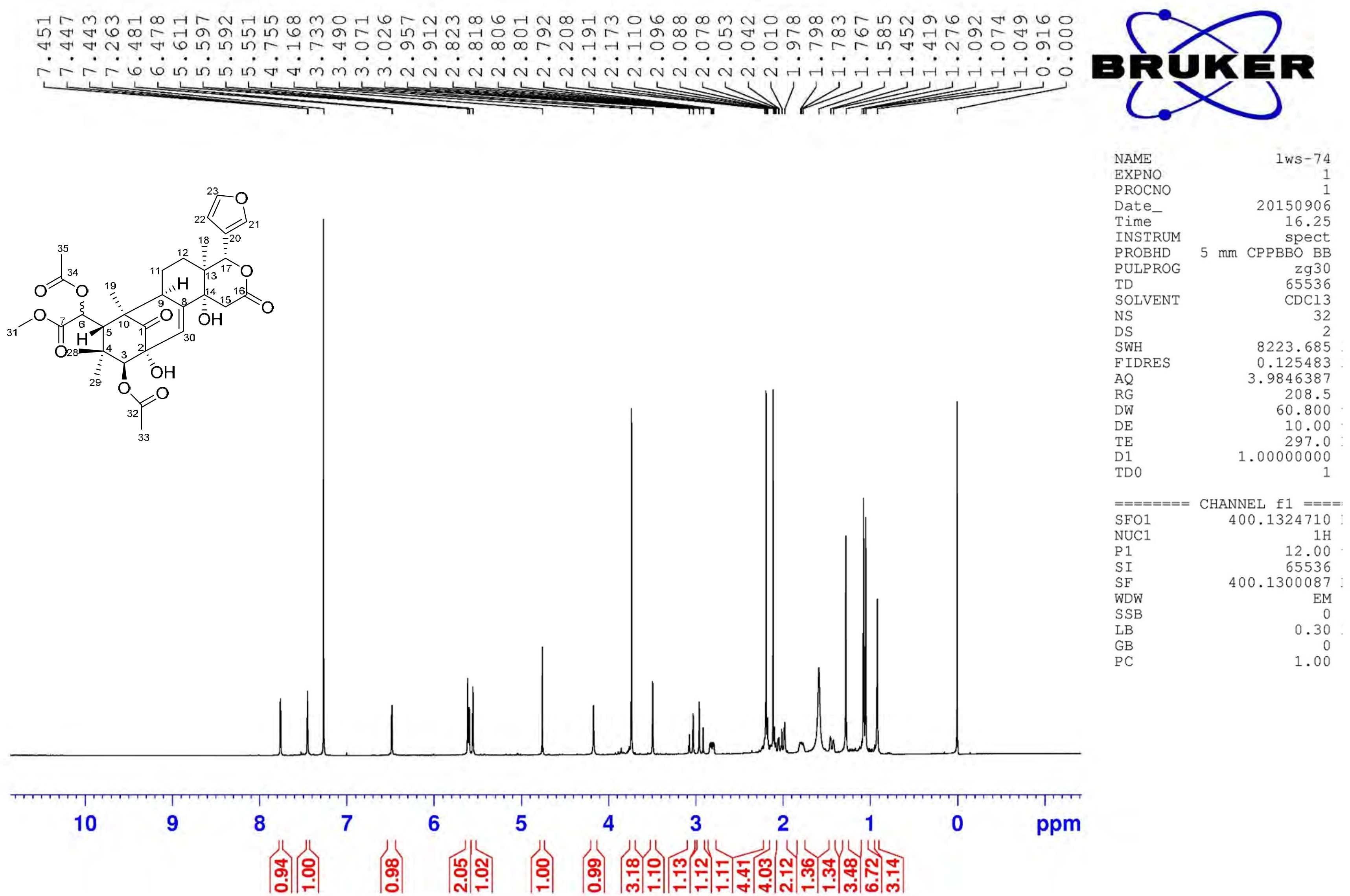

^{1}H NMR (400 MHz) spectrum of compound **13** in $CDCl_3$

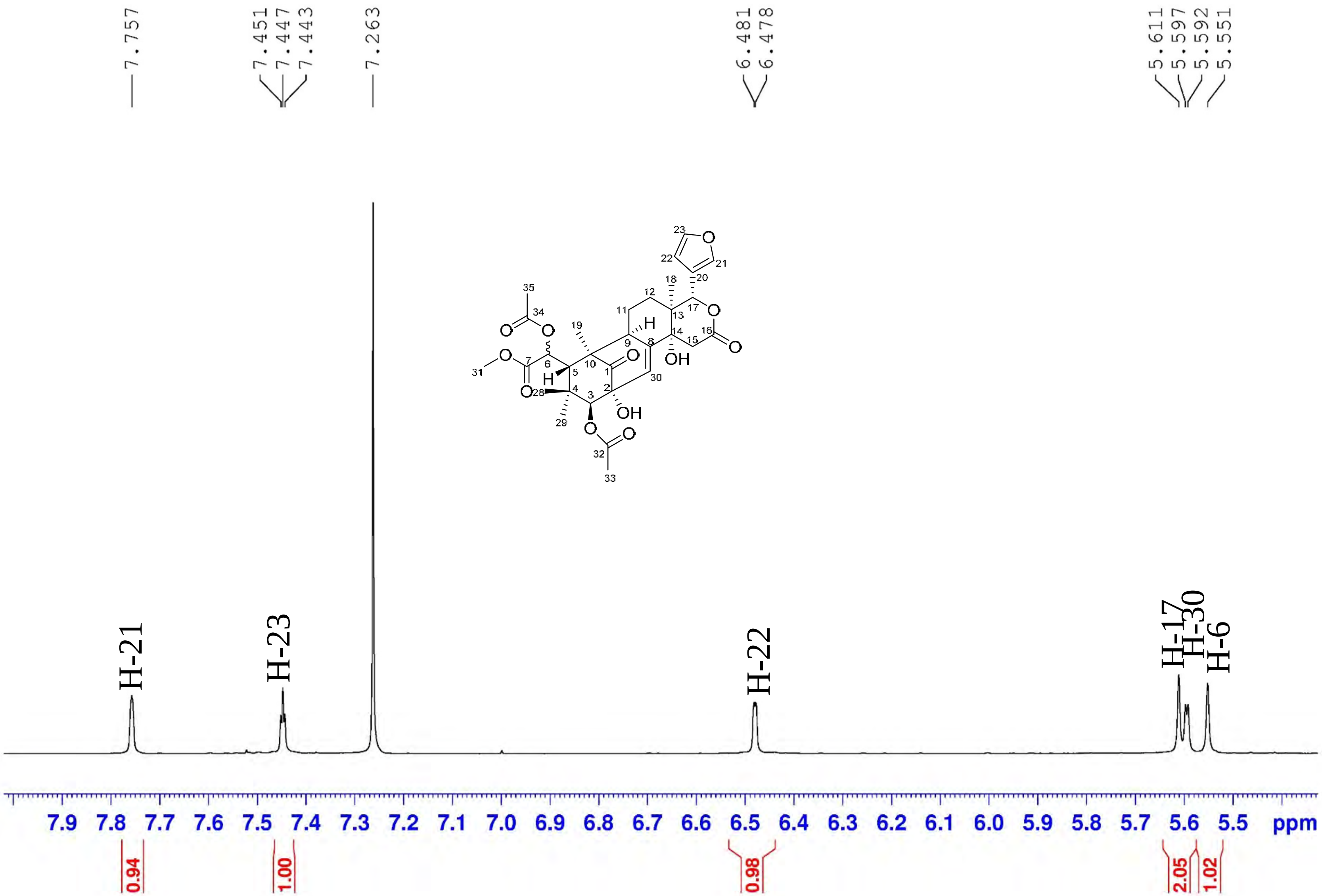

^{1}H NMR (400 MHz) spectrum of compound **13** in $CDCl_3$

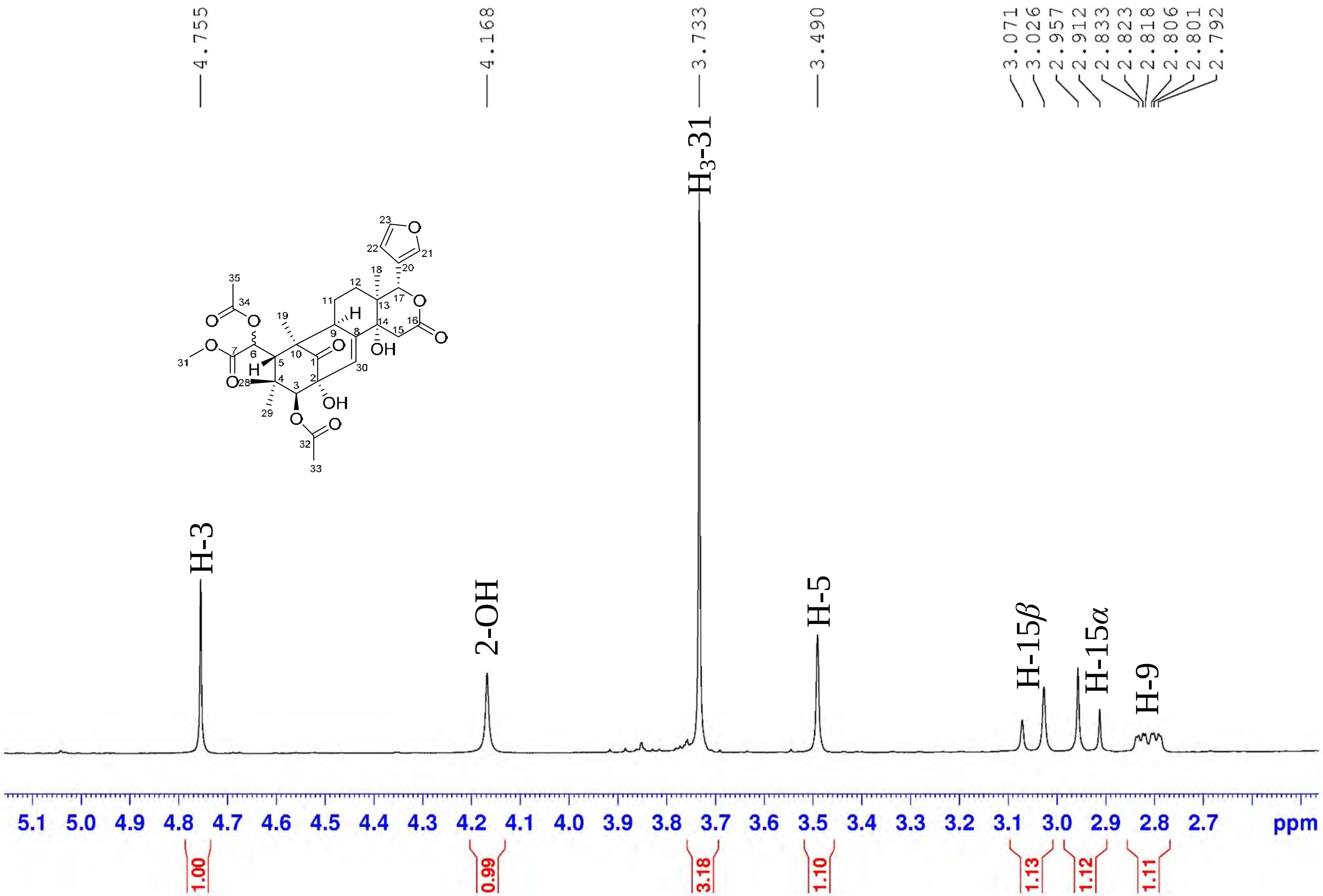

^{1}H NMR (400 MHz) spectrum of compound **13** in $CDCl_3$

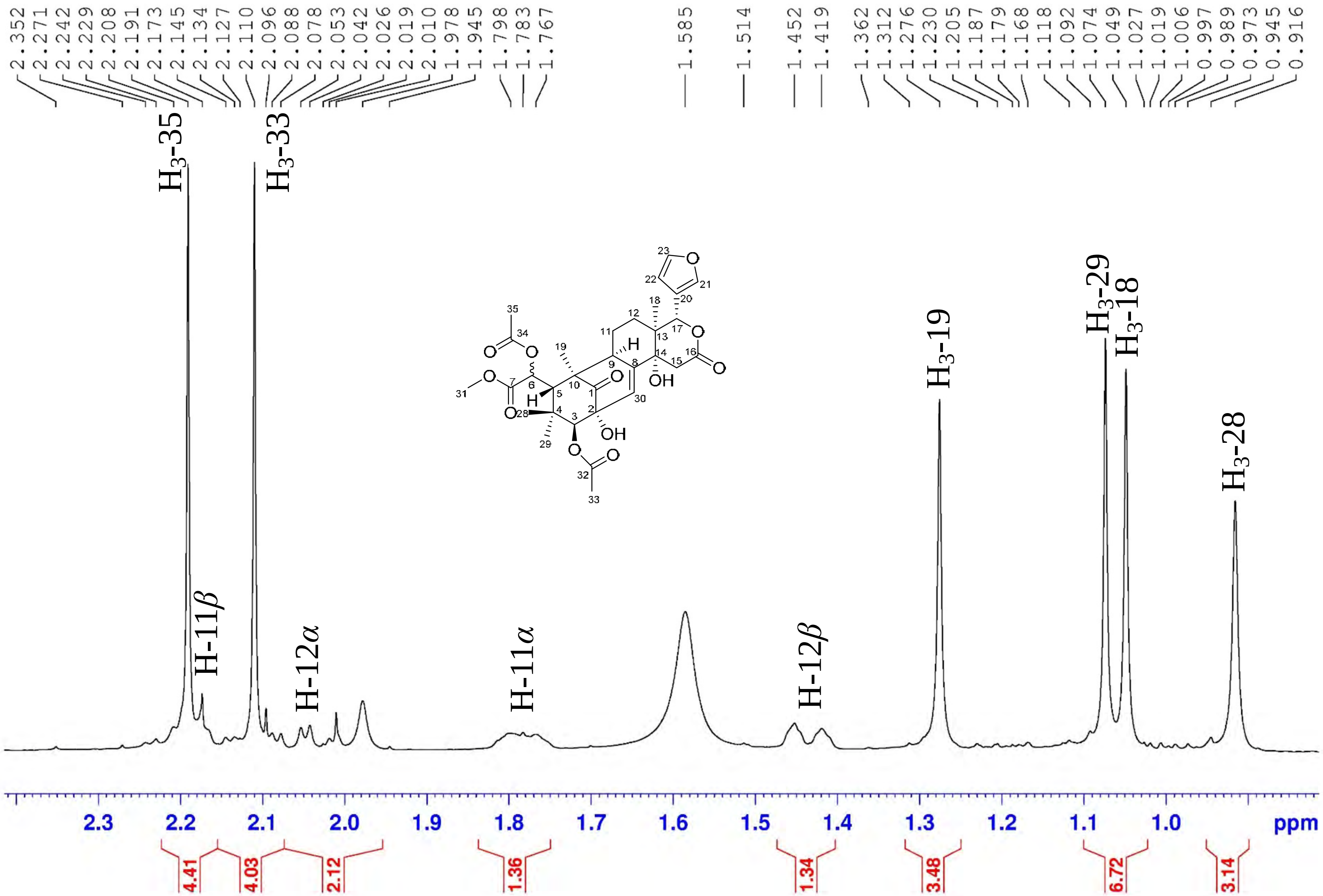

^{13}C NMR (100 MHz) spectrum of compound **13** in $CDCl_3$

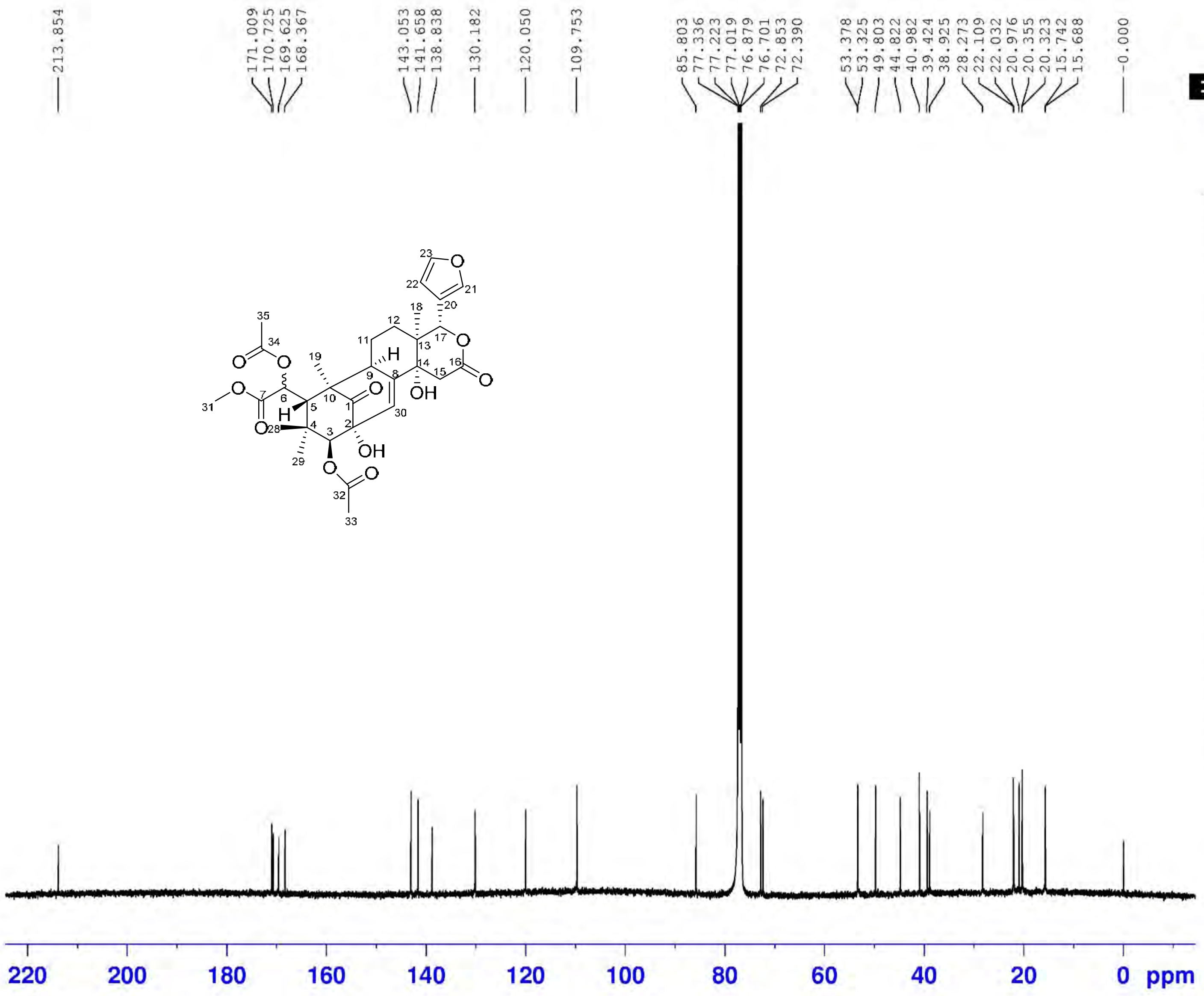

NAME 1ws-74
EXPNO 2
PROCNO 1
Date_ 20150907
Time 19.08
INSTRUM spect
PROBHD 5 mm CPPBBO BB
PULPROG zgpg30
TD 65536
SOLVENT CDCl3
NS 10000
DS 4
SWH 24038.461 H
FIDRES 0.366798 H
AQ 1.3631988 s
RG 147.94
DW 20.800 u
DE 18.00 u
TE 297.0 K
D1 2.00000000 s
D11 0.03000000 s
TD0 1

======== CHANNEL f1 =====
SFO1 100.6233324 M
NUC1 13C
P1 10.00 u
SI 32768
SF 100.6127692 M
WDW EM
SSB 0
LB 1.00 H
GB 0
PC 1.40

^{13}C NMR (100 MHz) spectrum of compound **13** in $CDCl_3$

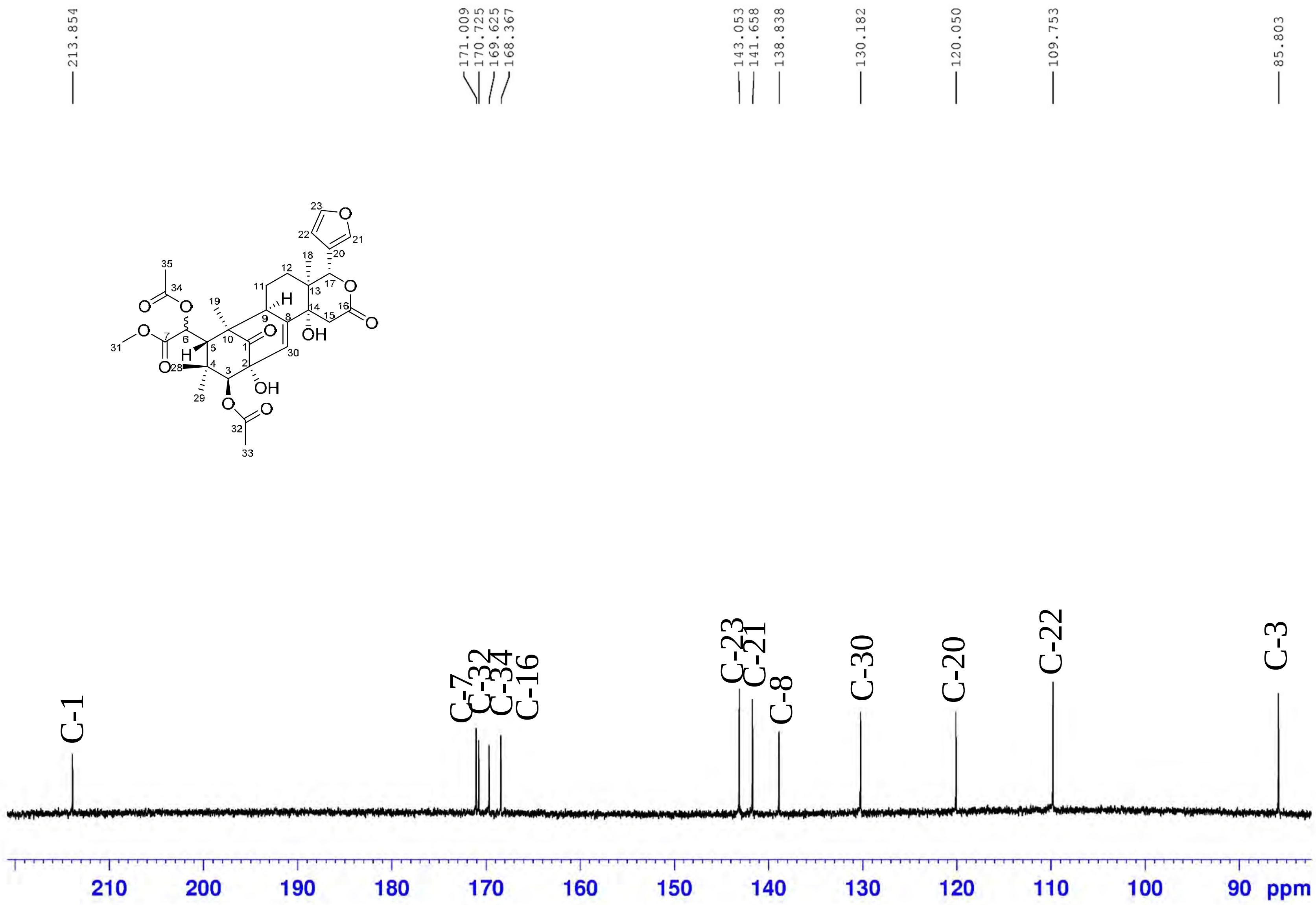

^{13}C NMR (100 MHz) spectrum of compound **13** in $CDCl_3$

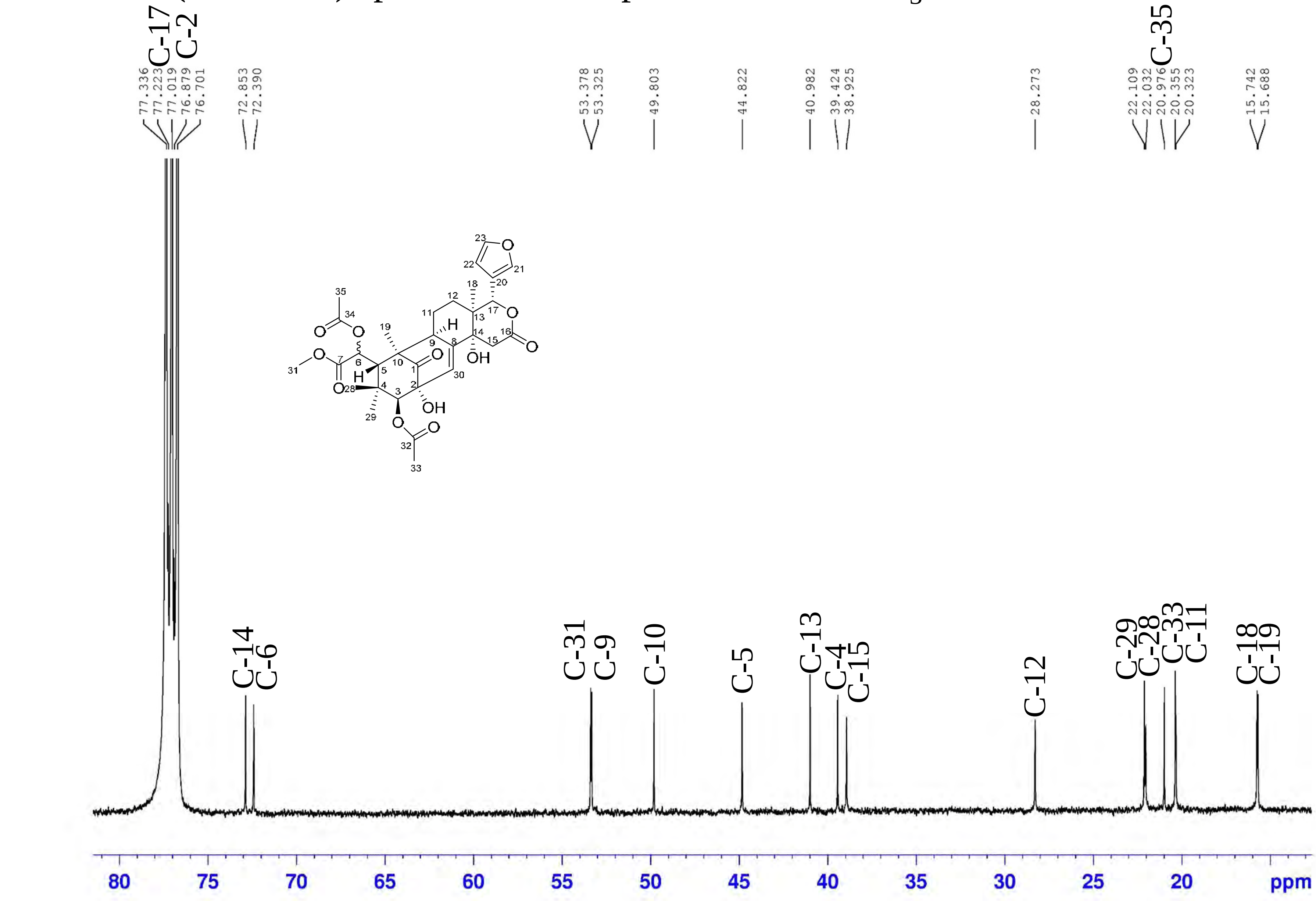

DEPT135 (100 MHz) spectrum of compound **13** in $CDCl_3$

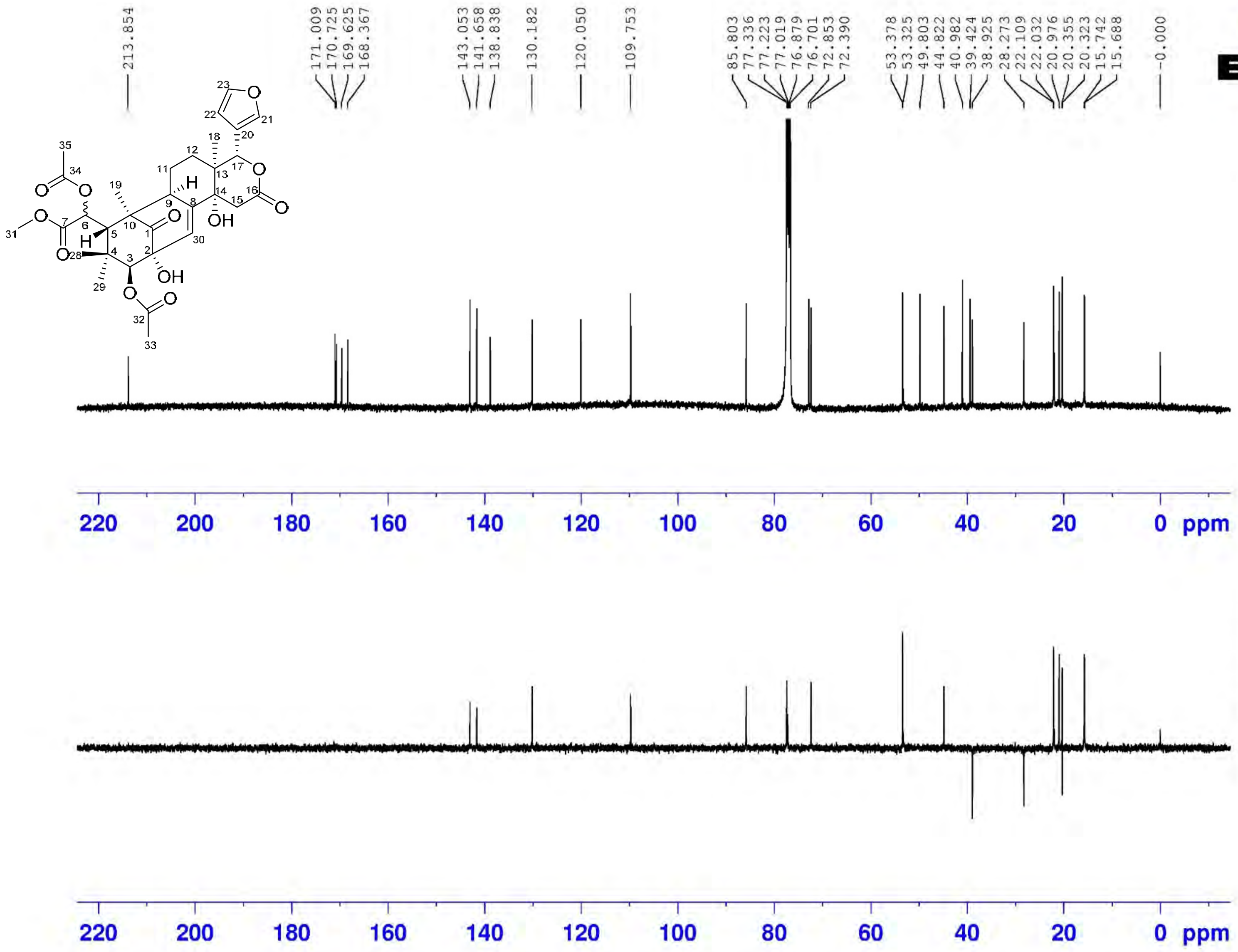

NAME	lws-74
EXPNO	3
PROCNO	1
Date_	20150907
Time	21.31
INSTRUM	spect
PROBHD	5 mm CPPBBO BB
PULPROG	deptsp135
TD	65536
SOLVENT	CDCl3
NS	2500
DS	4
SWH	24038.461
FIDRES	0.366798
AQ	1.3631988
RG	102.3
DW	20.800
DE	18.00
TE	297.0
CNST2	145.0000000
D1	2.00000000
D2	0.00344828
D12	0.00002000
TD0	1

======== CHANNEL f1 ===

SFO1	100.6233324
NUC1	13C
P1	10.00
P13	2000.00
SI	32768
SF	100.6127694
WDW	EM
SSB	0
LB	1.00
GB	0
PC	1.40

DEPT135 (100 MHz) spectrum of compound **13** in $CDCl_3$

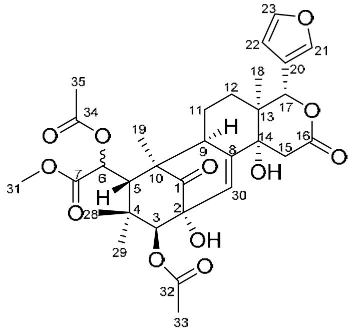

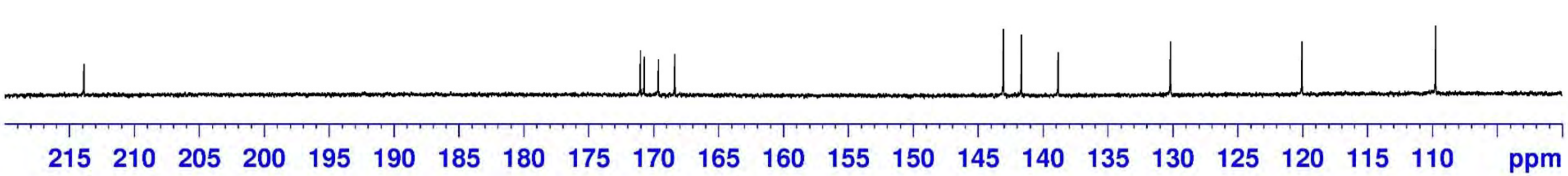

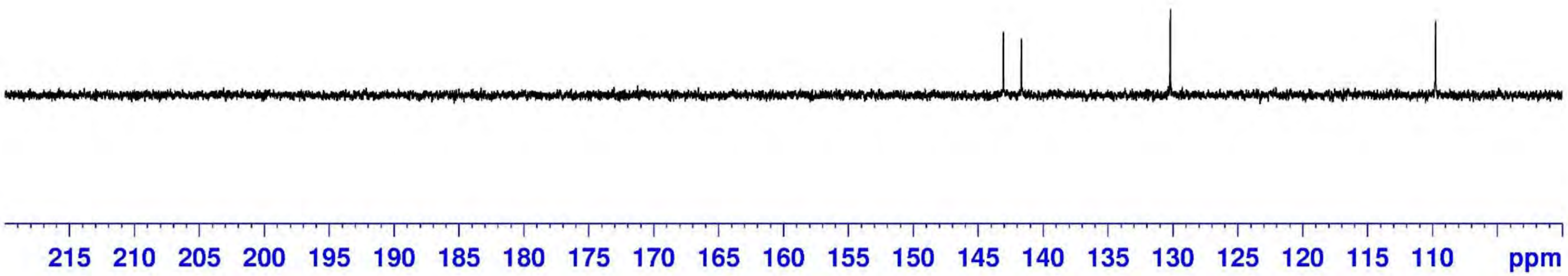

DEPT135 (100 MHz) spectrum of compound **13** in $CDCl_3$

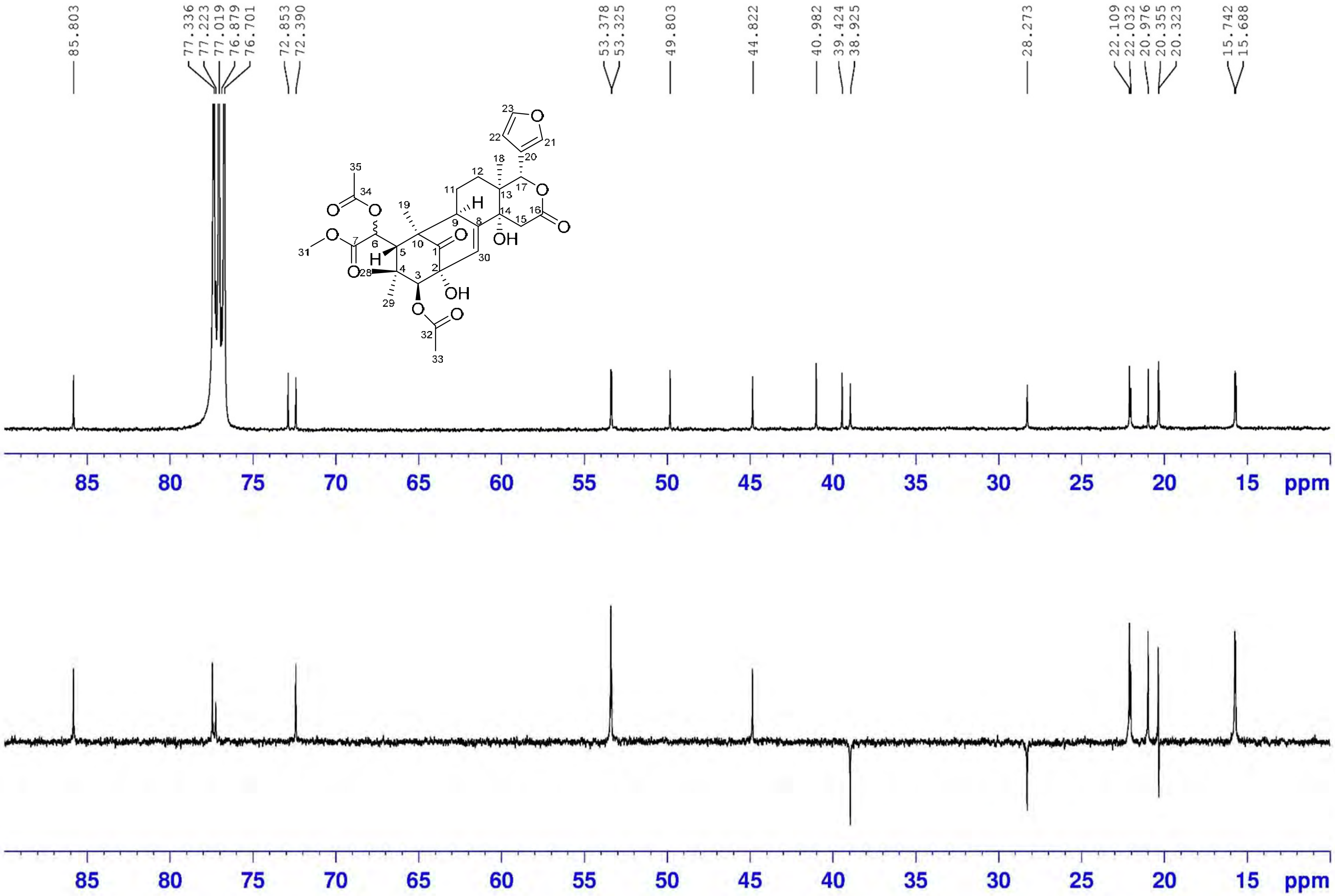

^1H-^1H COSY (400 MHz) spectrum of compound **13** in $CDCl_3$

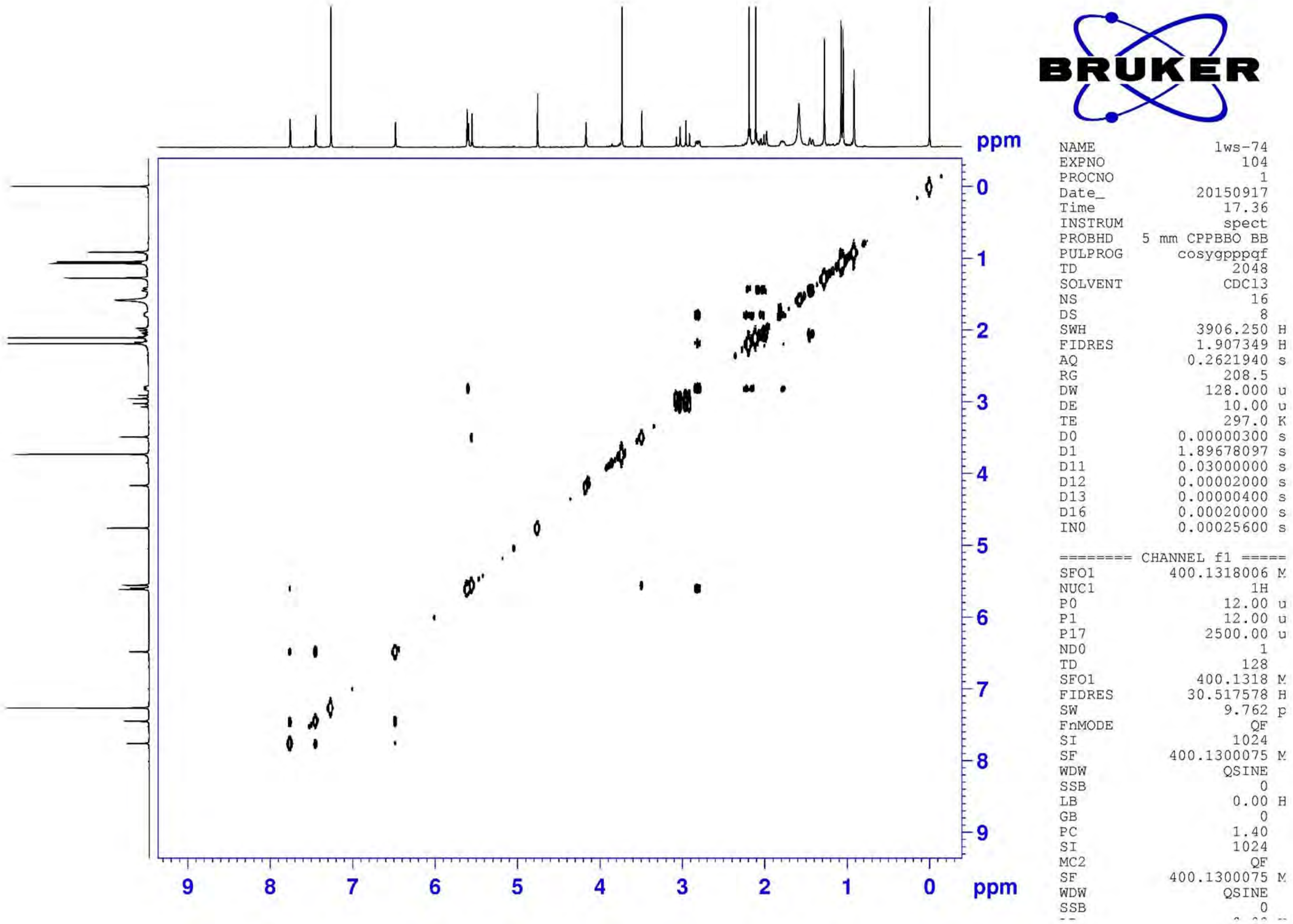

^{1}H-^{1}H COSY (400 MHz) spectrum of compound **13** in $CDCl_3$

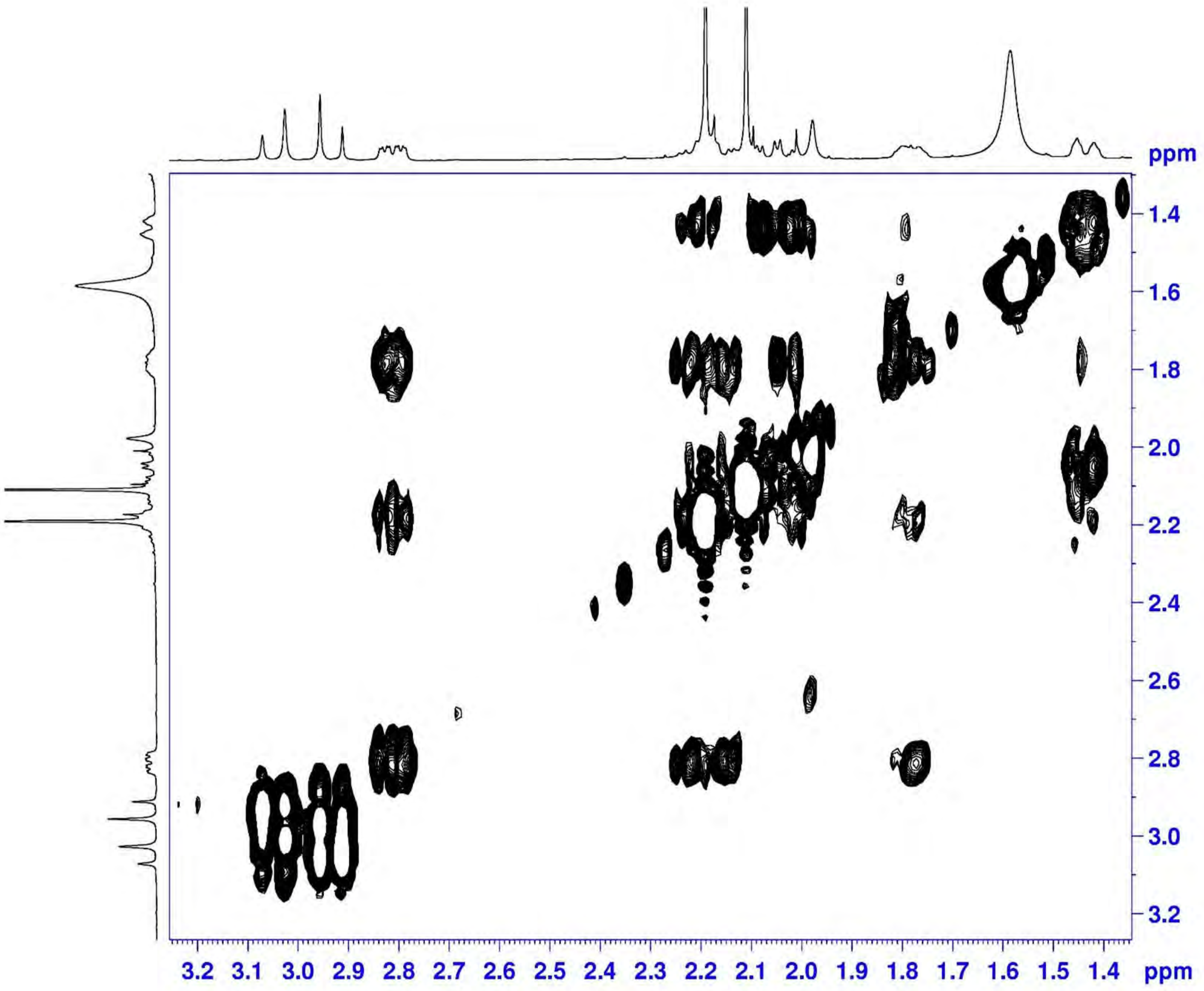

^{1}H-^{1}H COSY (400 MHz) spectrum of compound **13** in $CDCl_3$

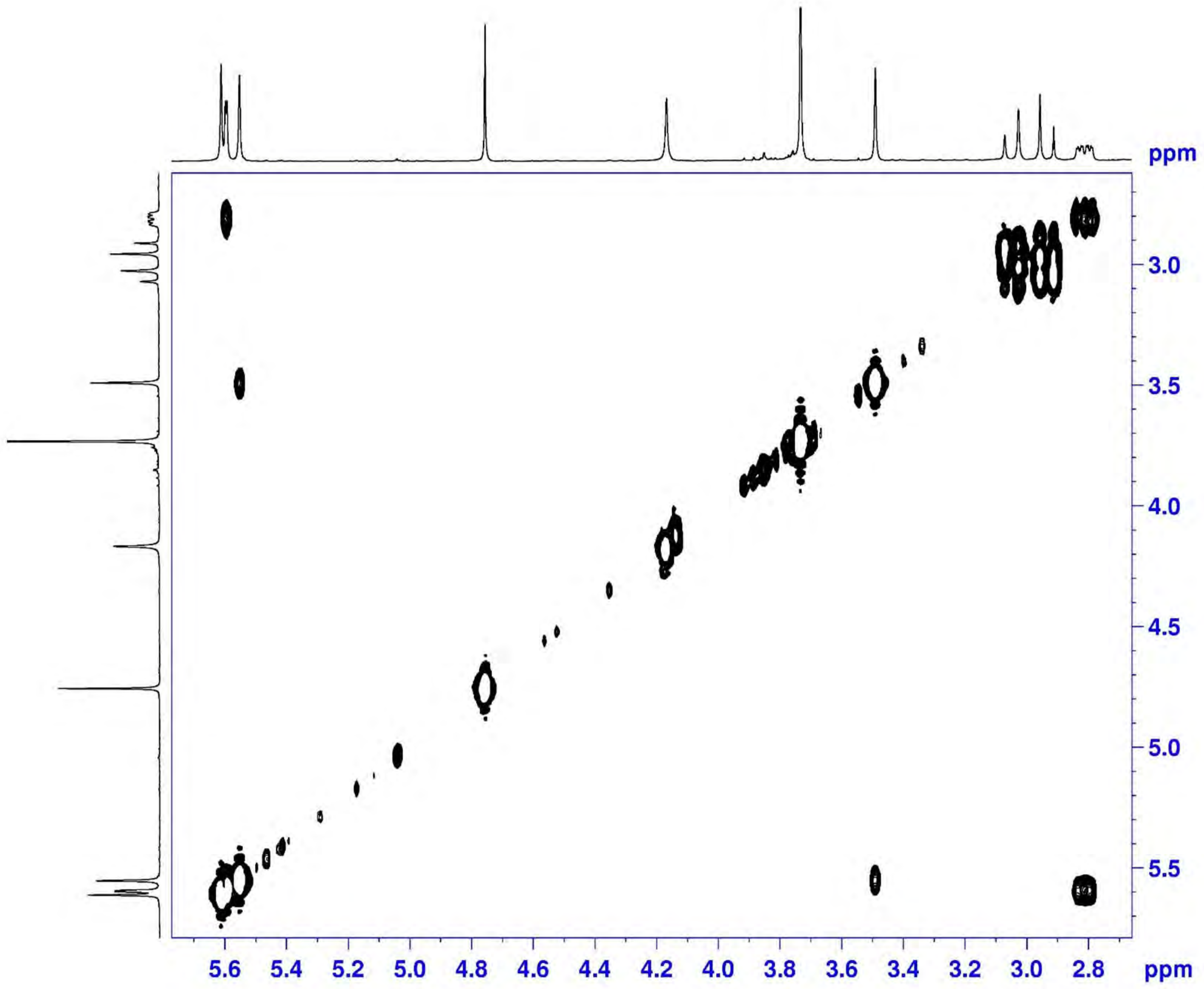

HSQC (400 MHz) spectrum of compound **13** in $CDCl_3$

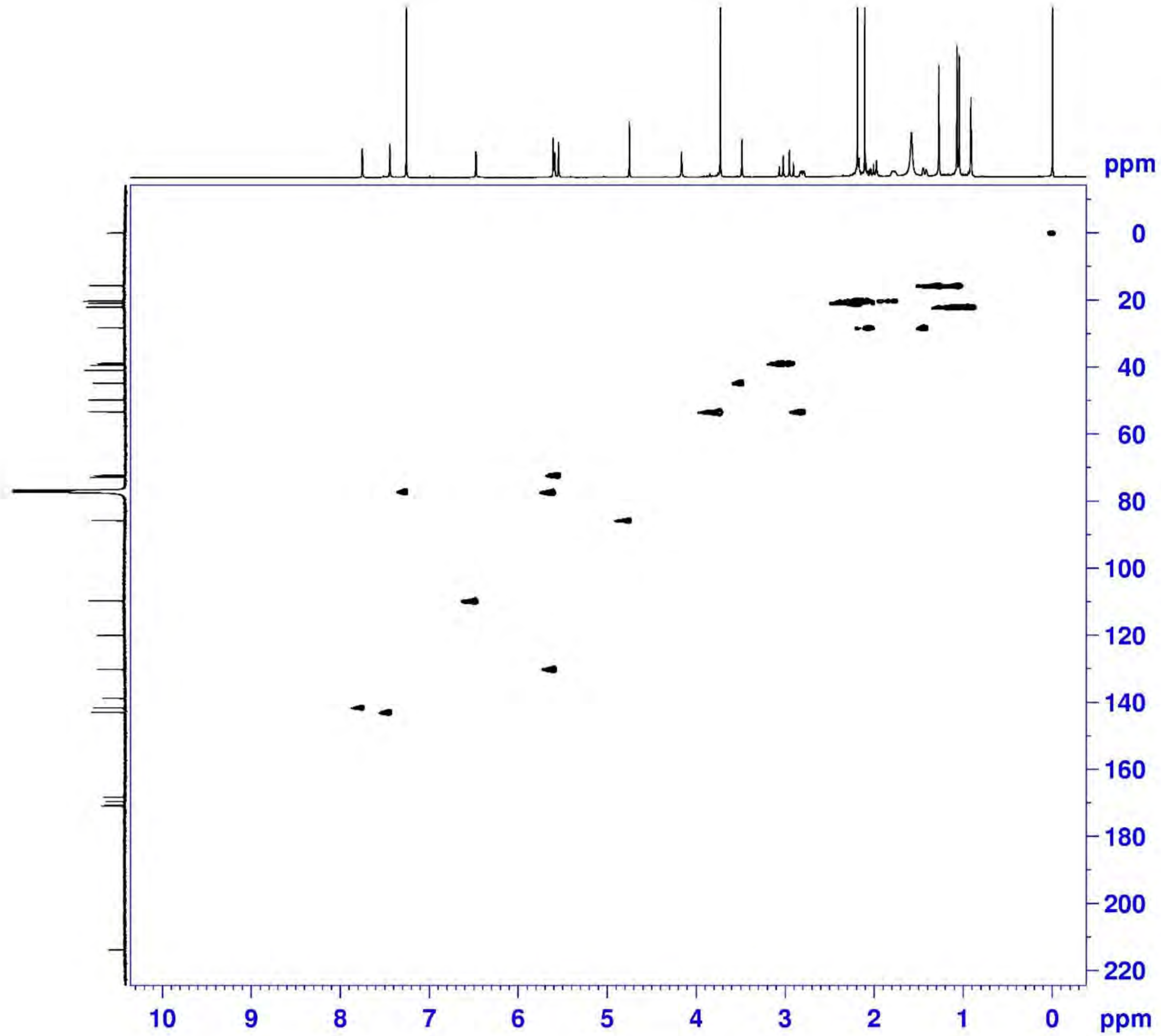


```
NAME              lws-74
EXPNO                  5
PROCNO                 1
Date_           20150907
Time               21.33
INSTRUM            spect
PROBHD   5 mm CPPBBO BB
PULPROG       hsqcetgpsi2
TD                  1024
SOLVENT            CDCl3
NS                    32
DS                    16
SWH             4302.926 Hz
FIDRES          4.202076 Hz
AQ             0.1190388 sec
RG                 208.5
DW               116.200 usec
DE                 10.00 usec
TE                 297.0 K
CNST2        145.0000000
D0            0.00000300 sec
D1            1.46497905 sec
D4            0.00172414 sec
D11           0.03000000 sec
D16           0.00020000 sec
D24           0.00086207 sec
IN0           0.00002080 sec
ZGOPTNS

======== CHANNEL f1 ========
SFO1         400.1320007 MHz
NUC1                  1H
P1                 12.00 usec
P2                 24.00 usec
P28                 0.00 usec
ND0                    2
TD                   256
SFO1            100.6233 MHz
FIDRES         93.900238 Hz
SW               238.896 ppm
FnMODE      Echo-Antiecho
SI                  1024
SF           400.1300079 MHz
WDW                QSINE
SSB                    2
LB                  0.00 Hz
GB                     0
PC                  1.40
SI                  1024
MC2         echo-antiecho
SF           100.6127685 MHz
WDW                QSINE
SSB                    [illegible]
```

HSQC (400 MHz) spectrum of compound **13** in $CDCl_3$

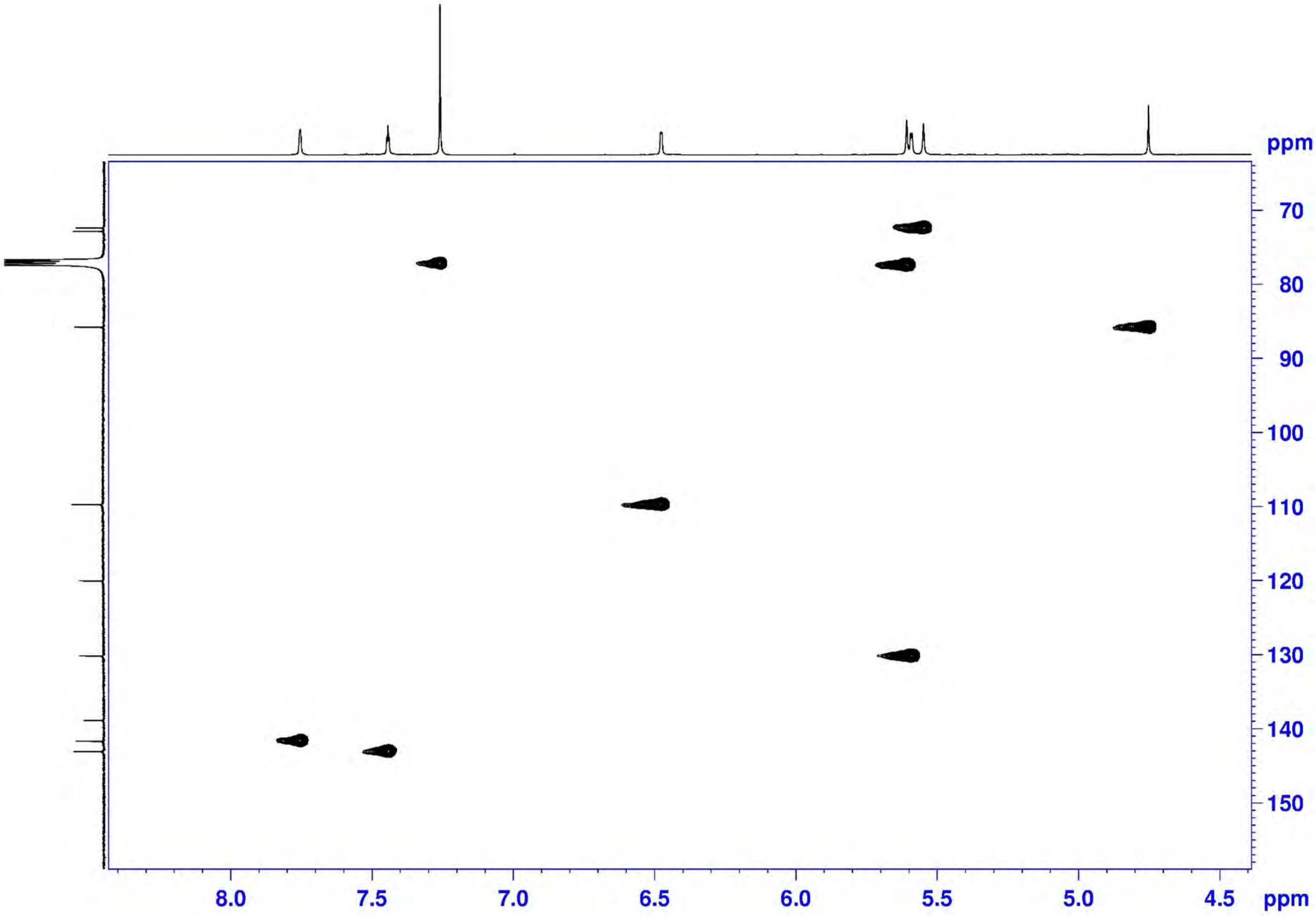

HSQC (400 MHz) spectrum of compound **13** in $CDCl_3$

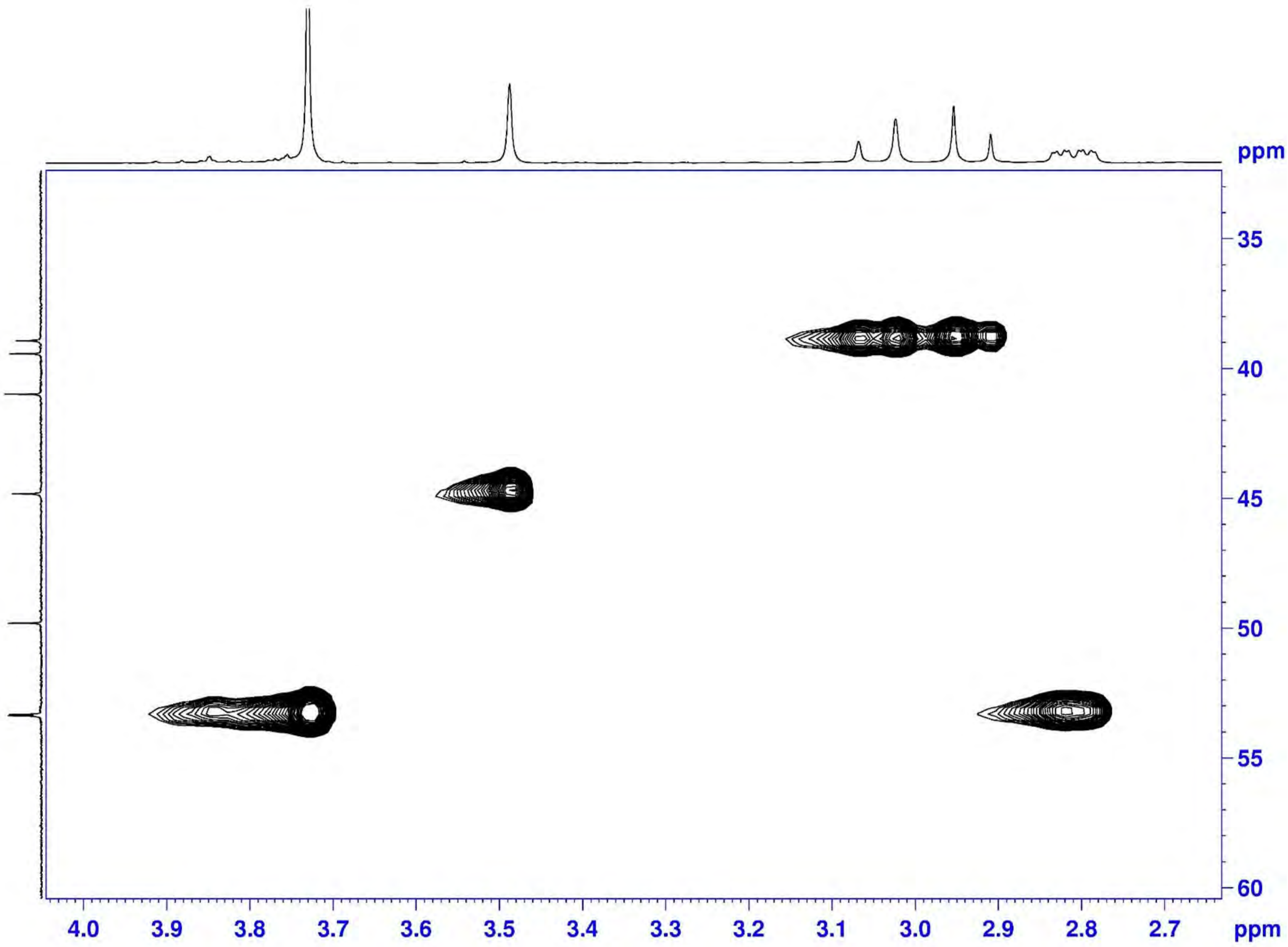

HSQC (400 MHz) spectrum of compound **13** in $CDCl_3$

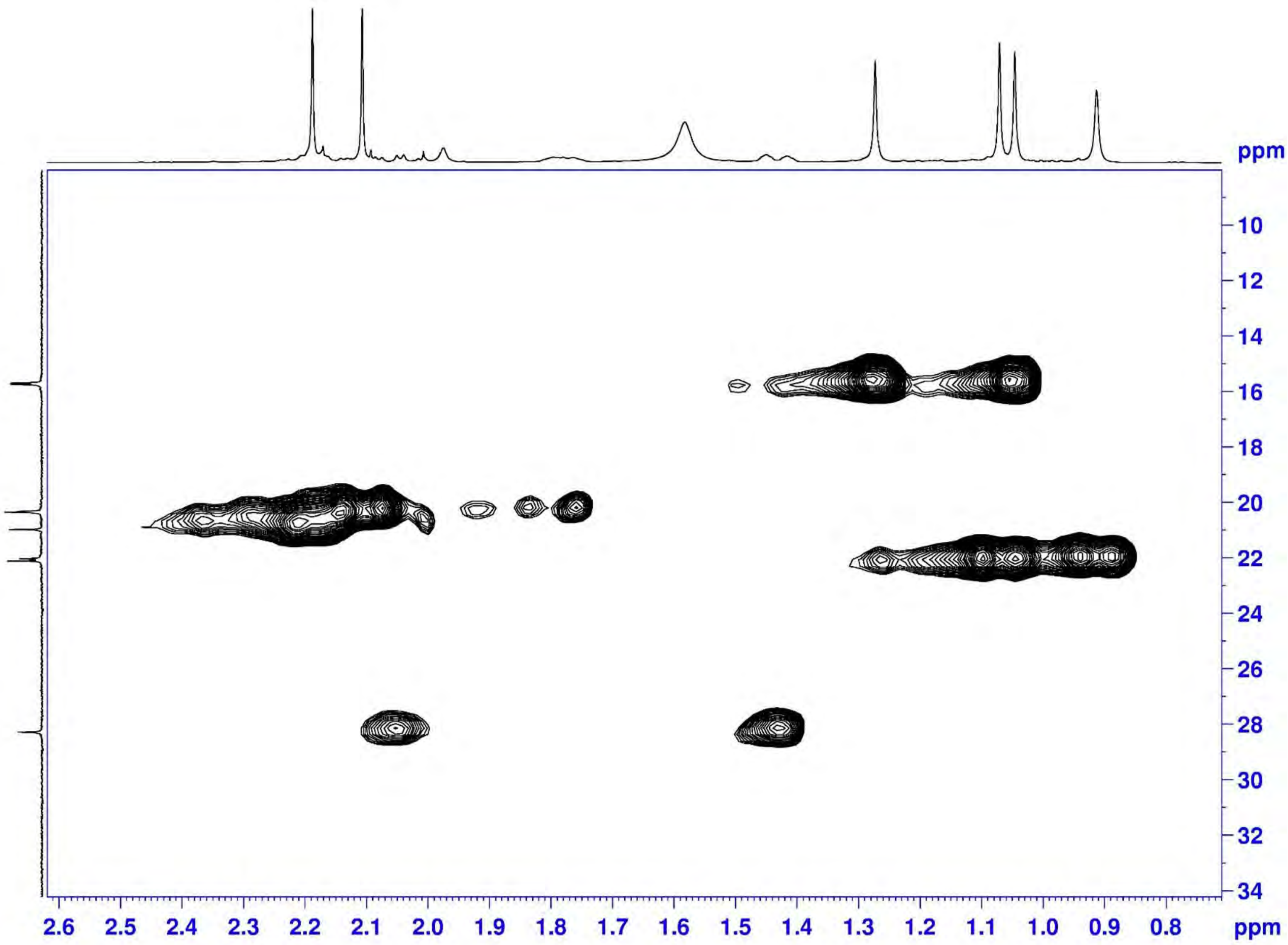

HMBC (400 MHz) spectrum of compound **13** in $CDCl_3$

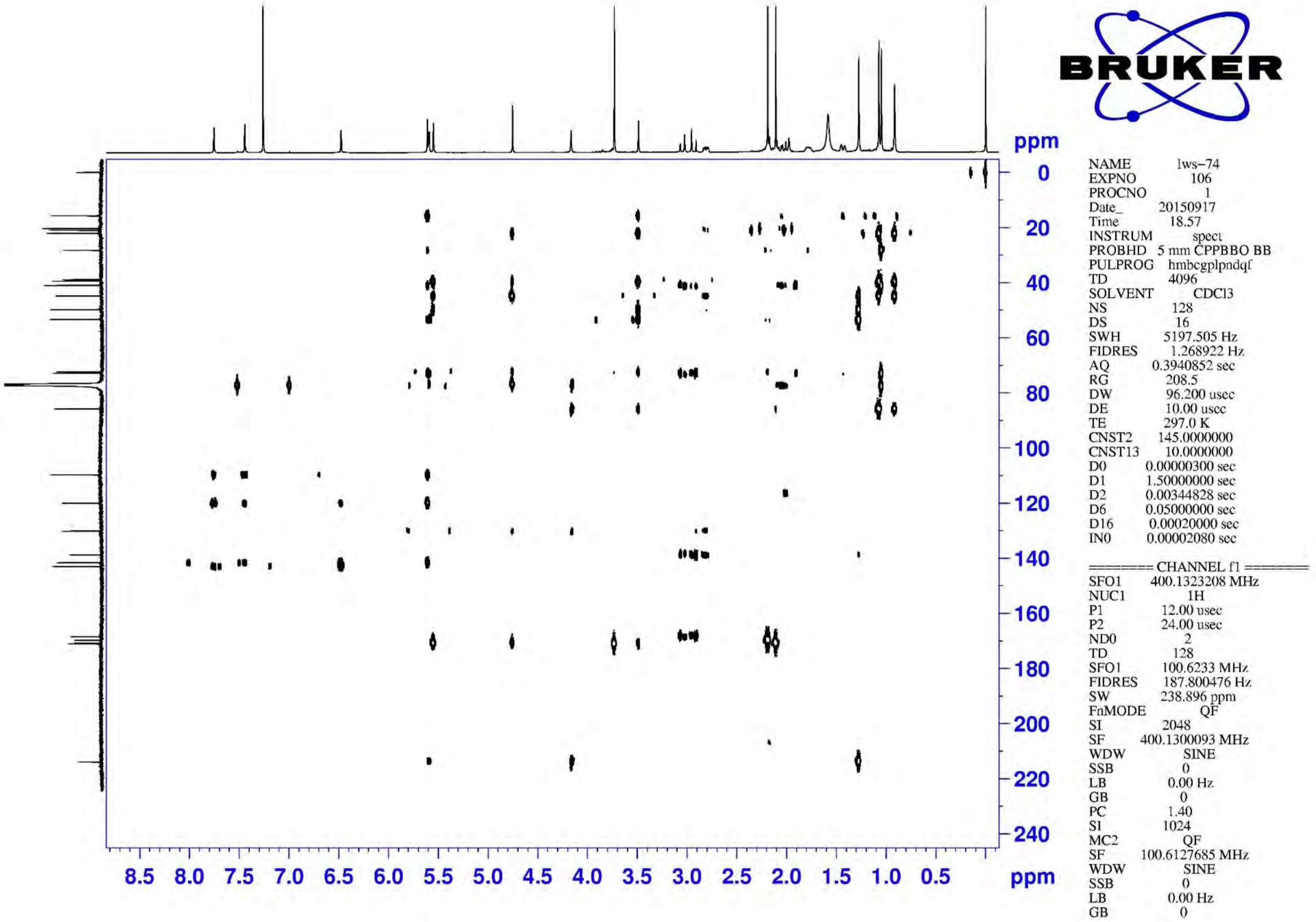

HMBC (400 MHz) spectrum of compound **13** in $CDCl_3$

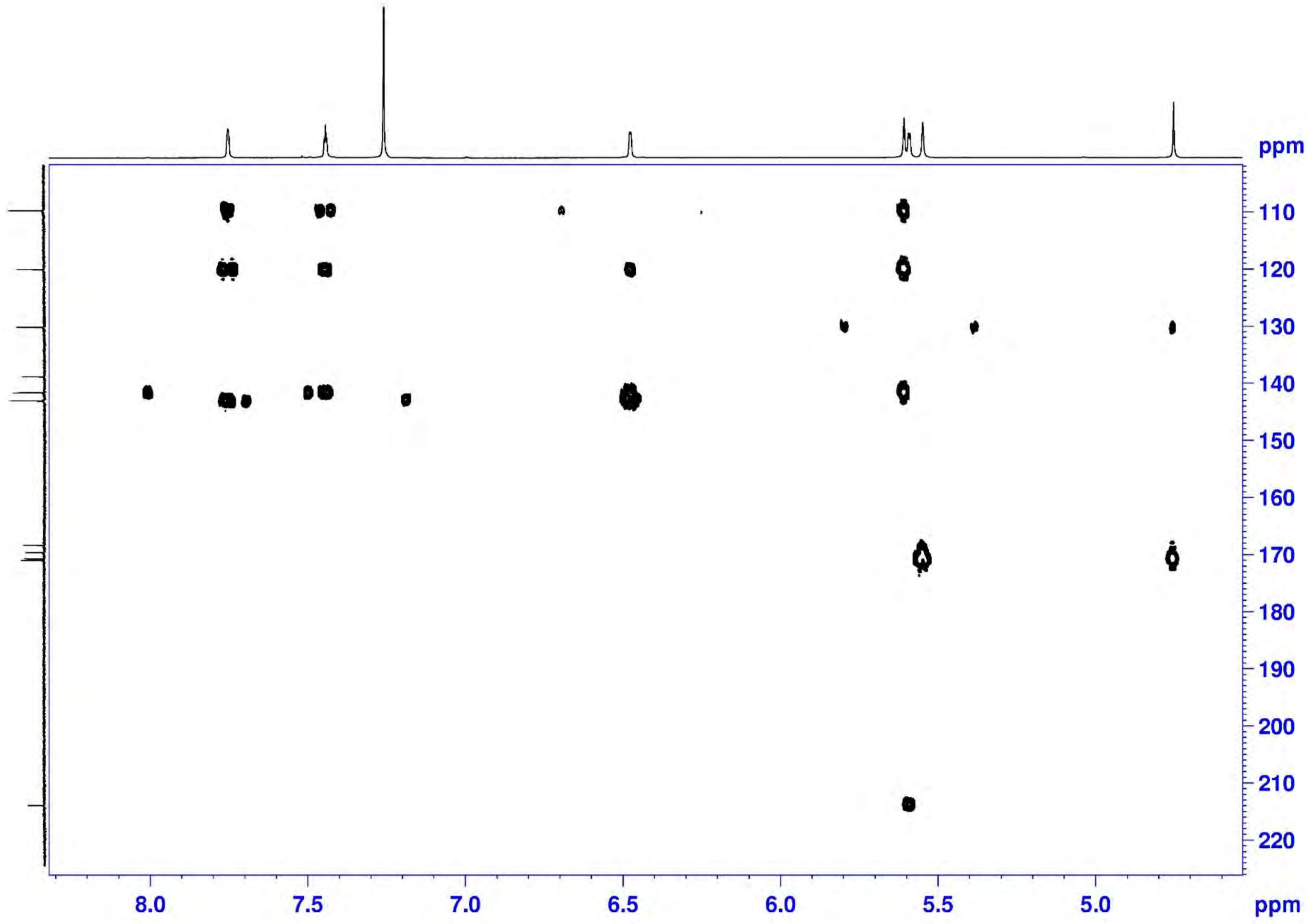

HMBC (400 MHz) spectrum of compound **13** in $CDCl_3$

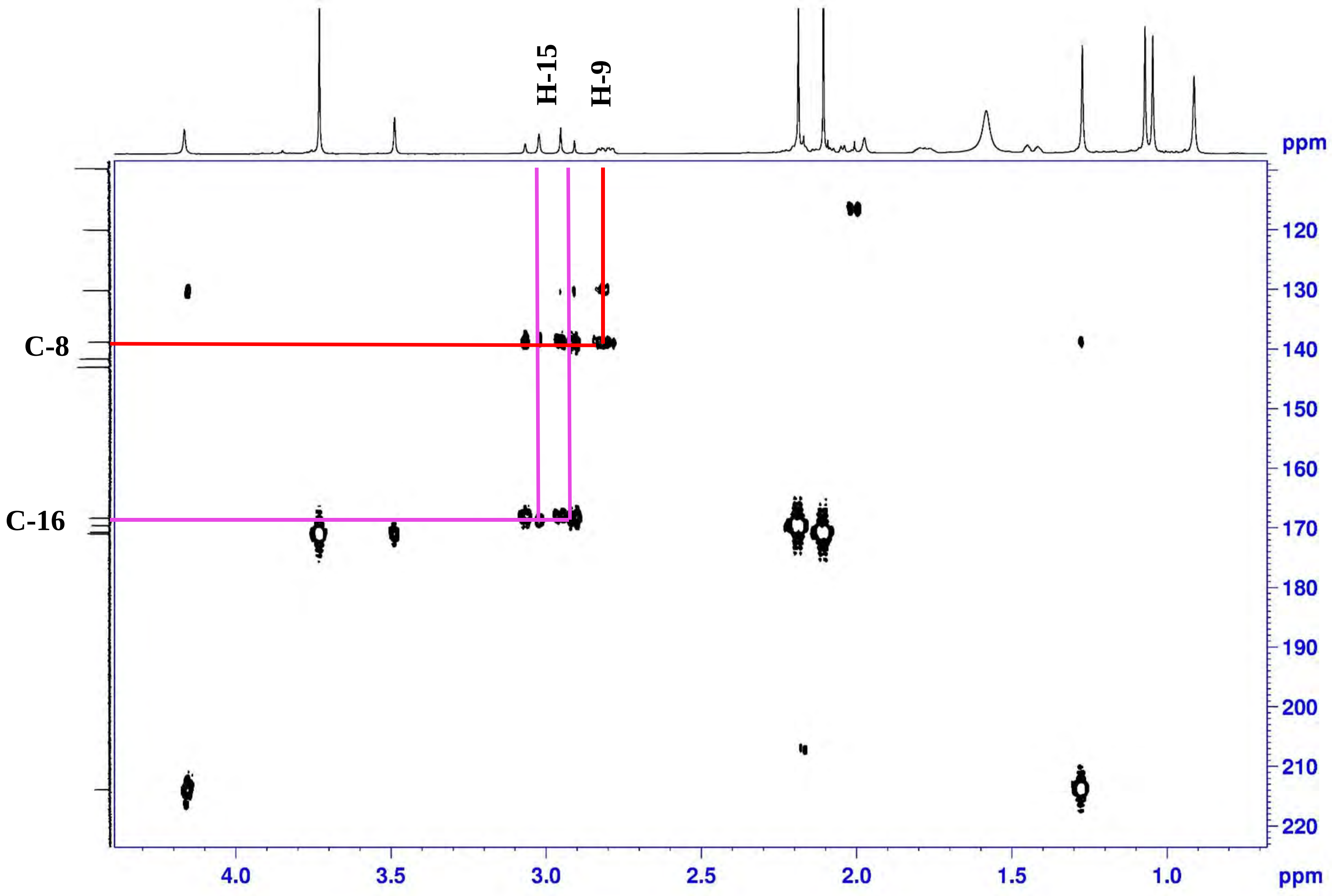

HMBC (400 MHz) spectrum of compound **13** in $CDCl_3$

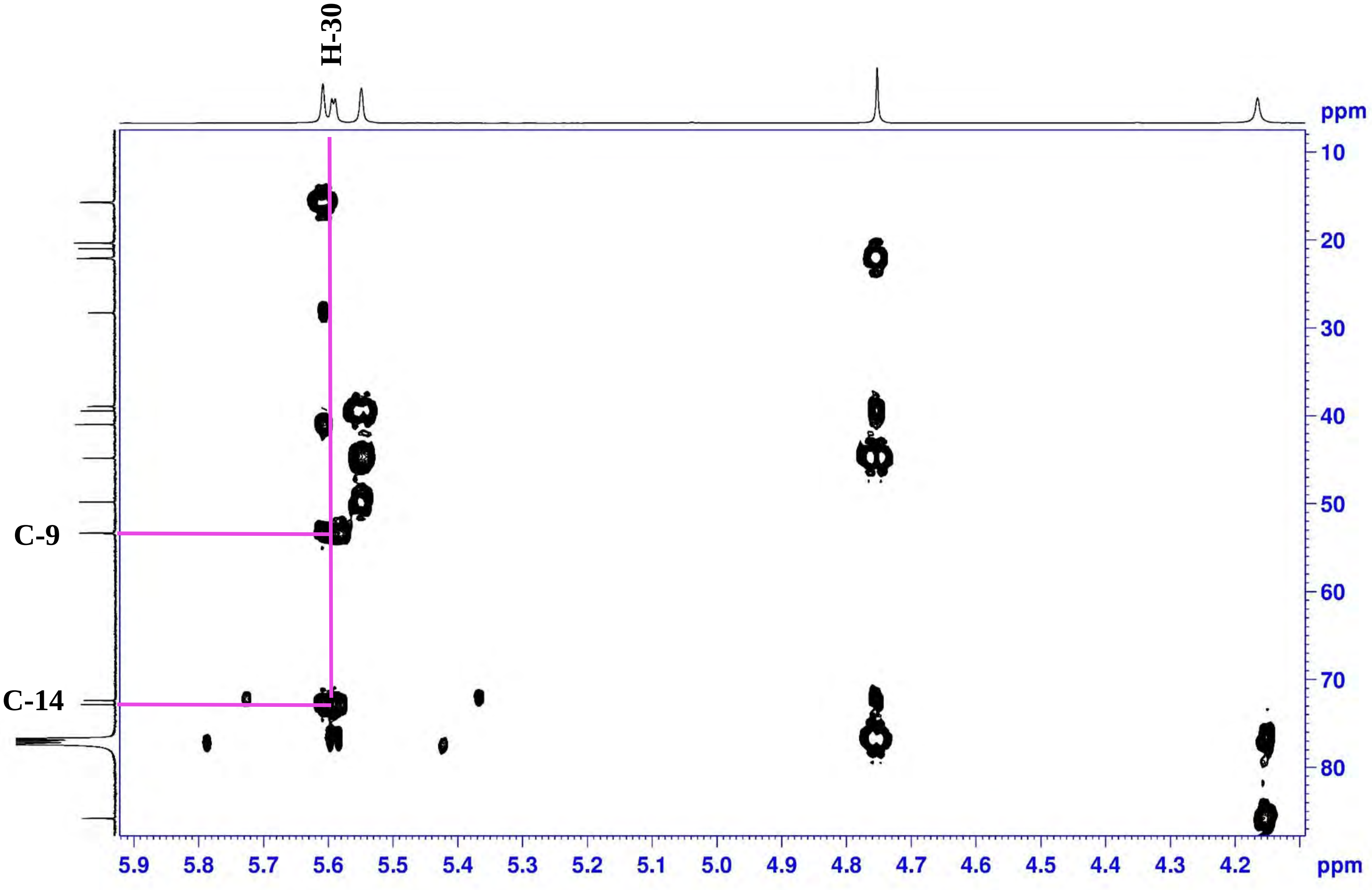

HMBC (400 MHz) spectrum of compound **13** in $CDCl_3$

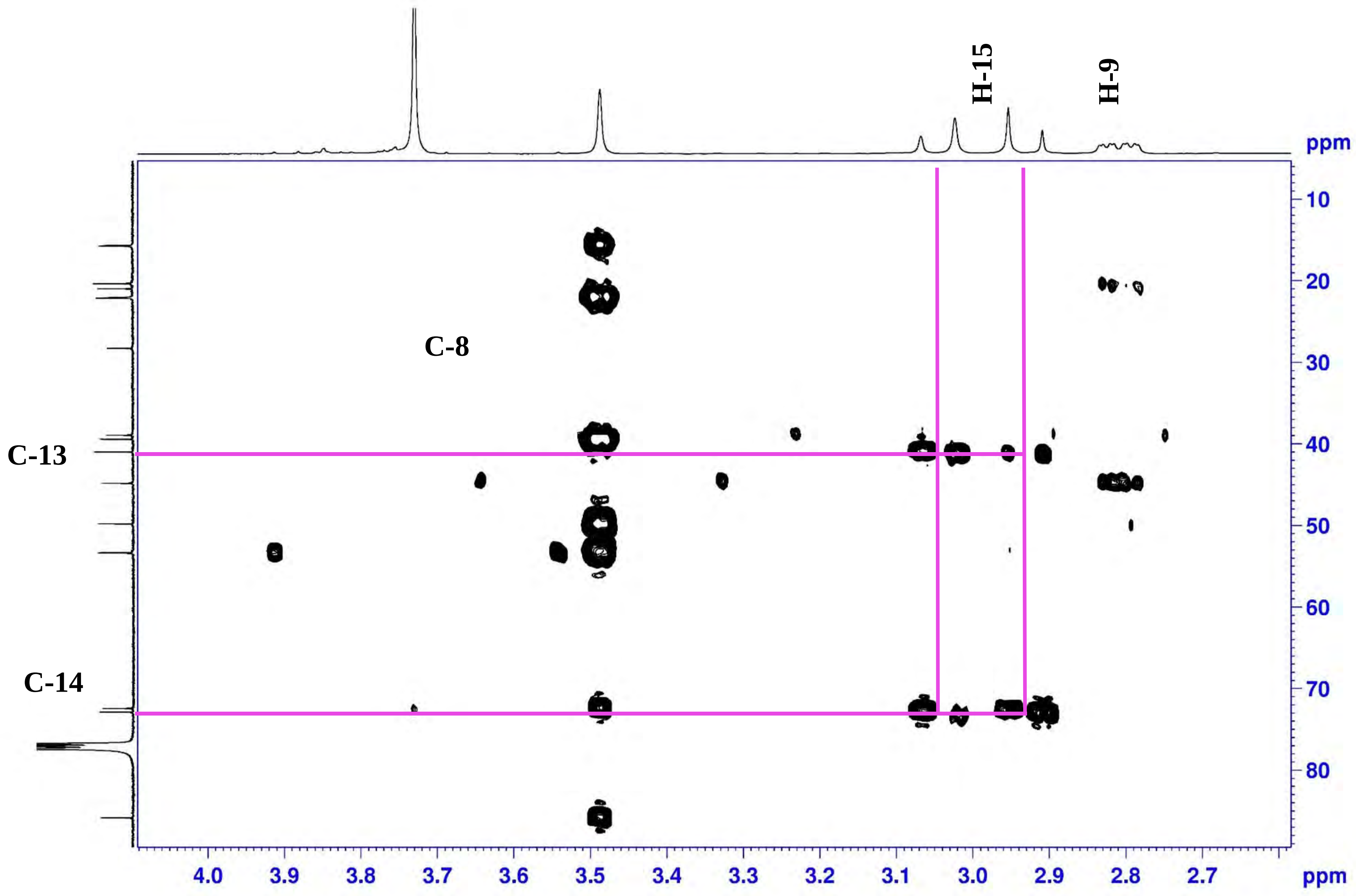

HMBC (400 MHz) spectrum of compound **13** in $CDCl_3$

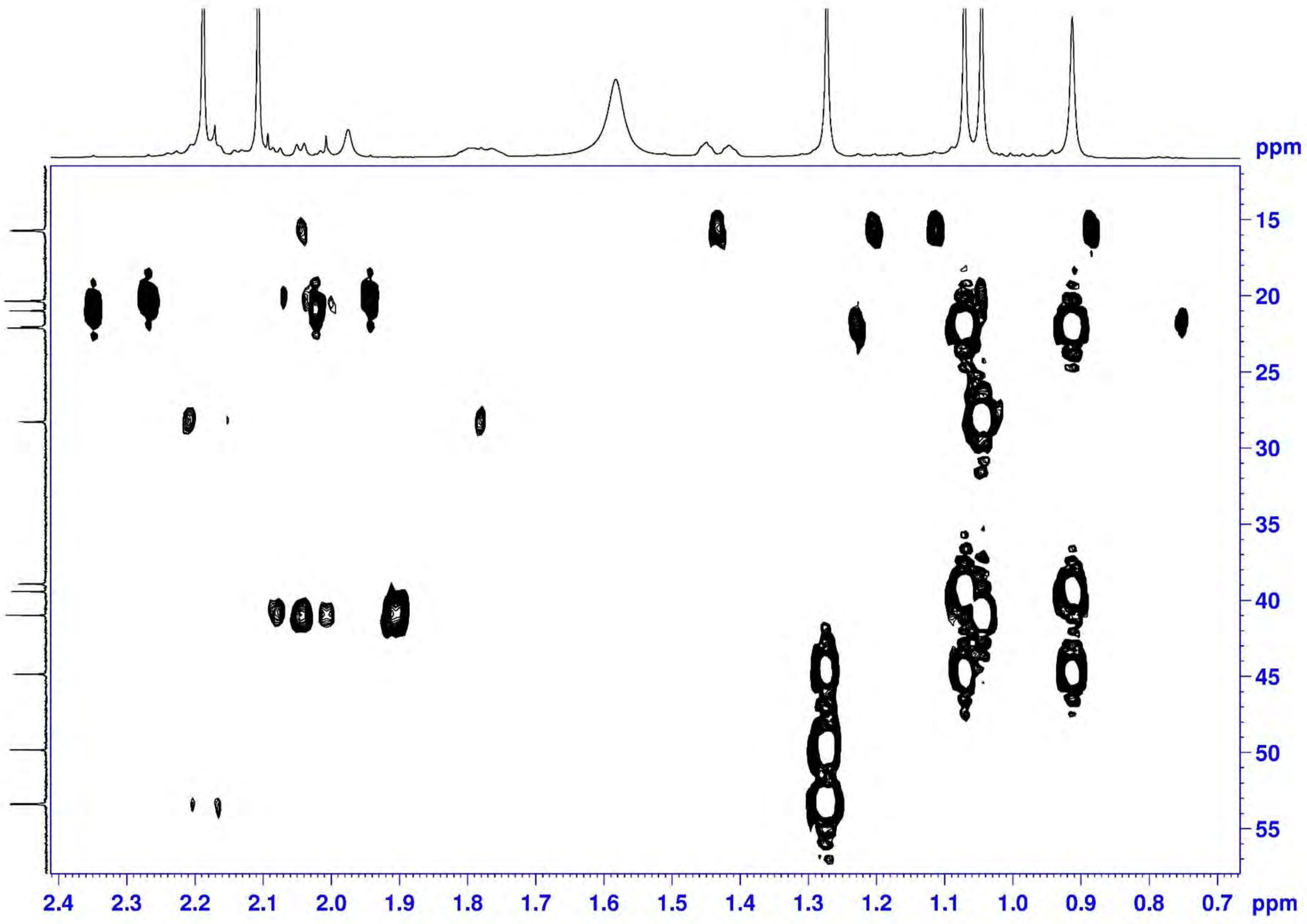

HMBC (400 MHz) spectrum of compound **13** in $CDCl_3$

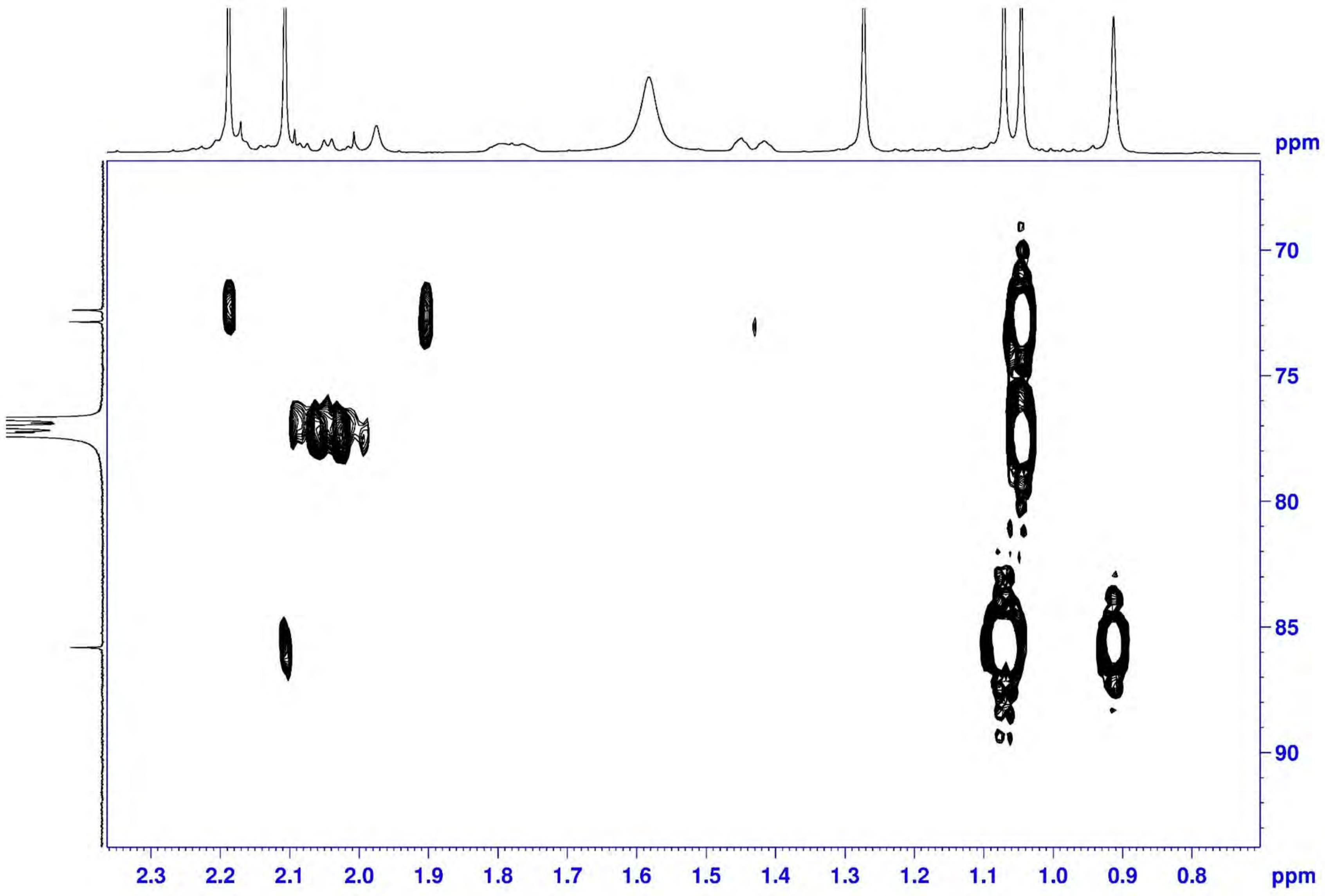

NOESY (400 MHz) spectrum of compound **13** in $CDCl_3$

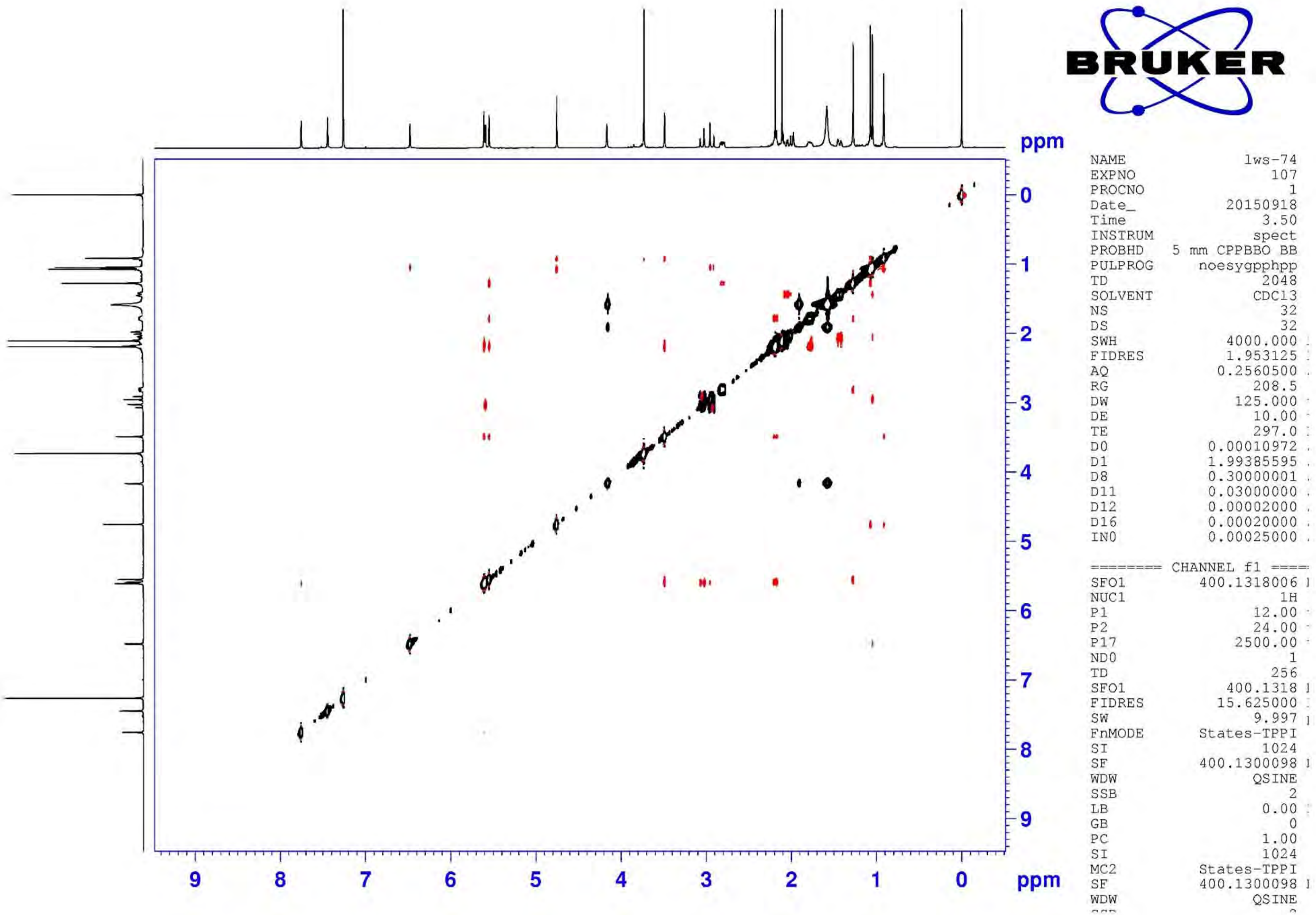

NOESY (400 MHz) spectrum of compound **13** in $CDCl_3$

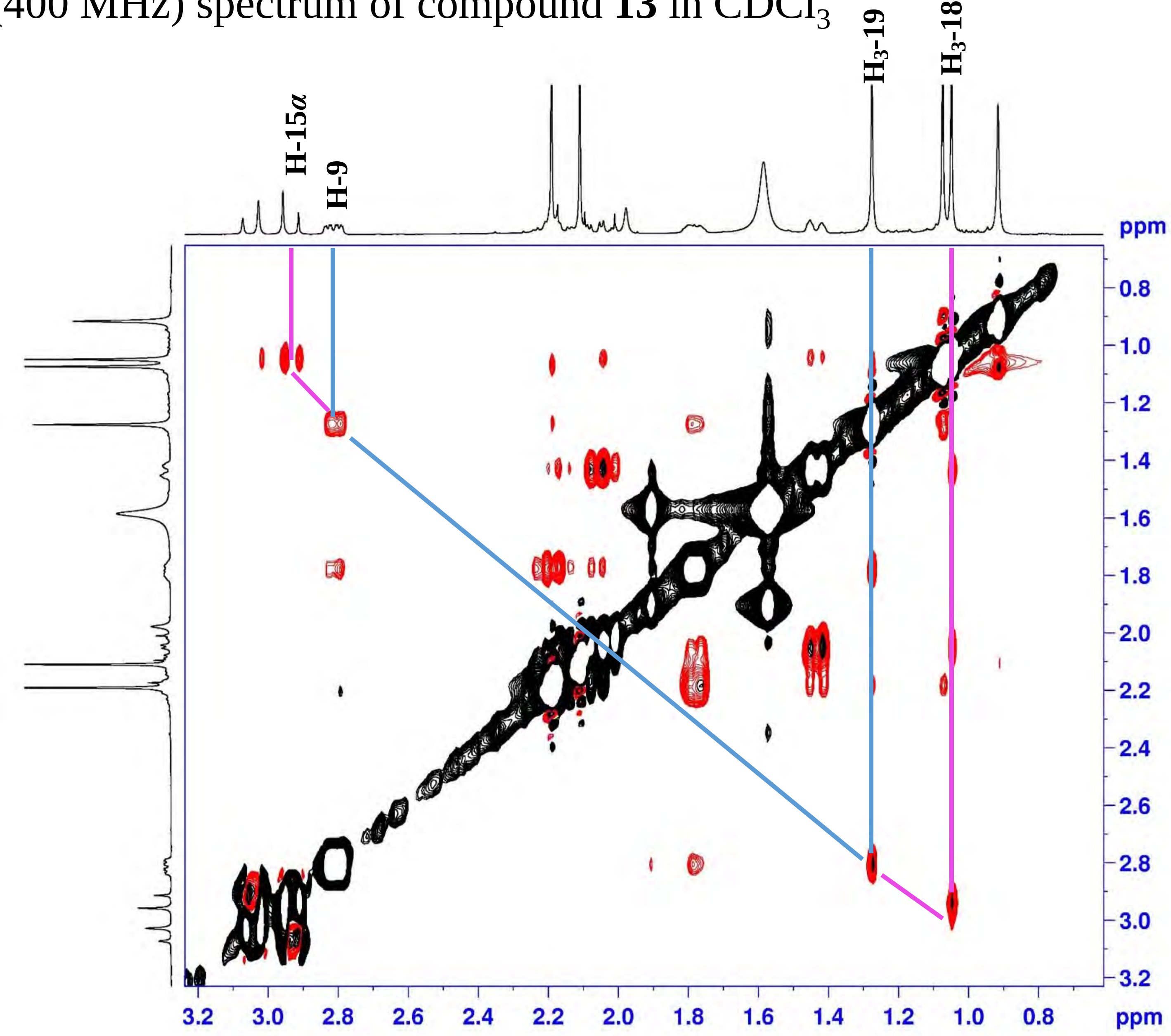

NOESY (400 MHz) spectrum of compound **13** in $CDCl_3$

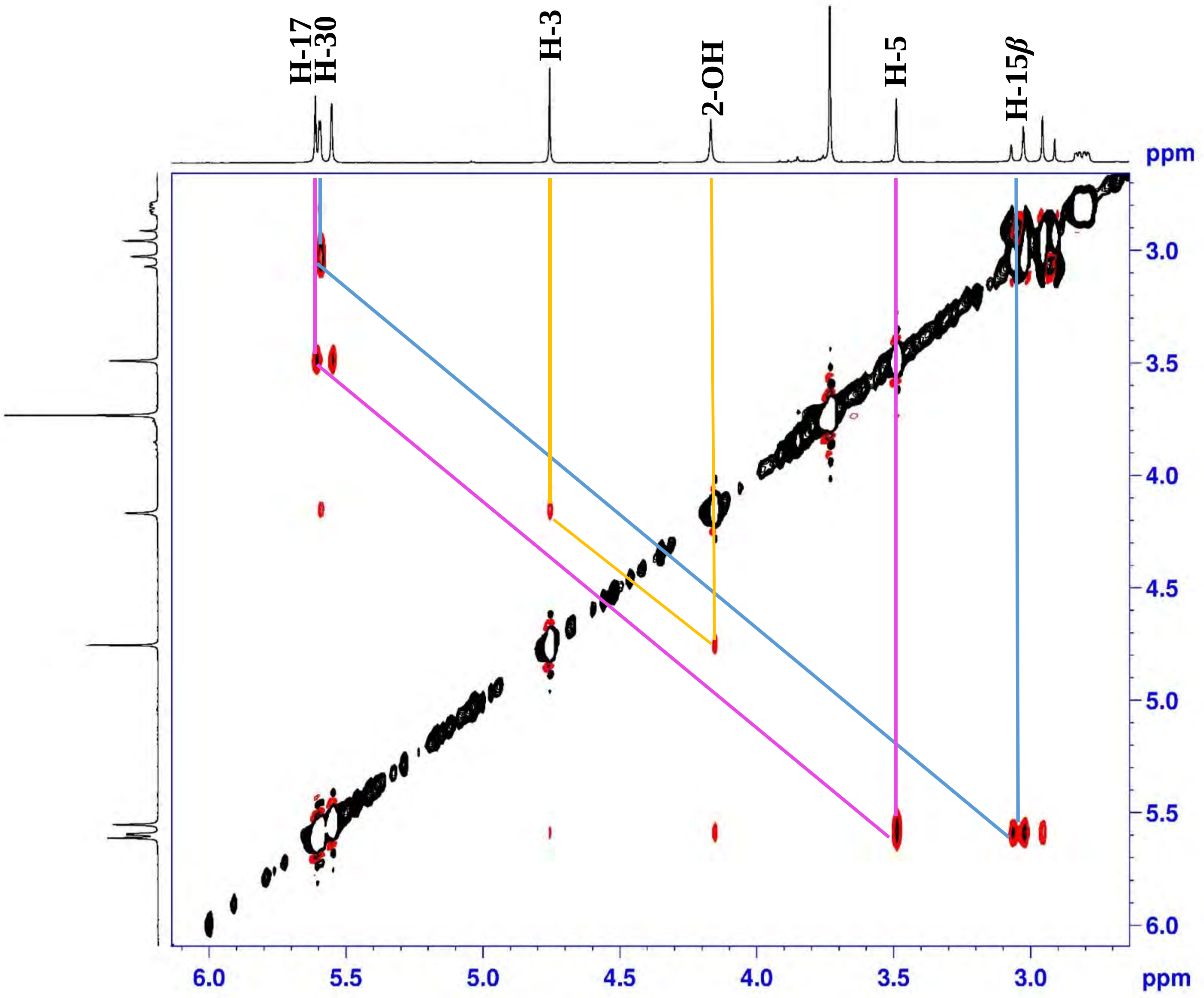

NOESY (400 MHz) spectrum of compound **13** in $CDCl_3$

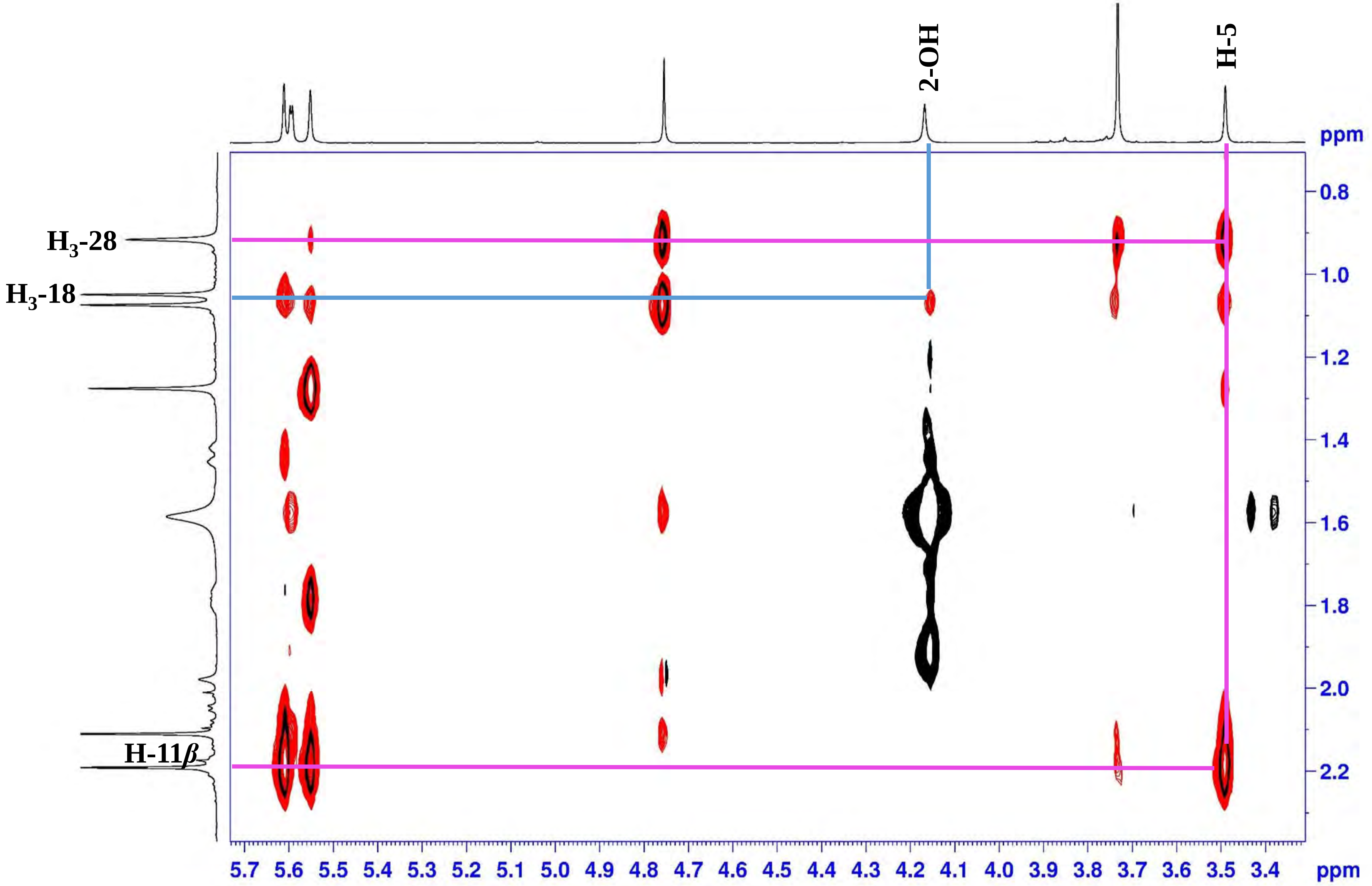

NOESY (400 MHz) spectrum of compound **13** in $CDCl_3$

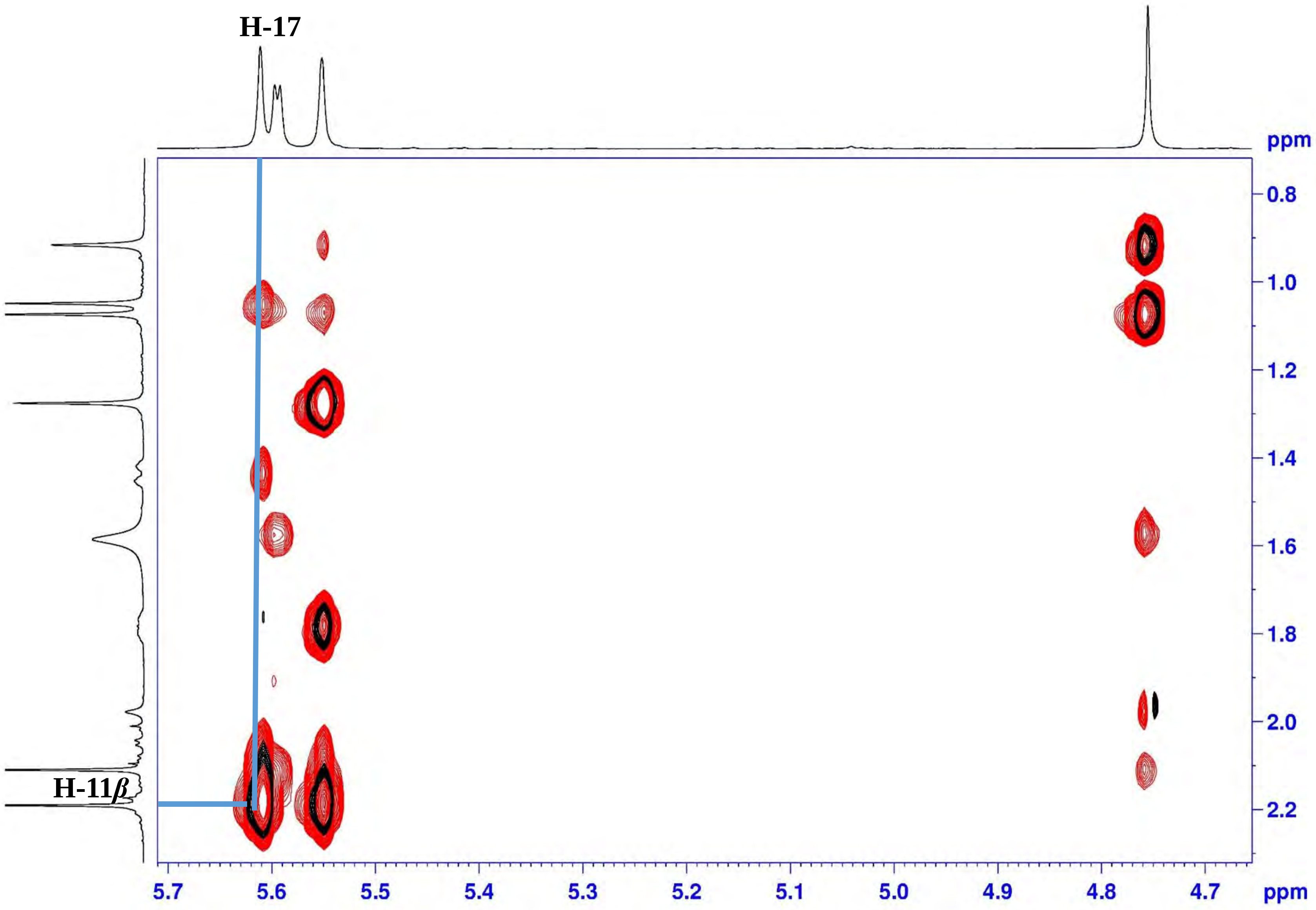

HR-ESIMS for compound **14**

Mass Spectrum SmartFormula Report

Analysis Info

Analysis Name	D:\Data\MS\data\201605\liwanshan_dgy-6_pos_2_01_1850.d	Acquisition Date	5/19/2016 8:24:06 AM
Method	LC_Direct Infusion_pos_100-1000mz.m	Operator	SCSIO
Sample Name	liwanshan_dgy-6_pos	Instrument / Ser#	maXis 29
Comment			

Acquisition Parameter

Source Type	ESI	Ion Polarity	Positive	Set Nebulizer	0.4 Bar
Focus	Active	Set Capillary	4500 V	Set Dry Heater	180 °C
Scan Begin	100 m/z	Set End Plate Offset	-500 V	Set Dry Gas	4.0 l/min
Scan End	2000 m/z	Set Collision Cell RF	800.0 Vpp	Set Divert Valve	Waste

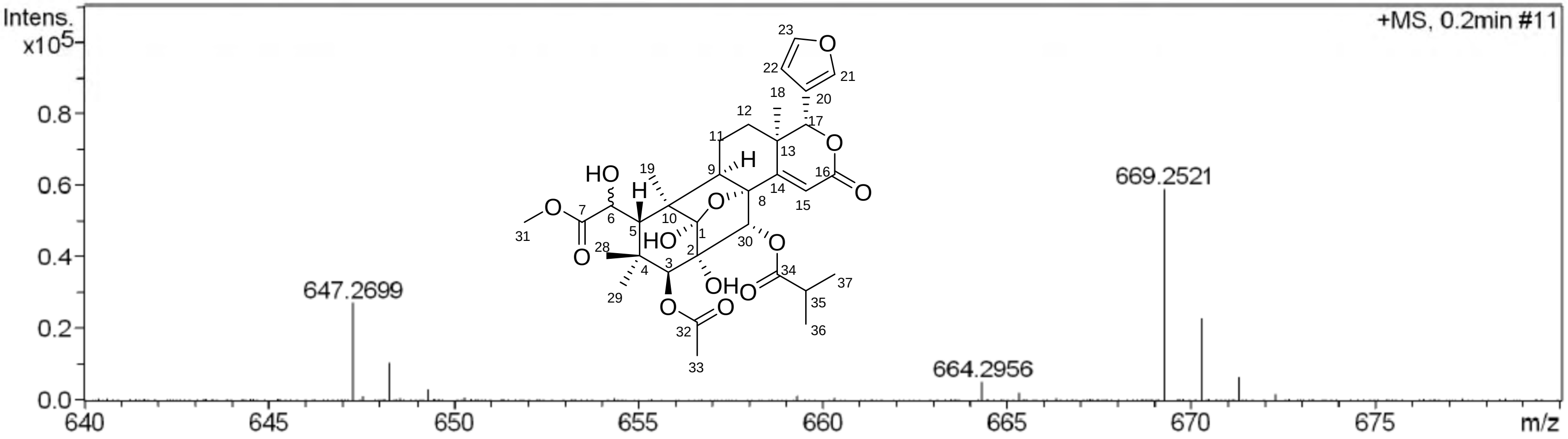

Meas. m/z	#	Formula	Score	m/z	err [ppm]	err [mDa]	mSigma	rdb	e⁻ Conf	N-Rule
647.2699	1	C 33 H 43 O 13	100.00	647.2698	-0.1	-0.0	9.3	12.5	even	ok
669.2521	1	C 33 H 42 Na O 13	100.00	669.2518	-0.5	-0.3	10.7	12.5	even	ok
1315.5159	1	C 66 H 84 Na O 26	44.81	1315.5143	-1.2	-1.5	58.3	24.5	even	ok

HR-ESIMS for compound **14**

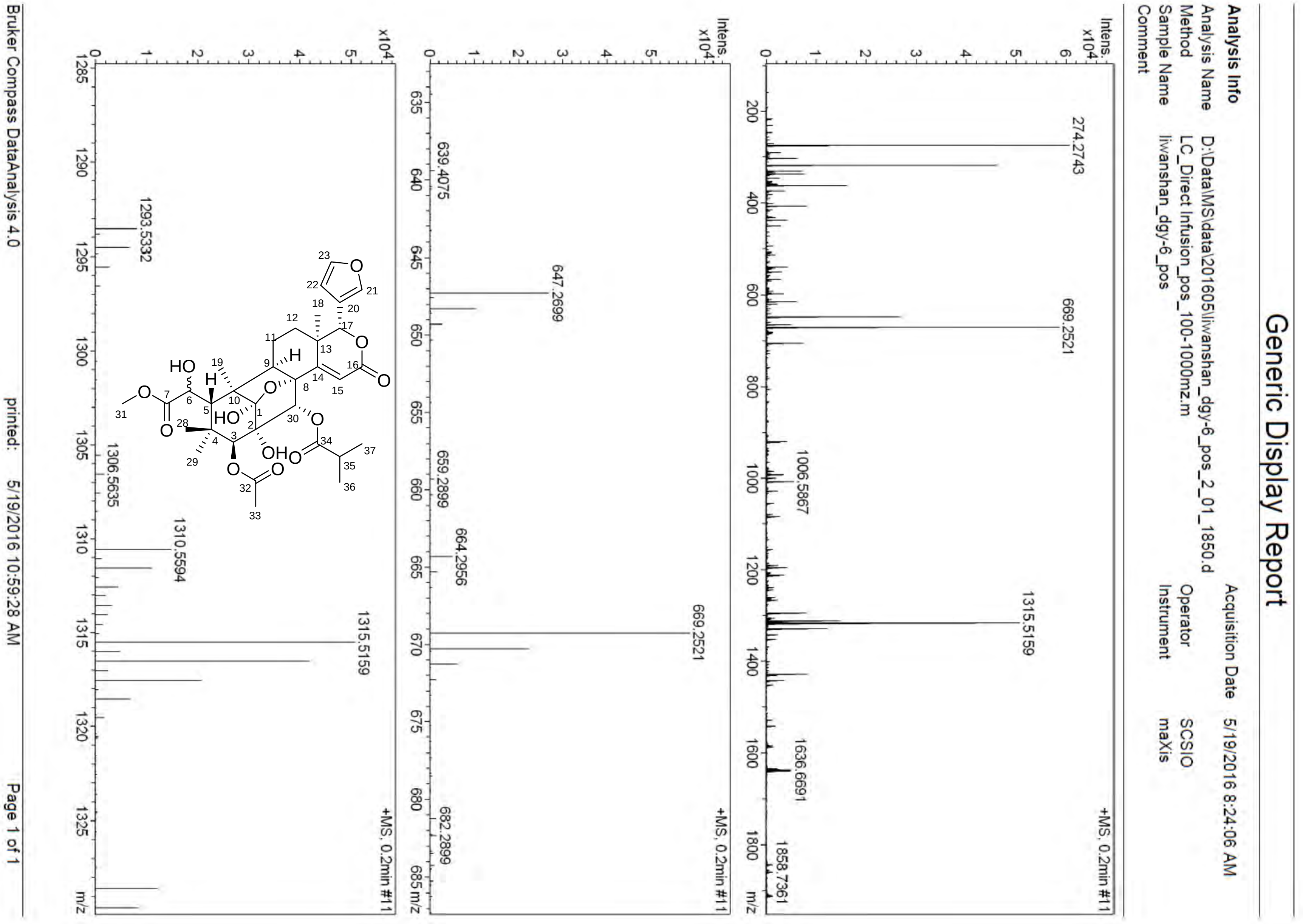

^{1}H NMR (400 MHz) spectrum of compound **14** in $CDCl_3$

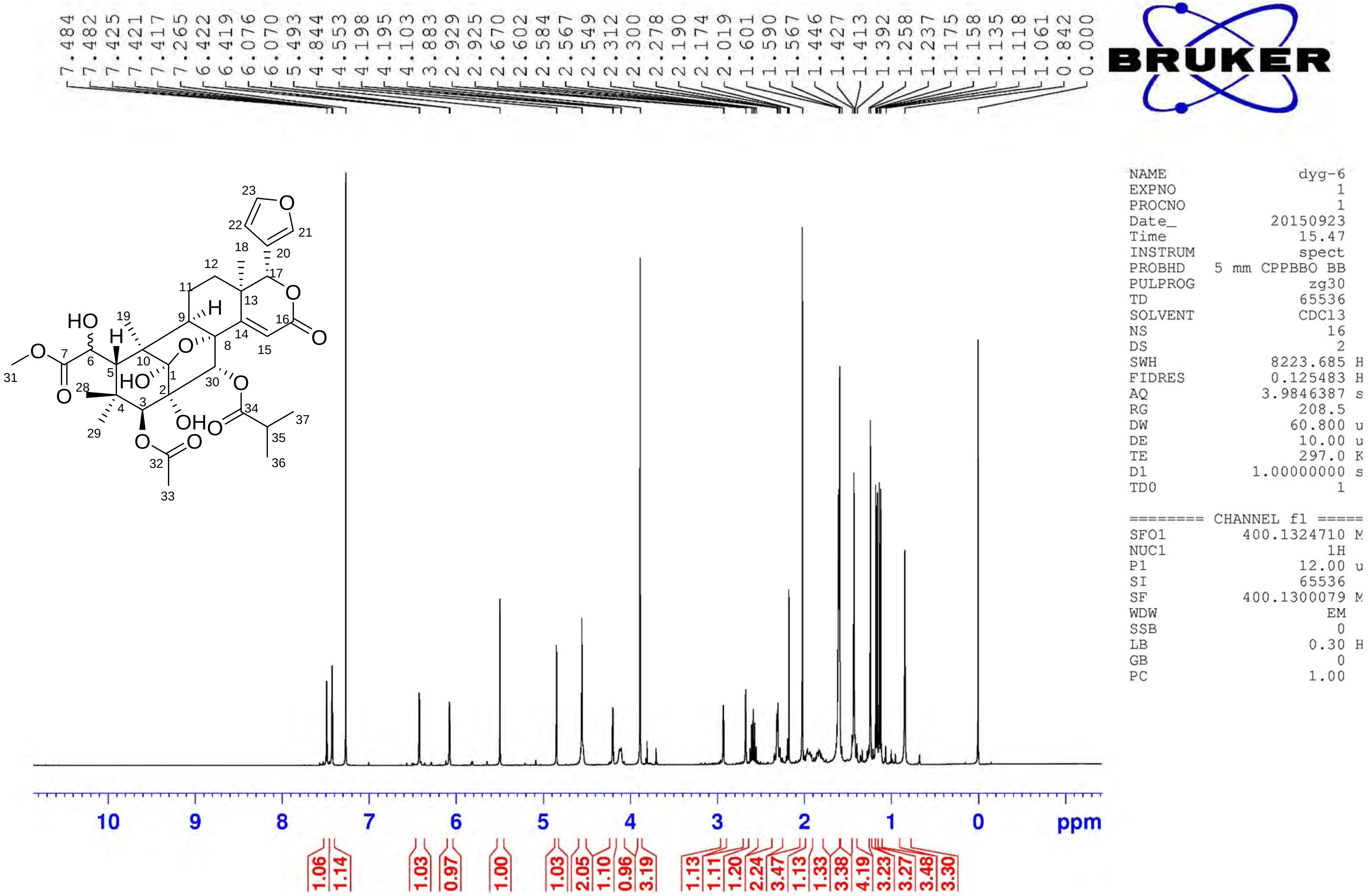

^{1}H NMR (400 MHz) spectrum of compound **14** in $CDCl_3$

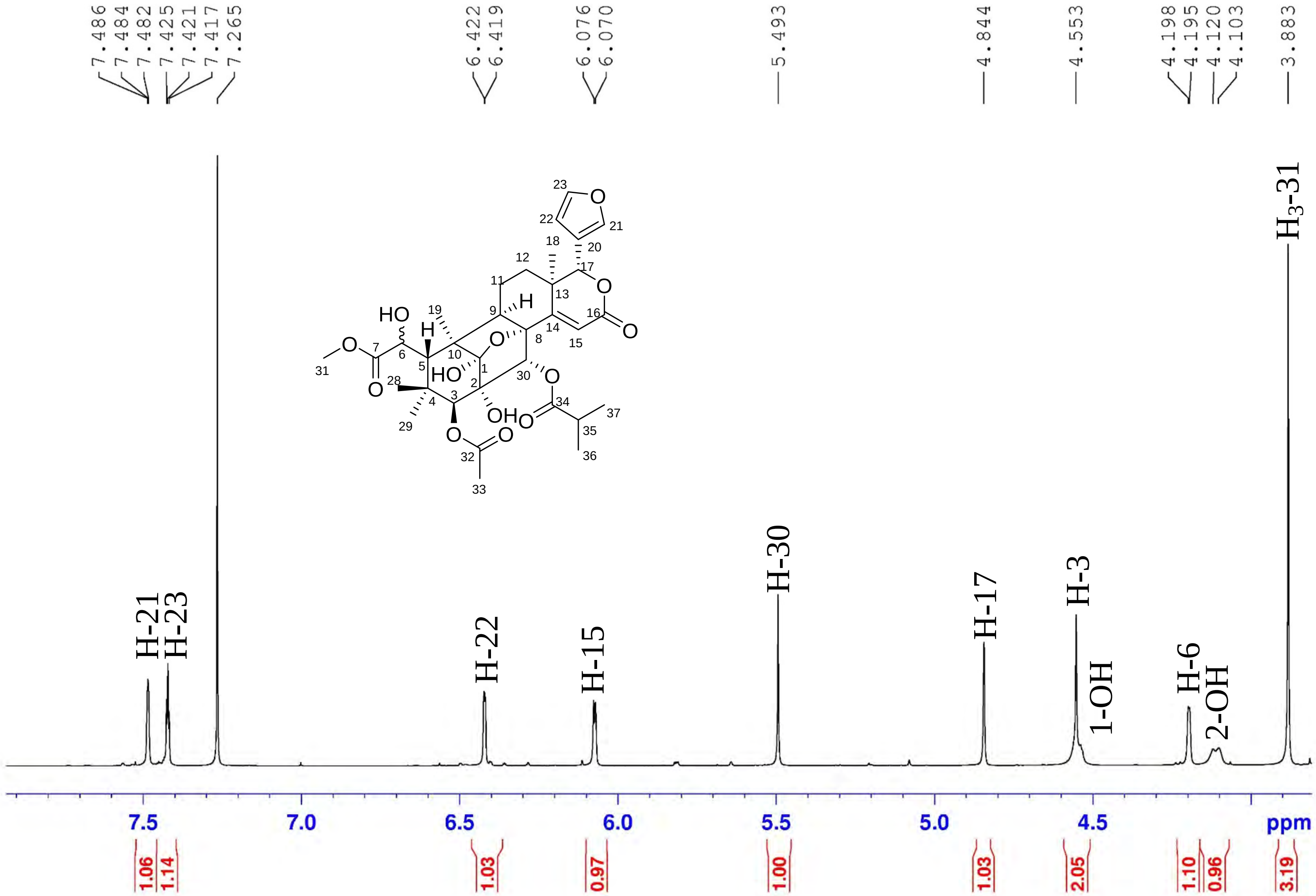

^{1}H NMR (400 MHz) spectrum of compound **14** in $CDCl_3$

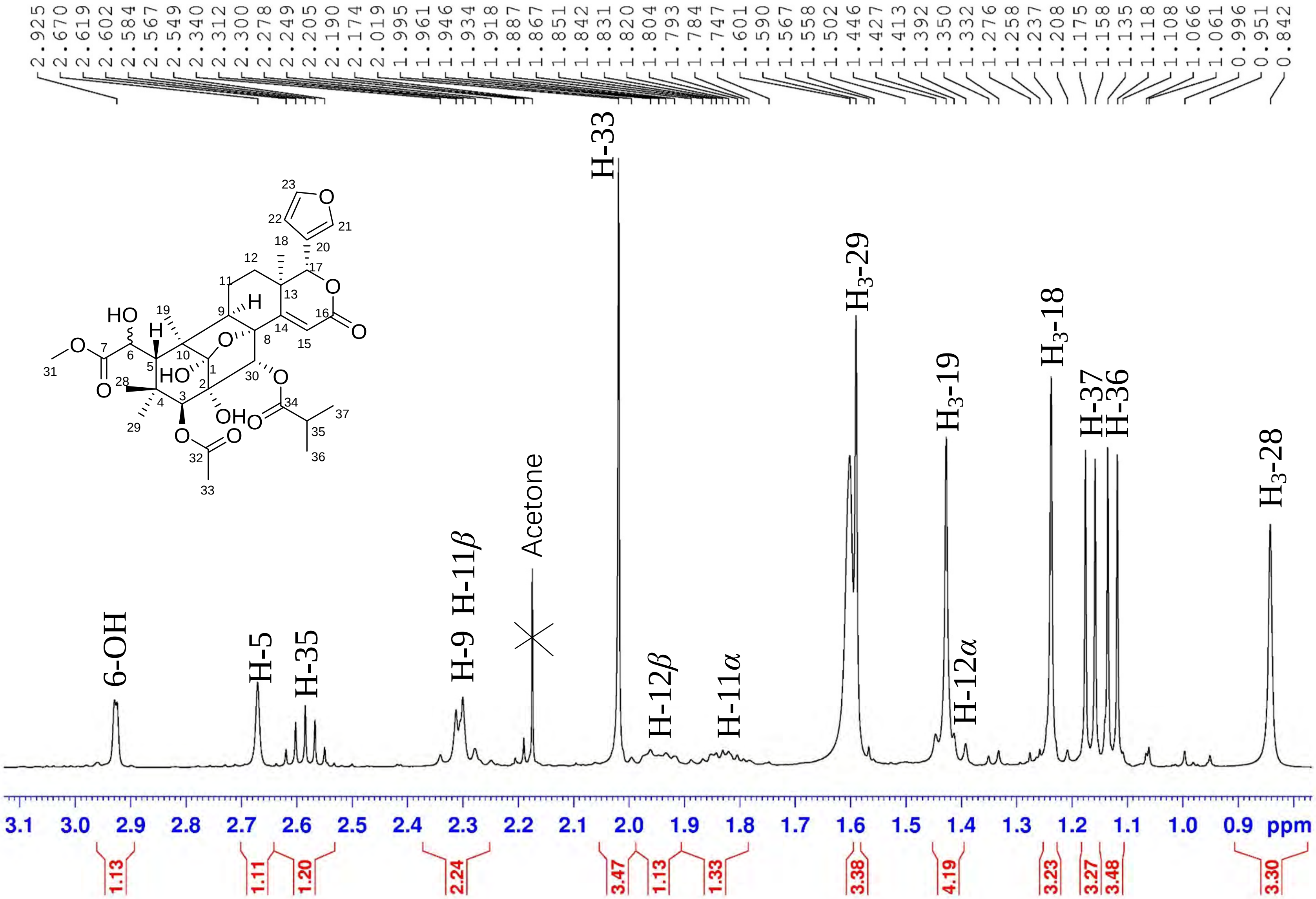

^{13}C NMR (100 MHz) spectrum of compound **14** in $CDCl_3$

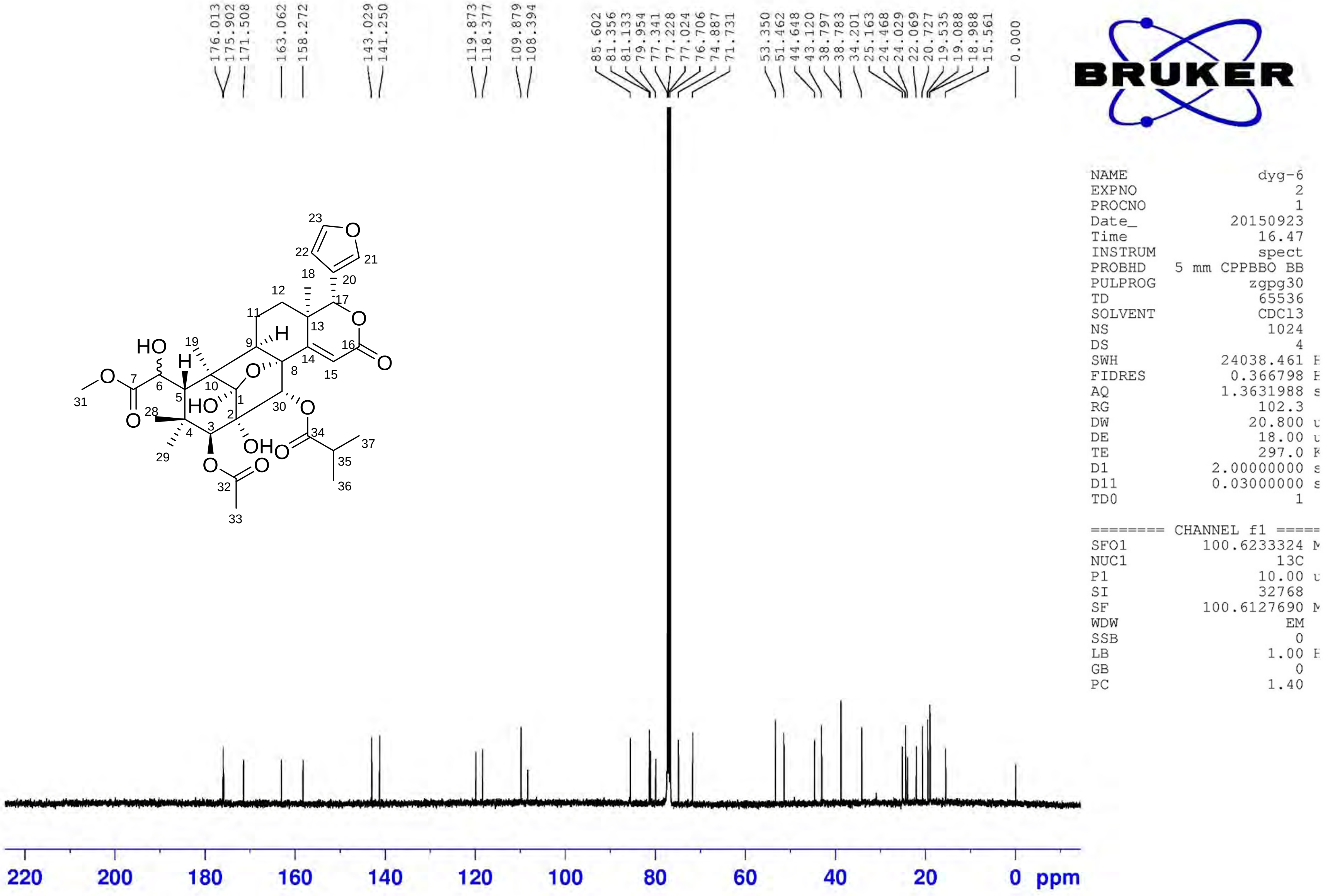

^{13}C NMR (100 MHz) spectrum of compound **14** in $CDCl_3$

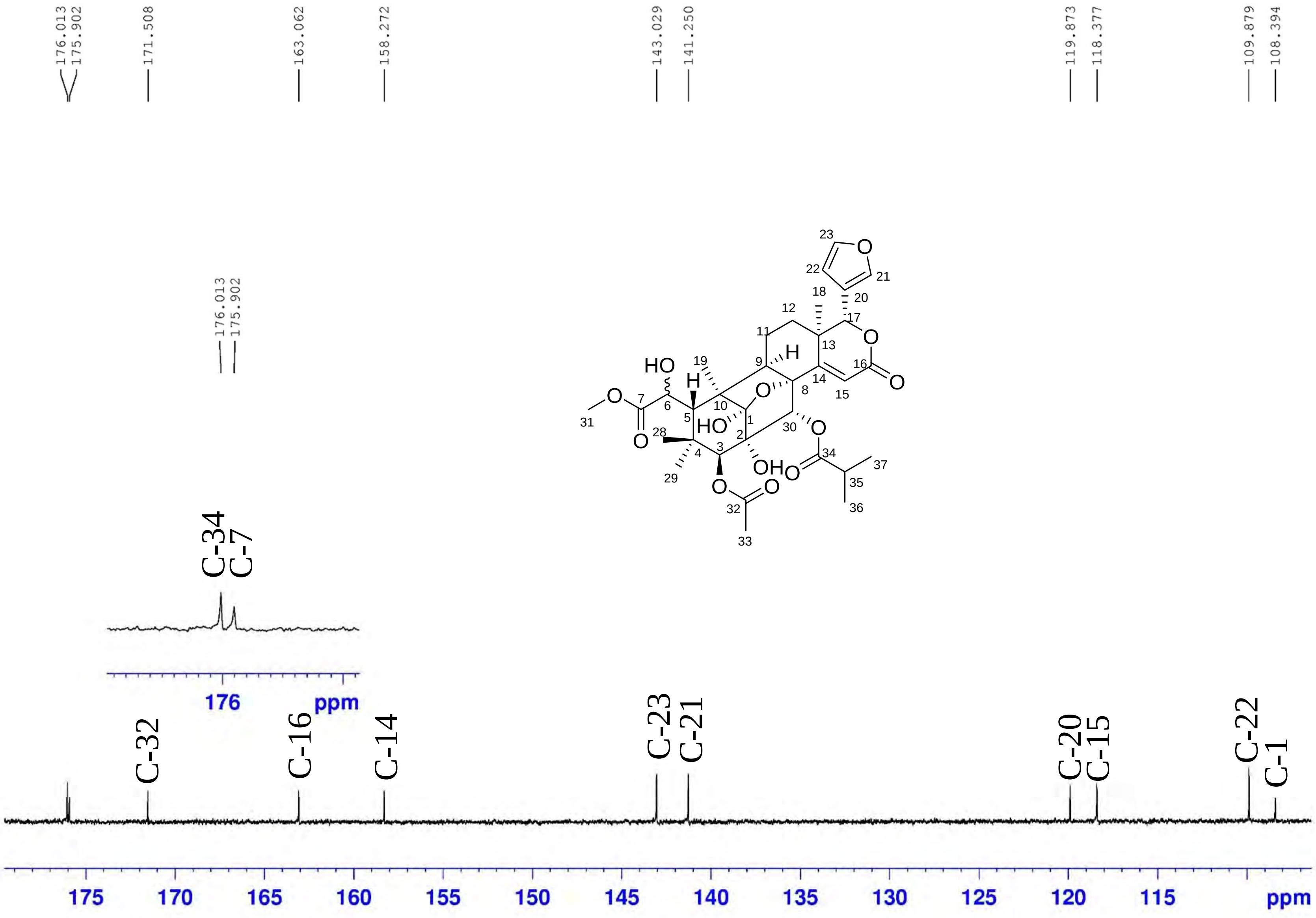

^{13}C NMR (100 MHz) spectrum of compound **14** in $CDCl_3$

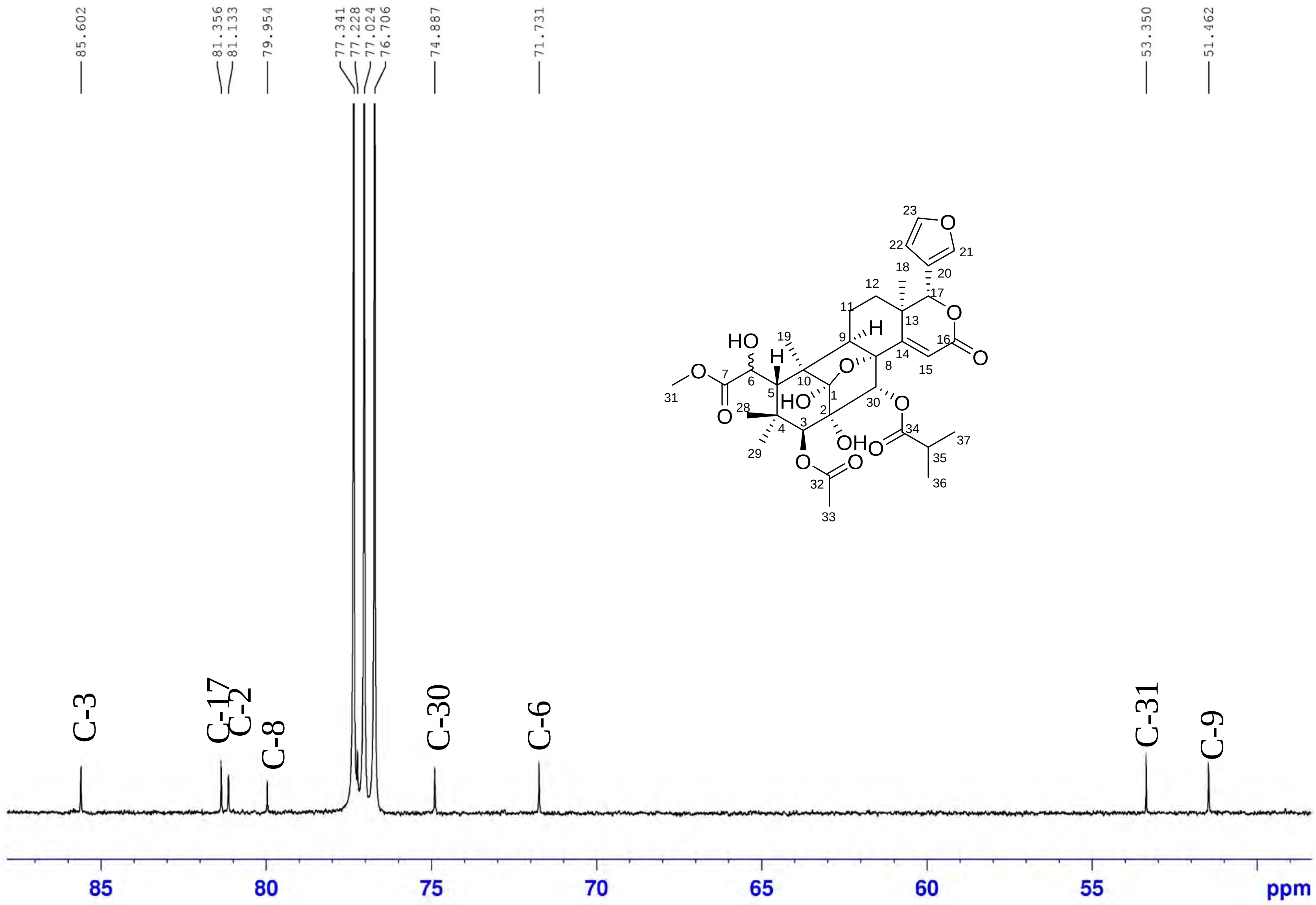

^{13}C NMR (100 MHz) spectrum of compound **14** in $CDCl_3$

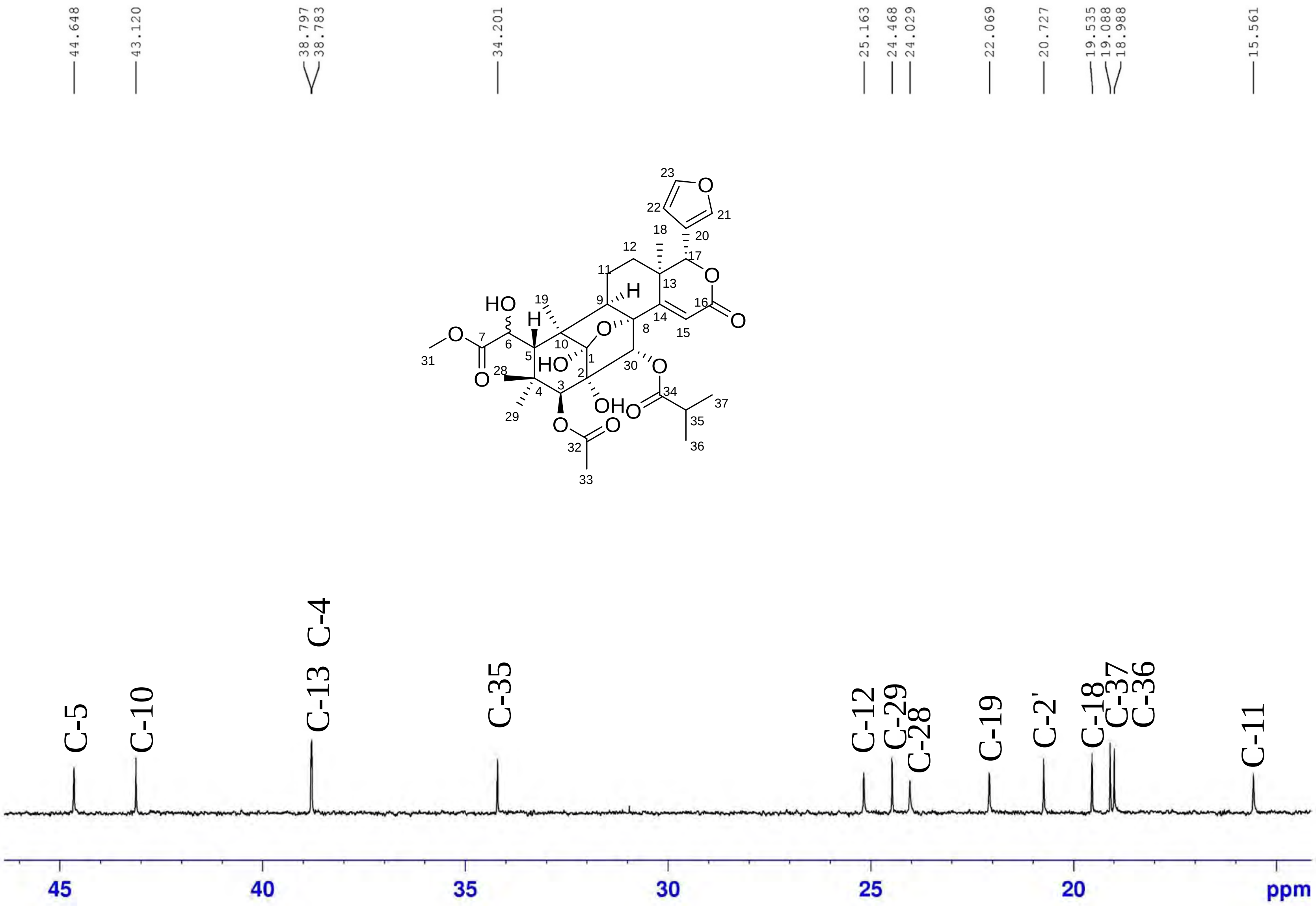

DEPT135 (100 MHz) spectrum of compound **14** in $CDCl_3$

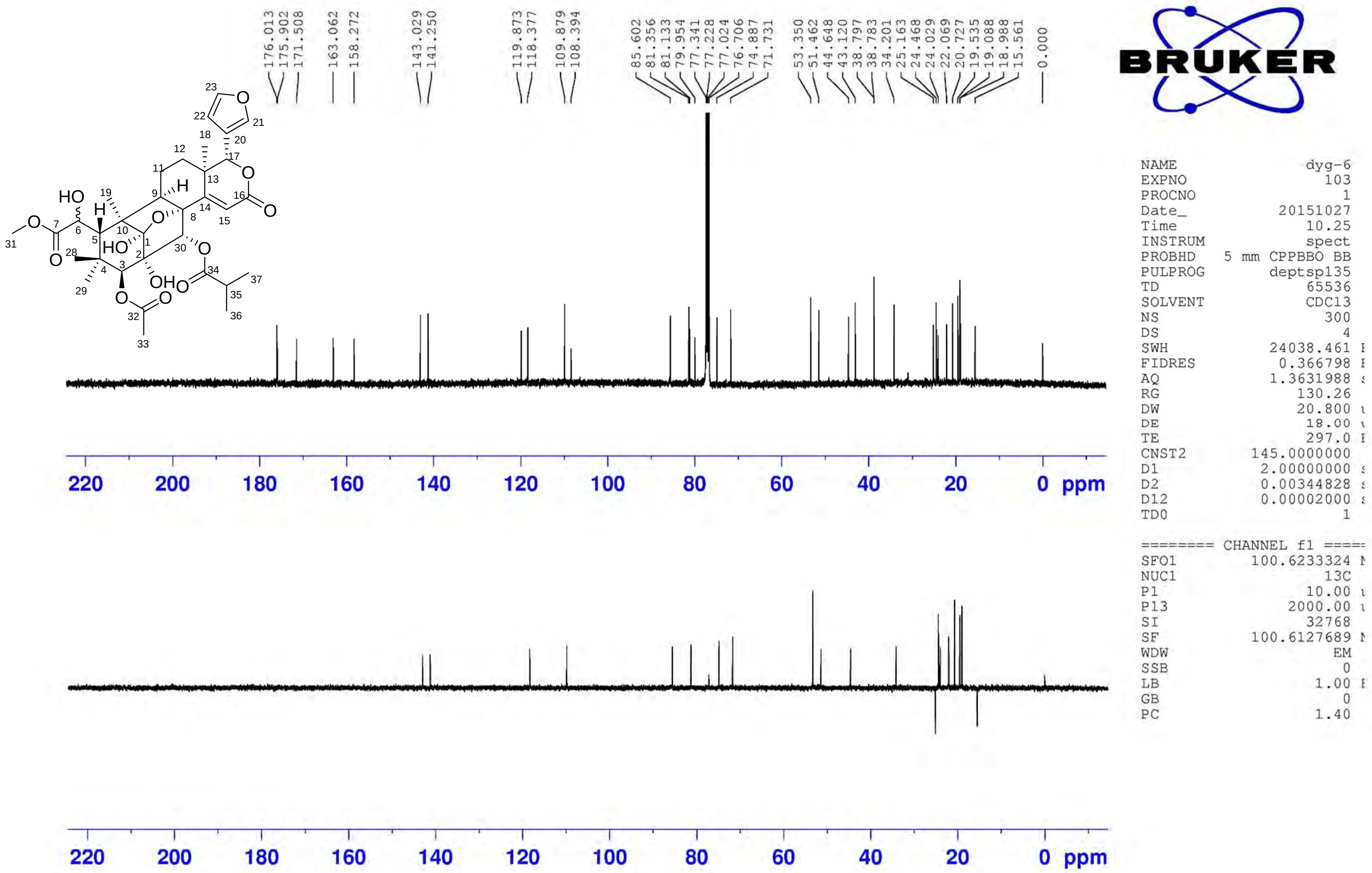

DEPT135 (100 MHz) spectrum of compound **14** in $CDCl_3$

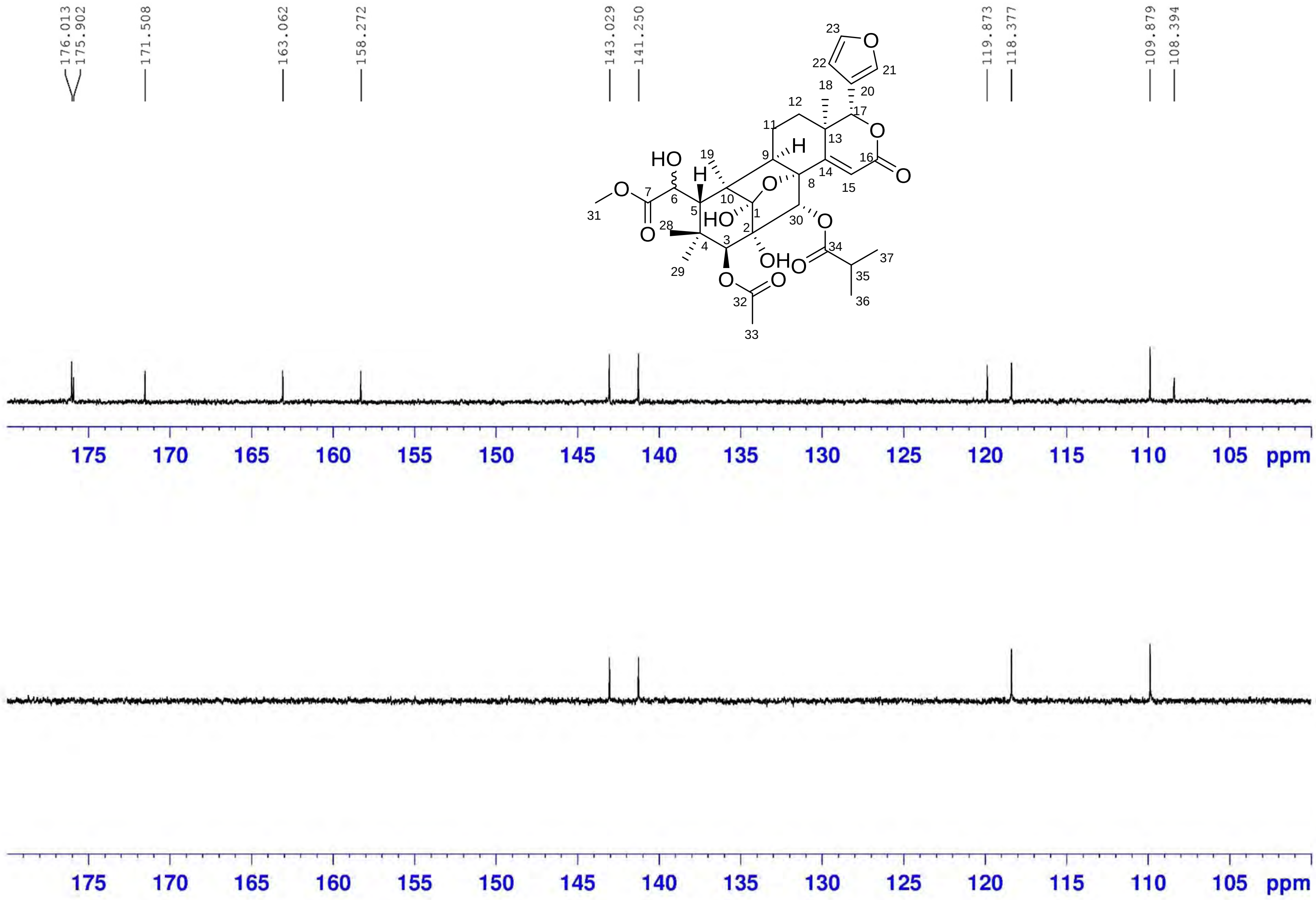

DEPT135 (100 MHz) spectrum of compound **14** in $CDCl_3$

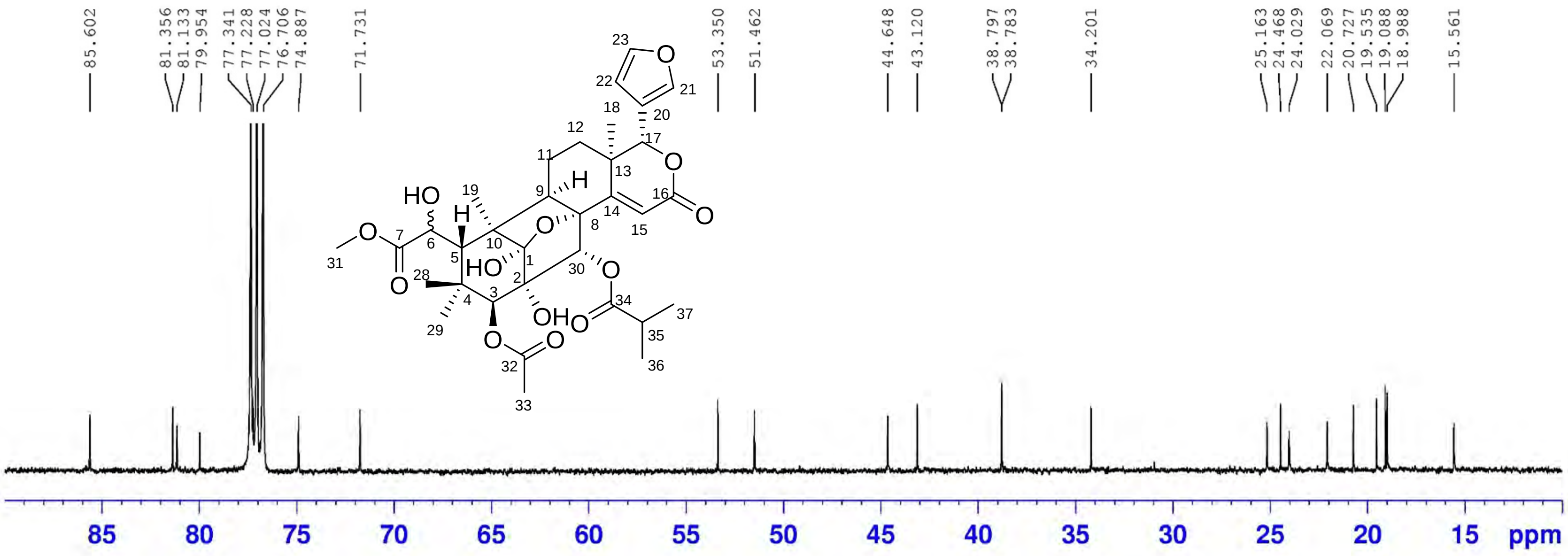

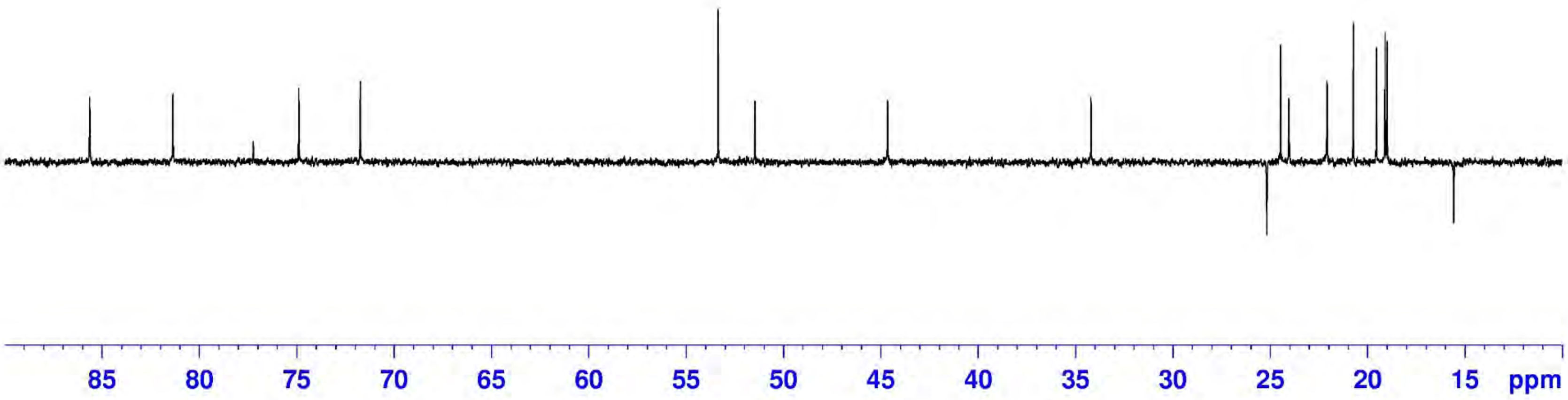

^{1}H-^{1}H COSY (400 MHz) spectrum of compound **14** in $CDCl_3$

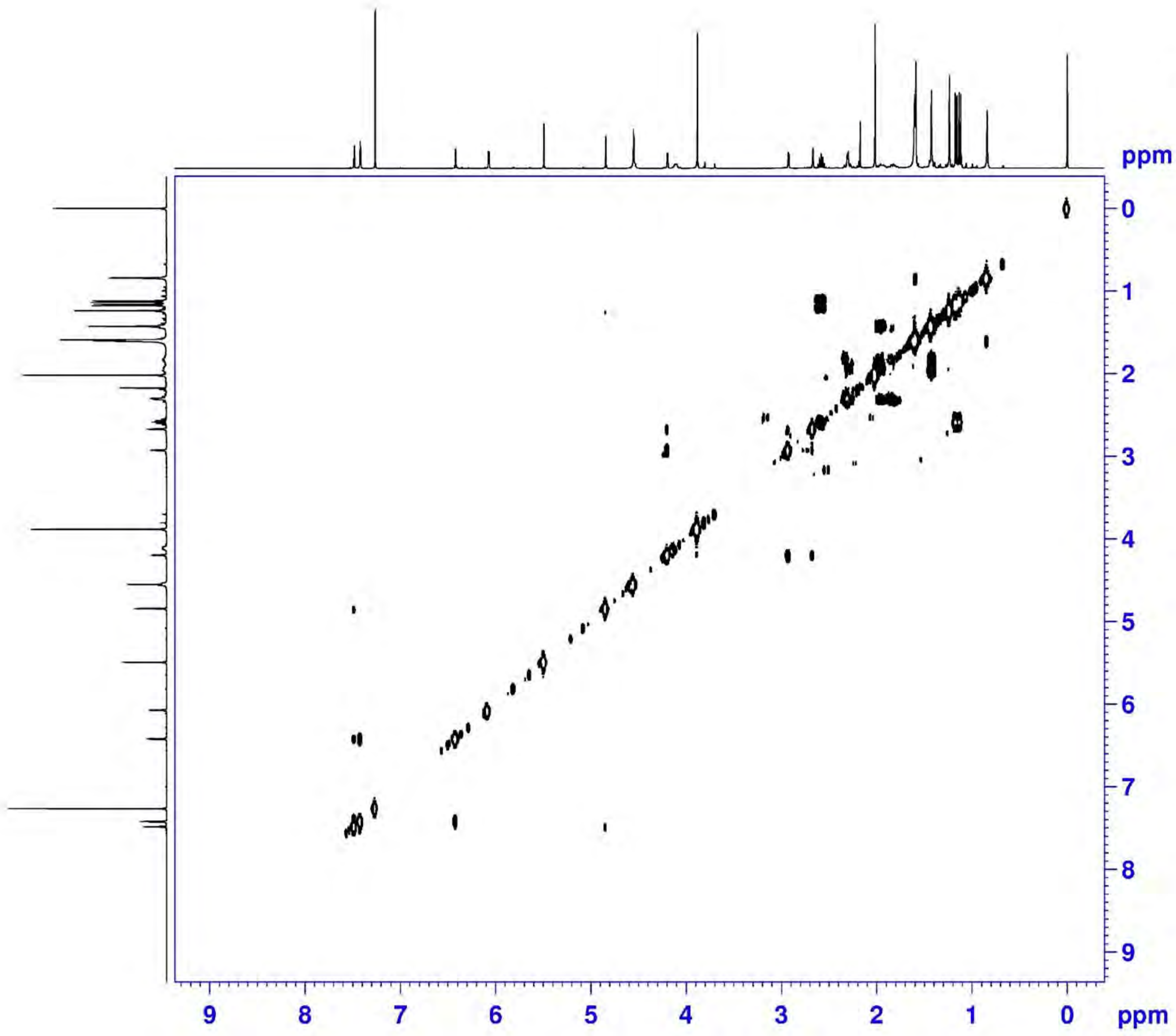

^{1}H-^{1}H COSY (400 MHz) spectrum of compound **14** in $CDCl_3$

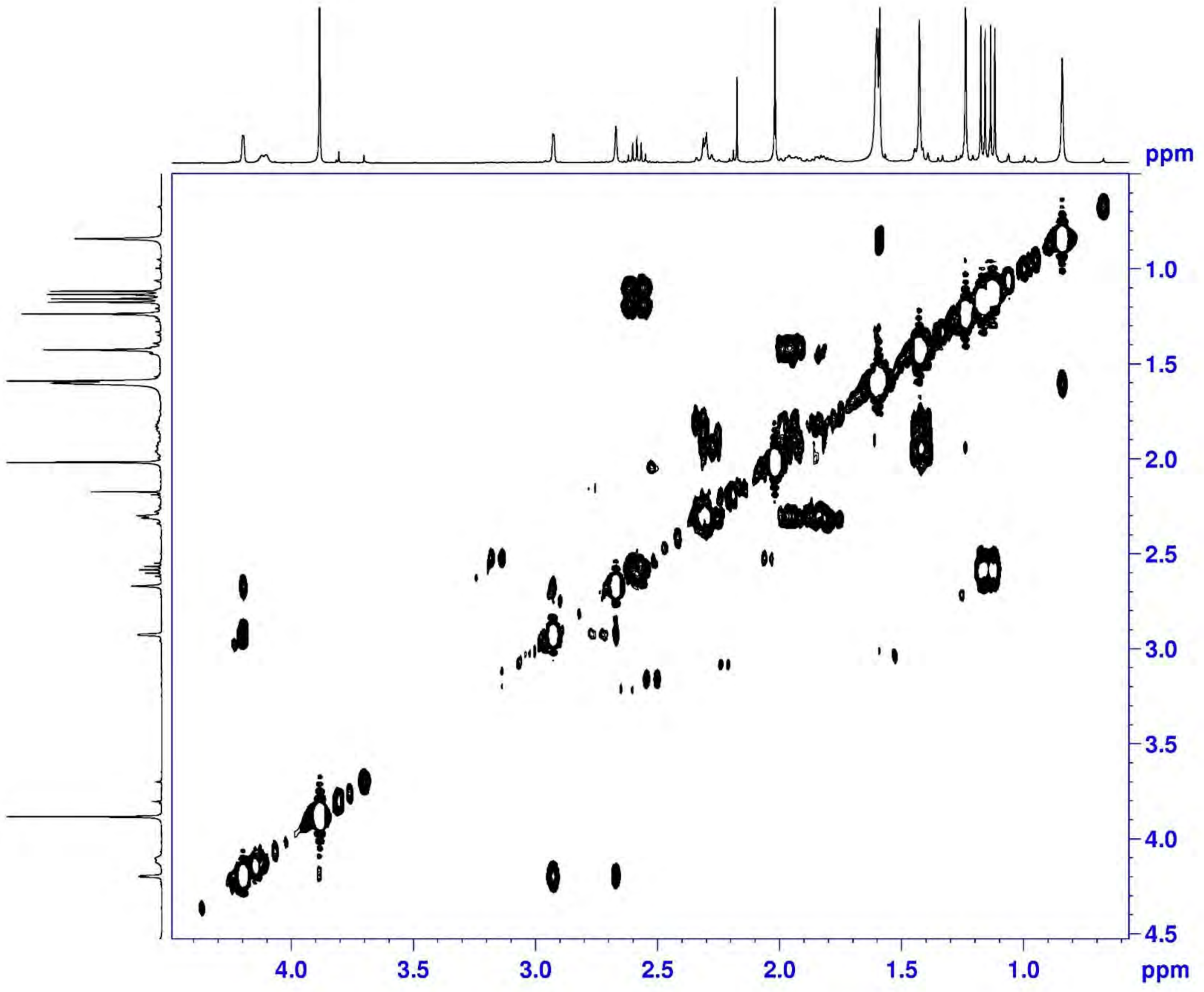

HSQC (400 MHz) spectrum of compound **14** in $CDCl_3$

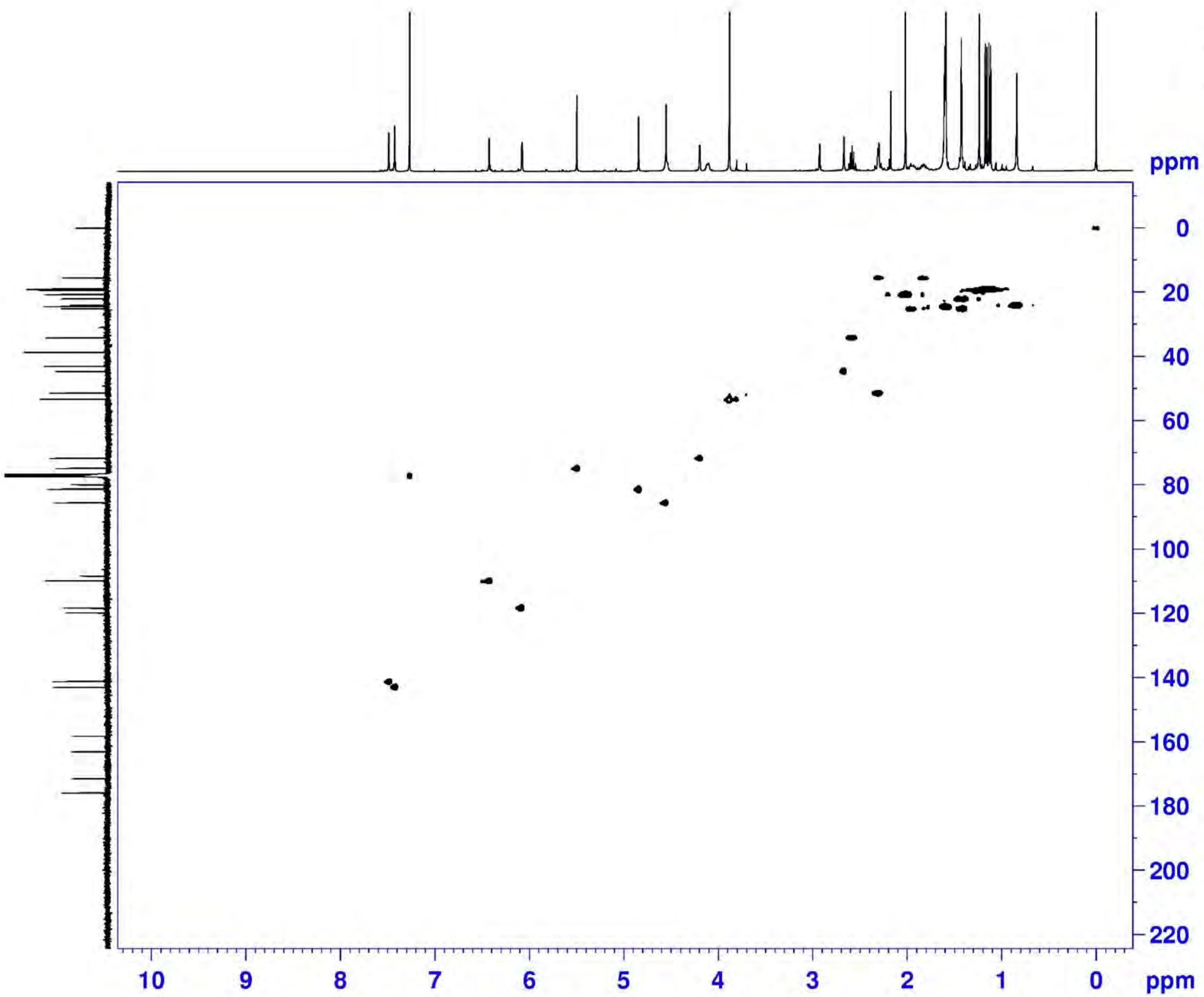

HSQC (400 MHz) spectrum of compound **14** in $CDCl_3$

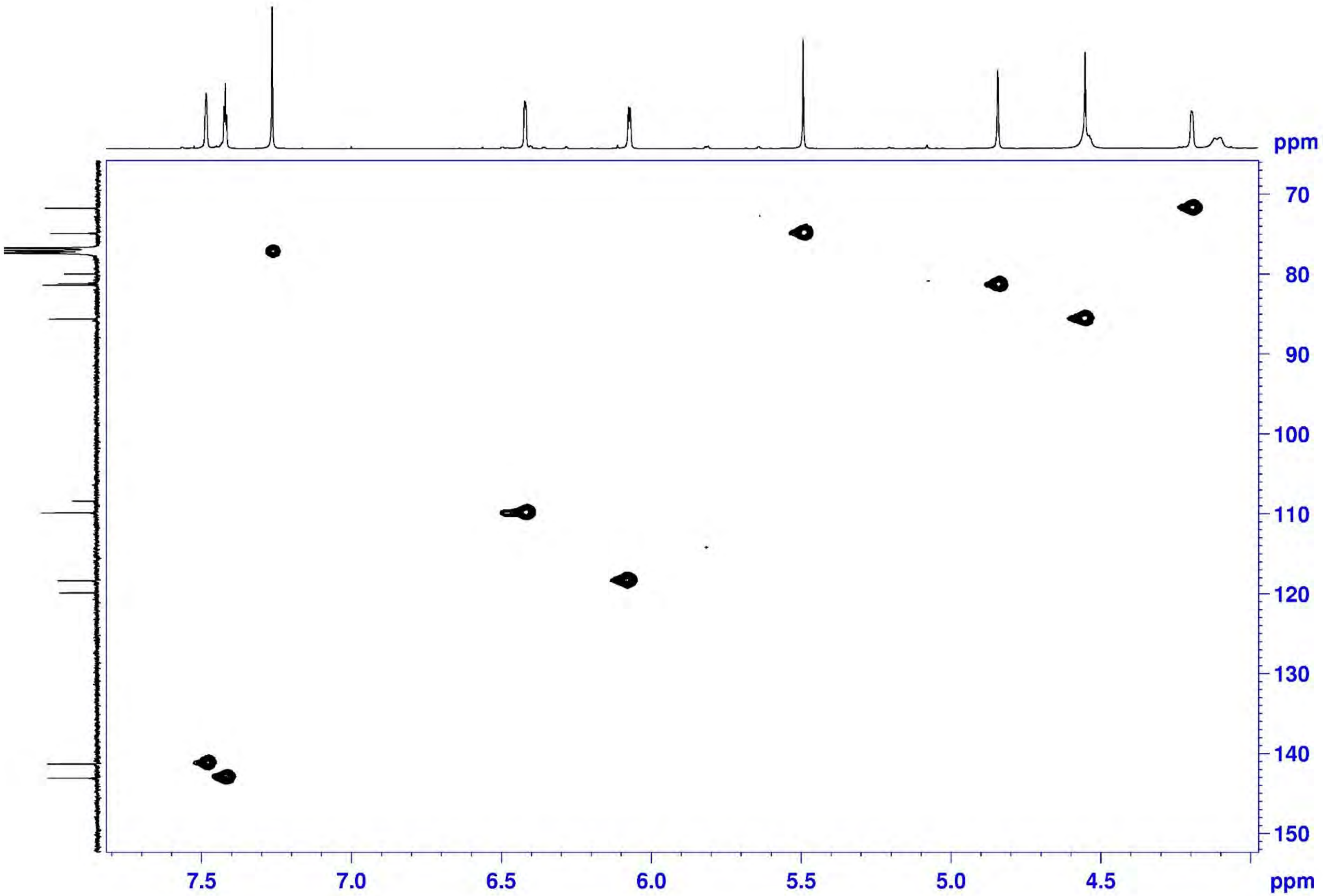

HSQC (400 MHz) spectrum of compound **14** in $CDCl_3$

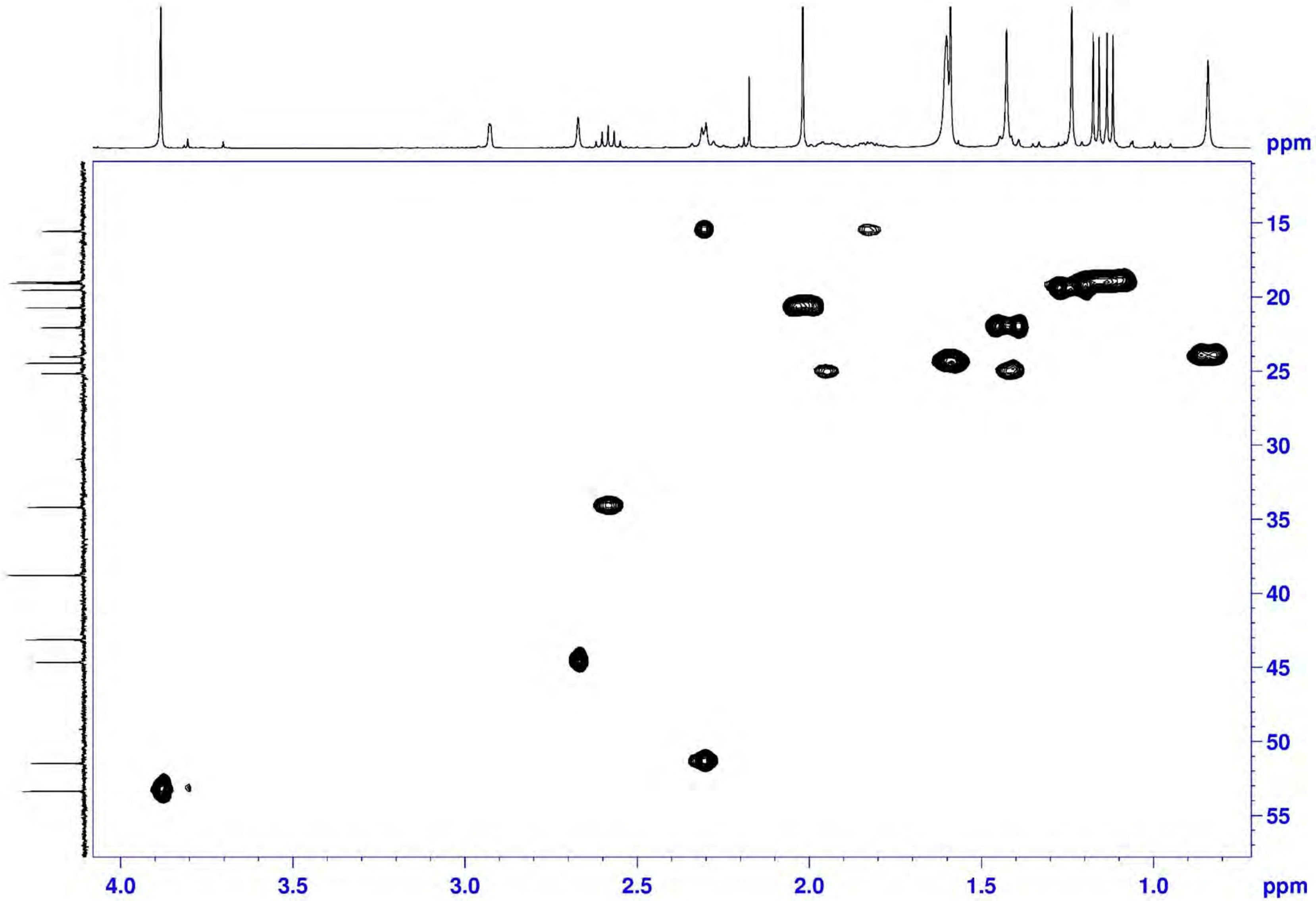

HMBC (400 MHz) spectrum of compound **14** in $CDCl_3$

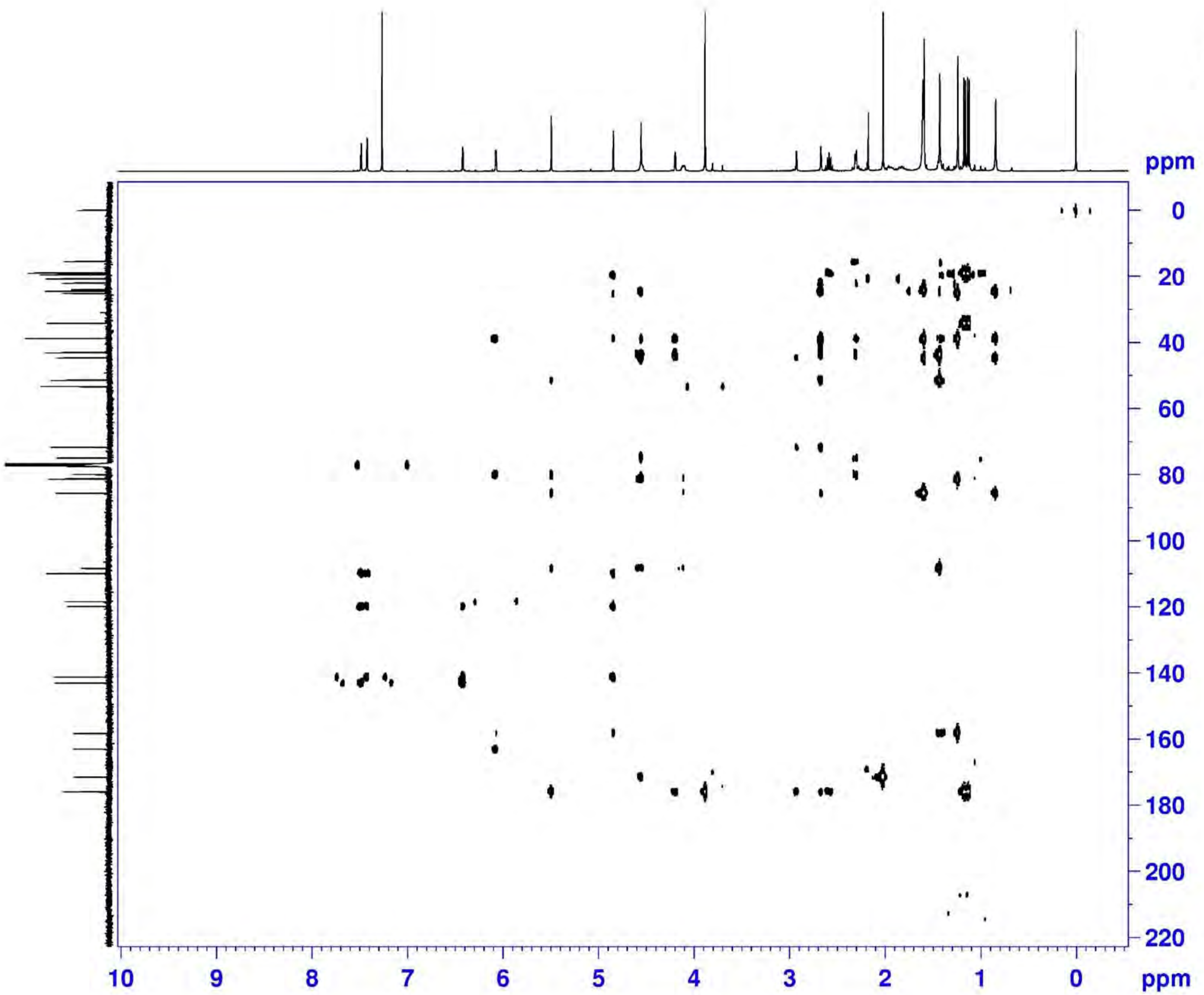

NAME	dyg-6
EXPNO	106
PROCNO	1
Date_	20151027
Time	12.57
INSTRUM	spect
PROBHD	5 mm CPPBBO BB
PULPROG	hmbcgplpndqf
TD	4096
SOLVENT	CDCl3
NS	32
DS	16
SWH	5197.505 Hz
FIDRES	1.268922 Hz
AQ	0.3940852 se
RG	208.5
DW	96.200 us
DE	10.00 us
TE	297.0 K
CNST2	145.0000000
CNST13	10.0000000
D0	0.00000300 se
D1	1.50000000 se
D2	0.00344828 se
D6	0.05000000 se
D16	0.00020000 se
IN0	0.00002080 se
======== CHANNEL f1 ======	
SFO1	400.1323208 MH
NUC1	1H
P1	12.00 us
P2	24.00 us
ND0	2
TD	128
SFO1	100.6233 MH
FIDRES	187.800476 Hz
SW	238.896 pp
FnMODE	QF
SI	2048
SF	400.1300079 MH
WDW	SINE
SSB	0
LB	0.00 Hz
GB	0
PC	1.40
SI	1024
MC2	QF
SF	100.6127690 MH
WDW	SINE
SSB	0

HMBC (400 MHz) spectrum of compound **14** in $CDCl_3$

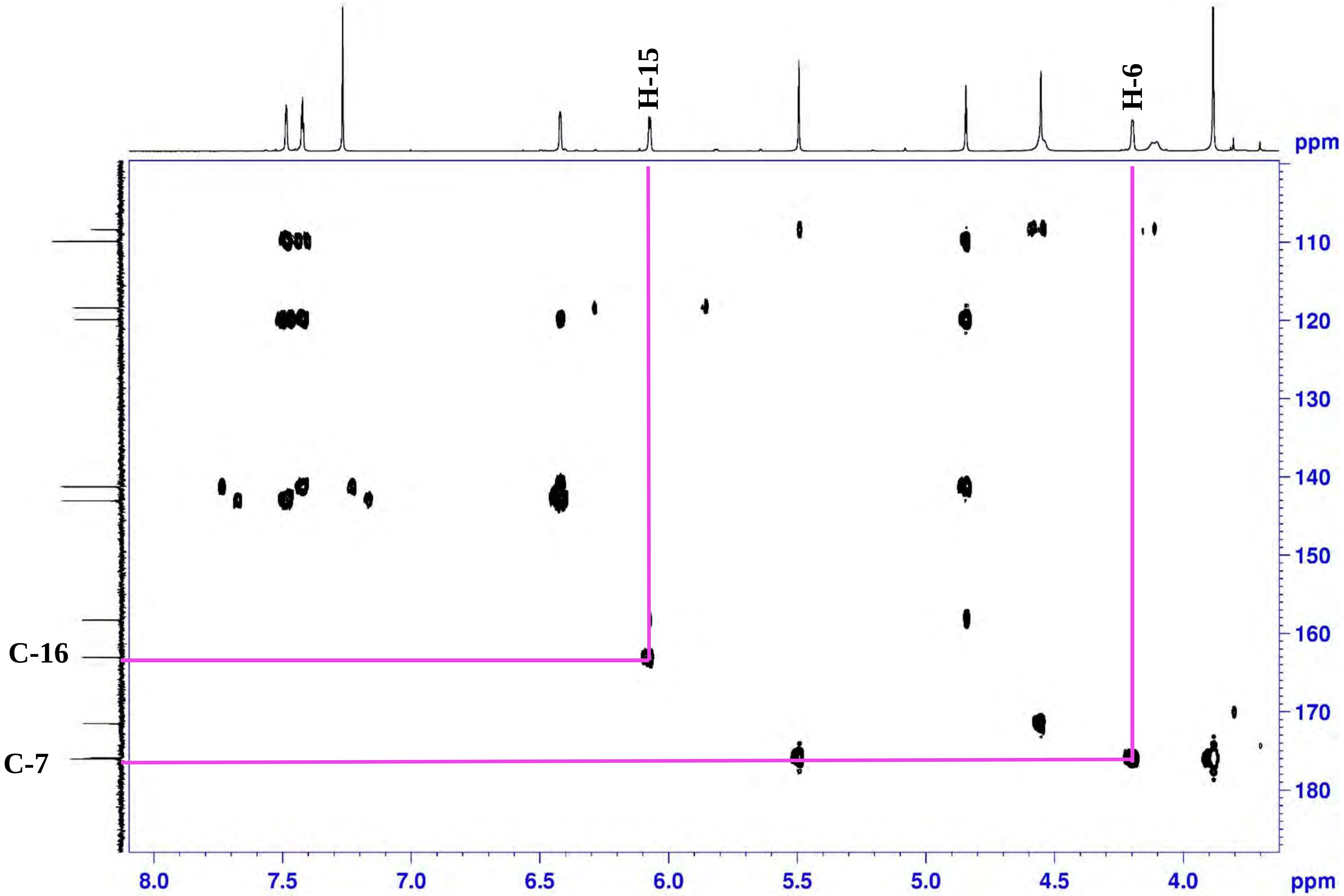

HMBC (400 MHz) spectrum of compound **14** in $CDCl_3$

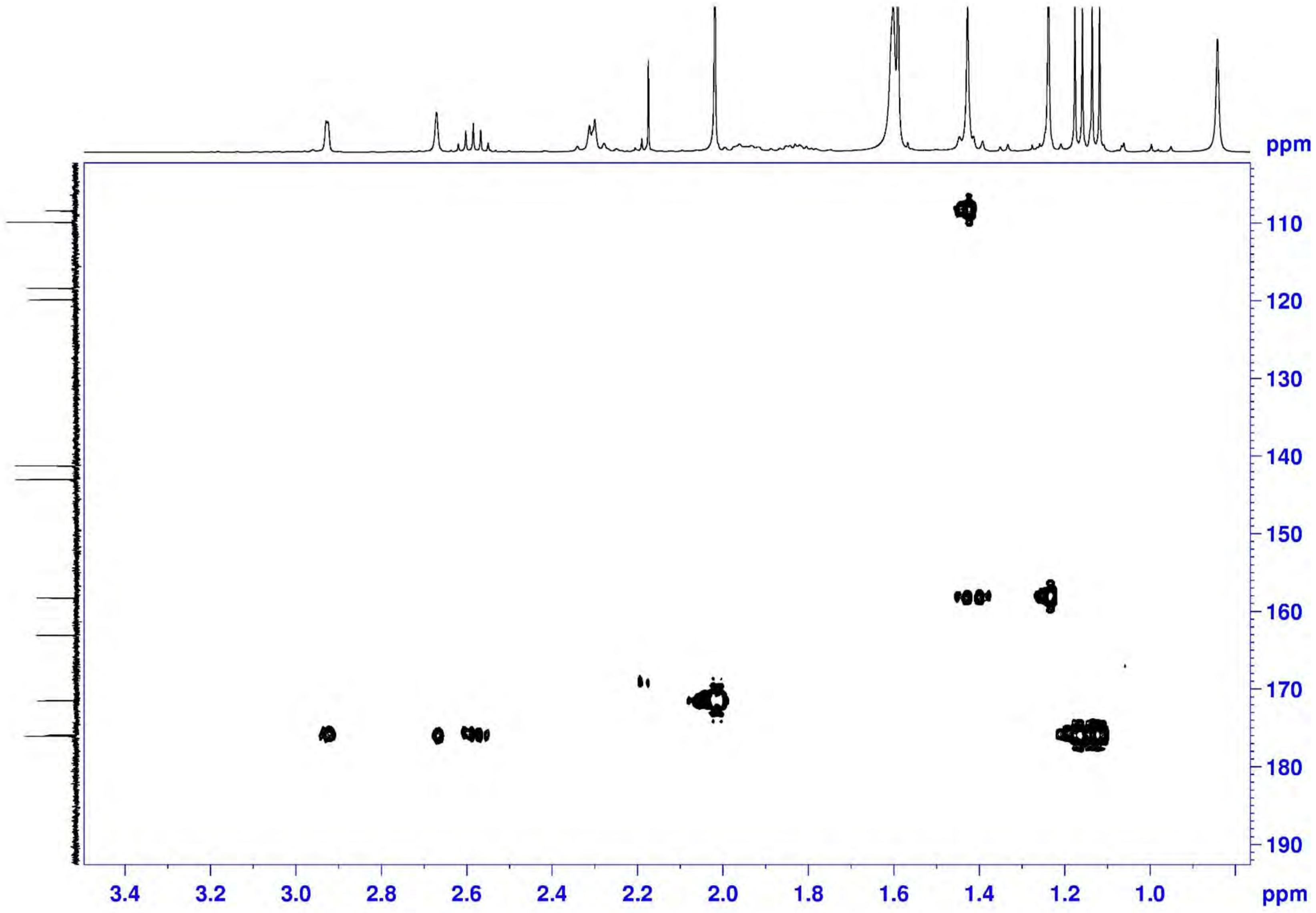

HMBC (400 MHz) spectrum of compound **14** in $CDCl_3$

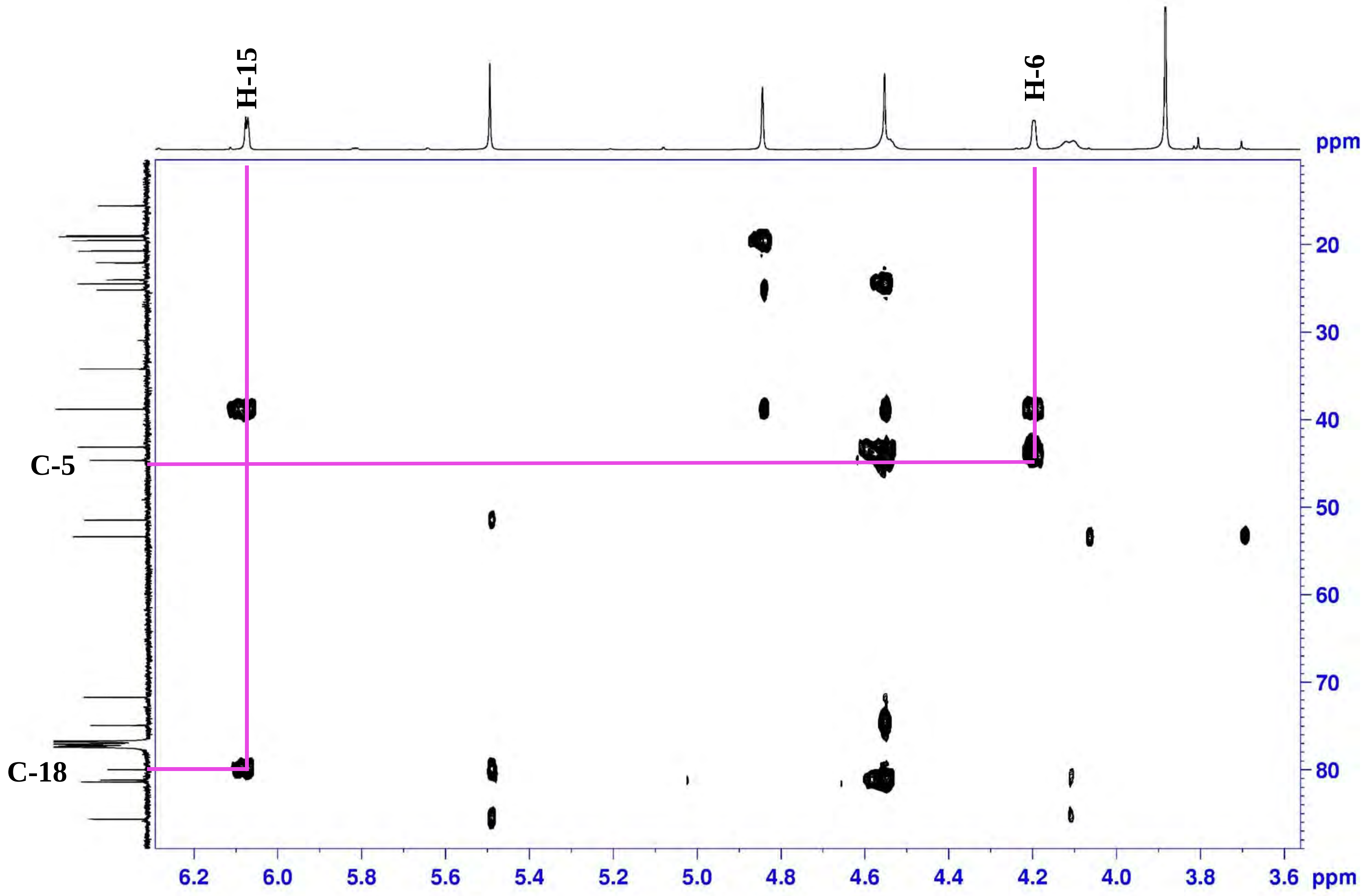

HMBC (400 MHz) spectrum of compound **14** in $CDCl_3$

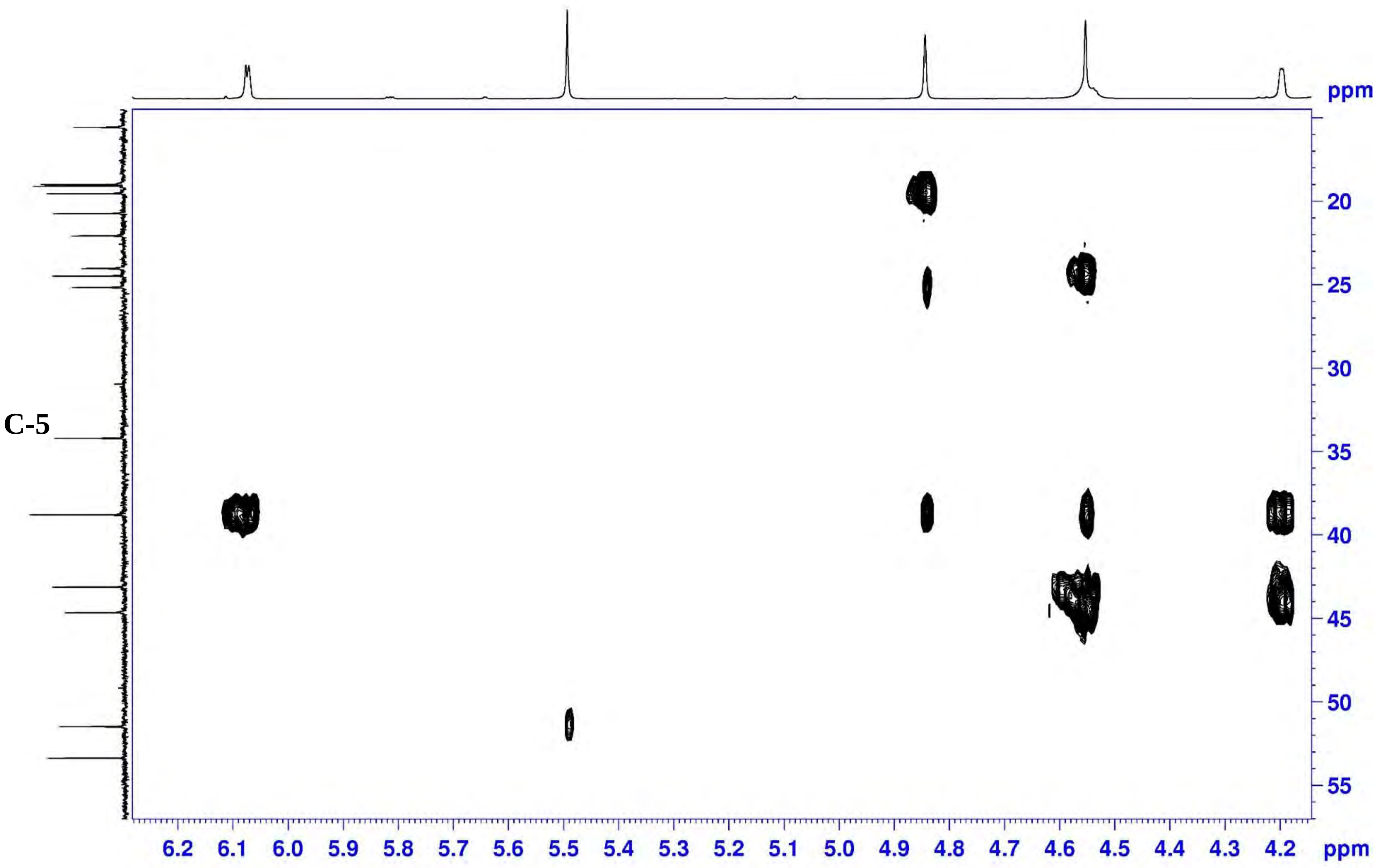

HMBC (400 MHz) spectrum of compound **14** in $CDCl_3$

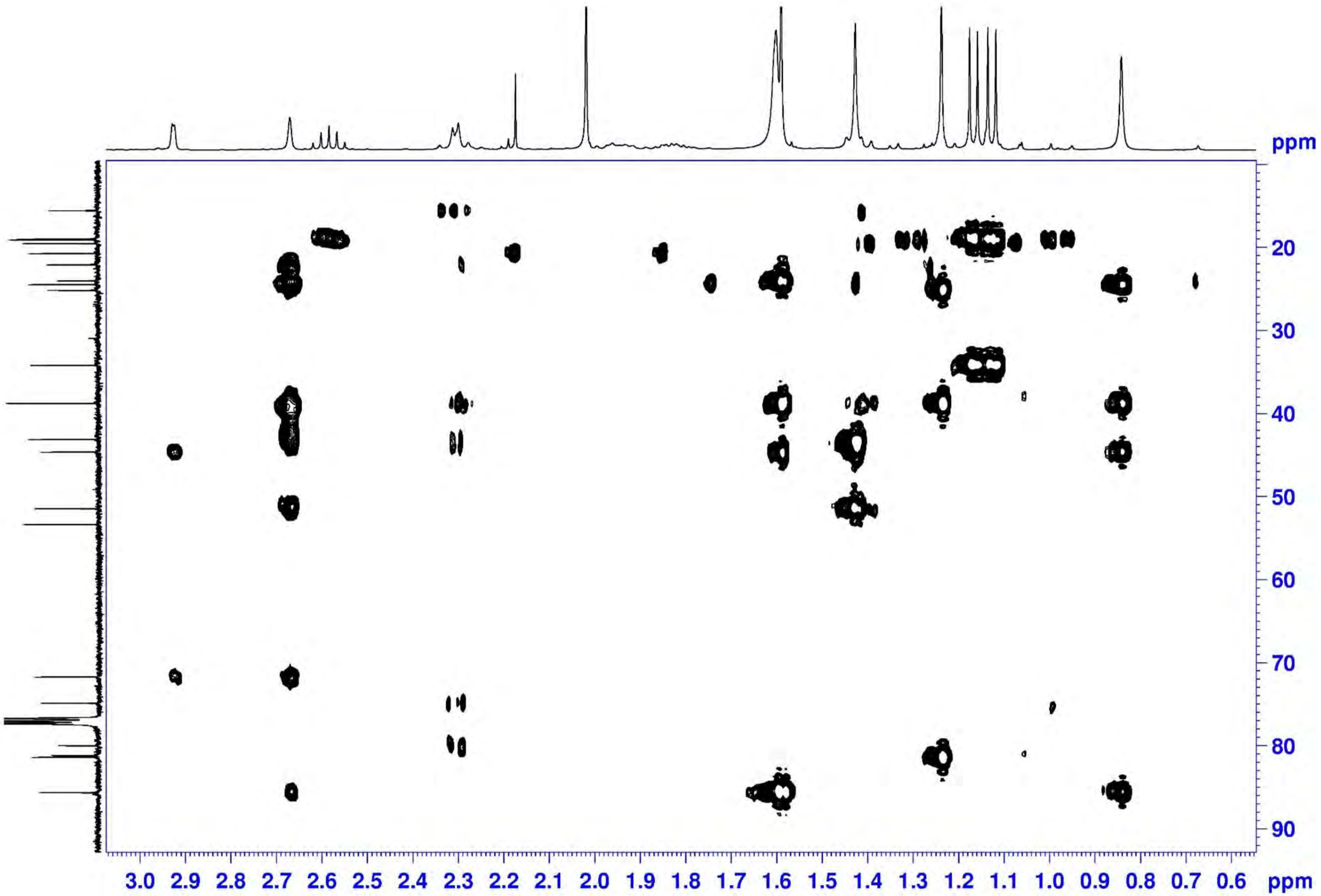

HMBC (400 MHz) spectrum of compound **14** in $CDCl_3$

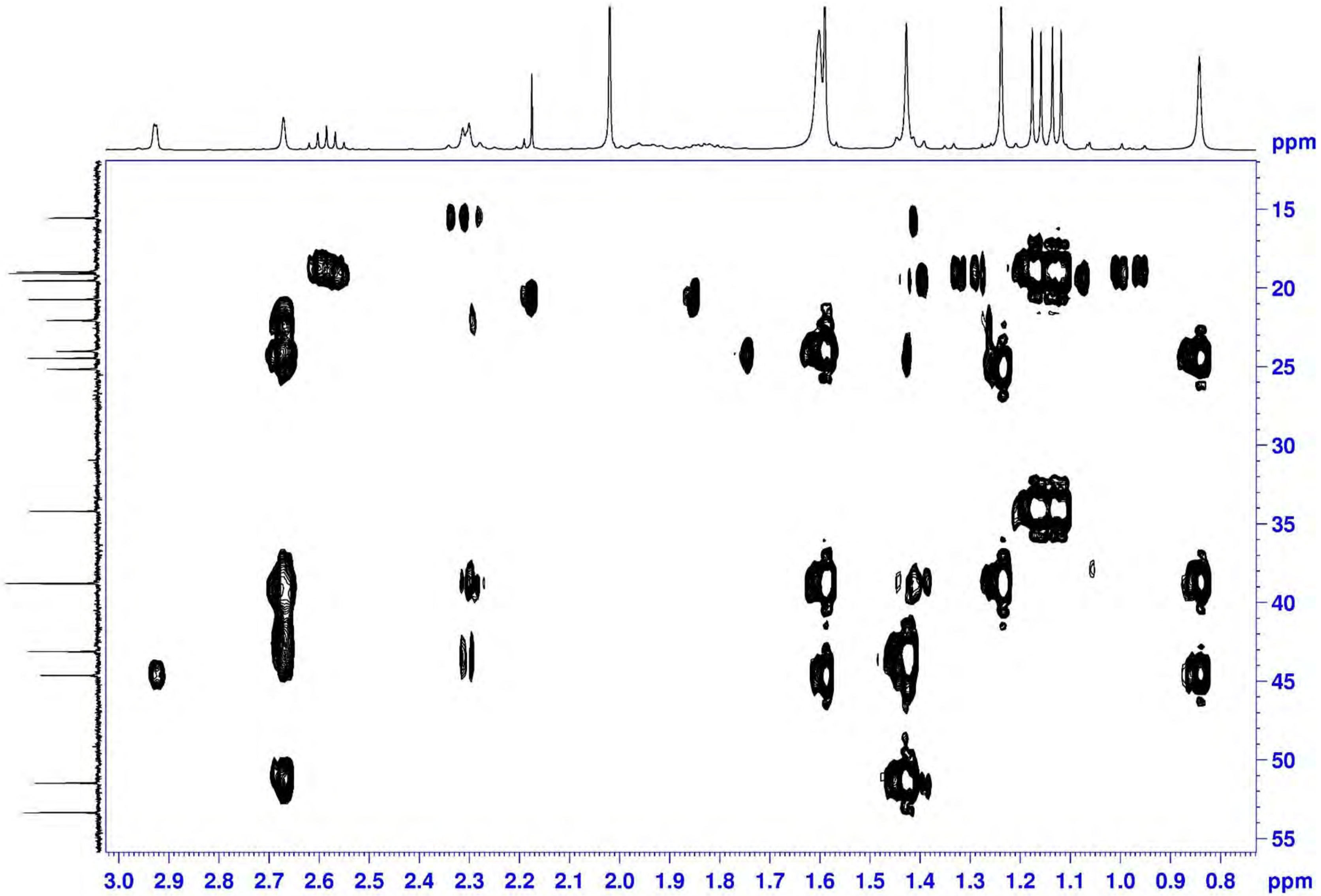

NOESY (400 MHz) spectrum of compound **14** in $CDCl_3$

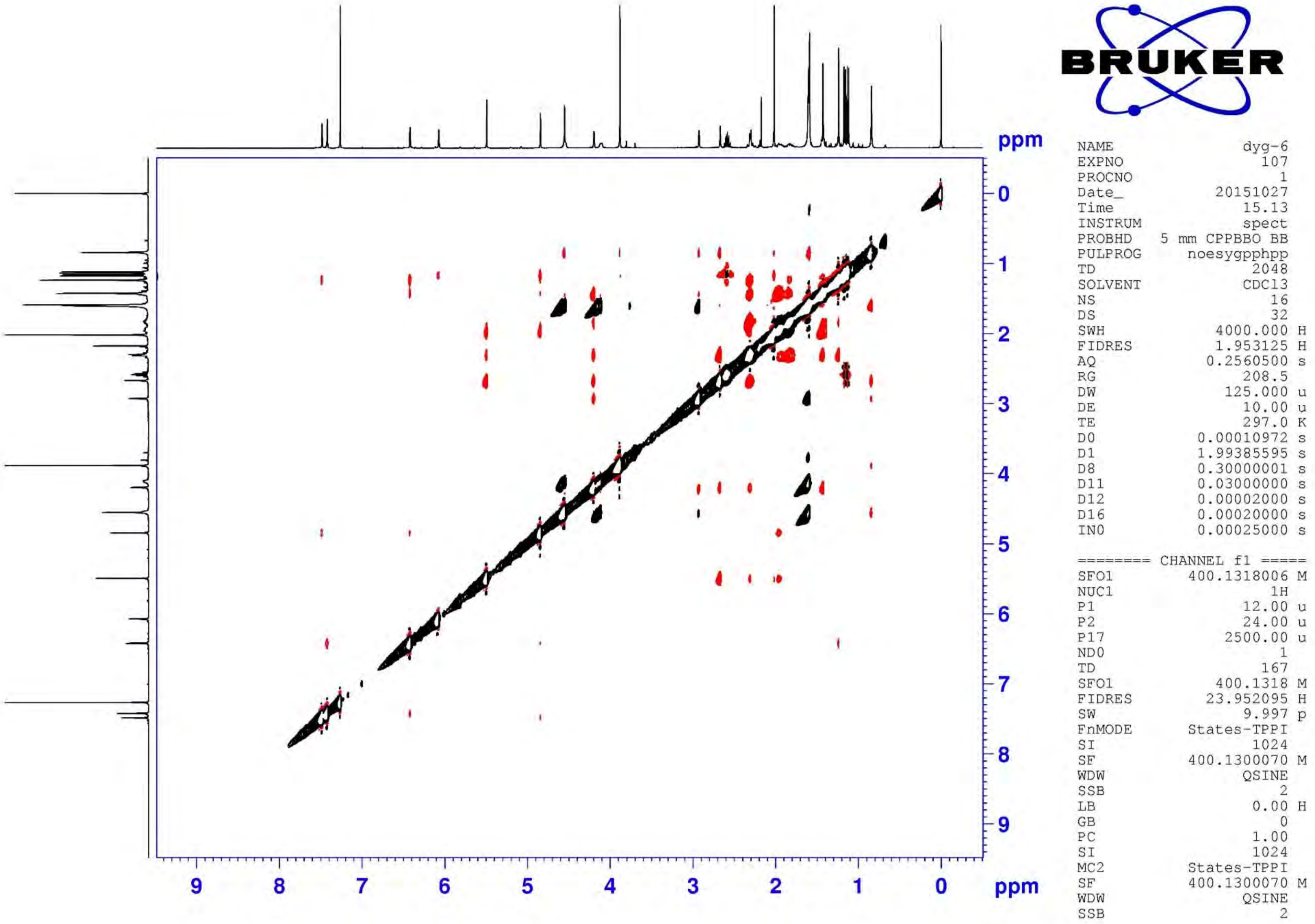

NOESY (400 MHz) spectrum of compound **14** in $CDCl_3$

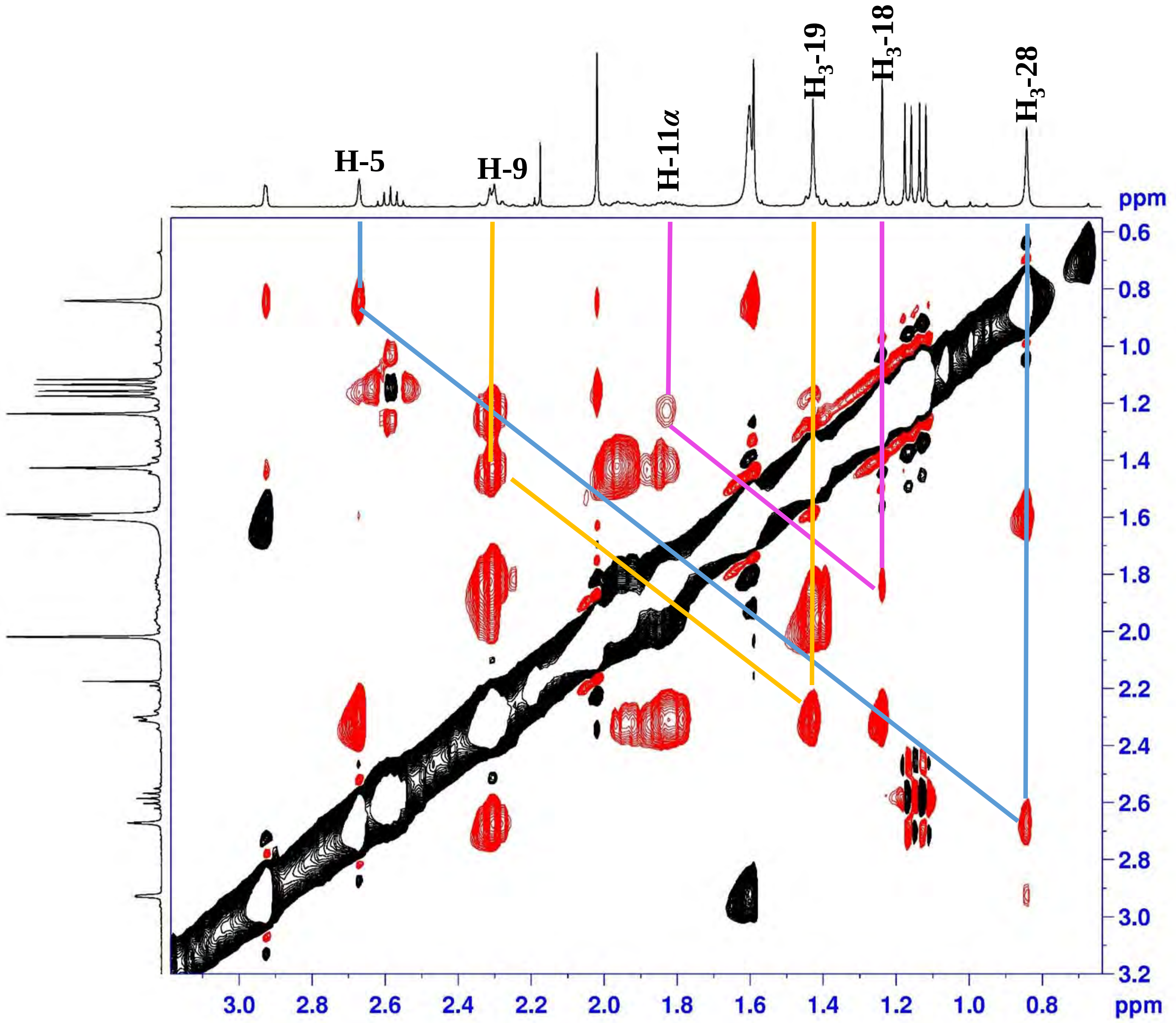

NOESY (400 MHz) spectrum of compound **14** in $CDCl_3$

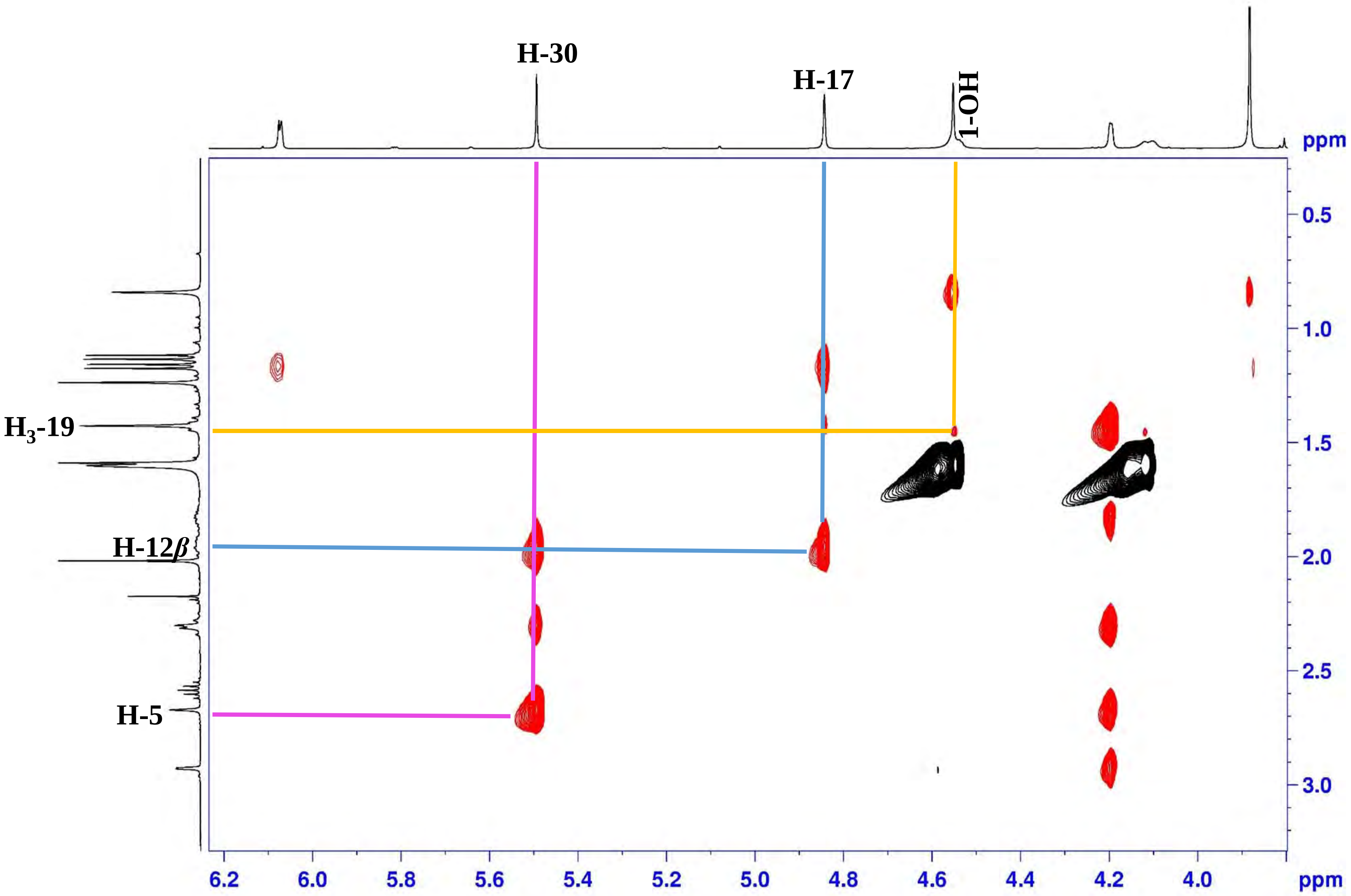

Supplement: Supplementary file 1 [file marinedrugs-16-00038-s001.zip › SI-for submission/SI-1.pdf]
