# Supplementary material for: Twenty-Nine New Limonoids with Skeletal Diversity from the Mangrove Plant, Xylocarpus moluccensis
Source: Mar Drugs. 2018 Jan 19;16(1):38. doi: 10.3390/md16010038 (PMC5793086; doi:10.3390/md16010038)

Supporting Information

Copies of HR-ESIMS, and 1D and 2D NMR spectra of Compounds 15-23.

Table of Contents:

1. HR-ESIMS for compound **15** ········· S6-S7
2. ^{1}H NMR (400 MHz) spectrum of compound **15** in $CDCl_3$ ········· S8-S12
3. ^{13}C NMR (100 MHz) spectrum of compound **15** in $CDCl_3$ ········· S13-S16
4. DEPT135 (100 MHz) spectrum of compound **15** in $CDCl_3$ ········· S17-S20
5. ^{1}H-^{1}H COSY (400 MHz) spectrum of compound **15** in $CDCl_3$ ········· S21-S24
6. HSQC (400 MHz) spectrum of compound **15** in $CDCl_3$ ········· S25-S28
7. HMBC (400 MHz) spectrum of compound **15** in $CDCl_3$ ········· S29-S37
8. NOESY (400 MHz) spectrum of compound **15** in $CDCl_3$ ········· S38-S42
9. HR-ESIMS for compound **16** ········· S43
10. ^{1}H NMR (400 MHz) spectrum of compound **16** in $CDCl_3$ ········· S44-S48
11. ^{13}C NMR (100 MHz) spectrum of compound **16** in $CDCl_3$ ········· S49-S51
12. DEPT135 (100 MHz) spectrum of compound **16** in $CDCl_3$ ········· S52-S55
13. ^{1}H-^{1}H COSY (400 MHz) spectrum of compound **16** in $CDCl_3$ ········· S56-S58
14. HSQC (400 MHz) spectrum of compound **16** in $CDCl_3$ ········· S59-S62
15. HMBC (400 MHz) spectrum of compound **16** in $CDCl_3$ ········· S63-S73
16. NOESY (400 MHz) spectrum of compound **16** in $CDCl_3$ ········· S74-S78
17. HR-ESIMS for compound **17** ········· S79
18. ^{1}H NMR (400 MHz) spectrum of compound **17** in $CDCl_3$ ········· S80-S82
19. ^{13}C NMR (100 MHz) spectrum of compound **17** in $CDCl_3$ ········· S83-S86
20. DEPT135 (100 MHz) spectrum of compound **17** in $CDCl_3$ ········· S87-S90
21. ^{1}H-^{1}H COSY (400 MHz) spectrum of compound **17** in $CDCl_3$ ········· S91-S93

22. HSQC (400 MHz) spectrum of compound **17** in $CDCl_3$ ················S94-S96
23. HMBC (400 MHz) spectrum of compound **17** in $CDCl_3$ ················S97-S102
24. NOESY (400 MHz) spectrum of compound **17** in $CDCl_3$ ··············S103-S107
25. HR-ESIMS for compound **18**···S108
26. ^{1}H NMR (400 MHz) spectrum of compound **18** in $CDCl_3$·············S109-S113
27. ^{13}C NMR (100 MHz) spectrum of compound **18** in $CDCl_3$·············S114-S118
28. DEPT135 (100 MHz) spectrum of compound **18** in $CDCl_3$ ···········S119-S123
29. ^{1}H-^{1}H COSY (400 MHz) spectrum of compound **18** in $CDCl_3$ ········S124-S126
30. HSQC (400 MHz) spectrum of compound **18** in $CDCl_3$ ················S127-S130
31. HMBC (400 MHz) spectrum of compound **18** in $CDCl_3$ ···············S131-S135
32. NOESY (400 MHz) spectrum of compound **18** in $CDCl_3$ ··············S136-S140
33. HR-ESIMS for compound **19**···S141
34. ^{1}H NMR (400 MHz) spectrum of compound **19** in $CDCl_3$·············S142-S144
35. ^{13}C NMR (100 MHz) spectrum of compound **19** in $CDCl_3$·············S145-S149
36. DEPT135 (100 MHz) spectrum of compound **19** in $CDCl_3$ ···········S150-S154
37. ^{1}H-^{1}H COSY (400 MHz) spectrum of compound **19** in $CDCl_3$ ········S155-S157
38. HSQC (400 MHz) spectrum of compound **19** in $CDCl_3$ ················S158-S161
39. HMBC (400 MHz) spectrum of compound **19** in $CDCl_3$ ···············S162-S166
40. NOESY (400 MHz) spectrum of compound **19** in $CDCl_3$ ··············S167-S171
41. HR-ESIMS for compound **20**····································S172-S173
42. ^{1}H NMR (400 MHz) spectrum of compound **20** in Acetone-*d6*·········S174-S176

43. ^{13}C NMR (100 MHz) spectrum of compound **20** in Acetone-*d6* ·······S177-S180
44. DEPT135 (100 MHz) spectrum of compound **20** in Acetone-*d6* ·······S181-S184
45. ^{1}H-^{1}H COSY (400 MHz) spectrum of compound **20** in Acetone-*d6* ···S185-S186
46. HSQC (400 MHz) spectrum of compound **20** in Acetone-*d6* ··········S187-S189
47. HMBC (400 MHz) spectrum of compound **20** in Acetone-*d6* ··········S190-S198
48. NOESY (400 MHz) spectrum of compound **20** in Acetone-*d6* ·········S199-S202
49. HR-ESIMS for compound **21**···S203-S204
50. ^{1}H NMR (400 MHz) spectrum of compound **21** in $CDCl_3$·············S205-S208
51. ^{13}C NMR (100 MHz) spectrum of compound **21** in $CDCl_3$·············S209-S212
52. DEPT135 (100 MHz) spectrum of compound **21** in $CDCl_3$ ···········S213-S217
53. ^{1}H-^{1}H COSY (400 MHz) spectrum of compound **21** in $CDCl_3$ ········S218-S219
54. HSQC (400 MHz) spectrum of compound **21** in $CDCl_3$················S220-S223
55. HMBC (400 MHz) spectrum of compound **21** in $CDCl_3$···············S224-S228
56. NOESY (400 MHz) spectrum of compound **21** in $CDCl_3$ ··············S229-S233
57. HR-ESIMS for compound **22**···S234
58. ^{1}H NMR (400 MHz) spectrum of compound **22** in $CDCl_3$·············S235-S237
59. ^{13}C NMR (100 MHz) spectrum of compound **22** in $CDCl_3$·············S238-S240
60. DEPT135 (100 MHz) spectrum of compound **22** in $CDCl_3$ ···········S241-S243
61. ^{1}H-^{1}H COSY (400 MHz) spectrum of compound **22** in $CDCl_3$ ·········S244-S247
62. HSQC (400 MHz) spectrum of compound **22** in $CDCl_3$················S248-S251
63. HMBC (400 MHz) spectrum of compound **22** in $CDCl_3$···············S252-S258

64. NOESY (400 MHz) spectrum of compound **22** in $CDCl_3$ ··············S259-S262
65. HR-ESIMS for compound **23**··S263
66. ^{1}H NMR (400 MHz) spectrum of compound **23** in $CDCl_3$·············S264-S266
67. ^{13}C NMR (100 MHz) spectrum of compound **23** in $CDCl_3$·············S267-S269
68. DEPT135 (100 MHz) spectrum of compound **23** in $CDCl_3$ ···········S270-S272
69. ^{1}H-^{1}H COSY (400 MHz) spectrum of compound **23** in $CDCl_3$ ········S273-S275
70. HSQC (400 MHz) spectrum of compound **23** in $CDCl_3$ ················S276-S279
71. HMBC (400 MHz) spectrum of compound **23** in $CDCl_3$ ···············S280-S287
72. NOESY (400 MHz) spectrum of compound **23** in $CDCl_3$ ··············S288-S292

HRESIMS for compound **15**

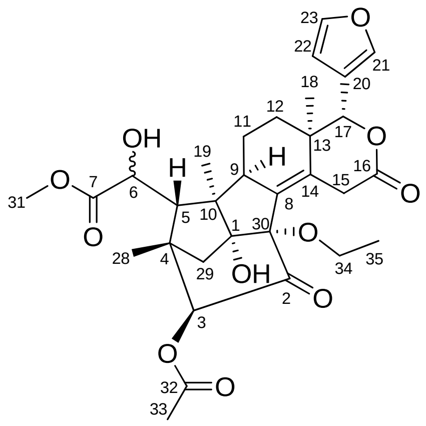

Elemental Composition Report

Single Mass Analysis

Tolerance = 5.0 mDa / DBE: min = -1.5, max = 50.0
Element prediction: Off
Number of isotope peaks used for i-FIT = 3

Monoisotopic Mass, Even Electron Ions
173 formula(e) evaluated with 1 results within limits (up to 50 best isotopic matches for each mass)
Elements Used:
C: 0-100 H: 0-200 O: 0-100
ZJZ-18
2016102403 158 (1.283)

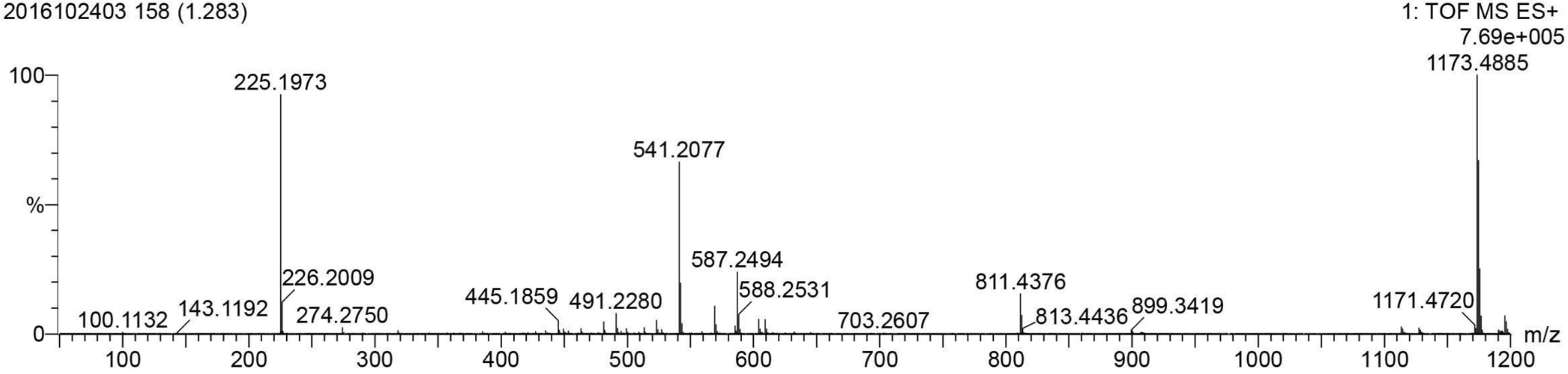

Minimum:				-1.5				
Maximum:		5.0	10.0	50.0				

Mass	Calc. Mass	mDa	PPM	DBE	i-FIT	Norm	Conf(%)	Formula
587.2494	587.2492	0.2	0.3	12.5	267.7	n/a	n/a	C31 H39 O11

HRESIMS for compound **15**

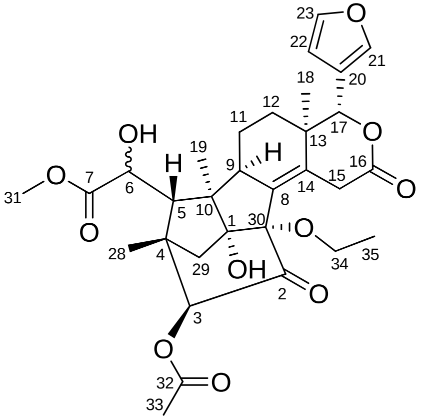

Elemental Composition Report

Single Mass Analysis

Tolerance = 5.0 PPM / DBE: min = -1.5, max = 50.0
Element prediction: Off
Number of isotope peaks used for i-FIT = 3

Monoisotopic Mass, Even Electron Ions
130 formula(e) evaluated with 1 results within limits (up to 50 best isotopic matches for each mass)
Elements Used:
C: 0-80 H: 0-100 O: 0-20
ZJZ-18
2016102403 158 (1.283)

1: TOF MS ES+
1.82e+005

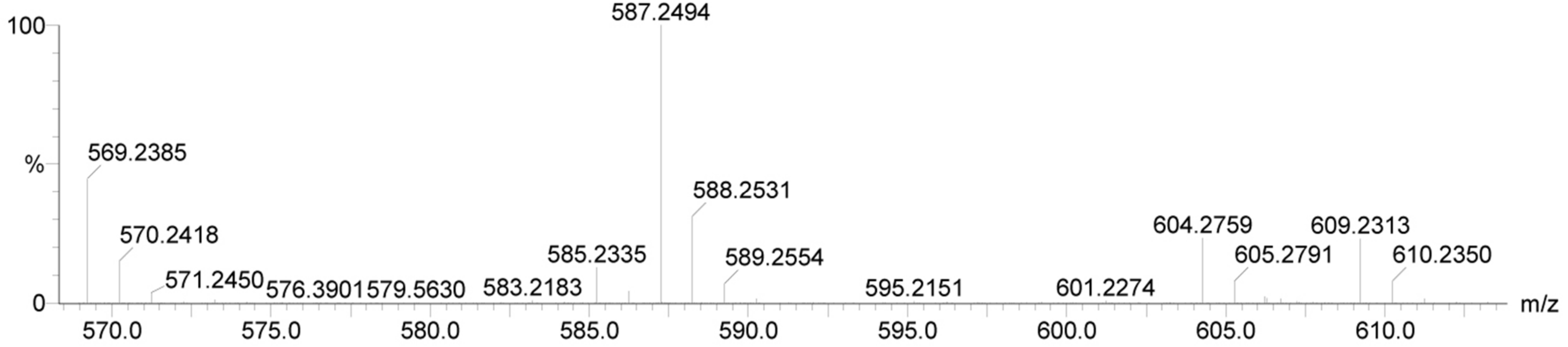

Minimum:				-1.5				
Maximum:		5.0	5.0	50.0				

Mass	Calc. Mass	mDa	PPM	DBE	i-FIT	Norm	Conf(%)	Formula
587.2494	587.2492	0.2	0.3	12.5	267.8	n/a	n/a	C31 H39 O11

^{1}H NMR (400 MHz) spectrum of compound **15** in $CDCl_3$

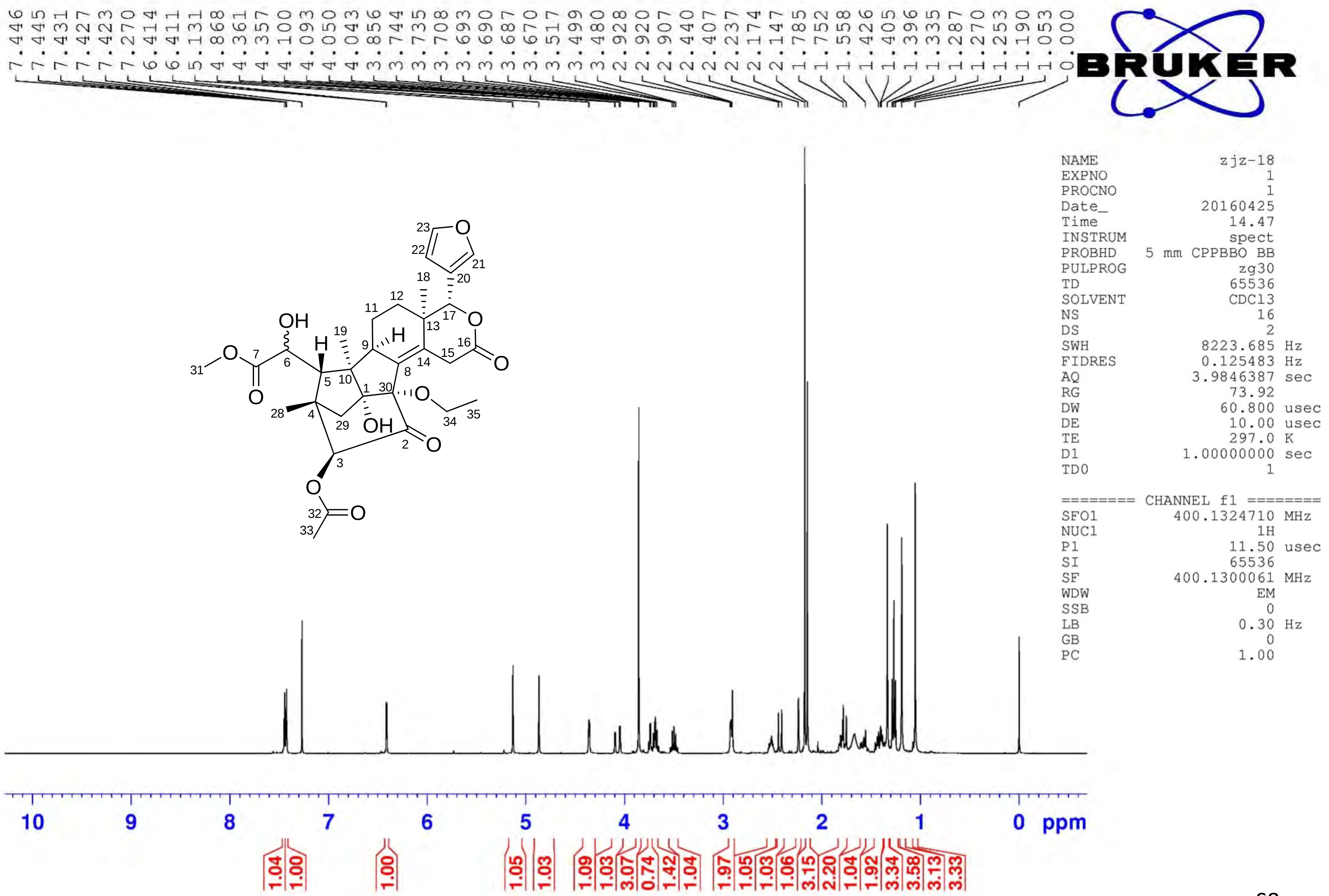

^{1}H NMR (400 MHz) spectrum of compound **15** in $CDCl_3$

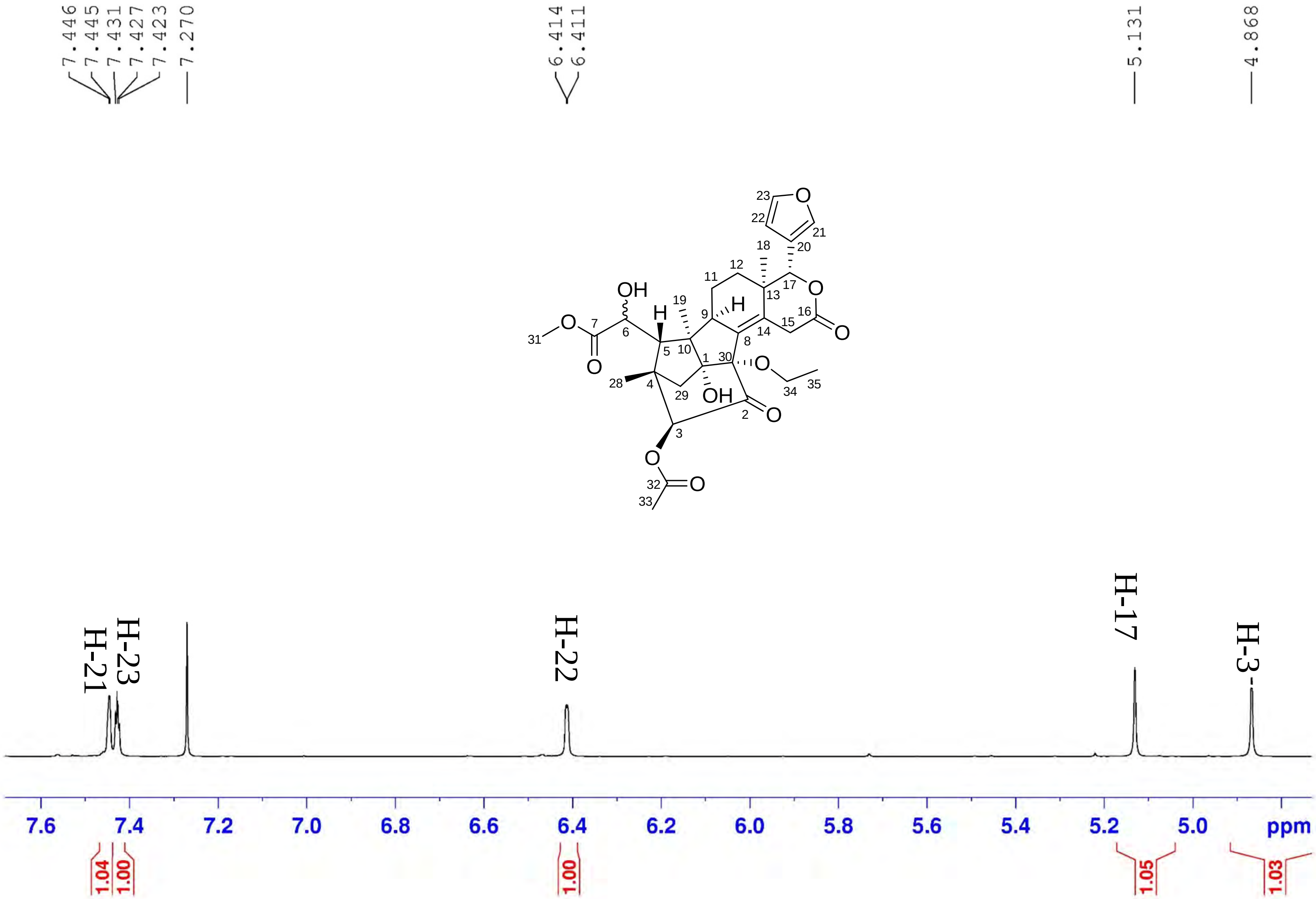

^{1}H NMR (400 MHz) spectrum of compound **15** in $CDCl_3$

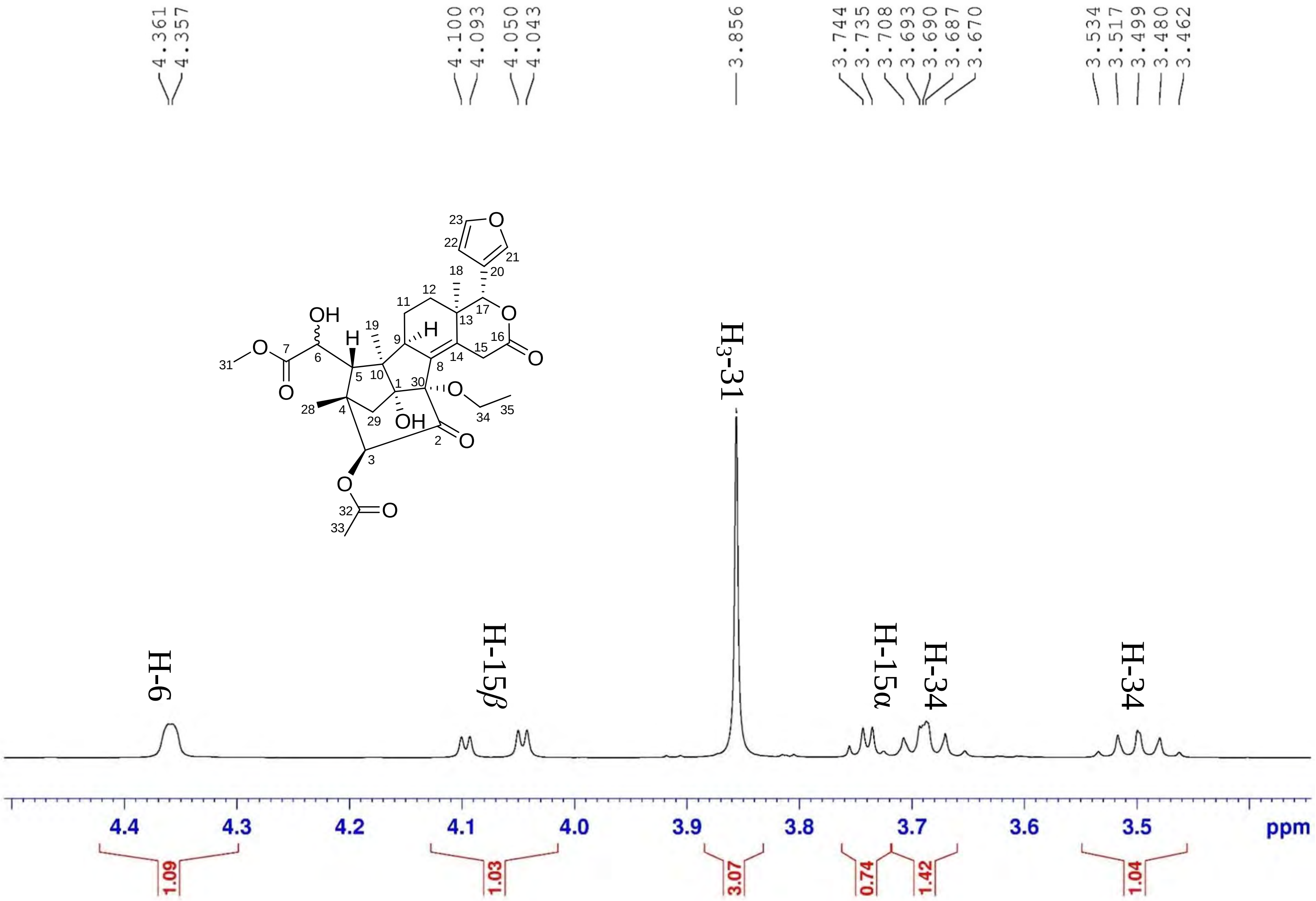

^{1}H NMR (400 MHz) spectrum of compound **15** in $CDCl_3$

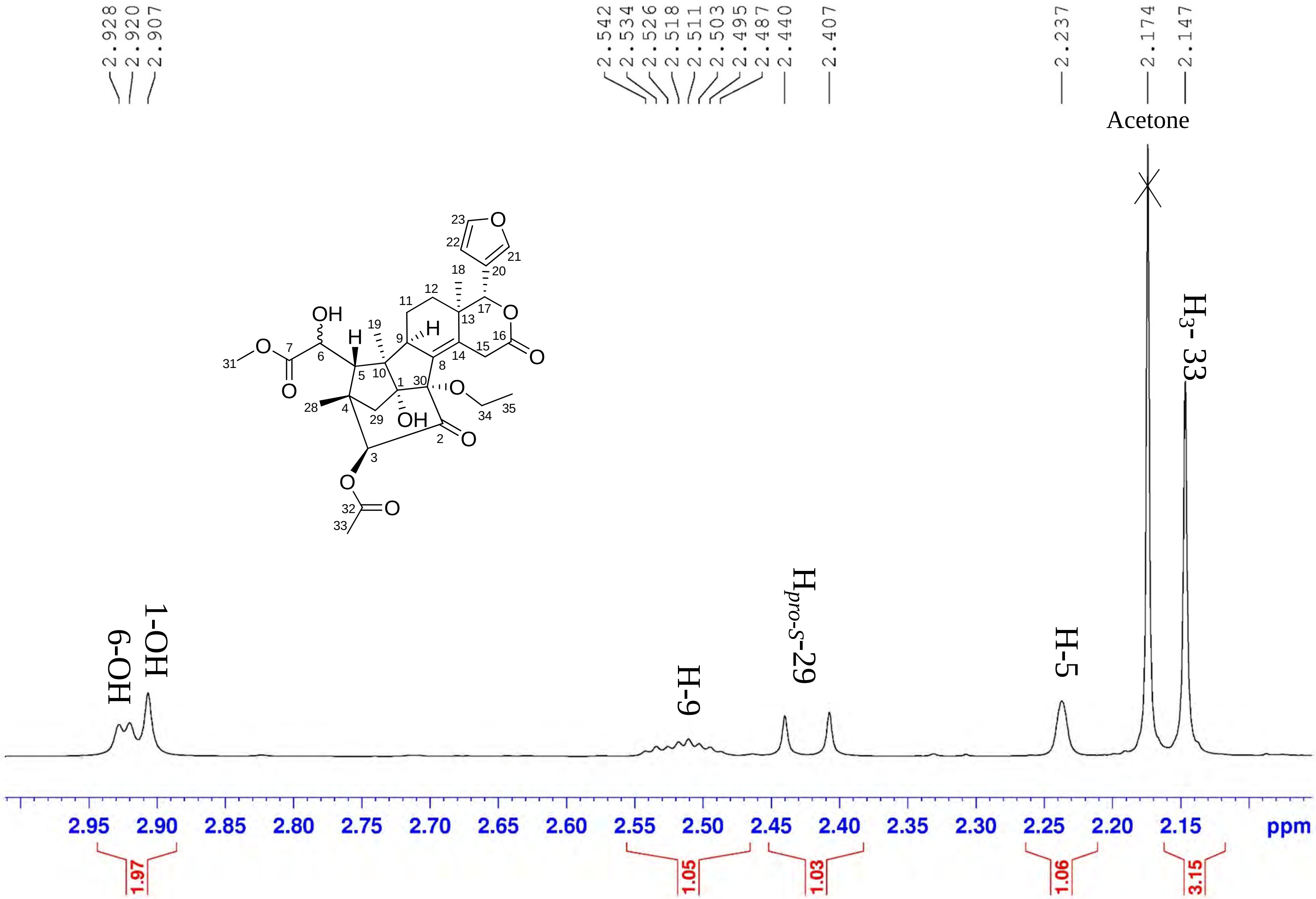

^{1}H NMR (400 MHz) spectrum of compound **15** in $CDCl_3$

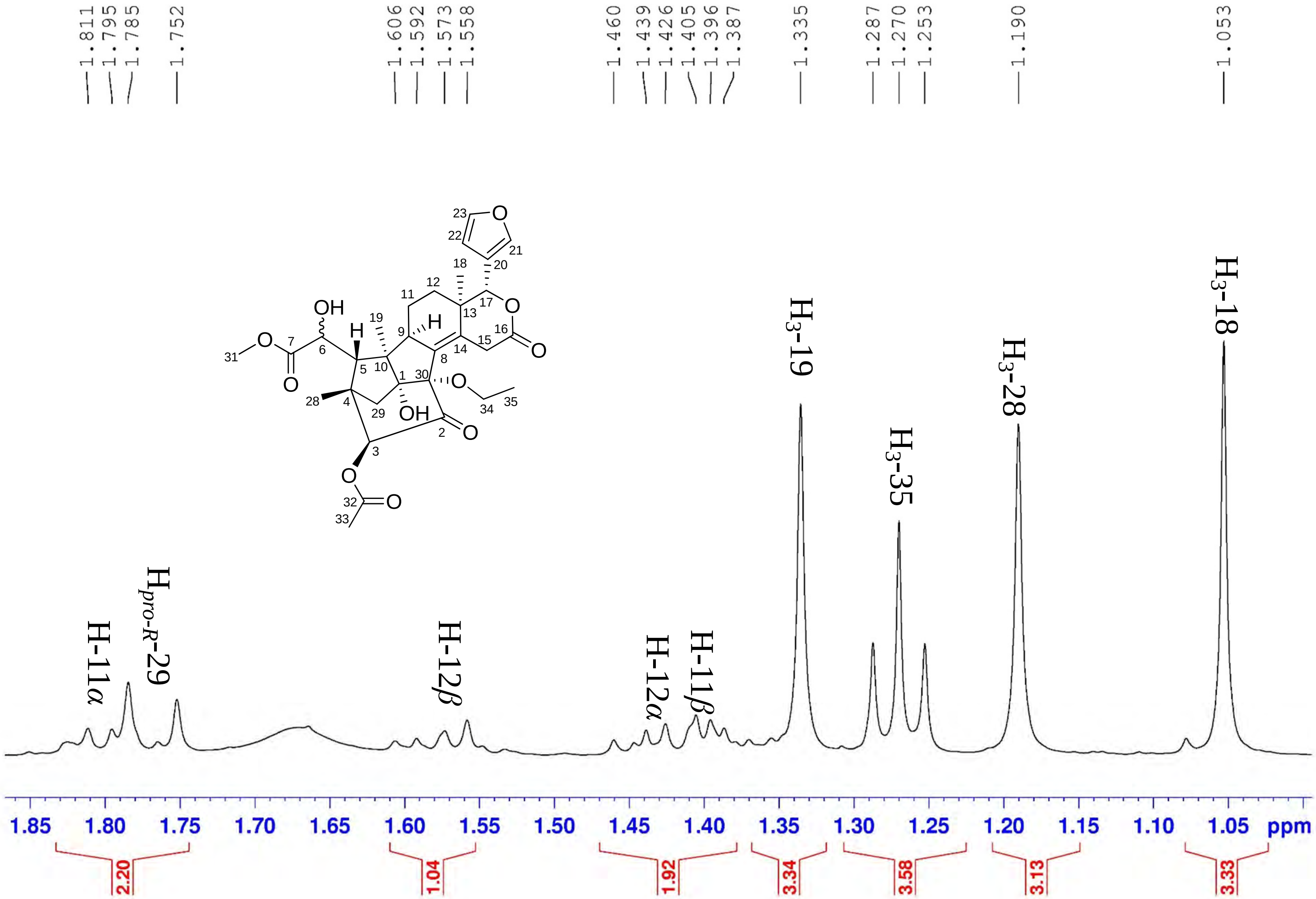

^{13}C NMR (100 MHz) spectrum of compound **15** in $CDCl_3$

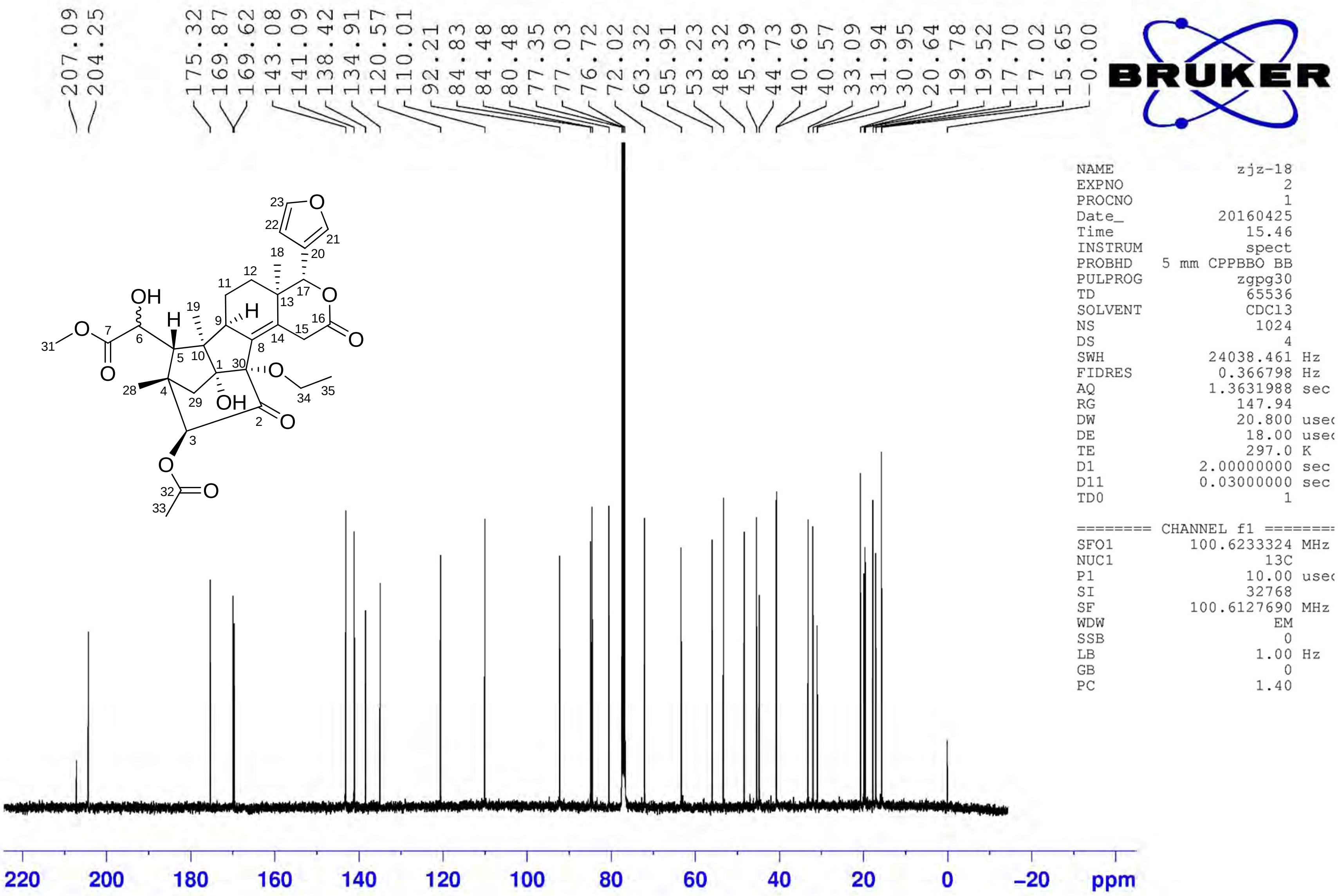

^{13}C NMR (100 MHz) spectrum of compound **15** in $CDCl_3$

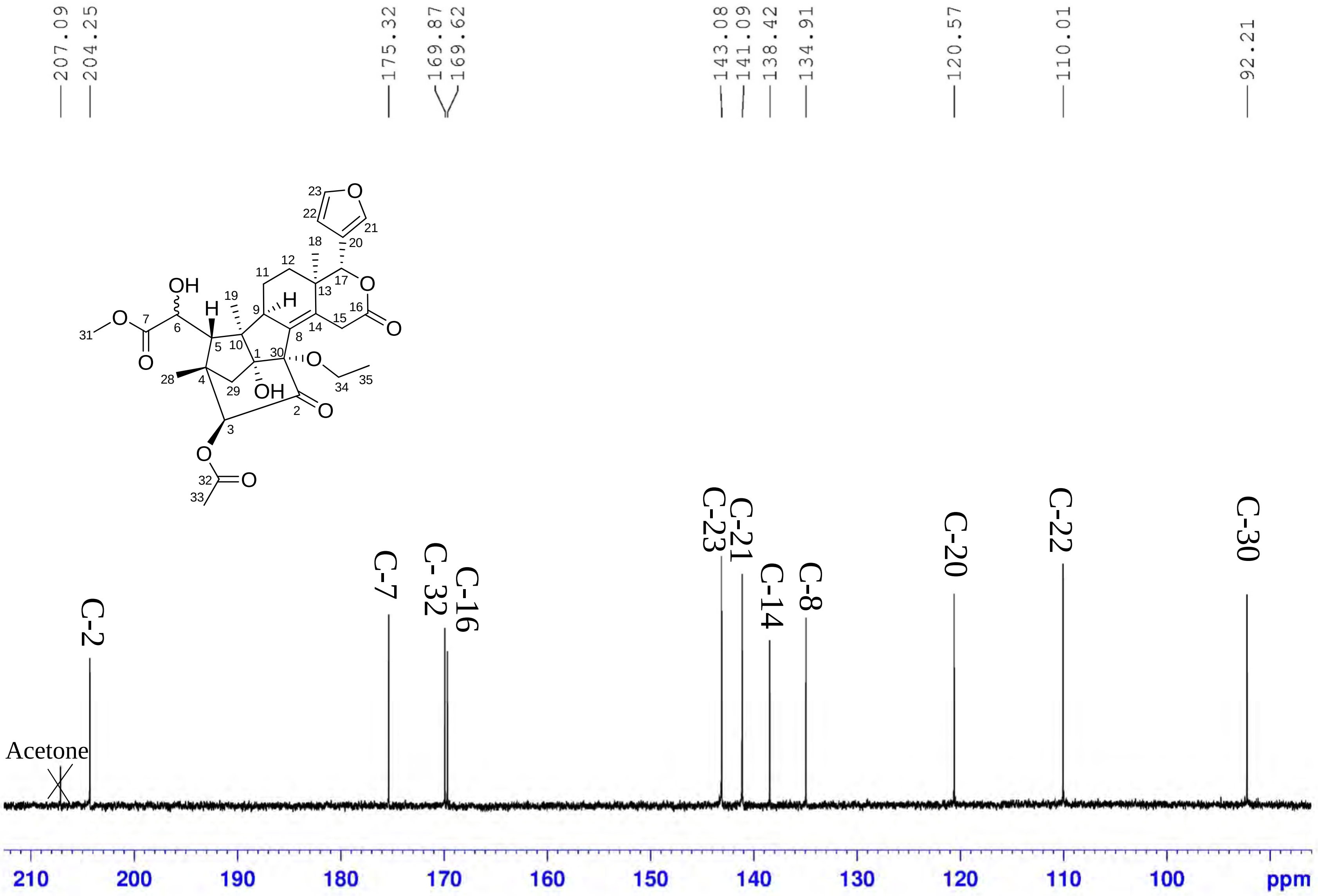

^{13}C NMR (100 MHz) spectrum of compound **15** in $CDCl_3$

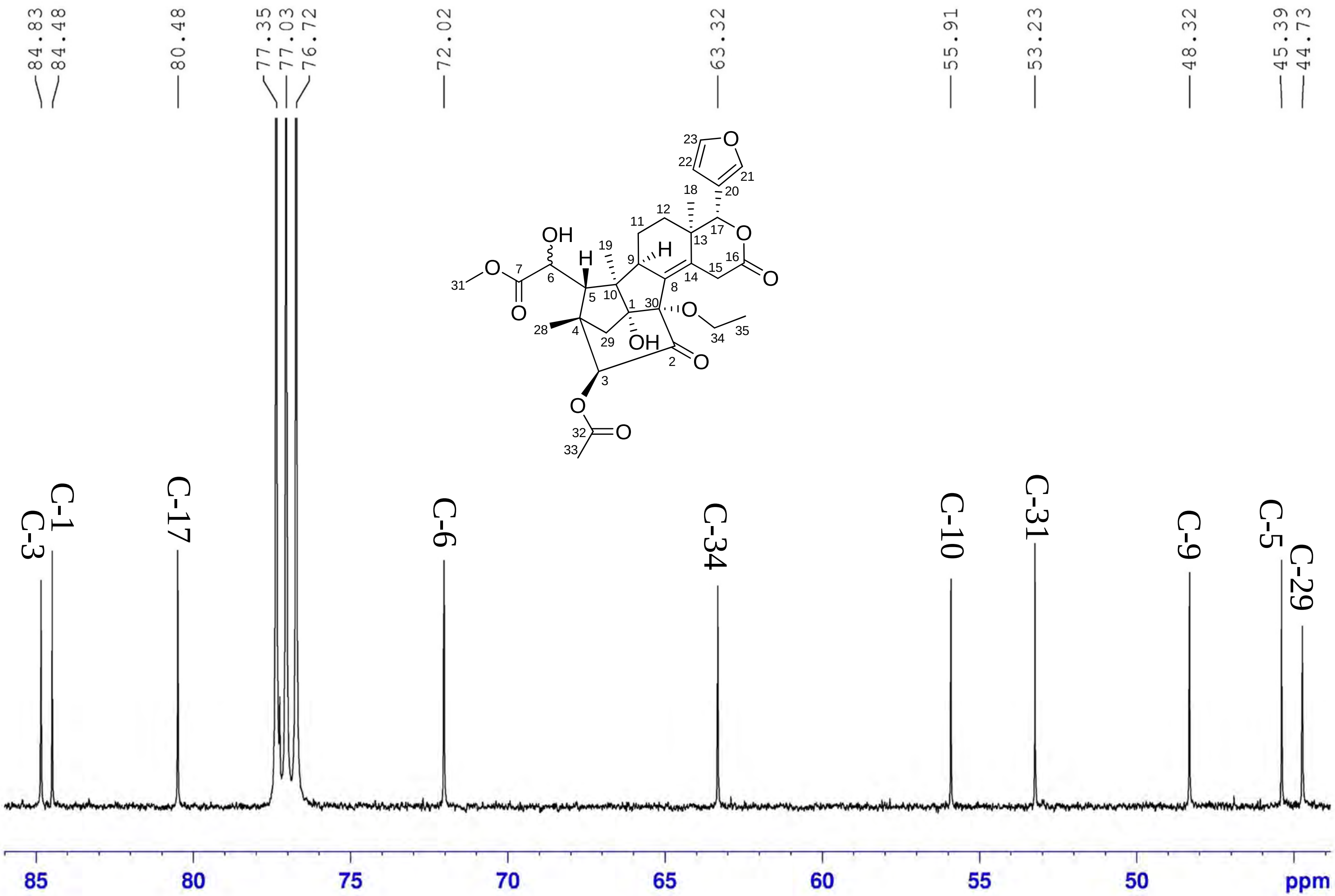

^{13}C NMR (100 MHz) spectrum of compound **15** in $CDCl_3$

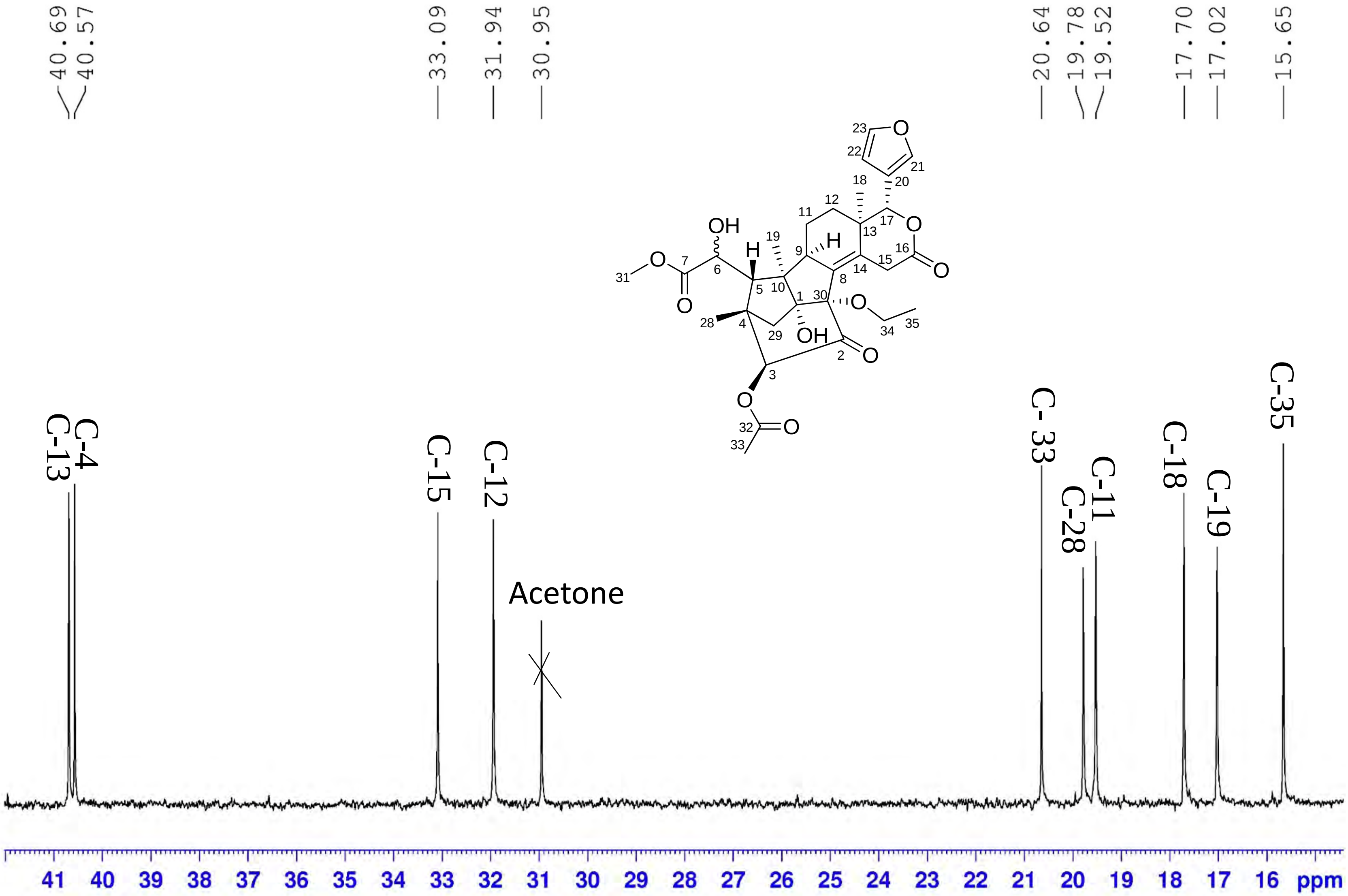

DEPT135 (100 MHz) spectrum of compound **15** in $CDCl_3$

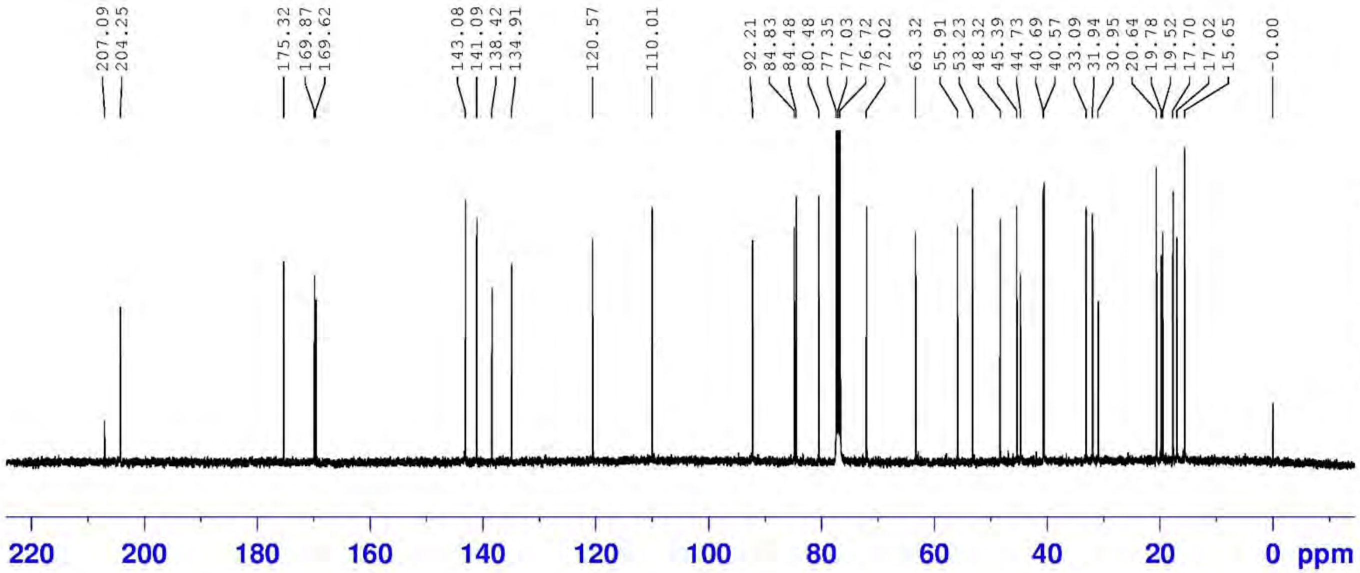

NAME	zjz-18	
EXPNO	3	
PROCNO	1	
Date_	20160425	
Time	16.04	
INSTRUM	spect	
PROBHD	5 mm CPPBBO BB	
PULPROG	deptsp135	
TD	65536	
SOLVENT	CDCl3	
NS	300	
DS	4	
SWH	24038.461	Hz
FIDRES	0.366798	Hz
AQ	1.3631988	sec
RG	130.26	
DW	20.800	usec
DE	18.00	usec
TE	297.0	K
CNST2	145.0000000	
D1	2.00000000	sec
D2	0.00344828	sec
D12	0.00002000	sec
TD0	1	

======== CHANNEL f1 ========

SFO1	100.6233324	MHz
NUC1	13C	
P1	10.00	usec
P13	2000.00	usec
SI	32768	
SF	100.6127690	MHz
WDW	EM	
SSB	0	
LB	1.00	Hz
GB	0	
PC	1.40	

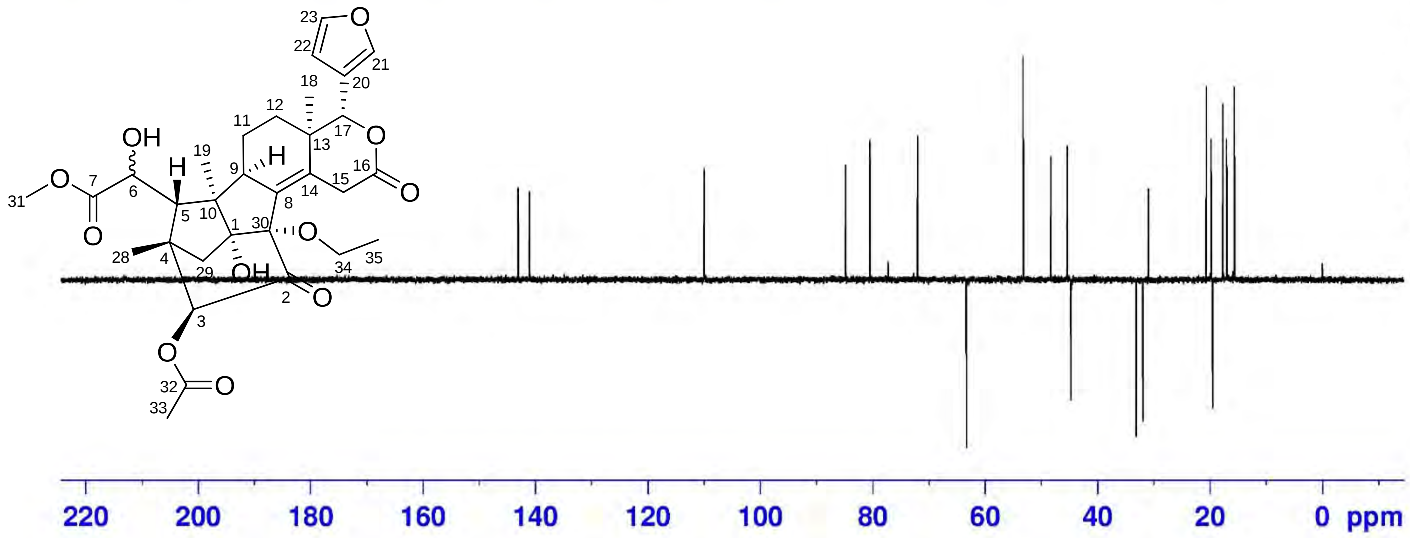

DEPT135 (100 MHz) spectrum of compound **15** in $CDCl_3$

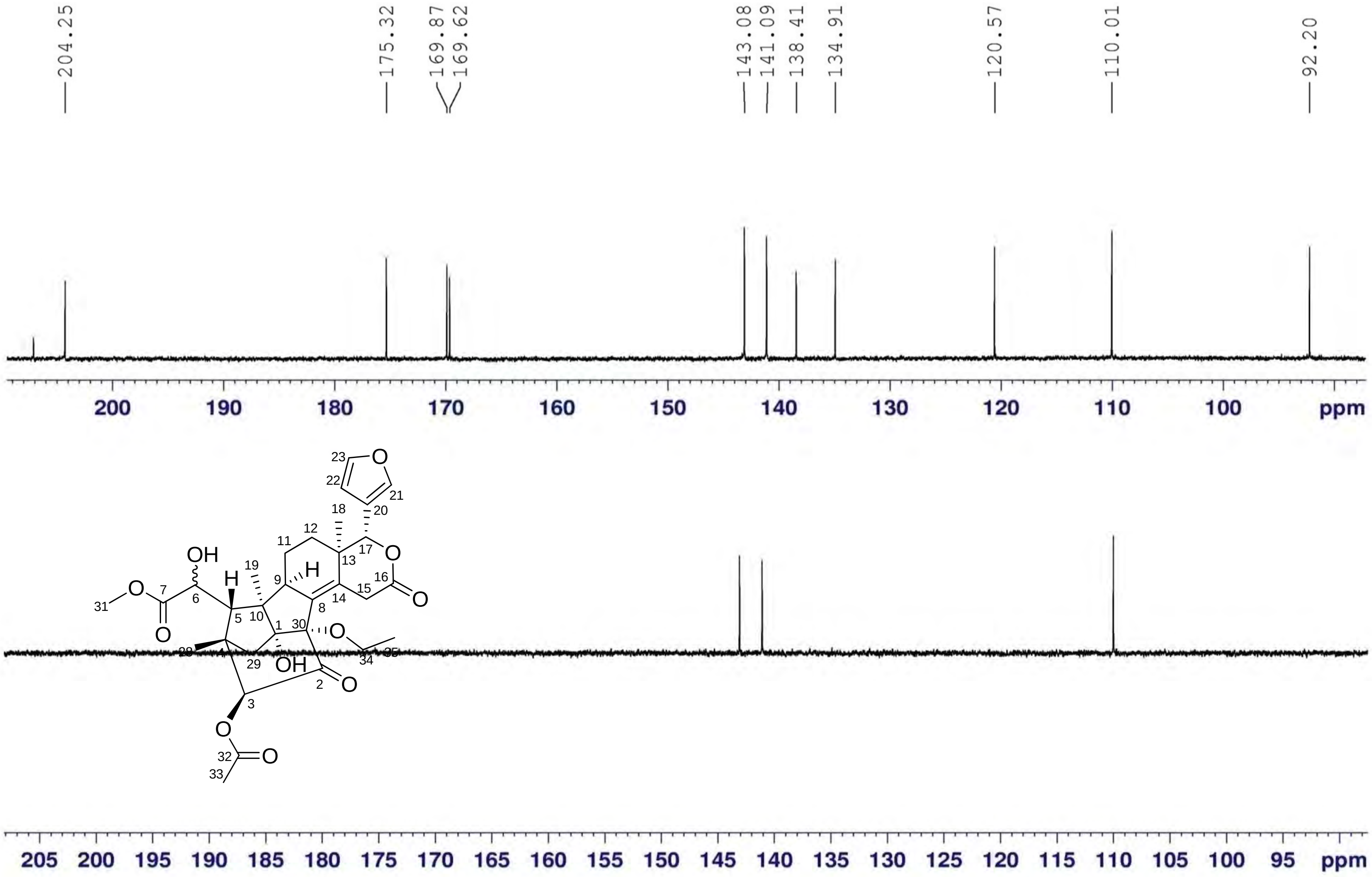

DEPT135 (100 MHz) spectrum of compound **15** in $CDCl_3$

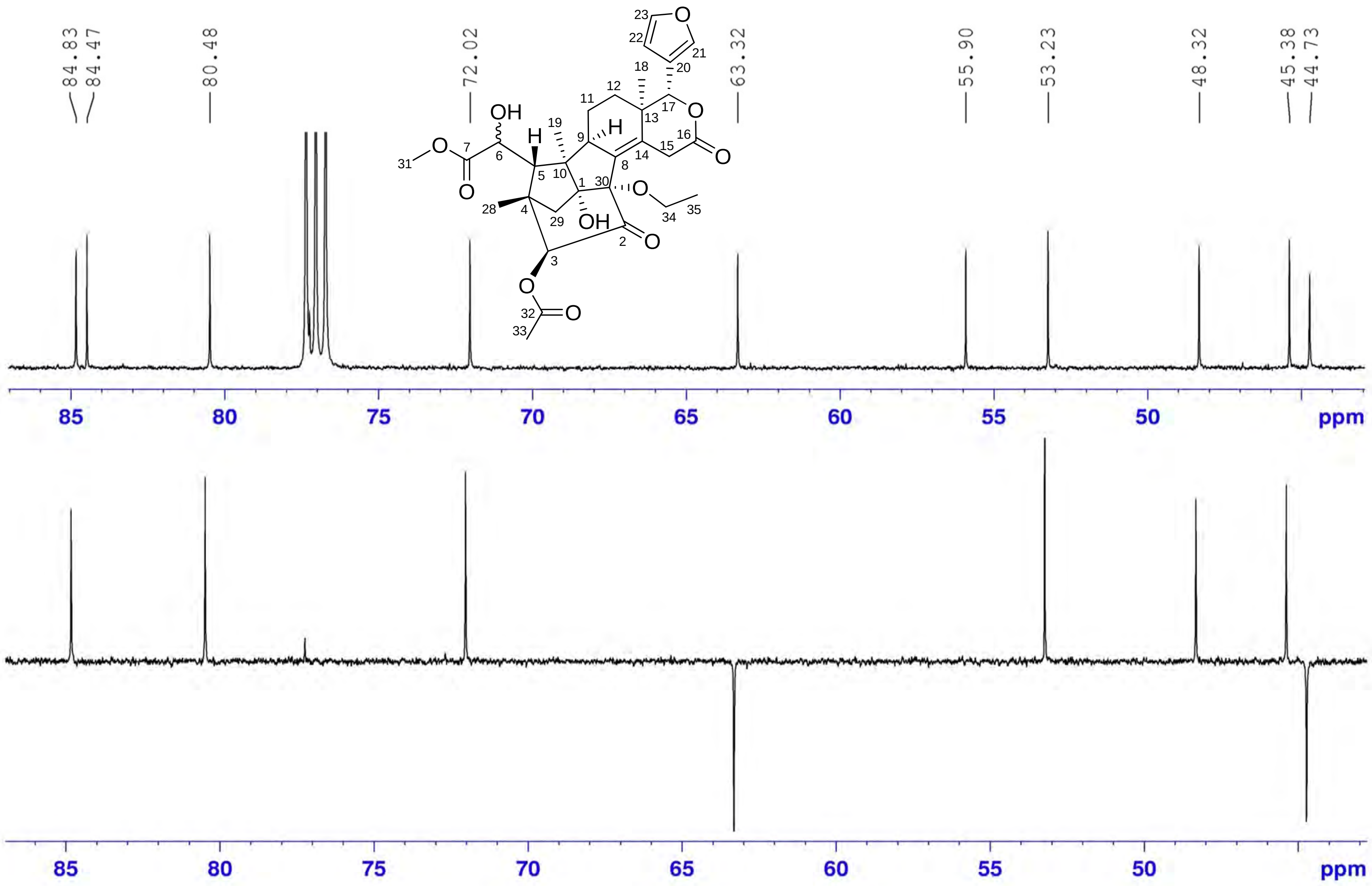

DEPT135 (100 MHz) spectrum of compound **15** in $CDCl_3$

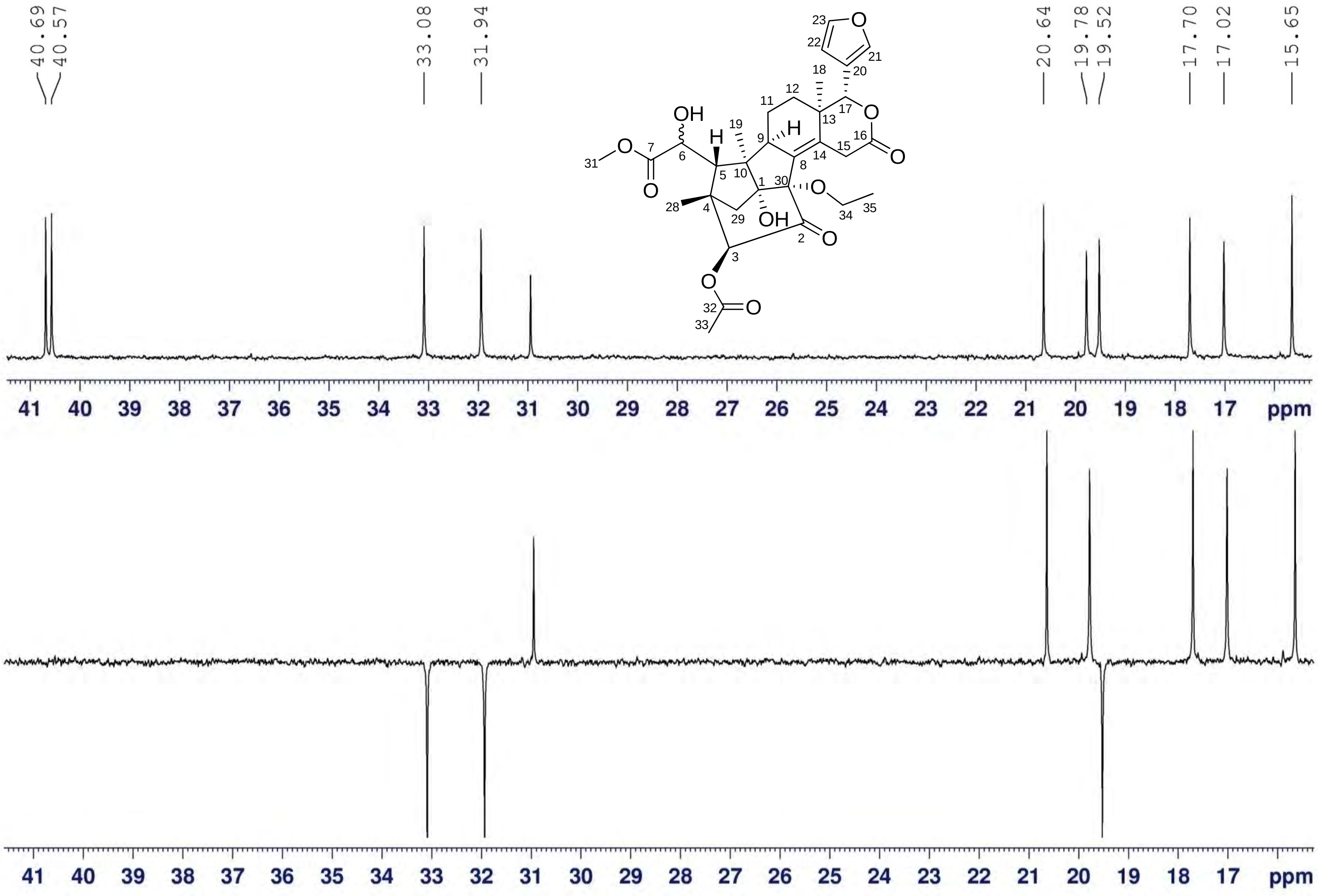

^{1}H-^{1}H COSY (400 MHz) spectrum of compound **15** in $CDCl_3$

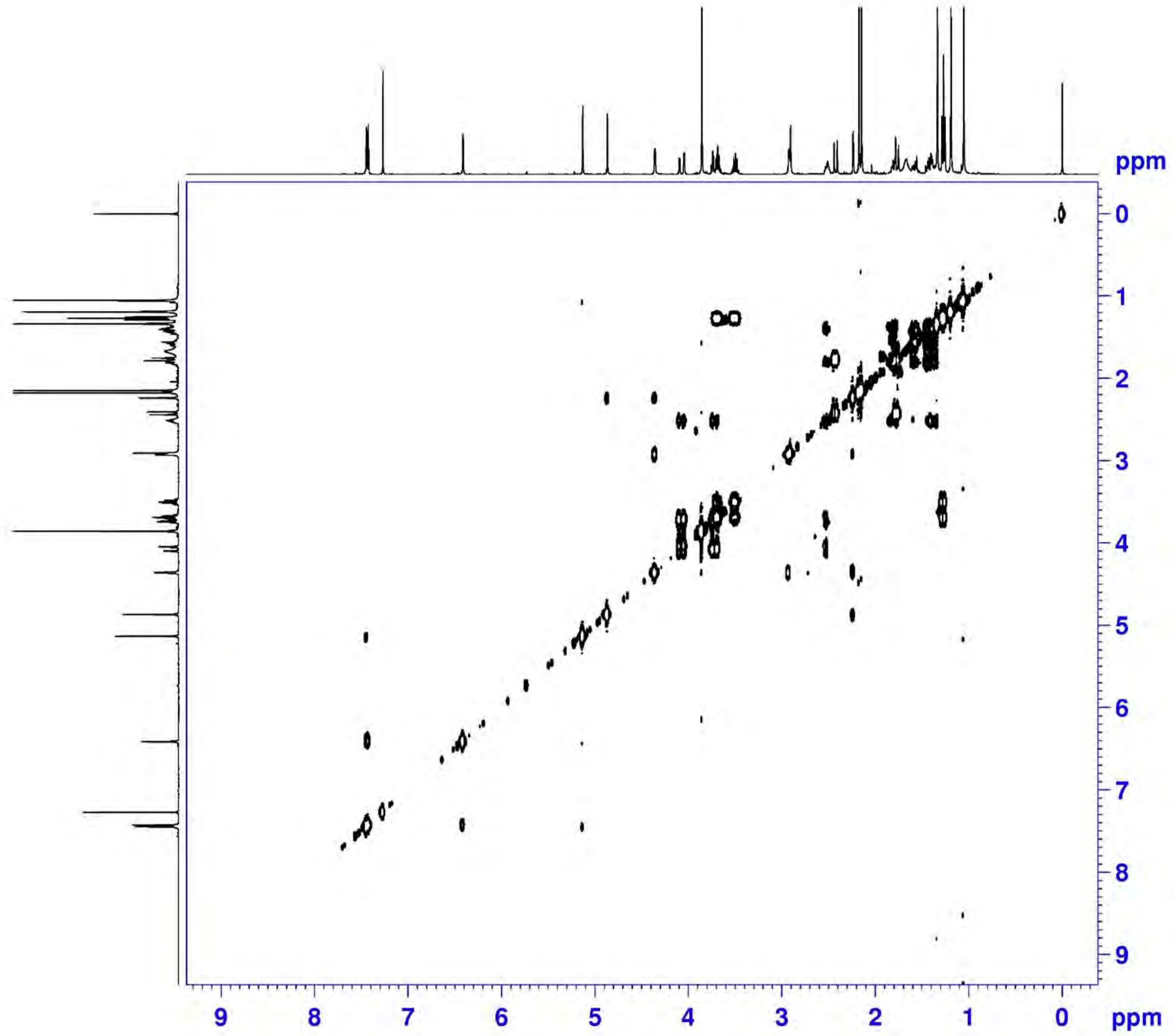

NAME	zjz-18	
EXPNO	4	
PROCNO	1	
Date_	20160426	
Time	13.54	
INSTRUM	spect	
PROBHD	5 mm CPPBBO BB	
PULPROG	cosygpppqf	
TD	2048	
SOLVENT	CDCl3	
NS	8	
DS	8	
SWH	3906.250	Hz
FIDRES	1.907349	Hz
AQ	0.2621940	sec
RG	208.5	
DW	128.000	usec
DE	10.00	usec
TE	297.0	K
D0	0.00000300	sec
D1	1.89678097	sec
D11	0.03000000	sec
D12	0.00002000	sec
D13	0.00000400	sec
D16	0.00020000	sec
IN0	0.00025600	sec
======== CHANNEL f1 ========		
SFO1	400.1318006	MHz
NUC1	1H	
P0	11.50	usec
P1	11.50	usec
P17	2500.00	usec
ND0	1	
TD	128	
SFO1	400.1318	MHz
FIDRES	30.517578	Hz
SW	9.762	ppm
FnMODE	QF	
SI	1024	
SF	400.1300051	MHz
WDW	QSINE	
SSB	0	
LB	0.00	Hz
GB	0	
PC	1.40	
SI	1024	
MC2	QF	
SF	400.1300053	MHz
WDW	QSINE	
SSB	0	
LB	0.00	Hz
GB	0	

^{1}H-^{1}H COSY (400 MHz) spectrum of compound **15** in $CDCl_3$

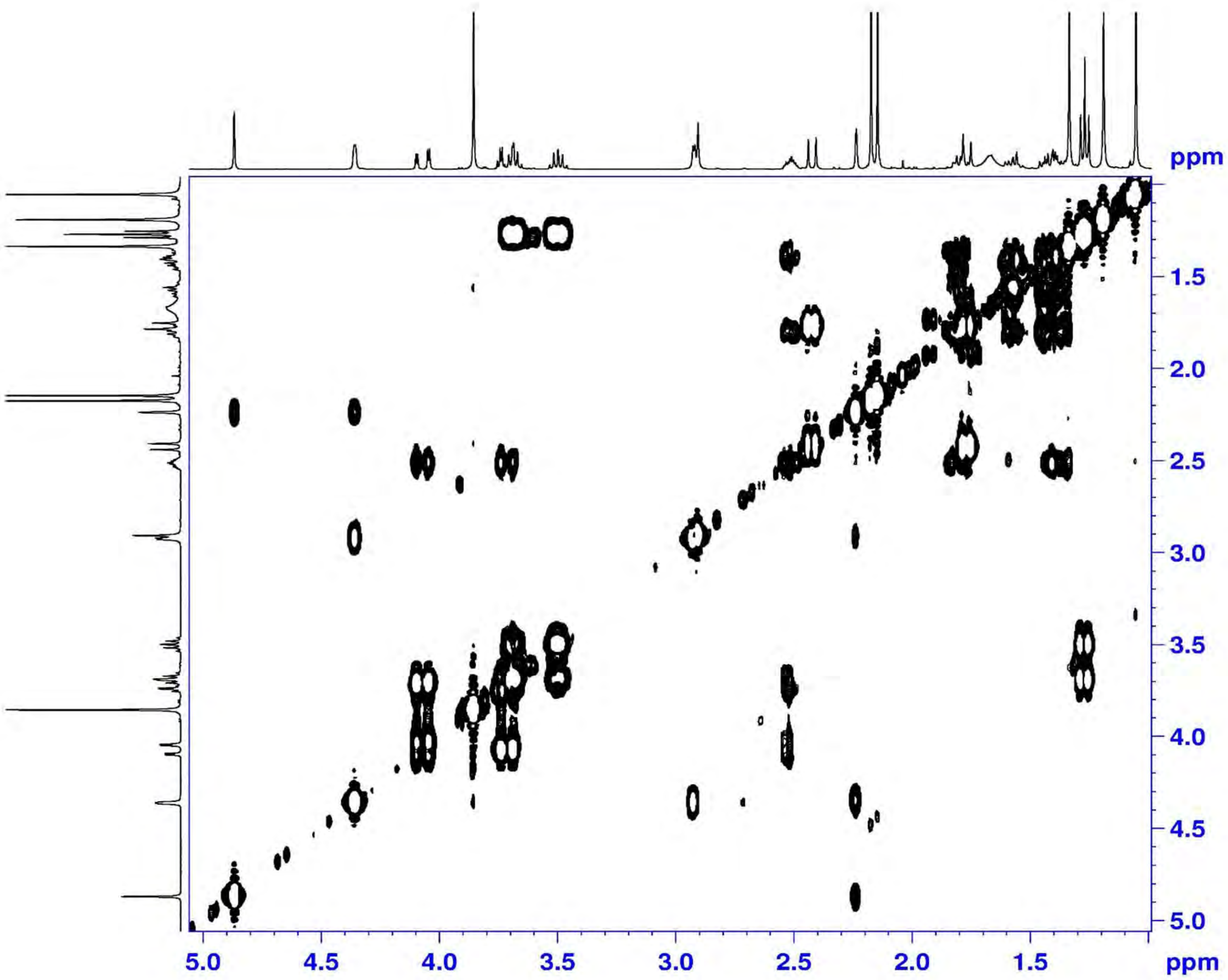

^{1}H-^{1}H COSY (400 MHz) spectrum of compound **15** in $CDCl_3$

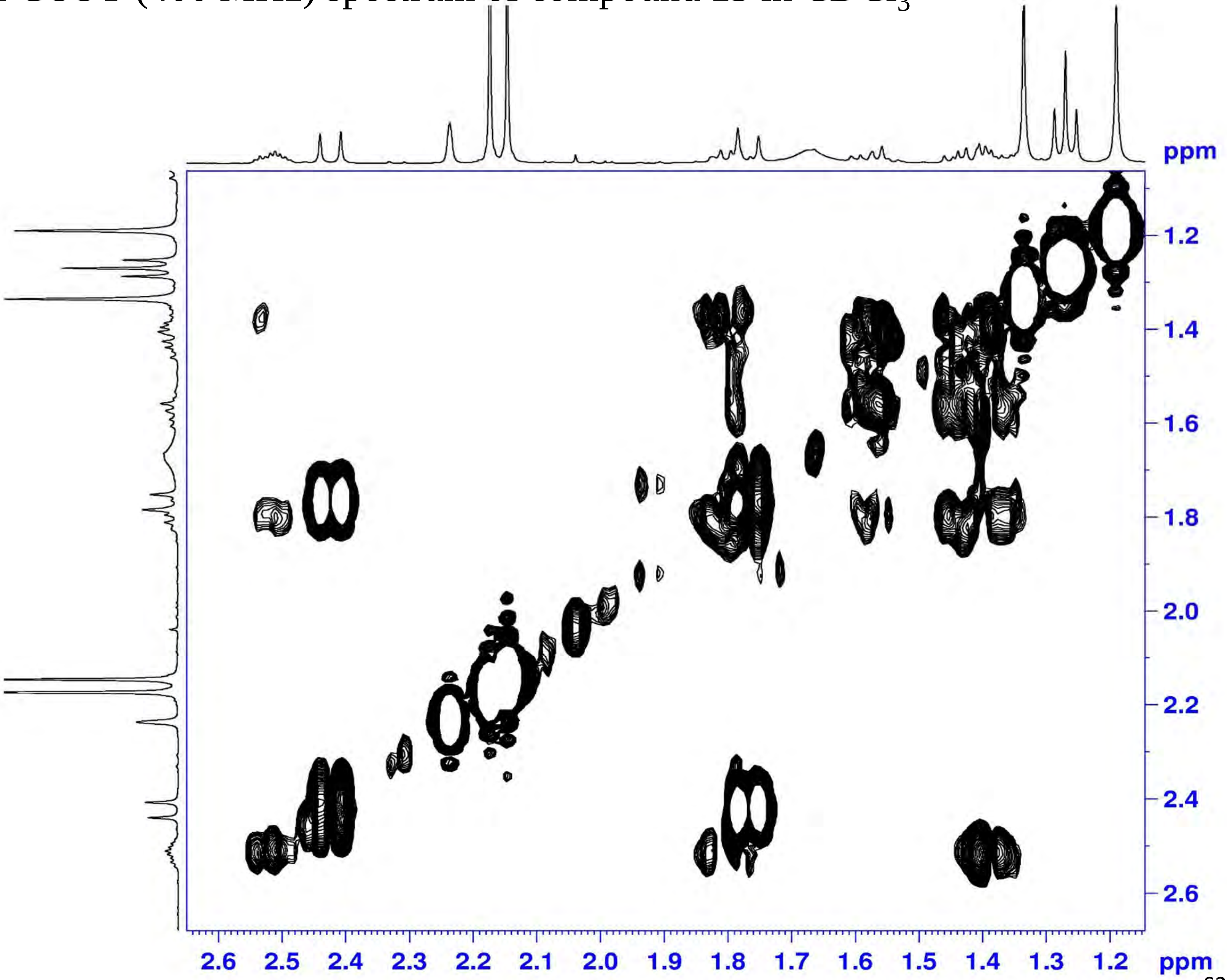

^{1}H-^{1}H COSY (400 MHz) spectrum of compound **15** in $CDCl_3$

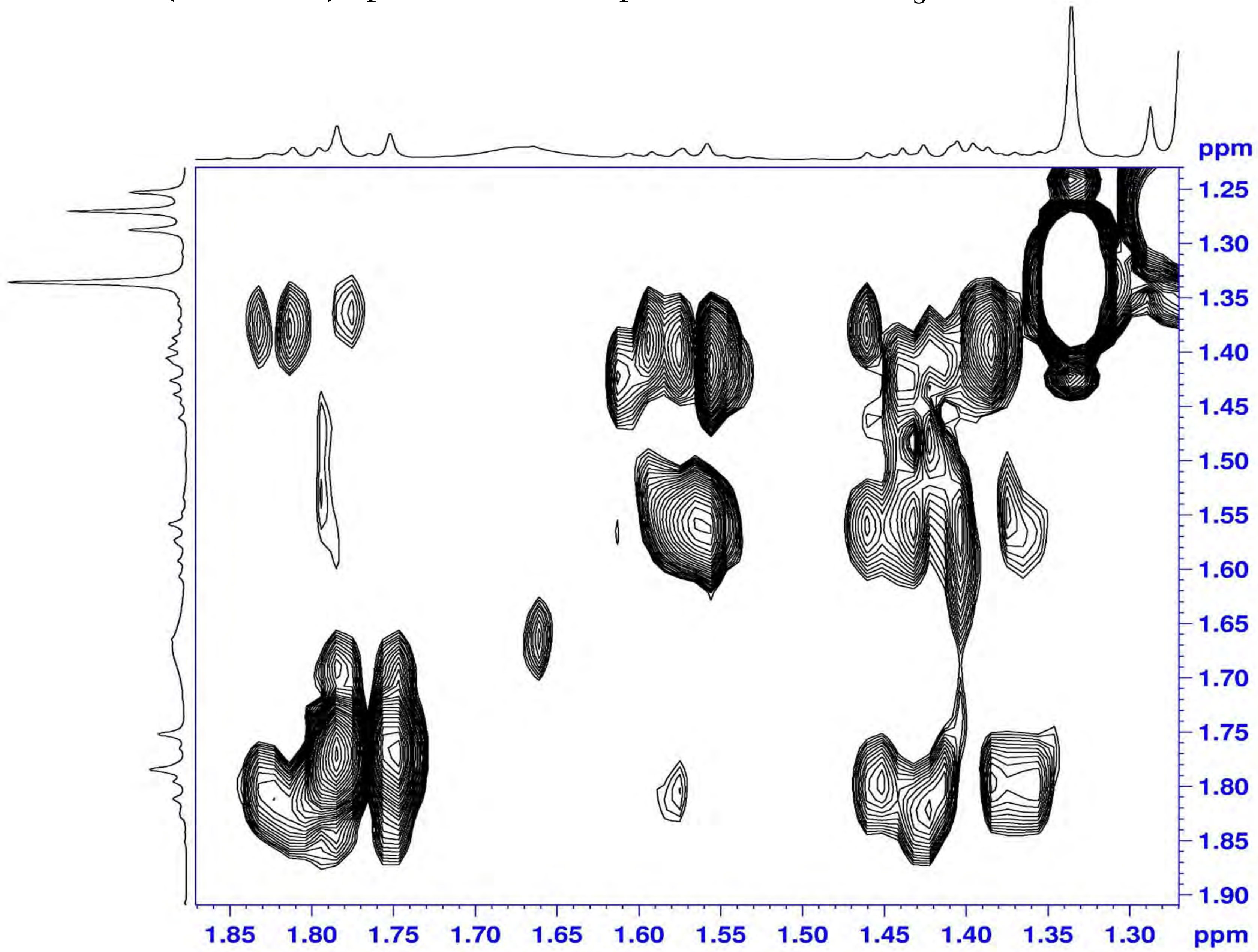

HSQC (400 MHz) spectrum of compound **15** in $CDCl_3$

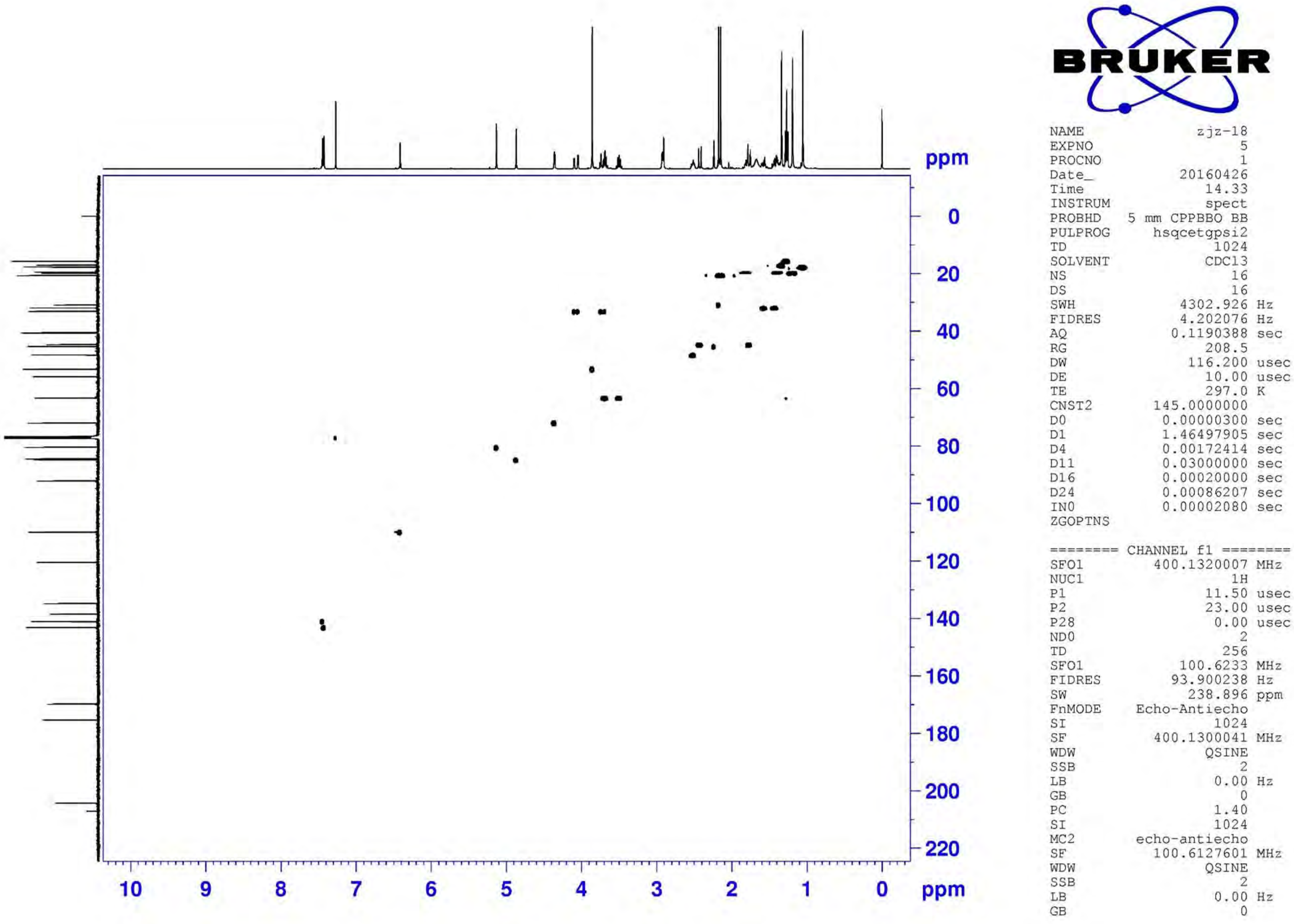

HSQC (400 MHz) spectrum of compound **15** in $CDCl_3$

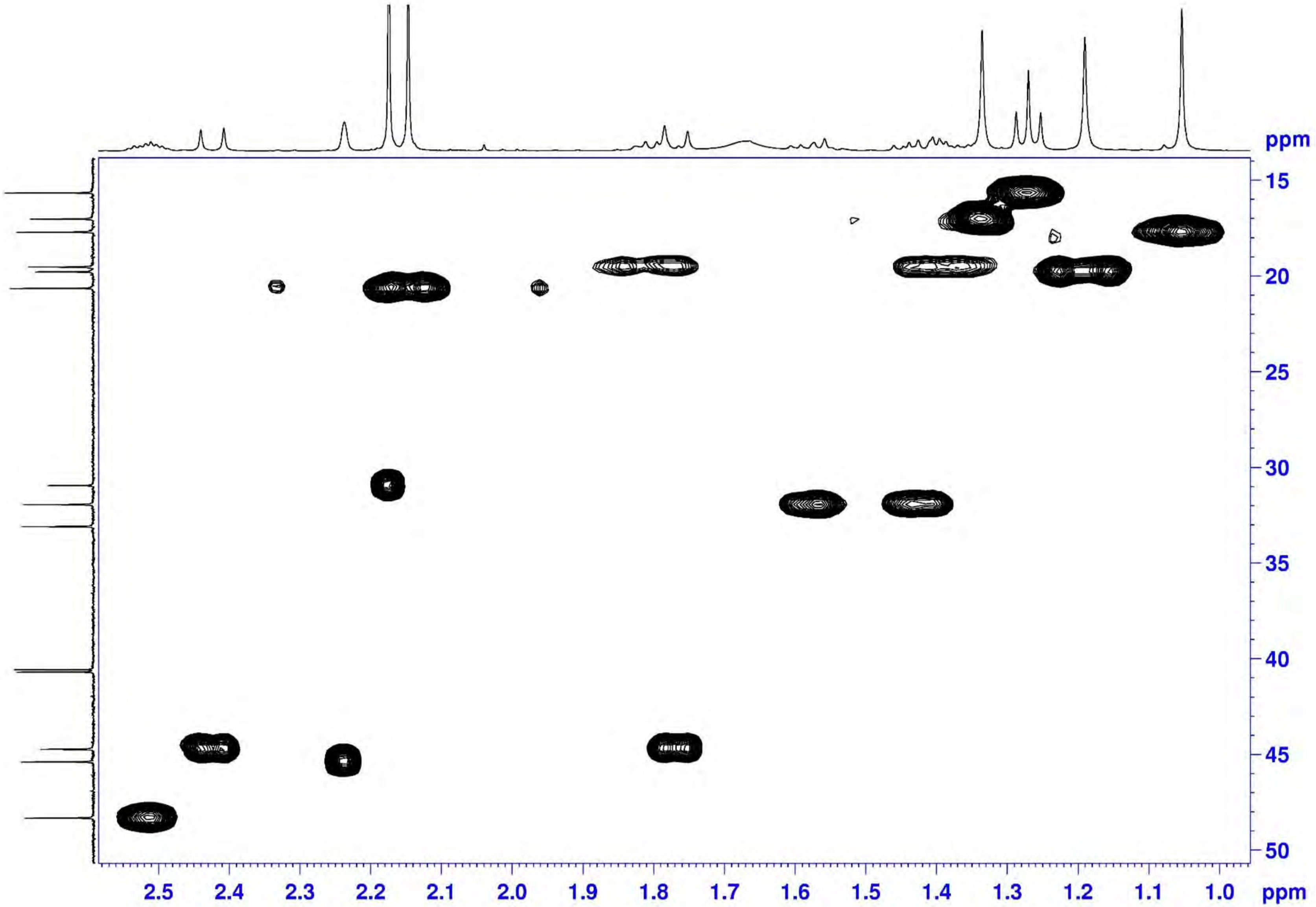

HSQC (400 MHz) spectrum of compound **15** in $CDCl_3$

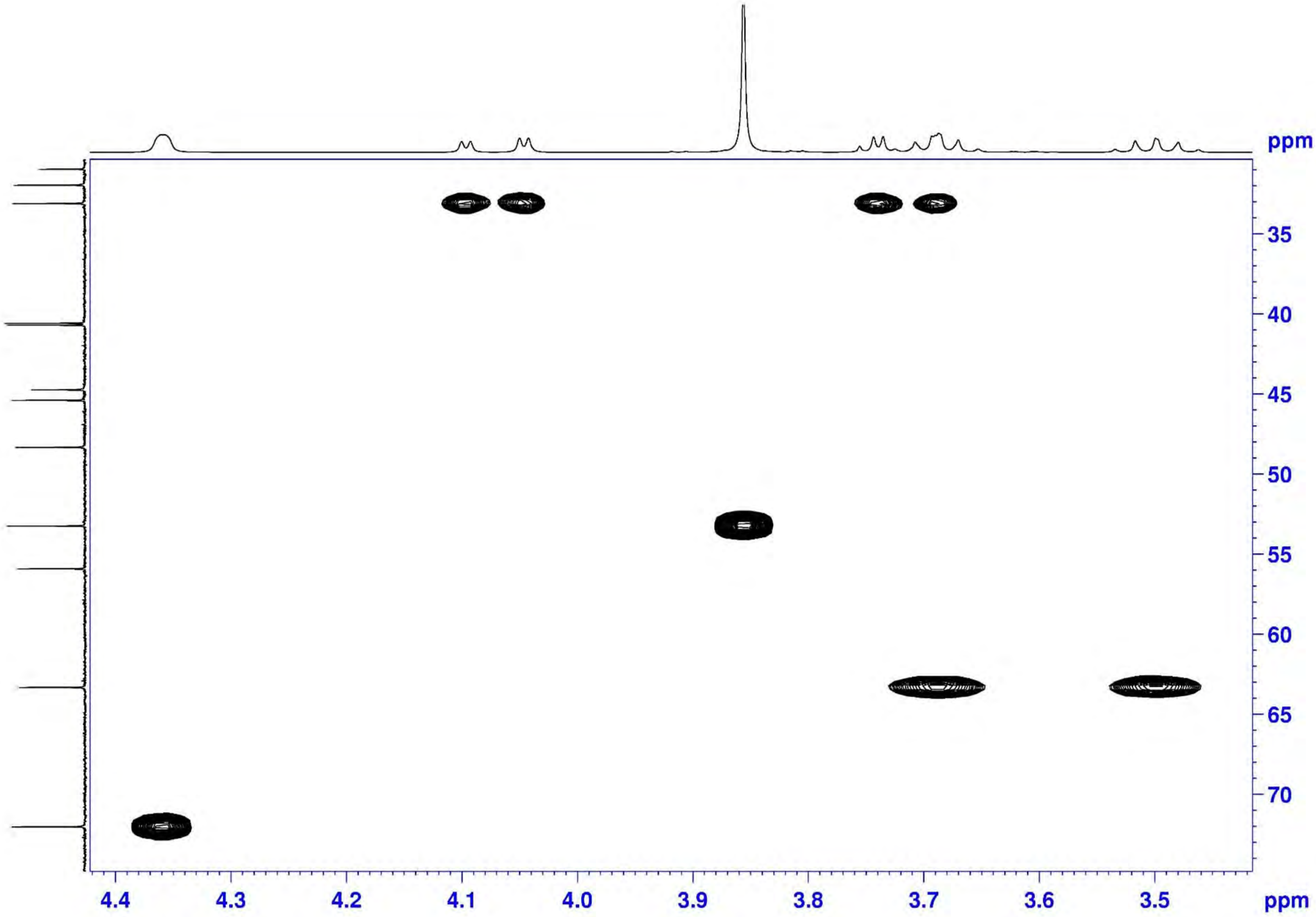

HSQC (400 MHz) spectrum of compound **15** in $CDCl_3$

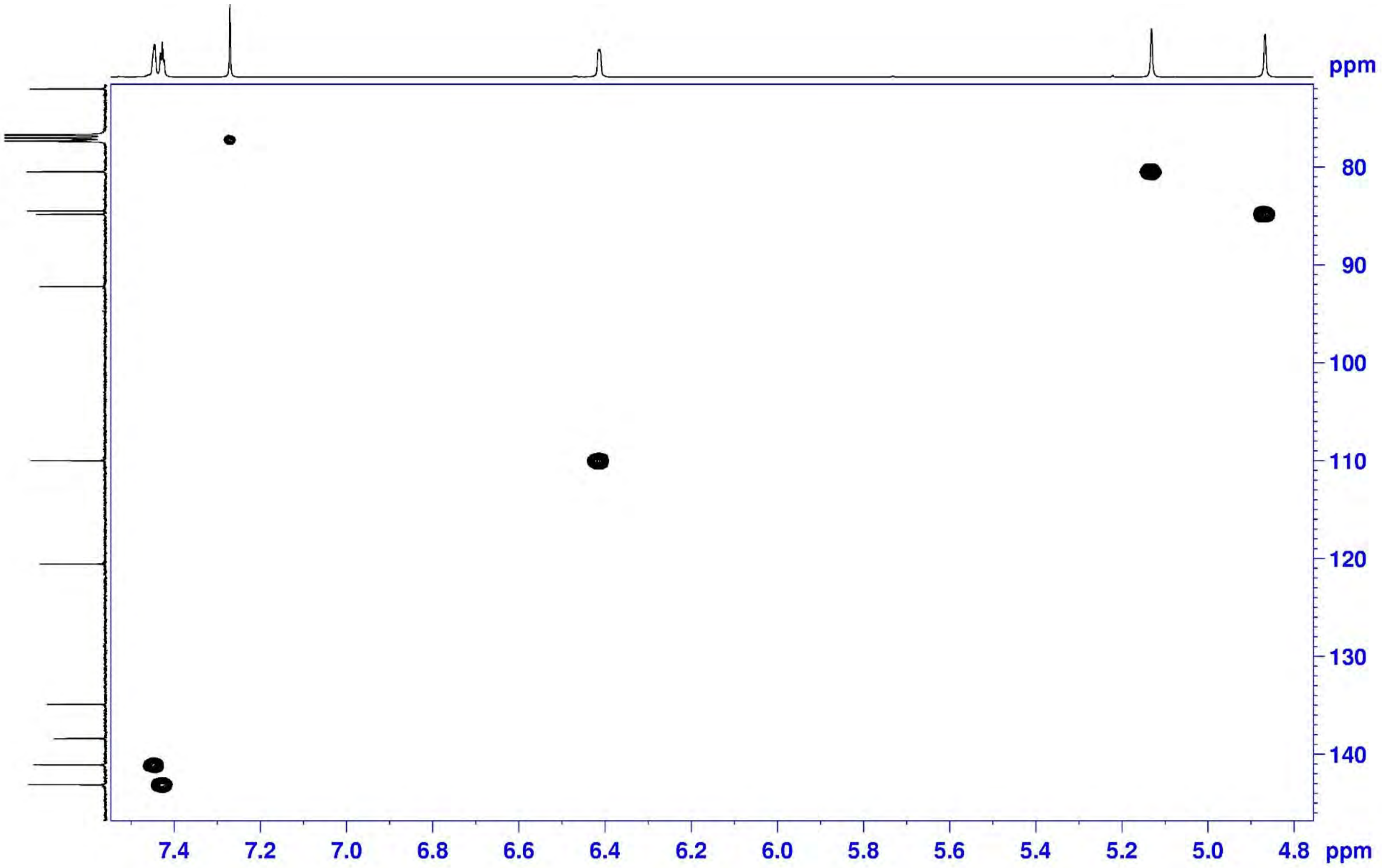

HMBC (400 MHz) spectrum of compound **15** in $CDCl_3$

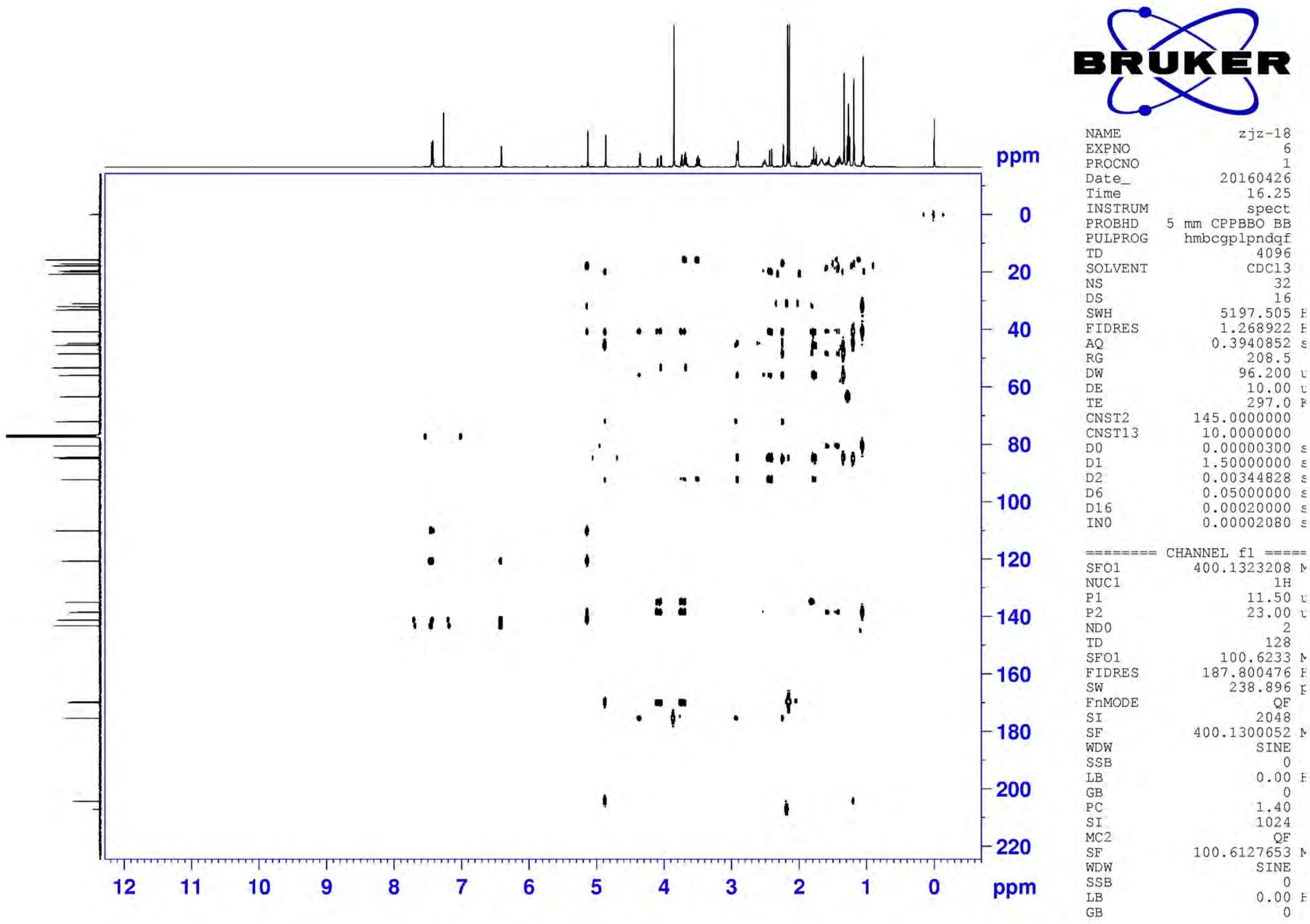

HMBC (400 MHz) spectrum of compound **15** in $CDCl_3$

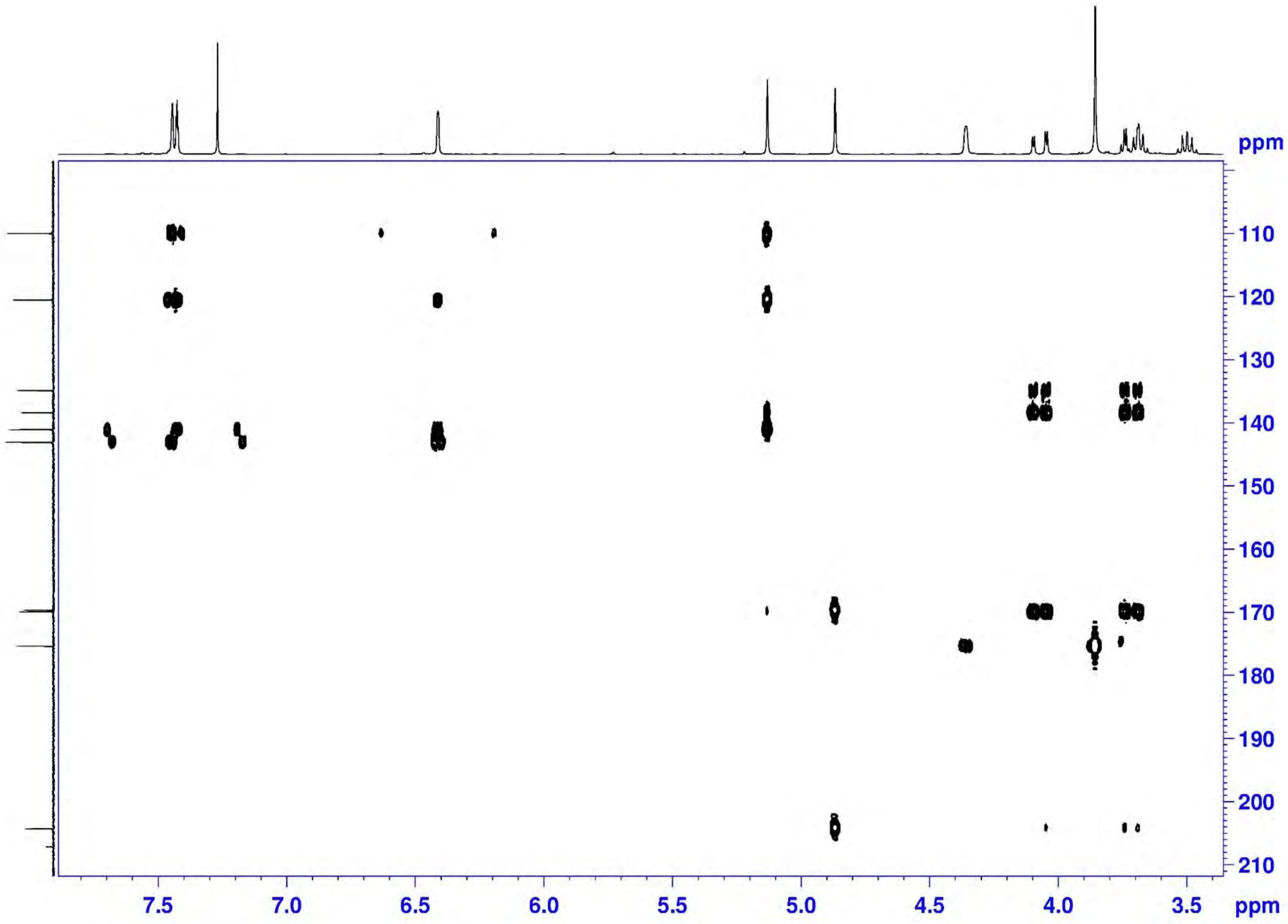

HMBC (400 MHz) spectrum of compound **15** in $CDCl_3$

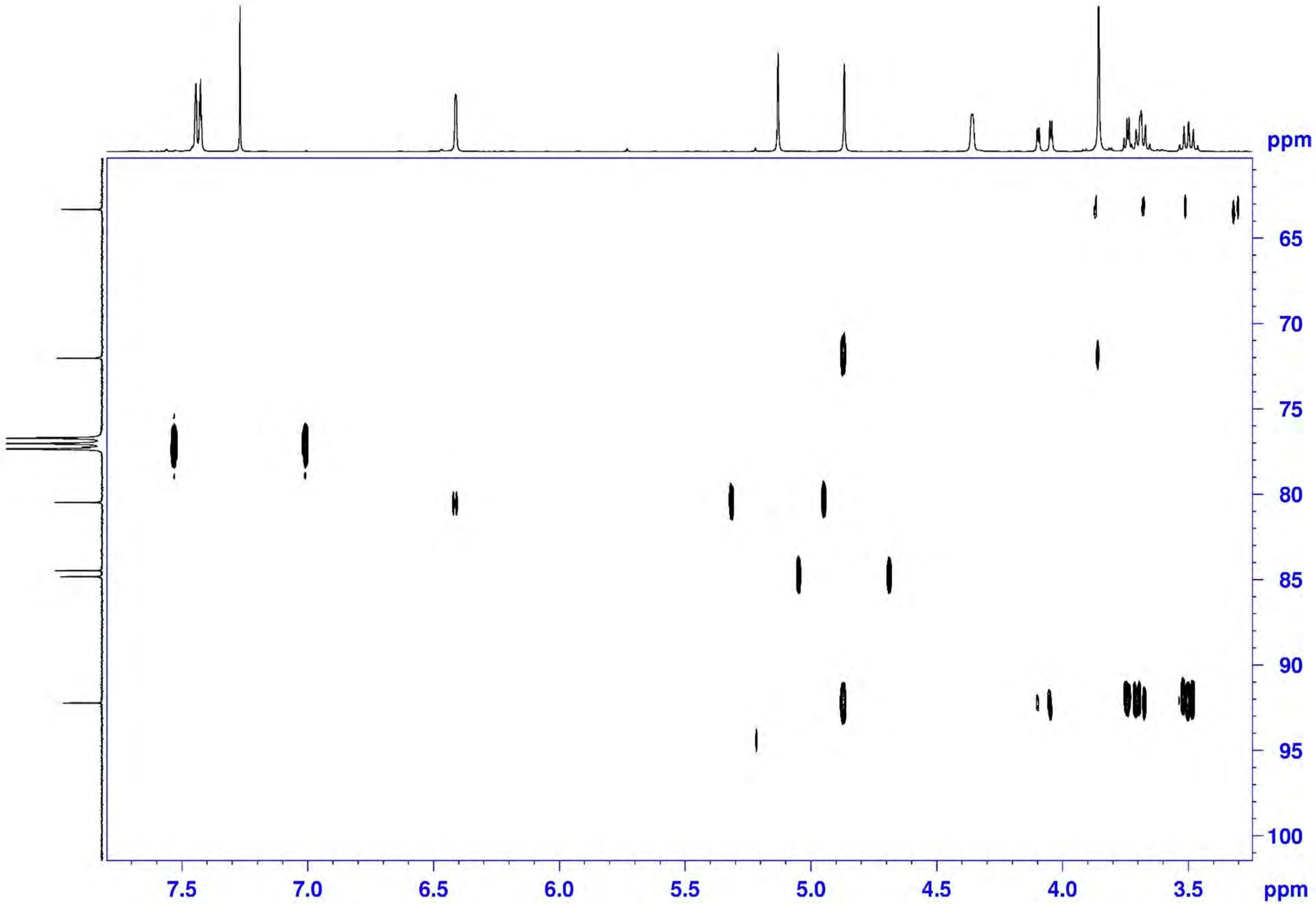

HMBC (400 MHz) spectrum of compound **15** in $CDCl_3$

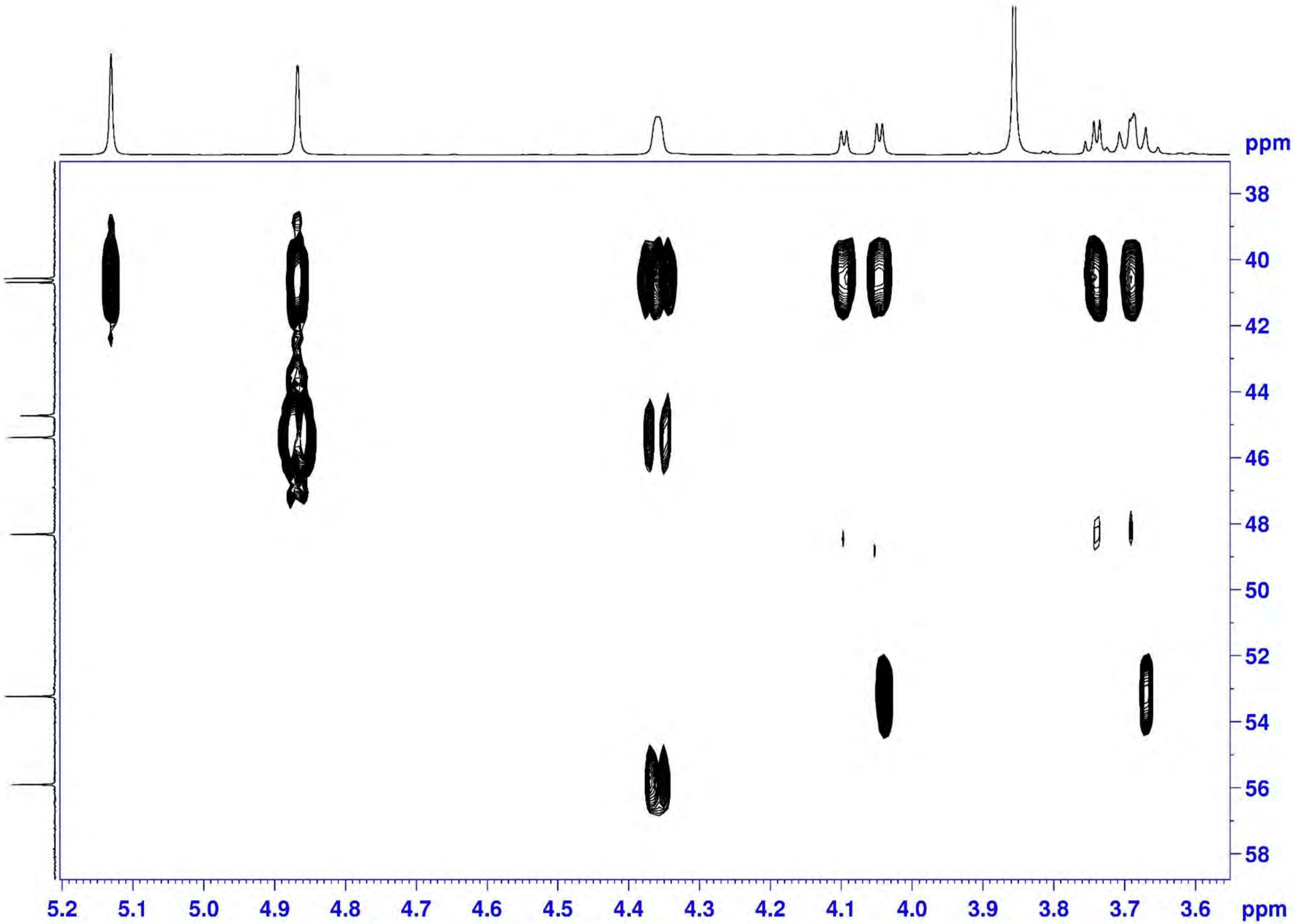

HMBC (400 MHz) spectrum of compound **15** in $CDCl_3$

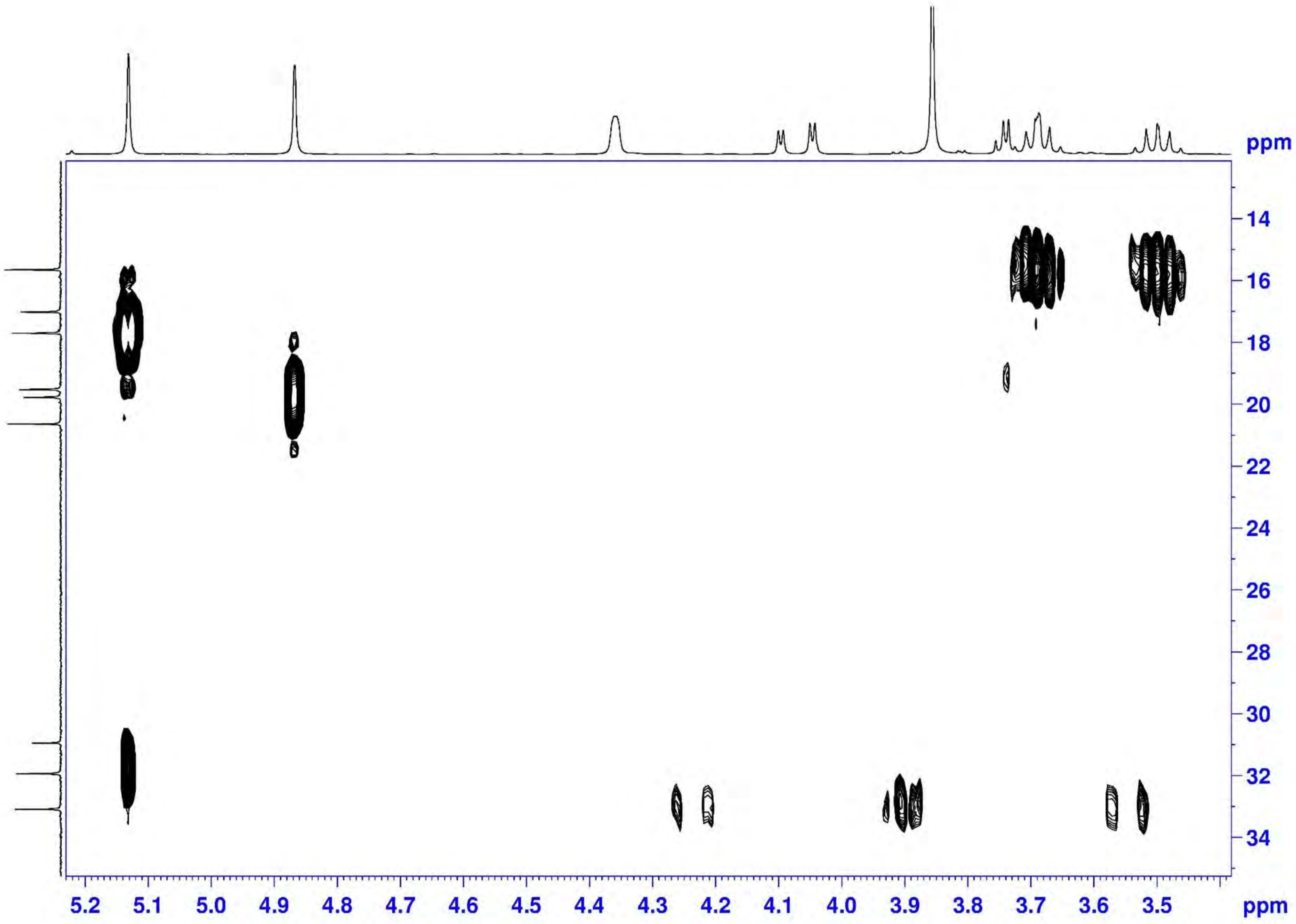

HMBC (400 MHz) spectrum of compound **15** in $CDCl_3$

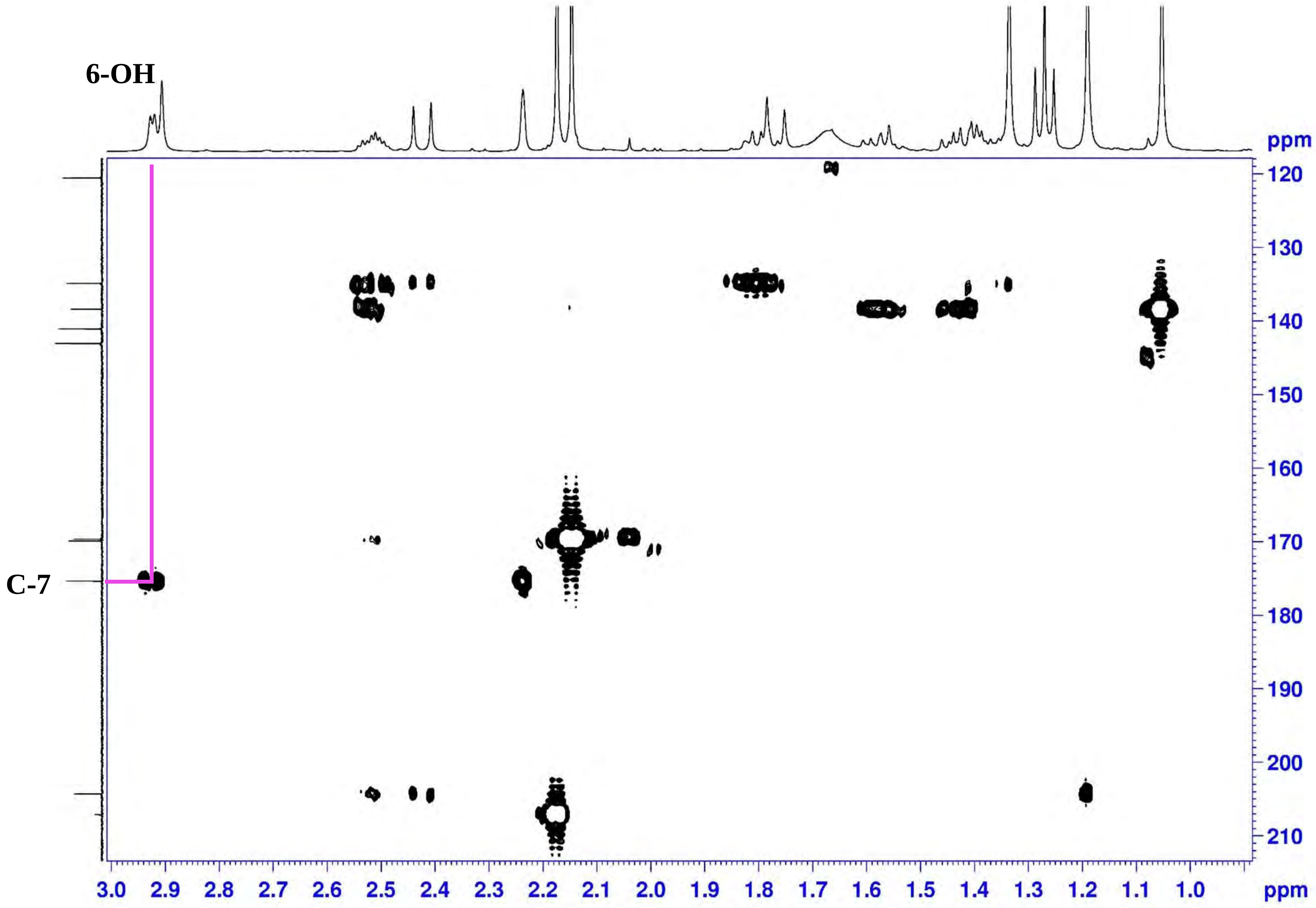

HMBC (400 MHz) spectrum of compound **15** in $CDCl_3$

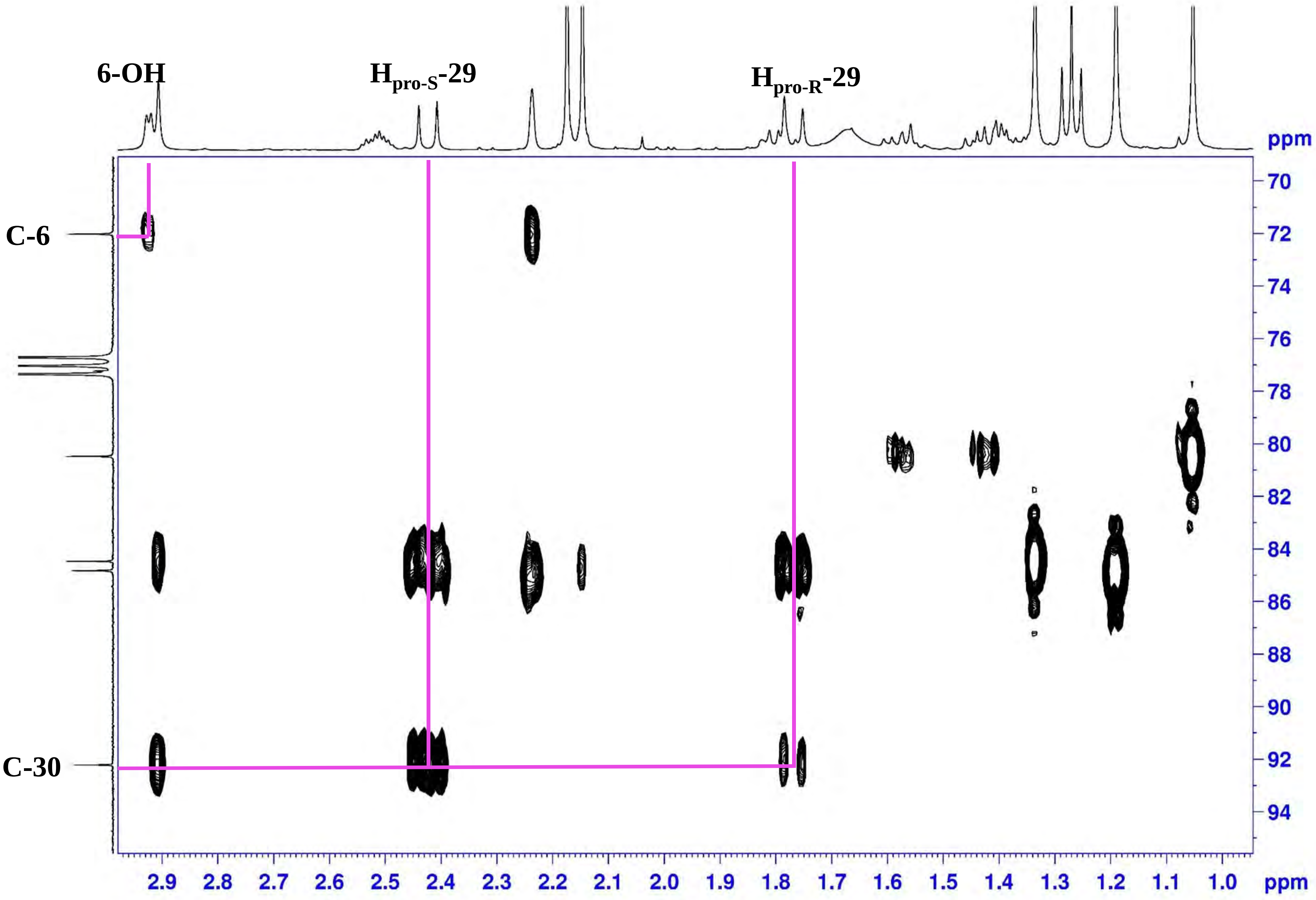

HMBC (400 MHz) spectrum of compound **15** in $CDCl_3$

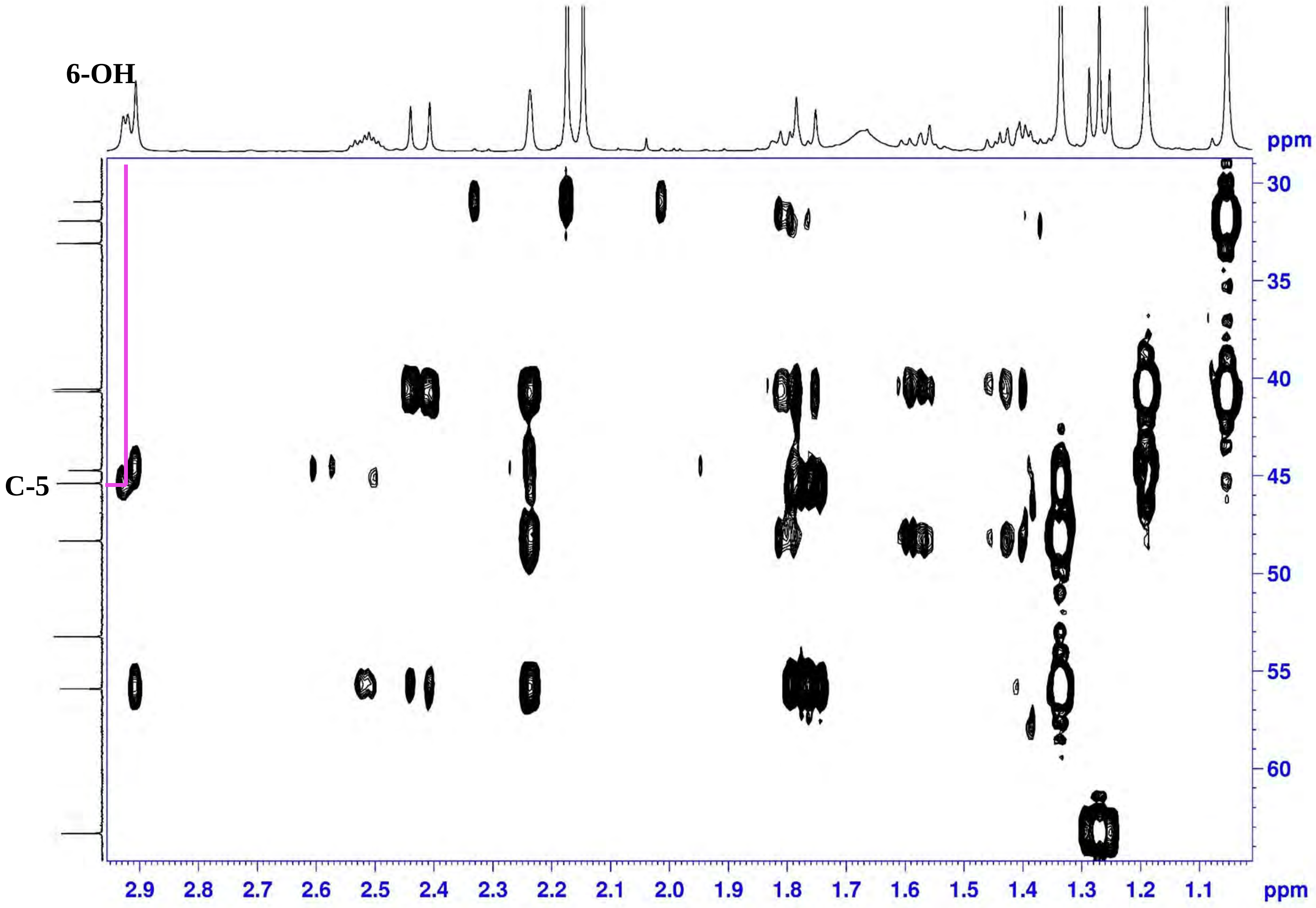

HMBC (400 MHz) spectrum of compound **15** in $CDCl_3$

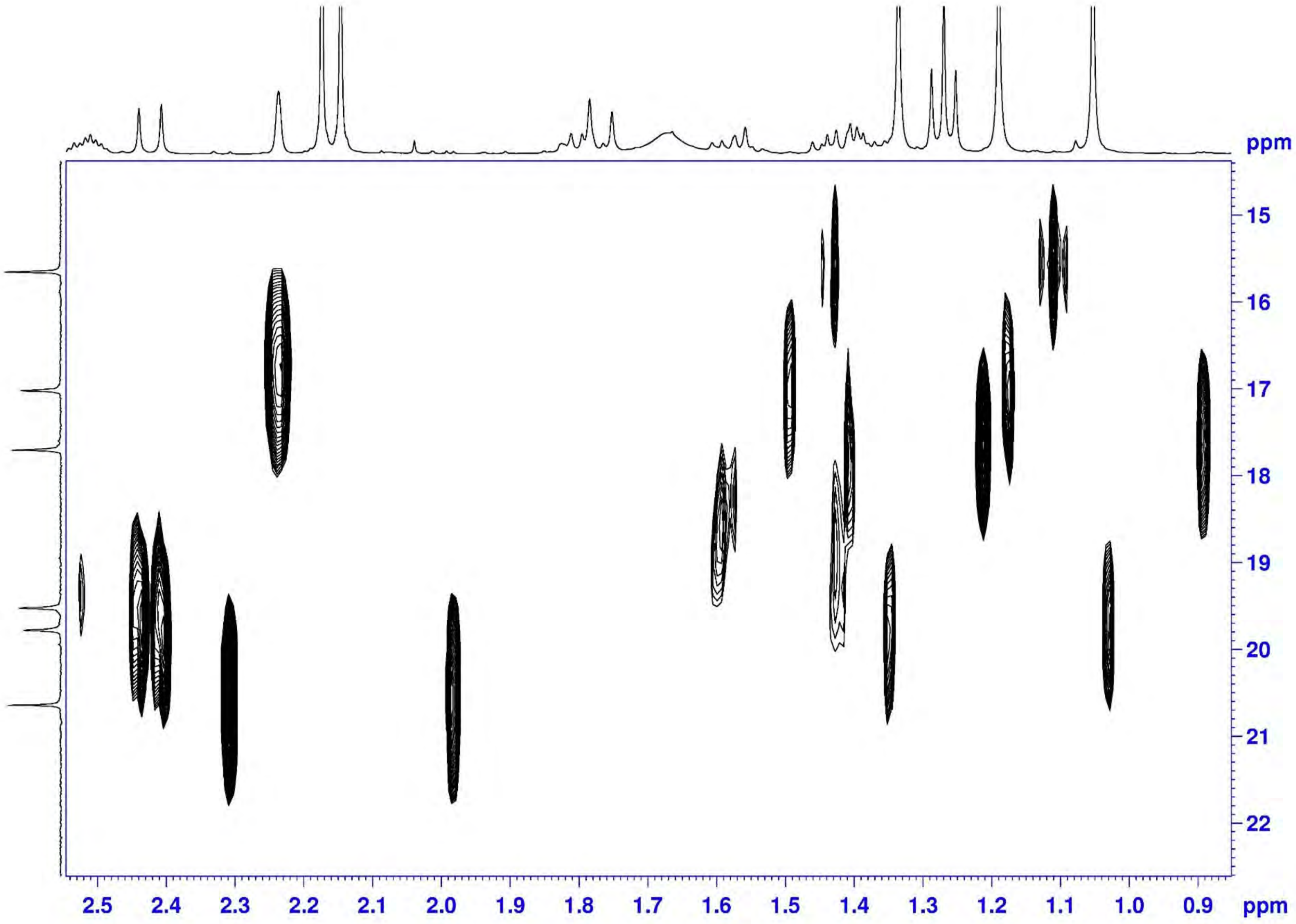

NOESY (400 MHz) spectrum of compound **15** in $CDCl_3$

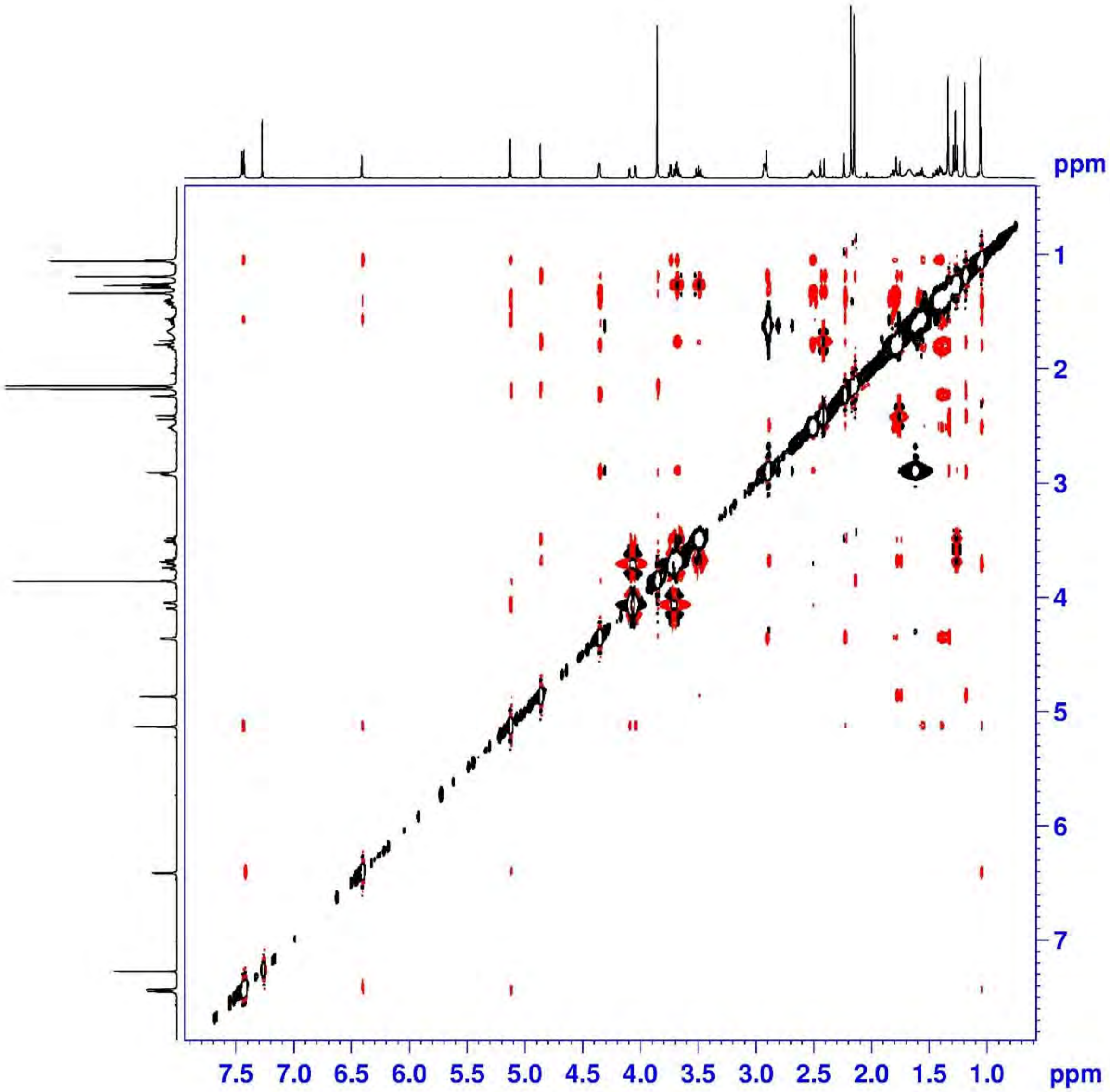

NAME	zjz-18	
EXPNO	7	
PROCNO	1	
Date_	20160517	
Time	17.14	
INSTRUM	spect	
PROBHD	5 mm CPPBBO BB	
PULPROG	noesygpphpp	
TD	2048	
SOLVENT	CDCl3	
NS	16	
DS	32	
SWH	4000.000	Hz
FIDRES	1.953125	Hz
AQ	0.2560500	sec
RG	208.5	
DW	125.000	usec
DE	10.00	usec
TE	297.0	K
D0	0.00011036	sec
D1	1.99385595	sec
D8	0.30000001	sec
D11	0.03000000	sec
D12	0.00002000	sec
D16	0.00020000	sec
IN0	0.00025000	sec
======== CHANNEL f1 ========		
SFO1	400.1318006	MHz
NUC1	1H	
P1	11.50	usec
P2	23.00	usec
P17	2500.00	usec
ND0	1	
TD	256	
SFO1	400.1318	MHz
FIDRES	15.625000	Hz
SW	9.997	ppm
FnMODE	States-TPPI	
SI	1024	
SF	400.1300105	MHz
WDW	QSINE	
SSB	2	
LB	0.00	Hz
GB	0	
PC	1.00	
SI	1024	
MC2	States-TPPI	
SF	400.1300098	MHz
WDW	QSINE	
SSB	2	
LB	0.00	Hz
GB	0	

NOESY (400 MHz) spectrum of compound **15** in $CDCl_3$

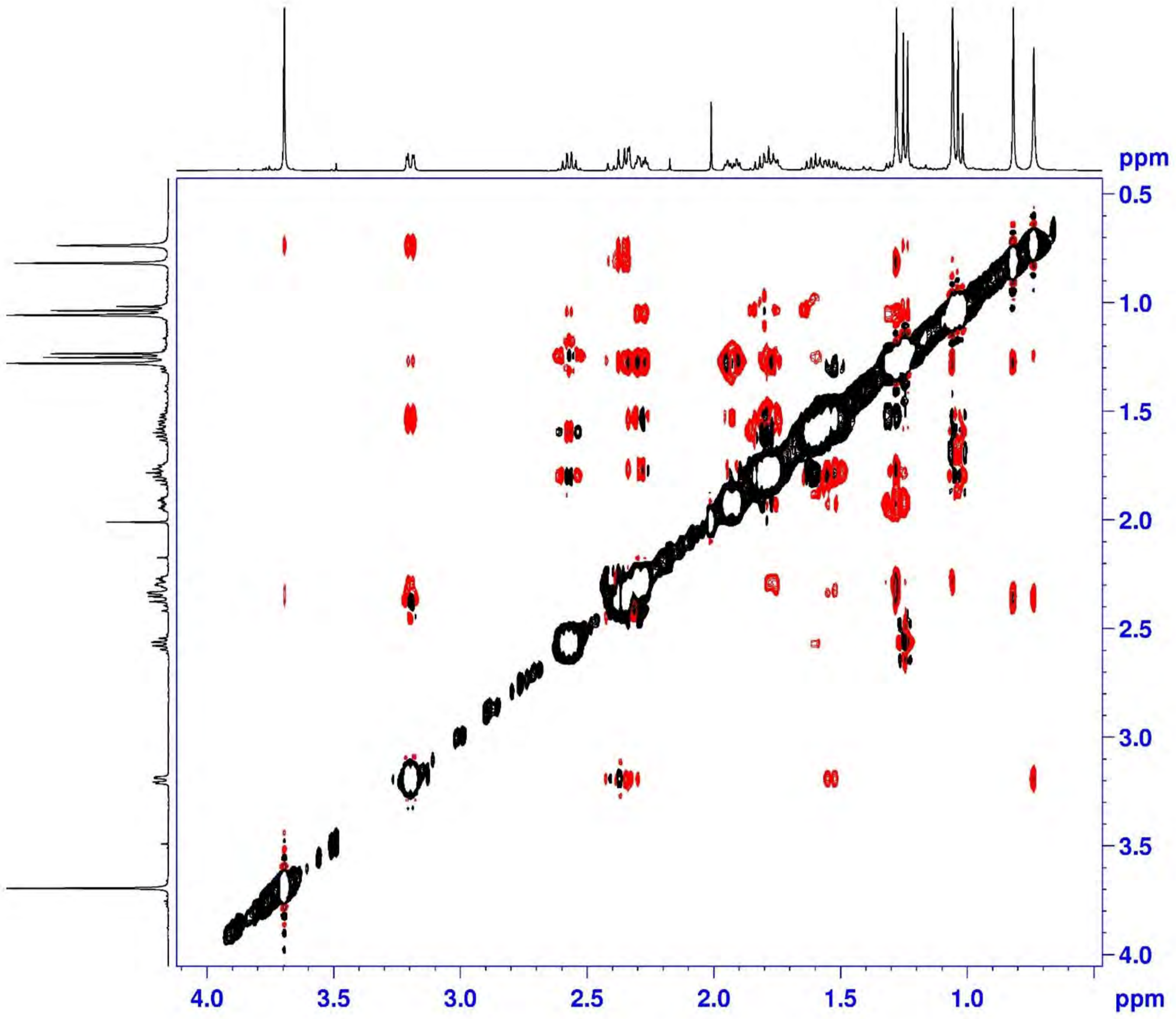

NOESY (400 MHz) spectrum of compound **15** in $CDCl_3$

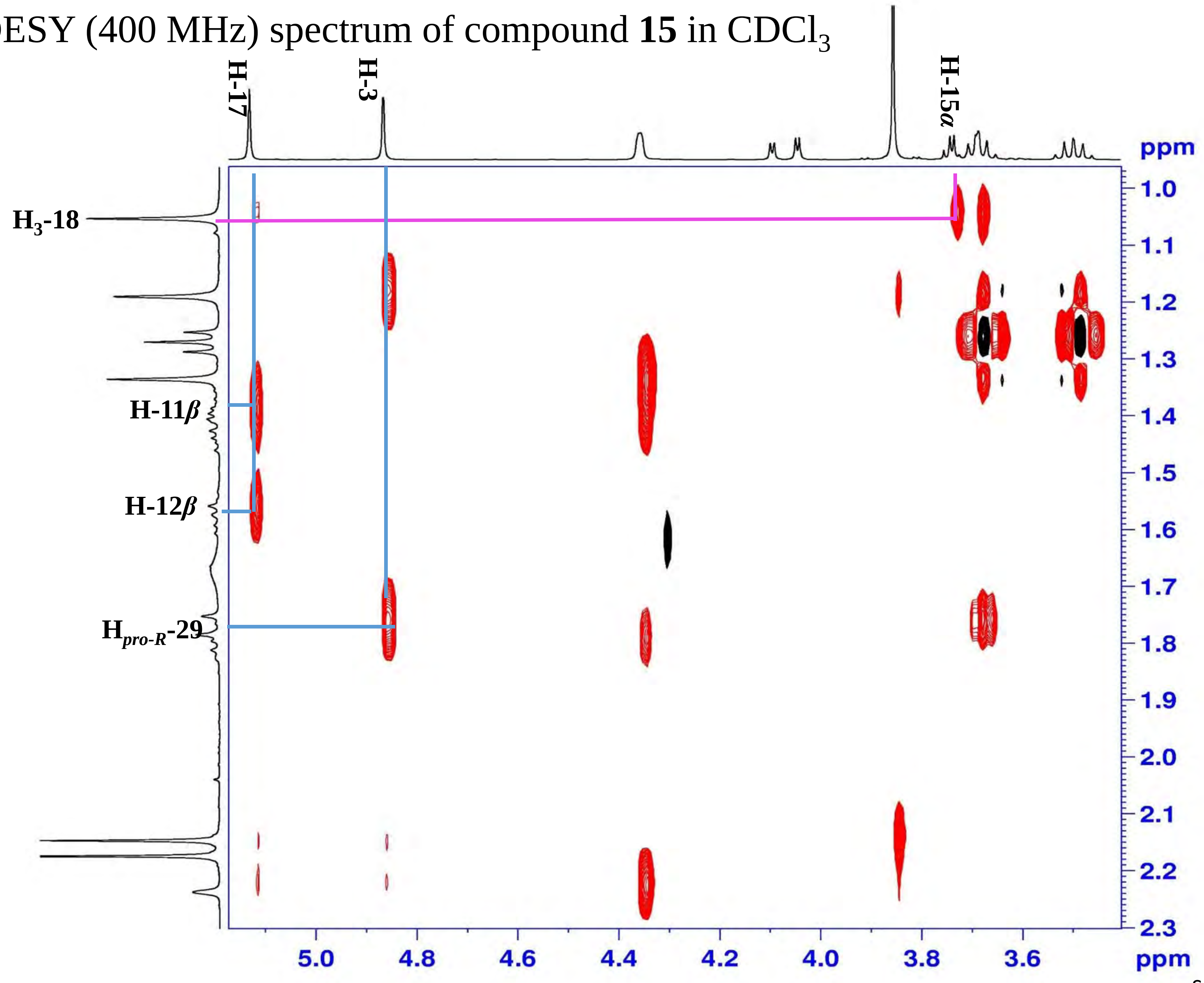

NOESY (400 MHz) spectrum of compound **15** in $CDCl_3$

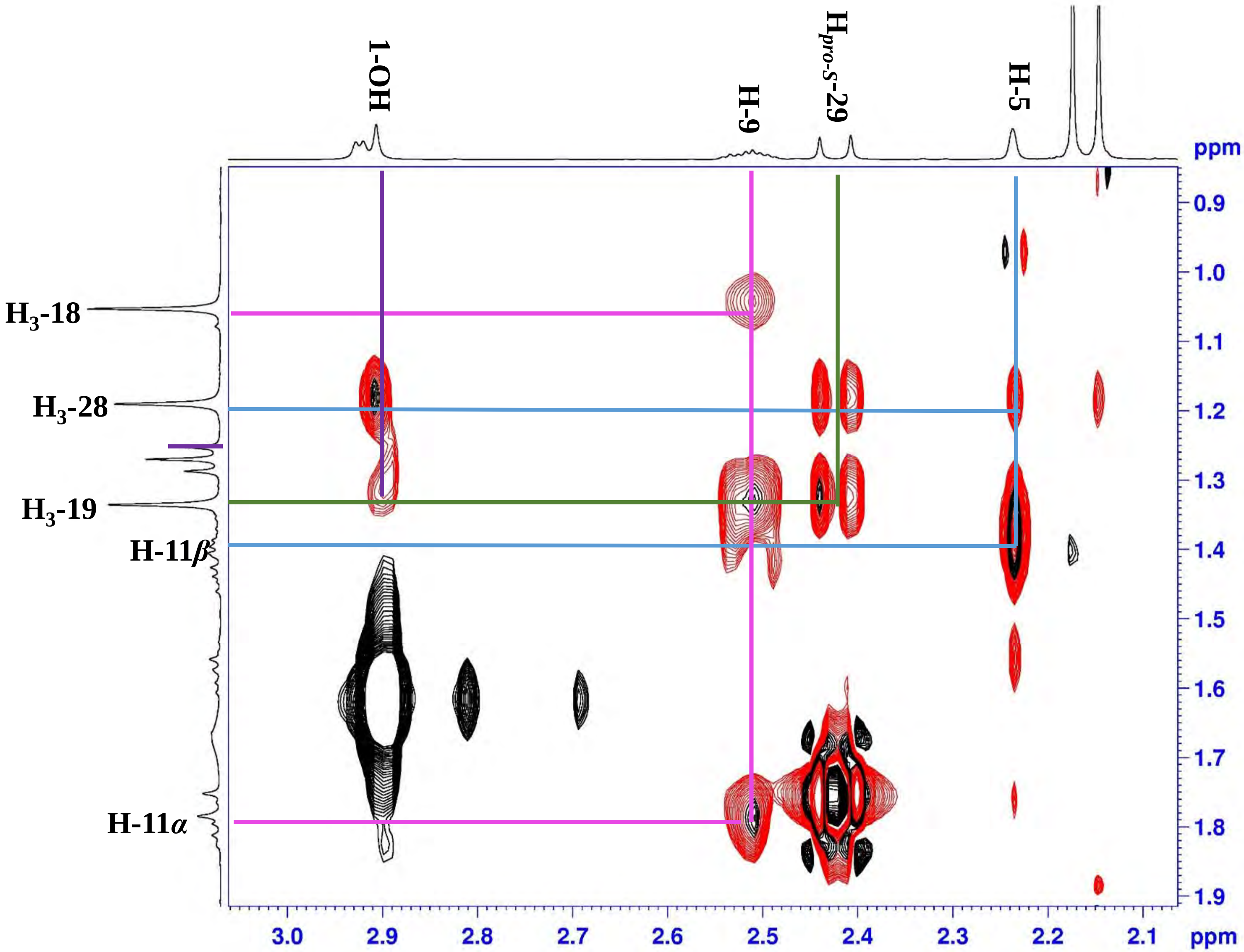

NOESY (400 MHz) spectrum of compound **15** in $CDCl_3$

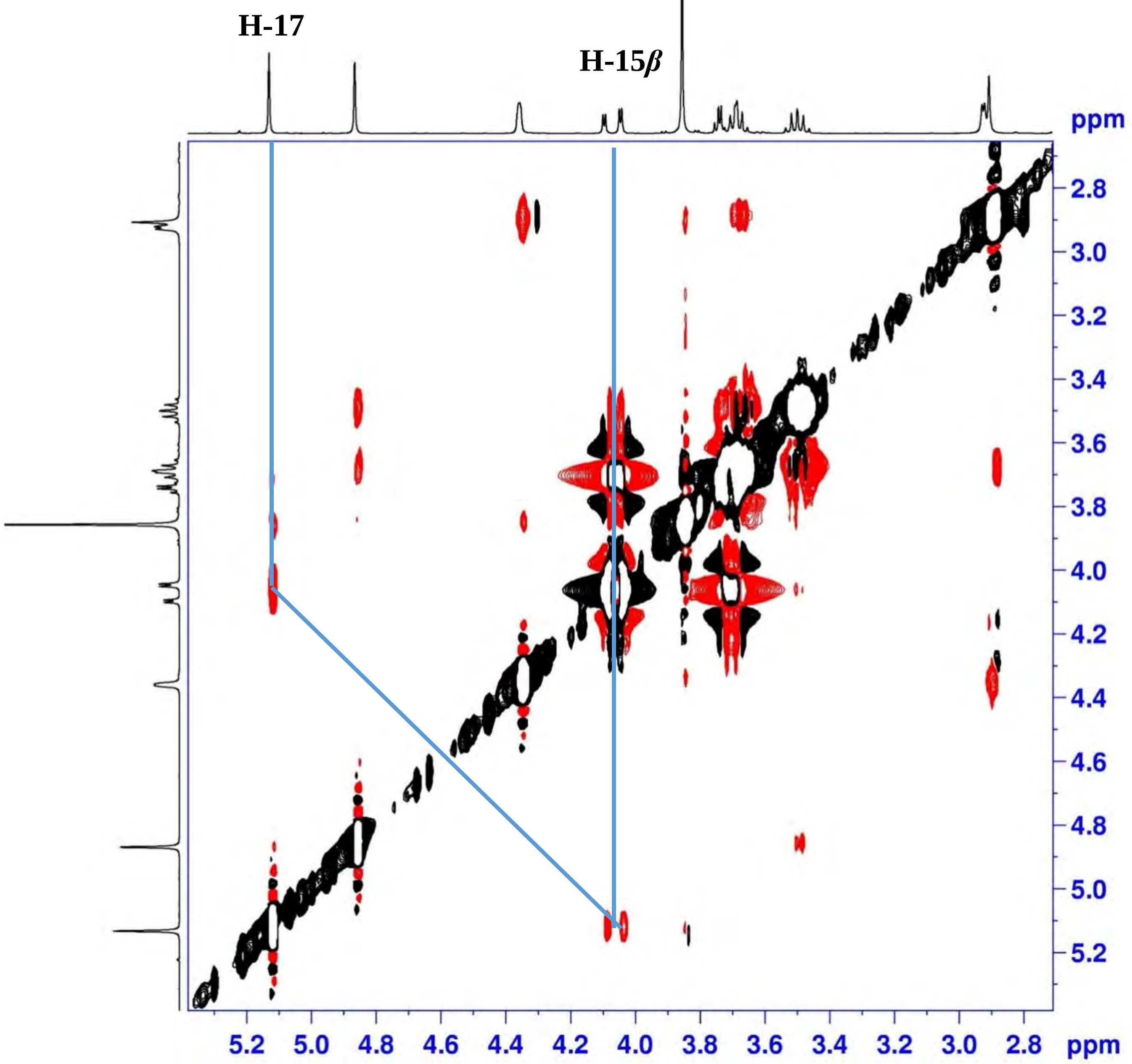

HRESIMS for compound **16**

Elemental Composition Report

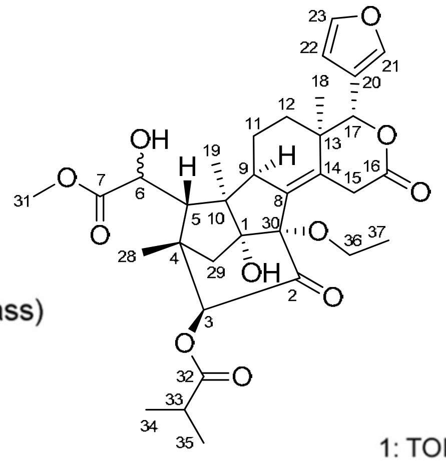

Single Mass Analysis

Tolerance = 10.0 mDa / DBE: min = -1.0, max = 50.0
Element prediction: Off
Number of isotope peaks used for i-FIT = 3

Monoisotopic Mass, Even Electron Ions
185 formula(e) evaluated with 5 results within limits (up to 10 closest results for each mass)
Elements Used:
C: 0-100 H: 0-200 O: 0-100
2J2-25
2016103108 175 (1.421)

1: TOF MS ES+
7.76e+005

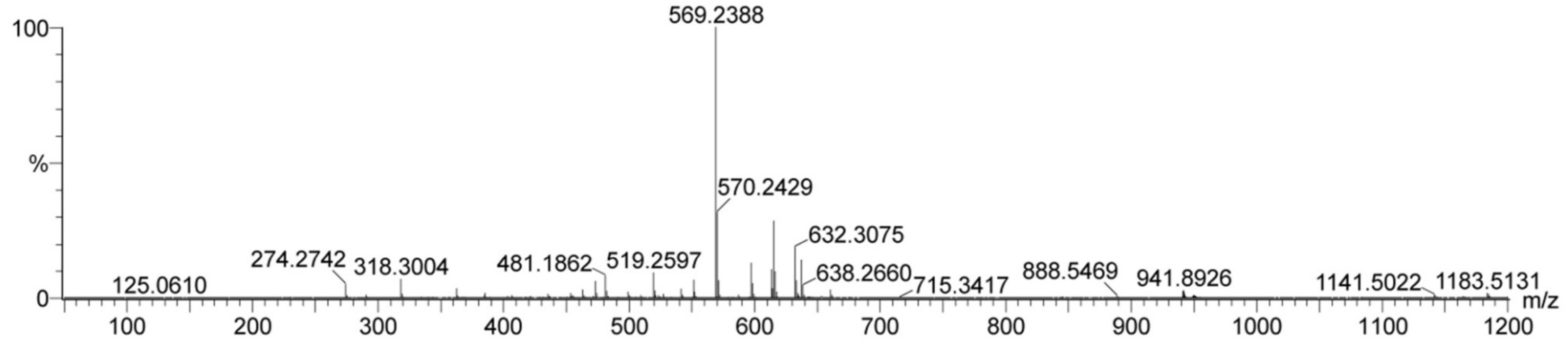

Minimum:				-1.0				
Maximum:		10.0	10.0	50.0				
Mass	Calc. Mass	mDa	PPM	DBE	i-FIT	Norm	Conf(%)	Formula
615.2809	615.2805	0.4	0.7	12.5	316.2	0.135	87.33	C33 H43 O11
	615.2864	-5.5	-8.9	3.5	318.2	2.106	12.17	C26 H47 O16
	615.2747	6.2	10.1	21.5	321.8	5.653	0.35	C40 H39 O6
	615.2712	9.7	15.8	-0.5	322.9	6.763	0.12	C22 H47 O19
	615.2899	-9.0	-14.6	25.5	324.2	8.139	0.03	C44 H39 O3

^{1}H NMR (400 MHz) spectrum of compound **16** in $CDCl_3$

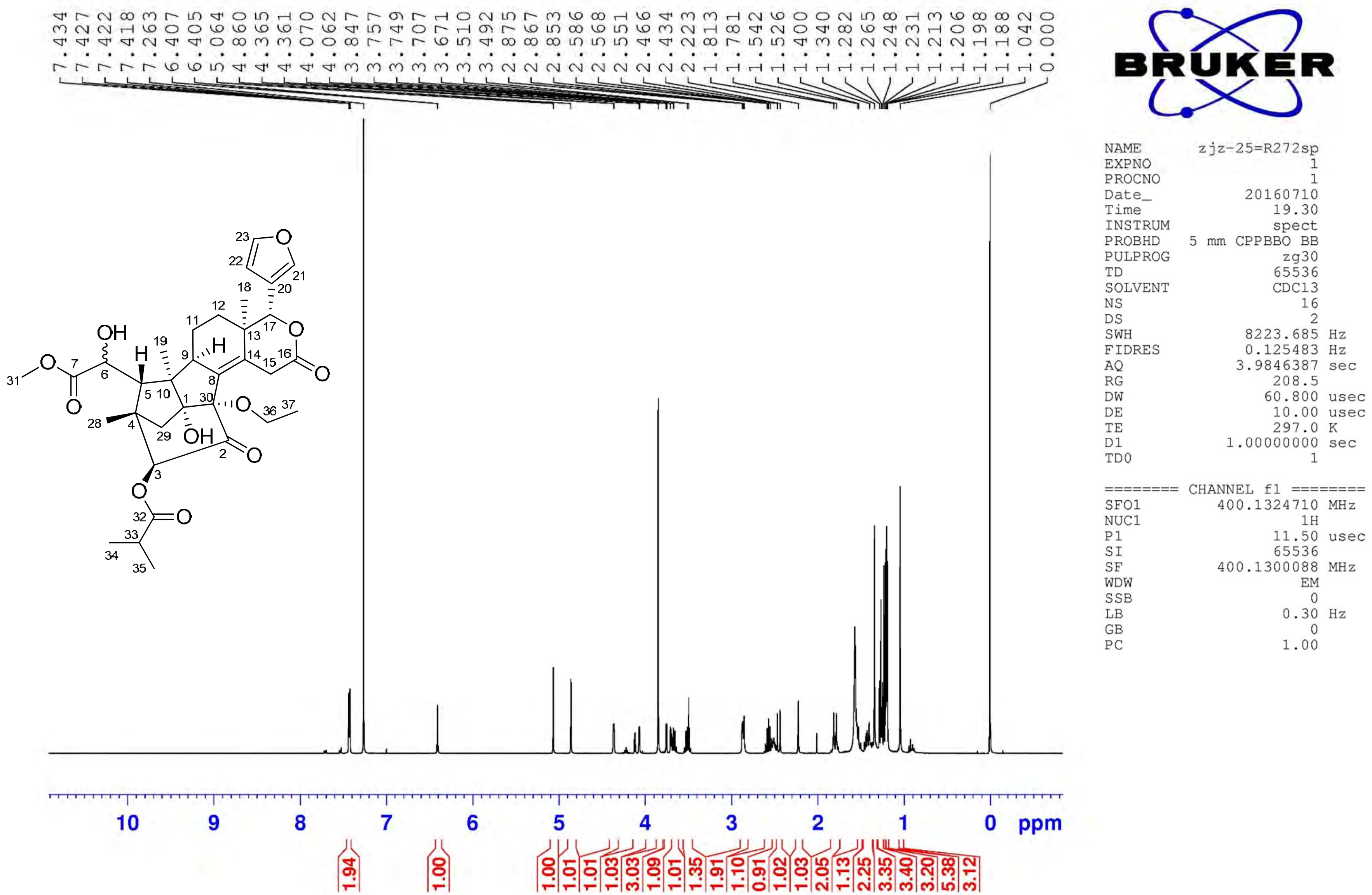

^{1}H NMR (400 MHz) spectrum of compound **16** in $CDCl_3$

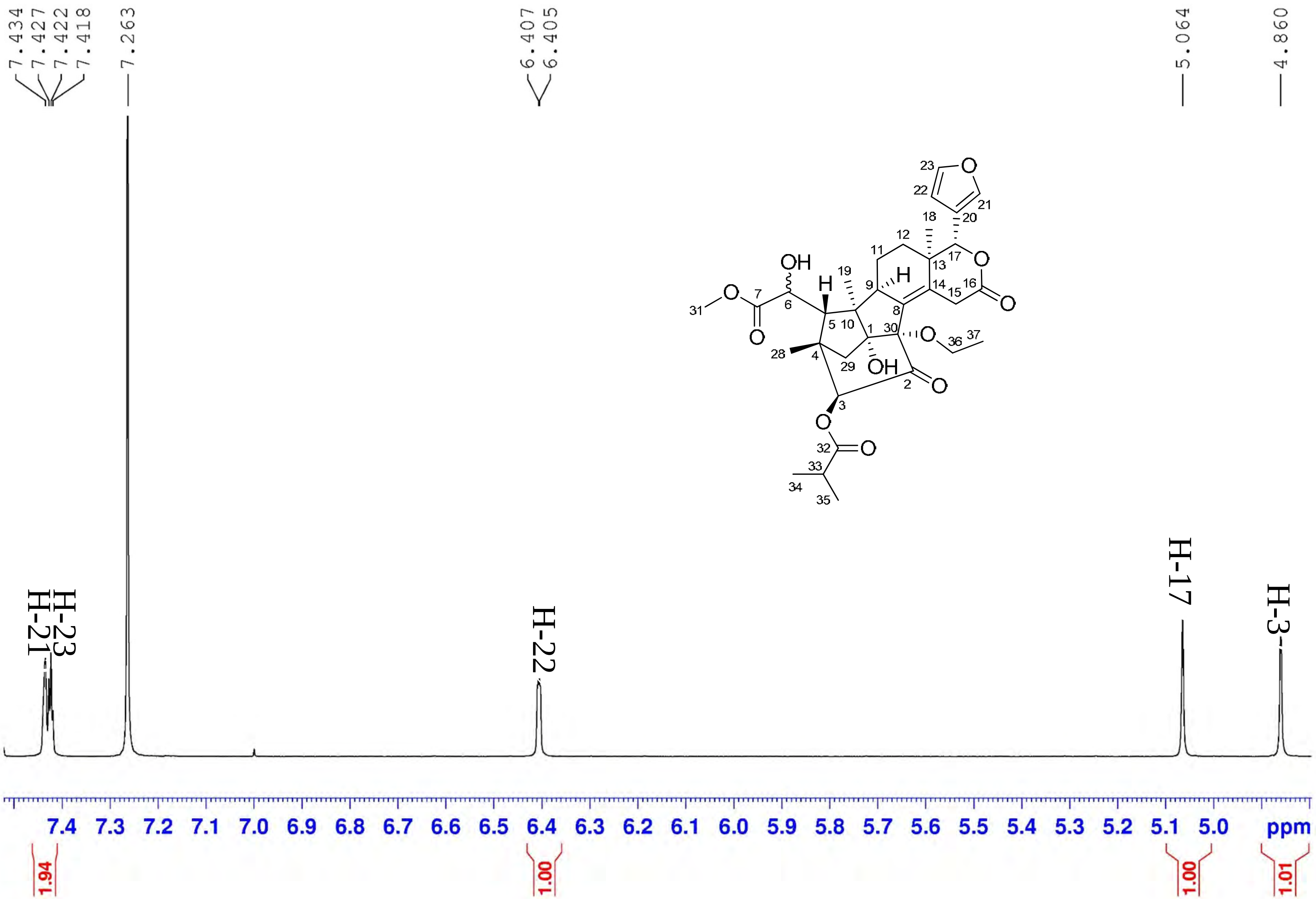

^{1}H NMR (400 MHz) spectrum of compound **16** in $CDCl_3$

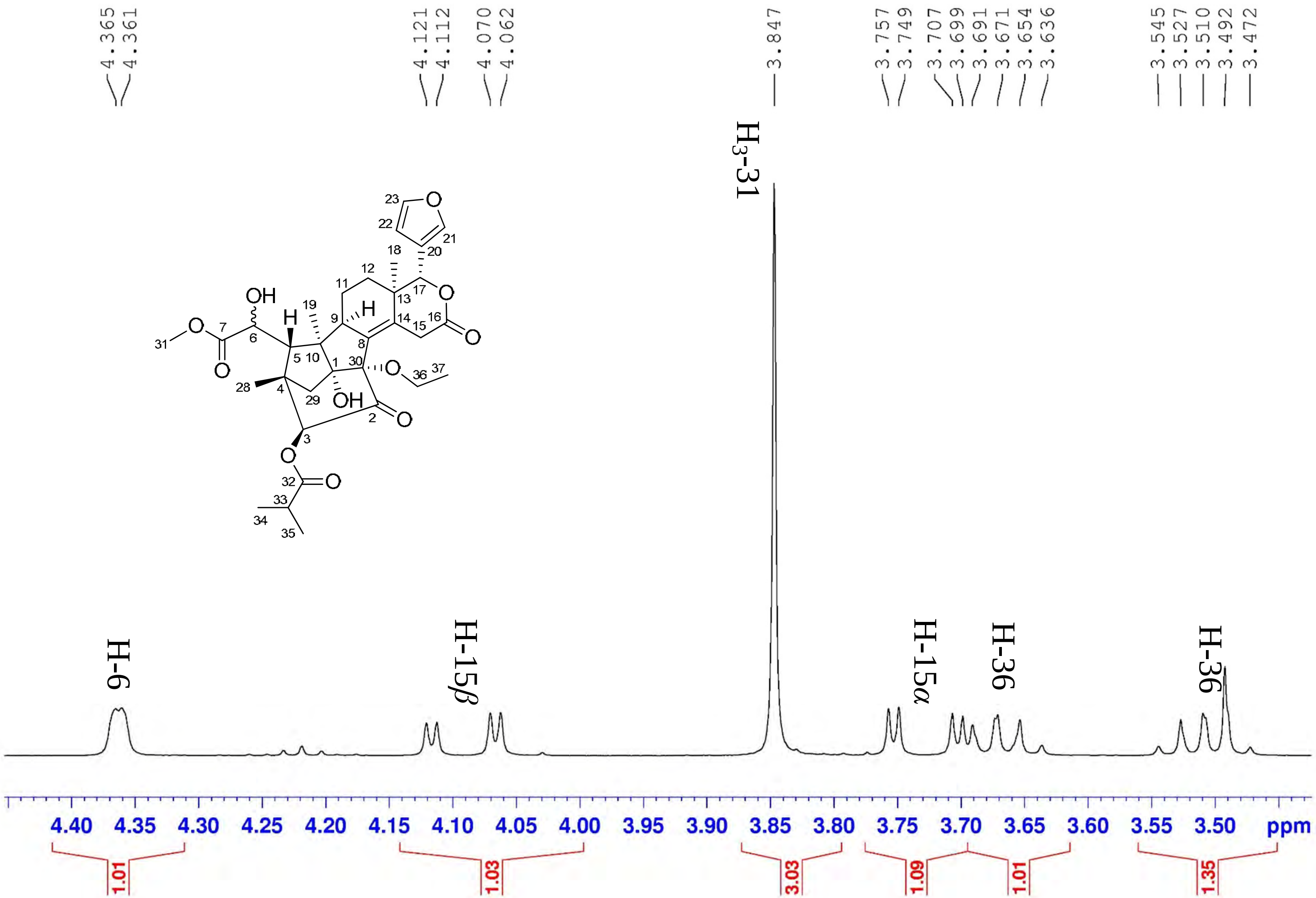

^{1}H NMR (400 MHz) spectrum of compound **16** in $CDCl_3$

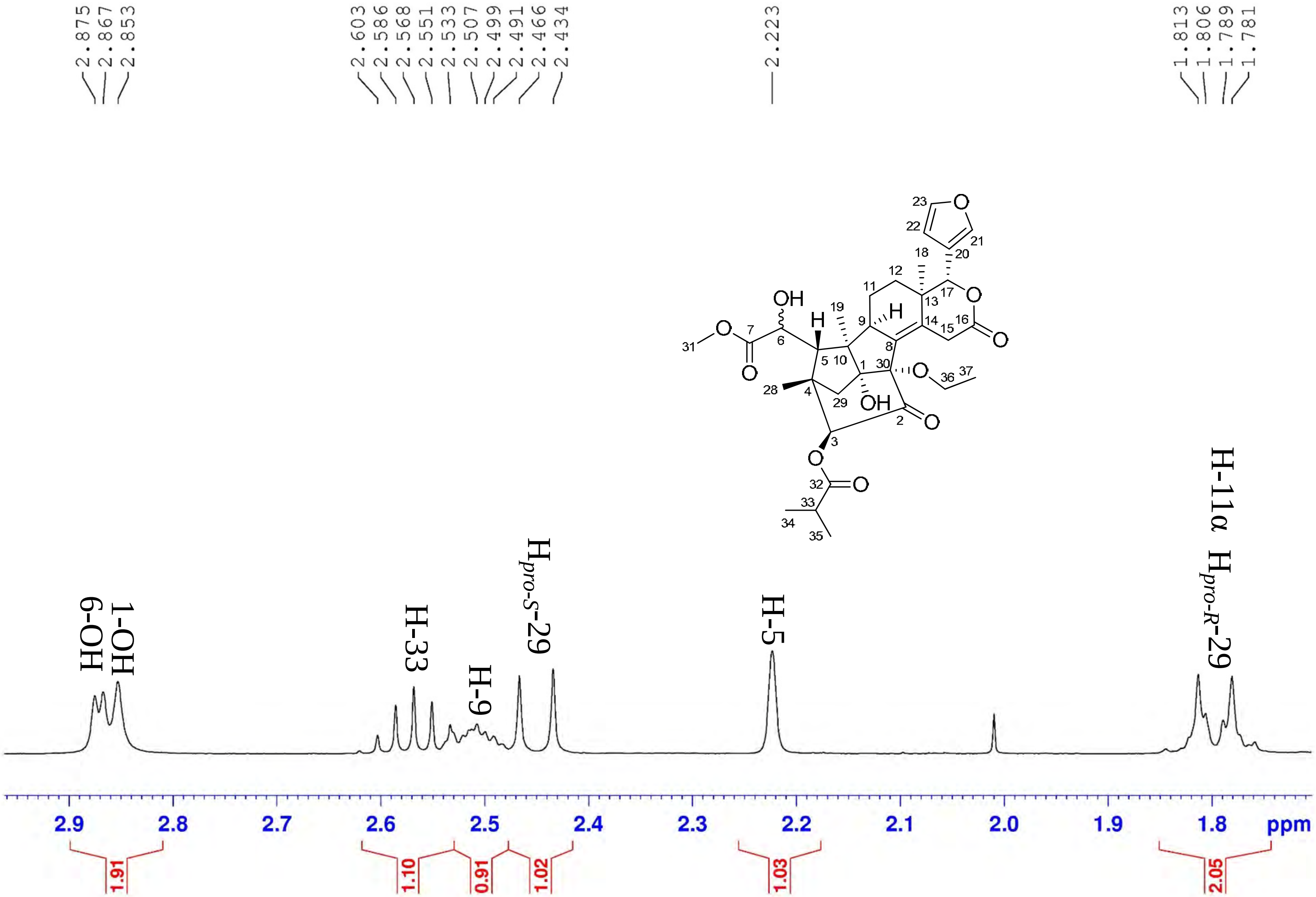

^{1}H NMR (400 MHz) spectrum of compound **16** in $CDCl_3$

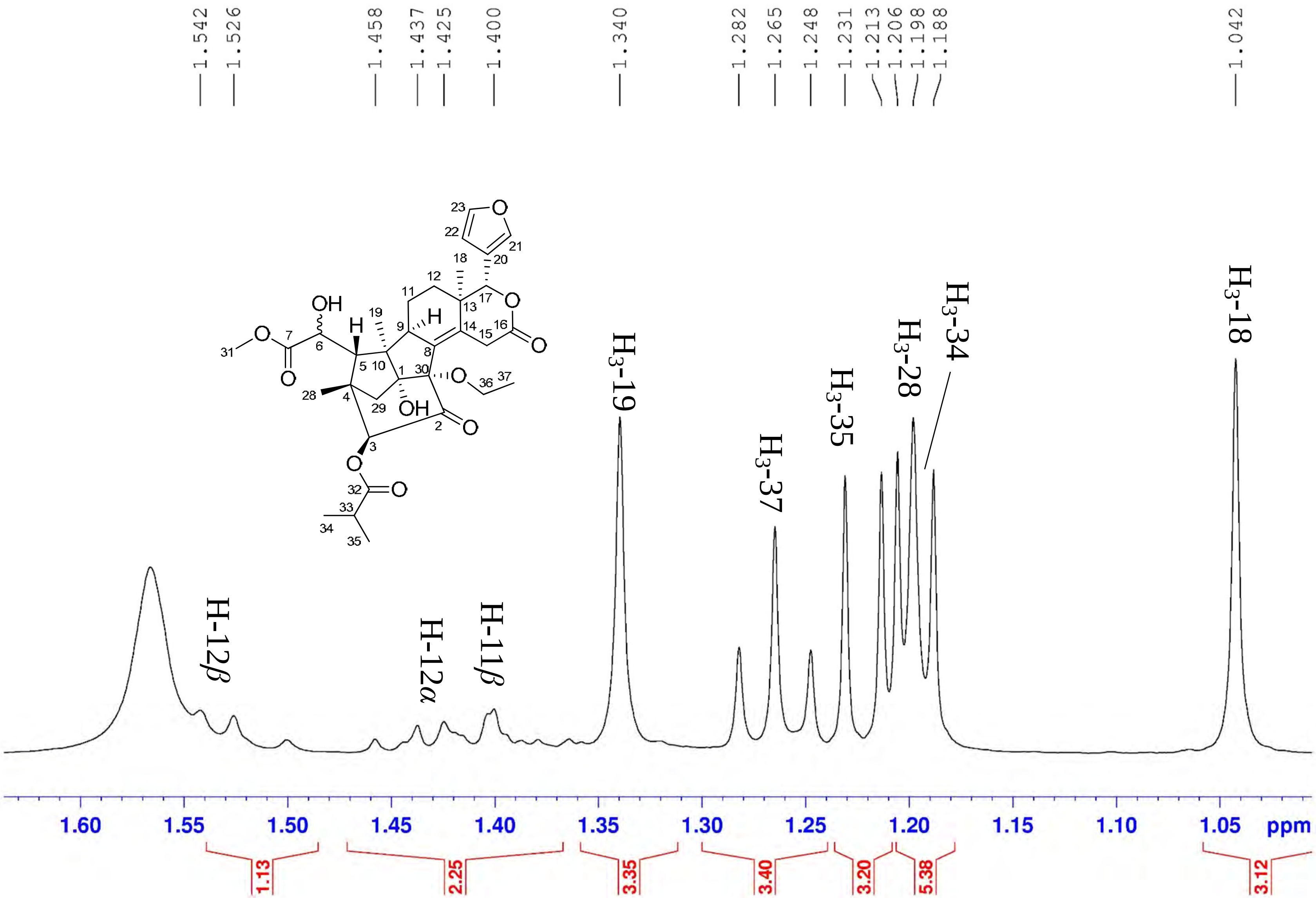

^{13}C NMR (100 MHz) spectrum of compound **16** in $CDCl_3$

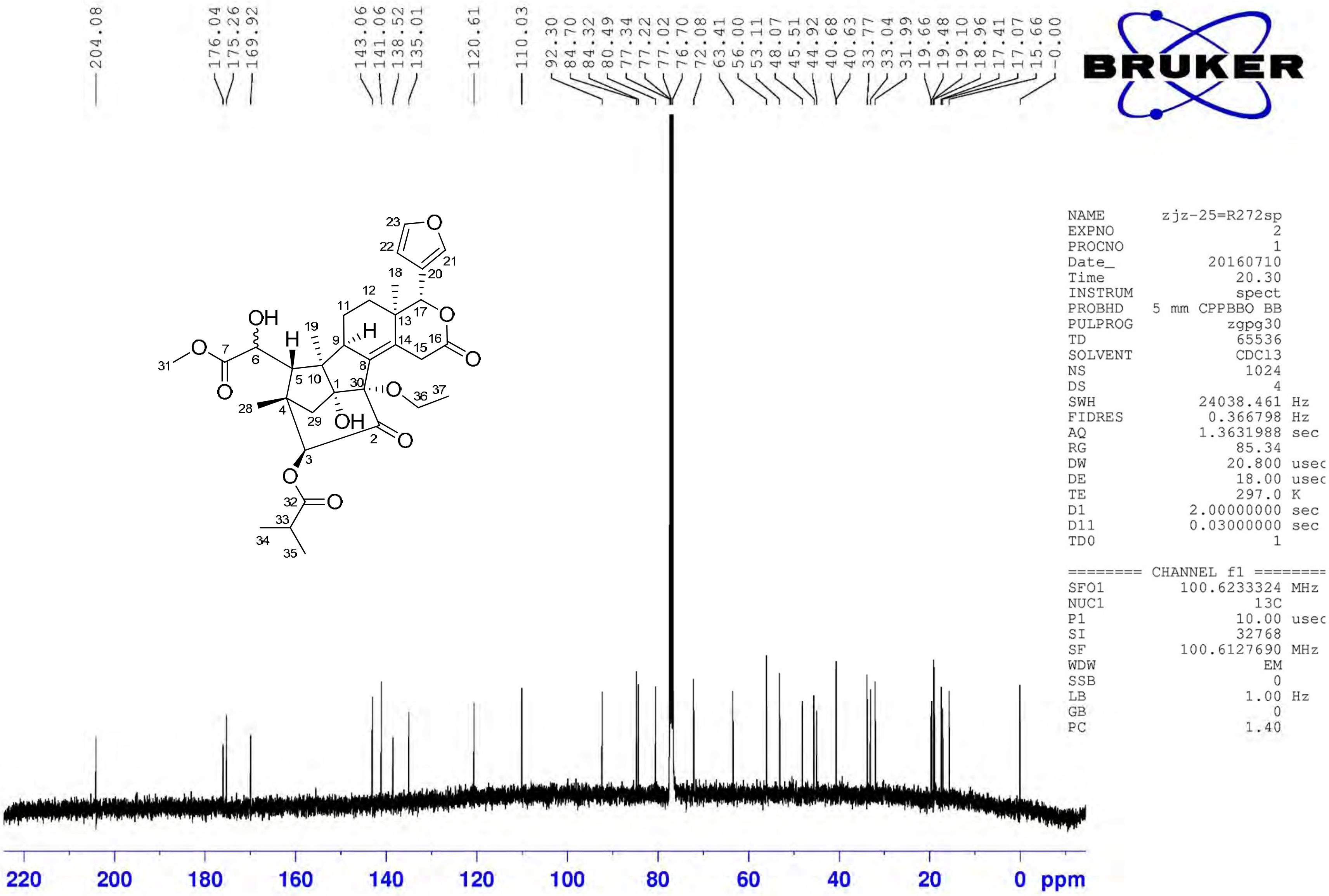

^{13}C NMR (100 MHz) spectrum of compound **16** in $CDCl_3$

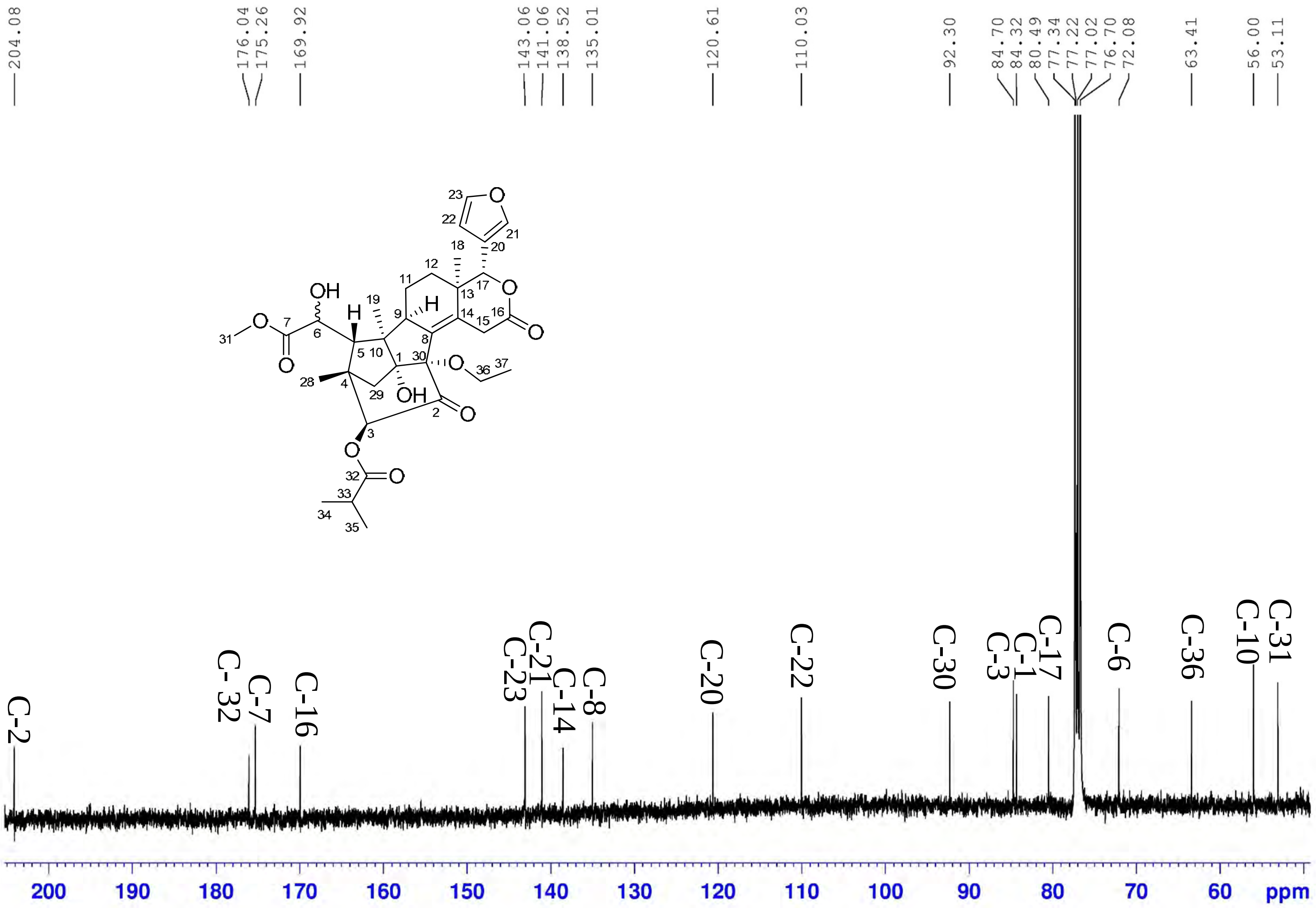

^{13}C NMR (100 MHz) spectrum of compound **16** in $CDCl_3$

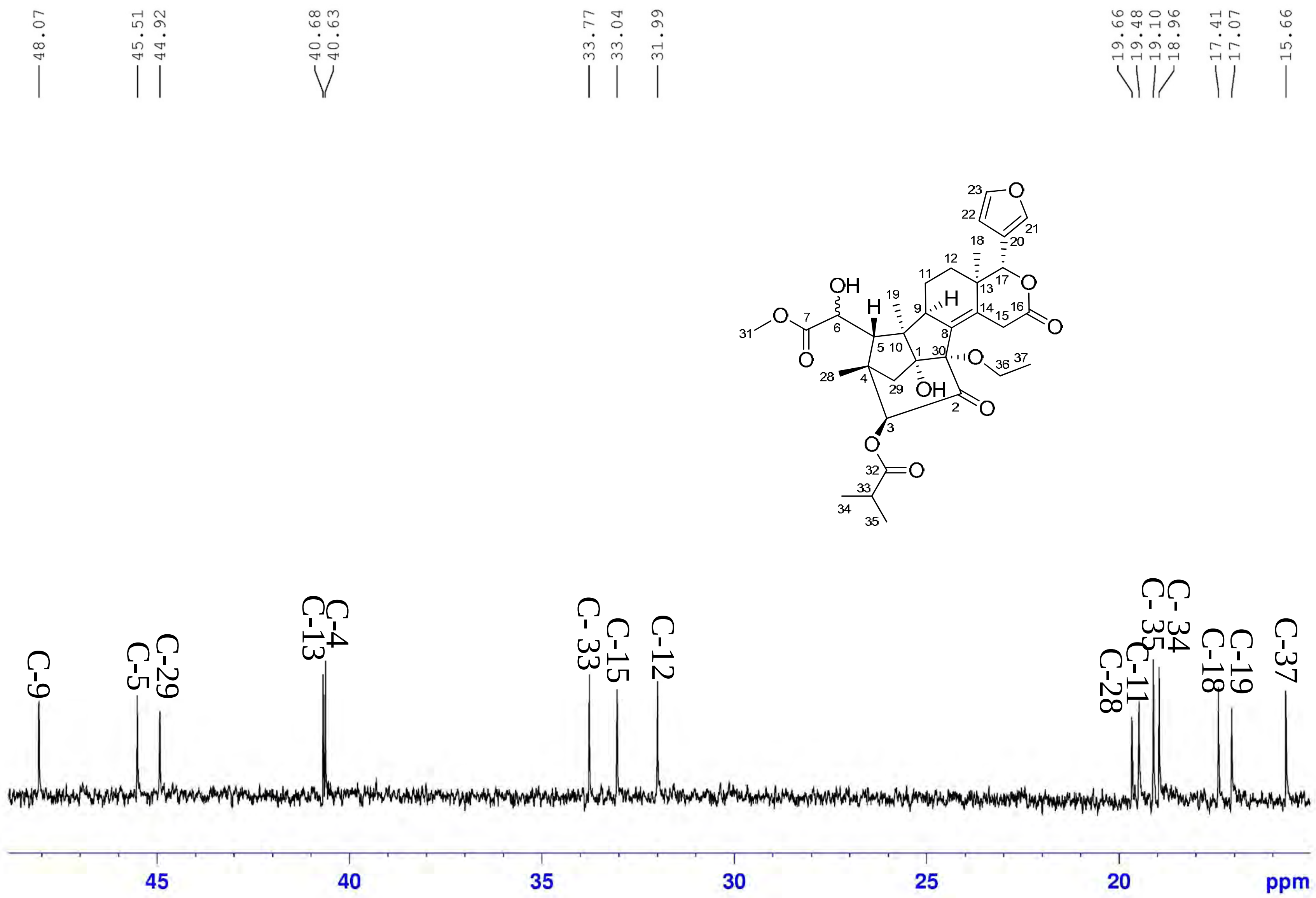

DEPT135 (100 MHz) spectrum of compound **16** in $CDCl_3$

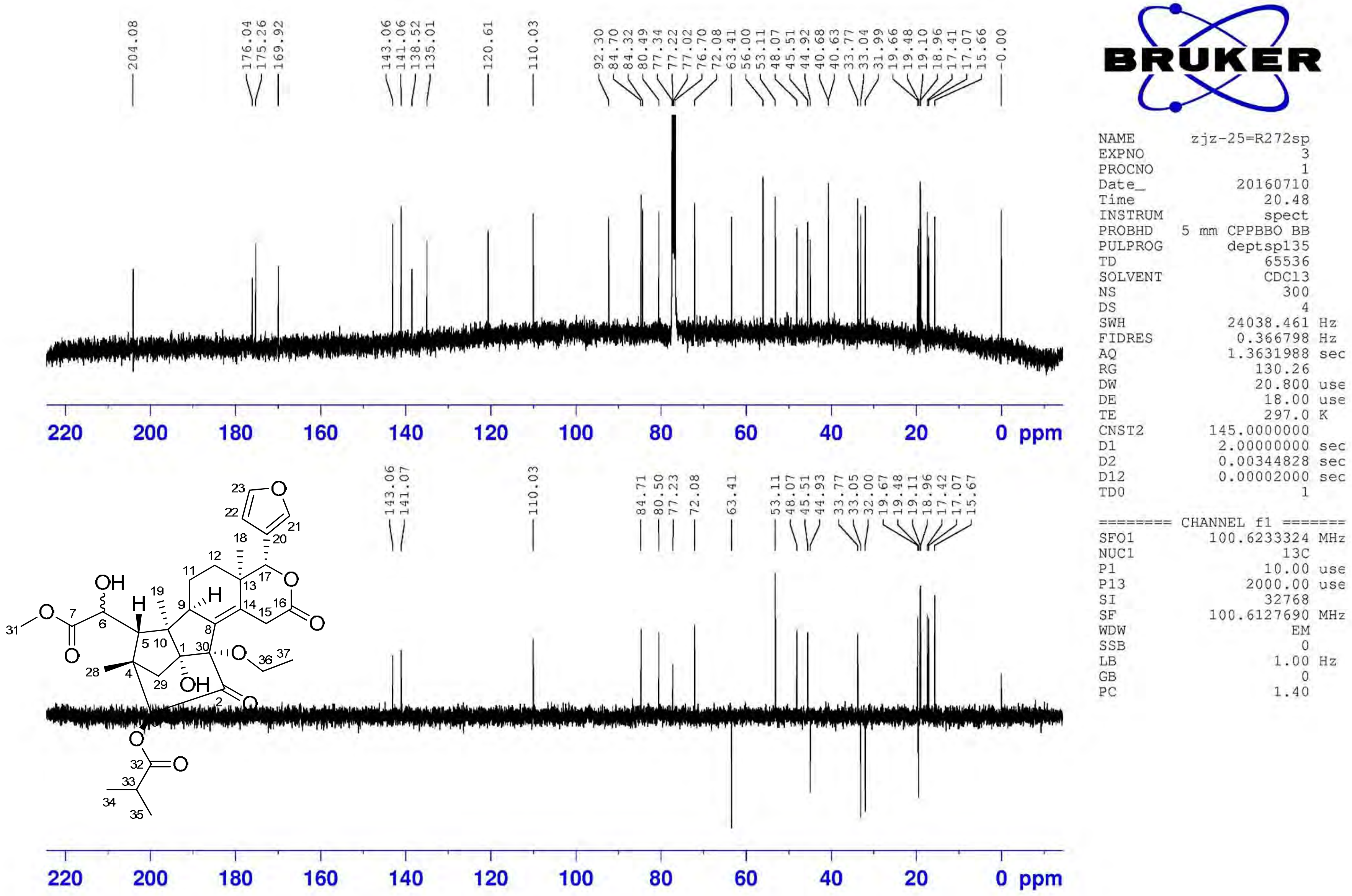

DEPT135 (100 MHz) spectrum of compound **16** in $CDCl_3$

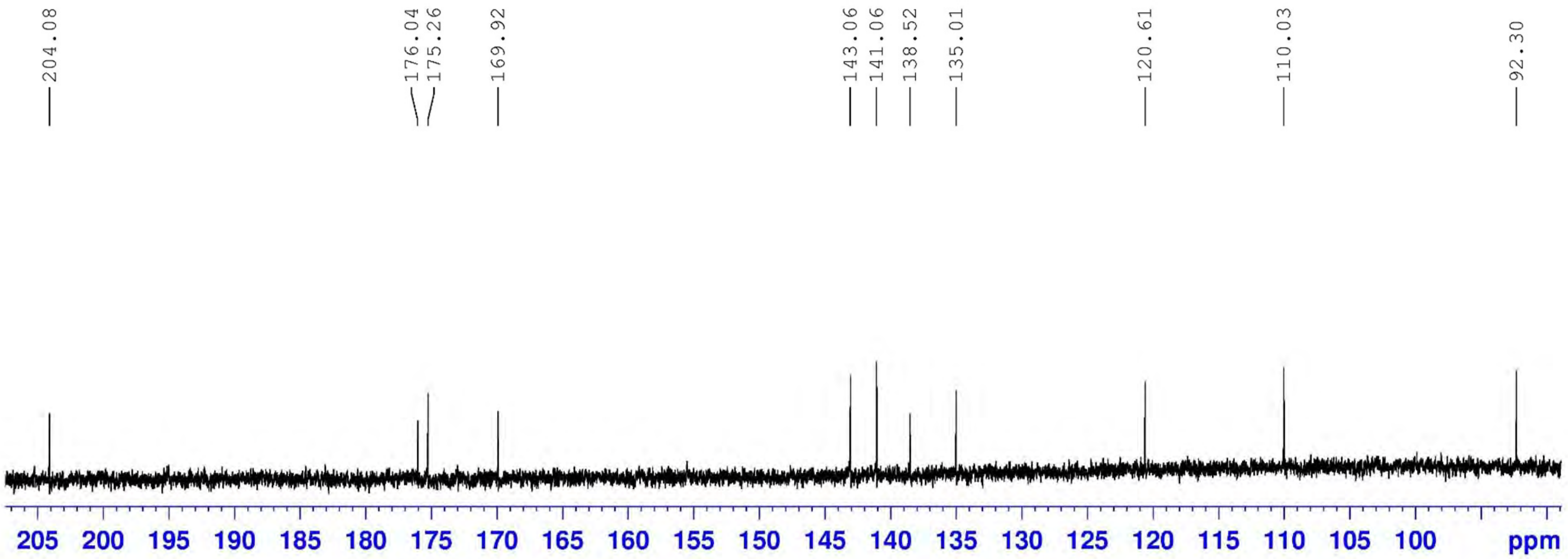

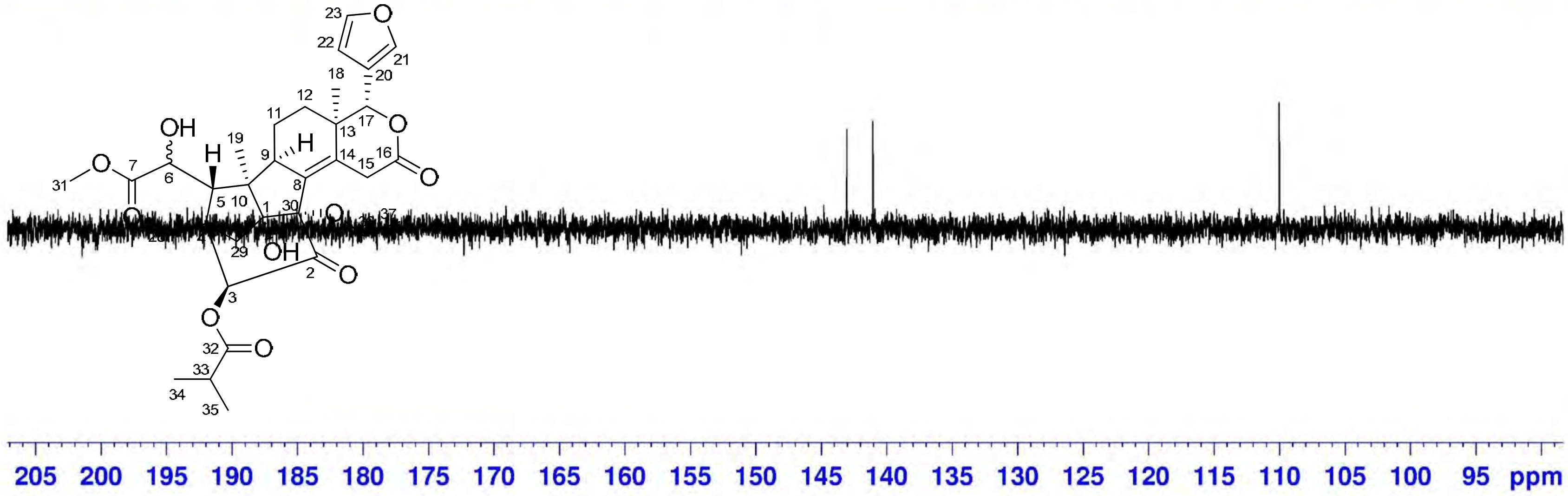

DEPT135 (100 MHz) spectrum of compound **16** in $CDCl_3$

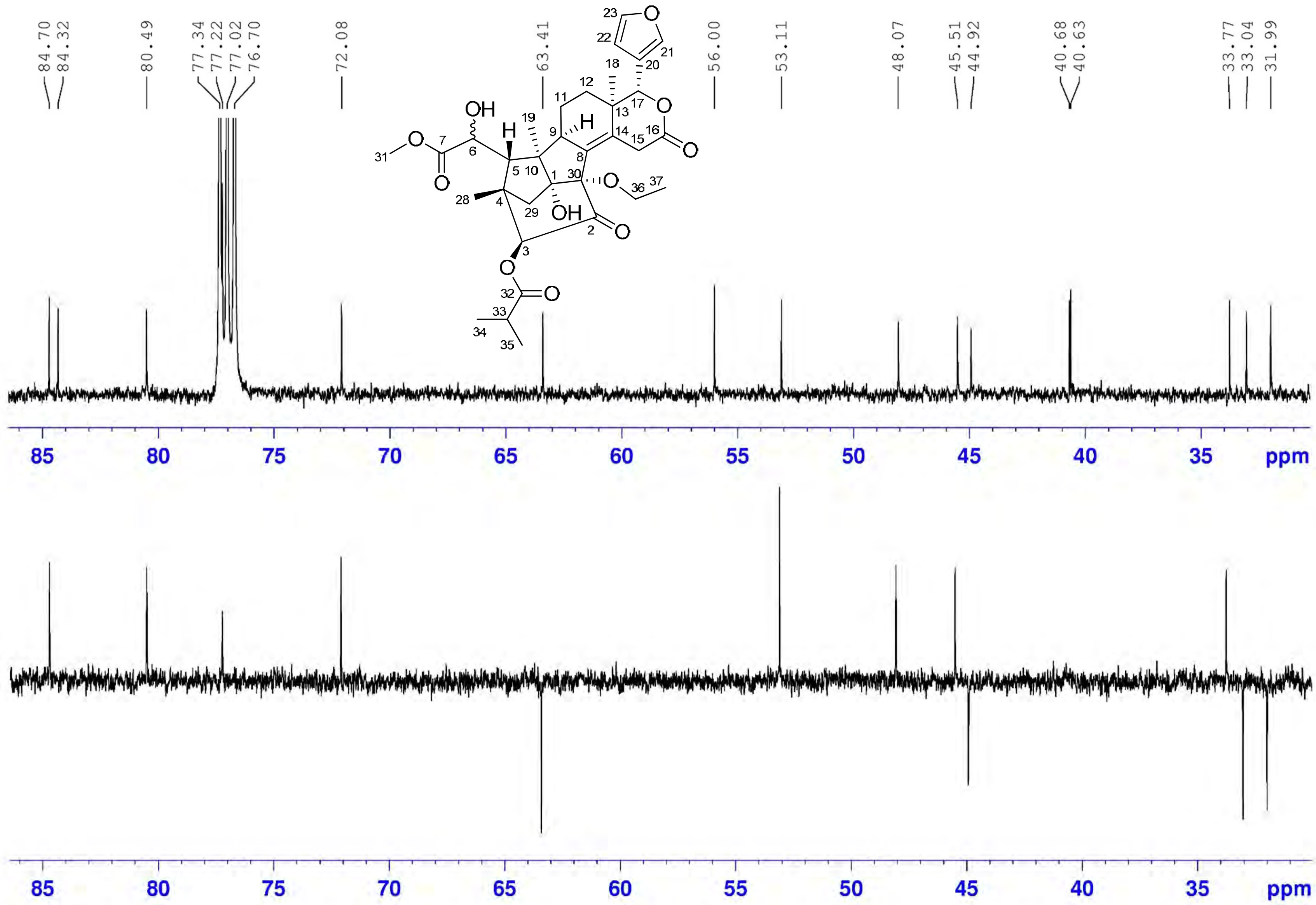

DEPT135 (100 MHz) spectrum of compound **16** in $CDCl_3$

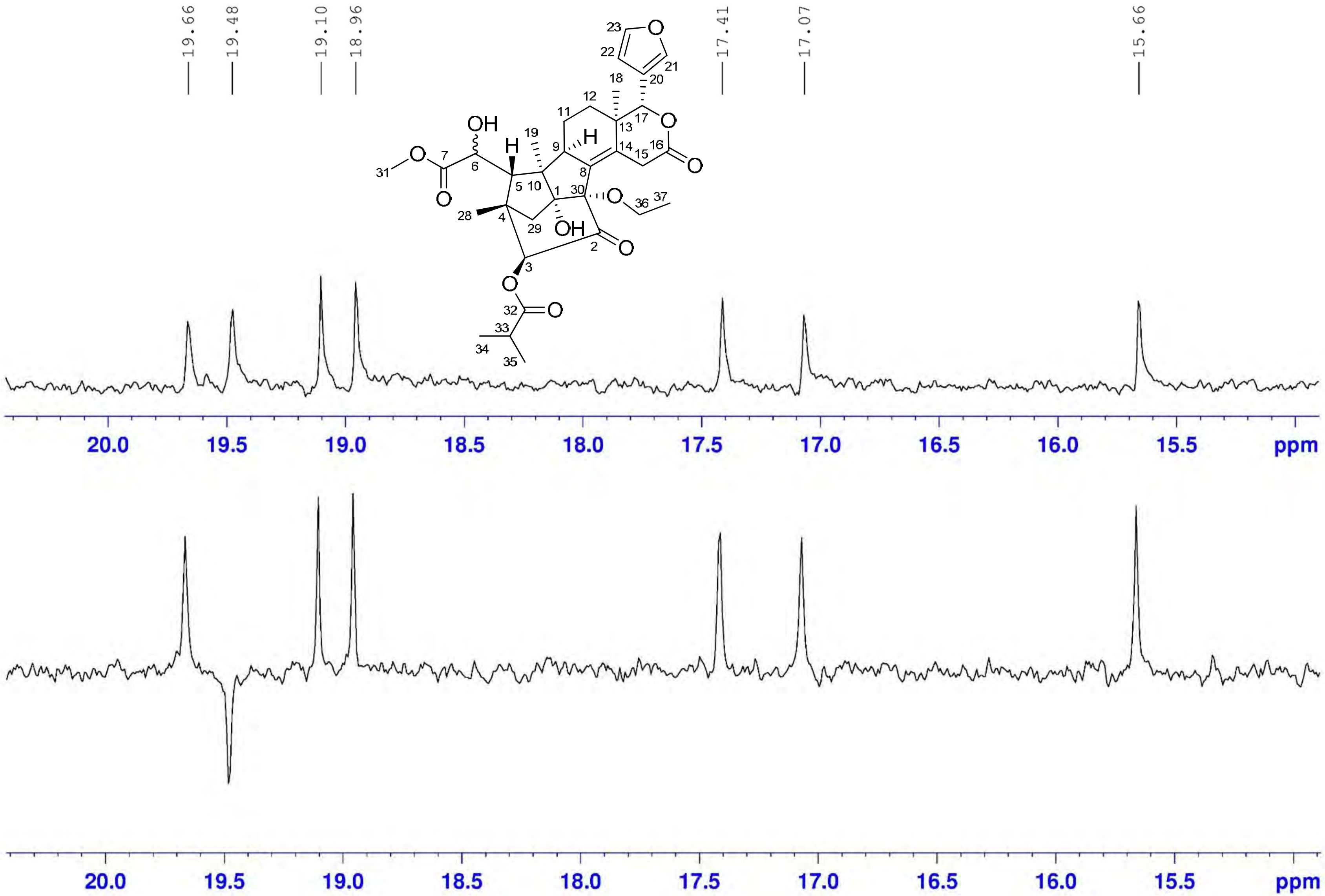

^{1}H-^{1}H COSY (400 MHz) spectrum of compound **16** in $CDCl_3$

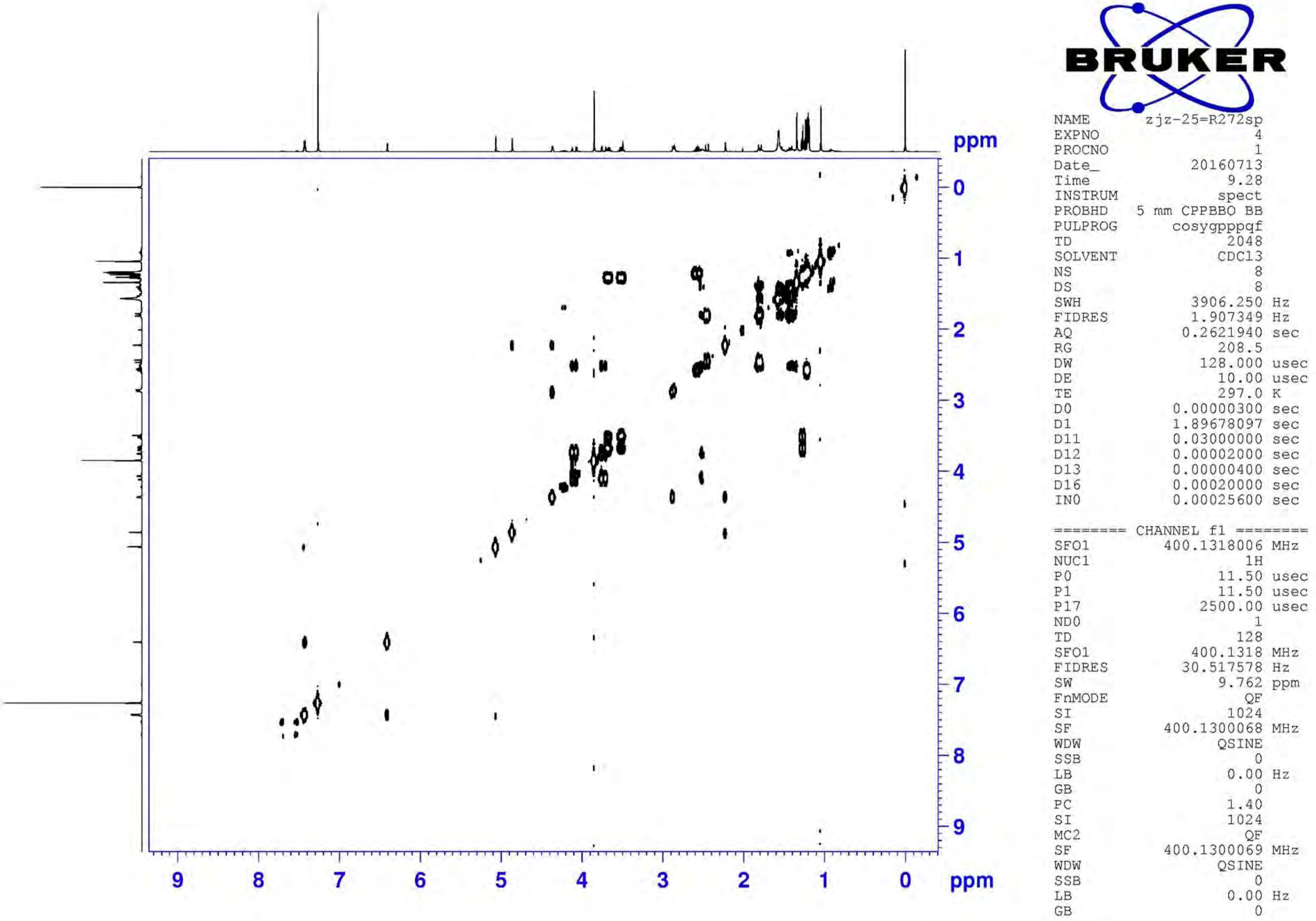

^1H-^1H COSY (400 MHz) spectrum of compound **16** in $CDCl_3$

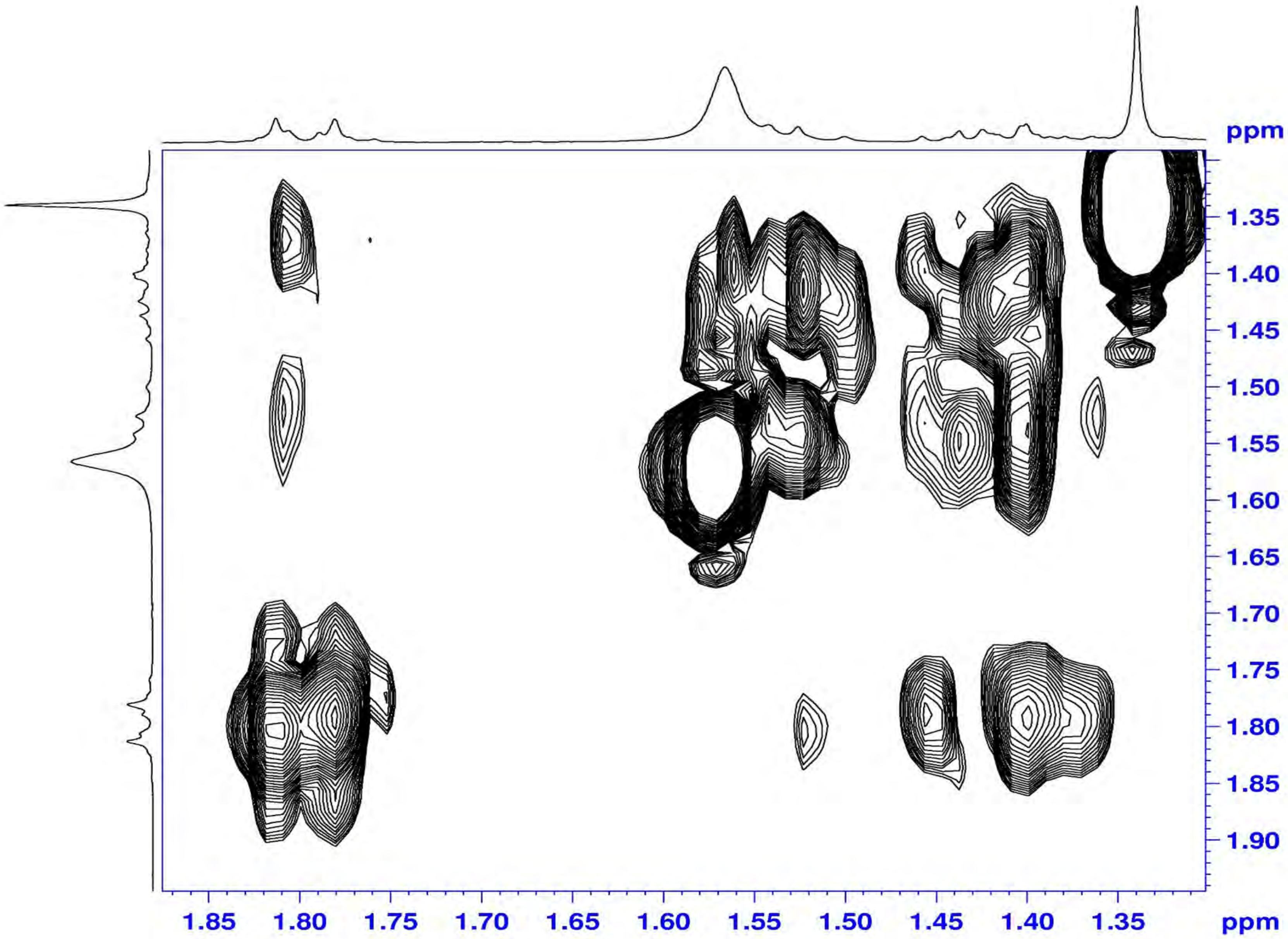

^{1}H-^{1}H COSY (400 MHz) spectrum of compound **16** in $CDCl_3$

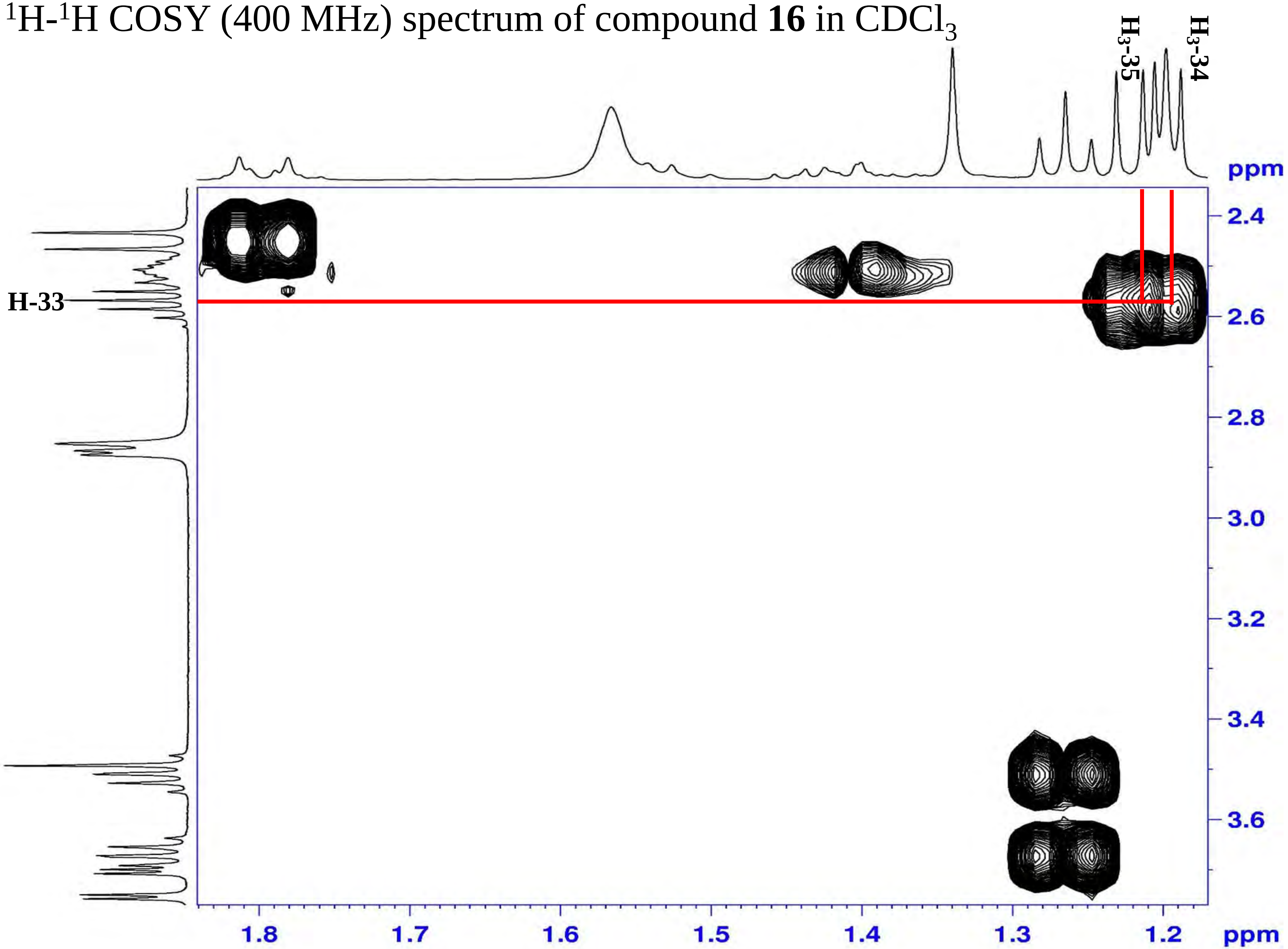

HSQC (400 MHz) spectrum of compound **16** in $CDCl_3$

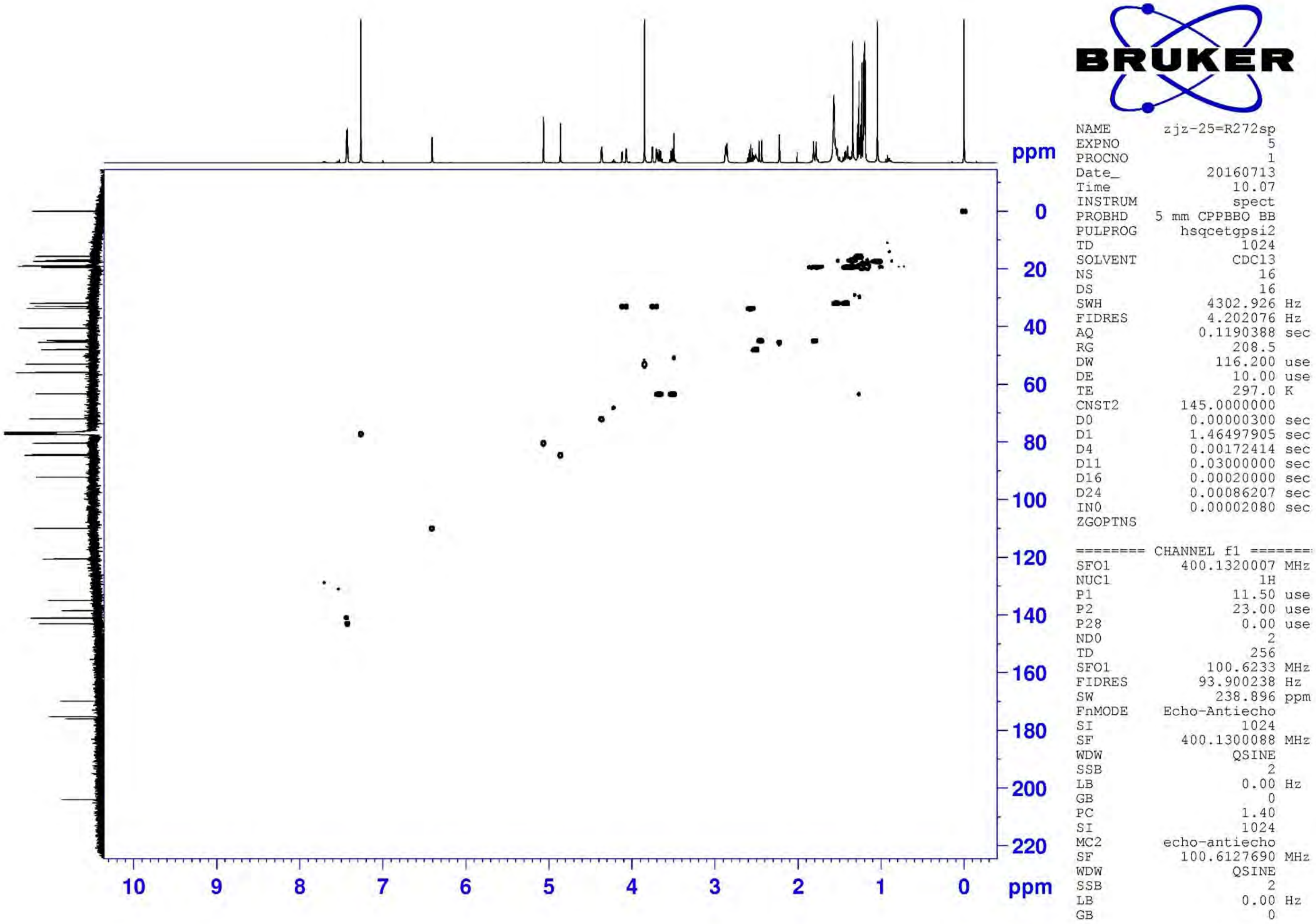

HSQC (400 MHz) spectrum of compound **16** in $CDCl_3$

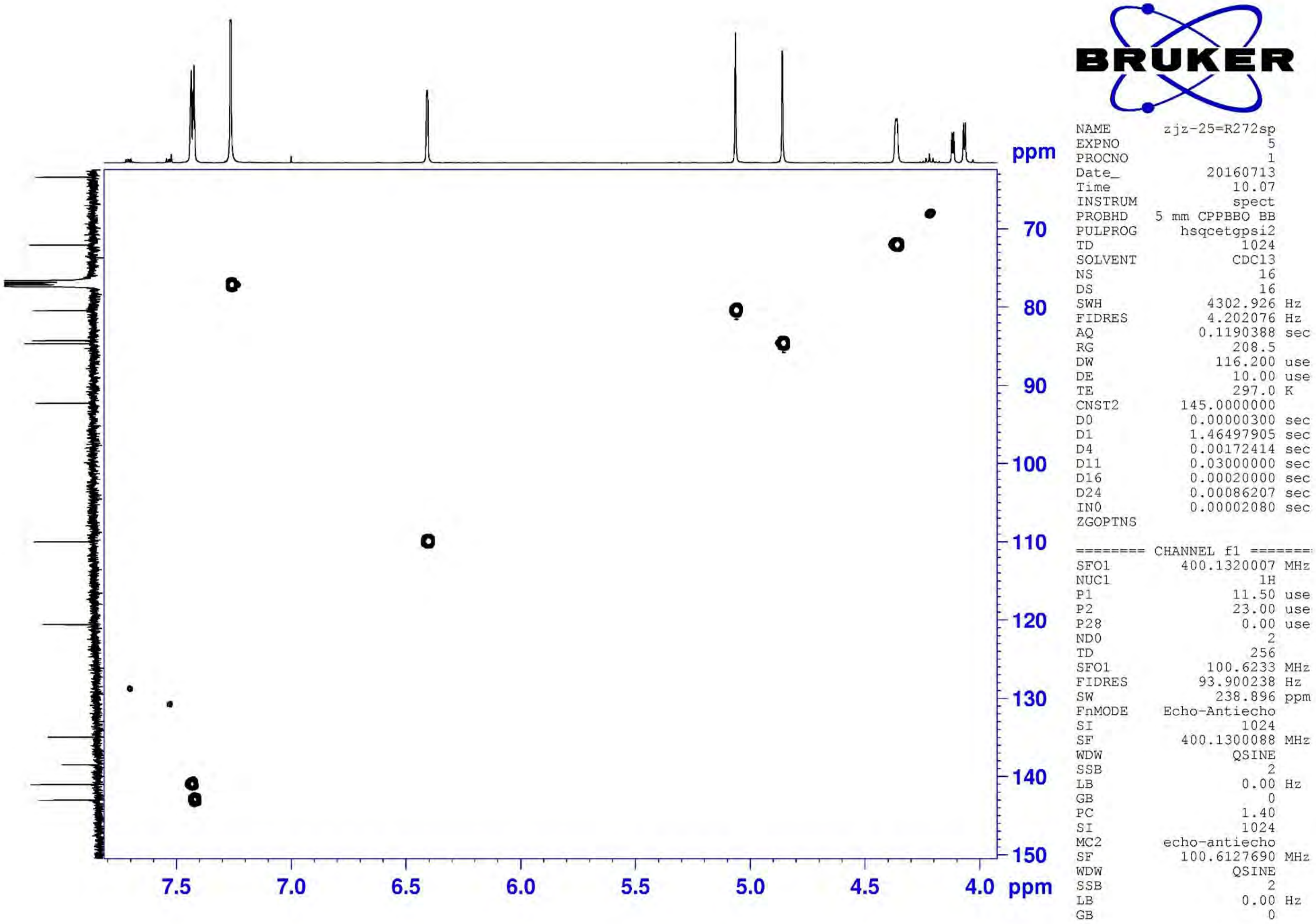

HSQC (400 MHz) spectrum of compound **16** in $CDCl_3$

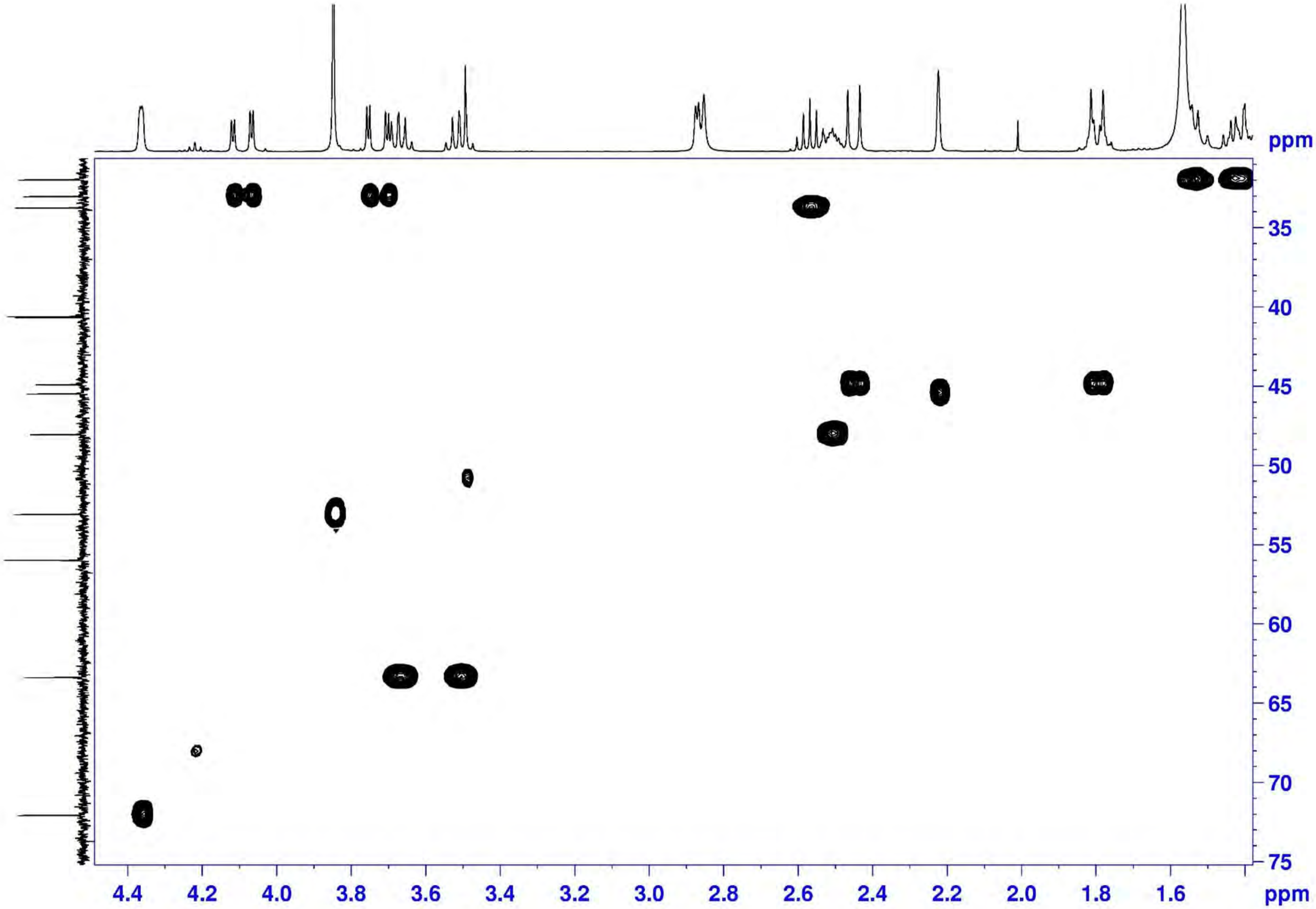

HSQC (400 MHz) spectrum of compound **16** in $CDCl_3$

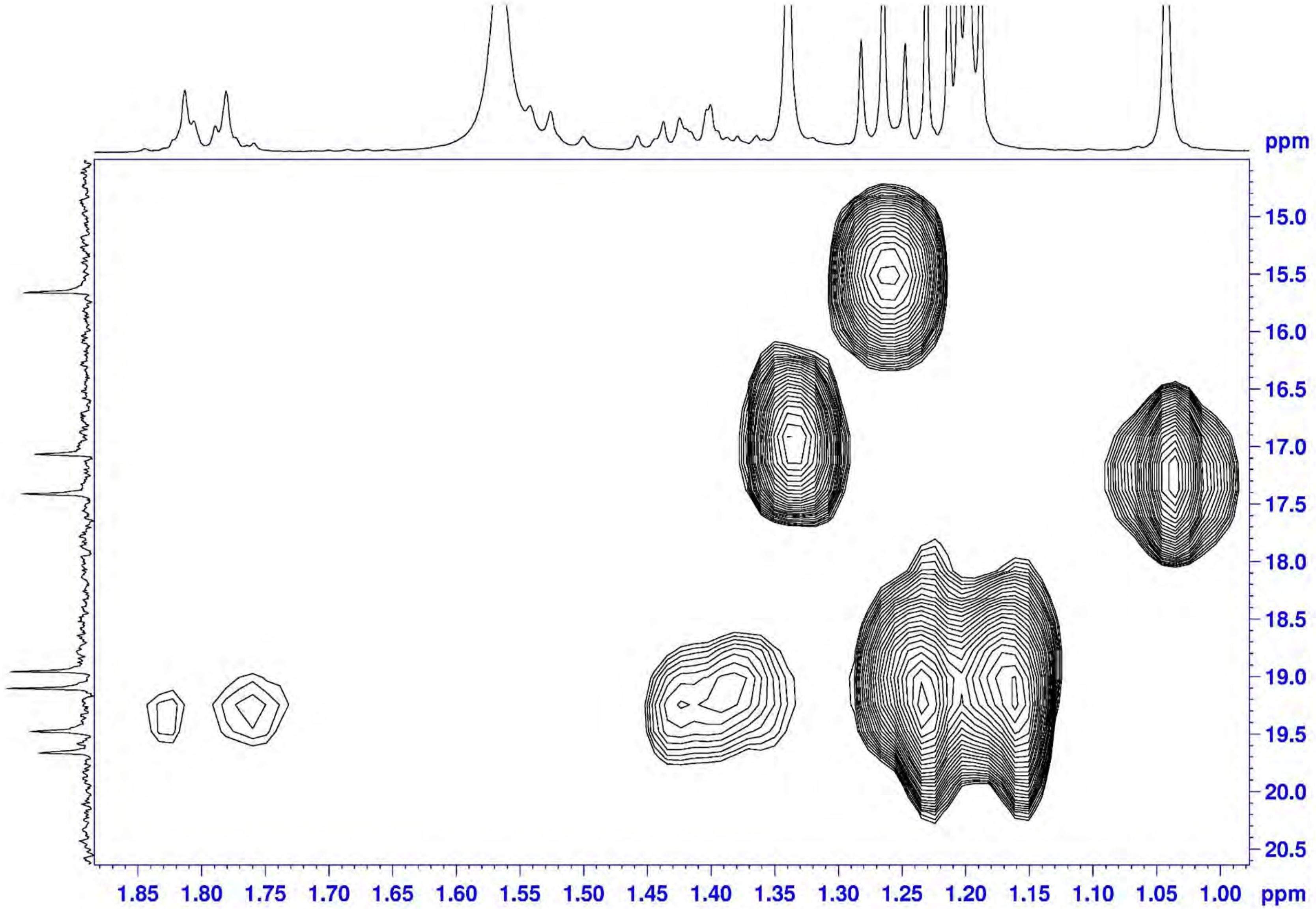

HMBC (400 MHz) spectrum of compound **16** in $CDCl_3$

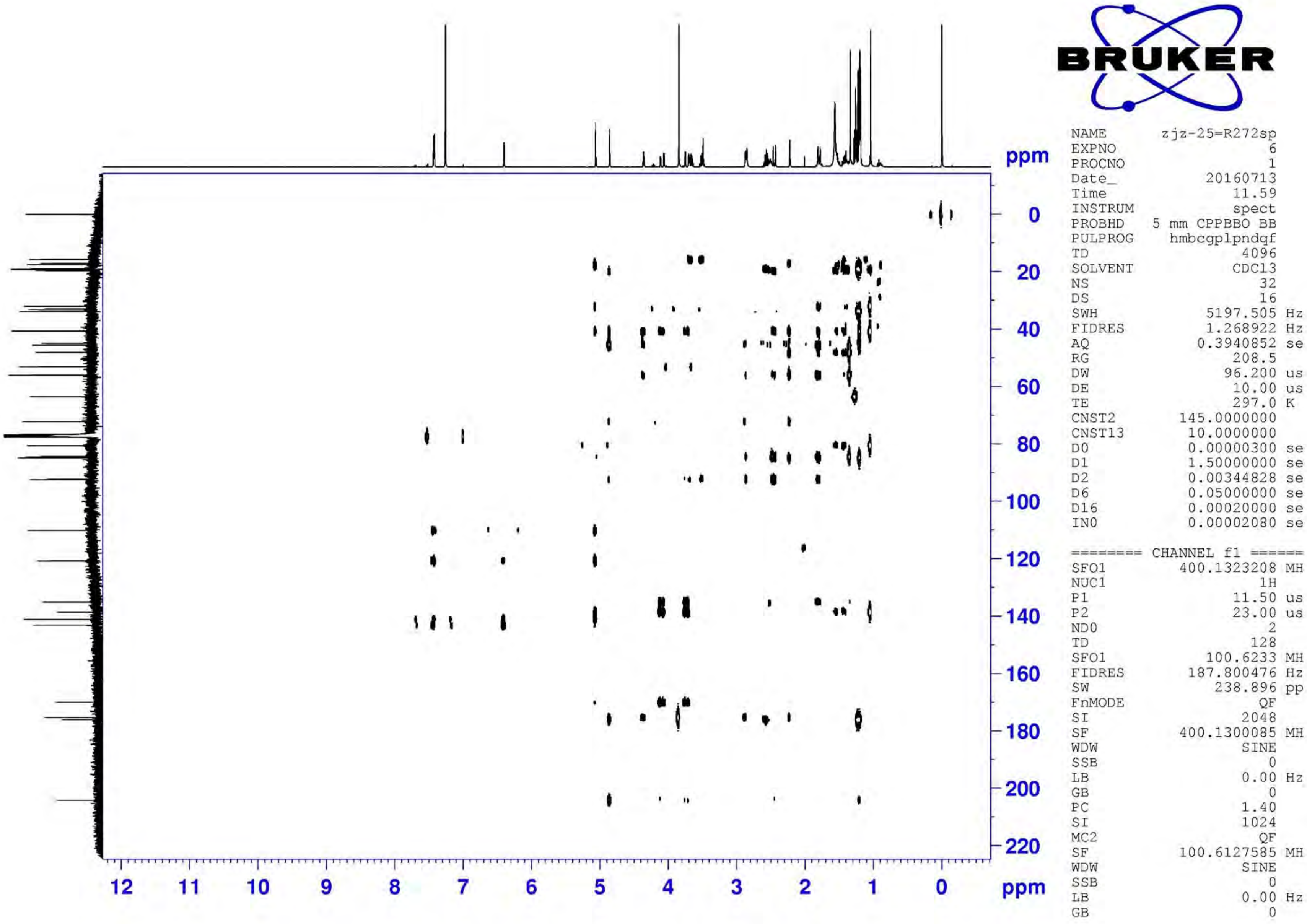

HMBC (400 MHz) spectrum of compound **16** in $CDCl_3$

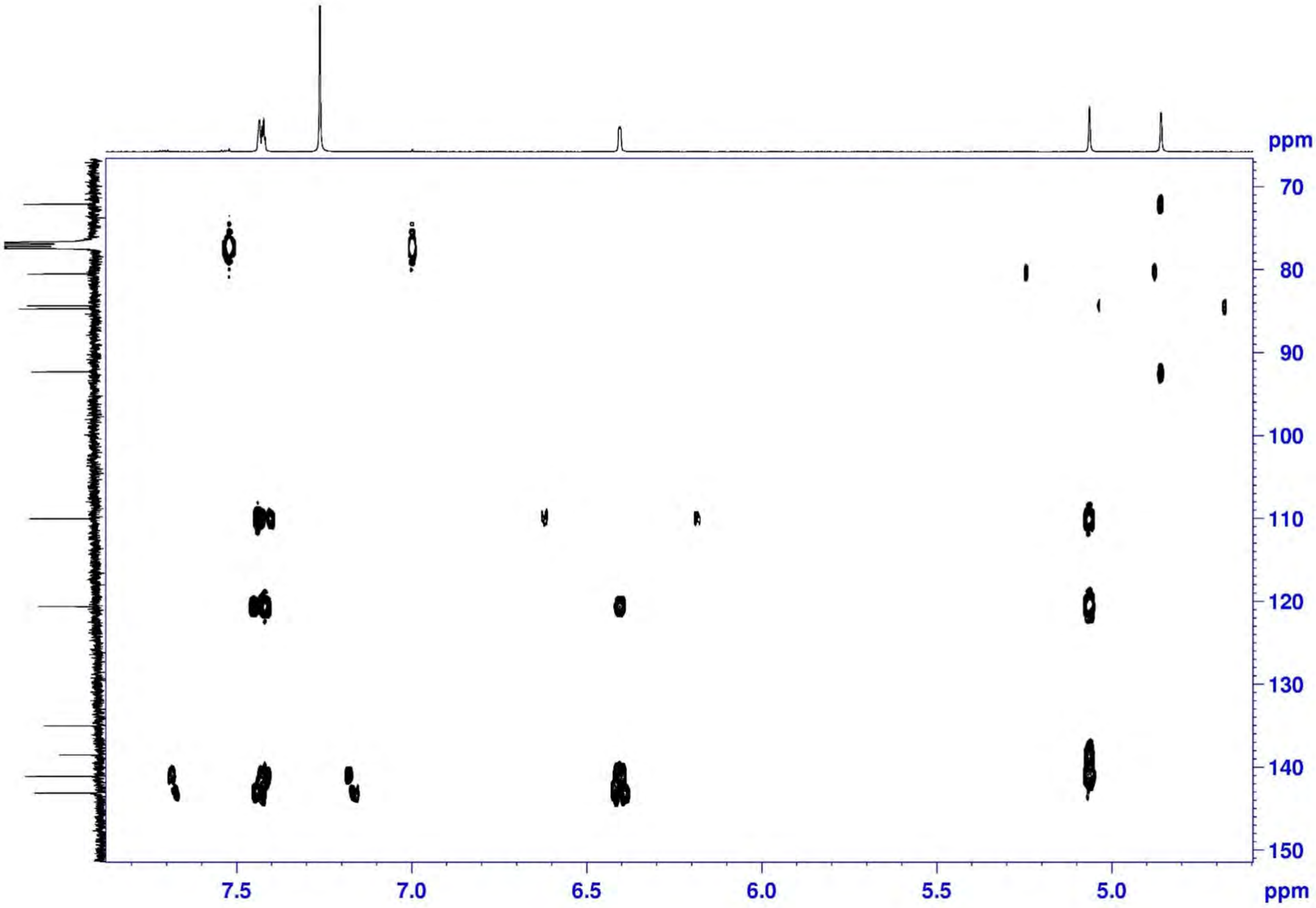

HMBC (400 MHz) spectrum of compound **16** in $CDCl_3$

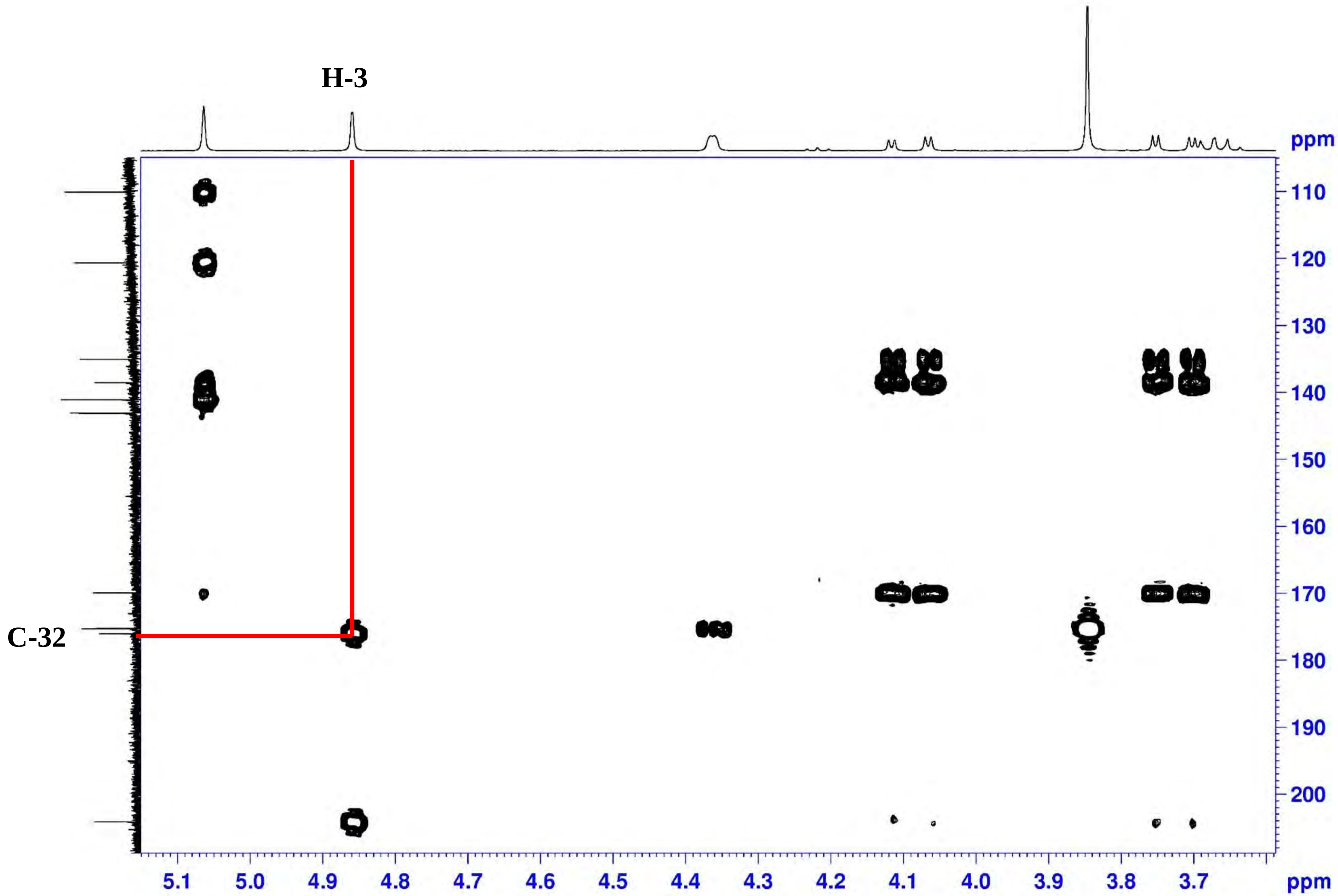

HMBC (400 MHz) spectrum of compound **16** in $CDCl_3$

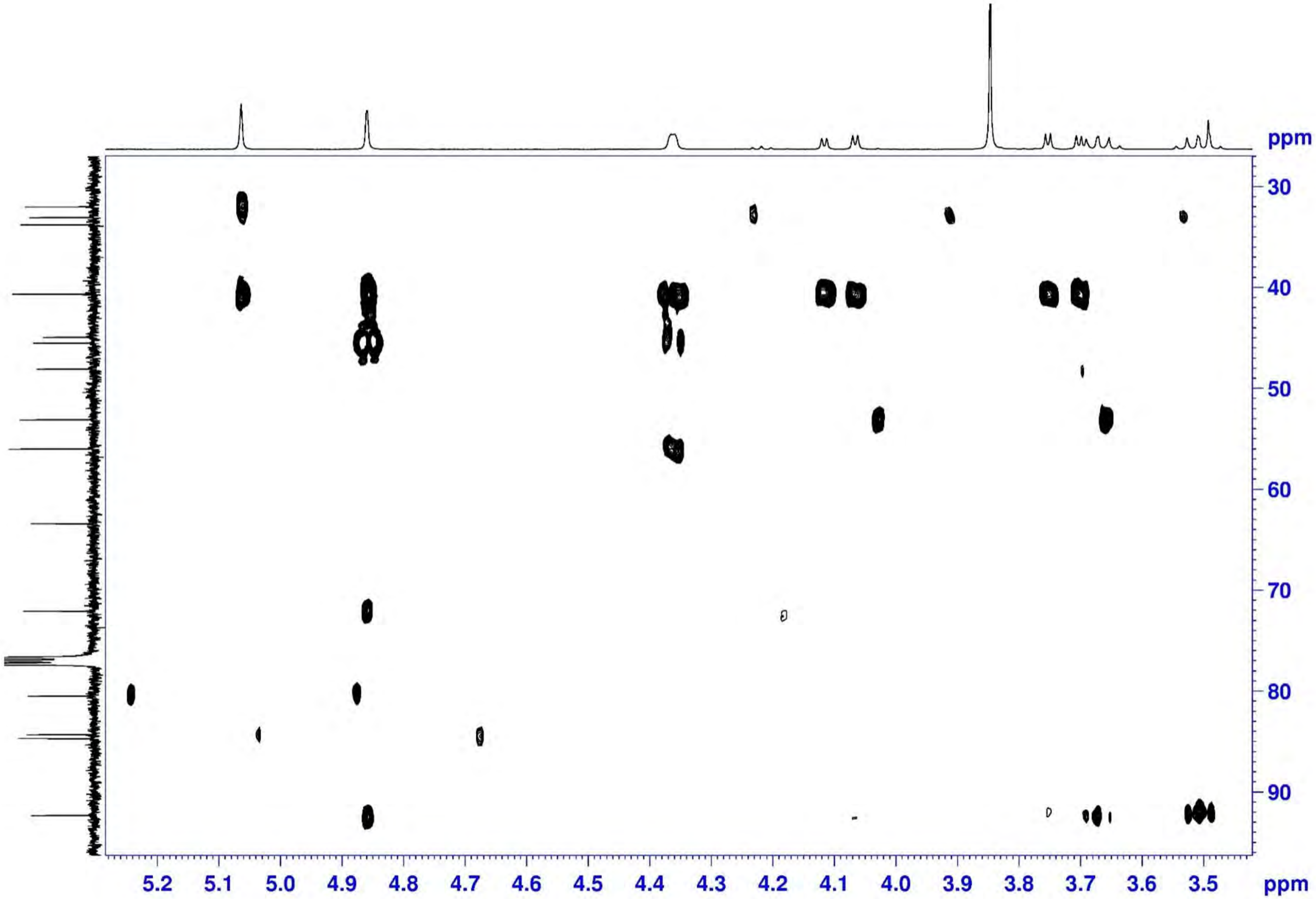

HMBC (400 MHz) spectrum of compound **16** in $CDCl_3$

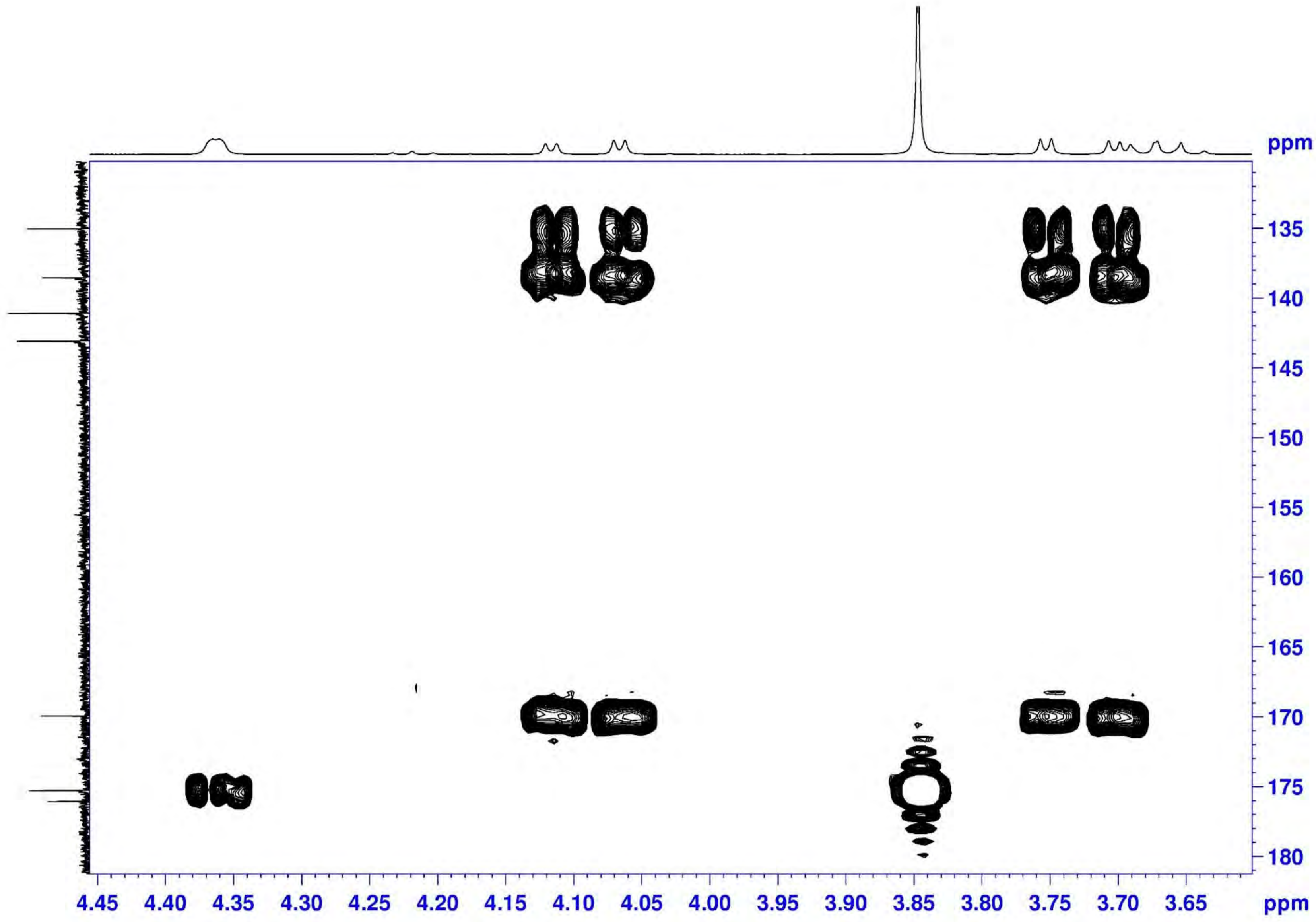

HMBC (400 MHz) spectrum of compound **16** in $CDCl_3$

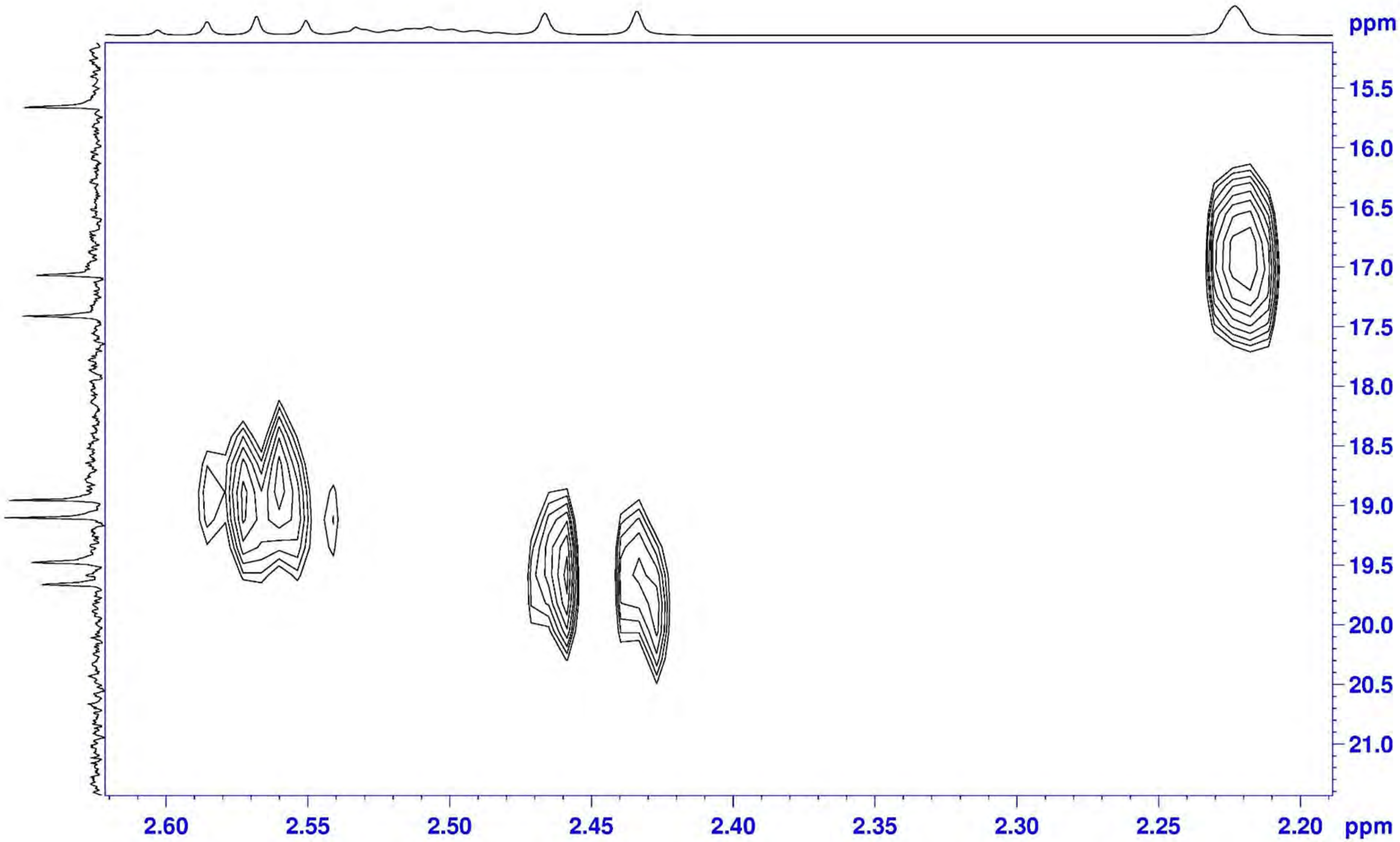

HMBC (400 MHz) spectrum of compound **16** in $CDCl_3$

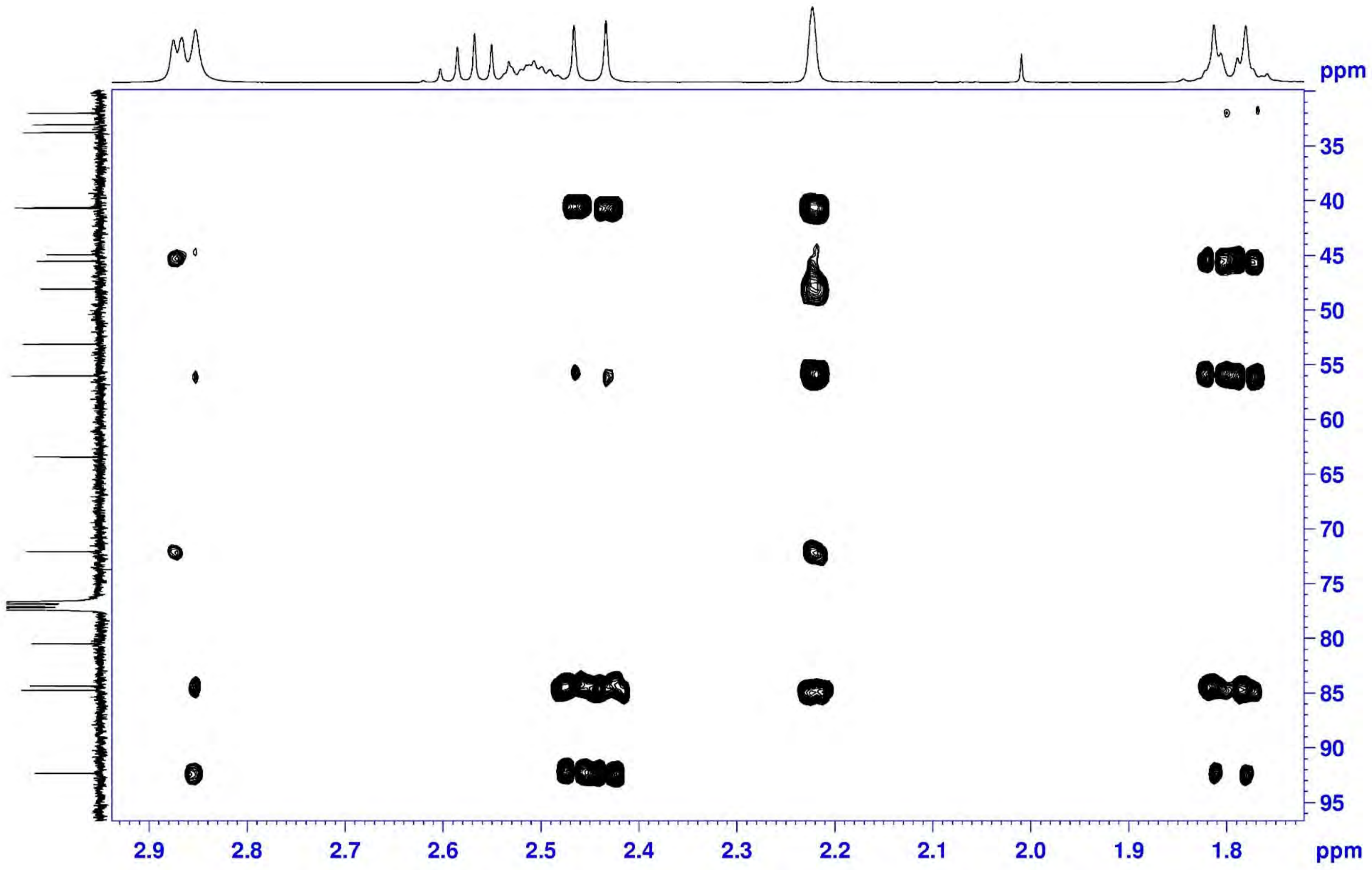

HMBC (400 MHz) spectrum of compound **16** in $CDCl_3$

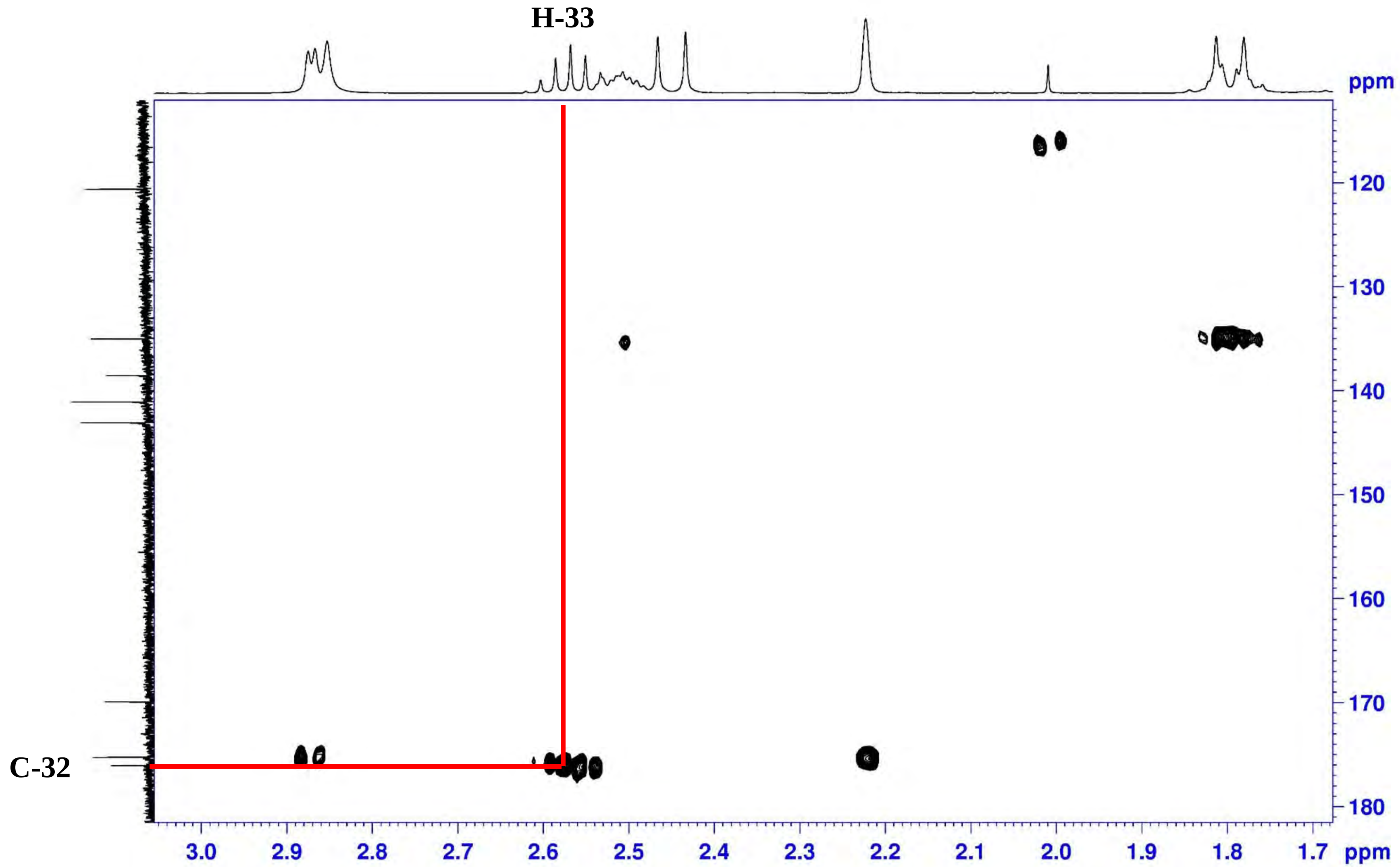

HMBC (400 MHz) spectrum of compound **16** in $CDCl_3$

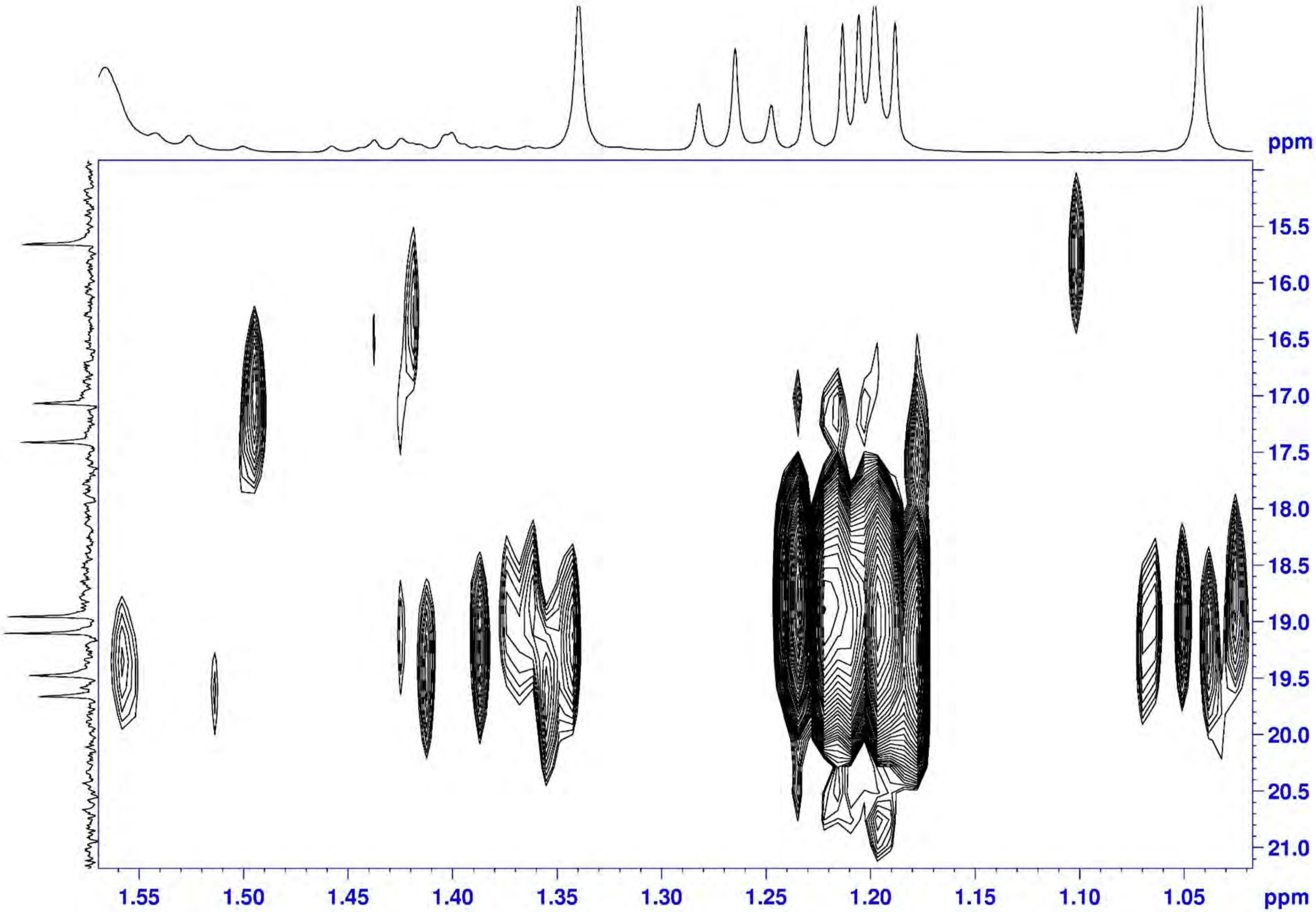

HMBC (400 MHz) spectrum of compound **16** in $CDCl_3$

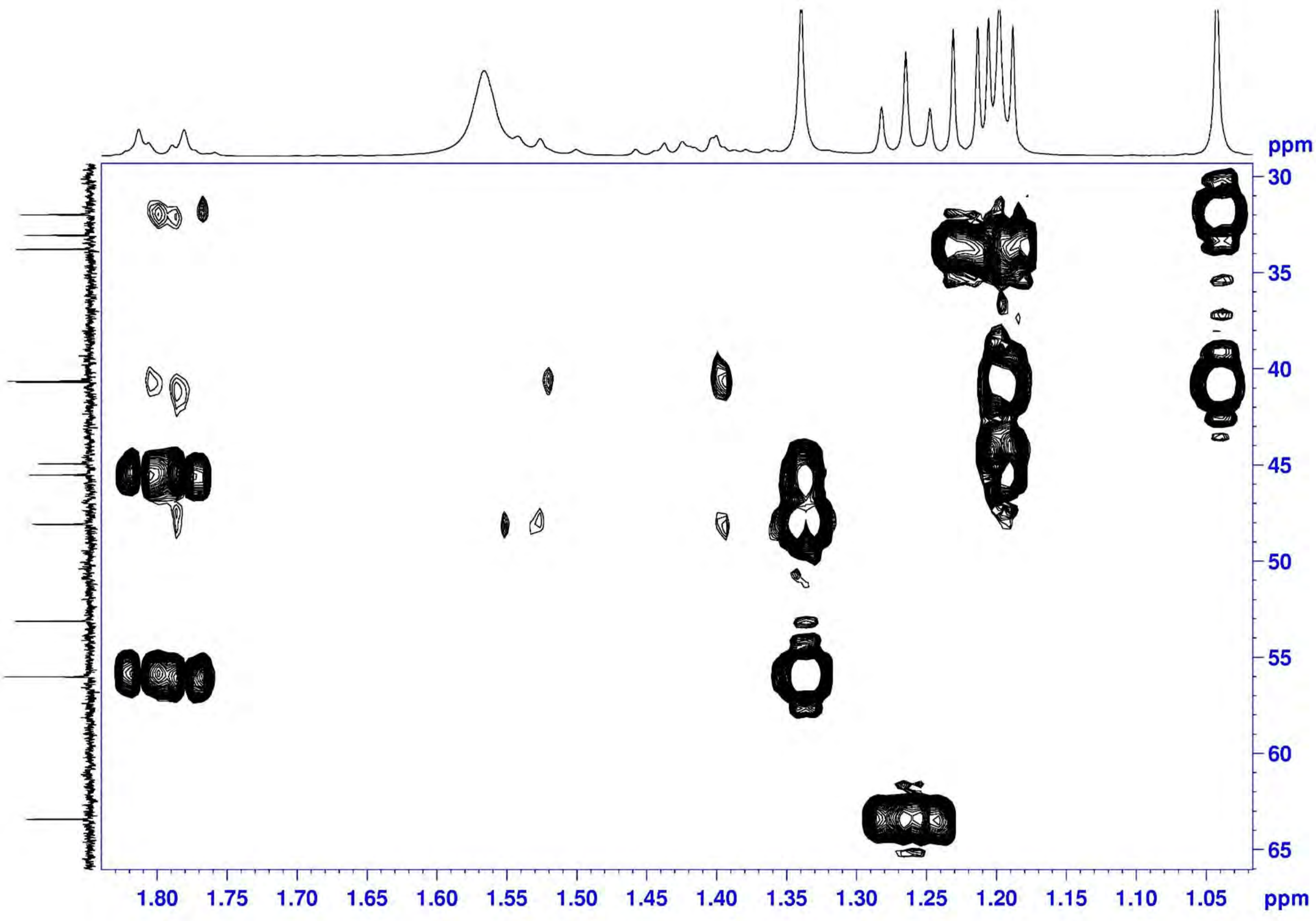

HMBC (400 MHz) spectrum of compound **16** in $CDCl_3$

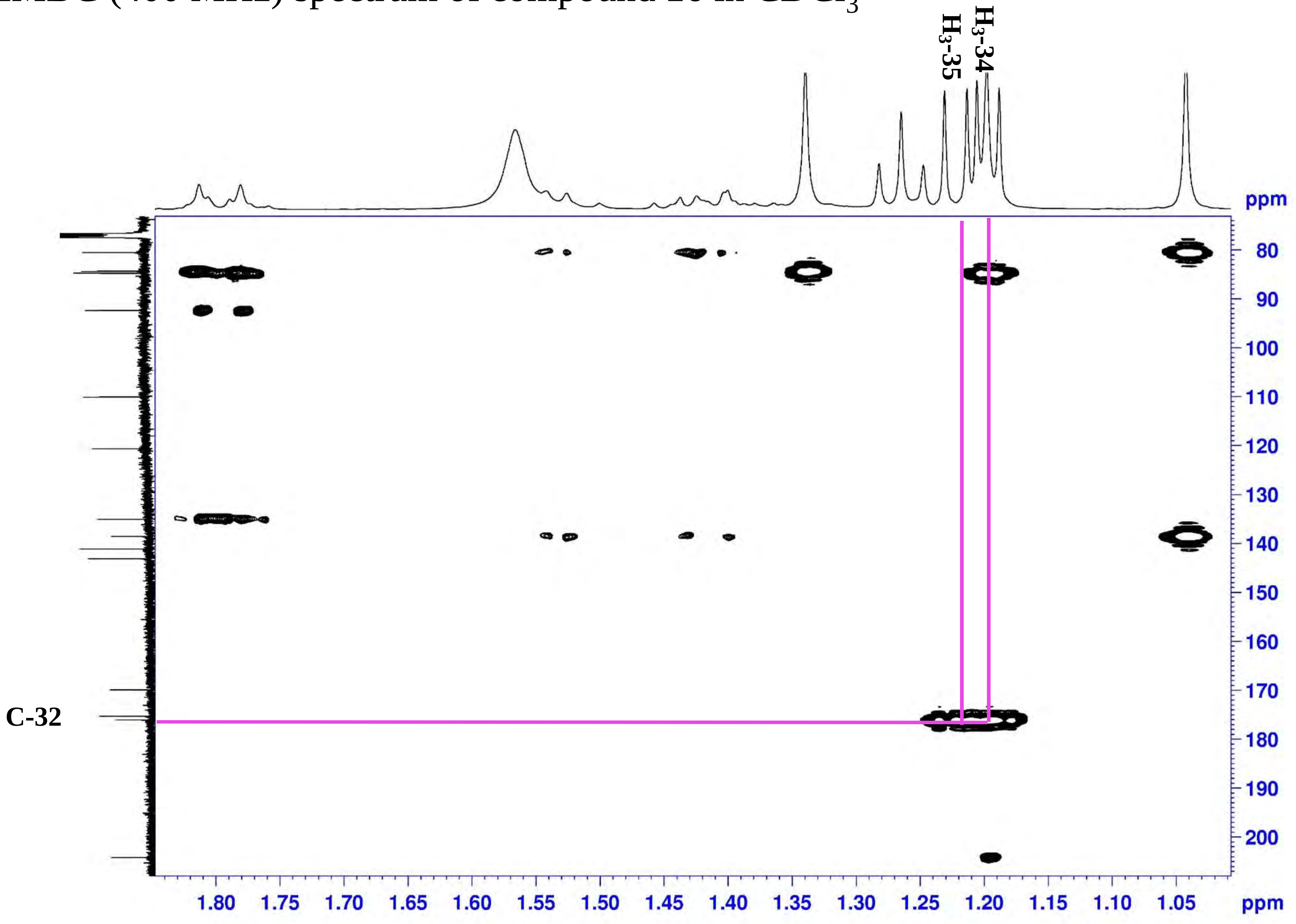

NOESY (400 MHz) spectrum of compound **16** in $CDCl_3$

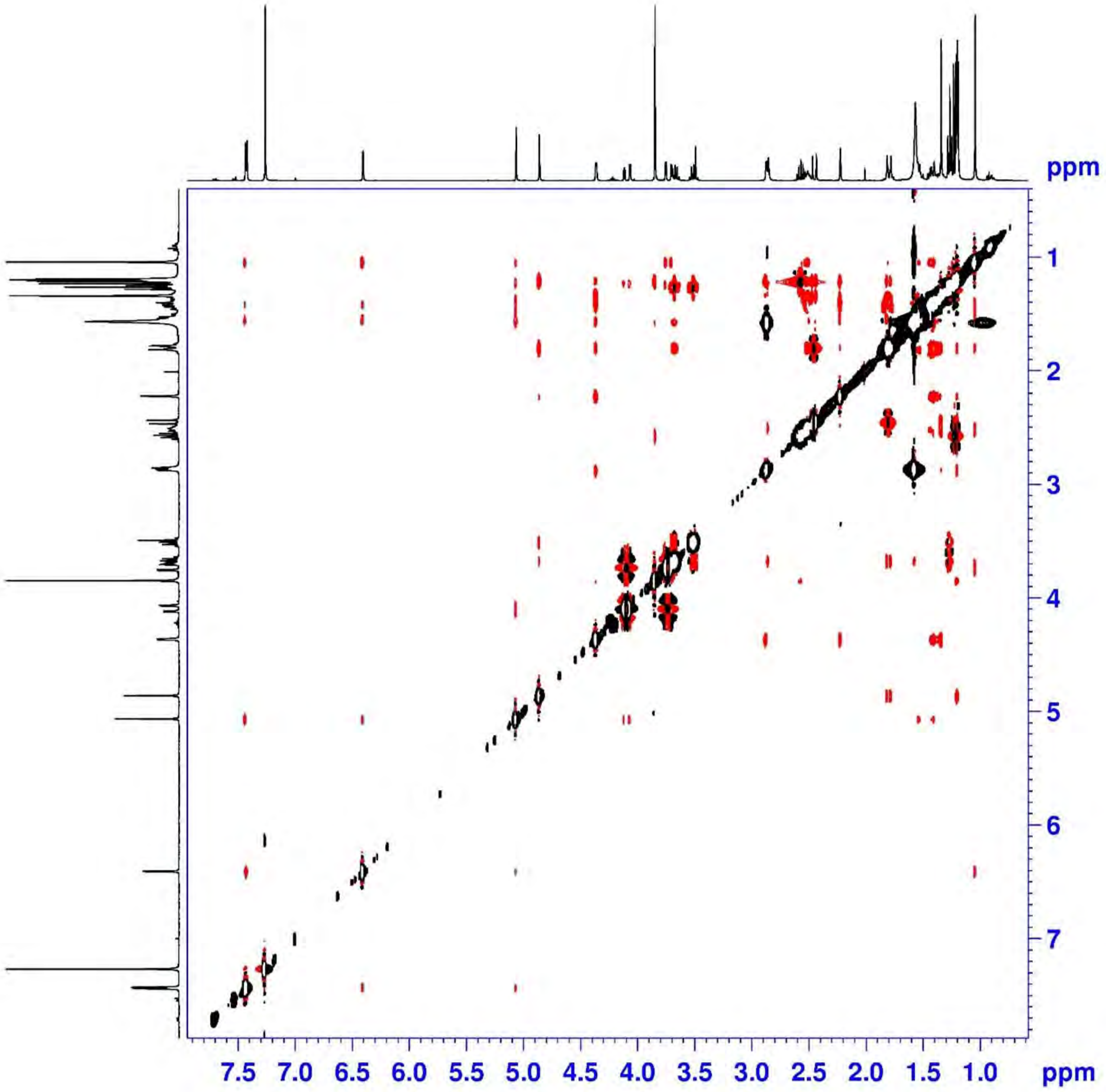

NAME	zjz-25=R272sp
EXPNO	7
PROCNO	1
Date_	20160713
Time	14.14
INSTRUM	spect
PROBHD	5 mm CPPBBO BB
PULPROG	noesygpphpp
TD	2048
SOLVENT	CDCl3
NS	16
DS	32
SWH	4000.000 Hz
FIDRES	1.953125 Hz
AQ	0.2560500 sec
RG	208.5
DW	125.000 usec
DE	10.00 usec
TE	297.0 K
D0	0.00011036 sec
D1	1.99385595 sec
D8	0.30000001 sec
D11	0.03000000 sec
D12	0.00002000 sec
D16	0.00020000 sec
IN0	0.00025000 sec
======== CHANNEL f1 ========	
SFO1	400.1318006 MHz
NUC1	1H
P1	11.50 usec
P2	23.00 usec
P17	2500.00 usec
ND0	1
TD	256
SFO1	400.1318 MHz
FIDRES	15.625000 Hz
SW	9.997 ppm
FnMODE	States-TPPI
SI	1024
SF	400.1300061 MHz
WDW	QSINE
SSB	2
LB	0.00 Hz
GB	0
PC	1.00
SI	1024
MC2	States-TPPI
SF	400.1300059 MHz
WDW	QSINE
SSB	2
LB	0.00 Hz
GB	0

NOESY (400 MHz) spectrum of compound **16** in $CDCl_3$

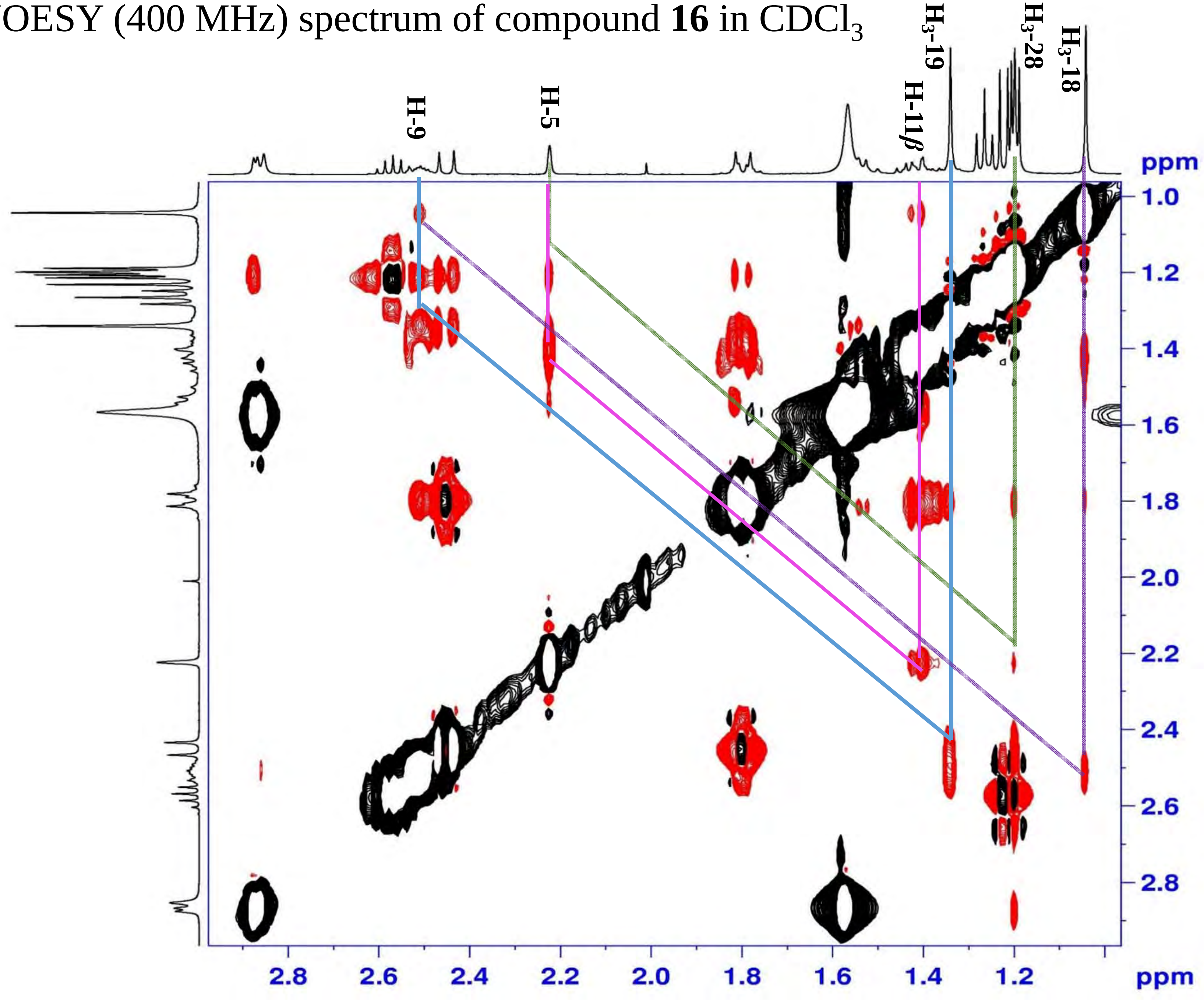

NOESY (400 MHz) spectrum of compound **16** in $CDCl_3$

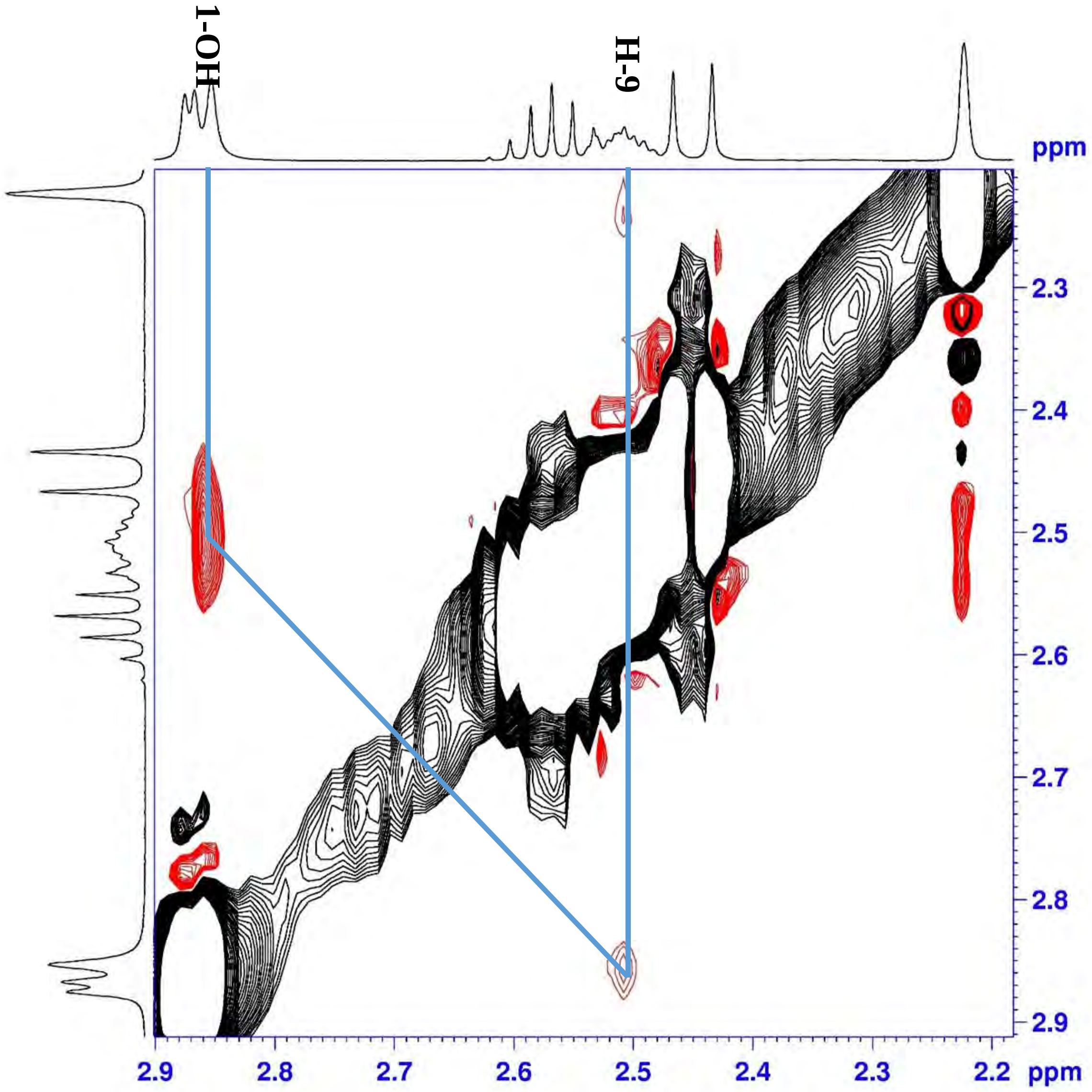

NOESY (400 MHz) spectrum of compound **16** in $CDCl_3$

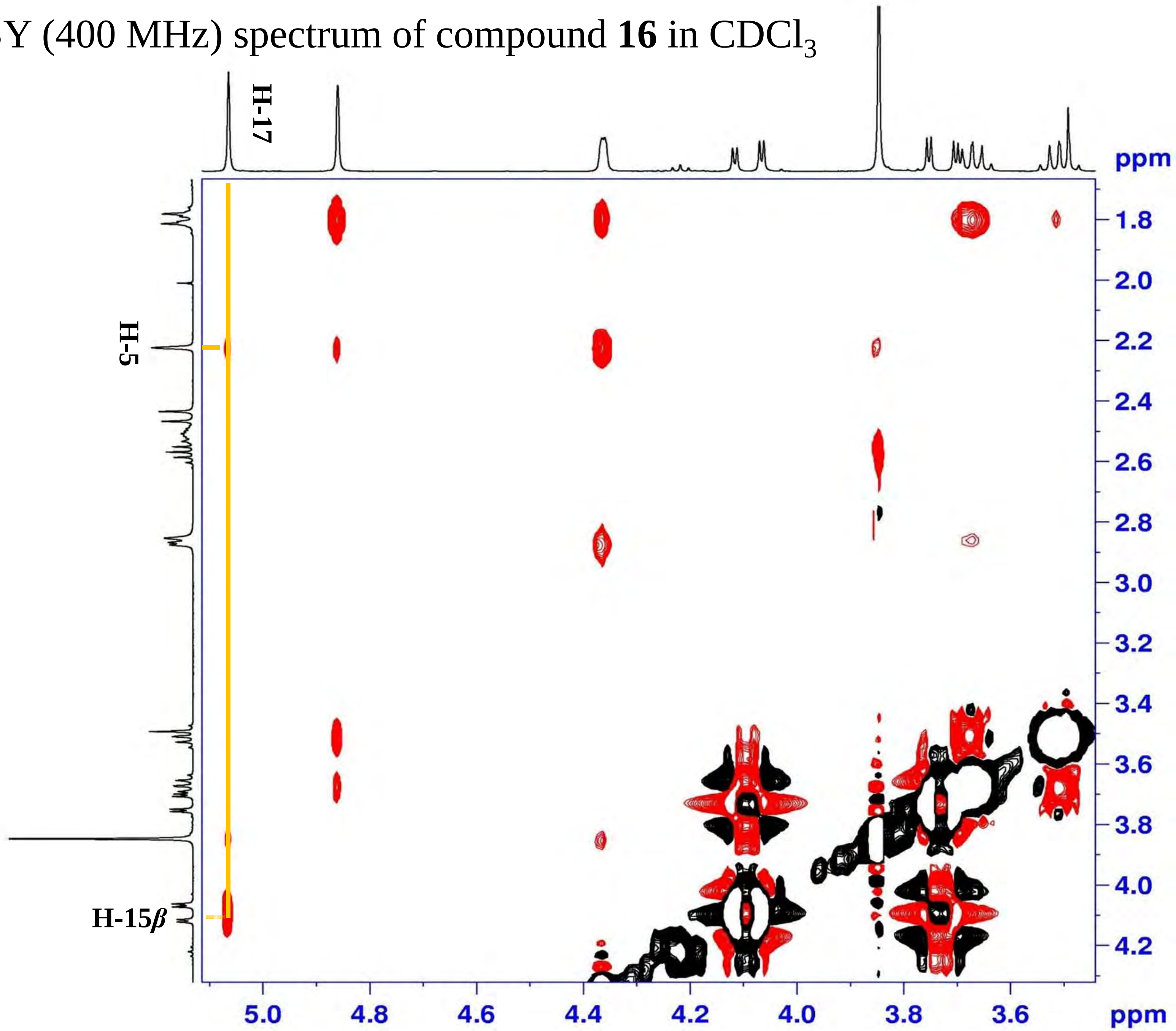

NOESY (400 MHz) spectrum of compound **16** in $CDCl_3$

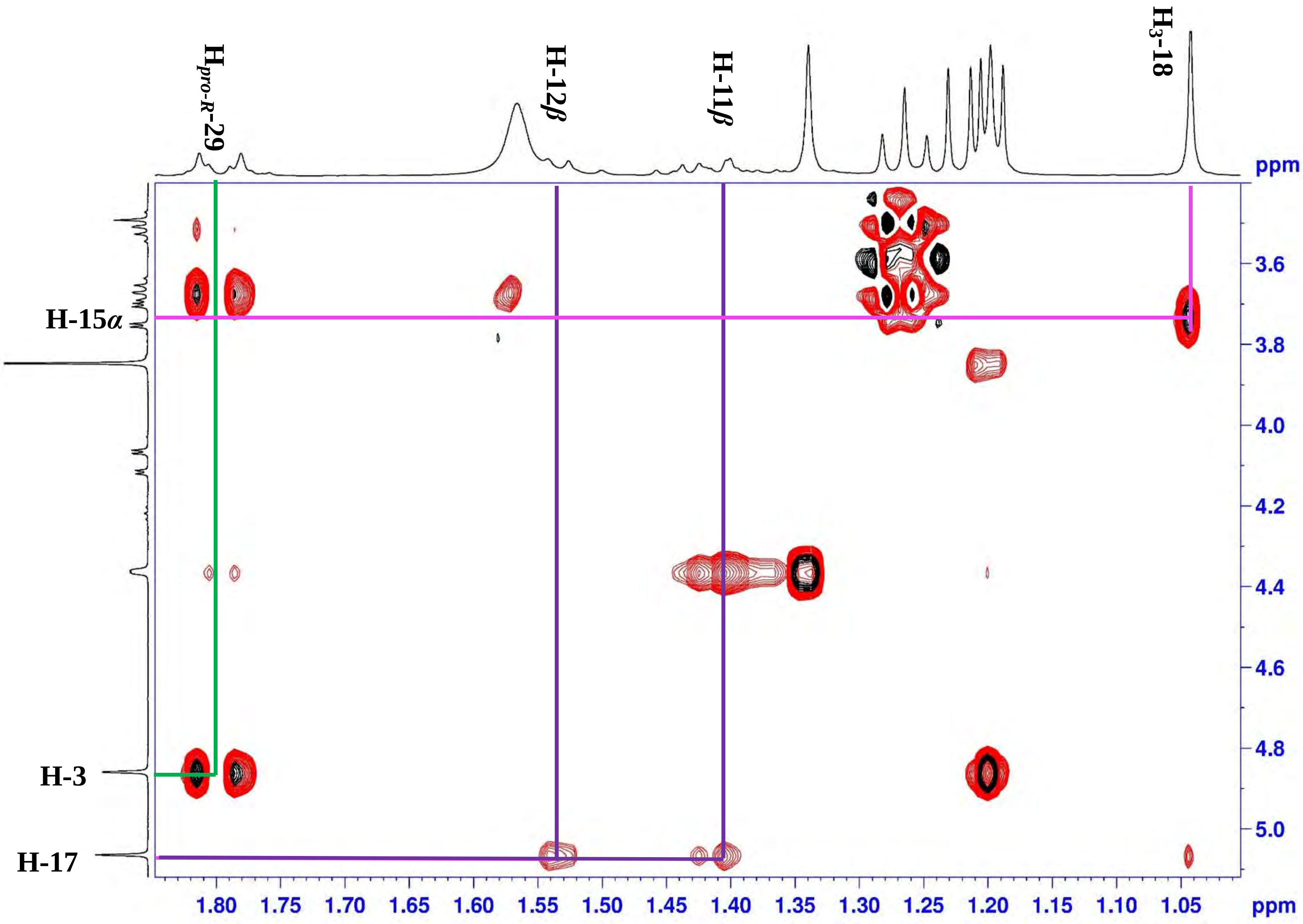

HRESIMS for compound **17**

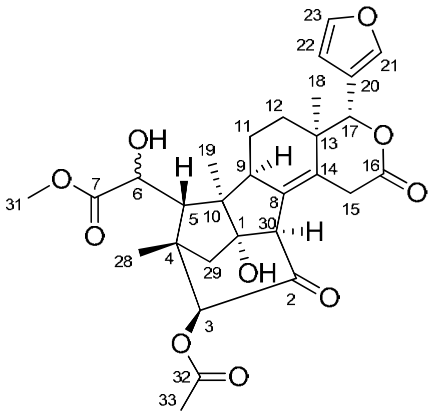

Elemental Composition Report

Page 1

Single Mass Analysis

Tolerance = 5.0 mDa / DBE: min = -1.5, max = 50.0
Element prediction: Off
Number of isotope peaks used for i-FIT = 3

Monoisotopic Mass, Even Electron Ions
151 formula(e) evaluated with 2 results within limits (up to 50 best isotopic matches for each mass)
Elements Used:
C: 0-100 H: 0-200 O: 0-100
S-3
2016102405 142 (1.152) Cm (142:143)

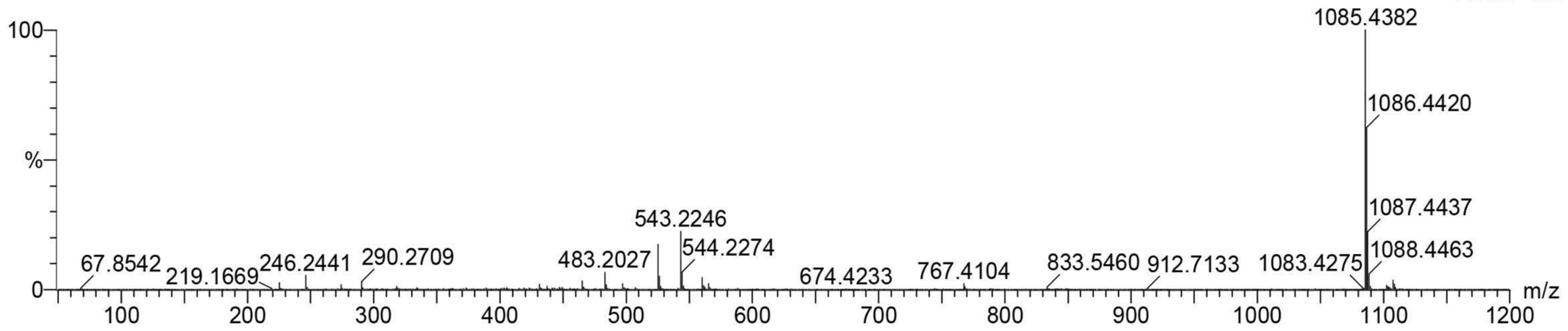

Minimum:				-1.5				
Maximum:		5.0	10.0	50.0				
Mass	Calc. Mass	mDa	PPM	DBE	i-FIT	Norm	Conf(%)	Formula
543.2246	543.2230	1.6	2.9	12.5	24.4	0.082	92.17	C29 H35 O10
	543.2289	-4.3	-7.9	3.5	26.8	2.547	7.83	C22 H39 O15

^{1}H NMR (400 MHz) spectrum of compound **17** in $CDCl_3$

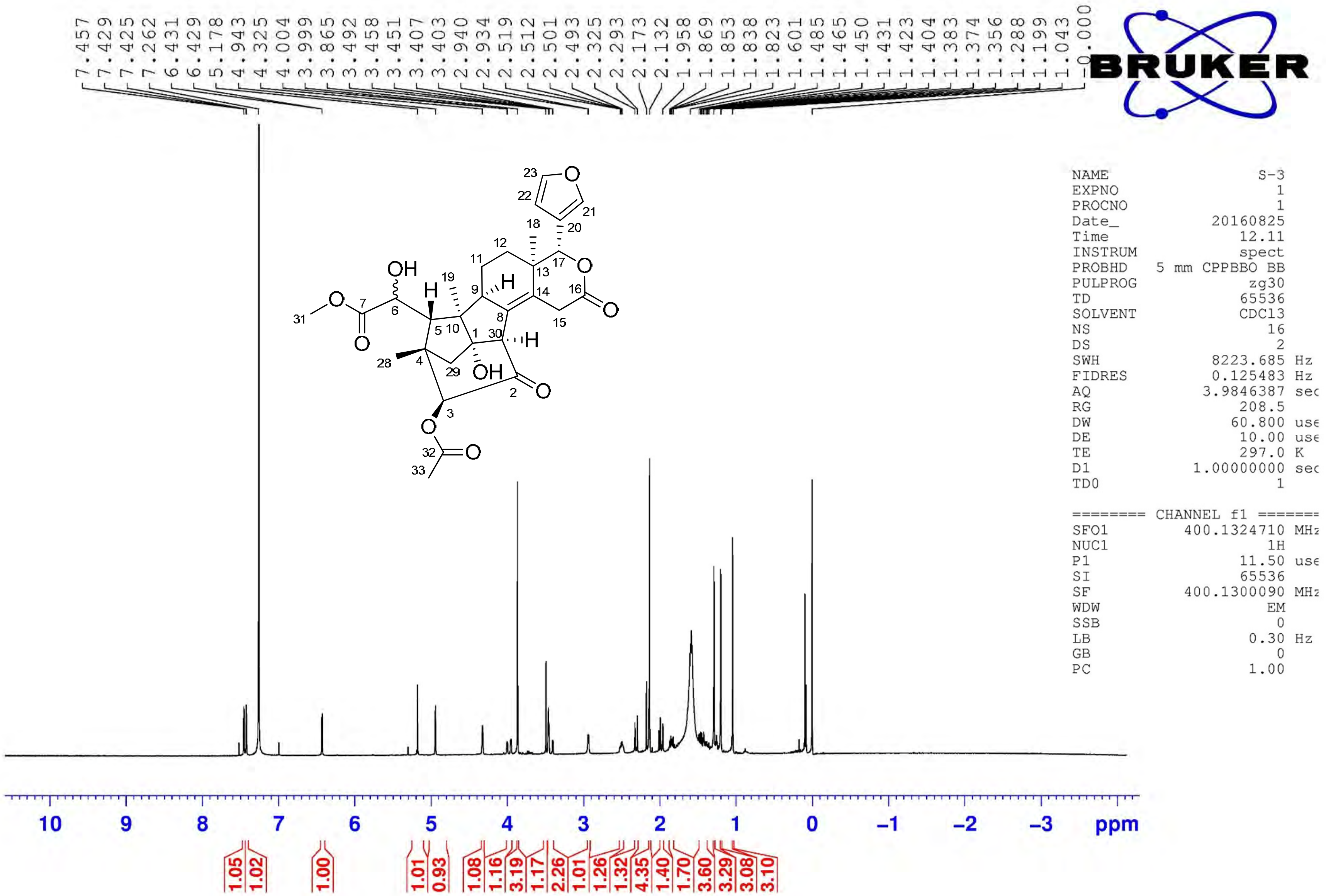

^{1}H NMR (400 MHz) spectrum of compound **17** in $CDCl_3$

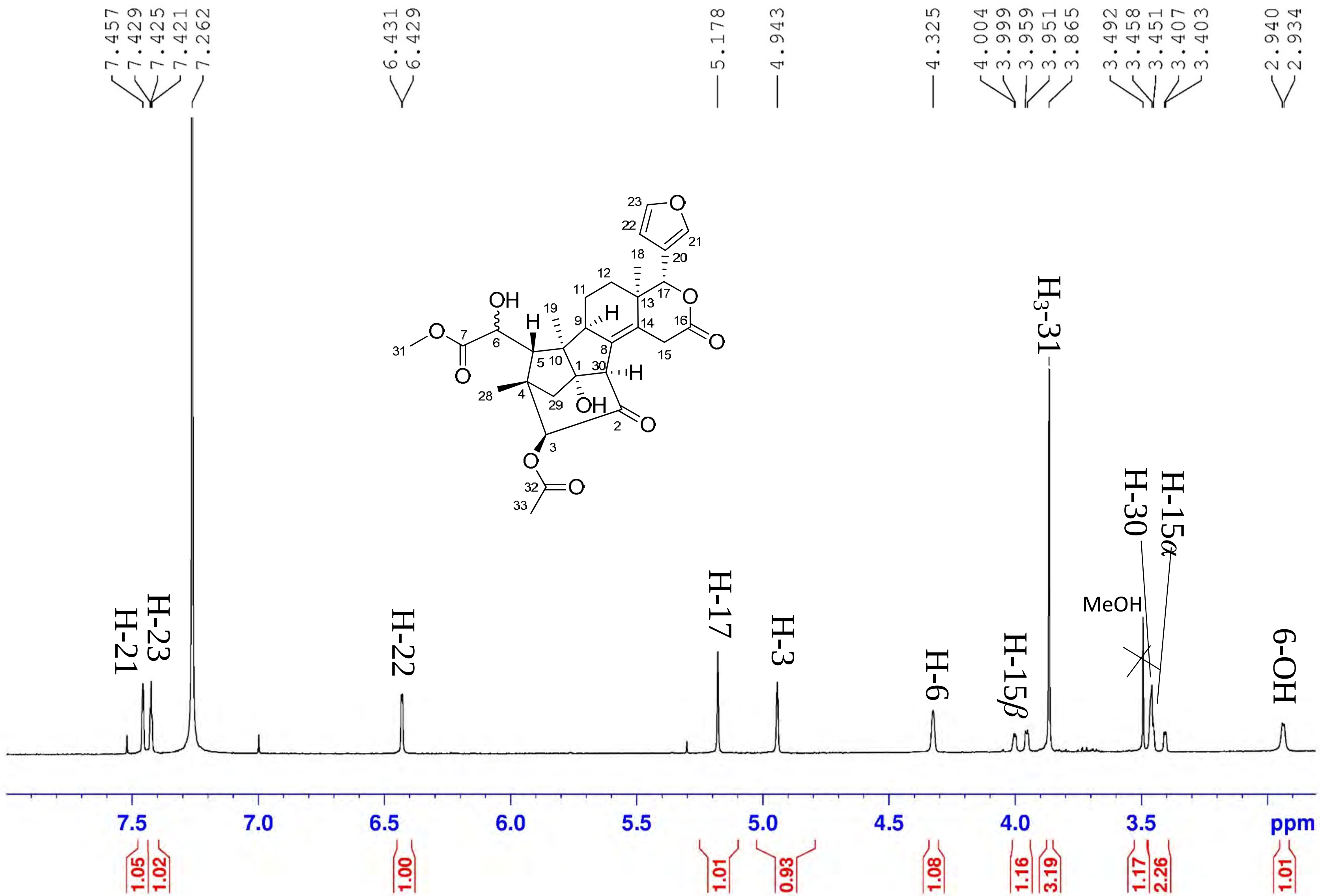

^{1}H NMR (400 MHz) spectrum of compound **17** in $CDCl_3$

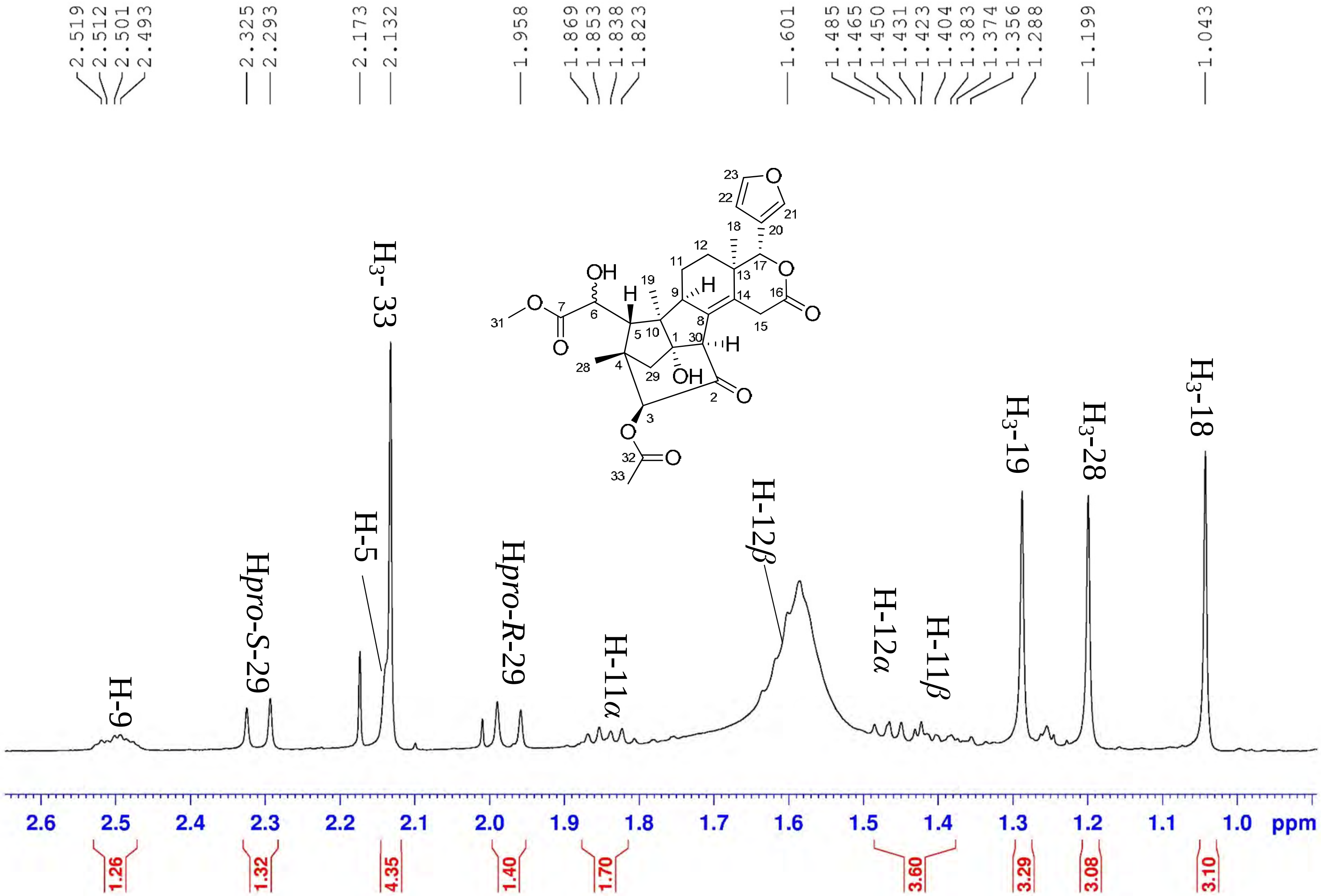

^{13}C NMR (100 MHz) spectrum of compound **17** in $CDCl_3$

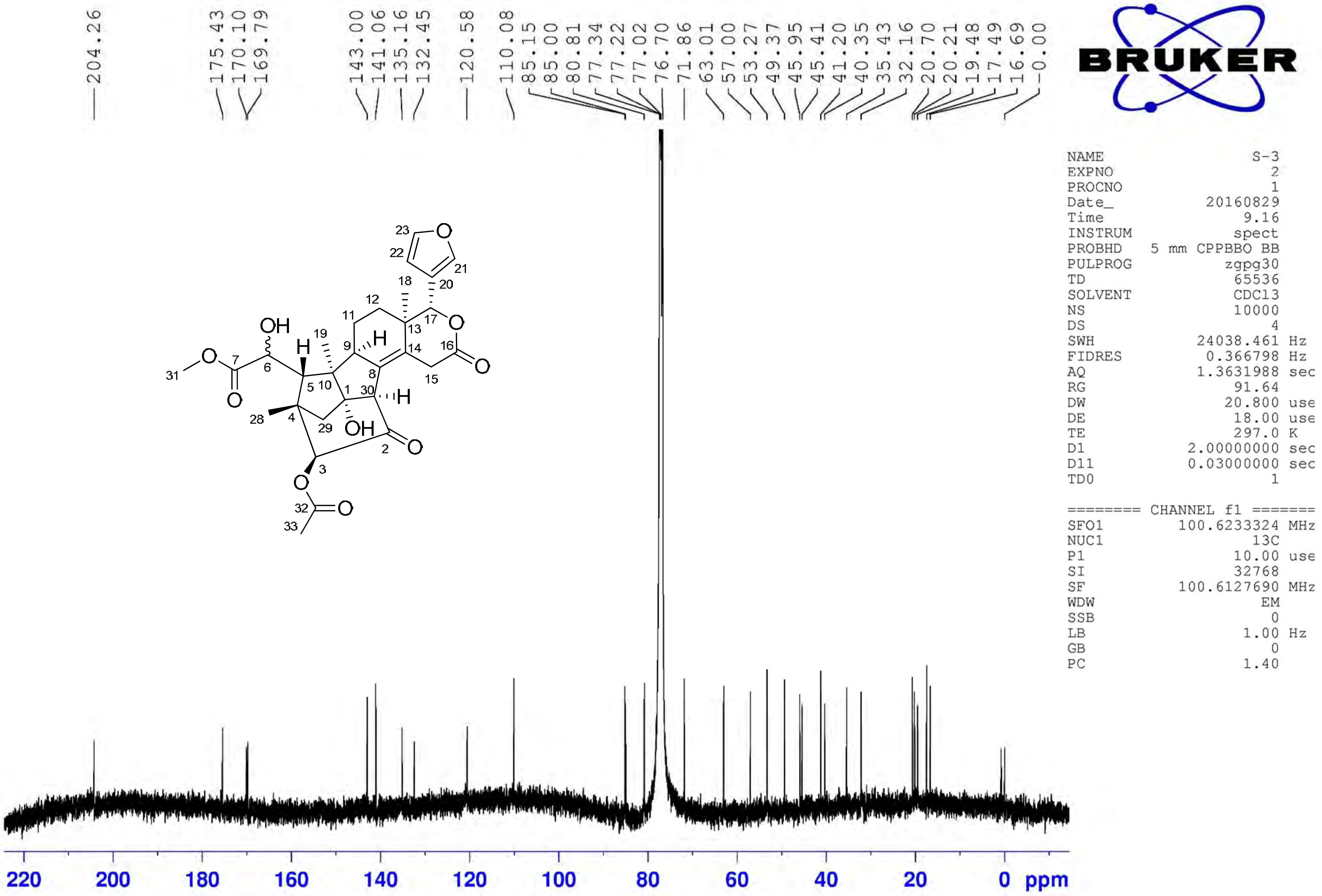

^{13}C NMR (100 MHz) spectrum of compound **17** in $CDCl_3$

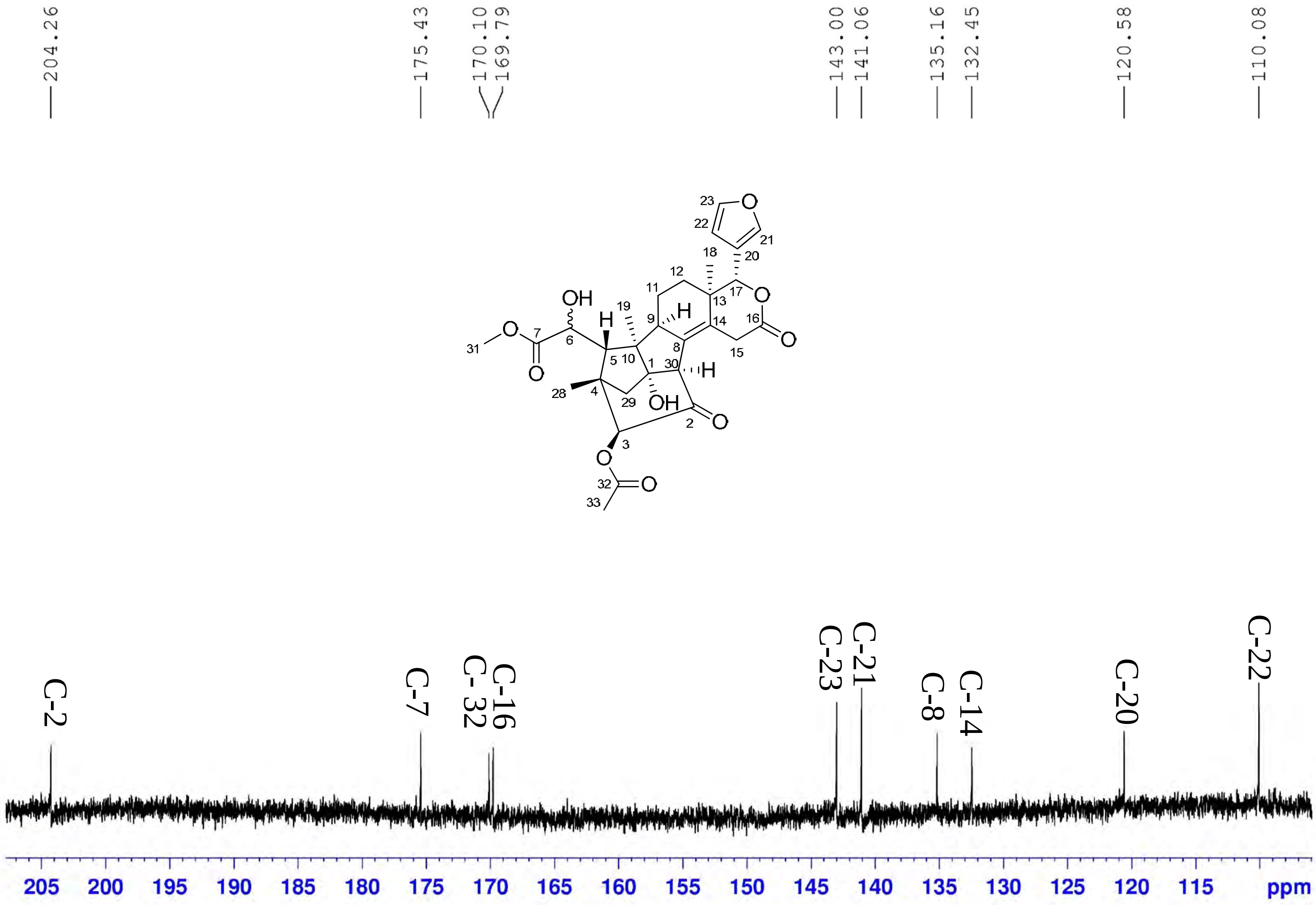

^{13}C NMR (100 MHz) spectrum of compound **17** in $CDCl_3$

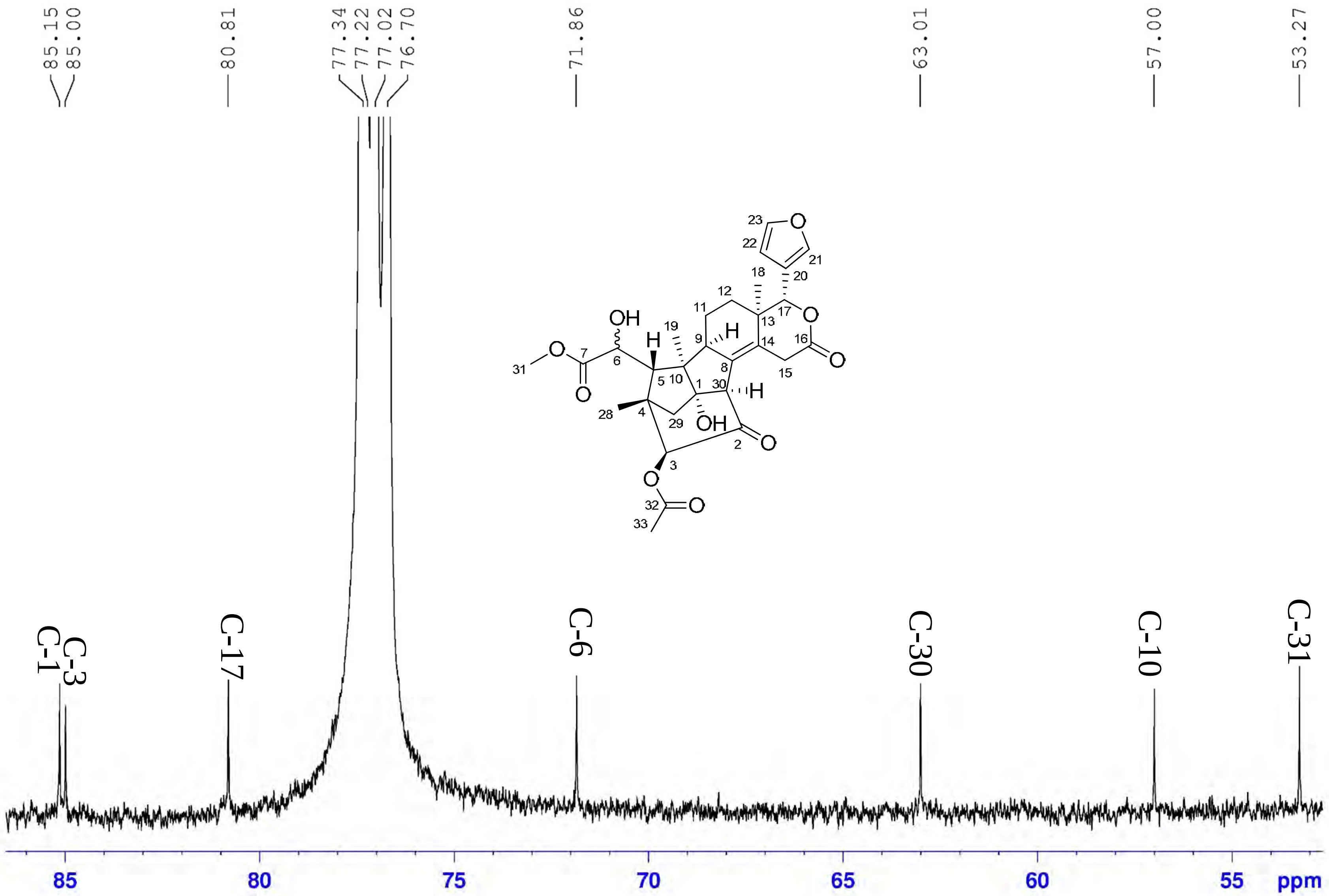

^{13}C NMR (100 MHz) spectrum of compound **17** in $CDCl_3$

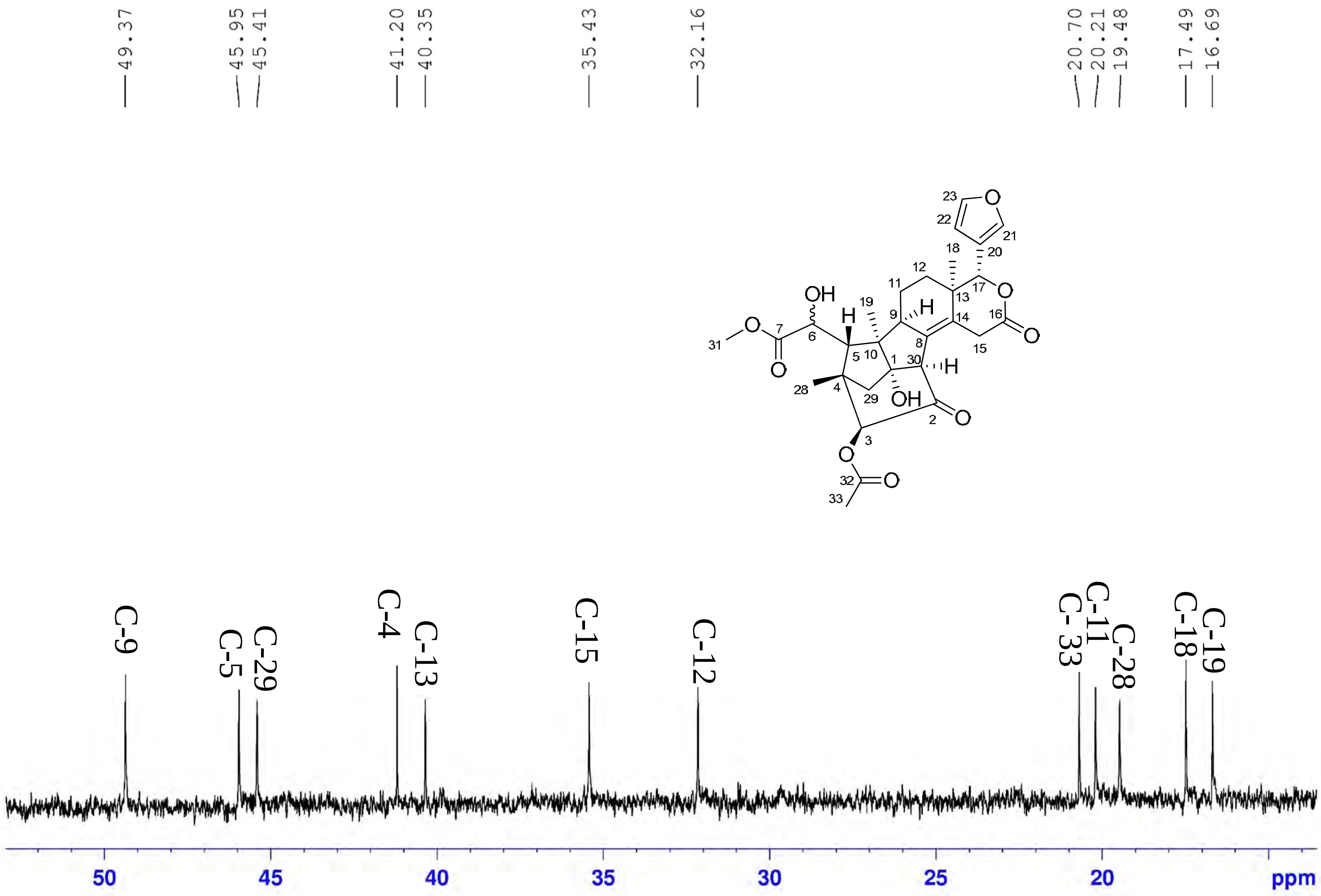

DEPT135 (100 MHz) spectrum of compound **17** in $CDCl_3$

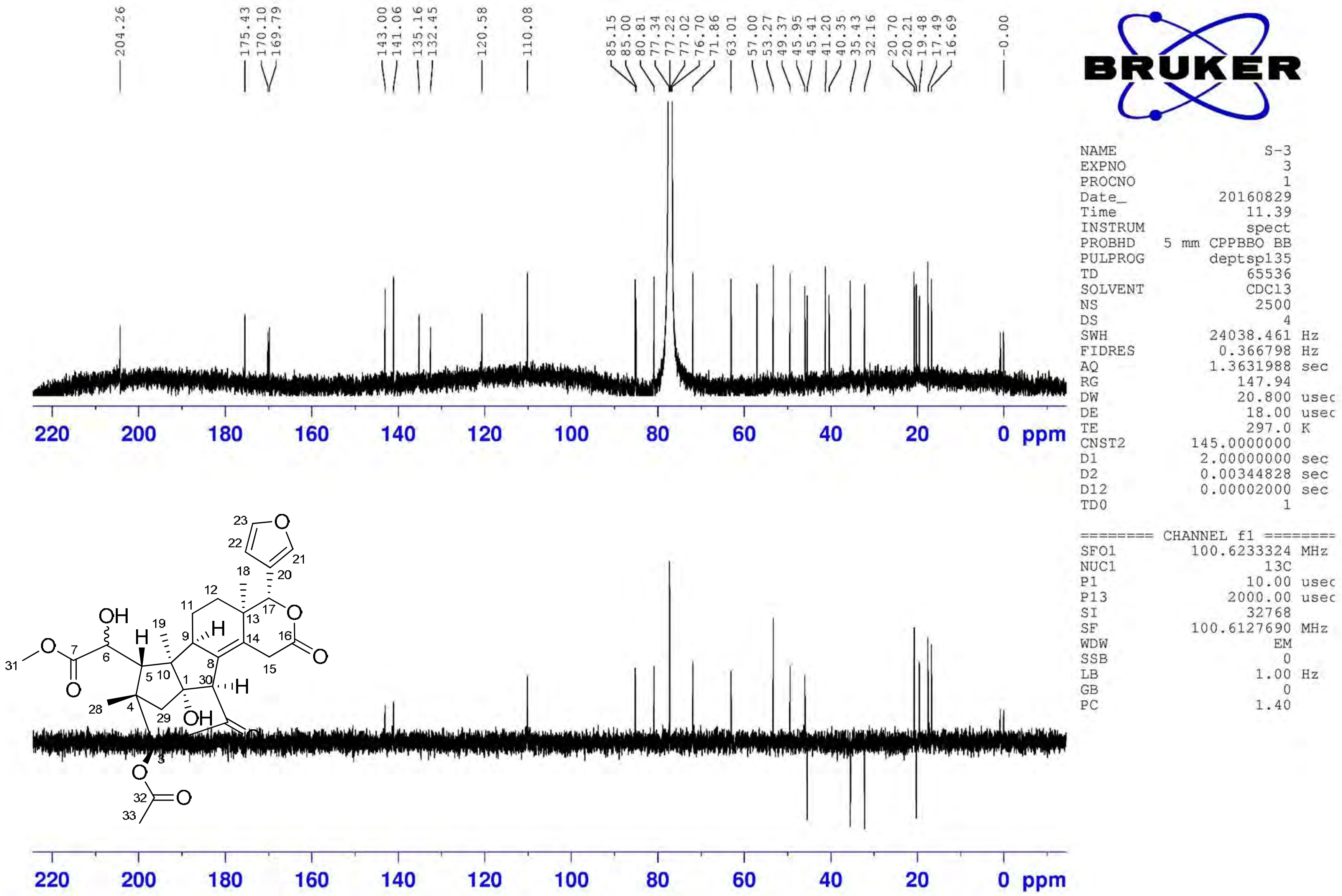

DEPT135 (100 MHz) spectrum of compound **17** in $CDCl_3$

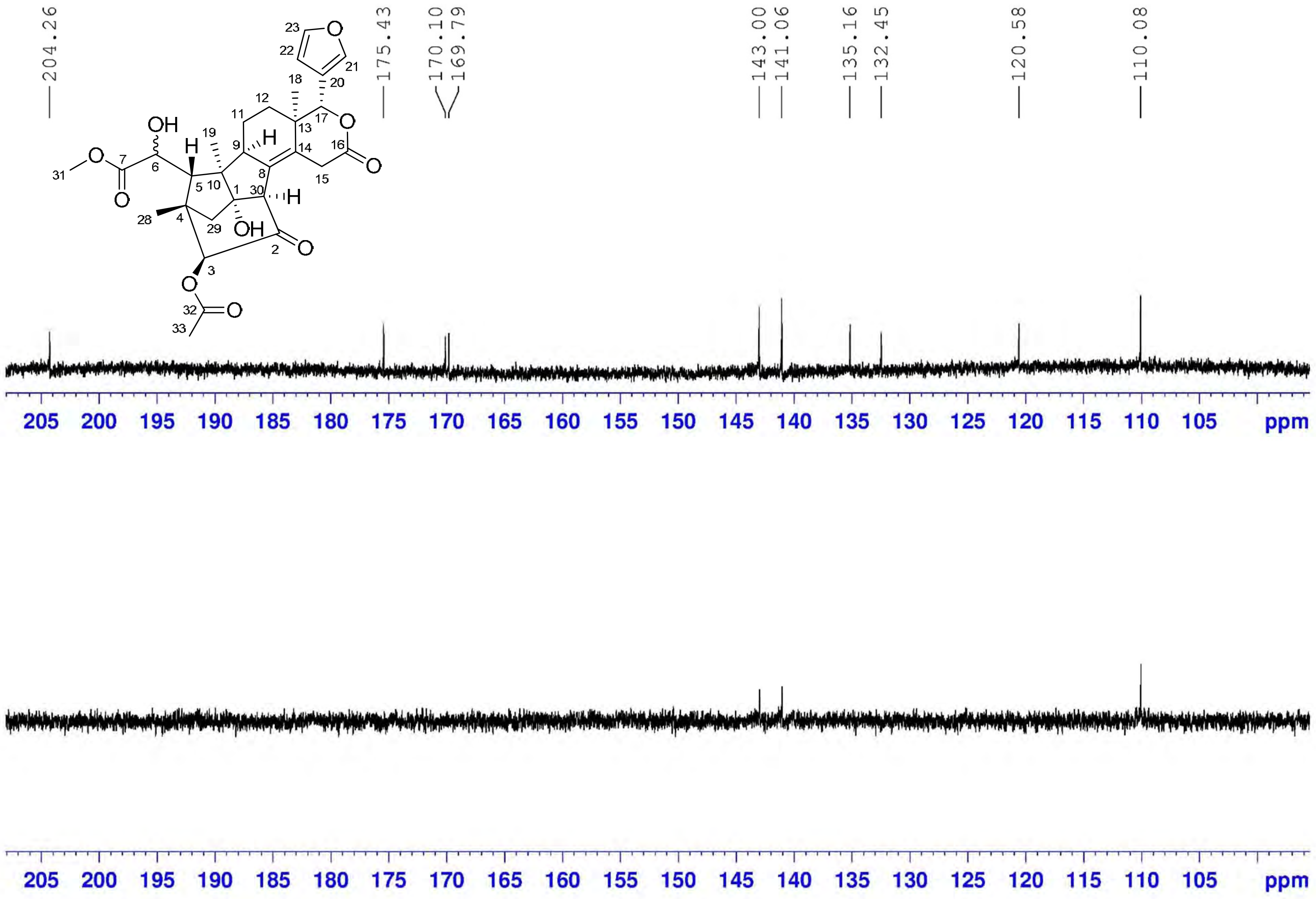

DEPT135 (100 MHz) spectrum of compound **17** in $CDCl_3$

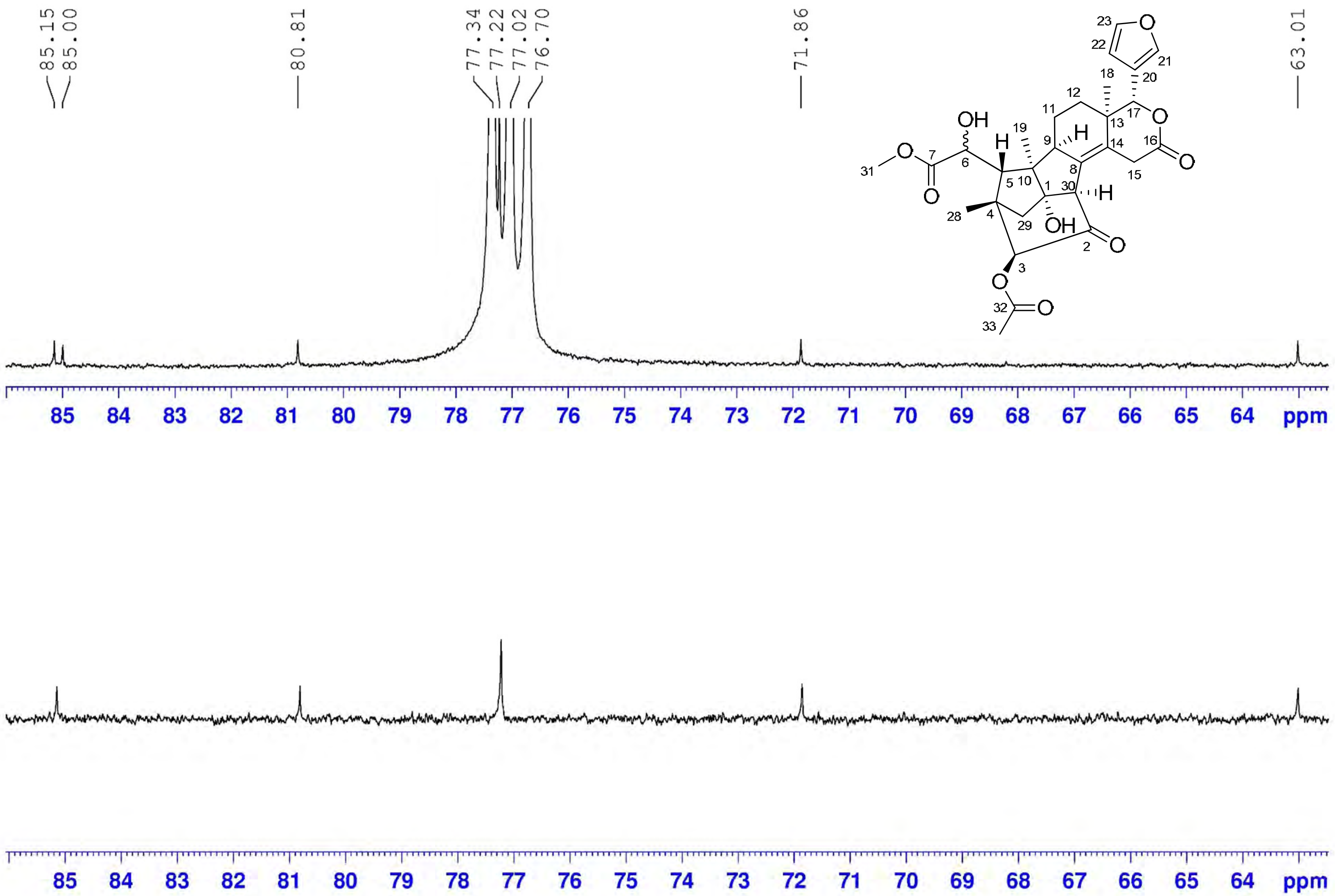

DEPT135 (100 MHz) spectrum of compound **17** in $CDCl_3$

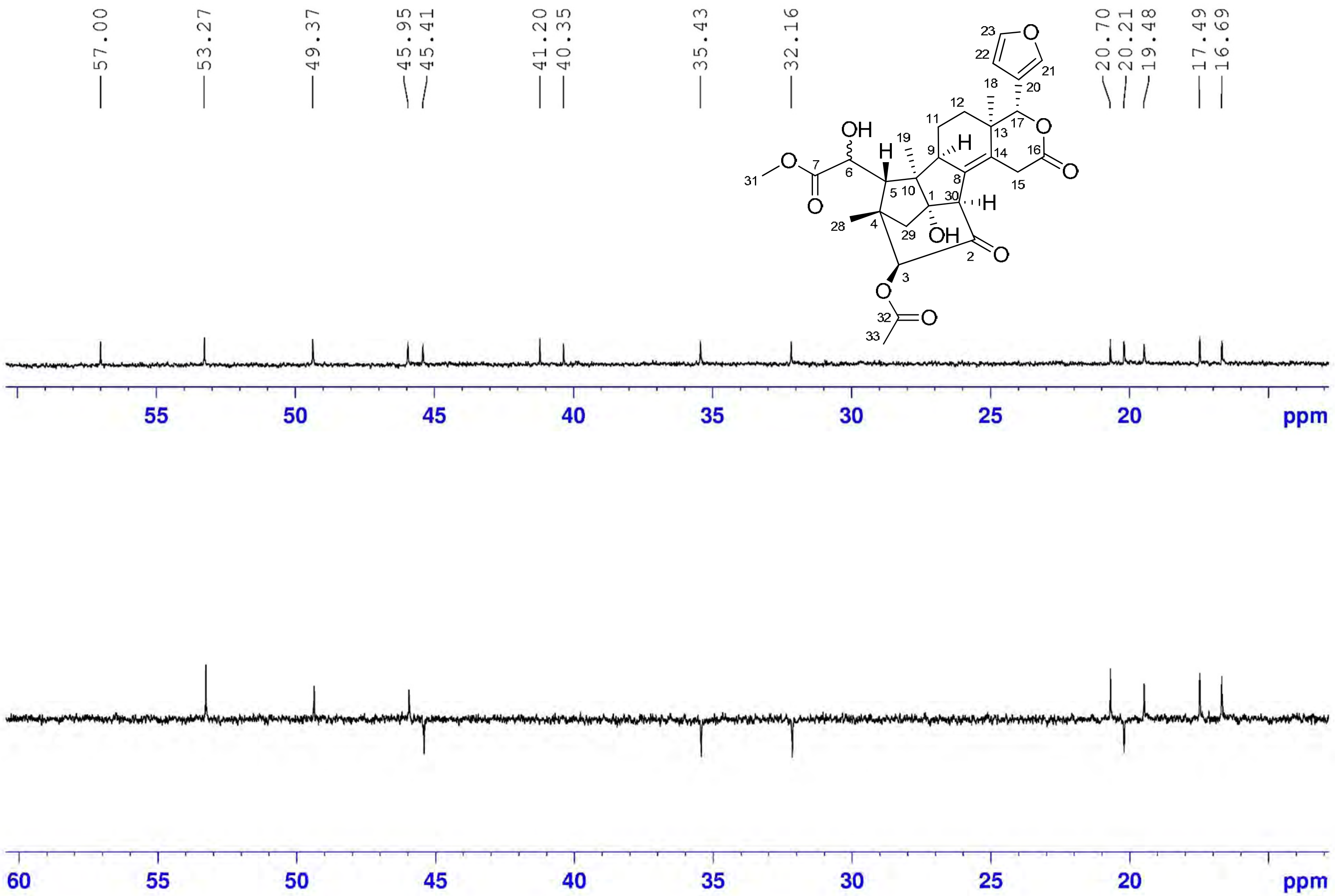

^1H-^1H COSY (400 MHz) spectrum of compound **17** in $CDCl_3$

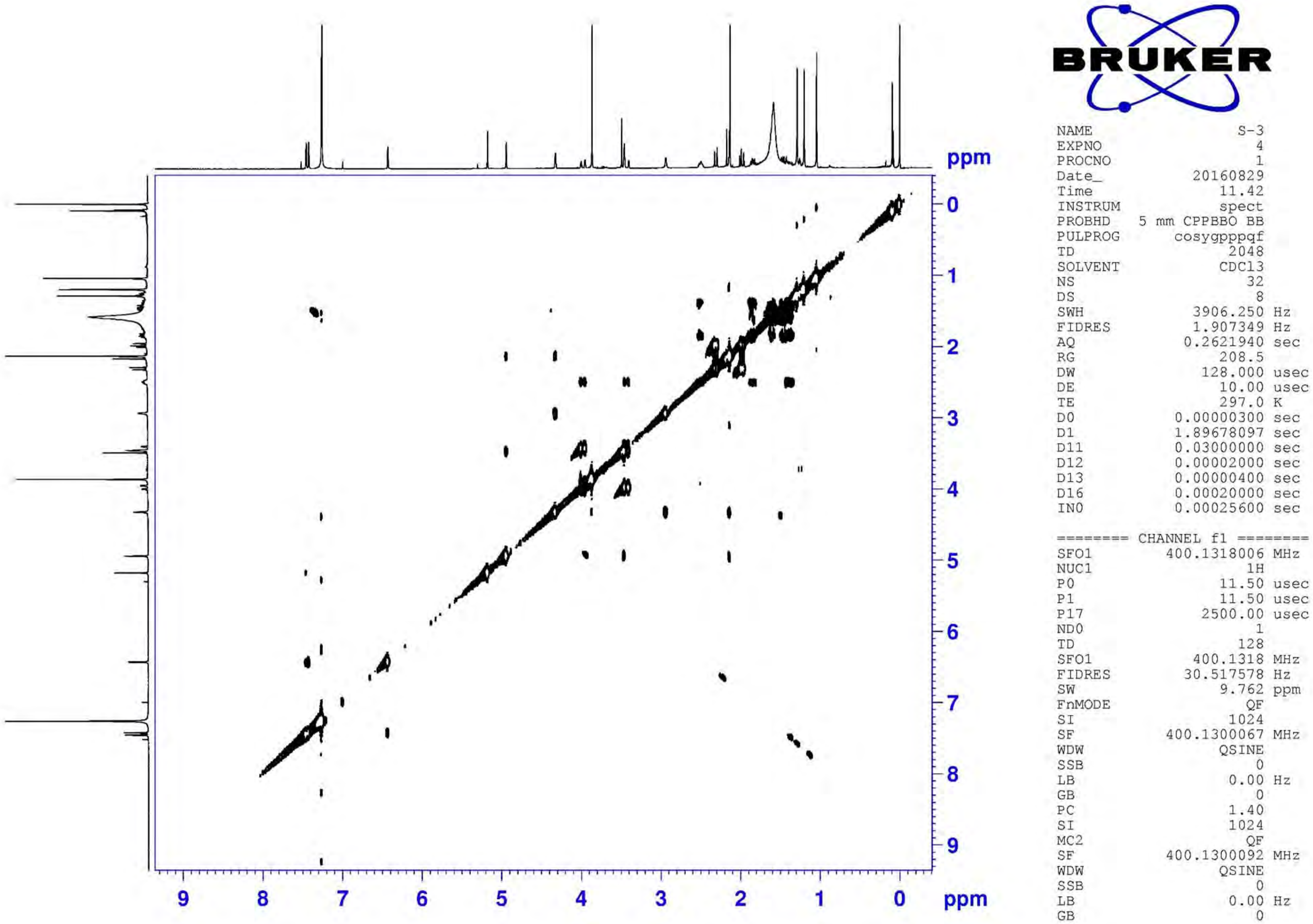

^1H-^1H COSY (400 MHz) spectrum of compound **17** in $CDCl_3$

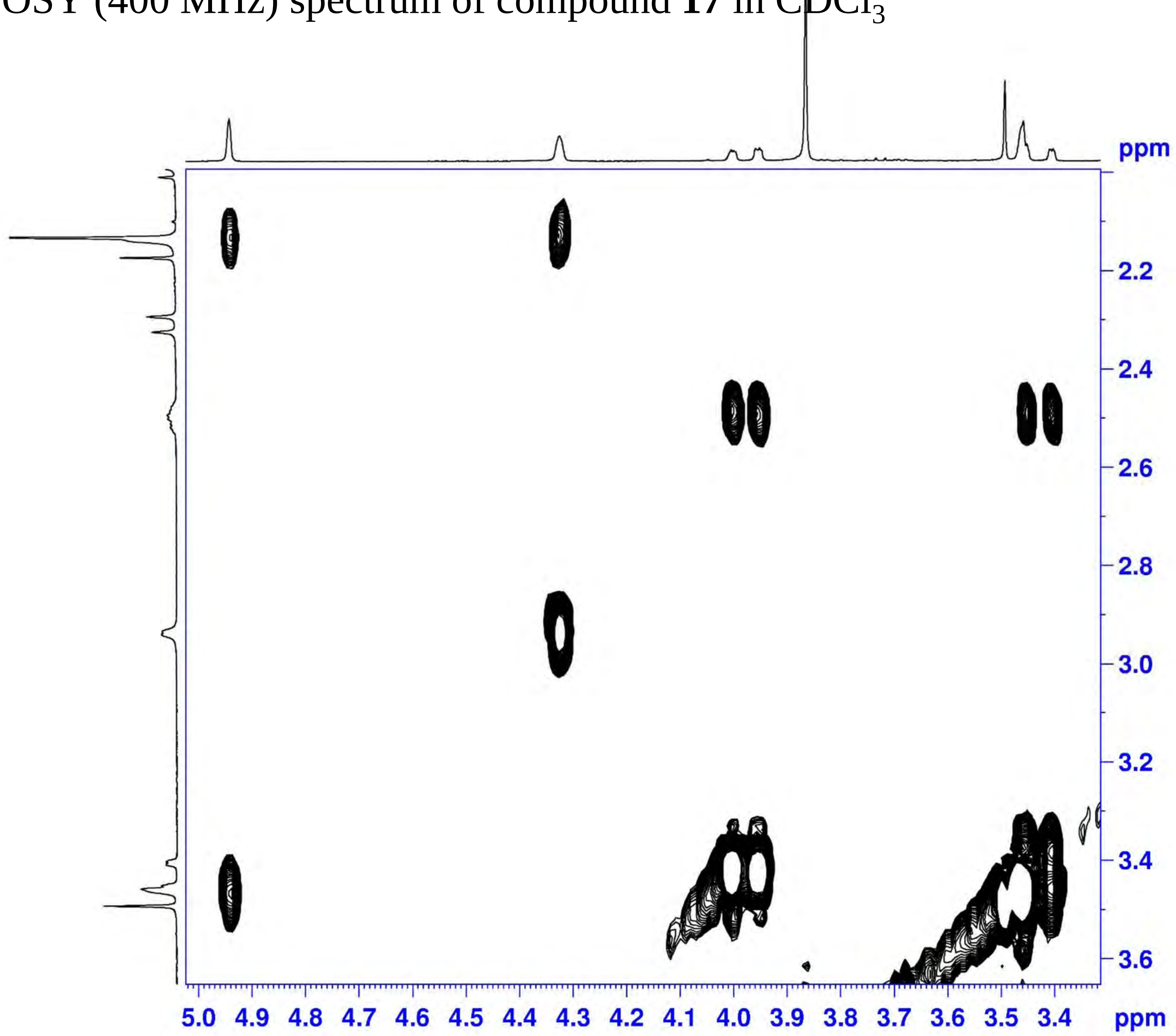

^{1}H-^{1}H COSY (400 MHz) spectrum of compound **17** in $CDCl_3$

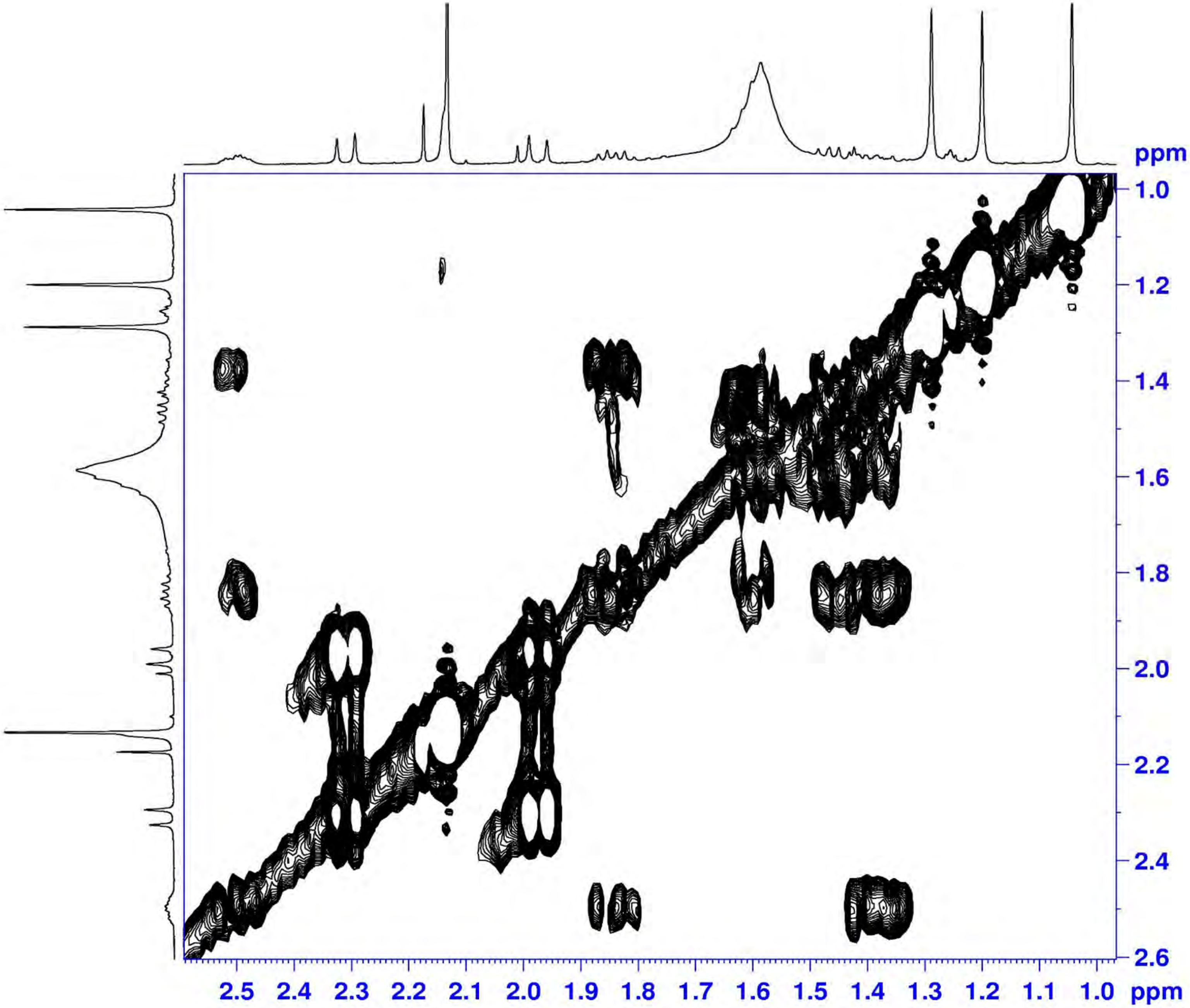

HSQC (400 MHz) spectrum of compound **17** in $CDCl_3$

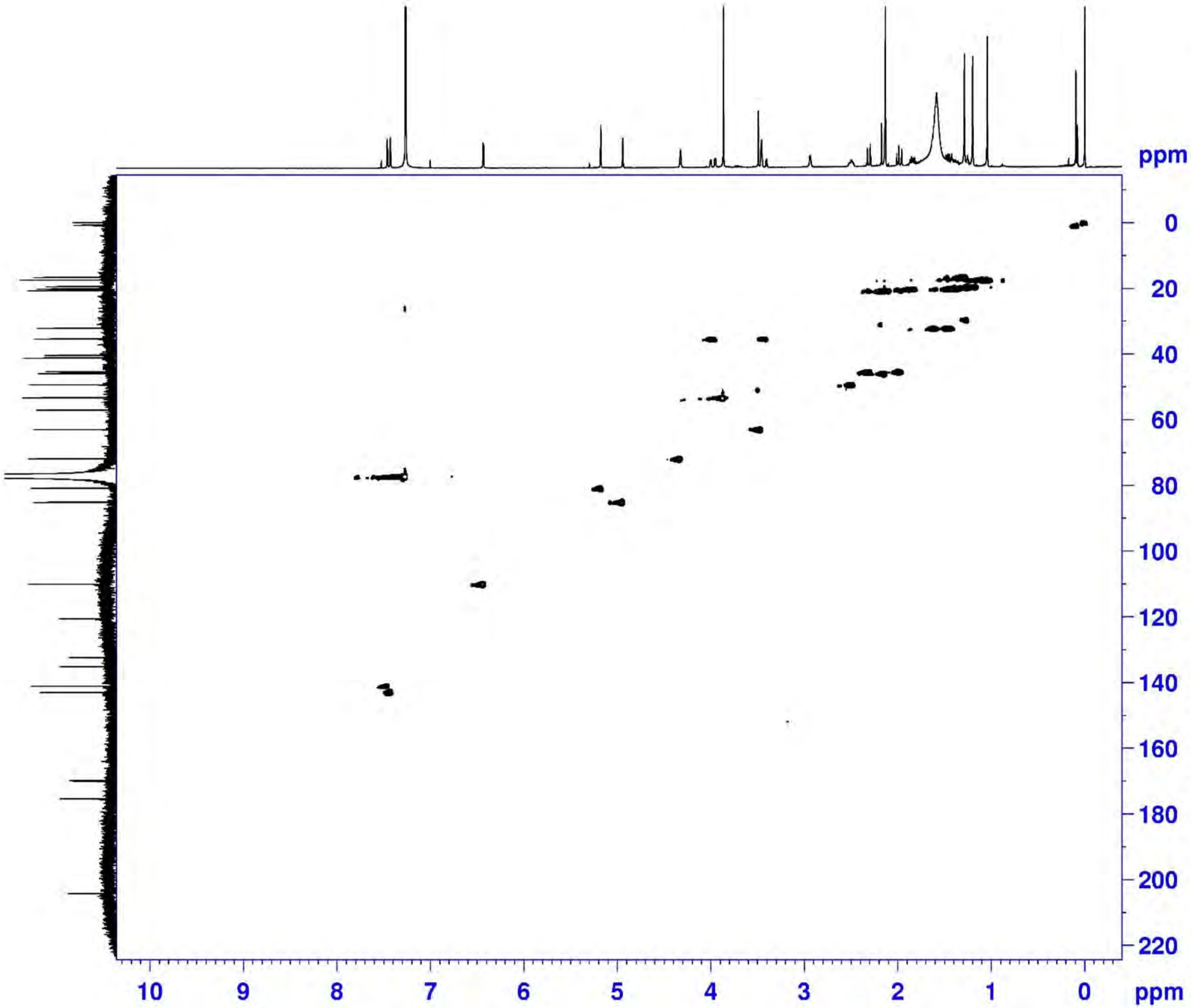


```
NAME                S-3
EXPNO                 5
PROCNO                1
Date_          20160829
Time              14.14
INSTRUM           spect
PROBHD   5 mm CPPBBO BB
PULPROG      hsqcetgpsi2
TD                 1024
SOLVENT           CDCl3
NS                   32
DS                   16
SWH            4302.926 Hz
FIDRES         4.202076 Hz
AQ            0.1190388 sec
RG                208.5
DW              116.200 usec
DE                10.00 usec
TE                297.0 K
CNST2       145.0000000
D0           0.00000300 sec
D1           1.46497905 sec
D4           0.00172414 sec
D11          0.03000000 sec
D16          0.00020000 sec
D24          0.00086207 sec
IN0          0.00002080 sec
ZGOPTNS

======== CHANNEL f1 ========
SFO1        400.1320007 MHz
NUC1                 1H
P1                11.50 usec
P2                23.00 usec
P28                0.00 usec
ND0                   2
TD                  256
SFO1        100.6233 MHz
FIDRES        93.900238 Hz
SW              238.896 ppm
FnMODE     Echo-Antiecho
SI                 1024
SF          400.1300068 MHz
WDW               QSINE
SSB                   2
LB                 0.00 Hz
GB                    0
PC                 1.40
SI                 1024
MC2        echo-antiecho
SF          100.6127587 MHz
WDW               QSINE
SSB                   2
LB                 0.00 Hz
GB                    0
```

HSQC (400 MHz) spectrum of compound **17** in $CDCl_3$

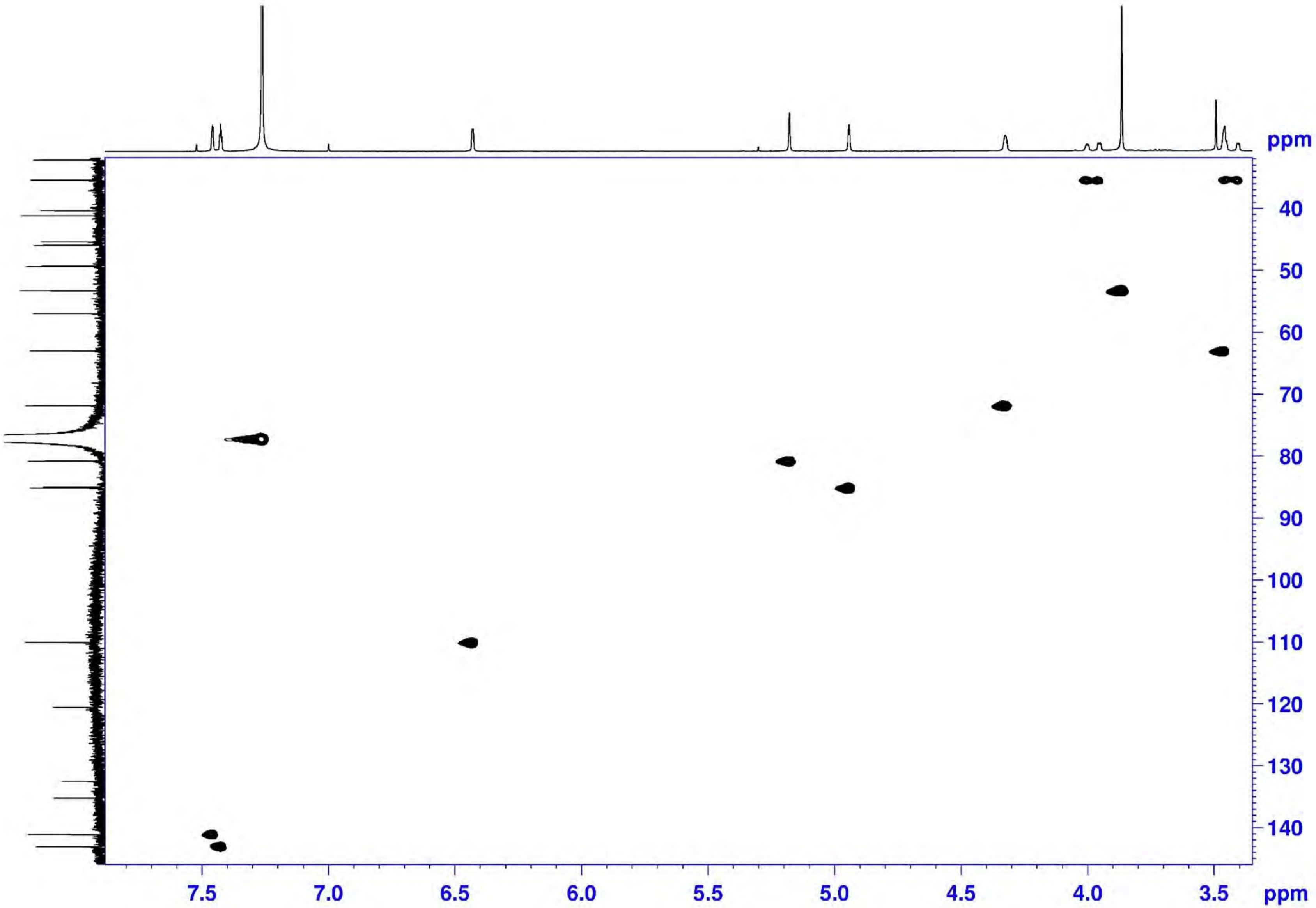

HSQC (400 MHz) spectrum of compound **17** in $CDCl_3$

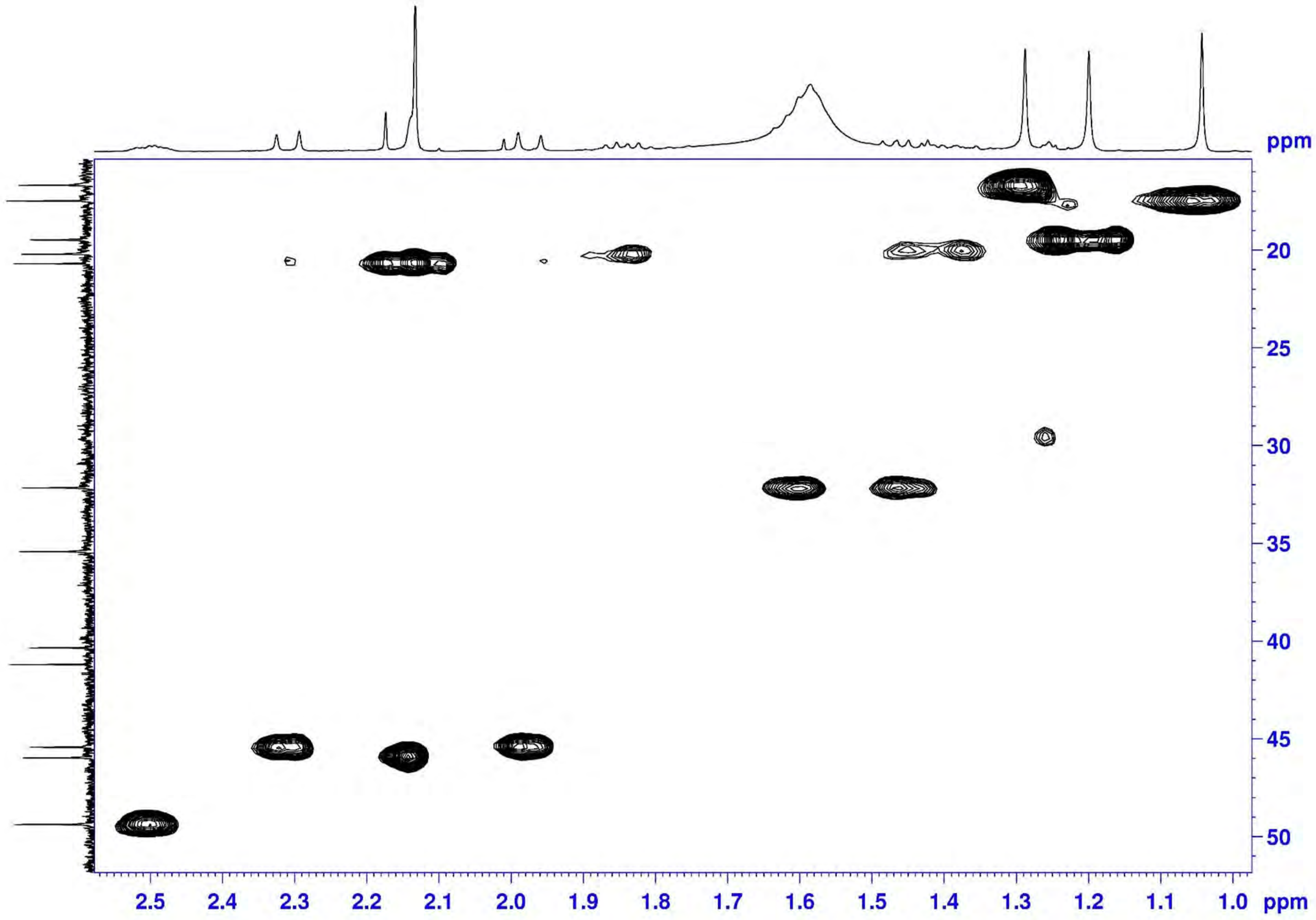

HMBC (400 MHz) spectrum of compound **17** in $CDCl_3$

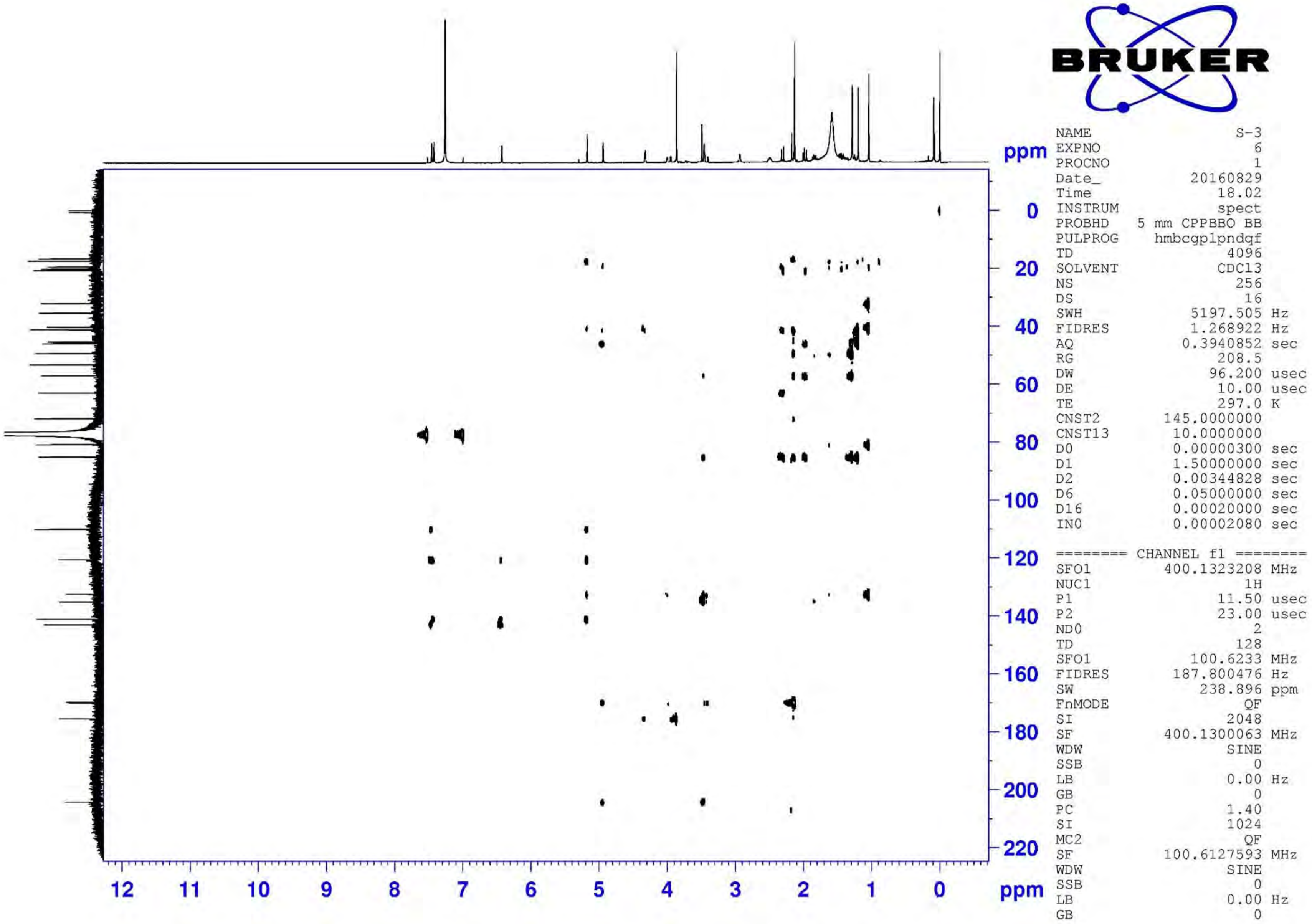

HMBC (400 MHz) spectrum of compound **17** in $CDCl_3$

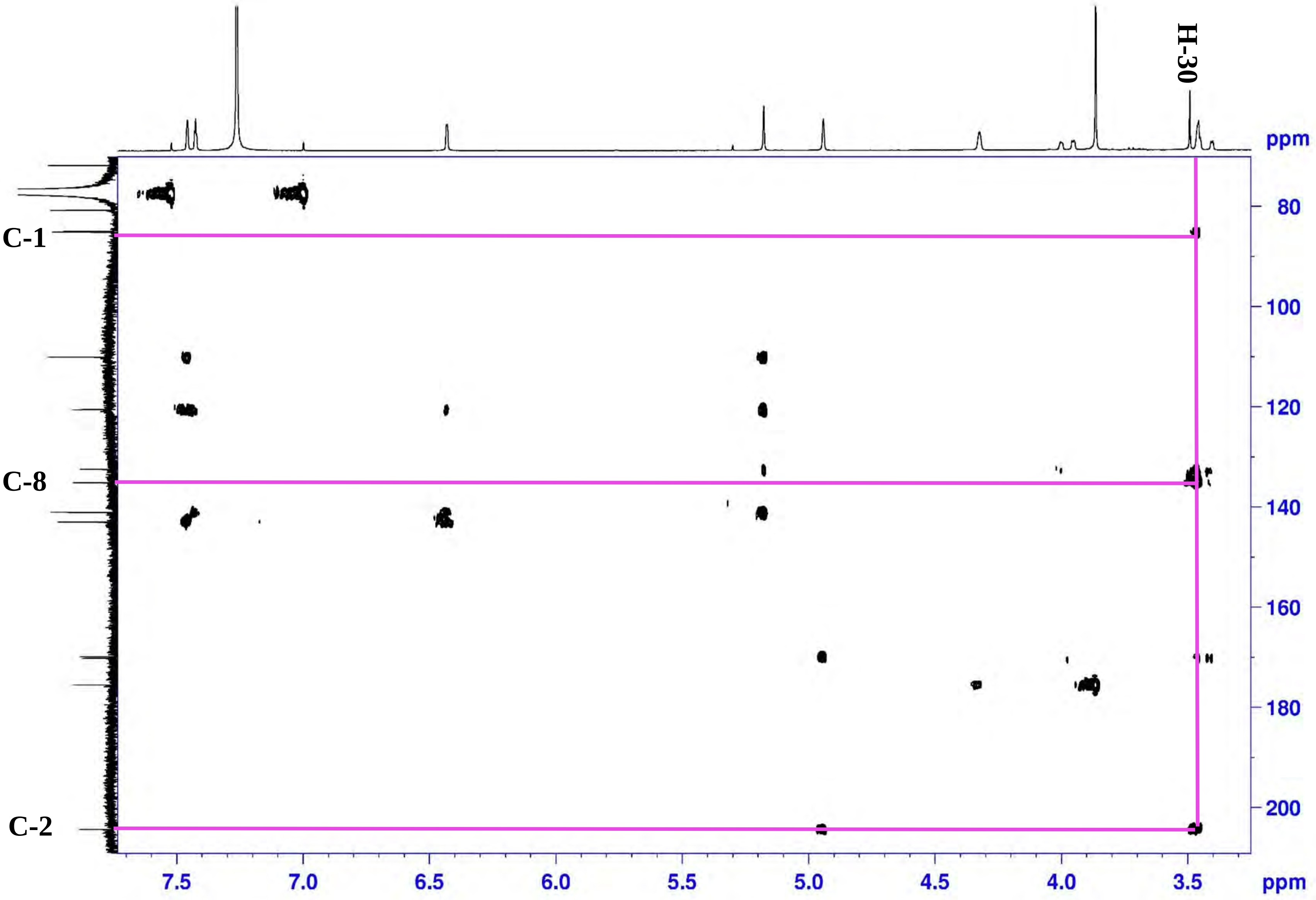

HMBC (400 MHz) spectrum of compound **17** in $CDCl_3$

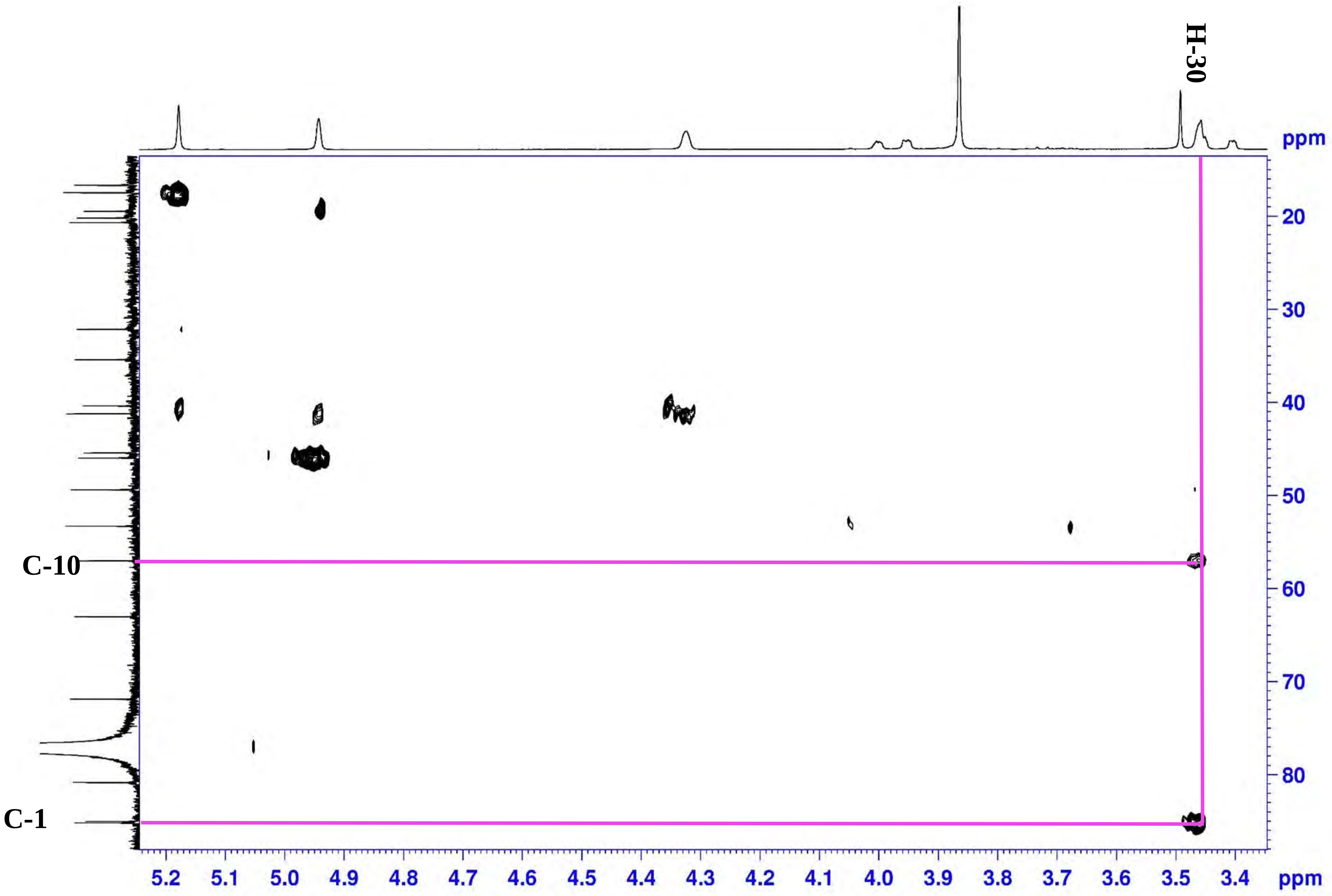

HMBC (400 MHz) spectrum of compound **17** in $CDCl_3$

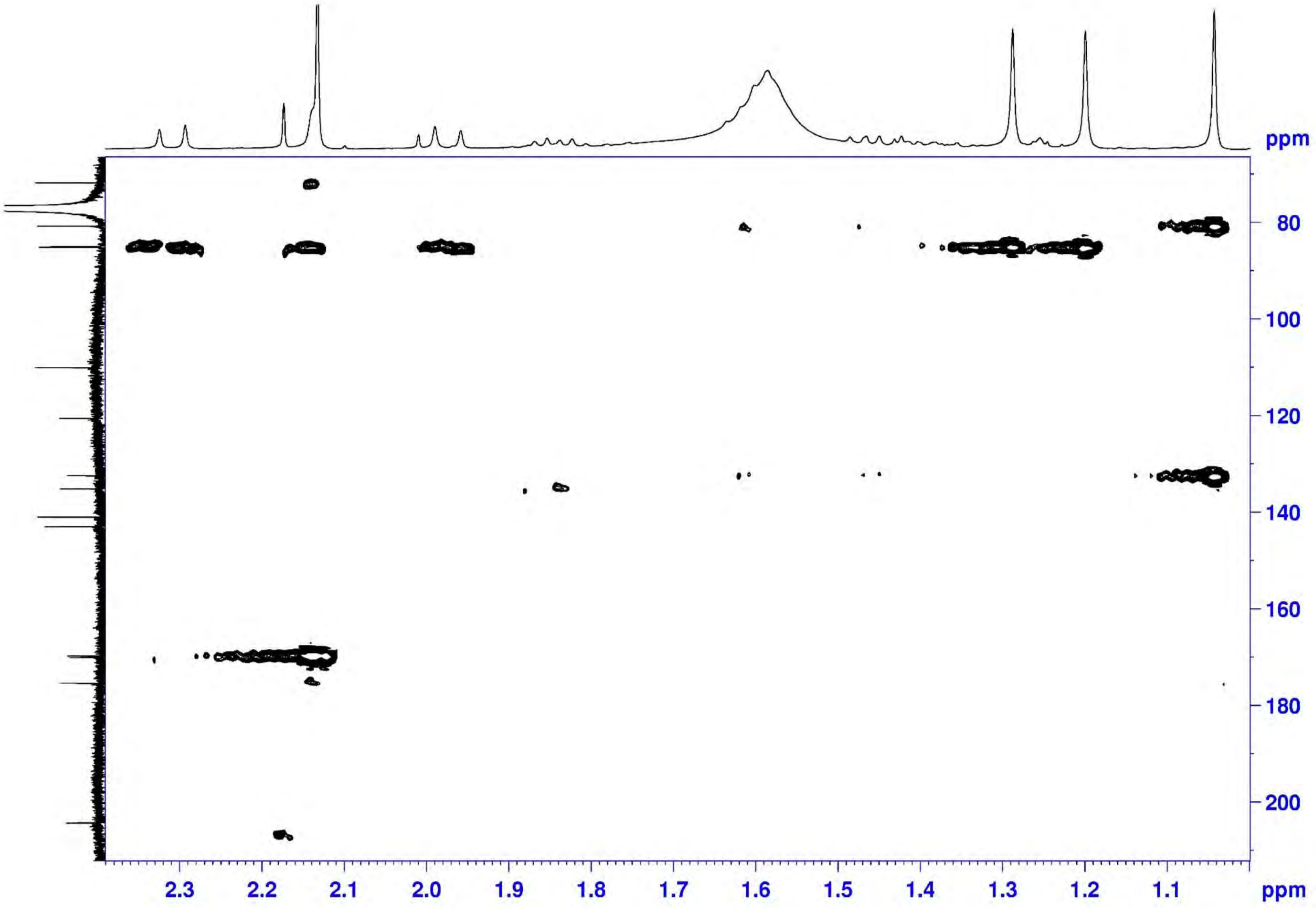

HMBC (400 MHz) spectrum of compound **17** in $CDCl_3$

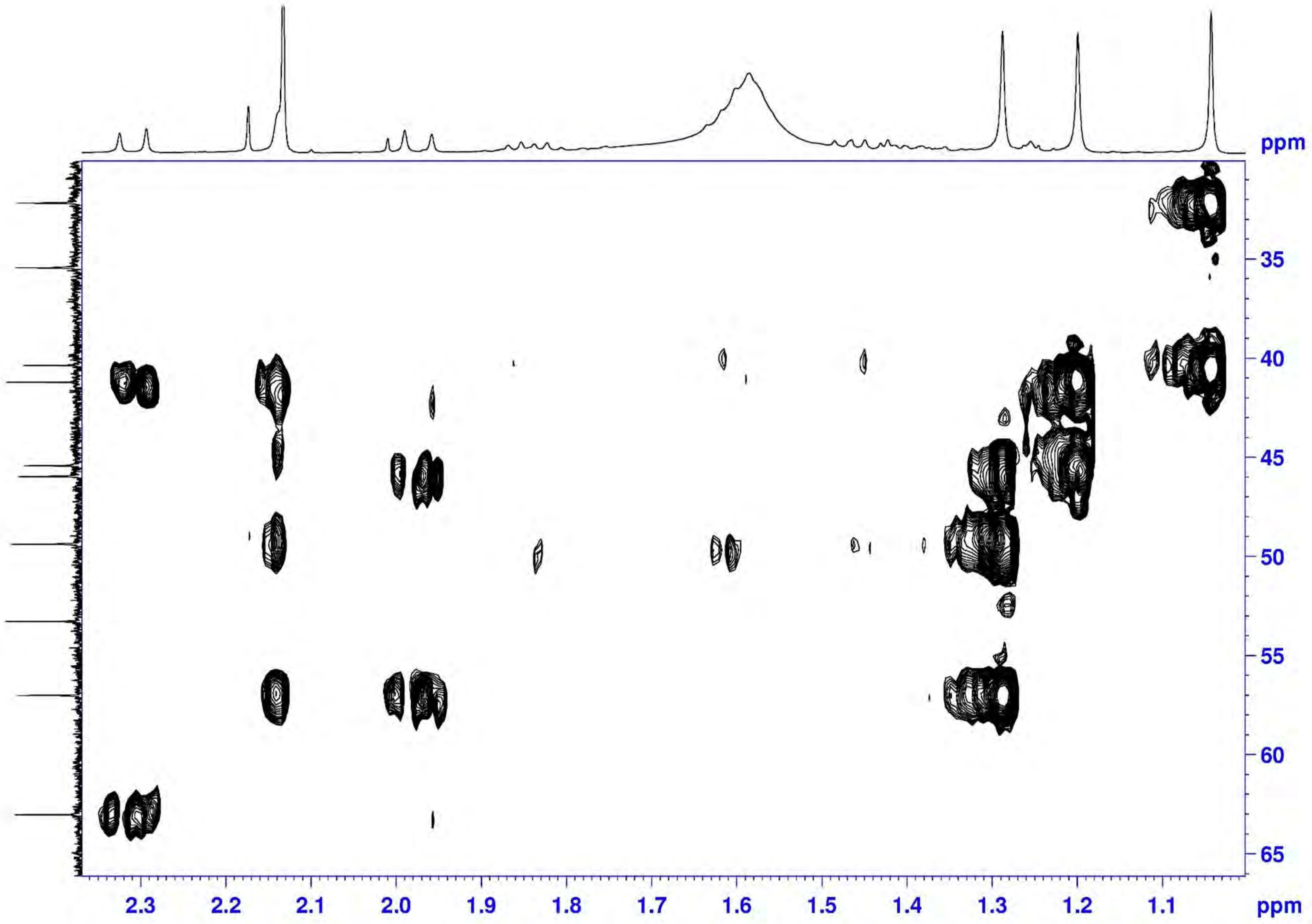

HMBC (400 MHz) spectrum of compound **17** in $CDCl_3$

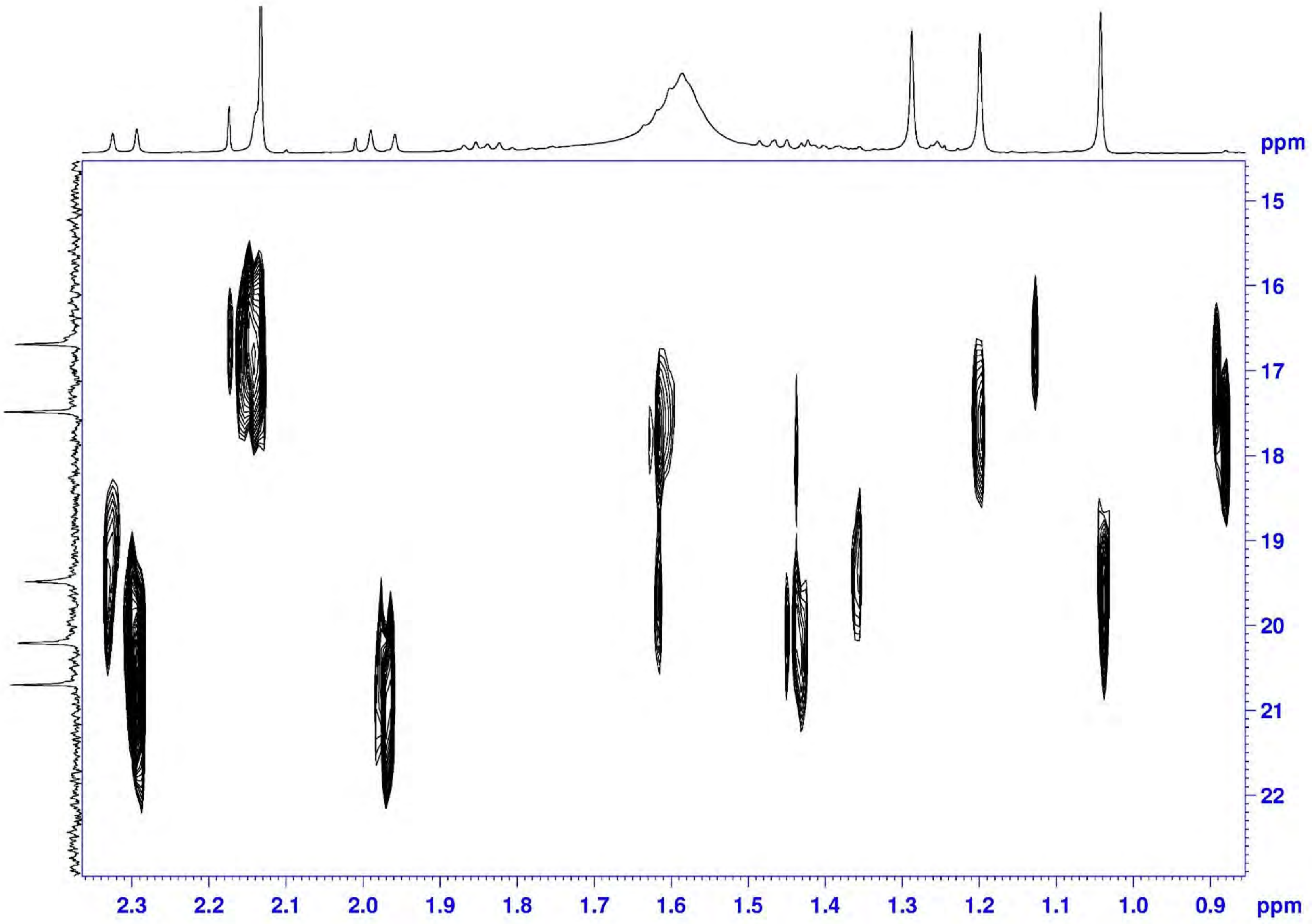

NOESY (400 MHz) spectrum of compound **17** in $CDCl_3$

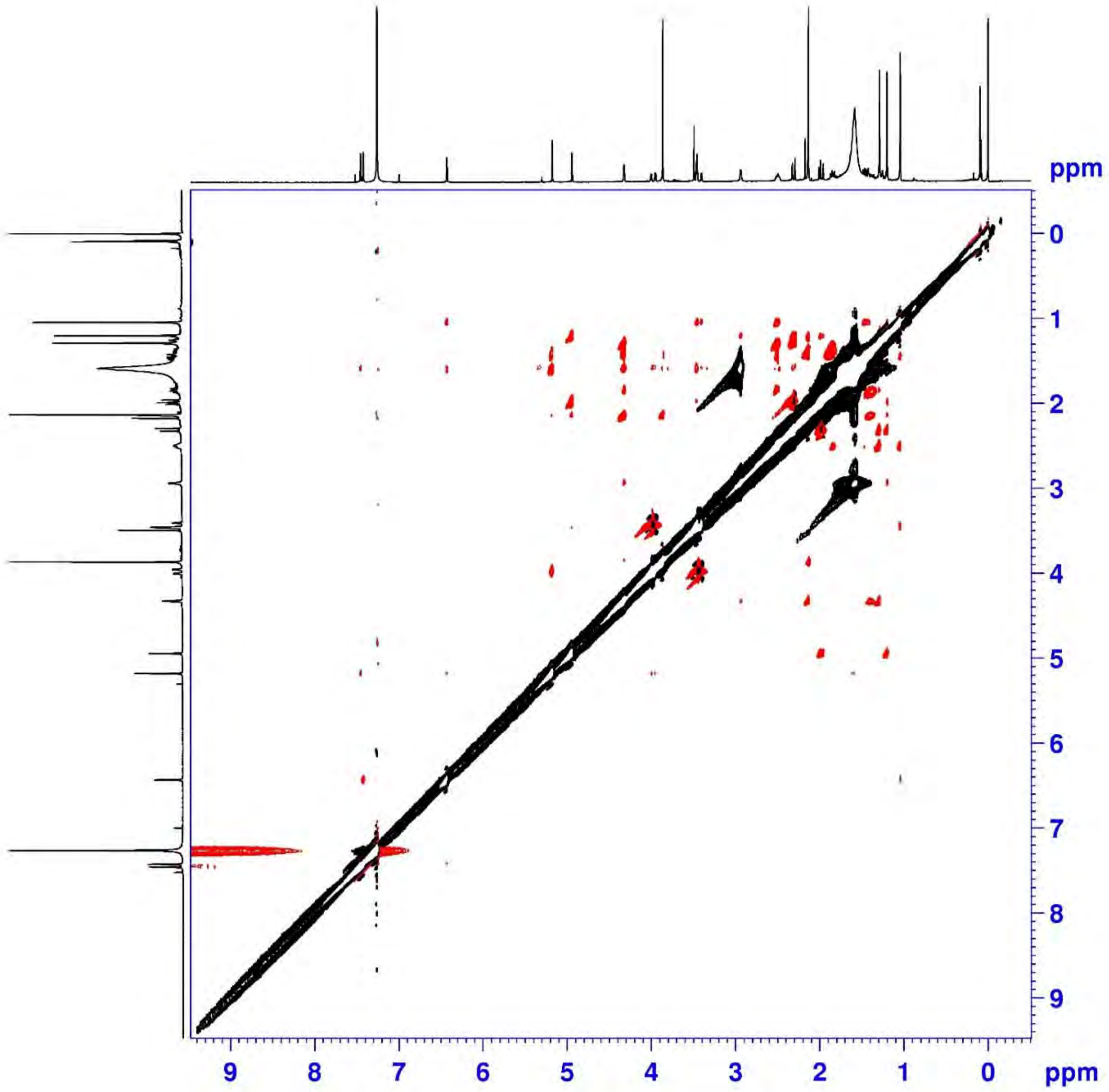

NAME	S-3
EXPNO	7
PROCNO	1
Date_	20160830
Time	11.46
INSTRUM	spect
PROBHD	5 mm CPPBBO BB
PULPROG	noesygpphpp
TD	2048
SOLVENT	CDCl3
NS	32
DS	32
SWH	4000.000 Hz
FIDRES	1.953125 Hz
AQ	0.2560500 sec
RG	208.5
DW	125.000 usec
DE	10.00 usec
TE	297.0 K
D0	0.00011036 sec
D1	1.99385595 sec
D8	0.30000001 sec
D11	0.03000000 sec
D12	0.00002000 sec
D16	0.00020000 sec
IN0	0.00025000 sec

======== CHANNEL f1 ========

SFO1	400.1318006 MHz
NUC1	1H
P1	11.50 usec
P2	23.00 usec
P17	2500.00 usec
ND0	1
TD	256
SFO1	400.1318 MHz
FIDRES	15.625000 Hz
SW	9.997 ppm
FnMODE	States-TPPI
SI	1024
SF	400.1300098 MHz
WDW	QSINE
SSB	2
LB	0.00 Hz
GB	0
PC	1.00
SI	1024
MC2	States-TPPI
SF	400.1300098 MHz
WDW	QSINE
SSB	2
LB	0.00 Hz
GB	0

NOESY (400 MHz) spectrum of compound **17** in $CDCl_3$

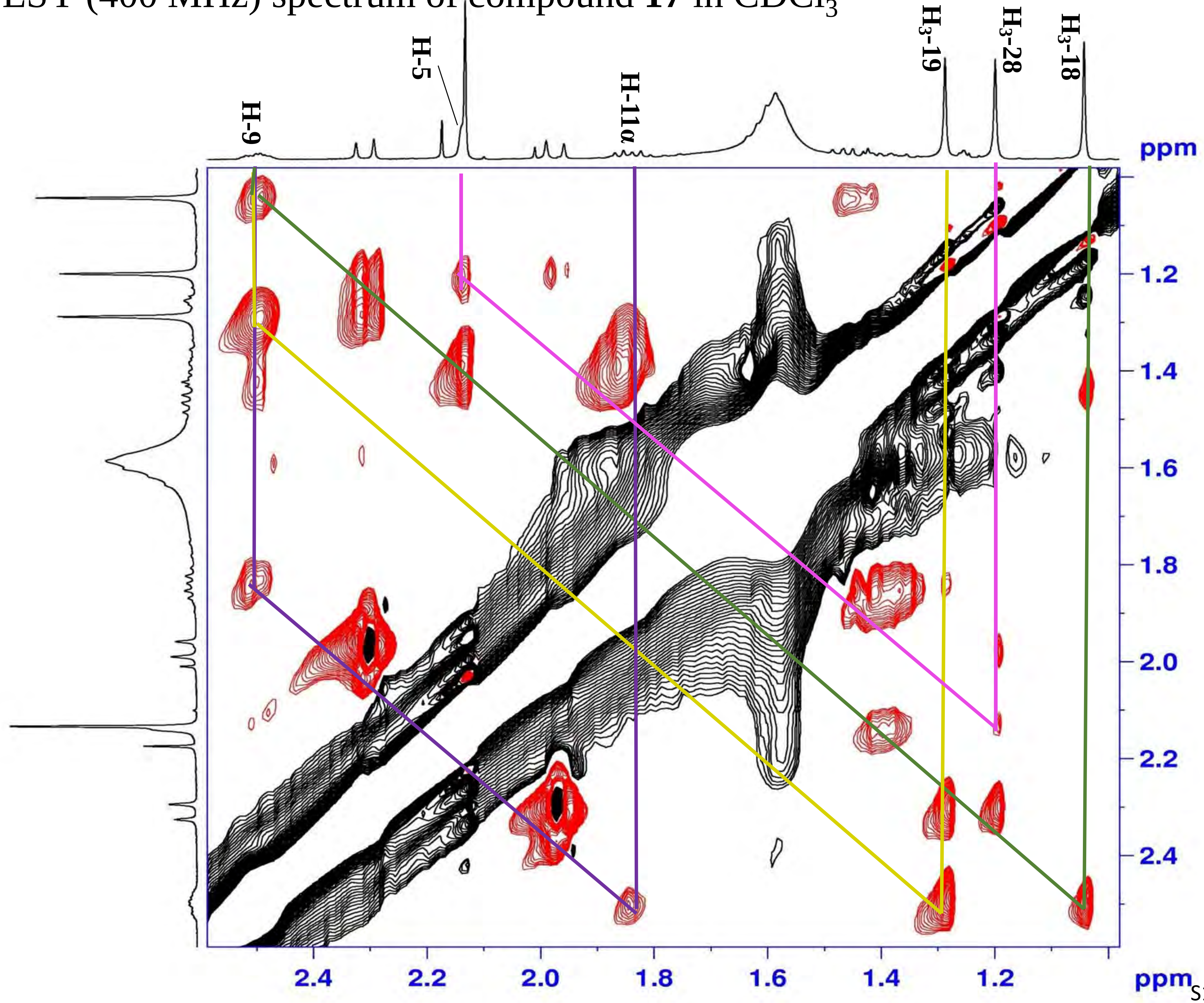

NOESY (400 MHz) spectrum of compound **5** in $CDCl_3$

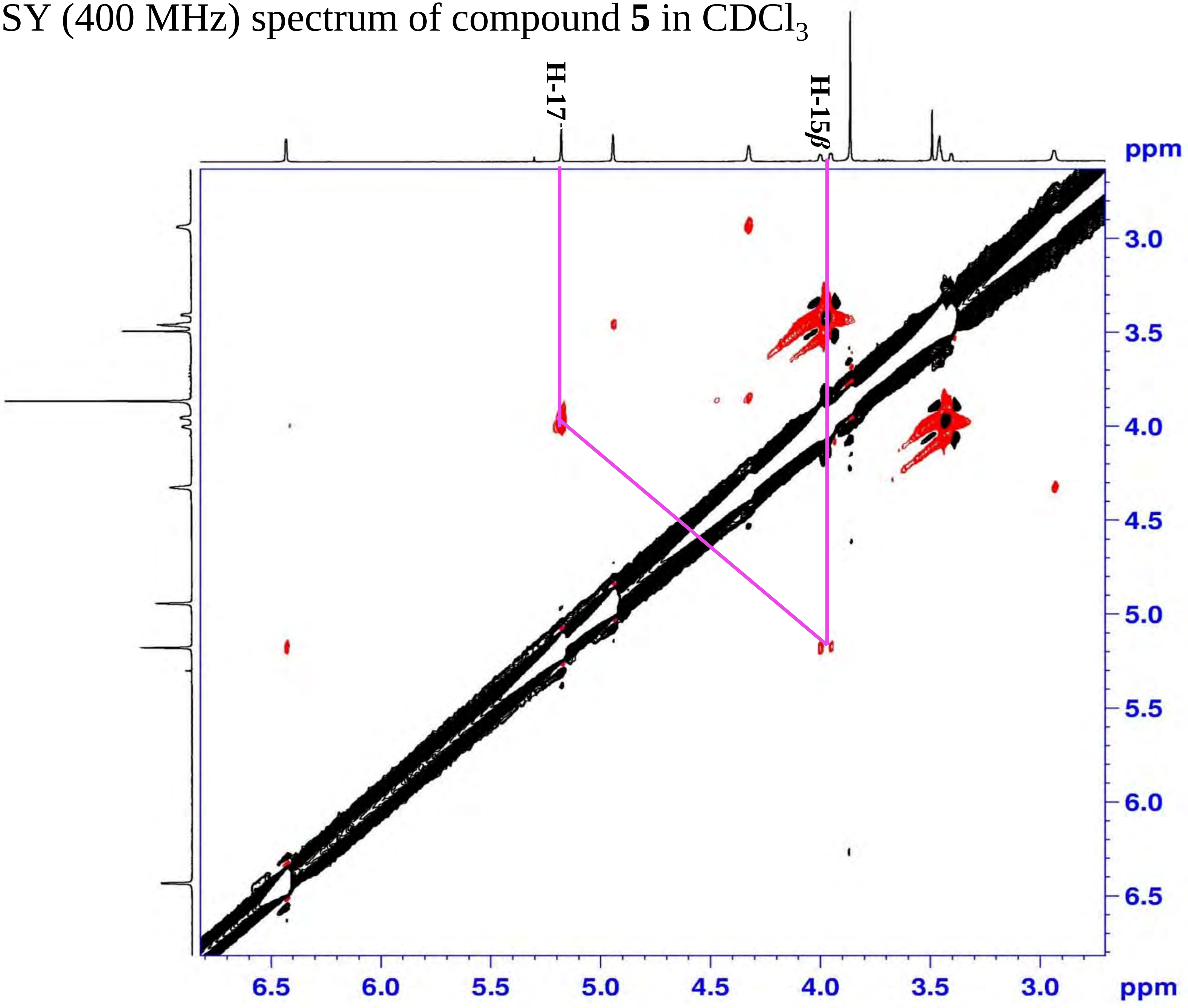

NOESY (400 MHz) spectrum of compound **17** in $CDCl_3$

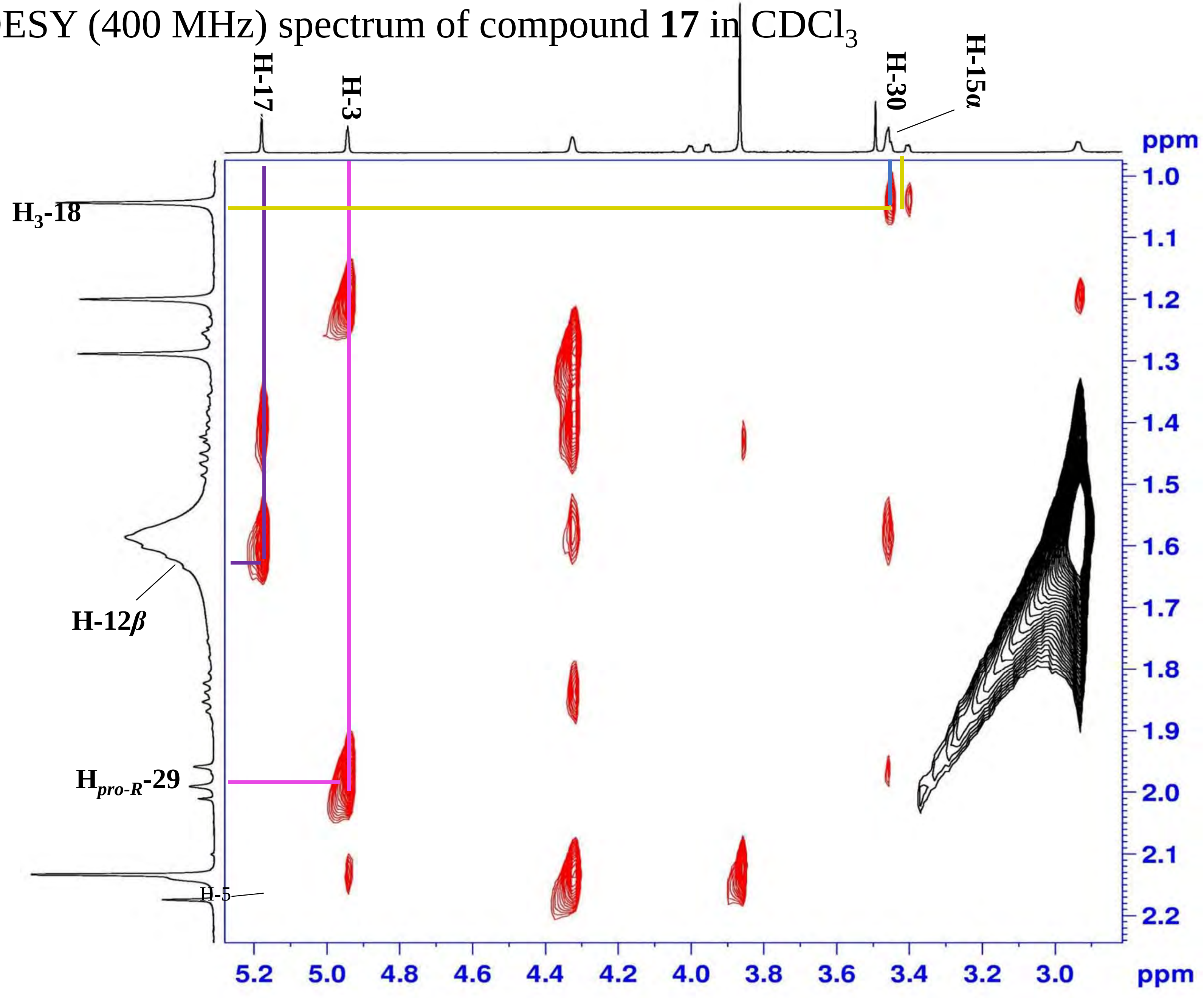

NOESY (400 MHz) spectrum of compound **17** in $CDCl_3$

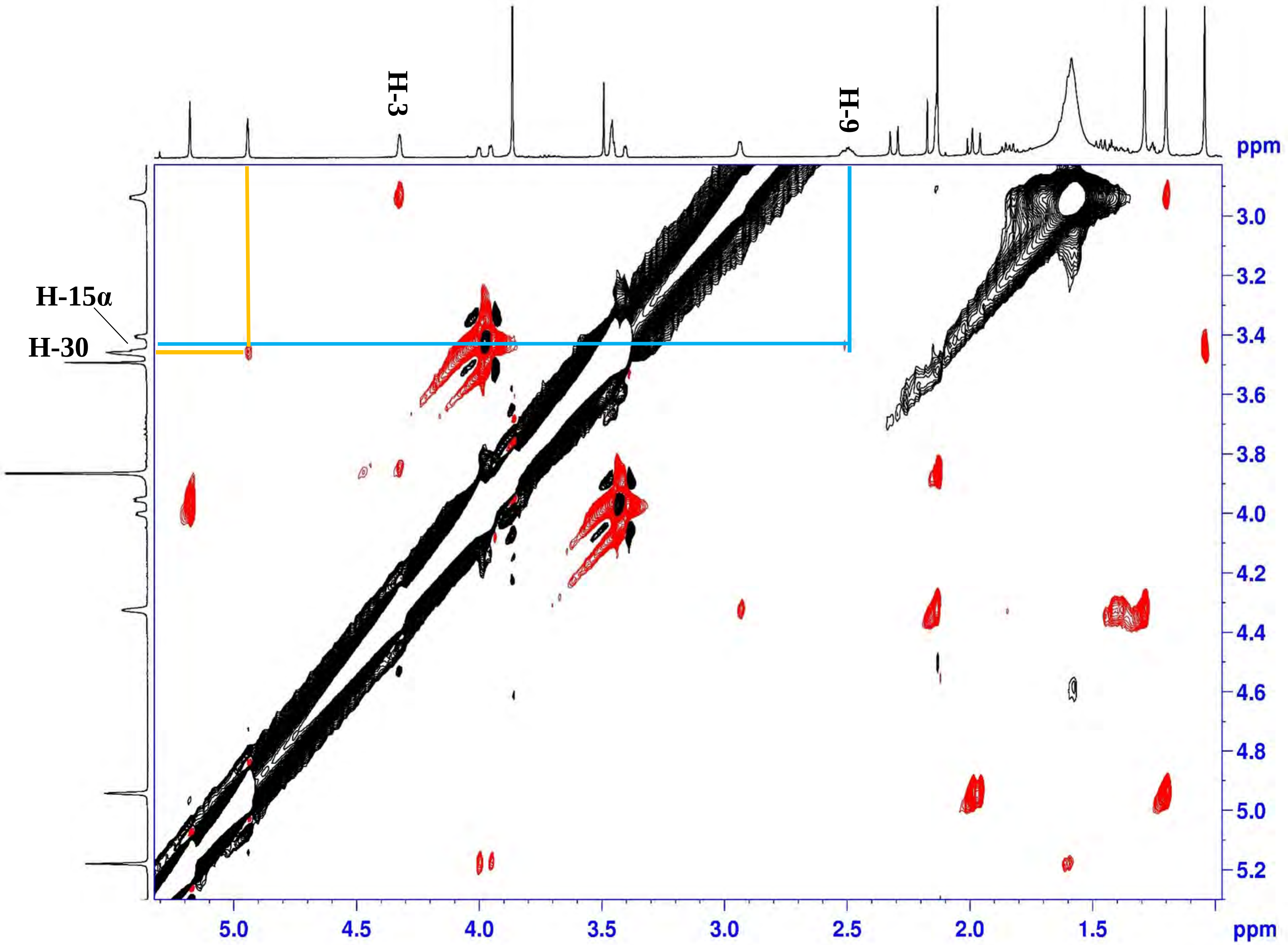

HRESIMS for compound **18**

Elemental Composition Report

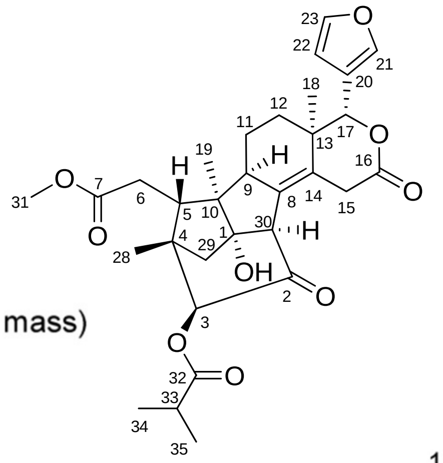

Single Mass Analysis

Tolerance = 10.0 mDa / DBE: min = -1.0, max = 50.0
Element prediction: Off
Number of isotope peaks used for i-FIT = 3

Monoisotopic Mass, Even Electron Ions
154 formula(e) evaluated with 5 results within limits (up to 10 closest results for each mass)
Elements Used:
C: 0-100 H: 0-200 O: 0-100
SYY-11
2016103106 189 (1.526)

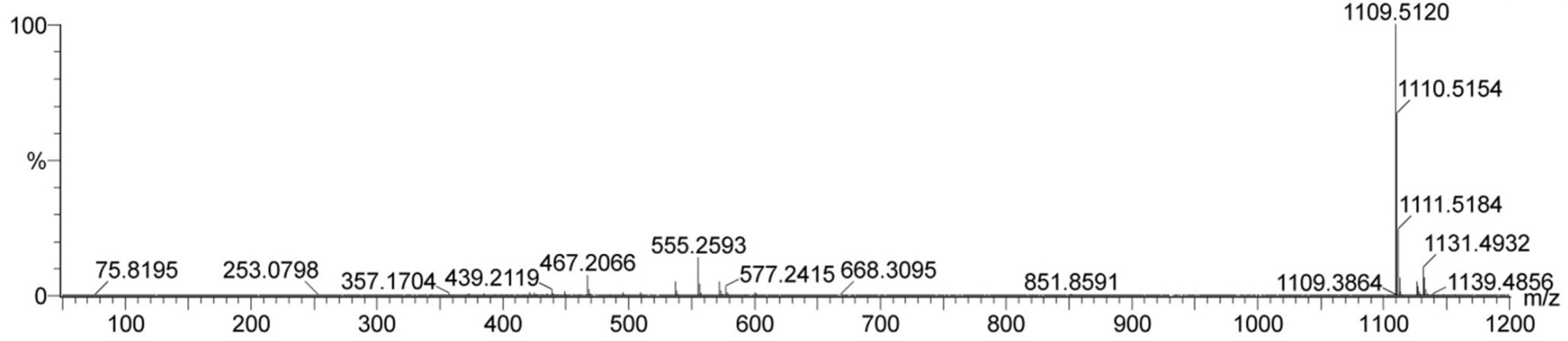

Minimum:				-1.0
Maximum:		10.0	10.0	50.0

Mass	Calc. Mass	mDa	PPM	DBE	i-FIT	Norm	Conf(%)	Formula
555.2593	555.2594	-0.1	-0.2	12.5	321.6	0.646	52.39	C31 H39 O9
	555.2535	5.8	10.4	21.5	326.7	5.768	0.31	C38 H35 O4
	555.2653	-6.0	-10.8	3.5	321.7	0.757	46.89	C24 H43 O14
	555.2500	9.3	16.7	-0.5	326.5	5.544	0.39	C20 H43 O17
	555.2688	-9.5	-17.1	25.5	329.5	8.523	0.02	C42 H35 O

^{1}H NMR (400 MHz) spectrum of compound **18** in $CDCl_3$

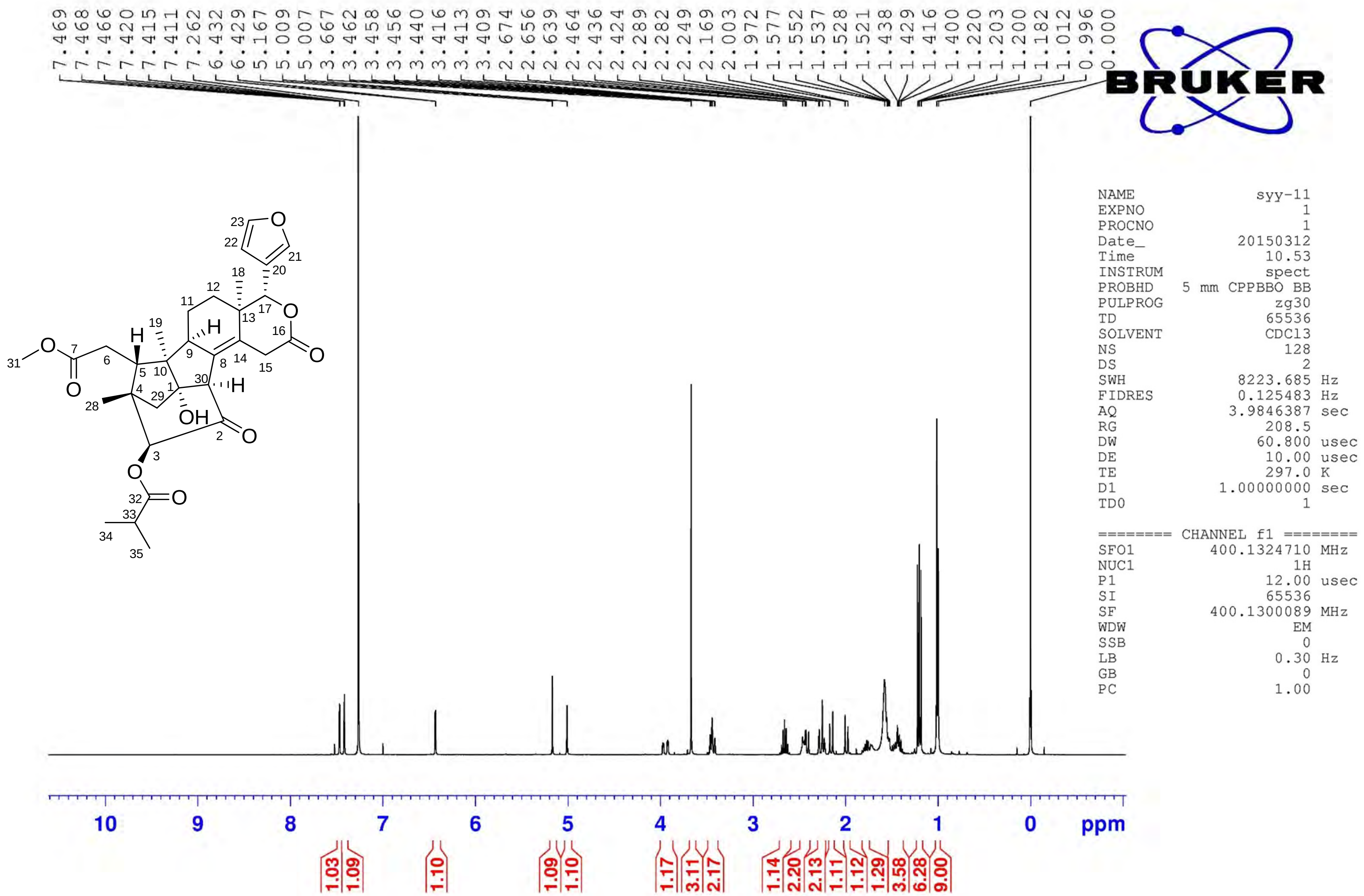

^{1}H NMR (400 MHz) spectrum of compound **18** in $CDCl_3$

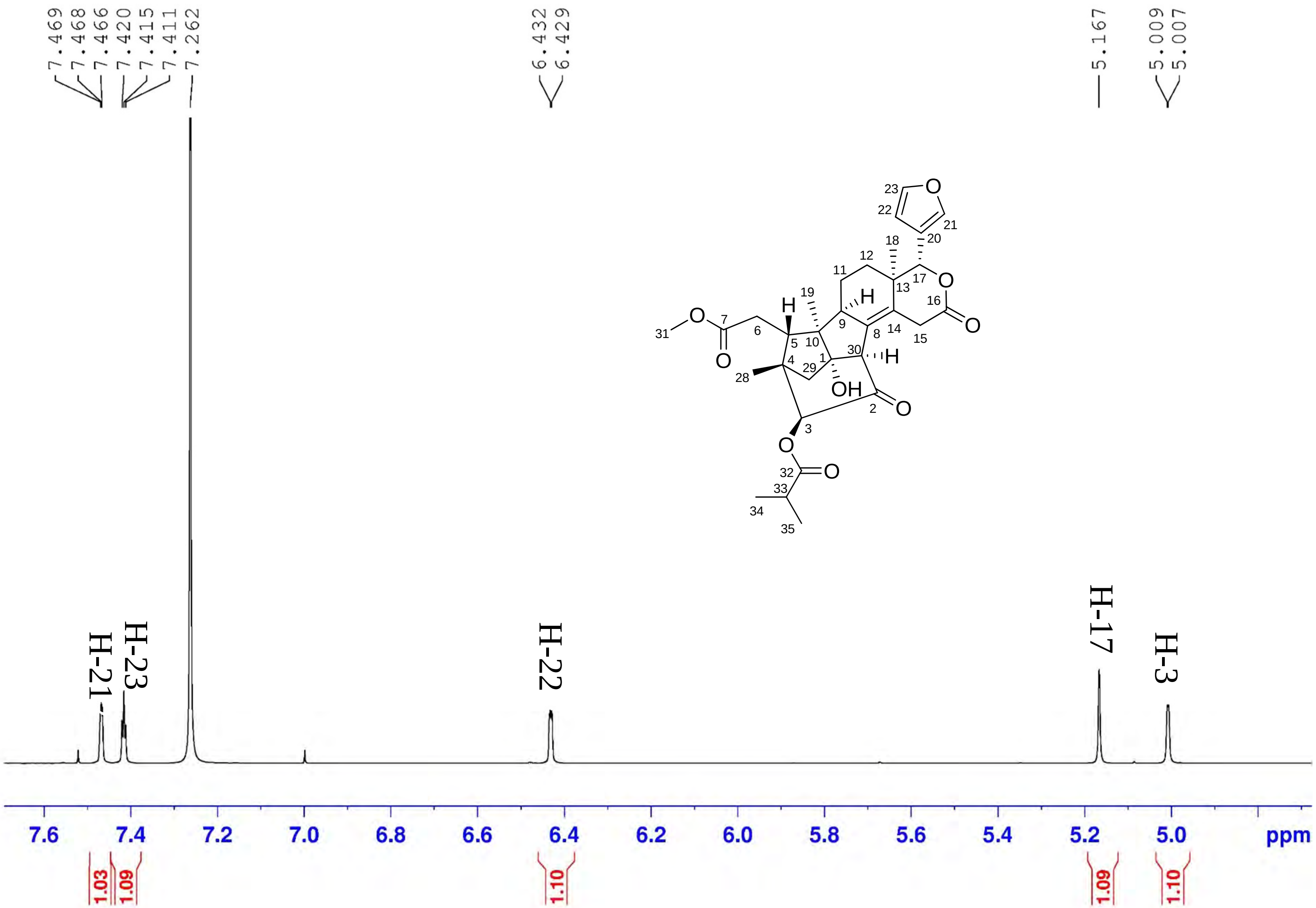

^{1}H NMR (400 MHz) spectrum of compound **18** in $CDCl_3$

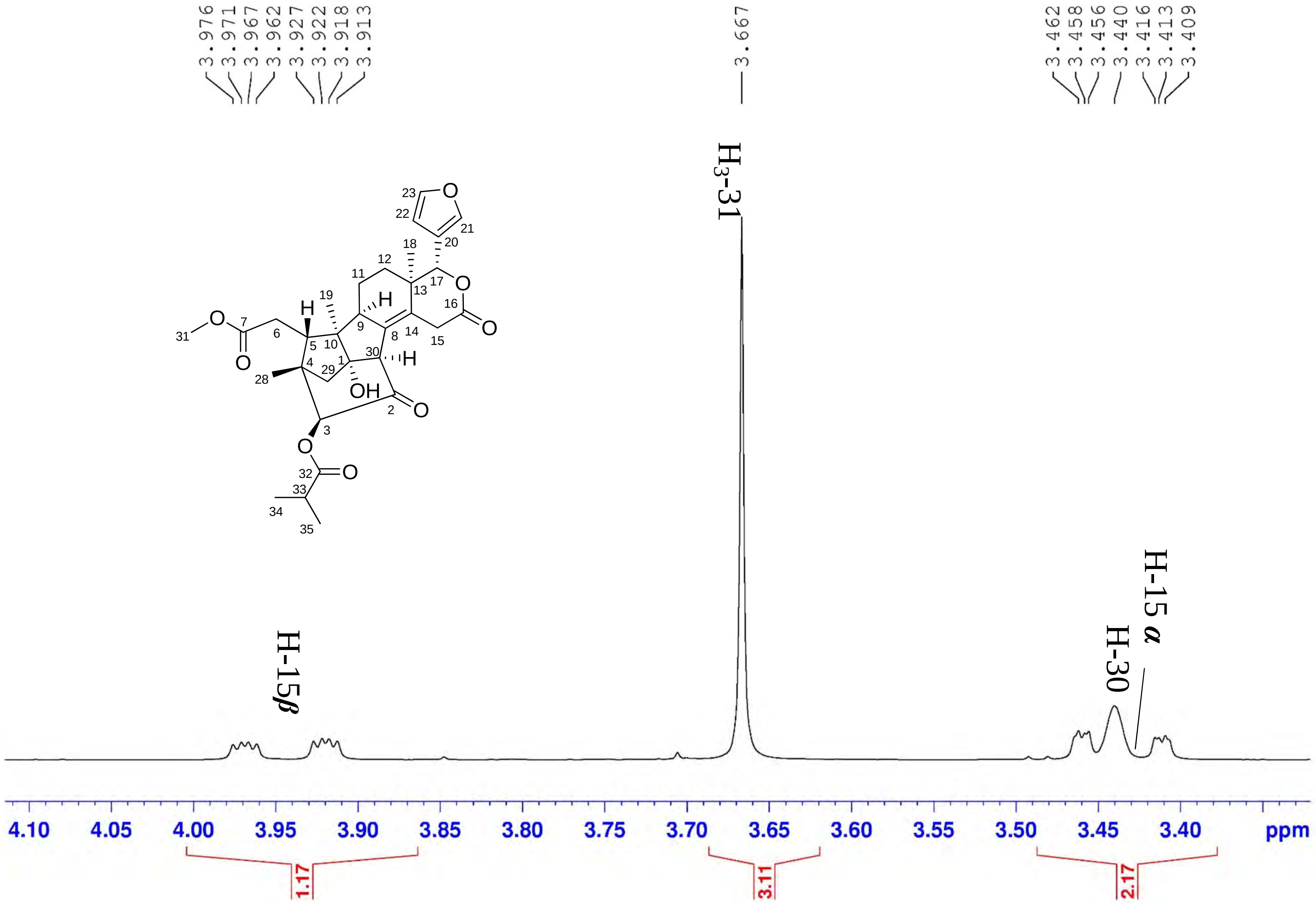

^{1}H NMR (400 MHz) spectrum of compound **18** in $CDCl_3$

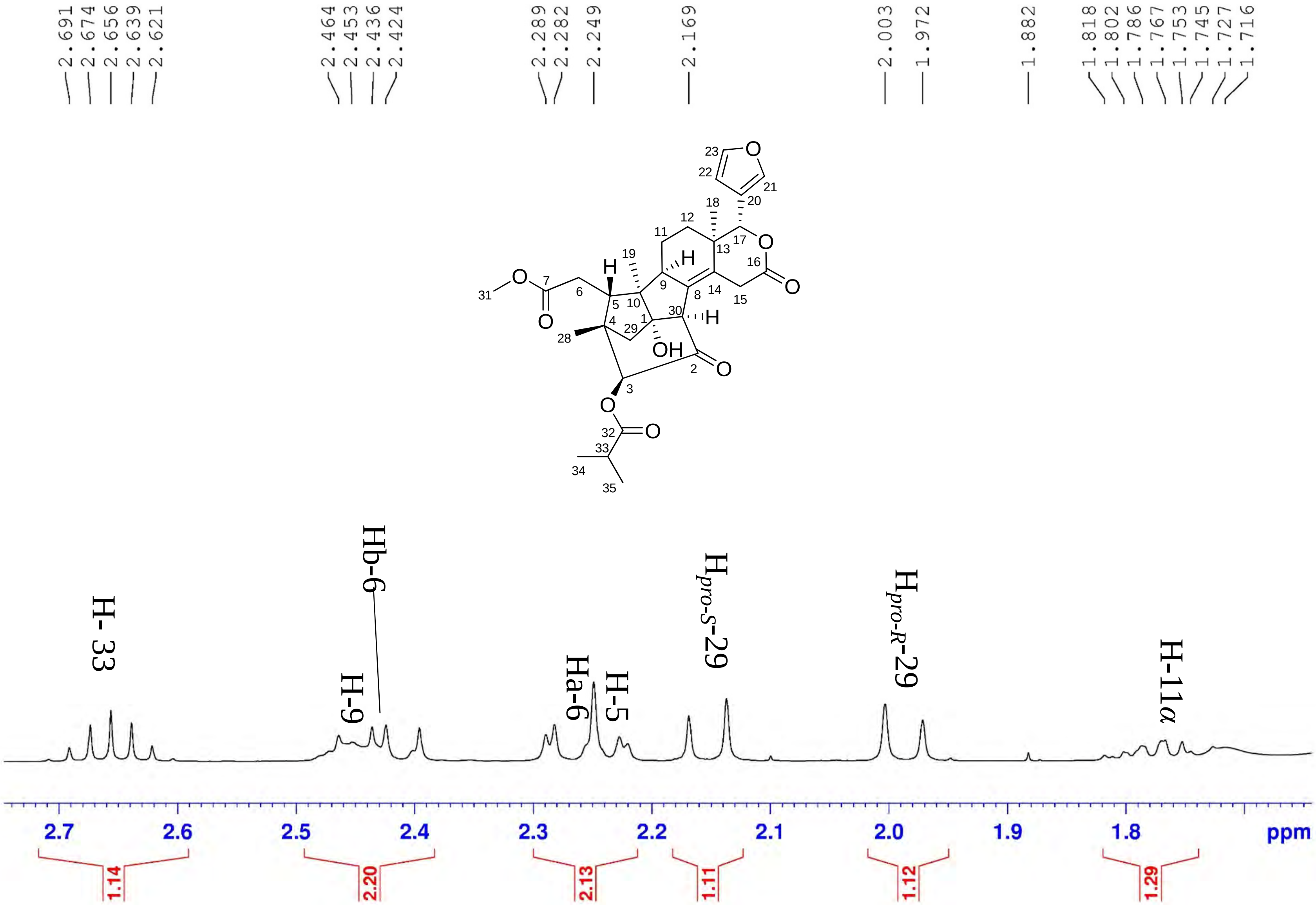

^{1}H NMR (400 MHz) spectrum of compound **18** in $CDCl_3$

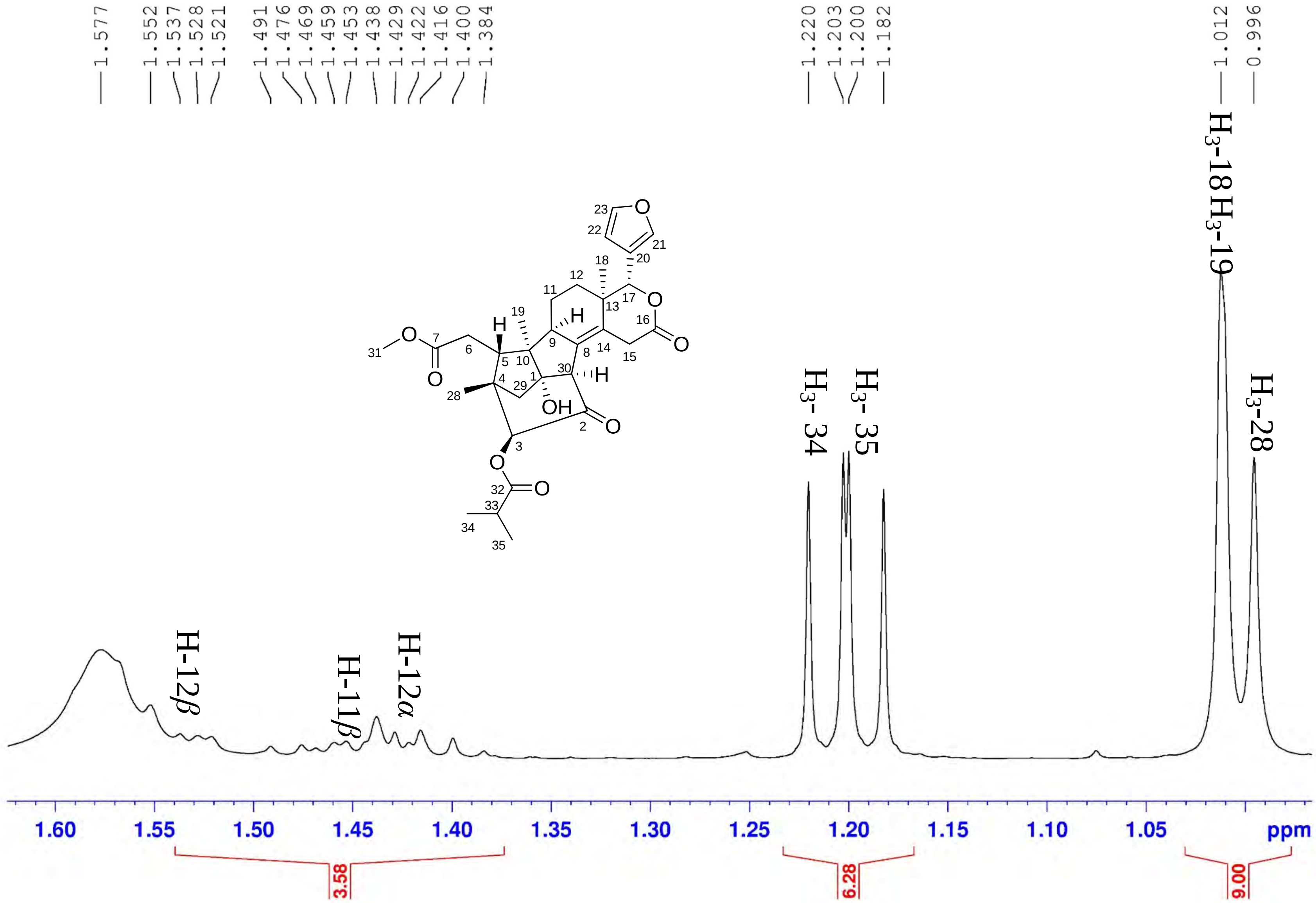

^{13}C NMR (100 MHz) spectrum of compound **18** in $CDCl_3$

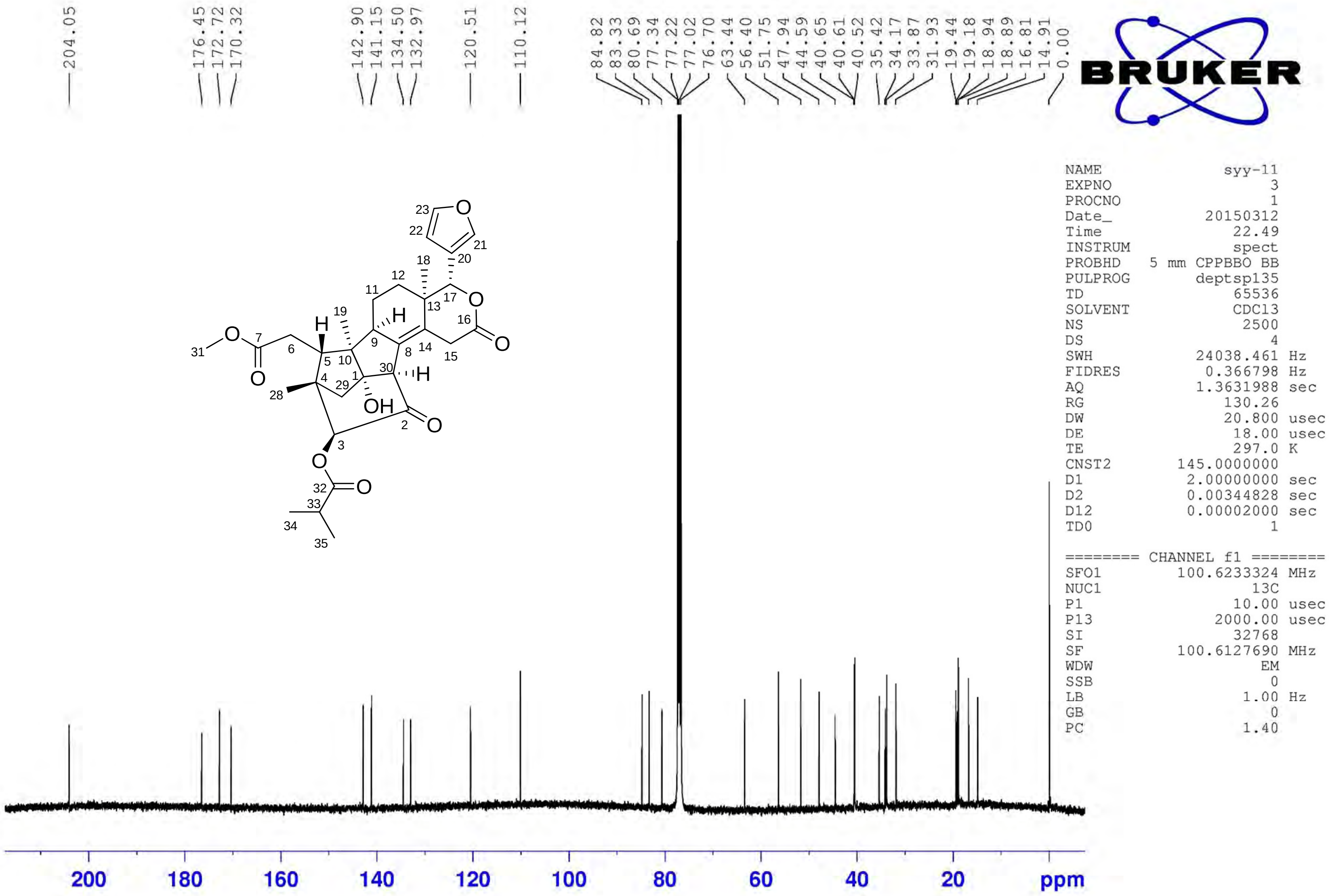

NAME	syy-11
EXPNO	3
PROCNO	1
Date_	20150312
Time	22.49
INSTRUM	spect
PROBHD	5 mm CPPBBO BB
PULPROG	deptsp135
TD	65536
SOLVENT	CDCl3
NS	2500
DS	4
SWH	24038.461 Hz
FIDRES	0.366798 Hz
AQ	1.3631988 sec
RG	130.26
DW	20.800 usec
DE	18.00 usec
TE	297.0 K
CNST2	145.0000000
D1	2.00000000 sec
D2	0.00344828 sec
D12	0.00002000 sec
TD0	1
======== CHANNEL f1 ========	
SFO1	100.6233324 MHz
NUC1	13C
P1	10.00 usec
P13	2000.00 usec
SI	32768
SF	100.6127690 MHz
WDW	EM
SSB	0
LB	1.00 Hz
GB	0
PC	1.40

^{13}C NMR (100 MHz) spectrum of compound **18** in $CDCl_3$

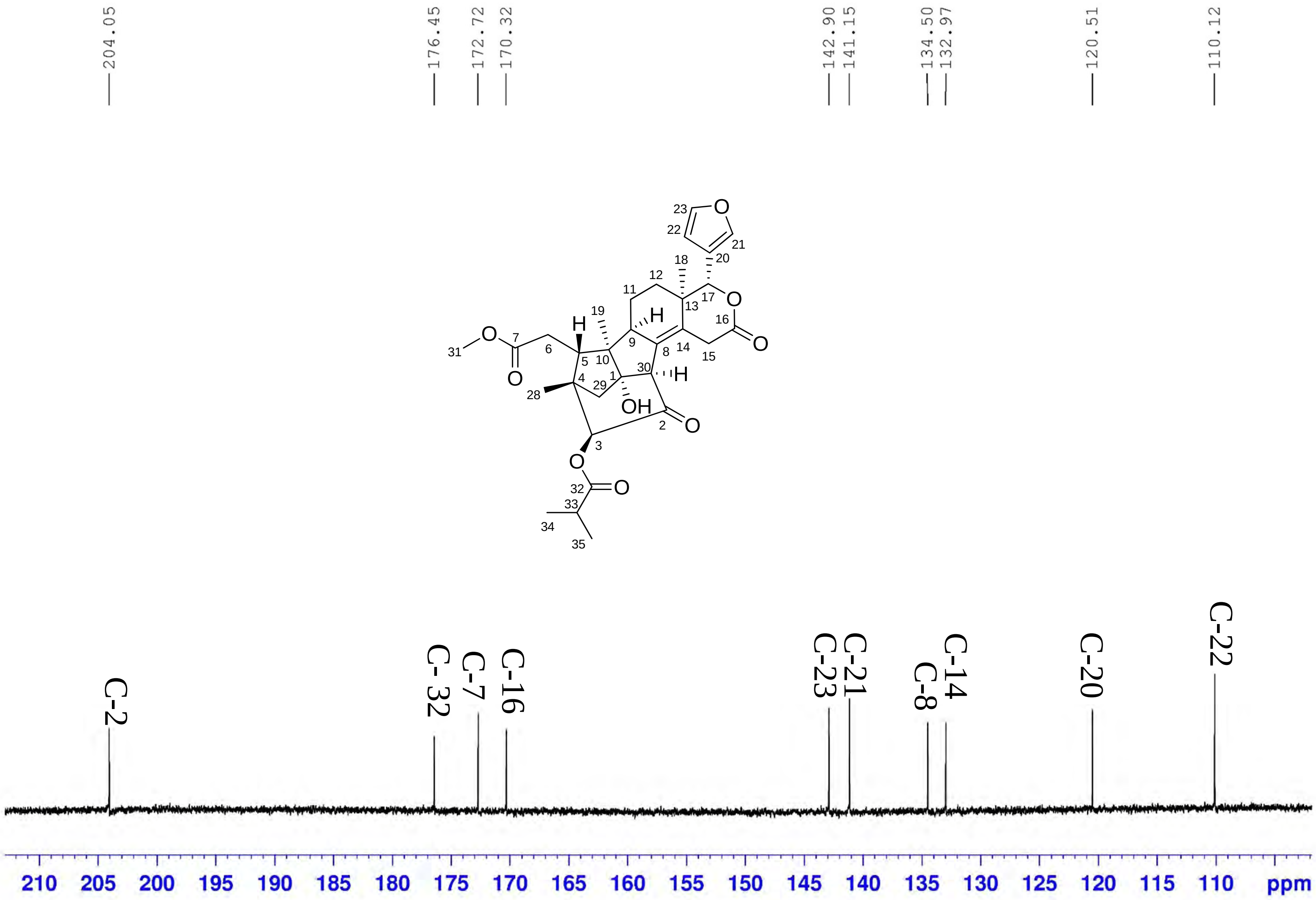

^{13}C NMR (100 MHz) spectrum of compound **18** in $CDCl_3$

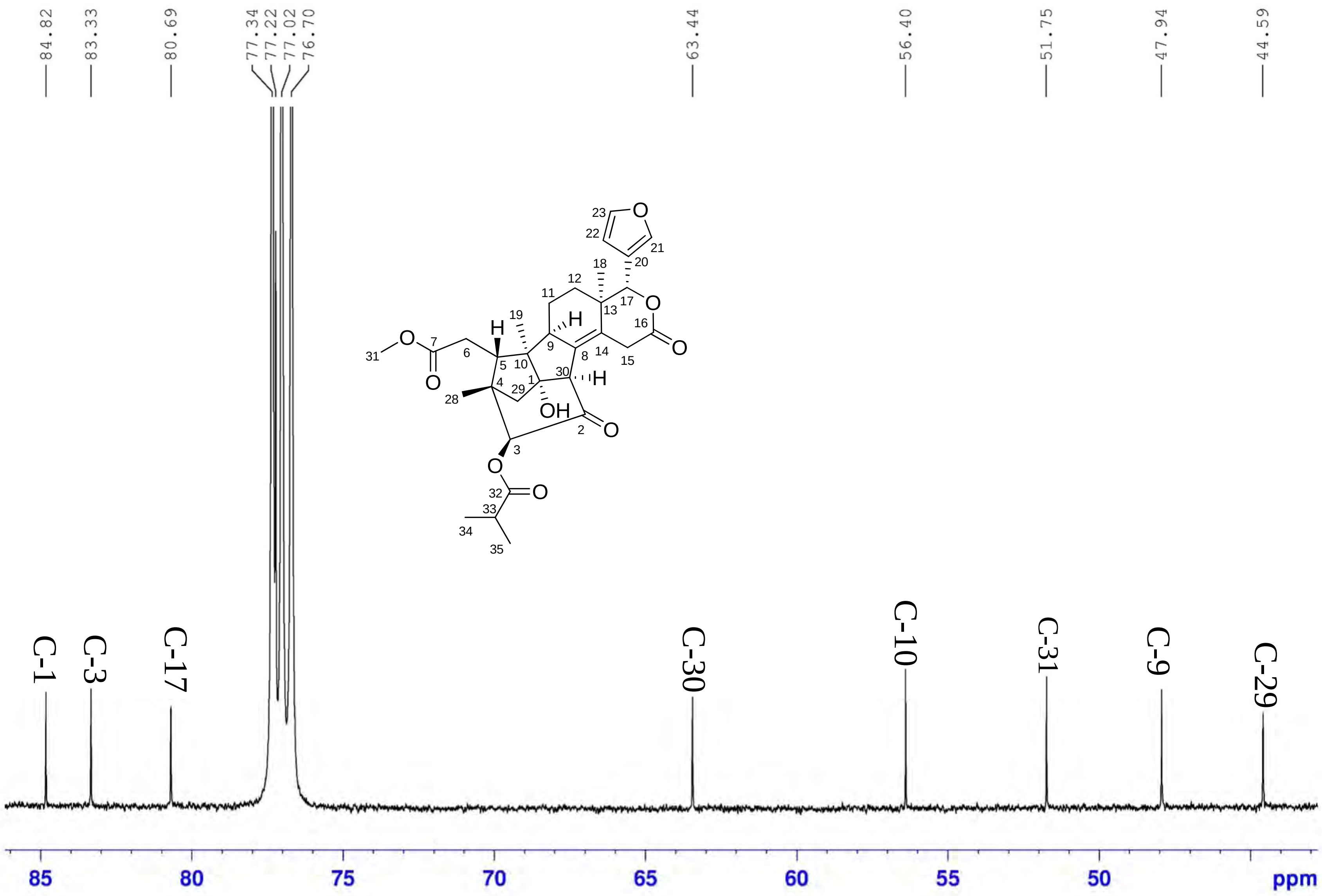

^{13}C NMR (100 MHz) spectrum of compound **18** in $CDCl_3$

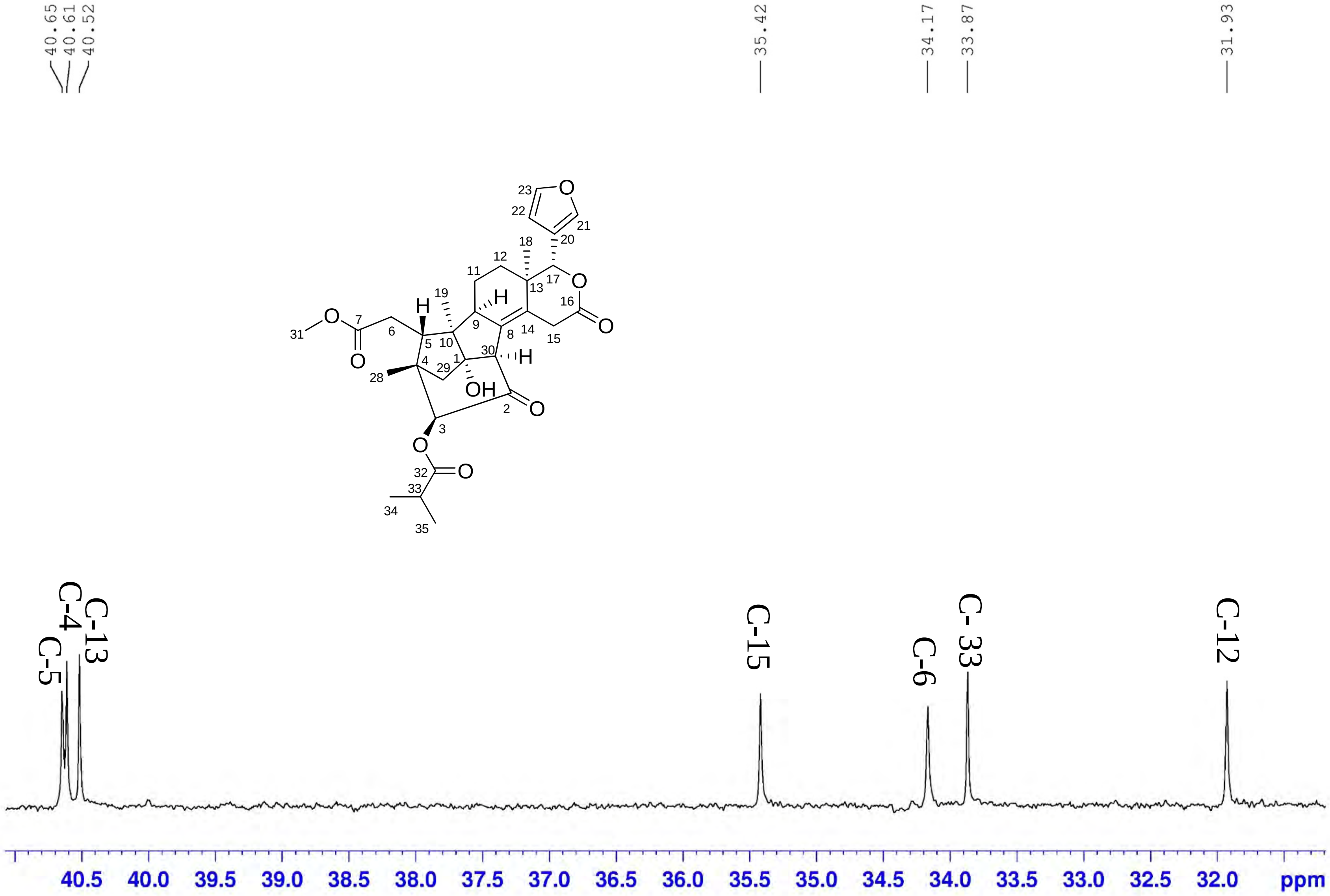

^{13}C NMR (100 MHz) spectrum of compound **18** in $CDCl_3$

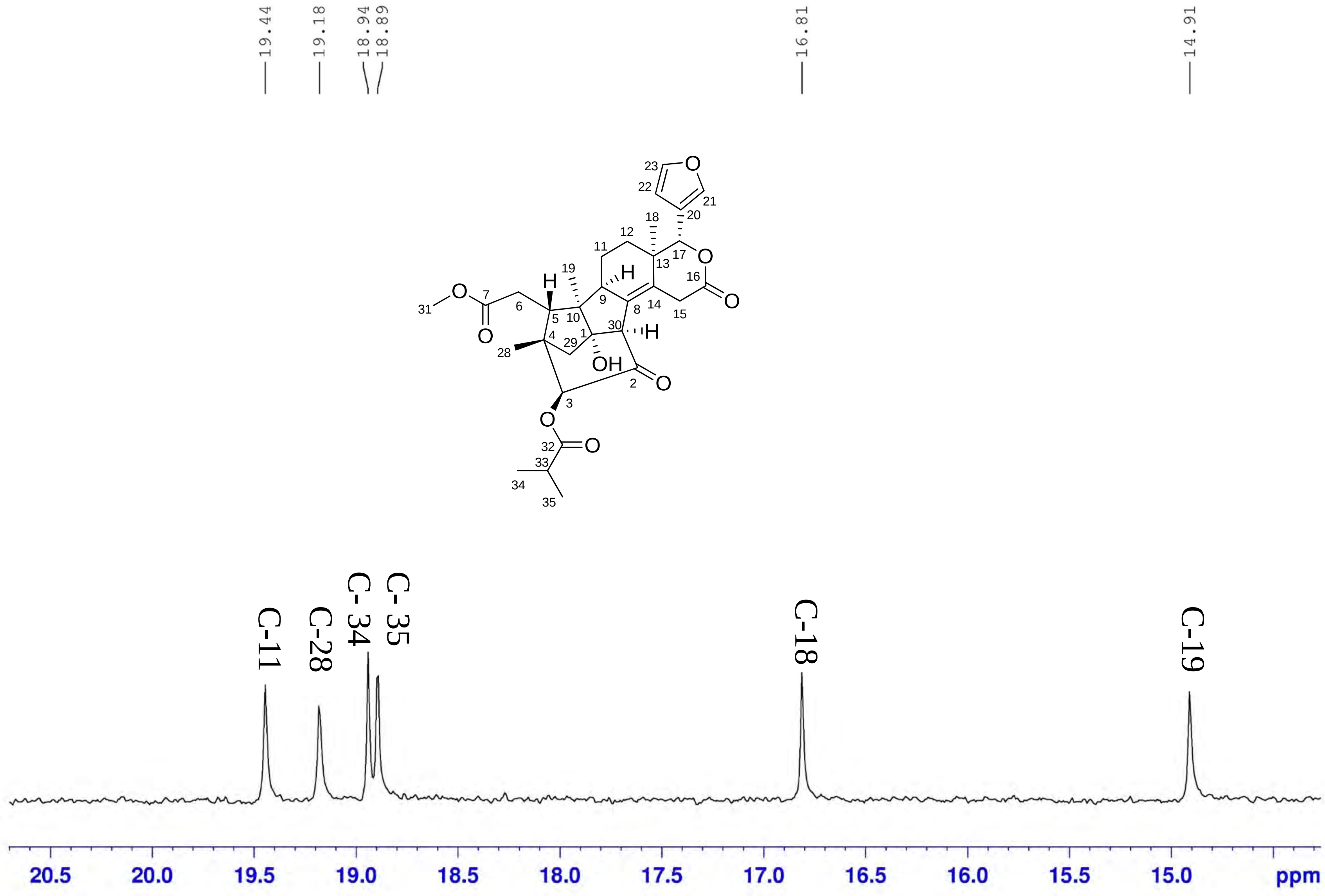

DEPT135 (100 MHz) spectrum of compound **18** in $CDCl_3$

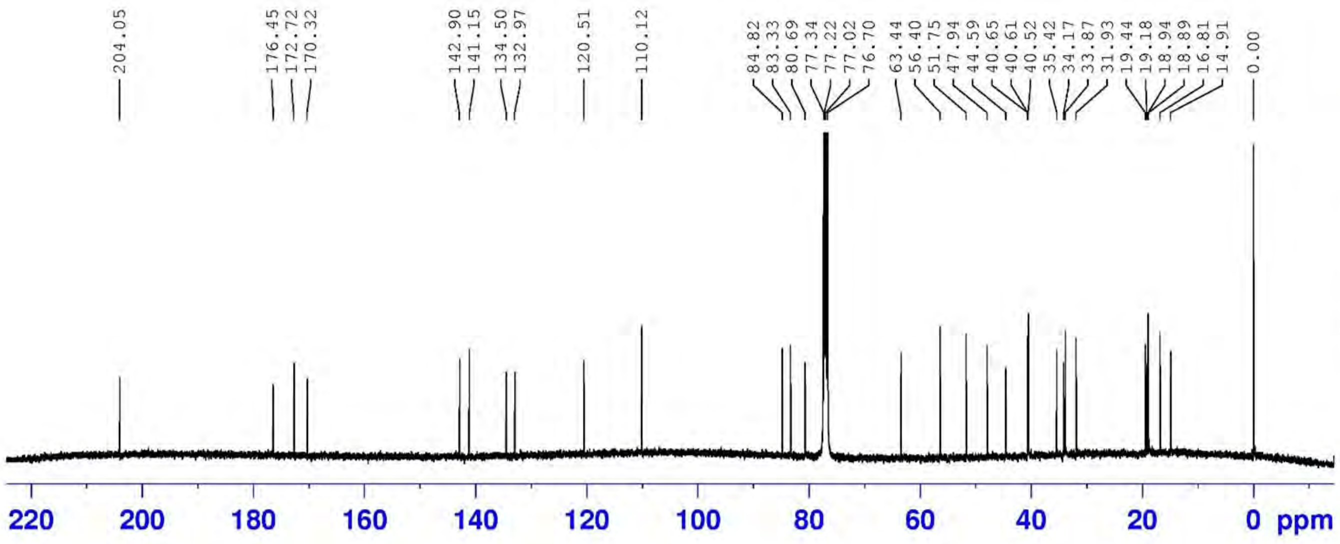

NAME	syy-11
EXPNO	3
PROCNO	1
Date_	20150312
Time	22.49
INSTRUM	spect
PROBHD	5 mm CPPBBO BB
PULPROG	deptsp135
TD	65536
SOLVENT	CDCl3
NS	2500
DS	4
SWH	24038.461 Hz
FIDRES	0.366798 Hz
AQ	1.3631988 sec
RG	130.26
DW	20.800 usec
DE	18.00 usec
TE	297.0 K
CNST2	145.0000000
D1	2.00000000 sec
D2	0.00344828 sec
D12	0.00002000 sec
TD0	1

======== CHANNEL f1 ========

SFO1	100.6233324 MHz
NUC1	13C
P1	10.00 usec
P13	2000.00 usec
SI	32768
SF	100.6127690 MHz
WDW	EM
SSB	0
LB	1.00 Hz
GB	0
PC	1.40

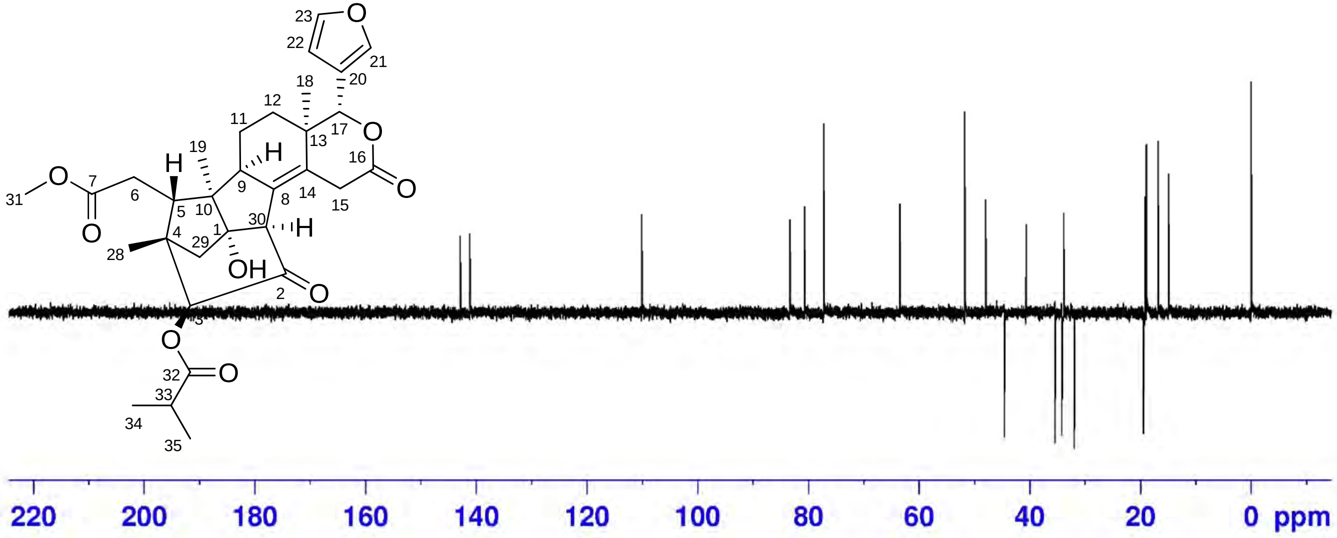

DEPT135 (100 MHz) spectrum of compound **18** in $CDCl_3$

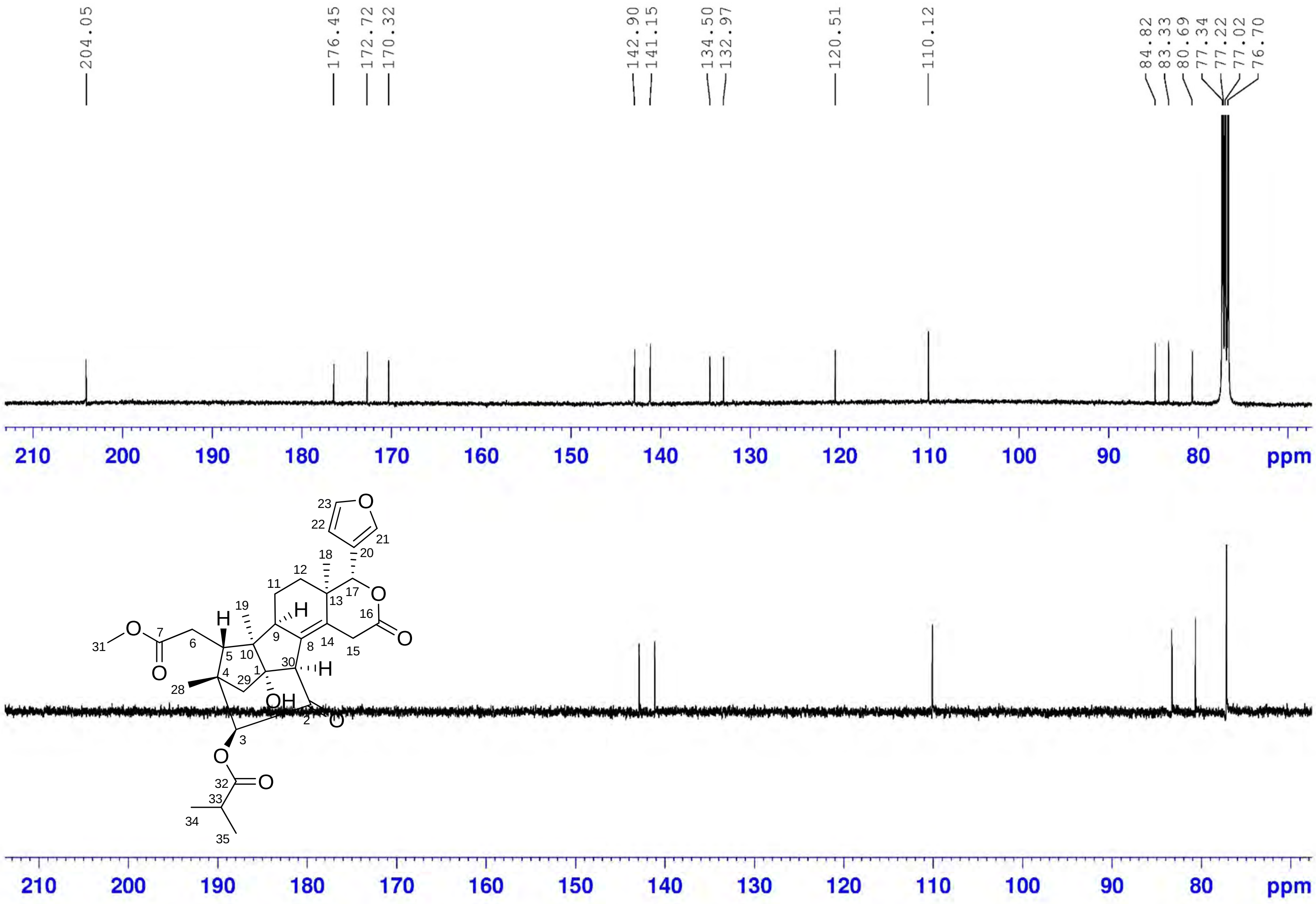

DEPT135 (100 MHz) spectrum of compound **18** in $CDCl_3$

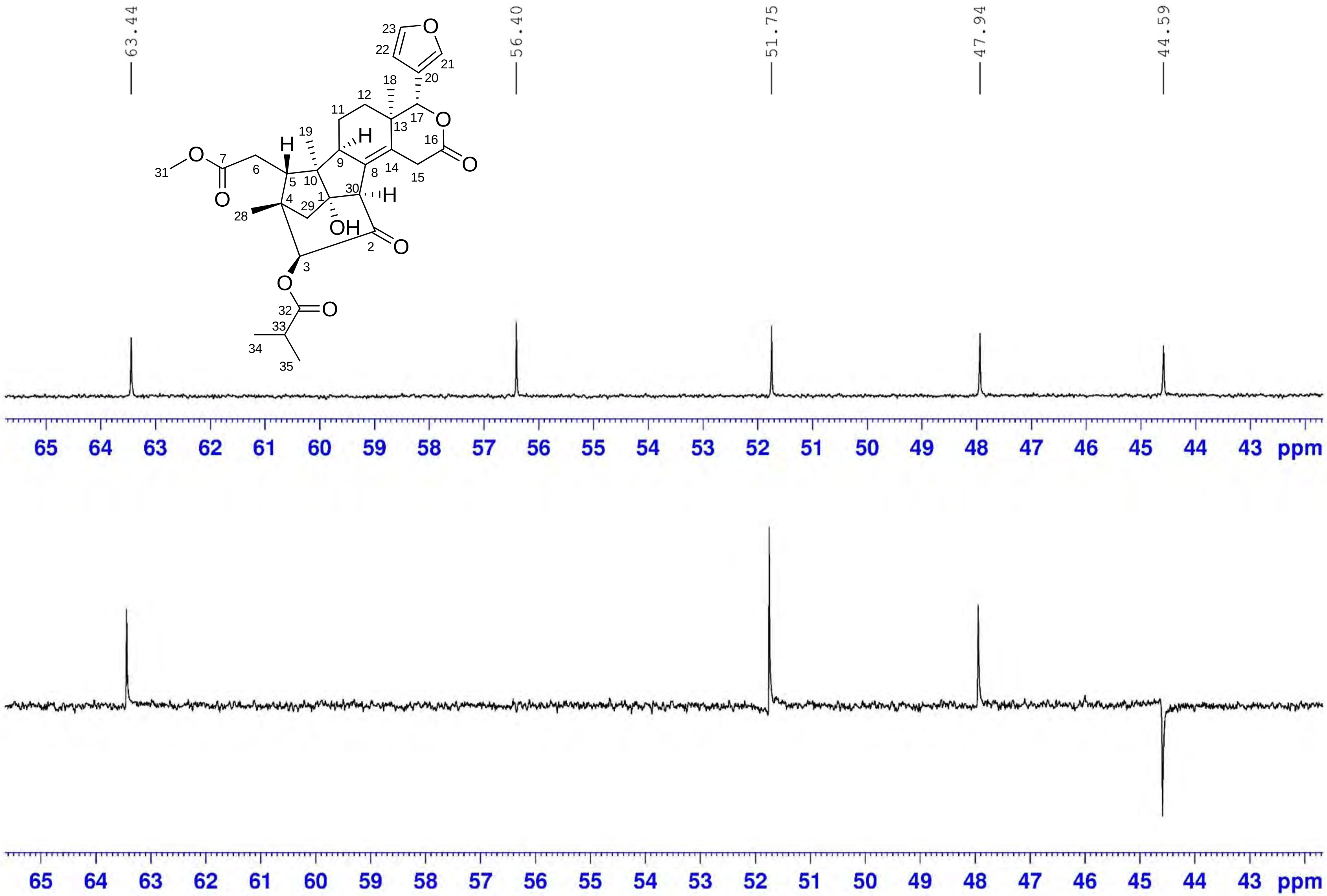

DEPT135 (100 MHz) spectrum of compound **18** in $CDCl_3$

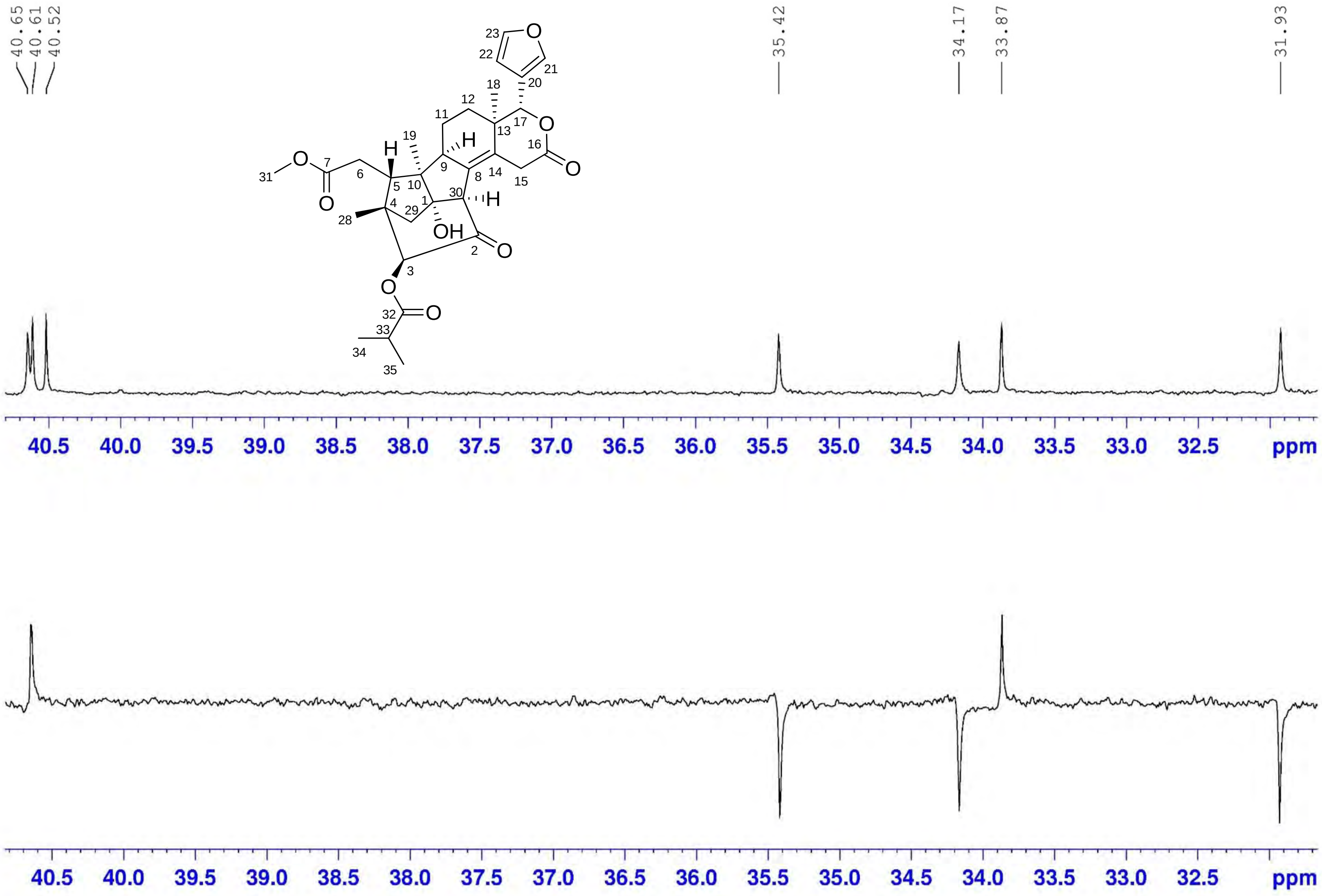

DEPT135 (100 MHz) spectrum of compound **18** in $CDCl_3$

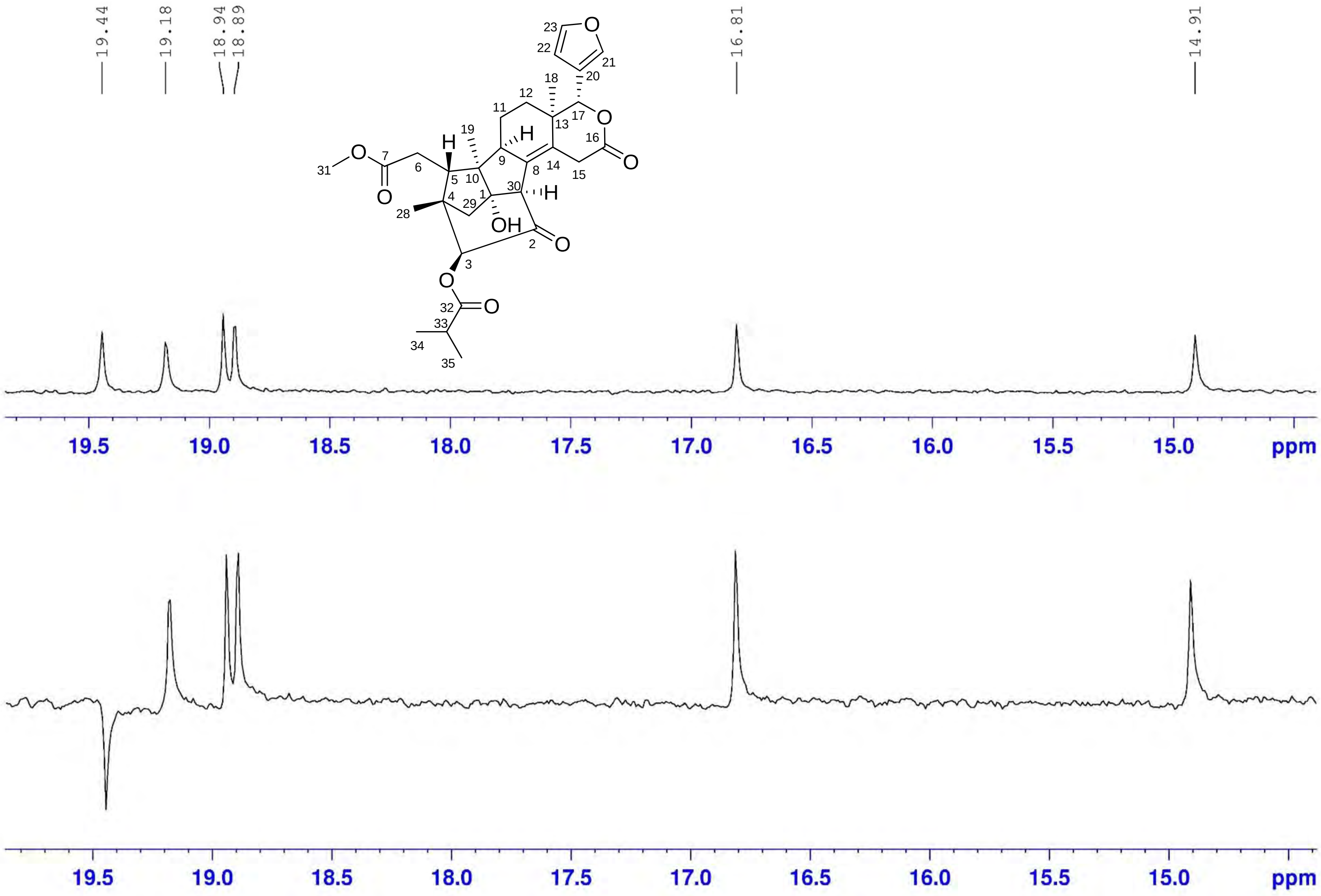

^{1}H-^{1}H COSY (400 MHz) spectrum of compound **18** in $CDCl_3$

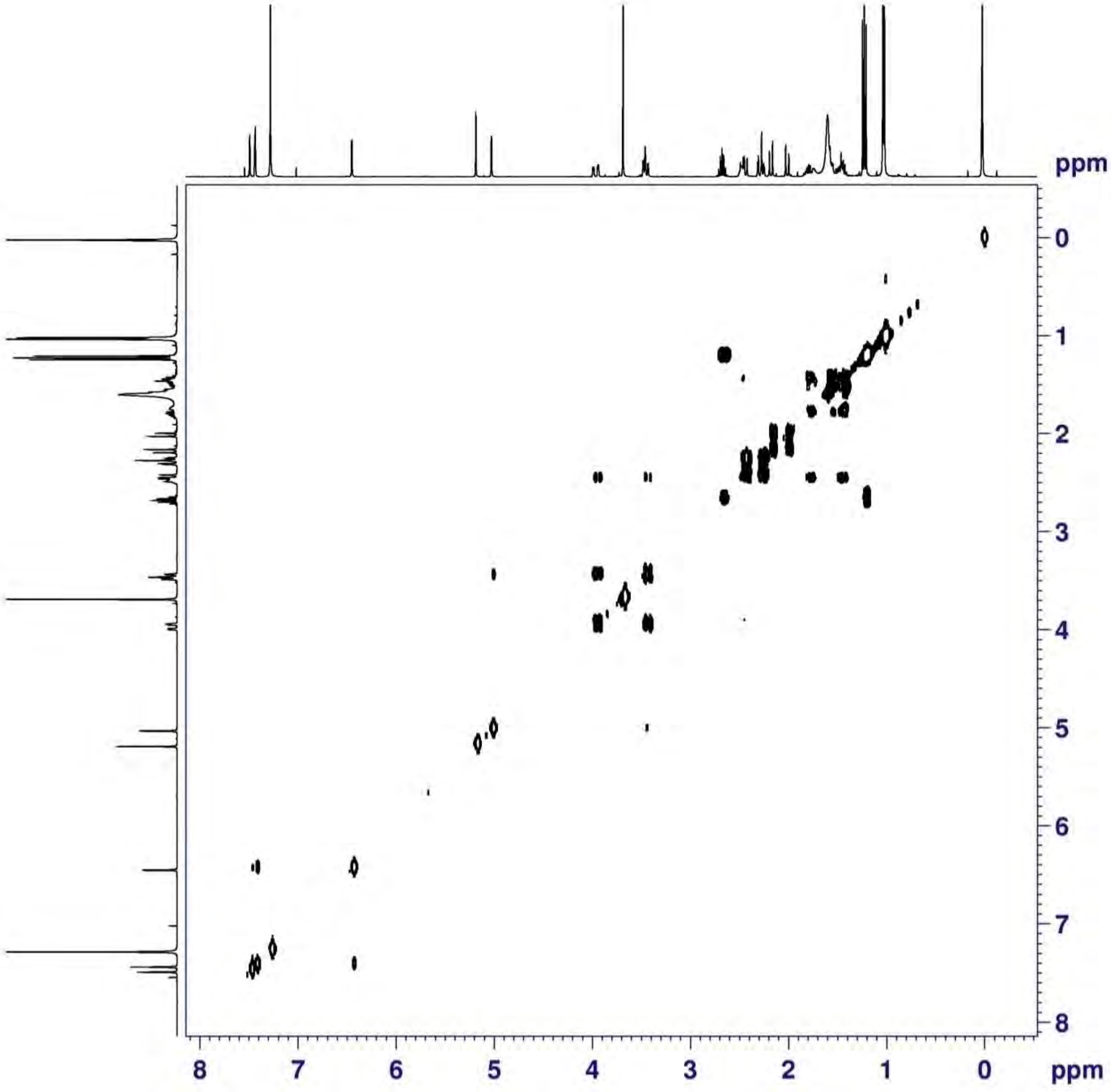


```
NAME              syy-11
EXPNO                  4
PROCNO                 1
Date_           20150810
Time                1.44
INSTRUM            spect
PROBHD   5 mm CPPBBO BB
PULPROG       cosygpppqf
TD                  2048
SOLVENT            CDCl3
NS                    16
DS                     8
SWH             3906.250 Hz
FIDRES          1.907349 Hz
AQ             0.2621940 sec
RG                 208.5
DW               128.000 usec
DE                 10.00 usec
TE                 297.0 K
D0            0.00000300 sec
D1            1.89678097 sec
D11           0.03000000 sec
D12           0.00002000 sec
D13           0.00000400 sec
D16           0.00020000 sec
IN0           0.00025600 sec

======== CHANNEL f1 ========
SFO1         400.1318006 MHz
NUC1                  1H
P0                 12.00 usec
P1                 12.00 usec
P17              2500.00 usec
ND0                    1
TD                   128
SFO1            400.1318 MHz
FIDRES         30.517578 Hz
SW                 9.762 ppm
FnMODE                QF
SI                  1024
SF           400.1300098 MHz
WDW                QSINE
SSB                    0
LB                  0.00 Hz
GB                     0
PC                  1.40
SI                  1024
MC2                   QF
SF           400.1300098 MHz
WDW                QSINE
```

^{1}H-^{1}H COSY (400 MHz) spectrum of compound **18** in $CDCl_3$

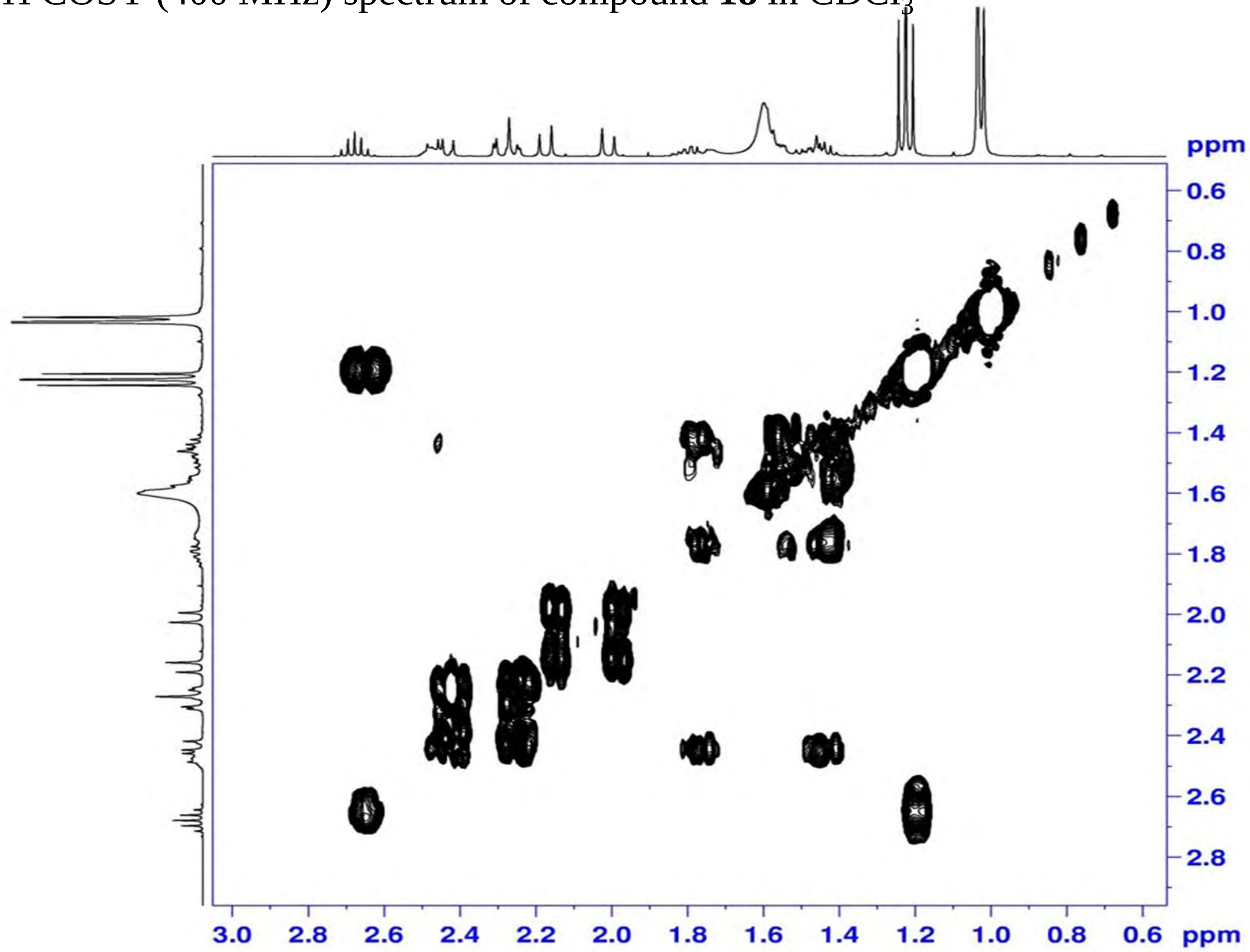

^{1}H-^{1}H COSY (400 MHz) spectrum of compound **18** in $CDCl_3$

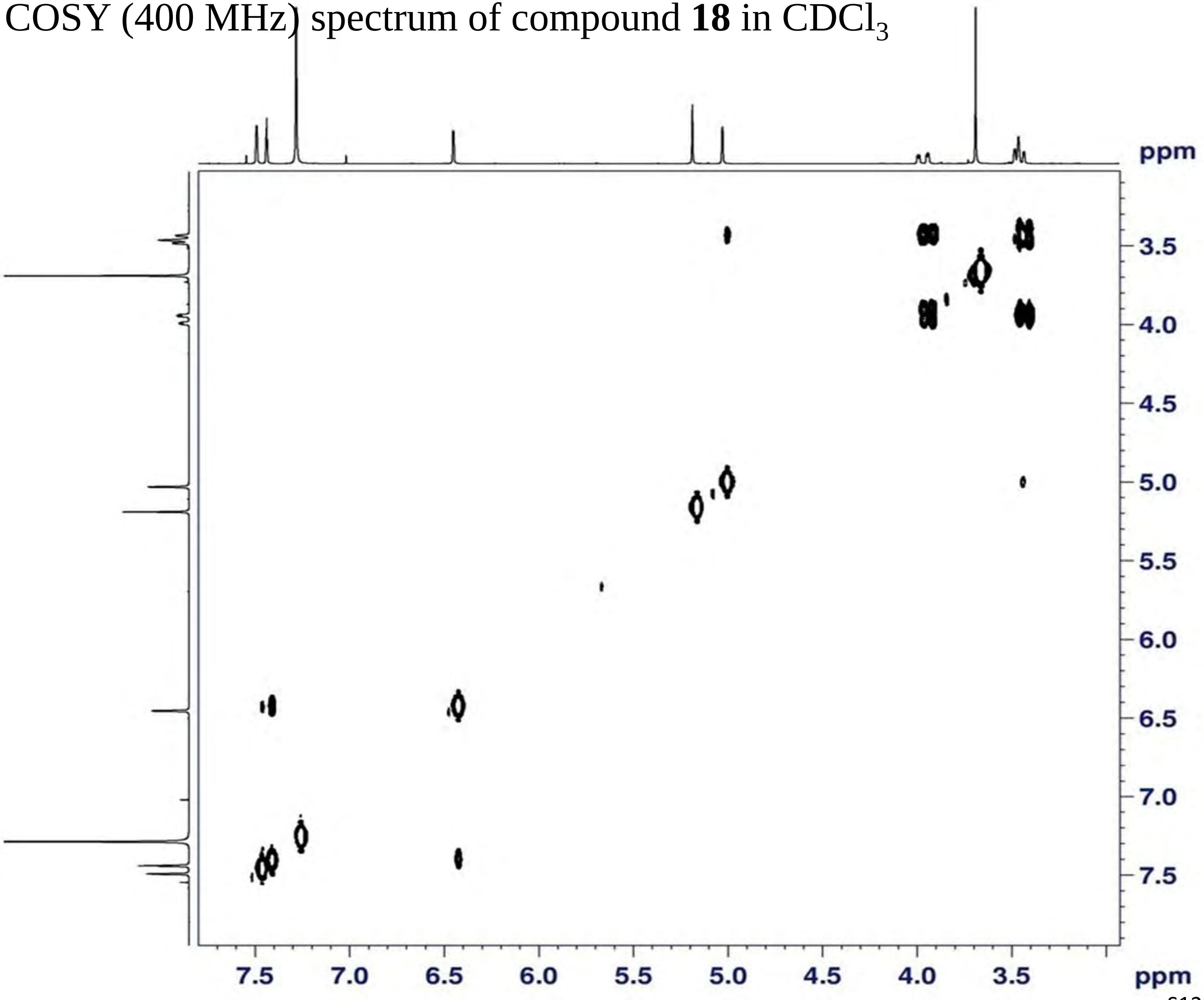

HSQC (400 MHz) spectrum of compound **18** in $CDCl_3$

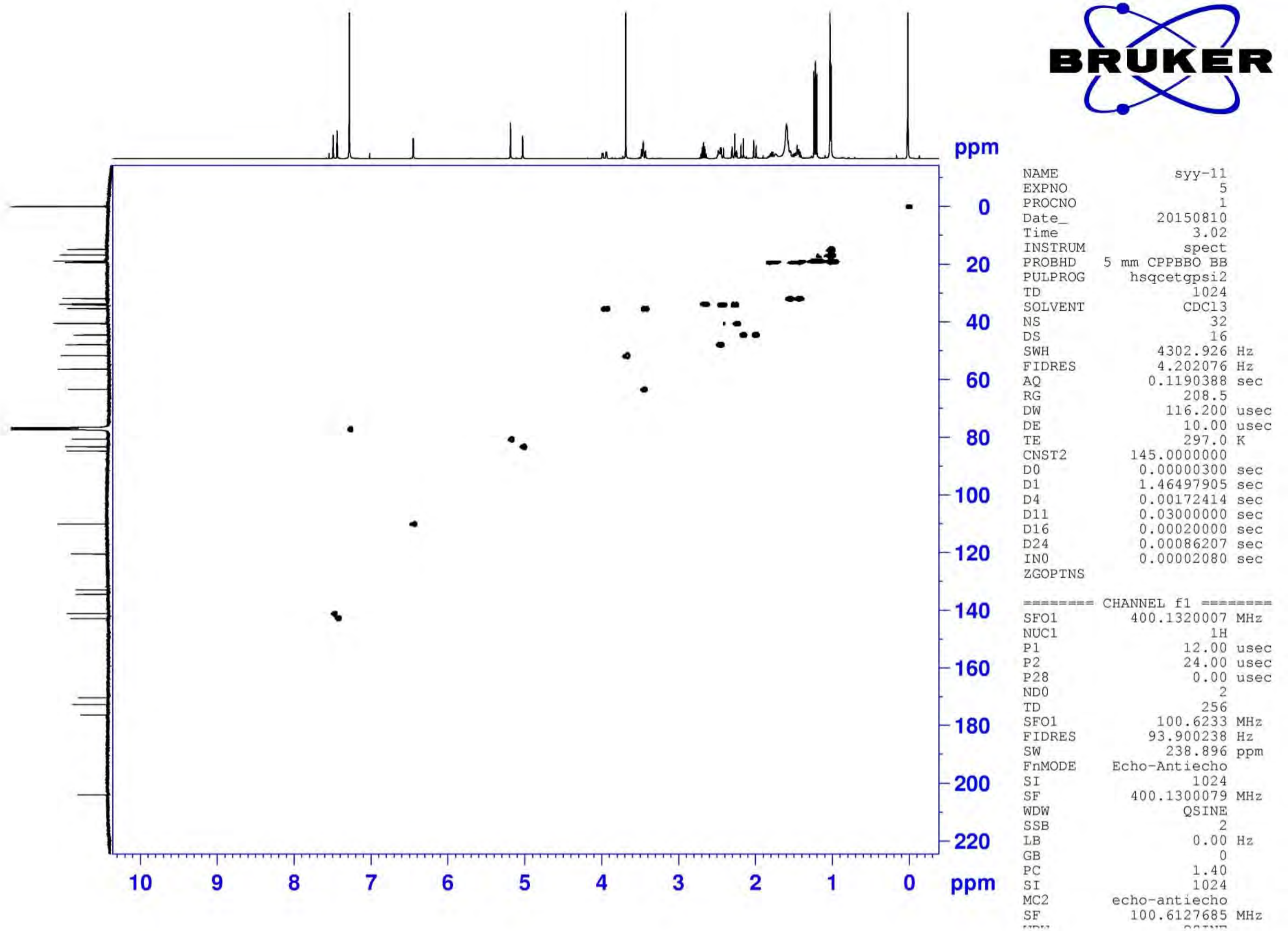

HSQC (400 MHz) spectrum of compound **18** in $CDCl_3$

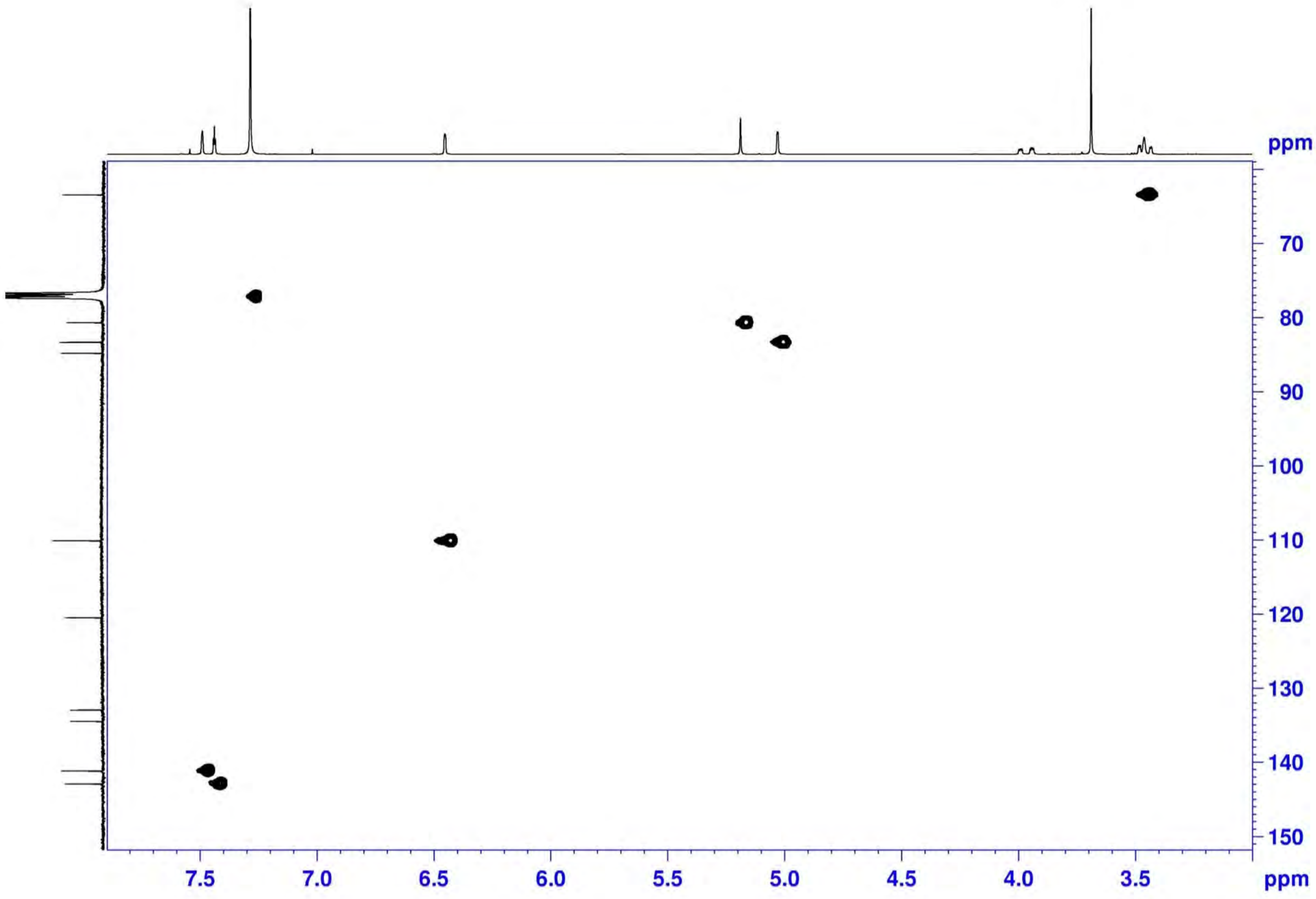

HSQC (400 MHz) spectrum of compound **18** in $CDCl_3$

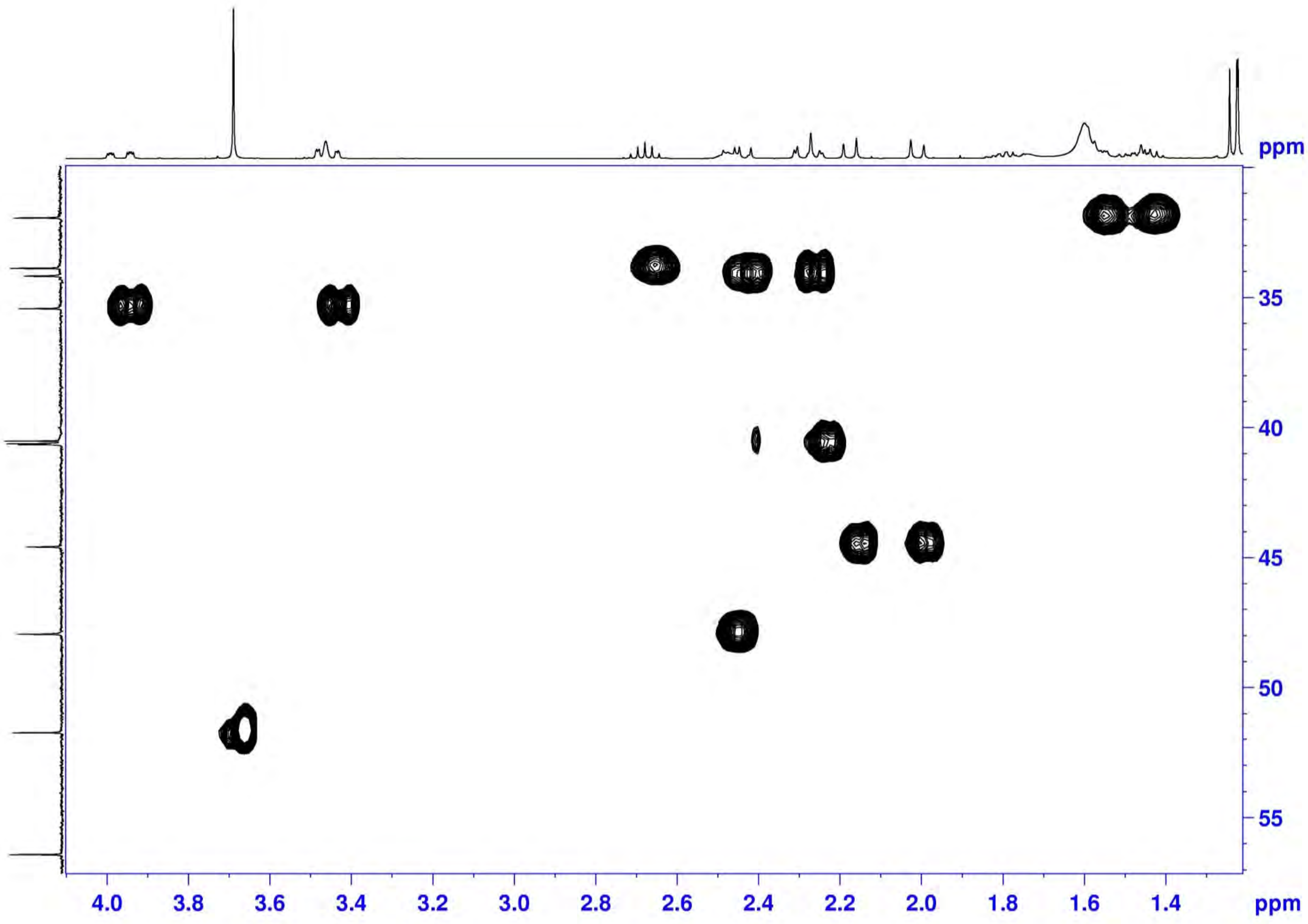

HSQC (400 MHz) spectrum of compound **18** in $CDCl_3$

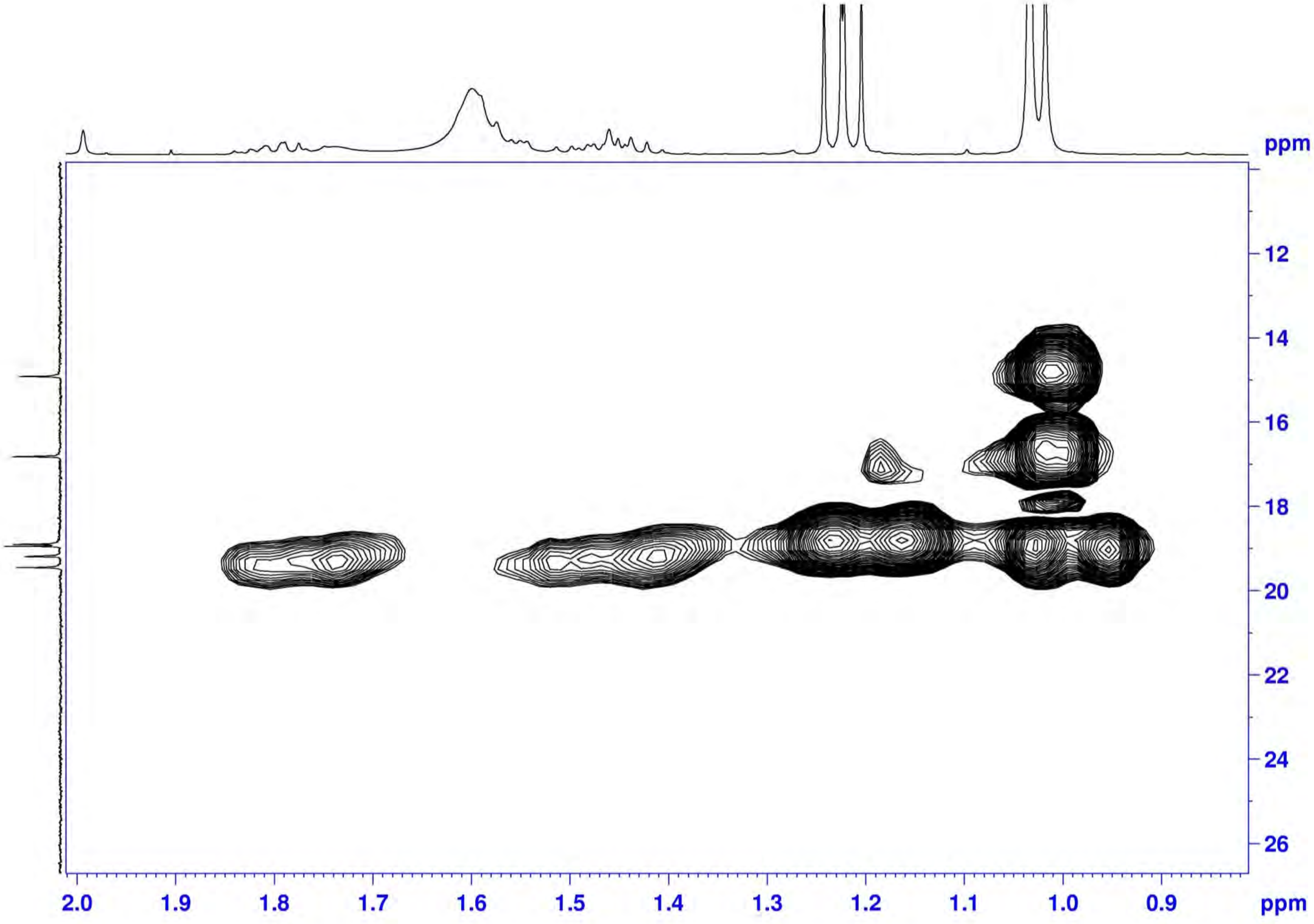

HMBC (400 MHz) spectrum of compound **18** in $CDCl_3$

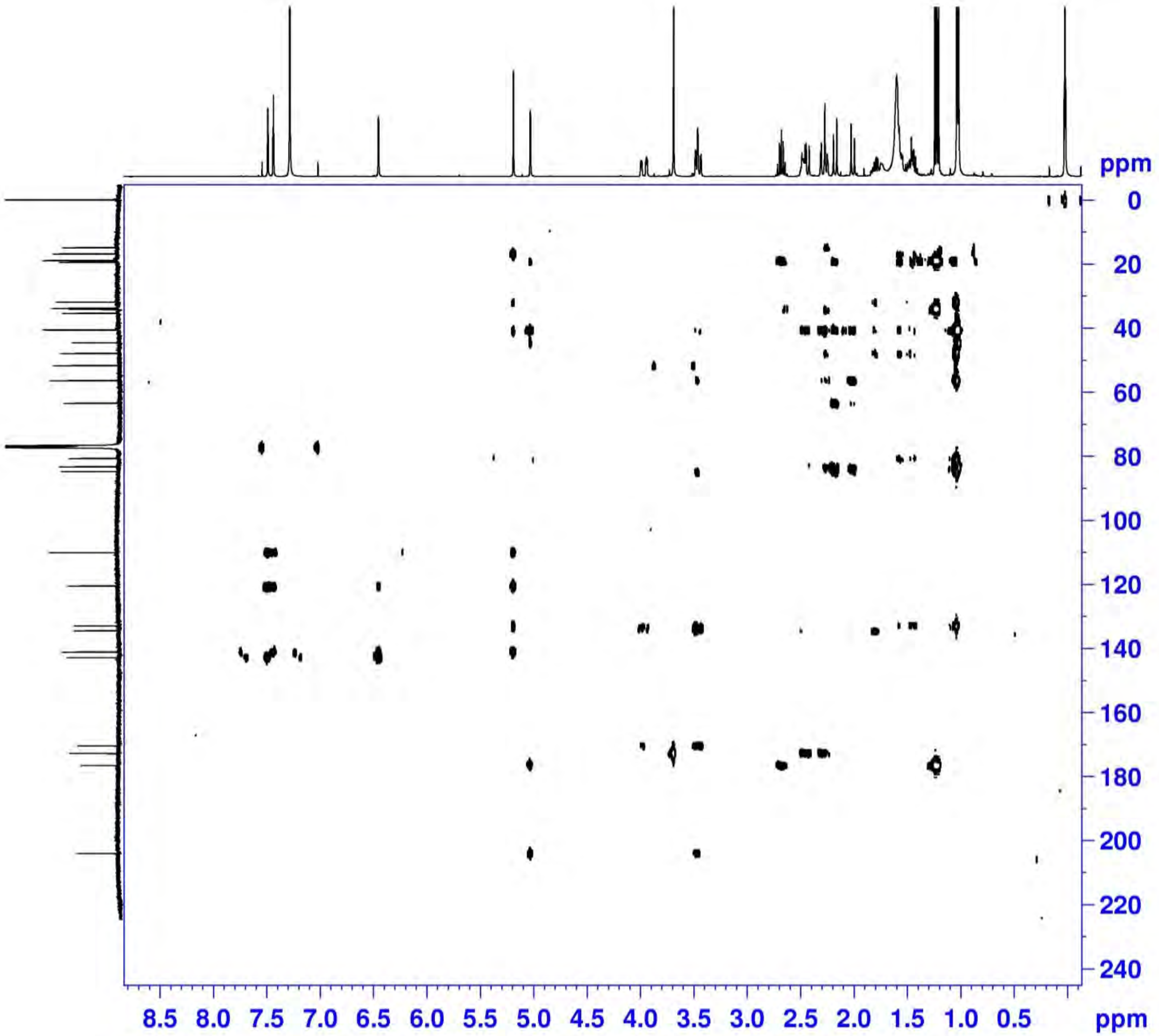

NAME syy−11
EXPNO 6
PROCNO 1
Date_ 20150810
Time 6.43
INSTRUM spect
PROBHD 5 mm CPPBBO BB
PULPROG hmbcgplpndqf
TD 4096
SOLVENT CDCl3
NS 64
DS 16
SWH 5197.505 Hz
FIDRES 1.268922 Hz
AQ 0.3940852 sec
RG 208.5
DW 96.200 usec
DE 10.00 usec
TE 297.0 K
CNST2 145.0000000
CNST13 10.0000000
D0 0.00000300 sec
D1 1.50000000 sec
D2 0.00344828 sec
D6 0.05000000 sec
D16 0.00020000 sec
IN0 0.00002080 sec

======== CHANNEL f1 ========
SFO1 400.1323208 MHz
NUC1 1H
P1 12.00 usec
P2 24.00 usec
ND0 2
TD 128
SFO1 100.6233 MHz
FIDRES 187.800476 Hz
SW 238.896 ppm
FnMODE QF
SI 2048
SF 400.1300000 MHz
WDW SINE
SSB 0
LB 0.00 Hz
GB 0
PC 1.40
SI 1024
MC2 QF
SF 100.6127685 MHz
WDW SINE
SSB 0
LB 0.00 Hz
GB 0

HMBC (400 MHz) spectrum of compound **18** in $CDCl_3$

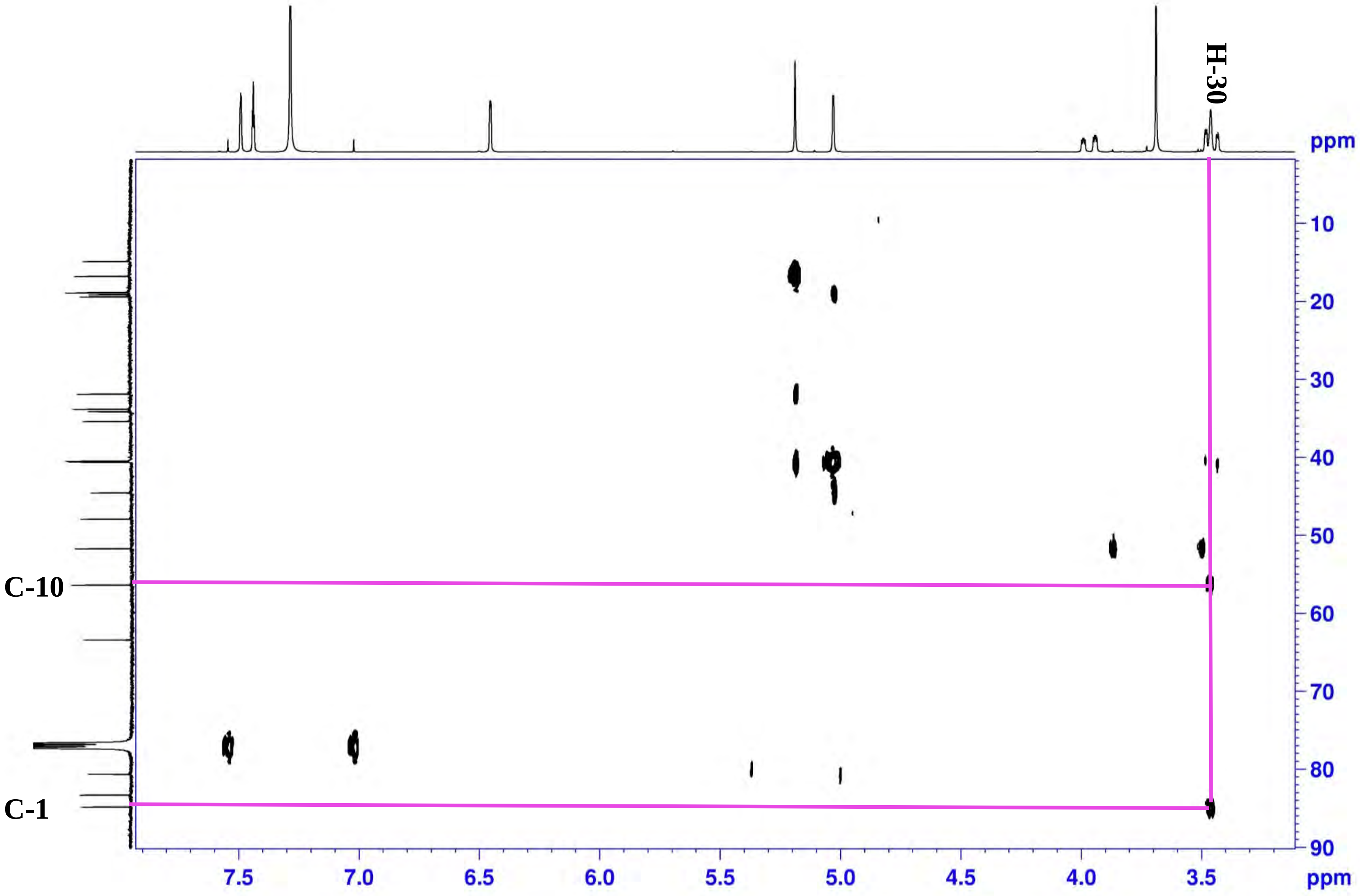

HMBC (400 MHz) spectrum of compound **18** in $CDCl_3$

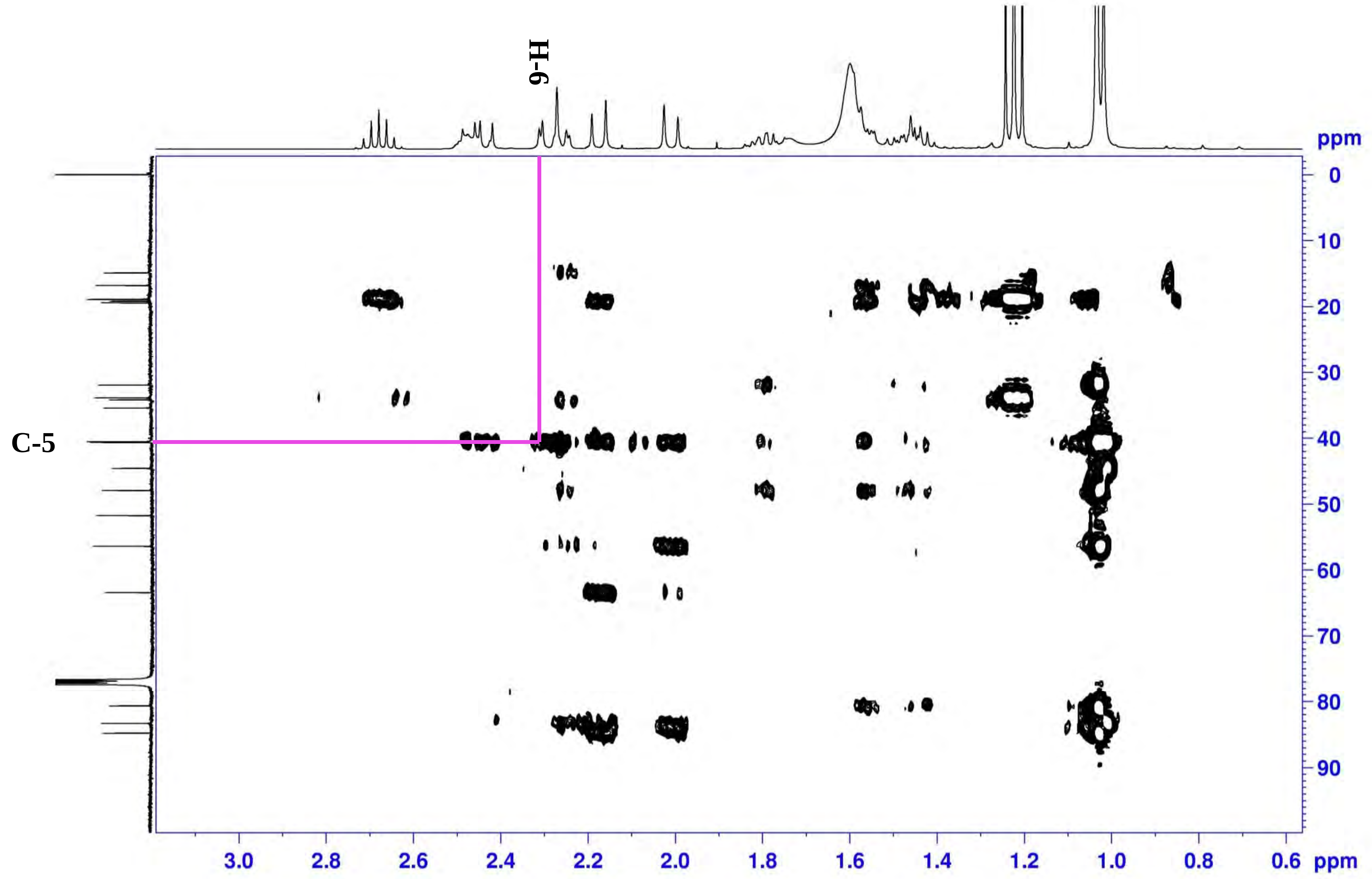

HMBC (400 MHz) spectrum of compound **18** in $CDCl_3$

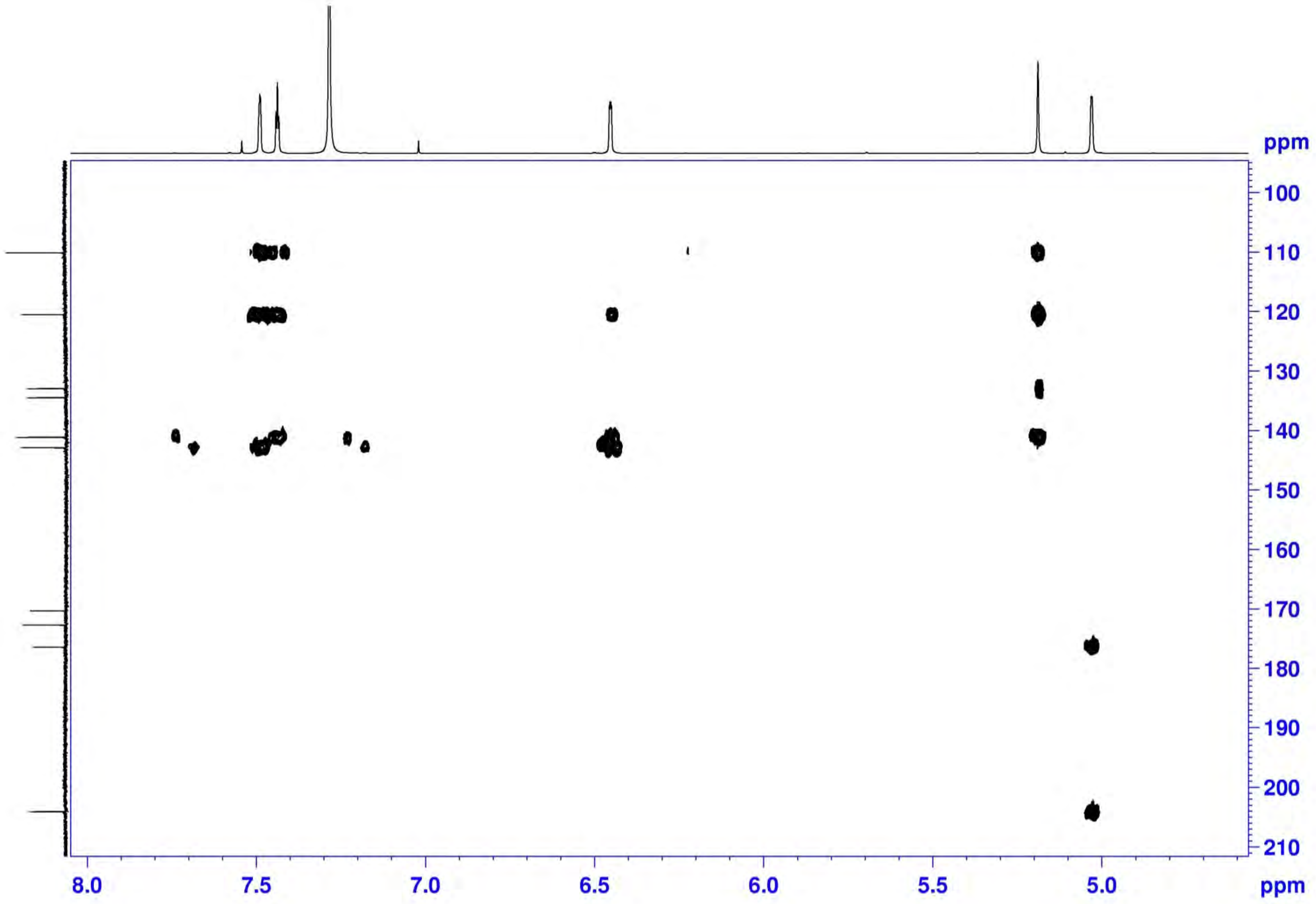

HMBC (400 MHz) spectrum of compound **18** in $CDCl_3$

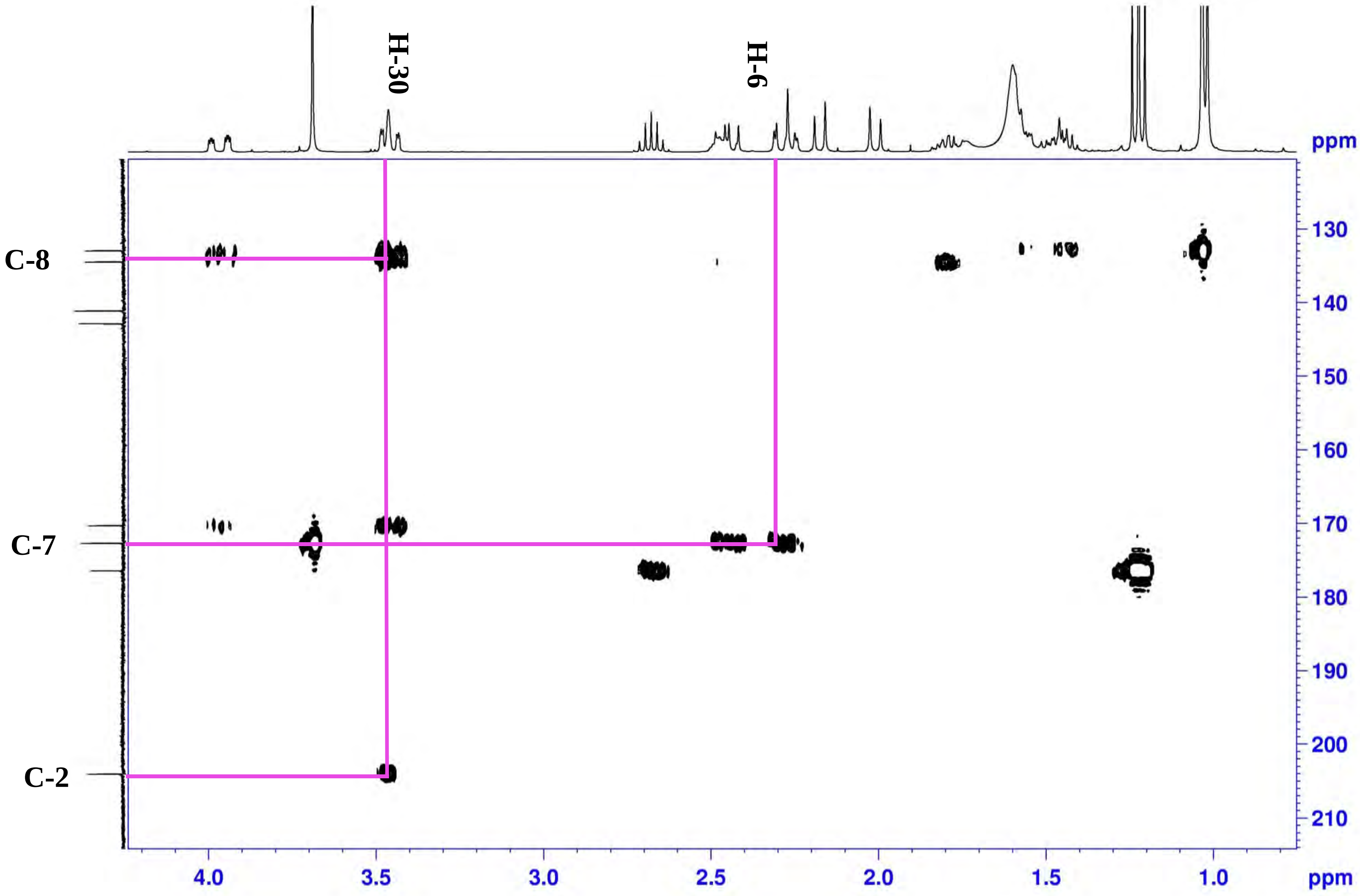

NOESY (400 MHz) spectrum of compound **18** in $CDCl_3$

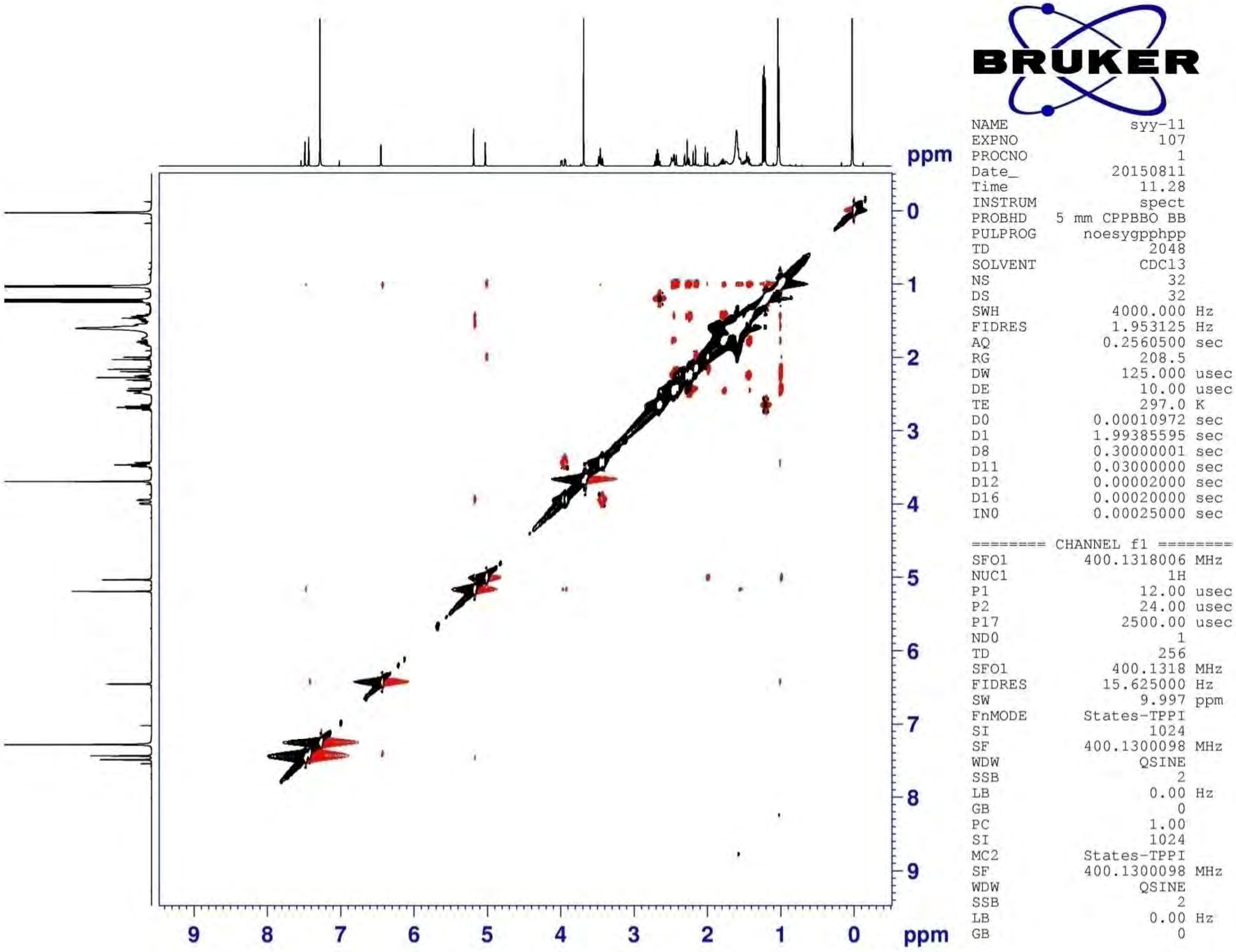

NOESY (400 MHz) spectrum of compound **18** in $CDCl_3$

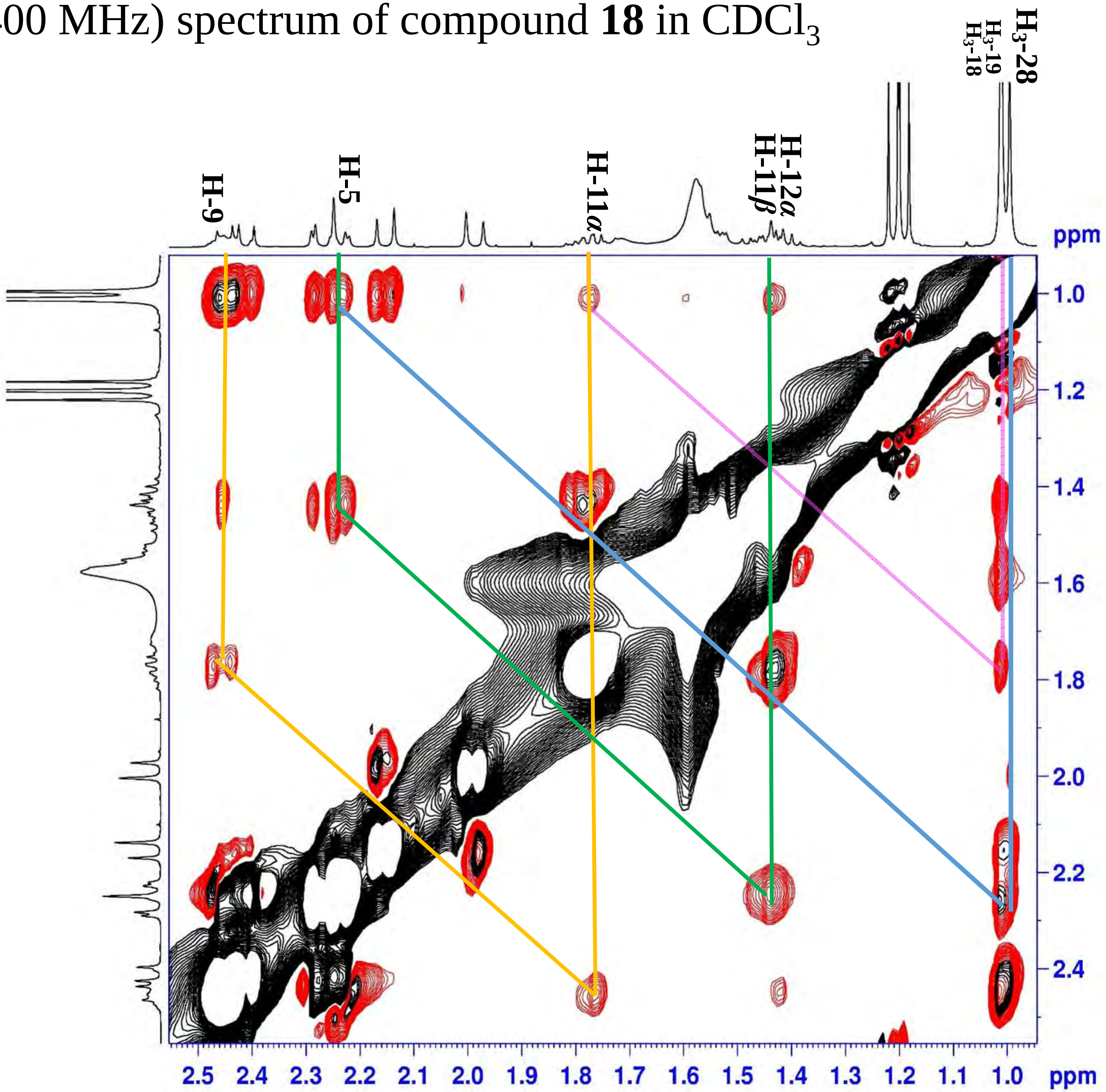

NOESY (400 MHz) spectrum of compound **18** in $CDCl_3$

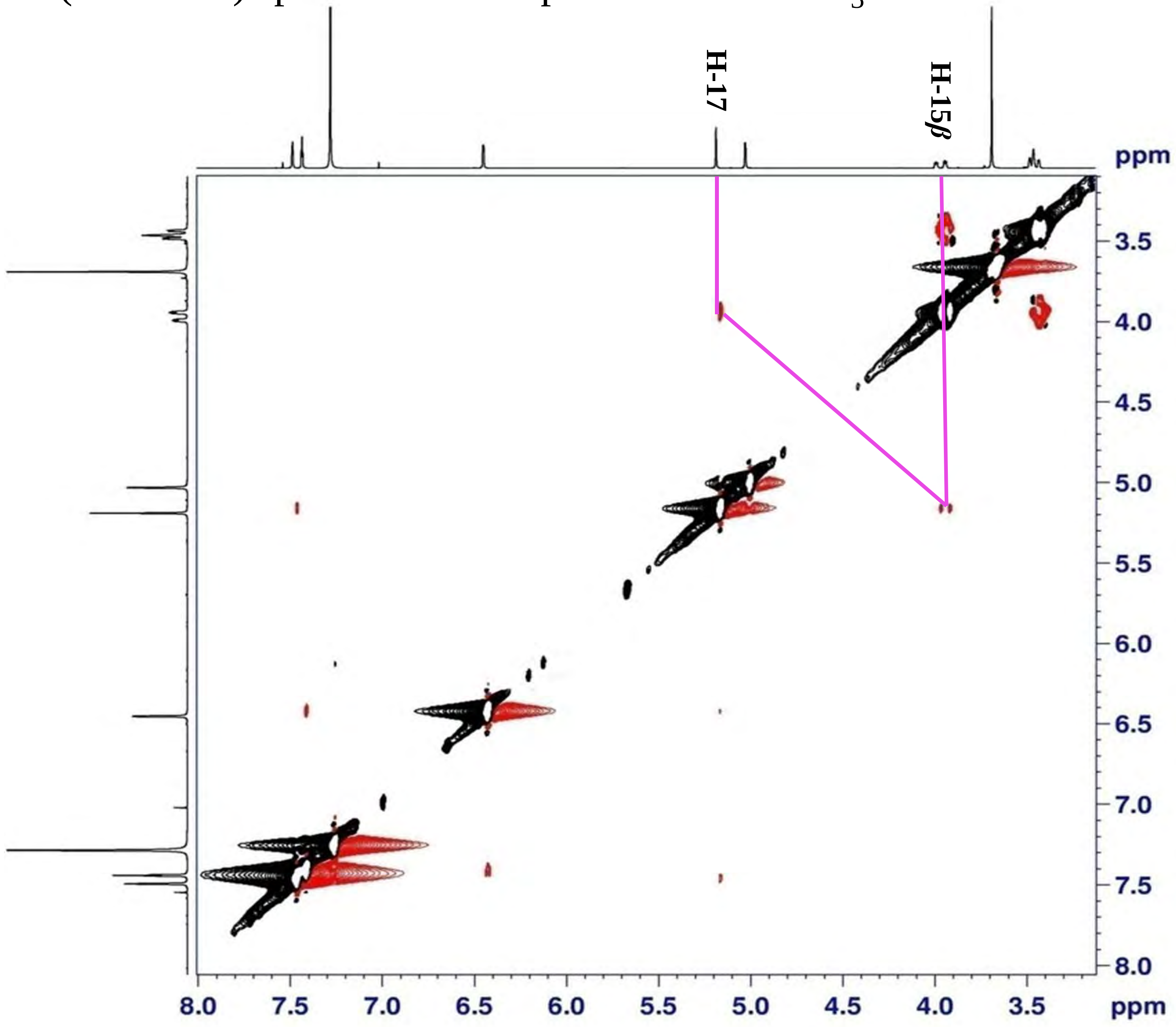

NOESY (400 MHz) spectrum of compound **18** in $CDCl_3$

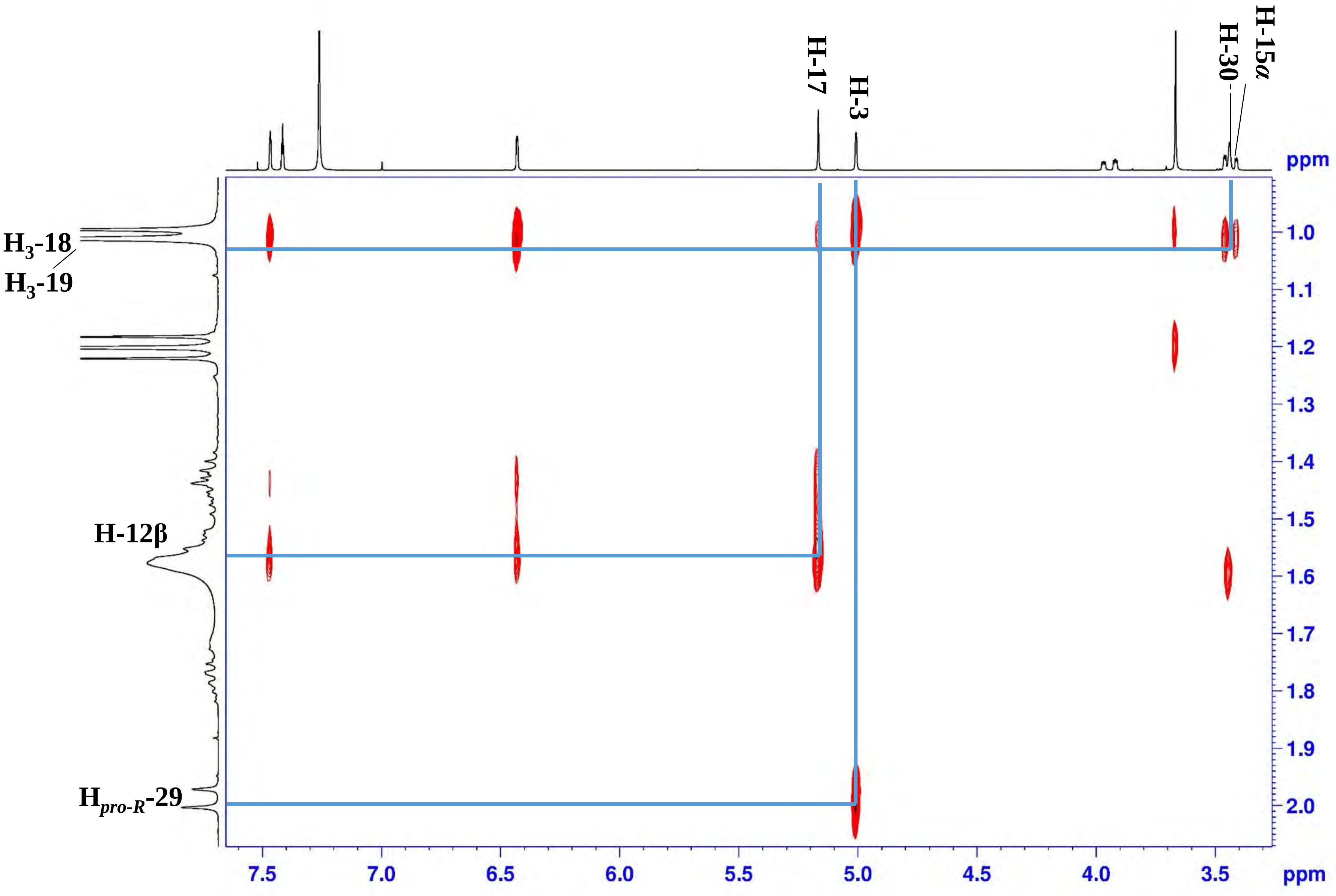

NOESY (400 MHz) spectrum of compound **18** in $CDCl_3$

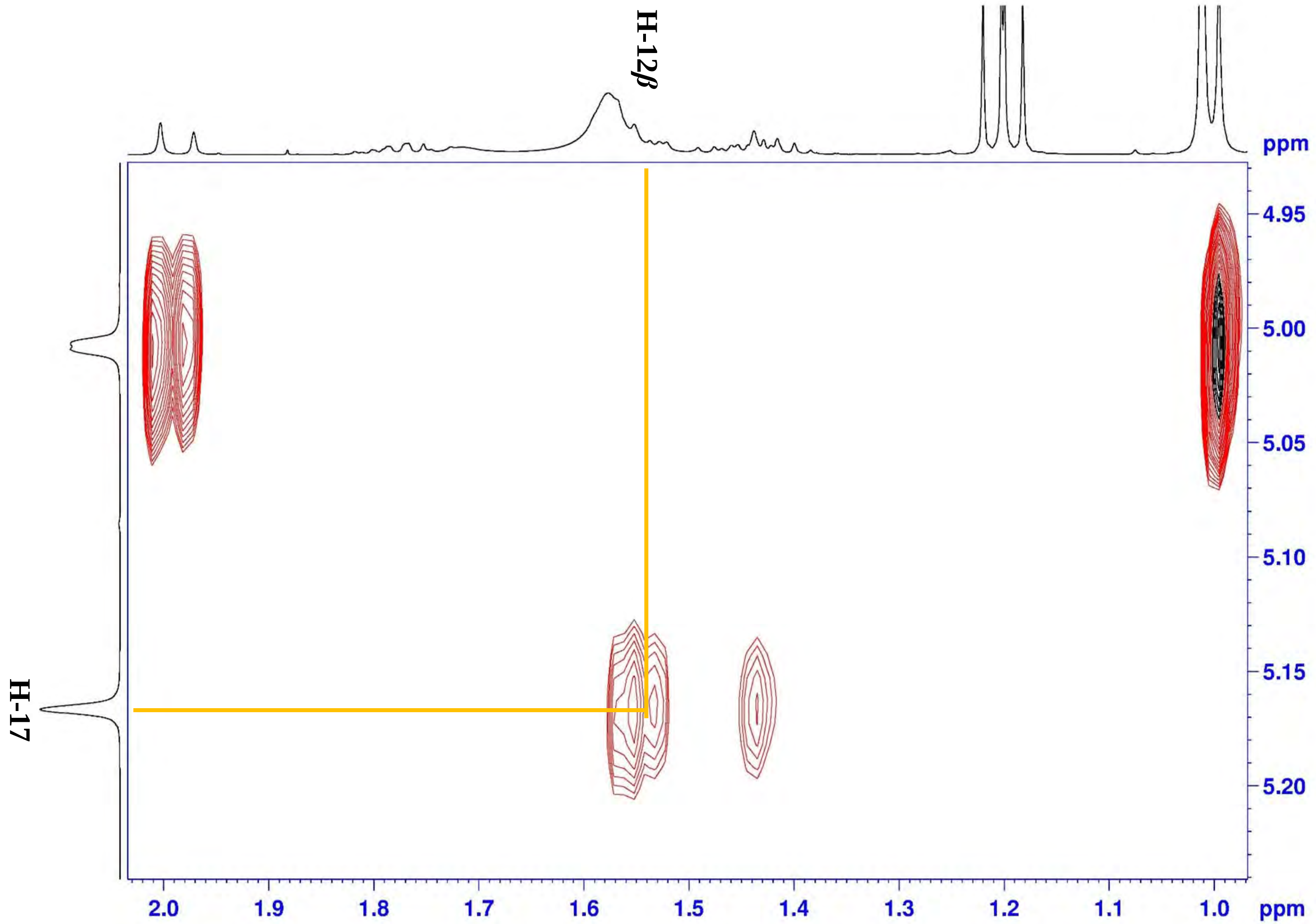

HRESIMS for compound **19**

Elemental Composition Report

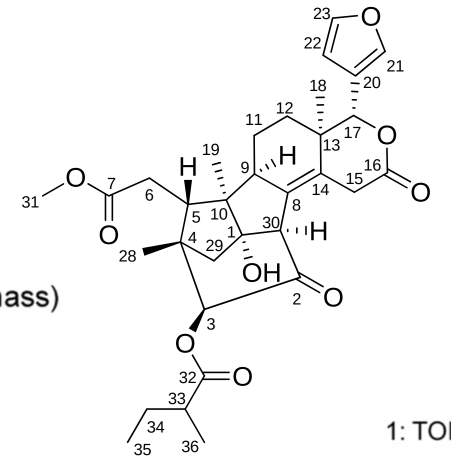

Single Mass Analysis

Tolerance = 10.0 mDa / DBE: min = -1.0, max = 50.0
Element prediction: Off
Number of isotope peaks used for i-FIT = 3

Monoisotopic Mass, Even Electron Ions
155 formula(e) evaluated with 5 results within limits (up to 10 closest results for each mass)
Elements Used:
C: 0-100 H: 0-200 O: 0-100
SYY-12
2016103107 197 (1.597)

1: TOF MS ES+
5.45e+005

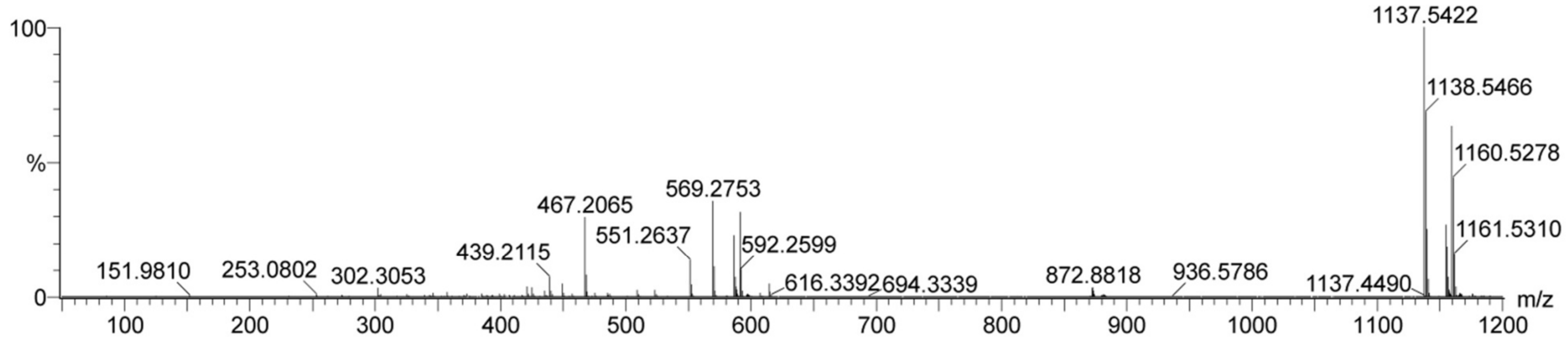

Minimum:				-1.0				
Maximum:		10.0	10.0	50.0				

Mass	Calc. Mass	mDa	PPM	DBE	i-FIT	Norm	Conf(%)	Formula
569.2753	569.2751	0.2	0.4	12.5	266.4	0.705	49.42	C32 H41 O9
	569.2809	-5.6	-9.8	3.5	266.4	0.697	49.81	C25 H45 O14
	569.2692	6.1	10.7	21.5	271.7	5.933	0.26	C39 H37 O4
	569.2844	-9.1	-16.0	25.5	274.2	8.428	0.02	C43 H37 O
	569.2657	9.6	16.9	-0.5	271.1	5.330	0.48	C21 H45 O17

^{1}H NMR (400 MHz) spectrum of compound **19** in $CDCl_3$

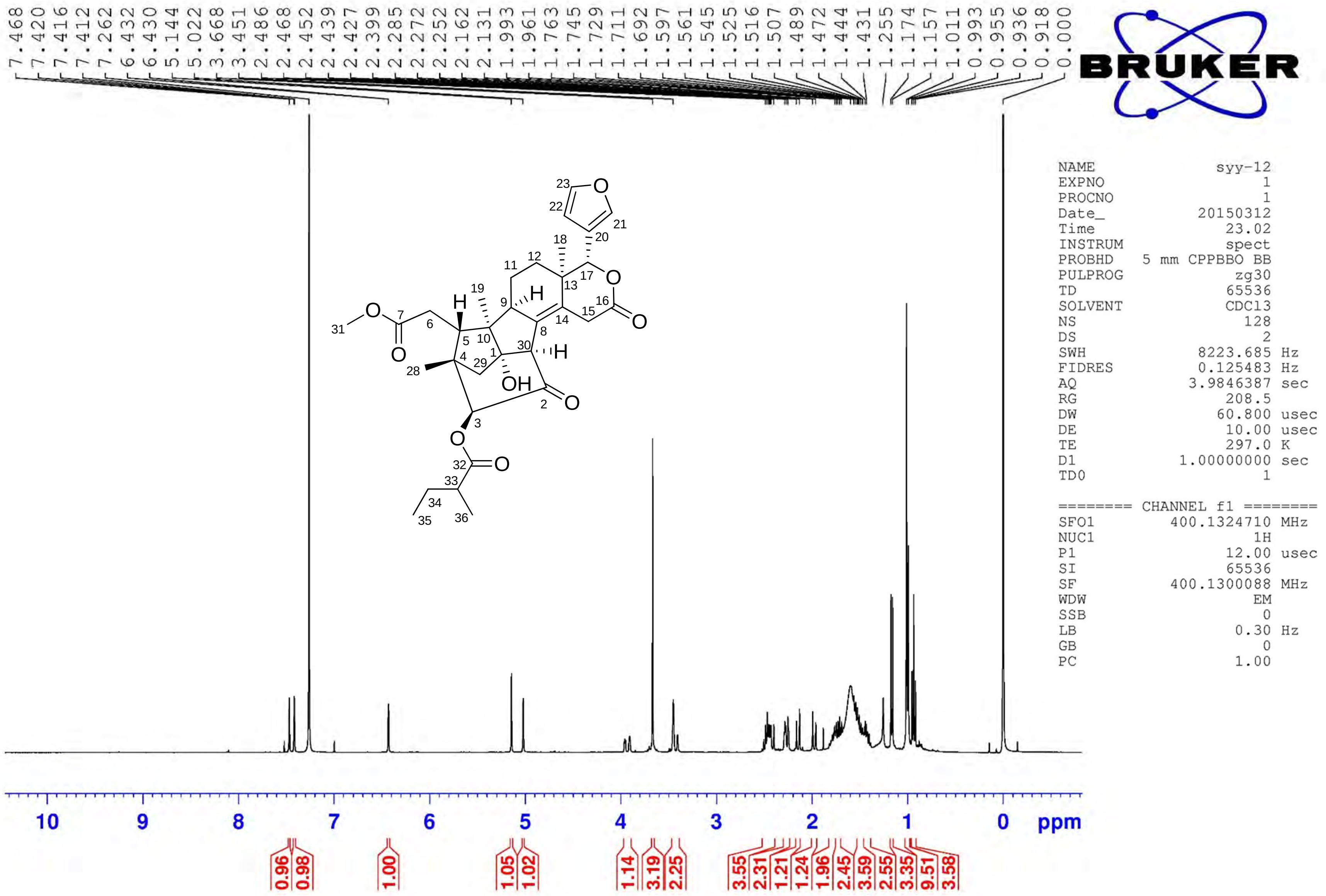

^{1}H NMR (400 MHz) spectrum of compound **19** in $CDCl_3$

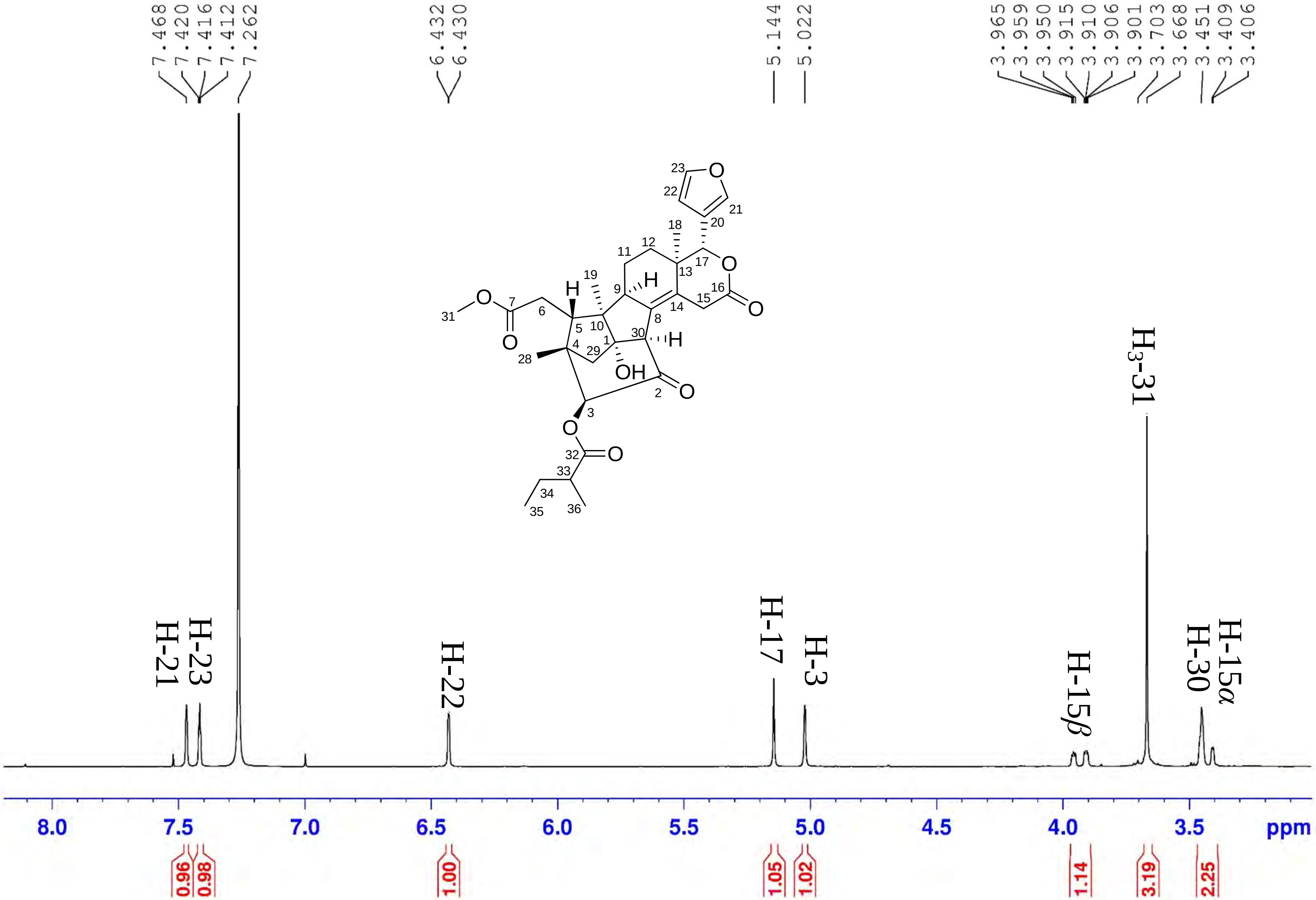

^{1}H NMR (400 MHz) spectrum of compound **19** in $CDCl_3$

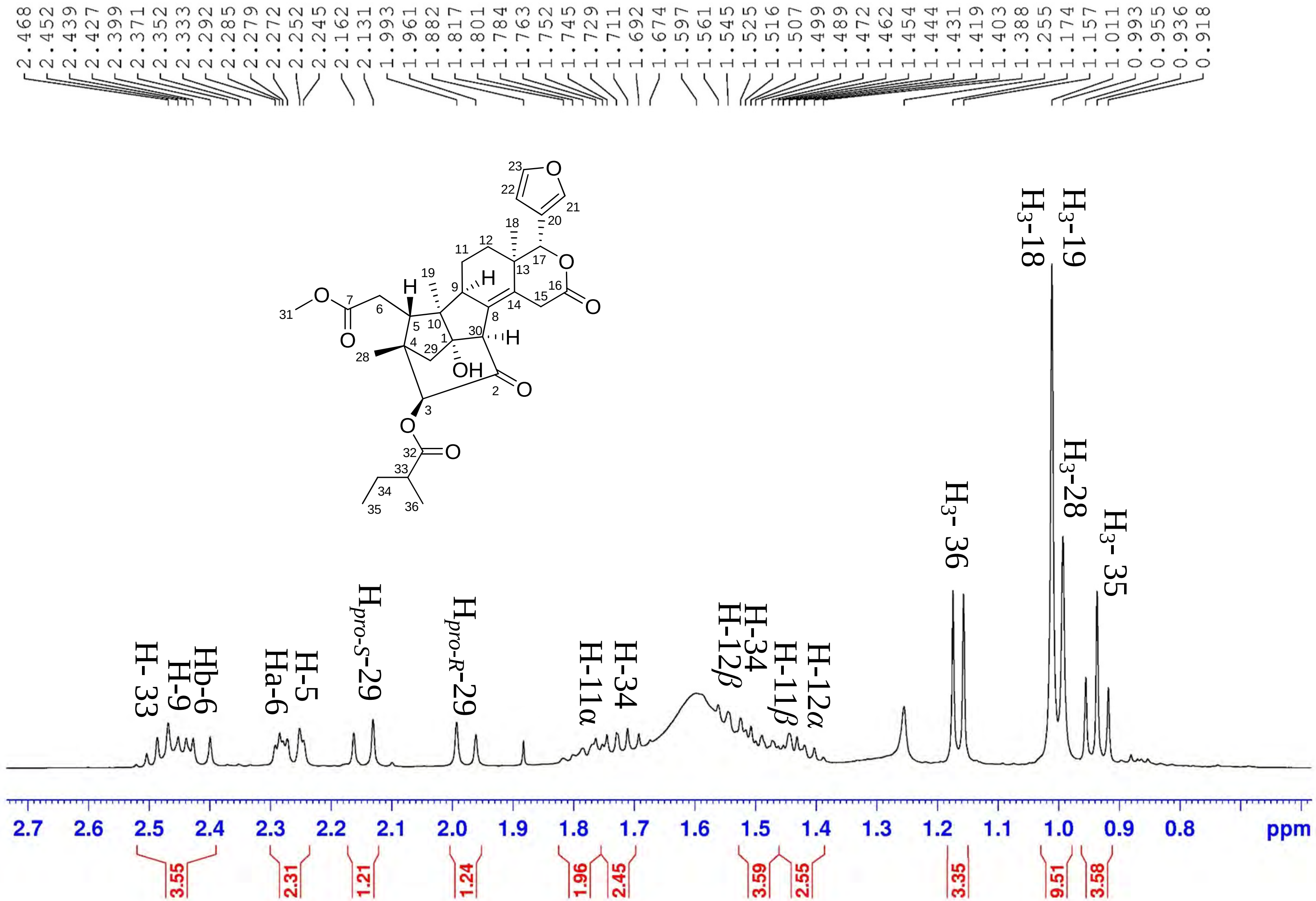

^{13}C NMR (100 MHz) spectrum of compound **19** in $CDCl_3$

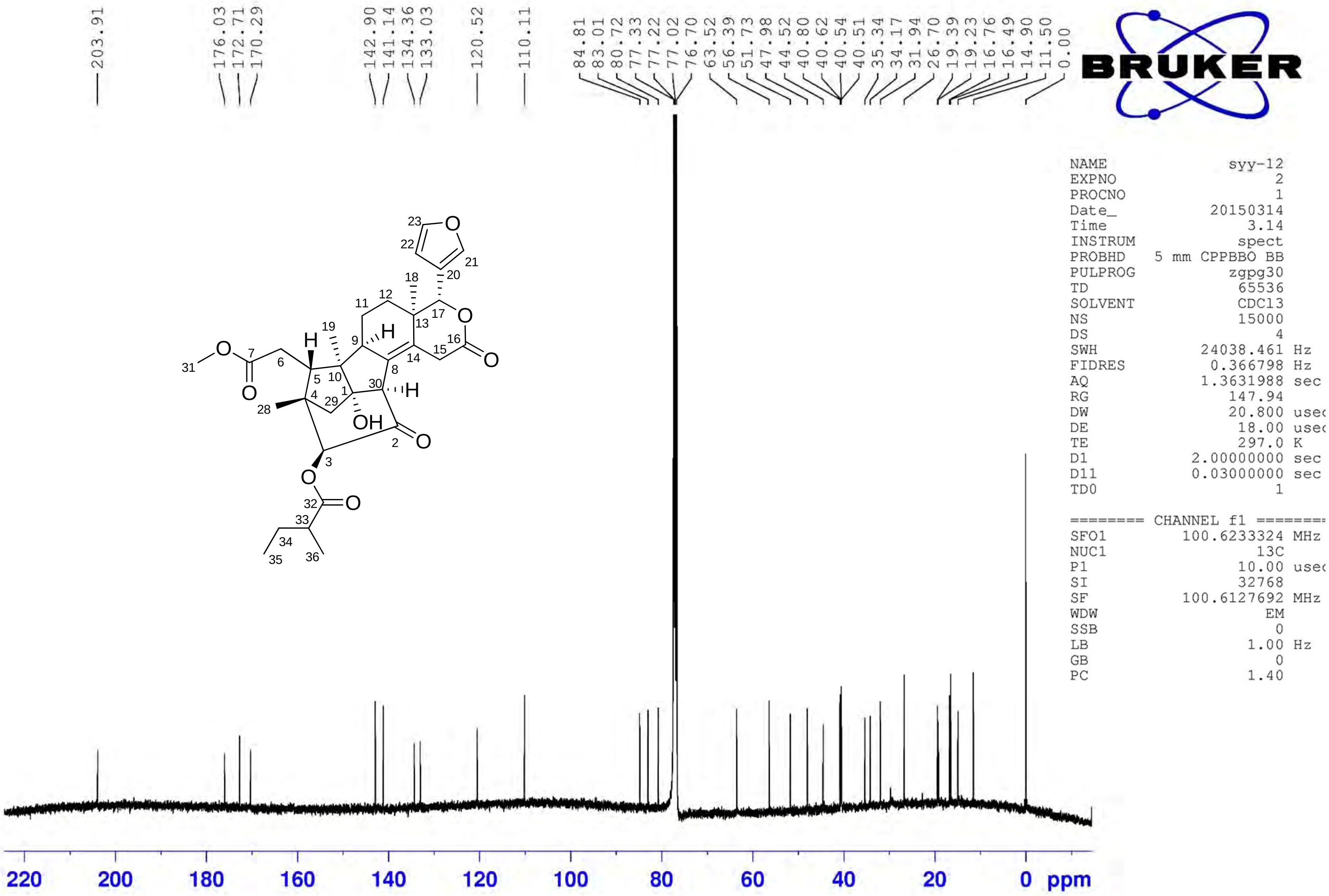

^{13}C NMR (100 MHz) spectrum of compound **19** in $CDCl_3$

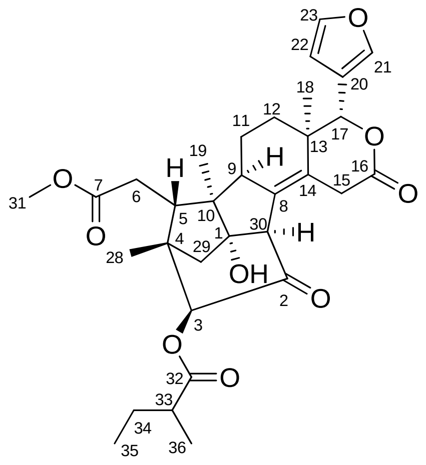

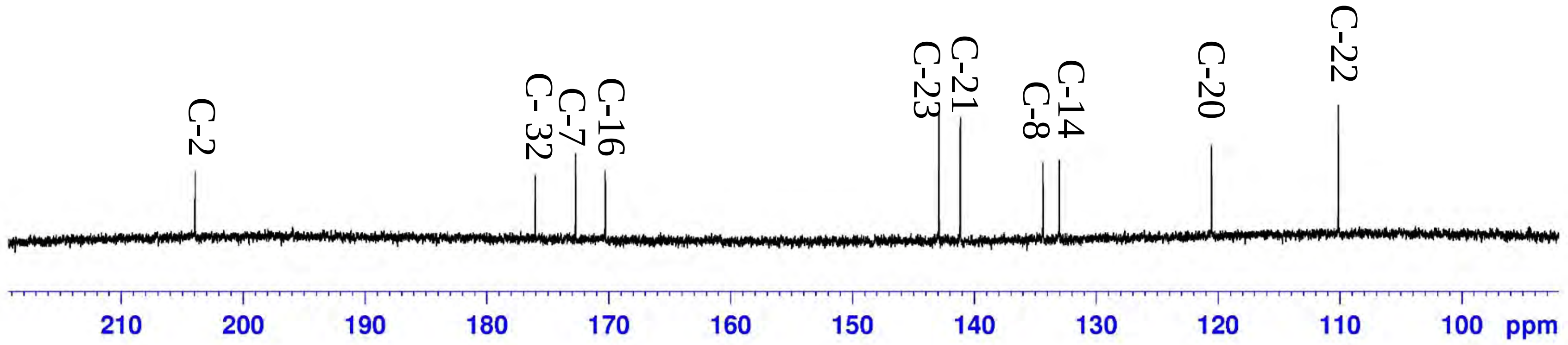

^{13}C NMR (100 MHz) spectrum of compound **19** in $CDCl_3$

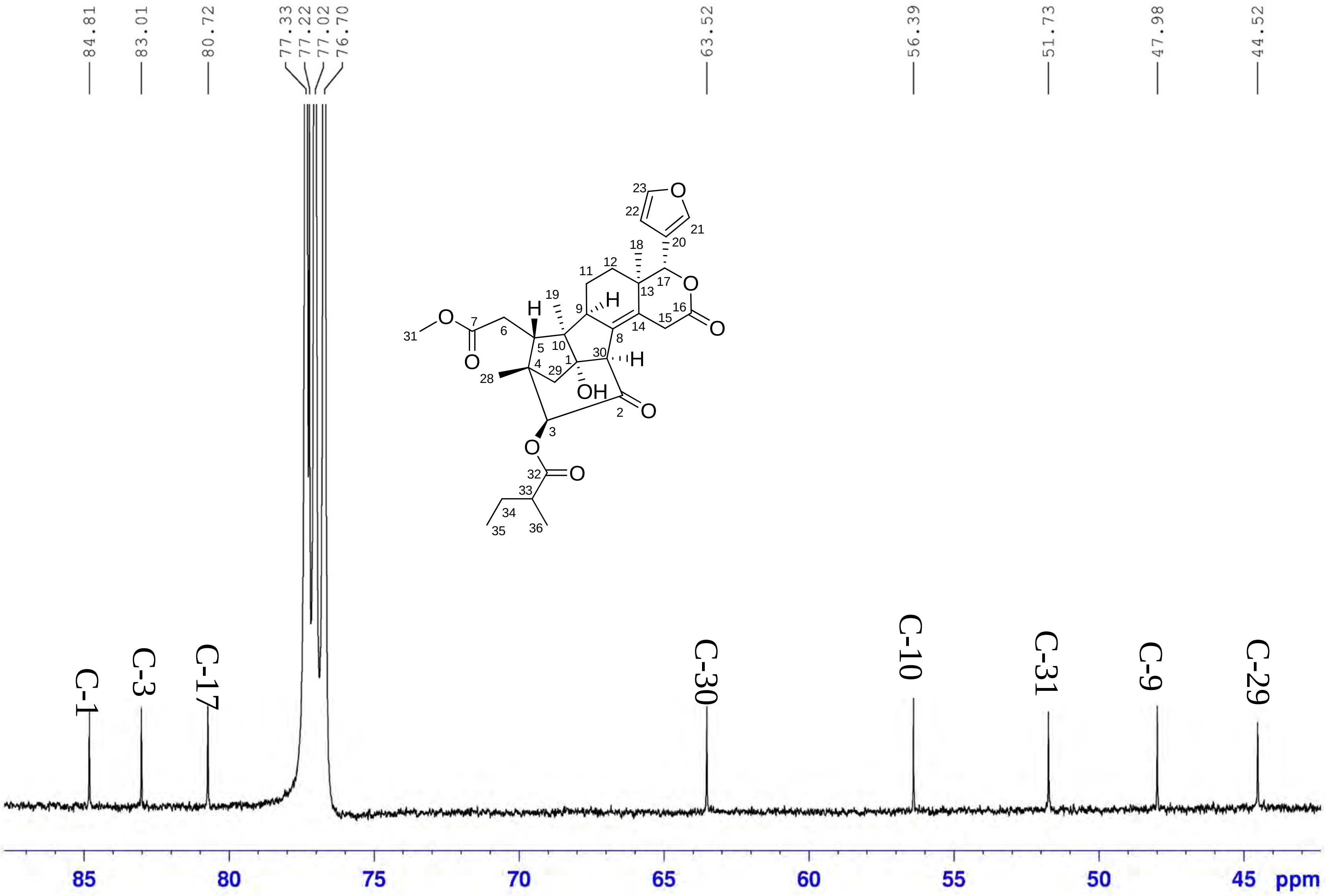

^{13}C NMR (100 MHz) spectrum of compound **19** in $CDCl_3$

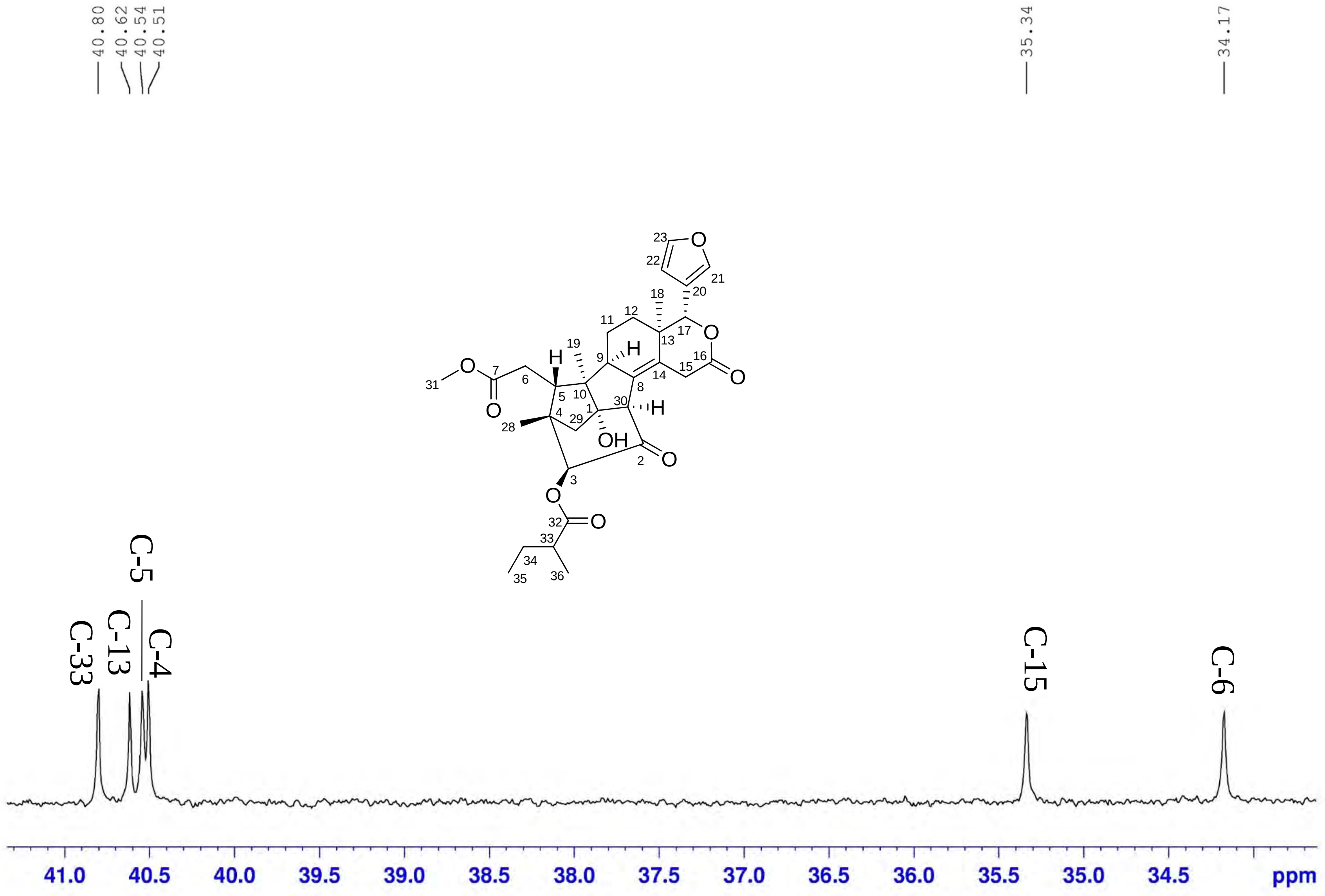

^{13}C NMR (100 MHz) spectrum of compound **19** in $CDCl_3$

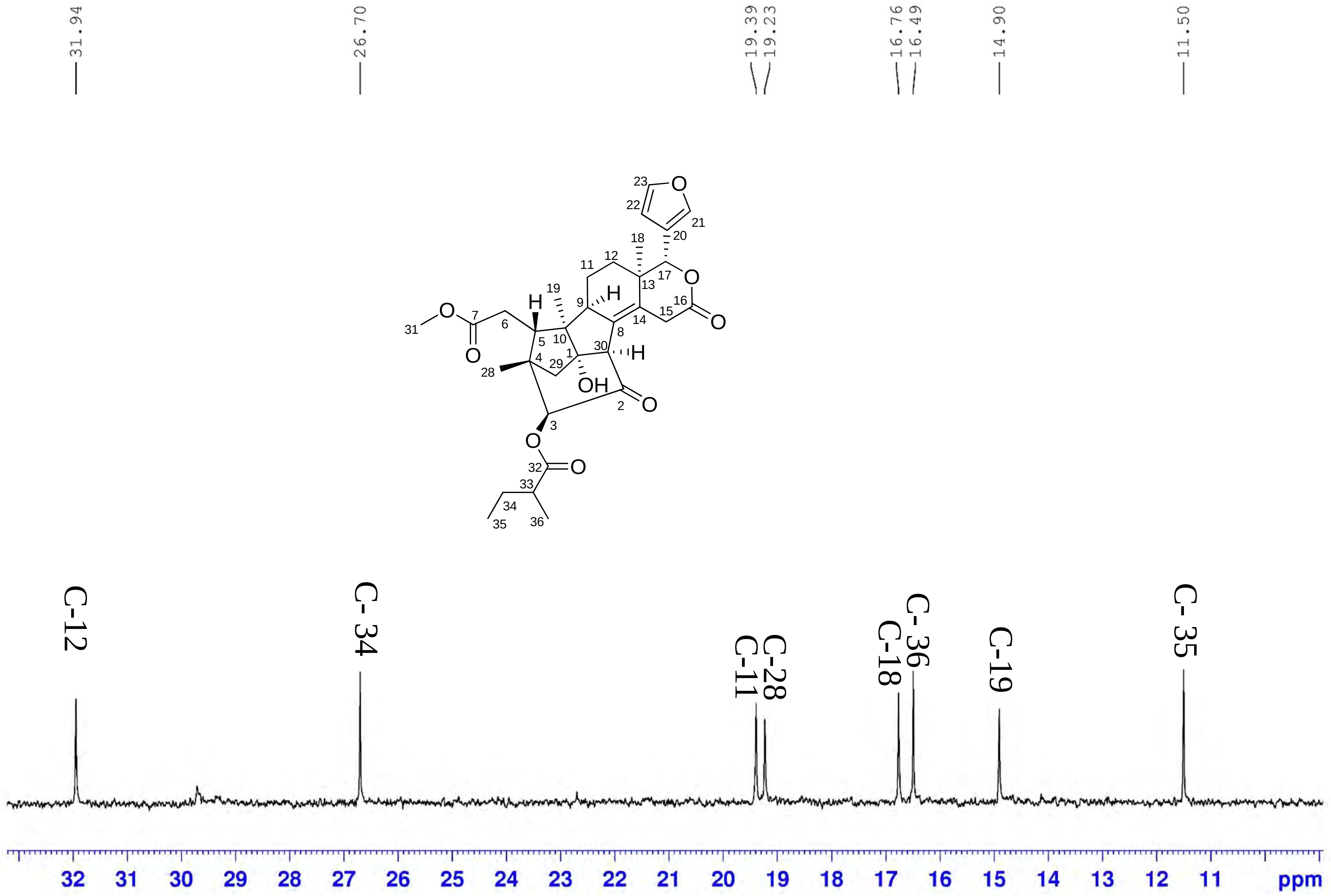

DEPT135 (100 MHz) spectrum of compound **19** in $CDCl_3$

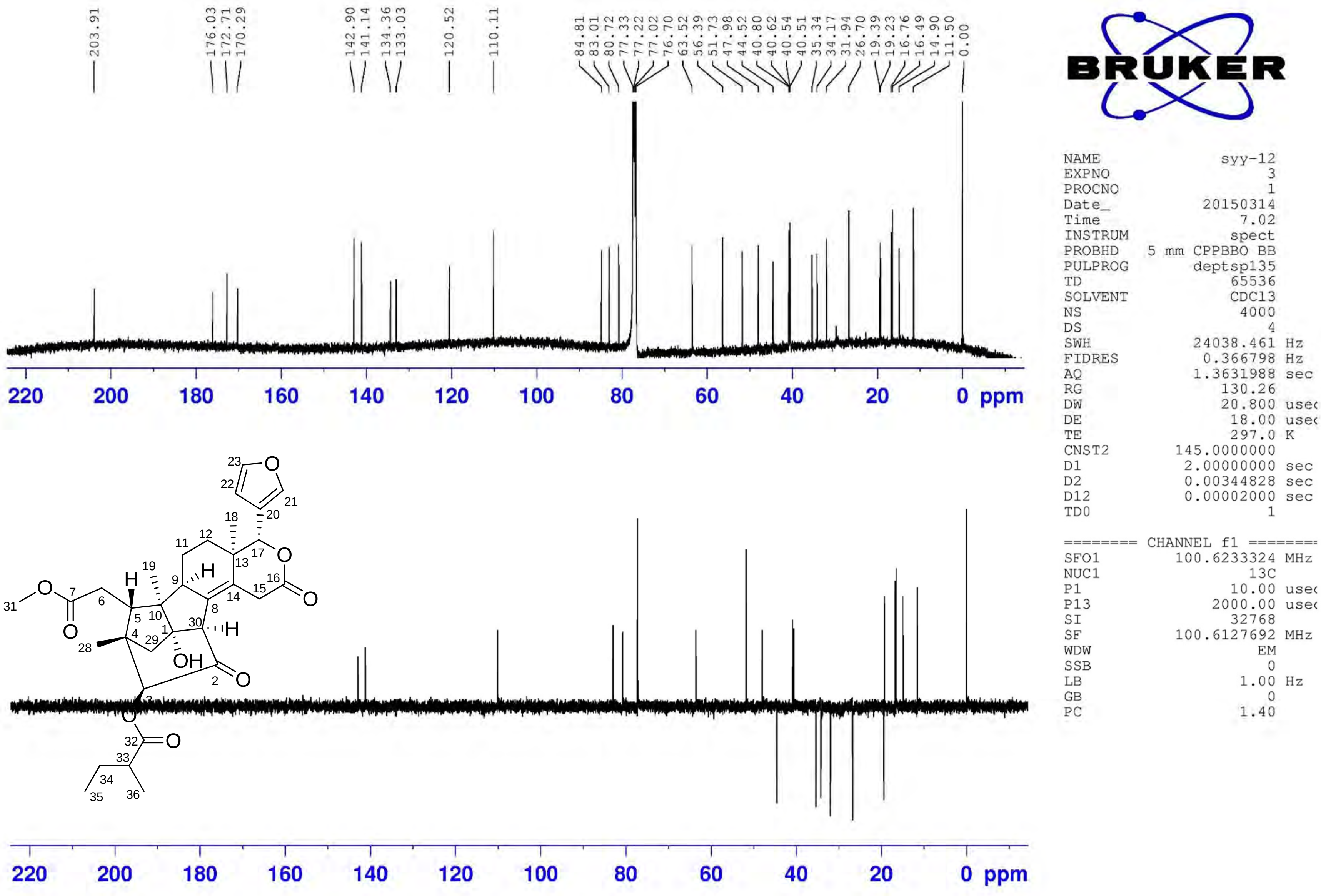

DEPT135 (100 MHz) spectrum of compound **19** in $CDCl_3$

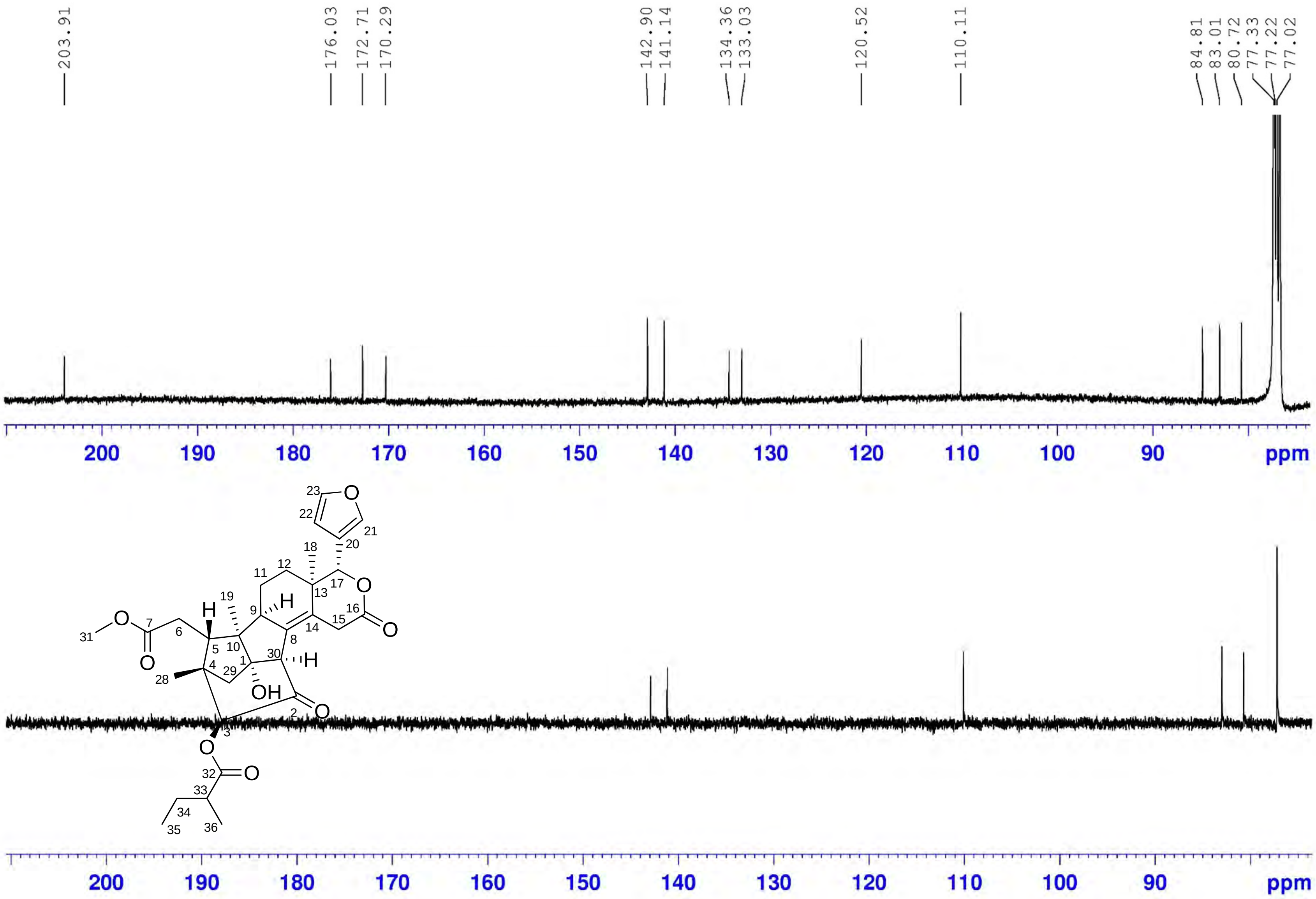

DEPT135 (100 MHz) spectrum of compound **19** in $CDCl_3$

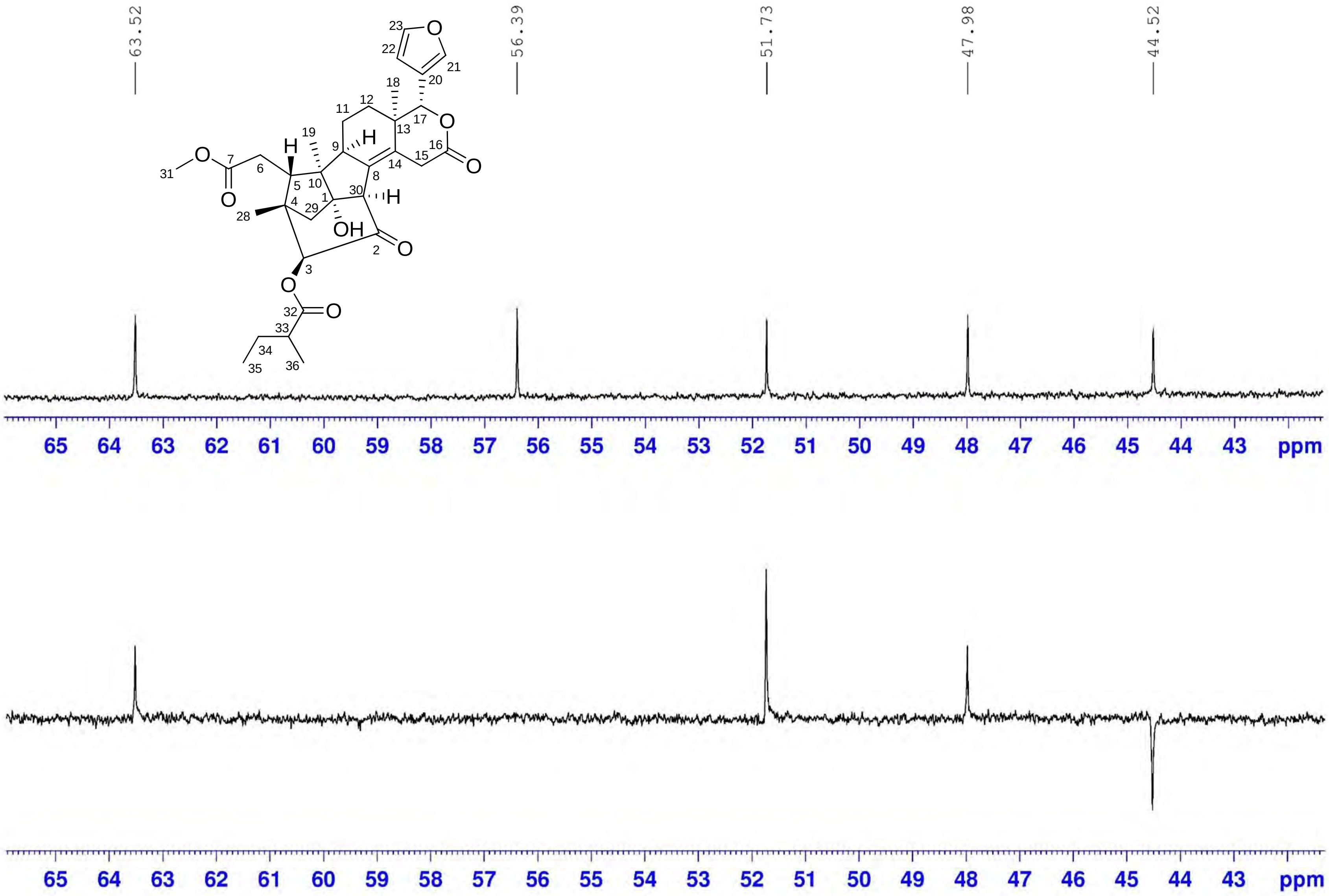

DEPT135 (100 MHz) spectrum of compound **19** in $CDCl_3$

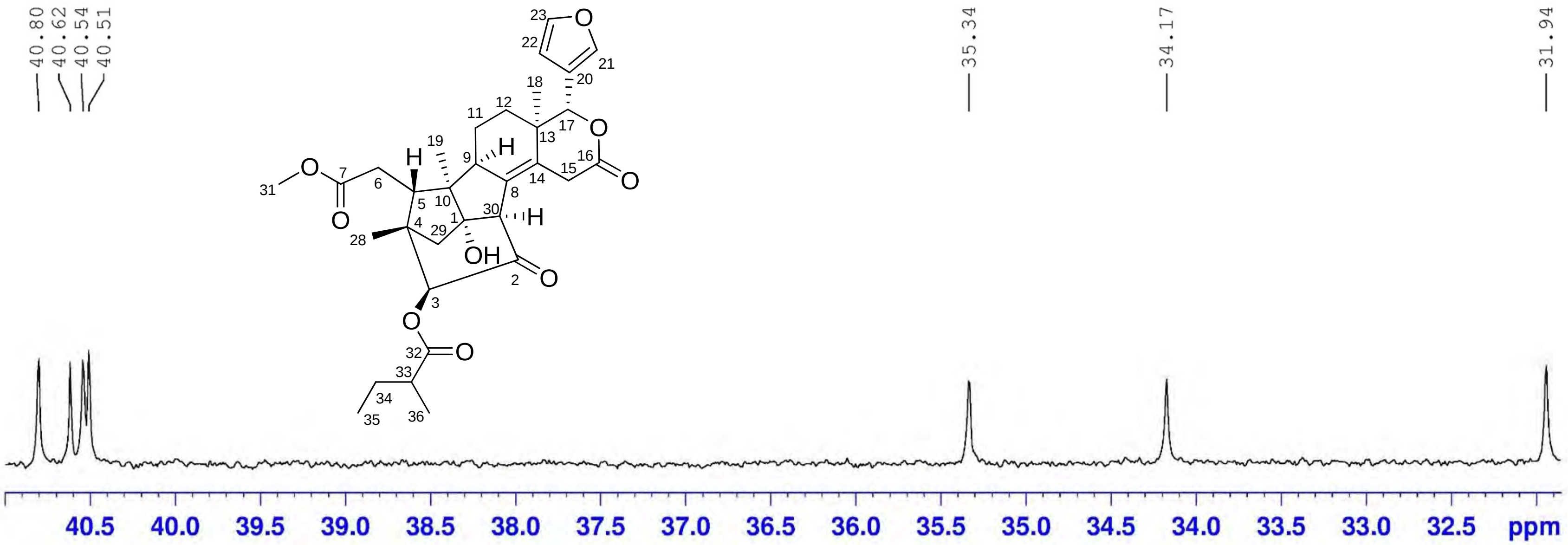

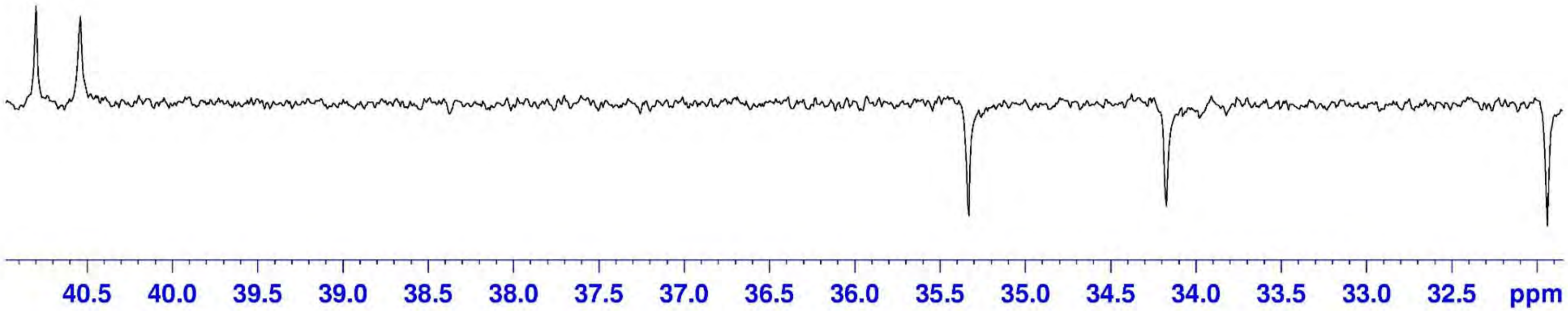

DEPT135 (100 MHz) spectrum of compound **19** in $CDCl_3$

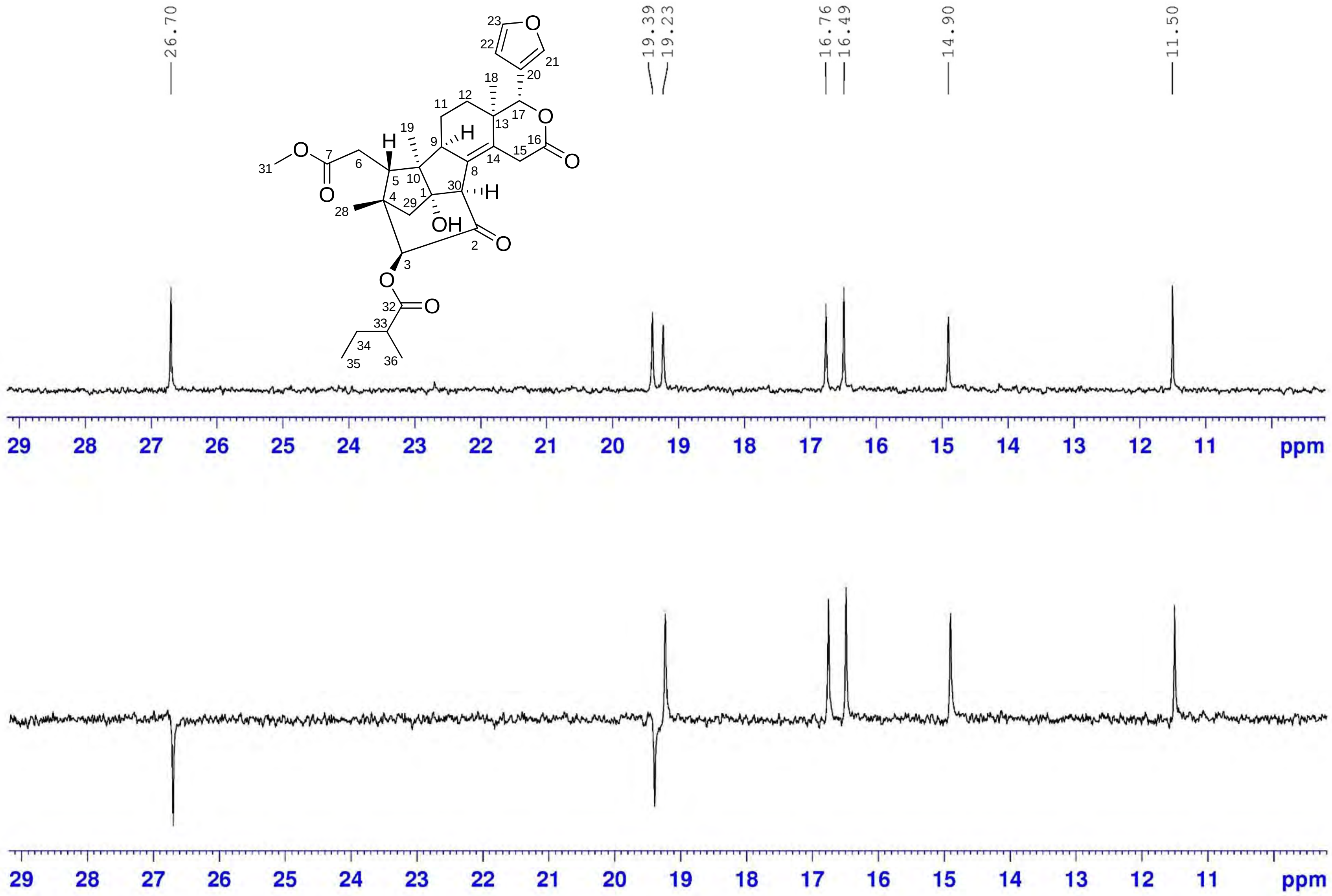

^1H-^1H COSY (400 MHz) spectrum of compound **19** in $CDCl_3$

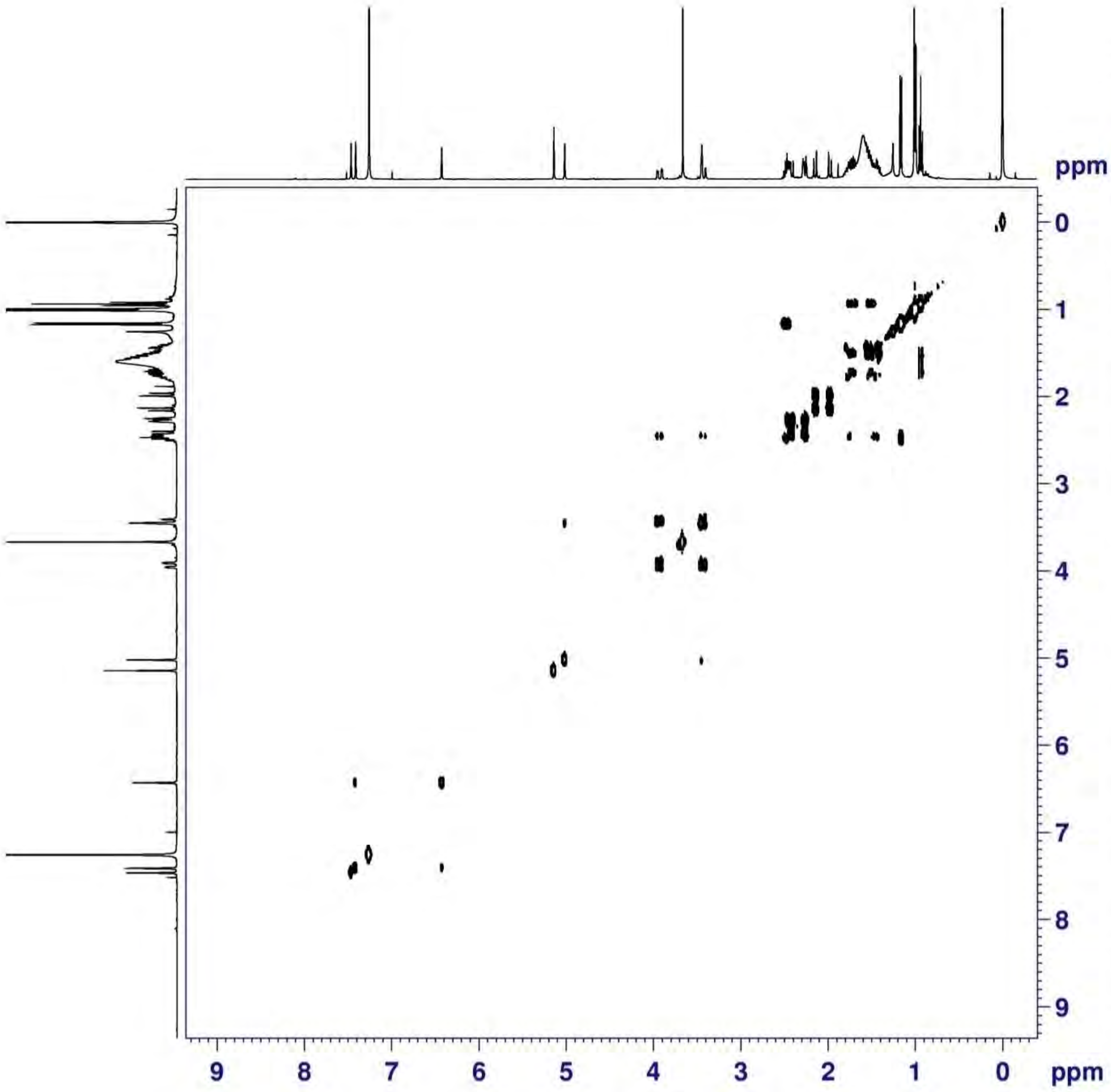

NAME	syy-12
EXPNO	4
PROCNO	1
Date_	20150801
Time	8.18
INSTRUM	spect
PROBHD	5 mm CPPBBO BB
PULPROG	cosygpppqf
TD	2048
SOLVENT	CDCl3
NS	16
DS	8
SWH	3906.250 Hz
FIDRES	1.907349 Hz
AQ	0.2621940 sec
RG	208.5
DW	128.000 usec
DE	10.00 usec
TE	297.0 K
D0	0.00000300 sec
D1	1.89678097 sec
D11	0.03000000 sec
D12	0.00002000 sec
D13	0.00000400 sec
D16	0.00020000 sec
IN0	0.00025600 sec
======== CHANNEL f1 ========	
SFO1	400.1318006 MHz
NUC1	1H
P0	12.00 usec
P1	12.00 usec
P17	2500.00 usec
ND0	1
TD	128
SFO1	400.1318 MHz
FIDRES	30.517578 Hz
SW	9.762 ppm
FnMODE	QF
SI	1024
SF	400.1300098 MHz
WDW	QSINE
SSB	0
LB	0.00 Hz
GB	0
PC	1.40
SI	1024
MC2	QF
SF	400.1300098 MHz
WDW	QSINE
SSB	0
LB	0.00 Hz
GB	0

^1H-^1H COSY (400 MHz) spectrum of compound **19** in $CDCl_3$

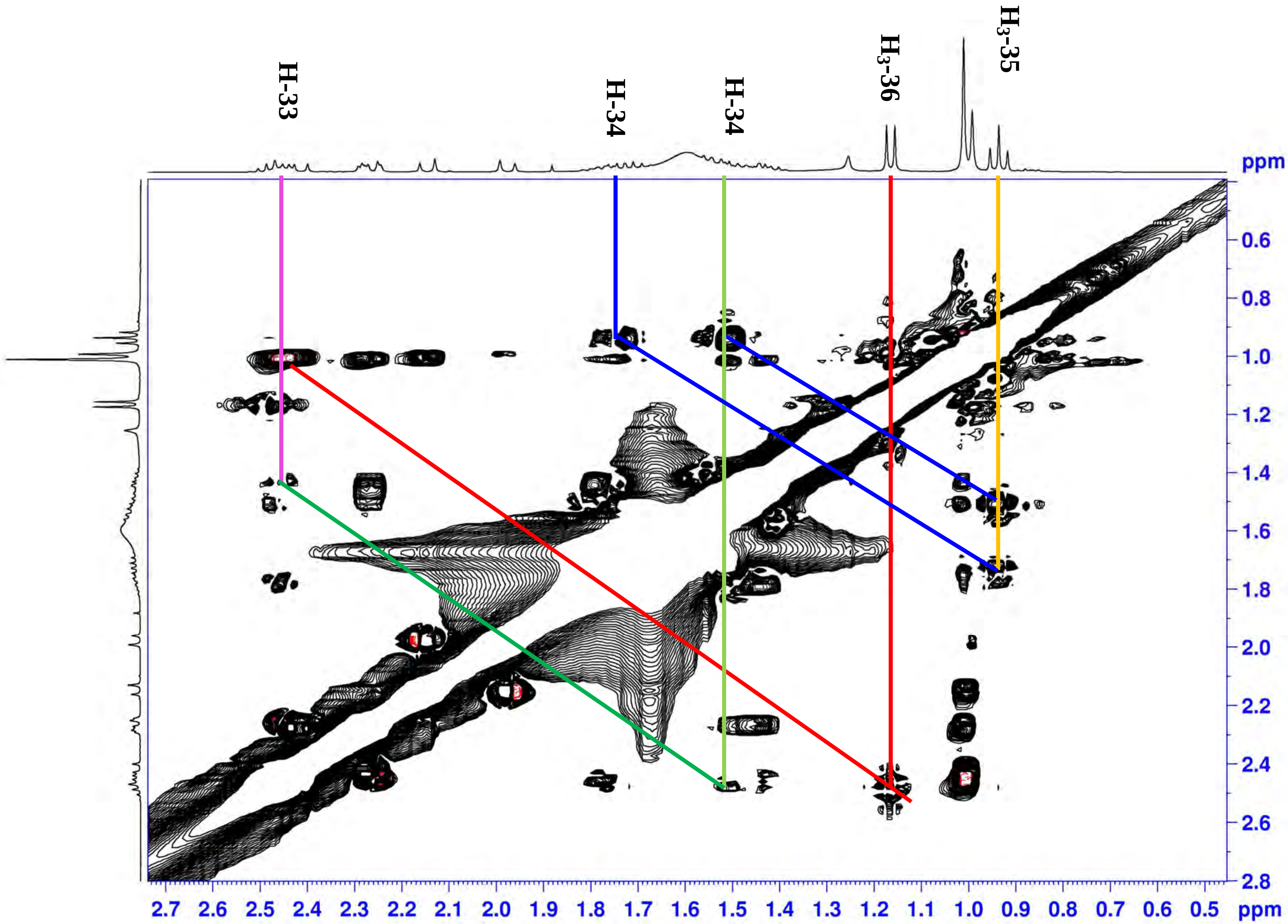

^{1}H-^{1}H COSY (400 MHz) spectrum of compound **19** in $CDCl_3$

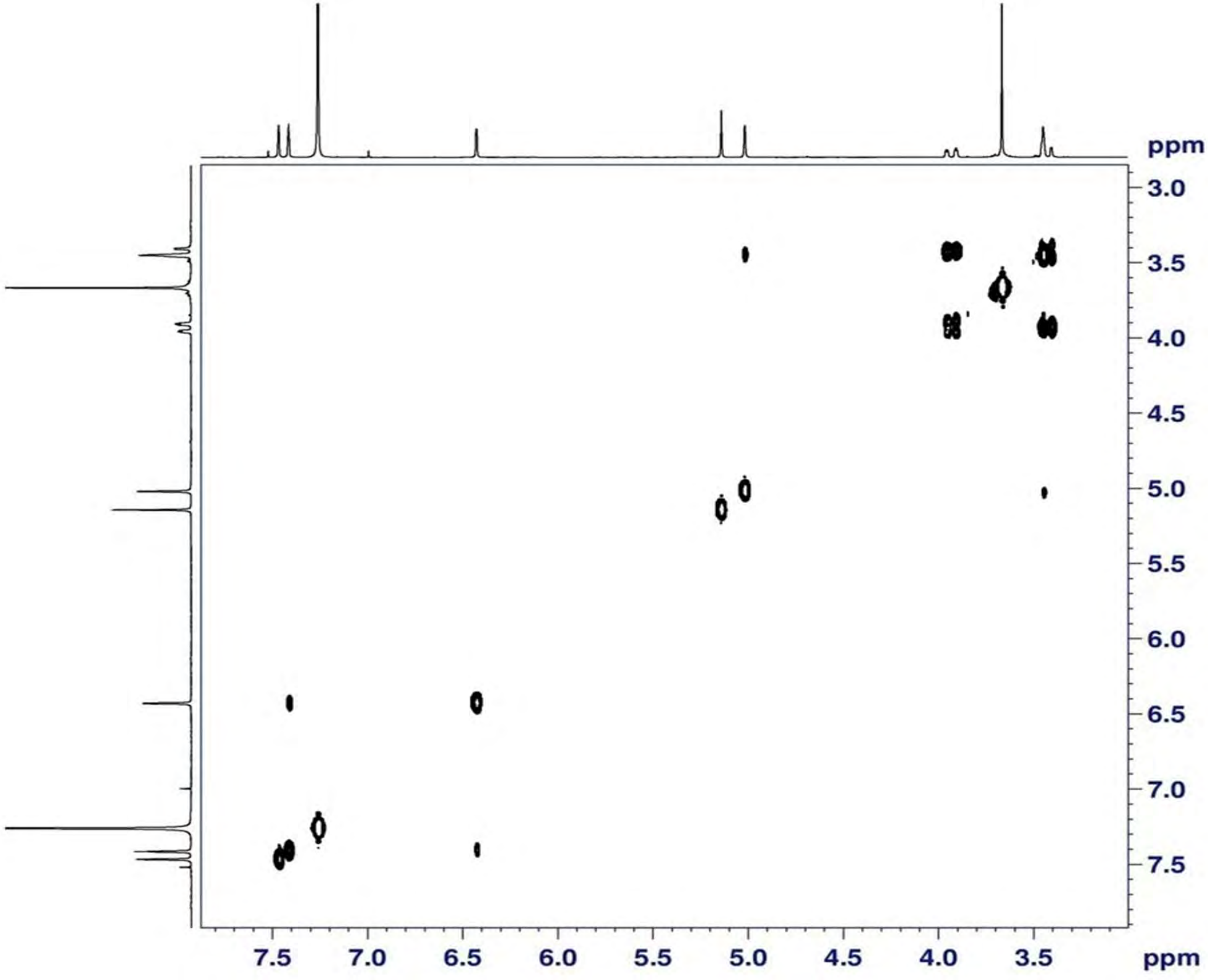

HSQC (400 MHz) spectrum of compound **19** in $CDCl_3$

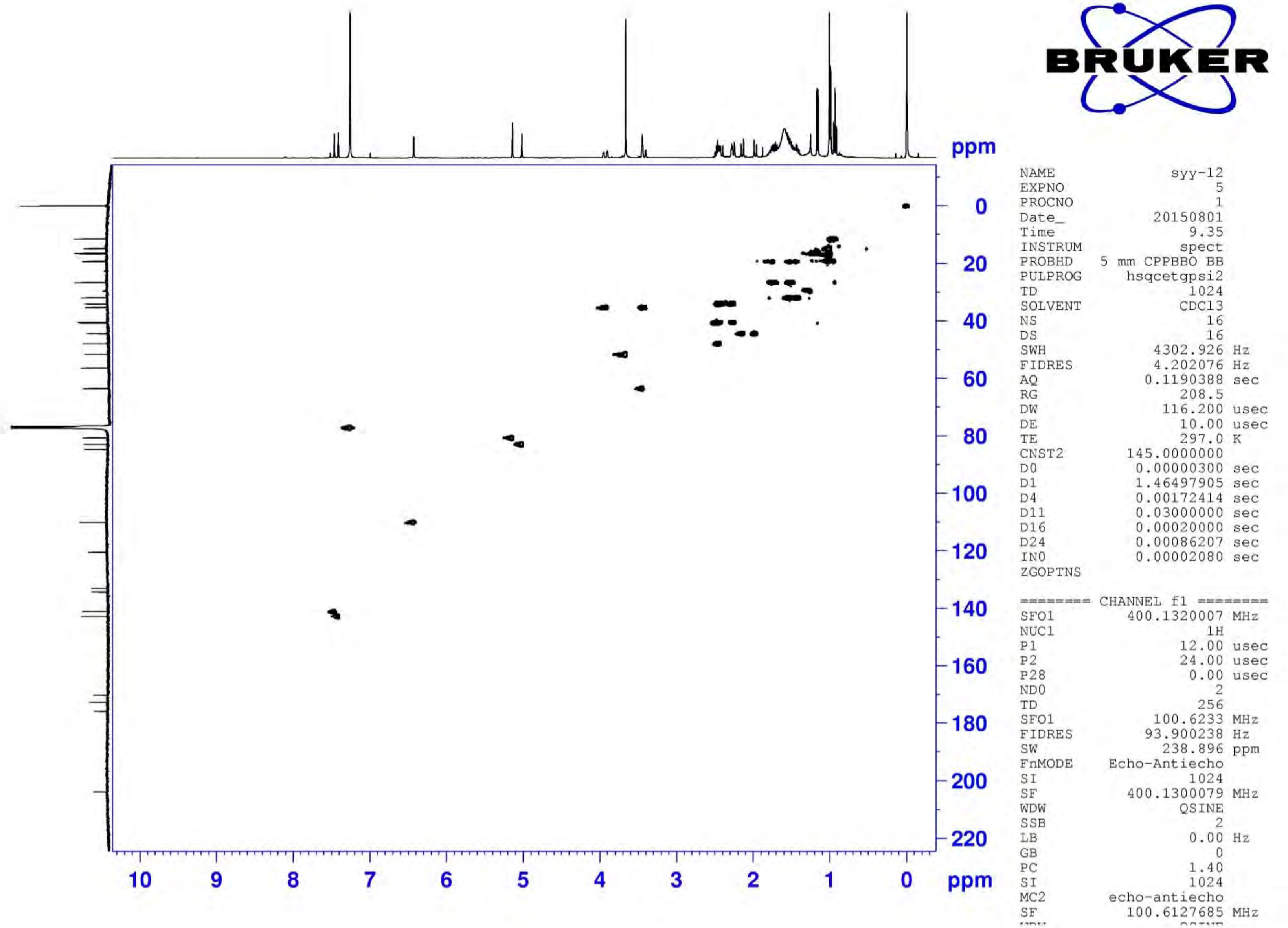

HSQC (400 MHz) spectrum of compound **19** in $CDCl_3$

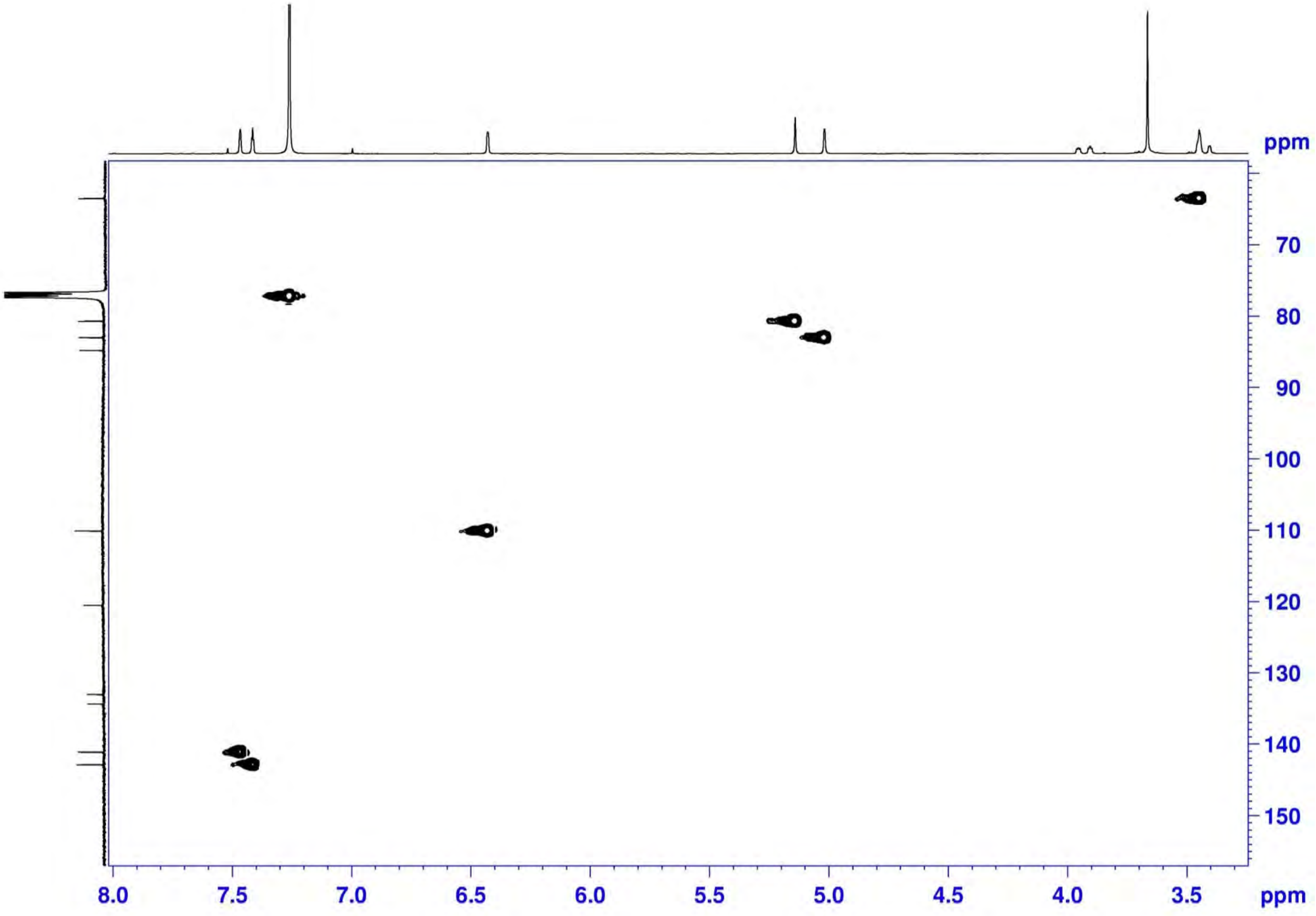

HSQC (400 MHz) spectrum of compound **19** in $CDCl_3$

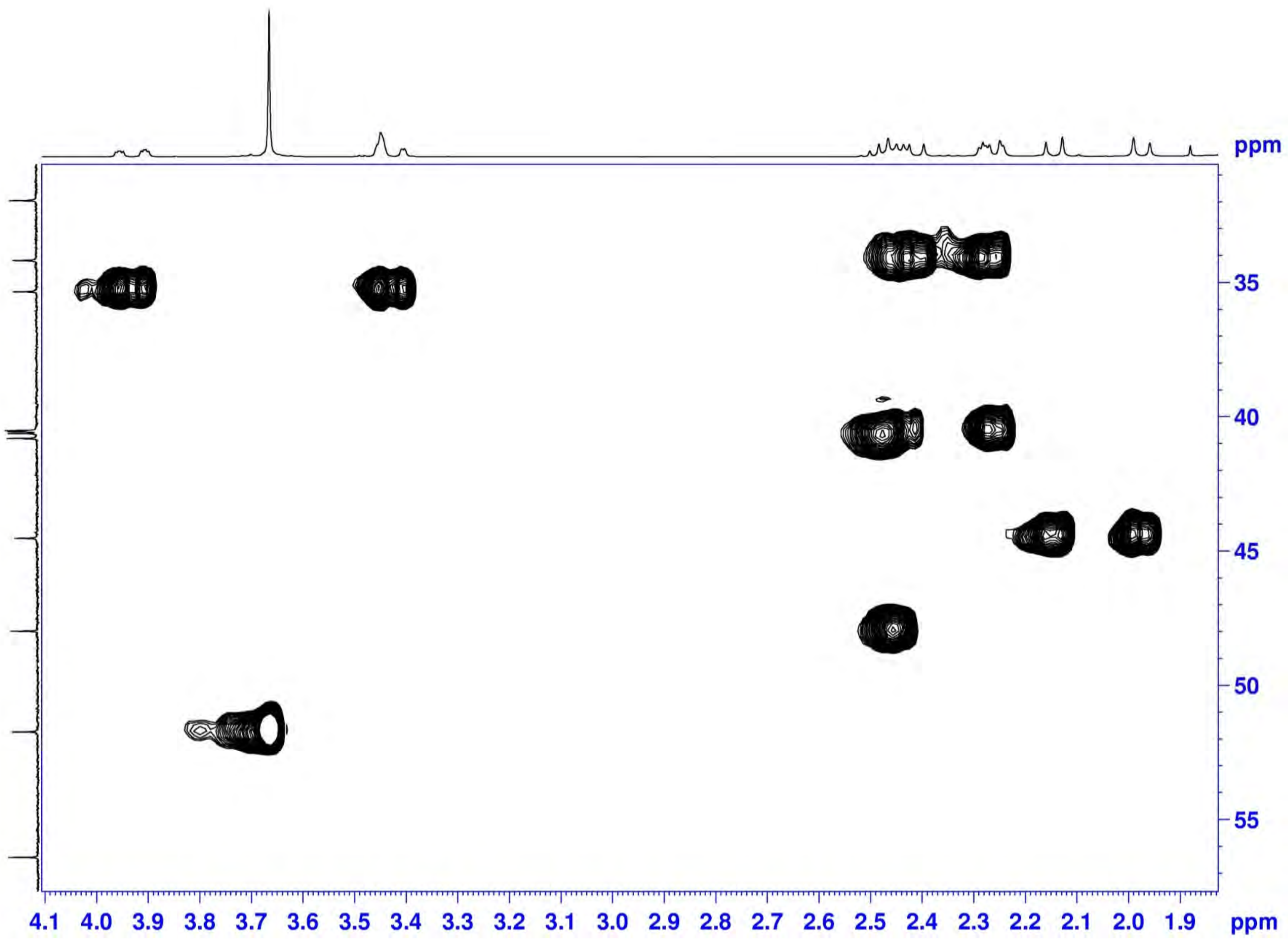

HSQC (400 MHz) spectrum of compound **19** in $CDCl_3$

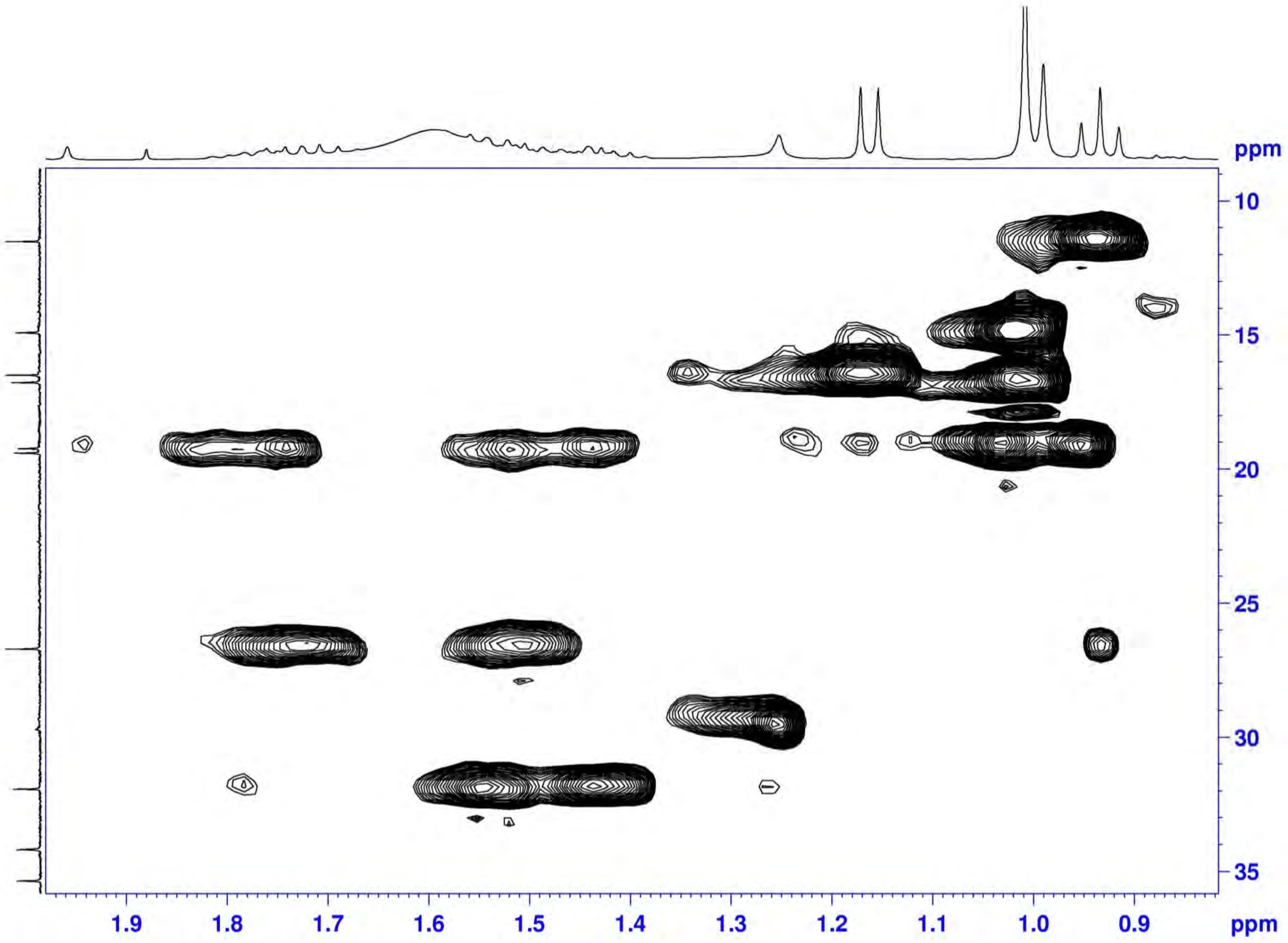

HMBC (400 MHz) spectrum of compound **19** in $CDCl_3$

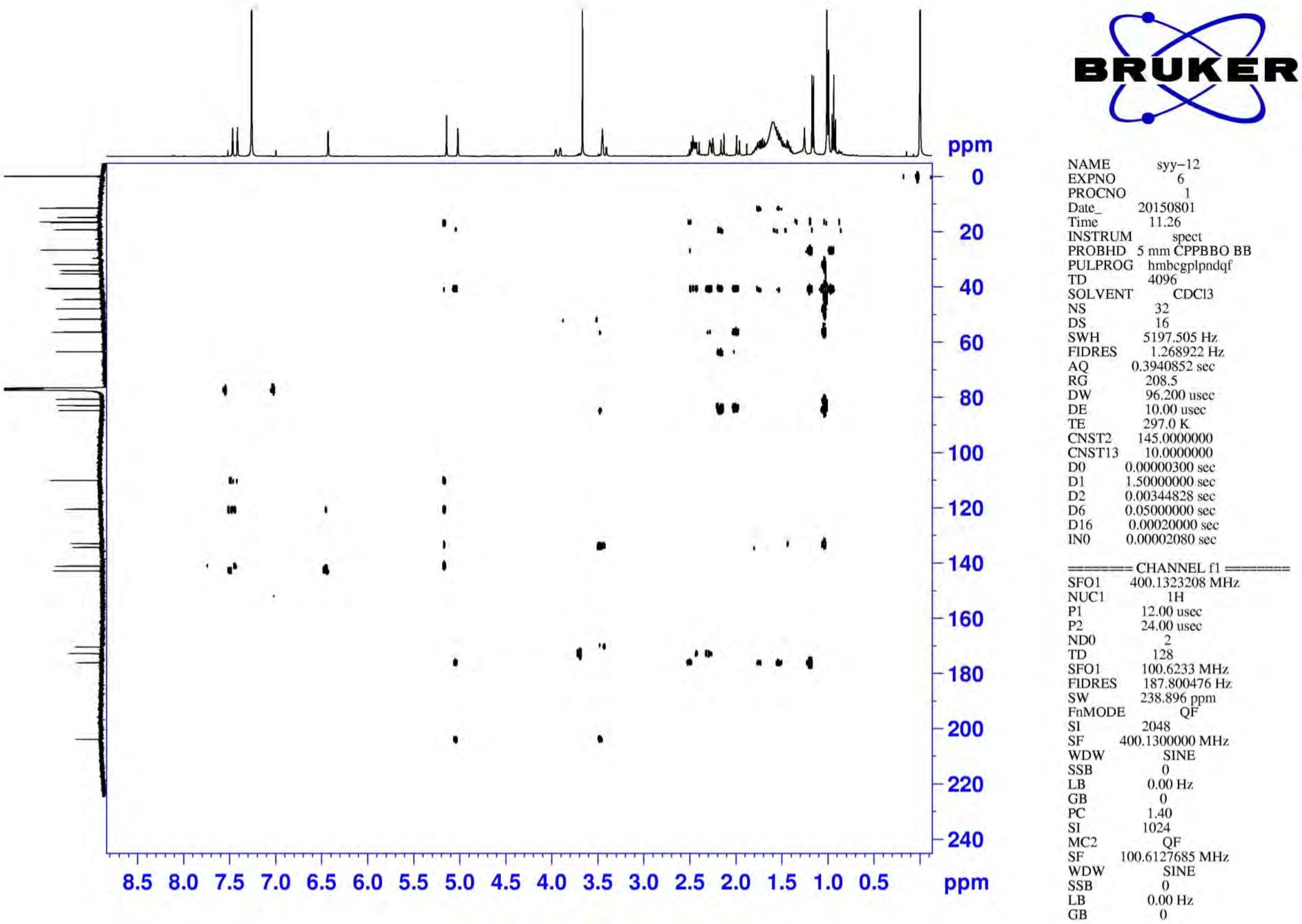

HMBC (400 MHz) spectrum of compound **19** in $CDCl_3$

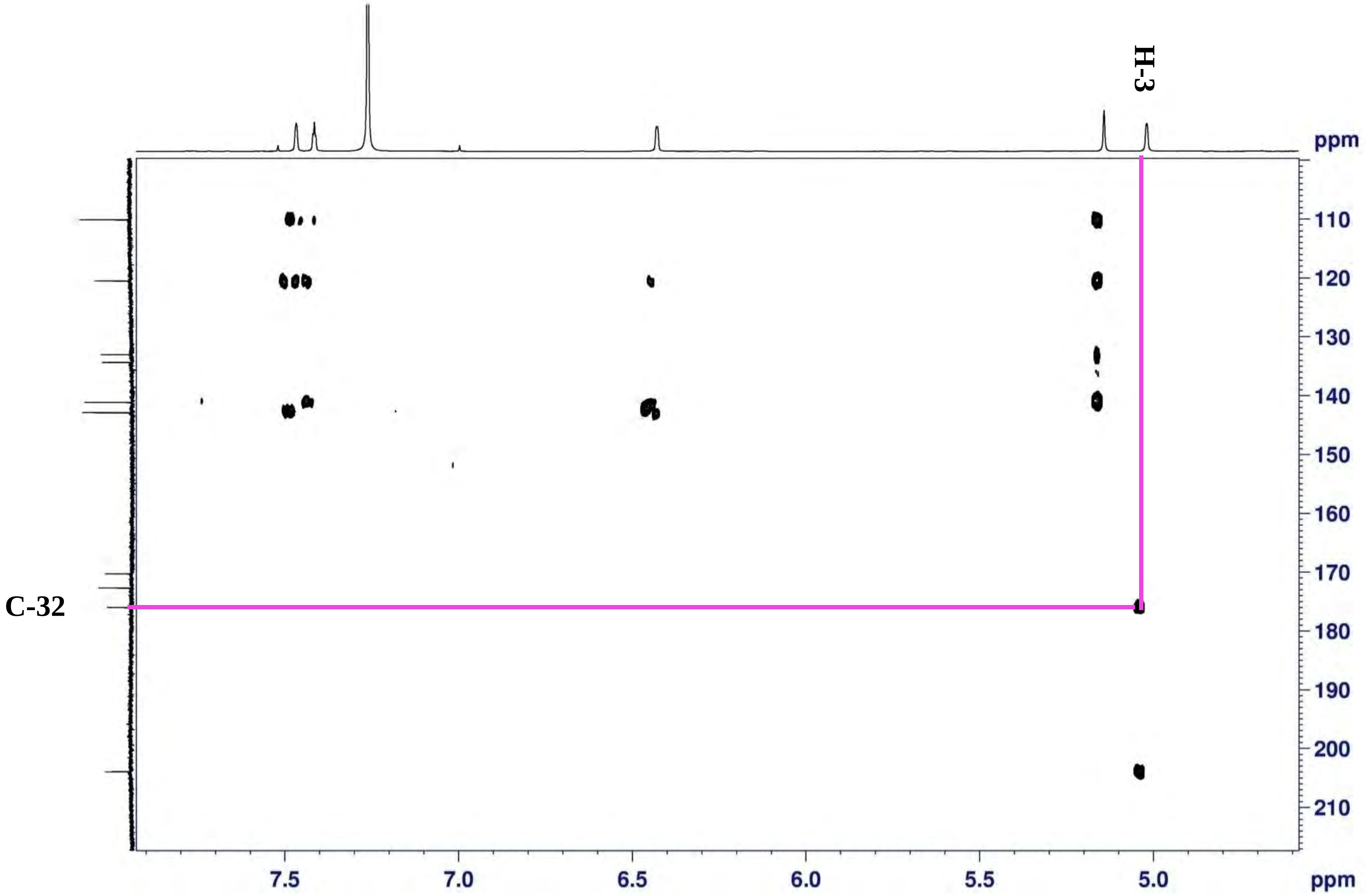

HMBC (400 MHz) spectrum of compound **19** in $CDCl_3$

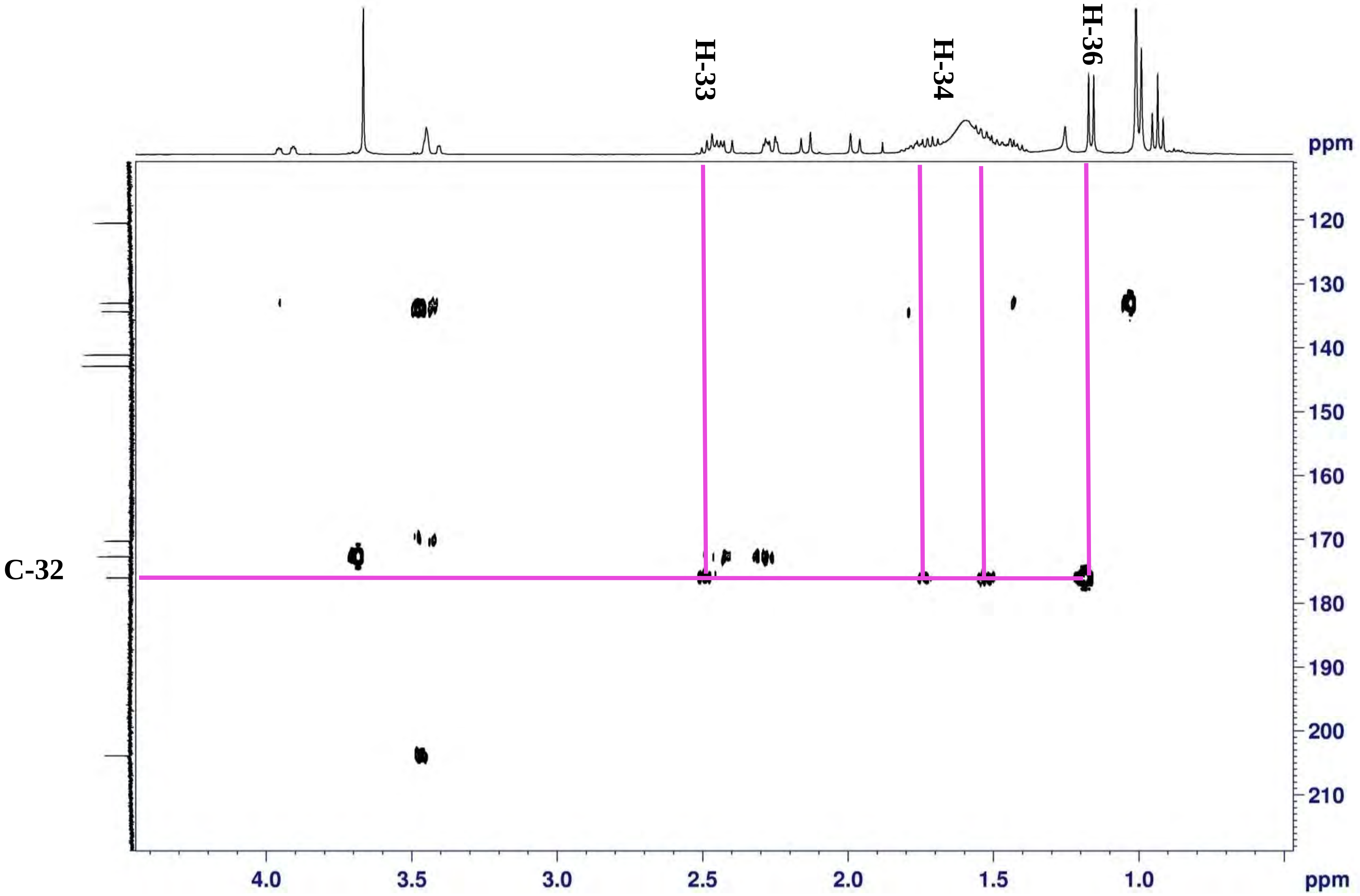

HMBC (400 MHz) spectrum of compound **19** in $CDCl_3$

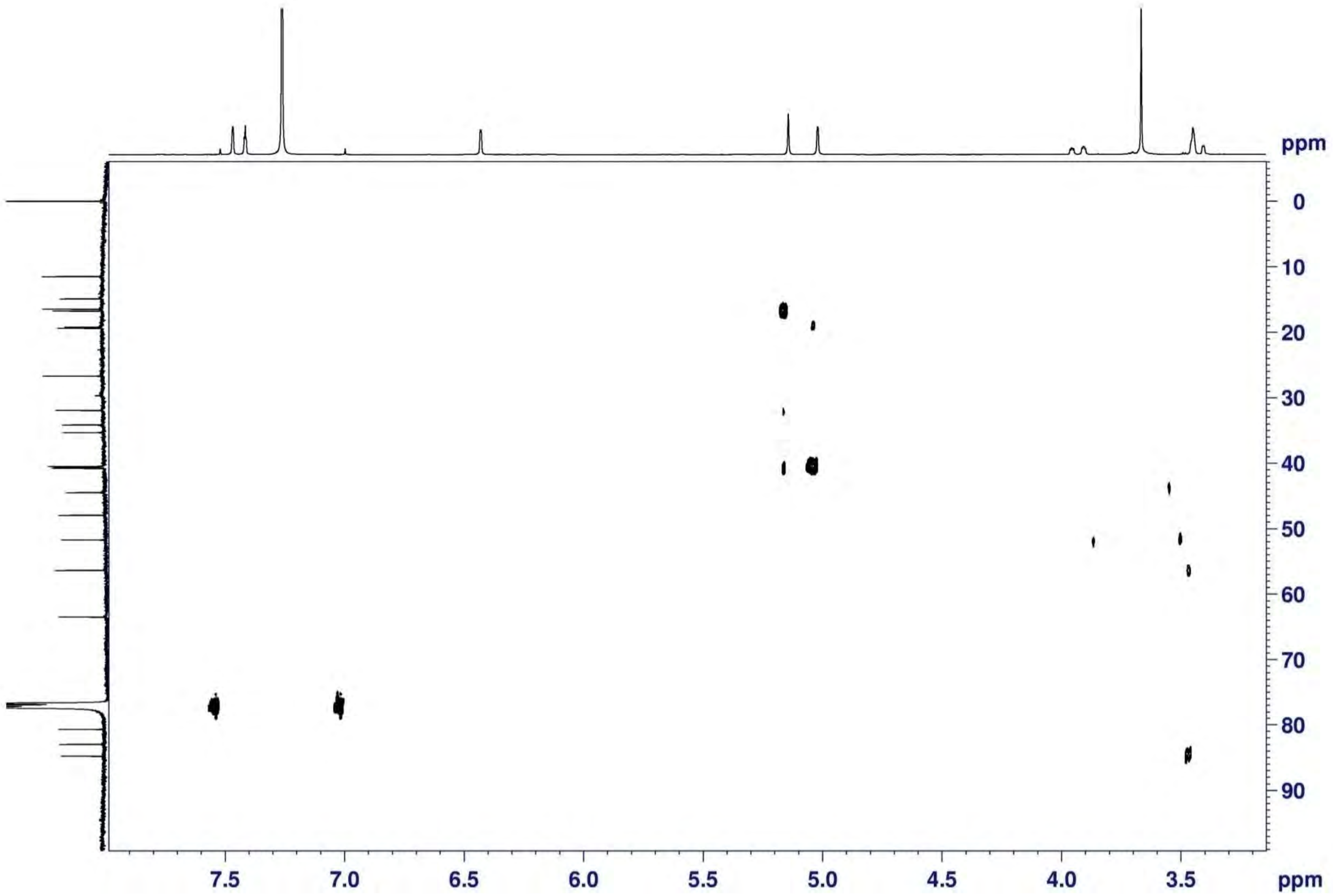

HMBC (400 MHz) spectrum of compound **19** in $CDCl_3$

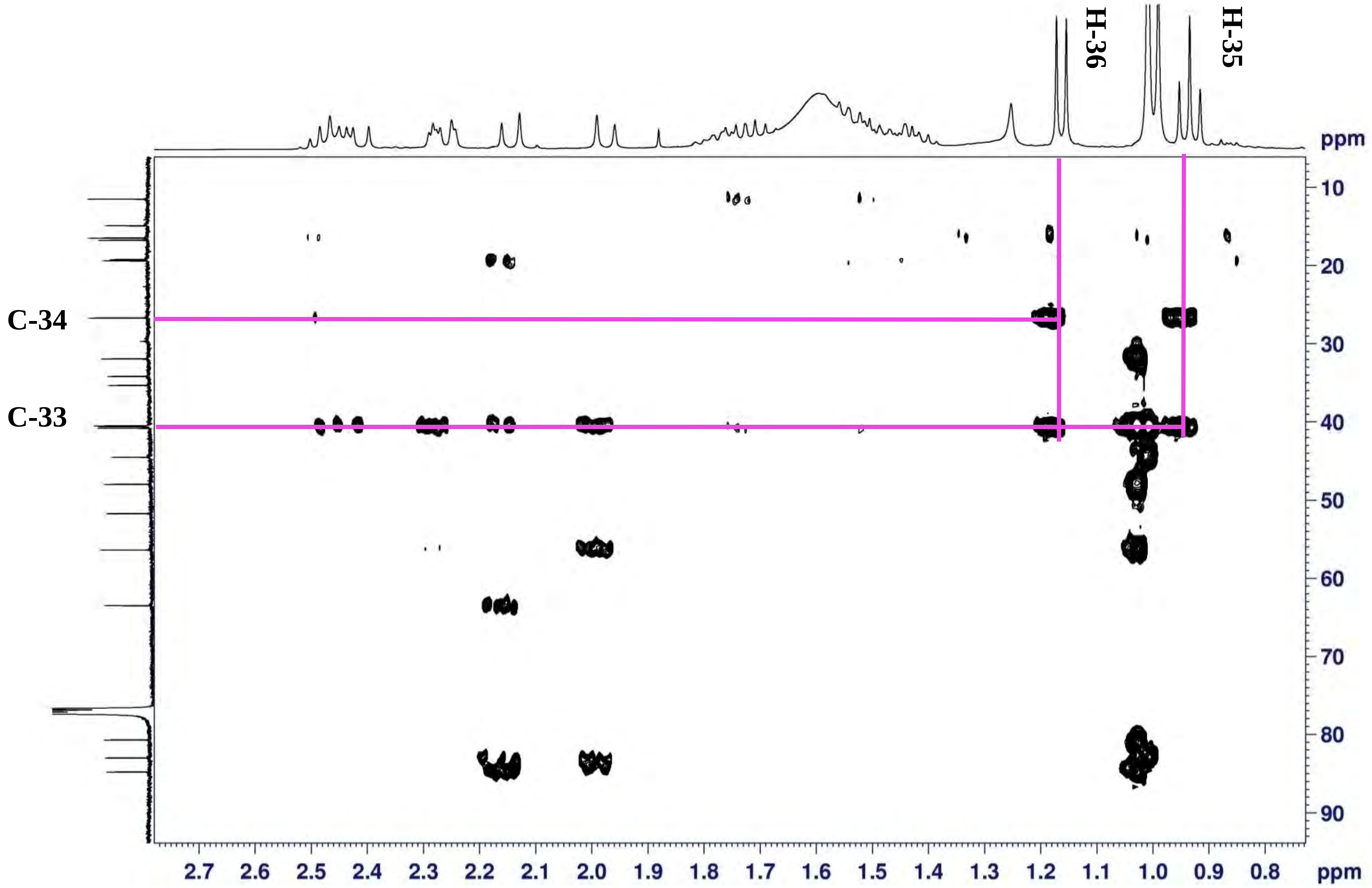

NOESY (400 MHz) spectrum of compound **19** in $CDCl_3$

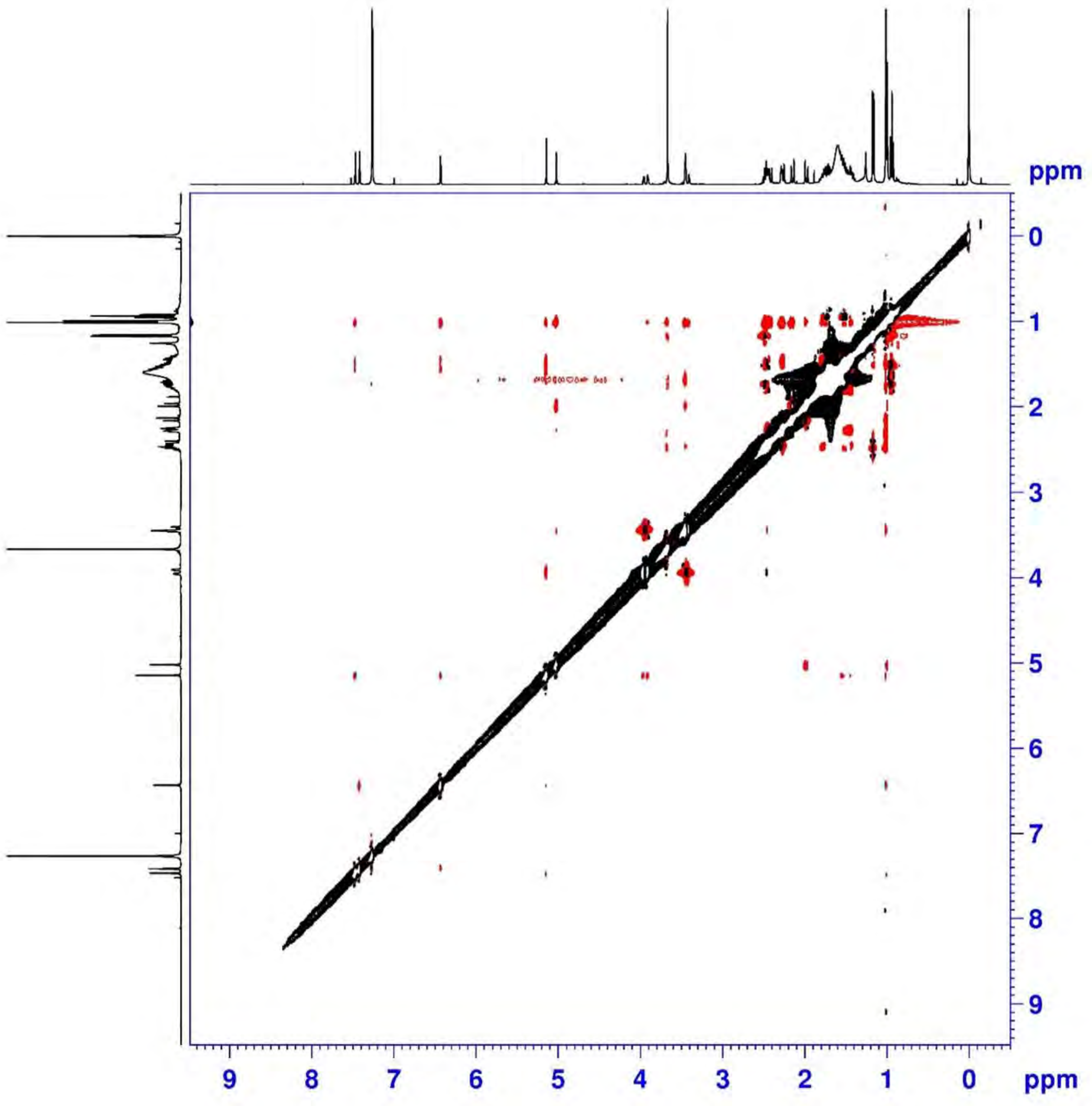

NAME	syy-12	
EXPNO	7	
PROCNO	1	
Date_	20150804	
Time	20.16	
INSTRUM	spect	
PROBHD	5 mm CPPBBO BB	
PULPROG	noesygpphpp	
TD	2048	
SOLVENT	CDCl3	
NS	16	
DS	32	
SWH	4000.000	Hz
FIDRES	1.953125	Hz
AQ	0.2560500	sec
RG	208.5	
DW	125.000	usec
DE	10.00	usec
TE	297.0	K
D0	0.00010972	sec
D1	1.99385595	sec
D8	0.30000001	sec
D11	0.03000000	sec
D12	0.00002000	sec
D16	0.00020000	sec
IN0	0.00025000	sec
======== CHANNEL f1 ========		
SFO1	400.1318006	MHz
NUC1	1H	
P1	12.00	usec
P2	24.00	usec
P17	2500.00	usec
ND0	1	
TD	256	
SFO1	400.1318	MHz
FIDRES	15.625000	Hz
SW	9.997	ppm
FnMODE	States-TPPI	
SI	1024	
SF	400.1300059	MHz
WDW	QSINE	
SSB	2	
LB	0.00	Hz
GB	0	
PC	1.00	
SI	1024	
MC2	States-TPPI	
SF	400.1300061	MHz
WDW	QSINE	
SSB	2	
LB	0.00	Hz
GB	0	

NOESY (400 MHz) spectrum of compound **19** in $CDCl_3$

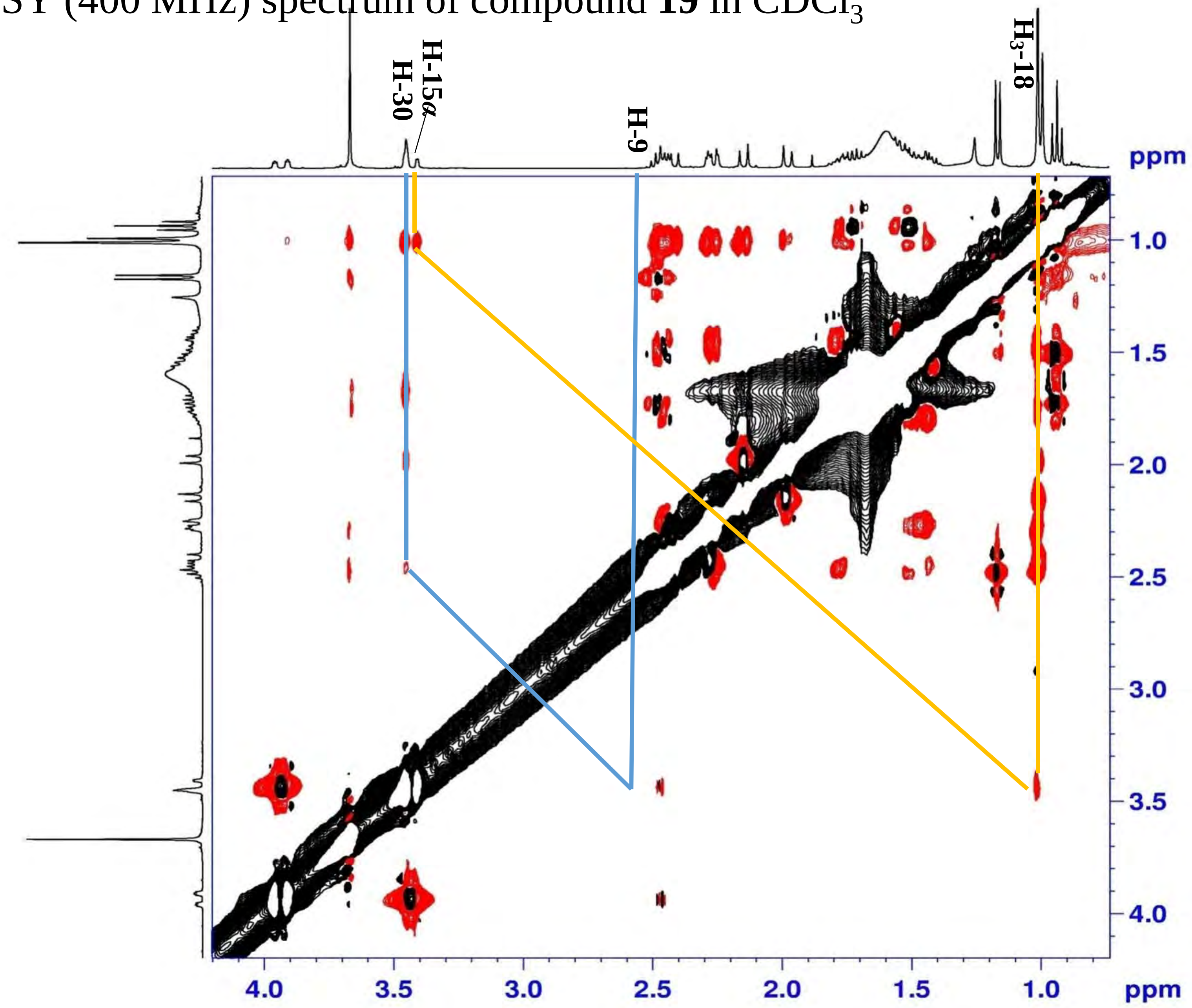

NOESY (400 MHz) spectrum of compound **19** in $CDCl_3$

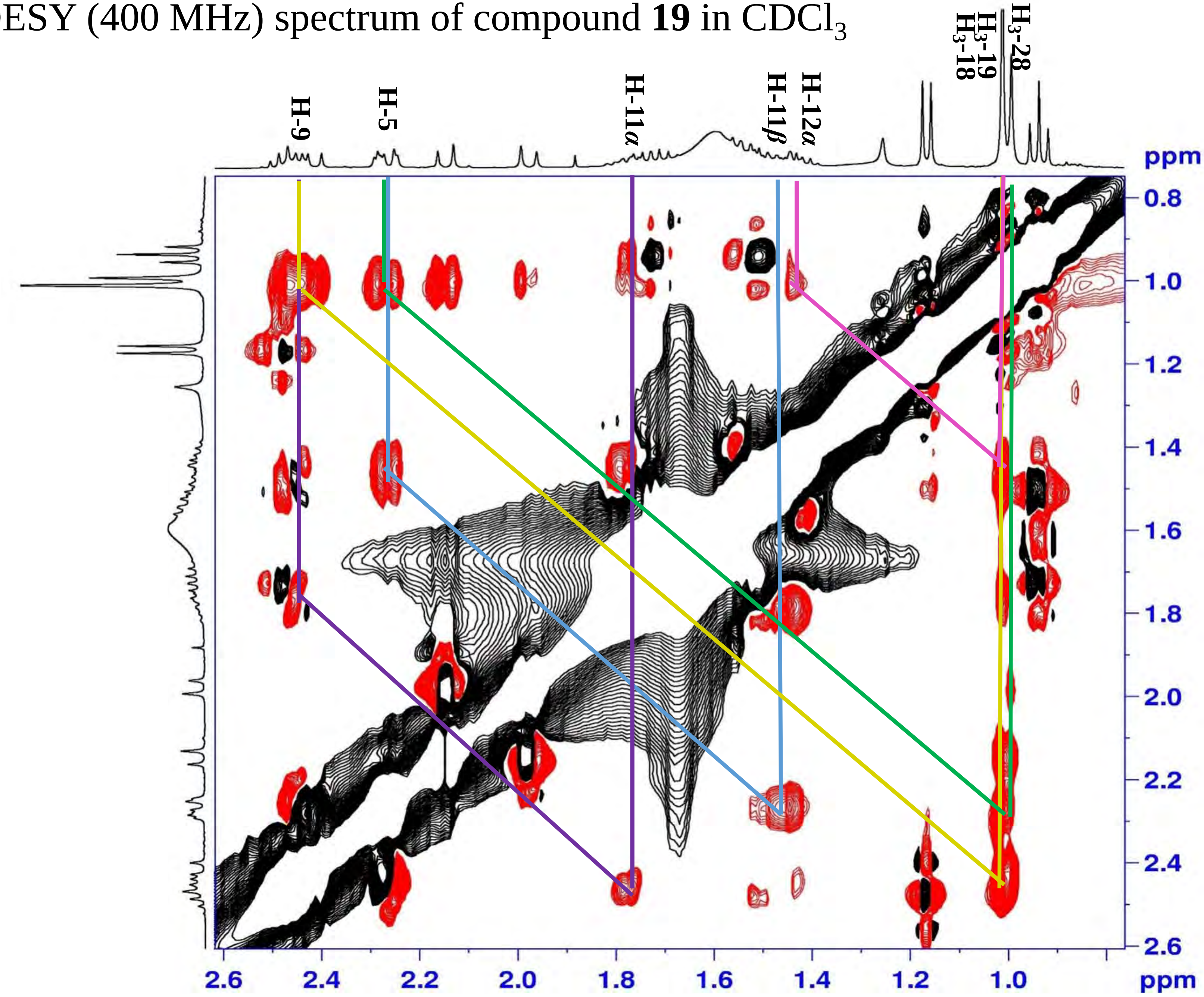

NOESY (400 MHz) spectrum of compound **19** in $CDCl_3$

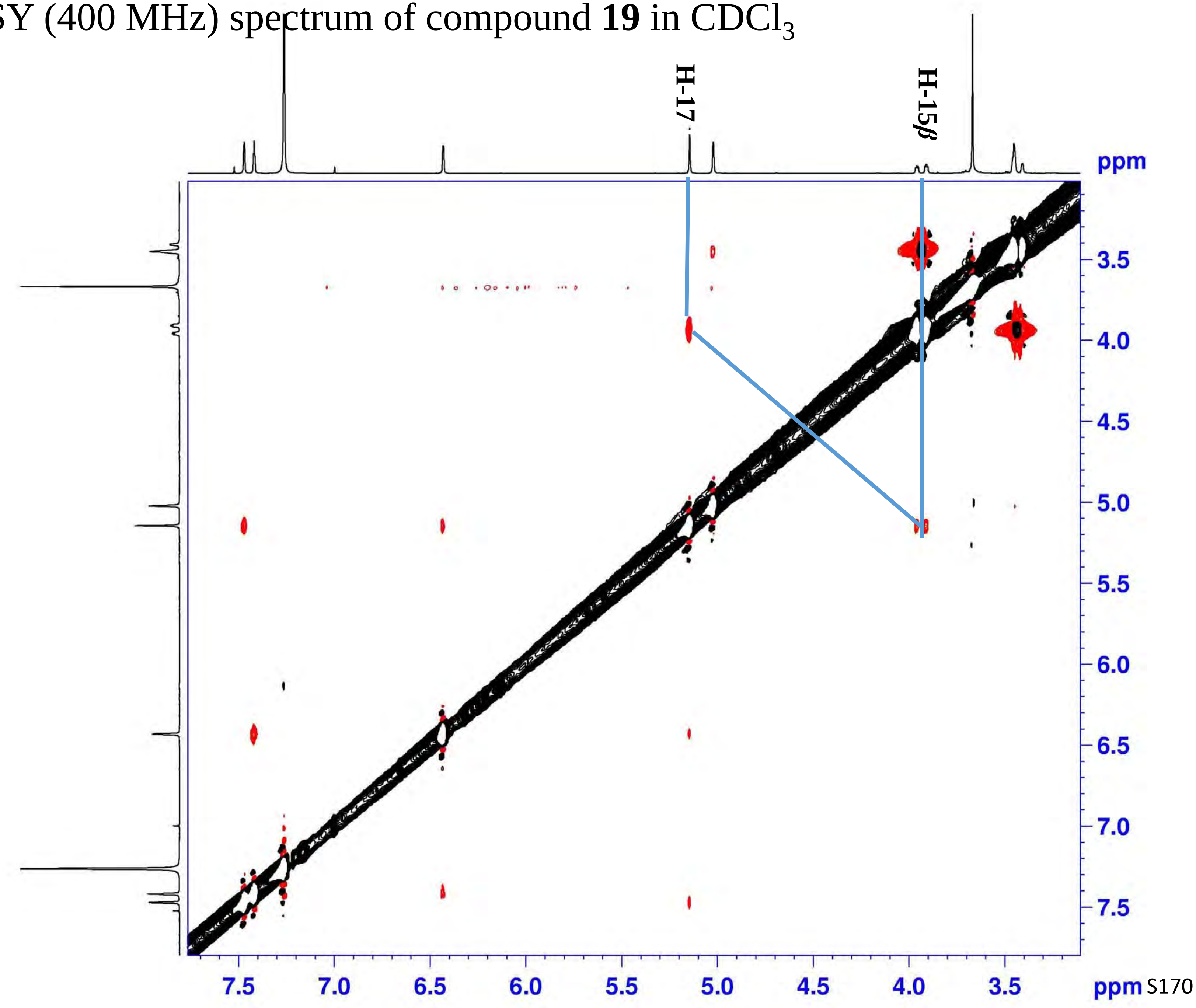

NOESY (400 MHz) spectrum of compound **19** in $CDCl_3$

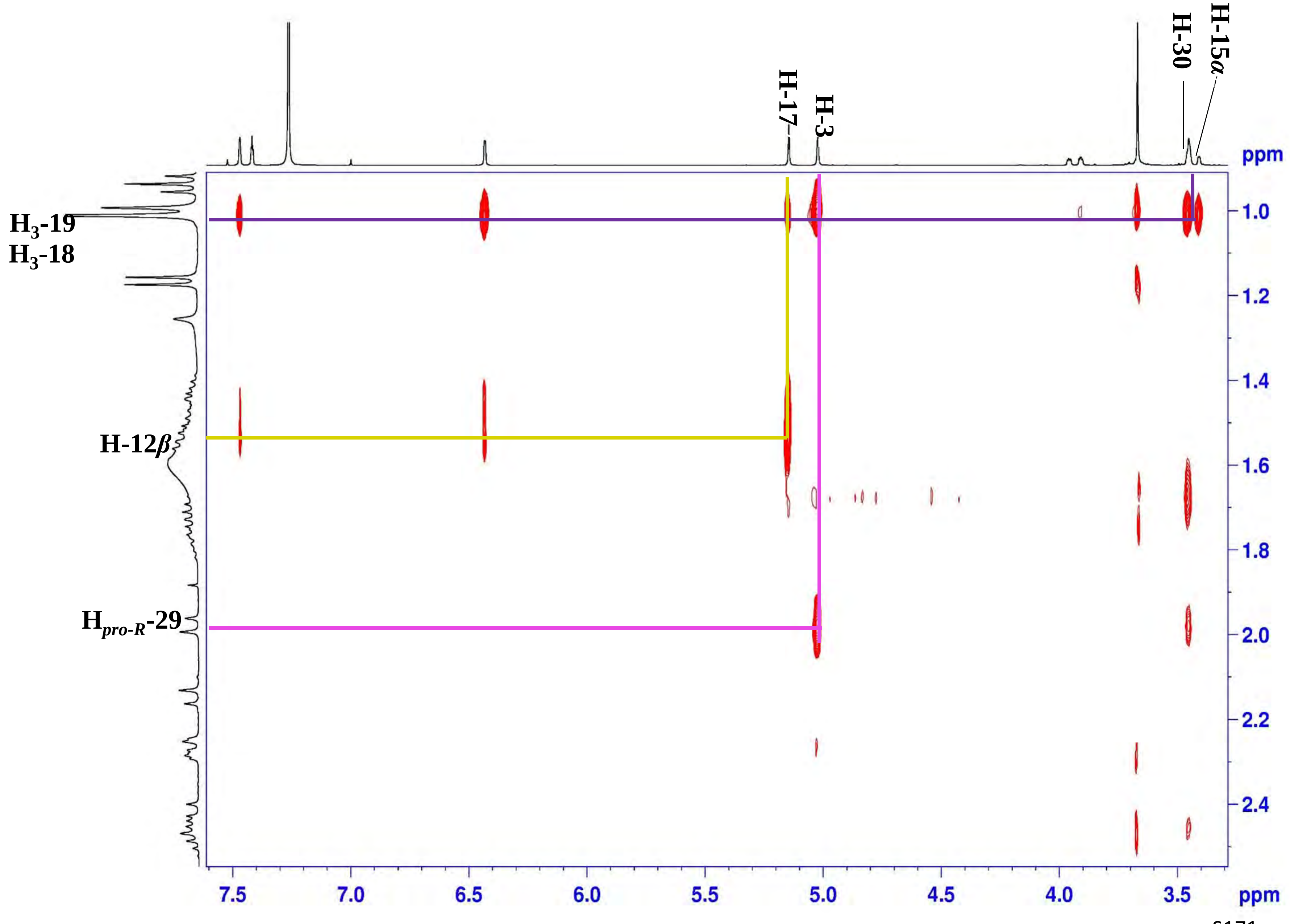

HRESIMS for compound **20**

Mass Spectrum SmartFormula Report

Analysis Info

Analysis Name	D:\Data\MS\data\201605\liwanshan_lws-60_pos_13_01_1860.d	Acquisition Date	5/19/2016 8:58:21 AM
Method	LC_Direct Infusion_pos_100-1000mz.m	Operator	SCSIO
Sample Name	liwanshan_lws-60_pos	Instrument / Ser#	maXis 29
Comment			

Acquisition Parameter

Source Type	ESI	Ion Polarity	Positive	Set Nebulizer	0.4 Bar
Focus	Active	Set Capillary	4500 V	Set Dry Heater	180 °C
Scan Begin	100 m/z	Set End Plate Offset	-500 V	Set Dry Gas	4.0 l/min
Scan End	2000 m/z	Set Collision Cell RF	800.0 Vpp	Set Divert Valve	Waste

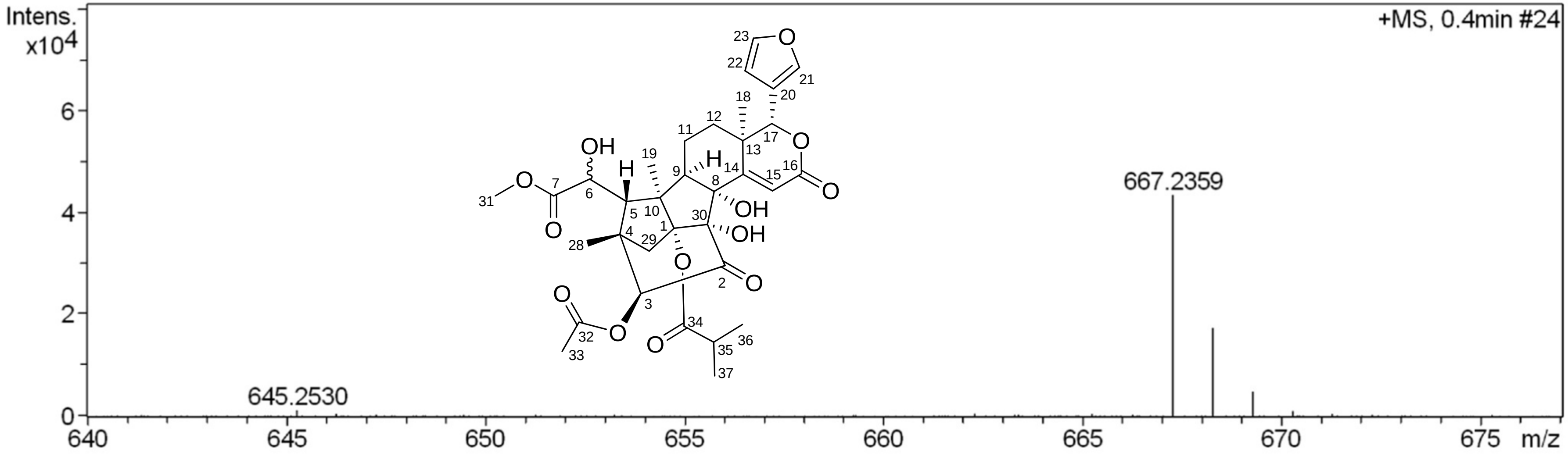

Meas. m/z	#	Formula	Score	m/z	err [ppm]	err [mDa]	mSigma	rdb	e⁻ Conf	N-Rule
645.2530	1	C 33 H 41 O 13	93.42	645.2542	1.8	1.2	26.5	13.5	even	ok
667.2359	1	C 33 H 40 Na O 13	100.00	667.2361	0.3	0.2	15.8	13.5	even	ok
1311.4852	1	C 66 H 80 Na O 26	22.18	1311.4830	-1.7	-2.2	46.0	26.5	even	ok

HRESIMS for compound **20**

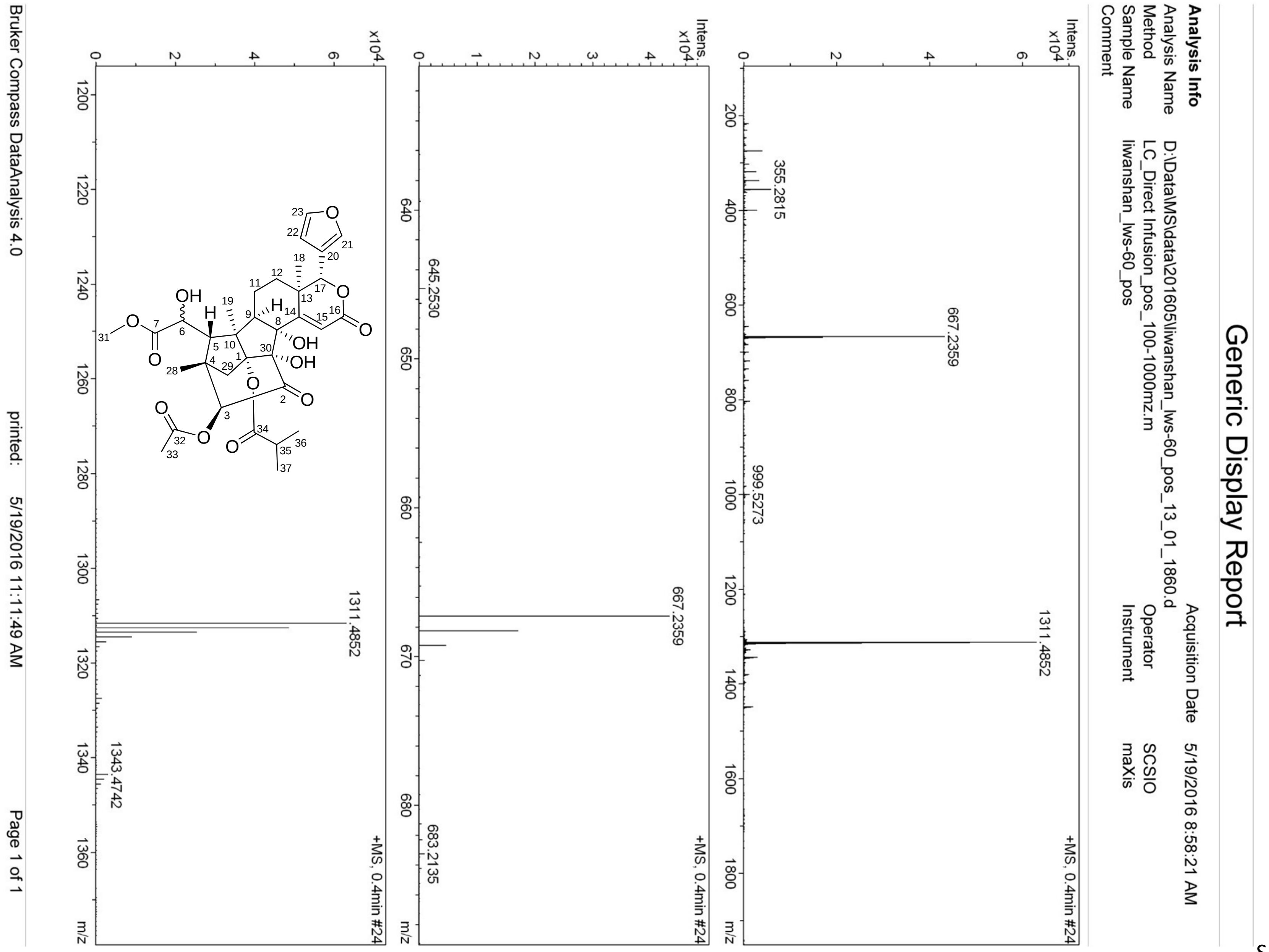

^{1}H NMR (400 MHz) spectrum of compound **20** in Acetone-d_6

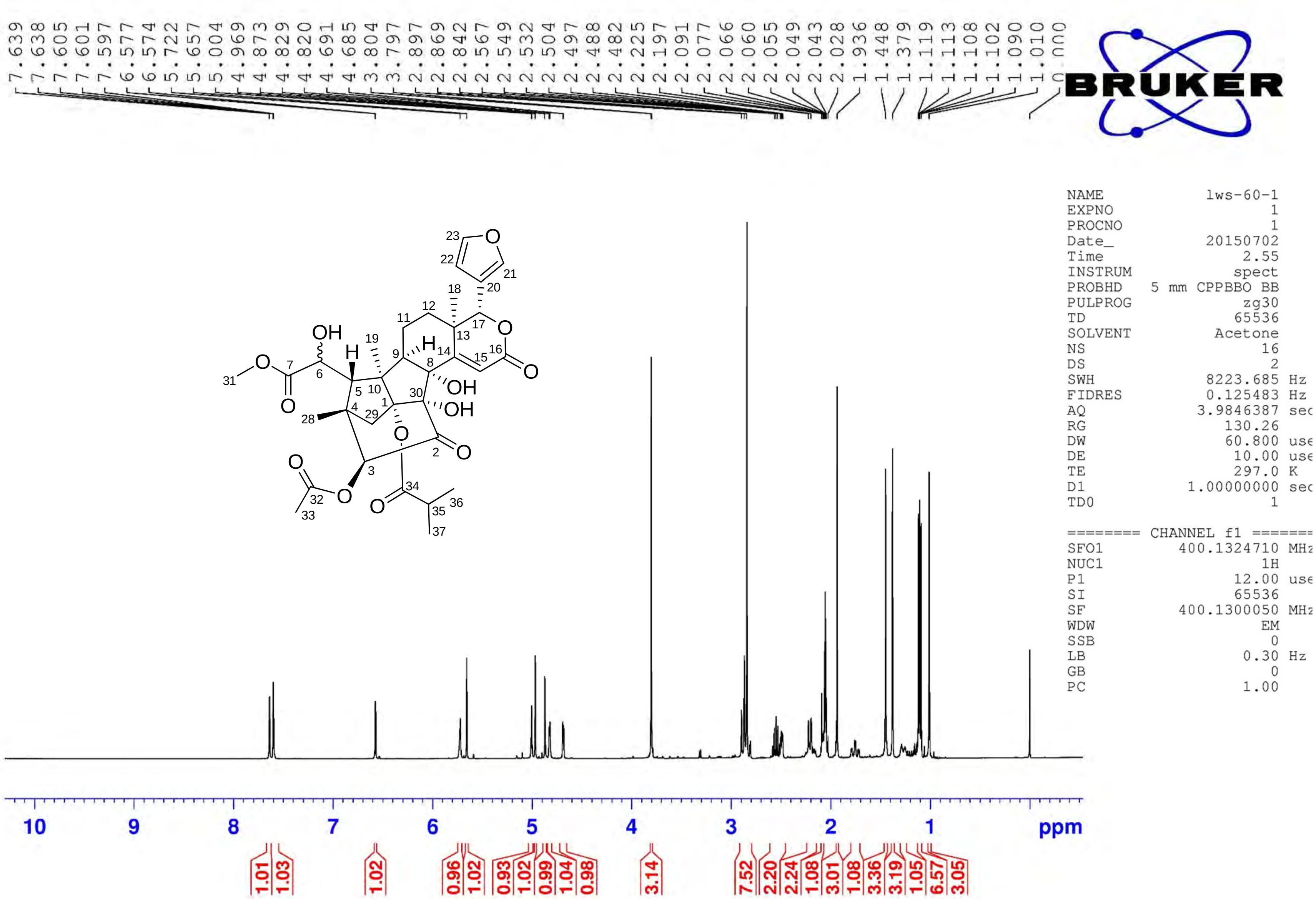

^{1}H NMR (400 MHz) spectrum of compound **20** in Acetone-d_6

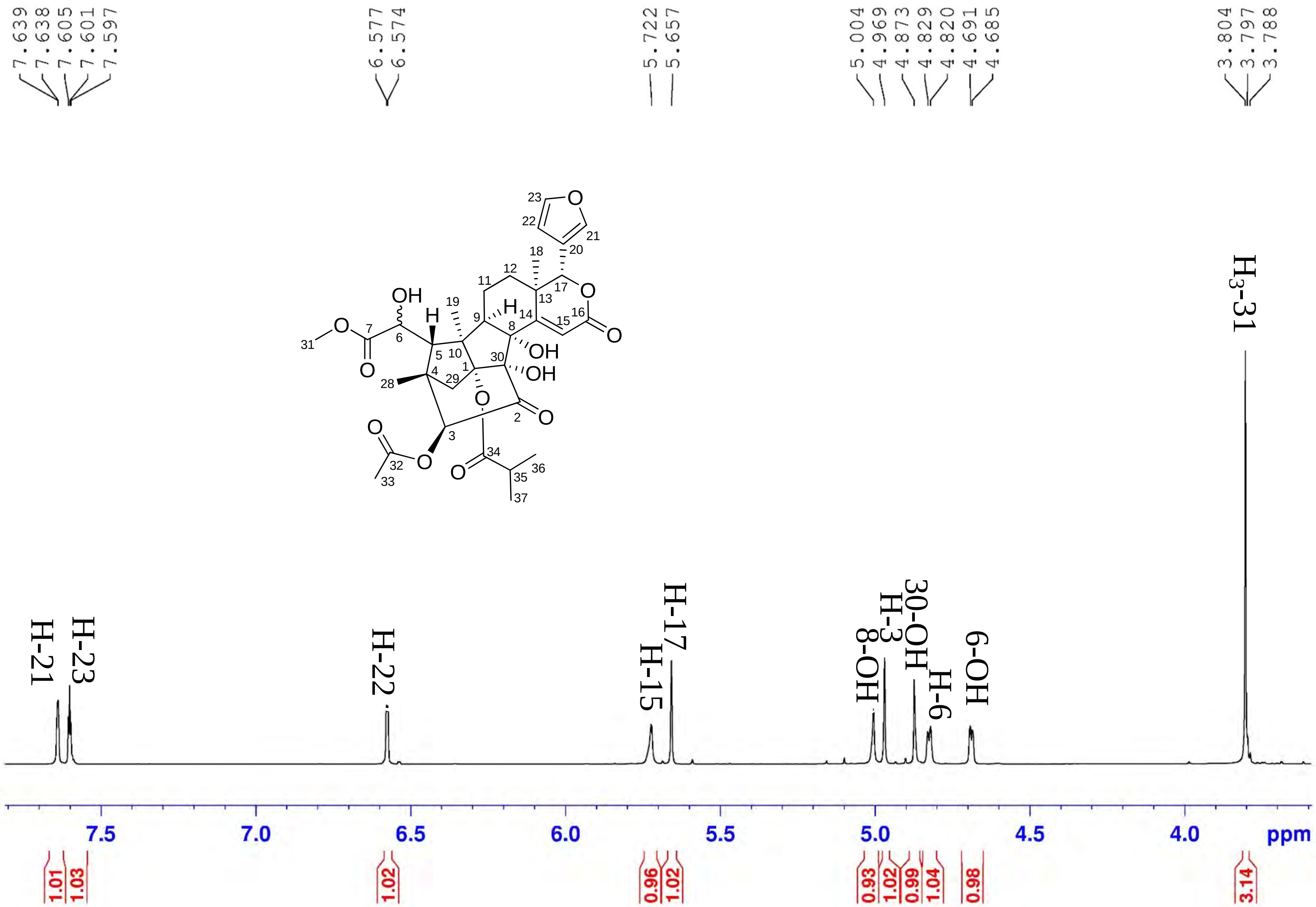

^{1}H NMR (400 MHz) spectrum of compound **20** in Acetone-d_6

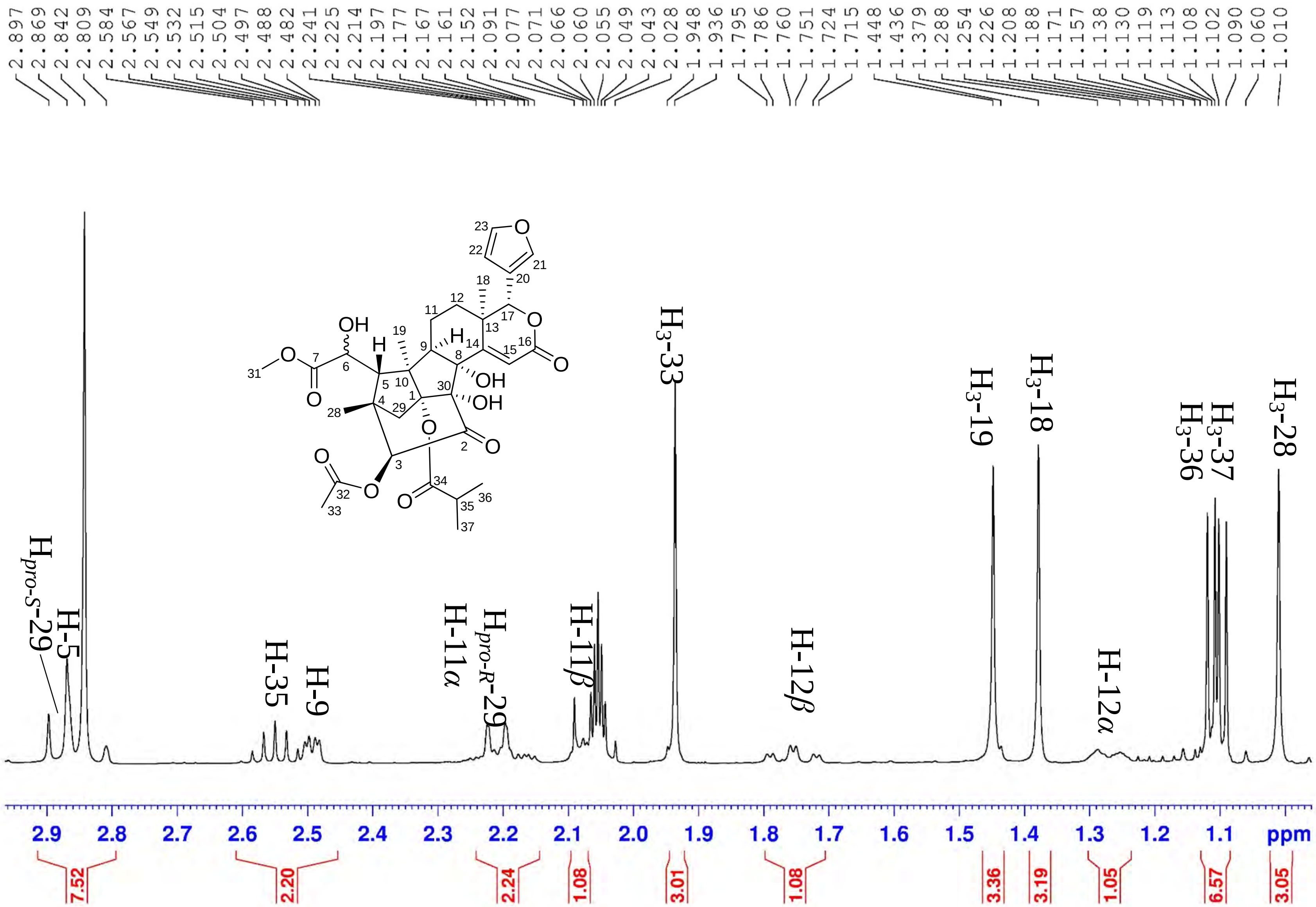

^{13}C NMR (100 MHz) spectrum of compound **20** in Acetone-d_6

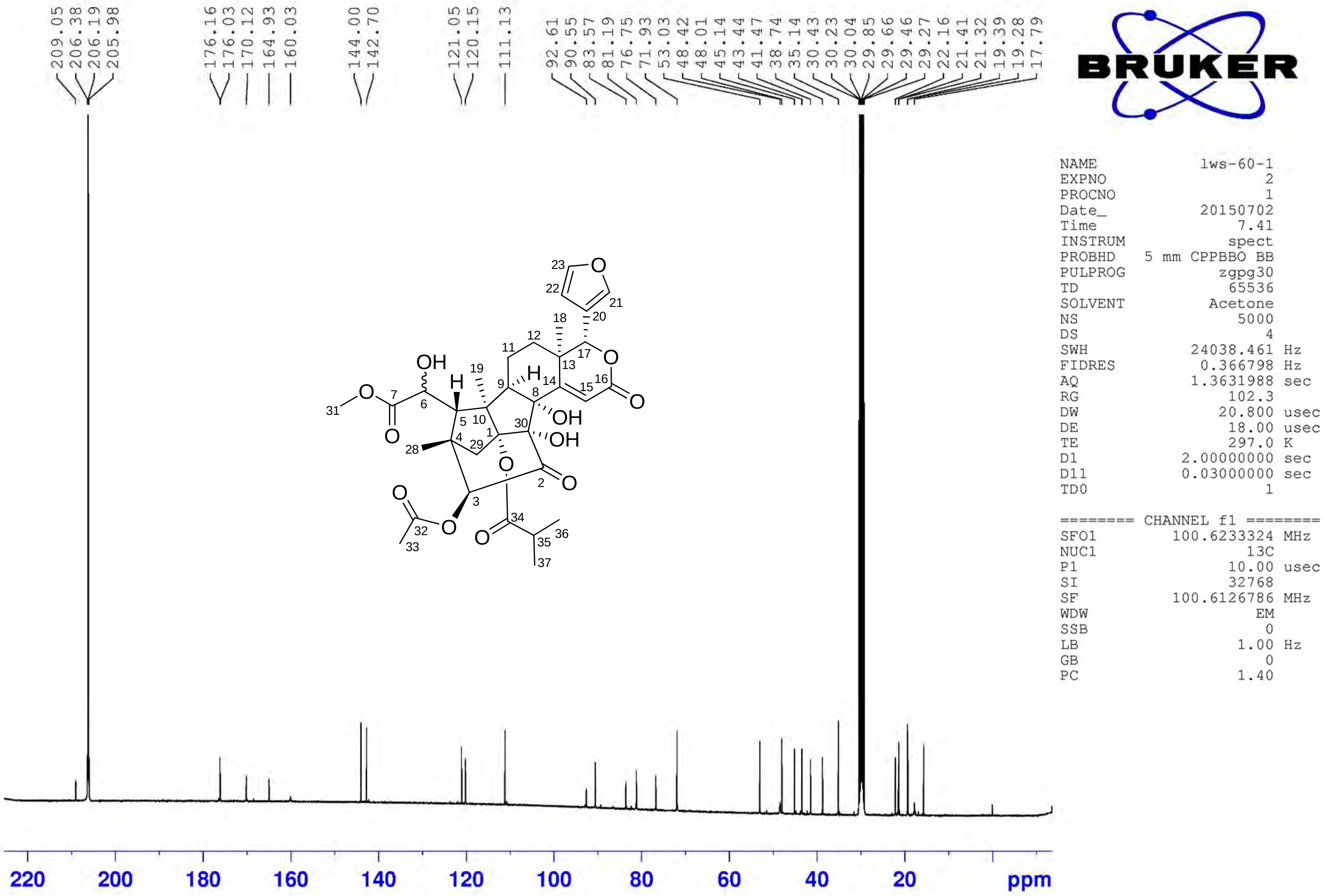

^{13}C NMR (100 MHz) spectrum of compound **20** in Acetone-d_6

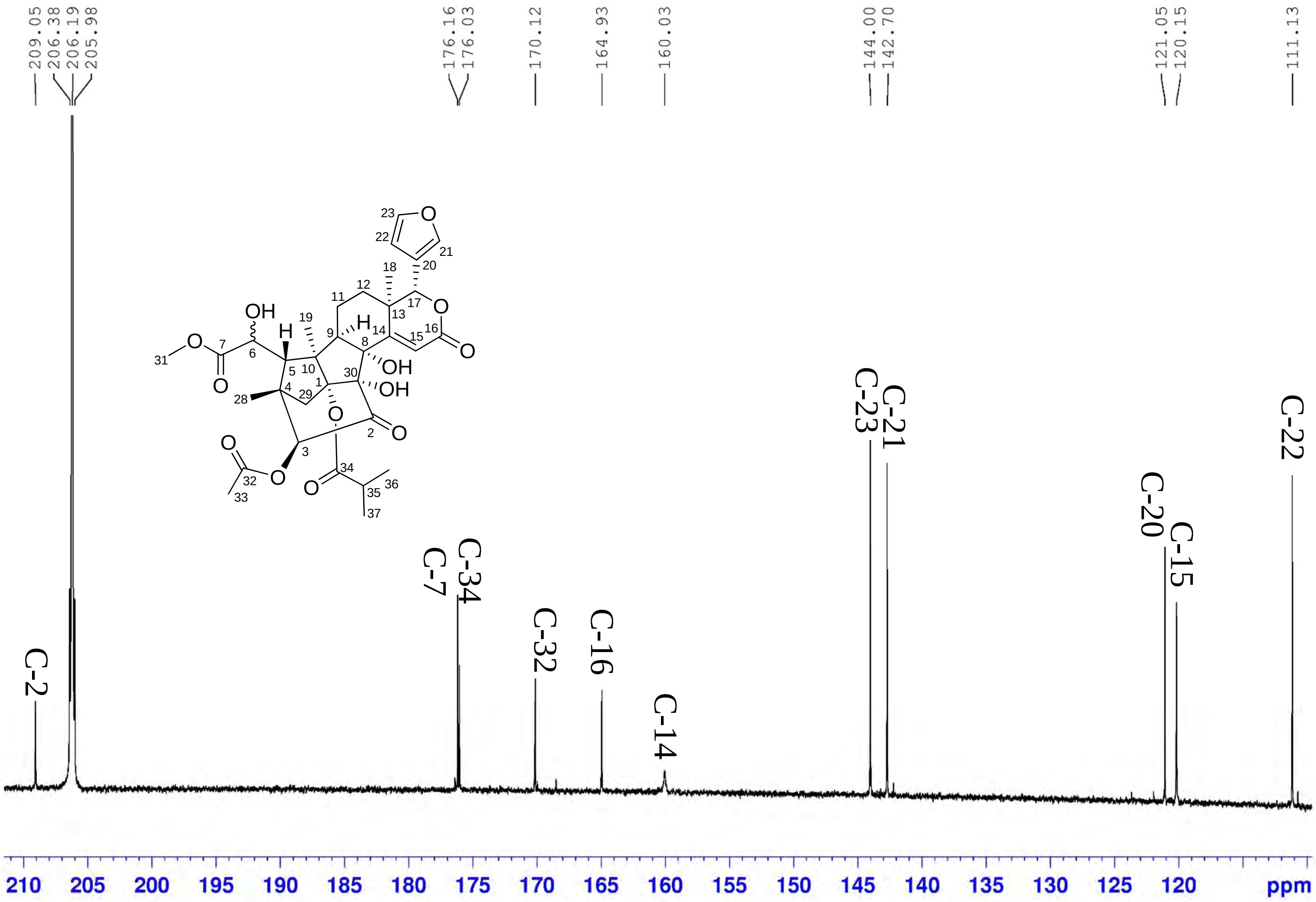

^{13}C NMR (100 MHz) spectrum of compound **20** in Acetone-d_6

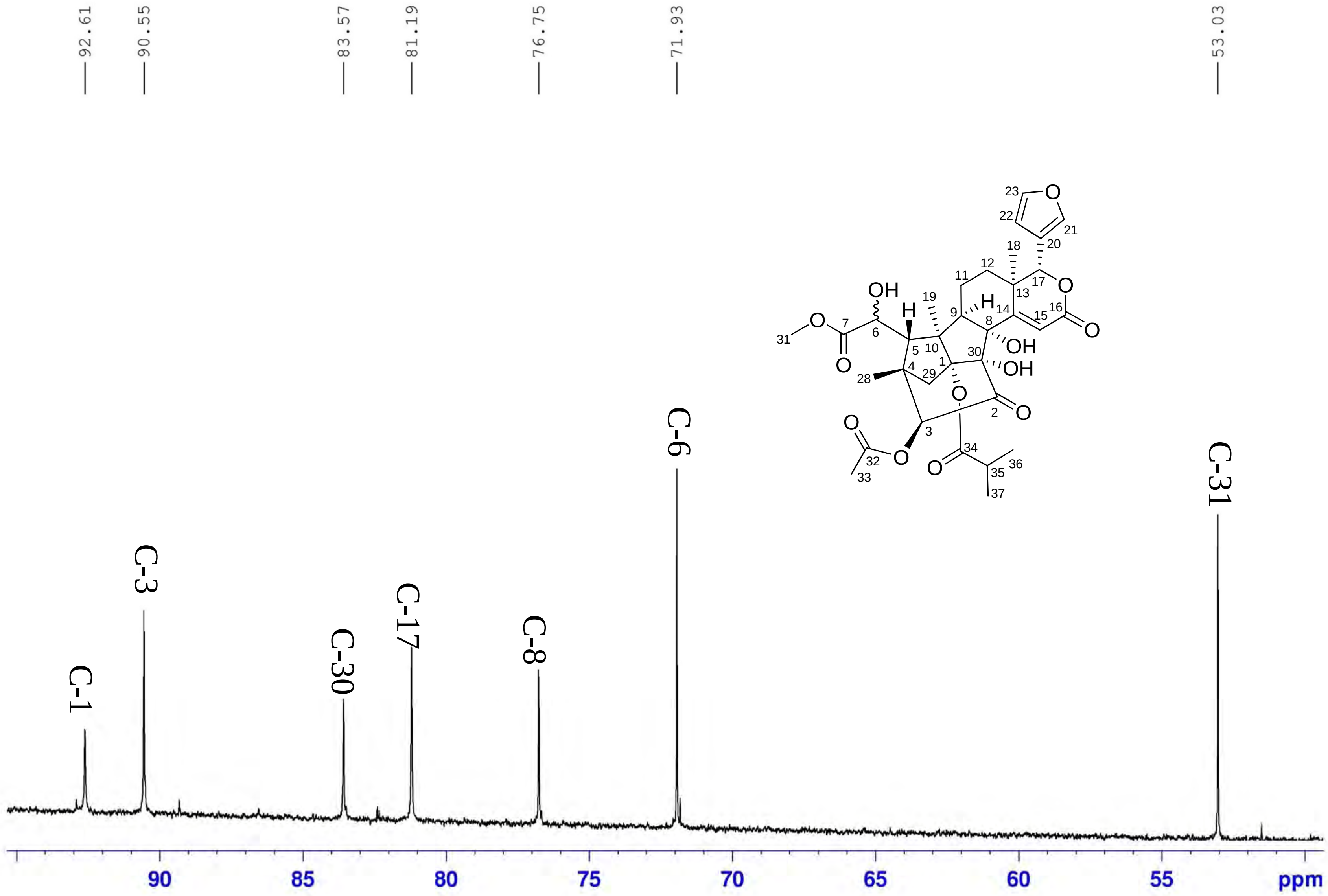

^{13}C NMR (100 MHz) spectrum of compound **20** in Acetone-d_6

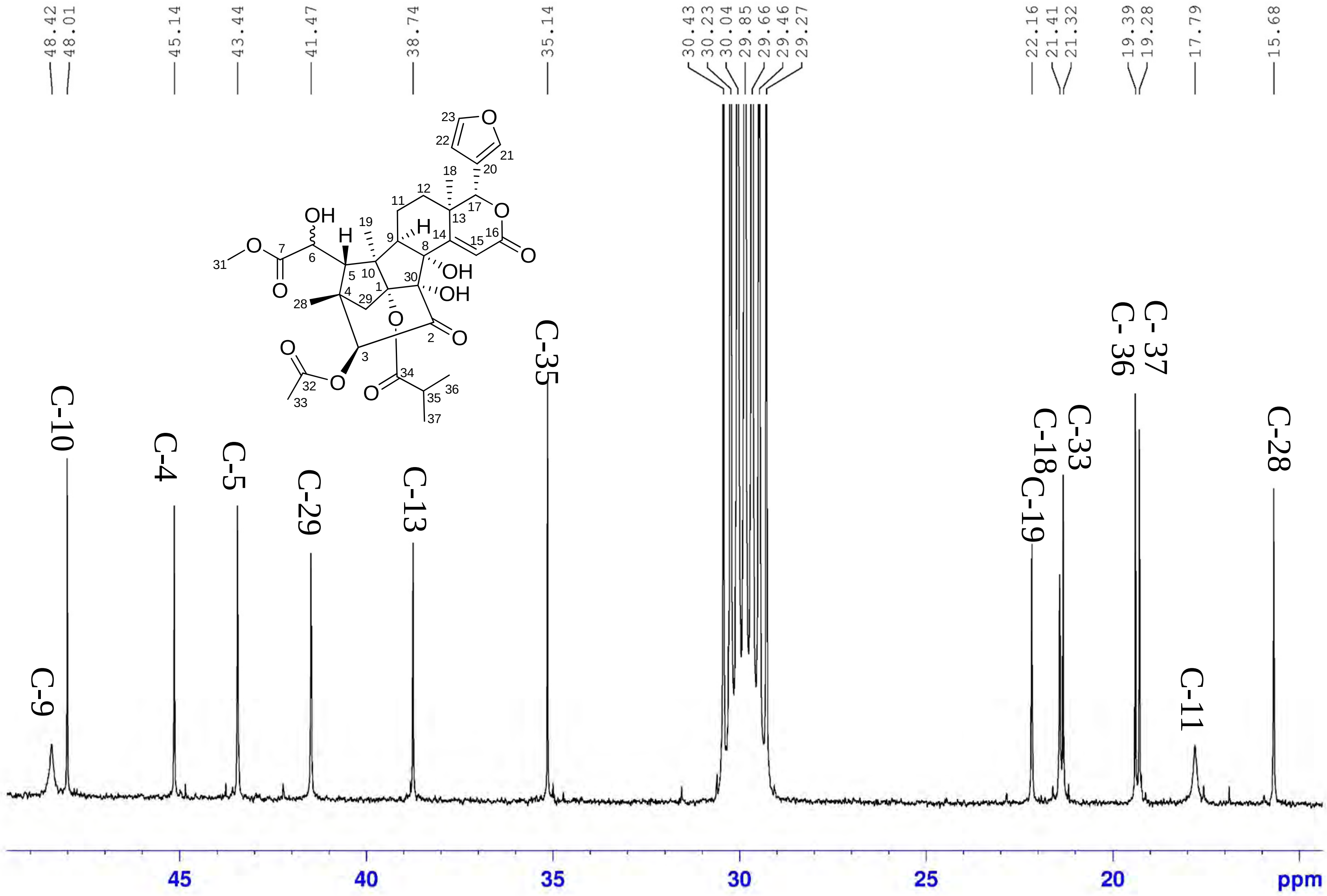

DEPT135 (100 MHz) spectrum of compound **20** in Acetone-d_6

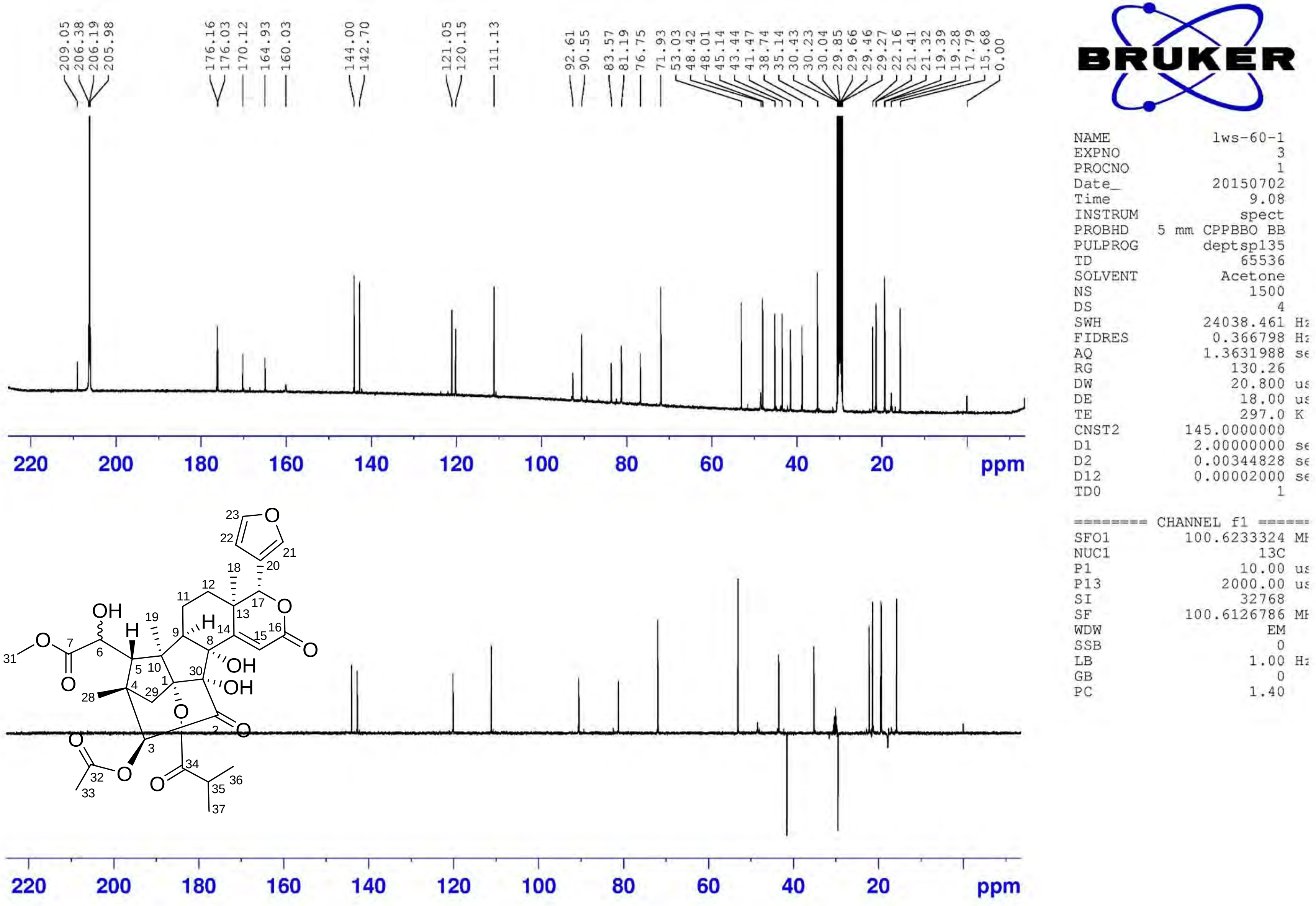

DEPT135 (100 MHz) spectrum of compound **20** in Acetone-d_6

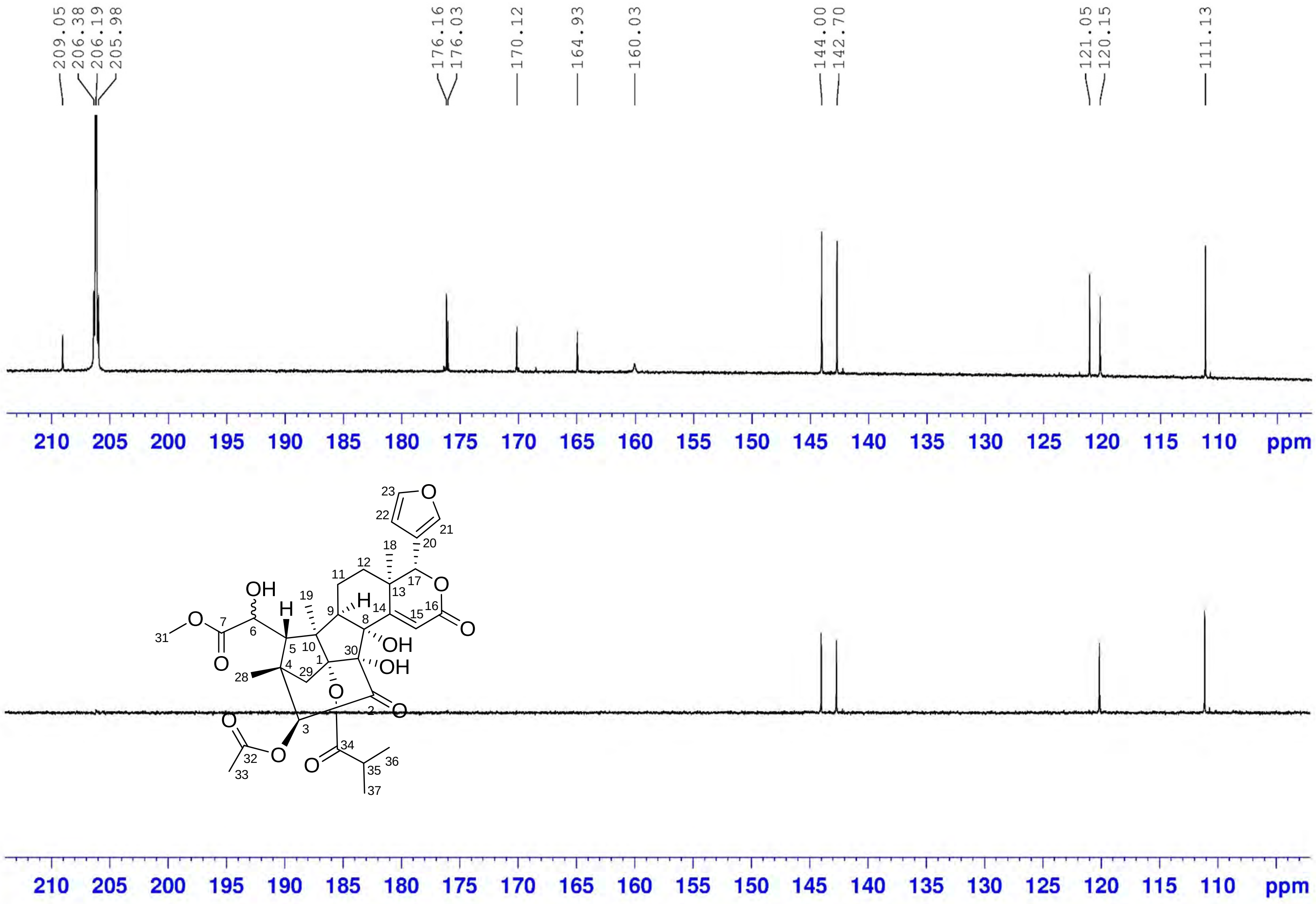

DEPT135 (100 MHz) spectrum of compound **20** in Acetone-d_6

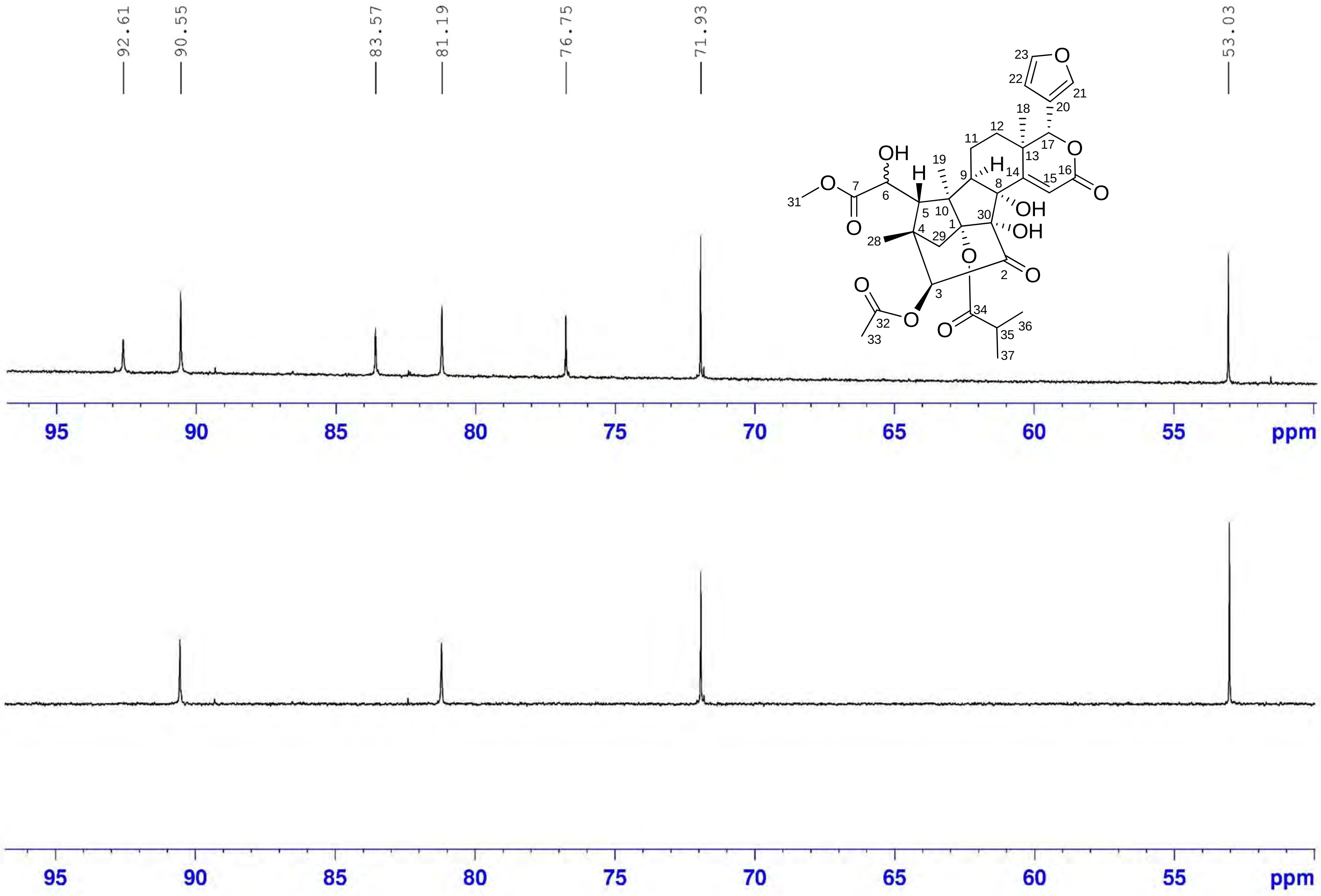

DEPT135 (100 MHz) spectrum of compound **20** in Acetone-d_6

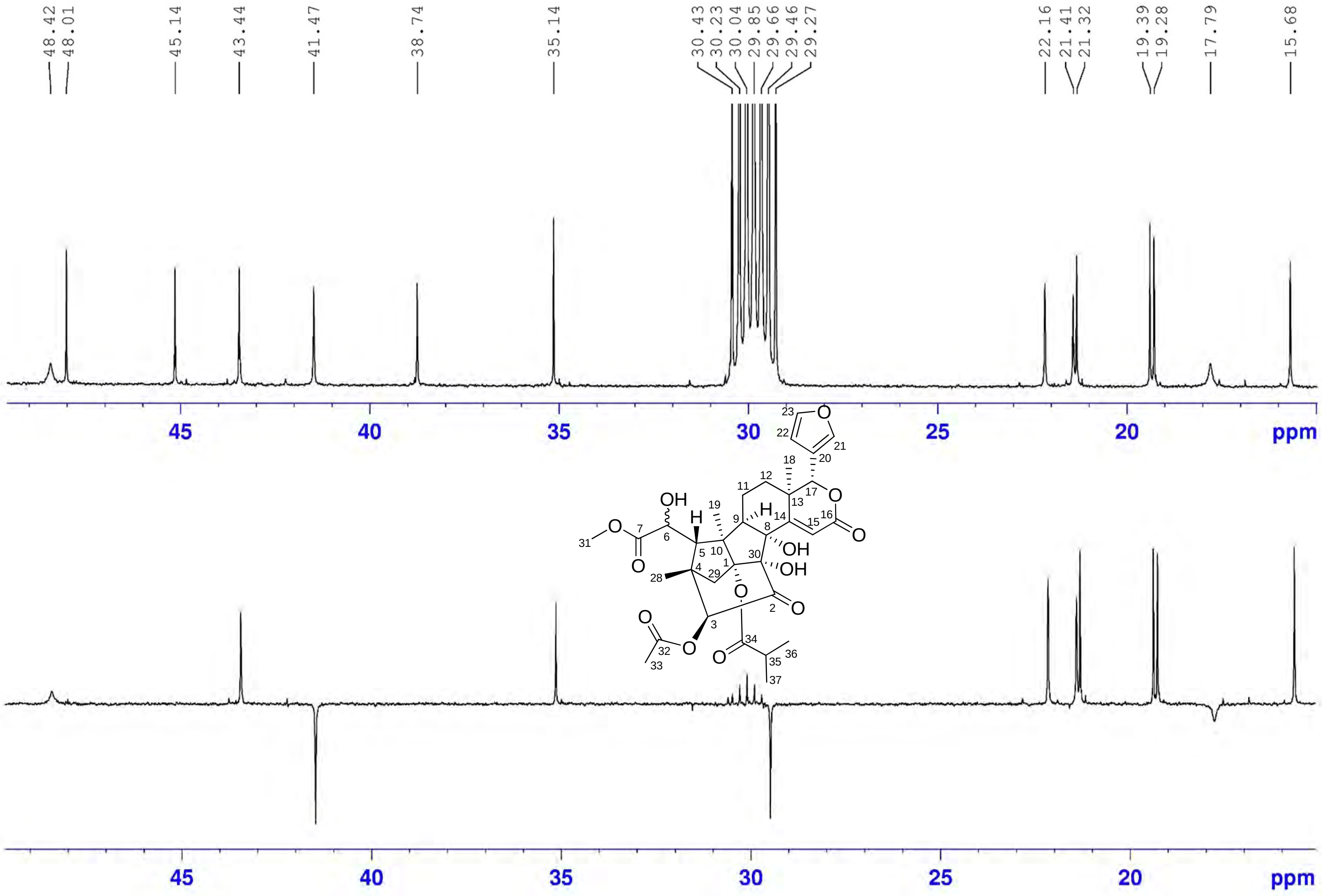

^{1}H-^{1}H COSY (400 MHz) spectrum of compound **20** in Acetone-d_6

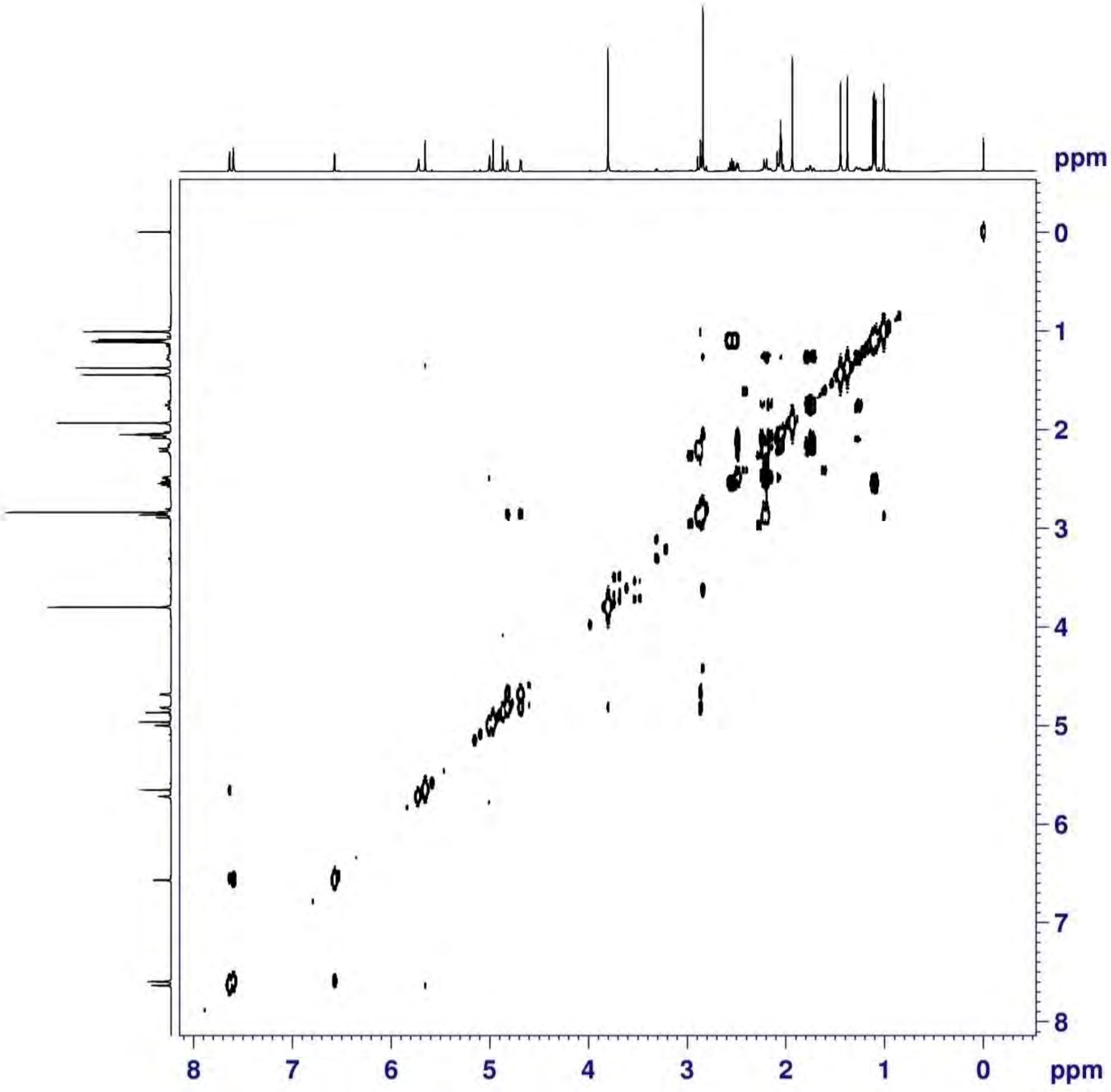

NAME	lws-60-1
EXPNO	104
PROCNO	1
Date_	20150703
Time	21.14
INSTRUM	spect
PROBHD	5 mm CPPBBO BB
PULPROG	cosygpppqf
TD	2048
SOLVENT	Acetone
NS	32
DS	8
SWH	3906.250 Hz
FIDRES	1.907349 Hz
AQ	0.2621940 sec
RG	208.5
DW	128.000 usec
DE	10.00 usec
TE	297.0 K
D0	0.00000300 sec
D1	1.89678097 sec
D11	0.03000000 sec
D12	0.00002000 sec
D13	0.00000400 sec
D16	0.00020000 sec
IN0	0.00025600 sec
======== CHANNEL f1 ========	
SFO1	400.1318006 MHz
NUC1	1H
P0	12.00 usec
P1	12.00 usec
P17	2500.00 usec
ND0	1
TD	128
SFO1	400.1318 MHz
FIDRES	30.517578 Hz
SW	9.762 ppm
FnMODE	QF
SI	1024
SF	400.1300068 MHz
WDW	QSINE
SSB	0
LB	0.00 Hz
GB	0
PC	1.40
SI	1024
MC2	QF
SF	400.1300068 MHz
WDW	QSINE

^{1}H-^{1}H COSY (400 MHz) spectrum of compound **20** in Acetone-d_6

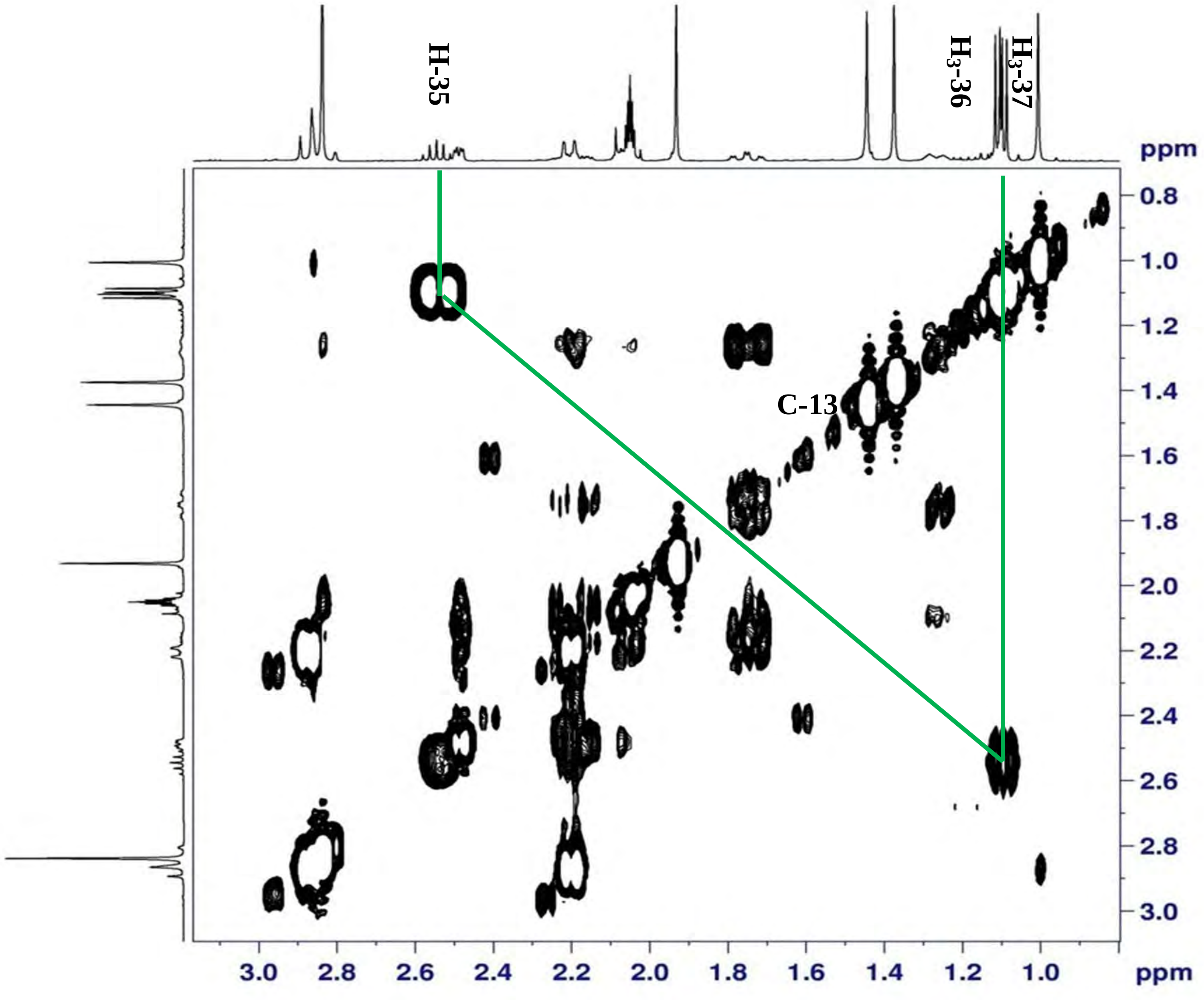

HSQC (400 MHz) spectrum of compound **20** in Acetone-d_6

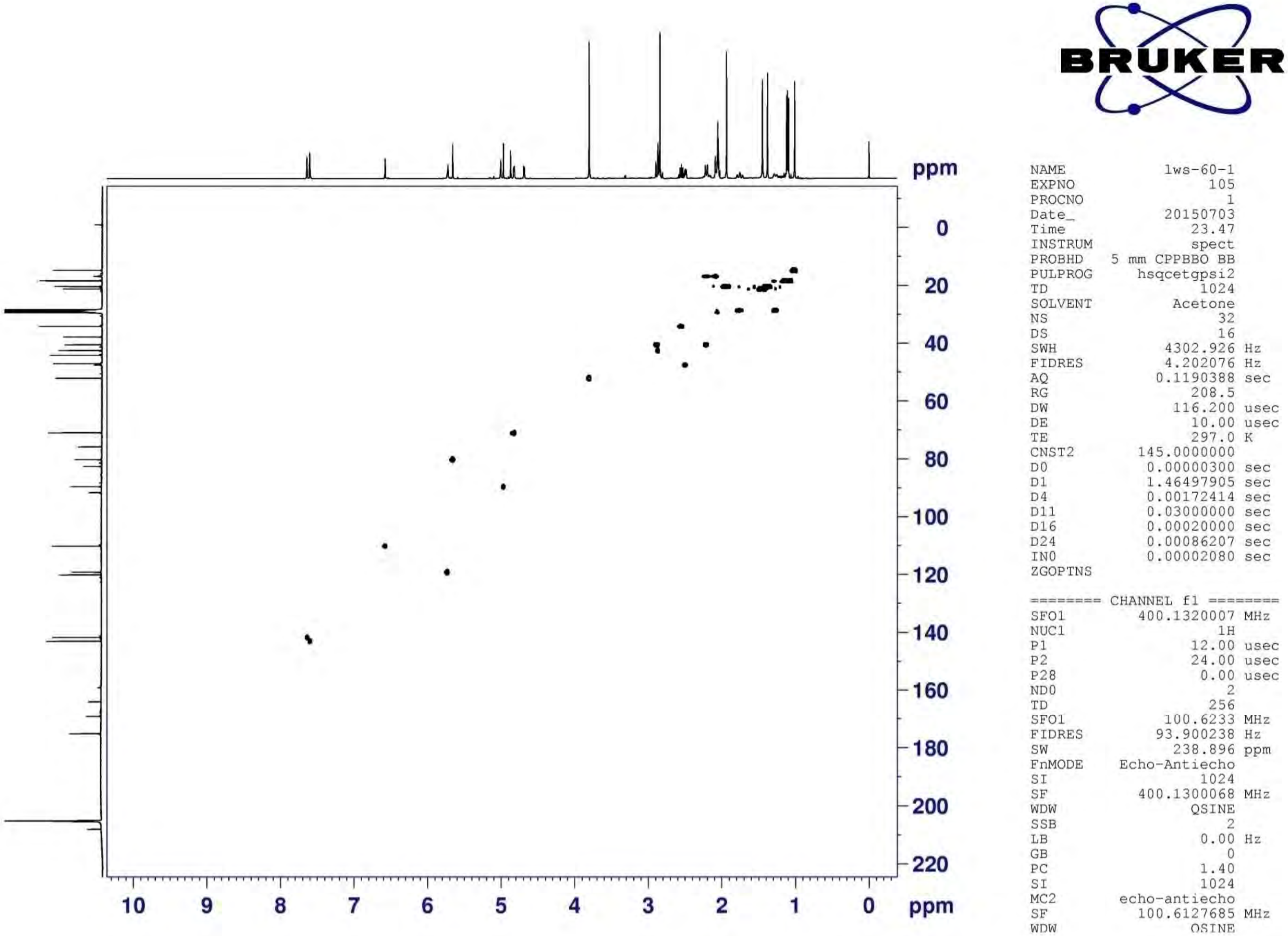

HSQC (400 MHz) spectrum of compound **20** in Acetone-d_6

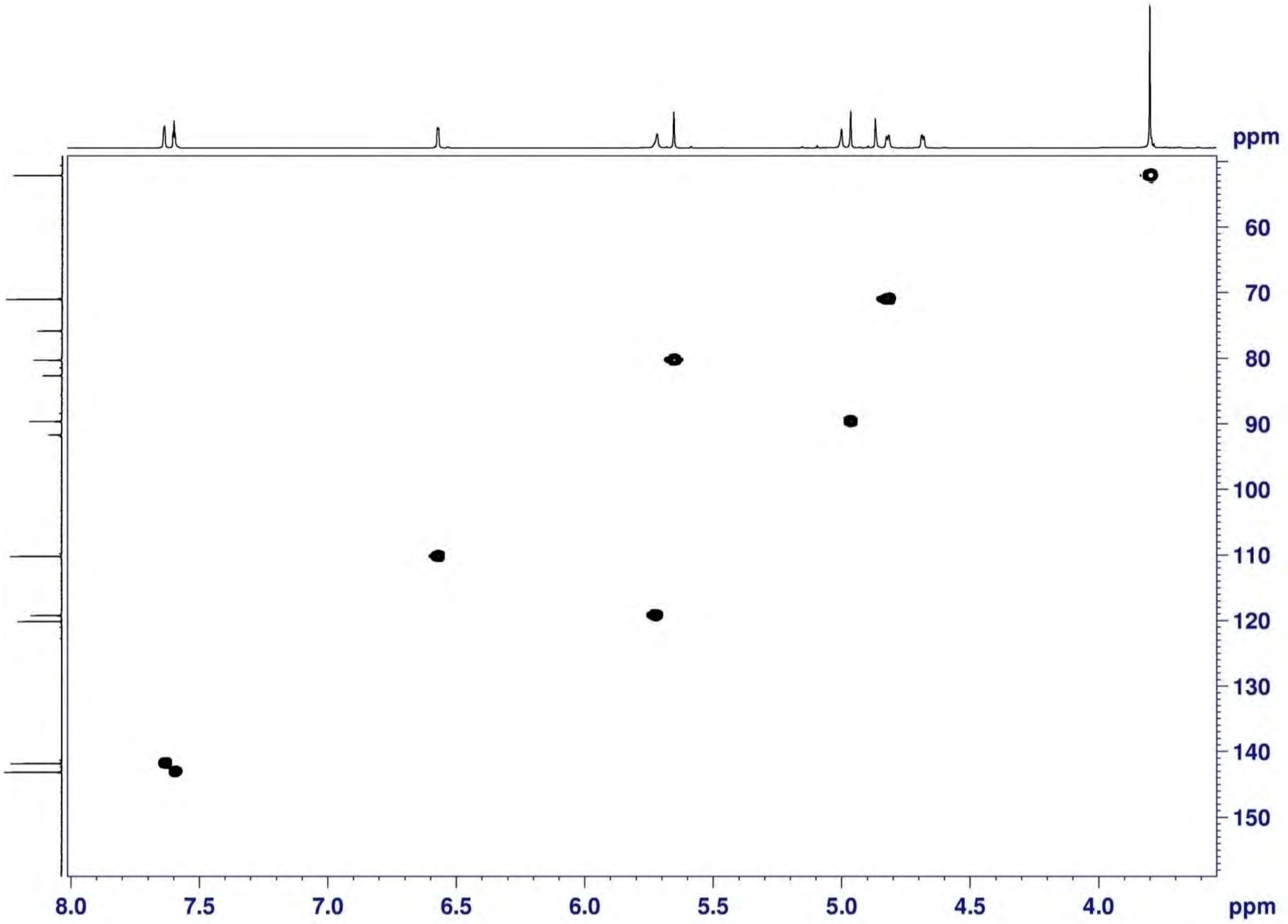

HSQC (400 MHz) spectrum of compound **20** in Acetone-d_6

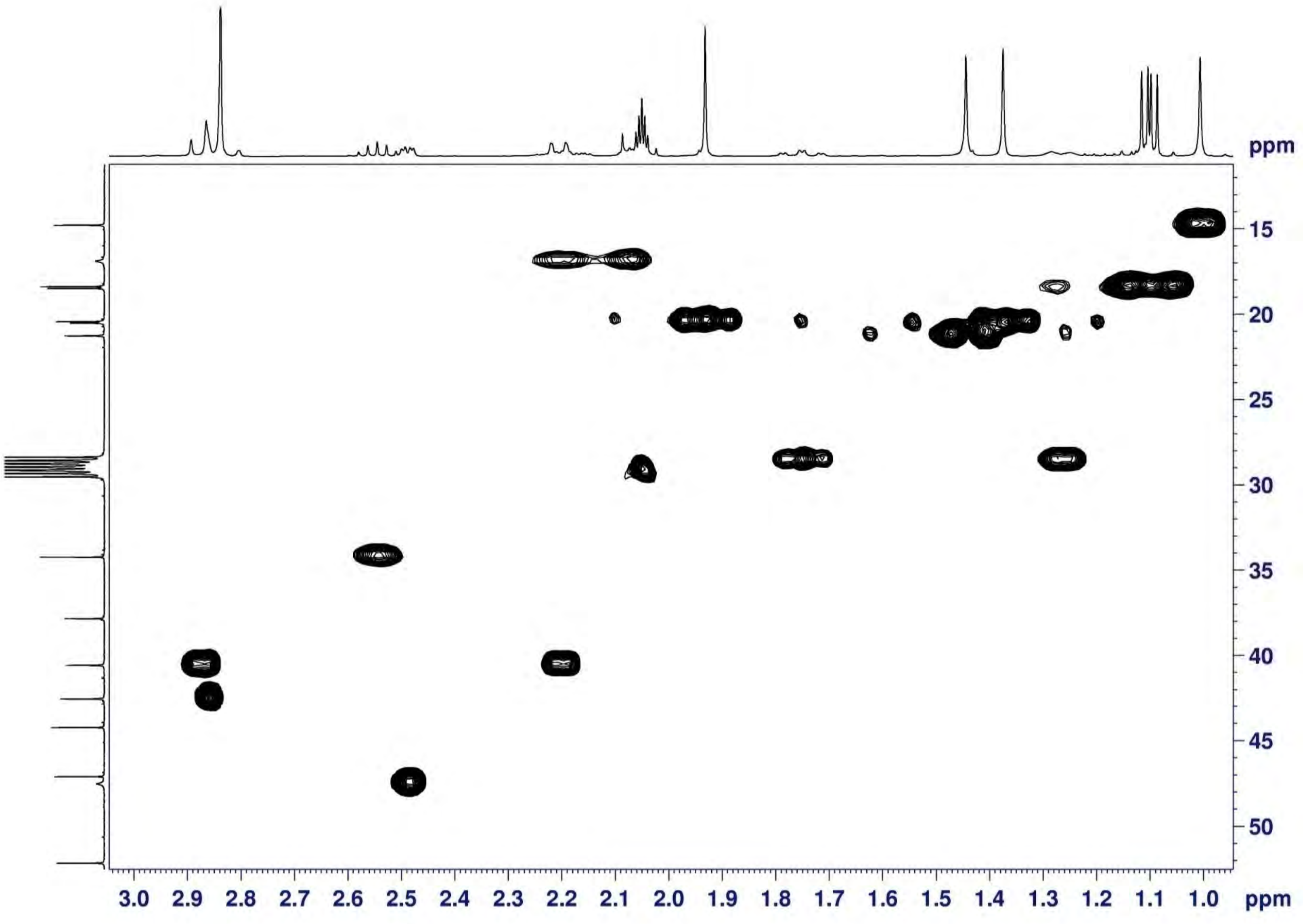

HMBC (400 MHz) spectrum of compound **20** in Acetone-d_6

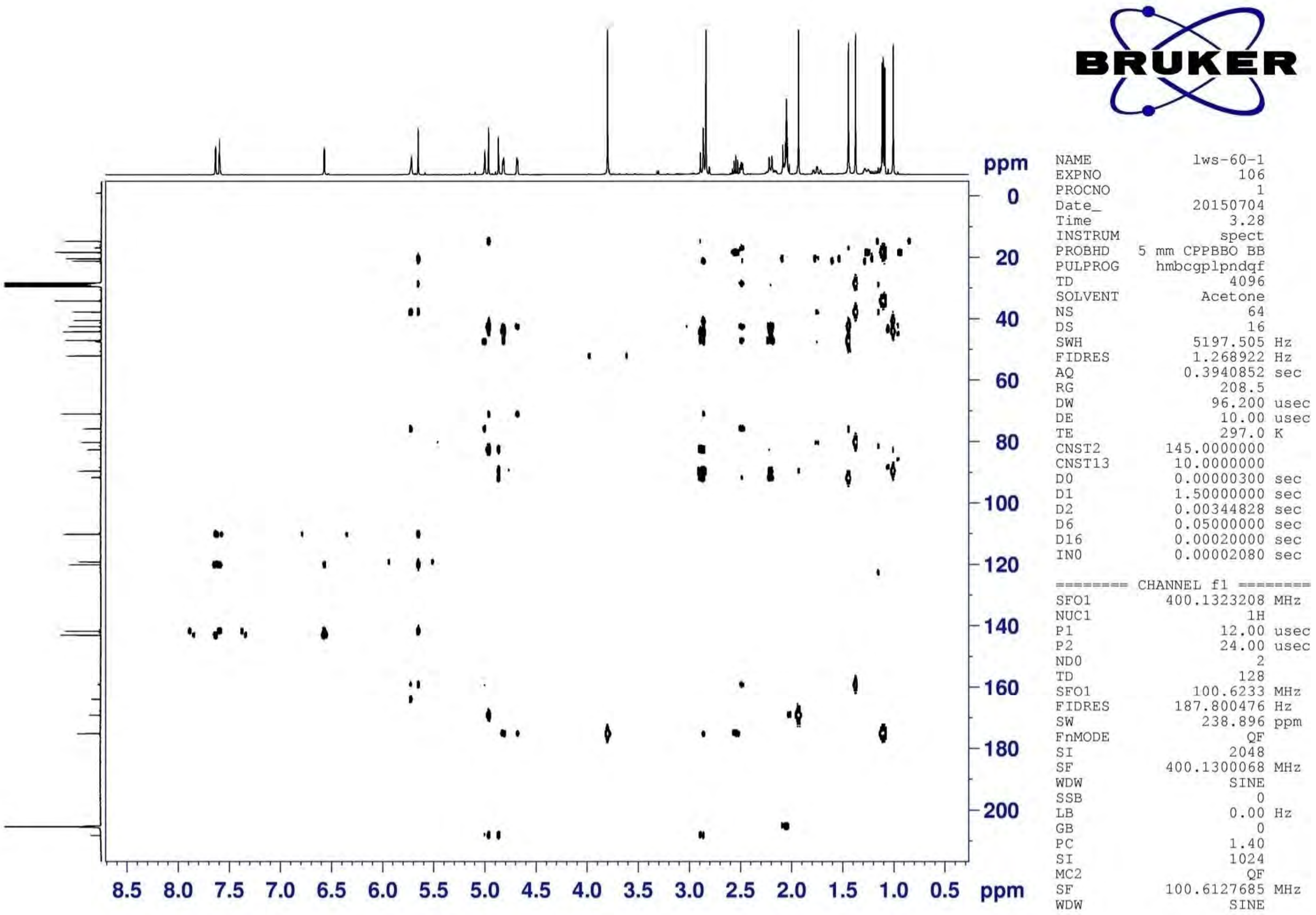

HMBC (400 MHz) spectrum of compound **20** in Acetone-d_6

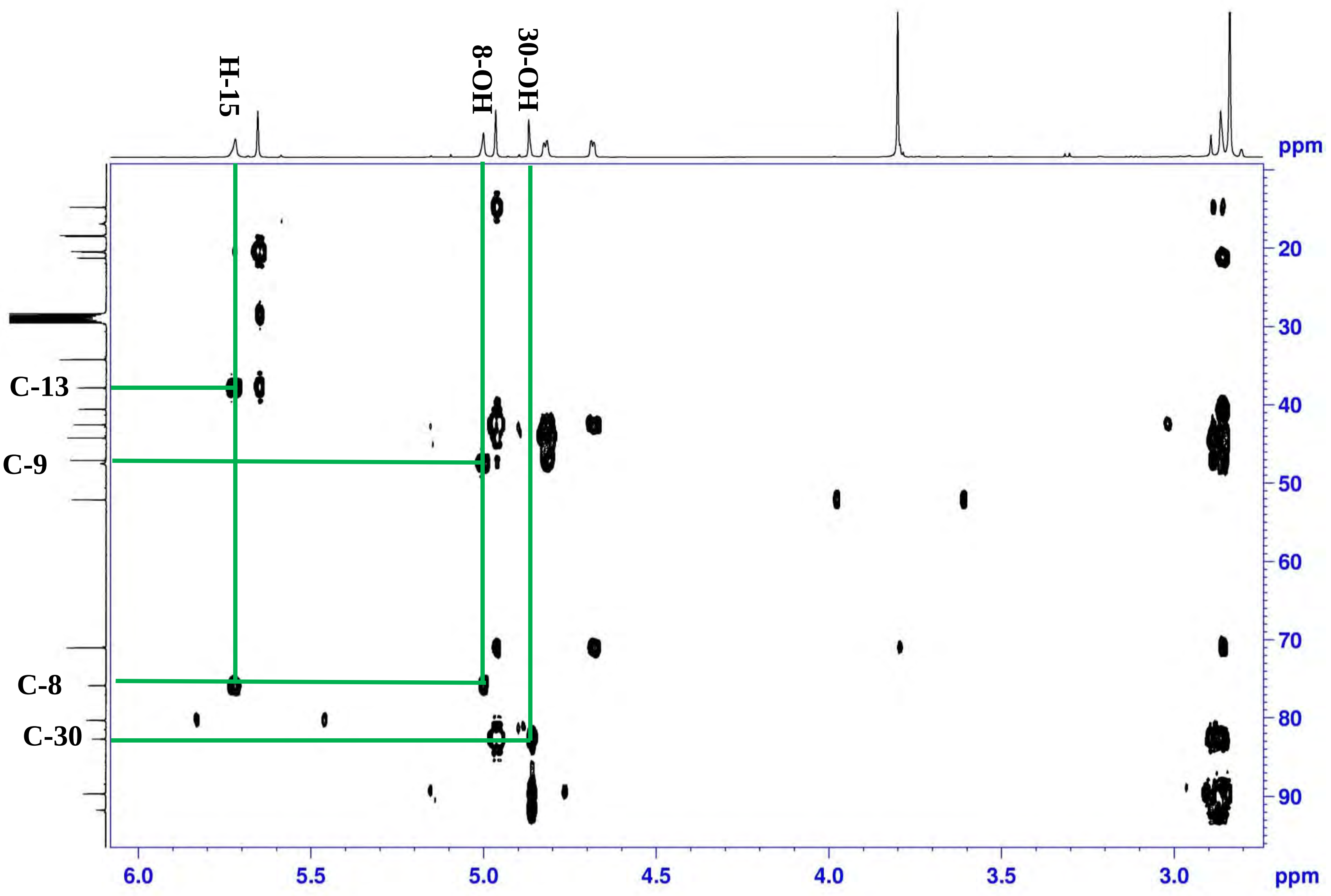

HMBC (400 MHz) spectrum of compound **20** in Acetone-d_6

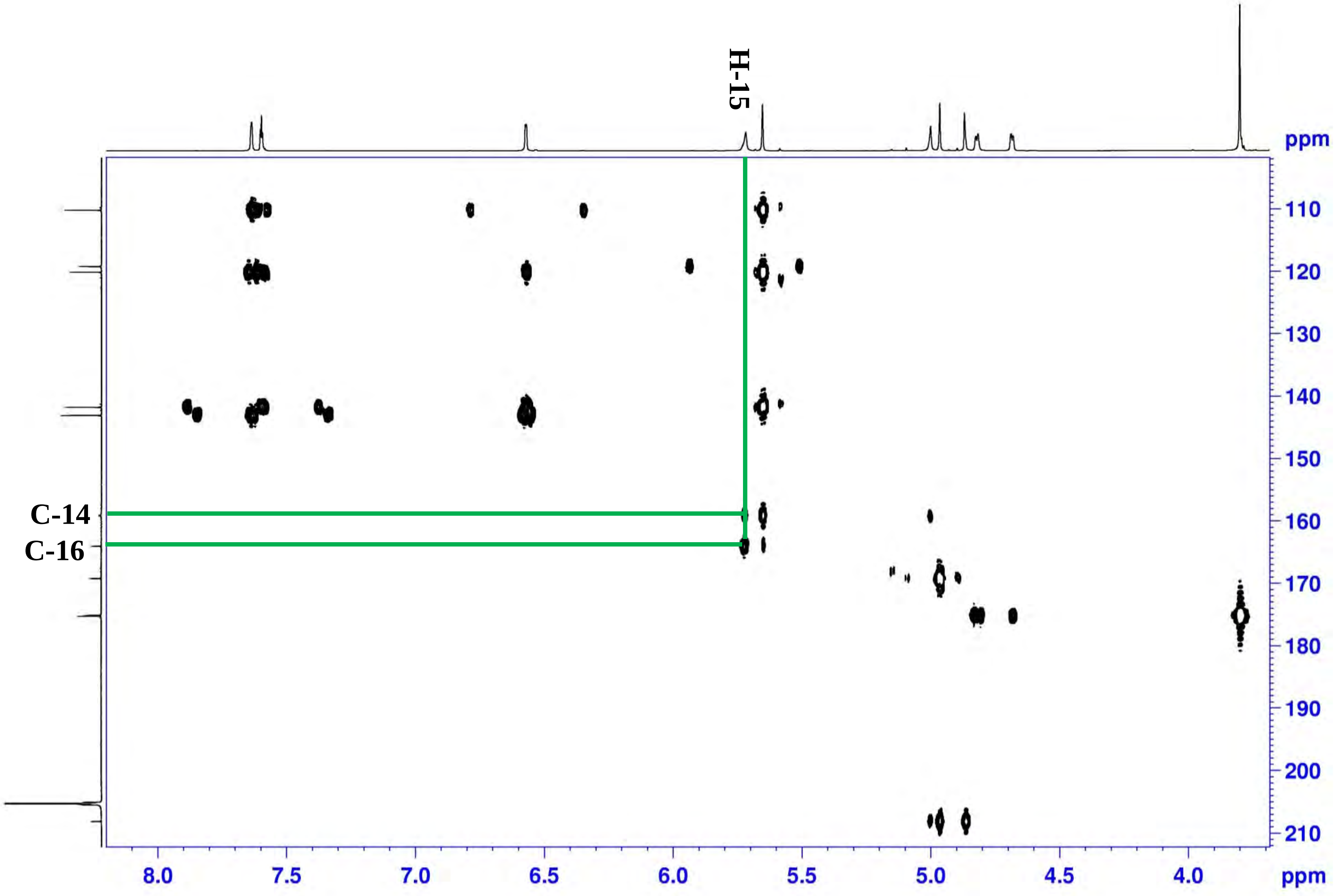

HMBC (400 MHz) spectrum of compound **20** in Acetone-d_6

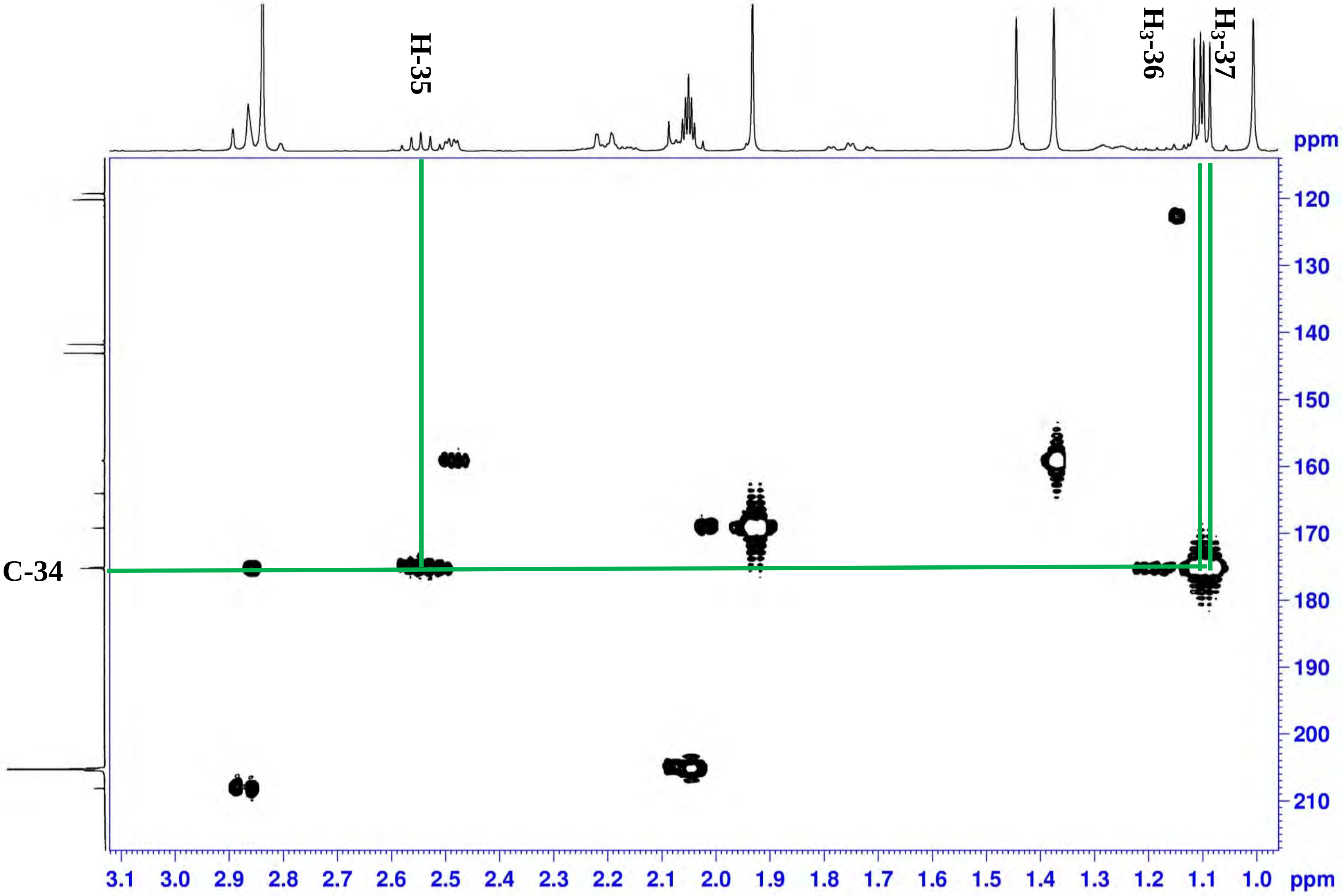

HMBC (400 MHz) spectrum of compound **20** in Acetone-d_6

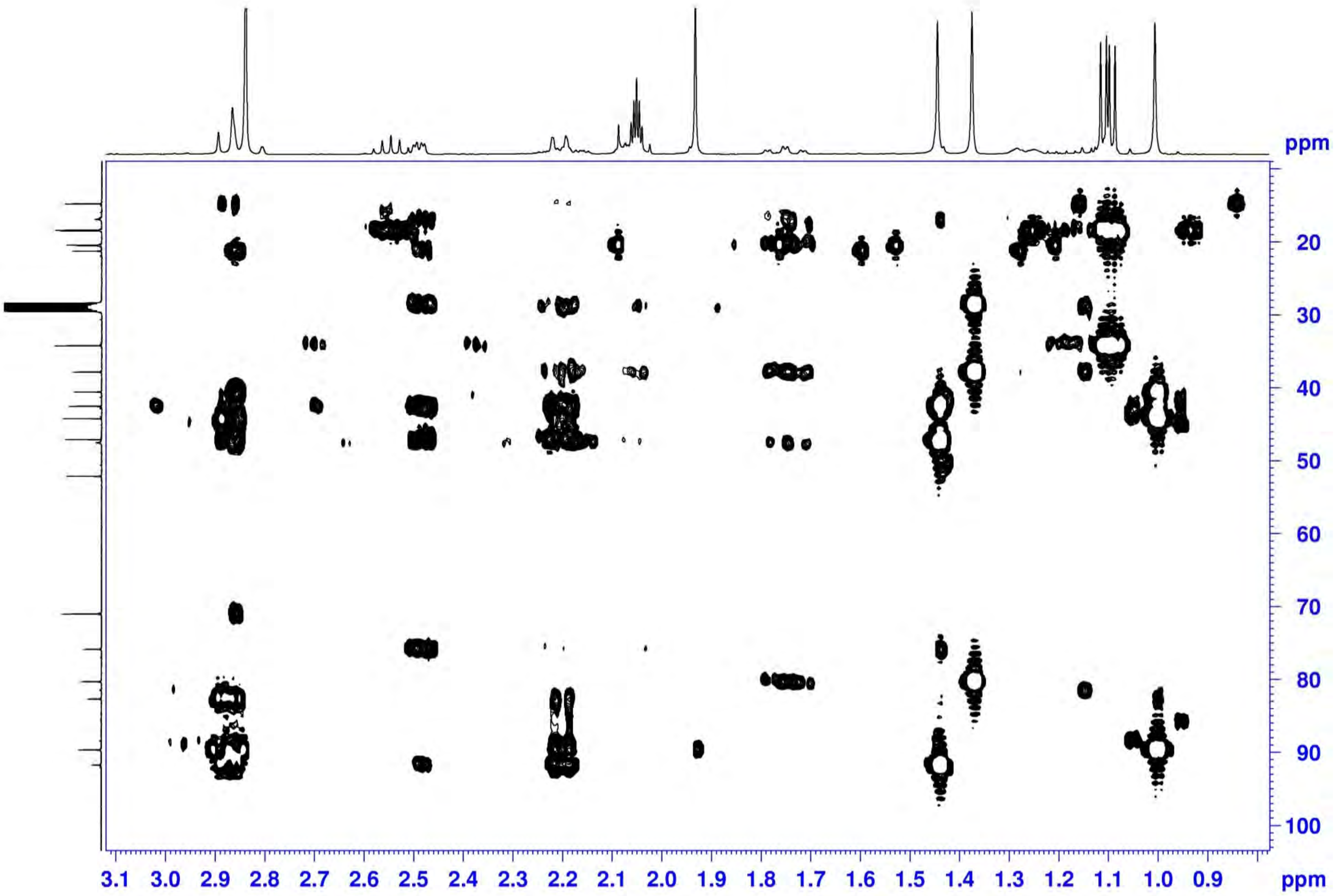

HMBC (400 MHz) spectrum of compound **20** in Acetone-d_6

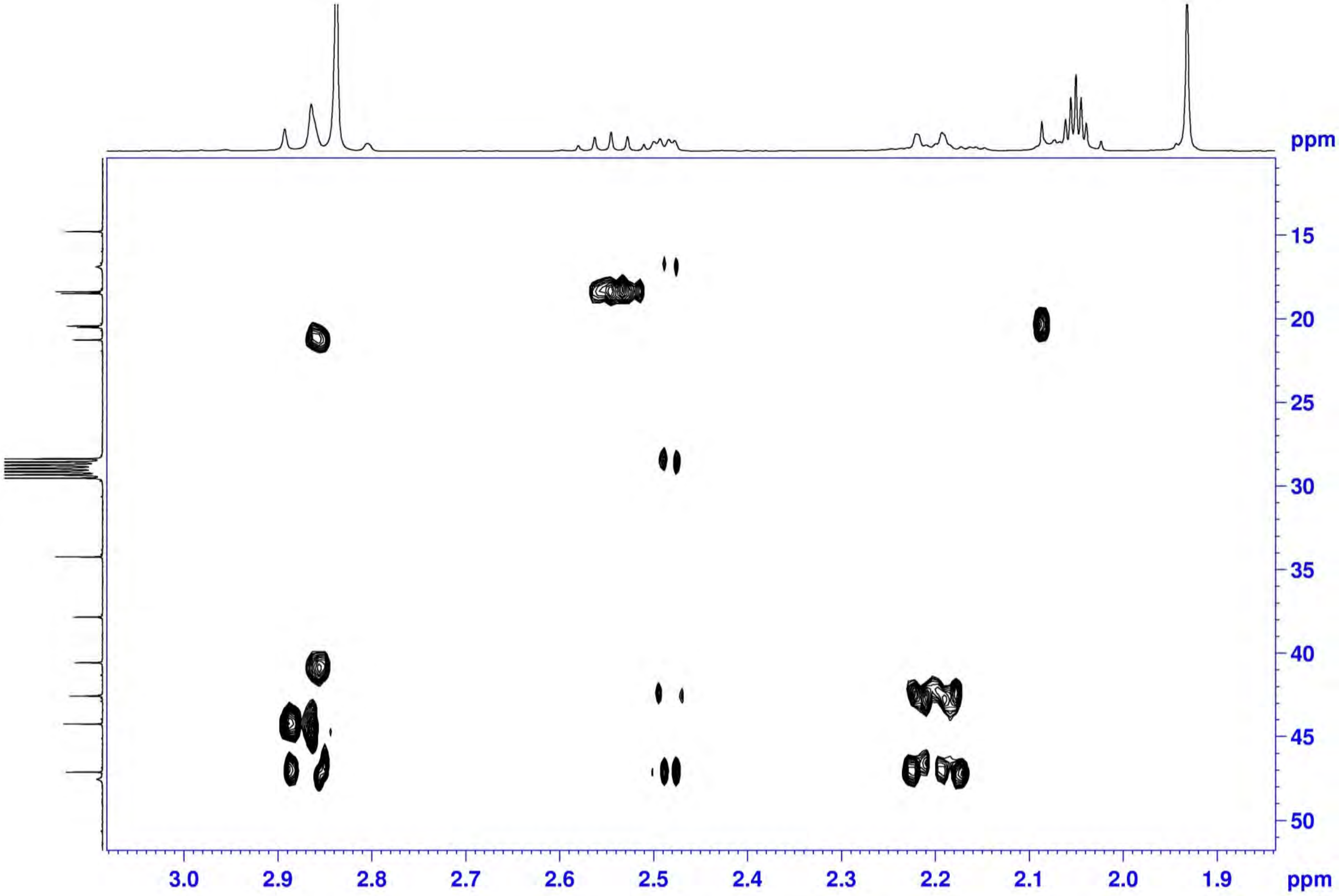

HMBC (400 MHz) spectrum of compound **20** in Acetone-d_6

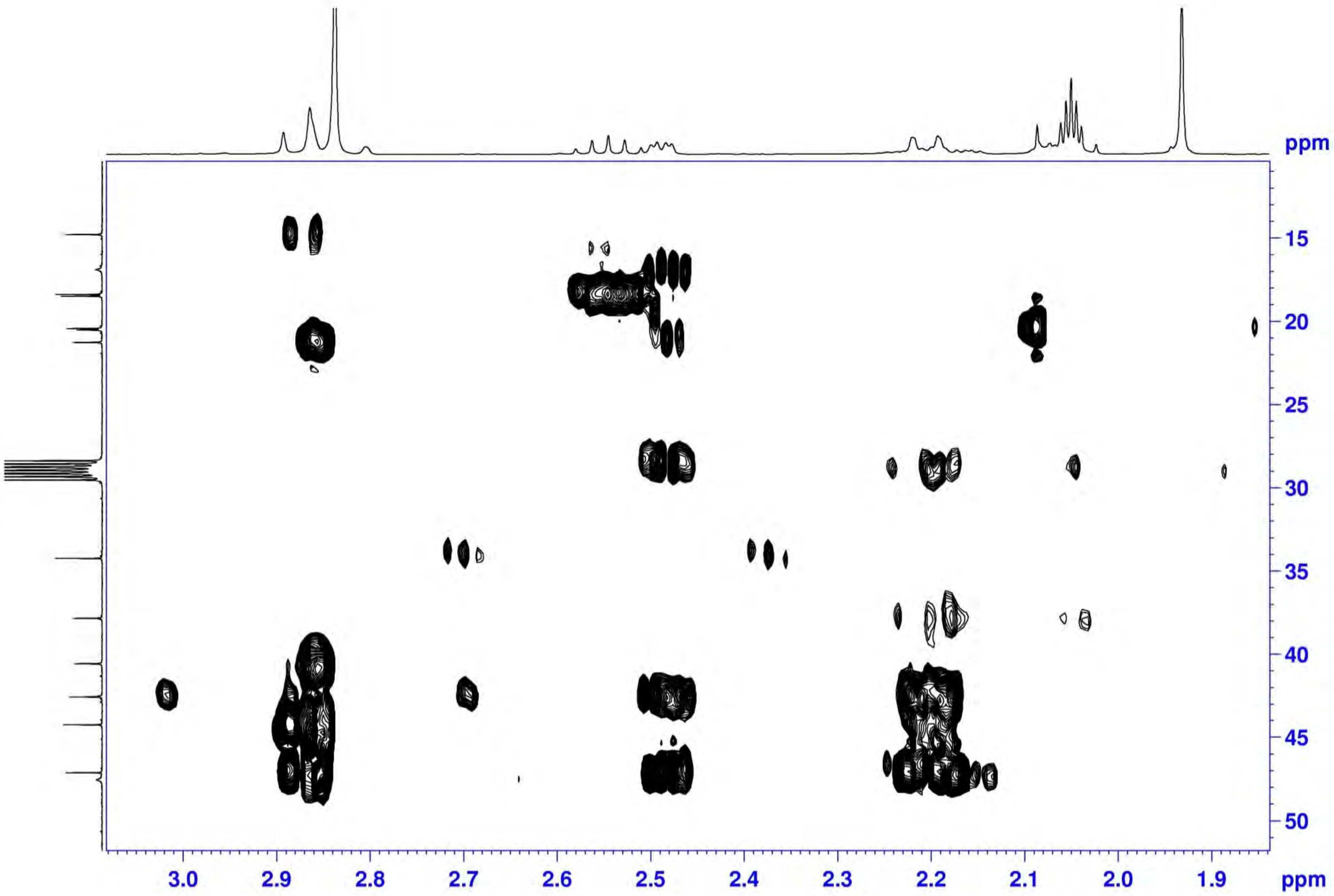

HMBC (400 MHz) spectrum of compound **20** in Acetone-d_6

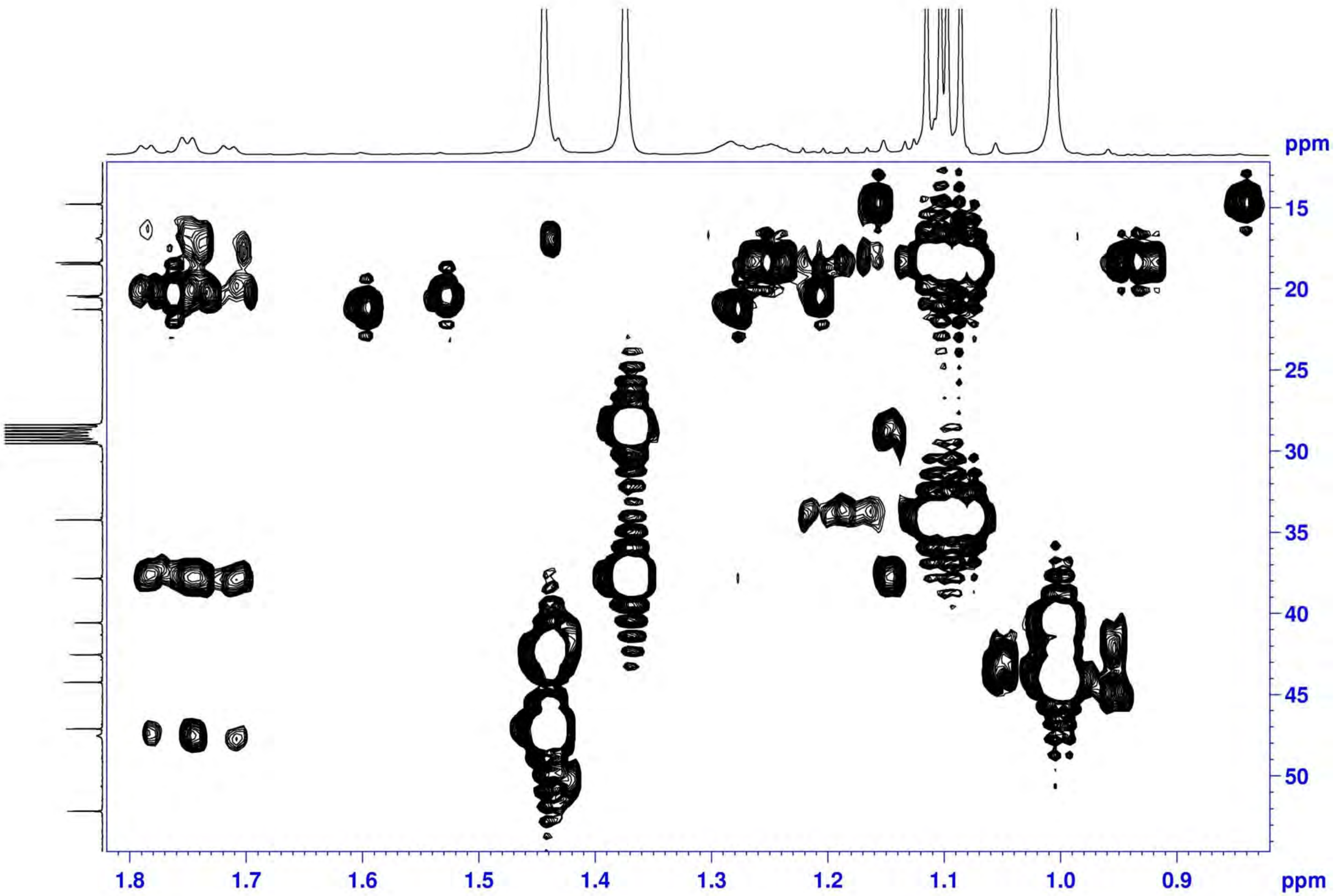

HMBC (400 MHz) spectrum of compound **20** in Acetone-d_6

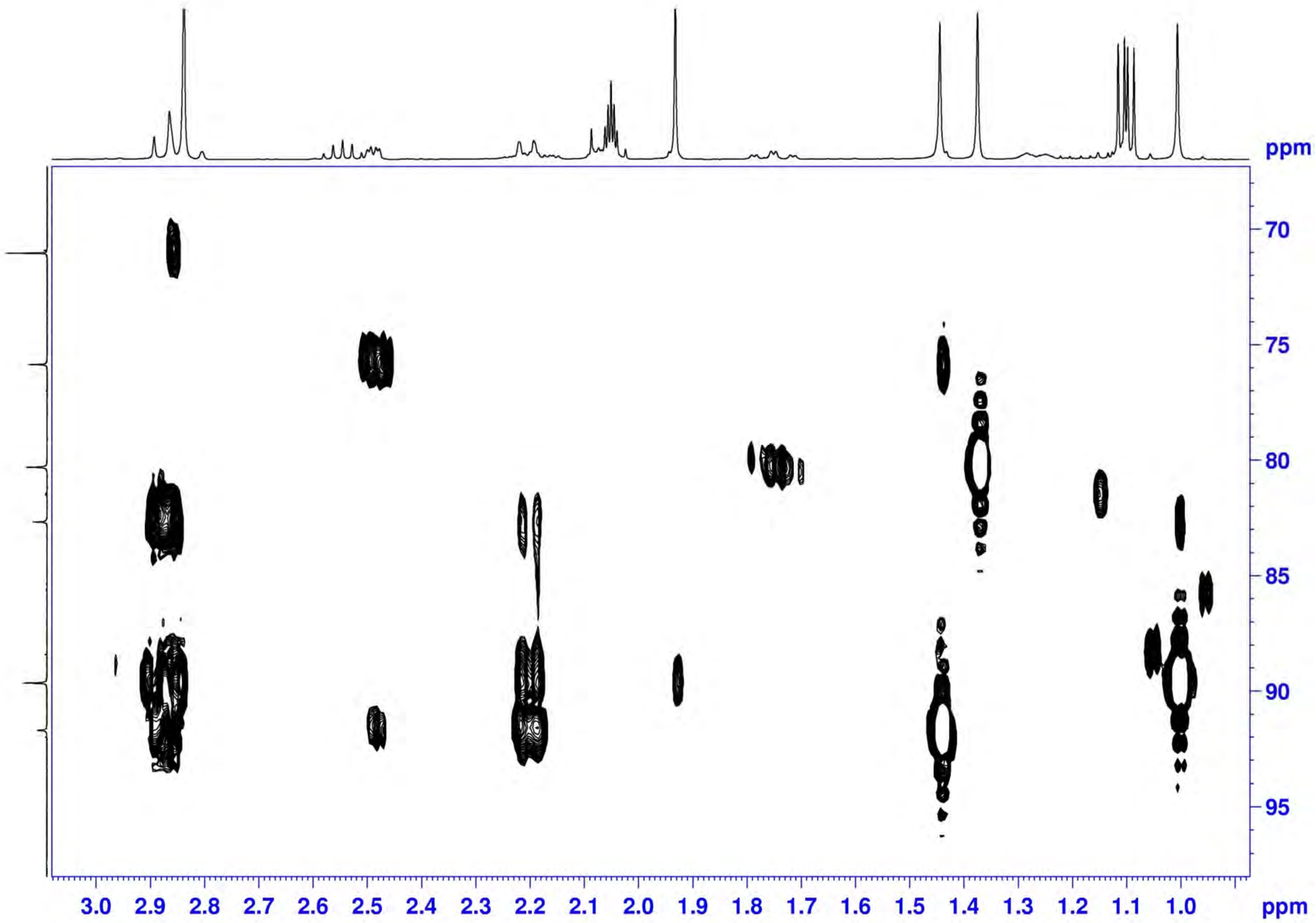

NOESY (400 MHz) spectrum of compound **20** in Acetone-d_6

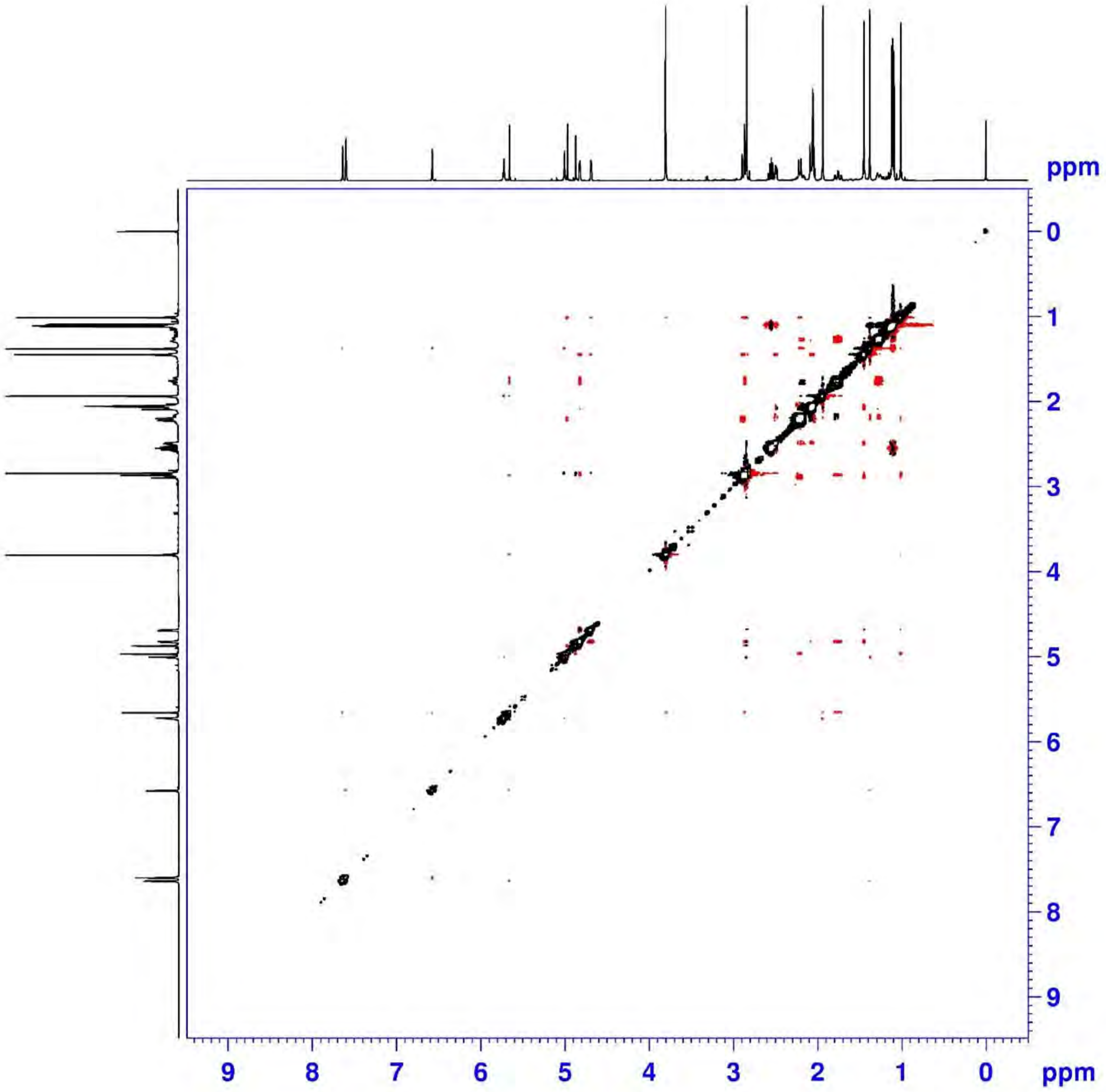

NAME	lws-60-1
EXPNO	107
PROCNO	1
Date_	20150704
Time	7.57
INSTRUM	spect
PROBHD	5 mm CPPBBO BB
PULPROG	noesygpphpp
TD	2048
SOLVENT	Acetone
NS	32
DS	32
SWH	4000.000 Hz
FIDRES	1.953125 Hz
AQ	0.2560500 sec
RG	208.5
DW	125.000 usec
DE	10.00 usec
TE	297.0 K
D0	0.00010972 sec
D1	1.99385595 sec
D8	0.30000001 sec
D11	0.03000000 sec
D12	0.00002000 sec
D16	0.00020000 sec
IN0	0.00025000 sec
======== CHANNEL f1 ========	
SFO1	400.1318006 MHz
NUC1	1H
P1	12.00 usec
P2	24.00 usec
P17	2500.00 usec
ND0	1
TD	256
SFO1	400.1318 MHz
FIDRES	15.625000 Hz
SW	9.997 ppm
FnMODE	States-TPPI
SI	1024
SF	400.1300050 MHz
WDW	QSINE
SSB	2
LB	0.00 Hz
GB	0
PC	1.00
SI	1024
MC2	States-TPPI
SF	400.1300050 MHz
WDW	QSINE
SSB	2
LB	0.00 Hz
GB	0

NOESY (400 MHz) spectrum of compound **20** in Acetone-d_6

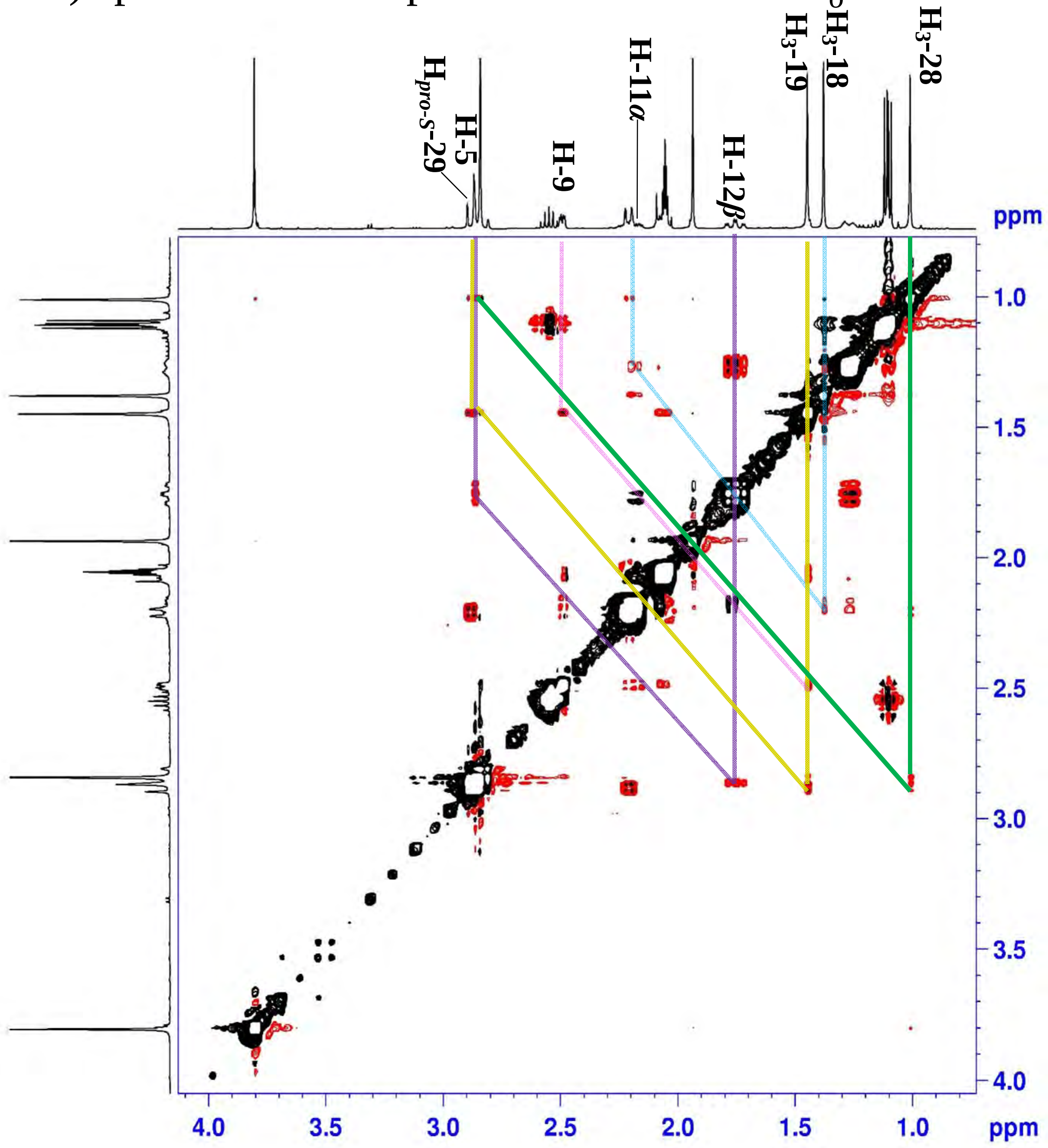

NOESY (400 MHz) spectrum of compound **20** in Acetone-d_6

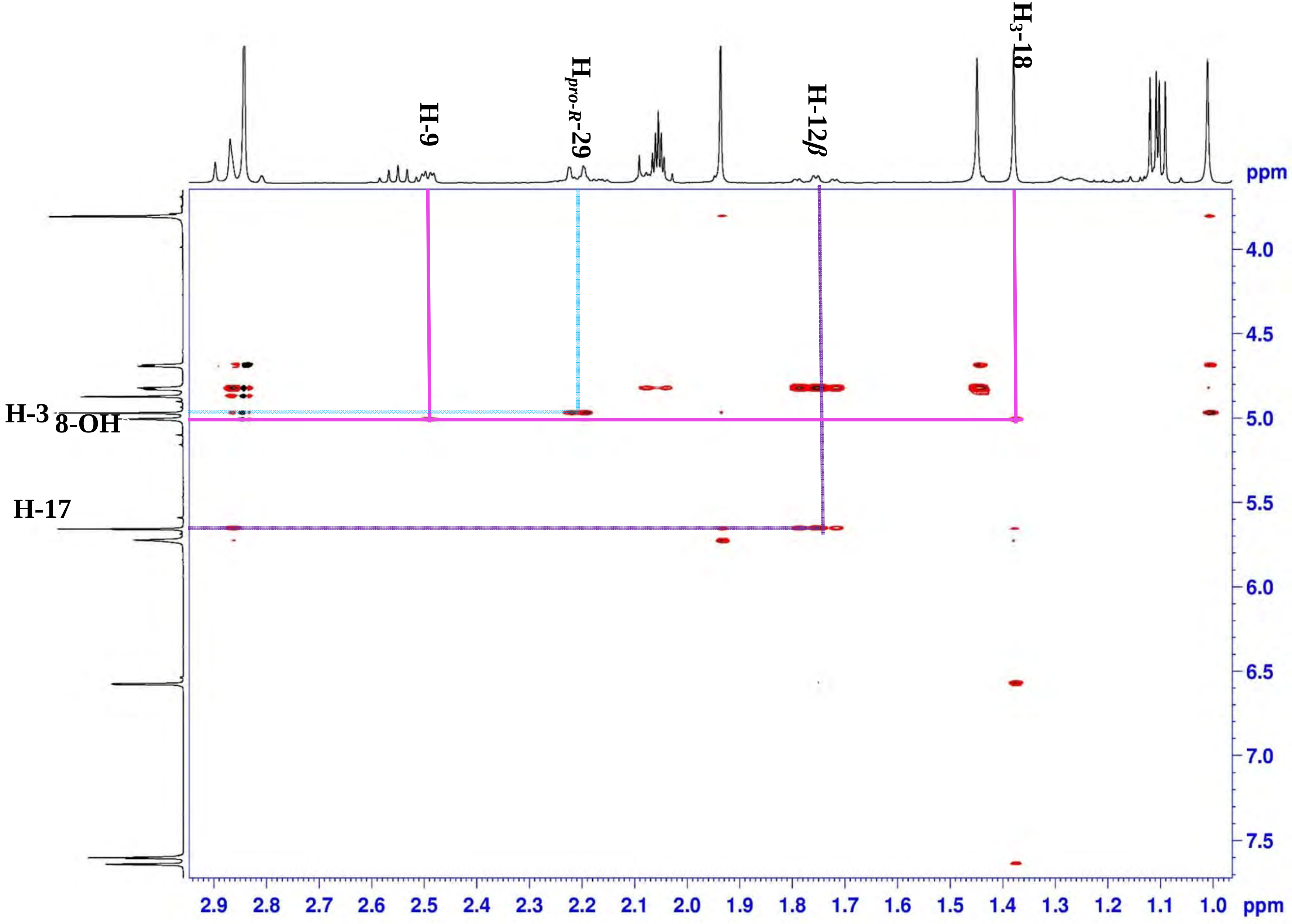

NOESY (400 MHz) spectrum of compound **20** in Acetone-d_6

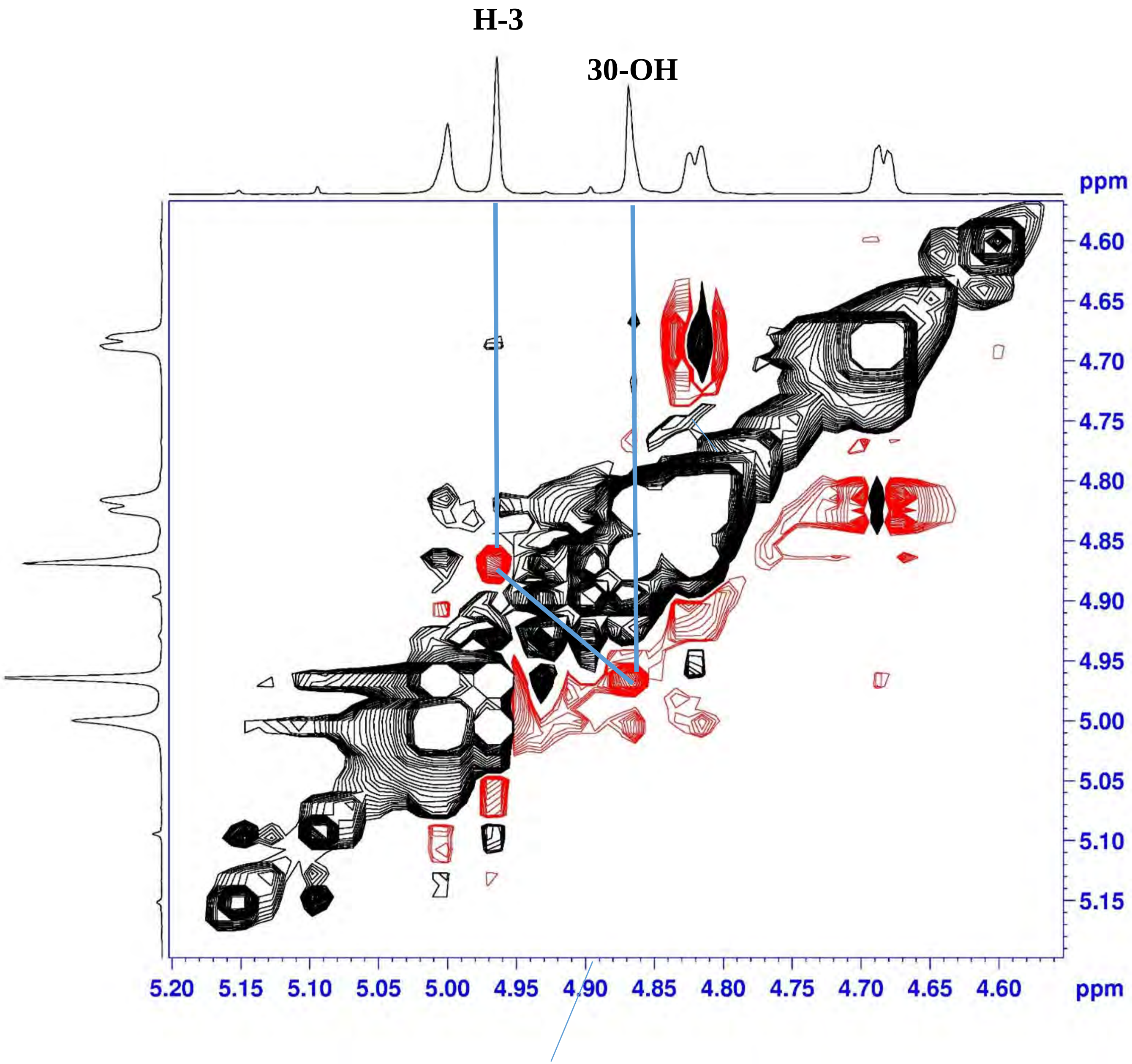

HRESIMS for compound **21**

Mass Spectrum SmartFormula Report

Analysis Info

Analysis Name	D:\Data\MS\data\201511\liwanshan_lws-73_pos_21_01_831.d	Acquisition Date	11/18/2015 10:00:16 PM
Method	LC_Direct Infusion_pos_100-1000mz.m	Operator	SCSIO
Sample Name	liwanshan_lws-73_pos	Instrument / Ser#	maXis 29
Comment			

Acquisition Parameter

Source Type	ESI	Ion Polarity	Positive	Set Nebulizer	0.4 Bar
Focus	Active	Set Capillary	4500 V	Set Dry Heater	180 °C
Scan Begin	100 m/z	Set End Plate Offset	-500 V	Set Dry Gas	4.0 l/min
Scan End	2000 m/z	Set Collision Cell RF	800.0 Vpp	Set Divert Valve	Waste

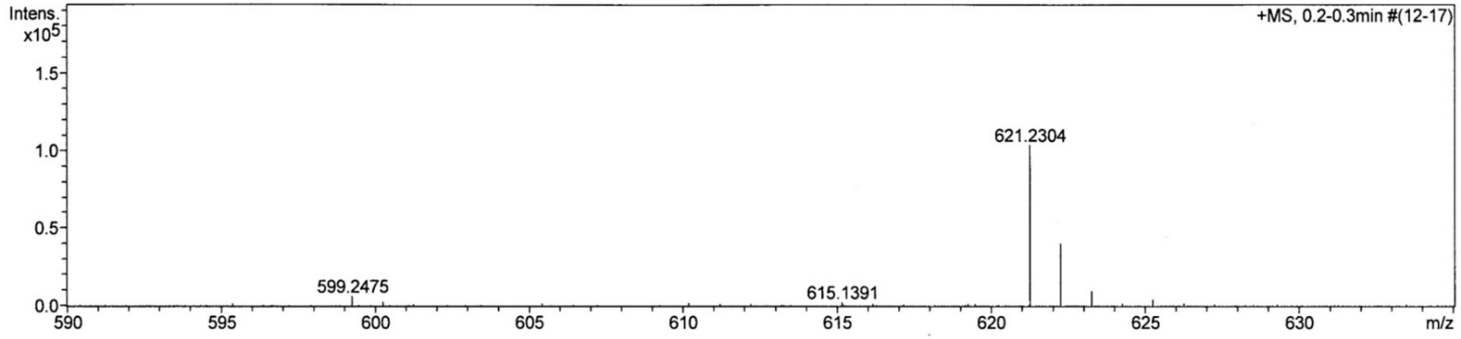

Meas. m/z	#	Formula	Score	m/z	err [ppm]	err [mDa]	mSigma	rdb	e⁻ Conf	N-Rule
599.2475	1	C 32 H 39 O 11	100.00	599.2487	2.0	1.2	23.1	13.5	even	ok
621.2304	1	C 32 H 38 Na O 11	100.00	621.2306	0.4	0.3	16.5	13.5	even	ok

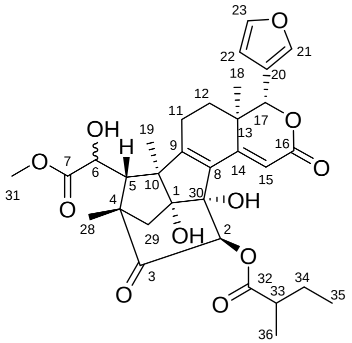

HRESIMS for compound **21**

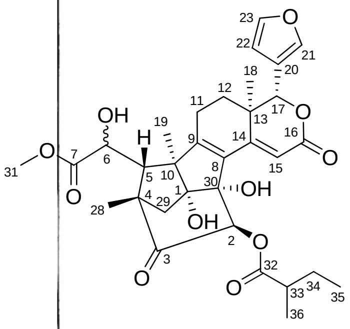

Generic Display Report

Analysis Info

Analysis Name	D:\Data\MS\data\201511\liwanshan_lws-73_pos_21_01_831.d	Acquisition Date	11/18/2015 10:00:16 PM
Method	LC_Direct Infusion_pos_100-1000mz.m	Operator	SCSIO
Sample Name	liwanshan_lws-73_pos	Instrument	maXis
Comment			

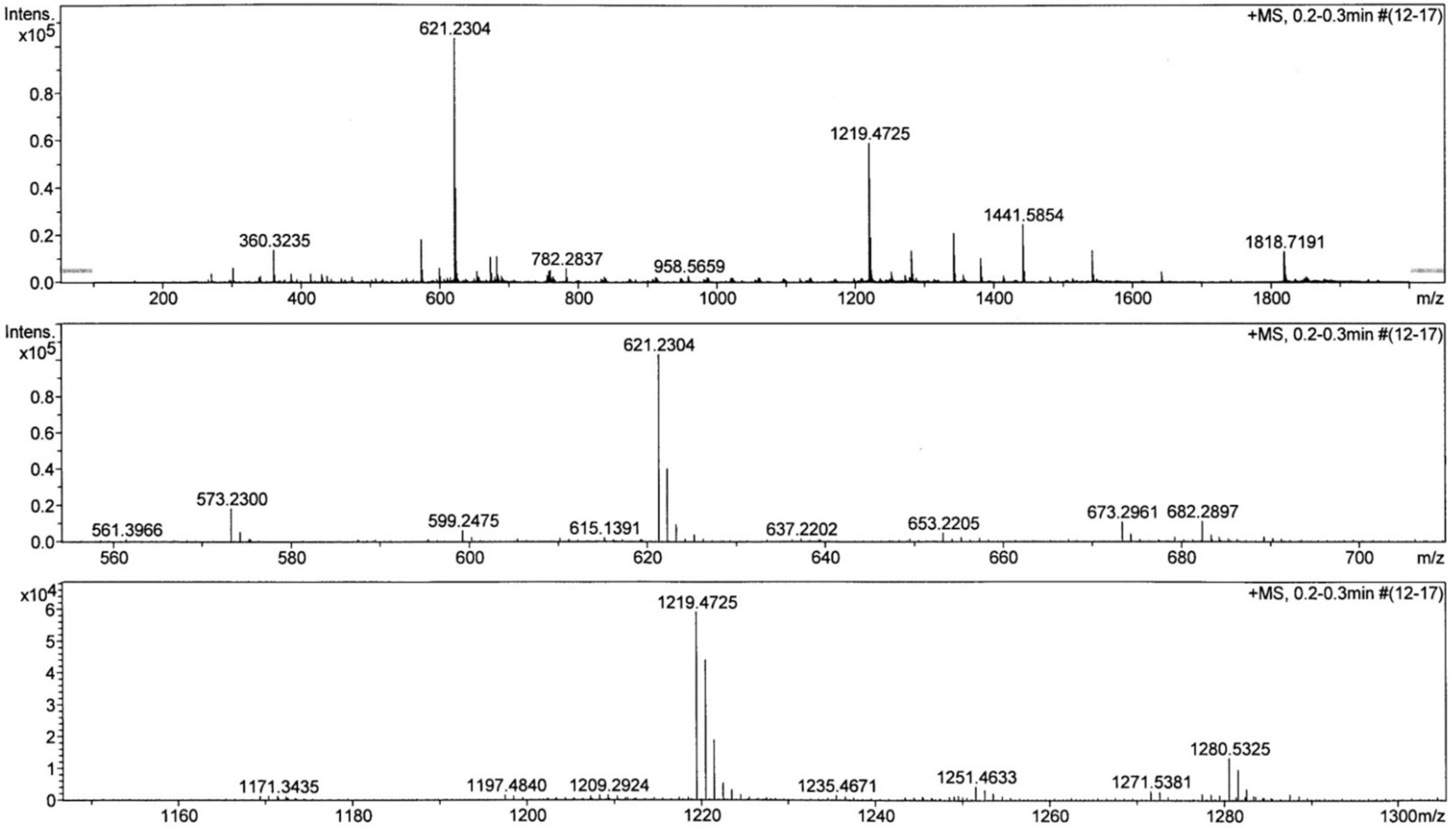

Bruker Compass DataAnalysis 4.0 printed: 11/18/2015 10:09:17 PM Page 1 of 1

^{1}H NMR (400 MHz) spectrum of compound **21** in $CDCl_3$

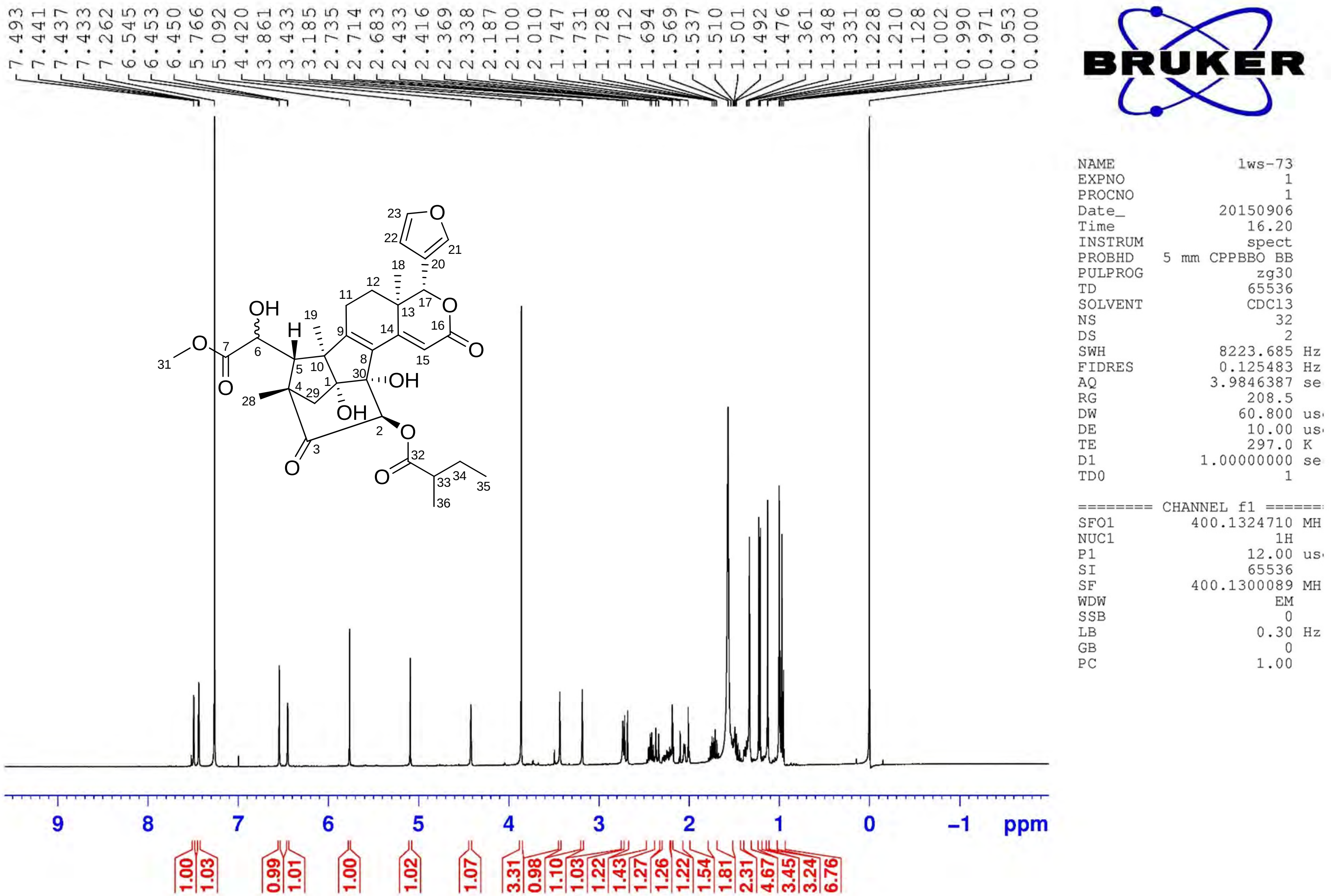

^{1}H NMR (400 MHz) spectrum of compound **21** in $CDCl_3$

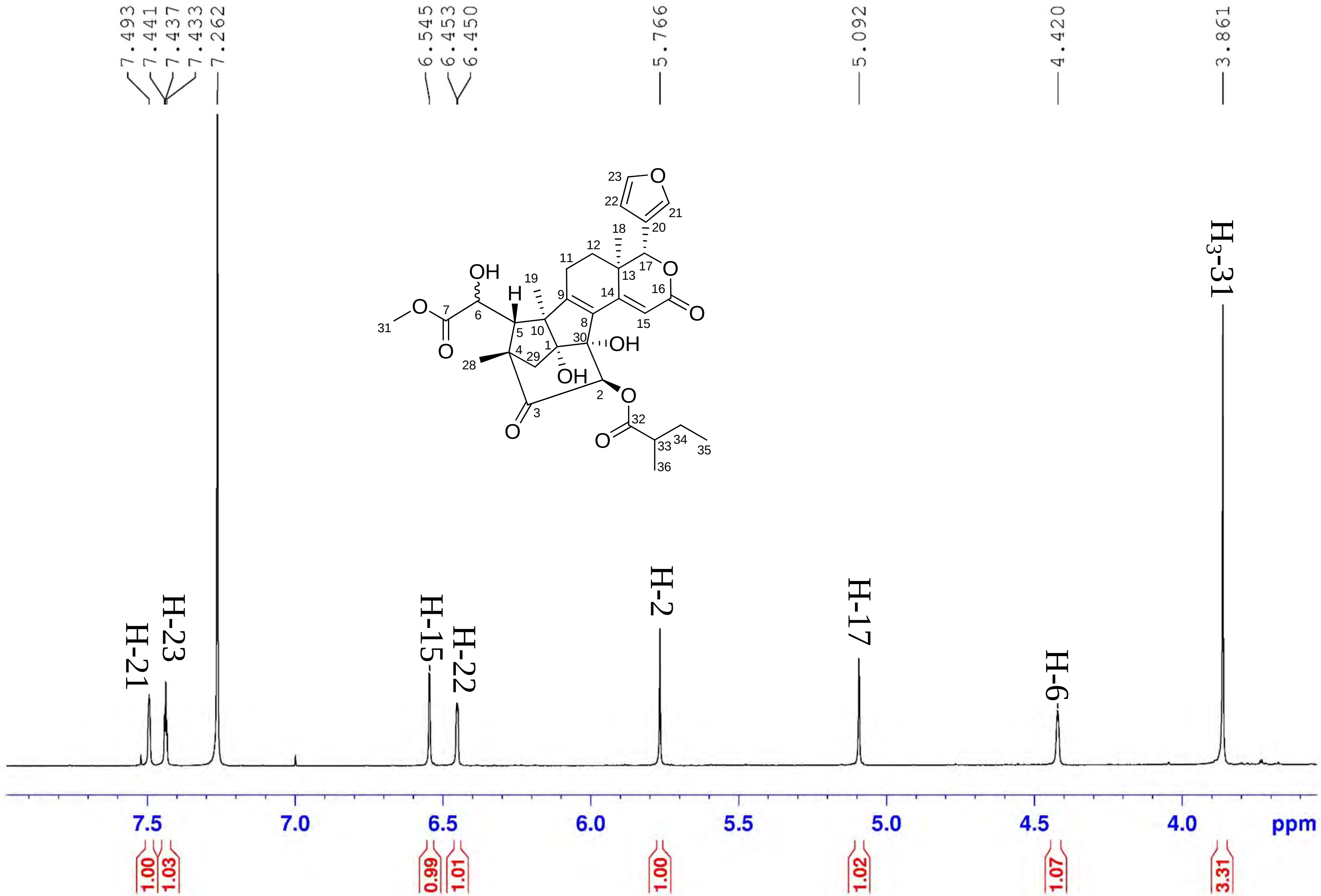

^{1}H NMR (400 MHz) spectrum of compound **21** in $CDCl_3$

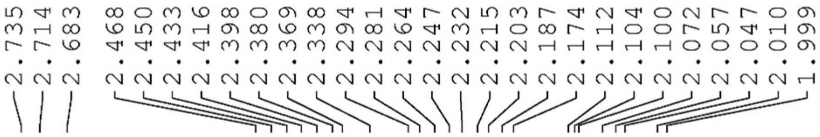

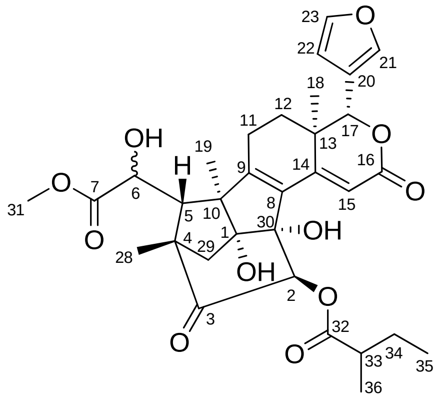

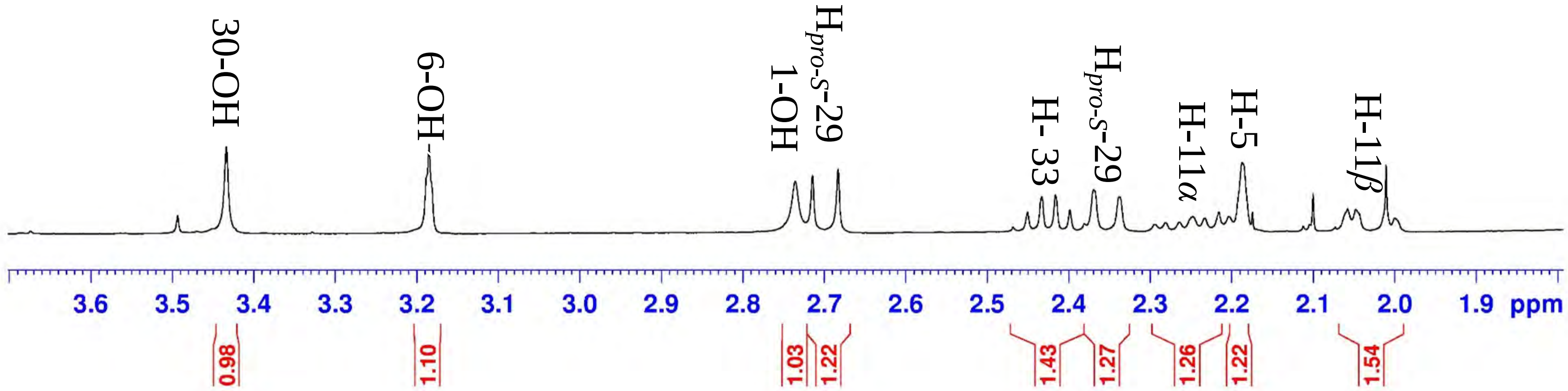

^{1}H NMR (400 MHz) spectrum of compound **21** in $CDCl_3$

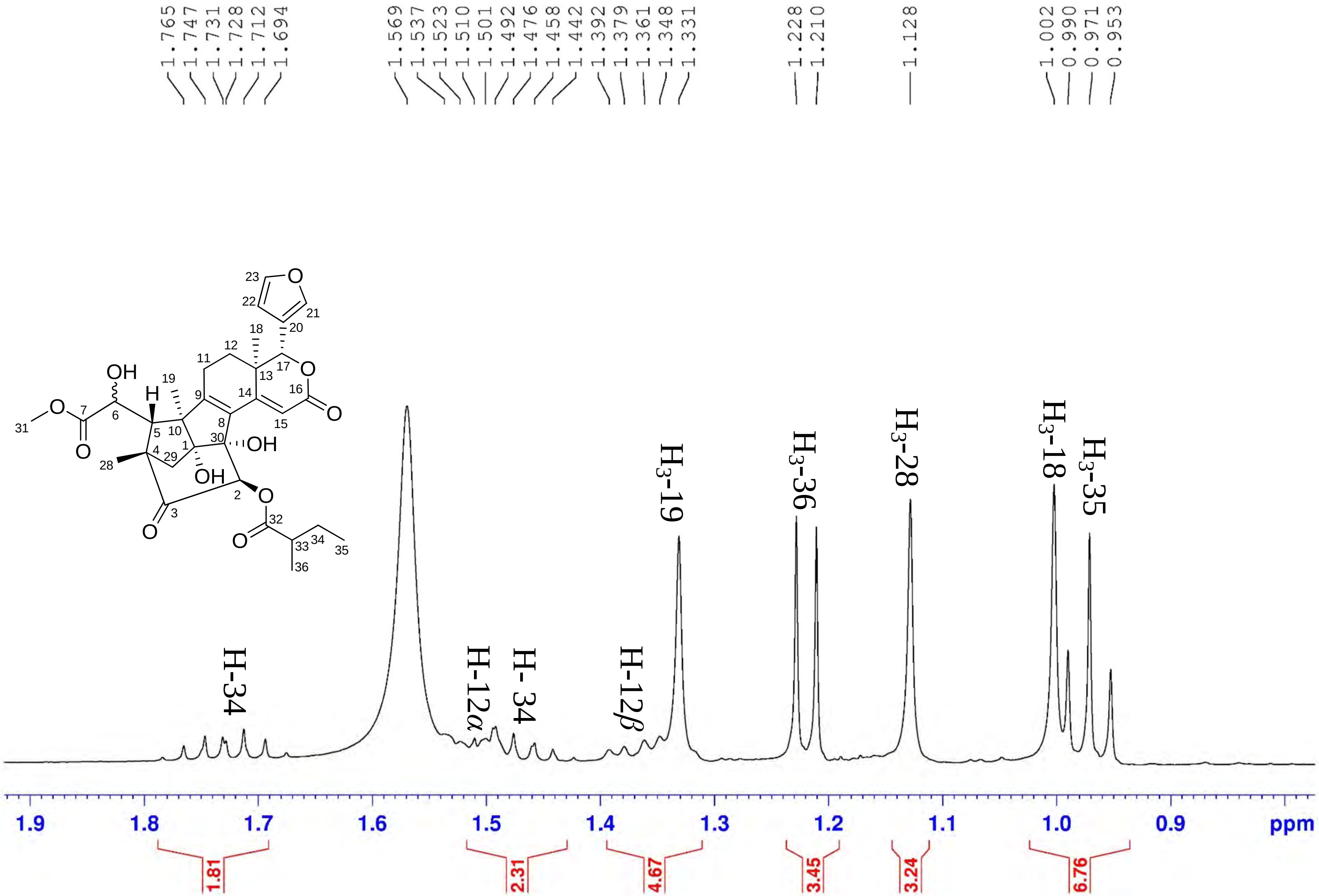

^{13}C NMR (100 MHz) spectrum of compound **21** in $CDCl_3$

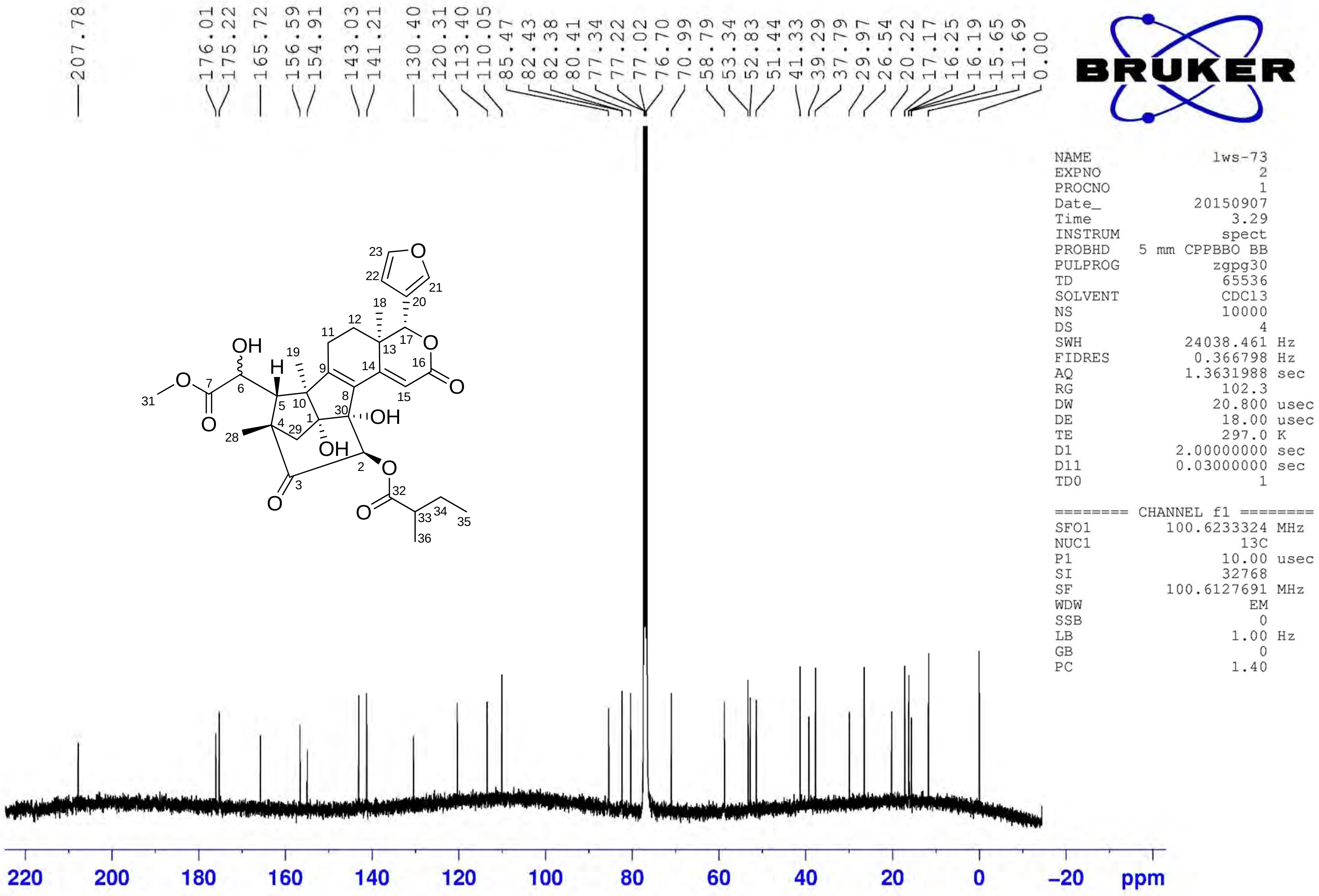

^{13}C NMR (100 MHz) spectrum of compound **21** in $CDCl_3$

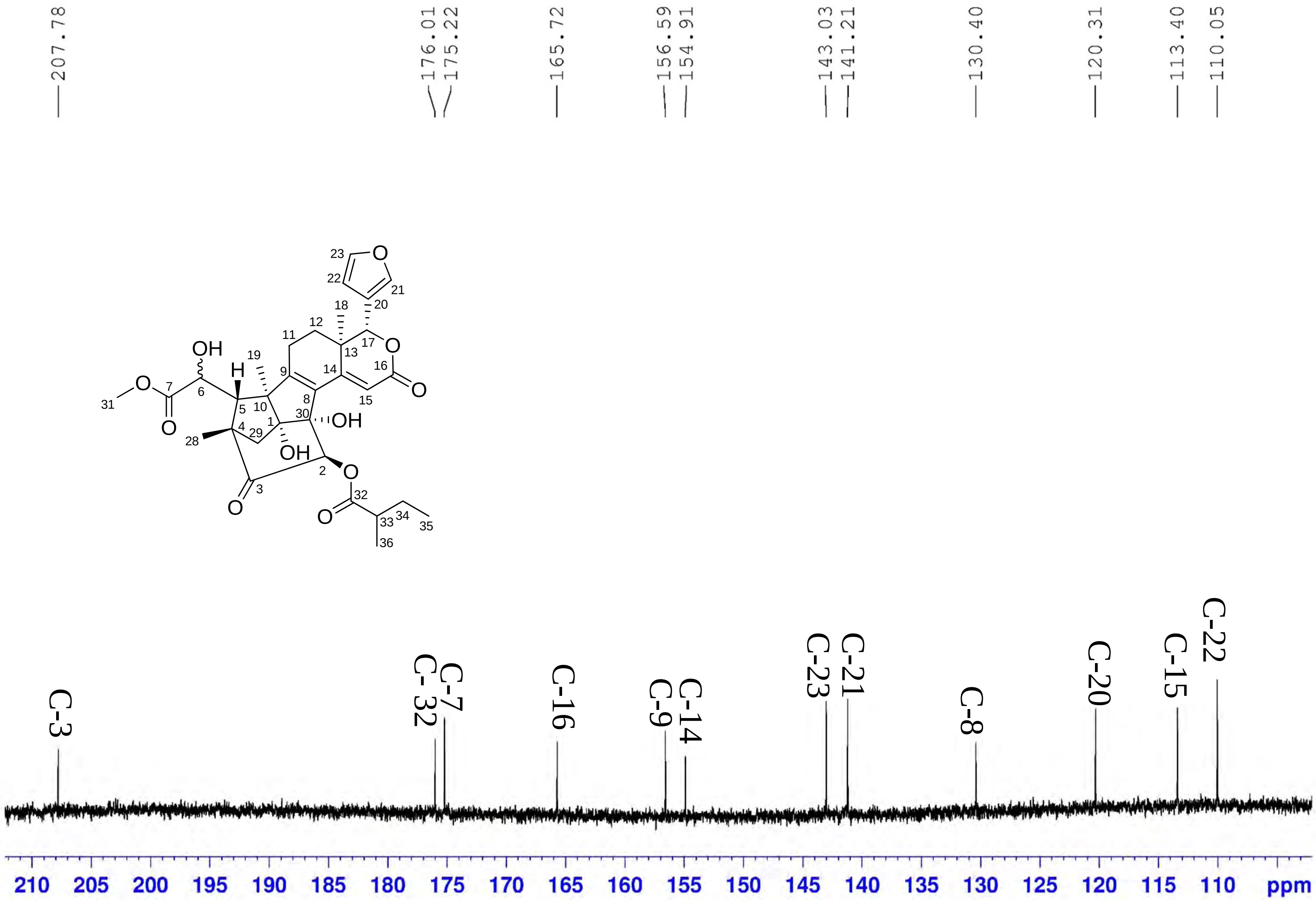

^{13}C NMR (100 MHz) spectrum of compound **21** in $CDCl_3$

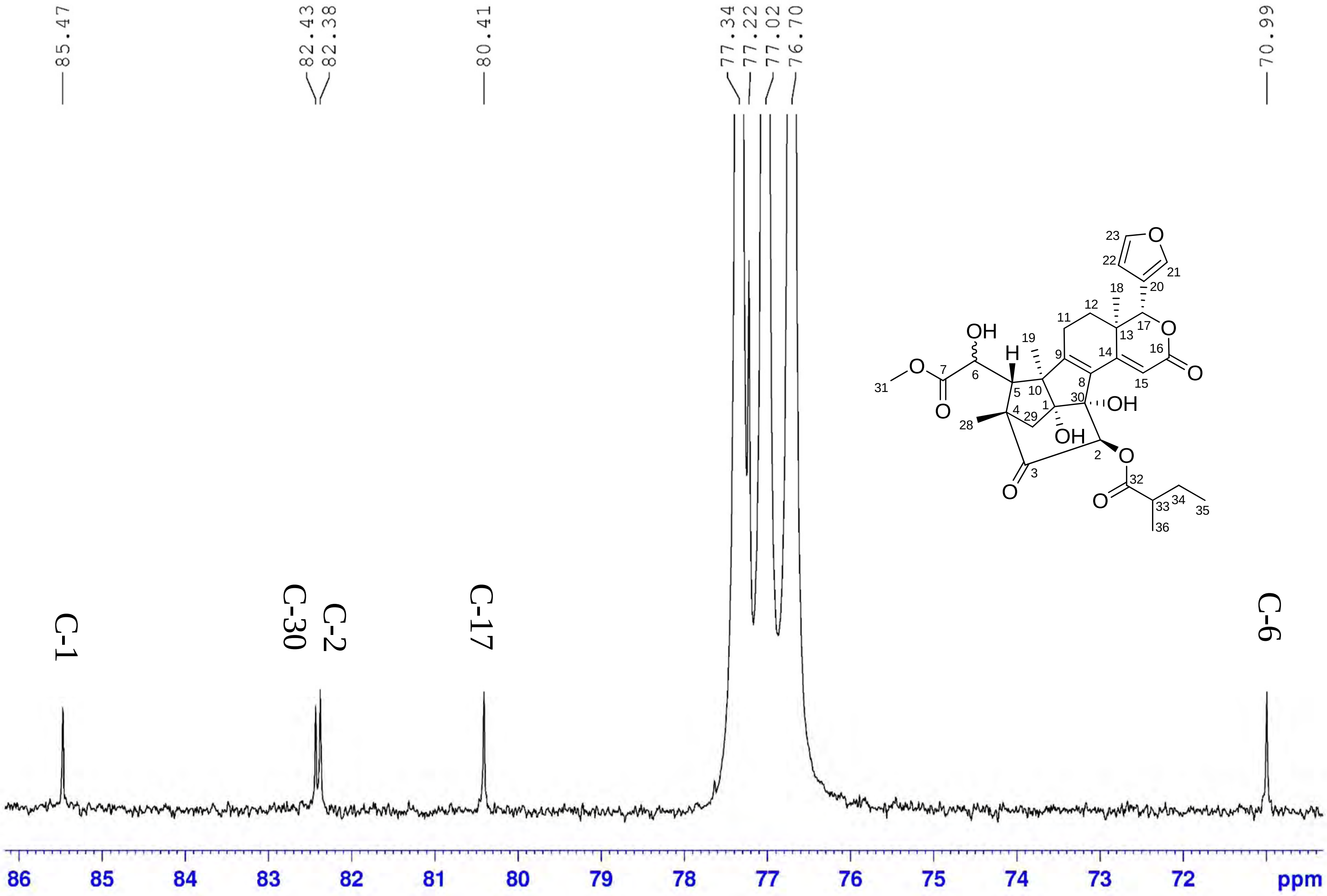

^{13}C NMR (100 MHz) spectrum of compound **21** in $CDCl_3$

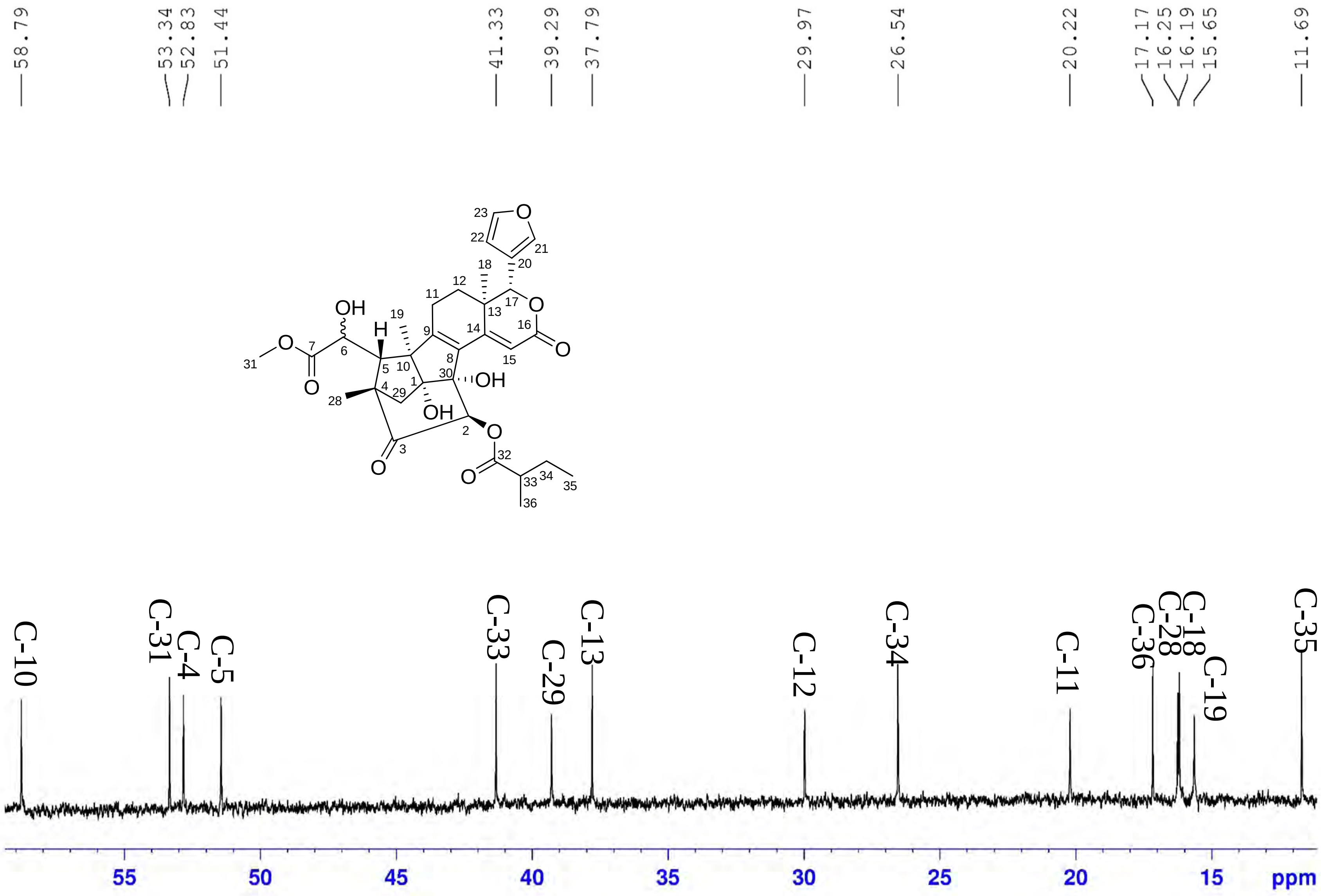

DEPT135 (100 MHz) spectrum of compound **21** in $CDCl_3$

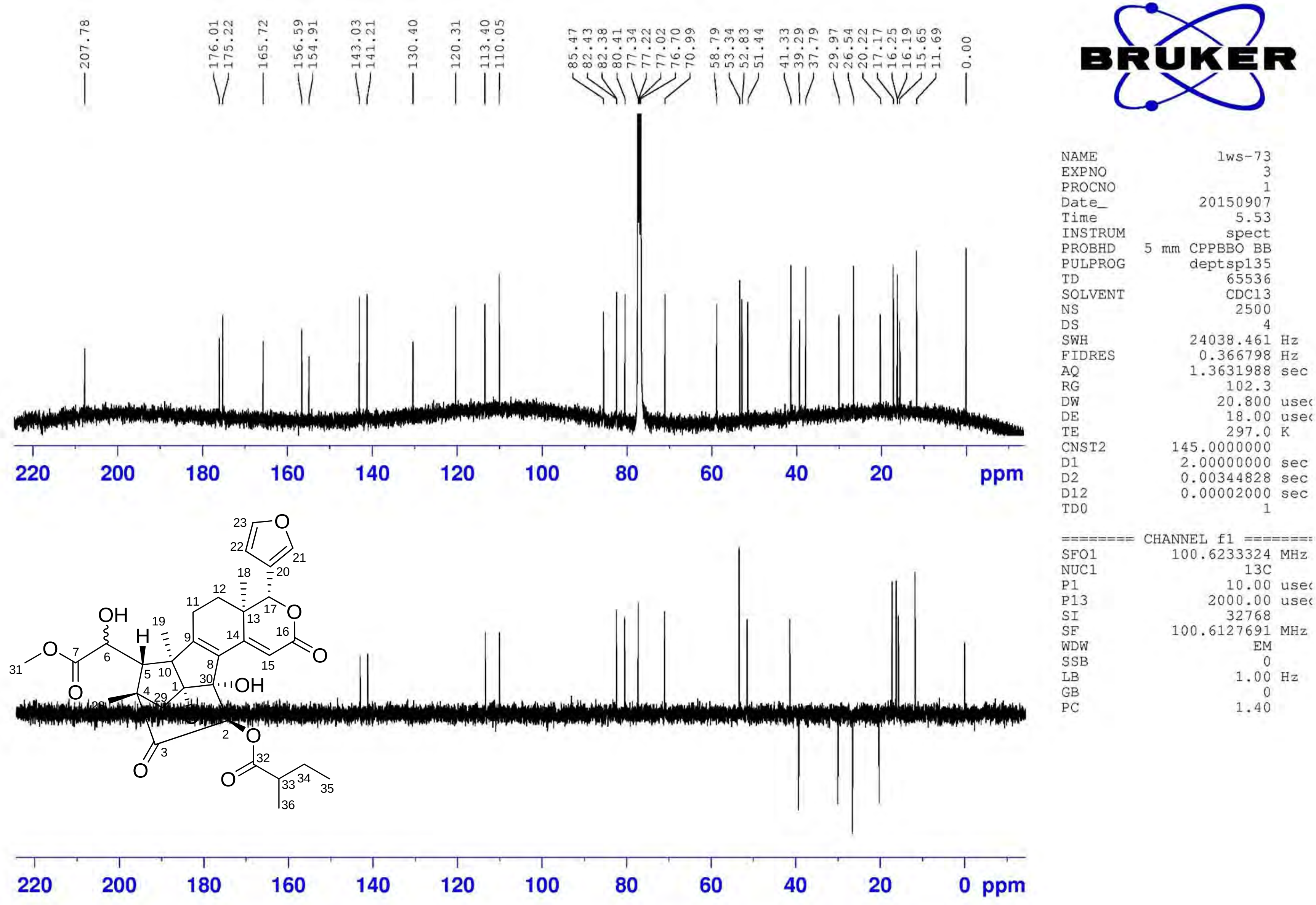

DEPT135 (100 MHz) spectrum of compound **21** in $CDCl_3$

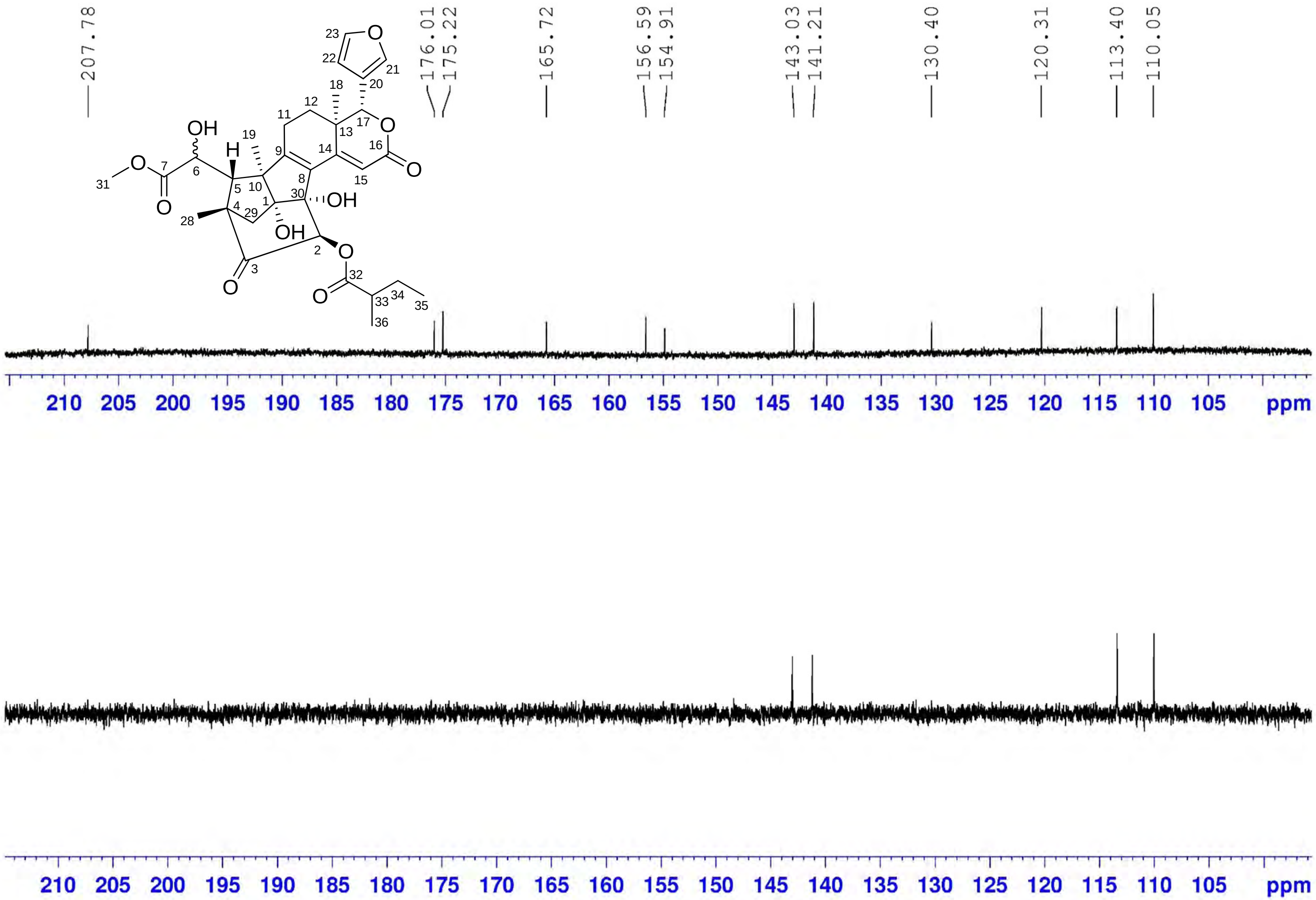

DEPT135 (100 MHz) spectrum of compound **21** in $CDCl_3$

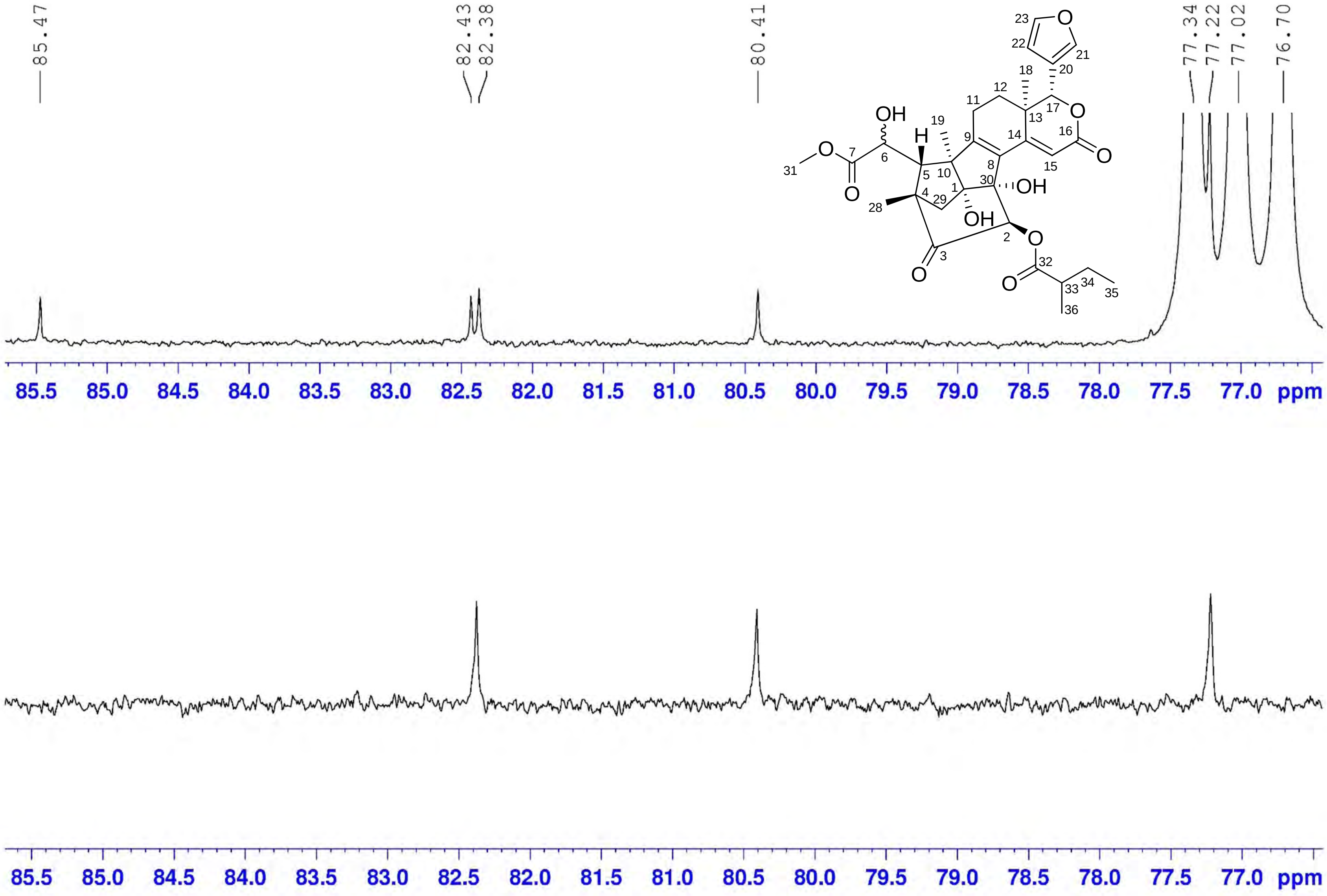

DEPT135 (100 MHz) spectrum of compound **21** in $CDCl_3$

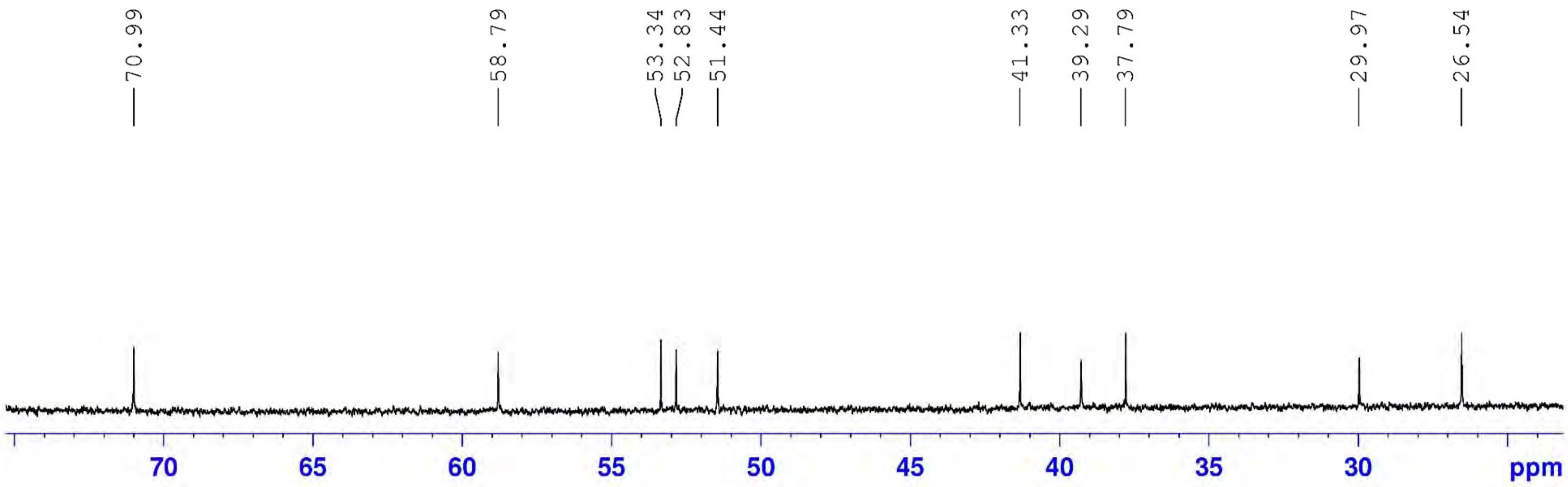

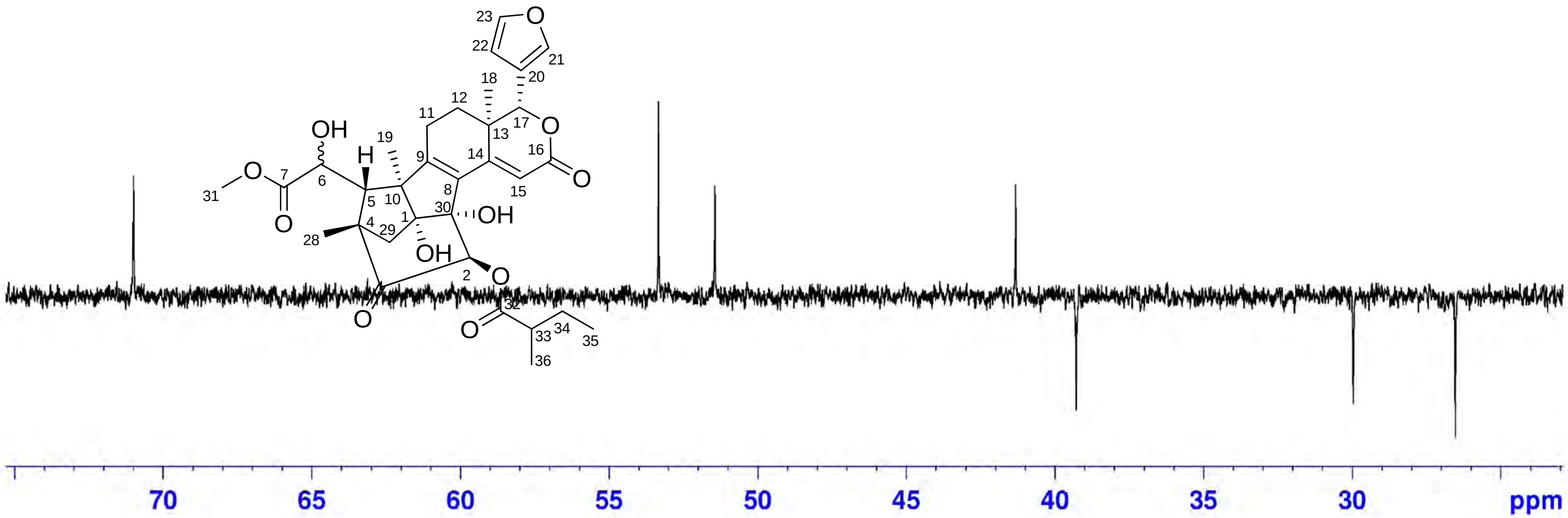

DEPT135 (100 MHz) spectrum of compound **21** in $CDCl_3$

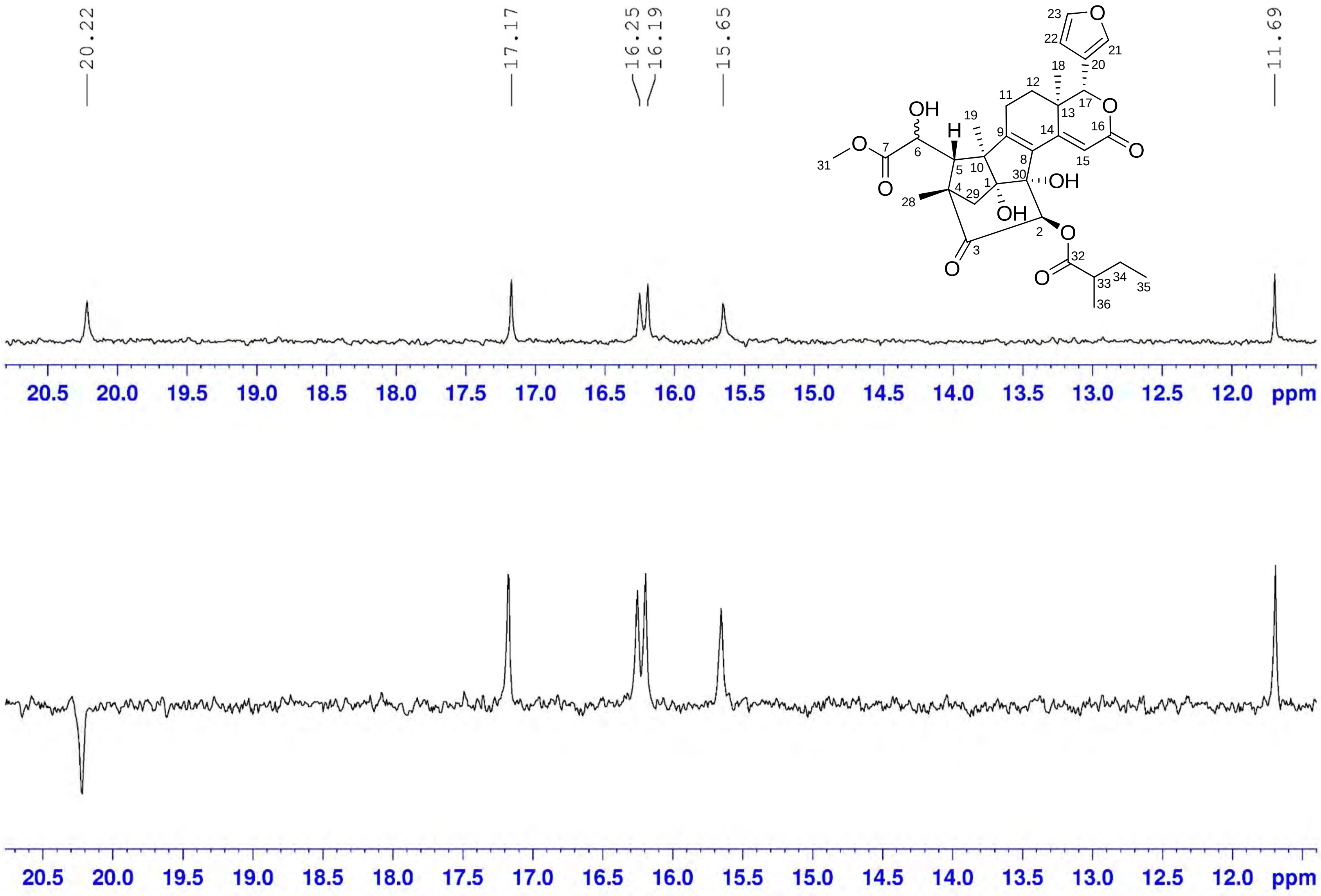

^{1}H-^{1}H COSY (400 MHz) spectrum of compound **21** in $CDCl_3$

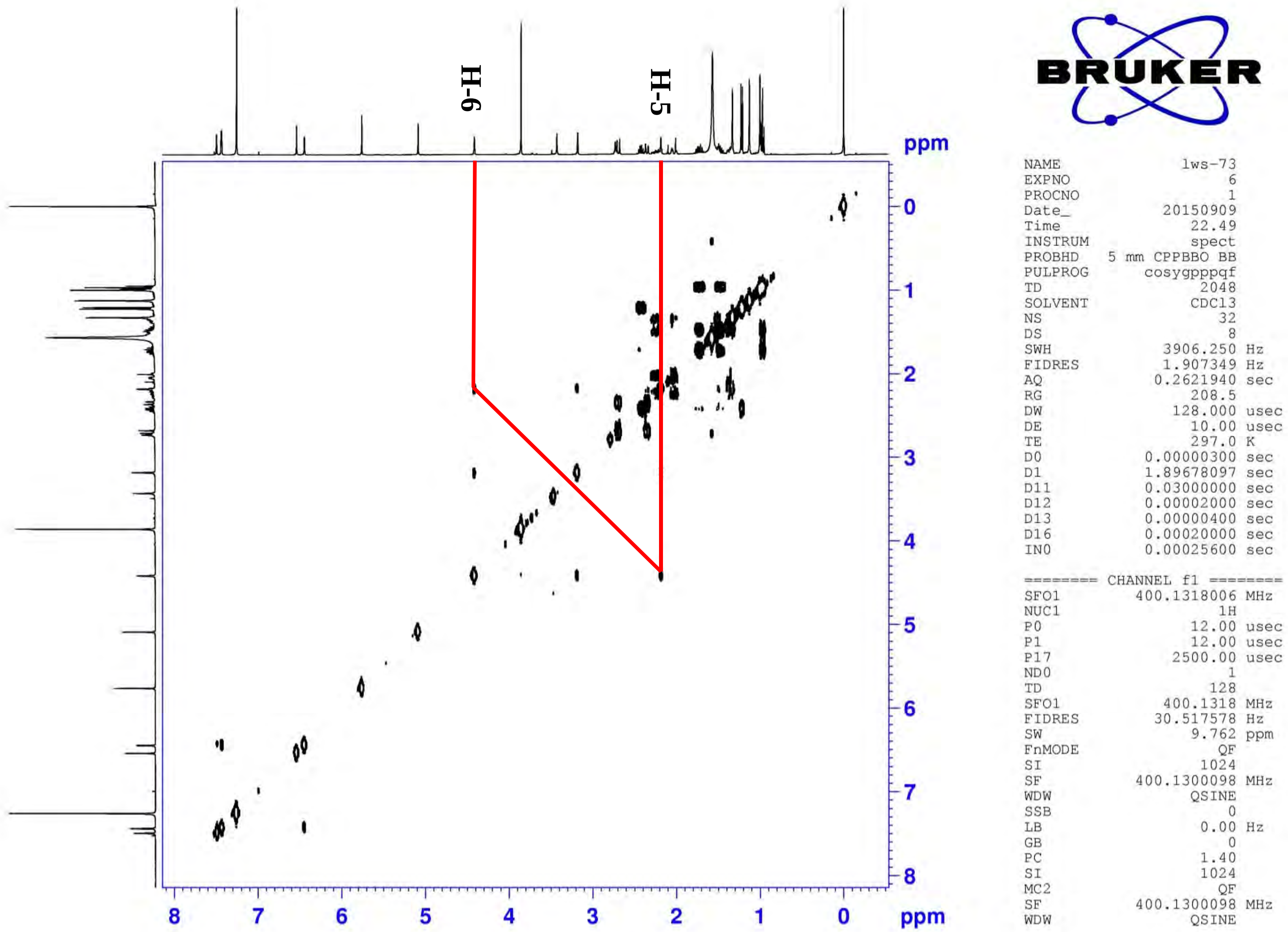

^{1}H-^{1}H COSY (400 MHz) spectrum of compound **21** in $CDCl_3$

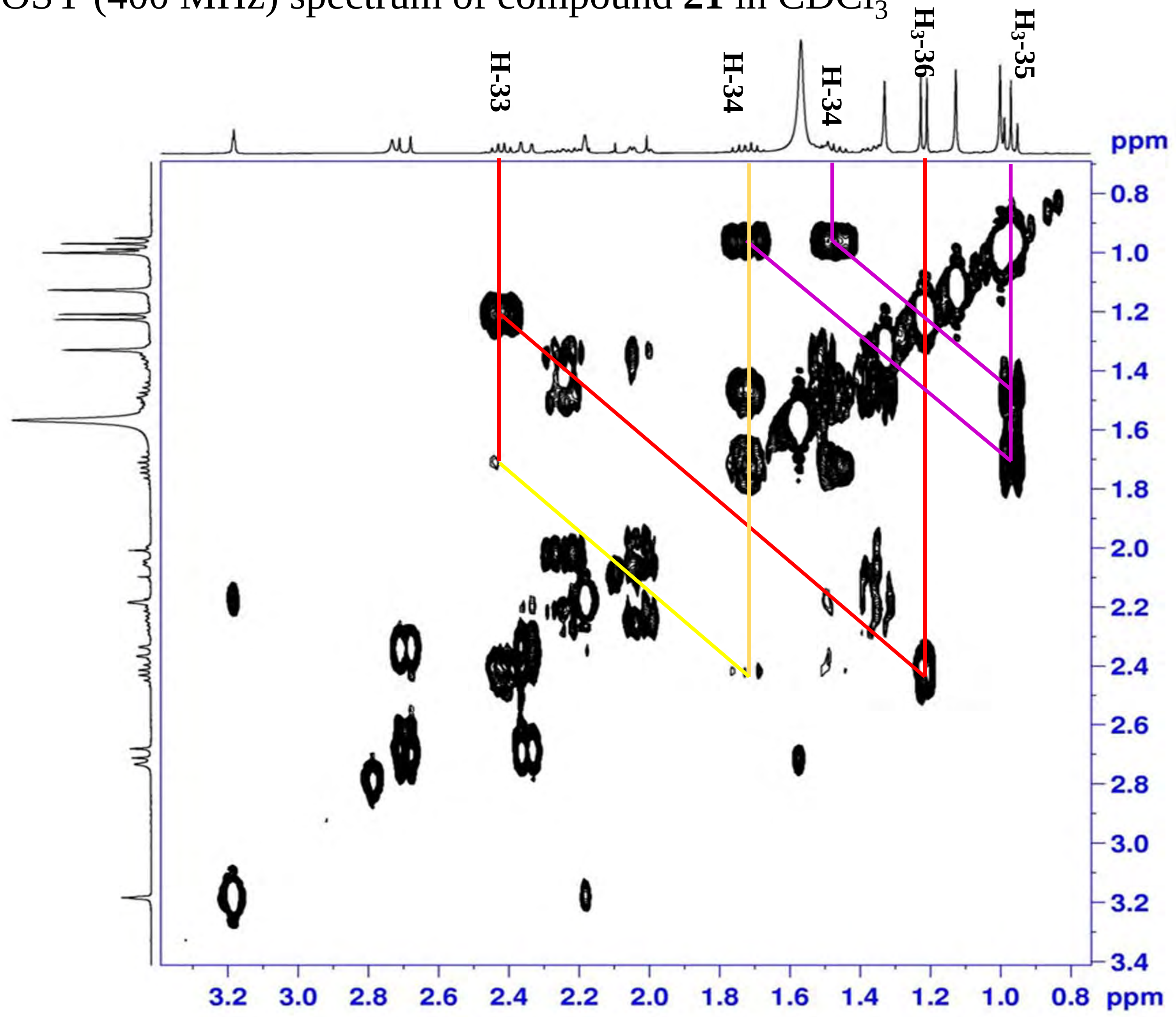

HSQC (400 MHz) spectrum of compound **21** in $CDCl_3$

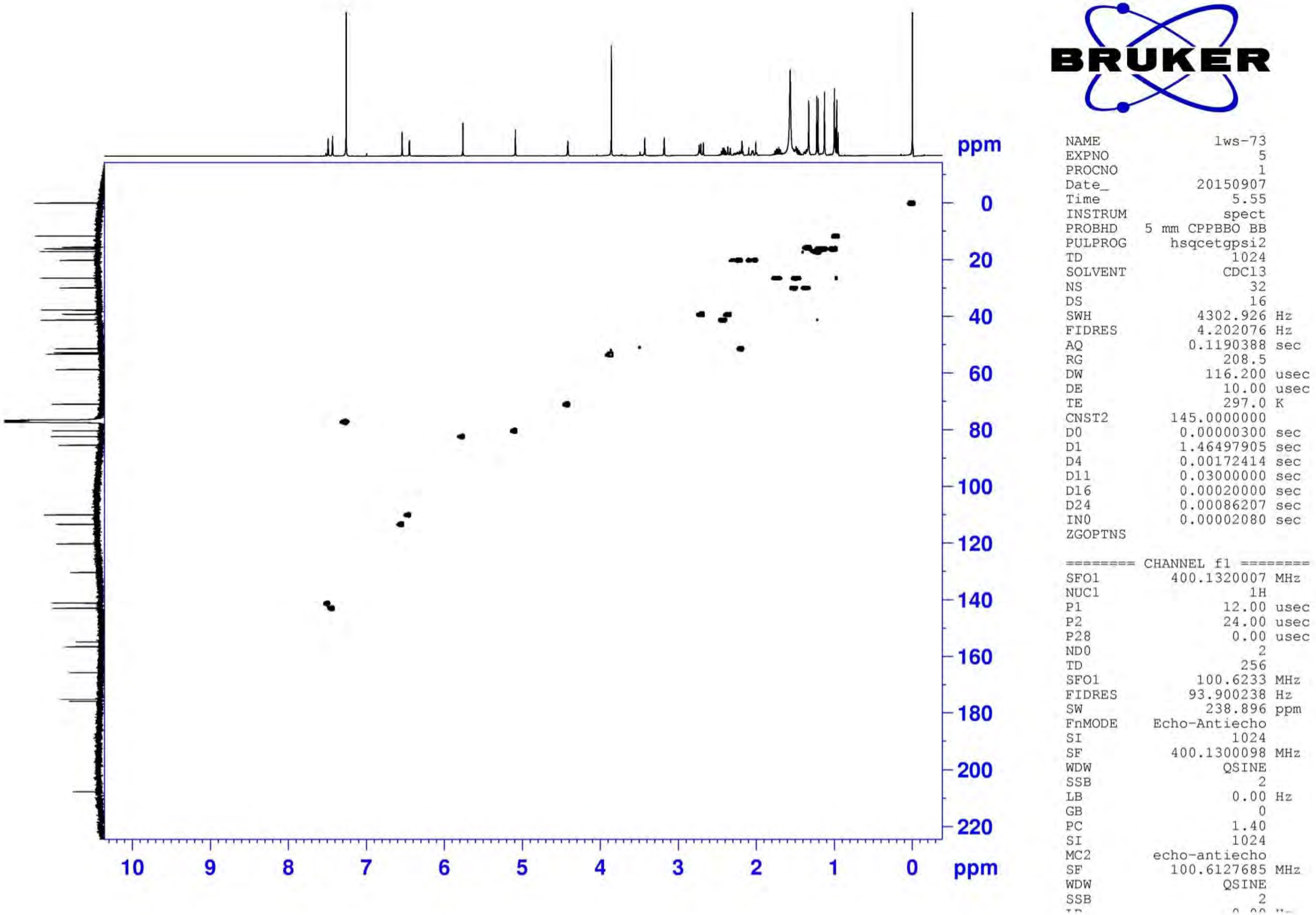

HSQC (400 MHz) spectrum of compound **21** in $CDCl_3$

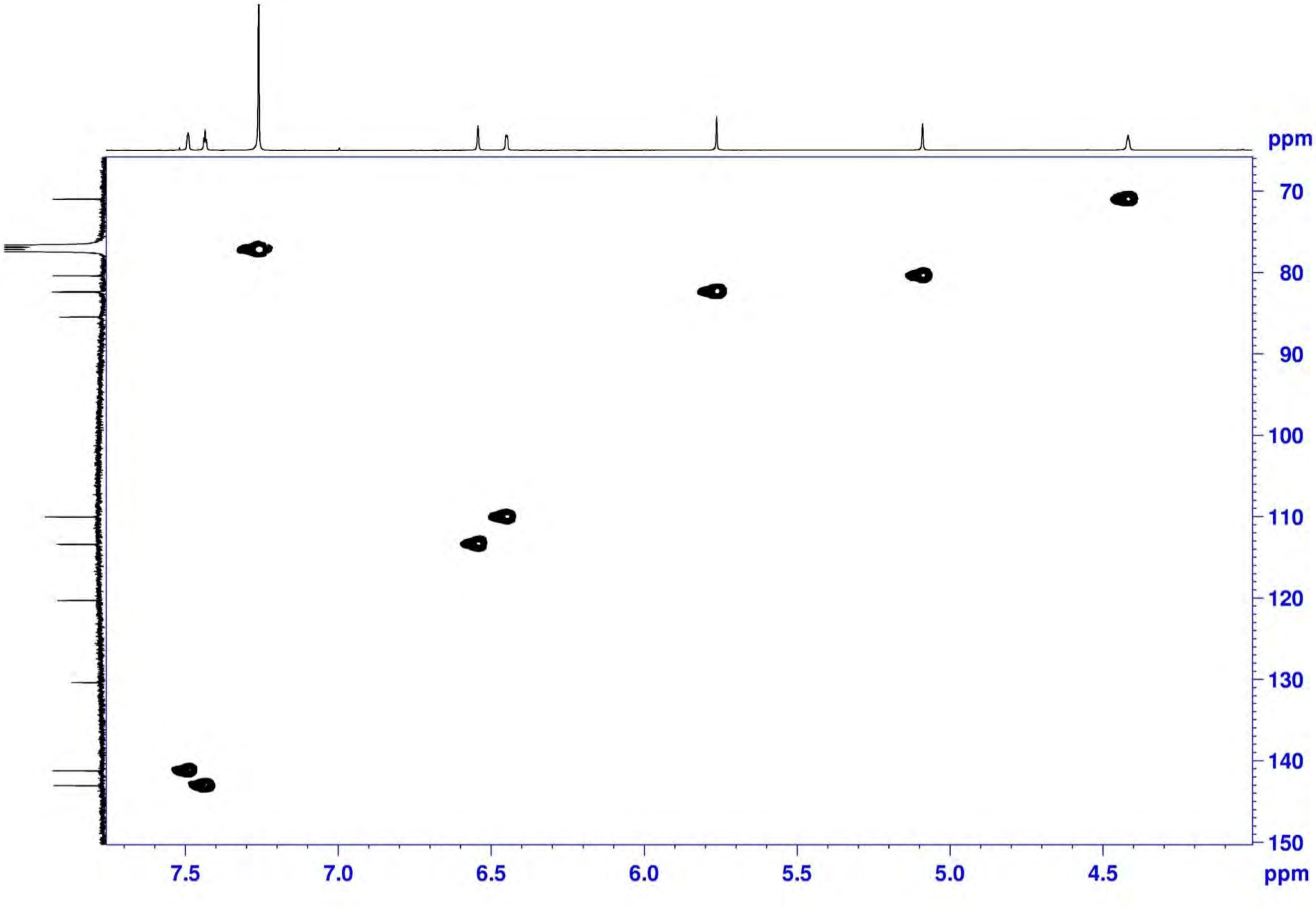

HSQC (400 MHz) spectrum of compound **21** in $CDCl_3$

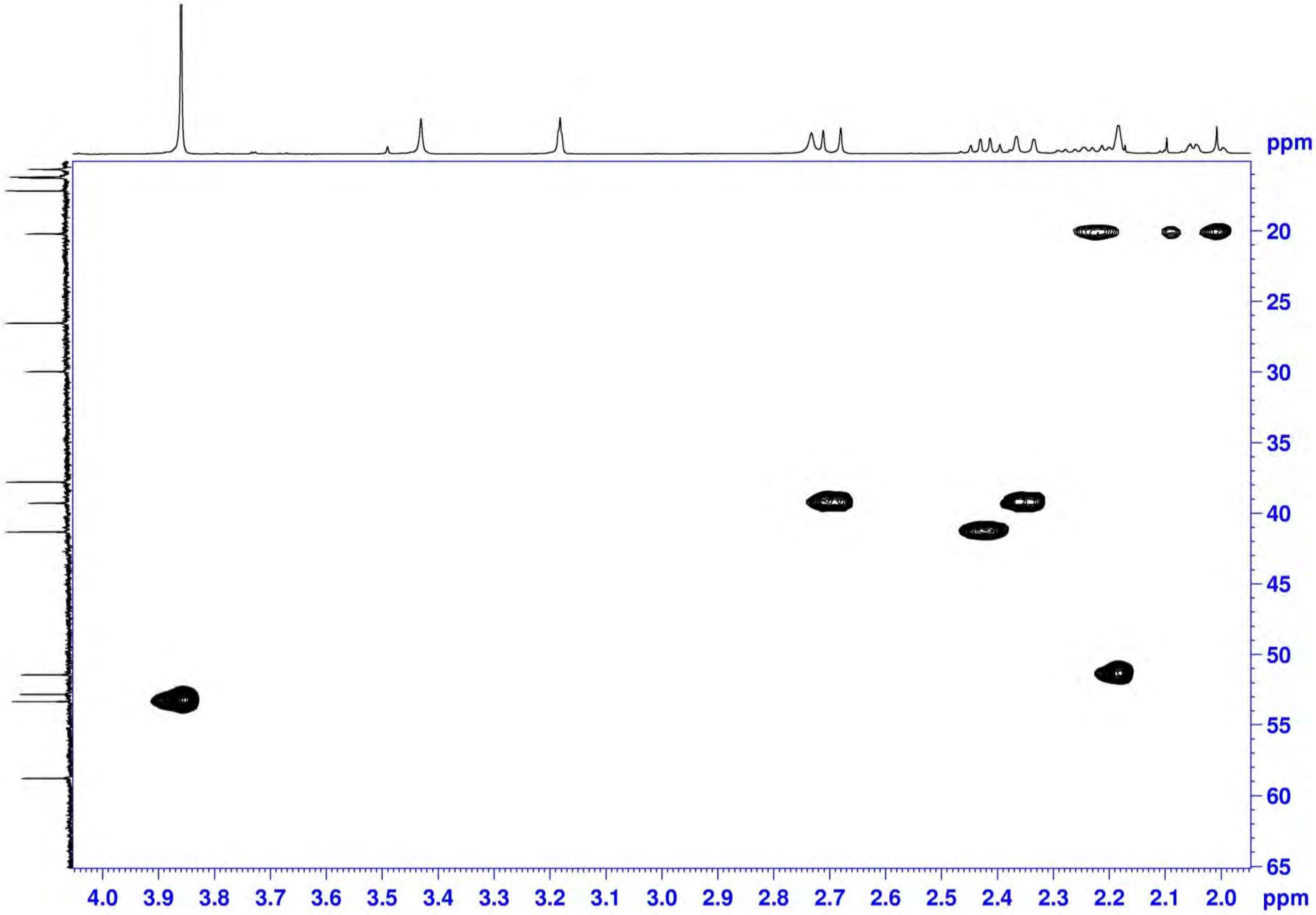

HSQC (400 MHz) spectrum of compound **21** in $CDCl_3$

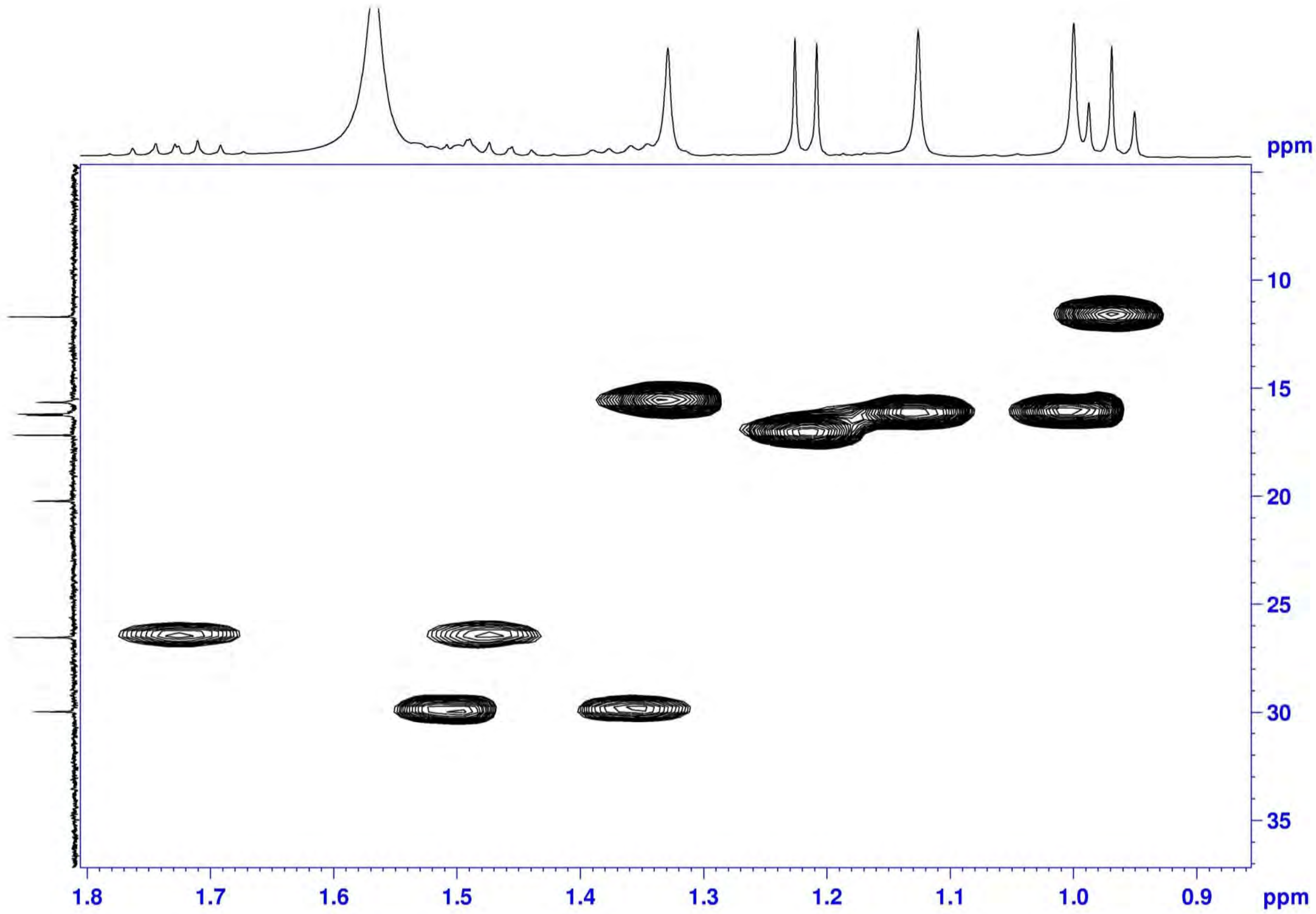

HMBC (400 MHz) spectrum of compound **21** in $CDCl_3$

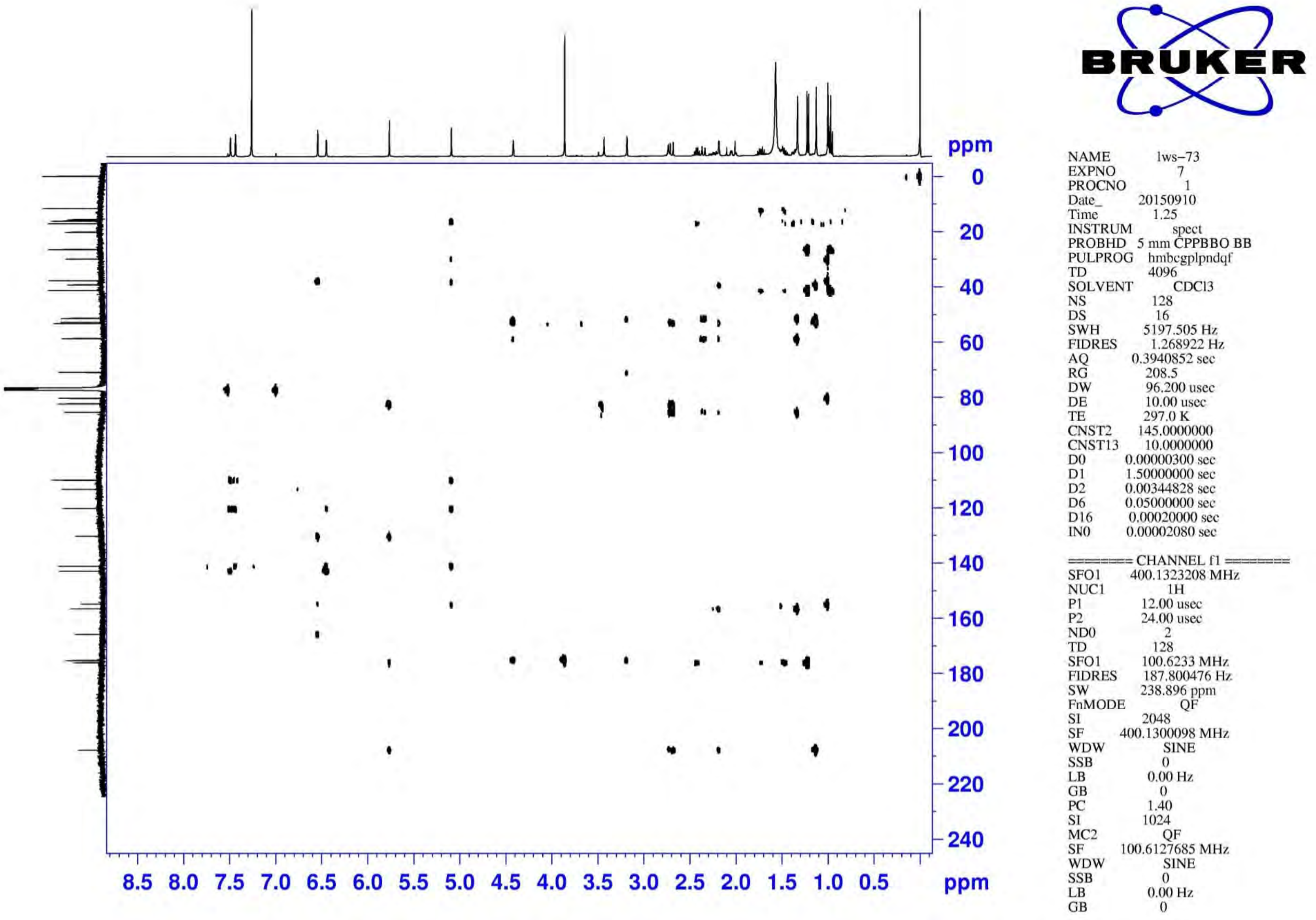

HMBC (400 MHz) spectrum of compound **21** in $CDCl_3$

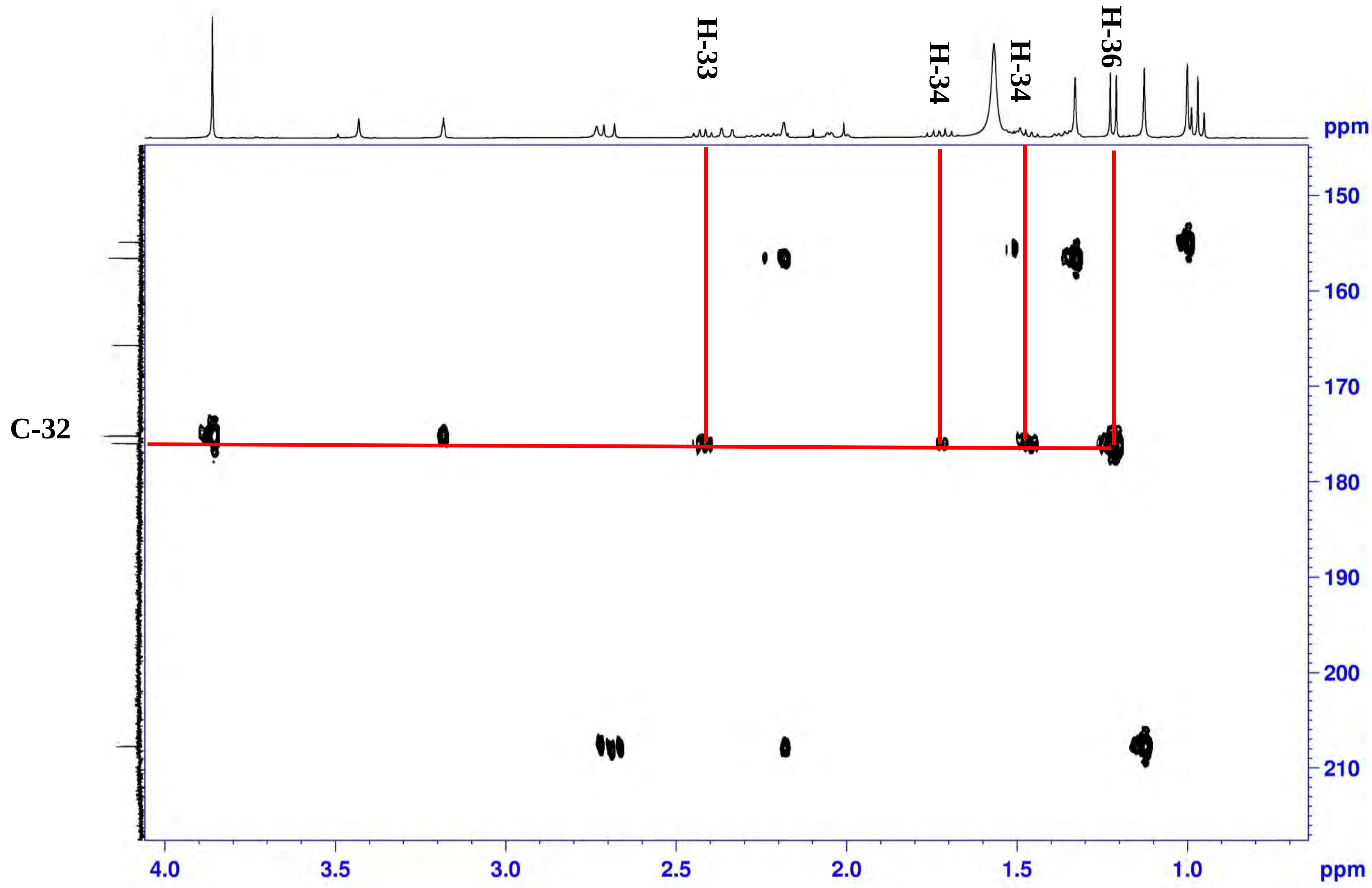

HMBC (400 MHz) spectrum of compound **21** in $CDCl_3$

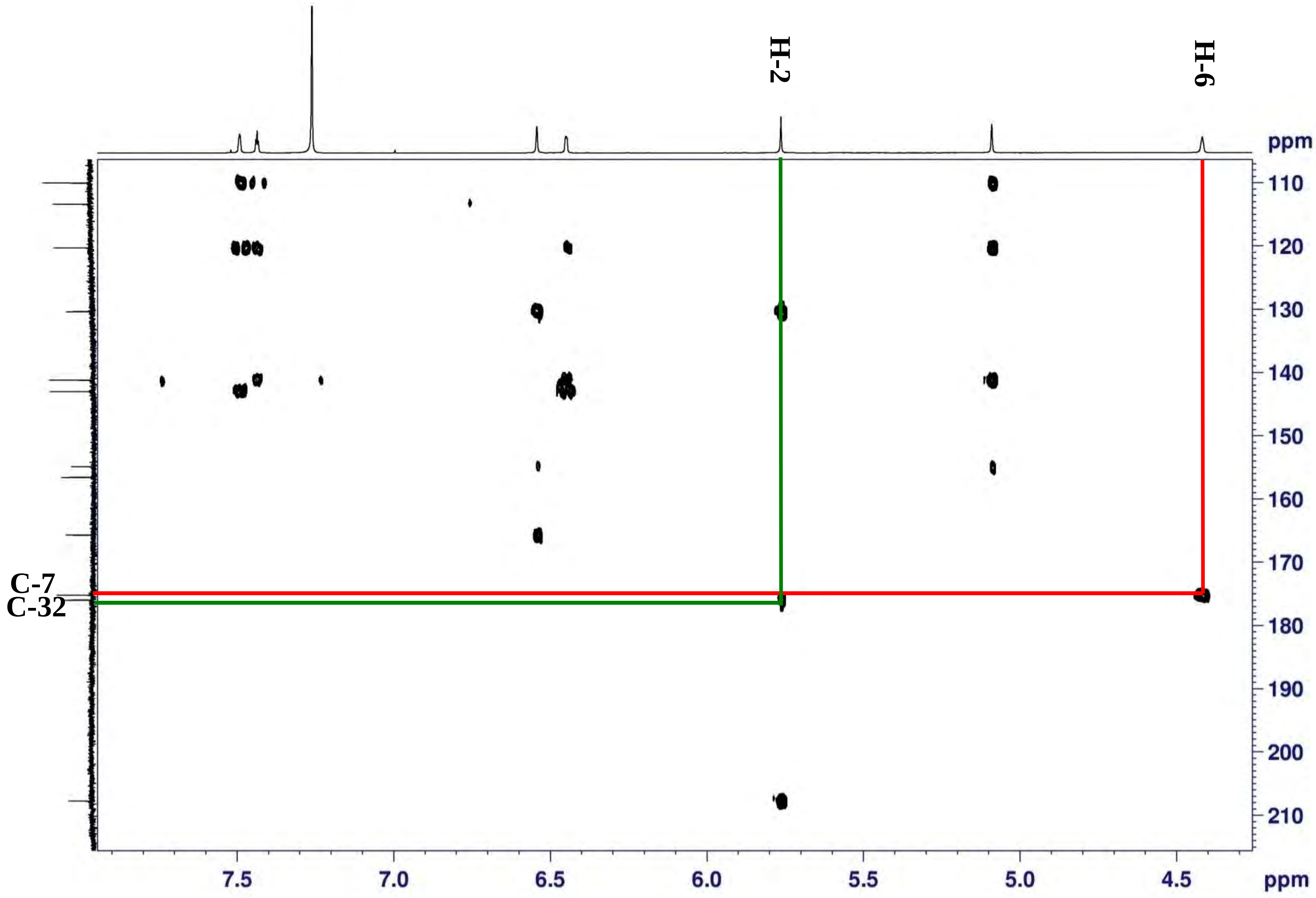

HMBC (400 MHz) spectrum of compound **21** in $CDCl_3$

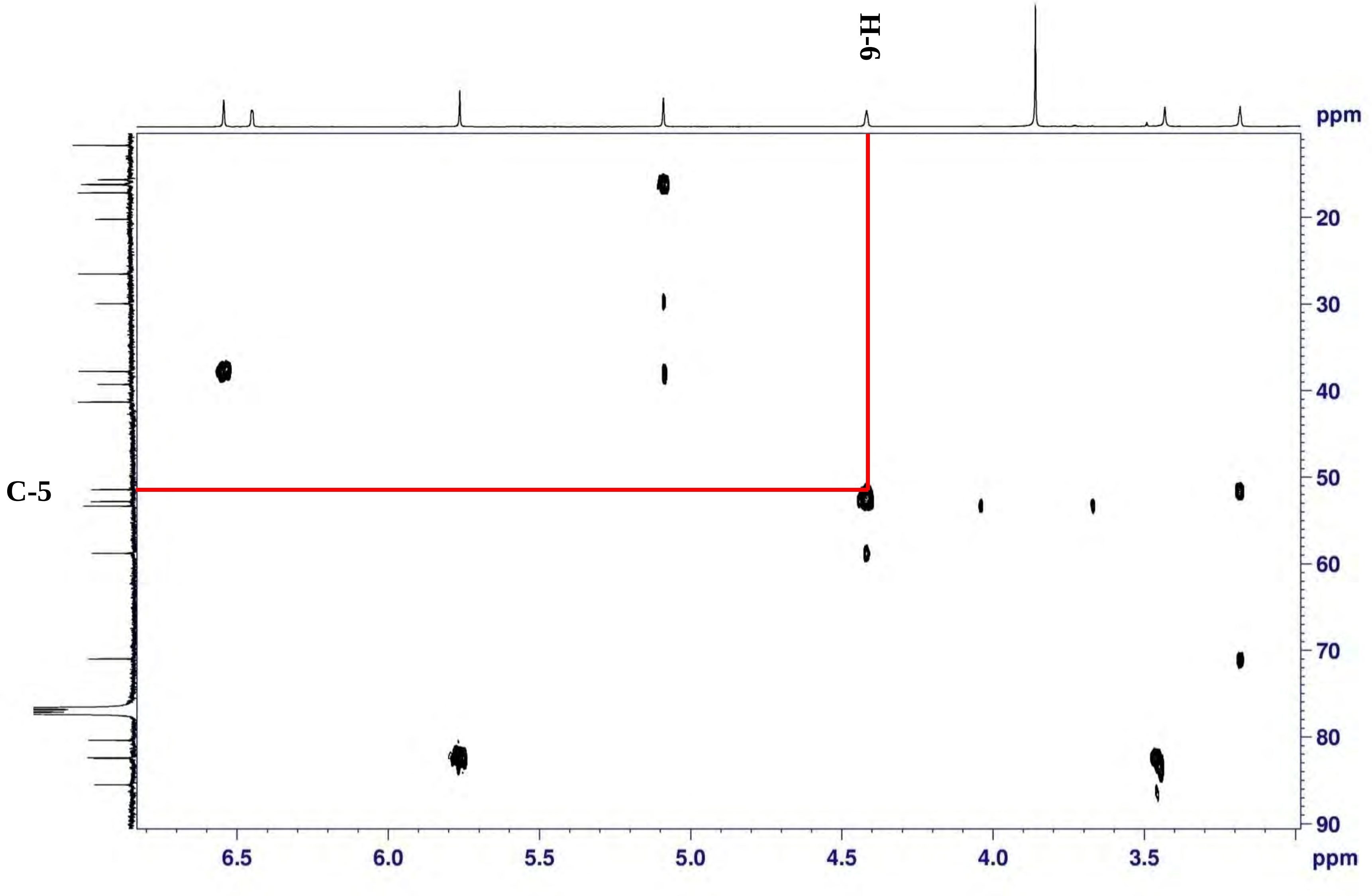

HMBC (400 MHz) spectrum of compound **21** in $CDCl_3$

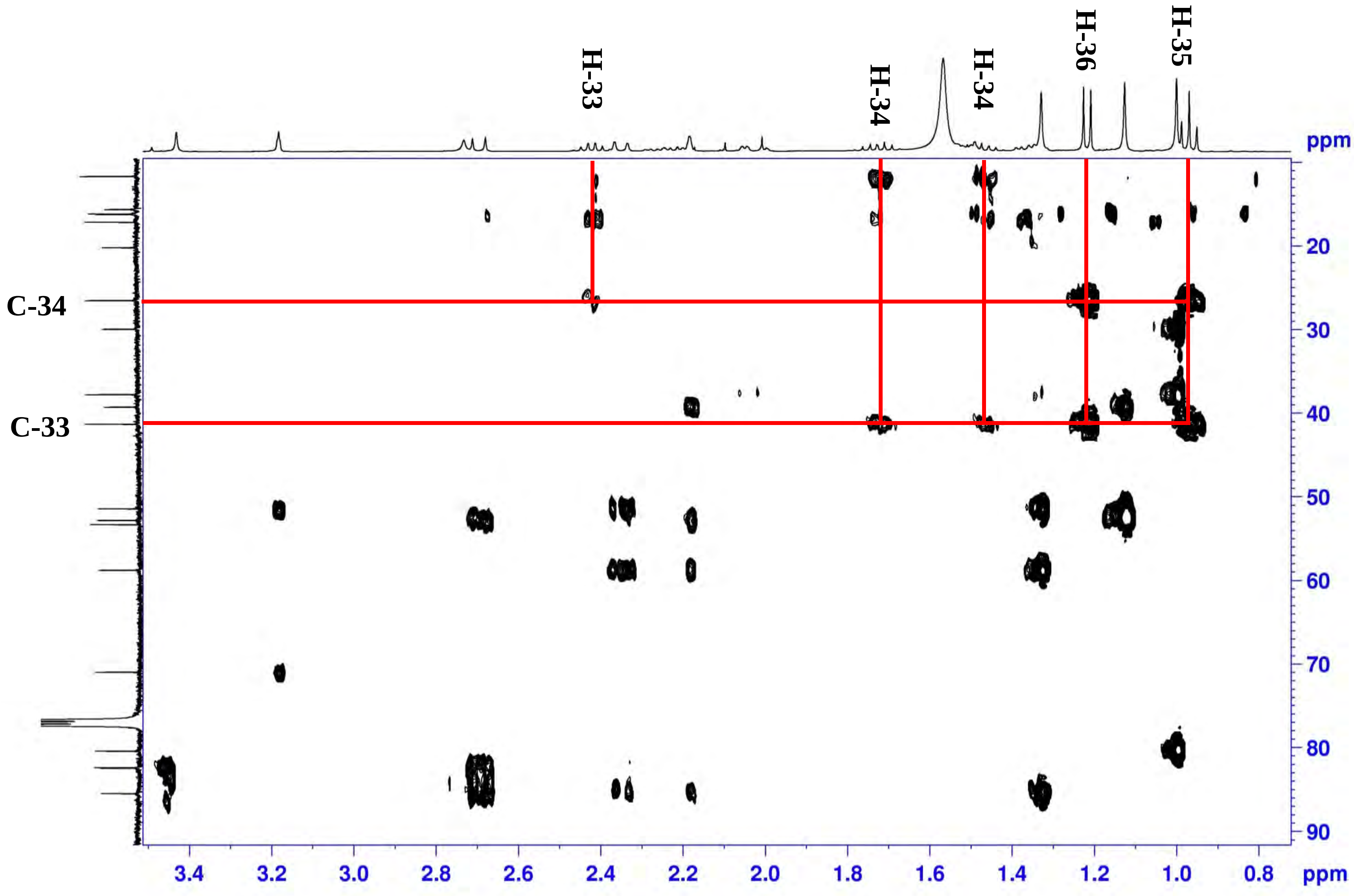

NOESY (400 MHz) spectrum of compound **21** in $CDCl_3$

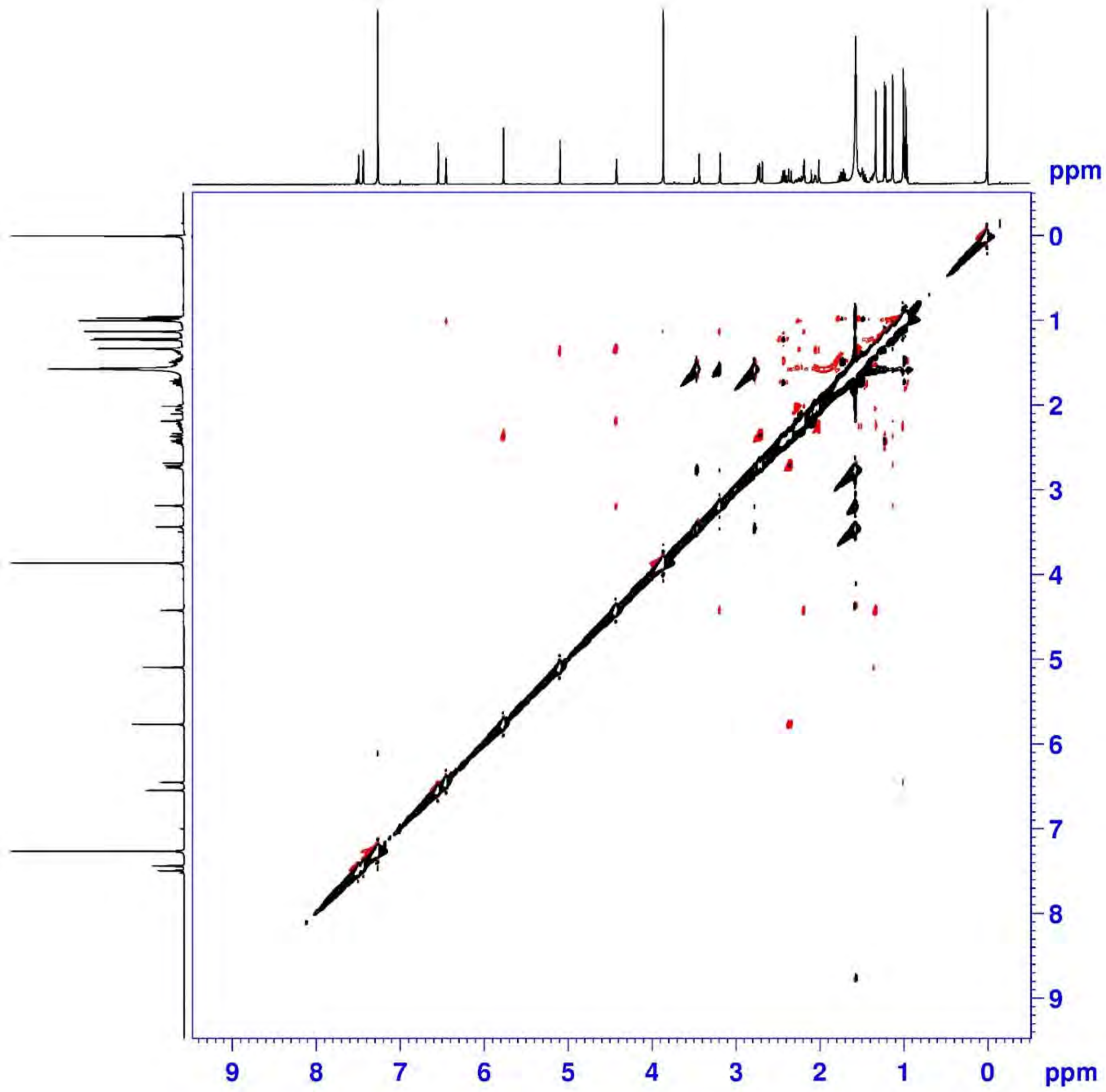

NAME	lws-73	
EXPNO	8	
PROCNO	1	
Date_	20150910	
Time	10.18	
INSTRUM	spect	
PROBHD	5 mm CPPBBO BB	
PULPROG	noesygpphpp	
TD	2048	
SOLVENT	CDCl3	
NS	32	
DS	32	
SWH	4000.000	Hz
FIDRES	1.953125	Hz
AQ	0.2560500	sec
RG	208.5	
DW	125.000	usec
DE	10.00	usec
TE	297.0	K
D0	0.00010972	sec
D1	1.99385595	sec
D8	0.30000001	sec
D11	0.03000000	sec
D12	0.00002000	sec
D16	0.00020000	sec
IN0	0.00025000	sec
======== CHANNEL f1 ========		
SFO1	400.1318006	MHz
NUC1	1H	
P1	12.00	usec
P2	24.00	usec
P17	2500.00	usec
ND0	1	
TD	256	
SFO1	400.1318	MHz
FIDRES	15.625000	Hz
SW	9.997	ppm
FnMODE	States-TPPI	
SI	1024	
SF	400.1300098	MHz
WDW	QSINE	
SSB	2	
LB	0.00	Hz
GB	0	
PC	1.00	
SI	1024	
MC2	States-TPPI	
SF	400.1300098	MHz
WDW	QSINE	
SSB	2	
LB	0.00	Hz
GB	0	

NOESY (400 MHz) spectrum of compound **21** in $CDCl_3$

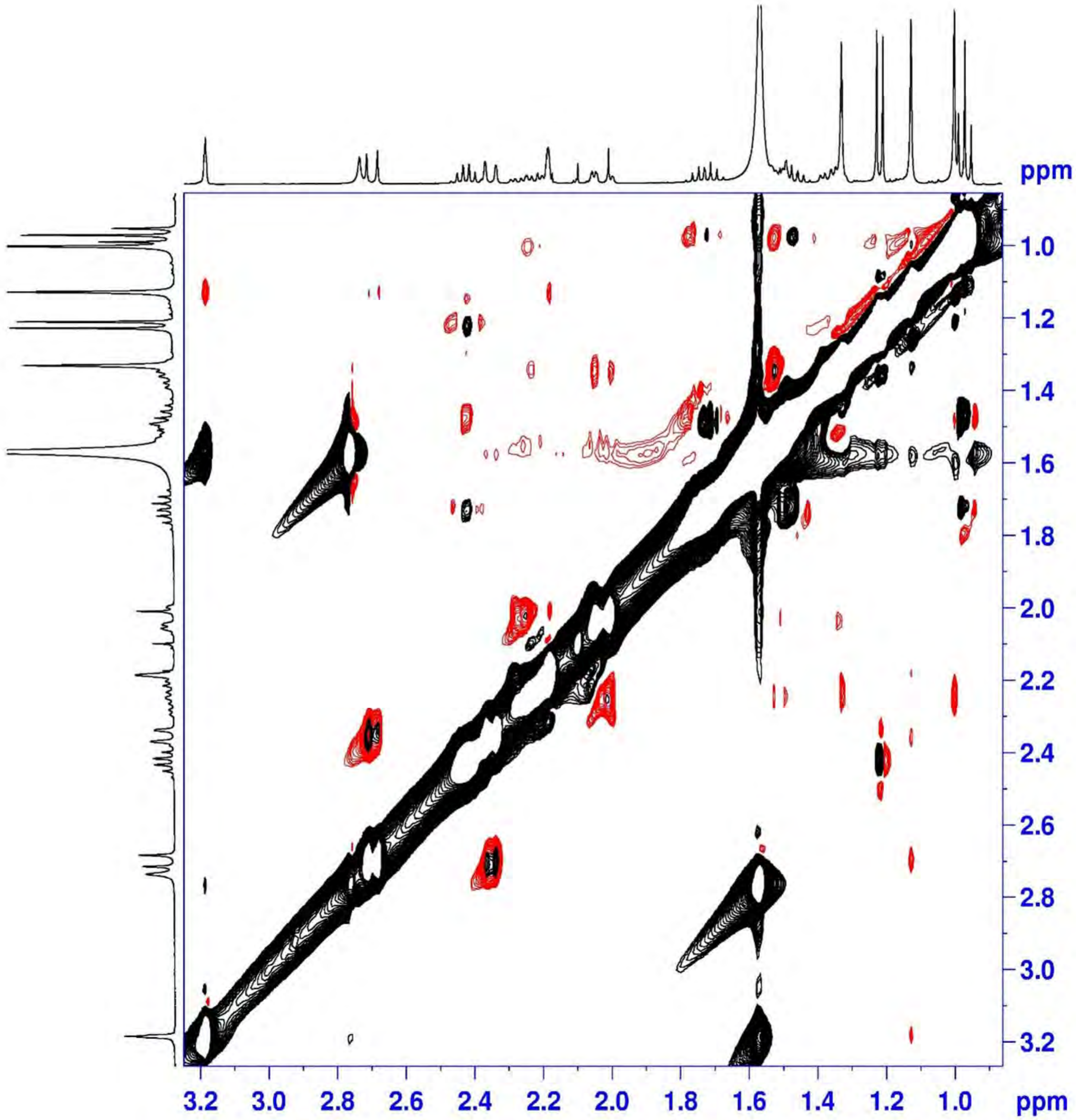

NOESY (400 MHz) spectrum of compound **21** in $CDCl_3$

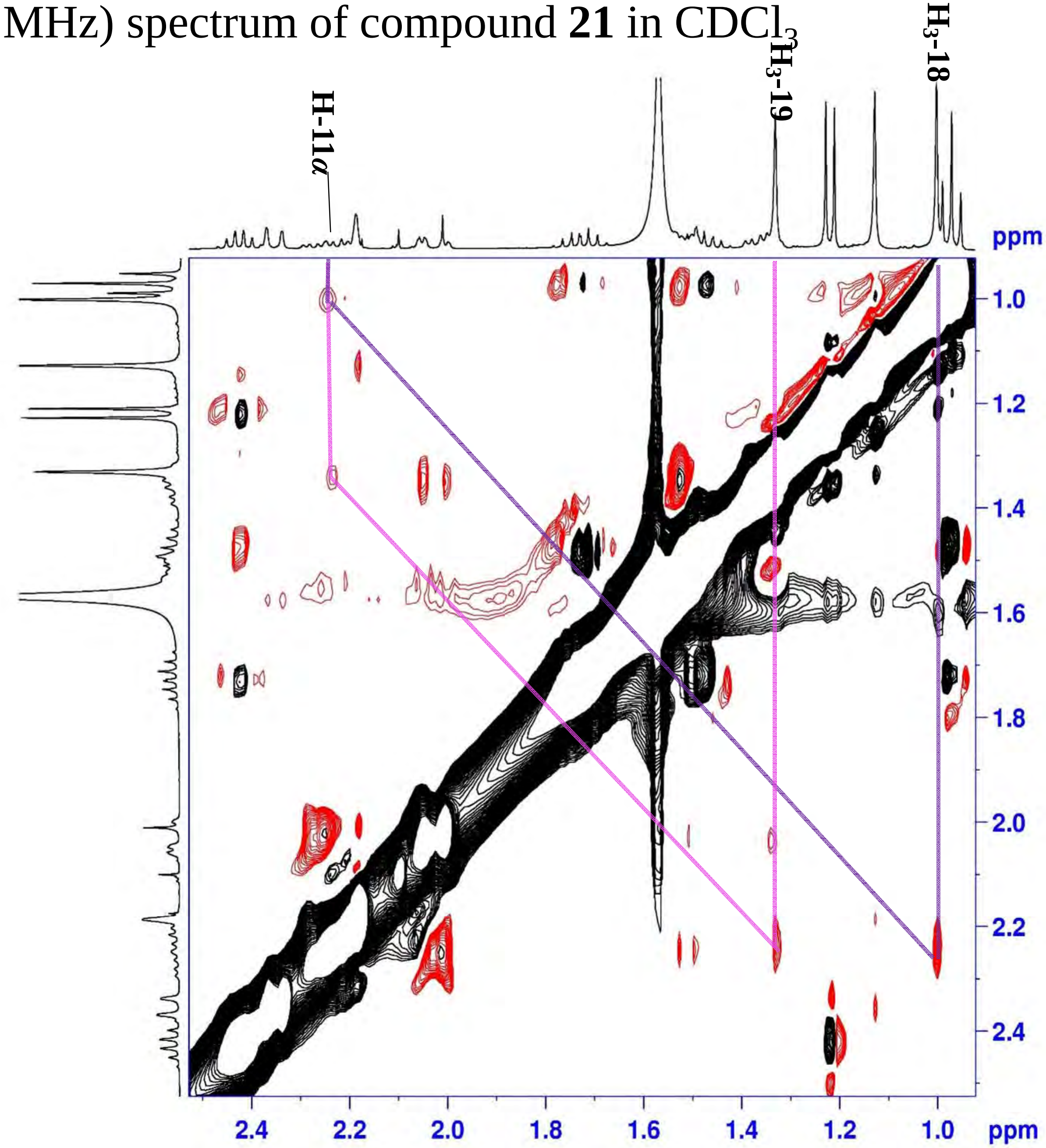

NOESY (400 MHz) spectrum of compound **21** in $CDCl_3$

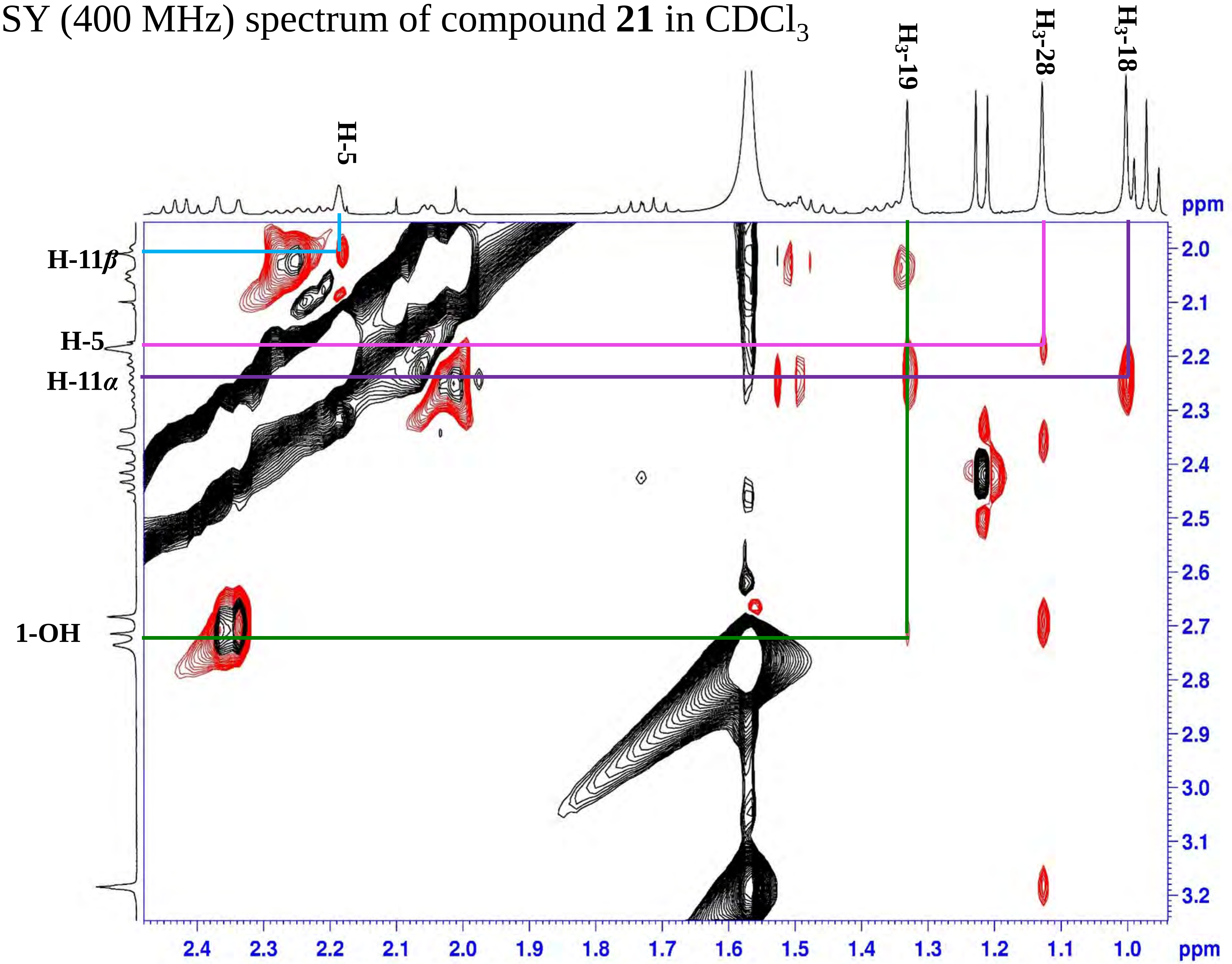

NOESY (400 MHz) spectrum of compound **21** in $CDCl_3$

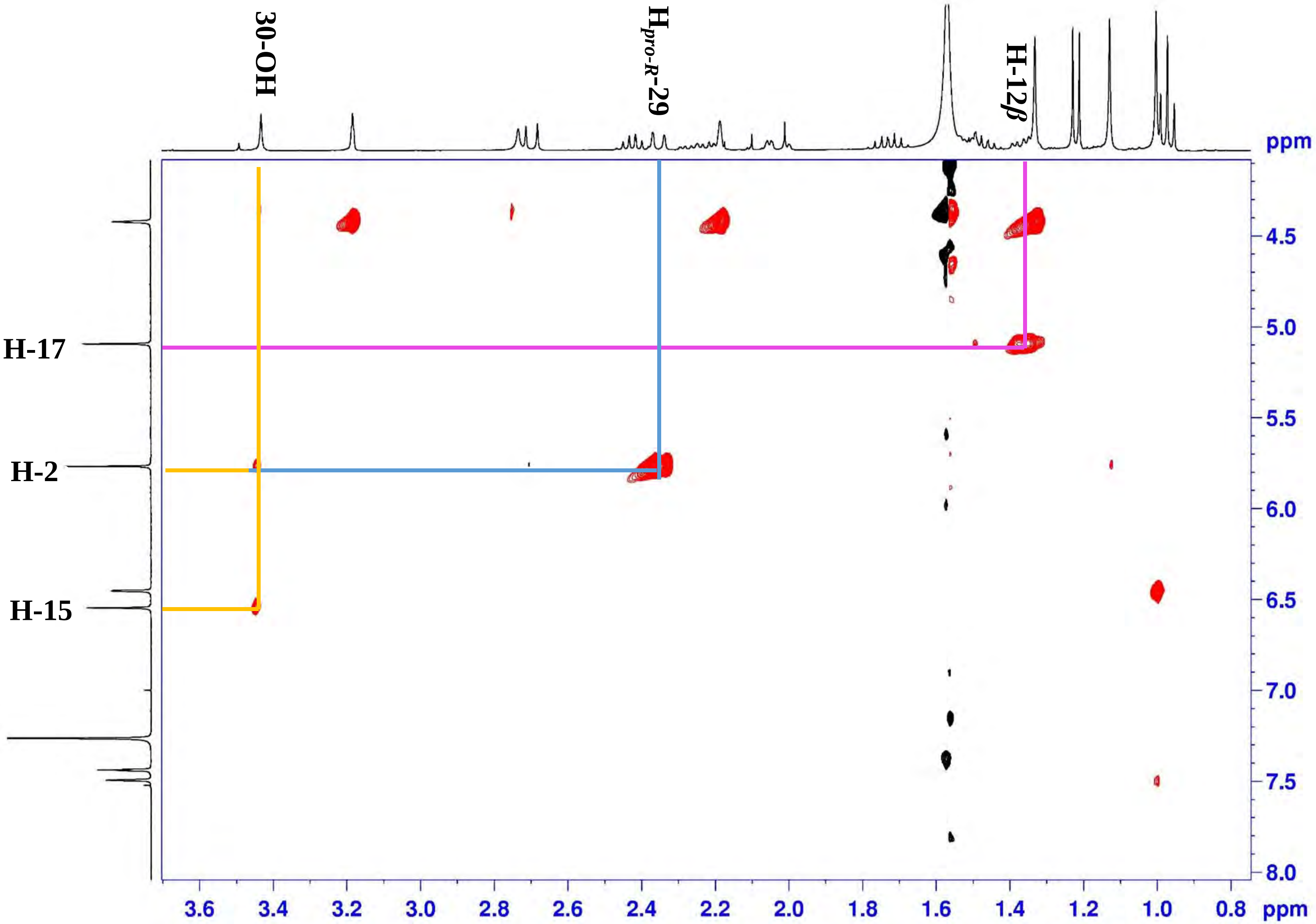

HRESIMS for compound **22**

Elemental Composition Report

Single Mass Analysis

Tolerance = 10.0 mDa / DBE: min = -1.0, max = 50.0
Element prediction: Off
Number of isotope peaks used for i-FIT = 3

Monoisotopic Mass, Even Electron Ions
142 formula(e) evaluated with 5 results within limits (up to 10 closest results for each mass)
Elements Used:
C: 0-100 H: 0-200 O: 0-100

252-27
2016112118 153 (1.246)
1: TOF MS ES+
1.46e+005

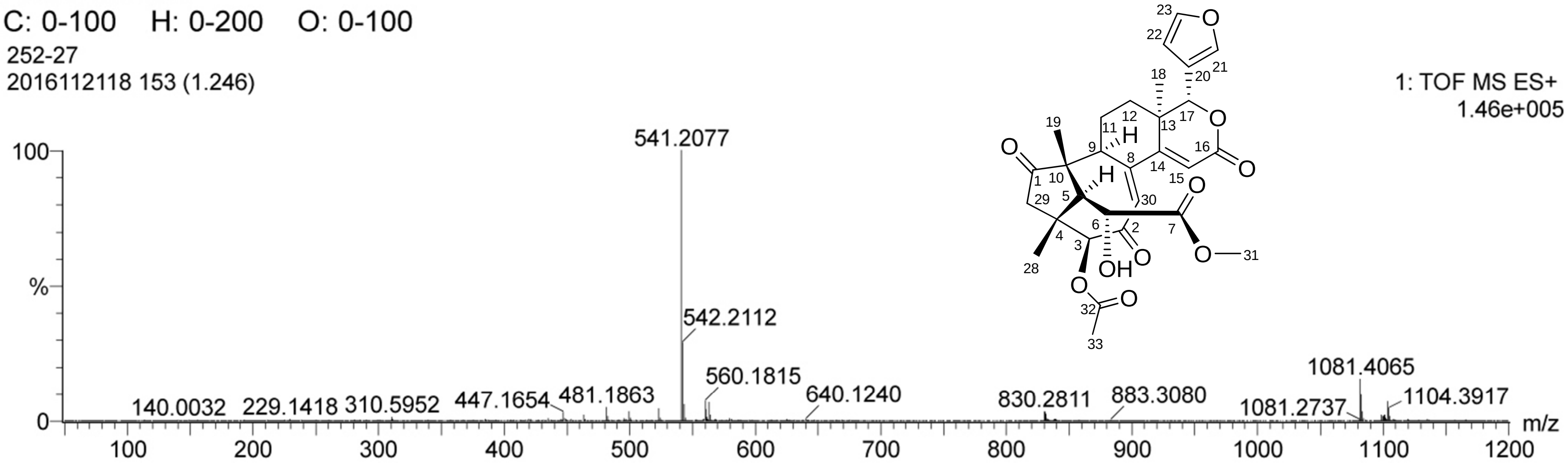

Minimum:				-1.0				
Maximum:		10.0	10.0	50.0				
Mass	Calc. Mass	mDa	PPM	DBE	i-FIT	Norm	Conf(%)	Formula
541.2077	541.2074	0.3	0.6	13.5	214.9	0.080	92.29	C29 H33 O10
	541.2132	-5.5	-10.2	4.5	217.4	2.591	7.49	C22 H37 O15
	541.2015	6.2	11.5	22.5	221.3	6.481	0.15	C36 H29 O5
	541.2168	-9.1	-16.8	26.5	223.9	9.106	0.01	C40 H29 O2
	541.1980	9.7	17.9	0.5	222.4	7.546	0.05	C18 H37 O18

^{1}H NMR (400 MHz) spectrum of compound **22** in $CDCl_3$

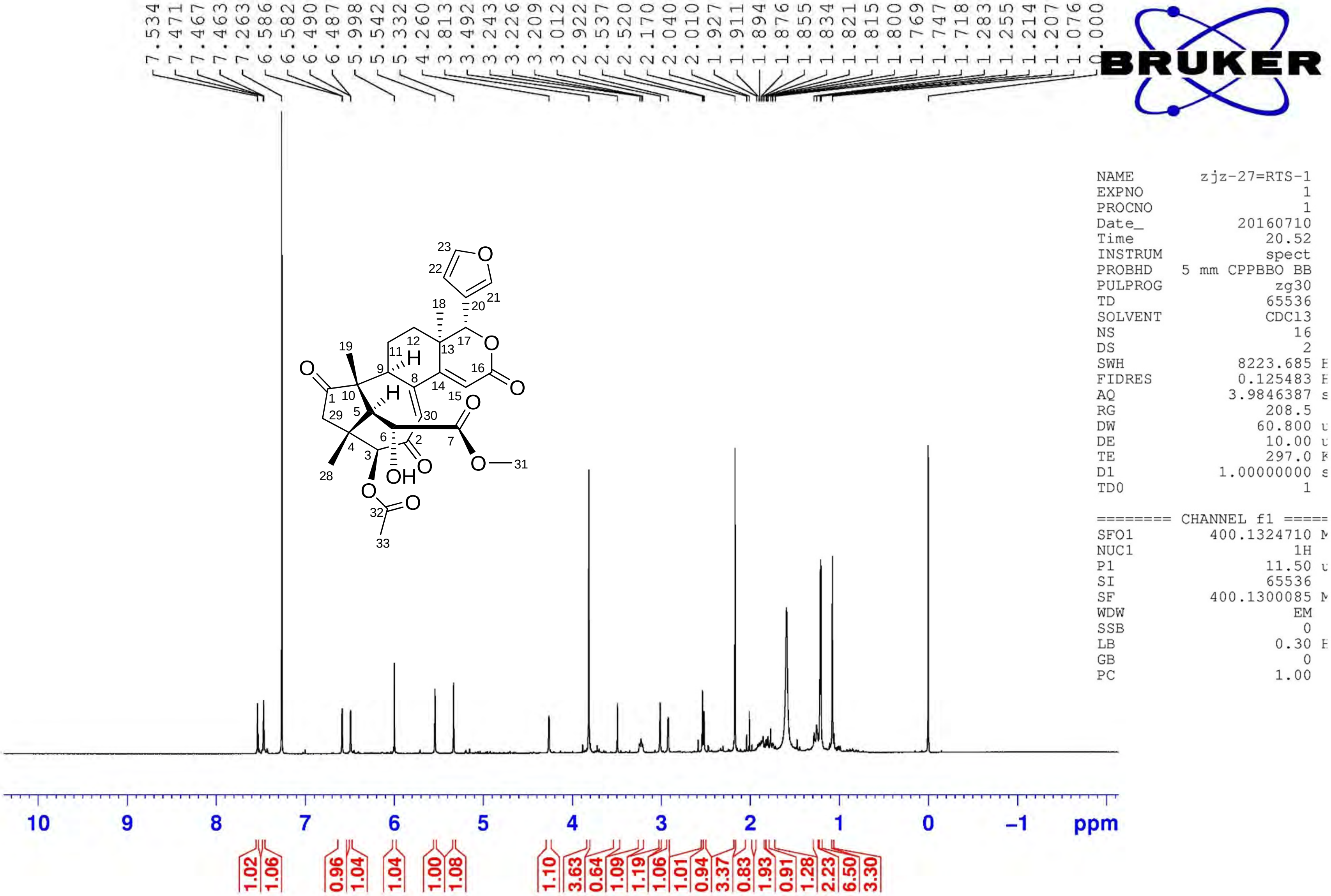

^{1}H NMR (400 MHz) spectrum of compound **22** in $CDCl_3$

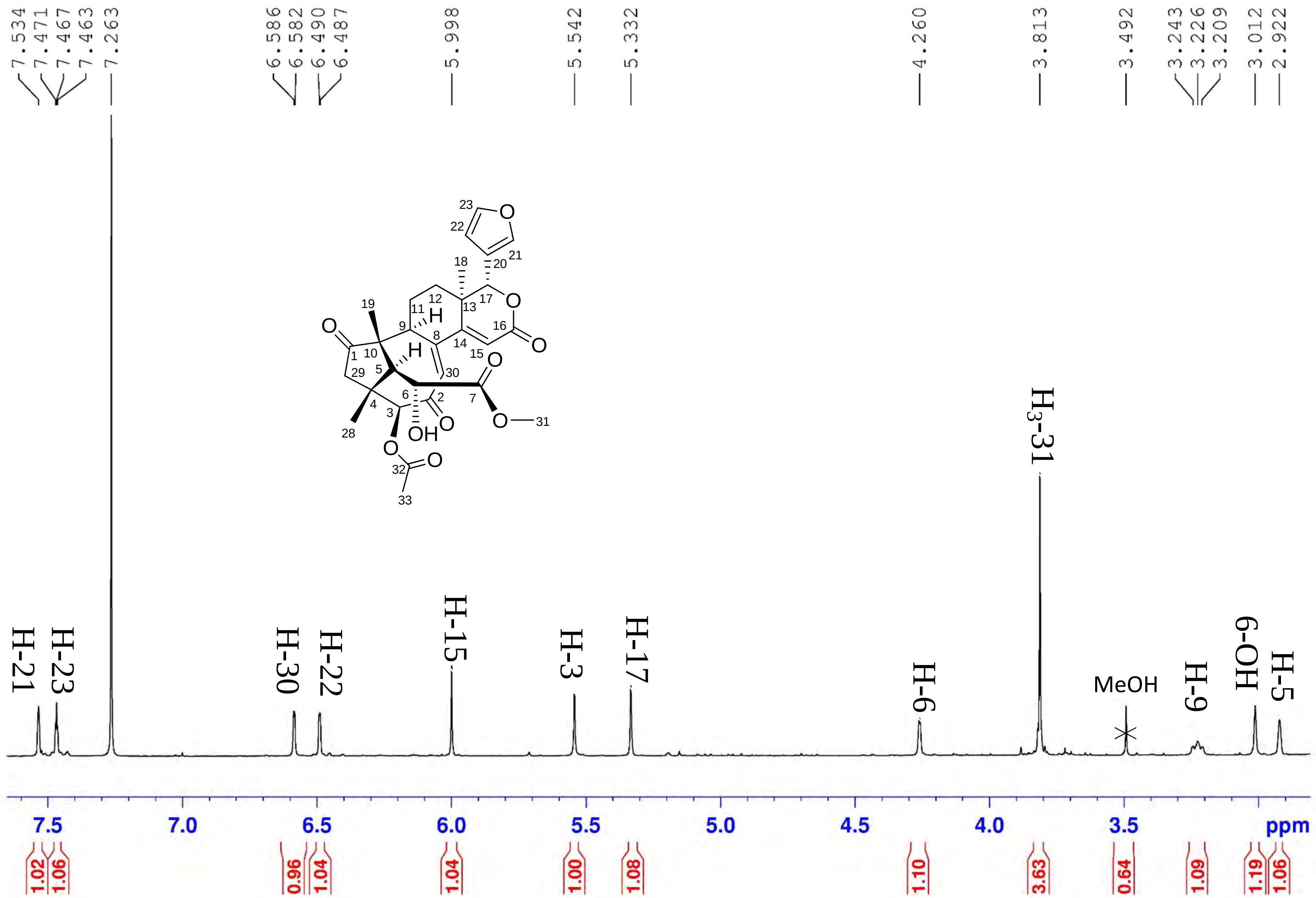

^{1}H NMR (400 MHz) spectrum of compound **22** in $CDCl_3$

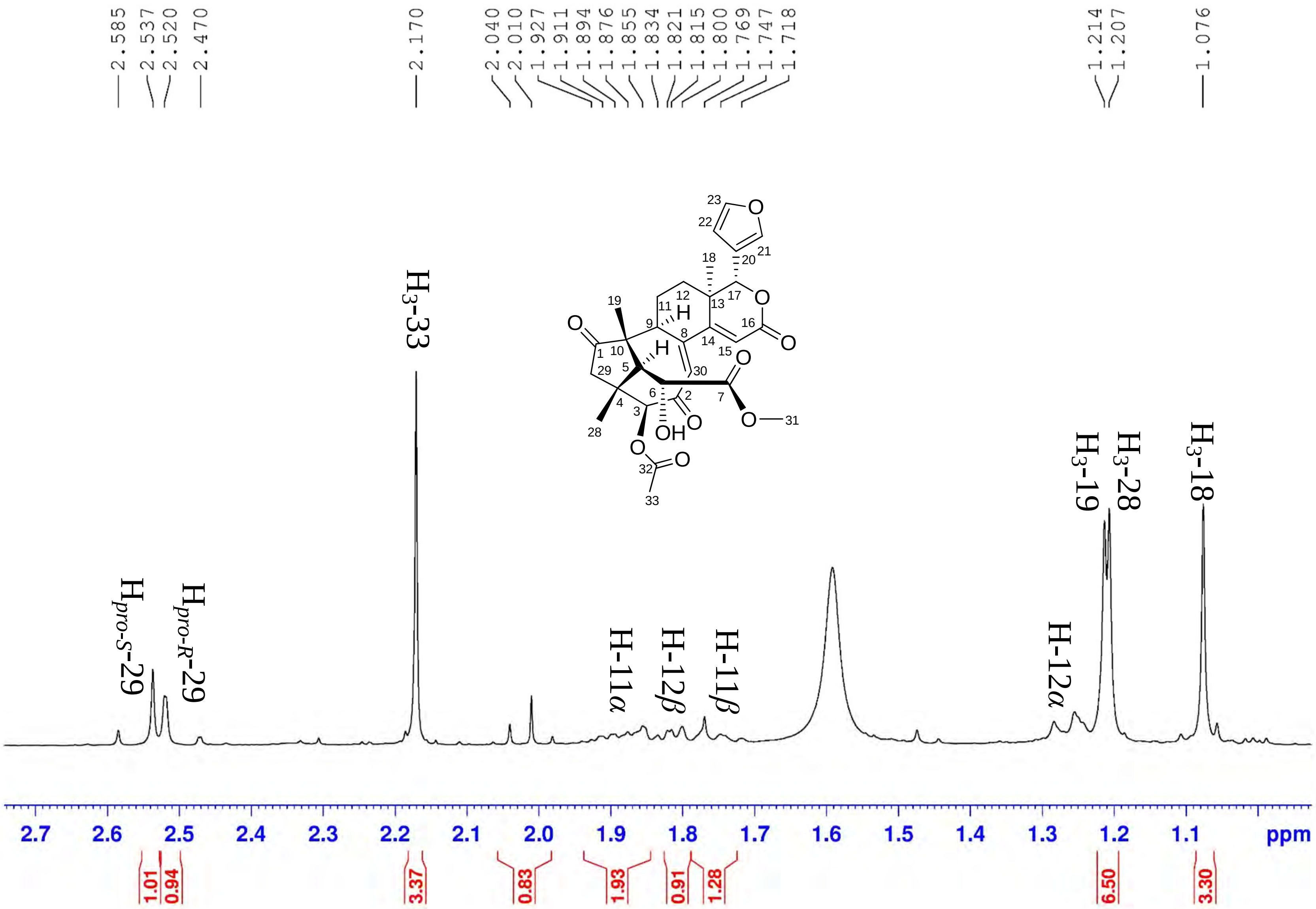

^{13}C NMR (100 MHz) spectrum of compound **22** in $CDCl_3$

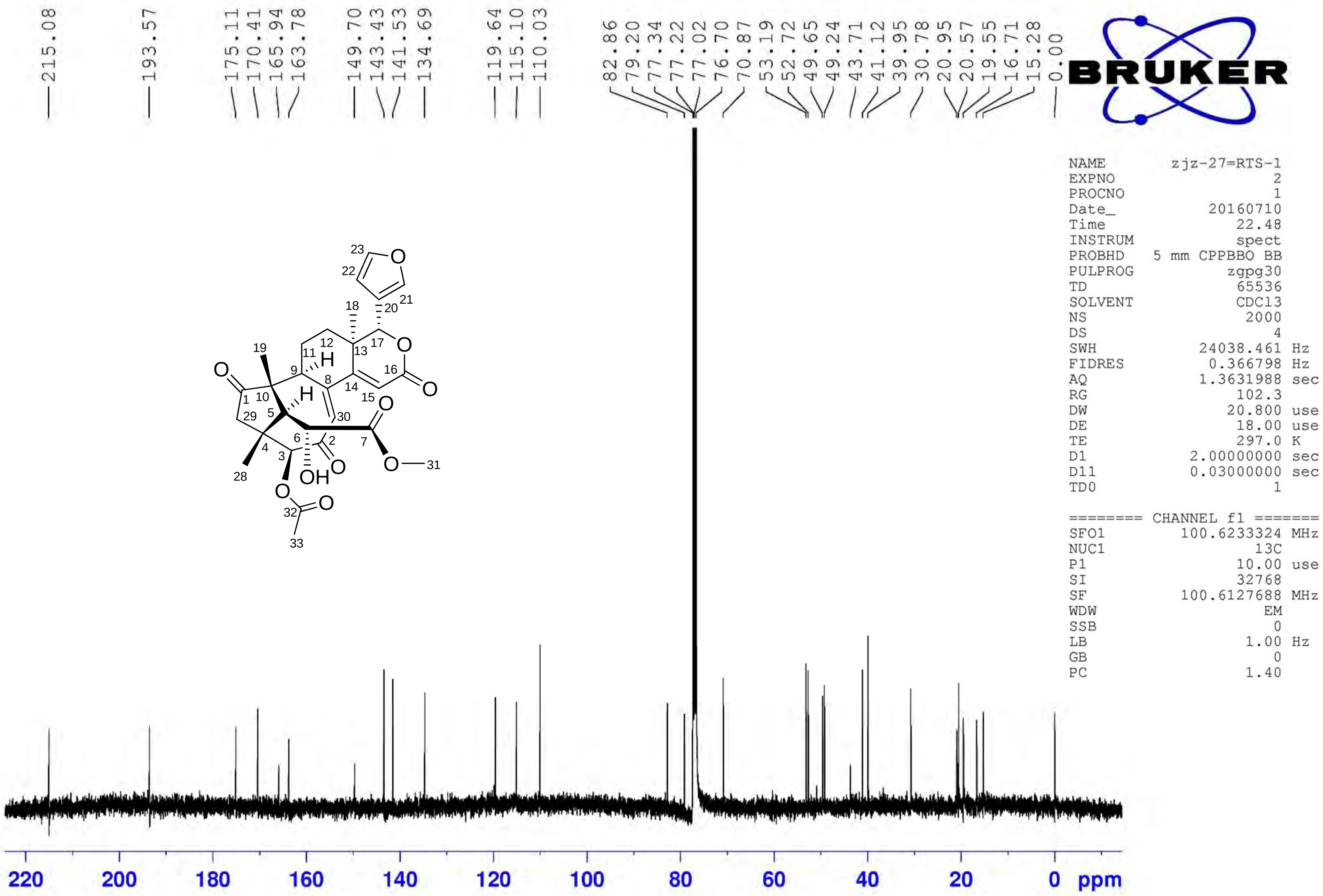

^{13}C NMR (100 MHz) spectrum of compound **22** in $CDCl_3$

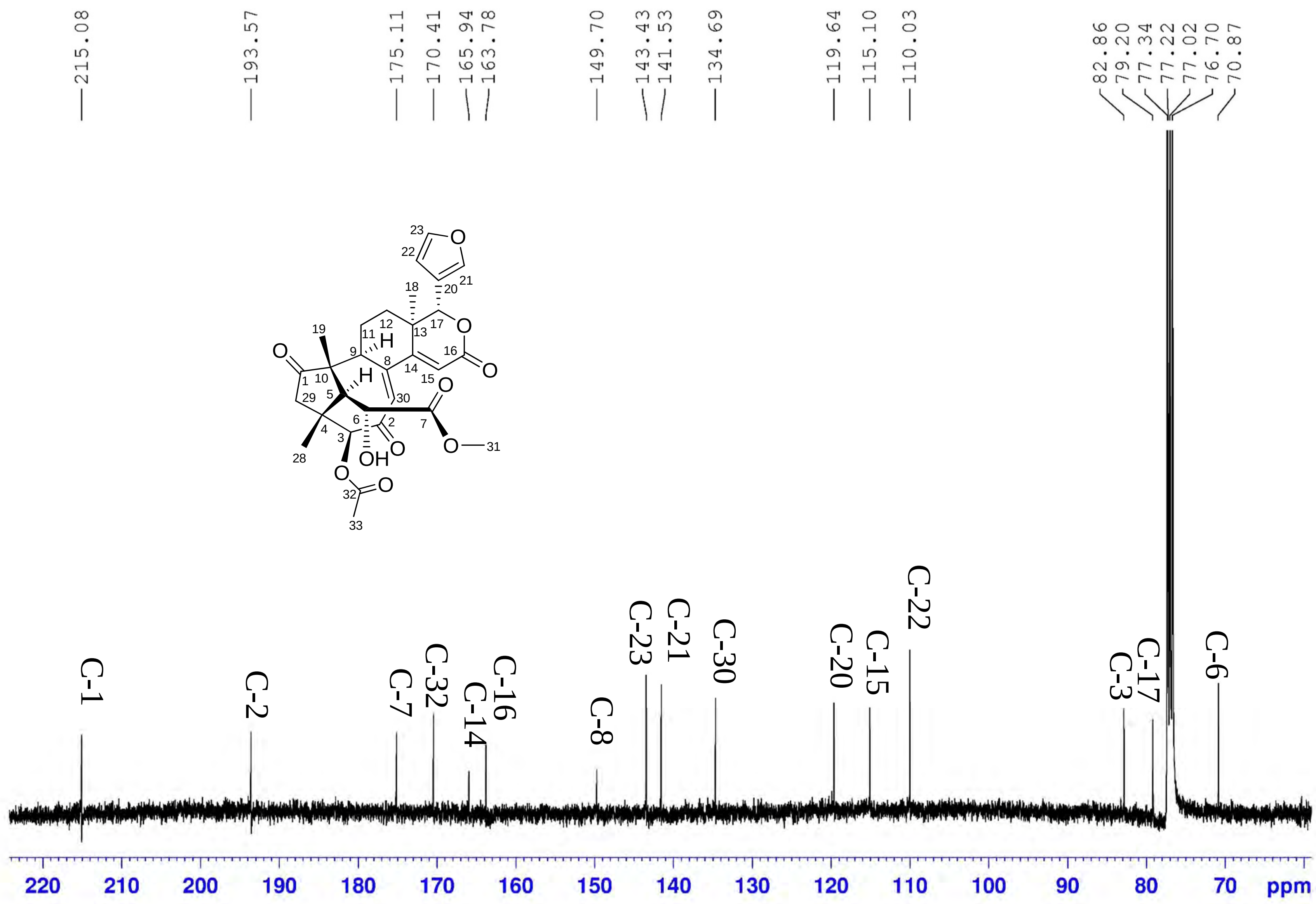

^{13}C NMR (100 MHz) spectrum of compound **22** in $CDCl_3$

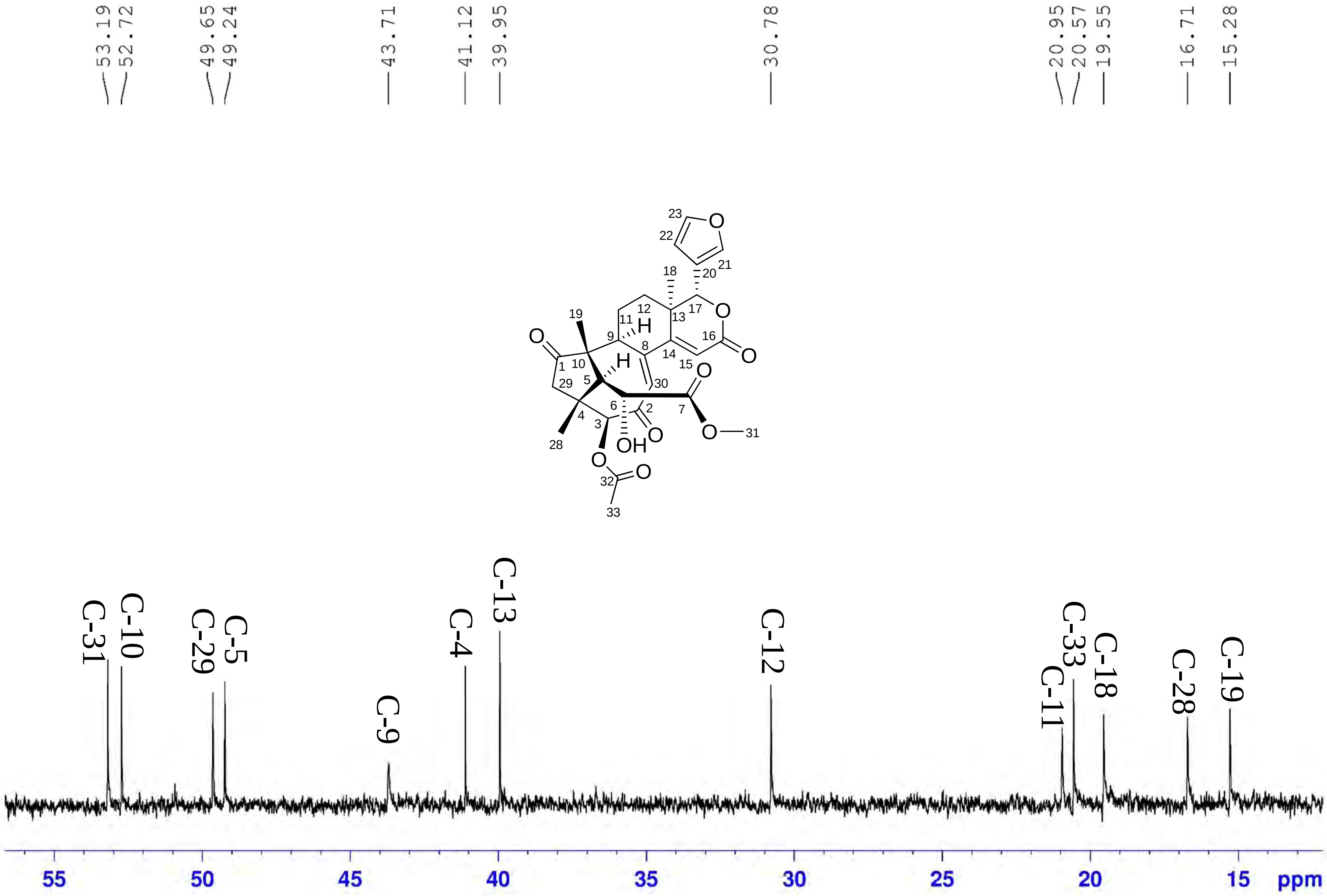

DEPT135 (100 MHz) spectrum of compound **22** in $CDCl_3$

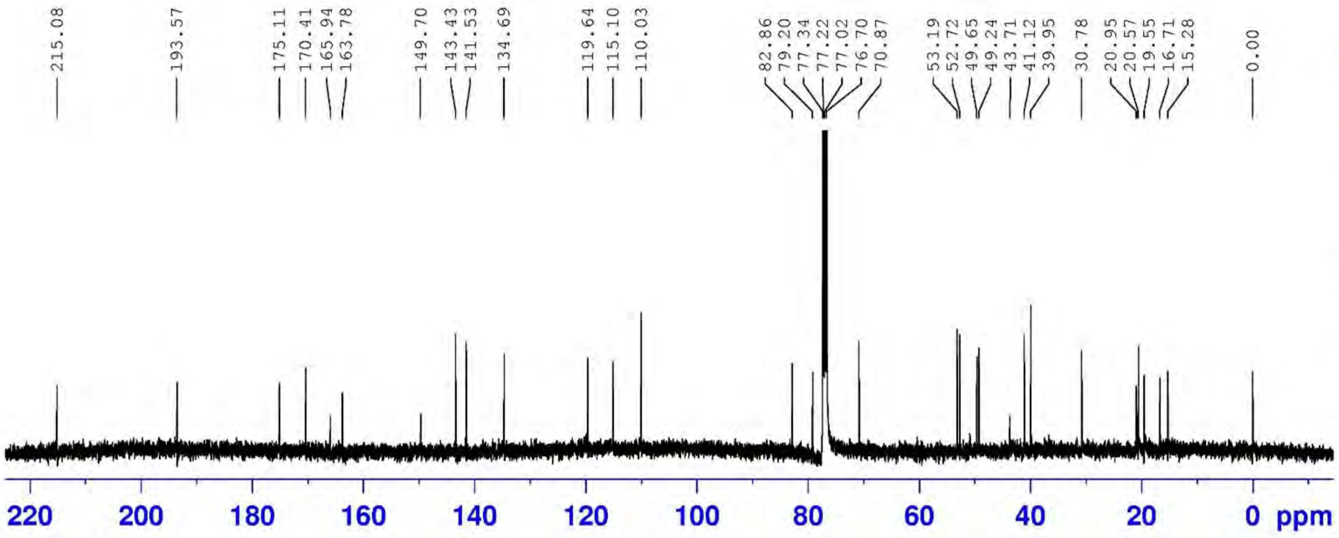


```
NAME          zjz-27=RTS-1
EXPNO                    3
PROCNO                   1
Date_             20160710
Time                 23.17
INSTRUM              spect
PROBHD   5 mm CPPBBO BB
PULPROG         deptsp135
TD                   65536
SOLVENT              CDCl3
NS                     500
DS                       4
SWH          24038.461 Hz
FIDRES        0.366798 Hz
AQ           1.3631988 sec
RG                  130.26
DW             20.800 usec
DE              18.00 usec
TE               297.0 K
CNST2         145.0000000
D1         2.00000000 sec
D2         0.00344828 sec
D12        0.00002000 sec
TD0                      1

======== CHANNEL f1 ========
SFO1       100.6233324 MHz
NUC1                   13C
P1              10.00 usec
P13           2000.00 usec
SI                   32768
SF         100.6127688 MHz
WDW                     EM
SSB                      0
LB                 1.00 Hz
GB                       0
PC                    1.40
```

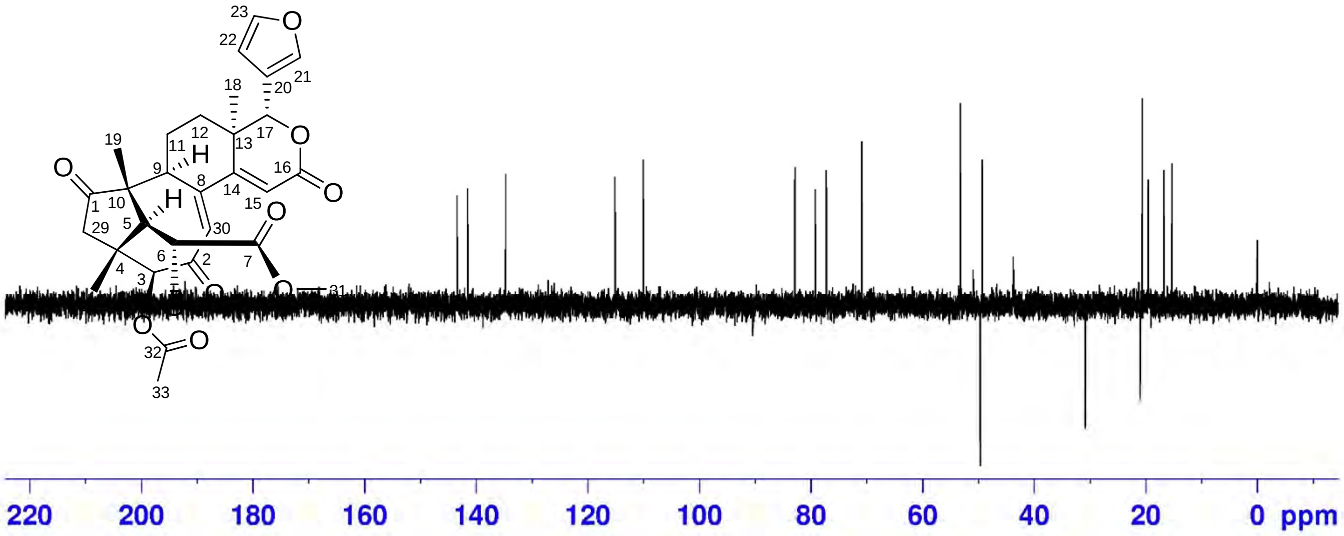

DEPT135 (100 MHz) spectrum of compound **22** in $CDCl_3$

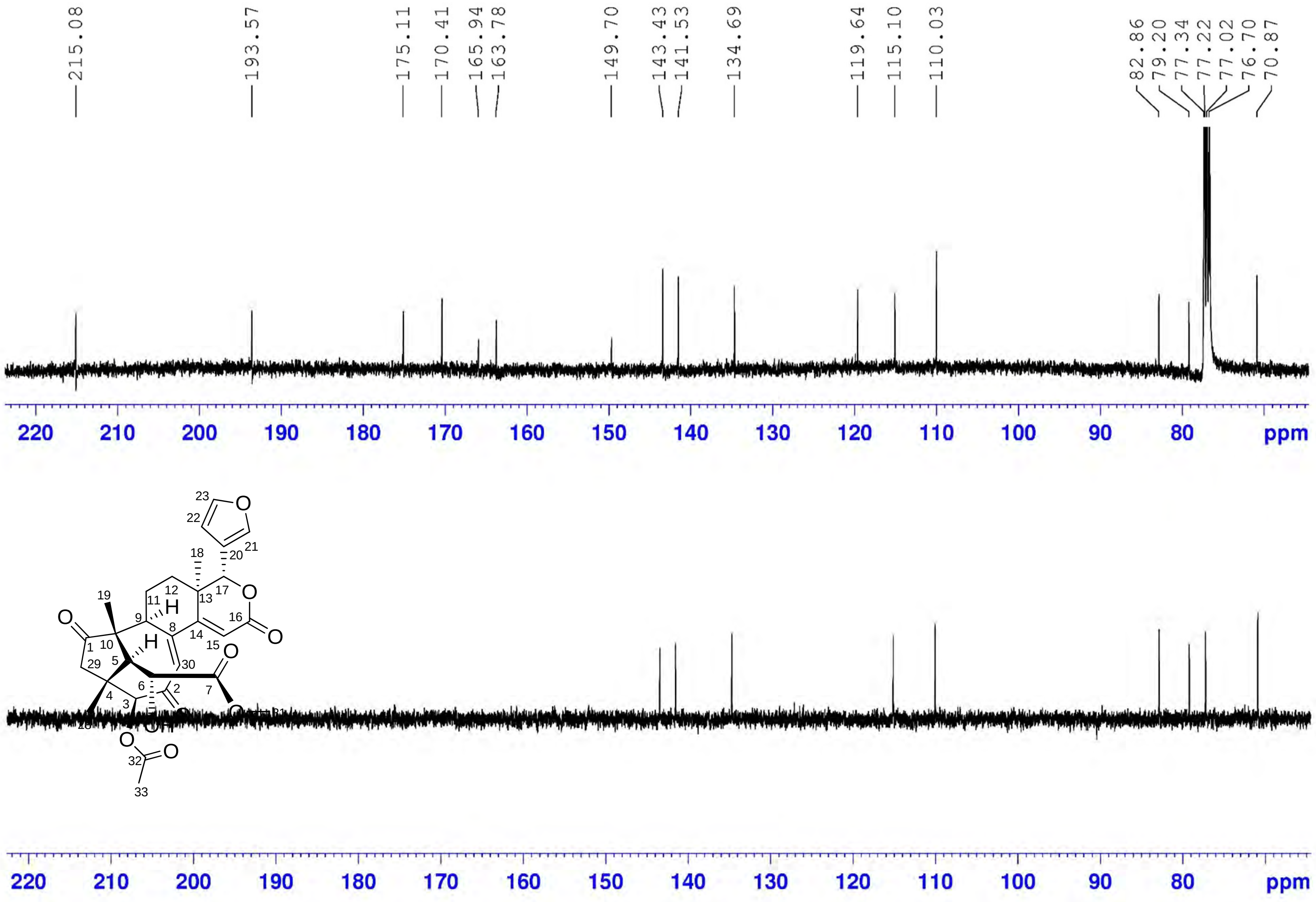

DEPT135 (100 MHz) spectrum of compound **22** in $CDCl_3$

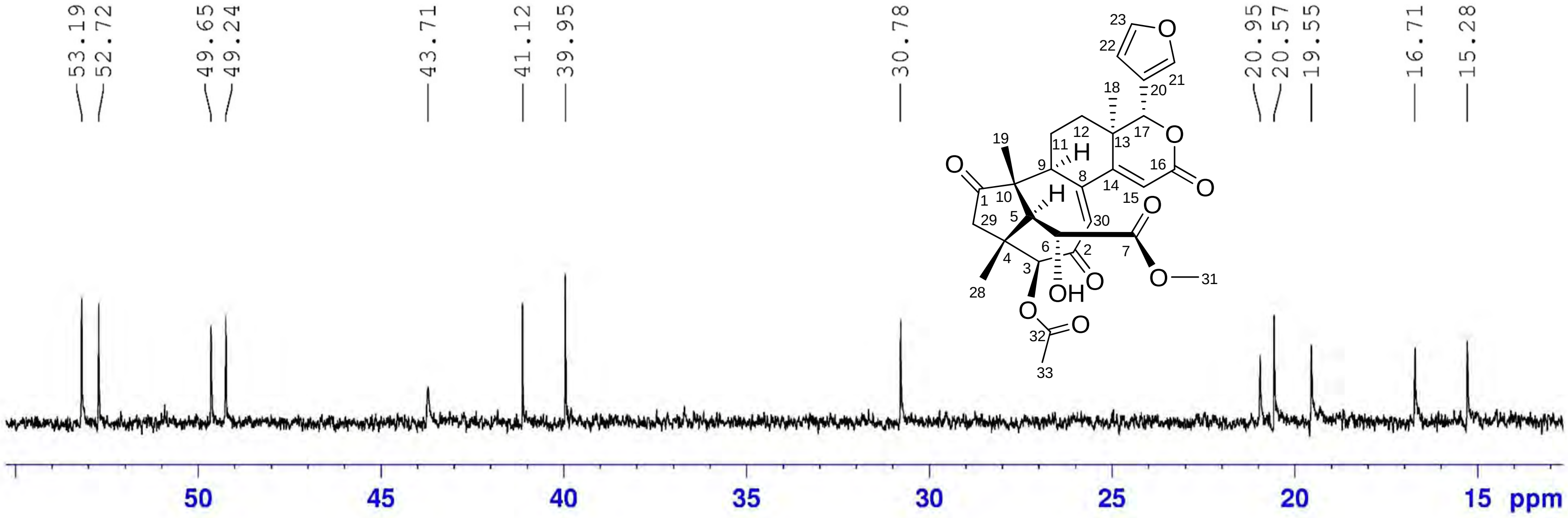

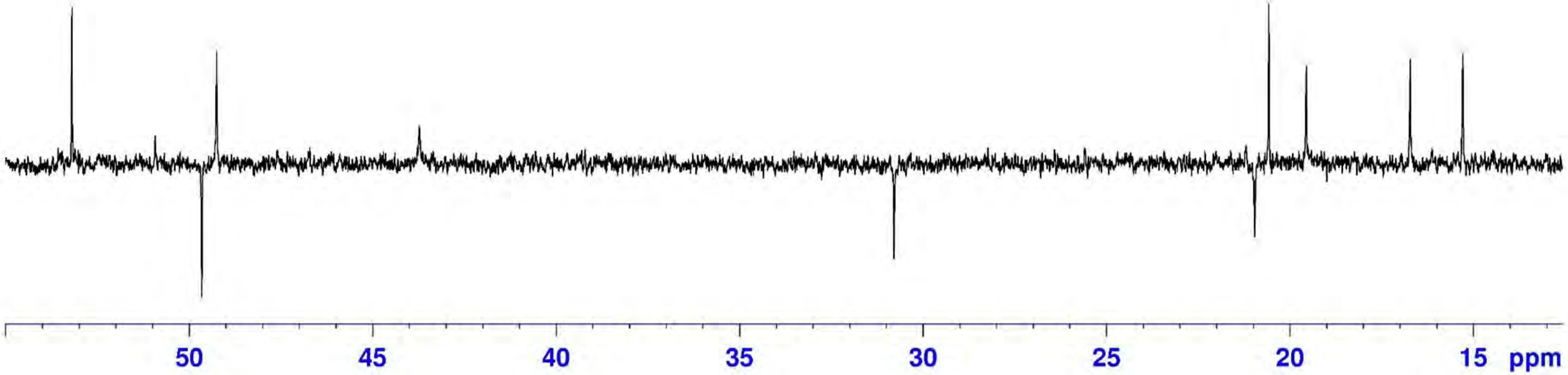

^{1}H-^{1}H COSY (400 MHz) spectrum of compound **22** in $CDCl_3$

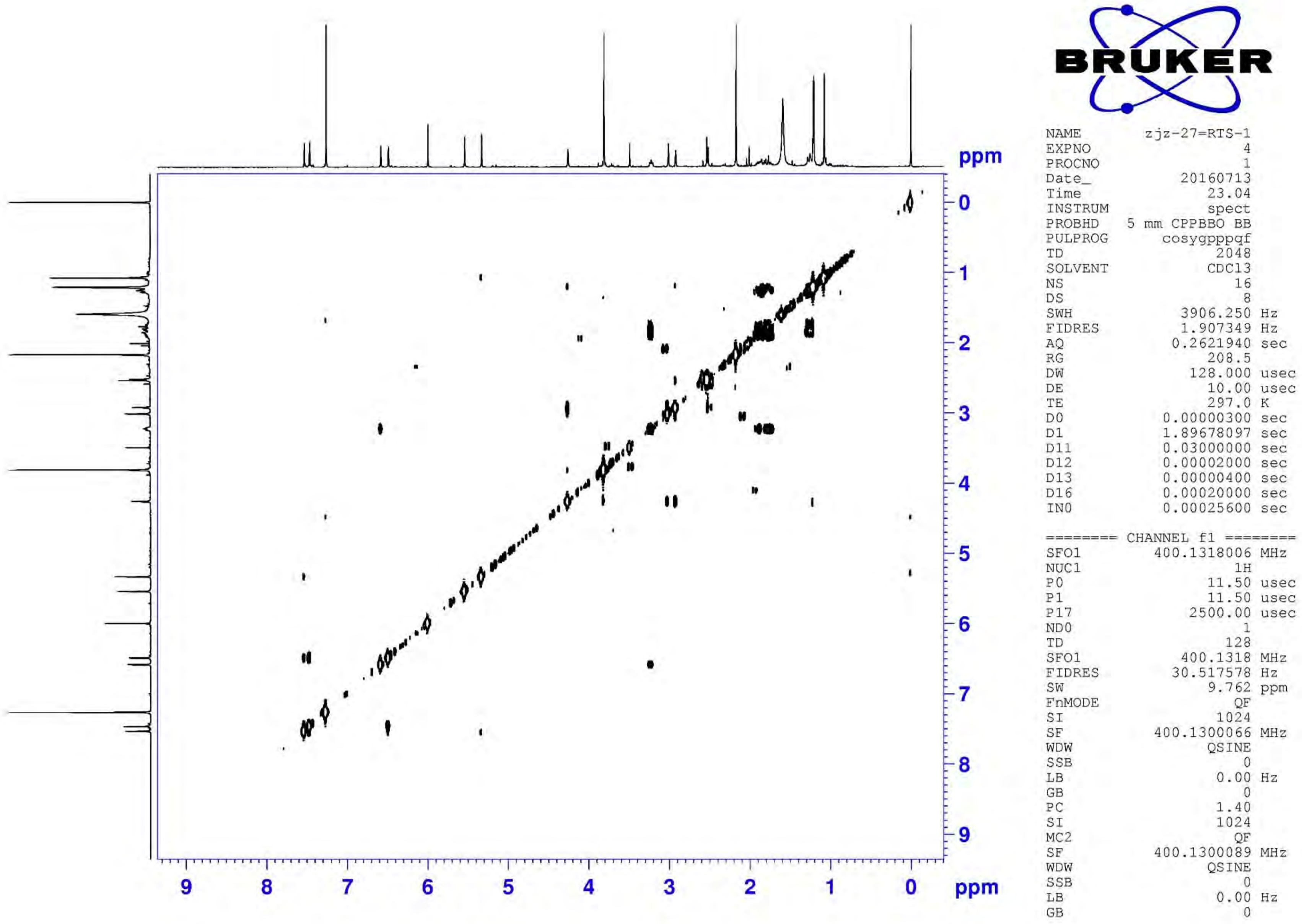

^{1}H-^{1}H COSY (400 MHz) spectrum of compound **22** in $CDCl_3$

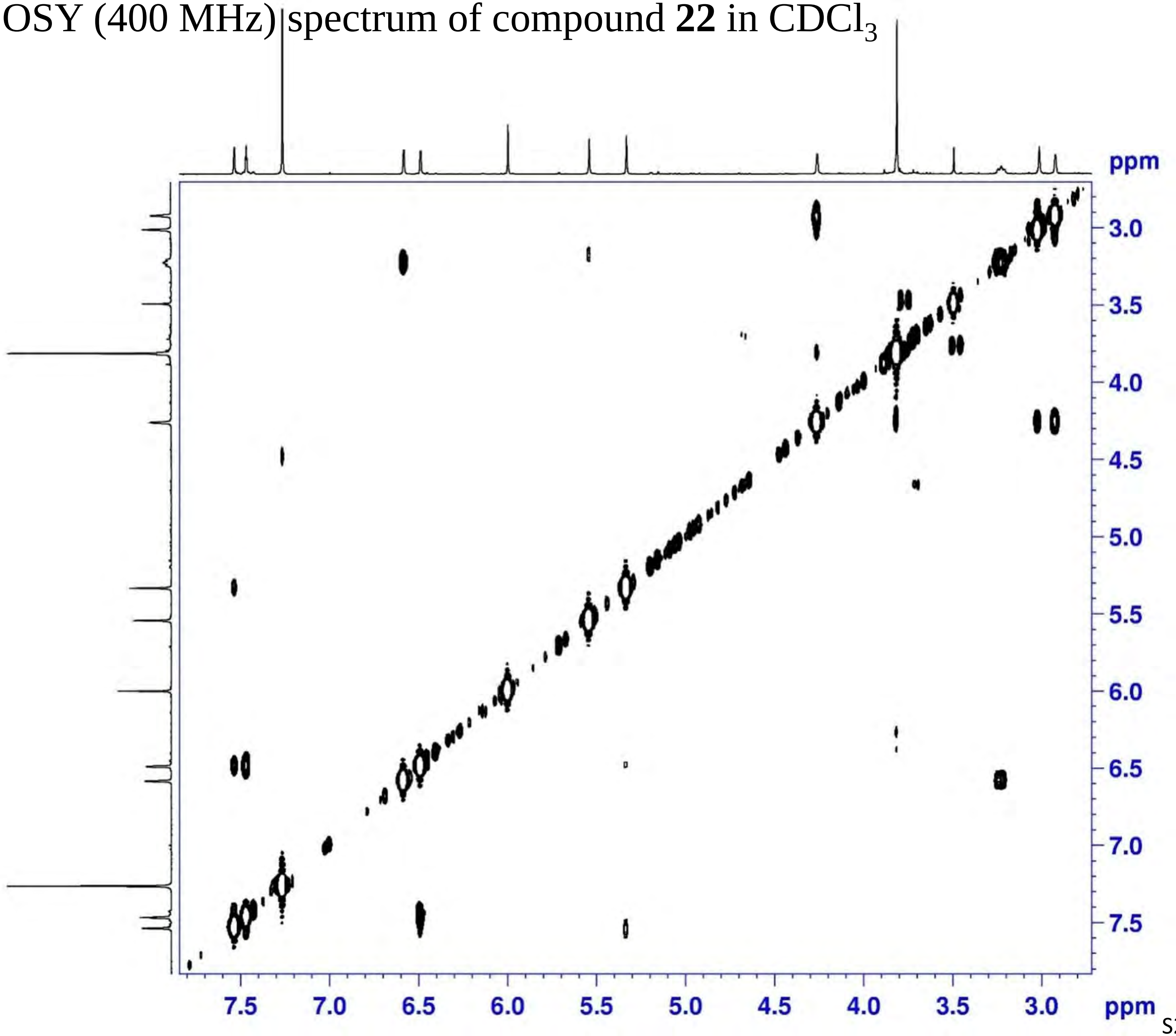

^1H-^1H COSY (400 MHz) spectrum of compound **22** in $CDCl_3$

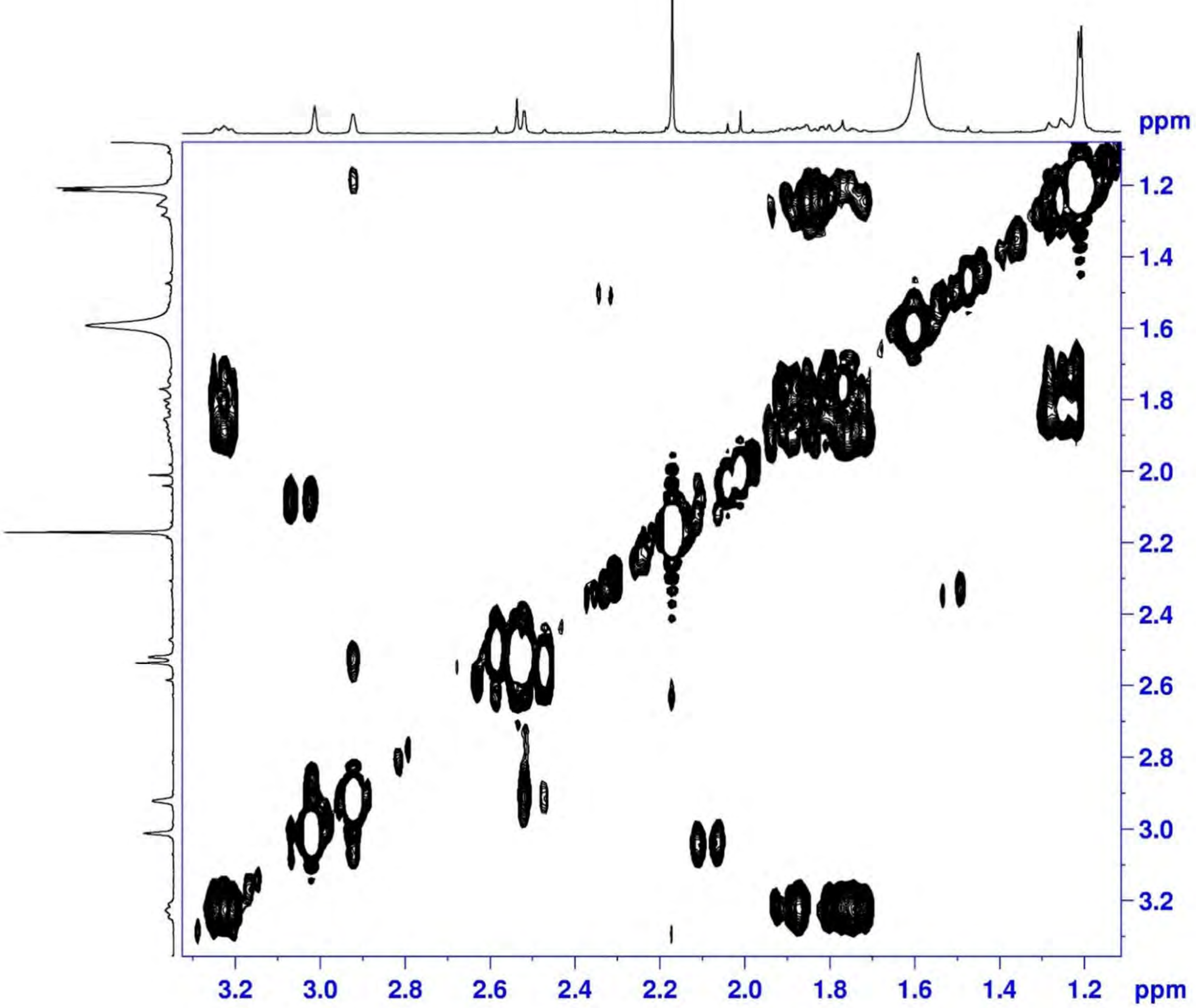

^{1}H-^{1}H COSY (400 MHz) spectrum of compound **22** in $CDCl_3$

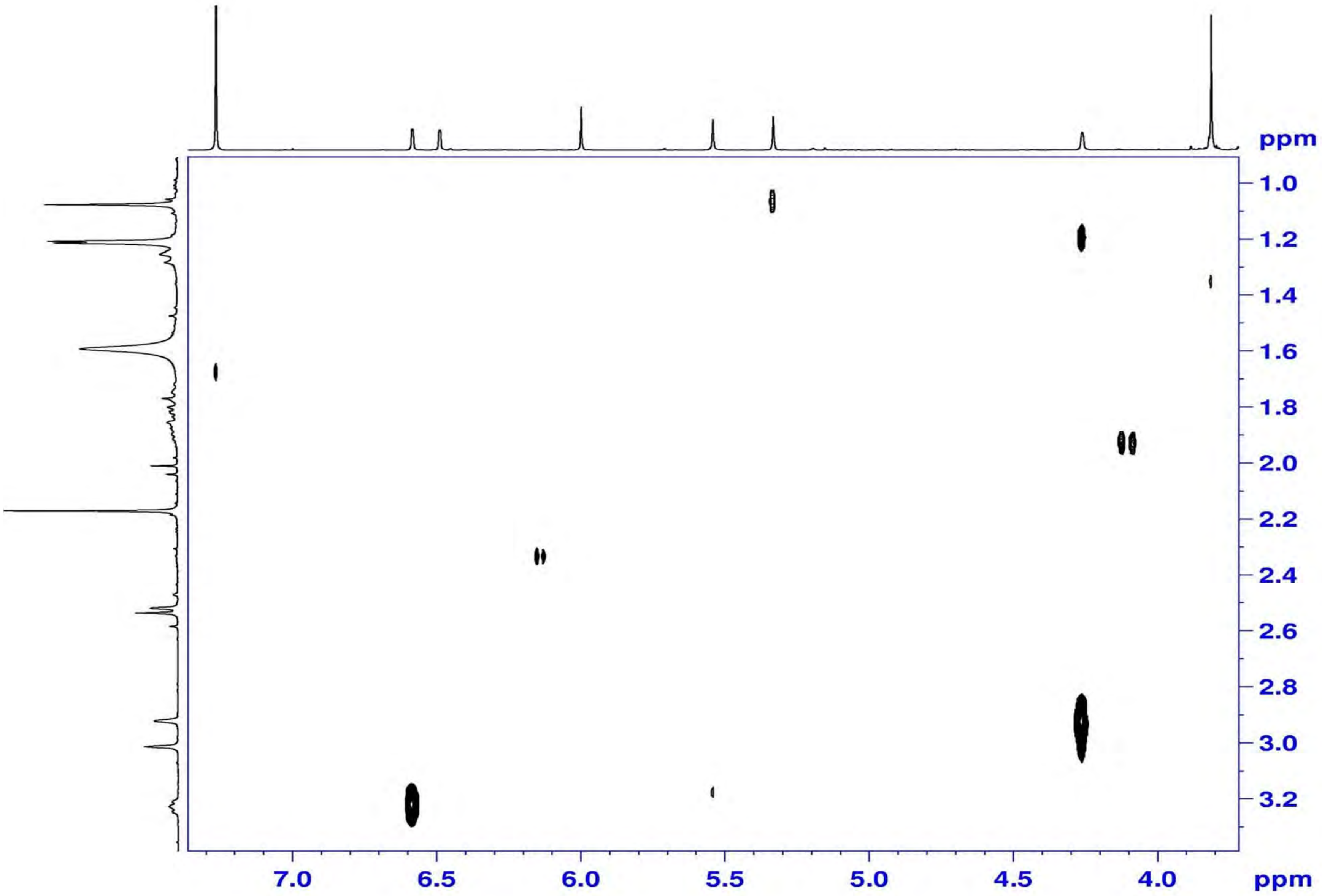

HSQC (400 MHz) spectrum of compound **22** in $CDCl_3$

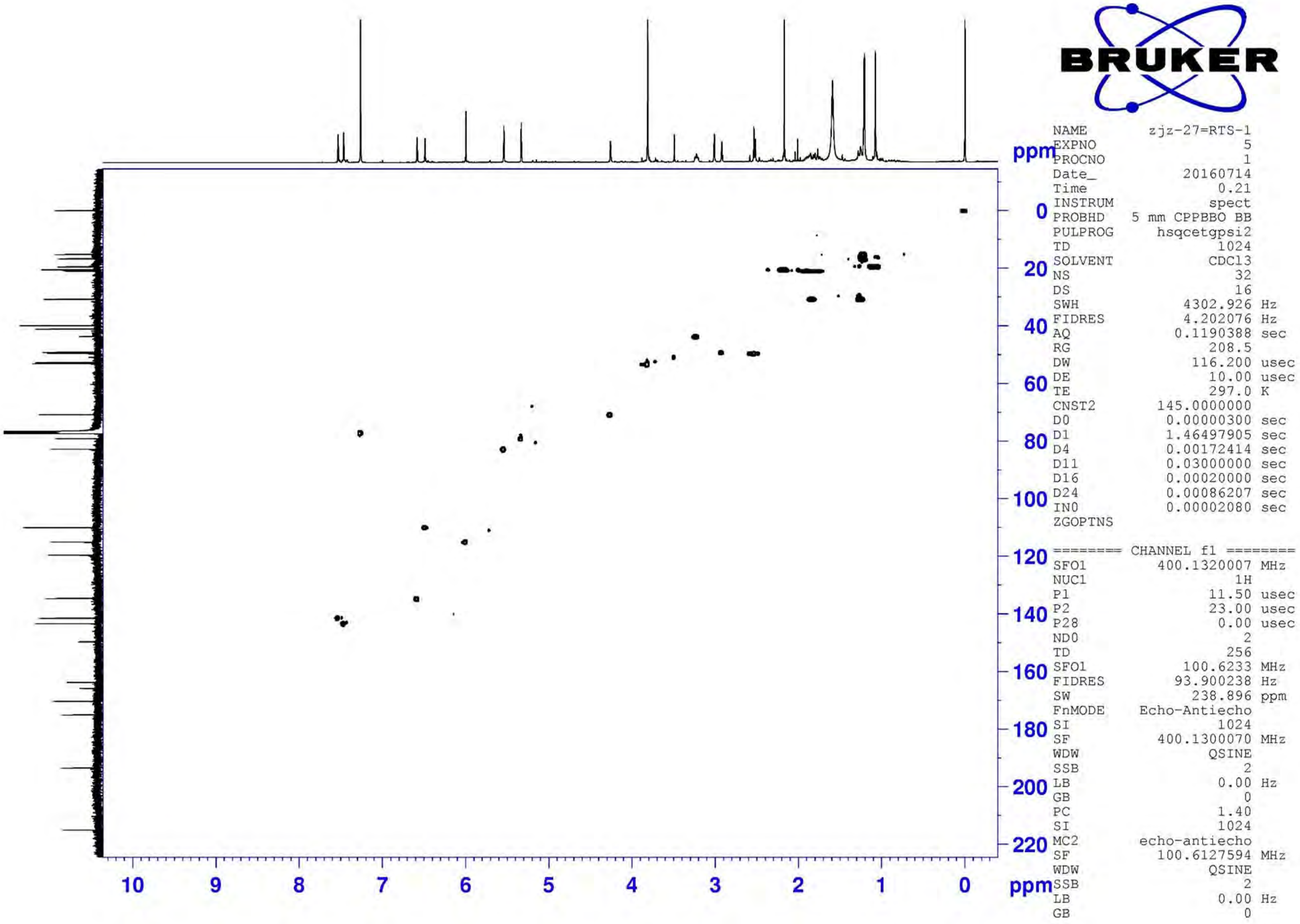

HSQC (400 MHz) spectrum of compound **22** in $CDCl_3$

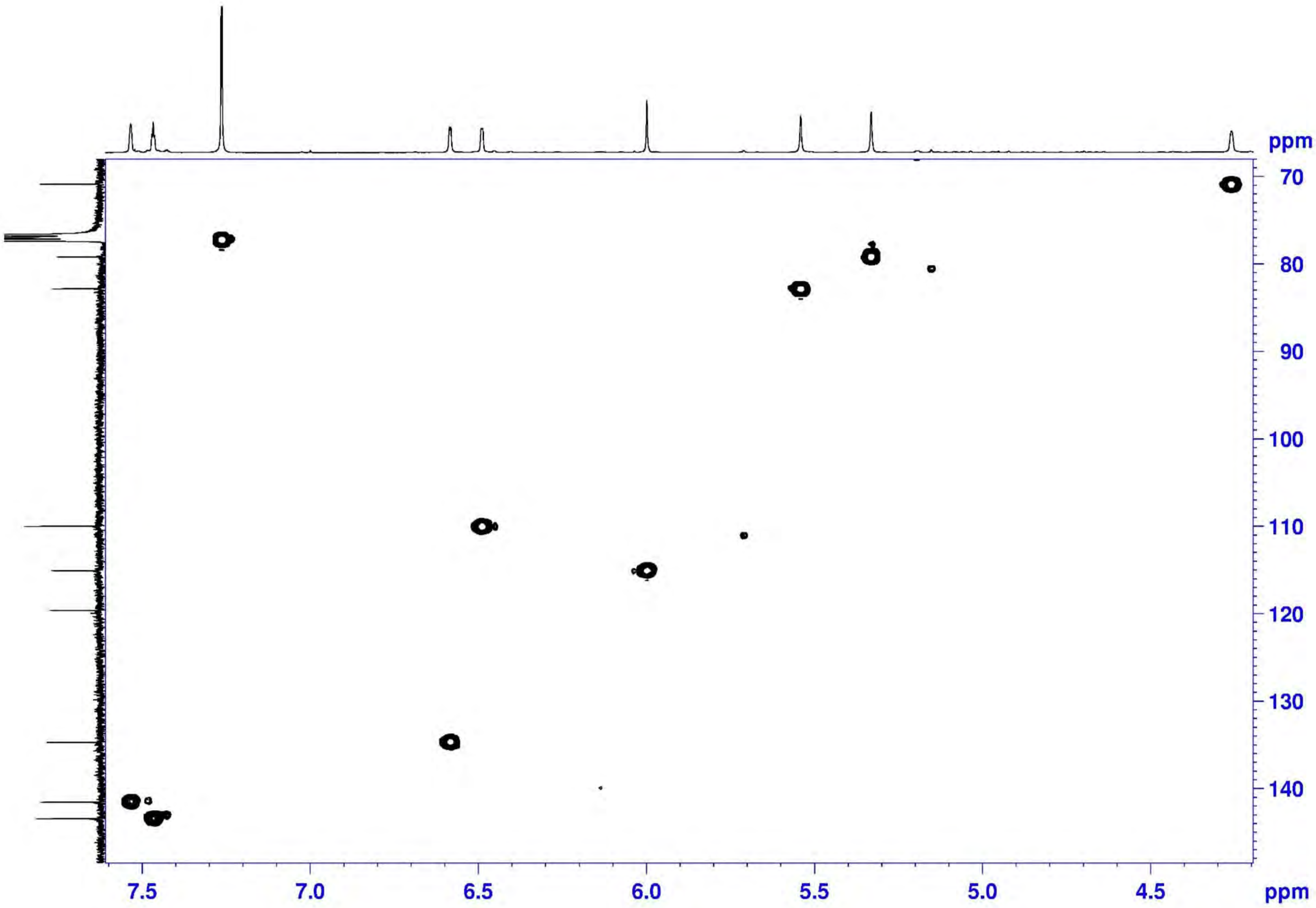

HSQC (400 MHz) spectrum of compound **22** in $CDCl_3$

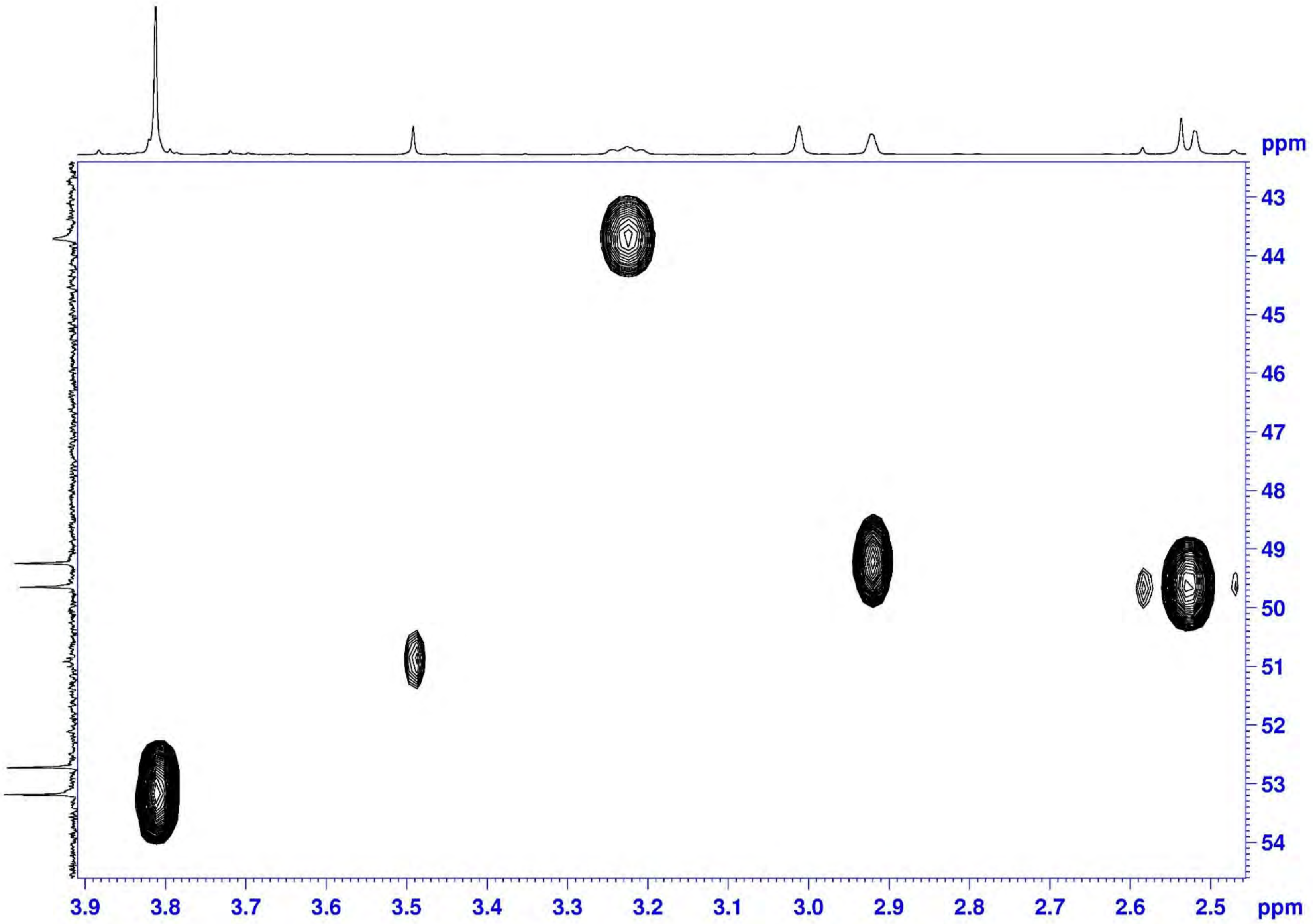

HSQC (400 MHz) spectrum of compound **22** in $CDCl_3$

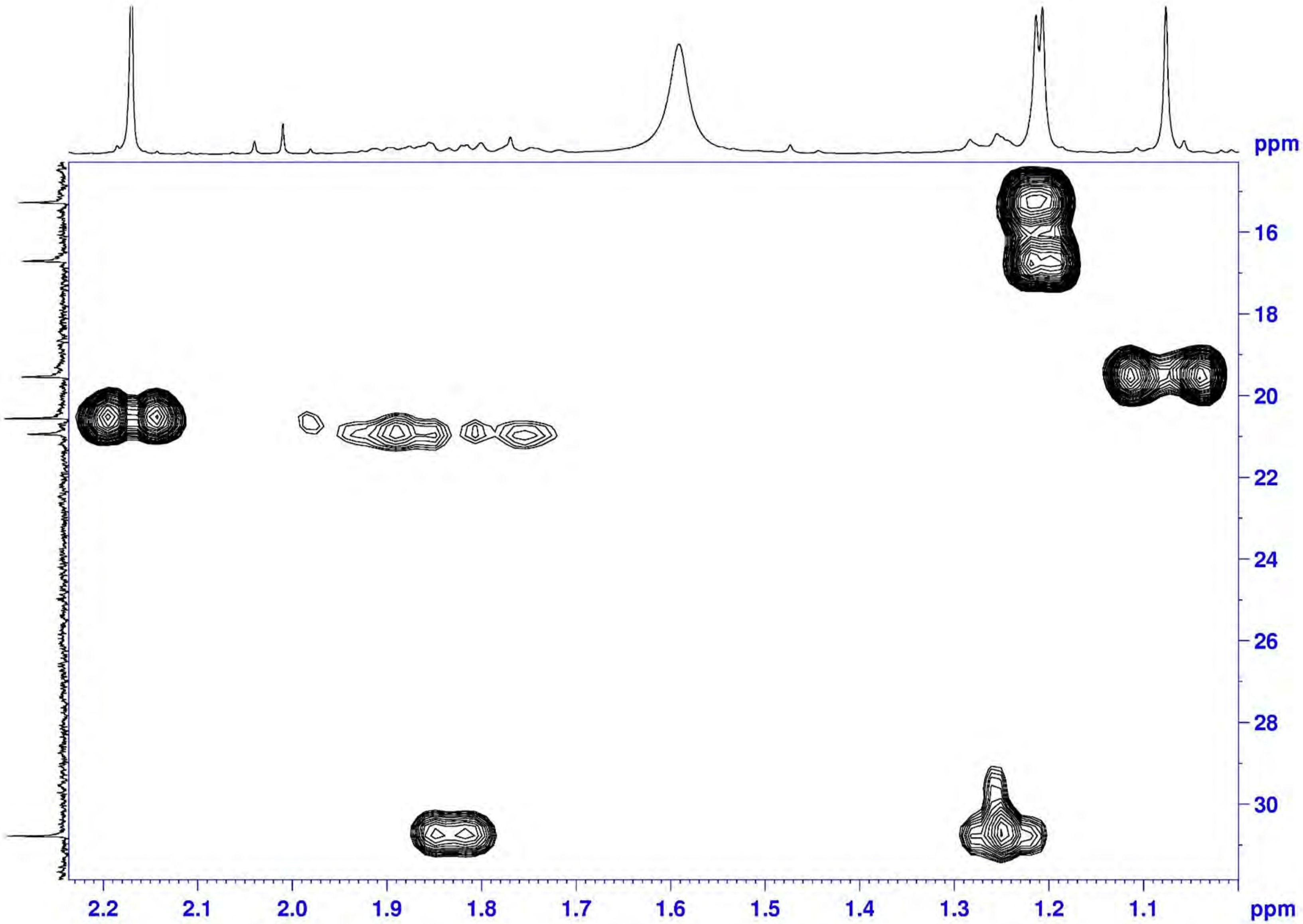

HMBC (400 MHz) spectrum of compound **22** in $CDCl_3$

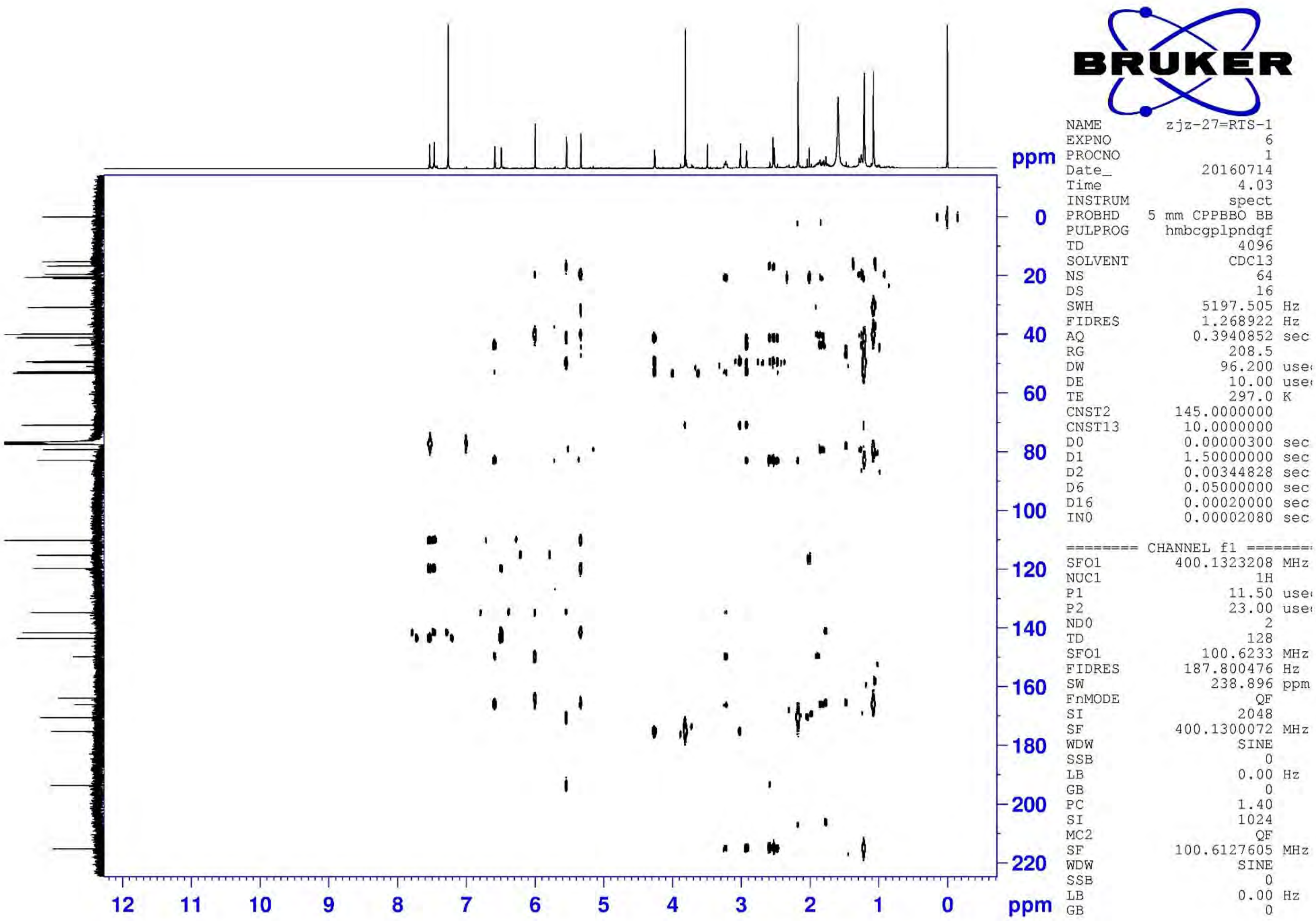

HMBC (400 MHz) spectrum of compound **22** in $CDCl_3$

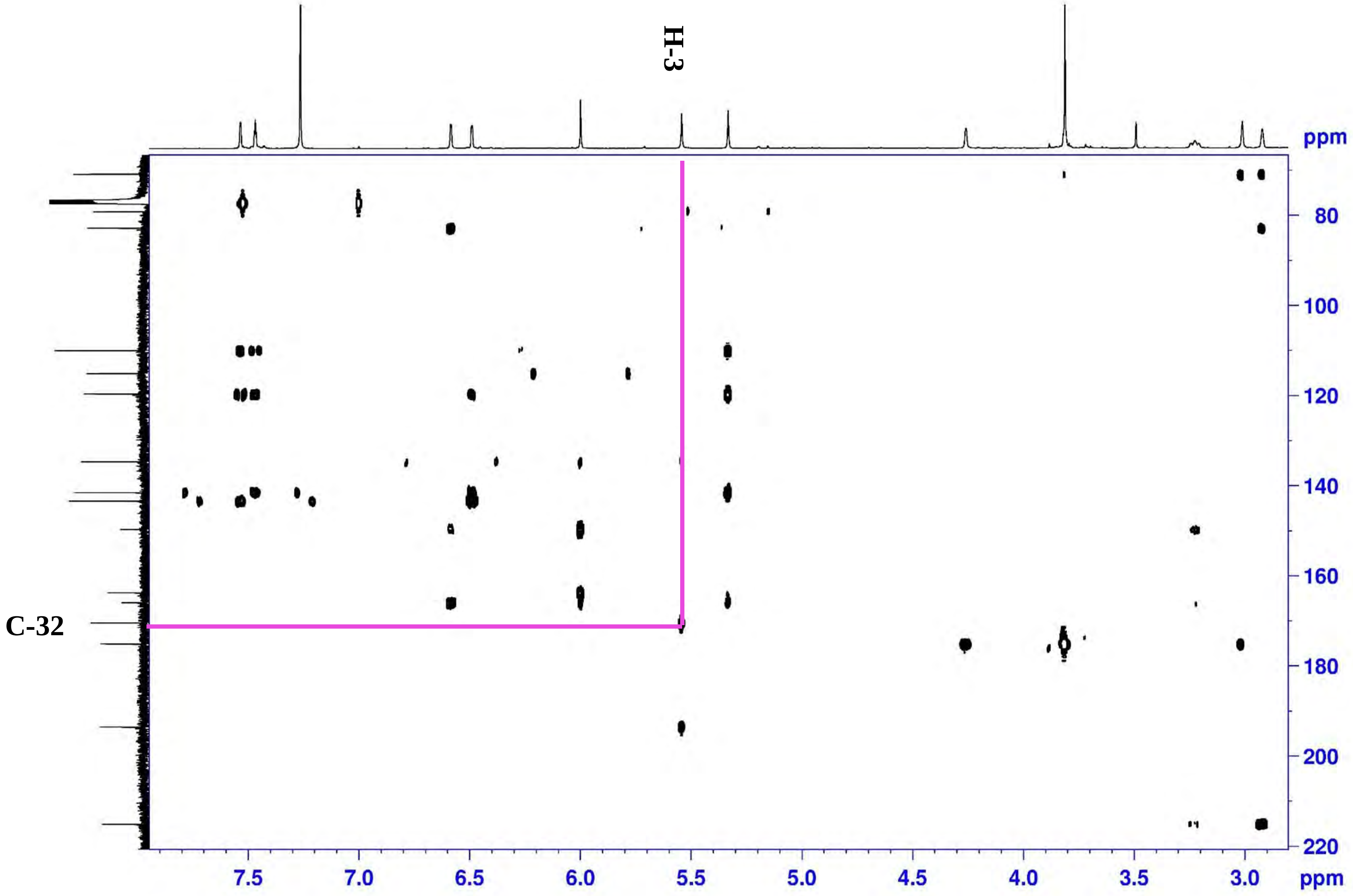

HMBC (400 MHz) spectrum of compound **22** in $CDCl_3$

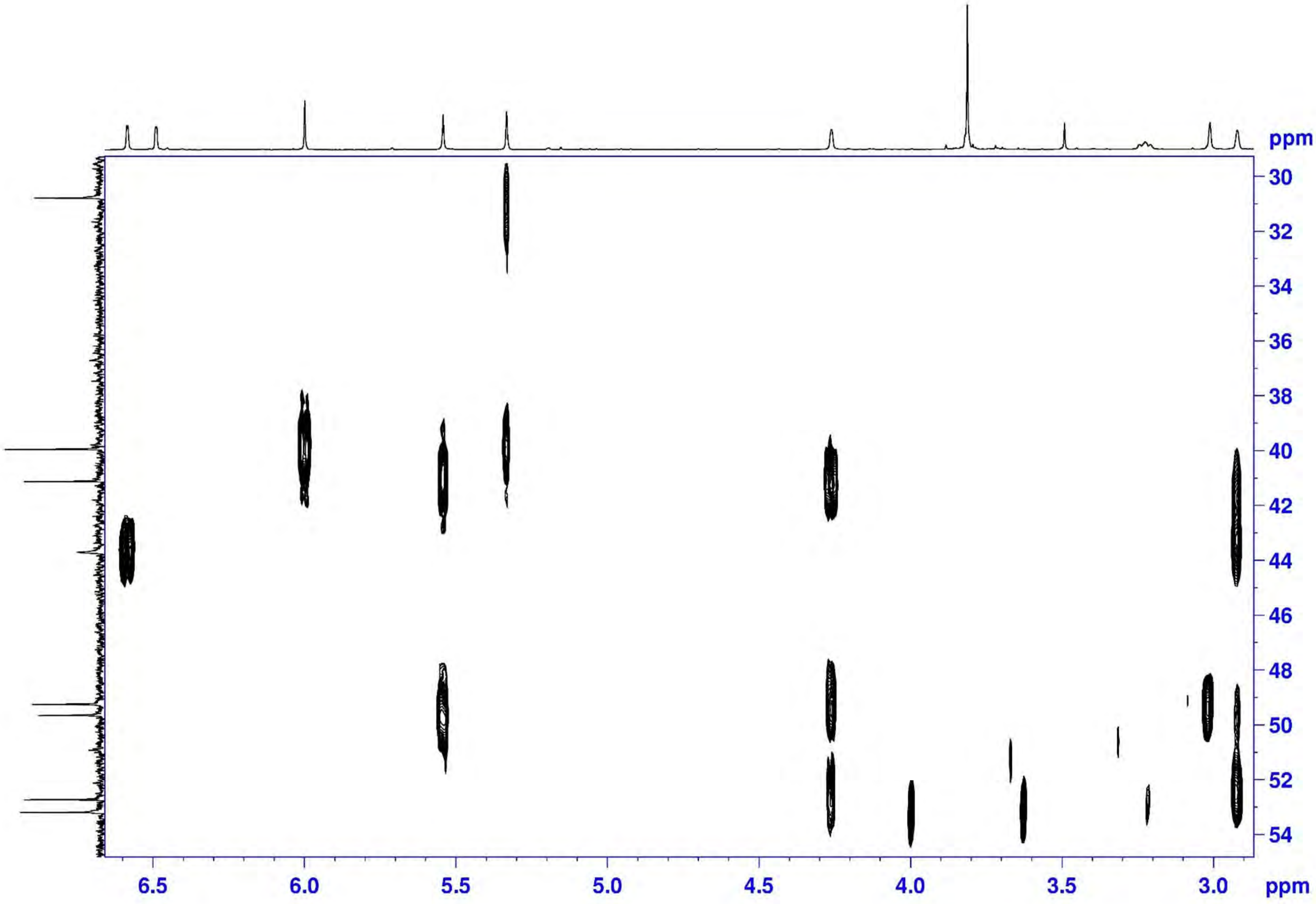

HMBC (400 MHz) spectrum of compound **22** in $CDCl_3$

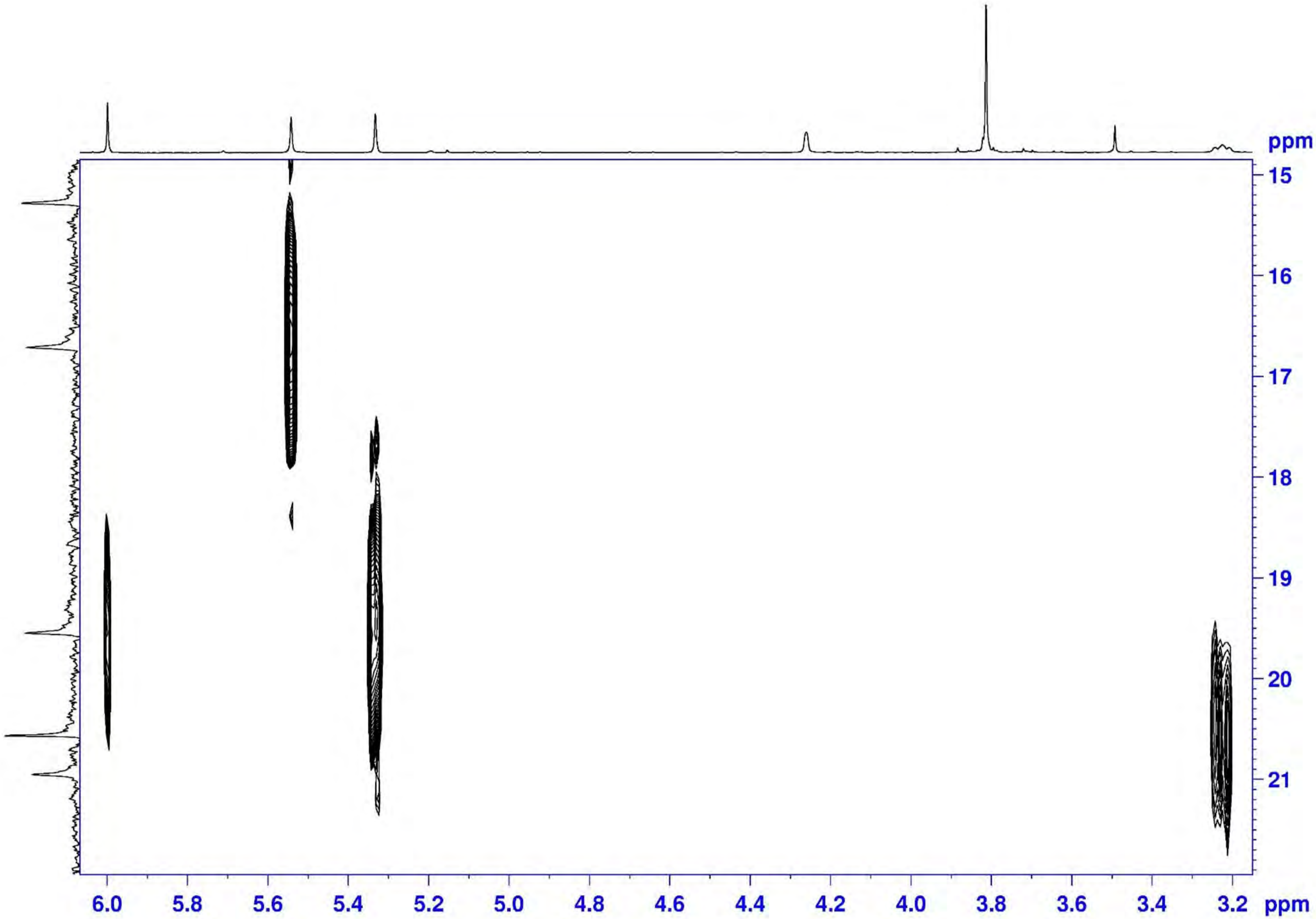

HMBC (400 MHz) spectrum of compound **22** in $CDCl_3$

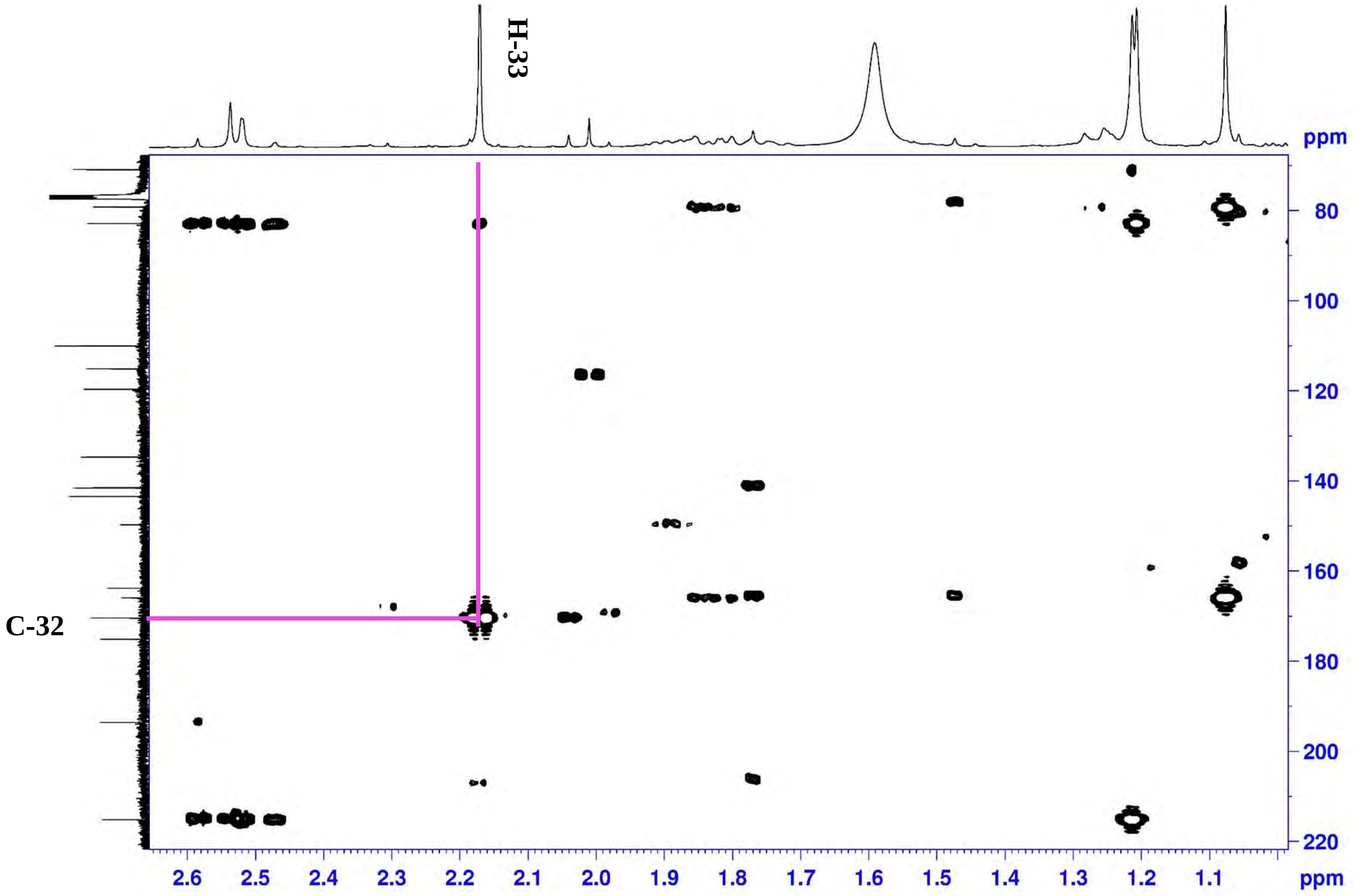

HMBC (400 MHz) spectrum of compound **22** in $CDCl_3$

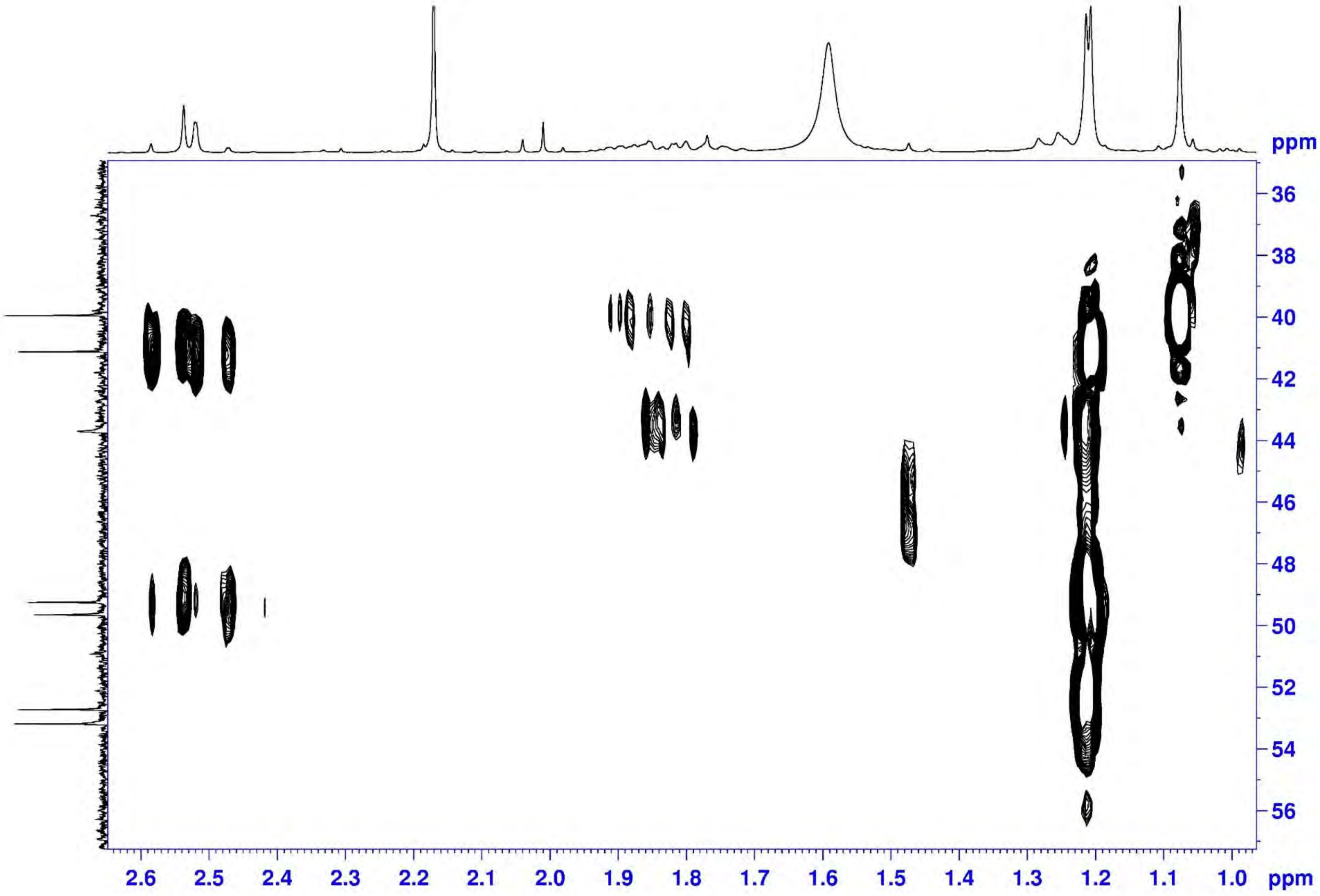

HMBC (400 MHz) spectrum of compound **22** in $CDCl_3$

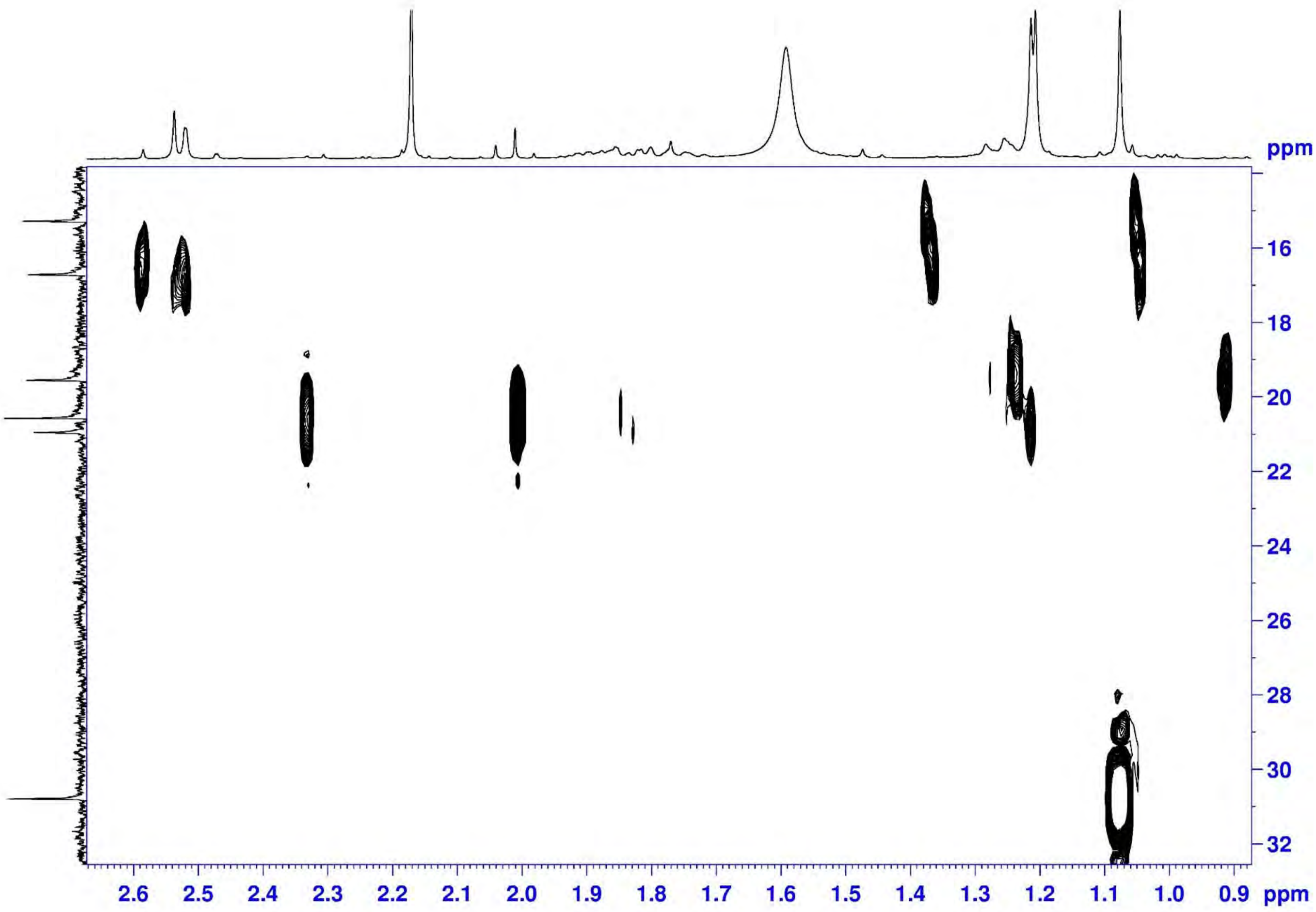

NOESY (400 MHz) spectrum of compound **22** in $CDCl_3$

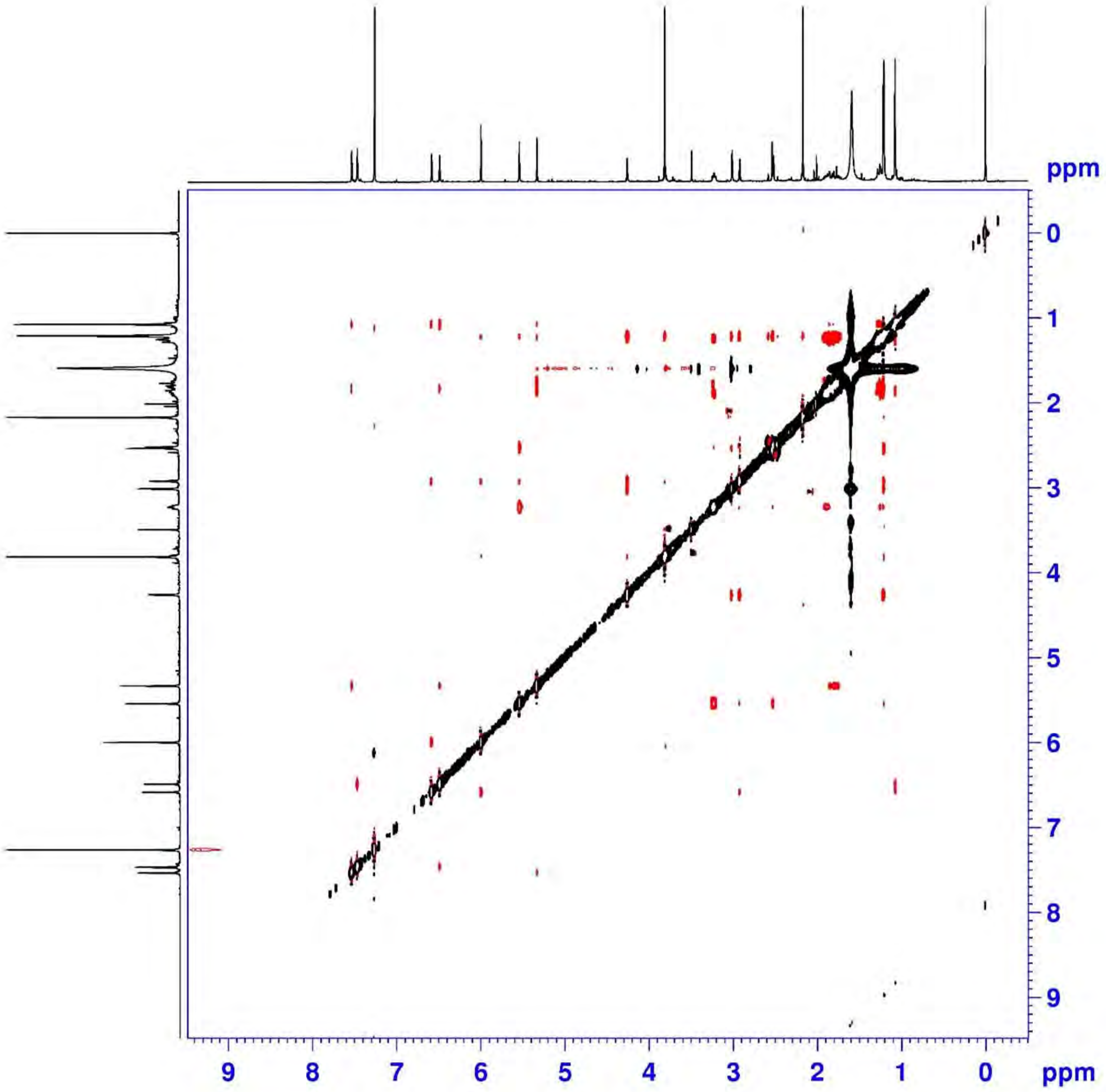

NAME	zjz-27=RTS-1
EXPNO	7
PROCNO	1
Date_	20160714
Time	8.32
INSTRUM	spect
PROBHD	5 mm CPPBBO BB
PULPROG	noesygpphpp
TD	2048
SOLVENT	CDCl3
NS	32
DS	32
SWH	4000.000 Hz
FIDRES	1.953125 Hz
AQ	0.2560500 sec
RG	208.5
DW	125.000 usec
DE	10.00 usec
TE	297.0 K
D0	0.00011036 sec
D1	1.99385595 sec
D8	0.30000001 sec
D11	0.03000000 sec
D12	0.00002000 sec
D16	0.00020000 sec
IN0	0.00025000 sec
======== CHANNEL f1 ========	
SFO1	400.1318006 MHz
NUC1	1H
P1	11.50 usec
P2	23.00 usec
P17	2500.00 usec
ND0	1
TD	256
SFO1	400.1318 MHz
FIDRES	15.625000 Hz
SW	9.997 ppm
FnMODE	States-TPPI
SI	1024
SF	400.1300070 MHz
WDW	QSINE
SSB	2
LB	0.00 Hz
GB	0
PC	1.00
SI	1024
MC2	States-TPPI
SF	400.1300068 MHz
WDW	QSINE
SSB	2
LB	0.00 Hz
GB	0

NOESY (400 MHz) spectrum of compound **22** in $CDCl_3$

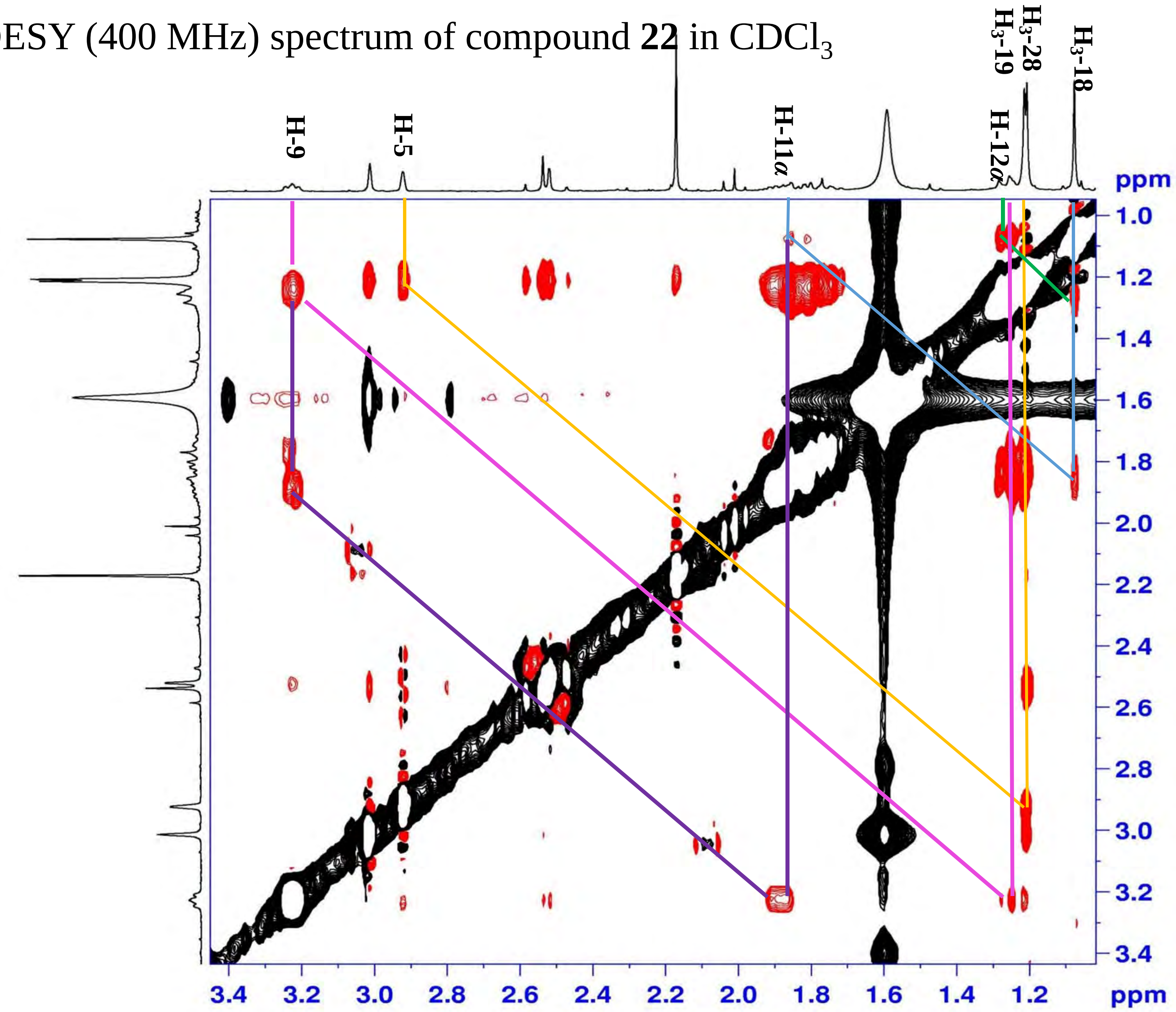

NOESY (400 MHz) spectrum of compound **22** in $CDCl_3$

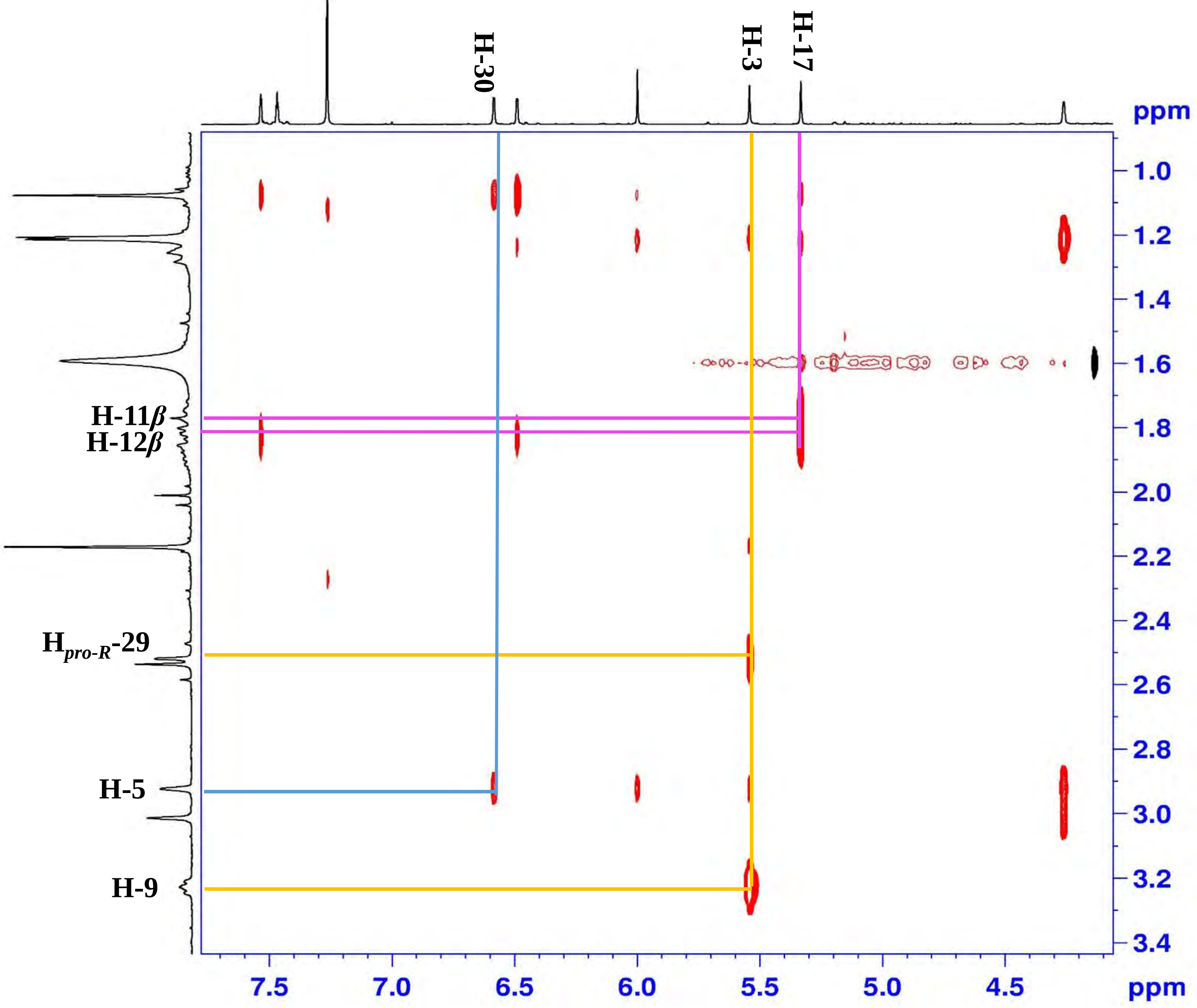

NOESY (400 MHz) spectrum of compound **22** in $CDCl_3$

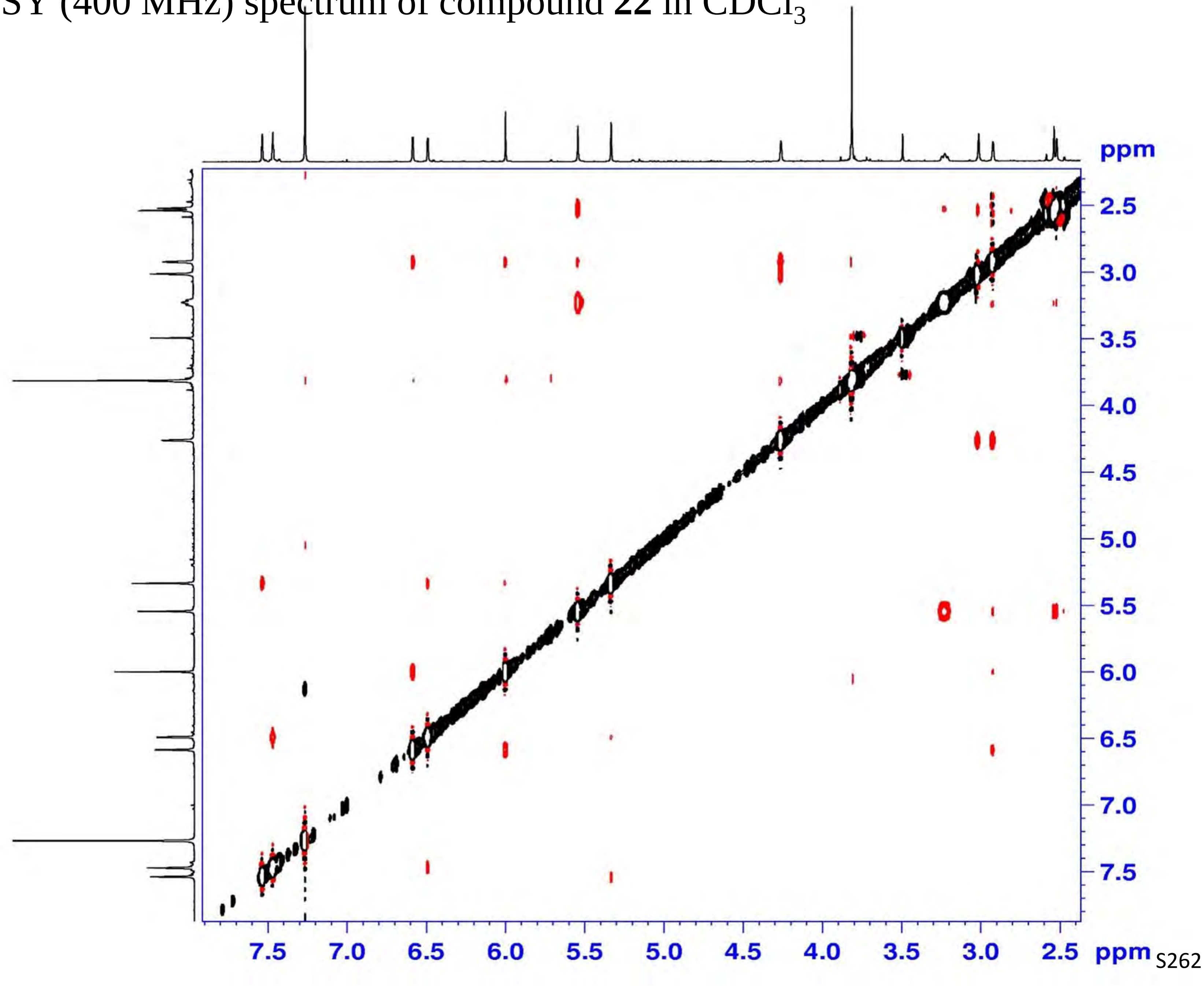

HRESIMS for compound **23**

Elemental Composition Report

Single Mass Analysis

Tolerance = 10.0 mDa / DBE: min = -1.0, max = 50.0
Element prediction: Off
Number of isotope peaks used for i-FIT = 3

Monoisotopic Mass, Even Electron Ions
168 formula(e) evaluated with 5 results within limits (up to 10 closest results for each mass)
Elements Used:
C: 0-100 H: 0-200 O: 0-100
252-35
2016112117 194 (1.563)

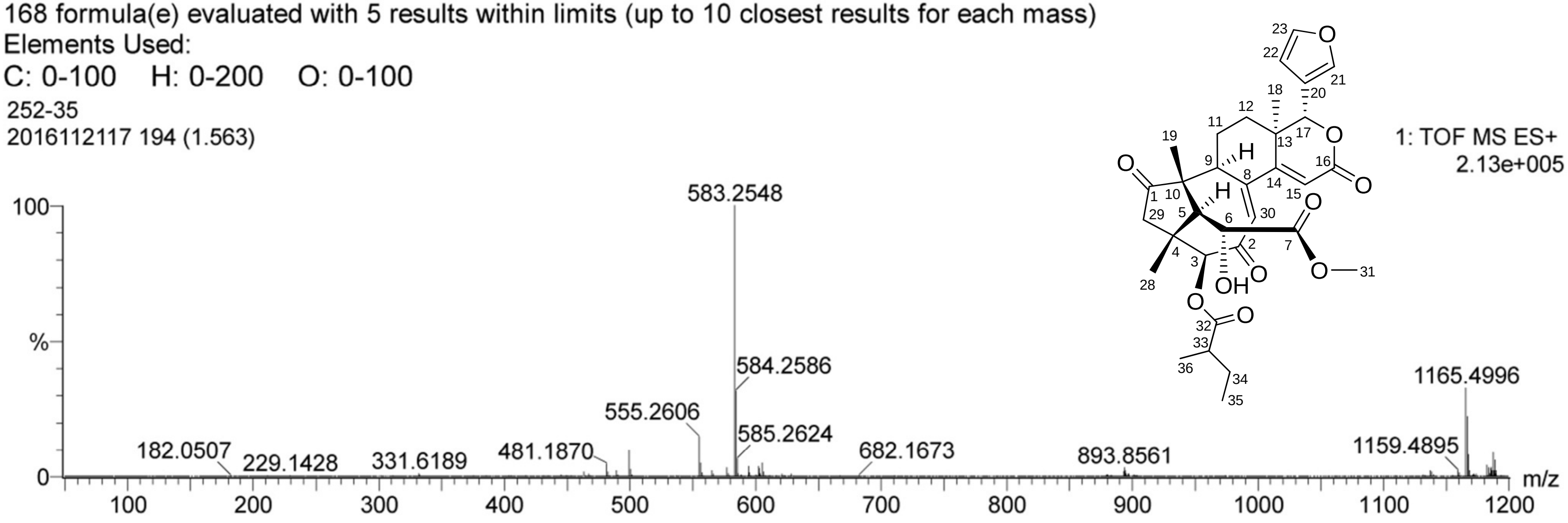

Minimum:				-1.0
Maximum:		10.0	10.0	50.0

Mass	Calc. Mass	mDa	PPM	DBE	i-FIT	Norm	Conf(%)	Formula
583.2548	583.2543	0.5	0.9	13.5	255.3	0.441	64.37	C32 H39 O10
	583.2602	-5.4	-9.3	4.5	256.0	1.073	34.19	C25 H43 O15
	583.2484	6.4	11.0	22.5	259.6	4.729	0.88	C39 H35 O5
	583.2449	9.9	17.0	0.5	260.5	5.585	0.38	C21 H43 O18
	583.2637	-8.9	-15.3	26.5	261.2	6.297	0.18	C43 H35 O2

^{1}H NMR (400 MHz) spectrum of compound **23** in $CDCl_3$

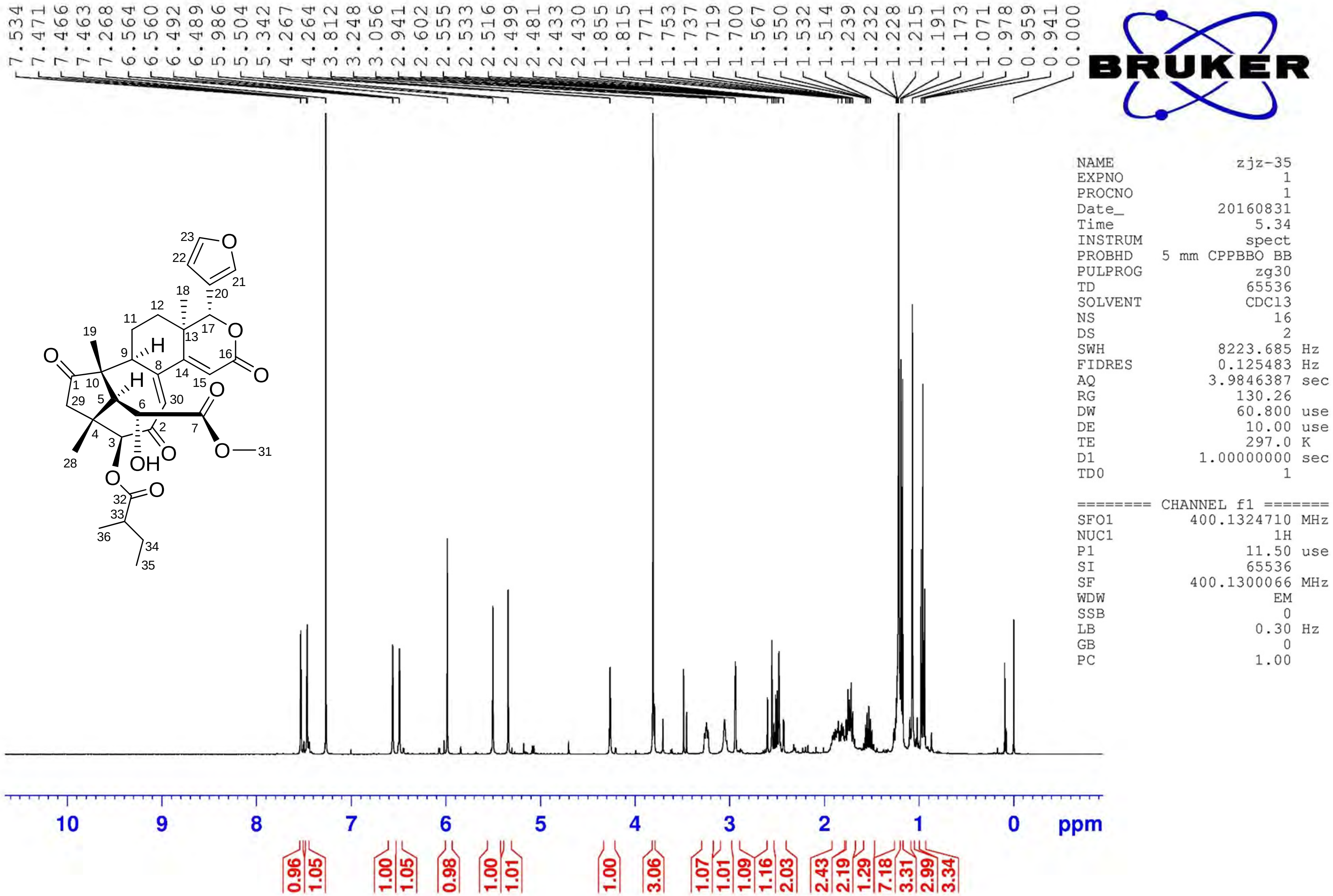

^{1}H NMR (400 MHz) spectrum of compound **23** in $CDCl_3$

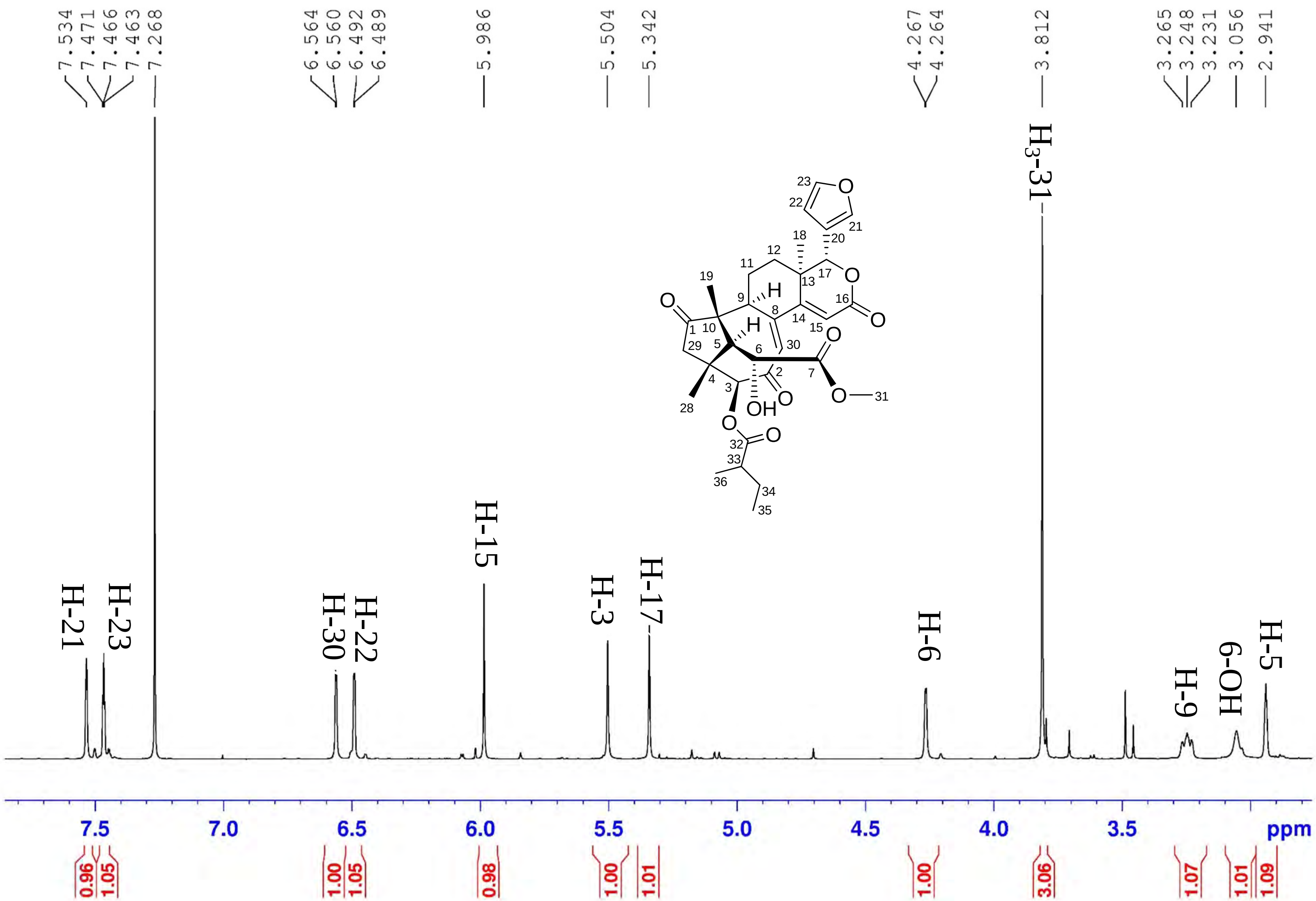

^{1}H NMR (400 MHz) spectrum of compound **23** in $CDCl_3$

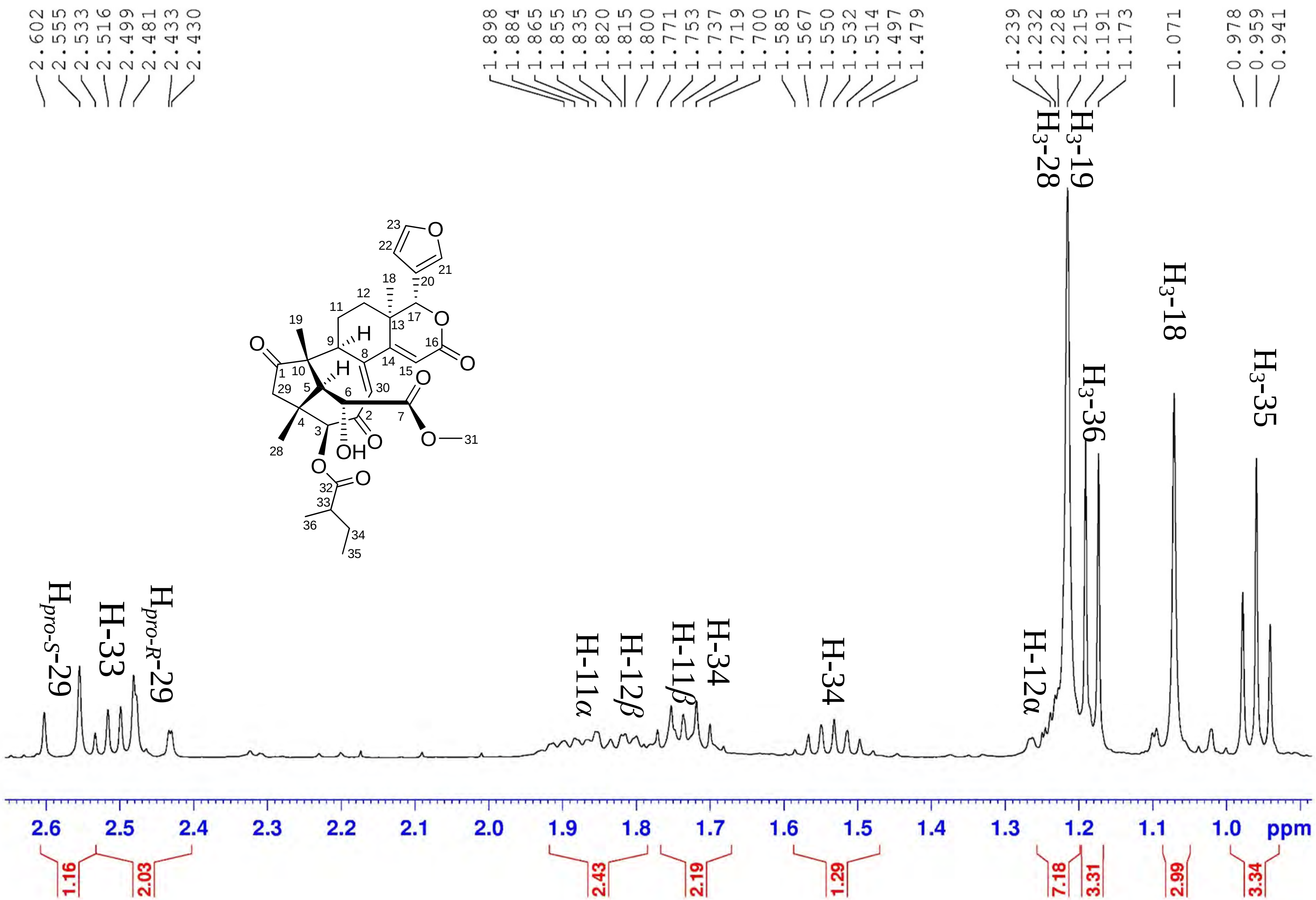

^{13}C NMR (100 MHz) spectrum of compound **23** in $CDCl_3$

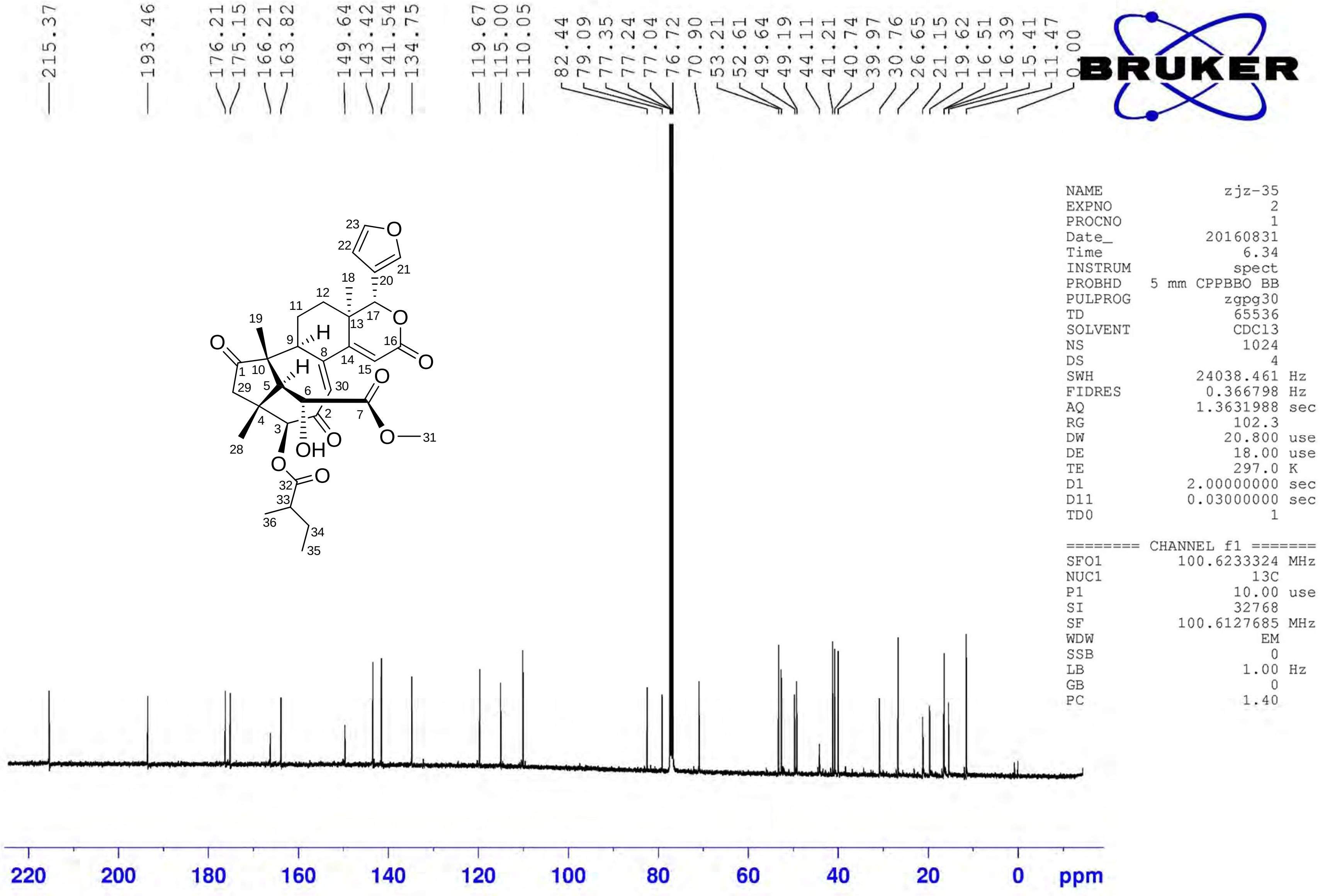

NAME	zjz-35
EXPNO	2
PROCNO	1
Date_	20160831
Time	6.34
INSTRUM	spect
PROBHD	5 mm CPPBBO BB
PULPROG	zgpg30
TD	65536
SOLVENT	CDCl3
NS	1024
DS	4
SWH	24038.461 Hz
FIDRES	0.366798 Hz
AQ	1.3631988 sec
RG	102.3
DW	20.800 use
DE	18.00 use
TE	297.0 K
D1	2.00000000 sec
D11	0.03000000 sec
TD0	1

======== CHANNEL f1 =======

SFO1	100.6233324 MHz
NUC1	13C
P1	10.00 use
SI	32768
SF	100.6127685 MHz
WDW	EM
SSB	0
LB	1.00 Hz
GB	0
PC	1.40

^{13}C NMR (100 MHz) spectrum of compound **23** in $CDCl_3$

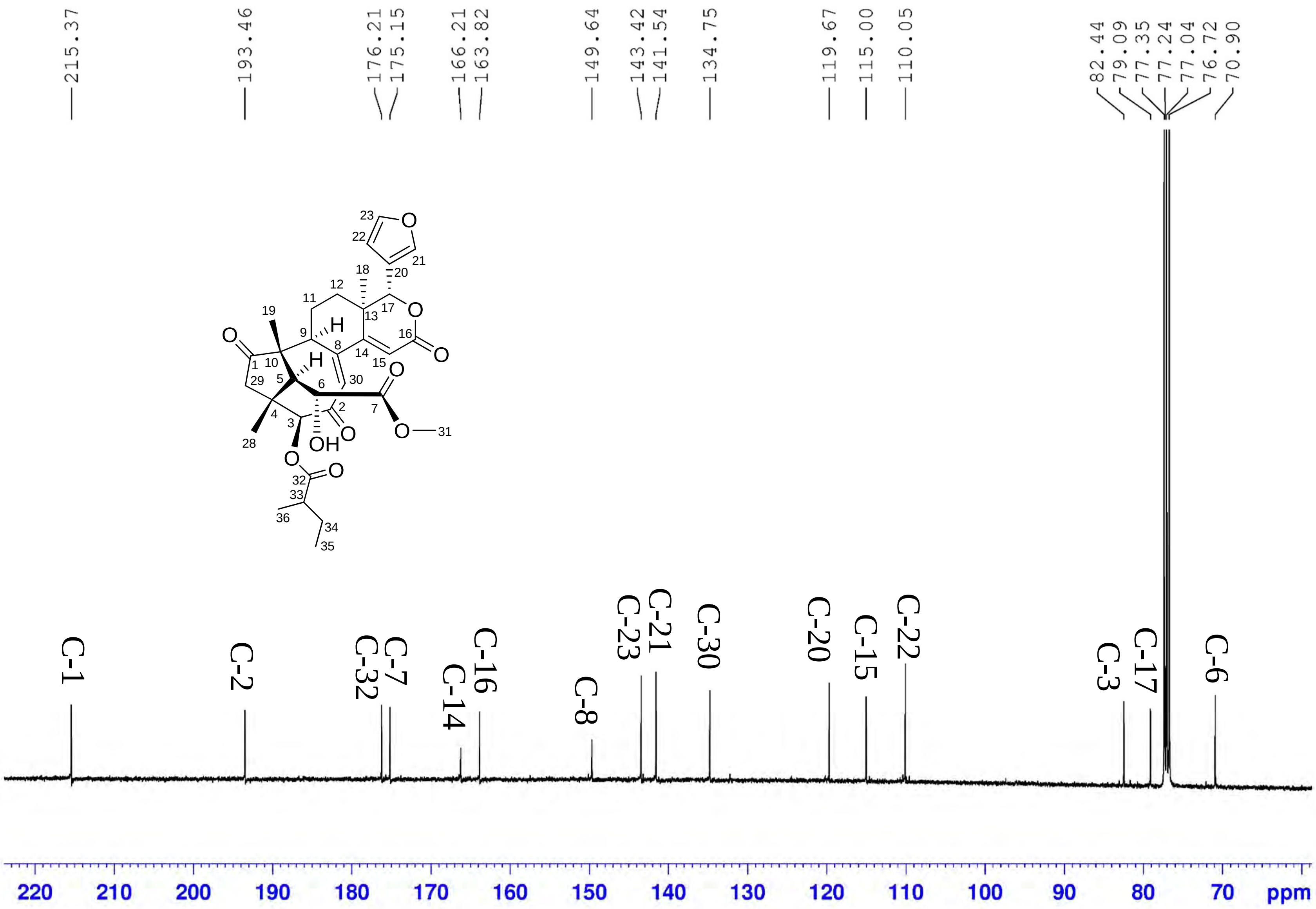

^{13}C NMR (100 MHz) spectrum of compound **23** in $CDCl_3$

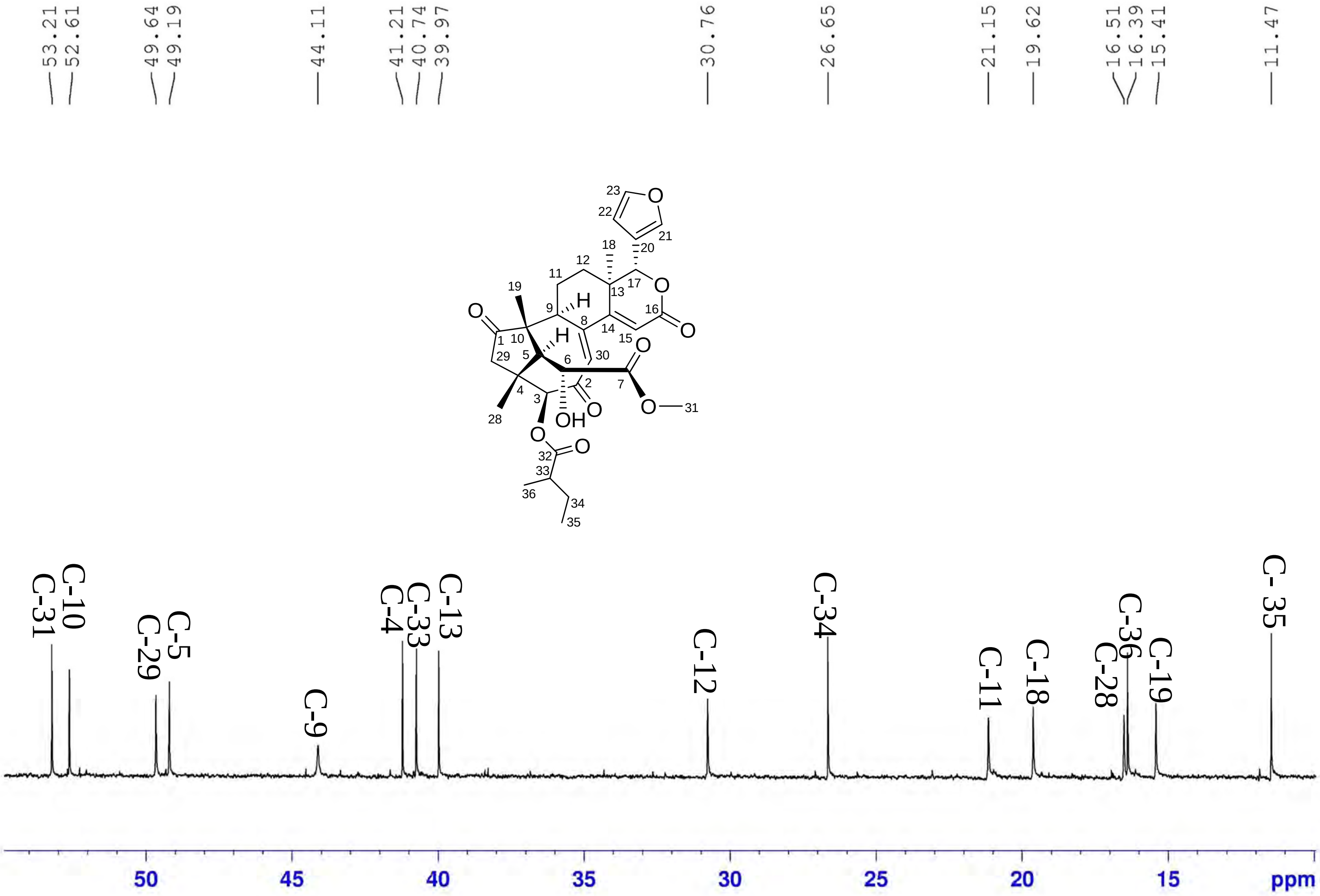

DEPT135 (100 MHz) spectrum of compound **23** in $CDCl_3$

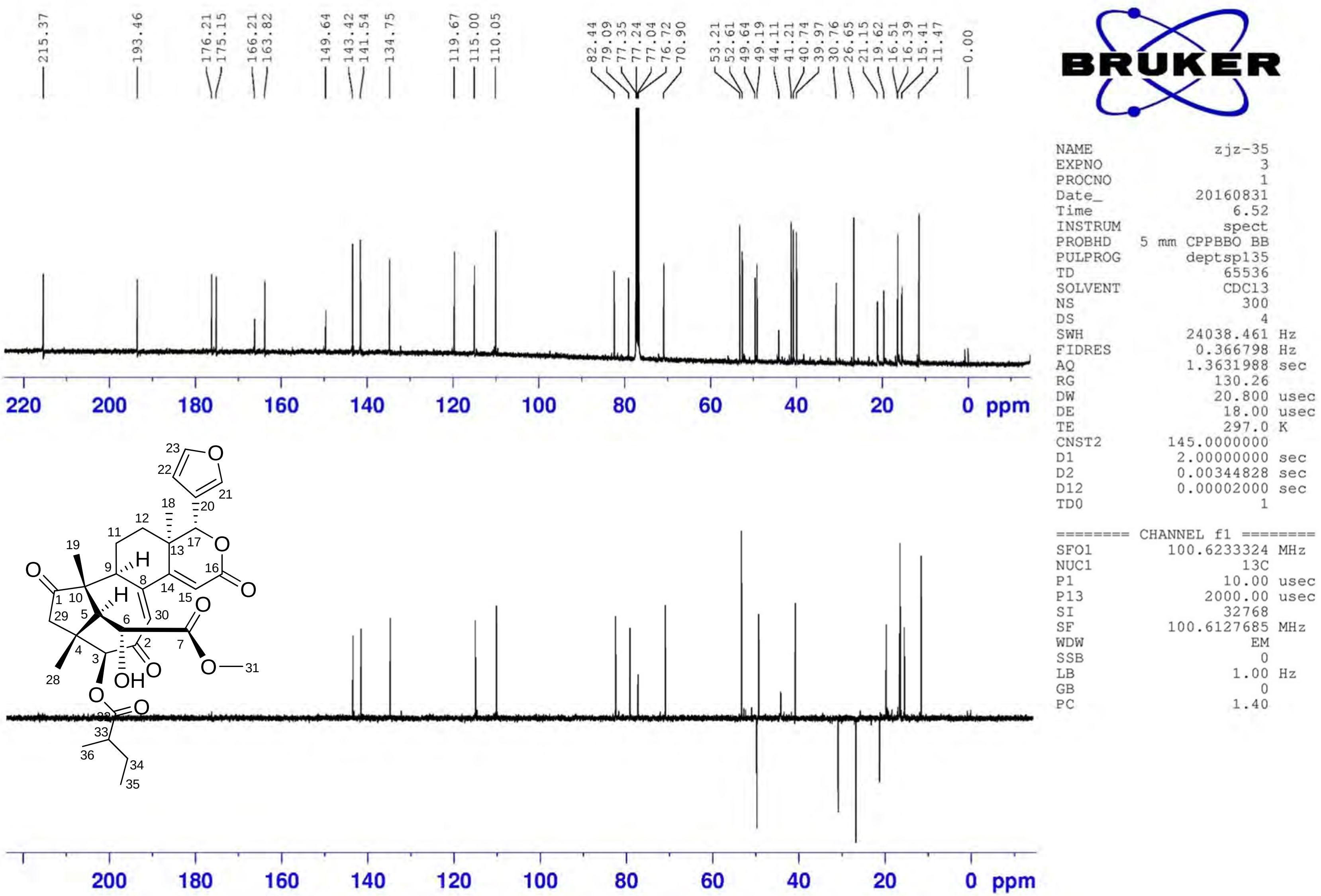

DEPT135 (100 MHz) spectrum of compound **23** in $CDCl_3$

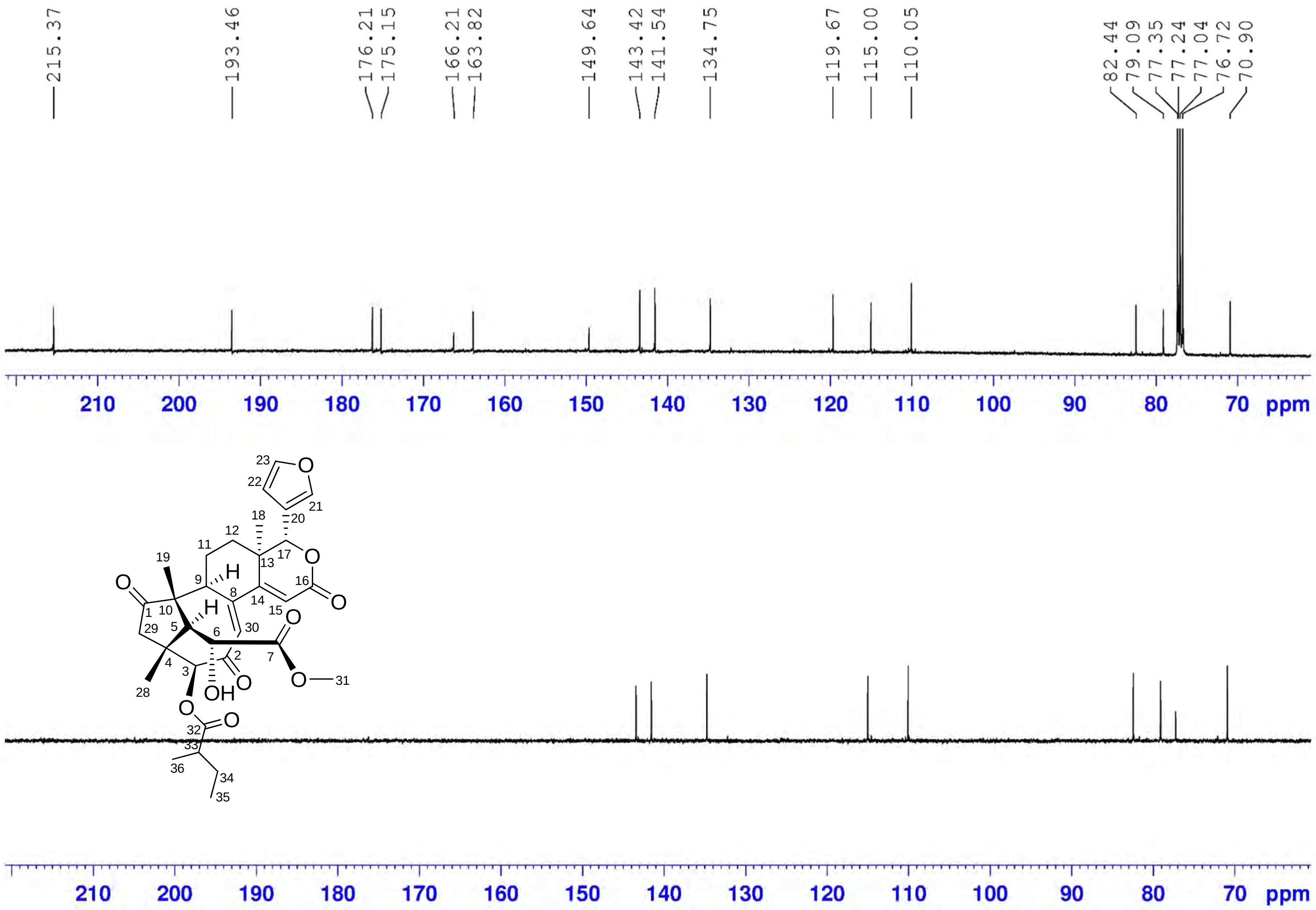

DEPT135 (100 MHz) spectrum of compound **23** in $CDCl_3$

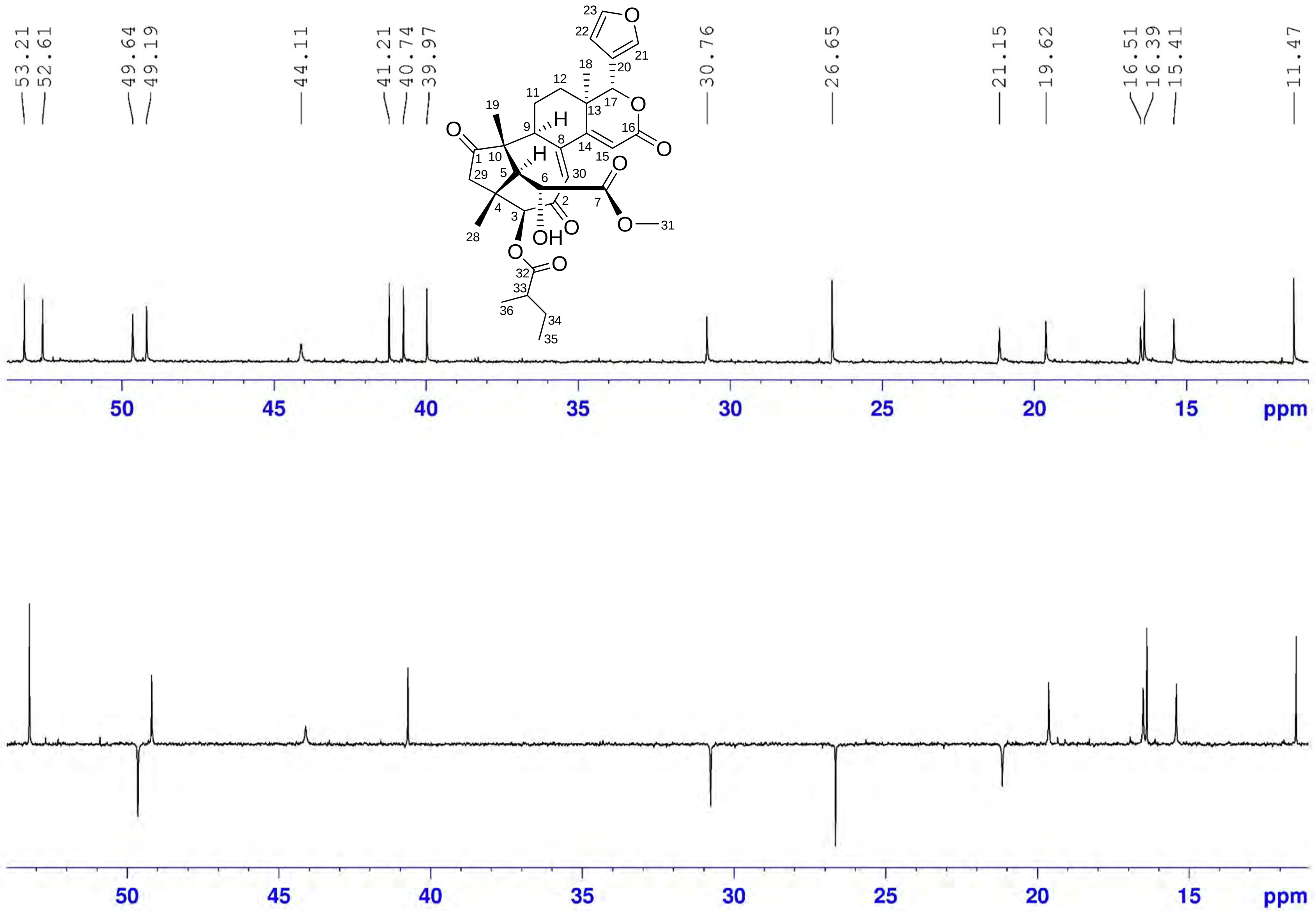

^{1}H-^{1}H COSY (400 MHz) spectrum of compound **23** in $CDCl_3$

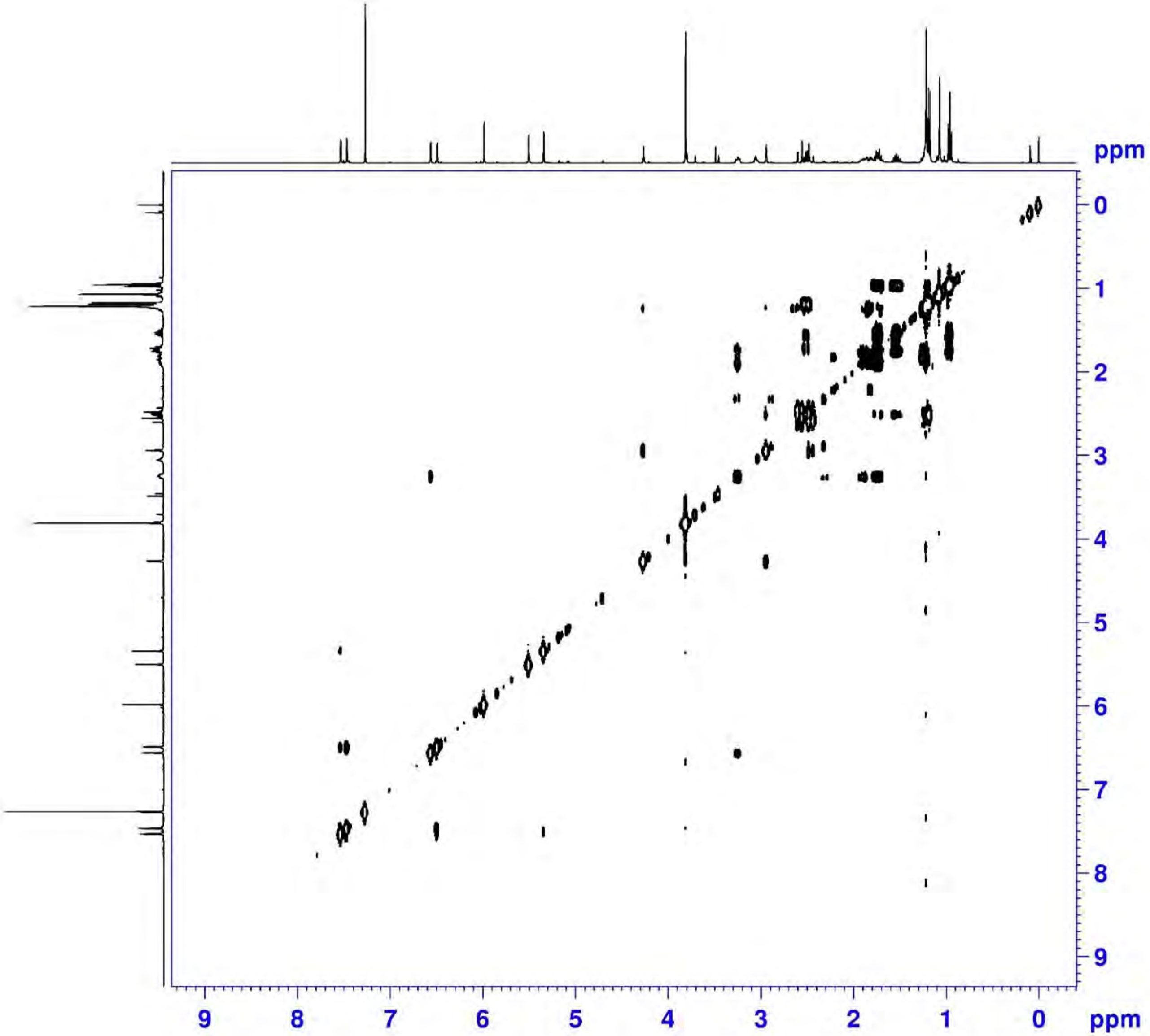

NAME	zjz-35
EXPNO	4
PROCNO	1
Date_	20160913
Time	14.44
INSTRUM	spect
PROBHD	5 mm CPPBBO BB
PULPROG	cosygpppqf
TD	2048
SOLVENT	CDCl3
NS	8
DS	8
SWH	3906.250 Hz
FIDRES	1.907349 Hz
AQ	0.2621940 sec
RG	208.5
DW	128.000 usec
DE	10.00 usec
TE	297.0 K
D0	0.00000300 sec
D1	1.89678097 sec
D11	0.03000000 sec
D12	0.00002000 sec
D13	0.00000400 sec
D16	0.00020000 sec
IN0	0.00025600 sec

======== CHANNEL f1 ========

SFO1	400.1318006 MHz
NUC1	1H
P0	11.50 usec
P1	11.50 usec
P17	2500.00 usec
ND0	1
TD	128
SFO1	400.1318 MHz
FIDRES	30.517578 Hz
SW	9.762 ppm
FnMODE	QF
SI	1024
SF	400.1300047 MHz
WDW	QSINE
SSB	0
LB	0.00 Hz
GB	0
PC	1.40
SI	1024
MC2	QF
SF	400.1300039 MHz
WDW	QSINE
SSB	0
LB	0.00 Hz
GB	0

^1H-^1H COSY (400 MHz) spectrum of compound **23** in $CDCl_3$

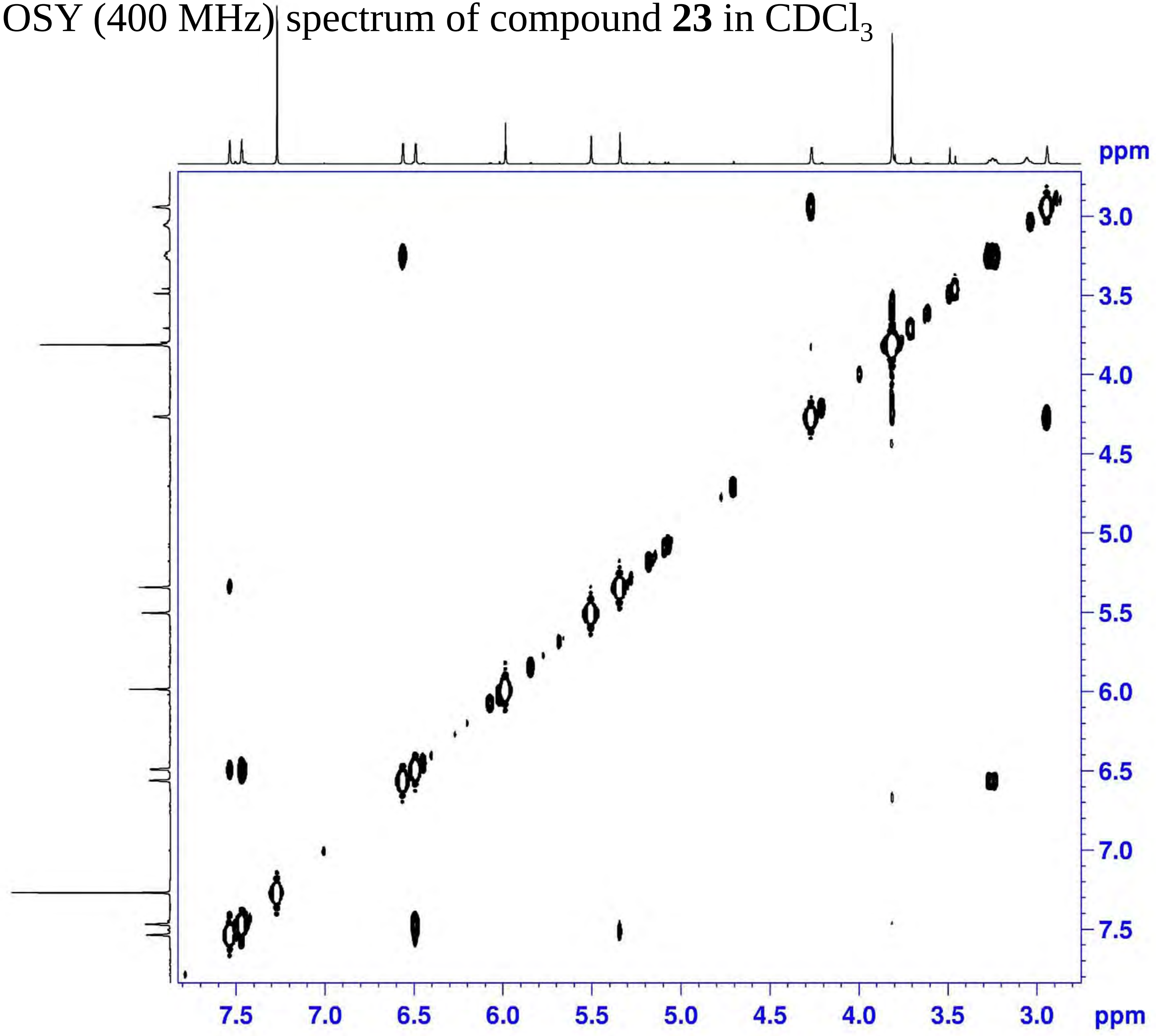

^{1}H-^{1}H COSY (400 MHz) spectrum of compound **23** in $CDCl_3$

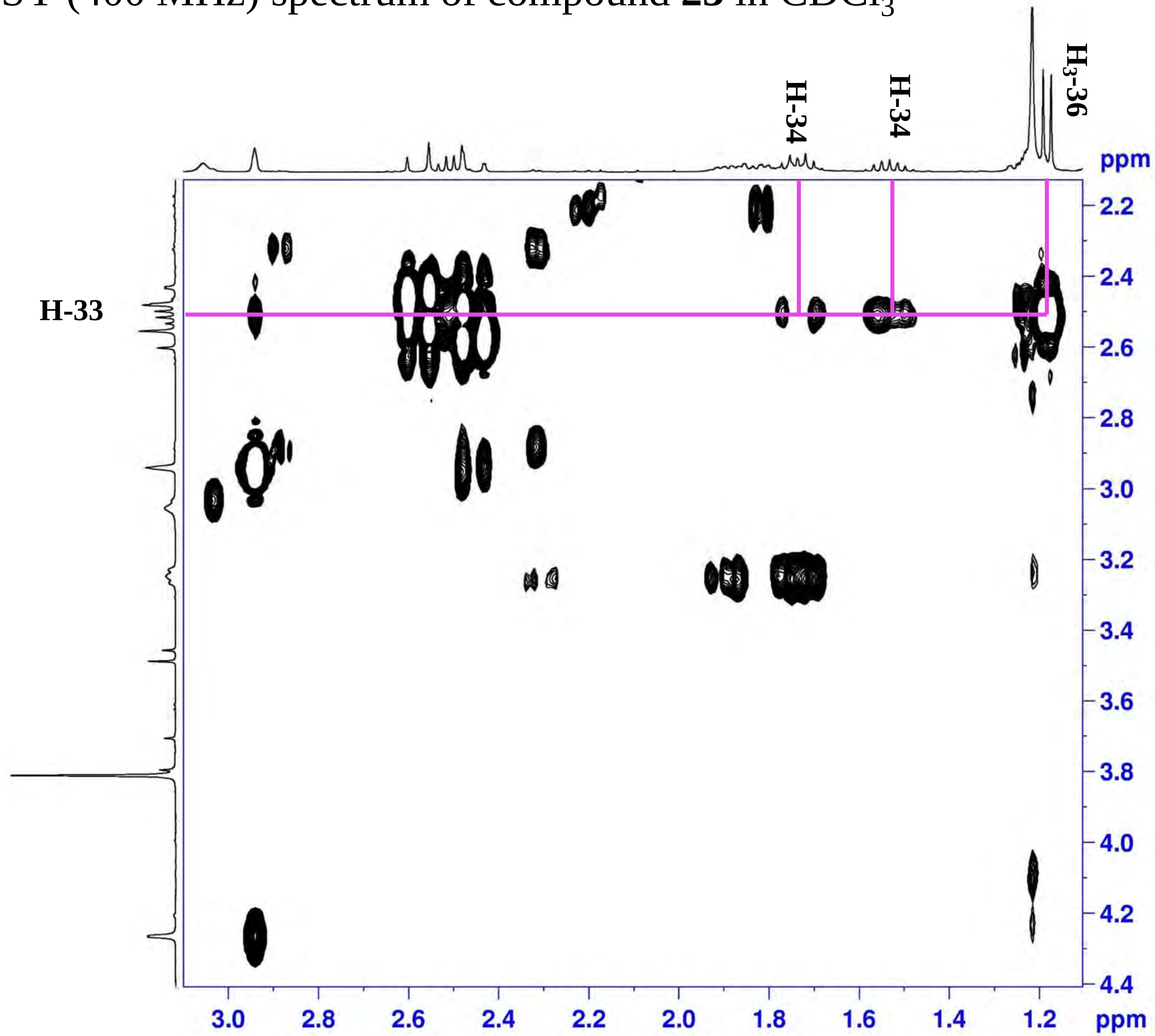

HSQC (400 MHz) spectrum of compound **23** in $CDCl_3$

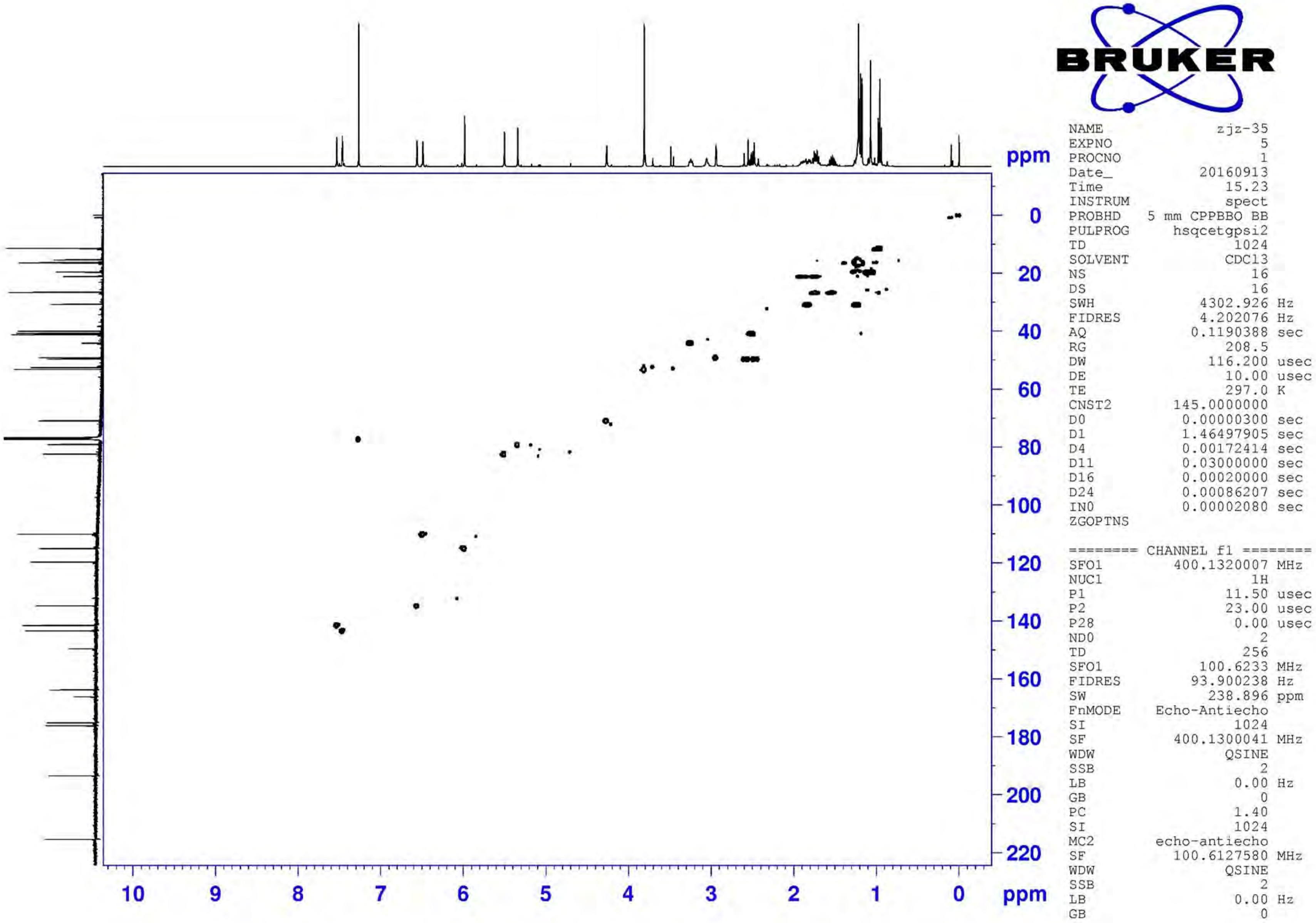

HSQC (400 MHz) spectrum of compound **23** in $CDCl_3$

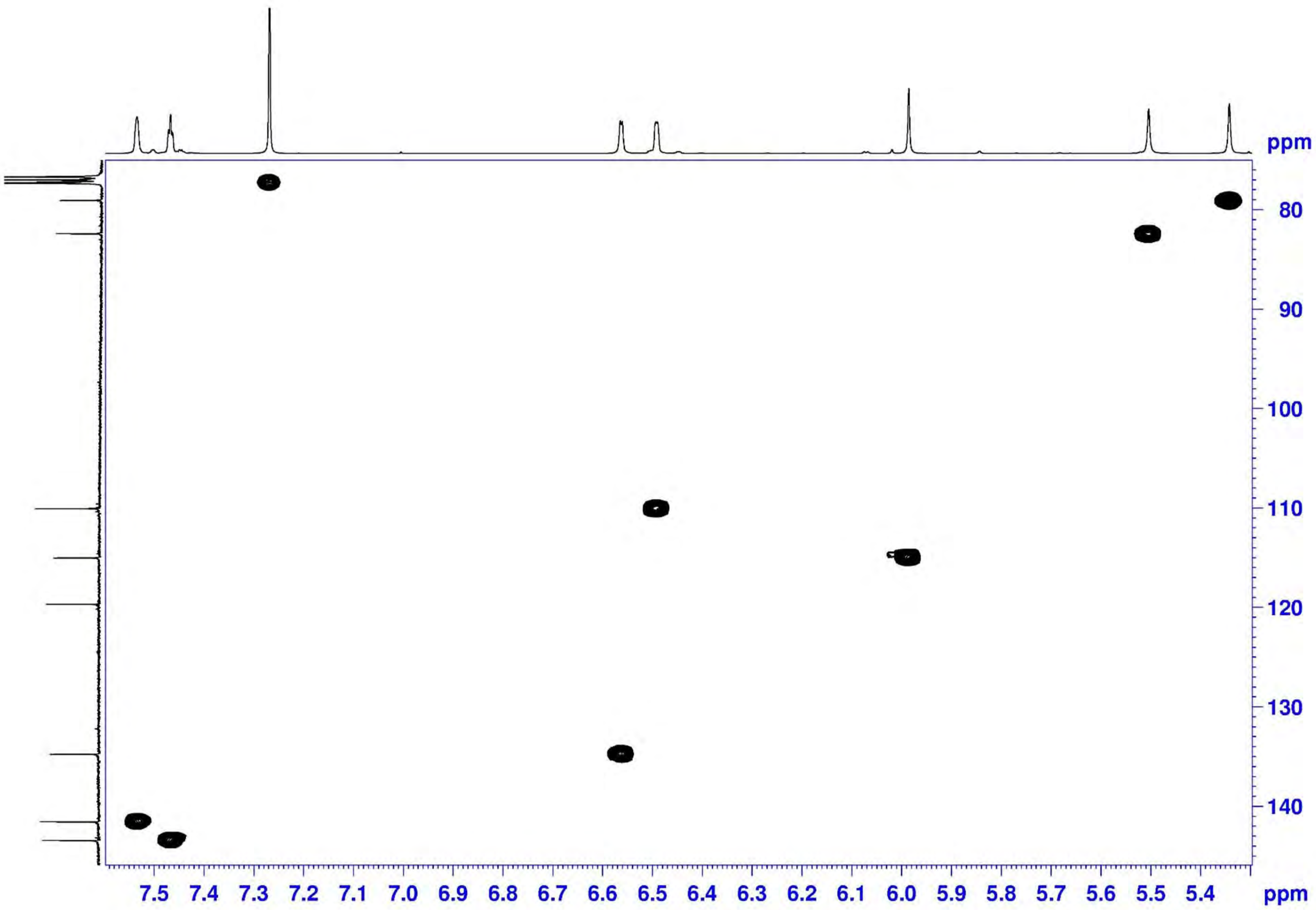

HSQC (400 MHz) spectrum of compound **23** in $CDCl_3$

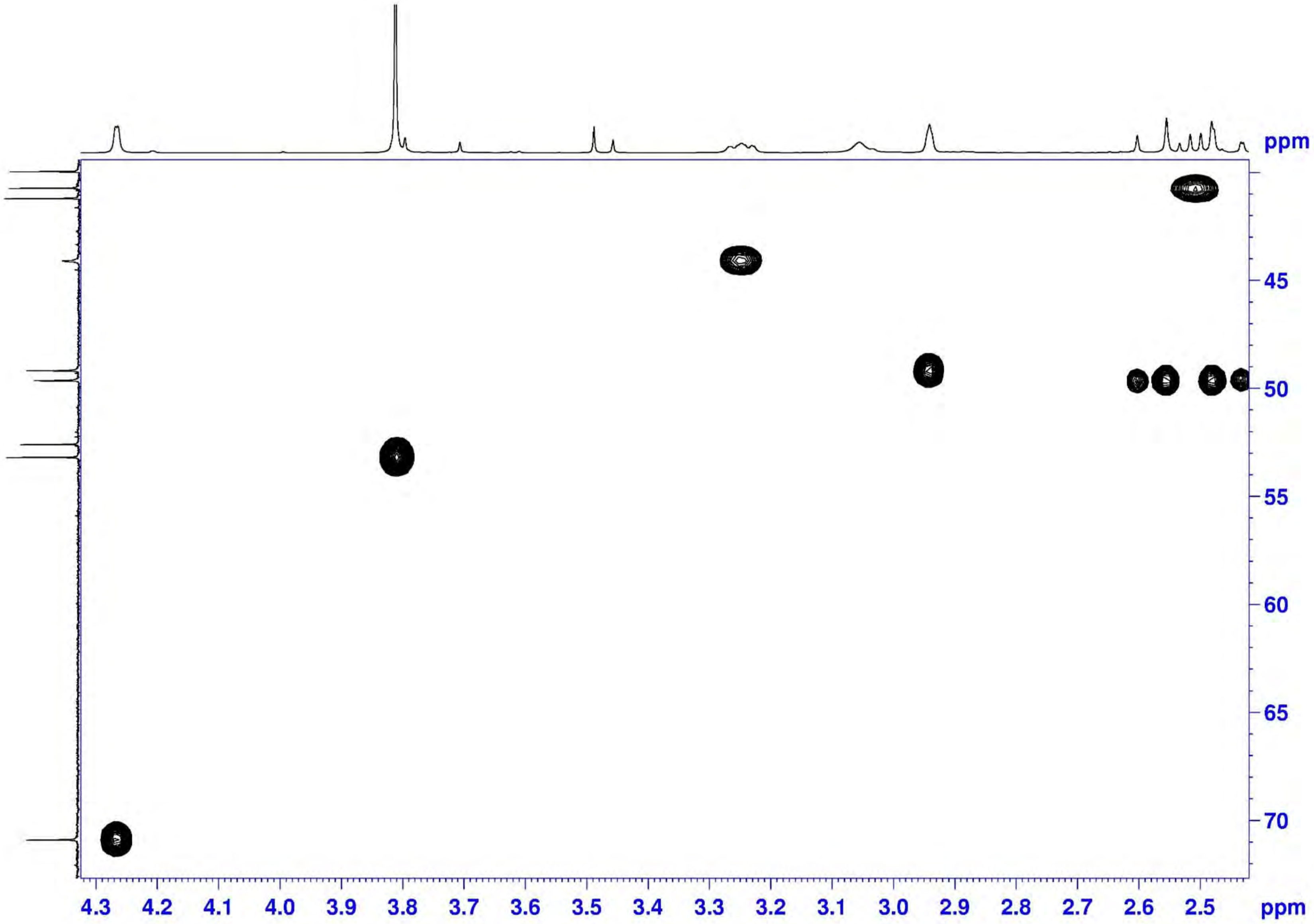

HSQC (400 MHz) spectrum of compound **23** in $CDCl_3$

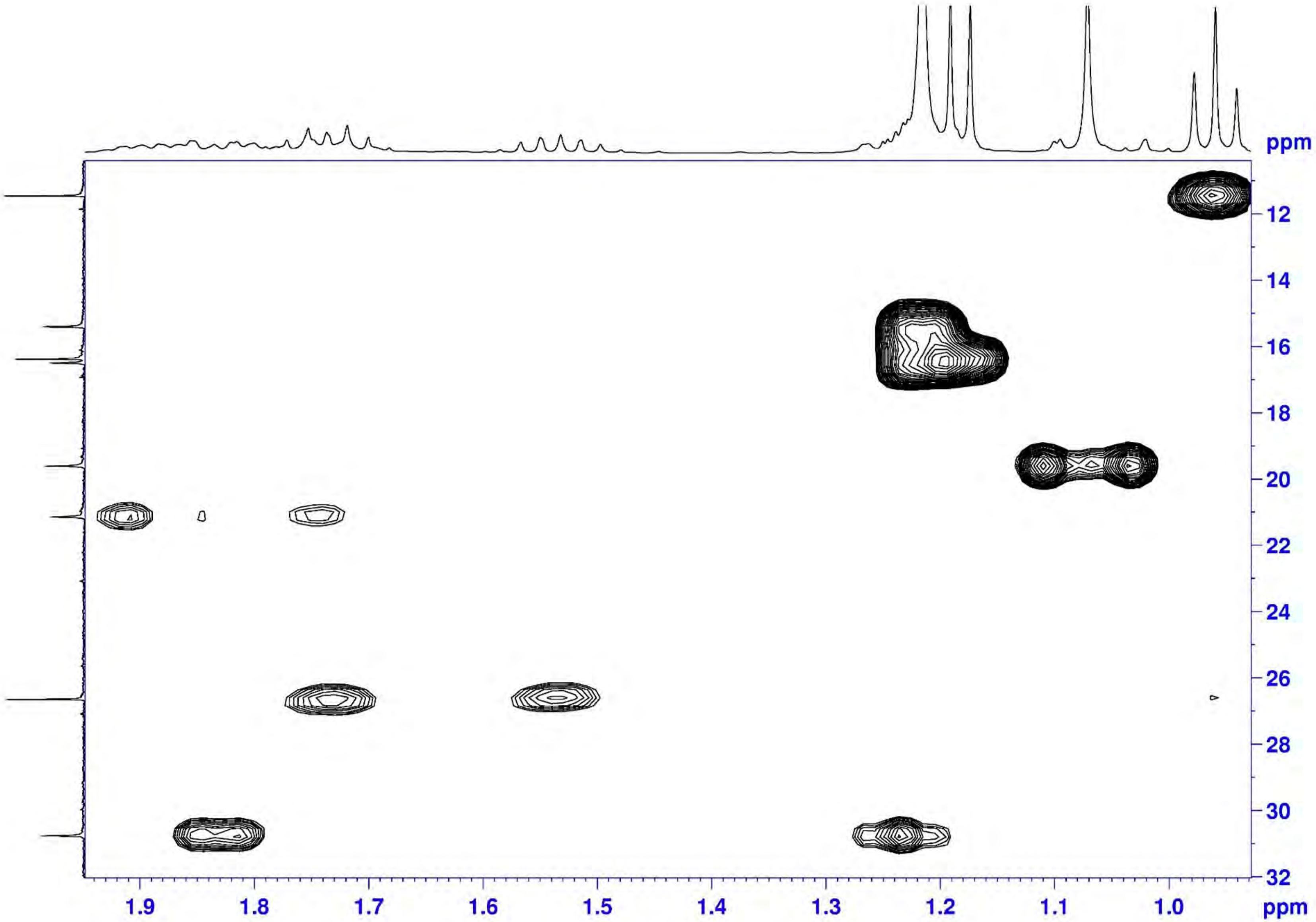

HMBC (400 MHz) spectrum of compound **23** in $CDCl_3$

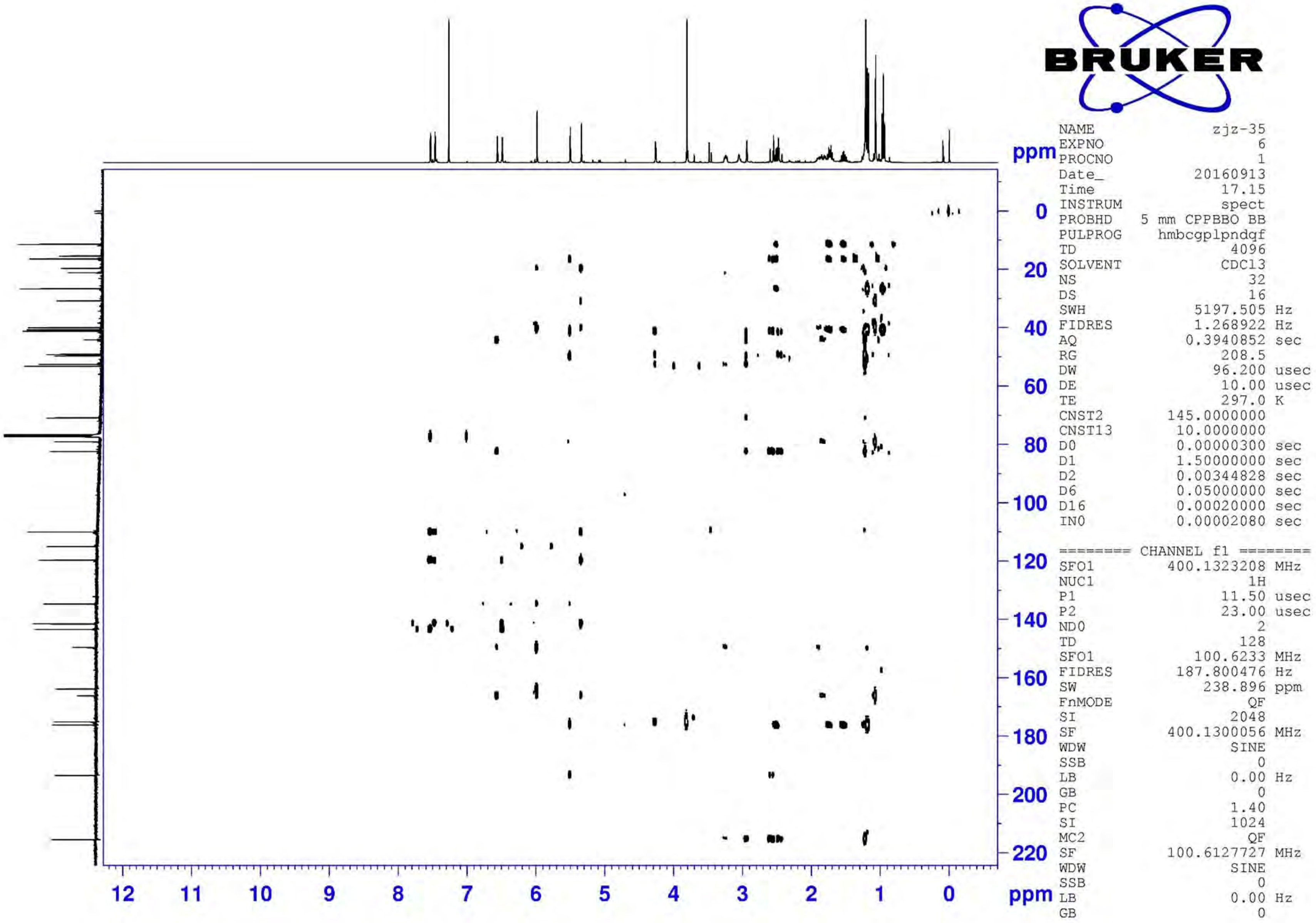

HMBC (400 MHz) spectrum of compound **23** in $CDCl_3$

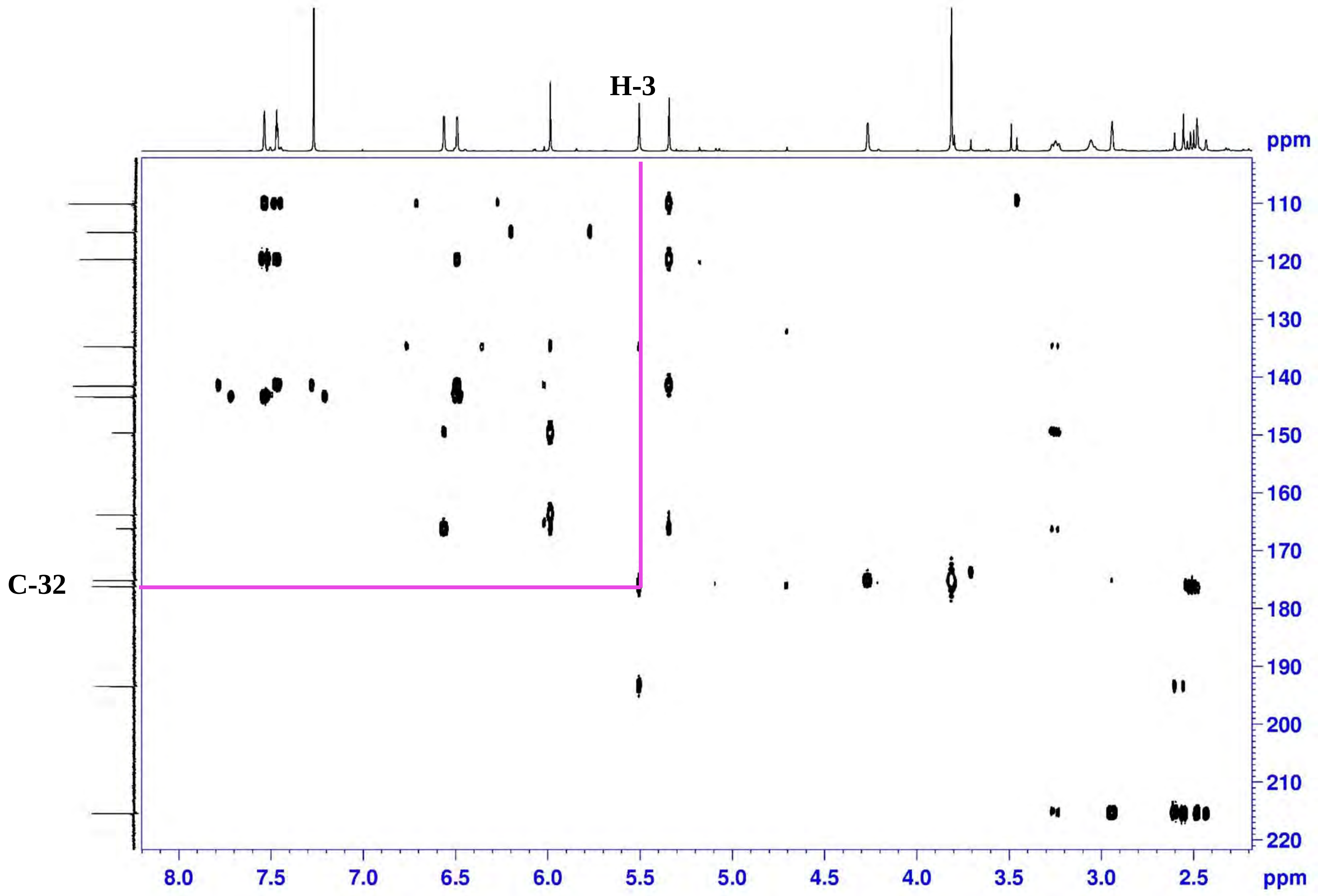

HMBC (400 MHz) spectrum of compound **23** in $CDCl_3$

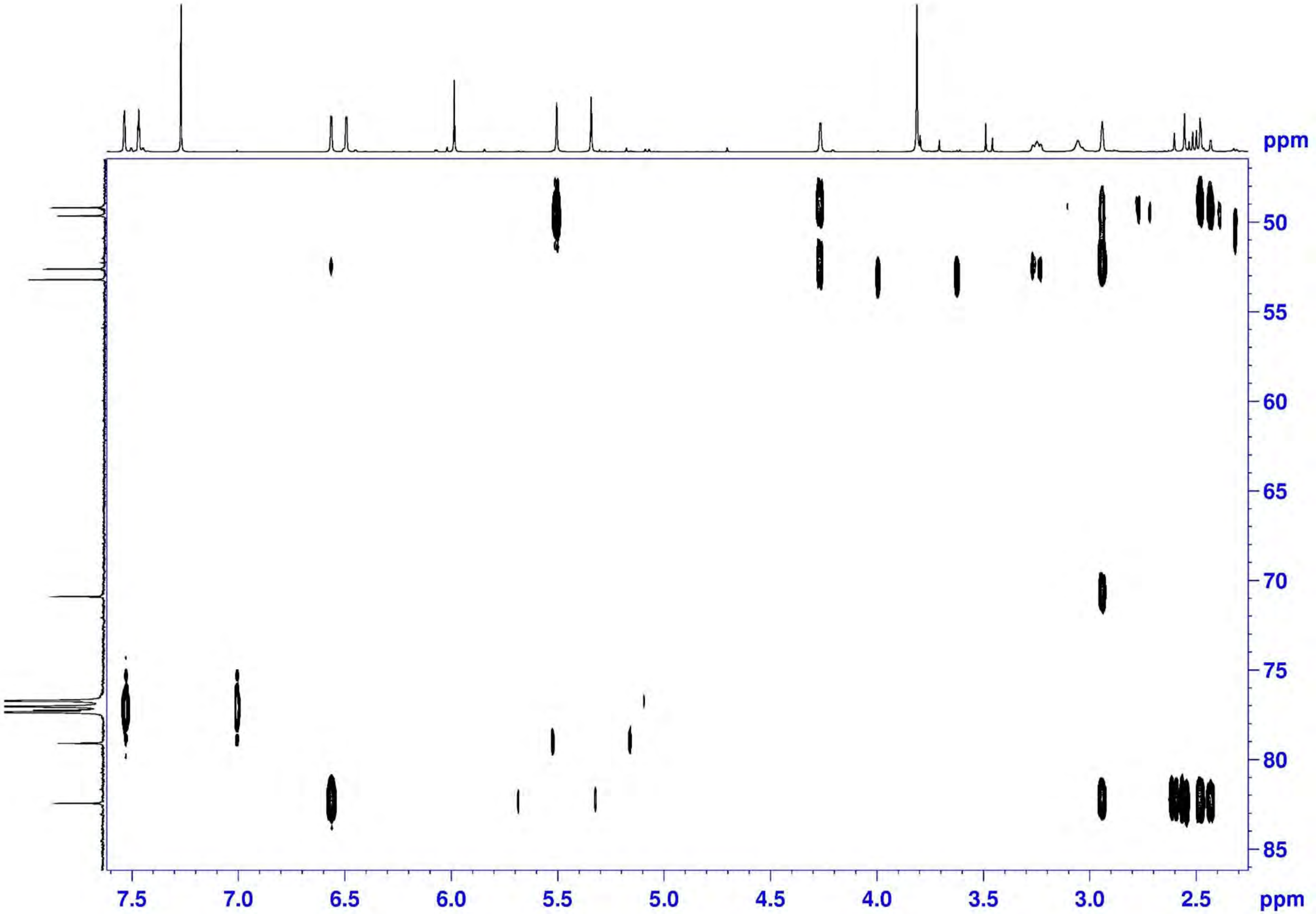

HMBC (400 MHz) spectrum of compound **23** in $CDCl_3$

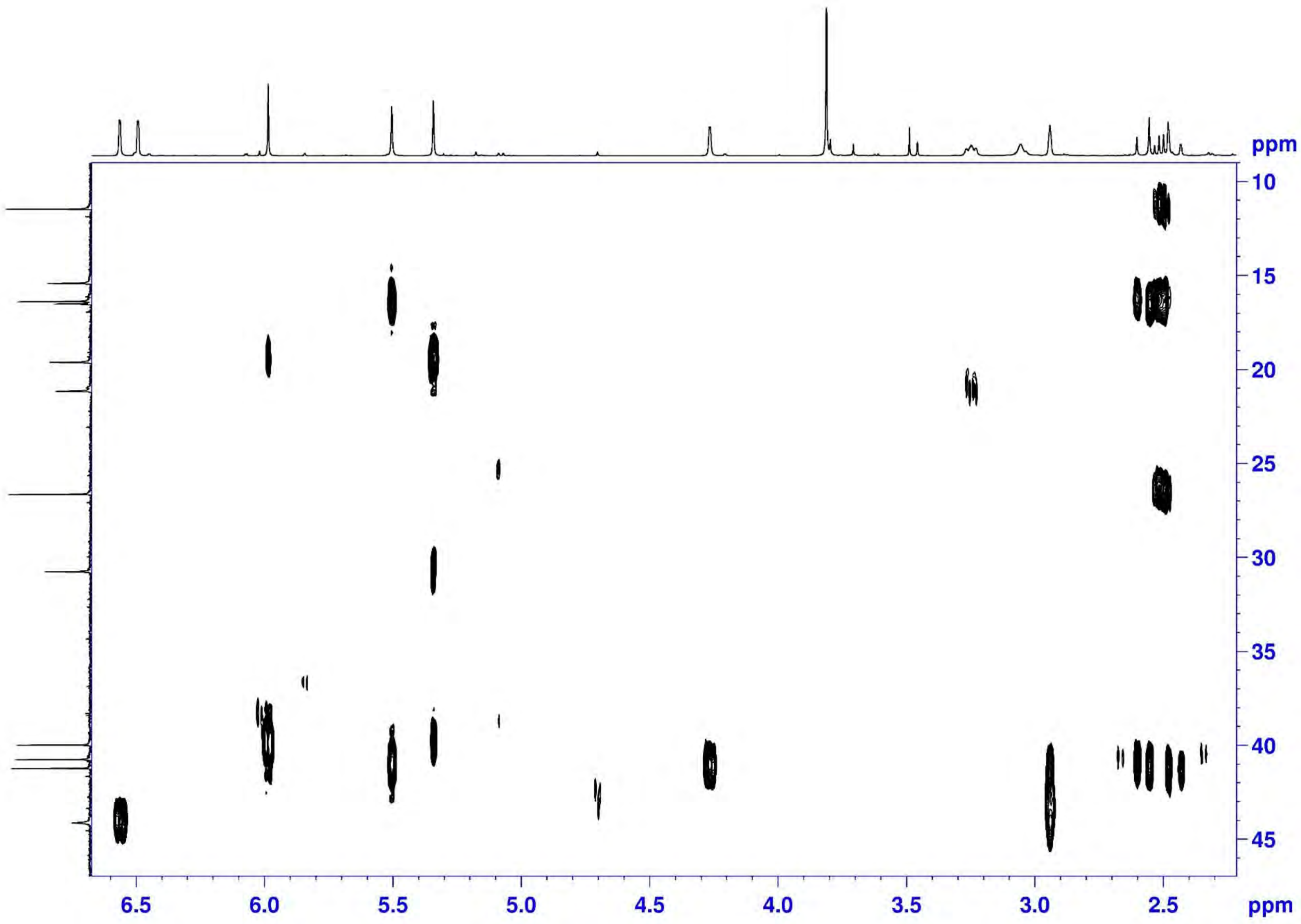

HMBC (400 MHz) spectrum of compound **23** in $CDCl_3$

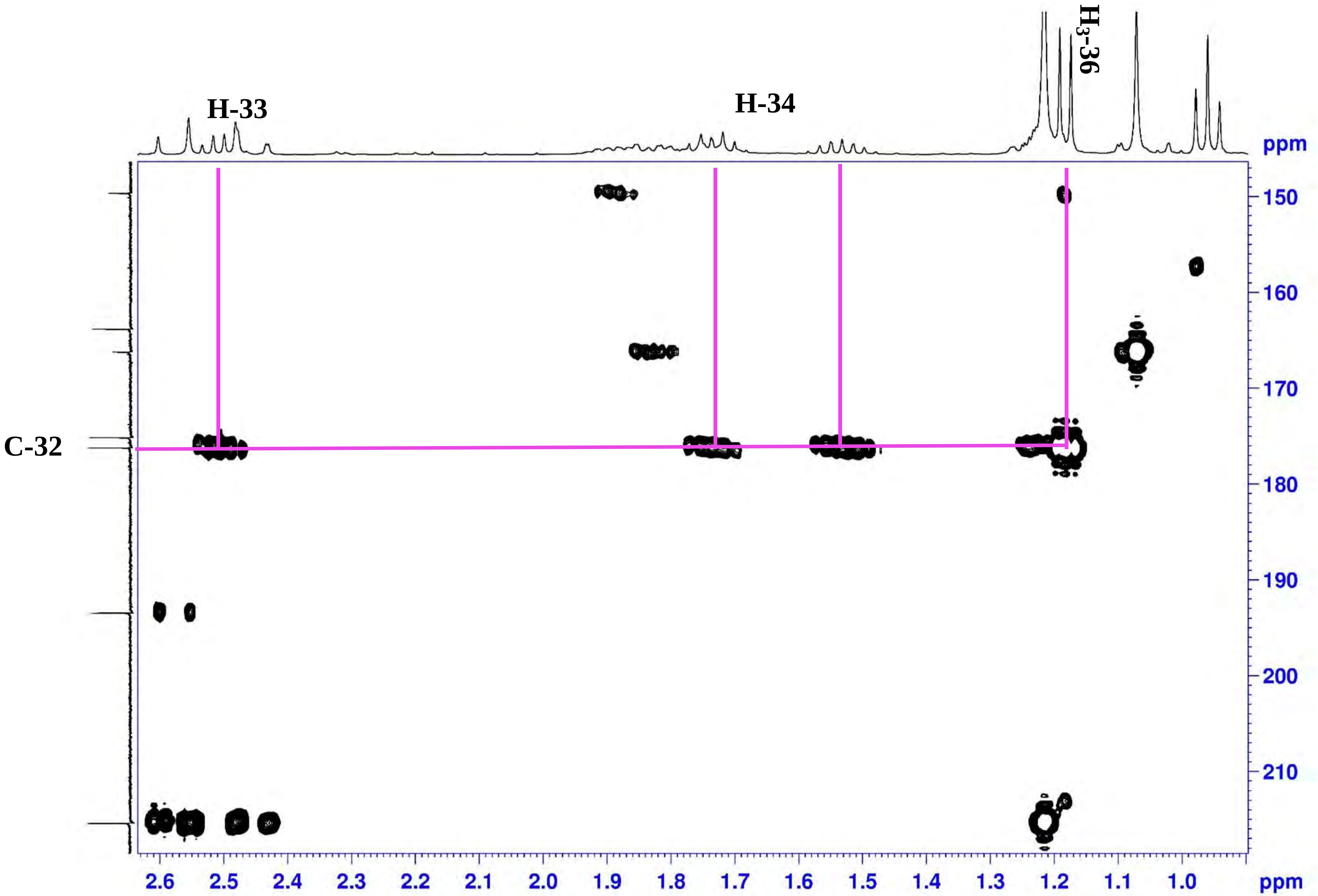

HMBC (400 MHz) spectrum of compound **23** in $CDCl_3$

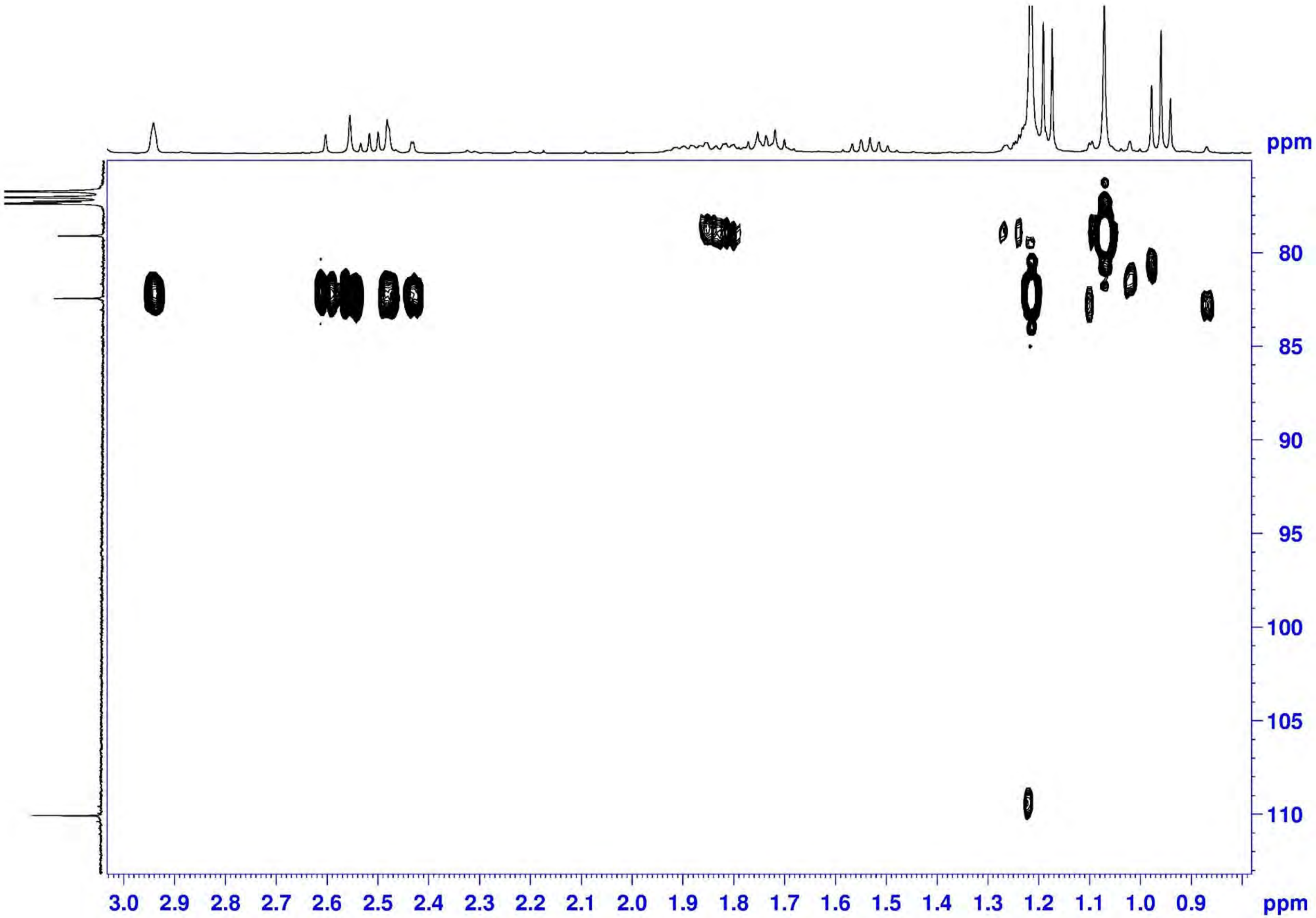

HMBC (400 MHz) spectrum of compound **23** in $CDCl_3$

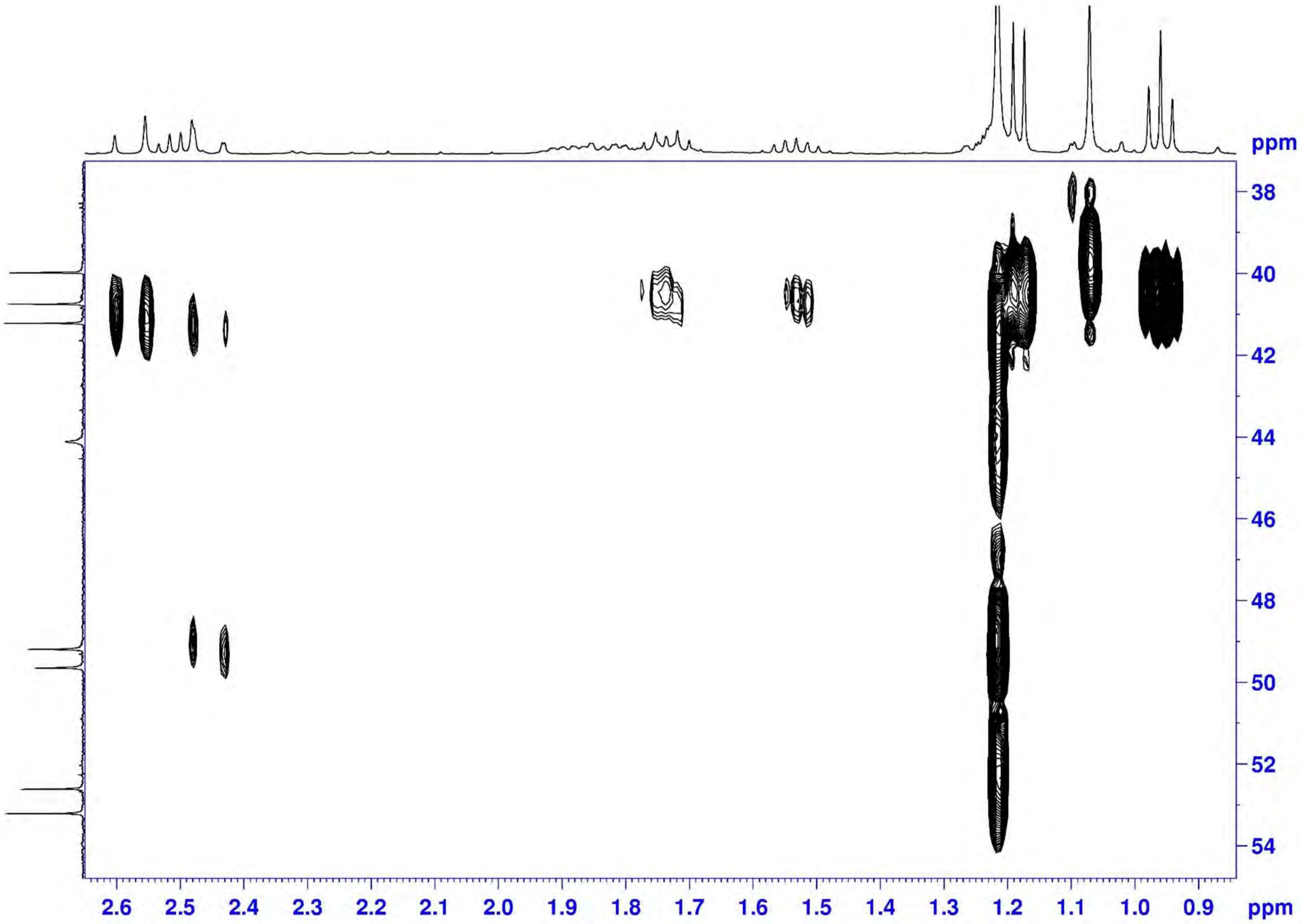

HMBC (400 MHz) spectrum of compound **23** in $CDCl_3$

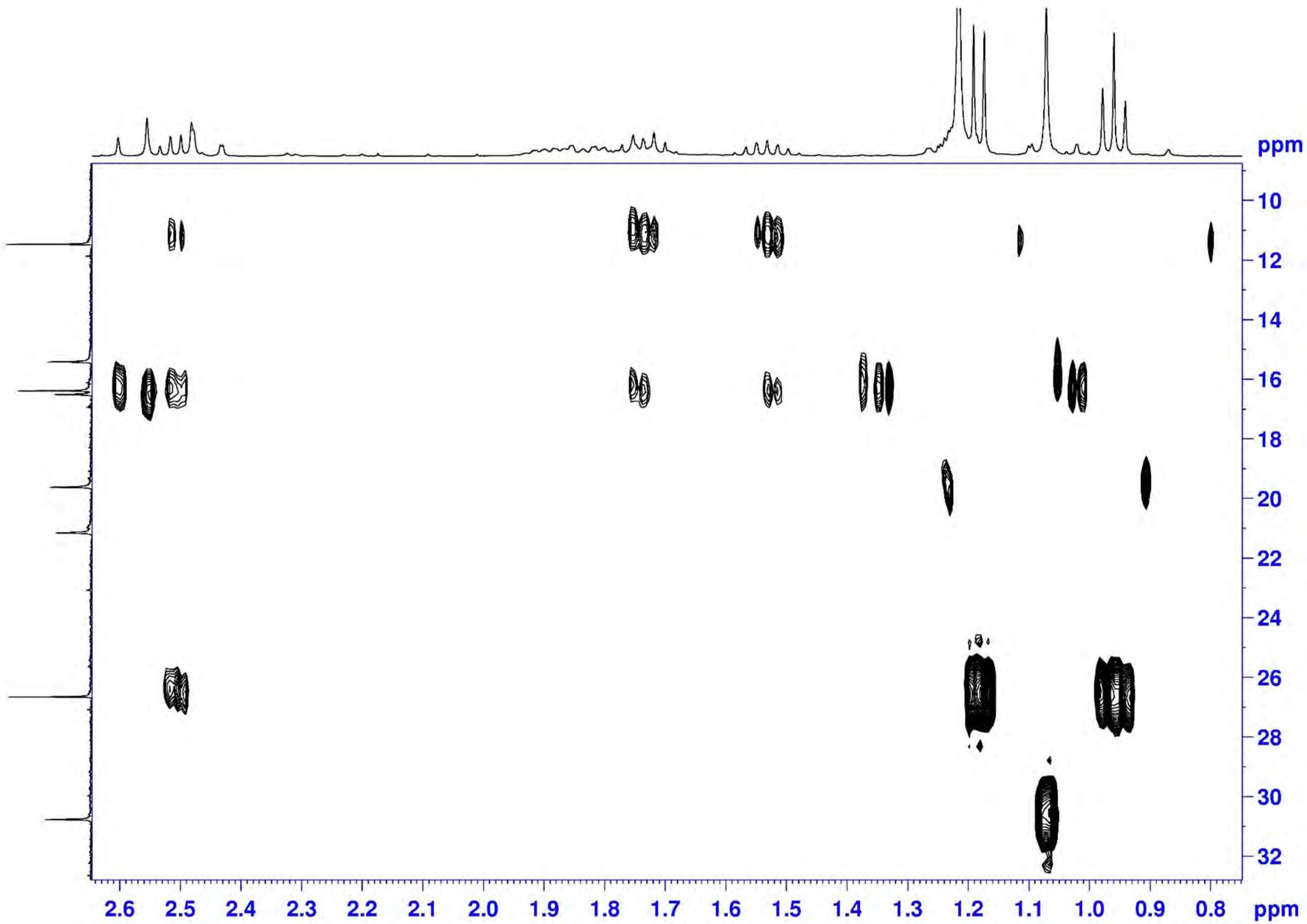

NOESY (400 MHz) spectrum of compound **23** in $CDCl_3$

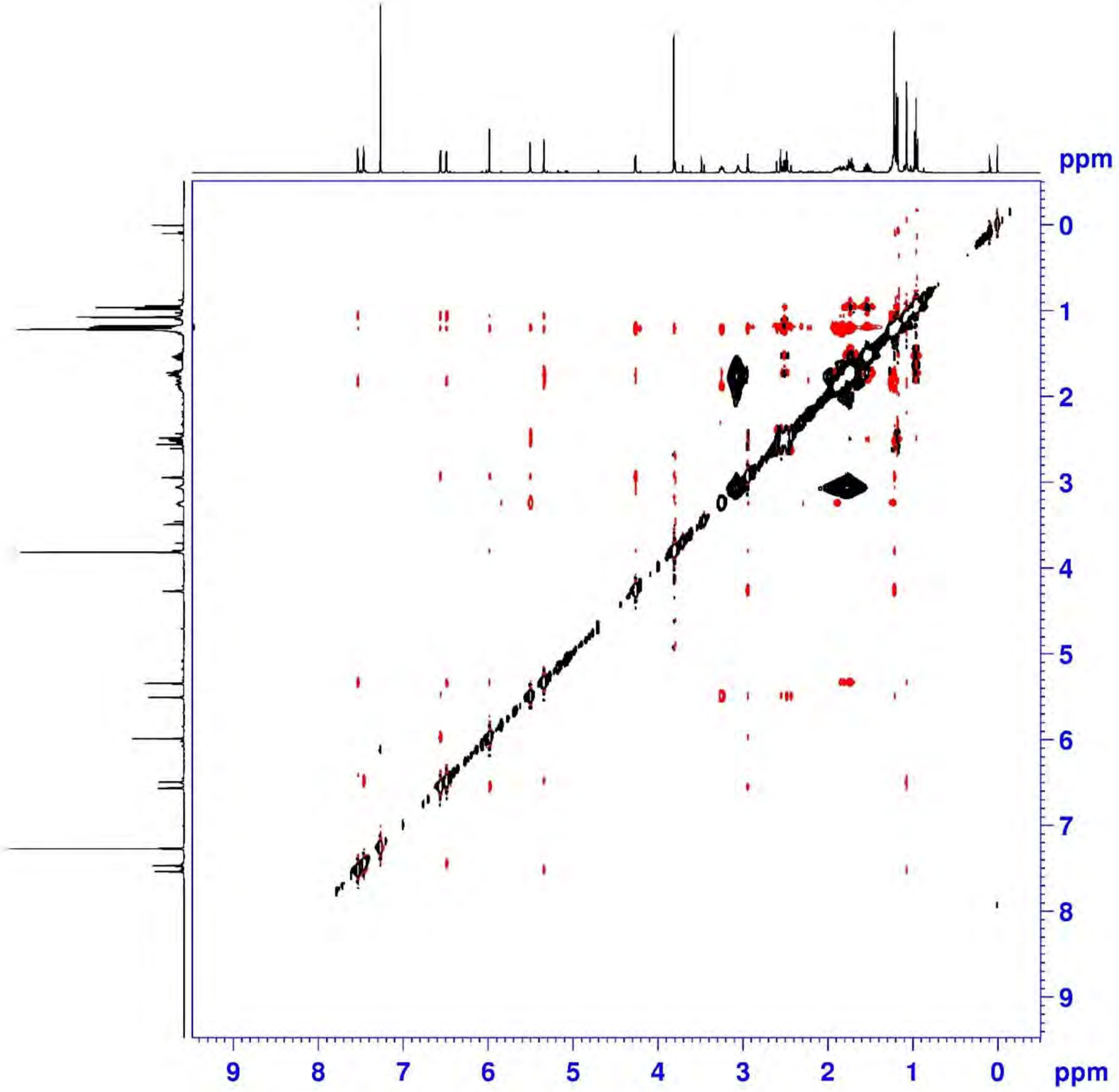

NAME	zjz-35
EXPNO	7
PROCNO	1
Date_	20160913
Time	19.30
INSTRUM	spect
PROBHD	5 mm CPPBBO BB
PULPROG	noesygpphpp
TD	2048
SOLVENT	CDCl3
NS	16
DS	32
SWH	4000.000 Hz
FIDRES	1.953125 Hz
AQ	0.2560500 sec
RG	208.5
DW	125.000 usec
DE	10.00 usec
TE	297.0 K
D0	0.00011036 sec
D1	1.99385595 sec
D8	0.30000001 sec
D11	0.03000000 sec
D12	0.00002000 sec
D16	0.00020000 sec
IN0	0.00025000 sec
======== CHANNEL f1 ========	
SFO1	400.1318006 MHz
NUC1	1H
P1	11.50 usec
P2	23.00 usec
P17	2500.00 usec
ND0	1
TD	256
SFO1	400.1318 MHz
FIDRES	15.625000 Hz
SW	9.997 ppm
FnMODE	States-TPPI
SI	1024
SF	400.1300070 MHz
WDW	QSINE
SSB	2
LB	0.00 Hz
GB	0
PC	1.00
SI	1024
MC2	States-TPPI
SF	400.1300102 MHz
WDW	QSINE
SSB	2
LB	0.00 Hz
GB	0

NOESY (400 MHz) spectrum of compound **9** in $CDCl_3$

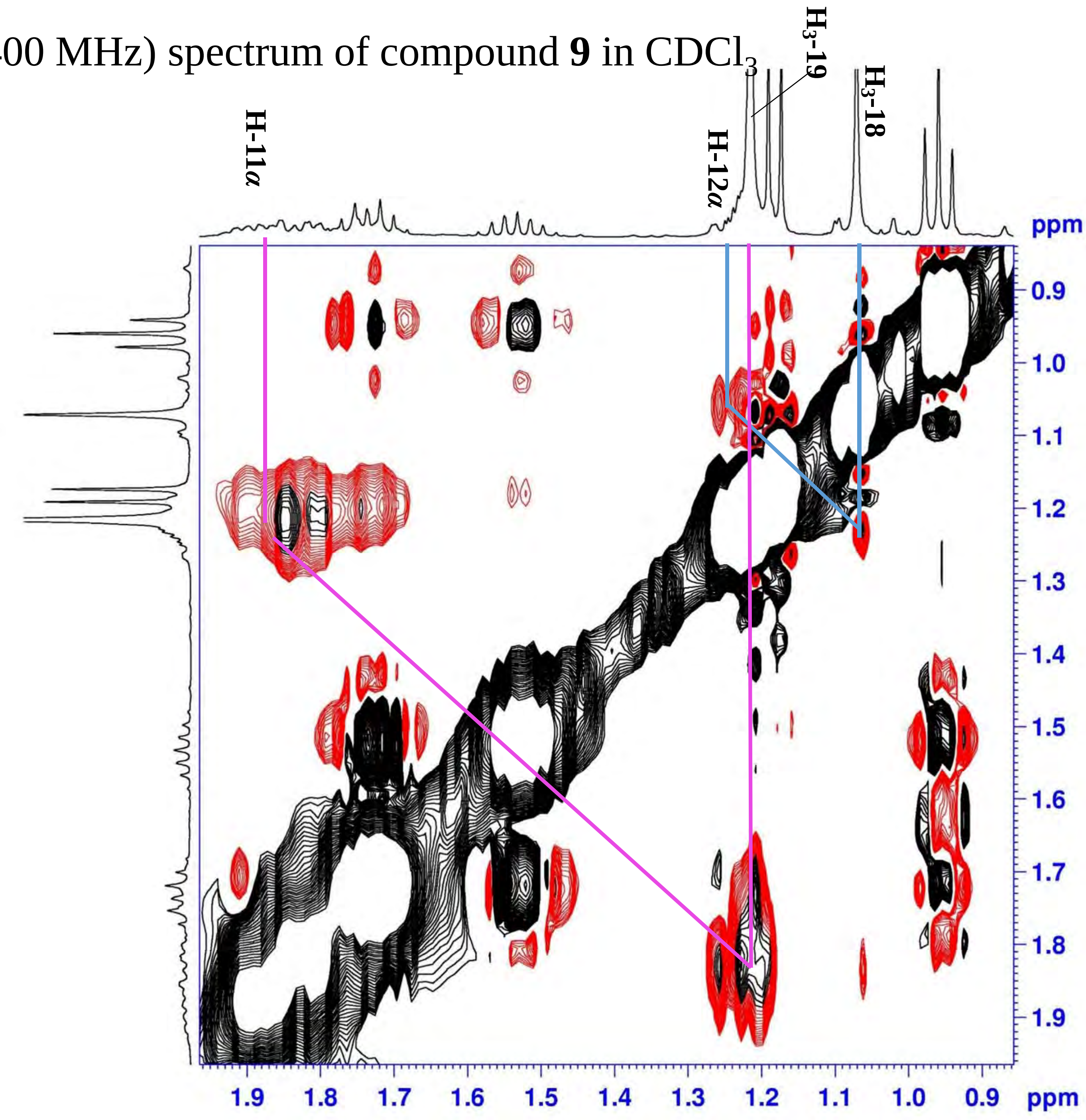

NOESY (400 MHz) spectrum of compound **23** in $CDCl_3$

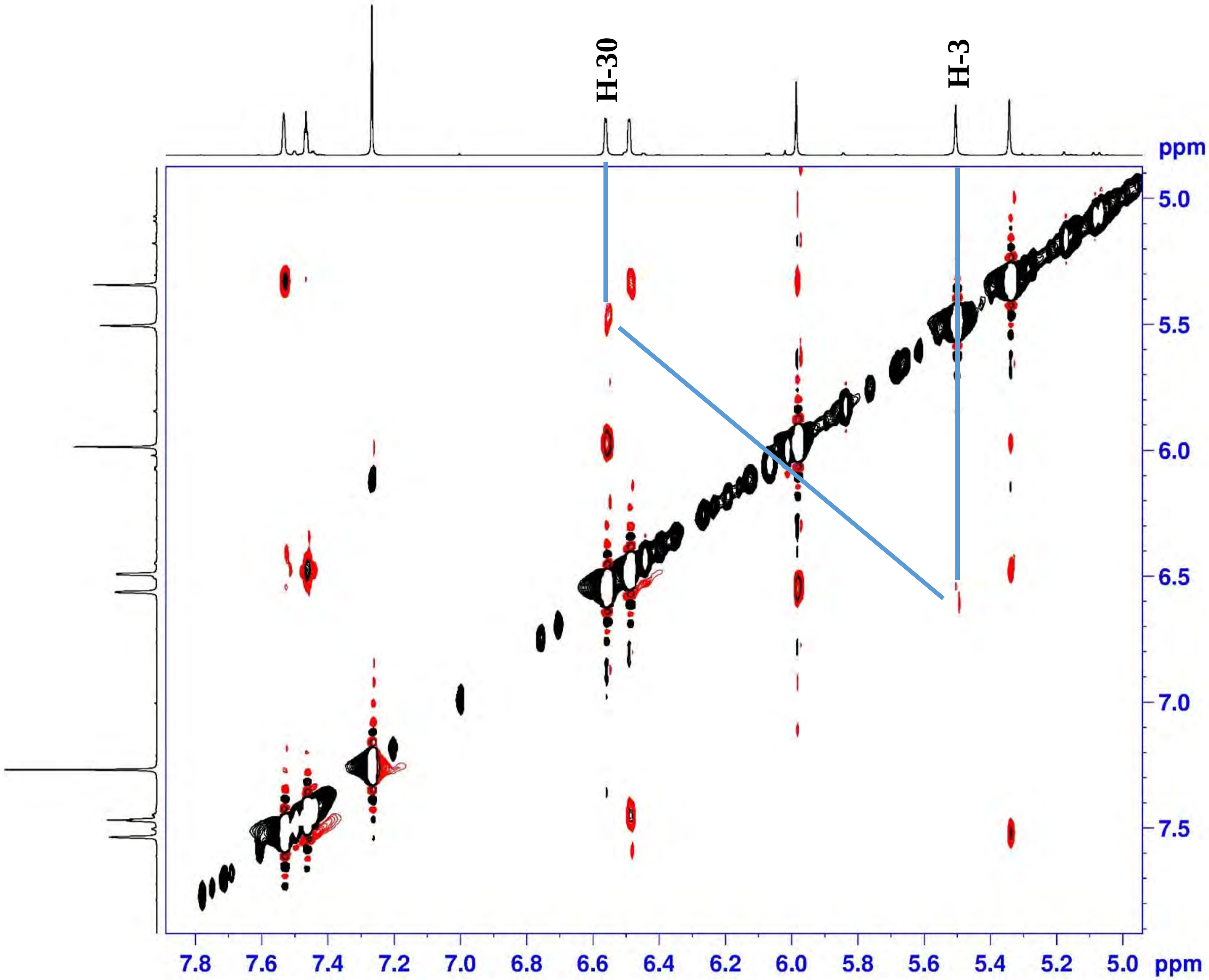

NOESY (400 MHz) spectrum of compound **23** in $CDCl_3$

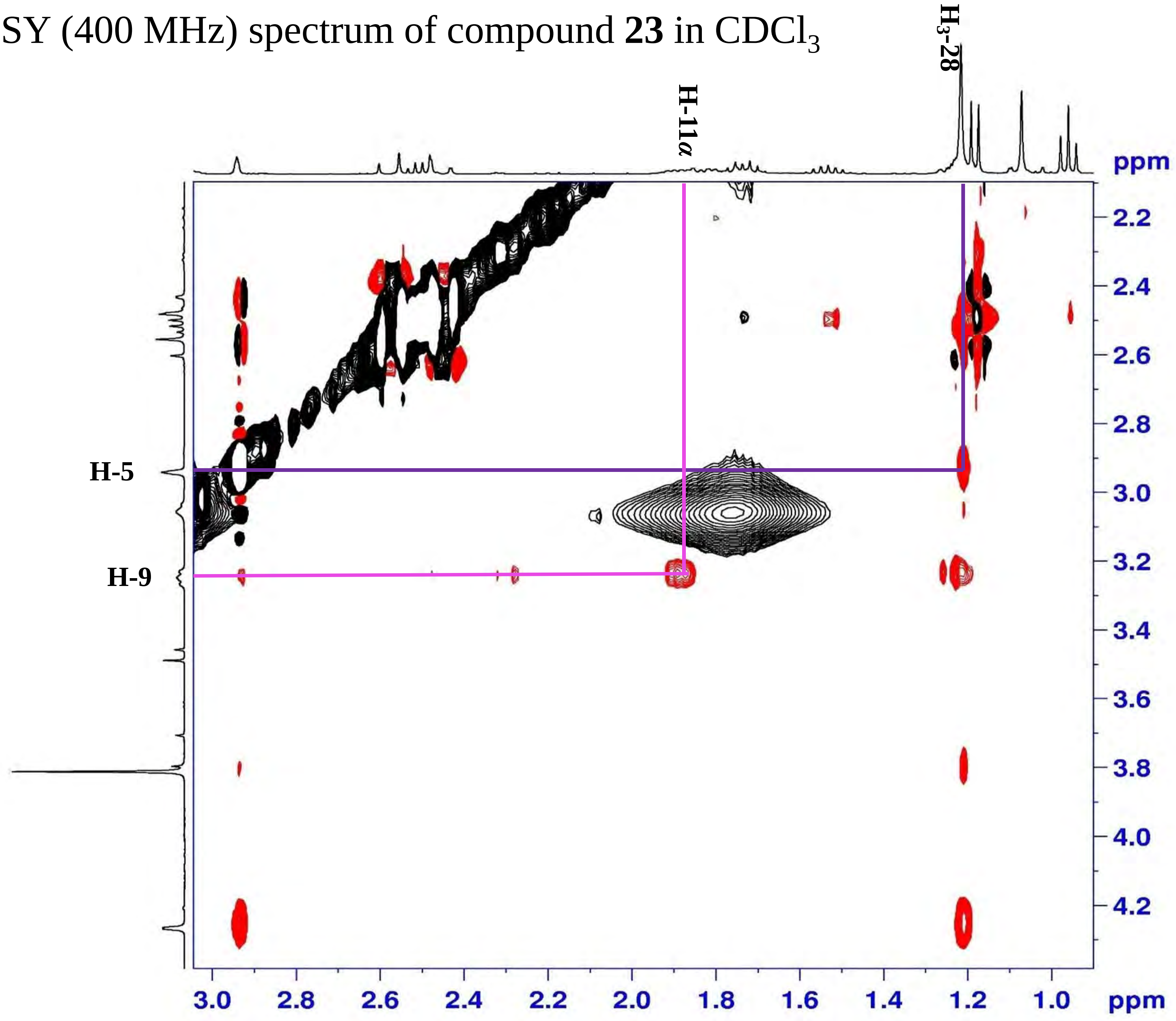

NOESY (400 MHz) spectrum of compound **23** in $CDCl_3$

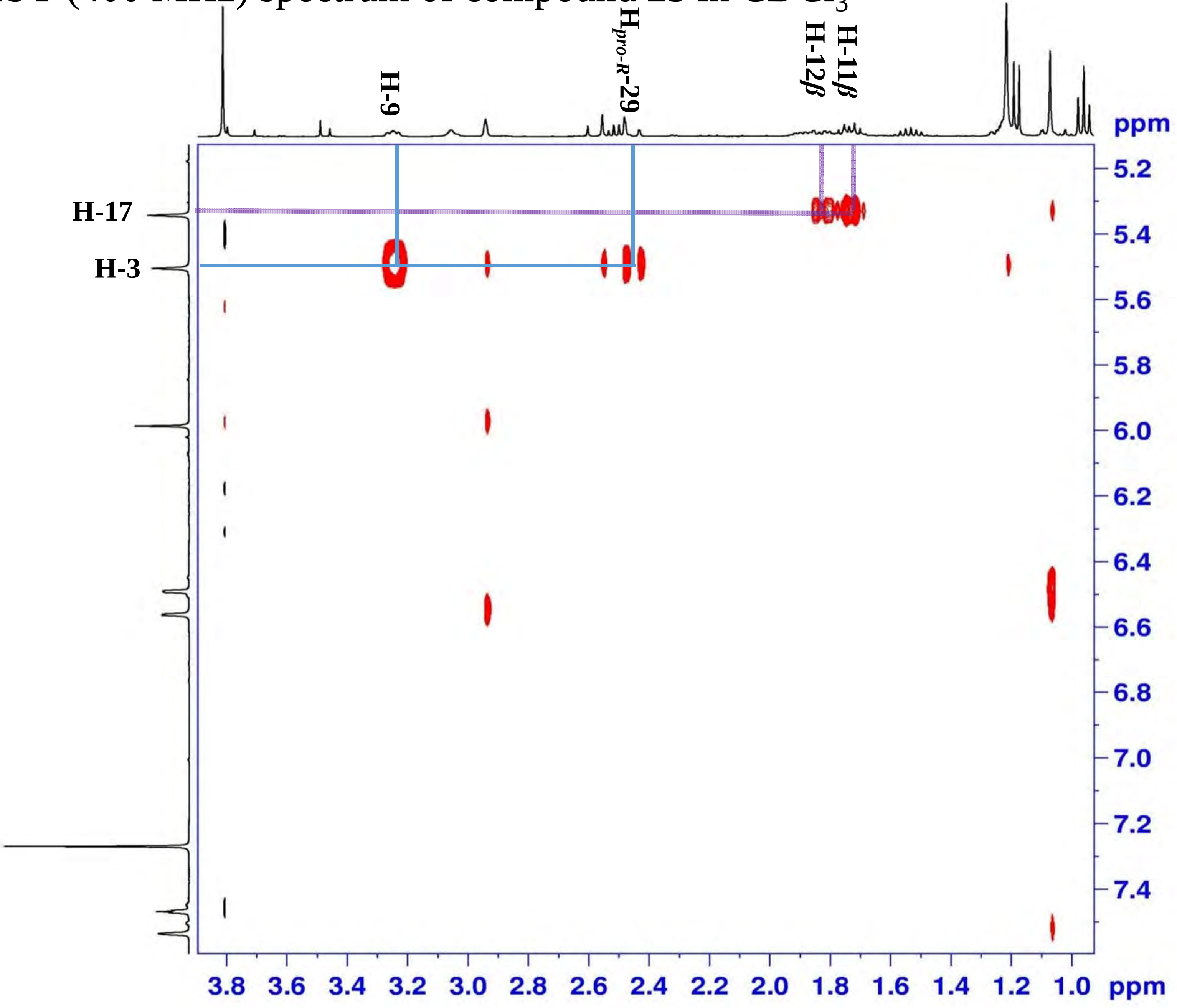

Supplement: Supplementary file 1 [file marinedrugs-16-00038-s001.zip › SI-for submission/SI-2.pdf]
