# Supplementary material for: Twenty-Nine New Limonoids with Skeletal Diversity from the Mangrove Plant, Xylocarpus moluccensis
Source: Mar Drugs. 2018 Jan 19;16(1):38. doi: 10.3390/md16010038 (PMC5793086; doi:10.3390/md16010038)

Supporting Information

Copies of HR-ESIMS, and 1D and 2D NMR spectra of Compounds 24-29.

Table of Contents:

1. HR-ESIMS for compound **24**··········S5-S6
2. ^{1}H NMR (400 MHz) spectrum of compound **24** in $CDCl_3$··········S7-S10
3. ^{13}C NMR (100 MHz) spectrum of compound **24** in $CDCl_3$··········S11-S13
4. DEPT135 (100 MHz) spectrum of compound **24** in $CDCl_3$··········S14-S16
5. ^{1}H-^{1}H COSY (400 MHz) spectrum of compound **24** in $CDCl_3$··········S17-S20
6. HSQC (400 MHz) spectrum of compound **24** in $CDCl_3$··········S21-S23
7. HMBC (400 MHz) spectrum of compound **24** in $CDCl_3$··········S24-S29
8. NOESY (400 MHz) spectrum of compound **24** in $CDCl_3$··········S30-S35
9. HR-ESIMS for compound **25**··········S36-S37
10. ^{1}H NMR (400 MHz) spectrum of compound **25** in $CDCl_3$··········S38-S40
11. ^{13}C NMR (100 MHz) spectrum of compound **25** in $CDCl_3$··········S41-S43
12. DEPT135 (100 MHz) spectrum of compound **25** in $CDCl_3$··········S44-S47
13. ^{1}H-^{1}H COSY (400 MHz) spectrum of compound **25** in $CDCl_3$··········S48-S50
14. HSQC (400 MHz) spectrum of compound **25** in $CDCl_3$··········S51-S54
15. HMBC (400 MHz) spectrum of compound **25** in $CDCl_3$··········S55-S62
16. NOESY (400 MHz) spectrum of compound **25** in $CDCl_3$··········S63-S66
17. HR-ESIMS for compound **26**··········S67-S68
18. ^{1}H NMR (400 MHz) spectrum of compound **26** in $CDCl_3$··········S69-S71
19. ^{13}C NMR (100 MHz) spectrum of compound **26** in $CDCl_3$··········S72-S74
20. DEPT135 (100 MHz) spectrum of compound **26** in $CDCl_3$··········S75-S77
21. ^{1}H-^{1}H COSY (400 MHz) spectrum of compound **26** in $CDCl_3$··········S78-S80

22. HSQC (400 MHz) spectrum of compound **26** in $CDCl_3$ ················ S81-S83
23. HMBC (400 MHz) spectrum of compound **26** in $CDCl_3$ ··············· S84-S88
24. NOESY (400 MHz) spectrum of compound **26** in $CDCl_3$ ·············· S89-S92
25. HR-ESIMS for compound **27**······································· S93-S94
26. ^{1}H NMR (400 MHz) spectrum of compound **27** in $CDCl_3$············ S95-S97
27. ^{13}C NMR (100 MHz) spectrum of compound **27** in $CDCl_3$············ S98-S100
28. DEPT135 (100 MHz) spectrum of compound **27** in $CDCl_3$ ··········· S101-S103
29. ^{1}H-^{1}H COSY (400 MHz) spectrum of compound **27** in $CDCl_3$ ········ S104-S105
30. HSQC (400 MHz) spectrum of compound **27** in $CDCl_3$ ················ S106-S109
31. HMBC (400 MHz) spectrum of compound **27** in $CDCl_3$ ··············· S110-S117
32. NOESY (400 MHz) spectrum of compound **27** in $CDCl_3$ ·············· S118-S121
33. HR-ESIMS for compound **28**······································· S122-S123
34. ^{1}H NMR (400 MHz) spectrum of compound **28** in $CDCl_3$············ S124-S129
35. ^{13}C NMR (100 MHz) spectrum of compound **28** in $CDCl_3$············ S130-S133
36. DEPT135 (100 MHz) spectrum of compound **28** in $CDCl_3$ ··········· S134-S136
37. ^{1}H-^{1}H COSY (400 MHz) spectrum of compound **28** in $CDCl_3$ ········ S137-S139
38. HSQC (400 MHz) spectrum of compound **28** in $CDCl_3$ ················ S140-S143
39. HMBC (400 MHz) spectrum of compound **28** in $CDCl_3$ ··············· S144-S150
40. NOESY (400 MHz) spectrum of compound **28** in $CDCl_3$ ·············· S151-S154
41. HR-ESIMS for compound **29**······································· S155-S156
42. ^{1}H NMR (400 MHz) spectrum of compound **29** in $CDCl_3$············ S157-S159

43. ^{13}C NMR (100 MHz) spectrum of compound **29** in $CDCl_3$ ············ S160-S162
44. DEPT135 (100 MHz) spectrum of compound **29** in $CDCl_3$ ·········· S163-S165
45. ^{1}H-^{1}H COSY (400 MHz) spectrum of compound **29** in $CDCl_3$ ········ S166-S168
46. HSQC (400 MHz) spectrum of compound **29** in $CDCl_3$ ················ S169-S172
47. HMBC (400 MHz) spectrum of compound **29** in $CDCl_3$ ··············· S173-S179
48. NOESY (400 MHz) spectrum of compound **29** in $CDCl_3$ ·············· S180-S183

HR-ESIMS for compound **24**

Mass Spectrum SmartFormula Report

Analysis Info

Analysis Name	D:\Data\MS\data\201605\liwanshan_dgy-23_pos_9_01_1857.d	Acquisition Date	5/19/2016 8:48:09 AM
Method	LC_Direct Infusion_pos_100-1000mz.m	Operator	SCSIO
Sample Name	liwanshan_dgy-23_pos	Instrument / Ser#	maXis 29
Comment			

Acquisition Parameter

Source Type	ESI	Ion Polarity	Positive	Set Nebulizer	0.4 Bar
Focus	Active	Set Capillary	4500 V	Set Dry Heater	180 °C
Scan Begin	100 m/z	Set End Plate Offset	-500 V	Set Dry Gas	4.0 l/min
Scan End	2000 m/z	Set Collision Cell RF	800.0 Vpp	Set Divert Valve	Waste

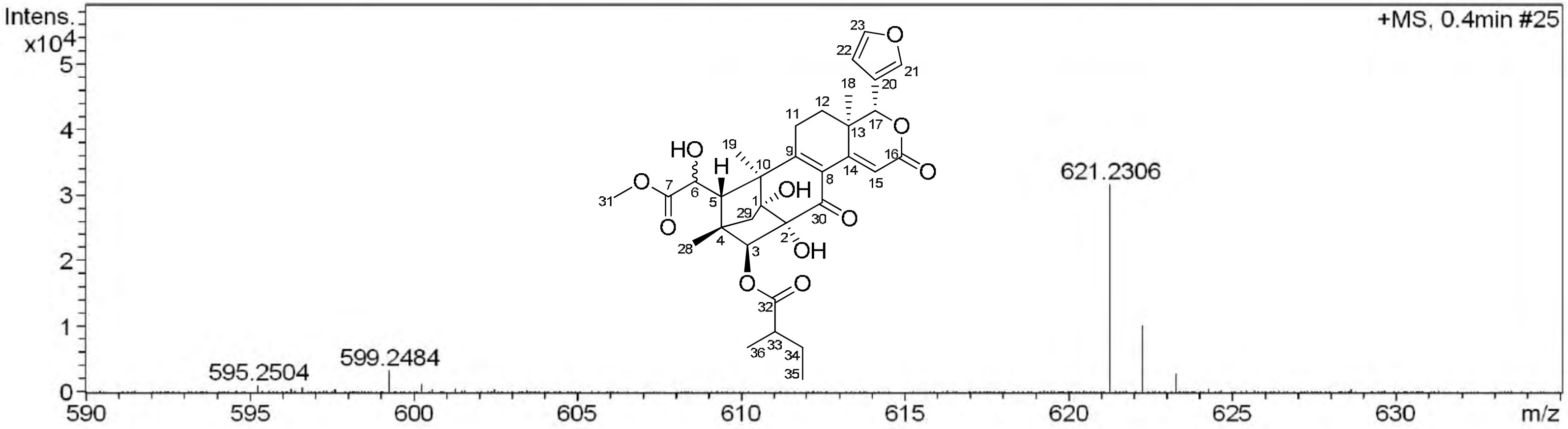

Meas. m/z	#	Formula	Score	m/z	err [ppm]	err [mDa]	mSigma	rdb	e⁻ Conf	N-Rule
599.2484	1	C 32 H 39 O 11	100.00	599.2487	0.5	0.3	16.2	13.5	even	ok
621.2306	1	C 32 H 38 Na O 11	100.00	621.2306	-0.0	-0.0	18.1	13.5	even	ok
1219.4732	1	C 64 H 76 Na O 22	84.10	1219.4720	-0.9	-1.2	19.8	26.5	even	ok

HR-ESIMS for compound **24**

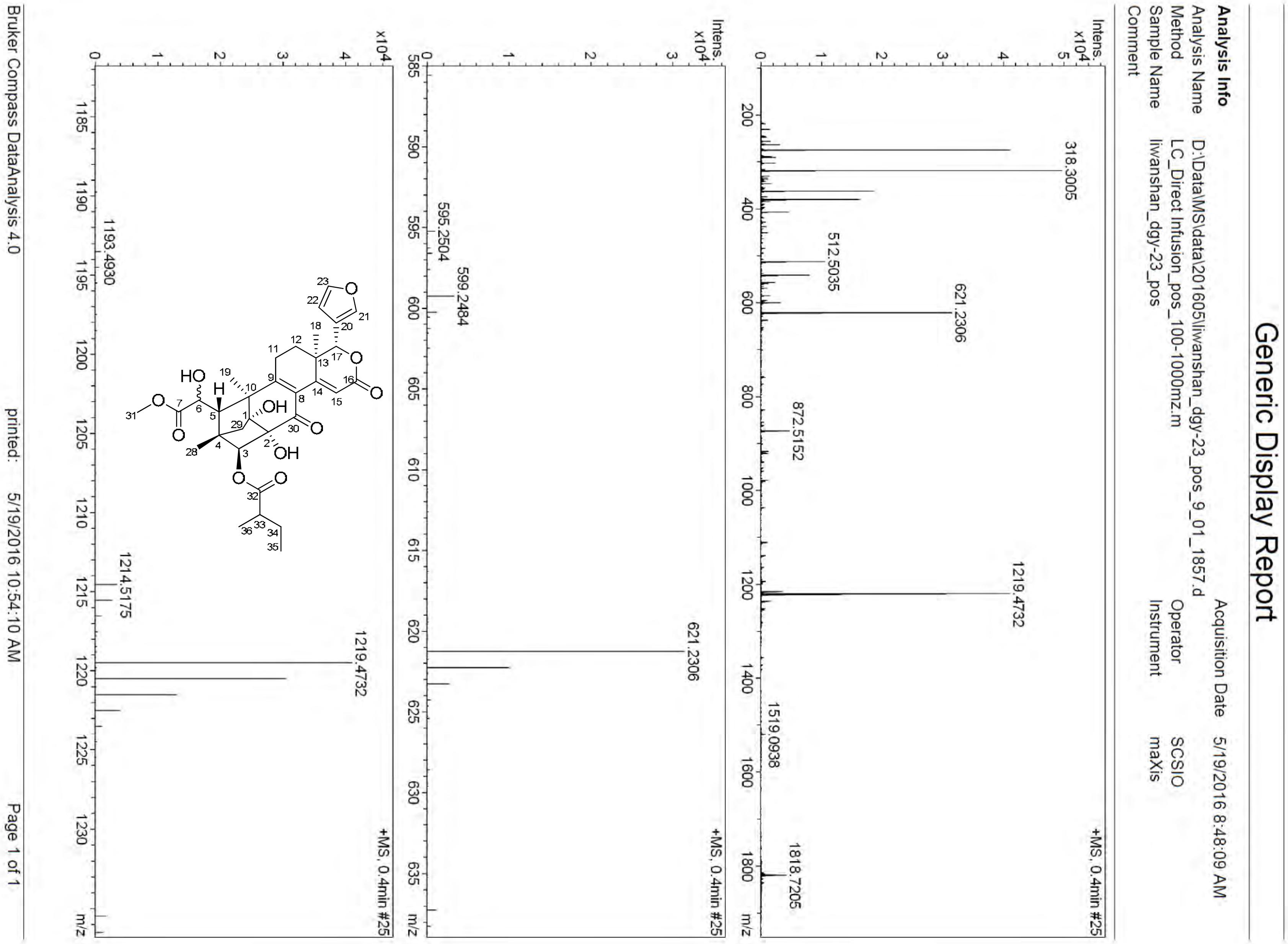

^{1}H NMR (400 MHz) spectrum of compound **24** in $CDCl_3$

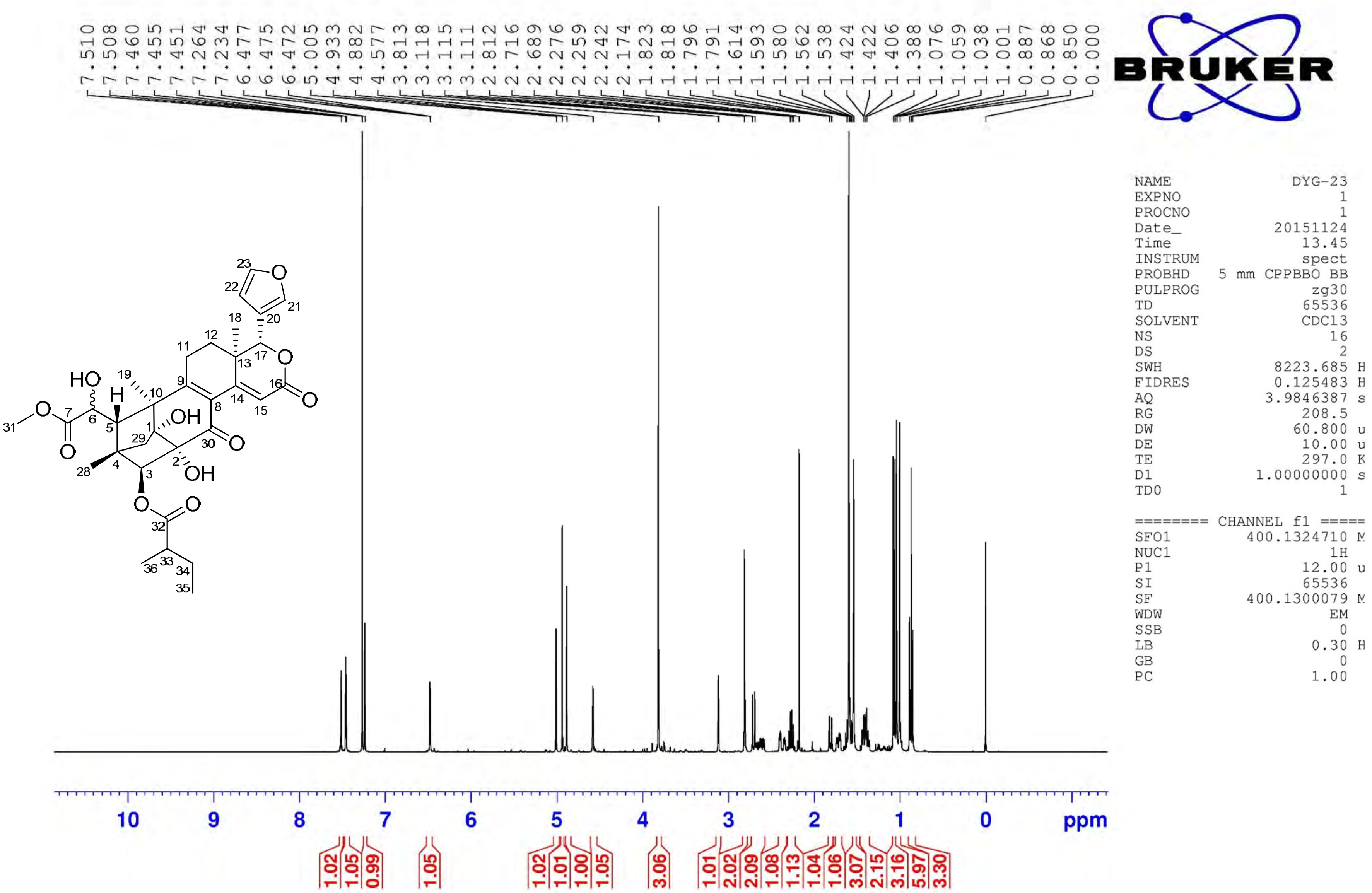

^{1}H NMR (400 MHz) spectrum of compound **24** in $CDCl_3$

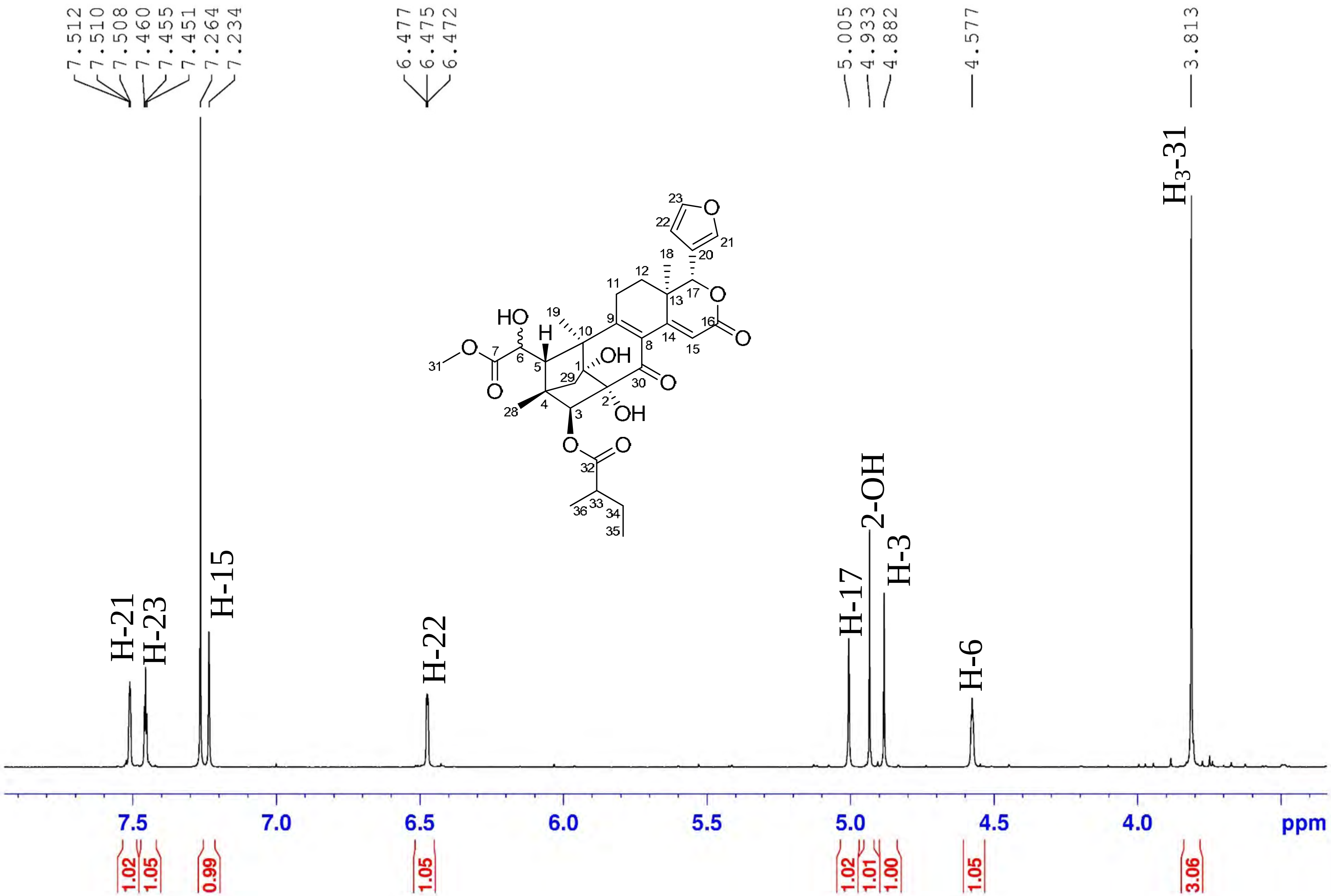

^{1}H NMR (400 MHz) spectrum of compound **24** in $CDCl_3$

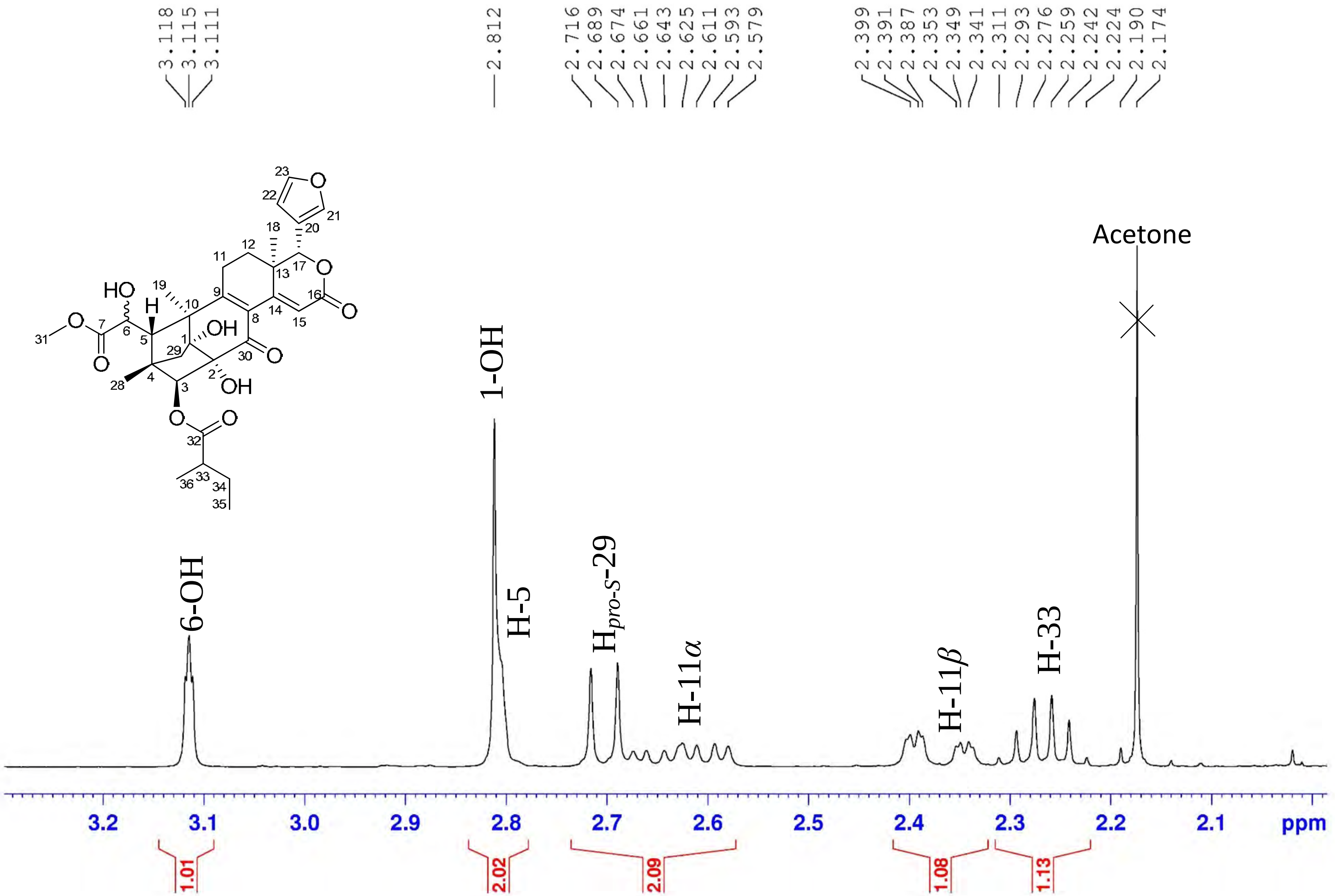

^1H NMR (400 MHz) spectrum of compound **24** in $CDCl_3$

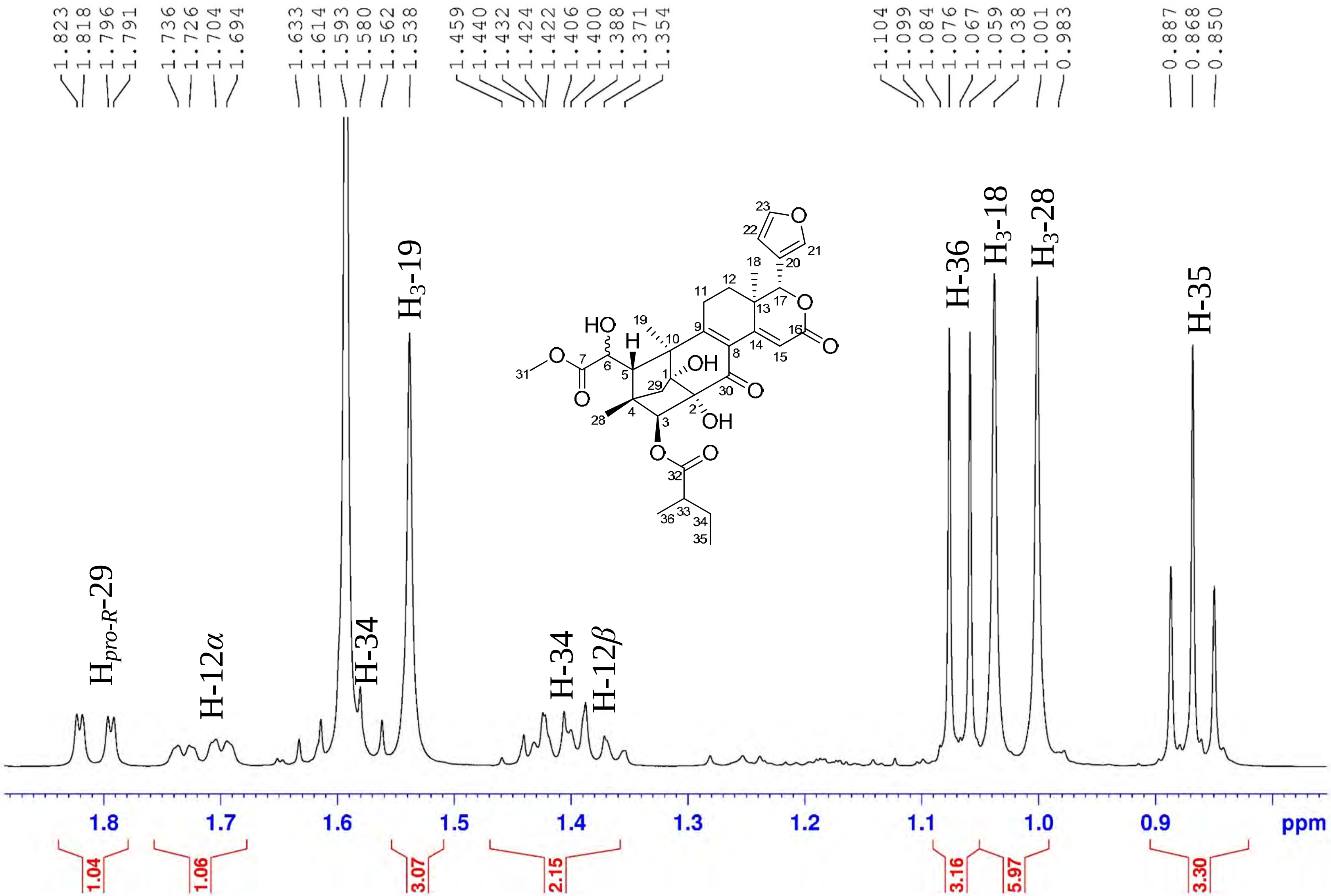

^{13}C NMR (100 MHz) spectrum of compound **24** in $CDCl_3$

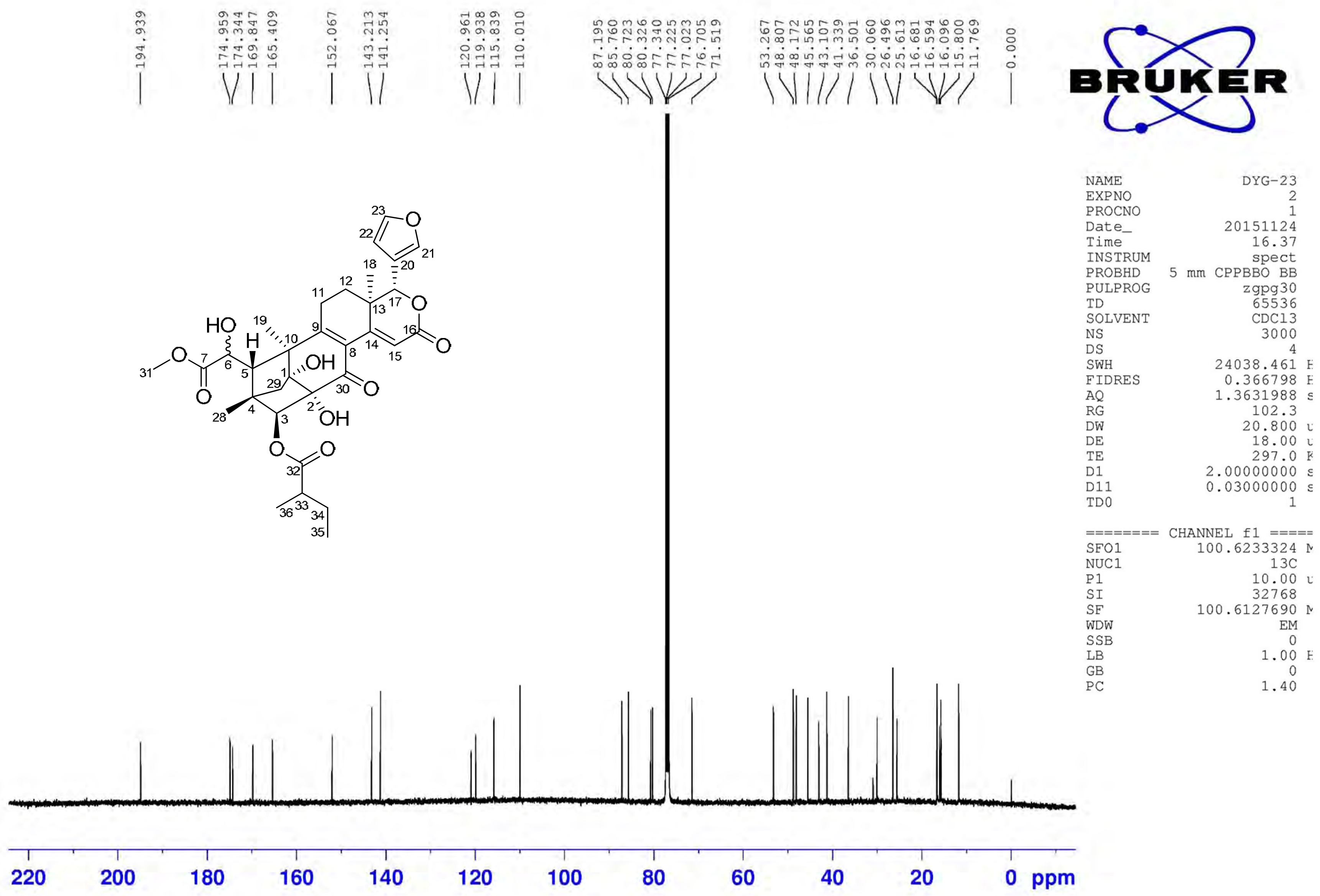

^{13}C NMR (100 MHz) spectrum of compound **24** in $CDCl_3$

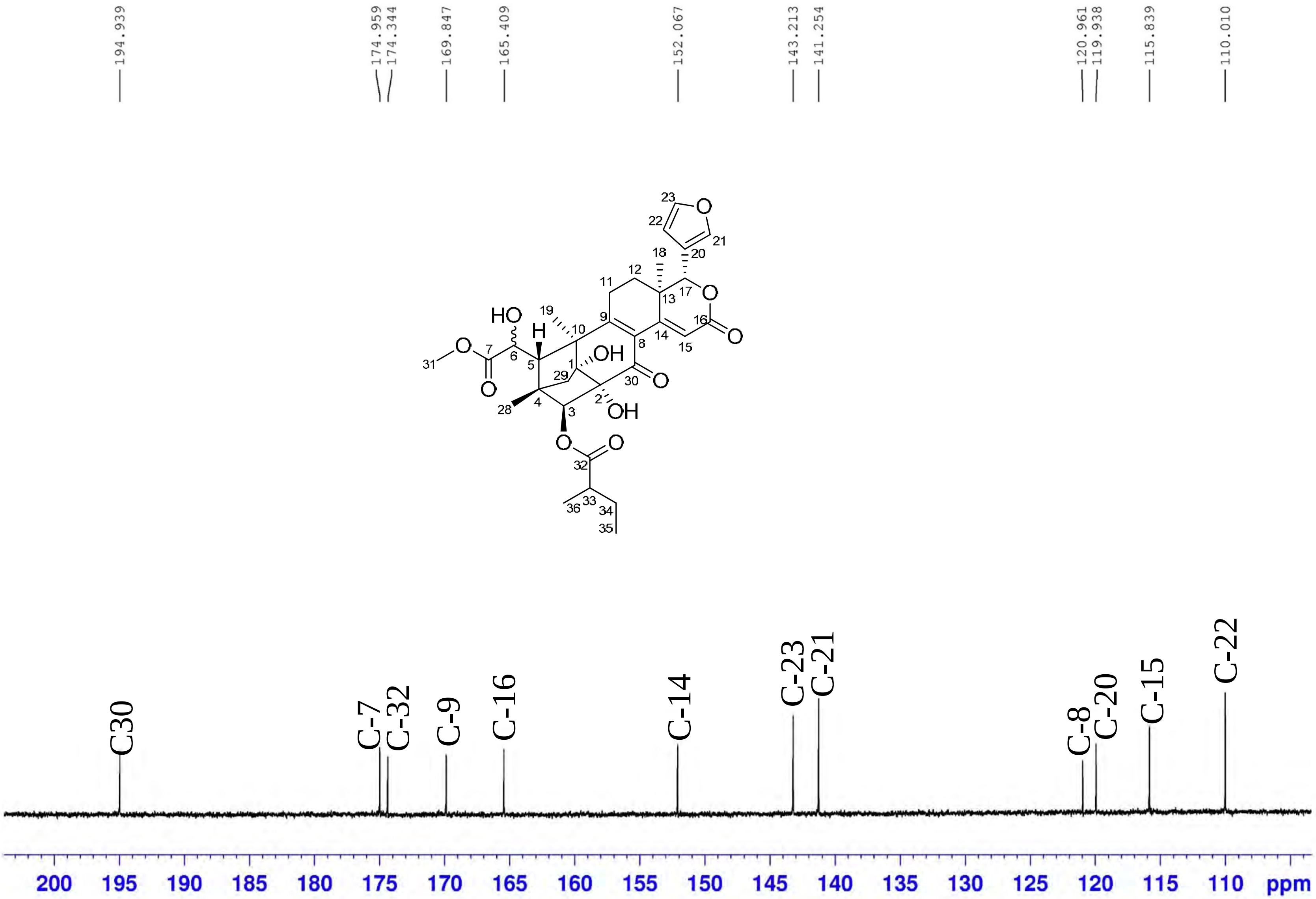

^{13}C NMR (100 MHz) spectrum of compound **24** in $CDCl_3$

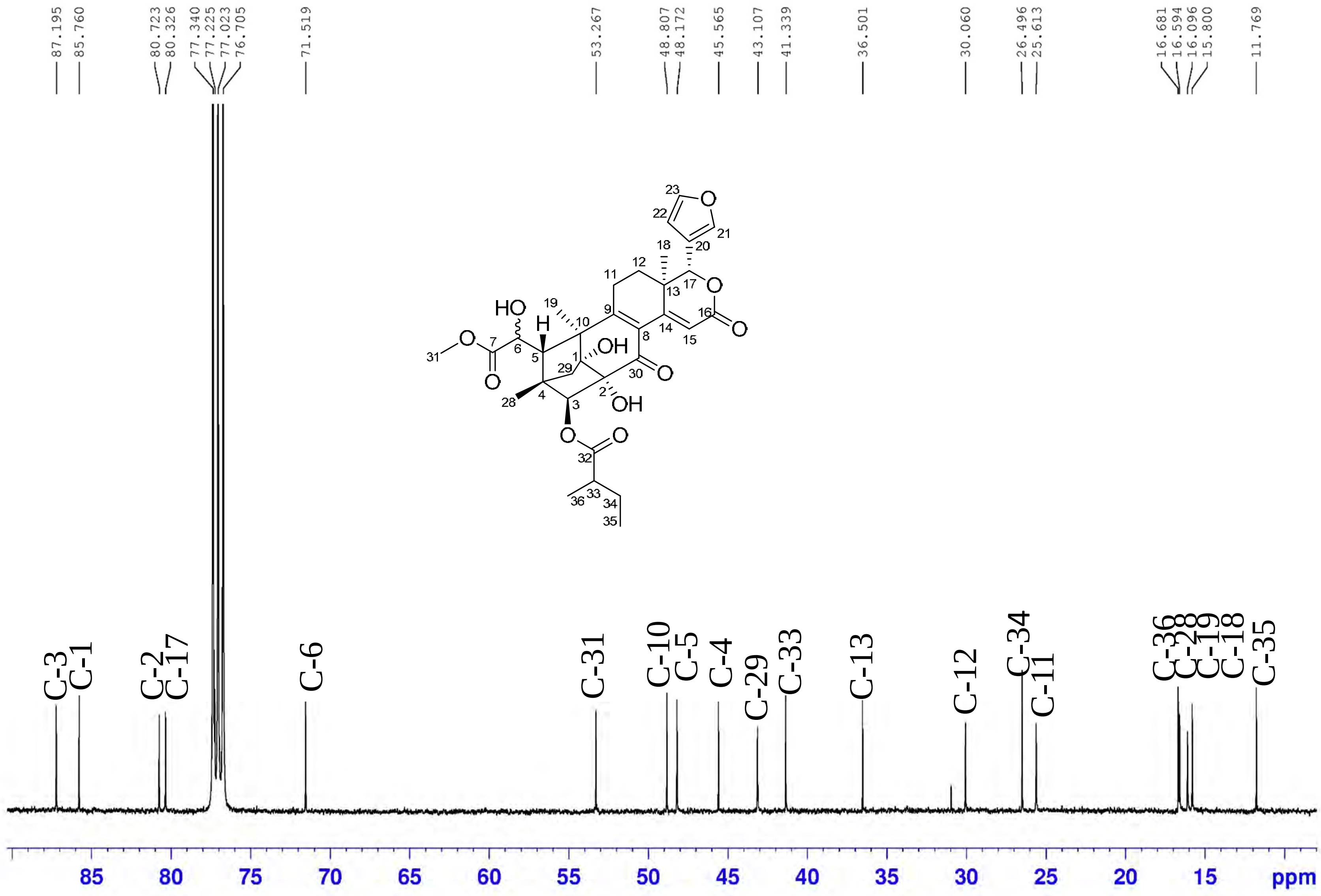

DEPT135 (100 MHz) spectrum of compound **24** in $CDCl_3$

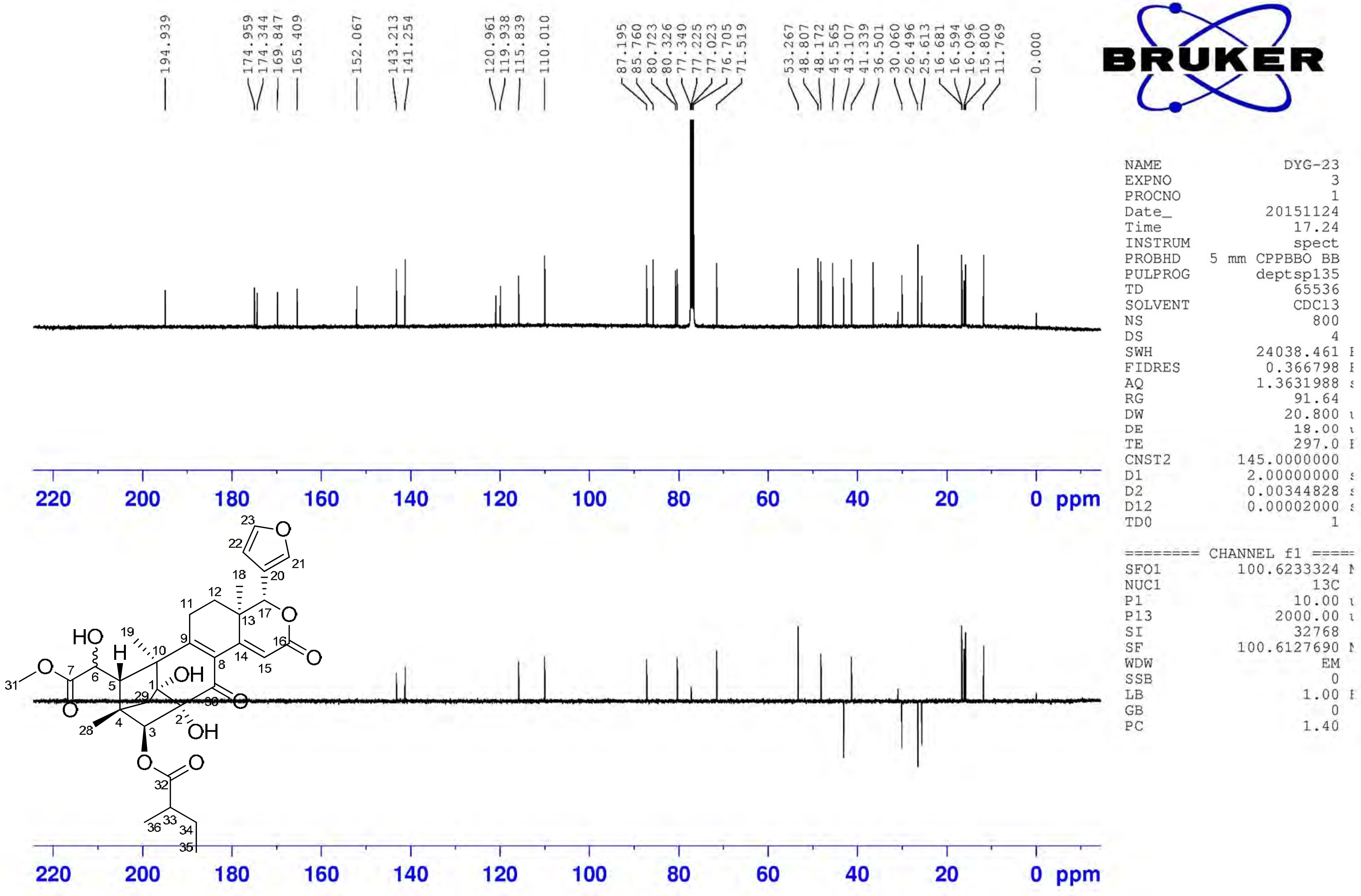

DEPT135 (100 MHz) spectrum of compound **24** in $CDCl_3$

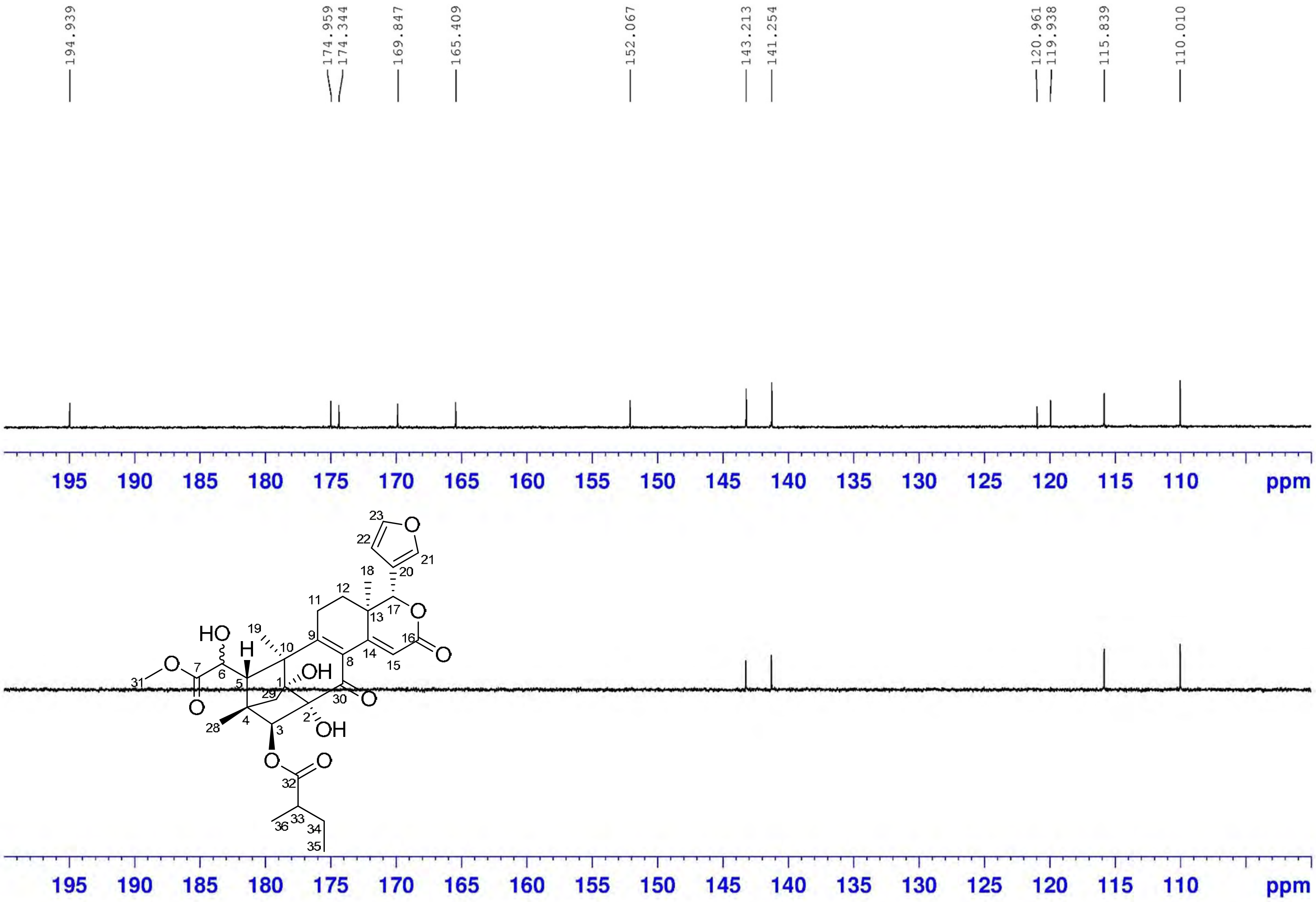

DEPT135 (100 MHz) spectrum of compound **24** in $CDCl_3$

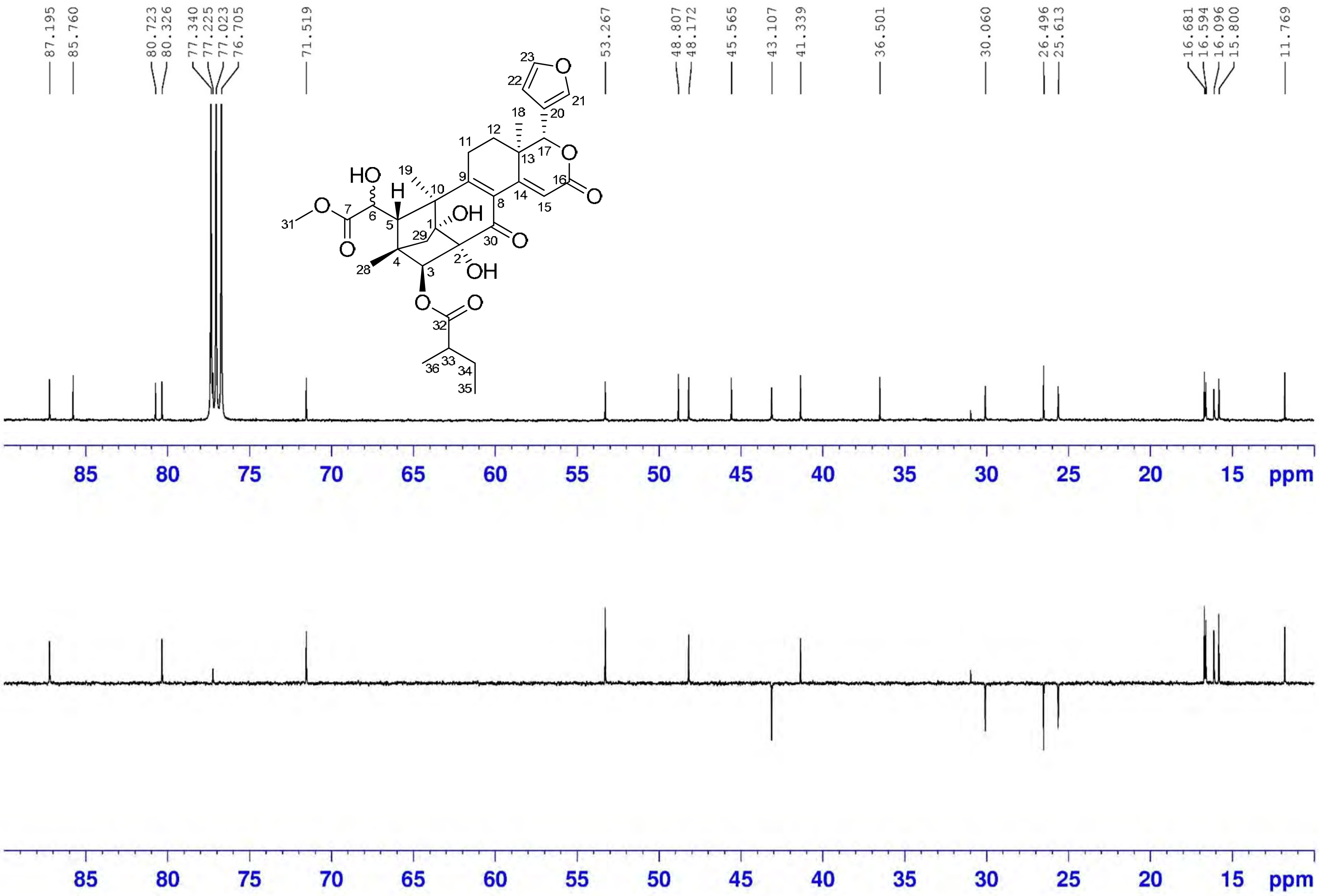

^{1}H-^{1}H COSY (400 MHz) spectrum of compound **24** in $CDCl_3$

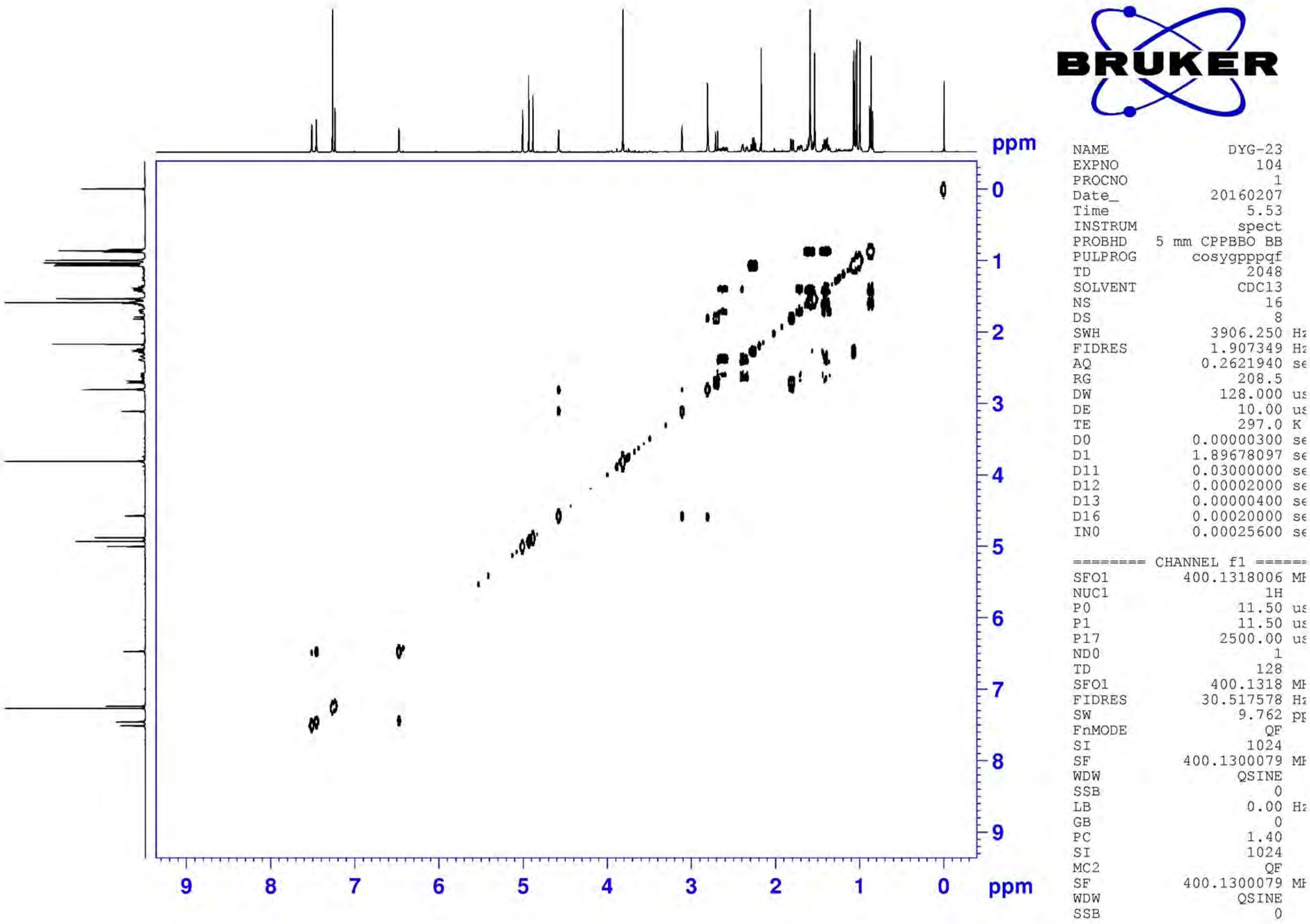

^{1}H-^{1}H COSY (400 MHz) spectrum of compound **24** in $CDCl_3$

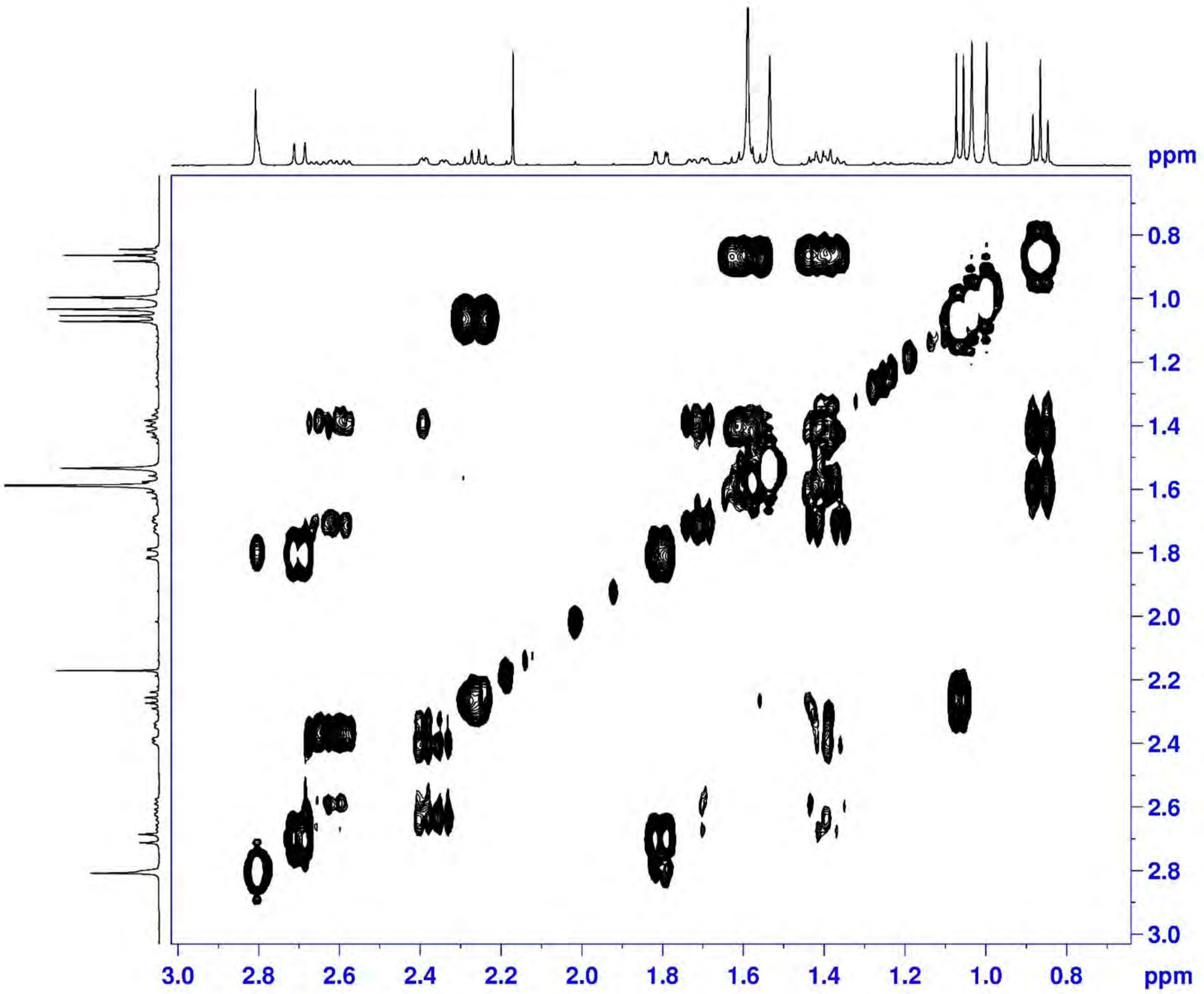

^{1}H-^{1}H COSY (400 MHz) spectrum of compound **24** in $CDCl_3$

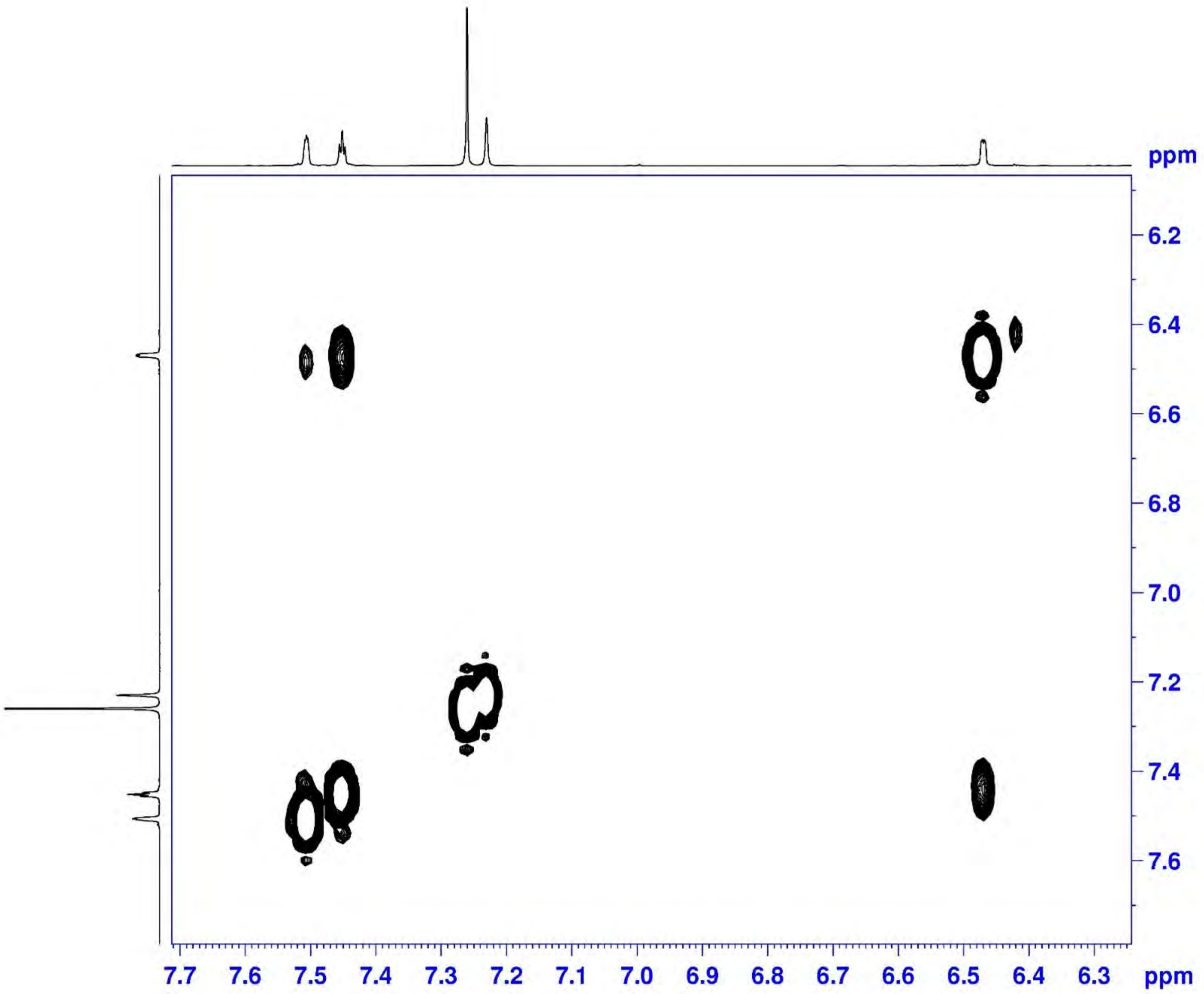

^1H-^1H COSY (400 MHz) spectrum of compound **24** in $CDCl_3$

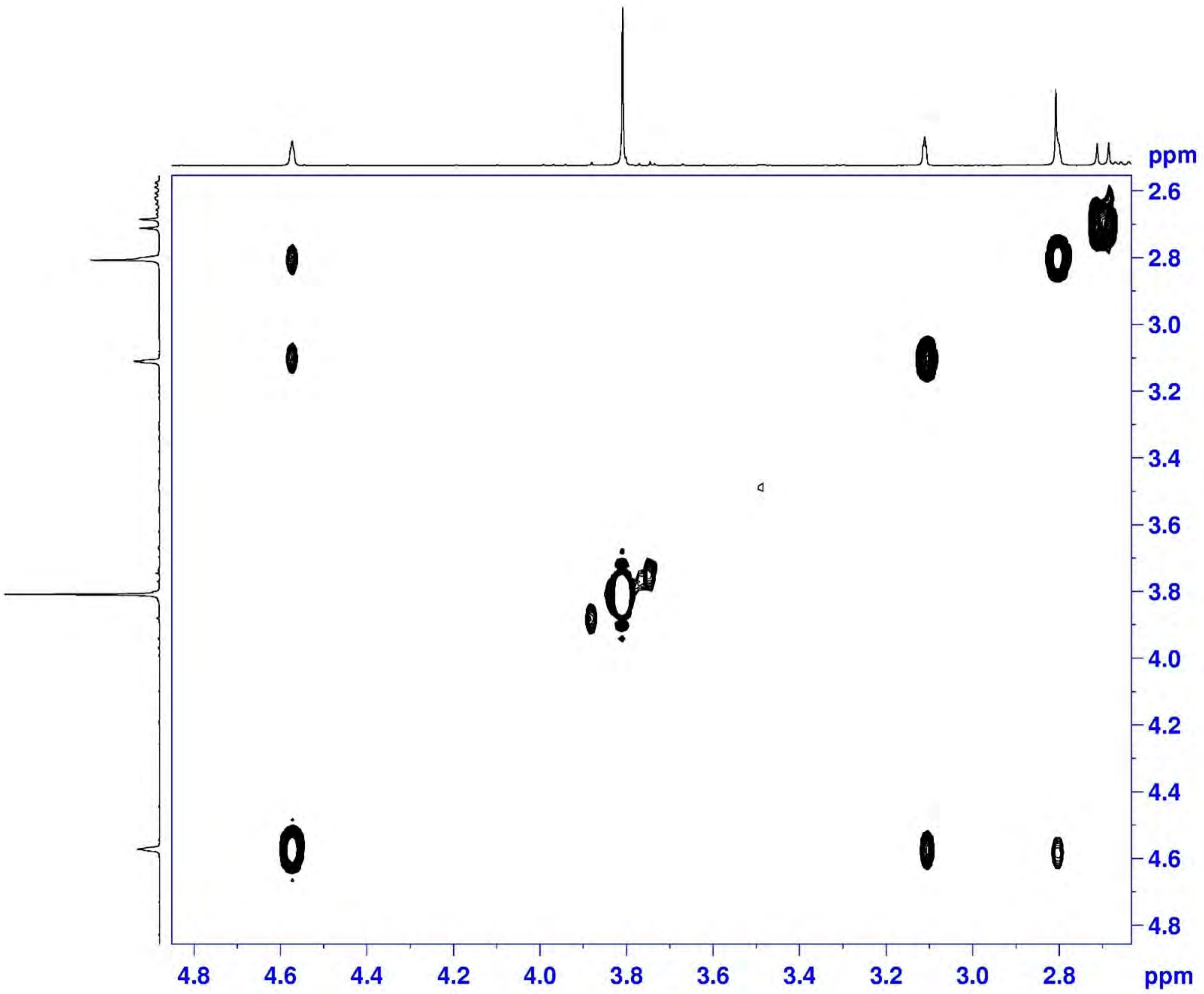

HSQC (400 MHz) spectrum of compound **24** in $CDCl_3$

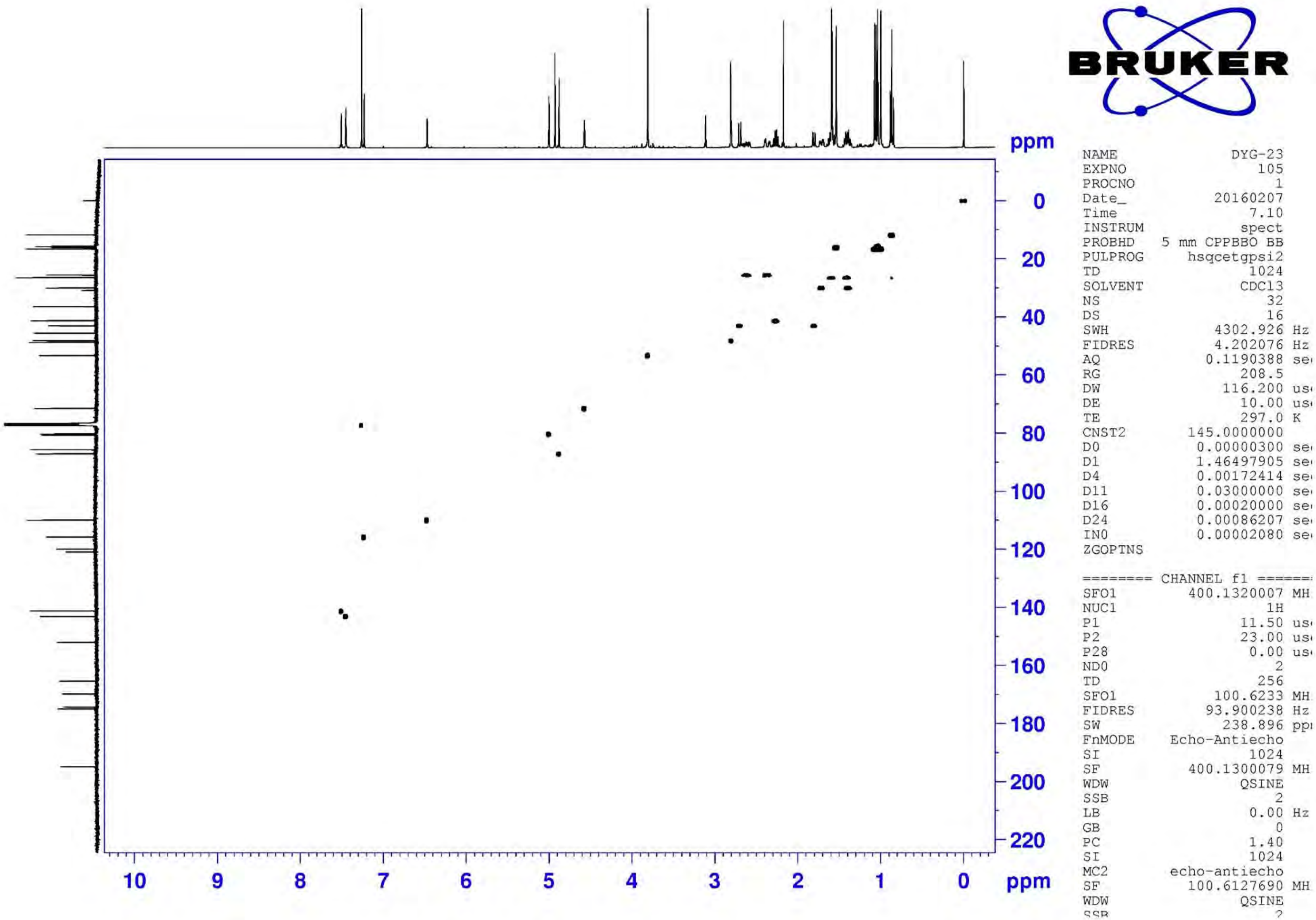

HSQC (400 MHz) spectrum of compound **24** in $CDCl_3$

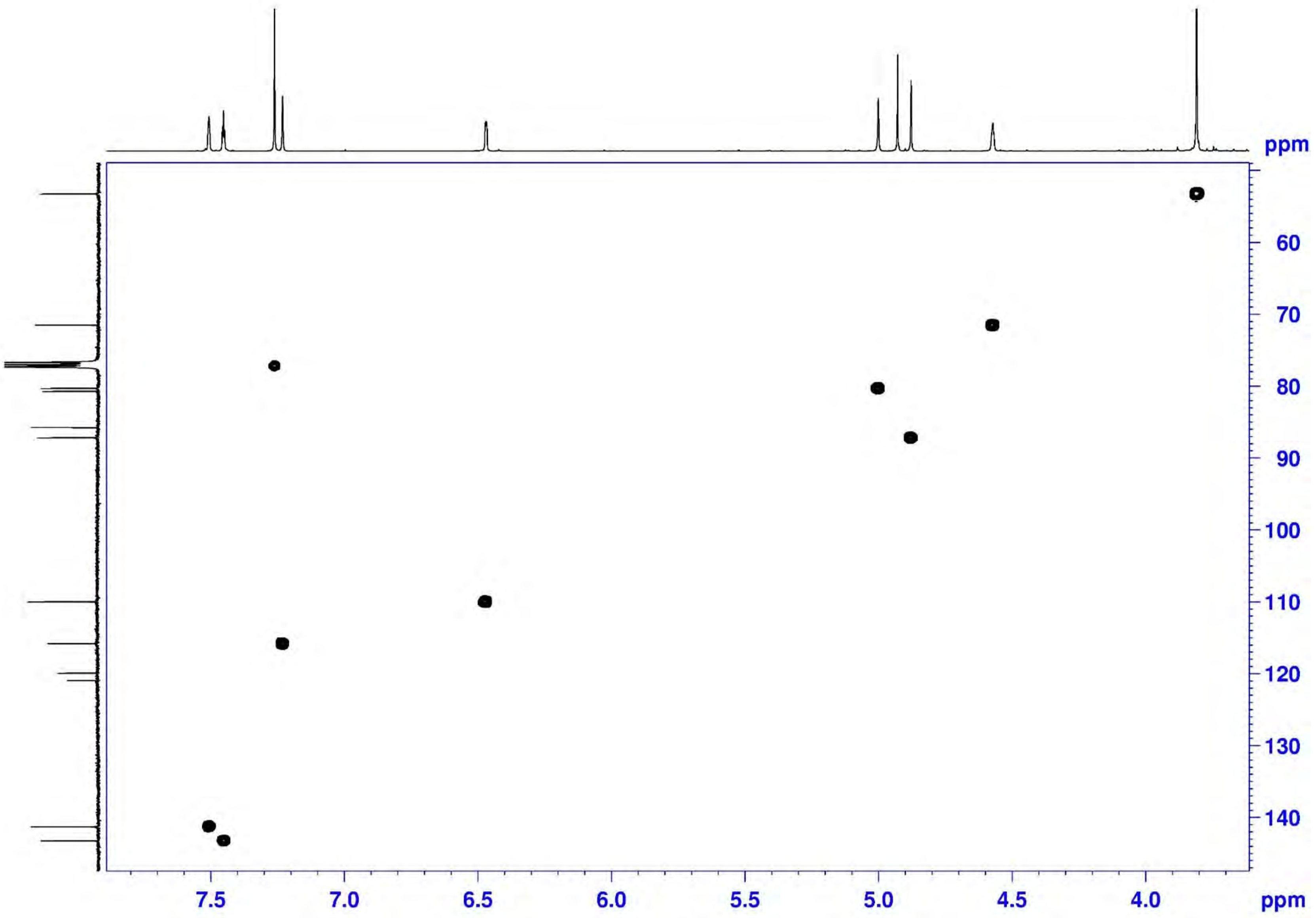

HSQC (400 MHz) spectrum of compound **24** in $CDCl_3$

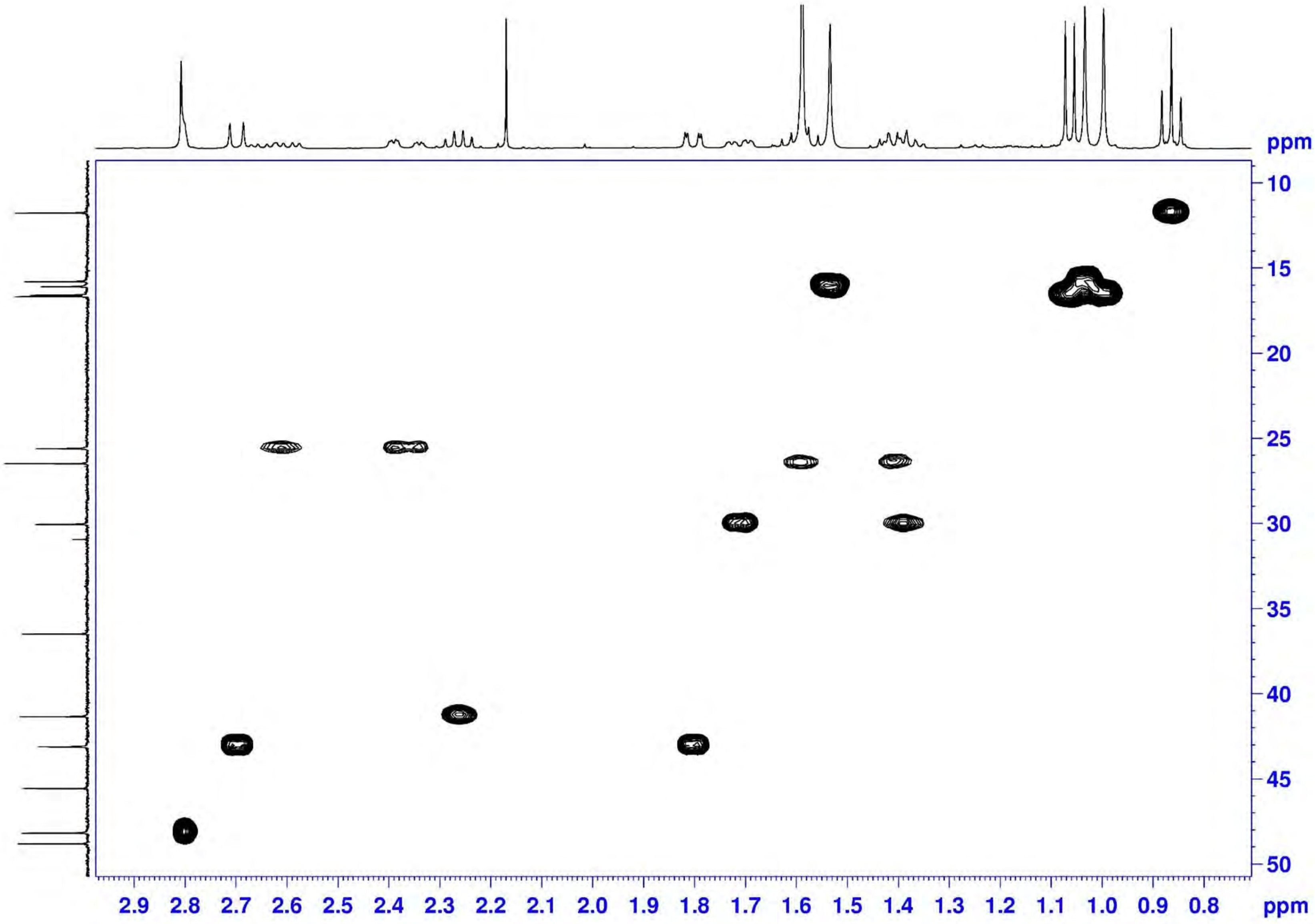

HMBC (400 MHz) spectrum of compound **24** in $CDCl_3$

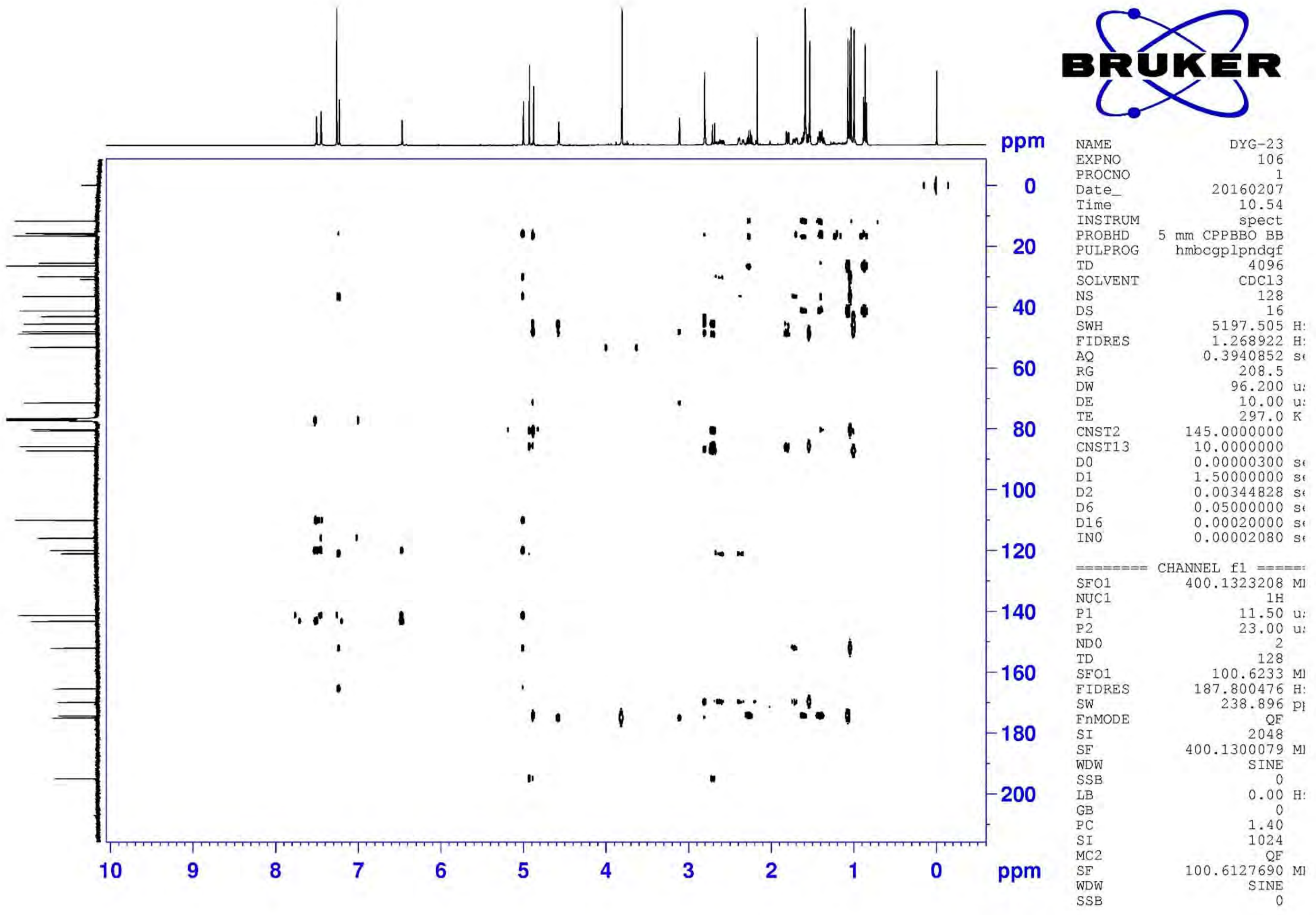

HMBC (400 MHz) spectrum of compound **24** in $CDCl_3$

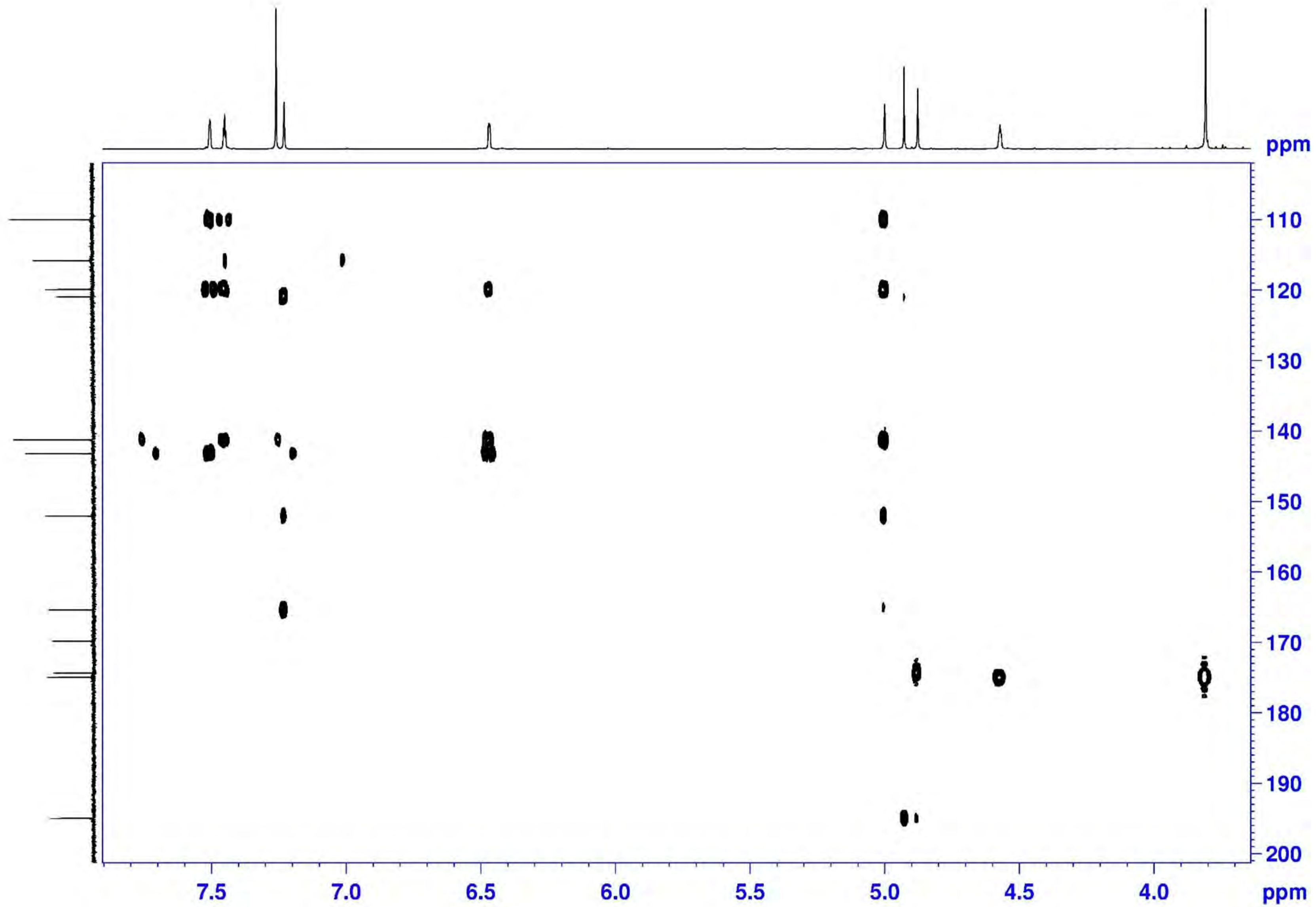

HMBC (400 MHz) spectrum of compound **24** in $CDCl_3$

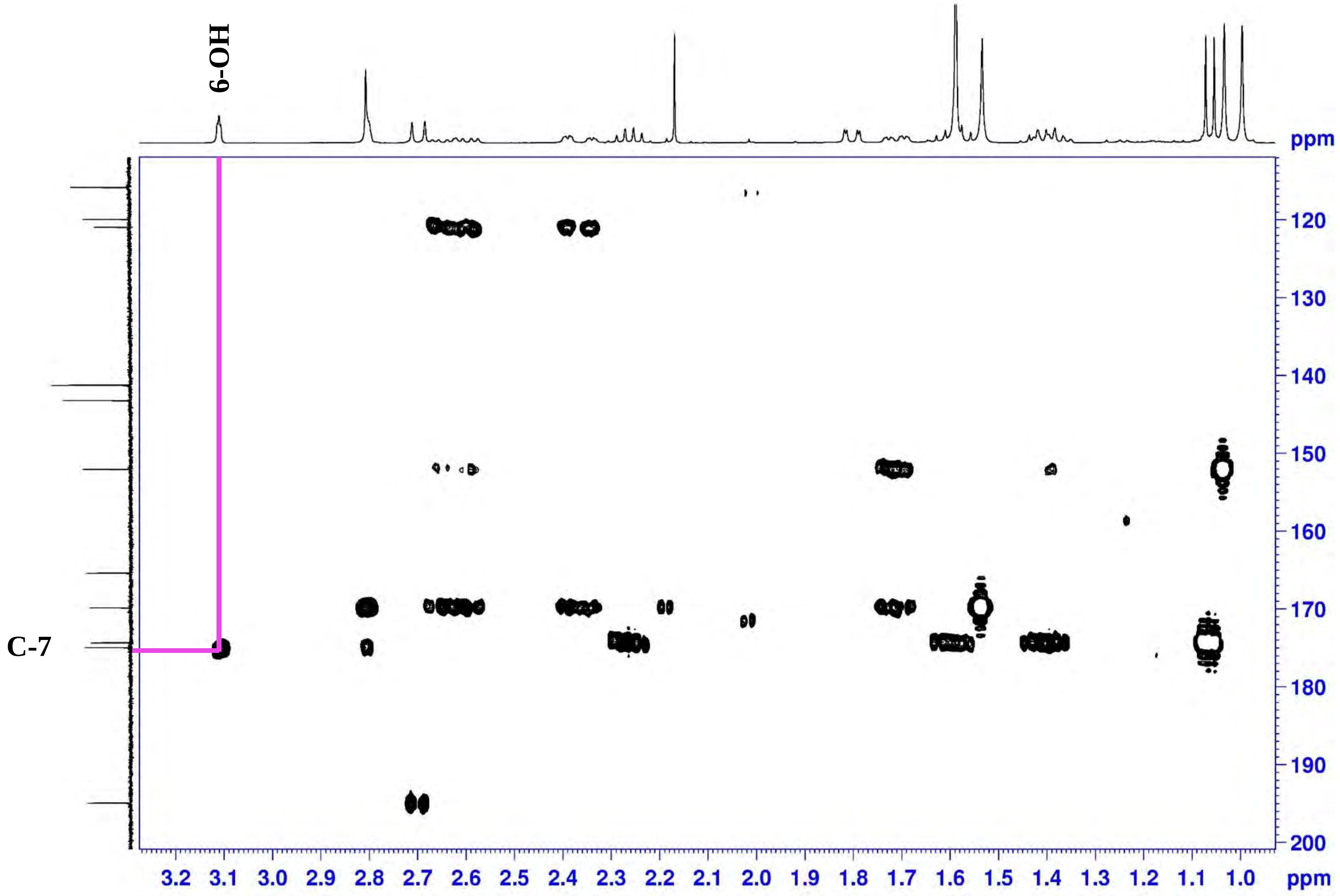

HMBC (400 MHz) spectrum of compound **24** in $CDCl_3$

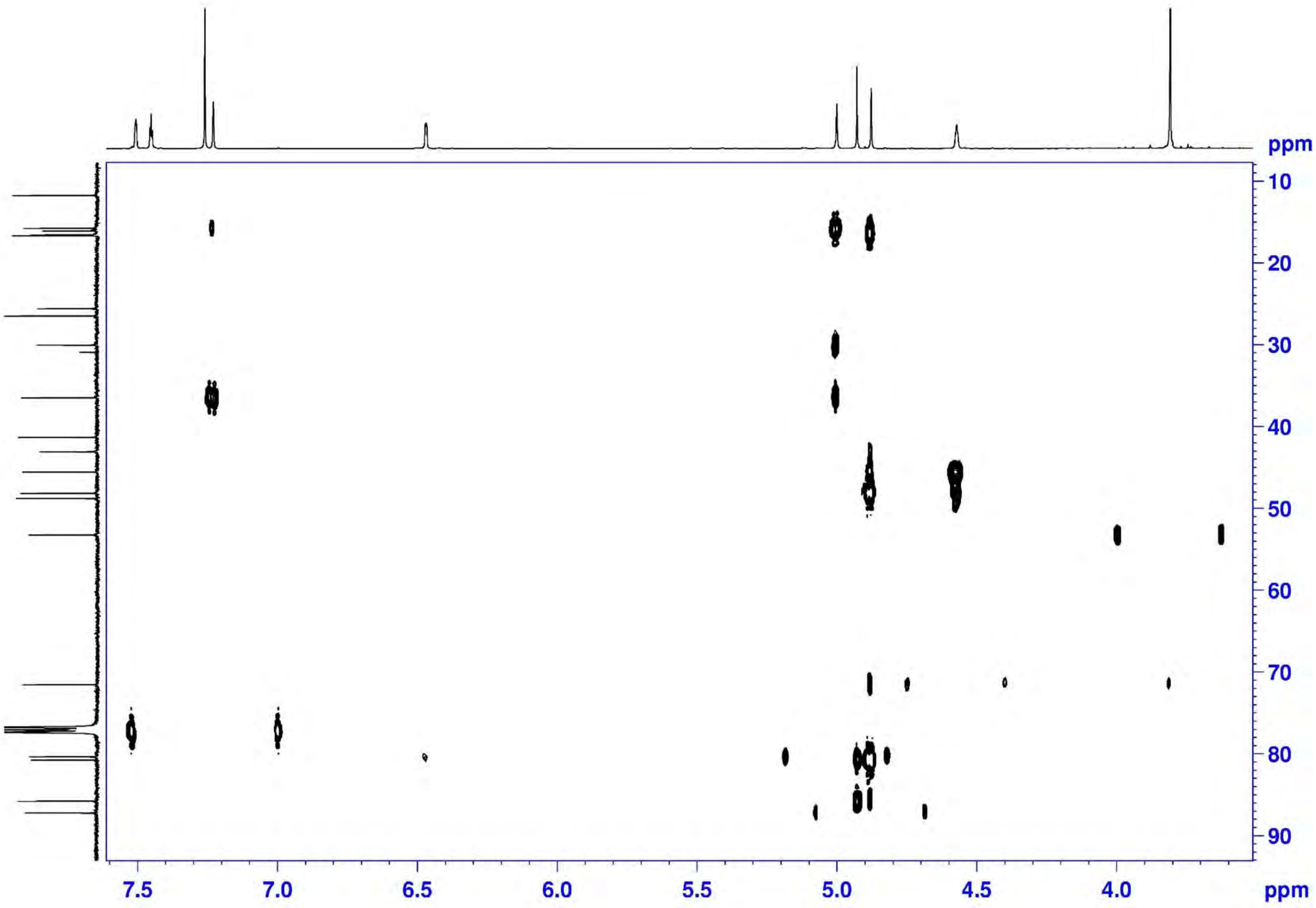

HMBC (400 MHz) spectrum of compound **24** in $CDCl_3$

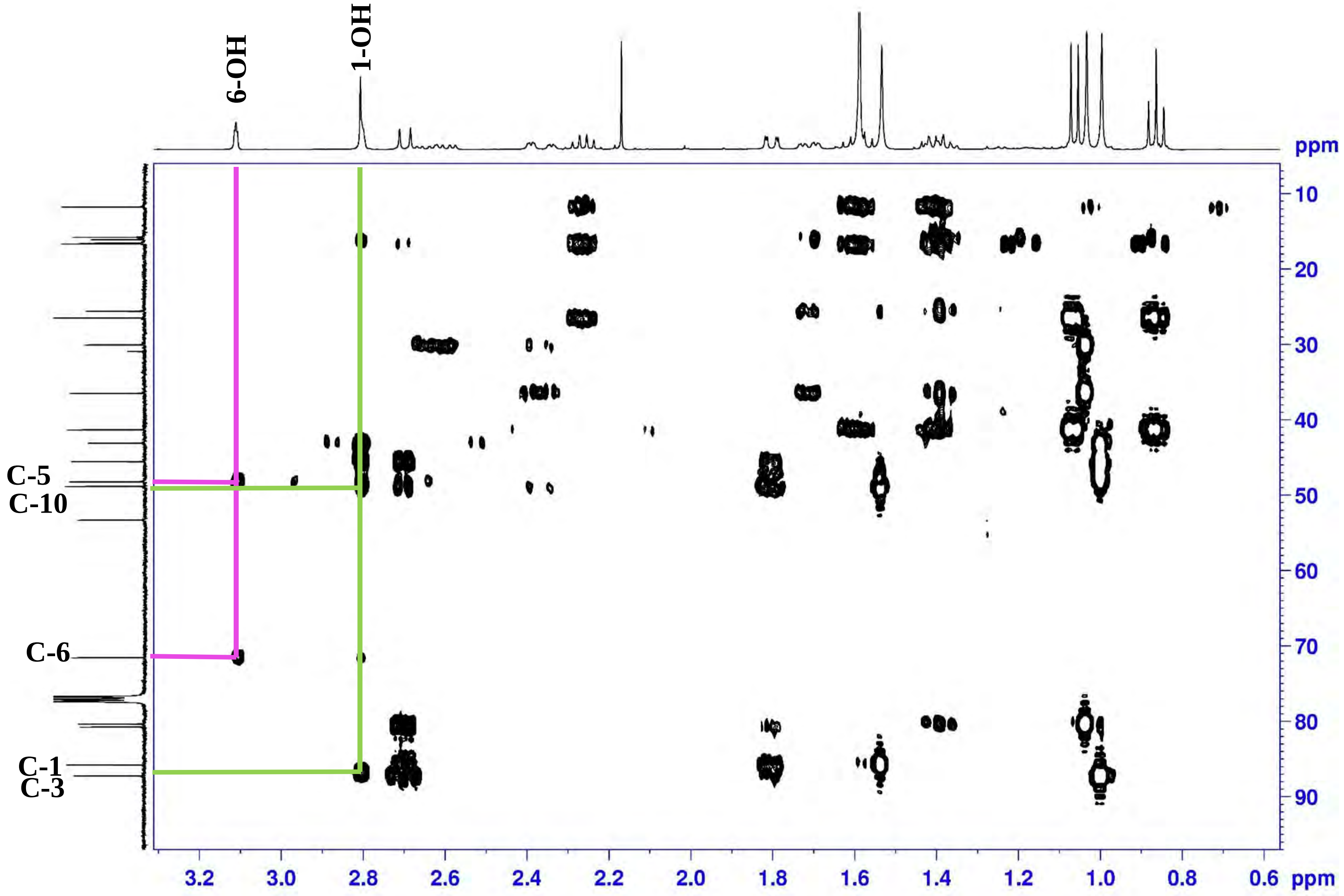

HMBC (400 MHz) spectrum of compound **24** in $CDCl_3$

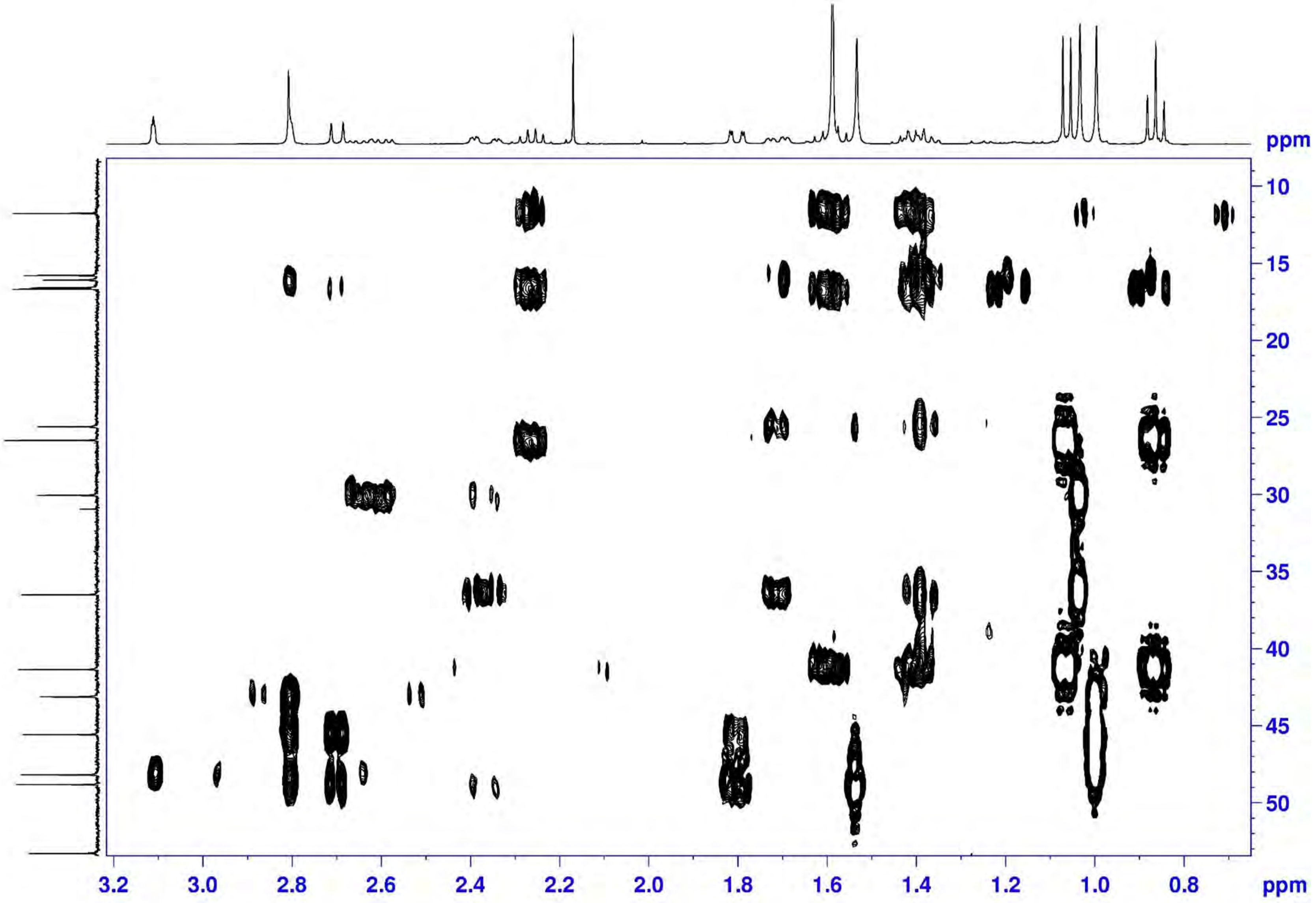

NOESY (400 MHz) spectrum of compound **24** in $CDCl_3$

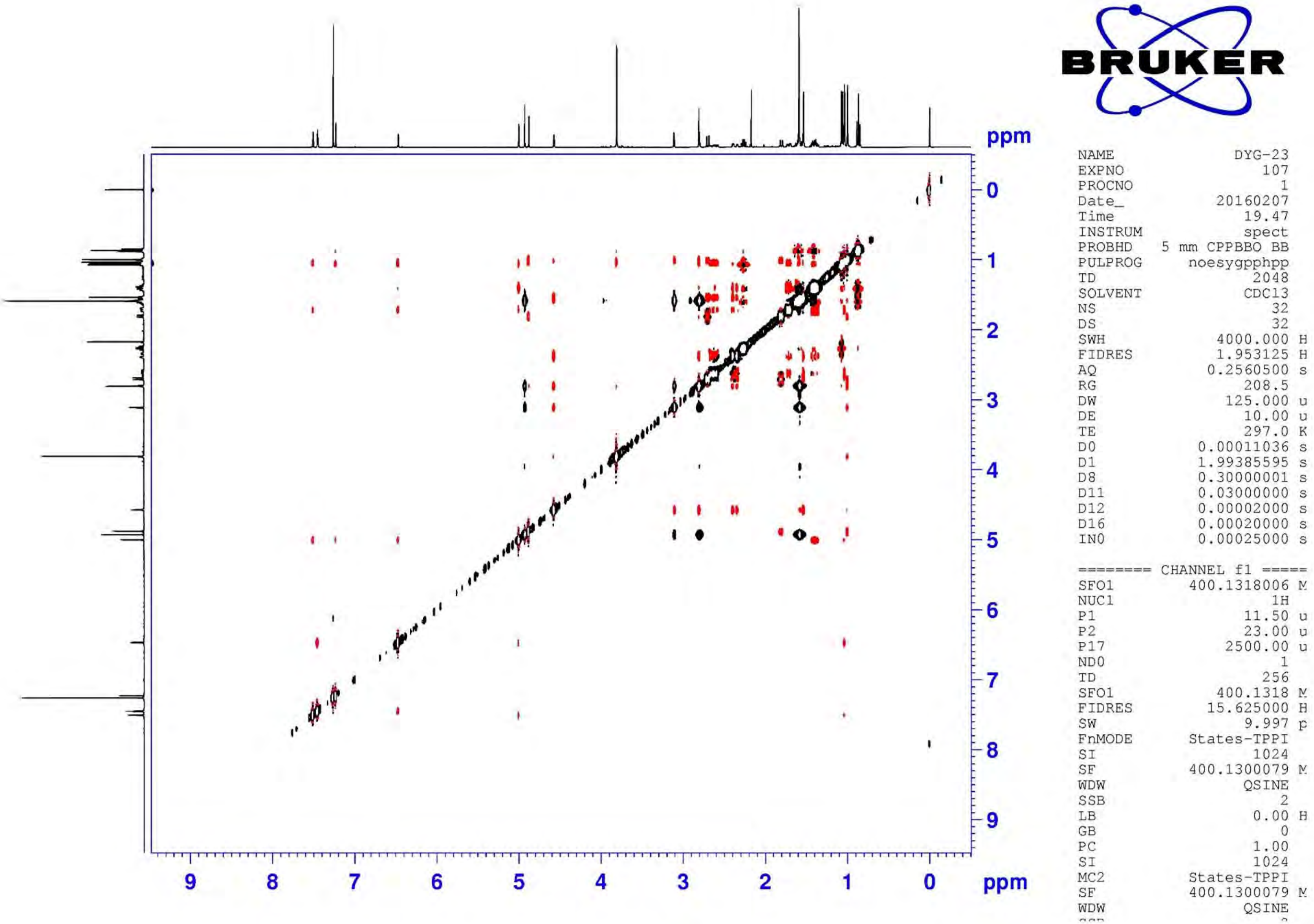

NOESY (400 MHz) spectrum of compound **24** in $CDCl_3$

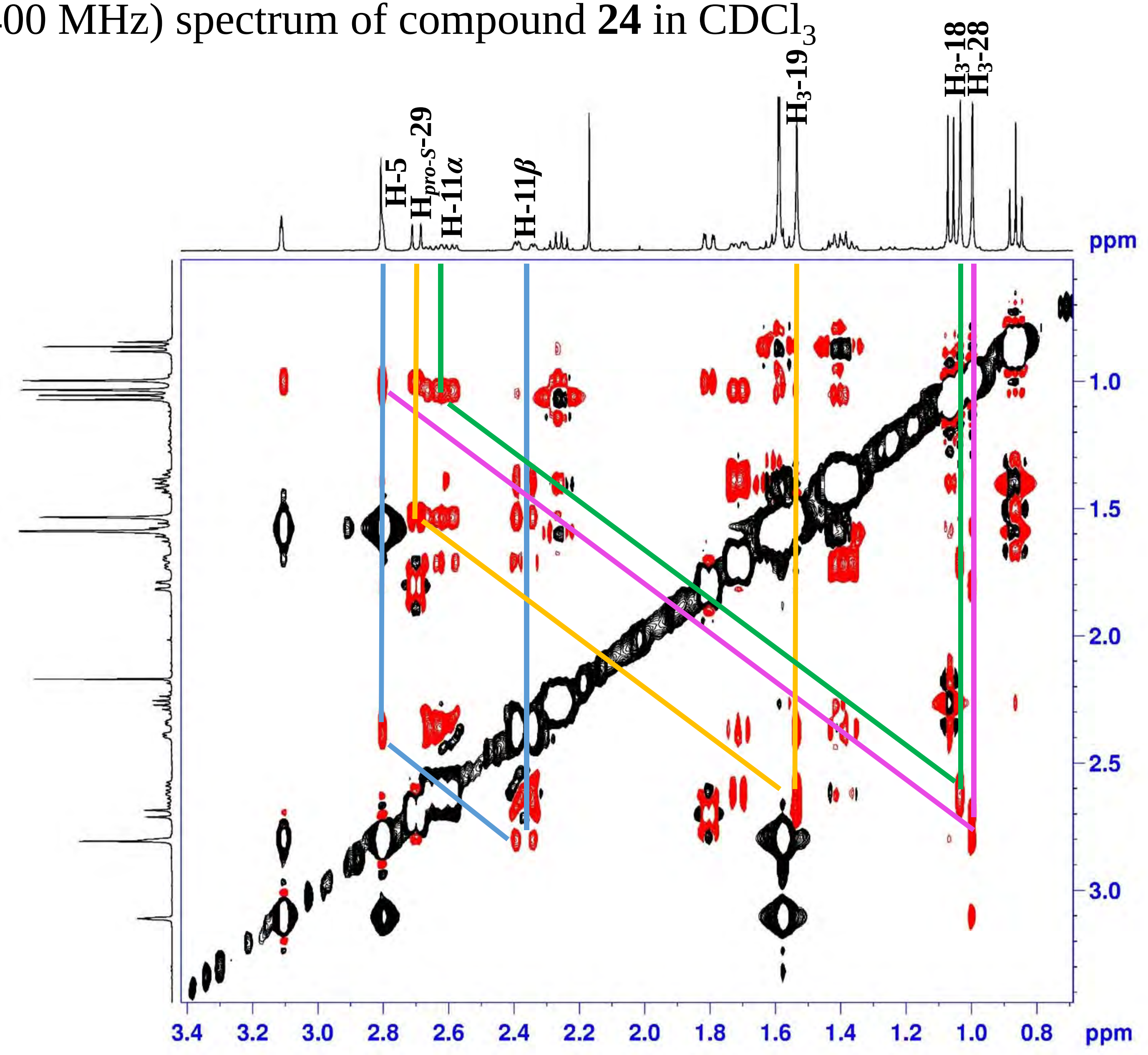

NOESY (400 MHz) spectrum of compound **24** in $CDCl_3$

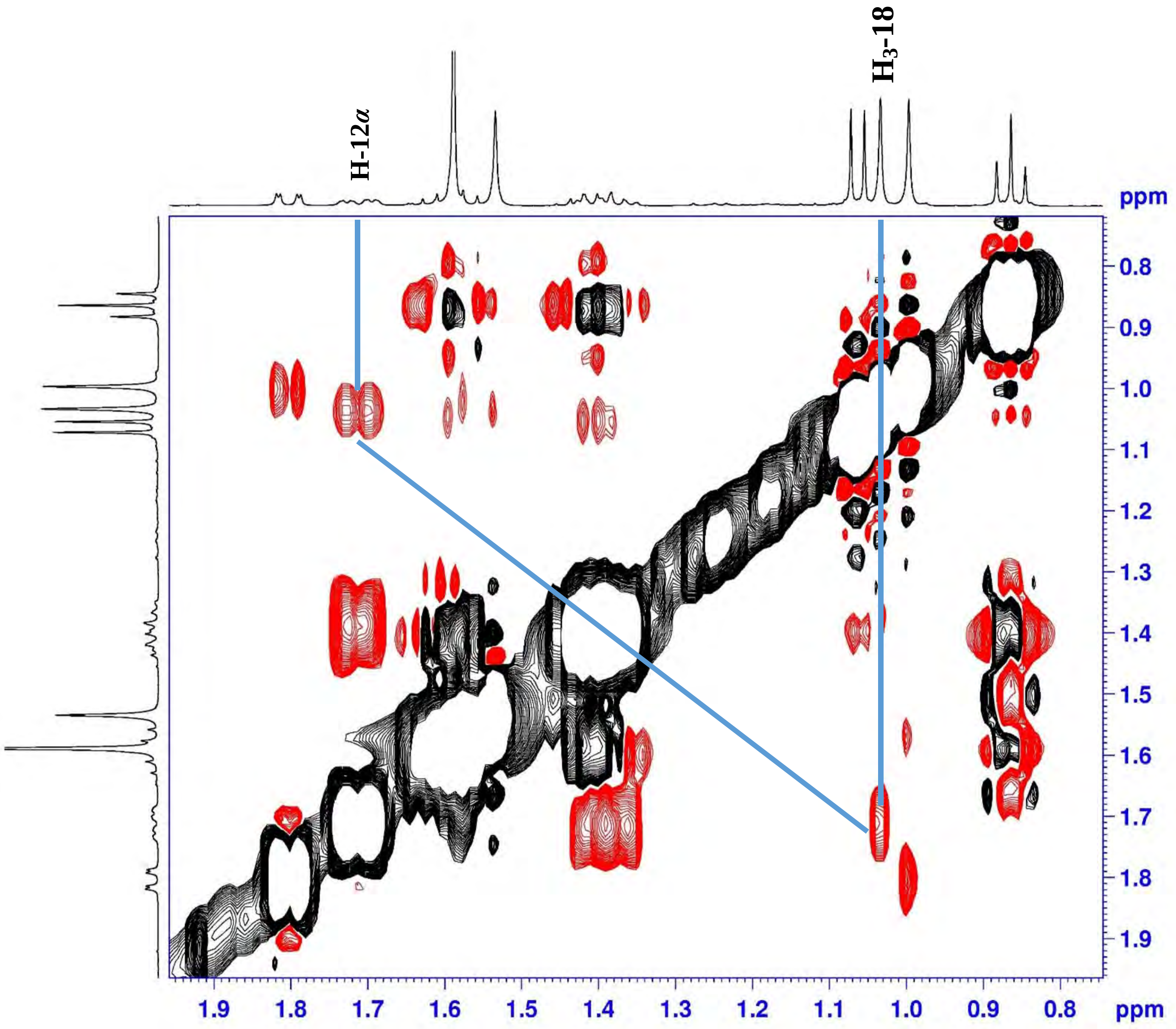

NOESY (400 MHz) spectrum of compound **24** in $CDCl_3$

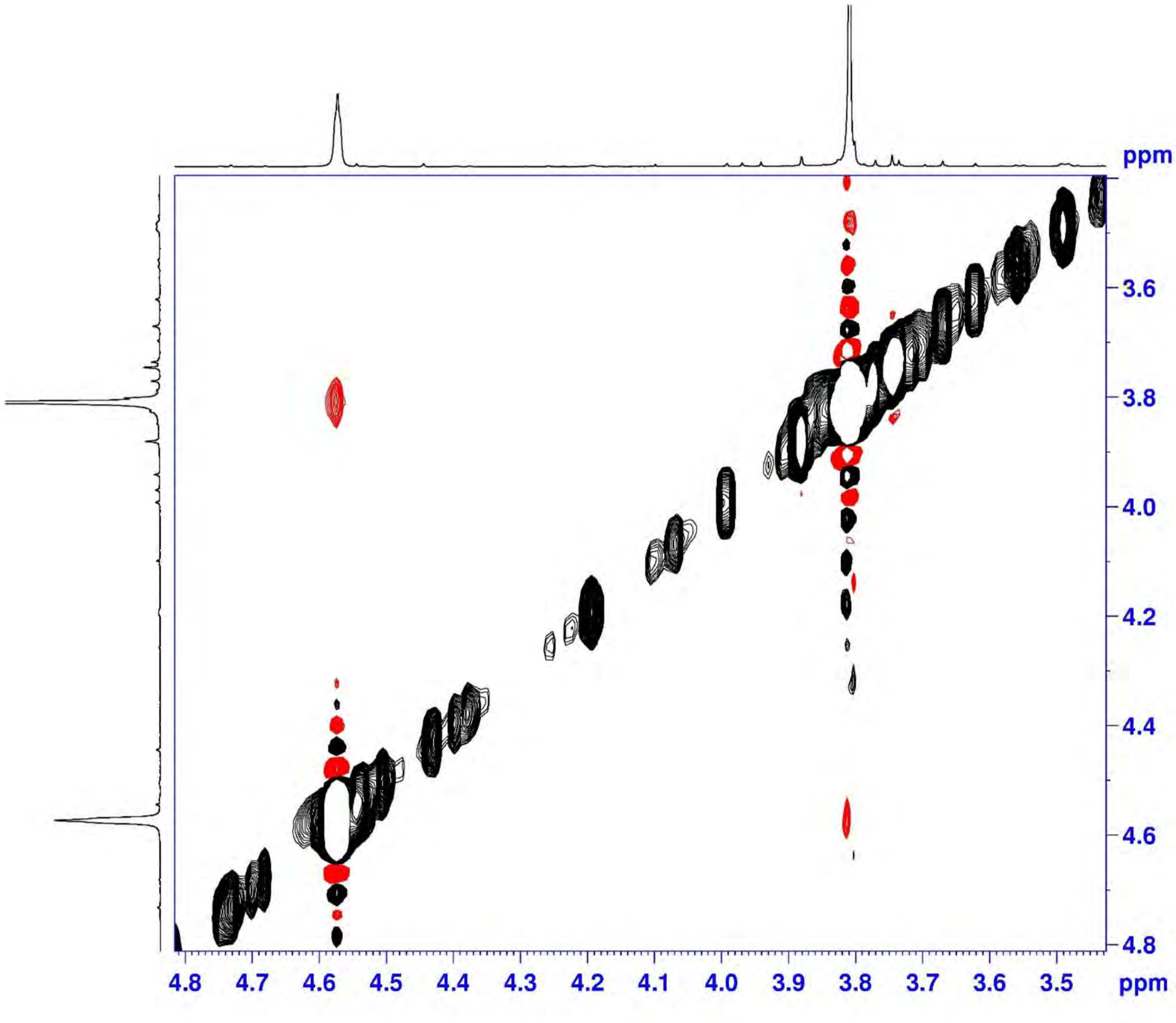

NOESY (400 MHz) spectrum of compound **24** in $CDCl_3$

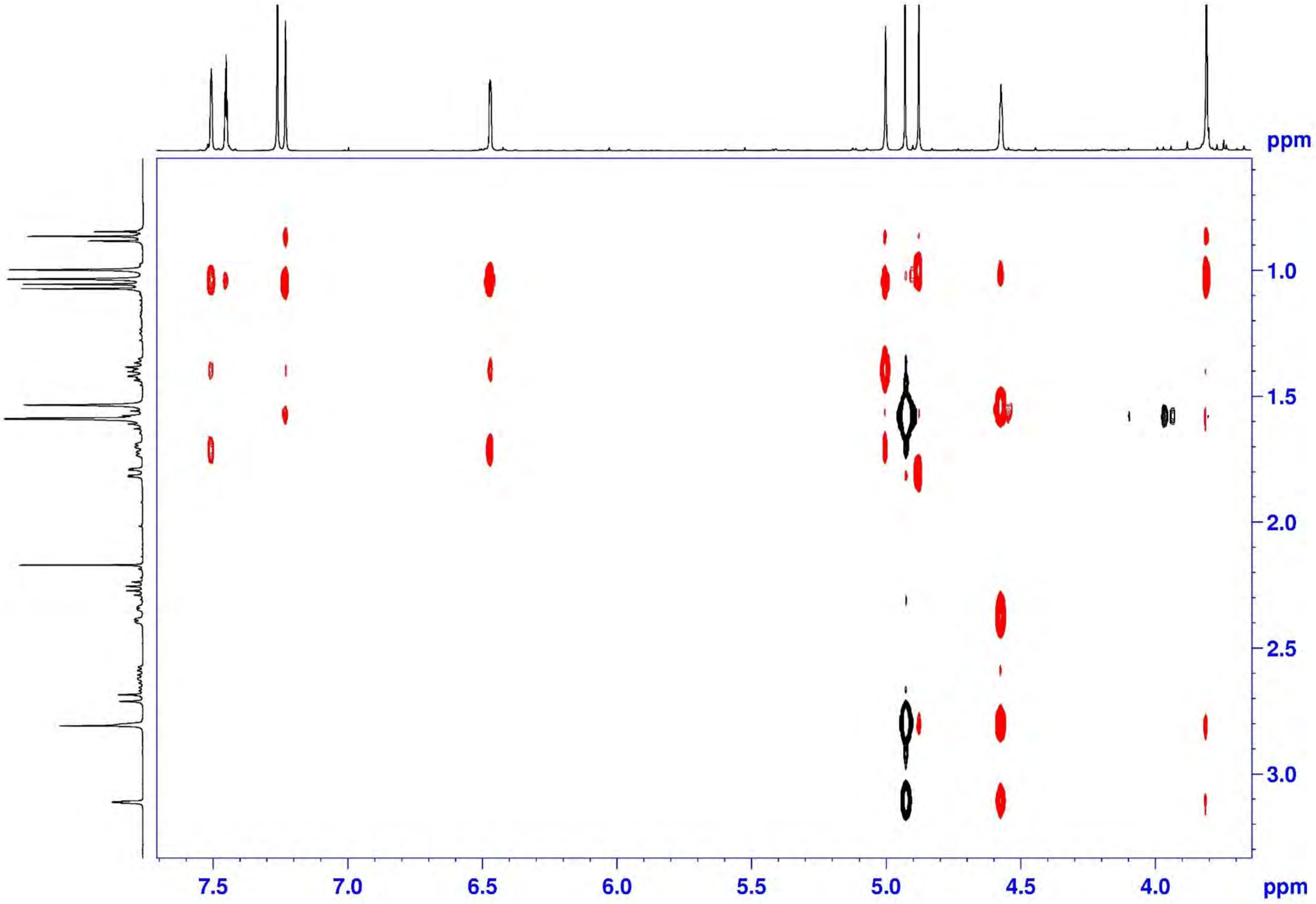

NOESY (400 MHz) spectrum of compound **24** in $CDCl_3$

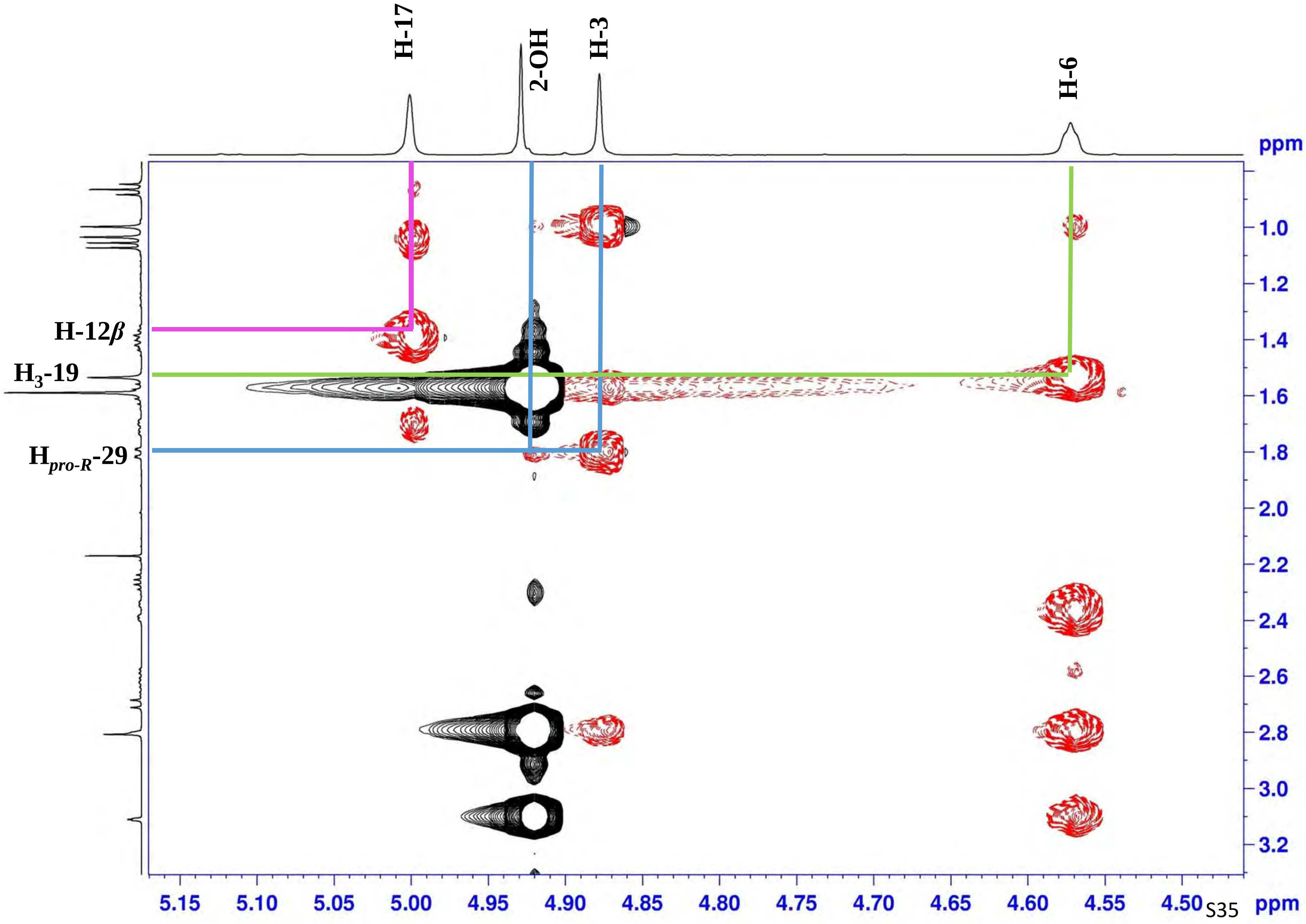

HRESIMS for compound **25**

Mass Spectrum SmartFormula Report

Analysis Info

Analysis Name	D:\Data\MS\data\201612\zhangjianzhi_zjz-16_pos_81_01_2757.d	Acquisition Date	12/14/2016 2:45:12 PM
Method	LC MS_Direct Infusion_pos_100-1000mz.m	Operator	SCSIO
Sample Name	zhangjianzhi_zjz-16_pos	Instrument	maXis 255552.00029
Comment			

Acquisition Parameter

Source Type	ESI	Ion Polarity	Positive	Set Nebulizer	0.4 Bar
Focus	Active	Set Capillary	4500 V	Set Dry Heater	180 °C
Scan Begin	100 m/z	Set End Plate Offset	-500 V	Set Dry Gas	4.0 l/min
Scan End	2000 m/z	Set Charging Voltage	0 V	Set Divert Valve	Waste
		Set Corona	0 nA	Set APCI Heater	0 °C

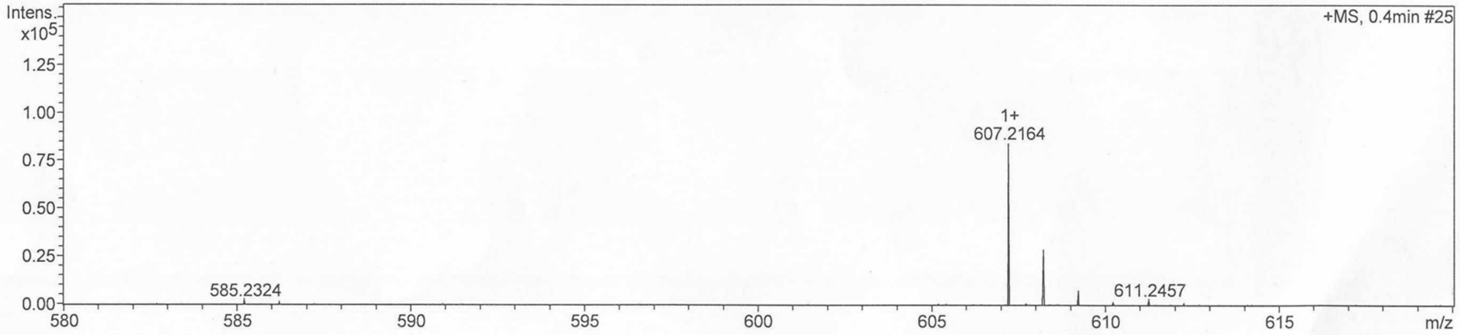

Meas. m/z	#	Ion Formula	Score	m/z	err [ppm]	err [mDa]	mSigma	rdb	e⁻ Conf	N-Rule
585.232407	1	C31H37O11	100.00	585.233038	1.1	0.6	63.5	13.5	even	ok
607.216367	1	C31H36NaO11	100.00	607.214983	-2.3	-1.4	5.6	13.5	even	ok
1191.442483	1	C62H72NaO22	100.00	1191.440745	1.5	1.7	24.0	26.5	even	ok

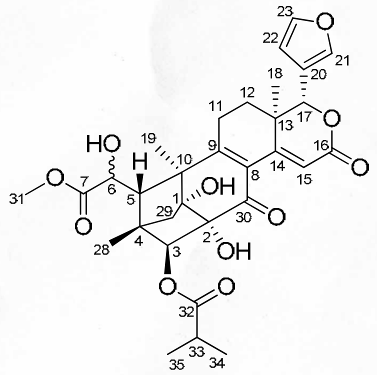

zhangjianzhi_zjz-16_pos_81_01_2757.d

Bruker Compass DataAnalysis 4.1 printed: 12/14/2016 2:49:58 PM by: SCSIO Page 1 of 1

HRESIMS for compound **25**

Generic Display Report

Analysis Info

Analysis Name	D:\Data\MS\data\201612\zhangjianzhi_zjz-16_pos_81_01_2757.d	Acquisition Date	12/14/2016 2:45:12 PM
Method	LC MS_Direct Infusion_pos_100-1000mz.m	Operator	SCSIO
Sample Name	zhangjianzhi_zjz-16_pos	Instrument	maXis
Comment			

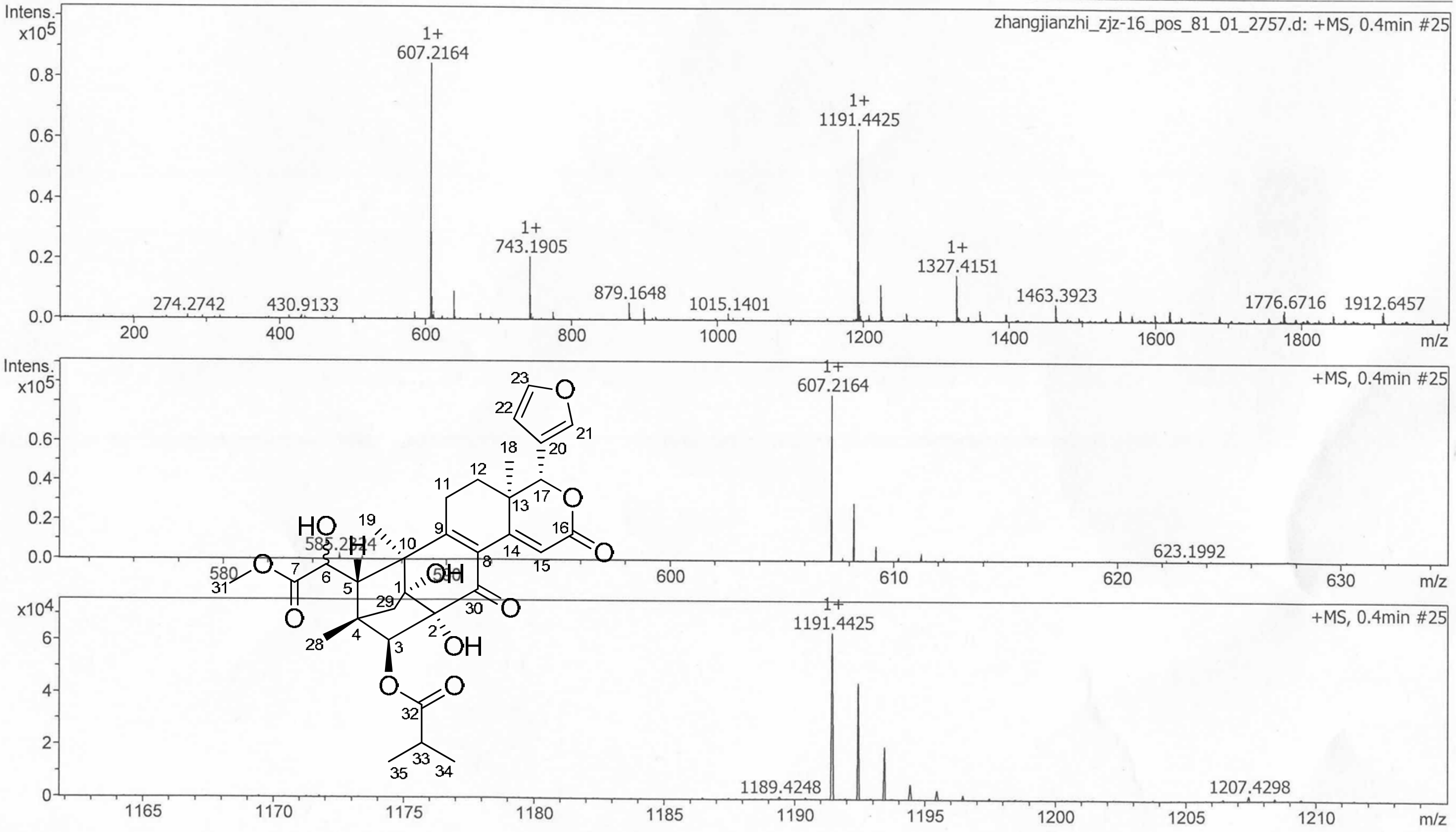

Bruker Compass DataAnalysis 4.1 printed: 12/14/2016 2:50:31 PM by: SCSIO Page 1 of 1

^{1}H NMR (400 MHz) spectrum of compound **25** in $CDCl_3$

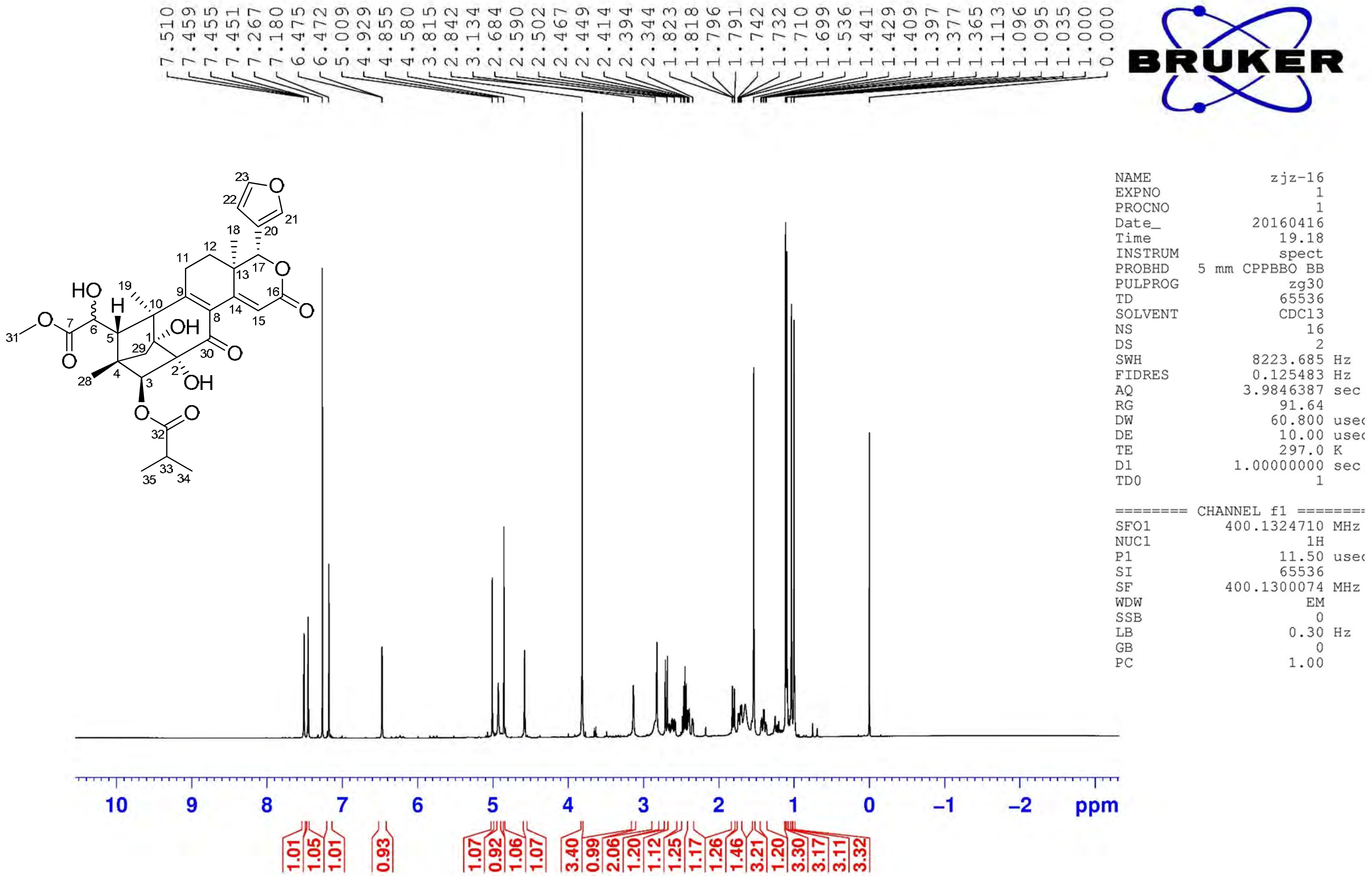

^{1}H NMR (400 MHz) spectrum of compound **25** in $CDCl_3$

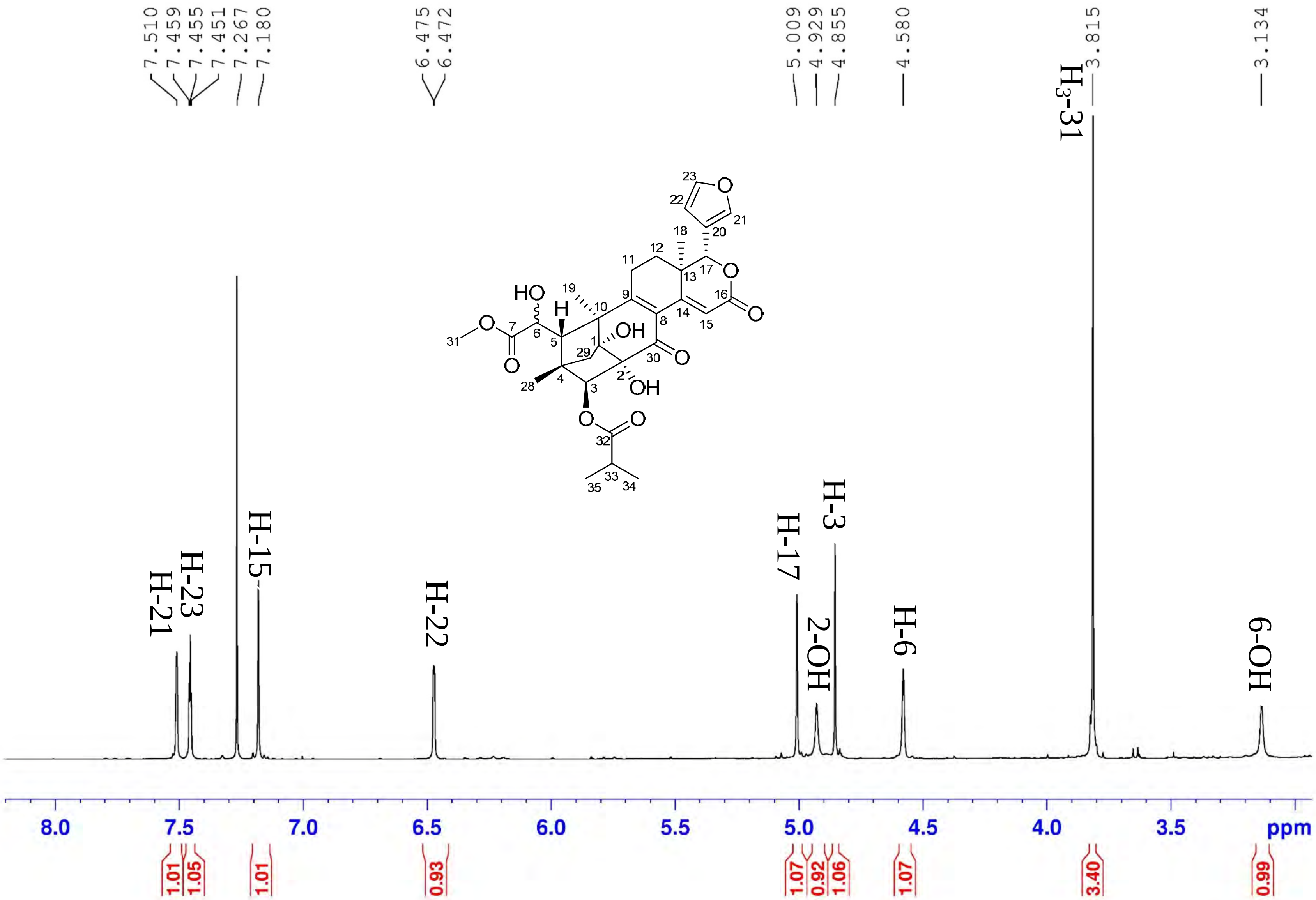

^{1}H NMR (400 MHz) spectrum of compound **25** in $CDCl_3$

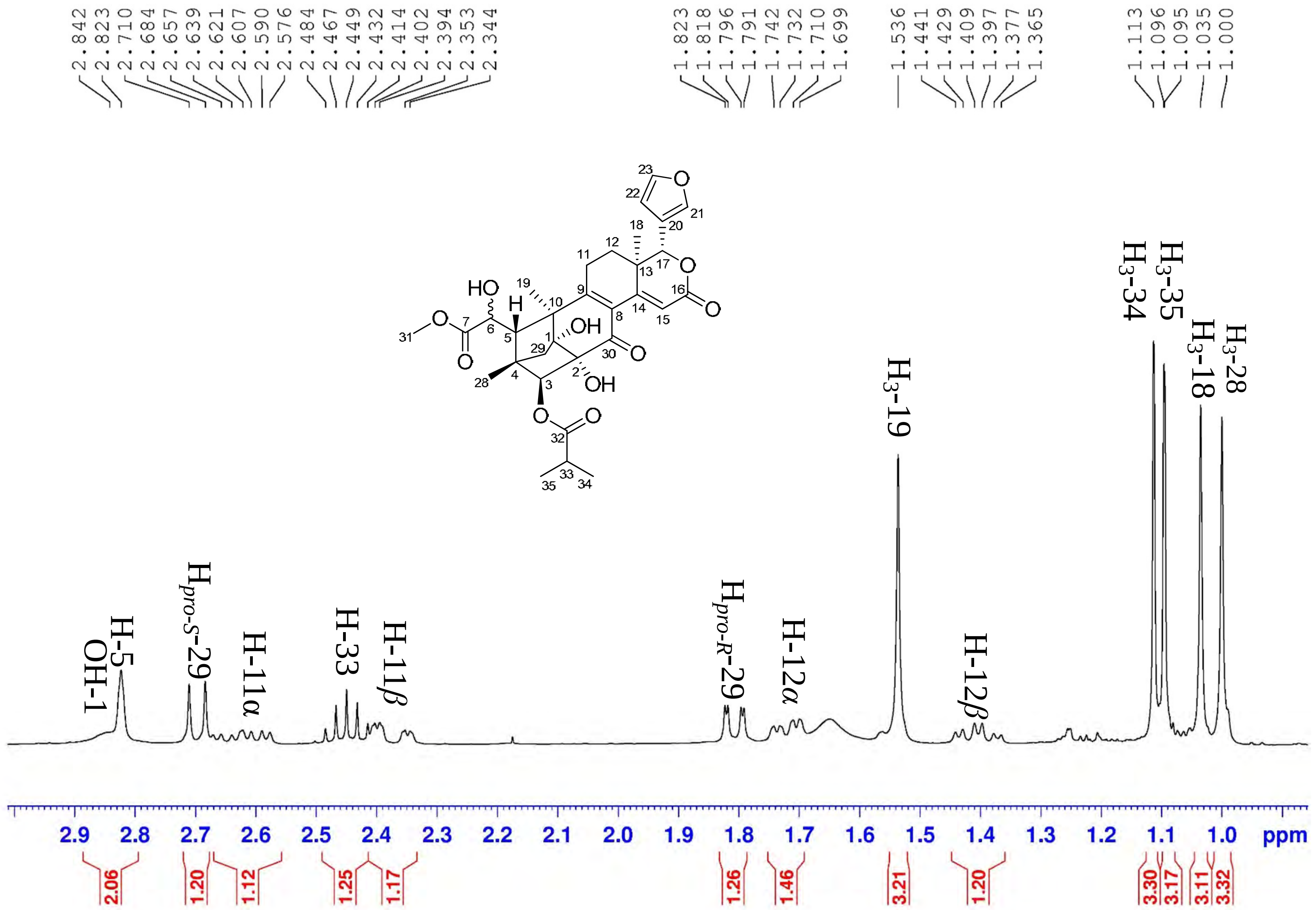

^{13}C NMR (100 MHz) spectrum of compound **25** in $CDCl_3$

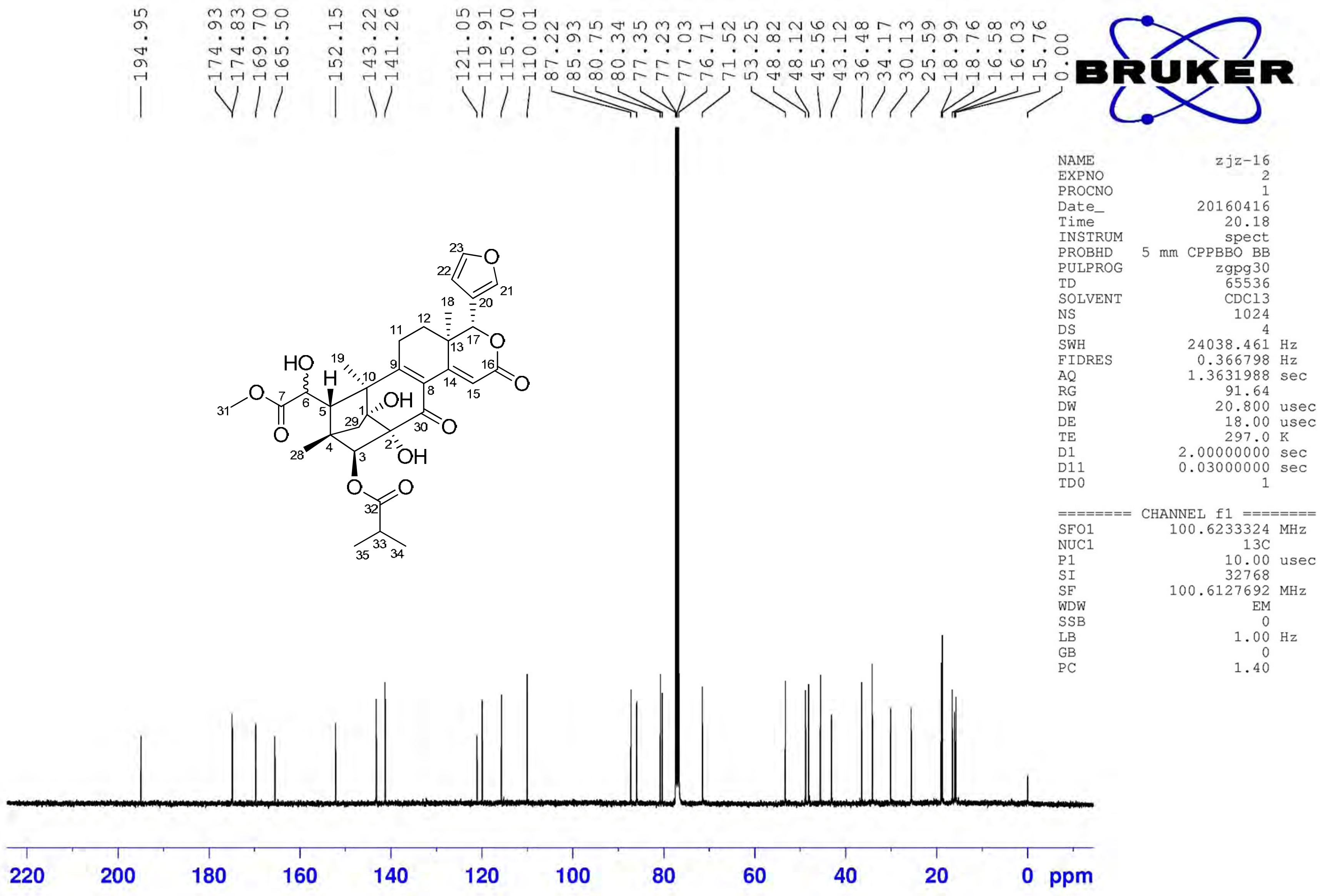


```
NAME              zjz-16
EXPNO                  2
PROCNO                 1
Date_           20160416
Time               20.18
INSTRUM            spect
PROBHD   5 mm CPPBBO BB
PULPROG          zgpg30
TD                 65536
SOLVENT            CDCl3
NS                  1024
DS                     4
SWH            24038.461 Hz
FIDRES          0.366798 Hz
AQ             1.3631988 sec
RG                 91.64
DW                20.800 usec
DE                 18.00 usec
TE                 297.0 K
D1            2.00000000 sec
D11           0.03000000 sec
TD0                    1

======== CHANNEL f1 ========
SFO1         100.6233324 MHz
NUC1                 13C
P1                 10.00 usec
SI                 32768
SF           100.6127692 MHz
WDW                   EM
SSB                    0
LB                  1.00 Hz
GB                     0
PC                  1.40
```

^{13}C NMR (100 MHz) spectrum of compound **25** in $CDCl_3$

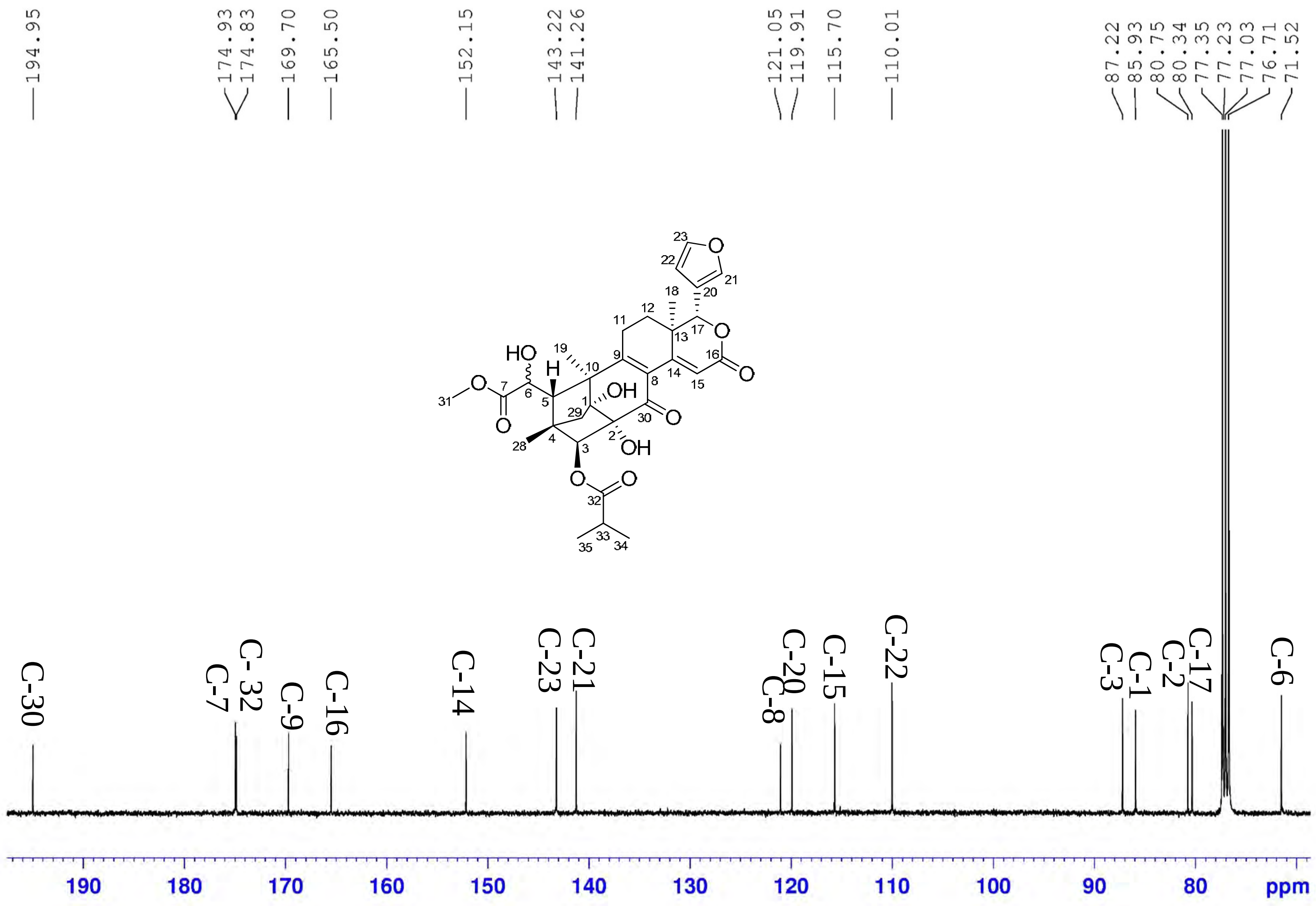

^{13}C NMR (100 MHz) spectrum of compound **25** in $CDCl_3$

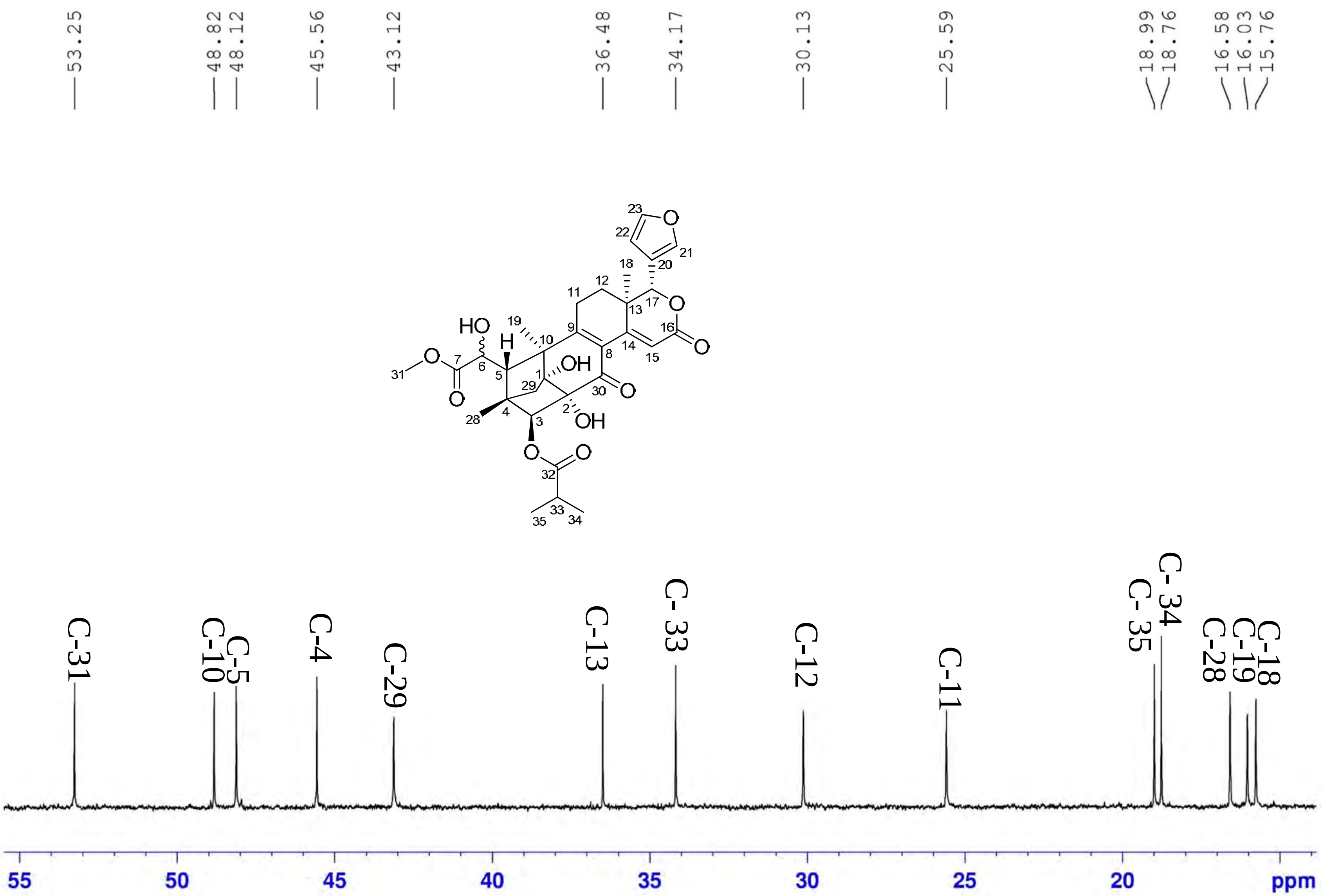

DEPT135 (100 MHz) spectrum of compound **25** in $CDCl_3$

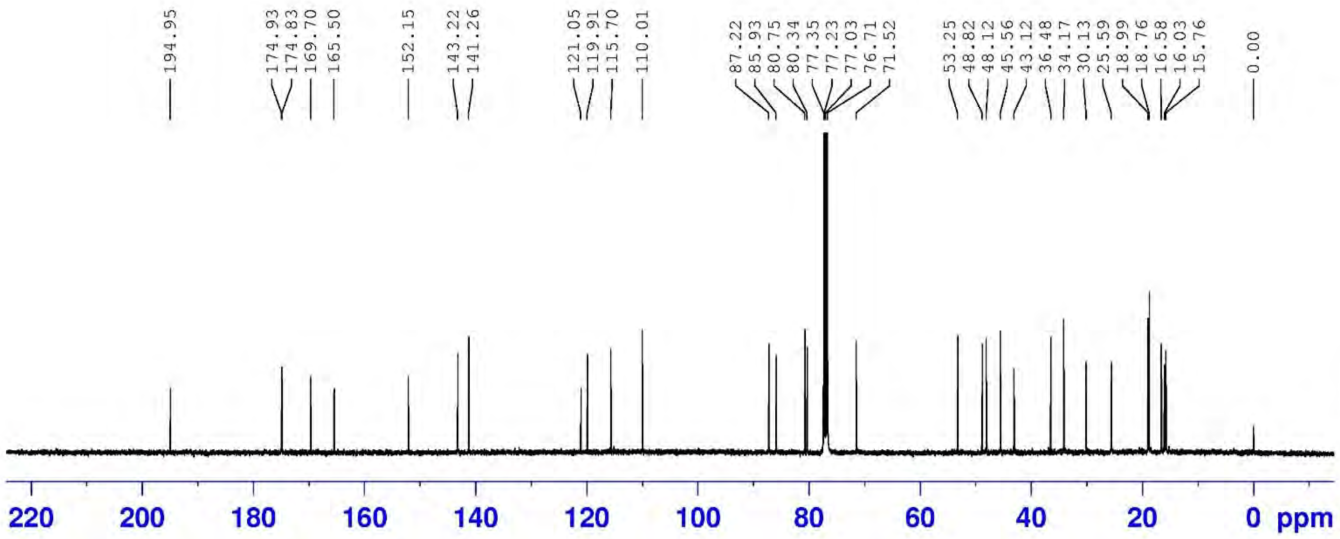

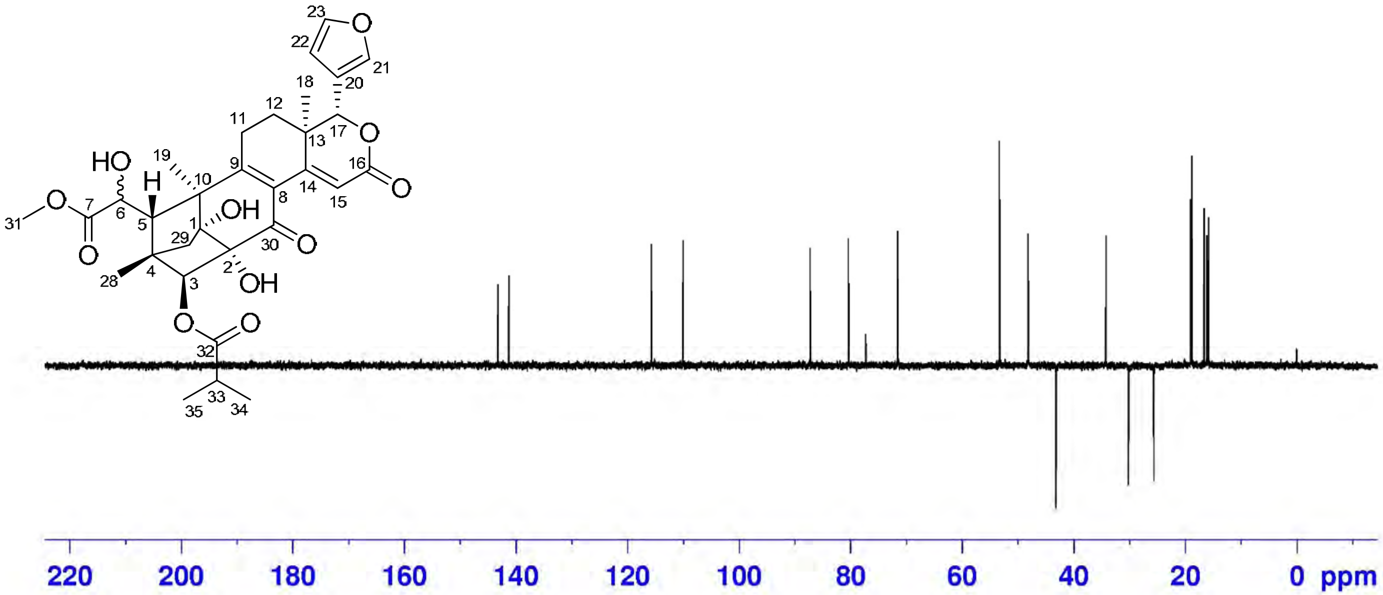

NAME	zjz-16
EXPNO	3
PROCNO	1
Date_	20160416
Time	20.36
INSTRUM	spect
PROBHD	5 mm CPPBBO BB
PULPROG	deptsp135
TD	65536
SOLVENT	CDCl3
NS	300
DS	4
SWH	24038.461 Hz
FIDRES	0.366798 Hz
AQ	1.3631988 sec
RG	130.26
DW	20.800 usec
DE	18.00 usec
TE	297.0 K
CNST2	145.0000000
D1	2.00000000 sec
D2	0.00344828 sec
D12	0.00002000 sec
TD0	1

======== CHANNEL f1 ========

SFO1	100.6233324 MHz
NUC1	13C
P1	10.00 usec
P13	2000.00 usec
SI	32768
SF	100.6127692 MHz
WDW	EM
SSB	0
LB	1.00 Hz
GB	0
PC	1.40

DEPT135 (100 MHz) spectrum of compound **25** in $CDCl_3$

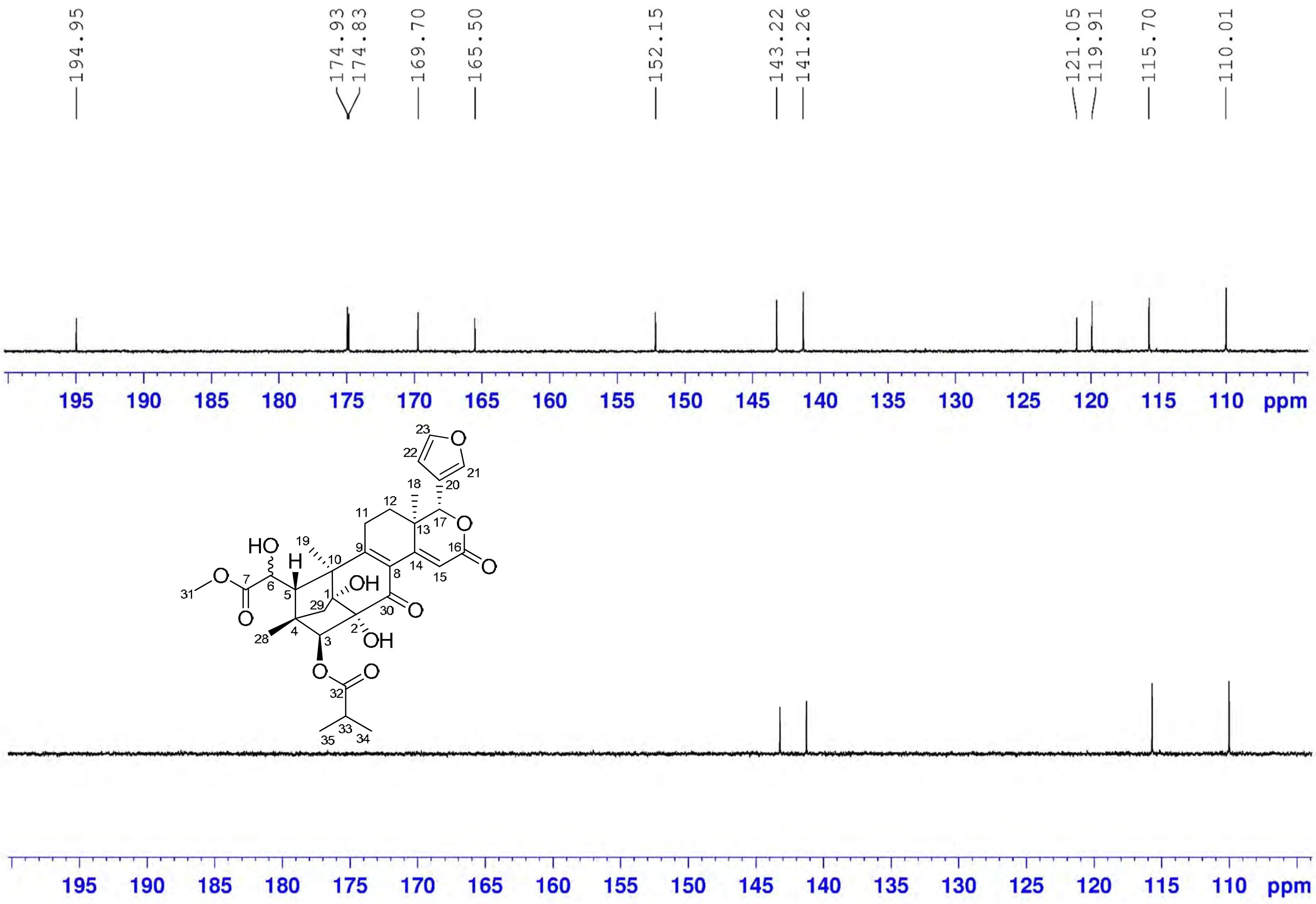

DEPT135 (100 MHz) spectrum of compound **25** in $CDCl_3$

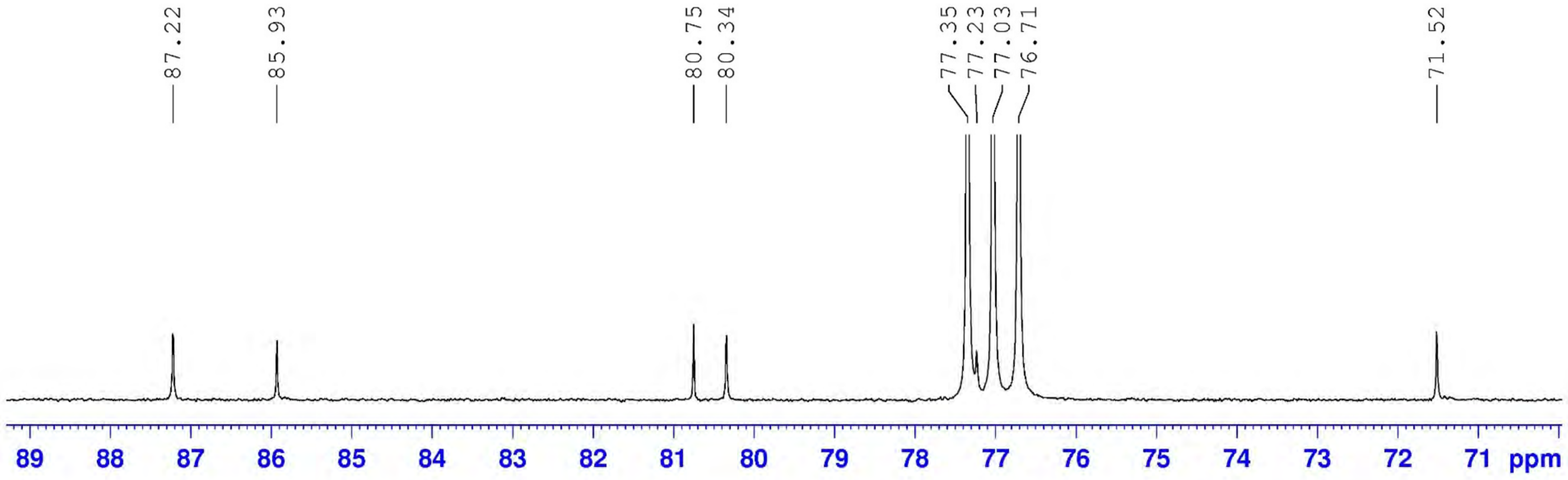

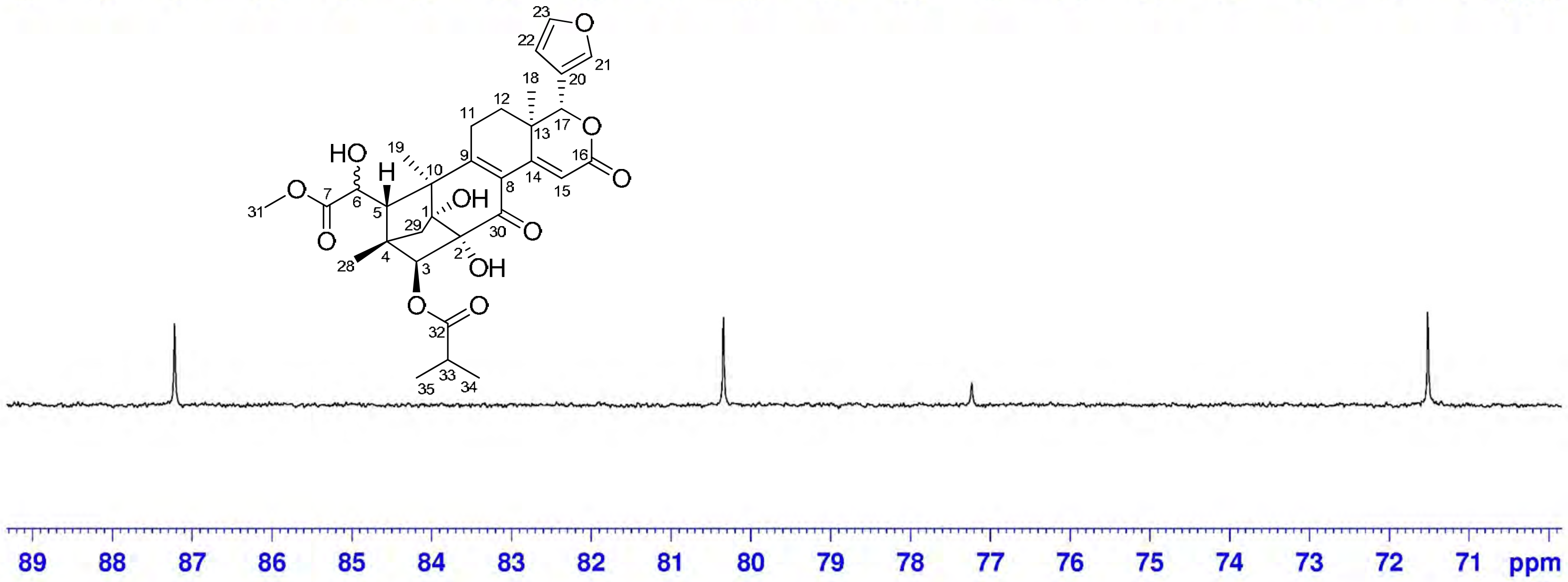

DEPT135 (100 MHz) spectrum of compound **25** in $CDCl_3$

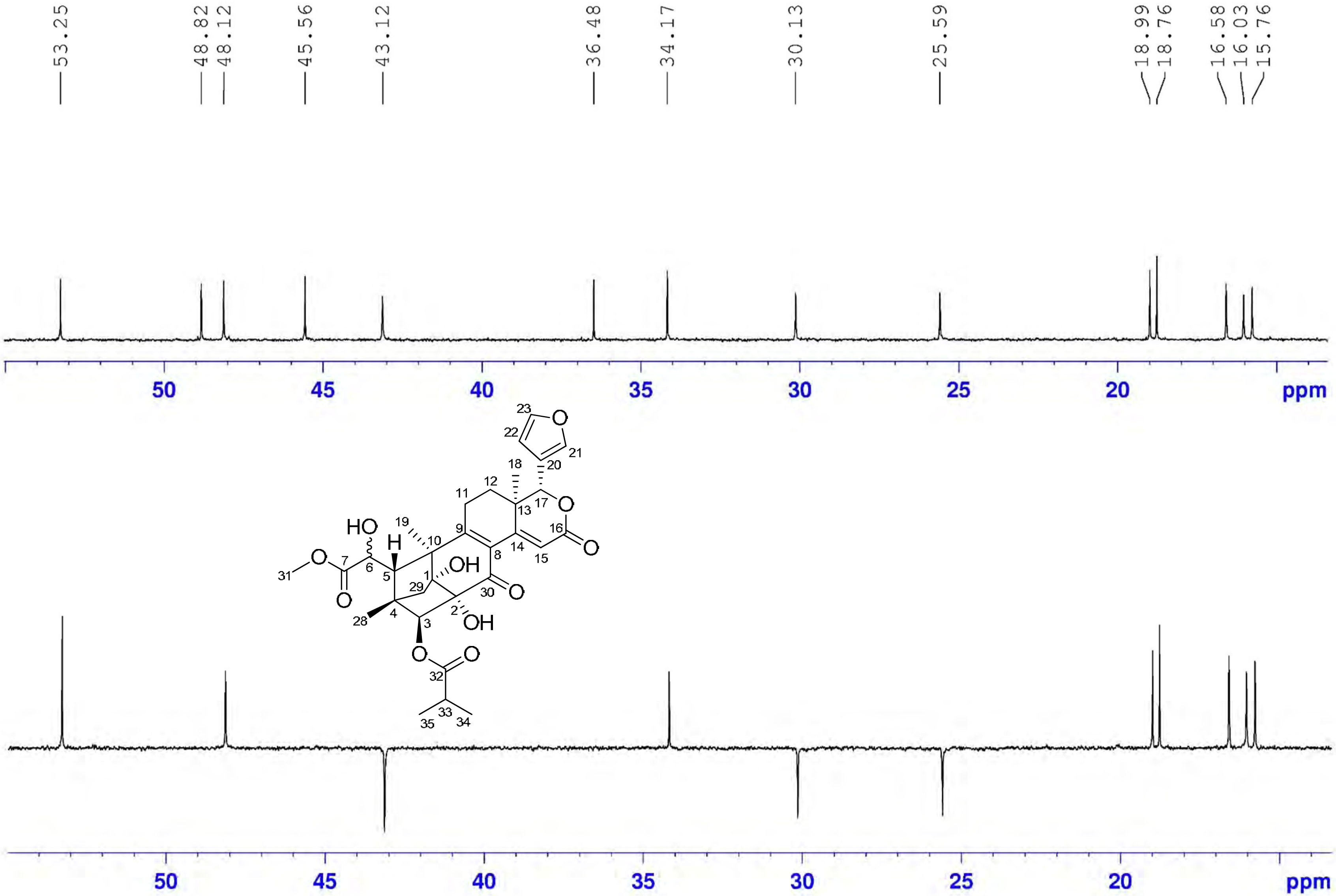

^{1}H-^{1}H COSY (400 MHz) spectrum of compound **25** in $CDCl_3$

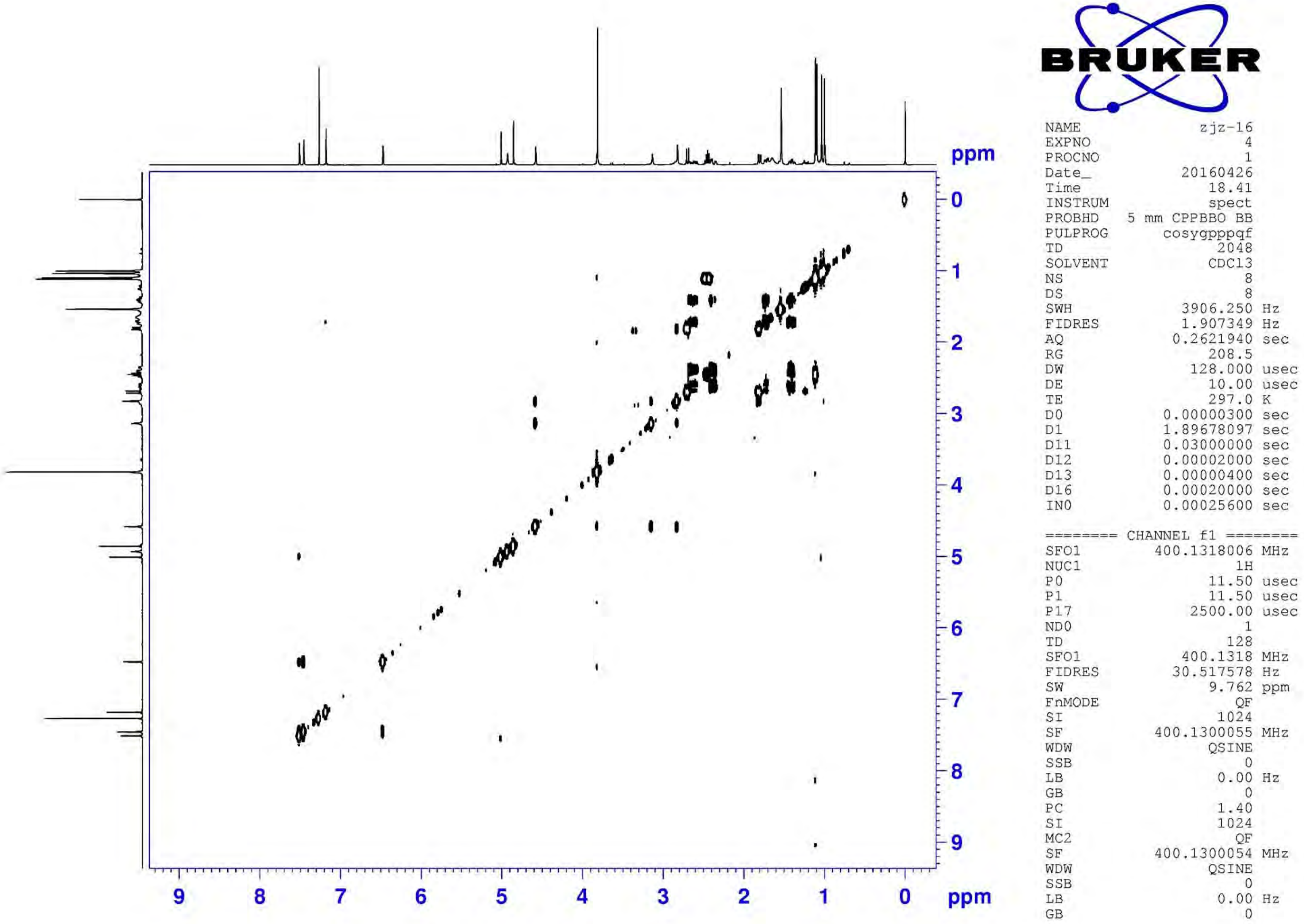

^{1}H-^{1}H COSY (400 MHz) spectrum of compound **25** in $CDCl_3$

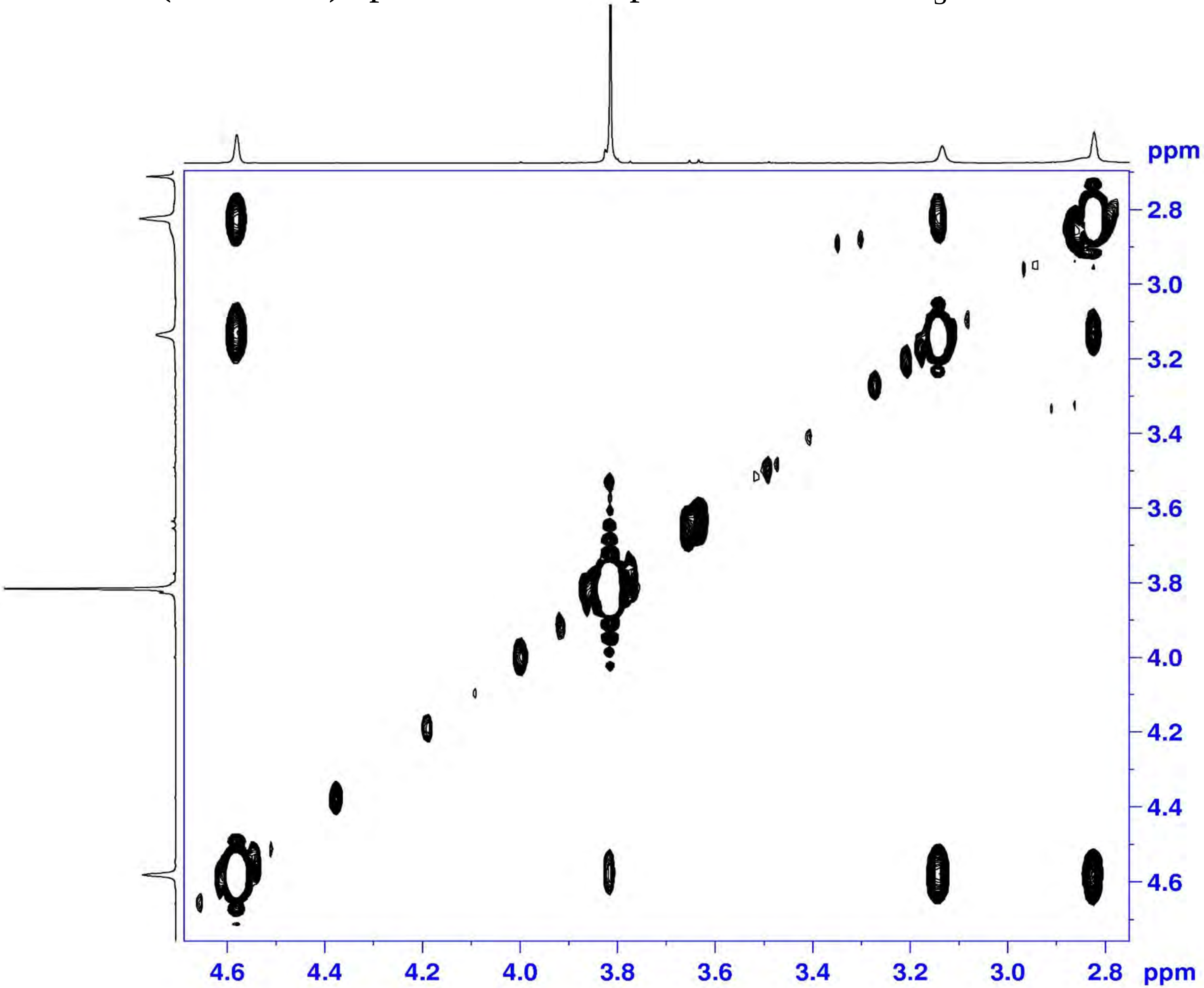

^{1}H-^{1}H COSY (400 MHz) spectrum of compound **25** in $CDCl_3$

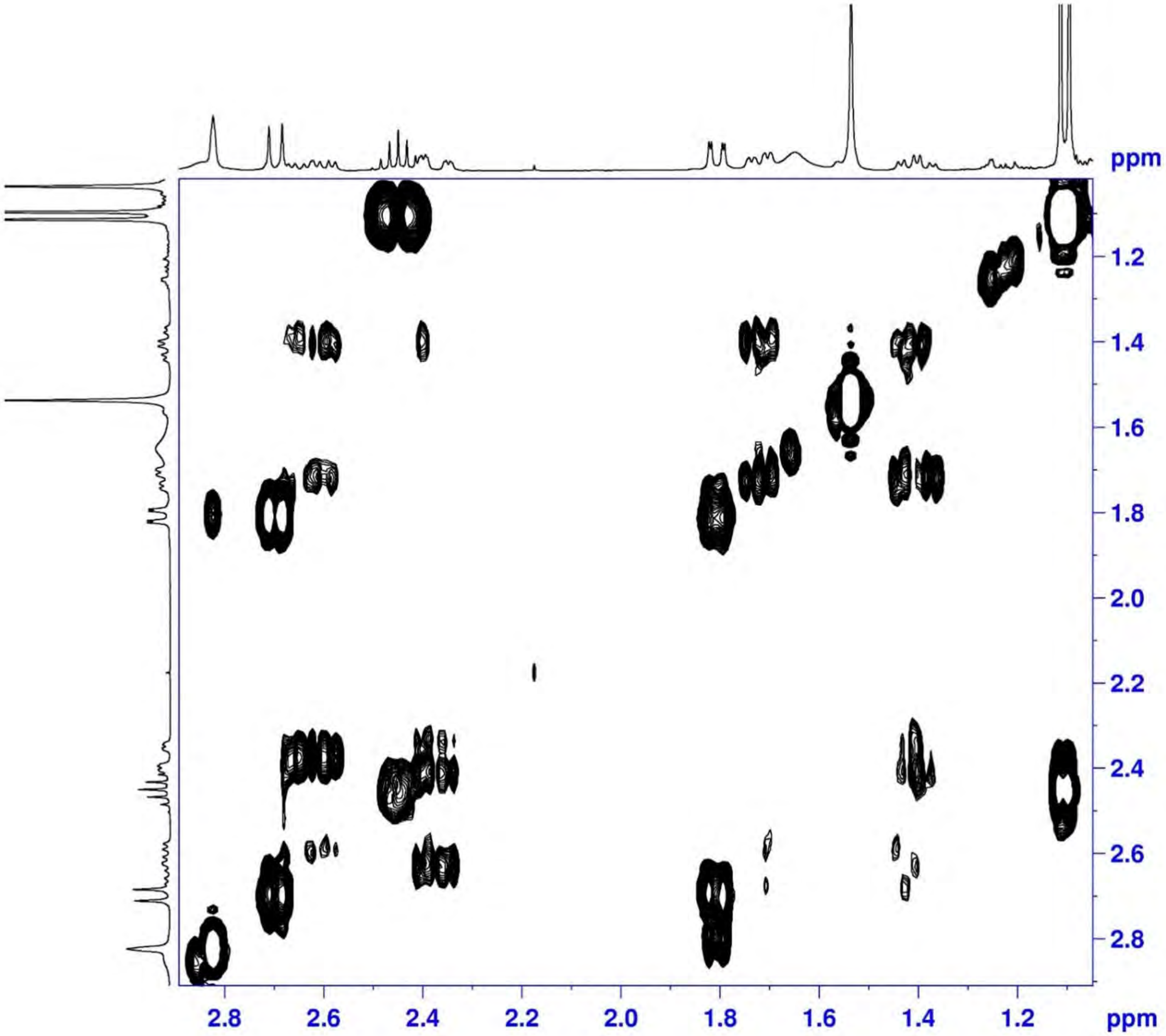

HSQC (400 MHz) spectrum of compound **25** in $CDCl_3$

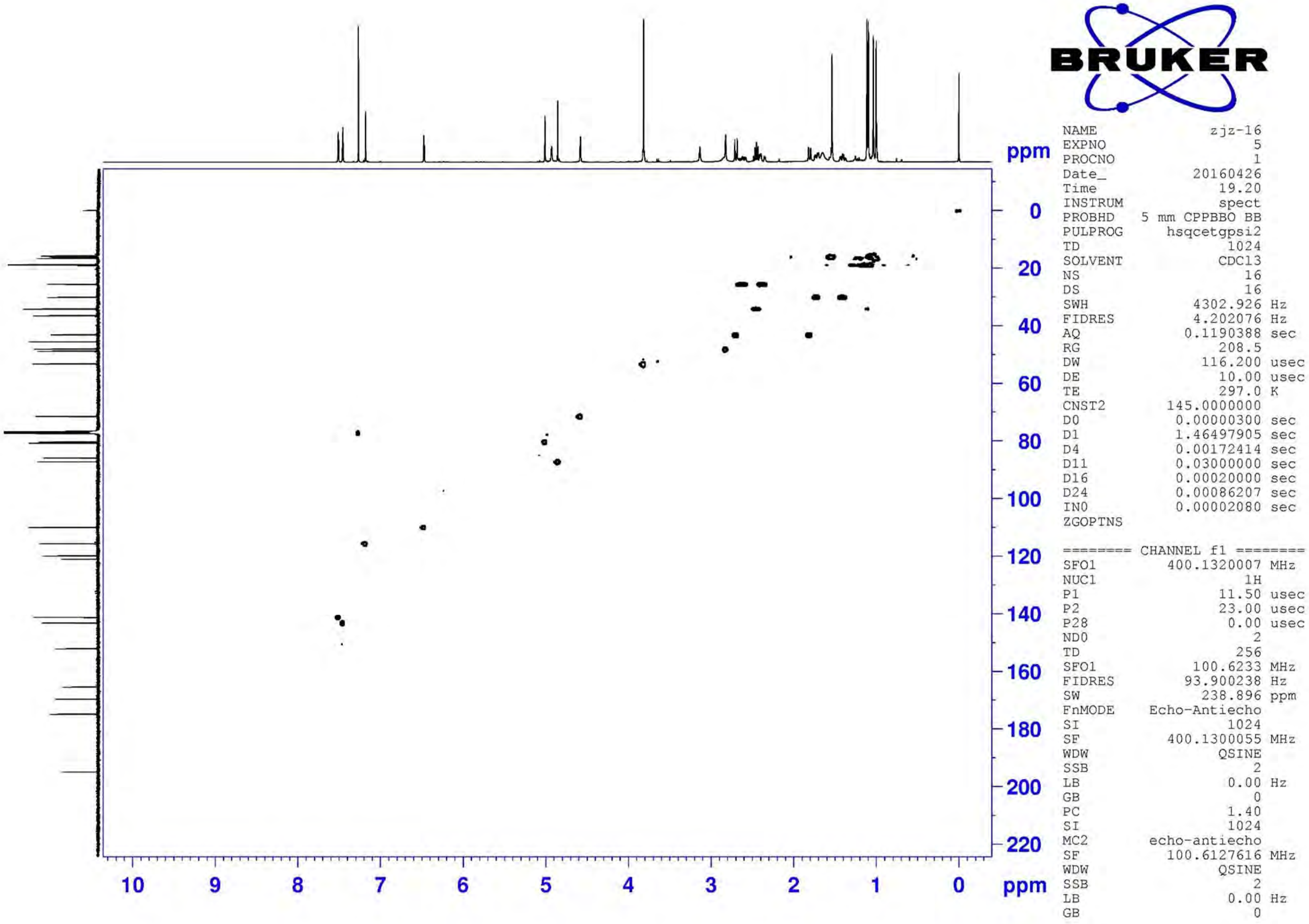

HSQC (400 MHz) spectrum of compound **25** in $CDCl_3$

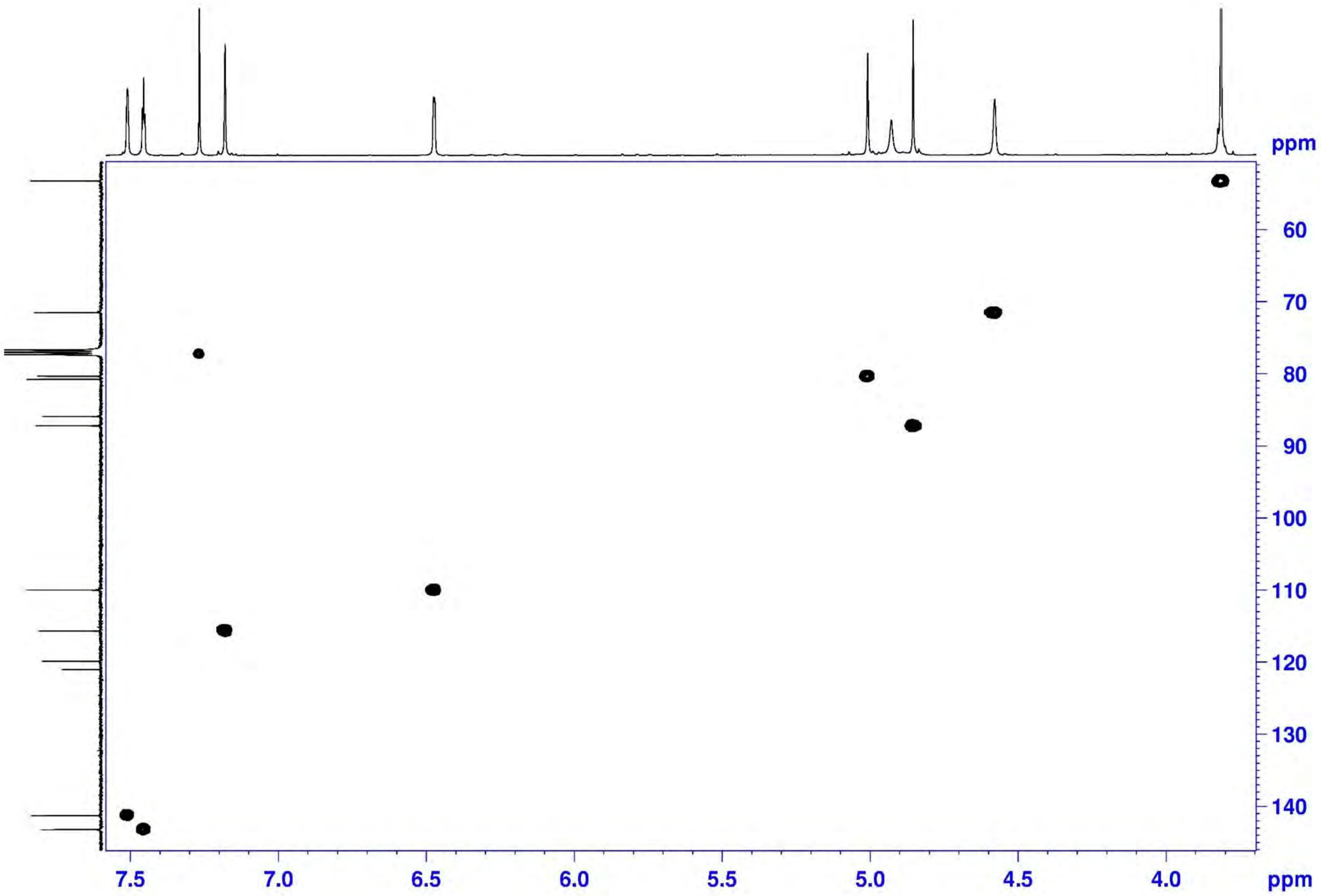

HSQC (400 MHz) spectrum of compound **25** in $CDCl_3$

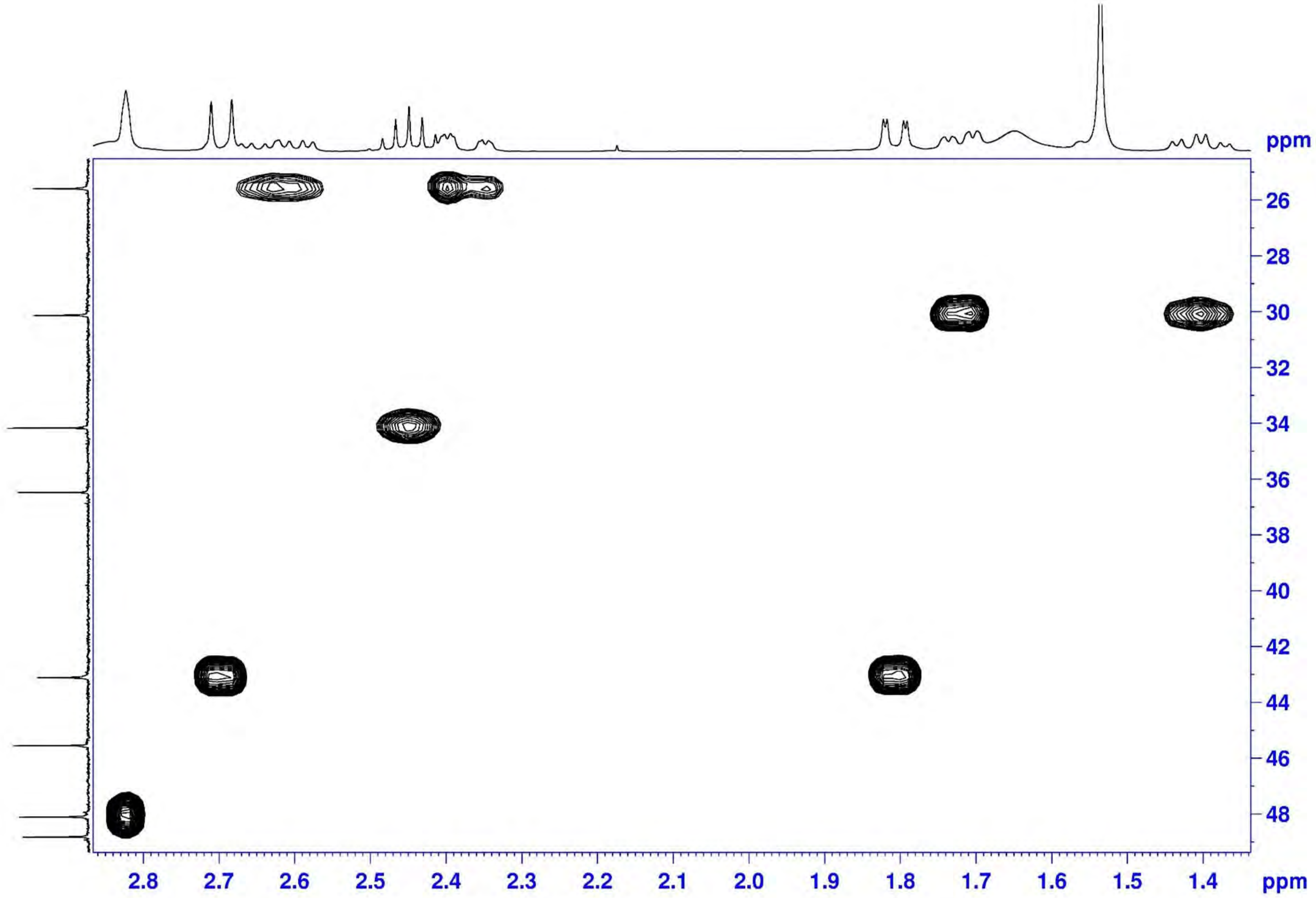

HSQC (400 MHz) spectrum of compound **25** in $CDCl_3$

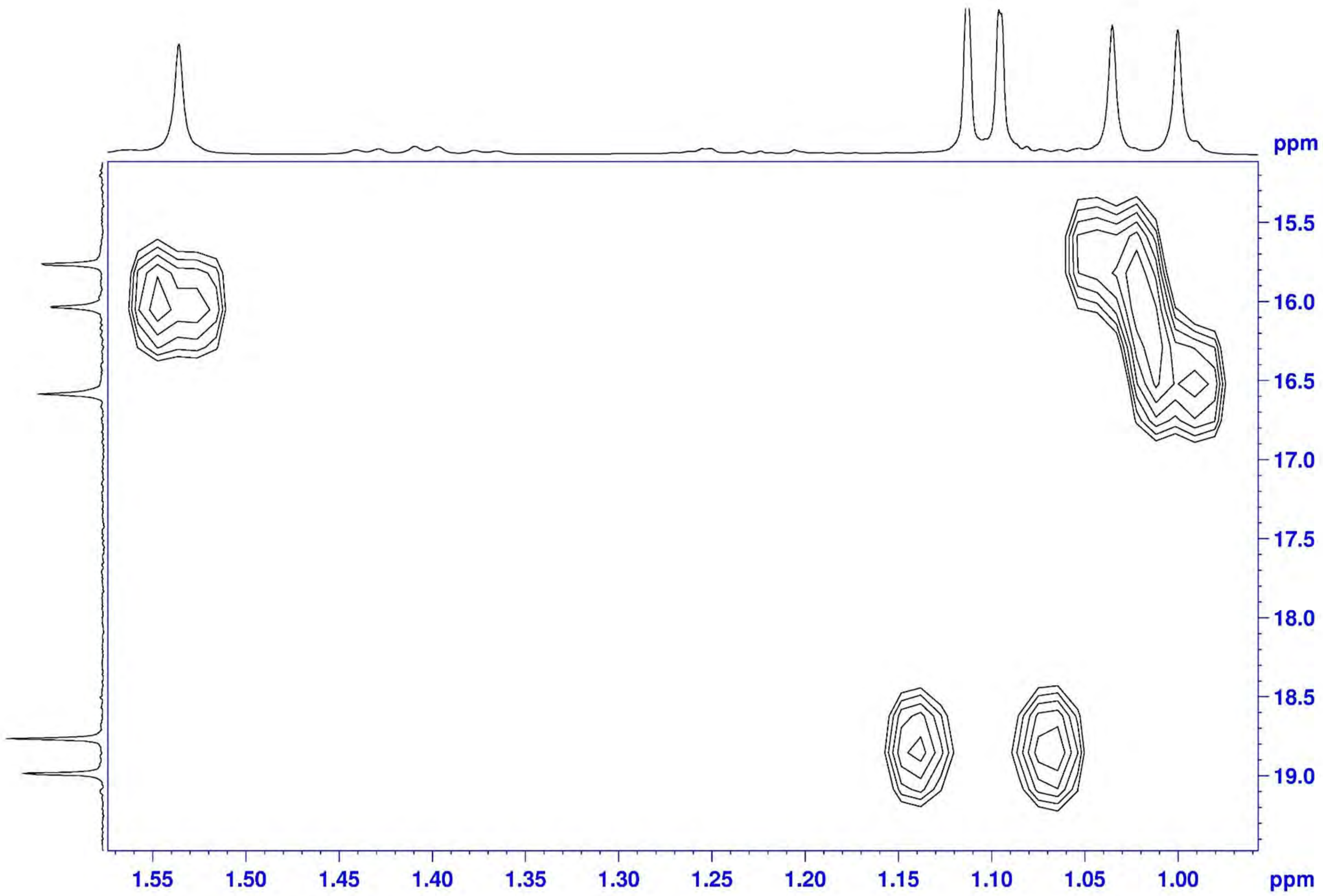

HMBC (400 MHz) spectrum of compound **25** in $CDCl_3$

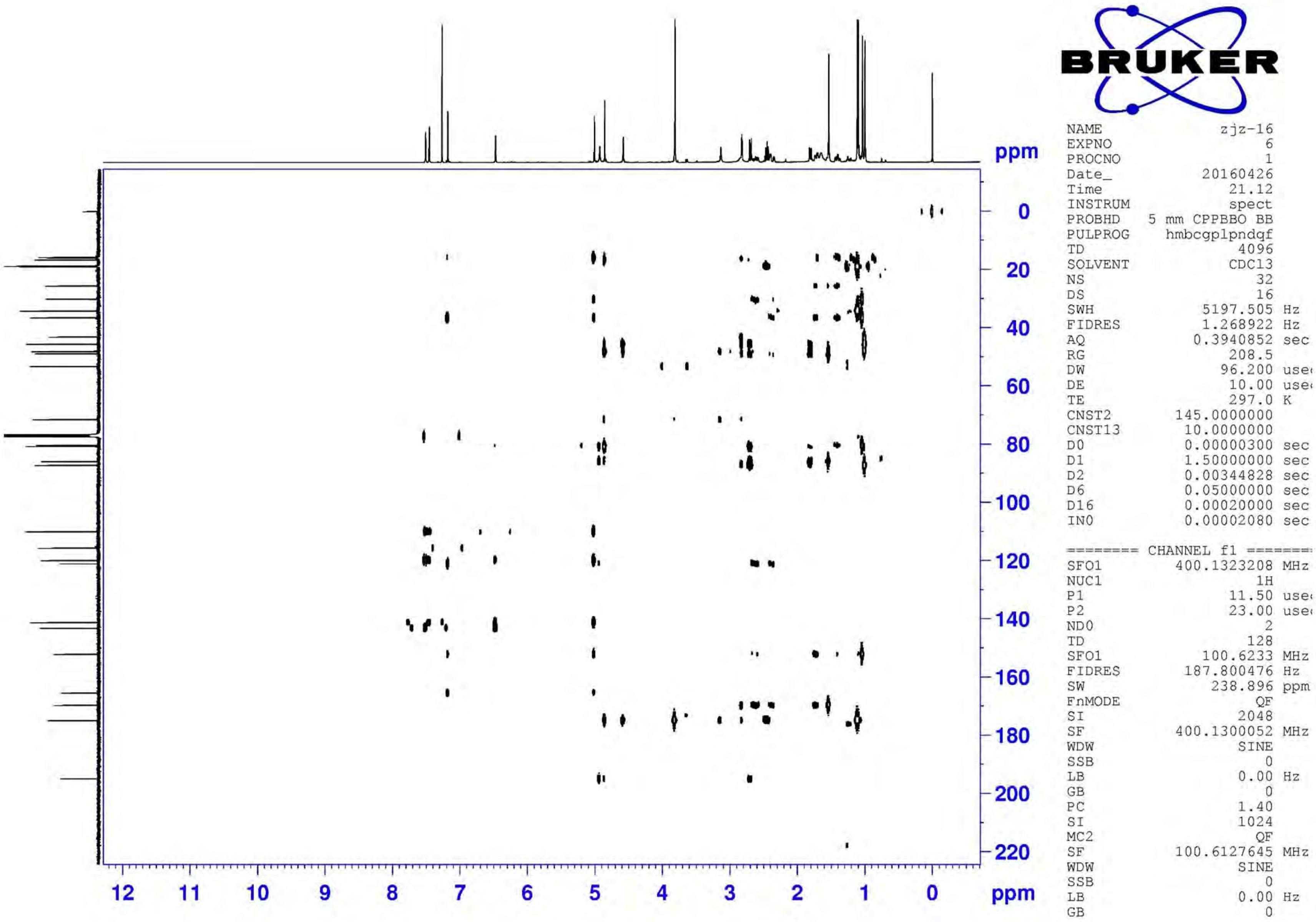

HMBC (400 MHz) spectrum of compound **25** in $CDCl_3$

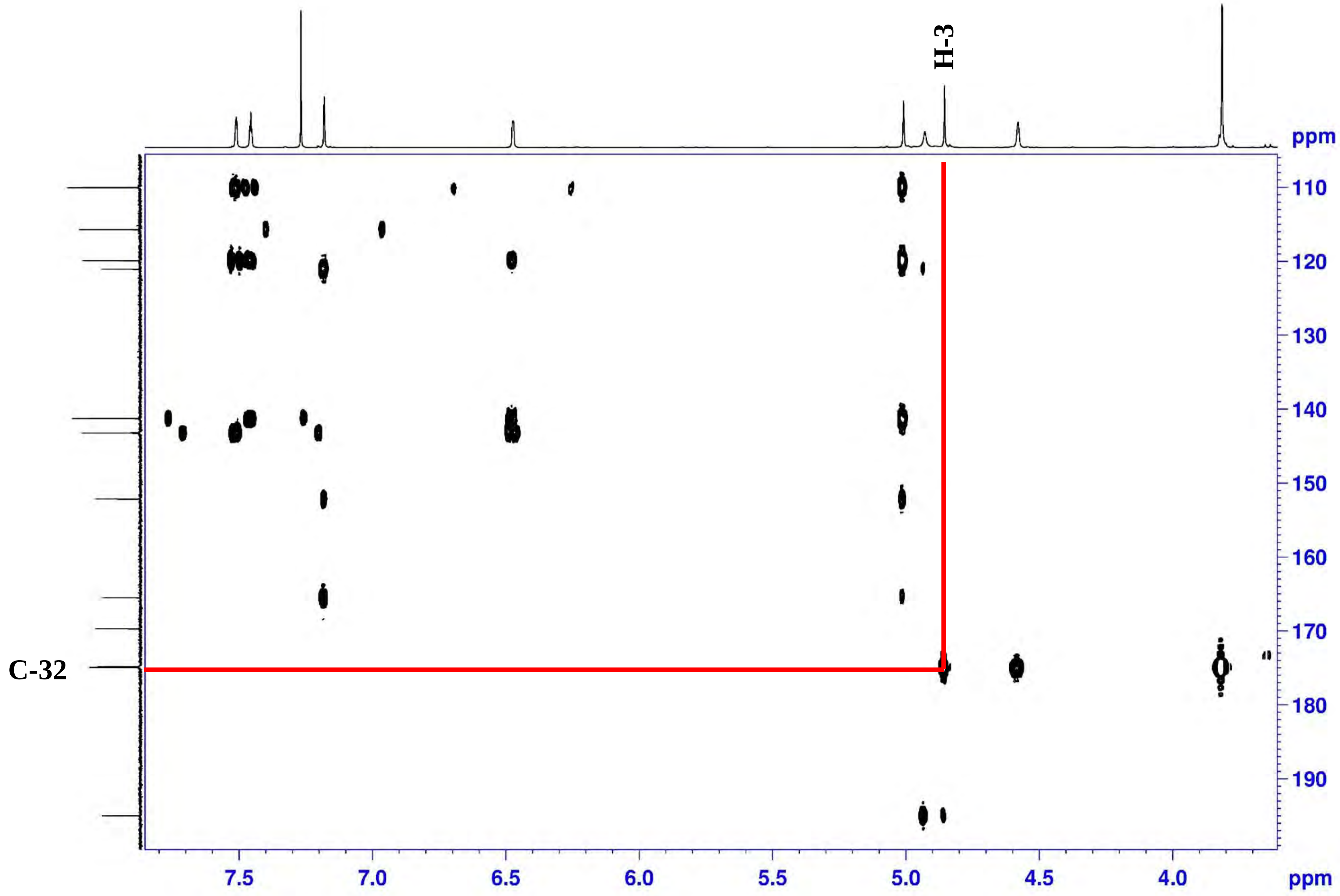

HMBC (400 MHz) spectrum of compound **25** in $CDCl_3$

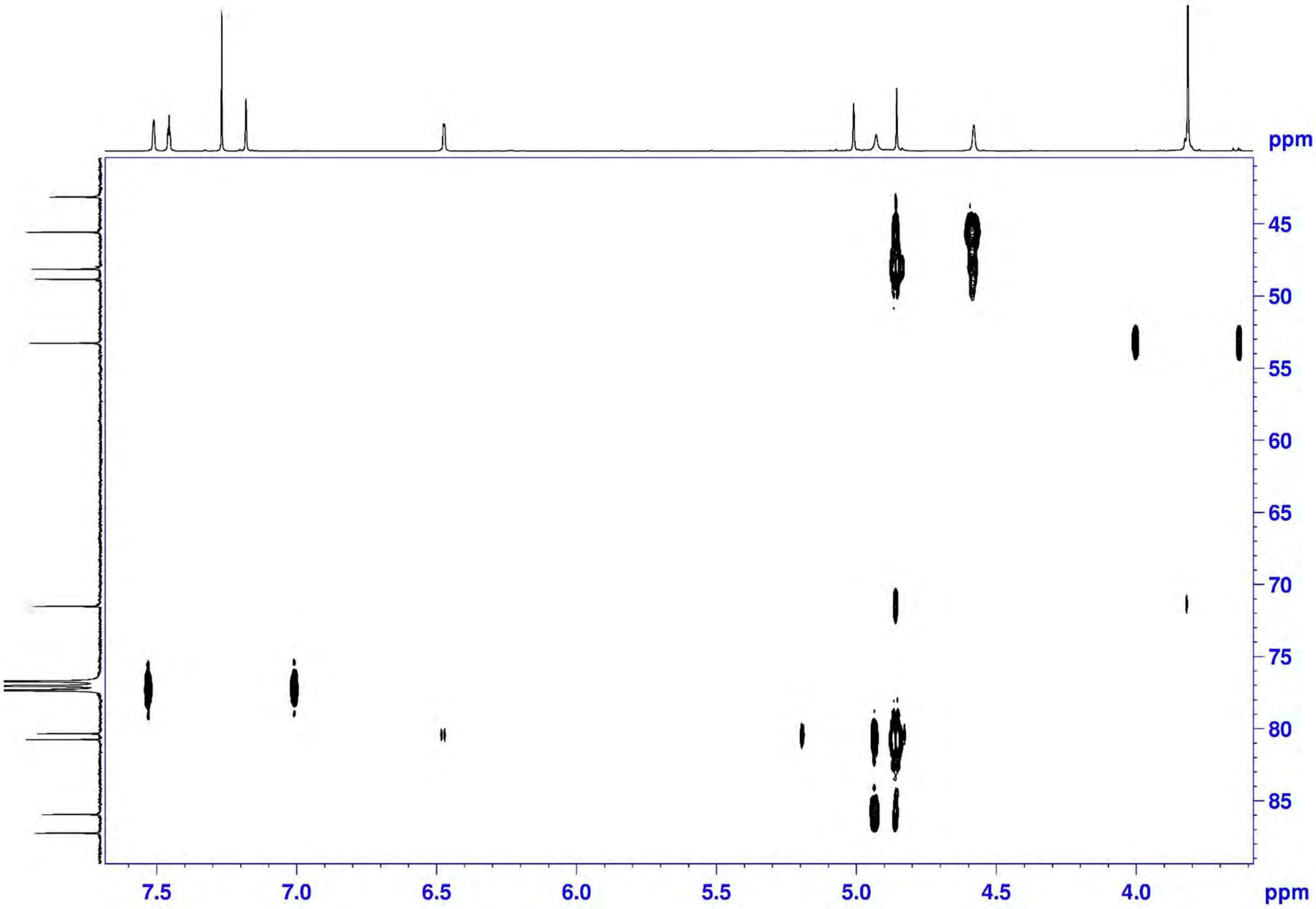

HMBC (400 MHz) spectrum of compound **25** in $CDCl_3$

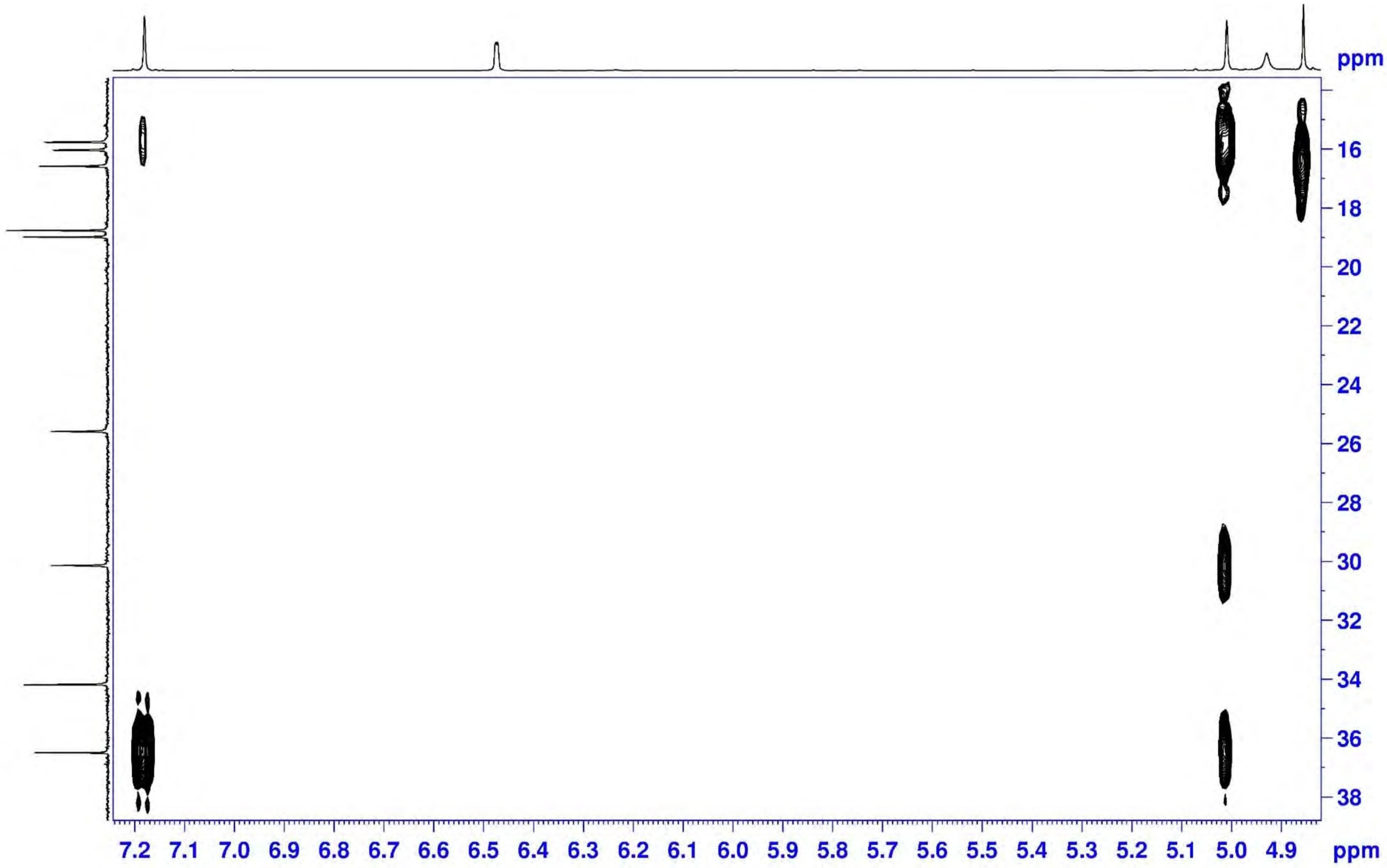

HMBC (400 MHz) spectrum of compound **25** in $CDCl_3$

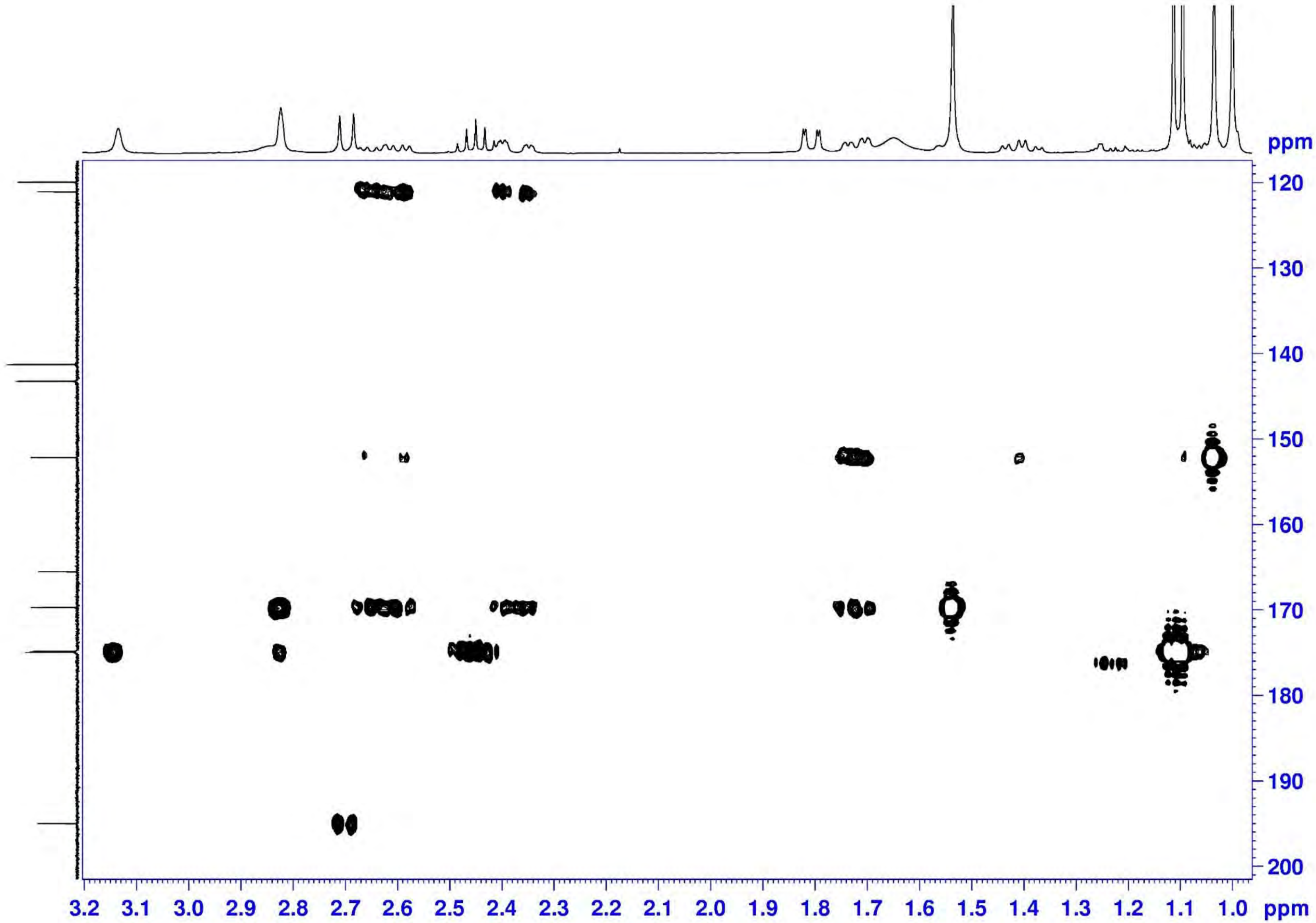

HMBC (400 MHz) spectrum of compound **25** in $CDCl_3$

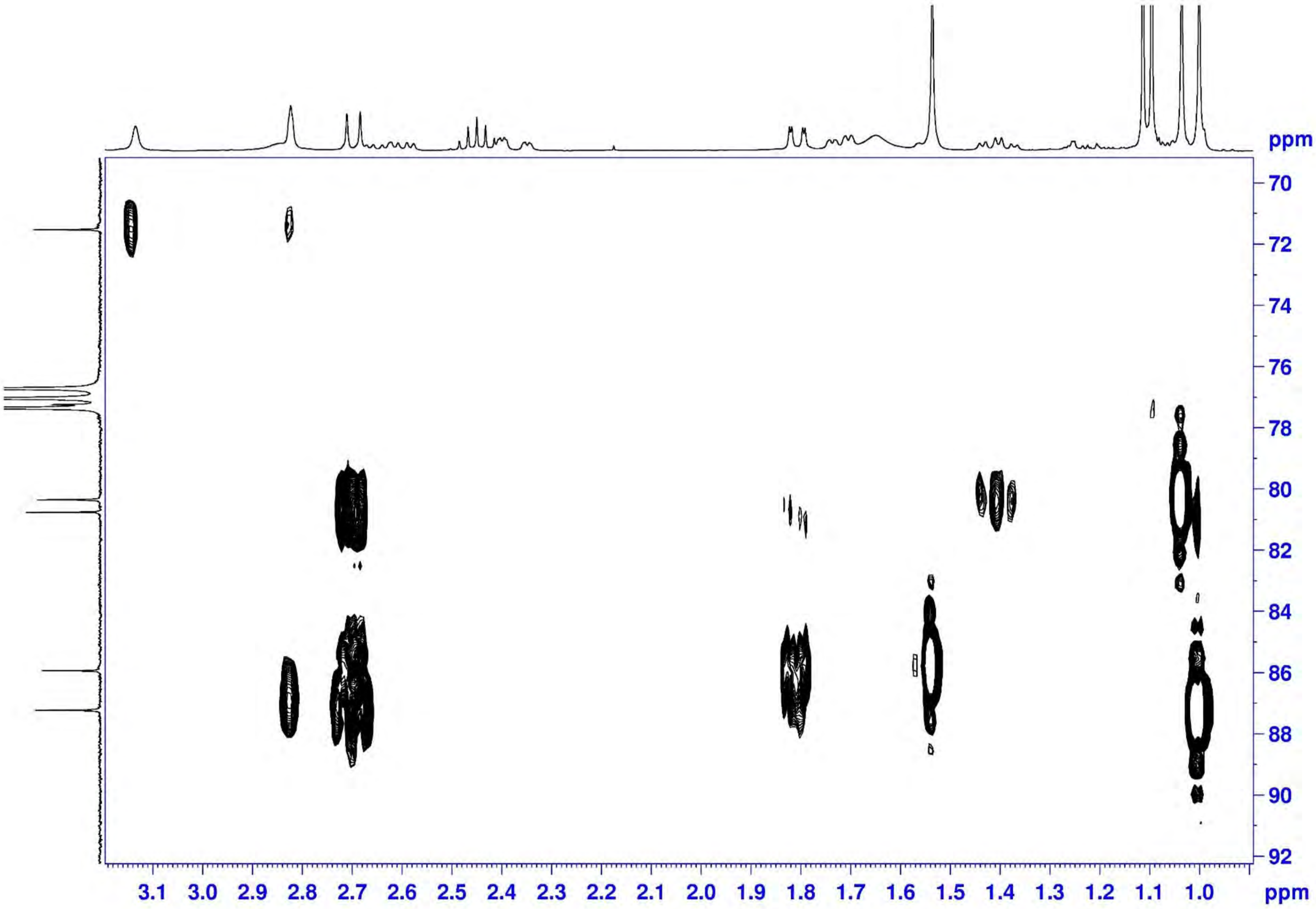

HMBC (400 MHz) spectrum of compound **25** in $CDCl_3$

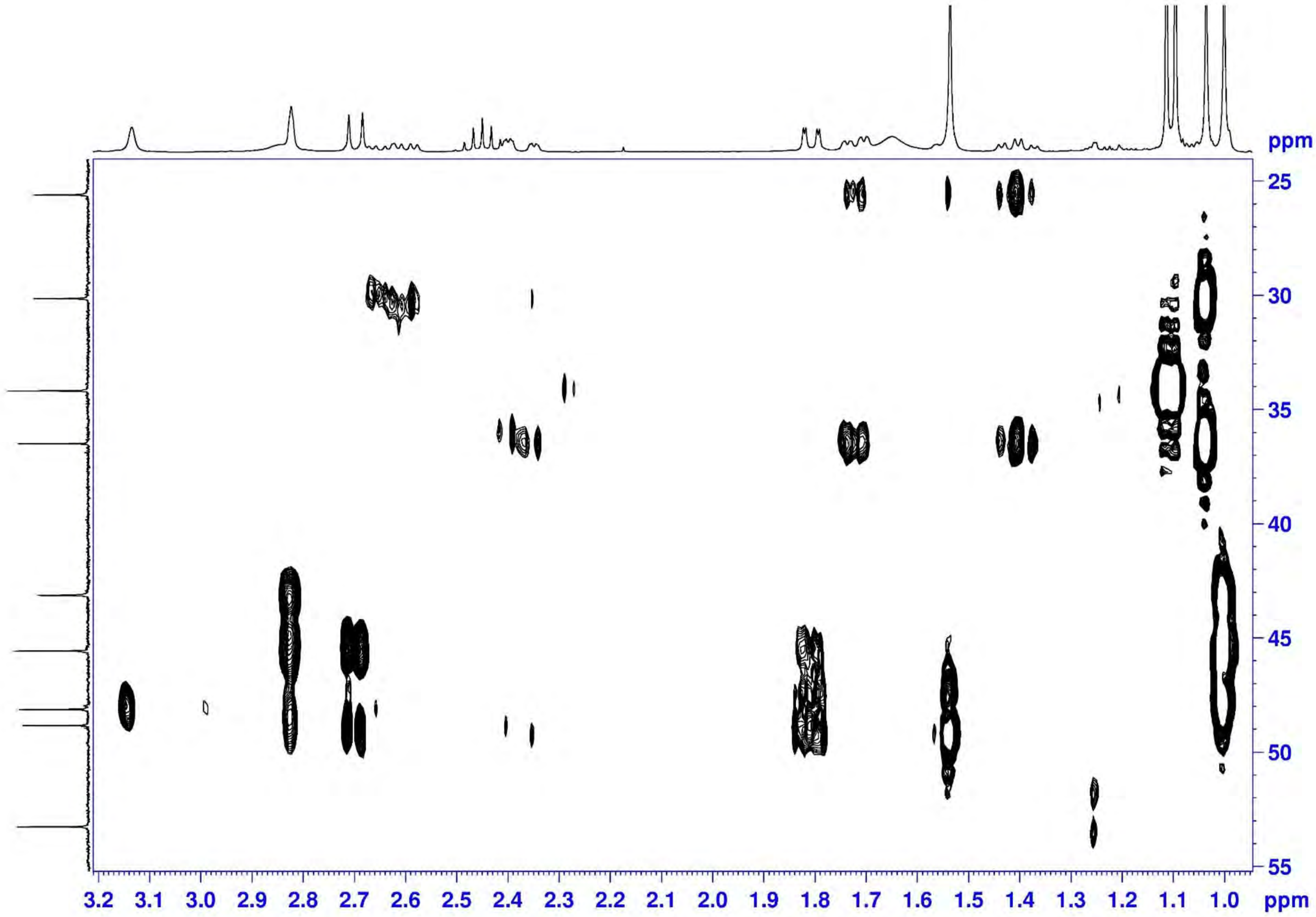

HMBC (400 MHz) spectrum of compound **25** in $CDCl_3$

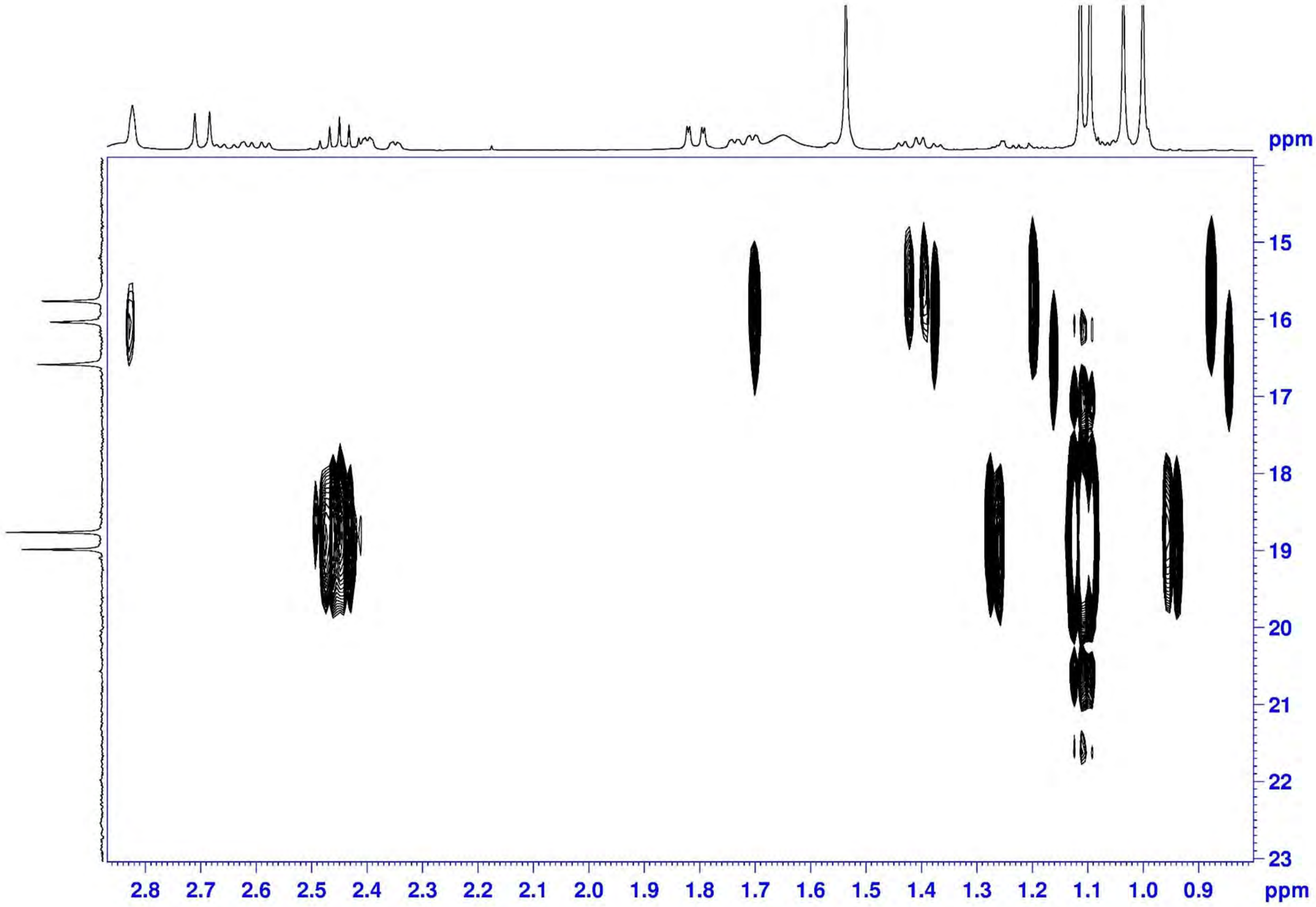

NOESY (400 MHz) spectrum of compound **25** in $CDCl_3$

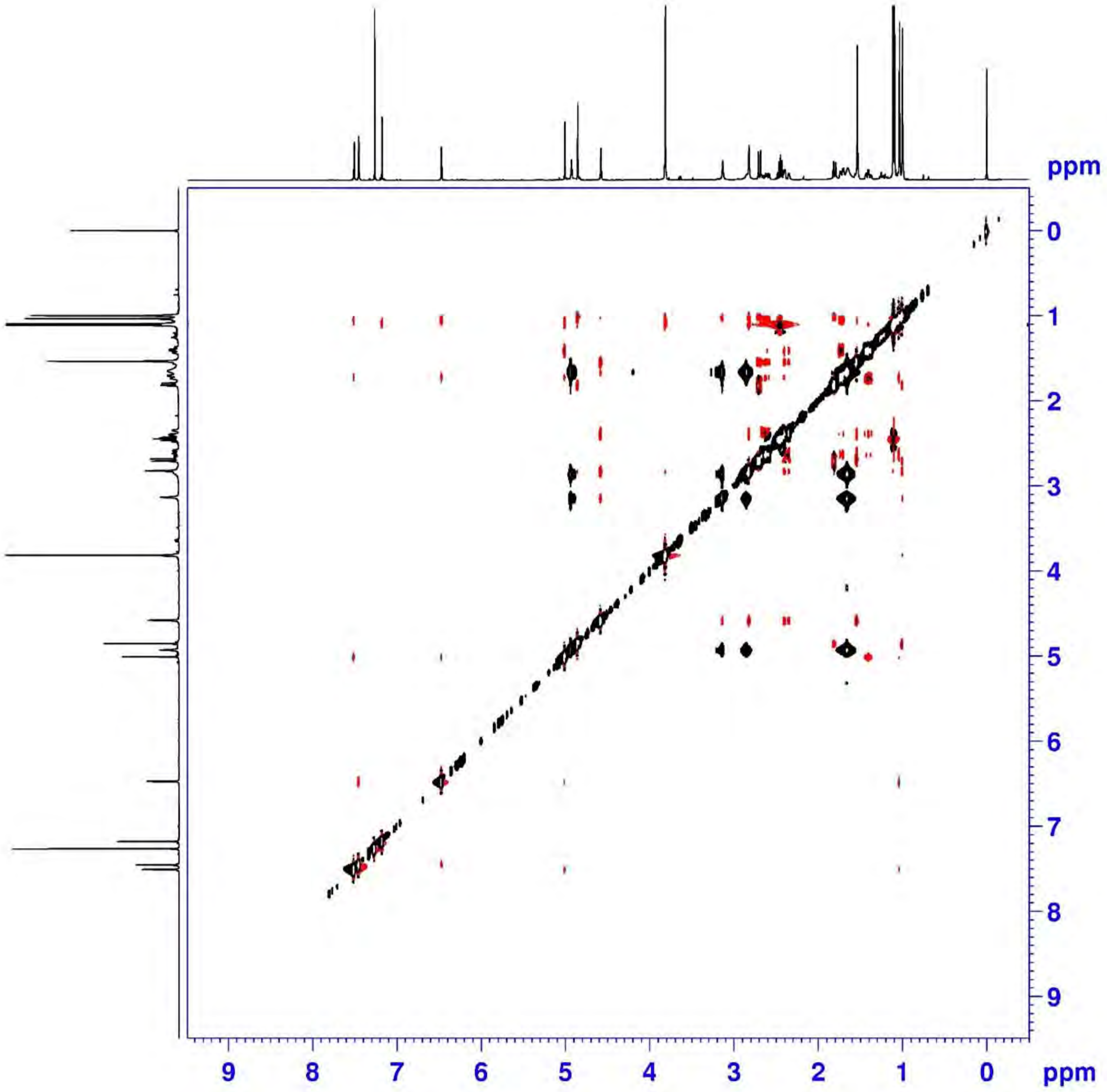

NAME	zjz-16
EXPNO	7
PROCNO	1
Date_	20160514
Time	19.55
INSTRUM	spect
PROBHD	5 mm CPPBBO BB
PULPROG	noesygpphpp
TD	2048
SOLVENT	CDCl3
NS	16
DS	32
SWH	4000.000 Hz
FIDRES	1.953125 Hz
AQ	0.2560500 sec
RG	117.37
DW	125.000 usec
DE	10.00 usec
TE	297.0 K
D0	0.00011036 sec
D1	1.99385595 sec
D8	0.30000001 sec
D11	0.03000000 sec
D12	0.00002000 sec
D16	0.00020000 sec
IN0	0.00025000 sec
======== CHANNEL f1 ========	
SFO1	400.1318006 MHz
NUC1	1H
P1	11.50 usec
P2	23.00 usec
P17	2500.00 usec
ND0	1
TD	256
SFO1	400.1318 MHz
FIDRES	15.625000 Hz
SW	9.997 ppm
FnMODE	States-TPPI
SI	1024
SF	400.1300051 MHz
WDW	QSINE
SSB	2
LB	0.00 Hz
GB	0
PC	1.00
SI	1024
MC2	States-TPPI
SF	400.1300043 MHz
WDW	QSINE
SSB	2
LB	0.00 Hz
GB	0

NOESY (400 MHz) spectrum of compound **25** in $CDCl_3$

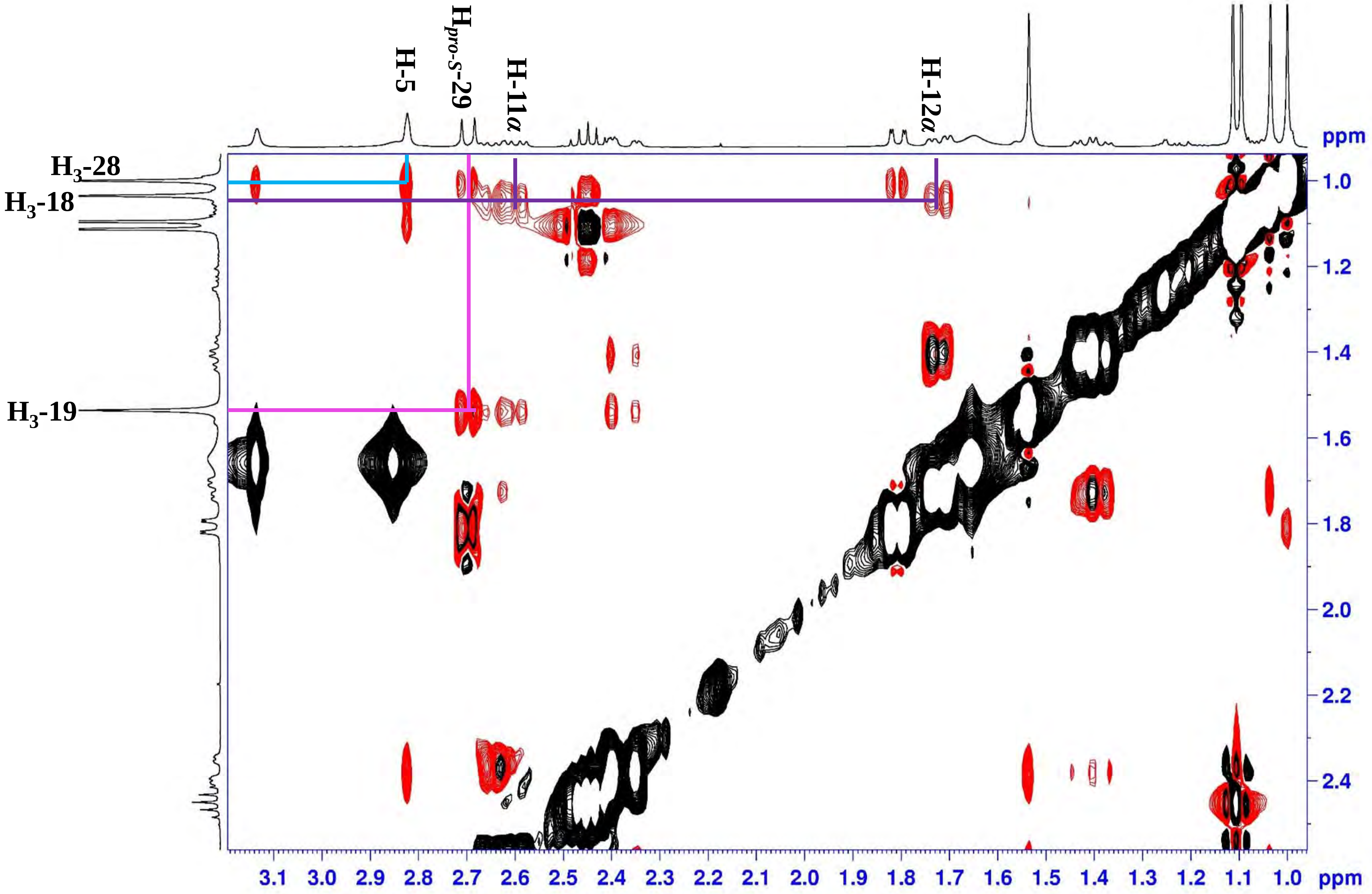

NOESY (400 MHz) spectrum of compound **25** in $CDCl_3$

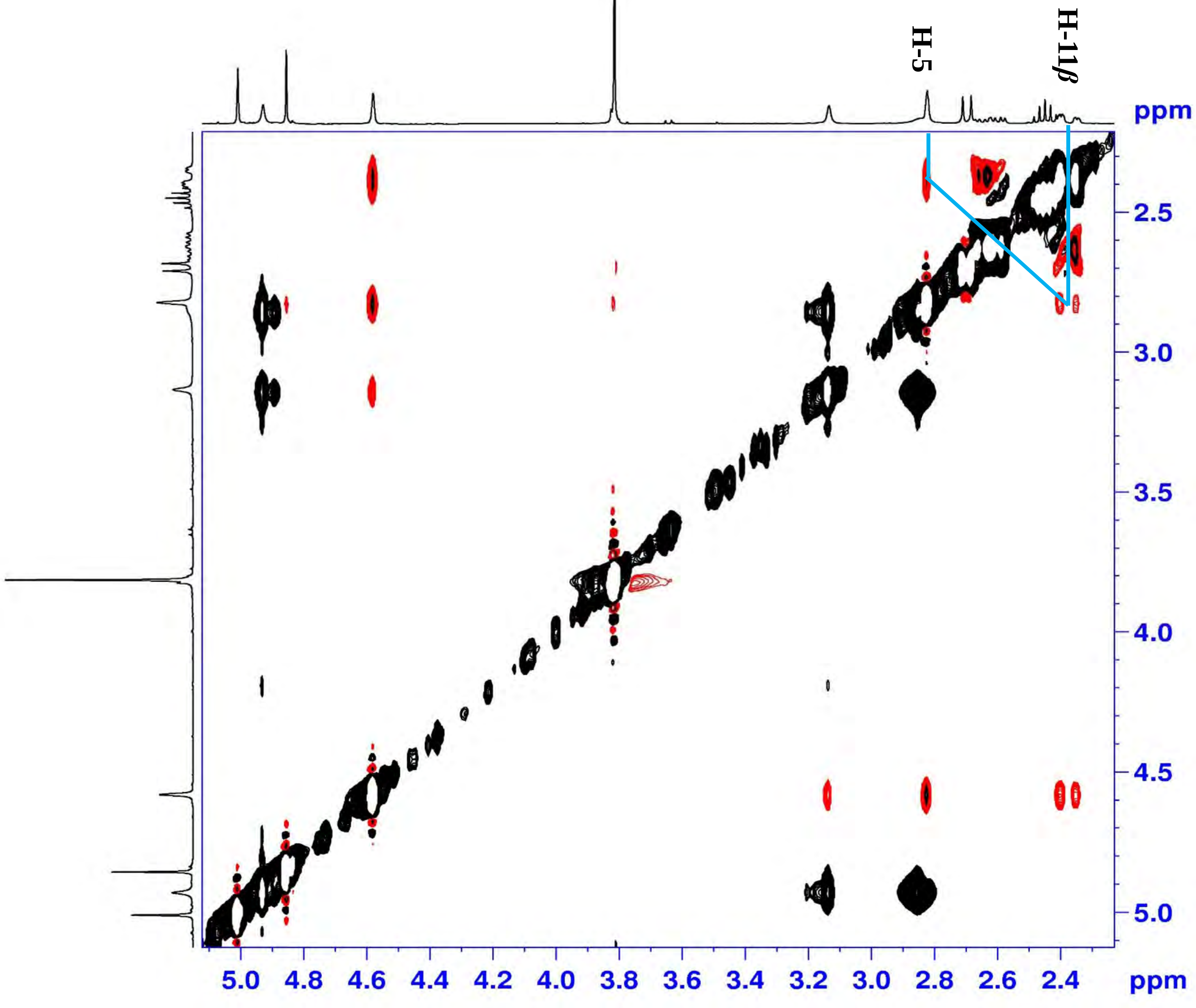

NOESY (400 MHz) spectrum of compound **25** in $CDCl_3$

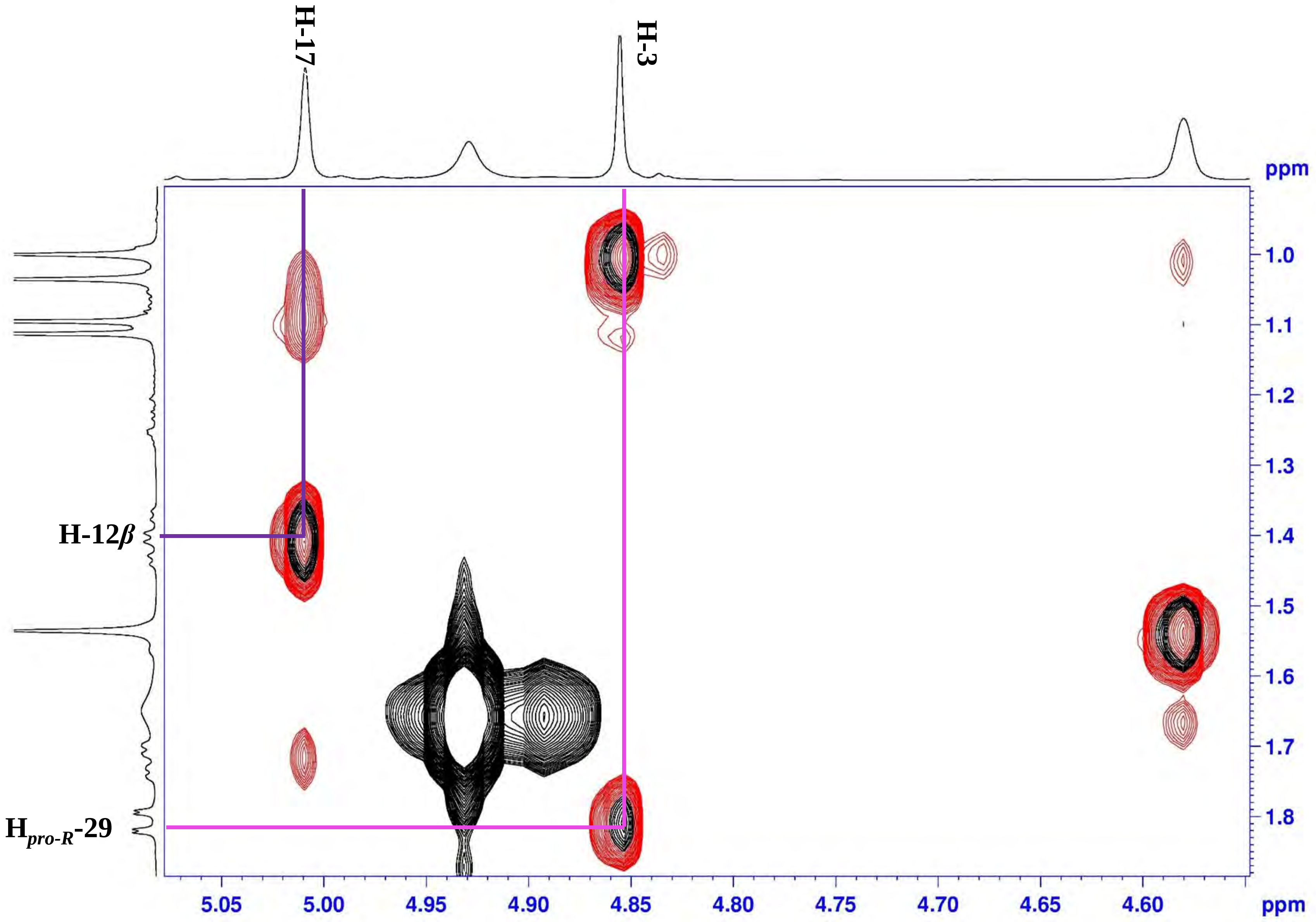

HR-ESIMS for compound **26**

Mass Spectrum SmartFormula Report

Analysis Info

Analysis Name	D:\Data\MS\data\201605\liwanshan_dgy-10_pos_4_01_1852.d	Acquisition Date	5/19/2016 8:30:55 AM
Method	LC_Direct Infusion_pos_100-1000mz.m	Operator	SCSIO
Sample Name	liwanshan_dgy-10_pos	Instrument / Ser#	maXis 29
Comment			

Acquisition Parameter

Source Type	ESI	Ion Polarity	Positive	Set Nebulizer	0.4 Bar
Focus	Active	Set Capillary	4500 V	Set Dry Heater	180 °C
Scan Begin	100 m/z	Set End Plate Offset	-500 V	Set Dry Gas	4.0 l/min
Scan End	2000 m/z	Set Collision Cell RF	800.0 Vpp	Set Divert Valve	Waste

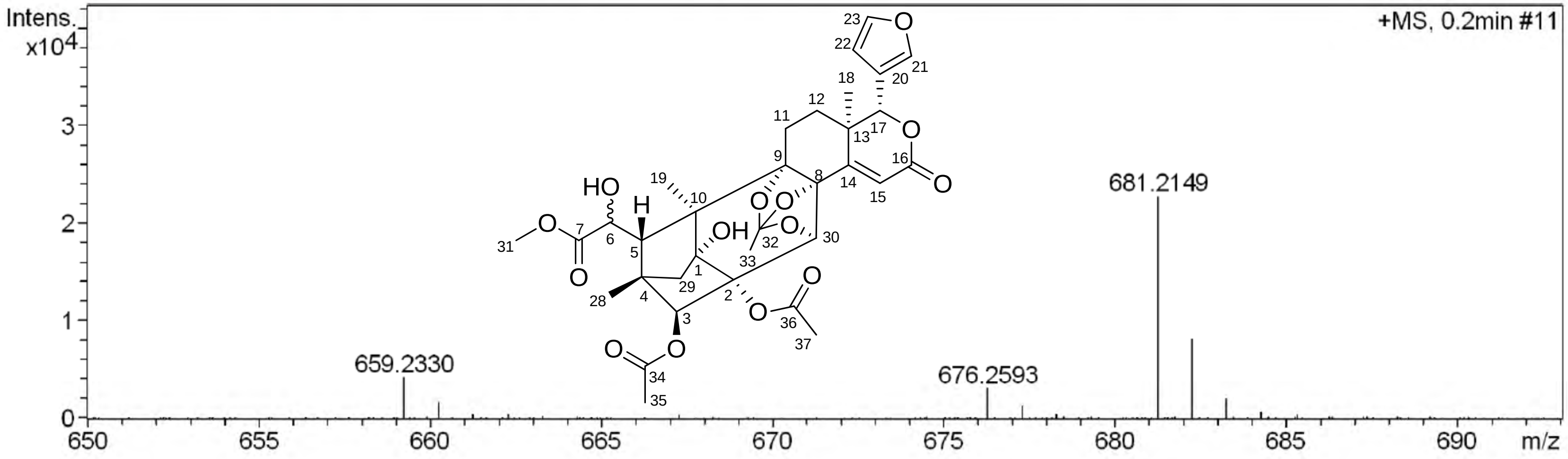

Meas. m/z	#	Formula	Score	m/z	err [ppm]	err [mDa]	mSigma	rdb	e⁻ Conf	N-Rule
659.2330	1	C 33 H 39 O 14	100.00	659.2334	0.6	0.4	11.2	14.5	even	ok
681.2149	1	C 33 H 38 Na O 14	100.00	681.2154	0.7	0.5	5.7	14.5	even	ok
1339.4422	1	C 66 H 76 Na O 28	100.00	1339.4415	-0.5	-0.6	44.9	28.5	even	ok

HR-ESIMS for compound **26**

Generic Display Report

Analysis Info

Analysis Name	D:\Data\MS\data\201605\liwanshan_dgy-10_pos_4_01_1852.d	Acquisition Date	5/19/2016 8:30:55 AM
Method	LC_Direct Infusion_pos_100-1000mz.m	Operator	SCSIO
Sample Name	liwanshan_dgy-10_pos	Instrument	maXis
Comment			

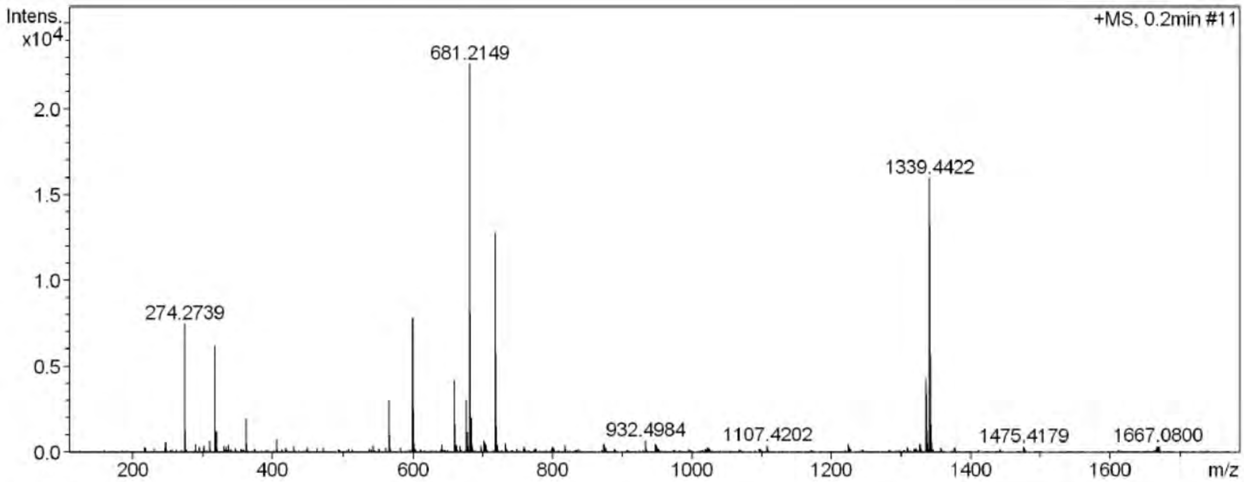

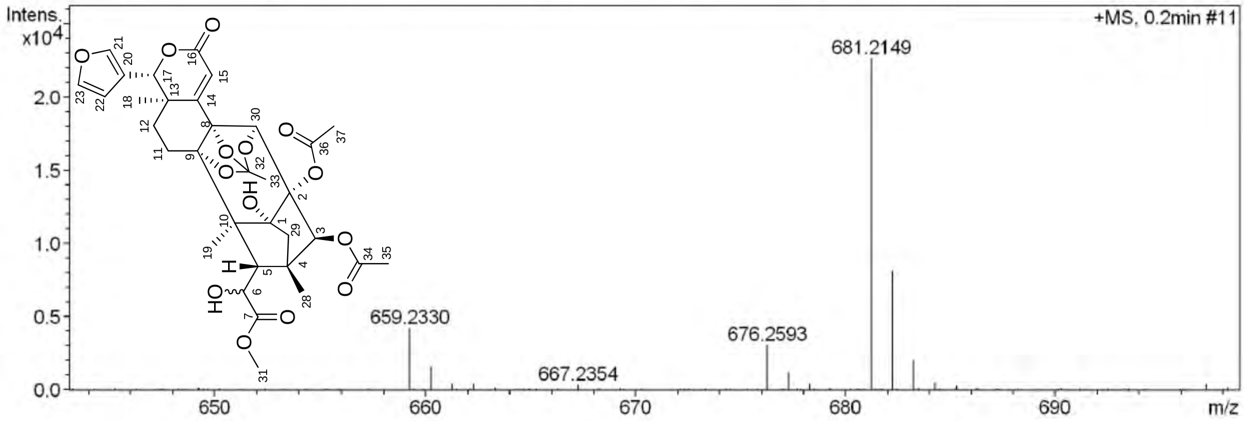

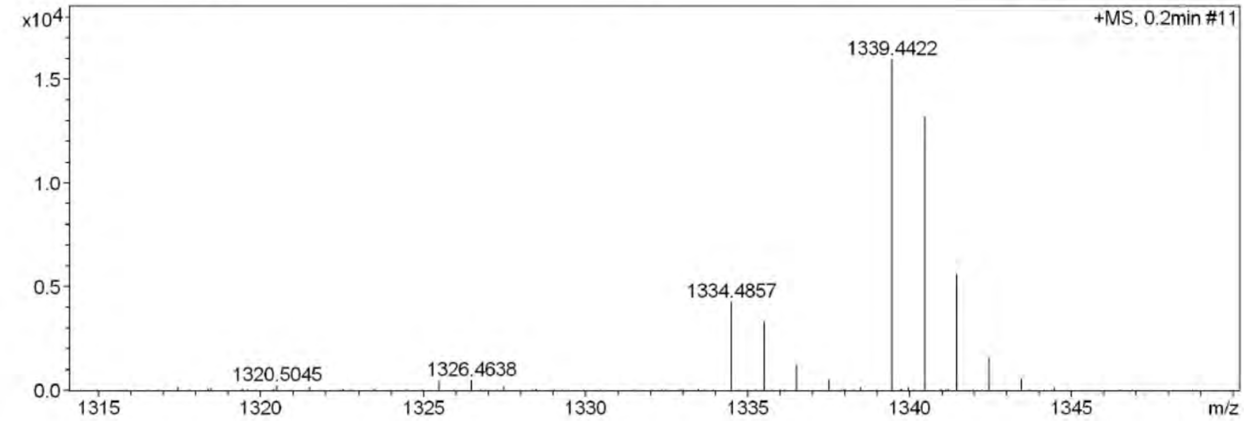

^{1}H NMR (400 MHz) spectrum of compound **26** in $CDCl_3$

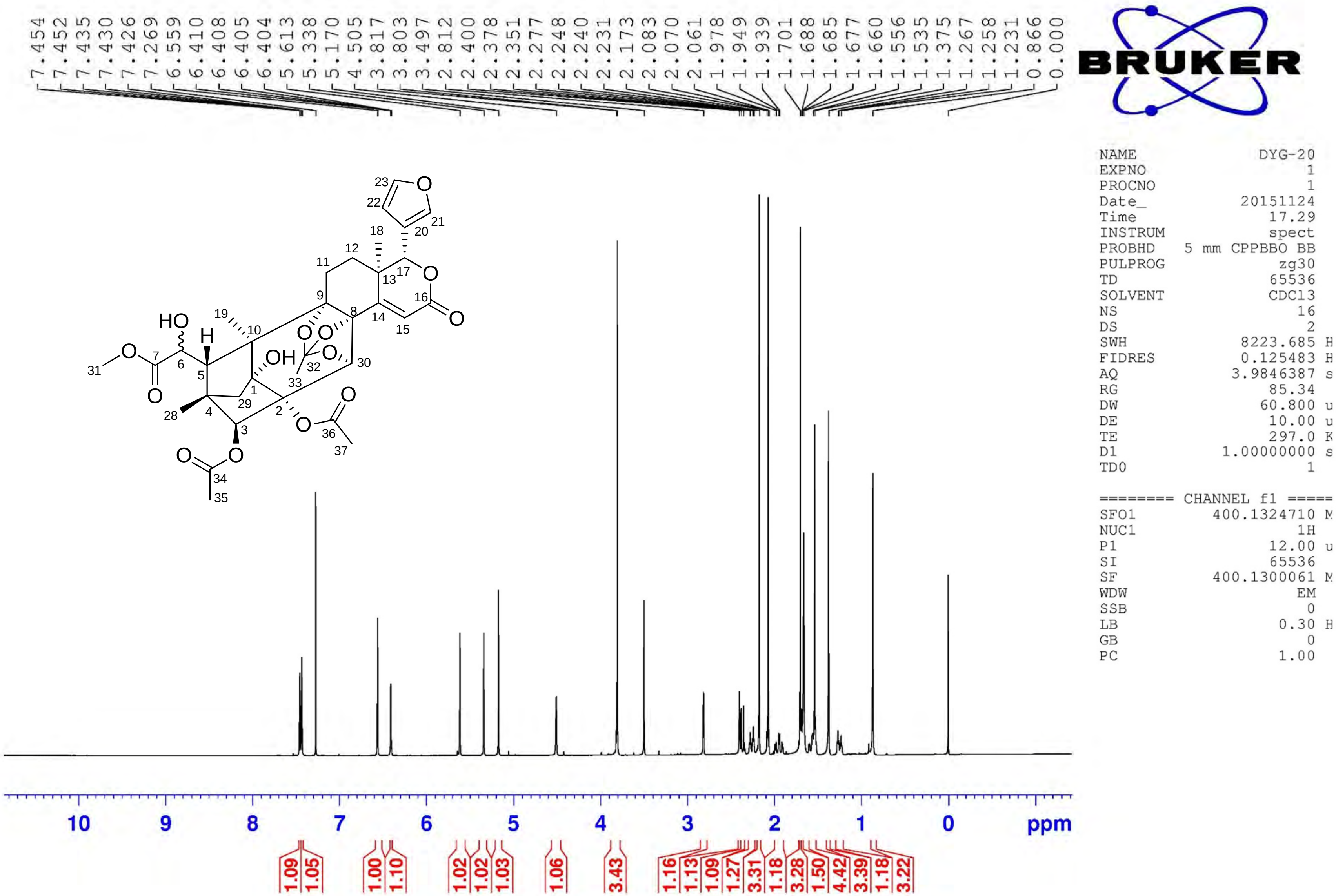

^1H NMR (400 MHz) spectrum of compound **26** in $CDCl_3$

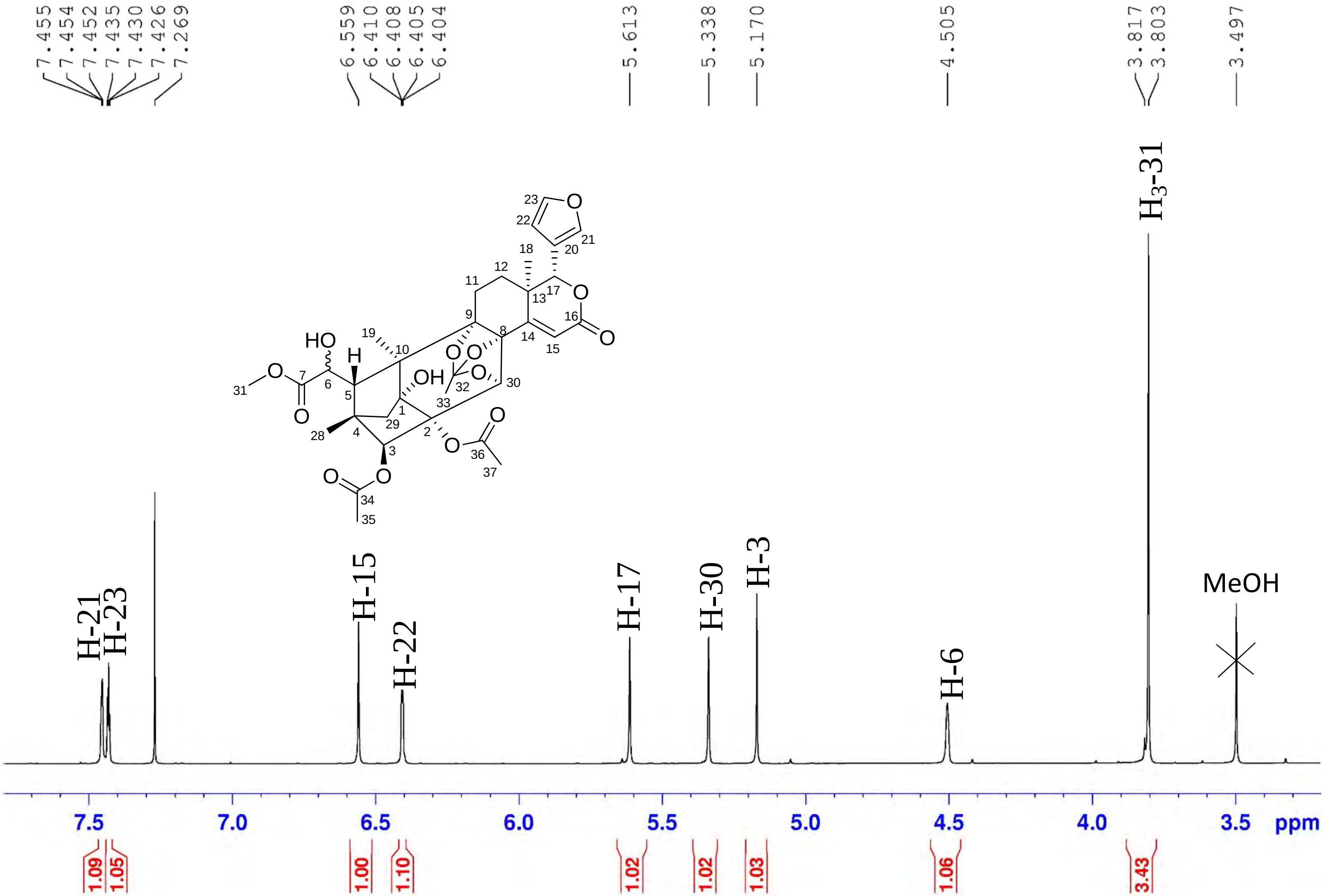

^{1}H NMR (400 MHz) spectrum of compound **26** in $CDCl_3$

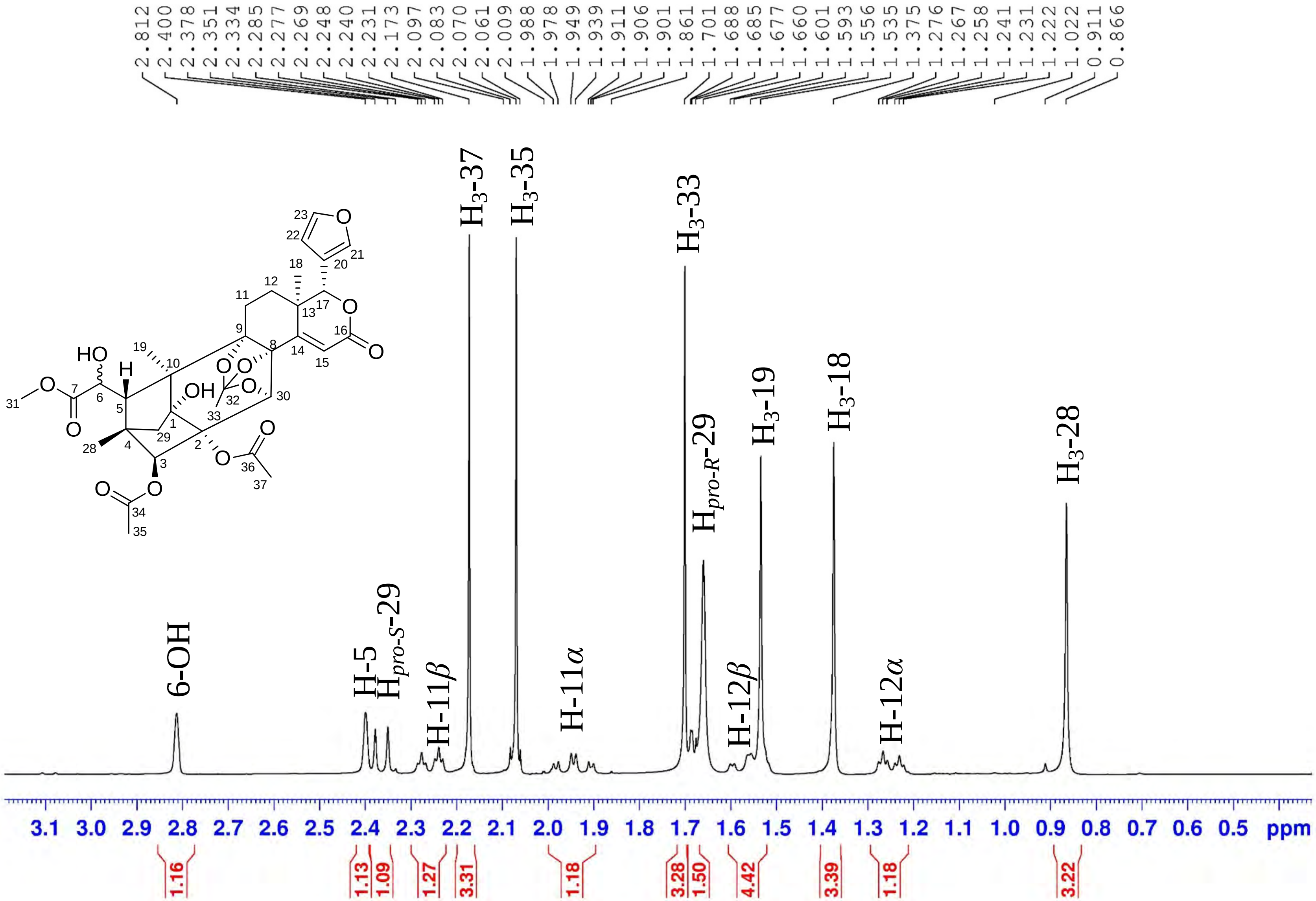

^{13}C NMR (100 MHz) spectrum of compound **26** in $CDCl_3$

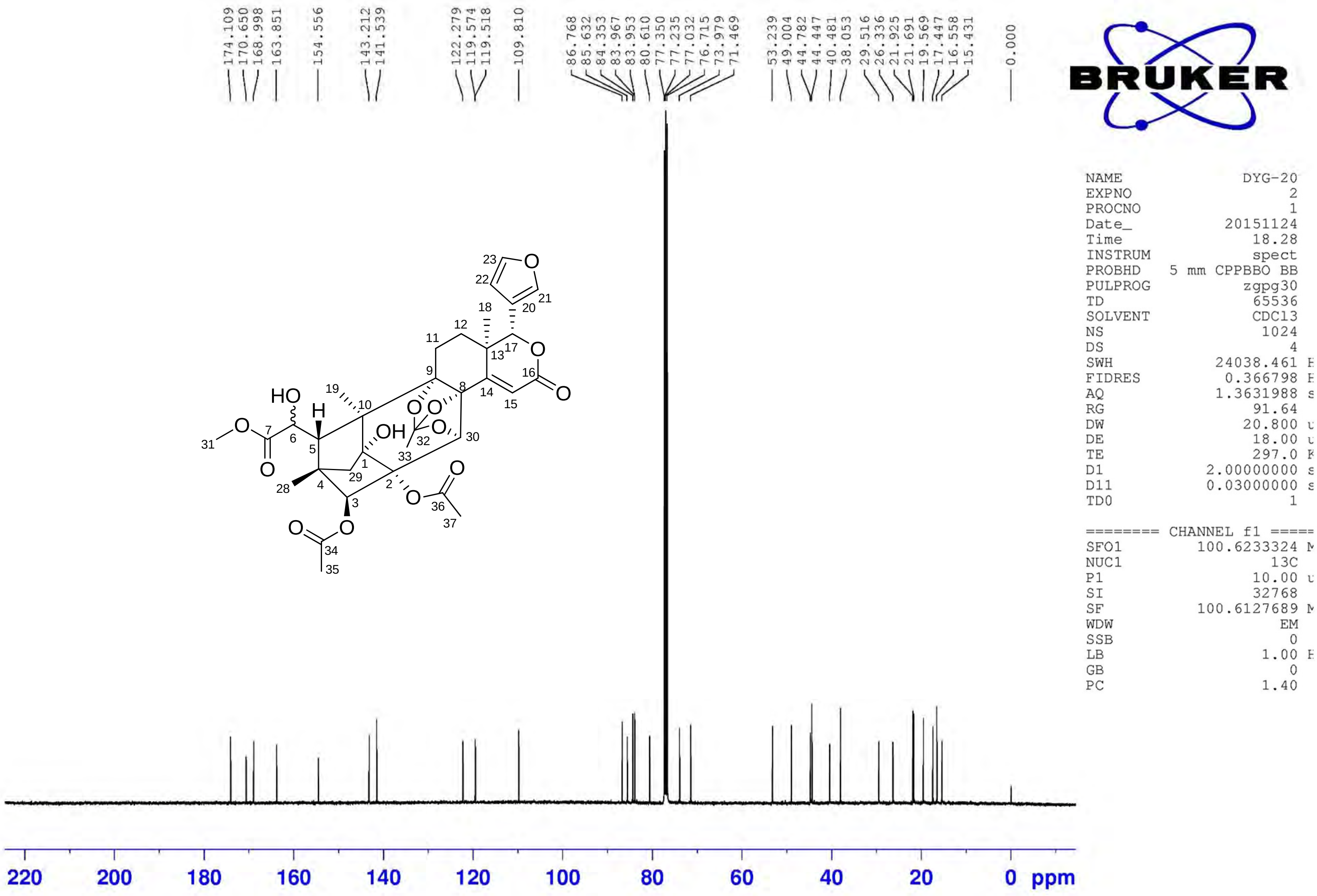

^{13}C NMR (100 MHz) spectrum of compound **26** in $CDCl_3$

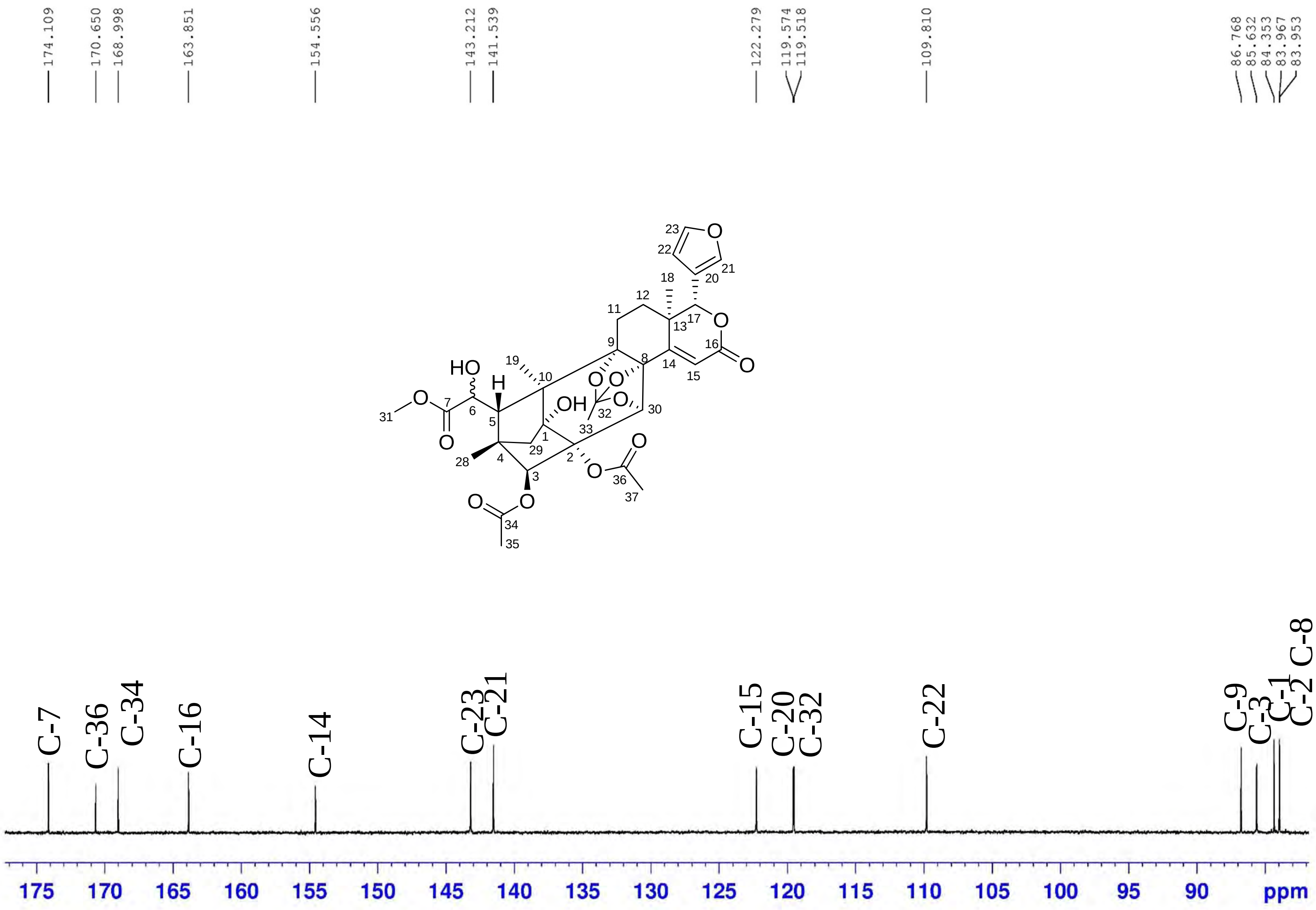

^{13}C NMR (100 MHz) spectrum of compound **26** in $CDCl_3$

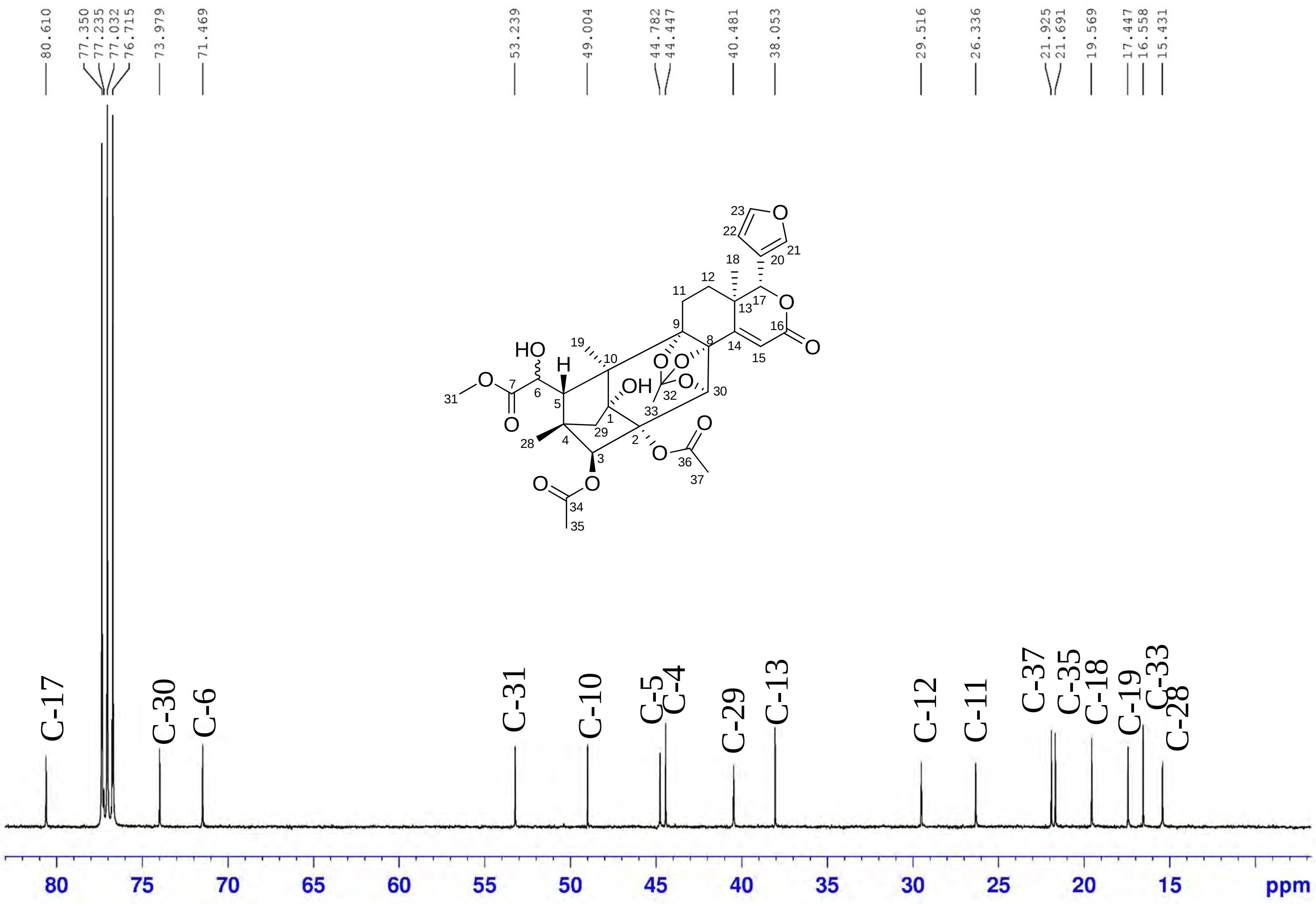

DEPT135 (100 MHz) spectrum of compound **26** in $CDCl_3$

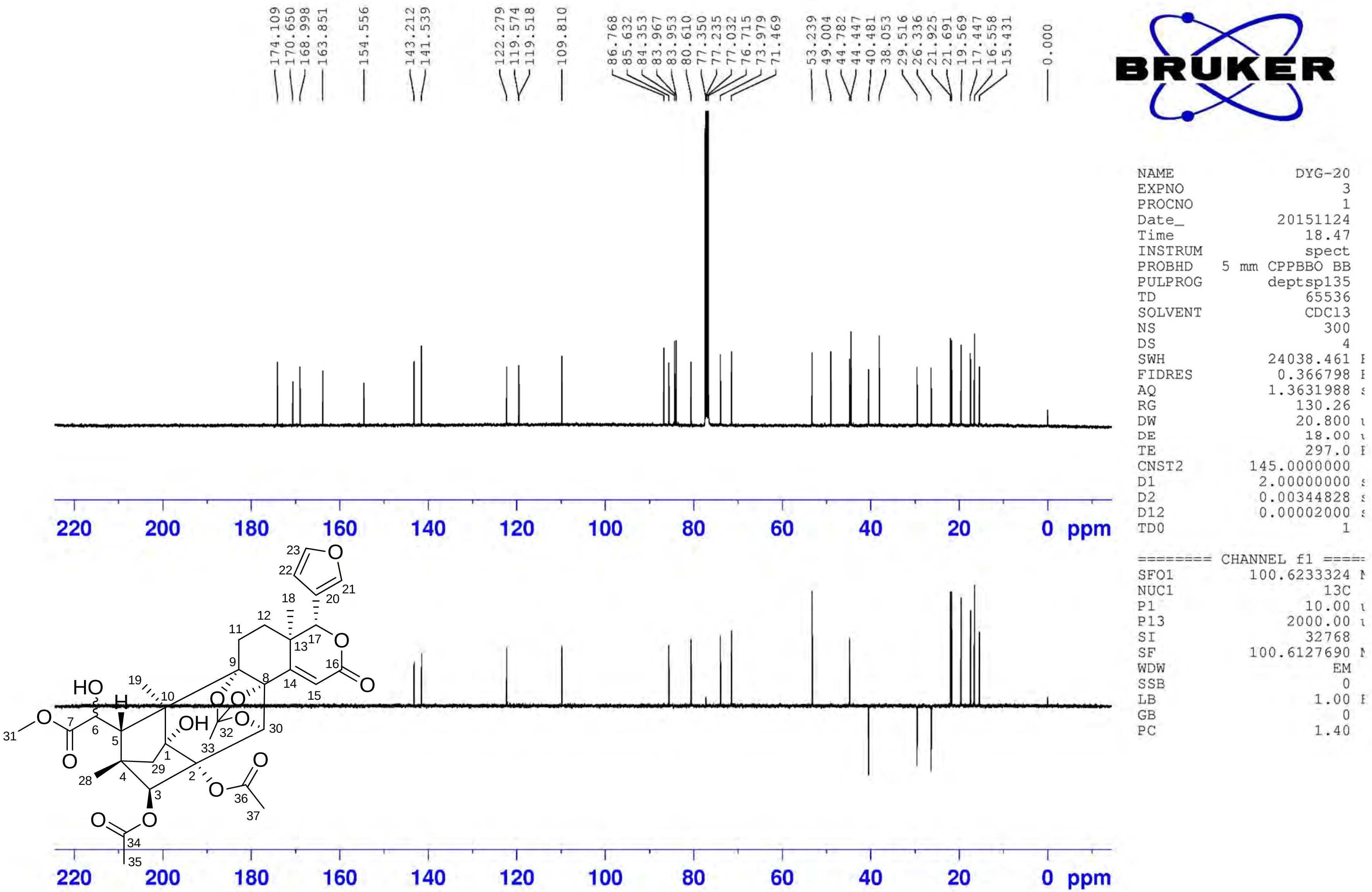

DEPT135 (100 MHz) spectrum of compound **26** in $CDCl_3$

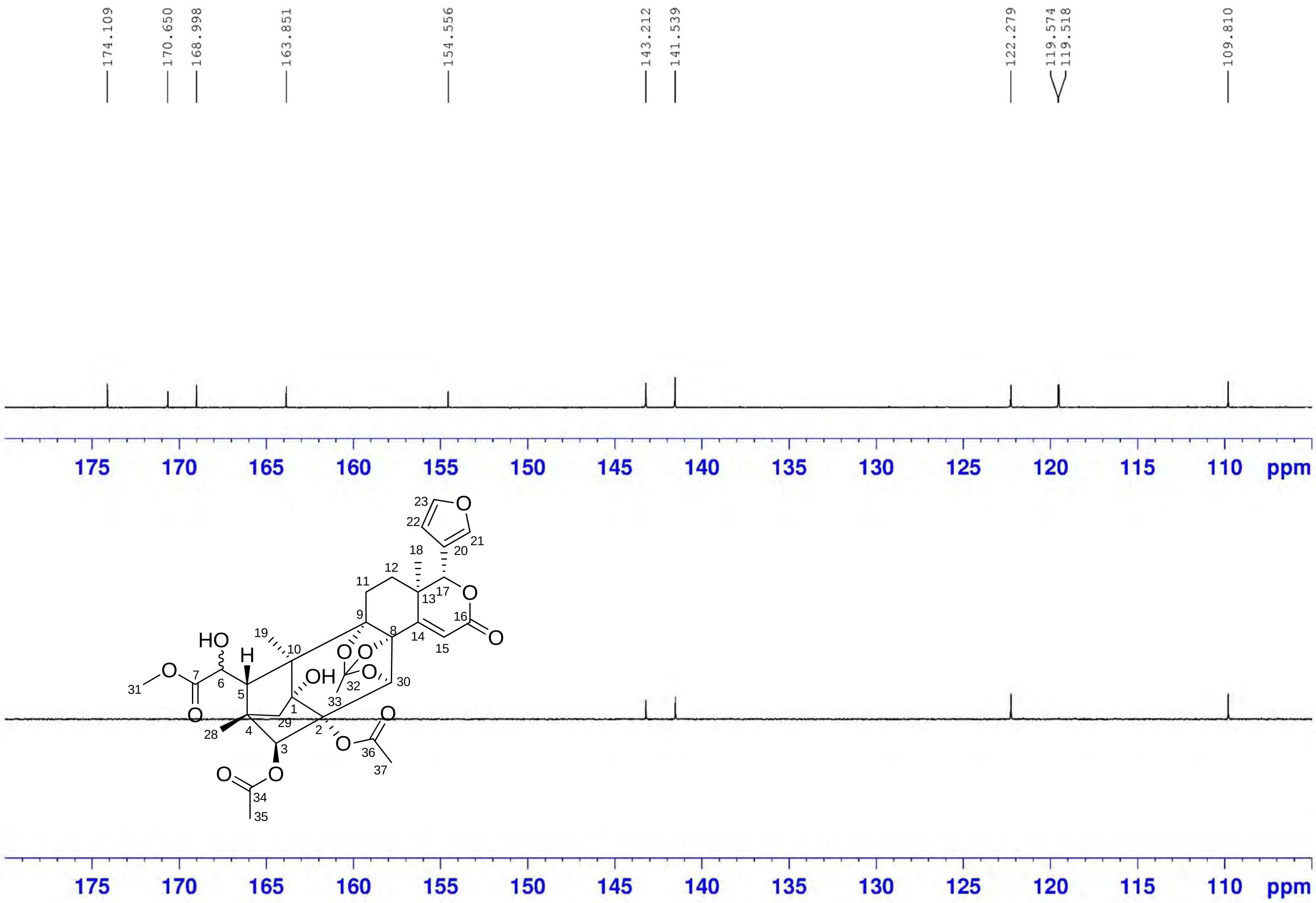

DEPT135 (100 MHz) spectrum of compound **26** in $CDCl_3$

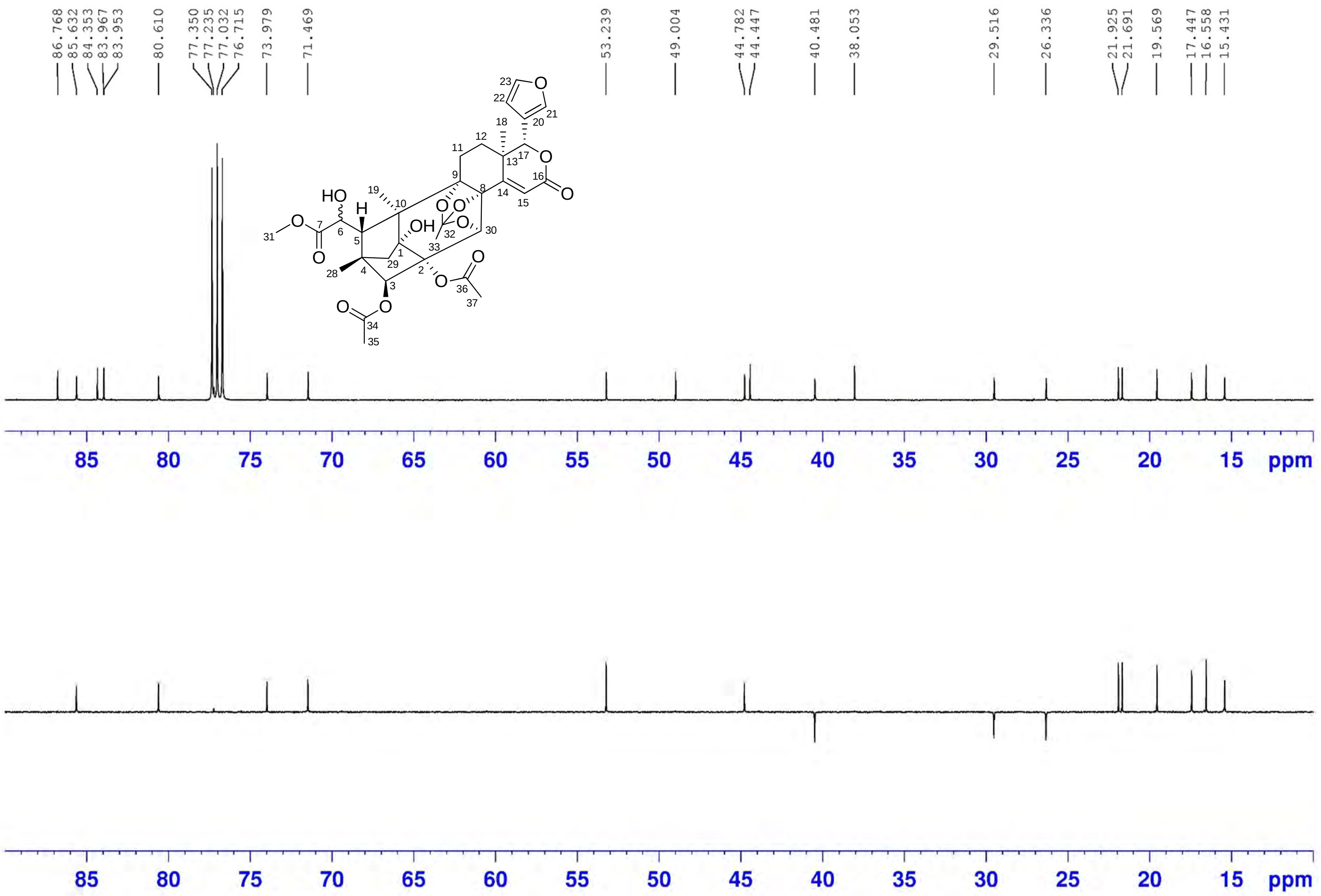

^{1}H-^{1}H COSY (400 MHz) spectrum of compound **26** in $CDCl_3$

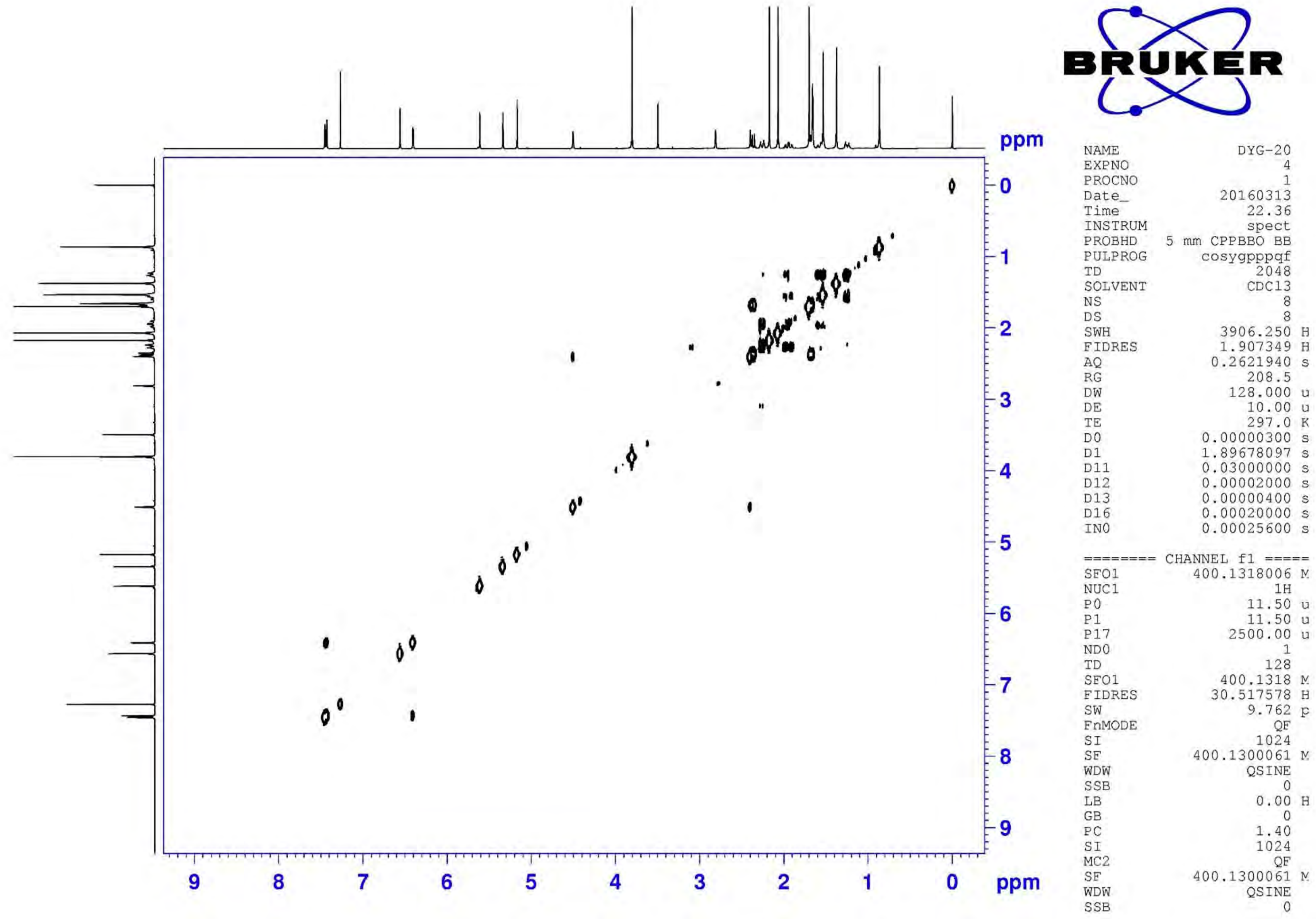

^{1}H-^{1}H COSY (400 MHz) spectrum of compound **26** in $CDCl_3$

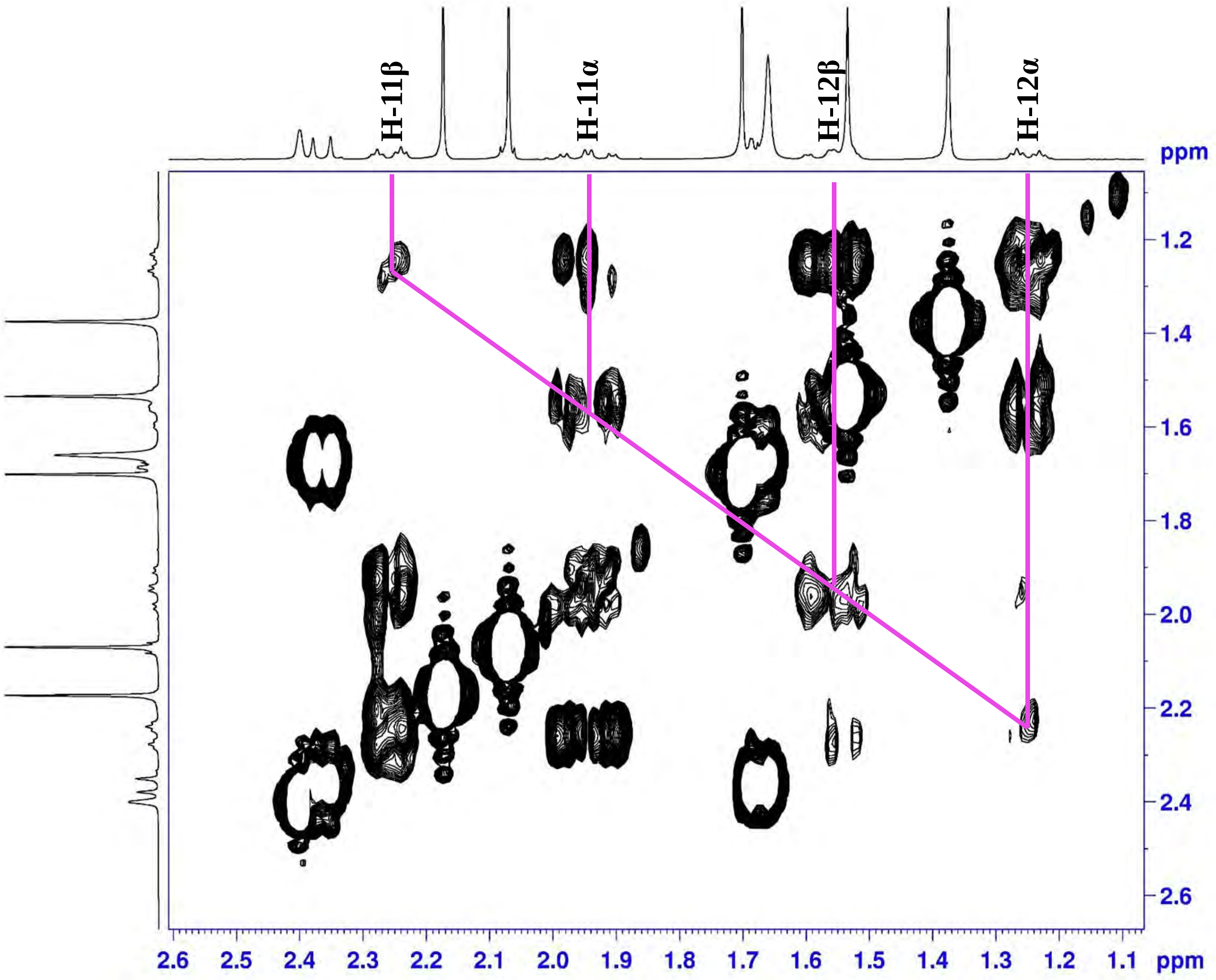

^{1}H-^{1}H COSY (400 MHz) spectrum of compound **26** in $CDCl_3$

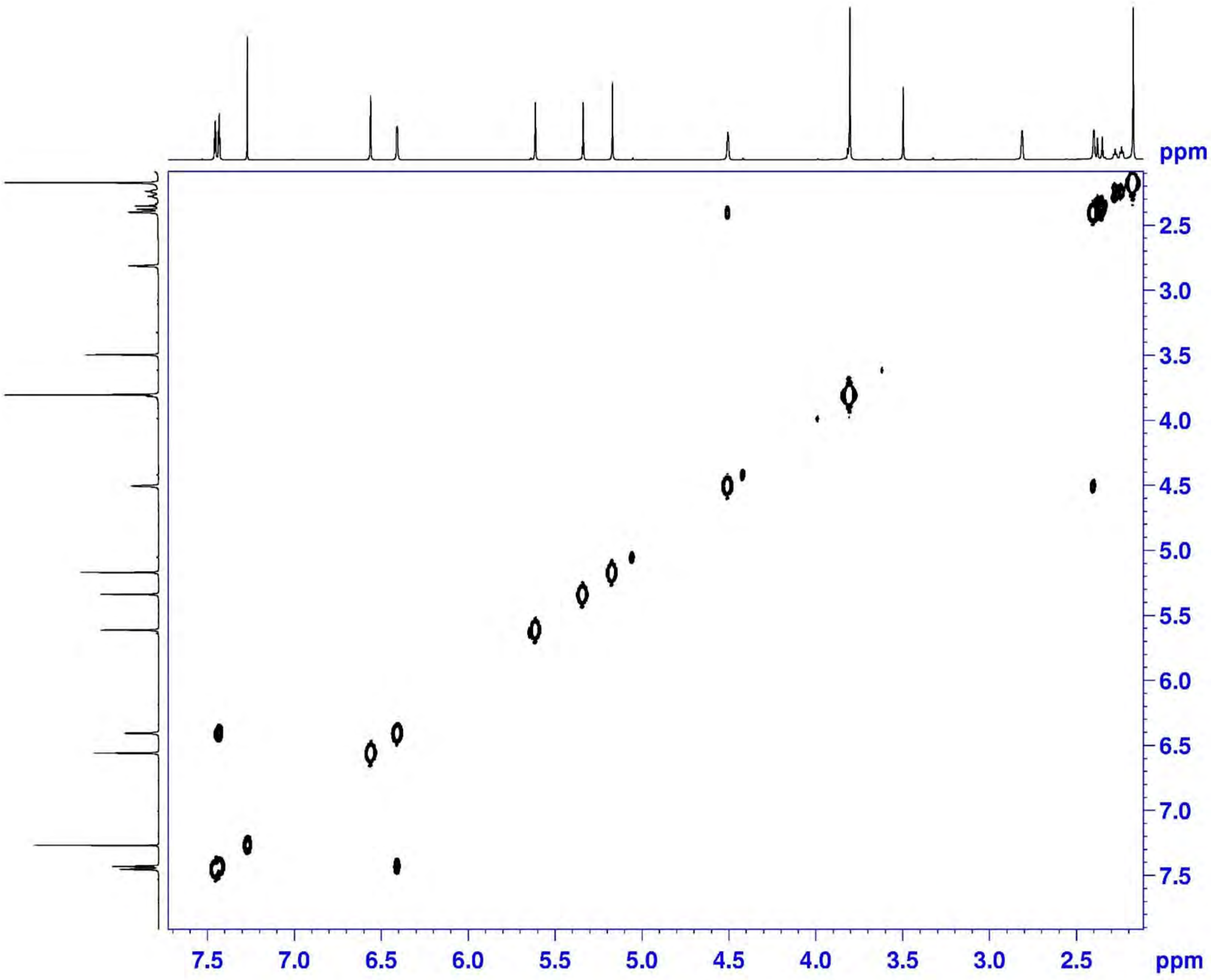

HSQC (400 MHz) spectrum of compound **26** in $CDCl_3$

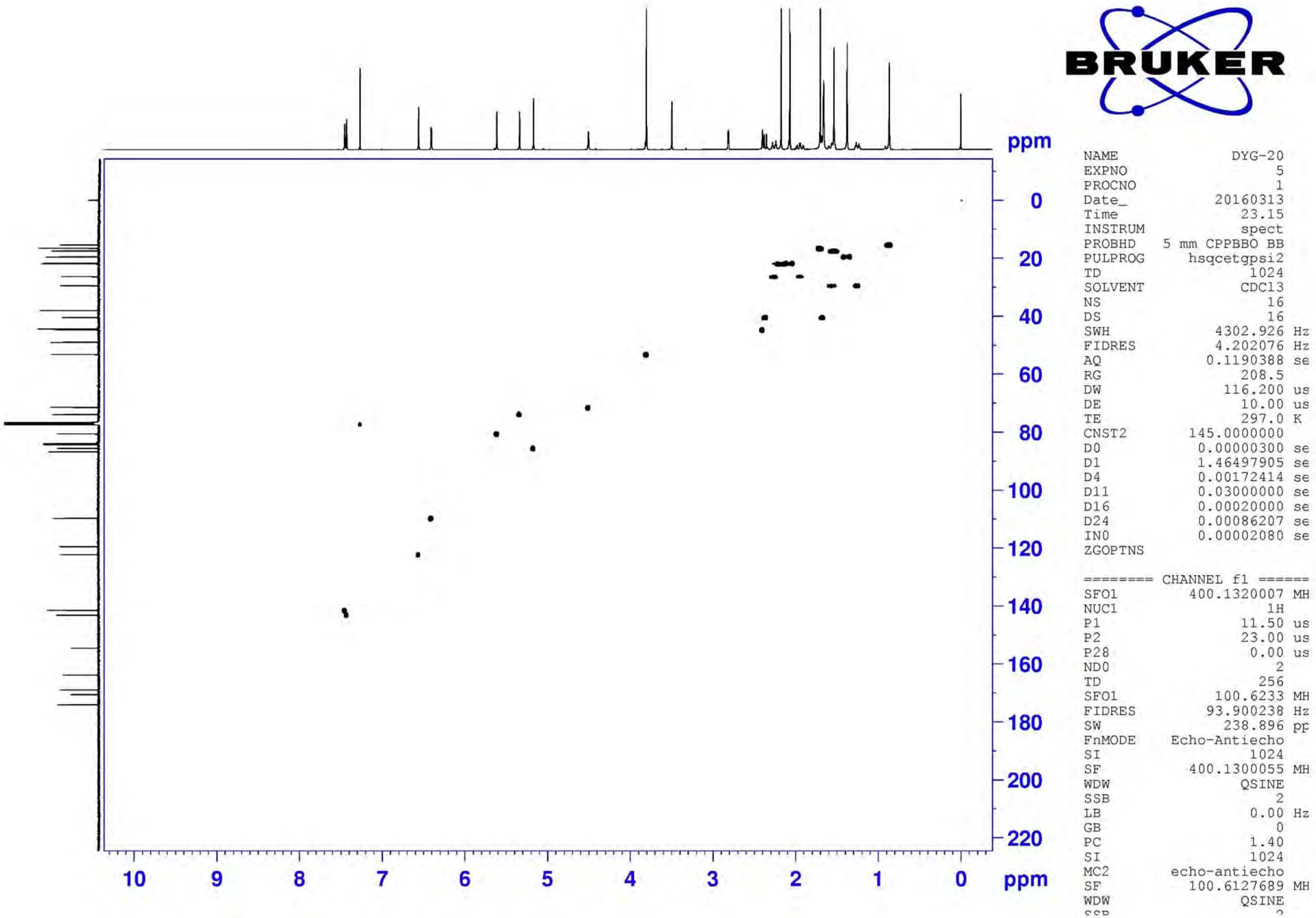

HSQC (400 MHz) spectrum of compound **26** in $CDCl_3$

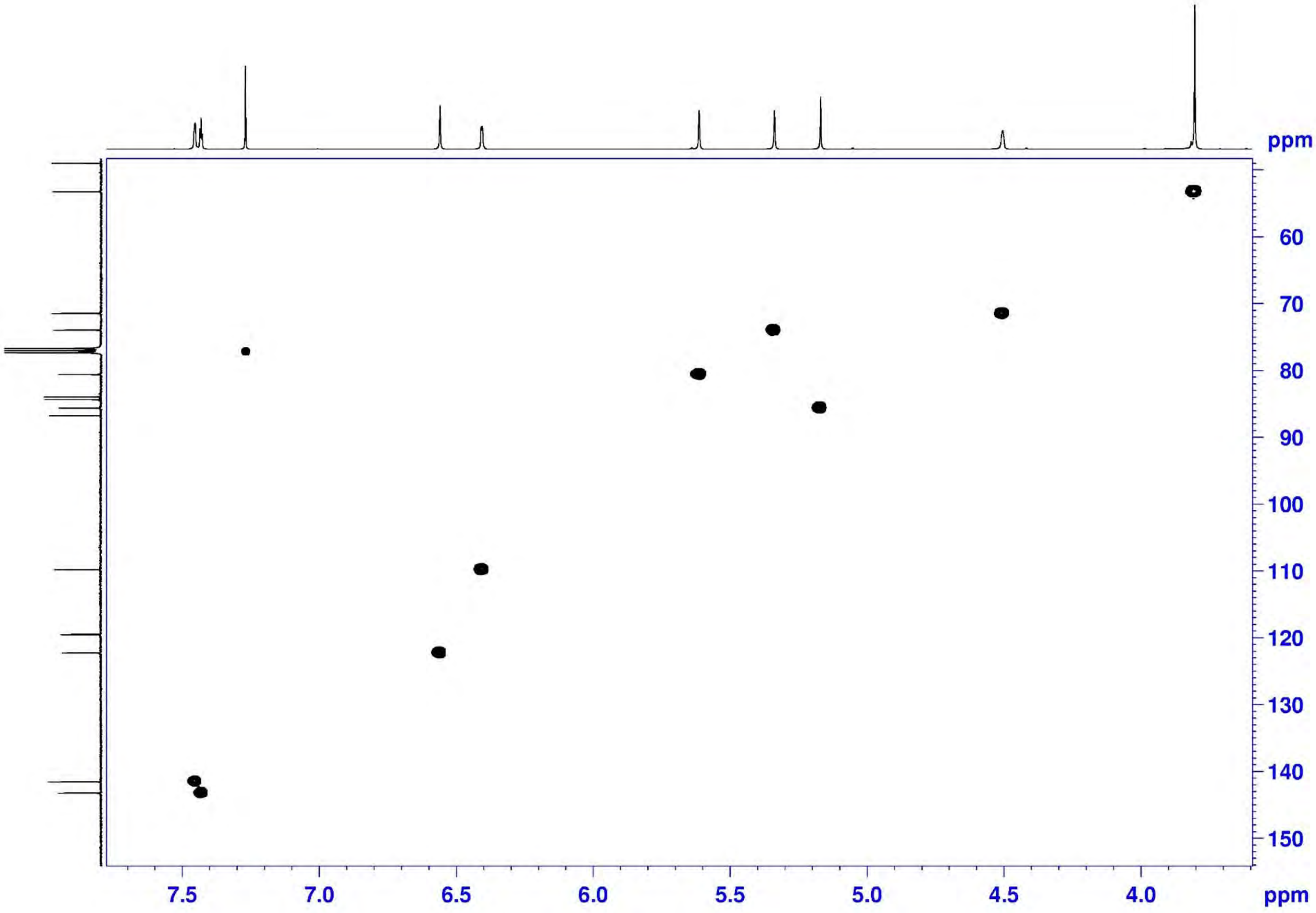

HSQC (400 MHz) spectrum of compound **26** in $CDCl_3$

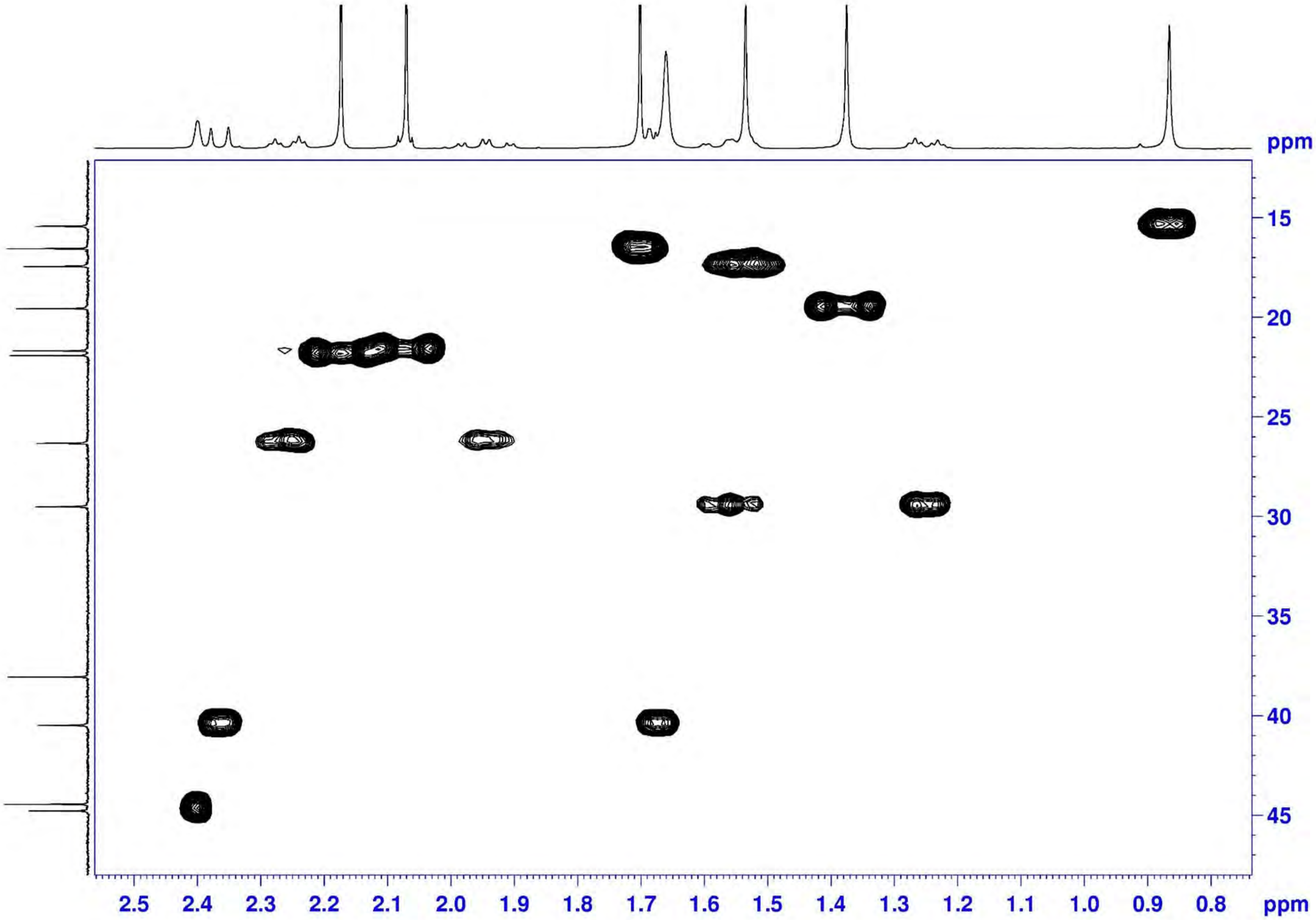

HMBC (400 MHz) spectrum of compound **26** in $CDCl_3$

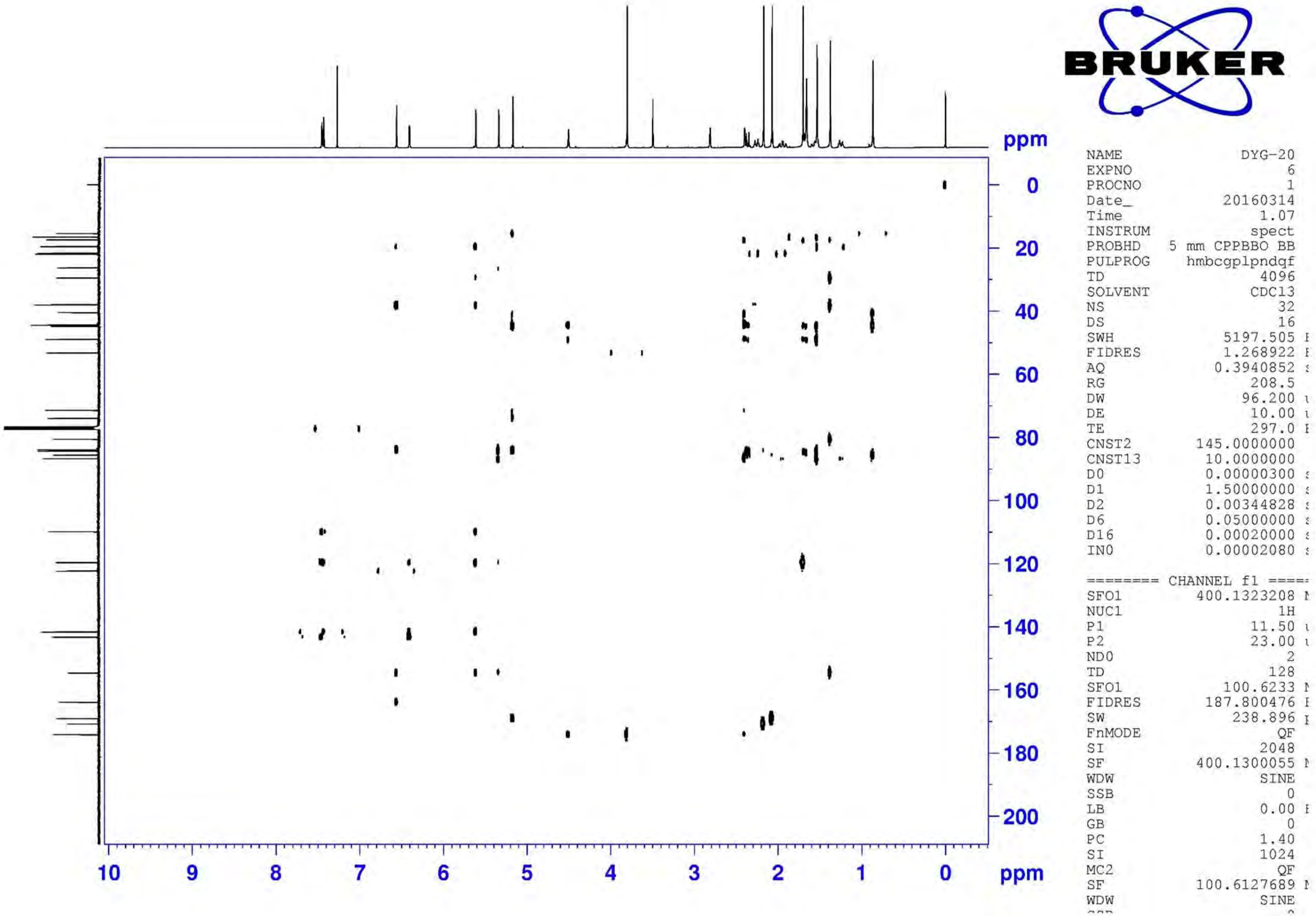

HMBC (400 MHz) spectrum of compound **26** in $CDCl_3$

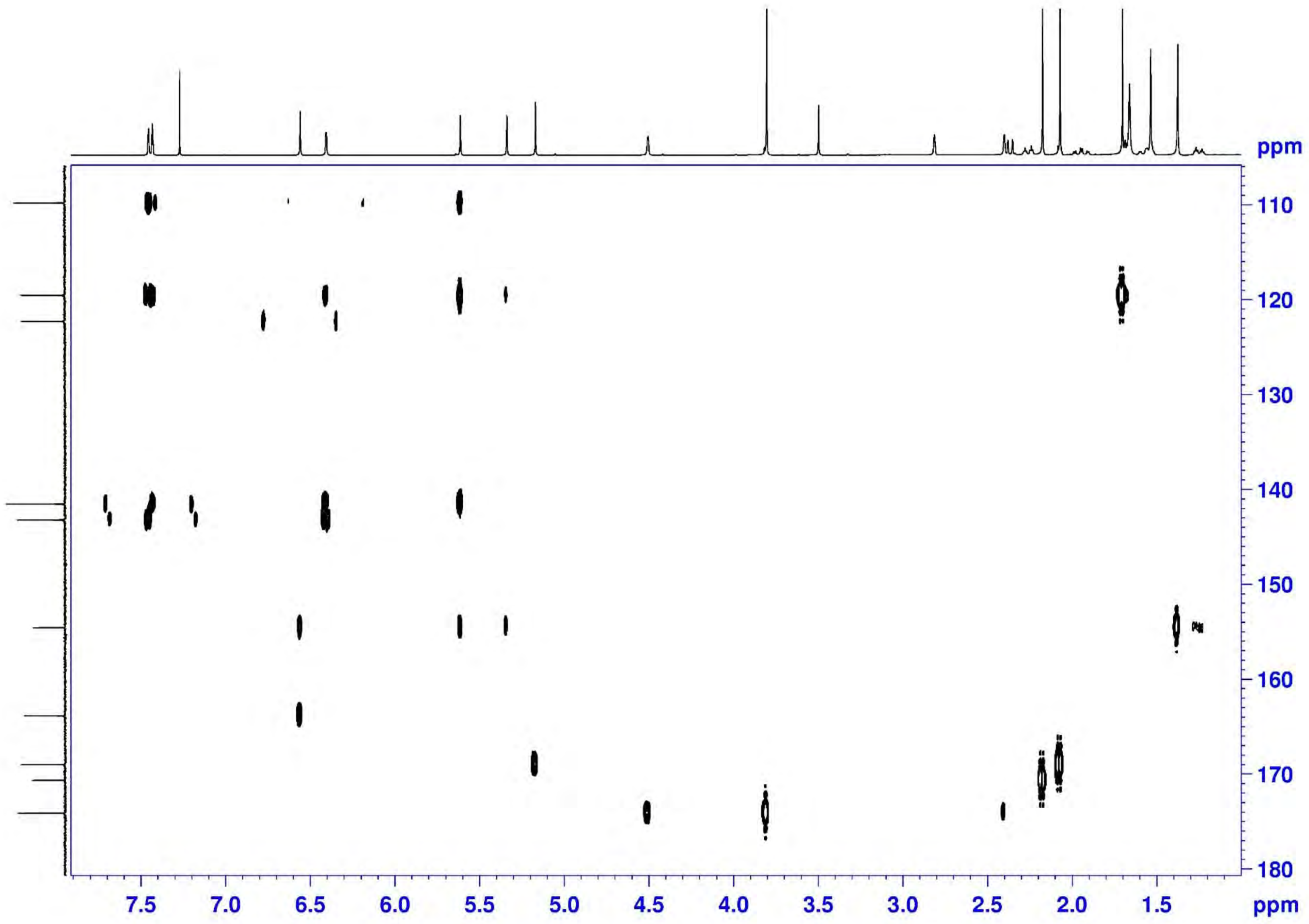

HMBC (400 MHz) spectrum of compound **26** in $CDCl_3$

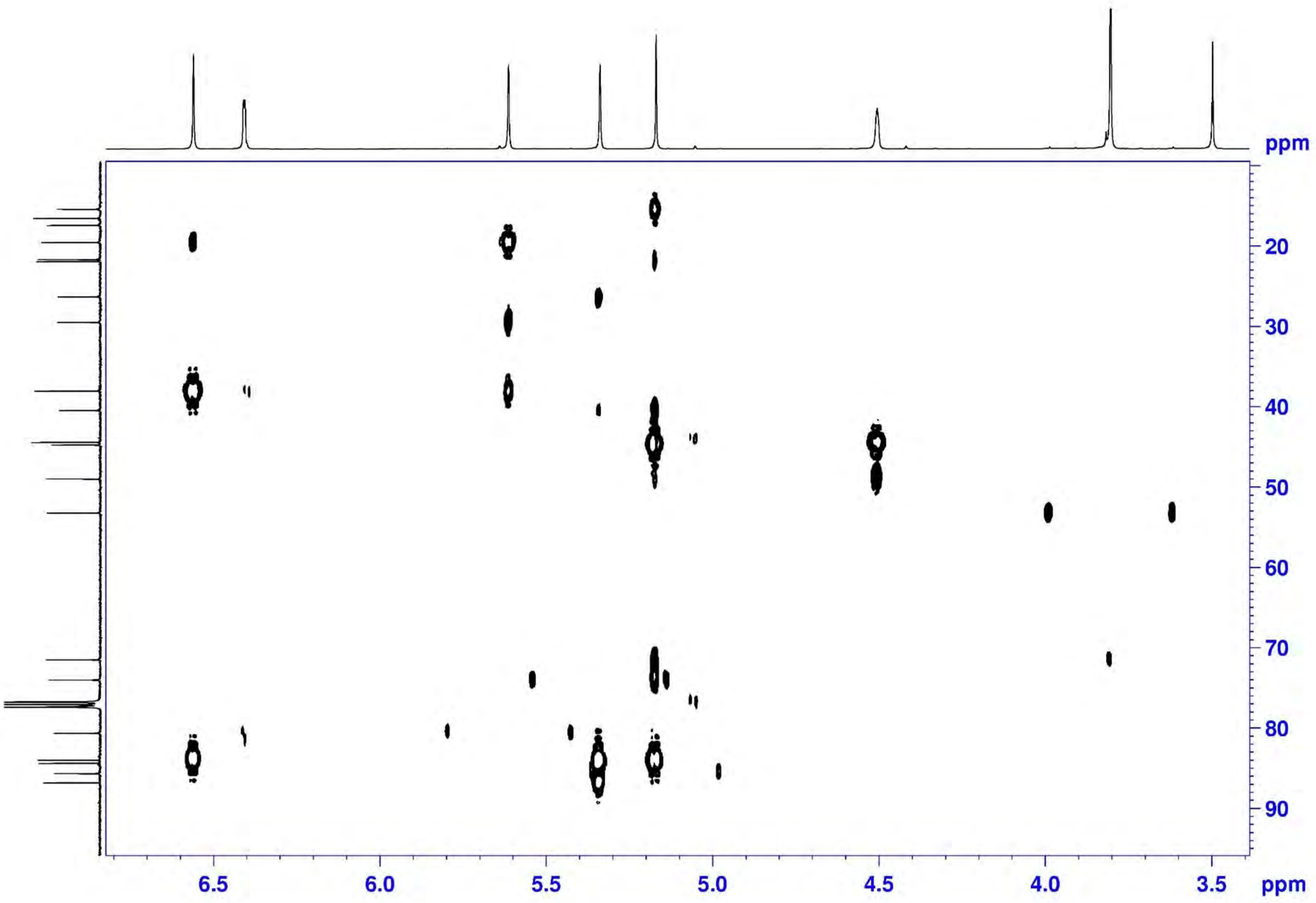

HMBC (400 MHz) spectrum of compound **26** in $CDCl_3$

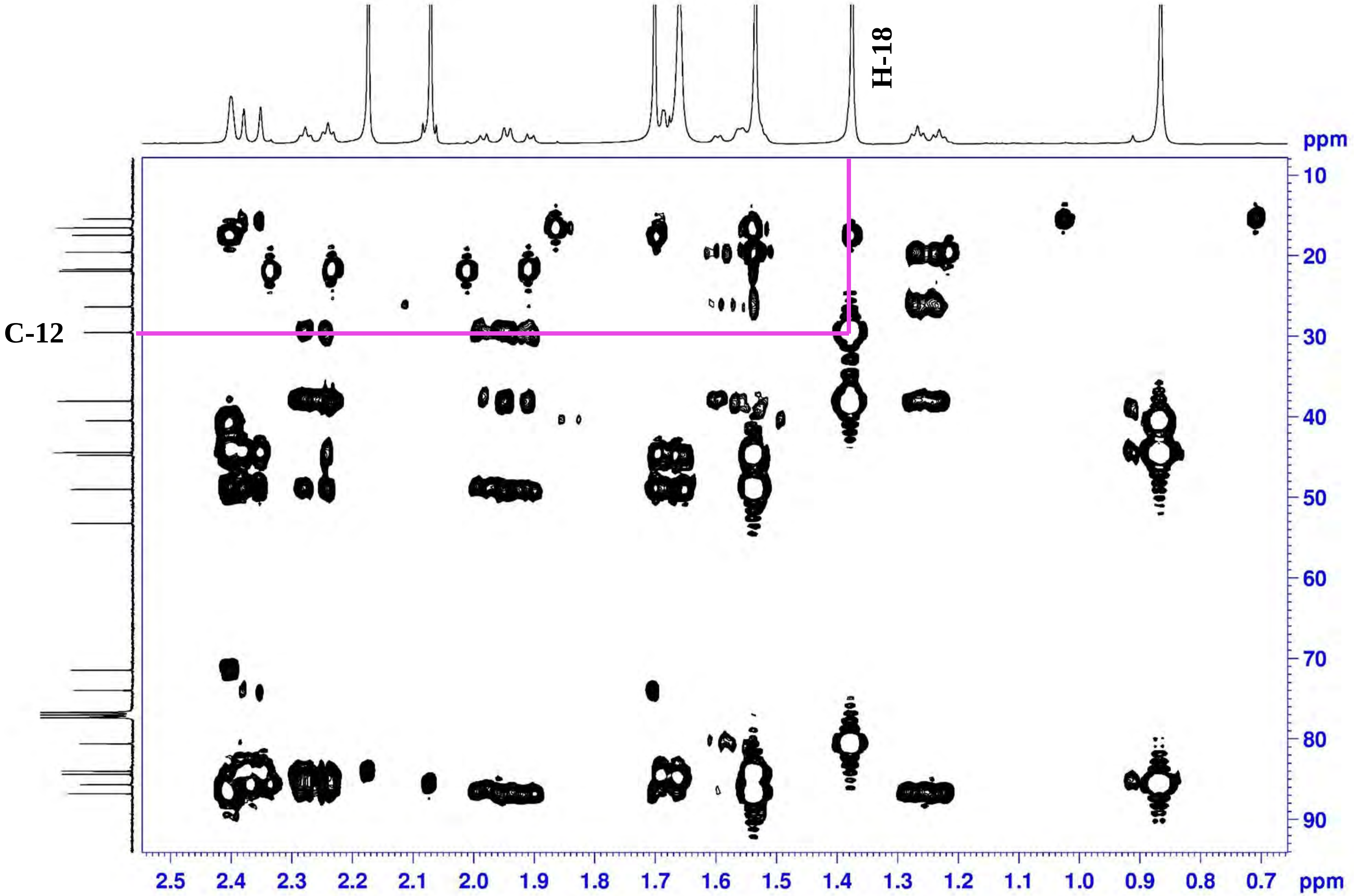

HMBC (400 MHz) spectrum of compound **26** in $CDCl_3$

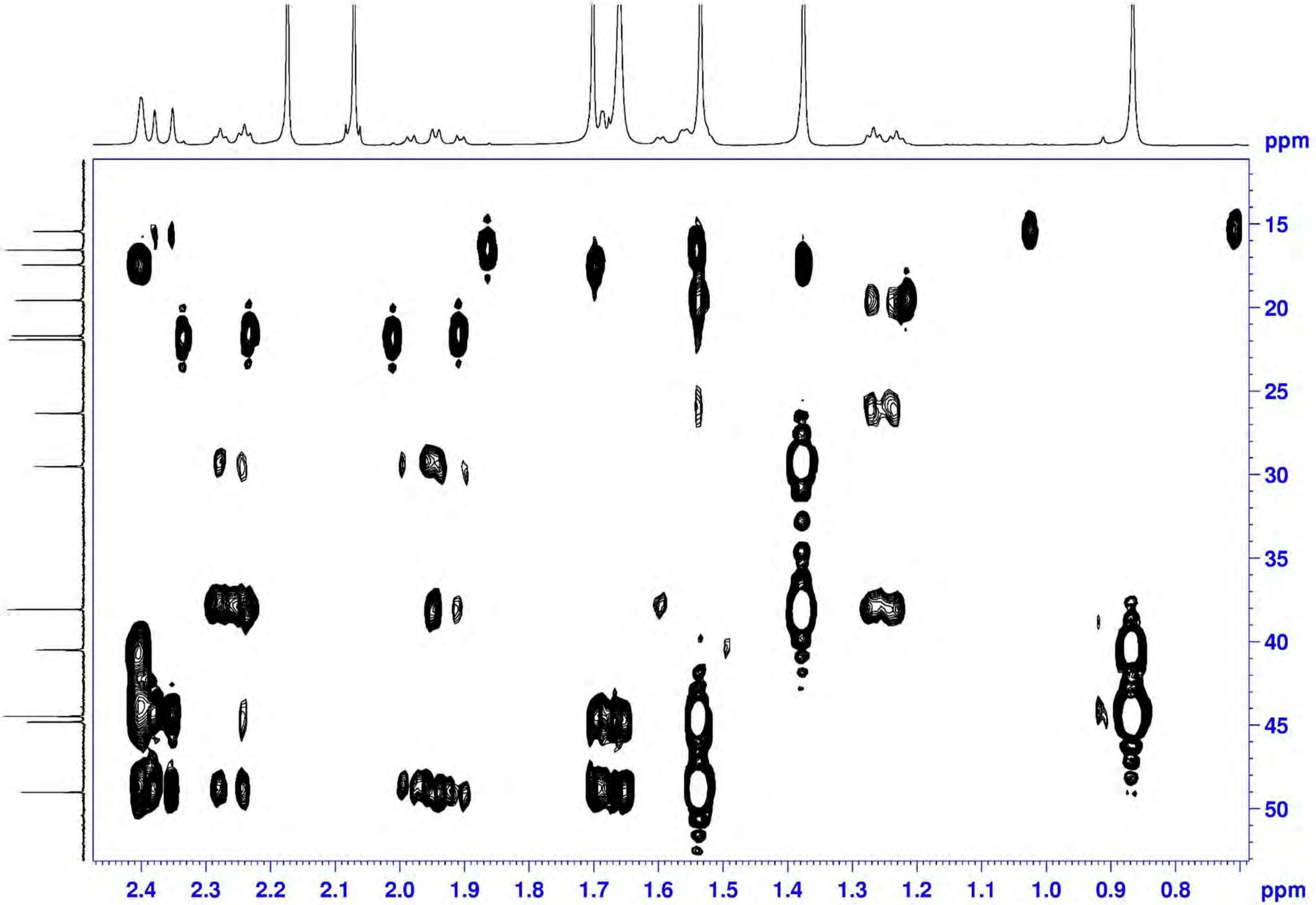

NOESY (400 MHz) spectrum of compound **26** in $CDCl_3$

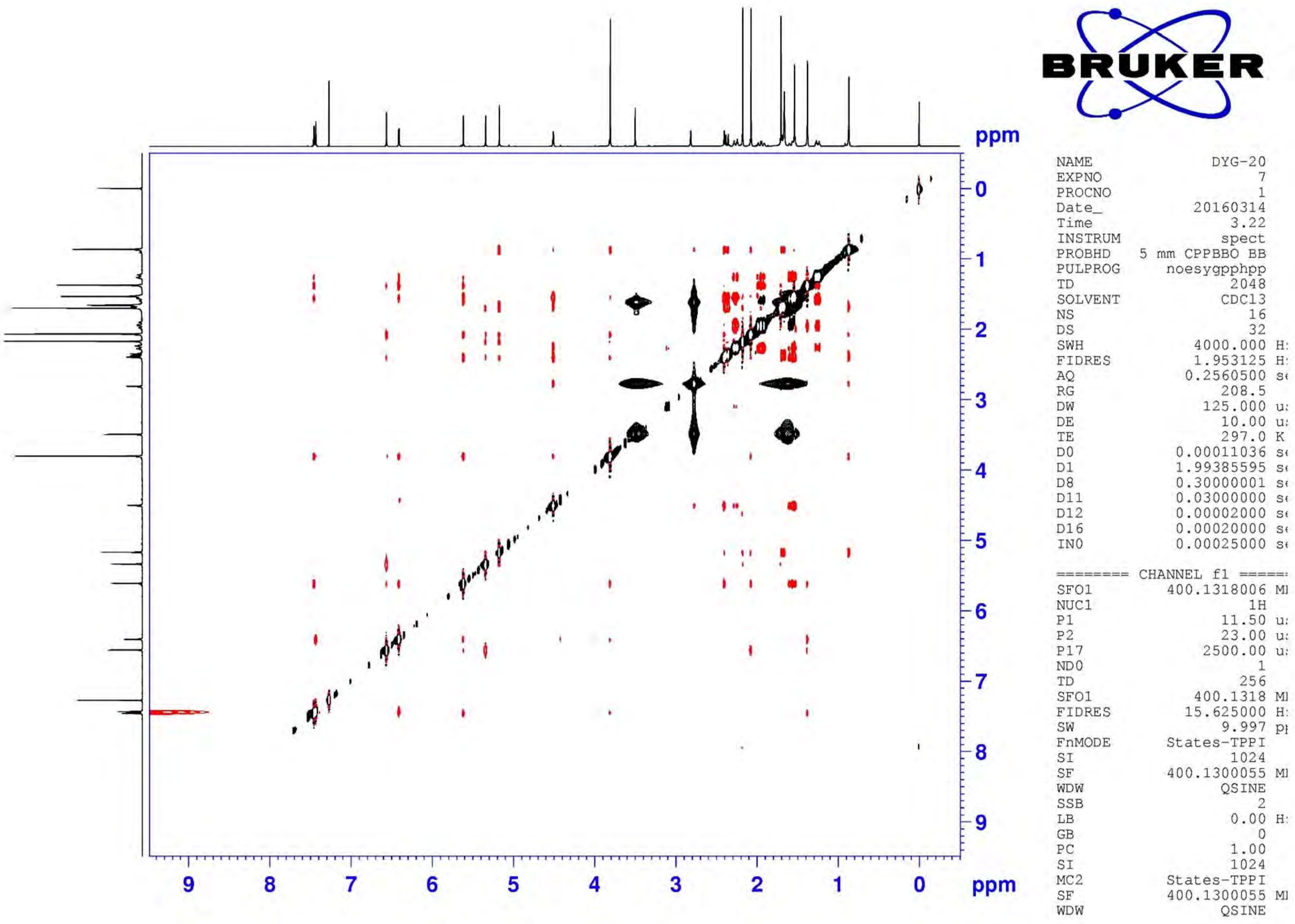

NOESY (400 MHz) spectrum of compound **26** in $CDCl_3$

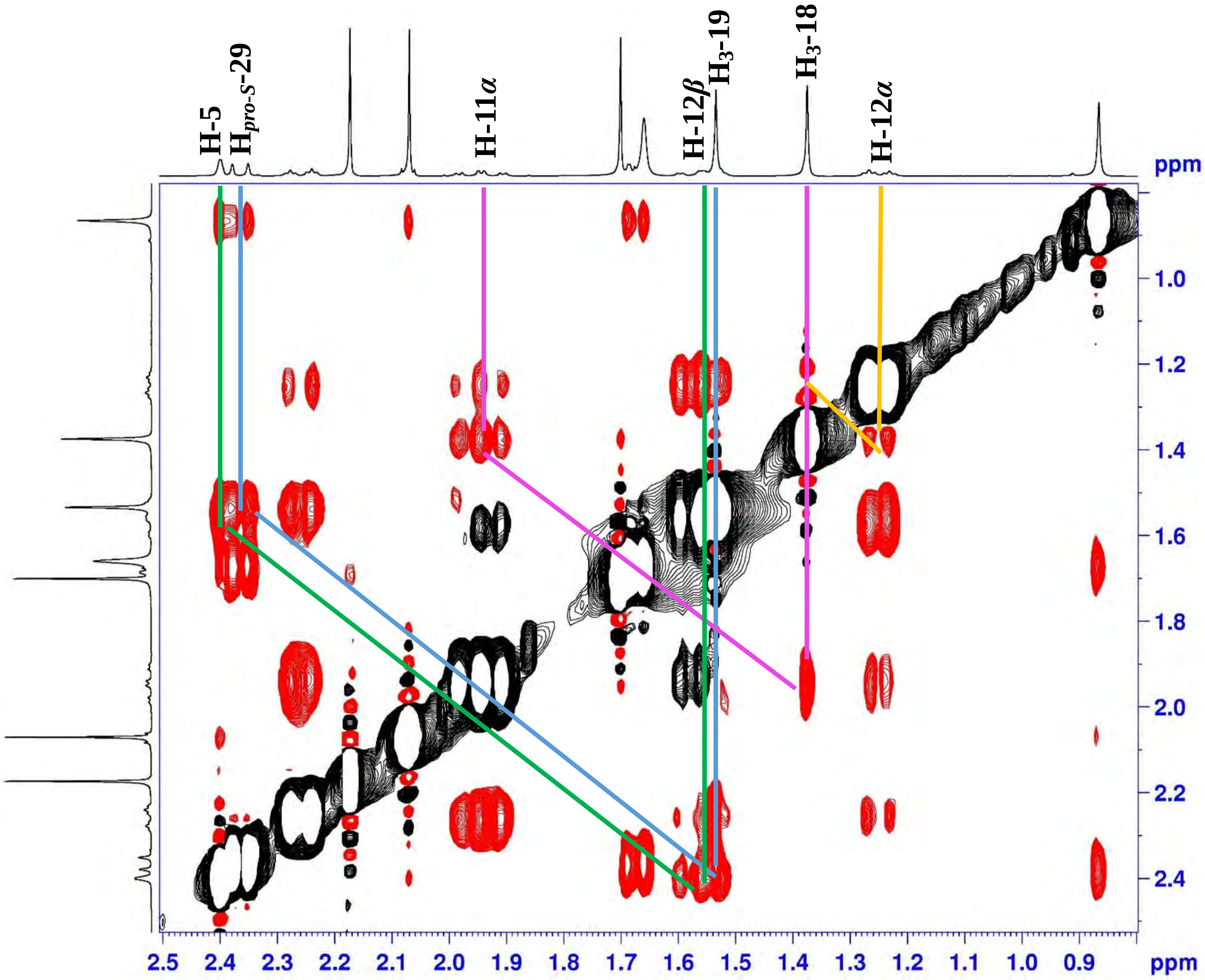

NOESY (400 MHz) spectrum of compound **26** in $CDCl_3$

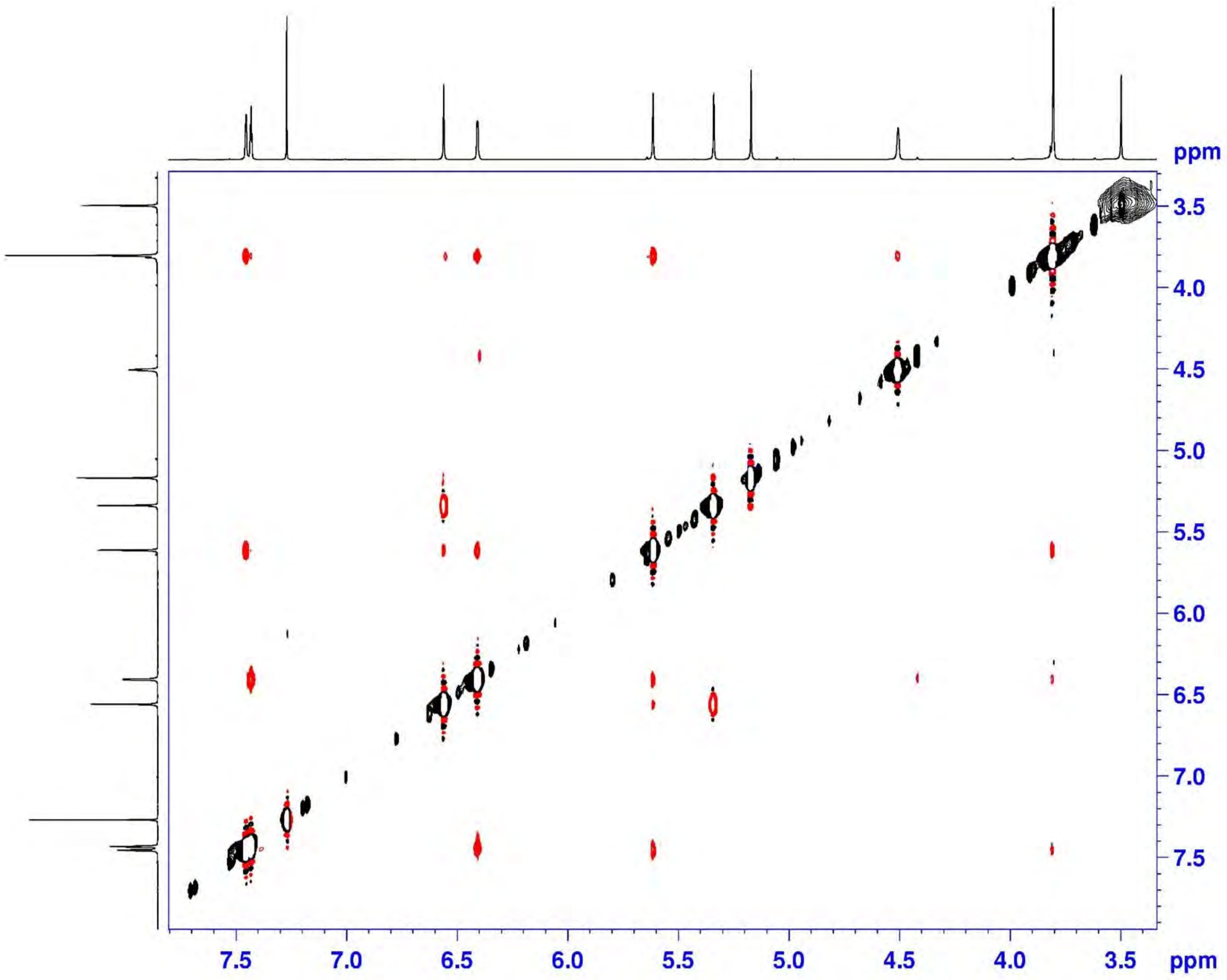

NOESY (400 MHz) spectrum of compound **26** in $CDCl_3$

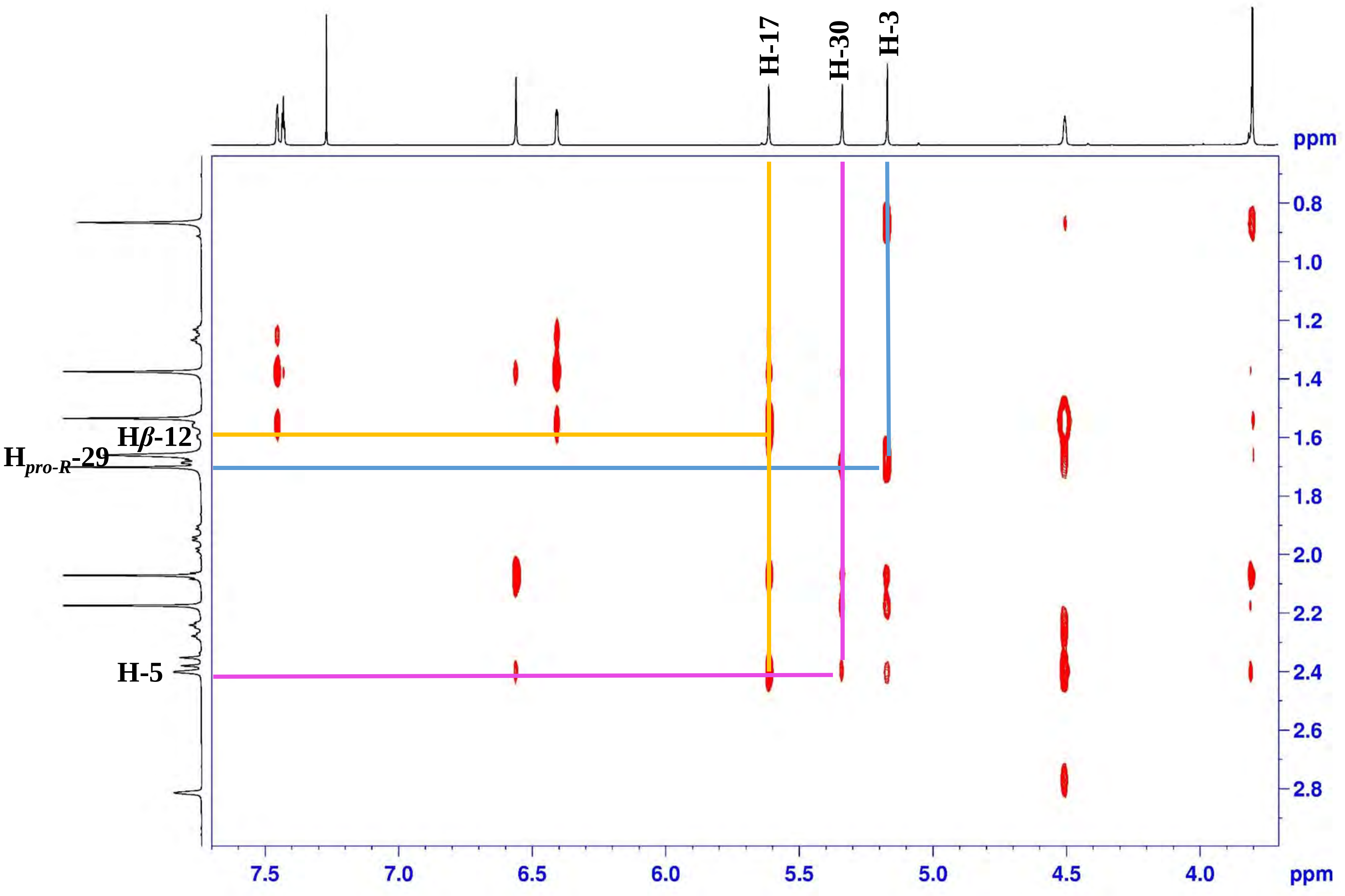

HR-ESIMS for compound **27**

Mass Spectrum SmartFormula Report

Analysis Info

Analysis Name	D:\Data\MS\data\201605\liwanshan_dgy-12_pos_5_01_1853.d	Acquisition Date	5/19/2016 8:34:21 AM
Method	LC_Direct Infusion_pos_100-1000mz.m	Operator	SCSIO
Sample Name	liwanshan_dgy-12_pos	Instrument / Ser#	maXis 29
Comment			

Acquisition Parameter

Source Type	ESI	Ion Polarity	Positive	Set Nebulizer	0.4 Bar
Focus	Active	Set Capillary	4500 V	Set Dry Heater	180 °C
Scan Begin	100 m/z	Set End Plate Offset	-500 V	Set Dry Gas	4.0 l/min
Scan End	2000 m/z	Set Collision Cell RF	800.0 Vpp	Set Divert Valve	Waste

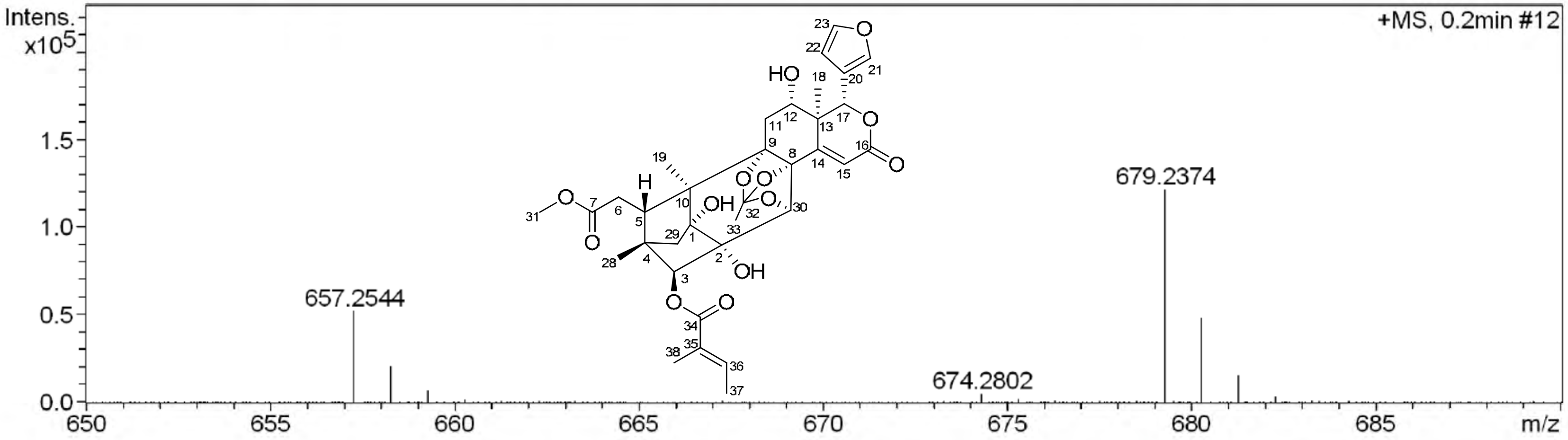

Meas. m/z	#	Formula	Score	m/z	err [ppm]	err [mDa]	mSigma	rdb	e⁻ Conf	N-Rule
657.2544	1	C 34 H 41 O 13	100.00	657.2542	-0.4	-0.3	15.3	14.5	even	ok
679.2374	1	C 34 H 40 Na O 13	79.62	679.2361	-1.9	-1.3	16.6	14.5	even	ok

HR-ESIMS for compound **27**

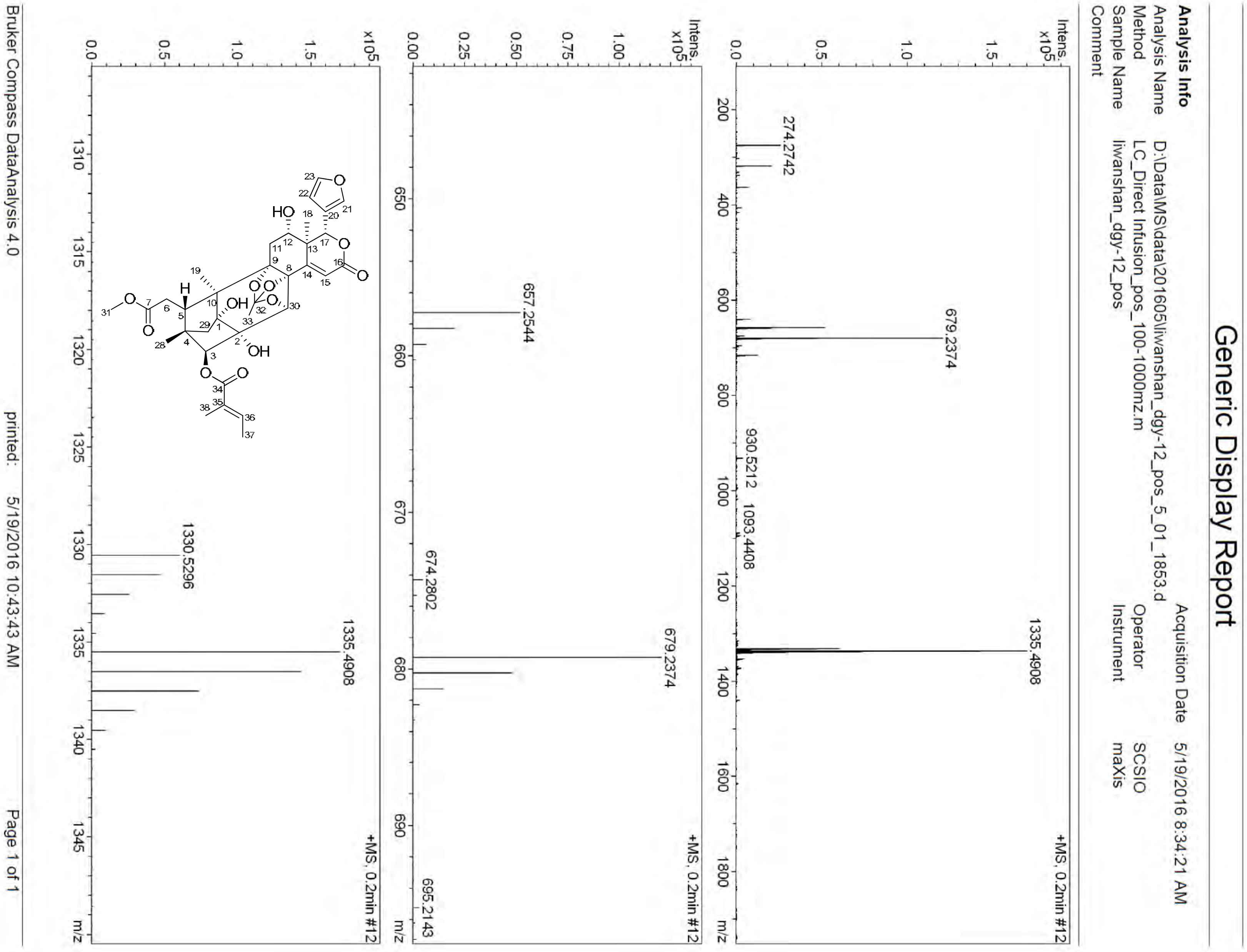

^{1}H NMR (400 MHz) spectrum of compound **27** in $CDCl_3$

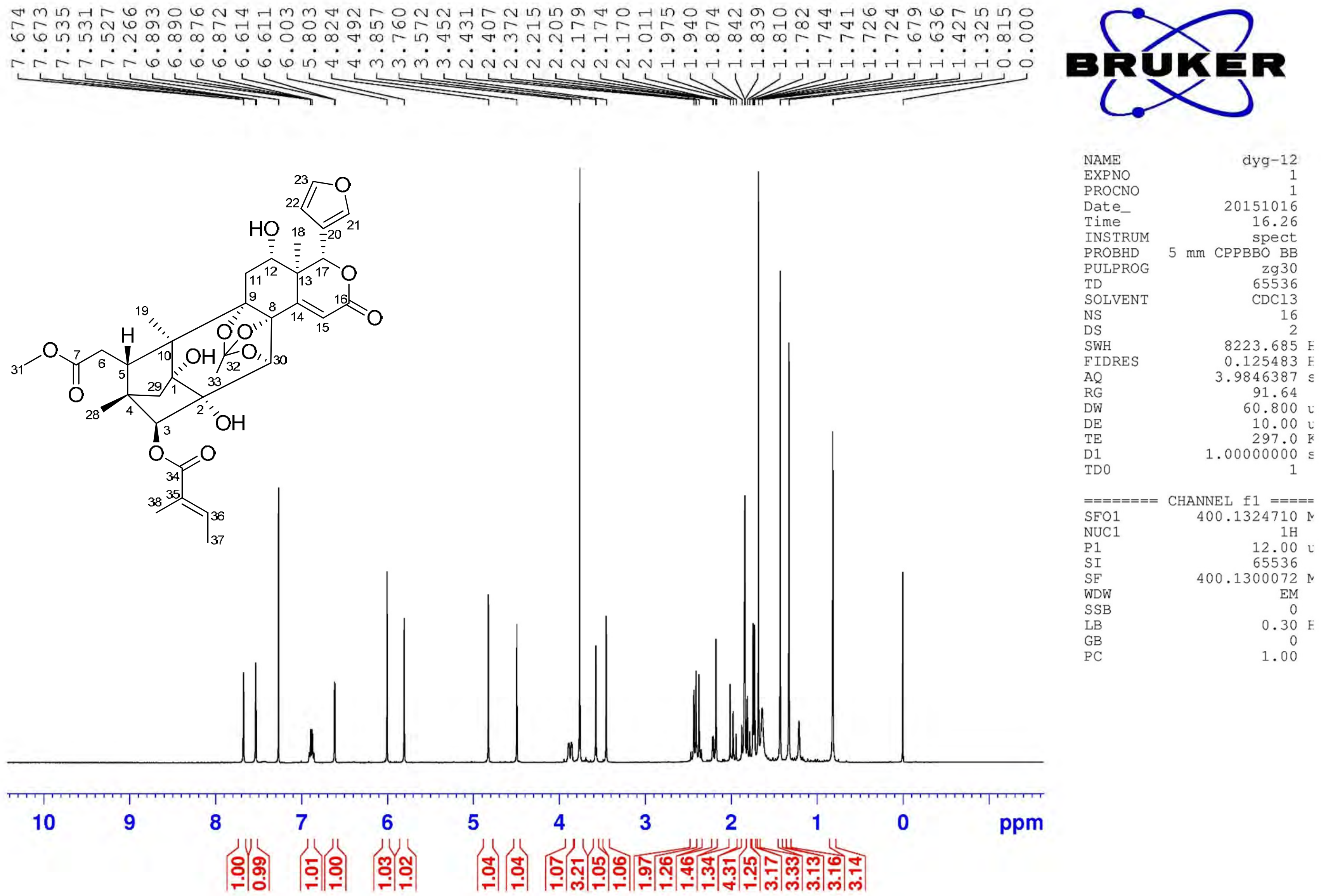

^{1}H NMR (400 MHz) spectrum of compound **27** in $CDCl_3$

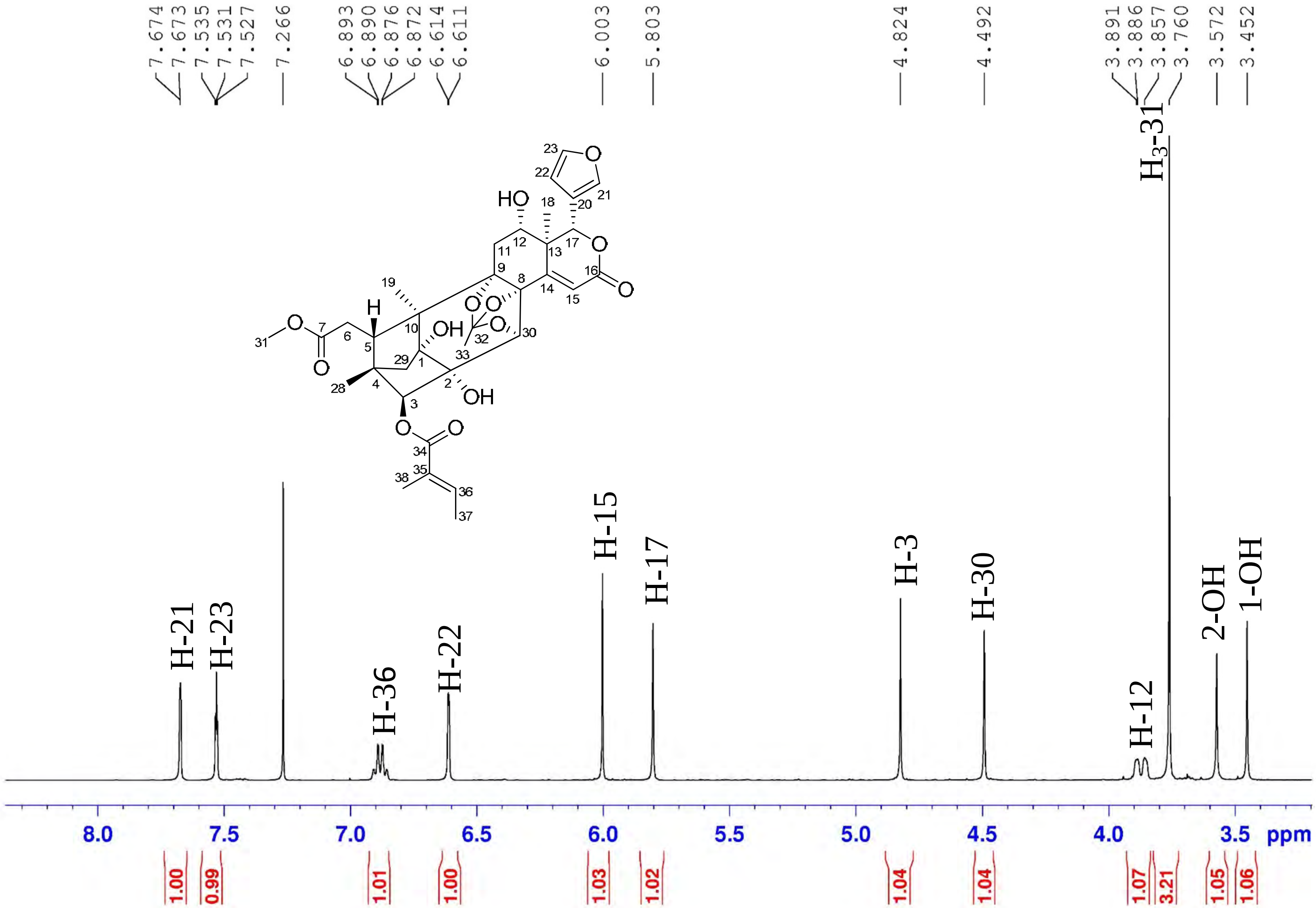

^{1}H NMR (400 MHz) spectrum of compound **27** in $CDCl_3$

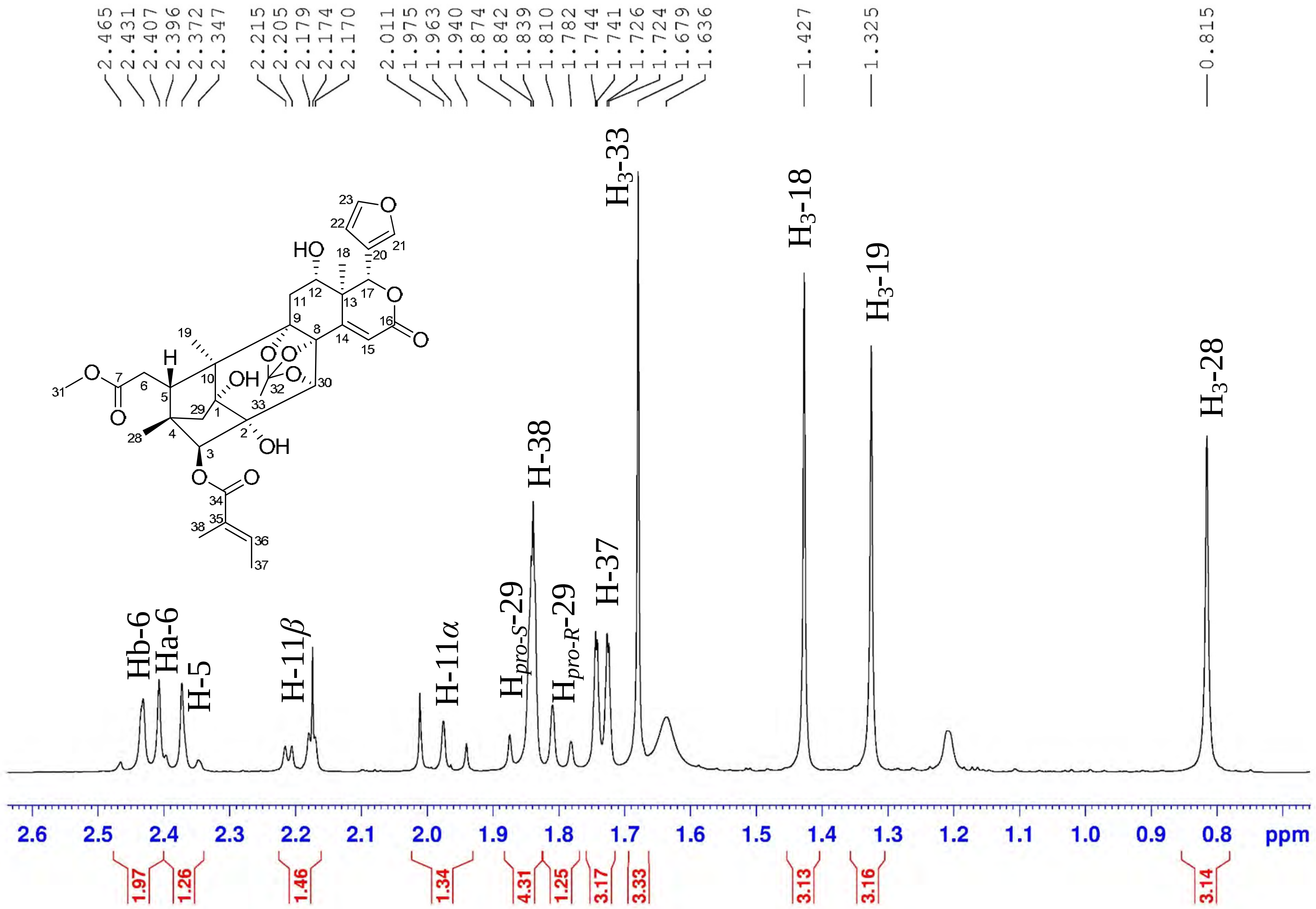

^{13}C NMR (100 MHz) spectrum of compound **27** in $CDCl_3$

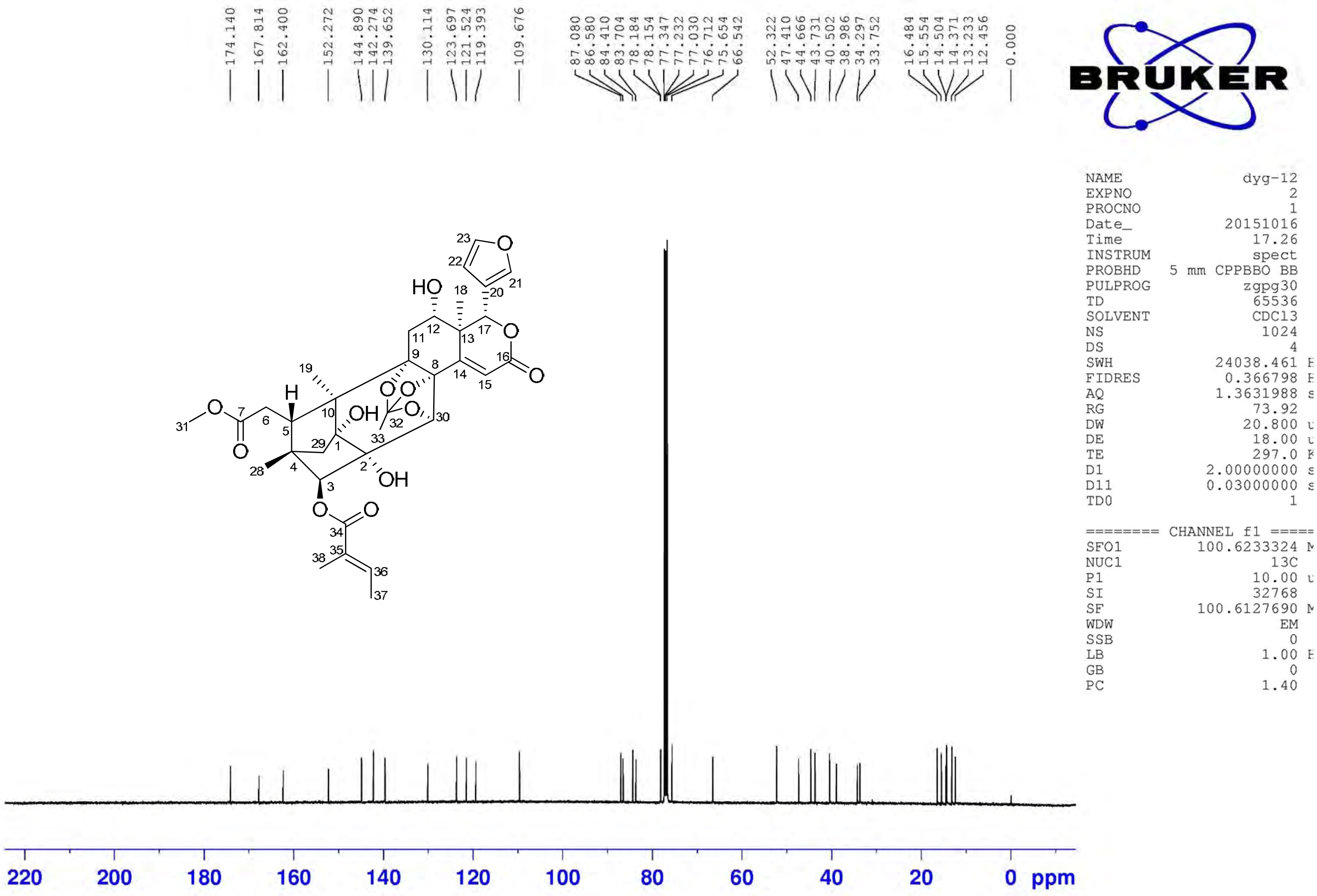

^{13}C NMR (100 MHz) spectrum of compound **27** in $CDCl_3$

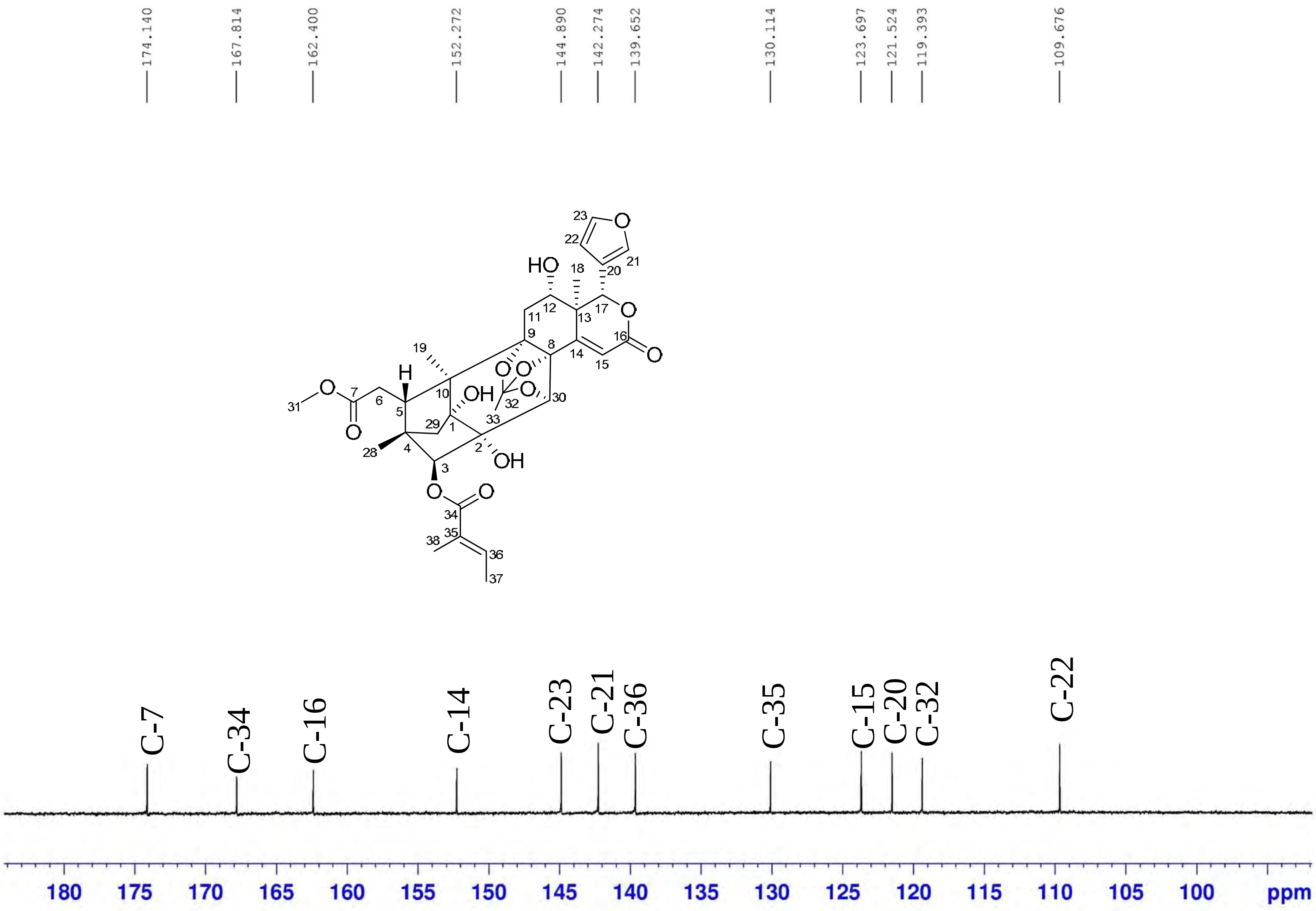

^{13}C NMR (100 MHz) spectrum of compound **27** in $CDCl_3$

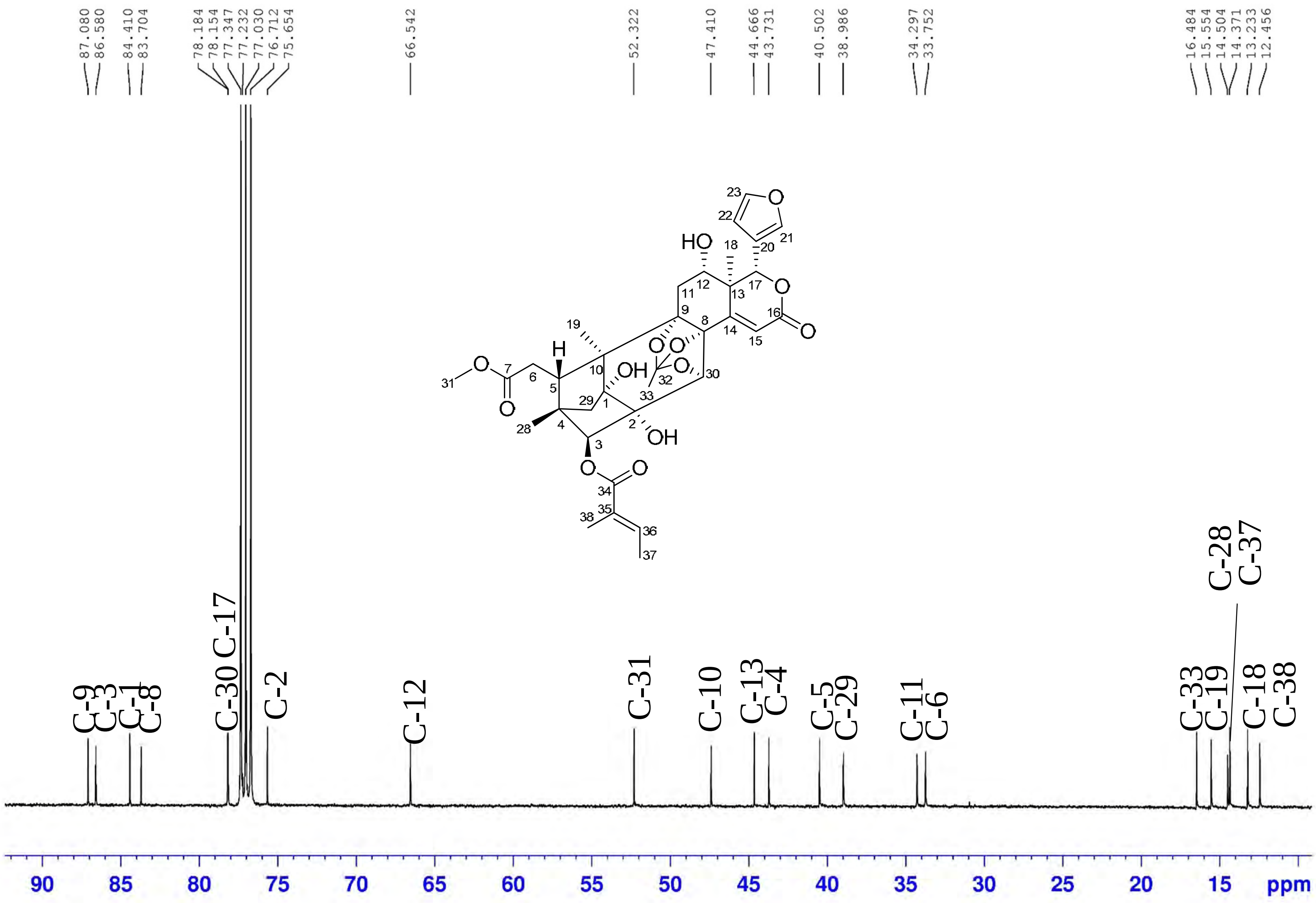

DEPT135 (100 MHz) spectrum of compound **27** in $CDCl_3$

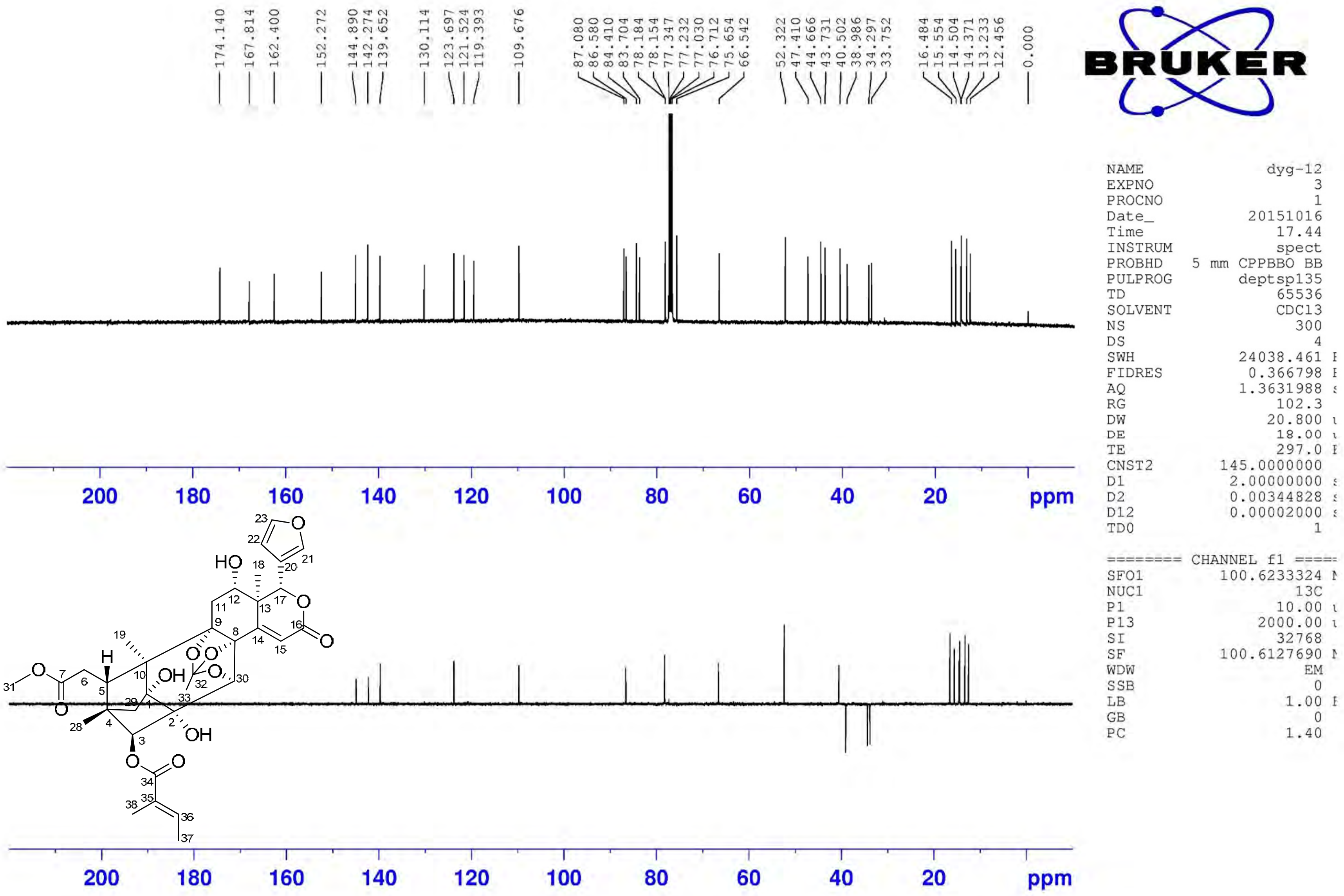

DEPT135 (100 MHz) spectrum of compound **27** in $CDCl_3$

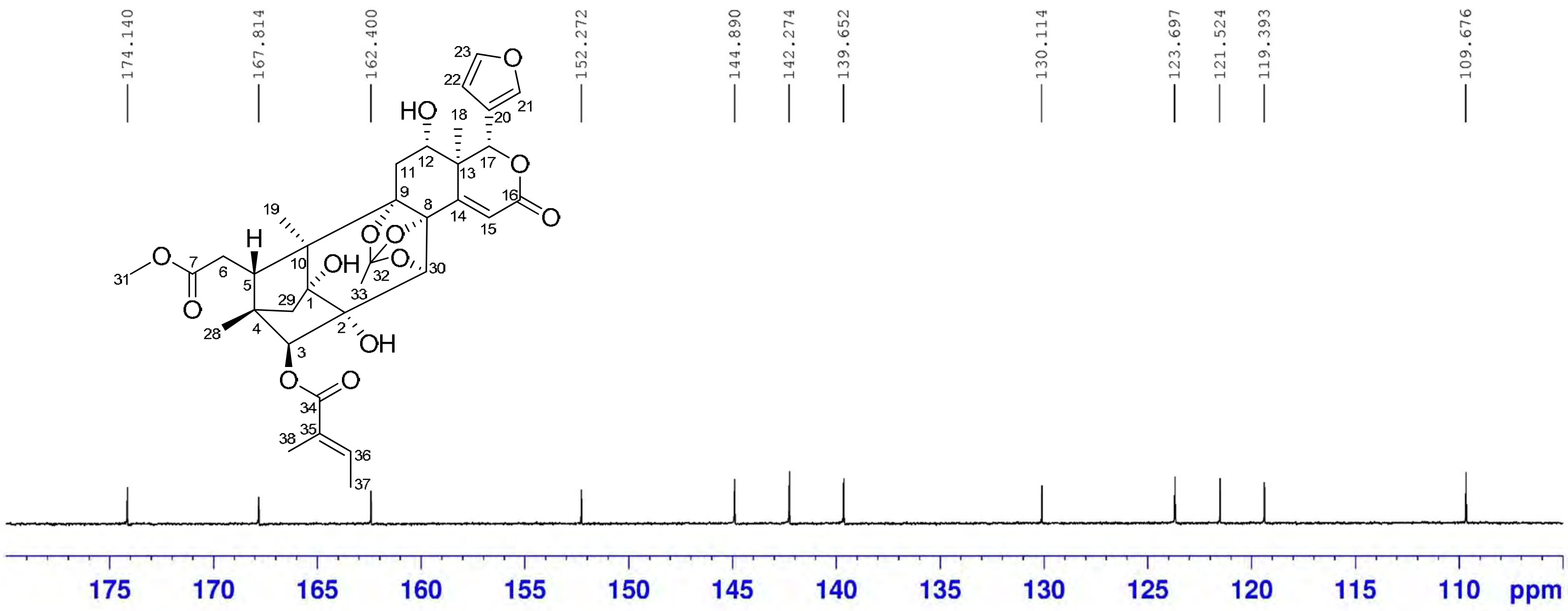

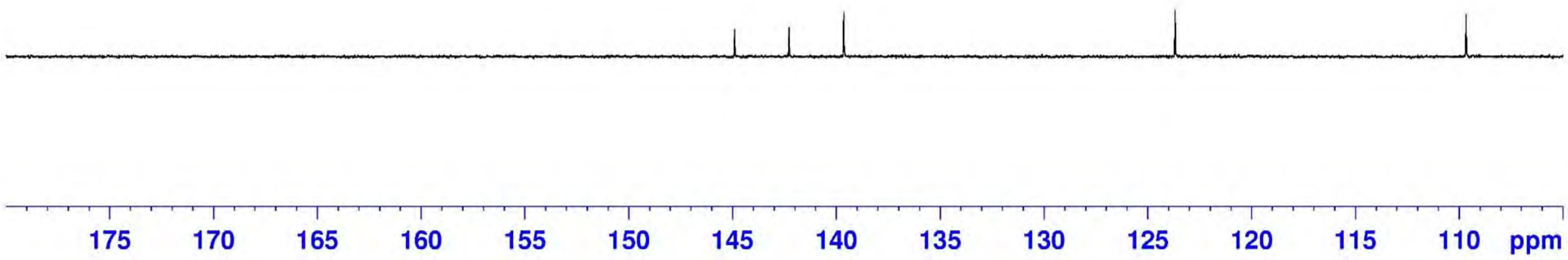

DEPT135 (100 MHz) spectrum of compound **27** in $CDCl_3$

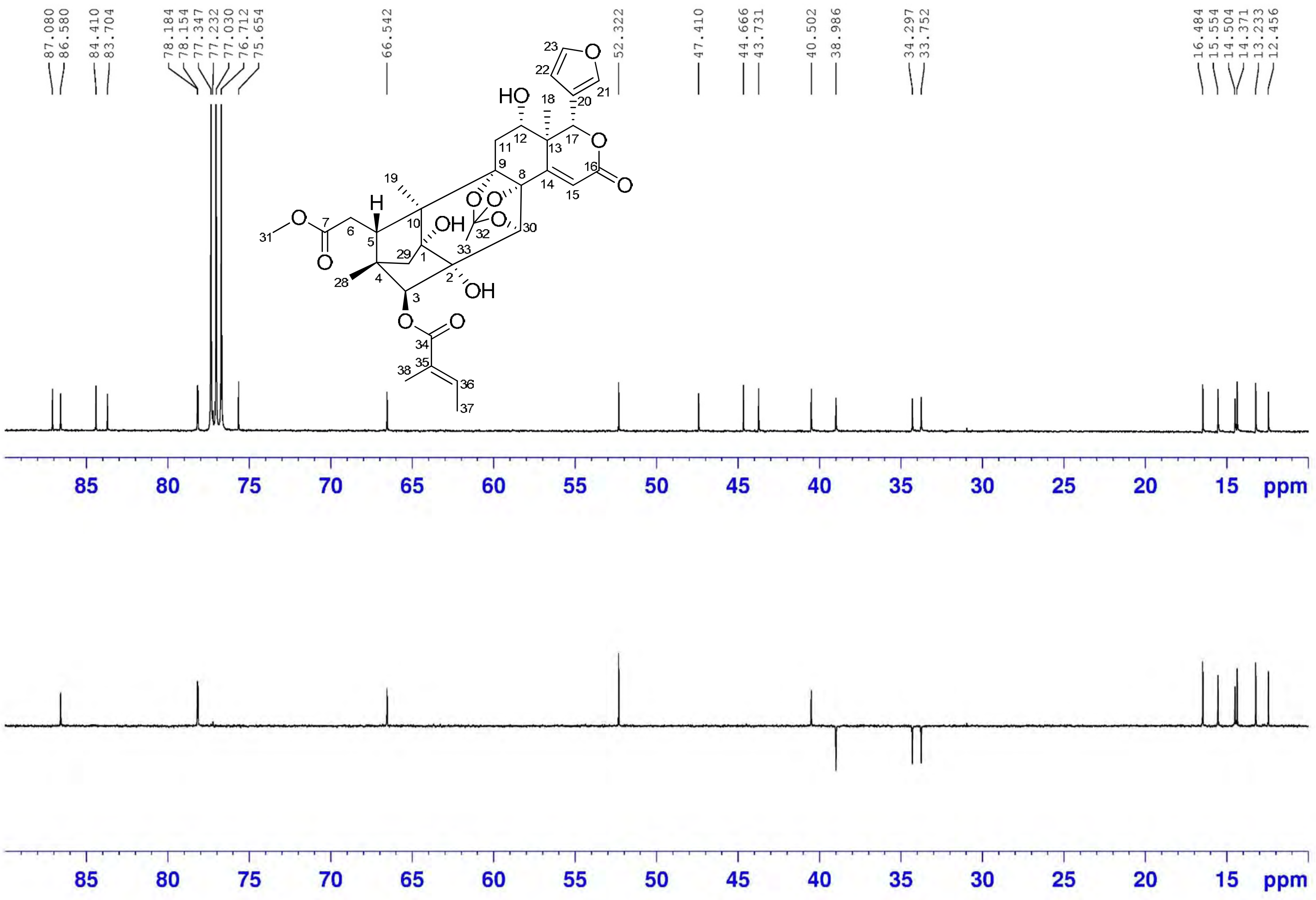

^{1}H-^{1}H COSY (400 MHz) spectrum of compound **27** in $CDCl_3$

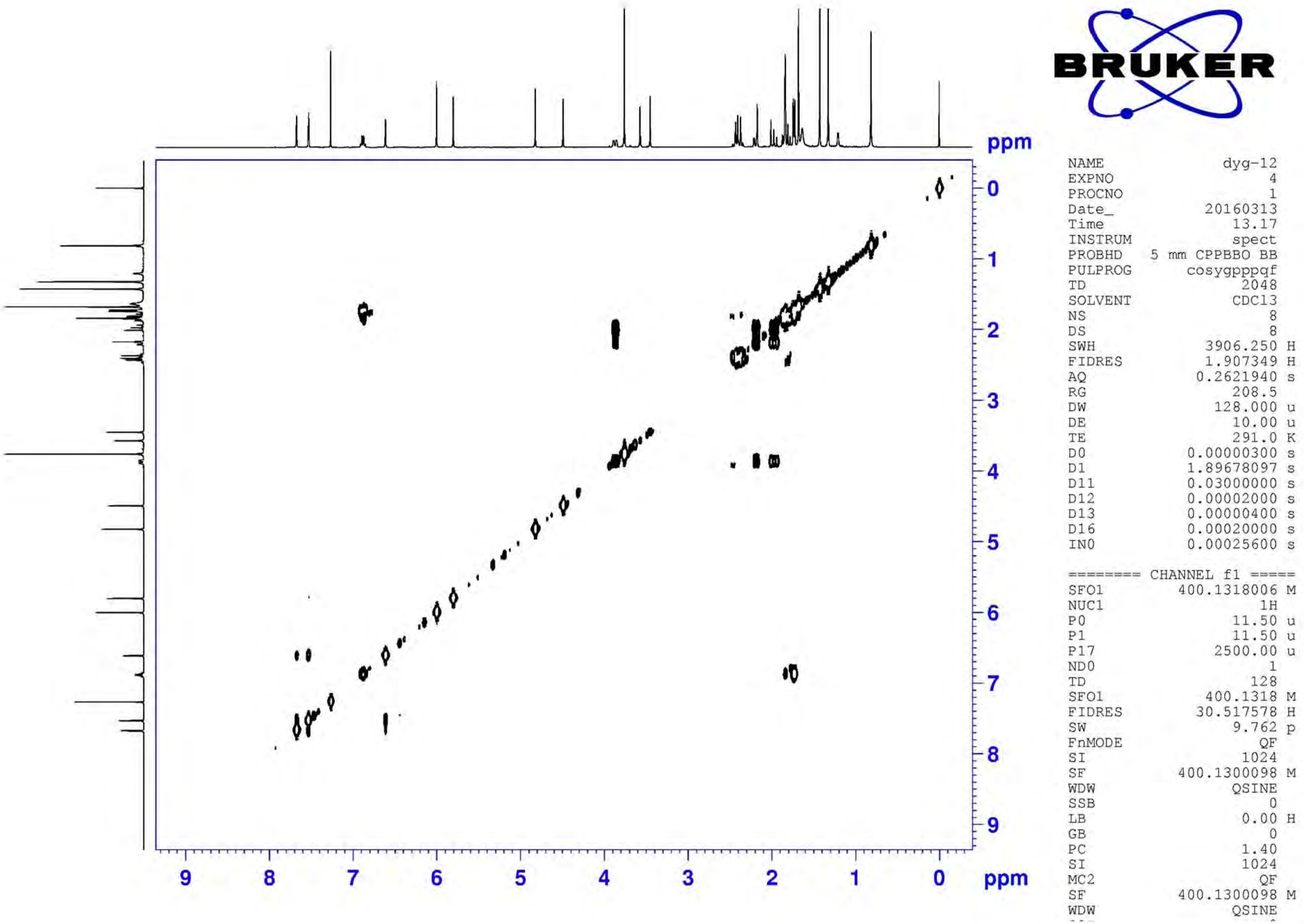

^{1}H-^{1}H COSY (400 MHz) spectrum of compound **27** in $CDCl_3$

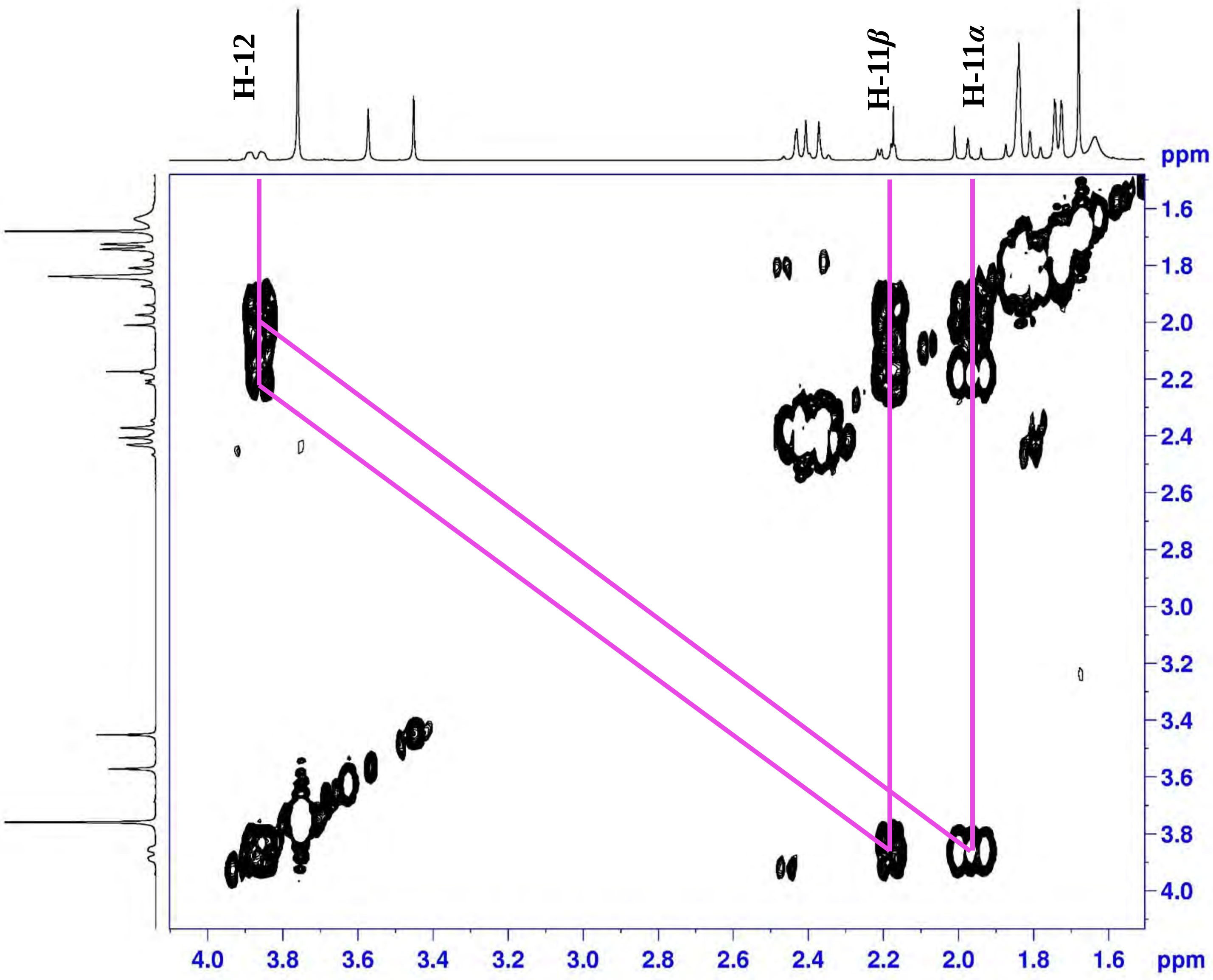

HSQC (400 MHz) spectrum of compound **27** in $CDCl_3$

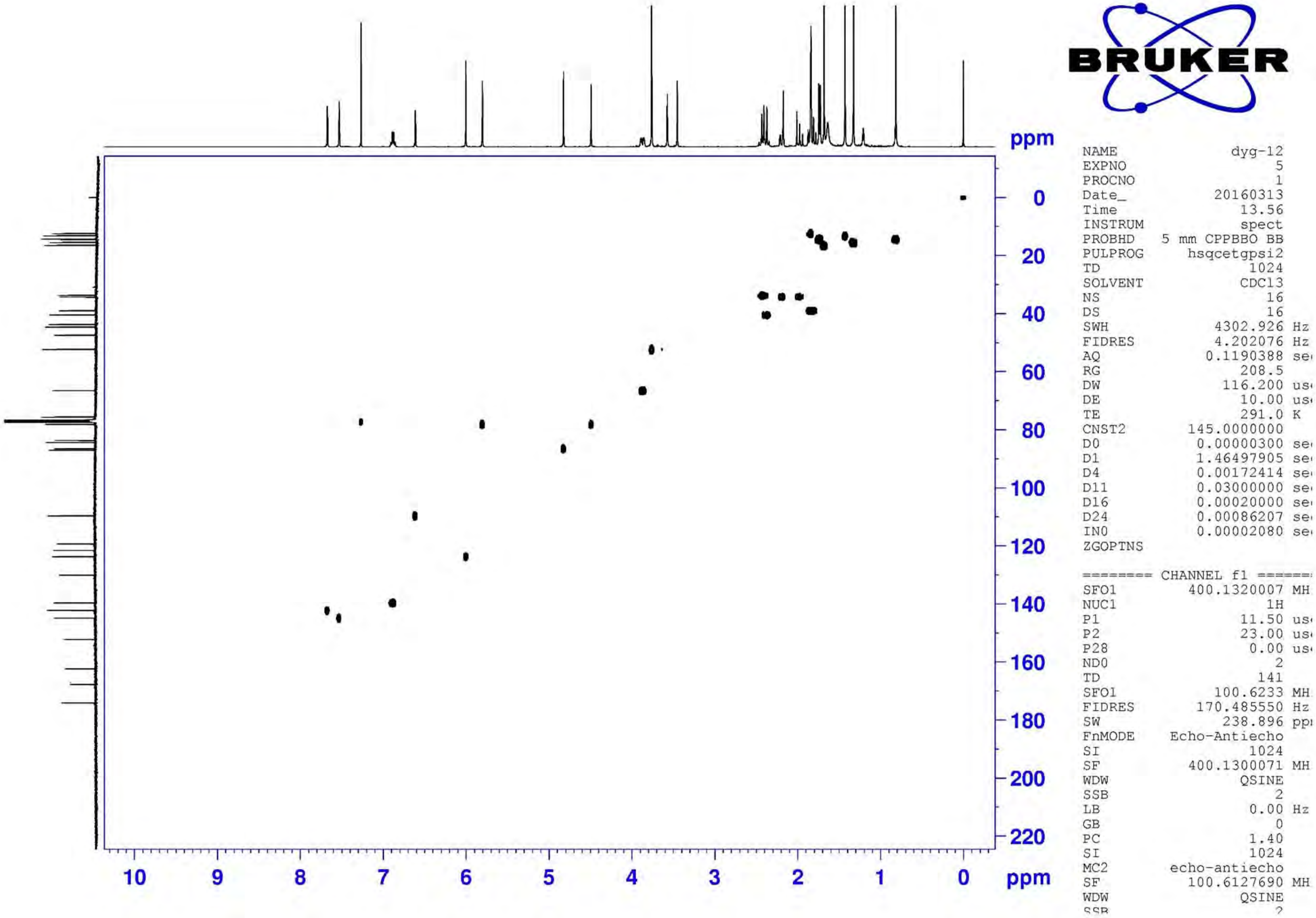

HSQC (400 MHz) spectrum of compound **27** in $CDCl_3$

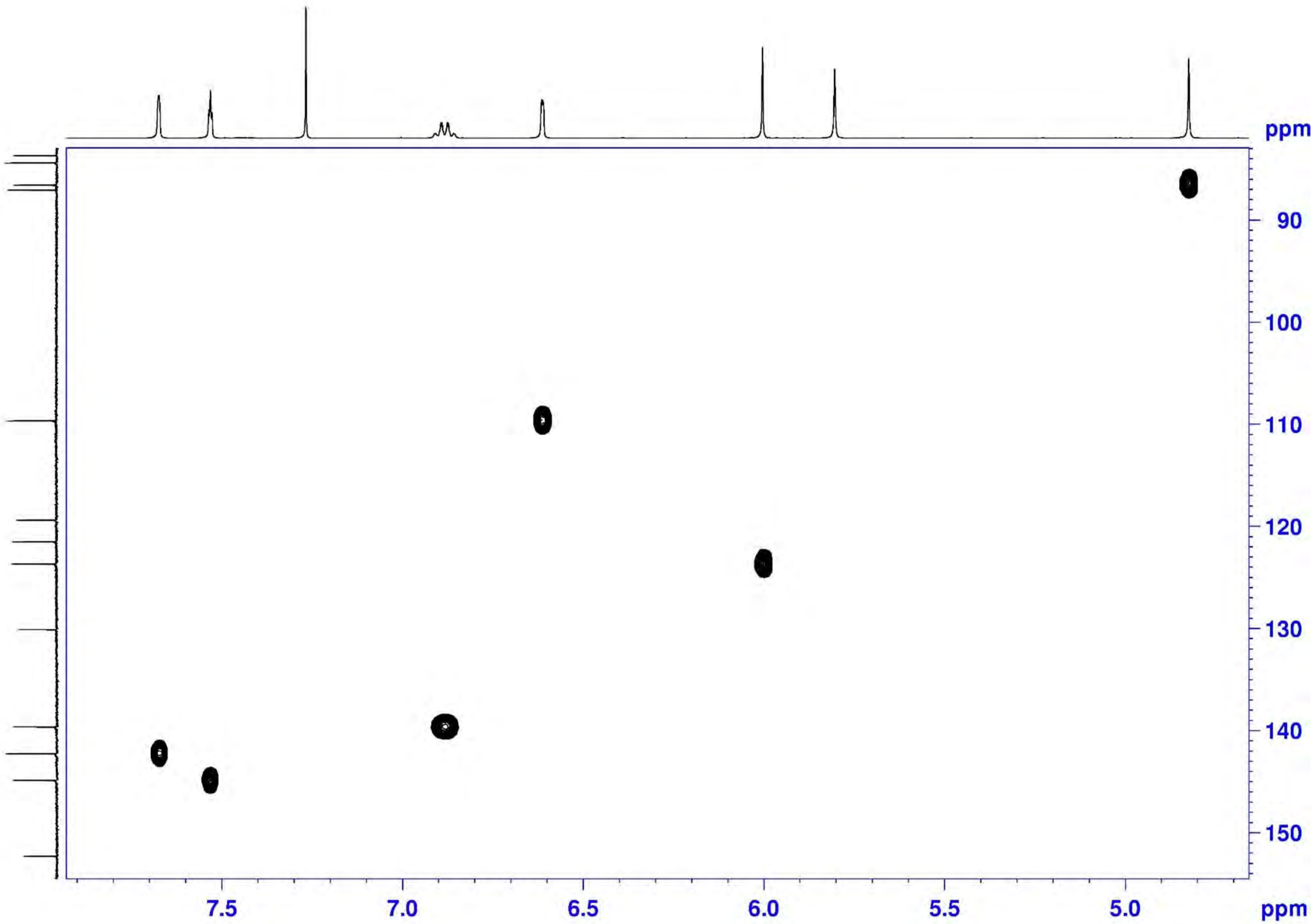

HSQC (400 MHz) spectrum of compound **27** in $CDCl_3$

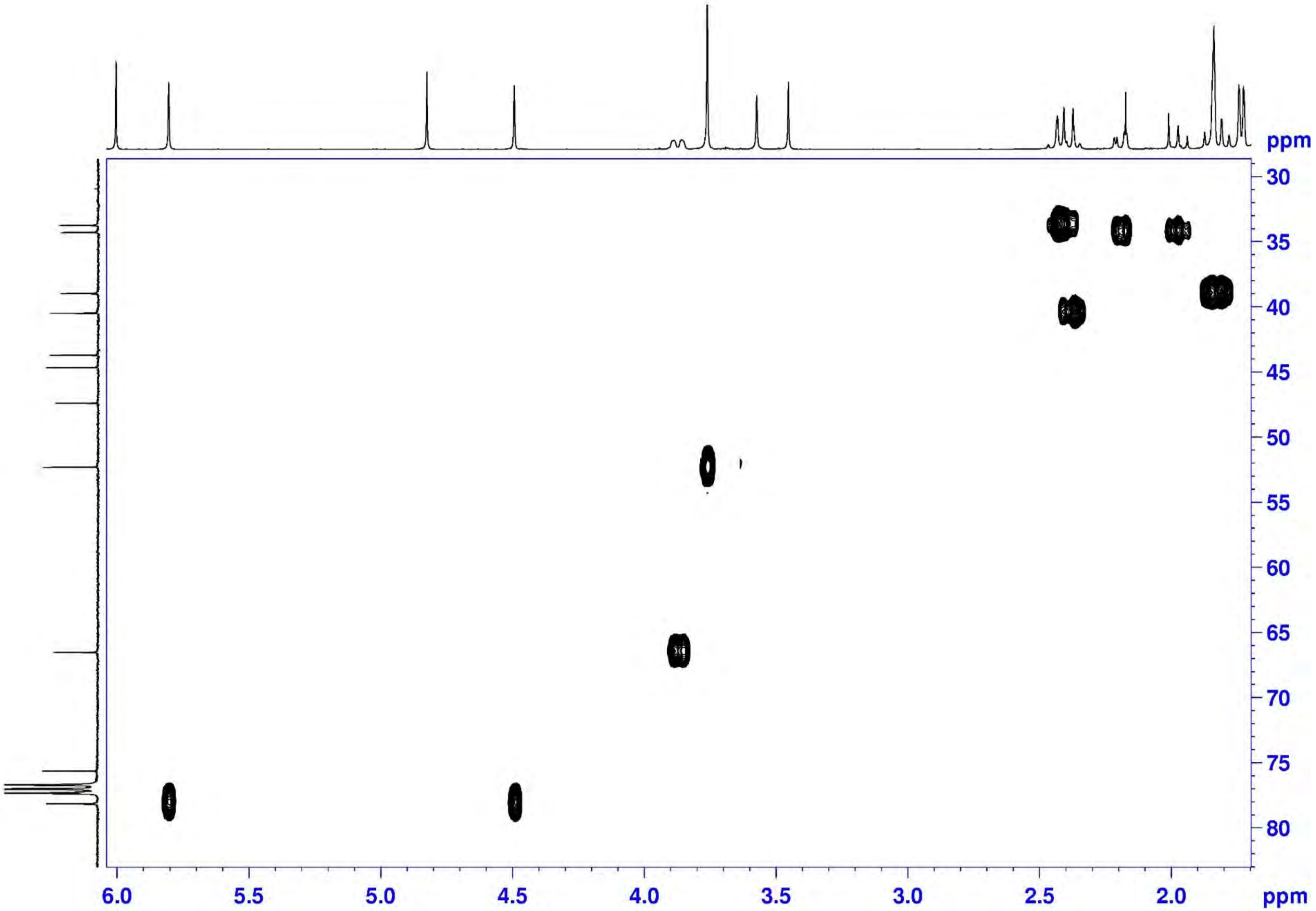

HSQC (400 MHz) spectrum of compound **27** in $CDCl_3$

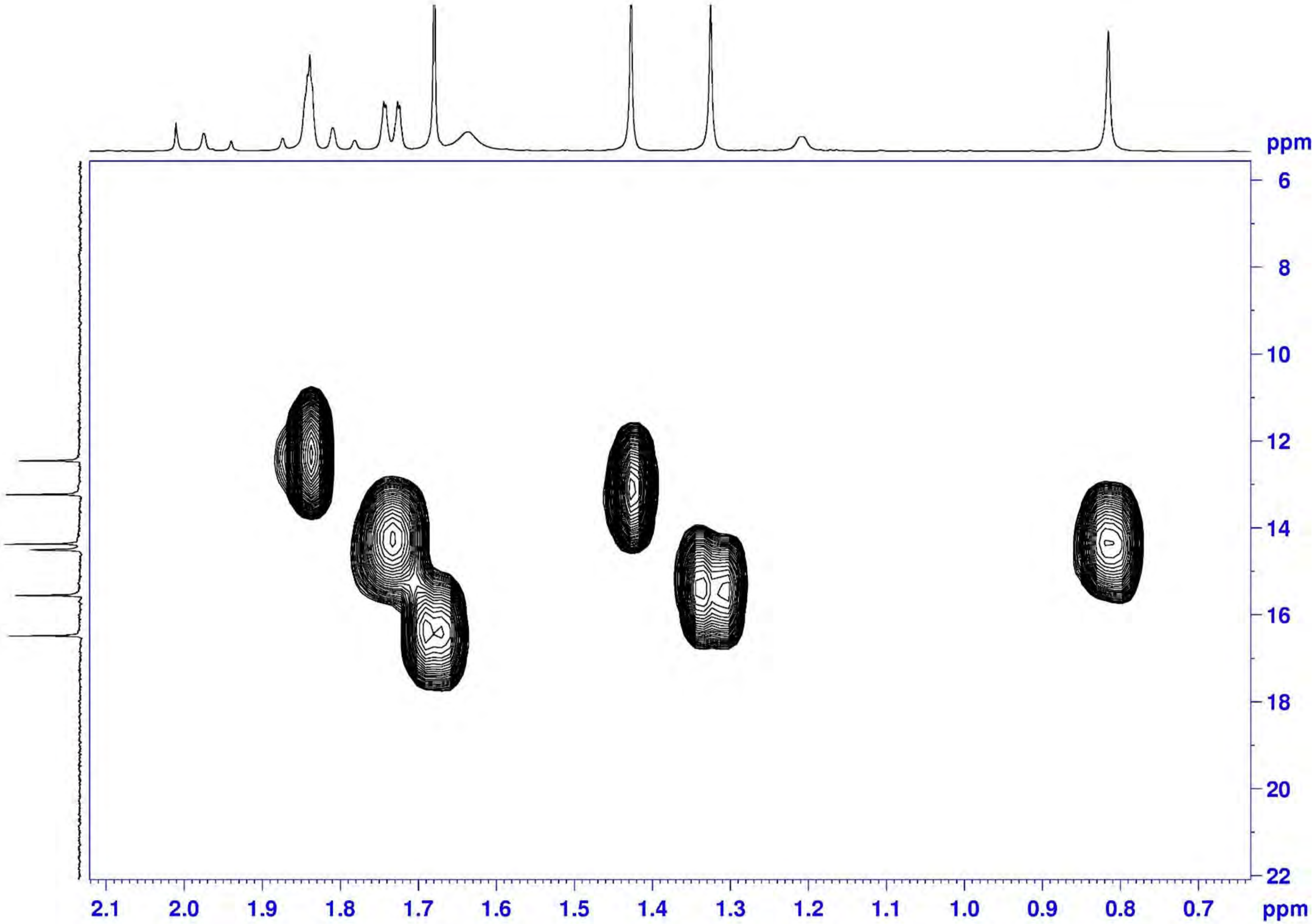

HMBC (400 MHz) spectrum of compound **27** in $CDCl_3$

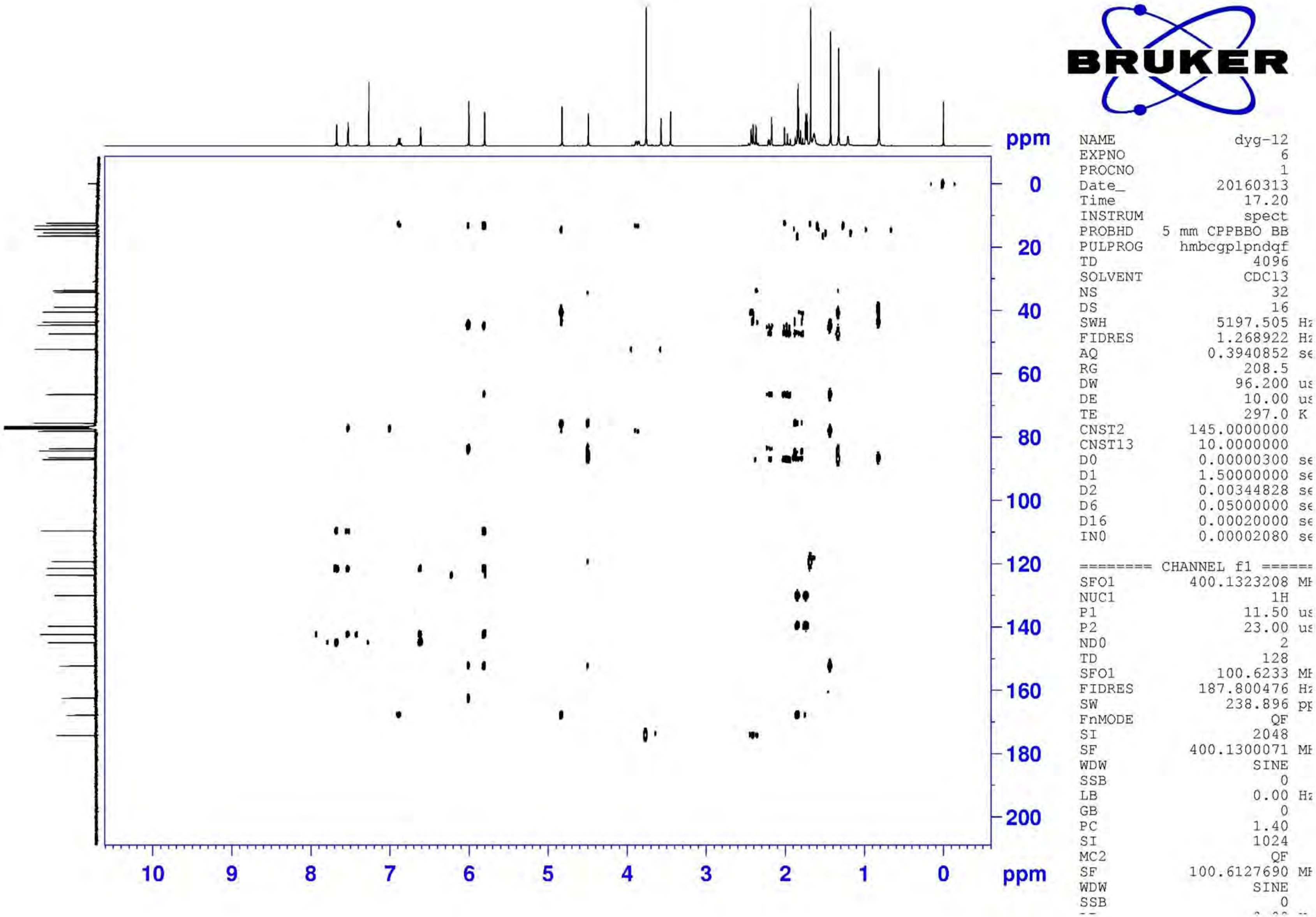

HMBC (400 MHz) spectrum of compound **27** in $CDCl_3$

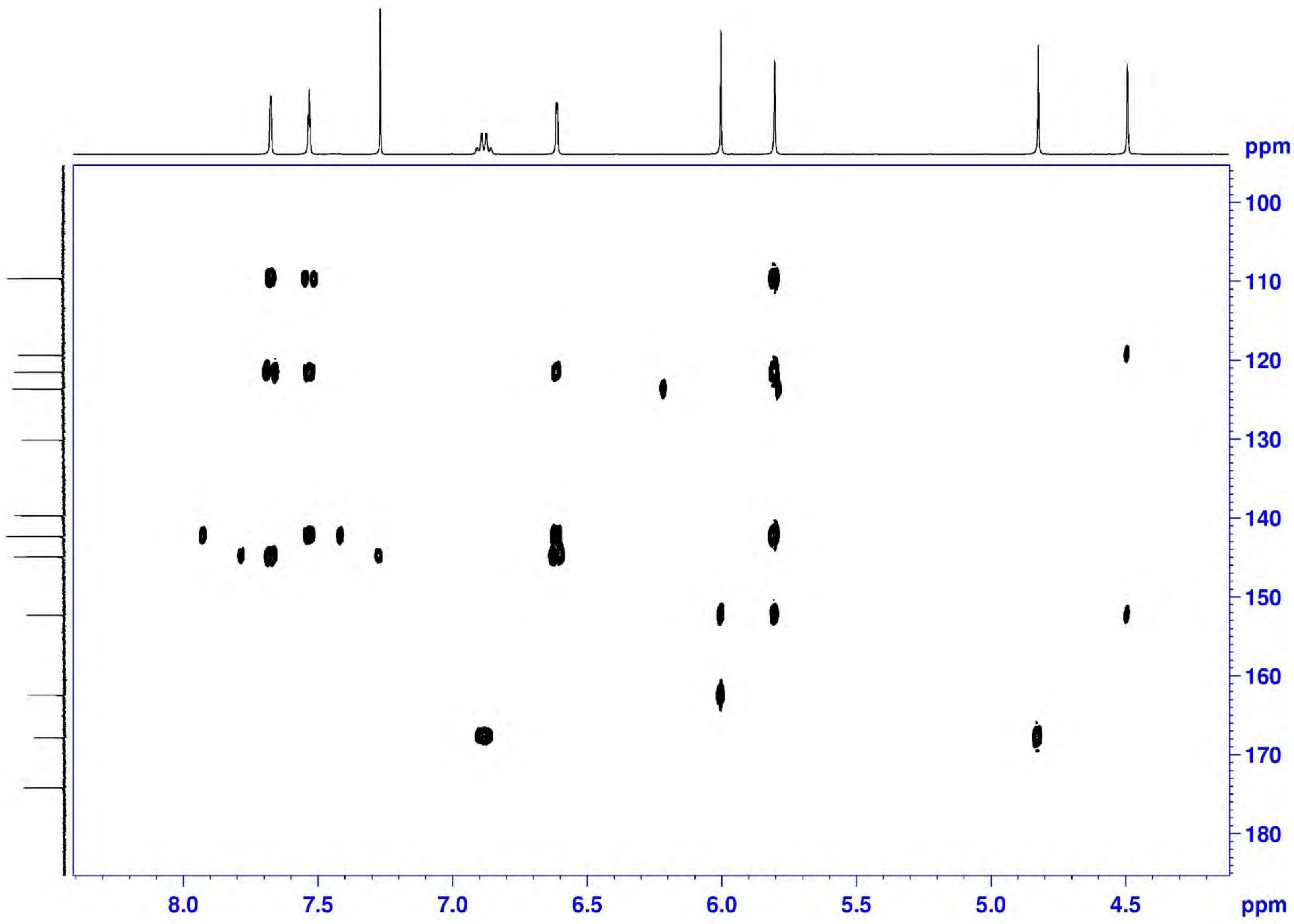

HMBC (400 MHz) spectrum of compound **27** in $CDCl_3$

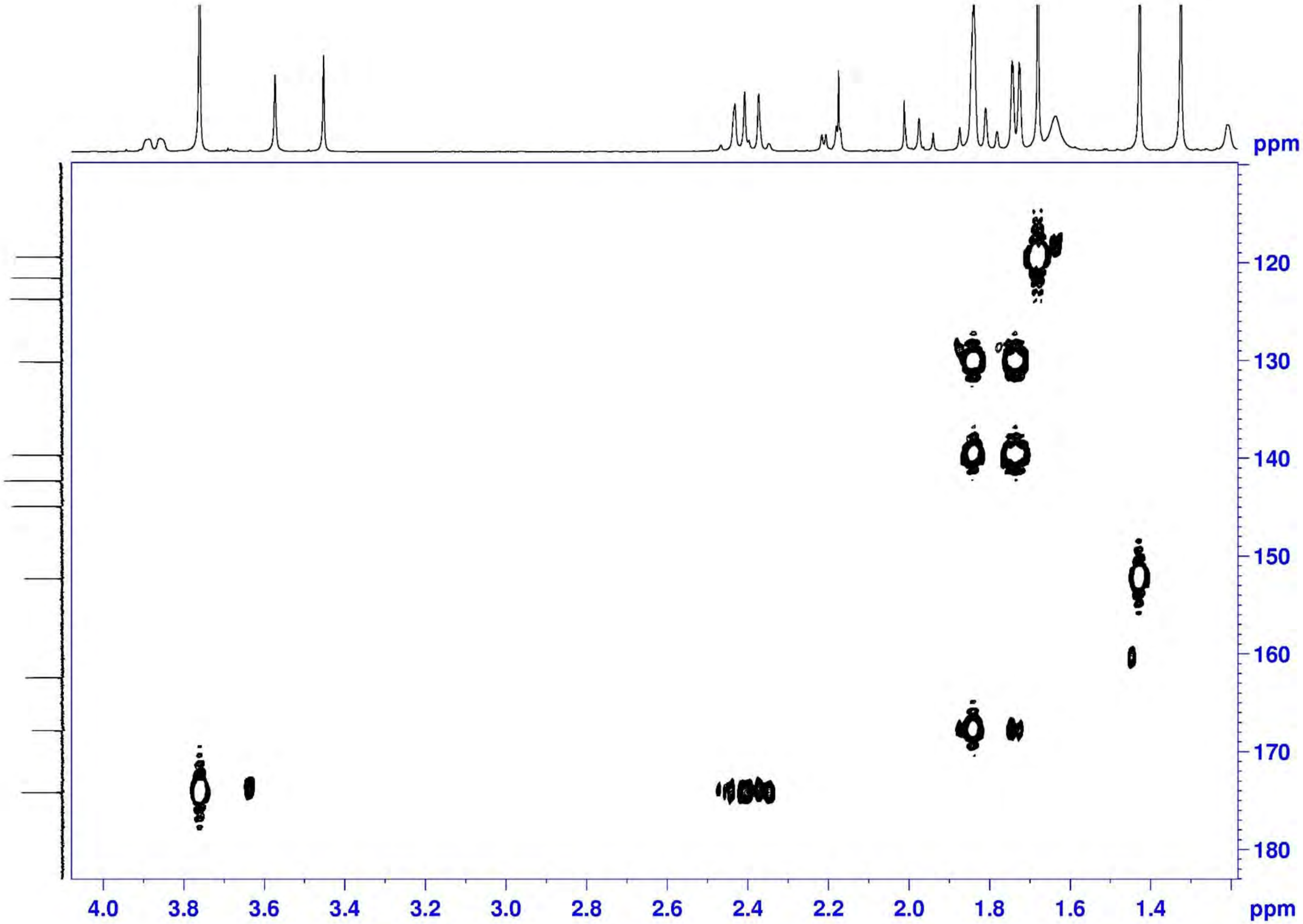

HMBC (400 MHz) spectrum of compound **27** in $CDCl_3$

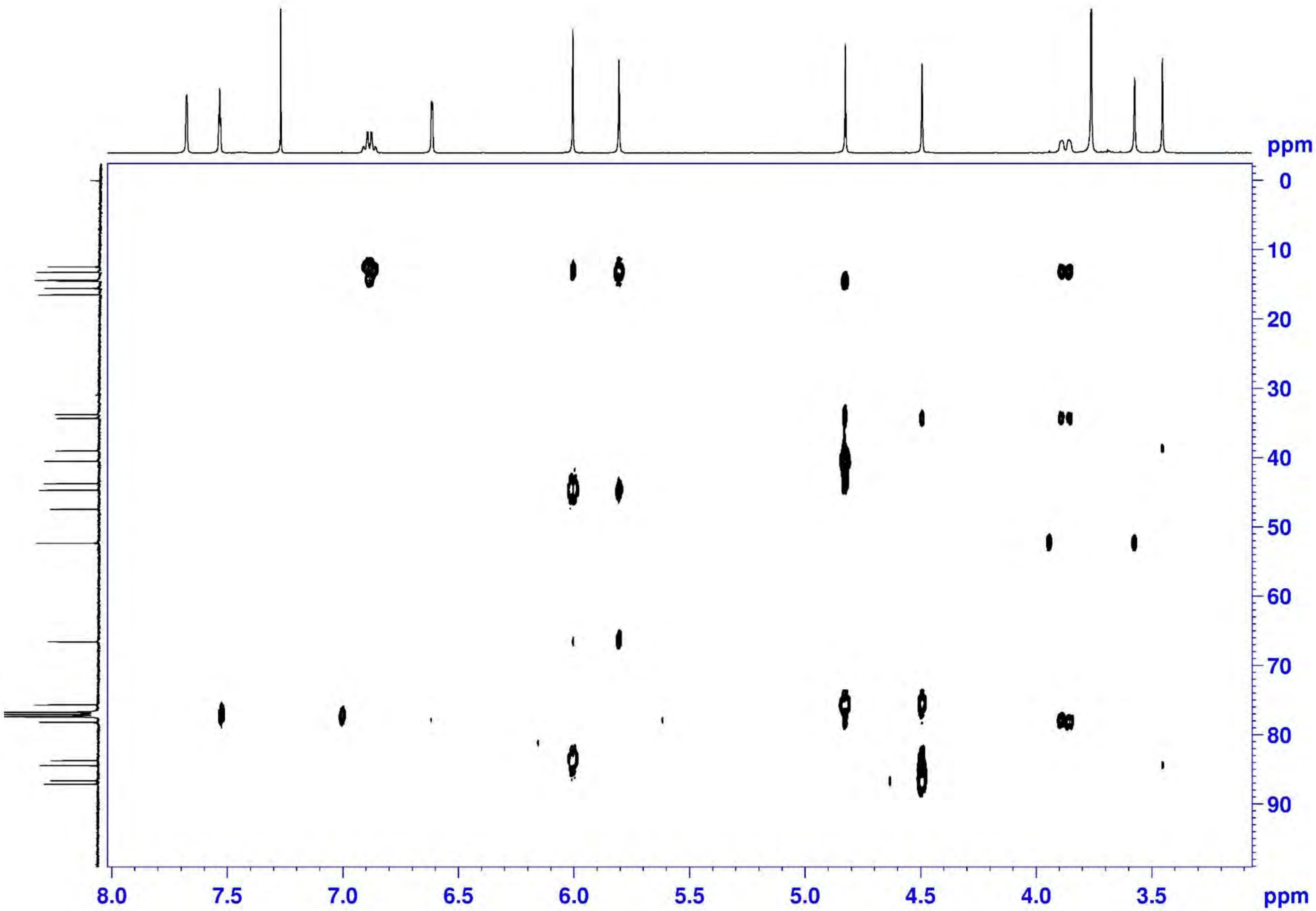

HMBC (400 MHz) spectrum of compound **27** in $CDCl_3$

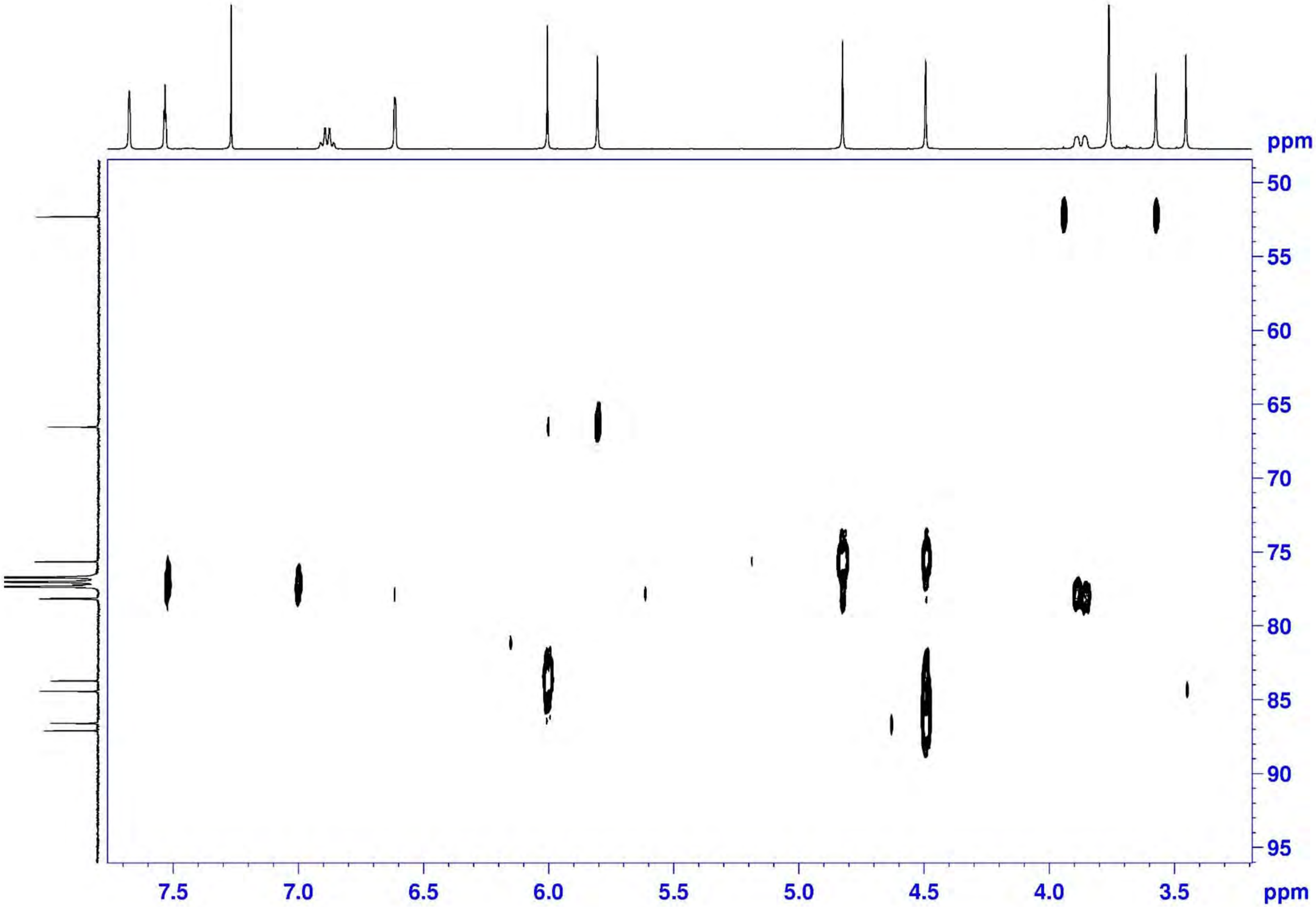

HMBC (400 MHz) spectrum of compound **27** in $CDCl_3$

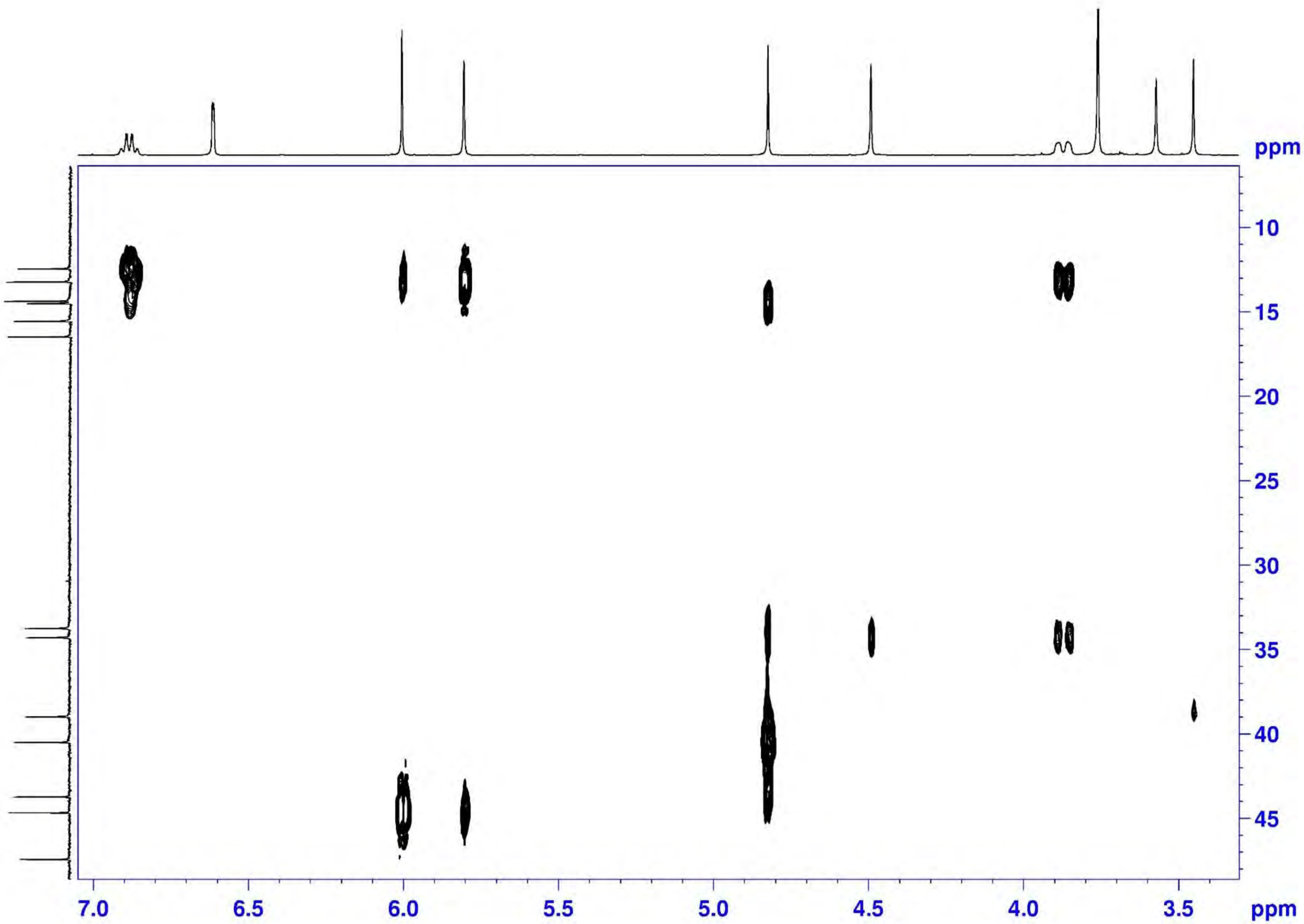

HMBC (400 MHz) spectrum of compound **27** in $CDCl_3$

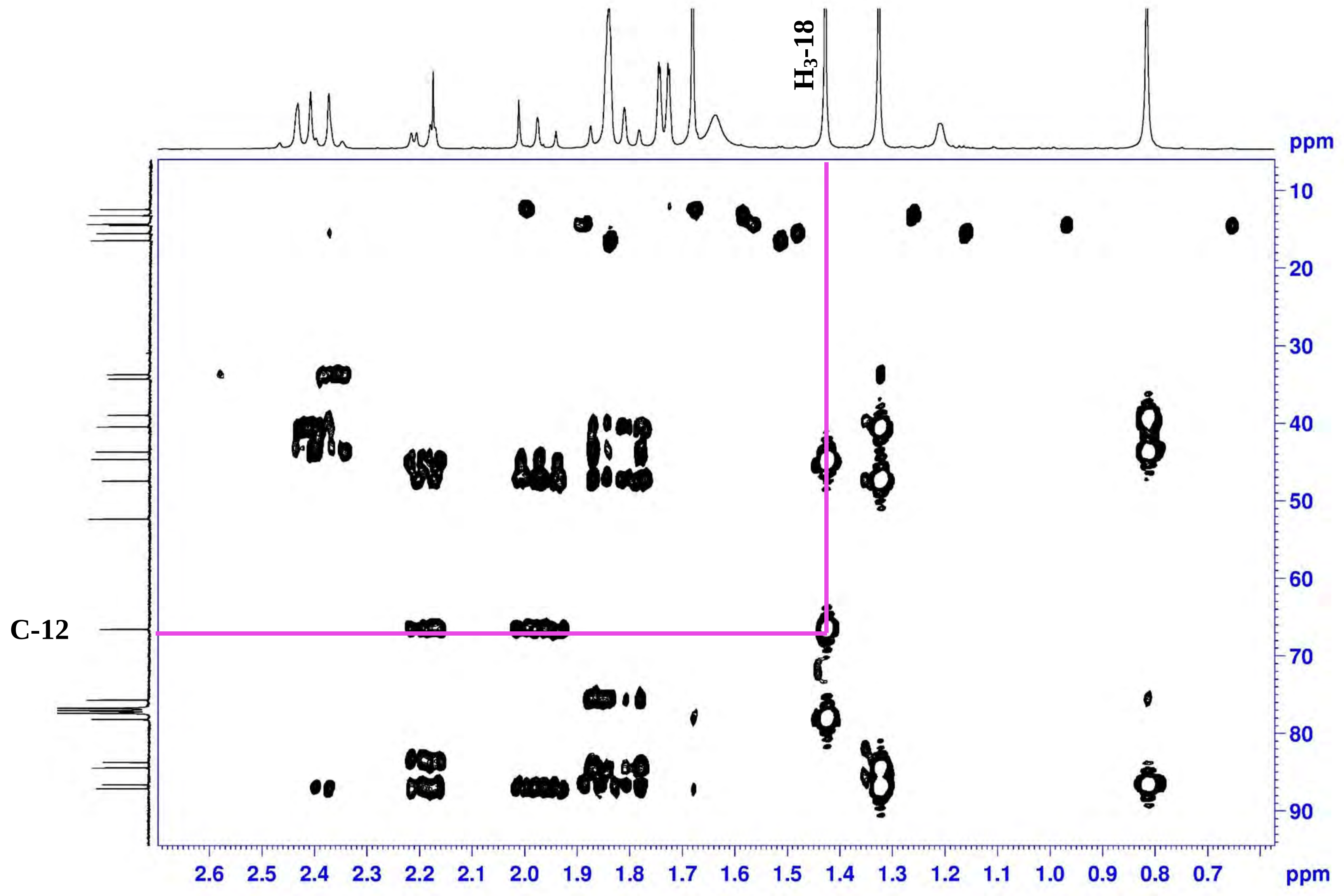

HMBC (400 MHz) spectrum of compound **27** in $CDCl_3$

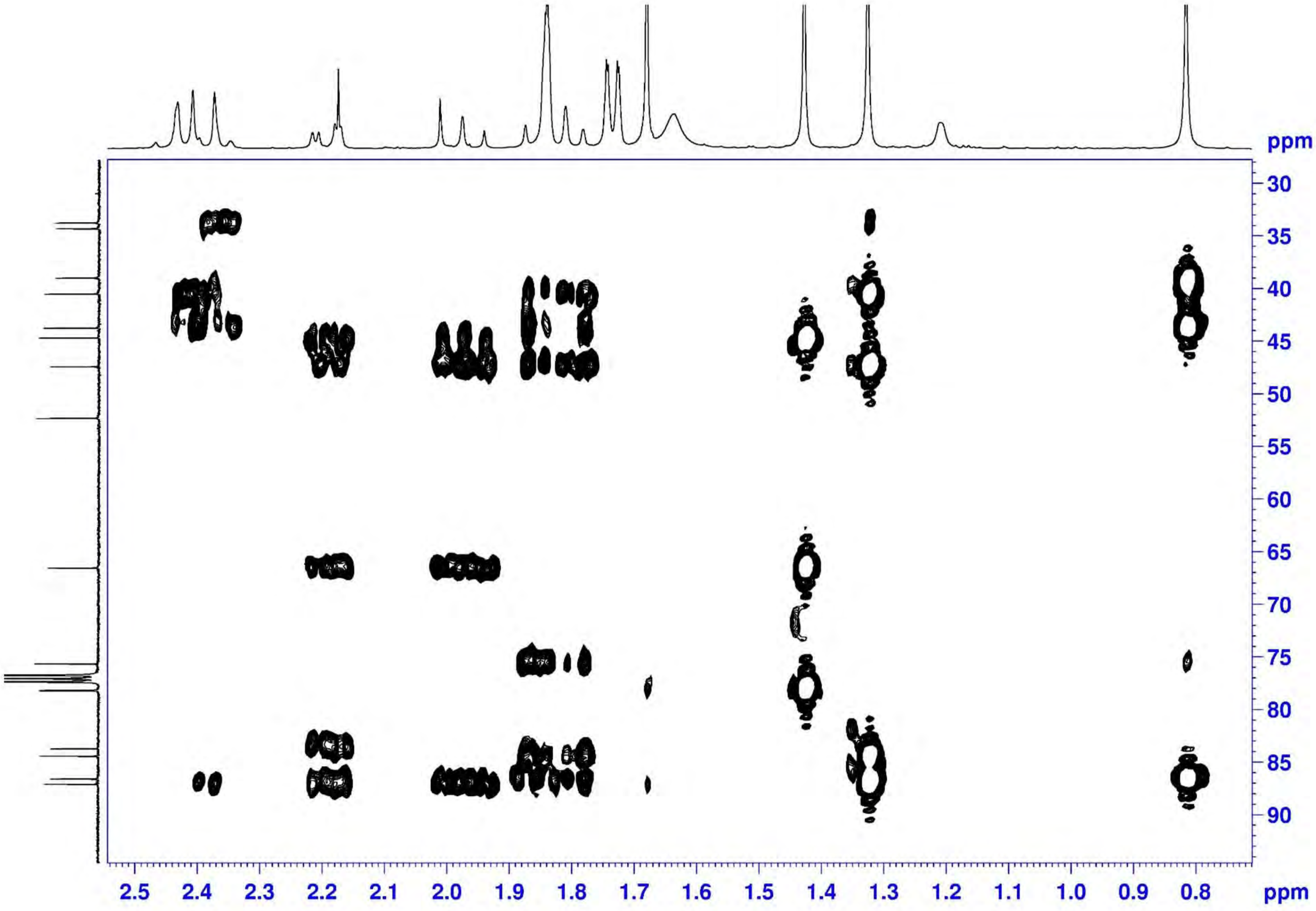

NOESY (400 MHz) spectrum of compound **27** in $CDCl_3$

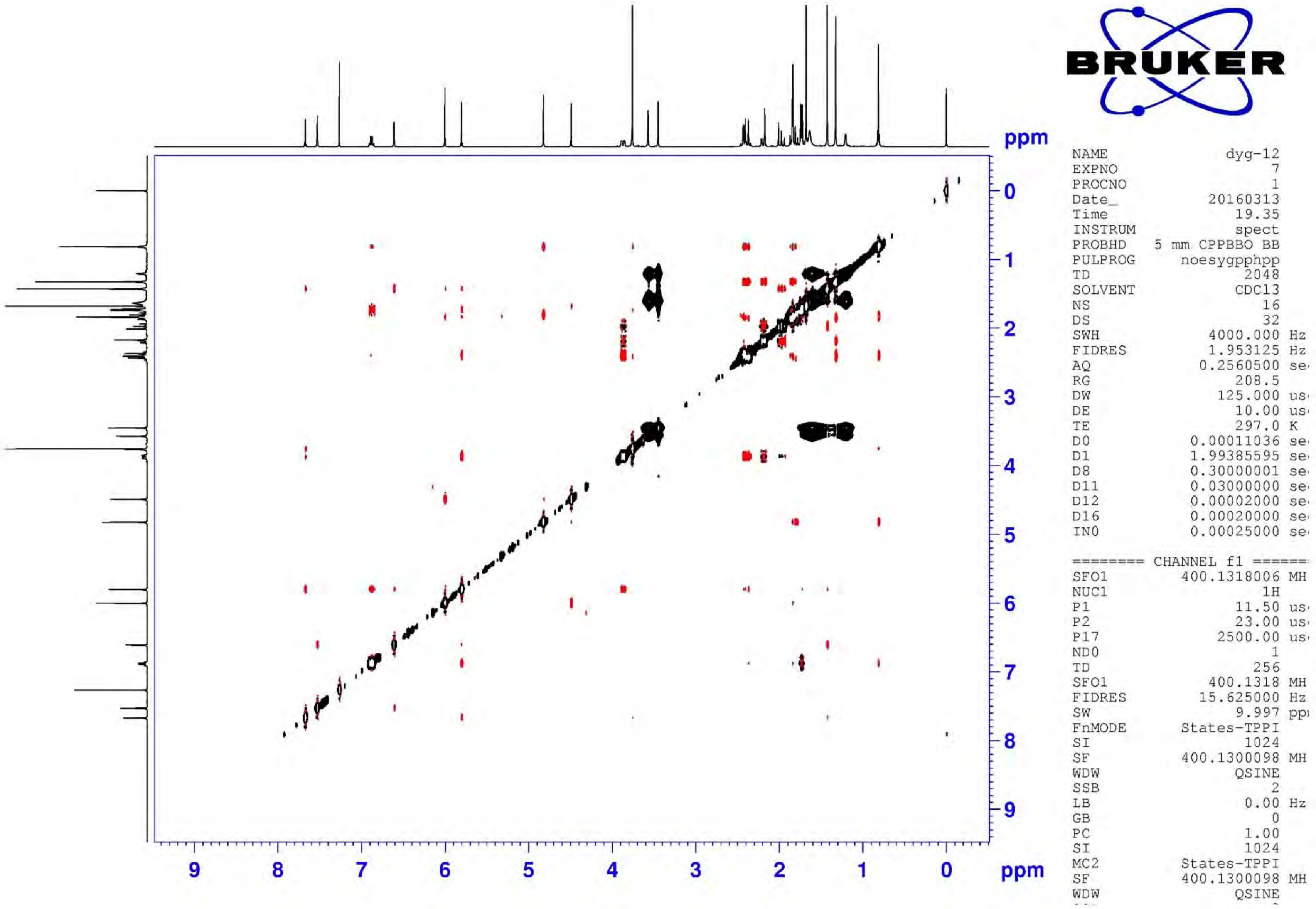

NOESY (400 MHz) spectrum of compound **27** in $CDCl_3$

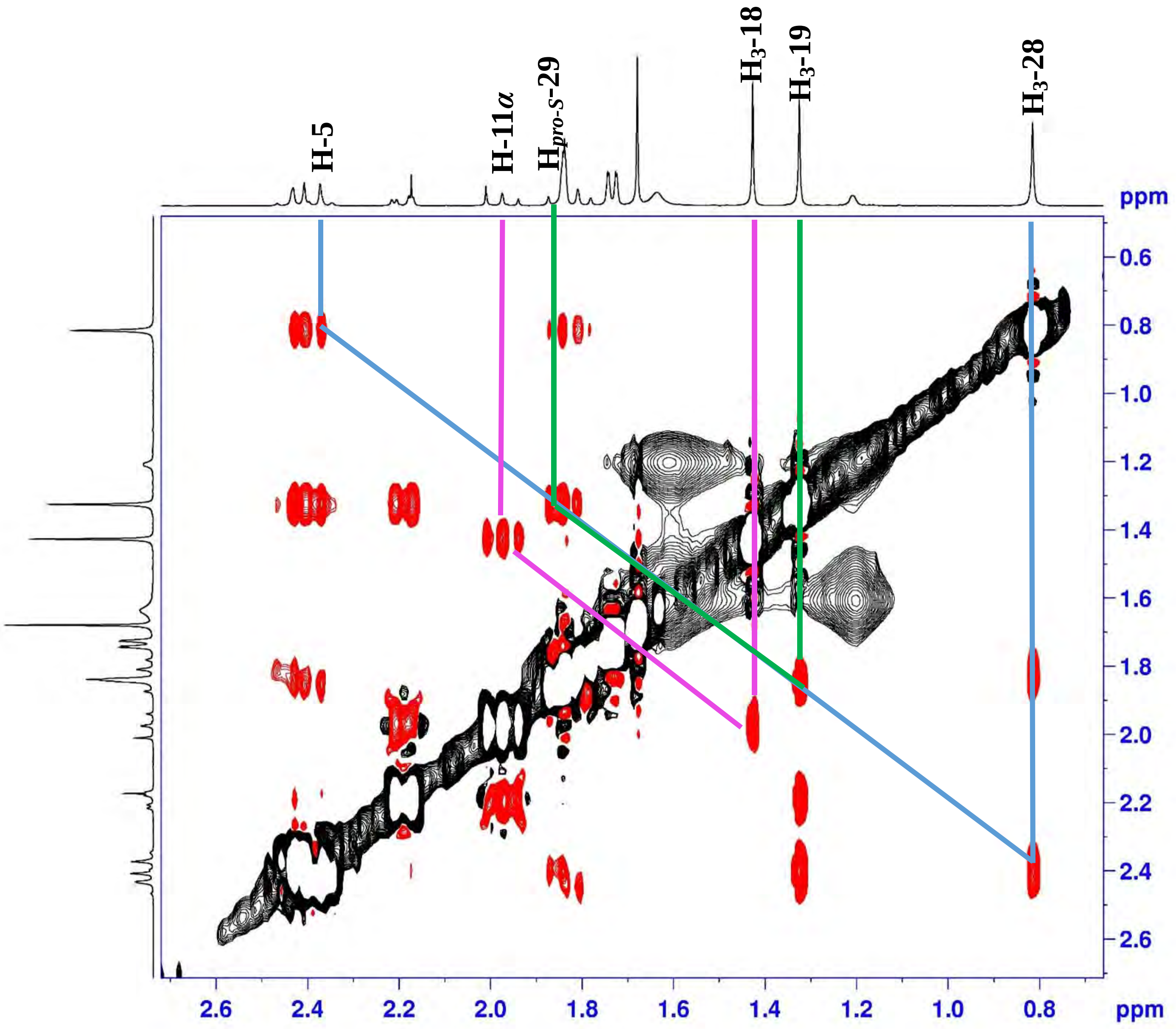

NOESY (400 MHz) spectrum of compound **27** in $CDCl_3$

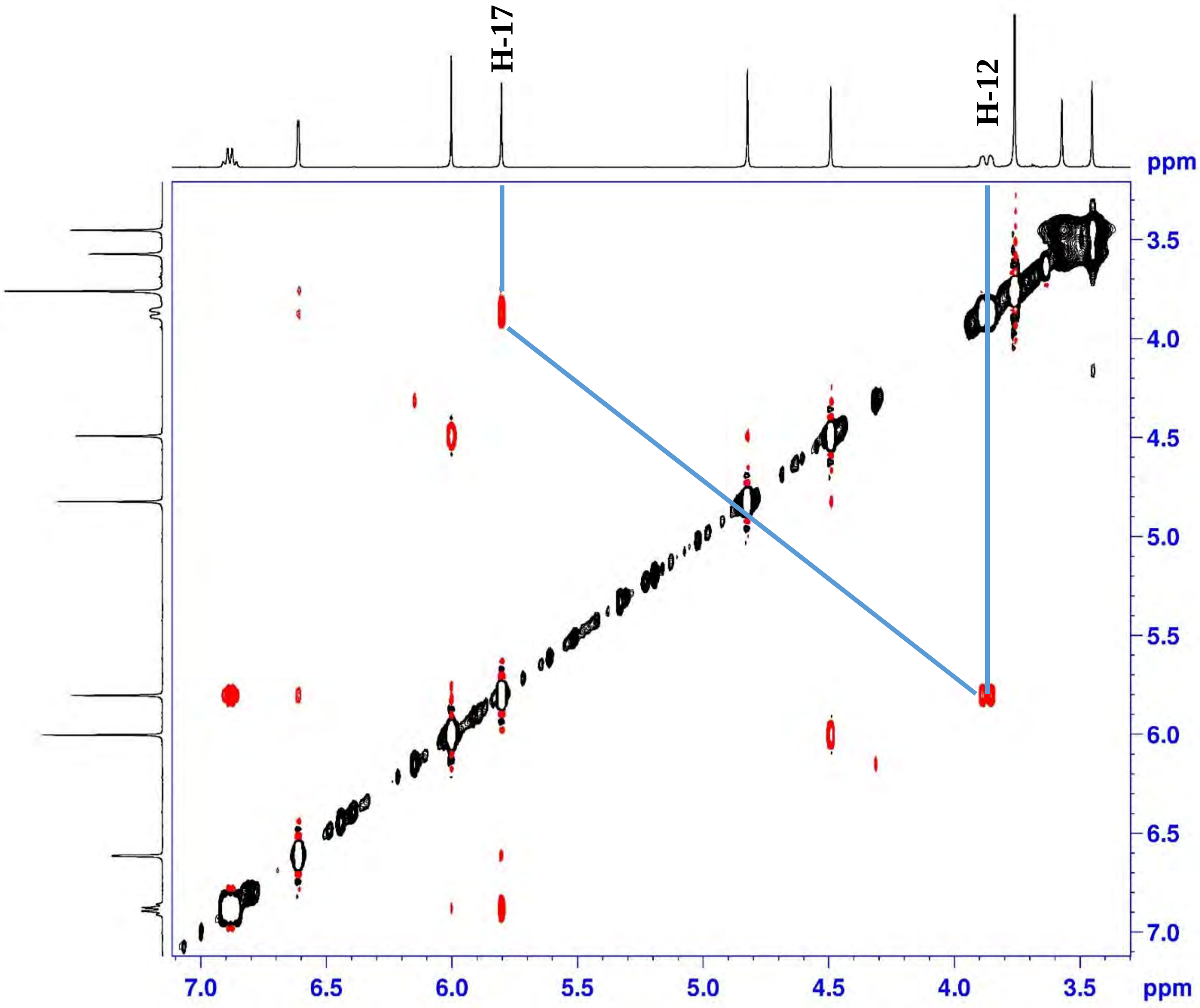

NOESY (400 MHz) spectrum of compound **27** in $CDCl_3$

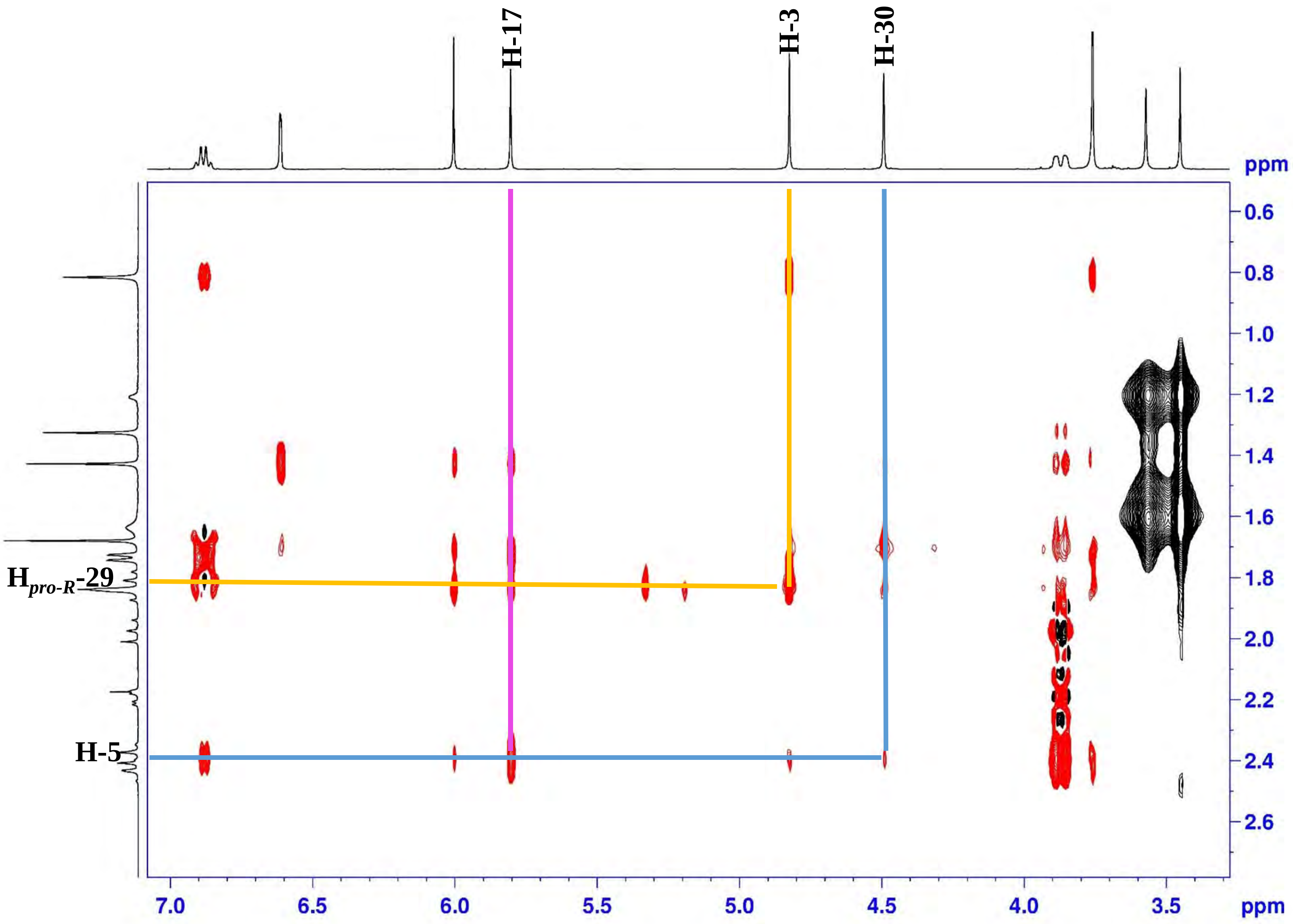

HRESIMS for compound **28**

Mass Spectrum SmartFormula Report

Analysis Info

Analysis Name	D:\Data\MS\data\201612\zhangjianzhi_zjz-23_pos_81_01_2734.d	Acquisition Date	12/12/2016 3:08:24 PM
Method	LC MS_Direct Infusion_pos_100-1000mz.m	Operator	SCSIO
Sample Name	zhangjianzhi_zjz-23_pos	Instrument	maXis 255552.00029
Comment			

Acquisition Parameter

Source Type	ESI	Ion Polarity	Positive	Set Nebulizer	0.4 Bar
Focus	Active	Set Capillary	4500 V	Set Dry Heater	180 °C
Scan Begin	100 m/z	Set End Plate Offset	-500 V	Set Dry Gas	4.0 l/min
Scan End	2000 m/z	Set Charging Voltage	0 V	Set Divert Valve	Waste
		Set Corona	0 nA	Set APCI Heater	0 °C

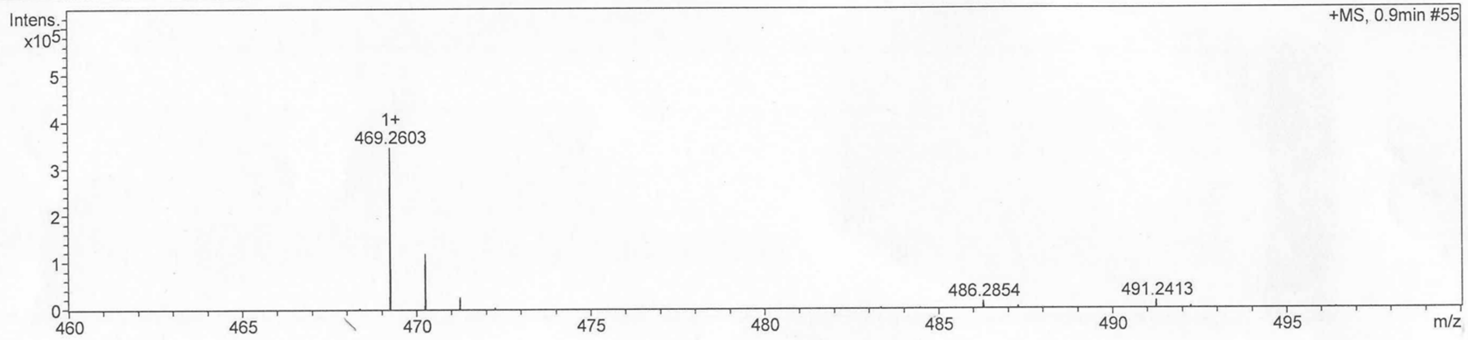

Meas. m/z	#	Ion Formula	Score	m/z	err [ppm]	err [mDa]	mSigma	rdb	e⁻ Conf	N-Rule
469.260338	1	C28H37O6	100.00	469.258465	4.0	1.9	19.5	10.5	even	ok
491.241289	1	C28H36NaO6	100.00	491.240410	1.8	0.9	7.2	10.5	even	ok
937.510657	1	C56H73O12	100.00	937.509654	-1.1	-1.0	31.6	20.5	even	ok
959.493824	1	C56H72NaO12	100.00	959.491598	-2.3	-2.2	42.8	20.5	even	ok

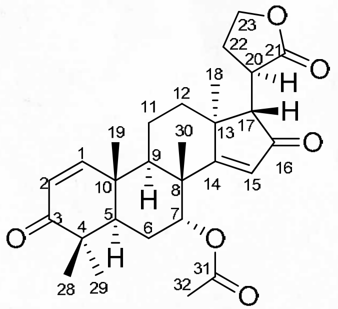

zhangjianzhi_zjz-23_pos_81_01_2734.d

Bruker Compass DataAnalysis 4.1 printed: 12/12/2016 3:13:36 PM by: SCSIO Page 1 of 1

HRESIMS for compound **28**

Generic Display Report

Analysis Info

Analysis Name	D:\Data\MS\data\201612\zhangjianzhi_zjz-23_pos_81_01_2734.d	Acquisition Date	12/12/2016 3:08:24 PM
Method	LC MS_Direct Infusion_pos_100-1000mz.m	Operator	SCSIO
Sample Name	zhangjianzhi_zjz-23_pos	Instrument	maXis
Comment			

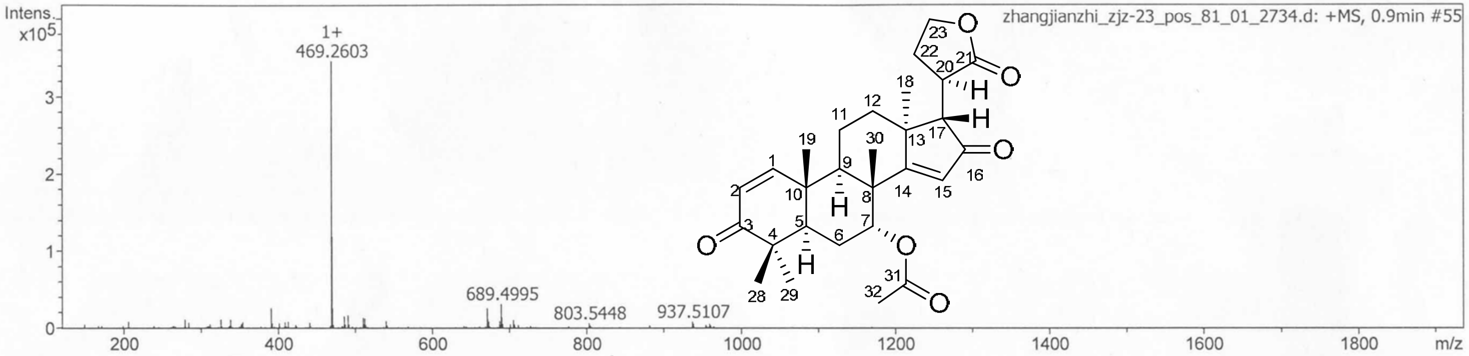

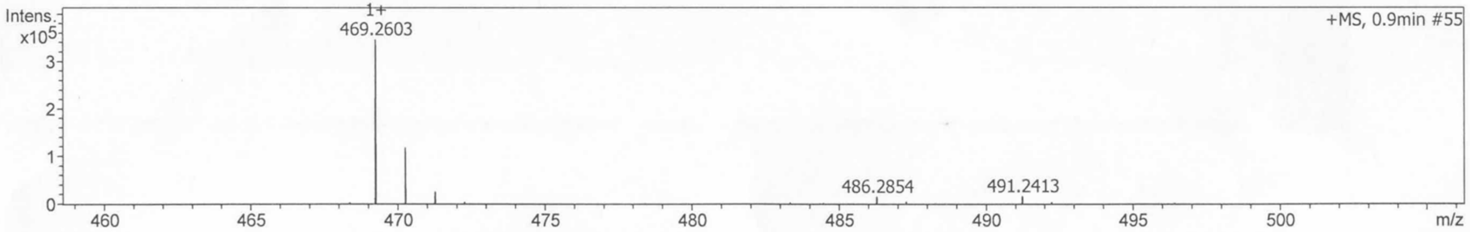

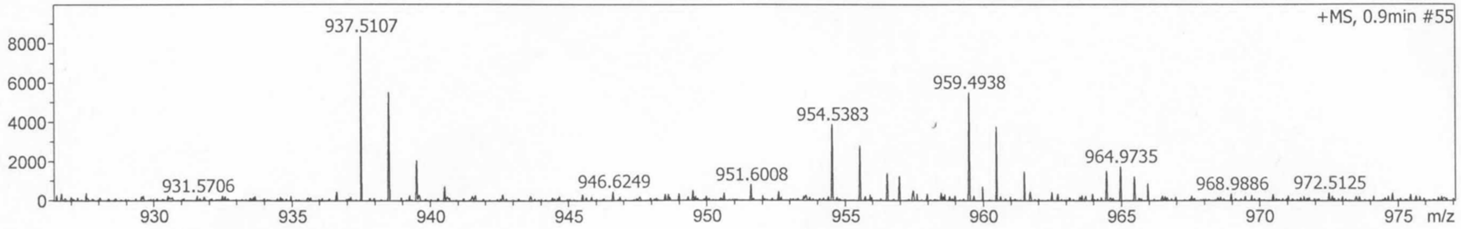

Bruker Compass DataAnalysis 4.1 printed: 12/12/2016 3:14:28 PM by: SCSIO Page 1 of 1

^{1}H NMR (400 MHz) spectrum of compound **28** in $CDCl_3$

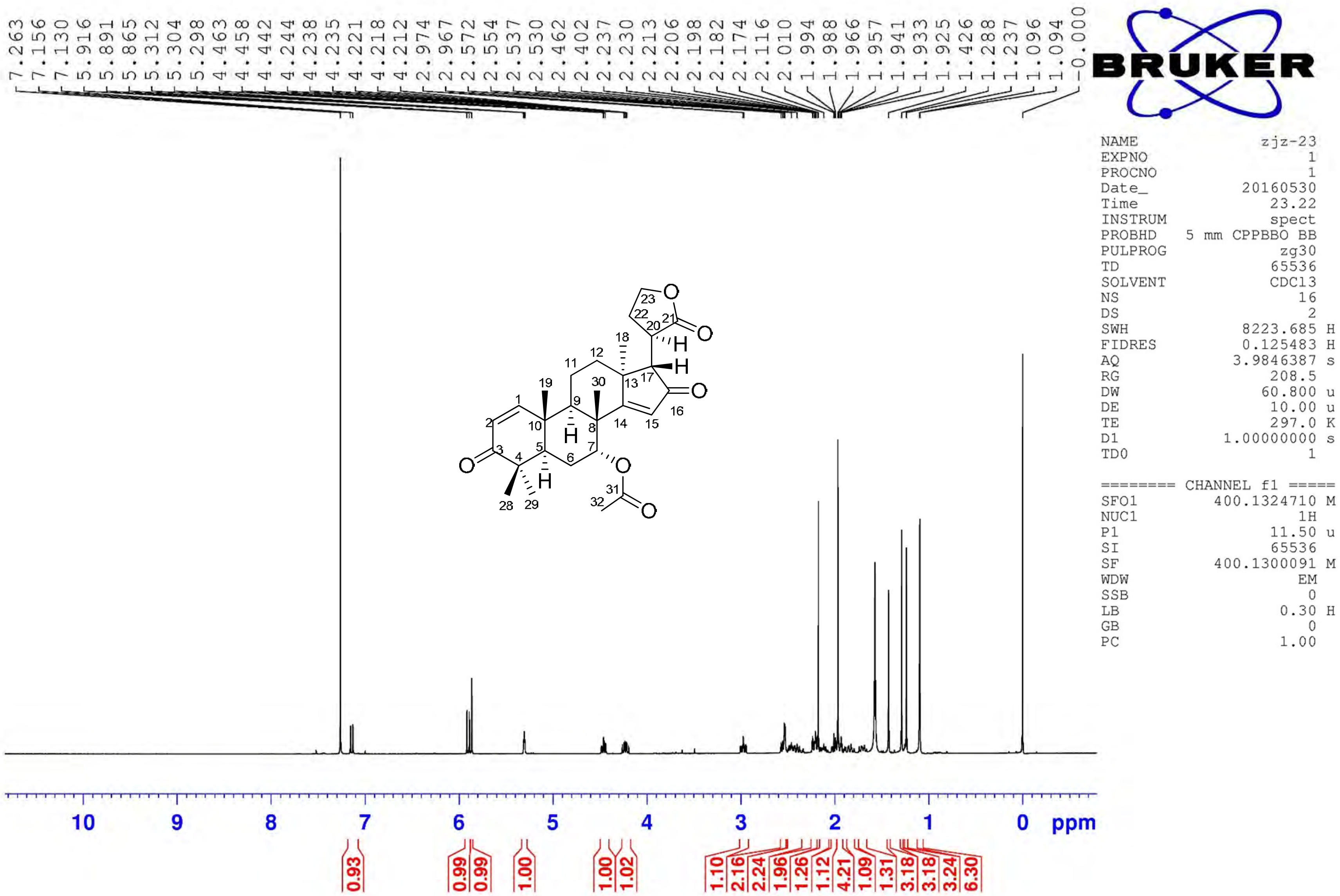

^{1}H NMR (400 MHz) spectrum of compound **28** in $CDCl_3$

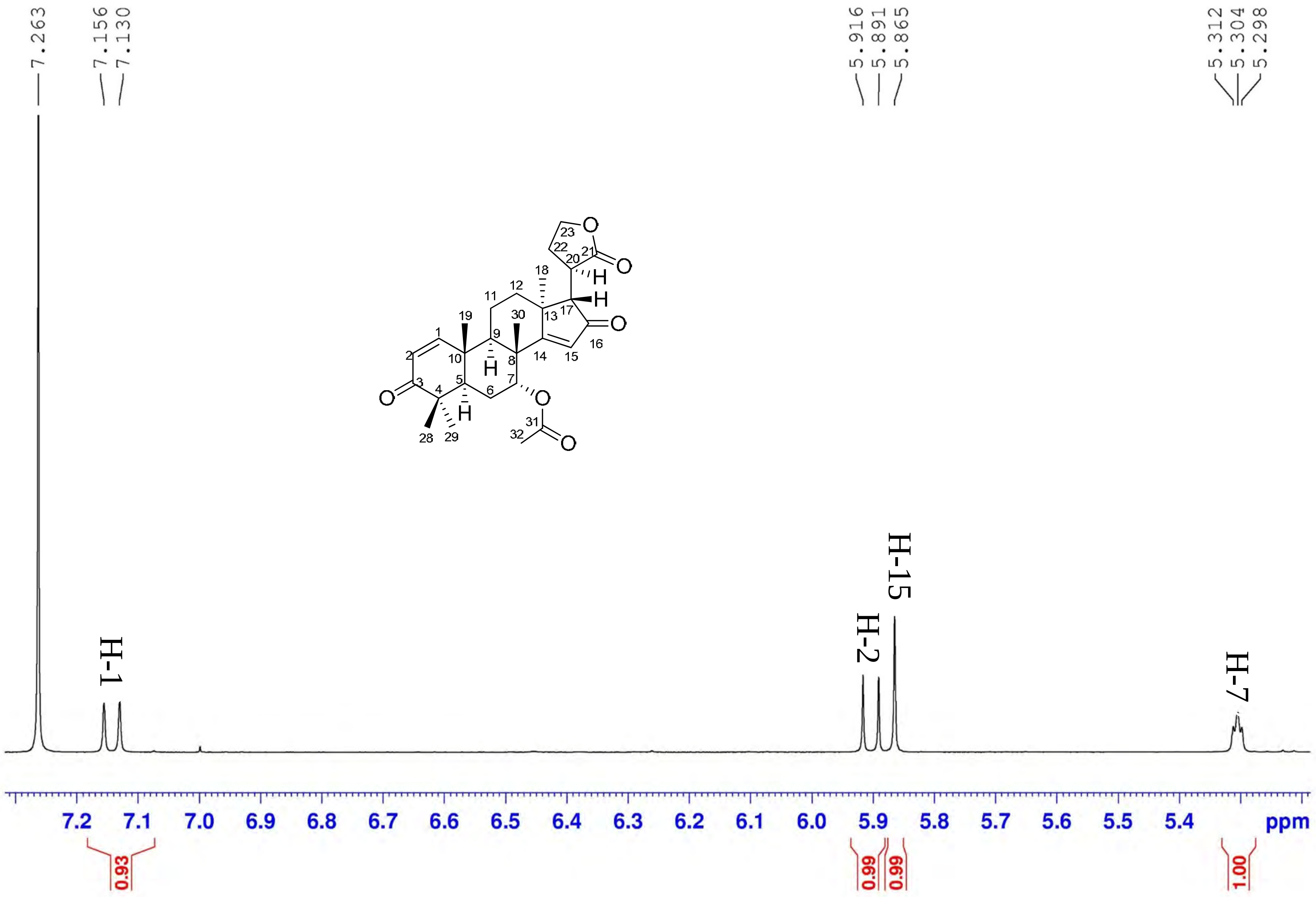

¹H NMR (400 MHz) spectrum of compound **28** in $CDCl_3$

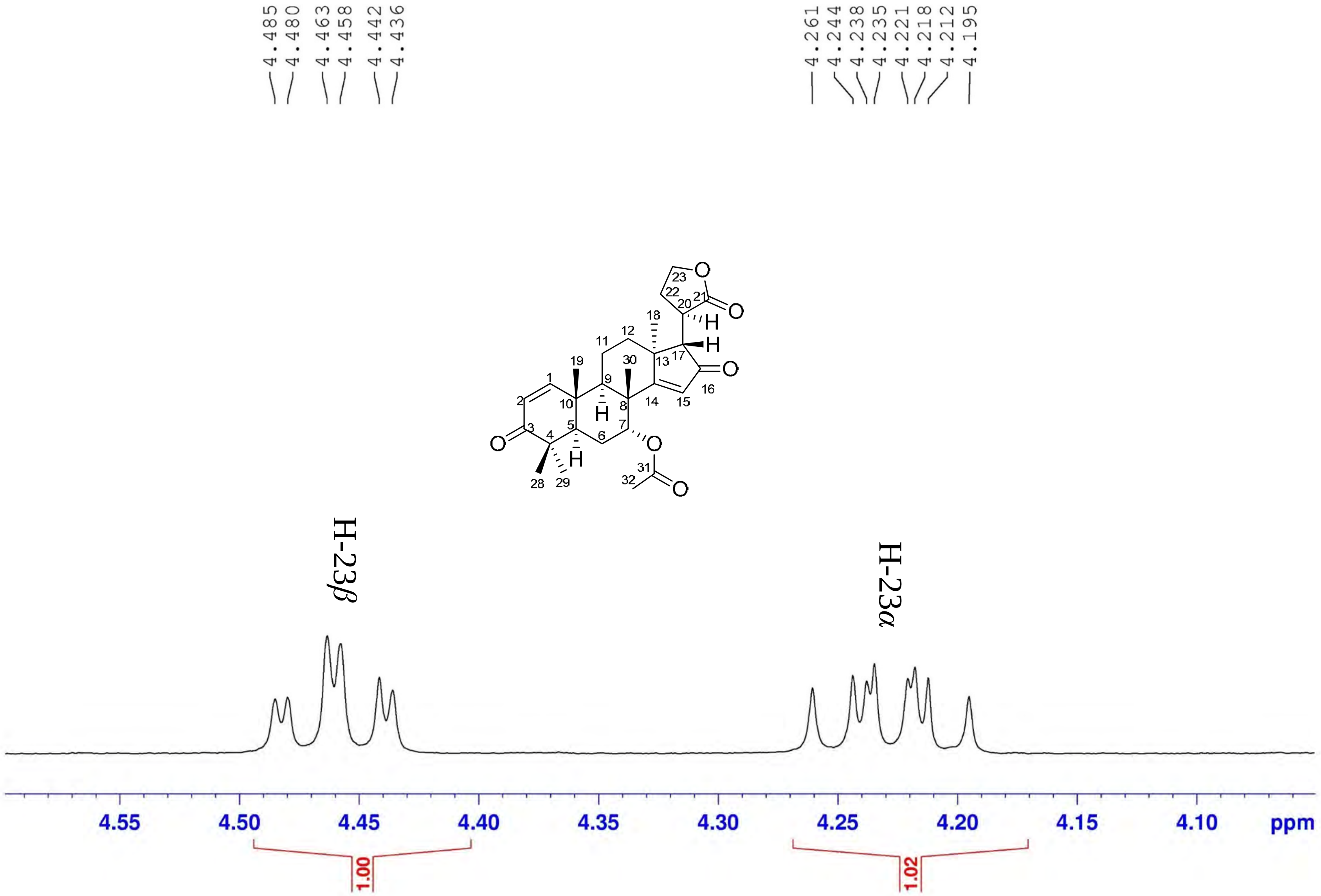

^1H NMR (400 MHz) spectrum of compound **28** in $CDCl_3$

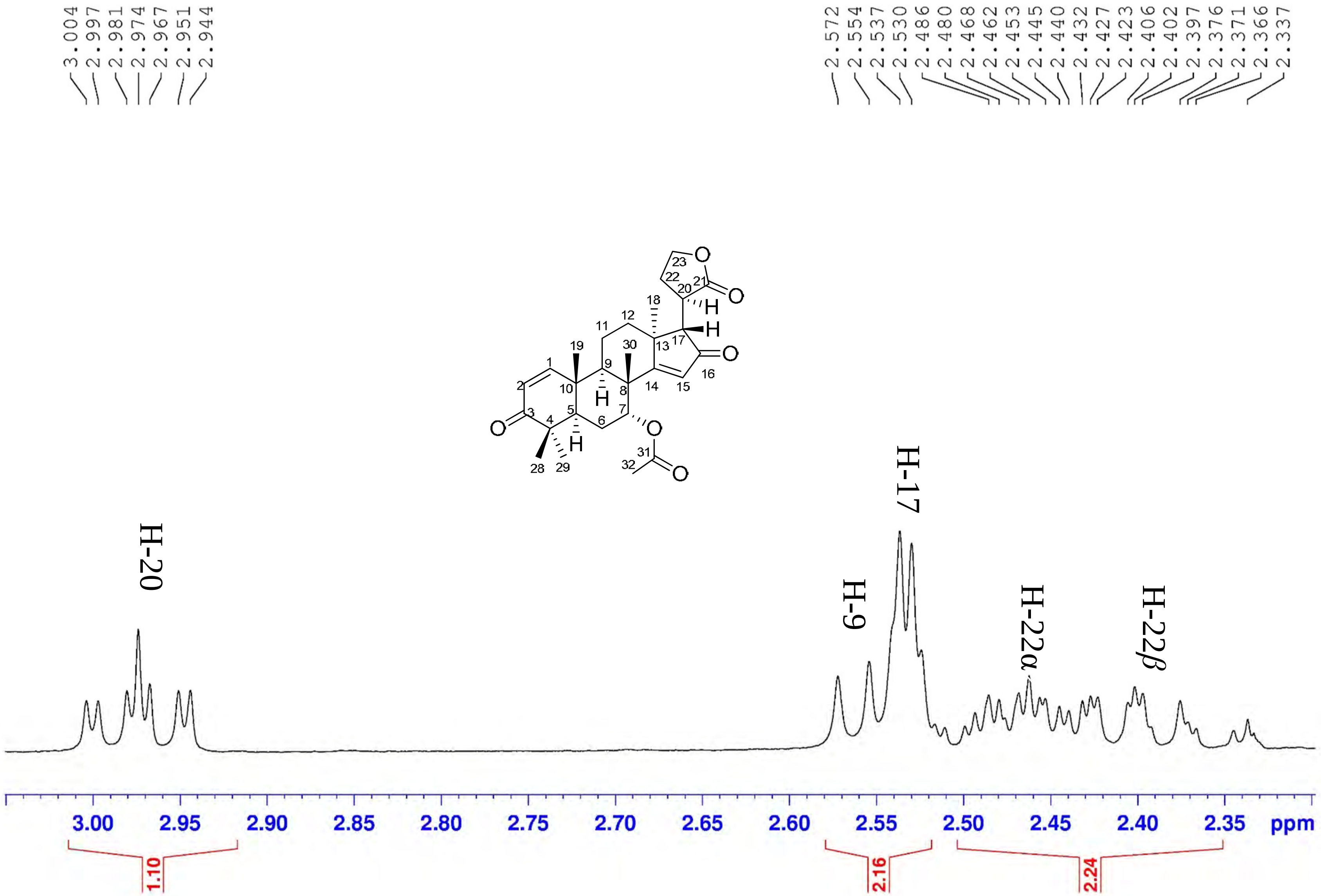

^{1}H NMR (400 MHz) spectrum of compound **28** in $CDCl_3$

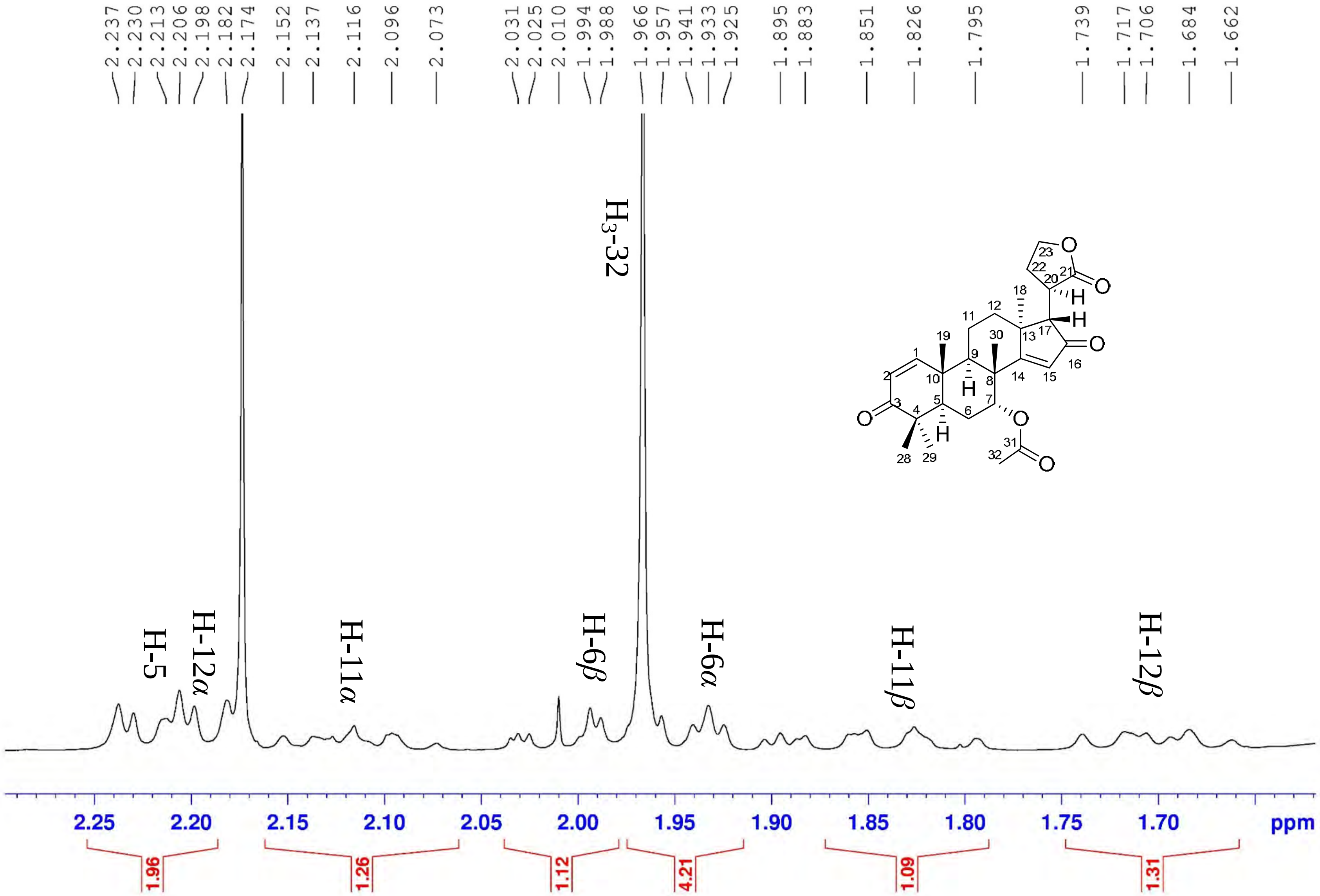

^{1}H NMR (400 MHz) spectrum of compound **28** in $CDCl_3$

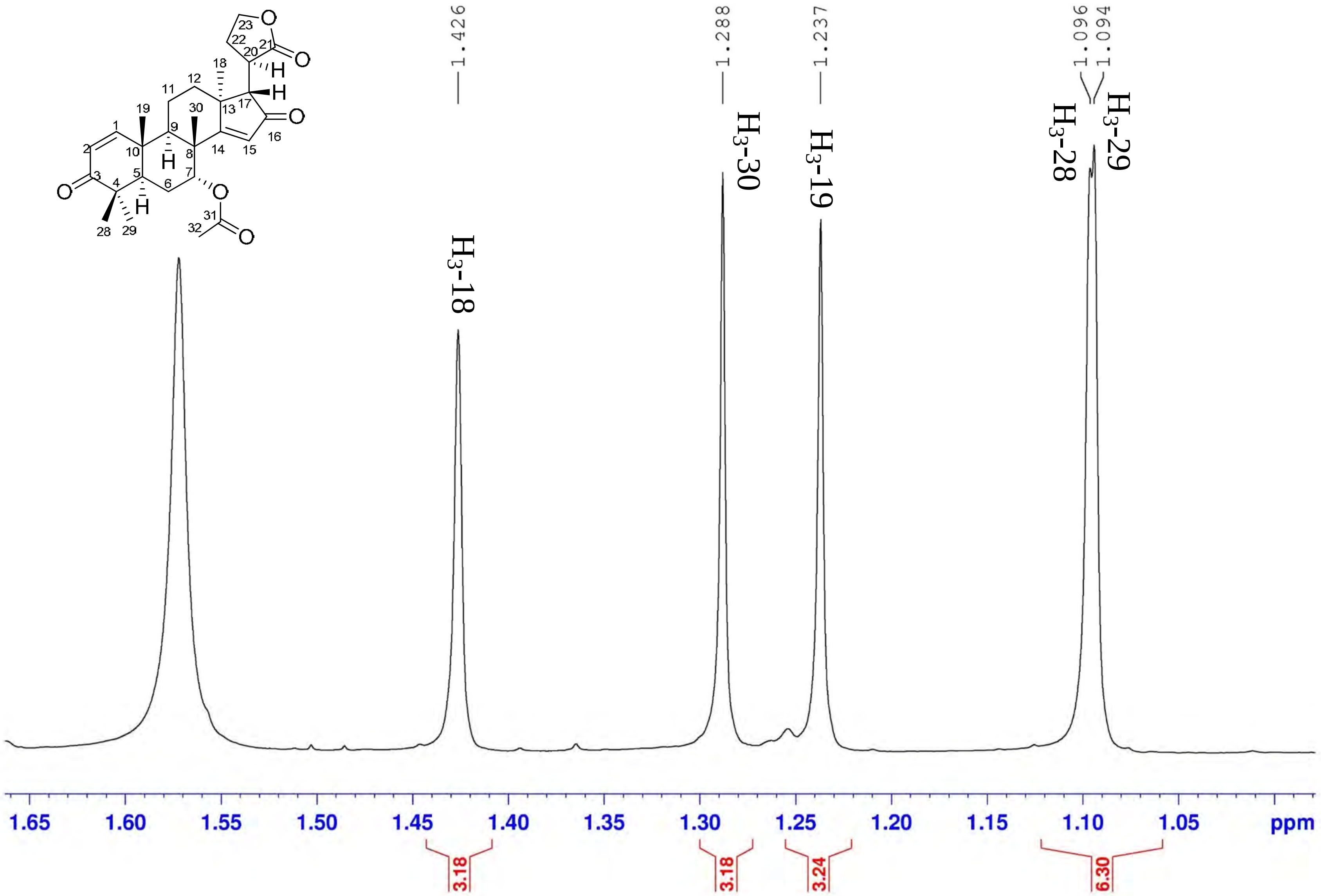

^{13}C NMR (100 MHz) spectrum of compound **28** in $CDCl_3$

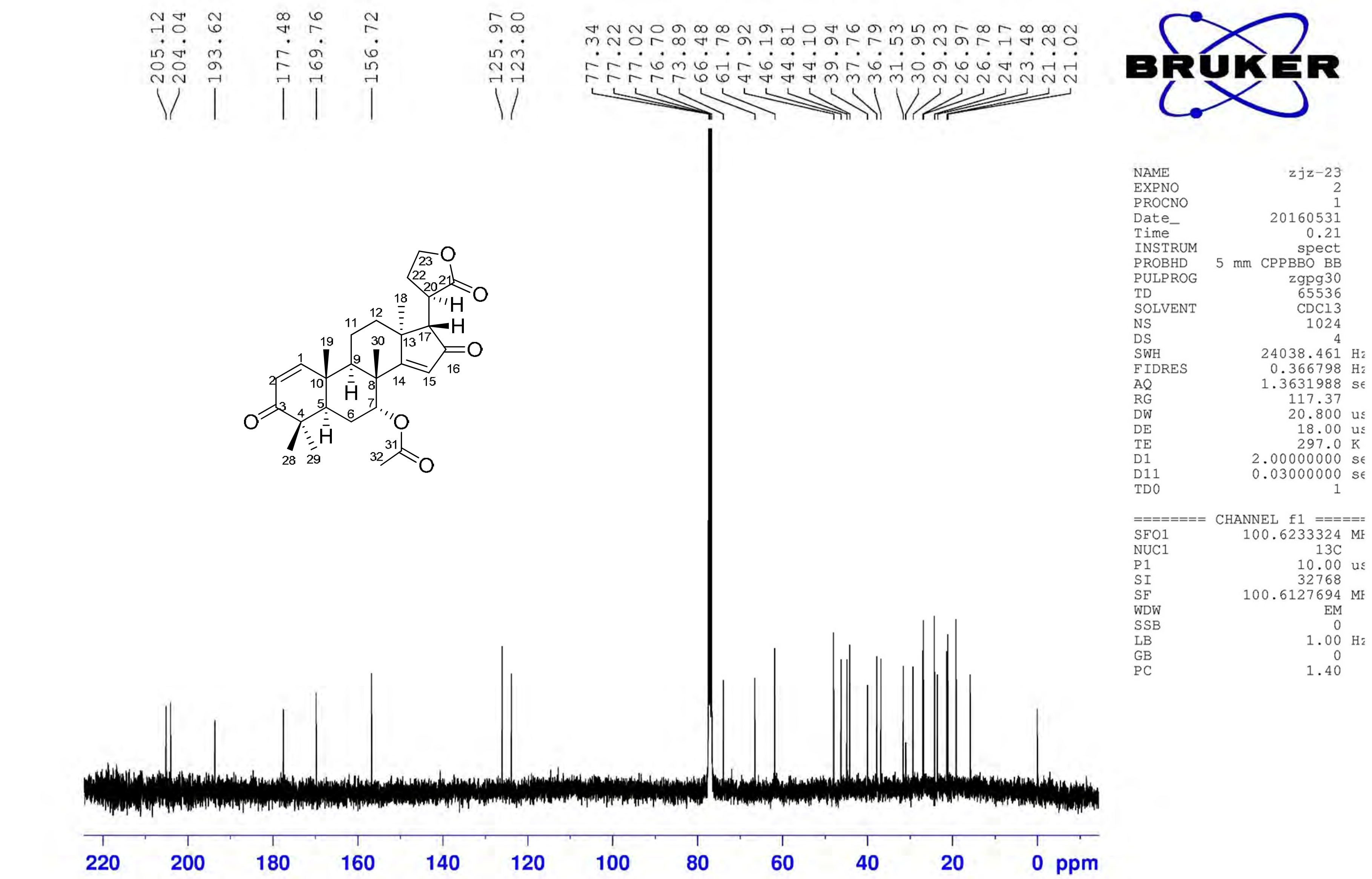

^{13}C NMR (100 MHz) spectrum of compound **28** in $CDCl_3$

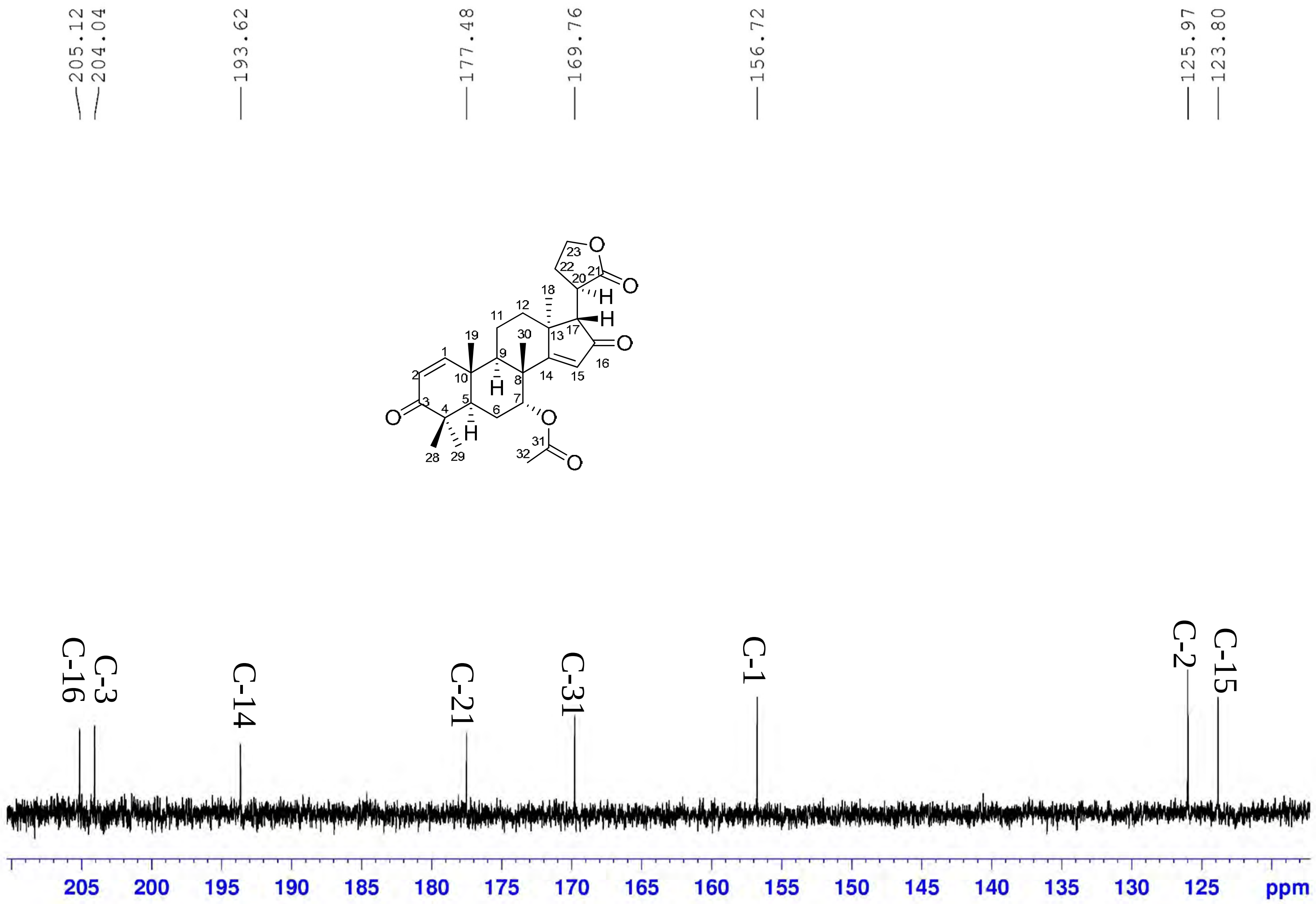

^{13}C NMR (100 MHz) spectrum of compound **28** in $CDCl_3$

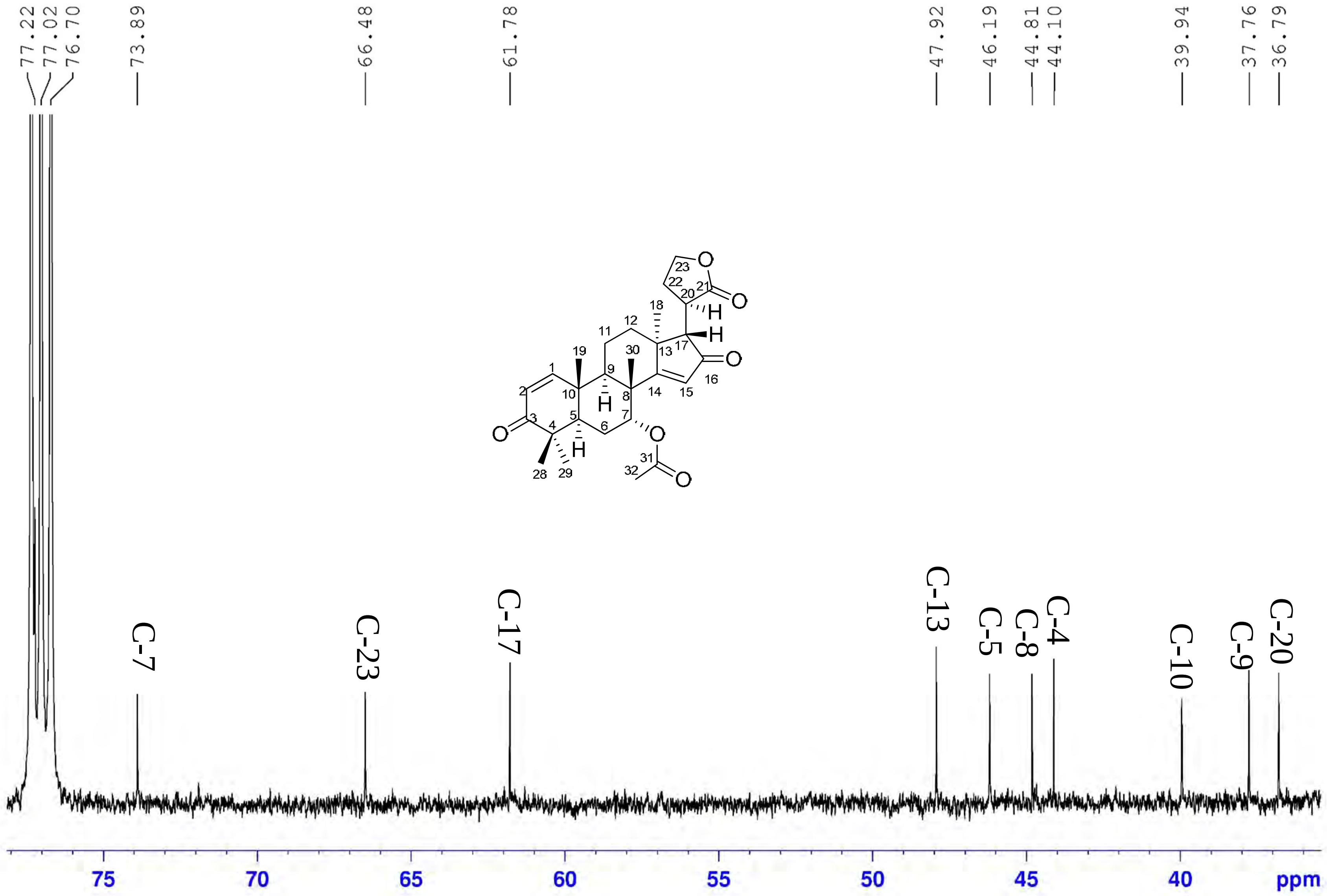

^{13}C NMR (100 MHz) spectrum of compound **28** in $CDCl_3$

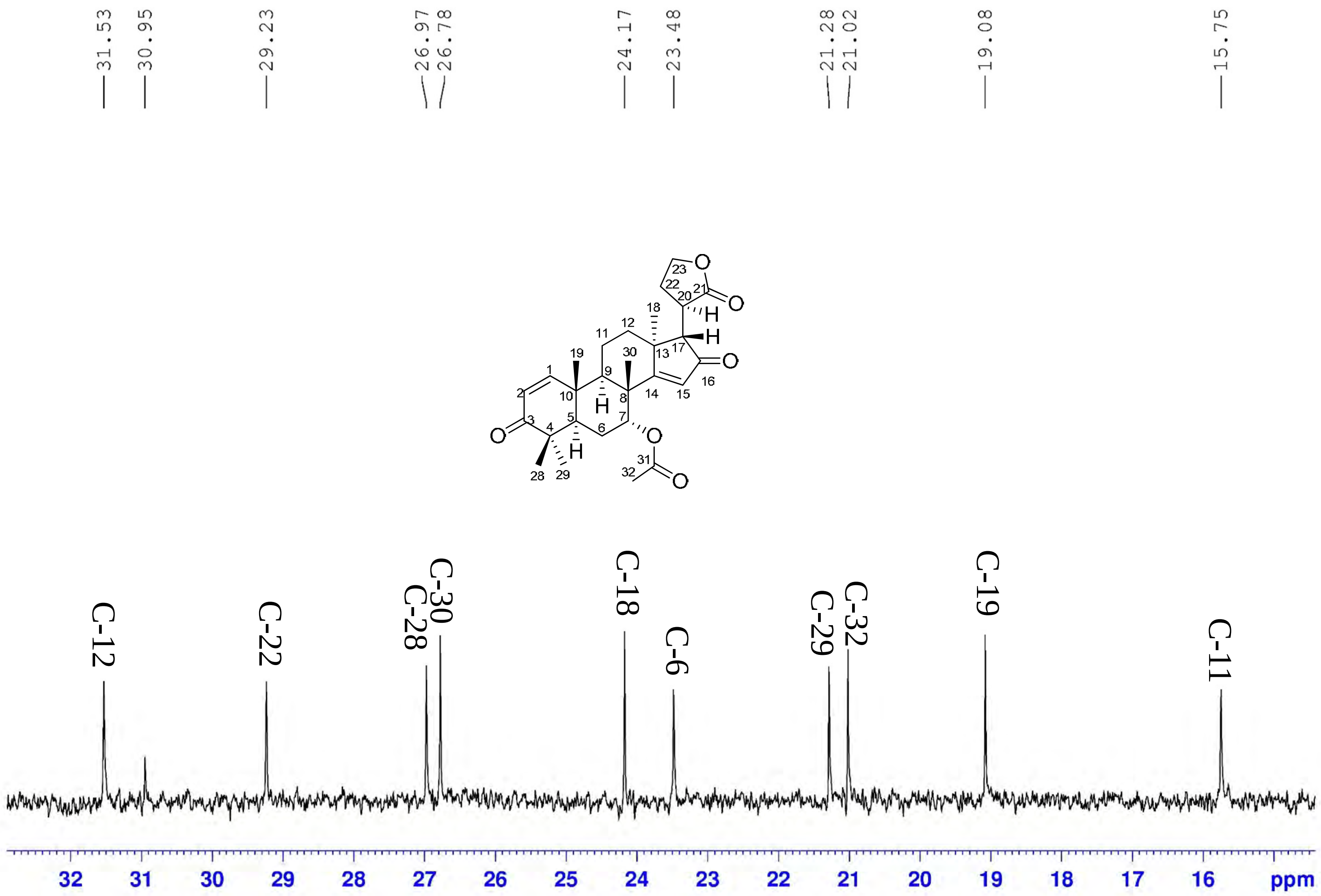

DEPT135 (100 MHz) spectrum of compound **28** in $CDCl_3$

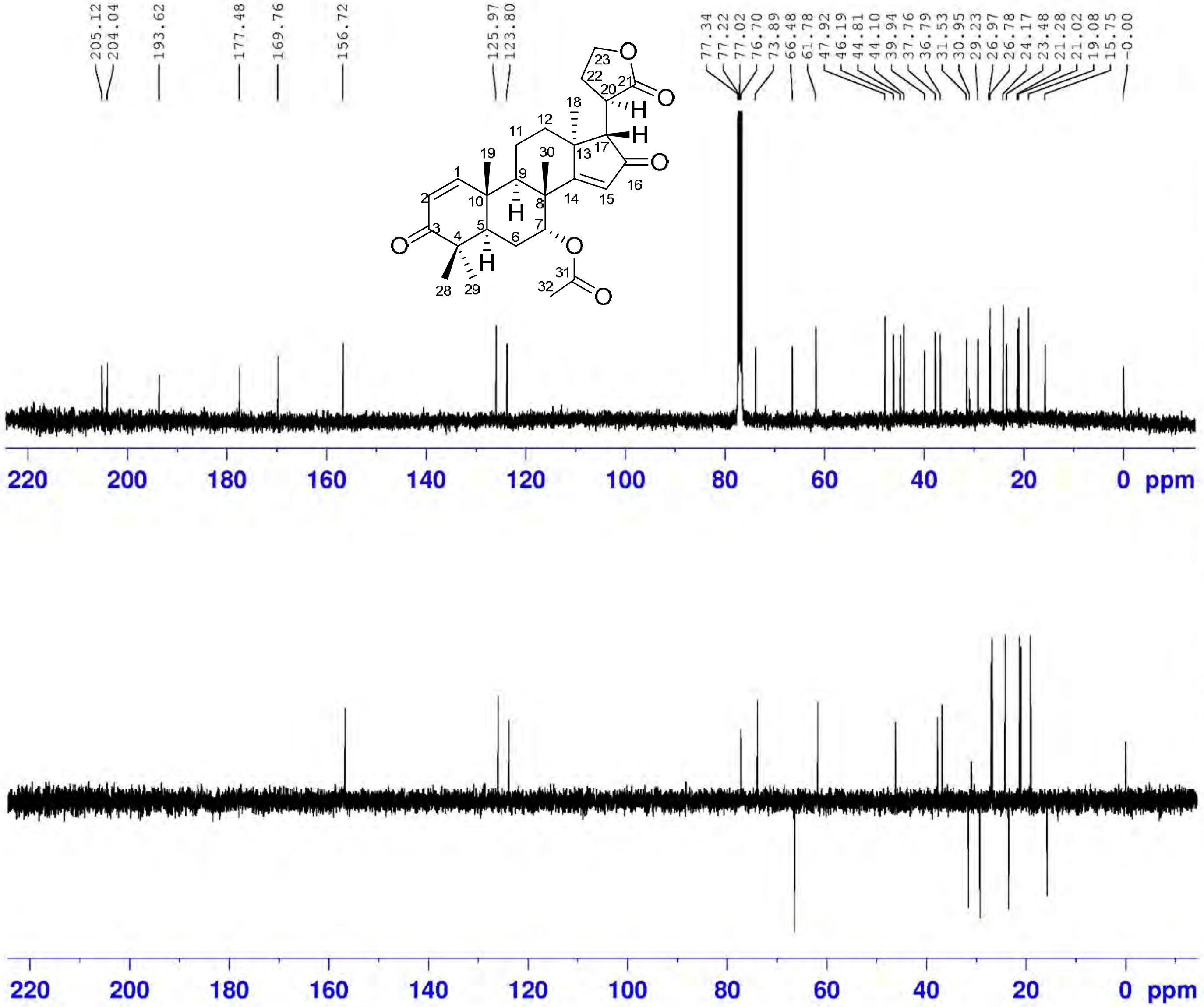

DEPT135 (100 MHz) spectrum of compound **28** in $CDCl_3$

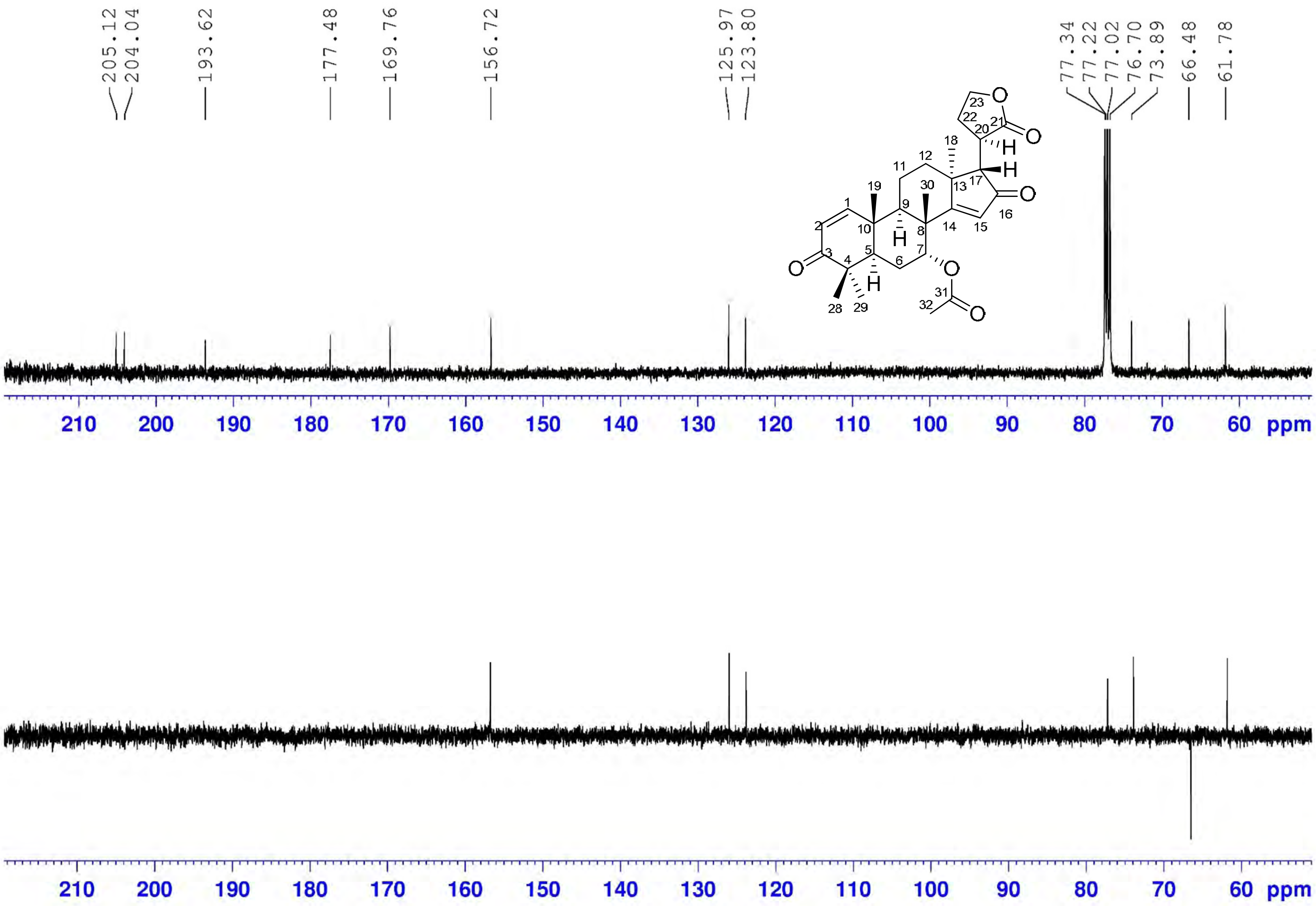

DEPT135 (100 MHz) spectrum of compound **28** in $CDCl_3$

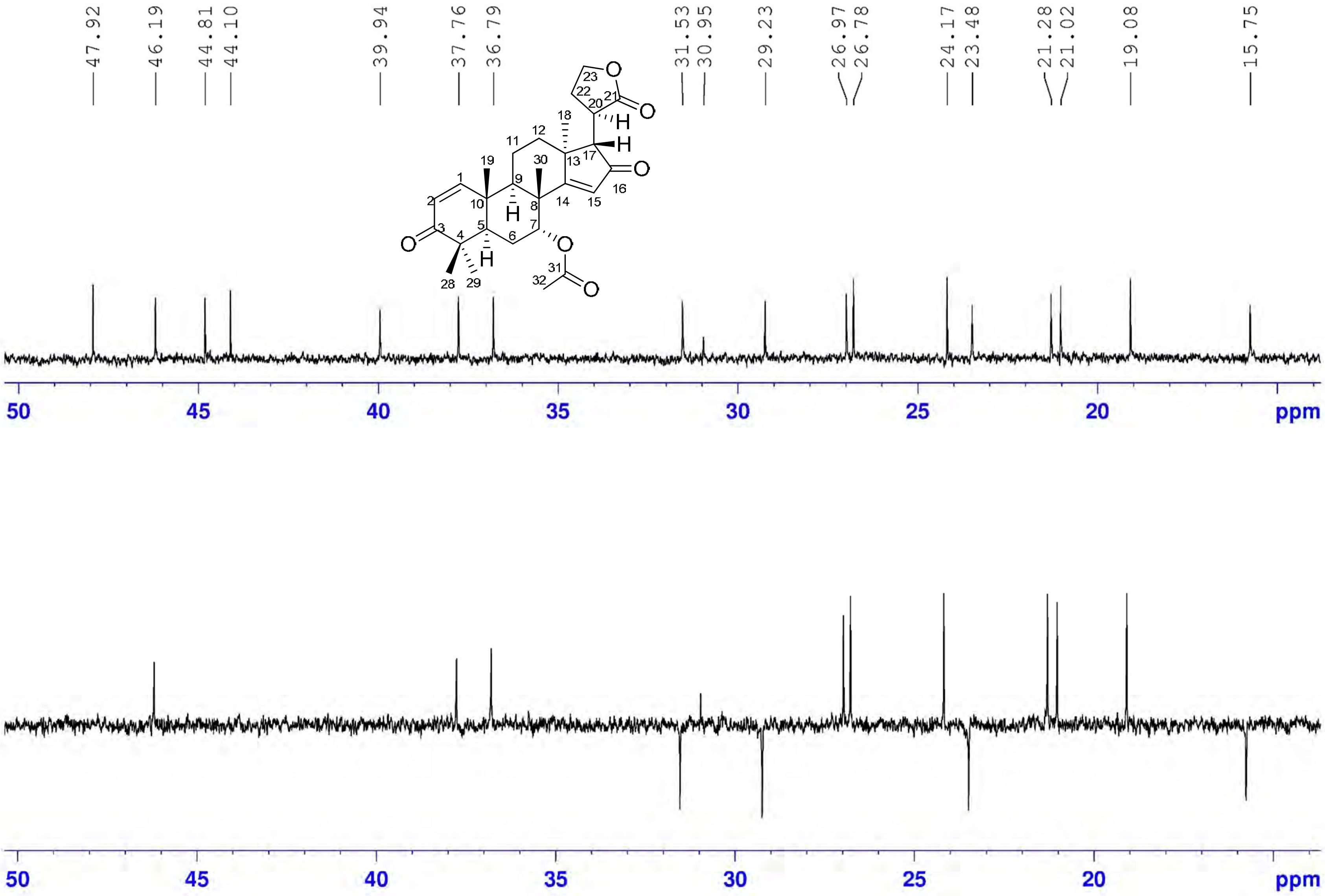

^{1}H-^{1}H COSY (400 MHz) spectrum of compound **28** in $CDCl_3$

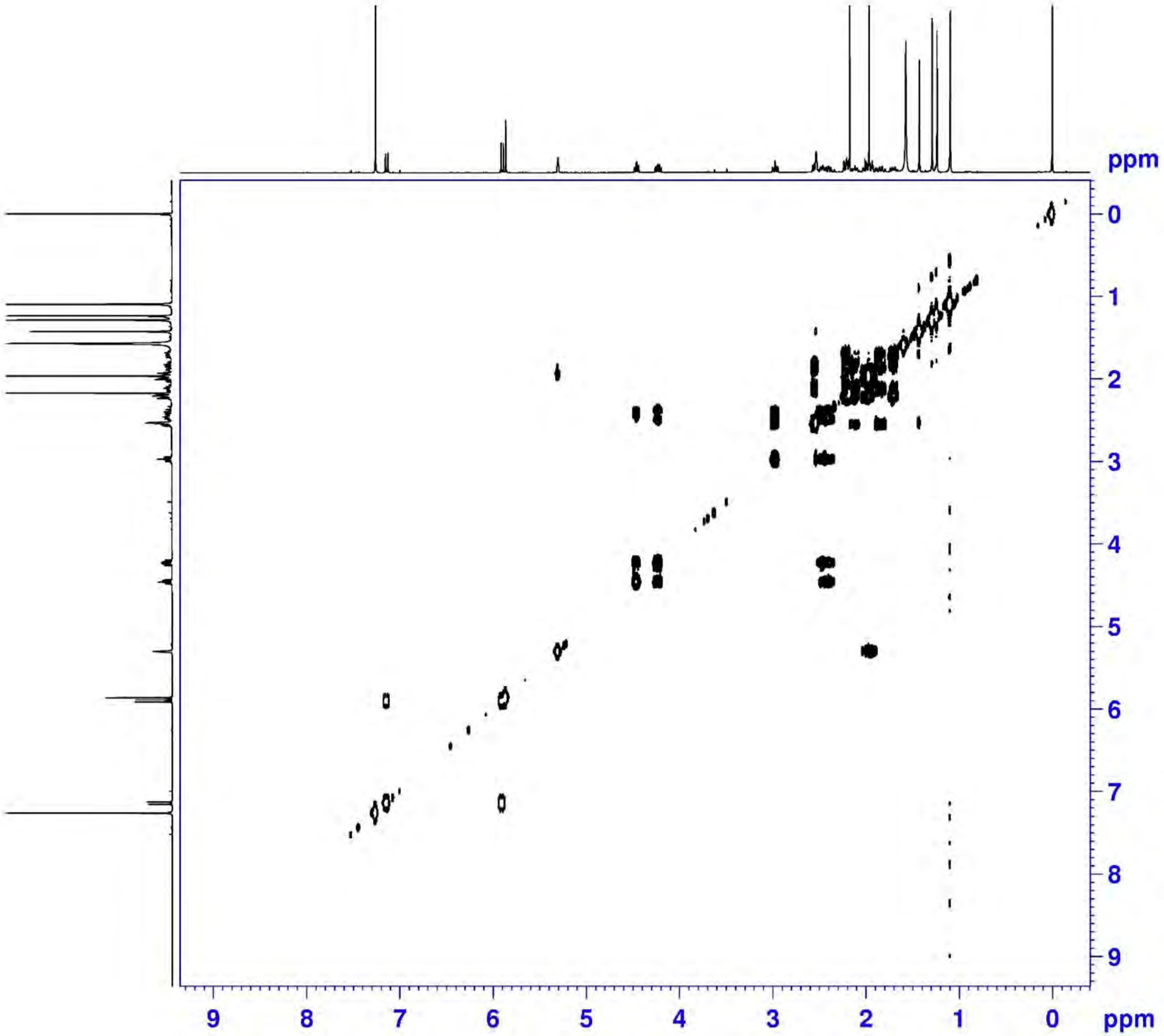

NAME	zjz-23
EXPNO	4
PROCNO	1
Date_	20160618
Time	0.02
INSTRUM	spect
PROBHD	5 mm CPPBBO BB
PULPROG	cosygpppqf
TD	2048
SOLVENT	CDCl3
NS	16
DS	8
SWH	3906.250 Hz
FIDRES	1.907349 Hz
AQ	0.2621940 sec
RG	208.5
DW	128.000 usec
DE	10.00 usec
TE	297.0 K
D0	0.00000300 sec
D1	1.89678097 sec
D11	0.03000000 sec
D12	0.00002000 sec
D13	0.00000400 sec
D16	0.00020000 sec
IN0	0.00025600 sec
======== CHANNEL f1 ========	
SFO1	400.1318006 MHz
NUC1	1H
P0	11.50 usec
P1	11.50 usec
P17	2500.00 usec
ND0	1
TD	128
SFO1	400.1318 MHz
FIDRES	30.517578 Hz
SW	9.762 ppm
FnMODE	QF
SI	1024
SF	400.1300089 MHz
WDW	QSINE
SSB	0
LB	0.00 Hz
GB	0
PC	1.40
SI	1024
MC2	QF
SF	400.1300089 MHz
WDW	QSINE
SSB	0
LB	0.00 Hz
GB	0

^{1}H-^{1}H COSY (400 MHz) spectrum of compound **28** in $CDCl_3$

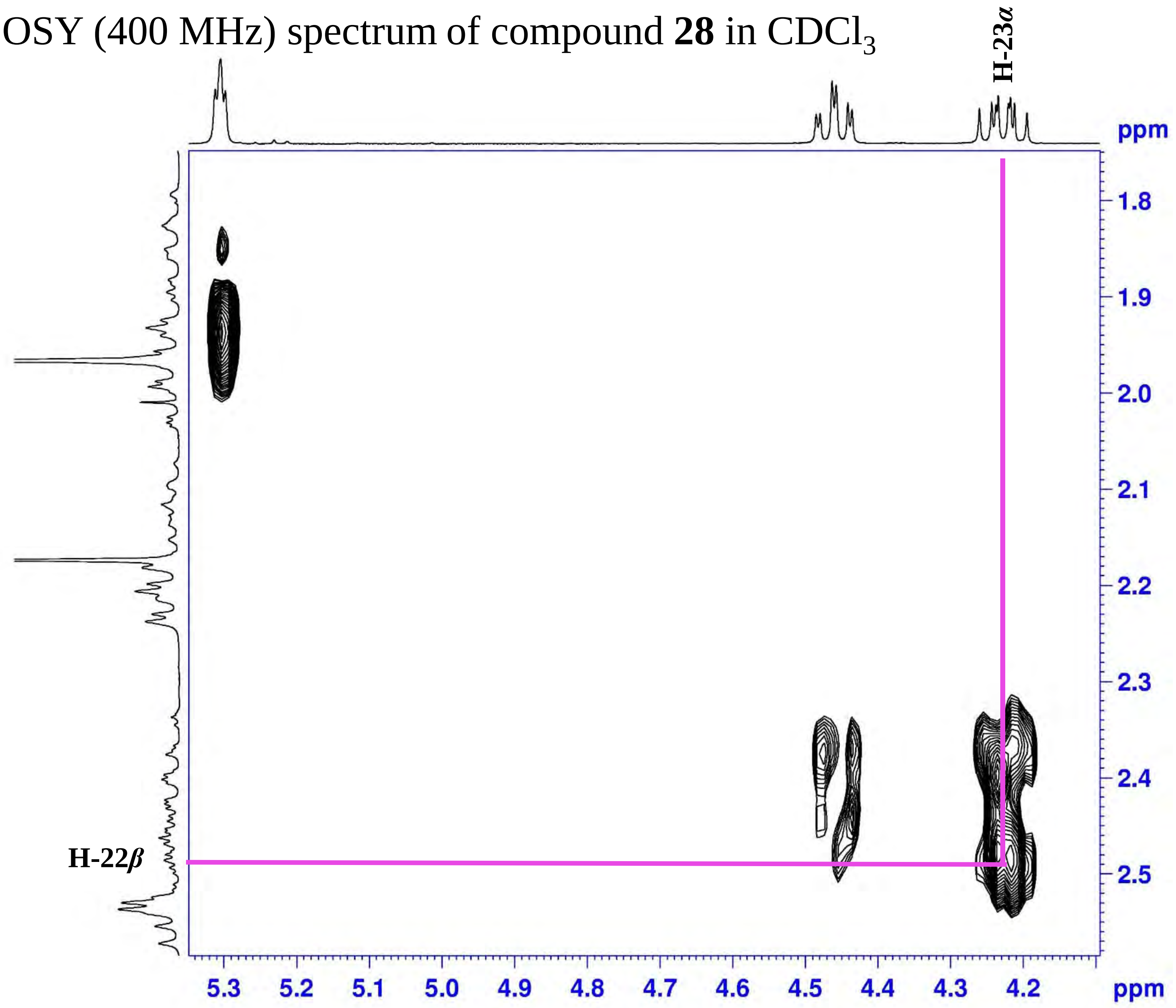

^{1}H-^{1}H COSY (400 MHz) spectrum of compound **28** in $CDCl_3$

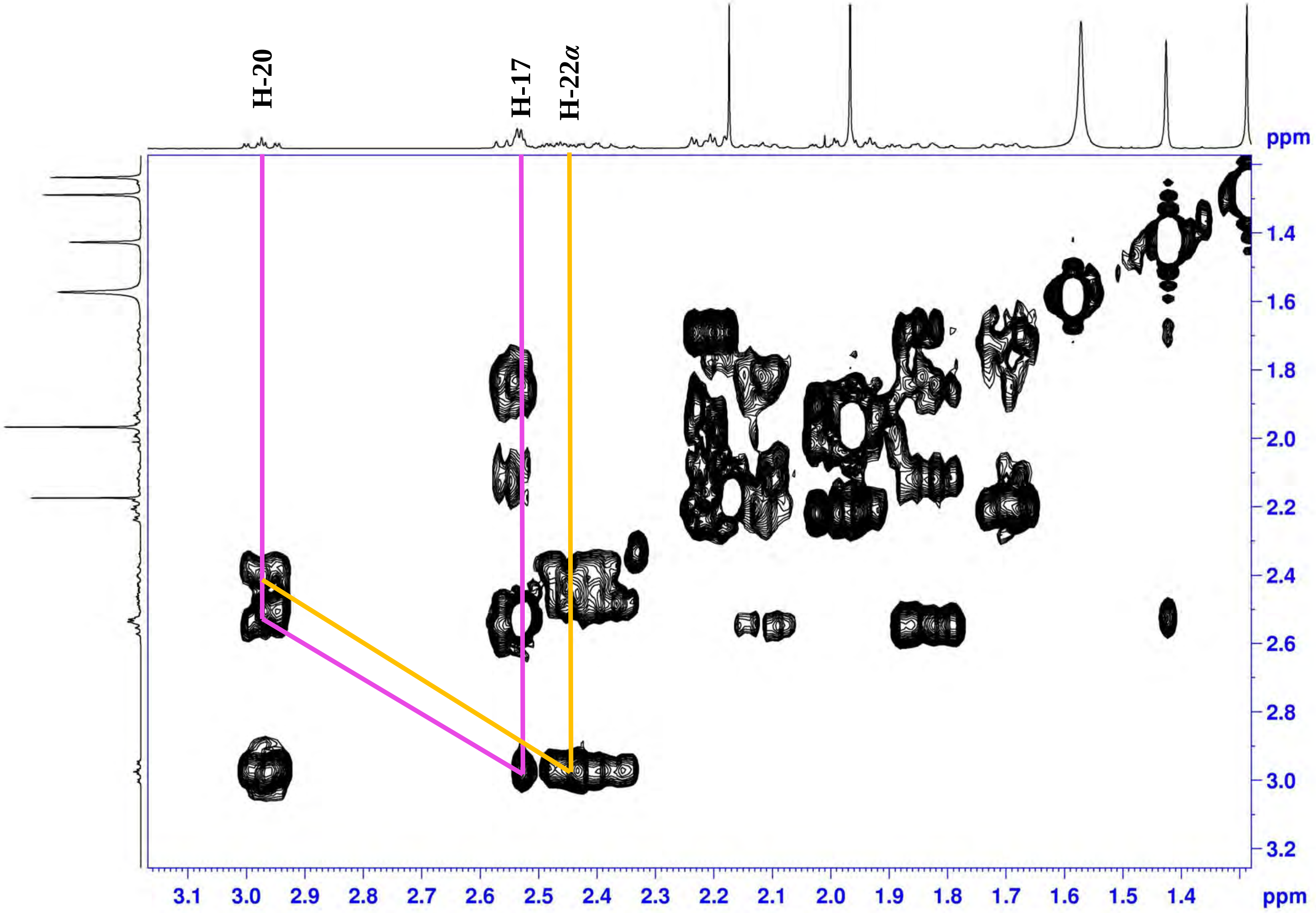

HSQC (400 MHz) spectrum of compound **28** in $CDCl_3$

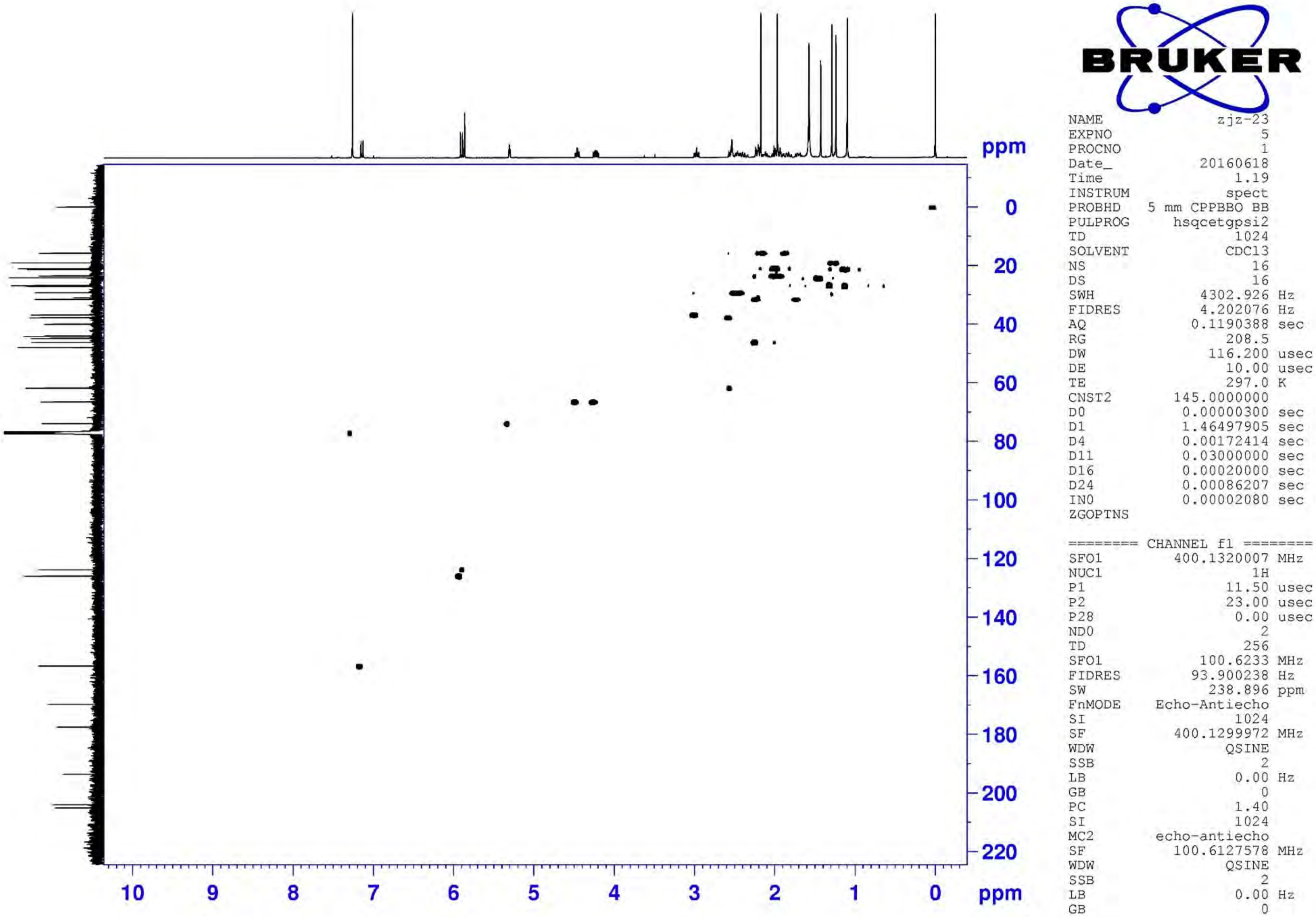

HSQC (400 MHz) spectrum of compound **28** in $CDCl_3$

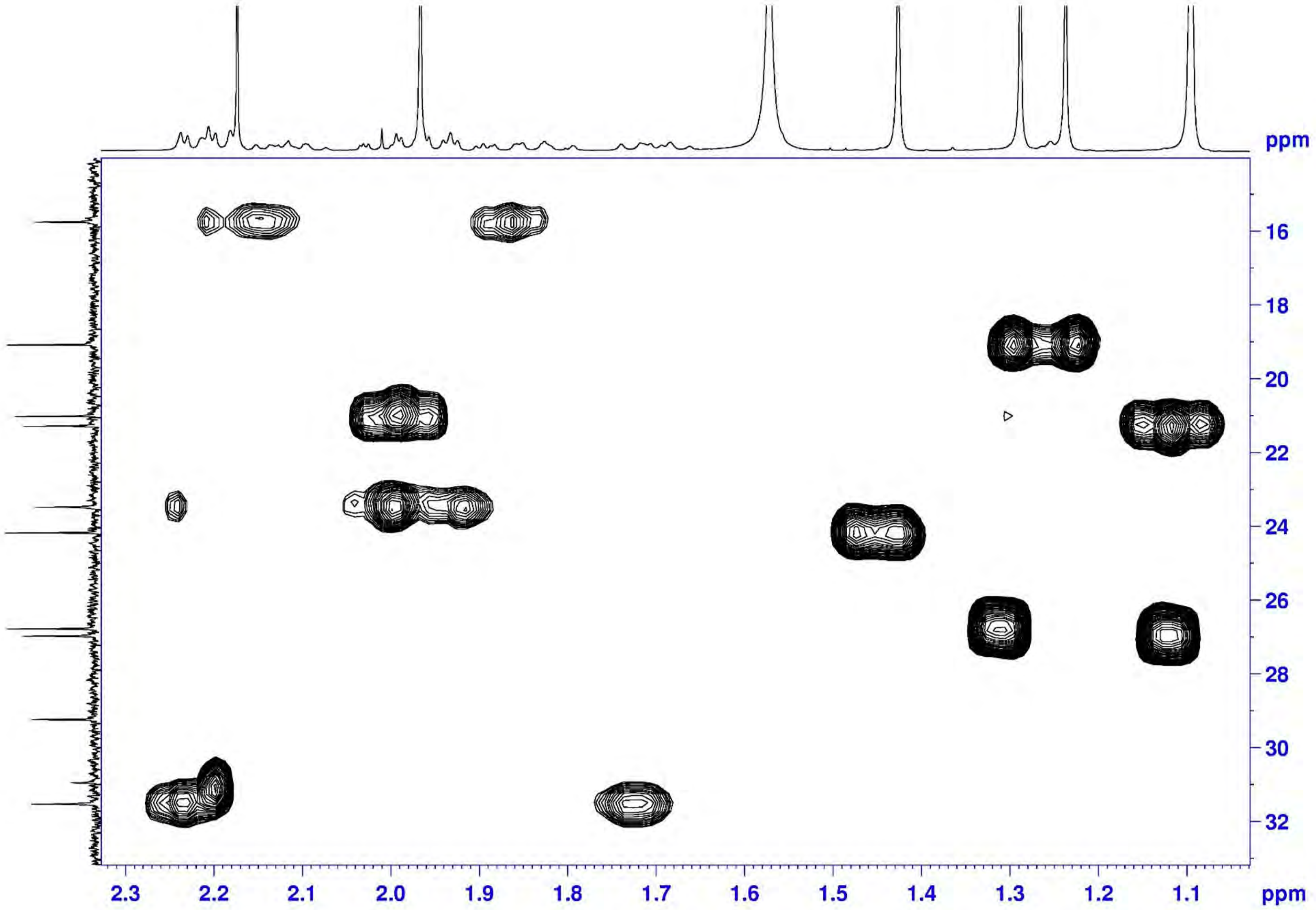

HSQC (400 MHz) spectrum of compound **28** in $CDCl_3$

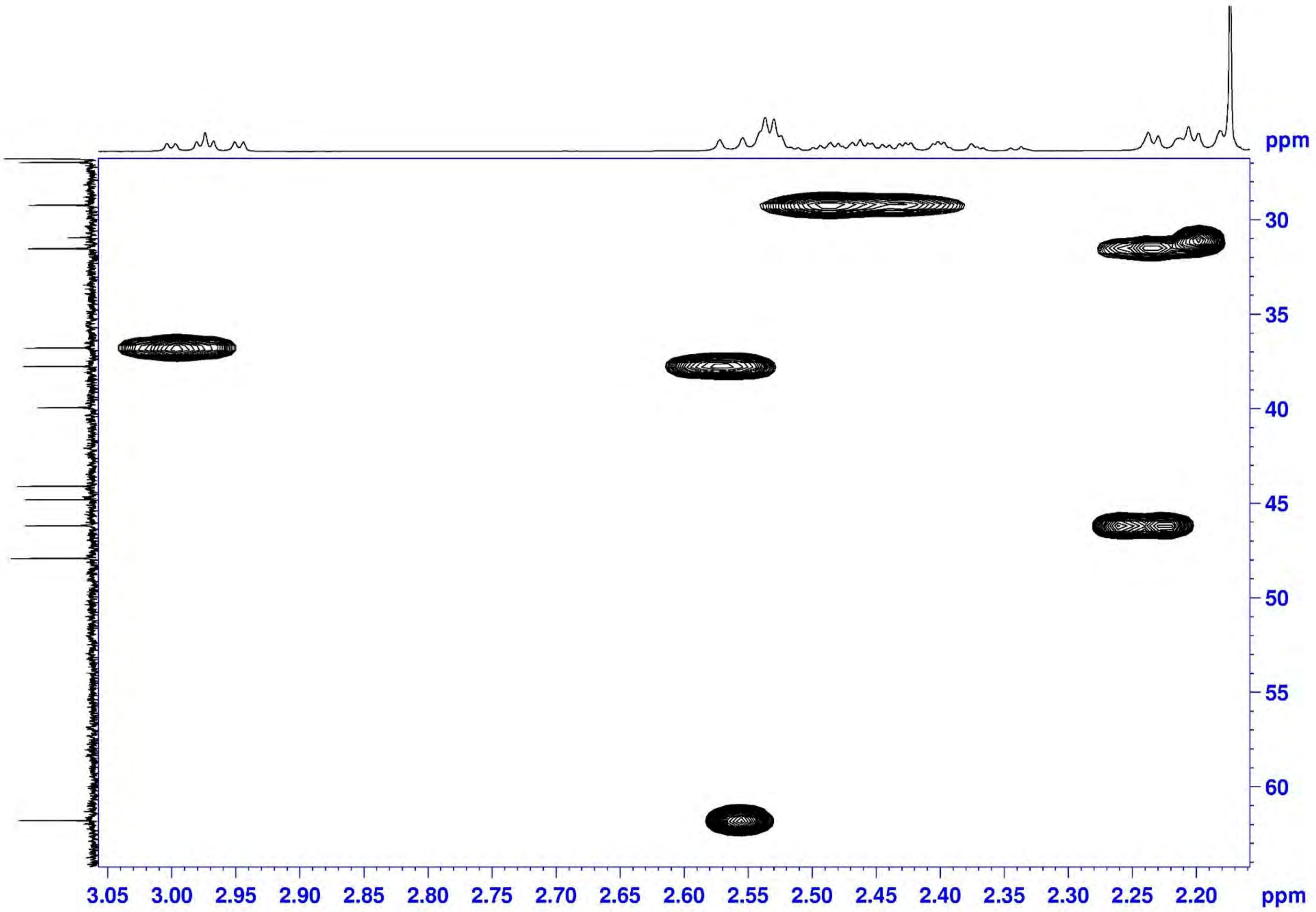

HSQC (400 MHz) spectrum of compound **28** in $CDCl_3$

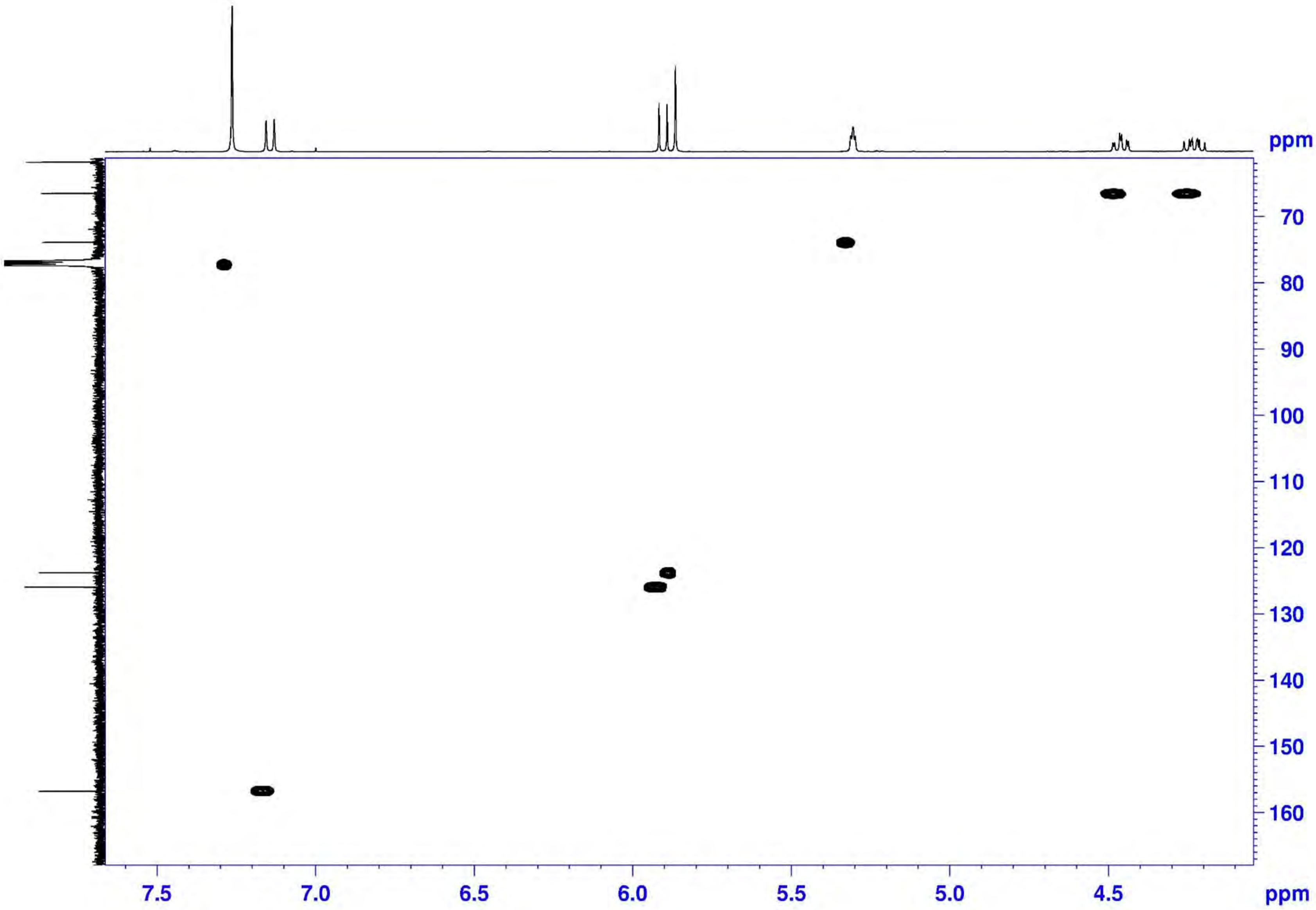

HMBC (400 MHz) spectrum of compound **28** in $CDCl_3$

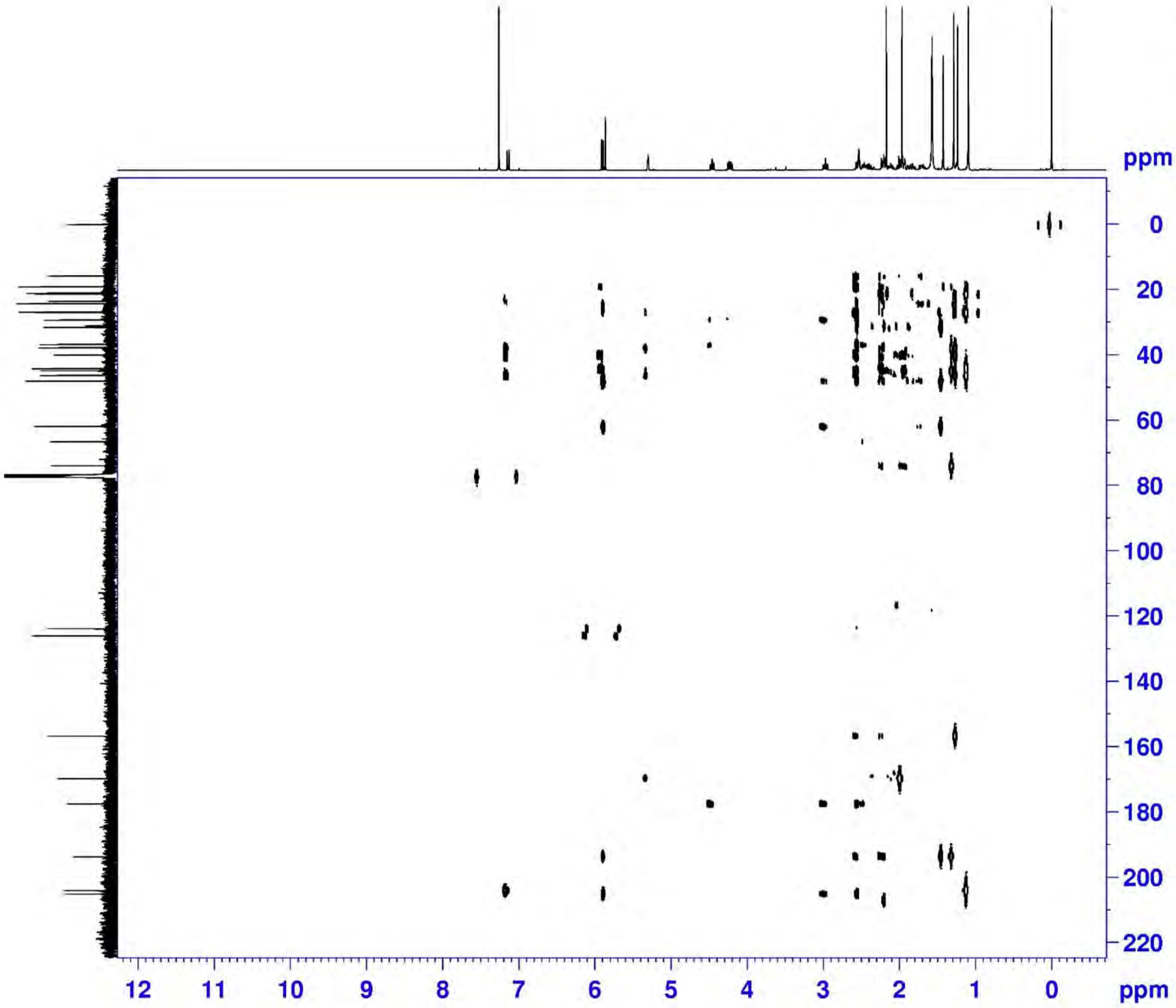

NAME zjz-23
EXPNO 6
PROCNO 1
Date_ 20160618
Time 3.14
INSTRUM spect
PROBHD 5 mm CPPBBO BB
PULPROG hmbcgplpndqf
TD 4096
SOLVENT CDCl3
NS 128
DS 16
SWH 5197.505 Hz
FIDRES 1.268922 Hz
AQ 0.3940852 sec
RG 208.5
DW 96.200 usec
DE 10.00 usec
TE 297.0 K
CNST2 145.0000000
CNST13 10.0000000
D0 0.00000300 sec
D1 1.50000000 sec
D2 0.00344828 sec
D6 0.05000000 sec
D16 0.00020000 sec
IN0 0.00002080 sec

======== CHANNEL f1 ========
SFO1 400.1323208 MHz
NUC1 1H
P1 11.50 usec
P2 23.00 usec
ND0 2
TD 128
SFO1 100.6233 MHz
FIDRES 187.800476 Hz
SW 238.896 ppm
FnMODE QF
SI 2048
SF 400.1299992 MHz
WDW SINE
SSB 0
LB 0.00 Hz
GB 0
PC 1.40
SI 1024
MC2 QF
SF 100.6127550 MHz
WDW SINE
SSB 0
LB 0.00 Hz
GB 0

HMBC (400 MHz) spectrum of compound **28** in $CDCl_3$

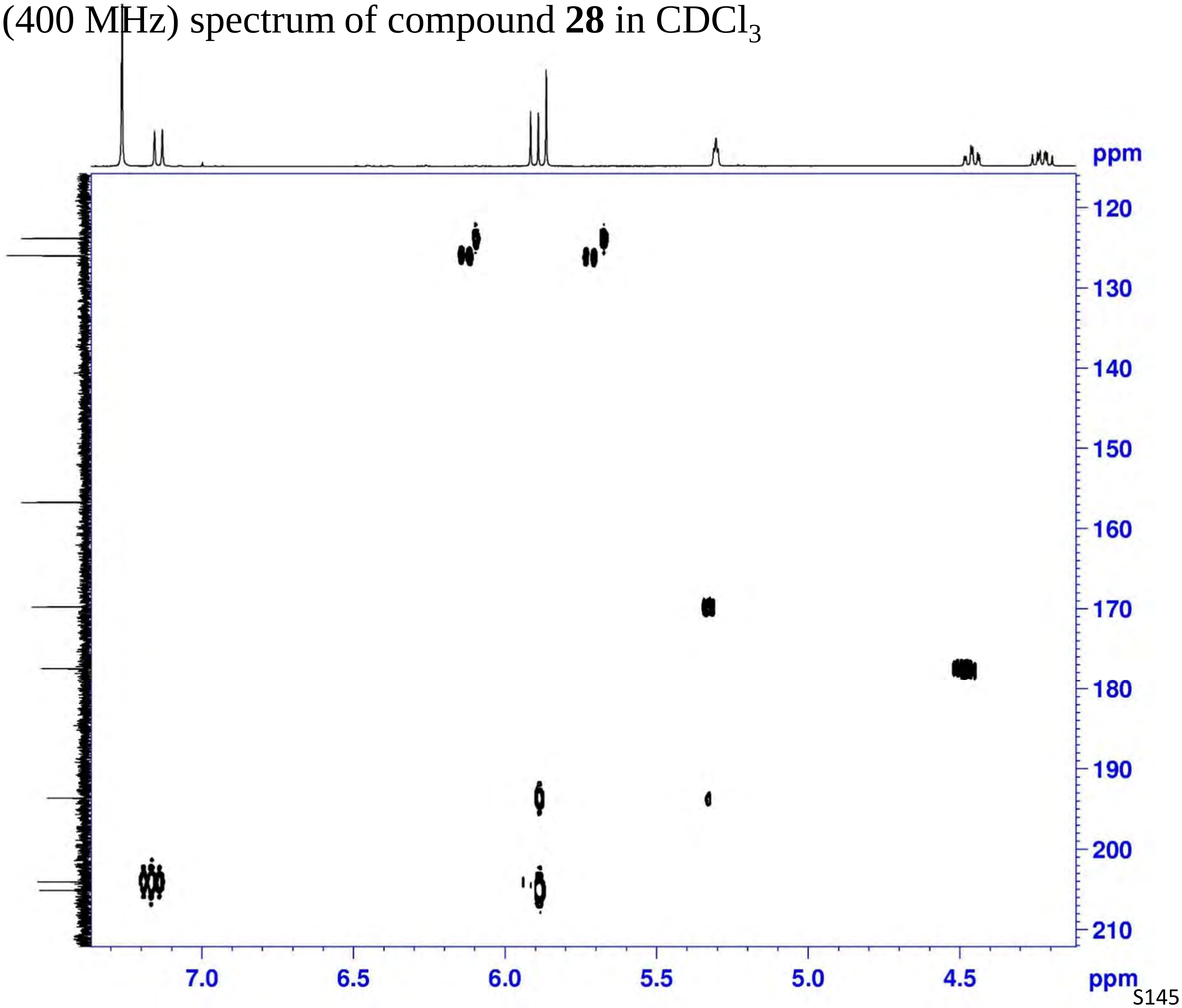

HMBC (400 MHz) spectrum of compound **28** in $CDCl_3$

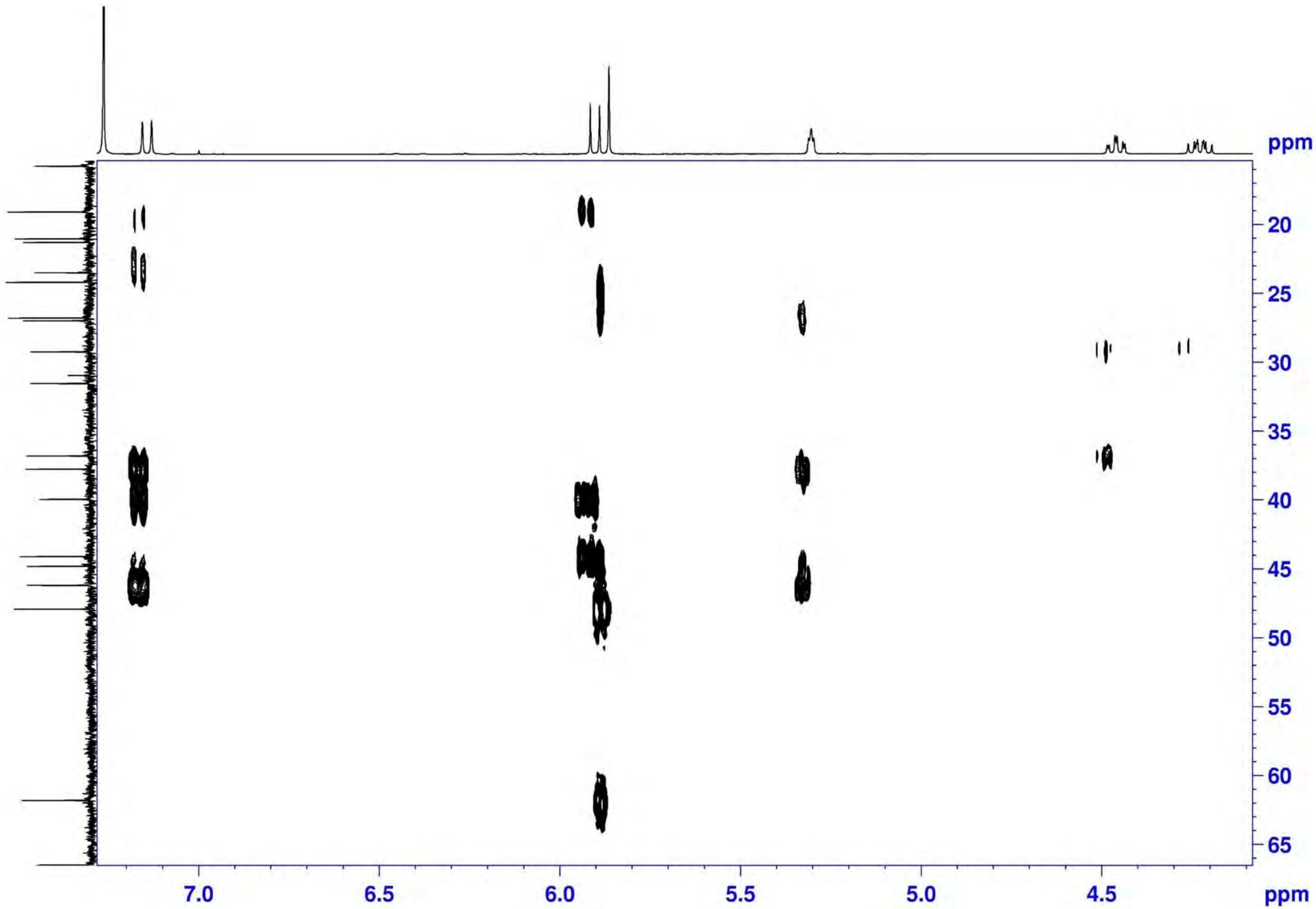

HMBC (400 MHz) spectrum of compound **28** in $CDCl_3$

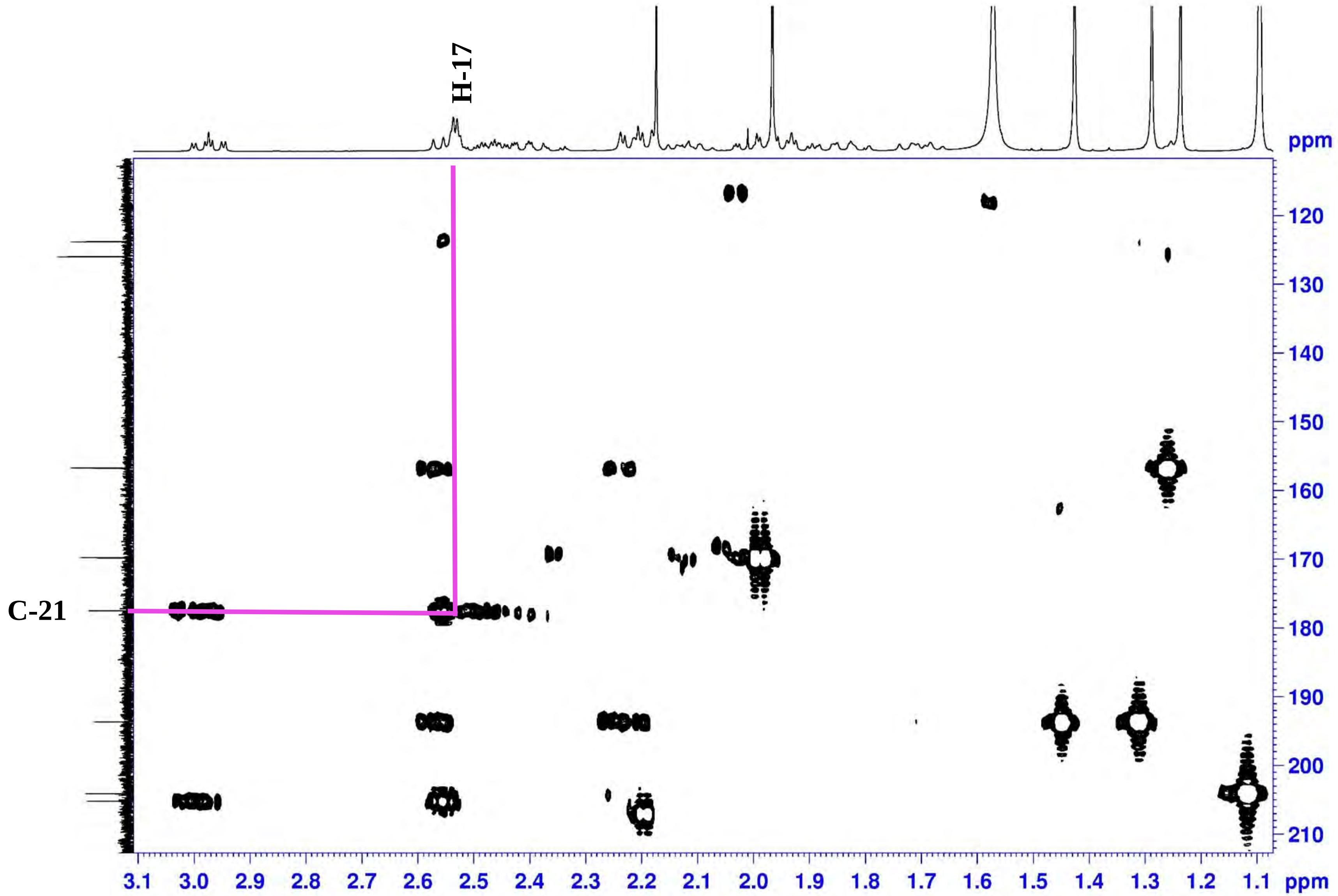

HMBC (400 MHz) spectrum of compound **28** in $CDCl_3$

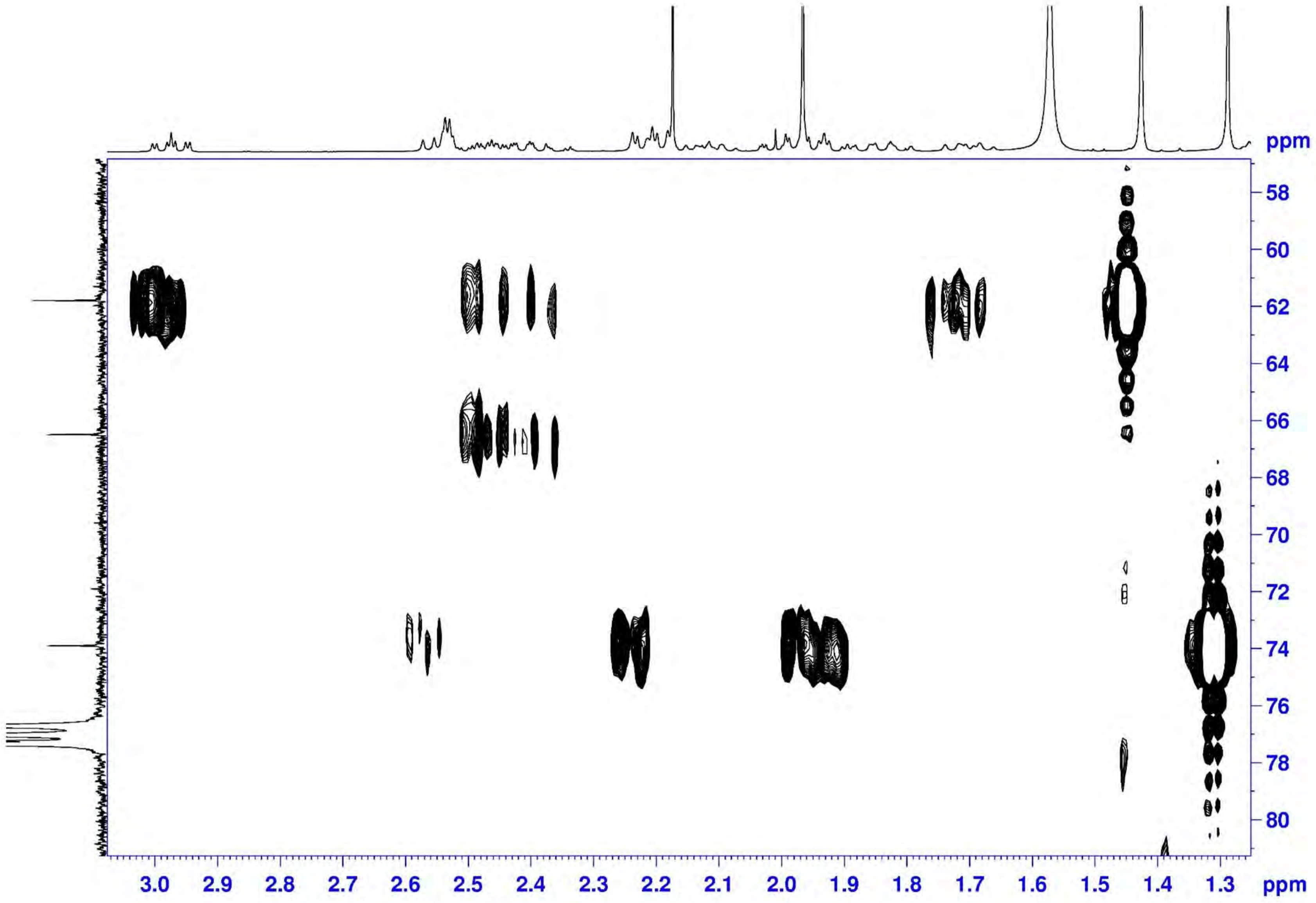

HMBC (400 MHz) spectrum of compound **28** in $CDCl_3$

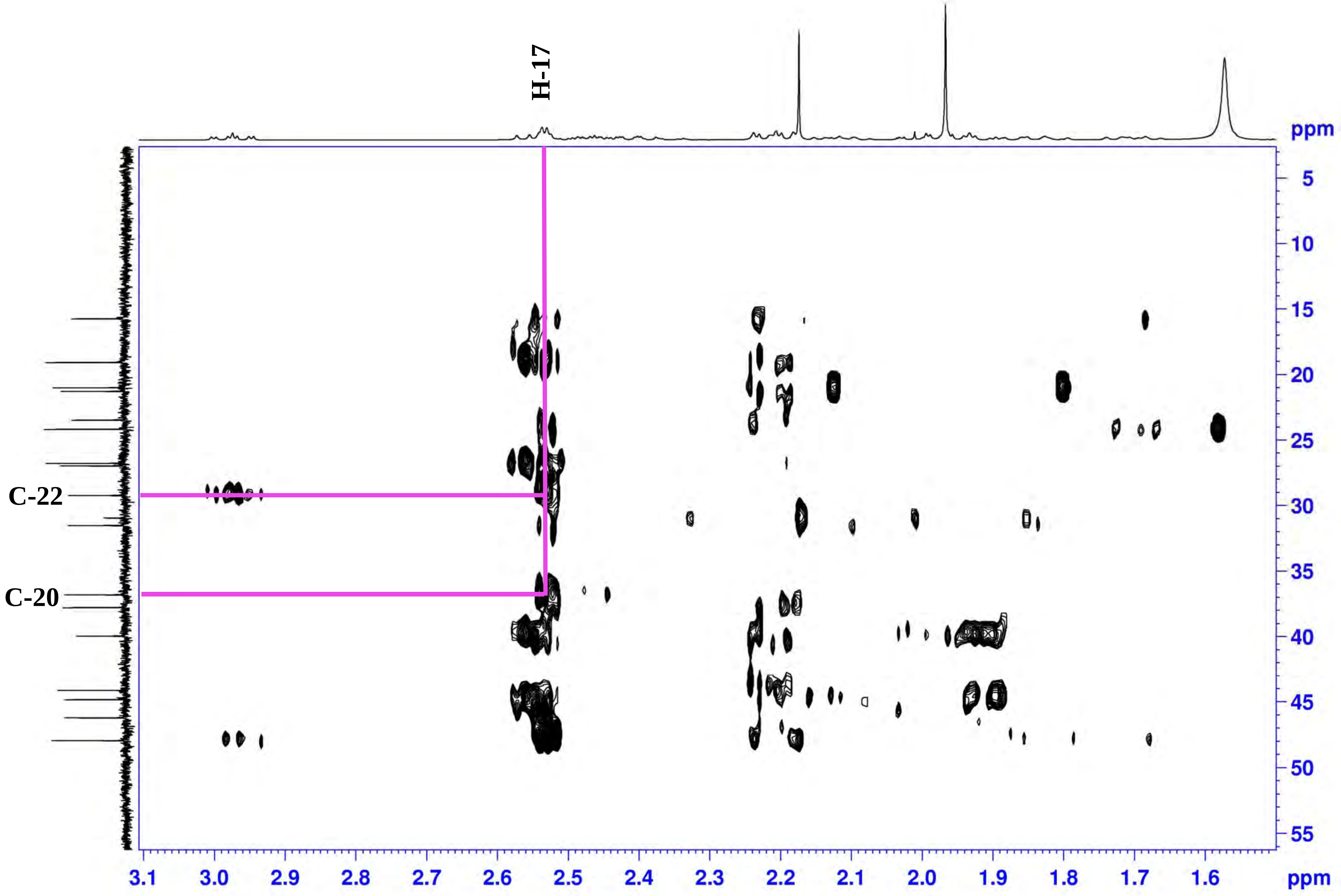

HMBC (400 MHz) spectrum of compound **28** in $CDCl_3$

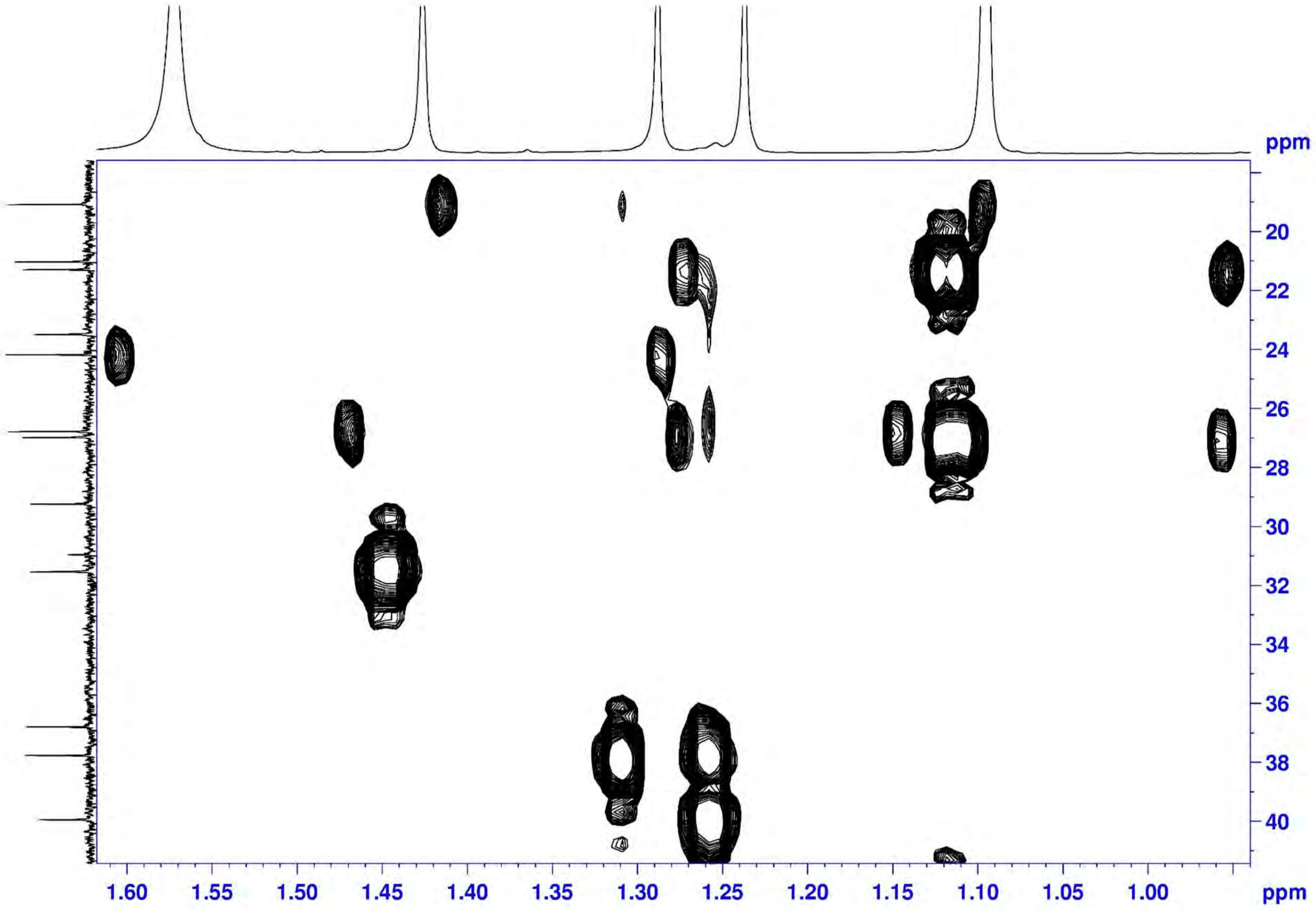

NOESY (400 MHz) spectrum of compound **28** in $CDCl_3$

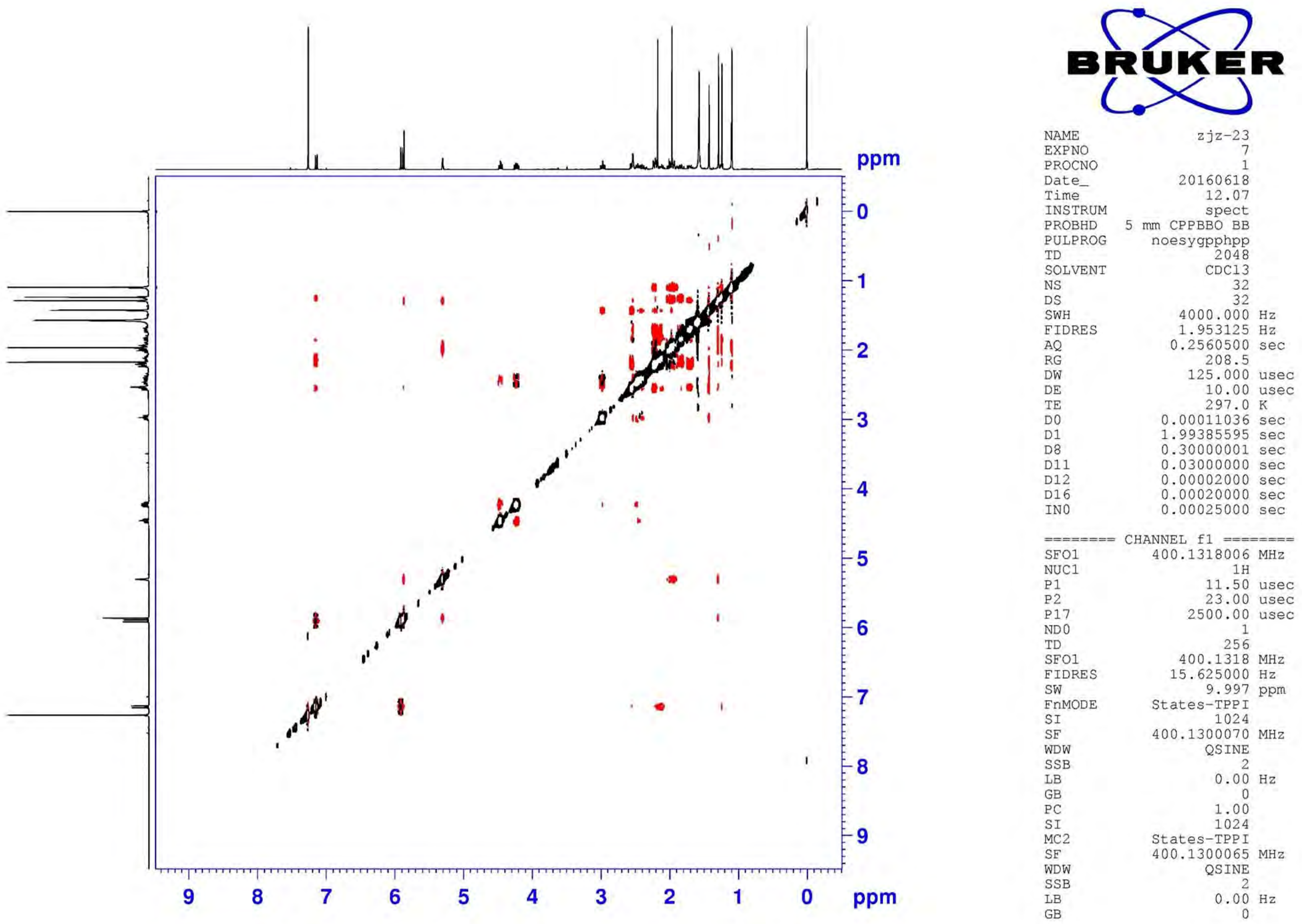

NOESY (400 MHz) spectrum of compound **28** in $CDCl_3$

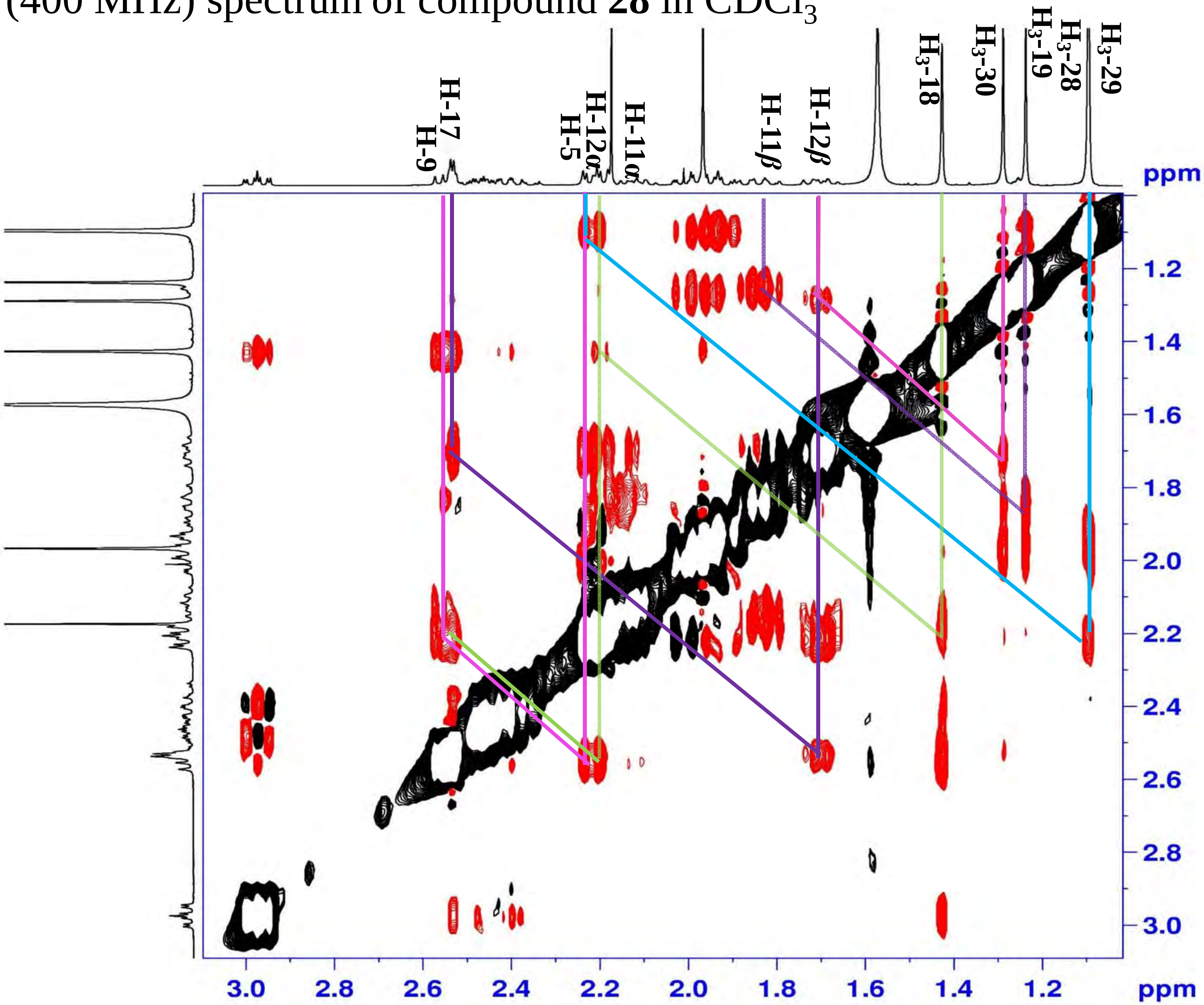

NOESY (400 MHz) spectrum of compound **28** in $CDCl_3$

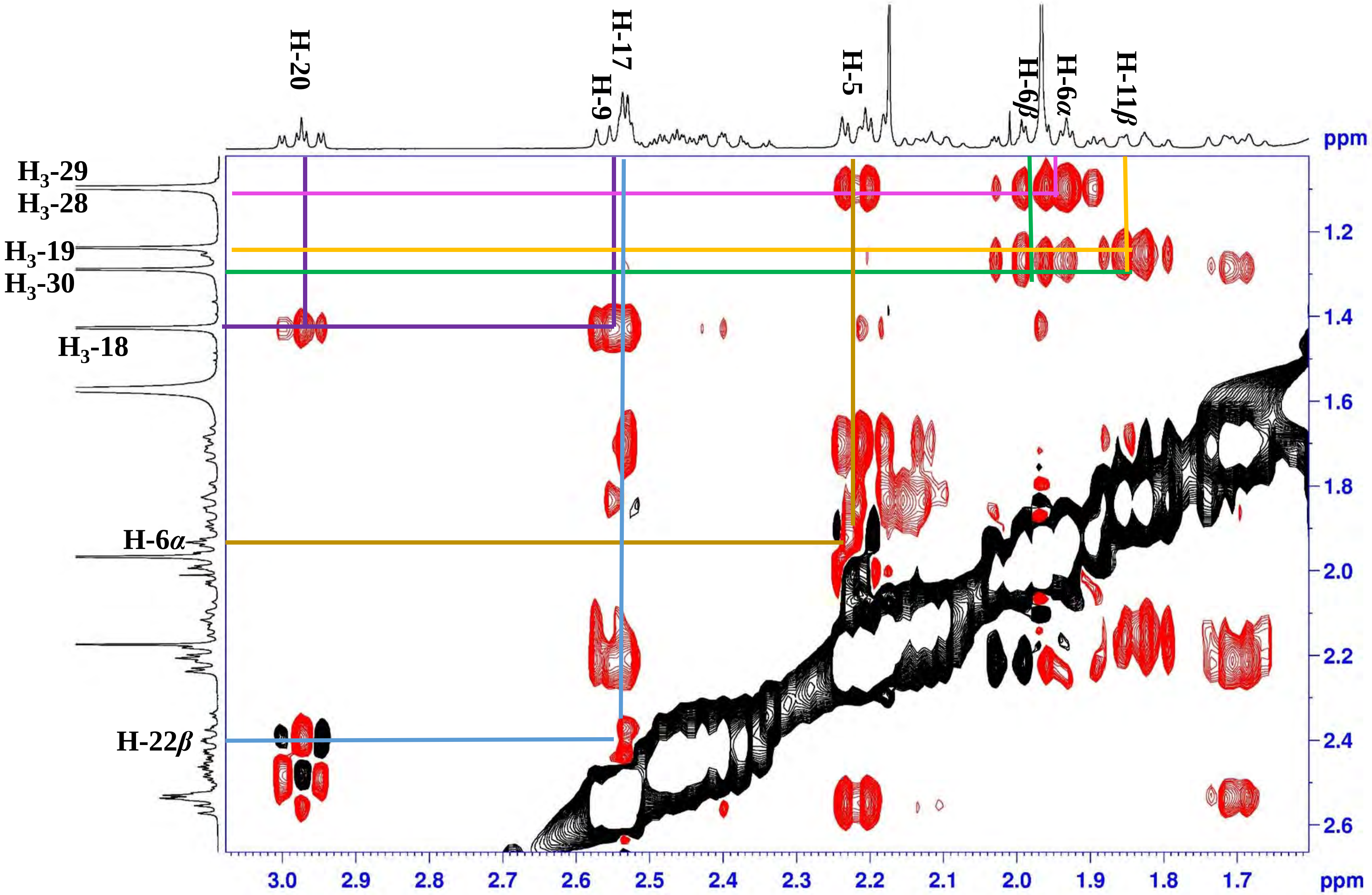

NOESY (400 MHz) spectrum of compound **28** in $CDCl_3$

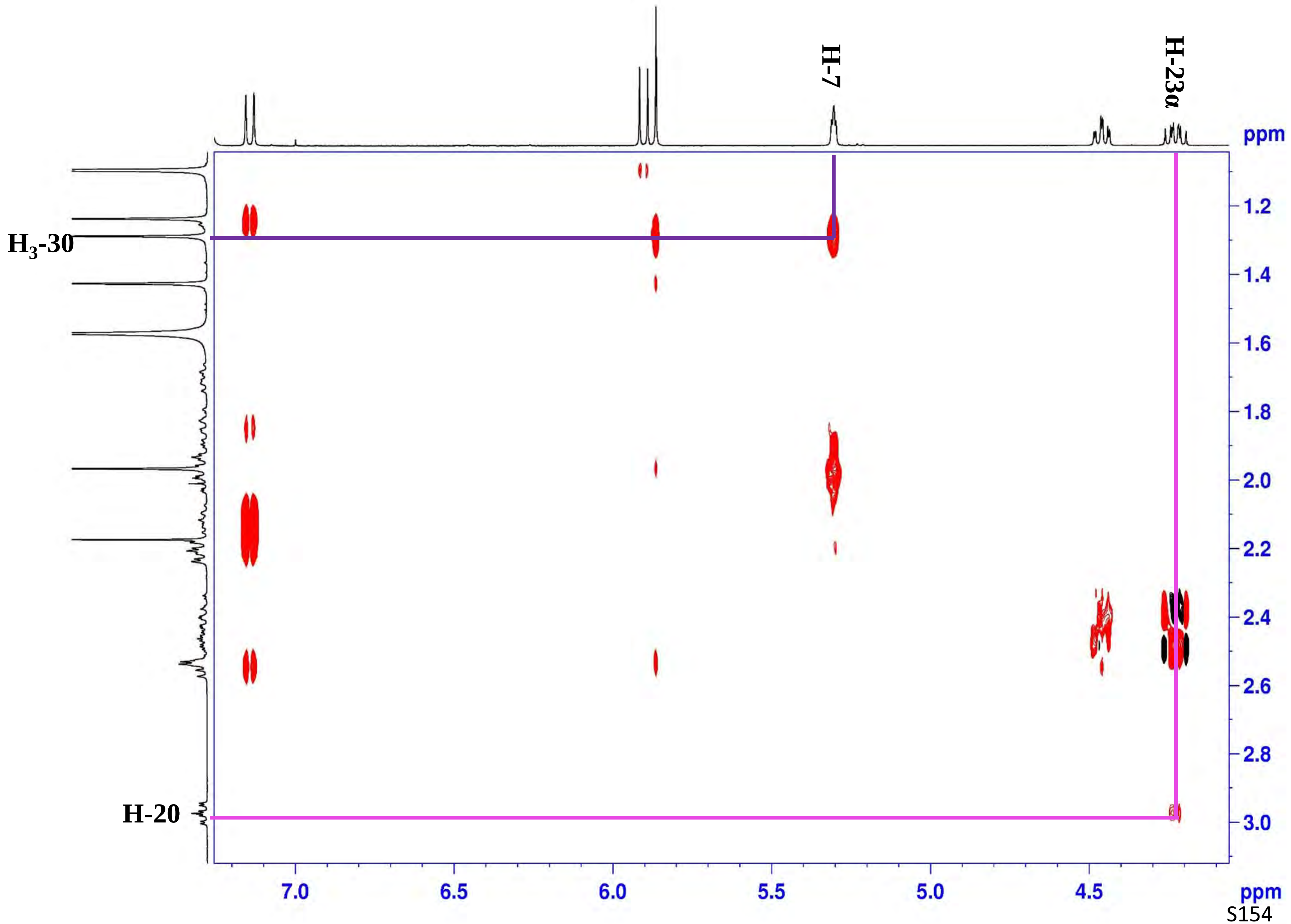

HR-ESIMS for compound **29**

Mass Spectrum SmartFormula Report

Analysis Info

Analysis Name	D:\Data\MS\data\201605\liwanshan_dgy-17_pos_6_01_1854.d	Acquisition Date	5/19/2016 8:37:47 AM
Method	LC_Direct Infusion_pos_100-1000mz.m	Operator	SCSIO
Sample Name	liwanshan_dgy-17_pos	Instrument / Ser#	maXis 29
Comment			

Acquisition Parameter

Source Type	ESI	Ion Polarity	Positive	Set Nebulizer	0.4 Bar
Focus	Active	Set Capillary	4500 V	Set Dry Heater	180 °C
Scan Begin	100 m/z	Set End Plate Offset	-500 V	Set Dry Gas	4.0 l/min
Scan End	2000 m/z	Set Collision Cell RF	800.0 Vpp	Set Divert Valve	Waste

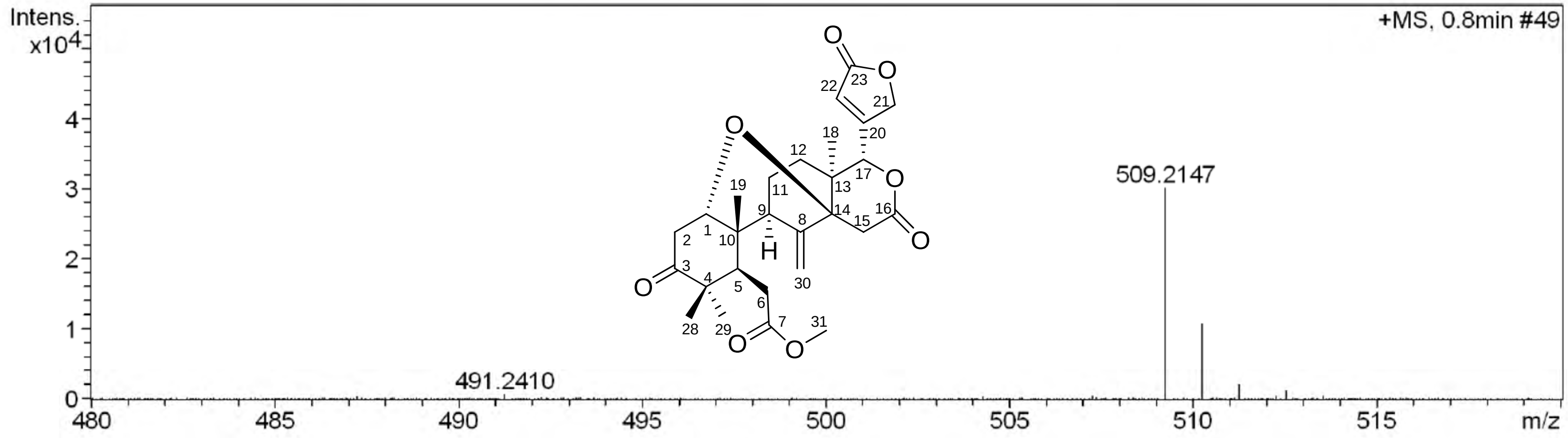

Meas. m/z	#	Formula	Score	m/z	err [ppm]	err [mDa]	mSigma	rdb	e⁻ Conf	N-Rule
487.2327	1	C 27 H 35 O 8	100.00	487.2326	-0.0	-0.0	85.1	10.5	even	ok
509.2147	1	C 27 H 34 Na O 8	100.00	509.2146	-0.2	-0.1	29.9	10.5	even	ok
995.4409	1	C 54 H 68 Na O 16	100.00	995.4400	-0.9	-0.9	71.3	20.5	even	ok

HR-ESIMS for compound **29**

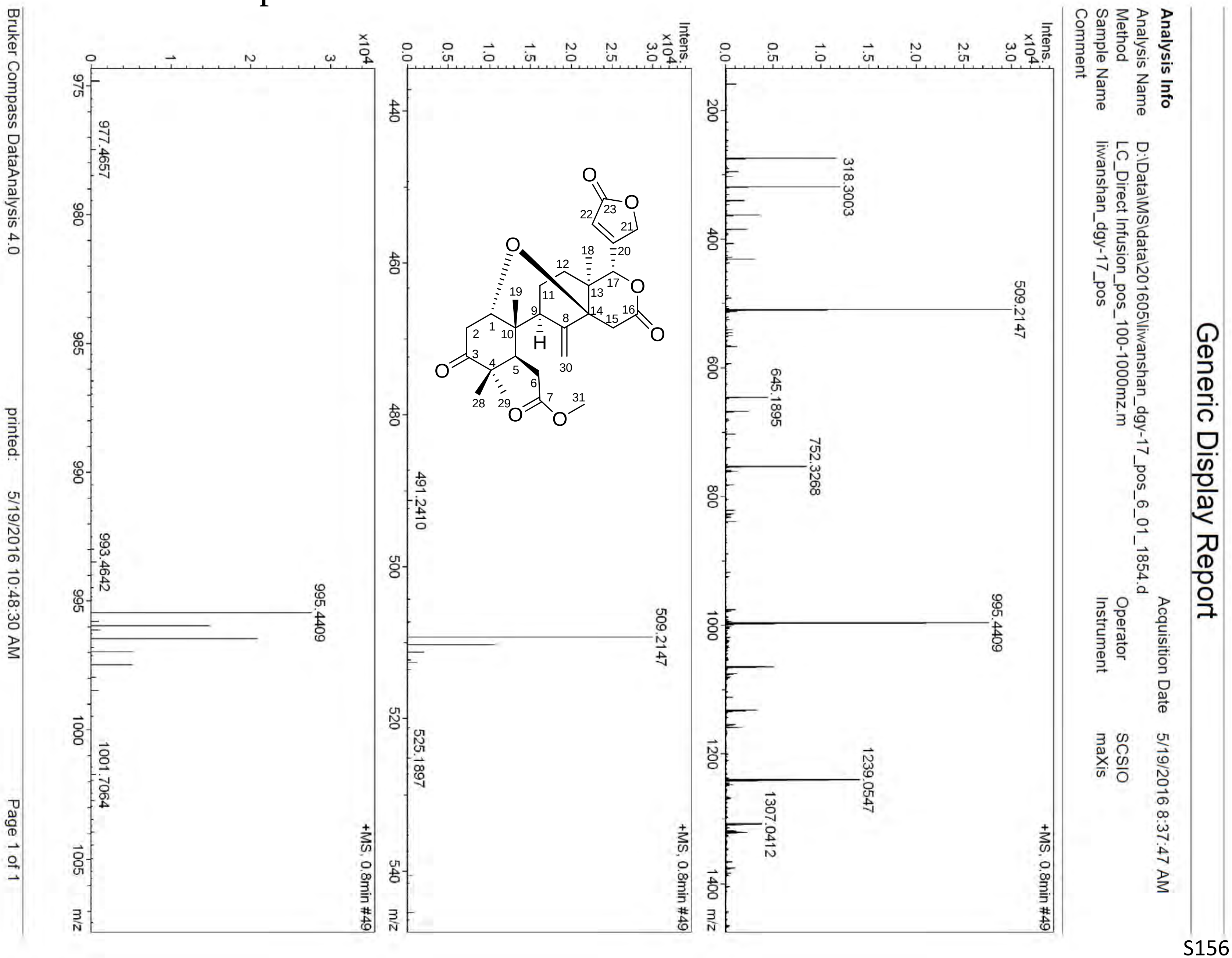

^{1}H NMR (400 MHz) spectrum of compound **29** in $CDCl_3$

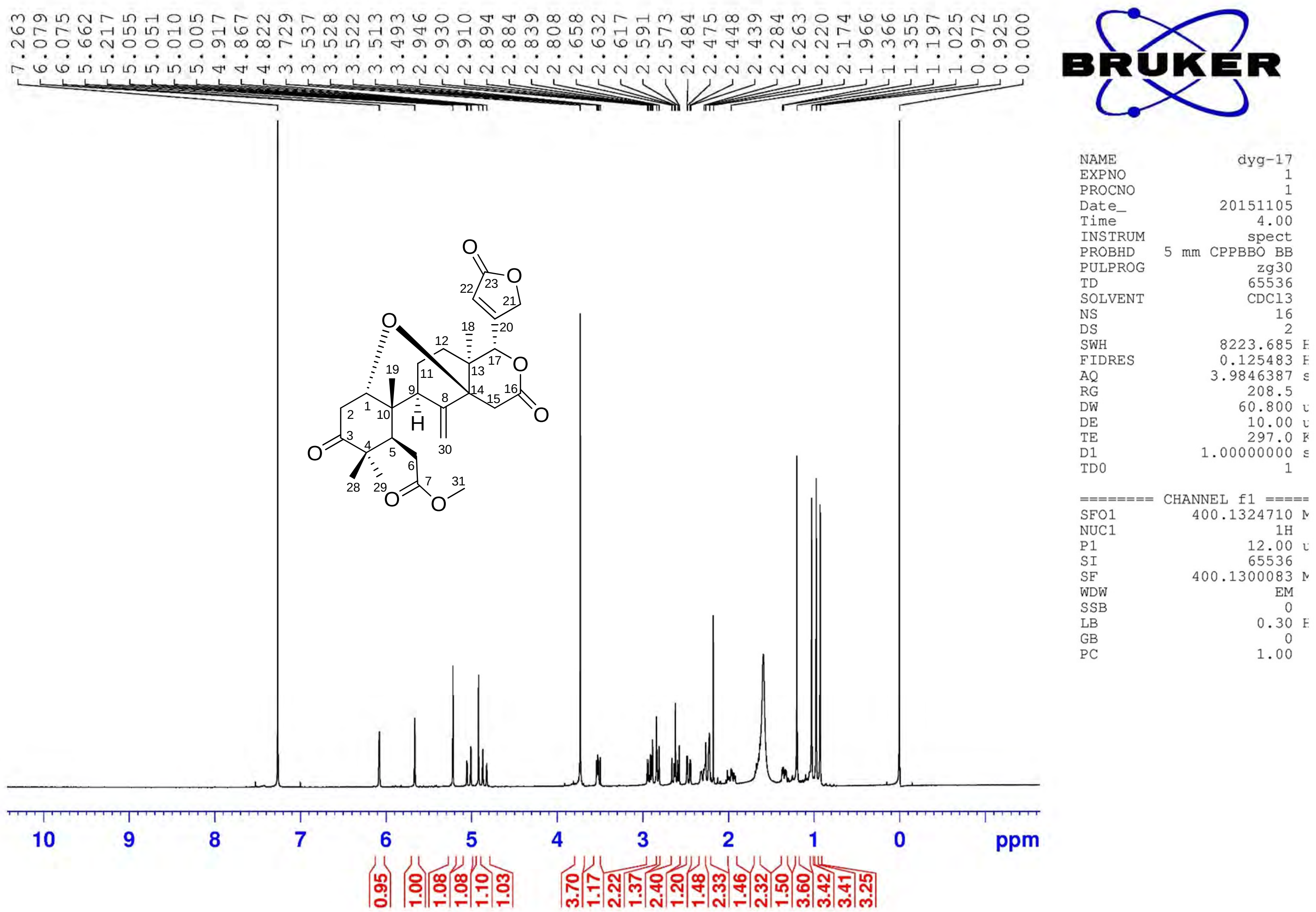

^{1}H NMR (400 MHz) spectrum of compound **29** in $CDCl_3$

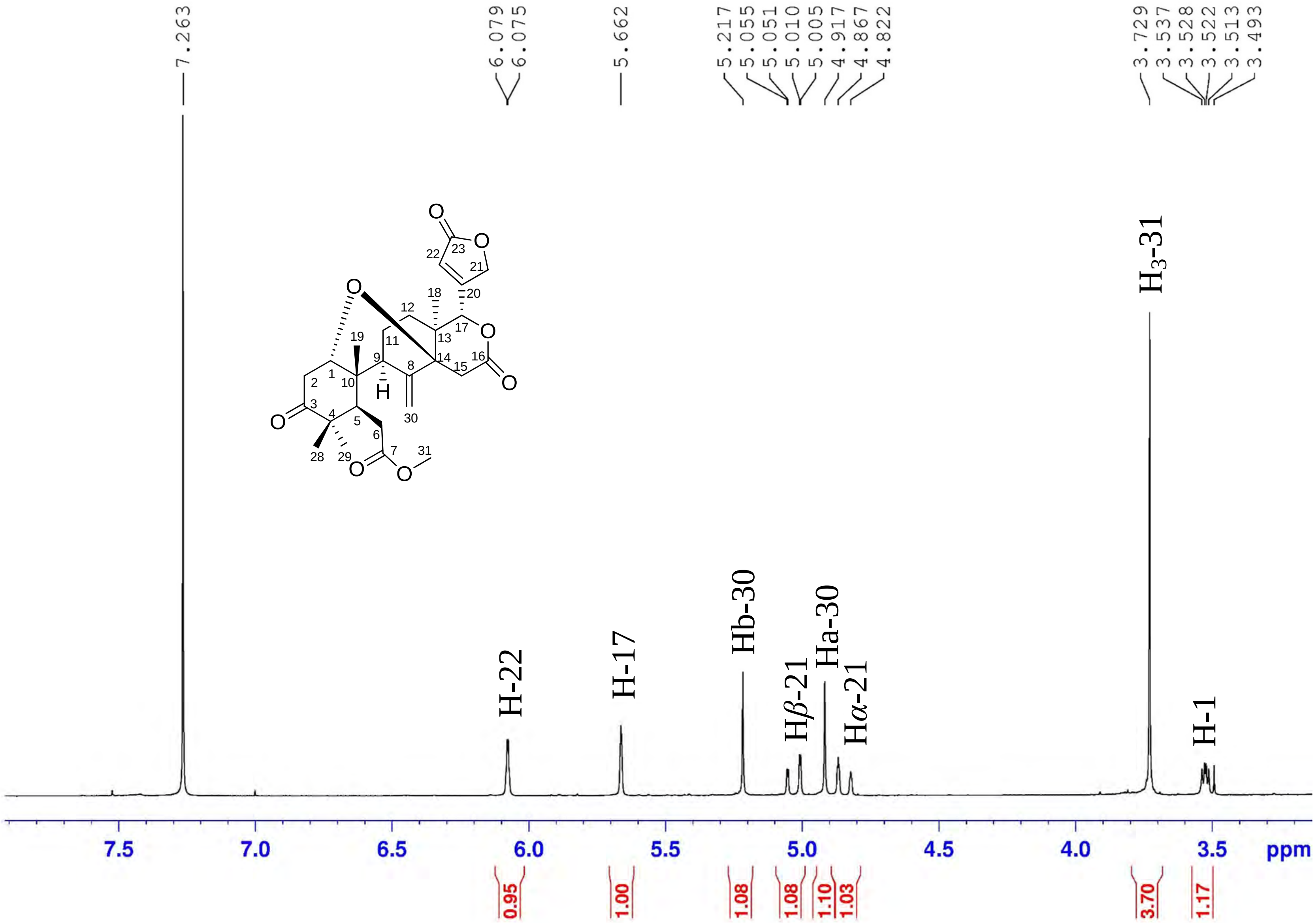

^{1}H NMR (400 MHz) spectrum of compound **29** in $CDCl_3$

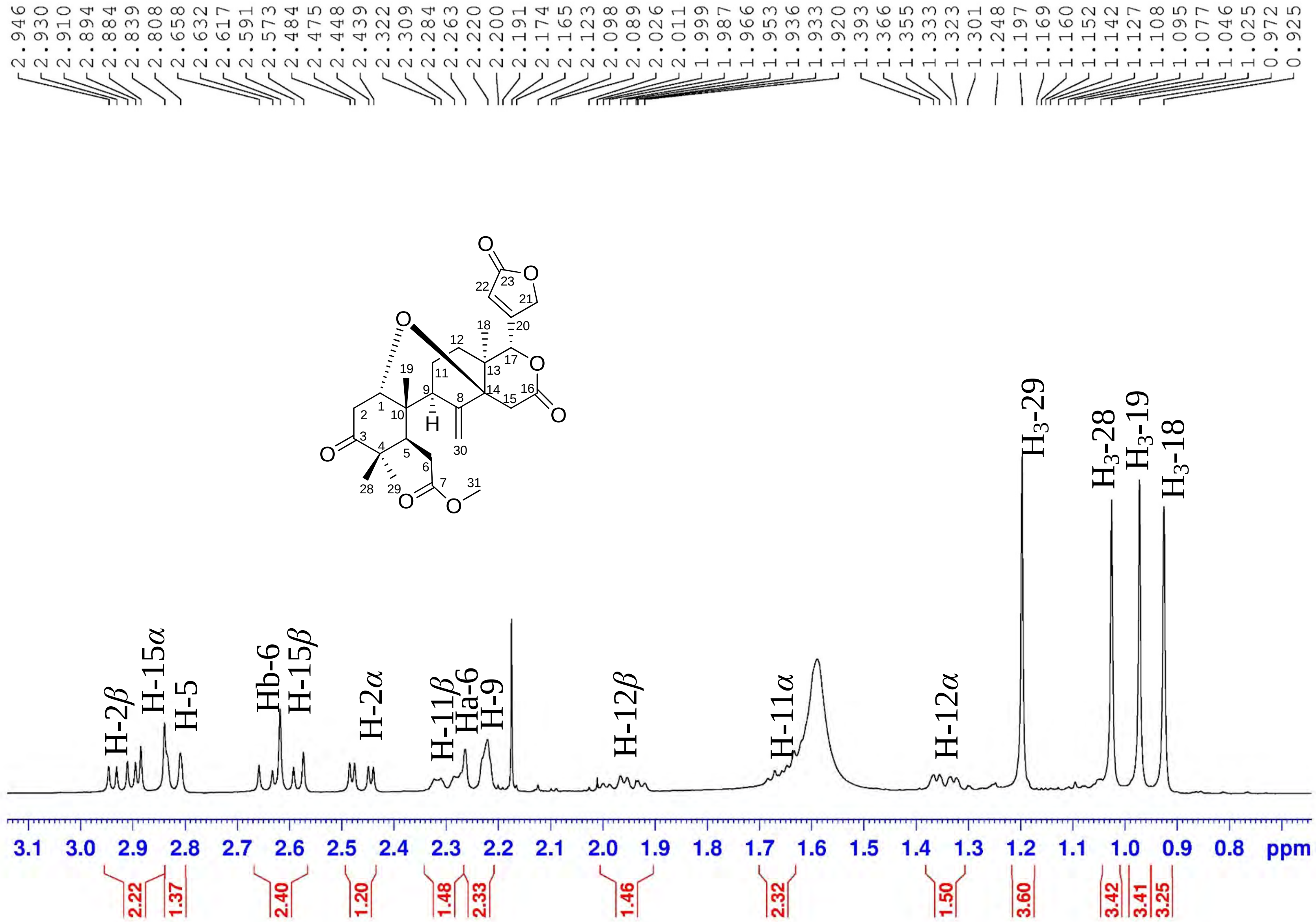

^{13}C NMR (100 MHz) spectrum of compound **29** in $CDCl_3$

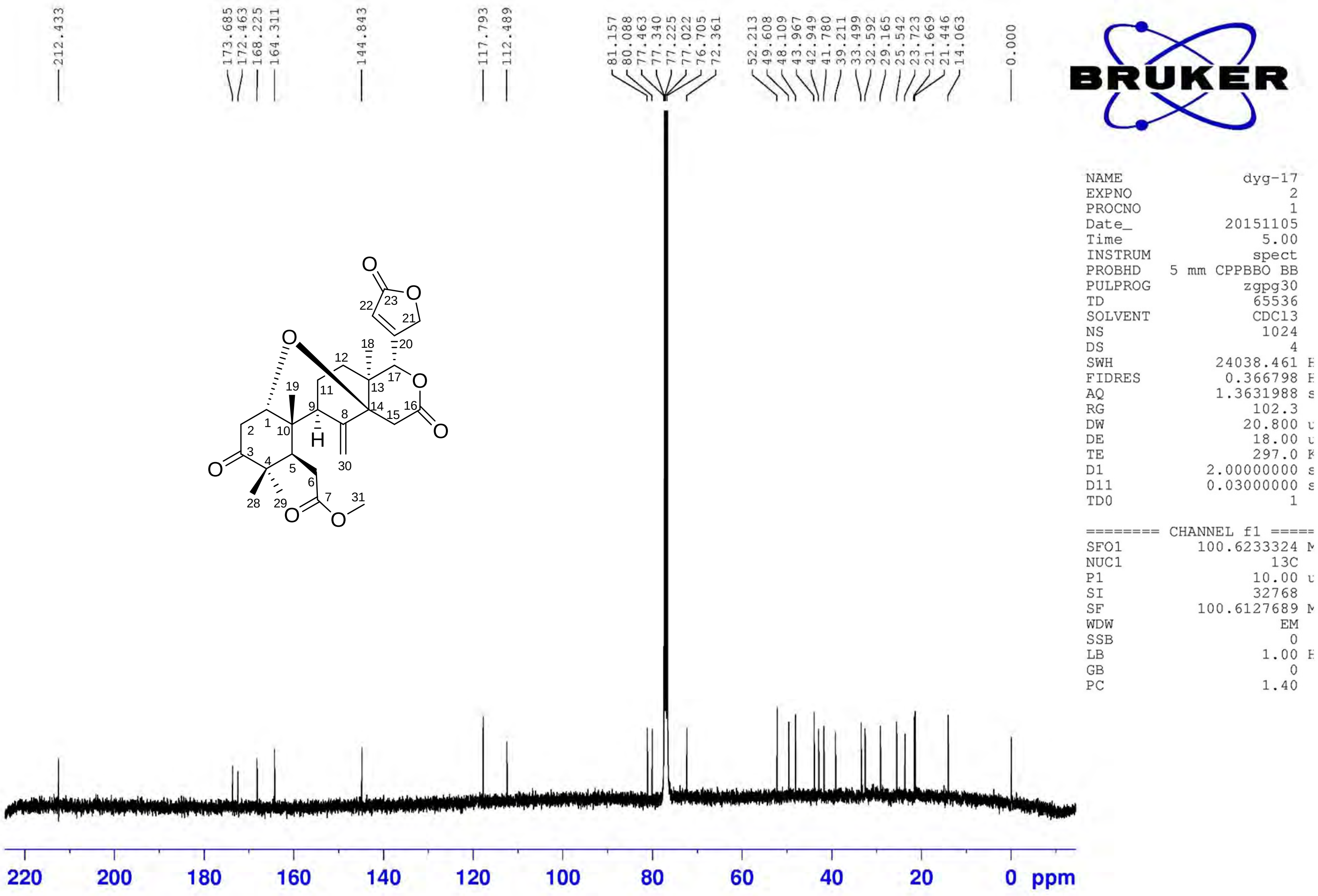

NAME	dyg-17
EXPNO	2
PROCNO	1
Date_	20151105
Time	5.00
INSTRUM	spect
PROBHD	5 mm CPPBBO BB
PULPROG	zgpg30
TD	65536
SOLVENT	CDCl3
NS	1024
DS	4
SWH	24038.461 H
FIDRES	0.366798 H
AQ	1.3631988 s
RG	102.3
DW	20.800 u
DE	18.00 u
TE	297.0 K
D1	2.00000000 s
D11	0.03000000 s
TD0	1
======== CHANNEL f1 =====	
SFO1	100.6233324 M
NUC1	13C
P1	10.00 u
SI	32768
SF	100.6127689 M
WDW	EM
SSB	0
LB	1.00 H
GB	0
PC	1.40

^{13}C NMR (100 MHz) spectrum of compound **29** in $CDCl_3$

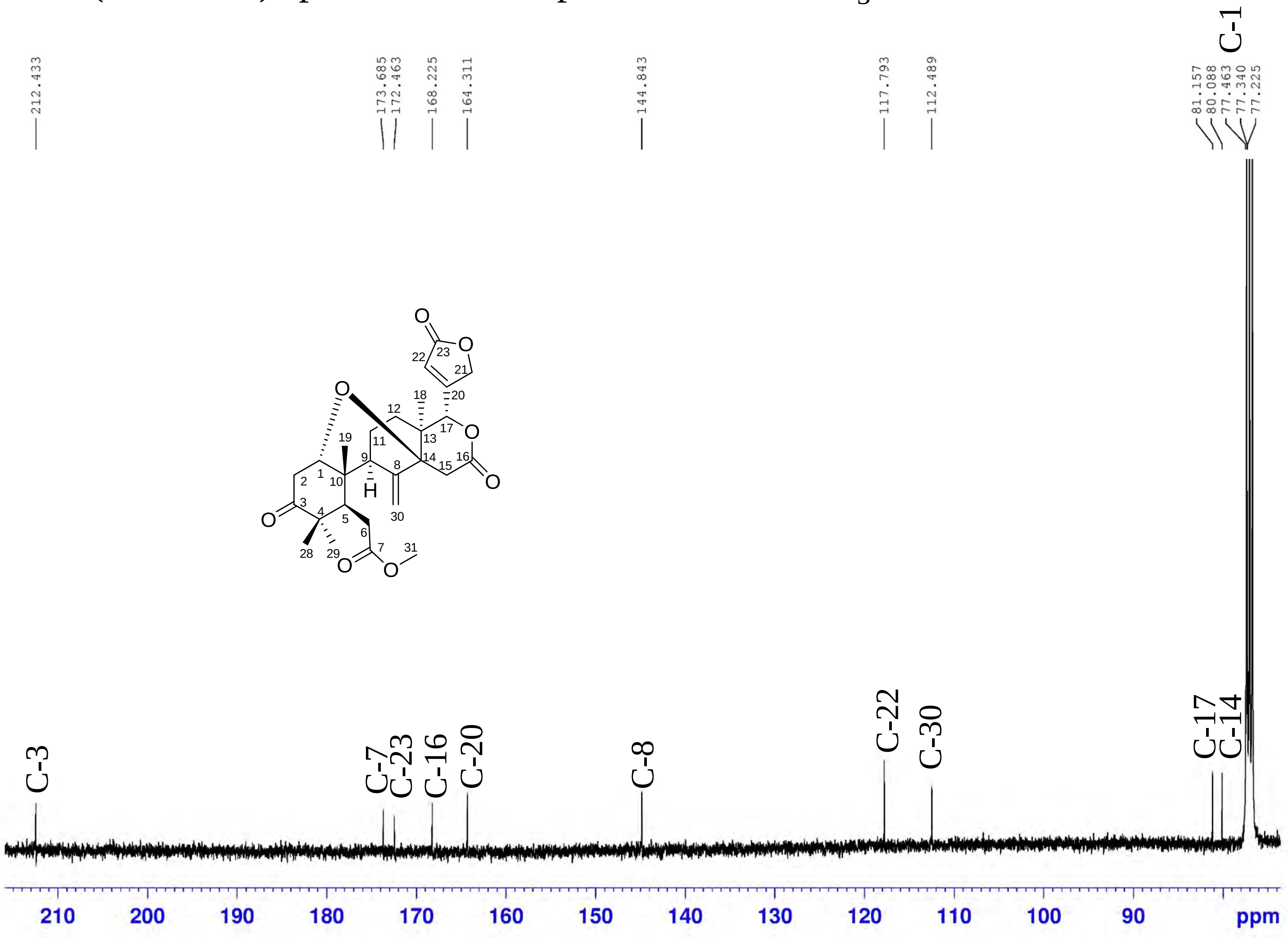

^{13}C NMR (100 MHz) spectrum of compound **29** in $CDCl_3$

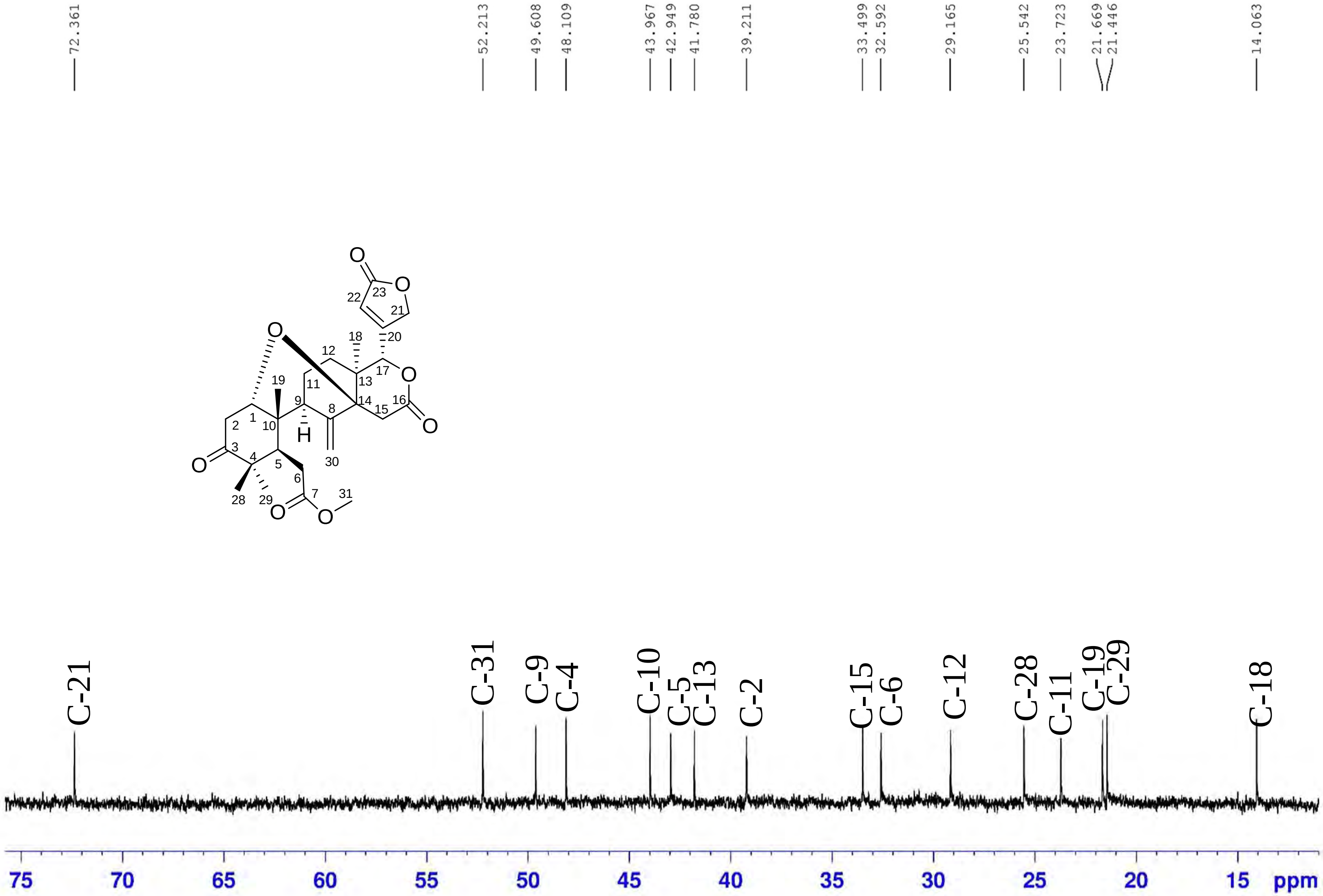

DEPT135 (100 MHz) spectrum of compound **29** in $CDCl_3$

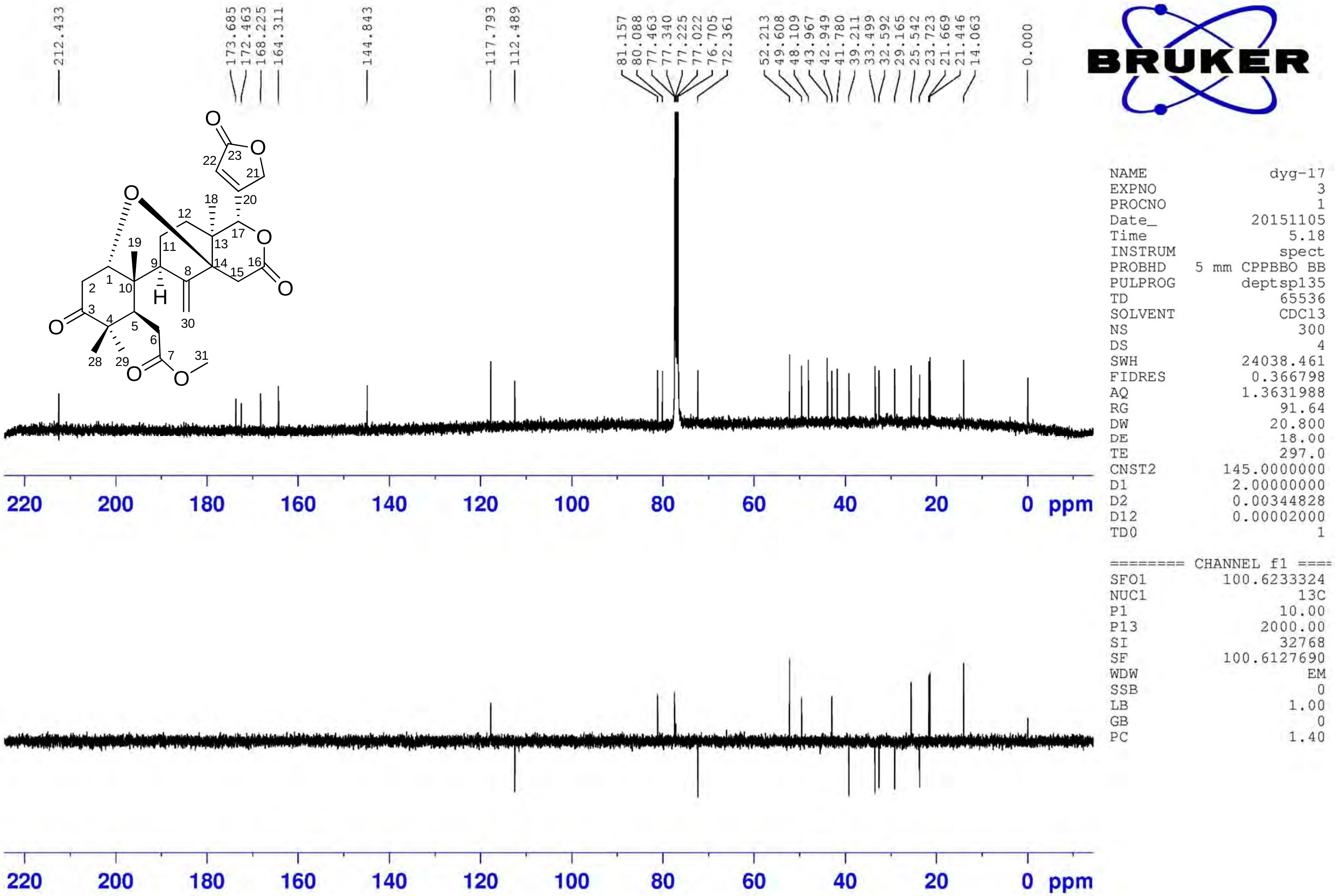

DEPT135 (100 MHz) spectrum of compound **29** in $CDCl_3$

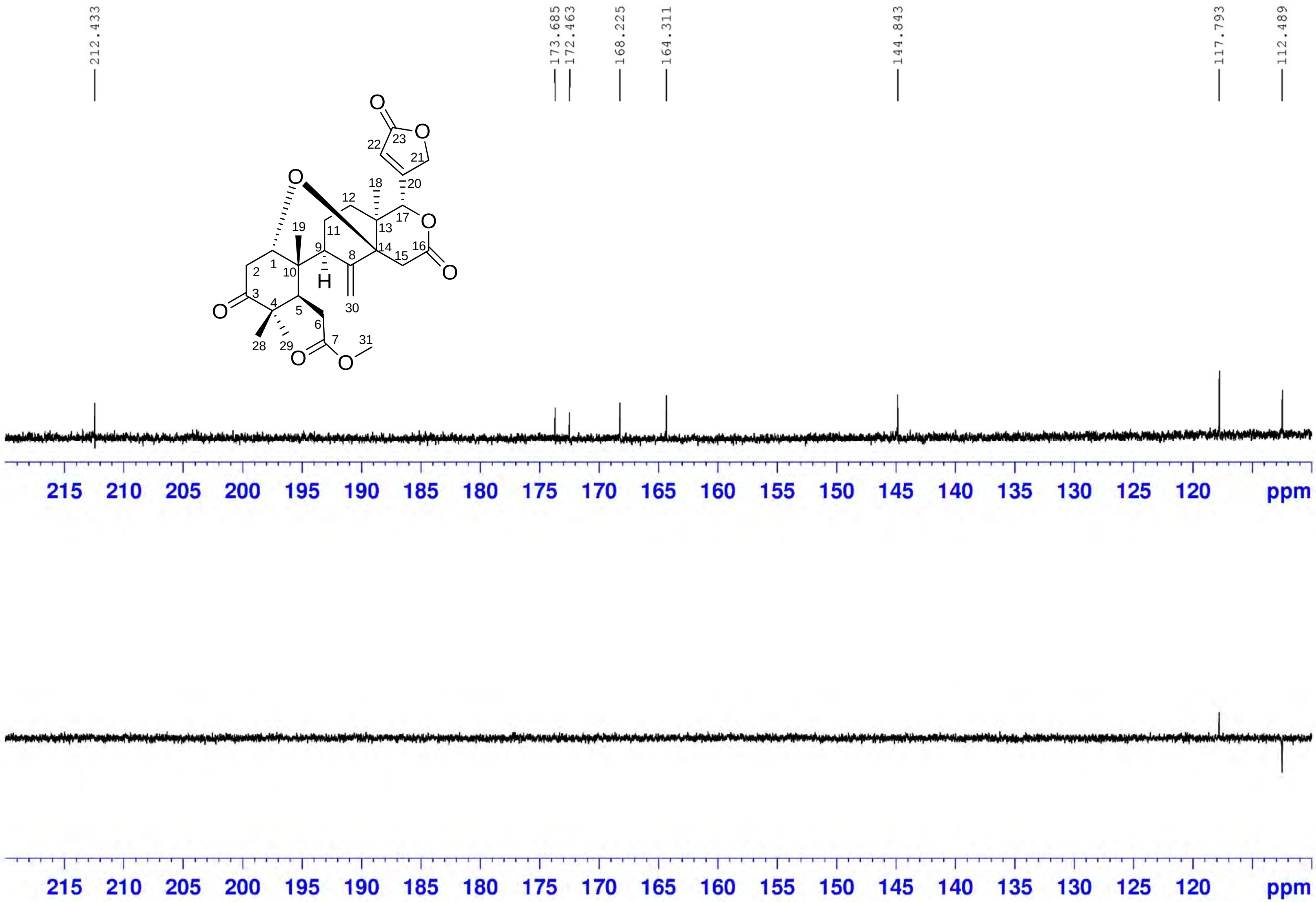

DEPT135 (100 MHz) spectrum of compound **29** in $CDCl_3$

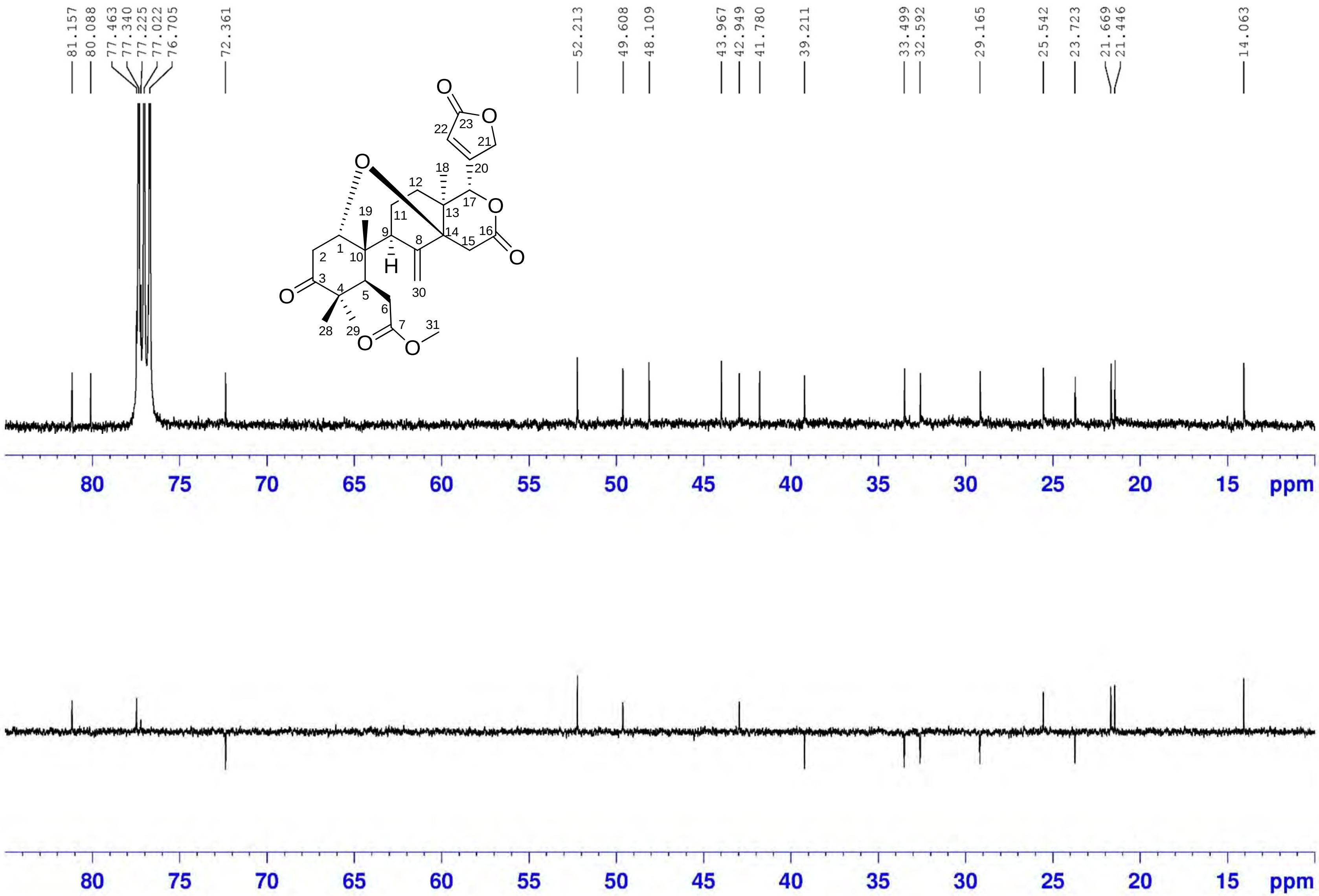

^{1}H-^{1}H COSY (400 MHz) spectrum of compound **29** in $CDCl_3$

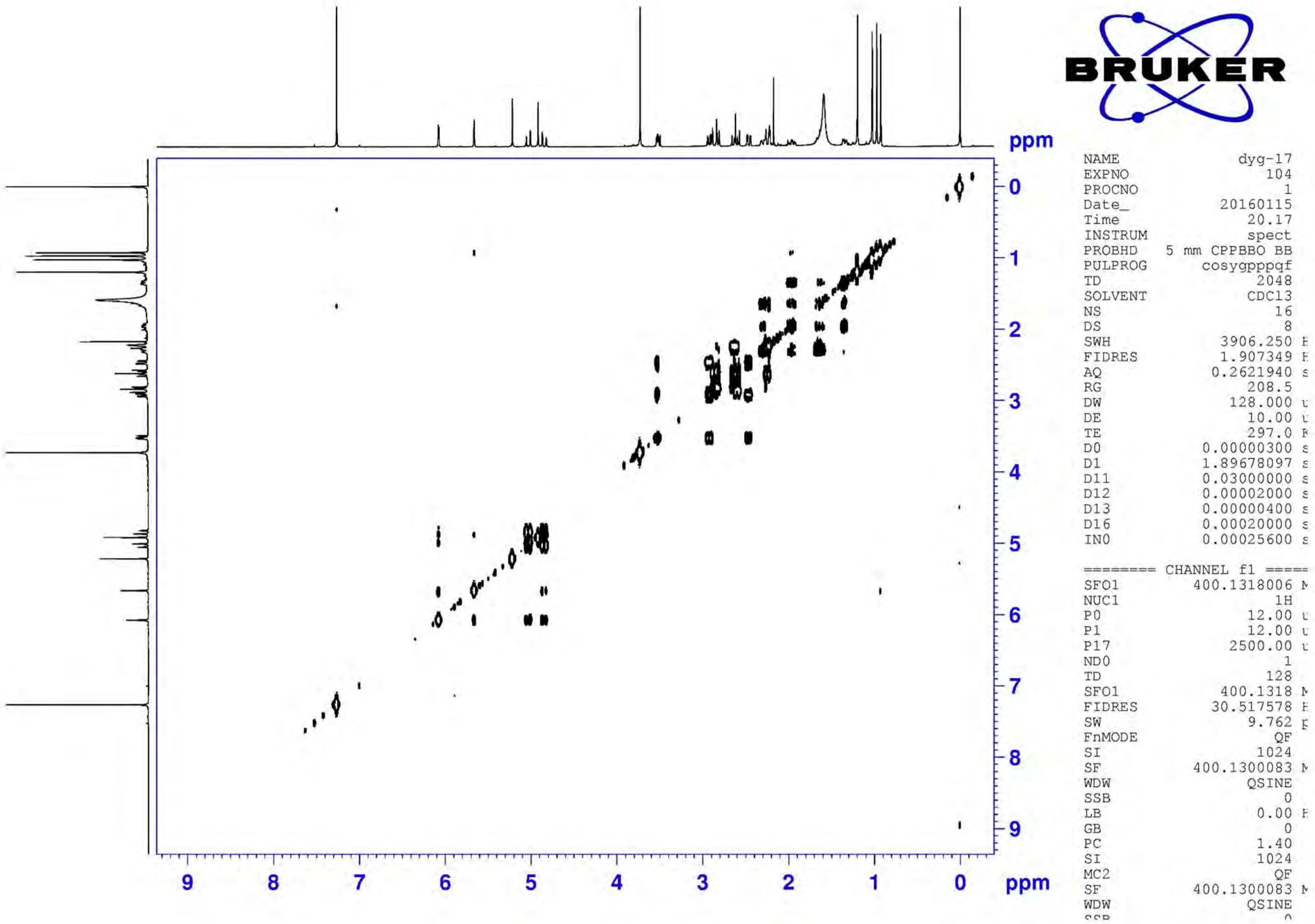

^{1}H-^{1}H COSY (400 MHz) spectrum of compound **29** in $CDCl_3$

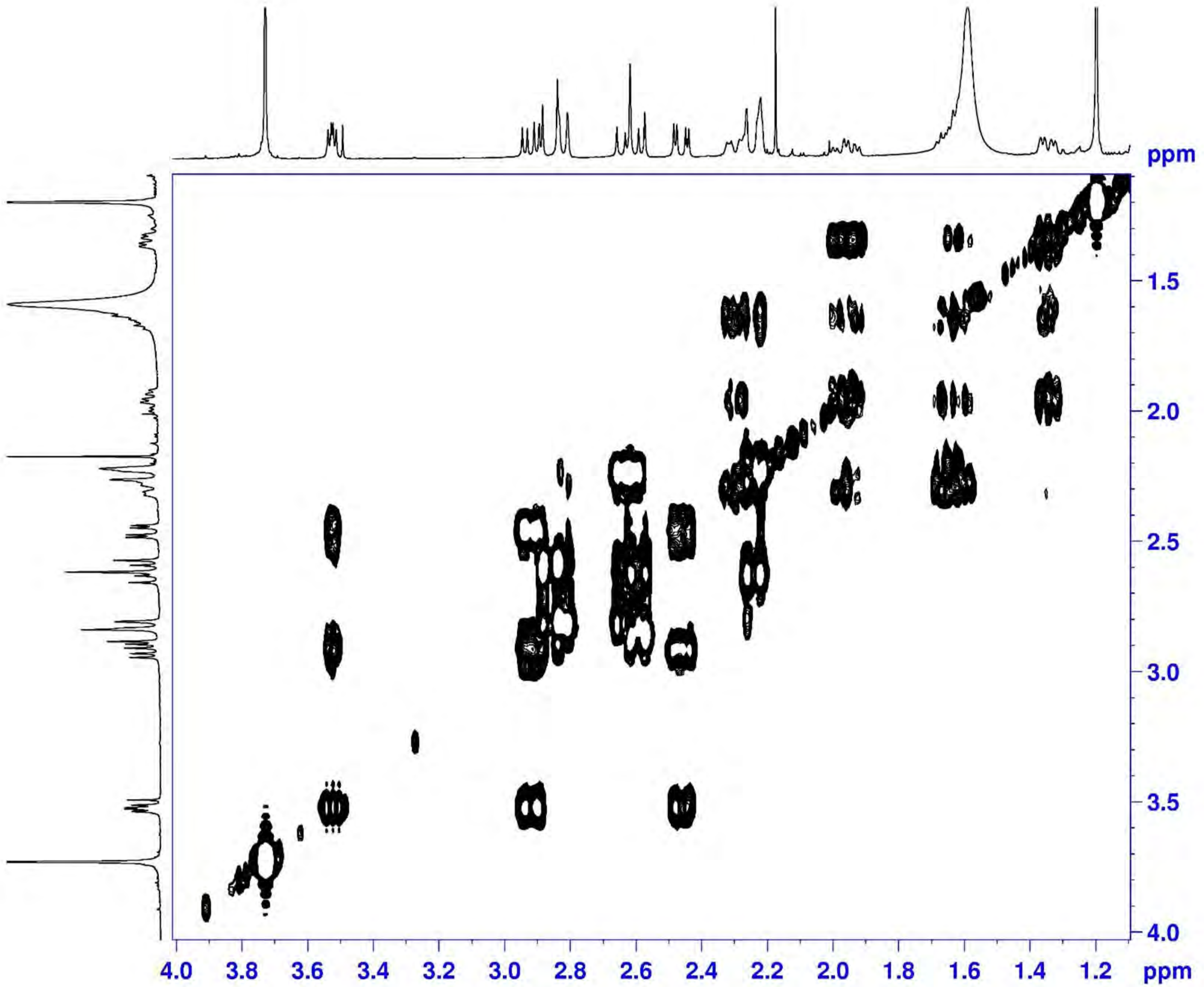

^{1}H-^{1}H COSY (400 MHz) spectrum of compound **29** in $CDCl_3$

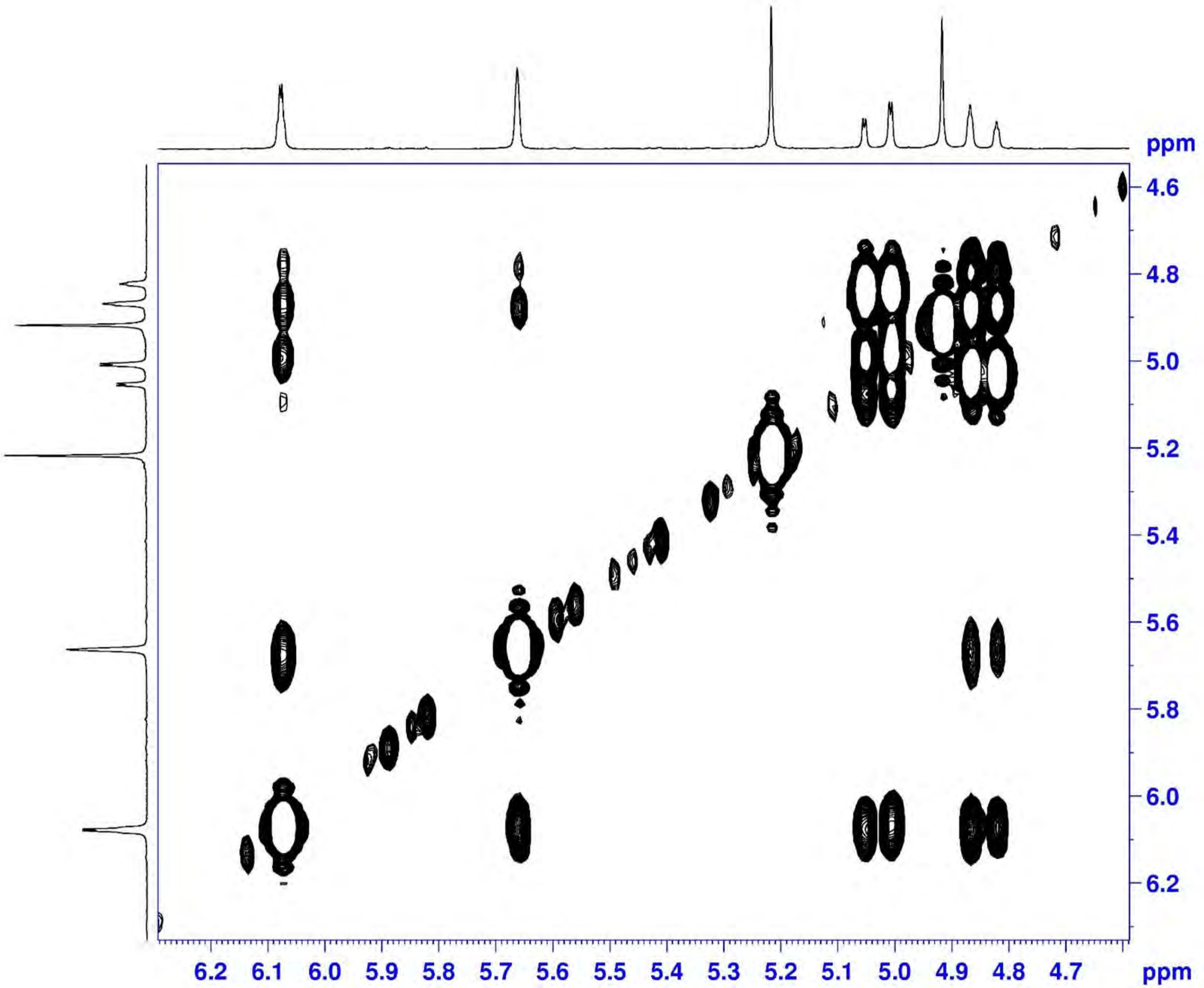

HSQC (400 MHz) spectrum of compound **29** in $CDCl_3$

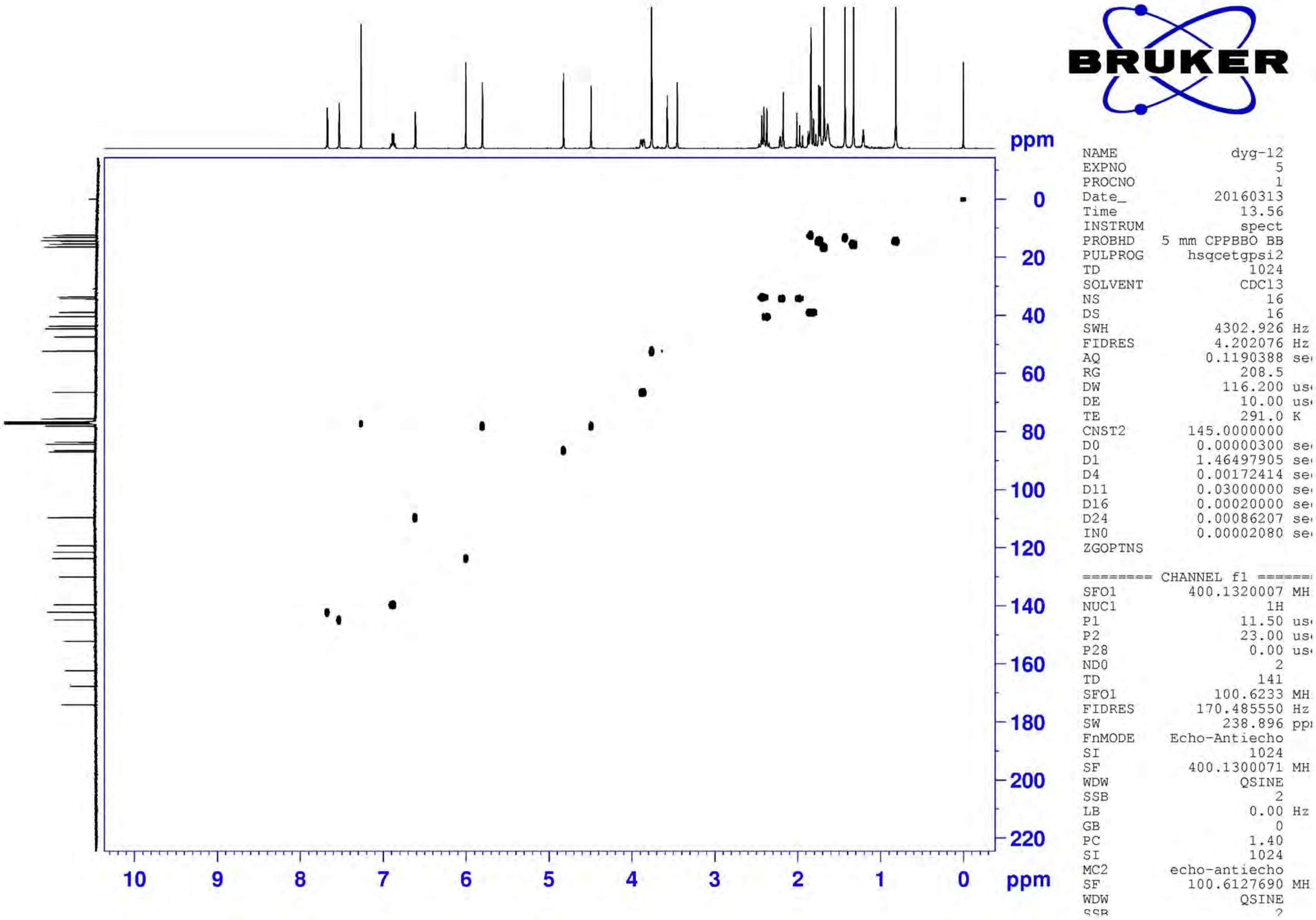

HSQC (400 MHz) spectrum of compound **29** in $CDCl_3$

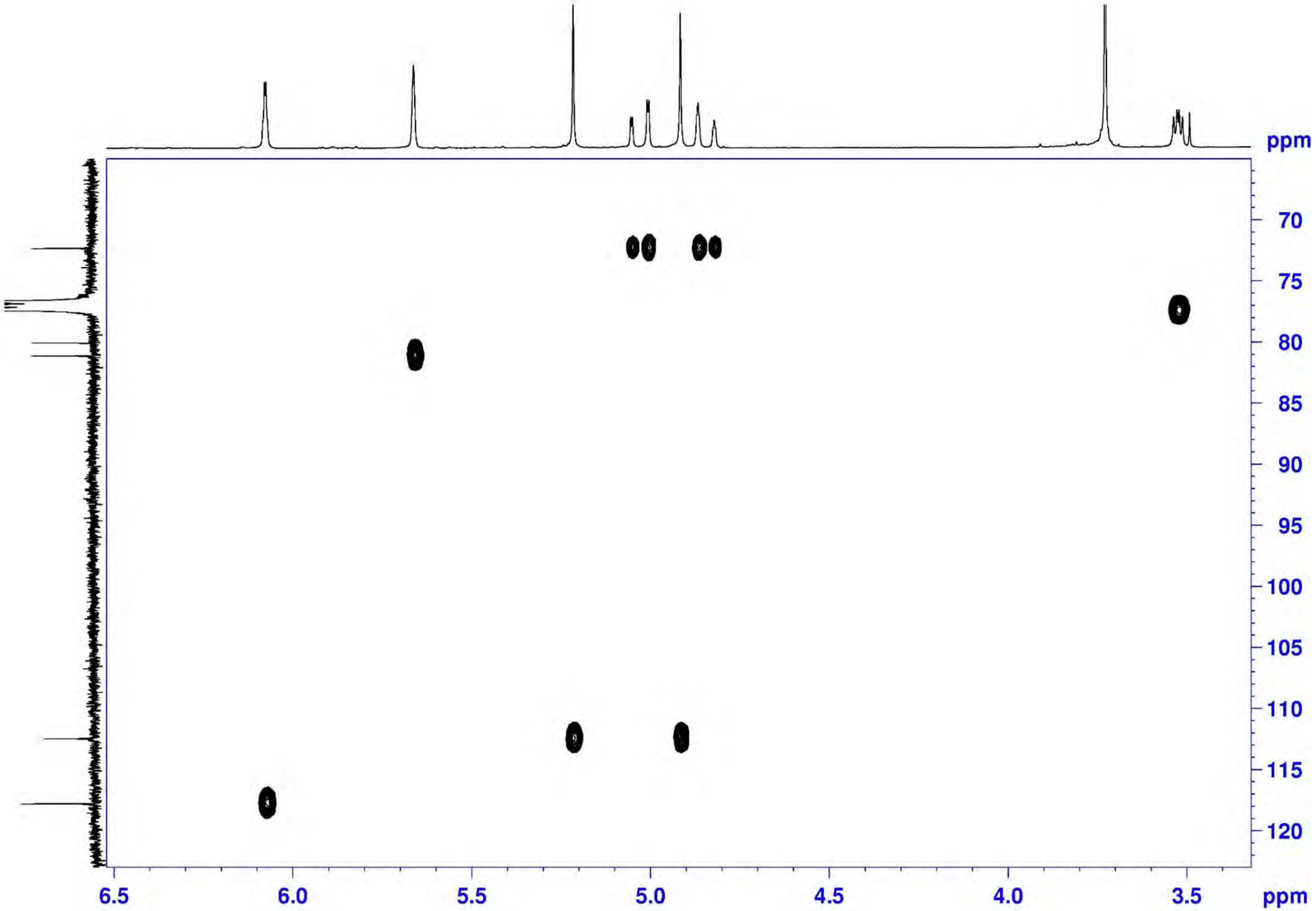

HSQC (400 MHz) spectrum of compound **29** in $CDCl_3$

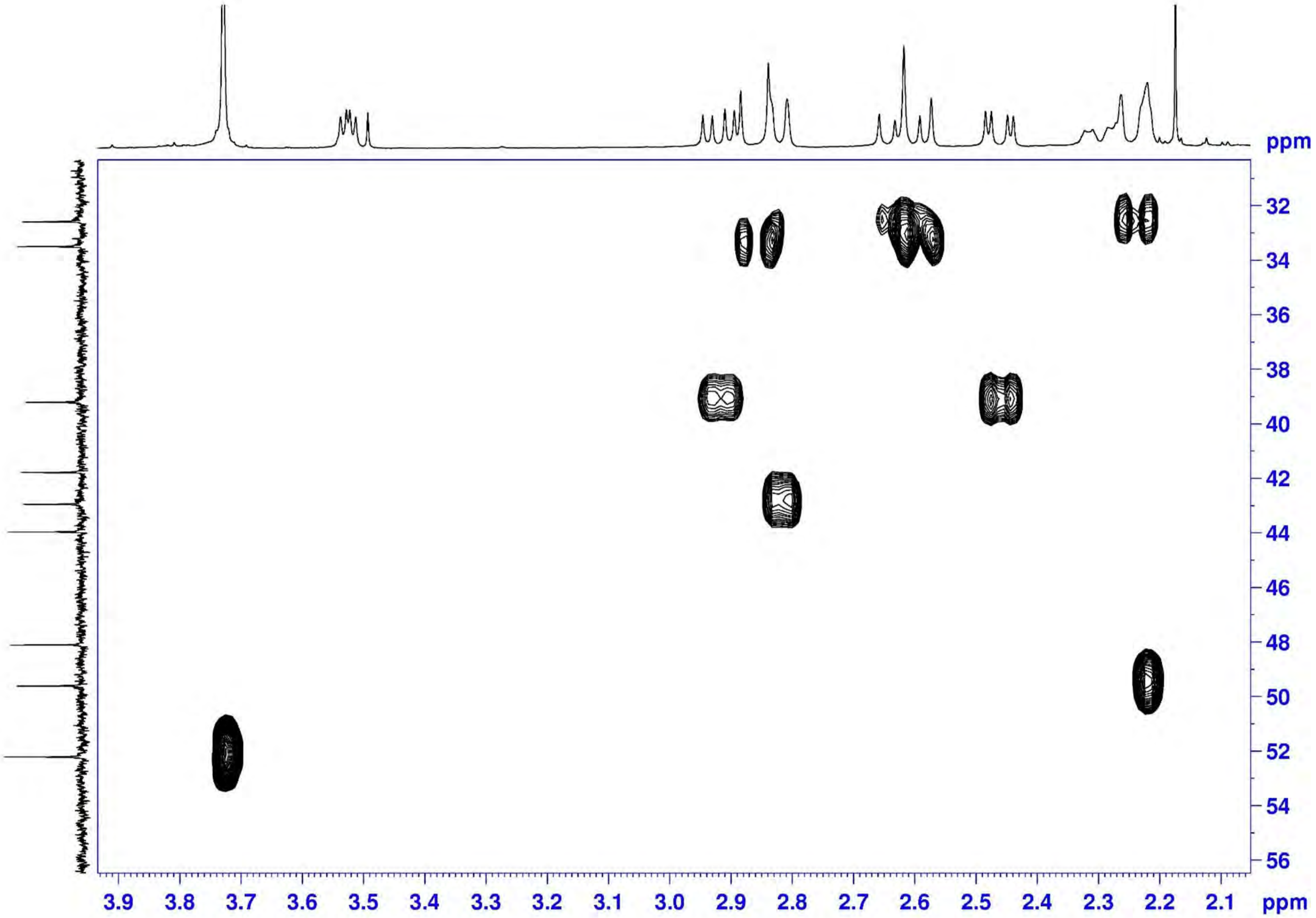

HSQC (400 MHz) spectrum of compound **29** in $CDCl_3$

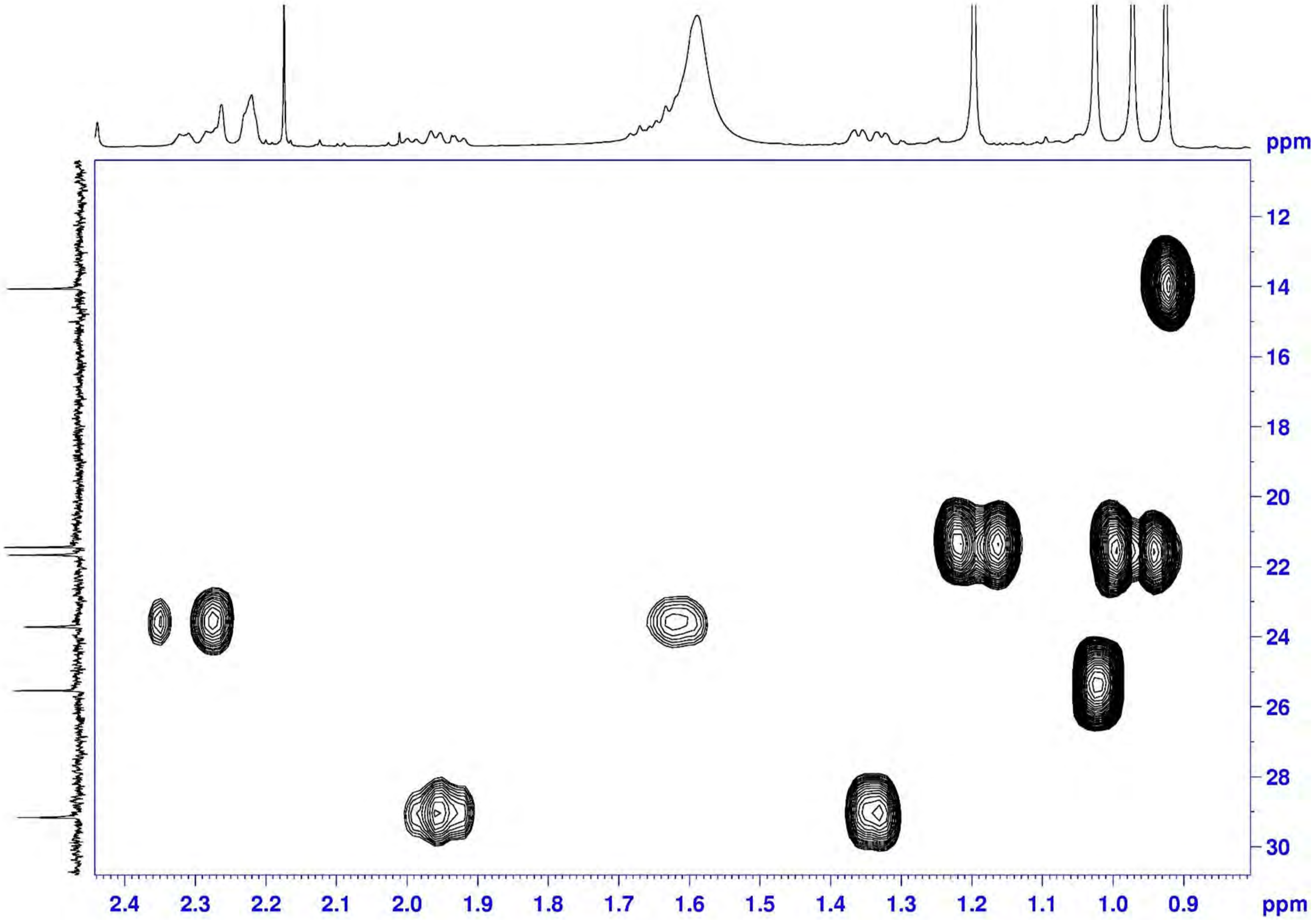

HMBC (400 MHz) spectrum of compound **29** in $CDCl_3$

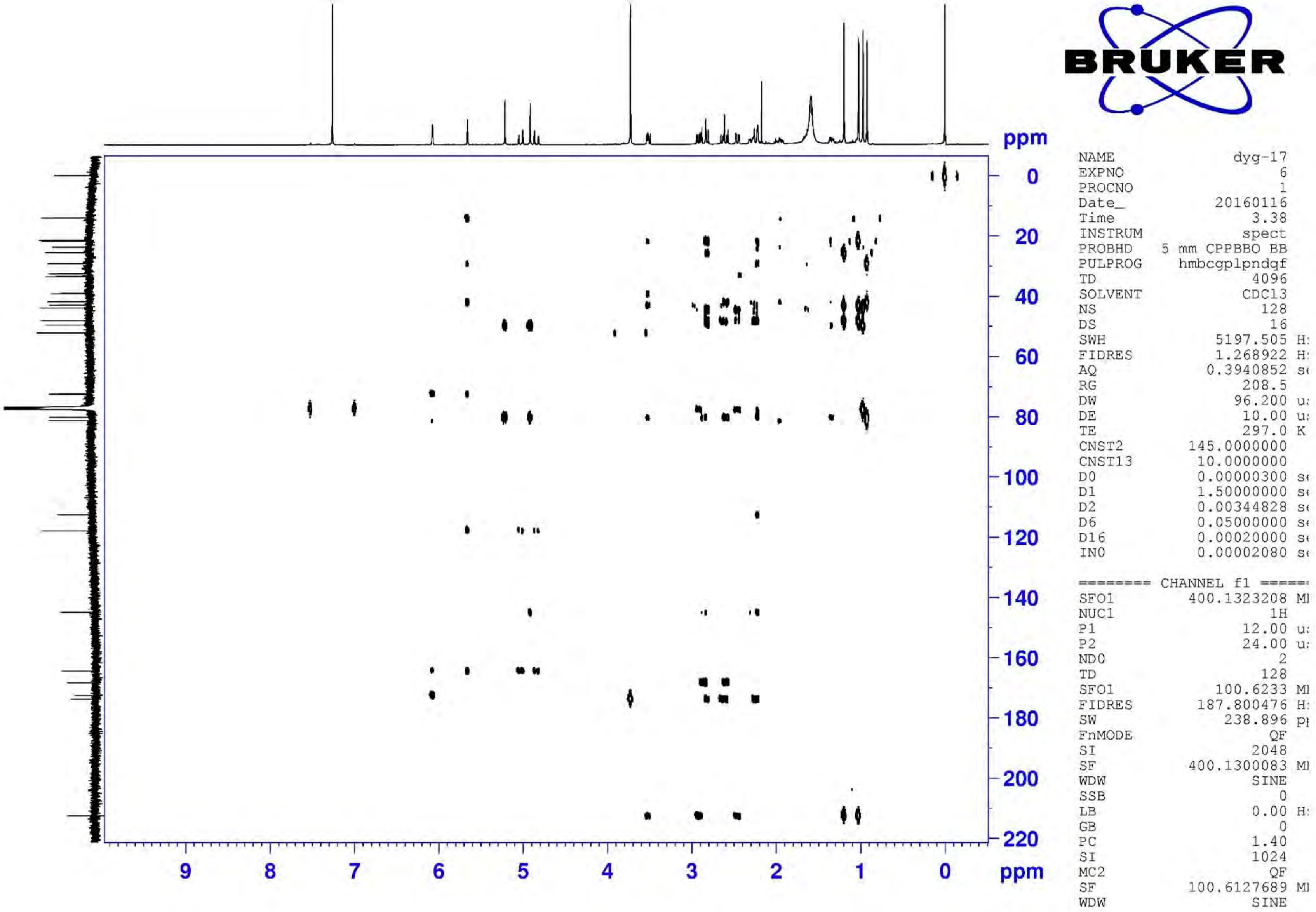

HMBC (400 MHz) spectrum of compound **29** in $CDCl_3$

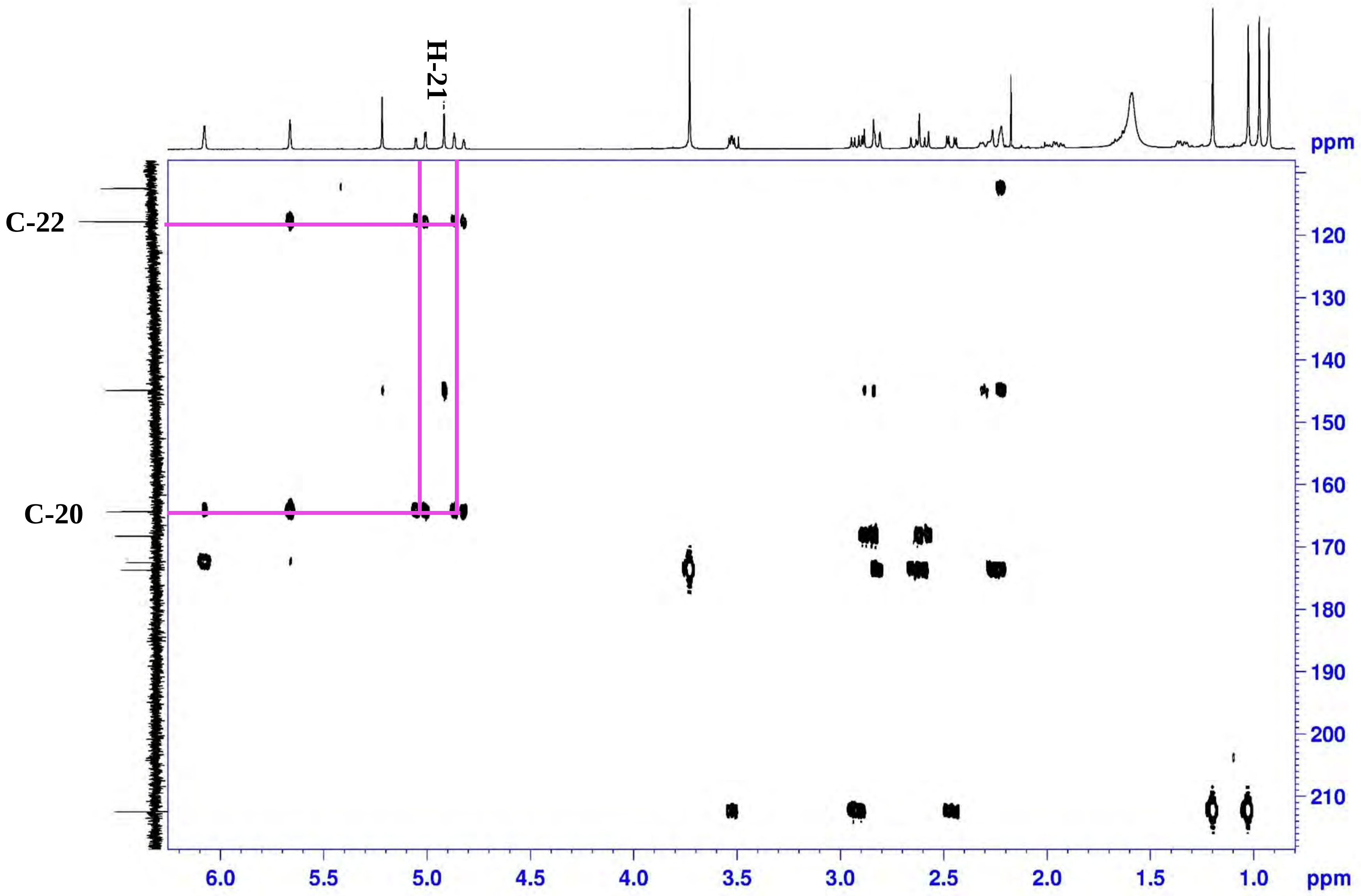

HMBC (400 MHz) spectrum of compound **29** in $CDCl_3$

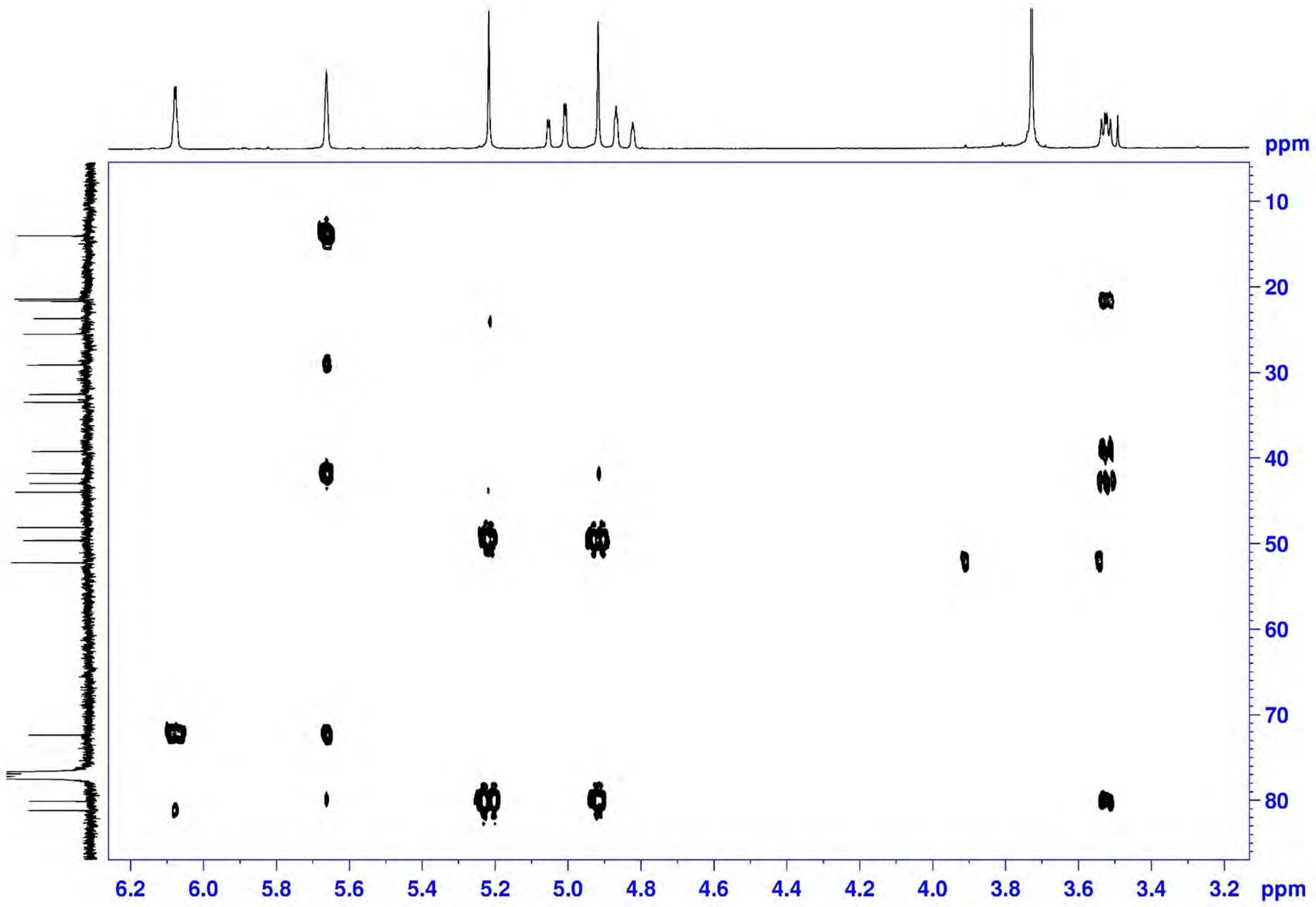

HMBC (400 MHz) spectrum of compound **29** in $CDCl_3$

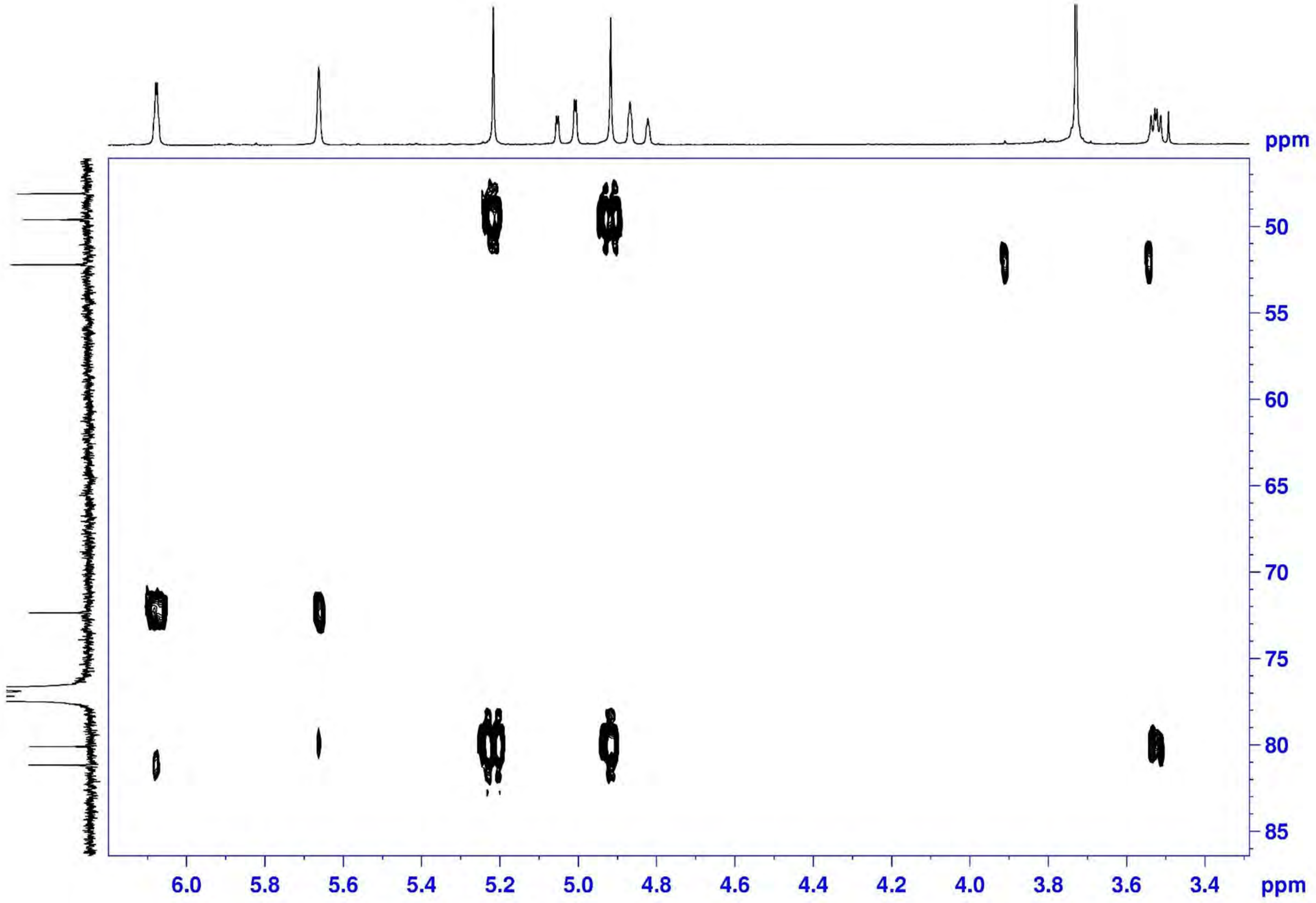

HMBC (400 MHz) spectrum of compound **29** in $CDCl_3$

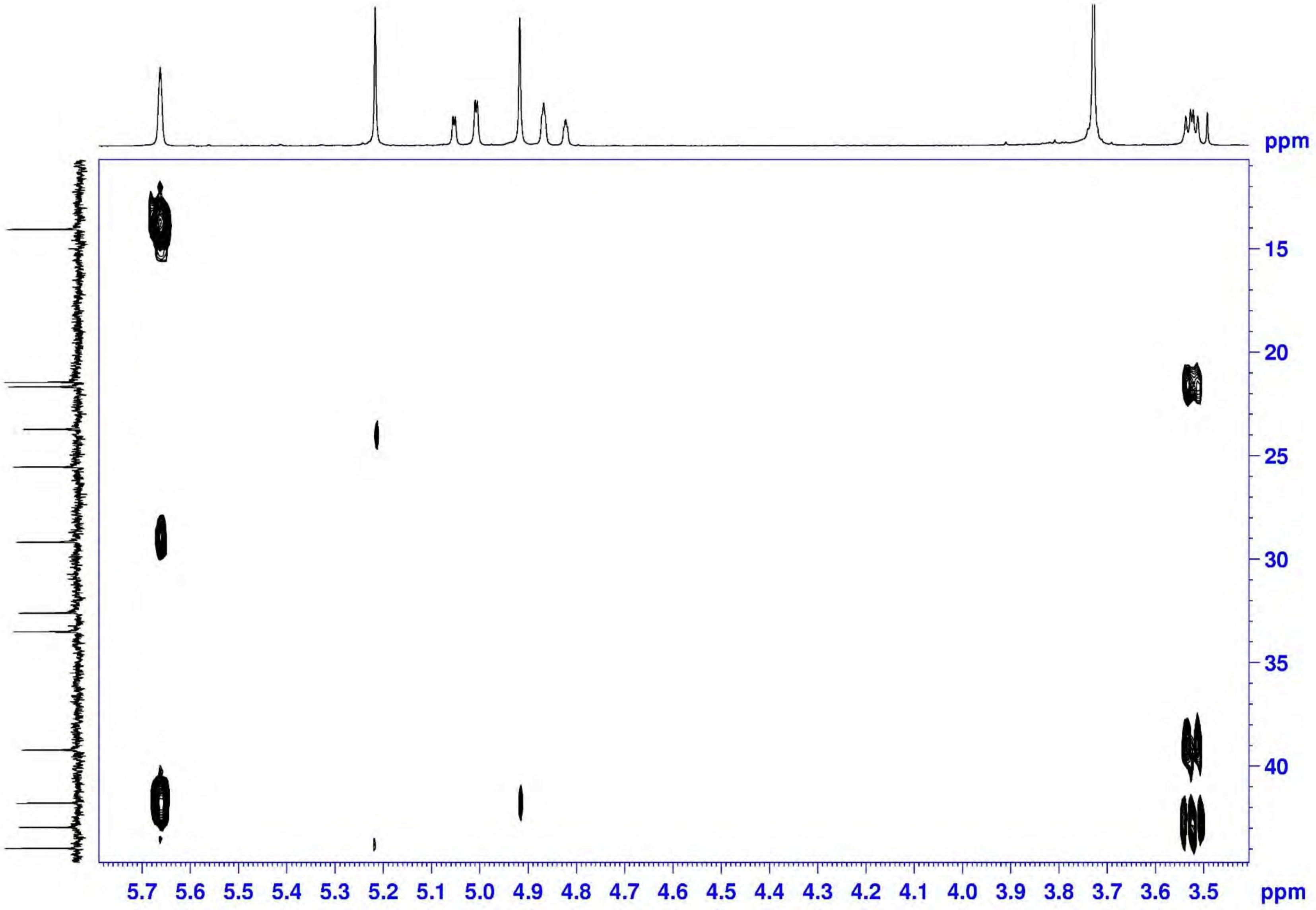

HMBC (400 MHz) spectrum of compound **29** in $CDCl_3$

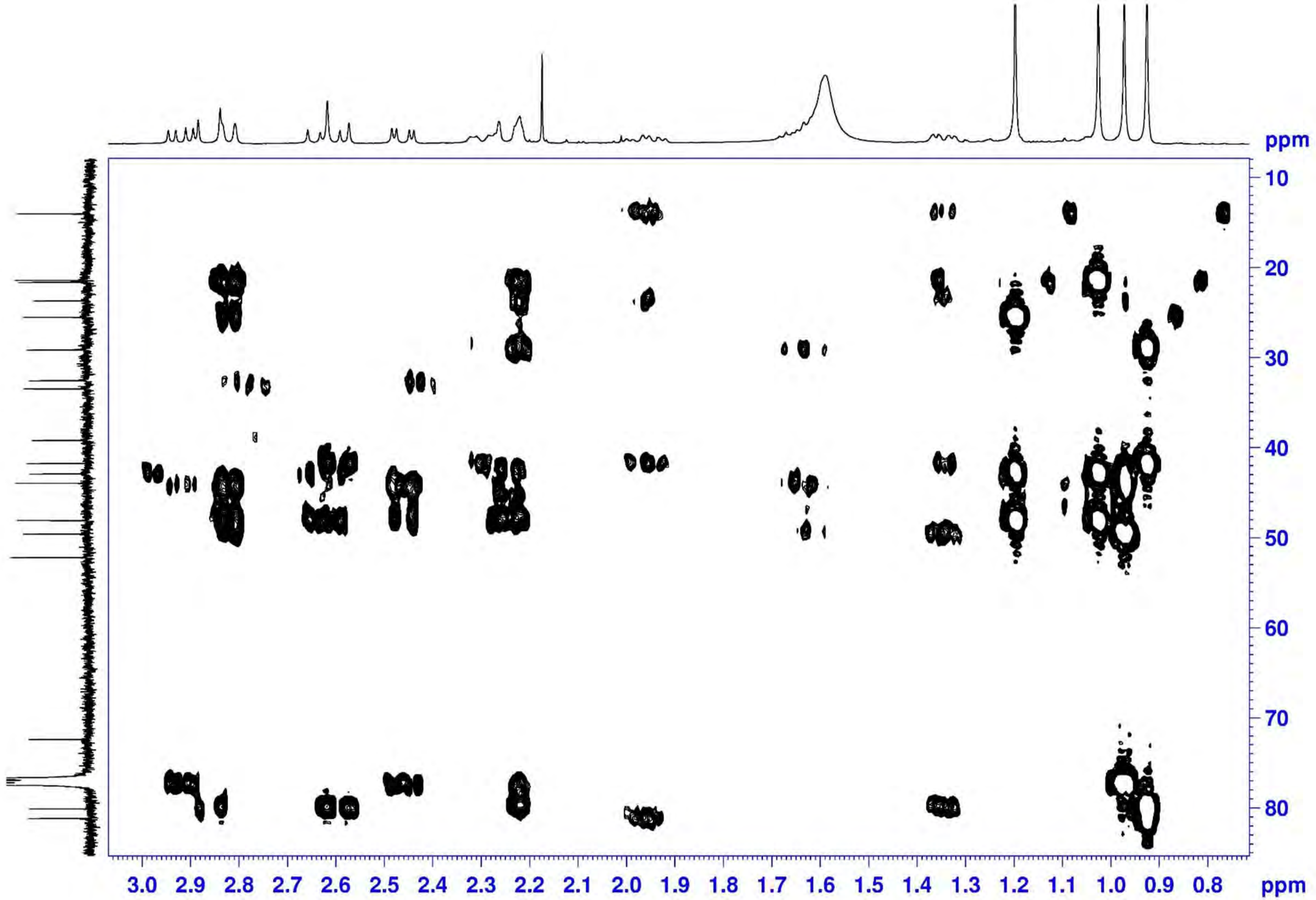

HMBC (400 MHz) spectrum of compound **29** in $CDCl_3$

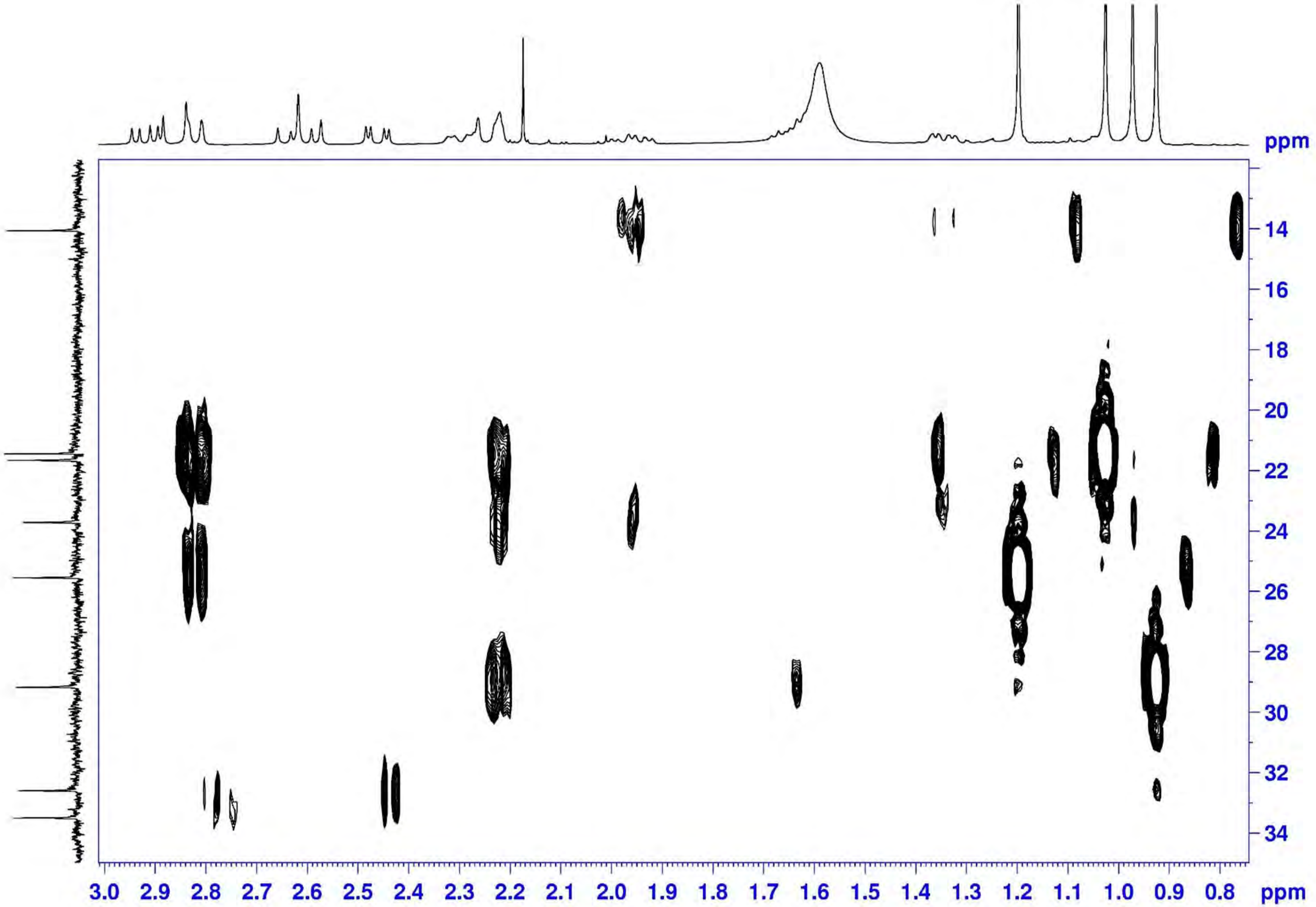

NOESY (400 MHz) spectrum of compound **29** in $CDCl_3$

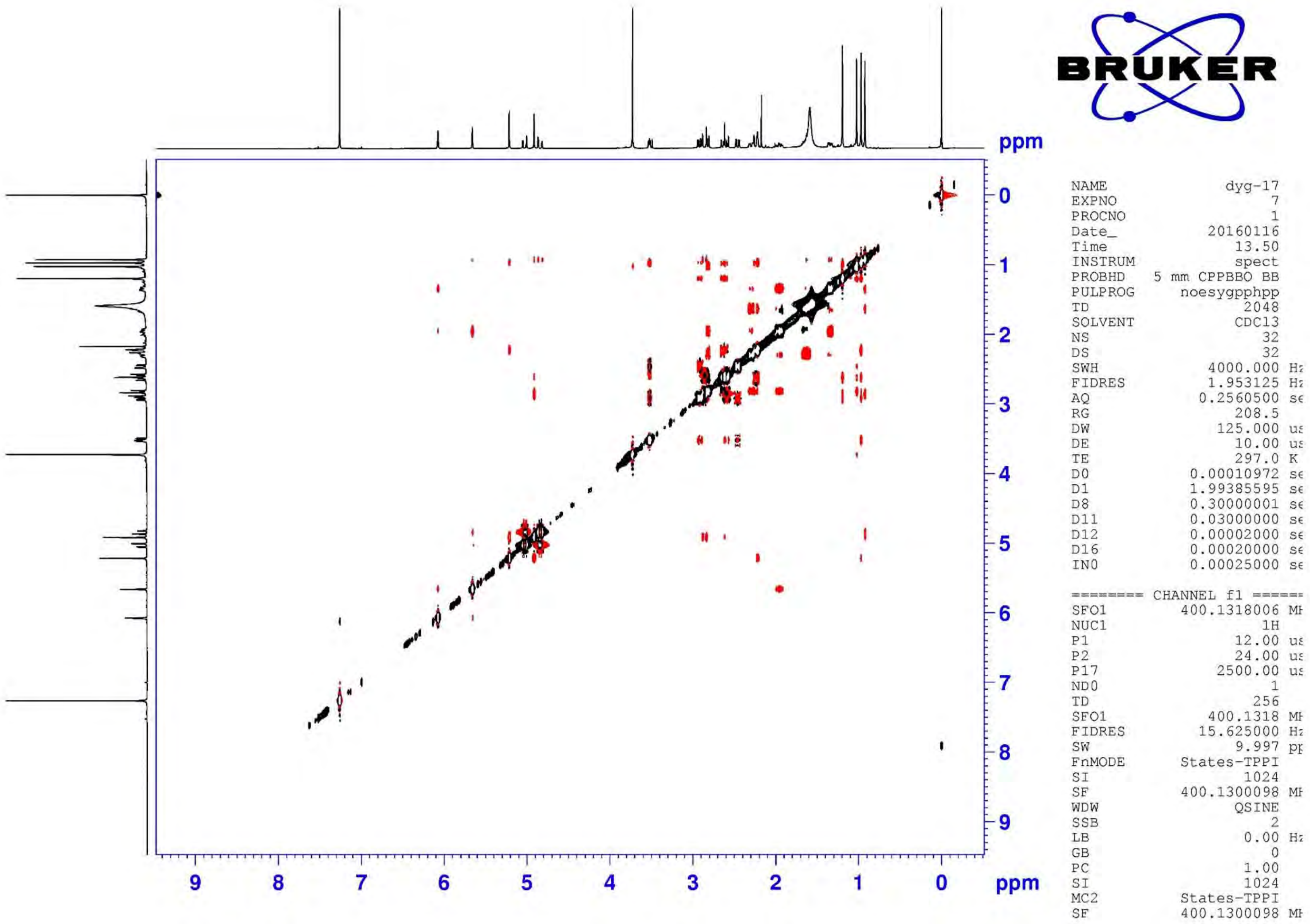

NOESY (400 MHz) spectrum of compound **29** in $CDCl_3$

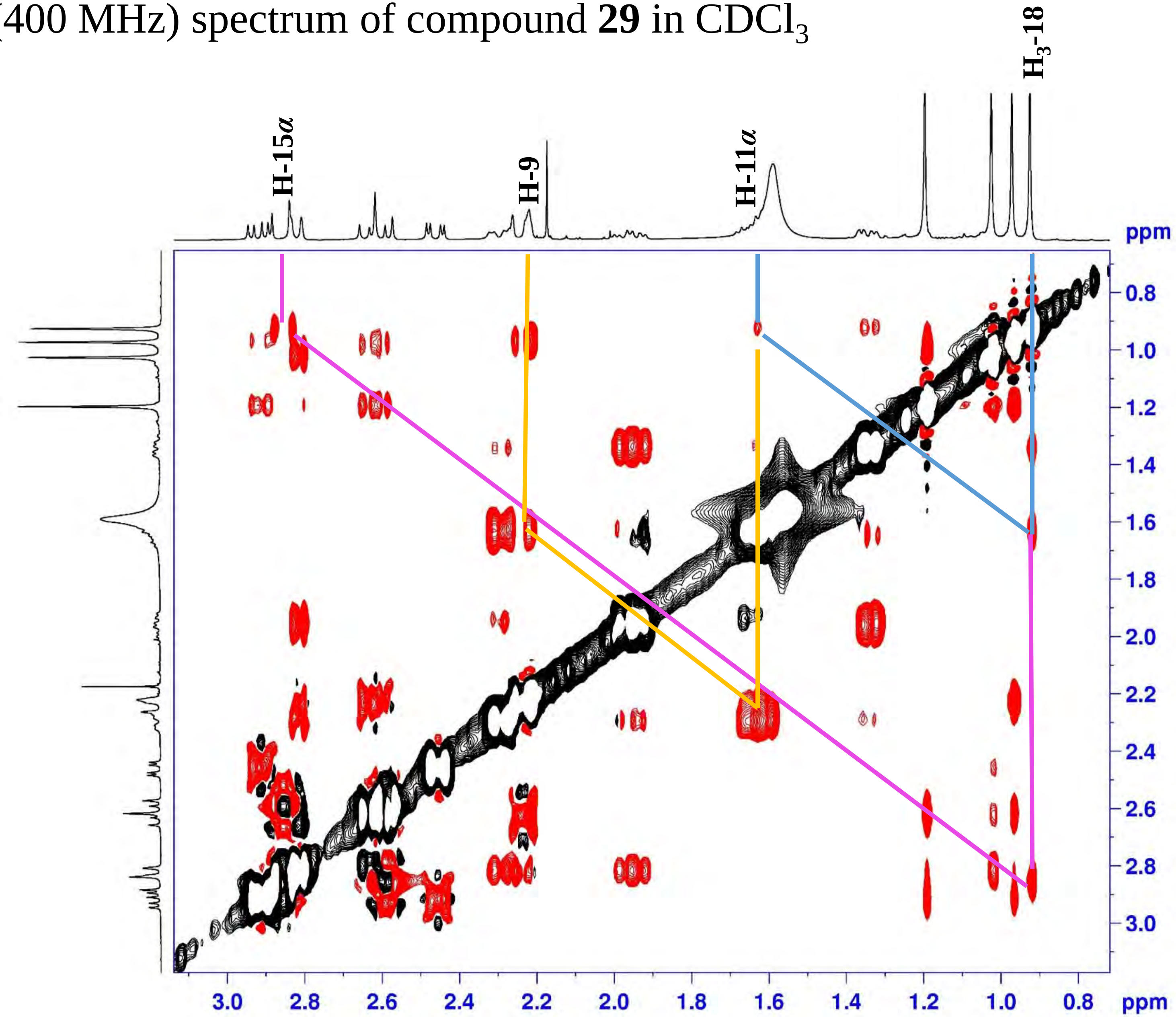

NOESY (400 MHz) spectrum of compound **29** in $CDCl_3$

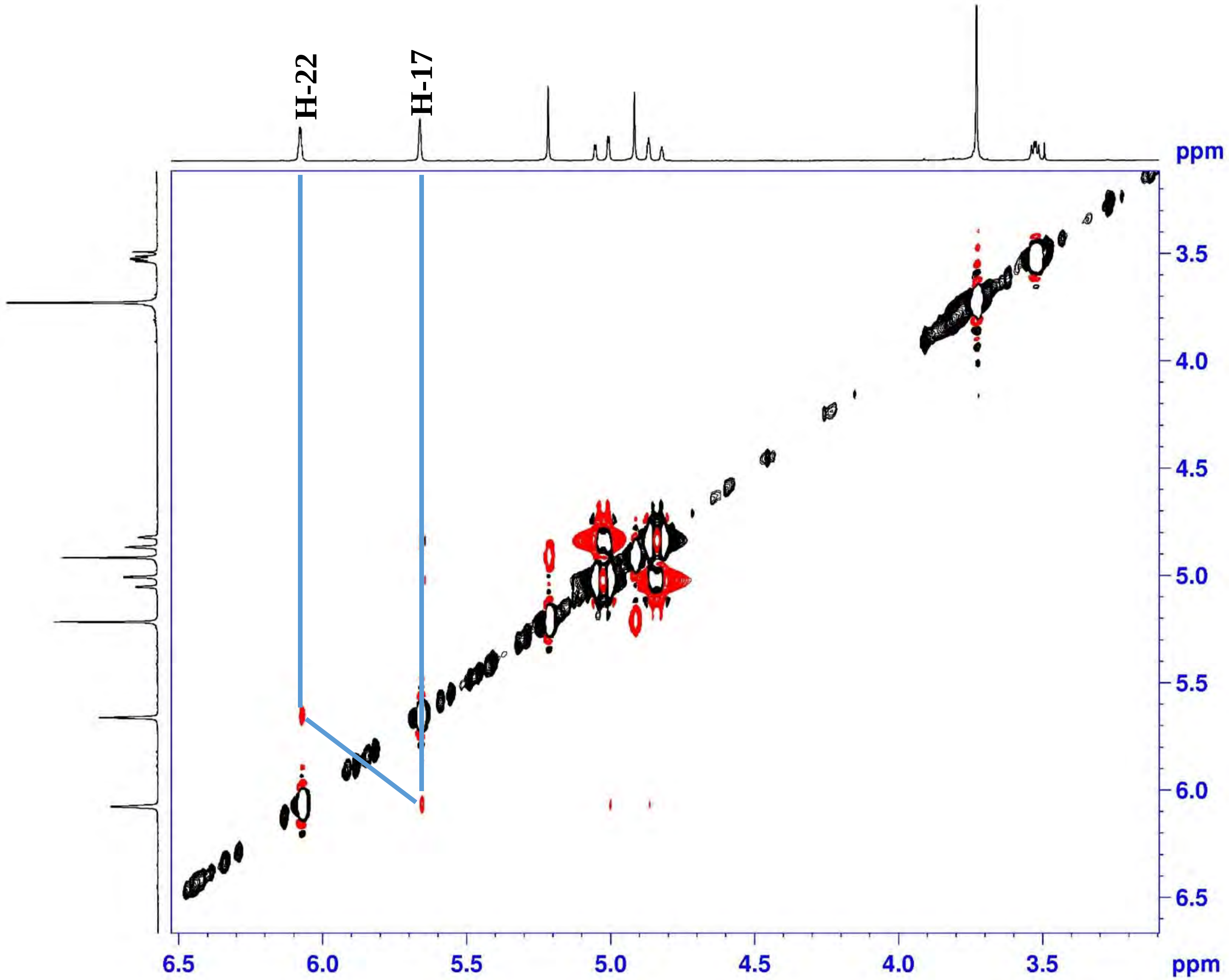

NOESY (400 MHz) spectrum of compound **29** in $CDCl_3$

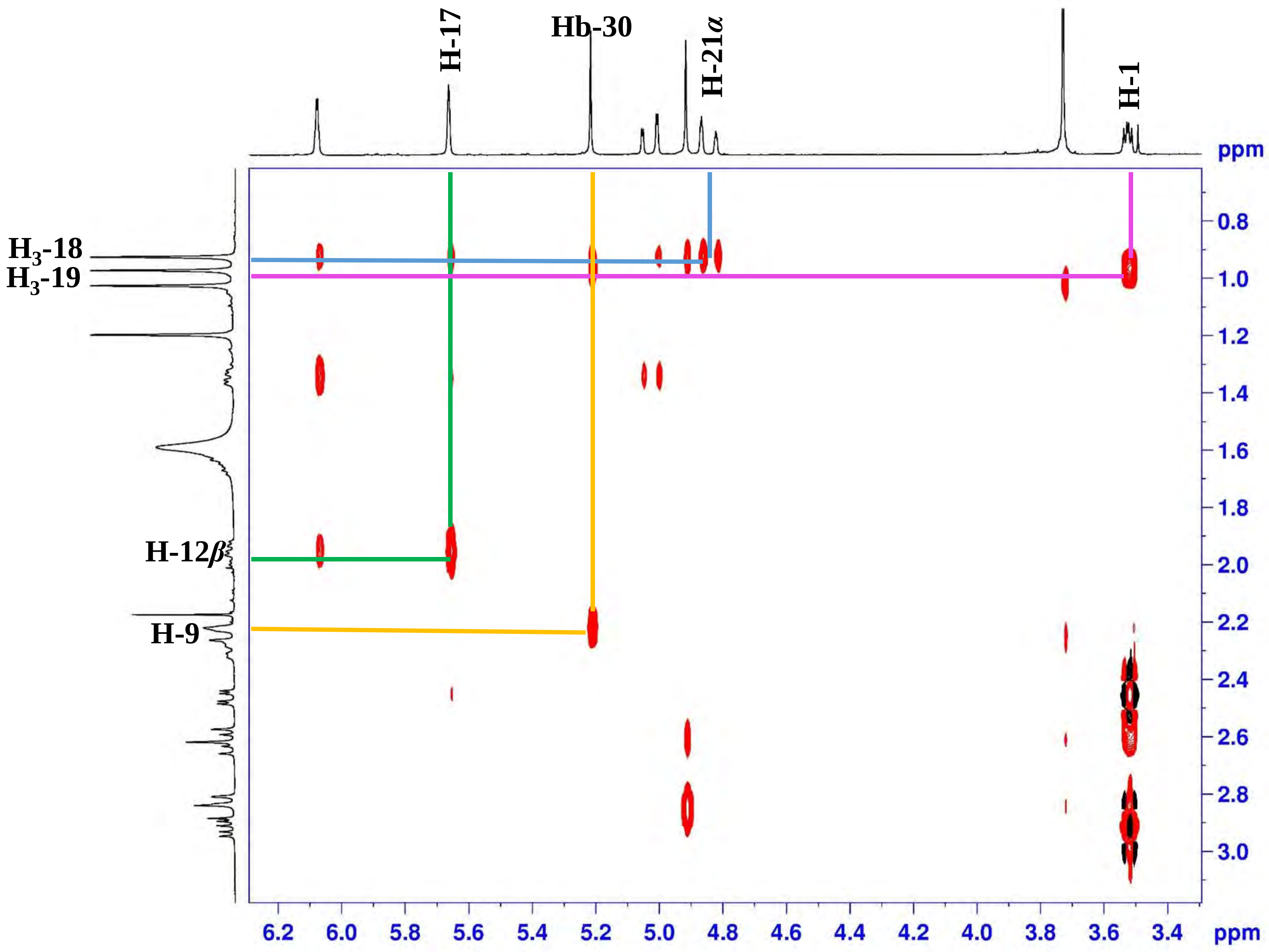

Supplement: Supplementary file 1 [file marinedrugs-16-00038-s001.zip › SI-for submission/SI-3.pdf]
